# Bone, dentin and cementum differentially influence the differentiation of osteoclast-like cells

**DOI:** 10.1038/s41598-025-04874-9

**Published:** 2025-06-05

**Authors:** Annika Both, Ghosn Ibrahim, Jana Marciniak, Birgit Rath-Deschner, Erika Calvano Küchler, Christian Kirschneck, Lina Gölz, Andreas Jäger, Svenja Beisel-Memmert

**Affiliations:** 1 Department of Orthodontics, Medical Faculty, University Hospital Bonn, Welschnonnenstr. 17, 53111 Bonn, Germany; 2 Private Practice, Euskirchen, Germany; 3 Department of Orthodontics and Orofacial Orthopedics, Friedrich-Alexander-University Erlangen-Nürnberg, Gluecksstrasse 11, 91054 Erlangen, Germany

**Keywords:** Orthodontic root resorption, Osteoclast differentiation, CXCL2, IGF-1, GDF15, HSPA1b, Gene expression, Biomarkers, Risk factors, Biomarkers, Genetics research

## Abstract

Our aim was to investigate how different oral hard tissues determine the differentiation of osteoclast-like cells. Murine macrophage cells were stimulated for 12 day with RANKL and M-CSF on dentin slices. Morphological changes of cells and hard tissues were examined by electron microscopy and toluidine blue staining. Cells were stimulated with RANKL and M-CSF on pulverized bone, dentin, cementum or polystyrene—with and without stimulation. TRAP staining was performed. To elucidate total gene expression, RNA sequencing was carried out. Four target genes (CXCL2, IGF-1, GDF15, HSPA1b) were selected and their expression was analyzed by RT-PCR and ELISA. Statistics comprised One-way ANOVA and Tukey’s test (*P* < 0.05). Stimulation induced differentiation of mouse macrophages into TRAP-positive osteoclast-like cells forming resorption pits on dentin. Gene expression analysis revealed that 1930, 446 and 87 genes were differentially regulated by cultivation on cementum, bone or dentin respectively compared to polystyrene. Culture on bone or dentin caused CXCL2 upregulation. In all stimulated groups IGF-1 was downregulated while GDF15 expression was elevated in cultures on dentin. Cultivation of cells on cementum resulted in an upregulated HSPA1b expression. Our results indicate that extracellular matrix of different oral hard tissues plays an important role in differentiation processes of osteoclast-like cells.

**Supplementary Information:**

The online version contains supplementary material available at 10.1038/s41598-025-04874-9.

## Introduction

Apical root resorption is among the most common and serious risks during orthodontic tooth movement. It can be assumed that there is a complex interplay of patient-specific and therapy related risk factors^1^. However, the biological etiology of this irreversible hard tissue loss of the tooth root by active osteoclast-like cells has not been adequately elucidated to date^2^.

During orthodontic treatment, forces initiate a complex metabolic process in the periodontal ligament (PDL)^3^. Proinflammatory cytokines, prostaglandins, chemokines and growth differentiation factors (such as CXCL2 and GDF15) are released which represent a sterile inflammatory reaction and initiate osteoclastic bone resorption^3–5^. At the same time, protective molecules are expressed to preserve tissue homeostasis in the PDL during the force induced stress reaction. In this regard, IGF-1 and HSP70 maintain cell physiology by inhibiting apoptosis and promoting proliferation, chemotaxis, differentiation and cell survival^6,7^.

Studies show that orthodontically induced root resorption is often associated with localized over-compression and sterile coagulation necrosis in the PDL which subsequently turns into a cell-free hyaline zone^8^. As a result, multinucleated cells with osteoclast character, TRAP-negative fibroblast-like cells and macrophages migrate from the adjacent vital PDL and the blood to initiate the removal of the necrotic and hyalinized tissue. In addition, those multinucleated cells with osteoclast character participate in the reorganization of the periodontal ligament^9,10^. However, it is mostly unavoidable that the degradation of the hyaline zones is accompanied by partial removal of the adjacent cementoid layer, an organic tissue covering the outer tooth root^10^. As a result, the mineralized root surface is denuded, allowing osteoclast-like cells to gain access and adhere to the dental hard tissues^11^. The recognition and attachment to mineral surfaces is mediated by integrins which interact with the extracellular matrix of the underlying substrate^12,13^. Thereby, the direct contact with certain extracellular matrix proteins causes activation of osteoclast-like cells and thus initiates the resorption of the mineralized root tissues^12^.

In the literature, these cells are called cementoclasts or odontoclasts/dentinoclasts which apparently originate from common hematopoietic precursor cells with osteoclasts^1^. Although they have been described to be smaller in size and to form smaller resorption pits on mineralized tissues, they share functional and morphological similarities with osteoclasts including the formation of ruffled borders and sealing zones, active secretion of acids and proteolytic enzymes as well as stimulation by the cytokines RANKL and M-CSF^14^.

So far, there is no evidence of possible influencing factors that control the differentiation pathway to the individual clast cells. Despite the frequently observed damage to their roots if teeth are orthodontically moved through bone, the extent of resorption in the areas of cementum and dentin is typically significantly lower than in the area of bone. Thus, the process of attachment and activation on the different hard tissues seems to take place to different degrees. It was the aim of this study to elucidate if the hard tissue extracellular matrix in contact, namely that of bone, dentin or cementum, may have an influence on the differentiation of maturing osteoclast-like cells. We hypothesize that the progenitor cells sense the respective hard tissue matrix and react with a corresponding differentiation which could explain the different susceptibility of the different hard tissues.

## Results

### Differentiation of osteoclast-like cells

**Fig. 1:**
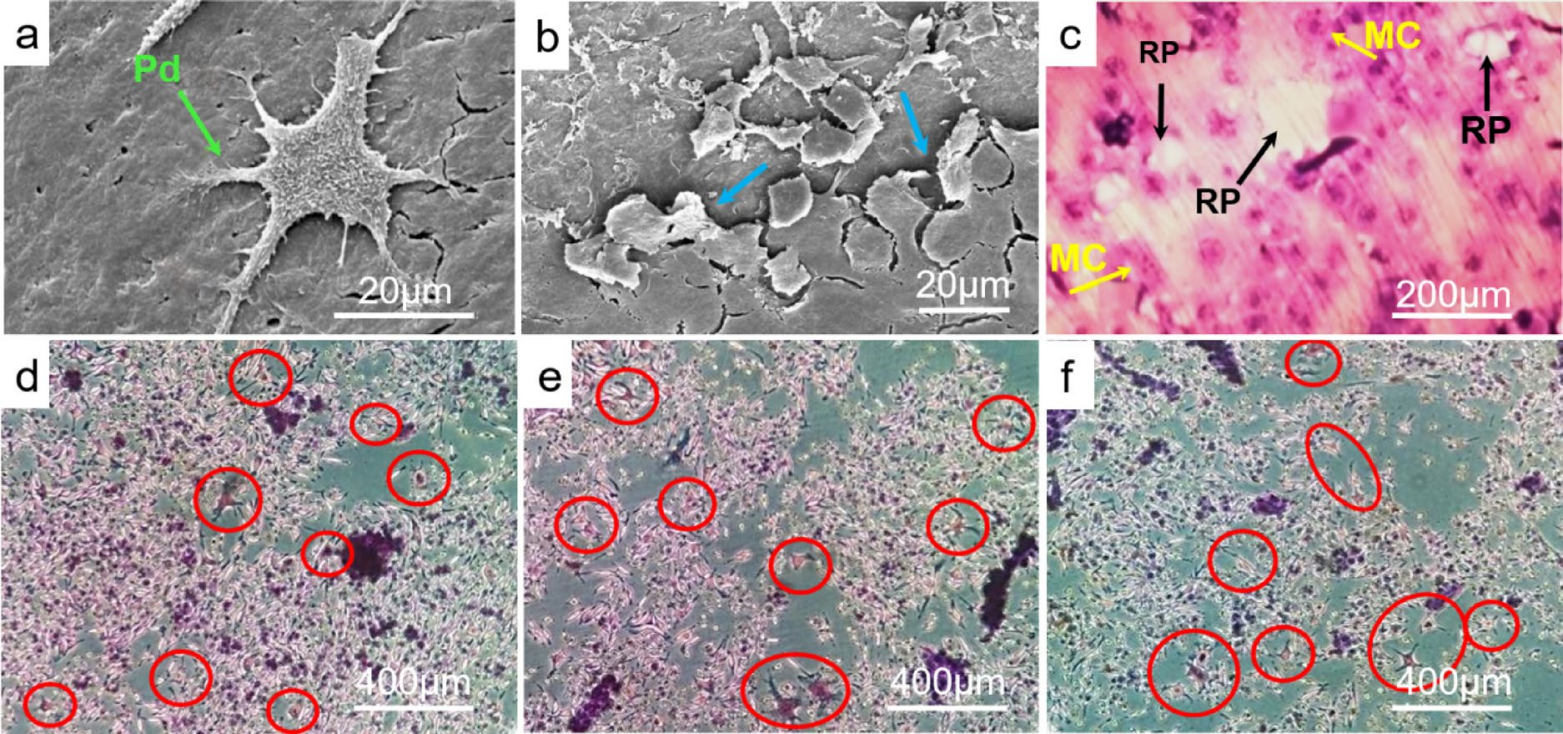
Differentiation of osteoclast-like cells. (**a**) Scanning electron microscopy of differentiated osteoclast-like cell on dentin disc with developed podosomes (green arrow) at 1600 × magnification; (**b**) Scanning electron microscopy of resorption pits formed by osteoclast like cells on dentin slices (blue arrows) at 1600 × magnification; (**c**) Toluidine blue staining of differentiated osteoclast-like cells on dentin disc visualized by light reflection microscopy at 30 × magnification. (RP) Resorption pits, (MC) multinucleated cells; TRAP-stained multinucleated osteoclast-like cells (circled in red) on bone (**d**), dentin (**e**) and cementum (**f**) powder at 10 × magnification; modified after^15^.

First, we wanted to evaluate the success of the chosen protocol to differentiate the murine macrophage cells into resorbing osteoclast-like cells. After 3 day of stimulation, attached cells were observed at the edge of dentin disks developing incipient cellular extensions. At the end of the incubation period (12 day), differentiated cells with characteristic podosomes and resorption pits in the surface of the dentin slices were visualized by scanning electron microscopy (Fig. 1a and b). Furthermore, multinucleated cells as well as multiple resorption pits were detected on dentine surface by toluidine blue staining (Fig. 1c).

Secondly, the murine macrophage cells were stimulated on pulverized bone, dentin and cementum tissue. After 12 day of stimulation, TRAP staining revealed numerous TRAP-positive cells in every hard tissue sample which corroborates the ability of differentiated murine macrophage cells to resorb the different hard tissue substrates they are cultured on (Fig. 1d–f).

### Genome-wide gene expression analysis

We aimed to investigate the effects of the three different hard tissue substrates on differentiating osteoclast-like cells at the transcriptional level. After RNA extraction and genome-wide gene expression analysis, differential gene expression analysis (DGE) was performed to analyze quantitative possible differentiated changes of gene expression between the different experimental groups (supplementary material Tables S1–S10, Fig. S21). In total, cells differentiated on cementum showed the highest number of significantly differentially expressed genes compared to the stimulation control group (1930 different transcripts) and to the negative control group (857 different trancripts), respectively (supplementary material Tables S11–S12). Furthermore, 446 transcripts were significantly differentially regulated on bone and 87 on dentin in comparison to stimulation control (supplementary material Tables S13–S14). Multiple comparisons between the different hard tissue groups revealed that there were 314 differentially expressed genes between the cementum and the dentin group, 252 between the bone and the cementum group and just one between the dentin and the bone group (supplementary material Tables S15–S17).

According to our sequencing results, we focused on four selected target genes for further investigation. The first gene was CXCL2, whose expression was significantly upregulated (by 5.2 fold) due to our stimulation protocol (supplementary material Table S18). We further analyze IGF-1 and GDF15 expression of the differentiated murine macrophage cells which were upregulated in every hard tissue sample in comparison to the negative control group (supplementary material Tables S12, S19–S20). Furthermore, HSPA1b was found to be an interesting gene, as it showed the strongest upregulation in the cementum group in comparison to stimulation (227 fold) and negative control (43,eightfold) (supplementary material Tables S11–S12).

### RT-PCR

**Fig. 2:**
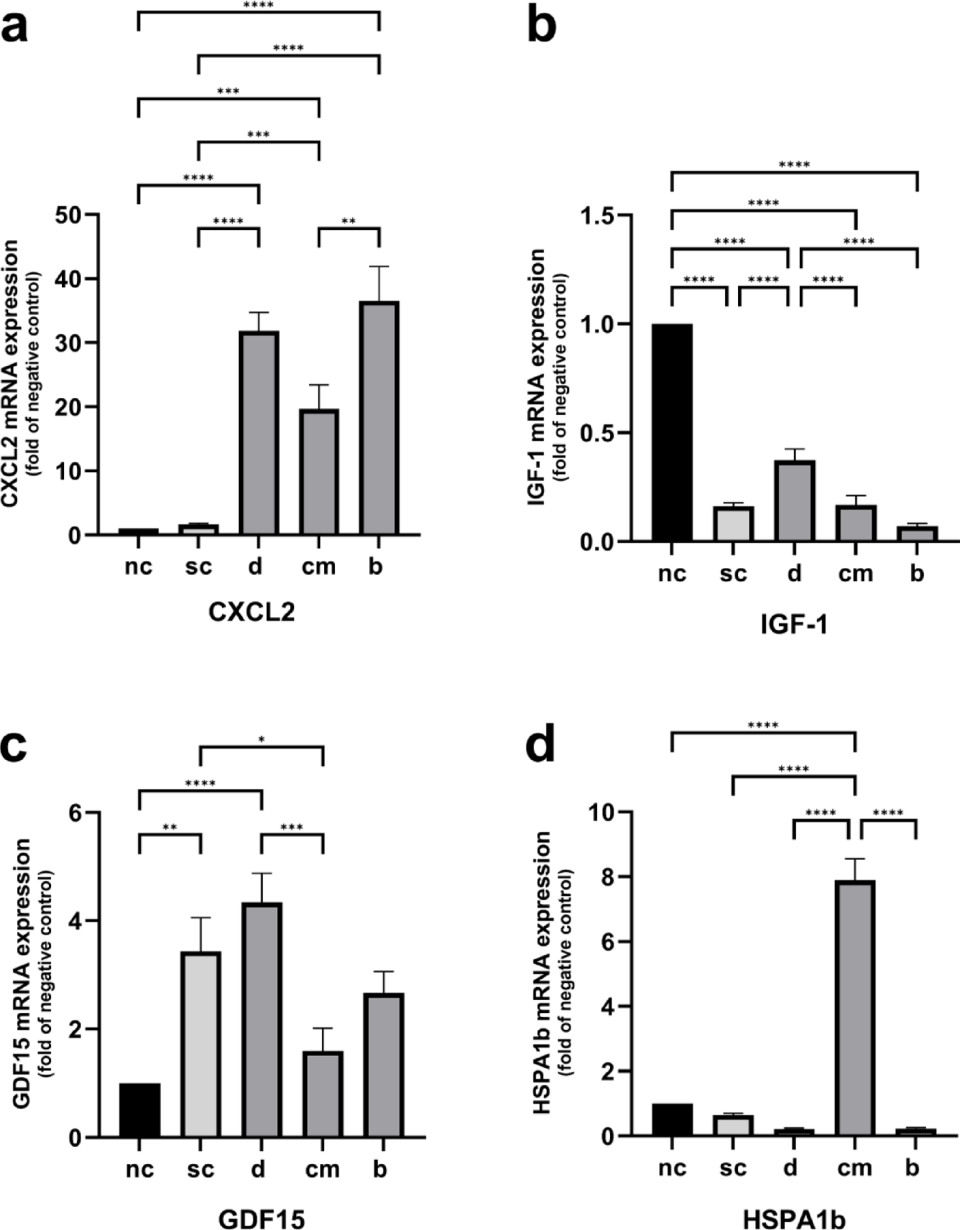
Quantitative gene expression of CXCL2 (**a**), IGF-1 (**b**), GDF15 (**c**) and HSPA1b (**d**). Statistically significant differences between experimental groups were determined using Tukey’s post hoc test. Data are represented as mean ± SEM; *n* = 18. * *p* < 0.05; ** *p* < 0.01; *** *p* < 0.001; **** *p* < 0.0001.

The CXCL2 expression was found to be significantly upregulated in cells stimulated on bone, dentin and cementum tissue compared to both control groups. CXCL2 expression was significantly higher in cells differentiated on bone tissue than in cells differentiated on cementum tissue. The comparison between the stimulation and negative control did not provide any significant differences in CXCL2 expression levels (Fig. 2a).

RT-PCR revealed that IGF-1 expression was significantly downregulated in the stimulation control as well as in each hard tissue sample as compared to the negative control. The IGF-1 expression of cells cultured on dentin was significantly higher expressed in comparison to the stimulation control and the other two hard tissues (Fig. 2b).

In comparison to unstimulated cells, stimulation of murine macrophage cells on polystyrene as well as on dentin tissue led to a significant upregulated GDF15 expression. Cells from the cementum group revealed a significantly decreased GDF15 expression in comparison to stimulation control (Fig. 2c).

As indicated by the genome-wide gene expression analysis, the differentiation of murine macrophage cells on cementum tissue resulted in a significantly elevated HSPA1b expression in comparison to the bone, dentin as well as both control groups (Fig. 2d).

### ELISA

**Fig. 3:**
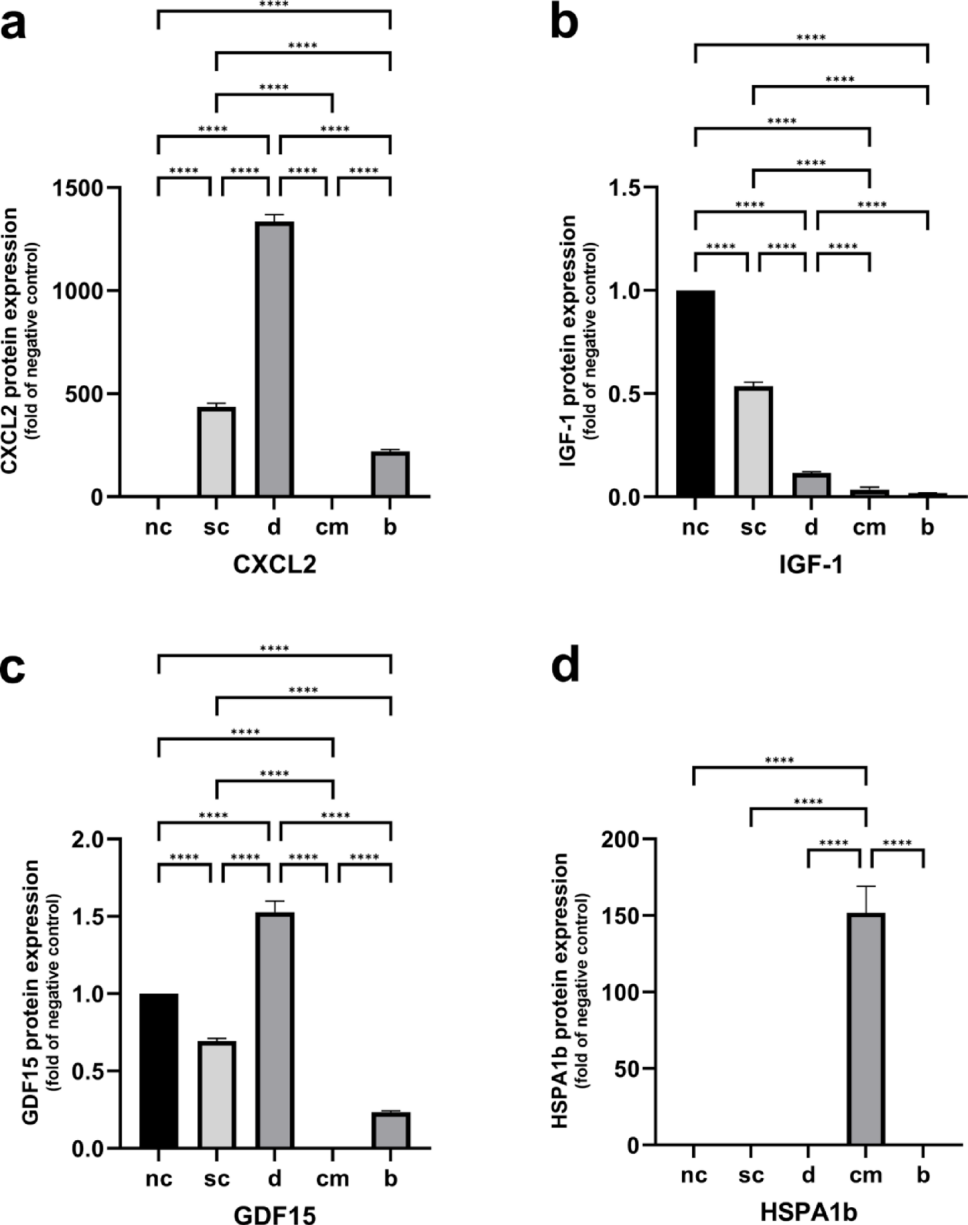
Quantitative protein formation of CXCL2 (**a**), IGF-1 (**b**), GDF15 (**c**) and HSPA1b (**d**). Statistically significant differences between experimental groups were determined using Tukey’s post hoc test. Data are represented as mean ± SEM; *n* = 18. * *p* < 0.05; ** *p* < 0.01; *** *p* < 0.001; ****p < 0.0001.

Stimulation of the cells on polystyrene alone led to a significant upregulation of CXCL2 protein concentration in the supernatant in comparison to the unstimulated control. On dentin powder, protein formation of CXCL2 was strongly increased in comparison to both controls as well as to the cementum and bone group. Cells stimulated on bone powder showed a significantly upregulated CXCL2 synthesis in comparison to the negative control and a significant lower one in comparison to the stimulation control (Fig. 3a).

The protein formation of IGF-1 was significantly downregulated due to stimulation with RANKL and M-CSF on polystyrene in comparison to the unstimulated cells. In addition, IGF-1 formation was significantly downregulated in every hard tissue group in comparison to both controls. The cells from the dentin cultures revealed a significant higher IGF-1 synthesis in comparison to the cementum and bone groups (Fig. 3b).

Cells from the stimulation control group showed a downregulation in GDF15 protein formation compared to the negative control. The protein synthesis of GDF15 was significantly increased in the dentin group in comparison to the unstimulated and stimulated control. Cells which were cultured on bone tissue as well revealed a significant downregulated GDF15 synthesis compared to both controls. In the cementum culture, no detectable GDF15 protein concentration was measurable (Fig. 3c).

Finally, a significant increased protein level of HSPA1b was found in the supernatant of the cementum cultures in comparison to all other experimental groups (Fig. 3d).

## Discussion

This study looked for a possible explanation for the different susceptibility of the 3 different oral hard tissues in the context of orthodontically induced periodontal remodeling processes. We provide evidence that the differentiation of osteoclast-like cells is affected by the hard tissue substrate to which they adhere. In particular, the different hard tissues clearly affected the expression of specific growth factors and inflammatory mediators such as CXCL2, IGF1-1 and GDF15. Additionally, our results suggest a particular importance of heat shock protein HSPA1b for resorptive cells cultivated on cementum as compared to the other hard tissues.

The resorption of dental hard tissues is caused by active osteoclast-like cells which are supposed to originate from a common hematopoietic progenitor cells with osteoclasts^16^.

The differentiation of osteoclasts is subject to a strict regulation by numerous cytokines and molecular factors. Especially cytokines M-CSF and RANKL are responsible for maturation and cell fusion into differentiated TRAP-positive multinuclear cells which adhere to mineralized tissues via podosomes and induce resorption^17,18^. In terms of in-vitro differentiation assays, two main criteria are described in the literature for the characterization of mature osteoclasts, namely positive TRAP staining and the presence of at least three nuclei per cell, which both served as important indicator for our cell cultivation^19^. Since we used a macrophage cell line in our study, we first wanted to investigate whether these murine macrophage cells can transform into mature osteoclast-like cells through cultivation with RANKL and M-CSF. Since the hard tissue powders produce an uneven surface, which makes it impossible to ensure the formation of resorption pits and therefore, the differentiation process, we initially used dentin discs for proof of principle. After 12 day of stimulation, we were able to confirm the differentiation of murine macrophage cells by detecting multinucleation, cell expansion and podosome formation as well as the appearance of resorption lacunae on the dentin surface representing the typical characteristics of active mature osteoclasts. Thus, the stimulation of murine macrophage cells by RANKL and M-CSF proved to be a suitable model for our further experiments on osteoclast differentiation.

Next, we wanted to investigate whether the different hard tissues to be resorbed have an influence on the differentiation pathway of maturing osteoclast-like cells. To complement and expand the existing knowledge on osteoclast differentiation mainly on bone and dentin^20–22^, we additionally used cementum tissue as an interesting growth matrix for differentiating osteoclast-like cells since the resorption of the cementum layer is described as a decisive factor of external root resorption^1,23^. Moreover, we were the first to pulverize the hard tissue samples in order to increase the contact area for the interacting adherent cells.

After cultivation and stimulation of murine macrophage cells on bone, dentin and cementum, we were able to detect TRAP-positive cells on all three tested hard tissue substrates indicating that our stimulation protocol proved to be suitable for cell stimulation on bone, dentin and cementum.

Differential gene expression analysis revealed significant quantitative changes in gene expression levels between the experimental groups confirming that osteoclastic differentiation was clearly affected by the different hard tissue substrates. Furthermore, the subsequent gene and protein expression analysis revealed a hard tissue-specific regulation of CXCL2, IGF-1, GDF15 and HSPA1b. Studies provide evidence that chemical and physical properties of the extracellular matrix influence the formation and activity of adherent osteoclasts^20,22,24^. Interestingly, Rumpler et al. demonstrated that the osteoclast formation rate was higher on dentin than on bone slices and hypothesized that the amount of non-collagenous proteins such as osteopontin as well as unknown osteocyte-derived proteins could influence osteoclast activity^21^. Important mediators in the interaction between the hard tissue matrix and osteoclasts are integrin signaling systems, such as integrin α_v_β_3_, which binds to the RGD-sequence of specific proteins^25^. These so-called SIBLING-proteins are an important component of the hard tissue matrix of bone, dentine and cementum, whereby each hard tissue has a different amount of these proteins. In addition, the hard tissues contain individual proteins, such as dentinsialophosphoprotein and cementum attachment protein, which may have an important influence on the specific gene regulation of the interacting osteoclasts^26,27^. The question of which signaling pathways are addressed by the different cultivations is a very interesting one and should be addressed by future studies.

CXCL2 is known as a stimulating factor on osteoclast differentiation by promoting proliferation, adhesion and migration of osteoclastic precursor cells^28^. During the early stage of orthodontic tooth movement, chemokines, like CXCL2, are released in the periodontal ligament as part of the acute inflammatory response. These chemokines activate and recruit cells from the murine macrophage line especially to the pressure side of periodontal ligament^29^ which mature and fuse into osteoclasts and exert their resorptive activity on alveolar bone as well as on the tooth root surface^4,30^. Interestingly it was shown that CXCL2 expression is differentially regulated by RANKL through JNK and NF-κB signaling pathways in mouse macrophagic precursor cells to promote osteoclast differentiation^28^. In accordance with the literature, our sequencing results revealed a significant upregulation of CXCL2 in osteoclast-like cells after stimulation by RANKL and M-CSF on polystyrene which was confirmed at the protein level. Furthermore, RT-PCR and ELISA test revealed that osteoclast-like cells which had been cultured on bone and dentin tissue, significantly upregulated CXCL2 gene expression and protein formation in comparison to the unstimulated control. This suggests that CXCL2 is involved in bone as well as in tooth root resorption. Moreover, our sequencing results showed that CXCL2 gene expression was significantly downregulated in osteoclast-like cells which were cultured on cementum tissue in comparison to the stimulation control. This finding was supported at the protein level. Therefore, we hypothesize that cementum may provide anti-inflammatory and thus anti-resorptive effects on differentiating osteoclast-like cells. On the other hand, the low CXCL2 protein concentration in the supernatant of the cementum cultures could also be influenced by the smaller number of differentiated cells grown on cementum. Although we performed a normalization of protein concentration for our analyses, the CXCL2 protein concentration in total was outside the optimal assay range. Further investigations are required to understand how CXCL2 protein expression is involved in clastic differentiation due to contact of the cells with different surfaces.

IGF-1 is known as an important growth factor in bone matrix and regulates distinct functions in oral biology including tooth development and growth^31,32^. During experimental tooth movement, increased IGF-1 expression levels have been detected in PDL-cells providing antiapoptotic and homeostatic effects on the periodontal ligament in response to mechanical strain^33–35^. Furthermore, previous studies reported on the stimulating effect of IGF-1 on osteoclast formation^36–38^. On the one hand, IGF-1 is released by osteoblasts and binds to the IGF-1 receptor which is expressed on osteoclast precursor cells to stimulate osteoclastogenesis^38^. On the other hand, studies have shown that IGF-1 is secreted by osteoclasts and thus acts in an autocrine manner on osteoclast differentiation^38–40^. Moreover, Götz et al.^33^ immunohistochemically detected IGF family members in cementum- and dentin-resorbing odontoclasts as well as in resorption lacunaes during external root resorption. Our genome-wide sequencing points into the direction of an upregulation of IGF-1 expression due to cultivation in the presence of RANKL and M-CSF. In contrast, validation by RT-PCR and ELISA test revealed that cell stimulation by RANKL and M-CSF demonstrated a decreased IGF-1 expression in the osteoclast-like cells in all groups as compared to the negative control. According to the latest literature, Ma et al. demonstrated that IGF-1 expression in RAW264.7 osteoclast-like cells was significantly downregulated at both RNA and protein levels due to stimulation with RANKL^41^. In addition, the reciprocal interaction between IGF-1 and RANKL expression was described in vivo by Xu et al.^42^ regarding to the OPG/RANKL/RANK/IGF-1 pathway. Although RNA sequencing has become the gold standard for transcriptome studies, it is a very complex and expensive technique that usually prevents several repetitions of the experiment. Thus, our results emphasize the importance of a repeated validation of the Seq based expression profiles by RT-PCR and the protein assays with respect to IGF-1.

Growth differentiator factor 15 (GDF15) is a member of the transforming growth factor (TGF-)β and bone morphogenic protein (BMP) superfamily. Initially detected in activated macrophages and described as an autocrine regulator of macrophage activation^43^, GDF15 is mainly expressed under pathological states such as tissue injury and inflammation^44^. With respect to orthodontic tooth movement, GDF15 was shown to be secreted by hPDL fibroblasts under mechanical stress and in the following stimulated the GDF15 expression of osteogenic marker genes to increase osteoblast differentiation^45^. Besides, studies proved that GDF15 acts as a proinflammatory promoter for osteoclast differentiation^5,46,47^. After RANKL induced stimulation of RAW264.7 cells in the presence of recombinant GDF15, Li et al. detected an increased number of TRAP-positive cells as well as a higher expression of osteoclast differentiation marker genes in comparison to control cells. Thereby, they were able to demonstrate that GDF15 contributes to the force-induced activation of NF-κB and ERK signaling pathways to promote osteoclast differentiation^48^. Regarding to its stress-induced expression and its different roles in regulating cell functions, development and survival, Symmank et al.^45^ proposed that GDF15 could be an interesting therapeutic approach for the treatment of bone and dental root resorptions.

Our sequencing data revealed a significant upregulation of GDF15 expression in every hard tissue group in comparison to the negative and to the stimulation control group. These results were completed and expanded by the results of the RT-PCR and by ELISA: GDF15 expression was upregulated by cultivation on dentin tissues on the gene and protein level in comparison to the stimulation and to the negative control. As already mentioned, cultivation on cementum resulted in fewer cells and therefore we suspect that the GDF15 protein expression was below the assay range.

The HSP70 family is highly conserved during evolution and has been extensively studied in the literature^49,50^. In the human genome, about 17 genes of the HSP70 family have been identified, including HSPA1b^51^. While some HSP70 genes are constitutively expressed and serve as housekeeping genes, others, like HSPA1b, are inductively expressed in response to environmental stresses^52,53^. Previous studies demonstrated the cytoprotective role of HSP70 in the periodontal ligament^54^. During orthodontic tooth movement, increased HSP70 levels were detected in the pressure zone^55^ and revealed anti-inflammatory effects to the mechanically loaded periodontal ligament. Thus, HSP70 is supposed to dampen the host’s inflammatory tissue response and to prevent excessive tissue loss in the hPDL during orthodontic tooth movement^7,56,57^. Furthermore, studies indicated a dampening effect on osteoclast formation which can be related to the suppression of the NF-κB and MAPK signaling pathways^57^. Inhibition of HSP70 clearly increased the number of osteoclasts in mechanically stimulated periodontal ligament cells^7,54^. Moreover, heat pre-treatment, which induces cytoplasmic upregulation of heat shock proteins, resulted in reduced osteoclast formation^58^. Our sequencing results revealed that culturing mouse macrophage cells on cementum tissue resulted in a strong upregulation of HSPA1b (227 fold) compared to the stimulation control which was confirmed by RT-PCR. The same results were obtained at the protein level.

In this context, the strong upregulation of HSPA1b which was induced by direct contact of the cells with the cementum matrix could indicate an autologous inhibition of cementum-resorbing osteoclast-like cells. As the outer root surface usually remains almost undamaged during physiological as well as pathological resorption of the alveolar bone, e.g. periapical periodontitis, the cementum layer is described in literature as a natural protective shield against external root resorption^59–61^. Our study suggests that the cementum matrix slows down the resorption process by upregulating HSPA1b expression in maturing osteoclast-like cells and thus acts as natural defense mechanism against the progression of external root resorption. Future studies must show whether this finding can be implemented as a clinical avoidance strategy in the sense of a translation process with local pharmacological induction of HSPA1b.

## Conclusion

In summary, the present results indicate an influence of the different oral hard tissue substrates bone, dentin and cementum on the activity and differentiation of osteoclast-like cells. We identified IGF-1, GDF15 and CXCL2 as significantly regulated target genes in bone-, dentin- and cementum-resorbing osteoclastic cells which represents a useful basis for further investigations elucidating the molecular mechanisms of external root resorption. In addition, we were the first to analyze the expression of HSPA1b in the context of dental root resorption. The clearly increased expression of HSPA1b, which was activated by the cells in contact with the cementum matrix, indicates an autoinhibitory effect in osteoclast-like cells which could attenuate the progressive degradation of the tooth root. This highlights HSPA1b as a possible target gene for therapeutic approaches.

## Materials and methods

### Cells and differentiation protocol

A murine macrophage cell line has been a widely accepted model for osteoclast maturation and function for 20 years and was used in our study^62^.

Firstly, we wanted to evaluate the success of the chosen protocol to differentiate the macrophages into resorbing osteoclast-like cells, as a proof of principle. Therefore, a murine macrophage cell line (American Type Culture Collection, #TIB-71, Manassas, VA, USA) was cultured on commercially available dentine discs (Immunodiagnostic Systems, #AE-8050, Boldon Colliery, UK) in 24-well plates (30.000 cells/well). The cells were incubated in Dulbecco’s Modified Eagle Medium (Thermo fisher scientific, #11965092, Waltham, MA, USA) supplemented with 10% FBS, 1% Penicillin/ Streptomycin, Plasmocin and Vitamin C at 37 °C in an atmosphere of 5% CO_2_. Osteoclast differentiation was induced by the addition of RANKL (Enzo, #ALX-522-131-C010, Farmingdale, NY, USA) and M-CSF (R&D Systems, # 416-ML, Minneapolis, MN, USA). The medium was replaced every 72 h. After 3 day of stimulation, incipient morphological changes were assessed under light reflection microscope. After 12 day, toluidine blue staining was used (Sigma Aldrich, #T3260, St. Louis, MO, USA) to identify the differentiated cells as well as formed resorption pits on the dentin slices.

In addition, scanning electron microscopy was performed to analyze the maturation process of the active osteoclast-like cells after 12 day of stimulation. For this purpose, the samples were fixed in glutaraldehyde (3%, pH 7.3) at room temperature for one hour, followed by dehydration in a graded ethanol series. Next, the samples were immersed in hexamethyldisilazane (HMDS) overnight and subsequently air-dried. Subsequently, the samples were coated with a thin layer of gold/palladium (Au/Pd) alloy using a sputtering process. The prepared samples were then scanned with a high-energy electron beam in a raster scan pattern and the emitted secondary electrons were detected by Everhart–Thornley detectors, enabling the generation of high-resolution, three-dimensional magnified images of the dentin surface.

To continue with the main question of our study, we further intended to analyze the osteoclast-like character of cells differentiated on three different hard tissues bone, dentin and cementum.

**Fig. 4:**
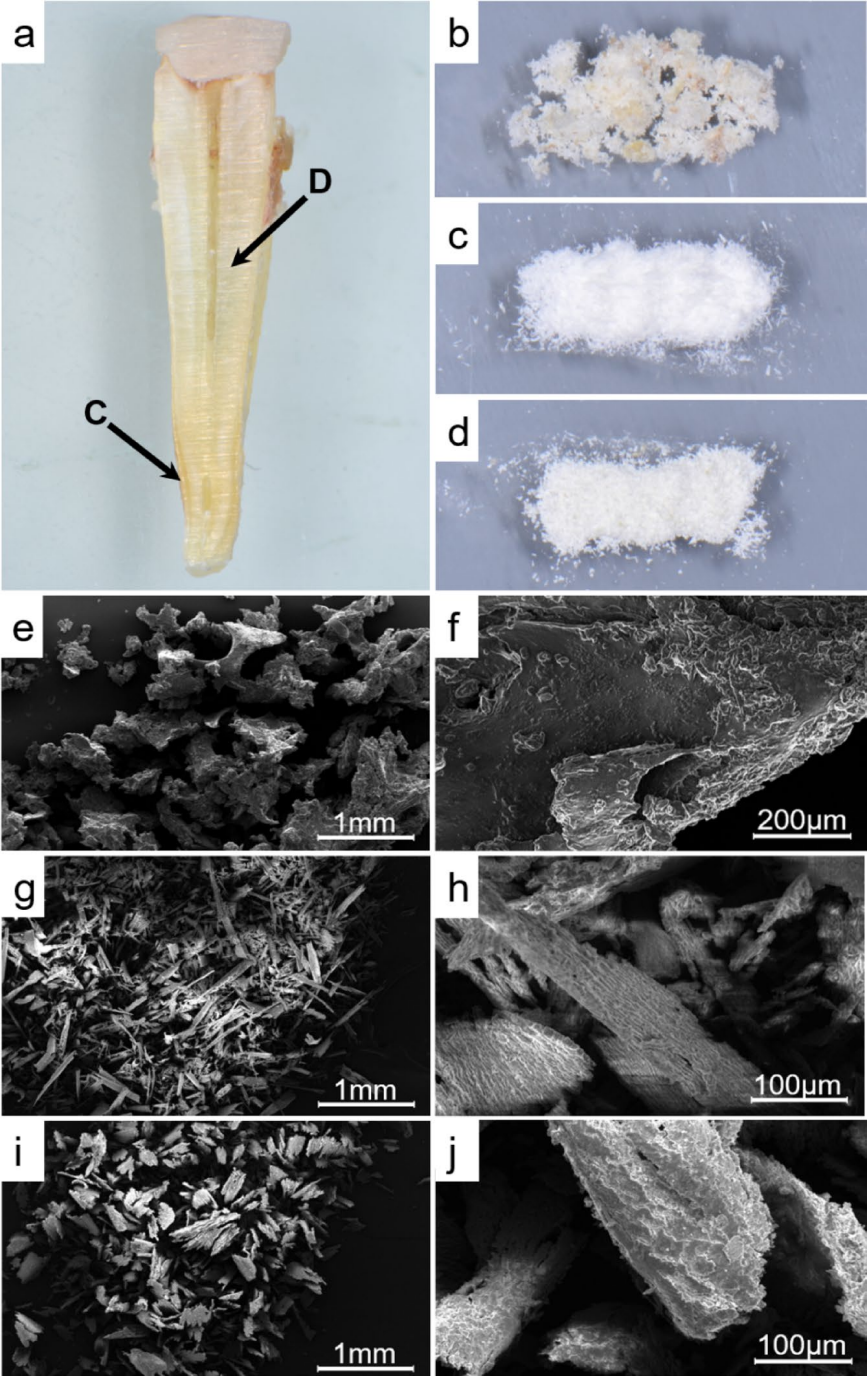
(**a**) Vertical section of a human tooth root for demonstrating the different hard tissue layers cementum (C) and dentin (D); Prepared hard tissue powders (**b**) iliac crest bone, (**c**) dentin and (**d**) cementum; Scanning electron microscopy to evaluate the morphology of different hard tissue powder particles: (**e**) Iliac crest bone at 25 × magnification, (**f**) Iliac crest bone at 400 × magnification, (**g**) dentin at 25 × magnification, (**h**) dentin at 250 × magnification, (**i**) cementum at 25 × magnification, (**j**) cementum at 400 × magnification.

After the approval of the Ethics Committee of the University of Bonn and written informed consent by the patients (# 458/22), extracted teeth (Fig. 4a) and human bone have been collected. In order to increase the contact surface between the hard tissue and the differentiating murine macrophage cells, the hard tissues were used in powder form. For the bone powder, excess human iliac crest bone (cortical and cancellous bone) was gained as residual autologous graft bone from orthognathic surgeries and reduced to powder particles using a surgical Lindemann bur (Komet Dental, Lemgo, Germany) (Fig. 4b). For the hard tissue powders human periodontally healthy extracted teeth were collected. Firstly, the periodontal fibrous tissue of the tooth root was removed by immersing the tooth in 0.5% NaOCl and subsequent mechanical scaling with a surgical scalpel. Afterwards, the teeth were split lengthwise to expose the dentin and cementum layer (Fig. 4a). The two different hard tissues were differentiated on the basis of their morphological differences using dental binocular magnifiers. For the dentin powder, the dentin core around the dental pulp was prepared with a surgical Lindemann bur (Fig. 4c). Subsequently, the most superficial layer of the tooth root was prepared circumferentially using also an identical surgical bur to obtain cementum powder (Fig. 4d). Thereby, we focused only on the apical third of the tooth root due to the increasing thickness of the cementum layer in this area. Afterwards, scanning electron microscopy was performed to visualize the different hard tissue powder particles (Fig. 4e–j). The estimated size of powder particles ranged from 400 to 1100 μm.

The murine macrophage cells were seeded into 6-well plates (n = 6) in a density of 30.000 cells per well and stimulated with 30 ng/mL RANKL (Enzo, #ALX-522-131-C010) and 20 ng/mL M-CSF (R&D Systems, # 416-ML) per well on the three different hard tissues bone, cementum and dentin under the same experimental conditions as described previously. Before cell seeding, 0.01 g of each different hard tissue powders was accurately measured using a precision balance (Merck, #Z741370, Darmstadt, Germany) and then added to the designated well. The plates containing the hard tissue powder were sterilized under UV light for one hour and subsequently 2 mL medium was added to each well. 

As stimulation control group, murine macrophage cells were stimulated with RANKL (Enzo, #ALX-522-131-C010) and 20 ng/mL M-CSF (R&D Systems, # 416-ML) on polystyrene without any hard tissue substrate. Cells cultivated on polystyrene without further stimulation served as negative control.

After 12 day, tartrate-resistant acid phosphatase (TRAP) staining was performed on the differentiated cells using the Acid Phosphatase Leukocyte Kit (Sigma Aldrich, #387A, St. Louis, MO, USA) regarding to manufacturer’s instructions. The stained cells were analyzed using reflected light microscopy.

### RNA extraction and genome-wide gene expression analysis

The murine macrophage cells were cultured under the same experimental conditions as described before. After 12 d, cells were lysed in RLT-buffer (QIAGEN, #79216, Hilden, Germany), RNA was extracted using the QIAshredder (QIAGEN, #79656, Hilden, Germany) and RNeasy Mini-Kit (QIAGEN, #74106, Hilden, Germany). RNA concentration and purity were measured with Nanodrop (PeqLab, Erlangen, Germany).

Afterwards, the extracted RNA was converted into a cDNA library using QuantSeq 3’mRNA-Seq Library Prep Kid FWD (Lexogen, #015.96, Vienna, Austria) for genome-wide gene expression analysis by sequencing according to manufacturer’s instructions. Cluster generation and the sequencing protocol was created by the Next Generation Sequencing (NGS) Core Facility of the Medical Faculty of the University of Bonn. Further information on this step can be found at www.illumina.com.

### Determination of gene and protein expression

The previous experiment was repeated three times for each hard tissue, and the RNA was extracted. Afterwards, the RNA was converted into cDNA with iScript™ Select cDNA.

Synthesis Kit (Bio-Rad Laboratories, #4106228, Munich, Germany) and PTC-200 Peltier Thermal Cycler (Bio-Rad Laboratories, Hercules, CA, USA).

Based on the sequencing results, the expression of conspicuous genes from the genome-wide analysis namely CXCL2, IGF-1, GDF15 and HSPA1b was analyzed by quantitative Realtime-PCR (RT-PCR). The primers used were commercially available standard primers. For this purpose, 1 µL of cDNA was used in a 25-µL reaction mixture containing 2.5 µL of QuantiTect Primer Assay (CXCL2 #QT00113253, IGF1 #QT02423379, GDf15 #QT00124481, HSPA1b #QT00254436, QIAGEN, Hilden, Germany), 12.5 µL of iQ SYBR Green Supermix (Bio-Rad Laboratories, #1708880, Hercules, CA, USA) and 9 µL of nuclease free water (QIAGEN #129117, Hilden, Germany).

For data normalization, a housekeeping gene (glyceraldehyde-3-phosphate dehydrogenase (GAPDH) (#QT01658692, QIAGEN, Hilden, Germany) was included in the plate setup and employed for comparative △△-CT analysis with the Software CFX-Manager (Bio-Rad Laboratories, Hercules, CA, USA) and iCycler (Bio-Rad Laboratories, Hercules, CA, USA) according to the manufacturer’s instruction. The applied protocol consisted of a heating phase at 95 °C for 5 min for enzyme activation, 40 cycles including a denaturation step at 95 °C for 10 s and a combined annealing/ extension step at 60 °C for 30 s per cycle with subsequent melting pot analysis after each run.

Finally, protein expression of CXCL2, IGF-1, GDF15 and HSPA1b was quantitatively detected from the supernatant of the cell cultures using commercially available enzyme-linked Immunosorbent Assay (ELISA) kits (CXCL2 #MM200, IGF1 #MG100, GDF15 #MGD15, R&D systems, Minneapolis, USA and HSPA1b #E2120Mo, BT LAB, Shanghai, China). For a detailed protocol description, we refer to the manufacturer’s instructions. The standard series were prepared and the microtiter plates were coated with the capture antibody. Next, the sample containing the target antigen was introduced into the wells and incubated. Any unbound antigen was then removed through multiple washes. For the detection of the bound antigens, an enzyme-conjugated detection antibody was added and the color reaction was initiated by adding a substrate solution. After sufficient incubation, the reaction was arrested by adding the stop solution. The photometric measurement was performed by the microplate reader PowerWave X (BioTek Instruments, Winooski, VT, USA) at absorbance of 450 nm. The DNA concentration of differentiated cells was used to normalize the measured protein concentrations.

### Statistical analysis

The transcriptional data from RNA-Seq were compared by differential gene expression analysis (DGE) using the statistical software R (www.r-project.org). The PCR and ELISA experiments were repeated at least three times. Descriptive analyses of data were presented as means ± standard errors of the mean (SEM). For statistical analysis, we used GraphPad Prism statistics software (Version 7.00 for Windows, GraphPad Software, San Diego, California, USA, www.graphpad.com). Normal distribution was examined using the Kolmogorov–Smirnov test. Multiple comparisons were conducted by ANOVA and Tukey’s multiple comparisons test. Differences with *P* < 0.5 were considered significant.

## Supplementary Information

Supplementary Information 1.

Supplementary Information 2.

Supplementary Information 3.

Supplementary Information 4.

Supplementary Information 5.

Supplementary Information 6.

Supplementary Information 7.

Supplementary Information 8.

Supplementary Information 9.

Supplementary Information 10.

Supplementary Information 11.

Supplementary Information 12.

Supplementary Information 13.

Supplementary Information 14.

Supplementary Information 15.

Supplementary Information 16.

Supplementary Information 17.

Supplementary Information 18.

Supplementary Information 19.

Supplementary Information 20.

Supplementary Information 21.

## Data Availability

The datasets presented in this study can be found in online repositories. The datasets generated and/or analysed during the current study are available in the Zenodo repository https://doi.org/10.5281/zenodo.14959164 .
