# Supplementary figures and images for: Bone, dentin and cementum differentially influence the differentiation of osteoclast-like cells
Source: Sci Rep. 2025 Jun 5;15:19857. doi: 10.1038/s41598-025-04874-9 (PMC12141432; doi:10.1038/s41598-025-04874-9)

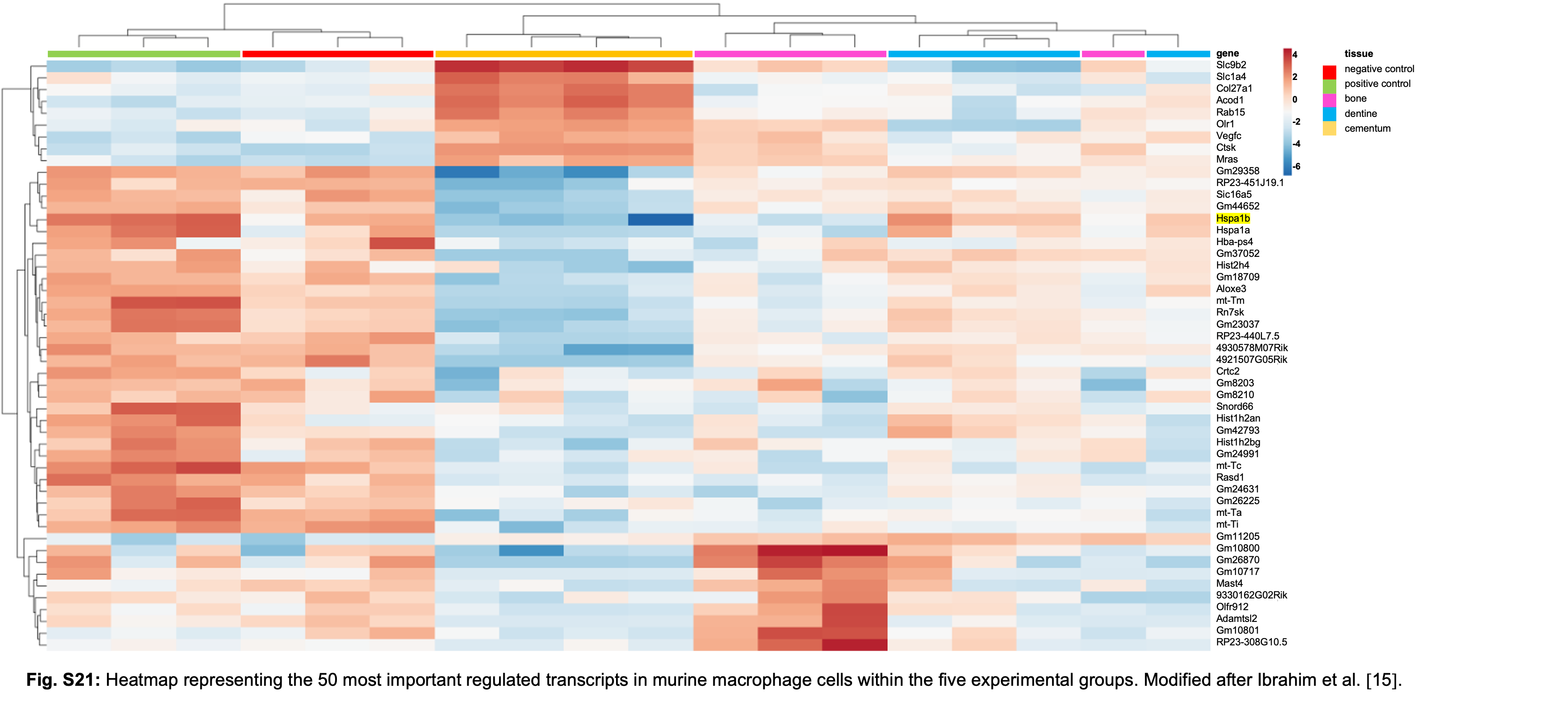

Supplement: Supplementary file 1 — Supplementary Information 1. [file 41598_2025_4874_MOESM1_ESM.png]
