# Supplementary material for: Bone, dentin and cementum differentially influence the differentiation of osteoclast-like cells
Source: Sci Rep. 2025 Jun 5;15:19857. doi: 10.1038/s41598-025-04874-9 (PMC12141432; doi:10.1038/s41598-025-04874-9)
Supplement: Supplementary file 2 — Supplementary Information 2. [file 41598_2025_4874_MOESM2_ESM.pdf]

Tab. S1:
Transcripts induced in murine macrophage cells stimulated on polystyrene (n=6), fold of stimulation control

gene name	regulation of expression	adj.P.Val
Usp27x	14,0286084	0,3525
Dmrt2	11,07550057	0,1272
Sfn	9,981108155	0,22757
Rbak	8,815240927	0,14522
Cbfa2t3	8,663800364	0,29186
Filip1l	7,578435249	0,2058
Ighd	7,119618809	0,64877
Gm7895	7,007015075	0,74788
Gm16740	6,797951175	0,60812
9130019O22Rik	6,42679743	0,2844
Mfsd9	6,162005628	0,22145
Zfp58	6,116897071	0,68407
Zfp790	5,922884693	0,54104
Trib1	5,913859628	0,001392
Gm29994	5,821536583	0,92604
BC024386	5,783328295	0,64669
Tmem44	5,749753073	0,93384
Rpl7l1-ps1	5,723509343	0,60286
C1ra	5,703707505	0,93384
Tmem204	5,618168911	0,45851
Zfp39	5,61233062	0,796
Gm5251	5,608053063	0,61316
Gm42850	5,457347836	0,82462
D17H6S53E	5,450920956	0,48118
Slc9a4	5,387440385	0,91431
Zfp408	5,364709311	0,1674
Gm6257	5,312165152	0,75518
A930015D03Rik	5,275105692	0,66731
Gm37234	5,249572713	0,91431
Ctla2b	5,192034609	0,80533
Cxcl2	5,151169454	0,030839
Zfp90	5,129435271	0,2844
Nedd9	5,119844524	0,93384
Slc9a3r1	5,089415578	0,42271
Gm43350	5,018304455	0,76361
Mrm2	4,989862288	0,096644
Zfp691	4,987787494	0,62264
Zfp41	4,98675042	0,92604
Zfp729b	4,976391538	0,1272
Egr1	4,966742664	0,023829
Csf2rb2	4,900741328	0,7434
Ppp1r26	4,900401646	1
Mettl18	4,829922366	0,38824
Zfp28	4,78261613	1
Ifi47	4,708932361	1
N4bp3	4,694918129	0,63881
Homez	4,677053722	1
Gm20632	4,674460931	0,80396

Speer9-ps1	4,622905911	1
Gm43024	4,622265085	0,63881
Oasl1	4,607550569	0,72746
Dnmt3b	4,60371971	0,648
Lcmt2	4,589700456	0,14522
Zfp65	4,588428097	0,11562
Tmem185b	4,569384987	0,038583
Zfp40	4,569384987	0,84725
Gm28071	4,569384987	1
1700052K11Rik	4,541912775	0,93372
Ppp1r10	4,511477529	0,0019331
Col7a1	4,470077982	0,99275
Tmem51	4,42261605	0,22757
Hoxa3	4,384766447	0,93436
Zfp747	4,331900182	0,35128
Gdpgp1	4,316613731	1
Zfp35	4,291851027	0,38824
D6Ertd527e	4,27789182	1
2010008C14Rik	4,254530692	1
Slfn8	4,225142501	0,93372
C3ar1	4,211400264	0,035008
Cnr2	4,198284688	0,2844
C8g	4,191596954	0,99275
Hdhd3	4,183469741	0,89432
Pygm	4,175937154	1
Slc10a3	4,167551421	0,62264
Il1b	4,135896262	1
Gm4262	4,134749707	1
Jrk	4,134749707	1
Tlcd2	4,126732717	1
Egfl8	4,11274037	1
Carnmt1	4,0948199	1
6430511E19Rik	4,093968494	1
Endov	4,081218637	0,61039
Fastkd5	4,061464403	0,95881
Gm9711	4,053308545	1
Gm10801	4,038725238	1
Nkrf	4,021127274	0,63765
Hoxb3	4,017505503	1
Tigd5	3,980638887	1
Kctd21	3,944110578	1
Foxd2	3,931554899	1
Ptafr	3,927469304	0,68407
Tagap	3,920669412	0,22757
Tnks1bp1	3,917952753	1
Zc3h12a	3,909543066	0,22757
Zfp689	3,899799628	0,82962
Prokr1	3,897367565	1
Arhgef17	3,895477009	1
Tspoap1	3,884960688	0,83388
Lysmd4	3,875547138	0,031957
2310001H17Rik	3,846376535	1

Gtf2h3	3,84291216	0,93384
Zfp189	3,83386623	0,60763
Sec22a	3,825106722	0,45264
Srl	3,80052836	1
Gm18860	3,786067153	1
Gramd1c	3,777416854	1
Gm14585	3,768525096	1
Thap6	3,716900324	0,81787
Ubiad1	3,714067409	0,33466
Mir22hg	3,702757325	0,0077196
Rab20	3,687901185	0,22757
Ccrl2	3,682026475	0,29438
Arid5a	3,658874673	0,81787
Gm13226	3,658874673	1
Nupl2	3,640912324	1
Zbtb45	3,624796493	1
Zfp719	3,607251469	1
RP23-440I21.3	3,605751567	1
Gm6743	3,601255603	1
Tmem37	3,597263897	0,81787
Zfp69	3,571425433	1
Armc5	3,56845604	0,46672
Trp53rka	3,564994868	0,32138
Gm42918	3,563759549	1
Gm11764	3,562030822	1
Gm17586	3,552168454	1
Limk1	3,539878885	1
9930120I10Rik	3,53179107	1
Creb5	3,504964908	1
Zfp974	3,500594613	1
RP24-84O13.9	3,499866761	0,9669
Gm12459	3,492112411	1
B130021K23Rik	3,476655215	1
Fam83d	3,473042343	0,91431
Ubox5	3,45695063	1
Zfp810	3,45575275	0,61837
Gm15824	3,449769572	1
Gm10842	3,443558056	1
Zfp738	3,44117199	1
Snip1	3,434499799	0,1272
Gm17430	3,427127819	1
Gm6450	3,422854578	1
2310043L19Rik	3,420957071	1
Nrros	3,419297615	0,0077196
Commd5	3,419297615	0,45851
Cttnbp2nl	3,415270858	0,014421
Gm20604	3,40463467	0,26239
Zfp248	3,392855529	1
Zfp27	3,389094817	1
Gm45445	3,387216025	1
Gpatch3	3,376199136	0,84725
Efna2	3,373158242	1

Kbtbd4	3,369652922	0,2628
Epop	3,367318066	1
Pus7l	3,365917929	1
Ajuba	3,35939165	1
Gm5578	3,35101931	1
Gm14013	3,349858136	1
Zfp111	3,338962753	1
Ubxn8	3,329025681	0,92604
Slfn10-ps	3,32741082	0,9969
Zfp94	3,326718976	1
Tuba1c	3,321189403	0,032407
Gm33142	3,311534756	1
Zfp119b	3,304884837	1
Gm45167	3,30373965	0,22757
Dusp5	3,300992819	0,14817
Gm10180	3,282283833	1
Irx5	3,275692653	1
Rinl	3,274557581	0,68407
RP24-93F20.12	3,270474578	1
Ttc30b	3,25825609	1
Elmod3	3,255547075	1
Rwdd3	3,251487776	0,45264
9130230N09Rik	3,250586397	1
Nr0b2	3,24226048	1
Irgm2	3,240687707	1
Ttc30a1	3,224554827	1
Zfp772	3,218748824	0,98206
Spsb3	3,196072201	1
Gm42463	3,195850674	1
Angptl4	3,194964719	1
Osgin1	3,183469628	0,45264
Lrrc14	3,165646144	0,84725
Exo1	3,165646144	1
Socs2	3,142036631	1
Mul1	3,137901356	0,52232
Gm38345	3,136161816	1
Zfp870	3,13464051	1
Gm38009	3,13464051	1
Hmgb1-ps8	3,124878291	1
Zfp703	3,123362458	0,14522
Gba2	3,122063758	1
Ptrf	3,121847361	1
Cfap126	3,114714652	1
2610044O15Rik8	3,107382846	1
Gm8818	3,106736751	1
Cage1	3,1058755	1
B3galt6	3,100498088	1
Gm43144	3,099853425	1
Gm7815	3,099853425	1
Cog1	3,097490806	1
Zfp866	3,095773668	1
Gm20302	3,092342248	1

Fosl1	3,089557021	0,29186
Zfp874a	3,0763081	0,42271
Fam222b	3,070769023	0,7434
Fam212a	3,070556181	1
Jup	3,068428571	1
D6Wsu163e	3,067365319	0,95893
Ctu1	3,066727545	0,45851
Pomt2	3,059932864	1
Gm8925	3,052941616	1
Gm19777	3,051249175	1
Plpp7	3,051037686	0,81968
3110082I17Rik	3,048500959	0,93372
Gm20219	3,047233387	1
Zfp12	3,042379241	0,93384
E230016M11Rik	3,039639014	1
Gm44851	3,035217706	1
Zfp951	3,034376282	1
Hps6	3,03080283	0,29902
Slc9b2	3,03080283	1
Ticam1	3,029962629	0,21879
Ier2	3,028492839	0,3031
RP24-316F13.7	3,014460865	1
Trim47	3,007781987	1
Ints5	2,998207016	0,3021
Cmtr2	2,995298949	0,037273
Zswim3	2,992393703	0,53543
Slx4	2,990320252	1
RP23-114G13.1	2,986798683	1
Gm26710	2,970076313	1
Rpp38	2,968429809	0,63881
Gm8885	2,964317543	1
CH25-309J2.1	2,958775018	1
Dlx1	2,957544754	0,45165
Casz1	2,953038161	0,53543
March4	2,951810282	1
Zfp868	2,94506607	1
5430427O19Rik	2,941801698	0,45264
Slc15a3	2,938744635	1
Champ1	2,93609775	0,0066202
Iba57	2,93345325	0,97048
Fbxo46	2,931623837	0,6184
E130311K13Rik	2,92573682	0,8071
Hemk1	2,925128493	0,93384
Dnah8	2,922899044	1
Cptp	2,920468856	1
Gm37642	2,91763619	1
1810062G17Rik	2,91157544	1
Gm13140	2,907340407	1
Ccdc130	2,891263305	0,6184
Fut7	2,889259929	1
Tfb2m	2,88725794	1
Snx19	2,884257558	1

Zfp623	2,883657856	0,97048
Rpusd2	2,872685388	1
Gm22	2,866121958	1
Zfp61	2,859177131	0,93384
Taf7	2,852249131	0,2058
Hoxa7	2,847902983	1
Gcc1	2,847705588	0,10794
2610203C20Rik	2,846324208	0,88288
Gm12151	2,844943498	1
Gm7666	2,837066564	1
Rmi2	2,83215456	1
AI467606	2,824704596	0,49891
Fbxo9	2,824313036	0,66711
Pde4b	2,820400419	0,76361
Hexim2	2,810642575	1
Mast4	2,809863407	1
Sdr42e1	2,804415279	1
Ankrd9	2,799365762	0,80919
Gm10463	2,795681481	1
Spata2l	2,794325337	1
Zfp748	2,792389129	1
Trmo	2,792195582	1
Gm4890	2,791808528	1
Bcdin3d	2,791615022	0,99275
Erbb3	2,79026085	1
Chst14	2,787554477	1
Gm16537	2,786202276	1
Isl2	2,785429883	1
Hpdl	2,78485073	1
Pih1d2	2,783885741	1
Gm43378	2,783885741	1
Cyb561d1	2,780414551	1
Zkscan5	2,774446532	1
2310015A10Rik	2,773869662	1
Dusp8	2,765039327	1
Zfp119a	2,764081203	1
A630072M18Rik	2,763314944	1
Car7	2,762931893	0,68407
Gm18969	2,752609584	1
Adora2b	2,7463205	1
Zfp850	2,741185575	1
Prss36	2,741185575	1
Gm15703	2,740805592	1
Gm5580	2,736249906	1
E430018J23Rik	2,730187436	1
Mir763	2,727161239	1
Gm4258	2,72527157	1
Trmt44	2,720553124	1
Tmem8	2,719421913	0,72615
Adgre5	2,714149144	1
Zbtb3	2,709825591	1
Zfp628	2,707009593	1

RP23-136K21.4	2,704196521	1
Sac3d1	2,703634258	0,45264
Fastkd2	2,703072111	0,91431
Gm14326	2,702697412	1
Zfp551	2,698766203	1
A830008E24Rik	2,686634379	1
Stra8	2,684214566	1
Txnl4b	2,681611052	1
Vps37d	2,681053484	1
5430421F17Rik	2,679381478	1
Mrap	2,679010062	1
Mok	2,676597117	1
Zfp11	2,676226088	1
Tlr13	2,67585511	1
Mdk	2,672889136	1
Zc3h10	2,671592557	0,19838
Shq1	2,671222221	1
6330408A02Rik	2,66881629	1
Zkscan6	2,668446339	0,2844
Armc7	2,656450813	0,68757
Nup160	2,655162208	1
Mlh3	2,653138519	1
Ccdc166	2,652219171	1
Gm14140	2,652035339	1
Crybb3	2,649095768	1
Gm44198	2,648177821	1
Gm6921	2,648177821	1
Adamts1	2,640296591	0,95893
Polr2k	2,636456147	1
Gm6682	2,635359903	1
Hmgb1-ps6	2,630067817	1
Stac3	2,630067817	1
Prag1	2,629703237	0,68113
Gm8973	2,627516821	1
Zfp768	2,623876833	0,31494
Zfp518a	2,62278582	0,9969
Gm12543	2,619152386	1
Mbip	2,617156143	0,841
Gpr183	2,613892845	0,35528
Traf6	2,610271731	1
Vegfc	2,609728996	1
Extl2	2,603044544	0,94545
Rbm12b1	2,602503313	1
Aldh1b1	2,60070002	1
Otud1	2,597457241	1
Nlrp3	2,597097182	0,53543
Gpr180	2,596197253	1
Card11	2,594578166	1
Gm8930	2,594038694	1
Kbtbd7	2,593679109	0,95638
Hgh1	2,58972696	0,91431
Gm15131	2,588829585	1

Lhx5	2,586497864	1
Pcdhb22	2,586497864	1
Gm38262	2,586497864	1
Pou6f2	2,586497864	1
Tssk6	2,583093741	1
Nkpd1	2,583093741	1
Evi5l	2,578978954	1
Phf11c	2,57629893	1
Zfp276	2,573978495	1
Gm9173	2,573443306	1
Gm12312	2,572016682	1
RP24-547N4.7	2,57183841	1
Tlr1	2,570947233	1
Arhgef18	2,570947233	1
Fos	2,570590849	0,93384
Gm42600	2,570234514	1
Peg12	2,570234514	1
Mthfr	2,568809668	0,93372
D230017M19Rik	2,568097542	1
Gm5054	2,565606653	1
Tsix	2,560277167	1
Gm44419	2,552657538	1
Gdf9	2,552303689	1
Gm44075	2,552303689	1
D2hgdh	2,551596138	1
Tfip11	2,549474662	0,38824
Mylip	2,547354951	0,53543
Gm13443	2,547354951	1
Gm16433	2,547178388	1
2210008F06Rik	2,545237001	1
Swsap1	2,542944543	1
Adal	2,542768286	1
Gm7224	2,542768286	1
Gm4754	2,539597741	1
Mgat2	2,535552243	0,30142
Vwf	2,535376498	1
Wdr73	2,533092921	0,93372
Mdm2	2,532917346	0,032407
Magee1	2,532741784	1
Zcchc10	2,530986829	0,052156
Gm8129	2,53046058	1
Rftn1	2,530285188	0,63881
Zfp455	2,528181431	1
Oas1g	2,52380422	1
2210406H18Rik	2,52310457	1
Rep15	2,521880649	1
Fsbp	2,521006783	1
Tlr4	2,51925996	1
Gm14126	2,519085344	1
Gm9434	2,516641994	1
Kdm1b	2,51106609	1
Tbc1d25	2,510021984	1

Rps15a-ps1	2,508978312	1
Mafb	2,508456639	0,030987
Tmem260	2,506544765	1
Cebpa	2,505155227	0,28509
Btbd6	2,503072364	1
Hhex	2,501164594	0,7434
Iffo2	2,500297907	0,60763
Rdh10	2,494412391	0,75518
Tlr6	2,494066616	0,76697
Ap5b1	2,494066616	1
Ccdc92b	2,487850856	1
Mir3091	2,487850856	1
Gm11772	2,486988782	1
Zfp202	2,478728055	1
Acot2	2,478212672	1
Epc1	2,477697396	0,66845
Mfsd4b4	2,47477952	1
Cyb561d2	2,469809896	1
Mtg2	2,469296366	1
Gm44545	2,463825362	1
1810055G02Rik	2,461094406	1
Gm6177	2,46075325	1
Gm24890	2,459048177	1
Gm45250	2,458877735	1
Det1	2,457003648	1
Ppcs	2,454450378	1
Zfp420	2,450030994	1
Gm42835	2,448672785	1
Gm38387	2,440370168	1
Usp42	2,439524547	0,38244
Spred1	2,438341169	0,22757
Ddx28	2,432938749	0,76697
4732491K20Rik	2,432264289	1
Hs1bp3	2,431927129	1
Zfp764	2,42738004	1
Gm37760	2,426202553	1
Gm14017	2,425193734	1
Snx20	2,421330477	0,28927
Pskh1	2,420491453	0,99275
Gm9722	2,414626421	1
Gm28731	2,414459058	1
Hlx	2,41228439	1
Srxn1	2,410445816	0,68113
Gm45718	2,405438643	1
Gm26730	2,403105523	1
Oxt	2,402772405	1
Gm37121	2,402272814	1
Gm37124	2,401773327	1
Zfp418	2,401107507	1
Tbcc	2,399776419	0,63576
Mybpc3	2,394957409	1
Gm5828	2,393795651	1

Nmb	2,391639594	1
Gm38380	2,391142318	1
Srf	2,388988651	0,12904
Gfod2	2,380558364	0,62264
Paqr5	2,380558364	1
Inpp5b	2,379073758	1
Bmf	2,378579095	1
Gm44829	2,377425281	1
Gm29539	2,370678459	1
Gm13776	2,370678459	1
Gm43351	2,370514142	1
Gm5910	2,367722491	1
Ftx	2,365753894	1
Gm16372	2,365589918	1
E130102H24Rik	2,356588726	1
Pramef8	2,356425385	0,48551
Tctn2	2,355935431	1
Trim16	2,349575293	1
Gm16061	2,343882096	1
Gm5544	2,342420362	1
Cebpd	2,339661797	0,45851
Yjefn3	2,338688961	1
Sik1	2,338526861	1
Rlf	2,337554497	0,38244
Gm7965	2,33577288	1
Gm11895	2,334963503	1
4921511C10Rik	2,334316204	1
Tada2b	2,333183861	1
Smg8	2,332052068	0,27173
Ankrd49	2,331405575	0,96418
Pcdhb16	2,330920824	1
Slc35f6	2,327530382	0,2844
Fdxacb1	2,32720774	1
Fam175a	2,327046436	1
Sh3rf1	2,326078846	1
Rab7b	2,324628215	0,29186
C1qtnf6	2,323500572	1
Gm13433	2,323500572	1
Osm	2,321890602	1
Gm26890	2,321568742	1
A430033K04Rik	2,318031225	1
Znhit2	2,316906782	1
Mex3c	2,316585612	0,68757
Ighmbp2	2,316425044	1
Gm43379	2,315140902	1
Gpr146	2,312093915	0,93384
Enho	2,311933659	1
Klhl18	2,303775285	1
Suox	2,299946018	1
Zfp3	2,298989696	1
Cbr3	2,298671011	0,2628
Mettl22	2,29787449	1

Zfp712	2,297715219	1
Bag5	2,295804828	0,38244
Gm12726	2,294850228	1
Cass4	2,294055031	1
Alkbh4	2,29373703	1
Tepsin	2,289607019	1
Gm6905	2,287544803	1
Tmem177	2,282160093	1
Etohd2	2,280578768	1
Fth-ps2	2,278524684	1
Zbtb24	2,278208836	0,63881
Zfp944	2,27599912	1
Maml2	2,275368167	1
Ptgir	2,274895066	0,42271
Gm42786	2,273318776	1
Pwp2	2,273003649	1
Srrd	2,269068244	1
Gm15787	2,269068244	1
Mrgpre	2,267338826	1
Gm13736	2,265924827	1
Hps4	2,26545369	0,38824
Gm43660	2,26545369	1
Urb1	2,260277646	0,93372
Gm45422	2,259494429	1
Lrrc8a	2,257459335	1
Kin	2,256989959	0,45264
Cdk5r1	2,256051498	0,52729
Gm10132	2,256051498	1
Dhx33	2,255738765	1
Gm14584	2,255113428	1
Grhl1	2,254644539	1
Gm7785	2,254488264	1
Zbtb2	2,251208998	0,81787
Ell	2,249337273	0,1674
Tnfrsf1b	2,249025471	0,60763
Fam161b	2,248246153	1
Gm26532	2,246376892	1
Lgals9	2,244975966	1
Ccdc71l	2,244509185	1
Ndor1	2,244353613	1
E330011M16Rik	2,242798484	1
Plk2	2,241089088	1
Gm17060	2,238760192	1
Irf2bp1	2,233799966	0,60763
Gm3362	2,233490317	1
Pkd2	2,231478645	1
Tsku	2,231323976	1
Ifi44	2,231323976	1
Gm43859	2,230396187	1
Gm14248	2,22885073	1
Gpr35	2,228232847	1
N6amt1	2,227769547	1

Xaf1	2,227151964	1
Vps25	2,225146	1
Zfp472	2,224837551	0,68757
Endog	2,223604181	1
Ccdc51	2,223450058	1
Zfp707	2,222833672	1
Gm37274	2,22206343	1
Zc3h4	2,221909414	0,56517
B3galt4	2,221755408	1
Il10ra	2,221293455	0,29902
Gm43961	2,218677535	1
Gm20223	2,218677535	1
Gm2885	2,218523753	1
Vil1	2,218523753	1
D7Bwg0826e	2,218523753	1
Ccdc116	2,218523753	1
Gm12430	2,218523753	1
Zfp563	2,217293884	1
Trim30a	2,215911095	1
Trim13	2,215603926	1
Gm26606	2,215603926	1
Evi2a	2,214529169	0,93384
Nop9	2,213915256	1
Sat2	2,213608364	1
Gm44916	2,212074539	1
Ifih1	2,211921215	1
Numbl	2,209010077	1
Mcat	2,208550774	1
2310011J03Rik	2,207020454	0,93384
Gm20699	2,204268549	1
Mcm10	2,202435852	1
Mettl2	2,198622626	1
Gm13453	2,198165483	1
Gm14034	2,197099185	1
Krcc1	2,196490105	1
1700017B05Rik	2,19375133	0,2844
Toe1	2,190712252	1
Zfp830	2,190560408	0,75854
Shb	2,190104942	0,95893
Gm13445	2,18980135	1
Tradd	2,189346041	1
Gm3724	2,188435706	1
RP23-213P10.2	2,188435706	1
Gm5276	2,187374128	1
Dhrs9	2,181469042	1
Depdc5	2,180108595	1
Adamts4	2,179957487	1
I830077J02Rik	2,178446979	1
Wdr24	2,177692118	1
Gm15798	2,177239326	1
Bccip	2,176484883	1
Gm11448	2,172565992	1

Gm8423	2,172114267	1
Zfp607a	2,171211098	1
Gm18867	2,171211098	1
Gm13268	2,170007456	1
Mblac1	2,169105163	1
Bcl9	2,166550703	1
Zfp251	2,165349645	1
Igf1	2,16519956	0,087381
Sema6b	2,163249396	1
Gm5576	2,162799608	1
Tgfbr2	2,159354337	0,53543
Gm14328	2,15546629	1
Clec2l	2,15546629	1
Dusp2	2,153524893	0,93372
Wdr53	2,153524893	1
Gm37747	2,153077127	1
Tefm	2,152927892	1
Fam212b	2,151436113	1
Utp23	2,149200383	0,44926
Efnb1	2,143992709	1
Arhgap35	2,141913175	1
Cx3cr1	2,141319393	0,50716
Irf4	2,139983985	1
Gm5873	2,138204736	1
Mov10	2,136130816	1
Slc39a2	2,13509461	1
Dido1	2,134354771	1
Gm37305	2,134058907	1
Fam57a	2,130659419	1
Ddias	2,130216407	1
Il15ra	2,130216407	1
Cstf2t	2,1271179	0,93384
6430573P05Rik	2,125644005	1
Pacsin2	2,122846418	0,93384
Btg2	2,121081418	0,81787
Ppard	2,120787394	1
Zfp811	2,118877232	1
3110001I22Rik	2,115648568	1
Sepsecs	2,115062067	1
Gm16181	2,114622298	1
C1rl	2,114622298	1
Ppp1r7	2,112571251	1
Rpl31-ps22	2,112132	1
Slc16a13	2,111546473	1
Gipc2	2,111546473	1
Rpl28-ps3	2,110375908	1
Rab43	2,109790869	1
Lig4	2,109352196	1
Knop1	2,108182847	0,29186
Gm37486	2,107598415	1
Celf5	2,107160198	1
Gm6162	2,107160198	1

Themis2	2,105992064	1
2510016D11Rik	2,105846093	1
Zbtb6	2,10540824	0,95881
Fam46c	2,1022001	1
Ripk1	2,099287851	1
Acot6	2,099142344	1
Mtrf1	2,097833239	1
Irgm1	2,097542438	1
Gm12791	2,096960956	1
Mrpl46	2,095943751	1
Mgam	2,095798477	1
Dnttip2	2,095217479	0,48118
Birc3	2,093330347	1
Zbtb1	2,0907202	1
Isg15	2,089271526	1
Gm38115	2,088258051	1
Dph2	2,087679143	1
Ankle1	2,086955734	1
Esrp2	2,086955734	1
1700007K09Rik	2,086955734	1
Sprtn	2,084064603	1
Rgs2	2,079447123	0,1272
Abca5	2,079014759	1
Gm20712	2,077574195	1
H3f3b	2,077286202	0,65559
Ptpn14	2,076566394	1
RP23-403E19.1	2,075990728	1
Maml1	2,075415221	1
Trim45	2,074839873	1
Nhlrc1	2,071965527	1
Zfp281	2,070816904	1
Gm45856	2,070242831	1
Zfp341	2,061650812	1
Gm12655	2,061507914	1
Dusp6	2,061222149	0,61316
Zhx1	2,060079481	0,84725
Gm8168	2,056370185	1
Exo5	2,054233245	1
Rnf170	2,053948488	1
B4galt6	2,053236767	1
Naif1	2,052098526	1
Zfp759	2,05081876	1
Mfsd2a	2,050108123	1
Med8	2,049255685	0,9969
RP23-312A24.1	2,0484036	1
Nsrp1	2,048261621	0,7037
Tbl3	2,043440165	1
Rundc1	2,042873682	1
Fam120b	2,042307356	1
C330018D20Rik	2,04018503	1
Slc26a6	2,039760829	1
Zfp1	2,038347465	1

Xylt2	2,037782393	0,93384
Slfn2	2,03764115	0,39543
Cxxc5	2,036088118	1
Wdr46-ps	2,035382586	1
Micall2	2,034395252	1
Gm43457	2,033549347	1
Dok4	2,031154525	1
Top3a	2,031013741	1
Zfp503	2,029747123	1
Cspg4	2,029747123	1
Gm42876	2,029747123	1
Gm12589	2,029325093	1
Socs3	2,029184436	1
Fancf	2,026092442	1
P2ry6	2,025109615	1
Rpl26-ps2	2,02496925	1
Phospho2	2,024407887	0,90711
Egr2	2,02426757	1
Svil	2,021463284	1
Gm26397	2,020482702	1
Gm12421	2,020482702	1
Gm37420	2,018243157	1
Rabif	2,015167835	1
Gipc1	2,012655149	1
Mppe1	2,011260566	1
Cebpe	2,010981765	1
AA986860	2,010563637	1
Fam19a2	2,009727641	1
RP24-175C20.10	2,009449053	1
Tnfaip8l1	2,009449053	1
Mocs3	2,00583092	1
Gm5566	2,005691891	1
Cdc42ep2	2,005413862	1
Tmem2	2,004857921	1
Gm12743	2,004441065	1
Rin2	2,003885393	0,46672
Rdh14	2,002635694	1
Dimt1	2,002635694	1
Csf2rb	2,001664245	1
Zfp319	2,000554595	1
Elf2	2,000415932	1
Arl11	1,998752724	1
Vdr	1,997921639	1
C130023A14Rik	1,997921639	1
Caskin2	1,997506226	1
Junb	1,996952477	0,92604
Brca2	1,995983784	1
Stx1a	1,993909597	1
Twf1	1,993080526	1
Mfap1b	1,993080526	1
Tmem129	1,991699506	1
Gm19287	1,989491865	1

5430420F09Rik	1,989078205	1
Elmo2	1,988664631	1
Vac14	1,987562187	1
Gm4880	1,987424424	1
Zfp235	1,987011194	1
Gm44254	1,986184991	1
Sh2d2a	1,98425852	1
Nif3l1	1,983708443	1
RP23-380K24.3	1,983708443	1
Gm37082	1,982196517	1
Ptpn7	1,980548458	1
Zfp799	1,978078938	1
Drg2	1,977941833	1
Zfp729a	1,977667651	1
Capn10	1,976297311	1
1600002H07Rik	1,976160329	1
4933440N22Rik	1,975338638	1
Gm10616	1,973422687	1
Dedd	1,971098674	1
Cdc42ep3	1,970552245	1
Epb41l1	1,970415661	1
Pigc	1,969050347	0,94985
Dqx1	1,968913867	0,93372
Pdss1	1,968913867	1
Taf1a	1,967549593	1
Cep19	1,966595164	1
Alkbh2	1,96577745	1
Saysd1	1,964415349	1
Dcp1b	1,962918128	1
Rhot2	1,962509993	1
Gm13840	1,95816183	1
Gnl3	1,957618988	0,49891
Wfs1	1,956669376	1
Arhgap4	1,955178058	1
Gm6304	1,955178058	1
Xirp1	1,953552463	1
Zfp46	1,952334154	1
Caml	1,952198833	1
Gm11222	1,951116604	1
Gm37776	1,951116604	1
AA914427	1,951116604	1
1700088E04Rik	1,950034975	1
Gm6564	1,94881886	1
Lat	1,946793685	1
Dyrk3	1,946523821	0,841
Arhgap25	1,945175061	0,93372
Fbxl15	1,945175061	1
Gm6794	1,945175061	1
Zbtb39	1,94342307	1
Bet1l	1,943153673	0,83389
Cenpu	1,942345706	1
Rraga	1,942211078	1

Gm45640	1,941941849	1
Fcnaos	1,939654909	1
Gpn1	1,937773569	1
Cbx2	1,937236378	1
Gm15964	1,936296653	1
Lnx2	1,936028244	1
Card9	1,933614236	1
Bbs10	1,932408361	1
Gpr19	1,932274421	1
Pqlc2	1,93200657	1
Fn3k	1,931738755	1
Gm26740	1,931069382	1
Adamtsl1	1,930667869	1
Gm26569	1,93053405	1
Zfp85	1,93053405	1
Snrk	1,927859615	1
Ccl4	1,927058008	0,39543
Coil	1,926256734	1
Il4ra	1,926256734	1
Mutyh	1,92612322	1
Gm14650	1,925322335	1
Cbarp	1,923588225	1
Gm7496	1,923321577	1
Gtf3c6	1,923054967	1
Fnip2	1,922122121	0,24767
Zfp619	1,922122121	1
Tmem201	1,92158927	1
Nek4	1,921322899	1
Slc6a4	1,920923413	1
Zfp30	1,919991601	1
Zfp568	1,919725452	1
Gm7292	1,919060241	1
Abcd2	1,918395261	1
Gm6913	1,918262293	1
Smco3	1,917996383	1
Klhl25	1,917863443	1
Frat1	1,917730511	1
Nedd4l	1,917597589	1
Pop7	1,916268872	1
Ranbp6	1,915206561	1
Gm20517	1,914277523	1
A930001C03Rik	1,912685934	1
Slpi	1,909109694	0,9669
Frg2f1	1,908448162	1
4933412L11Rik	1,908448162	1
Gm36930	1,908183613	1
Lrtm2	1,908183613	1
Wbscr22	1,907786859	1
Hsdl1	1,907786859	1
Ccdc62	1,907786859	1
Rsl1	1,907786859	1
Tac4	1,907786859	1

Mrps2	1,907654626	1
A830073O21Rik	1,906597091	1
Fam69b	1,906200666	1
Mfsd13b	1,906200666	1
Lrrc73	1,904087794	1
Hexim1	1,903559942	1
Gm12074	1,903164149	1
Gm12582	1,902768438	1
Nlrc4	1,901449996	1
Rangrf	1,900132468	1
Gspt2	1,898947474	1
Ifrd1	1,898421047	0,93372
RP23-359K10.9	1,897105617	1
Bc1-ps1	1,89605393	1
Selenos	1,895265548	0,66845
Cstad	1,895265548	1
Gm11343	1,894477493	1
Sbk3	1,894477493	1
RP24-325N9.5	1,894477493	1
Gm12663	1,894477493	1
Gm8574	1,892902366	1
Gm6206	1,89159076	1
Ndufs5	1,891328548	1
Rrs1	1,890673177	0,63881
RP23-88C11.5	1,890149044	1
Gm25596	1,888315722	1
Rhox5	1,888053964	1
BC003965	1,887268908	1
D030028A08Rik	1,885961206	1
Sec31b	1,88452378	1
Uprt	1,884262548	1
Zfp763	1,883870767	1
Zfp605	1,883740192	1
Gm7733	1,88334852	1
Bcl3	1,88256542	1
Gm6526	1,882173992	1
Slc39a1	1,882043534	1
Zfp984	1,882043534	1
Gm19552	1,881913085	1
Hoga1	1,881913085	1
Mesdc1	1,881260977	1
Ippk	1,879175748	1
Ppan	1,878915257	0,66845
Pcdhb15	1,878915257	1
Fastkd3	1,878654803	1
Zfp867	1,878394384	1
Chac2	1,877092831	1
Xpot	1,87579218	0,93384
S1pr1	1,874232589	1
Cdca7	1,873583142	1
Gm13094	1,873193582	1
Gpr65	1,873063746	1

Nip7	1,872804103	0,64877
Gm12778	1,871636151	1
Gm13022	1,871246996	1
Pard6b	1,870598583	1
Mtif3	1,870080015	1
Zbtb22	1,868654694	1
5830432E09Rik	1,868525173	1
mt-Nd2	1,867877704	0,99199
BC022687	1,867230459	1
Dpp3	1,866583438	1
Snora73b	1,866195333	1
Gm43672	1,865807309	1
Lsm10	1,865548671	1
Pla2g2d	1,865160782	1
Cdkl4	1,865160782	1
Plekhf1	1,860770332	1
Gm38247	1,859738788	1
Gabrd	1,859352106	1
Zfand4	1,858836656	1
Fbxw7	1,858578984	1
Pelo	1,858063748	0,81787
Zdhhc24	1,858063748	1
Gnl2	1,857677415	1
Rap1gap	1,857677415	1
Gm6322	1,857677415	1
Gm37125	1,857677415	1
Tmem71	1,857677415	1
Ccnb1	1,857548655	1
Bap1	1,857291162	1
Mir17hg	1,856390217	1
Spats1	1,856261547	1
Fmo5	1,856261547	1
Med4	1,856004232	1
Eid2b	1,85548971	1
A430105I19Rik	1,85548971	1
Zfp64	1,854461093	1
Tmie	1,854075509	1
Emc9	1,853304581	1
Rubcnl	1,851122025	1
4632427E13Rik	1,848045145	1
Gm2986	1,847276724	1
Rit1	1,847020655	1
Lonrf1	1,846892634	0,79888
Plag1	1,846508623	1
Rab3il1	1,845229164	1
Gm7990	1,843950592	1
Gm5801	1,843694984	1
Ythdf2	1,842672906	0,82462
Fus	1,842672906	1
Gm4468	1,842162079	1
Gm9521	1,842162079	1
Ahrr	1,840885632	1

Arid3a	1,839992646	1
C1galt1c1	1,839482562	1
Pex12	1,839227573	1
Dusp1	1,838590256	1
Cd3eap	1,838207972	1
Fam219a	1,837825767	1
Cables2	1,837188935	1
Gm14325	1,836552325	1
Gm20274	1,836297742	1
Sde2	1,836170464	0,41979
Zfp839	1,835279765	1
Gm28686	1,835025358	1
Gm9796	1,834262352	1
Zscan12	1,833880967	1
Sgms1	1,833499662	1
Olfm1	1,832610258	0,79711
Dhx32	1,831086569	1
Arsk	1,831086569	1
Tmub2	1,830578955	1
Dusp14	1,82918374	1
5730405O15Rik	1,82740955	1
Nudt7	1,82411919	1
Ecd	1,823739915	1
Wrnip1	1,822855245	1
Zfp526	1,822855245	1
Eepd1	1,822728899	1
Ddx42	1,822602561	1
Gm25007	1,822097299	1
Zfp579	1,821718444	1
RP23-225D5.4	1,819825349	1
Nelfa	1,818942579	1
Shpk	1,818816504	1
Ipo13	1,817934222	1
9530053A07Rik	1,817934222	1
Tab3	1,817304284	1
Nrbf2	1,81680049	1
Coq6	1,816548645	1
Chrac1	1,814032119	1
Gemin6	1,813780658	1
Gar1	1,813152158	1
Socs6	1,812649515	1
Cdk5rap1	1,811016888	1
1110059G10Rik	1,810891363	1
Zfp846	1,810640337	1
Dpys	1,810640337	1
Sirt4	1,810640337	1
Gm15892	1,810389347	1
Nudt8	1,810389347	1
Gm14277	1,810263864	1
Fhad1	1,810263864	1
Gm18913	1,808884133	1
Dnajc30	1,808758755	1

mt-Tt	1,808633386	1
Mrnip	1,807129632	1
3110070M22Rik	1,807129632	1
AW047730	1,807129632	1
Gldc	1,807129632	1
Ttll13	1,807129632	1
Tmem184c	1,80587746	1
Trim11	1,80575229	0,83388
Gm11737	1,805251699	1
Trmt5	1,805126572	1
Rnft1	1,805126572	1
Ccr1	1,804751246	1
Gm9568	1,804501072	1
Gpr162	1,803500721	1
Bcl2a1b	1,803000754	1
Gm3375	1,802750822	1
Irf5	1,802625869	0,68407
Aggf1	1,80237599	0,93384
Gm42636	1,802251063	1
Gm44178	1,80125196	1
Bend6	1,80025341	1
Gm9378	1,80000386	1
Tshz3	1,799006002	1
Runx2os1	1,798008698	1
Uchl4	1,797634851	1
Sf3a3	1,796264746	1
Gmeb2	1,796015748	1
Fuz	1,795891262	1
Hmgb1-rs16	1,795766784	1
Pomk	1,795642316	1
Cbx8	1,794771276	1
Ccdc32	1,793279048	1
Wdyhv1	1,792906185	1
Gm13835	1,791167182	1
Zfp639	1,790794758	0,93384
Nfkbie	1,790670633	1
Ppp1r13b	1,790050142	1
Gm7972	1,789429865	1
Card6	1,789181815	1
Tmem38a	1,789057802	1
Eif1a	1,788809804	0,6523
Rin1	1,787818152	1
Mrps36-ps1	1,787694234	1
Gm45212	1,785960286	1
Gm9774	1,785588945	1
Dtx4	1,784475384	0,91431
Cenpb	1,784104351	1
Mapk11	1,783115308	1
Pspc1	1,782868133	1
2310057M21Rik	1,782744558	1
Gm8762	1,782620992	1
mt-Ts2	1,782373885	1

Tmem104	1,782250345	1
Tnnc2	1,782003289	1
Ncdn	1,781879775	1
Ccdc86	1,781509282	1
Jagn1	1,781509282	1
Gmppb	1,781015411	1
Gm20056	1,780645098	1
Zfp961	1,779904702	1
Fen1	1,779164615	1
Gm44423	1,77854811	1
Gm8317	1,778424835	1
Gm10126	1,776946197	1
Kat6b	1,776699878	1
Zfp658	1,776699878	1
Gm4342	1,77509963	1
Gm11298	1,7737467	1
Sema4c	1,773500823	1
Nceh1	1,773009173	0,78566
Rabgef1	1,773009173	1
Enc1	1,772517658	1
Gm11363	1,771657836	1
2410022M11Rik	1,771289468	1
Ddx27	1,771043932	1
C5ar1	1,769816764	1
Pid1	1,769203498	1
Rpf2	1,768958252	1
Fez2	1,768590445	1
Zfp459	1,768590445	1
Gm10080	1,768345284	1
Dnajc19-ps	1,767977605	1
Exog	1,767487485	1
Simc1	1,767242476	1
Pde12	1,766630103	1
Brf2	1,766385213	1
Slc43a2	1,766017942	1
Scly	1,766017942	1
Mtmr12	1,764916586	1
Tldc1	1,764916586	1
Gm3617	1,764916586	1
RP24-550H10.4	1,764671934	1
Erp27	1,762471587	1
Gm43684	1,762471587	1
Tsc2	1,762349426	1
Ccdc138	1,761738748	1
Gm8326	1,761494536	1
Prps1l3	1,760396002	1
Snx33	1,760273984	1
Tsacc	1,759176211	1
Ppm1a	1,758444742	1
Zfp296	1,755643595	1
Tmem158	1,755278557	1
Gm38305	1,754913595	1

Tns2	1,754548709	1
Kmt5b	1,753940734	1
Polr3d	1,753576051	1
Tut1	1,753211443	1
Qrich1	1,752360985	1
Ccdc120	1,751510941	1
Ybx1-ps2	1,748842056	1
Polr3e	1,748720839	1
4930432K21Rik	1,747145792	1
Sephs2	1,747024693	1
9930022D16Rik	1,746903603	1
Elf4	1,745814167	1
Nfu1	1,745693161	1
Il17ra	1,744483558	1
Ptgr1	1,744120841	1
Pard6a	1,743879071	1
Arvcf	1,743637335	1
H60c	1,743153963	1
Lbp	1,742791522	1
Ltv1	1,742549936	1
Trim14	1,741946119	1
Rhob	1,741221815	1
Trmt12	1,741101127	1
Bcl2l12	1,740135923	1
Caprin2	1,740135923	1
Apoo	1,74001531	1
Prune1	1,739653522	1
Ovca2	1,739291808	1
Mon1a	1,738809641	1
Sh2d3c	1,738809641	1
Rps3a3	1,73820712	1
Rpp25l	1,737725253	1
Cacnb1	1,735558507	1
Tubb2b	1,735077374	1
Phykpl	1,734957112	1
Rbsn	1,734716613	1
Gm17251	1,734716613	1
Gm9530	1,734355926	1
Gm45454	1,734235714	1
Rap1b	1,733875127	0,7434
Rela	1,733754948	1
Pms2	1,73303405	1
Lpar1	1,731713181	1
Chac1	1,731713181	1
Lrp12	1,731353119	1
Gnmt	1,730273381	1
F7	1,730273381	1
Gm43364	1,730273381	1
Acacb	1,730273381	1
Mypop	1,730033531	1
Ppp4r1	1,72955393	1
Tk2	1,729194317	1

Lipt1	1,728115926	1
Gm44884	1,728115926	1
Gm5321	1,727397372	1
Polr3f	1,727157921	1
Gm26782	1,727038207	1
Mustn1	1,726559437	1
Tmem251	1,726200446	1
Riox1	1,725961161	1
Dcun1d4	1,72584153	1
Ltbr	1,725721908	1
Mical1	1,725243503	1
Dtx2	1,723809082	1
Hyls1	1,723331207	1
Snx4	1,721301716	1
AI987944	1,720705261	1
Nr4a3	1,720585995	1
Ndufaf6	1,719632164	1
Pom121	1,718917138	1
Fem1b	1,718678862	1
Klhl23	1,718559736	1
Exosc2	1,71832151	1
Med10	1,718202409	1
Wdr35	1,718083316	1
Stam2	1,717130873	1
Haus2	1,715941061	1
Bag2	1,715346464	1
Dapk1	1,715227569	1
Pinx1	1,713801476	1
Fance	1,71356391	1
Gm5900	1,713445138	1
Tbc1d30	1,713326376	1
Nfatc3	1,712376569	1
Tgif1	1,71225788	1
Gm12966	1,7121392	1
Zfp84	1,712020527	1
Gm7287	1,711901863	1
Phlda3	1,711308666	1
Egln3	1,711190051	1
Ldlrap1	1,710715675	0,93372
Nckap1	1,710122888	1
Maip1	1,709885831	1
Alg11	1,708582606	1
Gm5871	1,708345762	1
Prkch	1,707872174	1
Cdk9	1,707872174	1
D330041H03Rik	1,707398717	1
Dcstamp	1,706215648	1
Nlrp10	1,706097386	1
Matn1	1,705624421	1
Particl	1,705151587	1
Id2	1,70491522	1
Oip5	1,704324443	1

Acod1	1,703970076	1
Gm10167	1,703970076	1
Gm12186	1,703497701	1
Fzd9	1,703261562	1
RP23-168F21.4	1,703143505	1
Prdm4	1,702671359	1
1700030M09Rik	1,702317335	1
Tmem115	1,701845418	1
Nudc-ps1	1,701609508	1
1600029O15Rik	1,701609508	1
Fli1	1,701019878	1
Asb11	1,701019878	1
Zfp62	1,70054832	1
Usp38	1,700076893	1
Arhgap39	1,699959057	1
Tnip3	1,699605597	1
Ptpro	1,698427929	1
Bysl	1,698310207	1
Gm37033	1,697133436	1
6430590A07Rik	1,696310181	1
Tti2	1,696192606	1
Gm15007	1,696075039	1
Zfp473	1,695017302	1
Pggt1b	1,69466487	1
Tuba4a	1,694195074	1
Gtf2b	1,693490625	1
Tspan14	1,692903807	1
Sla	1,692786468	1
Piwil2	1,692551814	1
Ccr2	1,692551814	1
Gm37660	1,6924345	1
Gm42549	1,6924345	1
Fam195a	1,691379033	1
Gm5449	1,691379033	1
Dusp10	1,6912618	1
Dse	1,690675755	1
Slc4a11	1,69055857	1
Gm12529	1,689972769	1
Gm12990	1,689855633	1
Slc30a6	1,689621385	1
K230015D01Rik	1,689152988	1
Ly6g6d	1,689035909	1
Ythdf1	1,688216582	1
Zbtb5	1,687397653	1
Gm16755	1,68669603	1
BC005624	1,685760986	1
Rpl30-ps1	1,685293659	1
Zfpl1	1,684476148	1
Gm10605	1,683892454	1
Gm8250	1,683775739	1
Nasp	1,683659033	1
Snapc5	1,683659033	1

Grwd1	1,683542335	1
Gm13378	1,683425645	1
Gm14680	1,682958965	1
Exosc4	1,682609039	1
Sema6c	1,682492414	1
Gm33080	1,681909408	1
Zfp36	1,681210067	1
Pomgnt1	1,681093538	1
Mmaa	1,680394536	1
Ubn1	1,678415609	1
Rad17	1,677717721	1
Rasl2-9	1,677717721	1
Pla2g2e	1,676206625	1
Dnajb5	1,675393522	1
Rbm5	1,674580813	1
Midn	1,674348683	1
Platr3	1,674116585	1
Gm37726	1,674116585	1
Cog8	1,673768498	1
Ssh2	1,673420484	1
Gm7514	1,673304495	1
RP24-511J14.2	1,673072542	1
Rsad1	1,672029151	1
Tubg2	1,671913258	1
Twnk	1,67144977	1
Arl4c	1,670754778	1
Gm37795	1,670175839	1
Mboat1	1,670175839	1
Gm3355	1,6695971	1
Snx16	1,669249953	1
RP23-442M18.5	1,669018562	1
Gm10827	1,668555876	1
Cxx1a	1,668555876	1
Ccdc80	1,668208946	1
Myh11	1,668208946	1
Nol6	1,666937485	1
Dlg3	1,666706414	1
Pus3	1,666359868	1
Kcnk13	1,665897919	1
Eif2ak1	1,665551542	1
Pgf	1,665551542	1
Setmar	1,665205236	1
Msantd4	1,664974406	1
Ercc2	1,66462822	1
Il1rn	1,66462822	1
Ptcd1	1,663936064	1
Gm45833	1,663590095	1
BC060293	1,663590095	1
6030442K20Rik	1,663590095	1
Zdhhc7	1,662898371	1
Gm5764	1,662322154	1
Kmt2e	1,661630957	1

Eogt	1,661630957	1
Neurl1a	1,660019283	1
Med6	1,659789171	1
Afmid	1,659789171	1
Tsen34	1,659444064	1
Lrif1	1,658984032	1
Gm42970	1,658639092	1
Gm14593	1,658179283	1
Gm12251	1,65783451	1
Gm29155	1,657719602	1
Frmd4a	1,656915466	1
Mzb1	1,656915466	1
Lrrc8d	1,656456135	1
Adrb2	1,656226518	1
B130006D01Rik	1,656111721	1
Zfp217	1,655537856	1
Sag	1,655423107	1
Trim32	1,65496419	1
Dhx57	1,65496419	1
Alg2	1,65496419	1
Fbxw17	1,654276052	1
Tns4	1,653358981	1
Arl8a	1,653129793	1
RP23-48A24.3	1,65278607	1
Zp1	1,652442418	1
Tm9sf1	1,651984328	1
RP23-110E20.5	1,651869825	1
Gm6768	1,651068527	1
Gm14794	1,650725233	1
Gm4875	1,650496409	1
Rnmt	1,65038201	1
Gm9951	1,649009831	1
Gm42967	1,649009831	1
Igf2bp1	1,649009831	1
Gm44103	1,649009831	1
Slc39a8	1,649009831	1
Pou4f1	1,649009831	1
Gm23054	1,649009831	1
Mtcl1	1,649009831	1
Lin28b	1,649009831	1
Fancg	1,648895535	1
Klf9	1,648666965	1
Vsig10	1,647981446	1
Bloc1s6os	1,647981446	1
Zfp599	1,647981446	1
Adgb	1,647182035	1
Dusp16	1,646611264	1
Cwc25	1,646383011	1
Gm44913	1,646383011	1
Creb3l1	1,646268896	1
Hmbs	1,645698442	1
Ankrd26	1,644672122	1

Qsox2	1,644330157	1
Gm13398	1,643760375	1
Otub2	1,643304691	1
Gm12176	1,642735263	1
Gm31274	1,642507548	1
Pex11g	1,641028165	1
Leng9	1,640459526	1
Rgs20	1,640004758	1
Gm6395	1,639322841	1
Smn1	1,638868387	1
Gm10636	1,638868387	1
S1pr2	1,638073396	1
Des	1,638073396	1
Zbtb38	1,637505781	1
Tubgcp3	1,637505781	1
Pknox1	1,636598007	1
Ndst1	1,634897286	1
AU019823	1,634217493	1
RP23-447C2.2	1,633877702	1
Rflnb	1,633085132	1
Cox11	1,632745577	1
Klhl36	1,632406092	1
Mfsd5	1,631953556	1
Scamp1	1,631274987	1
Dusp9	1,631161919	1
Zfp775	1,630935808	1
Gtf2h2	1,630257662	1
Hspb6	1,629466848	1
Bcar3	1,62845065	1
Arl15	1,628224915	1
Mapre3	1,628112059	1
Supv3l1	1,627547896	1
Pfkfb4	1,627435087	1
Utp20	1,626420157	1
D5Ertd605e	1,624955264	1
Gm43223	1,624955264	1
Dhx38	1,624730013	1
Cpt2	1,624730013	1
Nmi	1,624617399	1
Keap1	1,624392195	1
Mrps22	1,624279604	1
Mtmr4	1,624167022	1
Depdc1b	1,624167022	1
Gm6123	1,623716769	1
Gm12799	1,623604226	1
Cmklr1	1,623604226	1
Gm45224	1,623604226	1
6430571L13Rik	1,623604226	1
Zfp820	1,623604226	1
Jmjd4	1,623379162	1
Gm37063	1,623266642	1
Tmem170	1,623154129	1

Hist1h4h	1,622929128	1
Gm4217	1,622816639	1
Suco	1,622704158	1
Gm5869	1,622704158	1
Gm13392	1,622366761	1
Fbxo32	1,622254311	1
Lpin3	1,622029435	1
BC030867	1,621354992	1
Fam175b	1,621242612	1
Kctd7	1,62079317	1
Rars	1,620456171	1
Dbr1	1,620231543	1
Ifngr1	1,619557848	1
Mtrf1l	1,619333345	1
Nr1h2	1,61899665	1
Srd5a1	1,618884433	1
Hoxa1	1,618772224	1
Gzf1	1,616865867	1
Asb8	1,616305601	1
Thap7	1,616081549	1
Cog2	1,616081549	1
Gad2	1,616081549	1
Tnfaip8l2	1,61540958	1
D3Ertd254e	1,614849818	1
1600020E01Rik	1,614849818	1
Igtp	1,614514055	1
Wipi2	1,613507183	1
Trmt6	1,613395347	1
Zfp637	1,612277414	1
Pogz	1,611942185	1
Bms1	1,610936917	1
Bbs5	1,610825259	1
Llgl2	1,610825259	1
Akna	1,610601967	1
Ubqln2	1,610490332	1
Susd3	1,609932275	1
Gm6155	1,607924872	1
Ugt1a7c	1,607813423	1
Mad1l1	1,607590548	1
Fut10	1,607590548	1
Mpv17l	1,606922109	1
Gpank1	1,606810729	1
Acsf3	1,606699358	1
Gm8722	1,606476637	1
Riok1	1,605474777	1
Mrpl22	1,605474777	1
Ccdc173	1,605474777	1
Shc4	1,605140963	1
Thap12	1,605029707	1
Gm29487	1,604918459	1
2310033P09Rik	1,604695985	1
Rap2b	1,603695236	1

Ddx56	1,603361792	1
Gm38067	1,603250659	1
Scfd1	1,603028416	1
Fam208b	1,603028416	1
Mfsd11	1,602584024	1
Panx1	1,602584024	1
Gm6919	1,602584024	1
Gm5786	1,602472945	1
Cited2	1,602361874	1
D11Wsu47e	1,602028707	1
Gm8806	1,601917667	1
Bid	1,601695609	1
Sfxn5	1,601584592	1
Hsh2d	1,601584592	1
Gm7351	1,6011406	1
Tnfrsf1a	1,60036391	1
Tgfbrap1	1,60036391	1
Tbp	1,599809362	1
Sphk2	1,599144159	1
Fbxo18	1,599033318	1
1810024B03Rik	1,599033318	1
4732440D04Rik	1,598811661	1
Lrsam1	1,598479232	1
Gm14279	1,598257651	1
Arhgap1	1,597260917	1
Elmod2	1,596818123	1
Clec7a	1,596264804	1
Lpar6	1,595158741	1
Zfp606	1,594827072	1
Arl4a	1,594716531	1
Gm2223	1,59361154	1
Gm16072	1,59361154	1
Fancm	1,593169759	1
Bloc1s4	1,592507316	1
Dolk	1,592507316	1
Agpat2	1,59195549	1
Gm4866	1,59195549	1
Gm5139	1,591403856	1
Atmin	1,591072968	1
Ribc1	1,590852413	1
Lilr4b	1,590742148	1
E430021H15Rik	1,590742148	1
Fas	1,5888688	1
Gm16106	1,588648551	1
Cish	1,588538439	1
Junos	1,588318236	1
Mfsd8	1,587107665	1
Usp12	1,58622783	1
Orai1	1,586117885	1
Fkbpl	1,585568273	1
Dnajc17	1,585458374	1
9930014A18Rik	1,585348482	1

Cltb	1,585238598	1
Gm13992	1,584689291	1
Pdcd11	1,584249983	1
Gm7936	1,584249983	1
Gnptab	1,584030374	1
Eif2b4	1,583810796	1
Praf2	1,583261985	1
Kif13a	1,583042513	1
Gm8463	1,582713363	1
Gm20257	1,582055268	1
Sh3bp5l	1,581835964	1
Mrps10	1,581835964	1
Cmtr1	1,581507064	1
Tirap	1,581178233	1
Supt7l	1,580849471	1
Gmfg	1,580739898	1
Mzf1	1,580630334	1
Ppil3	1,580411227	1
Haus4	1,579863593	1
Spr	1,578987773	1
Poli	1,57887833	1
Tysnd1	1,578550046	1
1110002L01Rik	1,577674954	1
Cnksr1	1,577456257	1
Ngrn	1,577018954	1
Rilpl1	1,576581772	1
Slc25a33	1,575161768	1
Irf8	1,574834256	1
Zfp180	1,574725101	1
Med26	1,57439768	1
Cbfa2t2	1,574179437	1
Bri3	1,573961225	1
U2surp	1,57385213	1
Rab33b	1,573743043	1
Fam35a	1,572979643	1
Maf1	1,572761597	1
Tob2	1,572434584	1
Zfp553	1,572216614	1
Ube2v2	1,571998673	1
Gm37482	1,57123612	1
Fbxo25	1,570691665	1
Vps37c	1,570365083	1
Ppp4r2	1,570038569	1
Rpl30-ps11	1,569929746	1
Cxcr4	1,569712122	1
Slc22a21	1,569712122	1
Pde6g	1,569603322	1
Polr3a	1,569276966	1
Rcan1	1,568080908	1
Kctd3	1,567972221	1
Fam187b	1,567972221	1
Zfp945	1,567537547	1

Nudt18	1,567428897	1
5031439G07Rik	1,566559971	1
Prorsd1	1,566017137	1
Gm43668	1,565800056	1
Rpl30-ps2	1,564715102	1
Surf2	1,564715102	1
Mysm1	1,564498201	1
Gm6166	1,564498201	1
Naa40	1,5636309	1
Als2	1,5636309	1
Myo10	1,56341415	1
Brpf1	1,562764079	1
Gm42479	1,562764079	1
Csrnp2	1,56222256	1
Tor4a	1,561464749	1
Zfp574	1,560815489	1
Axin1	1,560382799	1
E230029C05Rik	1,560382799	1
Aptx	1,560274645	1
RP23-138K22.2	1,560274645	1
Sp2	1,559950229	1
Gm12762	1,559842105	1
Tbc1d16	1,559517779	1
Elmsan1	1,559409685	1
Rpl3l	1,559301599	1
Mad2l1bp	1,558977385	1
Gm15877	1,558653238	1
Dclre1b	1,558545205	1
Spata24	1,558437178	1
Rps19-ps5	1,558329159	1
Traf2	1,558221148	1
Taf5l	1,558005148	1
Lat2	1,557357326	1
Ptpn21	1,557033516	1
Slu7	1,556601875	1
Pik3r4	1,556601875	1
Brix1	1,556493983	1
Nol4l	1,555954637	1
Gm43692	1,555954637	1
Cdc14a	1,55584679	1
Syvn1	1,555523295	1
Pirb	1,555523295	1
Dagla	1,555523295	1
Phldb3	1,555307668	1
Ccdc66	1,555307668	1
Pitpnb	1,554984285	1
Klf13	1,554768733	1
Nifk	1,554660968	1
Dnaaf2	1,554229985	1
Gm8116	1,554122257	1
Slc25a44	1,553906825	1
Zfp617	1,553906825	1

Tm9sf2	1,55379912	1
Sh3tc1	1,553583733	1
Faap100	1,55293775	1
Grpel1	1,552830112	1
Smad7	1,552830112	1
Alas1	1,552722482	1
Rnasel	1,552614859	1
Ppm1d	1,552399636	1
Cxcr3	1,55164659	1
Zfp953	1,55164659	1
Celsr1	1,55164659	1
Plcd1	1,55164659	1
Klhl40	1,55164659	1
Shisa3	1,55164659	1
Mrps35	1,551431501	1
Cep162	1,551001412	1
Rbm34	1,550893909	1
Gm44013	1,550893909	1
Zfp821	1,550786413	1
Cfap43	1,550678924	1
Gm14270	1,550356503	1
Mpc1	1,549819283	1
Tsr1	1,549711862	1
Ebag9	1,549604448	1
Mettl13	1,549497041	1
Rpl19-ps1	1,549174866	1
Cdk13	1,548316059	1
Gm42829	1,548316059	1
Gpalpp1	1,547028741	1
Plagl2	1,546385484	1
Tnfaip2	1,5462783	1
B630019K06Rik	1,5462783	1
Dnajc8	1,546171124	1
Psme2	1,546171124	1
3110009E18Rik	1,545742494	1
Lmbrd1	1,545313983	1
Tma16	1,545313983	1
Brca1	1,545313983	1
Wdr78	1,54488559	1
Znhit3	1,544564373	1
Adrm1	1,544457316	1
Trmt10c	1,544350266	1
Tmem203	1,544243224	1
Gm7535	1,544029161	1
Cped1	1,543494134	1
Gm20432	1,543387151	1
Ip6k3	1,543066246	1
Ptgs2os2	1,543066246	1
Xkr8	1,542317727	1
Zswim4	1,542210825	1
Gm13777	1,542103931	1
Hnrnpf	1,541142217	1

Ssh1	1,541035397	1
9930111J21Rik2	1,540394631	1
Atl2	1,540287863	1
RP24-175C20.18	1,540074348	1
Slc35c1	1,539967602	1
Gm6542	1,539967602	1
A930007I19Rik	1,539860864	1
Kcnn4	1,539647408	1
Arhgef3	1,539327281	1
Tgs1	1,538900548	1
Slfn5	1,538473933	1
Gm9246	1,537727642	1
Dffb	1,537514483	1
Acvr1b	1,537514483	1
Foxn2	1,537407914	1
Ahsa1	1,537301353	1
Chtf18	1,537301353	1
Clp1	1,537088252	1
Ubtd2	1,537088252	1
Adnp2	1,536981713	1
Ccdc71	1,53666214	1
Acad8	1,536449128	1
BC048403	1,536449128	1
Ccdc186	1,536129666	1
Gm26530	1,536023193	1
Ears2	1,53581027	1
Tfr2	1,53581027	1
Gm13998	1,535384512	1
Tm2d3	1,535065271	1
Smarca5	1,534958872	1
Nolc1	1,53485248	1
Ube2o	1,534746096	1
Gm7860	1,534639719	1
Gm8444	1,53453335	1
C78859	1,534001613	1
Sertad1	1,53368266	1
Fbxo34	1,53368266	1
Gm13186	1,533576357	1
Gm10060	1,533576357	1
Ublcp1	1,533576357	1
Zdhhc14	1,533470061	1
Amacr	1,532513731	1
Gm11918	1,532407509	1
Gm17511	1,531982694	1
Phf11d	1,531982694	1
Zfp523	1,531876509	1
Stat5b	1,531557997	1
Slc30a2	1,531451841	1
Gm13205	1,531345693	1
Aar2	1,531133418	1
D130007C19Rik	1,530178545	1
Kif13b	1,529542294	1

Gm9761	1,528694371	1
Rbm43	1,527635128	1
Mex3a	1,527423367	1
Zfp595	1,527317498	1
Hilpda	1,526894094	1
Scaf4	1,526576619	1
Tmem67	1,526259209	1
Usb1	1,525836099	1
Dtwd1	1,525836099	1
Decr2	1,525307376	1
Arl2bp	1,525095938	1
Gapdh-ps14	1,524778836	1
Hes7	1,524356136	1
Fosl2	1,523722306	1
Cecr5	1,523194316	1
Thumpd1	1,522877609	1
Depdc7	1,521822397	1
Fam214b	1,521716916	1
Kctd12	1,521505976	1
Tug1	1,521400517	1
Mettl3	1,521400517	1
Ddx58	1,521295065	1
A230028O05Rik	1,521295065	1
Il7r	1,521295065	1
Faf2	1,521084183	1
Dennd2c	1,521084183	1
Llph-ps2	1,521084183	1
Gm13349	1,520240949	1
Wdr43	1,520030213	1
Edc4	1,519714165	1
Sft2d3	1,519292869	1
Fam20c	1,518976974	1
Gtf3c3	1,517819253	1
Arid1b	1,517082981	1
Diexf	1,516977829	1
Med29	1,516347066	1
Fam136a	1,516136871	1
F8a	1,516031784	1
Mis12	1,515926704	1
Serac1	1,515401415	1
Sec13	1,514981315	1
Gm15694	1,514666316	1
Zfp688	1,514351383	1
Gtpbp8	1,513931573	1
Trip11	1,513721713	1
Recql5	1,513616793	1
Pias1	1,513197187	1
Fam118a	1,513092304	1
Tmem181b-ps	1,512043872	1
Zfp800	1,511729484	1
Sel1l	1,511519928	1
Cd14	1,511415161	1

Tax1bp3	1,510472586	1
Paqr4	1,510263204	1
E2f4	1,509844528	1
Mirlet7b	1,509635234	1
Gm42515	1,508693768	1
Atp6v1d	1,508275527	1
Fem1a	1,507439394	1
Tlr7	1,507125963	1
Uqcc1	1,507021501	1
Xbp1	1,506290467	1
Ccnk	1,504933773	1
Rpl30-ps3	1,504829462	1
Gm14541	1,504620863	1
Kctd6	1,504308018	1
1700021F05Rik	1,503995239	1
Ints10	1,503995239	1
Mllt1	1,503995239	1
Gm9727	1,502744771	1
Zfp141	1,502328179	1
Klc3	1,501911703	1
2610306M01Rik	1,501703509	1
Slc35f5	1,501495343	1
Top1mt	1,501079098	1
Nsmce2	1,500975054	1
Gm15032	1,500558953	1
2700062C07Rik	1,500350946	1
Gm19028	1,499831054	1
Nr2c1	1,499519205	1
Yif1a	1,49941527	1
Zfhx2	1,499311342	1
Bsn	1,499103508	1
Rwdd1	1,498895703	1
Mib2	1,498791811	1
Plekhm2	1,498480178	1
Blzf1	1,498480178	1
Gm9762	1,49816861	1
Gm8539	1,497545669	1
P2ry2	1,496819232	1
Sucla2	1,496715484	1
Mlycd	1,496715484	1
Rnd1	1,496715484	1
Daxx	1,496611743	1
Gm43137	1,496611743	1
Itpk1	1,496508009	1
Ddx51	1,496300563	1
Gm45855	1,496300563	1
Ntmt1	1,496196851	1
Tex2	1,495989449	1
Rnf139	1,495782075	1
Gm20554	1,495782075	1
Jrkl	1,495263766	1
Tjap1	1,495056493	1

Gm8330	1,494745637	1
Arhgdig	1,494642033	1
Znhit6	1,494434846	1
Cops8	1,493813457	1
Rab11fip3	1,493813457	1
Zbtb11os1	1,49350286	1
Gm22581	1,49350286	1
Eci1	1,492985341	1
Malsu1	1,492881858	1
E130201H02Rik	1,492674915	1
9130008F23Rik	1,492571455	1
Myo9b	1,492364555	1
Slc25a32	1,492261115	1
Ranbp1	1,492157683	1
4930453N24Rik	1,49184743	1
Tmem120b	1,49164063	1
Gm43761	1,49164063	1
Psmc1	1,491537241	1
Sco2	1,491537241	1
Rbm19	1,491433859	1
Eps8	1,490607062	1
Gm6293	1,490297131	1
Usp7	1,489987265	1
Fam58b	1,48957421	1
Kdelc1	1,48957421	1
Fbxo31	1,489470964	1
Strn3	1,489367726	1
RP23-38L16.4	1,489058052	1
Ttl	1,488954842	1
Ube2cbp	1,488954842	1
RP23-316F10.2	1,488542074	1
Lrrc8c	1,488438899	1
Tsg101-ps	1,488438899	1
Gas2l3	1,488026274	1
Gm42548	1,487820004	1
Rtn4	1,48771688	1
Cdc27	1,48771688	1
Spryd3	1,487510652	1
Sgpp1	1,48740755	1
Tnfrsf14	1,48740755	1
Gemin8	1,486582984	1
Tyms	1,486479946	1
Tbc1d8	1,48596486	1
Gm12497	1,485655895	1
Msra	1,485655895	1
Paqr7	1,485346994	1
Dhrs13	1,485346994	1
Hnrnpk	1,485244041	1
Rnf149	1,485141095	1
Gm43878	1,485141095	1
Nipa1	1,484729384	1
RP23-246F14.1	1,484523571	1

Gm9825	1,484317786	1
Brd2	1,48411203	1
Oxld1	1,48411203	1
Zfp626	1,483186482	1
Rnf41	1,483186482	1
Gm6198	1,483186482	1
Zfp952	1,48246701	1
Zrsr1	1,48205604	1
Gm13771	1,48205604	1
Arf4	1,481953315	1
Syf2	1,481542487	1
Dpp9	1,481542487	1
Sgk1	1,481337116	1
AI464131	1,481131773	1
Sec24a	1,480926459	1
Nom1	1,480926459	1
Lyrm2	1,480515916	1
Cdc45	1,480413298	1
Dnajb9	1,480310687	1
Eapp	1,480002897	1
Topors	1,47959261	1
Stard7	1,47959261	1
March9	1,479182437	1
Gm45133	1,479079911	1
Fgd3	1,478874881	1
Gm5898	1,478772377	1
Gm8995	1,478464907	1
St8sia4	1,478362431	1
Sars2	1,478259963	1
Wtap	1,478055046	1
Mrps7	1,477850159	1
Tmem131	1,477850159	1
Exoc8	1,47754288	1
Lhpp	1,476723784	1
Gm27039	1,476723784	1
Bod1	1,476519081	1
4930590J08Rik	1,476519081	1
RP24-295J1.1	1,47641674	1
Chmp7	1,476314406	1
Abcc3	1,476109759	1
Igf2bp2	1,475700551	1
Lysmd1	1,475291457	1
Mpeg1	1,474882476	1
Zfp239	1,474473609	1
Rbm41	1,474167032	1
Dopey2	1,47386052	1
2310039H08Rik	1,473247686	1
Utp15	1,47243097	1
Zbtb41	1,47243097	1
Gsg2	1,471920753	1
R3hcc1l	1,471104773	1
Gins1	1,471002807	1

Setd1b	1,470391162	1
Zfp959	1,470085434	1
Gm9712	1,469983539	1
Hirip3	1,46977977	1
Ankrd17	1,469677897	1
Rft1	1,469677897	1
Gm26533	1,469677897	1
Tbkbp1	1,46957603	1
Smcr8	1,469270472	1
Brox	1,469168633	1
Lima1	1,46876135	1
Gm5787	1,46876135	1
9130011E15Rik	1,468659546	1
Gm7384	1,468455961	1
Thoc3	1,468354179	1
Kctd11	1,468048875	1
Meiob	1,467641902	1
Uaca	1,467540176	1
Ftsj3	1,467336746	1
Dhps	1,467336746	1
Tamm41	1,467336746	1
Nr6a1	1,467336746	1
C730034F03Rik	1,467133344	1
Dnajc9	1,467031654	1
Cd83	1,46692997	1
Slirp	1,46692997	1
Emc4	1,466828294	1
Gm6598	1,466726625	1
Azin1	1,466624963	1
Tor1b	1,466421659	1
Cep85	1,46571032	1
A630033H20Rik	1,465608728	1
1700123O20Rik	1,465202431	1
Gm42851	1,465202431	1
Trmt61a	1,464897783	1
Mief1	1,464593197	1
Man2c1os	1,464187182	1
Adprhl2	1,46327406	1
Wbp4	1,462665627	1
Rbmxl1	1,462564247	1
Paxip1	1,462564247	1
Blvra	1,462462873	1
Zdhhc12	1,462260147	1
Tmem19	1,462158794	1
Gm12182	1,462057448	1
Nuak2	1,461550826	1
Tifa	1,461449522	1
Gtf2h1	1,461246936	1
Gm6028	1,461145654	1
Fbxl3	1,461044379	1
Gm17494	1,460841849	1
Fam13c	1,460639347	1

Haus3	1,460538107	1
Slc12a2	1,460436874	1
Hars	1,460335647	1
Sbk2	1,460335647	1
Naa25	1,460133216	1
Frs2	1,460032011	1
Fbrsl1	1,459930812	1
Gtpbp6	1,45962726	1
RP24-174I4.1	1,45962726	1
Psmb6	1,45952609	1
Cbll1	1,459323771	1
Notch1	1,459323771	1
Chpf2	1,459222621	1
Gm42508	1,459222621	1
Slc37a3	1,458919216	1
Gatad2a	1,458818095	1
Clpp	1,458818095	1
Strn	1,458716981	1
Stoml1	1,458312595	1
Gm9575	1,457908321	1
Sla2	1,457201111	1
Mdfic	1,456999114	1
Polr2l	1,456797145	1
Rps12-ps24	1,456696171	1
Gm9381	1,456090474	1
Gm10388	1,455989549	1
Trib3	1,455888631	1
Gm15484	1,455888631	1
A430035B10Rik	1,45578772	1
Selenop	1,455585919	1
Slc41a1	1,455485029	1
Phactr4	1,455081539	1
Lif	1,4543757	1
Gk	1,454174095	1
Sertad3	1,454073303	1
Nup214	1,453972517	1
Gm7964	1,453569446	1
Sf1	1,453367953	1
Taco1os	1,453367953	1
Pdgfb	1,453267217	1
Mrps12	1,45296505	1
Atxn7l2	1,452461578	1
Zfp446	1,452360904	1
Kbtbd3	1,452159578	1
Rgs9bp	1,452058925	1
Rfesd	1,451455157	1
Glipr1	1,451253956	1
Zfp324	1,451253956	1
Zfp386	1,451052784	1
Taok3	1,450348899	1
Gm37968	1,449645356	1
Cxcl14	1,449544878	1

H2-Q10	1,449544878	1
Cebpz	1,449444407	1
Gm5391	1,449444407	1
Ercc1	1,449143035	1
Ttc28	1,448640887	1
Snai2	1,448440076	1
Rtfdc1	1,448138913	1
Ppfia1	1,447938172	1
Skor1	1,447737459	1
Ap5s1	1,447637113	1
Avl9	1,447536774	1
Uri1	1,447536774	1
Abhd6	1,447436442	1
Sppl3	1,447336117	1
Usp36	1,447235799	1
Bcl7c	1,447235799	1
Rmnd5b	1,447135488	1
Soga1	1,447035183	1
Gdap2	1,446934886	1
Fam188b	1,446433503	1
Gm17039	1,446333248	1
Mat2a	1,44573186	1
Gm43511	1,445631653	1
Vps36	1,445531453	1
Itga7	1,445531453	1
Rps2-ps11	1,445531453	1
Kat6a	1,445331073	1
Slc41a2	1,445230894	1
Adat3	1,444529834	1
BC052040	1,44442971	1
Oas1c	1,44442971	1
Actr6	1,444029285	1
Angpt2	1,443929196	1
Fchsd1	1,443929196	1
Ffar4	1,443428855	1
Rpp14	1,443128733	1
Camsap2	1,442828674	1
Rps6kc1	1,442828674	1
Ino80c	1,442628669	1
Oit3	1,442628669	1
AW209491	1,442428693	1
Hivep3	1,441928871	1
5031425F14Rik	1,441928871	1
Gm15846	1,441629062	1
Gm12906	1,441629062	1
Setd4	1,441429224	1
Gm9333	1,441429224	1
Zfp74	1,441429224	1
Klhl12	1,441329315	1
Fig4	1,441329315	1
Gm7079	1,441229413	1
Rgs1	1,440730007	1

Sema4d	1,440630147	1
Tlk1	1,440530294	1
Gm8894	1,440330608	1
Gm37522	1,439931319	1
Dbf4	1,439731715	1
Ptger4	1,438833844	1
Snord35a	1,438634393	1
Rps10-ps4	1,438534678	1
Nrip1	1,43843497	1
Narfl	1,438235574	1
Parp12	1,437637553	1
Gm5244	1,437438268	1
Zfp943	1,436442257	1
Gm10425	1,435944511	1
Mrs2	1,435844982	1
Ctsc	1,435546437	1
Hoxb4	1,434850075	1
Rpl7l1	1,434551737	1
Stk17b	1,434253462	1
Sertad2	1,43415405	1
Slc38a7	1,433557726	1
Gm11263	1,433557726	1
Gpam	1,433458363	1
Gm11631	1,433458363	1
Wdr61	1,433359007	1
Gm11281	1,433359007	1
Rpp30	1,433259657	1
5430403G16Rik	1,432763013	1
Gm11966	1,432365822	1
Erbin	1,432266542	1
Gmps	1,432167268	1
Cnot11	1,431968741	1
Rpl35	1,431869488	1
Atxn7l3	1,43157177	1
Ehbp1	1,431075711	1
H1f0	1,43097652	1
Snx1	1,430877336	1
Mbd6	1,430877336	1
Gm4034	1,430877336	1
Eng	1,430678988	1
Gm13712	1,430678988	1
Pgrmc1	1,430480667	1
Ccar2	1,429885871	1
Ppp2cb	1,429786762	1
Ranbp9	1,42968766	1
1110037F02Rik	1,42968766	1
Chd1	1,429588565	1
Gtf2e1	1,429489477	1
Atg4d	1,428895093	1
Gm12732	1,428498974	1
St6gal1	1,428498974	1
Rpl3-ps1	1,428300956	1

Msi1	1,428201957	1
Zfp318	1,427410213	1
Hectd1	1,427311276	1
Snap29	1,427311276	1
A130050O07Rik	1,427212346	1
Esf1	1,42622342	1
Coro1c	1,426025717	1
Gm11964	1,425828042	1
P2rx4	1,42553158	1
Ercc6l2	1,425037614	1
Spi1	1,424445081	1
Gm13487	1,424346349	1
Zfyve1	1,423458073	1
Tubb2a	1,42257035	1
Zfp955a	1,422471749	1
Trak2	1,421880282	1
Snapc1	1,421486108	1
Ppat	1,421486108	1
Snrpa1	1,421190549	1
Nmnat3	1,421092043	1
Map4k3	1,420796566	1
Zfp770	1,420698087	1
Treml4	1,420698087	1
Cd101	1,420599616	1
Rab35	1,42050115	1
Gm19620	1,420402692	1
Smad3	1,420304241	1
Scyl1	1,420304241	1
Elp6	1,420304241	1
Prpsap2	1,420008928	1
Nos1	1,420008928	1
Mtmr9	1,419812086	1
Tspan15	1,419516875	1
Rnf113a2	1,419221726	1
Pik3cb	1,419123356	1
Rassf5	1,419024993	1
Gemin2	1,418533282	1
0610040B10Rik	1,41843496	1
Ehd4	1,417648631	1
Gm4734	1,41725563	1
Fcrl5	1,416371775	1
Zfp229	1,416273603	1
Spryd4	1,416175438	1
Ppp1r13l	1,41519416	1
Ppip5k2	1,414997986	1
Csnk1d	1,41480184	1
4930579K19Rik	1,41460572	1
Zbtb8os	1,414311592	1
Lrrc17	1,413527548	1
Tnfrsf4	1,413429573	1
Slc29a2	1,413135689	1
Phf13	1,413037742	1

Pelp1	1,413037742	1
Abl1	1,412743939	1
Fbxo38	1,412743939	1
Proser1	1,412743939	1
Chrna1os	1,412743939	1
Nlrx1	1,412646019	1
Rin3	1,411862899	1
Ddx24	1,41156934	1
Hgsnat	1,411373669	1
Gpr85	1,411178025	1
Ctbp1	1,410982407	1
Prr14l	1,410884609	1
Celsr3	1,410591254	1
Alkbh7	1,410493483	1
Hmgb3	1,410395718	1
Ostm1	1,41029796	1
Rpl10a	1,410200209	1
Pwwp2b	1,410004728	1
Prss44	1,409418445	1
Sh2d6	1,409418445	1
Fam32a	1,409027726	1
Gas8	1,408930063	1
Gm44957	1,408734757	1
Rps11-ps1	1,408637114	1
Gm42559	1,408637114	1
Trim26	1,408539478	1
Gm8738	1,408539478	1
Gm8822	1,408441849	1
Gm2962	1,408344227	1
Efl1	1,408149002	1
Ung	1,407856217	1
Gm27043	1,407563492	1
Gm12469	1,40746593	1
Gm43411	1,407368375	1
Parp10	1,406685682	1
Snord13	1,406685682	1
Derl2	1,406588181	1
Unc50	1,4063932	1
Mcm9	1,406100779	1
Gm7434	1,405613546	1
Zfp958	1,405321287	1
Esco1	1,405029089	1
D230025D16Rik	1,40434753	1
Rab12	1,404250192	1
Chchd4	1,40415286	1
Aph1c	1,403666302	1
Gspt1	1,403666302	1
Gm9732	1,403277177	1
Poglut1	1,403082655	1
Psmc4	1,402596468	1
Tpst2	1,402499251	1
Urgcp	1,402207639	1

Nrde2	1,402013266	1
4931414P19Rik	1,401916089	1
Pus1	1,401721756	1
Zhx3	1,401721756	1
Tmem140	1,401721756	1
Sumo1	1,401527449	1
Sesn2	1,400653403	1
Txnrd1	1,400556321	1
Ambp	1,400459245	1
Dynlt1-ps1	1,400459245	1
Mmp19	1,399973967	1
Gm5575	1,399973967	1
Tiprl	1,399876932	1
Ccdc69	1,399682881	1
1700001C19Rik	1,399488857	1
Osgin2	1,398616083	1
Ncbp1	1,398616083	1
Eif5	1,398616083	1
Pisd	1,398519141	1
Tyw5	1,398422207	1
Gm19566	1,398228358	1
Tmem98	1,398228358	1
Ube2q2	1,398131443	1
Adar	1,39784074	1
Nlrc5	1,397646972	1
Mpp3	1,397646972	1
Hnrnpab	1,397162668	1
Cd63-ps	1,397065828	1
Ufl1	1,397065828	1
Tmem138	1,396968994	1
Gm43728	1,396968994	1
Lemd2	1,396775346	1
Bvht	1,396678532	1
Gm43182	1,396581725	1
Marc2	1,396388132	1
3110002H16Rik	1,396194564	1
RP24-282C4.13	1,396194564	1
Gm12704	1,396097791	1
Orc6	1,396001024	1
Abt1	1,395807511	1
Nsun3	1,395807511	1
Zxdb	1,395710764	1
Snora21	1,395710764	1
Fam192a	1,395614024	1
Gm6134	1,395517291	1
Atg4b	1,395517291	1
Gm6612	1,395323845	1
Ebna1bp2	1,395227131	1
Leng1	1,394646993	1
Foxc1	1,394550327	1
Mios	1,394357015	1
Nol9	1,394260369	1

Zfp280b	1,393970471	1
Ada	1,393970471	1
Mgme1	1,393873851	1
Slc27a4	1,393680633	1
Gm37494	1,393101138	1
Mpdu1	1,393004579	1
6720427I07Rik	1,392714942	1
Pak1ip1	1,391942871	1
Rnf144b	1,391460544	1
Ampd3	1,391460544	1
Ndufaf1	1,391364098	1
Arhgap30	1,39126766	1
Gm8172	1,391171228	1
Cdk17	1,390881972	1
Dpy30	1,390592776	1
Tlk2	1,390592776	1
Rtf1	1,390592776	1
Kbtbd8	1,390592776	1
Dync1li1	1,390496391	1
Tmem171	1,39030364	1
Gm7832	1,390207275	1
Znfx1	1,390110917	1
Zfp263	1,38991822	1
Gm13391	1,389147699	1
Trdmt1	1,388858864	1
Gm12341	1,388666341	1
Lgals2	1,388666341	1
Gm12350	1,388570089	1
Casp1	1,388473844	1
Tigar	1,388377606	1
Zadh2	1,388088931	1
Ice1	1,388088931	1
Ptpn11	1,387992719	1
Gm38125	1,387800316	1
Itpripl2	1,387704124	1
Aasdhppt	1,387415589	1
Sun1	1,387223266	1
Gm11450	1,387223266	1
Gm29666	1,387127114	1
Gm10268	1,386934831	1
RP23-139H6.1	1,386550344	1
5031425E22Rik	1,386550344	1
Sdcbp	1,386262049	1
Coa7	1,386165964	1
Nsmce3	1,386069886	1
Nop14	1,385973814	1
Gm26759	1,385973814	1
Smim1	1,385589594	1
Rpusd4	1,385493555	1
Rwdd2a	1,385109468	1
Cmbl	1,385013463	1
Slc26a11	1,384341613	1

Nfs1	1,384245661	1
Ppp6r1	1,384053777	1
Wdr77	1,383957845	1
Phf20	1,383766001	1
Mrm3	1,383670089	1
Tgfbr1	1,383286508	1
Slc5a6	1,383190629	1
Siah2	1,383190629	1
4930404I05Rik	1,383190629	1
Pithd1	1,383094757	1
Ddx3x	1,383094757	1
Lhfpl2	1,382711335	1
Kcnk6	1,382423838	1
Zkscan14	1,382423838	1
Tubb6	1,382328019	1
Serpinc1	1,382328019	1
Gm5422	1,382040602	1
Tmem25	1,381849024	1
Cav2	1,381753244	1
Traf5	1,381465947	1
Usp11	1,381465947	1
Nbeal2	1,381178709	1
Tns1	1,381178709	1
Eftud2	1,381082976	1
Ybey	1,381082976	1
Wbp1l	1,380891531	1
Ick	1,380891531	1
Gm37963	1,380891531	1
Kif2c	1,380700112	1
Gm16286	1,380700112	1
Ncoa6	1,380700112	1
Gm13416	1,380413033	1
4930430E12Rik	1,38022168	1
Asxl1	1,379456535	1
Gm4994	1,379265315	1
Il10rb	1,379169715	1
Gpat4	1,378596254	1
Wipf1	1,378309613	1
RP23-193N1.2	1,378118551	1
Tubb4b	1,378023031	1
Mtss1	1,37783201	1
5830444B04Rik	1,377736509	1
Gm12254	1,377641015	1
Chst12	1,377545527	1
Cdkn1a	1,377259104	1
Ppp1r8	1,377163643	1
Eif1ad	1,376591016	1
Gfap	1,376591016	1
Mier2	1,376304792	1
Dgat2	1,376114009	1
Etfbkmt	1,376018627	1
Lyrm1	1,375827884	1

Sufu	1,375637167	1
Rab29	1,375541818	1
Rab11fip5	1,375255812	1
Gm4832	1,375065174	1
Hbs1l	1,374969865	1
Eif4a3	1,374874563	1
Zfp709	1,374779267	1
Ccdc61	1,374302888	1
Gm11224	1,374017139	1
Rnf219	1,373826673	1
Ttyh2	1,37373145	1
Spata5l1	1,373636233	1
Mrpl36	1,373636233	1
Rpl23a	1,373541024	1
Gtf2f2	1,373065073	1
Metrnl	1,37249415	1
Faah	1,37249415	1
Gm16288	1,37249415	1
Map3k11	1,372208778	1
Ammecr1l	1,371638211	1
Gm6543	1,371638211	1
Slc16a12	1,371543139	1
Polr2a	1,371448074	1
2210016F16Rik	1,371257964	1
Gm42522	1,371067881	1
Ube2l3	1,371067881	1
Pcyt1a	1,370782805	1
Car9	1,37030781	1
Erlin2	1,370117858	1
Tmem86a	1,370022892	1
Kdm4a	1,369927933	1
Gm7867	1,36983298	1
Pald1	1,369738034	1
Gm5312	1,369643094	1
Api5	1,369548161	1
Ccnyl1	1,369358314	1
Kif1bp	1,369073594	1
Ralgds	1,369073594	1
Tshz1	1,368694059	1
Tmem41a	1,368694059	1
Prkce	1,368599191	1
Nedd1	1,368409476	1
Cd300lb	1,368409476	1
Lemd3	1,368314629	1
Cnot2	1,367650879	1
RP24-233B16.6	1,367556084	1
Zfp940	1,367556084	1
Sf3b6	1,367366514	1
Ints12	1,36717697	1
Ppih	1,366987452	1
Rpap1	1,366892703	1
Nbr1	1,366892703	1

Rbm48	1,366419056	1
Gm5905	1,366324346	1
Gm8185	1,366229643	1
Osbpl8	1,366229643	1
Map2k3	1,366134947	1
Sorl1	1,366134947	1
Btbd10	1,365945573	1
Gm15541	1,365566905	1
Gm43421	1,365566905	1
Mepce	1,365282973	1
Ptp4a1	1,365282973	1
Lars	1,365188342	1
Rpl31-ps16	1,365188342	1
Hgs	1,364809884	1
Rad51	1,364526109	1
Klf3	1,364526109	1
Tns3	1,364526109	1
Reps2	1,364336959	1
Rab11fip4	1,364336959	1
Gltscr1l	1,364242394	1
Baz1a	1,364242394	1
Pcsk4	1,364053283	1
Dgat1	1,363958737	1
Klf2	1,363864198	1
Oxnad1	1,363675139	1
Adsl	1,363297101	1
Rpl27a-ps1	1,363202607	1
Slc46a3	1,362730239	1
Fibp	1,362635785	1
Flywch1	1,362541338	1
Ankrd54	1,362446897	1
Taf13	1,362446897	1
Dicer1	1,36197479	1
Spata5	1,361408479	1
Gm15513	1,361408479	1
Klrg2	1,361219761	1
Gm9403	1,361125411	1
Sap130	1,361031069	1
Mybl2	1,361031069	1
Dcun1d3	1,360936732	1
Zranb3	1,360936732	1
Mphosph10	1,36074808	1
6330418K02Rik	1,360653763	1
Gosr1	1,360088001	1
Jade2	1,359899465	1
Rcc1l	1,359899465	1
Herpud2	1,359710956	1
Neurl3	1,359710956	1
Psma2	1,359710956	1
Anp32-ps	1,359710956	1
Slc39a9	1,359616712	1
Lrrc47	1,359522474	1

Adgrl2	1,359145586	1
Pigyl	1,359051381	1
Nmnat1	1,358862989	1
Cul5	1,358862989	1
Usp10	1,358580451	1
Gm28041	1,358580451	1
Gpr132	1,358486285	1
Cyth4	1,358109684	1
Zcchc2	1,358015551	1
RP24-547N4.5	1,35754498	1
Psma4	1,35754498	1
Trnau1ap	1,357262716	1
Slc30a1	1,357074572	1
Zfp653	1,356792406	1
Gmeb1	1,356792406	1
Zc3h13	1,356604327	1
Ube2n	1,356416275	1
Psme2b	1,356228249	1
Gm6733	1,355664327	1
Gm12059	1,355664327	1
Rftn2	1,355570363	1
RP24-401G4.1	1,355476405	1
Gm11517	1,354818883	1
Snhg15	1,354818883	1
Slc22a4	1,354724977	1
Fkrp	1,354631078	1
Prpf18	1,354537186	1
Catip	1,35434942	1
Cep152	1,354255547	1
Rbm27	1,354255547	1
Nkiras2	1,354255547	1
Cutc	1,353317174	1
Dhodh	1,352848231	1
Rpl3-ps2	1,352754462	1
Zfp456	1,352754462	1
Etv6	1,352285714	1
Cenpo	1,352285714	1
Abhd17c	1,352285714	1
Dirc2	1,352285714	1
Gm4895	1,352098261	1
Blnk	1,352098261	1
A130010J15Rik	1,351817129	1
Ryk	1,351817129	1
Trappc12	1,351536056	1
Nop2	1,351536056	1
Gm9497	1,351348706	1
Cdca4	1,351348706	1
Lyl1	1,350974085	1
Mcrs1	1,350974085	1
Pprc1	1,350412347	1
Atg14	1,350412347	1
Kctd18	1,350037985	1

Rbmx2	1,349757282	1
Retn	1,349570179	1
Gdpd1	1,349476637	1
Gm13612	1,349289573	1
3110043O21Rik	1,34919605	1
Parp2	1,348915522	1
Sec61g	1,348448104	1
Erich1	1,348448104	1
Nxt1	1,348261183	1
Zfp668	1,348167732	1
Rpl28	1,347980849	1
Katnb1	1,347980849	1
Ercc4	1,347700573	1
Tceanc2	1,347607161	1
Mrpl2	1,347513755	1
Gm27046	1,346673395	1
Rars2	1,346486719	1
Pafah1b1	1,346393391	1
Tmsb4x	1,345833558	1
Ngdn	1,345833558	1
Zfp120	1,345740275	1
Gm4879	1,345367209	1
Oxsr1	1,345273958	1
Gm12501	1,344807802	1
BC002059	1,344714591	1
Gm12164	1,344434994	1
Apobec1	1,344434994	1
Hipk3	1,344341808	1
Sdad1	1,344248629	1
Mfsd14b	1,344062289	1
Htatsf1	1,343969129	1
Nrbp1	1,343689688	1
Nme6	1,343689688	1
Cd200r1	1,343410304	1
Nosip	1,343410304	1
Prmt2	1,34331719	1
Mrpl55	1,34331719	1
Mthfsl	1,342851712	1
Trim12c	1,342665566	1
Gm12380	1,342572503	1
Smim3	1,341828229	1
Iars	1,341363267	1
Cnnm4	1,340991414	1
Commd2	1,340898467	1
0610007P14Rik	1,340526742	1
Bag3	1,340433827	1
Dock9	1,340248017	1
Egf	1,340248017	1
Rpsa-ps4	1,340155121	1
Lrp5	1,340062232	1
Wdr62	1,339969349	1
Tmem39b	1,339876472	1

Exosc1	1,339876472	1
Xpo5	1,339690739	1
Atp23	1,339690739	1
1110038F14Rik	1,339597882	1
Zfp358	1,339505031	1
Ing3	1,339412187	1
Hspa14	1,339412187	1
Zmynd19	1,339319349	1
1700066M21Rik	1,339133693	1
Tpm3-rs7	1,338855257	1
Plekhm1	1,338855257	1
Eif3j2	1,338762458	1
Dcaf13	1,338762458	1
Gm5697	1,338669665	1
Slf1	1,338298559	1
Fam198b	1,338113044	1
Parp4	1,338020296	1
Adprm	1,338020296	1
Hba-ps4	1,338020296	1
Mitd1	1,337742091	1
Zfp513	1,337556654	1
Srp68	1,337278546	1
Gm6222	1,337093172	1
Rhno1	1,337093172	1
Zfp740	1,337093172	1
Sez6	1,337000495	1
Gm2076	1,337000495	1
Gm16053	1,337000495	1
Psmd6	1,336907825	1
Rpl38-ps1	1,336815161	1
Ube2d1	1,336629852	1
Acot10	1,336351936	1
Thap1	1,33625931	1
Mef2d	1,33625931	1
4921507G05Rik	1,336074078	1
Mob3c	1,335981472	1
Zbtb7a	1,335981472	1
Ppp1r16a	1,335888872	1
Plpp6	1,335703691	1
Ppp6r2	1,335518536	1
Dynlrb1	1,335425968	1
Taco1	1,335333407	1
1110003F10Rik	1,335240852	1
Il1rap	1,335148303	1
1700012D14Rik	1,334778173	1
Ier5	1,334315654	1
1600012H06Rik	1,33422317	1
Tbc1d15	1,333945755	1
Cc2d1a	1,333853296	1
RP23-349H12.3	1,333760843	1
Wdr5	1,333760843	1
Apbb1	1,333391098	1

Tmem175	1,333298677	1
Nelfb	1,333298677	1
Creb3	1,333298677	1
Wiz	1,333113855	1
Uba5	1,333113855	1
Arhgap27	1,332559544	1
Bcor	1,332282475	1
Gm45840	1,332097794	1
Clcc1	1,332097794	1
Prpf19	1,331913139	1
Gm13743	1,331820821	1
Cdpf1	1,331820821	1
Ifi207	1,33172851	1
Fam3c	1,331636204	1
Ss18l1	1,331359327	1
Psmc2	1,330713504	1
Traf3ip2	1,33062127	1
Lsg1	1,33062127	1
Gm37780	1,330436819	1
Fbxo36	1,330344603	1
Cherp	1,330344603	1
Gm5532	1,330344603	1
Atf3	1,330252394	1
Slc23a2	1,329699273	1
Fbxo28	1,329422798	1
Ppp1r12a	1,329330653	1
Tox4	1,329238514	1
Phb	1,328870022	1
Gm45716	1,328777915	1
Gm44667	1,328685814	1
Fam111a	1,328685814	1
Epc2	1,328501632	1
Atxn7l3b	1,328041288	1
RP23-350F7.3	1,327949238	1
Crtc1	1,327857195	1
Rab8a	1,327765158	1
Fam134b	1,327673128	1
Unc5b	1,327581104	1
Kank3	1,32730507	1
Klhl11	1,32730507	1
Arhgap21	1,327213071	1
4931406P16Rik	1,327213071	1
Rad9b	1,32656926	1
Heatr3	1,326477312	1
Scyl3	1,326201507	1
Mark3	1,326109585	1
Sco1	1,32592576	1
Ighm	1,325833857	1
Tmem173	1,325558187	1
H2afx	1,325466309	1
Myo1c	1,325282573	1
Map7d1	1,325098863	1

Gm8357	1,324639698	1
Dusp12	1,324639698	1
Polr2c	1,324547884	1
Gm11334	1,324456077	1
9330159M07Rik	1,324364276	1
Washc5	1,324272481	1
Fpgs	1,324180692	1
5730480H06Rik	1,32408891	1
Utp3	1,32408891	1
Vamp3	1,323997135	1
Cep250	1,323630096	1
Trappc2	1,323630096	1
Rffl	1,323263158	1
Gm43712	1,323263158	1
Lyz2	1,32317144	1
Nepro	1,323079728	1
Naca	1,322988022	1
Lrch1	1,32280463	1
Clk4	1,322712943	1
Slc3a2	1,322712943	1
Arrdc3	1,322621263	1
Gm14336	1,322621263	1
Gm16020	1,32234626	1
Gm9794	1,32234626	1
Trim65	1,322162957	1
Gskip	1,321796426	1
Mtmr3	1,321796426	1
Hmgxb3	1,321796426	1
Hras	1,321613199	1
Gm36189	1,321521595	1
Cdv3	1,321521595	1
Trmt61b	1,321429997	1
Zfp422	1,320788991	1
Pdzd8	1,320697444	1
Gm11362	1,320422841	1
Kat5	1,320331319	1
Wbp11	1,320239804	1
Atp1b3	1,319965297	1
Clec4d	1,319965297	1
Gm15542	1,319873807	1
Amz1	1,319873807	1
Usp4	1,319690846	1
Nudt12	1,319599375	1
Gstt1	1,319233555	1
Dnajc2	1,319233555	1
Hook2	1,319142116	1
Tbc1d7	1,318319449	1
Frrs1	1,318228073	1
Dab2	1,317679952	1
Trim25	1,317314664	1
Zfp865	1,317223358	1
Piezo1	1,317040765	1

Ppfia4	1,316949477	1
Mtx1	1,316949477	1
Cep350	1,316949477	1
Gm15151	1,316766922	1
Gm5644	1,316219408	1
Ythdc2	1,316219408	1
Mbp	1,316219408	1
Gm5879	1,316036954	1
Ppp1r15a	1,315672122	1
Gm37009	1,315489743	1
Ube2z	1,315489743	1
Nfe2l2	1,315489743	1
Tsr2	1,31530739	1
Teddm2	1,315216223	1
Mob4	1,315125063	1
Mospd1	1,314760483	1
Zfp236	1,314669354	1
2810001G20Rik	1,314578231	1
Rasa2	1,314487115	1
Gm6377	1,314396005	1
Usp25	1,314122712	1
Ubap1	1,313849476	1
Dnmt1	1,313849476	1
Spty2d1	1,31375841	1
Gm11989	1,31348525	1
Gm44777	1,31303011	1
Rbm6-ps1	1,31303011	1
Tars	1,31303011	1
Ift74	1,312939101	1
Mrps36-ps2	1,312939101	1
Gm10269	1,312848098	1
Tceanc	1,312757101	1
Rfc4	1,312757101	1
Crnde	1,312393178	1
Gorab	1,312302213	1
Arsg	1,312211255	1
Gm8919	1,312120302	1
Dennd4b	1,311847483	1
Xxylt1	1,311756556	1
Fcor	1,311756556	1
Plekha3	1,311665635	1
Mea1	1,311483812	1
Ginm1	1,311120242	1
6030460B20Rik	1,311029365	1
Ttc32	1,310938494	1
Pmp22	1,310938494	1
5430405H02Rik	1,31084763	1
Xab2	1,310756772	1
Rassf8	1,310756772	1
Trmt10b	1,310665921	1
Fam161a	1,310665921	1
Twistnb	1,310484237	1

Ctr9	1,310484237	1
Elovl5	1,310484237	1
Tstd3	1,310120944	1
Fbxl14	1,310030136	1
Akip1	1,30984854	1
Srgap2	1,30984854	1
Fam173b	1,309757751	1
Cd300ld	1,309576193	1
Dnajc11	1,309485423	1
Dmtf1	1,30939466	1
Lrp10	1,309303902	1
Tm4sf19	1,309303902	1
Zc3h8	1,309213152	1
Xkr5	1,309213152	1
Xpc	1,308940937	1
Gsr	1,308396677	1
Dtd2	1,308396677	1
Slc25a30	1,308396677	1
Txn1	1,308305989	1
Fcf1	1,308124631	1
Gm36936	1,307943299	1
Gm10260	1,307852643	1
Map3k4	1,307852643	1
Ring1	1,307761993	1
Ints2	1,307490079	1
Pdik1l	1,307490079	1
Slfn3	1,307399454	1
Comt	1,307127616	1
Zfp516	1,307037016	1
B4gat1	1,306855835	1
Pygo2	1,30658411	1
Gm15500	1,30658411	1
Gm15446	1,306493548	1
E2f3	1,306402992	1
Fam160b1	1,306312442	1
Rpl34-ps1	1,30604083	1
Nme4	1,305859787	1
Wdr3	1,305769275	1
Acp2	1,305678769	1
RP23-440L7.5	1,305678769	1
Tecpr1	1,305226334	1
Zxdc	1,305045403	1
9230111E07Rik	1,304954948	1
Tmem51os1	1,304683618	1
March11	1,304502763	1
Tbccd1	1,304502763	1
Phtf1	1,304502763	1
Rapgef1	1,304502763	1
Mxra8	1,303779594	1
Fbxo45	1,303689226	1
Ing2	1,303689226	1
Inpp4a	1,303418159	1

Adamts6	1,303327816	1
Mkl1	1,30323748	1
Gm12005	1,303147149	1
Cep131	1,303056825	1
Unk	1,303056825	1
Ddx10	1,302695591	1
5830487J09Rik	1,30224419	1
Tmem43	1,302153928	1
Ddx18	1,301522272	1
Med23	1,301341855	1
RP23-38L16.3	1,301161464	1
Dhrs7	1,301071277	1
Gm13436	1,300890923	1
P2rx7	1,300710594	1
Dag1	1,300620438	1
Prr12	1,300530289	1
Mtmr2	1,300530289	1
Gm3695	1,30035001	1
Gm20620	1,30025988	1
Dph5	1,30025988	1
Utp11	1,300169756	1
Slc10a7	1,300079638	1
Gm22748	1,299899421	1
Cep78	1,299809322	1
Nol11	1,299809322	1
Ptpra	1,299629143	1
Hus1	1,299539062	1
Dtnbp1	1,299539062	1
Rps6-ps3	1,299448988	1
Fbxo11	1,299448988	1
Ndufa5	1,299268859	1
Gm38355	1,299088755	1
Ccs	1,298998712	1
Gm15728	1,298908675	1
Gm5436	1,298818645	1
Vapa	1,298818645	1
CAAA01194877.2	1,298728621	1
1300002E11Rik	1,298548591	1
Mrpl44	1,297918684	1
Zeb2	1,297828722	1
Rpl21-ps5	1,297199165	1
Gm43794	1,296569912	1
Pik3r3	1,296120633	1
Zfp53	1,295581704	1
Pex3	1,295402111	1
Rnf8	1,29486348	1
Brip1	1,29477373	1
Mfsd3	1,294594249	1
Nln	1,294504518	1
Lrrc75a	1,294504518	1
Vta1	1,294145654	1
F2	1,293428227	1

Gm45286	1,293159294	1
Zfp335	1,293159294	1
Ormdl1	1,292980036	1
Supt16	1,292980036	1
Krr1	1,292890417	1
Rrp8	1,292890417	1
Rae1	1,292890417	1
Zfp316	1,292621596	1
Txnl4a	1,292621596	1
Arl13b	1,292442413	1
Rbm8a	1,292442413	1
Crtap	1,292263255	1
Rnf123	1,292263255	1
Zbtb11	1,292263255	1
Tfeb	1,292084121	1
Tec	1,291994564	1
Ifi204	1,291905013	1
Tmem267	1,29154687	1
Psmd12	1,29145735	1
RP23-162P10.2	1,291367836	1
Riox2	1,291278329	1
2810004N23Rik	1,291278329	1
Prkd2	1,291009843	1
Gm2991	1,290830883	1
Exosc9	1,290830883	1
Tgds	1,290741413	1
Gm8649	1,290651949	1
Cipc	1,29020472	1
Pptc7	1,29020472	1
Phactr1	1,290115293	1
Amigo3	1,290115293	1
Rpl36a-ps1	1,290115293	1
Tmem106a	1,289668251	1
Eef1e1	1,289578861	1
Gm26664	1,289489477	1
Qtrtd1	1,289310728	1
Rnf31	1,289221363	1
Psmb4	1,288863965	1
Gm32340	1,288595981	1
Otud6b	1,288506665	1
Atp6v1c1	1,288417356	1
Stard3nl	1,288328053	1
Zc3h15	1,288328053	1
Grpel2	1,287970902	1
Dnaja2	1,287970902	1
Vps72	1,287346126	1
Glmn	1,287078458	1
Ccdc115	1,287078458	1
Rdh11	1,286989247	1
Gm19325	1,286900043	1
Rps6-ps1	1,286810845	1
Gm15148	1,286810845	1

Tra2b	1,286632468	1
Wdr55	1,286454115	1
Trp53bp2	1,286364948	1
Dus1l	1,286364948	1
Supt6	1,286364948	1
Tax1bp1	1,286364948	1
Trpm2	1,286275787	1
Cpsf2	1,286275787	1
A430018G15Rik	1,286186632	1
Ano7	1,286097483	1
Col27a1	1,286097483	1
Gm15157	1,286097483	1
Rab15	1,286097483	1
Gm37675	1,286097483	1
Gm6977	1,285919205	1
Fhl3	1,285830075	1
Pip5k1a	1,285117257	1
Map4k5	1,284939114	1
Hist1h1c	1,284939114	1
Zwint	1,284939114	1
Bcar1	1,284582903	1
Nfkbib	1,284582903	1
Usp47	1,284404834	1
Gm42571	1,284315809	1
Gstm4	1,284315809	1
Gm10343	1,284048771	1
Snx14	1,284048771	1
St3gal5	1,28395977	1
Tmem62	1,283870776	1
Gm38192	1,283870776	1
Gm38377	1,283870776	1
Ing4	1,283514861	1
Mfsd6	1,283425898	1
Dhx30	1,28333694	1
Gm7504	1,283159044	1
Dok1	1,282892247	1
Gm17455	1,282803326	1
Gm45184	1,282714412	1
RP24-418P10.4	1,282714412	1
Tmcc2	1,282625505	1
Gm32856	1,282358818	1
Rassf2	1,282358818	1
Tox2	1,282269935	1
Katnbl1	1,282003322	1
March6	1,281825611	1
Gm14094	1,281647924	1
Mrpl49	1,281647924	1
Gm20667	1,281292625	1
Hira	1,281203816	1
Ficd	1,281203816	1
Vps37b	1,280671089	1
Cwc22	1,280404809	1

Fam110a	1,280404809	1
Borcs5	1,280227319	1
Ticam2	1,279872414	1
Herc3	1,279872414	1
Dhrs3	1,279694998	1
Rfc3	1,279694998	1
Acot8	1,2796063	1
Gm12183	1,2796063	1
Gm11598	1,279517607	1
Gm27219	1,279428921	1
Zfp788	1,279428921	1
Ccdc57	1,279428921	1
Gm6969	1,279428921	1
RP23-13B8.12	1,279428921	1
Hspa4	1,279428921	1
Gm45033	1,279251567	1
Pi4k2b	1,279074237	1
Hars2	1,278985581	1
Gm45495	1,278808289	1
Clk3	1,278808289	1
Plk3	1,27863102	1
Dnajb11	1,27863102	1
Ist1	1,27863102	1
Ints14	1,278453777	1
Ptpn12	1,278365164	1
Alkbh3	1,278276558	1
Rexo4	1,278187957	1
Eif2b1	1,278099363	1
Bcl2l11	1,278010775	1
Ubc	1,277656484	1
Mast2	1,277567927	1
Hpse	1,277036712	1
Riok3	1,277036712	1
Tpst1	1,276682691	1
Daam1	1,27641724	1
Ppfibp1	1,276240304	1
Slc20a2	1,276151844	1
Pik3r1	1,276063391	1
Bpgm	1,275974944	1
Rabl6	1,275886504	1
Larp4b	1,275886504	1
Naa30	1,275798069	1
Gm19705	1,27570964	1
Stard5	1,275621218	1
Slc38a1	1,275621218	1
Puf60	1,275444392	1
Gpbp1	1,275444392	1
Pdcd2l	1,27526759	1
Gm15644	1,274648976	1
Ddx5	1,274207293	1
Mrps21	1,274118975	1
Rab4a	1,273854057	1

Rpl38-ps2	1,273854057	1
B3galnt1	1,273677475	1
Emp1	1,273677475	1
Btbd7	1,273589194	1
Nt5m	1,273589194	1
Kdm2b	1,273589194	1
Trmu	1,273500918	1
Gm13675	1,273500918	1
Akr1c13	1,273412649	1
Nfatc2ip	1,273059633	1
Tmem156	1,272971394	1
Gm28530	1,272883162	1
Sh3bp5	1,272706715	1
Gm6863	1,272530293	1
Hpgds	1,272530293	1
Cul4a	1,272265705	1
Med25	1,272265705	1
Rpl36al	1,272177521	1
Zfand2a	1,271736694	1
Deaf1	1,271560406	1
Arpc1a	1,271560406	1
Foxo1	1,271472272	1
Cd300c2	1,271384143	1
Prmt6	1,27129602	1
Gm11633	1,27129602	1
Rrp15	1,271207904	1
Slc16a6	1,270767413	1
Spdl1	1,27059126	1
Yrdc	1,27059126	1
Gm8909	1,269710858	1
Vps13d	1,269622851	1
Gm16585	1,269534851	1
Tpd52l2	1,269534851	1
Dact3	1,269446857	1
Sin3b	1,269446857	1
Polr1b	1,269270886	1
Mrpl43	1,269270886	1
Grin1	1,26918291	1
Telo2	1,26909494	1
Slc25a53	1,268831066	1
Ss18	1,268303484	1
Plekhf2	1,268303484	1
Manbal	1,268303484	1
Zscan20	1,268215575	1
Abhd16a	1,268039775	1
2310022A10Rik	1,268039775	1
Thoc6	1,267951885	1
Nt5dc2	1,267776121	1
Terf2	1,267688249	1
Gm8186	1,267688249	1
Cdk1	1,267688249	1
Etf1	1,26733682	1

Actr5	1,267248978	1
Entpd6	1,267073312	1
Gm6501	1,267073312	1
Gm43430	1,267073312	1
Dctn6	1,267073312	1
1810030O07Rik	1,266985488	1
Notch2	1,266985488	1
Wdr47	1,266809859	1
Zpr1	1,266809859	1
D130020L05Rik	1,26654646	1
Mks1	1,266283117	1
Nek3	1,266195348	1
Rbks	1,266195348	1
Cd200r3	1,266195348	1
Papd7	1,266107585	1
Sep 02	1,266019828	1
Atp6v0d1	1,265844333	1
Ppp2r5a	1,265756594	1
Ubac2	1,265668861	1
Rnf113a1	1,265668861	1
Pnpo	1,265581135	1
Tfam	1,2654057	1
Vps33a	1,2654057	1
Mtpap	1,265317992	1
Fth1	1,265317992	1
Dyrk1a	1,265142594	1
E130309D02Rik	1,265054904	1
Fam91a1	1,26496722	1
Trak1	1,264704205	1
Gm37584	1,264353603	1
Wfdc17	1,264265968	1
Ipo11	1,264178339	1
Nsmce4a	1,263915487	1
Anxa7	1,263915487	1
Cd151	1,263740284	1
Rspry1	1,263740284	1
Plppr2	1,263565104	1
Rpl30-ps5	1,263565104	1
Rbm12	1,263477524	1
Gde1	1,263389949	1
Gm11273	1,262952167	1
2610528A11Rik	1,262864629	1
Asl	1,262864629	1
Atp5k-ps2	1,262864629	1
Samd10	1,262689571	1
Gm14173	1,26242703	1
Tubgcp5	1,26242703	1
Samsn1	1,262339528	1
Pex11a	1,262339528	1
Aff1	1,262077059	1
Siah1a	1,261989581	1
Pofut2	1,26190211	1

Eif1-ps1	1,261814645	1
Abcd1	1,261727185	1
Nars2	1,261727185	1
Fgfrl1	1,261639732	1
Ptrhd1	1,261552285	1
RP24-131G14.13	1,261464844	1
Gm8451	1,261464844	1
Mob3a	1,261377409	1
Ubash3b	1,26111514	1
Rassf3	1,261027729	1
Gm37569	1,260940324	1
Socs7	1,260852926	1
1700109H08Rik	1,260765533	1
Ngly1	1,259979272	1
Etaa1	1,259979272	1
H2afy	1,259804614	1
Emc3	1,259717294	1
Pik3c3	1,25962998	1
Myo19	1,25962998	1
Sav1	1,259542672	1
Gm7967	1,25945537	1
Scap	1,259368074	1
Stat5a	1,259368074	1
Tpgs1	1,259193501	1
Ncf4	1,258844427	1
Bcl7b	1,258669926	1
Egln2	1,258669926	1
Uqcrb	1,258582685	1
Nt5c	1,25849545	1
Slc25a16	1,25840822	1
1110004E09Rik	1,25840822	1
Tor1aip1	1,258059364	1
Gm38335	1,257884972	1
Paqr3	1,257884972	1
Mlf2	1,257710604	1
Rpl38	1,257710604	1
Rhebl1	1,257623429	1
Mknk2	1,257361941	1
Agtrap	1,257187646	1
Rbpj	1,257187646	1
Cep63	1,257100507	1
Sssca1	1,256926248	1
Pfdn6	1,256752013	1
RP23-371B13.3	1,256490706	1
Abhd1	1,256316532	1
Tmem65	1,255968255	1
Mia3	1,255968255	1
Degs1	1,255707111	1
Adam17	1,255271991	1
Tm2d2	1,254837023	1
Aff4	1,254837023	1
Wdr12	1,254663077	1

Fam63a	1,254489156	1
Jun	1,254402205	1
Kif21a	1,25422832	1
Rps19-ps7	1,254141386	1
Arhgef2	1,254141386	1
Polr1a	1,254054459	1
Gsk3b	1,253793712	1
Ssu72	1,253706809	1
Gm7730	1,253185515	1
Tmppe	1,253185515	1
Ralgapb	1,253185515	1
Tmem220	1,25292495	1
Fuk	1,252838107	1
Zkscan1	1,252838107	1
Gm43359	1,25275127	1
Pik3ca	1,25275127	1
Sesn1	1,252577614	1
Moap1	1,252577614	1
Med7	1,252577614	1
Tnfrsf13b	1,252490794	1
Cks1brt	1,252403981	1
Prr18	1,252317174	1
Trappc5	1,252317174	1
Fam220a	1,252317174	1
St14	1,252230373	1
Gm20689	1,252056789	1
Usp2	1,252056789	1
RP24-324J2.1	1,251970007	1
Sigmar1	1,251796459	1
Mcl1	1,251796459	1
Plekhg2	1,251709694	1
Gm15393	1,251622935	1
Gm11810	1,251275959	1
Eef1g	1,251102508	1
Setdb2	1,251015791	1
Foxj2	1,250582297	1
Tma7	1,250495616	1
Zfyve21	1,250495616	1
Reps1	1,250495616	1
Frmd8	1,250408942	1
Rab23	1,250322273	1
2010111I01Rik	1,250322273	1
Thyn1	1,25023561	1
Rpl5-ps1	1,250062303	1
Adh5	1,250062303	1
Yipf5	1,250062303	1
Cd200r4	1,249975658	1
Lasp1	1,249975658	1
Klhdc3	1,249889019	1
Terf1	1,249369313	1
H60b	1,249369313	1
Ccnf	1,249282716	1

Rock1	1,249282716	1
Lrrc25	1,248763262	1
BC002163	1,248244024	1
Timm50	1,248157506	1
Nop58	1,248157506	1
Zfp654	1,248070993	1
Mfsd7b	1,248070993	1
Stambp	1,247984486	1
Zbtb43	1,247897985	1
Naa50	1,247897985	1
Gm3555	1,247725002	1
Gm5590	1,247725002	1
Phf10	1,247725002	1
Gm8724	1,247638519	1
Anapc4	1,247638519	1
Gm23442	1,247638519	1
Tmem101	1,247552043	1
Tsfm	1,247379107	1
Rfx5	1,247292648	1
Ddx31	1,247119749	1
Gm20707	1,247119749	1
Chst10	1,246860445	1
Copb2	1,246860445	1
Tbc1d10a	1,246774022	1
Mrpl10	1,246428391	1
Gm8731	1,24616923	1
Tbc1d23	1,246082855	1
Siva1	1,246082855	1
Rpl18a	1,245910123	1
Pias2	1,245737416	1
RP23-444K20.4	1,245478399	1
Traf3	1,245392072	1
Pibf1	1,245305751	1
Pgm5	1,245219436	1
Kti12	1,245133127	1
Tspan13	1,245133127	1
Arhgap26	1,245046824	1
Metap2	1,245046824	1
Ccdc97	1,244960526	1
5730409E04Rik	1,244874235	1
Gm14006	1,244874235	1
Bola1	1,244874235	1
Abhd17b	1,24478795	1
Slc25a14	1,244701671	1
Mapkap1	1,24444287	1
Asb10	1,244270365	1
Gm8659	1,244270365	1
Emc7	1,244270365	1
Chchd10	1,244097885	1
Gpnmb	1,244011653	1
Gm11688	1,243839209	1
Vash2	1,243839209	1

Haus6	1,243839209	1
Arc	1,243752995	1
Pik3r5	1,243494391	1
Kctd5	1,243408201	1
Gm10169	1,243408201	1
Nub1	1,243408201	1
Adcy9	1,243322018	1
Cdc25a	1,24289119	1
Mtrr	1,242805042	1
Pex14	1,242632765	1
Ppp6r3	1,242632765	1
Gm17745	1,242546635	1
Ermard	1,242546635	1
Irf7	1,242460511	1
Uba3	1,242374394	1
Morc3	1,242288282	1
Rpl36a-ps2	1,242202176	1
Tmco1	1,242202176	1
Rdh5	1,242116076	1
Wdr33	1,242029982	1
Ccdc59	1,242029982	1
Gm10059	1,241943894	1
Gm11131	1,241943894	1
Sdcbp2	1,241857812	1
Rttn	1,241857812	1
Cox8a	1,241857812	1
Zic5	1,241685666	1
Med18	1,241599602	1
Slc1a5	1,241513544	1
Sc5d	1,241513544	1
Gm37465	1,241427492	1
Hmox2	1,241083342	1
Trappc6b	1,24099732	1
Alg5	1,24099732	1
Gm17259	1,240825293	1
Trafd1	1,240739289	1
Otulin	1,24039533	1
Hs6st1	1,240309356	1
9230116N13Rik	1,240223387	1
Gm20633	1,240223387	1
Thumpd3	1,240137424	1
Usp16	1,240137424	1
Cnot9	1,240051468	1
Cmc4	1,239793633	1
Phlpp1	1,239793633	1
Gm44168	1,2397077	1
Tgm2	1,2397077	1
Arnt	1,239621773	1
Dcaf5	1,239364028	1
Rnf216	1,239364028	1
Pgap1	1,239364028	1
Rce1	1,239192227	1

Gm3608	1,239192227	1
Tacc2	1,239106336	1
Gm16373	1,239020451	1
Adprh	1,239020451	1
Mrps11	1,238934571	1
Fam53a	1,238676969	1
Slc11a2	1,238676969	1
Hmx3	1,23824775	1
Gsto2	1,237732885	1
Tmem126a	1,237647095	1
Mtmr14	1,23756131	1
Gm5812	1,237475532	1
Secisbp2	1,23738976	1
Ilkap	1,23738976	1
Gm8869	1,237303994	1
Ppp1r15b	1,237132479	1
Ogfod2	1,23704673	1
Timm23	1,236703796	1
Ankrd55	1,236532364	1
Timm10	1,236532364	1
Uspl1	1,236532364	1
Slc25a19	1,236360956	1
Gm14253	1,236189572	1
Pcnx4	1,236189572	1
Rab18	1,236189572	1
Zbtb42	1,236018211	1
Slc17a5	1,236018211	1
Meis2	1,23593254	1
Gm45413	1,235846875	1
Gm6808	1,235675562	1
Cdk10	1,235589914	1
Ndufs3	1,235504273	1
Cfap36	1,235247384	1
Tmem144	1,234819354	1
Gm7658	1,234648183	1
Cdk7	1,234477037	1
Tspyl1	1,234477037	1
Kbtbd2	1,234391472	1
Fbxw5	1,234220361	1
Fth-ps3	1,234220361	1
Atg2b	1,234134814	1
Mtf1	1,23387821	1
Rpl31-ps17	1,233621659	1
Phf14	1,233536154	1
Bud13	1,233450654	1
Hint3	1,233365161	1
Mta2	1,233194192	1
Nupl1	1,233108717	1
Gm43588	1,233108717	1
1700120C14Rik	1,233023247	1
Dhx37	1,232937783	1
Sgk3	1,232852325	1

Oas2	1,232852325	1
Osbp2	1,232852325	1
9330162012Rik	1,232852325	1
Psmd7	1,232681428	1
Laptm5	1,232339703	1
Ifnar2	1,232254287	1
1110012L19Rik	1,232168876	1
Cul2	1,232168876	1
U2af1	1,232168876	1
Rabepk	1,231998073	1
Plekhg3	1,231912681	1
Gm15727	1,231827294	1
Snrnp27	1,231656538	1
Gm20091	1,231400449	1
Cpne2	1,231315098	1
Bcl10	1,231315098	1
Dffa	1,23105908	1
Cldn12	1,230973752	1
Zbtb25	1,230973752	1
Vps4a	1,230717805	1
Sdhb	1,230717805	1
Klf4	1,230120802	1
Pim2	1,229950283	1
Ssb	1,229950283	1
Rora	1,229694548	1
Slc46a1	1,229609315	1
Grb2	1,229609315	1
Nek8	1,229353652	1
Slc25a51	1,229268443	1
Phax	1,229268443	1
Wars2	1,229098042	1
Zik1	1,228757311	1
Siah1b	1,228672143	1
Snai1	1,22858698	1
Arhgap17	1,228501824	1
Cisd1	1,228501824	1
Gm45223	1,228161258	1
Gm28555	1,228161258	1
Gm44283	1,228076132	1
Irx2	1,227905896	1
Rev3l	1,227820787	1
Rpf1	1,227820787	1
Grk4	1,227735684	1
Msl2	1,227735684	1
Sap18	1,227650587	1
Minpp1	1,227565495	1
Mbnl1	1,227565495	1
Pin1	1,22748041	1
Acbd3	1,22739533	1
Anapc15	1,227140127	1
Gm14706	1,227140127	1
Gm5070	1,226884977	1

Rpl36a	1,226799939	1
Fbxo4	1,22662988	1
Tmem14c	1,22662988	1
Ccnh	1,22654486	1
Spata13	1,22654486	1
Tbk1	1,226459845	1
Ppp3r1	1,226374836	1
Whamm	1,226289833	1
Sypl	1,226119845	1
Gm45221	1,22603486	1
Gm20673	1,225864907	1
Rapgef2	1,225864907	1
Fbxw11	1,22577994	1
Ubald1	1,225694978	1
Rnf215	1,225610022	1
Ddx23	1,225610022	1
Gm45311	1,225525073	1
Ckap4	1,225525073	1
Gm10177	1,225440129	1
Rhbdd3	1,22535519	1
Prob1	1,22535519	1
Rreb1	1,225270258	1
Ggta1	1,225185332	1
Ap1s3	1,224930588	1
Casc3	1,224845685	1
Npat	1,224760789	1
Ift20	1,224675898	1
Kctd20	1,224675898	1
Mink1	1,224591012	1
Gm7299	1,224591012	1
C130071C03Rik	1,224506133	1
Ftsj1	1,22442126	1
Cryga	1,224336392	1
Elp2	1,224166675	1
Cxxc1	1,224081825	1
Gm11686	1,223996981	1
Il6st	1,223996981	1
A430005L14Rik	1,223996981	1
Afg3l2	1,223996981	1
Pxmp4	1,223912143	1
Rps29	1,223912143	1
Aak1	1,223912143	1
Ddi2	1,223912143	1
Nectin2	1,223827311	1
Clint1	1,223742485	1
Zc4h2	1,22357285	1
Rnf103	1,22357285	1
Gm26799	1,223488041	1
Snapin	1,22323365	1
Ube2b	1,223064086	1
RP23-226H21.3	1,222894545	1
Gstz1	1,222809783	1

Sirpa	1,222809783	1
Tcaf1	1,222725028	1
Abhd3	1,222640278	1
Poldip2	1,222301337	1
Il27	1,222216616	1
Tceal8	1,22196249	1
Wdr75	1,221877793	1
Aif1	1,221793102	1
Ankra2	1,221623737	1
Nup35	1,221285078	1
Dyrk2	1,221115784	1
Dhrs4	1,220946513	1
D830044I16Rik	1,220946513	1
Impact	1,220608041	1
Ncoa2	1,220608041	1
Dkc1	1,220608041	1
Zfp760	1,220523438	1
Nsa2	1,220523438	1
Trappc10	1,220354249	1
Gm6181	1,220269664	1
Furin	1,220269664	1
Mat2b	1,22010051	1
Lmna	1,22010051	1
Ccdc12	1,21993138	1
Tram2	1,219762273	1
Lrrc57	1,219677729	1
Rbm15b	1,219677729	1
Letm1	1,219677729	1
Ppil1	1,219508657	1
Zfp566	1,219339609	1
Arih2	1,219339609	1
Zmiz1	1,219255094	1
Asb7	1,218917092	1
Zfp24	1,218832606	1
Mnat1	1,218832606	1
1110059E24Rik	1,218748126	1
Zswim1	1,218663651	1
Fuca2	1,218663651	1
Usp46	1,218663651	1
Gtpbp4	1,218663651	1
Morc2a	1,218579183	1
Cog6	1,218410264	1
Scarf1	1,218241368	1
Scel	1,21739724	1
Cdc73	1,21739724	1
Ythdf3	1,217059752	1
Rcbtb2	1,216975395	1
Xlr	1,216891044	1
Rsl24d1	1,216806698	1
Ranbp3	1,216722359	1
Nr4a1	1,216638025	1
Eif5b	1,216638025	1

Nudt21	1,216553697	1
Arih1	1,216553697	1
Gm42890	1,216469375	1
Llph	1,216385058	1
Rplp2	1,216385058	1
Tango2	1,216300748	1
Nr1h3	1,216216443	1
Usp31	1,216216443	1
Tfpt	1,216132144	1
Sh3pxd2a	1,216132144	1
G3bp1	1,216132144	1
Pttg1ip	1,215963564	1
Rtca	1,215963564	1
Cyld	1,215963564	1
Lmbr1	1,215795008	1
Ptpre	1,215710738	1
Gm20768	1,215626474	1
Nipa2	1,215205243	1
Trim33	1,215036792	1
Fem1c	1,215036792	1
Angptl2	1,214952575	1
Cerk	1,214952575	1
Tmem231	1,214868364	1
Dapk3	1,214784158	1
Jkamp	1,214615765	1
Ctnnal1	1,214531577	1
Fam214a	1,214363219	1
Tmem55a	1,214110726	1
Rap2c	1,214110726	1
Wdr4	1,213858285	1
Zfp128	1,213774149	1
Gm44953	1,213774149	1
Fam83h	1,213774149	1
Gper1	1,213774149	1
Slain2	1,213774149	1
Gm13758	1,21369002	1
St7l	1,21369002	1
Ttf1	1,21369002	1
Smap2	1,21369002	1
Gm14830	1,213605896	1
Trpv4	1,213521778	1
Rrp1b	1,21335356	1
Rps2	1,21326946	1
Gm10557	1,213017194	1
Iqsec3	1,212933117	1
Snrnp35	1,212849046	1
Gm18943	1,212680921	1
Zfp330	1,212680921	1
Rrp1	1,212680921	1
Gm5297	1,212596867	1
Def8	1,21226071	1
Slc39a11	1,21226071	1

Akap11	1,212176686	1
Rrp12	1,211588677	1
Litaf	1,211588677	1
Rps11	1,211420726	1
Golim4	1,211084896	1
n-R5-8s1	1,211000953	1
Itpripl1	1,211000953	1
Wwp1	1,211000953	1
Gm11945	1,210917015	1
Zbed5	1,210833084	1
Hps5	1,210581324	1
Rasgrp3	1,210497416	1
Impdh1	1,210413514	1
Cmtm3	1,210329617	1
Mtmr6	1,210329617	1
Slc30a4	1,210245727	1
Mesdc2	1,210161842	1
4930461G14Rik	1,210077963	1
RP23-278O17.1	1,210077963	1
Gm42893	1,210077963	1
Gm37900	1,210077963	1
Gm13223	1,210077963	1
RP24-226A8.2	1,210077963	1
Slc20a1	1,209994089	1
Gm13461	1,209742504	1
Timm17a	1,209658654	1
Klhl15	1,20957481	1
Ttll1	1,209407139	1
Slk	1,209407139	1
Wrb	1,209323312	1
Actr8	1,209239491	1
Zfp655	1,209239491	1
Gm7600	1,209071866	1
Ndc1	1,208904265	1
Hebp2	1,208820473	1
Gm15013	1,208736687	1
Ift140	1,208736687	1
Pdcl	1,208652907	1
Rabggta	1,208485363	1
Pcbp1	1,208485363	1
RP24-550H10.6	1,208234092	1
Nfyc	1,208234092	1
Usp9x	1,208066607	1
Gid8	1,207982873	1
Rnf183	1,207899145	1
Mterf4	1,207731706	1
Emg1	1,207731706	1
Spag7	1,207647995	1
Vps26a	1,207647995	1
Gm16096	1,207564291	1
Gm5100	1,207564291	1
Gm13350	1,207564291	1

Gm20696	1,207396898	1
9430038I01Rik	1,207229529	1
Ccz1	1,207145853	1
Thumpd2	1,207062183	1
Trmt2a	1,206978519	1
Hsd17b11	1,20689486	1
Rell1	1,20689486	1
Nol8	1,206393031	1
Tnrc6a	1,206393031	1
Etfrf1	1,206309413	1
Dbt	1,206309413	1
Slco4a1	1,206309413	1
Trit1	1,206225801	1
BC005537	1,206058594	1
Secisbp2l	1,205975	1
Sec23b	1,205975	1
Slc35b3	1,205557113	1
Zfp91	1,205557113	1
Rdh1	1,205473553	1
Zfp93	1,205473553	1
Slc38a6	1,205473553	1
Usp33	1,205389999	1
Taldo1	1,205306451	1
Ulk2	1,205222908	1
A930005H10Rik	1,205139371	1
Exoc3	1,205139371	1
Pdap1	1,205139371	1
Gm5050	1,20505584	1
Gm9803	1,20505584	1
Klhl26	1,204888796	1
Prtg	1,204805282	1
Polr2g	1,204721774	1
Fnta	1,204638272	1
Mob2	1,204638272	1
Phf3	1,204638272	1
Syk	1,204471285	1
Sh2b2	1,204387801	1
Pum2	1,204304322	1
Smpd4	1,204220849	1
Dgke	1,204137381	1
Sp3	1,203970464	1
Ssx2ip	1,203887014	1
Cdkn2aip	1,203720131	1
Tmem161b	1,203636699	1
Gm9833	1,203386436	1
Arhgef19	1,203303026	1
Trappc1	1,203219622	1
Tex264	1,203136224	1
Mrpl14	1,203136224	1
Mtif2	1,203052832	1
Gm16638	1,202969446	1
Rps27a-ps1	1,20280269	1

Tmem87a	1,202469249	1
Zfp398	1,202302563	1
Mgat5	1,202302563	1
Smad2	1,202219228	1
Ext1	1,2021359	1
Fam204a	1,2021359	1
D830050J10Rik	1,202052577	1
Mfsd1	1,202052577	1
Magoh	1,201885949	1
Chmp2a	1,201802643	1
Prpf6	1,20163605	1
Atg101	1,201552761	1
Fdx1	1,201386202	1
Gm11652	1,201219666	1
Atf2	1,200803427	1
Eif3g	1,200553753	1
Hp1bp3	1,20047054	1
Gm2735	1,200387333	1
Psmb2	1,200304131	1
Dnmbp	1,200220935	1
B230208H11Rik	1,200137745	1
Gm43513	1,199971382	1
Clip1	1,199971382	1
Fam72a	1,19988821	1
Smyd5	1,19988821	1
Tle3	1,199805043	1
Nkiras1	1,199223035	1
Itgb1bp1	1,198973689	1
Ndufaf7	1,198890586	1
Slc7a5	1,198890586	1
Cd200r2	1,198807488	1
Atad1	1,198641309	1
Tmem181a	1,198641309	1
Rnh1	1,198558229	1
Abcc5	1,198392085	1
Thoc1	1,198225964	1
Eef1akmt1	1,197976826	1
Snrpert	1,197893791	1
Rbm6	1,197810762	1
Bach1	1,197810762	1
Ttpal	1,197727739	1
Gm6451	1,197727739	1
Trmt11	1,19756171	1
St3gal6	1,197395705	1
Gm5939	1,19731271	1
Fyn	1,197229722	1
Larp4	1,197229722	1
Pdcd7	1,197063762	1
Gm10031	1,197063762	1
Fosb	1,196814866	1
Gm15696	1,196648963	1
Gm15207	1,196566021	1

Rpl37rt	1,196483084	1
Gm8825	1,196400153	1
Cdc42ep4	1,196234308	1
Snrnp40	1,196234308	1
Bfar	1,196234308	1
3830403N18Rik	1,195902688	1
Card19	1,195736912	1
D5Ertd579e	1,195654032	1
Dnajc25	1,195488291	1
Rnf169	1,195488291	1
Brd1	1,195405429	1
Rpl21-ps14	1,195239722	1
Clcnkb	1,195156877	1
Camsap1	1,194908377	1
Hmga1-rs1	1,194659929	1
Exosc8	1,194659929	1
Utp4	1,194659929	1
Ankrd13c	1,194659929	1
Gatc	1,194577124	1
Etv5	1,194494325	1
Fbxo42	1,194494325	1
2310022B05Rik	1,194494325	1
Ccar1	1,194494325	1
Gm37452	1,194411532	1
Mybl1	1,194245963	1
Pla2g5	1,194245963	1
Psmd8	1,194245963	1
Mapk8	1,194163187	1
Wdr11	1,194080417	1
Commd3	1,194080417	1
Exoc3l4	1,193997652	1
Josd1	1,193997652	1
Set	1,193832141	1
Etfb	1,193749393	1
1110004F10Rik	1,193583916	1
Naxd	1,193418462	1
Hdac9	1,193418462	1
Arhgap18	1,193335743	1
Acbd4	1,19325303	1
Yeats4	1,193170323	1
Gm12854	1,193004926	1
Pum3	1,193004926	1
Grk6	1,192839552	1
Gm14586	1,192674201	1
Fam50a	1,192591534	1
Ppp1r21	1,192508872	1
Appl1	1,192508872	1
Fbxw2	1,192508872	1
Brap	1,192426217	1
Gm12280	1,192260923	1
Zfp646	1,192178284	1
Unc119b	1,192013025	1

Gm28438	1,191930404	1
Gm11977	1,191847788	1
Kifc3	1,191765179	1
Vapb	1,191765179	1
Rbm7	1,191682575	1
Rpsa-ps1	1,191434797	1
Rps16-ps2	1,191434797	1
Gtpbp3	1,191352216	1
Asb6	1,191352216	1
Nab1	1,191352216	1
Gm2950	1,191187071	1
Zdhhc5	1,191021949	1
Srp19	1,190939396	1
Cct6a	1,190939396	1
Zscan22	1,190774308	1
Eif3s6-ps1	1,19052672	1
Zfp930	1,190361689	1
RP23-115A18.3	1,190114186	1
Romo1	1,190031696	1
Fam71e1	1,189949212	1
Cers2	1,189866734	1
Mir6236	1,189784262	1
Pafah2	1,189784262	1
Zfp51	1,189701795	1
Gm45051	1,189701795	1
Lzts3	1,189454429	1
Taf11	1,189042267	1
Dph7	1,188795039	1
Cyp51	1,188795039	1
Gm12604	1,188630248	1
Tulp3	1,188630248	1
Gm7670	1,188547861	1
Gm13827	1,188383105	1
Pigm	1,188383105	1
Timm9	1,187888973	1
Rnf25	1,187806638	1
Mmadhc	1,187641984	1
Ccm2	1,187395047	1
Sirt1	1,187312746	1
Ccdc127	1,18723045	1
Acsl4	1,18723045	1
Fam118b	1,187065877	1
RP24-378K7.3	1,186901326	1
Gm13368	1,186819059	1
Ube2q1	1,186654542	1
Med16	1,186572292	1
Serbp1	1,186325577	1
Mtmr10	1,186161129	1
Vav1	1,186161129	1
Stil	1,186078913	1
Tbc1d20	1,185996704	1
Cst3	1,185996704	1

Ulk3	1,185914499	1
Mrps31	1,185832301	1
B4galt5	1,185832301	1
Zfp384	1,185667921	1
Cycs	1,185667921	1
Gadd45gip1	1,185667921	1
Prr13	1,185667921	1
Atp6v1e1	1,185667921	1
Eefsec	1,18558574	1
Gm15198	1,185503564	1
Pigw	1,185092771	1
AU040320	1,184846363	1
Zfp36l1	1,18468212	1
Prmt9	1,184600007	1
Myd88	1,1845179	1
Ubr2	1,1845179	1
Gm12222	1,184435798	1
A130048G24Rik	1,184271612	1
Fam216a	1,184189527	1
Nfkbil1	1,183943308	1
Gtpbp1	1,183943308	1
Tnks2	1,183943308	1
Gm4613	1,183861246	1
H2-D1	1,18377919	1
Gm43792	1,183533055	1
Rcc1	1,183533055	1
Ipmk	1,183451022	1
Thtpa	1,183286972	1
Irf1	1,183204956	1
Mynn	1,182958941	1
Ppp2r5e	1,182958941	1
Ttc7b	1,182876947	1
Supt5	1,182876947	1
Tcea3	1,182712977	1
Clns1a	1,182549029	1
Stx11	1,182467064	1
Zcchc8	1,182385105	1
Wfikkn1	1,182303151	1
Cd53	1,182303151	1
Utp6	1,182303151	1
Mtmr1	1,182221203	1
Rnf40	1,182221203	1
Atp5s	1,18213926	1
Rad54l2	1,18213926	1
Aqp11	1,182057323	1
Madd	1,182057323	1
1700031P21Rik	1,181975392	1
Skiv2l	1,181811547	1
Pink1	1,181811547	1
Sp110	1,181729633	1
Gm10073	1,181729633	1
Commd8	1,181647724	1

Asb13	1,181565822	1
Tprn	1,181402033	1
Zkscan4	1,181320148	1
Pag1	1,181320148	1
Gm12716	1,181156393	1
Otud7b	1,180992661	1
Gm11687	1,180910804	1
Zfp592	1,180910804	1
March2	1,180828952	1
Abcf3	1,180665266	1
Adssl1	1,180583431	1
Gm44116	1,180501603	1
Hnrnpul2	1,180337962	1
Rbbp6	1,180337962	1
Wdr81	1,180092543	1
Tmem184b	1,180092543	1
Abhd17a	1,180010748	1
March5	1,179847175	1
BC065397	1,179765397	1
Tlr2	1,179765397	1
Hif1an	1,179601858	1
Naf1	1,179601858	1
Thnsl1	1,179520097	1
Tas1r1	1,179438342	1
Slc13a2	1,179438342	1
Gab2	1,179356592	1
Mphosph8	1,179274848	1
Gm4859	1,17919311	1
Lias	1,179111377	1
Dot1l	1,178947929	1
Atp6v1g2	1,178784504	1
Srp14	1,178702799	1
Bpnt1	1,178621101	1
Prpf4	1,178621101	1
Gm11560	1,178457721	1
Csrnp1	1,178212693	1
Actl6a	1,178131028	1
Mapk1ip1l	1,177804426	1
Rhobtb2	1,17772279	1
Reep3	1,177641159	1
Chmp2b	1,177641159	1
Traf1	1,177559534	1
Otud5	1,177559534	1
Tomm70a	1,177314693	1
Senp5	1,177151494	1
Ypel1	1,177069902	1
Bcl9l	1,177069902	1
Mtfmt	1,176988317	1
G6pdx	1,176743595	1
Pank3	1,176743595	1
Mcm4	1,176335837	1
Apitd1	1,176254302	1

Ap1m1	1,176009733	1
Tdrd3	1,175683719	1
Cyb5a	1,175683719	1
Accs	1,175520746	1
Gm19739	1,175520746	1
Ppig	1,175520746	1
Trps1	1,175439268	1
Psma7	1,175357795	1
Slc26a9	1,175276328	1
Rpl4	1,175276328	1
Gm13039	1,175113412	1
Ercc3	1,174950518	1
Gm14780	1,174869079	1
Mier3	1,174869079	1
Ddhd1	1,174787646	1
Tmem186	1,174706219	1
Cd33	1,174543382	1
Arfgef1	1,174543382	1
Eif1	1,174543382	1
Gm19898	1,174299167	1
Gm15163	1,174299167	1
Lzts2	1,174299167	1
Lrp8	1,174136386	1
Rps11-ps4	1,174055004	1
Ccdc77	1,173810892	1
Vps33b	1,173729532	1
Esd	1,173648178	1
Gm4366	1,17356683	1
Eed	1,17356683	1
Derl1	1,17356683	1
Rrp7a	1,173485487	1
Gm45871	1,173485487	1
Zcchc14	1,173322819	1
Zcchc17	1,173160173	1
Smu1	1,172916247	1
Gm37510	1,172834949	1
Tusc2	1,172753657	1
Zfp651	1,172509815	1
Akap1	1,172428546	1
1500004A13Rik	1,172347282	1
Ap4b1	1,172347282	1
Gm16200	1,172266024	1
Gch1	1,172266024	1
AA474408	1,172184772	1
Zfp706	1,172184772	1
Ubxn4	1,172022284	1
Slc25a46	1,171778594	1
Man1c1	1,171616162	1
Nadsyn1	1,171534955	1
Sec23ip	1,171372557	1
Prkab1	1,171291366	1
Sgta	1,171291366	1

Gm18737	1,171129002	1
Sirt7	1,171129002	1
Gm10146	1,171047828	1
Gm7407	1,171047828	1
Jarid2	1,17096666	1
Gm12034	1,170885498	1
9330162G02Rik	1,170885498	1
Tcaim	1,170642044	1
Mad2l2	1,170560904	1
Mttp	1,17047977	1
Nf1	1,170317518	1
Aimp2	1,170317518	1
Gtf3c4	1,170155289	1
Nudt13	1,170074183	1
Farp2	1,169993082	1
Tab1	1,169911988	1
Hacd3	1,169830898	1
Prrc1	1,169830898	1
Phka1	1,169587664	1
Flad1	1,16926343	1
Olfr912	1,169182386	1
Psma1	1,169182386	1
Usmg5	1,169101347	1
Rcor2	1,168777249	1
Sec24b	1,168696238	1
Gm28875	1,168534233	1
Il12rb1	1,168372251	1
Kdsr	1,168372251	1
Ctdp1	1,168210292	1
Akt2	1,168210292	1
Abcd3	1,16812932	1
Pcgf2	1,167967395	1
Rbm38	1,167967395	1
Rrp9	1,16788644	1
Psmb8	1,16788644	1
Tspan33	1,167562678	1
Aftph	1,167562678	1
Dgcr2	1,167481752	1
Rpl21-ps1	1,167481752	1
Ywhag	1,167400831	1
Washc1	1,167077203	1
Neu3	1,166996311	1
Plin3	1,166996311	1
Slc7a6	1,166915423	1
Wdr18	1,166834542	1
Slc16a3	1,166834542	1
Ep300	1,166672795	1
Dcun1d5	1,166672795	1
Cbx4	1,166511071	1
Flnc	1,166511071	1
Gm9013	1,16634937	1
Rab1a	1,166268527	1

Pdcd5-ps	1,166187691	1
Tnip1	1,166187691	1
Atp9b	1,166106859	1
Pdhb	1,166026034	1
Srsf7	1,165945214	1
Timm8b	1,165945214	1
Srr	1,165783591	1
Chordc1	1,165783591	1
Pnp	1,16562199	1
D1Ertd622e	1,165541198	1
Galk1	1,165460412	1
Fbxo6	1,165298856	1
Actr1b	1,165218086	1
Pafah1b1-ps2	1,165056564	1
Gm9256	1,165056564	1
Trappc3	1,164814322	1
Gpr137b	1,164814322	1
Zrsr2	1,164814322	1
Pex11b	1,164572131	1
Eif4enif1	1,164410699	1
Gm43655	1,164329991	1
Dtwd2	1,164329991	1
Sdhaf1	1,164249288	1
Taf3	1,1640879	1
Tmem202	1,163442572	1
Zfp110	1,163442572	1
Taf1c	1,163281295	1
Ntn5	1,163200666	1
Gm12848	1,163120042	1
Dfna5	1,16295881	1
Ercc8	1,162878203	1
Gm4963	1,162636414	1
Ap2m1	1,162636414	1
Gm12967	1,162555829	1
Usp39	1,162555829	1
Sema4b	1,162314108	1
Dennd2d	1,162314108	1
Mrps23	1,162314108	1
Gm9435	1,16199189	1
Rsl1d1	1,16191135	1
Gm12338	1,161669762	1
Osbpl2	1,161669762	1
Pfdn2	1,161669762	1
Gfm2	1,161669762	1
Laptm4a	1,161669762	1
Wdr19	1,161508732	1
Gpr107	1,161508732	1
Faap24	1,161428225	1
Gcfc2	1,161267228	1
2700049A03Rik	1,161186738	1
Dnajb12	1,161186738	1
Plrg1	1,161186738	1

Cenpa	1,161106253	1
Ermap	1,161025774	1
Gm6341	1,160864833	1
Arf3	1,160864833	1
Aste1	1,160784371	1
Gm12751	1,160784371	1
Rnf138	1,160784371	1
Ufsp2	1,160703914	1
Hivep2	1,160462578	1
Slc2a3	1,160462578	1
Top3b	1,160301715	1
Glul	1,160301715	1
Rab10	1,160301715	1
Ryr1	1,159980055	1
9330102E08Rik	1,159980055	1
Gm37116	1,159980055	1
Ankle2	1,159819259	1
Fam96b	1,159738869	1
Wasl	1,159497734	1
Zranb1	1,159417366	1
Gm13422	1,159256648	1
Gm6493	1,159015612	1
Spg21	1,159015612	1
Orc3	1,158774627	1
Ccl25	1,158694309	1
Gm38200	1,158694309	1
Tnfsf12	1,158694309	1
Lactb	1,158694309	1
Harbi1	1,158613998	1
Stau1	1,158613998	1
Clptm1l	1,158613998	1
Vps4b	1,158533691	1
Rbm47	1,158533691	1
Ptpn1	1,158453391	1
Pcid2	1,158212522	1
Mrpl37	1,158212522	1
Lin52	1,158132244	1
Txn-ps1	1,157971704	1
Aars	1,157891442	1
Dip2b	1,157891442	1
Rad23a	1,157811186	1
Ankrd27	1,157811186	1
Gm6851	1,15765069	1
Snrpb2	1,157490217	1
Lrrc41	1,157329766	1
Aatf	1,157329766	1
Renbp	1,157249549	1
Dlat	1,157169337	1
Ccdc82	1,157089131	1
Gtf3c1	1,157089131	1
Rbm28	1,157089131	1
Ttc7	1,156928735	1

Smug1	1,156848546	1
Acsl3	1,156608011	1
N4bp2l2	1,156527844	1
Igsf3	1,156367526	1
Rab11fip1	1,156207231	1
Map1lc3b	1,156127091	1
Peg13	1,156046958	1
RP23-47A1.1	1,155966829	1
Ap3m1	1,155966829	1
Psen2	1,155966829	1
Mkrn2	1,155806589	1
Mapkapk2	1,155806589	1
Actb	1,155646372	1
Wdfy3	1,155566271	1
Impad1	1,155566271	1
Ikbkap	1,155165852	1
Kpna6	1,155085785	1
Ankrd28	1,154925667	1
Rrm2	1,154765571	1
Med17	1,154765571	1
Dzip1	1,154685532	1
Gon4l	1,154685532	1
Gm43524	1,154605498	1
Dhx36	1,154605498	1
Txlng	1,154525469	1
Ipo9	1,154525469	1
Zfp143	1,154285418	1
Poldip3	1,154285418	1
Gm7027	1,154205411	1
Tnfrsf26	1,154205411	1
Gm29284	1,154125411	1
Cdc34	1,154125411	1
Rgs10	1,154125411	1
Gm37733	1,154045416	1
Zkscan7	1,153885442	1
Ets2	1,153885442	1
Nudt16l1	1,153805464	1
Cndp2	1,153725491	1
Taf1b	1,153565561	1
Arl10	1,153405654	1
4933434E20Rik	1,153405654	1
Pkig	1,153405654	1
Glrx5	1,153405654	1
Ehbp1l1	1,153405654	1
Rps14	1,153325709	1
Mpv17	1,153245769	1
Cmas	1,153245769	1
Ddb2	1,153005984	1
Wdr5b	1,153005984	1
Gm44250	1,152926066	1
Acvr2a	1,152606451	1
Hsd17b4	1,152606451	1

Vcpip1	1,152606451	1
Anks3	1,152526562	1
Tecpr2	1,152286925	1
Cib2	1,152047339	1
Gm13328	1,151887642	1
Ddx20	1,151887642	1
Skil	1,151807802	1
Ubr1	1,151727968	1
Slc36a3os	1,151727968	1
Cnot3	1,151727968	1
Prss35	1,151408685	1
Accsl	1,151408685	1
Lctl	1,151408685	1
Galnt15	1,151408685	1
Oscar	1,151408685	1
Plekhs1	1,151408685	1
RP24-365A12.2	1,151408685	1
4930556M19Rik	1,151408685	1
Gm13657	1,151408685	1
RP23-268C22.3	1,151408685	1
Tmem17	1,151408685	1
A330074K22Rik	1,151408685	1
4930568A12Rik	1,151408685	1
Apba1	1,151408685	1
Gm43112	1,151408685	1
Gm22299	1,151408685	1
Fbxo10	1,151408685	1
Zfp964	1,151408685	1
Gm7769	1,151408685	1
Cldn15	1,151408685	1
Tnfsf8	1,151408685	1
Wisp1	1,151408685	1
RP23-308G10.5	1,151408685	1
Gpatch1	1,151328879	1
Gm29462	1,151328879	1
Gm11470	1,151249077	1
Gm15946	1,151009707	1
0610005C13Rik	1,150850154	1
Rps26-ps1	1,150850154	1
Gm5547	1,150610866	1
Cdo1	1,150610866	1
Prkrip1	1,150531115	1
Pafah1b1-ps1	1,150451369	1
Rnpc3	1,150291893	1
Armcx5	1,150291893	1
Strap	1,150291893	1
E2f7	1,150212164	1
Atp6v0a1	1,150212164	1
Usp18	1,15013244	1
Sgpl1	1,15013244	1
Ptp4a2	1,15013244	1
Donson	1,150052722	1

Gm12669	1,149973009	1
Cntnap1	1,149973009	1
Kdelr2	1,149973009	1
Hspa9	1,149973009	1
Marcksl1	1,149893302	1
Rbm15	1,1498136	1
Gnal	1,149733904	1
Rtcb	1,149733904	1
Psmg3	1,149654213	1
1110008F13Rik	1,149654213	1
Ccng1	1,149574528	1
Suclg1	1,149335505	1
Coa3	1,149255842	1
Tnfrsf18	1,149176185	1
Taok2	1,149176185	1
Osbpl11	1,148937245	1
Rcsd1	1,148857609	1
Gpatch2	1,148698355	1
Sap18b	1,148379912	1
Eif3b	1,148379912	1
Trpm1	1,148300315	1
Trub1	1,148300315	1
Csnk1e	1,148300315	1
Lman2	1,148220724	1
Tsen54	1,147981983	1
Cysltr1	1,147981983	1
Uck2	1,147981983	1
RP24-82M14.1	1,147902414	1
BC030499	1,147902414	1
Foxp1	1,147902414	1
Gabarapl1	1,147902414	1
9130401M01Rik	1,147584192	1
Pot1a	1,147425114	1
Gm15782	1,147186538	1
Gm11604	1,147186538	1
Map3k7	1,147027516	1
Zfp667	1,146948012	1
Patl1	1,146709536	1
Gm5805	1,146550579	1
Thap11	1,146550579	1
Znrd1	1,146391645	1
Zfp142	1,146312186	1
Ambra1	1,146312186	1
Chmp1b	1,146232732	1
Suds3	1,146232732	1
Sgsm1	1,146153284	1
Snx13	1,146073842	1
Phrf1	1,145835547	1
Rps13-ps1	1,145676711	1
Ctu2	1,145676711	1
Stard3	1,145676711	1
BC031181	1,145597302	1

Abcb7	1,145438499	1
Cacul1	1,145438499	1
Dcaf17	1,145279719	1
Mccc2	1,145279719	1
Vav2	1,145200337	1
RP23-187B11.16	1,145200337	1
Prep	1,145200337	1
Synj2	1,145200337	1
Fam160a2	1,144882864	1
Ppm1h	1,14480351	1
Rps9	1,14480351	1
Pstpip2	1,144724161	1
Zfp638	1,144724161	1
Fmc1	1,144644817	1
Bcl2l1	1,144644817	1
Ppp1cb	1,144486147	1
Ifit2	1,144327498	1
Akap7	1,144168872	1
Smpdl3a	1,144010267	1
Tnfaip1	1,143930973	1
Pum1	1,143930973	1
Dgkd	1,143851685	1
Gm2199	1,143772402	1
Cwf19l1	1,143693124	1
Atad3a	1,143613852	1
Rnf168	1,143613852	1
Tpgs2	1,143534586	1
Rpl36a-ps3	1,143455325	1
Mavs	1,143376069	1
Ninj1	1,143376069	1
Stk4	1,143296819	1
Nab2	1,143217574	1
Man2a1	1,143059102	1
Fes	1,143059102	1
Comtd1	1,143059102	1
Uqcrfs1	1,143059102	1
Txlna	1,142821434	1
Ergic2	1,142742223	1
Ncaph	1,142583816	1
Zfp777	1,142583816	1
Brwd3	1,142504621	1
Mettl14	1,142425431	1
Clcn7	1,142346247	1
mt-Nd1	1,142346247	1
Cse1l	1,142346247	1
Tm9sf4	1,142187895	1
Med19	1,142108727	1
Zbtb4	1,142029565	1
Gm28578	1,141950408	1
Tbc1d2	1,141871257	1
Fzd5	1,141792111	1
Rps15-ps2	1,141712971	1

Atad3aos	1,141554707	1
Actg1	1,141554707	1
Sfr1	1,141554707	1
Appbp2	1,141475583	1
Aifm1	1,141000956	1
Wdr36	1,14092187	1
Layn	1,14084279	1
Rbl2	1,140763716	1
Psmg2	1,140684647	1
Angel2	1,140684647	1
Paox	1,140684647	1
Psmc3ip	1,140526525	1
Rere	1,140368426	1
Gm26698	1,140289384	1
Becn1	1,140289384	1
Tmem268	1,140210348	1
Srek1ip1	1,140210348	1
Pdcd6ip	1,140210348	1
Ifngr2	1,140052292	1
Zfp26	1,139973273	1
Nuf2	1,139973273	1
Nap1l4	1,139973273	1
Htra2	1,139894259	1
Lin54	1,13981525	1
Trim27	1,13981525	1
Lins1	1,139578257	1
Cactin	1,139578257	1
Nsfl1c	1,13949927	1
Prpf38a	1,13949927	1
4930542C12Rik	1,139341313	1
Mak16	1,139262342	1
Gtf2a1	1,138946515	1
Fam26f	1,138867572	1
AY074887	1,138788635	1
Inpp5e	1,138788635	1
Ehmt1	1,138788635	1
Alg13	1,138709703	1
Nprl3	1,138551855	1
Setdb1	1,138551855	1
Cul3	1,138394029	1
Plekho1	1,138394029	1
Gm45050	1,138315124	1
Mydgf	1,138315124	1
Fam104a	1,138236225	1
Taf5	1,138078443	1
Aida	1,13799956	1
Hivep1	1,137920683	1
Trim23	1,137920683	1
Gm10237	1,137920683	1
Sirt3	1,137920683	1
Ttc33	1,137920683	1
Dcaf12	1,137841811	1

Plaa	1,137605228	1
Cmss1	1,137526378	1
Gm26982	1,137526378	1
Polr2m	1,137526378	1
Gm8805	1,137368694	1
Phf23	1,137368694	1
Uqcrc1	1,13697458	1
Gm15265	1,136895774	1
Acer3	1,136895774	1
Myo1e	1,136895774	1
Slc27a1	1,136816973	1
Dnajc27	1,136738178	1
Ccdc159	1,136659388	1
Dhx8	1,136659388	1
Cdc42bpb	1,136659388	1
Rps6ka4	1,136580603	1
Smndc1	1,136580603	1
Hn1	1,136423051	1
Psme4	1,136423051	1
Faim	1,136344283	1
Rab5b	1,136344283	1
Tfg	1,136344283	1
Dpf2	1,136344283	1
Fkbp11	1,13626552	1
Hccs	1,136186763	1
Gm16712	1,136108011	1
Trappc8	1,136029265	1
Rb1cc1	1,136029265	1
Gm2225	1,135950524	1
Rsbn1l	1,135950524	1
Mrpl54	1,135871789	1
Sqrdl	1,135793059	1
Capn5	1,135714334	1
Nudcd3	1,135556902	1
Tanc2	1,135399491	1
Cox6b1	1,135399491	1
Cyb5r3	1,135320794	1
Cstb	1,135242102	1
Mtm1	1,135163416	1
Gm10704	1,135084735	1
Alg1	1,135084735	1
Gm38340	1,134848725	1
Plekhh1	1,134770066	1
Gclm	1,134612765	1
Eif4a1	1,134612765	1
Zfp106	1,134534122	1
Rxrb	1,134455485	1
Gm42535	1,134376853	1
Mkks	1,134376853	1
Nr1d1	1,134376853	1
Rgl1	1,134376853	1
Zfp784	1,134298227	1

Gm29593	1,134140991	1
Cops3	1,134140991	1
Spata2	1,134062381	1
Mpp1	1,134062381	1
Trp53i13	1,133983776	1
Tmem161a	1,133983776	1
Gm45853	1,133905177	1
Med27	1,133905177	1
Uchl5	1,133826584	1
Tpd52	1,133826584	1
Hspa13	1,133747995	1
Traf7	1,133747995	1
1810041H14Rik	1,133433697	1
Ldah	1,133433697	1
Dcaf10	1,133433697	1
Cnbp	1,133355136	1
Gm8599	1,133276581	1
Gas6	1,133276581	1
Zmat2	1,133276581	1
Dnajc19	1,133198031	1
Rnaset2a	1,133119486	1
Polm	1,133119486	1
Ppm1g	1,133119486	1
Gm43715	1,132962414	1
Tbc1d13	1,132962414	1
Cep170b	1,132962414	1
Mettl21a	1,132805362	1
Inpp1	1,132726845	1
Slc22a5	1,132569827	1
A430027C01Rik	1,132569827	1
Zfp335os	1,132569827	1
Rpl36-ps3	1,132491326	1
Slc6a6	1,132491326	1
Ankrd33b	1,132177376	1
Trim28	1,132177376	1
Fanca	1,132020434	1
Pja1	1,132020434	1
Gm13822	1,131941971	1
Cdc40	1,131941971	1
Zbtb17	1,131863513	1
Prmt3	1,131314463	1
Uvrag	1,131314463	1
Mecp2	1,131236049	1
Pnpla8	1,13115764	1
Wbp2	1,131079237	1
Rpp40	1,13084406	1
Mmachc	1,130765679	1
Rabep2	1,130452207	1
Hikeshi	1,130295504	1
Noc4l	1,130295504	1
Csnk1g3	1,130295504	1
Gm5599	1,130217161	1

Adck5	1,13006049	1
Zfr	1,13006049	1
Tatdn3	1,129982163	1
Son	1,129825525	1
Rps8-ps4	1,129747215	1
Gm13680	1,129747215	1
Atad5	1,129355742	1
Ecel1	1,129355742	1
Gm7676	1,129199191	1
Tagap1	1,129199191	1
Lpar2	1,129120923	1
RP23-184H3.5	1,129120923	1
Gtf3c5	1,129042661	1
Hs3st3b1	1,129042661	1
Snrpd1	1,129042661	1
Psmd5	1,128964405	1
M1ap	1,128886154	1
Tinf2	1,128729668	1
RP23-324E2.11	1,128729668	1
Pms1	1,128651433	1
Cdc34b	1,128338548	1
Atp6v1a	1,128338548	1
Ccdc43	1,12826034	1
Slc7a1	1,128103941	1
Ptch1	1,128025749	1
Hcfc1r1	1,127947563	1
Nup50	1,127869382	1
Denr	1,127869382	1
Itfg2	1,127791207	1
Ephx1	1,127791207	1
Taf8	1,127634873	1
Anxa3	1,127634873	1
Smyd4	1,127556714	1
Gtse1	1,127556714	1
Dda1	1,12747856	1
Apbb2	1,12747856	1
Csk	1,127400412	1
Pgd	1,127322269	1
Spopl	1,127244132	1
Rhot1	1,127166	1
Taf4	1,127087874	1
Eloa	1,126697322	1
Rrn3	1,126697322	1
Dapp1	1,126619228	1
Plekha8	1,12654114	1
Ndufb6	1,12654114	1
Smad4	1,12654114	1
Phlpp2	1,126463057	1
Cbx3	1,126463057	1
BC004004	1,126384979	1
Lats1	1,126384979	1
Eif4ebp1	1,126384979	1

Ift81	1,126306907	1
Arid3b	1,126306907	1
Gm45113	1,12622884	1
Ncl	1,12622884	1
4930558J18Rik	1,126150778	1
Eif4e2	1,126150778	1
Mrpl48-ps	1,125994671	1
Srsf4	1,125916626	1
Unc45a	1,125916626	1
Slc36a1	1,125916626	1
Rasgef1a	1,125838586	1
Atg4c	1,125526481	1
Zfp597	1,125448468	1
Zbed3	1,125448468	1
Tmem8b	1,125448468	1
Mbtps2	1,125448468	1
Gins3	1,125448468	1
Abcg1	1,125058485	1
Gm1848	1,124980504	1
Gm4525	1,12482456	1
Rad18	1,124668637	1
Gm12174	1,124512735	1
Fut11	1,124512735	1
Trp53inp1	1,124434793	1
Ostc	1,124045161	1
Gm13015	1,123889346	1
Rab2a	1,123889346	1
Pigl	1,123811447	1
Dlst	1,123811447	1
Fabp5l2	1,123655664	1
Tmem189	1,123422031	1
Imp4	1,123344164	1
Mmgt2	1,123188446	1
Polr2b	1,123188446	1
Rnf20	1,123188446	1
Usp30	1,123110595	1
Gm4149	1,12303275	1
Cdt1	1,12295491	1
Vsir	1,12295491	1
Rxra	1,122877075	1
Tyw1	1,122721422	1
Nod2	1,122487983	1
Tvp23b	1,122487983	1
Eif2b3	1,122487983	1
Zfp975	1,122410181	1
Leo1	1,122176806	1
Rnf128	1,122176806	1
Zfp862-ps	1,122099026	1
Gas2l1	1,122099026	1
Hbb-bh3	1,12202125	1
Ufd1l	1,12202125	1
Fam114a2	1,12202125	1

Zfp160	1,121943481	1
Akirin1	1,121943481	1
Pa2g4	1,121865716	1
Kmt5a	1,121632455	1
Pip4k2a	1,121554712	1
Pcnx	1,121399242	1
Rnf220	1,121399242	1
Med13l	1,121321515	1
Prkar1a	1,121321515	1
Gm7863	1,121243794	1
Ppm1b	1,121243794	1
Smarce1	1,121166078	1
Gsto1	1,121166078	1
Ttc17	1,121166078	1
Eif2ak3	1,120932962	1
Havcr2	1,120932962	1
Rpl35a-ps5	1,120855268	1
Atp2b1	1,120777579	1
Gm6378	1,120699895	1
Clec4e	1,120622217	1
Ccnd1	1,120622217	1
Rapgef6	1,120544544	1
Ints3	1,120466876	1
BC037032	1,120389214	1
Gm16124	1,120311557	1
Nol10	1,120311557	1
Sike1	1,120233906	1
Srm	1,12015626	1
Rfk	1,120000984	1
4930526A20Rik	1,119768111	1
Iqcf1	1,119690497	1
Zcchc6	1,119690497	1
Gm11759	1,119535286	1
Ndel1	1,119302509	1
BC037039	1,119069781	1
Inpp5f	1,119069781	1
Mrpl48	1,118914656	1
Daglb	1,118759552	1
Acad12	1,118682008	1
Uhrf1bp1l	1,118682008	1
Tbce	1,118526937	1
Atp8a1	1,118449409	1
4931406C07Rik	1,118371887	1
Smim12	1,118216858	1
Aplf	1,118139352	1
Dmxl2	1,118139352	1
Pdcd4	1,118139352	1
Nectin1	1,118139352	1
Alg14	1,118061851	1
Atp5g3	1,118061851	1
Nek7	1,117984356	1
Pim1	1,117984356	1

Orc5	1,117751901	1
Wdr76	1,117596959	1
Gss	1,117519496	1
Alyref2	1,117442038	1
Med11	1,117442038	1
Gm27248	1,117442038	1
Shmt2	1,117442038	1
Sart3	1,117364585	1
Gm29228	1,117287138	1
H2-DMa	1,117287138	1
Gm7887	1,117209696	1
Necap1	1,117054829	1
Rasgef1b	1,116977403	1
Mgat4b	1,116822568	1
Tcf4	1,116822568	1
Cct4	1,116822568	1
Selenok	1,116590355	1
Akr1a1	1,116590355	1
Sar1a	1,116590355	1
Dhx58	1,116435574	1
Ube2w	1,116358191	1
Nostrin	1,116358191	1
Bzw1	1,116358191	1
Arid4b	1,116048714	1
Gm14567	1,115894007	1
Pan3	1,115894007	1
Alpk1	1,115816662	1
Orc2	1,115816662	1
Dok2	1,115816662	1
Rpl18-ps2	1,115739322	1
Calu	1,115584659	1
Snhg5	1,115584659	1
Ube2r2	1,115584659	1
Alg8	1,115507335	1
Kif5b	1,115507335	1
Tipin	1,115275396	1
Fads3	1,115198094	1
Fbxl6	1,115120797	1
Col4a6	1,114966219	1
Zfp451	1,114966219	1
Ddx46	1,114888938	1
Gm3511	1,114579868	1
Zyg11b	1,114579868	1
Slc16a1	1,114579868	1
Rab22a	1,114579868	1
Aim2	1,114579868	1
Surf4	1,114579868	1
Nup37	1,114425365	1
Tln1	1,114425365	1
Grasp	1,114348122	1
Isy1	1,114348122	1
Llgl1	1,114193651	1

Stap1	1,114193651	1
Arv1	1,114116423	1
Guk1	1,114116423	1
Gm6285	1,113961985	1
Gm12504	1,113884774	1
Ptgr2	1,113884774	1
Zfp7	1,113807568	1
Dazap2	1,113807568	1
Gm43681	1,113653172	1
Rps6kb1	1,113421618	1
Ddx3y	1,113344444	1
Gstp-ps	1,113267276	1
Egfl7	1,113267276	1
Gm26656	1,113112955	1
Hic2	1,113035803	1
Tcea1	1,113035803	1
Pik3cg	1,112958655	1
Cyp26b1	1,112958655	1
Serinc3	1,112958655	1
Gm37206	1,112804377	1
Hbegf	1,112727246	1
Itsn2	1,112650121	1
Lyn	1,112573	1
Uba2	1,112495885	1
Polg	1,112418776	1
Gm12165	1,112341671	1
Ppargc1b	1,112341671	1
Dgcr8	1,112264572	1
Orai3	1,112264572	1
Gm23127	1,112187479	1
Golga5	1,112187479	1
Gm13602	1,11211039	1
Retsat	1,11211039	1
Rpsa-ps10	1,11211039	1
Mrps30	1,112033307	1
Gm2058	1,111802091	1
Gm17150	1,111725029	1
Uqcrh-ps2	1,111725029	1
Tcf3	1,111725029	1
Zdhhc16	1,111647973	1
Mgrn1	1,111570922	1
Gm6563	1,111493876	1
Suz12	1,111493876	1
Npc1l1	1,111339801	1
Slc25a20	1,111262772	1
Nudcd1	1,111262772	1
Ibtk	1,111262772	1
Ddx52	1,111262772	1
1700096K18Rik	1,111185748	1
Asap1	1,111108729	1
Tmx1	1,111108729	1
Cox5a	1,111108729	1

Cdc16	1,110954707	1
Tada1	1,110954707	1
Gm4604	1,110723714	1
Pmepa1	1,110723714	1
Sec14l1	1,110723714	1
Idh1	1,110723714	1
L2hgdh	1,110569746	1
Lrrfip1	1,110569746	1
Atic	1,11049277	1
1600002K03Rik	1,110338834	1
Rps19bp1	1,110184919	1
Ggnbp2	1,110184919	1
Cand1	1,110031025	1
Mrpl23-ps1	1,109954086	1
Mon1b	1,109800225	1
Mapkbp1	1,109723302	1
Tjp2	1,109646384	1
Rbm17	1,109492565	1
Dph3	1,109492565	1
Acp1	1,109108111	1
AA465934	1,109108111	1
Hells	1,109108111	1
Pik3cd	1,109108111	1
Brf1	1,109031236	1
Tmod1	1,109031236	1
Nsun6	1,108954367	1
Lpcat3	1,108877502	1
Zfp146	1,108800644	1
Akt1s1	1,108800644	1
Parp9	1,108646942	1
Ksr1	1,108570099	1
Snx18	1,108570099	1
Lipa	1,108570099	1
Ints7	1,108493261	1
Rubcn	1,108493261	1
Gpx1	1,108493261	1
Zfp148	1,108262781	1
Gm7206	1,108185964	1
Abcb10	1,108032348	1
Cpsf3	1,108032348	1
Cytip	1,107955548	1
Atp5j	1,107801963	1
Edrf1	1,107801963	1
Emc8	1,107801963	1
Rabep1	1,107725179	1
Arl8b	1,107725179	1
Foxred1	1,1076484	1
Fbf1	1,107571626	1
Ubap2	1,107418095	1
Cdkn2aipnl	1,107341337	1
Hist1h2ae	1,107264584	1
Tnf	1,107264584	1

Gdpd3	1,107264584	1
Nudt22	1,107187837	1
Pcgf3	1,107111096	1
Zfand3	1,106727467	1
3110080O07Rik	1,106650757	1
Irf2bpl	1,106650757	1
Purg	1,106574052	1
Bnip3	1,106574052	1
Map3k1	1,106497353	1
Slc29a3	1,106420659	1
Pdss2	1,106190609	1
Pop4	1,106190609	1
Senp6	1,106190609	1
Aaed1	1,106113937	1
Gm9703	1,106037269	1
Sltm	1,105960607	1
Pdgfa	1,105883951	1
Gm44567	1,105807299	1
Pold3	1,105807299	1
Nfkbia	1,105807299	1
Olfml3	1,105730653	1
Chchd1	1,105654013	1
Zfp131	1,105424122	1
Usp1	1,105424122	1
Naip2	1,105347503	1
Selplg	1,105041078	1
Bud31	1,105041078	1
Fbxw9	1,104811315	1
Zmynd11	1,104811315	1
C530005A16Rik	1,104734738	1
Elovl6	1,104734738	1
Sos1	1,104734738	1
Bdp1	1,104734738	1
Agfg1	1,104734738	1
Ddx21	1,104658166	1
Foxm1	1,1045816	1
Aamp	1,1045816	1
Snw1	1,1045816	1
Gm5865	1,104351932	1
Gm12017	1,104275387	1
Kyat3	1,104122313	1
Metap1	1,104122313	1
Foxp4	1,103969259	1
Dennd3	1,103969259	1
Tes3-ps	1,103969259	1
Naip5	1,103969259	1
Psmd10	1,103892741	1
Gpr157	1,103816227	1
Cul4b	1,103816227	1
Mrpl3	1,103739719	1
Commd7	1,103739719	1
Zmym5	1,103663216	1

Mrpl17	1,103663216	1
Atg16l2	1,103663216	1
6030400A10Rik	1,103357258	1
Pax3	1,103357258	1
Hcfc1	1,103357258	1
Cadm1	1,103280782	1
Acbd5	1,103204311	1
Mtdh	1,103204311	1
Fkbp4	1,103051385	1
Star	1,102822036	1
Slc25a45	1,102745597	1
Frmd4b	1,102669163	1
Arl14ep	1,102669163	1
Bcap29	1,102592735	1
Nt5c3	1,102592735	1
Wdr89	1,102592735	1
Gm9645	1,102439893	1
Trappc4	1,102439893	1
Cisd3	1,102287073	1
Slc8b1	1,102287073	1
Map3k12	1,102287073	1
Xpnpep1	1,102287073	1
Ttc1	1,102287073	1
Vps54	1,102134274	1
Tmem176b	1,102134274	1
Stk11ip	1,101905116	1
Abhd13	1,101905116	1
Rgs3	1,101676005	1
Rfx1	1,101599645	1
Ppp2r2a	1,101523291	1
Gm8662	1,101370598	1
Gm20703	1,101294259	1
Trim24	1,101217926	1
Dynll1	1,101141598	1
Troap	1,100988958	1
Cept1	1,100760038	1
1810013L24Rik	1,100683741	1
Gm11675	1,100531165	1
Mbd1	1,100531165	1
Pnkp	1,100531165	1
Cgrrf1	1,100454884	1
Ptov1	1,100454884	1
Gng5	1,10030234	1
Lrfn4	1,100073562	1
Mettl16	1,099997313	1
Tor1aip2	1,099997313	1
Wdr7	1,09992107	1
Ptrh2	1,09992107	1
Snrpb	1,09992107	1
Gm9625	1,099844832	1
Zc3hc1	1,099844832	1
Zfp101	1,099768599	1

Slc26a2	1,099692372	1
Dhrs11	1,099616149	1
Nr4a2	1,099616149	1
Fam98b	1,099616149	1
Gm38235	1,099539932	1
Sh3gl1	1,099539932	1
Abce1	1,099539932	1
Mfap3	1,099463721	1
2510009E07Rik	1,099463721	1
Smarca4	1,099463721	1
Malt1	1,099311313	1
Tmem60	1,099311313	1
Gpt2	1,098854218	1
Plekho2	1,098854218	1
Ncoa5	1,098701895	1
Fxr1	1,098701895	1
Epb41	1,098625741	1
Txnrd2	1,098549593	1
Nars	1,09847345	1
B230369F24Rik	1,098397312	1
Mto1	1,098245052	1
Sipa1	1,098245052	1
Ccdc6	1,098245052	1
Snx10	1,09816893	1
Dnajc16	1,098092814	1
Gm14853	1,097864496	1
Rhpn2	1,097864496	1
Mum1	1,0977884	1
Rprd2	1,0977884	1
Maoa	1,09771231	1
Ccsap	1,097636225	1
Tsc22d4	1,097636225	1
Eif2b2	1,097636225	1
Tsc22d1	1,097560145	1
Snx11	1,097560145	1
Vps8	1,097484071	1
Ctnnd1	1,097408001	1
Serpinf2	1,097408001	1
Gm26826	1,097408001	1
Iscu	1,097408001	1
Spast	1,097331938	1
C1qbp	1,097331938	1
Bbs2	1,097255879	1
Lpcat2	1,097255879	1
Chaf1a	1,097255879	1
Rab32	1,097255879	1
Acap2	1,097255879	1
Igbp1	1,097255879	1
Pdhx	1,097179826	1
Mir703	1,097179826	1
Tram1	1,097027735	1
Pcnt	1,096951697	1

1810014B01Rik	1,096799638	1
Psme3	1,096723616	1
Sap30l	1,096495583	1
Eif1ax	1,096343587	1
Wsb1	1,096115632	1
Epg5	1,096039658	1
Fndc3b	1,096039658	1
2610507B11Rik	1,096039658	1
Hadhb	1,095887725	1
Capn3	1,095659865	1
Gm10320	1,095659865	1
Cstf1	1,095659865	1
2610021A01Rik	1,095583922	1
Pex5	1,095432053	1
Gm43547	1,095432053	1
Prelid3b	1,095356126	1
Herc6	1,095128377	1
Eif6	1,095052471	1
4930440I19Rik	1,09497657	1
5830454E08Rik	1,094824785	1
Tmem185a	1,094824785	1
Stat2	1,0947489	1
Ptar1	1,094521277	1
Pigo	1,094521277	1
Snapc2	1,094521277	1
Sult6b1	1,094445413	1
Fbrs	1,094445413	1
Adam8	1,094445413	1
Kif21b	1,094217853	1
Gm12696	1,094217853	1
Mgat1	1,094217853	1
Klhl22	1,094066173	1
Sms	1,094066173	1
Tcirg1	1,094066173	1
Ifi35	1,093990341	1
Cd40	1,093990341	1
Ptcd2	1,093838692	1
4930455G09Rik	1,093762875	1
Nt5dc3	1,093687064	1
Gm45420	1,093611258	1
Uqcrh-ps1	1,093535457	1
Rmrp	1,093535457	1
Casp8ap2	1,093459662	1
Mapk7	1,093459662	1
Plk1	1,093459662	1
Wdr45	1,093383872	1
Golm1	1,093156533	1
0610038B21Rik	1,093080763	1
Gm5963	1,093004999	1
Casd1	1,092929241	1
Npm3	1,092853487	1
Timm44	1,092853487	1

Cdan1	1,092777739	1
Mkrn1	1,092777739	1
Rbfa	1,092626258	1
Gm44890	1,092550526	1
2010015M23Rik	1,092550526	1
Tcof1	1,092550526	1
Cox18	1,092399077	1
Tomm5	1,09232336	1
Lrp6	1,09232336	1
Gm44291	1,09232336	1
Lcp2	1,09232336	1
Rnf34	1,092171942	1
Fam13b	1,092096241	1
Nras	1,092020546	1
Zfpm1	1,09186917	1
Slc39a10	1,09179349	1
Cbr4	1,091717815	1
Ankrd13b	1,091642146	1
Ube3c	1,091566482	1
Lmtk2	1,091490823	1
Gm45733	1,091490823	1
Zfp644	1,091415169	1
Slfn4	1,09133952	1
Slc39a6	1,09133952	1
Rab3ip	1,091263877	1
Tbc1d1	1,091263877	1
Cox10	1,091263877	1
Hbp1	1,091112606	1
Gm4943	1,091036979	1
Ikbkb	1,091036979	1
Med1	1,091036979	1
Psmd11	1,091036979	1
Btrc	1,090885739	1
Cab39	1,090885739	1
Celf2	1,090810128	1
Gns	1,090810128	1
Pim3	1,090810128	1
Zfp532	1,090583324	1
Atf4	1,090583324	1
Nsun5	1,090507733	1
Zfp672	1,090507733	1
Prox2	1,090507733	1
Alkbh5	1,090507733	1
Tpcn1	1,090356567	1
Abl2	1,090205422	1
Wdr59	1,090054298	1
Fgfbp3	1,090054298	1
Gm32175	1,090054298	1
Gm5262	1,090054298	1
Phtf1os	1,090054298	1
Gm43062	1,090054298	1
Taf6l	1,090054298	1

Rnf214	1,089978743	1
Spc25	1,089827651	1
Wdr60	1,089827651	1
Smpd2	1,089752112	1
Rnf185	1,089752112	1
Cnksr3	1,089601051	1
Jund	1,089601051	1
Map1lc3a	1,089525528	1
RP24-365N15.9	1,089374498	1
Aurkb	1,089298991	1
Socs5	1,089223489	1
Rpl23a-ps5	1,089147993	1
Gm16045	1,089147993	1
Gm43447	1,089147993	1
Olr1	1,088997015	1
Mettl9	1,088846059	1
Ddx1	1,088846059	1
Atp10d	1,088770588	1
Pnpla7	1,088695123	1
Zc3h7b	1,088468759	1
Hmga1	1,088393315	1
Yars2	1,088393315	1
Csgalnact2	1,088242442	1
Ythdc1	1,088242442	1
Irak2	1,08809159	1
Cfh	1,087865351	1
Gm18916	1,087789948	1
Ncbp3	1,087789948	1
Rmdn3	1,087639159	1
Prdx1	1,087639159	1
Rps21	1,087563772	1
Gale	1,087488391	1
Mtx2	1,087413015	1
Mrpl15	1,087337643	1
Ndst2	1,087262278	1
Mbtps1	1,087262278	1
H2-Oa	1,087036211	1
Elf1	1,087036211	1
Nop16	1,087036211	1
Fabp5	1,086960866	1
Dennd5a	1,086960866	1
Tm4sf5	1,086885526	1
Mrpl41	1,086810192	1
Bnip2	1,086810192	1
Rchy1	1,086734863	1
Nsmaf	1,086659538	1
Psen1	1,086508906	1
Gm10039	1,086433597	1
Tmem248	1,086433597	1
Rbm8a2	1,086433597	1
Pxylp1	1,086282996	1
Arid4a	1,086282996	1

Arhgap23	1,086207703	1
Ykt6	1,086207703	1
Vma21	1,085906584	1
Capzb	1,085906584	1
Gm9701	1,0856808	1
L3mbtl2	1,085605549	1
Tex30	1,085530303	1
Armc1	1,085530303	1
Ampd2	1,085530303	1
Tnfrsf12a	1,085455062	1
Farsb	1,085455062	1
Enpp4	1,085304597	1
Atat1	1,085154152	1
Rnf19b	1,085078938	1
Aig1	1,084928524	1
Prrg2	1,084853325	1
Gm6520	1,084778132	1
Sbno2	1,084702943	1
Parp1	1,08462776	1
Stk25	1,084552582	1
Oard1	1,084477409	1
Cep57	1,084477409	1
Map4k2	1,084327079	1
Opa3	1,084026481	1
RP23-123D6.12	1,083951345	1
Golph3	1,083951345	1
Pced1b	1,083801088	1
Pdp1	1,083801088	1
Sfswap	1,083801088	1
Dynll2	1,083801088	1
Uap1l1	1,083725967	1
Akap8	1,083650851	1
Chid1	1,083425536	1
Snf8	1,083350441	1
Ccdc84	1,083200267	1
Zmpste24	1,083200267	1
Pdp2	1,083200267	1
Por	1,083200267	1
Ube3a	1,083050114	1
Ctsb	1,083050114	1
Pml	1,082899982	1
Fpgt	1,082674823	1
Atp7a	1,082674823	1
Map4k4	1,08259978	1
Wbscr27	1,082524743	1
Olfr95	1,082524743	1
Ccdc93	1,082524743	1
Lsm3	1,082374683	1
Ppp1cc	1,082224645	1
RP23-304C21.3	1,082149633	1
Jmy	1,082149633	1
Clcn4	1,082074627	1

C1d	1,082074627	1
Edem3	1,08192463	1
Pkn3	1,081849639	1
Btbd3	1,081699673	1
Rnf19a	1,081624698	1
D630045J12Rik	1,081474763	1
Scaf8	1,081474763	1
Syap1	1,081249901	1
Uchl3	1,081249901	1
Fn1	1,081100018	1
2610037D02Rik	1,081025084	1
Gm4332	1,081025084	1
Brd4	1,081025084	1
Abcc1	1,080950156	1
Gorasp2	1,080950156	1
Fam78a	1,080875233	1
Ndufb7	1,080875233	1
Asnsd1	1,080875233	1
Xrn2	1,080800315	1
Sh2b3	1,080650494	1
Dhx29	1,080650494	1
Fyttd1	1,080650494	1
Get4	1,080575592	1
BC029214	1,080575592	1
Mon2	1,080575592	1
Plpp1	1,080276034	1
Scaf11	1,080276034	1
Thoc5	1,080126287	1
Bdh1	1,079901704	1
Runx1	1,079752008	1
Exosc7	1,079677168	1
Gm26737	1,079602333	1
Stx7	1,079527504	1
Cyth1	1,079452679	1
Cog7	1,07937786	1
Mob1a	1,07937786	1
Arel1	1,079303045	1
Fam149b	1,079153433	1
Lace1	1,079078634	1
Atp11a	1,079003841	1
Mrpl27	1,078929052	1
Setd2	1,078929052	1
Rab8b	1,078929052	1
Stk3	1,078779491	1
Dram2	1,078629951	1
Cwc15	1,078555189	1
Adamtsl4	1,078480432	1
Mettl8	1,078106724	1
Pnrc1	1,078106724	1
Gm7266	1,078031998	1
Psmc3	1,078031998	1
Gtf2f1	1,077957277	1

Ddx55	1,077882561	1
Ninl	1,07780785	1
Echs1	1,07780785	1
March7	1,07780785	1
Synj1	1,077733145	1
Galnt4	1,077658445	1
Bmp2k	1,077658445	1
Cd63	1,077434375	1
Sub1	1,077359696	1
Irf9	1,077285022	1
Zc3h18	1,077210353	1
Pter	1,07706103	1
Psmc5	1,07706103	1
Tasp1	1,076986376	1
Wac	1,076911728	1
Msr1	1,076687814	1
B230398E01Rik	1,076613186	1
Tbc1d10b	1,076613186	1
Suclg2	1,076538563	1
Cox4i1	1,076538563	1
Bahcc1	1,076463946	1
Traf4	1,076463946	1
Rpl41	1,076463946	1
Camk1	1,076389334	1
Arhgef6	1,076314727	1
Tnfaip8	1,076314727	1
Myo1f	1,076240125	1
Rsbn1	1,076240125	1
Ndufb9	1,076165528	1
Copb1	1,075941769	1
Osgepl1	1,075718056	1
Spryd7	1,075718056	1
Smarcd1	1,075643496	1
Mybbp1a	1,075568941	1
Rragc	1,07549439	1
Alyref	1,075047198	1
Mrps5	1,075047198	1
Evi5	1,075047198	1
Fnbp1	1,075047198	1
Lipt2	1,074823671	1
Tanc1	1,074823671	1
Atraid	1,074823671	1
Ubtd1	1,074749173	1
4930430F08Rik	1,07467468	1
Chd2	1,074600191	1
Tab2	1,074525708	1
Frg1	1,074525708	1
Mcmbp	1,07445123	1
Tapbpl	1,074376758	1
Fam129a	1,07430229	1
Gm15440	1,074227828	1
Nup62	1,074227828	1

Itpr1	1,074153371	1
Prex1	1,074153371	1
Arhgap9	1,074078919	1
Ccdc90b	1,074078919	1
Bicd2	1,074078919	1
Klhl41	1,07393003	1
Pmaip1	1,07393003	1
Arid1a	1,073706736	1
Taok1	1,073706736	1
Fancl	1,073632315	1
Gm15708	1,073483488	1
Tmem199	1,073483488	1
Zfp948	1,073334682	1
Trub2	1,073334682	1
Ndufab1-ps	1,073334682	1
Fadd	1,073334682	1
Fgd6	1,073334682	1
Noc2l	1,073334682	1
Hspd1	1,073260286	1
RP23-451J19.1	1,073185896	1
Mdc1	1,073111511	1
Rcl1	1,073037131	1
Rasip1	1,072814023	1
0610010F05Rik	1,072590961	1
Atpif1	1,072516617	1
Dnajc21	1,072442278	1
Mfng	1,072442278	1
Bicdl1	1,072219293	1
Pip4k2c	1,072070662	1
Gm45251	1,071847755	1
Tmem69	1,071699175	1
Naa10	1,071699175	1
Gm1840	1,071624893	1
Ntpcr	1,071624893	1
Zfp593	1,071624893	1
Spg20	1,071624893	1
Cela1	1,071476345	1
Ogfr	1,071476345	1
Acvr1	1,071402079	1
Unc13b	1,071253561	1
Ubl4a	1,071105064	1
Senp1	1,071105064	1
Fam8a1	1,070956588	1
Tcerg1	1,070882357	1
Tmem222	1,070808132	1
Ercc5	1,070808132	1
Tmbim4	1,070808132	1
Tmf1	1,070733912	1
Trim21	1,070659697	1
Cpeb2	1,070659697	1
Arpc4	1,070585487	1
Phb2	1,070585487	1

Ppp2r5c	1,070511282	1
Trim36	1,070437082	1
Pgp	1,070437082	1
Psmd2	1,070288698	1
Lancl2	1,070214514	1
Ptp4a3	1,070214514	1
Gab3	1,070140335	1
Cops5	1,070066161	1
Trim68	1,069917829	1
Cep68	1,069769517	1
Ecsit	1,069695369	1
9130221H12Rik	1,069621226	1
Ubac1	1,069621226	1
Gm8292	1,069472955	1
Vgll4	1,069398827	1
Gm14776	1,069324705	1
Zbtb40	1,069102368	1
Borcs6	1,068954169	1
Hmgn1	1,068880078	1
Scyl2	1,068805991	1
Cnot6	1,068805991	1
Mrpl47	1,06873191	1
Myl6b	1,068583762	1
Ndfip1	1,068583762	1
Ube4b	1,068583762	1
Ndufb5	1,068583762	1
Ccdc25	1,068435635	1
Slc25a15	1,06836158	1
Gm5380	1,068213484	1
Chml	1,068065408	1
Gm35106	1,067991378	1
Ubxn2b	1,067917353	1
Amfr	1,067917353	1
Chtop	1,067917353	1
Gm12231	1,067843333	1
Gins4	1,067695309	1
Atp6v1g1	1,067695309	1
Gm29243	1,067325338	1
Gm45110	1,067325338	1
Dpm2	1,067325338	1
Hspd1-ps3	1,067325338	1
Rpl6l	1,067251359	1
Myl6	1,067177386	1
Zfp36l2	1,067177386	1
Ptbp3	1,067103417	1
2610001J05Rik	1,067029454	1
Rpl23	1,067029454	1
Lin7c	1,067029454	1
Nsmf	1,066881542	1
2010320M18Rik	1,066881542	1
Slc35g1	1,066807594	1
Dnmt3a	1,066807594	1

1700003G18Rik	1,066733651	1
Mcm3ap	1,066733651	1
Hnrnpa0	1,066733651	1
Crybg3	1,066659713	1
Mrpl18	1,066659713	1
Lpxn	1,066659713	1
Sep 07	1,066659713	1
Gm11722	1,066585781	1
Hdac4	1,066585781	1
Gm21057	1,066511853	1
Abhd10	1,066511853	1
Larp7	1,066511853	1
Dcun1d1	1,066511853	1
Creg1	1,066511853	1
Spata7	1,066437931	1
Ptk2	1,066437931	1
Mkx	1,066364014	1
Ahcyl1	1,066216194	1
Zc3h6	1,066142293	1
Hnrnph1	1,066142293	1
Stk24	1,066068396	1
Prmt5	1,065994504	1
Smad5	1,065994504	1
Rap1a	1,065994504	1
Smim15	1,065920617	1
Ppp2r2d	1,065846736	1
Lhx1	1,06577286	1
Rabgap1	1,06577286	1
Gm20900	1,065551262	1
Rps10-ps2	1,065551262	1
Dcp1a	1,065477406	1
Ado	1,065329709	1
Ap4e1	1,065255869	1
Ndufb11	1,065255869	1
Ap1g1	1,065255869	1
Wdsub1	1,065182034	1
Cntd1	1,065182034	1
Itga11	1,065108203	1
Plekhh3	1,065108203	1
Cdc5l	1,065108203	1
Znrd1as	1,065034378	1
Hipk1	1,064812934	1
Ppp1r14b	1,064665329	1
Mcm3	1,064517746	1
Gopc	1,064517746	1
Tjp3	1,064296408	1
Tor1a	1,064075117	1
Nhp2	1,064075117	1
Rdh13	1,064001364	1
Gapvd1	1,064001364	1
Carm1	1,063927615	1
Rps27l	1,063927615	1

Gm8730	1,063853872	1
Pmm2	1,063853872	1
Miga1	1,063853872	1
Gm11826	1,063780134	1
RP24-84C23.4	1,063780134	1
Aph1b	1,063780134	1
Cfap97	1,063706401	1
Rnf166	1,063706401	1
Mst1	1,063632673	1
Diablo	1,063632673	1
BC005561	1,063632673	1
Gosr2	1,06341152	1
Cars	1,063337812	1
Ahctf1	1,06326411	1
Cep192	1,06311672	1
Hat1	1,063043033	1
Rps12	1,062969351	1
Slc35b2	1,062969351	1
Tcf7l2	1,062895674	1
Traf3ip1	1,062674674	1
Cdc26	1,062601018	1
Pgm1	1,06245372	1
Gm10762	1,062380079	1
Cflar	1,062306443	1
Ptpmt1	1,062232812	1
Irak1bp1	1,062232812	1
Pigf	1,062232812	1
Ing1	1,062232812	1
2410131K14Rik	1,062159186	1
Gm12020	1,062159186	1
Vdac2	1,06201195	1
Fam207a	1,06193834	1
Hdgf	1,06193834	1
Zfp346	1,061864734	1
Parp16	1,061791134	1
Snx8	1,061791134	1
Zfp950	1,061717539	1
Gm9385	1,061643949	1
Umps	1,061496784	1
Gtf2a2	1,061423209	1
Mrpl16	1,061423209	1
Rab21	1,061423209	1
Gtf3c2	1,061349639	1
Gltp	1,061349639	1
Glyctk	1,061276075	1
Arfgap3	1,061276075	1
Tollip	1,061276075	1
Tomm40	1,061202515	1
Gclc	1,061202515	1
Uhmk1	1,061055411	1
Lrrc59	1,060981867	1
Dguok	1,060981867	1

Ncor1	1,060981867	1
Mtap	1,060834794	1
Slc4a8	1,060761265	1
Ropn1l	1,060614223	1
Id3	1,060614223	1
Pccb	1,060540709	1
C630043F03Rik	1,060540709	1
Vcp-rs	1,060540709	1
Lats2	1,060540709	1
Taf1d	1,060540709	1
Fitm2	1,0604672	1
Brcc3	1,0604672	1
Gdpd5	1,0604672	1
Nit2	1,0604672	1
Ube2v1	1,060393697	1
Eya4	1,060393697	1
Rnf157	1,060246705	1
Ppil4	1,060246705	1
Surf6	1,060173217	1
Wdr82	1,060173217	1
Gm10335	1,060099734	1
Ulk1	1,060099734	1
Rab3d	1,060026256	1
Sec24c	1,059952783	1
Mvp	1,059952783	1
Klhl20	1,059732395	1
Lamb2	1,059732395	1
Gtf2ird2	1,059365184	1
Zufsp	1,059291757	1
Hectd3	1,059218335	1
Poll	1,059218335	1
Bex3	1,059218335	1
Nfyb	1,059218335	1
Ik	1,059218335	1
Setd3	1,058998099	1
Antxr2	1,058924698	1
Neu1	1,058924698	1
Gm8927	1,058851301	1
Lars2	1,05877791	1
S100a13	1,05877791	1
Brms1l	1,058484395	1
Itpa	1,058337668	1
Stoml2	1,058337668	1
Zfx	1,058264312	1
Crygn	1,058190961	1
Sbk1	1,058190961	1
Ndufa10	1,058044275	1
Smc6	1,057897609	1
Rpap3	1,057824284	1
Timm22	1,057750964	1
Sart1	1,057604338	1
Gm6030	1,057531033	1

Dctn5	1,057384439	1
Rnf4	1,057384439	1
Ttc4	1,057164585	1
Tcf12	1,057164585	1
Apbb1ip	1,057164585	1
Gm12468	1,05709131	1
Polr2f	1,05709131	1
Limd1	1,05709131	1
Mcee	1,057018041	1
Rps13-ps5	1,056944776	1
Pef1	1,056944776	1
Gm14323	1,056871517	1
Gtf2e2	1,056871517	1
Chic2	1,056798263	1
Ttbk2	1,056725014	1
Ppp2ca	1,056725014	1
2810428I15Rik	1,056651769	1
Lyar	1,056651769	1
Smim10l1	1,056578531	1
Tes	1,056578531	1
Zkscan3	1,056505297	1
Hint1	1,056432068	1
Taf12	1,056358844	1
Mfn2	1,056285625	1
Gm12583	1,056139203	1
Mrc1	1,056139203	1
Atp5a1	1,056139203	1
Gm11599	1,056066	1
Nufip1	1,055773237	1
Pcnx3	1,055700059	1
D17Wsu92e	1,055700059	1
Kdelr3	1,055626886	1
Gm14698	1,055407397	1
Nudt16	1,055334244	1
Bub3	1,055334244	1
4930524J08Rik	1,055261097	1
Casp8	1,055261097	1
Ppp2r5b	1,055187954	1
Las1l	1,055187954	1
Uqcrh	1,055114816	1
Bmpr1a	1,054822317	1
Mars	1,054822317	1
Fhod1	1,054749205	1
Epha2	1,054749205	1
Gng12	1,054749205	1
Gm26983	1,054676098	1
Aktip	1,054602996	1
Kif2a	1,054529899	1
Magi1	1,054456807	1
Snx24	1,05438372	1
Chmp5	1,054310638	1
Cript	1,054237562	1

Sdhc	1,054237562	1
Tmem127	1,054091423	1
Gusb	1,054091423	1
Tsen2	1,053799208	1
Gcsh	1,053726166	1
Zc3hav1l	1,05365313	1
Camk2n1	1,05365313	1
Gm11625	1,05365313	1
Hnrnpc	1,053215019	1
Ppp1r12c	1,053142018	1
Pkp4	1,053069023	1
Clptm1	1,053069023	1
Chuk	1,053069023	1
Tomm22	1,052923046	1
Eif4g1	1,052923046	1
Amdhd2	1,052850066	1
Fkbp5	1,052850066	1
Peli1	1,052850066	1
Ndufa6	1,052850066	1
Cdc42se1	1,05277709	1
Atg9b	1,05270412	1
Wdfy1	1,052631155	1
Rplp0	1,052631155	1
Tmem143	1,052558194	1
Tubd1	1,052485239	1
Limk2	1,052485239	1
Clec3b	1,052412289	1
Coq7	1,052193469	1
Gars	1,052193469	1
Polr2e	1,052120539	1
Gm17786	1,052047614	1
Specc1	1,052047614	1
Acp6	1,051974694	1
Cul1	1,051974694	1
Pdcd5	1,051755965	1
Gm13383	1,051537281	1
Gyg	1,051537281	1
Calm2	1,051537281	1
Mgst3	1,051464397	1
Gm42783	1,051318643	1
Med13	1,051245773	1
Stx2	1,051172909	1
Iah1	1,051172909	1
St6galnac4	1,051172909	1
Dsel	1,05110005	1
Dnajc4	1,050954347	1
Rbm18	1,050954347	1
Als2cr12	1,050881502	1
Scarb2	1,050881502	1
Fbxo7	1,050808663	1
Xrn1	1,050808663	1
Sec11a	1,050808663	1

Iffo1	1,050735829	1
Pex7	1,050663001	1
Ptpn2	1,050663001	1
Tomm34	1,050663001	1
Prkci	1,050517358	1
Naglu	1,050517358	1
Chd8	1,050517358	1
Rexo2	1,050444544	1
Rpia	1,050371735	1
Pip5k1c	1,050371735	1
Mtx3	1,050226133	1
Pdcd2	1,050226133	1
Mt2	1,050226133	1
Surf1	1,050007767	1
Triap1	1,049862215	1
Plxdc1	1,049789447	1
Ints11	1,049716684	1
Klhl9	1,049716684	1
Taf6	1,049643925	1
Sfpq	1,049643925	1
Tmem9b	1,049643925	1
Cltc	1,049643925	1
Tmem59	1,049643925	1
Vps18	1,049571172	1
Prpsap1	1,049498424	1
Ndufs7	1,049498424	1
Pcgf6	1,049425681	1
Rmnd1	1,049352943	1
Top1	1,049352943	1
Nfil3	1,049207481	1
Zc3h7a	1,049207481	1
G3bp2	1,049134758	1
Zdhhc13	1,04906204	1
Selenot	1,04906204	1
AI846148	1,048698526	1
Pwp1	1,048698526	1
Gsk3a	1,048698526	1
Psmg4	1,048698526	1
Srpr	1,048625839	1
Kat7	1,048553156	1
Washc4	1,048480478	1
Zranb2	1,048262476	1
Gm9409	1,048044518	1
Slc37a1	1,047971876	1
Jade1	1,047971876	1
Dlgap4	1,047971876	1
2510046G10Rik	1,047899238	1
Leprot	1,047826606	1
Bloc1s5	1,04746352	1
R3hcc1	1,047390918	1
Smpd1	1,047318321	1
Dolpp1	1,047173142	1

Smim14	1,047173142	1
Ofd1	1,04710056	1
Atp5l	1,04710056	1
Rps19-ps6	1,046955411	1
Pex26	1,046882844	1
Itgb5	1,046882844	1
Papd4	1,046737725	1
Phf20-ps	1,046737725	1
Gm42566	1,046737725	1
Dhrs7b	1,046665173	1
Golga3	1,046447548	1
Carf	1,046375016	1
Ctc1	1,046157451	1
Brat1	1,04608494	1
Ncoa3	1,04608494	1
Slc7a11	1,046012433	1
Zfp715	1,046012433	1
Epn1	1,046012433	1
Usp22	1,045939932	1
Azi2	1,045867435	1
Gsap	1,045867435	1
Rps3	1,045867435	1
Zfp995	1,045794944	1
Dohh	1,045722457	1
Gm9828	1,045577499	1
Preb	1,045577499	1
Cygb	1,045432562	1
Enkd1	1,045432562	1
Gamt	1,045287644	1
Cdc6	1,045070305	1
Pde4a	1,044997869	1
Nol7	1,044925438	1
Ric8b	1,044853011	1
Pno1	1,044853011	1
Rnf141	1,044853011	1
Trim35	1,044853011	1
Kdelr1	1,044853011	1
Nin	1,044708174	1
2610301B20Rik	1,044635763	1
Snrpd2	1,044563357	1
Polr1c	1,044563357	1
Zfp622	1,044563357	1
Gcnt1	1,04441856	1
Sdf4	1,04441856	1
Gm8624	1,044346168	1
Wee1	1,044346168	1
Srprb	1,044346168	1
Syngr2	1,044201401	1
Smarcd2	1,044129025	1
1110025M09Rik	1,043984288	1
Lyz1	1,043911927	1
Ciao1	1,043839571	1

Mdm1	1,043767221	1
0610012G03Rik	1,043767221	1
Pias4	1,043694875	1
Mthfd2l	1,043622534	1
Papola	1,043622534	1
Jade3	1,043550198	1
Coq5	1,043550198	1
Nudt9	1,043405541	1
Cdc42bpg	1,043405541	1
Mrpl57	1,043405541	1
Gm10863	1,04333322	1
Cnppd1	1,04333322	1
Tuft1	1,043260904	1
Hoxa5	1,043188594	1
Nubpl	1,043116288	1
Mocs2	1,043116288	1
Gm2796	1,043043987	1
Oas3	1,042971691	1
Fam105a	1,042971691	1
4833439L19Rik	1,042971691	1
Zmat5	1,0428994	1
Eno3	1,0428994	1
Islr2	1,0428994	1
Cnot8	1,0428994	1
Mrpl45	1,0428994	1
Rpl7-ps7	1,042754834	1
Tmem192	1,042682558	1
Washc2	1,042682558	1
Mtfp1	1,042610287	1
Rbm12b2	1,042610287	1
Zmym2	1,042538022	1
D030056L22Rik	1,042465761	1
Ubr7	1,042465761	1
Arpc5l	1,042393505	1
Dazap1	1,042321254	1
Trpm7	1,042249009	1
Tmem11	1,042249009	1
Gtf2h4	1,042176768	1
Ccne1	1,042104532	1
Fam129b	1,042104532	1
Dctn3	1,042104532	1
Pafah1b2	1,042104532	1
Zfp428	1,041960076	1
Gin1	1,041887855	1
9130023H24Rik	1,041887855	1
Baat	1,041887855	1
Neo1	1,041887855	1
Cyfip2	1,041887855	1
Dcaf8	1,04181564	1
Gm15800	1,041526827	1
Rfc2	1,041454636	1
Emd	1,041382451	1

Dync1i2	1,041165924	1
Matr3-ps2	1,040949442	1
Casp3	1,040877291	1
Nt5c3b	1,040805146	1
1110006O24Rik	1,040805146	1
Slc30a5	1,040805146	1
Ica1	1,040588739	1
Timm17b	1,040588739	1
Gm13567	1,040444493	1
Shcbp1	1,040444493	1
Homer3	1,040300267	1
Usp5	1,040300267	1
Necap2	1,040300267	1
Gm44152	1,040228161	1
Cby1	1,040228161	1
Pkn2	1,04015606	1
Gm12844	1,040083965	1
Zfp524	1,040083965	1
Tnni2	1,040083965	1
Dcaf15	1,040083965	1
Gm10923	1,040011874	1
Rnf44	1,040011874	1
Tigd2	1,039939788	1
Impa1	1,039939788	1
Samd8	1,039939788	1
Dnajb2	1,039867708	1
Afp	1,039795632	1
Pacs2	1,039795632	1
Prkag1	1,039723561	1
Rps5	1,039723561	1
Dock4	1,039651496	1
Ppp6c	1,039435329	1
Snx15	1,039363283	1
Jag1	1,039363283	1
Arf6	1,039363283	1
Slc35e1	1,039291243	1
Gm11520	1,039219207	1
Git2	1,039219207	1
Galnt3	1,039147176	1
Atg13	1,039147176	1
Htatip2	1,039147176	1
Mvk	1,03907515	1
Nmd3	1,03907515	1
Tgoln1	1,03907515	1
Ankib1	1,03900313	1
Tssc1	1,038931114	1
Gm45749	1,038859103	1
Gm37503	1,038859103	1
Cryl1	1,038787098	1
Pde8a	1,038787098	1
Exosc10	1,038715097	1
Coa5	1,038715097	1

Akirin2	1,03857111	1
Alg3	1,038499125	1
Top2b	1,038427144	1
Lamp1	1,038355168	1
Tsn	1,038355168	1
Spire2	1,037995364	1
Ciart	1,037923418	1
1700112E06Rik	1,037779541	1
Gm16310	1,037779541	1
Rad1	1,037779541	1
Uqcc3	1,037779541	1
2700060E02Rik	1,03770761	1
Picalm	1,03770761	1
Calm1	1,03770761	1
Ubxn1	1,037635684	1
Pld3	1,037419937	1
Sugt1	1,037348031	1
Isca1	1,037204234	1
Phf12	1,037132343	1
Upk1a	1,036844828	1
Gm37339	1,036844828	1
Sfxn1	1,036844828	1
Cyp20a1	1,036772962	1
Mrpl35	1,036772962	1
Mrpl23	1,036701101	1
Serf2	1,036629245	1
Cpsf6	1,036629245	1
Dis3	1,036557394	1
Tmem57	1,036485547	1
Cln8	1,036270039	1
Micu3	1,036198213	1
Siglec1	1,036126391	1
Gm15773	1,036054575	1
Dbndd2	1,036054575	1
Svbp	1,035982764	1
Kdm3b	1,035982764	1
Atp6v1f	1,035982764	1
Immp2l	1,035910957	1
Rhoc	1,035910957	1
Rps25-ps1	1,035767359	1
Rnf2	1,035695568	1
Gm13532	1,035623782	1
Cask	1,035623782	1
Bola2	1,035623782	1
Gm11868	1,035552	1
Sra1	1,035552	1
B230118H07Rik	1,035480224	1
Swt1	1,035336685	1
Aagab	1,035264924	1
Fam53c	1,035193167	1
2410004B18Rik	1,035193167	1
Ncln	1,035049669	1

Galc	1,034977927	1
Ptges3	1,034906191	1
Tmem246	1,034762732	1
Oprl1	1,034762732	1
Prss46	1,034762732	1
Gm11808	1,034762732	1
Pgpep1	1,03469101	1
Cggbp1	1,034619293	1
Sh3bp2	1,034619293	1
Lrig2	1,034475875	1
Gm7094	1,034404173	1
Mrps26	1,034404173	1
Lin7b	1,034260784	1
Pold2	1,034260784	1
Lcmt1	1,034260784	1
Lbr	1,034260784	1
Gm38213	1,034189097	1
Gpd1l	1,034189097	1
Gm43721	1,034117415	1
2900026A02Rik	1,034117415	1
Gm6088	1,034117415	1
Napsa	1,034045738	1
Mars2	1,033974066	1
Ndufb3	1,033974066	1
Gm13889	1,033830736	1
Dock8	1,03361578	1
Gm29736	1,033544137	1
Ppp1r11	1,033544137	1
Polr2j	1,033544137	1
Lcp1	1,033186	1
Rps16	1,033114388	1
Focad	1,033114388	1
Ppp3cc	1,032827987	1
Cpne8	1,032827987	1
Phldb1	1,032827987	1
Gnpda2	1,0327564	1
Zcchc9	1,032613239	1
Icmt	1,032613239	1
Nabp2	1,032470098	1
Hspa5	1,032470098	1
Crtc3	1,032398535	1
Dlg1	1,032398535	1
Kdm2a	1,032398535	1
Rpl26-ps4	1,032326978	1
Ndufaf8	1,032255425	1
Hexa	1,032255425	1
Spcs1	1,032183877	1
Dbnl	1,032112334	1
Bahd1	1,032112334	1
Ube2l6	1,032040795	1
Actr3	1,032040795	1
Rpl27a	1,032040795	1

Fcgr2b	1,031826211	1
Cfdp1	1,031826211	1
Fam134a	1,031826211	1
Wdr90	1,031754693	1
Sephs1	1,031754693	1
Flcn	1,031754693	1
Tmem87b	1,031754693	1
Rpl18	1,031683179	1
Gorasp1	1,031611671	1
Socs1	1,031397176	1
Stk10	1,031397176	1
Tmem205	1,031397176	1
Wipf2	1,031325687	1
Psmd3	1,031325687	1
Cct3	1,031254204	1
Mocos	1,031182725	1
Coasy	1,031182725	1
Mrpl11	1,031111251	1
Ndufb8	1,031111251	1
Mrps6	1,031111251	1
Atp6v1b2	1,031039783	1
Papd5	1,030968319	1
Pus10	1,030825406	1
Shoc2	1,030753957	1
Fbxl12	1,030753957	1
Gse1	1,030682513	1
Lpin1	1,030682513	1
Nr1d2	1,030682513	1
Washc3	1,030611074	1
Ube2g2	1,030611074	1
Slc35e4	1,03053964	1
Cops7b	1,030468211	1
Dera	1,030396787	1
Hcls1	1,030325368	1
Gm10602	1,030253954	1
Cluh	1,030253954	1
Sdccag3	1,030182544	1
Mrpl13	1,03011114	1
Bbof1	1,030039741	1
Atr	1,030039741	1
Kctd10	1,029968346	1
Zfp809	1,029968346	1
Oraov1	1,029896957	1
Zfp266	1,029896957	1
Gm10250	1,029754193	1
F630040K05Rik	1,029754193	1
Asrgl1	1,029754193	1
Tspan31	1,029682818	1
Ndufs4	1,029682818	1
Mrpl39	1,029611448	1
Akap17b	1,029468724	1
Gm12396	1,029468724	1

Alkbh6	1,029397369	1
Npc1	1,029254674	1
Rbm14	1,029254674	1
Chmp3	1,029254674	1
Pten	1,029254674	1
Pdzk1ip1	1,029111999	1
Psmc6	1,029040669	1
Crls1	1,028826708	1
Gm16556	1,028755398	1
Slc48a1	1,028755398	1
Tada3	1,028684092	1
Amz2	1,02839892	1
Spry2	1,028256363	1
Vkorc1l1	1,028185093	1
Klhl2	1,028185093	1
Fbxw8	1,028185093	1
Tex9	1,028113827	1
Sec16a	1,028042566	1
B4galt7	1,028042566	1
Gm14857	1,02797131	1
Plekhb2	1,027900059	1
Mapk8ip1	1,027828812	1
4930431P19Rik	1,027828812	1
Crybg3	1,027828812	1
Snrpe	1,027828812	1
Ric1	1,027757571	1
Sec61b	1,027686335	1
Pla2g15	1,027686335	1
Lrrc8b	1,027543877	1
Rps12-ps26	1,027472656	1
Entpd7	1,027472656	1
Garnl3	1,027472656	1
Strip1	1,027472656	1
Tdpx-ps1	1,027401439	1
Sqstm1	1,027187819	1
Kif16b	1,027116623	1
1110032A03Rik	1,027045431	1
Dhdds	1,027045431	1
Susd6	1,026974244	1
Stk35	1,026903062	1
Fndc3a	1,026831885	1
Cep120	1,026760713	1
Atp5f1	1,026760713	1
Knl1	1,026689546	1
Nxpe3	1,026689546	1
Pdpr	1,026618383	1
Ube2j1	1,026547226	1
Gm44791	1,026404926	1
Gemin7	1,026404926	1
Zmynd8	1,026404926	1
Tmed5	1,026262646	1
Bcas2	1,025907032	1

Gle1	1,025907032	1
Ssrp1	1,025907032	1
Kcnn1	1,02562263	1
Ctnnbl1	1,02562263	1
Git1	1,02562263	1
Maea	1,02562263	1
Sod2	1,02562263	1
Cdk19	1,025551542	1
Zmym1	1,025480458	1
Ccl5	1,025480458	1
Aspm	1,025480458	1
Cenpj	1,02540938	1
Rps3a1	1,02540938	1
Elovl1	1,02540938	1
Gm8770	1,025338306	1
Parp14	1,025338306	1
Hyi	1,025338306	1
Hltf	1,025267238	1
4930518I15Rik	1,025125116	1
Tpmt	1,024983013	1
Gm16437	1,024983013	1
Ktn1	1,024983013	1
Nxf1	1,024698867	1
Lonp2	1,024698867	1
Ano6	1,024698867	1
Pxk	1,024627842	1
Gm8566	1,024556823	1
Ikbkg	1,024556823	1
Cct7	1,024556823	1
Insl6	1,024485809	1
Sec62	1,024485809	1
Gm42670	1,024414799	1
Kmt2b	1,024414799	1
Kiss1r	1,024414799	1
Pstk	1,024343795	1
Hist3h2a	1,024343795	1
Pxn	1,024272795	1
Cant1	1,024059826	1
Rac1	1,023988846	1
Pigb	1,023917871	1
Cox19	1,023917871	1
Phf8	1,023917871	1
Ralgps1	1,0238469	1
1810044D09Rik	1,023492124	1
Spin1	1,023492124	1
Tnip2	1,023492124	1
Cyp2u1	1,023350247	1
Ndufv2	1,023350247	1
Larp1b	1,023350247	1
Rad50	1,023279317	1
Ss18l2	1,023279317	1
AI413582	1,023279317	1

Fundc2	1,023279317	1
Slc31a2	1,023208391	1
Tmub1	1,023208391	1
Snhg1	1,023208391	1
Srrm1	1,023208391	1
Mrps34	1,02313747	1
Rprd1b	1,022924736	1
Ppif	1,022924736	1
Ssr2	1,022924736	1
Mb21d2	1,022782939	1
Atxn7	1,022782939	1
Nbas	1,022782939	1
Grsf1	1,022782939	1
Rad23b	1,022782939	1
Cops9	1,022712047	1
Mif4gd	1,022641161	1
Smim4	1,022641161	1
Lrmp	1,022641161	1
Ikzf5	1,022641161	1
Zfp68	1,022570279	1
9530082P21Rik	1,022570279	1
Atp5d	1,022499402	1
Gm17827	1,022428531	1
Nubp2	1,022357664	1
Naa16	1,022357664	1
Ahnak2	1,022357664	1
Gla	1,022357664	1
Ifrd2	1,022286802	1
Rab19	1,022145093	1
Gm26514	1,022145093	1
Got1	1,021932566	1
Trappc13	1,021861733	1
Gm15159	1,021790905	1
Vcp	1,021790905	1
Nsun2	1,021790905	1
Ino80e	1,021720083	1
Kif24	1,021649265	1
Gm37108	1,021649265	1
Slc9a1	1,021649265	1
Vma21-ps	1,021578452	1
Adam10	1,021578452	1
Kif9	1,021153678	1
Gm17541	1,021153678	1
Ppa2	1,021153678	1
RP24-496O17.7	1,021082899	1
Gpat3	1,021012126	1
Psph	1,020941357	1
Zfp275	1,020941357	1
Dnph1	1,020799834	1
Chsy1	1,02072908	1
Cdadc1	1,020587587	1
Snu13	1,020587587	1

Ccdc174	1,020516848	1
Qsox1	1,020516848	1
Ncs1	1,020446113	1
Ctbs	1,020375384	1
Psmd1	1,020375384	1
Pole	1,020304659	1
Sdhaf4	1,020304659	1
Tmem110	1,02023394	1
Mogs	1,02023394	1
1810011H11Rik	1,020092515	1
2410015M20Rik	1,01995111	1
Csad	1,019880415	1
Tdrkh	1,019880415	1
1700047K16Rik	1,019809724	1
Mcm6	1,019809724	1
Bhlhe40	1,019809724	1
Trim39	1,019668359	1
Bcorl1	1,019456347	1
Stim2	1,019385686	1
Rap2a	1,019385686	1
Nsd3	1,019385686	1
9430034N14Rik	1,01931503	1
Nploc4	1,01931503	1
Nfic	1,01931503	1
Qars	1,01931503	1
Noa1	1,019244379	1
Ranbp10	1,019244379	1
Zfp317	1,019173732	1
Aldh9a1	1,019173732	1
Cdc42se2	1,019173732	1
Ccdc47	1,019103091	1
Slc38a10	1,019032455	1
Scamp5	1,018961823	1
Rhbdf2	1,018891197	1
Usf2	1,018820575	1
Rnf115	1,018820575	1
Tapt1	1,018749958	1
Trip12	1,018749958	1
Fhod3	1,018679346	1
Cir1	1,018608739	1
Gmpr	1,018538137	1
D2Bwg1423e	1,018396947	1
9530068E07Rik	1,018396947	1
Ebp	1,01832636	1
Vprbp	1,01832636	1
Elp3	1,01832636	1
Cbwd1	1,018255777	1
Tmbim1	1,018255777	1
Dus2	1,0181852	1
Rps7	1,0181852	1
Rrad	1,0181852	1
Xpa	1,0181852	1

Slmap	1,0181852	1
Gm5883	1,018114627	1
Gdi2	1,017691293	1
Srd5a3	1,017620755	1
Gm2810	1,017550221	1
Ndufb10	1,017550221	1
Ilk	1,017479692	1
Eif3f	1,017479692	1
Cln5	1,017409168	1
Osbpl9	1,017338649	1
Elac2	1,017268135	1
Abcf1	1,017056622	1
Prrc2c	1,017056622	1
Pycr2	1,016986128	1
Eprs	1,016986128	1
Slc7a4	1,016915638	1
Ehd1	1,016915638	1
Akt1	1,016915638	1
Nkap	1,016845153	1
Napg	1,016845153	1
Nipbl	1,016845153	1
BC028528	1,016774673	1
Snapc4	1,016704198	1
Lap3	1,016563263	1
Zbtb21	1,016492803	1
Rb1	1,016492803	1
Rps11-ps3	1,016422347	1
Pld4	1,016281451	1
Eml4	1,01621101	1
Golga2	1,01621101	1
Nde1	1,016140574	1
Arhgef7	1,015999717	1
Fau	1,015929296	1
Golga4	1,015929296	1
Gm15464	1,015858879	1
Eef2	1,015718061	1
Ahsa2	1,015647659	1
9430060I03Rik	1,015436483	1
Pctp	1,015366101	1
Ldlrad3	1,015366101	1
Coq9	1,015295723	1
Mvb12b	1,015295723	1
Rilpl2	1,015154983	1
Wdr26	1,015154983	1
Prkdc	1,015084621	1
Oaz1-ps	1,015084621	1
Tmem242	1,015084621	1
Arhgap22	1,01473288	1
Slc9a8	1,01473288	1
Atg7	1,014592218	1
Scamp2	1,014451575	1
Nudcd2	1,014310952	1

Gm42483	1,014240648	1
Arl14epl	1,014240648	1
A730062M13Rik	1,014240648	1
Bloc1s3	1,014170349	1
Slc9b1	1,014100054	1
1700008J07Rik	1,014100054	1
Hif1a	1,014029765	1
Hook3	1,01395948	1
Ubtf	1,013818925	1
Inf2	1,013818925	1
B230377A18Rik	1,013748655	1
Ndufb4	1,013678389	1
Ndufa12	1,013608129	1
Vipas39	1,013537874	1
Gga3	1,013537874	1
Tnnt3	1,013467623	1
Gm5921	1,013467623	1
Twf2	1,013467623	1
Atp5h	1,013397377	1
Pbrm1	1,013397377	1
Arfip2	1,013327136	1
Cdk18	1,013327136	1
Naa38	1,0132569	1
Actn4	1,013186669	1
Blcap	1,013186669	1
Sec11c	1,013186669	1
Eml3	1,013116443	1
2900009J06Rik	1,012905793	1
Tomm7	1,012765384	1
Cep95	1,012695187	1
Kif18a	1,012554807	1
Aacs	1,012484625	1
Incenp	1,012484625	1
Atp6v0a2	1,012414447	1
Slc45a4	1,012344274	1
Prdm2	1,012344274	1
Trpv2	1,012344274	1
9930021J03Rik	1,011993483	1
Vti1b	1,011993483	1
Elmo1	1,01192334	1
Psmd13	1,01192334	1
Zfand2b	1,011712938	1
Atp5c1	1,011712938	1
Dennd4a	1,011712938	1
Usp14	1,011712938	1
Wdr45b	1,011712938	1
Rbm10	1,011642814	1
Mnt	1,011572695	1
Ice2	1,011432471	1
Kcnb1	1,011292266	1
Srsf10	1,011292266	1
Samd9l	1,011222171	1

Nhej1	1,011222171	1
Prss50	1,011152081	1
Gm5113	1,011152081	1
Syncrip	1,011152081	1
Gm9920	1,01094184	1
Ddit4	1,01094184	1
Gm7618	1,010871769	1
Ireb2	1,010871769	1
Wnk1	1,010801703	1
Ttc27	1,010661586	1
Tchp	1,010661586	1
Hoxc4	1,010591535	1
Lamc2	1,010591535	1
Fbxo44	1,010521488	1
Gemin5	1,010521488	1
1190002N15Rik	1,010521488	1
Samm50	1,010521488	1
Itgb3bp	1,010451446	1
Adck2	1,010451446	1
Mrps25	1,010451446	1
Tubgcp6	1,01038141	1
Ctcf	1,01038141	1
Tfb1m	1,010241351	1
Map4k1	1,010171329	1
Jdp2	1,010101311	1
Bmyc	1,010031299	1
Ankrd40	1,010031299	1
Gm45109	1,009961291	1
Unc13a	1,009821291	1
Gnl1	1,009821291	1
Dgkq	1,009681309	1
Rpa2	1,009681309	1
Efcab7	1,009611326	1
Fam160b2	1,009611326	1
Gpr108	1,009541348	1
Nudc	1,009471374	1
Actr3b	1,009471374	1
Zfp426	1,009401405	1
Xrcc1	1,009401405	1
Ints13	1,009401405	1
Tm7sf3	1,009331441	1
Tmem230	1,009261482	1
Tmem167	1,009191528	1
Sppl2b	1,008981694	1
Tnfrsf11a	1,008981694	1
Parn	1,008841829	1
Ptdss1	1,008771904	1
mt-Ti	1,008701984	1
Ralbp1	1,008632068	1
Rps10-ps1	1,008632068	1
Gata3	1,008492252	1
Acot9	1,008492252	1

Mrpl34	1,008492252	1
Cxcl16	1,008422351	1
Gm5277	1,008352455	1
Ptdss2	1,008352455	1
Sec22b	1,008282564	1
Psap	1,008212677	1
Milr1	1,008072919	1
Wdr44	1,007863318	1
Sat1	1,007863318	1
2810474O19Rik	1,007863318	1
Polr2h	1,007793461	1
Epb41l2	1,007793461	1
Rnf11	1,007793461	1
Gpbp1l1	1,007723608	1
Tbc1d8b	1,007583918	1
Fgr	1,00751408	1
Slc29a1	1,007444246	1
Vps37a	1,007304595	1
Mtch2	1,007304595	1
Serpinb6b	1,007234776	1
Rpl31-ps14	1,007164962	1
Zic2	1,007025349	1
Tank	1,007025349	1
Psrc1	1,007025349	1
Mrpl32	1,00695555	1
Glud1	1,006885756	1
Gm36378	1,006815966	1
Cenph	1,006815966	1
Alad	1,006746181	1
Rps15a-ps5	1,006606626	1
Txndc9	1,006606626	1
Rbpsuh-rs3	1,006606626	1
Ube2f	1,006606626	1
Pcmt1	1,006606626	1
Ubqln1	1,006606626	1
Psmb6-ps2	1,006467091	1
Sass6	1,00639733	1
Snora30	1,006327574	1
Hyal2	1,006327574	1
Gm10074	1,006118336	1
Zyx	1,005978868	1
Gm25857	1,005978868	1
Etv3	1,005978868	1
Gramd1b	1,005978868	1
Fam122a	1,005909142	1
Fmr1	1,005909142	1
Snrnp70	1,005909142	1
Cap1	1,005909142	1
Nos3	1,00583942	1
Khnyn	1,005769703	1
Spsb1	1,005769703	1
Dip2c	1,00569999	1

Arf1	1,005630283	1
Apex1	1,00556058	1
Fam3a	1,00542119	1
Sri	1,005351502	1
Hsd17b10	1,005351502	1
Clip2	1,005281818	1
Atf1	1,005281818	1
Ubqln4	1,00521214	1
Tmem141	1,00521214	1
Zfp385a	1,005142466	1
Tspyl2	1,005142466	1
Ski	1,004724526	1
Dalrd3	1,004654887	1
Coq8b	1,004654887	1
Fam120a	1,004654887	1
Gpsm1	1,004585252	1
Tpm1	1,004585252	1
Gm29019	1,004515622	1
Frmd8os	1,004515622	1
Slc12a9	1,004515622	1
Gm15417	1,004515622	1
Anp32e	1,004515622	1
Rrp36	1,004445996	1
Itch	1,00430676	1
Angel1	1,004028346	1
Ccdc136	1,004028346	1
Elk3	1,004028346	1
Klc4	1,004028346	1
Psmd9	1,003958754	1
Tmem106b	1,003889167	1
Magi2	1,003819586	1
Psmf1	1,003750009	1
Ptk2b	1,003750009	1
Lin37	1,003680436	1
Uhrf2	1,003610869	1
Eif2s3x	1,003402196	1
Irak4	1,003402196	1
Helb	1,003332647	1
Usp19	1,003332647	1
2500002B13Rik	1,003193566	1
Dstyk	1,003193566	1
Nle1	1,003124032	1
Ino80b	1,003054503	1
Rnaseh2a	1,00291546	1
Eif4e	1,00291546	1
Ssfa2	1,002706931	1
Sbf1	1,002637431	1
Eif4ebp2	1,002637431	1
Elk1	1,002567936	1
Rnpepl1	1,002567936	1
Atp5g1	1,002567936	1
Med15	1,002498446	1

Ldlr	1,00242896	1
Gaa	1,00235948	1
Lrrc40	1,00235948	1
Ndufa11	1,00235948	1
Trp53bp1	1,00235948	1
Galnt7	1,00235948	1
Stat3	1,002290004	1
Ap4m1	1,002220533	1
Zfp449	1,002151066	1
Gm6265	1,002151066	1
Rps27-ps1	1,002012148	1
Map3k5	1,002012148	1
Psmb1	1,001942697	1
Gm6444	1,00187325	1
Dcps	1,00187325	1
Smarcb1	1,001803808	1
Stxbp5	1,001803808	1
Hnrnpm	1,001803808	1
Tmem55b	1,001803808	1
Abcf2	1,001664938	1
Fcho2	1,001664938	1
Prrc2b	1,00159551	1
E2f5	1,001526087	1
Fmnl1	1,001456669	1
Dnajc18	1,001317847	1
Sep 08	1,001109651	1
Snhg3	1,001040261	1
Mrps17	1,001040261	1
Hmgcl	1,000832123	1
Relb	1,000762753	1
Gm8667	1,000624027	1
Tstd1	1,000554672	1
Gm12097	1,000554672	1
Mllt10	1,000554672	1
Rpl10a-ps1	1,000554672	1
Rp9	1,000554672	1
Ube2d2a	1,000415975	1
Wdr37	1,000415975	1
Runx3	1,000415975	1
Slc11a1	1,000346634	1
Uqcrc2	1,000346634	1
Eif3j1	1,000277297	1
Ormdl3	1,000277297	1
Sema4a	1,000277297	1
Rcan3	1,000207966	1
Atxn7l1	1,000207966	1
Rnf122	1,000138639	1
Glyr1	1,000138639	1
Rassf4	1,000138639	1
Sbds	1,000069317	1
Rasa3	1	1
Gm42418	1	1

D3Ertd751e	-0,00018811	1
BC037034	-0,00023235	1
Gm14130	-0,00027313	1
Abi3	-0,00046306	1
Fam117a	-0,00046392	1
Ppie	-0,00065675	1
Casp6	-0,00089284	1
Steap3	-0,0010999	1
Cbr1	-0,0011894	1
Smim20	-0,0012036	1
1110019D14Rik	-0,0013356	1
Rogdi	-0,0012914	1
Sh3pxd2b	-0,0015056	1
Gm9294	-0,0015935	1
Rlim	-0,001699	1
Cdk11b	-0,0018416	1
Scmh1	-0,0018946	1
Gon7	-0,0018878	1
Naa15	-0,0018803	1
Ralb	-0,0021462	1
Gm24336	-0,0022466	1
Tmem135	-0,0022694	1
Tmem198b	-0,0023626	1
Nvl	-0,002435	1
Parvb	-0,0023554	1
Fam109a	-0,002694	1
Hmgcr	-0,0029789	1
Polr3k	-0,0030965	1
Prr14	-0,0030667	1
Gm24276	-0,003073	1
Carhsp1	-0,0030652	1
Lgals3bp	-0,003174	1
Vps53	-0,0033226	1
Cep290	-0,0034575	1
Zfyve28	-0,0035277	1
Sccpdh	-0,0035287	1
Snx17	-0,0037735	1
Atp5o	-0,0038418	1
Tfpi	-0,0040178	1
Fam185a	-0,0039725	1
Casp9	-0,0042654	1
Nob1	-0,0043188	1
Agpat5	-0,0042622	1
Atg16l1	-0,004502	1
Zbtb46	-0,0046181	1
Ylpm1	-0,0046788	1
Tnpo3	-0,004676	1
Clock	-0,0047518	1
Brd7	-0,0048373	1
Srcap	-0,0049374	1
Hmgxb4	-0,004958	1
Mafg	-0,0050132	1

Stx17	-0,0050869	1
Cd180	-0,005171	1
Slc47a2	-0,0054287	1
Prkaca	-0,0054112	1
Tyrobp	-0,0054788	1
Ivd	-0,0055905	1
Dgkz	-0,0055612	1
Saraf	-0,0057167	1
Nus1	-0,0057076	1
Gm4204	-0,0057042	1
Greb1	-0,0058066	1
March8	-0,0058515	1
Mcm2	-0,0059139	1
Micu2	-0,0059103	1
Nhlrc3	-0,0059463	1
Cyb5d2	-0,0059505	1
Ppp2r3c	-0,0060421	1
Zmym3	-0,0060863	1
Psma6	-0,0062783	1
Ctdsp1	-0,0064646	1
Acadm	-0,0065036	1
Kif3b	-0,0067425	1
Tm9sf3	-0,0066896	1
Mkln1	-0,0066677	1
Slc25a3	-0,0067008	1
Ywhae	-0,0066775	1
Med9	-0,0067817	1
Iws1	-0,0067665	1
Hsd17b7	-0,0071333	1
Ivns1abp	-0,0071514	1
Lsm14b	-0,0073069	1
Gm11478	-0,0075124	1
Krtcap3	-0,0075811	1
Tspan4	-0,0077046	1
Fam217b	-0,0078298	1
Ece1	-0,0078895	1
Rny3	-0,0079155	1
Slc4a1ap	-0,0080093	1
Vps16	-0,0079952	1
Rdx	-0,0080741	1
Cebpzos	-0,0083059	1
Znrf2	-0,0083117	1
Polr3c	-0,0082995	1
Spag9	-0,0082849	1
Dusp18	-0,0083695	1
Ecm1	-0,0083502	1
Aebp2	-0,0084986	1
Stard8	-0,0089317	1
Rnf14	-0,0089624	1
Ranbp2	-0,0090398	1
Txnl1	-0,0089732	1
Ncf2	-0,0091126	1

Actr10	-0,0092244	1
Rpl13-ps3	-0,0093733	1
Ddx47	-0,0095268	1
Kif3c	-0,0096778	1
Sf3b5	-0,0097143	1
B4galt1	-0,0096983	1
Ociad1	-0,010121	1
Gm14681	-0,010242	1
Bhlhe41	-0,010178	1
Ppwd1	-0,010183	1
Slc4a7	-0,010286	1
Coprs	-0,010418	1
Wdr6	-0,010454	1
Cox7a2	-0,010492	1
Fam171a2	-0,010601	1
Hscb	-0,010607	1
Thada	-0,010661	1
Mllt11	-0,010654	1
Hnrnpll	-0,010922	1
Zmat3	-0,010887	1
Snupn	-0,010868	1
Sh3glb1	-0,010906	1
Prnp	-0,010974	1
Erp29	-0,01104	1
Rcor1	-0,011097	1
Serf1	-0,011195	1
App	-0,011233	1
Nckap1l	-0,01124	1
Trip4	-0,011344	1
Cramp1l	-0,01139	1
Rnaseh2b	-0,011487	1
Gps2	-0,011452	1
Gm6472	-0,011563	1
Ndufa8	-0,011747	1
Ufm1	-0,01183	1
Tmem50a	-0,011844	1
Mid1ip1	-0,011907	1
Atp2a2	-0,012016	1
Mapk1	-0,012032	1
Fxr2	-0,012092	1
Eif3c	-0,012051	1
Zfp874b	-0,01217	1
Cyba	-0,012201	1
Mtg1	-0,012256	1
Klf11	-0,012311	1
Fkbp2	-0,012287	1
Epb41l4aos	-0,012343	1
Wars	-0,012537	1
Pola2	-0,012737	1
Gpr155	-0,01294	1
Cyc1	-0,012898	1
Atp1a1	-0,012925	1

Mrpl21	-0,013021	1
Gm12013	-0,013083	1
Pcif1	-0,01334	1
Sec61a2	-0,013418	1
Tmcc3	-0,013454	1
Cnpy4	-0,013497	1
Gm14138	-0,013598	1
Cep97	-0,013624	1
Pomp	-0,013646	1
BC030336	-0,013717	1
Rexo1	-0,013673	1
Atg12	-0,013663	1
Cat	-0,014074	1
Hexb	-0,014211	1
Rps15	-0,014188	1
Msh2	-0,01431	1
Fam168b	-0,01431	1
Clec5a	-0,014511	1
Tbc1d22b	-0,014583	1
Eif3d	-0,014617	1
Polrmt	-0,01471	1
Gm14620	-0,014735	1
Spcs2	-0,01483	1
Csnk1a1	-0,014842	1
Snord118	-0,014946	1
Saa3	-0,014852	1
Abhd5	-0,015212	1
Gm20342	-0,01543	1
Specc1l	-0,015511	1
Bptf	-0,015816	1
Atp5j2	-0,01582	1
Stat6	-0,015967	1
Rnf146	-0,016072	1
Ccl3	-0,016054	1
Baz2b	-0,01614	1
Opa1	-0,016154	1
Eci2	-0,016361	1
Oat	-0,016515	1
Ptprc	-0,016517	1
Rpl36-ps2	-0,016744	1
Appl2	-0,016688	1
2010315B03Rik	-0,016834	1
Trim44	-0,016833	1
Cntrl	-0,016813	1
Ndufaf4	-0,016827	1
Gm10916	-0,016893	1
Psmb5	-0,017031	1
Terf2ip	-0,017113	1
Aspscr1	-0,017268	1
Calcoco1	-0,017337	1
Cd300a	-0,017389	1
Arl1	-0,017381	1

Ndfip2	-0,017641	1
Tspo	-0,017628	1
Wapl	-0,017594	1
Tomm20	-0,018043	1
Tyw3	-0,018146	1
Dpy19l4	-0,018155	1
Mycbp2	-0,018447	1
Eif4b	-0,018391	1
Ankrd24	-0,018911	1
Mcur1	-0,019083	1
Sars	-0,019144	1
Thra	-0,019105	1
Usp3	-0,019125	1
Tmbim6	-0,019163	1
Gmcl1	-0,019316	1
Naa35	-0,019292	1
Gm15834	-0,019429	1
6230400D17Rik	-0,019371	1
Rab40c	-0,0196	1
Tatdn1	-0,019762	1
Ruvbl2	-0,019877	1
Cpped1	-0,019941	1
Fip1l1	-0,020009	1
Mcoln2	-0,020162	1
Eif2b5	-0,020222	1
Mettl6	-0,020293	1
Rida	-0,020405	1
Scnm1	-0,020564	1
Pdxk	-0,020897	1
Sgsh	-0,021113	1
Gm9442	-0,021423	1
Trp53inp2	-0,021397	1
Hs2st1	-0,021436	1
Myo9a	-0,021544	1
Diaph1	-0,0215	1
Fech	-0,021848	1
Ska3	-0,021765	1
Tcta	-0,021814	1
Mfsd10	-0,021862	1
Adss	-0,021893	1
Mmab	-0,021974	1
D330023K18Rik	-0,022041	1
Trim8	-0,022117	1
Als2cl	-0,02221	1
Exoc5	-0,022231	1
Tbl1xr1	-0,022345	1
Glod4	-0,022319	1
Gm19196	-0,022363	1
Per2	-0,022534	1
Zbtb8a	-0,022622	1
Hyal3	-0,022609	1
Alpk2	-0,022795	1

5730455P16Rik	-0,022787	1
Actn1	-0,022773	1
Phc2	-0,022824	1
Thap3	-0,022811	1
Tctex1d2	-0,022932	1
Ghitm	-0,022978	1
Nat14	-0,023092	1
Efhd2	-0,023189	1
Ctns	-0,023297	1
Trim41	-0,023313	1
Slc35c2	-0,023398	1
Asah2	-0,023623	1
Msmo1	-0,0236	1
Fam84b	-0,023659	1
Rps19	-0,023745	1
Gm7128	-0,023751	1
Gm8394	-0,023994	1
Mphosph6	-0,02396	1
Tle4	-0,024238	1
Gnl3l	-0,024312	1
Fyb	-0,024256	1
Mapre1	-0,02431	1
Cdyl	-0,024449	1
Csrp1	-0,02448	1
Tcp1	-0,024488	1
Ccpg1	-0,024721	1
Vps39	-0,02466	1
Csnk1g1	-0,024747	1
Gm9332	-0,024934	1
Fbxo22	-0,025042	1
Mzt1	-0,02501	1
Rnf114	-0,025138	1
Ddx6	-0,025175	1
Bbc3	-0,025297	1
S100a6	-0,02535	1
Ppp2r1b	-0,025417	1
Ccl9	-0,025523	1
Ell2	-0,025465	1
Rps6kb2	-0,025605	1
Ccndbp1	-0,025806	1
Arpp19	-0,025762	1
Gm7701	-0,025883	1
Gm5451	-0,025873	1
Trmt10a	-0,025862	1
1110046J04Rik	-0,025969	1
1500015A07Rik	-0,026072	1
Ccdc163	-0,026176	1
Farsa	-0,026181	1
C430042M11Rik	-0,026306	1
Zfp362	-0,026471	1
Stk16	-0,026489	1
Atg3	-0,02661	1

Acox3	-0,026623	1
Cast	-0,026676	1
Senp2	-0,026751	1
Hnrnph2	-0,026885	1
Hpcal1	-0,026897	1
Mpp6	-0,026999	1
Vasp	-0,027144	1
Rasa1	-0,027201	1
Drg1	-0,027211	1
Kdm1a	-0,027294	1
Ccnt1	-0,027324	1
Immt	-0,027535	1
Usf1	-0,027755	1
Cep89	-0,027775	1
Copg1	-0,027788	1
Gm6204	-0,027834	1
3110040N11Rik	-0,027831	1
Gm16238	-0,027882	1
Gm5835	-0,027859	1
Megf9	-0,027862	1
Arl6	-0,028015	1
C920021L13Rik	-0,028148	1
Mrpl30	-0,028057	1
Dtd1	-0,028195	1
Stamos	-0,028377	1
2610002M06Rik	-0,028639	1
Erfe	-0,0288	1
Smg5	-0,028925	1
Hk1	-0,028901	1
Klf8	-0,029073	1
Slc7a7	-0,02923	1
Nanos1	-0,029152	1
Ago2	-0,029211	1
Gm43275	-0,029416	1
Gmip	-0,029497	1
Ilvbl	-0,029569	1
Pvt1	-0,029568	1
Ankrd39	-0,029749	1
Gm11410	-0,029684	1
Acsl1	-0,029654	1
Ctbp2	-0,029796	1
Mgat4a	-0,029804	1
Ctss	-0,029758	1
Ube4a	-0,029927	1
Rpa1	-0,029876	1
Rpusd3	-0,030055	1
Cct2	-0,030206	1
Cux1	-0,030271	1
Slc6a13	-0,030498	1
Oma1	-0,030551	1
Gm13215	-0,030697	1
Aim1	-0,030706	1

Kansl2	-0,030791	1
Gm11605	-0,0309	1
Asf1b	-0,031048	1
Naa20	-0,031001	1
Ccny	-0,030976	1
Dctn4	-0,030951	1
Tmx3	-0,031078	1
Pak2	-0,031218	1
Mknk1	-0,031283	1
Gpatch4	-0,031402	1
Rgs19	-0,031408	1
Cenpc1	-0,031481	1
Nek6	-0,031544	1
Ube2d3	-0,031518	1
Fat1	-0,031574	1
Eif3a	-0,031698	1
Pam16	-0,031799	1
Itpkc	-0,031805	1
Hdac3	-0,031812	1
Polr3h	-0,031949	1
Ccdc126	-0,032044	1
Foxk1	-0,032038	1
Adipor1	-0,032015	1
Ctdnep1	-0,032112	1
Matr3	-0,032111	1
Hmcn2	-0,032357	1
Capza2	-0,032427	1
Phf20l1	-0,032589	1
Ran	-0,032611	1
Ing5	-0,032678	1
Itm2c	-0,03267	1
Slc4a2	-0,032849	1
Dtx3l	-0,032922	1
Gm1943	-0,032987	1
Ago4	-0,033148	1
Marveld1	-0,03307	1
Ocel1	-0,033447	1
Phc1	-0,033398	1
Gfm1	-0,033479	1
Wdr46	-0,033599	1
Tbc1d14	-0,033595	1
Pdcd6	-0,033666	1
Gpr89	-0,033748	1
Cnnm3	-0,034081	1
Rita1	-0,03419	1
Dnajc3	-0,034159	1
Gm6394	-0,034303	1
Rps26	-0,034347	1
Crk	-0,034415	1
Nelfe	-0,034483	1
Sclt1	-0,034932	1
Tmcc1	-0,035035	1

Gm7236	-0,035014	1
Psmb7	-0,034983	1
Yipf7	-0,035107	1
Bag1	-0,035149	1
Lgals8	-0,035186	1
Wdfy4	-0,035179	1
E2f6	-0,035363	1
Pes1	-0,035364	1
Gm38157	-0,035662	1
Ube2e1	-0,035673	1
Ndufa7	-0,03571	1
Tpr	-0,035675	1
Yars	-0,035899	1
Mrpl9	-0,036017	1
H2-K1	-0,036004	1
Tmem258	-0,035967	1
Gm13204	-0,036276	1
Atp13a2	-0,0363	1
Klhl28	-0,036363	1
Ctsd	-0,036433	1
Map2k1	-0,036476	1
Snx7	-0,036686	1
Gm9169	-0,036654	1
Mb21d1	-0,036741	1
Gm6560	-0,036846	1
Sp140	-0,036893	1
Slc7a6os	-0,036955	1
Eef1b2	-0,037025	1
Map1s	-0,037104	1
Rpl36-ps4	-0,03728	1
Reep4	-0,037436	1
Irgq	-0,037549	1
AI506816	-0,03761	1
Fopnl	-0,037743	1
Alkbh8	-0,037908	1
Ltc4s	-0,037987	1
Banp	-0,038128	1
Usp21	-0,038214	1
Pde4dip	-0,038274	1
Ak6	-0,038472	1
Timp1	-0,038553	1
Zbtb7b	-0,03857	1
Psd	-0,038894	1
Sdsl	-0,039023	1
Tmod3	-0,039073	1
Cpeb3	-0,03932	1
Crip1	-0,039274	1
Morf4l2	-0,039402	1
Pdia4	-0,039441	1
Timm29	-0,039498	1
Gm37677	-0,03958	1
Gm43707	-0,039569	1

Cd276	-0,039623	1
Atad2b	-0,039652	1
Igsf8	-0,039668	1
Gm22516	-0,039796	1
Hist1h4i	-0,039864	1
Bag4	-0,040028	1
Gm43106	-0,040123	1
Rps6ka1	-0,040051	1
Mff	-0,040114	1
Ctps	-0,040217	1
7330423F06Rik	-0,040369	1
Psmb9	-0,040405	1
Tspan3	-0,040419	1
Rab11a	-0,040425	1
Cpne3	-0,040454	1
Ufsp1	-0,040559	1
Gm15720	-0,040662	1
Serinc1	-0,04075	1
Chkb	-0,040884	1
Smagp	-0,041012	1
Rab3gap1	-0,040991	1
Slc25a13	-0,041058	1
Cyhr1	-0,041188	1
Foxn3	-0,041189	1
Kif23	-0,041195	1
Lclat1	-0,041338	1
Xpo1	-0,041445	1
2610008E11Rik	-0,041483	1
H2-Q6	-0,041502	1
Rpp21	-0,041502	1
St3gal3	-0,041577	1
Khsrp	-0,041727	1
Zswim8	-0,041965	1
Tdrd7	-0,042123	1
Zfp54	-0,042064	1
Gbf1	-0,042082	1
Ypel3	-0,042127	1
Baz1b	-0,042108	1
Ccnc	-0,0423	1
Cks1b	-0,042597	1
Klhdc1	-0,042893	1
Stam	-0,043505	1
Gm4374	-0,043627	1
Itm2b	-0,043626	1
Rcn1	-0,04391	1
Zfp445	-0,043868	1
Use1	-0,043904	1
Zbtb48	-0,043963	1
Tmem234	-0,044103	1
Mss51	-0,0442	1
Tmem39a	-0,044161	1
Pkn1	-0,044235	1

Plxnb2	-0,044281	1
Cmtm6	-0,044582	1
Homer1	-0,044748	1
Gm14769	-0,044989	1
Dgcr6	-0,045075	1
Gm4950	-0,045106	1
Map3k14	-0,045081	1
Gm12267	-0,045233	1
RP24-499N24.6	-0,045197	1
Dedd2	-0,045175	1
Rpl27a-ps2	-0,045499	1
Snrpg	-0,045512	1
Smurf2	-0,045664	1
Nfkbiz	-0,045815	1
Mrfap1	-0,045941	1
Net1	-0,046049	1
Rpn1	-0,046128	1
Clpx	-0,046196	1
Srsf3	-0,046248	1
2310035C23Rik	-0,046268	1
Ddb1	-0,046254	1
Gm15975	-0,046423	1
Prr3	-0,046376	1
Pik3c2a	-0,046477	1
Gm7363	-0,046882	1
Thap8	-0,046969	1
Thap2	-0,046979	1
Rala	-0,046957	1
Gm14048	-0,047136	1
Zdhhc20	-0,047109	1
Cd302	-0,047317	1
Utp18	-0,047527	1
Nsd2	-0,047525	1
Clec4a3	-0,047571	1
Rab11fip2	-0,04762	1
Aqr	-0,047598	1
Trappc2l	-0,047615	1
Olfr460	-0,047713	1
Grk2	-0,04786	1
1110038B12Rik	-0,047945	1
P4ha1	-0,048182	1
Gm10658	-0,048413	1
Calhm2	-0,048475	1
Gm24951	-0,048476	1
Ifitm3	-0,048537	1
Gm3571	-0,04864	1
Cct5	-0,048648	1
Mcts2	-0,048716	1
Dek	-0,048756	1
Zc3h14	-0,048932	1
Pls3	-0,048928	1
Zfp260	-0,049001	1

Aen	-0,049005	1
Emc2	-0,049129	1
D8Ertd738e	-0,049109	1
Cenpe	-0,049154	1
Canx	-0,049243	1
AW549877	-0,049347	1
Ptpn22	-0,049302	1
Dcaf4	-0,049572	1
Ndufs2	-0,049718	1
Fbxo30	-0,049891	1
Plaur	-0,050018	1
Cep83	-0,049962	1
Aprt	-0,049958	1
Ssr4	-0,050127	1
Prpf40a	-0,050187	1
Kri1	-0,050286	1
Idh3b	-0,0503	1
Cxx1b	-0,050377	1
Ulbp1	-0,050483	1
Atp5l-ps1	-0,050838	1
Slc30a9	-0,0508	1
Acin1	-0,050831	1
Gba	-0,050759	1
Dbp	-0,050916	1
Arpc5	-0,050868	1
Coq3	-0,050963	1
Lin9	-0,050993	1
Ruvbl1	-0,051102	1
Efcab14	-0,0513	1
Gm14303	-0,051264	1
Ash1l	-0,051384	1
Ctnnb1	-0,05135	1
Ltn1	-0,051452	1
Pigx	-0,051502	1
Cep76	-0,051645	1
Mrps15	-0,05158	1
Trabd	-0,051593	1
Dad1	-0,051669	1
Plcg2	-0,051759	1
Crebbp	-0,051788	1
Cbfb	-0,051785	1
Cenpi	-0,052043	1
Sap30bp	-0,05205	1
Them4	-0,051964	1
Scoc	-0,05198	1
Cfap74	-0,052327	1
Pigg	-0,052263	1
Fam206a	-0,052276	1
Gna11	-0,052325	1
Slc25a43	-0,052425	1
Gm17994	-0,052578	1
mt-Nd4	-0,0526	1

Gpatch11	-0,052868	1
Tprkb	-0,05319	1
Ddx11	-0,053306	1
Msh6	-0,053251	1
Foxo3	-0,053292	1
Rps8-ps3	-0,053415	1
Slc2a8	-0,053602	1
Sgol1	-0,053593	1
Heatr6	-0,053645	1
Zfp52	-0,053699	1
Gadd45a	-0,053733	1
Cbx1	-0,053667	1
Camk2d	-0,053813	1
Prmt1	-0,05383	1
Zfp560	-0,053989	1
Klc1	-0,05402	1
Pcna-ps2	-0,054332	1
Snhg17	-0,054251	1
Per1	-0,054281	1
Arrb2	-0,054394	1
Ccdc181	-0,054479	1
Inip	-0,054538	1
Plp2	-0,054657	1
Zcrb1	-0,05482	1
Lig3	-0,054873	1
Tmem147	-0,054916	1
Cks2	-0,054952	1
Eif2s1	-0,055015	1
Emc6	-0,055057	1
Pgk1	-0,055189	1
Gart	-0,055199	1
Isoc1	-0,055248	1
Rpl8	-0,055205	1
Gm10736	-0,055335	1
Tspyl3	-0,055263	1
Aldh7a1	-0,055263	1
Tmem209	-0,055435	1
Mrpl40	-0,055355	1
Copa	-0,05541	1
Rbms1	-0,055448	1
mt-Cytb	-0,055605	1
Sem1	-0,055605	1
Tmem159	-0,055901	1
Rhod	-0,055901	1
Jaml	-0,055925	1
Ska1	-0,05594	1
Dcakd	-0,055978	1
Ndufaf5	-0,056037	1
Klhl5	-0,056219	1
Gm6368	-0,056349	1
RP23-159E10.1	-0,056489	1
Irf2	-0,056531	1

Rab3a	-0,056726	1
Phf2	-0,056789	1
Timm21	-0,056869	1
Tle1	-0,057078	1
Dhx15	-0,057074	1
Mrps14	-0,057165	1
Gm20568	-0,057343	1
Pea15a	-0,057352	1
Tonsl	-0,057474	1
Atad2	-0,057728	1
Ppp4r3a	-0,057754	1
Ddx54	-0,057809	1
Card14	-0,057959	1
Prrc2a	-0,057995	1
Mrto4	-0,057958	1
Zfand5	-0,058111	1
Zfp467	-0,058245	1
Heatr1	-0,058175	1
Urb2	-0,058273	1
Tspyl4	-0,058497	1
Kifap3	-0,058505	1
Eif5a	-0,058544	1
Npm1	-0,058467	1
Gnpat	-0,058603	1
Klhl21	-0,058555	1
Far1	-0,058593	1
Mrpl50	-0,05863	1
Cep41	-0,058684	1
Fbxo21	-0,058739	1
Ctif	-0,05915	1
Tmem160	-0,059054	1
Edem1	-0,059147	1
Hal	-0,059435	1
6330403L08Rik	-0,059598	1
Stag2	-0,0596	1
Wasf2	-0,059585	1
Gm26881	-0,059689	1
Msrb1	-0,059695	1
Sirt2	-0,059847	1
Rhoq	-0,059898	1
Cdc42	-0,060046	1
Lyrm4	-0,06013	1
Mcts1	-0,060283	1
Zfyve9	-0,060387	1
Vps35	-0,060354	1
Scfd2	-0,060555	1
Lrrc61	-0,060614	1
Pnpla2	-0,060572	1
Tal1	-0,060678	1
Rnaseh2c	-0,060732	1
Prdx6	-0,060652	1
Gm10313	-0,060813	1

Gm16399	-0,060897	1
Tbrg4	-0,060866	1
Aimp1	-0,060986	1
Prc1	-0,06121	1
Pds5b	-0,061343	1
Cdk2	-0,061345	1
Imp3	-0,061326	1
Rfc5	-0,061409	1
Irf2bp2	-0,061381	1
Ube2k	-0,061406	1
Hdac6	-0,061512	1
Mrgbp	-0,061464	1
Tmem167b	-0,061523	1
Rps10	-0,061457	1
1110035H17Rik	-0,061594	1
Rtn3	-0,061581	1
Pcf11	-0,061738	1
1600014C23Rik	-0,061757	1
A430105J06Rik	-0,061757	1
Galnt1	-0,061763	1
Cpsf7	-0,062016	1
Lmbr1l	-0,062137	1
Otud4	-0,06208	1
Arhgef39	-0,062175	1
Pdxdc1	-0,062168	1
Polk	-0,062208	1
Vmac	-0,062373	1
Snhg9	-0,06246	1
Tmem79	-0,062625	1
Zbtb18	-0,062588	1
Rab11b	-0,062612	1
Gm4285	-0,062761	1
Rpap2	-0,062831	1
Zfand6	-0,062825	1
Lck	-0,062922	1
Dcbld2	-0,062933	1
Rab6a	-0,062946	1
Nrp2	-0,062918	1
Gm6159	-0,063127	1
Emilin2	-0,063222	1
Pet100	-0,063282	1
Bcl6	-0,063275	1
Taf15	-0,063258	1
Zfp512	-0,063586	1
Akr1b3	-0,063755	1
Slamf9	-0,063789	1
Psmb10	-0,063947	1
Dnm1l	-0,063917	1
F10	-0,063973	1
Ifi27l2a	-0,06418	1
Rab9	-0,064159	1
Dnm2	-0,064318	1

Rras2	-0,064481	1
Lfng	-0,064641	1
Pnisr	-0,06458	1
Mthfd1	-0,064729	1
Mier1	-0,064723	1
Smad6	-0,064766	1
Pphln1	-0,064906	1
Ubb	-0,064899	1
Tnrc18	-0,064999	1
Snx5	-0,065001	1
Tmed9	-0,065013	1
Zcchc7	-0,065127	1
Fundc1	-0,065233	1
Lyrm9	-0,065279	1
Dnajb14	-0,0653	1
Mfsd12	-0,065311	1
Gm12912	-0,065426	1
Elp5	-0,065487	1
Afg3l1	-0,065512	1
Dmap1	-0,065843	1
Gm15950	-0,065874	1
Nup133	-0,0659	1
Ankmy2	-0,065863	1
Psme1	-0,065885	1
Ndufs8	-0,066036	1
Tnks	-0,06601	1
Eny2	-0,06597	1
2200002J24Rik	-0,066119	1
Rufy2	-0,066139	1
Sdc3	-0,066147	1
Snx2	-0,066188	1
Nfe2l1	-0,066215	1
Slc8a1	-0,066432	1
Cenpw	-0,066367	1
Lrpap1	-0,066497	1
Fzd2	-0,066563	1
R74862	-0,066668	1
Plcd3	-0,066782	1
Tnfrsf10b	-0,066822	1
Nusap1	-0,06676	1
Dctn1	-0,066851	1
Gm15634	-0,066891	1
Tex261	-0,067126	1
Tor3a	-0,06717	1
Gga1	-0,067212	1
Nudt4	-0,067531	1
Gm12466	-0,067677	1
Slc22a17	-0,067654	1
Gm15530	-0,067653	1
Zfp59	-0,067653	1
Mllt6	-0,067863	1
Zmiz2	-0,067918	1

Gm7589	-0,068008	1
Gm15327	-0,068152	1
Gm10676	-0,068152	1
Hdc	-0,068152	1
Gnb5	-0,068343	1
Ikzf1	-0,06836	1
Gabpa	-0,068451	1
Atxn1	-0,068516	1
Pcgf1	-0,068699	1
Nipal3	-0,068805	1
Gm44269	-0,068757	1
Xylb	-0,068757	1
Gm14403	-0,068757	1
Vezt	-0,0688	1
Gm13341	-0,068897	1
Etfdh	-0,068924	1
Rbck1	-0,068868	1
Yy1	-0,068899	1
Fzr1	-0,069029	1
Snx3	-0,068954	1
Rps6ka3	-0,069106	1
Atp6v0b	-0,069148	1
Gm26917	-0,069071	1
Rbm45	-0,069267	1
Gm2214	-0,069373	1
Ncf1	-0,069357	1
Chpt1	-0,069495	1
Mt1	-0,069481	1
Tgif2	-0,069595	1
Rgl2	-0,069692	1
Cnih1	-0,069658	1
Fam76a	-0,070116	1
Tpm4	-0,07032	1
Herpud1	-0,070261	1
Vps50	-0,070453	1
Elob	-0,070537	1
Edf1	-0,070471	1
Impa2	-0,070577	1
Gm43756	-0,070971	1
Gdf15	-0,071104	1
2410080I02Rik	-0,071172	1
5430402O13Rik	-0,071154	1
Sf3b4	-0,071442	1
Tor2a	-0,071377	1
Mtf2	-0,071687	1
Zmym4	-0,071821	1
Lgals3	-0,071888	1
Lca5	-0,072001	1
mt-Nd6	-0,071977	1
Kpna3	-0,072037	1
Clcn3	-0,071951	1
Ube2d-ps	-0,072057	1

Ak1	-0,072311	1
Cd109	-0,072338	1
Gm11474	-0,072366	1
Ralgapa2	-0,072484	1
Gm45762	-0,072497	1
Wdr20	-0,072649	1
Gm43501	-0,072723	1
Pik3ip1	-0,072691	1
Polr1d	-0,072722	1
Mrpl51	-0,072909	1
Mus81	-0,073126	1
Cbx7	-0,073068	1
Fabp3	-0,073168	1
Pigk	-0,073486	1
Myo18a	-0,073611	1
4833418N02Rik	-0,073789	1
Nop10	-0,073926	1
Prdm15	-0,074133	1
Gli1	-0,074082	1
Aip	-0,074098	1
Gm42595	-0,074228	1
Aldh3b1	-0,074192	1
Xrcc6	-0,074187	1
Nufip2	-0,074232	1
Impdh2	-0,074268	1
Prosc	-0,074402	1
Gltscr2	-0,074403	1
Ufc1	-0,074581	1
Csnk2a2	-0,074569	1
Slc35b4	-0,074735	1
Ndufa1	-0,074808	1
Exoc2	-0,074874	1
Gm8618	-0,074981	1
Srpk2	-0,075143	1
Tmem29	-0,075174	1
Asns	-0,07518	1
Nsun4	-0,075258	1
Ppib	-0,075331	1
Pex16	-0,07536	1
Dnajc1	-0,075385	1
Erh	-0,075509	1
Zfp598	-0,075689	1
Endod1	-0,075822	1
Gm6210	-0,075879	1
Odc1	-0,075902	1
Utp14a	-0,075885	1
H2-Q5	-0,076162	1
1110008L16Rik	-0,07626	1
Helz	-0,076277	1
Cd99l2	-0,076387	1
Parp3	-0,076476	1
Rabac1	-0,076451	1

Scrib	-0,076588	1
Rnf167	-0,076552	1
Atg10	-0,076763	1
Habp4	-0,076825	1
Ankrd46	-0,076939	1
Gm6565	-0,077143	1
AI606181	-0,077093	1
Csnk2a1	-0,077266	1
Fads6	-0,077701	1
Fam83a	-0,077767	1
Ptges2	-0,077793	1
Shcbp1l	-0,077921	1
Mtus2	-0,078026	1
Ascl2	-0,078248	1
Ddn	-0,078244	1
Ap3s1	-0,078331	1
Dennd1a	-0,078414	1
Oxr1	-0,07838	1
Brk1	-0,078436	1
Fam96a	-0,078476	1
Tm2d1	-0,07861	1
Mapk1ip1	-0,078688	1
Spats2	-0,078748	1
Rps23-ps2	-0,078754	1
Prkaa1	-0,078807	1
Vegfa	-0,078817	1
Myl12a	-0,07893	1
Sin3a	-0,078922	1
Alg12	-0,078974	1
Gm15427	-0,079094	1
Mrps9	-0,079063	1
Gm5867	-0,079182	1
Trnt1	-0,079221	1
Ube2g1	-0,07915	1
Uap1	-0,07923	1
Prrg4	-0,079281	1
2810433D01Rik	-0,07942	1
2410002F23Rik	-0,079472	1
Stk40	-0,079592	1
Fam171b	-0,079714	1
Yipf4	-0,079668	1
Spata6	-0,079818	1
N4bp2l1	-0,079779	1
Gm43071	-0,079918	1
RP23-277D1.1	-0,079918	1
Rfxap	-0,079892	1
Prkcd	-0,0799	1
Anp32b-ps1	-0,079961	1
Maml3	-0,079995	1
Aurkaip1	-0,079977	1
Abr	-0,079973	1
Ift46	-0,080074	1

Cdca8	-0,080063	1
Pank1	-0,080194	1
Kdm3a	-0,080275	1
Mplkip	-0,080345	1
Qdpr	-0,080412	1
Unc13d	-0,080501	1
Hint2	-0,080533	1
Anapc1	-0,080632	1
Vhl	-0,080564	1
Ap1s1	-0,080616	1
Relt	-0,080653	1
Zdhhc3	-0,080822	1
Gm45902	-0,080928	1
Thoc7	-0,080865	1
Tmeff1	-0,081046	1
Ei24	-0,08102	1
Pigu	-0,081138	1
9030617O03Rik	-0,081117	1
Lsm6	-0,081172	1
Gstt3	-0,081285	1
Mapk9	-0,081333	1
Dnaja3	-0,08126	1
Lamtor1	-0,08138	1
Cep170	-0,081358	1
Eif3i	-0,081512	1
Trim59	-0,081615	1
Rc3h1	-0,081617	1
Gm43096	-0,081704	1
Rpl7a-ps5	-0,08167	1
RP23-261O3.5	-0,081698	1
Mpc2	-0,081781	1
Mib1	-0,081818	1
Mre11a	-0,081792	1
Prkd3	-0,081856	1
Rps11-ps2	-0,081986	1
Pole3	-0,082141	1
Ctsz	-0,082119	1
Tmem63a	-0,082169	1
Ate1	-0,082276	1
Sar1b	-0,082526	1
Slc12a7	-0,08247	1
Ensa	-0,082823	1
P3h1	-0,083186	1
Tap1	-0,083215	1
Mrpl20	-0,083325	1
Pfdn1	-0,083309	1
RP23-255F14.4	-0,083457	1
Zfp444	-0,083493	1
Arhgap12	-0,08354	1
Ip6k1	-0,083475	1
Stt3a	-0,083615	1
Mtor	-0,083611	1

Mrpl24	-0,083603	1
Stxbp1	-0,083931	1
Gm9769	-0,083954	1
Bcl2l13	-0,08397	1
Slc25a28	-0,084061	1
Traip	-0,084192	1
Bub1b	-0,084317	1
Gm19503	-0,084448	1
Bcl7a	-0,084355	1
Ethe1	-0,084368	1
1190005I06Rik	-0,084573	1
Cpox	-0,084614	1
Clk1	-0,085001	1
Gm4617	-0,085111	1
Atp8b2	-0,085461	1
Arl6ip1	-0,085511	1
Fntb	-0,085723	1
Lmo4	-0,085716	1
Ahnak	-0,085728	1
Fam63b	-0,085918	1
Kdm6b	-0,086023	1
Rnf10	-0,085986	1
Gm10443	-0,085973	1
Dbi	-0,086047	1
Pfn2	-0,086051	1
Ppp2r1a	-0,086121	1
Ier3ip1	-0,086094	1
Cog5	-0,086262	1
Rwdd4a	-0,08634	1
Sft2d2	-0,08638	1
Acot13	-0,086458	1
Rfx7	-0,086678	1
Ms4a6b	-0,086786	1
Ccdc88b	-0,086797	1
Coq10b	-0,086941	1
Avpi1	-0,086872	1
Ube2j2	-0,08694	1
Sf3b2	-0,086937	1
Tmem94	-0,087015	1
2700099C18Rik	-0,087013	1
M6pr	-0,086957	1
Gnai3	-0,086998	1
Sptlc1	-0,087105	1
Uty	-0,087282	1
Slc9a6	-0,087279	1
Gm13578	-0,087363	1
Gm9892	-0,08744	1
Hspa9-ps1	-0,087717	1
Eef1d	-0,087681	1
5330426L24Rik	-0,087885	1
Hsf2	-0,087903	1
Gm13397	-0,087997	1

Srbd1	-0,088117	1
Dcxr	-0,088064	1
Cd52	-0,088302	1
Hadha	-0,088463	1
1700029J07Rik	-0,088631	1
Yipf6	-0,08862	1
Iqgap1	-0,088737	1
Gm7722	-0,08883	1
Dennd6a	-0,088763	1
Idua	-0,088947	1
Odf2	-0,088858	1
Crnkl1	-0,088889	1
Hnrnph3	-0,088883	1
Tmem109	-0,089048	1
Smarca2	-0,089095	1
Isca2	-0,089172	1
Fut8	-0,089211	1
Hspa2	-0,089198	1
Aldh3a2	-0,089384	1
Kdm5a	-0,089456	1
Psmd4	-0,089704	1
Csrp2bp	-0,090064	1
Rbbp5	-0,090106	1
Ddx19a	-0,090149	1
Zfp746	-0,09023	1
Dctpp1	-0,090242	1
Sptlc2	-0,090317	1
Rpl36	-0,090298	1
Rfng	-0,090537	1
Sowahc	-0,090516	1
Rnf7	-0,090482	1
Icam1	-0,090617	1
Vamp5	-0,090661	1
Rpl34	-0,090665	1
Gm5614	-0,090817	1
Gm7984	-0,090836	1
Snora31	-0,09087	1
Tgfb1i1	-0,091198	1
Tti1	-0,091198	1
Bag6	-0,091178	1
Ankhd1	-0,091516	1
Kpna1	-0,091562	1
Rplp1	-0,091724	1
Ric8a	-0,091933	1
Smyd3	-0,091857	1
Rictor	-0,091947	1
Gm45806	-0,092299	1
D930015E06Rik	-0,092439	1
Soat1	-0,092621	1
Pla2g4a	-0,092717	1
Alkbh1	-0,092844	1
9230114K14Rik	-0,092908	1

Leprotl1	-0,092875	1
Atxn3	-0,092965	1
Eif3e	-0,093065	1
Gm7847	-0,093197	1
Tatdn2	-0,093161	1
Man1a2	-0,093438	1
Krtcap2	-0,093449	1
Higd2a	-0,093475	1
Ddx39	-0,093577	1
Creld1	-0,093749	1
Gm44950	-0,093921	1
Mrm1	-0,093947	1
Syngr1	-0,093861	1
Adat1	-0,094102	1
Rmdn1	-0,094093	1
Tbpl1	-0,094213	1
Rcc2	-0,094185	1
Kdm7a	-0,094339	1
Tbca	-0,094295	1
Hagh	-0,094407	1
Ptcd3	-0,094462	1
Ndufa4	-0,09455	1
Agpat4	-0,094575	1
Ogg1	-0,094807	1
Pla2g6	-0,094846	1
Strn4	-0,094834	1
Hsf2bp	-0,094941	1
Hdac5	-0,094923	1
Mir99ahg	-0,094953	1
Lta4h	-0,094995	1
Gm6548	-0,095183	1
Tesk1	-0,095179	1
Ddx49	-0,095331	1
A730071L15Rik	-0,095417	1
Ophn1	-0,095351	1
Slc25a24	-0,09541	1
Emp3	-0,095418	1
Atp13a3	-0,095353	1
Celf3	-0,095616	1
Slc25a2	-0,095733	1
Atp6v0e2	-0,095683	1
Gm15920	-0,095815	1
Gm6415	-0,095907	1
Spred2	-0,09593	1
Cct8	-0,095891	1
Errfi1	-0,09604	1
Eif3k	-0,096105	1
Uxt	-0,096263	1
Gm11942	-0,096369	1
4930532G15Rik	-0,096507	1
Ccdc191	-0,096478	1
Snrnp48	-0,096582	1

Spn	-0,096703	1
Gm45292	-0,096688	1
Ogfrl1	-0,096745	1
Itpr2	-0,096689	1
C130013H08Rik	-0,096755	1
Herc4	-0,096756	1
Arhgap45	-0,096763	1
Rny1	-0,09685	1
Atg5	-0,09693	1
Mrip-ps	-0,097002	1
Qrsl1	-0,097041	1
Rps24-ps2	-0,096995	1
Kyat1	-0,09727	1
Ccdc14	-0,097427	1
Cyp2c55	-0,09741	1
Cdyl2	-0,09744	1
Ptpn23	-0,09748	1
Zbtb44	-0,097497	1
Mief2	-0,097649	1
Sptbn1	-0,097648	1
Psd3	-0,097877	1
Nck1	-0,098048	1
Prelid2	-0,098237	1
Bbip1	-0,098241	1
Ap4s1	-0,098396	1
4833420G17Rik	-0,098382	1
Pts	-0,098532	1
Nfya	-0,09845	1
Nfx1	-0,098596	1
Zc2hc1a	-0,098675	1
Stk19	-0,09869	1
Nptxr	-0,098903	1
Crlf3	-0,098905	1
Prps1	-0,099028	1
Btg1	-0,09909	1
Nckipsd	-0,099168	1
Sort1	-0,099309	1
Ciapin1	-0,099443	1
Dxo	-0,09948	1
RP24-240E7.1	-0,09955	1
Ddit3	-0,099518	1
Pip4k2b	-0,099492	1
Ezr	-0,099455	1
Fnbp4	-0,09968	1
Tada2a	-0,099828	1
Vat1	-0,099874	1
Tmem219	-0,1	1
Tmem168	-0,10003	1
Slc15a4	-0,10012	1
Taf1	-0,10023	1
Fam129c	-0,10031	1
Pdrg1	-0,10035	1

Nemf	-0,10048	1
Ero1lb	-0,10076	1
Nudt3	-0,10078	1
Ncoa1	-0,10087	1
Bin1	-0,10087	1
Polr3gl	-0,10087	1
Tars2	-0,10099	1
Itga5	-0,10106	1
Chd7	-0,10124	1
Mnd1-ps	-0,10135	1
Ap5m1	-0,10177	1
Uso1	-0,10182	1
Mettl5	-0,10195	1
Gm26542	-0,10209	1
Btbd19	-0,1021	1
RP24-325P4.5	-0,10206	1
Baz2a	-0,10219	1
Zfp329	-0,10233	1
Gm37254	-0,10241	1
Gm15772	-0,10239	1
Slc36a4	-0,10243	1
Wrap73	-0,10254	1
Gm45836	-0,10258	1
Trip6	-0,10259	1
Erp44	-0,10256	1
Plek	-0,10282	1
Rpl37	-0,10291	1
Orc4	-0,10302	1
Gfpt1	-0,10296	1
Tfdp1	-0,10313	1
Gm11914	-0,10325	1
C920009B18Rik	-0,10321	1
Thap4	-0,10331	1
Rack1	-0,10329	1
Slc6a8	-0,10341	1
Gstm1	-0,10354	1
Agap3	-0,10348	1
Gng2	-0,10355	1
Stub1	-0,10358	1
Gm5881	-0,10384	1
Gm14539	-0,10384	1
Sash1	-0,10378	1
Ankrd11	-0,10384	1
Eea1	-0,10395	1
Gm6654	-0,1041	1
Prpf39	-0,10409	1
Cd72	-0,10409	1
Gm11221	-0,10423	1
Ccdc9	-0,10425	1
Bri3bp	-0,1043	1
Klhdc4	-0,10452	1
H2afv	-0,10464	1

Dcaf11	-0,10479	1
Fam102a	-0,1048	1
Slc45a3	-0,10512	1
Ggct	-0,10521	1
Rpsa-ps9	-0,10555	1
Rgs12	-0,1055	1
Tbc1d2b	-0,10563	1
Mark2	-0,10568	1
Glmp	-0,10565	1
Cnot7	-0,10568	1
2300009A05Rik	-0,10601	1
Setd7	-0,10608	1
Sord	-0,10623	1
Gm7123	-0,10644	1
Smim11	-0,10643	1
Dctn2	-0,1064	1
Flot2	-0,10649	1
Rara	-0,10652	1
Sgcb	-0,10679	1
Mr1	-0,10678	1
Fcer1g	-0,10685	1
Tom1l2	-0,107	1
Fkbp1a	-0,10716	1
Arfgap1	-0,10736	1
Lcor	-0,10741	1
Rfc1	-0,10739	1
Tprgl	-0,10749	1
Pik3r2	-0,10759	1
Gfod1	-0,10763	1
Inpp5d	-0,1077	1
Yeats2	-0,1079	1
Gm7634	-0,10797	1
Lancl1	-0,108	1
Pgrmc2	-0,10804	1
Gstp1	-0,1081	1
Gm11970	-0,10808	1
Clspn	-0,10823	1
A430010J10Rik	-0,10817	1
Tmem64	-0,10819	1
RP23-65M10.2	-0,10827	1
Agk	-0,10869	1
Stk38l	-0,10875	1
Gm6140	-0,10893	1
Pex6	-0,10904	1
Tsg101	-0,10906	1
Ube2h	-0,10919	1
Ripk2	-0,10934	1
Rpl22	-0,1095	1
Esyt1	-0,10979	1
Gatb	-0,10998	1
Cyth2	-0,11011	1
Vps29	-0,11011	1

Cinp	-0,11018	1
Gm9354	-0,11018	1
Mcph1	-0,1102	1
A330069E16Rik	-0,11039	1
Atp6v0c	-0,11058	1
Gm26830	-0,11064	1
Pycrl	-0,11074	1
Klf6	-0,11071	1
Zfp407	-0,11083	1
Cops6	-0,11101	1
Srsf6	-0,11105	1
Pak1	-0,11103	1
Vezf1	-0,11101	1
Ywhaq	-0,11095	1
Taf2	-0,11107	1
Hk2	-0,11132	1
Bloc1s6	-0,11136	1
Alox5	-0,1117	1
Ubr5	-0,11186	1
Kxd1	-0,112	1
Gm15730	-0,11205	1
Shisa5	-0,11207	1
Atf7ip	-0,11224	1
Wdr48	-0,1122	1
Pth1r	-0,11231	1
Lamtor5	-0,11254	1
Fgd2	-0,1126	1
Asna1	-0,11263	1
Rbm22	-0,11273	1
Hotairm1	-0,11289	1
Sbf2	-0,11303	1
Nap1l1	-0,11312	1
Map2k4	-0,11355	1
Brdt	-0,11365	1
Tmem256	-0,11367	1
Paf1	-0,11389	1
Gm43793	-0,11403	1
Rwdd2b	-0,11413	1
Hspbp1	-0,11406	1
Csde1	-0,11414	1
Mpg	-0,11427	1
Glo1	-0,1144	1
Nr2c2	-0,11461	1
Apoa1bp	-0,11463	1
Cenpv	-0,11467	1
Ints8	-0,11467	1
Echdc1	-0,11482	1
Gm28187	-0,11487	1
Tmem120a	-0,11503	1
Anapc7	-0,11523	1
Ep400	-0,11535	1
Tbl1x	-0,11548	1

Bin2	-0,11559	1
Cog4	-0,11565	1
Smarcal1	-0,11571	1
Cyb5rl	-0,11582	1
Usp40	-0,11596	1
Osbpl7	-0,11605	1
Csrp2	-0,11628	1
Kdm6a	-0,11627	1
Mpp5	-0,11632	1
Gm15903	-0,11636	1
Mri1	-0,11638	1
Gm43387	-0,11655	1
Rnf135	-0,11649	1
Ppox	-0,11659	1
Prdx5	-0,11657	1
Nemp1	-0,11667	1
Itga4	-0,11675	1
Sec61a1	-0,11678	1
Sfxn2	-0,11693	1
Ccnd2	-0,11695	1
Ccdc167	-0,11703	1
F830115B05Rik	-0,11697	1
H6pd	-0,11714	1
Fbxl20	-0,11709	1
Rabl3	-0,1172	1
Jtb	-0,11724	1
1600010M07Rik	-0,11737	1
Csf1r	-0,11747	1
Kcmf1	-0,11764	1
Elac1	-0,11769	1
Cd2bp2	-0,11767	1
Gm7102	-0,11781	1
Pepd	-0,11776	1
Gm12038	-0,11792	1
Eif3h	-0,11791	1
Cdr2	-0,11802	1
Uck1	-0,11799	1
Rbm4b	-0,11818	1
Tdp2	-0,11823	1
Alg6	-0,11854	1
Gm7452	-0,11845	1
Arl5b	-0,1185	1
Rps13-ps2	-0,11873	1
Hibadh	-0,1188	1
Snta1	-0,11877	1
Enox2	-0,11898	1
Tiparp	-0,11906	1
Rnf111	-0,11922	1
Nkapl	-0,1193	1
C2cd2	-0,11937	1
Gucd1	-0,11936	1
Pin4	-0,11943	1

Stx18	-0,11975	1
Cnot1	-0,11995	1
Sdc4	-0,1199	1
Zfp780b	-0,12022	1
Dnlz	-0,12024	1
Fubp3	-0,12023	1
Hmgcs1	-0,12016	1
Map3k20	-0,12019	1
Twsg1	-0,12031	1
Fbxw4	-0,12028	1
Gm6305	-0,12054	1
Phf5a	-0,12061	1
Nampt	-0,12075	1
Ipo5	-0,12074	1
Fam188a	-0,12084	1
Apmap	-0,12082	1
Mrpl12	-0,12102	1
Tmem208	-0,12102	1
Ubl7	-0,12112	1
Clec16a	-0,1211	1
D630023F18Rik	-0,1212	1
Nadk	-0,12125	1
Rps15a-ps6	-0,12131	1
Dhrs1	-0,12132	1
Plscr4	-0,12138	1
Eif2ak4	-0,12139	1
Cacna1b	-0,12152	1
Ino80	-0,12153	1
Gm7424	-0,12164	1
2310068J16Rik	-0,12172	1
Snx30	-0,12171	1
Rrm2b	-0,12179	1
Gm11353	-0,1219	1
Glt1d1	-0,12198	1
Tlr3	-0,12205	1
Tstd2	-0,12205	1
Eef2k	-0,12222	1
Fan1	-0,12224	1
Gm10784	-0,12221	1
Tbc1d4	-0,12261	1
Ppp1ca	-0,1226	1
Ncstn	-0,12292	1
Slc39a13	-0,12299	1
Rev1	-0,12302	1
Crbn	-0,12325	1
Nup188	-0,12333	1
Thrap3	-0,12333	1
Vamp8	-0,12327	1
Tbc1d17	-0,12335	1
Pbx3	-0,12343	1
D730045B01Rik	-0,12349	1
Eml5	-0,12356	1

Reep6	-0,12361	1
Olfr286	-0,12368	1
Gm17018	-0,12373	1
C2cd2l	-0,12368	1
Mapre2	-0,12383	1
Baiap2	-0,12392	1
Zfp174	-0,12409	1
Gm5601	-0,12406	1
Armc10	-0,12408	1
Rnpep	-0,12413	1
Fam126a	-0,1242	1
Rpl31-ps11	-0,12447	1
Zfp113	-0,12473	1
Yipf2	-0,12468	1
Fkbp1b	-0,12503	1
Fnip1	-0,12501	1
Katnal1	-0,12508	1
Eif3l	-0,12525	1
Gm7776	-0,12526	1
Capn7	-0,12535	1
Asf1a	-0,12557	1
Timm13	-0,12566	1
Zc3hav1	-0,12571	1
6430548M08Rik	-0,12585	1
Shmt1	-0,12577	1
Ercc6	-0,12586	1
Zfand1	-0,12602	1
Sec23a	-0,12597	1
Cuta	-0,12614	1
Morn2	-0,12624	1
Parl	-0,12633	1
Pi4ka	-0,12648	1
Tmed3	-0,1268	1
Cdca3	-0,12694	1
Zfyve27	-0,12694	1
Phlda1	-0,12702	1
Pdcd10	-0,12715	1
Dner	-0,12707	1
Gm9392	-0,12743	1
Anapc2	-0,1274	1
Xiap	-0,12744	1
Stx3	-0,12735	1
Inppl1	-0,12754	1
Rps15a-ps4	-0,12772	1
Snrnp25	-0,12768	1
Mrpl1	-0,12772	1
Fam134c	-0,12787	1
Caap1	-0,12793	1
AI837181	-0,12805	1
Zswim6	-0,12827	1
Gm7117	-0,12844	1
Gm2272	-0,12873	1

Fdft1	-0,12873	1
Fam219b	-0,12878	1
Cers4	-0,12885	1
Qtrt1	-0,12895	1
Sumo3	-0,12896	1
Gm12606	-0,12906	1
Prpf3	-0,12917	1
Fam117b	-0,12933	1
Cnst	-0,12938	1
Klf16	-0,12949	1
Txndc12	-0,1297	1
Gm38077	-0,1298	1
Fcgr1	-0,13007	1
Dus3l	-0,13015	1
Ubr3	-0,13035	1
Clta	-0,13031	1
Coro1a	-0,13035	1
Lst1	-0,13042	1
Txndc17	-0,13043	1
Acyp1	-0,13099	1
Mdrl	-0,13103	1
Fbxo33	-0,13096	1
Trpc4ap	-0,13113	1
Apc	-0,13135	1
Smim13	-0,13153	1
Atg4a-ps	-0,13159	1
2810025M15Rik	-0,1317	1
Hnrnpl	-0,13182	1
Cox7b	-0,13181	1
Mia2	-0,13193	1
Dars2	-0,13196	1
Armc2	-0,13202	1
Nat9	-0,13204	1
Abcb4	-0,13208	1
Arfgef2	-0,13243	1
Mrps18a	-0,1324	1
Ezh1	-0,13239	1
Trove2	-0,13252	1
Chd4	-0,13272	1
Mcfd2	-0,13294	1
Myg1	-0,13316	1
Dusp22	-0,13329	1
Asb1	-0,13333	1
Gm10689	-0,1334	1
Ndrg4	-0,13342	1
2010107E04Rik	-0,13336	1
Tmem123	-0,13354	1
Eif2s2	-0,13356	1
Dnaja1	-0,13371	1
Eri1	-0,13368	1
Ctso	-0,13382	1
Ndufc2	-0,13385	1

Pkd1	-0,13402	1
Adck1	-0,13411	1
Szt2	-0,13418	1
Rhof	-0,13416	1
Gm44093	-0,13431	1
Ppp1r2	-0,13459	1
Dhx35	-0,13471	1
Disp1	-0,13478	1
Zkscan17	-0,13479	1
4921524J17Rik	-0,13491	1
Acbd6	-0,13488	1
Ube2t	-0,13503	1
Tmem229b	-0,13516	1
Smap1	-0,13517	1
Jmjd1c	-0,13529	1
Pafah1b3	-0,1357	1
Faf1	-0,13575	1
Cd68	-0,13574	1
Nova2	-0,13585	1
Morc4	-0,13599	1
Sf3a2	-0,13624	1
Mien1	-0,1362	1
Gm43110	-0,13638	1
Gm16536	-0,13653	1
Polr2d	-0,1365	1
0610039K10Rik	-0,13658	1
Cox4i2	-0,13657	1
Ghdc	-0,13655	1
Icam4	-0,13694	1
3110062M04Rik	-0,13705	1
Rrbp1	-0,13702	1
Rassf7	-0,13712	1
Prkab2	-0,13725	1
Fam234b	-0,13733	1
Ppm1f	-0,13727	1
Nup54	-0,13763	1
Ciz1	-0,13775	1
Nfrkb	-0,13765	1
Cox14	-0,13767	1
Rrm1	-0,13781	1
Gm13181	-0,13792	1
Arhgap31	-0,13811	1
1700037H04Rik	-0,13806	1
Spsb2	-0,13824	1
Napa	-0,13817	1
Hmox1	-0,13832	1
Gm9517	-0,13835	1
Gm5131	-0,13844	1
Aup1	-0,13836	1
Ern1	-0,13868	1
Akap13	-0,13877	1
Aga	-0,13877	1

Hck	-0,13885	1
Btbd1	-0,13891	1
Gnb1	-0,13894	1
Npepps	-0,13895	1
Camta2	-0,13911	1
Pfdn4	-0,13909	1
Cdc7	-0,13918	1
Fkbp7	-0,13924	1
Gak	-0,13921	1
Fbxl12os	-0,13925	1
Rbbp7	-0,13933	1
Gramd2	-0,13941	1
Chd5	-0,13941	1
Morn1	-0,13941	1
F9	-0,13941	1
Eif4g2	-0,13936	1
Il11ra1	-0,13951	1
Spen	-0,13948	1
Nrf1	-0,13956	1
Gm16046	-0,13971	1
Map2k5	-0,13966	1
Tcf20	-0,13992	1
Rhoh	-0,14002	1
Mycbp	-0,13996	1
Ckap2l	-0,14	1
Gm9840	-0,14008	1
Rad54b	-0,14038	1
Rps4x	-0,14051	1
8430429K09Rik	-0,1405	1
Efr3a	-0,14048	1
Cdon	-0,1406	1
Cracr2b	-0,14076	1
Cnih4	-0,14083	1
Stambpl1	-0,1409	1
Pex10	-0,14103	1
Ap3d1	-0,14119	1
Ccdc28b	-0,14131	1
Mxd3	-0,14128	1
Cmip	-0,14125	1
Psmb3	-0,14164	1
Pot1b	-0,14183	1
Arid2	-0,14188	1
2810013P06Rik	-0,14216	1
Skp2	-0,14215	1
Rps18	-0,14235	1
Med20	-0,14233	1
Haus8	-0,14237	1
Lamp2	-0,14236	1
Gm4032	-0,14246	1
Zfp383	-0,14265	1
Cryz	-0,14274	1
Gm10941	-0,14277	1

Zfp292	-0,14289	1
Atp6v0e	-0,14291	1
Nol12	-0,14296	1
Pde4c	-0,14307	1
Ywhah	-0,14309	1
Cklf	-0,14315	1
Pbx2	-0,14322	1
Slc25a4	-0,1433	1
Ube2a	-0,14328	1
Gm45501	-0,14343	1
Gm5124	-0,14335	1
Plgrkt	-0,14341	1
Rcor3	-0,14341	1
Cdc25b	-0,14344	1
Pdzd11	-0,14354	1
Gas7	-0,14353	1
Fbxo3	-0,14349	1
Srpk1	-0,14355	1
Uggt2	-0,14385	1
Sdhaf2	-0,14382	1
Trp53	-0,14415	1
Sptan1	-0,14418	1
Rps19-ps12	-0,14434	1
Ccl6	-0,14441	1
Mast3	-0,14439	1
Tsr3	-0,14441	1
Zfp326	-0,14444	1
Ubxn6	-0,14479	1
Rad21	-0,14477	1
C87436	-0,14497	1
Naaa	-0,14508	1
Zfp287	-0,14526	1
Anks1	-0,14532	1
S100pbp	-0,14526	1
Gpatch2l	-0,14547	1
Abi1	-0,14549	1
Rel	-0,14547	1
Uba6	-0,14555	1
Ptbp1	-0,14548	1
Pla1a	-0,14561	1
Yif1b	-0,14565	1
9430015G10Rik	-0,14582	1
Lmnb2	-0,14611	1
Dnase2a	-0,14609	1
Bora	-0,14622	1
Zeb2os	-0,1463	1
Tet2	-0,14626	1
Camkk2	-0,14626	1
Glrx2	-0,14639	1
Gm8304	-0,14646	1
Pura	-0,14648	1
Nmt1	-0,14651	1

Hsbp1	-0,14664	1
Enpp1	-0,14665	1
Wdr91	-0,14676	1
Snrpd3	-0,1468	1
Sema5a	-0,147	1
Jak1	-0,14698	1
Zfr2	-0,14713	1
Pkp2	-0,14744	1
Ccdc88a	-0,14741	1
Gm4799	-0,14749	1
Cbr2	-0,14748	1
Aplp2	-0,14753	1
Tpp1	-0,14778	1
Vps11	-0,14789	1
Dcaf7	-0,14802	1
Chchd2	-0,14809	1
Gm27605	-0,14821	1
Purb	-0,14823	1
Paip2	-0,14834	1
Plxnc1	-0,14837	1
Tmem30a	-0,14838	1
Slc37a4	-0,14851	1
Skp1a	-0,14859	1
Ppp4r3b	-0,14857	1
Crebl2	-0,14885	1
Gpatch8	-0,14892	1
Cdk8	-0,14895	1
Gm44024	-0,14913	1
Zfp629	-0,1492	1
Gm3145	-0,14933	1
Med28	-0,14929	1
Safb	-0,14945	1
Cd44	-0,14939	1
Gm8508	-0,14962	1
Gm7332	-0,1497	1
Atpaf2	-0,14981	1
Nans	-0,1498	1
Cwf19l2	-0,14994	1
Ly9	-0,15023	1
Slc25a12	-0,15029	1
Cfl2	-0,15029	1
Gm5384	-0,15041	1
Rps12-ps19	-0,15048	1
Chchd7	-0,15058	1
Gigyf2	-0,15068	1
Scd2	-0,15087	1
Spop	-0,15086	1
Pomt1	-0,15101	1
Cds1	-0,15103	1
Gfer	-0,15103	1
Slc19a1	-0,15113	1
Zswim7	-0,15117	1

Jmjd6	-0,15116	1
Capg	-0,15119	1
Lsp1	-0,15131	1
Ssr1	-0,15133	1
Rps4x-ps	-0,15143	1
Erc1	-0,15152	1
Dscr3	-0,15146	1
AC149090.1	-0,15163	1
Pou2f1	-0,15163	1
Gm38375	-0,1523	1
Gm6433	-0,15233	1
Sugp1	-0,15234	1
D10Jhu81e	-0,15242	1
Ankrd44	-0,15268	1
Anp32a	-0,15269	1
Tceal9	-0,15271	1
Calr3	-0,15284	1
Ccdc137	-0,15311	1
Vps45	-0,15309	1
Snx6	-0,15311	1
Srgn	-0,15316	1
Snhg8	-0,1533	1
Gm2308	-0,15355	1
Man2a2	-0,15363	1
Ssna1	-0,1541	1
Kdm5c	-0,15413	1
Tmem35b	-0,15505	1
Atp5sl	-0,15502	1
Gm44775	-0,15525	1
Ncmap	-0,15525	1
Dmrta2	-0,15525	1
Six5	-0,15525	1
Gm43154	-0,15525	1
Slc24a5	-0,15525	1
Matn4	-0,15525	1
Arg1	-0,15525	1
Gm42486	-0,15525	1
Gm44510	-0,15525	1
9030624J02Rik	-0,15527	1
Mmp2	-0,15552	1
Gm5845	-0,15558	1
Rab5c	-0,15556	1
Gm6023	-0,15579	1
Exoc4	-0,15602	1
AB124611	-0,15597	1
Zfp661	-0,15616	1
Cirbp	-0,15628	1
Fah	-0,15654	1
S100a3	-0,15661	1
Snord92	-0,15663	1
Prtn3	-0,1566	1
A930018M24Rik	-0,1566	1

Pla2g16	-0,15701	1
Cox20	-0,15698	1
Dym	-0,15698	1
Ezh2	-0,15724	1
Pgm3	-0,15728	1
Srsf9	-0,15728	1
Anapc10	-0,15735	1
Klk8	-0,15755	1
Ubap2l	-0,15748	1
Mrrf	-0,15769	1
Pank2	-0,15775	1
Chtf8	-0,15782	1
Shkbp1	-0,15829	1
Herc2	-0,15839	1
Ubxn2a	-0,15887	1
Gtf2h5	-0,15892	1
Sgf29	-0,15912	1
Mrpl52	-0,15912	1
Gm6946	-0,15915	1
Tesk2	-0,15931	1
Adpgk	-0,15929	1
Cdk5rap2	-0,15942	1
Gm14165	-0,15948	1
Rab7	-0,15947	1
Zfp983	-0,15958	1
Gm13360	-0,15982	1
Nradd	-0,1598	1
Gm26520	-0,15978	1
Etnk1	-0,15984	1
Cotl1	-0,15977	1
Hsdl2	-0,16003	1
Gm8242	-0,15995	1
Gm10288	-0,1601	1
Eif4h	-0,16007	1
Gm43788	-0,1602	1
Clcn6	-0,16021	1
Mbd3	-0,16018	1
Ttyh3	-0,1603	1
Cd164	-0,1603	1
Gm10086	-0,16027	1
Rer1	-0,1603	1
Mir7078	-0,16041	1
C77080	-0,16049	1
Gm4742	-0,16057	1
CAAA01180111.2	-0,1608	1
Smchd1	-0,16089	1
Ifi30	-0,16094	1
2510039O18Rik	-0,16101	1
Atp5k	-0,16149	1
Btd	-0,16155	1
5430416N02Rik	-0,16164	1
Slfn9	-0,16162	1

Ogdh	-0,16184	1
Phc3	-0,16178	1
Lman1	-0,16189	1
Pqlc1	-0,16187	1
Gm7488	-0,16203	1
Fam65c	-0,16203	1
Rpl18a-ps1	-0,16204	1
Btbd2	-0,162	1
Rusc2	-0,16216	1
Txndc5	-0,16217	1
Crocc	-0,16236	1
Trrap	-0,16248	1
Txnip	-0,16275	1
Cd9	-0,16276	1
Galt	-0,16281	1
Ttll12	-0,16285	1
9330175E14Rik	-0,16285	1
Mettl4	-0,1629	1
Kif14	-0,16314	1
Mapk14	-0,16311	1
Zfas1	-0,1633	1
Lsm8	-0,16363	1
Rps24-ps3	-0,16363	1
Gas5	-0,16358	1
Gm1947	-0,1637	1
Tmem261	-0,16384	1
Sp1	-0,16383	1
Ndufa13	-0,16386	1
Hsd17b12	-0,16402	1
Ap3s2	-0,16411	1
Dnajc14	-0,16418	1
Gm9843	-0,16419	1
Sacm1l	-0,1643	1
Uqcrq	-0,16438	1
Lonrf3	-0,1646	1
Mllt3	-0,16462	1
Fcgr3	-0,16479	1
Rpl23a-ps14	-0,16489	1
Pdia5	-0,16495	1
Gm4540	-0,16501	1
Hcfc2	-0,165	1
Luc7l	-0,16532	1
Tcea1-ps1	-0,1654	1
Lsm12	-0,16536	1
Chd9	-0,16545	1
Kif3a	-0,16551	1
Usp32	-0,16556	1
Pex19	-0,1657	1
Qk	-0,1657	1
Aldh2	-0,1658	1
Letmd1	-0,16595	1
Ewsr1	-0,16614	1

Gm42547	-0,16617	1
Cyb5r1	-0,16644	1
Ppa1	-0,16635	1
Psmd14	-0,16636	1
Usp20	-0,16663	1
Blvrb	-0,16669	1
Gm27003	-0,16685	1
Ddhd2	-0,167	1
Snapc3	-0,16715	1
Kansl1	-0,16713	1
Prdx3	-0,16711	1
Scand1	-0,16734	1
Ppp3ca	-0,16732	1
Mical3	-0,16745	1
Fmn1	-0,16743	1
H2-T10	-0,16752	1
Unc119	-0,16755	1
Fads1	-0,16763	1
2410006H16Rik	-0,16761	1
H13	-0,16764	1
Trf	-0,16787	1
Ino80d	-0,16792	1
Mtbp	-0,168	1
Cpd	-0,16798	1
Exosc3	-0,16811	1
Hn1l	-0,16817	1
Mef2a	-0,16853	1
Yaf2	-0,16887	1
S100a1	-0,1689	1
Yme1l1	-0,16913	1
Arap1	-0,16915	1
Rpl37a	-0,16909	1
C1galt1	-0,16917	1
Cln3	-0,16924	1
Lrrc42	-0,16919	1
Nemp2	-0,16933	1
Ppp2r5d	-0,16935	1
Zfp933	-0,16935	1
Nsmce1	-0,16964	1
Bin3	-0,16976	1
Tmem70	-0,16984	1
Lsm1	-0,16994	1
Hnrnpu	-0,16989	1
Gm8276	-0,16997	1
Rcn2	-0,17014	1
Ubl3	-0,17025	1
Mdh2	-0,17019	1
Gm20045	-0,17028	1
Nup155	-0,17032	1
Hmg20a	-0,17034	1
Mtr	-0,17037	1
Nfkbid	-0,17039	1

Clec11a	-0,17053	1
Rpl30-ps9	-0,17064	1
Cenpn	-0,17069	1
Acy3	-0,1707	1
Acat2	-0,1707	1
Cops2	-0,17075	1
Cetn2	-0,1708	1
Golph3l	-0,1709	1
Smc3	-0,17103	1
Cers6	-0,17109	1
A930006K02Rik	-0,17123	1
Srp9	-0,17121	1
Ide	-0,17131	1
Rnf145	-0,17134	1
Rpl9-ps6	-0,17142	1
Ckap5	-0,17148	1
Dld	-0,17148	1
AW146154	-0,1716	1
Pde8b	-0,17163	1
Gm6344	-0,1717	1
Ccnl1	-0,17176	1
Clcn5	-0,17192	1
Mtfr1l	-0,17192	1
Upf3a	-0,17219	1
Anp32b	-0,1723	1
Slc35b1	-0,17229	1
Gm12355	-0,17271	1
Gm13421	-0,17273	1
Ppcdc	-0,17268	1
Ankrd50	-0,17267	1
Iqsec2	-0,17294	1
Tbc1d32	-0,17339	1
Sestd1	-0,17345	1
Zfp282	-0,17383	1
Il2rg	-0,17382	1
Rpl22-ps1	-0,17389	1
D130017N08Rik	-0,17398	1
Gm44822	-0,17404	1
Ugp2	-0,17413	1
Wdtc1	-0,17422	1
Setd5	-0,17425	1
Rad51ap1	-0,17428	1
Gm29358	-0,17437	1
Rangap1	-0,17437	1
Ppp1r35	-0,17447	1
Eif3m	-0,17456	1
2310061I04Rik	-0,17475	1
Stx16	-0,17496	1
Chmp4b	-0,17502	1
Nr3c1	-0,17513	1
Gm5239	-0,17526	1
Abca1	-0,17545	1

Aasdh	-0,17543	1
Mlxip	-0,17544	1
Scrn2	-0,17536	1
Rbbp4	-0,17551	1
1810058I24Rik	-0,1757	1
Eif1b	-0,17567	1
Stx8	-0,17588	1
Lilrb4a	-0,17598	1
Myo5a	-0,17609	1
Bbx	-0,17606	1
Gm4924	-0,17616	1
Rabggtb	-0,17616	1
Nr2f6	-0,17635	1
Zfp277	-0,17679	1
Uqcr10	-0,17689	1
Hebp1	-0,17705	1
Atp6ap2	-0,17702	1
Slc19a2	-0,17724	1
Rpl14-ps1	-0,17732	1
Mecr	-0,17753	1
Smarcc1	-0,1775	1
Caly	-0,17757	1
Pidd1	-0,17768	1
Wsb2	-0,17766	1
Borcs8	-0,1778	1
Dnm3	-0,17787	1
Bnip1	-0,17789	1
Cabin1	-0,17799	1
Bcl2l2	-0,17826	1
Recql4	-0,17843	1
Synrg	-0,17843	1
Nlrc3	-0,17864	1
Trio	-0,17868	1
Ppt2	-0,17892	1
BC029722	-0,17909	1
Crem	-0,17922	1
Syne3	-0,17929	1
Npc2	-0,17927	1
Klf7	-0,17951	1
Commd6	-0,17963	1
Smad1	-0,17974	1
Xpo6	-0,17968	1
Plekhg4	-0,17979	1
Armt1	-0,17976	1
H2afj	-0,17989	1
Gm42432	-0,17996	1
Fam107b	-0,17996	1
Spc24	-0,18021	1
Apba3	-0,18035	1
Selenon	-0,18032	1
Klf10	-0,18027	1
Mxd1	-0,18041	1

Ssbp3	-0,18073	1
Mbd2	-0,18068	1
Gm8606	-0,18087	1
Rad51d	-0,18094	1
Rnf6	-0,18088	1
Gm42690	-0,18103	1
Arhgdia	-0,18099	1
Lrrc51	-0,18116	1
Plcb4	-0,1812	1
Stxbp2	-0,18138	1
Zbed4	-0,18159	1
Mcub	-0,18156	1
Runx2	-0,18178	1
AI314180	-0,18176	1
Eml2	-0,18199	1
Pax6	-0,18208	1
Atp6v0d2	-0,1821	1
Hist1h2al	-0,18217	1
Wdr74	-0,18241	1
Dus4l	-0,18254	1
Nr2c2ap	-0,18264	1
Ndrg2	-0,18262	1
Stx6	-0,18284	1
Mroh1	-0,18293	1
Mta3	-0,18321	1
Gm9800	-0,1833	1
Gm6524	-0,18326	1
Zcchc24	-0,18341	1
Commd9	-0,18351	1
Ctdsp2	-0,1835	1
Emsy	-0,18372	1
Ctsh	-0,18403	1
Ptpru	-0,18413	1
Sptssa	-0,18411	1
B3gat3	-0,18413	1
Zfp949	-0,18417	1
Esrra	-0,1842	1
Srrm2	-0,18418	1
Prr7	-0,18428	1
Ago3	-0,18457	1
Eid1	-0,18462	1
C2cd3	-0,18493	1
Bcap31	-0,185	1
Arpc2	-0,18507	1
Skap2	-0,18512	1
Rnf38	-0,18522	1
Tyk2	-0,1852	1
Prdm9	-0,18528	1
Gm43309	-0,18534	1
Smg7	-0,18531	1
Ywhaz	-0,18543	1
Nhlrc2	-0,1855	1

Kpna4	-0,18556	1
Rpl36-ps10	-0,18574	1
Cds2	-0,18567	1
Gm11249	-0,18578	1
Ifnar1	-0,1858	1
4933404O12Rik	-0,18611	1
Insig2	-0,18613	1
St13	-0,18621	1
Ndufa3	-0,18643	1
Dclre1c	-0,18652	1
Scaper	-0,18646	1
Scp2	-0,18664	1
Gatsl3	-0,18676	1
Polr3g	-0,1868	1
Bad	-0,18691	1
Dnajb6	-0,18691	1
Dock10	-0,18697	1
Lmo2	-0,18716	1
Usp34	-0,18729	1
Akr1e1	-0,18737	1
Hmces	-0,18745	1
Snrnp200	-0,18743	1
Plcl2	-0,1876	1
Tmem26	-0,18768	1
Gm44609	-0,18784	1
Slc35a5	-0,188	1
Exoc7	-0,18834	1
Hcn2	-0,18849	1
4930550C14Rik	-0,18862	1
2810002D19Rik	-0,18857	1
Slc2a1	-0,1886	1
Eif4e3	-0,1889	1
Kif1b	-0,18885	1
Bzw2	-0,18902	1
Ten1	-0,18922	1
Rpl19-ps11	-0,1893	1
Sik3	-0,1897	1
Tmem237	-0,18991	1
Fam43a	-0,18994	1
Atpaf1	-0,19013	1
Nphp3	-0,19023	1
Plcg1	-0,1902	1
Bmpr2	-0,19038	1
Inpp5a	-0,19037	1
Cbl	-0,19071	1
Six1	-0,19081	1
Bsdc1	-0,19101	1
Gm8522	-0,19112	1
Atp2c1	-0,19117	1
Ube2e3	-0,19132	1
Cyp27a1	-0,1914	1
Mms22l	-0,19139	1

Serpinb9	-0,19168	1
Fra10ac1	-0,19188	1
Slc2a4rg-ps	-0,19202	1
Pced1a	-0,19199	1
Tpd52-ps	-0,1921	1
Atp5e	-0,1922	1
Mgea5	-0,19233	1
Rps25	-0,19247	1
Kif11	-0,19259	1
Blmh	-0,19287	1
Cldnd1	-0,19322	1
Gm7561	-0,19327	1
Gm5611	-0,19326	1
Camta1	-0,19375	1
Got2	-0,19368	1
Cd300lf	-0,19388	1
Gltscr1	-0,19412	1
Plscr3	-0,19412	1
Gm16973	-0,19422	1
Bcas3	-0,19446	1
Ly96	-0,19473	1
Bckdha	-0,19472	1
Clec12a	-0,19471	1
Cdc123	-0,19488	1
Qser1	-0,19492	1
Ppm1l	-0,19492	1
Stt3b	-0,19503	1
Melk	-0,19506	1
Mex3d	-0,19531	1
Pgs1	-0,19541	1
Tbc1d9b	-0,19559	1
Desi1	-0,1957	1
Ankfy1	-0,19583	1
Spice1	-0,19592	1
Mfsd14a	-0,19595	1
Akap9	-0,19588	1
Cic	-0,19587	1
Ostf1	-0,19586	1
Gm43213	-0,19609	1
Ttc37	-0,19621	1
Ywhab	-0,19619	1
Aurka	-0,19621	1
Man2b1	-0,19633	1
Spink10	-0,19638	1
Mad2l1	-0,19655	1
Opn3	-0,19688	1
Cenpp	-0,19691	1
Pi4kb	-0,19709	1
Chchd3	-0,19719	1
Paip2b	-0,1973	1
Spns1	-0,19738	1
Tcp11l2	-0,19738	1

Cdkn2d	-0,19742	1
Zfp397	-0,19751	1
Ilf3	-0,19746	1
Arhgap11a	-0,19756	1
Bak1	-0,19774	1
Nae1	-0,19787	1
Pstpip1	-0,19801	1
Scaf1	-0,1982	1
Wdr83os	-0,19817	1
Gatad1	-0,19832	1
Lrrc28	-0,19853	1
Hdac2	-0,1985	1
B4galt3	-0,19853	1
Apobr	-0,19868	1
Ccdc34	-0,19869	1
Man2c1	-0,19877	1
Apeh	-0,19884	1
Fam98a	-0,19905	1
Tpm3	-0,19918	1
Dnttip1	-0,1993	1
Kif1c	-0,19949	1
Tsta3	-0,2002	1
Snap23	-0,20023	1
Ndufa9	-0,2003	1
Brd9	-0,20044	1
Polr1e	-0,20054	1
Uevld	-0,20045	1
Gm13862	-0,20067	1
Gm7432	-0,20084	1
Mxi1	-0,20095	1
Fchsd2	-0,20102	1
Ndufv3	-0,20105	1
Ctdspl	-0,20131	1
Tnnc1	-0,20164	1
Trim37	-0,20179	1
Thop1	-0,20197	1
Babam1	-0,20219	1
Ifi27	-0,2026	1
Desi2	-0,2026	1
RP23-359K10.8	-0,20267	1
Srsf11	-0,20296	1
Gm37474	-0,20318	1
Mzt2	-0,20319	1
Btaf1	-0,20354	1
Slc6a12	-0,20353	1
Odf2l	-0,20359	1
Ccnt2	-0,20359	1
Uba1	-0,20364	1
Fxyd2	-0,2036	1
Gabarap	-0,20361	1
Arl3	-0,20379	1
Phf1	-0,20385	1

Foxd2os	-0,20403	1
Helz2	-0,20416	1
Igip	-0,20437	1
Dlg4	-0,20436	1
AA414768	-0,20454	1
Ppp5c	-0,20453	1
Mrps18c	-0,2047	1
Tulp4	-0,20473	1
Dusp4	-0,20468	1
Copz1	-0,20474	1
Lgi4	-0,20482	1
Car5b	-0,20499	1
Malat1	-0,20512	1
Abcb1b	-0,2051	1
Aldoart1	-0,20521	1
Gm38190	-0,20549	1
Jak2	-0,20551	1
Zcchc11	-0,20558	1
Birc5	-0,20572	1
Arhgap10	-0,20583	1
Lnpep	-0,2059	1
Siae	-0,20603	1
Cd82	-0,20613	1
0610009B22Rik	-0,20635	1
Rpsa-ps12	-0,20643	1
Gm42715	-0,20643	1
a	-0,20655	1
2210013O21Rik	-0,20648	1
Kcne3	-0,20659	1
Ctnnbip1	-0,20657	1
Lrp4	-0,20668	1
Npy	-0,20675	1
Oas1d	-0,20675	1
Plxna1	-0,20683	1
Add1	-0,20692	1
Acot7	-0,20708	1
Pgm2l1	-0,20718	1
Nup88	-0,20717	1
Gm10161	-0,20735	1
Pcnp	-0,20781	1
Thoc2	-0,2082	1
Ddt	-0,2084	1
Pja2	-0,20842	1
6820402A03Rik	-0,20849	1
Spout1	-0,20849	1
Gm12989	-0,20872	1
Gm11346	-0,20883	1
Ptprs	-0,20879	1
Anxa4	-0,20876	1
Abcd4	-0,20899	1
Abtb1	-0,20905	1
Uqcc2	-0,20902	1

BC025920	-0,20923	1
Ccdc22	-0,20926	1
Gm15506	-0,20937	1
Iars2	-0,20937	1
Ccnd3	-0,20945	1
Gm10051	-0,20948	1
Hdac10	-0,2096	1
Dpy19l1	-0,20963	1
Fiz1	-0,20966	1
Fhit	-0,20966	1
Gm7808	-0,20993	1
Gnai2	-0,21009	1
Nmt2	-0,21019	1
Nfkb1	-0,21031	1
Cmc1	-0,2104	1
Gid4	-0,21067	1
Gpsm2	-0,21095	1
Gm5523	-0,21095	1
Rprd1a	-0,2111	1
Tsga10ip	-0,21106	1
Gm12481	-0,21106	1
Slc44a1	-0,21108	1
Osbp	-0,21134	1
Erap1	-0,21129	1
Idh3g	-0,2113	1
Msl3	-0,21141	1
Ap3b1	-0,21143	1
Rab14	-0,21146	1
Ankrd12	-0,2118	1
Gm2a	-0,21189	1
Rnase4	-0,21222	1
Ak4	-0,21231	1
Dnajc5	-0,21234	1
Gm6297	-0,21242	1
Klkb1	-0,21259	1
Fam167b	-0,21264	1
Nqo2	-0,21266	1
Nubp1	-0,21271	1
Fbxo8	-0,21288	1
Gpr137	-0,2133	1
Clec10a	-0,21326	1
2900093K20Rik	-0,21361	1
Upf2	-0,21356	1
Lcorl	-0,21371	1
mt-Tv	-0,21387	1
Pon3	-0,21397	1
Hnrnpr	-0,21418	1
Dars	-0,21425	1
Fam135a	-0,21434	1
Dhcr7	-0,21432	1
Zfp87	-0,21455	1
Numa1	-0,21454	1

Entpd1	-0,21477	1
Fars2	-0,21531	1
Cpeb4	-0,21529	1
Exoc6	-0,21553	1
Rps15a-ps7	-0,21557	1
Aunip	-0,21565	1
Ehd2	-0,21572	1
Polr3b	-0,21584	1
Gna15	-0,21583	1
Clk2	-0,21585	1
Fbxl17	-0,21587	1
Ids	-0,21588	1
Tmem245	-0,21586	1
Gm28417	-0,21599	1
Ticrr	-0,21604	1
Brd3	-0,21603	1
Scn11a	-0,21624	1
Gm43727	-0,21624	1
Ablim1	-0,21624	1
Met	-0,21624	1
Zfp14	-0,21624	1
Ceacam10	-0,21624	1
RP24-91J7.1	-0,21624	1
Atp8b3	-0,21624	1
Gm44053	-0,21624	1
Rorc	-0,21624	1
Gnat2	-0,21624	1
C1rb	-0,21624	1
Xrra1	-0,21624	1
RP23-104D6.2	-0,21624	1
Tbxas1	-0,21624	1
Rgs8	-0,21624	1
Acot11	-0,21624	1
6720464F23Rik	-0,21624	1
Ssc5d	-0,21624	1
Colec12	-0,2163	1
Usp45	-0,21637	1
Ap1ar	-0,21666	1
Adap2	-0,21715	1
Arid5b	-0,21711	1
Plau	-0,21709	1
Ybx1	-0,21724	1
Fxyd5	-0,21736	1
E030030I06Rik	-0,21759	1
Npepl1	-0,21781	1
Usp6nl	-0,21818	1
Arpc3	-0,21823	1
Gm37959	-0,21844	1
Gm42731	-0,21846	1
Bod1l	-0,21856	1
Nrm	-0,21864	1
Gm42728	-0,21874	1

Rsrc1	-0,21876	1
Fcrl1	-0,21896	1
Tacc1	-0,21902	1
Ccdc36	-0,2191	1
Kifc1	-0,2191	1
Rptor	-0,21909	1
Gm5121	-0,21924	1
Plekhg5	-0,2192	1
Mob1b	-0,21919	1
Hpf1	-0,21928	1
Msh3	-0,21943	1
Hsd17b14	-0,21957	1
Hip1r	-0,21975	1
Sppl2a	-0,21982	1
Gm43294	-0,21988	1
Tmc6	-0,21993	1
Gm26384	-0,21994	1
Nedd8	-0,2202	1
Os9	-0,22042	1
Thbs3	-0,22064	1
Srp72	-0,22064	1
Akap8l	-0,22075	1
Cep104	-0,22079	1
Med21	-0,22083	1
Prkar2a	-0,22081	1
Slc31a1	-0,22089	1
Dnajb4	-0,22113	1
Oxsm	-0,22118	1
Gna12	-0,22116	1
Atxn10	-0,22117	1
Zdhhc4	-0,22168	1
Sh3bgrl3	-0,22169	1
Gm17807	-0,22232	1
Ptges3l	-0,22225	1
Mettl23	-0,22243	1
Slc50a1	-0,22242	1
Cdipt	-0,2224	1
Ap1b1	-0,22251	1
Mlh1	-0,22256	1
Ttc5	-0,22259	1
Hdgfrp2	-0,2227	1
Plekhm3	-0,22298	1
Polr2i	-0,22321	1
Prps2	-0,22325	1
Vamp7	-0,22331	1
Abhd11	-0,22329	1
Dok3	-0,22329	1
Gm37334	-0,2234	1
Sacs	-0,22361	1
Cwc27	-0,22374	1
Acox1	-0,22369	1
Gm5617	-0,22369	1

Cstf3	-0,22392	1
Xpo7	-0,22387	1
Cd2ap	-0,22395	1
Exoc3l2	-0,22413	1
Rpl14	-0,22421	1
Tube1	-0,2243	1
AI597479	-0,22445	1
Tcn2	-0,22445	1
Acadl	-0,2245	1
Dr1	-0,22459	1
Srek1	-0,22465	1
Dnal4	-0,22506	1
Dstn	-0,22512	1
Ppme1	-0,22523	1
Tsen15	-0,22532	1
Map4	-0,22548	1
Seh1l	-0,22559	1
3830406C13Rik	-0,22583	1
Slc39a3	-0,22593	1
Gm37238	-0,22602	1
Ttc3	-0,22601	1
Gm35315	-0,22612	1
Rngtt	-0,22612	1
Hist1h2bc	-0,22613	1
Cyb561	-0,22621	1
Dleu2	-0,22621	1
Stk38	-0,22634	1
Cpsf4	-0,22635	1
Cyp4v3	-0,22652	1
Pcyox1l	-0,22649	1
Ggps1	-0,22682	1
Cry1	-0,22685	1
Flii	-0,22694	1
Mcu	-0,22704	1
Lztr1	-0,22713	1
Catsper2	-0,22727	1
Kank2	-0,22734	1
Fam102b	-0,22727	1
Park7	-0,22753	1
Llph-ps1	-0,22771	1
Prkag2	-0,22774	1
Wdr41	-0,22807	1
Plod3	-0,22811	1
Ly6e	-0,22823	1
Abracl	-0,22826	1
Msn	-0,22829	1
Alms1	-0,22838	1
Tmem68	-0,22844	1
Lgalsl	-0,22837	1
Rab10os	-0,22848	1
Rsf1	-0,22863	1
Aph1a	-0,22911	1

Cab39l	-0,22919	1
Magt1	-0,22918	1
Pgam5	-0,22927	1
Slc25a5	-0,22961	1
Mmgt1	-0,22961	1
Tmem50b	-0,22959	1
Sparc	-0,2298	1
Prkacb	-0,22977	1
Pop5	-0,22994	1
Plekha2	-0,2302	1
Abhd4	-0,2303	1
Dennd2a	-0,23084	1
Fermt3	-0,23077	1
Szrd1	-0,23083	1
Scamp3	-0,23085	1
Tmpo	-0,23087	1
Kif20a	-0,23111	1
Asxl2	-0,23119	1
Erf	-0,23118	1
Gm37702	-0,23145	1
Atg2a	-0,23148	1
Tslp	-0,23163	1
Selenoo	-0,23158	1
Ppip5k1	-0,23179	1
Exoc1	-0,23187	1
Rpl13a	-0,23201	1
Ugcg	-0,23206	1
Eaf1	-0,23215	1
Akr1b10	-0,23218	1
Grhpr	-0,23241	1
Gm16523	-0,23249	1
Isg20	-0,23267	1
Faap20	-0,23277	1
Guca1a	-0,23275	1
Prune2	-0,23288	1
Noct	-0,23304	1
Anapc11	-0,23319	1
mt-Rnr2	-0,23325	1
Ybx3	-0,23328	1
Vbp1	-0,23338	1
Ppp1r18	-0,23352	1
Gm11694	-0,23356	1
Ank	-0,23366	1
Snhg12	-0,23379	1
Pcdh7	-0,23389	1
Ccdc50	-0,23387	1
Strada	-0,23417	1
Prkx	-0,23416	1
Gmfb	-0,23455	1
Lrrc27	-0,23456	1
Aven	-0,23474	1
Ap1g2	-0,23467	1

Irf3	-0,23469	1
Fam162a	-0,23474	1
2810006K23Rik	-0,23485	1
Gpt	-0,23486	1
Gm12389	-0,23518	1
Phkg1	-0,23518	1
Gm38376	-0,23524	1
Coq10a	-0,23539	1
Ifitm6	-0,2355	1
RP23-325K4.10	-0,23569	1
Cnot4	-0,23586	1
Ndufv1	-0,2359	1
Cacybp	-0,23589	1
1110034G24Rik	-0,23613	1
Prpf31	-0,23605	1
Larp1	-0,23612	1
Atox1	-0,23629	1
Pcm1	-0,23654	1
Tnpo1	-0,23697	1
Parp11	-0,23722	1
Mau2	-0,23733	1
Tbc1d22a	-0,23729	1
Gm13340	-0,23735	1
Papolg	-0,23742	1
Vrk2	-0,23743	1
Scrn3	-0,2375	1
Pdha1	-0,2378	1
Ppil2	-0,23788	1
Cenpm	-0,23795	1
Gm38055	-0,23802	1
Mark4	-0,2381	1
Uvssa	-0,23816	1
Idh2	-0,23829	1
Rhov	-0,23843	1
Gstcd	-0,23837	1
S100a10	-0,23842	1
Cdk4	-0,23855	1
Gm14673	-0,2386	1
St3gal1	-0,23895	1
Zgpat	-0,23906	1
2310034G01Rik	-0,23913	1
Pfas	-0,23921	1
Birc2	-0,23917	1
Plxna2	-0,23931	1
Slc35a3	-0,23938	1
Vps13a	-0,23982	1
Gm6640	-0,24025	1
Adipor2	-0,24034	1
Pqbp1	-0,24047	1
Bcl2	-0,24058	1
Cdc20	-0,24071	1
Grcc10	-0,24078	1

Cetn3	-0,24079	1
Gm25291	-0,24094	1
Mterf3	-0,24109	1
Coq8a	-0,24106	1
Zfp956	-0,24118	1
Gm13005	-0,2413	1
Slc33a1	-0,24146	1
Srsf5	-0,24164	1
Kras	-0,24163	1
Hexdc	-0,24167	1
Ppia	-0,24186	1
Pi4k2a	-0,24194	1
Mfap3l	-0,2422	1
Med22	-0,24229	1
Efcab11	-0,24245	1
Dpm3	-0,24262	1
Serpinb8	-0,24285	1
Atxn2	-0,24277	1
Adap1	-0,24277	1
Mvd	-0,24288	1
Rnf26	-0,24297	1
Foxred2	-0,24302	1
Dhx40	-0,24308	1
Lrch3	-0,24306	1
Eya3	-0,24324	1
RP23-2N7.4	-0,24334	1
Fuom	-0,24328	1
Pik3ap1	-0,24331	1
Gm28424	-0,24336	1
Uckl1	-0,24345	1
Ptpn6	-0,24354	1
Gpd2	-0,24345	1
Smc4	-0,2435	1
Cox6a2	-0,24364	1
Ap2a1	-0,24366	1
Lrrfip2	-0,24365	1
Ccdc124	-0,244	1
Pole4	-0,24402	1
Kdm5b	-0,24407	1
Sarnp	-0,24424	1
Gm6525	-0,2446	1
Vdac3	-0,24467	1
Crip2	-0,24474	1
Gm45456	-0,2449	1
4930563E22Rik	-0,2449	1
Gm4602	-0,2449	1
2810405F17Rik	-0,2449	1
Micall1	-0,24487	1
Fam199x	-0,24492	1
Slc35a2	-0,24503	1
Gm13270	-0,24504	1
Cdkl2	-0,24508	1

Pds5a	-0,24528	1
H2-Ob	-0,24554	1
Tbc1d12	-0,24572	1
BC003331	-0,24575	1
Plpp2	-0,24601	1
Fam195b	-0,24603	1
Bet1	-0,24615	1
Lypla2	-0,24606	1
Fam179b	-0,24614	1
Prcp	-0,24614	1
Glce	-0,24625	1
Nat10	-0,24615	1
Tssc4	-0,24656	1
Bclaf1	-0,24665	1
Dap3	-0,24674	1
Hoxb6	-0,24693	1
Psmg1	-0,24721	1
Ormdl2	-0,24732	1
Ift172	-0,24726	1
Gpaa1	-0,24728	1
1700030K09Rik	-0,24761	1
RP23-331E5.10	-0,24755	1
Phtf2	-0,24759	1
Nadk2	-0,24779	1
Rheb	-0,24802	1
Dtymk	-0,24797	1
Dcun1d2	-0,24836	1
Rpl18-ps1	-0,24836	1
Adgre1	-0,24848	1
Minos1	-0,2485	1
Prss42	-0,24861	1
RP23-476G10.1	-0,24861	1
A930016O22Rik	-0,24861	1
6720475M21Rik	-0,24861	1
Arhgef15	-0,24861	1
Prkar1b	-0,24866	1
Dhx34	-0,24868	1
Sys1	-0,24872	1
D330045A20Rik	-0,2488	1
RP24-286J14.3	-0,24898	1
Slc9a9	-0,24905	1
Cd81	-0,2491	1
Gatad2b	-0,24919	1
Fndc10	-0,24957	1
Cldn11	-0,24962	1
Cib1	-0,24985	1
Pcbp2	-0,24981	1
Nucks1	-0,24978	1
Myo7a	-0,24992	1
Stx5a	-0,25006	1
Mpnd	-0,25009	1
Galns	-0,25022	1

Tpra1	-0,25022	1
Eri2	-0,25033	1
Bbs1	-0,2503	1
Hmmr	-0,25037	1
Higd1a	-0,25046	1
Cdc37	-0,25063	1
Ppp1r9b	-0,25095	1
B9d2	-0,25103	1
Hdlbp	-0,25098	1
Arhgef40	-0,25109	1
Ddx39b	-0,25112	1
Gm15625	-0,25116	1
Kif22	-0,25133	1
Pmpca	-0,25125	1
Gga2	-0,25131	1
Ms4a6c	-0,25143	1
Firre	-0,25146	1
Srfbp1	-0,25168	1
Ddost	-0,25177	1
Mrps24	-0,25202	1
Arntl	-0,25221	1
Smdt1	-0,25236	1
Tcea2	-0,25282	1
Trap1	-0,25285	1
Snhg4	-0,25299	1
Rc3h2	-0,25326	1
Trim56	-0,2535	1
4931440P22Rik	-0,25374	1
Wdr83	-0,25368	1
Dhx16	-0,2538	1
Gigyf1	-0,25392	1
Safb2	-0,25401	1
Gm5257	-0,2541	1
Cdkal1	-0,25421	1
Arhgap5	-0,2543	1
Grap	-0,25437	1
Smc2	-0,25445	1
Slc22a13b-ps	-0,25447	1
Bcl6b	-0,25454	1
Rpl10	-0,25466	1
Asah1	-0,25469	1
H1fx	-0,25509	1
Gm14121	-0,25528	1
Tmco6	-0,25542	1
Gnb2	-0,25535	1
Elavl1	-0,25545	1
Crry-ps	-0,25546	1
D130051D11Rik	-0,25552	1
Hmg20b	-0,25573	1
Gm7308	-0,25617	1
Jam2	-0,25637	1
Tubgcp4	-0,25653	1

Gm29257	-0,25656	1
Rnps1	-0,25709	1
Neurl1b	-0,25705	1
Glipr2	-0,25756	1
Mmd	-0,25783	1
Rpain	-0,25819	1
Uhrf1bp1	-0,2582	1
Serpinb6a	-0,25819	1
Fam174a	-0,25825	1
1700003F12Rik	-0,25836	1
Slc25a1	-0,25842	1
Commd4	-0,25865	1
Mapk12	-0,25877	1
Coa6	-0,25897	1
Tbrg1	-0,25899	1
Msl1	-0,25915	1
Pqlc3	-0,2593	1
Tbcb	-0,25925	1
Gm9835	-0,25938	1
Myo1g	-0,25958	1
Sdha	-0,25966	1
Rac2	-0,25973	1
Nup107	-0,25981	1
Aco1	-0,25986	1
Prdx4	-0,2599	1
Mtpn	-0,26002	1
Csnk1g2	-0,26032	1
Zfp664	-0,26041	1
Cdk12	-0,26038	1
Ubp1	-0,26042	1
Pcdhb17	-0,26052	1
Gm38021	-0,26073	1
Chka	-0,26111	1
Dock5	-0,26121	1
Hddc3	-0,26124	1
Clic1	-0,26123	1
Fam49b	-0,26133	1
2310010J17Rik	-0,26166	1
Gm20072	-0,26179	1
RP24-497N7.2	-0,2618	1
Eef1a1	-0,26196	1
Chek2	-0,26203	1
Tusc3	-0,26196	1
Kidins220	-0,26214	1
Gcdh	-0,26216	1
Prkra	-0,26218	1
Dcp2	-0,26227	1
Ece2	-0,2624	1
Pick1	-0,26247	1
Borcs7	-0,26263	1
Fzd7	-0,26262	1
Pou2f2	-0,26272	1

Lsm4	-0,26294	1
Lmnb1	-0,26289	1
Erlin1	-0,26295	1
Sod1	-0,26297	1
Esyt2	-0,26324	1
Fcgr4	-0,26333	1
Gm4430	-0,2634	1
Gm18889	-0,26341	1
Pcbd2	-0,26346	1
Upf3b	-0,26357	1
Arap3	-0,26374	1
Cdca5	-0,26379	1
Gm44901	-0,26396	1
Copg2	-0,26422	1
Trmt112-ps2	-0,2643	1
Xdh	-0,26435	1
2810408I11Rik	-0,2644	1
Gm44237	-0,26436	1
Cr1l	-0,26453	1
Ndc80	-0,2647	1
Lsm14a	-0,2647	1
Sqle	-0,26471	1
Pfdn5	-0,26466	1
Rgcc	-0,26498	1
Mbnl2	-0,265	1
Supt4a	-0,26514	1
Tagln2	-0,26508	1
Dgkg	-0,26533	1
Cars2	-0,26539	1
Zfp367	-0,2654	1
Kif20b	-0,26548	1
Klhdc2	-0,26546	1
Snord65	-0,26571	1
Zfp382	-0,26582	1
Aldoc	-0,26582	1
Trem2	-0,26578	1
Usf3	-0,26594	1
Rbmx2-ps	-0,26603	1
Zfp942	-0,26608	1
Clic4	-0,26609	1
Ang	-0,26632	1
Maff	-0,26628	1
Poln	-0,26645	1
1700056N10Rik	-0,26645	1
Pyroxd1	-0,26649	1
Zfyve16	-0,2667	1
Tmem183a	-0,26712	1
Was	-0,2672	1
Bdh2	-0,26748	1
Gm12346	-0,26761	1
Gm44434	-0,26766	1
Usp48	-0,26783	1

Il16	-0,26809	1
Alox5ap	-0,26817	1
Spag4	-0,26844	1
Rpl5	-0,26855	1
Gm38162	-0,26856	1
Ceacam1	-0,26861	1
Ttc39b	-0,269	1
Nrd1	-0,26902	1
Slc12a4	-0,26919	1
Tubb4a	-0,2693	1
Cmtm4	-0,26925	1
Thg1l	-0,26962	1
Dvl3	-0,26994	1
Cyfip1	-0,26993	1
Gm5586	-0,27012	1
Gm43162	-0,27024	1
Mbd4	-0,27046	1
Chchd5	-0,27046	1
Gm12693	-0,27062	1
Gpn3	-0,27072	1
Hnrnpul1	-0,27071	1
Gm37390	-0,27086	1
B230322F03Rik	-0,27091	1
Zfp280c	-0,27095	1
Lrrk1	-0,27099	1
Tmem170b	-0,27112	1
4930579G24Rik	-0,27122	1
B3gntl1	-0,27129	1
Zfp932	-0,27134	1
Tmem263	-0,27128	1
Tmem241	-0,27136	1
Ipo7	-0,27138	1
Setd6	-0,27146	1
Zzz3	-0,27169	1
Ergic3	-0,27165	1
Amhr2	-0,27177	1
Pitpna	-0,27177	1
Nagk	-0,27215	1
Pdcl3	-0,2723	1
Pbdc1	-0,27237	1
Oxa1l	-0,27301	1
Senp7	-0,27322	1
Nlgn2	-0,27334	1
Gm37145	-0,2734	1
Map2k7	-0,2734	1
Rmi1	-0,27362	1
Rai1	-0,27363	1
Narf	-0,27363	1
Sf3b1	-0,27362	1
Spata33	-0,27372	1
Men1	-0,27372	1
Mycn	-0,2738	1

RP23-70B19.5	-0,27391	1
Gm5093	-0,27399	1
Zfp212	-0,27422	1
Gm16439	-0,27421	1
Abcc4	-0,27434	1
Ubfd1	-0,27435	1
Gm13886	-0,27449	1
Il18	-0,27465	1
Arl6ip5	-0,27466	1
Fam65a	-0,2752	1
Gm12857	-0,27527	1
Gm37183	-0,27542	1
E230032D23Rik	-0,27537	1
Pfkfb2	-0,27547	1
Nicn1	-0,27547	1
Slc25a25	-0,27583	1
Nfkb2	-0,27594	1
Ikbip	-0,27588	1
Chrnb2	-0,27598	1
Smim19	-0,27633	1
Sh3bp1	-0,27634	1
Ttf2	-0,27636	1
Mta1	-0,27645	1
Ppp1r12b	-0,27644	1
Lrrc58	-0,27649	1
Mrps16	-0,27651	1
Itgb1	-0,27654	1
Gnpda1	-0,27664	1
Csnk2b	-0,27679	1
Ubl5	-0,27676	1
Mdp1	-0,27689	1
Eda2r	-0,27707	1
Pogk	-0,27707	1
RP23-3F1.8	-0,27746	1
Zfp827	-0,27757	1
Fam133b	-0,27762	1
Fxn	-0,27799	1
Tmed10	-0,27823	1
Cops4	-0,27827	1
Pmm1	-0,27847	1
Gm12944	-0,27875	1
1500011B03Rik	-0,27877	1
Arhgdib	-0,27875	1
Nupr1l	-0,27888	1
Agpat3	-0,27898	1
Zbtb14	-0,27907	1
Cyth3	-0,27919	1
Arhgef10l	-0,27919	1
C130026I21Rik	-0,27924	1
Nrap	-0,27925	1
Gm11205	-0,27933	1
Ints6l	-0,27941	1

Slc7a8	-0,27939	1
Nme1	-0,27976	1
Klhdc10	-0,27993	1
Adcy7	-0,27994	1
Cblb	-0,28003	1
A130071D04Rik	-0,28001	1
Man1a	-0,28004	1
Mrpl4	-0,28007	1
Samd4b	-0,28006	1
Gm5822	-0,28033	1
Slc35e2	-0,28044	1
Mitf	-0,28045	1
Micu1	-0,28058	1
Fgfr1op	-0,28066	1
Tmem206	-0,28079	1
Rtn4ip1	-0,28085	1
Dhcr24	-0,28082	1
Racgap1	-0,28104	1
A330035P11Rik	-0,28119	1
Col4a5	-0,28117	1
Gngt2	-0,28137	1
Sbno1	-0,28136	1
Hnrnpa2b1	-0,28149	1
Mis18a	-0,28184	1
Nupr1	-0,2818	1
AV356131	-0,28184	1
Ppp3cb	-0,28211	1
C130050O18Rik	-0,28215	1
Hdac11	-0,28219	1
Gm13868	-0,28244	1
Heca	-0,28252	1
Tmem33	-0,28281	1
Ccdc112	-0,28291	1
Zdhhc2	-0,28296	1
Upf1	-0,28305	1
Ldha	-0,28312	1
Polg2	-0,28345	1
Snhg11	-0,28345	1
Capn2	-0,28336	1
Pkm	-0,28344	1
Fbxl5	-0,28367	1
Snhg18	-0,28378	1
Dmxl1	-0,28379	1
A330023F24Rik	-0,28394	1
Selenoh	-0,28402	1
Stim1	-0,28402	1
S100a4	-0,28446	1
Pold4	-0,28464	1
Gm10327	-0,28469	1
Sdf2l1	-0,28472	1
Fam178a	-0,28474	1
Pdk2	-0,28483	1

Xndc1	-0,28482	1
Casp7	-0,28477	1
Gm996	-0,28475	1
Ncbp2	-0,28492	1
Stx12	-0,28489	1
A430046D13Rik	-0,28518	1
Ddrgk1	-0,28515	1
Rps19-ps11	-0,28529	1
Agpat1	-0,28545	1
Sfxn3	-0,28548	1
Atp6v1h	-0,28567	1
Lage3	-0,28576	1
Ppm1m	-0,28593	1
Fignl1	-0,28614	1
Synpo	-0,28605	1
Parg	-0,28618	1
A930004J17Rik	-0,2863	1
Ckb	-0,28627	1
Xpnpep3	-0,28637	1
Eif4a2	-0,28646	1
Gm13373	-0,2867	1
Lamtor4	-0,28692	1
Khk	-0,28692	1
4930427A07Rik	-0,287	1
Ctnna1	-0,28696	1
Memo1	-0,28706	1
Heatr5b	-0,28716	1
Fcgrt	-0,28733	1
Dram1	-0,28742	1
Prmt7	-0,28743	1
Ermp1	-0,28752	1
Nelfcd	-0,28749	1
Zfp710	-0,28765	1
1810043G02Rik	-0,2877	1
Gprasp1	-0,28771	1
Elp4	-0,2879	1
Lpcat1	-0,28819	1
Bicd1	-0,2884	1
Gm15185	-0,28844	1
Gm10093	-0,28855	1
Il15	-0,2887	1
Coro1b	-0,28884	1
Zfp954	-0,28885	1
Col11a2	-0,28888	1
Amotl1	-0,28891	1
Nthl1	-0,289	1
Gm8181	-0,28912	1
Vmp1	-0,2893	1
Agbl3	-0,2894	1
Rps20	-0,28942	1
Snap47	-0,28969	1
U2af2	-0,28974	1

Rpl39	-0,28971	1
Usp37	-0,28967	1
Lsm11	-0,28971	1
Nagpa	-0,28981	1
Mbtd1	-0,28979	1
Rpn2	-0,28983	1
Zfp219	-0,2899	1
Miip	-0,29003	1
Efna1	-0,29023	1
Gm5745	-0,29028	1
Bckdk	-0,29025	1
D10Wsu102e	-0,29044	1
Chd3os	-0,29063	1
Nfatc1	-0,29065	1
Gm5619	-0,29091	1
Ccnb2	-0,29095	1
Phyh	-0,2909	1
Rnf13	-0,29095	1
Utp14b	-0,29108	1
Mipep	-0,29114	1
BC017158	-0,29122	1
Gm45137	-0,29143	1
Tmem151a	-0,29143	1
Birc6	-0,29139	1
Fam193a	-0,29154	1
Gm10076	-0,29196	1
G430095P16Rik	-0,29215	1
Stx4a	-0,29207	1
Batf2	-0,29242	1
Acap3	-0,2924	1
Laptm4b	-0,29256	1
Phf21a	-0,2926	1
Uimc1	-0,29284	1
Slc43a3	-0,29304	1
Dnajb1	-0,29296	1
Ndrg1	-0,29314	1
Gm6085	-0,29322	1
Acaa1a	-0,29317	1
Zfp322a	-0,29328	1
Paics	-0,29328	1
Heatr5a	-0,2935	1
Gtf3a	-0,29345	1
Dynlt3	-0,29364	1
Trpm4	-0,29379	1
Mcf2l	-0,29379	1
Acsl5	-0,29395	1
Inafm1	-0,2941	1
Rpl21-ps6	-0,29434	1
Ddx50	-0,29433	1
Mfge8	-0,29426	1
Tmem9	-0,2944	1
Paxbp1	-0,29437	1

Rps27a	-0,29454	1
Pip5k1b	-0,29458	1
Btbd9	-0,29501	1
Gm17971	-0,29515	1
Xpr1	-0,29515	1
Ube3b	-0,29545	1
1700025G04Rik	-0,29557	1
Sec63	-0,29561	1
Arglu1	-0,29567	1
Sumf1	-0,29584	1
Slc25a39	-0,29576	1
Tuba1b	-0,29601	1
Rpl22l1	-0,29602	1
Bloc1s2	-0,29611	1
Smc1a	-0,29628	1
2210016L21Rik	-0,2965	1
Uggt1	-0,29652	1
Cuedc2	-0,29694	1
Mertk	-0,29695	1
Gm43627	-0,29699	1
Tnfrsf17	-0,29712	1
Sdhaf3	-0,2972	1
Gm42872	-0,29738	1
Gpsm3	-0,29765	1
Kctd2	-0,29759	1
Limd2	-0,29778	1
Gm42632	-0,29789	1
Slc35d1	-0,29787	1
Katna1	-0,29815	1
Tob1	-0,29821	1
Slc2a6	-0,29826	1
Setd1a	-0,29854	1
Hba-a1	-0,29859	1
Man1b1	-0,29865	1
Haus1	-0,29866	1
Zdhhc6	-0,29892	1
E330009J07Rik	-0,29886	1
Edc3	-0,29888	1
Gm20442	-0,29915	1
Exosc5	-0,29911	1
Max	-0,29911	1
Lgmn	-0,29918	1
Aes	-0,29924	1
Zfp512b	-0,2995	1
Bmi1	-0,29959	1
Ak2	-0,29963	1
Commd1	-0,2998	1
Dennd1b	-0,30005	1
Rest	-0,29999	1
Hmga2	-0,30009	1
Rhoa	-0,30005	1
Zbtb12	-0,30017	1

Ccdc107	-0,30061	1
Hps3	-0,30057	1
Cnot6l	-0,30059	1
Mtfr2	-0,30078	1
Prcc	-0,30076	1
Map2k2	-0,30093	1
Hax1	-0,30101	1
Smpdl3b	-0,30096	1
Ctsl	-0,30099	1
Arcn1	-0,30112	1
Grn	-0,30114	1
Eif2a	-0,30125	1
Ubr4	-0,30119	1
Mapk3	-0,30129	1
Arl6ip6	-0,3014	1
Afdn	-0,30157	1
Cox7c	-0,30178	1
Pabpc1	-0,30205	1
Glrx	-0,30218	1
Osgep	-0,30219	1
Pdpn	-0,30237	1
Ola1	-0,30264	1
Wrap53	-0,30298	1
Crot	-0,30317	1
Vps41	-0,30362	1
Dnajc13	-0,30368	1
Kmt5c	-0,30372	1
Trim7	-0,30393	1
Tmed4	-0,30438	1
Plekhh2	-0,30451	1
Pemt	-0,30465	1
Manea	-0,30491	1
Suv39h1	-0,30493	1
Bola3	-0,30485	1
Gnaq	-0,30515	1
Synj2bp	-0,30545	1
Sdc1	-0,30555	1
Gm5944	-0,30569	1
Map3k2	-0,30571	1
Wdr13	-0,30566	1
Gins2	-0,30589	1
Mrpl28	-0,30596	1
Map3k3	-0,30616	1
Rhog	-0,30641	1
Celf1	-0,30658	1
Lonp1	-0,30665	1
Tnfaip3	-0,30688	1
Pddc1	-0,307	1
Lrpprc	-0,30714	1
Abca7	-0,30722	1
Tbx15	-0,30752	1
Zfp608	-0,30752	1

Rab24	-0,30745	1
Gm14513	-0,30759	1
Tmem165	-0,30771	1
Crcp	-0,30781	1
Mrpl33	-0,30802	1
Basp1	-0,30807	1
Kcnd1	-0,30822	1
Tlcd1	-0,3082	1
Rps23	-0,30826	1
Iqsec1	-0,30832	1
Unc93b1	-0,30863	1
Slc25a23	-0,30864	1
Gm16379	-0,30887	1
Gm28809	-0,30892	1
Ndufs1	-0,30892	1
Kifc5b	-0,30896	1
1110051M20Rik	-0,30909	1
Acly	-0,30918	1
Repin1	-0,30935	1
Gm5525	-0,3098	1
Hjurp	-0,30977	1
Phf6	-0,30992	1
Yipf1	-0,31012	1
Fgf11	-0,31017	1
Luzp1	-0,3102	1
Scpep1	-0,31028	1
Mpi	-0,31043	1
Sgsm3	-0,31049	1
Itsn1	-0,3105	1
Txn2	-0,31054	1
Fbxo2	-0,31059	1
Lamtor3	-0,31062	1
B3gnt2	-0,3107	1
E4f1	-0,31073	1
Acvrl1	-0,31077	1
Stbd1	-0,31093	1
Sfmbt1	-0,31102	1
Mef2c	-0,31122	1
Gtpbp10	-0,31131	1
Mprip	-0,31126	1
Crebrf	-0,31131	1
Lrig3	-0,31135	1
Cdk14	-0,3115	1
5530601H04Rik	-0,31156	1
Dpp8	-0,31159	1
Pcna	-0,31157	1
Tep1	-0,31163	1
Gm16177	-0,31169	1
Fam46a	-0,31168	1
Ptprj	-0,31209	1
Gm7799	-0,31219	1
Extl3	-0,31261	1

Gm7380	-0,31275	1
Gm37706	-0,3127	1
Hspa4l	-0,31284	1
Arpc1b	-0,31317	1
1110065P20Rik	-0,31327	1
Xk	-0,3134	1
Gm10499	-0,3134	1
Mettl15	-0,31351	1
Sipa1l3	-0,31388	1
Gm42466	-0,31387	1
Sh2b1	-0,31398	1
Vdac1	-0,31402	1
Nectin4	-0,31407	1
B2m	-0,31409	1
Ccser2	-0,3142	1
Rps6-ps4	-0,31428	1
Lactb2	-0,31446	1
Vps26b	-0,31452	1
Zc3h11a	-0,31468	1
Rps12-ps1	-0,31466	1
Hacl1	-0,3148	1
Dusp7	-0,31486	1
Zzef1	-0,31487	1
Gm7899	-0,31495	1
Hprt	-0,31495	1
Chek1	-0,31516	1
Skiv2l2	-0,31535	1
Ccnj	-0,31542	1
Gm45053	-0,31547	1
Pradc1	-0,3155	1
RP23-453B15.7	-0,31572	1
Htt	-0,31592	1
Gm5687	-0,31595	1
Plxnd1	-0,31598	1
Rab13	-0,31621	1
Nfxl1	-0,31626	1
Gm43714	-0,31641	1
Gm42941	-0,31662	1
Ndrg3	-0,3167	1
1500011K16Rik	-0,31677	1
Rps19-ps3	-0,31688	1
Rpl35a	-0,31699	1
Trip13	-0,31708	1
Abcb8	-0,31723	1
Ndufaf2	-0,3172	1
Adat2	-0,31725	1
Gm38120	-0,31751	1
D430042O09Rik	-0,31757	1
Tmem97	-0,31816	1
Kmt2c	-0,31825	1
Gm6419	-0,31828	1
Airn	-0,31844	1

Ndufs6	-0,31837	1
Gm43795	-0,31847	1
Clpb	-0,31852	1
Cenpl	-0,31851	1
Sdf2	-0,31863	1
Tmx4	-0,31856	1
Scd1	-0,31885	1
Bbs12	-0,31896	1
Ccdc91	-0,31915	1
Pmvk	-0,31909	1
Cox17	-0,31923	1
Capn15	-0,31935	1
Procr	-0,31936	1
Slc35a4	-0,31947	1
Galk2	-0,31973	1
Ttc14	-0,32	1
Hmbox1	-0,32021	1
Fkbp3	-0,32016	1
0610037L13Rik	-0,32036	1
Rab31	-0,32037	1
Phf7	-0,32064	1
Ndufab1	-0,32067	1
Ap2b1	-0,32073	1
Cyp4f13	-0,32083	1
Fgfr1op2	-0,32085	1
Slc25a36	-0,32077	1
Rbm39	-0,32088	1
Pikfyve	-0,32104	1
Ptgs2	-0,32122	1
Hnrnpd	-0,32134	1
Josd2	-0,3214	1
Gm5624	-0,32149	1
Idnk	-0,32153	1
Armc8	-0,32147	1
Gm38022	-0,32162	1
RP24-282C4.10	-0,322	1
C330013E15Rik	-0,32205	1
Tmem218	-0,32197	1
Gm4784	-0,32209	1
Slc16a5	-0,32209	1
Srgap3	-0,32263	1
Mboat7	-0,32277	1
Gm9531	-0,32288	1
Col4a3bp	-0,3232	1
Kctd9	-0,32317	1
Zmynd10	-0,32326	1
Drosha	-0,32332	1
Cox7a2l	-0,32326	1
Gm15575	-0,32344	1
Atp13a1	-0,32341	1
Arhgef12	-0,32346	1
Clu	-0,32346	1

Ankrd37	-0,32364	1
Ypel2	-0,32372	1
Mpv17l2	-0,32369	1
Nav2	-0,32389	1
Gm8719	-0,32395	1
Hps1	-0,32398	1
4930503L19Rik	-0,32404	1
Snx12	-0,32396	1
Nbn	-0,32422	1
Bloc1s1	-0,32419	1
Rpl29	-0,32419	1
Pdpk1	-0,32429	1
Gm7353	-0,32448	1
Elk4	-0,32448	1
Rnf181	-0,32464	1
Rpl32-ps	-0,32466	1
Gbas	-0,3249	1
Lpin2	-0,32486	1
Rhbdf1	-0,32501	1
Dennd1c	-0,32506	1
1190007I07Rik	-0,32524	1
Actr1a	-0,3252	1
Zfp687	-0,32531	1
Akr1b8	-0,32532	1
Gm17491	-0,32583	1
Pttg1	-0,32593	1
Ncaph2	-0,32591	1
Gm8623	-0,32632	1
Ankrd35	-0,32641	1
Hsp90b1	-0,32674	1
Ndufa2	-0,32673	1
Gm1976	-0,32694	1
Efcab2	-0,32694	1
Spag5	-0,32728	1
Elof1	-0,32727	1
Tmem128	-0,32758	1
Plin2	-0,32764	1
Vars2	-0,32773	1
Ptpa	-0,32772	1
Tmem238	-0,32784	1
St7	-0,3279	1
Cox6a1	-0,32789	1
Osbpl10	-0,32833	1
Nek9	-0,32852	1
Plec	-0,32854	1
Edem2	-0,32863	1
Dnase1l1	-0,32872	1
Usp53	-0,32865	1
Cenpk	-0,32879	1
Egln1	-0,32884	1
Rgl3	-0,32894	1
Dand5	-0,32909	1

Rps15a	-0,32934	1
Gpcpd1	-0,32944	1
Ebpl	-0,32937	1
Srebf2	-0,32947	1
Cebpg	-0,32953	1
Gm43343	-0,32956	1
Cdkn1b	-0,32961	1
Cep44	-0,32983	1
Cyb5b	-0,32985	1
Gxylt1	-0,3303	1
Otud3	-0,33047	1
Gm6142	-0,33055	1
Igf2r	-0,33066	1
Serp1	-0,33065	1
Gm14005	-0,33077	1
Dopey1	-0,33084	1
Ggh	-0,33086	1
Usp24	-0,33093	1
Creb1	-0,33098	1
Pias3	-0,33125	1
Slc5a3	-0,33128	1
0610030E20Rik	-0,33143	1
Tardbp	-0,33152	1
Ypel5	-0,33153	1
Mogat1	-0,33184	1
Slc16a10	-0,33185	1
Pnkd	-0,33192	1
Usp49	-0,33204	1
Ikbke	-0,33205	1
Cfl1	-0,33205	1
Arl5c	-0,33215	1
Zfp652	-0,33228	1
Flrt2	-0,33263	1
Ipo8	-0,33275	1
Snrpc	-0,33277	1
Zfa-ps	-0,33277	1
Gpn2	-0,33295	1
Rpsa-ps11	-0,33306	1
Ergic1	-0,33311	1
Tm6sf1	-0,33332	1
Dusp11	-0,33354	1
Rpl32	-0,33392	1
Tfe3	-0,33391	1
Neurl4	-0,33402	1
Nup93	-0,33443	1
Rnf213	-0,33451	1
Glrx3	-0,33464	1
Src	-0,3347	1
Lsr	-0,33466	1
Tet3	-0,33474	1
Anxa5	-0,33475	1
Rapgef5	-0,33475	1

Tcam1	-0,33478	1
Cdc23	-0,33486	1
Tuba1a	-0,33522	1
Zdhhc18	-0,33519	1
Ncor2	-0,33523	1
Slc30a7	-0,33585	1
Lipe	-0,33606	1
Gm23458	-0,33611	1
RP24-454N4.2	-0,33624	1
Map3k8	-0,33618	1
Rasal2	-0,33637	1
Ccdc92	-0,33648	1
Cnpy3	-0,33646	1
Ssbp1	-0,33675	1
Smurf1	-0,3367	1
Gm6272	-0,33668	1
E130307A14Rik	-0,33687	1
Nup153	-0,33715	1
Gm24507	-0,33733	1
Car6	-0,33725	1
Fam92a	-0,33745	1
Klhl6	-0,33769	1
Gm9143	-0,33783	1
Pak4	-0,33821	1
Tmtc3	-0,33835	1
Bst1	-0,33833	1
Fastk	-0,33859	1
Gm10382	-0,33919	1
Stab1	-0,33942	1
Snord104	-0,33943	1
Cs	-0,33944	1
Pnpt1	-0,3395	1
Tma7-ps	-0,3396	1
Def6	-0,33966	1
Atrn	-0,3398	1
4933427D14Rik	-0,33983	1
Pih1d1	-0,34005	1
Xrcc2	-0,34011	1
Tsc1	-0,34028	1
Lgals1	-0,3403	1
Rpl31-ps1	-0,34046	1
Apobec3	-0,34047	1
Tom1l1	-0,34056	1
Wdr1	-0,34065	1
Banf1	-0,34069	1
Colgalt1	-0,34077	1
Fam213b	-0,34092	1
Eif2d	-0,34127	1
Usp54	-0,34145	1
Ak3	-0,34242	1
Gm2895	-0,34262	1
Cep57l1	-0,34262	1

Ints9	-0,34262	1
Nup43	-0,34294	1
Ggact	-0,34292	1
BC017643	-0,34286	1
2310009B15Rik	-0,34313	1
Atp9a	-0,34356	1
Hsp90ab1	-0,34372	1
Rbbp8	-0,3439	1
AW046200	-0,34423	1
Golga1	-0,34443	1
Tctn3	-0,34454	1
Gm37199	-0,34499	1
Rnf187	-0,34507	1
Pkib	-0,34544	1
Pecr	-0,34535	1
Acads	-0,34563	1
Gm37352	-0,3457	1
Fry	-0,34569	1
Ttc38	-0,34579	1
Dpysl2	-0,34608	1
Timp2	-0,34623	1
Ptma	-0,34657	1
Xrcc5	-0,34673	1
Cisd2	-0,34669	1
Rnf32	-0,34696	1
Mrpl38	-0,34708	1
Zfp994	-0,34726	1
Gcc2	-0,34741	1
Mir5128	-0,34757	1
Ociad2	-0,34761	1
Entpd5	-0,34774	1
Slc17a7	-0,34768	1
Iqgap3	-0,3478	1
Rnf5	-0,3482	1
Mdm4	-0,34824	1
Tcf25	-0,34853	1
Prim2	-0,34858	1
Fam114a1	-0,34861	1
Pdlim2	-0,34886	1
H2-DMb1	-0,34901	1
Inafm2	-0,34922	1
2310036O22Rik	-0,3492	1
Gna13	-0,34921	1
Clec4n	-0,34974	1
Ppm1k	-0,34971	1
Gm44836	-0,34984	1
Tmed8	-0,34992	1
Dgcr14	-0,35046	1
Tcp11l1	-0,35058	1
Serpine1	-0,35056	1
Gsn	-0,35073	1
Arrdc4	-0,35072	1

Araf	-0,35117	1
Nek1	-0,35126	1
Calr	-0,35126	1
Arhgef10	-0,35137	1
Syt11	-0,35142	1
Apopt1	-0,35149	1
Abca3	-0,35161	1
Marf1	-0,35172	1
Sae1	-0,35168	1
Cox6c	-0,35166	1
Gm12115	-0,35178	1
Neurl2	-0,35195	1
Gm16418	-0,35204	1
Gm9776	-0,35203	1
Magohb	-0,35211	1
Ddx19b	-0,35211	1
Vim	-0,35227	1
Nudt2	-0,35236	1
Plbd2	-0,3524	1
Irak3	-0,35248	1
Zfp787	-0,35247	1
Gatm	-0,35263	1
Klhl42	-0,3527	1
Gm38104	-0,35288	1
Acoxl	-0,35308	1
Kars	-0,35312	1
Fbxl8	-0,3532	1
Gm14137	-0,35325	1
Zscan26	-0,35329	1
Pym1	-0,35348	1
Yod1	-0,35365	1
Kansl3	-0,35364	1
Ccdc28a	-0,35374	1
Cul9	-0,35372	1
Gm5453	-0,35383	1
Mvb12a	-0,35375	1
Atp5b	-0,35406	1
Gm3940	-0,35424	1
Ago1	-0,35432	1
Gm7809	-0,35434	1
Wdr70	-0,35428	1
Dck	-0,35436	1
Stac2	-0,35452	1
Mpst	-0,35468	1
Clec1a	-0,35484	1
Urm1	-0,35484	1
Gm8213	-0,35493	1
Mfn1	-0,35494	1
Calr-ps	-0,35523	1
Sun2	-0,35523	1
Zfyve19	-0,35551	1
Gm2830	-0,35577	1

Fasn	-0,35624	1
Tcte2	-0,35644	1
Ppp2r3d	-0,35669	1
H2-K2	-0,35674	1
Acy1	-0,35672	1
Brd8	-0,35709	1
Ccp110	-0,35729	1
Bmt2	-0,35734	1
4933421O10Rik	-0,35744	1
Mkl2	-0,35752	1
Gm9025	-0,35772	1
Rsu1	-0,35782	1
Slc25a11	-0,35788	1
Apaf1	-0,35807	1
St18	-0,35838	1
Ydjc	-0,35871	1
Raf1	-0,35947	1
Vrk1	-0,3595	1
Il23a	-0,3599	1
Tmem18	-0,36005	1
Ascc2	-0,36034	1
Lsm2	-0,3604	1
Zgrf1	-0,36056	1
Ttc9c	-0,36065	1
Zcchc4	-0,36123	1
BC055324	-0,36128	1
Hus1b	-0,36134	1
Manf	-0,36156	1
Pcca	-0,36181	1
Acaca	-0,36191	1
Fam19a3	-0,36195	1
Pdlim7	-0,36199	1
Rpph1	-0,36198	1
Bend3	-0,36218	1
Gramd1a	-0,36239	1
Gm14439	-0,36245	1
Slc25a17	-0,36255	1
Meg3	-0,363	1
Cep135	-0,36309	1
RP23-269H21.1	-0,36316	1
Rassf1	-0,36317	1
Eps15	-0,36319	1
Patz1	-0,3634	1
Arl16	-0,36359	1
Fkbp15	-0,36362	1
Gnpnat1	-0,36368	1
Foxj3	-0,36367	1
Hadh	-0,36373	1
Dvl1	-0,36366	1
Abhd14a	-0,36384	1
Tfap4	-0,36378	1
Hoxc6	-0,36382	1

Tef	-0,36376	1
Prelid1	-0,36386	1
AC133103.1	-0,36413	1
E130208F15Rik	-0,36414	1
Smim8	-0,36471	1
Mpzl1	-0,3648	1
Tmed7	-0,36523	1
Acvr2b	-0,36544	1
Fahd2a	-0,36547	1
Fignl2	-0,36561	1
Samhd1	-0,36575	1
Zfp414	-0,3662	1
Ppp1r18os	-0,3662	1
Agtpbp1	-0,36641	1
Itfg1	-0,36667	1
Rhobtb1	-0,36684	1
Ift52	-0,36695	1
6330562C20Rik	-0,367	1
Zc3h3	-0,36696	1
Cers5	-0,36734	1
Cox20-ps	-0,36767	1
Nprl2	-0,36779	1
Tspan17	-0,36808	1
Pbxip1	-0,36812	1
H2-T23	-0,36851	1
Nup205	-0,3686	1
Lmf1	-0,36902	1
Adi1	-0,36897	1
Ifitm2	-0,36914	1
Ccng2	-0,36917	1
Tbc1d24	-0,36926	1
3110045C21Rik	-0,36934	1
Rint1	-0,36941	1
Rab34	-0,36953	1
Tmem5	-0,36985	1
Idi1	-0,36982	1
Adk	-0,36977	1
Ssr3	-0,36986	1
Scarna2	-0,37005	1
Wwc2	-0,3703	1
Snx27	-0,37063	1
Gchfr	-0,37073	1
Pus7	-0,37089	1
Dnajc15	-0,371	1
E2f2	-0,37098	1
Polb	-0,37105	1
Timmdc1	-0,37108	1
Anxa1	-0,37118	1
Enoph1	-0,37124	1
Slx1b	-0,3713	1
Tecr	-0,37131	1
Bbs7	-0,37144	1

Rab28	-0,37155	1
Dnaaf3	-0,37171	1
Rrh	-0,37182	1
Pabpc4	-0,37178	1
Rock2	-0,37183	1
Stat1	-0,372	1
Snord82	-0,37206	1
Vps9d1	-0,37238	1
Trpt1	-0,37278	1
Mlx	-0,37285	1
Usp8	-0,3728	1
Tial1	-0,37311	1
Wipi1	-0,3733	1
Vars	-0,37351	1
Rab27a	-0,37362	1
Gm2467	-0,37358	1
Mlf1	-0,37375	1
Ebi3	-0,37387	1
Brms1	-0,37399	1
Slc38a2	-0,37402	1
Trmt1	-0,37399	1
Lyst	-0,37405	1
Myh9	-0,3742	1
Hspb11	-0,37438	1
Cracr2a	-0,37453	1
Rps12-ps10	-0,37483	1
Gm34121	-0,37487	1
Cep70	-0,37505	1
Acat1	-0,37503	1
Vav3	-0,3753	1
Sidt2	-0,37545	1
Selenof	-0,37567	1
Kat2a	-0,37613	1
Chmp1a	-0,3761	1
Pex1	-0,37622	1
9930012K11Rik	-0,37648	1
Gm5445	-0,37669	1
Rmnd5a	-0,37668	1
Stip1	-0,37666	1
Dpp7	-0,37698	1
Cebpb	-0,37721	1
Rpl10a-ps2	-0,37723	1
Tmed2	-0,37731	1
Syce2	-0,3773	1
Calcrl	-0,37764	1
R3hdm2	-0,37763	1
Ripk3	-0,3779	1
Ect2	-0,37791	1
Setx	-0,37832	1
Kdm5d	-0,37839	1
RP23-63H11.3	-0,37853	1
Prpf4b	-0,3787	1

Ppid	-0,37873	1
Ammecr1	-0,37886	1
Psip1	-0,3792	1
Abi2	-0,37929	1
B230307C23Rik	-0,37938	1
Xylt1	-0,3794	1
Smim7	-0,37953	1
Gm36266	-0,37994	1
Cnpy2	-0,38007	1
Amn1	-0,38034	1
Srrt	-0,38035	1
Stard4	-0,38034	1
Ccna2	-0,38043	1
Trp53rkb	-0,38087	1
Calm3	-0,38125	1
Rhd	-0,38138	1
Ska2	-0,38195	1
Cntln	-0,38242	1
Gm15050	-0,38236	1
Kitl	-0,38257	1
Wrn	-0,38274	1
Rbm26	-0,38314	1
Dut	-0,38329	1
Galnt10	-0,3835	1
Tmem176a	-0,38365	1
Plpp5	-0,38391	1
Cmc2	-0,38393	1
Otub1	-0,38409	1
Slc22a15	-0,38419	1
Mtfr1	-0,38454	1
Sep 10	-0,38448	1
Mrpl42	-0,38467	1
N4bp2	-0,38489	1
Bop1	-0,38502	1
Ncapg2	-0,38504	1
Gm38020	-0,38508	1
Sh3bgr	-0,38508	1
A630081D01Rik	-0,38508	1
Gm13196	-0,38531	1
P3h3	-0,38544	1
Wwox	-0,38574	1
Uxs1	-0,38596	1
Amd1	-0,38606	1
Padi2	-0,38627	1
Cybb	-0,38629	1
Ppt1	-0,38627	1
Xrcc4	-0,38646	1
Prim1	-0,38651	1
Fbl	-0,38651	1
Me1	-0,38646	1
Gm30329	-0,3866	1
Pon2	-0,38674	1

Kcnc3	-0,38691	1
Cc2d1b	-0,38692	1
Lrba	-0,38705	1
Tfdp2	-0,38721	1
Zfp580	-0,38721	1
Ston1	-0,3874	1
RP23-134M7.3	-0,3874	1
Flt3l	-0,38773	1
AI662270	-0,38779	1
Ift57	-0,38806	1
Mgst2	-0,38834	1
Gm37140	-0,38857	1
Ptbp2	-0,38877	1
Gk5	-0,38886	1
1810032O08Rik	-0,38912	1
Guf1	-0,38936	1
Fryl	-0,38939	1
Lrp1	-0,38941	1
Stxbp3	-0,38964	1
Fh1	-0,38963	1
Gm2756	-0,38968	1
Fbxo15	-0,38971	1
Trim46	-0,3898	1
Spef1	-0,38981	1
Foxk2	-0,38989	1
Prkar2b	-0,39013	1
Gm11451	-0,39016	1
Mrpl19	-0,39028	1
Gm22714	-0,39042	1
RP23-403D16.3	-0,39053	1
Gm28659	-0,39076	1
Cryzl1	-0,39078	1
Mthfd2	-0,3909	1
Pnrc2	-0,3913	1
Gdi1	-0,39144	1
Agps	-0,39136	1
Stk11	-0,39147	1
RP24-389J11.1	-0,39206	1
Ap5z1	-0,39218	1
Acadsb	-0,39216	1
Eloc	-0,39228	1
Map3k9	-0,39254	1
Phkg2	-0,39263	1
Psat1	-0,39313	1
Gm16580	-0,39325	1
Gm26930	-0,39325	1
Vwa7	-0,39343	1
Vcpkmt	-0,39348	1
Ralgapa1	-0,39414	1
Zdhhc9	-0,3941	1
Fam234a	-0,3942	1
Acadvl	-0,39422	1

Fkbp8	-0,39434	1
Cpt1c	-0,39436	1
Atxn1l	-0,39453	1
Erlec1	-0,39472	1
Alg10b	-0,39481	1
Arfip1	-0,39494	1
Il6ra	-0,39515	1
Sergef	-0,39542	1
Fam20b	-0,39538	1
Gm38299	-0,39551	1
Tmco4	-0,39573	1
Rnf126	-0,39578	1
4930578M07Rik	-0,39586	1
Inpp5k	-0,39597	1
Slx4ip	-0,39619	1
Fam53b	-0,39616	1
Med14	-0,39635	1
Cuedc1	-0,39644	1
Ddx17	-0,39636	1
Naga	-0,39687	1
Chpf	-0,39703	1
Zfp365	-0,39703	1
Cdnf	-0,39715	1
Bbs9	-0,39725	1
Snx29	-0,39751	1
Lrrc20	-0,39758	1
Gm17690	-0,39776	1
Dst	-0,39777	1
Dpagt1	-0,3979	1
Tapbp	-0,39803	1
Zwilch	-0,39814	1
Gm43300	-0,39809	1
Ptrh1	-0,39831	1
Hist1h1e	-0,3984	1
Atp2b4	-0,399	1
Mut	-0,39924	1
Med12	-0,39943	1
Hnrnpdl	-0,39942	1
9030407P20Rik	-0,39948	1
Gm10240	-0,39959	1
Ogfod1	-0,39967	1
Rps13-ps7	-0,39983	1
Gm5914	-0,39984	1
Ccdc50-ps	-0,39986	1
Porcn	-0,40008	1
Gm13050	-0,40014	1
5730508B09Rik	-0,40009	1
Sep 06	-0,40009	1
Vegfb	-0,40024	1
Ankzf1	-0,4005	1
Gm36445	-0,40052	1
Rps6ka5	-0,40062	1

Aldh4a1	-0,40072	1
1810037I17Rik	-0,4007	1
Pik3r6	-0,40084	1
Hcst	-0,40087	1
Ptpn18	-0,40152	1
Syngap1	-0,40155	1
Gm13464	-0,40166	1
Cdca7l	-0,40193	1
Loxl3	-0,40197	1
Cpsf1	-0,40199	1
Clasrp	-0,40222	1
Slc37a2	-0,40225	1
Usp15	-0,40244	1
D630024D03Rik	-0,40262	1
Gm13573	-0,40271	1
RP23-114G13.7	-0,40299	1
Mga	-0,40322	1
Rpl15	-0,40344	1
Ckap2	-0,40383	1
Tnfrsf9	-0,40376	1
Ascc3	-0,40387	1
RP23-164P21.3	-0,4041	1
Gm7132	-0,40456	1
Sft2d1	-0,4047	1
Ptpn9	-0,40487	1
Dap	-0,40512	1
Qpctl	-0,40566	1
Chfr	-0,40572	1
Aaas	-0,40581	1
Haghl	-0,40596	1
Klhl24	-0,40598	1
Me2	-0,40633	1
Ipp	-0,40648	1
Gm8268	-0,40657	1
Gm12090	-0,40656	1
Epn2	-0,40655	1
Fto	-0,40713	1
Akt3	-0,40748	1
Cdc14b	-0,40769	1
Myh7b	-0,40766	1
Myof	-0,40793	1
St3gal2	-0,4083	1
Dennd4c	-0,40871	1
Ube2i	-0,40877	1
Hes6	-0,40898	1
Snrpa	-0,40913	1
Acta2	-0,40992	1
Eva1b	-0,4101	1
Gm6382	-0,41016	1
Lztfl1	-0,41061	1
Chd3	-0,4107	1
Arl6ip4	-0,41093	1

Pxmp2	-0,411	1
Fam103a1	-0,41114	1
Gm5069	-0,4111	1
Strbp	-0,41124	1
Socs4	-0,41171	1
Gm7312	-0,4118	1
2610203C22Rik	-0,41184	1
Akr7a5	-0,41176	1
Mtch1	-0,41177	1
4933417C20Rik	-0,41183	1
Il13ra1	-0,41198	1
Isyna1	-0,41234	1
Rpusd1	-0,41235	1
Ncoa7	-0,41283	1
Peak1	-0,41281	1
Lacc1	-0,41286	1
Slc44a2	-0,41306	1
Wwp2	-0,4132	1
Gm9938	-0,41325	1
Grina	-0,4137	1
Slc9a5	-0,41404	1
Gatsl2	-0,41414	1
Mdh1	-0,41408	1
Spred3	-0,41428	1
Ccdc94	-0,41426	1
Rfwd3	-0,41437	1
Gpi1	-0,41471	1
Prpf38b	-0,41475	1
Pex13	-0,41498	1
Gm19353	-0,41519	1
Cd36	-0,41549	1
Aatk	-0,41551	1
Gm42979	-0,4156	1
Adm	-0,41563	1
Arl4d	-0,41557	1
Ccdc15	-0,41565	1
5033430I15Rik	-0,41566	1
Sf3a1	-0,41574	1
Wdhd1	-0,41581	1
Ppm1j	-0,41607	1
Fam210a	-0,41614	1
Gm12924	-0,41616	1
Hyou1	-0,41636	1
Foxo4	-0,41672	1
Gm13009	-0,41678	1
Ganab	-0,4169	1
Zbtb34	-0,41735	1
Gm5857	-0,41743	1
Vps13c	-0,41758	1
Txndc15	-0,41766	1
Gm9790	-0,41774	1
Hspe1	-0,41781	1

Bcl2a1d	-0,41781	1
Aldh18a1	-0,41786	1
Nfat5	-0,418	1
Triobp	-0,41821	1
Tifab	-0,41827	1
Gm15445	-0,41827	1
Ankrd13a	-0,41839	1
Vwa5a	-0,41843	1
Tubb5	-0,41838	1
Serhl	-0,41875	1
Tnrc6b	-0,41883	1
Pfn1	-0,41888	1
2010204K13Rik	-0,41913	1
Kif15	-0,4191	1
Epm2aip1	-0,4191	1
Zw10	-0,41909	1
Msto1	-0,4192	1
Mki67	-0,41976	1
Ttll3	-0,4199	1
Itpkb	-0,41992	1
Gpr137b-ps	-0,41986	1
Slc38a9	-0,41987	1
Pitrm1	-0,42057	1
Dna2	-0,42072	1
Gm7436	-0,42101	1
Tpi1	-0,42108	1
Mdn1	-0,42119	1
Mafk	-0,42144	1
Chm	-0,42148	1
Wnt6	-0,42194	1
Bscl2	-0,42218	1
Rpl9	-0,4223	1
Immp1l	-0,4224	1
Rpl39-ps	-0,42238	1
Kiz	-0,42267	1
Nenf	-0,42284	1
2900060B14Rik	-0,42299	1
Gnas	-0,42312	1
Sdccag8	-0,42352	1
Rps12-ps23	-0,42347	1
Rpl17	-0,42363	1
Tfrc	-0,42358	1
Ndufb2	-0,42364	1
Fbxl18	-0,42374	1
Fn3krp	-0,42394	1
BC024978	-0,4242	1
Ddr1	-0,4242	1
Mras	-0,4242	1
Gm19026	-0,4242	1
C230035I16Rik	-0,4242	1
Slc1a4	-0,4242	1
Gm4117	-0,4242	1

Gtf2i	-0,4244	1
Prpf8	-0,42469	1
Sil1	-0,42474	1
Blm	-0,42499	1
Smyd2	-0,42511	1
Trmt1l	-0,42515	1
Nfatc2	-0,42544	1
Sep 09	-0,4255	1
Camk1d	-0,42556	1
Tiam1	-0,42565	1
Dlgap5	-0,42575	1
Prkcsh	-0,42639	1
Ipo4	-0,42653	1
Usp28	-0,42666	1
Atm	-0,42696	1
RP23-128C4.4	-0,42728	1
Rpsa	-0,42774	1
Pebp1	-0,42772	1
Kpnb1	-0,42794	1
Rps24	-0,42795	1
C330007P06Rik	-0,42834	1
Cep295	-0,42827	1
Vamp4	-0,42827	1
Gm14292	-0,42861	1
Dync1li2	-0,4287	1
Abca2	-0,42884	1
Pdia6	-0,42882	1
Rasgrp4	-0,42888	1
Ddx41	-0,42895	1
Bax	-0,42894	1
Ctsa	-0,42929	1
D930030I03Rik	-0,42942	1
Cpne9	-0,42954	1
Itgav	-0,42957	1
Tgfb1	-0,42964	1
Ssbp4	-0,42992	1
Dock11	-0,43039	1
Zfp410	-0,43045	1
Pabpc1l	-0,43045	1
Treml1	-0,43073	1
Nacc1	-0,43074	1
Atrx	-0,43065	1
Pear1	-0,43116	1
Gm43010	-0,43118	1
Cdc37l1	-0,43152	1
Spire1	-0,43145	1
Gm42611	-0,43158	1
Clmp	-0,43167	1
Gm44623	-0,43194	1
Sirt5	-0,43203	1
Tnfrsf22	-0,43203	1
Gm4705	-0,43211	1

Cry2	-0,4322	1
Cspp1	-0,43246	1
Lrrc2	-0,43261	1
Smc5	-0,43256	1
2410089E03Rik	-0,4328	1
Scai	-0,43299	1
Macrod1	-0,43307	1
Gpx4	-0,43314	1
Znrf1	-0,43322	1
P4hb	-0,43328	1
Mrps36	-0,43347	1
Pde7a	-0,43361	1
Khdrbs1	-0,43377	1
Ash2l	-0,43402	1
Ttll4	-0,43396	1
Bckdhb	-0,4341	1
Sos2	-0,43407	1
Hip1	-0,43419	1
Gm10642	-0,43418	1
Mgarp	-0,4344	1
Gm12689	-0,43444	1
Fam168a	-0,43438	1
Gm5909	-0,43445	1
Gm10275	-0,4347	1
Asb3	-0,43488	1
Rpl31-ps10	-0,43521	1
Eif2s3y	-0,4353	1
Tnfrsf23	-0,43552	1
Uqcr11	-0,43552	1
2810402E24Rik	-0,43565	1
Slc25a26	-0,4358	1
Snora17	-0,43577	1
Ift43	-0,43605	1
Pla2g12a	-0,43649	1
Gm4017	-0,43659	1
Mir5136	-0,43669	1
Gm8210	-0,43684	1
Rad54l	-0,43688	1
Phf11b	-0,43688	1
Fam173a	-0,43711	1
Rras	-0,43717	1
B930036N10Rik	-0,43744	1
Arhgap27os2	-0,43778	1
Fer	-0,43776	1
Myadm	-0,438	1
Rps19-ps9	-0,43828	1
Tpp2	-0,43842	1
B3gnt3	-0,43847	1
Rpa3	-0,43848	1
Pgap2	-0,43849	1
Dusp3	-0,43846	1
Serinc5	-0,43919	1

Cope	-0,43927	1
Ddx59	-0,43945	1
Spcs3	-0,43947	1
Myl12b	-0,4398	1
Glrp1	-0,43977	1
Glg1	-0,43991	1
Smco4	-0,44002	1
Hist2h3c2	-0,44013	1
2810403D21Rik	-0,44046	1
Hmgb1-ps5	-0,4411	1
Rbx1	-0,44137	1
RP24-351I17.3	-0,44136	1
Rsrc2	-0,44171	1
RP24-282C4.9	-0,44184	1
Trappc6a	-0,44178	1
Vdac3-ps1	-0,44203	1
Cfap20	-0,44208	1
Cdkn3	-0,44212	1
Rps13	-0,44233	1
Aco2	-0,4423	1
Bcat1	-0,44259	1
Epb41l5	-0,44279	1
Rbm33	-0,44277	1
Ano8	-0,44289	1
E130317F20Rik	-0,4429	1
Taf4b	-0,44302	1
Ddx43	-0,44344	1
Arfrp1	-0,44387	1
Gm28557	-0,44388	1
Pyurf	-0,44418	1
Gm44694	-0,44463	1
Gm13450	-0,44476	1
Smarcad1	-0,44513	1
AU020206	-0,44515	1
Cpt1a	-0,44519	1
Btf3	-0,44526	1
Nexn	-0,44535	1
Gmds	-0,44535	1
Klhl8	-0,44548	1
Cacna1d	-0,4458	1
H2-Q7	-0,44582	1
Pou6f1	-0,44602	1
Tmed1	-0,44613	1
Naa60	-0,44651	1
Rps15a-ps8	-0,44682	1
Mxd4	-0,4468	1
Vti1a	-0,44686	1
Alcam	-0,44686	1
Cd320	-0,44703	1
Krit1	-0,44708	1
Gm37101	-0,44743	1
Ints1	-0,44757	1

Wdcp	-0,44763	1
Pcx	-0,44772	1
Gm8254	-0,44772	1
Rpl27	-0,44791	1
Fis1	-0,44794	1
Dvl2	-0,44793	1
2310058D17Rik	-0,4483	1
Cyp4f16	-0,44833	1
Lmbrd2	-0,44868	1
Golt1b	-0,4488	1
St3gal4	-0,44912	1
Rfwd2	-0,44931	1
Ctdspl2	-0,44942	1
Gm42659	-0,4495	1
Gm14822	-0,44969	1
Lpgat1	-0,44967	1
Kdm4c	-0,45009	1
Gm8318	-0,45023	1
Rhou	-0,45016	1
Slc41a3	-0,45056	1
Gm37566	-0,4507	1
Nbeal1	-0,45077	1
Gls	-0,45079	1
Pfkm	-0,45094	1
Ly86	-0,45088	1
Myom1	-0,45131	1
Prdm10	-0,45145	1
Klhl7	-0,45162	1
Gm38111	-0,45195	1
Zfp369	-0,45225	1
Gm38297	-0,45232	1
Tmem134	-0,45234	1
Nacc2	-0,45244	1
D830025C05Rik	-0,45263	1
Shtn1	-0,4532	1
Ovgp1	-0,45344	1
C030034I22Rik	-0,45351	1
Arl5a	-0,45402	1
Hinfp	-0,45409	1
Plcb2	-0,45462	1
Smox	-0,45471	1
Ppp4r1l-ps	-0,45488	1
Creb3l3	-0,45522	1
Tmem14a	-0,45522	1
A530072M11Rik	-0,45522	1
Vcl	-0,45526	1
Hypk	-0,45555	1
Gm16754	-0,45557	1
Ubn2	-0,45562	1
Rpl13	-0,45615	1
Srebf1	-0,45627	1
Tfcp2	-0,4566	1

Chchd6		-0,45668	1
Gm12577		-0,4569	1
Nabp1		-0,457	1
Tmco3		-0,45714	1
Saal1		-0,45714	1
Eps15l1		-0,45725	1
Srsf1		-0,45738	1
Mcm7		-0,4576	1
Gm10358		-0,45769	1
Golgb1		-0,45777	1
Cst7		-0,45789	1
Ccni		-0,45791	1
	Sep 01	-0,45793	1
Acaa1b		-0,45799	1
Rpl31-ps13		-0,4582	1
H2-M3		-0,45872	1
Paip1		-0,45876	1
Cenpq		-0,45894	1
Itgb2		-0,45904	1
Snx9		-0,45922	1
Ndufaf3		-0,45939	1
Ier3		-0,46001	1
Acss2		-0,46008	1
Lgals7		-0,46028	1
Aldh6a1		-0,46098	1
G6pc3		-0,46116	1
Dnm1		-0,46139	1
Pcyox1		-0,46171	1
Slc25a37		-0,46169	1
Rbm25		-0,46174	1
Clybl		-0,46181	1
Gm10110		-0,46186	1
Anapc16		-0,46201	1
Ech1		-0,46197	1
Ncoa4		-0,46205	1
Tspan5		-0,46231	1
Cmpk1		-0,46228	1
Gm1862		-0,46241	1
Cdk2ap1		-0,46249	1
1810022K09Rik		-0,46258	1
Iqcb1		-0,46284	1
Gm8203		-0,46284	1
Hacd1		-0,46295	1
Fdxr		-0,46325	1
Gm4997		-0,46319	1
Slc9a3r2		-0,46345	1
Pfkp		-0,46354	1
Ndufc1		-0,4635	1
Ptms		-0,46356	1
Pigq		-0,46395	1
1700086O06Rik		-0,46395	1
Ints6		-0,46407	1

Gng7	-0,46413	1
Gm6329	-0,46437	1
Ubald2	-0,46458	1
Gm9008	-0,46462	1
Plk4	-0,46481	1
Gm5112	-0,46508	1
Nipsnap1	-0,46517	1
Abhd2	-0,4655	1
Tbck	-0,46557	1
Cd9-ps	-0,46564	1
Bre	-0,46571	1
Ccdc17	-0,46592	1
Cdkl3	-0,46635	1
Gm14494	-0,46684	1
Gm23639	-0,467	1
Pcmtd2	-0,46715	1
Rpl23a-ps3	-0,46734	1
Tspan32	-0,46759	1
Zfp81	-0,46807	1
Abhd12	-0,46815	1
Mfap1a	-0,46834	1
Fbxo5	-0,46839	1
Hmgn5	-0,46875	1
Ccdc117	-0,46885	1
Gm44027	-0,46908	1
Add3	-0,46968	1
Etfa	-0,47003	1
Yae1d1	-0,47013	1
Plekha5	-0,4701	1
Gsdmd	-0,4701	1
Dclre1a	-0,47022	1
Nf2	-0,47025	1
E130308A19Rik	-0,47029	1
Isg20l2	-0,47038	1
Gm16062	-0,47062	1
Nxf7	-0,47072	1
Mtl5	-0,47071	1
Cox15	-0,47103	1
Spg7	-0,47167	1
Nfix	-0,47244	1
Tnni3	-0,47237	1
Miga2	-0,47238	1
Churc1	-0,4725	1
Lrrk2	-0,47302	1
Fmnl3	-0,4736	1
Zscan2	-0,47356	1
Rnf217	-0,47429	1
Kdelc2	-0,47431	1
Uhrf1	-0,47444	1
RP23-356P21.1	-0,47448	1
Amt	-0,47468	1
Ttc19	-0,47485	1

Mir155hg	-0,47487	1
Nisch	-0,47522	1
Efna3	-0,47591	1
Txndc16	-0,47598	1
Plekha1	-0,47623	1
Phka2	-0,47632	1
C330027C09Rik	-0,47686	1
Nucb2	-0,4771	1
Rhbdd1	-0,4775	1
S100a11	-0,47752	1
Tmem164	-0,47766	1
Klc2	-0,47791	1
Zfp771	-0,47802	1
Aamdc	-0,47853	1
Ldb3	-0,47847	1
Rpl21	-0,47899	1
Gm43499	-0,47896	1
Gm9347	-0,47903	1
Nfia	-0,47911	1
G730013B05Rik	-0,47924	1
Pde6d	-0,47934	1
Rpl26	-0,47945	1
Npl	-0,47937	1
Fblim1	-0,47964	1
Mthfd1l	-0,47966	1
Gm37598	-0,47966	1
Supt3	-0,48	1
Tnrc6c	-0,48032	1
Lrrcc1	-0,48053	1
Fam122b	-0,48069	1
Eral1	-0,48086	1
Gm11930	-0,48087	1
Vps13b	-0,4813	1
Orai2	-0,48128	1
Bnip3l	-0,48175	1
Tctn1	-0,4822	1
Gm37303	-0,48239	1
C430049E01Rik	-0,48288	1
Aldh16a1	-0,48302	1
Dnajc7	-0,48306	1
Gm22009	-0,48318	1
Gm5841	-0,48332	1
Chmp6	-0,48349	1
Fsd2	-0,48354	1
Gm7327	-0,48358	1
C2cd5	-0,48367	1
Hibch	-0,48379	1
Ap2a2	-0,48387	1
Rab3gap2	-0,48424	1
Gm21399	-0,48439	1
RP23-43M12.2	-0,48465	1
Gm6649	-0,48462	1

Stn1	-0,48469	1
Tmem126b	-0,48467	1
Kmt2a	-0,48474	1
9130230L23Rik	-0,48511	1
E330020D12Rik	-0,48561	1
Map3k10	-0,48589	1
2700097O09Rik	-0,48591	1
Pcmtd1	-0,48596	1
Hoxb7	-0,48613	1
Fbxl4	-0,48675	1
3110031N09Rik	-0,48685	1
Gabpb1	-0,48736	1
Lpl	-0,48753	1
Tpk1	-0,48761	1
Insr	-0,48773	1
Reep5	-0,48772	1
Lyrm7	-0,48792	1
Zbtb37	-0,48826	1
Ap3m2	-0,48842	1
Creb3l2	-0,48835	1
Fam131a	-0,48853	1
Gm22767	-0,48847	1
Gls2	-0,48847	1
Gm45407	-0,4886	1
Plekha7	-0,48856	1
Gm7099	-0,48904	1
B4galnt1	-0,48914	1
Gm13815	-0,48908	1
Fam210b	-0,48915	1
Dnpep	-0,48926	1
Gm7638	-0,48999	1
Gm13803	-0,49014	1
Gm17066	-0,49015	1
Gabarapl2	-0,49029	1
Chst3	-0,49041	1
Ypel4	-0,49051	1
Gm42937	-0,49053	1
Gm11889	-0,49084	1
Stradb	-0,49097	1
4932416K20Rik	-0,49127	1
Hsph1	-0,49139	1
Gmpr2	-0,49188	1
Engase	-0,49195	1
Nphp1	-0,49201	1
Nat2	-0,49203	1
Gm14633	-0,49222	1
Rps8	-0,49251	1
Fam120aos	-0,49245	1
Poc5	-0,49258	1
Tubg1	-0,49258	1
Rif1	-0,49308	1
Nop56	-0,49317	1

Mrps27	-0,49347	1
Gm8121	-0,49355	1
Klhl30	-0,4935	1
Arf5	-0,49365	1
Gm12230	-0,49378	1
Idh3a	-0,49397	1
Gm561	-0,49423	1
Mthfs	-0,49442	1
9230102O04Rik	-0,49477	1
Gm4978	-0,49496	1
Gm11427	-0,49524	1
Slc2a4	-0,49528	1
Gm9506	-0,49534	1
Chd6	-0,49543	1
Pbk	-0,49548	1
Zfp429	-0,49547	1
Lysmd3	-0,49573	1
Mblac2	-0,4957	1
Sh3bgrl	-0,49576	1
Gm11423	-0,49575	1
Anxa6	-0,49618	1
4932438A13Rik	-0,49663	1
Znhit1	-0,49665	1
Nxt2	-0,4968	1
Naip6	-0,49681	1
Topbp1	-0,49704	1
Yipf3	-0,49708	1
Fcna	-0,49714	1
Trp53cor1	-0,49708	1
Nit1	-0,49777	1
Camkmt	-0,4978	1
Zfp882	-0,49802	1
RP24-131G14.10	-0,49802	1
Nrg4	-0,49802	1
1700084J12Rik	-0,49802	1
Phf19	-0,49835	1
3300002I08Rik	-0,49846	1
Selenom	-0,49921	1
Gm6745	-0,49949	1
Cnot10	-0,49978	1
Ptpn4	-0,49996	1
Pde4d	-0,50002	1
Polh	-0,50028	1
Slc2a9	-0,50042	1
Napb	-0,5007	1
Lxn	-0,50101	1
Med31	-0,50154	1
Lnpk	-0,50183	1
Gm26549	-0,50199	1
Zbtb49	-0,50199	1
Adam15	-0,50241	1
Gm42748	-0,50245	1

Abhd14b	-0,50276	1
Metrn	-0,50294	1
Stard10	-0,50367	1
Arsb	-0,50466	1
Gm16630	-0,50471	1
Abhd8	-0,50492	1
Ccnl2	-0,50531	1
Cad	-0,50555	1
Flnb	-0,50574	1
Tnk2	-0,50592	1
Car13	-0,50585	1
Serpinf1	-0,50592	1
RP24-232D3.1	-0,50596	1
Ttc26	-0,50624	1
Gsg1	-0,50631	1
Nono	-0,50659	1
Gm15216	-0,50673	1
Mettl1	-0,50682	1
St6galnac6	-0,50701	1
Pmpcb	-0,50697	1
Cetn4	-0,50718	1
3300005D01Rik	-0,50764	1
Macrod2	-0,50798	1
Atg4a	-0,50825	1
2810403A07Rik	-0,50834	1
Insig1	-0,50855	1
Gm37978	-0,50861	1
Dctd	-0,5089	1
Dusp19	-0,50904	1
Cox5b	-0,50921	1
Tsc22d2	-0,50936	1
Glb1	-0,50948	1
Wwc1	-0,50946	1
1700086P04Rik	-0,50964	1
Hdhd2	-0,50967	1
Gm12943	-0,51005	1
Gm11956	-0,51024	1
Ankrd10	-0,51037	1
Gm43201	-0,51046	1
Ahi1	-0,51073	1
D730003I15Rik	-0,5109	1
Ugdh	-0,51115	1
Psma5	-0,51137	1
Gps1	-0,51141	1
Atp1a3	-0,51179	1
Gm45630	-0,51217	1
Ube2c	-0,5125	1
Tmx2	-0,51261	1
Jak3	-0,51298	1
Rpl6	-0,51312	1
Gm45884	-0,51314	1
A530013C23Rik	-0,51325	1

Ncapd2	-0,51332	1
Kansl1l	-0,51342	1
Abtb2	-0,5136	1
Gadd45b	-0,514	1
D330050G23Rik	-0,51404	1
Ucp2	-0,51424	1
Ubxn7	-0,51465	1
Nmrk1	-0,51484	1
Gm12643	-0,51497	1
Ehmt2	-0,51579	1
Papss1	-0,51632	1
Lss	-0,51633	1
Pign	-0,5164	1
Phip	-0,51646	1
Gm4707	-0,51646	1
Gabpb2	-0,51686	1
Gm10033	-0,51723	1
2610016A17Rik	-0,51717	1
Gm12258	-0,51722	1
Pdlim5	-0,51742	1
Adcy2	-0,51746	1
Arl2	-0,5178	1
Ip6k2	-0,51795	1
Wbp1	-0,51785	1
Rnd2	-0,5179	1
Stau2	-0,51786	1
Rpl9-ps7	-0,5183	1
Lrrc24	-0,51844	1
Gm15564	-0,51865	1
Gm11539	-0,51913	1
Mif	-0,51931	1
Gm6209	-0,52026	1
Lncpint	-0,52038	1
Cul7	-0,5205	1
Tm7sf2	-0,52081	1
Tpx2	-0,52097	1
Slc16a9	-0,52102	1
Gnb1l	-0,52126	1
Sec31a	-0,52185	1
Gm10131	-0,52205	1
Hace1	-0,52207	1
Gm4607	-0,52222	1
Nudt1	-0,523	1
Crtam	-0,52399	1
Kif5a	-0,52413	1
Braf	-0,52422	1
9530062K07Rik	-0,52418	1
Rad9a	-0,52438	1
B230219D22Rik	-0,52459	1
Mlec	-0,52484	1
Agmo	-0,52554	1
Diaph3	-0,52547	1

Nmral1	-0,52569	1
Crat	-0,52578	1
Plcb3	-0,52586	1
Gm12166	-0,52599	1
Glt8d1	-0,52603	1
9330160F10Rik	-0,52626	1
Gfi1	-0,52629	1
Brwd1	-0,52649	1
Snora57	-0,52649	1
Fam64a	-0,52698	1
Galm	-0,52698	1
Slc25a42	-0,52699	1
Zfyve26	-0,52724	1
Galnt6	-0,52733	1
Sik2	-0,5276	1
4632415L05Rik	-0,52765	1
Il20rb	-0,52802	1
Rbl1	-0,52812	1
Ubxn11	-0,52848	1
Caprin1	-0,52888	1
Gm15421	-0,52915	1
Ptchd1	-0,53004	1
Gm3283	-0,53014	1
1110008P14Rik	-0,53058	1
Rbms2	-0,53076	1
Dock1	-0,53076	1
Tsnax	-0,53185	1
Gm26800	-0,53187	1
Ccdc58	-0,532	1
Rpl31	-0,53266	1
Gm17034	-0,53294	1
Cacna1s	-0,53298	1
Gm43566	-0,53331	1
Car2	-0,53364	1
Clasp1	-0,53396	1
AI839979	-0,53454	1
Rcbtb1	-0,53481	1
Arhgef25	-0,53504	1
Vrk3	-0,53542	1
Mipol1	-0,53538	1
Auh	-0,5357	1
Pck2	-0,53575	1
Drap1	-0,53592	1
Aplp1	-0,53645	1
AC168977.1	-0,53671	1
Sgtb	-0,53718	1
Rab1b	-0,53749	1
Atp11c	-0,53747	1
E2f1	-0,53806	1
4933408B17Rik	-0,53812	1
Gramd4	-0,53822	1
Atf6b	-0,5383	1

Fuca1	-0,53838	1
Rpl31-ps8	-0,53862	1
Nt5dc1	-0,53857	1
Rarg	-0,53866	1
Ccdc114	-0,53925	1
R3hdm1	-0,53954	1
Tnpo2	-0,53955	1
Hipk2	-0,53957	1
Herc1	-0,53966	1
Myef2	-0,53967	1
Txlnb	-0,53966	1
L3mbtl3	-0,54024	1
Gm6807	-0,54059	1
Ocrl	-0,54073	1
Rab26os	-0,54085	1
Slc6a9	-0,54107	1
Emc1	-0,54158	1
Aldoa	-0,54217	1
Bsg	-0,54231	1
Sec16b	-0,54235	1
R3hdm4	-0,54252	1
Nsdhl	-0,54277	1
Tfcp2l1	-0,54282	1
Parvg	-0,5432	1
Nudt19	-0,54328	1
Pnpla6	-0,54406	1
Pwwp2a	-0,54426	1
Ahdc1	-0,54439	1
Gm18709	-0,54451	1
Smarca5-ps	-0,54452	1
Atl3	-0,54461	1
Trmt2b	-0,54469	1
Zbtb20	-0,54523	1
Pianp	-0,5457	1
Aifm2	-0,54596	1
Nrxn3	-0,54602	1
Scarna9	-0,54644	1
Them6	-0,54651	1
Pitpnm2	-0,54658	1
Vkorc1	-0,54666	1
Stom	-0,5471	1
Pvr	-0,54727	1
Lair1	-0,54738	1
Gcn1l1	-0,54764	1
Hoxb5	-0,54787	1
Wls	-0,54806	1
Ltbp4	-0,54808	1
Gm11488	-0,54817	1
Gm9484	-0,54846	1
Poc1b	-0,54928	1
Evl	-0,54936	1
Gm12902	-0,54966	1

Noc3l	-0,54987	1
Kbtbd11	-0,54997	1
Rrnad1	-0,55009	1
Zfp511	-0,55016	1
Ano10	-0,55046	1
Cbx6	-0,55127	1
Pygb	-0,55195	1
Tmem116	-0,55227	1
Copz2	-0,55236	1
Dexi	-0,5525	1
Grk5	-0,5527	1
Acd	-0,55312	1
B130034C11Rik	-0,55346	1
Fam126b	-0,55387	1
Arrdc2	-0,55392	1
Brpf3	-0,55393	1
Gm10399	-0,55393	1
Hacd4	-0,55398	1
Sap30	-0,55414	1
Ift27	-0,55415	1
Txndc11	-0,55428	1
Atp5g2	-0,55427	1
Rdh12	-0,55431	1
Oxct1	-0,55443	1
Gm12618	-0,55447	1
Fam208a	-0,5546	1
Dnajc10	-0,55502	1
Recql	-0,55497	1
Syne1	-0,55513	1
Gpkow	-0,55517	1
Cacna1a	-0,55542	1
Cox7a1	-0,55569	1
Etv4	-0,55566	1
Smg1	-0,55581	1
Lamc1	-0,556	1
Gm16380	-0,556	1
Il17rc	-0,55615	1
6030458C11Rik	-0,55622	1
Ilf2	-0,55631	1
Tarbp1	-0,55704	1
Adarb1	-0,55708	1
Gcat	-0,55713	1
Cd47	-0,55721	1
Tspan10	-0,55741	1
Lta	-0,55753	1
Oscp1	-0,55753	1
Ulk4	-0,5577	1
Pnn	-0,55838	1
Cstf2	-0,5585	1
Frat2	-0,55864	1
Cep55	-0,55876	1
mt-Rnr1	-0,55927	1

Zfp692	-0,55934	1
Agbl5	-0,55934	1
Slc25a22	-0,55932	1
Bst2	-0,55942	1
Gm15690	-0,55964	1
B330016D10Rik	-0,55971	1
Rps2-ps5	-0,55973	1
Clasp2	-0,55994	1
Macf1	-0,56022	1
Oaz2	-0,56084	1
Arrdc1	-0,56084	1
Pgm2	-0,56078	1
Irak1	-0,56089	1
Ube2m	-0,56124	1
Gm42869	-0,56152	1
Mapk6	-0,56169	1
Sgol2a	-0,56175	1
Ttll5	-0,562	1
Lig1	-0,56261	1
Syt8	-0,5628	1
Pitpnc1	-0,56294	1
Exd2	-0,56297	1
Taf10	-0,56313	1
Ddah2	-0,56358	1
Actr2	-0,56377	1
Gm14040	-0,56392	1
Zmat1	-0,56392	1
Dnmt3l	-0,56408	1
Pcgf5	-0,56479	1
Gm26670	-0,56475	1
Zfat	-0,56577	1
Rgmb	-0,56638	1
Gm17530	-0,56658	1
Otx1	-0,56783	1
Ahcy	-0,56806	1
Pop1	-0,56808	1
Gm14537	-0,56835	1
Agfg2	-0,56837	1
C030015A19Rik	-0,56869	1
Chst1	-0,56945	1
Gripap1	-0,56957	1
9630010A21Rik	-0,56959	1
Tdp1	-0,56969	1
Atp6ap1	-0,56989	1
Sharpin	-0,57035	1
Wdr34	-0,57047	1
Gm9396	-0,57114	1
Anln	-0,57165	1
Plekhj1	-0,57187	1
Zfp462	-0,57195	1
A630001G21Rik	-0,57192	1
Creld2	-0,57212	1

Cpq	-0,57228	1
Timm10b	-0,57302	1
Pdia3	-0,5734	1
Shc1	-0,57352	1
Tkt	-0,57363	1
Gm13092	-0,57358	1
Rpe	-0,57394	1
1700001G11Rik	-0,57391	1
Ascc1	-0,57403	1
Casp2	-0,57404	1
Mms19	-0,57413	1
Piga	-0,5741	1
Gm15210	-0,57426	1
Tpt1	-0,57443	1
Mir142hg	-0,57459	1
Pmf1	-0,57467	1
Calml4	-0,57496	1
Cacfd1	-0,57548	1
Fbxl19	-0,57559	1
Nectin3	-0,57581	1
Ggcx	-0,57579	1
Sh3glb2	-0,57607	1
Gm2000	-0,57671	1
Brip1os	-0,57674	1
Fam193b	-0,57704	1
Nptn	-0,57742	1
Ctsf	-0,57764	1
Zfhx4	-0,57757	1
Snx21	-0,57826	1
Mospd3	-0,57849	1
Gm4968	-0,57863	1
Sec22c	-0,57893	1
G6pd2	-0,57891	1
Xpo4	-0,57923	1
Ank2	-0,5792	1
Gm7783	-0,57978	1
Rpl10-ps2	-0,58	1
Gtdc1	-0,58042	1
Tmem214	-0,58041	1
Tmem107	-0,58046	1
C330006A16Rik	-0,58138	1
Itga6	-0,58143	1
H2-T22	-0,5815	1
Ctps2	-0,58166	1
Aim1l	-0,58185	1
C330011M18Rik	-0,58185	1
Mastl	-0,58238	1
Gm340	-0,58239	1
Tbcel	-0,5826	1
Fam98c	-0,58265	1
Gphn	-0,58301	1
Pigp	-0,583	1

Pclaf	-0,58313	1
Zfp207	-0,58457	1
Gm23935	-0,58482	1
Sp3os	-0,58482	1
2900076A07Rik	-0,5856	1
Amer1	-0,5859	1
Tia1	-0,58606	1
Fgf13	-0,58607	1
Rgp1	-0,58625	1
Gm28535	-0,58627	1
Camk2g	-0,58647	1
RP23-162P10.8	-0,58647	1
Usp50	-0,5865	1
Pank4	-0,58665	1
Plxnb3	-0,58697	1
Zfp78	-0,58697	1
Gm45206	-0,58697	1
Gm37578	-0,58697	1
Apol11b	-0,58697	1
BC085271	-0,58722	1
9230112E08Rik	-0,5875	1
Satb2	-0,58756	1
Eif4g3	-0,58782	1
Gm17100	-0,58793	1
Arpin	-0,58811	1
Cog3	-0,5889	1
Gm7160	-0,58916	1
C130089K02Rik	-0,58962	1
Swap70	-0,58996	1
RP23-354J5.3	-0,59012	1
Bivm	-0,59013	1
1600014C10Rik	-0,59023	1
Ap1s2	-0,59032	1
N4bp1	-0,59043	1
Pros1	-0,59055	1
Zfp9	-0,59059	1
Svip	-0,59092	1
Phpt1	-0,59146	1
Rai14	-0,59158	1
Gm36964	-0,59177	1
Pigv	-0,59208	1
Mettl25	-0,59232	1
Ttc13	-0,59244	1
Stag1	-0,59271	1
Slc25a38	-0,59339	1
Slc40a1	-0,59353	1
Syne2	-0,59362	1
Ccne2	-0,59363	1
Nomo1	-0,59382	1
Dgka	-0,59378	1
Gm8494	-0,59395	1
Tnfsf13b	-0,59398	1

Ccdc180	-0,59395	1
As3mt	-0,59414	1
Psma3	-0,5949	1
RP23-149L23.1	-0,59534	1
Fubp1	-0,59568	1
Nav1	-0,59587	1
Mrpl58	-0,59606	1
Gm43331	-0,5969	1
Kctd13	-0,59736	1
Nktr	-0,59789	1
Dip2a	-0,59825	1
Sirt6	-0,59861	1
Ap2s1	-0,59902	1
Tacc3	-0,5991	1
Mfsd13a	-0,59932	1
Hmgb2	-0,5996	1
Nup98	-0,59982	1
Gm21975	-0,59995	1
Adamts7	-0,60013	1
mt-Co1	-0,60016	1
Dnd1	-0,60015	1
Gmnn	-0,60052	1
Smg9	-0,60096	1
Gm43290	-0,6011	1
Kazald1	-0,60111	1
Dhfr	-0,60113	1
Gm6457	-0,60122	1
Deptor	-0,60144	1
Tubgcp2	-0,6018	1
Dhdh	-0,60209	1
Mlkl	-0,60219	1
Tkfc	-0,60264	1
Capns1	-0,60306	1
Top2a	-0,60375	1
Ctsk	-0,60379	1
Mapk8ip3	-0,60409	1
Dcaf6	-0,60426	1
Cd37	-0,60451	1
Sptbn4	-0,60455	1
mt-Tl1	-0,60476	1
1700061G19Rik	-0,60503	1
Zkscan8	-0,60509	1
Ltb	-0,60539	1
Gm43088	-0,60572	1
Ero1l	-0,6059	1
Sp100	-0,60605	1
Gm43147	-0,6068	1
Gm2383	-0,60695	1
Pgls	-0,60705	1
Exoc6b	-0,60736	1
Mlst8	-0,6076	1
2810021J22Rik	-0,60758	1

Tmem106c	-0,60855	1
Nod1	-0,60934	1
4930539J05Rik	-0,60932	1
Nat8f1	-0,60937	1
Tra2a	-0,60955	1
Gm11531	-0,60996	1
Tmem38b	-0,61014	1
Dis3l	-0,61006	1
Dync1h1	-0,61051	1
Btk	-0,61088	1
Chil6	-0,61092	1
Sgsm2	-0,61099	1
Zfp871	-0,61152	1
Mgmt	-0,61171	1
Sntb2	-0,61181	1
Dnaaf5	-0,61347	1
1700123M08Rik	-0,61367	1
Pars2	-0,61372	1
Knstrn	-0,61385	1
Gucy2g	-0,6138	1
Gpr179	-0,61435	1
Snn	-0,61475	1
Dynlt1f	-0,61471	1
Gm28373	-0,6154	1
Hk3	-0,61545	1
Gm43560	-0,61547	1
Trappc9	-0,61585	1
Orc1	-0,61617	1
Spp1	-0,61799	1
Gm35931	-0,61823	1
Raph1	-0,61839	1
6430531B16Rik	-0,61847	1
Dhx9	-0,61861	1
Eef2kmt	-0,61861	1
Gm14286	-0,61857	1
Dpm1	-0,61878	1
Scarb1	-0,61914	1
Bub1	-0,61926	1
Agl	-0,61959	1
Raly	-0,61961	1
D430001F17Rik	-0,6197	1
Cdk5rap3	-0,61984	1
Anapc13	-0,62002	1
Rnf130	-0,62001	1
Pole2	-0,62003	1
Znrf3	-0,62026	1
Cdk2ap2	-0,62041	1
Pde1b	-0,62049	1
E330034L11Rik	-0,62047	1
Selenoi	-0,62062	1
Zfp607b	-0,62071	1
Ift80	-0,62146	1

Rps12-ps4	-0,62202	1
Lpp	-0,62234	1
Ttc12	-0,62246	1
Gm44126	-0,62268	1
Wdr25	-0,62276	1
Gm37080	-0,62321	1
Gm45629	-0,62321	1
RP23-307F3.6	-0,62343	1
Fktn	-0,62382	1
Rufy3	-0,62418	1
A530041M06Rik	-0,62473	1
Anxa2	-0,62527	1
Cox16	-0,62547	1
Gm29340	-0,62577	1
Cd74	-0,62603	1
Rccd1	-0,62599	1
Gm8430	-0,62606	1
Pfkl	-0,62636	1
RP23-243B24.1	-0,62662	1
9330020H09Rik	-0,62728	1
Lamtor2	-0,62743	1
Nsf	-0,62749	1
Capza1	-0,6284	1
Pigs	-0,62869	1
Gm4987	-0,62867	1
Slc39a4	-0,62923	1
Alox8	-0,62923	1
2310009A05Rik	-0,62953	1
A930024E05Rik	-0,62972	1
Agrn	-0,63001	1
Arrb1	-0,63	1
Chp1	-0,63028	1
Tmsb10	-0,63031	1
RP24-550H10.3	-0,63028	1
Mbd5	-0,63041	1
Gm43362	-0,63075	1
Zfp873	-0,63106	1
Pltp	-0,63162	1
Slc12a6	-0,63167	1
Gm42640	-0,63237	1
Mcm5	-0,63291	1
9130024F11Rik	-0,63302	1
Gm44771	-0,63314	1
Gm14427	-0,63338	1
Rpgrip1l	-0,63368	1
Gm38248	-0,63379	1
Bcl2l14	-0,63461	1
Atn1	-0,6348	1
Phxr4	-0,63518	1
RP23-228B2.5	-0,63518	1
Gm37785	-0,63548	1
4831440E17Rik	-0,63549	1

Snord89	-0,63584	1
RP24-122E11.4	-0,63601	1
9530085L11Rik	-0,6361	1
2510002D24Rik	-0,63622	1
H3f3a	-0,63644	1
Zbtb33	-0,6364	1
Gm45342	-0,63687	1
Nyap1	-0,63713	1
G2e3	-0,6372	1
Usp35	-0,6372	1
Coa4	-0,63721	1
Zbtb26	-0,63738	1
Gnb4	-0,63744	1
Cdk6	-0,63833	1
Ppp4c	-0,6383	1
Pkmyt1	-0,63848	1
Gm24927	-0,63973	1
Gm9207	-0,63973	1
Cpeb1	-0,63973	1
Zdhhc21	-0,64025	1
Rpl3	-0,64066	1
Arfgap2	-0,64083	1
Tex10	-0,64132	1
Kcnab3	-0,64135	1
Riiad1	-0,64145	1
Pex2	-0,6416	1
Kdm4d	-0,64195	1
D630029K05Rik	-0,64195	1
Trip10	-0,642	1
Gm5735	-0,6425	1
Gm6987	-0,64281	1
Dnajc24	-0,64311	1
Smg6	-0,64332	1
Eno2	-0,64343	1
Aoc2	-0,64336	1
Dennd6b	-0,64358	1
8430408G22Rik	-0,64385	1
Commd10	-0,64388	1
Rpl17-ps4	-0,64406	1
Clec4a2	-0,64447	1
Id1	-0,64463	1
Prr11	-0,64483	1
Ier5l	-0,64486	1
Hddc2	-0,64502	1
Dusp28	-0,64517	1
Pycr1	-0,64577	1
Rpl17-ps8	-0,64616	1
Tpt1-ps3	-0,64619	1
Gnptg	-0,64634	1
Nup85	-0,64644	1
Pacs1	-0,64658	1
Txnrd3	-0,64716	1

Lix1l	-0,64743	1
Pitpnm1	-0,6479	1
Ncapg	-0,6482	1
Kat8	-0,64839	1
Gm15610	-0,64892	1
Palm	-0,64906	1
Gm9165	-0,64909	1
Gm19726	-0,64916	1
Rufy1	-0,64925	1
Ntan1	-0,64961	1
Depdc1a	-0,64989	1
4930520O04Rik	-0,65006	1
Tmem41b	-0,65038	1
Rps27rt	-0,65113	1
Samd1	-0,65137	1
Zc3h12c	-0,65148	1
Gne	-0,65224	1
Ptgs2os	-0,65225	1
9630013D21Rik	-0,65249	1
Gm15472	-0,65334	1
Sema4g	-0,65339	1
Zfp652os	-0,65342	1
Gm9320	-0,65387	1
Fam45a	-0,65393	1
Fam213a	-0,65399	1
Gm15753	-0,65445	1
Huwe1	-0,65461	1
Adam9	-0,65593	1
Tmigd3	-0,65586	1
Urod	-0,65603	1
Gm13864	-0,65603	1
Wdpcp	-0,65657	1
Apoe	-0,65658	1
Atp8b4	-0,65698	1
Tst	-0,65714	1
Gm43569	-0,65728	1
Luc7l2	-0,6578	1
Gm20186	-0,65796	1
Gm12758	-0,65816	1
Fam151b	-0,65845	1
Rps12-ps9	-0,65855	1
Pld1	-0,65916	1
Pold1	-0,6596	1
Zfp609	-0,66032	1
Mettl17	-0,66047	1
Sh3kbp1	-0,66092	1
Adgrl1	-0,66151	1
Lmntd2	-0,66156	1
B230354K17Rik	-0,66208	1
Prpf40b	-0,6624	1
Ms4a6d	-0,6625	1
Metap1d	-0,6627	1

B230217C12Rik	-0,66328	1
Gm9134	-0,66361	1
Bcs1l	-0,66366	1
Pola1	-0,66381	1
Pcbp4	-0,66522	1
Prr36	-0,66526	1
Gm14240	-0,66546	1
Rpgr	-0,66574	1
Tbc1d19	-0,66579	1
Plod1	-0,666	1
Prdx2	-0,66636	1
9330104G04Rik	-0,66665	1
Gm42481	-0,66681	1
Gm527	-0,66717	1
Itga2b	-0,66739	1
Lrrc1	-0,66755	1
Helq	-0,66849	1
Sh3bgrl2	-0,66857	1
Gm16425	-0,66857	1
RP23-151L20.5	-0,66864	1
Flot1	-0,66881	1
Chrnb1	-0,66901	1
Clstn1	-0,67051	1
Nrp1	-0,67062	1
Rpl12-ps1	-0,67144	1
Asph	-0,6725	1
Tmem132a	-0,67339	1
Flna	-0,67432	1
Pou5f2	-0,67453	1
Gm45728	-0,67453	1
Fahd1	-0,67493	1
Gm12834	-0,67512	1
Mcoln1	-0,67564	1
Gan	-0,67616	1
Rpl19-ps9	-0,67643	1
4930578M01Rik	-0,67658	1
Coro2a	-0,6767	1
Zfp157	-0,6768	1
8030453O22Rik	-0,6769	1
Rps27	-0,677	1
Nova1	-0,67716	1
AU022252	-0,6773	1
Sec24d	-0,67764	1
Gm12940	-0,67769	1
Srsf2	-0,67794	1
Gm15779	-0,6782	1
Mndal	-0,67849	1
Cenpf	-0,67897	1
Cdk16	-0,67901	1
Nckap5l	-0,67928	1
Tmem63b	-0,67991	1
Noxo1	-0,68001	1

Ctxn1	-0,68059	1
Nt5c2	-0,68065	1
4833417C18Rik	-0,6811	1
Gm21781	-0,68131	1
Dennd5b	-0,68143	1
Gmppa	-0,68159	1
Cluap1	-0,68242	1
1500002F19Rik	-0,6824	1
Mis18bp1	-0,68253	1
Oaz1	-0,6828	1
Gm10036	-0,68284	1
4921531C22Rik	-0,68329	1
Slc39a14	-0,68359	1
Rad51c	-0,68357	1
Camk2b	-0,68386	1
Gm28192	-0,68423	1
Sugp2	-0,68428	1
Pdk3	-0,68432	1
Rbm4	-0,68479	1
Letm2	-0,68487	1
Snord49b	-0,68519	1
Gm44557	-0,6857	1
Leng8	-0,68584	1
Rpl10-ps3	-0,68658	1
Hsd3b7	-0,68668	1
Atf6	-0,68709	1
B3gnt6	-0,68735	1
Gm26226	-0,68811	1
Glb1l	-0,68844	1
Nup210	-0,68881	1
Lypla1	-0,68926	1
Aldh1l1	-0,68928	1
Sh2d5	-0,68951	1
Suv39h2	-0,68969	1
RP23-413G8.2	-0,69008	1
S100a8	-0,69032	1
Rasa4	-0,69058	1
Napepld	-0,69118	1
Lmtk3	-0,69116	1
Gm9726	-0,69138	1
Wdr92	-0,69159	1
Atxn2l	-0,6919	1
Senp3	-0,69187	1
2210417A02Rik	-0,6919	1
RP23-356D13.11	-0,6921	1
Gm38399	-0,69218	1
Gm8019	-0,693	1
Lrwd1	-0,69331	1
Gm13611	-0,69427	1
Gm37010	-0,69471	1
Gm43178	-0,69496	1
Pam	-0,69522	1

mt-Nd5	-0,6955	1
Ttk	-0,69605	1
Tarsl2	-0,69596	1
5830408C22Rik	-0,69629	1
Vps51	-0,69658	1
Matk	-0,69675	1
Gm42972	-0,69675	1
Ifi203-ps	-0,6969	1
Iqcg	-0,69765	1
Gt(ROSA)26Sor	-0,69847	1
Numb	-0,69853	1
1700020I14Rik	-0,69973	1
Tfec	-0,69968	1
Gm10136	-0,69982	1
Ntrk3	-0,70017	1
Gm42856	-0,70113	1
A830080D01Rik	-0,70123	1
Col18a1	-0,70159	1
Tbl2	-0,70167	1
Col20a1	-0,70194	1
Igsf6	-0,70205	1
9330111N05Rik	-0,70262	1
Zdhhc17	-0,70286	1
2610020C07Rik	-0,70339	1
Natd1	-0,70377	1
RP23-6C18.6	-0,70398	1
Bard1	-0,70407	1
Pdk1	-0,70421	1
Sapcd1	-0,70494	1
Hspb7	-0,70517	1
Gm43323	-0,70524	1
Sp4	-0,7053	1
Rybp	-0,70534	1
Gm12309	-0,70549	1
RP24-275P22.2	-0,70563	1
4930522L14Rik	-0,70598	1
Tmem150a	-0,70615	1
Gm10717	-0,70628	1
Lockd	-0,70639	1
Gm6245	-0,70683	1
Gm44951	-0,70683	1
Sf3b3	-0,7074	1
Fyco1	-0,70791	1
Gm43737	-0,70786	1
Anpep	-0,70808	1
Neil3	-0,70872	1
Nnt	-0,70921	1
Rpl21-ps12	-0,70958	1
Uba7	-0,70969	1
Dtx3	-0,71038	1
Gm27477	-0,71063	1
Fnbp1l	-0,71076	1

Gkap1	-0,71202	1
Trmt13	-0,71208	1
Lims1	-0,71227	1
Hdac1	-0,71237	1
Zdhhc8	-0,71247	1
Cnep1r1	-0,71266	1
Psma8	-0,71301	1
Tbcd	-0,71306	1
1700084E18Rik	-0,7132	1
Gm27029	-0,7134	1
Rp2	-0,71372	1
Apool	-0,71389	1
Ggnbp1	-0,71402	1
Pate2	-0,71398	1
9930104L06Rik	-0,71496	1
Slc35a1	-0,71544	1
Prickle2	-0,71558	1
Zscan29	-0,71606	1
Hsf1	-0,71663	1
Gm13456	-0,71663	1
Snord7	-0,71859	1
Nucb1	-0,71885	1
Rap1gds1	-0,71889	1
Kremen1	-0,71912	1
Gm13604	-0,71956	1
Kantr	-0,71969	1
Pan2	-0,71995	1
Ift88	-0,71997	1
Lmf2	-0,72012	1
Icam5	-0,72041	1
4930581F22Rik	-0,72082	1
4930589L23Rik	-0,72089	1
Trmt112	-0,7211	1
Xrcc3	-0,72118	1
Gm43924	-0,7212	1
Zer1	-0,72164	1
Gm45828	-0,72156	1
Tsga10	-0,72213	1
Gm44509	-0,72246	1
RP23-288C18.3	-0,72297	1
4930426I24Rik	-0,72386	1
B230216N24Rik	-0,72466	1
Rdm1	-0,72521	1
Gm11878	-0,72643	1
Phkb	-0,72693	1
F730043M19Rik	-0,72769	1
Plekhn1	-0,72782	1
Gm24601	-0,72819	1
Fign	-0,72838	1
Gm10478	-0,72855	1
Pcdhgc4	-0,72873	1
Tnfsf9	-0,72944	1

Wdr54	-0,72961	1
Smarcc2	-0,73027	1
Mcm8	-0,73038	1
Gm14057	-0,7309	1
Il18rap	-0,73089	1
Gm9009	-0,73157	1
H2afz	-0,73202	1
Gm43848	-0,7325	1
Gm12981	-0,73248	1
Snd1	-0,73277	1
Gm29488	-0,73337	1
Nfam1	-0,73391	1
4632404H12Rik	-0,73395	1
RP23-182J19.2	-0,73434	1
Gm11110	-0,73474	1
Pld2	-0,73589	1
Spa17	-0,73648	1
Gm45222	-0,73679	1
Btf3l4	-0,7376	1
Casp4	-0,73889	1
2610318N02Rik	-0,73972	1
2210408I21Rik	-0,73988	1
Cyb5r4	-0,74015	1
Hist1h4a	-0,74018	1
Itpr3	-0,74035	1
Tbc1d31	-0,74095	1
Rpl11	-0,74125	1
Gm43533	-0,74126	1
Zfp72	-0,74142	1
Gm37699	-0,74198	1
Atp11b	-0,74255	1
Cdip1	-0,74256	1
Acrbp	-0,74271	1
Cystm1	-0,74297	1
Olfr920	-0,74336	1
Cit	-0,74351	1
Arhgap15	-0,74365	1
Nsl1	-0,74492	1
Ogfod3	-0,74492	1
Ank3	-0,74582	1
4932441J04Rik	-0,7459	1
Epsti1	-0,74611	1
Gm26772	-0,74605	1
Gm43462	-0,74629	1
Snord55	-0,7472	1
Ifi211	-0,74722	1
C130036L24Rik	-0,74728	1
Meaf6	-0,74826	1
Gen1	-0,74882	1
Nipsnap3b	-0,74902	1
4930402H24Rik	-0,74939	1
Gm12454	-0,7495	1

Hsp90aa1	-0,75009	1
H2-DMb2	-0,75144	1
Gm12096	-0,7518	1
Gm15535	-0,75209	1
Gm42551	-0,75232	1
Catsperg1	-0,75265	1
Sult2b1	-0,75306	1
Bbs4	-0,75323	1
Gm20430	-0,75339	1
Ccdc88c	-0,75339	1
Gm5045	-0,75351	1
Ifi213	-0,75364	1
Adamtsl5	-0,7539	1
Gm10263	-0,75469	1
Got2-ps1	-0,75469	1
Dpep2	-0,75537	1
Gm45568	-0,75539	1
Spaca6	-0,75553	1
Selenow	-0,75574	1
Ssh3	-0,75587	1
Gm10069	-0,7568	1
Rac3	-0,75696	1
Fdps	-0,75706	1
Hoxa4	-0,75763	1
Efr3b	-0,75807	1
Gm3650	-0,75877	1
Celf6	-0,75876	1
Soat2	-0,75876	1
Sympk	-0,75943	1
Kmt2d	-0,75952	1
Zfp938	-0,7595	1
Spidr	-0,7598	1
Npr1	-0,76033	1
Hdac8	-0,7604	1
mt-Ta	-0,76063	1
Crlf2	-0,76141	1
2700046G09Rik	-0,76163	1
Gm5776	-0,76217	1
D130019J16Rik	-0,76254	1
Gm15501	-0,76272	1
Zfp541	-0,76395	1
Polq	-0,76422	1
Gda	-0,76466	1
Gm13477	-0,76528	1
Aldh5a1	-0,76565	1
Car11	-0,76579	1
Lrrc45	-0,7662	1
Hnrnpa3	-0,76635	1
Gtf2ird1	-0,76636	1
Kif4	-0,76658	1
Mccc1	-0,76765	1
Gm14813	-0,76809	1

2810030D12Rik	-0,76863	1
Rpl36-ps8	-0,76873	1
Pask	-0,76898	1
Rnf24	-0,7691	1
Slc16a7	-0,76925	1
E230020A03Rik	-0,76954	1
Tctex1d4	-0,76983	1
Gm21816	-0,77006	1
Gm12276	-0,77006	1
Mettl26	-0,77031	1
Tmem81	-0,77036	1
Rbfox2	-0,77052	1
Neil1	-0,77049	1
Gm25008	-0,77059	1
Trappc11	-0,77123	1
Hcar2	-0,77166	1
Gm43466	-0,77238	1
Pcsk7	-0,77374	1
Zfp60	-0,77377	1
F11r	-0,77395	1
Rtel1	-0,77454	1
Sorbs1	-0,77509	1
Ceacam16	-0,77558	1
A930029G22Rik	-0,7756	1
Gm15796	-0,77574	1
Pigh	-0,77726	1
Svep1	-0,77805	1
Lgals4	-0,77827	1
Pgghg	-0,77878	1
Proscos	-0,77985	1
Rpl13a-ps1	-0,78048	1
Gm12816	-0,78064	1
Scp2-ps2	-0,78121	1
Parp6	-0,7817	1
Rnf225	-0,78284	1
A230050P20Rik	-0,78302	1
Med24	-0,78296	1
Fam229b	-0,78328	1
Mettl10	-0,78328	1
Gm3550	-0,78354	1
Rab11fip4os1	-0,78432	1
Ppm1e	-0,78465	1
Hacd2	-0,78495	1
Dync2h1	-0,78519	1
Haus7	-0,78545	1
Eif2ak2	-0,78648	1
RP23-58B7.2	-0,78682	1
Acp5	-0,78721	1
Crkl	-0,78742	1
Toporsos	-0,78758	1
Bcl2l15	-0,78772	1
Pxdn	-0,78772	1

Kifc2	-0,78786	1
Swi5	-0,78854	1
Mir124-2hg	-0,78848	1
Fgd4	-0,78905	1
Rpl27-ps3	-0,79103	1
Acaa2	-0,79156	1
1810021B22Rik	-0,79232	1
Speg	-0,79311	1
Lgr4	-0,79324	1
Rpgrip1	-0,7933	1
Rasal3	-0,79381	1
Rnf121	-0,79398	1
Gm6136	-0,79401	1
D16Ertd472e	-0,79412	1
Inca1	-0,79419	1
Ptpdc1	-0,79444	1
Kcnq1ot1	-0,7945	1
Dntt	-0,79476	1
Bend4	-0,79495	1
Kcnab2	-0,79511	1
Arhgef4	-0,79573	1
Dpy19l3	-0,7966	1
1700022N22Rik	-0,7966	1
Lzic	-0,797	1
Invs	-0,79709	1
Gm14056	-0,79717	1
Rps15a-ps3	-0,79835	1
Acad10	-0,79836	1
Rusc1	-0,79877	1
Gm43637	-0,79962	1
Gm43247	-0,80041	1
Galnt11	-0,80075	1
Adamts10	-0,80102	1
Gm42639	-0,80181	1
Mettl21b	-0,80234	1
Mocs1	-0,8034	1
Rplp1-ps1	-0,80347	1
Mettl7a1	-0,80387	1
Slc25a10	-0,80439	1
Ankrd13d	-0,80484	1
Alg9	-0,80518	1
Cnp	-0,80518	1
Gm5609	-0,80517	1
Cbx5	-0,80571	1
Gm5446	-0,8057	1
Snord15a	-0,80649	1
Cln6	-0,80663	1
Tbc1d5	-0,80659	1
Itgam	-0,80779	1
Rbpms	-0,80892	1
Cdca2	-0,80911	1
Dis3l2	-0,80917	1

Golga7	-0,80952	1
Gm43920	-0,81075	1
Cdk20	-0,81089	1
Arhgap27os1	-0,81291	1
Cenpt	-0,81335	1
Gm31166	-0,81414	1
Nudt5	-0,8147	1
Gm43581	-0,81476	1
Fam76b	-0,81488	1
Gm26244	-0,81487	1
Ppic	-0,81534	1
Gm43742	-0,81628	1
Arf2	-0,81736	0,68407
Gm44153	-0,81747	1
Mtus1	-0,81784	1
Gm23722	-0,81796	1
Ext2	-0,81876	1
Creb3l4	-0,82002	1
Atf7	-0,8208	1
Dnajc12	-0,8216	1
Npm3-ps1	-0,82178	1
Coro7	-0,82364	1
C030013C21Rik	-0,82405	1
RP24-225A16.3	-0,8244	1
Kptn	-0,82437	1
Arhgef11	-0,82475	1
Gm37354	-0,82495	1
Comp	-0,82517	1
Doc2g	-0,82766	1
Myo1d	-0,82839	1
Fastkd1	-0,8285	1
Jmjd7	-0,82863	1
Mrps33	-0,83034	1
Ppp1r37	-0,8304	1
Adh7	-0,83044	1
Rsph1	-0,83174	1
Gm5637	-0,83175	1
Igfbp4	-0,83186	0,63576
Gca	-0,83421	1
Lman2l	-0,83433	1
5031434O11Rik	-0,83454	1
Klf1	-0,83478	1
Gm45802	-0,83489	1
Osbpl1a	-0,83529	1
RP23-366E4.9	-0,83578	1
Ifi202b	-0,83612	1
5930420M18Rik	-0,83618	1
Selenbp1	-0,83635	1
AI661453	-0,8367	1
Tmem259	-0,83719	1
Utrn	-0,83799	1
Tmem216	-0,83846	1

1110020A21Rik	-0,83882	1
Prickle3	-0,83939	1
Lum	-0,84113	1
Hist1h1b	-0,8417	1
Grtp1	-0,84182	1
Jpx	-0,84284	1
Gm44552	-0,84281	1
Arsa	-0,84352	1
Rpl15-ps5	-0,84348	1
Gm37606	-0,84456	1
Cd59a	-0,84633	1
Carmil1	-0,8466	1
Acot1	-0,84683	1
Gm24916	-0,84711	1
AW554918	-0,84848	1
Cdkn2c	-0,84907	1
2900052L18Rik	-0,84931	1
Cntrob	-0,84971	1
Ccdc134	-0,85002	1
Rps12-ps5	-0,85031	1
Dnal1	-0,8508	1
Msi2	-0,8509	1
Nlk	-0,85129	1
Jmjd8	-0,85139	1
Inhbe	-0,85204	1
Lgr5	-0,8527	1
Spata1	-0,85349	1
Gm37788	-0,85487	1
Rad52	-0,85502	1
Ppfia3	-0,85591	1
Arhgef1	-0,85641	1
Zfp280d	-0,85656	1
Gm37962	-0,85677	1
6330403N20Rik	-0,85746	1
Gm37297	-0,85747	1
Apex2	-0,85761	1
Tmem243	-0,85797	1
Hapln3	-0,85903	1
4931428F04Rik	-0,85933	1
Angptl6	-0,86003	1
Gm37399	-0,86044	1
Cda	-0,86048	1
Gm11952	-0,86089	1
Ftl1	-0,86139	1
Rbmx	-0,86162	1
Nkain1	-0,86181	1
Dock6	-0,86198	1
Gm45380	-0,86218	1
Gm42986	-0,86247	1
Cnrip1	-0,86251	1
Rn7s6	-0,8633	1
Snord110	-0,86355	1

Rpl28-ps1	-0,86373	1
Gm11613	-0,86403	1
Ttc21b	-0,86465	1
Zcwpw1	-0,86504	1
Maz	-0,86535	1
BC051226	-0,86614	1
Ifi203	-0,86705	1
Abhd18	-0,86735	1
Gm37851	-0,86729	1
Gm9844	-0,86767	1
Dubr	-0,86779	1
Cdk5	-0,86823	1
Gm20628	-0,86843	1
Stard9	-0,86965	1
2700033N17Rik	-0,86996	1
Six4	-0,87011	1
Fam132a	-0,87026	1
Vwa8	-0,8705	1
3830408C21Rik	-0,8721	1
Luc7l3	-0,87407	0,9669
Gm6576	-0,8741	1
AK157302	-0,87493	1
Notch4	-0,87569	1
Stc2	-0,87587	1
Rps28	-0,87609	1
Isoc2a	-0,8782	1
Whrn	-0,87866	1
Abcg4	-0,87884	1
Upp2	-0,87921	1
Rragb	-0,87977	1
Gm37105	-0,88014	1
Gm15289	-0,88042	1
Tbx6	-0,88053	1
Rpl19	-0,88078	1
Gtpbp2	-0,88079	1
Azin2	-0,88102	1
Clgn	-0,88096	1
Gm13641	-0,88112	1
Gm16223	-0,88112	1
Tk1	-0,88127	1
Anapc5	-0,88158	0,68757
Sep 11	-0,88175	1
Gm44190	-0,88215	1
Rps18-ps1	-0,88235	1
2810414N06Rik	-0,88237	1
Gm10501	-0,88306	1
Apoo-ps	-0,88358	1
Gm37084	-0,88406	1
Tmem191c	-0,88425	0,93372
Mnd1	-0,88432	1
Gm21967	-0,88534	1
Gm37728	-0,88554	1

Lrp8os3	-0,88558	1
Car12	-0,88575	1
Gm26870	-0,88657	1
Gm26810	-0,88743	1
Gm14843	-0,88882	1
Dock7	-0,88908	1
Gm7285	-0,89029	1
Sipa1l2	-0,89072	1
Gm37065	-0,8918	1
Gm29650	-0,89237	1
Cadps	-0,89352	1
Mospd2	-0,89373	1
Pfkfb3	-0,89483	1
Trem3	-0,8957	1
Gm37670	-0,89625	1
Gm44935	-0,89629	1
Myo6	-0,89758	1
Gm13935	-0,89859	1
Frk	-0,8987	1
4930556M19Rik	-0,8987	1
Dgkh	-0,89975	1
Murc	-0,90093	1
Ganc	-0,9011	1
Poc1a	-0,90181	1
Defb25	-0,90211	1
Vamp7-ps	-0,90219	1
Gm7784	-0,90261	1
Amd2	-0,9028	1
Zfc3h1	-0,90297	1
Dph6	-0,9047	1
Gm10012	-0,90558	1
Gm30074	-0,9057	1
Msh5	-0,90663	1
Gm15459	-0,90691	1
Slc25a40	-0,90712	1
Rps19-ps8	-0,90709	1
Dtl	-0,90862	1
Mob3b	-0,90898	1
Gpr68	-0,90912	1
Osbpl3	-0,9097	1
Tomm40l	-0,91019	1
Neat1	-0,91042	1
Nrtn	-0,91084	1
Fam69a	-0,91128	1
Hist2h2be	-0,912	0,99275
Gm42747	-0,91201	1
Grik5	-0,91245	1
0610009L18Rik	-0,9128	1
Slc17a9	-0,914	1
Zfp169	-0,9141	1
Acap1	-0,91563	1
Mthfsd	-0,91732	1

Pif1	-0,91745	1
Coq4	-0,91886	1
Rpl30	-0,91905	1
4933439C10Rik	-0,91986	1
Cradd	-0,92008	1
Cd93	-0,92061	1
Vamp2	-0,92122	1
Gm23502	-0,92181	1
Dync2li1	-0,92269	1
Bphl	-0,92333	1
Ltbp2	-0,92356	1
8030462N17Rik	-0,92452	1
Vaultrc5	-0,92577	1
Csf2ra	-0,92583	1
Gm45266	-0,92583	1
Rab2b	-0,9261	1
Pigt	-0,92661	1
Gm28791	-0,92714	1
Arfgef3	-0,92733	1
Rps3a2	-0,9284	1
Ankrd34a	-0,92844	1
Ppp2r3a	-0,92868	1
Tpt1-ps5	-0,92919	1
Mturn	-0,92943	1
Zscan21	-0,93005	1
Gm43360	-0,93205	1
Hfe	-0,93213	1
Snrpf	-0,93245	1
H2-Ab1	-0,93236	1
Gm10800	-0,93353	1
Agap1	-0,93445	1
Mypopos	-0,93459	1
Gm8013	-0,93707	1
Sumf2	-0,93718	1
Fam71f2	-0,93731	1
Dnali1	-0,93732	1
Prepl	-0,93738	1
Sapcd2	-0,93855	1
Iqce	-0,93893	1
Gm45360	-0,93931	1
Gm42567	-0,93935	1
Nsa2-ps2	-0,94014	1
D230022J07Rik	-0,94197	1
D930016D06Rik	-0,94231	1
4932422M17Rik	-0,94304	1
3110056K07Rik	-0,9432	1
Slc35d2	-0,94331	1
Usp26	-0,94375	1
Snhg6	-0,94398	1
Wnk2	-0,94416	1
2810454H06Rik	-0,94441	1
Gm8093	-0,94461	1

Gm24924	-0,94539	1
Cd274	-0,9471	1
Cops7a	-0,94817	1
Sec14l2	-0,94849	1
Gm13498	-0,94861	1
4833412K13Rik	-0,94963	1
Gm5117	-0,94995	1
4933421A08Rik	-0,95025	1
Mfhas1	-0,95205	1
Rpl35a-ps4	-0,95258	1
Esco2	-0,95311	1
Megf8	-0,95316	1
Cfp	-0,95337	1
2310047D07Rik	-0,95374	1
Slc24a3	-0,95367	1
Gm43128	-0,9541	1
Dmwd	-0,95429	1
5330438D12Rik	-0,95471	1
Gm7909	-0,95499	1
Rapgef3	-0,95499	1
Aarsd1	-0,95639	1
Etv1	-0,95648	1
Gm43484	-0,95664	1
6430710M23Rik	-0,95658	1
Gm43571	-0,95776	1
Fendrr	-0,9597	1
1700037C18Rik	-0,96032	1
Armc9	-0,9623	1
Gm29759	-0,96394	1
Gm5302	-0,96421	1
Hoxb8	-0,96433	1
Gm2788	-0,96441	1
Vps52	-0,96515	1
A130014A01Rik	-0,96557	1
Slbp	-0,96581	1
Kdm4b	-0,96776	1
Map2k3os	-0,96797	1
Traf3ip3	-0,97251	1
Dzip3	-0,97261	1
Gm20492	-0,97284	1
Fam172a	-0,97324	1
Gm43329	-0,97354	1
Nat6	-0,97363	0,93384
Gm28404	-0,97366	1
Gbe1	-0,97408	0,84725
Cep83os	-0,97538	1
Gm44652	-0,97587	1
Gm29438	-0,97589	1
St5	-0,97623	1
Hist1h2be	-0,97724	1
Nudt6	-0,97734	1
n-R5s151	-0,97802	1

Psenen	-0,97826	1
Gm43336	-0,97922	1
Wdfy2	-0,97934	1
Gm37423	-0,98007	1
Chaf1b	-0,98024	1
Gm7846	-0,98146	1
Lsm5	-0,98261	1
Lsm7	-0,98373	1
Oas1b	-0,98428	1
RP24-366E11.4	-0,98477	1
Acad9	-0,9856	1
Dnajc22	-0,98556	1
Gm43775	-0,9863	1
Itgb3	-0,98641	1
Gm20156	-0,98681	1
Gm38366	-0,98687	1
Gm22716	-0,98777	1
Ccdc106	-0,98814	1
Gm12770	-0,98813	1
Spg11	-0,98903	1
Maged1	-0,9892	0,90711
Tln2	-0,99024	1
B3galnt2	-0,99261	1
Gm26652	-0,99411	1
Myoz1	-0,99504	1
L3hypdh	-0,99572	1
Gm25514	-0,99569	1
Tarbp2	-0,99654	1
Tango6	-1,0007	1
A730011C13Rik	-1,0012	1
Trim3	-1,0017	1
2810428J06Rik	-1,0019	1
Hoxaas3	-1,0021	1
Adcy6	-1,0024	1
Gm26912	-1,0028	1
Gm5075	-1,0034	1
Gm7565	-1,0035	1
Nudt14	-1,0045	1
Itpka	-1,0049	1
Psca	-1,005	1
Rbbp9	-1,0059	1
C230096K16Rik	-1,0062	1
Ift22	-1,0079	1
Gm16253	-1,0082	1
Fanci	-1,0085	1
Maged2	-1,0086	1
Meis3	-1,0107	1
Uros	-1,0107	1
Celf4	-1,0109	1
Rtkn	-1,0114	1
Tmlhe	-1,0126	1
Rabl2	-1,014	1

Proser3	-1,0152	1
Palld	-1,0155	1
Ints4	-1,0156	1
Chd1l	-1,0156	1
Hmx2	-1,0157	1
Ppdpf	-1,0162	1
Atp1b4	-1,0197	1
Gm15832	-1,02	1
Gm4459	-1,0204	1
Gm16201	-1,0221	1
Cnnm2	-1,0223	1
Snord71	-1,0229	1
Gm42482	-1,0231	1
Pcyt2	-1,0243	1
Sfi1	-1,0264	1
Ccdc63	-1,027	1
Zmym6	-1,0275	1
Gm43774	-1,0289	1
Tufm	-1,029	1
L1cam	-1,0295	1
Gm15829	-1,0314	1
Gm12517	-1,0326	1
Gm45153	-1,0341	1
Gm37490	-1,0354	1
Efemp2	-1,0356	1
Taz	-1,0357	1
Ccr10	-1,0363	1
Akap10	-1,0369	1
Frmd6	-1,0393	1
Itgb7	-1,0396	0,91431
Gm42895	-1,04	1
Gm45890	-1,0413	1
B230312C02Rik	-1,042	1
Gm15853	-1,0425	1
Park2	-1,0431	1
Ift122	-1,0452	1
RP24-370M23.1	-1,0454	1
Ube2e2	-1,0468	1
Zfp786	-1,0475	1
Primpol	-1,0476	1
Gm26610	-1,048	1
0610009O20Rik	-1,049	1
Gm43138	-1,0506	1
Tmtc4	-1,0512	1
Msantd3	-1,0513	1
Gm12444	-1,0525	1
Gm5778	-1,053	1
Eri3	-1,0534	1
Emc10	-1,0538	0,60286
Ahcyl2	-1,0539	1
Gm23346	-1,0554	1
Gzmm	-1,0554	1

Gm43133	-1,0559	1
Gm13423	-1,0575	1
Gm37696	-1,0588	1
Crebzf	-1,0597	1
Gja1	-1,0609	1
Atp2a3	-1,0612	0,48661
Gm12988	-1,0621	1
C230037L18Rik	-1,0626	1
Tle6	-1,0633	1
Rps17	-1,0635	1
Gm6418	-1,0648	1
Parpbp	-1,0679	1
Gm38257	-1,0683	1
Pofut1	-1,0686	1
Ttc25	-1,0695	1
Supt20	-1,0698	0,68757
Eid3	-1,0698	1
Armc6	-1,0699	1
Gm10138	-1,071	1
Gm4737	-1,0719	1
Cmtm7	-1,0727	0,69335
Iqcc	-1,0732	1
Serpini1	-1,0735	1
RP23-36H21.3	-1,0753	1
Gm12184	-1,0757	1
Gm24631	-1,0771	1
Maats1os	-1,0792	1
Gm23300	-1,0801	1
Gm26497	-1,0802	1
Atf5	-1,0818	0,46672
Gm10175	-1,0831	0,82541
Gm26620	-1,0831	1
4933433G15Rik	-1,0837	1
Olfr921	-1,085	1
Odf3l1	-1,0868	1
Gm11464	-1,0869	1
Gapdh	-1,0877	1
D430013B06Rik	-1,0886	1
Kif18b	-1,0888	1
Gm42798	-1,0893	1
Ncapd3	-1,0898	0,53517
Vamp1	-1,0899	0,9382
Gm44164	-1,0902	1
Cdh23	-1,0903	1
4930447F24Rik	-1,0913	1
Kat2b	-1,0921	1
Spaca9	-1,0925	1
Gm12089	-1,0929	1
1700124L16Rik	-1,0931	1
Eme1	-1,0934	1
RP23-426K2.3	-1,0945	1
AA386476	-1,0968	1

Prkcg	-1,0974	1
AI480526	-1,0985	1
Gm22973	-1,0988	1
Rps18-ps3	-1,1007	1
Mapkapk5	-1,1018	1
Mphosph9	-1,103	1
Nfatc4	-1,1031	1
Gm37914	-1,1047	1
Pi16	-1,1056	0,91431
Ptgs1	-1,1072	1
Fancc	-1,1077	1
Gm37349	-1,1081	1
B430305J03Rik	-1,1106	1
Echdc3	-1,1107	1
Prkca	-1,1108	1
Hist1h2aa	-1,1111	1
Gm14636	-1,1124	0,93384
Gm28727	-1,1126	1
Mtmr11	-1,1143	1
Gm23751	-1,1154	1
Gem	-1,1155	1
Gm43578	-1,1166	1
Med30	-1,1167	1
Vsig8	-1,1174	1
Tmem240	-1,1181	1
Psd2	-1,1183	1
Ifitm1	-1,1186	1
Fancd2	-1,1209	1
Slc27a3	-1,1232	1
Gm29170	-1,1233	0,78566
Gipr	-1,1237	1
Myc	-1,1239	1
Klhl35	-1,1239	1
H2-Q4	-1,1243	1
Gm20522	-1,1273	1
Gm43773	-1,1305	1
Msantd2	-1,1324	0,99275
Ttc39a	-1,1326	1
Hdac7	-1,133	1
Rabgap1l	-1,1357	1
9130604C24Rik	-1,1357	1
Acad11	-1,1375	1
4930448A20Rik	-1,1377	1
Lims2	-1,1378	1
RP23-402A24.3	-1,1403	1
Gm23100	-1,1446	1
Gm15785	-1,1447	1
Rilp	-1,1453	1
Klra2	-1,146	1
Tsc22d3	-1,1464	0,51847
2900055J20Rik	-1,1466	1
Rsrp1	-1,1495	0,32868

Camk2a	-1,1505	1
Gemin4	-1,1507	1
RP23-390D8.2	-1,1508	1
Dynlt1b	-1,1519	1
Zg16	-1,1544	1
Zfp182	-1,1549	1
Gm5577	-1,1557	1
5430434F05Rik	-1,1558	1
Gm13413	-1,1579	1
Bcat2	-1,1583	0,93372
Pde2a	-1,1585	1
Gm44258	-1,1595	1
Clcn2	-1,1617	1
Snx25	-1,1636	1
F830208F22Rik	-1,1638	1
Pomc	-1,166	1
DHRSX	-1,1667	1
Tmem42	-1,1679	1
Gm37738	-1,1686	1
Pkd1l2	-1,1688	1
Pygl	-1,1721	1
Gm42511	-1,1721	1
Gm15596	-1,1728	1
Kirrel3	-1,1735	1
Rab42	-1,1738	1
Gm4673	-1,1742	1
Hyal1	-1,1747	0,38824
Gm27010	-1,1769	1
RP24-460E12.3	-1,177	1
Ost4	-1,1791	0,90711
Cd80	-1,1803	1
Pyroxd2	-1,1812	1
Tpm2	-1,1841	1
Shprh	-1,1844	1
Gm12428	-1,1866	1
Gm37558	-1,1869	1
Ube2s	-1,1875	1
Per3	-1,1877	0,7434
Hist1h2bp	-1,1882	1
Uba52	-1,1892	0,9787
Diaph2	-1,1892	1
Phf21b	-1,1892	1
BC049715	-1,1897	1
Gm37383	-1,19	1
Gm15453	-1,1904	1
Tpcn2	-1,1911	1
Cxcl10	-1,1916	1
2610020H08Rik	-1,1918	1
Tiam2	-1,1925	1
Kif5c	-1,1925	1
Gm26631	-1,1925	1
Mroh2a	-1,1925	1

Hap1	-1,1932	1
Emp2	-1,1939	1
Gm37893	-1,1939	1
Fancb	-1,1958	1
Gys1	-1,196	0,09429
Gm37621	-1,1961	1
Gm44834	-1,1987	1
Gm43817	-1,2008	1
Gm45185	-1,2023	1
Gm5830	-1,2033	1
Cspg5	-1,2041	1
Mical2	-1,2046	1
Rfxank	-1,2051	1
Extl1	-1,2071	1
Zfp773	-1,2072	0,93372
E330037G11Rik	-1,2082	1
Gm42666	-1,2083	1
Gm25636	-1,2083	1
Gm23301	-1,2107	1
Tmem91	-1,2128	1
Gm44130	-1,2129	1
Gm43059	-1,2129	1
Stxbp4	-1,2132	1
Ccdc33	-1,2137	1
Gm16104	-1,2154	1
Peak1os	-1,2169	1
Cep164	-1,2191	1
Fabp7	-1,2213	1
Map3k15	-1,2223	1
Nrbp2	-1,223	1
Ankdd1a	-1,2238	1
E2f8	-1,2242	1
Gm45248	-1,2301	1
RP24-282C4.3	-1,231	1
Espl1	-1,2328	1
Gm13622	-1,2332	1
Ercc6l	-1,2337	1
Gm43011	-1,2339	1
Ccdc18	-1,2352	1
Hk1os	-1,237	1
Gm16199	-1,2374	1
9530078K11Rik	-1,2385	1
mt-Tp	-1,2386	0,68407
9330151L19Rik	-1,2401	1
Sipa1l1	-1,2408	1
Tmem80	-1,2414	1
Cdhr4	-1,2426	1
Jsrp1	-1,2429	1
Dock2	-1,243	0,66845
Senp8	-1,243	1
Ankrd52	-1,2444	1
Hist1h2bg	-1,2467	1

Gm30238	-1,2531	1
4933437G19Rik	-1,2547	1
Gm37052	-1,2556	1
Gm42820	-1,257	1
Masp2	-1,2584	1
Gm14239	-1,26	1
Gm42467	-1,2607	1
Lrrc49	-1,2624	1
Gm26132	-1,2624	1
Gm13890	-1,2626	1
2210408F21Rik	-1,2645	0,99275
Gm16845	-1,266	1
Slc52a2	-1,268	1
Epm2a	-1,2684	1
Gm42724	-1,2715	1
Ogt	-1,2719	0,45851
Eno1b	-1,2726	0,99275
4930529C04Rik	-1,2727	0,93384
4930412F12Rik	-1,2733	1
Eno1	-1,275	0,41052
Nek2	-1,2769	1
Gm33370	-1,277	1
Timeless	-1,2772	1
Rpl17-ps10	-1,2773	1
2810029C07Rik	-1,2774	1
C130083A15Rik	-1,2777	1
Gm26847	-1,2794	1
Gm15859	-1,2811	1
Gm12248	-1,2835	1
Snhg20	-1,2845	0,1272
Nxn	-1,2857	1
Gm26947	-1,2868	1
Rpl7a	-1,2887	0,60763
Zbtb32	-1,2894	1
Gm37718	-1,2894	1
Slc13a3	-1,2926	1
Gm12882	-1,2937	1
Serinc2	-1,2938	0,79888
Fads2	-1,2952	1
Rasd1	-1,2954	1
Hlcs	-1,2957	1
Coq2	-1,2985	0,93384
Asap3	-1,3002	1
C030037D09Rik	-1,3005	1
Kntc1	-1,3012	1
RP24-282C4.4	-1,3019	1
Trim2	-1,3024	1
Gm45084	-1,3026	1
Gm38082	-1,3058	0,93436
Gm37289	-1,3058	1
Tcf19	-1,3072	1
Ccdc171	-1,308	1

D530018E20Rik	-1,3085	1
Spink5	-1,3087	0,030839
Itgal	-1,3094	0,80278
Gdap10	-1,3099	0,98848
Hist1h3d	-1,3117	1
Gm26601	-1,3121	1
Gm6266	-1,3125	1
C530043K16Rik	-1,3129	0,95396
Snord87	-1,3132	0,93436
2900005J15Rik	-1,3133	1
Gm12240	-1,3152	1
Gm13408	-1,3177	1
Gm2011	-1,3177	1
Gadd45g	-1,319	0,23852
Gm38062	-1,3198	1
Gm11491	-1,3218	0,81787
Gm26935	-1,323	1
1700054M17Rik	-1,3261	1
Txk	-1,3265	1
5330406M23Rik	-1,3274	1
Apip	-1,329	1
Gm24959	-1,3294	1
Edil3	-1,3296	0,36402
Cdc25c	-1,3303	1
Rnu11	-1,3309	1
B9d1	-1,3322	1
Cc2d2a	-1,3328	1
Gm43200	-1,3342	1
Rab5a	-1,3343	1
Gm25541	-1,3357	1
Gm24339	-1,3364	1
Dtnb	-1,3365	1
Gm38365	-1,3406	1
Gm38036	-1,3407	1
2700038G22Rik	-1,3419	1
Fndc7	-1,3434	1
Ankrd16	-1,3444	1
Prelid3a	-1,3445	1
Gm17108	-1,3446	1
Rps19-ps4	-1,346	1
Timm8a1	-1,3473	1
Gm37333	-1,3485	1
C2	-1,3486	1
Ptges	-1,3514	1
Il1rl1	-1,3515	1
Kif7	-1,3525	1
Tap2	-1,354	1
RP24-183O8.6	-1,3541	1
Gm43006	-1,3551	1
Man2b2	-1,3567	0,83388
Rnaseh1	-1,3592	1
Gm23849	-1,3622	1

Nme7	-1,3627	1
Zfp931	-1,3634	1
1810010D01Rik	-1,3638	1
Gm7114	-1,3664	1
Gm24920	-1,3699	1
Gm37255	-1,3704	1
Gm43148	-1,3706	1
Exoc3l	-1,3721	1
Cenpx	-1,373	0,42835
Acsbg1	-1,3757	1
Manba	-1,3758	1
Abcb9	-1,3765	1
Ccdc85b	-1,3767	1
Gnrh1	-1,3775	1
C030014I23Rik	-1,379	1
Gas2	-1,3819	1
Gm42635	-1,3828	0,82462
Ldb1	-1,3828	1
Gm24876	-1,3866	1
Gm43482	-1,3899	0,93384
Izumo4	-1,3915	1
Elfn2	-1,3917	1
Gm13038	-1,3921	1
Gm43868	-1,3946	1
Rps6	-1,3965	1
Gm37589	-1,3971	1
P4ha2	-1,4012	0,44022
Robo3	-1,4054	1
Pgap3	-1,4055	1
RP24-310D17.9	-1,4067	0,84725
Rpl23a-ps2	-1,4079	1
Gm44044	-1,4087	1
Tnnt1	-1,4129	1
Plxna3	-1,413	1
Gm2367	-1,4149	1
Hrc	-1,419	1
3010003L21Rik	-1,4196	1
Gm15420	-1,4204	1
Gm37949	-1,4204	1
Gm7856	-1,4204	1
9430092D12Rik	-1,4204	1
Icosl	-1,4214	1
2010016I18Rik	-1,4217	1
Rasal1	-1,4234	1
Gm37357	-1,4238	1
Fsd1l	-1,4269	1
Shox2	-1,4281	1
Morf4l1	-1,4293	0,29438
Gm11516	-1,4302	1
RP23-55A6.4	-1,4326	1
Gm36963	-1,4379	1
Capn1	-1,4381	0,21586

Flt1	-1,4393	0,56295
Umad1	-1,4413	0,99275
Gm42480	-1,4424	1
Lmln	-1,4449	1
Bank1	-1,4449	1
Hspbap1	-1,4461	1
Gm10575	-1,4469	1
Pdcd1	-1,4483	1
Gm42819	-1,4484	0,96335
Prss53	-1,4498	1
Gm11944	-1,4508	1
Gm2531	-1,4537	0,84725
Gm6290	-1,4545	1
2310074N15Rik	-1,4554	1
1700007L15Rik	-1,4555	1
Amigo1	-1,4563	1
Zdhhc1	-1,4597	1
Dkkl1	-1,4597	1
Cd48	-1,4625	1
Htr2b	-1,4628	1
Aloxe3	-1,463	1
Rps2-ps10	-1,4633	1
Gm43149	-1,4677	1
Mdm4-ps	-1,471	0,66711
Stc1	-1,4735	1
Cep128	-1,4737	1
Gm7336	-1,4765	1
Camk2n2	-1,4816	1
1810026B05Rik	-1,4835	0,03978
Pgam1	-1,4836	1
Gm36989	-1,4841	1
Pbx1	-1,4843	1
Col15a1	-1,4977	1
Sigirr	-1,4979	1
Esr1	-1,4979	1
Akr1b7	-1,4994	1
A430110C17Rik	-1,5003	1
Gm43445	-1,5049	1
Cnbd2	-1,5058	1
Gm44090	-1,5068	0,93372
Gm45289	-1,5076	1
Dsn1	-1,5096	1
Gm37470	-1,51	1
Hnrnpa1	-1,5102	1
4921536K21Rik	-1,5105	1
Hist2h4	-1,5131	1
Eldr	-1,5131	1
Gm43544	-1,5196	1
Hmgb1	-1,5204	0,80396
2610524H06Rik	-1,5249	1
Gm5362	-1,5314	1
Stap2	-1,5371	1

Scarna17	-1,5388	1
Gm12833	-1,542	1
3110083C13Rik	-1,5432	1
Dmpk	-1,5436	0,81968
Dpf1	-1,5514	0,80278
Itgax	-1,557	0,44926
Gm45477	-1,5576	1
Gm42633	-1,5587	1
Mkln1os	-1,5599	1
Gm15441	-1,5601	1
Intu	-1,5614	1
Anxa9	-1,5646	1
Ptpn5	-1,5657	1
Gm38043	-1,5698	0,93372
Arhgap19	-1,5707	1
Gm45546	-1,5743	1
4833445I07Rik	-1,575	1
Ddr2	-1,5758	1
Ndufa4l2	-1,5758	1
Gng8	-1,5758	1
Gm5837	-1,5766	1
Gm15496	-1,5826	1
Cchcr1	-1,5832	1
Gm28151	-1,5836	1
Snx32	-1,5863	1
Gm37407	-1,5876	1
Gm26225	-1,588	1
Il13ra2	-1,5882	1
Gm3531	-1,5949	1
Gm42484	-1,5979	1
Gm44187	-1,5988	1
Gm43111	-1,5989	1
mt-Tc	-1,606	0,80278
Gm37519	-1,6111	1
Gm43420	-1,6112	1
Gm10029	-1,6205	1
Rbm3	-1,6218	0,90785
Gm8337	-1,6249	1
RP23-454I20.1	-1,6402	0,97048
1700034H15Rik	-1,6417	1
Cep72	-1,6546	1
Olfr933	-1,6551	1
Mfsd7a	-1,6564	1
Gm15503	-1,6575	0,68407
Gm16540	-1,6577	1
B230317F23Rik	-1,6611	1
Ttc9	-1,6709	1
Rpl12	-1,6753	0,0451
Sdhd	-1,6788	0,85941
Enpp5	-1,6821	0,44926
Cerkl	-1,7035	1
Spcs2-ps	-1,7054	1

Exd1	-1,707	1
RP24-75M13.2	-1,7087	1
Gm43061	-1,7099	1
Nt5e	-1,7129	1
Gm45873	-1,7171	1
Gm22513	-1,7172	0,93372
C630004M23Rik	-1,7184	1
Gm44699	-1,72	1
4833421G17Rik	-1,7276	1
Gm42576	-1,728	0,93384
Gm8550	-1,73	1
Gm24009	-1,7318	1
Gm45809	-1,7366	1
RP24-323H7.5	-1,738	1
Gm45534	-1,7438	1
Gm43800	-1,744	1
Smtn	-1,7449	1
Gm25517	-1,7563	1
Gm37531	-1,7568	1
Gm43628	-1,7591	1
Rgs14	-1,7612	0,43823
B3glct	-1,7634	0,61316
Rnft2	-1,7674	1
Palb2	-1,7686	1
Gm37906	-1,7757	0,7002
Lppos	-1,7777	1
Gm38319	-1,7812	1
Gm18284	-1,7859	1
Gm12479	-1,7924	1
Gm45358	-1,7934	1
1700020D05Rik	-1,7961	1
Mylpf	-1,7978	1
Kcnj2	-1,8012	0,60812
Gm20594	-1,8023	1
Gm44432	-1,8052	1
Gm43813	-1,8053	0,95396
Gm37204	-1,8168	1
Gm43696	-1,8182	0,70782
Pabpn1	-1,82	0,31637
Gm37060	-1,821	0,30142
RP23-23P9.3	-1,8227	1
Cd5l	-1,8233	0,93372
Mrps18b	-1,8283	1
Gm23037	-1,8286	0,39543
Gm37653	-1,8296	0,52428
Lekr1	-1,8371	1
B930086L07Rik	-1,8384	0,29186
BC055308	-1,8386	0,35241
2700029L08Rik	-1,8429	1
Bco2	-1,8526	1
Ccdc122	-1,8539	1
Gm42478	-1,8546	1

Snord72	-1,8571	0,99275
Gm19272	-1,8579	1
RP23-320D23.6	-1,8601	0,22757
Gm45343	-1,8618	0,53543
Gm26594	-1,862	1
Gm12522	-1,864	0,95893
Gstt2	-1,8677	0,72615
Gm10657	-1,8724	1
Gm26129	-1,8825	1
Gm43328	-1,884	0,64669
1700001P01Rik	-1,8852	1
Fkbp14	-1,8853	0,68113
Rgs11	-1,8882	0,46672
Gm37106	-1,8888	1
Gm12933	-1,8924	1
Gm45220	-1,9021	0,91431
C3	-1,9081	1
Gm45729	-1,9097	1
Hist2h2ac	-1,9156	1
Fam13a	-1,9212	1
Gm11716	-1,9234	0,89432
Rhbdd2	-1,955	1
Gm10698	-1,9652	0,82541
Plat	-1,9734	1
Gm43153	-1,9765	1
Gm42671	-1,9833	1
Gm42793	-1,9962	1
RP23-205H11.3	-2,0064	0,49891
Gm19967	-2,0137	0,45264
Gm45203	-2,0144	0,75599
Ifitm5	-2,0161	1
mt-Tq	-2,0162	0,2844
Aldh1l2	-2,0297	0,32138
Rn7sk	-2,0356	0,088944
Adora2a	-2,046	1
Gm37902	-2,0504	1
Gm37678	-2,0549	1
A530017D24Rik	-2,0874	0,81968
mt-Tm	-2,098	0,066107
Bok	-2,0991	0,79526
Gm37472	-2,1038	1
Dixdc1	-2,1068	0,82462
RP23-356D13.9	-2,1144	0,97048
Rpsa-ps2	-2,1165	1
Sit1	-2,1237	0,68407
Hist1h4d	-2,1558	0,45264
Plch2	-2,1581	1
Gm24991	-2,1611	0,83388
Gm15268	-2,172	1
C730045M19Rik	-2,1727	0,73222
Hist1h1a	-2,1869	1
AV099323	-2,2106	0,86485

Rpl7	-2,2194	0,016219
Mrps28	-2,2203	0,87095
Hist4h4	-2,2223	0,91431
Gm38220	-2,2437	1
Gm43273	-2,2489	1
Abcc10	-2,2708	0,93384
Gm26461	-2,2851	0,81368
Gm44292	-2,2948	0,25154
Gm43609	-2,3328	1
Crtc2	-2,3442	0,49891
Rgs16	-2,3503	0,1272
Gm42743	-2,3562	0,19588
Hspa1b	-2,3706	0,053356
Slc12a5	-2,3708	0,32138
Gm44639	-2,3798	0,7434
4930589O11Rik	-2,3808	0,66845
Il34	-2,3958	1
4930509H03Rik	-2,4052	0,53543
RP23-40D21.1	-2,4565	0,26239
Gm26202	-2,4938	0,3021
RP24-282K24.4	-2,5382	0,62264
Rbfox1	-2,5666	0,52232
Gm22980	-2,6272	0,3021
Gm8228	-2,6403	0,49891
Hist1h4n	-2,6417	0,59434
Mmp9	-2,6514	0,29902
Hspa8	-2,6946	0,03978
Gm3699	-2,709	0,78566
Gm17249	-2,7117	0,6184
Gm37219	-2,7547	0,7434
Hspa1a	-2,7657	0,28509
Rnf152	-2,7946	0,54398
Gm23344	-2,8535	0,32138
Snord59a	-2,8591	0,2844
Ccpg1os	-2,9197	0,30983
Vwa1	-2,9271	0,66353
Gm42908	-2,9468	0,46918
Snord66	-3,0538	0,45264
Gm15937	-3,3689	0,26842
Gm44270	-3,5664	0,19838
Hist1h1d	-3,7067	0,066107
Hist1h2an	-3,7338	0,22757
Gm45698	-3,7689	0,032407
Snord83b	-3,7928	0,030987
Gm23969	-4,172	0,031957
