# Supplementary material for: Bone, dentin and cementum differentially influence the differentiation of osteoclast-like cells
Source: Sci Rep. 2025 Jun 5;15:19857. doi: 10.1038/s41598-025-04874-9 (PMC12141432; doi:10.1038/s41598-025-04874-9)
Supplement: Supplementary file 3 — Supplementary Information 3. [file 41598_2025_4874_MOESM3_ESM.pdf]

Tab. S2:
Transcripts induced in murine macrophage cells stimulated on bone (n=6)
fold of negative control

gene name	regulation of expression	adj.P.Val
mt-Tc	15,4442724	0,039238
mt-Ts2	13,62420365	0,0090394
mt-Ti	10,27452647	0,0007572
mt-Tl1	9,069355302	4,11E-06
Rasd1	8,476683963	0,15963
Gdf15	7,756468787	4,11E-06
Gm8210	7,754856043	0,46978
mt-Ta	7,744112978	0,20396
Gm24631	7,680498758	0,092681
Gm42522	6,719714786	0,23517
Lgr5	6,46656695	0,80904
Hspa1b	6,431253691	0,15406
Gfap	6,405006346	0,58759
9930120I10Rik	6,388158019	0,28366
4921507G05Rik	6,286687277	0,069188
Gpr19	6,186399493	0,32705
March4	6,182969976	0,32705
Gm18709	6,098269886	0,0085007
Gm20186	5,945095561	0,32705
Gm26225	5,937271145	0,60972
mt-Tm	5,892173858	0,0097563
Gm9920	5,779721592	0,48612
Mpc1	5,626742726	0,80904
Gm6450	5,581295378	0,67177
D2hgdh	5,429050863	0,49081
Ighd	5,353194234	0,66523
RP23-440L7.5	5,333934097	0,15406
RP23-213P10.2	5,326914038	0,82245
Lbp	5,29525441	0,21571
Gm5100	5,282423613	0,59509
Gm7351	5,256490848	0,57935
Gm42670	5,171203214	0,30209
Hist1h4h	4,950588378	0,3836
Gm29358	4,862168866	0,010814
Gm42632	4,820223361	0,87488
Gm43714	4,798222304	0,48002
5730405O15Rik	4,708932361	1
Gm8203	4,666044285	1
mt-Tv	4,662811151	0,10479
1110035H17Rik	4,650545656	0,32021
Snord7	4,641850312	0,91264
Lgals7	4,61714167	0,49081
Hba-ps4	4,604677126	1
Gm11343	4,591927933	0,51121
Gm26759	4,585566579	0,77268
Depdc1a	4,584295366	1
Socs1	4,579531456	0,11171
Fam72a	4,570018483	0,31881

RP24-295J1.1	4,517422967	0,31881
Gm23127	4,387806789	1
mt-Tq	4,361123966	0,46978
Gm12940	4,305258907	1
2210406H18Rik	4,296017886	0,97341
Mcm8	4,28828265	0,069312
Sema6c	4,252466881	0,72091
1700030M09Rik	4,243045061	0,32705
Tsix	4,185500065	0,087738
Ppp2cb	4,163509166	0,46413
Fcrl5	4,106473534	1
Samd9l	4,103912581	1
Gm13226	4,095387602	0,60972
Fzd7	4,074434957	0,008065
Gm25008	4,071047346	0,31881
Trf	4,065689388	0,54539
Gm8909	4,063435516	0,66813
Gm43660	4,049096426	0,82245
Gm19028	4,038445304	1
RP23-226H21.3	4,026705606	0,6841
Gm42850	4,02475231	0,86681
Gm26810	4,024473345	0,5517
C8g	4,015835017	0,84623
Gm45167	4,011383782	0,010814
Gm37851	3,975124373	1
Fsbp	3,952868605	0,97341
Gm15787	3,937281873	0,96202
Tnfsf9	3,917952753	0,31429
A830008E24Rik	3,917952753	0,86681
RP23-451J19.1	3,887923952	0,19651
Hsh2d	3,876621817	0,87488
Hspa1a	3,870446481	1
Pam16	3,86080048	0,57935
Gm10069	3,851979433	1
Gm11631	3,848243261	1
4930589L23Rik	3,832537744	0,82245
5930420M18Rik	3,825637031	1
Mrc1	3,801845751	1
4930578M07Rik	3,785017577	0,10479
RP24-174I4.1	3,780036067	0,16637
Gm28404	3,767741534	0,60972
Rnf25	3,759914876	0,5517
S1pr1	3,753665258	0,0007572
Rps13-ps1	3,748725011	0,98528
Gm42728	3,727736798	1
D6Ertd527e	3,718961983	1
RP24-316F13.7	3,710465004	0,80904
Aloxe3	3,709179278	0,69659
Gm5578	3,707123042	1
Zfp619	3,69993519	1
Gm26664	3,696602712	0,51724
Gm14650	3,690714141	1

Gm16372	3,688668142	1
Slc16a5	3,67208639	0,60972
Med12	3,659381936	1
Kif18b	3,655325801	1
Gm15542	3,639398426	0,60972
Zp1	3,637380874	0,84623
Gm44901	3,624294024	1
Gm26397	3,614760342	0,96993
Gm10167	3,611754919	1
2310058D17Rik	3,609752692	0,2836
Snord82	3,586558096	0,87488
Notch1	3,585812369	0,98528
Lrrc17	3,575388468	0,023749
Gm15634	3,575388468	1
Gm4285	3,556849669	1
Klkb1	3,542333392	0,97341
Vps37d	3,534239969	1
4930578M01Rik	3,533505121	1
Gm14137	3,531056731	1
Gm36989	3,526165038	1
Adam9	3,524210259	0,48208
Carnmt1	3,517864708	0,90856
Gm6140	3,502536293	0,98528
A430035B10Rik	3,495260541	1
mt-Tp	3,467990629	0,16619
Sez6	3,446423518	0,052865
4930430E12Rik	3,434975955	0,15406
Erfe	3,430692928	1
Slc25a30	3,423566415	1
Gm26772	3,421668513	0,92561
Snord89	3,40628701	0,068506
Gm11759	3,40416272	0,77153
Gm4832	3,403454918	1
Xaf1	3,390504596	0,6607
4732440D04Rik	3,369185822	0,92466
Hmgb1-ps8	3,363352523	1
Zfp712	3,344058301	1
Gm45133	3,335492969	0,90856
Dmrt2	3,32395304	0,78503
Lgals9	3,317048255	1
Hist1h2ae	3,29962026	1
Olfr286	3,298248272	1
AY074887	3,283876793	0,1571
Il1b	3,274103662	1
Zfp335os	3,270927993	1
Snord110	3,270927993	1
H2-Q10	3,264133357	1
Gm37696	3,264133357	1
Ccrl2	3,259385511	0,15074
Gm10463	3,254870174	1
Tnfrsf13b	3,23440424	1
Foxd2	3,228356722	1

Stra8	3,220534172	1
C78859	3,206056833	1
Atp6v0c	3,203835334	1
Tob1	3,181043278	0,13206
Gm43566	3,168719597	0,097271
Rmi2	3,168499966	0,84623
Zfp69	3,166085026	1
Gm37522	3,161479797	1
Mok	3,150323536	1
Snord66	3,144869169	1
Meiob	3,13964186	1
Snord71	3,139206644	1
Gm3724	3,138553932	1
Snord15a	3,133337121	1
Phf11c	3,132468496	1
Plekhh1	3,13051497	1
Gm37334	3,129430204	1
Slc25a2	3,11752256	0,92561
Nr4a1	3,115362406	0,62652
5330426L24Rik	3,111909274	1
Gm13416	3,104799272	1
Gm16046	3,101787817	1
RP24-93F20.12	3,097490806	0,90856
1700054M17Rik	3,097276111	1
Gm42793	3,082284433	1
Lif	3,074176504	1
Vil1	3,070556181	1
Col4a6	3,069279438	1
Rflnb	3,062479103	1
Gm23442	3,061842345	1
Gm14326	3,043011951	1
Gm14279	3,042801033	0,092694
Gm45251	3,03774338	1
Gm6969	3,035007328	1
Gm44013	3,022411299	1
Gm13383	3,012580933	1
Gm22513	3,006531346	1
Hmx3	3,003615205	1
Cks2	2,999870036	1
Dgat2	2,992601127	1
Gm23037	2,990734827	0,97341
4632415L05Rik	2,973784286	0,082515
Igf1	2,972547781	3,35E-05
RP23-168F21.4	2,968224061	1
Tap2	2,960416167	1
Gm29994	2,960210973	1
Gm13349	2,959595479	1
Gm44283	2,948947217	1
Gm42483	2,935894242	1
Abcc3	2,933656588	1
Amd2	2,923709556	1
Gm6564	2,906332975	0,92561

Trpm2	2,905728682	1
Gm44198	2,905325891	0,98528
Tcea3	2,901703278	1
Gm17034	2,894872884	1
Gm28373	2,891263305	1
Gm8885	2,888659186	0,95857
RP23-162P10.2	2,881260294	1
Mterf3	2,880860894	0,60972
Dnd1	2,870296949	1
4930532G15Rik	2,86632063	1
Bard1	2,865128808	0,93127
Gm43331	2,862151422	1
Grin1	2,85620593	1
Platr3	2,85244684	1
Egr1	2,848297813	0,25743
1500004A13Rik	2,833529063	0,30209
Cbx2	2,831958256	0,1571
Hsd3b7	2,83019214	1
1700052K11Rik	2,829603679	1
0610005C13Rik	2,827839031	1
Gm43800	2,826075483	1
Pin4	2,825487879	1
Ppp1cc	2,823725797	1
Ahrr	2,823138681	1
Gm3550	2,821573634	0,56285
Snora17	2,817078957	1
Snord35a	2,809668649	1
Arc	2,805387384	0,56179
Gm42851	2,803054898	1
Tfr2	2,79878371	1
Snord49b	2,791034582	0,32021
Gm26226	2,789680692	1
Llgl2	2,782342455	1
Hes7	2,780992782	1
Gm42600	2,777140178	1
Gm12762	2,774638848	1
Gm42559	2,774446532	1
Spdl1	2,772524098	0,13243
Gm28187	2,77098711	1
Gm4607	2,77041096	0,71295
Gm5830	2,766573016	1
Gm45445	2,760826066	1
Khnyn	2,754518209	1
Snord87	2,750892952	0,90856
Cenpu	2,749177391	0,74604
Gm44957	2,747843806	1
Gm9008	2,737008661	1
Gm16181	2,735680978	1
Hoxa3	2,729430572	1
D430001F17Rik	2,724516069	0,60972
Socs2	2,720741705	1
Crtc2	2,71998746	1

Gm42918	2,716031101	1
Gm26656	2,715466377	1
Gm23301	2,714149144	1
Osgin1	2,713396727	0,32705
Zfp36l2	2,713208655	0,040849
Ccdc62	2,703821666	1
Trappc2	2,699514564	1
Tnfrsf12a	2,6983921	0,012785
Nr4a3	2,6983921	0,6994
Gdf9	2,698205069	1
Gm13433	2,695401147	1
Rnf122	2,689429181	0,32705
Gm9938	2,681796933	1
Snord65	2,671962944	1
Rpl27-ps3	2,671222221	0,91264
Pik3r3	2,667521687	1
Dusp8	2,665119089	1
Rn7sk	2,659767242	0,8145
Bbs5	2,643959357	1
Gm7099	2,643776098	0,80904
Ncoa4	2,640296591	1
Gm28041	2,63170905	1
Cxcl2	2,631161858	0,15115
C1qtnf6	2,604849463	1
Adgre5	2,599618644	1
Bicdl1	2,599618644	1
Gm15728	2,596377214	0,47526
Gm12165	2,596377214	1
Gm20517	2,595657445	1
Gm45250	2,595297635	1
Hmgb1-ps6	2,59439833	1
Rgmb	2,590265535	1
St5	2,586497864	1
Teddm2	2,585960073	1
RP24-496O17.7	2,578978954	1
Gm2885	2,578085303	1
Gm10132	2,575406207	1
Med16	2,5752277	0,067381
Gm10863	2,574335349	1
Nfyc	2,57166015	1
Spire2	2,567207661	0,7952
Acoxl	2,565073206	1
Gm5786	2,563828926	1
Gm43182	2,562940525	1
Mtfr2	2,559744828	1
H2-T10	2,557262041	1
Gm45185	2,555490096	1
Gm42535	2,554604584	1
Papd4	2,554427518	1
Gpr35	2,551242436	1
Gm43668	2,550888783	0,97341
Gm15421	2,549474662	1

RP24-175C20.10	2,548767896	0,33785
Gm5828	2,547354951	1
Trdmt1	2,54541343	1
Gm19287	2,544707789	1
Tsg101-ps	2,542768286	1
Gm12034	2,540830261	1
2510016D11Rik	2,539421716	1
Selenop	2,535903769	0,57935
Rangrf	2,535376498	1
Gm20091	2,533619717	1
Gm43878	2,529057784	1
Gm19777	2,527305382	1
Spata2l	2,526254524	1
Dusp10	2,52223028	1
Kif18a	2,520657322	0,2836
Thap6	2,519434588	1
Mmp2	2,509848008	1
Ckap2	2,509500094	0,32021
Pask	2,509152227	1
Ppia	2,504460747	1
Gm5131	2,503940013	1
Zfp563	2,499258278	1
Gm37206	2,497007233	1
Gm6501	2,496315011	1
Gtse1	2,495968972	0,22159
Gm6919	2,490956797	1
Celf5	2,490784143	1
D830025C05Rik	2,48750599	0,98528
Gm13776	2,48526553	1
Nup98	2,482338742	0,74604
Camk2b	2,480790661	1
Rps13-ps5	2,477182226	1
0610039K10Rik	2,474264957	1
Gm8818	2,473407591	1
Gm6612	2,472893314	1
Zfp36l1	2,465362855	0,03277
Gm44552	2,462459505	1
Fos	2,46075325	0,87488
Camk2n1	2,460241604	1
Gm42731	2,459048177	1
Mir124-2hg	2,455301173	1
Gm44567	2,452409673	1
A330069E16Rik	2,451899761	1
Gm43010	2,450370664	1
Tnfaip3	2,450030994	0,88761
Gm12355	2,449691371	1
Mxd3	2,449351795	1
Lpar6	2,448503062	0,067381
Gm18860	2,446976082	1
Gm37510	2,445280555	1
Rbm19	2,443247474	0,60972
Hsd17b14	2,441893025	1

Ccdc33	2,439693648	1
Gm28555	2,438003167	0,068506
Hus1b	2,435976136	1
Gm22767	2,430073584	1
Hnrnph2	2,429063155	1
Rpl28-ps3	2,426370731	1
Gm28438	2,42552996	1
Gm29228	2,424017308	1
Jun	2,419317308	0,097271
Gm10131	2,418311352	1
Gm10382	2,413120569	0,92561
Kbtbd8	2,411949999	1
C430049E01Rik	2,403438687	1
Hmgb1-rs16	2,403438687	1
Gm43059	2,399277452	1
Depdc1b	2,396618043	1
Donson	2,392800306	1
2900060B14Rik	2,392302789	1
Gm13736	2,389485479	1
Slc30a2	2,388657489	1
Osm	2,386340646	1
Gm13840	2,386175243	1
Gm12604	2,385679104	0,89201
Hist1h2be	2,383034767	0,60972
Gm16439	2,382869594	1
9930022D16Rik	2,382209014	1
Rybp	2,379073758	1
Rpl7a	2,375942628	0,83459
Ptpru	2,375613276	1
Gm26520	2,373473595	0,23021
A830073O21Rik	2,37281562	1
Gm25291	2,371171479	1
Gm44777	2,370185542	1
Gm27003	2,370185542	1
1810062G17Rik	2,36805075	1
Gm44178	2,367722491	1
6820402A03Rik	2,361985323	1
Gm29759	2,360839558	1
Gm20673	2,357078816	1
Kif24	2,356915441	1
Btf3	2,355935431	1
Stamos	2,352671674	1
Gm7488	2,351530428	1
Gm44168	2,350389735	1
Pigb	2,349738159	1
Gm45413	2,347947256	1
Arhgap26	2,344044567	0,60972
Efcab11	2,343882096	1
Gm4963	2,342420362	1
Zfp248	2,341121809	1
Itga11	2,337878573	1
Vamp7-ps	2,337392476	1

Hlx	2,33528722	1
Stac3	2,33269874	1
Gm22516	2,331567182	1
Aunip	2,331405575	1
Gm4613	2,331405575	1
Siah1b	2,330113128	0,57771
Dnm3	2,328337183	1
Insig1	2,327853069	0,19165
Btbd6	2,326562591	1
2410080I02Rik	2,321407829	0,97341
Gm44836	2,321086036	1
Hist2h4	2,319799309	1
Arhgap39	2,319477739	0,60972
Ppp1r15a	2,318352594	0,096514
Gm10636	2,314178262	1
Rnd1	2,313697092	0,94786
Gm22714	2,310491849	1
Gm17251	2,310011446	1
Rac3	2,309851334	1
Cytip	2,309531142	1
Gm8326	2,309210996	1
Rps12-ps10	2,309050939	0,62991
Gm10177	2,305212903	0,97341
Wwc1	2,304254391	0,11798
Dynlt1-ps1	2,303934976	1
Car7	2,303615605	0,77131
Gm43421	2,303455936	1
Pygm	2,302019413	1
Isg15	2,298033772	1
Gm14673	2,296759825	1
Gm14584	2,295804828	1
Timm23	2,295804828	1
Gm13567	2,292942219	1
Gm12902	2,292465464	1
Gm29170	2,291671094	0,7247
9930111J21Rik2	2,291194604	1
Pank4	2,290876999	1
Prps1l3	2,290718213	1
Slfn8	2,288179133	1
Gm9521	2,287703369	1
Gm4890	2,287703369	1
A930007I19Rik	2,286593638	0,9712
Gm12799	2,284850862	1
Ntn5	2,28437579	1
Asf1b	2,283584223	0,80904
E330011M16Rik	2,281369294	1
Gm26533	2,281053051	1
Dusp5	2,279314498	0,47526
Gm6768	2,278840577	1
Rny1	2,277893031	0,74763
Egfl7	2,275210456	0,94063
Ier5l	2,274422065	0,59183

Zfp607a	2,274264419	1
Gm5822	2,273949161	1
Dynlt1b	2,273476356	1
Gm11298	2,27253104	1
1500015A07Rik	2,271586117	0,64938
Hexim1	2,26985478	0,32021
Gm12770	2,269697451	1
Gm37677	2,268596454	1
Epha2	2,268124761	0,69659
Gm12924	2,259807683	1
Gm6905	2,259494429	1
Maff	2,25652068	0,3387
Enho	2,254957121	1
Gm9769	2,253238457	1
Rpl30-ps1	2,249181367	0,97341
Aif1	2,248090322	0,77946
Tgfb1i1	2,246999806	1
Gm45051	2,246844061	1
Pcdhb16	2,246065499	1
Slpi	2,245598492	0,1571
Snrpc	2,245287207	1
Gm45630	2,244198051	1
Gm14541	2,242643031	1
Gm43721	2,242332156	1
4930542C12Rik	2,242021324	1
Kctd6	2,240467812	0,32705
Gm5070	2,239846709	1
Rpl35a-ps4	2,236898818	1
Zfp866	2,236588739	1
Ccdc69	2,233335509	1
Cxxc5	2,232871148	1
RP24-418P10.4	2,230860033	1
Bbs10	2,22885073	1
Gm8623	2,22792397	1
Atp1b4	2,22792397	1
3110045C21Rik	2,227615135	1
Mafk	2,22499177	0,15406
1700007K09Rik	2,22499177	1
Rnft2	2,224683342	1
Scel	2,224066615	0,96568
Atxn7l2	2,222525543	1
Gm7887	2,220215938	1
Rps12-ps26	2,218677535	1
Gm10237	2,217601287	1
Shcbp1	2,214222191	1
Rpl10a	2,213454933	1
Rdh13	2,212687941	0,087406
C3ar1	2,211461307	0,49081
Itgb3bp	2,207785481	1
Gm7363	2,207479439	1
Hacd2	2,205796961	1
Arid5a	2,204268549	1

Dennd4c	2,204115766	0,51121
Gm5312	2,202588519	1
RP23-440I21.3	2,202435852	1
Cdan1	2,201214901	1
Otud1	2,200909769	1
Gm44652	2,200604679	0,89808
Atad5	2,200604679	1
Gm44851	2,198927441	1
Bloc1s3	2,197403789	1
Lzts3	2,197403789	1
Ubc	2,196033405	0,49081
RP23-182J19.2	2,19527245	1
Gm32175	2,194359651	1
Syt8	2,193903394	1
Npl	2,192991165	1
5830432E09Rik	2,188132346	1
Gm5277	2,187525751	1
Cdca7	2,187222516	0,83459
Gm11625	2,184192483	1
Snord59a	2,18358698	1
Gpat3	2,182679041	1
Cox4i2	2,181469042	1
Gm8337	2,180864294	1
Nuf2	2,179655301	1
Rps19-ps11	2,179202102	1
Prss35	2,178597983	1
Scn11a	2,178597983	1
Sgk1	2,178295986	0,94786
Kif11	2,171662635	0,60972
Nectin4	2,168954818	1
Gm7638	2,167602175	0,98528
Mafb	2,162499801	0,074487
Atf3	2,162499801	0,23139
Gm28659	2,162499801	1
Ciart	2,16100139	0,49549
Kbtbd7	2,157858105	1
RP24-84C23.4	2,156213447	0,53789
Gm7308	2,155765122	0,93127
4732491K20Rik	2,154271377	1
Gm16740	2,15412206	1
Gm45733	2,153375627	1
Rpl10-ps3	2,149945368	0,98528
Gm4875	2,149200383	1
Rpl36-ps2	2,147413471	1
Gm8423	2,143398351	1
Zbed3	2,143249787	1
Btg2	2,142655634	0,55555
Hist1h2bp	2,141764714	1
Inpp5e	2,139687341	1
Id2	2,139539034	0,15406
Gm17541	2,13894591	1
Sik1	2,136130816	1

Gm8624	2,135834706	1
Gm8019	2,133467303	1
Gm43672	2,133023707	1
Atr	2,132728027	0,74604
Gm8116	2,131841235	1
Gm12981	2,131397977	1
Fbxo33	2,130364067	0,40856
Gm42895	2,130216407	1
Rpl34-ps1	2,129625867	1
Kcnd1	2,127265346	1
Gm20257	2,125054733	1
Bc1-ps1	2,124907441	1
Gm5841	2,118730367	1
BC030867	2,118436669	1
Vps25	2,115648568	1
Gm15265	2,113743034	1
Rbm4b	2,112864136	0,83697
Gm12164	2,11096111	1
Gm24991	2,110814794	1
Pcdhb22	2,108036724	1
Gm9435	2,10657605	1
Dusp2	2,106284037	0,82245
6430511E19Rik	2,105116389	1
Ccl4	2,104532808	0,069188
Gm13204	2,101908693	1
Ceacam16	2,099724431	1
Rpl23a-ps14	2,098996848	1
Gm43178	2,098851361	1
Gm13456	2,097397053	1
Rell1	2,096815611	0,16619
Gm29736	2,096815611	1
Gm12504	2,096089036	1
Zfp473	2,095072254	1
A130014A01Rik	2,094781836	1
Rpl39-ps	2,093910822	0,66259
Ost4	2,09304017	1
Gm11491	2,092024866	1
Txlnb	2,091589887	1
Gm13140	2,091444914	1
Asb10	2,090575288	0,96993
Cxcl10	2,090430385	1
Gm20302	2,087823855	1
Gm6913	2,086811082	1
Gm23935	2,085798801	0,864
Slc15a3	2,085076043	1
Gm10827	2,083631279	0,60105
Rsl1	2,083053654	1
mt-Nd6	2,082909273	0,60972
Aurkb	2,082043195	0,78503
Gm11363	2,082043195	1
Ddit4	2,081321739	0,3662
Gm44075	2,079735416	1

Tbc1d30	2,079014759	1
4930522L14Rik	2,078150301	1
Gm28578	2,076710336	1
Gm7299	2,076710336	1
L1cam	2,074264685	1
Gm2308	2,074120913	1
Gm12017	2,070529848	1
Dtd2	2,069238587	0,77947
Tnf	2,064940197	0,49549
Snord55	2,064367753	1
Raf1	2,063938525	1
Rpl31-ps13	2,063509385	1
Gm5601	2,062937338	1
Gm43006	2,060936422	1
Sep 01	2,060793574	1
Zfp36	2,06022228	0,91211
Myc	2,059793913	1
Rad54b	2,059080167	1
Gm8925	2,058509348	1
Gm8649	2,055515143	0,18452
Tnfrsf1b	2,054660456	0,57935
Plekhh3	2,054233245	0,82952
Mybl2	2,054233245	1
Rnf144b	2,054090862	1
Gm15703	2,053379091	1
Rps6-ps3	2,049966026	1
Dnajb9	2,049255685	0,60972
Gm5112	2,048687589	1
Mpp3	2,047268039	1
Lrp5	2,045565877	1
Ly86	2,045282322	0,32021
Gm14057	2,044857061	1
Id1	2,043581811	0,46413
Wdhd1	2,043156904	0,96037
Gm20689	2,042590499	1
Ect2	2,042307356	1
Cog3	2,042024251	1
Uevld	2,041458161	1
Gm5124	2,040750768	1
Gm2986	2,038347465	1
Col11a2	2,03764115	1
Capn3	2,037499916	1
Troap	2,037217478	1
Gm11945	2,03693508	1
Gm23639	2,03566477	1
Cit	2,035100442	1
Gmnn	2,034959384	0,8145
Cox7c	2,033972255	1
Ube2cbp	2,033831276	1
Pnrc1	2,031717758	0,13206
Gm43148	2,031576935	1
Rbm48	2,030450702	0,60972

Gm12778	2,028481296	1
Rps19-ps6	2,0277784	1
Spata5	2,026232885	0,97341
H60c	2,026092442	1
Nemp1	2,02426757	0,57506
RP23-325K4.10	2,023706402	0,57771
Gm18943	2,023285628	1
Gm42508	2,022444342	1
Mrip-ps	2,021883679	1
Klf11	2,020202623	0,97341
Gm29019	2,019782577	1
Rnf130	2,018942748	1
Cntnap1	2,017264137	1
RP24-240E7.1	2,017124315	1
C920009B18Rik	2,016844702	0,96993
Gm26594	2,014469552	1
Zswim8	2,014329925	0,77153
Pole	2,013492363	1
9230111E07Rik	2,013492363	1
Rpl7l1-ps1	2,01279466	1
Arl4d	2,010981765	1
Iqgap3	2,00861352	0,91264
Mcm10	2,008474298	1
Prokr1	2,007082613	1
Ccdc36	2,006665295	1
Gm11686	2,004857921	1
Gadd45b	2,003607615	0,62652
Abt1	2,003329875	1
Zic2	2,002774511	1
Gm11737	2,001664245	1
Gm26782	2,000138634	1
Thap8	1,998337138	1
Pim1	1,997783159	0,21571
Hoxb6	1,996260506	1
Idi1	1,994186031	1
Rdh5	1,994047809	1
Gm10240	1,99321868	1
Pla2g2d	1,993080526	1
Hax1	1,993080526	1
Gm7353	1,990733362	1
Gm43364	1,990457407	1
Gm9833	1,99018149	1
Slc17a7	1,989905611	1
Gm26710	1,989767686	1
Bst2	1,988802479	0,60972
Rpl30-ps2	1,986873469	0,83459
Rin1	1,985221522	1
Ltb	1,984808749	0,87488
Hace1	1,983845948	1
Rgs12	1,982471327	1
Gm26244	1,981372316	1
Mxd1	1,980548458	0,60807

Ube2n	1,979862171	1
Tbc1d10a	1,978627454	0,49081
Gm4784	1,978627454	1
Sft2d1	1,978490311	1
Gm12280	1,977256449	1
Pigg	1,976023357	1
Cnot3	1,975338638	0,47526
Wdr20	1,974106742	1
Gm11989	1,973696281	1
Gm42856	1,973285905	1
Mapkapk5	1,971918601	1
Gm7224	1,970005968	1
Irf4	1,967276852	1
Cenpn	1,965913712	1
Gm17060	1,965232496	1
Rnf225	1,963870773	1
Hpse	1,960334706	1
Gm24924	1,960334706	1
Dusp1	1,959927108	0,83697
Gm8599	1,959519595	1
Gm43727	1,958026106	1
Prss42	1,958026106	1
Ncmap	1,958026106	1
Gm42479	1,95789039	1
Gm26461	1,957076296	1
Gm43011	1,956669376	1
Rfc4	1,955178058	1
Tns2	1,95504254	1
Gm12459	1,954771533	1
Dnajb5	1,954500563	1
Fam83d	1,953823302	1
Nup160	1,953417058	1
Tbc1d8b	1,953010898	1
Mospd1	1,952740172	1
Lnpep	1,952469484	1
Gm5787	1,952198833	1
RP24-225A16.3	1,952063522	1
Clec2l	1,950710923	1
Gm17259	1,948548715	1
Gm6257	1,947738504	1
Gm38299	1,947333526	1
Znrf3	1,945714453	1
Gm37383	1,944635819	1
Gli1	1,942480344	1
Gm30074	1,940596264	1
Gm42566	1,939386034	1
Rps12-ps9	1,939251611	1
Pard6b	1,937370662	1
Gm4525	1,937102104	1
Gm7117	1,936162444	1
Cd274	1,935491538	1
Zwilch	1,935089105	1

Creb3l1	1,934686757	1
Dffb	1,934016362	1
Gm45762	1,933346199	1
Bend6	1,932140491	1
4933421A08Rik	1,929597576	1
Gm43430	1,929463831	1
Snora30	1,929330096	1
H2-Q5	1,928928944	1
Gm38335	1,928126892	1
Fam46c	1,927859615	1
Mcts2	1,926924439	1
Isg20l2	1,92612322	1
Ece1	1,925856221	1
Ptgs1	1,925722736	1
Map3k12	1,925455793	1
Pabpc1	1,924521782	0,7952
Gm42432	1,923721562	1
Got2-ps1	1,923454897	1
Gm45223	1,921855677	0,97341
Rrad	1,921056566	1
Ccdc163	1,920790269	0,97341
2810013P06Rik	1,919725452	1
Gm42876	1,918927227	1
Pramef8	1,915073814	0,89201
Ang	1,915073814	1
Trim59	1,914808347	1
Epb41	1,914012166	0,5297
Oas1g	1,913879501	1
Basp1	1,912685934	0,83109
Gm13196	1,912685934	1
2410022M11Rik	1,912288243	1
Gm43637	1,91136062	1
Gm6565	1,911228139	1
Ppfia4	1,910830752	1
Cep192	1,91030103	1
Gm16020	1,91030103	1
RP23-324E2.11	1,908712747	1
N4bp2	1,908183613	1
Cmklr1	1,908051353	1
2610528A11Rik	1,907919101	1
Magohb	1,906068543	1
Txlng	1,904615792	1
Hoxa5	1,901977263	1
Rps6ka5	1,899737388	1
Ppp1r13l	1,896185359	1
Card14	1,895528305	1
Exoc6	1,894477493	1
Gm10762	1,891984147	1
Fas	1,891328548	1
Gm12184	1,891328548	1
Gm14620	1,891197456	0,58715
Gm8394	1,889232161	1

Rps15a-ps1	1,888446614	1
Gm34121	1,887268908	1
Gm14140	1,880609095	1
Rps15a-ps6	1,880348405	1
Gm5883	1,878654803	1
Pcdhb17	1,877613344	1
Adamts6	1,877613344	1
Atn1	1,877092831	1
Gm43411	1,876442393	1
Pmp22	1,875532158	0,31451
Supt7l	1,874232589	0,97652
Gm45137	1,87345328	1
Gm6640	1,872155151	1
Gm24507	1,871765887	1
Rpl30-ps5	1,870987604	1
Gm6543	1,870468928	1
Mogat1	1,869691183	1
Proscos	1,86904331	1
Hyls1	1,868913762	1
Bloc1s6os	1,868784224	1
Gm6341	1,86748933	0,74684
Gm16537	1,865031503	1
Gm10358	1,864902234	1
Abcg4	1,864772973	1
Wfdc17	1,86425602	1
Gm38297	1,863997597	1
Gm45220	1,863093399	1
Gm22009	1,862576911	1
Hist2h2ac	1,861028308	1
Dram1	1,860770332	1
Tslp	1,860770332	1
Abhd10	1,85922323	1
Atp11a	1,858965505	1
Gm14094	1,858836656	1
Gm5914	1,858321349	1
Zbtb21	1,856904989	1
Ccdc116	1,856904989	1
Nup205	1,856261547	1
Hs3st3b1	1,855232502	1
Kctd21	1,854461093	1
Spata2	1,853946998	1
Xkr8	1,853690005	1
Evi5l	1,853304581	1
Gm13094	1,852790807	1
Sh2d3c	1,852533973	1
Gm12663	1,851635336	1
Gm7514	1,851506995	1
Hpdl	1,849454746	1
Gm8930	1,848173246	1
Ranbp9	1,847020655	0,83549
Fem1c	1,846764621	0,60105
Syap1	1,845484985	1

Pdzk1ip1	1,84535707	1
9330175E14Rik	1,842928372	1
Fanca	1,842800635	1
Matr3-ps2	1,841906719	1
Strn	1,839865112	1
Gm15445	1,839737586	1
Psd2	1,837061596	1
Cpne9	1,833880967	1
Prr7	1,833372578	1
Hist1h1a	1,833372578	1
Aaed1	1,833118436	1
Gm27248	1,832356222	1
Gm43024	1,832229217	1
Gm20274	1,831086569	1
Mtmr12	1,830578955	1
Tmed8	1,830452073	1
Trim14	1,830071481	1
Mkrn2	1,828549903	1
Gm38365	1,828042992	1
Gm24916	1,827536221	1
Lilr4b	1,82740955	1
Bsdc1	1,82740955	1
Dnmt3b	1,82740955	1
Plag1	1,827282888	1
Pcdhb15	1,826902955	1
Gm26912	1,825130977	1
Gla	1,824625013	0,90856
Rpl30-ps3	1,824372084	1
Lilrb4a	1,823487108	1
6330403N20Rik	1,823487108	1
Gm15131	1,823360718	1
Gm37733	1,821971005	1
Ftsj1	1,818816504	1
Vps8	1,817934222	1
Gm43379	1,816674563	1
Snx13	1,816296835	1
Zc3h12a	1,815541616	1
Rep15	1,815038311	1
Efna3	1,815038311	1
Ercc1	1,814912506	1
Atg4c	1,81478671	1
Pnp	1,814283614	1
Trip10	1,814032119	1
9330151L19Rik	1,814032119	1
Gm26530	1,813780658	1
A730011C13Rik	1,812900819	1
Lin52	1,812398246	1
Naa20	1,811142423	0,93403
Gm16045	1,809260319	1
Zfp326	1,80825733	0,68172
Gm44890	1,806753889	1
Rnaset2a	1,806002638	1

Zfp101	1,80587746	1
Arvcf	1,804501072	1
Zfp811	1,804375997	1
Ano7	1,802750822	1
5430420F09Rik	1,802251063	1
Fanci	1,801876334	1
Gm44292	1,80100227	1
Gas8	1,80000386	1
Tmem120b	1,799754343	1
Slc4a8	1,798756624	1
Cdc42ep4	1,797510253	1
Apoo-ps	1,797261083	1
Gm19566	1,79713651	1
Ezr	1,796513778	0,60972
Cox19	1,795642316	1
Nup107	1,795642316	1
n-R5s151	1,795268961	1
Rpl27a-ps2	1,795144527	1
Zfp747	1,795020101	1
Rpl19-ps11	1,793651989	1
Slc22a13b-ps	1,793403353	1
Hnrnpa3	1,792906185	1
Gm16437	1,792906185	1
Gm45853	1,792284918	1
Hist2h3c2	1,791663867	1
Dlgap5	1,790794758	1
Arl14epl	1,790794758	1
Gadd45g	1,790298313	1
Gm12469	1,789802005	1
Rps12-ps4	1,789553903	1
Irx5	1,789429865	1
Apba3	1,788809804	1
Mcm2	1,788313909	1
Coq10b	1,787446424	1
Rasl2-9	1,785588945	1
Plk2	1,784351698	1
Gm9320	1,782868133	1
Gm7701	1,782868133	1
Gm26799	1,782620992	1
Cdc7	1,781879775	1
Jund	1,781632771	0,66523
Gm28151	1,781138866	1
Rps19-ps5	1,780274861	1
Ptpn14	1,780274861	1
Sparc	1,778424835	1
Gpn2	1,777931819	1
Zranb3	1,777808586	1
Gins1	1,775961122	1
Usp18	1,77546879	1
Gm15694	1,775345728	1
Stk17b	1,775222675	1
Rad51ap1	1,7737467	1

4930579K19Rik	1,772886281	1
Fmr1	1,771535038	0,90856
Cped1	1,770675693	1
Tmem170b	1,769816764	1
Rpl7a-ps5	1,769571432	1
Gm11764	1,768100156	1
Wwp1	1,767855062	0,94063
Rbm3	1,767119985	1
Slc19a2	1,766752561	1
Gm13758	1,765773137	1
Rps2-ps10	1,765405993	1
Gm12097	1,765283628	1
Marf1	1,764305018	1
RP23-320D23.6	1,763204731	1
Spty2d1	1,763082519	0,81876
Pea15a	1,762960316	1
Gm12791	1,762593757	1
Gm12428	1,762471587	1
Il10ra	1,762349426	0,98528
1110006O24Rik	1,762227274	1
Klhl12	1,761616638	1
D730045B01Rik	1,760762104	1
Gm7832	1,760151976	1
Gm6136	1,758932354	1
Rps6-ps4	1,757957265	1
Icosl	1,756739164	1
Gm7846	1,756373899	1
Gm16072	1,75625216	1
Ccl5	1,75625216	1
Rps2-ps11	1,754913595	1
Polr2k	1,754062313	1
mt-Rnr2	1,753940734	1
Rps16	1,753940734	1
Gm5910	1,753819165	1
C730034F03Rik	1,753576051	1
H2afz	1,753089923	1
Stom	1,75284691	1
Bub1	1,752118073	1
Neurl1b	1,751753769	0,97341
AW047730	1,751753769	1
Ccdc84	1,751268147	1
Traip	1,750904019	1
Med6	1,74956953	1
Kpna4	1,749327005	0,98528
Gm44090	1,749327005	1
Srfbp1	1,749205755	1
Gm14593	1,748478432	1
Piwil2	1,747993718	1
Gm26737	1,747751411	1
Zfp873	1,747024693	1
Patl1	1,746903603	1
Slc26a11	1,746782521	1

Zfp280b	1,746177237	1
Hist1h3d	1,746177237	1
Sesn1	1,745693161	1
2610037D02Rik	1,745693161	1
Gm6682	1,745088255	1
Cbwd1	1,744967299	1
Lmtk2	1,744725412	1
Gfod2	1,744483558	1
Nup35	1,743999952	1
Gm38200	1,743758199	1
Lncpint	1,742308384	1
Gm7863	1,741704651	1
Mfsd14a	1,741342511	1
Lonrf3	1,740859775	1
Gm9143	1,740256544	1
Cmtm6	1,74001531	0,83459
Ppard	1,74001531	1
Tfb2m	1,739774109	1
E230029C05Rik	1,739171254	1
Rpl13-ps3	1,73796617	1
Ppp1r18os	1,737725253	1
Crkl	1,73748437	1
Ugt1a7c	1,737243521	1
Snora31	1,73616011	1
Cep55	1,735919443	1
RP23-58B7.2	1,735558507	1
Moap1	1,735558507	1
Tecpr1	1,73411551	0,80904
Esco2	1,733634778	1
Hapln3	1,733154179	1
Fam188b	1,73303405	1
RP24-325P4.5	1,732913929	1
Gm7860	1,731593152	1
Gm25596	1,731473131	1
Dennd4a	1,731353119	0,98528
D330041H03Rik	1,730993131	1
Cep135	1,730873152	1
G2e3	1,730273381	1
Txnip	1,730153452	1
Nrap	1,729673818	1
Rgs9bp	1,729434051	1
Mcm4	1,72931418	1
Cbr2	1,728475315	1
Cpsf7	1,728235714	1
Nkpd1	1,728235714	1
Gadd45a	1,727996146	1
Gm10343	1,727636857	1
Gm43200	1,726439765	1
Esco1	1,725721908	1
5033430I15Rik	1,725721908	1
Secisbp2	1,725602295	1
Man1a	1,724645683	1

Gm37660	1,723450664	1
Dock4	1,72273405	1
Gm12174	1,722256473	1
Coa6	1,722137099	1
Sbk3	1,722017734	1
Crip2	1,721898377	1
Mat2a	1,721779028	0,97341
Cbx4	1,721182408	1
Prdm10	1,721182408	1
Hist1h2bg	1,720943818	1
Lrrc41	1,720347488	1
Nfkbiz	1,720109014	1
Suco	1,719989789	0,63229
2610044O15Rik8	1,718678862	1
4930503L19Rik	1,718202409	1
Papd5	1,718083316	1
Blm	1,717487977	1
Zbtb3	1,717487977	1
Prosc	1,717249899	1
Nedd4l	1,717249899	1
RP24-131G14.10	1,716416887	1
Nceh1	1,716297918	0,80674
2310015A10Rik	1,715584278	1
Ptcd3	1,715108683	1
Gm9434	1,714989805	1
Gm43350	1,71463322	1
Anp32-ps	1,714514375	1
Gm20667	1,713920272	1
Atl2	1,712970136	1
Svil	1,710478535	1
Rnf185	1,710241429	1
Mcm3	1,710122888	1
Gdap10	1,710122888	1
Phf11d	1,710122888	1
Pmaip1	1,709411816	1
Rps13	1,708345762	1
Gm42666	1,708345762	1
Bach1	1,707990559	1
6430573P05Rik	1,707398717	1
Gm13886	1,70680708	1
Nat6	1,706688777	1
Cfap126	1,705860887	1
Fam103a1	1,70491522	1
Gm4866	1,704797048	1
Rps19-ps12	1,704678884	1
Gm37204	1,704560729	1
Phf8	1,70408819	1
Zfp516	1,703261562	1
Tmem64	1,702553343	0,91264
Gm37738	1,702435335	1
Ddx11	1,701609508	1
Spred3	1,701373632	1

Cdc42ep3	1,700901976	1
Paqr3	1,700901976	1
4932416K20Rik	1,699605597	1
Zscan21	1,698781144	1
Gm9825	1,698310207	1
Rpl10a-ps2	1,698310207	1
Gm15446	1,69795709	1
Klhl15	1,697721719	1
Nod2	1,697015803	1
Slc20a1	1,696310181	0,80904
RP23-2N7.4	1,696192606	1
Fth-ps2	1,696192606	1
Trp53	1,696075039	1
St3gal4	1,69595748	1
Cysltr1	1,694547409	1
Gm12906	1,692903807	1
5430427O19Rik	1,692317193	1
Rpl21-ps6	1,690324225	1
Rcbtb2	1,690089913	1
Gm7895	1,689972769	1
Csrnp2	1,689972769	1
Pik3c2a	1,689504274	1
Dnajb4	1,689270075	1
Gm21781	1,687397653	1
Usp22	1,686345328	1
Serpinb6b	1,685994699	1
C030015A19Rik	1,685644142	1
9230116N13Rik	1,684242646	1
Iqcf1	1,684125907	1
Inpp5f	1,684009177	1
Susd6	1,683892454	1
Cd200r4	1,683192288	1
Celsr3	1,682842315	1
Gm6177	1,682375797	1
Trim13	1,681559701	1
Skp2	1,681443148	1
Anln	1,681093538	1
Cbfa2t2	1,680977017	1
2410004B18Rik	1,680744001	1
H2-T23	1,6801616	1
0610040B10Rik	1,680045144	1
Gm7964	1,679812257	1
Polr2l	1,679230179	1
Rpl28-ps1	1,679230179	1
Gm8268	1,679113788	1
Gm11868	1,678764662	1
Gm9530	1,678531952	1
Gm15575	1,678531952	1
Foxj2	1,678182947	1
Fam234b	1,677834015	1
Gm8463	1,677834015	1
Dact3	1,677020123	1

Gm18284	1,676903885	1
Ets2	1,676671432	1
Ska1	1,676090443	1
3110062M04Rik	1,675509655	1
D930030I03Rik	1,675161279	1
Zdhhc13	1,674929068	1
Lbr	1,673652485	0,97341
Zfp52	1,673652485	1
Stil	1,672840621	1
3110056K07Rik	1,672840621	1
Sdhd	1,672724673	1
Nsl1	1,671218074	1
Dhrs3	1,669249953	1
1810041H14Rik	1,668093318	1
Gm14286	1,667515302	1
Gm38248	1,666937485	1
Rpl13a-ps1	1,666590891	1
1110003F10Rik	1,666475376	1
Spsb3	1,666359868	1
Gm15289	1,666244369	1
Gm6493	1,666013395	1
Sh2d2a	1,665782452	1
BC024386	1,665551542	1
Gm7312	1,664512841	1
Sp4	1,664282106	1
Gm42576	1,663474787	1
Rpl17-ps8	1,663128913	1
Adh5	1,662552617	1
Socs4	1,662206935	1
Rccd1	1,662206935	1
Kdm3a	1,662091723	1
Sdc4	1,660940048	1
1600012H06Rik	1,660594701	1
Fchsd1	1,660594701	1
Zc3h12c	1,66036451	1
Unc13a	1,66036451	1
Traf3ip1	1,659789171	1
Med22	1,659099028	1
Rad51	1,659099028	1
Gm28424	1,658064351	1
Hyal1	1,657604701	1
Ier5	1,657489809	1
Ropn1l	1,657489809	1
Lin54	1,656685785	1
Edrf1	1,655308365	1
Itgam	1,655193632	1
Zfp367	1,654620085	1
Gm45266	1,654046737	1
Etohd2	1,653358981	1
Gm23849	1,653244383	1
Erbin	1,652671512	1
A930015D03Rik	1,652327884	1

Mad2l2	1,652213357	1
Ctdp1	1,651411893	1
Ndor1	1,65129743	1
Abca1	1,651182974	1
Ncapg2	1,650610817	1
Melk	1,65038201	1
Oas2	1,65038201	1
Phldb3	1,650267618	1
Dnali1	1,64981013	1
Nfil3	1,649467097	1
RP23-70B19.5	1,647867221	1
Gsk3a	1,647182035	1
2810433D01Rik	1,646611264	1
Ceacam1	1,645356264	1
Bcl10	1,64524222	1
Usp26	1,644672122	1
Gm38257	1,644444137	1
Slc25a32	1,644330157	1
Gnmt	1,643874315	1
Cldnd1	1,643760375	1
Vdr	1,643760375	1
Gm44884	1,643760375	1
Tfeb	1,642963011	1
Prkx	1,642963011	1
Gm7504	1,642052211	1
Kif15	1,640573238	1
Dqx1	1,63977742	1
Lmnb1	1,639663764	1
Gm4880	1,639095598	1
Smpd4	1,638868387	1
Lrig2	1,638641207	1
4933404O12Rik	1,638641207	1
Gm36936	1,638186942	1
Gm13445	1,638073396	1
Ccnl1	1,637505781	1
Cd300lf	1,636938363	1
Sema4c	1,636711451	1
Smco3	1,636371142	1
Satb2	1,636144308	1
Tob2	1,636030903	1
Gm8317	1,635690736	1
Hook2	1,634330772	1
Iffo1	1,634217493	1
Bvht	1,633537982	1
Gm37598	1,633537982	1
Diaph3	1,632858754	1
Gm7287	1,632406092	1
Rpl7	1,632292946	1
Irf1	1,632179808	1
4933417C20Rik	1,632179808	1
Tiparp	1,632066678	1
1110020A21Rik	1,632066678	1

RP23-268C22.3	1,632066678	1
Ftl1	1,631274987	1
Gm6305	1,631274987	1
4930529C04Rik	1,629805721	1
Gm17971	1,629466848	1
Usp35	1,628902215	1
Gm16380	1,628902215	1
Tgif2	1,62845065	1
Ccng2	1,62788637	1
Rbm47	1,626871158	1
Ier3	1,626645642	1
Slc45a4	1,626420157	1
Ubtd2	1,626194702	1
Ints6l	1,62596928	1
Nfkbid	1,625405859	1
H60b	1,625067901	1
RP23-350F7.3	1,624842634	1
Gm26569	1,624054447	1
Ada	1,62394188	1
Gnb2	1,623266642	1
Gm12005	1,623266642	1
Lysmd3	1,623154129	1
Gm8770	1,622929128	1
Tcf20	1,622704158	1
Sumo1	1,622591685	1
Gins3	1,622479219	1
Abcg1	1,622254311	1
Gnpda1	1,621804589	1
Dgkz	1,620568496	1
Accsl	1,620343853	1
Snord92	1,620343853	1
Rc3h1	1,620231543	1
Gm7292	1,620119241	1
9330162G02Rik	1,620006947	1
H2-Ob	1,619782382	1
Rpl23a-ps2	1,619782382	1
Gm6807	1,61899665	1
Ccnyl1	1,61899665	1
Rnf5	1,618884433	1
Ctsc	1,618323468	1
Atp5g1	1,617986982	1
Gm2962	1,61720212	1
Senp3	1,617090028	1
Gm13215	1,616641738	1
Zc3h7a	1,616305601	1
Nr0b2	1,616193571	1
Smarca5-ps	1,616193571	1
Rasgef1b	1,615857528	1
Gas6	1,6150737	1
1700031P21Rik	1,6150737	1
Naip5	1,614737889	1
Tmem8	1,614737889	1

Tnip3	1,613730878	1
Cbfa2t3	1,613507183	1
Vps13b	1,613171698	1
Gm15785	1,613171698	1
Strn4	1,613059885	1
Slc7a5	1,61294808	1
H3f3a	1,612612712	1
Mtx3	1,612277414	1
Sf3a2	1,611607026	1
Kifc1	1,611160255	1
Nars2	1,610825259	1
Pdgfb	1,610825259	1
Pag1	1,610601967	1
Id3	1,610267086	1
Eif2ak3	1,610155475	1
Gm24009	1,609820687	1
Zfp503	1,609262862	1
Trmt112	1,608593728	1
Slc31a1	1,608482233	1
Tia1	1,608482233	1
Usp28	1,608036328	1
Gm5869	1,608036328	1
Tra2a	1,607813423	1
Alox8	1,607701981	1
Rpl30-ps11	1,607256294	1
Gm10335	1,607033496	1
Uba3	1,606810729	1
Tmem71	1,606810729	1
Gm26202	1,606587994	1
4930589O11Rik	1,606587994	1
Rpl19	1,606476637	1
Vma21-ps	1,606365289	1
Lrrc57	1,606031289	1
Erp27	1,606031289	1
1700120C14Rik	1,605474777	1
2010008C14Rik	1,605474777	1
Cxx1a	1,605252227	1
Armc5	1,605140963	1
Gm15007	1,603917571	1
Gm11977	1,603695236	1
Gm8172	1,603695236	1
Zswim3	1,603472932	1
Adamts1	1,603361792	1
Gm37052	1,602917307	1
Zfp11	1,602584024	1
Micall2	1,602472945	1
Nhlrc3	1,602361874	1
Dis3	1,601917667	1
Ifnar1	1,601806634	1
Lamb2	1,601584592	1
Oat	1,601029621	1
Fzd5	1,600696731	1

Sap18	1,599587597	1
Shpk	1,599587597	1
Pde4b	1,599033318	1
Gosr1	1,599033318	1
Efcab7	1,598700843	1
Rps4x	1,598479232	1
Pabpc4	1,598368437	1
Gm8430	1,598257651	1
Cfh	1,598036101	1
Vps37b	1,597593092	1
Ypel2	1,597371634	1
Rps11-ps4	1,597260917	1
Cpeb2	1,596707444	1
Gng5	1,596264804	1
Gas2l3	1,59604353	1
Cks1brt	1,595601074	1
Mfsd2a	1,595048177	1
Tmem109	1,594716531	1
Rmnd5a	1,594274443	1
Tbc1d22b	1,59416394	1
Gm15975	1,59416394	1
Gm11687	1,594053445	1
Gm28727	1,593169759	1
Ric1	1,592838503	1
Tex30	1,592838503	1
Zfp281	1,592838503	1
Tmem151a	1,59206584	1
Fam118a	1,591845148	1
Ncaph	1,590962687	1
Ypel1	1,590962687	1
Gm45153	1,590190934	1
Gm43588	1,589860297	1
Gm12276	1,589089079	1
Ttc30b	1,588978936	1
2310022B05Rik	1,588758672	1
Zfp703	1,588648551	1
Rai1	1,588648551	1
Rsad1	1,588318236	1
Mtmr1	1,587877921	1
Adnp2	1,587437729	1
Gm15779	1,58688766	1
Btaf1	1,586667686	1
B4galt3	1,586447743	1
Snapc1	1,585788095	1
5430434F05Rik	1,585018852	1
Arhgap23	1,584140175	1
Inpp4a	1,584030374	1
Mynn	1,583920582	1
Rpl31-ps8	1,583920582	1
Zfp87	1,583481487	1
Ddx51	1,583152245	1
Usp33	1,582823072	1

Dmxl2	1,582493967	1
Fam53c	1,582055268	1
Wdr62	1,58161669	1
Fam69b	1,58161669	1
AW549877	1,581178233	1
Gm7206	1,581178233	1
Gm20492	1,580849471	1
Ube2t	1,579754089	1
Gm12543	1,578659466	1
Tbc1d7	1,578331227	1
Nhlrc1	1,578112439	1
Gm4430	1,577893682	1
8030453O22Rik	1,577565602	1
Cdo1	1,577456257	1
Pim3	1,576581772	1
Zfp948	1,576363227	1
Pggt1b	1,576035465	1
Uprt	1,576035465	1
Zxdc	1,575816996	1
4931406P16Rik	1,575707772	1
D5Ertd605e	1,575707772	1
Sh2d6	1,575598556	1
Ctnnd1	1,575489348	1
Slc25a25	1,575161768	1
Exosc3	1,575161768	1
Wdr74	1,574943419	1
Bud13	1,574834256	1
Med25	1,574834256	1
Hmgb1	1,574834256	1
Ptrf	1,57439768	1
Tac4	1,574179437	1
Klf4	1,574070327	1
Chek1	1,574070327	1
Sfn	1,574070327	1
Smg7	1,57385213	1
Sh3bgrl2	1,573633963	1
Csrp2	1,573306769	1
Sec24a	1,572870616	1
Blnk	1,572652585	1
Zbtb43	1,571998673	1
Etv5	1,571671819	1
Zdhhc18	1,571562883	1
Jade3	1,571562883	1
Ap2b1	1,571345033	1
Ddx23	1,57123612	1
B130034C11Rik	1,570800541	1
Pvr	1,570582797	1
Fam122a	1,570147399	1
Gm37795	1,56982093	1
Glipr1	1,569059433	1
Hs1bp3	1,569059433	1
Kti12	1,568950678	1

Brca1	1,568515733	1
Gm11810	1,567754869	1
Gm43133	1,567211621	1
Gtpbp2	1,567102993	1
Btg1	1,565908593	1
Gm16585	1,565908593	1
Upf2	1,565583005	1
Adgb	1,565257485	1
Dhx9	1,565148993	1
Zfpm1	1,565040509	1
Cldn11	1,564064491	1
Wee1	1,563739286	1
Ddias	1,563522521	1
Pcgf6	1,563522521	1
3830403N18Rik	1,563522521	1
Gm24276	1,563089081	1
Glt1d1	1,562872405	1
Kctd20	1,562439145	1
Gm42571	1,562006005	1
Etfdh	1,561572985	1
2010111I01Rik	1,56135652	1
Cdc6	1,560707305	1
Gm12176	1,560599129	1
Zcchc10	1,56049096	1
Pom121	1,559301599	1
Nr1d1	1,55876128	1
RP24-511J14.2	1,558329159	1
Taf2	1,558329159	1
Pole2	1,558329159	1
Mtpap	1,557897159	1
Efna2	1,557573237	1
Rnf19a	1,557465278	1
Gm20594	1,557357326	1
9530085L11Rik	1,557033516	1
Secisbp2l	1,556925595	1
Rnf38	1,556817681	1
9330159M07Rik	1,556709774	1
Gpcpd1	1,556170353	1
Gm38115	1,556062491	1
Ints12	1,555954637	1
Ndufb4	1,555738951	1
Rps3a3	1,555092072	1
4933437G19Rik	1,555092072	1
Lrtm2	1,554445462	1
C230035I16Rik	1,553260708	1
Gm5580	1,553153048	1
Dnmbp	1,551969279	1
Dip2b	1,551216442	1
Gm43859	1,551001412	1
Ambp	1,550893909	1
Rpl31-ps22	1,550786413	1
Cables2	1,550678924	1

Lpcat2	1,549926712	1
Mkln1os	1,549604448	1
A130050O07Rik	1,549497041	1
Fam26f	1,549389642	1
Phactr1	1,549389642	1
Gm15464	1,54928225	1
Sertad3	1,549067489	1
RP23-403D16.3	1,548638056	1
Srebf2	1,548530716	1
Gm43343	1,548530716	1
Gm16523	1,548423384	1
Foxm1	1,547135977	1
Klhl41	1,547135977	1
Ift80	1,546921513	1
Polq	1,546599873	1
Il27	1,546599873	1
Zbtb11os1	1,546492675	1
Tpt1	1,54584964	1
Slc30a5	1,545635355	1
Mxi1	1,545528223	1
Gm26730	1,545528223	1
Elavl1	1,545206873	1
Mad2l1	1,545206873	1
C030034I22Rik	1,54488559	1
Gm7785	1,54488559	1
Pdss1	1,54477851	1
Pias2	1,544564373	1
Pidd1	1,544136189	1
Wdcp	1,543815128	1
Dcp1a	1,543494134	1
Gm45749	1,543494134	1
Fig4	1,543494134	1
Adck2	1,542531552	1
Gm10923	1,542531552	1
Eif4g2	1,542103931	1
Sepsecs	1,541890165	1
Rabif	1,541783293	1
Gm14126	1,541676428	1
Snx18	1,541569571	1
Ppwd1	1,541355879	1
C230096K16Rik	1,541249044	1
Rrm1	1,540928584	1
Gm42547	1,540287863	1
Gm6987	1,540181102	1
Fuca2	1,539540692	1
Fn1	1,539433983	1
Gm26514	1,538793883	1
Cyth1	1,537834233	1
Traf4	1,537727642	1
Gm26826	1,537621059	1
Trappc8	1,537407914	1
4930579G24Rik	1,537301353	1

Gm43088	1,537194799	1
Tmem29	1,537088252	1
Gatm	1,53666214	1
Gm11604	1,53666214	1
Cxcr4	1,53655563	1
Dennd2c	1,536342633	1
Prox2	1,536236146	1
Mapre2	1,535916728	1
RP23-136K21.4	1,535916728	1
Med18	1,535703819	1
Abhd18	1,535597376	1
Ythdc2	1,534958872	1
Ptp4a1	1,534426988	1
Tsc22d2	1,534214286	1
Zbtb41	1,534214286	1
Ncapd2	1,533895288	1
Socs6	1,53368266	1
Mbip	1,533257491	1
Rpl3-ps2	1,533257491	1
Kif20b	1,533044952	1
Larp4	1,53261996	1
AI846148	1,532301294	1
Cd40	1,531876509	1
Tmed7	1,531770331	1
Gm10051	1,531451841	1
Atf4	1,531133418	1
Creb5	1,531133418	1
Bub1b	1,530921172	1
Serpinb9	1,530815061	1
Gpsm1	1,530602859	1
Zfp874a	1,529860386	1
Chac2	1,529860386	1
Phf20l1	1,529754348	1
Trmt10c	1,529542294	1
Abcb7	1,529224268	1
Ube2h	1,528588413	1
Trappc6b	1,528588413	1
Tmem171	1,528482463	1
Lonrf1	1,527846918	1
Dhx57	1,527741019	1
Tjp3	1,527741019	1
Oxt	1,527105781	1
Wdr47	1,526682437	1
Tmem185b	1,526576619	1
Atp5l-ps1	1,526576619	1
Ndrg1	1,526047639	1
Atp7a	1,525518843	1
Recql4	1,525518843	1
Dnajb1	1,525413106	1
Atp6v0a1	1,52499023	1
Tpmt	1,52499023	1
Gm38190	1,524884529	1

Anp32a	1,524778836	1
4930404I05Rik	1,524778836	1
Fam83a	1,524567472	1
Ccdc25	1,5244618	1
Kdm6b	1,523933554	1
7330423F06Rik	1,523933554	1
Arv1	1,52340549	1
Tet2	1,522349912	1
Pate2	1,521716916	1
Top2a	1,521505976	1
Vdac3-ps1	1,521400517	1
Eno1b	1,521084183	1
Hist1h2aa	1,520978753	1
Phactr4	1,519819507	1
Sesn2	1,519819507	1
Fbxo21	1,519714165	1
Gm19620	1,51960883	1
Snord83b	1,519503502	1
Mtcl1	1,519187563	1
Sdc3	1,518976974	1
Rps27rt	1,518766413	1
Arhgef17	1,518661144	1
Ddx20	1,518450628	1
P2rx7	1,51834538	1
Gm15216	1,51834538	1
Osgepl1	1,51824014	1
Rusc2	1,517924464	1
Gm5898	1,517924464	1
Gm8995	1,517398482	1
Gm12834	1,517398482	1
Gm12038	1,517082981	1
D7Bwg0826e	1,517082981	1
Ogfr	1,516767545	1
Syne2	1,516662415	1
Snx33	1,516452175	1
Iscu	1,516241965	1
Klf6	1,516031784	1
Rab33b	1,515926704	1
2310022A10Rik	1,515821632	1
Asb11	1,515821632	1
Tdrd7	1,515821632	1
Atg14	1,514981315	1
Gm37255	1,514876308	1
Hspa9-ps1	1,514351383	1
Mettl16	1,514246419	1
Gm43275	1,514246419	1
Col27a1	1,514246419	1
Rpl38-ps1	1,514141463	1
Gon7	1,513826639	1
Tk1	1,513721713	1
Dnm1	1,512463158	1
Rgs1	1,511415161	1

RP24-550H10.3	1,511100904	1
Cxcr3	1,511100904	1
Mirlet7b	1,510158524	1
Lctl	1,509635234	1
Lyst	1,509216732	1
Slfn9	1,509112125	1
9630010A21Rik	1,508902932	1
Hmgb1-ps5	1,508798346	1
Cygb	1,508484633	1
St14	1,508170985	1
Slc22a5	1,50806645	1
Tbc1d16	1,507021501	1
Themis2	1,506499297	1
Junos	1,506394878	1
E130102H24Rik	1,506186062	1
Gm13680	1,506186062	1
Gm5544	1,506186062	1
Gm45248	1,505142415	1
Ctnnal1	1,504933773	1
Irx2	1,504516574	1
Csnk1e	1,504412293	1
Haus3	1,504203751	1
Gm14537	1,503682524	1
Gm10916	1,503578301	1
Zbtb49	1,503474084	1
Ptger4	1,503265673	1
Gm10616	1,503265673	1
Adgre1	1,50305729	1
Gm38345	1,50305729	1
Zfp574	1,502848937	1
Tmppe	1,502848937	1
Mknk1	1,502744771	1
Suv39h2	1,502744771	1
Gm3531	1,502744771	1
Mzb1	1,502536461	1
Ankrd9	1,502119927	1
Clk4	1,502015812	1
Acacb	1,502015812	1
Mef2c	1,501599422	1
mt-Rnr1	1,501183148	1
Prpf8	1,500975054	1
Dpys	1,500975054	1
Ccdc9	1,500871018	1
Fam174a	1,500350946	1
Ddit3	1,500142968	1
RP24-324J2.1	1,499935018	1
Magee1	1,499623148	1
Fnip1	1,499519205	1
Socs3	1,499519205	1
Pop4	1,499519205	1
E130208F15Rik	1,49941527	1
E130309D02Rik	1,498791811	1

Spns1	1,498687926	1
Taf5	1,498480178	1
Gm15800	1,498272459	1
Gm11956	1,498272459	1
Gm37584	1,498272459	1
Qrich1	1,497960934	1
Gm42941	1,497441871	1
Pcgf5	1,49733808	1
Gnal	1,497234296	1
Ctr9	1,496922987	1
Rps27l	1,496508009	1
Kif2c	1,496508009	1
Brms1l	1,495989449	1
Dr1	1,495885758	1
Rpl21-ps14	1,495678399	1
Amigo3	1,49557473	1
Pclaf	1,495367414	1
Pmf1	1,495056493	1
D530018E20Rik	1,494745637	1
Rhov	1,494642033	1
Klf3	1,494434846	1
Pnpt1	1,494434846	1
Gt(ROSA)26Sor	1,493917004	1
Phc1	1,493813457	1
Ldlrad3	1,49350286	1
Gm10842	1,49350286	1
Zfp39	1,493295831	1
Gm7236	1,493192327	1
Pacs1	1,49308883	1
Gm38380	1,492778383	1
Pcnt	1,492571455	1
Gm10388	1,492054258	1
Sgcb	1,491950841	1
Lsm12	1,49164063	1
Med14	1,49164063	1
Gm16253	1,49164063	1
Ctns	1,491227117	1
Tas1r1	1,490813719	1
Chd5	1,490813719	1
Stxbp3	1,490400435	1
Zfp868	1,489780723	1
Rara	1,48957421	1
Gm12166	1,487923135	1
Incenp	1,487820004	1
Zfp800	1,487820004	1
RP24-84O13.9	1,48771688	1
Gm38247	1,487510652	1
2210417A02Rik	1,487510652	1
Gm37082	1,487201365	1
Ticam1	1,487098284	1
Smad7	1,487098284	1
Gm26947	1,487098284	1

1700066M21Rik	1,487098284	1
Pex5	1,486892143	1
Rab20	1,486582984	1
Slc16a1	1,486376915	1
Gm4017	1,486376915	1
Anxa3	1,48627389	1
Gm45833	1,486170873	1
Gm37706	1,48596486	1
Gm6344	1,48596486	1
Brip1	1,485244041	1
Ppp1r16a	1,484832301	1
Rassf2	1,484729384	1
Gm5121	1,484626474	1
Necap1	1,484420675	1
Ovgp1	1,484420675	1
Grasp	1,483906303	1
Tcn2	1,483700604	1
Dnajb6	1,483289292	1
Gm12583	1,483289292	1
Gm16580	1,483186482	1
Bbs2	1,483186482	1
AA474408	1,483083679	1
Ubb	1,482980883	1
Dhx8	1,482980883	1
Tbc1d31	1,482980883	1
BC028528	1,48256977	1
Slc7a7	1,481747887	1
Arl15	1,481131773	1
RP23-43M12.2	1,481029113	1
Arsg	1,480823813	1
Nxt2	1,480413298	1
Junb	1,480208083	1
Tmem39b	1,480105487	1
Als2cl	1,479900315	1
Kdm6a	1,479797739	1
Gm12230	1,47959261	1
Chmp1b	1,479079911	1
Smg5	1,47866988	1
E130201H02Rik	1,47856739	1
Gm29666	1,477850159	1
Gm9761	1,477645299	1
Gpr132	1,477235666	1
9330102E08Rik	1,477133275	1
Gm45053	1,476621429	1
Pla2g6	1,476212079	1
Gm11249	1,476212079	1
Mknk2	1,476109759	1
Rpl32	1,476109759	1
Sap30	1,476109759	1
Slc31a2	1,475802843	1
Gm22581	1,475700551	1
Gm2000	1,47539372	1

Gm6378	1,47539372	1
Hmox1	1,475291457	1
Gm7327	1,475291457	1
Nab2	1,474882476	1
Ptprj	1,474780249	1
Gm43144	1,474678028	1
Zfp341	1,474575815	1
Gm37009	1,474575815	1
Rcsd1	1,473145572	1
Gm9354	1,473043464	1
Bbc3	1,472737186	1
Slc46a3	1,472124819	1
Gm37399	1,471818731	1
Tmem176a	1,471410713	1
Iffo2	1,471308726	1
RP23-246F14.1	1,471104773	1
Zfp760	1,470493085	1
Gm9009	1,469983539	1
6030400A10Rik	1,469677897	1
Gk	1,46947417	1
Rnpc3	1,469168633	1
Ripk1	1,468964977	1
5330438D12Rik	1,468150636	1
Rdh1	1,467641902	1
Unc5b	1,467438458	1
Gm13350	1,467336746	1
Psmc3ip	1,467235042	1
Pbk	1,46692997	1
Gm9442	1,466828294	1
Zfp385a	1,466726625	1
Chuk	1,466624963	1
Gm7407	1,466320018	1
Tex2	1,466320018	1
Gm14427	1,466320018	1
Gm5576	1,466116757	1
Gpkow	1,466116757	1
Hmcn2	1,466015137	1
Tmem33	1,465811918	1
Pou6f1	1,465811918	1
AA986860	1,465608728	1
Usp53	1,464999325	1
Sptbn4	1,464999325	1
Tgds	1,464593197	1
Gm15484	1,463984217	1
Tmem248	1,463882745	1
Gm31274	1,463882745	1
Dnah8	1,463882745	1
Ighm	1,46378128	1
Gm4987	1,463679821	1
Abl1	1,463071221	1
H2-T22	1,463071221	1
Flywch1	1,462767015	1

Syngap1	1,462462873	1
Pdxk	1,461854778	1
Hba-a1	1,461550826	1
Gm42890	1,461246936	1
Gm17827	1,461246936	1
Kcnj2	1,461145654	1
Fancm	1,460639347	1
Gltp	1,460436874	1
Ip6k2	1,460335647	1
Scarna9	1,460335647	1
Rgcc	1,459829621	1
9930104L06Rik	1,459424927	1
Gm13890	1,459323771	1
Arhgap17	1,458919216	1
Cd300c2	1,458615874	1
Spc25	1,458514774	1
Hmmr	1,458413681	1
Arsb	1,458211516	1
Cntrob	1,458110444	1
Tpra1	1,457908321	1
a	1,457706226	1
Gm42908	1,457706226	1
Cep120	1,457403136	1
Gm7496	1,457403136	1
Nup153	1,457201111	1
Gm42611	1,457201111	1
Gm45084	1,456696171	1
Ormdl3	1,456595204	1
Gm13712	1,456494244	1
Cnksr3	1,456292345	1
Tprn	1,456090474	1
Gm15501	1,456090474	1
Atf1	1,455384146	1
Rpl12-ps1	1,45467816	1
Gm10499	1,4543757	1
Tmub2	1,453770968	1
Vps13d	1,453569446	1
Foxo3	1,453468696	1
Csrnp1	1,453367953	1
Mir155hg	1,453367953	1
Errfi1	1,453166487	1
Ccl3	1,452562258	1
Zdhhc24	1,452260238	1
Phlda3	1,452159578	1
Gigyf1	1,452159578	1
Gm12912	1,451857641	1
RP23-151L20.5	1,451555767	1
Mccc2	1,450952208	1
Rnf24	1,450851639	1
Ifi47	1,450851639	1
Cnnm4	1,450650522	1
Mier1	1,450449433	1

Plppr2	1,449846334	1
Gtf2h4	1,449745841	1
Aldh6a1	1,449645356	1
Gm5900	1,449444407	1
Med23	1,448942155	1
Prc1	1,448741302	1
H2-Q4	1,448540478	1
Pla2g15	1,448038539	1
Chst10	1,447737459	1
RP23-278O17.1	1,447637113	1
Wdr82	1,447436442	1
Elac1	1,447035183	1
Casp7	1,447035183	1
Hsf2bp	1,446734312	1
Otub1	1,446634036	1
Rfwd2	1,446333248	1
Trp53bp2	1,446132757	1
Litaf	1,446032522	1
Kctd13	1,446032522	1
Gm14013	1,44573186	1
Rnf216	1,445431259	1
Galnt11	1,445431259	1
Traf7	1,445331073	1
2610203C20Rik	1,445331073	1
Akap1	1,445130722	1
Micu3	1,445030556	1
2410089E03Rik	1,444730102	1
Uhrf2	1,444229483	1
Piezo1	1,444229483	1
Nab1	1,444229483	1
Gm43149	1,44412938	1
Rab3gap2	1,444029285	1
Gm29539	1,444029285	1
Rpl39	1,443729038	1
Taf15	1,44362897	1
Nadk2	1,44362897	1
Helq	1,443528909	1
Gm43684	1,442928687	1
Cpne2	1,442828674	1
Nup88	1,442628669	1
Tmod1	1,442428693	1
Zfp54	1,441928871	1
Gm13223	1,441928871	1
Zfp773	1,441828928	1
Rmnd1	1,441629062	1
Sde2	1,441529139	1
Tbk1	1,44102963	1
Rps28	1,440929749	1
Stx7	1,440230775	1
Nf1	1,44003113	1
Zkscan5	1,439731715	1
Capza1	1,43953214	1

Tspan13	1,439332592	1
Mif	1,439133072	1
2310035C23Rik	1,439133072	1
Mcm6	1,439033323	1
Fbxl15	1,438833844	1
Ptafr	1,438534678	1
Gm12943	1,43843497	1
Arpin	1,438135887	1
Gm15013	1,438036206	1
Fbxw11	1,437936533	1
Tsacc	1,437438268	1
3110043O21Rik	1,437139393	1
C330011M18Rik	1,437139393	1
Ifih1	1,437039781	1
Map3k8	1,436740988	1
Accs	1,436740988	1
Gm43756	1,436740988	1
Gm3940	1,436442257	1
Sp1	1,43574546	1
2210408I21Rik	1,435546437	1
Nufip1	1,435347442	1
Nt5dc3	1,435148475	1
Gm14005	1,434253462	1
Eif3m	1,43415405	1
Zfp119a	1,43415405	1
Ddhd1	1,434054646	1
Rbl2	1,433756473	1
Tax1bp3	1,433557726	1
Pbdc1	1,433359007	1
Slx4ip	1,433259657	1
Gm42748	1,43296165	1
Gm6794	1,43296165	1
9130011E15Rik	1,432663705	1
Gm4374	1,432266542	1
Tgfb1	1,432167268	1
Isca1	1,432167268	1
Zfyve26	1,431968741	1
Gm45716	1,431968741	1
Uck1	1,431770242	1
Mob2	1,431671003	1
Kifc5b	1,431174909	1
Gm24336	1,430778158	1
Gm9207	1,430778158	1
Enc1	1,430084109	1
Ccnj	1,430084109	1
Ugp2	1,429885871	1
Nfrkb	1,42968766	1
Gm14706	1,429489477	1
Gm13392	1,429489477	1
Gm14585	1,429291322	1
Fpgs	1,429093193	1
Gm15720	1,42899414	1

Qk	1,428796053	1
Tle4	1,428597993	1
Sgol2a	1,428597993	1
Hirip3	1,428498974	1
Cry1	1,428201957	1
Gm7634	1,428201957	1
Fam161b	1,42780603	1
M6pr	1,427608108	1
9630013D21Rik	1,427608108	1
Dcun1d4	1,427113422	1
Rnf2	1,427014506	1
Gnai3	1,427014506	1
Gpr162	1,426915596	1
Kansl1	1,426717797	1
Pgf	1,426618908	1
Aph1b	1,426322282	1
Zbtb34	1,426322282	1
Gm38262	1,425828042	1
U2af2	1,425729214	1
Gm29650	1,425729214	1
Kdm5c	1,425630394	1
Gltscr1l	1,42523518	1
Ier2	1,424938841	1
Tmtc3	1,424642565	1
Mir99ahg	1,424543819	1
Gmeb2	1,424445081	1
Hikeshi	1,424445081	1
Stt3a	1,424346349	1
Kdm3b	1,424050196	1
Gm2214	1,424050196	1
4833421G17Rik	1,423852794	1
Arl5b	1,423754103	1
Bccip	1,423754103	1
Gm13889	1,423556743	1
Gm3362	1,423458073	1
Rn7s6	1,42335941	1
Hspa8	1,423162104	1
Rpl23a	1,422767574	1
Gm3555	1,422767574	1
Gmps	1,422373154	1
Anapc15	1,422077411	1
Gm14769	1,421978843	1
Rhoh	1,421880282	1
Eif3s6-ps1	1,421683182	1
Mtmr14	1,421683182	1
Npm3-ps1	1,421683182	1
Mbnl1	1,421584642	1
1110059G10Rik	1,421486108	1
Rad21	1,421387582	1
Rbm12b1	1,421092043	1
Rbm12b2	1,420993544	1
Cd14	1,420796566	1

Gm24920	1,420796566	1
Nudt8	1,42050115	1
1700012D14Rik	1,420304241	1
Rps15a-ps4	1,420107359	1
Lzic	1,419910504	1
Bcl2l2	1,419812086	1
Flcn	1,419713676	1
Rnft1	1,419713676	1
Chmp4b	1,419615272	1
Kctd10	1,419615272	1
Fbrsl1	1,419615272	1
Sag	1,419123356	1
Cd83	1,419024993	1
Zfp119b	1,419024993	1
Terf2ip	1,418828288	1
Nusap1	1,418533282	1
Pitrm1	1,418533282	1
Bicd2	1,418140036	1
Zfp867	1,417943453	1
Gm12481	1,417845172	1
Gm15163	1,417845172	1
Golga5	1,41755037	1
Ly9	1,417452117	1
Dennd3	1,41725563	1
Fam43a	1,417157397	1
Prim1	1,416273603	1
Sertad1	1,416175438	1
Phf1	1,41607728	1
Cir1	1,415979128	1
Gm12074	1,415782845	1
Zfp459	1,41558659	1
Snip1	1,415488472	1
Zmynd10	1,41519416	1
Sh3bp2	1,414997986	1
Txndc5	1,414997986	1
Fabp5l2	1,41489991	1
Ube2s	1,41480184	1
Ccdc117	1,41460572	1
Chaf1a	1,414409628	1
Gm37140	1,414311592	1
Actr6	1,414213562	1
Mtbp	1,41411554	1
Pacsin2	1,414017524	1
Trp53rkb	1,414017524	1
Magoh	1,413821513	1
Celf1	1,413723518	1
Akt1s1	1,41362553	1
Per1	1,41362553	1
Runx1	1,412743939	1
AW046200	1,412743939	1
C1ra	1,412548105	1
Dnajc9	1,412254404	1

Gm6525	1,412156518	1
Rdh10	1,411862899	1
Piga	1,411862899	1
Map3k1	1,411765039	1
RP23-114G13.1	1,411765039	1
Slfn4	1,411667186	1
Got1	1,411667186	1
Acat1	1,411471501	1
Rraga	1,411373669	1
Cul4b	1,411178025	1
Mbd6	1,411080213	1
Gm35106	1,410982407	1
Glipr2	1,410982407	1
Rbmx	1,410982407	1
Gm37390	1,410884609	1
Trpm7	1,410689032	1
Yjefn3	1,410591254	1
Sirt1	1,410493483	1
Eya3	1,41029796	1
Gm45184	1,410200209	1
Fam187b	1,410200209	1
Rps13-ps2	1,409906997	1
Gm44913	1,409809273	1
Dph2	1,409711556	1
Fam135a	1,409613846	1
Nfatc2ip	1,409320755	1
Rhoa	1,409223072	1
Slc5a6	1,409125395	1
Zfp617	1,409125395	1
Bmpr1a	1,408930063	1
Dynlrb1	1,408832406	1
Lmbrd1	1,408734757	1
Gm10175	1,408441849	1
Tgoln1	1,408149002	1
Ncapd3	1,407953805	1
Phtf1	1,407856217	1
Lsp1	1,407368375	1
Asap1	1,407270827	1
Pdcl3	1,407075752	1
Spin1	1,406978224	1
Brcc3	1,406880703	1
Uhrf1	1,406685682	1
Mef2d	1,406685682	1
Tmem107	1,406490687	1
Tollip	1,40629572	1
Rps12-ps19	1,40629572	1
Tox2	1,406198246	1
Nampt	1,406100779	1
Pkn2	1,405808419	1
Ccm2	1,405710979	1
Tmem176b	1,4054187	1
Snrpd3	1,4054187	1

Rnf215	1,405126482	1
Stard5	1,405029089	1
Gm13005	1,405029089	1
Gm18867	1,405029089	1
Harbi1	1,404834325	1
2810001G20Rik	1,404834325	1
Mgst3	1,40434753	1
Elf1	1,404250192	1
N4bp3	1,404250192	1
Ptcd1	1,40415286	1
Gm7285	1,40415286	1
Nr6a1	1,404055535	1
Prkd2	1,403958217	1
Clk1	1,403860905	1
Helb	1,403666302	1
Kpna3	1,403374448	1
E230016M11Rik	1,40288816	1
Gm13368	1,402304836	1
Itga7	1,402110449	1
D230025D16Rik	1,402013266	1
Nbr1	1,401916089	1
Ern1	1,401818919	1
Gm9385	1,40133317	1
Brwd3	1,40123604	1
Efhd2	1,40123604	1
Maml1	1,401138917	1
Lpxn	1,400556321	1
Crls1	1,400265114	1
Tm4sf19	1,400168058	1
Zyx	1,399779903	1
Apmap	1,399585866	1
Gm6159	1,399488857	1
Pias4	1,399488857	1
Insig2	1,399391855	1
Lrp1	1,39929486	1
Gm8822	1,399197872	1
Smagp	1,39910089	1
Rps29	1,398906947	1
Zxdb	1,398713031	1
Rbm38	1,398713031	1
RP24-75M13.2	1,398616083	1
Acot9	1,398519141	1
A930005H10Rik	1,398519141	1
Nedd1	1,398422207	1
Agtpbp1	1,398325279	1
Cdc27	1,398228358	1
Uspl1	1,398131443	1
Knl1	1,397550098	1
Gpr137b	1,39735637	1
Fus	1,397065828	1
1600029O15Rik	1,396872167	1
Rhob	1,396678532	1

Ssfa2	1,396291345	1
Tmem168	1,396001024	1
Lsr	1,395904264	1
Gm4968	1,395710764	1
Ugcg	1,395420564	1
Gm38376	1,395323845	1
Cacnb1	1,395130425	1
Gm17511	1,394937032	1
Slc41a1	1,394743666	1
Fyttd1	1,394453668	1
Gm12380	1,394163729	1
Nbas	1,393873851	1
Ggh	1,393777239	1
Slc37a1	1,393487441	1
Sik2	1,393390855	1
Ltv1	1,393101138	1
Nuak2	1,392908026	1
Ctsh	1,392714942	1
Rps7	1,392618409	1
RP24-454N4.2	1,392232347	1
Upf1	1,392135848	1
Dynll2	1,391846392	1
Socs5	1,391556996	1
Fgfbp3	1,391460544	1
Scyl2	1,391364098	1
Btbd7	1,39126766	1
Gm15892	1,391074802	1
Slc9a3r1	1,390978384	1
Zgrf1	1,390785566	1
Pcna-ps2	1,390592776	1
Ppih	1,390110917	1
E230032D23Rik	1,389821881	1
Gm26983	1,389243991	1
Gm16061	1,389243991	1
Gm11531	1,389051414	1
Fam53b	1,388858864	1
Smg8	1,388858864	1
Ctc1	1,388473844	1
Dab2	1,388185149	1
Rnf145	1,387992719	1
Snapc3	1,387704124	1
P4ha1	1,387415589	1
Gm14633	1,387415589	1
Gm6649	1,387127114	1
Gm13864	1,386454239	1
Gna12	1,386262049	1
Dgke	1,386069886	1
Pml	1,385877749	1
Pigo	1,38578169	1
Iqsec2	1,38578169	1
Safb	1,38578169	1
A430027C01Rik	1,38578169	1

Hnrnpa1	1,385397523	1
Gpr157	1,385301498	1
Nr4a2	1,385301498	1
Cdc42	1,385301498	1
Tmx2	1,38520548	1
Bcor	1,38520548	1
Atg4a-ps	1,385013463	1
Fam13b	1,384917464	1
Tm2d1	1,384533537	1
Hist1h1c	1,384533537	1
Fbxo42	1,384341613	1
Zfp65	1,384245661	1
Gm26549	1,383957845	1
Pcid2	1,383766001	1
Mrs2	1,383478285	1
Ppp1r2	1,383382393	1
Fam71f2	1,383286508	1
Adap1	1,383094757	1
Ssx2ip	1,38280718	1
Zbtb11	1,382711335	1
1190002N15Rik	1,382615496	1
Fbxo18	1,382519664	1
Ifi204	1,382519664	1
Celsr1	1,382519664	1
Hbb-bh3	1,381849024	1
Gm43309	1,381274448	1
Foxk1	1,381082976	1
Tapbp	1,38098725	1
Slc25a3	1,380891531	1
Tor1aip1	1,380413033	1
Erf	1,380030354	1
Gm5873	1,379839055	1
Fam78a	1,379456535	1
Gm26982	1,379456535	1
RP23-162P10.8	1,379456535	1
Acadsb	1,379360922	1
Mob1b	1,379360922	1
Gna11	1,379169715	1
AW554918	1,378978535	1
Nlgn2	1,378691814	1
Gm27039	1,378691814	1
Srsf1	1,378596254	1
Morc3	1,378023031	1
Sp3	1,377927517	1
Plekho2	1,37783201	1
Riiad1	1,377736509	1
Rev1	1,377641015	1
Ythdf3	1,377545527	1
Arnt	1,377450046	1
Dnmt3l	1,377354572	1
Mir3091	1,376972741	1
Slc29a2	1,376781866	1

Tssk6	1,376686438	1
Taf7	1,376114009	1
Gm15950	1,375923252	1
Plcg2	1,375923252	1
RP23-442M18.5	1,375923252	1
Gm9711	1,375827884	1
Gm28417	1,375446476	1
Arid3b	1,37516049	1
Bag3	1,37516049	1
Hivep3	1,375065174	1
Msantd2	1,374969865	1
B230219D22Rik	1,374779267	1
Fbl	1,37439815	1
Cln6	1,374112382	1
Xirp1	1,374017139	1
Bcl6	1,373921903	1
Dpf2	1,373921903	1
C77080	1,37373145	1
Mrps11	1,372874739	1
Mfsd8	1,372779582	1
Cracr2a	1,372589287	1
Acin1	1,372399019	1
Pum1	1,372113667	1
Alkbh4	1,371923464	1
Ahctf1	1,371923464	1
Clic1	1,371828373	1
Gm17039	1,371828373	1
Gatad2b	1,371638211	1
Cd164	1,371448074	1
Gm8129	1,371257964	1
C130071C03Rik	1,371162919	1
Clec12a	1,370877824	1
Lsm7	1,370782805	1
Dot1l	1,370782805	1
Smc2	1,370687793	1
Snx2	1,370212831	1
Clec4n	1,370117858	1
Rhoq	1,36983298	1
Dedd2	1,369358314	1
Rsl1d1	1,369358314	1
Zfp296	1,369263401	1
Ptbp1	1,369263401	1
Sf3b4	1,369073594	1
Pstpip2	1,368788933	1
Pdcl	1,368788933	1
Gm42715	1,368694059	1
Rwdd1	1,368124953	1
Adm	1,368030125	1
Traf3	1,368030125	1
Zfp143	1,368030125	1
Mybpc3	1,367366514	1
Carm1	1,367366514	1

Gemin8	1,367366514	1
Klf13	1,367271739	1
Impact	1,367082208	1
Krr1	1,366892703	1
Smarca5	1,366608495	1
Sdcbp	1,366608495	1
A730071L15Rik	1,366513772	1
Tma7	1,366419056	1
Rnpep	1,366324346	1
Gm15159	1,366040257	1
Abcd2	1,365945573	1
Gm12582	1,365945573	1
Nt5c2	1,365756226	1
Smg6	1,365756226	1
Vezt	1,365661562	1
Snord104	1,364715286	1
2900076A07Rik	1,364431531	1
Ppp5c	1,364242394	1
Zfand2a	1,364242394	1
Depdc7	1,364053283	1
Gm10033	1,3633916	1
Gm6162	1,3633916	1
9130604C24Rik	1,3633916	1
Gpbp1l1	1,363202607	1
Map3k3	1,363108121	1
Zfand5	1,363108121	1
Aoc2	1,362919167	1
Kbtbd4	1,3628247	1
Gm10784	1,362730239	1
Camta1	1,362635785	1
Foxj3	1,362635785	1
C330006A16Rik	1,362541338	1
Gm45033	1,362541338	1
Cul1	1,362541338	1
Ankrd55	1,362258035	1
Fbxl3	1,362163613	1
Mb21d1	1,362163613	1
Alg6	1,361880389	1
Ethe1	1,361880389	1
Gm15834	1,361691605	1
Plcl2	1,361408479	1
Wdr37	1,361408479	1
Gm2223	1,361314116	1
Dcun1d3	1,361125411	1
Rab11fip4os1	1,360936732	1
Metap1d	1,360088001	1
Aste1	1,35999373	1
Rchy1	1,359710956	1
Gm23100	1,359334017	1
Lats2	1,359145586	1
Yars2	1,358768803	1
Srsf6	1,358674624	1

Zfp729b	1,358486285	1
Gm13998	1,358486285	1
Rps2	1,358392125	1
Zfp995	1,358297971	1
Pcif1	1,358203825	1
Rpl35	1,357921423	1
Hn1l	1,357827303	1
Naa60	1,357733189	1
Gm10941	1,357074572	1
Ercc4	1,35698051	1
Nfe2l2	1,356886455	1
Gabpa	1,356698363	1
Edem3	1,356698363	1
Zfand1	1,356510298	1
Urb1	1,356416275	1
Ptgs2	1,356416275	1
Racgap1	1,356416275	1
Pigt	1,356134246	1
Gm8618	1,355758298	1
Cd74	1,355570363	1
Gltscr1	1,355288509	1
Zc3h11a	1,355288509	1
Svbp	1,355194571	1
Pald1	1,355194571	1
Map3k2	1,355194571	1
Lpar2	1,355100639	1
Tsr2	1,354818883	1
Gm17745	1,354724977	1
Fam105a	1,35416168	1
Ints8	1,353973967	1
Sap130	1,35388012	1
Nol4l	1,353504796	1
Pikfyve	1,353504796	1
Oaz1	1,353317174	1
Gm16373	1,353317174	1
Mdm1	1,353129577	1
Samd10	1,353129577	1
Rpl26	1,353035789	1
Smc4	1,352942007	1
Gm9409	1,352848231	1
Gm37963	1,352191984	1
Etv3	1,352191984	1
Frat2	1,352004544	1
Col4a5	1,351817129	1
Zmym2	1,35162974	1
Lrrc2	1,351348706	1
Trmt1l	1,351348706	1
Hcfc1	1,351348706	1
Eme1	1,351161382	1
Rora	1,35106773	1
Ntrk3	1,350974085	1
Rap2c	1,350599567	1

4930461G14Rik	1,350505954	1
RP23-413G8.2	1,350131566	1
Hsd17b4	1,349850843	1
Cbx8	1,349570179	1
Ly6g6d	1,349570179	1
Pqlc2	1,349476637	1
Slc38a2	1,349476637	1
Tma7-ps	1,349383101	1
Gm42783	1,349289573	1
Cep44	1,34919605	1
L2hgdh	1,349102534	1
Hadhb	1,348915522	1
Rpp30	1,348822026	1
Gm1848	1,348822026	1
Cdkal1	1,348448104	1
Pwp1	1,34835464	1
Ubxn11	1,348261183	1
Gm21975	1,347980849	1
Gm12090	1,347887417	1
Tnfrsf26	1,347887417	1
Gm7336	1,346673395	1
Hspa5	1,346673395	1
Sf3b1	1,346486719	1
Prpf38a	1,346300069	1
Ifitm3	1,346206754	1
Lin9	1,345926847	1
Cnr2	1,345646999	1
Hoxb5	1,345460466	1
Nelfa	1,345367209	1
Dennd6b	1,345367209	1
Higd2a	1,345367209	1
Nectin1	1,345273958	1
Slc36a1	1,344994245	1
Gm5835	1,344901021	1
Neu3	1,344714591	1
Gm11772	1,344621385	1
Zic5	1,344155456	1
Srcap	1,344062289	1
Znrd1	1,344062289	1
Fzd2	1,344062289	1
Bhlhe41	1,343875976	1
Med15	1,343596554	1
Pkmyt1	1,343503426	1
Rpl18	1,343503426	1
Dimt1	1,343037884	1
Recql	1,342758636	1
Gm12716	1,342665566	1
3110031N09Rik	1,342665566	1
Lamc1	1,342386396	1
Spink10	1,341735224	1
Gm2225	1,341642225	1
Rpl26-ps2	1,341363267	1

Gm20604	1,341363267	1
Rbm14	1,341363267	1
Gm11362	1,341177328	1
Mrpl42	1,340712592	1
Tcp11l1	1,340619664	1
Cdkn1a	1,340619664	1
Selenbp1	1,339969349	1
Mta3	1,339597882	1
Zmym5	1,339226518	1
Brd2	1,339133693	1
5031425E22Rik	1,339133693	1
Gm21816	1,339040875	1
Eif4a3	1,338948063	1
Gm37670	1,338855257	1
Tor1aip2	1,338855257	1
Gm4617	1,338762458	1
Arpc3	1,338762458	1
Tnrc18	1,338298559	1
B3gnt2	1,338205798	1
Gm43359	1,338020296	1
Gm15727	1,337927555	1
Gm4258	1,33783482	1
Kctd5	1,337742091	1
Srsf4	1,337742091	1
Ubqln2	1,337556654	1
Ice2	1,337278546	1
Gm5251	1,337093172	1
Rps11-ps3	1,337000495	1
Gm29593	1,337000495	1
Pcnx3	1,336351936	1
2810403A07Rik	1,33625931	1
Gem	1,336166691	1
9430034N14Rik	1,335796278	1
Armc2	1,335333407	1
Uimc1	1,335240852	1
Prpsap2	1,335148303	1
Arl8a	1,334963225	1
Proser1	1,334963225	1
Fkbp7	1,334870696	1
Erbb3	1,334778173	1
Ndufaf1	1,334593146	1
Fam117b	1,334593146	1
Ipo11	1,334500642	1
Mcm7	1,334408145	1
Ap2s1	1,334315654	1
Kmt5c	1,33422317	1
Gm11722	1,33403822	1
Fam19a2	1,333945755	1
Snora73b	1,333853296	1
Clp1	1,333853296	1
Mgat4a	1,333853296	1
Yrdc	1,333668397	1

mt-Co1	1,333668397	1
Nup210	1,333298677	1
Ifi213	1,333206263	1
Acadm	1,333021454	1
Gm20620	1,332929059	1
Mybl1	1,332836671	1
Hdac5	1,332744289	1
Gm45902	1,332651913	1
Snu13	1,332651913	1
Oxr1	1,332467181	1
Armcx5	1,332374825	1
Rev3l	1,332282475	1
Rpusd2	1,332005464	1
Cdc45	1,331913139	1
Gm42511	1,331820821	1
Hspbp1	1,33172851	1
Slbp	1,331636204	1
Gm17455	1,331636204	1
Arhgef40	1,331543906	1
Skor1	1,330713504	1
Taf5l	1,330529041	1
Gm13328	1,330252394	1
Dda1	1,330252394	1
Zfp358	1,330160191	1
Pear1	1,330067995	1
G6pd2	1,329975805	1
Terf1	1,329883621	1
6720427I07Rik	1,329791444	1
Zfp174	1,329791444	1
Tsc22d3	1,329699273	1
Cactin	1,329699273	1
Gm29438	1,329607108	1
Sep 02	1,329422798	1
Zc3h18	1,329330653	1
Prpf3	1,328777915	1
Cenpc1	1,328685814	1
H2-Q6	1,328501632	1
Pcna	1,328225406	1
Klf10	1,328225406	1
Gm6472	1,327857195	1
Ctdspl2	1,327673128	1
Rpl37	1,327489086	1
Dyrk3	1,32730507	1
Gm4859	1,327213071	1
Ppp3cc	1,327121079	1
Gm10275	1,327029093	1
Med26	1,326937114	1
6330562C20Rik	1,326845141	1
Rps19-ps7	1,326385371	1
Arhgap11a	1,326385371	1
Fbxl20	1,326385371	1
Irak1bp1	1,326293436	1

Gm23054	1,326293436	1
Blcap	1,326201507	1
Ccdc22	1,326109585	1
Eloa	1,32601767	1
Tmem240	1,32592576	1
Gm4540	1,325741961	1
Adat1	1,325558187	1
Ccdc71l	1,325466309	1
Rab5b	1,325466309	1
Myef2	1,325282573	1
Pfn1	1,325007017	1
Gm14776	1,324639698	1
Thap1	1,324639698	1
Evi5	1,324456077	1
Vgll4	1,324364276	1
Ndc80	1,324272481	1
Grpel2	1,32408891	1
Mnt	1,32408891	1
Cebpb	1,323905365	1
Rpl6	1,323905365	1
B130006D01Rik	1,323813602	1
Dusp4	1,323538352	1
8030462N17Rik	1,323446614	1
Ddx3x	1,323446614	1
Gm11517	1,32317144	1
Nfix	1,32317144	1
Klhl28	1,323079728	1
Gm5276	1,32234626	1
Xiap	1,321796426	1
Siglec1	1,321613199	1
Slc16a10	1,321613199	1
Casp6	1,321429997	1
A930029G22Rik	1,320514369	1
4930412F12Rik	1,320422841	1
Col7a1	1,320056793	1
C5ar1	1,320056793	1
Cbx5	1,319873807	1
Naa15	1,319690846	1
Gm14513	1,319599375	1
Car11	1,319599375	1
Foxn2	1,319507911	1
Mcrs1	1,319416453	1
Stat2	1,318959257	1
Setdb1	1,318776423	1
H2afx	1,318593614	1
5430421F17Rik	1,31850222	1
Hltf	1,31850222	1
Rnf123	1,318136704	1
Mir7078	1,318045341	1
Usp2	1,31777129	1
Kctd3	1,31758862	1
Gm19353	1,317497295	1

Klhl9	1,317497295	1
Taf11	1,317497295	1
Gm45286	1,317405976	1
Cdc42bpb	1,317314664	1
RP24-226A8.2	1,317040765	1
Fcho2	1,317040765	1
Napsa	1,316675654	1
Rpl18a-ps1	1,316310644	1
RP23-288C18.3	1,315854525	1
Cib2	1,31576332	1
Phlda1	1,31576332	1
Pja2	1,31576332	1
Klhl7	1,315580929	1
Usp37	1,315580929	1
Rictor	1,315580929	1
Rhog	1,315398564	1
Ino80d	1,315398564	1
Pan3	1,315398564	1
Plin2	1,31530739	1
BC025920	1,315216223	1
Trp53inp1	1,31494276	1
Tstd1	1,314851618	1
Cmtm4	1,314851618	1
Slc25a5	1,314578231	1
Haus6	1,314396005	1
Ddr1	1,314396005	1
Elk4	1,314304901	1
E030030I06Rik	1,314122712	1
Gm18889	1,314122712	1
E430021H15Rik	1,313940549	1
Ppp1r37	1,313849476	1
Gm44419	1,31375841	1
Aldh9a1	1,31375841	1
Triobp	1,313121125	1
Gm37106	1,313121125	1
Stard4	1,31303011	1
Sub1	1,31303011	1
Gm18916	1,312939101	1
Clec4e	1,312848098	1
Yipf4	1,312757101	1
Gm8093	1,312211255	1
Sec14l1	1,312120302	1
Tpst2	1,311847483	1
Tec	1,311756556	1
Uba2	1,31157472	1
Dhcr7	1,311483812	1
Lemd3	1,311302014	1
Wbp4	1,311029365	1
Gm14794	1,310938494	1
Atp5g2	1,310211757	1
Eda2r	1,310211757	1
Gm16053	1,309939335	1

Rpl13	1,309939335	1
Nsmce3	1,309757751	1
Cmpk1	1,309576193	1
Ppp1r12c	1,309485423	1
Adcy6	1,30939466	1
Gm37486	1,30939466	1
Tbc1d8	1,308850211	1
Gtpbp10	1,308759491	1
Xpo1	1,308759491	1
Plekha7	1,308124631	1
Vapa	1,308033962	1
Pcnp	1,307943299	1
9130024F11Rik	1,307852643	1
Smarce1	1,307852643	1
Klc2	1,307580711	1
Ccng1	1,307580711	1
Mtm1	1,307490079	1
Nek3	1,307490079	1
Birc2	1,307308835	1
Pelo	1,307127616	1
Gtf2h3	1,307037016	1
Polr2h	1,306855835	1
Ldlr	1,306765254	1
Scrib	1,30658411	1
Cdk17	1,30658411	1
Gm32856	1,306312442	1
Gm6377	1,306131361	1
Gyg	1,306131361	1
Tmem185a	1,305950306	1
Rpf1	1,305950306	1
Cdk5r1	1,305769275	1
Gm11694	1,305678769	1
Polr1b	1,305588269	1
Bahd1	1,305407289	1
Jak2	1,305226334	1
Ppp2r5e	1,305226334	1
Cep76	1,305045403	1
2700038G22Rik	1,305045403	1
Gmeb1	1,304954948	1
Rapgef2	1,304774055	1
Mecp2	1,304593188	1
Atg16l1	1,304593188	1
Tipin	1,304321933	1
Rpl18a	1,304231528	1
Prdm15	1,304141128	1
Lig1	1,304141128	1
Kif14	1,304050735	1
Gm3511	1,304050735	1
Gm10126	1,303869968	1
Zcchc11	1,303869968	1
Asb13	1,303779594	1
Map4k2	1,303689226	1

Rab3il1	1,303689226	1
Kpna1	1,303689226	1
Raly	1,303598864	1
Hat1	1,303598864	1
Phf12	1,303327816	1
Cep170b	1,303327816	1
Gm7984	1,30323748	1
Jag1	1,30323748	1
Suv39h1	1,302966507	1
Sccpdh	1,302966507	1
St3gal6	1,302966507	1
Jup	1,302695591	1
AV356131	1,302605298	1
Gtf3c3	1,302334457	1
Atp6v0d1	1,302334457	1
Cebpg	1,30224419	1
Jmjd1c	1,302153928	1
Gm14328	1,301432061	1
Kdm1b	1,301432061	1
Gga1	1,301341855	1
Dstn	1,301161464	1
Efnb1	1,300710594	1
Rps15a-ps7	1,300620438	1
Oaz2	1,300620438	1
Ube2f	1,30025988	1
Nfkbib	1,300169756	1
Copb1	1,299989526	1
Atp6ap1	1,299899421	1
Luzp1	1,299629143	1
Hmgcr	1,299629143	1
Epg5	1,299539062	1
Mcm5	1,299539062	1
Gm10658	1,299358921	1
Ptprc	1,299358921	1
Gm6134	1,299178804	1
RP23-149L23.1	1,299178804	1
Fam134c	1,298998712	1
Epn2	1,298908675	1
Cenph	1,298728621	1
Rab26os	1,298458586	1
Ndst1	1,298368586	1
Slc2a3	1,298278594	1
Dnajc4	1,298188607	1
Ifit2	1,298098626	1
Ndufaf4	1,298008652	1
Gm7658	1,297828722	1
Gm10689	1,297738767	1
Atp1a3	1,297648818	1
Rps3a1	1,297558875	1
Eif4ebp2	1,297558875	1
Aldh2	1,297468938	1
Npat	1,297379007	1

Utp15	1,297199165	1
Fam212b	1,297109253	1
Eps8	1,296929448	1
Creld2	1,296749668	1
Ranbp10	1,296569912	1
Arrdc2	1,296480044	1
Gm38355	1,296480044	1
Gm45221	1,296390182	1
Gm37893	1,296210477	1
Gm16223	1,296030796	1
Luc7l3	1,295940965	1
Hist1h1b	1,295851141	1
Gorasp1	1,295851141	1
BC030499	1,295581704	1
Hpgds	1,295491904	1
Stam	1,295402111	1
Rrp12	1,295402111	1
Hdgfrp2	1,295312323	1
Gm13815	1,29486348	1
Zhx1	1,294594249	1
Prkar2a	1,294594249	1
Ermp1	1,294504518	1
Tanc2	1,294325074	1
Cnst	1,294145654	1
Midn	1,294145654	1
Itga6	1,294055954	1
Rab10os	1,29396626	1
Spg21	1,293697215	1
Tpx2	1,293428227	1
Tial1	1,293428227	1
Ctla2b	1,293338576	1
Arl2bp	1,293338576	1
H2-DMb1	1,293248932	1
Plxnd1	1,293248932	1
E2f5	1,292980036	1
Gm15877	1,292621596	1
Trpt1	1,292621596	1
Cenpk	1,292532001	1
Cwf19l1	1,292532001	1
Suz12	1,292532001	1
Pus10	1,292442413	1
Slc26a6	1,292352831	1
Tpp2	1,291994564	1
Gspt1	1,291994564	1
Agbl5	1,291636397	1
Mfsd7b	1,29154687	1
Alpk2	1,291278329	1
Sqstm1	1,291278329	1
Trim33	1,291009843	1
Ppp2r2d	1,291009843	1
Msrb1	1,291009843	1
A330023F24Rik	1,29092036	1

Serpinf1	1,290383593	1
Slc35f5	1,29020472	1
Plekhm2	1,290025872	1
Gm44116	1,289668251	1
Ncapg	1,289578861	1
Gspt2	1,289578861	1
Mmd	1,289489477	1
Mypop	1,2894001	1
Nr3c1	1,289132004	1
Fut10	1,288863965	1
4931440P22Rik	1,288863965	1
Zmat3	1,288863965	1
Kiz	1,288506665	1
Paxip1	1,288238756	1
Slc6a6	1,288238756	1
Rsrc2	1,288238756	1
Acss2	1,288149465	1
Pdcd7	1,28806018	1
Gm9722	1,28788163	1
Lgals2	1,28788163	1
Rab27a	1,287703104	1
Numb	1,28761385	1
Tubg1	1,287524602	1
Slc35g1	1,287524602	1
Pank3	1,287435361	1
Slc9a4	1,287346126	1
Ctdnep1	1,287346126	1
Kctd18	1,287256897	1
Pts	1,287167674	1
Prim2	1,287078458	1
Pdia6	1,286900043	1
C3	1,286721653	1
Ajuba	1,286632468	1
Sbno2	1,286454115	1
Kctd12	1,286364948	1
Gm43360	1,286186632	1
Hus1	1,286008341	1
1700088E04Rik	1,285919205	1
Zfr	1,285830075	1
Dtwd2	1,285651833	1
Wasl	1,285651833	1
Siah2	1,285651833	1
Psma5	1,285295424	1
RP24-389J11.1	1,285206337	1
Rassf3	1,285117257	1
Rpl31	1,285117257	1
H1f0	1,285028182	1
Dvl2	1,284939114	1
Golga3	1,284939114	1
Sh2b1	1,284760996	1
Nbeal1	1,284760996	1
Ift172	1,284671946	1

Lpin2	1,284493865	1
Fam13c	1,284404834	1
Mettl2	1,284315809	1
Adpgk	1,28422679	1
Smad4	1,28422679	1
Trib1	1,284137777	1
2900093K20Rik	1,284137777	1
Dgat1	1,284048771	1
Ppp2r1a	1,284048771	1
Caap1	1,28395977	1
Rngtt	1,28395977	1
Cycs	1,283870776	1
Coq6	1,283781788	1
Tspan31	1,283603831	1
Praf2	1,283514861	1
Pfkp	1,283514861	1
Trnt1	1,283247989	1
Cnot11	1,283159044	1
Taf4	1,283070106	1
Cnep1r1	1,283070106	1
Mfsd13b	1,283070106	1
Peli1	1,282981173	1
Naga	1,282625505	1
Rrm2	1,282536603	1
2610301B20Rik	1,282447707	1
Rps20	1,282447707	1
Nfkbil1	1,282358818	1
Myl12a	1,282358818	1
Fam214a	1,282269935	1
Hist1h2al	1,282092186	1
Hccs	1,282003322	1
Tgfbr1	1,281914463	1
Laptm4b	1,281736764	1
Gm7266	1,28155909	1
Kdm2b	1,28155909	1
Nop10	1,28155909	1
Pak4	1,281381441	1
Cdkn2c	1,281381441	1
Tbc1d23	1,281292625	1
Gm16973	1,281203816	1
Gm8806	1,281203816	1
Rragc	1,281203816	1
Tnfrsf17	1,281115013	1
Tmem184b	1,280937425	1
Rad51c	1,280759861	1
C87436	1,280759861	1
Treml4	1,280671089	1
Cdk2	1,280582323	1
Tab3	1,280493562	1
RP23-307F3.6	1,280404809	1
C1galt1c1	1,280404809	1
Supv3l1	1,280316061	1

Tctn1	1,280227319	1
Ss18l2	1,280227319	1
Fam120aos	1,280138584	1
Lgmn	1,279694998	1
Gm6123	1,279251567	1
Fam214b	1,279074237	1
Prpf39	1,279074237	1
Dpp9	1,278896932	1
Pgm3	1,278808289	1
Gm8250	1,278542395	1
Slc4a11	1,278453777	1
Gabpb1	1,278365164	1
Smarcal1	1,278276558	1
Slc2a8	1,278187957	1
Tmem131	1,278099363	1
Ppfia1	1,278099363	1
Acrbp	1,277922193	1
Dus4l	1,277833618	1
Rps23	1,277745048	1
Lnpk	1,277390831	1
Pgm1	1,277213759	1
Zfp639	1,276948198	1
Tmem106b	1,276505718	1
Arrdc3	1,27641724	1
Pdia3	1,27641724	1
N6amt1	1,276240304	1
Zw10	1,276240304	1
B230322F03Rik	1,275974944	1
Rnf146	1,275974944	1
Zbtb24	1,275886504	1
Gopc	1,275886504	1
Emc2	1,275798069	1
Lamtor3	1,275179198	1
Ccdc50	1,275179198	1
Scand1	1,275002433	1
Csnk1g2	1,27491406	1
Uchl4	1,274737331	1
Gm2058	1,274737331	1
Nln	1,274648976	1
Gatc	1,274118975	1
Copg2	1,274030663	1
Selenow	1,274030663	1
Tmod3	1,273765763	1
Orc6	1,273677475	1
Ckap5	1,273677475	1
Lancl2	1,273589194	1
Atpaf2	1,273412649	1
Gm43868	1,273059633	1
Dennd5a	1,273059633	1
Myh11	1,272706715	1
Nelfb	1,272706715	1
Gm37274	1,272442091	1

Wbp11	1,272265705	1
Gm38022	1,272001172	1
Usp14	1,272001172	1
Rdh11	1,271824848	1
Gm1976	1,271648547	1
Pim2	1,271560406	1
Gm5764	1,271472272	1
RP23-269H21.1	1,271472272	1
Naa30	1,271472272	1
Qsox1	1,271119794	1
Mex3c	1,270855499	1
Gm19726	1,270767413	1
Gm6142	1,270767413	1
Hjurp	1,270767413	1
Pip5k1a	1,270767413	1
Zfp784	1,270679333	1
Mysm1	1,270679333	1
Prkcd	1,270679333	1
Traf6	1,270503192	1
Ccsap	1,270503192	1
Mrpl22	1,270239026	1
Glul	1,270239026	1
Zfp652	1,270150983	1
Zfyve1	1,269622851	1
Arl4c	1,269622851	1
Cdc14a	1,269534851	1
Ptk2b	1,269446857	1
AI480526	1,26909494	1
Gm11652	1,26909494	1
Chfr	1,26909494	1
Xlr	1,269006976	1
Rps24	1,268919018	1
Osbp	1,268831066	1
Cln5	1,268655181	1
Slc25a36	1,268391399	1
Npm3	1,268303484	1
Ralgapa1	1,268127672	1
Slc3a2	1,268127672	1
Rars	1,268039775	1
Cdkn2aip	1,267951885	1
Hira	1,267776121	1
Gpr137b-ps	1,267776121	1
Foxn3	1,267688249	1
Smad5	1,267600382	1
Gm5139	1,267512522	1
March7	1,267512522	1
Yod1	1,267248978	1
Dhx40	1,267161142	1
Ralgds	1,267161142	1
Amhr2	1,267073312	1
Fam160a2	1,267073312	1
Gm7965	1,266985488	1

Snrpf	1,266634254	1
Gm43138	1,266458673	1
Grk4	1,266370892	1
Crnkl1	1,266195348	1
Rnf138	1,266195348	1
Vezf1	1,265932077	1
Cpeb4	1,265844333	1
D830050J10Rik	1,265756594	1
Wdr90	1,265581135	1
Spc24	1,265581135	1
Gm19503	1,265581135	1
Mapk7	1,265493415	1
Cad	1,265317992	1
Map1lc3a	1,265317992	1
Trappc10	1,26523029	1
Pgpep1	1,264704205	1
Rap1a	1,264616545	1
Ndel1	1,264528892	1
Mfsd11	1,264441244	1
Pcf11	1,264353603	1
Asxl1	1,264353603	1
Fam98b	1,264265968	1
Fam63b	1,264265968	1
Zc3h3	1,264178339	1
Dalrd3	1,264178339	1
Nsdhl	1,264003098	1
Sema4a	1,263915487	1
4930430F08Rik	1,263827883	1
Gm2011	1,263652691	1
Tlr1	1,263302381	1
Llph	1,263214818	1
Gm8181	1,263039712	1
Med20	1,262952167	1
Snrpb	1,262952167	1
Tnks1bp1	1,262864629	1
Card19	1,262864629	1
Ttpal	1,262777097	1
Eml2	1,262777097	1
Baat	1,26242703	1
Marcksl1	1,262252032	1
I830077J02Rik	1,262252032	1
Uba6	1,262164543	1
Rbm41	1,262077059	1
Gm37900	1,262077059	1
Hist1h2bc	1,262077059	1
Helz2	1,261989581	1
Hectd3	1,261989581	1
Ubxn4	1,261814645	1
Phc2	1,261639732	1
Cog6	1,261377409	1
Dctn1	1,26111514	1
Ska3	1,260852926	1

Ppm1a	1,260852926	1
Cmc4	1,260678147	1
Fbxo34	1,260678147	1
Banp	1,260678147	1
Gm28071	1,260590766	1
Map1lc3b	1,260503392	1
Gm11539	1,260416023	1
Rab22a	1,260416023	1
Gm38067	1,260328661	1
9330020H09Rik	1,260328661	1
Rpl23a-ps3	1,260328661	1
Arl1	1,260328661	1
Phf2	1,260328661	1
Plekha1	1,26006661	1
Fbxo5	1,25989194	1
Golm1	1,259717294	1
Eif1a	1,259542672	1
D230017M19Rik	1,25945537	1
Gm9774	1,259368074	1
Wtap	1,259280785	1
Osbpl8	1,259280785	1
Ipo13	1,259193501	1
Irgq	1,259106223	1
Adrb2	1,259018952	1
Rsrp1	1,259018952	1
Sppl3	1,259018952	1
Fat1	1,258844427	1
Oscar	1,258757174	1
Usp1	1,258757174	1
Top1	1,258757174	1
Sco1	1,258669926	1
Prrg4	1,25849545	1
CH25-309J2.1	1,258146569	1
Clock	1,258146569	1
Azin1	1,258146569	1
Chst14	1,258059364	1
A930004J17Rik	1,258059364	1
Cul3	1,257884972	1
Gm10110	1,257797785	1
Nova1	1,257710604	1
Nlrc3	1,257710604	1
B430305J03Rik	1,257710604	1
Cybb	1,257449098	1
Det1	1,257361941	1
Pla2g2e	1,25727479	1
Zbtb46	1,25727479	1
A630072M18Rik	1,257100507	1
Sf1	1,257100507	1
Chmp2b	1,256839128	1
Gm22980	1,256229453	1
Gm45222	1,256055315	1
Stk35	1,256055315	1

Csf2rb2	1,255881201	1
Crebrf	1,255794153	1
Wdsub1	1,255707111	1
Ccar1	1,255620075	1
Lmf2	1,255533045	1
mt-Tt	1,255271991	1
March5	1,255271991	1
Cdc42se1	1,255271991	1
Gm45855	1,255010992	1
Fam76b	1,254924004	1
Carhsp1	1,254924004	1
Zfand4	1,254663077	1
Yme1l1	1,254663077	1
Gm6946	1,254576114	1
Sash1	1,254576114	1
Urb2	1,254489156	1
Lmbrd2	1,254489156	1
Actr3	1,254489156	1
Cyp2c55	1,254402205	1
Hist1h4i	1,254402205	1
H3f3b	1,254315259	1
Mss51	1,254054459	1
Dazap1	1,254054459	1
E2f7	1,253967537	1
Frs2	1,253967537	1
C430042M11Rik	1,253793712	1
Gfpt1	1,253706809	1
4933439C10Rik	1,253619912	1
Kif23	1,25353302	1
BC029214	1,253359256	1
Mmaa	1,253272383	1
Casz1	1,253272383	1
Gm43213	1,253098654	1
Gm15644	1,253098654	1
Oprl1	1,253098654	1
Trim16	1,25292495	1
Pus7l	1,25275127	1
Acbd5	1,252664439	1
Acot2	1,252577614	1
Wdyhv1	1,252490794	1
Vcpip1	1,252317174	1
Ptms	1,252230373	1
Gm8667	1,252143578	1
Hnrnpu	1,252056789	1
Ssbp4	1,251709694	1
Stk24	1,251709694	1
Impdh1	1,251622935	1
Prune2	1,251449435	1
Lhpp	1,251189231	1
Mink1	1,250842375	1
Dnajc7	1,250755677	1
Ube2v2	1,250668984	1

Sart1	1,250668984	1
Bpnt1	1,250582297	1
Erh	1,250582297	1
Park2	1,250582297	1
Snhg4	1,250322273	1
Ing3	1,249975658	1
Kif4	1,249889019	1
Iba57	1,249802386	1
Rplp1-ps1	1,249542524	1
2810428J06Rik	1,249542524	1
Fam20b	1,249455916	1
Zfp235	1,249369313	1
Pex7	1,249196126	1
Atp8b2	1,249109541	1
Fam20c	1,249109541	1
Kif21b	1,248849823	1
Trib3	1,248763262	1
Zfp414	1,248676708	1
Isyna1	1,248676708	1
Ftx	1,24841708	1
A230028O05Rik	1,24841708	1
RP23-225D5.4	1,24841708	1
Gm44953	1,248070993	1
Gm23300	1,248070993	1
Prkcg	1,247984486	1
Creb3l3	1,247984486	1
Gm11470	1,247811491	1
Pigu	1,247465572	1
Ice1	1,247379107	1
Gm43737	1,247292648	1
Nkrf	1,247119749	1
Gabpb2	1,247119749	1
Mis18a	1,247033308	1
Hspa13	1,246860445	1
Mt1	1,246860445	1
Bdp1	1,246774022	1
Rbx1	1,246601194	1
Orc1	1,246514789	1
Rpia	1,246514789	1
Dazap2	1,246514789	1
Gm38192	1,246428391	1
2900055J20Rik	1,246255611	1
Gapdh	1,246255611	1
Mapk8ip3	1,246255611	1
Dynlt1f	1,24616923	1
Bmt2	1,24616923	1
Gm42786	1,245910123	1
Fbxo30	1,245651071	1
Aldh3a2	1,245478399	1
Cmtm7	1,245305751	1
Plxnc1	1,245305751	1
Zfp422	1,245219436	1

Mylip	1,245133127	1
Tjap1	1,245133127	1
Bcl7c	1,244960526	1
Tubg2	1,244356614	1
Zcchc8	1,244356614	1
Gm9762	1,244270365	1
Arf4	1,244270365	1
Zcchc14	1,244011653	1
Sestd1	1,243925428	1
Samd8	1,243925428	1
Zcchc9	1,243752995	1
Irf8	1,243494391	1
Usp50	1,243063503	1
Mxra8	1,243063503	1
Gps2	1,243063503	1
Sh3glb1	1,242977344	1
Rpl14	1,24289119	1
Fes	1,242632765	1
Cep85	1,242632765	1
Sbf2	1,242632765	1
R3hdm2	1,242546635	1
Nup214	1,242460511	1
Dck	1,242374394	1
Lcorl	1,242202176	1
Vrk2	1,242116076	1
Comp	1,242029982	1
Rgs3	1,241771736	1
Gtf3c2	1,241771736	1
Pex1	1,241599602	1
Qtrt1	1,241599602	1
Gm43848	1,241255405	1
Hist3h2a	1,241083342	1
4833445I07Rik	1,24099732	1
Pou2f1	1,240911304	1
Slc25a22	1,240825293	1
Bcas2	1,240825293	1
Arf2	1,240825293	1
Mcm9	1,240739289	1
Rps17	1,240739289	1
Amacr	1,24065329	1
Fbxo8	1,24065329	1
Slc8b1	1,24065329	1
Tbc1d25	1,240567298	1
Cops8	1,240567298	1
Gm37420	1,240481311	1
4930524J08Rik	1,240309356	1
Naa16	1,240309356	1
Csrp1	1,240223387	1
Ctbp2	1,240223387	1
Gm13777	1,240051468	1
Trrap	1,240051468	1
Cracr2b	1,239965517	1

Dut	1,239965517	1
Dhx38	1,239879572	1
Tarbp2	1,239793633	1
RP23-63H11.3	1,239793633	1
Gm37780	1,239621773	1
Rbm15	1,239621773	1
Gm11605	1,239192227	1
Ap1ar	1,239192227	1
Rab6a	1,239192227	1
Dcaf7	1,239106336	1
Hsp90aa1	1,239020451	1
Hspb6	1,238934571	1
Rpl15	1,238934571	1
Celf2	1,23876283	1
Dcp2	1,238676969	1
Rps6ka1	1,238591113	1
Yae1d1	1,23841942	1
Arl6ip1	1,238161925	1
Gm43817	1,238076105	1
Dse	1,237990291	1
Pcsk4	1,237904483	1
Fam168b	1,237732885	1
Srebf1	1,237647095	1
Ttc39b	1,237475532	1
Gm15207	1,23738976	1
Tcf7l2	1,23738976	1
Entpd6	1,237303994	1
Ube2j1	1,237132479	1
Tyms	1,236960988	1
Gtf2e1	1,236960988	1
Gm6395	1,236875251	1
Srl	1,23678952	1
Esrp2	1,23678952	1
Coprs	1,23678952	1
Gm6210	1,236618077	1
Mon1a	1,236618077	1
Eif5	1,236532364	1
Icmt	1,236360956	1
Tsc2	1,236018211	1
Lrrc28	1,236018211	1
Nck1	1,23593254	1
Gm44269	1,235846875	1
Plaur	1,235761215	1
Megf9	1,235504273	1
Sf3a3	1,235418637	1
1190005I06Rik	1,235333007	1
Ndc1	1,235161766	1
Swt1	1,235161766	1
Maea	1,235161766	1
Gm7079	1,234904948	1
Zfp266	1,234733765	1
Nxt1	1,234733765	1

Ap3d1	1,234648183	1
Cyp26b1	1,234562607	1
Tubgcp5	1,234391472	1
Grhl1	1,234305914	1
3110002H16Rik	1,234305914	1
Tbkbp1	1,234134814	1
Gtpbp1	1,234049273	1
Gm36964	1,233963739	1
Crlf3	1,233792687	1
Wdfy2	1,23370717	1
Gm3617	1,23370717	1
Ubr2	1,233621659	1
Rcor1	1,233450654	1
Brat1	1,233108717	1
Rtn4	1,232937783	1
Wdr45b	1,232852325	1
Bcl6b	1,232681428	1
Zfp472	1,232681428	1
Zfp655	1,232595988	1
Ybx1-ps2	1,232510554	1
Ccdc58	1,232510554	1
Cdca2	1,232425125	1
RP24-365N15.9	1,232254287	1
Pdpk1	1,232254287	1
Mmachc	1,232168876	1
Gm7561	1,232083472	1
Asb3	1,232083472	1
Far1	1,232083472	1
Prkd3	1,231827294	1
Rpl5	1,231571169	1
Cyth3	1,231485806	1
Psd3	1,231400449	1
Arglu1	1,231400449	1
Rabggta	1,23105908	1
Vamp3	1,230717805	1
Ubald2	1,230632501	1
Gm26610	1,230461911	1
Svip	1,230291345	1
1810026B05Rik	1,230291345	1
Anapc1	1,230035539	1
Gm17430	1,229865032	1
Gm38319	1,229694548	1
Gm4754	1,229609315	1
RP24-282K24.4	1,229438867	1
Dyrk1a	1,229438867	1
Fcnaos	1,229353652	1
Atf2	1,229268443	1
Cep350	1,229183239	1
Snn	1,229098042	1
Usp19	1,22901285	1
Gtf2h5	1,228927664	1
Stab1	1,228672143	1

Gm6560	1,22858698	1
Ybx1	1,22858698	1
Clu	1,228501824	1
Gm5801	1,228416674	1
5730508B09Rik	1,228246391	1
Gm12312	1,228161258	1
Gnl3l	1,228161258	1
Gm13341	1,227991011	1
Clspn	1,227991011	1
Klf2	1,227991011	1
Kat5	1,227905896	1
2200002J24Rik	1,227735684	1
BC055324	1,227735684	1
Brca2	1,227650587	1
Timm22	1,227650587	1
Tceal9	1,227650587	1
Ube2q2	1,227565495	1
Pinx1	1,22748041	1
Pla2g4a	1,22748041	1
Tpi1	1,227310257	1
Recql5	1,227225189	1
4930520O04Rik	1,226970021	1
Vkorc1	1,226970021	1
Rpl7-ps7	1,226970021	1
Dmtf1	1,226799939	1
Socs7	1,226799939	1
Trmt44	1,226714907	1
Thop1	1,22662988	1
Mrps31	1,22662988	1
Smim14	1,22662988	1
Wdr48	1,226459845	1
Plekhf2	1,226374836	1
Rps15a	1,226289833	1
Ttc9c	1,226204836	1
Utp14a	1,226204836	1
Clic4	1,22603486	1
1810013L24Rik	1,225949881	1
Snhg17	1,225864907	1
Rilpl2	1,225694978	1
Fam122b	1,225610022	1
Klhl21	1,225610022	1
Tle3	1,225610022	1
Chpf2	1,22535519	1
Zfp384	1,225185332	1
Rubcnl	1,225100411	1
Speer9-ps1	1,225100411	1
Mrps7	1,225100411	1
Gm44237	1,224930588	1
Rps11-ps1	1,224760789	1
Rpl37a	1,224760789	1
Rps25	1,224675898	1
Sbf1	1,224591012	1

Mast2		1,224251531	1
Papss1		1,224081825	1
Pip4k2b		1,224081825	1
Cep70		1,223827311	1
Gm44291		1,223827311	1
	Sep 08	1,223827311	1
Gm12643		1,223742485	1
Rpl28		1,223657664	1
Plek		1,223657664	1
Gm9835		1,223064086	1
Lamp2		1,223064086	1
Gm6285		1,222979313	1
Ccna2		1,222979313	1
Edem1		1,222979313	1
Tmx4		1,222894545	1
Hbp1		1,222809783	1
Atp6v1a		1,222809783	1
Dync1li1		1,222725028	1
Rlf		1,222640278	1
Ccnt2		1,222640278	1
Fam63a		1,222640278	1
Csnk2a1		1,222555534	1
Gm42467		1,222470796	1
Exoc2		1,222386063	1
Wasf2		1,222386063	1
Crocc		1,222301337	1
AI606181		1,222216616	1
Csnk1g3		1,222047193	1
Cxxc1		1,22196249	1
Cnot4		1,221877793	1
Gm15796		1,221793102	1
Atad2		1,221793102	1
Lta4h		1,221539063	1
Slc7a8		1,221539063	1
Mtpn		1,221539063	1
Ist1		1,221369734	1
Champ1		1,221285078	1
Desi2		1,221115784	1
Rpsa-ps4		1,220946513	1
Trim47		1,220777265	1
Mospd3		1,220777265	1
Xrcc2		1,22069265	1
Usp32		1,220438841	1
Rhebl1		1,220354249	1
Pigl		1,220269664	1
Ddx5		1,22010051	1
Manba		1,220015942	1
Sec23b		1,220015942	1
Rnf32		1,219762273	1
Hgsnat		1,21959319	1
Borcs7		1,219508657	1
Gm14680		1,21942413	1

Gm10093	1,219339609	1
Snrpert	1,219339609	1
Rel	1,219255094	1
4932438A13Rik	1,219255094	1
Tomm20	1,219170585	1
Vma21	1,219170585	1
Polr3f	1,219086081	1
Samsn1	1,219001583	1
Pibf1	1,218917092	1
Ginm1	1,218832606	1
Pfdn5	1,218832606	1
A330035P11Rik	1,218748126	1
Tmem259	1,218663651	1
Ankzf1	1,218579183	1
C2cd5	1,218410264	1
Tpst1	1,218325813	1
Nrm	1,218241368	1
Cpne3	1,218241368	1
Plpp6	1,218156929	1
Rpp38	1,218072495	1
Ndufc2	1,218072495	1
Gm25007	1,217988068	1
Fbxo45	1,217988068	1
Rps19bp1	1,217988068	1
Alkbh5	1,217903646	1
Orc4	1,217819231	1
Ephx1	1,217734821	1
Adprm	1,217650417	1
Crybb3	1,217566019	1
Gm42743	1,217566019	1
Uap1	1,217566019	1
Smad6	1,217481626	1
Hint3	1,217481626	1
Gm45640	1,217312859	1
Nkiras1	1,217228484	1
Rbm39	1,217144116	1
2300009A05Rik	1,217144116	1
Fxyd2	1,216975395	1
Cmip	1,216806698	1
Gtf3c1	1,216553697	1
Tmem251	1,216300748	1
Pde7a	1,216216443	1
Zfp949	1,215879283	1
Cnpy4	1,215879283	1
Mfsd14b	1,215879283	1
D430013B06Rik	1,215795008	1
E2f4	1,215795008	1
Tmem9b	1,215710738	1
Hist1h1e	1,215626474	1
Rbm18	1,215626474	1
Slc6a8	1,215373718	1
Bnip3	1,215289478	1

Kmt5b	1,215205243	1
Ppp1r21	1,214868364	1
Lfng	1,214784158	1
Zfp532	1,214784158	1
Nlrc4	1,214615765	1
Scpep1	1,214615765	1
Ak4	1,214615765	1
Ncstn	1,214363219	1
Synj1	1,214279049	1
Gm7899	1,214110726	1
Fgf11	1,214026573	1
Gm11826	1,213774149	1
Hoxc6	1,213774149	1
Arhgap5	1,213774149	1
Eif4b	1,21369002	1
Seh1l	1,213605896	1
Dkc1	1,213437666	1
Gm12704	1,21326946	1
Gm11464	1,21326946	1
Arntl	1,213017194	1
Fgfr1op	1,213017194	1
Hnrnph1	1,212933117	1
Pop7	1,21276498	1
Slc39a2	1,212596867	1
Eif4h	1,212512819	1
Ccnc	1,212344741	1
Ppa1	1,212344741	1
Cd36	1,212092667	1
Phc3	1,212092667	1
Med13	1,211924647	1
Zbtb1	1,211840646	1
Ccdc86	1,211840646	1
Sh3bp1	1,211840646	1
Gna13	1,211588677	1
Rpl21	1,211168845	1
Cgrrf1	1,211168845	1
March2	1,211084896	1
Gse1	1,211000953	1
Casp3	1,211000953	1
Qars	1,211000953	1
Dtx3l	1,210917015	1
Strip1	1,210833084	1
Hells	1,210749158	1
Utp23	1,210749158	1
Tab2	1,210749158	1
Washc5	1,210665238	1
Hnrnpll	1,210413514	1
Reps1	1,210161842	1
Tmem183a	1,210161842	1
Gm6198	1,209910222	1
Atp11c	1,209742504	1
Fgd2	1,209658654	1

Gm12844	1,20957481	1
Por	1,20957481	1
Gm13092	1,209490971	1
Gm13453	1,209407139	1
Rnf10	1,209407139	1
Osgin2	1,209323312	1
Traf1	1,209155676	1
Creb3	1,209155676	1
Trp53i13	1,208988063	1
5330406M23Rik	1,208904265	1
Gm8276	1,208904265	1
Trip12	1,208736687	1
Gm10169	1,208569132	1
Mapkbp1	1,208485363	1
Ube2j2	1,208485363	1
Phlpp1	1,208234092	1
Rps4x-ps	1,207982873	1
Nrbp1	1,207982873	1
Mettl3	1,207899145	1
Bax	1,207899145	1
Zfp397	1,207647995	1
Utp4	1,207647995	1
Pak2	1,207480591	1
Ngly1	1,207396898	1
Gm45856	1,207229529	1
Got2	1,207229529	1
Zfp53	1,207062183	1
Rwdd2a	1,20689486	1
Zfp131	1,20689486	1
Mdfic	1,206476655	1
Gm14270	1,206393031	1
BC065397	1,206309413	1
Nupl1	1,206225801	1
Bnip3l	1,206142195	1
Zfp626	1,205975	1
Phf13	1,205975	1
Rpl22l1	1,205975	1
B630019K06Rik	1,205807828	1
Ttc30a1	1,20572425	1
Nol11	1,20572425	1
Hal	1,205557113	1
D130020L05Rik	1,205473553	1
RP23-447C2.2	1,205473553	1
Vamp7	1,205473553	1
Rnf115	1,205389999	1
Rps10-ps2	1,205222908	1
Fnip2	1,205222908	1
Hmces	1,205139371	1
Gm23346	1,20505584	1
Zscan26	1,20505584	1
Vprbp	1,204888796	1
G3bp2	1,204888796	1

Krit1	1,204638272	1
Gm6166	1,204471285	1
Anapc5	1,204387801	1
Gm45358	1,204304322	1
Rbmxl1	1,204304322	1
Stx1a	1,203970464	1
Lacc1	1,20380357	1
Pfdn4	1,203636699	1
Zfp770	1,203553272	1
Cops2	1,203386436	1
Ddx19b	1,203219622	1
Arhgdia	1,203219622	1
Gm8213	1,203136224	1
Dtl	1,203052832	1
Rnf6	1,203052832	1
Sc5d	1,203052832	1
Gng12	1,203052832	1
Bmp2k	1,202969446	1
Gm8318	1,202886065	1
Gm12346	1,20280269	1
Gm5687	1,20280269	1
Coq5	1,20280269	1
Zfp637	1,202719321	1
Ddah2	1,202635958	1
Adamtsl4	1,202635958	1
Rlim	1,202635958	1
B3gnt6	1,202469249	1
Fosb	1,202469249	1
Hif1a	1,202469249	1
Bzw1	1,202302563	1
Ggta1	1,202052577	1
Gm9506	1,20196926	1
Rnf4	1,20196926	1
Gm20633	1,201802643	1
Spag9	1,201802643	1
Gm37914	1,201719343	1
Calm1	1,201719343	1
Scai	1,201552761	1
Nfkb1	1,201469479	1
Gm11353	1,201302931	1
Mark4	1,201302931	1
Rheb	1,201302931	1
Wbp2	1,201219666	1
Map3k15	1,201136407	1
Ilf3	1,201053154	1
Kif9	1,200969906	1
Gm12013	1,200886664	1
Tjp2	1,200803427	1
Eno1	1,200636972	1
Fxr1	1,200636972	1
Kcnc3	1,200553753	1
Gm5697	1,20047054	1

Exd2	1,200387333	1
Gm12882	1,200220935	1
Palm	1,200220935	1
Zgpat	1,200137745	1
Mtf2	1,199971382	1
Zfp932	1,19988821	1
Cfap74	1,199721881	1
Kyat3	1,199389294	1
Tmem86a	1,199389294	1
Tom1l1	1,199306161	1
Rps8-ps4	1,199139914	1
Zfp212	1,199139914	1
Sec62	1,199139914	1
Abca7	1,198973689	1
Lockd	1,198890586	1
Pax3	1,198807488	1
Pcm1	1,198641309	1
Ldb1	1,198475154	1
Rps6-ps1	1,198475154	1
Tank	1,198475154	1
Gm12589	1,198392085	1
Tmem63b	1,198392085	1
Rab1a	1,198309021	1
Washc2	1,198309021	1
Gm9169	1,198059866	1
Gm15530	1,198059866	1
Bri3	1,198059866	1
A430105I19Rik	1,197976826	1
4932422M17Rik	1,197976826	1
Ythdf1	1,197810762	1
Gm38235	1,197644722	1
Lrrc59	1,197644722	1
Ik	1,197644722	1
Klf1	1,19756171	1
Tex264	1,19756171	1
Clip2	1,19756171	1
Psme1	1,19756171	1
Cnot7	1,197478705	1
Leng8	1,197395705	1
Top1mt	1,197395705	1
R3hdm4	1,197229722	1
Pxn	1,197063762	1
Tecpr2	1,196980791	1
Deaf1	1,196980791	1
Lipe	1,196814866	1
Ssh2	1,196814866	1
Lamp1	1,196814866	1
Gm5845	1,196731911	1
Rpl10	1,196731911	1
Meis2	1,196566021	1
Spryd7	1,196566021	1
Eaf1	1,196317228	1

Ccdc61	1,196234308	1
Gm26542	1,196151394	1
Rbm8a2	1,196068486	1
Mical1	1,195736912	1
Inppl1	1,195571159	1
Cbl	1,195488291	1
Klhl18	1,195239722	1
Ergic2	1,195239722	1
Uqcrh	1,195239722	1
Zfp959	1,195074038	1
Ube2g1	1,195074038	1
Zfp595	1,194991205	1
Rad54l	1,194825555	1
Vamp2	1,194825555	1
Borcs8	1,194825555	1
Paics	1,194742739	1
Rps8-ps3	1,194577124	1
Aqr	1,194245963	1
Setd1a	1,194163187	1
B2m	1,194163187	1
Nudt16l1	1,194080417	1
Eif1b	1,194080417	1
5031439G07Rik	1,193749393	1
Malt1	1,193666652	1
Dnajc3	1,193666652	1
Nfatc2	1,193583916	1
Scd1	1,193583916	1
Ccdc82	1,19325303	1
Sh3gl1	1,19325303	1
2700097O09Rik	1,193087622	1
Dnaaf3	1,193087622	1
4833420G17Rik	1,193087622	1
Usp36	1,192756873	1
Ddx47	1,192756873	1
Zfp865	1,192674201	1
Ndfip1	1,192591534	1
Arhgef6	1,192343567	1
Gm11808	1,192343567	1
B130021K23Rik	1,192178284	1
Arsk	1,192013025	1
Cox20	1,191930404	1
Asna1	1,191599976	1
Klhl35	1,191517384	1
Plcb3	1,191517384	1
Trim23	1,191434797	1
Paip2	1,191434797	1
Gpr137	1,19126964	1
Eif4e	1,191187071	1
Tsga10ip	1,191104507	1
Psat1	1,191104507	1
Anp32e	1,191104507	1
Ubl7	1,190939396	1

Phldb1	1,190856849	1
Jmjd4	1,190774308	1
Gm12186	1,190774308	1
Rnf170	1,190774308	1
Sec22b	1,190774308	1
Snhg15	1,190691773	1
Snx20	1,190609243	1
Scarna2	1,19052672	1
Thg1l	1,19052672	1
Tcf12	1,190361689	1
Zfp638	1,190196681	1
Nrf1	1,189866734	1
Dohh	1,189866734	1
Tspyl3	1,189619334	1
Akirin2	1,189536879	1
Smyd5	1,189289547	1
Nfic	1,189124688	1
Gm38305	1,189042267	1
Elf2	1,188877442	1
Adcy9	1,188795039	1
Adi1	1,188795039	1
Hexim2	1,18871264	1
Inpp5b	1,188547861	1
Taok2	1,188547861	1
Aff4	1,188547861	1
Abhd13	1,188547861	1
Mtrf1	1,18846548	1
Fam46a	1,18846548	1
Anxa2	1,188383105	1
Crcp	1,188218372	1
Mre11a	1,188053661	1
Gm10036	1,187971314	1
Serinc1	1,187888973	1
Uqcrh-ps2	1,187806638	1
Rffl	1,187724308	1
Caml	1,187559666	1
Rpl36a	1,187559666	1
Ubqln4	1,187559666	1
Mettl17	1,187477354	1
Irf2	1,187395047	1
Cct2	1,187395047	1
Ttyh2	1,187312746	1
Mpp1	1,187312746	1
Dlg1	1,187148161	1
Hadha	1,187148161	1
Ipo5	1,187065877	1
Cdca5	1,186983598	1
Slc25a44	1,186983598	1
Ppp1r13b	1,186819059	1
Tax1bp1	1,186819059	1
Phkb	1,186736798	1
Gstz1	1,186490048	1

Hnrnpa0	1,186490048	1
Rab8b	1,18640781	1
Rapgef5	1,186325577	1
Aurka	1,186325577	1
Ppp4r2	1,186325577	1
Mbtps2	1,18624335	1
Prkacb	1,18624335	1
Elmsan1	1,186078913	1
Kdm5a	1,185996704	1
Snx5	1,185914499	1
Egln3	1,185667921	1
Cdk16	1,185667921	1
RP24-282C4.3	1,185503564	1
Eed	1,185421394	1
Mrpl50	1,18533923	1
Nek2	1,185257071	1
Yipf5	1,185174918	1
Rnf26	1,185010629	1
Ing5	1,184928494	1
Tmem104	1,18468212	1
Gm24339	1,184600007	1
Sat1	1,1845179	1
Calcrl	1,184353702	1
Rbm8a	1,184189527	1
Mapk8ip1	1,183943308	1
Atp5s	1,183943308	1
Hnrnpk	1,18377919	1
Usf1	1,183615094	1
Rttn	1,183615094	1
Zbtb17	1,183533055	1
Hps3	1,183451022	1
Mllt3	1,183451022	1
Cdc40	1,183368994	1
Hnrnpd	1,183286972	1
Top3b	1,183204956	1
Tagap	1,183122945	1
Vps72	1,183122945	1
Polr1e	1,18304094	1
Med27	1,18304094	1
E2f3	1,182794959	1
Oxsr1	1,182712977	1
Pde1b	1,182631	1
Xpot	1,182549029	1
Smcr8	1,182467064	1
Gm43681	1,182385105	1
Skiv2l2	1,182385105	1
Gm38375	1,182221203	1
Als2	1,18213926	1
Rnf34	1,182057323	1
Atp11b	1,182057323	1
Camsap1	1,182057323	1
Vps37c	1,181975392	1

Tsga10	1,181893467	1
Kat2a	1,181893467	1
Ninj1	1,181893467	1
Jmjd7	1,181565822	1
Fbxo9	1,181565822	1
Slc35b3	1,181565822	1
Efcab2	1,181483925	1
Kin	1,181483925	1
Metrnl	1,181483925	1
Mkx	1,181156393	1
Ncoa5	1,181156393	1
Ddx3y	1,180747106	1
Gm14034	1,180665266	1
Wdr44	1,180501603	1
Usp21	1,180501603	1
Smg1	1,180501603	1
Ddx6	1,180337962	1
Ell	1,18025615	1
Gnpda2	1,180174343	1
Igsf8	1,180174343	1
Tspyl1	1,180174343	1
Gm42466	1,180010748	1
Asns	1,180010748	1
Plekhg4	1,180010748	1
Sem1	1,179928958	1
1700123O20Rik	1,179847175	1
Lrif1	1,179765397	1
Ppp4r1	1,179765397	1
BC030336	1,179438342	1
Prkab1	1,17919311	1
Thrap3	1,17919311	1
Hspe1	1,17919311	1
Cox20-ps	1,178947929	1
Actn4	1,178947929	1
Gm5525	1,178866214	1
Gm28530	1,178784504	1
Gm10501	1,178702799	1
Rbm27	1,178702799	1
Snrpb2	1,178702799	1
Dmxl1	1,178621101	1
Api5	1,178294363	1
Slx1b	1,178131028	1
Tbp	1,178131028	1
Dock1	1,178049369	1
Pdlim7	1,177967716	1
RP23-390D8.2	1,177886068	1
Glyctk	1,177886068	1
Gm20342	1,177804426	1
Ints1	1,17772279	1
Cacna1s	1,17772279	1
Dusp16	1,17772279	1
Polg2	1,177396301	1

Gm44024	1,17723309	1
Acot6	1,177069902	1
Stac2	1,176988317	1
Otud4	1,176906737	1
Gm29488	1,176825163	1
Pola2	1,176743595	1
Fxr2	1,176417377	1
Cnot2	1,176254302	1
Nup93	1,175846714	1
Gemin6	1,175765214	1
Etnk1	1,175765214	1
Gm42636	1,175602229	1
Adam15	1,175602229	1
Gm14292	1,175520746	1
Rarg	1,175520746	1
Fndc3a	1,175520746	1
5830408C22Rik	1,175439268	1
Chst12	1,175439268	1
Htt	1,175439268	1
Mettl9	1,175357795	1
Smad3	1,175357795	1
Grtp1	1,175276328	1
Gm37558	1,175031962	1
Ccdc94	1,174461971	1
Dnttip2	1,174461971	1
Eloc	1,174299167	1
Vrk1	1,174299167	1
Nedd9	1,174217774	1
Gm14303	1,174217774	1
Uqcrb	1,174217774	1
Abhd4	1,174055004	1
Acap2	1,173973628	1
Asf1a	1,173892257	1
Selenok	1,173810892	1
St13	1,173810892	1
Chic2	1,173648178	1
Dclre1a	1,17356683	1
Akap8l	1,17356683	1
Usp39	1,17340415	1
Fam117a	1,173322819	1
RP23-48A24.3	1,173241493	1
Rab18	1,173078859	1
Mrpl32	1,17299755	1
Pcgf1	1,172916247	1
Mast3	1,172834949	1
Snrpg	1,172834949	1
Mttp	1,172753657	1
Gm37305	1,172753657	1
St6galnac6	1,172753657	1
Ifrd1	1,172672371	1
Khsrp	1,172672371	1
Mapk8	1,172509815	1

Mphosph6	1,172428546	1
Aga	1,172428546	1
Setmar	1,172347282	1
Mthfr	1,172184772	1
Slfn10-ps	1,172103525	1
Cenpe	1,172103525	1
1110012L19Rik	1,171859818	1
Gm5735	1,171778594	1
Chml	1,171453753	1
Utp14b	1,171372557	1
Git2	1,171210181	1
Jak1	1,171210181	1
Pcgf2	1,171129002	1
Fyn	1,171129002	1
Mrpl34	1,171129002	1
Nlrc5	1,171047828	1
Arhgap15	1,171047828	1
Ifi207	1,17096666	1
Gm6304	1,170804341	1
Ptpn2	1,170642044	1
2700029L08Rik	1,170560904	1
Reep4	1,170560904	1
Mitd1	1,170560904	1
A530041M06Rik	1,17047977	1
Zmym1	1,17047977	1
Tubb2a	1,17047977	1
Gm10269	1,170398641	1
Cecr5	1,170317518	1
Leng9	1,170236401	1
Mbd2	1,170236401	1
Gm10443	1,170155289	1
Dusp11	1,170155289	1
Slc9a9	1,170074183	1
Zfp58	1,170074183	1
Spop	1,170074183	1
Parp9	1,169993082	1
RP23-128C4.4	1,169749815	1
Slc35e4	1,169668737	1
Tbccd1	1,169587664	1
Gm11952	1,169506597	1
Daam1	1,169506597	1
Ifitm6	1,169506597	1
Fam179b	1,169506597	1
Gnas	1,169425536	1
Zmiz2	1,169425536	1
Ambra1	1,16934448	1
Ift88	1,169101347	1
Slc25a16	1,169101347	1
Pex13	1,169020314	1
Pcsk7	1,169020314	1
Gm13827	1,169020314	1
Itgb1	1,169020314	1

Poln	1,168939287	1
Rhod	1,168939287	1
Cdk8	1,168858265	1
Acer3	1,168534233	1
Pprc1	1,168372251	1
Arhgap12	1,168291269	1
Timm17a	1,168291269	1
D17Wsu92e	1,168291269	1
Rpa3	1,16812932	1
Gba2	1,167967395	1
Thbs3	1,16788644	1
Prss44	1,16764361	1
Gab3	1,16764361	1
Trim24	1,16764361	1
Arl6ip6	1,167319916	1
Ube2b	1,167239006	1
Otub2	1,167077203	1
RP23-205H11.3	1,166996311	1
Arhgap1	1,166996311	1
1810037I17Rik	1,166834542	1
Gramd1a	1,166753666	1
Rnf217	1,166672795	1
Alas1	1,166672795	1
Ubr7	1,166672795	1
Skiv2l	1,16634937	1
Gm13340	1,166268527	1
Ppp1cb	1,166268527	1
D3Ertd254e	1,165783591	1
Itpk1	1,165783591	1
Pum2	1,165783591	1
Ppif	1,165298856	1
Tubb5	1,165298856	1
Tube1	1,165218086	1
A630033H20Rik	1,165218086	1
Prcp	1,165056564	1
Rnf128	1,165056564	1
Gm3355	1,164895064	1
Dock6	1,164895064	1
Btbd10	1,164814322	1
Etf1	1,164814322	1
Rnf113a2	1,164733586	1
Msl2	1,164733586	1
Fam175a	1,164491412	1
Sgol1	1,164491412	1
Snhg6	1,164410699	1
2510039O18Rik	1,164410699	1
Neil3	1,164329991	1
Rab35	1,164329991	1
Sars2	1,164249288	1
Ankmy2	1,164168591	1
Taf6l	1,164168591	1
Polr1d	1,164168591	1

Cnih1	1,1640879	1
Dok2	1,16384586	1
Gm13397	1,163765191	1
Actr1a	1,163765191	1
Ttk	1,163684528	1
Mta1	1,163684528	1
Rps19-ps3	1,16360387	1
Med10	1,16360387	1
3110009E18Rik	1,163523218	1
Zfp142	1,163442572	1
Actb	1,163361931	1
Blzf1	1,163361931	1
Dnajc19-ps	1,163281295	1
Ddx43	1,163281295	1
Sos1	1,163281295	1
Mgea5	1,163281295	1
Ddx42	1,163200666	1
Hgs	1,163200666	1
Arhgap4	1,163039423	1
Zfp81	1,16295881	1
Gm5921	1,162878203	1
Zfp160	1,162878203	1
Cltc	1,162878203	1
Matn1	1,162797601	1
Fam120a	1,162797601	1
Prpf18	1,162797601	1
Mbtd1	1,16247525	1
Nfat5	1,16247525	1
Vcp	1,16247525	1
Pdcd5	1,162314108	1
Ensa	1,162314108	1
St6galnac4	1,162314108	1
Gdpd1	1,162233545	1
Fbxw4	1,162152988	1
Lca5	1,162072436	1
Shoc2	1,16191135	1
Gm27605	1,161830815	1
Edc4	1,161750286	1
0610030E20Rik	1,161589244	1
Cep83	1,161508732	1
Tmem50b	1,161347724	1
Nckipsd	1,161267228	1
Bckdha	1,161025774	1
Gltscr2	1,160945301	1
Gm42659	1,160784371	1
Nop56	1,160784371	1
Golga7	1,160703914	1
Tbrg4	1,160462578	1
Bak1	1,160301715	1
Pdia4	1,160221292	1
Bag4	1,160140874	1
Polr2a	1,160140874	1

Sart3	1,160060462	1
Lrrc51	1,159899655	1
Gm27029	1,159819259	1
Gm15903	1,159417366	1
Atf6	1,159417366	1
Rassf1	1,159417366	1
Nrbf2	1,159337004	1
Phtf1os	1,159256648	1
Elmod2	1,159095952	1
Bbof1	1,159095952	1
4930440I19Rik	1,159015612	1
Tmem60	1,158935278	1
Isoc1	1,15885495	1
Tnks	1,15885495	1
Rny3	1,158694309	1
Topors	1,158613998	1
Atg12	1,158613998	1
Pde12	1,158453391	1
Rps6	1,158373096	1
Hcfc1r1	1,158212522	1
Pank2	1,158132244	1
Trmt61b	1,158051971	1
Tmem63a	1,158051971	1
Vps29	1,157971704	1
Rnf19b	1,157971704	1
Hsdl1	1,157891442	1
Cdc42se2	1,157811186	1
Napg	1,157811186	1
Selenof	1,157730935	1
Szt2	1,15765069	1
Mthfs	1,157490217	1
Lhx1	1,157329766	1
Zc3hav1l	1,157249549	1
Gm32340	1,157249549	1
Bend3	1,157169337	1
Dirc2	1,157089131	1
Pdpr	1,15700893	1
Nolc1	1,15700893	1
Prss36	1,156928735	1
AI597479	1,156848546	1
1700017B05Rik	1,156848546	1
Pabpc1l	1,156688184	1
Lpcat1	1,156608011	1
Ube2d3	1,156447682	1
Arid1a	1,156367526	1
Rpl23a-ps5	1,156287376	1
Fli1	1,156287376	1
mt-Nd2	1,156287376	1
Ckb	1,156207231	1
Arf3	1,155966829	1
Unk	1,155726478	1
Trove2	1,155726478	1

Pdcd6ip	1,155726478	1
Pias1	1,155646372	1
Yy1	1,155646372	1
Mtss1	1,155646372	1
Gtf2h1	1,155566271	1
Ogdh	1,155566271	1
Lsm3	1,155486176	1
Sufu	1,155486176	1
Arel1	1,155406087	1
Bcas3	1,155326003	1
Sav1	1,155326003	1
Rassf5	1,155326003	1
Eif2b2	1,155326003	1
Zranb1	1,155245925	1
1700020D05Rik	1,155165852	1
Scrn2	1,155165852	1
Mrnip	1,155085785	1
Gm10320	1,155085785	1
Ttc5	1,154845616	1
Mettl23	1,154845616	1
Ypel5	1,154845616	1
Tm9sf2	1,154685532	1
Eef1b2	1,154685532	1
1700056N10Rik	1,154605498	1
Chd1	1,154605498	1
Phip	1,154605498	1
Copz2	1,154525469	1
Cep152	1,154445447	1
Map4k3	1,154365429	1
Bend4	1,154365429	1
Gapdh-ps14	1,154285418	1
Slc35a5	1,154205411	1
Rnf13	1,154205411	1
Slc25a23	1,154205411	1
Lactb2	1,153965426	1
Morf4l1	1,153885442	1
Ubap2l	1,153885442	1
Cers2	1,153805464	1
1700109H08Rik	1,153565561	1
Fzr1	1,153485605	1
Olfr95	1,153405654	1
C330027C09Rik	1,153405654	1
Bloc1s1	1,153085907	1
Cops7b	1,153085907	1
Ing2	1,153085907	1
Hdac9	1,153005984	1
Zfp790	1,152926066	1
Ndufs3	1,152926066	1
Epc1	1,152926066	1
Unc13b	1,152766248	1
Cog5	1,152766248	1
Mbnl2	1,152686347	1

Gm14813	1,152446677	1
Gm7967	1,152366799	1
Gm16200	1,152366799	1
Prag1	1,152286925	1
Zyg11b	1,152286925	1
Rbbp6	1,152286925	1
Cdkn3	1,152207058	1
Snap47	1,152127196	1
Dtymk	1,152127196	1
Zfp568	1,152047339	1
Tnfrsf14	1,152047339	1
Gm15513	1,151967488	1
Tmbim6	1,151967488	1
Cdk1	1,151887642	1
Dpy30	1,151807802	1
Topbp1	1,151727968	1
Gm5445	1,151648139	1
Washc4	1,151648139	1
Glrx3	1,151568316	1
Sin3a	1,151488498	1
Gpr155	1,151408685	1
Gm11599	1,151328879	1
4930427A07Rik	1,151169282	1
Slc7a6os	1,151169282	1
Gm28791	1,151009707	1
Rbm43	1,150929928	1
Tpt1-ps3	1,150850154	1
Ptar1	1,150850154	1
Nelfe	1,150770386	1
Gm37234	1,150610866	1
Kpna6	1,150531115	1
Tfe3	1,150531115	1
Amfr	1,150371628	1
Ints14	1,150291893	1
Tlcd1	1,150212164	1
Phka2	1,150212164	1
Shb	1,150212164	1
Tspan14	1,15013244	1
Polk	1,15013244	1
Gm37124	1,150052722	1
Katnbl1	1,150052722	1
Nnt	1,149973009	1
Scd2	1,149973009	1
Ltbp4	1,149654213	1
Ccdc57	1,149574528	1
Gm7867	1,149494848	1
Tfb1m	1,149415174	1
Tchp	1,149255842	1
Ino80	1,149255842	1
Armc8	1,149176185	1
Dennd6a	1,149096533	1
Cpt2	1,148937245	1

Mzt1	1,148857609	1
Cxcl14	1,148777979	1
Specc1	1,148777979	1
Gpatch2l	1,148698355	1
Mphosph10	1,148539123	1
Ddx19a	1,148379912	1
Gm23502	1,148220724	1
Ssb	1,148220724	1
Ubald1	1,148141138	1
Ormdl1	1,148061558	1
Pcgf3	1,147981983	1
Mars2	1,147663739	1
Eif2s3y	1,147584192	1
Mroh1	1,14750465	1
Mgat4b	1,147425114	1
Mpeg1	1,147345583	1
Dpm3	1,147266058	1
Thumpd3	1,147186538	1
Pttg1	1,146868515	1
Hdac2	1,146789023	1
A930001C03Rik	1,146709536	1
Cpsf1	1,146709536	1
Snx12	1,146709536	1
Vcl	1,146630055	1
Dek	1,146630055	1
Mios	1,146550579	1
Nxf1	1,146550579	1
Pik3r6	1,146471109	1
Nr1h2	1,146391645	1
Ccnb2	1,146391645	1
Unc93b1	1,146312186	1
Gm13611	1,146232732	1
Hdhd2	1,146153284	1
Cntrl	1,146153284	1
Tomm22	1,146153284	1
Atxn2	1,146153284	1
Sort1	1,146073842	1
Kat7	1,145994405	1
1700124L16Rik	1,145756126	1
Dph3	1,145756126	1
Gm8722	1,145676711	1
Mrpl16	1,145676711	1
Erlin1	1,145597302	1
Ap3m1	1,145597302	1
Gm37063	1,145517898	1
Ipo9	1,145279719	1
Nmrk1	1,145200337	1
Mrm2	1,145120961	1
Gm7799	1,14504159	1
Ikzf5	1,14504159	1
RP23-444K20.4	1,144882864	1
Rhou	1,144724161	1

Smarcb1	1,144724161	1
Gm6155	1,144644817	1
Cnot9	1,144644817	1
Mob3a	1,144565479	1
Chd3os	1,144486147	1
Arih2	1,144486147	1
Mtx2	1,14440682	1
Pold3	1,14440682	1
Arl8b	1,144327498	1
Ubxn8	1,144248182	1
Tanc1	1,144248182	1
Prpf4	1,144248182	1
Gm25541	1,144168872	1
Dbr1	1,144089567	1
Lsm14a	1,144089567	1
RP23-356D13.9	1,143930973	1
Nfya	1,143930973	1
Pgs1	1,143930973	1
Zbtb2	1,143772402	1
Pgk1	1,143693124	1
Cnot6l	1,143613852	1
Mrpl4	1,143534586	1
Pno1	1,143455325	1
Fau	1,143376069	1
Mvp	1,143376069	1
Lsg1	1,143376069	1
Akt1	1,143296819	1
Ankrd13a	1,143217574	1
Ndrg3	1,143217574	1
Cacybp	1,143217574	1
Gm45495	1,143138335	1
Smurf2	1,143138335	1
Rpl29	1,142979874	1
Tmem39a	1,142979874	1
Rhoc	1,142979874	1
Steap3	1,142900651	1
Sar1b	1,142900651	1
Rps26	1,142900651	1
Gm15846	1,142742223	1
Bcl3	1,142583816	1
Rhbdf2	1,142425431	1
Sp2	1,142267068	1
Chtf8	1,142267068	1
Ccnk	1,142187895	1
Pik3r5	1,142187895	1
Ebag9	1,142108727	1
Appbp2	1,142108727	1
Prune1	1,142108727	1
Nucb1	1,142029565	1
Exosc10	1,141950408	1
Edc3	1,141871257	1
Cipc	1,141871257	1

Hsp90ab1	1,141554707	1
Rpsa-ps12	1,141475583	1
Tomm34	1,141475583	1
Usp45	1,141475583	1
Uxs1	1,141475583	1
Iqcb1	1,141396465	1
Stbd1	1,141317352	1
Gm14253	1,141080047	1
4933427D14Rik	1,141080047	1
Gm4032	1,141000956	1
4930431P19Rik	1,141000956	1
Peg13	1,141000956	1
Cpsf2	1,14092187	1
Neurl3	1,14084279	1
Cntd1	1,140763716	1
Nudt7	1,140605583	1
4932441J04Rik	1,140526525	1
Lrp6	1,140447473	1
Nsd3	1,140447473	1
5430405H02Rik	1,140368426	1
Ywhag	1,140368426	1
Tacc3	1,140289384	1
Nek7	1,140052292	1
Cpd	1,140052292	1
Dcakd	1,139973273	1
Syncrip	1,139894259	1
Srrm1	1,13981525	1
Chek2	1,139657249	1
Rplp1	1,139657249	1
H2-K2	1,139578257	1
Immp1l	1,139578257	1
Trim41	1,139262342	1
Eif2ak4	1,139104418	1
Dleu2	1,139025464	1
Gemin2	1,139025464	1
Rfwd3	1,138946515	1
Gm42819	1,138867572	1
Mthfsl	1,138867572	1
Npc1	1,138867572	1
Kdm4b	1,138788635	1
Lats1	1,138709703	1
BC022687	1,138472939	1
Myo1f	1,138236225	1
Lamtor2	1,137920683	1
Cd82	1,137841811	1
Npepps	1,137684083	1
RP24-550H10.4	1,137605228	1
Zfp30	1,137605228	1
Cacna1d	1,137447533	1
Gm5865	1,137447533	1
Cby1	1,137447533	1
Hprt	1,137447533	1

Il6st	1,13728986	1
Klc1	1,13728986	1
Fam114a2	1,13713221	1
Ercc3	1,137053392	1
Zc3h13	1,13697458	1
Hnrnpa2b1	1,13697458	1
Brd4	1,136895774	1
Rps25-ps1	1,136895774	1
Rpl36-ps4	1,136659388	1
Cx3cr1	1,136659388	1
Fam220a	1,136501824	1
Ldah	1,136423051	1
Pi4kb	1,136344283	1
Ift57	1,136344283	1
Wac	1,13626552	1
1700001G11Rik	1,135950524	1
Il16	1,135871789	1
Hist4h4	1,135871789	1
Morc2a	1,135871789	1
Mob1a	1,135871789	1
Ankle2	1,135714334	1
Kbtbd2	1,135556902	1
Klk8	1,135478194	1
Spice1	1,135478194	1
Gm5384	1,135399491	1
Tfip11	1,135399491	1
Tyw5	1,135242102	1
Ywhah	1,135242102	1
Abhd3	1,135163416	1
Elovl1	1,135163416	1
Slc38a6	1,135084735	1
Gm13743	1,13500606	1
Cxcl16	1,13500606	1
Spast	1,13492739	1
Adora2a	1,134770066	1
Fam64a	1,134770066	1
Phrf1	1,134770066	1
E4f1	1,134691413	1
Gm15964	1,134612765	1
Gamt	1,134612765	1
Dlx1	1,134612765	1
Cep170	1,134612765	1
Serpinc1	1,134455485	1
Lcmt2	1,134455485	1
Thumpd2	1,134376853	1
Zfp667	1,134298227	1
Ppp3r1	1,134298227	1
Vps35	1,134219606	1
Gar1	1,134140991	1
Cds1	1,134140991	1
Dpm1	1,134062381	1
Slc4a7	1,134062381	1

Gtf3c5	1,133983776	1
Rrn3	1,133983776	1
Cenpf	1,133747995	1
Smim7	1,133669413	1
Atp6v1h	1,133590836	1
Ppp6c	1,133590836	1
Tti2	1,133512264	1
Pot1a	1,133512264	1
Apaf1	1,133433697	1
Rpl14-ps1	1,133433697	1
Lix1l	1,133119486	1
Ppp1r12a	1,132962414	1
Zfp526	1,132883885	1
Pex26	1,132648333	1
Gm43061	1,132569827	1
Wdr83os	1,132569827	1
Ndst2	1,132569827	1
Gm29243	1,132491326	1
Farp2	1,132177376	1
Gm5257	1,132098902	1
Gtf2a1	1,132098902	1
Pfkfb2	1,131941971	1
Gm8304	1,131941971	1
Stt3b	1,131941971	1
Ints4	1,131863513	1
Ptgs2os2	1,131785061	1
Ncoa3	1,131785061	1
Asah1	1,131706615	1
Map2k7	1,131628173	1
Pip4k2a	1,131628173	1
Plk3	1,131549738	1
Lpin1	1,131392882	1
Mkln1	1,13115764	1
Gabarap	1,13115764	1
Zfp382	1,131000839	1
Gm26740	1,131000839	1
Cstf3	1,13084406	1
Vhl	1,130765679	1
Gm16062	1,130687303	1
Ift140	1,130687303	1
2310009B15Rik	1,130687303	1
Rbms1	1,130687303	1
Man1c1	1,130608932	1
Lonp2	1,130608932	1
Kpnb1	1,130530567	1
Ccdc93	1,130452207	1
Tnpo2	1,130452207	1
Nol8	1,130373853	1
Igf2r	1,130295504	1
Gm9625	1,13006049	1
Zc3hc1	1,13006049	1
Taf8	1,129903842	1

Syt11	1,129903842	1
Map4k4	1,129903842	1
Gm9392	1,129825525	1
Bod1	1,129668909	1
Rbms2	1,129668909	1
Gm26606	1,129590609	1
Eif2s1	1,129434026	1
Hnrnpab	1,129434026	1
Cd302	1,129355742	1
Ccdc50-ps	1,129042661	1
Farsa	1,129042661	1
Map3k7	1,128886154	1
Sco2	1,128807908	1
Retn	1,128807908	1
Eif4a2	1,128494979	1
Pcnx	1,128494979	1
Abcb1b	1,128494979	1
Mgam	1,128338548	1
Alkbh1	1,12826034	1
Man2b2	1,128025749	1
Jak3	1,127947563	1
Dpy19l4	1,127947563	1
Tfg	1,127869382	1
Slc44a1	1,12747856	1
1700084E18Rik	1,127400412	1
Izumo4	1,127322269	1
Lsm11	1,127244132	1
Gdpd3	1,126931637	1
Vegfa	1,126931637	1
Zfp449	1,126853527	1
Kank3	1,126775422	1
Gm16399	1,126697322	1
Ppp6r2	1,126619228	1
Gzmm	1,126463057	1
Ippk	1,126463057	1
D030056L22Rik	1,126463057	1
Nup54	1,126384979	1
Sft2d3	1,126072722	1
Oxct1	1,126072722	1
Cstf2t	1,125994671	1
Pip4k2c	1,125994671	1
Hipk2	1,125994671	1
Ghdc	1,125838586	1
Rpap1	1,125760552	1
Agk	1,125682523	1
Nde1	1,125682523	1
Clpx	1,125682523	1
Mettl25	1,125604499	1
Myl6b	1,125604499	1
C330007P06Rik	1,125448468	1
Eif2b1	1,125448468	1
Rbmx2	1,12537046	1

Fdps	1,125292458	1
Cfap20	1,125214462	1
0610012G03Rik	1,125214462	1
Itch	1,125136471	1
Pik3ip1	1,125136471	1
Whrn	1,125058485	1
Tnfaip2	1,125058485	1
Gpnmb	1,124902529	1
Rb1	1,124902529	1
Dph7	1,12482456	1
Phka1	1,12482456	1
Ptch1	1,12482456	1
H2-Q7	1,124746595	1
Cdkn1b	1,124668637	1
Abi1	1,124512735	1
Mapre3	1,124434793	1
Aggf1	1,124434793	1
Pds5a	1,124356856	1
Pqbp1	1,124356856	1
Spag5	1,124278924	1
Gm42979	1,124200997	1
Fancl	1,124200997	1
Tbpl1	1,124123076	1
Dido1	1,124045161	1
Sbk2	1,123811447	1
Rogdi	1,123811447	1
Papolg	1,123577781	1
Ctso	1,123577781	1
RP23-312A24.1	1,123499903	1
Gnpnat1	1,123499903	1
A830080D01Rik	1,123422031	1
Galnt3	1,123344164	1
Galnt10	1,123266302	1
Gm4342	1,123266302	1
Ino80b	1,123266302	1
Exoc3	1,123110595	1
1810022K09Rik	1,12303275	1
Srsf7	1,12303275	1
Orc3	1,12295491	1
Gm17786	1,122877075	1
Gm13050	1,122721422	1
Rhot1	1,122643604	1
Ikbkap	1,122410181	1
Rcn2	1,122410181	1
Aak1	1,122332384	1
Alox5ap	1,122332384	1
Gm45840	1,122254592	1
Atxn7l1	1,122254592	1
Ppid	1,122176806	1
Srsf2	1,122099026	1
Esf1	1,12202125	1
Mcf2l	1,121865716	1

Zfp335	1,121710203	1
Sgpl1	1,121710203	1
Prr11	1,121632455	1
Ciz1	1,121632455	1
Gpr183	1,121632455	1
Hk2	1,121632455	1
Ube2k	1,121632455	1
Gm5963	1,121632455	1
Ube2w	1,121321515	1
Rnf11	1,121321515	1
Nipa2	1,121166078	1
Prdx6	1,121166078	1
Greb1	1,121088367	1
Gm20223	1,121088367	1
Gpi1	1,121088367	1
Dad1	1,121088367	1
Rpsa	1,120932962	1
Tmem199	1,120932962	1
Vcpkmt	1,120855268	1
Kmt5a	1,120855268	1
Scaf8	1,120777579	1
Suclg2	1,120622217	1
Zfp706	1,120622217	1
Tram2	1,120466876	1
Ccdc159	1,120311557	1
Leo1	1,120233906	1
Ptrhd1	1,120000984	1
4933434E20Rik	1,120000984	1
Mtmr6	1,120000984	1
Parp6	1,119923354	1
Hist1h4n	1,11984573	1
E330034L11Rik	1,11984573	1
Tbca	1,11984573	1
Ccdc136	1,119768111	1
Bet1	1,119768111	1
Gm15210	1,119768111	1
St3gal1	1,119768111	1
Zfp748	1,119690497	1
Cln8	1,119690497	1
Gale	1,119612889	1
2610021A01Rik	1,119535286	1
Zbtb18	1,119457688	1
Ltc4s	1,119457688	1
Map2k1	1,119380096	1
Cdk13	1,119224928	1
Rrs1	1,119224928	1
B230369F24Rik	1,118837101	1
2010015M23Rik	1,118837101	1
Fam206a	1,118837101	1
Thyn1	1,118837101	1
Gm16433	1,118526937	1
Dip2a	1,118449409	1

Otud7b	1,118371887	1
Flnc	1,11829437	1
Ppp4r3b	1,118216858	1
Dhx37	1,118061851	1
Pink1	1,118061851	1
Opa1	1,117829381	1
Ctsd	1,117829381	1
Ulk1	1,117596959	1
Ifnar2	1,117519496	1
Gm20696	1,117442038	1
BC037039	1,117442038	1
Tubgcp4	1,117364585	1
Tmem165	1,117364585	1
Cst3	1,117364585	1
Gm43300	1,117209696	1
4833439L19Rik	1,11713226	1
Rpl27	1,117054829	1
Mad2l1bp	1,117054829	1
Baz1a	1,116822568	1
Zfyve16	1,116745158	1
Vamp8	1,116745158	1
Matr3	1,116590355	1
Clec3b	1,116512962	1
Pde2a	1,116512962	1
Hnrnpdl	1,116512962	1
Pfkfb3	1,116435574	1
Eid3	1,116358191	1
Gm13436	1,116358191	1
Epsti1	1,116203442	1
Antxr2	1,116203442	1
Neurl1a	1,115894007	1
Rmnd5b	1,115816662	1
4921524J17Rik	1,115816662	1
Aplp2	1,115661988	1
Mak16	1,115584659	1
Trmo	1,115507335	1
Cdt1	1,115507335	1
Xylt2	1,115430017	1
Trim8	1,115430017	1
Fbxl5	1,115430017	1
Cdc5l	1,115430017	1
Pwwp2a	1,115352704	1
Gm16536	1,115275396	1
Rock1	1,115198094	1
Ccdc15	1,115120797	1
Kank2	1,115043505	1
Cox6c	1,115043505	1
Cep57	1,114811662	1
Srsf3	1,114811662	1
Gm15782	1,114734392	1
RP24-282C4.13	1,114734392	1
Fam92a	1,114734392	1

Kdm7a	1,114734392	1
Gm20072	1,114579868	1
Zfp943	1,114502614	1
Aspm	1,114425365	1
Grn	1,114425365	1
Arf5	1,114425365	1
Zbtb44	1,114425365	1
Ercc2	1,114348122	1
Tra2b	1,114348122	1
Gm13641	1,114270884	1
Gm43457	1,114116423	1
Dusp12	1,114039201	1
3110001I22Rik	1,113961985	1
Zfyve27	1,113961985	1
Rpl30	1,113884774	1
Bag6	1,113653172	1
Atad2b	1,113653172	1
Stambp	1,113653172	1
Atp10d	1,113575982	1
Nr1h3	1,113267276	1
Dgkd	1,113267276	1
Hnrnpr	1,113190113	1
Acsl3	1,113190113	1
Zswim4	1,113112955	1
Agpat2	1,113112955	1
Polr2j	1,113112955	1
Aacs	1,113035803	1
Gm2991	1,113035803	1
Atp2b1	1,113035803	1
Mrps18b	1,112958655	1
Gm14439	1,112881514	1
Prkci	1,112881514	1
Gdap2	1,112804377	1
Hist1h2an	1,112650121	1
Tigd2	1,112650121	1
Gm38009	1,112418776	1
Plau	1,112418776	1
Atxn1l	1,112264572	1
Atmin	1,112264572	1
Itm2c	1,112264572	1
Rps6kc1	1,112187479	1
Rsbn1l	1,11211039	1
Rnf103	1,111725029	1
Tnrc6a	1,111725029	1
N4bp2l2	1,111725029	1
Rpl22-ps1	1,111647973	1
Igfbp4	1,111647973	1
Esyt1	1,111570922	1
Ids	1,111416836	1
1700086P04Rik	1,111339801	1
Ocrl	1,111339801	1
Atic	1,111339801	1

Dynll1	1,111262772	1
Cuta	1,111185748	1
Rprd1a	1,111108729	1
Ppp1r8	1,111108729	1
Xpo5	1,110877704	1
Gm11675	1,110800707	1
H1fx	1,110800707	1
Ripk2	1,110800707	1
Arf6	1,110800707	1
Nmnat1	1,110723714	1
Rbm28	1,110723714	1
Nsun6	1,110646728	1
Camk2g	1,110646728	1
Rrp15	1,110415799	1
Gm12341	1,110338834	1
Cited2	1,110261873	1
Apobec1	1,110184919	1
Gm5867	1,110107969	1
Dennd1a	1,110107969	1
Cdc73	1,110107969	1
Fitm2	1,109954086	1
Irak3	1,109877153	1
Tet3	1,109877153	1
Rabgef1	1,109877153	1
Psenen	1,109800225	1
Herc6	1,109338768	1
Hdac11	1,109338768	1
Gak	1,109261877	1
Gm5321	1,109108111	1
Uvrag	1,109108111	1
Cdip1	1,109031236	1
RP23-187B11.16	1,108954367	1
Mrpl1	1,108954367	1
Eif1	1,108954367	1
Gnb1	1,108954367	1
Pgap1	1,108877502	1
Il12rb1	1,108646942	1
Arl6ip5	1,108646942	1
Uchl5	1,108646942	1
Rxra	1,108570099	1
2610203C22Rik	1,108493261	1
Itpkc	1,108416429	1
Zbtb42	1,108416429	1
Ifitm2	1,108416429	1
Chd4	1,108416429	1
Kdm4c	1,108339602	1
Gm7432	1,108109153	1
Maoa	1,108032348	1
Sirpa	1,107955548	1
Wipf2	1,107955548	1
2310001H17Rik	1,107878753	1
Zfp277	1,107878753	1

Acbd3	1,107878753	1
Nabp2	1,107801963	1
Cdc42bpg	1,107801963	1
Ltn1	1,107725179	1
D1Ertd622e	1,1076484	1
Tmf1	1,107571626	1
Cept1	1,107571626	1
Rasa4	1,107494858	1
Trim21	1,107418095	1
Prdm2	1,107418095	1
Samd4b	1,107418095	1
Kif16b	1,107341337	1
Mib2	1,107341337	1
Ttyh3	1,107341337	1
Spcs3	1,107264584	1
Mapk6	1,107187837	1
Stx3	1,107187837	1
Ccdc186	1,107034359	1
Plgrkt	1,106957628	1
Scoc	1,106957628	1
Armc10	1,106957628	1
Hmgcs1	1,106880902	1
Rbbp7	1,106880902	1
Zfp874b	1,106727467	1
Snx14	1,106727467	1
Ccdc71	1,106727467	1
Taok1	1,106650757	1
Zdhhc7	1,106497353	1
Gm5093	1,106420659	1
Gfod1	1,106420659	1
Rap2b	1,106420659	1
Rps5	1,106343971	1
Bcl9l	1,106267287	1
U2af1	1,106267287	1
Bcl7a	1,106190609	1
Nbeal2	1,106113937	1
Nifk	1,106113937	1
Ankrd50	1,106037269	1
F730043M19Rik	1,105960607	1
Nop2	1,105730653	1
Braf	1,105654013	1
Cnppd1	1,105577377	1
Gm12966	1,105424122	1
Tmem173	1,105347503	1
Endov	1,105270888	1
Man2a1	1,105270888	1
Mllt6	1,105270888	1
A430005L14Rik	1,105270888	1
Rasal1	1,10519428	1
Fcer1g	1,10519428	1
Mrap	1,105117676	1
Vps26a	1,105117676	1

Ccdc59	1,105117676	1
Tmem201	1,104887897	1
Akap11	1,104887897	1
Gm9645	1,104811315	1
Ttf1	1,104811315	1
Gm36189	1,104734738	1
Snx27	1,104734738	1
Tns3	1,104734738	1
Hdac1	1,104658166	1
Tspyl4	1,1045816	1
B230398E01Rik	1,104505039	1
Spata24	1,104428483	1
Car12	1,104428483	1
Crbn	1,104351932	1
Psmd9	1,104198847	1
Rhox5	1,104045783	1
Ufl1	1,104045783	1
Polr2d	1,103892741	1
Rpl35a	1,103816227	1
Cenpi	1,103739719	1
Fam76a	1,103739719	1
Synrg	1,103663216	1
Dnajc5	1,103663216	1
Ssh3	1,103586719	1
Vbp1	1,103586719	1
Plxna1	1,103510227	1
Mad1l1	1,10343374	1
Setdb2	1,103357258	1
Exog	1,103357258	1
9330104G04Rik	1,103357258	1
Prdx3	1,103357258	1
Manf	1,103280782	1
Cenpa	1,103204311	1
Pdcd10	1,103051385	1
Pigw	1,10297493	1
Galc	1,102898481	1
Setd2	1,102898481	1
2810029C07Rik	1,102822036	1
Rbm7	1,102745597	1
Gm25517	1,102669163	1
Slc9a8	1,102669163	1
Gm43628	1,102516311	1
Dusp22	1,102516311	1
Gm43445	1,102516311	1
Smchd1	1,102516311	1
Rps26-ps1	1,102516311	1
Npc2	1,102363481	1
Egfl8	1,102287073	1
Mfge8	1,102287073	1
Commd8	1,102210671	1
Gm45501	1,102134274	1
Ric8b	1,102134274	1

Pld4	1,102057883	1
Plekhg3	1,101676005	1
Smpdl3a	1,101676005	1
Cask	1,101599645	1
Fabp3	1,101523291	1
Aco1	1,101446942	1
Cd151	1,101446942	1
Cbfb	1,101446942	1
Capn15	1,101294259	1
Nf2	1,101141598	1
Abi2	1,101065275	1
Gm5451	1,100912646	1
Rangap1	1,100912646	1
Hip1r	1,100760038	1
Fam160b1	1,100760038	1
Txndc17	1,100760038	1
Tpd52-ps	1,100531165	1
Kat2b	1,100531165	1
Sqle	1,100531165	1
Snrpa	1,100454884	1
Gm6743	1,100378609	1
Gpr179	1,100378609	1
Msl1	1,100378609	1
Prmt9	1,10030234	1
Herpud2	1,10030234	1
Tmem87b	1,100226075	1
Gm7434	1,100149816	1
Ptchd1	1,100149816	1
Tbc1d12	1,100073562	1
Des	1,099997313	1
Pdxdc1	1,099997313	1
Gm18737	1,09992107	1
Map3k4	1,09992107	1
Irf2bp2	1,09992107	1
Rnase4	1,099844832	1
Rpl31-ps1	1,099768599	1
Akap10	1,099768599	1
Crry-ps	1,099692372	1
Tlr13	1,099616149	1
Fam151b	1,099616149	1
Nup62	1,099539932	1
4833417C18Rik	1,099463721	1
Pirb	1,099387514	1
Snord72	1,099387514	1
Il2rg	1,099387514	1
Gm15198	1,099311313	1
Akip1	1,099311313	1
Gm4997	1,099311313	1
Rab29	1,099235117	1
Akt3	1,099235117	1
Apoo	1,099158927	1
Tbce	1,099158927	1

Ncbp3	1,099082742	1
C230037L18Rik	1,099006562	1
Lrch3	1,099006562	1
Vps33a	1,099006562	1
Rps6ka4	1,098930387	1
Pdcd2l	1,098930387	1
Rpl9-ps6	1,098930387	1
Mief1	1,098854218	1
Hspa14	1,098701895	1
Fam134b	1,098549593	1
Ascc3	1,09847345	1
Atg4b	1,09847345	1
Mlxip	1,098397312	1
Msh3	1,09832118	1
Arfip1	1,09832118	1
Gm10288	1,09832118	1
Papola	1,09832118	1
Zufsp	1,098016702	1
Abl2	1,098016702	1
Mcl1	1,098016702	1
Pyurf	1,097940596	1
Casp2	1,097940596	1
Fam198b	1,097864496	1
Slc25a28	1,097864496	1
Sptan1	1,09771231	1
Rpl3l	1,097484071	1
Prdm4	1,097484071	1
Parp2	1,097484071	1
Mgrn1	1,097484071	1
Med31	1,097408001	1
Fnbp4	1,097408001	1
Chchd2	1,097255879	1
Eif3e	1,096951697	1
Gm13181	1,096875665	1
Hdac6	1,096799638	1
Usp42	1,096799638	1
Ppt2	1,096799638	1
Rab28	1,096723616	1
Gatad2a	1,096723616	1
Cadm1	1,096571589	1
Hipk3	1,096419582	1
Krtcap3	1,096343587	1
Camsap2	1,096267597	1
Skil	1,096267597	1
Gls	1,096267597	1
Ctss	1,096267597	1
Rmi1	1,096115632	1
Spire1	1,096115632	1
Ptma	1,096115632	1
Zfp689	1,096039658	1
Tcp11l2	1,095963689	1
Mthfd2	1,095963689	1

Rassf7	1,095887725	1
Zrsr2	1,095811766	1
Strap	1,095811766	1
Icam4	1,095659865	1
Sec22c	1,095583922	1
Fancd2	1,095356126	1
Fbxl4	1,095280204	1
Dhx34	1,095280204	1
Ick	1,095052471	1
Foxp4	1,094900675	1
Adal	1,0947489	1
4930558J18Rik	1,0947489	1
Snrpe	1,0947489	1
Fbxo11	1,09467302	1
Aph1c	1,094521277	1
Snx3	1,094521277	1
Knstrn	1,094521277	1
Mrps36	1,094369555	1
Mycbp2	1,094217853	1
Gm43499	1,09414201	1
Mia2	1,094066173	1
Khdrbs1	1,093838692	1
Tmed10	1,093838692	1
Rcor3	1,093687064	1
Cyb561d1	1,093459662	1
Slmap	1,093459662	1
Stip1	1,093383872	1
Fundc1	1,093383872	1
Mvd	1,093232307	1
Rbm34	1,093156533	1
Fkbp1a	1,093080763	1
Arf1	1,093004999	1
Tmem38a	1,092929241	1
Pi16	1,092929241	1
Gm24876	1,092853487	1
Mycn	1,092777739	1
Eif4a1	1,092777739	1
Cdca8	1,092701996	1
Tubgcp2	1,092171942	1
Kif5b	1,092171942	1
Rab2b	1,092096241	1
Gm6088	1,091944855	1
Snapc2	1,091944855	1
RP23-65M10.2	1,09186917	1
Gm43581	1,09186917	1
Gm12430	1,09186917	1
Rbbp5	1,09186917	1
Spopl	1,09179349	1
Brd9	1,09179349	1
Slc9a1	1,091490823	1
Gbe1	1,091188239	1
Wsb1	1,091188239	1

Gpbp1	1,091188239	1
Zfp511	1,091112606	1
Rnf7	1,091112606	1
Tbc1d15	1,091112606	1
Eif4enif1	1,091036979	1
Rbfox1	1,090885739	1
Psma3	1,090810128	1
Tpgs2	1,090810128	1
Slc39a10	1,090583324	1
Ranbp6	1,090507733	1
Rfx7	1,090507733	1
Smim13	1,090432147	1
Myo5a	1,090432147	1
Znhit3	1,090356567	1
Mki67	1,090356567	1
Rap2a	1,090356567	1
Rbm22	1,090129857	1
Gm9703	1,090054298	1
Gramd4	1,090054298	1
Gnpat	1,090054298	1
Cav2	1,089978743	1
Ppp2r5b	1,089903194	1
Mterf4	1,089903194	1
Csnk1a1	1,089903194	1
Baiap2	1,089827651	1
Abhd17a	1,089752112	1
Arpc1a	1,089676579	1
Zbtb7b	1,089676579	1
Gm24951	1,089601051	1
Hic2	1,089525528	1
Faap100	1,089298991	1
Ppp2r3d	1,088997015	1
Pmpca	1,088921534	1
Rpl10-ps2	1,088770588	1
Gm6290	1,088770588	1
Mapkapk2	1,088770588	1
Atp9b	1,088544208	1
Cmc1	1,088468759	1
Golga1	1,088393315	1
Ldlrap1	1,088317876	1
Sarnp	1,088242442	1
Ppp2ca	1,088242442	1
Numa1	1,088167013	1
Serp1	1,088167013	1
Ercc6	1,088016172	1
Taf1a	1,087789948	1
Yif1b	1,087789948	1
Hnrnph3	1,087714551	1
Ddi2	1,087639159	1
Otud3	1,087563772	1
Ewsr1	1,087413015	1
Mettl13	1,087262278	1

Psmd5	1,087111561	1
Arpp19	1,087036211	1
Gm45422	1,086885526	1
Gm13803	1,086885526	1
Calm2	1,086734863	1
Fbxo46	1,086659538	1
Phkg1	1,08658422	1
Arhgef18	1,086508906	1
Pik3cg	1,086508906	1
Igf2bp2	1,086433597	1
Ggcx	1,086358294	1
B930036N10Rik	1,086132416	1
RP24-547N4.7	1,086132416	1
Frg1	1,086132416	1
Borcs6	1,086057133	1
Nepro	1,085981856	1
Mob4	1,085981856	1
Pafah1b1-ps1	1,085906584	1
Ogfrl1	1,085756056	1
Cndp2	1,085756056	1
Rp9	1,085756056	1
Ccne1	1,085605549	1
Nptn	1,085605549	1
Srpr	1,085530303	1
Rps6kb2	1,085455062	1
Lrrc73	1,085379827	1
Trim35	1,085379827	1
Glrp1	1,085229372	1
Parvb	1,085229372	1
Aasdhppt	1,085154152	1
Snhg9	1,085154152	1
Ttc21b	1,085078938	1
Gm45568	1,085078938	1
Slc7a1	1,085003728	1
6330408A02Rik	1,084928524	1
Ahdc1	1,084928524	1
Tnfrsf10b	1,084928524	1
Gm9531	1,084928524	1
Rab3gap1	1,084778132	1
Clk3	1,084778132	1
Zfyve28	1,084552582	1
Gm43062	1,084552582	1
Csgalnact2	1,084552582	1
Pura	1,084552582	1
Tmem25	1,084477409	1
Ptpn9	1,084402241	1
Vps33b	1,084251922	1
2610001J05Rik	1,084026481	1
Gm4204	1,083951345	1
Gm12497	1,083876214	1
Lmo2	1,083876214	1
4930581F22Rik	1,083801088	1

Spcs1	1,083801088	1
Eef1a1	1,083725967	1
Ahcyl1	1,083650851	1
Thoc1	1,083575741	1
Tmem123	1,083575741	1
Mrpl9	1,083350441	1
Gm16199	1,083275352	1
Taco1os	1,083200267	1
Ankrd26	1,083125188	1
Polb	1,083125188	1
Adrm1	1,083050114	1
Vcp-rs	1,082975046	1
Luc7l	1,082749871	1
Atxn10	1,082524743	1
L3mbtl2	1,08244971	1
Cflar	1,08244971	1
Rras2	1,082374683	1
Mrps16	1,082224645	1
Tmem134	1,082149633	1
Plekhg2	1,082074627	1
Runx2	1,081999626	1
Rab9	1,08192463	1
Erp44	1,08192463	1
Snupn	1,081849639	1
Calr	1,081849639	1
Gpalpp1	1,081774654	1
Syne3	1,081774654	1
Hibadh	1,081774654	1
R3hdm1	1,081774654	1
Ctsb	1,081774654	1
Hilpda	1,081699673	1
Ncor2	1,081549728	1
Bbip1	1,081549728	1
Arid1b	1,081549728	1
Pcx	1,081474763	1
RP23-359K10.8	1,081474763	1
Dlgap4	1,081474763	1
Med19	1,081399804	1
Tusc3	1,081399804	1
Snhg11	1,08132485	1
Tm6sf1	1,08132485	1
Sptbn1	1,081249901	1
Slc12a6	1,081174957	1
Gm43290	1,081100018	1
Ube2e1	1,081100018	1
Itm2b	1,081100018	1
Rpsa-ps11	1,081025084	1
Cdc20	1,080950156	1
Ppp2r2a	1,080950156	1
Rita1	1,080875233	1
Gatsl2	1,080875233	1
Taf1	1,080875233	1

Zfp207	1,080725402	1
Fuom	1,080650494	1
Milr1	1,080650494	1
Mrps24	1,080650494	1
Unc45a	1,080575592	1
Mtmr3	1,080575592	1
Mturn	1,080500695	1
Plekhb2	1,080500695	1
Aff1	1,080350916	1
Grina	1,080350916	1
Zfp280d	1,080276034	1
Thap12	1,080276034	1
F7	1,080126287	1
Cyth2	1,080126287	1
Oas3	1,080051421	1
Slc25a17	1,080051421	1
Rnf111	1,07997656	1
Tmem143	1,079901704	1
Uhmk1	1,079826854	1
Cox14	1,079826854	1
Get4	1,079752008	1
Aplf	1,079677168	1
Ubl5	1,079677168	1
Prelid3b	1,079602333	1
Akap8	1,079527504	1
Gm7027	1,07937786	1
Snapin	1,079303045	1
2010016I18Rik	1,079003841	1
Pgam1	1,079003841	1
Ppip5k2	1,079003841	1
Aph1a	1,079003841	1
Gm15690	1,078929052	1
Zeb2	1,078854269	1
Simc1	1,078704719	1
Gm8662	1,078629951	1
Hmgb3	1,078555189	1
Gtf2e2	1,078480432	1
Gm13398	1,07840568	1
Orc5	1,07840568	1
Mpp6	1,078181455	1
Slfn2	1,078106724	1
Gm20568	1,078031998	1
Irak2	1,077957277	1
Heca	1,077957277	1
Wrnip1	1,077733145	1
Rpap3	1,077733145	1
Hps4	1,077733145	1
Lrrfip1	1,077658445	1
Cul7	1,07750906	1
Asl	1,07750906	1
Zdhhc14	1,07750906	1
Psmc4	1,077434375	1

Wdr33	1,077359696	1
Gm6382	1,077210353	1
Cdkn2d	1,077210353	1
Camkmt	1,077135689	1
Nlrp3	1,077135689	1
Naxd	1,077135689	1
RP24-378K7.3	1,07706103	1
Ggps1	1,07706103	1
Gm527	1,076986376	1
Gm10086	1,076986376	1
Rasip1	1,076911728	1
Matk	1,076911728	1
Hgh1	1,076911728	1
C2cd2l	1,076911728	1
Rplp0	1,076911728	1
Gpd1l	1,076837085	1
U2surp	1,076837085	1
Cage1	1,076613186	1
Dnm1l	1,076613186	1
Map2k3	1,076389334	1
C1galt1	1,076314727	1
Rfc5	1,076314727	1
Dna2	1,076240125	1
Crk	1,076240125	1
Zfp592	1,076165528	1
Gm37702	1,07601635	1
Ccser2	1,07601635	1
E2f6	1,07601635	1
Nol9	1,07601635	1
Ncor1	1,075941769	1
Eef2	1,075867193	1
Eif1-ps1	1,075792622	1
Rfxap	1,075792622	1
Ublcp1	1,075718056	1
Gm16379	1,075718056	1
Rnf114	1,075568941	1
4921511C10Rik	1,07549439	1
Gpr85	1,075419845	1
Snapc4	1,075419845	1
Ostm1	1,075419845	1
Mien1	1,075419845	1
Cnot8	1,075270771	1
Nckap1l	1,075270771	1
Abcd1	1,075196241	1
Zbed4	1,075121717	1
Pole4	1,075121717	1
Zfp598	1,075121717	1
Tmcc3	1,075047198	1
Top2b	1,075047198	1
Spcs2	1,074972684	1
Gm7733	1,074898175	1
Rps27a	1,074898175	1

4933421O10Rik	1,074898175	1
Ctdsp2	1,074898175	1
Saa3	1,074823671	1
Ifngr1	1,074823671	1
Rrnad1	1,074749173	1
Gm20707	1,07467468	1
Bloc1s4	1,07430229	1
Ubl3	1,07430229	1
Caskin2	1,074227828	1
Tnpo3	1,074227828	1
Msmo1	1,074227828	1
Gm996	1,074078919	1
Tmco1	1,074004472	1
Gm12231	1,07393003	1
Gm7783	1,073855593	1
Wdr76	1,073706736	1
Eif2d	1,073706736	1
Mboat7	1,073632315	1
Gm4459	1,073557899	1
Hps5	1,073409082	1
Dym	1,073409082	1
Stxbp1	1,073409082	1
Papd7	1,073334682	1
Zfr2	1,073185896	1
Cep250	1,073185896	1
Vps50	1,073185896	1
Ubxn7	1,073111511	1
Furin	1,073037131	1
Stk40	1,072962757	1
Gipc2	1,072665309	1
1500011K16Rik	1,072665309	1
Havcr2	1,072665309	1
Whamm	1,072590961	1
Fam126b	1,072590961	1
Mrpl35	1,072516617	1
Nsmaf	1,072442278	1
Msh6	1,072442278	1
RP23-138K22.2	1,071996355	1
Gm44027	1,071996355	1
Slc35e1	1,071996355	1
Ppp4r3a	1,071996355	1
B4galt1	1,071699175	1
Naa50	1,071624893	1
Bcl2	1,071476345	1
Atp1a1	1,071476345	1
Tpm3	1,071402079	1
Git1	1,071402079	1
Elob	1,071327817	1
Gm42551	1,071253561	1
Tgfbrap1	1,071105064	1
Rpa1	1,071030823	1
Alg13	1,070956588	1

Dhx35	1,070882357	1
Rab21	1,070808132	1
Rps12-ps24	1,070659697	1
Herpud1	1,070659697	1
Gm3608	1,070585487	1
Etfa	1,070511282	1
Sgms1	1,070437082	1
Ola1	1,070437082	1
Chd6	1,070362888	1
Cmtr1	1,070140335	1
Cpped1	1,069917829	1
Pigm	1,069695369	1
Map4k5	1,069547088	1
Rap1gds1	1,069472955	1
Gm6563	1,069398827	1
Abce1	1,069398827	1
Fabp5	1,069324705	1
Gm38111	1,069250588	1
Serinc3	1,069250588	1
Zbtb8a	1,069102368	1
5430402O13Rik	1,069102368	1
Akirin1	1,069102368	1
Ankrd17	1,069102368	1
Crnde	1,069028266	1
Atp5f1	1,068954169	1
Oxa1l	1,068805991	1
Rhobtb2	1,068583762	1
Wdr19	1,068509696	1
Gm44545	1,068435635	1
Plekha2	1,068435635	1
Fto	1,06836158	1
Nup50	1,06836158	1
Pigf	1,06836158	1
Tlk1	1,068139443	1
Bhlhe40	1,068139443	1
Zdhhc3	1,068065408	1
Vmp1	1,068065408	1
Dbt	1,067991378	1
Hsd17b12	1,067843333	1
Ier3ip1	1,067695309	1
Dcaf4	1,067621304	1
Stam2	1,067621304	1
Ube3c	1,067621304	1
Xrn1	1,067547305	1
Mettl14	1,067547305	1
RP23-356P21.1	1,067473311	1
Mat2b	1,067473311	1
Prex1	1,067399322	1
Msn	1,067399322	1
Daxx	1,067399322	1
9130019O22Rik	1,067103417	1
Ube2o	1,067029454	1

Lyn	1,067029454	1
Ip6k1	1,067029454	1
Tulp3	1,066881542	1
6430710M23Rik	1,066659713	1
Ube3a	1,066659713	1
Mrps10	1,066511853	1
Fbxo38	1,066511853	1
Zik1	1,066364014	1
Rps6kb1	1,066364014	1
9530082P21Rik	1,066290101	1
Fbxw8	1,066290101	1
Pdp1	1,066216194	1
Gins2	1,066142293	1
Sdccag3	1,066142293	1
Ift52	1,066142293	1
Yaf2	1,065994504	1
Cebpa	1,065920617	1
Snrnp40	1,065920617	1
Psmd8	1,065920617	1
C1qbp	1,06577286	1
Zfp91	1,06577286	1
Eif4e2	1,06577286	1
Ciao1	1,065698989	1
Aatk	1,065625123	1
Spg20	1,065625123	1
Eif2a	1,065625123	1
Ctps2	1,065551262	1
Smug1	1,065551262	1
Rbm5	1,065551262	1
Rexo1	1,065477406	1
Gpam	1,065403555	1
Exo5	1,065403555	1
Pigs	1,065403555	1
Car6	1,065182034	1
Adat2	1,065182034	1
Tefm	1,065108203	1
Prdx4	1,065108203	1
Acp1	1,065034378	1
Gldc	1,065034378	1
Gm16754	1,064886743	1
Cpeb3	1,064886743	1
Tmem94	1,064812934	1
Ythdf2	1,064812934	1
D8Ertd738e	1,064812934	1
Agfg1	1,064812934	1
Gm17994	1,064739129	1
Timp1	1,064665329	1
Rock2	1,064591535	1
Dock9	1,064370182	1
Itpr1	1,064370182	1
Gm37339	1,064296408	1
Tmem41a	1,064296408	1

Arpc2	1,064296408	1
Eef1e1	1,06422264	1
Nkiras2	1,06422264	1
Zfp7	1,064148876	1
Gm11346	1,064148876	1
Gm6433	1,064148876	1
Man2b1	1,064001364	1
Yars	1,064001364	1
Setd7	1,063927615	1
Casc3	1,063632673	1
Gm8330	1,06355895	1
Cox7a2l	1,06341152	1
Gm13573	1,06326411	1
Pwp2	1,06326411	1
Lsm8	1,06326411	1
Gm37785	1,063190412	1
Rab11a	1,063190412	1
Galnt4	1,06311672	1
Tmem128	1,06311672	1
Clcn6	1,06311672	1
Stag2	1,06311672	1
Sbno1	1,063043033	1
Mpp5	1,062895674	1
Ndufs1	1,062822003	1
Ttc7b	1,062748336	1
Gm8805	1,062674674	1
Sun2	1,062601018	1
Dst	1,062527367	1
Scfd2	1,06245372	1
Gm37949	1,06245372	1
Fmnl1	1,06245372	1
Terf2	1,06245372	1
Plk4	1,062380079	1
Dnajc18	1,062306443	1
Star	1,062085566	1
Ppme1	1,062085566	1
Pcdh7	1,06201195	1
Pmpcb	1,061791134	1
Atp13a1	1,061643949	1
Camk2d	1,061496784	1
Rrh	1,061423209	1
Slc12a9	1,061423209	1
Lyar	1,061423209	1
Capza2	1,061423209	1
Cdyl	1,061276075	1
Acsl1	1,061202515	1
Itpripl1	1,061055411	1
Klhl6	1,061055411	1
Srpk1	1,061055411	1
Gm15798	1,060981867	1
Spryd4	1,060981867	1
Kif21a	1,060981867	1

Vps51	1,060908328	1
Zfp788	1,060687741	1
Snap23	1,060687741	1
Isl2	1,060540709	1
Ehmt2	1,0604672	1
Cs	1,0604672	1
Elovl5	1,060393697	1
Lmtk3	1,060320199	1
Cstb	1,060320199	1
Gm8121	1,060099734	1
Xxylt1	1,060026256	1
Zfp275	1,059952783	1
Pih1d2	1,059879316	1
Mmgt1	1,059879316	1
Twistnb	1,059879316	1
Ubap1	1,059805853	1
Dnaja2	1,059658943	1
Zfp654	1,059585495	1
Cebpd	1,059512053	1
Mgme1	1,059512053	1
Rps27	1,059291757	1
Mtmr2	1,059291757	1
Psmg4	1,059218335	1
Tmem135	1,059218335	1
Ppp2r5a	1,059071506	1
Hdlbp	1,058998099	1
Cnih4	1,058924698	1
Gm42635	1,058851301	1
Tnfaip1	1,058851301	1
Emc7	1,058704523	1
Rtcb	1,058631142	1
Gm11221	1,058557766	1
Ppp1r15b	1,058557766	1
Pan2	1,058484395	1
Capns1	1,058484395	1
Nufip2	1,058484395	1
Gm12655	1,058411029	1
2700049A03Rik	1,058411029	1
Snhg20	1,058411029	1
Cenpw	1,058337668	1
RP23-88C11.5	1,058264312	1
Psmd4	1,058264312	1
Runx2os1	1,058117616	1
9230112E08Rik	1,058117616	1
Tmem129	1,058117616	1
Gm37357	1,058044275	1
2610002M06Rik	1,05797094	1
Klhl25	1,05797094	1
Nfxl1	1,05797094	1
Gm45718	1,057824284	1
Chka	1,057824284	1
Anxa5	1,057750964	1

Zfp420	1,057677648	1
Cct4	1,057677648	1
Scrn3	1,057604338	1
Pofut2	1,057604338	1
Mfng	1,057604338	1
Zfp628	1,057384439	1
Gm28731	1,057311149	1
Slc2a4	1,057164585	1
Sppl2a	1,057164585	1
Sec23ip	1,05709131	1
Slc33a1	1,057018041	1
Glg1	1,057018041	1
Srgn	1,056798263	1
Pxk	1,056651769	1
Ndufa1	1,056578531	1
Car5b	1,056578531	1
Chmp7	1,056578531	1
Cyb5r3	1,056578531	1
Gfm1	1,056505297	1
St7	1,056432068	1
Cd68	1,056432068	1
Gm8444	1,056358844	1
Tmem38b	1,056358844	1
Cbll1	1,056358844	1
Klf8	1,056139203	1
Klrg2	1,056139203	1
D130051D11Rik	1,056139203	1
Nrde2	1,056066	1
Angel2	1,055919608	1
Clcn2	1,05584642	1
Fbxo2	1,05584642	1
Cyp51	1,05584642	1
Gm42798	1,055700059	1
Sh2b2	1,055700059	1
Yipf6	1,055626886	1
Sass6	1,055553718	1
RP23-371B13.3	1,055480555	1
Mpzl1	1,055480555	1
Zmym3	1,055480555	1
Rabgap1	1,055407397	1
Alkbh8	1,055407397	1
Zfp551	1,055334244	1
5730480H06Rik	1,055187954	1
Cpox	1,055114816	1
Strn3	1,055114816	1
Lin7c	1,055041684	1
Tshz3	1,054968557	1
Ints9	1,054895434	1
Gm5619	1,054749205	1
Anapc2	1,054749205	1
Ctsz	1,054749205	1
Homer1	1,054676098	1

Trp53bp1	1,054676098	1
Ahcyl2	1,054602996	1
Sowahc	1,054602996	1
Pnn	1,054602996	1
Nap1l1	1,054602996	1
Ahcy	1,054529899	1
Gm11516	1,054529899	1
Ep300	1,054529899	1
Prkcsh	1,054529899	1
Iws1	1,054456807	1
Xrcc4	1,05438372	1
Mex3d	1,054237562	1
4632427E13Rik	1,054237562	1
Psmd12	1,054237562	1
Pes1	1,054237562	1
Tarbp1	1,054091423	1
Tecr	1,054091423	1
Hdgf	1,054091423	1
Mbtps1	1,054018362	1
Ccnt1	1,054018362	1
Nupl2	1,053872254	1
Mcoln1	1,053799208	1
Bin1	1,053799208	1
Ghitm	1,05365313	1
Trappc9	1,053580099	1
Naf1	1,053580099	1
Rnf113a1	1,053580099	1
Gm17491	1,053507073	1
Plekho1	1,053507073	1
Kif1c	1,053361036	1
Myo9a	1,053288025	1
Nit2	1,053288025	1
Tmem167b	1,053288025	1
Nup43	1,053215019	1
Gm37303	1,053142018	1
Abhd6	1,053069023	1
Tsc1	1,052996032	1
Ap3s1	1,052923046	1
Cfl2	1,052923046	1
Tapt1	1,05277709	1
Mcmbp	1,05270412	1
Tbcc	1,052631155	1
Gm27043	1,052558194	1
Chd9	1,052485239	1
Relt	1,052412289	1
Srm	1,052266404	1
Txlna	1,052266404	1
Rsf1	1,052193469	1
Usf2	1,052120539	1
Psmd14	1,052120539	1
Dhx33	1,052047614	1
Mfsd5	1,052047614	1

Ybx3	1,052047614	1
Phf5a	1,052047614	1
Bloc1s2	1,051901779	1
Gm6394	1,051755965	1
Mrpl23-ps1	1,051755965	1
Cggbp1	1,051755965	1
Fer	1,051683065	1
2310043L19Rik	1,051683065	1
Rhof	1,051683065	1
Lrrc42	1,051683065	1
Alkbh6	1,051610171	1
Zfp219	1,051610171	1
Gm6548	1,051464397	1
Uhrf1bp1	1,051391517	1
Hsd17b7	1,051391517	1
Pus3	1,051172909	1
Grb2	1,051027196	1
Arid5b	1,050954347	1
E130317F20Rik	1,050881502	1
Ccdc47	1,050808663	1
Gm45109	1,050663001	1
Men1	1,050590177	1
Ccnb1	1,050444544	1
Dars	1,050371735	1
Fndc3b	1,050371735	1
Slfn3	1,050298932	1
Hcn2	1,050298932	1
Egln1	1,050298932	1
Glmn	1,050153339	1
Rbmx2-ps	1,049934989	1
Shmt2	1,049862215	1
Naa25	1,049862215	1
Ppp1r35	1,049789447	1
Nacc1	1,049789447	1
Acot10	1,049571172	1
Tmem115	1,049571172	1
Itpr3	1,049571172	1
Lcp2	1,049571172	1
Rab7	1,049571172	1
Fkbp1b	1,049498424	1
Gdpd5	1,049425681	1
Kdsr	1,049425681	1
Ap2a2	1,049280209	1
Isg20	1,04906204	1
Irf2bpl	1,04906204	1
Zfat	1,048989328	1
Map3k9	1,04891662	1
Sec61b	1,048843917	1
9930021J03Rik	1,048698526	1
Casp8ap2	1,048625839	1
Wipi2	1,048480478	1
Sf3b6	1,048480478	1

Nmd3	1,048335138	1
Gm10425	1,048262476	1
Srsf5	1,048189818	1
Pcbp2	1,048189818	1
Spata6	1,048117166	1
Nup188	1,048117166	1
Golph3l	1,048117166	1
Ing1	1,047971876	1
Calcoco1	1,047899238	1
Ctnnbip1	1,047681357	1
Gm15730	1,047608739	1
Tcaim	1,047608739	1
Psmd11	1,047608739	1
Lipa	1,047536127	1
Rab5a	1,04746352	1
Ints13	1,04746352	1
Trappc2l	1,047390918	1
Tmem230	1,047318321	1
D10Wsu102e	1,047318321	1
Ei24	1,047245729	1
Usp47	1,047245729	1
Tubgcp3	1,04710056	1
Rpa2	1,04710056	1
Smc3	1,047027983	1
Polr3g	1,046955411	1
Zfp64	1,046955411	1
Gm43513	1,046810282	1
Med7	1,046810282	1
Ube2a	1,046810282	1
Klf9	1,046810282	1
Itgb5	1,046737725	1
Il6ra	1,046737725	1
Gpn3	1,046665173	1
Tiprl	1,046592627	1
Extl2	1,046447548	1
Smu1	1,04630249	1
Sf3a1	1,046157451	1
Gmfg	1,046012433	1
Trappc3	1,045939932	1
Rnf181	1,045794944	1
Ahsa1	1,045649976	1
Rbm33	1,045649976	1
Iqgap1	1,045649976	1
9330160F10Rik	1,045505028	1
Tox4	1,045505028	1
Trim65	1,045432562	1
B3galnt2	1,045432562	1
Psen1	1,045432562	1
Zfx	1,0453601	1
Ctsa	1,045215193	1
Slc19a1	1,045142746	1
Baz2a	1,044997869	1

Pot1b	1,044853011	1
Nudcd2	1,04478059	1
Atg16l2	1,044635763	1
Tcte2	1,044563357	1
Guca1a	1,044563357	1
Slc29a1	1,044490956	1
Arsa	1,044346168	1
Drg2	1,044273782	1
Smc5	1,044201401	1
Hipk1	1,044201401	1
Hsf2	1,044129025	1
C920021L13Rik	1,044056654	1
Saraf	1,044056654	1
Zeb2os	1,043984288	1
Klhl24	1,043984288	1
Rubcn	1,043984288	1
Dnaaf5	1,043984288	1
Vps4b	1,043984288	1
Mrpl48-ps	1,043911927	1
Tbcel	1,043911927	1
Paox	1,043839571	1
Oraov1	1,043767221	1
Lrch1	1,043767221	1
Eif4e3	1,043767221	1
D930015E06Rik	1,043694875	1
Dnajc15	1,043622534	1
1600020E01Rik	1,043550198	1
Alpk1	1,043405541	1
Slc6a9	1,04333322	1
Nyap1	1,043260904	1
Inafm1	1,043260904	1
Stk3	1,043116288	1
Zzz3	1,043116288	1
Agps	1,043116288	1
Gm44152	1,043043987	1
Diaph1	1,043043987	1
Ttc32	1,0428994	1
Gm14857	1,0428994	1
Tsta3	1,0428994	1
Asph	1,0428994	1
1110051M20Rik	1,042754834	1
Tmem242	1,042754834	1
Dgkh	1,042538022	1
Armc7	1,042538022	1
Imp3	1,042538022	1
Rilpl1	1,042465761	1
Hoxaas3	1,042321254	1
Ezh1	1,042321254	1
Tpd52	1,042249009	1
Tmem238	1,042176768	1
Nasp	1,042176768	1
Mrps17	1,042176768	1

Jpx	1,042104532	1
Car9	1,042032302	1
Kras	1,042032302	1
Vdac2	1,042032302	1
Nadk	1,041960076	1
Psme4	1,041960076	1
Psmb3	1,041887855	1
Paxbp1	1,04181564	1
Tbc1d9b	1,041671223	1
Caprin1	1,041526827	1
Tatdn3	1,041382451	1
Tnip1	1,04131027	1
Snhg8	1,041165924	1
Ywhaq	1,041093758	1
Fam57a	1,040949442	1
Btbd3	1,040805146	1
Def8	1,040805146	1
Slc35a3	1,040805146	1
Rpph1	1,04066087	1
Luc7l2	1,04066087	1
Snrnp70	1,040588739	1
Mrpl30	1,040588739	1
Pdp2	1,040444493	1
Cln3	1,040372377	1
Cops3	1,040372377	1
Leng1	1,040300267	1
Cpsf6	1,040300267	1
Adk	1,040228161	1
Ubn1	1,04015606	1
Tspoap1	1,040083965	1
Gm44791	1,040083965	1
Mrpl47	1,040011874	1
Hnrnpc	1,039795632	1
Ptk2	1,039723561	1
Abhd8	1,039579435	1
Serf2	1,039579435	1
Scmh1	1,039579435	1
Txn-ps1	1,03950738	1
Kmt2c	1,03950738	1
Shc4	1,039435329	1
Raph1	1,039363283	1
Gtpbp4	1,039219207	1
4931406C07Rik	1,039147176	1
Unc119	1,039147176	1
Arpc4	1,03907515	1
2310057M21Rik	1,03900313	1
Vta1	1,03900313	1
Dap3	1,03900313	1
Tpgs1	1,038931114	1
Pogz	1,038859103	1
Gm20900	1,038787098	1
Bcl9	1,038787098	1

Bag5	1,038787098	1
B230312C02Rik	1,038715097	1
Tapbpl	1,038643101	1
Cotl1	1,03857111	1
Unc119b	1,038499125	1
Rps16-ps2	1,038427144	1
Brd1	1,038427144	1
Ago3	1,038355168	1
Fez2	1,038355168	1
Mta2	1,038355168	1
Tcf19	1,038283197	1
Xdh	1,038211231	1
Abca3	1,038211231	1
Oasl1	1,038067315	1
Narf	1,037851477	1
F11r	1,03770761	1
Cdadc1	1,037491848	1
Zswim1	1,037419937	1
Snx6	1,037419937	1
Gcfc2	1,037204234	1
Morf4l2	1,037204234	1
Smap2	1,037060457	1
Gm16630	1,036988575	1
Trip11	1,036988575	1
Scamp2	1,036916699	1
Firre	1,036844828	1
Fkbp5	1,036772962	1
Fam188a	1,036701101	1
Zfp707	1,036629245	1
Snrk	1,036485547	1
Gng2	1,036485547	1
Srek1	1,036485547	1
Cdk11b	1,036485547	1
Adam8	1,036413706	1
Gm9294	1,03634187	1
Gm6654	1,03634187	1
Poc5	1,036198213	1
Lcor	1,036198213	1
Stau1	1,036126391	1
Kif13b	1,036054575	1
Mfsd4b4	1,035910957	1
Rsbn1	1,035839156	1
Fads3	1,035623782	1
Gtdc1	1,035552	1
Rac2	1,035552	1
Cxx1b	1,035480224	1
Gm8522	1,035408452	1
RP23-134M7.3	1,035336685	1
Akna	1,035336685	1
Ikzf1	1,035336685	1
Fam129b	1,035336685	1
H2-K1	1,035336685	1

Trim39	1,035193167	1
Zfyve21	1,035193167	1
Pdzd8	1,035193167	1
Supt3	1,035193167	1
Gm13391	1,035121416	1
Gm11224	1,035049669	1
Fundc2	1,035049669	1
Set	1,034977927	1
Ptp4a3	1,034906191	1
Ddx52	1,034906191	1
Decr2	1,034834459	1
Dpep2	1,034762732	1
Zfp236	1,034619293	1
Lrp12	1,034619293	1
Fubp1	1,034619293	1
Kcnk6	1,034547582	1
BC005624	1,034475875	1
Neurl4	1,034404173	1
Fam107b	1,034332476	1
Nup133	1,034260784	1
Dolpp1	1,034260784	1
Gm28809	1,034189097	1
Klhdc10	1,034189097	1
Anks3	1,033974066	1
Rabl3	1,033902398	1
Tgif1	1,033687427	1
Zfp668	1,03361578	1
Slc30a1	1,03361578	1
Gm10073	1,033544137	1
Ubap2	1,0334725	1
Glyr1	1,0334725	1
4930518I15Rik	1,033400868	1
Dnajc13	1,03332924	1
Rps21	1,033257618	1
Slc30a7	1,033186	1
Cd33	1,033114388	1
Fem1b	1,03304278	1
Gm17690	1,032971178	1
Ccnf	1,032971178	1
Sh3pxd2b	1,03289958	1
Gm14539	1,032827987	1
Mocs1	1,0327564	1
Mtrr	1,032684817	1
Gm4742	1,032613239	1
RP23-403E19.1	1,032613239	1
Mfsd13a	1,032541666	1
Tmem59	1,032541666	1
Larp7	1,032470098	1
Csnk1g1	1,032470098	1
Gmcl1	1,032398535	1
Gm12726	1,032326978	1
2410131K14Rik	1,032326978	1

Batf2	1,032255425	1
Surf6	1,032255425	1
Man2c1	1,032112334	1
Mllt11	1,032112334	1
Nos1	1,032040795	1
Plekha8	1,032040795	1
Pld3	1,032040795	1
Taf3	1,031969262	1
Rap1b	1,031969262	1
Tnfrsf11a	1,031897734	1
Cyld	1,031897734	1
Mia3	1,031754693	1
Brpf3	1,031683179	1
Sptlc2	1,031683179	1
Mms22l	1,031540168	1
Vegfb	1,031468669	1
Brd7	1,031397176	1
Zfp746	1,031182725	1
Ctps	1,031182725	1
Gm45477	1,031111251	1
Slc10a7	1,031111251	1
Nlrx1	1,031039783	1
Cltb	1,031039783	1
Syvn1	1,030968319	1
Vps13a	1,030968319	1
Wdr75	1,030825406	1
Nek1	1,030682513	1
Hspa4l	1,030682513	1
Commd4	1,030682513	1
Malat1	1,030611074	1
Rnf220	1,03053964	1
Rpl27a-ps1	1,030468211	1
Gtf2ird2	1,030396787	1
Myd88	1,030396787	1
Chst1	1,030325368	1
Arl4a	1,030253954	1
Kdm2a	1,030253954	1
Ccnl2	1,03011114	1
Gm37968	1,029825572	1
Zfp317	1,029754193	1
Gm37065	1,029611448	1
Zbed5	1,029611448	1
E2f2	1,029611448	1
Dpy19l3	1,029540083	1
Fgr	1,029540083	1
Zc3h10	1,029540083	1
Adipor2	1,029540083	1
Bmpr2	1,029468724	1
Clcn3	1,029397369	1
Acta2	1,029326019	1
Fam178a	1,029254674	1
Wiz	1,029183334	1

Zfpl1	1,029183334	1
Brix1	1,029183334	1
Cic	1,029183334	1
Cog1	1,029040669	1
Plxnb2	1,028969343	1
Uba1	1,028755398	1
Kremen1	1,028541496	1
Dclre1b	1,028470206	1
Lsm4	1,028470206	1
Tmem97	1,02839892	1
Btbd9	1,028327639	1
Gfm2	1,028256363	1
Cox5a	1,028113827	1
Kmt2d	1,028042566	1
Med4	1,027828812	1
Gm43420	1,027757571	1
Slco4a1	1,027757571	1
B230217C12Rik	1,027686335	1
Gm43560	1,027615104	1
Tsc22d1	1,027543877	1
Rbm12	1,027543877	1
Gm12501	1,027472656	1
Epc2	1,027472656	1
Gm37653	1,027401439	1
Snhg3	1,027401439	1
Rin2	1,027401439	1
Prpf6	1,027401439	1
Gm5586	1,027330228	1
Qpctl	1,027330228	1
Mrps30	1,027259021	1
Rapgef6	1,027259021	1
Cdca3	1,027187819	1
Zfp597	1,027116623	1
Ythdc1	1,027116623	1
M1ap	1,027045431	1
Glrx	1,027045431	1
Map3k14	1,026974244	1
Fbxo3	1,026974244	1
Rnf141	1,026903062	1
Acp2	1,026760713	1
Igip	1,026689546	1
Higd1a	1,026689546	1
Eps15l1	1,026689546	1
Dag1	1,026689546	1
Zfp951	1,026547226	1
Tlr6	1,026547226	1
Aftph	1,026547226	1
Wdr26	1,026547226	1
Ngdn	1,026476074	1
Akr1b10	1,026404926	1
Bcl2l13	1,026404926	1
Paqr5	1,026262646	1

Ccdc66	1,026049263	1
Plp2	1,026049263	1
Fam133b	1,026049263	1
Snw1	1,026049263	1
Anapc16	1,025978145	1
Nudt9	1,025978145	1
Birc5	1,025978145	1
Taf13	1,025907032	1
Gm45407	1,025835924	1
Actr1b	1,025835924	1
Plod3	1,02562263	1
Gm37465	1,025551542	1
Tnks2	1,025551542	1
Gm14636	1,025480458	1
Rnf126	1,025480458	1
Ints7	1,02540938	1
Ppp2r1b	1,025267238	1
Stk11	1,025267238	1
Zswim6	1,025196174	1
Cryl1	1,025125116	1
Asnsd1	1,025125116	1
Tcerg1	1,025125116	1
Cd47	1,024983013	1
Rps15a-ps5	1,02484093	1
Arl16	1,02484093	1
Skp1a	1,02484093	1
Gm6206	1,024769896	1
Trit1	1,024698867	1
Gigyf2	1,024698867	1
Cd53	1,024556823	1
Telo2	1,024485809	1
Haus2	1,024485809	1
Denr	1,024485809	1
Gm12577	1,024414799	1
Zfp955a	1,024343795	1
Slc39a3	1,024343795	1
Tmx3	1,024272795	1
Anxa1	1,0242018	1
Hnrnpul1	1,0242018	1
Cbr1	1,02413081	1
Casp8	1,023988846	1
Pi4k2b	1,023988846	1
Mrpl15	1,023917871	1
Slx4	1,0238469	1
Ap5m1	1,023704975	1
Atg13	1,02363402	1
Macrod2	1,023563069	1
Gm26698	1,023492124	1
2410002F23Rik	1,023421183	1
Mpst	1,023279317	1
Senp5	1,023208391	1
Trps1	1,02313747	1

Pgrmc1	1,023066554	1
Traf3ip2	1,022995643	1
Apitd1	1,022853835	1
Gm5523	1,022782939	1
Ube2c	1,022782939	1
Nicn1	1,022641161	1
Phlpp2	1,022570279	1
Vdac3	1,022570279	1
Snrnp27	1,022570279	1
Rac1	1,022499402	1
Tgs1	1,022428531	1
Elof1	1,022428531	1
Mapk1ip1l	1,022286802	1
Usp7	1,022286802	1
Baz1b	1,022215945	1
Ilf2	1,022074245	1
Tmx1	1,022003403	1
Gm42829	1,021790905	1
Uba7	1,021720083	1
Senp2	1,021649265	1
Wdr83	1,021649265	1
Myo9b	1,021578452	1
Hmbs	1,021507644	1
Mgarp	1,021507644	1
Cnot1	1,021507644	1
Nudcd3	1,021507644	1
Npm1	1,021507644	1
Ddb1	1,021507644	1
Eci2	1,021224461	1
Sh3bgrl	1,021224461	1
A530013C23Rik	1,021012126	1
Hbs1l	1,020941357	1
Parp11	1,020799834	1
Gnai2	1,020799834	1
Snx16	1,02072908	1
Rad18	1,02072908	1
Akt2	1,020658331	1
Naa38	1,020658331	1
Btbd1	1,020587587	1
Gm42986	1,020516848	1
Fam193b	1,020516848	1
Dis3l2	1,020446113	1
Gtf3a	1,020446113	1
Cep89	1,020375384	1
Bex3	1,020375384	1
Cks1b	1,020375384	1
Kansl2	1,020304659	1
Slc30a6	1,02023394	1
Sema4d	1,02023394	1
Mapre1	1,020163225	1
Gm14248	1,020092515	1
Usp24	1,020092515	1

Ifi30	1,020092515	1
2810403D21Rik	1,02002181	1
Plrg1	1,02002181	1
Faah	1,019880415	1
Mgat2	1,019880415	1
Cdc37	1,019809724	1
Gm6222	1,019668359	1
Arhgap21	1,019668359	1
Tcea1	1,019668359	1
Prps2	1,019668359	1
Foxo1	1,019597683	1
1110034G24Rik	1,019527012	1
Tmem229b	1,019456347	1
Exo1	1,019385686	1
Gchfr	1,019385686	1
Cpsf4	1,019385686	1
Rrbp1	1,019385686	1
Nkapl	1,01931503	1
Tcam1	1,01931503	1
Pola1	1,01931503	1
Mrfap1	1,01931503	1
Slc11a2	1,019244379	1
Plekhf1	1,019173732	1
Commd1	1,019173732	1
Mvk	1,019173732	1
Mnat1	1,019103091	1
Anxa4	1,018961823	1
Hoxb8	1,018891197	1
Ppm1b	1,018891197	1
Tulp4	1,018891197	1
Nfx1	1,018820575	1
Patz1	1,018749958	1
Zc3h14	1,018679346	1
Creb1	1,018538137	1
Slk	1,018538137	1
Arid3a	1,01846754	1
Epop	1,018396947	1
Axin1	1,018396947	1
Clip1	1,018396947	1
Plpp7	1,01832636	1
Zdhhc5	1,01832636	1
Msi2	1,0181852	1
Cblb	1,018114627	1
Usp34	1,018044059	1
Zfp553	1,017973496	1
March6	1,017973496	1
Atp9a	1,017902938	1
Hn1	1,017832385	1
Minos1	1,017761836	1
Gm14325	1,017550221	1
Tnip2	1,017550221	1
Ankra2	1,017550221	1

Mrpl3	1,017479692	1
Gtf2a2	1,017479692	1
Fam91a1	1,017479692	1
Gm15503	1,017409168	1
Pycr1	1,017409168	1
RP23-354J5.3	1,017409168	1
Szrd1	1,017409168	1
Uba52	1,017338649	1
Rad17	1,017338649	1
Rpl18-ps2	1,017268135	1
Angpt2	1,017268135	1
Gm12151	1,017127122	1
Atf7	1,016915638	1
Epn1	1,016774673	1
Hk1	1,016774673	1
Ift22	1,016704198	1
0610037L13Rik	1,016704198	1
Kantr	1,016563263	1
Dennd1c	1,016492803	1
Xpnpep1	1,016492803	1
Inip	1,016492803	1
Nelfcd	1,016422347	1
Tpp1	1,016422347	1
Gm37199	1,016351897	1
Sirt3	1,016281451	1
Gmfb	1,016281451	1
Gm36266	1,016070143	1
Ctcf	1,016070143	1
Tardbp	1,015999717	1
Amz2	1,015999717	1
Ebi3	1,015999717	1
Ilvbl	1,015929296	1
Nol7	1,015929296	1
Dnajc19	1,015788468	1
Ube2e3	1,015788468	1
Fam49b	1,015788468	1
Pik3c3	1,015718061	1
Dctn4	1,015647659	1
Eif3l	1,015647659	1
Stambpl1	1,015577262	1
Nip7	1,015577262	1
Nsun3	1,01550687	1
Rab31	1,01550687	1
Ube2q1	1,01550687	1
Alg2	1,015436483	1
Lypla1	1,015436483	1
Gm12183	1,015436483	1
Gm5244	1,015295723	1
Rpl21-ps5	1,015154983	1
Prmt5	1,015154983	1
Actl6a	1,015084621	1
Gsg1	1,015014263	1

Ints2	1,015014263	1
Dopey2	1,015014263	1
P3h3	1,01494391	1
Dtx2	1,014803218	1
Efr3a	1,014803218	1
Pou2f2	1,01473288	1
Atp1b3	1,014592218	1
4632404H12Rik	1,014521894	1
Tcf4	1,014521894	1
Lrrc40	1,014451575	1
Gm43329	1,014310952	1
Mov10	1,014310952	1
Slc2a1	1,014310952	1
Rnf167	1,014310952	1
Tmem170	1,014240648	1
Pabpn1	1,014100054	1
Ap1m1	1,014100054	1
Rin3	1,014029765	1
Atf7ip	1,014029765	1
Notch4	1,01395948	1
Hist1h4a	1,0138892	1
Cdk12	1,013748655	1
Cry2	1,013608129	1
Soat1	1,013608129	1
Ptprs	1,013608129	1
Hdac3	1,013467623	1
5530601H04Rik	1,0132569	1
Fyb	1,0132569	1
Bzw2	1,0132569	1
Fam84b	1,013186669	1
Gm5944	1,013116443	1
Faf1	1,013116443	1
Cct5	1,013116443	1
Zfp646	1,012976005	1
Tmem263	1,012976005	1
Clec4a3	1,012835586	1
Tep1	1,012835586	1
Ddt	1,012695187	1
Rab5c	1,012695187	1
Noc3l	1,012624995	1
Chd7	1,012624995	1
Brk1	1,012624995	1
Smad2	1,012274106	1
C130083A15Rik	1,012203943	1
Fcor	1,011993483	1
Klhl23	1,01192334	1
Pold2	1,01192334	1
Scaf1	1,011853201	1
Polr3b	1,011853201	1
1110008L16Rik	1,011783067	1
Eftud2	1,011642814	1
Trmt13	1,011432471	1

Sirt2	1,011432471	1
Zbtb37	1,011292266	1
Toe1	1,011222171	1
Arhgef39	1,011152081	1
Strbp	1,011152081	1
Tatdn2	1,011081996	1
Acvr2a	1,011011915	1
Tinf2	1,01094184	1
Wdr81	1,01094184	1
Zkscan6	1,01094184	1
Mff	1,01094184	1
Abtb1	1,010871769	1
Epb41l2	1,010731642	1
Tspan33	1,010661586	1
4930526A20Rik	1,010661586	1
Vps39	1,010591535	1
B4galt5	1,010591535	1
Rpp21	1,010521488	1
Thoc5	1,010521488	1
Gm15453	1,01038141	1
Gm37726	1,010241351	1
B4galt7	1,010171329	1
Fbxw9	1,010101311	1
Hsph1	1,009891289	1
Txndc11	1,009821291	1
A630001G21Rik	1,009751298	1
A930006K02Rik	1,009751298	1
Atad3a	1,009751298	1
Tmem126a	1,009471374	1
Rwdd2b	1,009401405	1
Rab3a	1,009191528	1
Ccdc127	1,009191528	1
Ap1g1	1,008981694	1
Iars	1,008841829	1
Siva1	1,008701984	1
Atp6v1d	1,008701984	1
Peg12	1,008632068	1
Arl13b	1,008632068	1
Alyref	1,008562158	1
Ccdc32	1,008422351	1
Dhx15	1,008282564	1
Wdr43	1,008282564	1
Gm42869	1,008142796	1
Ppargc1b	1,008142796	1
Msantd4	1,008142796	1
Gm5575	1,008072919	1
Maip1	1,008072919	1
Lrrfip2	1,008072919	1
Bub3	1,00793318	1
Epb41l4aos	1,00793318	1
Dcaf5	1,007723608	1
Pak1	1,007723608	1

S100a3	1,00765376	1
Alg12	1,00765376	1
Prrc2a	1,00765376	1
Nprl2	1,00765376	1
Pcmtd1	1,00765376	1
Pnpla2	1,007583918	1
Gm44250	1,007374418	1
Thada	1,007234776	1
Ano8	1,007164962	1
Fn3k	1,00695555	1
Cox6a1	1,00695555	1
Arfgap2	1,006815966	1
Aebp2	1,006815966	1
Tm9sf3	1,006815966	1
Shcbp1l	1,006676401	1
Atp13a2	1,006536856	1
Cep97	1,006467091	1
Supt6	1,006467091	1
Hsp90b1	1,00639733	1
Dcaf8	1,006327574	1
Cabin1	1,006257823	1
Gm22	1,006188077	1
Rab19	1,006118336	1
Aen	1,006118336	1
Adar	1,006118336	1
Zfp787	1,005909142	1
Selenot	1,00583942	1
Arfgef1	1,00569999	1
Gm8508	1,005630283	1
Arhgef11	1,00556058	1
Rap1gap	1,00556058	1
Gpaa1	1,005490883	1
Rpl9	1,00542119	1
Fcgr3	1,00542119	1
Eif2ak1	1,005351502	1
Colgalt1	1,005072798	1
H2-DMa	1,005003134	1
Mtr	1,004933475	1
Cerkl	1,004724526	1
Enoph1	1,004724526	1
Cdc25a	1,004654887	1
Zfc3h1	1,004585252	1
Polr3d	1,004515622	1
B3galt4	1,004445996	1
Znrf1	1,004445996	1
Wdr91	1,004445996	1
Rpl32-ps	1,004376376	1
Tex9	1,004376376	1
Gm11450	1,004376376	1
Prr14	1,00430676	1
Triap1	1,00430676	1
Gm42549	1,004237149	1

Rpl17	1,004237149	1
Ppp1r7	1,004167543	1
Dopey1	1,004097942	1
Rdx	1,004097942	1
Cops4	1,004097942	1
Nt5dc2	1,004028346	1
S100pbp	1,004028346	1
Zfp984	1,003958754	1
Syngr1	1,003889167	1
Cbx1	1,003819586	1
Ccpg1	1,003610869	1
Oaz1-ps	1,003541306	1
Dnm2	1,003471749	1
Yipf7	1,003402196	1
Gm5362	1,003402196	1
Flii	1,003402196	1
Tnpo1	1,003402196	1
N4bp1	1,003402196	1
Tkt	1,003332647	1
Hmox2	1,003263104	1
Gtf2f1	1,003124032	1
Rpp25l	1,003124032	1
CAAA01194877.2	1,003054503	1
Mfap1b	1,003054503	1
Abhd2	1,003054503	1
Rps11	1,003054503	1
RP24-366E11.4	1,00291546	1
Rab14	1,00291546	1
Bbx	1,002776436	1
Kcne3	1,002637431	1
Ddx54	1,002637431	1
Ubqln1	1,002498446	1
Gm13532	1,00242896	1
Hmbox1	1,002290004	1
Chrac1	1,002220533	1
Zbtb26	1,002081605	1
Slc5a3	1,002012148	1
Zkscan7	1,001942697	1
Gm13477	1,001942697	1
Trappc1	1,001942697	1
1700025G04Rik	1,001942697	1
Maz	1,00187325	1
Paip1	1,00187325	1
Trim37	1,00187325	1
Pak1ip1	1,001803808	1
Cct8	1,001803808	1
Fuk	1,00173437	1
Vwa5a	1,00173437	1
Med17	1,001664938	1
Bud31	1,001664938	1
Tln1	1,00159551	1
Ppil4	1,001456669	1

Atp6v1g1	1,001387256	1
Cutc	1,001317847	1
Rp2	1,001248444	1
Usp15	1,001248444	1
Abhd17b	1,001109651	1
Erich1	1,000901497	1
Hinfp	1,000693387	1
Dusp9	1,000624027	1
Wdr53	1,000624027	1
Brf2	1,000624027	1
Zfp148	1,000554672	1
C130013H08Rik	1,000485321	1
Rpsa-ps9	1,000415975	1
Ncaph2	1,000415975	1
Gm43328	1,000346634	1
Gm16540	1,000346634	1
Rbm17	1,000346634	1
Vps36	1,000207966	1
Rnf44	1,000069317	1
Gm42418	1	1
Nek4	-0,00010669	1
Tspan15	-7,72E-05	1
Lrrc8a	-0,00020935	1
Nmt2	-0,00022029	1
Sik3	-0,0002506	1
Rcbtb1	-0,00043285	1
A430018G15Rik	-0,00042239	1
Bscl2	-0,00041543	1
Wdr70	-0,00057855	1
Hmgxb3	-0,00057421	1
Parp10	-0,00082662	1
Armt1	-0,00079008	1
Gnl3	-0,00083205	1
Nudt2	-0,00086405	1
Cfap43	-0,001048	1
Slc15a4	-0,0010225	1
Slc12a7	-0,0010811	1
Pbrm1	-0,0011365	1
Sptssa	-0,0012187	1
Tlk2	-0,0013219	1
Cd300lb	-0,0013132	1
Emc6	-0,0012533	1
Gm13822	-0,0013915	1
Golph3	-0,0014589	1
Prps1	-0,0015551	1
Smc6	-0,0016514	1
Vash2	-0,0018255	1
Pcyox1l	-0,0018492	1
Dtwd1	-0,0018867	1
Pds5b	-0,001939	1
Lmo4	-0,001868	1
Xpo6	-0,0019849	1

Ccne2	-0,0022313	1
Selenon	-0,0022203	1
Mob3c	-0,0024095	1
Rpl31-ps17	-0,0024854	1
Psmd2	-0,0025797	1
Timm8a1	-0,0026555	1
Paip2b	-0,0030384	1
Vps11	-0,003	1
Gm13578	-0,0032221	1
Sipa1l3	-0,0031957	1
Ap4e1	-0,0032403	1
Gm18969	-0,003435	1
Memo1	-0,0033989	1
Pmm2	-0,0035726	1
Clec10a	-0,0036813	1
H2afv	-0,0039749	1
Dynlt3	-0,0039557	1
Lrrc8c	-0,0040658	1
Rpl21-ps1	-0,0041003	1
Uty	-0,0042156	1
Stat3	-0,0041916	1
Nus1	-0,0042048	1
Dpm2	-0,0045232	1
Ckap2l	-0,0047344	1
Psmd7	-0,0049859	1
Pex3	-0,0051335	1
Dnajc21	-0,0054068	1
Chtf18	-0,0056287	1
Tmem144	-0,0056003	1
Fam126a	-0,0055593	1
Pphln1	-0,0057295	1
Amz1	-0,005697	1
Sec24b	-0,0057905	1
Emc4	-0,0058607	1
Fkbp3	-0,0058999	1
Gm27010	-0,0062279	1
Gstcd	-0,0061605	1
Ptpre	-0,0063072	1
Mrps14	-0,0064757	1
Ndufb6	-0,0064821	1
Leprot	-0,0068405	1
Phkg2	-0,0067884	1
Slc16a3	-0,0068059	1
Taldo1	-0,0067878	1
Blvrb	-0,0069188	1
Hmgb2	-0,0070193	1
Twf1	-0,0070693	1
Rida	-0,0072293	1
Pdcd11	-0,0073336	1
Zfp287	-0,0074309	1
Gm5566	-0,0074434	1
Gm43137	-0,0075461	1

Gm15832	-0,0074885	1
Diexf	-0,0075079	1
Mapk3	-0,0075643	1
1700096K18Rik	-0,0076886	1
Aprt	-0,0076722	1
Prpf40a	-0,0077811	1
Ndufab1	-0,0079114	1
Zfp740	-0,0079129	1
Lemd2	-0,0079207	1
Angel1	-0,0080264	1
Gm8762	-0,0080372	1
Gprasp1	-0,0079679	1
Amn1	-0,0079923	1
Ran	-0,0080346	1
N4bp2l1	-0,0083091	1
Nav2	-0,008422	1
AB124611	-0,008437	1
Ptgir	-0,0084805	1
Xpc	-0,0084721	1
Nek8	-0,0087038	1
Pdhb	-0,0087217	1
Heatr5b	-0,0088185	1
Scyl1	-0,0088608	1
Gm8242	-0,0090138	1
Coil	-0,008969	1
Mtf1	-0,008952	1
Hoxb4	-0,0089871	1
Cdkl3	-0,0091028	1
Slc25a26	-0,009078	1
Mbd4	-0,0090854	1
Rragb	-0,0092141	1
Ccnd1	-0,0091841	1
Bri3bp	-0,0092539	1
Mettl21b	-0,0094456	1
Prmt7	-0,0096788	1
Slc35b2	-0,0097422	1
Ndufaf6	-0,0098014	1
Samd1	-0,0099333	1
Kdelr1	-0,0099481	1
Lyrm9	-0,010028	1
Gm6457	-0,010219	1
Jam2	-0,010218	1
Chp1	-0,010249	1
Tug1	-0,010426	1
Urgcp	-0,010478	1
Hnrnpm	-0,010548	1
Spry2	-0,010903	1
Mlh1	-0,010888	1
Calr-ps	-0,010947	1
2700046G09Rik	-0,011109	1
Cfap97	-0,011138	1
Ccar2	-0,011174	1

Dok4	-0,011329	1
Washc3	-0,011453	1
Ago4	-0,011545	1
Zfp944	-0,011554	1
Gm45836	-0,011661	1
Stim1	-0,011701	1
Elmo2	-0,012134	1
Grsf1	-0,012077	1
Rpl35a-ps5	-0,01218	1
Fubp3	-0,0122	1
Rasa2	-0,012483	1
Cherp	-0,01246	1
Arhgap10	-0,012504	1
Mrps23	-0,012526	1
Txndc12	-0,012615	1
Mrpl38	-0,012782	1
Zkscan14	-0,012891	1
Galnt6	-0,012943	1
Blmh	-0,012958	1
Gpsm3	-0,013065	1
Gm16288	-0,01331	1
Eif3d	-0,01325	1
Pcbp4	-0,013394	1
Ctbs	-0,013358	1
Mbd3	-0,013424	1
4933412L11Rik	-0,013456	1
Adat3	-0,013498	1
Zc3hav1	-0,013616	1
A530017D24Rik	-0,013679	1
Vps53	-0,013654	1
Mkrn1	-0,013663	1
Csnk1d	-0,013764	1
Med21	-0,01394	1
Polr2b	-0,013878	1
Dapk1	-0,014239	1
Ikbke	-0,014342	1
Fam207a	-0,014373	1
Ccdc174	-0,014534	1
RP23-380K24.3	-0,014799	1
Gm44950	-0,014911	1
Gm13015	-0,015164	1
Rras	-0,015222	1
Nhlrc2	-0,015265	1
Fam208b	-0,015402	1
Rc3h2	-0,015388	1
Vps18	-0,015618	1
Usp25	-0,015722	1
Mdh2	-0,015714	1
Echs1	-0,015818	1
Klhdc4	-0,0158	1
Cse1l	-0,015989	1
Gm6520	-0,016195	1

Dicer1	-0,016542	1
Hmgcl	-0,016545	1
Gm37238	-0,016669	1
Sbds	-0,01671	1
Pten	-0,01666	1
Rbpj	-0,016717	1
Klc3	-0,017009	1
Pgap2	-0,017079	1
Nubpl	-0,017229	1
Arl5a	-0,017152	1
1700037H04Rik	-0,017205	1
Atp2a2	-0,017301	1
Tbc1d20	-0,017402	1
AI314180	-0,017362	1
Acyp1	-0,01768	1
9530068E07Rik	-0,017773	1
Dnajb2	-0,017863	1
RP23-6C18.6	-0,018023	1
Gm38157	-0,018027	1
Aamp	-0,017988	1
K230015D01Rik	-0,01816	1
Churc1	-0,018278	1
Ticrr	-0,018407	1
Gm24959	-0,018524	1
Dusp3	-0,018474	1
Slc25a13	-0,018736	1
1700001P01Rik	-0,0188	1
2900026A02Rik	-0,019106	1
Mapkap1	-0,019075	1
Polh	-0,019239	1
Tsn	-0,019387	1
Rpl38-ps2	-0,019469	1
Rufy2	-0,019599	1
Gm12059	-0,019684	1
Gm5391	-0,019898	1
Avl9	-0,019882	1
Zkscan8	-0,019957	1
Gm561	-0,020029	1
Gm43511	-0,020126	1
Aida	-0,020062	1
Ndufa3	-0,02014	1
Mr1	-0,020245	1
Slc25a1	-0,020316	1
Wdr3	-0,020482	1
Gpr65	-0,020782	1
Atp2c1	-0,020811	1
Zfp729a	-0,020918	1
A930018M24Rik	-0,020898	1
Fam131a	-0,021151	1
5830487J09Rik	-0,021209	1
St3gal5	-0,0212	1
mt-Nd4	-0,021215	1

R3hcc1l	-0,021193	1
Srp19	-0,021211	1
Fam175b	-0,021336	1
Ywhaz	-0,021331	1
Npr1	-0,021376	1
Pkib	-0,02138	1
Zscan2	-0,021612	1
Gm15417	-0,022048	1
Kdm1a	-0,022009	1
Trim25	-0,021996	1
Arhgap27os2	-0,022086	1
Snx32	-0,022157	1
Naif1	-0,022305	1
Gm5871	-0,022407	1
Mir22hg	-0,022431	1
Dusp6	-0,02238	1
Tcp1	-0,02241	1
Copb2	-0,022484	1
Man1a2	-0,022567	1
Psd	-0,022703	1
Ccny	-0,022698	1
Arhgef2	-0,022743	1
Nucks1	-0,022699	1
Ncoa1	-0,022973	1
Gtf2ird1	-0,023058	1
Slc29a3	-0,023237	1
Orai1	-0,02316	1
Aar2	-0,023152	1
Rsrc1	-0,023151	1
Eif3h	-0,023239	1
Dip2c	-0,023312	1
Mon2	-0,023253	1
Purb	-0,023393	1
8430408G22Rik	-0,023529	1
Fbxl14	-0,023697	1
Spag7	-0,023731	1
Gm4994	-0,02398	1
Tmed4	-0,02402	1
Actr2	-0,024122	1
Serpinf2	-0,024212	1
Ttc14	-0,024334	1
Gm15157	-0,024541	1
Tap1	-0,024507	1
Rbpsuh-rs3	-0,024522	1
Sf3b3	-0,024626	1
Fam210a	-0,024751	1
Rpe	-0,024858	1
Rabggtb	-0,024909	1
Atg3	-0,025118	1
Bmi1	-0,025107	1
Srp9	-0,025135	1
Hsbp1	-0,025083	1

Ccdc120	-0,025299	1
Sgk3	-0,025538	1
Zfp110	-0,025541	1
4930455G09Rik	-0,0255	1
Nol10	-0,025503	1
Cebpe	-0,025618	1
Ehbp1	-0,025941	1
Nectin2	-0,025856	1
Tdp2	-0,025931	1
Slc39a9	-0,026012	1
Lsm1	-0,026081	1
Nisch	-0,026118	1
Dpp7	-0,026342	1
Cct6a	-0,026648	1
Tbcd	-0,026828	1
Slc35c2	-0,026821	1
Pvt1	-0,026933	1
Itgav	-0,027137	1
Lypla2	-0,027165	1
Dnaja1	-0,027323	1
Ralb	-0,027369	1
Nfatc3	-0,027396	1
Ift46	-0,027457	1
9230102O04Rik	-0,027611	1
Fam234a	-0,027716	1
B230307C23Rik	-0,027777	1
Xk	-0,027834	1
Coq8b	-0,027802	1
Rgs2	-0,027938	1
Tmub1	-0,028123	1
Fasn	-0,028158	1
Gabarapl1	-0,028159	1
Me2	-0,028317	1
Ecm1	-0,028257	1
Aip	-0,028537	1
Copz1	-0,028657	1
C1rl	-0,028813	1
Rbm6	-0,028887	1
Rpl22	-0,028966	1
Nat10	-0,029144	1
Zfp772	-0,029151	1
Rnf166	-0,029228	1
Wdr11	-0,02928	1
Mcur1	-0,029362	1
Ogt	-0,02975	1
Impdh2	-0,029848	1
Sms	-0,029817	1
Txnl1	-0,029889	1
Eepd1	-0,030009	1
Fam111a	-0,029974	1
Phax	-0,03005	1
Gm13186	-0,030244	1

Usp31	-0,03016	1
Gosr2	-0,030215	1
Gapvd1	-0,030189	1
Mecr	-0,030321	1
Rtf1	-0,030331	1
Angptl4	-0,030377	1
Gm37519	-0,030434	1
Dbf4	-0,030457	1
Srsf11	-0,030473	1
Sap18b	-0,030642	1
Rnf40	-0,030562	1
Mafg	-0,030746	1
Trio	-0,030786	1
Cars2	-0,031072	1
Srsf10	-0,031436	1
Prpsap1	-0,031545	1
Mospd2	-0,031646	1
Rusc1	-0,031613	1
Afg3l2	-0,031596	1
Ube2l3	-0,031688	1
Ppm1k	-0,03184	1
Svep1	-0,031934	1
Dnpep	-0,032027	1
Dnajc30	-0,032235	1
Nsmce2	-0,032162	1
Gmip	-0,032341	1
Trim28	-0,032469	1
Gm43387	-0,032621	1
Gm37145	-0,032617	1
Xylb	-0,03268	1
Mdm4-ps	-0,032816	1
Cystm1	-0,032944	1
Fam185a	-0,033079	1
Sntb2	-0,033293	1
Fbxw2	-0,033341	1
Itpka	-0,033398	1
Srrt	-0,033369	1
Gas5	-0,033436	1
Hist1h4d	-0,033978	1
Pex11a	-0,034038	1
Eefsec	-0,034185	1
Fh1	-0,034408	1
Prrc2b	-0,03453	1
Nek9	-0,034511	1
Cd52	-0,034482	1
Tmem186	-0,034634	1
Gdpgp1	-0,034639	1
Kcnb1	-0,034677	1
Rpl12	-0,034895	1
Gm38043	-0,034956	1
Nfyb	-0,034956	1
Immp2l	-0,035132	1

Tuft1	-0,035368	1
Exosc2	-0,035616	1
Flna	-0,035579	1
Gm2531	-0,03569	1
Gm12240	-0,035825	1
Pdcd4	-0,035871	1
Ilkap	-0,035907	1
Vps13c	-0,035911	1
Siae	-0,035979	1
Trim46	-0,036105	1
Tmem62	-0,03605	1
Sephs1	-0,036206	1
Apobr	-0,036162	1
Tcof1	-0,036193	1
B9d2	-0,036259	1
Sidt2	-0,03627	1
Tbl1x	-0,036298	1
Msra	-0,036632	1
Acad10	-0,036743	1
Dgcr2	-0,03677	1
D2Bwg1423e	-0,037083	1
Capn7	-0,037144	1
Rpl36	-0,037158	1
Vps41	-0,037249	1
Emp1	-0,037348	1
Gm13039	-0,037355	1
Trim36	-0,037457	1
Arhgap25	-0,037479	1
Anapc7	-0,037609	1
Med13l	-0,037756	1
Polr3k	-0,037924	1
Pnpla8	-0,037901	1
Laptm5	-0,038269	1
Gm43484	-0,038527	1
Ctxn1	-0,038508	1
Fam217b	-0,038669	1
Atxn7l3	-0,03874	1
Med30	-0,038878	1
Wdr92	-0,039005	1
Khk	-0,038988	1
Hyal2	-0,039111	1
Gmds	-0,039301	1
Cd72	-0,039276	1
Pex6	-0,039419	1
Aktip	-0,039453	1
Gm43323	-0,039684	1
Cops5	-0,039719	1
Ncs1	-0,039803	1
Cnksr1	-0,039971	1
Hspd1-ps3	-0,040023	1
Dcps	-0,040028	1
Sdhaf2	-0,040148	1

Tcf3	-0,040224	1
Lcp1	-0,040316	1
Gm5045	-0,04041	1
Nub1	-0,040363	1
Gm5611	-0,040496	1
2810428I15Rik	-0,04053	1
Mier3	-0,040527	1
Gm12751	-0,04059	1
2900052L18Rik	-0,04065	1
Pick1	-0,040646	1
Rfc3	-0,040572	1
Acadvl	-0,040574	1
Trip4	-0,040659	1
Gm7565	-0,040783	1
Gm10031	-0,040819	1
Fbxl19	-0,040859	1
1700003G18Rik	-0,040897	1
Eif2b5	-0,040874	1
Azi2	-0,041131	1
Rpl18-ps1	-0,041118	1
Arhgap27os1	-0,04125	1
Slc25a45	-0,041306	1
Tmem87a	-0,041293	1
Mllt10	-0,041315	1
Tmem9	-0,041506	1
Fam149b	-0,04158	1
Zfp455	-0,041748	1
Tor1a	-0,041742	1
Kdm4a	-0,041749	1
Zfp623	-0,041752	1
Trmt1	-0,041807	1
Slc30a4	-0,041837	1
Gm19898	-0,041994	1
Cdyl2	-0,042045	1
Gm14843	-0,042064	1
Ipo7	-0,042063	1
Pced1b	-0,042248	1
Tab1	-0,04217	1
Gm6733	-0,04233	1
Slc30a9	-0,04233	1
Gm1943	-0,042432	1
Dhps	-0,04244	1
RP24-282C4.9	-0,042514	1
Llgl1	-0,042483	1
Ttc4	-0,042756	1
Ppp1r10	-0,042858	1
Taf1d	-0,042923	1
Mrpl23	-0,042955	1
Tada1	-0,043	1
Dcaf12	-0,042961	1
Lace1	-0,043142	1
Lin28b	-0,0431	1

Nono	-0,04306	1
Gtf3c4	-0,043063	1
Scaper	-0,04312	1
Lgals3	-0,043103	1
Ndfip2	-0,043226	1
Emilin2	-0,043226	1
Tmpo	-0,043319	1
Lgals1	-0,043272	1
Ap4b1	-0,043409	1
Sep 06	-0,043554	1
Gga3	-0,043662	1
Fth-ps3	-0,043821	1
Ubxn2a	-0,043889	1
Pam	-0,043935	1
Pdcd6	-0,043981	1
Idh3a	-0,044129	1
Hacd4	-0,044139	1
Ppm1d	-0,044091	1
Nom1	-0,044097	1
Zdhhc9	-0,044176	1
Mipep	-0,044397	1
Zbtb5	-0,044593	1
Ddx50	-0,044636	1
Fopnl	-0,044585	1
Ccdc77	-0,044691	1
Txn1	-0,044713	1
Parl	-0,044784	1
Txnrd1	-0,044966	1
Trp53inp2	-0,045109	1
Idh3g	-0,045136	1
Ryk	-0,04511	1
Zc3h15	-0,045118	1
Cenpl	-0,045417	1
Gm6085	-0,045541	1
Klhl42	-0,04584	1
Mlkl	-0,045928	1
1110025M09Rik	-0,046102	1
Nae1	-0,046112	1
0610009B22Rik	-0,046207	1
Cdk19	-0,046311	1
Gm1862	-0,046542	1
Psma8	-0,046538	1
Mark3	-0,046509	1
Pafah1b1	-0,046573	1
Gclm	-0,04668	1
Jmjd6	-0,0467	1
Ccr2	-0,046758	1
Ddx24	-0,047058	1
Mrto4	-0,047222	1
Lysmd1	-0,047339	1
Pomt2	-0,047528	1
Rbm6-ps1	-0,047527	1

Stat6	-0,047515	1
Hnrnpul2	-0,047463	1
Tlr2	-0,047628	1
Tle1	-0,047779	1
Rab43	-0,048074	1
Pitpnb	-0,048142	1
Kif1b	-0,048153	1
Ncl	-0,04817	1
Ccdc167	-0,04827	1
Arcn1	-0,048323	1
Snx8	-0,048374	1
Zfp346	-0,048574	1
Larp1	-0,048557	1
Arrdc1	-0,048739	1
Mpdu1	-0,048714	1
Prpf38b	-0,048712	1
Ube4b	-0,049009	1
Gm11273	-0,049198	1
St3gal3	-0,04921	1
Ofd1	-0,049339	1
Gm15829	-0,049435	1
Gm44126	-0,049537	1
Tmem11	-0,049634	1
Atxn2l	-0,049779	1
Cenpm	-0,049874	1
Ulbp1	-0,04987	1
Ireb2	-0,049941	1
Fbrs	-0,049899	1
Cox16	-0,050144	1
Mpi	-0,050182	1
Trim44	-0,050227	1
2310036O22Rik	-0,050274	1
Tmem41b	-0,050403	1
Ubiad1	-0,050638	1
Cbx6	-0,05076	1
Chchd6	-0,050869	1
Siah1a	-0,050995	1
Zfp622	-0,050979	1
Trim12c	-0,051092	1
Kat6a	-0,051202	1
Gde1	-0,051224	1
Nckap5l	-0,051276	1
Gm11964	-0,051367	1
Rae1	-0,051355	1
Mir142hg	-0,051501	1
Dctn6	-0,051649	1
Ube2r2	-0,05168	1
Tfdp1	-0,05185	1
Itsn2	-0,051948	1
Ssr1	-0,052048	1
Guf1	-0,052104	1
Slc17a5	-0,052323	1

Cnot6	-0,05231	1
Fam102b	-0,052411	1
Acp6	-0,052361	1
H2-D1	-0,052406	1
Pnpla6	-0,052518	1
Nmral1	-0,052527	1
Pisd	-0,052518	1
Aasdh	-0,052558	1
Crip1	-0,052563	1
Gm37566	-0,052746	1
Slc25a53	-0,052714	1
Tamm41	-0,052832	1
P4hb	-0,05308	1
Ap1s3	-0,053226	1
Mgat1	-0,053449	1
Ubxn2b	-0,053568	1
Fam96a	-0,053592	1
Slc25a39	-0,05383	1
Lrp10	-0,05391	1
Ubn2	-0,054118	1
Dzip1	-0,054201	1
Atg5	-0,054244	1
Yeats4	-0,054243	1
Smim15	-0,054232	1
Gm38021	-0,054414	1
Nemf	-0,05449	1
Eapp	-0,054744	1
Tmem69	-0,054767	1
Eif3j1	-0,054768	1
Itpkb	-0,054897	1
Gm12816	-0,054986	1
Atp5e	-0,055085	1
Rfesd	-0,055184	1
Gm11448	-0,055337	1
Gm6451	-0,055373	1
Snai2	-0,055534	1
Ski	-0,055524	1
Man2a2	-0,05614	1
Zfp566	-0,056329	1
Psmb2	-0,056313	1
Myo1g	-0,056381	1
Cyb5b	-0,056362	1
Ndufs4	-0,05646	1
Gm5069	-0,056694	1
Arid2	-0,056782	1
Tmed2	-0,056774	1
Tacc1	-0,056883	1
Cd200r1	-0,056968	1
AA465934	-0,05716	1
Flot2	-0,057246	1
Efl1	-0,057169	1
Frmd4b	-0,057243	1

Rbm15b	-0,057446	1
Eml5	-0,057463	1
Eno2	-0,057571	1
Nqo2	-0,057741	1
Katna1	-0,057691	1
Timm21	-0,057776	1
Stard10	-0,057791	1
Stk38	-0,057759	1
Nedd8	-0,057773	1
Rpl31-ps11	-0,058048	1
P2ry2	-0,058042	1
Pnrc2	-0,057981	1
Phf23	-0,058033	1
E130307A14Rik	-0,058103	1
Tlcd2	-0,058216	1
Sema4g	-0,058221	1
Irf5	-0,058233	1
Canx	-0,058334	1
Nmt1	-0,058383	1
Rps24-ps2	-0,058519	1
Snx17	-0,058583	1
Zfp451	-0,05857	1
Rbsn	-0,058657	1
Atrn	-0,058748	1
Arhgef10l	-0,058663	1
1110004E09Rik	-0,058665	1
Mir703	-0,058873	1
Atf6b	-0,058858	1
Gm8825	-0,058986	1
Gm5547	-0,058964	1
Tor1b	-0,058964	1
Eif1ax	-0,059177	1
Pycrl	-0,059272	1
Camk1	-0,059264	1
Gm9134	-0,059356	1
Zfp62	-0,059498	1
Polm	-0,059584	1
Alg11	-0,059721	1
2810402E24Rik	-0,059812	1
Ufm1	-0,059846	1
Swap70	-0,059877	1
Xpo7	-0,060266	1
Gm43096	-0,060352	1
Nsd2	-0,060449	1
9130230N09Rik	-0,060488	1
Gm14130	-0,060608	1
Fyco1	-0,060615	1
Mrpl41	-0,060566	1
Edf1	-0,060703	1
Tor2a	-0,060751	1
Pmvk	-0,060772	1
Gch1	-0,060829	1

Dcaf13	-0,06079	1
Gm30329	-0,061091	1
Ipmk	-0,061207	1
Wrn	-0,061336	1
Magi1	-0,06145	1
Birc6	-0,061351	1
Lrp8	-0,061433	1
Tmem57	-0,061428	1
Pitpnc1	-0,061573	1
Aco2	-0,061638	1
Srbd1	-0,061662	1
Wbscr22	-0,061669	1
Lman1	-0,062018	1
Gorab	-0,062091	1
Clcn4	-0,062185	1
Fosl1	-0,062188	1
Scaf4	-0,062205	1
Agpat5	-0,062226	1
Cd63	-0,062353	1
Psmd3	-0,062801	1
Mybbp1a	-0,062948	1
Kif3a	-0,062976	1
Mtor	-0,063087	1
Hdac8	-0,063164	1
Cdca4	-0,063176	1
Hspa4	-0,063223	1
Shisa5	-0,063355	1
Hspa9	-0,06337	1
Acox1	-0,06353	1
Gm45884	-0,06359	1
Atg4a	-0,06355	1
Polr2e	-0,063576	1
Nop16	-0,063819	1
Gm5422	-0,063987	1
Clasp1	-0,064056	1
Mfsd1	-0,064053	1
Snap29	-0,064164	1
Agmo	-0,064328	1
Wdr1	-0,064401	1
Sfpq	-0,06444	1
Esd	-0,064413	1
Ppig	-0,064375	1
Pdlim5	-0,06455	1
Mfsd9	-0,064604	1
Gm43223	-0,064554	1
G3bp1	-0,064638	1
Tubgcp6	-0,064689	1
Srp72	-0,064743	1
Gm44254	-0,064874	1
Crtc1	-0,065055	1
Zfp189	-0,065148	1
Ubfd1	-0,06529	1

Nudcd1	-0,065272	1
Snai1	-0,065454	1
Gm42970	-0,065512	1
Slc41a3	-0,065521	1
Isy1	-0,065547	1
Babam1	-0,065626	1
Usp40	-0,065785	1
Cox7a2	-0,065846	1
Zfp606	-0,065926	1
Zbtb40	-0,065875	1
Pdk2	-0,066033	1
Clk2	-0,066008	1
Ascc1	-0,065994	1
Gm44164	-0,066117	1
3830406C13Rik	-0,066121	1
Letm1	-0,066145	1
Rft1	-0,066167	1
Sorl1	-0,06622	1
Rwdd3	-0,066193	1
Arhgef1	-0,066246	1
Ccnh	-0,066166	1
Pcbd2	-0,066172	1
Herc3	-0,066409	1
Slc25a38	-0,066387	1
Srd5a3	-0,066529	1
Gstt1	-0,066465	1
Ppp3cb	-0,066507	1
Srgap2	-0,066467	1
Zfp141	-0,066643	1
Cdk9	-0,066683	1
Rad54l2	-0,066811	1
Gm11474	-0,066905	1
Fbxo4	-0,066927	1
Tubb2b	-0,066957	1
Bbs4	-0,067001	1
Gm45113	-0,067143	1
Ripk3	-0,067124	1
Sf3b2	-0,067074	1
Crebzf	-0,067219	1
Renbp	-0,067303	1
Wdfy3	-0,067473	1
Nr2c2ap	-0,067541	1
Fam53a	-0,067594	1
Gm13835	-0,067863	1
Tmed5	-0,068144	1
Zbtb7a	-0,068075	1
Myo19	-0,068248	1
Smyd4	-0,068218	1
Eps15	-0,068241	1
Rad52	-0,068292	1
D030028A08Rik	-0,068515	1
Gm16556	-0,068478	1

Polr2i	-0,068741	1
Apoa1bp	-0,068815	1
Caly	-0,068966	1
Fam50a	-0,068966	1
Prkaca	-0,069083	1
Haus4	-0,069244	1
Tut1	-0,069174	1
Mdm2	-0,069296	1
Ecd	-0,069637	1
Serf1	-0,069645	1
Tmc6	-0,069753	1
Cd37	-0,069771	1
Yeats2	-0,069922	1
Rexo2	-0,070029	1
Psmc1	-0,069952	1
Cyp27a1	-0,070234	1
2310033P09Rik	-0,070161	1
Rcc1l	-0,070286	1
Gpn1	-0,070405	1
Gm43774	-0,070491	1
Gm10059	-0,070486	1
Gm5054	-0,070853	1
Fsd1l	-0,070977	1
Phf20-ps	-0,071007	1
Mex3a	-0,071056	1
Mrpl45	-0,071086	1
Lhx5	-0,071267	1
Map1s	-0,071318	1
Gm38055	-0,071353	1
Abracl	-0,071592	1
Fam98c	-0,07195	1
Gm43924	-0,07199	1
Thap2	-0,072007	1
Scamp3	-0,072019	1
Psmc6	-0,072002	1
Purg	-0,072087	1
Ptdss1	-0,072095	1
Ccz1	-0,072233	1
Ddx39	-0,072409	1
Brap	-0,07252	1
March8	-0,072535	1
Stx16	-0,072522	1
Cramp1l	-0,072614	1
Josd2	-0,072653	1
Thap4	-0,072692	1
Noct	-0,072845	1
Vav3	-0,072869	1
Neo1	-0,072988	1
Zfp644	-0,073091	1
Ptbp3	-0,073238	1
Tmem159	-0,073296	1
Nipbl	-0,07334	1

Zfp692	-0,073355	1
Snora21	-0,073533	1
Gin1	-0,073462	1
Fpgt	-0,073685	1
Gm21967	-0,073778	1
Ssh1	-0,073787	1
Ppp2r3c	-0,073768	1
Snx1	-0,07379	1
Ano6	-0,073857	1
Ash1l	-0,073867	1
Gm6576	-0,073962	1
Cdc23	-0,074019	1
Eny2	-0,0743	1
2010107E04Rik	-0,074355	1
2310068J16Rik	-0,074504	1
Alg1	-0,074592	1
Atp5sl	-0,074556	1
Ccdc181	-0,07459	1
Slc48a1	-0,0748	1
Zfp446	-0,074994	1
Chchd7	-0,07509	1
Prelid1	-0,075088	1
Dld	-0,07517	1
Slain2	-0,075228	1
Cox7b	-0,075301	1
Synj2	-0,075444	1
Gm12693	-0,075458	1
Rnh1	-0,075485	1
Rpp40	-0,075746	1
2210013O21Rik	-0,075719	1
Banf1	-0,07573	1
2810454H06Rik	-0,075798	1
Gm43351	-0,075912	1
Fam204a	-0,076002	1
Sh3bgrl3	-0,076048	1
Rpl36a-ps2	-0,076133	1
Mbp	-0,076262	1
Rfc1	-0,07648	1
Mxd4	-0,076462	1
Aaas	-0,076622	1
Atg2a	-0,07691	1
Chtop	-0,076896	1
Gm8894	-0,077081	1
Gm11970	-0,077105	1
Gm13422	-0,077268	1
Prkce	-0,077415	1
Tmem189	-0,077389	1
Nsun2	-0,077538	1
Ttc17	-0,077725	1
Fam110a	-0,077786	1
Abhd16a	-0,077904	1
Trim32	-0,077862	1

Prpf4b	-0,077935	1
Ciapin1	-0,077894	1
Klhl26	-0,077965	1
Erap1	-0,078072	1
Heatr6	-0,078224	1
Grhpr	-0,078305	1
Dhx36	-0,078438	1
G6pc3	-0,078639	1
Atp6v0e	-0,078661	1
Usp16	-0,078805	1
Tmed9	-0,078934	1
Tbl1xr1	-0,079136	1
Ppm1m	-0,079072	1
Itfg2	-0,07923	1
Btk	-0,079285	1
Spata1	-0,079519	1
Tirap	-0,079597	1
Cep78	-0,079787	1
Scarb2	-0,079789	1
Lss	-0,079887	1
Arl5c	-0,079951	1
Zfp827	-0,079985	1
Eif3f	-0,079992	1
Prob1	-0,080219	1
Bclaf1	-0,080244	1
Sae1	-0,080168	1
Tyrobp	-0,080215	1
Smim11	-0,080322	1
Poldip3	-0,080466	1
Camk1d	-0,080605	1
Zfp263	-0,080809	1
Clint1	-0,080795	1
Cmtm3	-0,080878	1
Kansl3	-0,080974	1
Swi5	-0,081142	1
Hscb	-0,081234	1
Stk4	-0,081296	1
Ube2d1	-0,0814	1
D130007C19Rik	-0,081467	1
Itfg1	-0,081733	1
Sprtn	-0,081796	1
Rpl19-ps1	-0,081924	1
Mrpl14	-0,081856	1
Atp5a1	-0,081904	1
Rnaseh1	-0,082005	1
Gm4879	-0,082028	1
Rexo4	-0,082274	1
Mdh1	-0,082307	1
Gm44829	-0,082417	1
Fastkd3	-0,082359	1
Ssr3	-0,08237	1
Map2k5	-0,08274	1

Cbx3	-0,082784	1
Slc6a13	-0,083027	1
Twsg1	-0,082981	1
Poldip2	-0,083148	1
Pcmt1	-0,083127	1
3010003L21Rik	-0,08318	1
Xrn2	-0,083204	1
Exoc4	-0,083347	1
Manbal	-0,083494	1
Gm11895	-0,083691	1
Ctsl	-0,084079	1
Clcn5	-0,084207	1
Oard1	-0,084444	1
Lims1	-0,084448	1
Arhgap27	-0,084383	1
Gm5745	-0,084578	1
Gm12251	-0,084651	1
A630081D01Rik	-0,084689	1
Stk11ip	-0,084951	1
Cbarp	-0,085322	1
Rnd2	-0,08544	1
Dtx3	-0,085429	1
Ube2l6	-0,085451	1
Clec4d	-0,085503	1
Rhot2	-0,085866	1
Mtmr10	-0,085898	1
Adipor1	-0,085862	1
Spidr	-0,086033	1
Uggt1	-0,086002	1
Hectd1	-0,086063	1
Mthfd1	-0,086175	1
Mdm4	-0,086346	1
Gm20554	-0,086404	1
Sympk	-0,086393	1
Adam17	-0,086553	1
Ncf4	-0,086745	1
Timm29	-0,08683	1
9430060I03Rik	-0,086909	1
Tpr	-0,086962	1
Spred2	-0,087124	1
Tmem35b	-0,087177	1
Elac2	-0,087241	1
Ncbp1	-0,087171	1
Eif5a	-0,087199	1
9530053A07Rik	-0,087315	1
Cwc15	-0,08732	1
Mt2	-0,087391	1
Ubxn1	-0,087384	1
Ptpn23	-0,087489	1
Sec11a	-0,087466	1
Eif4g1	-0,087543	1
Tes3-ps	-0,087568	1

Rmdn3	-0,087618	1
Ampd2	-0,08757	1
Unc50	-0,087673	1
BC037032	-0,087813	1
Ccndbp1	-0,088027	1
Ccdc18	-0,088126	1
Brdt	-0,088055	1
Zmat2	-0,088075	1
Zfp24	-0,088315	1
Gm9013	-0,088368	1
Necap2	-0,088502	1
Pex2	-0,088597	1
Dpysl2	-0,088707	1
Rpl15-ps5	-0,088821	1
Jrkl	-0,088755	1
Gm11263	-0,088879	1
Bcorl1	-0,089038	1
Zmiz1	-0,088988	1
Lsm2	-0,089181	1
Zmynd11	-0,089166	1
Cenpp	-0,089322	1
Commd2	-0,089274	1
Senp1	-0,089623	1
Pnkp	-0,089732	1
Lst1	-0,089659	1
Chd3	-0,090021	1
Tes	-0,089957	1
Hoxb7	-0,090116	1
Gm42724	-0,090242	1
Twnk	-0,090244	1
Fbxw5	-0,09019	1
Sft2d2	-0,090362	1
Gm42820	-0,090546	1
Zmpste24	-0,090596	1
Arpc5	-0,090569	1
Arhgef25	-0,090747	1
Ppp6r3	-0,090737	1
Ext1	-0,090909	1
Gm20703	-0,091019	1
F8a	-0,091139	1
Gon4l	-0,091082	1
Ubtf	-0,091079	1
Nmb	-0,091214	1
Acbd6	-0,091221	1
Lars2	-0,091337	1
Arl2	-0,091348	1
Slc35a4	-0,091515	1
P2rx4	-0,091613	1
Ubp1	-0,091723	1
Gm2788	-0,091884	1
Zfp85	-0,091883	1
Spg7	-0,092064	1

RP24-497N7.2	-0,092087	1
Hdac4	-0,092234	1
Ctnna1	-0,092358	1
Mmadhc	-0,092443	1
Sfr1	-0,092646	1
Dubr	-0,092659	1
Ppp1r11	-0,092844	1
Sgpp1	-0,092792	1
Pgls	-0,092839	1
Slc4a1ap	-0,093221	1
Clec16a	-0,093417	1
Acvr2b	-0,093419	1
Grk2	-0,093365	1
Gm13270	-0,093523	1
Zfp609	-0,09353	1
Thumpd1	-0,093538	1
Gm11942	-0,093601	1
Dntt	-0,093738	1
RP23-366E4.9	-0,093775	1
Gsg2	-0,094156	1
Asrgl1	-0,094309	1
Atrx	-0,094317	1
Timm50	-0,094504	1
Phospho2	-0,094512	1
Wdr46-ps	-0,094592	1
Dagla	-0,094599	1
Gm9828	-0,094733	1
Cmtr2	-0,094813	1
Gid4	-0,094885	1
Lysmd4	-0,094986	1
Dnmt1	-0,095048	1
Lmf1	-0,095061	1
Spats2	-0,095095	1
Fchsd2	-0,09523	1
Prmt1	-0,095436	1
Setd5	-0,095502	1
Ifrd2	-0,095697	1
Pik3ca	-0,095679	1
Gm5380	-0,095844	1
Dnajc17	-0,095822	1
Rab40c	-0,09576	1
Chmp5	-0,095951	1
Col18a1	-0,096108	1
BC049715	-0,096202	1
Tyw1	-0,09618	1
Nif3l1	-0,096166	1
Uqcrfs1	-0,096229	1
Ints5	-0,096346	1
Drap1	-0,096323	1
Gm9790	-0,096354	1
Upf3b	-0,096432	1
Rce1	-0,096511	1

Cript	-0,096486	1
Atp13a3	-0,096476	1
Itsn1	-0,096596	1
Tram1	-0,097107	1
Hcfc2	-0,097214	1
Psmb1	-0,097394	1
Tmem267	-0,097621	1
Gm8869	-0,097736	1
Pms2	-0,097829	1
Rasa1	-0,097753	1
Zdhhc17	-0,097943	1
Itgb2	-0,097911	1
Rpl34	-0,097863	1
Arid4b	-0,097883	1
Gm8451	-0,098019	1
Hivep2	-0,098023	1
Wnk1	-0,097989	1
Ap1b1	-0,09797	1
Atg4d	-0,098129	1
Otulin	-0,098252	1
Gxylt1	-0,098413	1
Kcmf1	-0,098379	1
Cops7a	-0,098671	1
Qtrtd1	-0,098769	1
Supt5	-0,09889	1
Umad1	-0,099123	1
Rnf183	-0,099336	1
Wdtc1	-0,099342	1
Stat5a	-0,09943	1
Nploc4	-0,09947	1
4931428F04Rik	-0,099641	1
Mfsd7a	-0,099634	1
Rpsa-ps1	-0,099567	1
Spen	-0,099678	1
Rps24-ps3	-0,099694	1
Apex2	-0,10001	1
Gabarapl2	-0,10003	1
Rasgef1a	-0,10021	1
Ibtk	-0,10022	1
Gm15564	-0,1003	1
Gm45729	-0,10044	1
Ppat	-0,10051	1
Pias3	-0,10051	1
Mipol1	-0,10064	1
4930453N24Rik	-0,10073	1
Limd2	-0,10069	1
Brd8	-0,10066	1
Zfp513	-0,10095	1
Dhrs9	-0,10117	1
Ly6e	-0,10118	1
Timm10b	-0,10142	1
Cox4i1	-0,10144	1

S100a11	-0,10178	1
Plk1	-0,10192	1
Dnaja3	-0,10187	1
Gm15393	-0,10231	1
Gas7	-0,10243	1
Rbak	-0,10279	1
Hhex	-0,10289	1
Osbpl3	-0,10294	1
Snrnp200	-0,10296	1
Acap3	-0,10315	1
Gm12848	-0,10325	1
Ascl2	-0,10332	1
Trim56	-0,10332	1
B3gat3	-0,10341	1
Setd3	-0,10338	1
Kdelc1	-0,10356	1
Rbpms	-0,10373	1
Aim1	-0,10377	1
Sgf29	-0,10376	1
Nr2c2	-0,10392	1
Zfp59	-0,10402	1
Usp9x	-0,10404	1
Anp32b	-0,1041	1
Cers6	-0,10424	1
Tctn3	-0,10441	1
2610318N02Rik	-0,10455	1
A130010J15Rik	-0,10452	1
Ndufs5	-0,1046	1
Rps15-ps2	-0,10479	1
Fbxl6	-0,10476	1
Ddx39b	-0,10491	1
Tmsb4x	-0,10499	1
Ipo4	-0,10502	1
Rpl8	-0,10497	1
Gm4705	-0,10529	1
Rbm25	-0,1053	1
Slc26a2	-0,10541	1
Rcor2	-0,10545	1
Ecel1	-0,10594	1
BC037034	-0,1061	1
Fam8a1	-0,10612	1
Nme1	-0,10613	1
Nop58	-0,10607	1
Lrrk2	-0,10618	1
Gm27046	-0,10627	1
E130311K13Rik	-0,10643	1
Gm7618	-0,10661	1
Bcl2l1	-0,1066	1
9130230L23Rik	-0,10668	1
Nbn	-0,10678	1
Top3a	-0,10677	1
Slc9a6	-0,10678	1

Gm11966	-0,10686	1
Tbc1d5	-0,10696	1
Nsfl1c	-0,10727	1
Echdc1	-0,10728	1
Gm44609	-0,10736	1
Gskip	-0,10741	1
Ppil3	-0,1077	1
Rnf139	-0,10768	1
Ilk	-0,10782	1
1110059E24Rik	-0,10778	1
Impad1	-0,10804	1
Acad11	-0,10813	1
Dgkg	-0,10807	1
Pde6g	-0,10826	1
Smarca4	-0,10843	1
Zfp974	-0,10848	1
Wdr5	-0,10849	1
Nxf7	-0,10859	1
Lzts2	-0,10857	1
Ago2	-0,10856	1
Cab39	-0,10875	1
Mrps2	-0,10876	1
9330162012Rik	-0,10938	1
Copg1	-0,10963	1
Chchd1	-0,10956	1
Mks1	-0,10971	1
Repin1	-0,10976	1
Ube2g2	-0,10979	1
Odf2	-0,10991	1
Gm12396	-0,11001	1
Cldn12	-0,11016	1
Mycbp	-0,11022	1
Larp4b	-0,11043	1
Myo7a	-0,11064	1
1110004F10Rik	-0,11056	1
Cdk5rap2	-0,11076	1
Bcl2l11	-0,11111	1
Dusp14	-0,11133	1
Lonp1	-0,11131	1
Rps10	-0,11145	1
Plekhm1	-0,11137	1
Fam212a	-0,11152	1
Tcea2	-0,11151	1
Lxn	-0,11164	1
Clcn7	-0,11171	1
Zfp830	-0,1119	1
Mrps9	-0,11223	1
Rab10	-0,11234	1
Itga4	-0,11236	1
Nfs1	-0,11245	1
Nudt4	-0,11237	1
Stx8	-0,11247	1

Lsm6	-0,11283	1
Ppp1r14b	-0,1129	1
Cklf	-0,11323	1
Eef1g	-0,1132	1
Poc1a	-0,11337	1
Tmem44	-0,11344	1
Arpc5l	-0,11345	1
Pacs2	-0,11359	1
Mpc2	-0,11365	1
Peak1	-0,11382	1
Rpl41	-0,11381	1
Stxbp5	-0,11391	1
Riox1	-0,11398	1
Atp5j2	-0,11403	1
Ndufa6	-0,11396	1
Zfp652os	-0,11422	1
Abca2	-0,1144	1
Plekhh2	-0,11443	1
Tctex1d2	-0,11463	1
Plcd1	-0,1149	1
Prmt6	-0,11486	1
Skap2	-0,11496	1
Mrpl21	-0,1151	1
Zfp1	-0,11516	1
D6Wsu163e	-0,11532	1
Plxnb3	-0,11527	1
Ero1lb	-0,11534	1
Emsy	-0,11531	1
Tmem161b	-0,11539	1
Rpl10a-ps1	-0,1154	1
Slc9a5	-0,11547	1
Mapk14	-0,11546	1
Coa5	-0,11592	1
Hexdc	-0,11596	1
Homez	-0,11609	1
Ccdc88a	-0,11613	1
Phf6	-0,11609	1
Ankfy1	-0,11623	1
Snrnp48	-0,11621	1
Gm4468	-0,11629	1
Aifm1	-0,11641	1
Ccdc14	-0,11651	1
Pcmtd2	-0,11645	1
Hspd1	-0,11671	1
Gm22748	-0,1169	1
Nudt19	-0,11694	1
Akr7a5	-0,11697	1
Gm43466	-0,11708	1
Dock10	-0,11713	1
Elp6	-0,11722	1
Sun1	-0,11724	1
Phpt1	-0,11721	1

9130008F23Rik	-0,11734	1
Snrnp35	-0,11726	1
Gars	-0,11729	1
Efna1	-0,11739	1
Dcun1d5	-0,1174	1
Plcb2	-0,11754	1
mt-Nd1	-0,11747	1
Zfp523	-0,11759	1
Bloc1s6	-0,11766	1
Thap3	-0,11785	1
Fndc10	-0,11793	1
Sec31b	-0,11796	1
Thoc2	-0,11803	1
Mcee	-0,11811	1
Gm12758	-0,11831	1
Lipt1	-0,11828	1
Rps15	-0,11829	1
Tmem30a	-0,11841	1
Stc1	-0,11857	1
Gm9701	-0,11871	1
Zfp579	-0,11883	1
Rpl6l	-0,1189	1
App	-0,11886	1
Dsel	-0,11904	1
Ube2z	-0,11903	1
Gm6444	-0,11914	1
Flad1	-0,11911	1
Nabp1	-0,11924	1
Gm4149	-0,11915	1
Tstd2	-0,11929	1
Sec24c	-0,1194	1
Ctnnbl1	-0,11942	1
Soga1	-0,11955	1
Slc6a12	-0,11945	1
Pif1	-0,11988	1
Stk10	-0,11989	1
Appl2	-0,12028	1
Myo1e	-0,12037	1
Clptm1	-0,12047	1
Igbp1	-0,12059	1
Tceal8	-0,12066	1
Ncbp2	-0,12071	1
Anks1	-0,12073	1
Sfswap	-0,12065	1
Egr2	-0,12081	1
Gm12696	-0,12076	1
Zkscan3	-0,1209	1
Gm43773	-0,12101	1
Gm45212	-0,12113	1
Sypl	-0,12112	1
Zcwpw1	-0,12116	1
Psmc2	-0,1212	1

Cfdp1	-0,12117	1
D630023F18Rik	-0,12125	1
Gm16106	-0,1214	1
Tsen54	-0,12136	1
Slc36a3os	-0,12157	1
Map2k4	-0,12163	1
Fnbp1	-0,12163	1
Gm10074	-0,12168	1
Lman2l	-0,12177	1
Vat1	-0,12179	1
Gm6368	-0,12186	1
Cox8a	-0,12194	1
Cptp	-0,12197	1
Ifngr2	-0,12199	1
Cdv3	-0,12205	1
Daglb	-0,12214	1
Slc25a51	-0,12221	1
Cenpo	-0,12233	1
Ctif	-0,12234	1
Hist2h2be	-0,12244	1
Slc39a6	-0,12235	1
Ppp2r5c	-0,12264	1
Odc1	-0,12272	1
Bysl	-0,12272	1
Arfgef3	-0,12282	1
Oxnad1	-0,12289	1
Eif3i	-0,12302	1
Slc45a3	-0,12315	1
1810058I24Rik	-0,12327	1
Plpp5	-0,12339	1
1700001C19Rik	-0,12344	1
Cab39l	-0,12362	1
Zfand6	-0,1236	1
Gm29462	-0,12374	1
Gatsl3	-0,12379	1
Nsmce4a	-0,12379	1
1600002K03Rik	-0,12391	1
Acvr1b	-0,12402	1
Cyb5r1	-0,12412	1
Cenpj	-0,12419	1
Asb6	-0,12423	1
Exosc9	-0,12423	1
Arid4a	-0,12437	1
Gm37407	-0,12455	1
Pex12	-0,12451	1
Psma6	-0,12448	1
Aars	-0,1246	1
Gdi1	-0,12491	1
Ttc3	-0,125	1
Myo10	-0,12496	1
Fcf1	-0,12504	1
Myh9	-0,12498	1

Parn	-0,12522	1
Zfp810	-0,12526	1
Ube2d2a	-0,12529	1
Lrrcc1	-0,12549	1
Lztfl1	-0,12554	1
Fbxo28	-0,12563	1
Gm45224	-0,12591	1
Ap3s2	-0,12591	1
Ttc33	-0,12603	1
Psmd1	-0,12599	1
Lpp	-0,1261	1
Gm43788	-0,12635	1
Zfp956	-0,12631	1
Kctd9	-0,12651	1
Lamtor5	-0,12651	1
Fam104a	-0,12646	1
Tpcn2	-0,12657	1
Uaca	-0,12667	1
Chmp1a	-0,12678	1
Glo1	-0,12757	1
Ccdc138	-0,12772	1
Gm6598	-0,1278	1
Slc35a1	-0,12791	1
Aldh16a1	-0,12787	1
Smpd1	-0,12791	1
Hacd3	-0,12798	1
Ammecr1l	-0,12798	1
Smn1	-0,12798	1
Fen1	-0,12816	1
Txndc9	-0,12824	1
Tsc22d4	-0,12827	1
Tomm7	-0,12829	1
Gm42548	-0,12855	1
Slc22a17	-0,12862	1
Add3	-0,12884	1
Gm26384	-0,12885	1
Rad23b	-0,12889	1
Scfd1	-0,12901	1
Sugp2	-0,12923	1
Gtpbp3	-0,1293	1
Sltm	-0,12934	1
Tvp23b	-0,12939	1
Ppib	-0,12941	1
Sec61g	-0,12975	1
Gm9575	-0,12988	1
Atxn3	-0,13007	1
Supt4a	-0,13022	1
S1pr2	-0,13022	1
Fignl1	-0,13026	1
Ryr1	-0,13033	1
Gm2796	-0,13027	1
Snx4	-0,13034	1

Gm16638	-0,13039	1
Ankrd52	-0,13049	1
Gm9712	-0,13063	1
Rnf14	-0,13073	1
Anapc10	-0,13098	1
Mapk9	-0,13096	1
Gm15920	-0,13101	1
Pianp	-0,1311	1
Taf6	-0,13114	1
Fgd3	-0,13124	1
Arap1	-0,13125	1
Mef2a	-0,13154	1
Haus8	-0,1316	1
Emp3	-0,13161	1
Ebna1bp2	-0,13174	1
Dhx58	-0,1318	1
Hsd17b10	-0,13203	1
Ubtd1	-0,13196	1
Rrp7a	-0,13209	1
Phf3	-0,13207	1
Arrb2	-0,13217	1
Zcchc6	-0,13228	1
Ankrd13c	-0,13239	1
AI662270	-0,13253	1
Gps1	-0,13257	1
Rps27a-ps1	-0,13257	1
Mcu	-0,13259	1
Jade2	-0,13267	1
Bpgm	-0,13288	1
Zmym4	-0,13287	1
Rfx1	-0,13304	1
Trpm1	-0,13311	1
Gm12989	-0,13314	1
Lamc2	-0,13322	1
Acd	-0,13323	1
Entpd1	-0,13333	1
Ctsf	-0,13335	1
Plekha3	-0,13346	1
Lrrc8d	-0,1336	1
Atox1	-0,13361	1
Zfp560	-0,13374	1
Atg2b	-0,13372	1
D230022J07Rik	-0,13376	1
Lrrk1	-0,13383	1
Slc20a2	-0,13395	1
2900009J06Rik	-0,13389	1
Pgrmc2	-0,13403	1
Sap30l	-0,1341	1
Kif2a	-0,13408	1
Dscr3	-0,13421	1
Cspp1	-0,13419	1
4933408B17Rik	-0,13427	1

Loxl3	-0,13438	1
Zfp518a	-0,13442	1
Mfap3	-0,13451	1
Hspb11	-0,13451	1
Atp8a1	-0,13461	1
Ppan	-0,13458	1
Uqcrh-ps1	-0,13473	1
B3gnt3	-0,13467	1
Trappc13	-0,13465	1
Nvl	-0,13467	1
Lrrc25	-0,13481	1
RP24-131G14.13	-0,13495	1
Gtf2f2	-0,13487	1
Usp11	-0,13496	1
2310034G01Rik	-0,13513	1
Pgm2l1	-0,13509	1
Acsl4	-0,13513	1
Mtfr1l	-0,13514	1
Srek1ip1	-0,13526	1
Gtf2b	-0,13553	1
Akap13	-0,13551	1
Eif2s2	-0,13557	1
Plcd3	-0,1358	1
Ide	-0,13578	1
Ptpmt1	-0,13575	1
Gpx1	-0,13591	1
Hnrnpf	-0,13619	1
Stag1	-0,13622	1
AU019823	-0,13624	1
Ccdc115	-0,13645	1
Irgm1	-0,13673	1
BC002059	-0,13668	1
Gm37084	-0,13679	1
Arhgap30	-0,13678	1
Ankrd37	-0,13693	1
Eif5b	-0,13716	1
Snhg1	-0,13732	1
Ptpn18	-0,13741	1
Zdhhc8	-0,13751	1
Zfp950	-0,13749	1
Hars	-0,13753	1
Gm38125	-0,13757	1
Mtmr4	-0,13764	1
Mepce	-0,13761	1
Ubr3	-0,13772	1
Tmem258	-0,13774	1
Fgd6	-0,13785	1
Sdf2	-0,13777	1
Gm35315	-0,13787	1
Dctpp1	-0,13808	1
Pspc1	-0,13816	1
Gm4217	-0,13835	1

Mrrf	-0,13834	1
Hmga1-rs1	-0,13842	1
Dhdh	-0,13842	1
Gm17586	-0,13855	1
Gm9800	-0,13861	1
Ighmbp2	-0,13859	1
1700029J07Rik	-0,13871	1
Lrsam1	-0,13867	1
Ablim1	-0,13876	1
Cers5	-0,13891	1
Cog2	-0,13897	1
Cwc25	-0,13914	1
Gm43482	-0,13916	1
Aim2	-0,13918	1
Thoc6	-0,13932	1
Snapc5	-0,13938	1
Sec61a2	-0,13947	1
Hypk	-0,13964	1
Ddx27	-0,13969	1
Gm8574	-0,13981	1
Asb1	-0,13994	1
Lrwd1	-0,13993	1
Usmg5	-0,14011	1
Eif2b4	-0,1401	1
Pard6a	-0,14024	1
Bivm	-0,14031	1
Tagln2	-0,1403	1
RP23-453B15.7	-0,14038	1
Myg1	-0,14045	1
Pqlc3	-0,14066	1
Mapk1	-0,14071	1
Afdn	-0,14066	1
Scaf11	-0,14079	1
4933440N22Rik	-0,14086	1
Slc27a1	-0,14094	1
Emd	-0,14103	1
Picalm	-0,14108	1
Nfkb2	-0,14127	1
Macrod1	-0,14126	1
B330016D10Rik	-0,1414	1
Tssc4	-0,14136	1
Gm15625	-0,14145	1
Tdpx-ps1	-0,14161	1
Akr1a1	-0,14157	1
Mrps12	-0,14167	1
Srprb	-0,14167	1
Rab11fip2	-0,14187	1
F830115B05Rik	-0,14199	1
Cars	-0,14204	1
Olfr460	-0,14227	1
Sod2	-0,14238	1
Atl3	-0,14253	1

Bms1	-0,14245	1
Mthfd1l	-0,14263	1
Rxrb	-0,14264	1
1700020I14Rik	-0,14272	1
Hexb	-0,14268	1
Csde1	-0,14265	1
Rgs19	-0,14298	1
Emg1	-0,14297	1
Taok3	-0,143	1
Degs1	-0,14296	1
Sdhaf1	-0,14306	1
Abcc5	-0,14306	1
Gm5939	-0,1433	1
Faim	-0,14329	1
Fbxo22	-0,14335	1
Aldoa	-0,14354	1
Csf2rb	-0,1437	1
Pik3cd	-0,14377	1
Coq7	-0,144	1
Abcd3	-0,14416	1
Bsn	-0,14432	1
Ufd1l	-0,1443	1
Wdr61	-0,14436	1
RP24-275P22.2	-0,14452	1
Pcyt1a	-0,14456	1
Psmg1	-0,14471	1
Cenpq	-0,14469	1
Nkap	-0,1447	1
Myadm	-0,14466	1
Mettl18	-0,14519	1
Parp4	-0,14527	1
Sacm1l	-0,14543	1
Rfng	-0,14561	1
Zfp777	-0,14571	1
Vwa8	-0,14582	1
Klhl20	-0,14588	1
Kars	-0,14601	1
Fbxl12os	-0,14614	1
Crem	-0,14612	1
Stx18	-0,14619	1
2700060E02Rik	-0,14616	1
Gm43712	-0,14628	1
Itgb1bp1	-0,14662	1
Snx30	-0,14659	1
Dcun1d1	-0,14674	1
Dcaf15	-0,14692	1
Ube2v1	-0,14709	1
Hs2st1	-0,14711	1
Ubac1	-0,14715	1
Rsl24d1	-0,14717	1
Gemin5	-0,14726	1
Smarcc2	-0,14727	1

Chchd5	-0,14735	1
Zfp280c	-0,14753	1
Gm37105	-0,1475	1
Mfn1	-0,14764	1
2510046G10Rik	-0,14777	1
2310010J17Rik	-0,14797	1
Sucla2	-0,14806	1
Gm13378	-0,1482	1
Hoxc4	-0,14833	1
Hibch	-0,14834	1
Tcaf1	-0,14845	1
Tbc1d19	-0,14847	1
Gm9165	-0,14876	1
Atxn1	-0,14876	1
Tbc1d2	-0,14891	1
Sat2	-0,14897	1
Gm37962	-0,14903	1
Cd300ld	-0,14931	1
Arl14ep	-0,14934	1
Pi4ka	-0,14926	1
RP24-401G4.1	-0,14943	1
Pik3ap1	-0,14961	1
Traf5	-0,14967	1
Ypel3	-0,14973	1
Ddx49	-0,1498	1
Sel1l	-0,14978	1
Nudt21	-0,1499	1
Exosc1	-0,15012	1
Ankib1	-0,15016	1
B4galt6	-0,15056	1
Fis1	-0,15064	1
Tmem70	-0,15062	1
Gm5297	-0,15074	1
Med8	-0,15084	1
Ppp1r18	-0,15087	1
Fads6	-0,15111	1
Spsb2	-0,15107	1
Fhod1	-0,15123	1
Ranbp2	-0,15117	1
Sra1	-0,15124	1
Vapb	-0,15116	1
Atp8b3	-0,15133	1
Cstf2	-0,15129	1
Ak2	-0,15126	1
Heatr3	-0,15137	1
Fermt3	-0,15151	1
Otud5	-0,15155	1
Ngrn	-0,15158	1
Nol6	-0,15169	1
Galk2	-0,15172	1
Appl1	-0,15168	1
Serpinb6a	-0,15166	1

C530005A16Rik	-0,1518	1
Kifc3	-0,15176	1
Cox11	-0,15187	1
S100a6	-0,1519	1
Ift27	-0,15198	1
Bora	-0,15203	1
RP24-460E12.3	-0,15252	1
Rtca	-0,15247	1
Mcub	-0,1525	1
Suox	-0,15256	1
Tmem184c	-0,15258	1
Mprip	-0,15256	1
Polrmt	-0,15272	1
Tspo	-0,15269	1
Rab11b	-0,15282	1
Ddx21	-0,15283	1
Crybg3	-0,15301	1
Slirp	-0,15311	1
Park7	-0,15329	1
Gm36378	-0,15363	1
Gm13487	-0,15366	1
Spi1	-0,15381	1
Slu7	-0,15382	1
Notch2	-0,15412	1
Gm6023	-0,15416	1
Zbtb33	-0,15449	1
Timm10	-0,1545	1
Tcf25	-0,15446	1
Rrp1	-0,15474	1
Nomo1	-0,15477	1
Ccdc134	-0,15489	1
Riok3	-0,15491	1
Trmt10b	-0,15496	1
Them4	-0,15521	1
Rreb1	-0,15534	1
Usp3	-0,15532	1
Eri3	-0,15538	1
Zwint	-0,15538	1
Gm12854	-0,15551	1
Zfp72	-0,15548	1
Trnau1ap	-0,15551	1
Srrd	-0,15563	1
Timm8b	-0,15567	1
Rnps1	-0,15583	1
Ctnnb1	-0,15594	1
Dcbld2	-0,15611	1
Ap1s2	-0,15611	1
Gaa	-0,15621	1
Gm42478	-0,15643	1
Gm6028	-0,15649	1
Atf5	-0,15656	1
Elf4	-0,15669	1

Dennd1b	-0,15672	1
Larp1b	-0,15684	1
Slc39a14	-0,15688	1
Qdpr	-0,15707	1
Dpf1	-0,15734	1
Gm7776	-0,1573	1
Prpf40b	-0,15733	1
Ube2i	-0,15734	1
AU020206	-0,15736	1
Mcoln2	-0,15793	1
Gm13205	-0,15788	1
Slc27a3	-0,15798	1
Pus1	-0,15795	1
Itgal	-0,15823	1
Cdk2ap2	-0,15823	1
Cdon	-0,15849	1
Ggct	-0,15855	1
Rgs10	-0,15852	1
Tmem110	-0,15905	1
Nsa2-ps2	-0,15906	1
Pex10	-0,15916	1
Tbc1d1	-0,15922	1
Ezh2	-0,15931	1
Ralgps1	-0,15951	1
Csnk2b	-0,15946	1
Cdkn2aipnl	-0,15962	1
Fcgr4	-0,15981	1
Sfxn5	-0,15993	1
Tex261	-0,16009	1
Enkd1	-0,16017	1
Shq1	-0,16026	1
Ksr1	-0,16038	1
Gm14494	-0,1604	1
Ncdn	-0,16042	1
Eid1	-0,16052	1
Srf	-0,16058	1
RP23-114G13.7	-0,16074	1
Zbtb6	-0,161	1
D130017N08Rik	-0,16113	1
Tnnt1	-0,16109	1
Cryz	-0,16106	1
Kctd2	-0,16122	1
Tceanc2	-0,16136	1
Agrn	-0,16136	1
Irf9	-0,16152	1
Pef1	-0,16161	1
Dync2li1	-0,16166	1
Myo1c	-0,16167	1
Bckdhb	-0,16178	1
Cds2	-0,16176	1
Mzf1	-0,16192	1
Ttf2	-0,16187	1

Mppe1	-0,16195	1
Homer3	-0,16199	1
Usp30	-0,16208	1
Htatip2	-0,16221	1
Clcnkb	-0,16225	1
Gm8606	-0,1624	1
Fam199x	-0,16246	1
Gab2	-0,16254	1
B4galnt1	-0,16269	1
Pik3r4	-0,16284	1
Relb	-0,16292	1
Trappc11	-0,16298	1
Letm2	-0,16307	1
Tmem204	-0,16322	1
Mettl22	-0,16321	1
Gm37121	-0,16335	1
Ssc5d	-0,16351	1
Amd1	-0,16373	1
Gm7847	-0,16384	1
Gm7102	-0,16399	1
Surf1	-0,16407	1
Ptdss2	-0,16414	1
Sp140	-0,16425	1
Rcc2	-0,16419	1
1110065P20Rik	-0,16471	1
Vasp	-0,16472	1
Znrf2	-0,16476	1
Wdr89	-0,16495	1
Ogfod1	-0,16506	1
Arhgdib	-0,16514	1
Nhp2	-0,16507	1
Ndufs2	-0,16511	1
Stxbp2	-0,16544	1
Trip13	-0,1656	1
Rad9a	-0,16565	1
Adarb1	-0,16559	1
Lyrm4	-0,16567	1
Tmem43	-0,1657	1
Gm29257	-0,16577	1
Rspry1	-0,16592	1
Inca1	-0,16596	1
Tmem50a	-0,16598	1
Tada3	-0,16613	1
Frg2f1	-0,16617	1
Rpl21-ps12	-0,16626	1
Gata3	-0,16642	1
Metrn	-0,16649	1
Gss	-0,16654	1
Rpl36-ps10	-0,16653	1
Huwe1	-0,16662	1
Rabep1	-0,16656	1
Atraid	-0,16668	1

Etaa1	-0,16677	1
9430015G10Rik	-0,16697	1
Foxk2	-0,1671	1
Dexi	-0,16716	1
Sirt4	-0,16728	1
Gm6524	-0,16734	1
Dnajc27	-0,16739	1
Gm8731	-0,1674	1
Paf1	-0,16739	1
Gm44103	-0,16753	1
Ubox5	-0,16758	1
Metap1	-0,16778	1
Ranbp3	-0,16794	1
Jade1	-0,16815	1
Ero1l	-0,1681	1
Ggnbp2	-0,16813	1
Mum1	-0,16821	1
Ap2m1	-0,16854	1
Gm15032	-0,16858	1
Gm5239	-0,16896	1
Impa1	-0,16908	1
Usp27x	-0,16939	1
Hpcal1	-0,16951	1
Atp5d	-0,16951	1
Gm13935	-0,16989	1
Pros1	-0,16987	1
Gm14586	-0,17001	1
Gm2076	-0,17011	1
Dnmt3a	-0,17009	1
Serbp1	-0,17023	1
Cyhr1	-0,1703	1
Mnd1-ps	-0,17045	1
Snx29	-0,17066	1
Mtl5	-0,17082	1
Bcdin3d	-0,17077	1
Txndc16	-0,17083	1
Lum	-0,17097	1
F2	-0,17103	1
Zfp871	-0,17108	1
Bcl7b	-0,17116	1
Mrps28	-0,17133	1
Guk1	-0,17126	1
Il10rb	-0,17132	1
Wapl	-0,17129	1
Klhdc3	-0,1715	1
Enpp4	-0,1716	1
Srd5a1	-0,17165	1
Mut	-0,17175	1
Cr1l	-0,17174	1
Cdc26	-0,1718	1
Bsg	-0,17177	1
Ccdc34	-0,17224	1

Inafm2	-0,17228	1
Gm10080	-0,17244	1
Specc1l	-0,1725	1
Chsy1	-0,17249	1
Scly	-0,17256	1
Cacna1a	-0,1726	1
Dvl1	-0,1727	1
Ssbp3	-0,17293	1
Anp32b-ps1	-0,17293	1
Ptp4a2	-0,17293	1
Ncln	-0,17312	1
Exoc6b	-0,17322	1
Mrps5	-0,17322	1
9330111N05Rik	-0,17334	1
Wdr34	-0,17335	1
Mrpl58	-0,17342	1
Vipas39	-0,17351	1
Nr1d2	-0,17377	1
Aven	-0,1739	1
Psme3	-0,17388	1
Dstyk	-0,17392	1
Zfp672	-0,17402	1
Lap3	-0,17414	1
Agap3	-0,17417	1
Ndufv3	-0,17443	1
Blvra	-0,1745	1
Sumo3	-0,17455	1
Gm7990	-0,17463	1
Gm12743	-0,17462	1
Stx6	-0,17456	1
Rdm1	-0,1746	1
Nt5c3	-0,17466	1
Stx4a	-0,17472	1
Ncoa2	-0,17485	1
Gm9844	-0,1752	1
Pfdn2	-0,17518	1
Riok1	-0,17519	1
Brd3	-0,17529	1
Baz2b	-0,17543	1
Pigyl	-0,17555	1
Josd1	-0,17555	1
Lias	-0,17548	1
Sike1	-0,17562	1
Gm37760	-0,17576	1
Fam65a	-0,17579	1
Zbtb45	-0,17604	1
Nap1l4	-0,17604	1
Utp11	-0,17604	1
Ptpn4	-0,17606	1
Jtb	-0,17657	1
Txn2	-0,17661	1
Ralbp1	-0,17655	1

Fam3a		-0,17667	1
Sin3b		-0,17665	1
Nsa2		-0,17681	1
Adamtsl1		-0,17678	1
Ccdc124		-0,17677	1
2510009E07Rik		-0,17679	1
Eif3g		-0,17678	1
Hcar2		-0,1769	1
Gm10602		-0,17705	1
Il13ra1		-0,17719	1
Gm43362		-0,17733	1
Gm8973		-0,1773	1
	Sep 09	-0,17734	1
Slc35f6		-0,17732	1
Mcfd2		-0,17726	1
Fkbp4		-0,17745	1
Cetn2		-0,17748	1
Tceanc		-0,17765	1
Nop14		-0,17768	1
Fam134a		-0,17768	1
Ska2		-0,17781	1
Tmem208		-0,17777	1
Lrpap1		-0,1778	1
1110046J04Rik		-0,17795	1
Icam1		-0,17806	1
Atp6v0e2		-0,17809	1
RP23-331E5.10		-0,17824	1
Lhfpl2		-0,17843	1
Fut11		-0,17854	1
Gm2272		-0,17861	1
Ulk2		-0,17876	1
Sugt1		-0,17899	1
Llph-ps1		-0,17928	1
Cdk5		-0,17953	1
Arhgap22		-0,17962	1
Ube3b		-0,1796	1
Scp2		-0,17961	1
Cbx7		-0,17971	1
Eva1b		-0,17979	1
Cyfip1		-0,17978	1
Snf8		-0,17981	1
Gm11560		-0,17997	1
Emc3		-0,18019	1
Slfn5		-0,18028	1
mt-Nd5		-0,18033	1
Tmem222		-0,1803	1
Orai2		-0,18036	1
Prnp		-0,18045	1
Tprgl		-0,18064	1
Sephs2		-0,18075	1
Ndufb11		-0,18068	1
Gm7600		-0,1808	1

Ywhae	-0,18094	1
Tsen34	-0,18105	1
Cep290	-0,18114	1
1810032O08Rik	-0,18106	1
Dctn5	-0,18118	1
Gm13862	-0,18137	1
Gm38377	-0,18154	1
Lmbr1	-0,1816	1
Gm44258	-0,18172	1
Casp9	-0,18185	1
Dtx4	-0,1818	1
Nras	-0,18197	1
Iah1	-0,18196	1
Anxa6	-0,18214	1
Psrc1	-0,18222	1
Apba1	-0,18243	1
Erc1	-0,18263	1
AI506816	-0,18267	1
Mrm1	-0,18272	1
Focad	-0,18292	1
Cdk5rap1	-0,18301	1
Crot	-0,18304	1
Gm44694	-0,18315	1
Rhbdd3	-0,18316	1
Gm7670	-0,1833	1
Fip1l1	-0,18325	1
Usp38	-0,1836	1
Gm15708	-0,18361	1
Map3k20	-0,18372	1
Safb2	-0,18379	1
Vkorc1l1	-0,18386	1
Psip1	-0,18387	1
Gm5881	-0,18398	1
Gm17066	-0,18395	1
Gm43147	-0,18407	1
Trmt61a	-0,18408	1
Gm45380	-0,18419	1
Utp18	-0,18426	1
Usb1	-0,1843	1
Vars	-0,18437	1
Slc38a10	-0,18447	1
Vti1b	-0,18453	1
Tnnc1	-0,18456	1
Sap30bp	-0,18472	1
Wdr18	-0,18469	1
Reep3	-0,18473	1
Gstm1	-0,18476	1
Osbpl9	-0,18482	1
Gm37578	-0,18511	1
Dhrs7	-0,18526	1
Lasp1	-0,18552	1
H2-M3	-0,18561	1

Cep295	-0,1856	1
Hcls1	-0,18559	1
1110037F02Rik	-0,18574	1
Gm18913	-0,18593	1
Kmt2e	-0,18592	1
Hagh	-0,18602	1
Slc25a14	-0,18608	1
Cnbp	-0,18616	1
Gpank1	-0,18627	1
Desi1	-0,1864	1
Gnl1	-0,18636	1
Trim30a	-0,1865	1
Gcsh	-0,18648	1
Psmb6	-0,18661	1
Ears2	-0,18668	1
Ccdc137	-0,18667	1
Dpy19l1	-0,18673	1
Idh1	-0,1867	1
Unc13d	-0,18692	1
Ssrp1	-0,18693	1
Lrrc58	-0,18734	1
Sf3b5	-0,18733	1
5730455P16Rik	-0,18737	1
Fbxo25	-0,18789	1
Coq8a	-0,18803	1
Tshz1	-0,18801	1
Uchl3	-0,18801	1
Prorsd1	-0,188	1
Ddx56	-0,18825	1
Gripap1	-0,18829	1
Gm12115	-0,18844	1
Gm37470	-0,18889	1
Olfr920	-0,18891	1
Zfp952	-0,189	1
Smox	-0,18896	1
Trappc5	-0,18933	1
Ecsit	-0,18995	1
Gm27477	-0,19038	1
Tldc1	-0,19043	1
Smarcad1	-0,1904	1
Dnajc14	-0,19041	1
Exoc1	-0,1905	1
Trim7	-0,19055	1
Dus3l	-0,19062	1
Rnf213	-0,19057	1
Ddx18	-0,19065	1
Gm9381	-0,19074	1
Tgm2	-0,1907	1
Iqsec1	-0,19077	1
Cryga	-0,19086	1
Atm	-0,19089	1
Gtf2h2	-0,19105	1

Pax6	-0,19134	1
Syf2	-0,1915	1
Rmrp	-0,19158	1
Dync1i2	-0,19157	1
Nova2	-0,19182	1
Ehd2	-0,19212	1
Sdhaf3	-0,19211	1
AC133103.1	-0,19241	1
Fance	-0,19243	1
Brox	-0,19236	1
Wdfy1	-0,19265	1
Gm6265	-0,19291	1
Exoc5	-0,19288	1
Inpp1	-0,19296	1
Tm9sf4	-0,19304	1
Plcg1	-0,19308	1
9030617O03Rik	-0,19309	1
Ugdh	-0,19326	1
Gbf1	-0,19332	1
Gnb5	-0,19358	1
Usp4	-0,19372	1
Entpd5	-0,19389	1
Ubr5	-0,194	1
Ntpcr	-0,19408	1
Rela	-0,19405	1
Pcbp1	-0,19421	1
Rcc1	-0,1943	1
Trafd1	-0,19433	1
Micu1	-0,19452	1
Atp5g3	-0,19453	1
Epb41l5	-0,19461	1
Gm2383	-0,1947	1
Psma4	-0,19469	1
Cdipt	-0,19467	1
Epm2aip1	-0,19477	1
Rab3ip	-0,19491	1
Rps12	-0,195	1
Nudc	-0,19519	1
Xpa	-0,19535	1
Klhdc1	-0,19547	1
Gid8	-0,1956	1
Mfsd12	-0,1958	1
Dock8	-0,19602	1
Ccdc43	-0,19605	1
Ptgr2	-0,19628	1
Ccr10	-0,19636	1
Cebpz	-0,1964	1
Polr2m	-0,19644	1
Dhx30	-0,19662	1
2610008E11Rik	-0,19667	1
Tarsl2	-0,19702	1
Rpl5-ps1	-0,19711	1

Pigx	-0,19714	1
Med9	-0,19722	1
Pbx3	-0,1972	1
Bin2	-0,19753	1
Knop1	-0,19748	1
Ankrd28	-0,19757	1
Fmo5	-0,19781	1
Glod4	-0,19792	1
2810408I11Rik	-0,19803	1
Dctn3	-0,19823	1
Nrros	-0,19838	1
Sugp1	-0,19852	1
Utp6	-0,19845	1
Ankrd10	-0,19857	1
Pebp1	-0,19878	1
2210008F06Rik	-0,19889	1
mt-Cytb	-0,19889	1
Snta1	-0,19892	1
Mtfmt	-0,19901	1
Gm26129	-0,1991	1
Hif1an	-0,19913	1
Puf60	-0,19905	1
Gm43294	-0,1992	1
Mbd1	-0,19918	1
Pdap1	-0,19922	1
Gm10736	-0,19945	1
Agl	-0,19939	1
Clpb	-0,19952	1
Srp14	-0,19967	1
AV099323	-0,20011	1
Rpl36al	-0,20015	1
Mcm3ap	-0,2003	1
Ndufb2	-0,20031	1
Ppp6r1	-0,20027	1
Gm20219	-0,20042	1
Ormdl2	-0,20037	1
Tomm40l	-0,20058	1
Gm4604	-0,20069	1
Trim11	-0,20072	1
Cdc42ep2	-0,20075	1
Gatad1	-0,20083	1
Egln2	-0,20087	1
Fam193a	-0,2011	1
Foxp1	-0,20121	1
Ndufb10	-0,20146	1
Mrpl36	-0,20164	1
Vav2	-0,20166	1
Tfpt	-0,20183	1
Sp100	-0,20177	1
Gm13443	-0,20189	1
Anpep	-0,20214	1
Rprd1b	-0,20218	1

Trim26	-0,20238	1
Rab2a	-0,20248	1
Cap1	-0,20257	1
Nradd	-0,20289	1
Slc4a2	-0,203	1
C030037D09Rik	-0,20309	1
Rpl3-ps1	-0,20321	1
Pon3	-0,20337	1
Ttll4	-0,2035	1
Cdc16	-0,20346	1
Gm12338	-0,20357	1
Ifi27l2a	-0,20362	1
Mrps22	-0,20388	1
Pip5k1c	-0,20397	1
Utrn	-0,20431	1
Aimp2	-0,20426	1
Nphp3	-0,20436	1
Aspscr1	-0,20463	1
Fbxl18	-0,20494	1
Tyk2	-0,20505	1
Zfp445	-0,2051	1
Hmg20a	-0,20515	1
Tmem138	-0,20517	1
Mfsd10	-0,20528	1
Sep 11	-0,20528	1
Gm15148	-0,20528	1
Pdgfa	-0,20544	1
Fhl3	-0,20563	1
Irak1	-0,2056	1
Herc4	-0,20565	1
Ndufb5	-0,2056	1
Fdxacb1	-0,20572	1
Vdac1	-0,20583	1
Gm13009	-0,20589	1
Lmbr1l	-0,20594	1
Fdft1	-0,20586	1
Rwdd4a	-0,20588	1
Fam229b	-0,20604	1
Rpf2	-0,20615	1
Ncf1	-0,20617	1
Abcf1	-0,2063	1
Usp5	-0,20663	1
Phb2	-0,20655	1
Ubr1	-0,2068	1
Zfp954	-0,20675	1
Srp68	-0,20683	1
Micall1	-0,20693	1
Nin	-0,20704	1
Fam129a	-0,20702	1
2010320M18Rik	-0,20711	1
Gtf3c6	-0,20709	1
Brpf1	-0,20717	1

Zfand3	-0,20723	1
Efcab14	-0,2073	1
Atg101	-0,20745	1
Psma1	-0,20735	1
Drg1	-0,20756	1
Armc1	-0,20762	1
Rab11fip5	-0,20793	1
Cisd3	-0,20795	1
Mrpl18	-0,20797	1
Wdr41	-0,20821	1
Selenoi	-0,20831	1
Rad51d	-0,20831	1
Thra	-0,20827	1
Ehd4	-0,20829	1
Il17rc	-0,20854	1
Gm16096	-0,20853	1
Tpd52l2	-0,2086	1
Utp3	-0,20856	1
Pkig	-0,2088	1
Zfp512b	-0,20907	1
Srrm2	-0,20947	1
Ppie	-0,20962	1
Ppp4r1l-ps	-0,20986	1
Deptor	-0,2099	1
AI413582	-0,20994	1
Gpd2	-0,20993	1
Galt	-0,21001	1
Eml4	-0,21031	1
Cd81	-0,21042	1
Ralgapb	-0,21047	1
Scarb1	-0,21062	1
Map7d1	-0,2107	1
Ppil2	-0,21081	1
Uck2	-0,21093	1
Pign	-0,21107	1
Mrps15	-0,21111	1
Rcl1	-0,21127	1
Exosc7	-0,2114	1
Tle6	-0,21148	1
Pcyt2	-0,21153	1
Tmem256	-0,21159	1
Gpatch8	-0,21187	1
Cyp20a1	-0,21235	1
Gm11930	-0,21248	1
Slc25a42	-0,21247	1
Rpn2	-0,21251	1
Ak6	-0,21284	1
Nr2c1	-0,21285	1
Casd1	-0,21292	1
Morc4	-0,21289	1
Rps23-ps2	-0,21314	1
Farsb	-0,21315	1

Mir5136	-0,21332	1
Gm6851	-0,21349	1
Gm12967	-0,21348	1
Map3k11	-0,2135	1
Rpl37rt	-0,21347	1
Dedd	-0,21376	1
Ssr2	-0,2138	1
Gm7666	-0,21393	1
Dctd	-0,21394	1
Ccdc126	-0,21397	1
Brms1	-0,21398	1
Mrpl57	-0,21402	1
Tmco3	-0,21409	1
Sac3d1	-0,21405	1
Tmem261	-0,21414	1
Chil6	-0,21428	1
Map4	-0,21445	1
RP23-13B8.12	-0,21449	1
Adss	-0,21454	1
Zmynd8	-0,21455	1
Zbtb8os	-0,21475	1
Zfp330	-0,21477	1
Gm9803	-0,2149	1
Hsf1	-0,21505	1
Ing4	-0,21519	1
Coq10a	-0,21534	1
Htatsf1	-0,2154	1
Zfp715	-0,21559	1
Fendrr	-0,21566	1
Rnf219	-0,2157	1
Arl3	-0,21571	1
Ttc12	-0,21578	1
Zcchc2	-0,21577	1
Pigk	-0,21584	1
Prr18	-0,21586	1
Flt3l	-0,21604	1
Hmg20b	-0,21606	1
Wisp1	-0,21622	1
Pms1	-0,21638	1
Faap24	-0,21665	1
Pknox1	-0,21659	1
Slc37a2	-0,21674	1
Uhrf1bp1l	-0,21674	1
Mlh3	-0,21711	1
Poll	-0,21705	1
Aurkaip1	-0,21719	1
Srsf9	-0,21725	1
Dnaaf2	-0,21741	1
Pnpla7	-0,21753	1
Mark2	-0,21768	1
Dcaf17	-0,21789	1
Qrsl1	-0,21794	1

Gm8186	-0,21815	1
Comtd1	-0,21834	1
Uqcrc2	-0,21826	1
Trabd	-0,21836	1
Gm12020	-0,21868	1
Cryzl1	-0,21869	1
Gclc	-0,21876	1
Dus1l	-0,21891	1
Tpcn1	-0,2189	1
Ttc38	-0,21913	1
Stap1	-0,21919	1
Ybey	-0,21932	1
Rapgef3	-0,21939	1
Pold4	-0,21954	1
Nfe2l1	-0,2196	1
Atp5b	-0,21978	1
Mastl	-0,22025	1
Fuz	-0,22016	1
Afp	-0,22023	1
Uqcrc1	-0,2203	1
Gm5449	-0,22036	1
Them6	-0,2205	1
D330045A20Rik	-0,22055	1
Gm4895	-0,22075	1
Gm26830	-0,22075	1
Syngr2	-0,22077	1
Cmbl	-0,22093	1
Max	-0,22097	1
Gm5879	-0,22173	1
Fbxo7	-0,22175	1
Olfr921	-0,22214	1
Ddx46	-0,22207	1
Car13	-0,22222	1
1110008P14Rik	-0,22219	1
Kbtbd11	-0,22232	1
Fbxw17	-0,22241	1
Mri1	-0,22246	1
Gorasp2	-0,22249	1
Tesk1	-0,22263	1
Mlec	-0,22286	1
Nat14	-0,22298	1
Ptrh2	-0,22372	1
Mib1	-0,2237	1
Pex19	-0,2238	1
Clgn	-0,22395	1
Gm27219	-0,22389	1
Naa40	-0,22393	1
Rps10-ps1	-0,2239	1
Atxn7l3b	-0,22397	1
Ino80e	-0,22412	1
Mettl5	-0,22418	1
Mettl10	-0,22428	1

Slc7a6	-0,22428	1
RP23-139H6.1	-0,22462	1
Gsk3b	-0,22463	1
Rpl7l1	-0,22473	1
Jarid2	-0,22467	1
Idnk	-0,22487	1
Tpm3-rs7	-0,22486	1
Rpl3	-0,22505	1
Znfx1	-0,22517	1
Bola1	-0,2252	1
Traf3ip3	-0,22532	1
Hoga1	-0,22532	1
Myh7b	-0,22527	1
Cyb5a	-0,22535	1
Supt16	-0,2253	1
Sharpin	-0,22537	1
Mon1b	-0,22561	1
5031434O11Rik	-0,22592	1
Pitpnm1	-0,22612	1
0610038B21Rik	-0,22621	1
Snhg12	-0,2262	1
Uap1l1	-0,22638	1
Pkm	-0,22638	1
Smad1	-0,22651	1
Shkbp1	-0,22668	1
Rest	-0,22679	1
Chd8	-0,22676	1
Tmem101	-0,22685	1
RP23-426K2.3	-0,22698	1
Pth1r	-0,22696	1
Erlec1	-0,227	1
Clns1a	-0,22709	1
Galnt7	-0,22708	1
Sec13	-0,22724	1
Wipf1	-0,2273	1
Prr3	-0,22755	1
Ndufs6	-0,22759	1
Mrpl49	-0,22765	1
Plekhm3	-0,22772	1
Eri1	-0,22777	1
Gm4366	-0,22795	1
Elk1	-0,22803	1
Ppp1r9b	-0,22796	1
Arhgef7	-0,22811	1
Mpv17l	-0,22831	1
Gm7384	-0,2283	1
Yipf1	-0,22873	1
Fxn	-0,22893	1
Hpf1	-0,22889	1
Pja1	-0,22916	1
Xylt1	-0,2292	1
Abhd14b	-0,22951	1

Gm5617	-0,22954	1
Glce	-0,22983	1
Pym1	-0,23005	1
Lclat1	-0,23057	1
Gm15151	-0,2306	1
Rrp1b	-0,23058	1
RP23-47A1.1	-0,23066	1
Kat8	-0,2307	1
Cdk4	-0,23065	1
Zfp418	-0,23103	1
Mrps21	-0,23102	1
Commd3	-0,23103	1
Acads	-0,23109	1
St3gal2	-0,23123	1
Nosip	-0,23148	1
H2afj	-0,2315	1
Lactb	-0,2316	1
Rad50	-0,23174	1
Ftsj3	-0,23165	1
Tmem81	-0,2319	1
Idh3b	-0,23195	1
Gm2a	-0,2321	1
Tti1	-0,23228	1
Chrnb1	-0,23245	1
9030407P20Rik	-0,23256	1
Rbks	-0,23256	1
Prrc2c	-0,2327	1
Fhad1	-0,23276	1
Bnip1	-0,23279	1
Pfdn6	-0,2329	1
Etfbkmt	-0,23321	1
Slc37a3	-0,23337	1
AA914427	-0,23341	1
Med29	-0,23348	1
St6gal1	-0,23353	1
Tdp1	-0,23346	1
Igsf6	-0,23355	1
Eprs	-0,23371	1
Cbr3	-0,23378	1
Limk1	-0,23412	1
Sri	-0,23428	1
Dnajb11	-0,23437	1
Zfp593	-0,23463	1
Smarca2	-0,23464	1
Gm15753	-0,23467	1
RP23-359K10.9	-0,23471	1
Gpatch1	-0,23475	1
Pbx2	-0,2348	1
Tbcb	-0,23494	1
Snx11	-0,23487	1
Ociad1	-0,23491	1
Tradd	-0,23503	1

Nsrp1	-0,23499	1
Nthl1	-0,2351	1
Plagl2	-0,2351	1
Celf3	-0,23518	1
Zfp850	-0,23528	1
Naa35	-0,23533	1
Ptbp2	-0,2354	1
Tbc1d10b	-0,23538	1
C1d	-0,2355	1
Espl1	-0,23556	1
Usp10	-0,23557	1
Actr10	-0,23573	1
Kif5a	-0,23586	1
Pex14	-0,23609	1
1110038B12Rik	-0,23614	1
Spata33	-0,23624	1
Hyou1	-0,23627	1
Gm10263	-0,23638	1
Smc1a	-0,23643	1
Mis18bp1	-0,23651	1
Maml2	-0,2367	1
Hexa	-0,23667	1
Gm6977	-0,23703	1
9530078K11Rik	-0,23696	1
Nipsnap3b	-0,23715	1
Crebbp	-0,23731	1
Pdha1	-0,2373	1
Gm37349	-0,23744	1
Tef	-0,23749	1
Tspyl2	-0,23762	1
D830044I16Rik	-0,23764	1
Arfgef2	-0,23756	1
Adcy7	-0,23756	1
Cetn3	-0,23769	1
Cd99l2	-0,23784	1
Bola2	-0,23794	1
Gm43524	-0,23806	1
Fuca1	-0,23832	1
Gm16124	-0,23835	1
Mcts1	-0,23841	1
Fam171b	-0,23862	1
Rundc1	-0,23887	1
Rpl31-ps16	-0,23903	1
3830408C21Rik	-0,23896	1
Adh7	-0,23899	1
2610507B11Rik	-0,23926	1
Haghl	-0,23947	1
Syk	-0,23955	1
Ppfia3	-0,23974	1
Tpm4	-0,2397	1
Hivep1	-0,23981	1
Kitl	-0,23979	1

Tmbim4	-0,23978	1
Il15	-0,23989	1
Msl3	-0,24001	1
Ppt1	-0,24015	1
Nsun5	-0,24045	1
Neu1	-0,24035	1
Gm37116	-0,24059	1
Gcdh	-0,2408	1
Dap	-0,24076	1
Gm44187	-0,24126	1
Bap1	-0,2413	1
Bcl2a1b	-0,24151	1
Gpr89	-0,24147	1
Ado	-0,24178	1
Lnx2	-0,2421	1
Gins4	-0,24221	1
Trem2	-0,24231	1
Znhit1	-0,2424	1
Nfu1	-0,24248	1
Glrx2	-0,24251	1
Dolk	-0,24257	1
Dcaf6	-0,24298	1
Sfxn1	-0,243	1
Vps54	-0,24323	1
Pold1	-0,24322	1
Acad8	-0,24325	1
Tmem191c	-0,24323	1
Gm5050	-0,24352	1
Csnk2a2	-0,24354	1
Gm12466	-0,24365	1
Mrpl44	-0,24367	1
Rfk	-0,24371	1
Mrm3	-0,24377	1
Rpl36-ps8	-0,2439	1
Gm9794	-0,24394	1
Zfp945	-0,24402	1
Mrpl10	-0,24409	1
Txnrd2	-0,24421	1
Gm23751	-0,24428	1
Chkb	-0,24447	1
Mpv17l2	-0,24453	1
Chd2	-0,24455	1
Rpl36-ps3	-0,2447	1
Polr3a	-0,24475	1
Mmp19	-0,24477	1
Nrd1	-0,24478	1
Casp4	-0,24489	1
Ovca2	-0,24513	1
Tmco6	-0,24529	1
Noxo1	-0,24527	1
Suds3	-0,24527	1
Lig4	-0,24542	1

Dbp	-0,24537	1
Gm16286	-0,24555	1
Gm5599	-0,24573	1
Cox18	-0,2459	1
Trpc4ap	-0,24622	1
Ccdc51	-0,24637	1
Kmt2b	-0,24674	1
Bag1	-0,24685	1
RP24-351I17.3	-0,24699	1
Fiz1	-0,24704	1
Smarcd1	-0,24713	1
Agtrap	-0,24715	1
Zfp599	-0,2473	1
Aplp1	-0,24727	1
Gcn1l1	-0,24734	1
Psmd13	-0,24741	1
Nrip1	-0,24774	1
9430038I01Rik	-0,24781	1
Evi2a	-0,24792	1
Pygo2	-0,24797	1
Gm9396	-0,24814	1
Taf10	-0,24807	1
Ptrh1	-0,24827	1
Tfam	-0,2487	1
Smarcd2	-0,24876	1
Rps19-ps9	-0,24886	1
Osbpl1a	-0,24899	1
Atg7	-0,24904	1
C330013E15Rik	-0,24917	1
Nupr1	-0,24921	1
Marc2	-0,24918	1
2410006H16Rik	-0,24921	1
Gm26532	-0,24933	1
Ccnd2	-0,24926	1
Cluh	-0,24995	1
Eif3c	-0,2499	1
Btf3l4	-0,25002	1
Nrbp2	-0,2501	1
Eral1	-0,25008	1
Dnajc10	-0,25019	1
Gm16425	-0,25033	1
Gm12857	-0,2503	1
Heatr5a	-0,25039	1
Gm43201	-0,25073	1
Filip1l	-0,25067	1
Cda	-0,25075	1
Dguok	-0,25104	1
Gm37183	-0,25107	1
Tmem55b	-0,25118	1
Rab11fip3	-0,2513	1
Zfp383	-0,25137	1
Nostrin	-0,25154	1

Zfp839	-0,25178	1
Atp6v1f	-0,25185	1
Clasrp	-0,25187	1
Stk19	-0,25198	1
Sord	-0,25201	1
Usp12	-0,25214	1
Trp53cor1	-0,25237	1
Mgst2	-0,25248	1
Rnf20	-0,25247	1
Mettl4	-0,25266	1
BC031181	-0,2527	1
BC055308	-0,2528	1
Tnrc6b	-0,25279	1
Gm37101	-0,25301	1
Faf2	-0,25332	1
Ccnd3	-0,25339	1
Parp1	-0,25338	1
Gm11914	-0,25347	1
Gramd1b	-0,25355	1
Gm13464	-0,25359	1
Ech1	-0,25361	1
Pomt1	-0,2537	1
Phf21a	-0,25371	1
Fastkd2	-0,25392	1
Dtnbp1	-0,25401	1
Mtch2	-0,25413	1
Gm43110	-0,25422	1
Gm24927	-0,25418	1
Ddx58	-0,25443	1
Ddhd2	-0,25441	1
Psmd6	-0,25436	1
Cdkl4	-0,25459	1
Gm30238	-0,25457	1
Gnaq	-0,25464	1
0610009O20Rik	-0,25488	1
Uqcr10	-0,25488	1
Elp5	-0,25505	1
Fam171a2	-0,25508	1
Nubp1	-0,25508	1
Kdelr2	-0,25509	1
Gys1	-0,2553	1
Gm15946	-0,25543	1
Ndufaf5	-0,25549	1
Stn1	-0,25568	1
Gm11633	-0,25581	1
Mcph1	-0,25587	1
Mfn2	-0,25592	1
Klhdc2	-0,25588	1
Cisd1	-0,25587	1
Cep131	-0,25602	1
Rsu1	-0,256	1
Prkra	-0,25605	1

Sh3pxd2a	-0,25615	1
Limk2	-0,25642	1
Ndufa7	-0,25642	1
Glud1	-0,25639	1
Gm7972	-0,25645	1
Gm44623	-0,25656	1
Uri1	-0,25659	1
Pik3r1	-0,25677	1
Rnf169	-0,25679	1
Rps8	-0,25701	1
Osbp2	-0,25712	1
Il15ra	-0,25725	1
Anapc11	-0,25733	1
Kri1	-0,25744	1
Helz	-0,25737	1
Gsap	-0,25758	1
Mid1ip1	-0,25768	1
Plin3	-0,25797	1
Ssna1	-0,25806	1
Ercc5	-0,25825	1
Dand5	-0,2585	1
Ccdc28a	-0,25864	1
Cdc14b	-0,25868	1
Nexn	-0,25898	1
Nhej1	-0,25931	1
Tmem234	-0,25933	1
Rps2-ps5	-0,25946	1
Mrpl55	-0,25945	1
Shc1	-0,2596	1
Gm8539	-0,25967	1
Gm14165	-0,2597	1
Zfp68	-0,25969	1
Rit1	-0,25977	1
Derl1	-0,26005	1
Rab34	-0,26021	1
Gm7589	-0,26028	1
Gm5812	-0,26035	1
Rptor	-0,26048	1
Ccdc88b	-0,26064	1
Chrnb2	-0,26072	1
Apbb2	-0,26067	1
Xndc1	-0,26083	1
Clasp2	-0,26079	1
Abhd12	-0,26081	1
Zfp362	-0,26088	1
Fbxo31	-0,26085	1
Inpp5a	-0,26089	1
Sar1a	-0,26109	1
Nme7	-0,26115	1
RP23-349H12.3	-0,26142	1
Gm5075	-0,26142	1
Gm10704	-0,26145	1

Mustn1	-0,26146	1
Parp14	-0,26174	1
Zc3h8	-0,26178	1
Gm19196	-0,26191	1
Gpr146	-0,26201	1
Kcnk13	-0,26204	1
Osbpl2	-0,26197	1
Npepl1	-0,26215	1
Zrsr1	-0,26223	1
Gm5778	-0,26243	1
Gm10039	-0,26262	1
Lmnb2	-0,26279	1
St7l	-0,2628	1
Dhx16	-0,26288	1
Zfas1	-0,26285	1
Bptf	-0,26312	1
Mdk	-0,26327	1
Wrb	-0,26345	1
Lyrm2	-0,26349	1
Atp6v1c1	-0,26349	1
Rabepk	-0,26367	1
Fbxo10	-0,26378	1
Mkks	-0,2638	1
Oip5	-0,26411	1
Dbi	-0,26415	1
Riox2	-0,2642	1
Sdha	-0,2643	1
Psmf1	-0,26442	1
Alyref2	-0,26445	1
Mnd1	-0,26466	1
Gm6419	-0,2647	1
Stradb	-0,2649	1
3110040N11Rik	-0,26505	1
Dcaf10	-0,26506	1
Gm12454	-0,26517	1
Coq9	-0,26529	1
Clcc1	-0,26526	1
Trmt5	-0,26555	1
Zfp217	-0,26566	1
Zfp90	-0,26584	1
Msr1	-0,26583	1
Gm7809	-0,26587	1
Trmt12	-0,26601	1
Cacul1	-0,26599	1
Gm37452	-0,26625	1
Uckl1	-0,26623	1
Aldh7a1	-0,26634	1
Rasgrp4	-0,26659	1
Gm6204	-0,26657	1
Klf7	-0,26662	1
Gm20712	-0,26681	1
Mtif3	-0,26682	1

Bcap31	-0,26687	1
Gm3145	-0,26697	1
Herc1	-0,267	1
Gm9246	-0,26729	1
Dhx29	-0,26764	1
Gm5262	-0,26792	1
Pccb	-0,26799	1
Zdhhc20	-0,26828	1
Mrpl39	-0,26845	1
BC003965	-0,26837	1
C030013C21Rik	-0,26851	1
Wdr24	-0,26854	1
Fbxl12	-0,26866	1
Pwwp2b	-0,26882	1
Gtpbp6	-0,26892	1
Gm11613	-0,2689	1
Rbl1	-0,26892	1
Dnttip1	-0,26892	1
Wls	-0,2691	1
Gm7114	-0,26918	1
Rpsa-ps10	-0,26918	1
Trappc4	-0,26916	1
Pygb	-0,2693	1
Tsr1	-0,26935	1
Pi4k2a	-0,26947	1
Agfg2	-0,2697	1
Gm23344	-0,26991	1
Akr1c13	-0,26993	1
Toporsos	-0,26998	1
Osbpl11	-0,27009	1
Maml3	-0,27011	1
Klhl36	-0,27026	1
Elmo1	-0,2703	1
Mrgpre	-0,27056	1
Etfrf1	-0,27056	1
Ptges2	-0,2706	1
Ndufa12	-0,2706	1
Plod1	-0,27059	1
Fam118b	-0,27074	1
Ufc1	-0,27067	1
Fcgr2b	-0,27077	1
Eid2b	-0,27084	1
Mlycd	-0,2708	1
Nol12	-0,27089	1
Ninl	-0,27102	1
Hdac10	-0,27128	1
Ccdc112	-0,27133	1
Cand1	-0,27142	1
Il17ra	-0,27145	1
Ptpra	-0,27175	1
Ccdc90b	-0,27175	1
Calm3	-0,27203	1

Mettl6	-0,27206	1
Gm12254	-0,27226	1
Fahd1	-0,27226	1
Csk	-0,27246	1
Rbbp8	-0,27259	1
Jagn1	-0,27264	1
Gm17018	-0,2726	1
Foxred1	-0,27305	1
Slc38a1	-0,27314	1
Ddx31	-0,27332	1
Uqcc1	-0,27346	1
Ppip5k1	-0,27369	1
Sssca1	-0,27374	1
Lmna	-0,2737	1
Wdr7	-0,27375	1
Stx5a	-0,27404	1
Pik3cb	-0,27401	1
Arpc1b	-0,27403	1
Psmb4	-0,27416	1
Slc25a24	-0,27435	1
Zfp961	-0,27473	1
Lrba	-0,27466	1
Ehmt1	-0,27484	1
Mast4	-0,27495	1
Prepl	-0,27501	1
Spsb1	-0,27511	1
Bet1l	-0,2752	1
Ss18l1	-0,27541	1
Tnfaip8l1	-0,27573	1
Acaca	-0,27567	1
Gm16755	-0,27585	1
Vamp1	-0,27584	1
Fam102a	-0,27581	1
Rrm2b	-0,2762	1
Copa	-0,27619	1
Mphosph8	-0,27634	1
Tm7sf3	-0,27638	1
Gm45289	-0,27663	1
Iqcg	-0,27675	1
Casp1	-0,27694	1
Cd2ap	-0,27689	1
Mea1	-0,27705	1
Hacl1	-0,2772	1
Mbd5	-0,27723	1
Gm19272	-0,27715	1
Atp23	-0,27771	1
B230317F23Rik	-0,27782	1
Gm37494	-0,27787	1
Dlg4	-0,278	1
Krtcap2	-0,2785	1
Selenom	-0,27856	1
Dcxr	-0,27885	1

Tfpi	-0,27889	1
Sgsm3	-0,27903	1
Bid	-0,27896	1
Rgp1	-0,27952	1
Atxn7	-0,27965	1
Jkamp	-0,27962	1
Ndufc1	-0,27977	1
Rbm26	-0,27992	1
Rpl23	-0,28031	1
Cast	-0,28028	1
Clta	-0,28028	1
Zmynd19	-0,28039	1
Zfp608	-0,28046	1
Auh	-0,28051	1
Tmem167	-0,28054	1
Evl	-0,28062	1
Adgrl1	-0,2806	1
Mettl7a1	-0,28066	1
Txnl4a	-0,28074	1
Hip1	-0,28083	1
C130026I21Rik	-0,2808	1
Tuba4a	-0,28102	1
Cfap36	-0,28107	1
Scarna17	-0,28122	1
Twf2	-0,28144	1
Ap5s1	-0,28172	1
Gm12732	-0,28184	1
Letmd1	-0,28199	1
Hmga1	-0,28224	1
Insr	-0,28223	1
Eea1	-0,28219	1
Sirt7	-0,28235	1
Srr	-0,28248	1
Gstp-ps	-0,28265	1
Mlf2	-0,28288	1
Gm2810	-0,28304	1
Tmem14c	-0,28323	1
Tma16	-0,28332	1
Syce2	-0,2833	1
Bbs12	-0,28346	1
Gnl2	-0,28347	1
Myo18a	-0,28374	1
Nob1	-0,28372	1
Gpat4	-0,2837	1
Rer1	-0,28383	1
Sclt1	-0,28386	1
Arap3	-0,28409	1
Tmem245	-0,28407	1
Ahi1	-0,28428	1
Ptges3	-0,28453	1
Ulk3	-0,28469	1
Aldh1l1	-0,28471	1

Fntb	-0,28493	1
Tmem150a	-0,28542	1
Dock11	-0,28552	1
Xrcc6	-0,28545	1
Tbrg1	-0,28551	1
Gm16712	-0,28559	1
Surf4	-0,28572	1
Ankle1	-0,28581	1
Usp46	-0,28581	1
Aes	-0,28581	1
Ttbk2	-0,28585	1
Calu	-0,28605	1
Prcc	-0,28631	1
Lpgat1	-0,28659	1
Abcc1	-0,28663	1
Kif20a	-0,2867	1
Mkl1	-0,2869	1
4831440E17Rik	-0,28698	1
Rfc2	-0,28709	1
Zfp821	-0,28731	1
Aptx	-0,28732	1
Gcc1	-0,2876	1
Gm6297	-0,28769	1
5430416N02Rik	-0,28781	1
Cul4a	-0,28778	1
Madd	-0,28783	1
Arhgap35	-0,28792	1
Lrmp	-0,288	1
Gm9843	-0,28834	1
Atp5k	-0,28844	1
Gm14173	-0,28864	1
Isca2	-0,28863	1
Gramd1c	-0,28872	1
Tsfm	-0,28883	1
Gm6526	-0,28879	1
Usp8	-0,28887	1
Primpol	-0,2893	1
Zfp994	-0,28934	1
Pdcd5-ps	-0,28929	1
Hsd17b11	-0,28934	1
Cldn15	-0,28951	1
Ogfod2	-0,28955	1
Bbs7	-0,28968	1
Cebpzos	-0,28974	1
Pkp2	-0,28981	1
Depdc5	-0,28998	1
Gpsm2	-0,29001	1
Gm14017	-0,29013	1
Gm20432	-0,29007	1
Tmem37	-0,29024	1
Gnb1l	-0,29022	1
Megf8	-0,29063	1

Prpf19	-0,2907	1
Col4a3bp	-0,29088	1
Tmem106a	-0,29093	1
Add1	-0,29096	1
Spa17	-0,29118	1
Hmgn5	-0,2913	1
Tnfrsf22	-0,29139	1
Psmg3	-0,29141	1
Laptm4a	-0,29148	1
Serpinb8	-0,29156	1
Rbfox2	-0,29169	1
Dok3	-0,29172	1
Nfkbia	-0,29171	1
Smndc1	-0,29178	1
Gna15	-0,29216	1
Mlst8	-0,29228	1
Orc2	-0,29232	1
Paqr4	-0,29275	1
Galm	-0,2927	1
Aagab	-0,29266	1
Gm38120	-0,29288	1
BC085271	-0,29303	1
Crybg3	-0,2931	1
Tonsl	-0,29314	1
Rpl27a	-0,29324	1
RP23-36H21.3	-0,29337	1
Fnbp1l	-0,29342	1
Leprotl1	-0,29362	1
Gcat	-0,2938	1
Taz	-0,29379	1
Ddx1	-0,29395	1
Sec23a	-0,29401	1
Tnnt3	-0,29458	1
Gm10250	-0,29463	1
Ptpn11	-0,29464	1
Cisd2	-0,29468	1
Ttll1	-0,2948	1
Slc38a9	-0,29513	1
Rps19	-0,29531	1
Mapk1ip1	-0,29565	1
Gpt	-0,29576	1
Uso1	-0,296	1
Phb	-0,29613	1
Ptgr1	-0,29625	1
Dram2	-0,29618	1
Sep 10	-0,29625	1
Cd48	-0,29661	1
Tnrc6c	-0,29689	1
Rps15a-ps8	-0,29697	1
Gm15773	-0,29714	1
Pus7	-0,29709	1
Vps16	-0,29722	1

Ankhd1	-0,29716	1
Zfp120	-0,29752	1
Ddn	-0,29769	1
Ruvbl1	-0,2978	1
Gm10605	-0,29791	1
Arfrp1	-0,29802	1
Pafah1b2	-0,29855	1
Tcea1-ps1	-0,299	1
Mrpl43	-0,29896	1
Foxc1	-0,29906	1
Mb21d2	-0,29912	1
Rpl9-ps7	-0,29923	1
Gm14780	-0,29925	1
Prkaa1	-0,29938	1
Jrk	-0,29993	1
Mrpl51	-0,29991	1
Senp6	-0,30001	1
Rabl6	-0,3002	1
Ikbkg	-0,30033	1
Rab3d	-0,30036	1
Cwc22	-0,30054	1
Gm19739	-0,30062	1
Pfdn1	-0,30068	1
Psme2	-0,30087	1
Abhd14a	-0,30089	1
Fastkd1	-0,30104	1
Tbc1d24	-0,30095	1
Gm45311	-0,30115	1
Dgcr14	-0,30127	1
Ago1	-0,30125	1
Capn5	-0,30134	1
Agpat3	-0,30131	1
Gm29155	-0,30146	1
Bola3	-0,30153	1
Dnajc2	-0,30168	1
Snx10	-0,30182	1
Tfrc	-0,30252	1
Gm42463	-0,30267	1
Slc23a2	-0,30289	1
2810474O19Rik	-0,30302	1
Gm16310	-0,30314	1
Tmem203	-0,3031	1
Snrpd1	-0,30307	1
BC052040	-0,3034	1
Dhx32	-0,30343	1
Btbd2	-0,30344	1
Afg3l1	-0,30348	1
Tmem147	-0,30349	1
Ubr4	-0,30365	1
Sep 07	-0,30367	1
Tubb4a	-0,30383	1
Tusc2	-0,30393	1

Msh2	-0,30407	1
Gm37254	-0,30435	1
Kif1bp	-0,3043	1
Mtx1	-0,30431	1
Mier2	-0,30428	1
Nemp2	-0,30449	1
Tfap4	-0,30452	1
Gm11889	-0,30463	1
Mocos	-0,30458	1
Pa2g4	-0,30465	1
Sdad1	-0,30466	1
Ttc37	-0,30482	1
1700061G19Rik	-0,30481	1
Usf3	-0,3051	1
Eci1	-0,30521	1
Xrcc1	-0,30531	1
Rpp14	-0,30532	1
Nit1	-0,30541	1
Tns4	-0,30546	1
Sdr42e1	-0,30575	1
BC002163	-0,30577	1
Becn1	-0,30585	1
Panx1	-0,30591	1
Fam192a	-0,306	1
Gm13038	-0,30605	1
Gm5909	-0,30623	1
Klhl11	-0,30629	1
Zfp930	-0,30628	1
Zfp318	-0,30653	1
Gm6542	-0,30663	1
Pid1	-0,30661	1
Sec63	-0,30665	1
Zfp605	-0,30671	1
Narfl	-0,30668	1
Tmem132a	-0,30684	1
Gm4332	-0,3068	1
Psap	-0,30677	1
Prdx2	-0,30689	1
Tpk1	-0,307	1
Masp2	-0,30701	1
Rab1b	-0,30708	1
Trap1	-0,30719	1
Phykpl	-0,30743	1
Ywhab	-0,30751	1
Nectin3	-0,30772	1
Pex11b	-0,30773	1
2510002D24Rik	-0,3078	1
Slc25a12	-0,30781	1
Fam216a	-0,30784	1
H13	-0,30776	1
Srpk2	-0,30783	1
Snrnp25	-0,30785	1

Dnajb14	-0,30788	1
Vps26b	-0,30796	1
Tbx6	-0,30812	1
Emc1	-0,30806	1
Asb8	-0,30843	1
Ppm1g	-0,30845	1
Wdr54	-0,30859	1
Zfp282	-0,30864	1
Rps6ka3	-0,30859	1
Ndufb3	-0,30869	1
Gm14006	-0,30881	1
Cct7	-0,30885	1
Pcca	-0,30903	1
Mocs2	-0,30897	1
Tacc2	-0,30927	1
Bad	-0,30938	1
Nktr	-0,30953	1
Ankrd40	-0,30959	1
Ring1	-0,30974	1
Prkar1a	-0,30974	1
Rad9b	-0,30994	1
Rps19-ps4	-0,31005	1
Plekhn1	-0,3101	1
Mrpl33	-0,31062	1
Pnisr	-0,3106	1
Commd7	-0,31072	1
H2afy	-0,3107	1
Adamts10	-0,3109	1
Slc35c1	-0,31109	1
Ahsa2	-0,31143	1
Scarf1	-0,31155	1
Insl6	-0,31157	1
Pkn1	-0,31158	1
Dnajc16	-0,31175	1
Dnal4	-0,31194	1
Zfp40	-0,31198	1
Slc41a2	-0,31211	1
Prr13	-0,31222	1
Lyz2	-0,31234	1
Ankrd13d	-0,31254	1
Rpl11	-0,31254	1
Ccdc92b	-0,31248	1
Pigq	-0,31248	1
Zfp35	-0,31262	1
Ufsp1	-0,31265	1
Arl6	-0,31272	1
Pafah1b3	-0,31284	1
Cog8	-0,31287	1
Mga	-0,31328	1
Commd10	-0,31337	1
Sfxn3	-0,31341	1
Dusp7	-0,31347	1

4930402H24Rik	-0,31349	1
Net1	-0,31388	1
Gm9727	-0,31388	1
Fam71e1	-0,31408	1
Pdk3	-0,31421	1
Smg9	-0,31423	1
Golga4	-0,31435	1
Tada2b	-0,31453	1
Os9	-0,31487	1
Hoxa1	-0,315	1
Tbc1d22a	-0,31495	1
Rprd2	-0,3152	1
Ehbp1l1	-0,31525	1
Actr5	-0,31536	1
Hnrnpl	-0,31596	1
Cc2d1b	-0,31609	1
Ogg1	-0,31625	1
Tex10	-0,31652	1
Zc3h6	-0,31645	1
Dbndd2	-0,31674	1
Hap1	-0,31673	1
Grk6	-0,31688	1
Alg8	-0,31698	1
Sertad2	-0,31713	1
Golga2	-0,31734	1
Hotairm1	-0,31773	1
Irf3	-0,3179	1
Lars	-0,31793	1
Thnsl1	-0,31796	1
Son	-0,31798	1
Ntan1	-0,31813	1
Limd1	-0,31816	1
Tlr7	-0,31834	1
Akr1e1	-0,3183	1
Gm4943	-0,31869	1
Rorc	-0,31893	1
Nxpe3	-0,31903	1
Eif3k	-0,31912	1
Galnt1	-0,31923	1
Epm2a	-0,31931	1
Smyd3	-0,31928	1
Dhfr	-0,31951	1
Vps9d1	-0,31945	1
Las1l	-0,31946	1
Tigar	-0,31952	1
Lamtor1	-0,31957	1
Gm5436	-0,31969	1
Plscr3	-0,31977	1
Tmem156	-0,31986	1
Gm43761	-0,32012	1
Fgf13	-0,32028	1
Ccdc92	-0,3203	1

Alg10b	-0,32038	1
Scnm1	-0,32051	1
Mmgt2	-0,32045	1
Ogfod3	-0,32059	1
Rps13-ps7	-0,32062	1
Dtd1	-0,32056	1
Abca5	-0,32074	1
Mto1	-0,3209	1
Ccl6	-0,32101	1
Rplp2	-0,32099	1
Gm19705	-0,32124	1
Heatr1	-0,32144	1
A430105J06Rik	-0,32139	1
Gm43106	-0,3215	1
Uros	-0,32158	1
Ylpm1	-0,3216	1
Prdx1	-0,32158	1
Acly	-0,32179	1
Gm4034	-0,32192	1
Zfp46	-0,32187	1
Dnase2a	-0,32212	1
Samhd1	-0,32229	1
Dhrs1	-0,32244	1
8430429K09Rik	-0,32256	1
P4ha2	-0,32256	1
Rtn3	-0,32256	1
Tmeff1	-0,3228	1
Phf14	-0,32296	1
Rnf41	-0,32314	1
Tmem68	-0,3234	1
Bfar	-0,32336	1
Nagk	-0,32347	1
Ndufa8	-0,32357	1
Zfp710	-0,3238	1
Eef1d	-0,32406	1
Gm11520	-0,32422	1
C330018D20Rik	-0,32424	1
Gm37678	-0,3242	1
Napb	-0,32482	1
Clptm1l	-0,32491	1
Gm17150	-0,32504	1
Brwd1	-0,32522	1
Rps12-ps1	-0,32529	1
Pole3	-0,3258	1
Setd4	-0,32592	1
Tgfbr2	-0,32586	1
Smim20	-0,32586	1
Uba5	-0,32592	1
Fastk	-0,32595	1
Vps37a	-0,32603	1
Mpv17	-0,32609	1
Wdr36	-0,32615	1

Ptpn1	-0,32612	1
Rgs20	-0,32622	1
Vac14	-0,32637	1
5031425F14Rik	-0,3268	1
Trappc12	-0,32689	1
Nfia	-0,32704	1
Zfyve9	-0,32723	1
RP23-159E10.1	-0,32722	1
Zfp386	-0,3273	1
Tssc1	-0,32728	1
March9	-0,32737	1
Cd2bp2	-0,32757	1
Mlx	-0,32767	1
Haus1	-0,32785	1
Rasgrp3	-0,32783	1
Ptpn22	-0,32789	1
Eif3a	-0,32801	1
Gm43795	-0,3282	1
Gm43533	-0,32818	1
Cul2	-0,32831	1
Gm11281	-0,32835	1
Rdh14	-0,32844	1
4933433G15Rik	-0,32853	1
Scamp5	-0,32855	1
Dnal1	-0,32878	1
Adora2b	-0,32883	1
Pafah1b1-ps2	-0,32892	1
Mis12	-0,32891	1
Gnat2	-0,32896	1
Gm23458	-0,32911	1
Cd9-ps	-0,32922	1
Phf20	-0,32918	1
Myl12b	-0,32955	1
Ppp4c	-0,32969	1
Stoml2	-0,3297	1
Gm38387	-0,33004	1
Gm42967	-0,33003	1
Tuba1b	-0,33	1
Zfp26	-0,33011	1
Sla2	-0,33049	1
Mrpl27	-0,33064	1
Eif4g3	-0,33127	1
Fam96b	-0,33135	1
Minpp1	-0,33155	1
Zfp691	-0,3318	1
Atp5j	-0,33184	1
Ktn1	-0,33188	1
Prr14l	-0,33208	1
Ncf2	-0,33224	1
Gm4737	-0,33228	1
Uxt	-0,33237	1
RP23-3F1.8	-0,33252	1

Gba	-0,33249	1
Mrpl52	-0,33249	1
Slc6a4	-0,33262	1
Tsen15	-0,33265	1
Was	-0,33281	1
Kiss1r	-0,33278	1
Eif3j2	-0,33279	1
Hddc3	-0,33289	1
Fktn	-0,33295	1
Tmem161a	-0,33297	1
Sacs	-0,33312	1
Mical3	-0,33309	1
Mthfd2l	-0,33324	1
Oma1	-0,33371	1
Mtap	-0,33372	1
Gm4262	-0,3341	1
Rgl2	-0,33422	1
Rere	-0,33469	1
Zbtb48	-0,33477	1
Mlf1	-0,33487	1
B230216N24Rik	-0,33499	1
Scyl3	-0,33502	1
Supt20	-0,33506	1
Dlst	-0,33514	1
Cuedc1	-0,33535	1
BC051226	-0,33537	1
Tmem120a	-0,33541	1
Ptges3l	-0,3354	1
Wdr78	-0,33547	1
Yipf3	-0,33551	1
Gm16238	-0,33574	1
Dgkq	-0,33567	1
Ankrd49	-0,33581	1
Fam210b	-0,33576	1
Pltp	-0,33591	1
Fra10ac1	-0,33588	1
Snx15	-0,336	1
Gm44834	-0,33631	1
Mvb12b	-0,33664	1
Exoc3l4	-0,3367	1
As3mt	-0,33684	1
Zfp12	-0,33681	1
Rpl38	-0,33681	1
Gm4734	-0,33695	1
Ankrd24	-0,33712	1
Arfgap1	-0,33718	1
AI987944	-0,33722	1
Ndufa10	-0,33722	1
Slc35b1	-0,33721	1
Polr3gl	-0,33734	1
Apex1	-0,33736	1
Tifab	-0,33774	1

Gm9840	-0,33813	1
Rabac1	-0,33808	1
Gm11688	-0,33817	1
Aup1	-0,33818	1
Tns1	-0,33837	1
Mdp1	-0,33836	1
Creld1	-0,33868	1
Rpl13a	-0,33875	1
Gm12606	-0,33906	1
Gm37080	-0,33923	1
Gns	-0,33919	1
Fam35a	-0,33934	1
Inpp5d	-0,33949	1
Gm43920	-0,33962	1
Hps6	-0,3396	1
Ndrg2	-0,33969	1
Rnu11	-0,33986	1
Mrpl48	-0,34005	1
Strada	-0,34006	1
Gm37474	-0,3402	1
Timeless	-0,3402	1
Miip	-0,3402	1
Ddx10	-0,34021	1
Zfp398	-0,34029	1
Ddx55	-0,34029	1
Gm11427	-0,34034	1
Mkl2	-0,34051	1
Gm14853	-0,34049	1
Mrps33	-0,34057	1
Ccdc6	-0,34064	1
Itpripl2	-0,34074	1
Trak2	-0,341	1
Pstk	-0,34124	1
Mrps25	-0,34123	1
Atp5c1	-0,3412	1
Trmt2b	-0,3414	1
Tfdp2	-0,34148	1
RP23-356D13.11	-0,34161	1
Mogs	-0,34197	1
Dhcr24	-0,34229	1
Ndrg4	-0,34232	1
Atad1	-0,34259	1
Rpap2	-0,34265	1
Gm26652	-0,34266	1
Zfp975	-0,34267	1
Tmem268	-0,34276	1
Gm19552	-0,34296	1
Bcap29	-0,34296	1
Plpp2	-0,34302	1
Adsl	-0,34349	1
Lrpprc	-0,34372	1
Naaa	-0,34383	1

Romo1	-0,3438	1
Imp4	-0,34399	1
Rrp8	-0,34425	1
Stat5b	-0,34441	1
Zc2hc1a	-0,34446	1
Sgsm2	-0,34462	1
Tmem205	-0,34473	1
Pls3	-0,34473	1
Gm1840	-0,34482	1
Atp6v0a2	-0,34492	1
1810014B01Rik	-0,34516	1
Cep162	-0,34544	1
Glmp	-0,34544	1
Trem3	-0,34555	1
Exosc8	-0,34549	1
March11	-0,34569	1
Stard3nl	-0,34583	1
C730045M19Rik	-0,34591	1
Itpr2	-0,34586	1
1600014C10Rik	-0,34644	1
Mau2	-0,34647	1
Lrrc24	-0,34665	1
A430046D13Rik	-0,34711	1
Klhl30	-0,34722	1
Nat2	-0,34735	1
Apeh	-0,34744	1
Comt	-0,34759	1
2310061I04Rik	-0,34759	1
Tmigd3	-0,34794	1
AC168977.1	-0,34805	1
Gm43462	-0,34819	1
Herc2	-0,34826	1
Lck	-0,34843	1
Stx2	-0,34847	1
Gm7128	-0,34868	1
Ergic3	-0,3487	1
Galnt15	-0,34879	1
Ankrd11	-0,34878	1
Gadd45gip1	-0,34926	1
Slc16a13	-0,34946	1
Tbl3	-0,34954	1
Lztr1	-0,3495	1
Abcb8	-0,34959	1
Fam3c	-0,34958	1
Gm10076	-0,34971	1
Kxd1	-0,35007	1
Gm14048	-0,35047	1
Hddc2	-0,35067	1
Cux1	-0,3509	1
Tnfrsf18	-0,35109	1
Apobec3	-0,35112	1
Selenos	-0,35111	1

RP23-316F10.2	-0,35125	1
Ndufv2	-0,35136	1
Lppos	-0,35178	1
Gm13360	-0,35177	1
Gm15268	-0,35178	1
E230020A03Rik	-0,35191	1
Sh2b3	-0,35216	1
Gm7380	-0,35263	1
Cyb5d2	-0,35264	1
Sigmar1	-0,3526	1
Tmem164	-0,35287	1
Derl2	-0,35293	1
Fam195a	-0,35316	1
Fdx1	-0,3534	1
Alkbh3	-0,35356	1
Hars2	-0,35361	1
Qser1	-0,35372	1
Gm13868	-0,35377	1
Mrps27	-0,35441	1
Zfp580	-0,3544	1
Sumf1	-0,35446	1
Gm8730	-0,3547	1
Med28	-0,3547	1
Ints10	-0,35511	1
Cnot10	-0,35516	1
Vamp4	-0,35522	1
Npy	-0,3553	1
Rhno1	-0,35529	1
Gm13373	-0,35538	1
Zfp688	-0,35556	1
Ranbp1	-0,3557	1
Gm13413	-0,3558	1
Wsb2	-0,35587	1
Rmdn1	-0,35604	1
Zfp799	-0,35607	1
Gm7123	-0,35619	1
Gm35931	-0,35622	1
Ep400	-0,35615	1
Zfhx2	-0,35632	1
Mpg	-0,3564	1
Phyh	-0,35642	1
Gusb	-0,35649	1
Gm21057	-0,3568	1
Ica1	-0,35691	1
Parpbp	-0,35722	1
Ddost	-0,35717	1
Gm6418	-0,35733	1
Ostf1	-0,35729	1
Wdr60	-0,35741	1
RP23-38L16.3	-0,3576	1
RP24-282C4.10	-0,35803	1
Ndufa11	-0,35809	1

Inpp5k	-0,35824	1
Pank1	-0,35848	1
Osbpl10	-0,35851	1
Abcc4	-0,3587	1
Wwp2	-0,35866	1
D5Ertd579e	-0,35884	1
Polr2f	-0,35883	1
Pelp1	-0,35908	1
Rnf31	-0,35916	1
Tatdn1	-0,35928	1
Zcchc24	-0,35952	1
Cd180	-0,35953	1
Snora57	-0,35971	1
Ints6	-0,35968	1
Rapgef1	-0,35983	1
Ttc1	-0,35996	1
Ptov1	-0,36064	1
Thtpa	-0,36068	1
Psma2	-0,36075	1
Il4ra	-0,36087	1
Gm15427	-0,36095	1
Dvl3	-0,36133	1
Grwd1	-0,36129	1
Slc36a4	-0,36127	1
Polr1a	-0,36135	1
Lpcat3	-0,36137	1
Kdm5d	-0,36151	1
Cyb5r4	-0,36169	1
Osbpl7	-0,36174	1
Actr3b	-0,36168	1
Foxred2	-0,36169	1
Btrc	-0,36194	1
RP23-304C21.3	-0,36199	1
Gm13675	-0,3621	1
Rab8a	-0,36212	1
Cyp4v3	-0,36245	1
Tuba1c	-0,36239	1
Xkr5	-0,36254	1
Fgfr1op2	-0,36256	1
Fam168a	-0,36261	1
Nars	-0,36285	1
RP23-115A18.3	-0,36299	1
2410015M20Rik	-0,36321	1
Lrp4	-0,36346	1
Cdkl2	-0,36362	1
Stx12	-0,36358	1
Wdfy4	-0,36363	1
2700062C07Rik	-0,36386	1
Prkag1	-0,364	1
Nat9	-0,36402	1
Mir17hg	-0,36412	1
Wdr77	-0,36445	1

Gle1	-0,36458	1
Gm8719	-0,36467	1
Cnnm3	-0,36478	1
Gnb4	-0,36476	1
Ralgapa2	-0,36499	1
Gm9892	-0,36507	1
Vps4a	-0,36529	1
Gm37788	-0,3654	1
Upf3a	-0,36544	1
Ercc8	-0,36547	1
Cttnbp2nl	-0,36583	1
Nudt18	-0,36596	1
Gm10138	-0,36621	1
RP23-123D6.12	-0,36629	1
Rps11-ps2	-0,36634	1
Esrra	-0,3665	1
Smim3	-0,36661	1
Mettl15	-0,36676	1
Prpf31	-0,36697	1
Nup37	-0,36713	1
Tmem8b	-0,36724	1
Psmd10	-0,3677	1
Alox5	-0,36781	1
Utp20	-0,36793	1
Phtf2	-0,36801	1
Rbm10	-0,36804	1
Golgb1	-0,36799	1
Gm7535	-0,36851	1
Gm45546	-0,36854	1
Plpp1	-0,36877	1
Neurl2	-0,36902	1
Kat6b	-0,36962	1
Tmem55a	-0,3696	1
Cuedc2	-0,36962	1
Ddb2	-0,36985	1
Hint1	-0,36988	1
Reep5	-0,37004	1
Rack1	-0,36999	1
Aatf	-0,3702	1
Pcnx4	-0,37048	1
Thap11	-0,37047	1
Ercc6l2	-0,37061	1
Slc9b1	-0,37063	1
Usp49	-0,37075	1
H2-Ab1	-0,37072	1
Gm45809	-0,37066	1
Nacc2	-0,37083	1
Atp5l	-0,37078	1
Aqp11	-0,37094	1
Gas2	-0,37101	1
Abcb10	-0,37121	1
Zkscan1	-0,37123	1

Bin3	-0,37131	1
Pkp4	-0,37151	1
1810030O07Rik	-0,3717	1
Soat2	-0,37178	1
Ap3b1	-0,37221	1
Vps45	-0,3722	1
Gm15500	-0,3724	1
Calhm2	-0,37258	1
Crtc3	-0,37256	1
Wfs1	-0,37268	1
Gm15824	-0,37271	1
Fsd2	-0,37285	1
Dnajc25	-0,37279	1
Rinl	-0,37289	1
Zfp146	-0,37306	1
Dusp18	-0,37358	1
Dera	-0,37356	1
Tmem2	-0,37377	1
RP23-184H3.5	-0,37376	1
Pxmp4	-0,37397	1
Ndufv1	-0,37432	1
Nup155	-0,37464	1
Gm9732	-0,37456	1
Ppp2r5d	-0,3747	1
Plxna2	-0,3748	1
Gm43792	-0,37499	1
Adprhl2	-0,37514	1
Dnajb12	-0,37518	1
Hyal3	-0,37516	1
Cdk10	-0,37528	1
Dok1	-0,3753	1
Pigc	-0,3754	1
Noa1	-0,37561	1
Ap3m2	-0,37574	1
Ttll3	-0,37601	1
Poglut1	-0,37603	1
Rfx5	-0,37613	1
Slc38a7	-0,37619	1
A430010J10Rik	-0,37629	1
Smurf1	-0,37665	1
Olfml3	-0,37663	1
Gm8659	-0,37673	1
Fhit	-0,37674	1
2310047D07Rik	-0,37684	1
Gm38077	-0,37693	1
Gm15541	-0,37693	1
Adam10	-0,37697	1
Tk2	-0,3772	1
Rgl3	-0,37731	1
Stk16	-0,37747	1
E2f8	-0,37757	1
Zfp771	-0,37757	1

Gm7332	-0,37768	1
Ccdc97	-0,37783	1
Rtfdc1	-0,37799	1
Cd101	-0,37823	1
Gm29340	-0,37819	1
Gemin7	-0,3782	1
Bst1	-0,37839	1
Napepld	-0,37862	1
Camta2	-0,37868	1
Ddx28	-0,37886	1
Rrp36	-0,37887	1
Gm43571	-0,37898	1
Pfkm	-0,37911	1
Cib1	-0,37922	1
Flot1	-0,37954	1
Gm26620	-0,37984	1
Gm9403	-0,37992	1
Tomm40	-0,38015	1
Yipf2	-0,38019	1
Ankrd33b	-0,38048	1
Tmem14a	-0,38049	1
St8sia4	-0,3805	1
Plbd2	-0,38055	1
Ptcd2	-0,3805	1
Atat1	-0,38062	1
Agpat1	-0,3806	1
Smarcc1	-0,38064	1
Rab11fip4	-0,38074	1
Gm6030	-0,38087	1
Nfkbie	-0,38116	1
Ldha	-0,3813	1
Gm2756	-0,38147	1
Arl11	-0,38157	1
Capzb	-0,38163	1
Spata13	-0,38182	1
Gm37033	-0,38194	1
Ifitm5	-0,38251	1
Hs6st1	-0,38259	1
Slc11a1	-0,38287	1
Pithd1	-0,38301	1
Stk38l	-0,38304	1
Mrps36-ps2	-0,38333	1
Rassf8	-0,38344	1
Wbp1	-0,38346	1
Mndal	-0,38378	1
Nme4	-0,38439	1
Gm45420	-0,3845	1
Nme6	-0,38446	1
Polr2c	-0,38446	1
Eif3b	-0,38451	1
P3h1	-0,38457	1
Ankrd13b	-0,38474	1

1810043G02Rik	-0,38483	1
Wipi1	-0,38475	1
Rnpepl1	-0,38481	1
Polg	-0,38486	1
BC005537	-0,38491	1
Tor3a	-0,38493	1
Pptc7	-0,38501	1
Prickle3	-0,38524	1
Gm43547	-0,38518	1
Myof	-0,38522	1
Zc3h7b	-0,38542	1
Pyroxd2	-0,38551	1
Fam173a	-0,38555	1
Ndufa2	-0,3855	1
Icam5	-0,38563	1
Rnf168	-0,38574	1
E330009J07Rik	-0,38595	1
Gm12089	-0,386	1
Gm10012	-0,38596	1
Sdc1	-0,38605	1
Fam162a	-0,38634	1
Neat1	-0,38649	1
Gcc2	-0,38658	1
Lgals3bp	-0,38667	1
Ccl9	-0,3867	1
Tmem141	-0,38695	1
Mul1	-0,38747	1
Ifi35	-0,38779	1
Nckap1	-0,38799	1
Med11	-0,38811	1
Slc7a4	-0,38818	1
Phf11b	-0,38832	1
Eng	-0,38835	1
Prrc1	-0,38835	1
Gm5614	-0,38853	1
Dhodh	-0,38883	1
Trmt10a	-0,38891	1
Cep83os	-0,38905	1
Adck5	-0,38944	1
Mertk	-0,39015	1
Ndufs7	-0,39025	1
Pum3	-0,39019	1
Zbtb38	-0,39034	1
Gipr	-0,39046	1
Cops9	-0,39062	1
Parp12	-0,39077	1
Dmap1	-0,39093	1
Pkd2	-0,39095	1
Tmem237	-0,39115	1
Rab24	-0,39107	1
Gm4707	-0,39124	1
Gda	-0,39123	1

Pnpo	-0,39139	1
Synpo	-0,39194	1
Zfp428	-0,39222	1
Ext2	-0,39222	1
Gm16177	-0,39232	1
Polr3c	-0,39259	1
B230118H07Rik	-0,3927	1
Erp29	-0,39269	1
Gm12944	-0,39305	1
Alkbh7	-0,39309	1
Pkd1	-0,39326	1
Eif4ebp1	-0,3935	1
Abcb4	-0,39369	1
Fastkd5	-0,39381	1
6430590A07Rik	-0,39382	1
Acot13	-0,39376	1
Mgat5	-0,39389	1
Mrpl24	-0,39409	1
E130308A19Rik	-0,39425	1
Snrpd2	-0,39432	1
Cnnm2	-0,39456	1
C2cd3	-0,3947	1
Zc4h2	-0,39467	1
2900005J15Rik	-0,3951	1
1810011H11Rik	-0,39522	1
Tmem243	-0,39543	1
Arih1	-0,39577	1
Zfp780b	-0,3959	1
Ttc19	-0,39586	1
Gm14567	-0,39618	1
Apc	-0,39623	1
Smpdl3b	-0,39634	1
Cwf19l2	-0,39645	1
Trak1	-0,39652	1
Zfp524	-0,39692	1
Gm45828	-0,39714	1
Znrd1as	-0,39732	1
Stim2	-0,39732	1
Tomm70a	-0,39739	1
Dnajc24	-0,39791	1
Gm6322	-0,39831	1
Stard3	-0,39836	1
2610020C07Rik	-0,39837	1
Gfer	-0,39843	1
Pdik1l	-0,3987	1
Cope	-0,39913	1
2310011J03Rik	-0,39917	1
Zfp846	-0,39938	1
Alcam	-0,39937	1
Plekhj1	-0,39937	1
Urm1	-0,39952	1
Gm14277	-0,39962	1

Ppdpf	-0,39956	1
Ndufaf2	-0,39961	1
Gm10146	-0,39977	1
Nt5m	-0,39987	1
Ten1	-0,40035	1
Kdm5b	-0,40036	1
Gm38104	-0,40062	1
Tnfsf12	-0,40082	1
Fam129c	-0,40084	1
Wdr45	-0,40095	1
Chm	-0,4011	1
Ahnak	-0,40149	1
Bcs1l	-0,40179	1
Gm13602	-0,40197	1
Fam32a	-0,402	1
Fbxw7	-0,4023	1
Cct3	-0,40233	1
Mms19	-0,40278	1
Elovl6	-0,40303	1
Mif4gd	-0,40306	1
Pou6f2	-0,40312	1
Washc1	-0,40347	1
Dhrs13	-0,40359	1
Dxo	-0,40401	1
Capg	-0,40437	1
Tmcc2	-0,40475	1
Wbp1l	-0,40475	1
Zfp410	-0,4049	1
Sil1	-0,40529	1
Dffa	-0,40539	1
Gm13450	-0,4055	1
Tspan17	-0,40549	1
Tmem216	-0,40576	1
Ankrd27	-0,40584	1
Sptlc1	-0,40592	1
Idua	-0,40612	1
C2	-0,40645	1
Rbm4	-0,40662	1
Mrpl11	-0,40661	1
Zdhhc2	-0,40691	1
Dnph1	-0,40703	1
Rassf4	-0,40722	1
Wwox	-0,40748	1
1700021F05Rik	-0,4075	1
Ndufa13	-0,40763	1
Rnf8	-0,40796	1
Hook3	-0,40859	1
Dgcr8	-0,40891	1
Nenf	-0,40919	1
G6pdx	-0,40936	1
Gm28686	-0,40948	1
Atp2a3	-0,40947	1

RP23-454I20.1	-0,40965	1
Tnfrsf1a	-0,40957	1
Gpatch2	-0,40972	1
Ndufs8	-0,4097	1
Fam172a	-0,4098	1
Gm13992	-0,40979	1
AU022252	-0,41011	1
Cdk6	-0,41019	1
Sh3bp5	-0,41019	1
Gm6808	-0,4109	1
Tmem177	-0,41097	1
Meaf6	-0,41097	1
Zcchc17	-0,41122	1
Slc40a1	-0,41128	1
Acadl	-0,41127	1
BC029722	-0,4118	1
Gm15859	-0,41222	1
Gng7	-0,41243	1
Gm13461	-0,41249	1
Zfand2b	-0,41246	1
Gm7132	-0,41249	1
Magt1	-0,41259	1
Lrrc8b	-0,41297	1
Ssr4	-0,41302	1
Tagap1	-0,4132	1
Phf10	-0,4134	1
Zdhhc16	-0,41345	1
Sec31a	-0,4135	1
Dner	-0,41364	1
Rab23	-0,41383	1
Pmm1	-0,41393	1
Itga5	-0,41411	1
Pdk1	-0,41431	1
Gm44130	-0,41447	1
Acat2	-0,41459	1
RP24-232D3.1	-0,41481	1
Emc8	-0,41488	1
Cul5	-0,41495	1
Pgghg	-0,41495	1
Cdk18	-0,41508	1
Gm3375	-0,41529	1
Acsl5	-0,4153	1
Rpl30-ps9	-0,41536	1
Timm9	-0,41543	1
Meg3	-0,41558	1
Psen2	-0,41567	1
Pepd	-0,41566	1
Klhl2	-0,41596	1
Bcar1	-0,4163	1
Dennd4b	-0,41646	1
Zfp292	-0,41649	1
Cd44	-0,41648	1

Mrpl54	-0,41679	1
Slf1	-0,41689	1
1500002F19Rik	-0,41705	1
Pcyox1	-0,41709	1
Cpne8	-0,41722	1
Tspan10	-0,41763	1
Hp1bp3	-0,41763	1
Plekhs1	-0,41773	1
Gm26132	-0,41772	1
Rabep2	-0,41787	1
1110002L01Rik	-0,41797	1
Gpatch11	-0,41795	1
Gm11478	-0,41798	1
Cdk7	-0,41817	1
Bag2	-0,4182	1
Hps1	-0,4183	1
9230114K14Rik	-0,41854	1
Rnasel	-0,41867	1
Tubb4b	-0,41885	1
Capn2	-0,41956	1
Jmjd8	-0,41969	1
Pde6d	-0,41967	1
Rps27-ps1	-0,41971	1
9130023H24Rik	-0,42033	1
Sec61a1	-0,42058	1
Malsu1	-0,42074	1
Rnmt	-0,42097	1
Psmb10	-0,42109	1
Tbck	-0,42116	1
Trip6	-0,42131	1
Cbr4	-0,42186	1
Rpl36a-ps3	-0,42214	1
Rps18	-0,42268	1
Nle1	-0,42278	1
Arfip2	-0,42292	1
Slc25a19	-0,42298	1
Tmem175	-0,42313	1
Taco1	-0,42307	1
Meis3	-0,42314	1
Ndufa5	-0,42311	1
Rab11fip1	-0,42319	1
Gm2367	-0,42337	1
Psmc5	-0,42337	1
Tmem158	-0,42356	1
Bcar3	-0,4236	1
Klhl22	-0,42398	1
Gm11598	-0,42418	1
Anxa7	-0,42416	1
Ivns1abp	-0,42435	1
Stard7	-0,42451	1
Zfp78	-0,42464	1
Ankrd46	-0,42524	1

BC003331	-0,42519	1
Ndufaf8	-0,42524	1
Gm6272	-0,42538	1
Reps2	-0,42537	1
D130019J16Rik	-0,42543	1
Gm13268	-0,4256	1
Scamp1	-0,42562	1
Fxyd5	-0,42565	1
Cd320	-0,42626	1
Lcmt1	-0,42631	1
RP24-310D17.9	-0,42636	1
Atp5o	-0,42642	1
Otud6b	-0,42648	1
Tbc1d14	-0,42666	1
Immt	-0,42675	1
Spp1	-0,42691	1
Slc2a6	-0,42701	1
Ikbkb	-0,42701	1
Gm37531	-0,42703	1
Gm9333	-0,42707	1
D16Ertd472e	-0,42726	1
Grcc10	-0,42732	1
Mutyh	-0,42728	1
Xbp1	-0,42744	1
Wars	-0,42771	1
Rbm45	-0,42797	1
Psmb5	-0,42808	1
Tnfrsf23	-0,42808	1
Vwa7	-0,42807	1
Cdca7l	-0,42812	1
Trappc6a	-0,42844	1
Kif22	-0,4286	1
Tbc1d4	-0,42869	1
Rad23a	-0,42877	1
Gm9497	-0,42892	1
Rtel1	-0,42926	1
Gm8254	-0,42955	1
Gm20442	-0,42945	1
Ctbp1	-0,42952	1
Cc2d1a	-0,4298	1
Lgals8	-0,42998	1
Gm14681	-0,43008	1
Zcrb1	-0,43006	1
Trmu	-0,43059	1
Abcc10	-0,43063	1
Gm45890	-0,43058	1
Fech	-0,4312	1
Tbc1d17	-0,43127	1
Rpl17-ps10	-0,43133	1
Zbtb4	-0,43174	1
Fmc1	-0,43178	1
Gm6921	-0,43183	1

Tsg101	-0,43189	1
Gca	-0,43227	1
Ap4m1	-0,43226	1
Islr2	-0,43226	1
Txndc15	-0,43238	1
Bmyc	-0,43247	1
Gm37621	-0,43248	1
Cyb561d2	-0,43258	1
Hbegf	-0,43273	1
Miga1	-0,43314	1
1190007I07Rik	-0,43322	1
Zfp512	-0,4333	1
Ptpa	-0,43326	1
Timm17b	-0,43353	1
AU040320	-0,43372	1
Fam222b	-0,43383	1
Slc17a9	-0,43398	1
Gm1947	-0,43432	1
1810024B03Rik	-0,43463	1
Dpp8	-0,43461	1
Il23a	-0,43468	1
Adssl1	-0,43471	1
Hcst	-0,43483	1
Layn	-0,43483	1
Gm14138	-0,43501	1
Gm5805	-0,43525	1
Polr2g	-0,43568	1
Sh3bp5l	-0,43582	1
A230050P20Rik	-0,43595	1
Slc7a11	-0,43596	1
Plec	-0,43608	1
Spag4	-0,4362	1
Scap	-0,43635	1
Ddx41	-0,43632	1
Mrps26	-0,43632	1
1700047K16Rik	-0,43689	1
Gm37482	-0,43697	1
Slc12a4	-0,43716	1
Pskh1	-0,43734	1
Mars	-0,4378	1
Acot7	-0,43798	1
Gm42595	-0,43831	1
Otx1	-0,43845	1
Opa3	-0,4385	1
Scp2-ps2	-0,43908	1
Fmnl3	-0,43911	1
Saal1	-0,43916	1
Zc3h4	-0,43923	1
Gpr107	-0,43937	1
Gm13022	-0,4399	1
Cerk	-0,44006	1
Mitf	-0,44016	1

Cpt1c	-0,44074	1
Tars	-0,44084	1
Pogk	-0,44105	1
Nsmf	-0,44143	1
Prmt2	-0,4419	1
Thoc3	-0,44217	1
Atp6v1b2	-0,44223	1
Ppp3ca	-0,44233	1
Tm2d2	-0,44239	1
Gm10161	-0,44259	1
Slc27a4	-0,44277	1
Csad	-0,44305	1
Tmsb10	-0,44321	1
Zfp940	-0,44334	1
B9d1	-0,44361	1
Gm43961	-0,44371	1
Oas1c	-0,444	1
Hoxb3	-0,4447	1
Zcchc4	-0,44477	1
Tmem181a	-0,44488	1
Lig3	-0,44508	1
Nrg4	-0,44579	1
Lrrc45	-0,44614	1
Stxbp4	-0,44609	1
Usp48	-0,44619	1
Snx21	-0,44651	1
Ttc27	-0,44698	1
Pip5k1b	-0,44706	1
Maats1os	-0,44715	1
Tsku	-0,44755	1
Rhbdd1	-0,44769	1
BC048403	-0,44775	1
Lamtor4	-0,44765	1
D17H6S53E	-0,44776	1
Dusp28	-0,44788	1
Cul9	-0,44812	1
Frk	-0,44807	1
Gm7808	-0,44822	1
Hes6	-0,4482	1
Stard9	-0,44825	1
Plekha5	-0,4484	1
Mplkip	-0,44838	1
Mesdc2	-0,44844	1
Ptpn12	-0,44883	1
Ate1	-0,44879	1
Gm7424	-0,44949	1
Trmt6	-0,44961	1
Gm19967	-0,44965	1
Tlr4	-0,44997	1
Zfp94	-0,44997	1
Commd6	-0,44995	1
Tbl2	-0,45019	1

Hemk1	-0,45028	1
Dusp19	-0,45057	1
2010315B03Rik	-0,45067	1
Ece2	-0,45108	1
RP23-193N1.2	-0,45123	1
Abhd5	-0,45135	1
1700008J07Rik	-0,45191	1
Araf	-0,45196	1
Gm9378	-0,45216	1
Gm43794	-0,45253	1
Tcirg1	-0,45274	1
Zbtb25	-0,4533	1
Gm33080	-0,45325	1
Zcchc7	-0,45365	1
Gm43715	-0,45366	1
Ankrd44	-0,45379	1
Prkrip1	-0,45395	1
Pon2	-0,45386	1
4921531C22Rik	-0,45397	1
Dock7	-0,45423	1
Zbtb22	-0,45429	1
Slc10a3	-0,45442	1
Fbxo44	-0,45448	1
Ift81	-0,45476	1
Gm42671	-0,45479	1
Npc1l1	-0,45504	1
Abhd11	-0,45506	1
Pex11g	-0,45527	1
Etv1	-0,45551	1
Akr1b8	-0,4556	1
Gpatch3	-0,45577	1
Odf2l	-0,45582	1
Wrap73	-0,45591	1
Cela1	-0,45593	1
Trmt11	-0,45601	1
Alms1	-0,45601	1
Dync1h1	-0,45605	1
Rufy3	-0,45611	1
Ptpn6	-0,45628	1
Cdk5rap3	-0,4567	1
Chac1	-0,45708	1
Fkbp2	-0,45735	1
Ubac2	-0,45743	1
Kif3c	-0,45737	1
Elp2	-0,45762	1
Noc4l	-0,4578	1
Zfp329	-0,45814	1
Mavs	-0,45826	1
Zfp319	-0,45847	1
G730013B05Rik	-0,45874	1
Tars2	-0,45896	1
Slc50a1	-0,45921	1

Gm11334	-0,45975	1
Cep57l1	-0,4598	1
Zfp51	-0,45978	1
Cox5b	-0,45979	1
Pbxip1	-0,45989	1
Morn2	-0,46014	1
Wrap53	-0,46051	1
Spaca6	-0,46095	1
1700086O06Rik	-0,4616	1
Zer1	-0,4618	1
Med1	-0,46198	1
Abcf2	-0,4621	1
Tmbim1	-0,46232	1
Bcat2	-0,46287	1
Gm17494	-0,46287	1
Gm12618	-0,46287	1
Gm17108	-0,46328	1
Bahcc1	-0,46351	1
Parvg	-0,46365	1
Kbtbd3	-0,46362	1
Arhgap18	-0,46355	1
Zfp964	-0,46357	1
Runx3	-0,46365	1
Gm38082	-0,46376	1
Pxmp2	-0,4638	1
Slc12a5	-0,46378	1
Hadh	-0,46408	1
Hmgn1	-0,46411	1
Cirbp	-0,46441	1
A130048G24Rik	-0,46436	1
Rpl4	-0,46455	1
Cd109	-0,46456	1
Zfp260	-0,46482	1
Kmt2a	-0,46483	1
Aifm2	-0,46495	1
Gm37352	-0,4651	1
Ppm1f	-0,46517	1
Zfp74	-0,46545	1
Nfatc1	-0,46551	1
Gm8927	-0,46552	1
Gm20768	-0,46561	1
Kcnab3	-0,46622	1
Zfp651	-0,46646	1
Rpn1	-0,46653	1
Wdr6	-0,46674	1
Snhg5	-0,46746	1
Cox17	-0,46752	1
Mrpl46	-0,46771	1
Ppa2	-0,46778	1
Napa	-0,46778	1
Gm43071	-0,46791	1
Dyrk2	-0,46861	1

Smyd2	-0,4688	1
Zfp664	-0,46929	1
AC149090.1	-0,46932	1
Cchcr1	-0,4697	1
Borcs5	-0,47021	1
Gm4978	-0,47027	1
2210016L21Rik	-0,47053	1
Gm45050	-0,47057	1
Fgd4	-0,47101	1
AI837181	-0,47131	1
Slc25a37	-0,47126	1
Cmss1	-0,47202	1
Ddrgk1	-0,47208	1
Fam195b	-0,47205	1
Lgalsl	-0,47222	1
Fth1	-0,47224	1
Akap7	-0,47264	1
Lsm10	-0,47275	1
Gm13771	-0,47299	1
Gm3650	-0,4731	1
Mapk11	-0,4731	1
Fgfrl1	-0,47331	1
Trim3	-0,47389	1
Senp7	-0,47389	1
Tysnd1	-0,47402	1
Crat	-0,47406	1
Zfp687	-0,47446	1
Gm11110	-0,47449	1
Sys1	-0,47451	1
Gm28557	-0,47462	1
Nek6	-0,47479	1
Mdc1	-0,47488	1
Wdr55	-0,47517	1
Ssbp1	-0,47535	1
Cdc37l1	-0,47538	1
Uqcrq	-0,47539	1
Grpel1	-0,47548	1
Zfp444	-0,476	1
Map3k5	-0,476	1
Cyfip2	-0,47651	1
Atp6v1e1	-0,47668	1
Tmem160	-0,47684	1
Gart	-0,47678	1
Ccdc130	-0,4769	1
Preb	-0,47712	1
Arhgef19	-0,47715	1
Il1rn	-0,47722	1
Rftn1	-0,47725	1
Mrpl28	-0,47725	1
Eogt	-0,4773	1
Mrps18c	-0,47729	1
Apool	-0,47753	1

Samm50	-0,47752	1
Fam98a	-0,47761	1
Dhdds	-0,47778	1
Zfp759	-0,47792	1
Ctu1	-0,47816	1
Ncoa6	-0,47824	1
Slc16a12	-0,47821	1
Mrps34	-0,4783	1
Slc25a33	-0,47871	1
Sh3bgr	-0,47867	1
Slc16a6	-0,47877	1
Gm44093	-0,47878	1
Sh3tc1	-0,47893	1
Catsperg1	-0,47913	1
Fdxr	-0,47961	1
Ggnbp1	-0,47968	1
Slc43a2	-0,4797	1
Sos2	-0,47981	1
Ccdc12	-0,48018	1
Prkag2	-0,48019	1
Gm45871	-0,48033	1
Fibp	-0,48075	1
Rpl26-ps4	-0,48077	1
Gm5532	-0,48079	1
RP23-23P9.3	-0,48109	1
Mtch1	-0,48117	1
Acox3	-0,48142	1
Tsen2	-0,48149	1
Chpt1	-0,48151	1
Gcnt1	-0,48182	1
1110008F13Rik	-0,48193	1
Rnf214	-0,4827	1
Ttc28	-0,48269	1
Pigh	-0,48269	1
Zdhhc6	-0,48289	1
Shmt1	-0,48344	1
Rps18-ps3	-0,48368	1
Zfp467	-0,48417	1
Naca	-0,48418	1
Chchd3	-0,48415	1
Rps18-ps1	-0,48449	1
Rps10-ps4	-0,48474	1
Mesdc1	-0,48491	1
Agpat4	-0,48499	1
Fancf	-0,48513	1
Pomp	-0,48536	1
Mettl8	-0,48558	1
Uqcc2	-0,48621	1
Eif2s3x	-0,48642	1
Ganab	-0,48658	1
Gm26890	-0,48685	1
Zfp369	-0,48697	1

Snrpa1	-0,48715	1
Gngt2	-0,48733	1
Selenoo	-0,48729	1
Aldoart1	-0,48777	1
Setd1b	-0,48791	1
Fam114a1	-0,4881	1
S100a10	-0,48818	1
Gm44775	-0,48827	1
Prkab2	-0,48861	1
Zfp764	-0,48859	1
Tada2a	-0,48902	1
Gm43627	-0,48913	1
Slc26a9	-0,48939	1
Sdhc	-0,48973	1
Cdc34	-0,49014	1
Zfp958	-0,49043	1
Fahd2a	-0,49048	1
Slc39a8	-0,49051	1
Gm7094	-0,49102	1
Ruvbl2	-0,49115	1
Zfp106	-0,49114	1
Mphosph9	-0,49124	1
Cdpf1	-0,49131	1
Llph-ps2	-0,49128	1
Kptn	-0,49129	1
2610016A17Rik	-0,49128	1
Sdf4	-0,49144	1
Znhit6	-0,49159	1
Cep19	-0,49181	1
Gm8357	-0,49192	1
Ptpn21	-0,492	1
Gm42972	-0,49213	1
Acbd4	-0,49251	1
2700099C18Rik	-0,49329	1
Adprh	-0,49439	1
Psca	-0,49455	1
Cish	-0,49467	1
Glrx5	-0,4949	1
Rab4a	-0,49552	1
Clpp	-0,49559	1
Pla2g12a	-0,4957	1
Lrrc49	-0,49582	1
Pet100	-0,49593	1
Stk25	-0,49592	1
Saysd1	-0,49599	1
Gm43692	-0,49611	1
Srgap3	-0,49655	1
Gm44667	-0,49651	1
Macf1	-0,49666	1
Pdrg1	-0,49711	1
Ascc2	-0,49729	1
Esyt2	-0,49751	1

Zdhhc21	-0,49785	1
Abcf3	-0,49831	1
Chchd4	-0,4984	1
Coasy	-0,49857	1
RP24-91J7.1	-0,4992	1
Synj2bp	-0,49983	1
Osgep	-0,49978	1
Ppp1ca	-0,49989	1
Gm5113	-0,50014	1
Arrdc4	-0,50017	1
A430033K04Rik	-0,50023	1
2310039H08Rik	-0,50046	1
Mir6236	-0,50067	1
Cep63	-0,50085	1
Htra2	-0,50116	1
Mrps36-ps1	-0,50139	1
Tmem19	-0,50161	1
Ppm1j	-0,50171	1
Mfap3l	-0,50168	1
Wdr13	-0,50222	1
Setx	-0,50228	1
Tmie	-0,50263	1
Alg3	-0,50257	1
Rpusd1	-0,50262	1
Timm44	-0,50294	1
Gm45456	-0,50291	1
Tmem127	-0,50301	1
Tifa	-0,50333	1
Cep104	-0,50331	1
Atp6ap2	-0,50338	1
R3hcc1	-0,50337	1
Timmdc1	-0,50336	1
Kdelc2	-0,50348	1
Igtp	-0,50377	1
Prdx5	-0,504	1
Pitpna	-0,5041	1
Rab13	-0,5041	1
Gm20699	-0,50408	1
Dctn2	-0,50441	1
Gm15772	-0,50437	1
Sgta	-0,50453	1
Tsr3	-0,50465	1
Sphk2	-0,50492	1
Naglu	-0,50503	1
Dpagt1	-0,5058	1
Gphn	-0,506	1
Slc35a2	-0,50622	1
Eml3	-0,50647	1
Gm38162	-0,50651	1
Creb3l2	-0,50677	1
Acad9	-0,50682	1
Gm9332	-0,50705	1

Vsir	-0,50749	1
Ss18	-0,50761	1
Gas2l1	-0,50794	1
Acvr1	-0,50786	1
Cntln	-0,50821	1
Kifap3	-0,5083	1
Gm43247	-0,50886	1
Gm9173	-0,50959	1
Gdi2	-0,50971	1
Atp5h	-0,50968	1
Anapc4	-0,50967	1
Gm13612	-0,51028	1
Map2k3os	-0,51055	1
1500011B03Rik	-0,51073	1
Pgam5	-0,51107	1
Ints3	-0,51124	1
Aimp1	-0,51118	1
5830454E08Rik	-0,51128	1
Cd9	-0,51195	1
5830444B04Rik	-0,51196	1
Rab12	-0,51217	1
Clstn1	-0,51218	1
Smim8	-0,513	1
Entpd7	-0,51297	1
Bcl2a1d	-0,5132	1
Gucy2g	-0,51335	1
Manea	-0,51339	1
Cstf1	-0,5135	1
Slc12a2	-0,51345	1
Cox6b1	-0,51362	1
Gm26497	-0,5136	1
Arhgdig	-0,51389	1
Mtrf1l	-0,51413	1
Carf	-0,51417	1
Mir5128	-0,5142	1
Gsto1	-0,51434	1
Ykt6	-0,51437	1
Usp54	-0,51484	1
Tspan32	-0,51507	1
Pde4dip	-0,51522	1
Camkk2	-0,5154	1
Rab7b	-0,51536	1
Map4k1	-0,51589	1
Bop1	-0,51651	1
Arl10	-0,51665	1
Phf19	-0,51662	1
Ahnak2	-0,51691	1
Pop1	-0,51688	1
Hdhd3	-0,51733	1
Gm2735	-0,51727	1
Snd1	-0,51742	1
Ung	-0,51736	1

Pdss2	-0,51798	1
Rps3	-0,51838	1
Prr12	-0,51865	1
Rbfa	-0,51955	1
Zfp180	-0,51991	1
Exosc4	-0,51988	1
Amt	-0,52009	1
Ptpro	-0,5202	1
2810002D19Rik	-0,52025	1
Bcl2l12	-0,52029	1
Aldoc	-0,52057	1
Rtkn	-0,52064	1
Tmem218	-0,5211	1
Lgr4	-0,52106	1
Dlat	-0,52152	1
Gm6863	-0,52184	1
Slc25a4	-0,52203	1
Mrpl40	-0,52223	1
Lins1	-0,52224	1
Zfp239	-0,5222	1
Eya4	-0,52264	1
Wdr12	-0,52274	1
Creg1	-0,52291	1
Tmcc1	-0,5229	1
Nudt13	-0,52295	1
Vav1	-0,52333	1
Rps9	-0,52363	1
Dhrs4	-0,52363	1
Prtg	-0,52387	1
Rasal3	-0,52417	1
1600002H07Rik	-0,52471	1
Mrpl2	-0,52483	1
Lancl1	-0,52532	1
Slc35d1	-0,5255	1
Cd93	-0,52559	1
Gm8185	-0,52579	1
BC005561	-0,5262	1
Gm4950	-0,52635	1
Pld2	-0,52649	1
Ppfibp1	-0,52673	1
Lrrc75a	-0,52694	1
Lage3	-0,52768	1
Ggact	-0,52765	1
Etv6	-0,52811	1
Oxsm	-0,52811	1
Zfp276	-0,52823	1
Aldh1b1	-0,52852	1
Extl3	-0,52862	1
Slc25a46	-0,52874	1
F830208F22Rik	-0,52872	1
2010204K13Rik	-0,52907	1
Ccs	-0,52917	1

Apbb1ip	-0,53001	1
Jmy	-0,53001	1
Gzf1	-0,53026	1
4930550C14Rik	-0,53029	1
Tm9sf1	-0,53037	1
Smco4	-0,53052	1
Dfna5	-0,53097	1
Endod1	-0,53109	1
2500002B13Rik	-0,53118	1
Rcan3	-0,53133	1
B930086L07Rik	-0,53155	1
Srxn1	-0,53171	1
Cadps	-0,53168	1
Abhd17c	-0,53208	1
Myo6	-0,53212	1
Selenoh	-0,53282	1
BC017643	-0,53278	1
Bank1	-0,53304	1
Cog4	-0,5332	1
Sdhaf4	-0,53328	1
Chchd10	-0,53353	1
Xab2	-0,53375	1
Birc3	-0,5338	1
Tal1	-0,53399	1
Plaa	-0,53414	1
Kidins220	-0,53419	1
Rif1	-0,53429	1
Tomm5	-0,53449	1
Gm44432	-0,53501	1
Ift74	-0,5352	1
Ubl4a	-0,53538	1
Stub1	-0,53563	1
Gm5644	-0,53566	1
Tango2	-0,53614	1
Gm9951	-0,53653	1
Gm44153	-0,5367	1
Ttl	-0,53703	1
Snx25	-0,53704	1
Man1b1	-0,53718	1
Noc2l	-0,53776	1
Per3	-0,53785	1
Zfp28	-0,53788	1
Pgp	-0,53808	1
Gm7676	-0,53832	1
Gbas	-0,5386	1
Ankrd35	-0,53868	1
Gm8550	-0,53899	1
Gnptab	-0,53958	1
Nphp1	-0,53971	1
Zdhhc4	-0,53989	1
Gga2	-0,54018	1
Ndufaf7	-0,54044	1

Mtus2	-0,54075	1
Ticam2	-0,54135	1
Maged1	-0,54161	1
Mfap1a	-0,5416	1
C130023A14Rik	-0,54184	1
Ston1	-0,54181	1
Mzt2	-0,54185	1
Ap4s1	-0,54187	1
Eif6	-0,54206	1
Gm26670	-0,54206	1
Gm4799	-0,54228	1
Fam208a	-0,54226	1
Surf2	-0,543	1
Xpr1	-0,54327	1
Garnl3	-0,54342	1
Pxylp1	-0,54351	1
Arfgap3	-0,54364	1
Arhgap45	-0,54368	1
Zswim7	-0,5439	1
Tsnax	-0,54404	1
Tmem219	-0,54436	1
Sp110	-0,54448	1
Diaph2	-0,54469	1
Lman2	-0,54564	1
Coro1c	-0,54596	1
Tdrkh	-0,54596	1
Ppp2r3a	-0,546	1
Sult6b1	-0,54629	1
Pdcd2	-0,54637	1
Tigd5	-0,54641	1
Zkscan17	-0,54658	1
4930539J05Rik	-0,54661	1
Atpif1	-0,54671	1
D730003I15Rik	-0,54676	1
Tnfaip8l2	-0,54681	1
Asxl2	-0,54695	1
Cnpy3	-0,54716	1
Psmg2	-0,54717	1
Coro2a	-0,54721	1
Mtif2	-0,54751	1
Ipp	-0,5475	1
Gm42481	-0,54757	1
Cpt1a	-0,54775	1
RP24-499N24.6	-0,54787	1
Ergic1	-0,54797	1
Pttg1ip	-0,54823	1
Spcs2-ps	-0,54839	1
Kdelr3	-0,54847	1
Sipa1	-0,54868	1
Igf2bp1	-0,54866	1
Hras	-0,54879	1
Pla2g16	-0,54887	1

Rhd	-0,54914	1
Gm37490	-0,54921	1
Eif2b3	-0,54934	1
Gm11410	-0,54936	1
Grik5	-0,54947	1
Trmt2a	-0,5496	1
6230400D17Rik	-0,55004	1
Bmf	-0,55031	1
Rab32	-0,55059	1
Acsf3	-0,55079	1
Zfp182	-0,55091	1
Ly96	-0,55102	1
Kcnn4	-0,55114	1
Cfl1	-0,55134	1
Lgi4	-0,55128	1
Foxo4	-0,55137	1
Gm43112	-0,55144	1
Gm45728	-0,55144	1
Ermard	-0,55189	1
E2f1	-0,55222	1
Man2c1os	-0,55217	1
Klf16	-0,55254	1
Akr1b3	-0,55251	1
Gm12389	-0,55252	1
Myom1	-0,55248	1
Dmpk	-0,55256	1
Smdt1	-0,55273	1
Ydjc	-0,55266	1
Slc2a4rg-ps	-0,55281	1
Psmb8	-0,55314	1
S100a8	-0,55338	1
Smap1	-0,55364	1
Tuba1a	-0,55364	1
Zfp202	-0,5539	1
Bcat1	-0,55462	1
Ucp2	-0,55576	1
Rnf149	-0,55644	1
Slc1a5	-0,55719	1
C630043F03Rik	-0,55718	1
Ppp1r12b	-0,55728	1
Rnaseh2b	-0,55744	1
Gm15441	-0,55741	1
Six1	-0,55843	1
Mapk12	-0,5588	1
Fign	-0,55925	1
Rpusd4	-0,55951	1
Tmem67	-0,55945	1
Spryd3	-0,55999	1
Fnta	-0,56013	1
Pgd	-0,56036	1
Maf1	-0,56056	1
Gm44190	-0,56061	1

Sqrdl	-0,56074	1
Kansl1l	-0,5608	1
Abcd4	-0,56097	1
Dync1li2	-0,56136	1
Elp3	-0,56143	1
Slc1a4	-0,5614	1
Gm7160	-0,56159	1
Cpq	-0,56173	1
1700030K09Rik	-0,56181	1
Vsig8	-0,5618	1
Gemin4	-0,56204	1
Xpnpep3	-0,56208	1
Gpr180	-0,56208	1
Sfmbt1	-0,56233	1
Taf4b	-0,56246	1
Zfp763	-0,56253	1
AW209491	-0,56346	1
Fam219b	-0,56378	1
Cdc123	-0,56424	1
Tm7sf2	-0,56427	1
Jsrp1	-0,56455	1
Spata7	-0,56455	1
CAAA01180111.2	-0,56455	1
Atpaf1	-0,56468	1
RP24-122E11.4	-0,56471	1
Arhgap9	-0,56476	1
Smim10l1	-0,56488	1
Cacna1b	-0,56538	1
Gm9484	-0,56558	1
Klc4	-0,56594	1
Atg9b	-0,56592	1
Tm2d3	-0,56666	1
Fars2	-0,56696	1
4921536K21Rik	-0,56703	1
S100a1	-0,56709	1
Csrp2bp	-0,56728	1
Gm20628	-0,56769	1
Gm20632	-0,56787	1
Slc35e2	-0,56798	1
Fcna	-0,56818	1
Cacfd1	-0,5683	1
Gan	-0,56853	1
1700003F12Rik	-0,56866	1
Efr3b	-0,56894	1
Ints11	-0,569	1
Emc10	-0,56906	1
Pfkl	-0,5693	1
Il18	-0,5697	1
Zfp84	-0,56983	1
Cat	-0,57047	1
Pygl	-0,57047	1
Nipsnap1	-0,57058	1

Trp53rka	-0,57065	1
Tkfc	-0,57062	1
Zfp113	-0,57061	1
Smpd2	-0,57146	1
Gsr	-0,57164	1
RP24-233B16.6	-0,57184	1
Pex16	-0,57257	1
Pde8a	-0,57289	1
Dnajc8	-0,57296	1
Pradc1	-0,57297	1
Smim19	-0,57332	1
9430092D12Rik	-0,57336	1
Tpm1	-0,57365	1
Cox10	-0,57375	1
Eno3	-0,57388	1
Parg	-0,57428	1
Coro1a	-0,57438	1
Gm8738	-0,57474	1
Ikbip	-0,57499	1
Zfp408	-0,57552	1
Frat1	-0,5755	1
Rbck1	-0,57605	1
Gm12309	-0,57609	1
Ppox	-0,57663	1
Per2	-0,57692	1
Zfp820	-0,57695	1
Gm37906	-0,57696	1
Ap1s1	-0,57717	1
Gm25636	-0,57737	1
Zscan12	-0,57757	1
Gabrd	-0,57758	1
Tmem51	-0,57801	1
Slc37a4	-0,5783	1
Sdcbp2	-0,57835	1
Porcn	-0,57837	1
Gm5609	-0,57864	1
Pgm2	-0,57919	1
Gm10180	-0,57931	1
Tmem80	-0,57933	1
Msi1	-0,58074	1
Cyp4f13	-0,5813	1
Mrgbp	-0,58145	1
Zbtb39	-0,58157	1
Cd3eap	-0,58178	1
RP24-370M23.1	-0,5821	1
Pqlc1	-0,58227	1
Gm23722	-0,58228	1
Parp16	-0,58245	1
BC004004	-0,58287	1
Defb25	-0,58288	1
Gm42872	-0,58311	1
Vmac	-0,58316	1

Cmc2	-0,58336	1
E330020D12Rik	-0,58349	1
Timm13	-0,5838	1
Asap3	-0,58438	1
Engase	-0,58447	1
Tmem231	-0,58457	1
Thap7	-0,5847	1
Tesk2	-0,5848	1
Arhgef3	-0,58503	1
2310074N15Rik	-0,5851	1
Zfp407	-0,5853	1
9130401M01Rik	-0,58542	1
Ttll5	-0,58539	1
Mrpl13	-0,58546	1
Ano10	-0,58558	1
Gfi1	-0,58561	1
Zfp658	-0,5857	1
Ell2	-0,58619	1
Trmt112-ps2	-0,58618	1
Rpl31-ps10	-0,58643	1
Uqcc3	-0,58682	1
Vamp5	-0,58689	1
Trub1	-0,587	1
Ms4a6c	-0,58715	1
Psmb6-ps2	-0,58771	1
Tcta	-0,58801	1
Wdr4	-0,58808	1
Arhgef15	-0,58835	1
Sars	-0,58845	1
Oas1b	-0,58873	1
Arhgef12	-0,58885	1
Gm44916	-0,58883	1
Map3k10	-0,5891	1
Fbf1	-0,58936	1
Pyroxd1	-0,58947	1
Gm12267	-0,59005	1
Ptpdc1	-0,59029	1
Tnfrsf9	-0,59046	1
Elp4	-0,59104	1
Il1rap	-0,59108	1
Gm12529	-0,5917	1
Kctd7	-0,59213	1
Rars2	-0,59263	1
Gk5	-0,59261	1
Gm10268	-0,59276	1
Zfp60	-0,59295	1
Gm12421	-0,59377	1
1300002E11Rik	-0,59407	1
Rbbp4	-0,59422	1
Katnal1	-0,59421	1
Wdr59	-0,59436	1
Ndufb7	-0,59478	1

Txnl4b	-0,5948	1
Dnajc1	-0,59554	1
Prelid2	-0,59559	1
B230208H11Rik	-0,59586	1
Ltbr	-0,59606	1
Lsm5	-0,59625	1
Cant1	-0,59668	1
Cwc27	-0,59666	1
Crtap	-0,59774	1
Ap2a1	-0,59789	1
Hspa2	-0,59808	1
Zranb2	-0,59843	1
Kirrel3	-0,59836	1
Cdc34b	-0,59852	1
Mrps18a	-0,59866	1
Dmwd	-0,59923	1
Rpusd3	-0,59932	1
Matn4	-0,59968	1
Map2k2	-0,5999	1
B4gat1	-0,60004	1
Ak1	-0,60013	1
Fads1	-0,60044	1
Hmgxb4	-0,60044	1
Pigp	-0,60038	1
Gnptg	-0,60043	1
Suclg1	-0,60081	1
Polr1c	-0,60101	1
Vps52	-0,60095	1
D430042O09Rik	-0,60115	1
Gm13408	-0,60123	1
Wdr73	-0,60184	1
Sergef	-0,60196	1
Fmn1	-0,60214	1
Abi3	-0,60243	1
Zzef1	-0,60274	1
Plxna3	-0,60395	1
Gm37125	-0,6039	1
Cenpb	-0,60406	1
Apopt1	-0,60429	1
Mrpl20	-0,60512	1
Prep	-0,60549	1
Gm7730	-0,60583	1
Use1	-0,60591	1
Cers4	-0,6059	1
Isoc2a	-0,60618	1
Cox6a2	-0,60621	1
Gm37569	-0,60618	1
Capn10	-0,60674	1
Nt5dc1	-0,60681	1
Slc47a2	-0,60676	1
Tmem260	-0,60721	1
Slc8a1	-0,6072	1

Micu2	-0,60768	1
Cep128	-0,60793	1
Ap1g2	-0,60827	1
Cdk14	-0,60845	1
Il18rap	-0,6085	1
Gm26881	-0,60858	1
Acot8	-0,60856	1
Tmem5	-0,60858	1
Dzip3	-0,60863	1
2810004N23Rik	-0,60891	1
Snx19	-0,60885	1
Gm12222	-0,60903	1
Ckap4	-0,60911	1
Psme2b	-0,60933	1
Golt1b	-0,60946	1
Zbtb14	-0,60966	1
Taf12	-0,60987	1
Ccdc180	-0,61035	1
Hspbap1	-0,61078	1
Rnf187	-0,61199	1
Mettl21a	-0,61195	1
Rb1cc1	-0,61239	1
Irgm2	-0,61255	1
G430095P16Rik	-0,61275	1
0610007P14Rik	-0,61275	1
Zfp322a	-0,61291	1
Gm43501	-0,61288	1
Gm2950	-0,61307	1
1700112E06Rik	-0,61366	1
D630045J12Rik	-0,61378	1
Gm28875	-0,6141	1
Fosl2	-0,61434	1
Eef2k	-0,61429	1
1110038F14Rik	-0,61453	1
Tnni2	-0,6146	1
Ccdc166	-0,61499	1
Dis3l	-0,61506	1
Nlk	-0,61538	1
Itpa	-0,61536	1
Ap5z1	-0,61553	1
Rpl31-ps14	-0,61574	1
Gm2467	-0,6157	1
Tdrd3	-0,61584	1
Gm14830	-0,61654	1
Nrp1	-0,61671	1
Cinp	-0,61681	1
Gm5624	-0,61693	1
Ddx17	-0,61701	1
Slc35b4	-0,61747	1
Yif1a	-0,61769	1
Gm4673	-0,6178	1
Stau2	-0,61799	1

Cyb561	-0,61814	1
1810044D09Rik	-0,61826	1
Ehd1	-0,61848	1
Stat1	-0,61883	1
Wfikkn1	-0,61988	1
Zfp738	-0,62036	1
Elk3	-0,6205	1
Mrpl19	-0,62075	1
Mvb12a	-0,62077	1
Tmem192	-0,62082	1
4930563E22Rik	-0,62093	1
Lair1	-0,62096	1
Smim12	-0,62104	1
Ipo8	-0,62097	1
Chd1l	-0,62118	1
Zfp456	-0,62124	1
Fam45a	-0,62156	1
Uggt2	-0,62161	1
Ctu2	-0,62184	1
3110083C13Rik	-0,62175	1
Tmed1	-0,62272	1
Gm10060	-0,6228	1
Gm10557	-0,62326	1
Alg9	-0,62353	1
Gm43775	-0,62398	1
2700033N17Rik	-0,62402	1
6030460B20Rik	-0,62465	1
4930590J08Rik	-0,62505	1
Sec22a	-0,62508	1
Pdzd11	-0,62515	1
Chordc1	-0,62535	1
Mtmr11	-0,62543	1
B230377A18Rik	-0,62598	1
Tprkb	-0,62618	1
Faap20	-0,62702	1
Dhrs7b	-0,62713	1
Bod1l	-0,62722	1
Tom1l2	-0,6273	1
Pkd1l2	-0,62738	1
Acod1	-0,62742	1
Rgs11	-0,62747	1
Dnlz	-0,6277	1
Gm7722	-0,62783	1
Gm43696	-0,62793	1
Csf2ra	-0,62847	1
Slc39a4	-0,62861	1
Nfam1	-0,62872	1
Traf2	-0,62912	1
Tubd1	-0,62925	1
Gm7909	-0,62927	1
Fzd9	-0,62938	1
Usp6nl	-0,62953	1

Gad2	-0,62953	1
Nsmce1	-0,62957	1
D630024D03Rik	-0,63014	1
Ccp110	-0,63006	1
Gm11488	-0,63015	1
4930426I24Rik	-0,63024	1
3300002I08Rik	-0,63064	1
Selplg	-0,63125	1
Coro7	-0,63153	1
Chmp3	-0,63167	1
S100a4	-0,63249	1
Shisa3	-0,63251	1
Sdhb	-0,63274	1
Gm15459	-0,63267	1
Gm6745	-0,63276	1
Lrrc47	-0,63294	1
Oxld1	-0,6329	1
RP23-255F14.4	-0,63285	1
Enpp1	-0,63298	1
Gm44044	-0,63297	1
Gm10642	-0,63318	1
Brip1os	-0,63333	1
Gm12248	-0,63328	1
Aldh4a1	-0,63419	1
Fancb	-0,63441	1
Vrk3	-0,63449	1
Rps12-ps23	-0,63454	1
Gm11878	-0,63457	1
Etfb	-0,63472	1
Gm5302	-0,63498	1
Dbnl	-0,63562	1
Ocel1	-0,63567	1
Gm14336	-0,636	1
Ttc26	-0,63598	1
Lmntd2	-0,63636	1
Sdf2l1	-0,63681	1
Fam58b	-0,63755	1
Prtn3	-0,63817	1
Cdr2	-0,63869	1
Reep6	-0,63915	1
2610020H08Rik	-0,64004	1
Nkain1	-0,64061	1
Rgl1	-0,64071	1
Lat	-0,64083	1
Ccni	-0,64092	1
Nrxn3	-0,64112	1
Ssu72	-0,6418	1
Lpl	-0,64189	1
Lrfn4	-0,64199	1
Ifi27	-0,643	1
Spata5l1	-0,64335	1
Dhrs11	-0,64389	1

Sec11c	-0,64404	1
Taf1c	-0,64404	1
Dnajc22	-0,64505	1
Tspan5	-0,64562	1
Gm14403	-0,64572	1
Pced1a	-0,64707	1
Dnase1l1	-0,64716	1
Rgs14	-0,6476	1
Stx17	-0,64805	1
Clec11a	-0,64807	1
Slc25a40	-0,6482	1
Ltbp2	-0,64819	1
Serpine1	-0,64865	1
Irf2bp1	-0,64905	1
Gm43742	-0,64983	1
Nudt12	-0,64984	1
Dclre1c	-0,65008	1
Gm15535	-0,65093	1
Tasp1	-0,65114	1
Dapk3	-0,65121	1
Tspan3	-0,65133	1
6330418K02Rik	-0,65143	1
Prrg2	-0,65155	1
Tm4sf5	-0,65172	1
Dkkl1	-0,65243	1
Gm7815	-0,65352	1
Commd9	-0,65362	1
Cd300a	-0,65459	1
Retsat	-0,65476	1
Cfp	-0,65505	1
Lrrc27	-0,65506	1
Snx24	-0,65558	1
B3galt6	-0,65583	1
AW146154	-0,65631	1
Prr36	-0,65672	1
Invs	-0,65678	1
Nop9	-0,65686	1
Fkbp8	-0,65723	1
Asb7	-0,65732	1
Gmppa	-0,6582	1
Pfkfb4	-0,6591	1
Csf1r	-0,65932	1
Coq3	-0,65932	1
Gm9256	-0,6597	1
6430571L13Rik	-0,65987	1
Slc2a9	-0,66025	1
Zfp653	-0,66058	1
Psmb9	-0,66071	1
Nr2f6	-0,661	1
Hsdl2	-0,66127	1
Gtpbp8	-0,66147	1
Atp6v1g2	-0,66152	1

Chmp2a	-0,6616	1
Chst3	-0,66172	1
Fcgrt	-0,66255	1
Alg5	-0,6627	1
Arhgef10	-0,66275	1
Sh2d5	-0,66351	1
Ifi44	-0,66383	1
Rala	-0,66449	1
Umps	-0,66448	1
Avpi1	-0,66551	1
Coro1b	-0,66551	1
Mrpl37	-0,66584	1
Ubxn6	-0,66602	1
Bco2	-0,66605	1
Glt8d1	-0,6662	1
Celf4	-0,66631	1
Mccc1	-0,66642	1
Zmat5	-0,66694	1
C530043K16Rik	-0,66692	1
Lrrc14	-0,6678	1
Fancg	-0,66779	1
Vim	-0,66794	1
Mief2	-0,66799	1
Kif13a	-0,66866	1
Myl6	-0,66984	1
Zfp809	-0,67027	1
Rpgrip1l	-0,67055	1
Wdr46	-0,67078	1
Ccl25	-0,67101	1
Cd63-ps	-0,67115	1
Nprl3	-0,67177	1
Fam136a	-0,67192	1
Tepsin	-0,67199	1
Gm3571	-0,67223	1
Ube2d-ps	-0,67233	1
Rpl36a-ps1	-0,67315	1
Nudt3	-0,67344	1
Kif3b	-0,67381	1
Alkbh2	-0,67379	1
Kcnq1ot1	-0,67405	1
Msto1	-0,67397	1
Poli	-0,6741	1
Ndufb9	-0,67455	1
Tstd3	-0,67455	1
Exoc3l2	-0,67538	1
Sh3glb2	-0,67551	1
Snord13	-0,67606	1
Upp2	-0,67644	1
Zfp983	-0,67649	1
Gm37675	-0,67677	1
Gm14822	-0,67716	1
1810021B22Rik	-0,67741	1

Fbxo6	-0,67752	1
Mllt1	-0,67749	1
Gm14698	-0,67774	1
Tfcp2	-0,67779	1
Mtfr1	-0,67791	1
Tmem17	-0,67804	1
Fem1a	-0,67816	1
Intu	-0,67881	1
Rps14	-0,67954	1
2810414N06Rik	-0,68009	1
Gm42633	-0,68009	1
Dph5	-0,68039	1
Dcun1d2	-0,68087	1
Spout1	-0,68142	1
Coq4	-0,6814	1
RP24-547N4.5	-0,68184	1
Fam160b2	-0,68248	1
Wars2	-0,68256	1
Ndufa9	-0,68325	1
Alg14	-0,68349	1
Gm43336	-0,68396	1
H2-DMb2	-0,68401	1
Ncoa7	-0,68425	1
Fbxl17	-0,68436	1
Ostc	-0,68516	1
Serac1	-0,68518	1
Myoz1	-0,68525	1
Fcrl1	-0,68527	1
A930024E05Rik	-0,68581	1
4930568A12Rik	-0,68602	1
Shprh	-0,68655	1
Pih1d1	-0,68662	1
Trpv4	-0,68699	1
Ercc6l	-0,68715	1
Slc43a3	-0,6876	1
Gpx4	-0,68799	1
Btd	-0,68805	1
Gm37108	-0,68849	1
Gm14239	-0,68881	1
Alad	-0,68901	1
Chmp6	-0,68907	1
Ppcdc	-0,68932	1
Gm43578	-0,68946	1
Actn1	-0,69011	1
Ndufaf3	-0,69025	1
Metap2	-0,69048	1
Lrrc61	-0,69065	1
Card11	-0,69057	1
Mtdh	-0,69108	0,90856
Wdpcp	-0,69183	1
Cenpv	-0,69197	1
Ficd	-0,69247	1

Cep164	-0,69273	1
Gm43655	-0,69294	1
Zfp768	-0,69305	1
RP23-402A24.3	-0,69371	1
Ttc39a	-0,69394	1
Nudc-ps1	-0,69407	1
Clec1a	-0,69419	1
Akap9	-0,69494	1
Dars2	-0,69585	1
Fam161a	-0,696	1
Cog7	-0,69605	1
Ntmt1	-0,6968	1
Ndufa4	-0,69699	1
Tspan4	-0,69739	1
Plekhg5	-0,69758	1
Aig1	-0,69832	1
Lat2	-0,69853	1
Gm37728	-0,69847	1
AI839979	-0,6986	1
Nubp2	-0,69868	1
Atp6v0b	-0,69893	1
Gm37902	-0,69934	1
Sapcd1	-0,69959	1
Mfsd6	-0,70022	1
Ndufab1-ps	-0,7003	1
Nav1	-0,70044	1
Klhl8	-0,70042	1
Gm15050	-0,7007	1
Gm10676	-0,70084	1
Stard8	-0,70115	1
Psmc3	-0,70187	1
4930509H03Rik	-0,70191	1
Prmt3	-0,70239	1
Nrp2	-0,70262	1
Trpv2	-0,70355	1
Eri2	-0,7045	1
Slc9a3r2	-0,70473	1
Crlf2	-0,70487	1
Cox7a1	-0,70518	1
Gm43153	-0,70565	1
Ampd3	-0,70596	1
Irak4	-0,70608	1
Lekr1	-0,70608	1
Zfp316	-0,70632	1
P2ry6	-0,70688	1
Rpain	-0,70695	1
Gm9796	-0,70701	1
Rtn4ip1	-0,70712	1
Gm5905	-0,70706	1
Disp1	-0,70716	1
Gm31166	-0,70731	1
Xpo4	-0,70868	1

9030624J02Rik	-0,70878	1
Tmem206	-0,70885	1
Naa10	-0,70922	1
Gm9726	-0,70923	1
Gm6209	-0,7093	1
Tubb6	-0,70955	1
Prelid3a	-0,70957	1
A930016O22Rik	-0,7097	1
Nt5c	-0,7104	1
Cdc25b	-0,71055	1
Gm8292	-0,71073	1
2210016F16Rik	-0,71088	1
Lyz1	-0,71101	1
Wbscr27	-0,71096	1
Zfp365	-0,7113	1
Mtg1	-0,712	1
Ebpl	-0,71206	1
Tufm	-0,71234	1
RP24-183O8.6	-0,71255	1
Sfi1	-0,71275	1
Ankrd12	-0,71306	1
Edem2	-0,71325	1
Gm37503	-0,71337	1
Spaca9	-0,71339	1
Tmem26	-0,71487	1
A430110C17Rik	-0,71485	1
Trim27	-0,71562	1
Fkbpl	-0,71573	1
Zbtb20	-0,71613	1
Kcnab2	-0,71658	1
Gm37354	-0,71664	1
Zfp541	-0,71671	1
Tst	-0,717	1
Thoc7	-0,71748	1
Fam120b	-0,71751	1
AA414768	-0,71769	1
Snord118	-0,71776	1
Ccdc91	-0,7186	1
Gm43162	-0,7188	1
Actr8	-0,71886	1
Il11ra1	-0,7192	1
Mydgf	-0,71965	1
Adck1	-0,71978	1
9130221H12Rik	-0,71981	1
Gpr108	-0,71993	1
Pycr2	-0,72036	1
Ndufb8	-0,72051	1
Ufsp2	-0,72061	1
Pfas	-0,72152	1
Urod	-0,72191	1
Prkch	-0,72371	1
Znhit2	-0,7243	1

Kctd11	-0,7245	1
Lsm14b	-0,72484	1
Dock5	-0,7248	1
Keap1	-0,72488	1
Frrs1	-0,72522	1
Drosha	-0,72617	1
Ppm1h	-0,7262	1
Acad12	-0,7262	1
2810030D12Rik	-0,7265	1
Abr	-0,72665	0,89201
Uqcr11	-0,72739	1
Bnip2	-0,7276	1
4930447F24Rik	-0,72783	1
Fam219a	-0,72804	1
Frmd4a	-0,72858	1
Snx9	-0,72926	1
Aldh18a1	-0,72955	1
Bloc1s5	-0,73006	1
Gm45343	-0,73077	1
Nptxr	-0,73126	1
Ttc13	-0,73169	1
D930016D06Rik	-0,73193	1
Cyc1	-0,73201	1
Tmem51os1	-0,73213	1
1600010M07Rik	-0,73296	1
Fryl	-0,73309	1
Diablo	-0,7334	1
Gm29284	-0,73341	1
Pnkd	-0,73376	1
Sec24d	-0,73386	1
Fam213a	-0,73465	1
Zfp251	-0,7353	1
Impa2	-0,73589	1
Mcat	-0,73585	1
Gm45629	-0,73657	1
3300005D01Rik	-0,73749	1
Habp4	-0,73806	1
Iars2	-0,73825	1
Nans	-0,73846	1
Dock2	-0,73856	1
Clybl	-0,73871	1
Gmpr	-0,73889	1
Tmed3	-0,73967	1
Gm44509	-0,74012	1
Gm12990	-0,74022	1
Rnaseh2a	-0,74065	1
Dcp1b	-0,74195	1
1810010D01Rik	-0,74206	1
Syne1	-0,74223	1
Tbc1d13	-0,7423	1
Pgap3	-0,74281	1
Armc9	-0,7432	1

Pdia5	-0,74329	1
Tlr3	-0,74332	1
Amer1	-0,74338	1
Ebp	-0,74357	1
Gm28192	-0,74417	1
Olfr912	-0,74419	1
Gm14121	-0,74445	1
Ammecr1	-0,74457	1
Ccdc173	-0,74464	1
Irf7	-0,74523	1
Rad1	-0,74527	1
Galns	-0,7459	1
Nlrp10	-0,74599	1
Ribc1	-0,74711	1
Gm20430	-0,74747	1
Enox2	-0,74777	1
Coa7	-0,7483	1
Wnt6	-0,74846	1
Fn3krp	-0,74878	1
Slc13a2	-0,74924	1
Mdrl	-0,74924	1
Gm5590	-0,75003	1
Ifitm1	-0,75051	1
Aamdc	-0,75064	1
1700022N22Rik	-0,75084	1
Mtg2	-0,75099	1
Gm6329	-0,75135	1
Gm6266	-0,75145	1
Parp3	-0,75161	1
Inf2	-0,75166	0,98528
Sbk1	-0,75226	1
Mus81	-0,75264	1
D330023K18Rik	-0,75275	1
Gm13498	-0,75303	1
Cmas	-0,75348	1
Spr	-0,7537	1
Exoc8	-0,75373	1
Zfp128	-0,75431	1
Gm10327	-0,75461	1
n-R5-8s1	-0,75474	1
Slc39a1	-0,75639	1
Kntc1	-0,75757	1
Zfp931	-0,75844	1
Stoml1	-0,75885	1
Pde4d	-0,75891	1
Uvssa	-0,75907	1
Ccdc17	-0,7594	1
Ttll13	-0,75949	1
D11Wsu47e	-0,75971	1
Smim4	-0,76002	1
Mettl1	-0,76029	1
Adgrl2	-0,7611	1

Ankrd39	-0,76125	1
Tmlhe	-0,76122	1
Zscan20	-0,76203	1
Neil1	-0,76208	1
Susd3	-0,76256	1
Zfp157	-0,76354	1
Zfp775	-0,76361	1
Fkbp11	-0,76444	1
Ptgs2os	-0,76486	1
Rhobtb1	-0,76501	1
Jaml	-0,76518	1
6430548M08Rik	-0,76521	1
Gm5577	-0,76537	1
RP24-550H10.6	-0,76585	1
Lin7b	-0,76603	1
Snhg18	-0,7673	1
Pigv	-0,76732	1
Gm33142	-0,76736	1
Slc22a21	-0,76754	1
Exoc7	-0,76777	1
Dennd2d	-0,7687	1
Prdm9	-0,76889	1
Hint2	-0,76895	1
Gm43378	-0,76899	1
Gm29487	-0,76914	1
Brf1	-0,76925	1
Tor4a	-0,77062	1
Hacd1	-0,77092	1
Pdlim2	-0,77134	1
Gsdmd	-0,77141	1
Tmem65	-0,77181	1
RP23-55A6.4	-0,77265	1
Rasa3	-0,77292	1
Dnajc11	-0,77301	1
Gm7436	-0,77332	1
Gm42937	-0,77332	1
Polr3e	-0,77367	1
Amotl1	-0,77401	0,98528
Dgka	-0,77403	1
Fignl2	-0,7741	1
C130036L24Rik	-0,77431	1
Mdn1	-0,77458	1
Tnfaip8	-0,77518	0,83697
R74862	-0,77524	1
Gtf2i	-0,77612	1
Gm24601	-0,77607	1
Fkrp	-0,77639	1
Ric8a	-0,77651	1
Colec12	-0,77704	1
Cep95	-0,7771	1
Ganc	-0,77717	1
Coa4	-0,77733	1

Mocs3	-0,77738	1
Plxdc1	-0,77806	1
Gm43544	-0,7783	1
Plcb4	-0,77927	1
Ankrd54	-0,77974	1
Gm45360	-0,77999	1
Ermap	-0,78059	1
Zfp9	-0,78093	1
Gm6181	-0,78188	1
Oscp1	-0,78201	1
Pecr	-0,7831	1
Gm8724	-0,7833	1
Gstp1	-0,78327	1
Fam173b	-0,78359	1
Ccr1	-0,78413	1
Tmem220	-0,78417	1
Dmrta2	-0,7858	1
Tmem241	-0,78593	1
Nanos1	-0,78619	1
Nipa1	-0,78777	1
Mtfp1	-0,7879	1
Nfatc4	-0,78792	1
Pde8b	-0,78821	0,80904
Peak1os	-0,78862	1
Zfp429	-0,78866	1
Psmb7	-0,7889	1
Sdccag8	-0,78969	1
Kyat1	-0,79009	1
Gm2199	-0,79052	1
Gm12350	-0,79103	1
Lrig3	-0,79161	1
Eef2kmt	-0,79176	1
Adamtsl5	-0,79194	1
H6pd	-0,79198	1
Snx7	-0,79209	1
Acaa1a	-0,79259	0,60972
Arrb1	-0,79257	1
Gm37959	-0,7933	1
Gm42893	-0,79378	1
RP23-228B2.5	-0,79394	1
L3mbtl3	-0,79439	1
Tmem209	-0,79568	1
Gm16104	-0,79675	1
Dapp1	-0,79714	1
Endog	-0,79737	1
Gm45802	-0,79768	1
Gm37642	-0,79781	1
Acap1	-0,79817	1
Ppcs	-0,79865	1
Gm9517	-0,7988	1
Fcgr1	-0,79928	1
Grk5	-0,79934	1

Zfp607b	-0,7993	1
Galk1	-0,79973	1
Ppil1	-0,7998	1
RP23-40D21.1	-0,79989	1
Sgtb	-0,80007	1
Zadh2	-0,80028	1
Sipa1l2	-0,80046	1
Gm10260	-0,80194	1
Gm8168	-0,80195	1
Gm43813	-0,80197	1
Gm45806	-0,80233	1
Gm21399	-0,80257	1
Tctn2	-0,80299	1
Nupr1l	-0,80322	1
Vsig10	-0,80345	1
Olfm1	-0,80363	0,89634
Acvrl1	-0,80424	1
Cdnf	-0,80429	1
Mtus1	-0,80463	1
Upk1a	-0,8049	1
Atg10	-0,80579	1
Rnaseh2c	-0,80678	1
Ube2e2	-0,80734	1
Erlin2	-0,80762	1
A330074K22Rik	-0,80767	1
Bphl	-0,80785	1
Gm26800	-0,80877	1
Rps3a2	-0,81007	1
Amdhd2	-0,81028	1
Slc44a2	-0,81077	0,98528
Cst7	-0,81197	1
Rpl19-ps9	-0,8138	1
Atp8b4	-0,81409	1
Pddc1	-0,81425	1
Vwf	-0,81562	1
Lipt2	-0,816	1
Hdc	-0,81625	1
Zfp629	-0,81635	1
Sh3rf1	-0,81643	1
Pfn2	-0,81644	1
Nsf	-0,81648	1
Carmil1	-0,81662	1
Serhl	-0,81729	1
Pkn3	-0,81738	1
Slc24a5	-0,81874	1
Phf7	-0,81876	1
Gm5453	-0,81877	1
Gm37747	-0,81921	1
Atp5k-ps2	-0,82045	1
Hdac7	-0,82079	1
Fam167b	-0,82127	0,81232
Tmco4	-0,82127	1

4930448A20Rik	-0,82227	1
Tnnc2	-0,82305	1
Zfp41	-0,82409	1
Catip	-0,82483	1
Gm12182	-0,82529	1
Cops6	-0,82548	1
Gm14323	-0,82658	1
Gm7856	-0,82946	1
Pofut1	-0,82976	1
Gm45454	-0,83003	1
Pdhx	-0,83064	1
Tmem106c	-0,83058	1
C130089K02Rik	-0,83148	1
Gm12689	-0,83201	1
Gm14056	-0,83208	1
AI467606	-0,8326	1
Hebp1	-0,83285	1
Aim1l	-0,83276	1
Pmepa1	-0,83322	1
Slc25a10	-0,83347	1
Tnk2	-0,8336	1
Mthfsd	-0,83383	1
Tbc1d2b	-0,83426	0,82833
Golim4	-0,8346	1
Nudt16	-0,83473	1
Stap2	-0,83477	1
Rbbp9	-0,83542	1
Cyth4	-0,83621	0,83697
Cyp4f16	-0,83696	1
Ulk4	-0,83758	1
Frmd8	-0,83776	0,90856
Gm8013	-0,83813	1
Ccdc85b	-0,83819	1
Hk1os	-0,8382	1
Gm43154	-0,83863	1
Apoe	-0,83898	1
Tbxas1	-0,83924	1
Nsun4	-0,83965	1
Sema6b	-0,84191	1
Natd1	-0,84211	1
Il7r	-0,84248	1
Gm19325	-0,8427	1
Gm5117	-0,84358	1
Xrcc5	-0,84388	1
Ccdc28b	-0,84397	1
2210408F21Rik	-0,84401	1
Gm36930	-0,84472	1
Trub2	-0,84488	1
Fan1	-0,84723	1
Ank3	-0,8473	1
Nod1	-0,84737	1
Zfp661	-0,8485	1

Zfp786	-0,84863	1
Mrpl12	-0,84947	1
Chaf1b	-0,84946	1
RP23-476G10.1	-0,85024	1
RP23-104D6.2	-0,85024	1
Cyb5rl	-0,85077	1
Slc25a11	-0,85107	0,83459
Zfp61	-0,85125	1
Gm6415	-0,85254	1
Nudt22	-0,85263	1
0610009L18Rik	-0,85275	1
Ift20	-0,85389	1
Azin2	-0,85489	1
Acy3	-0,8549	1
Sod1	-0,85524	0,46413
Coq2	-0,85631	1
S100a13	-0,85648	1
Bre	-0,85773	1
Taf1b	-0,85783	1
Gpt2	-0,8589	1
Morn1	-0,86	1
Cd80	-0,86082	1
Lyrm7	-0,86143	1
Gm22716	-0,86203	1
Rhbdf1	-0,86252	1
Eldr	-0,86321	1
Gm11222	-0,86349	1
Kcnn1	-0,8637	1
Esr1	-0,86371	1
Rufy1	-0,86391	1
Vars2	-0,8653	1
Tmem79	-0,86617	1
Ift122	-0,86685	1
Clec4a2	-0,8674	1
Gstm4	-0,86748	1
Gm38036	-0,86749	1
Cnpy2	-0,86772	1
Gm42486	-0,86782	1
Kifc2	-0,86805	1
Mgmt	-0,86835	1
Itgb7	-0,86957	1
Atp2b4	-0,8706	0,94433
Gm43707	-0,8708	1
5430403G16Rik	-0,87087	1
Gm37589	-0,87102	1
Gm6245	-0,87141	1
1110019D14Rik	-0,8717	1
Gm38213	-0,8718	1
Gucd1	-0,87189	1
Fhod3	-0,87236	1
Coa3	-0,8739	1
Paqr7	-0,87392	1

2310009A05Rik	-0,87413	1
Ip6k3	-0,87428	1
Gstt3	-0,87622	1
Polr3h	-0,87671	0,90856
Slc25a20	-0,87683	1
Afmid	-0,87736	1
Gm44822	-0,87755	1
Kdm4d	-0,87785	1
Rhpn2	-0,8779	1
Med24	-0,87845	1
DHRSX	-0,87891	1
Pomk	-0,87941	1
Ccdc191	-0,88133	1
Cep68	-0,88204	1
Pop5	-0,88225	1
Dus2	-0,88228	1
Hyi	-0,8829	0,97341
Gm12669	-0,8838	1
Hoxa7	-0,88602	1
Tln2	-0,88625	1
Gm10717	-0,88664	1
Dpp3	-0,88697	1
Tpt1-ps5	-0,8871	1
Gm43569	-0,88942	1
Proser3	-0,8894	1
Gm38340	-0,8906	1
Gm43793	-0,89165	1
Crtam	-0,89192	1
Aldh5a1	-0,89218	1
E330037G11Rik	-0,89321	1
Nrtn	-0,89413	1
Ube4a	-0,89459	1
B230354K17Rik	-0,8948	1
Apbb1	-0,89646	1
Trpm4	-0,89736	1
Gm45292	-0,89741	1
Hk3	-0,89778	1
Arhgap19	-0,89814	1
Sit1	-0,89823	1
Tyw3	-0,89832	1
Vaultrc5	-0,89929	1
Tmtc4	-0,89981	1
Sp3os	-0,90081	1
6430531B16Rik	-0,90092	1
Rpl17-ps4	-0,90106	1
Olfr933	-0,90159	1
Foxd2os	-0,9019	1
Hebp2	-0,90217	1
Spred1	-0,90278	0,84115
Padi2	-0,90276	1
4930432K21Rik	-0,90445	1
Zdhhc12	-0,90533	1

Ifi202b	-0,90547	1
RP24-82M14.1	-0,90591	1
Tbc1d32	-0,90658	1
Chid1	-0,90684	1
Fah	-0,9073	1
Gm8494	-0,90787	1
Haus7	-0,90801	1
Dph6	-0,90829	1
Speg	-0,9084	1
Arl6ip4	-0,91237	0,86892
Mrps35	-0,9146	0,97345
Miga2	-0,91511	1
Gm7452	-0,91542	1
Dennd5b	-0,91542	1
Ankrd16	-0,916	1
Fam132a	-0,9165	1
Pik3r2	-0,91736	1
Gm37718	-0,91737	1
Zfp709	-0,91824	1
Nos3	-0,91853	1
Zmat1	-0,91867	1
Fadd	-0,9193	1
Nipal3	-0,91953	1
Atad3aos	-0,91971	1
Zfa-ps	-0,92076	1
Pde4a	-0,92099	1
Gen1	-0,92171	1
Clmp	-0,92189	1
Cd276	-0,92277	1
Ank2	-0,92295	1
Prss46	-0,92673	1
Car2	-0,92695	0,97341
Ceacam10	-0,9283	1
Btbd19	-0,93081	1
Ldb3	-0,93098	1
Fbxl8	-0,93211	1
Gm12522	-0,93264	1
Fam65c	-0,93373	1
Pemt	-0,93395	1
Mob3b	-0,93501	1
Zg16	-0,93587	1
Sec16a	-0,93658	1
Oit3	-0,93687	1
0610010F05Rik	-0,93837	1
Gm25857	-0,9386	1
Bckdk	-0,93917	1
Qsox2	-0,93917	1
Swsap1	-0,93935	1
D330050G23Rik	-0,93949	1
6720475M21Rik	-0,93998	1
Klhl40	-0,94039	1
Opn3	-0,94068	1

Frmd8os	-0,94125	1
Tmem126b	-0,94149	1
Cox15	-0,94178	1
Ttll12	-0,94255	0,64938
Iqcc	-0,94288	1
A130071D04Rik	-0,943	1
Sirt5	-0,94313	1
Zfp27	-0,94407	1
Inhbe	-0,94547	1
Orai3	-0,94563	1
Card9	-0,94616	1
Rint1	-0,94734	1
Rasal2	-0,94993	0,73208
1700123M08Rik	-0,95005	1
Sfxn2	-0,95022	1
Ccdc106	-0,95039	1
Agap1	-0,95057	1
Mst1	-0,95086	1
Def6	-0,9521	1
Trim68	-0,95344	1
Gm12833	-0,95343	1
Pin1	-0,95417	1
Rnf121	-0,95421	1
Cluap1	-0,95434	1
Mrpl17	-0,95445	0,49549
Gm4924	-0,9545	1
RP23-277D1.1	-0,95505	1
Prkdc	-0,95552	1
Akap17b	-0,95559	1
BC060293	-0,95583	1
2610306M01Rik	-0,95595	1
Rrp9	-0,95682	1
4930556M19Rik	-0,95738	1
Pstpip1	-0,9596	0,15115
Setd6	-0,96132	0,82245
Nudt5	-0,96237	1
Slc25a15	-0,9628	1
Clec5a	-0,96405	1
Ttc7	-0,96396	1
Prkar1b	-0,9643	1
AK157302	-0,96456	1
D10Jhu81e	-0,96589	0,81876
Poc1b	-0,9662	0,98528
Gm10801	-0,96631	1
Sirt6	-0,96657	1
Wdr5b	-0,96714	1
Pla2g5	-0,96757	0,7952
Gm7936	-0,96769	1
Edil3	-0,96824	1
Naip6	-0,96851	1
Fam13a	-0,96892	1
Gm5637	-0,96977	1

Lyl1	-0,97046	1
Zfp870	-0,97141	1
Fbxo32	-0,97331	1
Fut8	-0,97402	1
Gm44510	-0,97419	1
RP23-243B24.1	-0,97478	1
Lima1	-0,97587	0,77131
Tmem140	-0,97588	1
Sema5a	-0,97623	0,87657
Lyrm1	-0,97785	1
Gsn	-0,97938	0,26694
Cyba	-0,9799	0,25445
Gm37472	-0,97996	1
Gm20056	-0,98027	1
Cdc25c	-0,98059	1
Gm26601	-0,98083	1
Nagpa	-0,981	0,92561
Serpini1	-0,9812	1
Oas1d	-0,98118	1
Bdh1	-0,98261	1
Psma7	-0,98383	0,28971
Cnp	-0,98416	1
Ccdc88c	-0,98426	1
Gpr68	-0,98475	1
Fkbp15	-0,98621	0,49419
Ash2l	-0,98618	0,97341
Abcb9	-0,98637	1
Hmx2	-0,98708	1
Gm38062	-0,98707	1
1810055G02Rik	-0,98798	1
Glb1	-0,98931	0,30209
Gm38366	-0,98965	1
Elmod3	-0,98977	1
B3gntl1	-0,99087	1
Gm15440	-0,99314	1
Treml1	-0,99452	0,87488
Zfhx4	-0,99462	1
Mettl26	-0,99511	0,6607
Hoxa4	-0,99514	1
A530072M11Rik	-0,99571	1
Gls2	-0,99571	1
Calr3	-0,99651	1
Ms4a6b	-0,9969	1
Fry	-0,99697	1
4833412K13Rik	-0,99711	1
Gm14240	-0,99737	1
Als2cr12	-0,99809	1
Klhl5	-0,99868	0,91464
Gm45110	-0,9989	1
Dync2h1	-0,99917	0,59509
F9	-0,99935	1
Gm26847	-0,99943	1

H2-Oa	-0,99972	1
Mboat1	-1,0009	1
AI464131	-1,0011	1
Hmga2	-1,0013	0,17097
St18	-1,0016	1
Cdk2ap1	-1,0024	1
Acaa1b	-1,0038	1
Dgcr6	-1,004	1
Txnrd3	-1,0041	1
Zfp719	-1,0043	1
Gkap1	-1,005	1
Gm8919	-1,0055	1
Stx11	-1,0058	1
Procr	-1,0063	1
Etv4	-1,0067	1
Marveld1	-1,0075	0,57935
RP23-110E20.5	-1,0079	1
Mrps6	-1,0082	0,25445
Smim1	-1,0085	1
Rftn2	-1,0087	1
Gm9025	-1,0091	1
Ubash3b	-1,0094	0,32534
Jdp2	-1,0096	1
Gm45698	-1,0102	1
Pck2	-1,0105	1
Psph	-1,0106	1
Clec7a	-1,0108	0,86317
Gm5446	-1,0114	1
Gpatch4	-1,0119	0,8145
Cenpt	-1,0125	1
Abtb2	-1,0127	1
Gm45534	-1,0133	1
Mtmr9	-1,0139	1
Cd59a	-1,0143	1
Creb3l4	-1,0147	1
Ank	-1,0148	0,069188
Fblim1	-1,0152	0,97341
E430018J23Rik	-1,0152	1
Gm37978	-1,0161	1
Gm42515	-1,0169	1
Lgals4	-1,0174	1
Gne	-1,0178	1
Tnfsf13b	-1,02	1
Gm12096	-1,021	1
Tmem18	-1,0211	1
Ube2m	-1,0212	0,87232
1110032A03Rik	-1,0222	1
Adcy2	-1,0229	1
Vti1a	-1,023	1
Lrrc1	-1,024	1
Gnrh1	-1,0245	1
Xrra1	-1,0249	1

Odf3l1	-1,0257	1
Chrna1os	-1,0264	1
Gm10136	-1,0264	1
Gm44639	-1,0265	1
Krcc1	-1,0269	1
Capn1	-1,0276	0,86892
Kif7	-1,0276	1
Sorbs1	-1,0286	1
Katnb1	-1,0293	1
Ivd	-1,0296	1
Itga2b	-1,0303	1
Rcn1	-1,0305	0,57037
Zhx3	-1,0311	1
Prkca	-1,0311	1
Trim45	-1,0312	1
Cspg4	-1,0312	1
Ptpn7	-1,0325	1
Gm11423	-1,0334	1
Cenpx	-1,0341	1
RP23-261O3.5	-1,0342	1
Usp20	-1,0344	0,45584
Rdh12	-1,0349	1
Met	-1,0351	1
F10	-1,0362	0,65283
Sapcd2	-1,0378	1
Ddx59	-1,0379	1
Prss50	-1,039	1
Adamts4	-1,0401	1
Ccdc107	-1,0407	1
Celf6	-1,041	1
Actg1	-1,0424	1
Gm37776	-1,0425	1
Ms4a6d	-1,0432	1
Zpr1	-1,045	0,10479
Arhgef4	-1,0451	1
Anapc13	-1,0468	0,57935
Maged2	-1,0475	1
Wdr25	-1,0486	1
Exosc5	-1,0492	0,60105
Nucb2	-1,0506	0,47008
Anxa9	-1,0517	1
Naip2	-1,0519	1
Gm22299	-1,052	1
6030458C11Rik	-1,0522	0,94063
Rai14	-1,0524	0,51724
Mblac1	-1,053	1
Gm42484	-1,053	1
Aarsd1	-1,0534	1
Efemp2	-1,0542	1
6030442K20Rik	-1,0544	1
Doc2g	-1,0546	1
Gm44053	-1,0547	1

Gm11131	-1,0557	1
Pla1a	-1,0561	1
Bbs1	-1,0565	1
Gm42482	-1,0576	1
Numbl	-1,0581	1
Flrt2	-1,0586	0,83109
Zfp938	-1,0588	1
Six4	-1,0592	1
Eef1akmt1	-1,0593	1
Tnni3	-1,0604	1
Msh5	-1,0615	1
Gatb	-1,062	1
Caprin2	-1,0633	1
Acot1	-1,0634	1
Rabl2	-1,0635	1
Crygn	-1,0638	1
Slc39a11	-1,0642	0,11798
Nmi	-1,0655	1
Card6	-1,066	1
Slc39a13	-1,0668	0,61177
RP24-286J14.3	-1,0679	1
Cep41	-1,0681	1
Gm6293	-1,0702	1
Gm16418	-1,0714	1
Emc9	-1,0723	1
Gm5776	-1,0732	1
Ifi203	-1,0754	1
Ctdsp1	-1,0759	1
Mpnd	-1,0768	0,31881
Ifi211	-1,0775	1
Gm2895	-1,0778	1
RP23-38L16.4	-1,0789	1
Slc16a9	-1,0796	1
Cstad	-1,0816	1
Gm37289	-1,0819	1
Tfcp2l1	-1,082	1
BC017158	-1,0823	1
Nxn	-1,0832	1
Fam109a	-1,0849	0,88178
Ttc25	-1,0852	1
Bcl2l14	-1,088	1
Nt5c3b	-1,0883	1
Sgsm1	-1,0887	0,069552
Arg1	-1,0933	1
Grap	-1,0934	1
5730409E04Rik	-1,0938	1
Sigirr	-1,094	1
RP24-282C4.4	-1,0959	1
Gm26917	-1,0975	0,014849
Cpsf3	-1,0978	0,67036
Gm43128	-1,0985	1
Tmem198b	-1,0986	1

Slc25a43	-1,0991	1
Angptl6	-1,1007	1
Tiam1	-1,1015	0,97341
2810025M15Rik	-1,1041	0,046754
Rcan1	-1,105	0,23021
Gm15185	-1,1067	1
Mmab	-1,1072	1
Gm45203	-1,1076	1
Fancc	-1,1079	1
Flt1	-1,1089	1
Abhd1	-1,109	1
Htr2b	-1,1094	1
Tmem181b-ps	-1,1107	1
Adap2	-1,1114	1
Gm2830	-1,1115	0,89414
Gm15472	-1,112	1
Palb2	-1,1126	1
Sppl2b	-1,115	0,91211
Col20a1	-1,1161	1
Gm37423	-1,1167	1
Rps12-ps5	-1,1172	1
C1rb	-1,1178	1
Fam69a	-1,1182	1
Xrcc3	-1,1199	1
Robo3	-1,12	1
Lpin3	-1,1221	1
Timp2	-1,1229	0,45856
Zscan22	-1,1252	1
Eif1ad	-1,1285	0,33785
Gng8	-1,129	1
Frmd6	-1,1323	1
Mfsd3	-1,1352	1
Lmln	-1,1376	1
Cass4	-1,1376	1
Gm12468	-1,1405	1
Sh3kbp1	-1,1418	0,62652
Gm17530	-1,1436	1
Slc22a4	-1,148	1
Gm13421	-1,1482	1
Wwc2	-1,1483	1
1700034H15Rik	-1,1506	1
Mfhas1	-1,152	1
Murc	-1,1558	1
Slamf9	-1,157	1
Zdhhc1	-1,1584	1
Rpgr	-1,1608	1
Shtn1	-1,161	0,35151
Sema4b	-1,1615	1
Fbxo36	-1,1615	1
Aldh3b1	-1,1617	0,60972
Rpgrip1	-1,1618	1
Pomc	-1,1631	1

Rps19-ps8	-1,1631	1
Rnf157	-1,1633	0,31451
Zbtb12	-1,1641	0,83459
Ppm1l	-1,1645	0,082515
Egf	-1,1646	1
Gm10657	-1,1648	1
Pde4c	-1,1655	1
C630004M23Rik	-1,1659	1
Gsto2	-1,166	1
C2cd2	-1,1665	1
Me1	-1,1703	0,15115
Lpar1	-1,173	1
Nup85	-1,1742	1
9930012K11Rik	-1,1747	1
Slc16a7	-1,1752	1
Idh2	-1,1756	0,097271
Gm11918	-1,1771	1
Gm15696	-1,1773	1
Tfec	-1,1781	1
Sec16b	-1,1792	0,60105
Ppp1r26	-1,1797	1
Slc22a15	-1,18	1
Iqsec3	-1,18	1
Sdsl	-1,1804	1
Commd5	-1,1805	0,60972
Pter	-1,1813	1
Gm3283	-1,1818	1
Flnb	-1,1823	0,082515
Gm44423	-1,1834	1
Ino80c	-1,185	0,096092
Tmem214	-1,1873	0,98528
Fam213b	-1,1883	1
Gm9347	-1,189	1
Plch2	-1,1903	1
Myo1d	-1,1905	0,15406
Gm42835	-1,1925	1
Fut7	-1,1929	1
Mblac2	-1,1932	1
Plscr4	-1,1933	1
Ak3	-1,1937	1
Chpf	-1,194	0,67177
Gm15853	-1,1951	1
Ptpn5	-1,1953	1
Cdhr4	-1,1956	1
Rab15	-1,1956	1
Gm15420	-1,196	1
Klra2	-1,1961	1
Gm15596	-1,1974	1
Extl1	-1,1981	1
Gm38020	-1,1997	1
Bcl2l15	-1,1997	1
3110080O07Rik	-1,2028	1

Gm42567	-1,2056	1
3110082I17Rik	-1,2062	0,83459
Gm44270	-1,2079	1
Prickle2	-1,2088	1
Gm24890	-1,2103	1
6720464F23Rik	-1,211	1
Hrc	-1,2124	1
Gm13657	-1,2128	1
Gmpr2	-1,2133	0,92561
Echdc3	-1,2142	1
Pitpnm2	-1,2155	1
Gm20045	-1,218	1
Camk2a	-1,2182	1
Vwa1	-1,2187	1
Pomgnt1	-1,2215	0,83459
Kif5c	-1,2231	1
Zfp462	-1,2237	1
Pafah2	-1,2244	1
Prkar2b	-1,227	1
Bdh2	-1,2279	0,24456
Dcaf11	-1,2279	0,77281
Pcdhgc4	-1,2279	1
Gm11451	-1,2292	1
Prss53	-1,23	1
Gm45206	-1,2305	1
Hfe	-1,2308	1
Airn	-1,233	1
Gm9568	-1,2332	1
Gm11944	-1,2345	1
Zmym6	-1,2371	1
Nudt1	-1,2379	1
D3Ertd751e	-1,2385	1
Sipa1l1	-1,2442	1
Dsn1	-1,2473	1
Gm12988	-1,2476	1
Dnajc12	-1,2507	1
Agbl3	-1,2515	1
Zkscan4	-1,2522	1
Gm37060	-1,2537	1
Gm44434	-1,254	1
Ap5b1	-1,2569	1
C130050O18Rik	-1,2579	0,97341
Gm4602	-1,2595	1
Dtnb	-1,2599	1
Zfp324	-1,26	1
Catsper2	-1,2601	1
Gm44935	-1,2612	1
Gm26935	-1,2634	1
Zfp426	-1,2639	1
Zfp111	-1,264	1
RP24-365A12.2	-1,2642	1
Gm23969	-1,2647	1

4931414P19Rik	-1,2655	1
Crebl2	-1,2685	1
Nmnat3	-1,269	1
Cd200r3	-1,2703	1
Gm12517	-1,2722	1
Exoc3l	-1,2744	1
Gm8228	-1,2788	1
Smtn	-1,2789	1
Hist1h1d	-1,28	1
Gm10698	-1,2807	1
Fabp7	-1,2827	1
Akr1b7	-1,2838	1
Spats1	-1,2903	1
Nat8f1	-1,2978	0,97341
Rps15a-ps3	-1,298	1
Aldh1l2	-1,2982	1
Spg11	-1,2982	1
Gm340	-1,2991	0,98887
Rsph1	-1,3036	1
Gm15327	-1,3036	1
Ndufa4l2	-1,3056	1
Shox2	-1,3063	1
F630040K05Rik	-1,3132	1
Tctex1d4	-1,3165	1
9530062K07Rik	-1,3187	0,83697
Arhgap31	-1,3196	0,87657
Cetn4	-1,3202	1
Lrrc20	-1,3216	0,49172
Gm12444	-1,3241	1
Calml4	-1,3243	1
Cspg5	-1,3248	1
Sgsh	-1,3253	0,71474
Ccdc63	-1,3285	1
Rilp	-1,3293	1
Ypel4	-1,3314	1
Eif2ak2	-1,3321	0,97341
Kazald1	-1,3331	1
Cdk20	-1,335	1
Rabgap1l	-1,3366	0,97341
Pou5f2	-1,3408	1
Cyp2u1	-1,3411	1
Zfp953	-1,3416	1
Gm16845	-1,343	0,83697
Ppic	-1,3434	0,034765
Acot11	-1,344	1
Cdh23	-1,3452	1
Gm5857	-1,3467	1
RP24-175C20.18	-1,3481	1
Dennd2a	-1,3515	0,44209
Ccdc114	-1,3535	1
Gm10399	-1,3539	1
Nudt6	-1,3541	0,98528

Zscan29	-1,3545	1
Gm42747	-1,3594	1
B3galnt1	-1,361	1
Slc13a3	-1,3629	1
Gm10575	-1,3632	1
Gm13604	-1,3649	1
Zfp93	-1,3662	1
RP24-325N9.5	-1,3669	1
Gm44771	-1,3689	1
6330403L08Rik	-1,3705	1
Pbx1	-1,3711	1
Gm26631	-1,372	1
Fkbp14	-1,3724	1
Slc24a3	-1,3742	1
Tnfrsf4	-1,3744	1
Fads2	-1,376	1
Ociad2	-1,3775	1
Palld	-1,3784	1
Gmppb	-1,3808	0,53664
1700037C18Rik	-1,3816	1
1700007L15Rik	-1,3824	1
Tmem42	-1,3849	1
Ctdspl	-1,3875	1
4833418N02Rik	-1,3911	1
Wdr35	-1,3915	1
Adamts7	-1,3943	1
Gm37606	-1,3954	1
Zfp169	-1,4014	1
Gm45873	-1,4015	1
Fndc7	-1,4016	1
Tmem246	-1,4019	1
Gm15506	-1,4037	1
Lta	-1,4046	1
Gm14040	-1,405	1
Gm20522	-1,4054	1
Ophn1	-1,4068	1
Acaa2	-1,408	0,25445
Gm43447	-1,4081	1
D630029K05Rik	-1,4085	1
Dcstamp	-1,4117	0,20396
Mical2	-1,4141	0,6607
Sult2b1	-1,4152	1
Sumf2	-1,416	1
Bicd1	-1,4171	0,81876
Phf21b	-1,422	1
Ppm1e	-1,4259	1
Fam19a3	-1,4285	1
Acy1	-1,439	1
Nudt14	-1,4421	0,78733
Serinc5	-1,4421	1
Gm13622	-1,4441	1
Zfp942	-1,4488	1

Lin37	-1,4493	1
Gm17807	-1,4505	1
RP24-323H7.5	-1,4505	1
Tmem91	-1,451	1
Gm45342	-1,4532	1
Armc6	-1,4549	0,58759
Senp8	-1,4553	1
Gper1	-1,4565	1
Tmem98	-1,4584	1
Rnf135	-1,4588	1
Zfp14	-1,4605	1
2810405F17Rik	-1,4646	1
Cd200r2	-1,4658	0,3993
Igsf3	-1,4677	0,58759
Wnk2	-1,4699	1
Glb1l	-1,4707	1
Magi2	-1,4722	1
Cpeb1	-1,4729	1
Ifi203-ps	-1,4735	1
RP23-164P21.3	-1,474	1
Elfn2	-1,4745	1
Gm8566	-1,4759	0,60972
Lims2	-1,4762	0,87488
Mir763	-1,4813	1
Spef1	-1,4831	1
Trim2	-1,4852	0,90856
Zfp3	-1,4853	0,8145
Gm3695	-1,4862	1
Zfp229	-1,487	1
Ccdc171	-1,4873	1
4930556M19Rik	-1,4907	1
Pgm5	-1,4927	1
Gm17100	-1,4933	1
Zfyve19	-1,4967	0,994
Pdpn	-1,5097	1
L3hypdh	-1,5178	0,5517
Gm10313	-1,5199	1
Gm12479	-1,5203	1
Camk2n2	-1,521	0,94786
Zfp882	-1,5224	1
Ift43	-1,5235	0,67469
3110070M22Rik	-1,529	1
Gm16201	-1,5369	0,29534
Gm37333	-1,54	1
Bbs9	-1,5476	0,80904
Tbx15	-1,5476	1
Pld1	-1,5488	0,80904
Spn	-1,5492	0,96993
Gm9776	-1,556	1
Iqce	-1,5599	1
Gipc1	-1,5607	0,58759
Lrp8os3	-1,5619	1

Gm26930	-1,5684	1
Hspb7	-1,5714	0,60105
Gm12933	-1,5716	1
Six5	-1,5716	1
Gm37010	-1,5733	1
Gm43111	-1,5792	1
AA386476	-1,5811	1
Amigo1	-1,5816	1
Col15a1	-1,5827	1
Gm42639	-1,5841	0,67469
Gm43273	-1,5927	1
Gm26870	-1,5949	1
Gm15937	-1,5979	1
Gm28535	-1,6009	1
Gm7769	-1,6039	1
Gm36445	-1,6045	1
Src	-1,6088	0,6841
Ptges	-1,6095	0,94786
Slc52a2	-1,6124	0,60362
Cnrip1	-1,6131	0,69976
2610524H06Rik	-1,6131	0,87488
Gm7784	-1,6164	1
Serinc2	-1,6201	0,1571
2810006K23Rik	-1,6214	1
Gm5837	-1,6214	1
Gm15610	-1,6315	1
C030014I23Rik	-1,6332	1
Gm43728	-1,6409	0,96432
Gm38220	-1,6501	1
Gm11716	-1,658	1
Gm33370	-1,663	1
Txk	-1,6675	1
Gm12258	-1,6693	1
Ffar4	-1,6703	0,91464
A730062M13Rik	-1,6722	1
Hlcs	-1,6761	1
Gstt2	-1,6766	0,83109
Rnf152	-1,6954	1
Apol11b	-1,7015	1
Rgs8	-1,7084	1
Sla	-1,7137	0,067381
Tmem116	-1,7155	0,75189
Asah2	-1,7165	1
Ankdd1a	-1,7182	1
Slc46a1	-1,7184	1
Nadsyn1	-1,7199	1
Gm38399	-1,7303	1
Angptl2	-1,7314	0,0074793
Gm42690	-1,7314	1
Pars2	-1,7316	1
Gramd2	-1,7362	1
Zfp862-ps	-1,7403	0,90856

Gm4117	-1,7494	1
Gm37297	-1,7511	1
Apip	-1,7583	0,44721
Ankrd34a	-1,7606	1
Tmem202	-1,7622	0,89201
Gm44699	-1,7754	1
Cnbd2	-1,7756	1
Dixdc1	-1,7769	1
Gm17249	-1,7778	1
Zfp933	-1,7799	1
Phxr4	-1,787	1
Itgb3	-1,794	1
Olr1	-1,8017	0,97341
Fbxo15	-1,8085	1
Cc2d2a	-1,8139	1
Gm42480	-1,8189	0,51527
Rfxank	-1,8516	0,96374
Tiam2	-1,8588	1
Plat	-1,8608	1
Hck	-1,8619	0,33432
Rab42	-1,8702	1
Pctp	-1,8729	0,33278
1700084J12Rik	-1,8744	1
Vegfc	-1,8753	1
Gm10478	-1,8833	0,92561
Mroh2a	-1,8851	1
Tango6	-1,8915	1
Fam83h	-1,8968	1
Ccdc80	-1,9043	1
Dlg3	-1,9062	0,32705
Gm44951	-1,9063	1
Gm13423	-1,9077	1
Spink5	-1,9106	4,11E-06
Gm37219	-1,9371	1
Mylpf	-1,9381	1
9930014A18Rik	-1,9449	0,83109
Enpp5	-1,9461	0,10479
Exd1	-1,9485	0,89201
Gja1	-1,9505	0,96037
Atp6v0d2	-1,954	4,84E-05
Tpm2	-1,9572	0,20955
Gm19026	-1,9703	1
Rhbdd2	-1,9749	0,90856
Acp5	-1,9771	0,0097563
Gm20156	-1,9883	1
Zbtb32	-1,9905	0,33785
B3glct	-2,0044	0,21571
Ddr2	-2,0228	0,90856
Epb41l1	-2,0307	0,60972
Tnfsf8	-2,0371	1
Gm10029	-2,0385	0,90856
Pou4f1	-2,0443	1

Mypopos	-2,0516	1
Rpsa-ps2	-2,068	1
Il13ra2	-2,0753	0,84131
BC024978	-2,0763	0,7952
Cep72	-2,0767	0,80904
2810021J22Rik	-2,0781	0,90856
Particl	-2,0786	0,5517
Slc35d2	-2,082	0,63156
Gm37699	-2,0895	0,60972
Cradd	-2,0931	0,29132
Gm43609	-2,0942	1
Gm36963	-2,111	0,80904
Gm44557	-2,1204	1
Ttc9	-2,196	0,57935
Gm15496	-2,2189	0,94082
Cd5l	-2,2418	0,32705
Emp2	-2,2453	0,082515
AI661453	-2,2983	0,31881
Pxdn	-2,3417	0,51527
1600014C23Rik	-2,3551	0,62484
Stc2	-2,3835	0,60972
Ccdc122	-2,3847	0,55555
Msantd3	-2,3904	0,020257
Sec14l2	-2,3987	0,2667
Gm42640	-2,4009	0,48002
Gm25514	-2,4141	0,60972
Gm22973	-2,4406	0,22184
Mmp9	-2,4409	0,32021
Slc9b2	-2,4919	0,48208
RP23-308G10.5	-2,519	1
Itgax	-2,6087	0,0013074
Gm3699	-2,6804	0,64938
Il1rl1	-2,722	0,80904
Acsbg1	-2,7244	0,31451
Pdcd1	-2,8059	0,23139
Il20rb	-3,0296	0,041429
Ccpg1os	-3,0311	0,1571
Nt5e	-3,0699	0,046754
Rgs16	-3,0972	0,0035645
Gm10800	-3,1253	1
Bok	-3,2962	0,039238
Mras	-3,3958	0,096514
Il34	-3,4734	0,24456
Gm11205	-3,8202	0,012339
Ctsk	-3,8572	5,41E-06
