# Supplementary material for: Bone, dentin and cementum differentially influence the differentiation of osteoclast-like cells
Source: Sci Rep. 2025 Jun 5;15:19857. doi: 10.1038/s41598-025-04874-9 (PMC12141432; doi:10.1038/s41598-025-04874-9)
Supplement: Supplementary file 4 — Supplementary Information 4. [file 41598_2025_4874_MOESM4_ESM.pdf]

Tab. S3:
Transcripts induced in murine macrophage cells stimulated on dentin (n=6), fold of negative control

gene name	regulation of expression	adj.P.Val
mt-Ti	13,07555247	0,0025879
mt-Tc	9,897059829	0,22229
Gm13226	9,358647186	0,26235
mt-Ta	8,215224496	0,3804
mt-Ts2	7,997227892	0,10055
Ighd	7,445661351	0,66335
Gm43878	7,065540852	0,39257
Carnmt1	6,519222884	0,52583
RP24-316F13.7	6,276672783	0,53484
Gm20302	6,17954236	0,91083
Gm8885	6,165423517	0,29245
Gm26759	6,161578525	0,83534
Mast4	5,986032037	1
Il1b	5,95994906	1
Gm42850	5,820326155	0,82117
Trf	5,763319499	0,44451
Gm26225	5,691070297	0,98935
Gm16372	5,644712098	0,98935
RP24-295J1.1	5,588263311	0,38316
Adamtsl1	5,584391164	1
Socs2	5,550431756	0,6714
Gm7351	5,481231241	0,85058
Gm11631	5,462267621	1
mt-Tl1	5,404645529	0,00046663
4921507G05Rik	5,195274579	0,23643
Gfap	5,026311209	1
Olfr95	5,0116998	0,90498
Pla2g2d	4,966398407	0,52583
Lbp	4,94133203	0,53484
4930430E12Rik	4,818553088	0,082885
Gm45250	4,769043691	1
Ccdc33	4,692315435	1
Slc25a2	4,683217364	0,7043
D2hgdh	4,676081257	0,98935
Hsh2d	4,609467194	1
Gm17060	4,60052976	0,85058
Gm13433	4,56811826	1
Hist1h2bg	4,56558586	1
Rrh	4,500233939	1
Foxd2	4,467909613	0,98935
Gm6450	4,467290272	1
RP23-451J19.1	4,459864875	0,26235
Hist1h4h	4,445359205	0,85058
Hist1h2ae	4,437662655	1
Gm18969	4,418020154	1
mt-Tv	4,396330967	0,28334
Gm10463	4,371110956	1
Gm8818	4,341519308	0,96117

Hba-ps4	4,340014913	1
Cxxc5	4,321104138	1
Rasd1	4,282045127	1
Gpr19	4,252172133	1
Gm4034	4,214612521	0,83534
RP23-440L7.5	4,190144508	0,55152
RP23-134M7.3	4,169285018	1
Adgb	4,117018696	1
Gm24507	4,108466491	1
RP24-174I4.1	4,098227292	0,26235
Tsix	4,081501535	0,22826
Gm20517	4,0278222	1
Gm26569	4,024194399	1
Gm9711	3,996397258	1
Gm7132	3,987542789	1
4933412L11Rik	3,97815641	1
Gapdh-ps14	3,954512898	1
Slc4a8	3,950403446	1
Hist1h2be	3,937008971	0,10408
Gm15542	3,910898245	0,90306
Gpat3	3,910356117	1
Arhgap27os1	3,903585854	1
Prokr1	3,896017074	1
Gm20223	3,842379456	1
Rps13-ps5	3,824311396	0,85058
Gm43714	3,815044803	1
Gm19028	3,753144925	1
Rhoh	3,743791266	1
Zp1	3,716127497	1
A430035B10Rik	3,714324858	1
Gm45733	3,687645568	1
Gm14137	3,651780371	1
Gm11343	3,643436887	1
Sfn	3,642426852	0,96117
Gm17430	3,641417097	1
Igf1	3,628315724	4,18E-06
Stra8	3,624294024	1
Gm6564	3,624042816	0,92663
March4	3,615261489	1
AY074887	3,613006875	0,23008
Gm5578	3,60825175	1
Gm19566	3,549953187	0,51652
Tnfrsf14	3,549461093	1
Gdf15	3,541105926	0,011022
Tmem71	3,530811985	1
Gm6768	3,528610037	1
Gm42670	3,525920632	1
RP23-213P10.2	3,503264701	1
Arhgap26	3,499624177	0,19115
Hoxa3	3,494291584	1
Gm5873	3,492112411	0,91083
Gm5544	3,479065884	1

Gm26530	3,456471428	1
Arid5a	3,455513223	0,85058
Zfp54	3,44857418	1
Tubb2b	3,445229286	1
RP23-136K21.4	3,414797433	1
Gm12466	3,414560746	1
Gm4742	3,406050912	1
Gm29462	3,391209704	1
Gm15484	3,38932974	1
9130230N09Rik	3,388155291	1
Gm8181	3,384634386	1
Gm44957	3,357063904	1
Gm26397	3,34962595	1
Gm42548	3,319118183	1
Fam72a	3,315209403	1
Polr2k	3,295734442	1
Gm12791	3,294820797	1
Gm10636	3,285926021	1
Gm11895	3,284787403	1
Gm14650	3,277736773	1
Tbc1d30	3,265491154	1
Gm9381	3,246533284	1
Gm7799	3,237320047	0,85058
Gm29759	3,231715057	1
2210406H18Rik	3,224778344	1
Ccdc92b	3,215181096	1
Smco3	3,214958244	1
Gm3724	3,213398715	1
Plekhh1	3,207612799	1
Kyat3	3,20561241	1
Gm5251	3,196736875	1
Gm5828	3,196515302	1
Runx2os1	3,193636247	1
Rnf122	3,183910981	0,28334
Gm5764	3,1777376	1
Gm12017	3,1746554	1
Gm36936	3,17135636	1
Gm13416	3,163014128	1
E230016M11Rik	3,15469384	1
C8g	3,152944992	1
Gm42918	3,152289423	1
Etohd2	3,14879536	1
Zfp563	3,146613538	1
Gm8172	3,144433227	1
Sorl1	3,130731968	1
Gm14584	3,119035558	1
Gdf9	3,112124983	1
Tsg101-ps	3,110615336	1
Gm24631	3,106306096	1
Gm11764	3,10609079	1
Gm7895	3,102002824	1
Dnali1	3,101572825	1

Plscr4	3,099423724	1
Selenop	3,098994082	0,45248
Zfp119b	3,095344533	1
Gm9920	3,08977118	1
Gm9521	3,087630256	1
Gm2986	3,086132492	1
1700054M17Rik	3,083566586	1
Lgals9	3,080789262	1
Gm44884	3,074815828	1
Gm7965	3,074602705	1
Gm18860	3,074176504	1
Gm10180	3,0690667	1
Gm6682	3,059296635	1
Spata2l	3,051672197	1
5430421F17Rik	3,046177479	1
Enho	3,037532827	1
Gm13736	3,031012916	1
Myh11	3,013625195	1
Med12	3,007781987	1
Hist1h3d	2,993430967	1
Gm43379	2,991356798	1
Spsb3	2,990527533	1
RP23-324E2.11	2,986384653	1
Hlx	2,982454234	0,94714
Gm8973	2,981420774	1
Lamb2	2,978116105	1
Gm15163	2,975640008	1
Fcnaos	2,975021305	1
Gm15131	2,973165969	1
Gm25008	2,970899907	1
Tcea3	2,966167357	1
4933437G19Rik	2,965756188	1
Ptafr	2,961442347	1
Gm8203	2,953447567	1
A830073O21Rik	2,938744635	1
Gm8909	2,936504808	1
Slc30a2	2,934063308	1
Gm5822	2,93345325	1
Gm14248	2,933249925	1
Platr3	2,931623837	1
Gm12583	2,928983366	1
2610044O15Rik8	2,926345273	1
Gm9435	2,91925452	1
9330162G02Rik	2,894270975	1
Gm43721	2,891664147	1
Gm13094	2,891263305	1
Gm13445	2,887858391	1
Ctla2b	2,885857374	1
Gm8317	2,884257558	0,53484
Fzd7	2,88265863	0,21774
Pygm	2,879263849	1
Gm14794	2,877468231	1

Gm11298	2,874677272	1
Depdc1b	2,862151422	1
Gm28071	2,858186387	1
Ncoa4	2,841396175	1
Unc13b	2,841396175	1
Ccrl2	2,838443451	0,53484
Gm8129	2,835493797	1
Irx5	2,833725475	1
Rpl34-ps1	2,823138681	1
Dusp2	2,81590761	0,22229
Rpl30-ps1	2,812201558	0,79467
Nr0b2	2,804998501	1
Slc9a3r1	2,80480408	1
Nr4a3	2,792002048	0,98935
Zfp532	2,786395407	1
Trappc2	2,773100688	1
Cacnb1	2,769642945	1
Gm5131	2,767148368	1
Ntn5	2,73435394	1
Gm28659	2,733406449	1
Rhox5	2,730565946	1
C3ar1	2,72527157	0,22229
E330011M16Rik	2,725082675	1
Gm29243	2,719798931	1
Lin28b	2,719798931	1
Dnm3	2,714337281	1
Rpl30-ps2	2,700824694	0,22229
Sgk1	2,699701686	0,7043
Isg15	2,698205069	1
Rpl30-ps5	2,69708315	1
E130201H02Rik	2,695027511	1
Slc25a30	2,691107458	1
Olr1	2,687751951	1
Rpl19-ps11	2,687193107	1
Gm14326	2,684958892	1
Gm14279	2,680124463	0,39257
Gm45167	2,677896125	0,38316
Gm43182	2,673074413	1
4930578M07Rik	2,668446339	0,85058
H2-DMb2	2,659398545	1
Gm8250	2,657740044	1
Gm45051	2,653690281	1
Gm23639	2,653138519	1
A230028O05Rik	2,651116373	1
Gm13998	2,650932618	1
C78859	2,64964669	1
Jup	2,648728551	1
Gm7079	2,647260191	1
Kbtbd8	2,643592852	1
Slc16a5	2,643226398	1
Gm13758	2,641577981	1
Gm6905	2,637918514	1

Gm11464	2,637187229	1
Gm7867	2,635907968	1
Gm13443	2,632803775	1
Gm10237	2,632256355	1
Dusp8	2,630614781	1
0610039K10Rik	2,615886599	1
Gm38248	2,613892845	1
Gm5869	2,613349358	1
Rybp	2,610452667	1
Gm13453	2,600159276	1
Gm16585	2,593858896	1
Gm26710	2,58972696	1
Rpsa-ps9	2,585243192	1
Gm14140	2,583272793	1
A830008E24Rik	2,580051744	1
Otud1	2,578800199	1
Rpl23a-ps14	2,578085303	1
mt-Tp	2,575941804	0,98935
Cytip	2,574513794	1
Acoxl	2,572016682	1
Gm8463	2,568631618	1
Gm8623	2,56169728	1
Svil	2,560809617	1
RP23-278O17.1	2,559212599	1
Klhl40	2,559212599	1
Lgi4	2,559212599	1
mt-Tq	2,558148474	1
Cenpu	2,556375915	1
Tmem267	2,550181625	1
Egr1	2,549121255	0,78649
Gm10177	2,542944543	1
Gm12778	2,539245703	1
Gm6969	2,538013952	1
Gm11652	2,536079551	1
Rps13-ps7	2,533970976	1
Gm12312	2,53221517	1
Gm42731	2,531513188	1
Tmcc3	2,525729258	1
S1pr1	2,522929688	0,13105
Treml4	2,52223028	1
Usp18	2,519958544	1
Gm12430	2,517514347	1
St5	2,514723882	1
Rnaset2a	2,512981412	1
Snord82	2,509152227	1
Ccdc69	2,508282772	1
Olfr912	2,504981589	1
Vps25	2,502378461	1
5031425F14Rik	2,49943152	1
Faah	2,497699646	1
Snord65	2,496661098	1
Gm26870	2,496488048	1

Gm13383	2,495277039	1
Zfp36l1	2,478556249	0,05679
Gm44075	2,477869142	1
Slc47a2	2,473579041	1
Xk	2,468782944	1
Gm12469	2,466901309	1
Gm44836	2,466901309	1
Rps12-ps19	2,465021107	1
Rny1	2,462288827	0,90306
Abcd2	2,46160623	1
Gm28373	2,456152263	1
Gm7099	2,453940043	1
Usp27x	2,451899761	1
Rps12-ps26	2,448163651	1
Gm24991	2,44748497	1
Gm42851	2,443078127	1
Aif1	2,439524547	0,90306
Rhob	2,439017315	0,90306
Gm13889	2,435469642	1
Ppp2cb	2,435469642	1
Rgs12	2,435300834	1
4930589L23Rik	2,434119505	1
Slc9b2	2,434119505	1
Gpr35	2,43395079	1
Rps15a-ps6	2,432432886	0,85058
Gm8806	2,430747437	1
Snord15a	2,430578956	1
Gm13712	2,424017308	1
Gm29019	2,417976126	1
Rell1	2,417138264	0,065205
Spdl1	2,416635687	0,60976
Rn7s6	2,409276544	1
Gm23442	2,406439245	1
Zfp811	2,405271916	1
Saa3	2,405105201	1
Chtf18	2,4042718	1
2410022M11Rik	2,403605286	0,73611
Gm9409	2,392634455	1
Notch4	2,390976582	1
Gm6525	2,389816754	1
Gm8649	2,385183067	0,053116
Gm7701	2,382704432	1
Ypel1	2,379733469	1
Gm10863	2,37841423	1
Hist1h2al	2,376436743	1
Mapkapk5	2,376107322	1
Zfp790	2,375119332	1
mt-Tm	2,374131753	1
Gm6322	2,374131753	1
Arhgap39	2,373144585	0,91083
Gm29736	2,371664602	1
Gm29228	2,369692727	1

Gm5871	2,369200014	1
Mfsd13b	2,368379054	1
Arl4c	2,367558379	0,049241
Gm14094	2,367394278	1
Lif	2,366737988	1
Socs1	2,365098059	1
Rpsa-ps4	2,362312786	1
Gm8894	2,362312786	1
Rmi2	2,35969435	1
Tmem151a	2,359203716	1
Gm16106	2,357242202	1
B230377A18Rik	2,352019465	1
Teddm2	2,351204459	1
Gm20492	2,347784514	1
1500004A13Rik	2,34713366	1
Gm42535	2,342907506	1
Mrap	2,340148366	1
Pdzk1ip1	2,337230466	1
Tnfsf9	2,337068467	1
4930578M01Rik	2,330920824	1
Tbc1d10a	2,328982824	0,21774
Plk2	2,325272828	0,98935
Ccdc62	2,323339525	1
Fam187b	2,321246927	1
Gm29358	2,319799309	0,98935
Gpr162	2,316425044	1
2310043L19Rik	2,315782884	1
Abca5	2,314819977	1
Cygb	2,312574752	1
Gm11810	2,309851334	1
Gm43684	2,309210996	1
Gm20673	2,307291046	1
Gm6210	2,305692308	1
Gm11346	2,301381246	1
Insig1	2,29787449	0,39257
Gm10762	2,292942219	1
H2-Q5	2,292942219	1
Gm15877	2,292465464	1
Gm37486	2,289765728	1
RP23-246F14.1	2,289765728	1
Gm13776	2,289607019	1
Gm2223	2,287069171	1
Gm8927	2,285959748	1
Taf7	2,282951167	0,47425
Gm5100	2,280420696	1
Gm10801	2,279946545	1
Gm11989	2,2796305	1
Gm28731	2,278840577	1
Csrnp2	2,27757727	1
Pla1a	2,276945879	1
RP24-175C20.10	2,271901048	0,98935
Tac4	2,270326832	1

Atp1b4	2,270012119	1
Ubox5	2,267967553	1
mt-Nd6	2,267810354	0,60525
Snord71	2,266081894	1
RP23-2N7.4	2,265767771	1
D430001F17Rik	2,26466868	1
Anp32-ps	2,263727026	1
Ccl6	2,262942614	1
Pigb	2,261061134	1
Gm12164	2,260591009	1
5930420M18Rik	2,260591009	1
Gm7863	2,25792881	1
Hexim2	2,255426074	1
Gm29593	2,250116969	1
Gm42786	2,247934501	1
RP23-187B11.16	2,247467105	1
RP24-418P10.4	2,246688327	1
Gm16380	2,246688327	1
Gm14585	2,24575415	1
Dhrs11	2,244975966	1
Abhd3	2,244975966	1
Kif18a	2,241089088	0,91083
Gm5883	2,240157239	1
Gm12981	2,238139562	1
Gm5786	2,23225215	1
Gm18709	2,231788015	1
Setdb2	2,228387302	1
Poln	2,227151964	1
Notch1	2,225146	1
mt-Tt	2,22499177	1
D230017M19Rik	2,224220781	1
Gm12459	2,222217457	1
Olfml3	2,221755408	1
Slc15a3	2,22098554	1
Gm6543	2,220677667	1
Gm37696	2,218216222	1
5830432E09Rik	2,217908733	1
Gm42522	2,217601287	1
Rpl7l1-ps1	2,215450357	1
Maff	2,214989715	0,72131
Mrm2	2,197860774	1
Gm7504	2,197251482	1
Pcdhb22	2,193447232	1
1810062G17Rik	2,1932952	1
Bbs10	2,192535193	1
Gm16439	2,191167845	1
Gm24336	2,190560408	1
RP24-84O13.9	2,189194292	1
Rpl26-ps4	2,185858482	1
Slc9b1	2,185555478	1
Tigd5	2,182679041	1
Snord89	2,182527754	1

E230029C05Rik	2,181620255	1
Gm6177	2,180108595	1
Mpc1	2,178748997	1
Lpar6	2,178145003	0,38316
Gm15216	2,177994031	1
Rps19-ps5	2,176032343	1
Meiob	2,176032343	1
Gm13935	2,175730702	1
Gins1	2,175579897	1
Rps15a-ps4	2,174373834	1
Chchd10	2,172867195	1
Ubtd2	2,171963713	1
Mafb	2,1713616	0,13105
Gm20689	2,1713616	1
Nrap	2,171211098	1
Rps6-ps3	2,165799964	1
Gm7327	2,164299266	1
Sik1	2,163999251	1
Trim14	2,163849259	1
Gm9434	2,161151184	1
Gm44851	2,159653707	1
Btbd6	2,158157268	1
Rpl35	2,156063995	1
Mzb1	2,154868751	1
Gm14005	2,152927892	1
Gm15703	2,1520327	1
Taco1os	2,151734386	1
Zfp36l2	2,150541542	0,59291
Gm37108	2,148902461	1
Mfsd2a	2,147711187	1
Slc7a7	2,147413471	1
Gm8930	2,146074263	1
Clec4n	2,144141325	1
Trim13	2,141170973	1
Rps2-ps11	2,140725776	1
Gm10358	2,138797655	1
Gm9575	2,138797655	1
Gm44791	2,138501175	1
Hes7	2,137908339	1
Gm7785	2,136278887	1
Rpl29	2,135538637	1
Stamos	2,134798644	1
Snord35a	2,134798644	1
Ang	2,133910991	0,98935
K230015D01Rik	2,133319427	1
Gm8326	2,132875862	1
Gm15575	2,130511738	1
Camk2b	2,129921117	1
A930007I19Rik	2,123876679	1
Gm42728	2,123729469	1
Cbx2	2,122110823	1
Hax1	2,121963734	1

Tsku	2,120199468	1
Rhov	2,117996199	1
D5Ertd605e	2,11755582	1
Id2	2,115795218	0,33081
Gba2	2,115355297	1
Gm13822	2,11418262	1
9230116N13Rik	2,113743034	1
Gm19777	2,113450027	1
Tnfrsf1b	2,113010593	0,78649
Gm42970	2,110083368	1
Tmem37	2,10949841	1
Gm13140	2,10949841	1
Gm17786	2,108621276	1
Decr2	2,10657605	1
Tma7	2,106284037	1
Gm5778	2,104678688	1
Gm16062	2,104386937	1
Mok	2,104095228	1
Gm10382	2,103074562	1
Gm14057	2,102637285	1
Gnpda1	2,102491547	1
Pqlc2	2,102054391	1
Tfr2	2,101617327	1
Rps11-ps4	2,100161102	1
Gm3940	2,098269517	1
RP24-550H10.4	2,096815611	1
Gm43660	2,093765688	1
Clec2l	2,089706023	1
Tssk6	2,089706023	1
Gm12751	2,088402803	1
Gm7292	2,087823855	1
Csf2rb2	2,087534441	1
Gm28686	2,085798801	1
Ada	2,085509668	1
D6Ertd527e	2,082909273	1
Gm38247	2,08262054	1
Nos1	2,081754583	1
Hba-a1	2,081754583	1
Dhrs3	2,08146601	1
Gm5523	2,08146601	1
5330426L24Rik	2,080312121	1
Zfp69	2,079735416	1
Gm6155	2,078150301	1
Gm8019	2,075702954	1
Gm8423	2,074839873	1
Gm12604	2,072396425	1
Gm6162	2,07210915	1
Gm4285	2,069668918	1
Sertad1	2,06938202	0,90306
Fcor	2,06938202	1
Med16	2,069095163	0,79467
Kctd6	2,069095163	0,9607

Gm27039	2,067804797	1
Gm4890	2,06608556	1
Thap6	2,065942355	1
Dmrt2	2,064653955	1
Gm26730	2,064367753	1
Rpl10a-ps2	2,063080335	1
Ube2cbp	2,062651374	1
Gm6794	2,060936422	1
Gm44419	2,060507907	1
Bbs5	2,059936692	1
Rbm48	2,059508385	0,91083
Gm13204	2,057938687	1
Gm22767	2,056512727	1
4930589O11Rik	2,056512727	1
9330102E08Rik	2,056512727	1
Galnt10	2,054660456	1
Gm15787	2,054375639	1
Gm8210	2,052809853	1
Nedd4l	2,052098526	1
Slc36a3os	2,051814065	1
8030453O22Rik	2,050960917	1
1110006O24Rik	2,050250231	1
Wdyhv1	2,048687589	1
Rpl36-ps2	2,047835741	1
4930427A07Rik	2,047835741	1
Pcdhb15	2,047693801	1
Gm1848	2,046700495	1
Mcm8	2,04570767	1
Gm14593	2,044998805	1
Matr3-ps2	2,044290185	1
Creb5	2,043865131	1
Zfand1	2,041882714	1
Zfp459	2,038771371	1
1700030M09Rik	2,037782393	1
Gm14292	2,035382586	1
RP24-282C4.3	2,034113244	1
Gm43006	2,033972255	1
9530053A07Rik	2,033690306	1
Car7	2,033408397	1
Gm26226	2,031576935	1
Meg3	2,031436122	1
6430511E19Rik	2,031295318	1
Spata2	2,031013741	1
Khnyn	2,030309967	1
Gm44777	2,02763785	1
Dntt	2,02749731	1
Gm5580	2,025249989	1
Gm9392	2,02384668	1
Gm12902	2,023706402	1
F830208F22Rik	2,023425876	1
4930404I05Rik	2,022444342	1
Asb10	2,021883679	1

Gm16537	2,021042976	1
Gm26606	2,02076282	1
Gm13022	2,020202623	1
Gm37851	2,018942748	1
Gm12280	2,017823519	1
Sema6c	2,014609189	1
Gm28187	2,014329925	1
Pin4	2,012934181	1
Tnfrsf12a	2,011539405	0,7027
Snord7	2,009727641	1
Gm12182	2,009449053	1
N4bp3	2,009170503	1
Osgin1	2,008752751	1
Rps3a3	2,008195884	1
Gm12013	2,008195884	1
RP23-413G8.2	2,008056691	1
Gm11694	2,007917508	1
Gm10069	2,007221738	1
Gm31274	2,006665295	1
Gm11759	2,006109006	1
Leng9	2,004580008	1
Fos	2,004024297	1
Slc5a6	2,003052174	1
Gm43010	2,001109343	1
Cped1	1,999584155	1
Gm10658	1,999584155	1
Cdca7	1,999445559	1
Gm37234	1,997921639	1
Oas1g	1,997921639	1
Rpl7a	1,997644688	1
Rps15a-ps1	1,996675659	1
Gm10343	1,994877283	1
Gm14541	1,992942381	1
Gm42432	1,992666119	1
Xaf1	1,990871354	1
Ubb	1,989353969	0,53484
Gm6344	1,988940337	1
Gm16181	1,988664631	1
RP24-324J2.1	1,987286671	1
Gm5566	1,986322667	1
Crip2	1,98425852	1
Rac3	1,983158518	1
Bcl2a1d	1,982196517	1
RP23-350F7.3	1,982059127	1
Samd9l	1,980685744	1
Rpl27a-ps1	1,979999409	1
Nedd9	1,979862171	1
Gm16061	1,979587723	1
Gm5312	1,979313313	1
Gm10335	1,978901769	1
Gm26656	1,978627454	1
Psca	1,977667651	1

Gm9246	1,975064817	1
Gm9294	1,973696281	1
AA474408	1,973559479	1
Btg2	1,972875614	1
Rab11fip4os1	1,972465409	1
Slpi	1,971235305	0,90498
Gm13368	1,969459842	1
Cox4i2	1,968913867	1
C1qtnf6	1,968913867	1
Gm6649	1,968913867	1
Hpse	1,968368044	1
Gm10916	1,965096281	1
Sh2d2a	1,964279191	1
Mmp2	1,963054192	1
Gm13215	1,962782073	1
Gm12151	1,962373967	1
Nectin4	1,961014224	1
Rflnb	1,960878302	1
Gm26782	1,957483301	1
Fsbp	1,957347623	1
Arsg	1,956805006	1
Rps6-ps1	1,953958735	1
Ctsc	1,95192822	1
Gm9530	1,950710923	1
Zfp712	1,950575715	1
Gm4032	1,949494386	1
Alpk2	1,948548715	1
Gm6123	1,947603502	1
Oxt	1,947333526	1
Gm45133	1,946793685	1
Nhlrc1	1,945849324	1
Gm15013	1,944770615	1
Gm5449	1,944635819	1
Gm4832	1,944366254	1
Rbm19	1,944096726	1
Gm13675	1,943557782	1
Gm45718	1,943153673	1
5033430I15Rik	1,943018989	1
Fam19a2	1,942211078	1
Tmem29	1,941134386	1
H2-Ab1	1,941134386	1
Trpm2	1,940461756	1
Mcm10	1,93790789	1
Gm14130	1,937102104	1
Fbxl15	1,935759872	1
Gm18943	1,932408361	1
Rsad1	1,932274421	1
C430049E01Rik	1,931337103	1
Gm25007	1,930266439	1
Wwc1	1,92973133	0,98935
B3gnt2	1,92973133	1
Clec3b	1,928928944	1

Pnp	1,926657329	1
Gm7847	1,926523788	1
Iba57	1,925856221	1
Gm17150	1,925322335	1
Gm12059	1,923988265	1
Rpl26-ps2	1,923721562	1
Lcmt2	1,923588225	1
Fam71e1	1,923188268	1
Ciart	1,92052401	1
Rps19-ps7	1,918528239	1
Gm3695	1,917331772	1
Ppp1r15a	1,915472084	0,98935
Gm12704	1,9136142	1
Hist2h3c2	1,912023162	1
Gm45445	1,911625609	1
Gm2076	1,911095668	1
Apoo-ps	1,908712747	1
RP23-168F21.4	1,907786859	1
Atf3	1,906597091	1
Hexim1	1,90646494	1
Gm16020	1,905804324	1
Gm28555	1,905275996	1
Wfdc17	1,904351775	1
Rpl31-ps22	1,904351775	1
Gm44198	1,903428002	1
Gm6166	1,902768438	1
2700046G09Rik	1,899737388	1
Gm15446	1,899210742	1
Prss35	1,899079103	1
Accsl	1,899079103	1
Gm44775	1,899079103	1
Ceacam10	1,899079103	1
Il13ra2	1,899079103	1
Rorc	1,899079103	1
Arhgef15	1,899079103	1
Rassf7	1,898157888	1
Nlrc4	1,898026322	1
Gm45629	1,895396922	1
Gm15975	1,891328548	1
Klf11	1,890542131	1
Gm13777	1,888839347	1
Rasgef1b	1,888184838	1
Gm7308	1,887399728	1
Ier2	1,887268908	1
Dusp1	1,887138097	1
Il27	1,887007295	1
Jun	1,885961206	1
Gm17827	1,885961206	1
Kbtbd4	1,885699774	1
Gadd45a	1,885046354	1
Acvr2a	1,885046354	1
Zfp503	1,882043534	1

Ambp	1,881913085	1
Gm4217	1,880739453	1
Gm12577	1,880087751	1
Tmem171	1,878785025	1
Gm13360	1,878654803	1
Gm4342	1,877743495	1
Snn	1,875662165	1
Gm5898	1,875142193	1
Cxcl14	1,874882261	1
Riiad1	1,873842894	1
Gm13567	1,872025387	1
Gm26912	1,872025387	1
Cxcr4	1,871636151	0,73611
Scel	1,870468928	1
Soat2	1,86904331	1
RP24-93F20.12	1,868395661	1
Srd5a1	1,86735989	1
Gm6493	1,866583438	1
Pxylp1	1,866195333	1
Gm20594	1,866195333	1
Greb1	1,865936642	1
Gm12912	1,865290069	1
Gm43364	1,863868399	1
Gm6088	1,863868399	1
Gm14048	1,861802448	1
Lrrc17	1,861028308	1
Gm26737	1,861028308	1
Dok4	1,859480991	1
Gm15032	1,859094363	1
Gm23127	1,857806184	1
Gm10827	1,856132885	1
Pmaip1	1,854332556	1
Gm8599	1,853047676	1
Gm15720	1,852662385	1
Hist1h4a	1,852277175	1
Gm32340	1,852020413	1
Ppard	1,850352325	1
Gm8925	1,850095829	1
Tagap	1,84983937	1
Zfp637	1,849711153	1
Zfp607a	1,849454746	1
Mical1	1,849454746	1
Haus3	1,849326556	1
Eif1-ps1	1,849326556	1
Gm12543	1,848173246	1
Tob1	1,847148685	1
Rps19-ps3	1,846764621	1
Irf4	1,846380637	1
Gas8	1,846124693	1
Gm15728	1,845101267	1
Rpusd2	1,844461915	1
Gm5575	1,844206236	1

Gm38299	1,842672906	1
n-R5s151	1,842162079	1
2610528A11Rik	1,840247741	1
Gm27046	1,839482562	1
1500015A07Rik	1,837316284	1
5430402O13Rik	1,837061596	1
Gm5910	1,836934265	1
Gm43137	1,836806942	1
Yjefn3	1,835788683	1
4921524J17Rik	1,835152557	1
Gm15393	1,835152557	1
RP23-88C11.5	1,834898169	1
Map3k12	1,833118436	1
Rpl13-ps3	1,832991378	1
Cd300lf	1,832102221	1
Gm9169	1,831848255	1
Cdc6	1,831467373	1
Gm9332	1,829690968	1
Nbas	1,82918374	1
BC024386	1,82918374	1
Jund	1,828930179	0,82117
Cks2	1,828803412	1
Gm12504	1,826902955	1
1700031P21Rik	1,825890186	1
4930412F12Rik	1,825763629	1
Gm45856	1,824625013	1
Mtrf1	1,82411919	1
Hist1h1b	1,823866331	1
Gm45749	1,823613507	1
Arhgap4	1,823613507	1
Efna3	1,822349912	1
Gm44187	1,82184472	1
B430305J03Rik	1,820203811	1
Ksr1	1,819699213	1
Gm12176	1,818942579	1
RP24-84C23.4	1,818690437	1
Ly6g6d	1,818060236	1
Zbtb3	1,817178322	1
Gm44291	1,817052369	1
Ube2v1	1,816926425	1
Gm7363	1,815541616	1
Rep15	1,815415776	1
Gm12230	1,814157862	1
Rcbtb2	1,814032119	1
Lrtm2	1,813780658	1
Bvht	1,813654941	1
4930461G14Rik	1,813654941	1
Gm5900	1,813277841	1
D830025C05Rik	1,812523877	1
Sez6	1,811895812	1
Atg4a-ps	1,811267966	1
Gm7722	1,810891363	1

Gm7285	1,810263864	1
Gm6341	1,809762022	1
Pafah1b1-ps2	1,809636583	1
Gm42571	1,808758755	1
Zfa-ps	1,808131996	1
Gm43350	1,807630745	1
Gm12097	1,807380171	1
Sec24a	1,806753889	1
Gm6290	1,806628659	1
Gm15950	1,806002638	1
Gm7287	1,80587746	1
Gm5112	1,80575229	1
Gm7887	1,80575229	1
Gm44913	1,804876346	1
Gm11222	1,803500721	1
Snord87	1,803375716	1
Adm	1,802875784	1
Polr2l	1,802500925	1
Gm28578	1,802251063	1
Rab20	1,800877439	1
Homez	1,800128631	1
Rpsa-ps12	1,80000386	1
RP24-401G4.1	1,799754343	1
5430427O19Rik	1,799255415	1
Rraga	1,799006002	1
Gm10110	1,799006002	1
F2	1,798881309	1
Mafk	1,797759458	1
Sep 02	1,797759458	1
Gm3608	1,796513778	1
Park2	1,795517856	1
Insig2	1,795393404	1
Gm16045	1,795144527	1
Pim1	1,794025007	1
Gng5	1,793900659	1
Ppfia4	1,793154752	1
Tma7-ps	1,792906185	1
Ccdc159	1,792906185	1
Gm4875	1,792533399	1
Rps10-ps2	1,791912262	1
Gm12854	1,79129134	1
Ier5l	1,787942078	1
Gm8242	1,787694234	1
Utp23	1,787570325	1
Rps15a-ps5	1,787322532	1
Rps12-ps24	1,786950907	1
Fhit	1,78682705	1
Arvcf	1,786455528	1
Gm16373	1,786455528	1
Gm26664	1,786331705	1
Gm2058	1,785960286	1
2010015M23Rik	1,785341427	1

Nup160	1,784475384	1
1700003G18Rik	1,784475384	1
Fem1c	1,78422802	1
4930542C12Rik	1,782744558	1
Gm11249	1,779904702	1
Zfp747	1,779657972	1
Mxd3	1,776946197	1
Abcc3	1,776453592	1
Mzf1	1,776084227	1
Cacna1s	1,775838026	1
Zranb3	1,775345728	1
Gm11362	1,774607536	1
Tbc1d16	1,774484534	1
Gm17745	1,7737467	1
Gm14013	1,7737467	1
Gm11737	1,773377898	1
Arl14epl	1,772394801	1
Tnf	1,772271952	1
Sparc	1,772149112	1
Depdc1a	1,768958252	1
Atad5	1,767855062	1
Dusp5	1,767487485	1
Wdr62	1,766752561	1
Gm7964	1,766630103	1
Smarce1	1,765405993	1
Gm14253	1,764794256	1
Gon7	1,764427315	1
Rdh13	1,76418273	1
Mtcl1	1,762349426	1
Pank4	1,762227274	1
Btf3	1,761738748	1
4831440E17Rik	1,761616638	1
Gm10717	1,761250358	1
Usp11	1,761006214	1
Rpa3	1,761006214	1
1700052K11Rik	1,760640061	1
Coq6	1,759907984	1
Tnnc2	1,759298152	1
Gm6913	1,758200987	1
Gm12074	1,757713578	1
Gm26520	1,757591746	1
Ddx51	1,757591746	1
6430573P05Rik	1,756495645	1
Gm11868	1,756495645	1
D7Bwg0826e	1,756495645	1
Reps2	1,756373899	1
Slc25a25	1,755643595	1
Cd83	1,755156895	1
Gm6257	1,753819165	1
H60c	1,753454506	1
Gm7899	1,753211443	1
Gm4784	1,752968413	1

Fth-ps2	1,752360985	1
Gm45223	1,751632351	1
2810013P06Rik	1,750904019	1
Tmco4	1,748236058	1
Nuf2	1,74787256	1
RP24-82M14.1	1,747266899	1
Zgpat	1,746177237	1
Phf11d	1,745935182	1
Gm27043	1,745693161	1
Siglec1	1,745330192	1
Gm45251	1,745088255	1
Slc41a1	1,743999952	1
Tas1r1	1,743879071	1
Gm21057	1,743033141	1
Spata5	1,742912327	1
Gm4468	1,742066866	1
Mthfsl	1,741704651	1
Gm26826	1,741221815	1
Gm12848	1,741221815	1
Aplf	1,740618458	1
Gm45456	1,739532942	1
Adora2b	1,73893017	1
Gm8822	1,738448103	1
Zfyve28	1,738448103	1
Gm2214	1,73820712	1
Col4a6	1,73820712	1
Hspa9-ps1	1,738086641	1
Serpinb9	1,738086641	1
Rpl5-ps1	1,73796617	1
B3galt4	1,737725253	1
Ppp1r13l	1,737363941	1
Rps3a1	1,735799123	1
Tmem38a	1,735799123	1
1190005I06Rik	1,735678811	1
Zfp85	1,735197645	1
Gm6501	1,73411551	1
Gm10132	1,733634778	1
Pramef8	1,73303405	1
Mturn	1,732673713	1
Gm13827	1,731713181	1
Gpr137	1,731713181	1
Kif21a	1,731593152	1
C030034I22Rik	1,729913618	1
Hbb-bh3	1,72955393	1
Gm13461	1,728595128	1
Gm8722	1,727157921	1
Itgb3bp	1,726918502	1
Dnajb9	1,726798806	1
Proscos	1,726080799	1
Gm9761	1,725602295	1
Gm16124	1,725123922	1
Pgf	1,724884786	1

Wdr53	1,724406613	1
Gm37522	1,721301716	1
Gm12165	1,721182408	1
Gm37733	1,718917138	1
Gm10051	1,718797996	1
Tmem120b	1,718559736	1
Ticrr	1,717964232	1
Gm6285	1,717487977	1
Rps19-ps9	1,717368934	1
Orc1	1,717249899	1
Llph-ps1	1,716773843	1
Smad7	1,716654849	1
2900093K20Rik	1,714157888	1
Epop	1,714039076	1
Rps23-ps2	1,712851407	1
RP23-447C2.2	1,710359978	1
Rubcnl	1,709885831	1
Gm6159	1,709648807	1
Cdc34b	1,709056391	1
Cdkal1	1,707635429	1
RP23-226H21.3	1,707162037	1
Sesn2	1,706570483	1
Brms1l	1,706215648	1
C430042M11Rik	1,705387988	1
Rangrf	1,704797048	1
Dnajc17	1,704560729	1
Gm15564	1,704324443	1
Gm12355	1,704206313	1
mt-Rnr2	1,703970076	1
BC030499	1,703733872	1
Fam118a	1,703497701	1
Rpl10a	1,703379627	1
Nceh1	1,702789383	1
Itga7	1,702671359	1
Gm27219	1,702671359	1
2010111I01Rik	1,702199343	1
Gm8919	1,701609508	1
Magee1	1,701255705	1
Gm14680	1,70031259	1
Gm5830	1,698663397	1
Rwdd1	1,698310207	1
Nek3	1,6978394	1
Klhl25	1,696898179	1
Rps7	1,696192606	1
Igf2bp1	1,696075039	1
Macrod2	1,695722387	1
9930022D16Rik	1,695487326	1
Gm11687	1,695252298	1
Rpl31-ps11	1,695252298	1
Id3	1,695134796	1
Gm22581	1,69466487	1
Slc9a4	1,694195074	1

Gm5262	1,694077646	1
Zc3h12a	1,693842813	1
Gm10941	1,693725409	1
Rnf19a	1,693255873	1
H2-T10	1,692903807	1
Cir1	1,6924345	1
Fcrl5	1,692082604	1
Rps12-ps10	1,690910149	1
Leng1	1,690207065	1
Gm14034	1,690089913	1
4930579K19Rik	1,689855633	1
Zfp341	1,688918838	1
Gm43566	1,688801775	1
Gm3362	1,687982562	1
Gm15530	1,687748574	1
Cbfa2t3	1,687163746	1
A930015D03Rik	1,686812947	1
A930006K02Rik	1,686812947	1
2410080I02Rik	1,685994699	1
Gm28424	1,684592911	1
Gm25291	1,684592911	1
3110062M04Rik	1,684359393	1
Mcm9	1,683775739	1
Pnrc1	1,683192288	1
Vps8	1,682958965	1
Tnks1bp1	1,682958965	1
Supt7l	1,682842315	1
Rpl3l	1,682609039	1
Tec	1,682375797	1
Gspt2	1,681093538	1
Gm23037	1,680627504	1
Gm19726	1,680627504	1
Cib2	1,679346578	1
Cfh	1,678764662	1
Nfil3	1,678531952	1
Ccdc36	1,677834015	1
Hnrnph2	1,677485156	1
RP23-43M12.2	1,677136369	1
Gm32175	1,674348683	1
Armc5	1,674116585	1
Zfp553	1,673188515	1
Gm11273	1,673072542	1
Pard6b	1,672956578	1
Gm45833	1,672608732	1
Rpl17-ps8	1,67156563	1
Gm6987	1,671218074	1
Gm8304	1,670638974	1
Lsm12	1,670523178	1
Atr	1,67040739	1
Ints12	1,668671536	1
Csrp2	1,668671536	1
Ndufs5	1,668208946	1

Adamts1	1,668093318	1
Gm15824	1,667284151	1
Gadd45b	1,666821946	1
Ropn1l	1,666475376	1
Fbxo33	1,665666993	1
A430018G15Rik	1,665436099	1
Rasl2-9	1,665436099	1
Rpl39-ps	1,665205236	1
Ltb	1,664859002	1
Papd4	1,664859002	1
RP23-138K22.2	1,664743607	1
Gm9009	1,664282106	1
Zfp707	1,664166751	1
1810024B03Rik	1,663936064	1
RP24-547N4.5	1,66370541	1
H2-T23	1,663244197	1
Gm12529	1,662783111	1
Gm5525	1,662091723	1
Gm8116	1,661515786	1
4933417C20Rik	1,66036451	1
Stac3	1,660249426	1
Itga11	1,659904223	1
Ndufs3	1,659559091	1
Foxn2	1,659214032	1
Rpl30-ps3	1,658984032	1
Hmgb1-ps6	1,655996932	1
Rgs9bp	1,655193632	1
Snord49b	1,65496419	1
Arl4d	1,65496419	1
Dyrk3	1,654390722	1
Myc	1,654161391	1
Lin52	1,653473587	1
Pex5	1,653129793	1
Nfkbib	1,652442418	1
Nlrc3	1,652327884	1
Gm12186	1,651984328	1
Bloc1s6os	1,651640843	1
H60b	1,651068527	1
Gm18889	1,650496409	1
Tlcd2	1,650153234	1
Mrps11	1,64981013	1
Chrna1os	1,649581434	1
Gm7488	1,649009831	1
Spryd4	1,648666965	1
1110035H17Rik	1,64832417	1
Gm23751	1,64832417	1
Rpl35a-ps5	1,647638794	1
Gmfg	1,647182035	1
C230037L18Rik	1,646497133	1
Lgals7	1,646383011	1
H2-Ob	1,646268896	1
Pik3r3	1,645584374	1

Zfp788	1,645584374	1
Mkrn2	1,64524222	1
Zbtb24	1,64524222	1
Dennd5a	1,644786126	1
Rps29	1,644558126	1
Arc	1,644444137	1
Spty2d1	1,64410222	1
Fbxo34	1,643760375	1
Tnfrsf17	1,643760375	1
4930522L14Rik	1,643532517	1
Gm12726	1,643532517	1
Ntrk3	1,642621402	1
Tgif2	1,64182459	1
1700001G11Rik	1,641597001	1
Gm4994	1,641483218	1
Fam188b	1,641369443	1
Aurkb	1,641255676	1
Gm15694	1,641141916	1
Sult2b1	1,640345822	1
Rnf185	1,640232126	1
Rab11fip4	1,640118438	1
Fgfbp3	1,639891085	1
Fam107b	1,639095598	1
D130020L05Rik	1,638868387	1
Pou6f2	1,638641207	1
Ccl4	1,638414059	1
Ndfip1	1,638073396	1
Casz1	1,637165307	1
Sbk3	1,636598007	1
Rin1	1,635690736	1
Gm5865	1,634897286	1
Gm12762	1,634557354	1
Serpinf2	1,634104221	1
Gm7224	1,633990958	1
Rpl36	1,633877702	1
Ptgs2os2	1,633651214	1
A930029G22Rik	1,633651214	1
Shcbp1	1,633311541	1
Gm12454	1,633198333	1
Prr3	1,632858754	1
Pmp22	1,632519246	1
Tmem170	1,632292946	1
Nrbf2	1,632179808	1
Ranbp9	1,631840441	1
Gm12844	1,631840441	1
Gm6457	1,631840441	1
Sumo1	1,631614236	1
Upf2	1,629128045	1
Slc31a2	1,629128045	1
Kbtbd7	1,628676417	1
Litaf	1,627773538	1
Ppp1r21	1,627773538	1

Ptrf	1,627435087	1
Ankrd55	1,626871158	1
Gm14769	1,626081987	1
Crnde	1,62596928	1
Nup98	1,624392195	1
Gm26983	1,624392195	1
Gm10080	1,624167022	1
Hmgb1-ps8	1,624167022	1
Gm6733	1,62394188	1
Cage1	1,623716769	1
Mbip	1,623604226	1
Uba3	1,623266642	1
RP24-370M23.1	1,622929128	1
Zfp39	1,622479219	1
Ccl5	1,621579775	1
Gm15453	1,621017875	1
Rnf31	1,62079317	1
Gm11686	1,620343853	1
Fbxl3	1,620006947	1
Gm12589	1,619221106	1
Zfyve1	1,618435645	1
Zcchc10	1,617874835	1
Gm7436	1,616977944	1
Gm7990	1,616753799	1
Lgr5	1,616529684	1
Zfp36	1,615633539	1
Cxcl2	1,614737889	1
Aunip	1,614290251	1
Gm37082	1,614178361	1
Ticam1	1,613954604	1
Pop7	1,613842737	1
Mospd1	1,613842737	1
Vdr	1,611830457	1
RP23-48A24.3	1,610936917	1
Snora17	1,610601967	1
Gm14017	1,610155475	1
Brip1	1,610043871	1
Cd200r4	1,609932275	1
Gm12341	1,609597534	1
Txlng	1,609039787	1
Gm44552	1,609039787	1
Gm11964	1,608816742	1
Zfp703	1,608482233	1
Nup35	1,608259265	1
Atp5l-ps1	1,608259265	1
Gm8394	1,607367704	1
Jrk	1,607144891	1
Armcx5	1,606922109	1
Harbi1	1,606476637	1
Hsf2bp	1,605252227	1
Gm17541	1,605140963	1
Mettl3	1,605029707	1

Rpl19-ps1	1,604918459	1
Psmc3ip	1,604139936	1
Lrp5	1,603917571	1
9530085L11Rik	1,603139534	1
Bicd2	1,602917307	1
Pstpip2	1,602361874	1
Trib1	1,602250811	1
Gm45113	1,601806634	1
Zfp689	1,601362581	1
9330162012Rik	1,6011406	1
Gm3555	1,600807687	1
Esrp2	1,600474843	1
Gfod2	1,60036391	1
Mir3091	1,599809362	1
2900009J06Rik	1,599698476	1
Necap1	1,598922486	1
Gm12799	1,598922486	1
Gm7496	1,598922486	1
A930005H10Rik	1,598811661	1
Gm26810	1,597482359	1
Mybl2	1,597260917	1
Pgap3	1,596596773	1
Hist3h2a	1,596486109	1
Gm45413	1,596375453	1
Ssc5d	1,59604353	1
Gmnn	1,595932905	1
RP23-151L20.5	1,595932905	1
Ccdc25	1,594716531	1
Trpm1	1,594384953	1
Rpl18a	1,594053445	1
RP23-349H12.3	1,593832477	1
Brca1	1,593722005	1
Rps4x-ps	1,593501083	1
Rpl30-ps11	1,592617704	1
Inhbe	1,592507316	1
Phlda3	1,592286563	1
Zic2	1,592286563	1
Cyp26b1	1,592176198	1
Cgrrf1	1,591514168	1
Dynlt1b	1,591514168	1
Arl4a	1,59063189	1
Gm3511	1,590521639	1
Gm38262	1,589639911	1
Rabif	1,589419556	1
Nt5m	1,589309389	1
5830487J09Rik	1,588758672	1
Csrnp1	1,588428333	1
Thap8	1,588208146	1
Gm8121	1,587877921	1
Ppp6r2	1,58765781	1
Gm43351	1,587547766	1
Tmem44	1,58677767	1

Anapc15	1,586557711	1
Hook2	1,585458374	1
Gm5093	1,585458374	1
Ahrr	1,585128721	1
Gm6394	1,584140175	1
Fchsd1	1,583701019	1
RP24-511J14.2	1,582384281	1
Oip5	1,582164931	1
Tspan33	1,581726323	1
H2-DMb1	1,58161669	1
Cbx4	1,581397446	1
Tapbpl	1,581287836	1
Rgs1	1,581178233	1
Gm17034	1,581178233	1
RP23-403D16.3	1,580301685	1
Speer9-ps1	1,579863593	1
E2f4	1,579535104	1
Gm12643	1,579316149	1
Pea15a	1,579206683	1
Hsd3b7	1,578768894	1
Gm5050	1,577784314	1
Ybx1-ps2	1,577784314	1
Zmym1	1,57734692	1
Mterf3	1,577237591	1
Sav1	1,576691056	1
Junos	1,576581772	1
Lum	1,576472496	1
Serpinc1	1,576363227	1
Tjp3	1,575161768	1
Hsd17b14	1,574615953	1
Ptger4	1,574179437	1
Tsacc	1,573961225	1
Fbxo18	1,573415826	1
Kif11	1,573306769	1
Cenpp	1,573088677	1
Gm37339	1,572652585	1
N6amt1	1,57210764	1
Gm26698	1,571998673	1
Bcl7c	1,571889714	1
Zfp101	1,570909424	1
Hs1bp3	1,570800541	1
Stil	1,570691665	1
Gm16200	1,570365083	1
Dnajc19-ps	1,569712122	1
Gm5121	1,569276966	1
Pofut2	1,568624458	1
Adgre5	1,568298305	1
mt-Nd2	1,567754869	1
Cd200r1	1,567646204	1
Uggt2	1,567537547	1
Plekhh3	1,567211621	1
Zeb2os	1,567211621	1

Zfp772	1,567211621	1
Ormdl3	1,56666856	1
Mgst3	1,566017137	1
C330013E15Rik	1,566017137	1
Gm13391	1,565365984	1
Clp1	1,565257485	1
Icam4	1,565257485	1
Zfp595	1,564823563	1
Dusp10	1,564389762	1
Gm7733	1,564281331	1
Cfap126	1,563739286	1
H2-Q10	1,5636309	1
2410004B18Rik	1,563522521	1
Hmgb1-rs16	1,562764079	1
Gm10126	1,56265576	1
Klhl18	1,56265576	1
Gm10388	1,56222256	1
Rpph1	1,561897739	1
Skor1	1,560707305	1
Rpp38	1,560599129	1
Gm42666	1,56005836	1
Gm13743	1,559517779	1
Tyw5	1,559409685	1
Coq10b	1,558977385	1
Rnf25	1,558653238	1
Gm7434	1,558329159	1
Dqx1	1,557789177	1
5730405O15Rik	1,557681203	1
Gm19287	1,557465278	1
Stard5	1,557033516	1
4732491K20Rik	1,556817681	1
Gm9506	1,556709774	1
2310058D17Rik	1,556493983	1
Mgam	1,556493983	1
Prim1	1,556386099	1
Atp6v0c	1,556062491	1
Fig4	1,554768733	1
C1ra	1,554229985	1
Gm4607	1,55379912	1
Hist1h4i	1,553583733	1
Gm7266	1,553583733	1
Lctl	1,55347605	1
RP24-91J7.1	1,55347605	1
RP24-365A12.2	1,55347605	1
Sec61g	1,553368375	1
Epc1	1,553260708	1
Fuca2	1,552722482	1
Spsb2	1,552184443	1
Gpr157	1,55164659	1
Syap1	1,551108923	1
8430429K09Rik	1,551108923	1
Ptp4a1	1,550893909	1

Hdac11	1,550893909	1
Gosr1	1,550678924	1
Bicdl1	1,550678924	1
Ndufb4	1,550571443	1
Tslp	1,550141593	1
Hist1h1c	1,550034149	1
Gm9796	1,549389642	1
Plekhf2	1,54928225	1
Cryga	1,549067489	1
Hps4	1,548423384	1
Gm5576	1,547994129	1
Zfp326	1,547672266	1
4632415L05Rik	1,547564994	1
Gm26533	1,547564994	1
Slfn2	1,54735047	1
Rps27a	1,54724322	1
Gm16238	1,547135977	1
Sowahc	1,546385484	1
C87436	1,546171124	1
Gm5239	1,545956794	1
Gm9762	1,545313983	1
Plekhg3	1,545206873	1
Sh2d3c	1,545206873	1
Gm5601	1,544992677	1
Slfn10-ps	1,54488559	1
Bri3	1,544564373	1
Mir124-2hg	1,544243224	1
Gm5276	1,543066246	1
Adat2	1,542959292	1
Rps13-ps1	1,542852346	1
Iffo2	1,542745408	1
1700120C14Rik	1,542745408	1
Ms4a6b	1,542638476	1
Prps1l3	1,542424636	1
2610002M06Rik	1,541890165	1
Ywhah	1,541676428	1
RP23-114G13.1	1,541462722	1
Gm9385	1,541249044	1
Gm16712	1,541249044	1
Gm4617	1,541142217	1
Tob2	1,540714981	1
4930579G24Rik	1,540501407	1
Gins3	1,540394631	1
Prrg4	1,540287863	1
Irgm1	1,540287863	1
Gm37677	1,540287863	1
Rpl27a-ps2	1,539754132	1
Gm4374	1,53900722	1
Gm12834	1,538473933	1
Gm9835	1,53815405	1
Gm13840	1,538047437	1
Map4k2	1,537727642	1

Klhl15	1,537194799	1
Rad51	1,536981713	1
Asf1b	1,53666214	1
Adh5	1,53655563	1
Etfdh	1,536449128	1
0610005C13Rik	1,536449128	1
Gm14328	1,535171677	1
Ppwd1	1,53485248	1
Ap3s1	1,53485248	1
Wtap	1,534746096	1
Suco	1,534107946	1
Get4	1,534107946	1
Gm14620	1,534001613	1
Hmgb3	1,534001613	1
Phldb3	1,533895288	1
9130019O22Rik	1,533363772	1
Gm14513	1,533151218	1
Stx1a	1,532513731	1
Cdc45	1,532195087	1
Gm6451	1,532088887	1
Pgam1	1,531876509	1
Tspan15	1,531027292	1
Comp	1,53049677	1
Smagp	1,530284613	1
Rpl23a-ps3	1,529966432	1
Gtpbp10	1,529966432	1
Pafah1b1-ps1	1,529860386	1
Mad2l2	1,529436278	1
Gldc	1,528906308	1
Rbmx2	1,528694371	1
Cebpe	1,528588413	1
Mrps22	1,528270585	1
RP23-288C18.3	1,527846918	1
RP23-312A24.1	1,527635128	1
Gcat	1,527211636	1
Wwp1	1,527105781	1
Zfp984	1,526047639	1
Gm15421	1,526047639	1
RP23-268C22.3	1,526047639	1
Acta2	1,526047639	1
Slc1a4	1,526047639	1
Gm45640	1,525941865	1
Rpl36-ps3	1,525730339	1
Fam134c	1,525201653	1
Gm7658	1,5244618	1
Gm15159	1,5244618	1
Acad12	1,5244618	1
Tnfaip3	1,523827926	1
Tmem185b	1,523616694	1
Pacsin2	1,523511088	1
Fam214a	1,522772055	1
Rnf215	1,522666508	1

Inpp5f	1,522666508	1
Gtse1	1,522033381	1
Zbtb42	1,521716916	1
Hcn2	1,52118962	1
Adrb2	1,521084183	1
Cep120	1,519503502	1
Creb3l3	1,519503502	1
Il10ra	1,518976974	1
Ptpn14	1,518661144	1
Gm7730	1,518555882	1
Kif24	1,51824014	1
Morn2	1,51824014	1
Plaur	1,518029682	1
Rps19-ps12	1,518029682	1
Gm42559	1,517714049	1
Mynn	1,517293308	1
Gm5321	1,517293308	1
Fabp3	1,517082981	1
RP23-63H11.3	1,515401415	1
Zfp280b	1,51519135	1
Gm8995	1,513197187	1
Gm7407	1,512987428	1
Gm20219	1,51288256	1
Rfc4	1,512672844	1
Gm6377	1,512043872	1
Donson	1,512043872	1
Bach1	1,511939069	1
Klf6	1,511205649	1
Phf13	1,510996166	1
Mad2l1	1,510996166	1
Amacr	1,510996166	1
Gm8444	1,510577287	1
Gm13270	1,509949186	1
Oas1d	1,509949186	1
Fam103a1	1,509635234	1
Rps2	1,508484633	1
Gm45454	1,508380077	1
Osgepl1	1,508170985	1
Rn7sk	1,50806645	1
Gm44168	1,507752889	1
Pcgf1	1,506917046	1
Pop4	1,506603723	1
Mknk2	1,506186062	1
Pink1	1,506081665	1
Bard1	1,505455433	1
Gm2962	1,504620863	1
Bsdc1	1,504308018	1
St14	1,504203751	1
Gm13578	1,504203751	1
Gm43096	1,504203751	1
Ppp1cc	1,504203751	1
Tmod1	1,503682524	1

Xlr	1,503682524	1
Gm10736	1,503369875	1
Rpl3-ps2	1,503161478	1
Rmnd1	1,50222405	1
CH25-309J2.1	1,501911703	1
Tecpr1	1,500246953	1
Srfbp1	1,500246953	1
Rars	1,500142968	1
H3f3a	1,499935018	1
Pced1b	1,499727097	1
Gm6378	1,49941527	1
Gm5921	1,49941527	1
Rps3a2	1,49941527	1
Cyp2c55	1,499311342	1
Gemin4	1,498895703	1
BC002163	1,498687926	1
Dusp16	1,498480178	1
Sac3d1	1,498376315	1
Satb2	1,498376315	1
Gm5644	1,498272459	1
Tspan13	1,498064769	1
Marcksl1	1,498064769	1
Atf1	1,497753287	1
Pelo	1,497649475	1
Hspb6	1,497649475	1
Gm43859	1,49733808	1
Papd5	1,497026749	1
2310074N15Rik	1,496922987	1
Pald1	1,495782075	1
Zfp874a	1,495678399	1
Spire2	1,495678399	1
Kin	1,495263766	1
Gm13436	1,495160126	1
Mir6236	1,494952867	1
Cnr2	1,494849249	1
Lysmd3	1,494745637	1
Pcif1	1,494227688	1
Fcgr2b	1,494227688	1
Zfp119a	1,493606385	1
Cpeb2	1,493295831	1
Esco1	1,493295831	1
Gm7783	1,493295831	1
Gm11605	1,493192327	1
Ypel2	1,49308883	1
Gm10059	1,49308883	1
Ring1	1,492985341	1
Gm6565	1,492985341	1
C030015A19Rik	1,492674915	1
Cenpn	1,492674915	1
Itgam	1,49164063	1
Gm38345	1,49164063	1
1110003F10Rik	1,491227117	1

Klf1	1,490503745	1
Lsm3	1,490297131	1
BC022687	1,490297131	1
Secisbp2	1,489883991	1
Gm45546	1,489780723	1
Klf4	1,489367726	1
Erbin	1,489264494	1
Svbp	1,489264494	1
Med22	1,48916127	1
Gm13477	1,48885164	1
Gm16072	1,488748444	1
Lmtk2	1,488438899	1
Gm42856	1,488026274	1
Kdm2b	1,487923135	1
Gm4880	1,48771688	1
Mphosph10	1,487613762	1
2310001H17Rik	1,487613762	1
Snx18	1,487304454	1
Tstd1	1,486892143	1
Rpl28-ps3	1,486376915	1
Btbd7	1,48596486	1
C920021L13Rik	1,485346994	1
Qpctl	1,485346994	1
Gm34121	1,484935225	1
RP24-454N4.2	1,484317786	1
Sdc4	1,483392109	1
C630043F03Rik	1,483186482	1
Zfp189	1,482775312	1
9130024F11Rik	1,482672538	1
Gm14138	1,482364257	1
Lnpep	1,48226151	1
Rps8-ps3	1,48205604	1
Aacs	1,481850597	1
Zbtb43	1,481645183	1
Hikeshi	1,481337116	1
Dedd2	1,480618541	1
Snora31	1,480618541	1
Smpd4	1,480002897	1
Klhl12	1,479900315	1
Qrich1	1,479490056	1
Gm12696	1,479079911	1
Acot8	1,479079911	1
Mrps7	1,478977393	1
Mcm3	1,478772377	1
Gm13771	1,478772377	1
Gm17690	1,47856739	1
Gm38335	1,478362431	1
BC029214	1,478157501	1
Hpdl	1,477952599	1
Lonrf3	1,477747725	1
Osbp2	1,477645299	1
Ccne2	1,477338064	1

Mrpl48-ps	1,477133275	1
Gm9497	1,477133275	1
H2-Q6	1,476928515	1
Atg4c	1,476826146	1
Acot9	1,476621429	1
Kctd12	1,476314406	1
RP24-389J11.1	1,476212079	1
Pggt1b	1,476109759	1
Tex2	1,475700551	1
Gm9333	1,475291457	1
E130317F20Rik	1,475291457	1
Efcab2	1,475291457	1
D930030I03Rik	1,475086952	1
Mthfs	1,474882476	1
Tubb2a	1,474780249	1
Gm8254	1,474575815	1
E130102H24Rik	1,474473609	1
Gm43110	1,474473609	1
Gm12020	1,474371409	1
Ftsj1	1,474064854	1
Vgll4	1,473451935	1
Gyg	1,473349807	1
Med26	1,473349807	1
Gm11826	1,473247686	1
Manbal	1,472839271	1
Gm10131	1,47243097	1
Gm12940	1,471920753	1
RP23-47A1.1	1,471614708	1
Aen	1,471512707	1
Gm10269	1,471410713	1
Vps37b	1,471104773	1
Dennd4c	1,471104773	1
Dnah8	1,471002807	1
Ddx23	1,470289246	1
Gna12	1,470187336	1
Brcc3	1,470187336	1
Gm19196	1,470187336	1
Rpl36a	1,470085434	1
Slc26a11	1,469881651	1
Eif1a	1,469270472	1
Gm10240	1,469270472	1
Atl2	1,468964977	1
Unc5b	1,468354179	1
Bcl2a1b	1,466828294	1
Ddit4	1,466523307	1
Errfi1	1,466320018	1
Rpl38-ps1	1,466116757	1
Gm42508	1,465811918	1
Mex3c	1,465303995	1
Krtcap3	1,465303995	1
Vil1	1,465202431	1
Rpl7a-ps5	1,465100875	1

Gm14173	1,464694719	1
Rabgef1	1,463476926	1
Gm44254	1,463476926	1
Gm17511	1,462361506	1
Gemin6	1,462260147	1
Gm7312	1,462158794	1
Prss44	1,462158794	1
Zfp784	1,46195611	1
6230400D17Rik	1,461550826	1
Slfn9	1,461550826	1
Gmps	1,461348226	1
Mtpap	1,461246936	1
Gm15634	1,461246936	1
Dpys	1,461145654	1
Gm12501	1,46094311	1
Nxt1	1,459930812	1
Rpl21-ps6	1,45962726	1
Hip1r	1,459424927	1
Maml1	1,459323771	1
Sap30	1,458818095	1
Dtd2	1,458818095	1
Pla2g2e	1,458211516	1
Gm6520	1,457908321	1
Slc17a7	1,45780727	1
Ube2q2	1,457403136	1
Rps15a-ps7	1,45730212	1
Cdk9	1,456494244	1
Stk35	1,456090474	1
Gm14056	1,455989549	1
Gm5614	1,45578772	1
Gm11599	1,455585919	1
Tex264	1,455384146	1
Nadk2	1,45528327	1
Dennd2c	1,455081539	1
Phlda1	1,455081539	1
Bbs2	1,455081539	1
Gnmt	1,455081539	1
9330020H09Rik	1,4543757	1
4930431P19Rik	1,453972517	1
Kif20b	1,453770968	1
Fabp5l2	1,453770968	1
Ugt1a7c	1,453770968	1
Ncaph	1,453670204	1
Exosc2	1,453670204	1
Lrrc2	1,453670204	1
mt-Rnr1	1,453569446	1
Dalrd3	1,453569446	1
3110043O21Rik	1,452662945	1
Atp6v0a1	1,452461578	1
Nsl1	1,452058925	1
E230032D23Rik	1,452058925	1
Bod1	1,45195828	1

Gm6198	1,451455157	1
Mtfr2	1,450851639	1
Gm43672	1,450851639	1
Hist4h4	1,450449433	1
Lrig2	1,450248372	1
Egfl7	1,450248372	1
Olfr286	1,450248372	1
Iscu	1,449946833	1
Gm12380	1,449645356	1
Gm8719	1,449444407	1
Ndufaf1	1,449343942	1
Gm13398	1,449143035	1
Gm36189	1,449042591	1
Hat1	1,448942155	1
Gm9517	1,448942155	1
Pdcl	1,448339682	1
Rbm43	1,448138913	1
Rrp7a	1,448038539	1
Vhl	1,447637113	1
Junb	1,447637113	1
Gm8574	1,447436442	1
Atf4	1,447035183	1
Hist1h2bc	1,446533766	1
Gla	1,446533766	1
Gm45184	1,446433503	1
Tmem35b	1,446433503	1
Tmem204	1,445932295	1
4933404O12Rik	1,445531453	1
Dact3	1,445130722	1
Fas	1,444930398	1
Gm15727	1,444329593	1
Cycs	1,444229483	1
Gm45716	1,44412938	1
Inca1	1,44412938	1
Tmem41a	1,443929196	1
Gadd45g	1,443428855	1
Lfng	1,443228767	1
Gm12174	1,442528678	1
Gm44652	1,442328714	1
Gm13680	1,442128779	1
RP23-325K4.10	1,442028822	1
Carhsp1	1,441928871	1
Mad2l1bp	1,441928871	1
Klkb1	1,441828928	1
Gm13487	1,441629062	1
Slc26a6	1,441629062	1
F8a	1,441529139	1
2610021A01Rik	1,441429224	1
Gps1	1,441429224	1
Gamt	1,44102963	1
Gm14857	1,44003113	1
Zfand5	1,439931319	1

Gm37470	1,439931319	1
Tsga10ip	1,439931319	1
Gm12038	1,439332592	1
Cox19	1,439133072	1
Gm27248	1,439133072	1
Cox20	1,439033323	1
2310022B05Rik	1,43893358	1
Gm4525	1,43893358	1
Wdsub1	1,438833844	1
Nck1	1,43843497	1
RP24-232D3.1	1,43843497	1
Cdc42ep4	1,438335269	1
Rps15-ps2	1,438335269	1
Gm14427	1,437338636	1
Retn	1,437139393	1
Gm38380	1,436541827	1
Gm11675	1,435446936	1
Aste1	1,435049001	1
Gpatch3	1,434452305	1
Gm43447	1,434054646	1
Rpl28	1,433756473	1
Gm10167	1,433756473	1
Calr-ps	1,433657096	1
Cspg4	1,433458363	1
Gm9625	1,433359007	1
Ly86	1,433259657	1
Gstz1	1,43296165	1
Ccdc9	1,432862328	1
Abhd17a	1,432862328	1
Adnp2	1,431671003	1
Rdh10	1,43157177	1
Col11a2	1,43157177	1
Gm8731	1,431373326	1
Pgk1	1,430778158	1
Pla2g15	1,430381517	1
Cebpd	1,430381517	1
Zfp623	1,430084109	1
Gm6548	1,429984986	1
Syf2	1,429885871	1
1810044D09Rik	1,429885871	1
Gm12231	1,429588565	1
2310022A10Rik	1,429489477	1
Prdm10	1,429390396	1
Gm7832	1,429390396	1
Gm5881	1,429192254	1
Gm6368	1,429192254	1
Cish	1,428895093	1
E2f3	1,428796053	1
Spc24	1,428399961	1
Gm7936	1,428399961	1
Mob2	1,427707065	1
Bag3	1,427707065	1

Gm6640	1,427311276	1
Zfp90	1,426520026	1
Naip5	1,426124565	1
Psmc4	1,425432773	1
Pam16	1,425333973	1
1700001C19Rik	1,42523518	1
Ezr	1,424938841	1
Gm6851	1,424642565	1
Arl15	1,424247625	1
Gm11977	1,423458073	1
Hapln3	1,423260753	1
Chic2	1,422866196	1
Hist1h1a	1,422668959	1
Arhgef17	1,422175985	1
Gm8330	1,421683182	1
Nt5dc2	1,421289062	1
Gm42890	1,421190549	1
Mcts2	1,420796566	1
Depdc7	1,420205796	1
Gm30074	1,420205796	1
Top1mt	1,419812086	1
Creb3	1,419615272	1
Gorasp1	1,419615272	1
9930120I10Rik	1,419418485	1
Uqcrh-ps2	1,418631611	1
Gm16433	1,418533282	1
Borcs8	1,418140036	1
Mob3a	1,41705917	1
Cdan1	1,41705917	1
Spag7	1,41696095	1
Gm6807	1,416862738	1
Zwilch	1,416666332	1
Gm5599	1,416666332	1
Gm6140	1,416666332	1
Spata24	1,41656814	1
Rgs2	1,416175438	1
Gps2	1,415979128	1
Crnkl1	1,415390361	1
Stk17b	1,414997986	1
Ripk1	1,41450767	1
D030056L22Rik	1,414409628	1
Rchy1	1,414311592	1
Abhd6	1,41411554	1
Gm5944	1,41411554	1
Fau	1,414017524	1
Hyal1	1,413919516	1
4833417C18Rik	1,413527548	1
Cd200r3	1,412450198	1
Siva1	1,412352298	1
Coa6	1,412352298	1
Trmt10b	1,412254404	1
Steap3	1,412156518	1

Gm5735	1,411960765	1
Nkapl	1,411862899	1
Rab27a	1,411765039	1
4930440I19Rik	1,411765039	1
Npm3	1,411667186	1
Zfp729b	1,411373669	1
E2f7	1,411275843	1
Gm4859	1,411080213	1
Zfand2a	1,410884609	1
Chmp1b	1,410884609	1
Zkscan6	1,410200209	1
Mrs2	1,410004728	1
Blnk	1,409613846	1
Nkpd1	1,409613846	1
Snip1	1,409516142	1
RP23-440I21.3	1,409320755	1
Mxd1	1,408832406	1
Gm14336	1,408832406	1
Cd40	1,408149002	1
Panx1	1,407758635	1
Gm12254	1,40746593	1
AV356131	1,40746593	1
Zbtb11	1,407075752	1
Tns2	1,407075752	1
Tpst2	1,406978224	1
Slc35f5	1,406783189	1
Ddias	1,406588181	1
Celf5	1,406490687	1
Timm22	1,406198246	1
Kifc1	1,405613546	1
Ccng2	1,405613546	1
Phf20l1	1,405613546	1
Adrm1	1,405321287	1
March2	1,405029089	1
Gm4968	1,405029089	1
Lilrb4a	1,404931704	1
Tcte2	1,404834325	1
Gm8762	1,404834325	1
Rpl35a-ps4	1,404736952	1
C920009B18Rik	1,404639587	1
Tmem104	1,404444876	1
Gm37670	1,40434753	1
Spata6	1,404250192	1
Tgfbr1	1,404055535	1
Gm5787	1,404055535	1
Oprl1	1,404055535	1
Polm	1,403958217	1
Ubr7	1,403860905	1
Nars2	1,403374448	1
Taf5l	1,402790922	1
Als2cl	1,40240204	1
Sart1	1,40240204	1

Paqr3	1,402207639	1
Cenph	1,402110449	1
Sbk2	1,402110449	1
Gm11353	1,401818919	1
Mfsd13a	1,401721756	1
Gm13611	1,401721756	1
Nfkb2	1,401624599	1
Usp50	1,401624599	1
Gm42876	1,401527449	1
Med25	1,40123604	1
Rpl36a-ps2	1,400944691	1
Gm42479	1,400944691	1
Irx2	1,400653403	1
Pdss1	1,400556321	1
Cdk5r1	1,400362176	1
Ccdc167	1,400362176	1
Gm20604	1,399973967	1
Cenpk	1,399779903	1
Rpl31-ps17	1,399779903	1
Psmb2	1,399682881	1
Bloc1s1	1,399488857	1
Gm5384	1,39929486	1
Erich1	1,39910089	1
Shb	1,398906947	1
Dnajb6	1,398325279	1
Chst10	1,398228358	1
Gm5905	1,39784074	1
Gm10169	1,39784074	1
Eif3s6-ps1	1,39784074	1
Gm8318	1,39735637	1
4931406P16Rik	1,396968994	1
Gm12924	1,395033725	1
B930036N10Rik	1,394550327	1
Cndp2	1,394357015	1
Thap1	1,394163729	1
3830408C21Rik	1,394163729	1
Leo1	1,394067097	1
Gm5069	1,393970471	1
Gm2810	1,393873851	1
4930532G15Rik	1,393584034	1
Ercc1	1,393197704	1
Gm45212	1,393101138	1
Cbr2	1,393004579	1
Foxp4	1,393004579	1
Gm15964	1,392618409	1
Tex30	1,392328853	1
Kpna4	1,392328853	1
Snrpert	1,391653454	1
Gpsm1	1,391556996	1
Gm7160	1,391460544	1
Atg14	1,391364098	1
Selenok	1,391171228	1

Gm17259	1,391074802	1
Gm44178	1,390881972	1
Grpel2	1,390785566	1
Zfp472	1,390785566	1
Tmem251	1,390785566	1
Rpl18	1,390689168	1
Fhod1	1,390496391	1
Mettl16	1,39030364	1
Txnl4a	1,390207275	1
Mars2	1,390207275	1
Sort1	1,390207275	1
Lhpp	1,389821881	1
Gm7815	1,38972555	1
Napsa	1,389147699	1
Gm20274	1,388955136	1
Gad2	1,388858864	1
Socs3	1,388762599	1
Gm22513	1,388570089	1
Gm12743	1,388473844	1
Gm5139	1,388281374	1
Gm13890	1,388185149	1
Ccnj	1,387511761	1
Gm9803	1,387319424	1
Efemp2	1,387223266	1
Gm43430	1,387030969	1
Shpk	1,386934831	1
Gm12758	1,386838699	1
Tk1	1,386358141	1
Aloxe3	1,386262049	1
Gm37334	1,386262049	1
Nkrf	1,385877749	1
Sema4c	1,385877749	1
3110031N09Rik	1,385877749	1
Rbl2	1,385685639	1
Gpr85	1,385589594	1
Rpl31-ps1	1,385301498	1
Gm7128	1,384725487	1
Ctdspl2	1,384533537	1
Gm27029	1,384533537	1
Vps37d	1,384149716	1
Slfn8	1,38386192	1
Lzts3	1,383286508	1
Upp2	1,383190629	1
Crem	1,382615496	1
D8Ertd738e	1,382615496	1
Dnajb2	1,382519664	1
Rpl36al	1,382423838	1
Ankrd9	1,382328019	1
Dnttip2	1,382232207	1
Wdr78	1,381849024	1
Gm7638	1,381753244	1
Baz1a	1,381753244	1

Gm13328	1,381370194	1
Wbp4	1,381082976	1
Gm29155	1,381082976	1
Fam214b	1,380508719	1
Gm20620	1,380126014	1
Vps37c	1,380030354	1
Traf3	1,379934701	1
Gm8522	1,379839055	1
Xpo5	1,379839055	1
Card9	1,379839055	1
Gm13641	1,379552155	1
Gm6472	1,379456535	1
Rpl26	1,379456535	1
Zfp455	1,379360922	1
Usp33	1,379265315	1
Exosc3	1,379074122	1
2510046G10Rik	1,378882955	1
Gm11942	1,378596254	1
Gm11363	1,378118551	1
Gtf2f2	1,378118551	1
Rps19-ps6	1,377927517	1
Med10	1,37783201	1
D830050J10Rik	1,377641015	1
Tubgcp5	1,377641015	1
Ccdc50-ps	1,377545527	1
Gm8357	1,377545527	1
Gm16638	1,377545527	1
Ppp2r2d	1,377163643	1
Plekha7	1,377163643	1
Gm4963	1,377068189	1
A430105J06Rik	1,376781866	1
Gm15846	1,376114009	1
Top3a	1,376018627	1
Gm10036	1,376018627	1
Gm38375	1,375446476	1
Ghdc	1,375351141	1
Plp2	1,375255812	1
Aldh9a1	1,375255812	1
Gm29487	1,375255812	1
Zfp800	1,374588696	1
Gm13186	1,373921903	1
Gm6134	1,37373145	1
Gm12497	1,37373145	1
Hmmr	1,37344582	1
Ranbp6	1,37316025	1
Ip6k2	1,37316025	1
Etfbkmt	1,373065073	1
Gm43655	1,372779582	1
Tbc1d8b	1,372399019	1
Gm5867	1,372208778	1
Il4ra	1,372208778	1
Hmgb1-ps5	1,371923464	1

Neurl3	1,371638211	1
Gm8185	1,371543139	1
1700084E18Rik	1,371448074	1
Kcnd1	1,371448074	1
Ect2	1,371257964	1
Isoc1	1,371162919	1
Snrnp35	1,371067881	1
Nfkbiz	1,370972849	1
Rilpl1	1,370782805	1
Xkr8	1,370592787	1
Ttc30b	1,370592787	1
Uba7	1,370402796	1
Rbm34	1,370402796	1
Slc25a32	1,37030781	1
Gm9354	1,369548161	1
Gm20554	1,369358314	1
Nme4	1,369073594	1
Gm9320	1,3689787	1
Gss	1,3689787	1
Pnpt1	1,368694059	1
Gm27003	1,368694059	1
Fabp5	1,368219788	1
Jtb	1,367840489	1
Mrc1	1,367556084	1
C730034F03Rik	1,367461296	1
Zfp516	1,367366514	1
Dusp4	1,367366514	1
Rps12-ps9	1,367271739	1
Pdcd2l	1,366608495	1
Crk	1,366229643	1
Lrsam1	1,365945573	1
Gm11224	1,365945573	1
Cnot3	1,365756226	1
St3gal5	1,365566905	1
Psmb6-ps2	1,364620694	1
Bahd1	1,364620694	1
Zfp87	1,364431531	1
Midn	1,363958737	1
Taf1a	1,363769665	1
Rpl17	1,363769665	1
Rbm4b	1,363675139	1
Gm4895	1,36358062	1
Sh3bgrl2	1,36358062	1
Dnajc4	1,363486107	1
Snx13	1,363486107	1
Gm43149	1,3633916	1
Pkmyt1	1,3633916	1
Rpl36-ps10	1,363297101	1
Cep83	1,36301364	1
Polr2j	1,362730239	1
Naa20	1,362730239	1
Rps6-ps4	1,362635785	1

Hacd2	1,362635785	1
Gm11281	1,362541338	1
Ctr9	1,362352462	1
Gm5117	1,362258035	1
Gm14706	1,362163613	1
Gm8825	1,362163613	1
Smg8	1,361880389	1
Slc35a2	1,361691605	1
Oxr1	1,361691605	1
Pde12	1,361691605	1
Gm6542	1,361597223	1
Hoxa5	1,361502848	1
Gm7117	1,361502848	1
Rrm1	1,361502848	1
Mllt10	1,361219761	1
Gm6304	1,361219761	1
Acot2	1,361125411	1
Irak1bp1	1,361125411	1
Gm5547	1,360936732	1
Peg12	1,36074808	1
Rad54b	1,360653763	1
Ddx11	1,360559453	1
Kdm3a	1,360370852	1
BC037032	1,360370852	1
D230025D16Rik	1,360276562	1
Cul4b	1,359805208	1
Neurl1a	1,359805208	1
Batf2	1,359710956	1
Cyb561d1	1,359239798	1
Ankle2	1,359051381	1
Trp53bp2	1,358862989	1
Spata5l1	1,358862989	1
Krr1	1,358486285	1
Clspn	1,358392125	1
Lpar1	1,358392125	1
Glipr1	1,358297971	1
Vmac	1,358109684	1
Socs6	1,358109684	1
Setmar	1,358109684	1
Mogat1	1,357921423	1
Rpia	1,357921423	1
Smim3	1,357639081	1
Alox8	1,357639081	1
Ctdp1	1,357262716	1
Gm45109	1,357168641	1
Palld	1,35698051	1
Lonp2	1,356792406	1
Oaz1	1,356604327	1
Rbm41	1,356134246	1
Osm	1,355758298	1
Pate2	1,355382454	1
Serpinf1	1,355382454	1

Rpl18-ps2	1,355194571	1
Gm5745	1,355100639	1
Zfp263	1,355100639	1
Mapre2	1,355100639	1
Atp11a	1,355006714	1
Zfp874b	1,354631078	1
4932416K20Rik	1,354631078	1
Acot10	1,354631078	1
Ctsh	1,354631078	1
Anxa3	1,3544433	1
Tprn	1,35434942	1
RP24-550H10.6	1,35416168	1
Topors	1,35406782	1
Fam83a	1,353973967	1
Tldc1	1,35388012	1
Rpl21	1,35388012	1
Gm7670	1,353692445	1
Snrpc	1,353129577	1
Crkl	1,352942007	1
Islr2	1,352942007	1
Gm11808	1,352566943	1
Alkbh6	1,352285714	1
D730045B01Rik	1,352191984	1
Gm13392	1,352191984	1
Myg1	1,352004544	1
Slc2a4	1,352004544	1
Gm13422	1,351723431	1
Gm10602	1,35162974	1
Gm11560	1,35162974	1
Zfp948	1,351255041	1
Csrp1	1,351161382	1
Cyth1	1,350974085	1
Nmnat1	1,350505954	1
Tceal8	1,350412347	1
Rpl32-ps	1,350037985	1
BC055324	1,349944411	1
Dnajc30	1,349850843	1
Elmod2	1,349663727	1
Tmem176b	1,349663727	1
Dusp6	1,349383101	1
Rnf2	1,34919605	1
Plcl2	1,349102534	1
Npr1	1,349102534	1
Ssx2ip	1,349009025	1
Fosl1	1,349009025	1
Gm15464	1,348635052	1
2610306M01Rik	1,348261183	1
Mustn1	1,348261183	1
C130013H08Rik	1,348261183	1
Pomt2	1,348074287	1
2200002J24Rik	1,348074287	1
Shq1	1,347887417	1

Ythdc2	1,347793992	1
Traf3ip1	1,347793992	1
Phf8	1,347700573	1
Ccr1	1,347700573	1
Dynlrb1	1,347700573	1
Kti12	1,347420356	1
Gm12267	1,347140197	1
Rpl10-ps2	1,347140197	1
Trmt10c	1,346860096	1
Tax1bp3	1,346860096	1
Gm28530	1,346860096	1
Slc13a2	1,346860096	1
Rnf7	1,346673395	1
Fcf1	1,346673395	1
Gm14325	1,346486719	1
Gm45050	1,346486719	1
Gm10031	1,346393391	1
Spry2	1,346206754	1
Dcakd	1,346113445	1
Scn11a	1,345833558	1
Cldn15	1,345833558	1
Tnfsf8	1,345833558	1
Wisp1	1,345833558	1
Lrrc73	1,345460466	1
Gngt2	1,345273958	1
Rpp25l	1,345273958	1
Tgoln1	1,345273958	1
Rpl21-ps14	1,345180714	1
Traip	1,344994245	1
Eif1	1,344901021	1
Gm10250	1,344714591	1
Gm2383	1,344434994	1
4932441J04Rik	1,344248629	1
Mex3d	1,344155456	1
Gm37305	1,343875976	1
Rps19-ps11	1,343782828	1
Gm5054	1,343782828	1
Myh7b	1,343596554	1
Def8	1,343503426	1
Cbfa2t2	1,34331719	1
Rpl36-ps4	1,343130979	1
Smarcal1	1,343037884	1
Ssfa2	1,343037884	1
Esco2	1,342944795	1
Clk3	1,342944795	1
Phactr4	1,342851712	1
BC030867	1,342851712	1
AA465934	1,342665566	1
Gm15782	1,342665566	1
Snhg15	1,342665566	1
Lmo2	1,342014259	1
Gm6919	1,341921241	1

Sdr42e1	1,341828229	1
Gm11945	1,341456247	1
Pfdn6	1,341177328	1
Atf2	1,341177328	1
Fn1	1,340433827	1
Elf1	1,340248017	1
Tuba4a	1,340248017	1
Lpcat2	1,339969349	1
Uchl5	1,339969349	1
Nsa2	1,339783602	1
Ing2	1,339690739	1
Irf1	1,339226518	1
Atg12	1,339133693	1
Zfp626	1,338948063	1
Tcea1-ps1	1,338948063	1
Dis3	1,338576879	1
C1d	1,338576879	1
Zkscan5	1,338484099	1
Gm14240	1,338113044	1
Ccar1	1,338113044	1
Atp6v1g1	1,337927555	1
Aspscr1	1,337742091	1
Cnst	1,337649369	1
Ccdc59	1,337649369	1
Mycbp	1,337556654	1
Gm5909	1,337463945	1
Med19	1,337185856	1
Rpl31-ps13	1,337093172	1
Tfip11	1,337000495	1
Slc25a16	1,336907825	1
Nfe2l2	1,336537207	1
Gm13349	1,336444568	1
Gm9769	1,336444568	1
Nt5dc3	1,336166691	1
Mecp2	1,335981472	1
Wdfy2	1,335703691	1
Rpl18a-ps1	1,335611111	1
Abl1	1,335611111	1
Rpl21-ps1	1,335518536	1
Snapc1	1,335148303	1
Gm42600	1,335055761	1
Pcsk4	1,334870696	1
Coprs	1,334778173	1
Fn3k	1,334685656	1
Apoo	1,334593146	1
Sft2d1	1,334593146	1
Ablim1	1,334408145	1
Ss18l2	1,334315654	1
Zdhhc18	1,33422317	1
Ndufaf6	1,334130692	1
Bbc3	1,333853296	1
Pias2	1,333853296	1

Ifi213	1,333760843	1
Fez2	1,333760843	1
Hyal3	1,333668397	1
Unc13a	1,333575958	1
Lamtor3	1,333483524	1
Ccdc138	1,333206263	1
Il16	1,333113855	1
Rnf139	1,332929059	1
Zfp746	1,332836671	1
Zbtb18	1,332744289	1
Nfkbid	1,332744289	1
Prob1	1,332651913	1
Asns	1,332559544	1
Gapdh	1,332467181	1
Slc39a4	1,332467181	1
Tcf20	1,332374825	1
Gm13612	1,332190132	1
Alkbh4	1,331913139	1
Hgs	1,331636204	1
Crry-ps	1,331359327	1
Zfpm1	1,331359327	1
Tnfrsf18	1,331082508	1
Foxm1	1,331082508	1
Gm20072	1,330805746	1
Adgre1	1,330805746	1
RP23-354J5.3	1,330529041	1
9130008F23Rik	1,330436819	1
Gm8624	1,330436819	1
Snord59a	1,330160191	1
4930524J08Rik	1,330067995	1
Sap18b	1,329975805	1
Gm8659	1,329699273	1
Ext1	1,329607108	1
Cdkn2c	1,329607108	1
Rpl31-ps16	1,329422798	1
Gm7776	1,329146381	1
Tuba1c	1,329054255	1
Jmjd4	1,328962135	1
Map3k8	1,328962135	1
Zfp868	1,32859372	1
Ipo13	1,32859372	1
Serpinb6b	1,32859372	1
Paqr4	1,32840955	1
Hras	1,32840955	1
Smim13	1,328317475	1
Cdkn1a	1,328317475	1
Dmxl2	1,328225406	1
Nbr1	1,328041288	1
Clk4	1,327765158	1
Zfp599	1,327489086	1
Traf3ip2	1,327397075	1
Trp53	1,327397075	1

Tor1aip2	1,32730507	1
Mrpl9	1,327029093	1
Lin54	1,326753174	1
Clec12a	1,326753174	1
Mea1	1,326661214	1
Rras2	1,32656926	1
Supt4a	1,326477312	1
4930430F08Rik	1,32592576	1
Zdhhc24	1,325741961	1
Elac1	1,325741961	1
Pabpc4	1,325558187	1
Rpl41	1,325466309	1
Fam234b	1,325007017	1
Gm9143	1,325007017	1
Llph	1,325007017	1
Surf6	1,324915178	1
Gem	1,324915178	1
Tmem184b	1,324915178	1
Akna	1,324639698	1
Zfp65	1,324456077	1
Gm6395	1,324456077	1
Gm14126	1,324456077	1
Arl2bp	1,324364276	1
Timm8a1	1,324272481	1
Gm10616	1,32408891	1
Nme6	1,32408891	1
Slc8b1	1,323997135	1
Hcfc1r1	1,323813602	1
Gm4604	1,323721846	1
Hps6	1,323721846	1
C230096K16Rik	1,323538352	1
Pcnp	1,323446614	1
Zfp30	1,323446614	1
Rpl6l	1,323354883	1
Gm9703	1,323354883	1
Akirin1	1,323354883	1
Cd74	1,32317144	1
Gm15625	1,32280463	1
Wbp11	1,322621263	1
Mettl21b	1,32234626	1
Gm11450	1,321796426	1
Mnd1-ps	1,321704809	1
Gm9403	1,321521595	1
Foxj3	1,321521595	1
Gm42829	1,321429997	1
Prcc	1,321155242	1
Hint3	1,321155242	1
Mtmr14	1,32106367	1
Med4	1,32106367	1
Zfp296	1,320788991	1
Gm44013	1,320697444	1
Abcb7	1,320239804	1

Foxo3	1,319599375	1
Cmc4	1,319416453	1
Rassf3	1,319325001	1
Psd3	1,319233555	1
Cbx8	1,319142116	1
Tgm2	1,319050683	1
Gm38297	1,318959257	1
Usp1	1,318867837	1
Unc13d	1,318685016	1
Gm8724	1,318593614	1
Gas2l3	1,318593614	1
Got1	1,318593614	1
Nepro	1,318319449	1
Gm33080	1,318319449	1
Nudt16l1	1,318228073	1
Gm6136	1,318045341	1
Gm28438	1,317953984	1
Gm12481	1,31777129	1
Epha2	1,317679952	1
Ptpru	1,317497295	1
Ube2n	1,317405976	1
Vat1	1,317405976	1
Cxx1a	1,317314664	1
Gm15772	1,317040765	1
Cebpzos	1,316949477	1
Zfp667	1,316493137	1
Itga6	1,316128178	1
Zfp143	1,315580929	1
Mrps21	1,31530739	1
Hyls1	1,31530739	1
Eif1b	1,31530739	1
Zfp418	1,315216223	1
Nudt7	1,315125063	1
Pdgfa	1,315125063	1
Uhrf2	1,315125063	1
Hn1l	1,314122712	1
Gstt1	1,314031627	1
H1f0	1,314031627	1
Prss36	1,313940549	1
Fgd2	1,31375841	1
Rpl37a	1,31375841	1
Ppcs	1,313576297	1
Gm14277	1,31303011	1
Gm38305	1,31303011	1
Lockd	1,312939101	1
AW047730	1,312939101	1
Gm20257	1,312757101	1
Ccdc163	1,312757101	1
Gm15800	1,312666111	1
Plcd1	1,312666111	1
Srpr	1,312393178	1
Lancl2	1,312302213	1

Tbc1d8	1,312302213	1
Rhod	1,312211255	1
Stard3nl	1,312120302	1
BC005624	1,312120302	1
Ankrd35	1,311938416	1
Prkd2	1,311756556	1
Gm37009	1,311483812	1
Caml	1,31139291	1
Slc31a1	1,311029365	1
Itpkc	1,310756772	1
Zfp566	1,310756772	1
Cltb	1,310756772	1
Champ1	1,310756772	1
Zc3h12c	1,310665921	1
Tmub1	1,310484237	1
Traf2	1,310211757	1
Rps12-ps4	1,310120944	1
Zrsr1	1,310030136	1
Gpr137b	1,310030136	1
Uqcrh-ps1	1,309939335	1
Gm8550	1,30984854	1
Sdc3	1,30984854	1
Praf2	1,309757751	1
Tmem201	1,309303902	1
Pdia4	1,309122407	1
Ube2f	1,309122407	1
Cuta	1,308940937	1
Gm18737	1,308850211	1
Gm14494	1,308668778	1
Sgol1	1,308396677	1
Cbwd1	1,308305989	1
Fam13b	1,307852643	1
Siah1b	1,307580711	1
Gm15417	1,307580711	1
Snai2	1,307490079	1
Plekhh2	1,306946423	1
Gm13039	1,306855835	1
Usf2	1,306855835	1
Ier5	1,306855835	1
Mylip	1,306493548	1
Galnt15	1,306493548	1
Ubap2	1,306493548	1
Cul7	1,306402992	1
Ppia	1,306221898	1
Nsun6	1,306221898	1
A730011C13Rik	1,305950306	1
Gm16288	1,305678769	1
Foxd2os	1,305226334	1
Rpl5	1,305045403	1
Ubxn4	1,305045403	1
Gm12183	1,304954948	1
Gm13015	1,304593188	1

Ulk1	1,304593188	1
Kat5	1,304502763	1
Nsa2-ps2	1,304502763	1
Zfp867	1,304321933	1
Rnf34	1,304321933	1
Fam20c	1,304050735	1
Slc46a3	1,303960348	1
Naif1	1,303869968	1
E130311K13Rik	1,303779594	1
Rps17	1,303779594	1
Gm8869	1,303598864	1
Ndst1	1,303508509	1
Usp42	1,303508509	1
Lilr4b	1,303418159	1
Prpf4	1,303418159	1
Txlnb	1,303147149	1
Cnksr3	1,302876196	1
Zfp655	1,302876196	1
Ccnb1	1,302695591	1
Fbxo30	1,302695591	1
Smap2	1,302695591	1
Fam83d	1,302515012	1
Rffl	1,302515012	1
Zkscan14	1,302515012	1
Xylt2	1,302424731	1
Arhgap25	1,302424731	1
Zfp568	1,302334457	1
Sep 01	1,302153928	1
Gm11410	1,301973424	1
Gm6272	1,301883181	1
Polr3g	1,301792944	1
Nmrk1	1,301702714	1
Sec23b	1,301702714	1
Tmem158	1,301432061	1
Mfsd9	1,301251656	1
Eif4a2	1,301161464	1
Gm14681	1,300890923	1
Gm15198	1,300800755	1
Klf2	1,300620438	1
Ggps1	1,300440147	1
Erp27	1,299989526	1
Hoxaas3	1,299719229	1
Fanca	1,299539062	1
Zfp654	1,299539062	1
Grasp	1,299448988	1
Trit1	1,299448988	1
Mttp	1,299358921	1
Stk25	1,299268859	1
Rab33b	1,299268859	1
Gm12669	1,299088755	1
Hdac2	1,298908675	1
Nr2c2ap	1,298368586	1

Atp5g1	1,298278594	1
Fbxo9	1,298008652	1
Zfp932	1,297738767	1
Tbp	1,297468938	1
Gm12990	1,297109253	1
Crebrf	1,297019347	1
5730508B09Rik	1,296659787	1
Rap2b	1,296659787	1
AB124611	1,296569912	1
Eif2b1	1,296210477	1
Gm12468	1,295940965	1
Klk8	1,295851141	1
Mvk	1,29567151	1
3110009E18Rik	1,295491904	1
Tatdn2	1,295491904	1
Qars	1,295402111	1
Pgm1	1,295132768	1
Themis2	1,294953236	1
Snx33	1,29486348	1
RP23-316F10.2	1,29477373	1
Abhd10	1,294235361	1
Nop14	1,294145654	1
Csnk1e	1,294145654	1
Cdc7	1,293876572	1
1110059G10Rik	1,293876572	1
Mybpc3	1,293786891	1
Psmb6	1,293786891	1
Cebpa	1,293517883	1
Zfp53	1,293338576	1
P2ry2	1,293338576	1
Cyb561d2	1,292890417	1
2700062C07Rik	1,292532001	1
Mapre3	1,292263255	1
Tmem176a	1,292084121	1
Dpy30	1,292084121	1
Rps27a-ps1	1,291905013	1
Tnip3	1,291815468	1
Npm3-ps1	1,291725929	1
Mef2c	1,291725929	1
2010008C14Rik	1,291636397	1
Mir7078	1,291099332	1
Pirb	1,291099332	1
Gm12943	1,291009843	1
Zfp759	1,291009843	1
Rpl23a-ps5	1,29092036	1
Bzw1	1,290651949	1
Mrps36-ps1	1,290473039	1
Gnl2	1,290473039	1
Rfxap	1,290294153	1
Gm32856	1,290115293	1
Cks1brt	1,290025872	1
8430408G22Rik	1,290025872	1

Ythdf1	1,290025872	1
Gm10146	1,289757647	1
Trp53rkb	1,289668251	1
D17H6S53E	1,289310728	1
B4galt7	1,289310728	1
Chmp2b	1,289310728	1
Gm23935	1,288863965	1
Trim24	1,28877463	1
Strn4	1,288685303	1
Hspd1-ps3	1,288595981	1
Ogfr	1,288595981	1
Tax1bp1	1,288595981	1
Gm8770	1,288506665	1
Gm19898	1,288506665	1
Gemin2	1,288417356	1
Tusc2	1,288417356	1
Patl1	1,288238756	1
Ska1	1,288238756	1
Otub1	1,288149465	1
Gm26800	1,287970902	1
Tomm20	1,287970902	1
Per1	1,287970902	1
Tlk1	1,287703104	1
Gm11474	1,287524602	1
Gm20432	1,287435361	1
Gm6293	1,287435361	1
Mib2	1,287435361	1
Pinx1	1,287435361	1
Brix1	1,287435361	1
Tcn2	1,287078458	1
Dpf2	1,287078458	1
Pigo	1,286810845	1
Slx1b	1,286632468	1
Cysltr1	1,286632468	1
Prc1	1,286454115	1
Uhrf1	1,286364948	1
Fundc1	1,286275787	1
Deaf1	1,285919205	1
2410131K14Rik	1,285919205	1
Fam46c	1,285830075	1
Gm42972	1,285740951	1
Dlx1	1,285295424	1
Gm7535	1,285117257	1
Klf8	1,284939114	1
Fes	1,284939114	1
Gm8168	1,284850052	1
Atg101	1,284671946	1
Sdhaf2	1,284582903	1
Sf3a3	1,284493865	1
Gna11	1,28422679	1
Gm4204	1,28422679	1
Dcun1d4	1,284137777	1

Calr3	1,284048771	1
Traf4	1,28395977	1
Mob1b	1,28395977	1
Fyttd1	1,28395977	1
Tcf7l2	1,283870776	1
Ell	1,283870776	1
Grk4	1,283781788	1
Fmr1	1,283692806	1
Gm9774	1,283603831	1
Cby1	1,28333694	1
Wdr20	1,28333694	1
Epc2	1,28333694	1
Gm10842	1,283247989	1
Rcsd1	1,283159044	1
Gm43309	1,282892247	1
Rps2-ps10	1,282625505	1
Itpk1	1,282625505	1
Coro1a	1,282447707	1
BC031181	1,282092186	1
Zfpl1	1,282092186	1
Zcchc9	1,281914463	1
Gm15753	1,281736764	1
Fzd5	1,281736764	1
Gm5070	1,281470262	1
Nlgn2	1,281381441	1
Prosc	1,281381441	1
S100a3	1,281292625	1
Ppp1r16a	1,281203816	1
Rps10-ps4	1,281115013	1
Gm8662	1,281026216	1
Qtrtd1	1,280759861	1
Rps4x	1,280759861	1
Ginm1	1,280759861	1
Fem1b	1,280759861	1
Mrpl54	1,280582323	1
Cptp	1,280316061	1
Gm13862	1,280316061	1
Tdpx-ps1	1,280316061	1
Gm13341	1,279872414	1
Ifi27l2a	1,279428921	1
Prdm2	1,279428921	1
Dnaja2	1,279251567	1
Cebpz	1,279074237	1
Gm24276	1,278896932	1
Mb21d2	1,278808289	1
Lat	1,27863102	1
Rps19bp1	1,27863102	1
Cox6a2	1,278453777	1
Wdr83os	1,278365164	1
Ddx47	1,278365164	1
Zfp551	1,278099363	1
Erap1	1,278010775	1

Pkn2	1,278010775	1
Trp53rka	1,277922193	1
Rrm2	1,277922193	1
Pdxk	1,277922193	1
Hexdc	1,277745048	1
Gm44567	1,277479376	1
Rnf144b	1,277479376	1
Galnt3	1,277390831	1
Higd1a	1,277302292	1
Dhcr7	1,277036712	1
Esf1	1,277036712	1
Abcg1	1,276948198	1
Vdac3-ps1	1,276859689	1
Zfp84	1,276859689	1
Egfl8	1,276771187	1
Arl1	1,276682691	1
Arpp19	1,276682691	1
G6pc3	1,276594202	1
Cdc42ep3	1,27641724	1
Ccdc84	1,276240304	1
RP23-380K24.3	1,276151844	1
Gm4149	1,275886504	1
Bcl7a	1,275798069	1
Iqcf1	1,27570964	1
Phkg1	1,275621218	1
Rplp2	1,275532802	1
Arhgap1	1,275444392	1
Ubfd1	1,275444392	1
Gm9800	1,275355988	1
Il7r	1,275355988	1
Ankzf1	1,275179198	1
Elovl1	1,275002433	1
Cox7c	1,27491406	1
Dda1	1,27491406	1
Gm5801	1,274737331	1
Cldn11	1,274737331	1
Foxn3	1,274560627	1
Gtf2f1	1,274560627	1
Psmg4	1,274560627	1
Gm15903	1,274472285	1
Gm29650	1,274295617	1
1700123M08Rik	1,274295617	1
Pigl	1,274207293	1
Whrn	1,273942357	1
Lamc2	1,273765763	1
Tmub2	1,273589194	1
Cbr1	1,273589194	1
Gm10086	1,273589194	1
Metrnl	1,273589194	1
Mrpl34	1,273589194	1
Anp32a	1,273412649	1
Arpc1a	1,273324386	1

Slc39a2	1,273236129	1
Srl	1,272971394	1
Rps23	1,272971394	1
Kctd21	1,272883162	1
Eif4enif1	1,272794935	1
Gpkow	1,272618501	1
Znrd1	1,272530293	1
Taf4	1,272442091	1
Fancm	1,272353895	1
Rpl14-ps1	1,272089344	1
Ryr1	1,272001172	1
9530082P21Rik	1,271913007	1
Rpsa-ps1	1,271913007	1
Rnf32	1,271824848	1
Trim33	1,271824848	1
Rabggta	1,271648547	1
Mpdu1	1,271384143	1
Gm22516	1,27129602	1
Tbc1d20	1,271119794	1
Gm29170	1,271031689	1
Samd8	1,271031689	1
Ccsap	1,270943591	1
Gm16379	1,270943591	1
Naa30	1,270943591	1
Irf2bpl	1,270767413	1
D830044I16Rik	1,27059126	1
Nuak2	1,27059126	1
Pias1	1,27059126	1
Isl2	1,269974914	1
9330160F10Rik	1,269798871	1
Iqgap3	1,269710858	1
Gm8508	1,269622851	1
Gm8276	1,269622851	1
1110012L19Rik	1,269534851	1
Rbm38	1,269446857	1
Dbr1	1,269358868	1
5330438D12Rik	1,269006976	1
Rad51ap1	1,269006976	1
Gm37780	1,269006976	1
Rft1	1,268831066	1
Gm6028	1,268831066	1
Rab26os	1,268831066	1
Gsg1	1,268655181	1
Zswim3	1,268655181	1
Gm3355	1,268567248	1
Socs5	1,268479321	1
Fam76a	1,268303484	1
Dph3	1,268215575	1
Gm7634	1,268127672	1
Mllt1	1,268039775	1
BC030336	1,267864	1
Pcgf5	1,267776121	1

Taf8	1,267776121	1
Pld4	1,267688249	1
Psma4	1,267600382	1
Nsun3	1,267424668	1
Ahsa1	1,266985488	1
Rnf146	1,266985488	1
Hspa13	1,26654646	1
Lsm11	1,266458673	1
Abhd18	1,266370892	1
Fbxo45	1,266370892	1
Sertad3	1,266283117	1
B230322F03Rik	1,266019828	1
Magoh	1,265932077	1
Nsmce2	1,265844333	1
Eepd1	1,265844333	1
Snx8	1,265756594	1
Mboat7	1,265581135	1
Nfatc2	1,2654057	1
Fign	1,265317992	1
Larp7	1,265317992	1
Rprd1a	1,26523029	1
Tmem161a	1,265142594	1
Mvp	1,264791871	1
Uprt	1,264704205	1
Hyal2	1,264704205	1
Pla2g6	1,264704205	1
Gm12090	1,264704205	1
Agpat2	1,264616545	1
Epb41	1,264441244	1
Zfp738	1,264265968	1
Dnajc9	1,264178339	1
Tmem138	1,264178339	1
Sp140	1,264090716	1
Dlgap4	1,264003098	1
Tbc1d7	1,263740284	1
Gm45248	1,263652691	1
Rps27rt	1,263477524	1
Ubl7	1,263389949	1
Rnf103	1,263389949	1
Zfp639	1,263302381	1
Cnnm4	1,263214818	1
Gpr65	1,263214818	1
Haus6	1,263039712	1
Whamm	1,262952167	1
Cwf19l1	1,262602051	1
Zcwpw1	1,262514537	1
Slfn4	1,262339528	1
Gm11966	1,262164543	1
Sag	1,262077059	1
Mcm6	1,261989581	1
Gm13005	1,26190211	1
Got2-ps1	1,26190211	1

Mnat1	1,26190211	1
Trib3	1,261639732	1
4933440N22Rik	1,261552285	1
Gm18284	1,261464844	1
Zbtb41	1,261202557	1
Gm11956	1,260503392	1
Utp15	1,260503392	1
Slc25a44	1,260328661	1
Clec4e	1,260241305	1
Pprc1	1,260153954	1
Gm13886	1,26006661	1
Med18	1,26006661	1
RP23-110E20.5	1,259979272	1
Arhgap23	1,25989194	1
Dynll2	1,25989194	1
Coil	1,259280785	1
Arsk	1,259193501	1
Pisd	1,259193501	1
Arid5b	1,259106223	1
Ppp2r5e	1,259106223	1
RP23-114G13.7	1,259018952	1
Zbtb45	1,259018952	1
Asap1	1,259018952	1
Zfp574	1,259018952	1
Edc4	1,25849545	1
Ccdc117	1,25840822	1
Mrpl32	1,258320997	1
Cxx1b	1,25823378	1
Furin	1,25823378	1
Exoc6	1,258146569	1
Bet1	1,258059364	1
Mtl5	1,257972165	1
Mrpl22	1,257884972	1
Lrrc41	1,257797785	1
Snx2	1,257797785	1
Gm12732	1,257623429	1
Gm28557	1,257623429	1
Ptar1	1,25753626	1
Nr1d1	1,25753626	1
A130010J15Rik	1,25727479	1
Gm36378	1,257187646	1
Trmt12	1,256839128	1
Sap18	1,256577802	1
Cxcl16	1,256490706	1
2210008F06Rik	1,256403616	1
Snx20	1,256316532	1
Cmtr2	1,256142381	1
Gm10260	1,256055315	1
Fam105a	1,256055315	1
Crcp	1,255707111	1
Siah2	1,255359003	1
Ralb	1,255359003	1

Rilpl2	1,255359003	1
Fxn	1,255184985	1
Smad2	1,255184985	1
Pabpc1	1,255097986	1
Med6	1,255097986	1
Gm4866	1,255010992	1
Fanci	1,255010992	1
Hist1h1e	1,255010992	1
Mrpl47	1,254924004	1
Mtmr1	1,254837023	1
Erf	1,254750047	1
Mettl18	1,254663077	1
Sp4	1,254489156	1
Acsl3	1,254315259	1
Dcaf4	1,25422832	1
Nbn	1,254054459	1
Ing5	1,254054459	1
Sar1b	1,254054459	1
Smcr8	1,253967537	1
A330074K22Rik	1,253706809	1
Apol11b	1,253706809	1
Pfdn4	1,25353302	1
Slc25a3	1,253272383	1
Snrpg	1,253185515	1
Rab10os	1,253185515	1
Arid3b	1,253011799	1
9130011E15Rik	1,25292495	1
Ptgir	1,25292495	1
Mtmr3	1,252838107	1
Ppil4	1,252838107	1
Eif4e	1,252577614	1
Ube2h	1,252490794	1
Washc5	1,252317174	1
Mettl2	1,252143578	1
Ints8	1,252143578	1
Stk40	1,252056789	1
Elovl5	1,25188323	1
Atp7a	1,251796459	1
Lonrf1	1,251796459	1
Mtmr12	1,251709694	1
Lmbrd1	1,251536182	1
Dgke	1,251449435	1
Gm43578	1,251362694	1
Mcrs1	1,251275959	1
Gm26610	1,251189231	1
Tpd52l2	1,251189231	1
Tyms	1,251102508	1
Wdr48	1,251015791	1
Inpp4a	1,250842375	1
Mt1	1,250755677	1
Cep57	1,250582297	1
Gm9833	1,250322273	1

Rps26-ps1	1,250148953	1
Tmem64	1,250148953	1
Cnih1	1,249975658	1
Gm8805	1,249802386	1
Hjurp	1,249802386	1
Hscb	1,24971576	1
Nr4a1	1,249629139	1
Ube2o	1,249629139	1
Ptrhd1	1,249629139	1
Slc37a1	1,249542524	1
Rnf181	1,249542524	1
Diaph3	1,249369313	1
Rabl3	1,249369313	1
Aarsd1	1,249369313	1
Impdh2	1,249109541	1
Eloa	1,249022962	1
Slc30a1	1,249022962	1
Cyp51	1,24893639	1
Brf2	1,248849823	1
Mkln1os	1,248676708	1
6820402A03Rik	1,248676708	1
Emc7	1,248590159	1
Dab2	1,248590159	1
Stard4	1,248503616	1
Gm14270	1,24841708	1
8030462N17Rik	1,248070993	1
F7	1,248070993	1
Slc16a1	1,248070993	1
Gpbp1l1	1,247897985	1
Ing3	1,247897985	1
Rps16	1,247725002	1
Ypel5	1,247638519	1
Gm23054	1,247206196	1
Ric8b	1,247119749	1
Arrdc2	1,247033308	1
Ipo5	1,246860445	1
Rora	1,246774022	1
Phospho2	1,246687605	1
Gm16177	1,246601194	1
Mmgt1	1,246428391	1
Psmb5	1,24616923	1
Selenow	1,24616923	1
Gltscr2	1,245996486	1
Trappc1	1,245910123	1
Pim2	1,245823766	1
Mettl23	1,245737416	1
Zfp428	1,245564732	1
RP24-351I17.3	1,245564732	1
Zbtb2	1,245564732	1
Bag5	1,245564732	1
Bub1b	1,245564732	1
Ski	1,245564732	1

Dip2b	1,245133127	1
Snrnp27	1,245046824	1
Kctd18	1,244874235	1
Dusp9	1,24478795	1
Aph1a	1,244615398	1
Gm3617	1,244529131	1
Rnd1	1,24444287	1
Atn1	1,24444287	1
Gm6142	1,24444287	1
Casp6	1,244356614	1
Tceanc2	1,244356614	1
Hspa5	1,244356614	1
Blm	1,244184122	1
Rnf113a1	1,244184122	1
Abt1	1,244097885	1
Ptgs2	1,244097885	1
Asb11	1,243925428	1
Ddx42	1,243839209	1
Yif1b	1,243580586	1
Il12rb1	1,243408201	1
Gm5277	1,243322018	1
Taf13	1,24323584	1
Saraf	1,24323584	1
Dhx8	1,243149669	1
Psme1	1,242977344	1
Ccdc22	1,242805042	1
Dhrs7	1,242718901	1
Inpp5a	1,242718901	1
Tiprl	1,242718901	1
Cblb	1,242546635	1
Rdh1	1,242546635	1
Map4k3	1,242460511	1
Wdr37	1,242460511	1
Mapk1ip1l	1,242460511	1
Taf1d	1,242460511	1
Hexb	1,242374394	1
Gm26799	1,242288282	1
Mcoln2	1,242288282	1
2310068J16Rik	1,242202176	1
Cipc	1,242202176	1
Mad1l1	1,242116076	1
Stat2	1,242116076	1
Bcl2	1,242116076	1
Pfdn2	1,242116076	1
Slc16a10	1,242116076	1
Ccnk	1,242116076	1
Rxra	1,242029982	1
Gltp	1,242029982	1
Ifitm3	1,241943894	1
Arl8a	1,241685666	1
Ndufs2	1,241341445	1
Sap130	1,241083342	1

Gm12967	1,240911304	1
Uspl1	1,240911304	1
Adh7	1,240825293	1
Rnf11	1,240825293	1
Nudt9	1,240739289	1
Bloc1s4	1,240739289	1
Sep 08	1,240739289	1
Bcl2l2	1,240567298	1
Gm24951	1,240567298	1
Malsu1	1,240309356	1
Tmem50b	1,240223387	1
Micu3	1,239965517	1
Azin1	1,239965517	1
Trim47	1,239793633	1
Vezt	1,239793633	1
Gm9712	1,239621773	1
Ube2l6	1,239535852	1
Havcr2	1,239449937	1
Gm2272	1,239278124	1
Gm9701	1,239278124	1
Ccdc15	1,239020451	1
Gm14813	1,239020451	1
Gm8268	1,238934571	1
Kctd10	1,238848698	1
Fbxw11	1,238505264	1
Egln3	1,23824775	1
Amhr2	1,23824775	1
Ap3s2	1,23824775	1
Ric1	1,237990291	1
Tsc22d1	1,237904483	1
Cables2	1,237647095	1
Gm11334	1,23756131	1
Tspyl1	1,23756131	1
Msrb1	1,23756131	1
Rgs20	1,237475532	1
Dad1	1,237218233	1
Zxdc	1,237132479	1
Gm2467	1,236960988	1
Tgfb1	1,236703796	1
Hoxb8	1,236446657	1
Ccdc17	1,236275261	1
Rpl38-ps2	1,235846875	1
Tmem129	1,235333007	1
4931440P22Rik	1,235247384	1
Stk11ip	1,235161766	1
Sik2	1,234990548	1
Tsr2	1,234990548	1
Vcpip1	1,234904948	1
Gtf2h5	1,234477037	1
Aph1b	1,234391472	1
Kpna3	1,234391472	1
0610012G03Rik	1,234305914	1

Gorab	1,234220361	1
Slc35e4	1,234220361	1
Car11	1,234049273	1
Vcpkmt	1,23387821	1
Tmem8	1,233792687	1
Cd302	1,233792687	1
Mcl1	1,233792687	1
Frat2	1,23370717	1
Scyl2	1,233621659	1
Gm4943	1,233450654	1
4933421O10Rik	1,233279674	1
Rpf1	1,233279674	1
Fbxo5	1,233194192	1
Vps33a	1,233194192	1
2610203C22Rik	1,233023247	1
Dpp9	1,233023247	1
Aqr	1,232852325	1
Zfp346	1,232766874	1
Mss51	1,232681428	1
Polg2	1,232681428	1
Ift57	1,232595988	1
Prpf3	1,232510554	1
Eif2s3x	1,232425125	1
Arl11	1,232425125	1
Rccd1	1,232339703	1
Yrdc	1,232339703	1
Mrps23	1,232168876	1
Gm6808	1,232083472	1
Dhx38	1,231912681	1
Poli	1,231741913	1
Utp18	1,231741913	1
Gm12350	1,231656538	1
Eda2r	1,231571169	1
RP23-403E19.1	1,231400449	1
Mfsd11	1,231400449	1
Vma21	1,231229753	1
Osgin2	1,231144413	1
Hgsnat	1,230888431	1
Ogdh	1,230803115	1
Zfp760	1,230632501	1
Gm12396	1,230632501	1
Pyurf	1,230547203	1
Gm13868	1,230376625	1
Skp2	1,230376625	1
Txnrd2	1,230291345	1
Fosb	1,23020607	1
Mzt1	1,23020607	1
Mrip-ps	1,230120802	1
Murc	1,230035539	1
Ercc6l2	1,229950283	1
Pgpep1	1,229865032	1
Rpl10a-ps1	1,229865032	1

Tsc22d2	1,229609315	1
Sap30l	1,229098042	1
Rrn3	1,228927664	1
Bbof1	1,228842484	1
Ndrg1	1,228842484	1
Asnsd1	1,228842484	1
Med8	1,228757311	1
Prune2	1,22858698	1
Dpep2	1,228501824	1
Lpxn	1,228416674	1
Klhl35	1,22833153	1
Rab3a	1,228246391	1
RP23-149L23.1	1,228076132	1
Etv5	1,227991011	1
Pik3c3	1,227820787	1
Rbmxl1	1,227820787	1
Akip1	1,227650587	1
Gm5845	1,227565495	1
Gdap2	1,227310257	1
4932422M17Rik	1,227140127	1
Daam1	1,227140127	1
Prpsap2	1,227055071	1
H2afz	1,226799939	1
Nemp1	1,226714907	1
Flad1	1,22662988	1
Gm27605	1,22654486	1
Orc6	1,22654486	1
Tpgs2	1,22654486	1
Kdm3b	1,22654486	1
Eif3l	1,226374836	1
2810408I11Rik	1,226289833	1
Pycrl	1,226289833	1
Stam2	1,226289833	1
Prkab1	1,226204836	1
Bnip3l	1,226204836	1
Eif4b	1,22603486	1
Mipep	1,225949881	1
Fiz1	1,22577994	1
Ick	1,225694978	1
Serf2	1,225610022	1
Zfp974	1,225270258	1
Inafm1	1,225270258	1
Gm7860	1,224930588	1
Gm3550	1,224845685	1
Mapk1ip1	1,224845685	1
Sco1	1,224506133	1
Myo19	1,22442126	1
Tdp2	1,22442126	1
Hilpda	1,224336392	1
Zfp212	1,224251531	1
Nat6	1,224251531	1
Gsk3a	1,224251531	1

Hbp1	1,224251531	1
Pcnx4	1,223912143	1
Spire1	1,223827311	1
Foxj2	1,223827311	1
9330159M07Rik	1,223742485	1
Zfyve27	1,223742485	1
Nhlrc3	1,223742485	1
Tuba1a	1,223403238	1
Naa60	1,223403238	1
Stxbp3	1,223148865	1
Mcm4	1,223148865	1
Setd4	1,223064086	1
Gm43524	1,222894545	1
Sp3	1,222894545	1
Dpp7	1,222809783	1
Polq	1,222640278	1
Rpap3	1,222301337	1
Tmem248	1,222301337	1
Zfp386	1,222047193	1
Rpl23a	1,222047193	1
Gm37274	1,221877793	1
Gm15459	1,221877793	1
Gins2	1,221454396	1
Zfp235	1,221369734	1
Rps12	1,221115784	1
B4galt3	1,221031145	1
Tra2b	1,221031145	1
Gm3375	1,220946513	1
Klhl41	1,220861886	1
Med14	1,220861886	1
Cep44	1,220861886	1
Zfr	1,220861886	1
Ptprc	1,220777265	1
Bcl2l12	1,22069265	1
Wdr83	1,220438841	1
Gm6204	1,220354249	1
Abca7	1,220269664	1
Ndrg3	1,220185084	1
Vps72	1,220185084	1
Gm6560	1,22010051	1
March5	1,220015942	1
Elf2	1,219762273	1
Rai1	1,21959319	1
Ndc1	1,21959319	1
Adam9	1,219508657	1
Ssh2	1,219339609	1
Itpa	1,219170585	1
Golga3	1,219086081	1
Zyx	1,219001583	1
Bcas2	1,218917092	1
Pdxdc1	1,218917092	1
Taf3	1,218832606	1

Phc1	1,218748126	1
Mta3	1,218748126	1
Gm16104	1,218748126	1
Isca1	1,218663651	1
Pdgfb	1,218325813	1
Gm10076	1,218325813	1
Pigm	1,218156929	1
Gm11478	1,218072495	1
Eno1b	1,217650417	1
Gabarapl1	1,217650417	1
Gm9008	1,217566019	1
Thyn1	1,217566019	1
Gm9013	1,21739724	1
Eps8	1,21739724	1
Rfc3	1,217312859	1
Egr2	1,217228484	1
Ccdc93	1,217228484	1
Tmem126a	1,217059752	1
Amfr	1,217059752	1
Api5	1,217059752	1
Dnaaf3	1,216638025	1
Rcc1l	1,216385058	1
Sp2	1,216300748	1
Sccpdh	1,216300748	1
Supv3l1	1,216300748	1
Plppr2	1,216216443	1
Ccng1	1,216047852	1
Zfp248	1,215963564	1
Mier1	1,215879283	1
Xpnpep1	1,215710738	1
Dip2c	1,215626474	1
Acadsb	1,215626474	1
Coq8a	1,215626474	1
Cmbl	1,215542217	1
Leprot	1,215289478	1
Tmed7	1,215121015	1
Bend3	1,214952575	1
Armc7	1,214868364	1
AW549877	1,214784158	1
Rab6a	1,214784158	1
Sub1	1,214784158	1
Asxl1	1,214699959	1
Wipi2	1,214615765	1
Map1lc3b	1,214615765	1
Usp53	1,214531577	1
Pi4kb	1,214447395	1
Morc3	1,214447395	1
Ugp2	1,214363219	1
D130017N08Rik	1,214279049	1
Slmap	1,214194884	1
Aktip	1,214194884	1
Gm14537	1,214110726	1

Sart3	1,214026573	1
Nckipsd	1,213858285	1
Nacc1	1,213774149	1
Naa10	1,213605896	1
Galc	1,213605896	1
Fam204a	1,213521778	1
Prpf38a	1,213521778	1
Mknk1	1,213437666	1
Wdr82	1,213437666	1
Gm6085	1,213437666	1
Asrgl1	1,21335356	1
H2-K2	1,21326946	1
Ccdc116	1,21326946	1
Vprbp	1,21326946	1
Bdp1	1,21326946	1
Higd2a	1,213185365	1
Ikzf5	1,213101277	1
Card14	1,21276498	1
Gm12034	1,21276498	1
Wasl	1,21276498	1
Cebpb	1,212596867	1
Toe1	1,212596867	1
St6galnac4	1,212512819	1
Sgpl1	1,212512819	1
Gm13450	1,212428777	1
Pot1a	1,212428777	1
Gtf2e1	1,212176686	1
Cdkl3	1,212092667	1
Rab1a	1,212008654	1
Thap7	1,211924647	1
Tpst1	1,21175665	1
2510016D11Rik	1,211504699	1
Socs4	1,211504699	1
Gng2	1,211504699	1
1110025M09Rik	1,211420726	1
Ccnh	1,21133676	1
Snx12	1,211252799	1
Smpd1	1,210917015	1
Usp2	1,210833084	1
Fam53c	1,210749158	1
2810001G20Rik	1,210665238	1
Rnf113a2	1,210581324	1
Gm5297	1,210581324	1
Pim3	1,210497416	1
Ddb2	1,210329617	1
Usp39	1,210329617	1
Gm37760	1,210245727	1
Crls1	1,210245727	1
Psmd8	1,210245727	1
Rps28	1,210161842	1
H2-Q7	1,210077963	1
Sdf2l1	1,209994089	1

Hnrnph1	1,209994089	1
Mitd1	1,209994089	1
Aggf1	1,209742504	1
Fkbp7	1,209490971	1
Tmx4	1,209323312	1
Denr	1,209239491	1
Smg5	1,209155676	1
Mtor	1,209071866	1
Rnf26	1,208904265	1
Ecm1	1,208904265	1
Gm12005	1,208652907	1
Mrpl49	1,208150347	1
Ndufaf5	1,208066607	1
Tmod3	1,207982873	1
3110056K07Rik	1,207899145	1
Zfp384	1,207899145	1
Pacs1	1,207899145	1
Gpat4	1,207899145	1
Oard1	1,207815423	1
Ifngr2	1,207815423	1
Cdkn2aip	1,207815423	1
Gm1976	1,207564291	1
Gtf3c5	1,207396898	1
Gm8451	1,207062183	1
Crbn	1,207062183	1
Guk1	1,206727561	1
Actr10	1,206727561	1
Fam46a	1,20664392	1
Rpl9	1,20664392	1
Chd1	1,20664392	1
Ube2w	1,206476655	1
Abi2	1,206393031	1
Spns1	1,206309413	1
Zfp706	1,206309413	1
Aven	1,206142195	1
6430571L13Rik	1,206142195	1
Rdh14	1,206142195	1
Incenp	1,206058594	1
Timp1	1,205975	1
Copb1	1,205975	1
Hdac6	1,205891411	1
Piga	1,205891411	1
Hmgcs1	1,205807828	1
Ceacam16	1,20572425	1
4833445I07Rik	1,205640679	1
Zc3h10	1,205473553	1
Apmap	1,205306451	1
Dennd5b	1,205222908	1
Nrf1	1,20505584	1
Pdp1	1,204888796	1
Mcee	1,204888796	1
Med23	1,204721774	1

Ppp1r18os	1,204638272	1
Rtf1	1,204638272	1
Kcne3	1,204554776	1
Klhl6	1,204554776	1
Gm6023	1,204471285	1
Cox5b	1,204387801	1
Txnl1	1,204137381	1
Swt1	1,20405392	1
Nudc-ps1	1,203970464	1
Zxdb	1,203970464	1
Rps13	1,203970464	1
Gm4366	1,203720131	1
Skiv2l	1,203636699	1
Ighmbp2	1,203386436	1
2900060B14Rik	1,203303026	1
Wdcp	1,203136224	1
Rnf216	1,203136224	1
Polr2i	1,203136224	1
Nup50	1,203052832	1
Slirp	1,203052832	1
Cln8	1,202719321	1
Hpcal1	1,202635958	1
Top1	1,202469249	1
Rpl37rt	1,202385903	1
Zfp281	1,202302563	1
Dram1	1,202219228	1
Atp5g2	1,20196926	1
Nostrin	1,20196926	1
Calm1	1,201719343	1
Erh	1,20163605	1
Spsb1	1,201552761	1
Ube2v2	1,201386202	1
Srp9	1,201386202	1
Aldh6a1	1,201302931	1
RP23-371B13.3	1,201302931	1
Dhx57	1,201053154	1
Tbc1d22b	1,201053154	1
Dok2	1,200886664	1
Tmem131	1,200803427	1
Arhgap27os2	1,200803427	1
Flt3l	1,200803427	1
Gm12389	1,200720197	1
Pax3	1,200720197	1
Rpsa	1,200720197	1
Bcl7b	1,200636972	1
Gm9173	1,200387333	1
Syt11	1,200387333	1
Caap1	1,200304131	1
Rbm14	1,200137745	1
Cops9	1,200137745	1
Dgat2	1,199971382	1
Ilf3	1,19988821	1

Ttyh2	1,19988821	1
Irgq	1,199805043	1
Evi5l	1,199721881	1
Lig1	1,199721881	1
E030030I06Rik	1,199638726	1
Prdx4	1,199638726	1
Ccl3	1,199472432	1
Fdxacb1	1,199389294	1
Usp30	1,199389294	1
Larp4	1,199389294	1
Hdac5	1,199306161	1
Stab1	1,199223035	1
Ephx1	1,199139914	1
Ahcy	1,198973689	1
Snord104	1,198973689	1
Nelfe	1,198890586	1
Gm17251	1,198807488	1
Cep70	1,198724396	1
Igbp1	1,198724396	1
Cracr2b	1,198558229	1
Map1lc3a	1,198475154	1
Reps1	1,198392085	1
RP23-454I20.1	1,198309021	1
Fam212a	1,198142912	1
Gm38257	1,198142912	1
Agtrap	1,198142912	1
Gm10689	1,198142912	1
Gspt1	1,198142912	1
Pxmp2	1,198059866	1
Ptcd1	1,198059866	1
Trappc8	1,197976826	1
Cox14	1,197976826	1
Rpl22-ps1	1,197893791	1
Rpl13	1,197810762	1
Clu	1,197810762	1
Ddx27	1,197810762	1
Gm7666	1,19756171	1
AI597479	1,197478705	1
Hif1a	1,197395705	1
Sco2	1,19731271	1
Gm11889	1,197146739	1
Arhgap22	1,197063762	1
Ctps2	1,196980791	1
Gm13092	1,196897825	1
Mif	1,196897825	1
Cops8	1,196897825	1
Pag1	1,196814866	1
Tjp2	1,196731911	1
Xrcc6	1,196648963	1
Cmtm6	1,196648963	1
Atp5j	1,196566021	1
Ccdc43	1,196483084	1

Brap	1,196317228	1
Micall2	1,196317228	1
Cops2	1,196317228	1
Gm5257	1,196151394	1
Ubald1	1,196151394	1
Zfp59	1,196068486	1
Gnpda2	1,195819797	1
1600012H06Rik	1,195819797	1
Tiparp	1,195736912	1
Rpp40	1,195654032	1
Fam174a	1,195654032	1
Aip	1,195488291	1
Pola2	1,195405429	1
Gm12966	1,195322573	1
Mgat2	1,195239722	1
Kdm6a	1,195156877	1
Ccr2	1,194908377	1
Rc3h1	1,194908377	1
Gm6305	1,194742739	1
H1fx	1,194742739	1
Fut11	1,194742739	1
Sprvd7	1,194742739	1
Ubap1	1,194742739	1
Fpgs	1,194659929	1
Adprhl2	1,194659929	1
Gm7380	1,194494325	1
RP23-184H3.5	1,194328745	1
Rps27l	1,194328745	1
Gng12	1,194328745	1
Anp32b-ps1	1,194245963	1
Ndel1	1,194163187	1
Kdm1a	1,193914894	1
Zfp131	1,193914894	1
Insl6	1,193749393	1
Zc3hc1	1,193749393	1
Rsl1	1,193666652	1
Mrpl23	1,193583916	1
Pbdc1	1,193583916	1
Lbr	1,19325303	1
Ppih	1,193087622	1
Tbpl1	1,192922236	1
Sesn1	1,192839552	1
Ube2g1	1,192508872	1
Mrpl55	1,192426217	1
Gpbp1	1,192426217	1
Prox2	1,192178284	1
Adarb1	1,192095652	1
Map3k1	1,191847788	1
Ciao1	1,191765179	1
Gm24920	1,191352216	1
Rubcn	1,191352216	1
Zbtb7b	1,191352216	1

Mdrl	1,19126964	1
Nudt19	1,19126964	1
Ppp4r3a	1,191187071	1
Babam1	1,191021949	1
Cct5	1,191021949	1
Gm11516	1,190939396	1
Gm5445	1,190774308	1
Npat	1,190609243	1
Eif4e2	1,190609243	1
Vamp3	1,190361689	1
Gm28417	1,190279182	1
Zfp949	1,190279182	1
Zfp622	1,190196681	1
Sugt1	1,190031696	1
Btaf1	1,189866734	1
Ap4m1	1,189866734	1
Zfp91	1,189784262	1
Ttk	1,189701795	1
Gm7102	1,189371986	1
C3	1,189371986	1
Rpl18-ps1	1,189371986	1
A730071L15Rik	1,189289547	1
Surf1	1,189289547	1
Abca1	1,189207115	1
Lrrc57	1,189124688	1
Lrrc28	1,189042267	1
Gm43668	1,188959852	1
Exoc3l4	1,188959852	1
B230219D22Rik	1,188959852	1
Spink10	1,188877442	1
Hsdl1	1,18871264	1
Gm8618	1,188547861	1
Uhmk1	1,188547861	1
Arf6	1,188547861	1
Dennd3	1,188053661	1
Ifi204	1,188053661	1
Tcp1	1,188053661	1
Asf1a	1,187971314	1
Sirpa	1,187724308	1
Mrps31	1,187559666	1
Gpr132	1,187477354	1
RP23-225D5.4	1,187477354	1
Phax	1,187312746	1
Sec62	1,187312746	1
Rps8-ps4	1,187065877	1
Ercc4	1,187065877	1
Rasgrp4	1,186983598	1
Rassf2	1,186901326	1
0610009O20Rik	1,186819059	1
Gm37125	1,186736798	1
Gins4	1,186736798	1
Zfp511	1,186490048	1

Mthfr	1,18640781	1
Cenpe	1,18640781	1
Basp1	1,186325577	1
Psen1	1,186325577	1
Cpt1c	1,186161129	1
Galnt4	1,186078913	1
Cacybp	1,186078913	1
Gm8494	1,185914499	1
Gm45630	1,185914499	1
Rpl10-ps3	1,185667921	1
Sdccag3	1,185667921	1
Ccdc63	1,18558574	1
Naa16	1,18558574	1
Ifih1	1,185421394	1
Cdca5	1,18533923	1
Zrsr2	1,18533923	1
Cript	1,185174918	1
Snapc5	1,185010629	1
Aldh2	1,184928494	1
Birc2	1,184846363	1
Dennd4a	1,184846363	1
Polr3d	1,184600007	1
Rpl9-ps6	1,184600007	1
Rapgef5	1,1845179	1
Nsmce3	1,184271612	1
Ap1s3	1,184189527	1
Ltv1	1,184107448	1
Gm5879	1,183861246	1
Ppp2r1a	1,183615094	1
Trmt61a	1,183533055	1
Ppig	1,183368994	1
Il15	1,183286972	1
Eva1b	1,183204956	1
Gm15007	1,183122945	1
Ncapg2	1,18304094	1
Arel1	1,18304094	1
Polr1d	1,182958941	1
Tmed8	1,182794959	1
Clec4d	1,182712977	1
Acap1	1,182631	1
Slamf9	1,182549029	1
Ccm2	1,182549029	1
Thnsl1	1,182467064	1
Dhrs4	1,182385105	1
Ttc7b	1,182385105	1
Sdhaf1	1,182385105	1
Cnot9	1,182303151	1
Cdadc1	1,182221203	1
Prkd3	1,182221203	1
Golm1	1,182221203	1
Ndufb11	1,18213926	1
Ambra1	1,182057323	1

Bap1	1,181975392	1
Mr1	1,181893467	1
Zfp35	1,181729633	1
Mettl13	1,181729633	1
Cd164	1,181729633	1
Kbtbd3	1,181647724	1
B3gat3	1,181565822	1
Msl2	1,181565822	1
Endov	1,181483925	1
Gm44283	1,181483925	1
Psmd12	1,181483925	1
Exoc3	1,181402033	1
RP23-13B8.12	1,181238267	1
Srp19	1,181238267	1
Banp	1,181156393	1
Pwp1	1,181156393	1
H2afv	1,181156393	1
Urm1	1,181074524	1
Xpa	1,180992661	1
Adprm	1,180992661	1
D130051D11Rik	1,180583431	1
Ubald2	1,180583431	1
Scaf4	1,180419779	1
Phlpp1	1,180337962	1
Cenpo	1,18025615	1
Gnal	1,180092543	1
Gm13456	1,180092543	1
Gm6419	1,180092543	1
Por	1,180092543	1
Bud13	1,180010748	1
Sat1	1,180010748	1
Clcn6	1,179928958	1
Arl13b	1,179847175	1
Cep76	1,179765397	1
Gm16973	1,179683625	1
Kif14	1,179601858	1
Ndc80	1,179356592	1
Timm17a	1,179356592	1
Pex26	1,179029651	1
Rap2a	1,178947929	1
Cd274	1,178866214	1
Rpp30	1,178866214	1
Acadm	1,178866214	1
Cmss1	1,178621101	1
Slc6a8	1,178621101	1
Mycn	1,178621101	1
Adap1	1,178621101	1
Kctd5	1,178457721	1
Gm11539	1,178457721	1
Chaf1a	1,178294363	1
Orai1	1,178049369	1
Gm15730	1,177967716	1

Sqstm1	1,177886068	1
Sin3b	1,177804426	1
Rab22a	1,17772279	1
Gnl3	1,177641159	1
Gm7589	1,177396301	1
Bccip	1,177314693	1
Gm10923	1,177069902	1
Gm18913	1,177069902	1
Map2k7	1,177069902	1
Gm6563	1,176988317	1
Gm13803	1,176825163	1
Rbm47	1,176825163	1
Rpl13a-ps1	1,176662032	1
Ethe1	1,176580475	1
Gpank1	1,176417377	1
Rbm8a	1,176417377	1
Mrrf	1,176335837	1
Cog2	1,176335837	1
Tmem242	1,176172774	1
Usf1	1,17609125	1
Gm14673	1,176009733	1
Tnfsf12	1,176009733	1
Ctbp2	1,176009733	1
Tmem185a	1,175846714	1
Gm7236	1,175683719	1
Rnf38	1,175683719	1
Gm12428	1,175602229	1
Ss18l1	1,175602229	1
Nabp2	1,175602229	1
Klf3	1,175520746	1
Tjap1	1,175520746	1
Eif3m	1,175276328	1
Rab3ip	1,174869079	1
Gtpbp4	1,174787646	1
Adck2	1,174706219	1
Filip1l	1,174624798	1
Xkr5	1,174624798	1
Efhd2	1,174299167	1
Sirt1	1,174217774	1
Mri1	1,174055004	1
Etf1	1,174055004	1
Ddx31	1,173973628	1
Timm50	1,173485487	1
Cntd1	1,173322819	1
Acbd5	1,173160173	1
Spcs1	1,173078859	1
Cmtm4	1,17299755	1
Mir5128	1,172916247	1
Asb6	1,172916247	1
Tmx2	1,172834949	1
Akap1	1,172672371	1
Ubc	1,172509815	1

Usp22	1,172347282	1
Prpf6	1,172347282	1
Gm22009	1,172184772	1
Fbxo31	1,172184772	1
Pip4k2b	1,172103525	1
Arsb	1,171941048	1
Chrac1	1,171859818	1
Kctd20	1,171697375	1
Vac14	1,171616162	1
Tnfrsf26	1,171616162	1
Aasdhppt	1,171453753	1
Shoc2	1,171372557	1
Prelid1	1,171372557	1
Bc1-ps1	1,171291366	1
Anapc2	1,171129002	1
Ifitm2	1,171129002	1
Camk2n1	1,17096666	1
Gm10642	1,17096666	1
Phf12	1,170885498	1
Ccnl1	1,170885498	1
Smc4	1,170804341	1
Tnks2	1,170804341	1
Ddx3x	1,170804341	1
Rab3il1	1,17072319	1
Man1a	1,170642044	1
9330104G04Rik	1,170560904	1
Nudt22	1,170560904	1
Ap3d1	1,170560904	1
Cep135	1,17047977	1
Zfp786	1,17047977	1
Pigyl	1,17047977	1
Zscan21	1,170398641	1
Zfp11	1,170398641	1
Aldh3a2	1,170398641	1
Gm26244	1,170317518	1
Gm16286	1,170317518	1
Capn15	1,170236401	1
Parp9	1,170155289	1
Ostm1	1,170155289	1
Ube2j2	1,170155289	1
Zbtb21	1,170074183	1
Aim1l	1,169993082	1
Edrf1	1,169993082	1
Gatad2a	1,169993082	1
Snora30	1,169911988	1
Gm12816	1,169830898	1
Ywhag	1,169830898	1
Gm8730	1,169668737	1
Ppfia1	1,16934448	1
Blzf1	1,16926343	1
Hspe1	1,169182386	1
Pmvk	1,169101347	1

Ccdc126	1,168939287	1
Nlrx1	1,168939287	1
Hmx3	1,168777249	1
Apitd1	1,168777249	1
Rgcc	1,168615233	1
Rps15a	1,168615233	1
Nkiras2	1,16845324	1
Rplp1-ps1	1,168372251	1
Rad18	1,168372251	1
Fcgr3	1,168210292	1
Cbll1	1,168048355	1
Dlgap5	1,167967395	1
Zfp952	1,167967395	1
Gm6222	1,16788644	1
Zfand6	1,16788644	1
Mnt	1,167805491	1
Zbtb8a	1,16764361	1
Uqcc2	1,167481752	1
Dkc1	1,166915423	1
Bub1	1,166834542	1
Hdac3	1,166511071	1
Cmpk1	1,166511071	1
Prkx	1,16634937	1
Psat1	1,16634937	1
Klf13	1,166268527	1
Prcp	1,166187691	1
Angpt2	1,166026034	1
Itgb1bp1	1,1658644	1
Fads3	1,165702788	1
Zfp850	1,16562199	1
Bud31	1,165541198	1
Gm45422	1,165460412	1
Suz12	1,165460412	1
Vamp8	1,165379631	1
Cenpw	1,165298856	1
Brdt	1,165218086	1
Nsrp1	1,165137322	1
Tgds	1,165056564	1
Exosc9	1,164895064	1
Zfp787	1,164814322	1
Rap2c	1,164814322	1
Mtf1	1,164652856	1
Utp14a	1,164491412	1
Rps11-ps3	1,164329991	1
AA986860	1,164249288	1
Fbxo38	1,164168591	1
Sppl3	1,1640879	1
Tox2	1,163926534	1
Kif3a	1,163926534	1
Tspan14	1,16384586	1
Gm44103	1,163684528	1
Rps21	1,163442572	1

Napg	1,163281295	1
RP23-70B19.5	1,163200666	1
Rdh11	1,163200666	1
Fpgt	1,163039423	1
Ttf1	1,162797601	1
Cdc27	1,162797601	1
Trappc10	1,162797601	1
Dubr	1,162555829	1
Pdcd5-ps	1,162555829	1
Rps25	1,162555829	1
Dnajc18	1,162555829	1
Gm29257	1,16247525	1
Zfp606	1,162394676	1
Car13	1,162152988	1
Meis2	1,162152988	1
Axin1	1,16191135	1
Slc39a10	1,161830815	1
Zranb1	1,161750286	1
E2f6	1,161669762	1
Tbc1d2	1,161508732	1
Rps11	1,161428225	1
Pafah1b1	1,161347724	1
Prdx3	1,161267228	1
Tfeb	1,161267228	1
Med7	1,161186738	1
Gm42793	1,161106253	1
C330006A16Rik	1,161106253	1
Dazap2	1,161025774	1
Mul1	1,160784371	1
Flcn	1,160784371	1
Cdc5l	1,160784371	1
Alas1	1,160623463	1
Ccna2	1,160623463	1
Gm11604	1,160382144	1
Ahdc1	1,160301715	1
Nkiras1	1,160301715	1
Hhex	1,160221292	1
9330151L19Rik	1,160140874	1
Pdcd5	1,160140874	1
Gm10320	1,160140874	1
Gmcl1	1,159980055	1
Ppp1r12a	1,159980055	1
Gm15773	1,159899655	1
Jade3	1,159899655	1
Rpl35a	1,159899655	1
Cldnd1	1,159899655	1
Abhd17c	1,159899655	1
Sla2	1,159819259	1
Smpdl3a	1,159738869	1
Nolc1	1,159738869	1
Thumpd3	1,159658485	1
1700123O20Rik	1,159578107	1

Ap2b1	1,159578107	1
Cdc42se2	1,159497734	1
RP24-547N4.7	1,159256648	1
Suv39h1	1,159256648	1
Klc2	1,159176297	1
Rmnd5b	1,159095952	1
Nop10	1,159095952	1
G3bp2	1,159015612	1
Pabpc1l	1,158935278	1
Hmgcr	1,158935278	1
Casp8ap2	1,15885495	1
Psmc5	1,158774627	1
Ttc4	1,158774627	1
Gm6863	1,158694309	1
Gm26881	1,158453391	1
Dctpp1	1,158453391	1
Nr1h3	1,158373096	1
Gm5244	1,158373096	1
Ankrd37	1,158373096	1
Rragc	1,158292806	1
D330023K18Rik	1,158051971	1
Nicn1	1,158051971	1
Atp6v0d1	1,158051971	1
Lrrfip1	1,158051971	1
Rnft1	1,157971704	1
Prmt9	1,157891442	1
Gm6444	1,157811186	1
Gmeb2	1,157811186	1
Wdr45b	1,157811186	1
Fdps	1,157730935	1
Ddx20	1,157730935	1
Traf7	1,157730935	1
Gm7114	1,15765069	1
Pgm3	1,15765069	1
Spin1	1,157490217	1
Kdelr2	1,157409989	1
Pgs1	1,157329766	1
Rps15	1,157329766	1
Il17ra	1,157249549	1
Prune1	1,157249549	1
Arnt	1,157169337	1
Sec14l1	1,15700893	1
Dusp11	1,156768362	1
Slc8a1	1,156688184	1
Psmb4	1,156688184	1
Ptrh1	1,156608011	1
Ccdc18	1,156527844	1
Fam78a	1,156367526	1
Zfp810	1,156287376	1
Klrg2	1,156207231	1
Rtn4	1,156207231	1
Cd82	1,156127091	1

Tomm22	1,155966829	1
Zfp28	1,155646372	1
Patz1	1,155646372	1
Fam53b	1,155566271	1
Ccdc174	1,155486176	1
Trappc6b	1,155406087	1
Tnfaip8l2	1,155406087	1
Gm1862	1,155245925	1
Gm6743	1,155165852	1
Nsmce4a	1,155165852	1
RP23-115A18.3	1,155005723	1
3830403N18Rik	1,154845616	1
Ezh2	1,154845616	1
Naca	1,154765571	1
E130208F15Rik	1,154685532	1
Gpr155	1,154605498	1
Zc3h11a	1,154525469	1
Exo5	1,154525469	1
Mrps18c	1,154445447	1
Tlr6	1,154445447	1
Nab1	1,154365429	1
Nol8	1,154205411	1
Mob4	1,154205411	1
Gm12251	1,154125411	1
Mrpl21	1,154045416	1
Zfp81	1,153965426	1
Tnnt1	1,153965426	1
Ip6k1	1,153965426	1
Rhot1	1,153885442	1
Rpl31-ps8	1,153565561	1
Zfp830	1,153565561	1
Tchp	1,153485605	1
Cnppd1	1,153405654	1
Gm9378	1,153165835	1
Sf3a2	1,153085907	1
Gm14006	1,152766248	1
Ckap2	1,152686347	1
Metrn	1,152686347	1
Crip1	1,152526562	1
Arl8b	1,152526562	1
Tbrg4	1,152446677	1
Hoxb6	1,152127196	1
Secisbp2l	1,152047339	1
Morf4l2	1,151727968	1
Cactin	1,151727968	1
D330045A20Rik	1,151568316	1
Zc3h15	1,151568316	1
Zfp748	1,151328879	1
Pex7	1,151328879	1
Rps16-ps2	1,151328879	1
Smad5	1,151328879	1
Vcp-rs	1,151249077	1

Sema4d	1,151249077	1
Gpatch2l	1,151169282	1
Tfg	1,150850154	1
Ccdc32	1,150770386	1
Gm11625	1,150690623	1
Prim2	1,150371628	1
Fam195a	1,150371628	1
Mre11a	1,150371628	1
Cog5	1,150291893	1
Arf2	1,150052722	1
Mmaa	1,1498136	1
Pld3	1,1498136	1
Ctnnbl1	1,149733904	1
Rps24-ps3	1,149733904	1
Mterf4	1,149654213	1
Mat2b	1,149574528	1
Ddx52	1,149574528	1
Lamtor5	1,149415174	1
Nr3c1	1,149335505	1
Ift80	1,149255842	1
Gm35106	1,149255842	1
Cog6	1,149176185	1
Fkbp5	1,149016886	1
Mdfic	1,149016886	1
Taco1	1,148937245	1
Gabrd	1,148937245	1
Tubb4b	1,148777979	1
Sdcbp	1,148698355	1
Eef1d	1,148539123	1
Lemd2	1,148539123	1
Tmem33	1,148459515	1
Hnrnpf	1,148220724	1
Lrrc59	1,148220724	1
Ift140	1,147981983	1
Hoga1	1,147981983	1
Bloc1s2	1,147981983	1
Golph3	1,147902414	1
Exog	1,14782285	1
Snx4	1,147743292	1
Nasp	1,147743292	1
Fbxl6	1,147584192	1
Gm17994	1,14750465	1
Calm3	1,14750465	1
Eef1g	1,147425114	1
Hspbp1	1,147345583	1
Gm12693	1,147266058	1
Cep350	1,147266058	1
Scaper	1,147186538	1
Sugp1	1,147027516	1
Selenoh	1,147027516	1
St3gal6	1,146948012	1
Ist1	1,146948012	1

Bcl6	1,146789023	1
2300009A05Rik	1,146630055	1
Mmd	1,146471109	1
Mat2a	1,146471109	1
Eci1	1,146232732	1
Psme2	1,146073842	1
2010320M18Rik	1,146073842	1
Mrpl23-ps1	1,146073842	1
Txndc12	1,145914973	1
Rad17	1,145914973	1
Gm20707	1,145835547	1
Cdc34	1,145756126	1
Pja2	1,145756126	1
Gm8186	1,145676711	1
Cbx1	1,145597302	1
Rps27	1,145517898	1
Scyl1	1,145279719	1
Zmym2	1,145279719	1
Cul3	1,145279719	1
Etaa1	1,145200337	1
Nub1	1,145120961	1
Dym	1,14504159	1
Akt2	1,14504159	1
Rps9	1,144962224	1
Slc24a5	1,144882864	1
Wee1	1,14480351	1
Dhcr24	1,14480351	1
Kbtbd2	1,144644817	1
Gm13532	1,144565479	1
Raf1	1,144565479	1
Ndor1	1,14440682	1
Lrrc51	1,144327498	1
Gm5687	1,144327498	1
Wdr44	1,144248182	1
Pibf1	1,144168872	1
Rps11-ps1	1,144089567	1
Cdon	1,143930973	1
Gltscr1	1,143851685	1
Rheb	1,143851685	1
Hoxb4	1,143851685	1
Rp9	1,143851685	1
Fam26f	1,143693124	1
Gm5611	1,143613852	1
Kpna6	1,143613852	1
Sirt2	1,143613852	1
Fxr1	1,143613852	1
Gm43712	1,143534586	1
Scaf1	1,143455325	1
Zfp524	1,143455325	1
Cdc16	1,143455325	1
Lsm14a	1,143455325	1
Matr3	1,143455325	1

Usmg5	1,143376069	1
Ndufv3	1,143296819	1
RP23-65M10.2	1,143217574	1
Renbp	1,143217574	1
Rmdn3	1,143217574	1
Gm5586	1,143138335	1
Casc3	1,143138335	1
6330403N20Rik	1,143059102	1
Selenbp1	1,143059102	1
Elmsan1	1,143059102	1
Gm14165	1,142979874	1
Ppp1r12c	1,142979874	1
Reep4	1,142742223	1
Zbtb1	1,142663016	1
Tsr1	1,142663016	1
Plpp5	1,142583816	1
Bola2	1,142583816	1
H2-DMa	1,142504621	1
Camsap2	1,142504621	1
Nipa2	1,142504621	1
Rpl23	1,142504621	1
Fam175a	1,142346247	1
Tmem60	1,142346247	1
Mrpl18	1,142267068	1
Ino80b	1,142108727	1
Wdr46-ps	1,142029565	1
Sf3b6	1,142029565	1
Tspan31	1,141950408	1
Eapp	1,141950408	1
Rwdd2a	1,141871257	1
Mettl9	1,141871257	1
Specc1	1,141871257	1
Dhx40	1,141792111	1
2610016A17Rik	1,141712971	1
Gtpbp1	1,141712971	1
Gm45053	1,141633836	1
Nfix	1,141633836	1
4930453N24Rik	1,141554707	1
Fgd6	1,141475583	1
Fam171b	1,141317352	1
Gm26830	1,141238245	1
Lsm7	1,141159143	1
Cd36	1,14084279	1
Dot1l	1,14084279	1
Fam171a2	1,140684647	1
Gas6	1,140605583	1
Asl	1,140447473	1
Tmem170b	1,140447473	1
Syncrip	1,140447473	1
Bcl6b	1,140289384	1
Runx3	1,140289384	1
Naa50	1,140210348	1

Plek	1,139973273	1
Ccdc86	1,139894259	1
Cenpa	1,13981525	1
Nup43	1,139736247	1
Rps6ka5	1,139736247	1
Rbx1	1,139578257	1
B130006D01Rik	1,13949927	1
E2f5	1,139420288	1
Gm19353	1,139262342	1
Chchd7	1,139025464	1
Unc119b	1,138946515	1
Ppm1a	1,138946515	1
Chek2	1,138867572	1
Immp2l	1,138788635	1
Mrpl50	1,138788635	1
Ssna1	1,138709703	1
Tia1	1,138551855	1
Nfatc2ip	1,138472939	1
Cul1	1,138394029	1
Rap1a	1,138394029	1
Prorsd1	1,138236225	1
Ifitm6	1,138157331	1
Tmem106a	1,138157331	1
Nup205	1,138078443	1
Macrod1	1,13799956	1
Mgat4a	1,13799956	1
Ppp1r13b	1,137920683	1
Gm5835	1,137605228	1
Pole4	1,137605228	1
Plcg2	1,137526378	1
Trp53inp2	1,137447533	1
1810013L24Rik	1,137447533	1
Zbtb39	1,137368694	1
Akt1s1	1,137368694	1
Gm4459	1,137211032	1
Ddx28	1,137211032	1
Plau	1,137211032	1
B630019K06Rik	1,13697458	1
Rrp1	1,13697458	1
Rpl37	1,13697458	1
5031439G07Rik	1,13697458	1
S100a1	1,136895774	1
Atp1b3	1,136895774	1
Yae1d1	1,136816973	1
Igfbp4	1,136816973	1
Vps35	1,136580603	1
Stam	1,136501824	1
Gale	1,136344283	1
Rabep1	1,136344283	1
Cox8a	1,13626552	1
Nusap1	1,136186763	1
Rad51d	1,136108011	1

March11	1,136029265	1
St3gal4	1,136029265	1
Gm12222	1,135950524	1
Selenon	1,135871789	1
Cadm1	1,135871789	1
Gm12944	1,135793059	1
2210013O21Rik	1,135714334	1
Snrnp48	1,135635615	1
Gdpd3	1,135556902	1
Dnajc15	1,135478194	1
Il18rap	1,135320794	1
Ifnar1	1,135320794	1
Lats2	1,135163416	1
Gm5914	1,135084735	1
Rgs10	1,135084735	1
Plpp7	1,13500606	1
Gm5812	1,13500606	1
Ost4	1,134770066	1
2310036O22Rik	1,134770066	1
Fbxo11	1,134770066	1
2610203C20Rik	1,134691413	1
Cds1	1,134534122	1
Cwc15	1,134534122	1
Nfyc	1,134455485	1
Taf5	1,134455485	1
Klf10	1,134376853	1
Pcnt	1,134298227	1
Pnrc2	1,134298227	1
Atp5h	1,134062381	1
Zfp579	1,133983776	1
1700112E06Rik	1,133905177	1
Prss42	1,133905177	1
1110004E09Rik	1,133905177	1
Ltc4s	1,133826584	1
Amigo3	1,133747995	1
Gm4430	1,133512264	1
Ldlrap1	1,133512264	1
Nkap	1,133355136	1
Sertad2	1,133355136	1
Stx8	1,133198031	1
Ercc2	1,132962414	1
Fam134b	1,132883885	1
Cnot8	1,132805362	1
RP23-162P10.2	1,132648333	1
Gm10263	1,132491326	1
Dirc2	1,132491326	1
Gm10175	1,13233434	1
Bscl2	1,13233434	1
Rpl31	1,13233434	1
Hadhb	1,132255855	1
Gm4705	1,132177376	1
Ndufab1-ps	1,132020434	1

Dcun1d3	1,132020434	1
Sf3b1	1,131785061	1
4833439L19Rik	1,131628173	1
6330408A02Rik	1,131471307	1
Eef1a1	1,131471307	1
Gm14539	1,131236049	1
Cwc22	1,131236049	1
Ppme1	1,13115764	1
Sh3glb1	1,131079237	1
Med20	1,131000839	1
Nip7	1,131000839	1
Rpl9-ps7	1,130922447	1
Efcab7	1,130922447	1
Hipk2	1,13084406	1
Brwd3	1,13084406	1
Cnih4	1,13084406	1
Rpl28-ps1	1,130765679	1
Itch	1,130687303	1
Hsd17b10	1,130687303	1
Tbcb	1,130608932	1
Snhg5	1,130530567	1
Zcchc14	1,130452207	1
Slc35g1	1,130452207	1
Cox11	1,130373853	1
Gm13340	1,130217161	1
RP23-269H21.1	1,130217161	1
Fhod3	1,130138823	1
Mrpl16	1,130138823	1
AI846148	1,13006049	1
Acbd3	1,13006049	1
Chd3os	1,129982163	1
Gnb1l	1,129982163	1
Rbm5	1,129982163	1
RP24-499N24.6	1,129903842	1
Mrpl46	1,129903842	1
Cep290	1,129825525	1
Bhlhe40	1,129825525	1
Rpl22l1	1,129668909	1
Cela1	1,129590609	1
Pik3ap1	1,129590609	1
Rassf1	1,129355742	1
Hbegf	1,129277464	1
Rarg	1,129120923	1
Fam198b	1,128964405	1
P4ha1	1,128886154	1
Rbm7	1,128651433	1
Cdca4	1,128573203	1
Aplp2	1,128573203	1
Mkks	1,128494979	1
Triobp	1,128338548	1
Atmin	1,128338548	1
Lacc1	1,12826034	1

Pkig	1,12826034	1
Gm43756	1,128182137	1
Car6	1,128182137	1
Arhgap35	1,128103941	1
Gm11633	1,128025749	1
Zyg11b	1,127947563	1
Alkbh5	1,127947563	1
Ptbp1	1,127869382	1
Tpra1	1,127791207	1
Cox7a1	1,127634873	1
Eif4e3	1,127634873	1
Zfp358	1,127556714	1
Ddit3	1,127556714	1
Eif3e	1,127556714	1
Drg1	1,12747856	1
Mov10	1,127400412	1
Osbpl10	1,127166	1
Hccs	1,127166	1
Dtx2	1,126931637	1
Nlrc5	1,126931637	1
Dnajc21	1,126619228	1
Wrb	1,12654114	1
Sc5d	1,12654114	1
Ccdc71	1,126463057	1
Capza2	1,126384979	1
Ctnnal1	1,12622884	1
Caskin2	1,126072722	1
Exoc2	1,125916626	1
Rpl38	1,125916626	1
Pigx	1,125838586	1
Rnf4	1,125838586	1
Trappc3	1,125682523	1
Rps13-ps2	1,125682523	1
S1pr2	1,125682523	1
Rapgef2	1,125604499	1
Cep192	1,125526481	1
Pex12	1,125448468	1
Gm26202	1,125292458	1
B9d2	1,125214462	1
Ccnyl1	1,125136471	1
Pwwp2b	1,125136471	1
Ccdc166	1,125058485	1
Nsdhl	1,125058485	1
Smarca5	1,125058485	1
Dyrk1a	1,124980504	1
Prdm4	1,124902529	1
Tmem97	1,124902529	1
Ypel3	1,124902529	1
Capza1	1,12482456	1
Haghl	1,124746595	1
Strn	1,124668637	1
Nfkbil1	1,124668637	1

Rfesd	1,124590683	1
Hotairm1	1,124590683	1
Rhot2	1,124512735	1
Ptpn2	1,124512735	1
Arhgap17	1,124512735	1
Rps8	1,124512735	1
Dld	1,124434793	1
Plod3	1,124434793	1
Rbm8a2	1,124434793	1
Psmb1	1,124356856	1
Bmpr1a	1,124278924	1
Arhgap15	1,124200997	1
Herc6	1,124045161	1
Pcbp1	1,124045161	1
Rhog	1,123889346	1
Pik3ip1	1,123889346	1
Snord110	1,123811447	1
Tle3	1,123733553	1
Spast	1,123577781	1
Eno2	1,123577781	1
Fis1	1,123499903	1
1110004F10Rik	1,123499903	1
1810037I17Rik	1,123422031	1
C5ar1	1,123422031	1
Cdc26	1,123188446	1
Mrpl48	1,123188446	1
Pik3r4	1,123188446	1
Sh2b1	1,12303275	1
Gm11531	1,122799246	1
Nf1	1,122721422	1
5031425E22Rik	1,122721422	1
Isyna1	1,122643604	1
Upf1	1,122565791	1
Matk	1,122410181	1
Git1	1,122332384	1
Gm5436	1,122254592	1
Irak2	1,122176806	1
Rpl15	1,122099026	1
Pcna-ps2	1,121943481	1
Itpripl1	1,121943481	1
Znhit3	1,121710203	1
Hira	1,121710203	1
Cdyl	1,121710203	1
Rpsa-ps10	1,121632455	1
Naa25	1,121554712	1
Xpc	1,121476974	1
Fyb	1,121321515	1
Itga5	1,121166078	1
2900076A07Rik	1,121166078	1
Rnd2	1,121166078	1
Cbr3	1,121088367	1
Isg20	1,121010662	1

Als2cr12	1,121010662	1
Paip2b	1,120855268	1
Tmem183a	1,120855268	1
Tnfrsf1a	1,120777579	1
Ntpcr	1,120699895	1
Taf11	1,120622217	1
Ccdc186	1,120622217	1
Nup214	1,120544544	1
Kif5b	1,120544544	1
Gm11520	1,120233906	1
Tmem168	1,120078619	1
Vps26a	1,120078619	1
Bcl10	1,119768111	1
Rnf19b	1,119768111	1
Rrp36	1,119690497	1
Haus8	1,119690497	1
Rab3gap1	1,119690497	1
Cox20-ps	1,119612889	1
Rnf40	1,119612889	1
Mfsd5	1,119612889	1
Rnf166	1,119612889	1
Cmklr1	1,119380096	1
Rnh1	1,119380096	1
Trim21	1,119302509	1
Dnajb4	1,119069781	1
Klhl28	1,118992216	1
Zwint	1,118992216	1
Lzic	1,118914656	1
Mcur1	1,118914656	1
Tmem86a	1,118682008	1
Runx1	1,11860447	1
H2afx	1,118526937	1
Map3k2	1,118449409	1
Ranbp10	1,118449409	1
Fcgr4	1,11829437	1
Gm16755	1,11829437	1
Rab42	1,11829437	1
Dtx3	1,11829437	1
Ptpn1	1,11829437	1
Terf2ip	1,118216858	1
Tatdn3	1,118139352	1
Ncbp2	1,118139352	1
Icmt	1,118139352	1
Mrpl1	1,118061851	1
Ndufaf4	1,118061851	1
Dolk	1,117984356	1
Tox4	1,117984356	1
Cic	1,117984356	1
Zfp882	1,117829381	1
Map3k9	1,117751901	1
Tmsb10	1,117751901	1
Llph-ps2	1,117751901	1

Triap1	1,117674427	1
Hnrnpk	1,117596959	1
Calr	1,117596959	1
Ddt	1,117519496	1
Pbk	1,117442038	1
Ythdf2	1,117442038	1
Lsg1	1,117364585	1
Sult6b1	1,117287138	1
Kpna1	1,117287138	1
Tmem219	1,117209696	1
Nt5c3b	1,11713226	1
Gatsl3	1,11713226	1
Trp53inp1	1,117054829	1
Gm12989	1,116977403	1
Wdr91	1,116899983	1
Vma21-ps	1,116745158	1
Cd300c2	1,116667754	1
Ufl1	1,116512962	1
Pou6f1	1,116435574	1
Ndufb10	1,116435574	1
Tm4sf19	1,116126075	1
Ccl9	1,116126075	1
Arhgap21	1,115894007	1
Kdelr3	1,115816662	1
Gm17039	1,115739322	1
Snap47	1,115739322	1
Gm16754	1,115739322	1
Gm44024	1,115661988	1
Ctns	1,115661988	1
Ppp1r26	1,115584659	1
AK157302	1,115584659	1
Tmem144	1,115584659	1
Psmd9	1,115584659	1
Zfp277	1,115507335	1
5830454E08Rik	1,115352704	1
Rbm18	1,115352704	1
Fam160a2	1,115275396	1
Irf2bp2	1,115275396	1
Gtf2a2	1,115198094	1
Uqcrb	1,115198094	1
1600020E01Rik	1,115120797	1
Gm16437	1,115120797	1
Ctcf	1,115120797	1
Gm13573	1,115043505	1
Pom121	1,115043505	1
Uqcrfs1	1,115043505	1
Egln2	1,114966219	1
Aff4	1,114966219	1
Smad4	1,114888938	1
Mettl22	1,114811662	1
Ipmk	1,114811662	1
Lats1	1,114734392	1

Tes3-ps	1,114657127	1
Ttc30a1	1,114657127	1
Eftud2	1,114657127	1
Pltp	1,114579868	1
Proser3	1,114348122	1
Anapc7	1,114348122	1
4933434E20Rik	1,114039201	1
Papd7	1,113961985	1
Tcof1	1,113961985	1
Recql5	1,113884774	1
Zfp275	1,113884774	1
Gm7600	1,113807568	1
Clgn	1,113807568	1
Gm10704	1,113807568	1
Acp2	1,113653172	1
5430416N02Rik	1,113653172	1
Tnfrsf9	1,113653172	1
Tmsb4x	1,113575982	1
Gm9825	1,113498797	1
Myd88	1,113421618	1
Mrpl4	1,113267276	1
Heca	1,113267276	1
1110034G24Rik	1,113190113	1
Tmem109	1,113190113	1
Cox4i1	1,113190113	1
RP23-193N1.2	1,113035803	1
Tcerg1	1,113035803	1
Atl3	1,112958655	1
Necap2	1,112881514	1
Alkbh7	1,112727246	1
Rpl10	1,112727246	1
Dnph1	1,112727246	1
1700061G19Rik	1,112727246	1
Gm16418	1,112573	1
4930556M19Rik	1,112495885	1
Med9	1,112495885	1
Ythdc1	1,112495885	1
Gm42515	1,112418776	1
Zfp335os	1,112341671	1
Gm9722	1,112264572	1
Psmd11	1,112264572	1
Slc35a4	1,112187479	1
Nubp1	1,112187479	1
Zfp24	1,112187479	1
Iffo1	1,11211039	1
Gm4540	1,11211039	1
Rbm27	1,11211039	1
Rsrc2	1,11211039	1
Polr1e	1,111879158	1
Gm9843	1,111879158	1
Tra2a	1,111802091	1
Snx1	1,111802091	1

Pip5k1a	1,111725029	1
Kif2c	1,111647973	1
Commd9	1,111493876	1
Tgif1	1,111493876	1
Gm10784	1,111339801	1
Clip2	1,111262772	1
0610037L13Rik	1,111108729	1
Arpc5l	1,111108729	1
Lemd3	1,111031715	1
Fam96a	1,111031715	1
Phldb1	1,111031715	1
Snrpb2	1,111031715	1
Layn	1,110954707	1
Maea	1,110877704	1
Osbpl1a	1,110800707	1
Gm11221	1,110723714	1
Gm2991	1,110569746	1
Bckdha	1,110569746	1
Zc3h7a	1,110569746	1
AI839979	1,11049277	1
Pold3	1,11049277	1
Ets2	1,11049277	1
Slc20a1	1,110415799	1
Psmd7	1,110338834	1
Chchd6	1,110261873	1
Ube2k	1,110261873	1
Cdc42	1,110184919	1
E430018J23Rik	1,110107969	1
Bcar3	1,110107969	1
Ppm1d	1,110031025	1
Nfic	1,110031025	1
Gm15207	1,109954086	1
Gm12338	1,109877153	1
Actr3	1,109877153	1
Gm14303	1,109723302	1
Gtf3c6	1,109646384	1
Sgms1	1,109646384	1
4921511C10Rik	1,109569472	1
B4galt6	1,109569472	1
Nup62	1,109569472	1
Kras	1,109415664	1
Casp3	1,109338768	1
Alg10b	1,109261877	1
Eif5	1,109261877	1
Rspry1	1,109108111	1
Nkain1	1,109031236	1
E4f1	1,109031236	1
Zfp120	1,108877502	1
Rnf138	1,108877502	1
Dnajc3	1,108877502	1
H3f3b	1,108800644	1
Yy1	1,108800644	1

Nhlrc2	1,10872379	1
Frs2	1,108646942	1
Dtnbp1	1,108646942	1
Gm5939	1,108570099	1
Mettl5	1,108493261	1
Clint1	1,108493261	1
Ubxn8	1,108416429	1
Qsox1	1,108416429	1
Mier3	1,108339602	1
Gde1	1,108262781	1
Snrpe	1,108185964	1
Thoc5	1,108109153	1
Impact	1,108032348	1
Ddx18	1,108032348	1
Ubqln4	1,108032348	1
Amd2	1,107878753	1
Hsd17b4	1,107801963	1
Mdm2	1,107801963	1
Trim65	1,107725179	1
Gars	1,107725179	1
Wdr18	1,1076484	1
Brk1	1,1076484	1
Arl5a	1,107571626	1
Ifrd1	1,107571626	1
Gm10499	1,107494858	1
Rce1	1,107494858	1
Atxn10	1,107341337	1
Rps24	1,107264584	1
Kmt5a	1,107187837	1
Tmem2	1,107111096	1
Pias4	1,107111096	1
Fitm2	1,107034359	1
Zfp688	1,107034359	1
Ik	1,107034359	1
Naa15	1,106804182	1
Stt3b	1,106804182	1
Ebag9	1,106727467	1
Lpin1	1,106727467	1
Nosip	1,106650757	1
Gm11517	1,106497353	1
Qk	1,106420659	1
Ftl1	1,106267287	1
Tbcc	1,106267287	1
Haus2	1,106267287	1
Mtfp1	1,106037269	1
Gm6206	1,105883951	1
Hivep3	1,105883951	1
Dek	1,105807299	1
BC028528	1,105730653	1
Nup153	1,105730653	1
Gm8213	1,105654013	1
Uvrag	1,105654013	1

Ogfrl1	1,105654013	1
Calcrl	1,105577377	1
Arfip2	1,105577377	1
Stt3a	1,105500747	1
Spc25	1,105347503	1
Kdm5c	1,105270888	1
Atad2	1,105117676	1
Atg4d	1,105041078	1
Epsti1	1,104964485	1
Rab11fip5	1,104964485	1
Lsp1	1,104887897	1
Uimc1	1,104811315	1
Gnas	1,104734738	1
Tmem106b	1,104658166	1
Ap2a1	1,1045816	1
Rpl11	1,104428483	1
Ugcg	1,104428483	1
Ripk3	1,104275387	1
Pigf	1,104275387	1
Tceal9	1,104198847	1
Phf5a	1,104122313	1
Gm8430	1,104045783	1
Ctsb	1,104045783	1
Parn	1,103892741	1
Mkrn1	1,103892741	1
Zfp959	1,103739719	1
Tmem263	1,103739719	1
Ppp1r2	1,103663216	1
Kpnb1	1,103586719	1
Gm44901	1,103510227	1
Cdc40	1,10343374	1
Slc30a5	1,103204311	1
Ubl5	1,103204311	1
Ubl3	1,103051385	1
Pgm2l1	1,10297493	1
Cdk17	1,10297493	1
Clip1	1,10297493	1
Rhbdf2	1,102898481	1
Actr1a	1,102822036	1
Abhd8	1,102516311	1
Poldip2	1,102516311	1
Uck1	1,102439893	1
Fam50a	1,102439893	1
Rbm22	1,102363481	1
Carm1	1,102287073	1
Rfc2	1,102210671	1
Cpsf2	1,102057883	1
Atxn7l1	1,10182874	1
Nelfb	1,10182874	1
Gm2735	1,10175237	1
Pml	1,101599645	1
Blcap	1,101599645	1

Polr2b	1,101446942	1
Rab18	1,101446942	1
Gm5619	1,101370598	1
Atp5c1	1,101370598	1
Plin2	1,101217926	1
Hadha	1,101217926	1
Ndufs4	1,101217926	1
Ptges3l	1,101141598	1
Dhrs13	1,100988958	1
Pold4	1,100988958	1
Gm45568	1,100683741	1
Snu13	1,10060745	1
Sptlc2	1,100454884	1
Cyb5a	1,100454884	1
Efnb1	1,100378609	1
Mien1	1,100378609	1
mt-Co1	1,100378609	1
Blvrb	1,100378609	1
Borcs6	1,10030234	1
Oraov1	1,100149816	1
Sars	1,100073562	1
Hist2h4	1,09992107	1
Rpl19	1,09992107	1
Arl5b	1,099844832	1
RP23-123D6.12	1,099768599	1
Papss1	1,099387514	1
Pak1	1,099387514	1
Rab21	1,099387514	1
Zfp78	1,099235117	1
Spred3	1,099158927	1
Tnfaip1	1,099158927	1
Pvr	1,099082742	1
Cyth2	1,099082742	1
Tmco1	1,099082742	1
Tcta	1,098930387	1
Utp11	1,098854218	1
Lmnb1	1,098778053	1
Vps13d	1,098778053	1
Hoxc6	1,098625741	1
Agtpbp1	1,098549593	1
Eid2b	1,098397312	1
RP23-139H6.1	1,098397312	1
Il1rn	1,098245052	1
Wrnip1	1,098245052	1
Nlrp10	1,09816893	1
Eif2b4	1,098092814	1
Rpl7-ps7	1,098016702	1
Tnnc1	1,097940596	1
Gm5451	1,097940596	1
Vezf1	1,097940596	1
Traf3ip3	1,097864496	1
Hbs1l	1,09771231	1

Ecsit	1,097636225	1
Atg4b	1,097636225	1
Sephs2	1,097560145	1
Zhx1	1,097560145	1
Tmcc2	1,097484071	1
Yeats2	1,097484071	1
Rnf169	1,097484071	1
Wbp2	1,097331938	1
Oat	1,097179826	1
Borcs7	1,096951697	1
Nod2	1,096951697	1
Pard6a	1,096875665	1
Trappc5	1,096875665	1
RP24-282C4.13	1,096799638	1
Dusp12	1,096799638	1
Pclaf	1,096723616	1
Tns1	1,096571589	1
Prr11	1,096495583	1
Trnau1ap	1,096495583	1
Fndc3a	1,096419582	1
Gm20667	1,096343587	1
Pvt1	1,096343587	1
Zbtb17	1,096267597	1
Plaa	1,096267597	1
Arf3	1,096191612	1
Casp7	1,095963689	1
Lyst	1,095963689	1
Rpl27a	1,095963689	1
Wsb1	1,095887725	1
Golga7	1,095735813	1
Fuk	1,095735813	1
Kcnk13	1,095735813	1
Ifi207	1,095735813	1
Map3k4	1,095735813	1
Cdkn1b	1,095735813	1
Glo1	1,095659865	1
Gm12346	1,095583922	1
5430403G16Rik	1,095432053	1
Urb2	1,095432053	1
Desi2	1,095432053	1
Taf6	1,095128377	1
Fam43a	1,095052471	1
Ywhaz	1,095052471	1
Mettl7a1	1,094900675	1
RP24-365N15.9	1,094900675	1
Tomm7	1,094824785	1
Rab9	1,0947489	1
Evi5	1,0947489	1
Pik3r5	1,0947489	1
Usp28	1,09467302	1
Cenpl	1,094597146	1
Riox2	1,094521277	1

Alox5ap	1,094521277	1
P2rx7	1,094445413	1
Gm17586	1,094369555	1
Ube2g2	1,094369555	1
Gpam	1,094293701	1
Ssb	1,094293701	1
Tma16	1,094217853	1
Ammecr1l	1,09414201	1
Faf1	1,09414201	1
Atxn7l2	1,093990341	1
Glmn	1,093990341	1
Adcy9	1,093990341	1
Khk	1,093914514	1
Ppt2	1,093838692	1
3110001I22Rik	1,093687064	1
Socs7	1,093687064	1
Zfp420	1,093611258	1
Osbp	1,093611258	1
Glipr2	1,093535457	1
RP24-275P22.2	1,093459662	1
Gm23301	1,093383872	1
Ccser2	1,093232307	1
Pex14	1,093004999	1
Imp3	1,092853487	1
2700060E02Rik	1,092701996	1
Mrps9	1,092626258	1
Rps27-ps1	1,092474799	1
Cdk2ap1	1,092474799	1
Nemf	1,092474799	1
Fam63b	1,092474799	1
Zmynd10	1,092399077	1
Saysd1	1,092399077	1
Sestd1	1,092399077	1
Eif4h	1,092399077	1
Rpl39	1,092171942	1
Atpaf2	1,092020546	1
Ndufa5	1,091944855	1
Chchd3	1,09186917	1
Gm16253	1,09179349	1
9430038I01Rik	1,09179349	1
Gm17100	1,091717815	1
Pus3	1,091717815	1
Nab2	1,091566482	1
Ice2	1,091490823	1
Vps51	1,091490823	1
Agpat4	1,091415169	1
Rrp8	1,091263877	1
Irf2	1,091263877	1
Tfpt	1,091188239	1
Snx6	1,091188239	1
B130034C11Rik	1,091112606	1
Rhobtb2	1,091112606	1

Eif2ak3	1,091112606	1
Gm15427	1,091036979	1
Paip2	1,091036979	1
Tnfaip8l1	1,090961356	1
Dedd	1,090885739	1
Ppp2r5a	1,090885739	1
Gm9645	1,090810128	1
Tmem57	1,090810128	1
Smim7	1,090810128	1
Cst3	1,090583324	1
Creld2	1,090507733	1
Clptm1	1,090356567	1
Mfsd14b	1,090280992	1
Supt6	1,090205422	1
Cdc25a	1,090129857	1
Npm1	1,090129857	1
Rps6kb2	1,090054298	1
Cdyl2	1,090054298	1
Ei24	1,090054298	1
Enpp4	1,089978743	1
4933408B17Rik	1,089903194	1
Trmt61b	1,089903194	1
Rpl3-ps1	1,089903194	1
Riox1	1,089827651	1
Ercc5	1,089827651	1
Adipor1	1,089752112	1
Minos1	1,089601051	1
RP23-162P10.8	1,089525528	1
Paics	1,089374498	1
Slc25a38	1,089298991	1
Pphln1	1,089298991	1
Clic1	1,089298991	1
Jrkl	1,089223489	1
1110037F02Rik	1,089223489	1
Ngrn	1,089223489	1
Megf9	1,089147993	1
Mcm2	1,089147993	1
Gm19705	1,089072501	1
Pigu	1,089072501	1
Gm10073	1,089072501	1
Tbc1d22a	1,088997015	1
Rel	1,088921534	1
Jak2	1,088921534	1
Gm15210	1,088921534	1
E230020A03Rik	1,088846059	1
St7l	1,088695123	1
Abi1	1,088695123	1
Narf	1,088695123	1
Gopc	1,088619663	1
Piezo1	1,088619663	1
Gtf2h3	1,088468759	1
Ndufa6	1,088468759	1

Usp12	1,088393315	1
9930021J03Rik	1,088317876	1
Ube2a	1,088317876	1
Fancl	1,088242442	1
Extl2	1,088167013	1
Gm11970	1,088167013	1
Elmo2	1,088016172	1
Ppp6c	1,088016172	1
Rbm12b1	1,087940759	1
Smim15	1,087940759	1
Zfp541	1,087865351	1
Rrs1	1,087789948	1
Gm11930	1,087639159	1
Pfdn1	1,087639159	1
Plekhg2	1,087563772	1
Sep 07	1,087563772	1
Ccdc77	1,087488391	1
Idh3b	1,087488391	1
Ggct	1,087413015	1
March6	1,087413015	1
RP23-58B7.2	1,087337643	1
Tmem101	1,087337643	1
Cd200r2	1,087262278	1
Gm19503	1,087036211	1
Cnot11	1,087036211	1
Mtmr4	1,087036211	1
Idi1	1,086885526	1
Mef2d	1,086885526	1
Nupl2	1,086659538	1
Creb3l1	1,086659538	1
Frg1	1,086659538	1
Cit	1,08658422	1
Pcf11	1,08658422	1
Snhg17	1,086508906	1
Cry1	1,086358294	1
Cd9	1,086282996	1
Gm13464	1,086282996	1
Ndufb6	1,086282996	1
Snap29	1,086207703	1
Slc29a2	1,086057133	1
Commd1	1,086057133	1
Mrpl14	1,086057133	1
Tnfrsf10b	1,085981856	1
Gpn1	1,085831318	1
Galnt6	1,085831318	1
Ak2	1,085831318	1
Cdc42se1	1,085831318	1
Gm8606	1,085756056	1
Scarf1	1,085756056	1
Aff1	1,0856808	1
Rpn2	1,0856808	1
1700066M21Rik	1,085605549	1

Ighm	1,085605549	1
Ppp1r35	1,085379827	1
Cyhr1	1,085304597	1
Gm44609	1,085229372	1
Rpl6	1,085229372	1
Pip4k2a	1,085229372	1
Pa2g4	1,085229372	1
Oxld1	1,085154152	1
Tprgl	1,085078938	1
Cdt1	1,085003728	1
Gm45855	1,084928524	1
Dctn1	1,084853325	1
Mydgf	1,084853325	1
Gm5590	1,084778132	1
Stard3	1,084778132	1
Gm9794	1,084778132	1
Mta2	1,084702943	1
Smad3	1,084477409	1
H2-K1	1,084477409	1
Foxo1	1,084402241	1
Dohh	1,084402241	1
Gm20768	1,084251922	1
Slc35c2	1,084251922	1
Pmepa1	1,08417677	1
Adk	1,08417677	1
Ptdss2	1,084101623	1
Rpl8	1,084026481	1
Plcg1	1,083876214	1
Luzp1	1,083876214	1
Mbd3	1,083801088	1
Tmem59	1,083801088	1
Gabpa	1,083725967	1
Gm43569	1,083650851	1
Mocs2	1,083650851	1
A430005L14Rik	1,083650851	1
2310034G01Rik	1,083500636	1
Tcp11l1	1,083500636	1
Zfp995	1,083425536	1
Il6st	1,083350441	1
Ranbp3	1,083350441	1
Stau1	1,083350441	1
Gm1947	1,083200267	1
Chmp4b	1,083200267	1
Sra1	1,083200267	1
Zfp593	1,083125188	1
Cep250	1,083050114	1
Tsc22d3	1,083050114	1
Exosc8	1,083050114	1
Vps29	1,082975046	1
Tmem30a	1,082975046	1
Mapk8	1,082824924	1
Lztr1	1,082749871	1

Rbm39	1,082749871	1
Pde2a	1,082674823	1
Syngr1	1,082674823	1
Lyz2	1,082524743	1
Sun2	1,082524743	1
Pgrmc2	1,082524743	1
Gm23300	1,08244971	1
Zfp385a	1,082374683	1
Tpx2	1,082374683	1
Wasf2	1,082374683	1
Gm10275	1,082299661	1
BC025920	1,082224645	1
Cenpj	1,082149633	1
Tusc3	1,082074627	1
Fgfr1op	1,082074627	1
A530013C23Rik	1,08192463	1
Abhd14a	1,08192463	1
Pak1ip1	1,08192463	1
Smyd5	1,081849639	1
Dpm3	1,081849639	1
Gga1	1,081849639	1
Cnpy4	1,081699673	1
Gm13196	1,081624698	1
Clcnkb	1,081624698	1
Polr2d	1,081624698	1
Bex3	1,081549728	1
Zfp40	1,08132485	1
Slc15a4	1,08132485	1
Thoc1	1,081100018	1
Gm15500	1,081100018	1
Gtf2ird1	1,081025084	1
Nmt1	1,081025084	1
Bahcc1	1,080950156	1
Eif4g2	1,080950156	1
Mrpl11	1,080800315	1
Atp5e	1,080800315	1
Eif4ebp2	1,080575592	1
Neo1	1,080500695	1
Fcer1g	1,080500695	1
Tpt1	1,080425803	1
Gcdh	1,080201158	1
Hist2h2ac	1,079901704	1
Lrrc8a	1,079826854	1
Zswim1	1,079752008	1
Arl10	1,079752008	1
Rab13	1,079752008	1
Ddx6	1,079752008	1
Sec22a	1,079677168	1
Moap1	1,079602333	1
Dcps	1,079602333	1
Wipf2	1,079602333	1
Rpl13a	1,079527504	1

Ift81	1,079303045	1
Dhx33	1,079303045	1
Med13	1,079303045	1
Gm13009	1,079153433	1
Dvl2	1,079153433	1
Ybx1	1,079153433	1
Trim36	1,079003841	1
Tesk1	1,078854269	1
Mrps24	1,078854269	1
Sgta	1,078779491	1
Commd2	1,078779491	1
Snw1	1,078779491	1
Gna15	1,07840568	1
Vbp1	1,07840568	1
Tpd52-ps	1,078256191	1
Lrpap1	1,078256191	1
Ccdc120	1,078181455	1
Fgr	1,078031998	1
Car12	1,078031998	1
Gm12663	1,077882561	1
Sec61b	1,07780785	1
Pdzd8	1,077658445	1
Rap1b	1,07758375	1
U2af1	1,07750906	1
Mrpl42	1,07750906	1
Pdap1	1,077434375	1
Raly	1,077359696	1
Acadl	1,077359696	1
Fut10	1,077285022	1
Ubn1	1,077285022	1
Pcnx	1,077210353	1
Cep162	1,077135689	1
Arid1b	1,07706103	1
Hmces	1,076986376	1
Snx14	1,076837085	1
Zfp598	1,076837085	1
Fnta	1,076762446	1
Ssr4	1,076762446	1
Fdft1	1,076687814	1
Synj1	1,076687814	1
Aprt	1,076613186	1
Atp6v1a	1,076538563	1
Gm13835	1,076463946	1
Mthfd2	1,076463946	1
Sufu	1,076314727	1
Myo9b	1,076240125	1
Phf11c	1,076090937	1
Stoml2	1,076090937	1
Lyn	1,075941769	1
RP23-304C21.3	1,075867193	1
Yeats4	1,075792622	1
Casp8	1,075718056	1

Nelfa	1,075718056	1
Strap	1,075718056	1
Gm6433	1,075643496	1
Ensa	1,075643496	1
Snord55	1,07549439	1
Trappc2l	1,07549439	1
Stbd1	1,075345306	1
Tmem115	1,075345306	1
Stx18	1,075345306	1
Rev3l	1,075270771	1
Tmed3	1,075196241	1
Ppp3cc	1,075121717	1
Rnf225	1,075047198	1
Gstcd	1,075047198	1
Slc6a4	1,074972684	1
Hectd3	1,074972684	1
Map2k1	1,074972684	1
Tbk1	1,074898175	1
Ddhd1	1,074823671	1
Rab35	1,074823671	1
Prnp	1,07467468	1
Rangap1	1,07467468	1
Slc39a3	1,074600191	1
Psmc1	1,074600191	1
Ube2s	1,074525708	1
Eif2a	1,074525708	1
Mrnip	1,07445123	1
Tradd	1,07445123	1
Mpst	1,07445123	1
Tbc1d12	1,074376758	1
Ndufv1	1,07430229	1
Ccdc127	1,07430229	1
Chst12	1,074227828	1
Rbks	1,074153371	1
L3mbtl2	1,074153371	1
Aph1c	1,074153371	1
Gm14830	1,074078919	1
Sec22c	1,074004472	1
Ier3	1,07393003	1
Enc1	1,073855593	1
Rpl31-ps14	1,073781162	1
Strip1	1,073706736	1
Repin1	1,073632315	1
RP23-359K10.9	1,073557899	1
Efna1	1,073557899	1
AI662270	1,073557899	1
Ttc39a	1,073409082	1
Asb8	1,073334682	1
Rttn	1,073260286	1
Yipf4	1,073185896	1
Cln6	1,073185896	1
Arl6ip1	1,073185896	1

Spg21	1,073111511	1
Ncapg	1,072962757	1
Icosl	1,072888387	1
Eif2ak1	1,072888387	1
Fzd9	1,072814023	1
Gosr2	1,072739664	1
Orc4	1,072665309	1
Phf23	1,072590961	1
Gzmm	1,072516617	1
Gm10425	1,072516617	1
Rhebl1	1,072516617	1
Pak2	1,072442278	1
Acbd6	1,072442278	1
Cpsf7	1,072293616	1
Herpud1	1,072293616	1
Mgat1	1,072293616	1
N4bp2l2	1,072219293	1
Nup133	1,072144975	1
Ptpmt1	1,072144975	1
St6galnac6	1,072070662	1
Rpf2	1,072070662	1
Slc30a6	1,071996355	1
Fdx1	1,071922052	1
Akap11	1,071922052	1
Umad1	1,071847755	1
Usp36	1,071847755	1
Mrpl39	1,071773463	1
Fth-ps3	1,071773463	1
Atg16l1	1,071773463	1
Wdr74	1,071699175	1
Gm26542	1,071476345	1
Riok3	1,071476345	1
Arpc4	1,071476345	1
Nckap1l	1,071476345	1
Zfp592	1,071327817	1
Rpl14	1,071327817	1
Hal	1,071253561	1
Fnip2	1,07117931	1
Mrps10	1,071030823	1
Fbrsl1	1,070956588	1
Fus	1,070956588	1
Camk1	1,070956588	1
Adamts6	1,070882357	1
Thap12	1,070882357	1
Tes	1,070882357	1
Gm44237	1,070808132	1
Trim8	1,070808132	1
Polr2m	1,070808132	1
Gm6654	1,070659697	1
AV099323	1,070659697	1
Rnf13	1,070659697	1
Mgea5	1,070585487	1

Car5b	1,070511282	1
Csnk1g3	1,070511282	1
Rock1	1,070437082	1
Gpn3	1,070362888	1
Baz2a	1,070362888	1
Gmeb1	1,070214514	1
Fyn	1,070140335	1
Dcun1d5	1,070140335	1
Dnajb1	1,070066161	1
Tmem268	1,070066161	1
Igsf8	1,070066161	1
Erfe	1,069991993	1
Fam175b	1,069991993	1
Xab2	1,06984367	1
Pet100	1,069769517	1
Zfp513	1,069769517	1
Maz	1,069695369	1
Mrpl20	1,069695369	1
Acad8	1,069695369	1
2610001J05Rik	1,069695369	1
Hmbs	1,069472955	1
Ddn	1,069472955	1
Ing1	1,069472955	1
Fopnl	1,069398827	1
Usp47	1,069398827	1
Mrpl41	1,069324705	1
Gm5841	1,069250588	1
Ermp1	1,069250588	1
Gm43547	1,069176475	1
Ankrd13a	1,069102368	1
Cln5	1,068954169	1
Tmem9b	1,068954169	1
Rbmx	1,068805991	1
Ajuba	1,068805991	1
Nenf	1,068805991	1
Odc1	1,06873191	1
Gm2000	1,068657834	1
Nufip1	1,068657834	1
Dut	1,068435635	1
Atp6v1d	1,068435635	1
Nif3l1	1,06836158	1
Naga	1,068213484	1
Tlr2	1,068139443	1
Rab29	1,068065408	1
Camta1	1,068065408	1
D630023F18Rik	1,068065408	1
Plk3	1,067917353	1
Ggh	1,067843333	1
Klhdc1	1,067769318	1
Id1	1,067769318	1
Dstyk	1,067769318	1
Tigd2	1,067769318	1

Ippk	1,067547305	1
Hnrnpa3	1,067473311	1
Stambp	1,067473311	1
Fbrs	1,067473311	1
Rnf126	1,067325338	1
Tstd3	1,067103417	1
Usp32	1,066955495	1
Eif3f	1,066881542	1
Unc119	1,066807594	1
Pde4b	1,066733651	1
Gm4754	1,066585781	1
Mrto4	1,066585781	1
Slc25a23	1,066511853	1
Ubac1	1,066437931	1
Psmb8	1,066437931	1
Gm16399	1,066364014	1
Ldlrad3	1,066142293	1
Sepsecs	1,066142293	1
Trim23	1,066068396	1
Cdca3	1,065994504	1
Pdk2	1,065846736	1
Gm16580	1,065846736	1
Marc2	1,06577286	1
Cpne9	1,065625123	1
Fbxo42	1,065625123	1
Alyref2	1,065551262	1
Eif2s1	1,065477406	1
Stac2	1,065403555	1
Gm5963	1,065403555	1
9130023H24Rik	1,065329709	1
Mak16	1,065329709	1
0610038B21Rik	1,065034378	1
Ppif	1,065034378	1
Rpp14	1,065034378	1
Adat1	1,064960558	1
Galt	1,064960558	1
Znrf1	1,064886743	1
Eid1	1,064812934	1
Zfp839	1,064739129	1
Sf3b4	1,064739129	1
9130230L23Rik	1,064665329	1
2310009B15Rik	1,064665329	1
Pdcd7	1,064591535	1
Nek7	1,064591535	1
Kiz	1,064517746	1
Myo1f	1,064443962	1
Pik3r6	1,064370182	1
Snrpa	1,064370182	1
Rnf5	1,06422264	1
Cox7a2l	1,06422264	1
Hmox1	1,064148876	1
Sin3a	1,064148876	1

Rps26	1,064148876	1
Mir703	1,064148876	1
Gnl1	1,064075117	1
C330027C09Rik	1,064001364	1
Kif2a	1,064001364	1
Bak1	1,063927615	1
Fxr2	1,063927615	1
Nfe2l1	1,063853872	1
Pef1	1,063706401	1
Tpd52	1,063632673	1
Nucb1	1,06355895	1
Laptm4b	1,06341152	1
Cxxc1	1,06326411	1
Rpl27	1,063190412	1
Ehmt1	1,06311672	1
Kif9	1,063043033	1
Guca1a	1,063043033	1
Yars2	1,062969351	1
Asah1	1,062969351	1
Cdk1	1,062969351	1
Slc10a3	1,062895674	1
Dera	1,062895674	1
Eef1e1	1,062895674	1
Tor2a	1,062895674	1
Fance	1,062895674	1
Spg20	1,062822003	1
Nupl1	1,062748336	1
Tle4	1,062748336	1
Ints14	1,062674674	1
Stk24	1,062674674	1
Eif3j2	1,062601018	1
Kctd3	1,06245372	1
Plxna1	1,062232812	1
Bhlhe41	1,062232812	1
Iah1	1,062232812	1
Gtf2e2	1,062232812	1
Mrpl43	1,062232812	1
Gabarapl2	1,06193834	1
Pus10	1,061864734	1
Adamtsl4	1,061791134	1
Mcfd2	1,061791134	1
Naa38	1,061570364	1
Eif3g	1,061570364	1
Brd4	1,061496784	1
Edf1	1,061496784	1
Car9	1,061423209	1
Zfp367	1,061349639	1
Ubtd1	1,061349639	1
Fam134a	1,061349639	1
Gpx4	1,061276075	1
Snapc4	1,061202515	1
Smc3	1,061202515	1

Pcna	1,061055411	1
Emilin2	1,061055411	1
Rhoq	1,060981867	1
Fer	1,060908328	1
Atp5s	1,060908328	1
Pdlim7	1,060908328	1
Lrrc40	1,060908328	1
Qdpr	1,060908328	1
Tshz1	1,060834794	1
Actr3b	1,060761265	1
Grcc10	1,060614223	1
Ceacam1	1,060614223	1
Ppp1r14b	1,060540709	1
Lactb	1,060540709	1
Sgcb	1,0604672	1
Vamp2	1,0604672	1
Plrg1	1,0604672	1
Tbl1xr1	1,060393697	1
5430405H02Rik	1,060320199	1
Atp9a	1,060320199	1
Echs1	1,060246705	1
Hmox2	1,059952783	1
Mrps30	1,059879316	1
Tbc1d15	1,059879316	1
Gatad1	1,059805853	1
Scarb2	1,059732395	1
Tle1	1,059658943	1
Mpp6	1,059585495	1
Mrpl52	1,059438616	1
Traf5	1,059218335	1
Gatad2b	1,059071506	1
Daglb	1,059071506	1
Uevld	1,058998099	1
Nrm	1,058998099	1
Ergic2	1,058998099	1
Mtmr6	1,058998099	1
Map4k5	1,058924698	1
Kdelc1	1,05877791	1
Hmga1-rs1	1,05877791	1
Fgf11	1,05877791	1
Bag1	1,05877791	1
2900055J20Rik	1,058631142	1
Zfp473	1,058631142	1
Washc1	1,058631142	1
Cenpc1	1,058557766	1
Phka1	1,058557766	1
Jmjd7	1,058484395	1
Ripk2	1,058484395	1
Clec4a2	1,058411029	1
Prkaa1	1,058411029	1
Serinc1	1,058411029	1
Xrcc1	1,058337668	1

Ube2t	1,058264312	1
Helb	1,058264312	1
Eif2b2	1,058190961	1
Gm29994	1,058117616	1
Gm9727	1,058044275	1
Got2	1,058044275	1
Arhgap12	1,05797094	1
Arf1	1,05797094	1
Neurl2	1,057897609	1
Me2	1,057824284	1
Vti1b	1,057824284	1
Zfp644	1,057750964	1
Ubr2	1,057750964	1
Gm12421	1,057677648	1
Ero1lb	1,057677648	1
Polr3b	1,057604338	1
Fam229b	1,057531033	1
Troap	1,057457733	1
Clic4	1,057457733	1
Tpi1	1,057457733	1
Ldlr	1,057384439	1
S100a13	1,057311149	1
Eif3k	1,057237864	1
Zfp820	1,057164585	1
Herpud2	1,057164585	1
Cnot2	1,057164585	1
A930004J17Rik	1,057018041	1
Pank2	1,057018041	1
Flnc	1,057018041	1
Spag5	1,056944776	1
Uchl4	1,056871517	1
Prpf19	1,056725014	1
Dazap1	1,056725014	1
Fam212b	1,056651769	1
Flii	1,056651769	1
St13	1,056651769	1
Gm7846	1,056578531	1
Slc44a1	1,056432068	1
Eef1b2	1,056432068	1
Rps5	1,056432068	1
4930558J18Rik	1,056358844	1
Gm7027	1,056066	1
Mrpl45	1,056066	1
Zfhx2	1,055992801	1
Atp5j2	1,055919608	1
Polr1c	1,05584642	1
Klhl9	1,05584642	1
Bnip3	1,05584642	1
Rab1b	1,055700059	1
Gm28041	1,055626886	1
Inip	1,055626886	1
Gm15798	1,055553718	1

Plxnc1	1,055553718	1
Pdhb	1,055480555	1
Stn1	1,055407397	1
RP24-240E7.1	1,055187954	1
Mfsd7b	1,055187954	1
Chchd5	1,055114816	1
Nhp2	1,055114816	1
Arhgdia	1,055114816	1
Nubpl	1,055041684	1
Mrps5	1,055041684	1
Slc25a17	1,055041684	1
Ift88	1,054968557	1
Bola1	1,054822317	1
Uqcrh	1,054749205	1
Dhx30	1,054676098	1
Gadd45gip1	1,054602996	1
Usp5	1,054602996	1
Ints5	1,054529899	1
Gtf3c3	1,054456807	1
Zfp944	1,05438372	1
Ap1b1	1,054310638	1
1500011B03Rik	1,054237562	1
Fbxw8	1,05416449	1
Slc25a19	1,054091423	1
Reep5	1,054091423	1
Sdc1	1,054018362	1
Rps18-ps1	1,053945305	1
Wdr43	1,053945305	1
Stx7	1,053945305	1
Mon1b	1,053872254	1
Ptges2	1,053799208	1
Mtrr	1,053799208	1
Gtf3c4	1,053726166	1
Msra	1,05365313	1
Zfp383	1,05365313	1
Mrps16	1,05365313	1
Comtd1	1,053580099	1
Gas5	1,053507073	1
Nedd1	1,053434052	1
Ndufb5	1,053434052	1
Usp14	1,053288025	1
Zfp141	1,053215019	1
RP23-366E4.9	1,053069023	1
Rnf114	1,052996032	1
Itm2c	1,052923046	1
Pdcd10	1,052850066	1
Tmem256	1,052850066	1
Ak4	1,052850066	1
Kif18b	1,05277709	1
B3gnt6	1,05270412	1
Cisd1	1,05270412	1
Msmo1	1,052631155	1

Tpgs1	1,052558194	1
Srek1ip1	1,052558194	1
Eme1	1,052412289	1
Psmb7	1,052412289	1
Glrx3	1,052412289	1
Slc3a2	1,052412289	1
Gm16523	1,052339344	1
Gm4987	1,052193469	1
Tmem11	1,052193469	1
Kifc5b	1,052120539	1
Gm1840	1,052120539	1
Tbc1d31	1,052120539	1
Nrde2	1,051974694	1
Hmcn2	1,051828869	1
Gm2950	1,051828869	1
Bcas3	1,051828869	1
Pan3	1,051755965	1
Nek8	1,051683065	1
Tpt1-ps3	1,051683065	1
Slu7	1,051683065	1
Ctsz	1,051683065	1
Pum1	1,051537281	1
Gm2756	1,051464397	1
Smyd4	1,051464397	1
Pdia3	1,051391517	1
Gm43331	1,051172909	1
RP24-325P4.5	1,051027196	1
Rab5b	1,051027196	1
Ifi35	1,050808663	1
Nudcd3	1,050808663	1
Tm2d3	1,050663001	1
Tef	1,050663001	1
D130007C19Rik	1,050590177	1
Zfp422	1,050590177	1
Mvd	1,050590177	1
Knop1	1,050517358	1
M1ap	1,050444544	1
Timm10	1,050444544	1
Gm43637	1,050226133	1
Rpl36a-ps3	1,050226133	1
Rer1	1,049789447	1
Mcat	1,049716684	1
Naxd	1,049716684	1
Ccdc124	1,049643925	1
Scd1	1,049643925	1
Lsm1	1,049571172	1
Gm10443	1,049571172	1
Ywhae	1,049571172	1
Nol6	1,049425681	1
Ten1	1,049280209	1
Atxn2l	1,049280209	1
Ccdc137	1,049134758	1

Atp6v1h	1,049134758	1
Man1a2	1,04906204	1
Vav3	1,04891662	1
Rnf115	1,04891662	1
Elp6	1,048843917	1
Gm44126	1,048843917	1
Pfdn5	1,048553156	1
Gm10161	1,048480478	1
Gpsm3	1,048480478	1
Dagla	1,048407806	1
Rnf10	1,048407806	1
Lamtor4	1,048262476	1
Mast2	1,048189818	1
Nr4a2	1,048189818	1
Snhg3	1,048189818	1
Ppp1r7	1,047899238	1
Coa5	1,047826606	1
Gabarap	1,047826606	1
Entpd6	1,047608739	1
2310061I04Rik	1,047608739	1
Scoc	1,047608739	1
Fbxo21	1,047608739	1
Gpatch1	1,047536127	1
Trmt112	1,047536127	1
Cyb5r1	1,047536127	1
Ndufb2	1,04746352	1
Klhl22	1,047318321	1
Smc6	1,047318321	1
Lxn	1,047245729	1
Gm7809	1,047173142	1
Tor3a	1,047173142	1
Fam53a	1,047173142	1
Ppox	1,04710056	1
Kif15	1,04710056	1
Celf3	1,046955411	1
Stambpl1	1,046882844	1
Dtx3l	1,046882844	1
Cdca8	1,046882844	1
Smim20	1,046810282	1
Dnal4	1,046810282	1
Chuk	1,046665173	1
Gm12606	1,046665173	1
Ccdc115	1,046665173	1
Samsn1	1,046592627	1
Hemk1	1,046520085	1
Edem1	1,046520085	1
Hnrnpc	1,046520085	1
Vwa7	1,046447548	1
Nr1d2	1,046447548	1
Slx4ip	1,046375016	1
D17Wsu92e	1,046375016	1
Gpr183	1,046157451	1

Emc8	1,04608494	1
Cab39	1,04608494	1
Cnot4	1,046012433	1
Thap4	1,045939932	1
Vapa	1,045939932	1
Cisd3	1,045867435	1
Cacul1	1,045867435	1
Itgb1	1,045867435	1
Ptgr2	1,045794944	1
Zfx	1,045794944	1
Stim1	1,045794944	1
Nupr1	1,045722457	1
Fam192a	1,045722457	1
H2afj	1,045722457	1
Rps2-ps5	1,045649976	1
Brox	1,045649976	1
Mutyh	1,045577499	1
P2rx4	1,045577499	1
Eif2s2	1,045577499	1
Ulk3	1,045505028	1
Sar1a	1,045505028	1
Qtrt1	1,045432562	1
Skp1a	1,0453601	1
Phf2	1,045215193	1
Dstn	1,045215193	1
Tmem107	1,045142746	1
Rsbn1l	1,045142746	1
Ccdc61	1,045070305	1
Cpt2	1,045070305	1
Abtb1	1,044925438	1
Atg13	1,044925438	1
Epn1	1,044925438	1
Fbxl20	1,04478059	1
Sfr1	1,044708174	1
Mark4	1,044490956	1
Fam110a	1,044490956	1
1700088E04Rik	1,044273782	1
Lta4h	1,044273782	1
2610301B20Rik	1,044129025	1
Smc2	1,043984288	1
Gm15501	1,043911927	1
Nap1l4	1,043911927	1
Tbccd1	1,043839571	1
Cd14	1,043839571	1
Lmf1	1,043767221	1
Ddx56	1,043694875	1
Fam207a	1,043622534	1
Psmc2	1,043405541	1
Jmjd6	1,04333322	1
Prr7	1,043043987	1
Ccne1	1,042971691	1
Ebp	1,042971691	1

Gm14843	1,0428994	1
Tmem9	1,0428994	1
Metap1d	1,0428994	1
Dennd6a	1,042827115	1
Nifk	1,042827115	1
Traf1	1,042754834	1
Itm2b	1,042754834	1
Fam219a	1,042682558	1
Poglut1	1,042682558	1
Mafg	1,042610287	1
Nudcd2	1,042610287	1
Tsc2	1,042538022	1
Gm15148	1,042538022	1
Lyrm2	1,042465761	1
Pex10	1,042393505	1
Gm7123	1,042321254	1
Det1	1,042249009	1
Zbtb5	1,042249009	1
Prep	1,042249009	1
N4bp2	1,042249009	1
Gm14586	1,042249009	1
M6pr	1,042176768	1
Zfp9	1,042104532	1
Mrps25	1,042032302	1
Chmp2a	1,042032302	1
Diaph1	1,042032302	1
Acp1	1,041887855	1
Sec22b	1,041887855	1
Mark3	1,04181564	1
Neu3	1,041743429	1
Snrnp40	1,041671223	1
Snx10	1,041599023	1
Rsl24d1	1,041526827	1
Arfip1	1,041454636	1
Srf	1,041454636	1
Klhdc4	1,041454636	1
Card19	1,041454636	1
Noa1	1,04131027	1
Zc3h14	1,04131027	1
Cryz	1,04131027	1
Elof1	1,04131027	1
6720427I07Rik	1,041238095	1
Lysmd1	1,041093758	1
Abcf2	1,041093758	1
Ndufa3	1,041093758	1
Rogdi	1,041021598	1
Irf8	1,040805146	1
Ccdc94	1,04066087	1
Cdkn2aipnl	1,04066087	1
Ift27	1,040588739	1
Aldoart1	1,040516613	1
Urb1	1,040516613	1

Tbc1d23	1,040516613	1
Slc35f6	1,040516613	1
Nek6	1,040372377	1
Esyt1	1,040372377	1
Atp6ap1	1,040372377	1
Gnpnat1	1,040228161	1
Nudcd1	1,04015606	1
Anapc16	1,04015606	1
Mettl6	1,04015606	1
RP24-496O17.7	1,040083965	1
Aar2	1,040083965	1
Arhgap11a	1,040083965	1
Gfm2	1,040011874	1
Fkbp2	1,039939788	1
Icam1	1,039939788	1
Mrpl35	1,039939788	1
Brd9	1,039867708	1
Rpl21-ps5	1,039795632	1
Fnip1	1,039795632	1
Taldo1	1,039795632	1
Gm7384	1,039723561	1
Parl	1,039723561	1
Tmem134	1,039723561	1
Fam104a	1,039651496	1
Eif3i	1,039651496	1
1600014C10Rik	1,03950738	1
Mllt3	1,039435329	1
Afp	1,039219207	1
Polr3c	1,039219207	1
Parp2	1,03900313	1
Mbtps2	1,03900313	1
Svip	1,038931114	1
Dus4l	1,038931114	1
Scfd1	1,038931114	1
Mapkap1	1,038931114	1
Cuedc2	1,038715097	1
Mia3	1,03857111	1
Actr6	1,038499125	1
Sde2	1,038427144	1
Rnf149	1,038355168	1
Apobec1	1,038355168	1
Sat2	1,038283197	1
Inpp1	1,038283197	1
Abce1	1,038283197	1
Zfp142	1,038211231	1
Ptcd3	1,038139271	1
Hic2	1,038139271	1
Vav2	1,038067315	1
R3hcc1l	1,038067315	1
Cfl1	1,037995364	1
Anp32b	1,037851477	1
Chrnb2	1,037779541	1

Txndc17	1,037779541	1
Mbd2	1,03770761	1
Ptp4a3	1,03770761	1
Slc2a1	1,037635684	1
Csgalnact2	1,037563763	1
Cox16	1,037491848	1
Slc2a8	1,03727613	1
Tnni2	1,03727613	1
Slc35b2	1,03727613	1
Kdm1b	1,037204234	1
Rrp12	1,037204234	1
Ctnnbip1	1,037204234	1
Mrpl44	1,037132343	1
Lin9	1,037132343	1
Slc25a5	1,037060457	1
Atp23	1,037060457	1
Hist2h2be	1,037060457	1
Gaa	1,037060457	1
Otud7b	1,037060457	1
Dicer1	1,036916699	1
Vps36	1,036916699	1
Cops7b	1,036844828	1
Zfyve16	1,036844828	1
Exosc10	1,036701101	1
Ttc33	1,036701101	1
Pdcd4	1,036629245	1
Rpsa-ps11	1,036557394	1
Wdr4	1,036557394	1
Selenom	1,036557394	1
Tmem234	1,036485547	1
Abl2	1,03634187	1
Dtx4	1,03634187	1
Rabgap1	1,036270039	1
Gm12184	1,036198213	1
Fndc10	1,036198213	1
Gt(ROSA)26Sor	1,036198213	1
Cfdp1	1,036198213	1
Xrn2	1,036198213	1
Chfr	1,036126391	1
Grsf1	1,036126391	1
Dock1	1,035910957	1
Gm43681	1,035839156	1
Letm1	1,035839156	1
1700021F05Rik	1,035767359	1
Kat7	1,035767359	1
Tifa	1,035695568	1
Dcaf12	1,035695568	1
Vkorc1	1,035552	1
Aimp1	1,035480224	1
Rnps1	1,035336685	1
4930455G09Rik	1,035336685	1
Zfp444	1,035264924	1

Brd1	1,035193167	1
Aftph	1,035193167	1
Hacd4	1,035121416	1
Dusp28	1,035049669	1
Eif2s3y	1,035049669	1
Lysmd4	1,034977927	1
Cpsf4	1,034977927	1
Smim11	1,034906191	1
Ifi47	1,034834459	1
Edc3	1,034834459	1
Ankmy2	1,034762732	1
Trnt1	1,03469101	1
Cecr5	1,034619293	1
Oaz2	1,034475875	1
Rps19	1,034475875	1
Coq5	1,034404173	1
Snord72	1,034260784	1
Ppp5c	1,034260784	1
Otud3	1,034189097	1
Slc45a3	1,034045738	1
Rbpsuh-rs3	1,034045738	1
Xxylt1	1,033974066	1
Tcea1	1,033974066	1
Gm43138	1,033902398	1
Gm2199	1,033830736	1
A130050O07Rik	1,03361578	1
Fbxo7	1,03361578	1
Rpl19-ps9	1,033544137	1
Gm5422	1,0334725	1
Dtwd1	1,0334725	1
Gm16053	1,033400868	1
Gm24916	1,033400868	1
Ddx49	1,033400868	1
Rpa1	1,033400868	1
Zfp408	1,033257618	1
Tollip	1,033257618	1
Skil	1,033186	1
Thop1	1,033114388	1
Il2rg	1,033114388	1
Ubqln2	1,032971178	1
Pdlim5	1,032971178	1
Fam168b	1,032971178	1
Relt	1,03289958	1
Scyl3	1,03289958	1
Stom	1,03289958	1
Nol4l	1,032827987	1
Adi1	1,0327564	1
Stag2	1,032684817	1
Gin1	1,032613239	1
Pdha1	1,032613239	1
Cct2	1,032541666	1
Mfsd8	1,032470098	1

Rhou	1,032470098	1
Lsm6	1,032470098	1
Snapc2	1,032326978	1
Sptssa	1,032255425	1
Clk1	1,032183877	1
Anks3	1,032040795	1
Kansl3	1,032040795	1
Gm9892	1,031969262	1
Exd2	1,031897734	1
Hoxa1	1,031897734	1
Met	1,031754693	1
Gatc	1,031754693	1
Dusp14	1,031754693	1
Snhg9	1,031754693	1
Ccdc71l	1,031683179	1
Lars2	1,031683179	1
Hcfc1	1,031611671	1
Metap1	1,031468669	1
2410015M20Rik	1,031468669	1
Fbl	1,031397176	1
Rpl4	1,031397176	1
Rac2	1,031397176	1
Rhoc	1,031397176	1
Gm45286	1,031325687	1
Gm11914	1,031325687	1
Emc2	1,031325687	1
Ankrd13c	1,031254204	1
Acer3	1,031111251	1
BC029722	1,03089686	1
Gm4673	1,030825406	1
Pmf1	1,030825406	1
Mapk8ip3	1,030825406	1
Cdkn2d	1,030825406	1
Ogfod2	1,030682513	1
Mpp1	1,030682513	1
Csnk2b	1,030682513	1
Tor1a	1,030682513	1
Lyz1	1,030682513	1
Nudt18	1,030611074	1
Chka	1,030611074	1
Usb1	1,030611074	1
Gm9840	1,03053964	1
Atp5sl	1,030468211	1
Trim7	1,030325368	1
Elp5	1,030325368	1
Eif2d	1,030253954	1
Gm9790	1,030253954	1
Tspo	1,030253954	1
Sephs1	1,030182544	1
Txn2	1,030182544	1
Nxf1	1,029968346	1
Timm8b	1,029968346	1

Hnrnpul1	1,029896957	1
Mrpl51	1,029825572	1
Rlf	1,029825572	1
Zbed3	1,029540083	1
Osbpl8	1,029540083	1
Lrig3	1,029468724	1
Rgmb	1,029397369	1
Rps24-ps2	1,029397369	1
Aup1	1,029397369	1
Dnajc2	1,029326019	1
D1Ertd622e	1,029326019	1
Smu1	1,029326019	1
Mbd6	1,029254674	1
A630033H20Rik	1,029183334	1
Gm8539	1,029111999	1
Aimp2	1,029111999	1
Tigar	1,029111999	1
Pla2g4a	1,029111999	1
Mpc2	1,029040669	1
B4galt5	1,028969343	1
Ubap2l	1,028969343	1
Slc11a2	1,028826708	1
Sdhaf3	1,028755398	1
Uqcrc2	1,028755398	1
Mybl1	1,028684092	1
Rplp1	1,028684092	1
2810433D01Rik	1,028541496	1
Cdc73	1,028541496	1
Mtpn	1,028541496	1
Uqcrc1	1,028470206	1
Puf60	1,028470206	1
Ppp2r2a	1,028470206	1
Itgal	1,028256363	1
Ubiad1	1,028256363	1
Atp5a1	1,028185093	1
Usp45	1,028113827	1
Slc2a3	1,028113827	1
Bcor	1,028042566	1
Yme1l1	1,028042566	1
Oxct1	1,028042566	1
Gtf3a	1,02797131	1
Tyrobp	1,02797131	1
Gm11772	1,027900059	1
Ppid	1,027757571	1
Spag9	1,027757571	1
Atox1	1,027757571	1
Ccdc82	1,027543877	1
Klhl7	1,027472656	1
Thumpd1	1,027401439	1
Mrpl15	1,027401439	1
Ddr1	1,027259021	1
Mios	1,027116623	1

Gtpbp6	1,027116623	1
Echdc1	1,027116623	1
Gcc1	1,027116623	1
Tmem192	1,027045431	1
Ccdc58	1,027045431	1
Mxi1	1,027045431	1
Srsf4	1,026831885	1
Rps25-ps1	1,026831885	1
Ncdn	1,026760713	1
1700037H04Rik	1,026760713	1
Cd99l2	1,026760713	1
Ube2b	1,026760713	1
Tor4a	1,026689546	1
Mff	1,026618383	1
Dync1li1	1,026618383	1
Snx3	1,026547226	1
Ufm1	1,026547226	1
Creld1	1,026476074	1
Urgcp	1,026404926	1
Alkbh1	1,026404926	1
Ifi30	1,026404926	1
Gm15829	1,026333784	1
Dtymk	1,026333784	1
Ndufs8	1,026333784	1
Gm4737	1,026262646	1
Rbbp6	1,026262646	1
Fundc2	1,026262646	1
Utp3	1,026262646	1
Dpysl2	1,026191514	1
Dpm2	1,026120386	1
Sarnp	1,026049263	1
Gm3145	1,025907032	1
Rbmx2-ps	1,025907032	1
Fip1l1	1,025907032	1
Gm42547	1,025764821	1
Dpp3	1,025764821	1
Pole3	1,025764821	1
Galk2	1,025693723	1
Gm36266	1,02562263	1
Emc6	1,025551542	1
Ggnbp2	1,025551542	1
Nop2	1,025480458	1
Rpl32	1,02540938	1
Cfl2	1,02540938	1
Mrfap1	1,02540938	1
Gm42566	1,025338306	1
Arf5	1,025267238	1
Kxd1	1,025267238	1
Lsm4	1,025196174	1
Pikfyve	1,025196174	1
Fabp7	1,025125116	1
Fam220a	1,025125116	1

Commd8	1,025054062	1
Hsbp1	1,024983013	1
Lpcat1	1,024911969	1
Cyld	1,024769896	1
Gm12716	1,024698867	1
Gm4924	1,024627842	1
Dcp2	1,024627842	1
Csf2rb	1,024343795	1
Rpl12-ps1	1,024272795	1
Nono	1,024272795	1
Hspa4	1,024272795	1
R3hdm2	1,0242018	1
Pcyt1a	1,0242018	1
Mkln1	1,024059826	1
Micu1	1,023988846	1
Mfng	1,023988846	1
RP23-277D1.1	1,023917871	1
Pnkp	1,0238469	1
Gm2796	1,023775935	1
March7	1,023775935	1
Chchd2	1,023775935	1
Narfl	1,023563069	1
N4bp2l1	1,023563069	1
2810428I15Rik	1,023492124	1
Dnmbp	1,023421183	1
Wbscr22	1,023421183	1
Gm12582	1,023279317	1
Zfp397	1,023208391	1
Zfp64	1,023208391	1
Nrbp1	1,02313747	1
Sord	1,02313747	1
2010107E04Rik	1,02313747	1
Bcl2l13	1,023066554	1
Pik3cb	1,023066554	1
Ssr2	1,023066554	1
Gm26532	1,022924736	1
Scrn3	1,022924736	1
Tep1	1,022924736	1
Ndufa4	1,022853835	1
Dr1	1,022853835	1
Susd6	1,022853835	1
Nol7	1,022853835	1
Ckap2l	1,022782939	1
Zmynd19	1,022641161	1
Tbce	1,022641161	1
Tsta3	1,022570279	1
Gm44950	1,022499402	1
Trim59	1,022499402	1
Nampt	1,022357664	1
Btrc	1,022286802	1
Terf1	1,022286802	1
Gm1943	1,022215945	1

Klhl21	1,022215945	1
Atg7	1,022145093	1
Cd2bp2	1,022145093	1
Sbno1	1,022145093	1
Vars	1,022074245	1
Eif2b5	1,022074245	1
Myo1e	1,022074245	1
Rnf217	1,022003403	1
Amz2	1,021932566	1
Sep 06	1,021861733	1
Etv3	1,021790905	1
Zfp764	1,021720083	1
Angel1	1,021578452	1
Tap1	1,021578452	1
Gm44152	1,021436841	1
Nek4	1,02129525	1
St3gal2	1,021224461	1
Gm43154	1,021153678	1
RP23-308G10.5	1,021153678	1
Mief2	1,021012126	1
Ppp1r8	1,020941357	1
Tm2d1	1,020870593	1
Rasgef1a	1,020870593	1
Tmem208	1,020870593	1
Lasp1	1,020870593	1
Scaf8	1,020870593	1
Mrm3	1,020799834	1
Papola	1,02072908	1
Rida	1,020658331	1
Camsap1	1,020658331	1
Ifnar2	1,020658331	1
Prr13	1,020658331	1
1110008L16Rik	1,020587587	1
Map3k10	1,020516848	1
Lrp10	1,020516848	1
Rps12-ps1	1,020446113	1
Grn	1,020446113	1
Scand1	1,020375384	1
Hdgfrp2	1,020375384	1
Pebp1	1,020304659	1
Mcm7	1,02023394	1
Mtbp	1,020163225	1
Ier3ip1	1,020163225	1
Heatr1	1,020092515	1
Atp9b	1,01995111	1
Racgap1	1,019880415	1
Gm4332	1,019880415	1
Txnrd1	1,019880415	1
Nelfcd	1,019739039	1
Unk	1,019668359	1
Med15	1,019597683	1
Pcbd2	1,019597683	1

Mipol1	1,019527012	1
Prpf18	1,019527012	1
Txlna	1,019456347	1
Sh3bp1	1,019385686	1
Nop58	1,019385686	1
Ap4s1	1,01931503	1
H2-T22	1,01931503	1
Mb21d1	1,01931503	1
Mapk7	1,019244379	1
Ndufa10	1,019244379	1
Gm6977	1,019173732	1
Gclm	1,019173732	1
Gorasp2	1,019173732	1
Gm43011	1,019032455	1
2610318N02Rik	1,019032455	1
Borcs5	1,018961823	1
Stx3	1,018961823	1
Thap3	1,018961823	1
Ralgds	1,018749958	1
Dffb	1,018679346	1
Ern1	1,018608739	1
Abhd13	1,018608739	1
Arhgef10l	1,018538137	1
Gm9531	1,018538137	1
Zfp382	1,01846754	1
Sos1	1,01846754	1
Gtf2a1	1,018396947	1
Psmb3	1,01832636	1
Tfb2m	1,018255777	1
Vps13b	1,018255777	1
Tipin	1,018255777	1
Itgb5	1,018044059	1
Snhg12	1,018044059	1
Maip1	1,017902938	1
Cmip	1,017902938	1
Rptor	1,017691293	1
Aldh1l1	1,017620755	1
Cab39l	1,017550221	1
Slc6a13	1,017479692	1
Tmem167b	1,017479692	1
Ccdc50	1,017409168	1
Sprtn	1,017268135	1
Zfp68	1,017268135	1
Gid4	1,017268135	1
Pip4k2c	1,017127122	1
Lnpk	1,017056622	1
Milr1	1,017056622	1
Kremen1	1,016986128	1
Ndufa1	1,016986128	1
Melk	1,016915638	1
Grpel1	1,016915638	1
Clec4a3	1,016845153	1

Commd7	1,016704198	1
Bloc1s6	1,016704198	1
Entpd1	1,016633728	1
Gpr179	1,016633728	1
Rplp0	1,016633728	1
Srprb	1,016563263	1
Uap1	1,016492803	1
Rps18	1,016422347	1
Gm17491	1,016422347	1
U2af2	1,016351897	1
Mis12	1,016351897	1
Rhoa	1,016351897	1
Tram2	1,01621101	1
Cpped1	1,01621101	1
Ckap5	1,01621101	1
Ngdn	1,01621101	1
Ctc1	1,016140574	1
Cdipt	1,016140574	1
Akap8	1,015999717	1
Fkbp1a	1,015999717	1
Glul	1,015929296	1
Lcmt1	1,015929296	1
Gm42636	1,015858879	1
Katnbl1	1,015858879	1
Ddx5	1,015858879	1
Ctu1	1,015788468	1
Arfgap3	1,015718061	1
Rnaseh2b	1,015647659	1
Actr1b	1,015647659	1
Slc12a9	1,015577262	1
Shmt2	1,01550687	1
Srp14	1,01550687	1
Nfxl1	1,015366101	1
Gata3	1,015295723	1
Dgkd	1,015295723	1
Daxx	1,015295723	1
Mkx	1,015225351	1
Gm17108	1,015084621	1
Gm9938	1,015084621	1
Kcnk6	1,015084621	1
Ywhaq	1,015084621	1
Blmh	1,015084621	1
Zfp51	1,015014263	1
Ift22	1,014873562	1
Ahctf1	1,014803218	1
A330069E16Rik	1,01473288	1
Taf2	1,014662547	1
Commd3	1,014662547	1
Dnajb5	1,014451575	1
Nln	1,014451575	1
Bmpr2	1,014451575	1
1600002K03Rik	1,014381261	1

Sec61a2	1,014381261	1
Cutc	1,014310952	1
Lyrm4	1,014240648	1
1110059E24Rik	1,014240648	1
Gm45033	1,014170349	1
Mink1	1,014170349	1
Tbkbp1	1,014170349	1
Rb1	1,014170349	1
Hmg20b	1,014170349	1
Prdx6	1,014170349	1
Rab4a	1,014100054	1
Als2	1,014029765	1
Mpp5	1,014029765	1
Polh	1,01395948	1
Gm20633	1,01395948	1
Diexf	1,01395948	1
H6pd	1,0138892	1
Slc4a1ap	1,0138892	1
Gm20900	1,013818925	1
Nde1	1,013748655	1
3110040N11Rik	1,013748655	1
Ndufs7	1,013608129	1
Rpl34	1,013608129	1
Plekhf1	1,013537874	1
Capn3	1,013467623	1
Ubac2	1,013327136	1
Hace1	1,0132569	1
Telo2	1,013116443	1
Cotl1	1,013116443	1
Col27a1	1,012976005	1
Dus3l	1,012976005	1
C77080	1,012695187	1
Ndufa13	1,012695187	1
Gm45840	1,012624995	1
Proser1	1,012624995	1
Atg9b	1,012554807	1
mt-Nd4	1,012554807	1
Cdk13	1,012414447	1
Azi2	1,012414447	1
Utp14b	1,012414447	1
Coq7	1,012414447	1
Szrd1	1,012414447	1
Yaf2	1,012344274	1
Rapgef6	1,012274106	1
Sp110	1,012203943	1
Nexn	1,012203943	1
Abhd14b	1,012203943	1
Suclg2	1,012203943	1
Trim41	1,012203943	1
Hagh	1,012203943	1
Erbb3	1,012133785	1
Gse1	1,012063632	1

Ankrd50	1,011993483	1
Itfg1	1,01192334	1
Gm10501	1,011783067	1
Dag1	1,011783067	1
Snx5	1,011642814	1
Epg5	1,01150258	1
Kif21b	1,01150258	1
Eny2	1,01150258	1
Eef2	1,01150258	1
Rbak	1,011432471	1
Txndc5	1,011432471	1
Gm11598	1,011362366	1
Wdr47	1,011292266	1
Sh3bgr	1,011292266	1
Rpa2	1,011292266	1
Gm45311	1,011222171	1
Gltscr1l	1,011222171	1
Zcchc11	1,011222171	1
Mrpl36	1,011222171	1
Fbxo46	1,011081996	1
Otud6b	1,011081996	1
Wdr73	1,011011915	1
Prdm15	1,011011915	1
Psmf1	1,011011915	1
Rpl31-ps10	1,01094184	1
Aifm1	1,010731642	1
Ufd1l	1,010661586	1
4930447F24Rik	1,010591535	1
Sys1	1,010591535	1
Washc2	1,010521488	1
Ndufv2	1,010521488	1
Dgat1	1,010451446	1
Gm11952	1,01038141	1
Copb2	1,010241351	1
Rgs3	1,010171329	1
Brat1	1,010171329	1
Llgl2	1,010171329	1
Sfxn1	1,010171329	1
Cap1	1,010101311	1
Dph7	1,009961291	1
Prpf40a	1,009751298	1
Zfand4	1,009681309	1
Eif4a3	1,009681309	1
Alg12	1,009611326	1
Pdp2	1,009611326	1
Sqle	1,009611326	1
Fam126a	1,009541348	1
Rfk	1,009541348	1
Nap1l1	1,009541348	1
1700012D14Rik	1,009471374	1
Cers6	1,009401405	1
Adat3	1,009261482	1

Mmadhc	1,009191528	1
Nectin1	1,009121578	1
Clpx	1,009121578	1
Kcmf1	1,009121578	1
Pid1	1,009051634	1
Arpc5	1,008981694	1
Cd81	1,008911759	1
Htatsf1	1,008911759	1
Zeb2	1,008911759	1
Agk	1,008841829	1
Ankrd17	1,008841829	1
Gm29666	1,008771904	1
Wdr33	1,008701984	1
Angptl4	1,008492252	1
Parp10	1,008492252	1
Pigq	1,008492252	1
Hist1h2bp	1,008352455	1
Mppe1	1,008352455	1
Fam120aos	1,008212677	1
Lcorl	1,008212677	1
Rps6ka1	1,008072919	1
Zkscan1	1,008003047	1
Recql	1,00793318	1
Stk4	1,007793461	1
Pex11g	1,007723608	1
Dusp7	1,00765376	1
Sumf1	1,00765376	1
Pomp	1,00765376	1
Trappc9	1,007374418	1
Trak1	1,007374418	1
Gm26384	1,007164962	1
Fry	1,007095153	1
Pnn	1,007095153	1
Tmem218	1,007025349	1
Zdhhc7	1,007025349	1
Ift172	1,007025349	1
Nsmaf	1,007025349	1
Fbxo22	1,00695555	1
Dnm1	1,006885756	1
Pitpnb	1,006885756	1
Tcf12	1,006815966	1
Bst2	1,006676401	1
Amz1	1,006676401	1
Msh3	1,006606626	1
Cryl1	1,006536856	1
2310015A10Rik	1,006536856	1
Dis3l2	1,006536856	1
Immp1l	1,006467091	1
Acap2	1,006467091	1
Aaed1	1,00639733	1
Zfp12	1,00639733	1
Psmd1	1,00639733	1

Gchfr	1,006327574	1
Apeh	1,006327574	1
Rit1	1,006327574	1
Stk19	1,006327574	1
Spag4	1,006257823	1
Gm15151	1,006257823	1
Atp5f1	1,0060486	1
Slc2a9	1,005909142	1
Ubl4a	1,00569999	1
Vps53	1,005630283	1
Uchl3	1,005630283	1
Armc10	1,00556058	1
Mxra8	1,005351502	1
Glrx5	1,005351502	1
Klhl26	1,005281818	1
Tmbim6	1,005281818	1
Lgmn	1,005142466	1
Inppl1	1,005142466	1
0610040B10Rik	1,005072798	1
Ttll1	1,005003134	1
Gm14780	1,005003134	1
Arglu1	1,005003134	1
Plekhm2	1,005003134	1
Tulp4	1,004933475	1
Zswim4	1,00486382	1
Snrpa1	1,00486382	1
Nt5c3	1,004794171	1
Ptgs1	1,004724526	1
Ppp4r2	1,004724526	1
Ncoa1	1,004654887	1
1500011K16Rik	1,004585252	1
Mrpl33	1,004515622	1
1700096K18Rik	1,004445996	1
Fbxo2	1,004445996	1
Dennd6b	1,004376376	1
Tab1	1,004376376	1
Tmem199	1,00430676	1
Ovgp1	1,004167543	1
Gm24601	1,004167543	1
Bmp2k	1,004167543	1
Ttc32	1,004097942	1
Gm6265	1,004097942	1
Gm5697	1,003958754	1
Syne3	1,003889167	1
Prkra	1,003610869	1
Dclre1a	1,003541306	1
Ltbr	1,003471749	1
Med11	1,003402196	1
Dvl3	1,003124032	1
Dlg1	1,003124032	1
Rexo1	1,003054503	1
Glyr1	1,003054503	1

Rpl21-ps12	1,00291546	1
Gm2225	1,002845945	1
Six1	1,002776436	1
Dolpp1	1,002776436	1
Gcsh	1,002776436	1
Nol9	1,002776436	1
RP23-261O3.5	1,002706931	1
Tspyl4	1,002706931	1
Rpl27-ps3	1,002567936	1
Ly9	1,002567936	1
Parp12	1,002567936	1
Ctdnep1	1,002567936	1
Pex11b	1,002498446	1
Agap3	1,002498446	1
Ddah2	1,00242896	1
Hist1h2aa	1,00242896	1
Ano6	1,00235948	1
Bfar	1,002290004	1
Hint1	1,002290004	1
Rpl3	1,002220533	1
A330035P11Rik	1,002151066	1
Gm7984	1,002151066	1
Rpap2	1,002081605	1
Nop56	1,002081605	1
Rras	1,002081605	1
Gm44434	1,002012148	1
Mtss1	1,002012148	1
Stxbp2	1,001942697	1
Rest	1,00187325	1
Ipo11	1,001803808	1
Exo1	1,00173437	1
Grin1	1,00173437	1
Cnep1r1	1,00173437	1
Cpd	1,00173437	1
Polrmt	1,001664938	1
Gm43588	1,00159551	1
Rps11-ps2	1,00159551	1
Gnai3	1,001456669	1
Gm26982	1,001248444	1
Pdhx	1,001179045	1
Ppp2r5c	1,001109651	1
Smc1a	1,000970877	1
Hmgxb3	1,000762753	1
Specc1l	1,000624027	1
Rab8b	1,000554672	1
Nucks1	1,000485321	1
Rmnd5a	1,000415975	1
Kifc2	1,000346634	1
Wdhd1	1,000277297	1
Zfp1	1,000138639	1
Atp8a1	1,000069317	1
Tmed9	1,000069317	1

Kmt5b	1	1
Fbxw2	1	1
Ube2r2	1	1
Gm42418	1	1
Bcl3	-7,13E-05	1
Smndc1	-6,54E-05	1
Prkag1	-0,0003784	1
Arhgap10	-0,0004154	1
Neu1	-0,00038565	1
Uqcr10	-0,00041068	1
Lace1	-0,0006123	1
Neurl1b	-0,00061427	1
Cs	-0,00058515	1
Gm16556	-0,00074213	1
Usp16	-0,00084681	1
Laptm4a	-0,00080079	1
Pth1r	-0,00092835	1
Psmd3	-0,00085832	1
Gm44027	-0,0011264	1
2310039H08Rik	-0,0010586	1
Slc48a1	-0,0010642	1
Pgrmc1	-0,001316	1
Cdk18	-0,0012883	1
Rala	-0,0012813	1
Cks1b	-0,001374	1
Pole	-0,0014588	1
Suox	-0,0014655	1
Mocos	-0,0014729	1
Tars2	-0,001521	1
Vegfa	-0,0014722	1
Fhad1	-0,0015734	1
Mto1	-0,0015775	1
Fmnl3	-0,0015659	1
Zfp646	-0,0017915	1
Tinf2	-0,0021117	1
Wdr70	-0,0021139	1
1810026B05Rik	-0,0020987	1
Ssbp3	-0,0021929	1
Ptch1	-0,0021986	1
Msl3	-0,0022512	1
Baiap2	-0,0022729	1
Tssc4	-0,0023071	1
Scpep1	-0,0023001	1
Ybey	-0,0023932	1
Ints6	-0,0023506	1
Otud4	-0,0023932	1
Xpo1	-0,002503	1
Ttc1	-0,0027052	1
Crtap	-0,0028305	1
Numb	-0,0030181	1
Parvb	-0,0029988	1
Lrp6	-0,0031017	1

Gm12857	-0,003102	1
Zmat3	-0,0031015	1
Tti2	-0,0031821	1
Opa1	-0,0032405	1
Cdk10	-0,0033392	1
Slbp	-0,0032753	1
Snx16	-0,003298	1
Ska3	-0,0034113	1
Vamp7	-0,0034177	1
Snrpb	-0,0033555	1
Zmat2	-0,0034192	1
Copg1	-0,0035006	1
Acd	-0,0037494	1
Ddx54	-0,0037181	1
Pxk	-0,0037689	1
Wrap53	-0,0038906	1
Ptges3	-0,0038596	1
Slc25a39	-0,0040583	1
Cpeb4	-0,0044299	1
Tor1aip1	-0,0044598	1
4930526A20Rik	-0,0045816	1
Atg5	-0,0046226	1
Gm6181	-0,0046992	1
Wiz	-0,0046742	1
Cebpg	-0,0047445	1
Supt3	-0,0046584	1
Dhrs7b	-0,0048228	1
Heatr5b	-0,0048398	1
Sh3bp2	-0,0048628	1
Zdhhc4	-0,0049592	1
Lsm2	-0,0049977	1
Tmbim4	-0,0050312	1
Guf1	-0,0050713	1
Snf8	-0,0052722	1
Vegfb	-0,0053255	1
Tanc2	-0,0053636	1
Fam160b1	-0,0054282	1
Aplp1	-0,0055111	1
Gm13408	-0,0055532	1
Etnk1	-0,0055988	1
Tmem25	-0,0057265	1
Lca5	-0,0056676	1
Fastkd3	-0,0056978	1
Rbfa	-0,005917	1
1700086P04Rik	-0,0060012	1
Idnk	-0,0061268	1
Smdt1	-0,0060967	1
Sptan1	-0,0062319	1
Grb2	-0,0062106	1
Zfas1	-0,0062009	1
Enoph1	-0,006333	1
Gk	-0,0062539	1

Rcn2	-0,0063208	1
Zfp652	-0,0063539	1
Scaf11	-0,0064161	1
Max	-0,0064065	1
Rbm6-ps1	-0,006546	1
Tmed5	-0,006507	1
Alyref	-0,0065807	1
Pura	-0,0065948	1
Mcmbp	-0,0066361	1
RP24-550H10.3	-0,0066861	1
Fkbp3	-0,0067163	1
Commd6	-0,0068161	1
Hk1	-0,0067909	1
Zfp975	-0,0072276	1
Gm43106	-0,0072634	1
Ppat	-0,0072941	1
Selenot	-0,0074154	1
Gm19739	-0,0073567	1
Zcchc8	-0,0074389	1
Rab2a	-0,0073559	1
Cops3	-0,0075014	1
Zfp319	-0,0076029	1
Paqr5	-0,0075869	1
Oscp1	-0,007622	1
Ddx19a	-0,0076111	1
Myl12b	-0,0076563	1
Arfgap2	-0,0078189	1
Gm37660	-0,0078808	1
Mepce	-0,0083308	1
Anln	-0,0084007	1
Ctso	-0,0087889	1
Mocs3	-0,0088558	1
Ninj1	-0,0089321	1
Tubb4a	-0,0090958	1
Mastl	-0,0091403	1
Pex13	-0,0090505	1
Mdk	-0,0091712	1
Cbr4	-0,0091612	1
Gm45380	-0,0093863	1
Cept1	-0,0094109	1
Mrps12	-0,0094813	1
Ung	-0,0095831	1
Fhl3	-0,0095686	1
A930024E05Rik	-0,0097002	1
Hdgf	-0,0096933	1
Cyp20a1	-0,0097966	1
Acbd4	-0,009823	1
Nol11	-0,0097861	1
Rtcb	-0,0098388	1
2310035C23Rik	-0,0098819	1
Eed	-0,010047	1
Sep 10	-0,010061	1

Gm10327	-0,010243	1
Kank2	-0,010198	1
Izumo4	-0,010263	1
Ilvbl	-0,01036	1
Maoa	-0,01065	1
Serpinb8	-0,010744	1
Cwc25	-0,010793	1
Wdr89	-0,010776	1
Smg6	-0,010911	1
Coq8b	-0,010961	1
Comt	-0,011042	1
Cers4	-0,010986	1
C2cd2l	-0,010981	1
Hnrnpa0	-0,011097	1
Rps19-ps8	-0,011536	1
Parp1	-0,011517	1
Naf1	-0,0116	1
Man1c1	-0,011701	1
Cyth3	-0,011924	1
E130309D02Rik	-0,01193	1
Msn	-0,012033	1
Rin2	-0,012143	1
Top2a	-0,012092	1
Slc35b1	-0,012086	1
4921536K21Rik	-0,012215	1
Gm37893	-0,012215	1
Zfp958	-0,012218	1
Zfp110	-0,012296	1
Ddost	-0,012376	1
Uap1l1	-0,012492	1
Rpl30	-0,012648	1
Ttc38	-0,012623	1
Timm9	-0,012644	1
Gm14439	-0,01269	1
Nfrkb	-0,012653	1
Map7d1	-0,012764	1
Slc29a1	-0,012894	1
Irf9	-0,012917	1
Hsp90aa1	-0,012939	1
Atp6v0e	-0,012888	1
Psmd14	-0,012928	1
Gm12906	-0,013121	1
Mta1	-0,01313	1
Plekhm1	-0,013116	1
Dnajc7	-0,013242	1
Pes1	-0,013195	1
Zgrf1	-0,013316	1
Rdh5	-0,013496	1
Cd63-ps	-0,013625	1
Rela	-0,013631	1
Uqcrq	-0,013597	1
Gm12166	-0,013691	1

Polr2h	-0,013675	1
Dok1	-0,013679	1
Akr1a1	-0,013663	1
Camk2d	-0,013829	1
Tmem39a	-0,013874	1
Peli1	-0,013869	1
Zc3hav1l	-0,014064	1
Mrps15	-0,014075	1
Vdac1	-0,014133	1
Ifi44	-0,014255	1
Ebi3	-0,014457	1
Zcchc17	-0,014584	1
Fam71f2	-0,014728	1
Scp2	-0,014674	1
Slc7a5	-0,014677	1
Sil1	-0,014741	1
Elob	-0,014876	1
Wdr5b	-0,01502	1
Thumpd2	-0,015043	1
Gpd1l	-0,014984	1
Cct8	-0,014982	1
Stk3	-0,015056	1
Iqsec2	-0,01515	1
Cox6b1	-0,015067	1
Mllt11	-0,015121	1
Nus1	-0,015128	1
Nploc4	-0,01526	1
Park7	-0,015279	1
Dynlt1f	-0,015364	1
Atf7	-0,015414	1
Dctn5	-0,015481	1
Tmx1	-0,015478	1
Vps11	-0,015614	1
Lrch1	-0,015852	1
Tomm5	-0,015934	1
Cc2d1a	-0,016027	1
Vapb	-0,015953	1
Fastkd2	-0,016059	1
Pop5	-0,016095	1
Erp29	-0,016333	1
Bin1	-0,016426	1
Btbd1	-0,016373	1
Snhg1	-0,016437	1
H2-M3	-0,016467	1
Sh2d6	-0,016488	1
Osbpl11	-0,016601	1
Gm20568	-0,016677	1
Gm10288	-0,016789	1
Ccnd3	-0,016809	1
Jag1	-0,017004	1
Lgals1	-0,01695	1
Sgf29	-0,01712	1

Slc36a1	-0,017148	1
Acy3	-0,017345	1
1700109H08Rik	-0,017272	1
Smim14	-0,017293	1
Kif1c	-0,017325	1
Eif5b	-0,017287	1
Hps5	-0,01742	1
Klf9	-0,017366	1
Gm12882	-0,017453	1
Gm19272	-0,017541	1
2310010J17Rik	-0,017736	1
Ccnb2	-0,017739	1
Hivep2	-0,017722	1
1810032O08Rik	-0,017788	1
Mlf2	-0,017808	1
Zfp335	-0,01784	1
Irf5	-0,017783	1
Gm11688	-0,017928	1
Fam20b	-0,018024	1
Usp7	-0,018061	1
Dock4	-0,018153	1
Tmem51	-0,018239	1
Cox5a	-0,018247	1
Sptbn4	-0,01825	1
Rgs19	-0,018256	1
Zscan12	-0,01841	1
Sirt3	-0,018498	1
Aga	-0,018549	1
Erlec1	-0,018874	1
Rabggtb	-0,018895	1
Gm8093	-0,019217	1
Pcm1	-0,019187	1
Gtf3c2	-0,01925	1
Sdf4	-0,019276	1
Dnmt3b	-0,019395	1
Gas2	-0,019357	1
Ran	-0,019396	1
Bcorl1	-0,019465	1
Mcph1	-0,019646	1
Copz1	-0,01958	1
A430010J10Rik	-0,019693	1
Gm3571	-0,019849	1
Map2k3	-0,019816	1
Tmf1	-0,020004	1
Ahcyl1	-0,019972	1
Fam234a	-0,020106	1
Bcl2l11	-0,020091	1
Gm8566	-0,020307	1
Abhd4	-0,020293	1
Commd4	-0,02035	1
A430033K04Rik	-0,020397	1
H2-D1	-0,02039	1

Ctsd	-0,020594	1
Limk2	-0,020725	1
Hspd1	-0,020705	1
Mtmr10	-0,020765	1
Ckb	-0,020815	1
Idua	-0,020887	1
Tmem63a	-0,02087	1
Pddc1	-0,020985	1
Ddx1	-0,021139	1
Abcc5	-0,021065	1
Abracl	-0,021225	1
Ap2s1	-0,021454	1
Gpsm2	-0,021624	1
Twistnb	-0,021619	1
Akt3	-0,021672	1
Lrrc75a	-0,021777	1
Lhx5	-0,021904	1
Ccdc34	-0,022061	1
Gm20186	-0,022173	1
Ddx58	-0,022222	1
Zfyve21	-0,022198	1
Snhg4	-0,022213	1
Slc19a2	-0,022317	1
Gm16425	-0,022457	1
Gm43378	-0,022645	1
Ndufaf2	-0,022579	1
D730003I15Rik	-0,022573	1
Il13ra1	-0,022654	1
Txndc11	-0,022766	1
mt-Cytb	-0,022769	1
Rnf183	-0,023026	1
Rab5c	-0,023009	1
Fbxl4	-0,023166	1
Riok1	-0,023197	1
Thtpa	-0,023303	1
Osbpl9	-0,023314	1
Antxr2	-0,023404	1
Sgk3	-0,023437	1
Pcdhb16	-0,023463	1
Nxt2	-0,023606	1
Psmc6	-0,023633	1
Cyb5r3	-0,023658	1
Fam98b	-0,023772	1
Ncbp3	-0,023996	1
Mrpl30	-0,023966	1
Cox7b	-0,02402	1
Mmachc	-0,024068	1
Dynlt1-ps1	-0,024083	1
Prelid3b	-0,024111	1
Cfap43	-0,024224	1
Slc35b3	-0,024274	1
Pnpo	-0,024433	1

Ahi1	-0,024385	1
Hgh1	-0,024472	1
Amigo1	-0,02456	1
Stk16	-0,024558	1
Exosc4	-0,024564	1
Poldip3	-0,024603	1
Gm11918	-0,024653	1
Ptgr1	-0,024689	1
Cd151	-0,0248	1
Zdhhc14	-0,024832	1
Impdh1	-0,024771	1
Tmem261	-0,024777	1
Samd4b	-0,02485	1
Ddx24	-0,024786	1
Emp3	-0,024971	1
Atxn2	-0,025009	1
Mrps17	-0,02502	1
Ptms	-0,025056	1
Ccdc80	-0,025245	1
Acap3	-0,025287	1
Fchsd2	-0,02544	1
Derl2	-0,025413	1
Sfxn3	-0,025385	1
Arl16	-0,025538	1
Rita1	-0,025533	1
Foxk2	-0,025581	1
Agpat3	-0,025603	1
Tada2b	-0,025698	1
Tcf3	-0,025836	1
Plpp1	-0,025928	1
Canx	-0,025865	1
Eif2ak4	-0,025956	1
Mrpl57	-0,026126	1
Hsp90b1	-0,02614	1
Phyh	-0,026224	1
Caprin1	-0,026227	1
Bzw2	-0,026187	1
Prr18	-0,026252	1
Zc3h13	-0,026275	1
Ncf4	-0,026358	1
Slc9a1	-0,026497	1
1110002L01Rik	-0,026594	1
Kitl	-0,02665	1
Cd300a	-0,026694	1
Tomm34	-0,027017	1
Hltf	-0,027048	1
Yif1a	-0,02713	1
Fkbp4	-0,027145	1
Lst1	-0,027096	1
Fbxw4	-0,027245	1
Cmc2	-0,027315	1
Phrf1	-0,027266	1

Hoxb7	-0,027337	1
Ubxn1	-0,027328	1
Dnajc19	-0,027356	1
Tuft1	-0,027362	1
Zdhhc3	-0,02749	1
1810022K09Rik	-0,027636	1
Akt1	-0,027557	1
Prps1	-0,027592	1
Ppp1cb	-0,027735	1
Mief1	-0,027809	1
Mettl4	-0,027926	1
Eif1ax	-0,027886	1
Yipf5	-0,027977	1
Ube2c	-0,028115	1
Fnbp4	-0,028411	1
Calm2	-0,028397	1
RP24-378K7.3	-0,028529	1
Tmem184c	-0,028456	1
Gnpat	-0,028507	1
Utp6	-0,02871	1
Psrc1	-0,028703	1
Tex9	-0,028908	1
Vps50	-0,028886	1
Uaca	-0,028932	1
Acss2	-0,029088	1
Aida	-0,029107	1
Gm7972	-0,029309	1
Akr1b10	-0,029272	1
Dnajb11	-0,029335	1
Gstp-ps	-0,029427	1
Tefm	-0,029477	1
Brd2	-0,02969	1
Txn1	-0,029657	1
Ube2d3	-0,029732	1
Fam208b	-0,029778	1
Ccdc134	-0,030115	1
A130048G24Rik	-0,030154	1
Nudt2	-0,030173	1
Hk2	-0,030175	1
Trim30a	-0,030264	1
Zmym5	-0,030259	1
Mettl14	-0,030299	1
Zc2hc1a	-0,030495	1
Pno1	-0,030534	1
Cep170b	-0,030626	1
Cox7a2	-0,030556	1
Hist1h4d	-0,030674	1
Cds2	-0,030703	1
Ppie	-0,030819	1
Derl1	-0,030774	1
Rbm15	-0,030921	1
Tmem135	-0,031109	1

Apopt1	-0,031157	1
Cbx5	-0,031207	1
Cct4	-0,031164	1
Eif3c	-0,031297	1
Iqcb1	-0,031455	1
Synj2	-0,031531	1
Ncoa5	-0,031727	1
Sms	-0,031651	1
Hnrnpll	-0,031755	1
Btbd10	-0,031886	1
Oxa1l	-0,032011	1
Snhg8	-0,032078	1
Rps6kc1	-0,032105	1
Trak2	-0,032155	1
Tnip1	-0,032225	1
Flot2	-0,032322	1
Dnm1l	-0,032264	1
Fmnl1	-0,032377	1
Twf1	-0,03249	1
Smarca4	-0,032476	1
Gm38376	-0,032621	1
Tmem143	-0,032622	1
Ppargc1b	-0,032619	1
1810058I24Rik	-0,032637	1
L1cam	-0,032692	1
Ppa1	-0,032785	1
Sf1	-0,03296	1
Haus1	-0,033095	1
Arih2	-0,033119	1
Rabac1	-0,033158	1
Bsn	-0,033333	1
Rlim	-0,033253	1
Akirin2	-0,033401	1
Asna1	-0,033537	1
Rab32	-0,033454	1
Hnrnpab	-0,033534	1
Rfc1	-0,033567	1
Chmp7	-0,0337	1
Rps10	-0,0337	1
Hsd17b12	-0,033683	1
Rab3d	-0,03377	1
Smco4	-0,033938	1
Romo1	-0,033887	1
Tspoap1	-0,034014	1
B4galt1	-0,034	1
Ppp2r5d	-0,034127	1
Sec13	-0,034146	1
Nat9	-0,034127	1
Ndufa7	-0,034145	1
Emd	-0,03433	1
Mgarp	-0,034437	1
Pla2g12a	-0,034423	1

Cltc	-0,034374	1
1190002N15Rik	-0,034457	1
Rdm1	-0,034553	1
Scamp5	-0,034797	1
Ank3	-0,034909	1
2510039O18Rik	-0,034856	1
Cstf2t	-0,035024	1
2700033N17Rik	-0,035062	1
BC004004	-0,035218	1
Ube2e1	-0,035237	1
Atp5k	-0,035342	1
Tapbp	-0,03542	1
Lamp1	-0,03536	1
Ppp2ca	-0,035548	1
Tnfaip2	-0,035823	1
Zfp526	-0,035897	1
Orc3	-0,035985	1
Fam91a1	-0,036002	1
Dusp3	-0,03602	1
4931428F04Rik	-0,036058	1
Nprl2	-0,036069	1
Aim2	-0,036103	1
Plpp6	-0,036235	1
Srsf7	-0,036213	1
Ccdc181	-0,036256	1
Hars2	-0,036436	1
Otulin	-0,036404	1
Csnk1g2	-0,036462	1
Hcst	-0,03656	1
Med21	-0,036644	1
Eif3h	-0,036585	1
1110038B12Rik	-0,036743	1
Srsf6	-0,036839	1
Sgpp1	-0,036924	1
Mapre1	-0,036912	1
1110051M20Rik	-0,036995	1
Mgme1	-0,037049	1
Smg7	-0,03715	1
Mms22l	-0,037376	1
Ino80	-0,037366	1
Dmtf1	-0,037449	1
Psmd4	-0,03753	1
Rnf14	-0,037604	1
Sucla2	-0,037564	1
Ptp4a2	-0,037624	1
Slc25a33	-0,037746	1
Acadvl	-0,037775	1
Tom1l1	-0,037882	1
Snrpf	-0,037952	1
Anapc11	-0,037982	1
Gm13815	-0,03848	1
Arf4	-0,038548	1

Aamp	-0,038523	1
Hacl1	-0,039016	1
Usp3	-0,039023	1
Trip10	-0,039142	1
Lncpint	-0,039089	1
Myo1c	-0,039175	1
Srp68	-0,039214	1
Rnase4	-0,039221	1
2700097O09Rik	-0,039305	1
Pold2	-0,039323	1
Mid1ip1	-0,039288	1
Rnf219	-0,039389	1
Yars	-0,039401	1
Trem2	-0,039447	1
Plpp2	-0,039533	1
Calml4	-0,03962	1
5730455P16Rik	-0,039572	1
Tmed10	-0,039594	1
Phc2	-0,039561	1
Mapk8ip1	-0,039706	1
Fam63a	-0,039842	1
Wdr36	-0,0399	1
2310057M21Rik	-0,040088	1
Mtif3	-0,040147	1
Malt1	-0,040212	1
Epb41l2	-0,040335	1
Hnrnpd	-0,040294	1
Sh2b2	-0,04045	1
Mpeg1	-0,040484	1
Serbp1	-0,040646	1
mt-Nd1	-0,040752	1
Cstf3	-0,040899	1
Dmrta2	-0,041011	1
Sel1l	-0,041074	1
Rwdd3	-0,04116	1
Gcnt1	-0,041159	1
Mxd4	-0,041151	1
Hn1	-0,041278	1
Ccdc6	-0,041493	1
Ehbp1	-0,041658	1
Ulk2	-0,041773	1
Gm6921	-0,042014	1
Ift52	-0,041975	1
Eci2	-0,042011	1
Cd52	-0,04202	1
Tsen34	-0,04207	1
Zfand2b	-0,042092	1
Klhl11	-0,042301	1
Atp5l	-0,042338	1
Lipa	-0,042417	1
Nectin2	-0,042495	1
Ola1	-0,042505	1

Ncbp1	-0,042451	1
Arid4b	-0,042565	1
Nek1	-0,04268	1
Elavl1	-0,042712	1
Pbx3	-0,0427	1
Degs1	-0,042671	1
Tmem14c	-0,042769	1
Fcho2	-0,04284	1
Ppil1	-0,04305	1
Tpm3-rs7	-0,043052	1
E430021H15Rik	-0,04336	1
Sfpq	-0,043412	1
Rbm28	-0,043484	1
Senp5	-0,043665	1
Ptprj	-0,044007	1
Sap30bp	-0,044035	1
Col7a1	-0,044059	1
Slc22a17	-0,044052	1
Pts	-0,044133	1
Sec23ip	-0,04407	1
Larp1b	-0,044275	1
Ncl	-0,044344	1
Snx32	-0,044399	1
Gm17018	-0,0444	1
Gm7909	-0,044621	1
Sh3gl1	-0,044638	1
Pmm1	-0,044641	1
Hars	-0,044704	1
Elp3	-0,044757	1
Fam120b	-0,045022	1
Parp6	-0,04498	1
Slain2	-0,044984	1
Bri3bp	-0,04498	1
Jade1	-0,0451	1
Gm42466	-0,04519	1
Mki67	-0,045365	1
Tmem39b	-0,045523	1
Inpp5b	-0,045502	1
Gm45420	-0,04556	1
Pxn	-0,045588	1
Fam49b	-0,045633	1
Fbxl12os	-0,04583	1
Tmem62	-0,045756	1
Ppm1m	-0,045845	1
Ebna1bp2	-0,045771	1
Epb41l4aos	-0,045756	1
Fbxw9	-0,045943	1
Rab11fip1	-0,045947	1
Tamm41	-0,046042	1
Gm12309	-0,046117	1
Fuom	-0,046086	1
Tulp3	-0,046143	1

Hnrnpa2b1	-0,046052	1
Ndufb9	-0,046302	1
Agpat5	-0,046254	1
Pcgf6	-0,0464	1
Cdc14a	-0,046629	1
Zfp827	-0,04663	1
Cklf	-0,046882	1
Rbbp5	-0,046982	1
Tmem87b	-0,047021	1
Abcd1	-0,047133	1
Ado	-0,047069	1
Hs3st3b1	-0,047189	1
Ero1l	-0,047395	1
Gm30329	-0,047463	1
Smpd2	-0,047492	1
Arpc3	-0,047553	1
Gnb1	-0,047572	1
Gdpd5	-0,047791	1
Spop	-0,047929	1
Dock6	-0,04796	1
Fam98c	-0,04805	1
Syngr2	-0,048023	1
Mon2	-0,048243	1
Gramd1a	-0,04825	1
Fbxo6	-0,04834	1
Ndufb7	-0,048624	1
Cox6c	-0,048552	1
Slc37a3	-0,04874	1
Usp10	-0,048696	1
Rac1	-0,048704	1
Ulbp1	-0,048848	1
Tuba1b	-0,048769	1
Ifi211	-0,04894	1
Rrm2b	-0,048935	1
Peg13	-0,048944	1
Ifngr1	-0,048949	1
Bbip1	-0,048901	1
Bpgm	-0,048959	1
Ints6l	-0,04896	1
Lamc1	-0,048979	1
R3hdm4	-0,048959	1
Usp4	-0,049016	1
Tnrc18	-0,048997	1
Setd3	-0,049043	1
Gm45185	-0,049056	1
Ociad2	-0,049109	1
Gm12770	-0,049193	1
Gm11263	-0,049213	1
Zfand3	-0,049188	1
Znrd1as	-0,049397	1
Tssc1	-0,049424	1
Hprt	-0,049502	1

Cog4	-0,04959	1
Zfp961	-0,049762	1
Zbtb22	-0,049801	1
Otud5	-0,049857	1
St3gal1	-0,04997	1
9530068E07Rik	-0,050037	1
Il6ra	-0,050135	1
Camk2g	-0,050339	1
Tmed4	-0,05038	1
G2e3	-0,050496	1
Rab8a	-0,050481	1
Gm43511	-0,05065	1
Ndufb3	-0,05057	1
Rpp21	-0,050716	1
Fam122b	-0,050831	1
Mirlet7b	-0,050899	1
Ubxn11	-0,050943	1
Tet2	-0,051042	1
Zfp873	-0,051316	1
Prpf8	-0,051271	1
Acsf3	-0,051438	1
Lnx2	-0,051362	1
Nfx1	-0,051434	1
Snord66	-0,051469	1
Stat5a	-0,051513	1
Hsd17b11	-0,051497	1
1110008P14Rik	-0,051517	1
Kat6a	-0,051517	1
Stap1	-0,051463	1
Slc7a6os	-0,051611	1
Zfp940	-0,051736	1
Tpmt	-0,052029	1
Csnk1d	-0,052106	1
Dctn3	-0,052159	1
Pdia6	-0,052241	1
Armc8	-0,052399	1
Tbl3	-0,052361	1
Slc25a4	-0,052437	1
D630029K05Rik	-0,052557	1
Nudt4	-0,052561	1
Prkar2a	-0,052577	1
Pitpnc1	-0,052846	1
Uba2	-0,052823	1
Zfp414	-0,052858	1
Mrps35	-0,052871	1
Ndufa12	-0,052884	1
Snupn	-0,052948	1
Gba	-0,052874	1
Srsf5	-0,053024	1
Ppib	-0,05308	1
Ankrd54	-0,053228	1
Ptk2	-0,053343	1

Gtf2h2	-0,053538	1
Dnaaf2	-0,053839	1
Tmem189	-0,05384	1
Otub2	-0,053909	1
Ubqln1	-0,053944	1
Apbb1	-0,053965	1
2410006H16Rik	-0,053958	1
Tlr13	-0,054051	1
Srebf2	-0,054105	1
Ncaph2	-0,054129	1
4933433G15Rik	-0,054178	1
Pde1b	-0,054211	1
Rbm3	-0,054309	1
Pigc	-0,054266	1
Atp10d	-0,054411	1
Pak4	-0,054413	1
Uqcr11	-0,054522	1
Bcdin3d	-0,054642	1
Eral1	-0,054683	1
D330041H03Rik	-0,054792	1
Coa4	-0,054863	1
Lpp	-0,054977	1
Stxbp1	-0,055011	1
Alg2	-0,055177	1
Polk	-0,055244	1
Trmt44	-0,055279	1
Pgp	-0,055266	1
Cybb	-0,055407	1
Tsfm	-0,055489	1
Ciz1	-0,055621	1
Bnip1	-0,055874	1
Dnajc25	-0,055856	1
Ccpg1	-0,055966	1
Nrros	-0,056044	1
Arhgef6	-0,055981	1
Lmnb2	-0,056103	1
Glt1d1	-0,056258	1
Hmgn1	-0,056503	1
U2surp	-0,056625	1
Rpusd3	-0,056741	1
Pcdhb17	-0,056719	1
Gm20703	-0,056686	1
Mdm1	-0,056787	1
Fbxo25	-0,056815	1
Prkcsh	-0,056819	1
Tbcel	-0,056939	1
Anxa5	-0,057034	1
Elac2	-0,05716	1
Rnaseh2c	-0,057231	1
Glmp	-0,057192	1
Ube3c	-0,057489	1
Phf20-ps	-0,057551	1

Ppp4r3b	-0,057592	1
BC037034	-0,057748	1
Aco1	-0,057948	1
Cfap74	-0,058014	1
Paox	-0,058094	1
Ccnt1	-0,058052	1
Ube2d1	-0,058152	1
Pafah1b3	-0,058154	1
Zfp217	-0,058292	1
Snx30	-0,058302	1
Tube1	-0,058402	1
Hdhd3	-0,058615	1
Psmd5	-0,058727	1
Rexo2	-0,058792	1
Sp1	-0,058824	1
Smug1	-0,058889	1
Cpq	-0,058915	1
Tcp11l2	-0,058922	1
Lhfpl2	-0,058859	1
Gm7424	-0,059055	1
Myo10	-0,059249	1
Gm2011	-0,059508	1
Asb13	-0,059499	1
Birc5	-0,05953	1
Gm15289	-0,059603	1
2310011J03Rik	-0,059682	1
Slc38a10	-0,059667	1
Tmem160	-0,05983	1
Zfp950	-0,060125	1
Ubxn6	-0,060298	1
St3gal3	-0,060371	1
Acyp1	-0,060596	1
Rbl1	-0,060663	1
Tfb1m	-0,060781	1
Slc25a22	-0,060938	1
Rpusd4	-0,060943	1
Tmem231	-0,061031	1
Ddb1	-0,060991	1
Zc3h3	-0,061087	1
Rfwd2	-0,061243	1
AC133103.1	-0,061263	1
Map4k4	-0,061277	1
Efl1	-0,061383	1
1600002H07Rik	-0,061516	1
Fxyd2	-0,061607	1
Wipi1	-0,062102	1
Tial1	-0,062131	1
Gm15445	-0,062214	1
Dpy19l4	-0,062418	1
Chek1	-0,062451	1
Hexa	-0,062466	1
Ptrh2	-0,062607	1

C2cd5	-0,062733	1
Emc1	-0,062745	1
Bbx	-0,062659	1
RP24-310D17.9	-0,062754	1
Ggta1	-0,062816	1
Heatr6	-0,063036	1
Lap3	-0,062952	1
Fnbp1	-0,063047	1
Rara	-0,063104	1
Gm43457	-0,063214	1
Trip11	-0,063208	1
Micall1	-0,063215	1
Pik3c2a	-0,063182	1
Tmem238	-0,063323	1
Trim26	-0,063296	1
Tnip2	-0,063344	1
Ak1	-0,063386	1
Baat	-0,063906	1
Tmem222	-0,063894	1
Cast	-0,063911	1
Gpr146	-0,064028	1
Fkbpl	-0,064077	1
Mysm1	-0,064051	1
Timm29	-0,064095	1
Kdm4b	-0,064242	1
Plcd3	-0,064177	1
Ube2z	-0,064196	1
Edem3	-0,064239	1
Oxsr1	-0,06421	1
Hspa14	-0,064232	1
Sft2d3	-0,064478	1
Ppm1b	-0,064526	1
Rab11a	-0,064478	1
1300002E11Rik	-0,064552	1
1700056N10Rik	-0,064702	1
Marf1	-0,064677	1
Dnajc5	-0,064686	1
Slc7a1	-0,064712	1
Rgl3	-0,064882	1
Ppp4r1	-0,065003	1
Bad	-0,064998	1
Nme1	-0,064971	1
Dbf4	-0,064991	1
Timm10b	-0,065426	1
1110008F13Rik	-0,06559	1
Gm15946	-0,065828	1
Pqbp1	-0,065997	1
Pole2	-0,066063	1
Bet1l	-0,066073	1
Epn2	-0,066082	1
Sf3b5	-0,066113	1
Man2c1os	-0,066243	1

Ttc9c	-0,066173	1
Exosc1	-0,066306	1
Tfe3	-0,066298	1
Gng7	-0,066388	1
Lrch3	-0,066402	1
Dnajb12	-0,066494	1
Wsb2	-0,066618	1
Ankra2	-0,066747	1
Gm7332	-0,066915	1
Pick1	-0,067104	1
Evl	-0,067417	1
Mrps14	-0,067358	1
Cers5	-0,067356	1
Bckdhb	-0,067541	1
Snhg18	-0,067489	1
Morc4	-0,067514	1
Supt5	-0,06749	1
Pank3	-0,067637	1
Ap2m1	-0,067697	1
2810402E24Rik	-0,067752	1
Isg20l2	-0,067896	1
Paf1	-0,067952	1
Tfam	-0,068128	1
Pds5b	-0,06811	1
Scamp3	-0,068102	1
AI413582	-0,068142	1
Ptpn18	-0,068083	1
Mpv17l2	-0,068338	1
Ppfia3	-0,068484	1
Apoa1bp	-0,068508	1
Aars	-0,068554	1
Pfkfb3	-0,068748	1
Ndufa11	-0,068737	1
Bbs12	-0,068755	1
Ankrd49	-0,068783	1
Rps20	-0,068902	1
Nars	-0,068896	1
Sbno2	-0,069029	1
Arl2	-0,069272	1
Ppp6r1	-0,069496	1
Fars2	-0,069582	1
Elf4	-0,069789	1
Ascc1	-0,069779	1
Cdc42bpb	-0,069788	1
Vsir	-0,06988	1
Rps6kb1	-0,069999	1
Prkce	-0,070061	1
Wdr77	-0,070054	1
Tspan5	-0,070263	1
Ppp2r3c	-0,070268	1
Cnnm3	-0,070651	1
Slfn5	-0,070819	1

Zfp580	-0,070884	1
Acox1	-0,070909	1
D3Ertd254e	-0,070957	1
Serf1	-0,071089	1
Phf1	-0,071148	1
Zfp148	-0,071066	1
Ddx10	-0,071217	1
Emc3	-0,07125	1
Svep1	-0,071344	1
Fam206a	-0,07128	1
Klc3	-0,071391	1
Rngtt	-0,071416	1
Csnk2a1	-0,071539	1
Ppan	-0,071634	1
Txndc15	-0,071671	1
Ormdl1	-0,071784	1
Lamtor1	-0,071949	1
Cmtr1	-0,071983	1
Ahsa2	-0,071975	1
Chpf2	-0,071969	1
Xiap	-0,072049	1
Alg6	-0,072143	1
Snora21	-0,072816	1
Cct6a	-0,072771	1
Mdh1	-0,072855	1
Tpr	-0,072864	1
Mapkbp1	-0,073029	1
Eps15	-0,073036	1
Cops5	-0,073544	1
Itgav	-0,073633	1
Rexo4	-0,073621	1
Bax	-0,073562	1
Atp2a2	-0,073822	1
Rbm15b	-0,074026	1
Atp5b	-0,074158	1
Gm4879	-0,074431	1
Arhgef2	-0,07441	1
Hmga1	-0,074558	1
Cd47	-0,074652	1
Clpb	-0,07478	1
Trim35	-0,074833	1
Nle1	-0,074852	1
Spopl	-0,074964	1
Nxf7	-0,075109	1
Fbxo4	-0,075077	1
Gfer	-0,075122	1
Vdac2	-0,075238	1
Rfc5	-0,075328	1
Polr2g	-0,075401	1
4632404H12Rik	-0,075468	1
Uba6	-0,075748	1
Phkg2	-0,075814	1

Pbx2	-0,075814	1
Fcgrt	-0,075945	1
Abhd16a	-0,075945	1
Hpgds	-0,075909	1
Cdc37	-0,075884	1
Kif22	-0,076056	1
Polr2c	-0,07619	1
Znhit1	-0,076168	1
Tfdp1	-0,076275	1
1110046J04Rik	-0,076499	1
Aurkaip1	-0,076495	1
Zfp638	-0,076694	1
Sptbn1	-0,076717	1
Nemp2	-0,076783	1
C1galt1c1	-0,07678	1
Mcts1	-0,077042	1
Arl3	-0,076954	1
Ctss	-0,076965	1
Lias	-0,077004	1
Cmc1	-0,077086	1
Pcdhgc4	-0,077319	1
Clpp	-0,077438	1
Tsr3	-0,077392	1
Pygo2	-0,07742	1
Cr1l	-0,077588	1
Ruvbl2	-0,077674	1
A930018M24Rik	-0,077676	1
Vipas39	-0,077668	1
Them4	-0,077734	1
Senp2	-0,07767	1
Ap1m1	-0,07765	1
Slc12a2	-0,077776	1
Irf2bp1	-0,077805	1
Trim32	-0,077854	1
Cdk19	-0,077937	1
Ntan1	-0,078139	1
Mrps34	-0,078144	1
Lmna	-0,078071	1
Acin1	-0,078219	1
Snrpd1	-0,078214	1
Ndufab1	-0,078314	1
Ndufs1	-0,078267	1
Snx21	-0,078411	1
Utp4	-0,078411	1
Acad10	-0,078625	1
Arhgap27	-0,078649	1
Mon1a	-0,078749	1
Alad	-0,078932	1
Trim3	-0,078998	1
Chp1	-0,079025	1
Cln3	-0,079051	1
Smn1	-0,079052	1

Lipe	-0,079205	1
Lgals3	-0,079377	1
Kank3	-0,079508	1
Eif4a1	-0,079516	1
Inafm2	-0,079585	1
Trpm7	-0,079622	1
Kdm2a	-0,07965	1
Tmem70	-0,079643	1
Men1	-0,079766	1
3110045C21Rik	-0,080036	1
Tmem159	-0,07998	1
4933427D14Rik	-0,079953	1
Ccny	-0,079967	1
Lrrc8c	-0,080331	1
Rassf5	-0,080373	1
Wdr60	-0,080436	1
Kdm5a	-0,080408	1
Prrc1	-0,080398	1
Gm37747	-0,080613	1
Zfp512	-0,080663	1
Ctps	-0,080749	1
Pomc	-0,08081	1
Anp32e	-0,080802	1
Jsrp1	-0,080941	1
Dhx37	-0,080869	1
Os9	-0,080889	1
Pfkp	-0,080911	1
Map3k14	-0,080984	1
Glrp1	-0,081152	1
Pcbp4	-0,081186	1
Uqcc1	-0,081157	1
Polr2f	-0,081163	1
Armc1	-0,081327	1
Tmed1	-0,081386	1
Igsf6	-0,081531	1
Fam135a	-0,081482	1
Nob1	-0,081495	1
Dynlt3	-0,08154	1
Phtf1	-0,081692	1
Siah1a	-0,081675	1
Rpl7l1	-0,081672	1
Fam109a	-0,081909	1
Rbpj	-0,082349	1
Bsg	-0,082383	1
Trappc4	-0,082634	1
Tbca	-0,082735	1
1700034H15Rik	-0,082781	1
Rab40c	-0,08277	1
Polr2e	-0,082907	1
Dbi	-0,083032	1
Nmt2	-0,083064	1
Dxo	-0,083334	1

Rnpepl1	-0,08335	1
Pdpk1	-0,08328	1
Arid3a	-0,083414	1
Pex3	-0,083513	1
Hipk3	-0,083478	1
Smim12	-0,083536	1
Lgr4	-0,083605	1
Eif5a	-0,083675	1
Plag1	-0,083933	1
BC003965	-0,083951	1
Ints9	-0,084055	1
Ctsf	-0,084073	1
Gstm4	-0,084239	1
Tmc6	-0,084192	1
Fam133b	-0,084175	1
Klhl23	-0,084342	1
Runx2	-0,084386	1
Gm5617	-0,084394	1
Gabpb1	-0,084493	1
Snora73b	-0,084502	1
Trp53bp1	-0,084472	1
Ints2	-0,084625	1
Taok3	-0,084745	1
Wdr81	-0,084919	1
Nnt	-0,084924	1
Psap	-0,08494	1
Tbl1x	-0,084899	1
Hdlbp	-0,084876	1
Enpp1	-0,085021	1
Ctnna1	-0,084996	1
Tmem161b	-0,085007	1
Rfng	-0,085058	1
Dnajb14	-0,085121	1
Blvra	-0,085232	1
Mars	-0,085277	1
Pus1	-0,085403	1
Senp3	-0,085501	1
Itpr1	-0,085521	1
Sem1	-0,085706	1
Vps41	-0,085767	1
Mocs1	-0,085885	1
Lss	-0,085879	1
Lrp12	-0,085877	1
Abcb1b	-0,085871	1
Psma5	-0,086204	1
Crlf3	-0,086446	1
B130021K23Rik	-0,086452	1
Cerkl	-0,086529	1
Tlr4	-0,086487	1
Ap3m1	-0,086524	1
Auh	-0,086486	1
Scmh1	-0,086582	1

Mphosph8	-0,086661	1
Srp72	-0,086879	1
Mrpl40	-0,087019	1
1600029O15Rik	-0,087289	1
Terf2	-0,087258	1
Fam117b	-0,087412	1
Tab3	-0,087491	1
Zfp62	-0,08758	1
Mbnl1	-0,087586	1
Acads	-0,087814	1
Gm5446	-0,087901	1
Adora2a	-0,08787	1
P4hb	-0,087972	1
Cdk12	-0,088089	1
Rc3h2	-0,088071	1
Stk11	-0,088084	1
Srsf3	-0,088114	1
Ssr3	-0,088178	1
Tmem258	-0,088157	1
Amdhd2	-0,088252	1
Arsa	-0,088441	1
Rcor1	-0,088546	1
Slc35a1	-0,088728	1
Fkbp1b	-0,088662	1
Cep152	-0,08865	1
Uba5	-0,088793	1
Cpne3	-0,088801	1
Khdrbs1	-0,089086	1
Sec31b	-0,089518	1
Dap3	-0,08955	1
Cad	-0,089591	1
Ppm1k	-0,089565	1
Slc19a1	-0,08966	1
Plekhs1	-0,089833	1
Emc10	-0,089887	1
Slc37a4	-0,089953	1
Kif23	-0,090149	1
A430046D13Rik	-0,090162	1
Ufc1	-0,09024	1
Slc16a13	-0,090289	1
Acp6	-0,090712	1
Tubb5	-0,090841	1
Fbxl17	-0,090899	1
Dck	-0,090902	1
Rap1gds1	-0,091041	1
Gm44890	-0,091343	1
Pou4f1	-0,091318	1
Arap3	-0,091296	1
Synrg	-0,091576	1
Fam114a1	-0,091742	1
Esrra	-0,091664	1
Apobec3	-0,091905	1

Rsrc1	-0,091987	1
Anxa7	-0,092119	1
Gm16046	-0,092155	1
Toporsos	-0,092217	1
Snrk	-0,092273	1
Setd2	-0,09225	1
Zc3h18	-0,092518	1
Myo18a	-0,092638	1
1700022N22Rik	-0,092709	1
Lyar	-0,093061	1
Snapc3	-0,093159	1
Sssca1	-0,093165	1
Snrpd2	-0,093203	1
Ift46	-0,09321	1
Snx17	-0,093306	1
Ghitm	-0,093345	1
Sdhb	-0,093489	1
Safb	-0,093709	1
Iqsec3	-0,093913	1
Prag1	-0,093932	1
Atp6v1c1	-0,093974	1
Rpl22	-0,093994	1
Ykt6	-0,094071	1
4930550C14Rik	-0,094238	1
Trappc6a	-0,094179	1
Idh3g	-0,094447	1
Avpi1	-0,094521	1
Ppp4r1l-ps	-0,094592	1
Zswim8	-0,094682	1
Trmt5	-0,095055	1
Arhgef19	-0,095077	1
Btbd9	-0,095071	1
Trafd1	-0,095102	1
Gna13	-0,095106	1
Rnasel	-0,095309	1
Cggbp1	-0,095433	1
Slc9a6	-0,095514	1
Leprotl1	-0,095452	1
Mpp3	-0,095715	1
Tut1	-0,095824	1
Knstrn	-0,095845	1
Isy1	-0,09593	1
Dcaf7	-0,095915	1
Grina	-0,095901	1
Rhno1	-0,09599	1
Actn4	-0,096228	1
Gtpbp3	-0,096336	1
Dusp22	-0,096427	1
Grwd1	-0,096421	1
Ddx39	-0,096369	1
Gnai2	-0,09638	1
Preb	-0,096573	1

Rab14	-0,096593	1
Arl6ip5	-0,09682	1
Bcar1	-0,096871	1
Ptchd1	-0,096855	1
Wdr55	-0,097018	1
Mmgt2	-0,097112	1
Ndufc2	-0,097063	1
Ltbp4	-0,097204	1
Fam129b	-0,09718	1
Pdcd6ip	-0,097167	1
Gm5391	-0,097303	1
Rnpep	-0,097303	1
Copz2	-0,097434	1
Lrrc25	-0,097397	1
Cfap97	-0,097355	1
Siae	-0,097357	1
Syne2	-0,097452	1
Snrnp70	-0,09755	1
Aes	-0,097462	1
Ptpn11	-0,097742	1
Bag4	-0,097671	1
Nedd8	-0,097652	1
Ogfod3	-0,097889	1
Kctd13	-0,098036	1
Zfp628	-0,098216	1
Gigyf1	-0,098226	1
Pogz	-0,098224	1
Shc4	-0,098349	1
Appbp2	-0,098286	1
Prmt7	-0,098431	1
Gm7618	-0,098475	1
1810041H14Rik	-0,098513	1
Chmp5	-0,098475	1
Dcaf5	-0,098615	1
Rusc1	-0,098726	1
Stard7	-0,098726	1
Glrx2	-0,098942	1
Hsf2	-0,099038	1
Coq9	-0,099052	1
Usp37	-0,099157	1
Npc2	-0,099192	1
1700025G04Rik	-0,099331	1
Bysl	-0,099488	1
Pdcd2	-0,099625	1
Txndc9	-0,099587	1
Gm2885	-0,09972	1
Mrps36	-0,099686	1
Orc5	-0,099731	1
Pithd1	-0,099709	1
Tor1b	-0,099678	1
Jak1	-0,099697	1
Sf3a1	-0,099696	1

Dhx32	-0,0998	1
Nup188	-0,099755	1
Ptma	-0,0998	1
Shkbp1	-0,099962	1
Ilkap	-0,10005	1
Nabp1	-0,10006	1
Gstm1	-0,10014	1
Traf6	-0,10018	1
Fbxl5	-0,10025	1
Gm43223	-0,10031	1
Mthfd1l	-0,10037	1
Gch1	-0,10035	1
Gm4613	-0,10051	1
Nckap1	-0,10055	1
Ppp1r10	-0,10062	1
Rad23a	-0,1008	1
Usp34	-0,10097	1
Pla2g16	-0,10114	1
Unc50	-0,10108	1
Cpne8	-0,10106	1
Tifab	-0,10118	1
Zfp560	-0,10132	1
Pxmp4	-0,10136	1
Nit2	-0,10141	1
Atp6v1g2	-0,10153	1
Katna1	-0,10145	1
Bmt2	-0,10156	1
Lipt1	-0,10169	1
Cystm1	-0,10177	1
Arfgef1	-0,10176	1
L2hgdh	-0,10201	1
Pitrm1	-0,102	1
Clcn4	-0,10208	1
Slc25a46	-0,10212	1
Ap5s1	-0,10217	1
Zbtb6	-0,10216	1
Fam111a	-0,10216	1
Memo1	-0,10228	1
Kdm6b	-0,10228	1
Anapc1	-0,10229	1
Wdr12	-0,1024	1
Cmtm7	-0,10249	1
Aptx	-0,10253	1
Trove2	-0,10258	1
Zfp318	-0,10298	1
Lcor	-0,10306	1
Mgst2	-0,10309	1
Pcdh7	-0,10321	1
Gapvd1	-0,10319	1
Dapk1	-0,1033	1
Mrpl12	-0,10358	1
Fuz	-0,10366	1

Fam210b	-0,1037	1
Gm2788	-0,10384	1
Ube2j1	-0,10389	1
Rdx	-0,10395	1
Pnisr	-0,10398	1
Gm43737	-0,10405	1
Dnd1	-0,10421	1
Adam17	-0,1043	1
Mrps26	-0,10474	1
Ndst2	-0,10478	1
Ppm1g	-0,10487	1
Usp31	-0,10495	1
Smox	-0,10501	1
Psma1	-0,10511	1
Golph3l	-0,10515	1
Wdr61	-0,10515	1
Colgalt1	-0,10529	1
Rnf145	-0,10534	1
Gkap1	-0,10537	1
Atp5o	-0,10544	1
Bcat2	-0,10551	1
Mtx2	-0,10564	1
Gle1	-0,10577	1
Rad9b	-0,10595	1
Vrk2	-0,10594	1
Gm26461	-0,10615	1
Dzip1	-0,10621	1
Abca3	-0,10625	1
Zbtb7a	-0,10626	1
Zufsp	-0,10639	1
Ints10	-0,10654	1
Gtf3c1	-0,10647	1
Speg	-0,10663	1
Hs2st1	-0,10667	1
Trim25	-0,10675	1
Polb	-0,10669	1
Bcl2l14	-0,10684	1
Timp2	-0,10684	1
Hsph1	-0,10681	1
Psmg3	-0,10695	1
Hsp90ab1	-0,1069	1
Eml4	-0,10696	1
Sik3	-0,10704	1
Rbm45	-0,10731	1
Vcp	-0,10734	1
Mrpl24	-0,10739	1
Gm43178	-0,10751	1
Golga4	-0,10751	1
Ddx43	-0,10765	1
Mfsd14a	-0,10769	1
Nbeal1	-0,10784	1
Zfp597	-0,10788	1

Bend6	-0,10789	1
Slc41a3	-0,10793	1
Cbx3	-0,10814	1
Dnmt1	-0,10812	1
3300002I08Rik	-0,10815	1
Gm37738	-0,10825	1
Icam5	-0,10823	1
Creb3l2	-0,10825	1
Ddx3y	-0,10833	1
Anxa4	-0,10835	1
Nipbl	-0,10833	1
Lin7c	-0,10839	1
Nomo1	-0,10852	1
Alpk1	-0,10865	1
Clcc1	-0,10862	1
Josd1	-0,10859	1
Dcun1d1	-0,10865	1
Ints4	-0,10876	1
Mpv17l	-0,10903	1
Drap1	-0,10896	1
Wdr3	-0,10907	1
Cdca2	-0,1092	1
Rad21	-0,10922	1
Capns1	-0,10924	1
Suds3	-0,10917	1
Sypl	-0,10933	1
Rusc2	-0,10967	1
Lyrm9	-0,1099	1
Trip12	-0,11001	1
Rrp15	-0,11019	1
Arid1a	-0,11021	1
Fam114a2	-0,11019	1
Psmb10	-0,11037	1
Slc20a2	-0,11051	1
Wfikkn1	-0,11048	1
Serp1	-0,11074	1
Ppp3r1	-0,11075	1
Scnm1	-0,11081	1
Gm38115	-0,11086	1
Cd53	-0,11101	1
Aagab	-0,11101	1
Dync1i2	-0,11107	1
Dnaja1	-0,11122	1
Akr1c13	-0,1112	1
Ostc	-0,11126	1
Rsf1	-0,11134	1
Josd2	-0,11147	1
Plekha1	-0,11168	1
Ptcd2	-0,11167	1
Rnpc3	-0,11176	1
Bdh2	-0,11176	1
Aldh16a1	-0,11194	1

Mtch1	-0,11207	1
Chkb	-0,11216	1
Pi16	-0,11233	1
Itpripl2	-0,1123	1
Pgap1	-0,11243	1
Arrdc4	-0,11244	1
Net1	-0,11263	1
Tmem127	-0,11293	1
Lzts2	-0,11292	1
Emg1	-0,11287	1
Ergic3	-0,11305	1
Mcoln1	-0,11308	1
Clec10a	-0,11309	1
Wdtc1	-0,11313	1
Fam179b	-0,11314	1
Fam122a	-0,11324	1
Bst1	-0,1132	1
Tsen15	-0,11324	1
9930111J21Rik2	-0,1133	1
Gm10313	-0,1133	1
Map4	-0,11335	1
Ttyh3	-0,11342	1
Rnmt	-0,11341	1
Erp44	-0,11339	1
Zfp821	-0,11345	1
Brca2	-0,11361	1
Mtfmt	-0,11355	1
Wdr5	-0,11363	1
Gm38377	-0,11368	1
Slc4a11	-0,1138	1
Slc38a6	-0,11378	1
Myo5a	-0,11377	1
Grhpr	-0,11389	1
Plekhj1	-0,11394	1
Tshz3	-0,11395	1
Ppa2	-0,11409	1
Cd33	-0,11418	1
Cdv3	-0,11422	1
Kif13b	-0,11434	1
BC060293	-0,11438	1
Gm5113	-0,11455	1
Mpzl1	-0,11452	1
Copg2	-0,11446	1
Vps18	-0,1146	1
Zfp954	-0,11465	1
Ndufa2	-0,1147	1
Eif3j1	-0,11478	1
Gm10012	-0,11497	1
Hint2	-0,11511	1
Tacc3	-0,11525	1
Clta	-0,11518	1
Gm42715	-0,11545	1

Oas3	-0,11536	1
Npc1	-0,11541	1
Trmt1	-0,11549	1
Slc22a13b-ps	-0,11564	1
Ss18	-0,11557	1
Dctn6	-0,11558	1
Rae1	-0,11562	1
Gm38213	-0,11575	1
Cdk2	-0,11573	1
Prkacb	-0,11567	1
Mfsd1	-0,11572	1
Cdkn3	-0,11584	1
Vwa8	-0,11582	1
Gtf2h1	-0,1158	1
Rcan1	-0,11582	1
Tcaim	-0,11576	1
Oas2	-0,11594	1
Hdac10	-0,11599	1
Ap2a2	-0,11595	1
Rnf20	-0,11595	1
Cep55	-0,11605	1
Avl9	-0,11608	1
Tank	-0,11617	1
Atp11c	-0,11632	1
Phf6	-0,11628	1
Gemin7	-0,11641	1
Tmco6	-0,11658	1
Nfu1	-0,11661	1
Ehd4	-0,11659	1
Sh3bgrl3	-0,11666	1
Aldh7a1	-0,11694	1
Gm15265	-0,11694	1
Srgn	-0,11701	1
Rsl1d1	-0,11731	1
Zfp777	-0,1174	1
Ucp2	-0,11743	1
Ldah	-0,11764	1
Gm10138	-0,11767	1
Dgcr2	-0,11783	1
Far1	-0,11785	1
Cd68	-0,11788	1
Pms2	-0,11804	1
Cog7	-0,11798	1
Rasip1	-0,1181	1
Desi1	-0,11815	1
Cdc42bpg	-0,11809	1
Rabep2	-0,11836	1
B3gnt3	-0,11853	1
Rpl17-ps10	-0,11846	1
Ccnt2	-0,11851	1
Ube2q1	-0,11845	1
Stip1	-0,11863	1

Btg1	-0,11868	1
Sf3b2	-0,1187	1
Setd7	-0,11876	1
Gm6030	-0,11878	1
Gm24959	-0,119	1
Haus7	-0,11909	1
Pdcd11	-0,11914	1
Zc3h8	-0,11927	1
Ifrd2	-0,11942	1
Jam2	-0,11941	1
Cnot7	-0,1194	1
Ctif	-0,11952	1
Gab2	-0,11954	1
Dgkg	-0,11961	1
Chd5	-0,11971	1
Larp1	-0,11975	1
Gm43466	-0,11994	1
Ppp2r5b	-0,1199	1
Dse	-0,1202	1
Isca2	-0,12017	1
Mbnl2	-0,12047	1
Pdik1l	-0,12079	1
Znfx1	-0,12079	1
BC052040	-0,12093	1
2210408I21Rik	-0,12115	1
Psmd10	-0,1212	1
Slc37a2	-0,12125	1
Fra10ac1	-0,12129	1
D10Wsu102e	-0,1213	1
Gm13421	-0,12141	1
Rev1	-0,12142	1
Zfyve26	-0,12141	1
Thoc3	-0,12142	1
E130307A14Rik	-0,12151	1
Slc27a1	-0,12146	1
Umps	-0,1215	1
4930556M19Rik	-0,12156	1
Mast3	-0,12173	1
Chsy1	-0,12171	1
4933421A08Rik	-0,12192	1
Gm10039	-0,12216	1
Hspa9	-0,12223	1
Ptk2b	-0,12239	1
Zfp202	-0,12262	1
Skiv2l2	-0,12257	1
Ap1g1	-0,12277	1
Fam57a	-0,12291	1
Apex1	-0,12286	1
Gatm	-0,12315	1
Ccdc88a	-0,12311	1
Psmd2	-0,12323	1
Slc4a2	-0,12327	1

Arpc1b	-0,12335	1
Pcx	-0,12337	1
Clcn7	-0,12353	1
Exoc4	-0,12347	1
Tcam1	-0,12357	1
Abcg4	-0,12356	1
Smurf1	-0,12363	1
Ntmt1	-0,12362	1
Chac1	-0,12373	1
Lrwd1	-0,12374	1
Raph1	-0,12386	1
Psma2	-0,12394	1
Ptpre	-0,12398	1
Trim12c	-0,1241	1
Wdfy1	-0,12418	1
Brd7	-0,12416	1
Cstb	-0,12417	1
Dcp1a	-0,12426	1
Twnk	-0,12427	1
Mlec	-0,12429	1
Zfp14	-0,12438	1
Arl5c	-0,12435	1
Chtop	-0,12448	1
Piwil2	-0,12463	1
Tmeff1	-0,12462	1
Tlk2	-0,12458	1
Gm15610	-0,12483	1
Ifi27	-0,12491	1
Ttc5	-0,12497	1
Usp38	-0,12499	1
Atraid	-0,12504	1
Cenpi	-0,12509	1
Tubg1	-0,12533	1
Faf2	-0,12536	1
Tmem167	-0,12539	1
Dnajc16	-0,12551	1
Chst14	-0,12568	1
Pttg1ip	-0,12569	1
Lrrc42	-0,12573	1
Rasa2	-0,126	1
Thap2	-0,12602	1
Nptn	-0,12602	1
Tm9sf2	-0,12607	1
Scp2-ps2	-0,12619	1
Adar	-0,12621	1
Fam35a	-0,12625	1
Tlr7	-0,1263	1
Gm37776	-0,12637	1
Cbfb	-0,12662	1
Ostf1	-0,12662	1
C330011M18Rik	-0,12674	1
4930503L19Rik	-0,12694	1

Rbms2	-0,12692	1
Vav1	-0,12697	1
Rab7	-0,12712	1
4931406C07Rik	-0,12722	1
Pwwp2a	-0,1275	1
Cops4	-0,12759	1
Csde1	-0,12757	1
Myl12a	-0,12769	1
Pnkd	-0,12776	1
Hnrnpl	-0,12792	1
2310033P09Rik	-0,12788	1
Dctn4	-0,12795	1
Bloc1s5	-0,12817	1
Lmo4	-0,12834	1
Ddi2	-0,12848	1
Ppp1r11	-0,12862	1
Chd3	-0,12863	1
Ankrd13b	-0,12872	1
Ccdc90b	-0,12866	1
Erc1	-0,12879	1
Bola3	-0,12893	1
Slc30a9	-0,12892	1
Pls3	-0,12911	1
Gm7514	-0,12924	1
Pigk	-0,12916	1
Tnpo3	-0,12933	1
Morc2a	-0,12941	1
Baz1b	-0,12951	1
Mia2	-0,12964	1
Rcan3	-0,12967	1
Prpf39	-0,12966	1
Chmp1a	-0,12969	1
B230217C12Rik	-0,12992	1
3110002H16Rik	-0,12992	1
Abhd17b	-0,12994	1
Tmem177	-0,12999	1
Fam65a	-0,13	1
Plcb3	-0,12996	1
Pptc7	-0,13022	1
Gm7432	-0,13031	1
Btbd3	-0,1304	1
Jmjd1c	-0,1305	1
Ube2l3	-0,1305	1
Grtp1	-0,13098	1
Gm11427	-0,13097	1
Rbm17	-0,13097	1
Set	-0,13113	1
Cdc20	-0,13112	1
Fahd1	-0,13131	1
Cfap36	-0,13144	1
Fbxo28	-0,13162	1
Hes6	-0,13166	1

Dner	-0,13177	1
Pop1	-0,13202	1
Mertk	-0,13213	1
Rnf44	-0,13223	1
Psen2	-0,13233	1
Ube3a	-0,13227	1
Fasn	-0,13232	1
Hells	-0,1324	1
Eif6	-0,13237	1
Gm4258	-0,13261	1
Pqlc1	-0,13275	1
Gpnmb	-0,13276	1
Mfge8	-0,13281	1
Oscar	-0,13292	1
Mbd4	-0,13293	1
Apba3	-0,13323	1
Gm2a	-0,13325	1
Clk2	-0,13326	1
Gstp1	-0,13354	1
Myo1g	-0,13345	1
Gm5075	-0,13358	1
Tfdp2	-0,13368	1
Topbp1	-0,13369	1
Npepl1	-0,13389	1
Mvb12a	-0,13389	1
Zcrb1	-0,13393	1
Cflar	-0,13389	1
Them6	-0,13397	1
Dhps	-0,13414	1
9430034N14Rik	-0,13421	1
Gm37598	-0,13416	1
Plekha3	-0,13431	1
Arrb2	-0,13452	1
Ibtk	-0,13462	1
Lpin2	-0,13463	1
Ascc3	-0,135	1
A130014A01Rik	-0,13505	1
Cd2ap	-0,1351	1
Atp5g3	-0,13511	1
Zmym3	-0,13524	1
Mis18a	-0,13526	1
Prmt6	-0,13535	1
Limd1	-0,13544	1
Picalm	-0,13545	1
Tnfrsf13b	-0,13555	1
Uba1	-0,13546	1
Orai3	-0,13563	1
Cyb5d2	-0,13585	1
Ptprs	-0,13587	1
Selplg	-0,136	1
Msantd4	-0,13603	1
Mrpl2	-0,13614	1

Gm7808	-0,13633	1
Eloc	-0,13634	1
Thap11	-0,13639	1
Nrxn3	-0,13661	1
Klhl24	-0,13656	1
Fen1	-0,13668	1
Hnrnph3	-0,13667	1
Sfxn5	-0,13678	1
Lrp8	-0,1368	1
Chchd1	-0,13685	1
Kdm4c	-0,13686	1
Kiss1r	-0,13704	1
Itgb2	-0,13704	1
Slc41a2	-0,13705	1
Serpinb6a	-0,13727	1
Gm7565	-0,13742	1
Tmco3	-0,13753	1
Cirbp	-0,13757	1
Mrgpre	-0,13766	1
Ogg1	-0,13768	1
Manf	-0,13766	1
Ino80d	-0,13792	1
Cbl	-0,13795	1
Trap1	-0,13803	1
Cfp	-0,13819	1
Reep3	-0,13821	1
Slc7a8	-0,13821	1
Gm37795	-0,13828	1
Gsr	-0,13862	1
Arhgef40	-0,13858	1
BC051226	-0,13886	1
Atg16l2	-0,13891	1
Ndufc1	-0,13903	1
Gm6415	-0,13911	1
Purb	-0,13922	1
Pex11a	-0,13928	1
Yipf7	-0,13954	1
Ttc17	-0,13952	1
Cbx6	-0,1397	1
Bcat1	-0,13986	1
Actr2	-0,13987	1
B330016D10Rik	-0,13999	1
Aldh5a1	-0,14001	1
AA414768	-0,14027	1
Vps26b	-0,14035	1
Man2c1	-0,14049	1
Psmd13	-0,14056	1
Mfsd12	-0,14056	1
Atp5d	-0,14068	1
Nfkbie	-0,14079	1
Alkbh2	-0,14083	1
Fh1	-0,14101	1

Adamts10	-0,14108	1
Baz2b	-0,14109	1
Gm4978	-0,14119	1
Tspan17	-0,1412	1
Zfp94	-0,14125	1
Mrpl28	-0,14134	1
Glrx	-0,14147	1
Adpgk	-0,14157	1
Gm14822	-0,14168	1
Pdk3	-0,14168	1
Ctsa	-0,14168	1
Slc6a6	-0,14182	1
Pcbp2	-0,1418	1
AU019823	-0,14192	1
Ralbp1	-0,14191	1
Eif3d	-0,14205	1
Tmem165	-0,14209	1
Gm20056	-0,14225	1
Mtmr2	-0,14221	1
Ptdss1	-0,14243	1
Ddx55	-0,14255	1
Gm15920	-0,14248	1
Psma6	-0,14247	1
Fam117a	-0,14265	1
Selenos	-0,1426	1
Dhx34	-0,14267	1
Wwox	-0,14275	1
Slc25a28	-0,14277	1
Gm14567	-0,14288	1
Papolg	-0,14288	1
Elk1	-0,14289	1
Dscr3	-0,14308	1
5530601H04Rik	-0,14323	1
Aebp2	-0,14359	1
Fuca1	-0,14359	1
Bub3	-0,14355	1
Ap4e1	-0,14379	1
Gm16740	-0,14389	1
C1rb	-0,14399	1
Ddx19b	-0,14402	1
Ece2	-0,14401	1
Ncapd3	-0,14412	1
Ctbs	-0,14419	1
Ncln	-0,14427	1
Pcca	-0,14441	1
Slc25a51	-0,14448	1
Gm20632	-0,1449	1
Gm18867	-0,14487	1
Gdi2	-0,14505	1
Gon4l	-0,14511	1
Ncf2	-0,14516	1
Acvr2b	-0,14524	1

Tns3	-0,14516	1
St7	-0,1453	1
Gm4799	-0,14533	1
Ssr1	-0,14528	1
Mospd3	-0,14553	1
Gm42567	-0,14569	1
Hpf1	-0,14573	1
Tceanc	-0,14588	1
Acat2	-0,14599	1
Swap70	-0,14595	1
Suclg1	-0,14603	1
Cd109	-0,14603	1
Cpox	-0,14608	1
Ska2	-0,14624	1
Ptpn23	-0,14633	1
Kif20a	-0,14634	1
Sri	-0,14637	1
Dnmt3a	-0,14654	1
Tmem206	-0,1466	1
Bmyc	-0,14658	1
9130401M01Rik	-0,14664	1
Tbck	-0,14689	1
Ppip5k1	-0,14703	1
Oxnad1	-0,14706	1
Pi4k2b	-0,14725	1
Trmt10a	-0,14733	1
Zfp729a	-0,14745	1
Rpe	-0,14741	1
Pkp4	-0,14765	1
Tmem110	-0,1477	1
Gramd4	-0,14787	1
Gmds	-0,14796	1
Tln1	-0,14798	1
Noxo1	-0,14809	1
Tmem19	-0,14811	1
Zcchc2	-0,14815	1
D11Wsu47e	-0,14842	1
Snrnp25	-0,1484	1
Arhgap5	-0,14836	1
2700038G22Rik	-0,14879	1
Upf3b	-0,14882	1
Pih1d1	-0,14889	1
Tm2d2	-0,149	1
Ncor2	-0,14913	1
Rftn1	-0,14919	1
Usp46	-0,1492	1
Zbtb34	-0,14926	1
Gm4997	-0,14933	1
B4gat1	-0,14946	1
Ikbkap	-0,14953	1
Vrk1	-0,14962	1
Aurka	-0,14984	1

Cerk	-0,1498	1
Hectd1	-0,14986	1
Vps54	-0,15013	1
Erlin1	-0,15009	1
Ccdc97	-0,15007	1
Rabepk	-0,15017	1
Tgfbrap1	-0,1505	1
Psmc3	-0,15045	1
Snd1	-0,15048	1
Khsrp	-0,15064	1
Bora	-0,15083	1
Crlf2	-0,15094	1
Gm37383	-0,15089	1
Maml3	-0,15089	1
Xpot	-0,15095	1
Gm11110	-0,15125	1
Chd2	-0,15123	1
Armc2	-0,1515	1
Nsfl1c	-0,15158	1
Cdk4	-0,15161	1
Zfp316	-0,15199	1
Lrrc8d	-0,15206	1
Zfp740	-0,15205	1
Nop9	-0,15224	1
2900026A02Rik	-0,15216	1
Gm14633	-0,15236	1
Etfb	-0,15236	1
Syvn1	-0,15238	1
Fastk	-0,15259	1
Fbxw5	-0,1527	1
Golga5	-0,15273	1
Srsf11	-0,15279	1
Prdx2	-0,15286	1
Tmbim1	-0,15294	1
Ptpn21	-0,15314	1
Lrp4	-0,15306	1
Gm5380	-0,15318	1
Ccdc66	-0,15321	1
Rps19-ps4	-0,15357	1
Arv1	-0,15373	1
Cul4a	-0,15373	1
Plekho1	-0,15382	1
Hspa8	-0,15391	1
Ccdc85b	-0,15389	1
Cd300ld	-0,15395	1
Agfg1	-0,15398	1
Cyp27a1	-0,15411	1
Tbc1d9b	-0,15406	1
Hus1	-0,15423	1
Tmem55b	-0,15421	1
Txn-ps1	-0,15435	1
Wdr34	-0,15437	1

Plekhb2	-0,15446	1
Mfn2	-0,15484	1
Luc7l	-0,1548	1
Pdcl3	-0,15534	1
Gm21399	-0,15539	1
Zfp715	-0,15551	1
AU022252	-0,15554	1
Ap1s2	-0,15561	1
Uqcc3	-0,15562	1
Dhx15	-0,15572	1
Srm	-0,15576	1
Vps13a	-0,15583	1
Tnpo2	-0,15597	1
Sod2	-0,15602	1
Rab11b	-0,15607	1
Atp6v1e1	-0,1562	1
1110065P20Rik	-0,15625	1
Mphosph6	-0,15632	1
G3bp1	-0,15657	1
Gm37352	-0,15666	1
Ipo7	-0,15674	1
Polr3f	-0,15692	1
Prdx5	-0,15729	1
Cnot6	-0,15729	1
1810043G02Rik	-0,15743	1
Rbsn	-0,15737	1
Tab2	-0,15746	1
Cav2	-0,15765	1
Nr2f6	-0,1576	1
Tmx3	-0,15758	1
Gm42783	-0,15775	1
Wdr41	-0,15779	1
Fibp	-0,15787	1
Fmc1	-0,15795	1
Nbeal2	-0,158	1
Zmpste24	-0,15798	1
Ncoa6	-0,15804	1
Ldha	-0,15814	1
Nvl	-0,15819	1
Skap2	-0,15817	1
Pwp2	-0,15833	1
Trim11	-0,15841	1
Nsun2	-0,15843	1
Arpin	-0,15866	1
Chtf8	-0,15869	1
Tbc1d17	-0,159	1
Tada1	-0,15895	1
Srsf9	-0,15915	1
Pex19	-0,15924	1
Yipf2	-0,15927	1
Cars	-0,15926	1
Atpif1	-0,1593	1

Ube2e3	-0,15945	1
Tmem203	-0,15947	1
Psme3	-0,1595	1
Unc45a	-0,15956	1
Rassf8	-0,1598	1
Gnl3l	-0,15982	1
Acacb	-0,15992	1
Ankrd26	-0,16003	1
Slc7a4	-0,15996	1
Atf6	-0,16008	1
Atp13a2	-0,16027	1
Msl1	-0,16035	1
Cbarp	-0,16037	1
Ppp3cb	-0,16043	1
Sec11a	-0,16041	1
Lmntd2	-0,16046	1
Aoc2	-0,16063	1
Slc25a1	-0,16059	1
Fzr1	-0,16062	1
Gm13050	-0,1609	1
Spg7	-0,16086	1
Zswim6	-0,16092	1
Hirip3	-0,16109	1
Acot7	-0,16126	1
Taf10	-0,16148	1
Surf4	-0,16154	1
Nup210	-0,16161	1
Gnb5	-0,16165	1
Sh3bgrl	-0,16156	1
Tmem50a	-0,1616	1
Med28	-0,16166	1
Anapc5	-0,16179	1
Vps16	-0,1619	1
Stk38l	-0,16189	1
Gm24890	-0,16197	1
Sugp2	-0,16195	1
Luc7l3	-0,16202	1
Uxs1	-0,162	1
Washc4	-0,16203	1
Acot13	-0,16232	1
Tpp1	-0,16226	1
Zfp773	-0,16249	1
Mvb12b	-0,16254	1
Gprasp1	-0,16257	1
Bag6	-0,16257	1
Ccr10	-0,16295	1
Gm11470	-0,16298	1
Trim16	-0,16306	1
Aco2	-0,16314	1
Polg	-0,16331	1
AI987944	-0,16341	1
Rufy2	-0,16351	1

Gm17971	-0,16367	1
Coro1b	-0,16384	1
Seh1l	-0,16382	1
Mfap1b	-0,16391	1
Gfpt1	-0,1639	1
Gm43144	-0,16404	1
Dcaf13	-0,16399	1
Rnf168	-0,16411	1
Gm16199	-0,16417	1
Hdac9	-0,16434	1
Fam162a	-0,16473	1
Gm44269	-0,16476	1
Fbxo8	-0,16488	1
Kctd2	-0,16491	1
Nsd2	-0,16514	1
Gm13602	-0,16521	1
Rsbn1	-0,16524	1
Camkmt	-0,16536	1
Gm13498	-0,16548	1
Lmbr1	-0,16557	1
Mtx1	-0,16573	1
Pdcd6	-0,16578	1
B230369F24Rik	-0,16588	1
Ythdf3	-0,16592	1
Gm13622	-0,16605	1
Selenof	-0,16624	1
Trip6	-0,16619	1
Map2k5	-0,16631	1
Akap10	-0,16643	1
Cetn2	-0,16636	1
Mtrf1l	-0,16663	1
Fbxl14	-0,1667	1
Fam161b	-0,16667	1
Vcl	-0,16666	1
Zfp955a	-0,16677	1
Sash1	-0,16693	1
Hnrnpdl	-0,16694	1
Cd44	-0,16693	1
Rcbtb1	-0,167	1
Tsen54	-0,16702	1
Ofd1	-0,16714	1
Wbp1l	-0,16723	1
Gm26549	-0,16731	1
Gm44116	-0,1673	1
Scarb1	-0,16734	1
Creb1	-0,16734	1
Slx4	-0,1675	1
Cnbp	-0,16761	1
Pskh1	-0,16795	1
Arrdc3	-0,16793	1
Atp2a3	-0,16791	1
Lman2	-0,16795	1

Tnfrsf11a	-0,16801	1
Ctu2	-0,16809	1
Ercc6	-0,16813	1
Pgap2	-0,16808	1
Mtch2	-0,16823	1
Dhrs9	-0,16833	1
Htatip2	-0,1683	1
Stat6	-0,16836	1
Fam32a	-0,1684	1
Ece1	-0,16849	1
Gm43059	-0,16854	1
Vdac3	-0,16858	1
Tkt	-0,16858	1
4833420G17Rik	-0,16866	1
Thoc6	-0,1687	1
Psme4	-0,16882	1
Cldn12	-0,16895	1
Pfn1	-0,16898	1
Bcs1l	-0,16909	1
Zc3hav1	-0,16907	1
Gm9442	-0,16916	1
Dcaf8	-0,16921	1
Srrd	-0,16932	1
Tcf25	-0,1693	1
Scamp2	-0,16961	1
Lmbr1l	-0,16973	1
Tomm40	-0,16976	1
Homer3	-0,16983	1
2810002D19Rik	-0,16994	1
Atp8b2	-0,16989	1
Arl14ep	-0,16998	1
Nae1	-0,17005	1
Smarcd2	-0,17015	1
Hoxa7	-0,1702	1
Dbp	-0,17032	1
Atp6v1b2	-0,17028	1
Lman1	-0,17037	1
Ino80e	-0,17049	1
Wdr90	-0,17072	1
C030037D09Rik	-0,17079	1
Mbp	-0,17078	1
Ppp1r15b	-0,17095	1
Zc3h6	-0,17097	1
Trip13	-0,17118	1
Fadd	-0,17119	1
Mapk3	-0,1713	1
Trps1	-0,17139	1
Igf2bp2	-0,17141	1
Cct7	-0,17143	1
Ap1ar	-0,17149	1
Rictor	-0,17151	1
Ppp1r37	-0,17177	1

Il10rb	-0,1718	1
Gm24339	-0,17192	1
Yipf6	-0,17189	1
Gm44090	-0,17235	1
Mob3c	-0,17236	1
Tm4sf5	-0,17264	1
Pik3ca	-0,17259	1
Evi2a	-0,17284	1
Atp6v0b	-0,17278	1
Rabl6	-0,17275	1
Lamp2	-0,17297	1
Atp2b1	-0,17299	1
Vkorc1l1	-0,17314	1
Pkib	-0,17333	1
Zfp668	-0,1735	1
Srek1	-0,17363	1
Gsg2	-0,17379	1
Ilk	-0,17383	1
Map3k11	-0,17394	1
Mbtps1	-0,17391	1
4732440D04Rik	-0,17395	1
Nipsnap3b	-0,17414	1
Snap23	-0,1741	1
Gatsl2	-0,17425	1
Twsg1	-0,17426	1
Irf7	-0,17463	1
Prmt5	-0,17462	1
Gm43533	-0,17477	1
Tm7sf3	-0,17483	1
Larp4b	-0,17479	1
Zfp775	-0,1749	1
Trip4	-0,17489	1
Trpv2	-0,17493	1
Rsph1	-0,17509	1
Chml	-0,17507	1
Krtcap2	-0,1753	1
Noct	-0,17536	1
Rundc1	-0,17549	1
Zdhhc5	-0,17549	1
Gpi1	-0,17547	1
Rhof	-0,17573	1
Gm6946	-0,17589	1
2810474O19Rik	-0,17588	1
Tmppe	-0,17604	1
Prrg2	-0,17596	1
Ftx	-0,17615	1
Mcm5	-0,17606	1
Gemin5	-0,17634	1
Dnmt3l	-0,17645	1
Efr3a	-0,17655	1
Tanc1	-0,1766	1
Rbm12	-0,17657	1

Rad9a	-0,17679	1
E330020D12Rik	-0,17688	1
Hnrnpu	-0,17692	1
Gm22	-0,17707	1
Mapk1	-0,17718	1
Atxn7l3b	-0,17722	1
Mypop	-0,17736	1
Use1	-0,17746	1
Atf7ip	-0,17774	1
Ybx3	-0,17773	1
Llgl1	-0,17777	1
Ankib1	-0,1779	1
Timm44	-0,17792	1
Rnf130	-0,17809	1
Atg3	-0,17812	1
Tomm70a	-0,17814	1
C130026I21Rik	-0,17828	1
Srebf1	-0,17842	1
Ninl	-0,17843	1
Fam96b	-0,17844	1
Celf1	-0,17839	1
Exoc5	-0,17873	1
Mesdc1	-0,17882	1
Soat1	-0,17894	1
Dnaja3	-0,17896	1
Gmfb	-0,179	1
Usp19	-0,17913	1
Cntln	-0,17923	1
Nmral1	-0,1792	1
Map3k3	-0,1792	1
Alg11	-0,17919	1
Abcb8	-0,1793	1
B2m	-0,17954	1
Xndc1	-0,1796	1
Alg1	-0,17976	1
Tmem123	-0,17979	1
Lck	-0,17988	1
Csnk1a1	-0,17987	1
Ccnc	-0,17998	1
S100a11	-0,18002	1
Mfap3l	-0,18023	1
Fam168a	-0,18023	1
Pros1	-0,18033	1
Rps3	-0,18039	1
Gigyf2	-0,18041	1
Sec24b	-0,18049	1
Ankfy1	-0,18052	1
Rps10-ps1	-0,18061	1
Plin3	-0,18088	1
Sltm	-0,18097	1
Ago3	-0,18109	1
Cep19	-0,18119	1

Zfp236	-0,18146	1
Rhd	-0,18156	1
1700017B05Rik	-0,1816	1
Gm7676	-0,18172	1
Map3k7	-0,18182	1
Pnpla8	-0,1818	1
Mlycd	-0,18215	1
Rgl1	-0,18219	1
BC003331	-0,18239	1
Mob1a	-0,1825	1
Pfkfb4	-0,18257	1
Orc2	-0,18275	1
Stxbp5	-0,18293	1
Nudc	-0,18296	1
Mir99ahg	-0,18314	1
Cxcr3	-0,18345	1
Snx15	-0,18355	1
Zfp467	-0,18372	1
4930539J05Rik	-0,18382	1
Pkm	-0,1839	1
Zbtb14	-0,18403	1
AW554918	-0,1841	1
Arid4a	-0,18441	1
Sec24c	-0,18452	1
Iars	-0,18466	1
Atic	-0,18466	1
Arap1	-0,18484	1
Lonp1	-0,18476	1
Dlst	-0,18488	1
2810403D21Rik	-0,18529	1
Usp9x	-0,18531	1
Rnf141	-0,18532	1
Rpl30-ps9	-0,18548	1
Gxylt1	-0,18555	1
Nfatc3	-0,18546	1
Flot1	-0,18561	1
Engase	-0,18557	1
Pcgf3	-0,18558	1
Fignl2	-0,18573	1
Atp11b	-0,18567	1
Chd9	-0,18585	1
Amn1	-0,18587	1
Afg3l2	-0,1859	1
Ktn1	-0,18589	1
Gm4707	-0,18598	1
Dennd1a	-0,18599	1
F10	-0,18611	1
Gm13864	-0,18617	1
Cbx7	-0,18626	1
D10Jhu81e	-0,18633	1
Spryd3	-0,1864	1
Dcxr	-0,18635	1

Dnm2	-0,18653	1
Ewsr1	-0,18681	1
Mdp1	-0,18681	1
Usp35	-0,18689	1
Brd8	-0,18689	1
Fdxr	-0,18704	1
Mpg	-0,18708	1
Vps39	-0,18728	1
Ggact	-0,18747	1
Mthfd1	-0,18746	1
Kdelr1	-0,18748	1
Gm21975	-0,18761	1
Trp53cor1	-0,18759	1
Zc3h4	-0,18759	1
Sun1	-0,1881	1
Atxn1l	-0,18815	1
Taf6l	-0,18822	1
Tyw1	-0,18831	1
Kars	-0,18826	1
Cox6a1	-0,18829	1
Eprs	-0,18828	1
Xdh	-0,18843	1
Mrps2	-0,18843	1
Fam210a	-0,18836	1
Suv39h2	-0,18847	1
Trim44	-0,18855	1
Crebbp	-0,18863	1
Rcor2	-0,18873	1
Nsmf	-0,18895	1
Vta1	-0,18892	1
C1galt1	-0,18905	1
Mesdc2	-0,18897	1
RP23-159E10.1	-0,18911	1
Fam3a	-0,18931	1
Chd6	-0,18928	1
Laptm5	-0,18933	1
Fam98a	-0,18952	1
Ptpn4	-0,18954	1
Msh6	-0,18949	1
Tardbp	-0,18954	1
Arhgdib	-0,18956	1
Srd5a3	-0,18972	1
Gm13268	-0,18966	1
Ercc3	-0,18972	1
Kif1b	-0,18968	1
Mrpl3	-0,18983	1
Tmem173	-0,18987	1
Ssbp1	-0,18992	1
Supt16	-0,18992	1
Glod4	-0,18986	1
Hadh	-0,19008	1
Ube2m	-0,19046	1

Pdpr	-0,19054	1
Polr2a	-0,19047	1
Wfs1	-0,19069	1
Ate1	-0,19086	1
Frat1	-0,19108	1
Pi4k2a	-0,19121	1
Tspan3	-0,19125	1
Pradc1	-0,19142	1
Zswim7	-0,1914	1
Tpm3	-0,19135	1
Zfp330	-0,19155	1
Pcmt1	-0,19148	1
Ncstn	-0,19158	1
Bcl9l	-0,19167	1
Manba	-0,19181	1
Adal	-0,19193	1
Fahd2a	-0,19197	1
Dvl1	-0,19202	1
Gm9025	-0,19211	1
Hmgcl	-0,19213	1
Ap1g2	-0,19222	1
Cpne2	-0,19239	1
Klhdc10	-0,19258	1
Alkbh8	-0,19257	1
Minpp1	-0,19274	1
Myadm	-0,1927	1
Rcor3	-0,19283	1
Slc39a8	-0,19302	1
Prmt2	-0,19329	1
Cc2d1b	-0,19329	1
Ranbp1	-0,19358	1
Ccdc28b	-0,19386	1
Slc35e1	-0,19391	1
Gm9828	-0,19396	1
Simc1	-0,19431	1
Gm9165	-0,1944	1
Hmgb2	-0,19437	1
Fli1	-0,19454	1
Zfp446	-0,19456	1
Npepps	-0,19462	1
Osbpl7	-0,19484	1
Tnrc6a	-0,19489	1
Hinfp	-0,1951	1
Atp6v1f	-0,19529	1
Cdpf1	-0,19537	1
Znrf3	-0,19544	1
Atad3a	-0,19546	1
Nmd3	-0,1955	1
Pik3cd	-0,19551	1
Des	-0,19587	1
Celf6	-0,19585	1
Slc25a45	-0,19588	1

Smarca5-ps	-0,19602	1
Gpr137b-ps	-0,19599	1
Zfp61	-0,19614	1
Cep170	-0,19608	1
Fam217b	-0,19643	1
Rtfdc1	-0,19641	1
Ccdc112	-0,19655	1
Rnf6	-0,19654	1
Taf15	-0,19653	1
Mybbp1a	-0,19647	1
Tubgcp4	-0,19662	1
Zdhhc13	-0,19704	1
Prr14	-0,19713	1
Palm	-0,19729	1
E2f2	-0,19731	1
Nudt8	-0,19741	1
Ogfod1	-0,19739	1
Tnks	-0,19737	1
Ints13	-0,19763	1
Faim	-0,1982	1
Hnrnpr	-0,19818	1
D630024D03Rik	-0,19842	1
Rrnad1	-0,1984	1
Srpk1	-0,19842	1
Tm7sf2	-0,19846	1
Rock2	-0,19856	1
Tmtc3	-0,19868	1
Zfyve9	-0,19872	1
Smim4	-0,19877	1
Trmt1l	-0,19903	1
Cttnbp2nl	-0,19895	1
Paxip1	-0,19909	1
Trmt2a	-0,19907	1
Ap4b1	-0,19916	1
Brd3	-0,19921	1
Rxrb	-0,19925	1
Tirap	-0,19936	1
Cyb5b	-0,1996	1
Tonsl	-0,19973	1
Procr	-0,19985	1
Gm5045	-0,1999	1
Dexi	-0,20008	1
Ica1	-0,20018	1
Mtf2	-0,2003	1
Phlpp2	-0,20042	1
Vasp	-0,20044	1
Zfp287	-0,20055	1
Pdrg1	-0,20057	1
Gm9732	-0,20067	1
Man1b1	-0,20113	1
Rtn4ip1	-0,20136	1
Phf10	-0,20155	1

Bend4	-0,20163	1
Slc26a2	-0,20163	1
P3h3	-0,20175	1
AI314180	-0,20202	1
Gm13378	-0,20223	1
Slc16a12	-0,2022	1
Zfp605	-0,20234	1
Hddc2	-0,20226	1
3830406C13Rik	-0,20243	1
Akap7	-0,20248	1
Rab7b	-0,20249	1
Rnf135	-0,20269	1
Gskip	-0,20269	1
Map1s	-0,20271	1
Timm13	-0,20285	1
Kctd9	-0,20289	1
Slfn3	-0,20315	1
D5Ertd579e	-0,20323	1
Ssbp4	-0,20316	1
Stard10	-0,20327	1
Arl6ip6	-0,20351	1
Zbtb25	-0,20346	1
Ehbp1l1	-0,20348	1
Ccs	-0,20363	1
Gcfc2	-0,20365	1
Tfrc	-0,20373	1
Pip5k1c	-0,20409	1
Zfp276	-0,20434	1
Coro1c	-0,20431	1
C330007P06Rik	-0,20436	1
H2-Q4	-0,20465	1
Capzb	-0,20462	1
P4ha2	-0,20483	1
Madd	-0,20487	1
Pam	-0,20494	1
Cul2	-0,20505	1
Zfp608	-0,20506	1
Mtif2	-0,20506	1
Rps14	-0,20517	1
Tmem240	-0,20525	1
Gm13657	-0,2053	1
Eno1	-0,20552	1
Pex6	-0,20558	1
F9	-0,20566	1
Gpalpp1	-0,2057	1
Coq10a	-0,2057	1
Rack1	-0,20581	1
Rab5a	-0,20594	1
Zbtb40	-0,20594	1
Coasy	-0,20615	1
Dnase2a	-0,20623	1
Smad6	-0,20637	1

Kmt2e	-0,20662	1
Braf	-0,20668	1
Jpx	-0,20693	1
Gpcpd1	-0,20692	1
N4bp1	-0,20686	1
Uck2	-0,20712	1
Cd101	-0,20735	1
Abi3	-0,20745	1
Gm15853	-0,20743	1
Noc3l	-0,20749	1
4921531C22Rik	-0,20786	1
Fam92a	-0,20789	1
RP24-497N7.2	-0,20793	1
Gm19620	-0,20805	1
Tmed2	-0,20795	1
Gm43501	-0,20824	1
Hs6st1	-0,20822	1
9030617O03Rik	-0,20833	1
Pcid2	-0,20842	1
Srsf10	-0,20842	1
Man2a2	-0,20841	1
Leng8	-0,20849	1
Slc38a7	-0,20846	1
Ikbkg	-0,20855	1
Cdk11b	-0,2086	1
Alg13	-0,20878	1
Abcf1	-0,20883	1
Med17	-0,20893	1
Txnl4b	-0,20908	1
Tmem229b	-0,20914	1
Cited2	-0,20918	1
Zmiz2	-0,20928	1
Cenpm	-0,20962	1
Prelid2	-0,20984	1
Chchd4	-0,2101	1
Las1l	-0,21053	1
1110038F14Rik	-0,21049	1
Usp25	-0,21058	1
Angel2	-0,2108	1
Rnf187	-0,21099	1
Irf3	-0,21111	1
Nfkbia	-0,21106	1
1600010M07Rik	-0,2112	1
Tal1	-0,21117	1
Txnip	-0,21134	1
Ahcyl2	-0,21141	1
Man2b1	-0,21139	1
Nit1	-0,21162	1
Wac	-0,21158	1
Oas1b	-0,21167	1
Arhgef39	-0,21173	1
Adcy2	-0,21193	1

Sumo3	-0,2119	1
Xpo6	-0,21242	1
Slc35a5	-0,21267	1
Ipo4	-0,21322	1
Ercc8	-0,21335	1
Rpn1	-0,21333	1
Dpf1	-0,21342	1
Qrsl1	-0,21336	1
Sigmar1	-0,21351	1
Dynll1	-0,21355	1
Tvp23b	-0,21383	1
Aamdc	-0,21387	1
Kif4	-0,21415	1
Ccdc180	-0,21422	1
H2-Oa	-0,21416	1
3010003L21Rik	-0,21422	1
Ap3b1	-0,21432	1
Lrrc27	-0,21445	1
Foxred1	-0,21451	1
Pou2f1	-0,21447	1
Lmf2	-0,21465	1
Coq3	-0,21493	1
Tmem230	-0,21505	1
Zbtb44	-0,21512	1
Dennd4b	-0,21518	1
Cryzl1	-0,21523	1
Wdr24	-0,21527	1
Hspa4l	-0,21529	1
Cherp	-0,21545	1
Fubp3	-0,21565	1
7330423F06Rik	-0,21576	1
Med13l	-0,21579	1
Tsn	-0,21601	1
Pbrm1	-0,21609	1
Trp53i13	-0,21616	1
Clasrp	-0,21615	1
Lsm8	-0,21624	1
Elmo1	-0,21634	1
Pum3	-0,21651	1
Acaa1b	-0,21656	1
0610030E20Rik	-0,21659	1
Kifc3	-0,2166	1
Ccz1	-0,21663	1
Tet3	-0,21669	1
Pcmtd1	-0,21675	1
Cnot6l	-0,21676	1
Pck2	-0,21722	1
D530018E20Rik	-0,2173	1
Dhx16	-0,21742	1
Scrib	-0,21784	1
Impad1	-0,21781	1
Zfp146	-0,21807	1

Kif16b	-0,21815	1
Gm20091	-0,21831	1
Dock9	-0,21846	1
Dpy19l3	-0,21863	1
Slc7a6	-0,21874	1
AC168977.1	-0,21875	1
Hoxc4	-0,21891	1
Zfp692	-0,21887	1
Cacna1d	-0,219	1
Ovca2	-0,21897	1
Gm5124	-0,21911	1
Ddx21	-0,21941	1
Fcgr1	-0,21954	1
Gm7206	-0,21964	1
Mrpl58	-0,21964	1
G6pd2	-0,21982	1
Dnpep	-0,21981	1
Clec16a	-0,2202	1
Prkar1a	-0,22023	1
Mlxip	-0,22031	1
Kcnab3	-0,22038	1
Msantd2	-0,22053	1
Psmd6	-0,22072	1
Ift20	-0,22081	1
Rad50	-0,22077	1
Gclc	-0,2208	1
Srsf2	-0,22079	1
Fbxo3	-0,22082	1
Ndrg2	-0,22076	1
Xrcc2	-0,22094	1
Tprkb	-0,22088	1
Deptor	-0,22088	1
Med27	-0,22102	1
Lrp1	-0,22098	1
Armt1	-0,22111	1
Rcc1	-0,22107	1
RP23-55A6.4	-0,2212	1
Cdk2ap2	-0,22123	1
Tram1	-0,22119	1
Ago4	-0,22138	1
Sike1	-0,22151	1
Cnnm2	-0,22155	1
Zfp451	-0,22181	1
Dhx35	-0,22186	1
Dhx36	-0,22189	1
Cog3	-0,22202	1
Acat1	-0,22202	1
Limd2	-0,22201	1
Srcap	-0,22222	1
Nat10	-0,22219	1
Ctdsp2	-0,2222	1
Anxa2	-0,22218	1

Sntb2	-0,2223	1
Banf1	-0,2223	1
Aaas	-0,2224	1
Vps13c	-0,22243	1
Tbx6	-0,22268	1
Gm13992	-0,22283	1
Gbe1	-0,22286	1
Hnrnpul2	-0,22299	1
Gm37785	-0,22307	1
Coq2	-0,22319	1
1190007I07Rik	-0,22333	1
Rad23b	-0,22342	1
Lrpprc	-0,22352	1
Slk	-0,22357	1
Med29	-0,22366	1
Tm6sf1	-0,22368	1
Esd	-0,22374	1
Gm5453	-0,22378	1
Nfya	-0,22388	1
Ttl	-0,2239	1
Mettl8	-0,22407	1
Znhit2	-0,22408	1
Slc25a12	-0,22423	1
Cope	-0,22434	1
Tsga10	-0,22437	1
Iqcg	-0,22445	1
Cnpy2	-0,22442	1
Gm17455	-0,22442	1
Ears2	-0,22447	1
Zfat	-0,22458	1
Gak	-0,22459	1
Nsd3	-0,22463	1
Yod1	-0,22468	1
Appl2	-0,22467	1
Opa3	-0,22498	1
Dffa	-0,22508	1
Sec63	-0,22516	1
Actb	-0,22528	1
Slc17a5	-0,2254	1
Plbd2	-0,22541	1
Ccdc47	-0,22559	1
Dcbld2	-0,2256	1
Ikbkb	-0,22567	1
Slc39a6	-0,22572	1
Rab24	-0,22568	1
5730480H06Rik	-0,2258	1
Scamp1	-0,22594	1
Ptbp3	-0,22589	1
Fam161a	-0,22604	1
Ganab	-0,22599	1
Pelp1	-0,22608	1
Stk10	-0,22624	1

Mccc2	-0,22631	1
Gm11722	-0,22641	1
Gtpbp2	-0,22644	1
Gm43961	-0,22662	1
Fam151b	-0,22671	1
Gdpd1	-0,22694	1
Top2b	-0,22739	1
Cpeb3	-0,22767	1
Zmynd11	-0,22786	1
Mycbp2	-0,2279	1
Nfyb	-0,22796	1
Galns	-0,22838	1
Tbc1d10b	-0,22836	1
Rbms1	-0,22841	1
Fam13c	-0,2285	1
Pigs	-0,22864	1
Gm45222	-0,22872	1
Sh3bp5l	-0,22869	1
Adipor2	-0,22875	1
Tmem141	-0,22881	1
2810403A07Rik	-0,22902	1
Becn1	-0,22902	1
Dok3	-0,22907	1
Tex261	-0,2291	1
Mogs	-0,2291	1
Scrn2	-0,22916	1
Pkd1	-0,22931	1
Dand5	-0,22944	1
Mapkapk2	-0,22938	1
Sema4g	-0,22959	1
Upf3a	-0,22969	1
2310047D07Rik	-0,22985	1
Pcsk7	-0,22984	1
Fbxl12	-0,22977	1
Ccar2	-0,2299	1
Fntb	-0,22992	1
Pik3r1	-0,23014	1
Chd4	-0,23012	1
Slc10a7	-0,23038	1
Foxk1	-0,23048	1
Cd72	-0,23046	1
Acvrl1	-0,23076	1
Rfx7	-0,23075	1
Snx27	-0,23077	1
D16Ertd472e	-0,23106	1
Ppil3	-0,23117	1
Rb1cc1	-0,23116	1
Nt5c	-0,23143	1
Tgs1	-0,23136	1
Kidins220	-0,23137	1
Rwdd4a	-0,23136	1
Gm11878	-0,23145	1

Dip2a	-0,23151	1
Gm23502	-0,23157	1
Cox17	-0,23164	1
Spcs2	-0,23168	1
Gm33142	-0,23182	1
Flywch1	-0,23205	1
Dopey2	-0,23213	1
Sh3pxd2b	-0,23213	1
Mcub	-0,23224	1
Nin	-0,23226	1
Anks1	-0,23233	1
Rnf41	-0,23246	1
AI606181	-0,2325	1
Fam102a	-0,23254	1
Fcrl1	-0,23257	1
Zcchc6	-0,23268	1
Gm8292	-0,23281	1
Micu2	-0,23275	1
Gm12655	-0,23292	1
Eif4ebp1	-0,23286	1
Fkbp11	-0,2331	1
Zfp207	-0,23305	1
Cspp1	-0,2333	1
Trrap	-0,23337	1
Naa35	-0,23341	1
Timmdc1	-0,23353	1
Nr6a1	-0,23353	1
Nova2	-0,23375	1
Tubgcp3	-0,23366	1
Sae1	-0,23366	1
1810014B01Rik	-0,23396	1
Ncor1	-0,23397	1
Ccdc136	-0,23414	1
Mrps18a	-0,23411	1
Klc1	-0,23408	1
Smarcb1	-0,23421	1
Mtx3	-0,2343	1
Mlst8	-0,2343	1
Rnf121	-0,23436	1
Nipal3	-0,23461	1
Ak6	-0,2346	1
Gusb	-0,235	1
Srgap2	-0,23513	1
Prkrip1	-0,23524	1
Nol10	-0,2352	1
Strn3	-0,23521	1
Prrc2c	-0,2353	1
Fan1	-0,23537	1
Gpt	-0,23554	1
Snrpd3	-0,23556	1
Phb2	-0,23564	1
Ecel1	-0,23574	1

Gm28727	-0,23582	1
Agps	-0,23584	1
Stk38	-0,23578	1
Amd1	-0,23587	1
Phf3	-0,23587	1
Ice1	-0,23597	1
Gpx1	-0,23612	1
Rpap1	-0,23623	1
Pccb	-0,23644	1
Gm35315	-0,23652	1
Kdm5d	-0,23666	1
Itpkb	-0,23714	1
Psme2b	-0,23714	1
Prpsap1	-0,23713	1
Rasal1	-0,23733	1
Mnd1	-0,23729	1
Ptpn12	-0,23742	1
Gramd1c	-0,23751	1
Ccnf	-0,23777	1
Kif13a	-0,23788	1
Arfrp1	-0,23793	1
Atp1a1	-0,23791	1
Prpf4b	-0,2381	1
Tspan4	-0,23812	1
Pola1	-0,23826	1
Xpo7	-0,23834	1
Rmi1	-0,23839	1
Usp15	-0,23854	1
Gm45762	-0,23859	1
Gm37052	-0,23862	1
Osgep	-0,23859	1
Wapl	-0,23868	1
Rprd1b	-0,23888	1
Nop16	-0,23893	1
Nme7	-0,23898	1
Gm16630	-0,23904	1
Nrd1	-0,23913	1
Ccndbp1	-0,23913	1
Map4k1	-0,23931	1
Mgat5	-0,23926	1
Cdk5rap1	-0,23961	1
Hcfc2	-0,23959	1
Ccdc107	-0,23972	1
Ndufaf8	-0,23971	1
Cd320	-0,2398	1
Vps4a	-0,2398	1
Stag1	-0,23989	1
Ccdc88b	-0,23994	1
Kcnc3	-0,24009	1
Cdk8	-0,2403	1
Trmt6	-0,24028	1
Strbp	-0,24058	1

Dars	-0,24069	1
Kdm7a	-0,24068	1
Ankrd46	-0,24078	1
Dpm1	-0,24094	1
Zfp317	-0,24088	1
Tpm4	-0,24097	1
Gm42511	-0,24129	1
Dnajc1	-0,24127	1
Nudt21	-0,24156	1
Commd10	-0,24181	1
Prkcd	-0,24189	1
Fbxl8	-0,24206	1
Srr	-0,24209	1
Fam195b	-0,24213	1
1110019D14Rik	-0,24217	1
Exoc1	-0,24229	1
Atp1a3	-0,24234	1
Pcgf2	-0,24237	1
Snta1	-0,24235	1
Ndufa8	-0,24236	1
Trmt2b	-0,24262	1
Stradb	-0,24287	1
Ube4b	-0,24311	1
Stat5b	-0,24325	1
Tacc1	-0,24322	1
Lamtor2	-0,24332	1
Dst	-0,24338	1
Gzf1	-0,24362	1
Nfatc1	-0,24357	1
Slc33a1	-0,24377	1
Eri1	-0,24377	1
Mapk14	-0,24435	1
Gm21781	-0,24438	1
Gm13223	-0,2445	1
Syce2	-0,24446	1
Yipf1	-0,24462	1
4930590J08Rik	-0,24472	1
Gm43411	-0,24481	1
Adam15	-0,24483	1
Cd63	-0,24482	1
Tle6	-0,24501	1
Fndc3b	-0,24511	1
Tnpo1	-0,24521	1
Gm7299	-0,24528	1
Phactr1	-0,2453	1
Hist1h4n	-0,24554	1
Casp2	-0,24558	1
Kctd7	-0,24568	1
Gm37482	-0,24568	1
Tug1	-0,24582	1
Chaf1b	-0,24585	1
Coa7	-0,24599	1

RP23-128C4.4	-0,24599	1
1810011H11Rik	-0,24603	1
Akr1b8	-0,2462	1
Col4a5	-0,24631	1
Clstn1	-0,24679	1
Cask	-0,24676	1
Phtf2	-0,24692	1
Grk2	-0,24692	1
Gm45836	-0,2471	1
Wdr45	-0,24729	1
Chac2	-0,24748	1
Shcbp1l	-0,24746	1
Ncoa3	-0,24757	1
Rragb	-0,24789	1
Cse1l	-0,24798	1
Map2k4	-0,24812	1
Csnk1g1	-0,24808	1
Fam193b	-0,24821	1
Notch2	-0,24821	1
Ddx46	-0,24824	1
Fto	-0,24833	1
Klhl36	-0,24835	1
Impa2	-0,24841	1
Dgkz	-0,24843	1
Tctex1d2	-0,24846	1
Lcp1	-0,24857	1
Cstf2	-0,24876	1
Pten	-0,24884	1
4930402H24Rik	-0,24891	1
D3Ertd751e	-0,24897	1
Nfs1	-0,249	1
Cluh	-0,24903	1
Zfp7	-0,24909	1
Oasl1	-0,24919	1
Ssrp1	-0,24917	1
Tmem259	-0,2493	1
Dph2	-0,24957	1
Vamp7-ps	-0,2497	1
Pum2	-0,24984	1
6430710M23Rik	-0,24991	1
Clns1a	-0,24988	1
Ap5m1	-0,24998	1
Eea1	-0,24998	1
6430590A07Rik	-0,25021	1
P3h1	-0,2502	1
Il11ra1	-0,25032	1
Ywhab	-0,25041	1
Slc50a1	-0,25036	1
Ydjc	-0,25038	1
Star	-0,25048	1
9330111N05Rik	-0,25064	1
Zbtb8os	-0,25067	1

Atad2b	-0,25072	1
Dph5	-0,25093	1
Focad	-0,25088	1
Ireb2	-0,25094	1
Nup54	-0,25096	1
Exosc7	-0,25099	1
Cstf1	-0,25096	1
9230111E07Rik	-0,25116	1
Gm38111	-0,25139	1
Snai1	-0,25151	1
Gm15596	-0,25147	1
Cdc42ep2	-0,25151	1
Syk	-0,25161	1
Il15ra	-0,25175	1
Padi2	-0,25181	1
Prkaca	-0,25181	1
Rbm10	-0,25195	1
Gpatch11	-0,25192	1
Tmem164	-0,25199	1
Rsrp1	-0,25211	1
Samd1	-0,25247	1
Spice1	-0,25256	1
Slc40a1	-0,25272	1
Cd300lb	-0,2527	1
Jagn1	-0,2529	1
Parp4	-0,25353	1
Smad1	-0,25352	1
Zfp943	-0,25409	1
Gm19552	-0,25425	1
Trappc13	-0,25433	1
Clec1a	-0,25446	1
Gm43800	-0,2545	1
Stoml1	-0,25449	1
Pspc1	-0,25464	1
Ahnak	-0,25459	1
Prkar1b	-0,25471	1
Ppm1j	-0,25482	1
Sp100	-0,25492	1
Atp2c1	-0,25502	1
Cyp4f13	-0,25514	1
Epb41l5	-0,2552	1
Dgkh	-0,25532	1
Uxt	-0,25525	1
Atp6v0a2	-0,25539	1
Nradd	-0,25542	1
Cd180	-0,2554	1
Glud1	-0,25548	1
Rpain	-0,25557	1
Smarca2	-0,25562	1
Rpl36-ps8	-0,25594	1
Commd5	-0,25609	1
Sec11c	-0,25621	1

Hibadh	-0,25629	1
Smchd1	-0,25646	1
Mmab	-0,25673	1
Ubxn2a	-0,25666	1
Dnttip1	-0,25667	1
Ehmt2	-0,25666	1
mt-Nd5	-0,25669	1
Sfswap	-0,2568	1
Pnpla2	-0,25694	1
Lrfn4	-0,257	1
Zfp607b	-0,25697	1
Gpaa1	-0,2572	1
Phip	-0,25752	1
Csad	-0,25765	1
Mfap3	-0,2578	1
Ttc39b	-0,25787	1
Dlat	-0,25794	1
Afdn	-0,2579	1
Thra	-0,25791	1
Cdip1	-0,25797	1
Rtn3	-0,25797	1
Akap13	-0,25806	1
Glg1	-0,25815	1
Ezh1	-0,25806	1
Gm13205	-0,2583	1
Dkkl1	-0,25828	1
Atg2a	-0,25835	1
Sipa1	-0,25854	1
Rbm33	-0,25846	1
Fam199x	-0,25865	1
Gm9484	-0,25887	1
Ltn1	-0,25894	1
Pmpca	-0,25892	1
Mir22hg	-0,25891	1
Plgrkt	-0,259	1
Wrn	-0,25911	1
Tecr	-0,25912	1
Ppp1ca	-0,25913	1
Taf1	-0,25909	1
Dnajc14	-0,25943	1
Tctn1	-0,25953	1
Rtca	-0,25953	1
Acly	-0,25949	1
Sdad1	-0,25949	1
S100a6	-0,25964	1
RP23-426K2.3	-0,25976	1
Ddx41	-0,25981	1
Fam193a	-0,25981	1
Pdlim2	-0,25977	1
4930520O04Rik	-0,25985	1
Tmem69	-0,2601	1
Rnf123	-0,26022	1

Hcar2	-0,26034	1
Abhd5	-0,26053	1
Shisa5	-0,2605	1
Ccnd1	-0,26053	1
Hmg20a	-0,26068	1
Plxnb2	-0,26089	1
Asb3	-0,2612	1
Eno3	-0,26121	1
Aldoa	-0,26132	1
Idh3a	-0,2615	1
Grhl1	-0,26171	1
Gm5532	-0,26167	1
Plxdc1	-0,26192	1
Mcm3ap	-0,26202	1
Pask	-0,26228	1
Lsm14b	-0,26231	1
Anapc4	-0,26231	1
Fam178a	-0,26239	1
Tars	-0,26241	1
Wdr26	-0,26241	1
Gm43088	-0,2625	1
Mapk11	-0,26262	1
Plekha8	-0,26261	1
Ankrd28	-0,2629	1
Zbtb4	-0,26292	1
Znrf2	-0,26297	1
Dctn2	-0,26301	1
Dnajc8	-0,26311	1
Hacd3	-0,26319	1
Tapt1	-0,26331	1
4932438A13Rik	-0,26326	1
Lig4	-0,26346	1
Nrbp2	-0,26378	1
Efcab11	-0,26381	1
Gm7094	-0,26382	1
Plekho2	-0,2638	1
Grk6	-0,26392	1
Tbc1d1	-0,26398	1
Sidt2	-0,26396	1
Ecd	-0,26413	1
Ctsl	-0,26409	1
Plxnd1	-0,26421	1
RP23-182J19.2	-0,26435	1
Eri3	-0,26432	1
Smap1	-0,26462	1
Cwf19l2	-0,2646	1
Pgd	-0,26471	1
Prex1	-0,26476	1
Sclt1	-0,26542	1
Tpk1	-0,26541	1
Hdac4	-0,26556	1
Akap8l	-0,26563	1

Ankrd40	-0,26584	1
Spata13	-0,26576	1
Bcl9	-0,26586	1
9230112E08Rik	-0,26598	1
Ptpra	-0,26608	1
Ciapin1	-0,26606	1
Map2k2	-0,26621	1
Wdfy3	-0,2663	1
Dusp19	-0,26631	1
Snord92	-0,2665	1
Tmem132a	-0,26684	1
Arpc2	-0,26688	1
Ube2d-ps	-0,267	1
Nom1	-0,26737	1
Aak1	-0,26741	1
C1qbp	-0,26741	1
Brpf1	-0,26771	1
Pidd1	-0,26785	1
Tada3	-0,26775	1
Tsc1	-0,26781	1
Nans	-0,26793	1
Snapin	-0,26812	1
Gm15157	-0,26819	1
Man2b2	-0,26841	1
Ccdc12	-0,2684	1
Smarcd1	-0,2684	1
Nup107	-0,26865	1
Scd2	-0,26857	1
D930015E06Rik	-0,26918	1
C130036L24Rik	-0,26928	1
Xpnpep3	-0,26935	1
Asap3	-0,26938	1
Slc2a6	-0,26949	1
Mrpl13	-0,26951	1
Emp1	-0,26948	1
Limk1	-0,2696	1
Rbbp7	-0,26958	1
Gm7452	-0,26985	1
Gm561	-0,27011	1
Rreb1	-0,27011	1
Trim28	-0,27031	1
Mdh2	-0,2703	1
Fancg	-0,2706	1
Jak3	-0,27058	1
Lactb2	-0,27074	1
Nmi	-0,27078	1
Atg2b	-0,27083	1
Ociad1	-0,2708	1
Gm5362	-0,27106	1
Pepd	-0,27107	1
Clcn2	-0,2712	1
Fam64a	-0,27116	1

Sirt7	-0,27128	1
Cntrl	-0,27134	1
Caly	-0,27144	1
Zzz3	-0,27149	1
Mndal	-0,27172	1
Itsn2	-0,27215	1
Dmap1	-0,27229	1
Hipk1	-0,27228	1
Pkp2	-0,27246	1
Kctd11	-0,27269	1
Calu	-0,27285	1
Gnb2	-0,27282	1
Cdk5	-0,2731	1
Zbtb38	-0,27323	1
Dtwd2	-0,27334	1
Slc16a3	-0,27343	1
Rnf214	-0,27371	1
Gas2l1	-0,27381	1
Rps6ka4	-0,27393	1
Cdc123	-0,27392	1
Capn2	-0,27389	1
Tbc1d25	-0,27406	1
Hspb11	-0,27432	1
Aig1	-0,2743	1
Tspyl3	-0,27461	1
Ccnl2	-0,27474	1
B3galt6	-0,27477	1
Trmu	-0,27488	1
Rnf128	-0,27503	1
Ide	-0,27512	1
RP24-183O8.6	-0,27521	1
Epm2aip1	-0,27517	1
Prpf38b	-0,27523	1
Tbcd	-0,27545	1
Bbs4	-0,27554	1
Mcf2l	-0,27554	1
Cant1	-0,27545	1
Mfsd4b4	-0,27574	1
Rnf24	-0,27572	1
Mcu	-0,27586	1
Gbf1	-0,27613	1
Gm43213	-0,27623	1
Ipo9	-0,27621	1
Gns	-0,27622	1
Gm45806	-0,27639	1
E330009J07Rik	-0,27635	1
Cdk5rap2	-0,27639	1
Gm37510	-0,27637	1
Lrrc58	-0,27682	1
Paxbp1	-0,27713	1
Iws1	-0,27707	1
Plagl2	-0,27716	1

Relb	-0,27721	1
Syngap1	-0,27783	1
Gtf2h4	-0,27781	1
Mapk6	-0,27801	1
Lgals2	-0,2781	1
Mgat4b	-0,27809	1
Slc25a53	-0,27824	1
Tysnd1	-0,27865	1
Mgrn1	-0,2786	1
Fam219b	-0,27864	1
Sppl2a	-0,27858	1
Crocc	-0,2788	1
2410089E03Rik	-0,27893	1
Tfcp2	-0,27912	1
Usp21	-0,27924	1
Gm23100	-0,27919	1
Abcb10	-0,27932	1
Foxp1	-0,27928	1
Mir17hg	-0,27937	1
Stim2	-0,27945	1
Gm37962	-0,27954	1
Scarna9	-0,27957	1
Dnal1	-0,27969	1
Mex3a	-0,27984	1
Gm16096	-0,28002	1
Tfap4	-0,28007	1
Bptf	-0,28015	1
Phf20	-0,28029	1
Ankrd24	-0,28042	1
Wrap73	-0,2805	1
Ncapd2	-0,28053	1
Safb2	-0,28057	1
Eif2b3	-0,28062	1
Gm27477	-0,28078	1
Trim37	-0,28098	1
Tmem147	-0,28099	1
Rab11fip2	-0,28107	1
Mtfr1l	-0,28111	1
Gm25541	-0,28118	1
Ddx50	-0,28132	1
Prps2	-0,28145	1
Zfp160	-0,28148	1
Psip1	-0,28147	1
Psma8	-0,28158	1
Plekha2	-0,28163	1
1110020A21Rik	-0,28167	1
Slc9a5	-0,28215	1
Mir5136	-0,28208	1
Mrpl10	-0,28221	1
Slc38a2	-0,28229	1
Acox3	-0,28259	1
CAAA01180111.2	-0,28286	1

Btbd2	-0,28294	1
Zfp46	-0,28298	1
Nectin3	-0,28308	1
Vamp5	-0,28325	1
Cep97	-0,28321	1
Acsl4	-0,28322	1
Drg2	-0,28348	1
Nr1h2	-0,28353	1
Ube2d2a	-0,28353	1
Accs	-0,28356	1
Crot	-0,28367	1
Mfap1a	-0,2837	1
Arrdc1	-0,28367	1
Tcaf1	-0,28391	1
Eif3a	-0,28389	1
Mef2a	-0,28405	1
Ids	-0,28413	1
9430015G10Rik	-0,28422	1
Egln1	-0,28423	1
Gpd2	-0,28415	1
Ndufa9	-0,28435	1
Zkscan17	-0,28441	1
2700049A03Rik	-0,28448	1
Plk4	-0,28456	1
Zranb2	-0,28463	1
Xirp1	-0,28485	1
Rab15	-0,28491	1
Vps33b	-0,28503	1
Setd1a	-0,28521	1
Cfap20	-0,28534	1
Sdf2	-0,2854	1
Pcmtd2	-0,28554	1
Lrrcc1	-0,28568	1
Anapc10	-0,28605	1
Gm15779	-0,28633	1
BC017643	-0,28626	1
Fam173a	-0,28638	1
Kat8	-0,28672	1
Stx16	-0,28685	1
Phf7	-0,28703	1
Ttc12	-0,28702	1
Mzt2	-0,28713	1
Zfp771	-0,28716	1
Hook3	-0,28729	1
Trabd	-0,28725	1
Cyb5r4	-0,28735	1
Gtf2ird2	-0,28751	1
Myl6	-0,28765	1
Srsf1	-0,28774	1
Sdha	-0,28791	1
Twf2	-0,28799	1
Gm4734	-0,28835	1

Nudt13	-0,2884	1
Def6	-0,2886	1
Ralgapa1	-0,28857	1
Msto1	-0,28861	1
Fbxw7	-0,28875	1
Pds5a	-0,28887	1
Mrpl37	-0,28898	1
Washc3	-0,28923	1
Ccni	-0,2893	1
Isoc2a	-0,28938	1
Tatdn1	-0,28963	1
Naa40	-0,2896	1
G6pdx	-0,28965	1
Bid	-0,28958	1
Coro2a	-0,28984	1
Sacm1l	-0,28984	1
Sdhc	-0,28976	1
Zfp609	-0,2899	1
Usp48	-0,29003	1
Thoc2	-0,28996	1
Zw10	-0,29014	1
Hyou1	-0,29029	1
Atf5	-0,2905	1
Trim39	-0,2908	1
Mmp19	-0,29076	1
Rbbp4	-0,29084	1
Zfp429	-0,29091	1
Lppos	-0,2909	1
Tomm40l	-0,29111	1
Dnlz	-0,29118	1
Farsb	-0,29154	1
Adsl	-0,29162	1
Dhx9	-0,2917	1
Pdzd11	-0,29201	1
Otx1	-0,2921	1
Cdk7	-0,29208	1
Tmem205	-0,29212	1
Pttg1	-0,29219	1
Lsm5	-0,29237	1
Fam172a	-0,29253	1
Cul5	-0,29249	1
Fam8a1	-0,29263	1
Cep295	-0,29337	1
Cdk5rap3	-0,2936	1
Kansl1	-0,2937	1
Wdr1	-0,29366	1
Mus81	-0,29396	1
5830444B04Rik	-0,294	1
Disp1	-0,29414	1
Dnase1l1	-0,29405	1
Catip	-0,29437	1
Gm23458	-0,29435	1

Gm5577	-0,29456	1
Zfp768	-0,29463	1
Zfp930	-0,29474	1
Nadk	-0,29473	1
Fam216a	-0,29481	1
Rgl2	-0,29496	1
Hip1	-0,29523	1
Odf2	-0,29516	1
Fxyd5	-0,29518	1
Gm20712	-0,29529	1
Apbb2	-0,29526	1
Gm44423	-0,29539	1
Fbf1	-0,29558	1
Gm3531	-0,29573	1
Ankhd1	-0,29574	1
Spr	-0,29589	1
Lta	-0,29598	1
Stat3	-0,296	1
Gm22714	-0,29623	1
Kcnn4	-0,29622	1
Apobr	-0,29643	1
Prpf31	-0,29649	1
Zfp280d	-0,29647	1
Rasa1	-0,29652	1
Arhgap9	-0,29672	1
0610007P14Rik	-0,29667	1
Immt	-0,29667	1
Capg	-0,29667	1
Adss	-0,29686	1
4930529C04Rik	-0,29697	1
Tbc1d2b	-0,29696	1
Tmem186	-0,29709	1
Cdnf	-0,2971	1
Nfkb1	-0,29707	1
Tpp2	-0,2971	1
Mllt6	-0,29716	1
Fam167b	-0,2973	1
B9d1	-0,29758	1
Crat	-0,29774	1
Gm6297	-0,29781	1
Selenoo	-0,29775	1
Gm13604	-0,29843	1
9130221H12Rik	-0,29873	1
Slc39a9	-0,29881	1
Gm7561	-0,29887	1
Thrap3	-0,2989	1
Rps15a-ps3	-0,29927	1
Slc30a4	-0,29939	1
Cenpb	-0,2995	1
Mrpl19	-0,29969	1
Dimt1	-0,29998	1
Cyfip2	-0,30004	1

a	-0,30021	1
Dbt	-0,30025	1
Dapk3	-0,30046	1
Pym1	-0,30051	1
Dpy19l1	-0,30053	1
Hmgn5	-0,30075	1
Capn5	-0,30078	1
Tubb6	-0,30082	1
Dus2	-0,301	1
Rilp	-0,30099	1
Acsl1	-0,30103	1
Zfp865	-0,30108	1
Mfn1	-0,30115	1
Cep85	-0,30115	1
Adam8	-0,30118	1
Hyi	-0,3013	1
Abcd3	-0,30136	1
Smg9	-0,30138	1
Iqgap1	-0,30145	1
Pld2	-0,30149	1
Alkbh3	-0,30163	1
Fam126b	-0,30163	1
Pigg	-0,30165	1
Sars2	-0,30171	1
Ankrd27	-0,3018	1
Lsm10	-0,3019	1
Jkamp	-0,30203	1
Mettl17	-0,30221	1
Bcap31	-0,30226	1
Gm7967	-0,30244	1
Wdr75	-0,30243	1
Lat2	-0,30253	1
Tmem55a	-0,30261	1
Polr3k	-0,30266	1
Rnf220	-0,30271	1
Zkscan8	-0,30321	1
Bcl2l1	-0,30317	1
Hoxb5	-0,30333	1
Atp8b3	-0,30339	1
Nrg4	-0,30339	1
Crybb3	-0,3035	1
Serpini1	-0,30357	1
Slc25a36	-0,30361	1
Gm8667	-0,30366	1
E2f8	-0,30376	1
AI837181	-0,30376	1
C130083A15Rik	-0,30403	1
Rab2b	-0,30408	1
Mkl1	-0,30413	1
Magi1	-0,30422	1
Dennd1b	-0,30425	1
Cyp4v3	-0,30451	1

Cmtm3	-0,30451	1
Nup88	-0,30462	1
Klhdc3	-0,30473	1
Shmt1	-0,30484	1
Nptxr	-0,30497	1
Bcap29	-0,30546	1
Ssh1	-0,30564	1
Scarna2	-0,30565	1
Hddc3	-0,30571	1
Gm37206	-0,30576	1
Cst7	-0,30576	1
Faap20	-0,30577	1
Samm50	-0,30575	1
Mroh1	-0,30612	1
Eml2	-0,30606	1
Afg3l1	-0,30622	1
Pafah1b2	-0,3062	1
Rmrp	-0,30627	1
Gsto1	-0,30656	1
Bclaf1	-0,30691	1
Fam129a	-0,30703	1
Gm5805	-0,3071	1
Zfp763	-0,30725	1
Sympk	-0,30741	1
Tmie	-0,30758	1
Scap	-0,30771	1
Camta2	-0,30775	1
Pkn1	-0,30787	1
Lrmp	-0,30796	1
Gm43343	-0,30808	1
Rab3gap2	-0,30835	1
Fgd3	-0,30839	1
Napepld	-0,30854	1
Txnrd3	-0,30856	1
Nol12	-0,30863	1
Atp13a1	-0,3086	1
Gm37199	-0,30868	1
Atxn7l3	-0,30876	1
Lage3	-0,30886	1
Dusp18	-0,30906	1
Synj2bp	-0,30906	1
Kdelc2	-0,30917	1
2510002D24Rik	-0,30922	1
Mpi	-0,3093	1
Rad51c	-0,30948	1
Unc93b1	-0,30946	1
Csf1r	-0,30949	1
Gm15892	-0,30959	1
Tubgcp6	-0,30967	1
Efna2	-0,30966	1
Zscan2	-0,30969	1
Gm38067	-0,31004	1

Tyw3	-0,31032	1
Cog1	-0,31038	1
Rad54l2	-0,31039	1
Farsa	-0,3108	1
Atp5k-ps2	-0,31093	1
Zfp518a	-0,31097	1
Cry2	-0,31099	1
Mrm1	-0,31125	1
Usp40	-0,31131	1
Trdmt1	-0,31143	1
Slc4a7	-0,31142	1
Ttc3	-0,31185	1
Xrra1	-0,31187	1
Polr3e	-0,31198	1
Mlf1	-0,31205	1
Celf2	-0,31204	1
Eya3	-0,31216	1
Cog8	-0,3128	1
Emc4	-0,31294	1
Tlcd1	-0,31313	1
Dgcr6	-0,31328	1
Smyd3	-0,31331	1
Osbpl2	-0,31354	1
Qser1	-0,31365	1
Tdrd7	-0,31368	1
Hdhd2	-0,31369	1
Nmb	-0,31382	1
Rpl15-ps5	-0,31378	1
Miip	-0,31378	1
Cd37	-0,31423	1
Mblac1	-0,31434	1
Slc25a13	-0,31435	1
Etfa	-0,31442	1
Tbrg1	-0,31466	1
Chpt1	-0,31485	1
Pigt	-0,31499	1
Abhd11	-0,31497	1
Pear1	-0,3151	1
Gm20442	-0,31507	1
Zmym4	-0,31506	1
Sh3pxd2a	-0,31506	1
Maml2	-0,31525	1
6720475M21Rik	-0,31517	1
Npl	-0,31533	1
Tm9sf4	-0,31544	1
Gm14776	-0,31553	1
Lix1l	-0,31567	1
Zbed4	-0,31628	1
Appl1	-0,31626	1
Sec61a1	-0,31628	1
Ttc21b	-0,3164	1
Cisd2	-0,31641	1

Anxa6	-0,31658	1
RP23-164P21.3	-0,3167	1
Ptov1	-0,31671	1
Gid8	-0,31682	1
Eefsec	-0,31689	1
Gm29539	-0,31702	1
Etfrf1	-0,31701	1
1700086O06Rik	-0,31711	1
Slc29a3	-0,31709	1
Aldh1b1	-0,31722	1
Dock8	-0,31733	1
Gm36964	-0,31737	1
Gm13373	-0,31749	1
D6Wsu163e	-0,31753	1
Zfp651	-0,31762	1
Rab34	-0,31769	1
Arhgef3	-0,31775	1
Sbf1	-0,31784	1
Ubr3	-0,31776	1
Trim56	-0,31777	1
Zfp866	-0,31787	1
Slc6a12	-0,31786	1
Git2	-0,31788	1
Phtf1os	-0,31804	1
Timm17b	-0,31802	1
RP23-331E5.10	-0,31819	1
Myo7a	-0,31823	1
6030400A10Rik	-0,31819	1
Slc13a3	-0,31815	1
Pde7a	-0,3183	1
Rab10	-0,31857	1
Trmt112-ps2	-0,31884	1
Vamp4	-0,31877	1
Atp6ap2	-0,31881	1
Srpk2	-0,31893	1
Gdi1	-0,319	1
Rhobtb1	-0,3191	1
Lin7b	-0,31917	1
Zkscan7	-0,31926	1
Ikbke	-0,31949	1
Slc35a3	-0,31946	1
Pogk	-0,31955	1
2900052L18Rik	-0,31972	1
Rassf4	-0,31986	1
Ngly1	-0,32006	1
Dus1l	-0,32014	1
Fam149b	-0,32022	1
Gm37503	-0,32017	1
Trim46	-0,32032	1
Phf14	-0,32033	1
Mrgbp	-0,32047	1
Gm7353	-0,32061	1

Ppp2r1b	-0,32073	1
Ppp6r3	-0,32096	1
Aim1	-0,32112	1
Rhbdd1	-0,32149	1
Brpf3	-0,32167	1
Mdm4-ps	-0,3218	1
Vps4b	-0,32184	1
Gm37702	-0,32195	1
Mpv17	-0,32222	1
Sft2d2	-0,32226	1
Lmbrd2	-0,32249	1
Gdpgp1	-0,32267	1
Ep300	-0,32266	1
Ly6e	-0,32275	1
4933439C10Rik	-0,32295	1
Gm43359	-0,32305	1
Rab28	-0,32309	1
Ftsj3	-0,3231	1
C2	-0,32315	1
Elk4	-0,32332	1
Eng	-0,32337	1
Il1rap	-0,3234	1
Tcf4	-0,32341	1
Gm6598	-0,32352	1
Sgol2a	-0,32347	1
Tmem38b	-0,32347	1
Zfp687	-0,32355	1
Kmt5c	-0,32397	1
Bop1	-0,32406	1
Usp54	-0,32425	1
Mical3	-0,32417	1
Chd8	-0,32434	1
Utp20	-0,32477	1
Htt	-0,32485	1
Pgam5	-0,32492	1
Wipf1	-0,3251	1
1600014C23Rik	-0,32519	1
Lztfl1	-0,32555	1
Gab3	-0,3256	1
5031434O11Rik	-0,32568	1
Dpagt1	-0,32576	1
Zdhhc6	-0,32592	1
Kdsr	-0,32614	1
AC149090.1	-0,32636	1
Tstd2	-0,3265	1
Mecr	-0,32656	1
Ttc28	-0,32672	1
Gm9726	-0,32673	1
Msr1	-0,32707	1
Ccdc88c	-0,3273	1
Rfx1	-0,32729	1
Vwa5a	-0,32727	1

Pacs2	-0,32748	1
Ncf1	-0,32763	1
Klc4	-0,32768	1
Trpm4	-0,32788	1
Casp9	-0,32792	1
Synpo	-0,32797	1
Dis3l	-0,32815	1
6330562C20Rik	-0,32817	1
Ampd2	-0,32823	1
Nod1	-0,32826	1
Gm45110	-0,32842	1
Klhdc2	-0,32855	1
Gm43868	-0,32898	1
Elovl6	-0,32918	1
Ppil2	-0,32916	1
Zbtb37	-0,32949	1
Zfp964	-0,32973	1
Gm43024	-0,33007	1
Dnajc24	-0,3305	1
Tagln2	-0,33053	1
Senp1	-0,33072	1
D2Bwg1423e	-0,3309	1
RP23-476G10.1	-0,33098	1
Scly	-0,33111	1
Taf12	-0,33111	1
Zfp251	-0,33141	1
Parp11	-0,33166	1
Eml3	-0,33186	1
Pik3cg	-0,33218	1
Gpr89	-0,33221	1
Gm7336	-0,33229	1
Gm45495	-0,33255	1
2810029C07Rik	-0,33247	1
Zfp266	-0,33253	1
Fosl2	-0,3325	1
Plod1	-0,33273	1
Msh2	-0,3328	1
Cetn3	-0,33299	1
Exoc3l2	-0,33312	1
Frmd4b	-0,33323	1
Gfod1	-0,33324	1
Helq	-0,33332	1
Dctd	-0,33371	1
Noc4l	-0,33368	1
Eif4g1	-0,33427	1
Dcaf6	-0,33436	1
Golt1b	-0,33446	1
Eif3b	-0,33449	1
Mlh1	-0,33459	1
Tmpo	-0,33464	1
2210016L21Rik	-0,33474	1
Zfp770	-0,33478	1

Crtc2	-0,33489	1
Rpl12	-0,33496	1
Gm37675	-0,33495	1
2010204K13Rik	-0,33506	1
Mt2	-0,33509	1
Fam118b	-0,33523	1
Prkab2	-0,33535	1
Kat2a	-0,33542	1
Eaf1	-0,33548	1
Gls	-0,33554	1
Rad54l	-0,33574	1
Snx19	-0,33581	1
Arhgef25	-0,33575	1
Rny3	-0,33583	1
Hnrnpa1	-0,33592	1
Zfp523	-0,33603	1
RP23-356P21.1	-0,33613	1
Fgf13	-0,33622	1
Lgalsl	-0,33646	1
Gm45153	-0,33668	1
Mtdh	-0,33684	1
Vps45	-0,33692	1
Sbds	-0,33702	1
Ralgps1	-0,33725	1
Gar1	-0,33729	1
Oaz1-ps	-0,3374	1
Thbs3	-0,33749	1
Hps3	-0,33759	1
Tmem120a	-0,33757	1
Rrp1b	-0,33755	1
Gm38104	-0,33777	1
Ptpn6	-0,33779	1
Lgals8	-0,33807	1
Spats2	-0,33821	1
Parpbp	-0,33845	1
Tmem243	-0,33859	1
Srrm1	-0,33863	1
Kazald1	-0,3387	1
Gm42941	-0,33875	1
Pigw	-0,33891	1
Gpr108	-0,33898	1
Tk2	-0,33897	1
Ints7	-0,33911	1
Fermt3	-0,33913	1
Vash2	-0,33924	1
Uso1	-0,33923	1
Gm11491	-0,33929	1
Gm24924	-0,33944	1
Pcyox1l	-0,3396	1
Rad52	-0,3397	1
Osbpl3	-0,3397	1
Ap3m2	-0,33978	1

RP23-228B2.5	-0,34029	1
Pex1	-0,34044	1
Ormdl2	-0,34043	1
Ccdc51	-0,34089	1
Acrbp	-0,3411	1
Jmjd8	-0,34111	1
Gm10268	-0,34107	1
Selenoi	-0,34126	1
Tspyl2	-0,34152	1
Ivns1abp	-0,34147	1
Smim10l1	-0,34155	1
Zkscan3	-0,34169	1
Ndufs6	-0,34209	1
Ttll4	-0,34216	1
Itfg2	-0,34234	1
Dcaf10	-0,34255	1
Rfwd3	-0,34285	1
Tmem128	-0,34293	1
Dmxl1	-0,34286	1
Loxl3	-0,3432	1
Fgfr1op2	-0,34324	1
Senp6	-0,34323	1
Zfp956	-0,3434	1
Usp26	-0,34354	1
RP23-255F14.4	-0,34361	1
Kmt2c	-0,34375	1
Cramp1l	-0,34384	1
Gm22716	-0,34392	1
Serinc3	-0,34392	1
Rhbdd3	-0,34412	1
C1rl	-0,34407	1
Lpgat1	-0,34409	1
Kifap3	-0,34421	1
Cnot10	-0,34429	1
Gm13350	-0,3443	1
Abca2	-0,34438	1
Atm	-0,34453	1
Tarbp1	-0,34476	1
Myo9a	-0,34487	1
Copa	-0,34493	1
Atp6v0e2	-0,3449	1
Golga1	-0,34514	1
A230050P20Rik	-0,34564	1
Ptpa	-0,34563	1
Zfp938	-0,34577	1
Zfp27	-0,3458	1
Nat2	-0,34578	1
Saal1	-0,34583	1
S100a10	-0,34586	1
Cdk16	-0,34604	1
Pih1d2	-0,34606	1
Lpl	-0,34635	1

Galm	-0,34655	1
Phf11b	-0,34678	1
Katnal1	-0,34698	1
Samhd1	-0,34726	1
RP24-131G14.13	-0,34744	1
Klf7	-0,34766	1
Zbtb46	-0,34783	1
Fam65c	-0,34776	1
Pan2	-0,34777	1
Parvg	-0,34789	1
Zfp512b	-0,34813	1
Slc9a3r2	-0,34812	1
Ttll13	-0,34807	1
Arhgap30	-0,34811	1
Tmem106c	-0,34855	1
Tnfrsf22	-0,34861	1
Fbxo44	-0,34874	1
Dlg4	-0,3487	1
Cand1	-0,34884	1
Gm22748	-0,34915	1
Itpr2	-0,34907	1
Polr3a	-0,34954	1
RP24-225A16.3	-0,34958	1
Rapgef1	-0,35011	1
Snhg20	-0,35025	1
Actl6a	-0,35041	1
Gm12689	-0,3505	1
Usp24	-0,35054	1
Tmem43	-0,3506	1
Idh1	-0,35068	1
Klf16	-0,35086	1
Agbl5	-0,35106	1
Rnf170	-0,35116	1
March8	-0,35116	1
Pgls	-0,35153	1
Tnni3	-0,35167	1
Szt2	-0,35202	1
Foxc1	-0,35199	1
Agpat1	-0,3523	1
Clock	-0,35274	1
Asb1	-0,35284	1
9630013D21Rik	-0,35301	1
Ndfip2	-0,35308	1
Gm996	-0,3534	1
Ndufb8	-0,3534	1
BC065397	-0,35358	1
Fam213b	-0,35382	1
Apex2	-0,35393	1
Gpn2	-0,35409	1
Lrrfip2	-0,35422	1
Akr1e1	-0,35433	1
Gpr107	-0,35432	1

Cnot1	-0,35444	1
Dtl	-0,35453	1
Ell2	-0,35461	1
Cct3	-0,35509	1
Cpsf1	-0,35532	1
Wdr59	-0,35541	1
Gm12089	-0,35551	1
Cmas	-0,3555	1
Nqo2	-0,35583	1
Ccdc130	-0,35601	1
Rpl7	-0,35621	1
Col18a1	-0,35627	1
Zbtb49	-0,35645	1
Homer1	-0,35647	1
Zfp180	-0,35662	1
Fcna	-0,35672	1
Knl1	-0,35679	1
Supt20	-0,35687	1
Pbxip1	-0,3571	1
Srbd1	-0,35719	1
Adam10	-0,3574	1
Cdr2	-0,35775	1
Cadps	-0,35833	1
Phb	-0,359	1
Slc26a9	-0,3591	1
Myh9	-0,35924	1
Paip1	-0,35974	1
Pcyt2	-0,35974	1
Dcp1b	-0,35996	1
Rasa4	-0,36003	1
Msi1	-0,3601	1
Purg	-0,36018	1
Apool	-0,36039	1
Ctbp1	-0,36047	1
Abcf3	-0,36061	1
Myef2	-0,36063	1
Capn7	-0,36104	1
Sep 11	-0,36127	1
Arl6ip4	-0,36151	1
Gpatch2	-0,36158	1
Gm44093	-0,36168	1
Sppl2b	-0,36189	1
Phkb	-0,36196	1
Camkk2	-0,36215	1
Taok1	-0,36229	1
Hcls1	-0,36256	1
Ruvbl1	-0,36291	1
Crybg3	-0,36309	1
Rin3	-0,36372	1
Pax6	-0,36384	1
Rmdn1	-0,36381	1
Tmem191c	-0,36375	1

Gm11131	-0,3639	1
Wars	-0,36392	1
Tnrc6b	-0,36392	1
Galnt7	-0,36391	1
Kantr	-0,36403	1
Iqsec1	-0,3642	1
Magohb	-0,3647	1
R3hdm1	-0,36472	1
Kmt2b	-0,36483	1
Gm12096	-0,36494	1
2700099C18Rik	-0,36501	1
Gm42835	-0,36518	1
Map3k5	-0,36519	1
Extl3	-0,36521	1
Hif1an	-0,36593	1
Xylt1	-0,36606	1
Yipf3	-0,36648	1
Ubtf	-0,36647	1
Mtfr1	-0,3667	1
Imp4	-0,36679	1
Spcs3	-0,36692	1
Zfp52	-0,36694	1
Card11	-0,36687	1
Nacc2	-0,36717	1
Trim45	-0,36732	1
Gm6418	-0,36744	1
Kif3c	-0,3674	1
Ip6k3	-0,36748	1
Pygl	-0,36745	1
Scfd2	-0,36781	1
Cd9-ps	-0,36825	1
Prkch	-0,36865	1
Susd3	-0,36873	1
Rbm12b2	-0,36897	1
Rere	-0,369	1
Gm33370	-0,36914	1
Slc12a7	-0,36909	1
Gm15050	-0,36921	1
Ssu72	-0,36918	1
Gli1	-0,36942	1
Ash1l	-0,36936	1
Cep78	-0,37002	1
Csk	-0,37018	1
Hsd17b7	-0,37038	1
Oas1c	-0,37092	1
C130071C03Rik	-0,37092	1
Bin2	-0,37108	1
Rab31	-0,37112	1
2610507B11Rik	-0,37121	1
Nsmce1	-0,37134	1
Adcy7	-0,37142	1
Tmem220	-0,37147	1

Shc1	-0,37165	1
Sfi1	-0,3718	1
As3mt	-0,37189	1
Rcn1	-0,37187	1
Polr3gl	-0,37214	1
Mir155hg	-0,37216	1
Trio	-0,3723	1
Snrnp200	-0,37234	1
Tnfaip8	-0,37235	1
Tasp1	-0,3727	1
Pfkm	-0,37274	1
Zadh2	-0,3728	1
Atrn	-0,37321	1
Ublcp1	-0,37319	1
Ascc2	-0,37356	1
Tagap1	-0,37362	1
Fam58b	-0,37442	1
Thg1l	-0,37473	1
March9	-0,37494	1
Anxa1	-0,37516	1
AW209491	-0,37541	1
Alg5	-0,37547	1
Uhrf1bp1	-0,37572	1
Spef1	-0,37572	1
Fn3krp	-0,37578	1
Mtm1	-0,37587	1
Firre	-0,3759	1
Dnajc27	-0,37606	1
Iqcc	-0,37628	1
Zbed5	-0,37656	1
Pitpnm1	-0,37692	1
Eps15l1	-0,37696	1
Uty	-0,37695	1
Pdia5	-0,37713	1
Megf8	-0,37712	1
Tspan10	-0,37742	1
Cox18	-0,37745	1
2610008E11Rik	-0,3775	1
AU020206	-0,37772	1
E2f1	-0,37791	1
Ift74	-0,37807	1
Wbp1	-0,37806	1
Zfp229	-0,37809	1
Lypla2	-0,37814	1
Tbc1d5	-0,37825	1
Col4a3bp	-0,37847	1
Abcc4	-0,37855	1
Fancd2	-0,37879	1
Kansl1l	-0,37884	1
Coa3	-0,37884	1
Gtf2i	-0,3788	1
Cenpq	-0,37891	1

Wdr13	-0,37888	1
Ssh3	-0,37899	1
Klhl2	-0,37905	1
Sgsm3	-0,37908	1
Fam76b	-0,37909	1
Hibch	-0,37922	1
Taf4b	-0,37928	1
Maged1	-0,37926	1
Rpl36a-ps1	-0,37934	1
Akr1b3	-0,3794	1
Tm9sf3	-0,37938	1
F11r	-0,37954	1
Gm9347	-0,37945	1
Mprip	-0,37951	1
Zbtb26	-0,37966	1
Stard8	-0,37979	1
Tnrc6c	-0,37981	1
Uggt1	-0,37983	1
Dgka	-0,38006	1
Slc25a11	-0,38006	1
Exoc6b	-0,38028	1
Lrrc14	-0,38045	1
Nubp2	-0,38067	1
Klhl8	-0,38131	1
Mtap	-0,38136	1
Pstk	-0,38146	1
Gm37963	-0,38154	1
Gabpb2	-0,38156	1
Gm9396	-0,38181	1
Bin3	-0,38208	1
Gm37145	-0,38217	1
Pmpcb	-0,38235	1
Klhl42	-0,38239	1
Slc45a4	-0,38238	1
Tmem68	-0,38249	1
Nthl1	-0,3829	1
Slc12a6	-0,38302	1
Vmp1	-0,38298	1
A630072M18Rik	-0,38334	1
Gm5609	-0,3833	1
Kat2b	-0,3834	1
Ugdh	-0,38336	1
Zfp931	-0,38351	1
Wdr19	-0,38357	1
Fbxl19	-0,38368	1
Gm38022	-0,38368	1
Lmtk3	-0,38406	1
Pcyox1	-0,38422	1
Zfp282	-0,38453	1
Jaml	-0,38469	1
Rnf167	-0,3848	1
Ckap4	-0,38487	1

Actr8	-0,38497	1
Arcn1	-0,38509	1
Hsdl2	-0,38522	1
Tacc2	-0,38547	1
Mtg1	-0,38583	1
Afmid	-0,38588	1
Ergic1	-0,38589	1
Txndc16	-0,38604	1
Spats1	-0,38612	1
Cyc1	-0,3861	1
Dclre1c	-0,38623	1
Cdh23	-0,38636	1
S100pbp	-0,38649	1
Ly96	-0,38663	1
Casp1	-0,38658	1
Poc5	-0,38669	1
Ufsp2	-0,38669	1
Sbf2	-0,38677	1
Phc3	-0,38683	1
Poc1a	-0,38707	1
Tst	-0,38714	1
Tsg101	-0,38708	1
Rufy3	-0,38738	1
Chmp3	-0,38745	1
Prkag2	-0,38779	1
Kdm4a	-0,38785	1
Zfp629	-0,38826	1
Lipt2	-0,3883	1
Dtd1	-0,38856	1
Fads1	-0,38878	1
Pmm2	-0,38898	1
Ehd1	-0,38902	1
Tarsl2	-0,38906	1
Wnk1	-0,38932	1
Cyba	-0,38952	1
Ncoa7	-0,38959	1
Chd7	-0,38979	1
H13	-0,38977	1
Diablo	-0,38992	1
Hypk	-0,39015	1
Rab11fip3	-0,39018	1
Gpt2	-0,39019	1
Lcp2	-0,39016	1
Golga2	-0,39035	1
Hivep1	-0,39047	1
Tdrkh	-0,39059	1
Kansl2	-0,39081	1
Gm44829	-0,39092	1
C230035I16Rik	-0,39092	1
Nek9	-0,39088	1
BC005561	-0,39103	1
Ttc37	-0,39156	1

Gpr180	-0,39201	1
Acaca	-0,39219	1
Trmt13	-0,39245	1
Rprd2	-0,39237	1
Malat1	-0,39281	1
Clasp2	-0,39298	1
Usp6nl	-0,39299	1
RP24-122E11.4	-0,39333	1
Rasa3	-0,39343	1
Nfam1	-0,39372	1
Tmem140	-0,39405	1
Tubgcp2	-0,39426	1
Gm4950	-0,39431	1
Ap1s1	-0,39429	1
Alox5	-0,39446	1
Ctnnd1	-0,39463	1
Fbxl18	-0,39456	1
Cda	-0,39469	1
Lins1	-0,39484	1
Zc4h2	-0,39494	1
Tcirg1	-0,39495	1
Habp4	-0,39526	1
0610009B22Rik	-0,39536	1
Ubxn2b	-0,39564	1
Lrrc49	-0,39557	1
Ppp3ca	-0,39562	1
Hp1bp3	-0,39579	1
Zfp219	-0,39601	1
Slc38a1	-0,39596	1
Porcn	-0,39605	1
Matn1	-0,3961	1
Zcchc7	-0,39647	1
Atf6b	-0,39655	1
Prss46	-0,39674	1
Mier2	-0,39672	1
Gmip	-0,39676	1
Ppfibp1	-0,39681	1
Cox10	-0,39691	1
Fam120a	-0,39704	1
Tmem181a	-0,39732	1
Fsd2	-0,39737	1
Acsl5	-0,39737	1
Adprh	-0,39758	1
Kcnab2	-0,39771	1
Churc1	-0,39779	1
Gm43924	-0,39819	1
Apc	-0,39816	1
Tkfc	-0,39863	1
R3hcc1	-0,39878	1
Mtg2	-0,39913	1
Zdhhc16	-0,3991	1
Gm43148	-0,39918	1

Ranbp2	-0,39932	1
Emsy	-0,39947	1
Lrrk2	-0,39956	1
Aspm	-0,39966	1
Gm44916	-0,39983	1
Slc11a1	-0,40046	1
Lrif1	-0,40066	1
Uba52	-0,40106	1
Ccdc57	-0,4015	1
Gm36989	-0,40167	1
Csrp2bp	-0,40189	1
Faap100	-0,40188	1
Anapc13	-0,40211	1
Numa1	-0,40209	1
Mettl25	-0,40218	1
2810025M15Rik	-0,40219	1
Gm42893	-0,40231	1
Spi1	-0,40267	1
Mtus2	-0,40276	1
2810004N23Rik	-0,40295	1
Xrn1	-0,40324	1
Slc1a5	-0,40352	1
Eml5	-0,40363	1
Pon3	-0,40381	1
Taok2	-0,40394	1
Itsn1	-0,40394	1
Aldoc	-0,40391	1
9130604C24Rik	-0,40424	1
Fmn1	-0,40451	1
Dguok	-0,40455	1
Gm15420	-0,4047	1
Ddrgk1	-0,40496	1
2010016I18Rik	-0,40597	1
Setd5	-0,40614	1
Gm26947	-0,40634	1
Gsdmd	-0,40626	1
Dyrk2	-0,40634	1
Olfr460	-0,40631	1
Zbtb11os1	-0,40639	1
Nup93	-0,4064	1
Atrx	-0,4068	1
4632427E13Rik	-0,40687	1
Pank1	-0,40709	1
Gm42611	-0,40717	1
Vamp1	-0,40783	1
Abcc1	-0,4078	1
Adamts4	-0,40802	1
Tmem260	-0,40814	1
Il18	-0,40845	1
Tbl2	-0,40846	1
Mbd1	-0,4085	1
1810030O07Rik	-0,40858	1

Fbxo32	-0,40871	1
Msi2	-0,40896	1
Gcn1l1	-0,40904	1
Slc39a1	-0,40908	1
Sh3rf1	-0,40915	1
Zfp846	-0,40941	1
Ermap	-0,40952	1
Pja1	-0,40959	1
Slc30a7	-0,40981	1
Glb1	-0,40994	1
Plcb2	-0,41004	1
Mlh3	-0,41017	1
Ankrd11	-0,41066	1
Chm	-0,41128	1
Frg2f1	-0,4115	1
Nras	-0,41151	1
Cers2	-0,41146	1
Spata1	-0,41158	1
Dnajc13	-0,41182	1
Gm28809	-0,41192	1
Ttpal	-0,41204	1
Pianp	-0,41216	1
Eef2k	-0,4124	1
Plk1	-0,41261	1
Zpr1	-0,4126	1
Mrps27	-0,4127	1
Fnbp1l	-0,41276	1
3110082I17Rik	-0,4134	1
Slco4a1	-0,41339	1
Sqrdl	-0,41347	1
Sep 09	-0,41363	1
1500002F19Rik	-0,414	1
Rps15a-ps8	-0,41404	1
Ppm1f	-0,41402	1
Gm38021	-0,41397	1
Smarcc1	-0,41413	1
Sec23a	-0,41435	1
Asph	-0,41438	1
Gm45802	-0,41457	1
Cd3eap	-0,41472	1
Gm15185	-0,41498	1
Rps18-ps3	-0,41561	1
Mrpl38	-0,41603	1
Paqr7	-0,41611	1
Ppm1e	-0,4163	1
Mlkl	-0,41627	1
Atp13a3	-0,41636	1
Zdhhc17	-0,41688	1
Inpp5d	-0,41686	1
Chid1	-0,41744	1
Parg	-0,41763	1
Ubr1	-0,41781	1

Ryk	-0,41787	1
Med31	-0,4181	1
Pyroxd2	-0,41823	1
Tlr1	-0,41817	1
Arhgef7	-0,41904	1
Rinl	-0,4192	1
Lhx1	-0,41922	1
Sdccag8	-0,41996	1
Fubp1	-0,41998	1
Cx3cr1	-0,42044	1
Ptpn22	-0,42105	1
Psma7	-0,42106	1
Pon2	-0,4211	1
Fancb	-0,42131	1
Cdc25b	-0,4215	1
Odf2l	-0,42176	1
Tctn3	-0,42232	1
Prmt1	-0,42234	1
Phpt1	-0,42238	1
Asb7	-0,42262	1
Zfp58	-0,42265	1
Spata7	-0,42314	1
F730043M19Rik	-0,42314	1
Gm12240	-0,42331	1
Tufm	-0,42343	1
Gm43133	-0,42354	1
Adgrl1	-0,42405	1
Cib1	-0,42405	1
Ggnbp1	-0,42456	1
Gm37254	-0,42468	1
Serhl	-0,42472	1
Xrcc4	-0,42491	1
6330418K02Rik	-0,4249	1
Gm44258	-0,42496	1
Lrrc61	-0,42505	1
Rnaseh2a	-0,42518	1
Hoxb3	-0,42525	1
Gm38157	-0,42568	1
Dapp1	-0,42581	1
Brwd1	-0,42586	1
Adgrl2	-0,42594	1
Map3k20	-0,42616	1
Usf3	-0,42645	1
Maf1	-0,42653	1
Sharpin	-0,42659	1
Add1	-0,42661	1
Trpc4ap	-0,42681	1
Rcl1	-0,42728	1
Swi5	-0,42747	1
Cntnap1	-0,42764	1
Aatk	-0,42786	1
Ube3b	-0,42802	1

Dleu2	-0,42812	1
A530072M11Rik	-0,42807	1
1700003F12Rik	-0,42824	1
Gmppa	-0,42817	1
AI480526	-0,42861	1
Slc35d1	-0,42926	1
Bpnt1	-0,42957	1
Pou2f2	-0,42985	1
Rbbp8	-0,43002	1
Ralgapb	-0,43006	1
Drosha	-0,43035	1
A430105I19Rik	-0,43057	1
Scai	-0,43072	1
Olfr921	-0,43069	1
Clcn3	-0,4309	1
Rbm25	-0,43101	1
Flna	-0,43135	1
Fam3c	-0,43174	1
Rbm4	-0,43178	1
Srrt	-0,43219	1
Fam188a	-0,43248	1
Psmg1	-0,43258	1
Smg1	-0,43262	1
Ttc27	-0,43277	1
Tmem41b	-0,43293	1
Atxn3	-0,43302	1
Sapcd2	-0,43307	1
Abhd12	-0,43308	1
Ccdc91	-0,43329	1
RP23-38L16.3	-0,43363	1
Snx11	-0,43383	1
Pnpla7	-0,43382	1
Mga	-0,43391	1
Ipo8	-0,43402	1
Gm37566	-0,43417	1
Ubn2	-0,43435	1
Gm8013	-0,43446	1
Pias3	-0,43465	1
Setdb1	-0,43502	1
Stx12	-0,4351	1
Zfp672	-0,43529	1
Cars2	-0,43543	1
AW046200	-0,43543	1
Nxpe3	-0,43588	1
Gsap	-0,43619	1
Cracr2a	-0,4364	1
Tom1l2	-0,43659	1
Tecpr2	-0,43688	1
Zfp329	-0,43748	1
Ptpn9	-0,43779	1
Urod	-0,43834	1
Zfp60	-0,43839	1

Mrps36-ps2	-0,43877	1
Nanos1	-0,43884	1
Psd2	-0,43884	1
Pi4ka	-0,43882	1
Miga1	-0,43894	1
Rbm6	-0,43889	1
Entpd5	-0,43905	1
Ppt1	-0,43924	1
B230118H07Rik	-0,43948	1
Fkbp8	-0,43955	1
Ufsp1	-0,43973	1
Mib1	-0,4397	1
Defb25	-0,43983	1
Gcc2	-0,44004	1
Rfx5	-0,44001	1
Sergef	-0,4404	1
Gm37204	-0,4404	1
Ankrd13d	-0,44049	1
Zfp799	-0,4405	1
Smurf2	-0,44063	1
Ints11	-0,4409	1
Pbx1	-0,44148	1
Brf1	-0,4417	1
Cenpx	-0,44208	1
Zfp449	-0,44211	1
Ilf2	-0,44222	1
Atg10	-0,44253	1
Tmem126b	-0,44257	1
Abhd2	-0,44261	1
Dfna5	-0,44265	1
Lancl1	-0,44281	1
Shox2	-0,44349	1
Spa17	-0,44357	1
Gm11613	-0,4437	1
Enox2	-0,44409	1
Mettl26	-0,44406	1
Irgm2	-0,44417	1
S100a4	-0,44417	1
Zfp951	-0,44437	1
Rnf213	-0,44459	1
Creg1	-0,44466	1
Zfp809	-0,44481	1
Gm25596	-0,44489	1
Prss50	-0,445	1
Sh2b3	-0,44524	1
Uhrf1bp1l	-0,44578	1
Chmp6	-0,44611	1
Man2a1	-0,44637	1
Samd10	-0,44639	1
Zfp369	-0,44645	1
Mdm4	-0,44682	1
Tns4	-0,44692	1

2500002B13Rik	-0,447	1
Neurl4	-0,44723	1
Lypla1	-0,44727	1
Bco2	-0,44743	1
Arntl	-0,44808	1
Wdr76	-0,44822	1
Sphk2	-0,44842	1
Slc25a26	-0,44856	1
Fads6	-0,44872	1
Lrrk1	-0,44866	1
Pygb	-0,44883	1
Gm12115	-0,44887	1
Gys1	-0,44887	1
Fem1a	-0,449	1
Gm37900	-0,44897	1
Pkn3	-0,44909	1
Tgfbr2	-0,44915	1
Letmd1	-0,44919	1
Acod1	-0,44972	1
Pitpnm2	-0,44993	1
Ubxn7	-0,44986	1
Gm44545	-0,45011	1
Wdr54	-0,45032	1
Zfp106	-0,45033	1
Gm45884	-0,45071	1
Slc35c1	-0,45065	1
Nup155	-0,45092	1
Ikzf1	-0,45096	1
Mms19	-0,45123	1
Fam84b	-0,45214	1
Crybg3	-0,45213	1
Cyfip1	-0,45235	1
Zdhhc2	-0,45275	1
Smarcc2	-0,45283	1
Herc2	-0,45282	1
Adap2	-0,45329	1
Zfp619	-0,45337	1
Fth1	-0,4537	1
Agmo	-0,45419	1
Lig3	-0,4542	1
Mitf	-0,4542	1
Ppcdc	-0,45439	1
Nf2	-0,45442	1
A930001C03Rik	-0,45447	1
Tspan32	-0,45531	1
Endod1	-0,45553	1
Gtf2b	-0,45558	1
Slc5a3	-0,45642	1
Wnt6	-0,45657	1
Dennd2d	-0,45659	1
BC005537	-0,45722	1
H2afy	-0,45721	1

Dcaf15	-0,45753	1
Aifm2	-0,45751	1
Nt5c2	-0,45767	1
Tepsin	-0,45793	1
Gm37558	-0,45792	1
Ptgs2os	-0,45846	1
Impa1	-0,45915	1
Ubr4	-0,45939	1
Ulk4	-0,45945	1
Gm10060	-0,45957	1
Mbtd1	-0,46006	1
Mfsd7a	-0,46046	1
Casp4	-0,46055	1
Krit1	-0,46098	1
Stx5a	-0,4611	1
Pex2	-0,46114	1
Gm35931	-0,46182	1
Keap1	-0,46181	1
Btd	-0,46188	1
Meis3	-0,46187	1
Apaf1	-0,46185	1
Ttll5	-0,46216	1
Tbc1d24	-0,46219	1
Nufip2	-0,46231	1
Gm9844	-0,46236	1
Mpnd	-0,46291	1
Ubr5	-0,46288	1
Tmem81	-0,46297	1
Fech	-0,46309	1
Poll	-0,46326	1
Cdc23	-0,46327	1
Tmem65	-0,46353	1
Jarid2	-0,46362	1
Tyk2	-0,46393	1
Magt1	-0,46404	1
Zmat5	-0,46406	1
Pgghg	-0,46412	1
Gm45729	-0,46474	1
Vwf	-0,46493	1
Helz	-0,46502	1
Slc36a4	-0,46527	1
Gpr68	-0,46538	1
E330034L11Rik	-0,46581	1
RP24-366E11.4	-0,46583	1
Mgmt	-0,46592	1
Lpar2	-0,46616	1
Klhl20	-0,46651	1
Ptbp2	-0,46669	1
Bms1	-0,46692	1
Ccnd2	-0,46735	1
Trpt1	-0,46737	1
Gipc2	-0,46735	1

Sh3bp5	-0,46808	1
Gm28875	-0,46823	1
Pced1a	-0,46835	1
Sipa1l2	-0,46849	1
Tpt1-ps5	-0,46857	1
Ppp1r9b	-0,46884	1
Fam185a	-0,46878	1
Birc6	-0,46891	1
Rwdd2b	-0,46943	1
Clec7a	-0,46988	1
Ctnnb1	-0,47	1
Ndrg4	-0,47012	1
Napa	-0,47009	1
Herc3	-0,47058	1
Dopey1	-0,47103	1
Pde6d	-0,47158	1
Kmt2d	-0,47158	1
Spen	-0,47174	1
Zfp322a	-0,47183	1
Sapcd1	-0,47191	1
Xpr1	-0,47194	1
Uri1	-0,47205	1
Akr7a5	-0,47261	1
AA914427	-0,47308	1
Edem2	-0,47316	1
Wars2	-0,4732	1
Slc27a4	-0,47342	1
Gm16536	-0,47356	1
Tfpi	-0,47381	1
Tmem216	-0,47428	1
Nova1	-0,47429	1
Rasgrp3	-0,47457	1
Gtdc1	-0,47467	1
Rps12-ps23	-0,47472	1
Nhej1	-0,47466	1
Zfp870	-0,47531	1
Morf4l1	-0,47553	1
Pus7l	-0,47547	1
Cd48	-0,47553	1
Usp49	-0,47583	1
Gm15541	-0,47576	1
Cwc27	-0,47597	1
2510009E07Rik	-0,47597	1
Ubash3b	-0,47615	1
Cdca7l	-0,47644	1
1700047K16Rik	-0,47635	1
Cenpf	-0,47679	1
Wdr7	-0,47699	1
Dcun1d2	-0,47713	1
Tex10	-0,47716	1
Gm14323	-0,47748	1
Cat	-0,47761	1

Eef2kmt	-0,47784	1
1810055G02Rik	-0,47794	1
Ldb1	-0,4782	1
Gripap1	-0,47832	1
Gm42632	-0,47835	1
Mroh2a	-0,47827	1
Dido1	-0,47844	1
Btf3l4	-0,47854	1
Espl1	-0,47857	1
Mrpl27	-0,47865	1
Tmem14a	-0,47866	1
Mir142hg	-0,47902	1
Kcnj2	-0,47901	1
Ppip5k2	-0,47936	1
Cep95	-0,4797	1
Dhdds	-0,47975	1
Haus4	-0,47983	1
Sod1	-0,48	1
Stau2	-0,48014	1
Pitpna	-0,48008	1
Mthfd2l	-0,48014	1
Zdhhc9	-0,48017	1
Prdx1	-0,48015	1
Prtg	-0,48053	1
Mis18bp1	-0,48065	1
Agfg2	-0,4808	1
Hsf1	-0,48104	1
Ncs1	-0,48116	1
Son	-0,48123	1
Rnf8	-0,48198	1
Prr14l	-0,48241	1
Gm10800	-0,48247	1
Atxn1	-0,48273	1
Tmem63b	-0,48287	1
Foxo4	-0,48302	1
Rrbp1	-0,48299	1
Dhx58	-0,48306	1
Golgb1	-0,4831	1
Adssl1	-0,48388	1
Rdh12	-0,48428	1
Prmt3	-0,48446	1
Dhx29	-0,48504	1
Tbc1d14	-0,4851	1
Alg9	-0,48513	1
Acaa1a	-0,48509	1
Tdrd3	-0,48522	1
Celsr1	-0,4852	1
A830080D01Rik	-0,48544	1
Gm45902	-0,48542	1
Pomt1	-0,48561	1
Chordc1	-0,48579	1
9930104L06Rik	-0,4859	1

Gucy2g	-0,48622	1
Taz	-0,48616	1
Trmo	-0,4863	1
Slc25a24	-0,48647	1
Gnb4	-0,48703	1
Dap	-0,48707	1
Gm37420	-0,48712	1
Gatb	-0,4872	1
Gm45224	-0,48747	1
Ttc19	-0,48774	1
Ubp1	-0,48789	1
Gm15832	-0,48871	1
Gm38200	-0,48879	1
Mplkip	-0,48891	1
Ddhd2	-0,48893	1
Srxn1	-0,48897	1
Pign	-0,48906	1
Ankle1	-0,48928	1
Cacna1a	-0,48937	1
C130023A14Rik	-0,48965	1
Gm37065	-0,48995	1
Aasdh	-0,49012	1
Gm13181	-0,4904	1
Smyd2	-0,4906	1
Glce	-0,49065	1
Pqlc3	-0,49077	1
Rcc2	-0,4908	1
Gm43362	-0,49097	1
Pold1	-0,49137	1
Grap	-0,49141	1
5830408C22Rik	-0,49151	1
Gipr	-0,49163	1
Dhodh	-0,49221	1
Parp14	-0,49223	1
Sacs	-0,49232	1
Myo6	-0,49232	1
Was	-0,49252	1
Epm2a	-0,49258	1
Gm43201	-0,49261	1
Prkci	-0,49274	1
Cep63	-0,49282	1
1700030K09Rik	-0,49292	1
Ebpl	-0,49308	1
Trem3	-0,4931	1
2410002F23Rik	-0,49325	1
Cdk6	-0,49335	1
Tap2	-0,49327	1
Wwc2	-0,49338	1
Timm23	-0,49342	1
Gm36445	-0,49345	1
Ylpm1	-0,49371	1
Gm28151	-0,49378	1

Sirt4	-0,49432	1
Pcnx3	-0,49443	1
Pyroxd1	-0,49446	1
Sh3tc1	-0,49457	1
Arg1	-0,49467	1
Hebp2	-0,49497	1
Gm11423	-0,49514	1
Cep128	-0,49544	1
Dhrs1	-0,49564	1
Tmem245	-0,4959	1
Ocrl	-0,49596	1
Tmem237	-0,49613	1
Cdhr4	-0,4961	1
Dram2	-0,49629	1
Thoc7	-0,49641	1
Ifi203	-0,4966	1
Polr3h	-0,49668	1
Gm15440	-0,49674	1
Aatf	-0,49783	1
Arhgef11	-0,49794	1
Cpsf6	-0,49798	1
Snx9	-0,49809	1
Dync1h1	-0,49839	1
B4galnt1	-0,4985	1
RP23-359K10.8	-0,49939	1
Uckl1	-0,49952	1
Lpcat3	-0,49947	1
Gpatch8	-0,50012	1
Sdcbp2	-0,50013	1
Spout1	-0,50025	1
Crebzf	-0,50027	1
Ppp1r18	-0,50044	1
Csnk2a2	-0,50049	1
Ago1	-0,50112	1
Sos2	-0,50127	1
Gm10557	-0,50143	1
Phka2	-0,50171	1
Cd59a	-0,50191	1
Atg4a	-0,5022	1
9430060I03Rik	-0,50235	1
Eef1akmt1	-0,50237	1
Pot1b	-0,50257	1
Pstpip1	-0,5029	1
Slc25a37	-0,50292	1
Nudt3	-0,50296	1
Utrn	-0,50321	1
Rps6	-0,50325	1
Heatr3	-0,50346	1
Rnu11	-0,50355	1
Kdm5b	-0,50441	1
Rgp1	-0,5047	1
Gtpbp8	-0,50481	1

Atp2b4	-0,50508	1
2010315B03Rik	-0,50545	1
Atat1	-0,50556	1
Ttf2	-0,50589	1
Hacd1	-0,50597	1
Gphn	-0,50654	1
Agl	-0,50673	1
RP23-320D23.6	-0,50672	1
Crtc3	-0,50703	1
Ndufaf7	-0,50713	1
Snx7	-0,50719	1
Gm37399	-0,50788	1
Me1	-0,50798	1
Ifi202b	-0,50824	1
Rsu1	-0,50834	1
Strada	-0,50826	1
Vps37a	-0,50861	1
Slc35b4	-0,50884	1
Nagk	-0,50913	1
Hoxa4	-0,5091	1
Vrk3	-0,50945	1
Dgkq	-0,50956	1
Kif5c	-0,51012	1
Gm26631	-0,51012	1
Ms4a6d	-0,51023	1
Ddx39b	-0,51035	1
Fam213a	-0,51042	1
Aldh18a1	-0,51037	1
Ptpn7	-0,51052	1
Celf4	-0,51091	1
Tarbp2	-0,51144	1
Igf2r	-0,51146	1
Cdo1	-0,51154	1
Hdc	-0,51155	1
Slc43a2	-0,51161	1
Lrrc47	-0,51166	1
Alcam	-0,51171	1
Dhfr	-0,51173	1
Sdhaf4	-0,51175	1
Fam136a	-0,51188	1
Fbxw17	-0,51189	1
3110083C13Rik	-0,51205	1
2610037D02Rik	-0,51259	1
Tmem87a	-0,51285	1
Zhx3	-0,51289	1
Sf3b3	-0,51323	1
Slc6a9	-0,51327	1
Colec12	-0,51343	1
Bmi1	-0,51349	1
Gm24876	-0,51374	1
Kat6b	-0,51428	1
Nudt6	-0,5146	1

Gm10093	-0,51469	1
Rai14	-0,51488	1
Birc3	-0,51509	1
Pex16	-0,51514	1
Abcd4	-0,51518	1
Gga2	-0,51534	1
Mbd5	-0,51558	1
Tm9sf1	-0,51574	1
Naglu	-0,51579	1
Clec11a	-0,51631	1
Gm5624	-0,5168	1
Abr	-0,51714	1
9330175E14Rik	-0,5174	1
Fam222b	-0,5175	1
Spidr	-0,51826	1
Slc9a9	-0,51827	1
Kcnn1	-0,51857	1
Gm9134	-0,51893	1
Gm37914	-0,51911	1
Kbtbd11	-0,51924	1
RP23-390D8.2	-0,51932	1
Hmbox1	-0,51941	1
Gm43581	-0,5195	1
Sass6	-0,51954	1
Atxn7	-0,5197	1
Dnaaf5	-0,51993	1
Arfgef2	-0,51994	1
Cacna1b	-0,52008	1
Lair1	-0,52019	1
Gm20628	-0,52037	1
Bmf	-0,5208	1
Stx4a	-0,52101	1
Mrps33	-0,52098	1
Cul9	-0,5212	1
St8sia4	-0,52138	1
Cass4	-0,52139	1
Hspa1b	-0,52163	1
Meaf6	-0,52167	1
Pde8b	-0,52242	1
Mrpl17	-0,52253	1
Calcoco1	-0,52246	1
Fancc	-0,52284	1
Ctxn1	-0,52302	1
Ints3	-0,52326	1
Clptm1l	-0,52366	1
Lims1	-0,52389	1
Gm20342	-0,52397	1
Lrrc45	-0,5241	1
Zc3h7b	-0,52418	1
Cpsf3	-0,52458	1
Gm4262	-0,52481	1
Sema6b	-0,52479	1

Lyrm1	-0,52486	1
Ccdc28a	-0,52495	1
Fam102b	-0,52555	1
Emc9	-0,52581	1
Tsen2	-0,52596	1
Znhit6	-0,52613	1
Oma1	-0,52659	1
Mettl21a	-0,52683	1
Gm26772	-0,52701	1
Herc4	-0,52768	1
Nup37	-0,52775	1
Tpcn1	-0,52787	1
Prrc2b	-0,52809	1
Zfp871	-0,52827	1
Celsr3	-0,52857	1
1110032A03Rik	-0,52883	1
Cops6	-0,5289	1
Trappc12	-0,52891	1
Timm21	-0,52886	1
Gm14853	-0,529	1
Cd276	-0,52913	1
Zdhhc20	-0,52916	1
Wdr11	-0,52927	1
RP23-3F1.8	-0,5297	1
Arid2	-0,52978	1
Zdhhc12	-0,52982	1
Naaa	-0,52993	1
2210417A02Rik	-0,53003	1
Gm43360	-0,53019	1
Rasal3	-0,53047	1
Zfp664	-0,5313	1
Rrp9	-0,53138	1
Sh3glb2	-0,53179	1
Gm2895	-0,53193	1
Tmem5	-0,53204	1
Gm42895	-0,53196	1
Tmem8b	-0,53196	1
Rbck1	-0,53221	1
Zfp407	-0,53286	1
Clybl	-0,53297	1
Eri2	-0,53319	1
Pdk1	-0,5333	1
Mtus1	-0,53349	1
Vim	-0,53357	1
Dock2	-0,53418	1
Bag2	-0,53433	1
Agrn	-0,53435	1
Kif1bp	-0,53454	1
Adck5	-0,5349	1
Gsk3b	-0,53516	1
Zfp74	-0,53527	1
Ints1	-0,53542	1

Snhg11	-0,53536	1
Ocel1	-0,53572	1
Igip	-0,53586	1
Ano10	-0,53598	1
Hdac1	-0,536	1
Zfyve19	-0,53617	1
Nek2	-0,53698	1
Nphp1	-0,53707	1
Rab43	-0,53723	1
Acvr1b	-0,53728	1
Nrip1	-0,53762	1
Insr	-0,53798	1
Gm24009	-0,53797	1
Itgb7	-0,53807	1
Zfp710	-0,53824	1
Aldh4a1	-0,53878	1
Smpdl3b	-0,53886	1
Gm45871	-0,53893	1
Gga3	-0,5391	1
Vti1a	-0,53964	1
Slc22a5	-0,53972	1
Slc12a4	-0,54082	1
Tmem94	-0,54079	1
Gart	-0,54125	1
Nudt16	-0,54196	1
Gm11488	-0,54203	1
Gm38387	-0,54259	1
Kif3b	-0,54291	1
Lrba	-0,54302	1
Apbb1ip	-0,54324	1
Elk3	-0,54403	1
Edil3	-0,54411	1
Nupr1l	-0,54423	1
Bloc1s3	-0,54445	1
Gfm1	-0,54548	1
Pabpn1	-0,54588	1
Tbc1d4	-0,546	1
Gas7	-0,54612	1
Gm15796	-0,54624	1
Gm38235	-0,54634	1
Hnrnpm	-0,54659	1
Ech1	-0,54694	1
Nisch	-0,54695	1
Idh2	-0,5473	1
Zfc3h1	-0,5476	1
Wwp2	-0,54791	1
Zmiz1	-0,54806	1
Acot11	-0,54806	1
Btk	-0,54845	1
Faap24	-0,54843	1
Gnptg	-0,54837	1
Eif4g3	-0,54849	1

Gm9568	-0,54868	1
Tpm1	-0,54891	1
St6gal1	-0,54939	1
Pla2g5	-0,54956	1
Zik1	-0,55	1
Wdr46	-0,55011	1
Hap1	-0,55016	1
Gm43792	-0,55035	1
Herc1	-0,55038	1
Actr5	-0,55045	1
Frrs1	-0,55068	1
Rnf111	-0,55105	1
Stx6	-0,55141	1
Trub2	-0,55165	1
AU040320	-0,55263	1
Stx17	-0,5527	1
Plec	-0,55293	1
Metap2	-0,55288	1
Gm42986	-0,55312	1
Inf2	-0,55339	1
Olfm1	-0,55335	1
Endog	-0,55343	1
G730013B05Rik	-0,55372	1
Tango2	-0,55397	1
Gm43328	-0,55397	1
Brip1os	-0,55413	1
Gm8337	-0,55475	1
Nxn	-0,55493	1
Luc7l2	-0,55521	1
Tmem79	-0,55543	1
Akr1b7	-0,5555	1
Gm28404	-0,55559	1
Golim4	-0,55586	1
Zcchc24	-0,55588	1
2810428J06Rik	-0,55595	1
Camk1d	-0,55598	1
Eif1ad	-0,55616	1
RP23-453B15.7	-0,55681	1
Dennd1c	-0,55783	1
1700008J07Rik	-0,55786	1
Adck1	-0,55796	1
Mtr	-0,55833	1
C330018D20Rik	-0,55849	1
Vps9d1	-0,55941	1
1700037C18Rik	-0,55936	1
Fah	-0,55957	1
Enkd1	-0,55974	1
Zmym6	-0,55967	1
Slc25a43	-0,55985	1
Cacfd1	-0,56041	1
Plekhn1	-0,5609	1
Gm42659	-0,5612	1

Zmynd8	-0,5615	1
Psma3	-0,56167	1
Per2	-0,56182	1
Eya4	-0,56203	1
Swsap1	-0,56226	1
Med30	-0,56295	1
Lsr	-0,56301	1
Zfp945	-0,56322	1
Efcab14	-0,56391	1
Gm20430	-0,56409	1
Gm44250	-0,56537	1
Ppp4c	-0,56554	1
Cabin1	-0,56558	1
Sh2d5	-0,56564	1
Tsc22d4	-0,56603	1
Nr2c1	-0,56604	1
Gm10033	-0,56603	1
Gm6612	-0,5665	1
D030028A08Rik	-0,56684	1
Galnt11	-0,56709	1
Ggcx	-0,56731	1
Sec31a	-0,56753	1
Mettl1	-0,56762	1
Zfp93	-0,56757	1
Gm37494	-0,5677	1
Lman2l	-0,56784	1
Tnfrsf23	-0,56822	1
Gm38190	-0,56817	1
Gm42551	-0,56822	1
Huwe1	-0,56866	1
9230114K14Rik	-0,56892	1
BC085271	-0,56888	1
2810030D12Rik	-0,56942	1
Tmem156	-0,56959	1
Mettl15	-0,56985	1
Ttc13	-0,56991	1
Clcn5	-0,57032	1
Gm19325	-0,5703	1
Ttc7	-0,57054	1
Rpusd1	-0,57072	1
Igtp	-0,57116	1
Gm10605	-0,57126	1
Mif4gd	-0,57149	1
Jdp2	-0,57169	1
1700124L16Rik	-0,57213	1
Ino80c	-0,57218	1
AI467606	-0,57303	1
Ccdc173	-0,57324	1
Gm10029	-0,57344	1
Casd1	-0,57354	1
P2ry6	-0,57388	1
Psmg2	-0,57401	1

Orai2	-0,57502	1
Rp2	-0,57502	1
Inpp5e	-0,57519	1
Brms1	-0,5756	1
Zfp658	-0,57565	1
Cdkl2	-0,57638	1
Stx2	-0,57651	1
Gm29284	-0,57652	1
Cep104	-0,57736	1
Lrrc24	-0,57743	1
Napb	-0,57753	1
Slc44a2	-0,57755	1
Prkcg	-0,57758	1
Cinp	-0,57771	1
Ago2	-0,57788	1
Hmgb1	-0,57789	1
Neil3	-0,57893	1
Clasp1	-0,57921	1
Plxna2	-0,58008	1
Rtkn	-0,58017	1
Dock10	-0,58054	1
Nsun5	-0,58081	1
Prrc2a	-0,58095	1
D630045J12Rik	-0,58169	1
Rbm26	-0,58202	1
Ifit2	-0,58245	1
Ercc6l	-0,58277	1
Adcy6	-0,58323	1
Nr2c2	-0,58327	1
R74862	-0,5835	1
Serac1	-0,58395	1
Gm6329	-0,58401	1
Zfp691	-0,58407	1
Stap2	-0,58417	1
Retsat	-0,58442	1
Ep400	-0,58464	1
Klra2	-0,5855	1
Smim19	-0,58564	1
Ttc14	-0,58569	1
Lars	-0,58656	1
Itpr3	-0,58678	1
Rnft2	-0,58676	1
Fam132a	-0,58712	1
Fignl1	-0,58728	1
Plekhm3	-0,58738	1
Top3b	-0,58736	1
Tpcn2	-0,58752	1
Galk1	-0,58862	1
Arhgap45	-0,58868	1
Rab12	-0,58973	1
Mut	-0,59008	1
Ttll12	-0,59034	1

Cenpv	-0,59043	1
Phf21a	-0,59063	1
Elp2	-0,5907	1
Phykpl	-0,59089	1
BC002059	-0,5916	1
Wls	-0,59187	1
Gdap10	-0,59192	1
Psd	-0,59197	1
Prelid3a	-0,59211	1
Gm37452	-0,59206	1
Timeless	-0,59213	1
Ttbk2	-0,59231	1
Tmem26	-0,59296	1
Gm23849	-0,59297	1
Kif5a	-0,5937	1
Pfkl	-0,59388	1
Xbp1	-0,59409	1
Ankrd44	-0,59408	1
Irak1	-0,5942	1
Tdp1	-0,59428	1
Polr1a	-0,59425	1
Mfsd10	-0,5947	1
Dhdh	-0,59515	1
Htra2	-0,5953	1
Gm14040	-0,59549	1
Nipsnap1	-0,59657	1
Senp7	-0,59657	1
G430095P16Rik	-0,59665	1
Prr36	-0,59679	1
Nos3	-0,59707	1
Wdpcp	-0,59729	1
Gm2830	-0,59875	1
Galnt1	-0,59985	1
Dbnl	-0,60018	1
Pknox1	-0,60037	1
Snx29	-0,60053	1
Ext2	-0,60063	1
B3galnt2	-0,60084	1
Zfp260	-0,60091	1
Gm37584	-0,60138	1
Taf1c	-0,60168	1
Capn1	-0,60178	1
Ncmap	-0,60204	1
Gm5637	-0,60208	1
Gnptab	-0,60235	1
Abcb4	-0,60323	1
Noc2l	-0,6034	1
Angptl6	-0,60376	1
Pkd1l2	-0,604	1
Chst3	-0,60429	1
Wdr92	-0,60488	1
Frmd4a	-0,60546	1

Dgcr14	-0,60584	1
Gca	-0,60594	1
Lpin3	-0,6069	1
Fastkd5	-0,60735	1
Arih1	-0,60778	1
Gm12276	-0,60781	1
Letm2	-0,60791	1
Spata33	-0,60795	1
Ift122	-0,60817	1
Gm11448	-0,60817	1
Sgsm2	-0,60874	1
Acad9	-0,60955	1
Cpt1a	-0,61015	1
Card6	-0,61019	1
B230307C23Rik	-0,61033	1
Zbtb33	-0,61079	1
Gm31166	-0,6111	1
Helz2	-0,61141	1
Il23a	-0,61154	1
Gm19026	-0,6115	1
Ankrd12	-0,61189	1
F830115B05Rik	-0,61187	1
Stub1	-0,61243	1
Sfmbt1	-0,61247	1
Acot1	-0,61263	1
Nat8f1	-0,61308	1
Tbxas1	-0,61382	1
Wbscr27	-0,6152	1
Fsd1l	-0,61538	1
Slc9a8	-0,6155	1
Snhg6	-0,61612	1
Dmwd	-0,61681	1
Crygn	-0,61678	1
Gm45853	-0,61705	1
Hps1	-0,61733	1
Soga1	-0,6176	1
Vsig8	-0,61772	1
Srgap3	-0,61788	1
Pgm2	-0,61815	1
Inpp5k	-0,61841	1
Kmt2a	-0,61857	1
Dph6	-0,61862	1
Trappc11	-0,61905	1
Tfcp2l1	-0,61916	1
Nyap1	-0,61932	1
Ncoa2	-0,61973	1
Mlx	-0,61977	1
Oit3	-0,62	1
Ccdc191	-0,62028	1
Gm43153	-0,62045	1
Zfp398	-0,62095	1
Coq4	-0,62101	1

Fgd4	-0,62156	1
Ankrd10	-0,62199	1
Gfi1	-0,62203	1
Trim27	-0,62228	1
Ppp1r12b	-0,62263	1
Atad1	-0,62306	1
Arhgap18	-0,62341	1
0610010F05Rik	-0,62349	1
Plat	-0,62422	1
Eogt	-0,62426	1
6030460B20Rik	-0,62431	1
Zscan20	-0,62461	1
Pomk	-0,62473	1
Zfp410	-0,62482	1
Psmb9	-0,62486	1
Map2k3os	-0,62521	1
Jmy	-0,62542	1
Alg3	-0,62626	1
Reep6	-0,62735	1
Sgtb	-0,62747	1
Slc7a11	-0,62823	1
Gm15513	-0,6284	1
Myl6b	-0,62862	1
2810414N06Rik	-0,62884	1
Snord83b	-0,62877	1
Parp16	-0,62893	1
Gm42743	-0,62949	1
Mst1	-0,62995	1
Bbs7	-0,6303	1
Cux1	-0,63039	1
Bod1l	-0,63049	1
Sorbs1	-0,63089	1
Cep83os	-0,63121	1
Wdfy4	-0,6314	1
Fat1	-0,63207	1
Slc43a3	-0,6322	1
Gm26514	-0,63218	1
Kyat1	-0,63238	1
Zfp780b	-0,63283	1
Myof	-0,63336	1
0610009L18Rik	-0,63422	1
Gm10399	-0,63428	1
Gucd1	-0,63467	1
Zscan26	-0,63489	1
Hspa1a	-0,63503	1
Mavs	-0,63522	1
Gm43162	-0,63518	1
Pin1	-0,6372	1
Primpol	-0,63725	1
Zdhhc8	-0,63764	1
Dcaf17	-0,63806	1
Sdhd	-0,63811	1

Cep131	-0,63857	1
A630001G21Rik	-0,63859	1
Smim8	-0,63954	1
Irak4	-0,63967	1
Dock11	-0,64002	1
Ms4a6c	-0,64006	1
Gm13038	-0,64024	1
Tada2a	-0,64037	1
Slc25a20	-0,64057	1
Ticam2	-0,64101	1
Mark2	-0,64111	1
Gm43290	-0,6414	1
Smim1	-0,64148	1
Numbl	-0,64156	1
Capn10	-0,64175	1
Tmlhe	-0,64213	1
App	-0,64268	1
Xylb	-0,64289	1
Chd1l	-0,64313	1
Mrps18b	-0,64307	1
Rftn2	-0,64321	1
Slc16a6	-0,64343	1
Fam131a	-0,64368	1
Kcnb1	-0,64473	1
Pycr2	-0,64482	1
Ap5z1	-0,64489	1
Gm16223	-0,64489	1
1700001P01Rik	-0,645	1
Prepl	-0,64559	1
Prickle3	-0,64571	1
Sgsm1	-0,64587	1
Itpka	-0,64599	1
Gm42549	-0,64609	1
Akap17b	-0,64659	1
Gm37726	-0,64663	1
Zfp994	-0,64685	1
Fndc7	-0,64692	1
Med1	-0,64709	1
Pdss2	-0,64714	1
Gm527	-0,64753	1
Sec24d	-0,64768	1
Gm10676	-0,64824	1
Gm43273	-0,6482	1
Ficd	-0,64871	1
Prpf40b	-0,64934	1
Rab23	-0,64964	1
5730409E04Rik	-0,64994	1
Amotl1	-0,65045	1
Dclre1b	-0,65057	1
Zfp617	-0,65102	1
Plxna3	-0,65173	1
Cnpy3	-0,6519	1

Gm37706	-0,65273	1
Pycr1	-0,65334	1
Irak3	-0,65347	1
Tln2	-0,6535	1
Gm43387	-0,65345	1
Cd93	-0,65433	1
1810010D01Rik	-0,65437	1
Etv6	-0,65459	1
Hus1b	-0,65456	1
Trmt11	-0,65468	1
Depdc5	-0,65537	1
Gne	-0,65559	1
Tnfsf13b	-0,65583	1
Smc5	-0,65715	1
Prdm9	-0,65741	1
Hspb7	-0,65846	1
Sema4a	-0,65893	1
Rgs8	-0,65903	1
Dbndd2	-0,65954	1
Morn1	-0,65951	1
Ifi203-ps	-0,66062	1
Hdac8	-0,66103	1
2210408F21Rik	-0,66097	1
Gmpr2	-0,66129	1
Heatr5a	-0,66148	1
Aqp11	-0,66222	1
Vars2	-0,66287	1
Thada	-0,66341	1
Zfp41	-0,66386	1
Ube2e2	-0,66457	1
Mettl10	-0,66513	1
D430042O09Rik	-0,66515	1
Ipp	-0,66522	1
Ndufaf3	-0,66542	1
A930016O22Rik	-0,6658	1
Araf	-0,66622	1
Efr3b	-0,66645	1
Trub1	-0,66686	1
Acad11	-0,667	1
Tmem17	-0,66728	1
Add3	-0,66743	1
2310009A05Rik	-0,66751	1
Gramd1b	-0,66788	1
Frk	-0,66818	1
Cluap1	-0,66866	1
Bre	-0,66906	1
Rufy1	-0,6696	1
Gm38077	-0,66958	1
Nfat5	-0,66975	1
Foxred2	-0,66992	1
Ctdsp1	-0,66989	1
Gm4117	-0,66986	1

Tcf19	-0,67058	1
Lin37	-0,67083	1
Rab19	-0,67121	1
Frmd8os	-0,67124	1
Mfsd6	-0,67132	1
Zfp128	-0,67135	1
2210016F16Rik	-0,67148	1
Ifitm1	-0,67162	1
2810405F17Rik	-0,67156	1
Nucb2	-0,67165	1
Cdc37l1	-0,67176	1
Pde4a	-0,67186	1
Psenen	-0,67274	1
Fut7	-0,67301	1
Tbc1d13	-0,67334	1
Opn3	-0,67332	1
Dcstamp	-0,67351	1
Fbxo36	-0,67349	1
Gm43200	-0,67352	1
Gm44935	-0,67352	1
4930448A20Rik	-0,67398	1
Shisa3	-0,67453	1
Spred2	-0,675	1
S100a8	-0,67503	1
Chrnb1	-0,6758	1
Srrm2	-0,6767	1
Gm37303	-0,67717	1
D130019J16Rik	-0,67722	1
Akap9	-0,67789	1
Pde8a	-0,67812	1
Gm43742	-0,67817	1
Acvr1	-0,6789	1
Slc22a4	-0,67896	1
Alg14	-0,67948	1
Dpp8	-0,68003	1
BC049715	-0,68038	1
Gm9951	-0,68159	1
Serinc5	-0,68252	1
Peak1os	-0,68287	1
Gm9207	-0,68314	1
Wdr6	-0,68365	1
Robo3	-0,68381	1
Mapk9	-0,68402	1
Hdac7	-0,68434	1
Flt1	-0,68445	1
Zfp292	-0,68478	1
DHRSX	-0,68539	1
Prr12	-0,6859	1
Ppdpf	-0,68605	1
Mtmr9	-0,68621	1
Gm17807	-0,68658	1
Syt8	-0,68682	1

Gm15535	-0,68692	1
Slc25a14	-0,68716	1
Farp2	-0,68729	1
Fendrr	-0,68856	1
Ccl25	-0,68906	1
Tmem181b-ps	-0,68973	1
B3galnt1	-0,69311	1
Elp4	-0,69338	1
Cpeb1	-0,69362	1
Pigv	-0,69391	1
Tmem209	-0,69428	1
Cops7a	-0,69456	1
Ric8a	-0,69458	1
Gm15441	-0,69464	1
Iars2	-0,69504	1
Gm44190	-0,69526	1
Gm44771	-0,69573	1
Npc1l1	-0,69649	1
Cdkl4	-0,69667	1
Gm6745	-0,69696	1
Ikbip	-0,69751	1
Fam160b2	-0,69779	1
Cyb561	-0,69784	1
2810454H06Rik	-0,69828	1
Zfr2	-0,6985	1
Usp8	-0,69897	1
Cdc14b	-0,69905	1
Gmpr	-0,69983	1
Bnip2	-0,70111	1
Fgfrl1	-0,70119	1
Exosc5	-0,70164	1
Diaph2	-0,70191	1
Slc22a21	-0,70218	1
1700020I14Rik	-0,70234	1
Tmem175	-0,7029	1
Dsel	-0,70292	1
Zfp652os	-0,70308	1
Htr2b	-0,70341	1
Plekhg5	-0,70402	1
Prtn3	-0,70436	1
Xrcc5	-0,70517	1
Cdk14	-0,70579	1
Calhm2	-0,70636	1
Cnksr1	-0,70674	1
Ash2l	-0,70696	1
C030013C21Rik	-0,70733	1
Ppp2r3d	-0,70848	1
Gm5857	-0,7085	1
Pms1	-0,70904	1
Gm14121	-0,70947	1
Mkl2	-0,70958	1
RP24-75M13.2	-0,70978	1

Zfp26	-0,71091	1
Mospd2	-0,71102	1
Gm26594	-0,71099	1
Ankrd33b	-0,71107	1
Gm44623	-0,71182	1
Kptn	-0,71192	1
C2cd2	-0,71196	1
Lrrc8b	-0,7127	1
Zfp445	-0,71313	1
Nrp1	-0,71367	1
Polr1b	-0,71407	1
A130071D04Rik	-0,71425	1
I830077J02Rik	-0,71432	1
Acot6	-0,71563	1
Gan	-0,71584	1
Zbtb48	-0,71627	1
Rbfox2	-0,71645	1
Setx	-0,71687	1
Ppm1h	-0,71709	1
Rnf157	-0,71734	1
Cep68	-0,71726	1
Pemt	-0,71915	1
Setd1b	-0,7192	1
Gm5302	-0,71916	1
Nfatc4	-0,71917	1
Sptlc1	-0,72064	1
Nav2	-0,72127	1
Cntrob	-0,72132	1
Ccdc114	-0,72176	1
Nrp2	-0,72214	1
Vwa1	-0,72283	1
Zcchc4	-0,72299	1
3300005D01Rik	-0,72422	1
Pgm5	-0,72437	1
Nlrp3	-0,72558	1
Gm18916	-0,72561	1
Plekha5	-0,72568	1
Rrad	-0,72574	1
Ptpro	-0,72581	1
Sipa1l3	-0,72584	1
Syne1	-0,72635	1
Gm38192	-0,72637	1
Natd1	-0,72661	1
Gm43300	-0,72655	1
Ube2i	-0,72684	1
Fkrp	-0,727	1
Slc38a9	-0,72803	1
Arfgef3	-0,72872	1
Gm15690	-0,72873	1
4931414P19Rik	-0,72894	1
Cenpt	-0,72903	1
Mum1	-0,73104	1

Tubd1	-0,73318	1
Dnajc11	-0,73411	1
Vps52	-0,73421	1
BC037039	-0,73635	1
Snx24	-0,73646	1
Anpep	-0,73689	1
Pip5k1b	-0,7375	1
Bivm	-0,73792	1
Tiam2	-0,73857	1
Rps6ka3	-0,73874	1
Plscr3	-0,73989	1
Peak1	-0,74193	1
Actn1	-0,742	1
Parp3	-0,74275	1
Nckap5l	-0,74325	1
Nfia	-0,74337	1
Gmppb	-0,74398	1
Pnpla6	-0,74492	1
Zzef1	-0,74646	1
Nsun4	-0,74658	1
Gm10657	-0,74671	1
Arhgef18	-0,74753	1
Zfp174	-0,74778	1
RP23-23P9.3	-0,74815	1
Gm26670	-0,74862	1
Dnajc10	-0,7491	1
Arhgef12	-0,74971	1
Sh3kbp1	-0,74982	1
Gm42463	-0,75057	1
Tbx15	-0,75088	1
Zbtb12	-0,75096	1
Zfp709	-0,75178	1
Mau2	-0,75194	1
Dna2	-0,75269	1
Sirt5	-0,75287	1
Alms1	-0,75305	1
Uros	-0,75303	1
Gm37349	-0,75417	1
Zfp365	-0,75422	1
Gm14286	-0,75507	1
Maged2	-0,75527	1
Gm17066	-0,7556	1
Elmod3	-0,75561	1
Gm37116	-0,75576	1
Gm42724	-0,75597	1
Pik3r2	-0,75686	1
A730062M13Rik	-0,757	1
Serpine1	-0,75707	1
Gnaq	-0,75731	1
Hmgxb4	-0,7573	1
Dync1li2	-0,75775	1
Mrps6	-0,75783	1

Stxbp4	-0,7578	1
Hebp1	-0,75835	1
Setd6	-0,75915	1
Dgcr8	-0,76042	1
Rps12-ps5	-0,7607	1
Rpl23a-ps2	-0,76181	1
Xrcc3	-0,76253	1
Jade2	-0,76275	1
Gm43707	-0,76268	1
Rapgef3	-0,7627	1
Gm42747	-0,76326	1
Myo1d	-0,76349	1
Gm26129	-0,76396	1
Snord118	-0,76406	1
Sirt6	-0,76478	1
Gm42869	-0,76499	1
Gja1	-0,76523	1
Gm37474	-0,7653	1
Tbc1d19	-0,76558	1
Arhgef1	-0,7661	1
Glyctk	-0,76626	1
Slf1	-0,76738	1
Arhgef4	-0,76759	1
Flnb	-0,76785	1
Ahnak2	-0,76875	1
Fblim1	-0,76897	1
Zer1	-0,76923	1
Lyrm7	-0,7697	1
Entpd7	-0,76983	1
RP24-282C4.10	-0,77028	1
Rpl17-ps4	-0,77034	1
Gm8738	-0,77113	1
Slc39a14	-0,77261	1
Itga4	-0,77312	1
Pde6g	-0,77325	1
Gm38340	-0,77353	1
Rif1	-0,7739	1
Sema5a	-0,77463	1
Fyco1	-0,77511	1
Gm15785	-0,77512	1
Itga2b	-0,77517	1
Dzip3	-0,77593	1
Mfsd3	-0,77599	1
Nt5dc1	-0,77657	1
Gsn	-0,77763	1
Flrt2	-0,77794	1
Recql4	-0,77921	1
Hspa2	-0,77919	1
Gm12618	-0,77925	1
Gm38043	-0,77938	1
Ogt	-0,77997	1
Gm44292	-0,78072	1

Zfp456	-0,78071	1
5430434F05Rik	-0,78164	1
Poc1b	-0,78175	1
Gemin8	-0,78203	1
n-R5-8s1	-0,78215	1
Gm10074	-0,7845	1
Zmat1	-0,78503	1
Zfp719	-0,78581	1
Clec5a	-0,7866	1
Trpv4	-0,78659	1
Tgfb1i1	-0,78668	1
Gm45501	-0,78791	1
Gm12933	-0,78812	1
Rars2	-0,78829	1
Mccc1	-0,78886	1
Doc2g	-0,78922	1
Tmem202	-0,7892	1
Atp6v0d2	-0,7897	1
Olfr920	-0,79	1
Btbd19	-0,79032	1
Ppic	-0,79107	1
Gm45360	-0,7918	1
Pars2	-0,7919	1
Spp1	-0,79269	1
Arrb1	-0,79317	1
Kri1	-0,7944	1
Gda	-0,79455	1
Gm19967	-0,79463	1
Gm2367	-0,79498	1
Plcb4	-0,7955	1
Slc39a11	-0,79752	1
Gm43128	-0,79803	1
4930581F22Rik	-0,79881	1
4930568A12Rik	-0,79898	1
Cyb5rl	-0,79963	1
Zfp157	-0,79998	1
Zdhhc21	-0,80013	1
B230317F23Rik	-0,80008	1
Slc23a2	-0,80065	1
Acaa2	-0,80158	1
Ndufa4l2	-0,80179	1
Tmem150a	-0,80213	1
Angptl2	-0,80217	1
Hk1os	-0,80249	1
Pus7	-0,80258	1
Esr1	-0,80274	1
Ap5b1	-0,80364	1
B230208H11Rik	-0,80502	1
Ston1	-0,80551	1
Zfp362	-0,80617	1
Ccp110	-0,80694	1
Tubg2	-0,80698	1

Gm37033	-0,80726	1
Dnajc12	-0,80752	1
Sigirr	-0,80808	1
Mks1	-0,80846	1
6330403L08Rik	-0,8086	1
Rasal2	-0,80929	1
6430548M08Rik	-0,81066	1
Gm43062	-0,81073	1
Exoc7	-0,81193	1
Ak3	-0,81225	1
4930518I15Rik	-0,8123	1
Spg11	-0,8133	1
Pigp	-0,81336	1
Pecr	-0,81358	1
Nprl3	-0,81387	1
Pif1	-0,81455	1
Zfp280c	-0,81498	1
Nup85	-0,81612	1
Ascl2	-0,81612	1
C2cd3	-0,81731	1
Fancf	-0,81878	1
Gm26847	-0,81877	1
Ttc25	-0,81992	1
Zfp661	-0,82038	1
Rap1gap	-0,82037	1
Smarcad1	-0,82201	1
Nlk	-0,82218	1
Aldh1l2	-0,82251	1
9530062K07Rik	-0,82403	1
Slc25a15	-0,82537	1
Clmp	-0,82571	1
Myoz1	-0,82594	1
Surf2	-0,82757	1
Gm14403	-0,82844	1
Gm42820	-0,82899	1
Gm9256	-0,82902	1
Arhgdig	-0,82981	1
Rbfox1	-0,83009	1
Zfp72	-0,83019	1
Zfp462	-0,8306	1
Tmem18	-0,83079	1
Zg16	-0,83078	1
Gipc1	-0,83152	1
Nav1	-0,83242	1
Adamtsl5	-0,83367	1
Fryl	-0,83435	1
Lgals3bp	-0,83469	1
C030014I23Rik	-0,83482	1
Slc17a9	-0,83532	1
Armc6	-0,83653	1
9030624J02Rik	-0,83657	1
Usp20	-0,83767	1

Esyt2	-0,83968	1
Npy	-0,8398	1
Tmem42	-0,84102	1
Cep89	-0,84359	1
Gm3699	-0,84391	1
BC017158	-0,8443	1
Ermard	-0,84569	1
Gm4017	-0,84622	1
Arfgap1	-0,84658	1
Gm43323	-0,84758	1
Trim68	-0,84888	1
Zfp113	-0,84935	1
Gm42483	-0,85015	1
Gbas	-0,85068	1
Abcb9	-0,85085	1
Cuedc1	-0,85094	1
Pfkfb2	-0,85122	1
Ttc26	-0,85266	1
Ifitm5	-0,85423	1
Psph	-0,85434	1
Gm37968	-0,85474	1
Creb3l4	-0,85593	1
Hist1h2an	-0,85619	1
Gm15834	-0,85662	1
Cd80	-0,85665	1
Fam129c	-0,85676	1
Rfxank	-0,85721	1
Gk5	-0,85733	1
RP23-6C18.6	-0,85738	1
Bckdk	-0,85757	1
Zfp653	-0,8583	1
Tmem246	-0,85892	1
Gm43294	-0,85936	1
Ammecr1	-0,85987	1
9630010A21Rik	-0,86015	1
Gm43773	-0,86028	1
Plekhg4	-0,86225	1
Ttll3	-0,86479	1
Eid3	-0,86579	1
Ypel4	-0,86594	1
9230102O04Rik	-0,86605	1
Gm17494	-0,86646	1
Nadsyn1	-0,86681	1
Fktn	-0,86702	1
Gm12517	-0,86723	1
Mapk12	-0,86818	1
Gm38055	-0,86886	1
Nat14	-0,86958	1
Gm37101	-0,86996	1
Ppm1l	-0,87018	1
Macf1	-0,87049	0,92354
Gm6526	-0,87141	1

Manea	-0,87195	1
Rpgr	-0,87227	1
Invs	-0,87456	1
Gm26620	-0,87483	1
Fkbp15	-0,8759	1
Glt8d1	-0,87653	1
Arhgap31	-0,8772	1
Myom1	-0,87825	1
Ophn1	-0,8787	1
Gm43571	-0,87914	1
Ank	-0,87981	0,52583
Pkd2	-0,88025	1
Gm17530	-0,8803	1
Sgsh	-0,88174	1
Mdc1	-0,88321	1
Miga2	-0,88332	1
Gm23346	-0,88387	1
Aldh3b1	-0,88406	1
2610020C07Rik	-0,88449	1
Slc27a3	-0,88463	1
A330023F24Rik	-0,88617	1
Gm45221	-0,88615	1
RP23-356D13.9	-0,88798	1
Ehd2	-0,88815	1
Fam173b	-0,88824	1
Gm13397	-0,88817	1
Gm43499	-0,88938	1
Gm10136	-0,89015	1
Vsig10	-0,89061	1
1700020D05Rik	-0,89077	1
Rbpms	-0,89206	1
Ffar4	-0,89245	1
Asxl2	-0,89257	1
4930432K21Rik	-0,89312	1
Gm38125	-0,89327	1
Gm22980	-0,89368	1
Gm45407	-0,89452	1
Gm26890	-0,89478	1
Gm12833	-0,89628	1
Gm43788	-0,89737	1
Cep41	-0,89788	1
9030407P20Rik	-0,89897	1
Gm20522	-0,89947	1
Nphp3	-0,89961	1
Rpgrip1l	-0,9002	1
Gm44130	-0,90066	1
Neil1	-0,90086	1
Tmem214	-0,90175	1
Dixdc1	-0,90192	1
Taf1b	-0,90332	1
Rad1	-0,90424	1
Dtnb	-0,90462	1

E130308A19Rik	-0,90744	1
Coro7	-0,90866	1
B230312C02Rik	-0,90882	1
Zfp983	-0,90892	1
Spred1	-0,90942	1
Pter	-0,90947	1
Caprin2	-0,90993	1
Gm29340	-0,91004	1
Gpatch4	-0,91122	1
4930426I24Rik	-0,91194	1
Nagpa	-0,9124	1
Gm37121	-0,91269	1
Arl6	-0,91468	1
1700029J07Rik	-0,91483	1
Mthfsd	-0,91508	1
Sec16b	-0,91533	1
Kirrel3	-0,91783	1
Zfp3	-0,92035	1
Slc52a2	-0,92045	1
Fam45a	-0,92082	1
Cep57l1	-0,92152	1
Bbs9	-0,92149	1
Lrrc20	-0,92232	1
Cyp4f16	-0,92233	1
Sbk1	-0,92229	1
Sema4b	-0,9225	1
Dmpk	-0,92281	1
5430420F09Rik	-0,9228	1
Gm42748	-0,92466	1
Dcaf11	-0,9249	1
Gm20156	-0,92514	1
Frmd8	-0,92554	0,85058
Tnnt3	-0,92577	1
Nktr	-0,92733	0,79467
Gm15708	-0,92743	1
Gm28791	-0,92852	1
Eif2ak2	-0,92866	1
Slc2a4rg-ps	-0,92974	1
Dock5	-0,92982	1
Gm42798	-0,93008	1
Ift43	-0,93027	1
Six4	-0,93232	1
Gm45266	-0,93244	1
Cyp2u1	-0,93257	1
Gm2308	-0,93369	1
RP24-282C4.9	-0,93381	1
Cxcl10	-0,93383	1
Pomgnt1	-0,93452	1
Igsf3	-0,93515	1
C130050O18Rik	-0,93608	1
Zfp182	-0,93739	1
Vegfc	-0,93741	1

Ccdc92	-0,938	1
Tbc1d32	-0,9381	1
C730045M19Rik	-0,93863	1
Slc16a7	-0,93877	1
Mphosph9	-0,93887	1
Gm38366	-0,94029	1
Sec16a	-0,94178	1
Ampd3	-0,94197	1
Gm37140	-0,94226	1
D230022J07Rik	-0,94291	1
Gstt3	-0,94466	1
Gm14698	-0,9449	1
CAAA01194877.2	-0,94583	1
Gm42635	-0,94632	1
Gm28192	-0,94718	1
AW146154	-0,94788	1
Slc39a13	-0,94901	1
Zbtb20	-0,94937	0,98935
Dennd2a	-0,94935	1
Lclat1	-0,94953	1
Gm43147	-0,94983	1
Chst1	-0,95082	1
Ano8	-0,95191	1
Klhl30	-0,95279	1
Gm38009	-0,95309	1
Ppp2r3a	-0,95652	1
Ralgapa2	-0,95666	1
Cep164	-0,95743	1
Lyl1	-0,95788	1
Sdsl	-0,96037	1
Per3	-0,96128	1
RP24-282K24.4	-0,96183	1
Gnat2	-0,96323	1
Gm26652	-0,96395	1
Cyth4	-0,9645	0,54405
Nsf	-0,96451	1
Prkar2b	-0,96462	1
Gm44153	-0,96576	1
Gm43420	-0,96609	1
Ivd	-0,9664	1
Gm42576	-0,96644	1
Xpo4	-0,9681	1
1700007K09Rik	-0,97034	1
RP23-307F3.6	-0,97179	1
Zfp426	-0,97226	1
Ankrd52	-0,97283	1
Gm43247	-0,97278	1
Lims2	-0,97307	1
Csf2ra	-0,97442	1
Fam69a	-0,97446	1
Pfn2	-0,97531	1
Gm45137	-0,97574	1

Ltbp2	-0,97625	1
Gm22299	-0,97647	1
Gm38319	-0,97696	1
Alg8	-0,97723	1
Gm43848	-0,97803	1
Gm5776	-0,97825	1
3110080O07Rik	-0,9788	1
Pde4dip	-0,97909	1
Il17rc	-0,97949	1
Nipa1	-0,98057	1
Slc16a9	-0,98118	1
Ube4a	-0,98129	1
Atpaf1	-0,98206	1
Odf3l1	-0,98247	1
Rgs11	-0,98258	1
Egf	-0,98271	1
Slc35e2	-0,98283	1
Gm20045	-0,98276	1
6720464F23Rik	-0,98324	1
Gm3283	-0,98387	1
RP23-444K20.4	-0,98398	1
Chpf	-0,98467	1
Gm13413	-0,98496	1
Gm12479	-0,98588	1
Pigh	-0,98615	1
Gm43513	-0,98786	1
Gm37465	-0,98845	1
Gm45292	-0,98932	1
Gm43329	-0,9896	1
Prickle2	-0,99047	1
Zbtb32	-0,992	1
L3mbtl3	-0,99281	1
Gm44053	-0,99323	1
Armc9	-0,99362	1
Gm20696	-0,99369	1
Rint1	-0,99426	1
Snx25	-0,99491	1
2610020H08Rik	-0,99514	1
Apip	-0,99614	1
Gm43061	-0,99641	1
Gm37238	-0,99673	1
Gm37653	-0,99694	1
Spcs2-ps	-0,99707	1
Gm20699	-0,99814	1
Gm43774	-0,99832	1
Acp5	-0,99918	1
Map3k15	-0,9995	1
Fbxo15	-0,99955	1
Cnp	-1,0006	1
Zscan29	-1,0017	1
Gm16845	-1,0018	1
Fmo5	-1,0025	1

Gm38120	-1,003	1
Gm11944	-1,0038	1
Gm43817	-1,0052	1
Erlin2	-1,0056	1
Gm37578	-1,0057	1
Naip6	-1,0064	1
6030442K20Rik	-1,0097	1
Trim2	-1,0123	1
Garnl3	-1,0126	1
Gm6209	-1,0136	1
Fam69b	-1,0141	1
Nudt12	-1,0161	1
Gm44834	-1,0162	1
Gm38162	-1,0169	1
Ankrd39	-1,0181	1
Rhbdf1	-1,0185	1
Rabgap1l	-1,0188	1
Gm26740	-1,0188	1
BC048403	-1,0196	1
St18	-1,0199	1
Chil6	-1,0215	1
Hrc	-1,0221	1
Snord13	-1,0227	0,98935
1810021B22Rik	-1,0247	1
2610524H06Rik	-1,0268	1
Gm7784	-1,0268	1
Gm7769	-1,0269	1
Rtel1	-1,0271	1
Sit1	-1,028	1
Mtmr11	-1,0305	1
5330406M23Rik	-1,0319	1
Gm37010	-1,0322	1
Zfp953	-1,0337	1
A430027C01Rik	-1,0344	1
RP24-226A8.2	-1,0346	1
RP23-356D13.11	-1,0361	1
2810006K23Rik	-1,037	1
D430013B06Rik	-1,038	1
Gm45873	-1,038	1
Gm14636	-1,0392	1
Gm44164	-1,0398	1
Gm6382	-1,0427	1
Exoc8	-1,0436	1
A530041M06Rik	-1,0439	1
Epb41l1	-1,046	1
Sp3os	-1,0468	1
Ptpdc1	-1,0475	1
Dars2	-1,0476	1
Gm6576	-1,0477	1
Abhd1	-1,0482	1
RP24-131G14.10	-1,0496	1
Gm21967	-1,0511	1

Agap1	-1,0513	1
Gen1	-1,0514	1
Gm16310	-1,0516	1
Apba1	-1,0532	1
Gm43275	-1,0539	1
4930563E22Rik	-1,0539	1
Ptges	-1,0554	1
6430531B16Rik	-1,0562	1
Rhpn2	-1,0562	1
C630004M23Rik	-1,0571	1
A530017D24Rik	-1,0607	1
Zfp324	-1,061	1
2900005J15Rik	-1,0612	1
Stat1	-1,0617	1
Eldr	-1,0623	1
Ank2	-1,0637	1
Dock7	-1,0642	1
Etv1	-1,0649	0,98935
Smtn	-1,0663	1
Hfe	-1,0665	1
Dync2h1	-1,0676	0,64463
Kif7	-1,0682	1
4833421G17Rik	-1,0687	1
Gstt2	-1,0701	1
A630081D01Rik	-1,0706	1
9930012K11Rik	-1,0709	1
Fam83h	-1,0711	1
Ldb3	-1,0713	1
Adamts7	-1,0729	1
Gm37124	-1,0746	1
Amer1	-1,0748	1
Oxsm	-1,0751	1
1700084J12Rik	-1,0757	1
Cdc25c	-1,0783	1
Gm4602	-1,0786	1
Six5	-1,0796	1
B230398E01Rik	-1,0797	1
Slc46a1	-1,0797	1
Tmem80	-1,0804	1
Sumf2	-1,0809	1
Gm15472	-1,0825	1
Tmem98	-1,0828	1
Crtc1	-1,0831	1
Mfhas1	-1,0835	1
Cetn4	-1,084	1
Bphl	-1,0854	1
Rnaseh1	-1,0855	1
Gm23969	-1,0858	1
Nudt1	-1,0862	1
Shtn1	-1,0866	0,86005
Gm37255	-1,0879	1
Ccdc106	-1,0888	1

Gm42595	-1,0894	1
Phf21b	-1,0919	1
Tmcc1	-1,0926	1
RP23-442M18.5	-1,0948	1
Abtb2	-1,0952	1
Gm42937	-1,098	1
Gm3650	-1,0981	1
Stard9	-1,0999	0,72131
Gm37357	-1,1003	1
Zfp239	-1,1018	1
E330037G11Rik	-1,1038	1
Gm43794	-1,1039	1
Gm44667	-1,1047	1
Cox15	-1,1055	1
Tsnax	-1,1058	1
Gm43421	-1,1071	1
Bdh1	-1,1079	1
Gm43775	-1,1085	1
Phf19	-1,1109	1
Gm42467	-1,111	1
9430092D12Rik	-1,1124	1
Atad3aos	-1,1129	1
RP24-325N9.5	-1,1129	1
Uvssa	-1,113	1
Dlg3	-1,1132	1
Gm2531	-1,1135	1
Gm43692	-1,1135	1
4833418N02Rik	-1,1135	1
Dnajc22	-1,114	1
Gm15268	-1,1167	1
Crtam	-1,1172	1
Dync2li1	-1,1175	1
Gm42671	-1,1176	1
Gm6524	-1,1184	1
Intu	-1,1186	1
Ccdc14	-1,1229	1
Prkca	-1,1245	1
Pafah2	-1,1246	1
Gm43560	-1,1267	1
Rbbp9	-1,1271	1
Tcea2	-1,1271	1
Gm37621	-1,1288	1
Naip2	-1,1319	1
Lima1	-1,1322	0,53279
Frmd6	-1,1328	1
F630040K05Rik	-1,1328	1
Hist1h1d	-1,1337	1
Spaca6	-1,1352	1
Cdk20	-1,1354	1
Pld1	-1,1356	1
Wdr25	-1,1357	1
Gm43795	-1,1371	1

D330050G23Rik	-1,1374	1
Mblac2	-1,1381	1
Snora57	-1,1389	1
Neat1	-1,1406	0,44451
Gm36930	-1,141	1
Stx11	-1,1428	1
Gm37407	-1,1435	1
Gm42690	-1,146	1
Spaca9	-1,1461	1
Slc24a3	-1,1466	1
Palb2	-1,1479	1
Gm28535	-1,1504	1
Gm42872	-1,1539	1
Zfp111	-1,1546	1
Grk5	-1,1548	1
Hlcs	-1,1557	1
Ctdspl	-1,1563	1
Nrtn	-1,1581	1
Gm44557	-1,1594	1
Nmnat3	-1,1599	1
Mdn1	-1,1612	0,28394
Qsox2	-1,1615	1
Camk2a	-1,1616	1
Slc25a40	-1,1624	0,76817
Ddr2	-1,1654	1
Gsto2	-1,1665	1
Gm45220	-1,1679	1
Gm340	-1,169	1
Gm44822	-1,1696	1
Hmx2	-1,17	1
Tmem51os1	-1,1703	1
Ddx17	-1,1704	0,22229
Abcc10	-1,1716	1
RP24-233B16.6	-1,1718	1
Grik5	-1,1733	1
Apoe	-1,1755	1
6030458C11Rik	-1,1795	0,94714
Acy1	-1,1811	1
Slc25a42	-1,1817	1
2700029L08Rik	-1,1828	1
Gm26917	-1,1867	0,007978
Klhl5	-1,1869	0,7027
Gm37183	-1,1872	1
Bicd1	-1,1895	1
Tiam1	-1,1951	1
Gramd2	-1,1958	1
Bank1	-1,1959	1
Gm37949	-1,1963	1
Sipa1l1	-1,2002	1
Gm38355	-1,2031	1
Zkscan4	-1,2039	1
Gm43484	-1,2045	1

Gm42979	-1,2055	1
Gm43920	-1,2055	1
Fut8	-1,2064	1
Slc25a10	-1,2071	1
Gm16540	-1,2076	1
Rpgrip1	-1,2136	1
Msh5	-1,2138	1
Azin2	-1,2144	1
Glb1l	-1,215	1
9530078K11Rik	-1,2205	1
Zic5	-1,2211	1
Particl	-1,2223	1
Gm21816	-1,2224	1
Gm37589	-1,2228	1
Fam13a	-1,2234	1
Gm44044	-1,2293	1
C530005A16Rik	-1,2295	1
Gm42478	-1,2316	1
AI506816	-1,234	1
Pdcd1	-1,2347	1
Matn4	-1,2363	1
Gm42819	-1,2376	1
Gm38365	-1,2376	1
Gm37063	-1,2391	1
Marveld1	-1,24	0,22229
Rgs14	-1,2406	1
Gm12988	-1,2407	1
Gm12248	-1,2419	1
Gm6266	-1,2435	1
Nudt14	-1,2481	1
Fbxo10	-1,2489	1
Gm43761	-1,2498	1
RP23-205H11.3	-1,25	1
Atp8b4	-1,2502	1
Cchcr1	-1,2535	1
Hk3	-1,2559	1
Rpsa-ps2	-1,2559	1
Gm37678	-1,2559	1
Gm45477	-1,2585	1
Gm25857	-1,259	1
Car2	-1,2591	0,28525
Pofut1	-1,2594	1
Gm37354	-1,2652	1
Gm15503	-1,2699	1
Gm37959	-1,2701	1
Arhgef10	-1,2728	1
Zdhhc1	-1,2729	1
Tlr3	-1,2733	1
RP24-286J14.3	-1,2741	1
Cnrip1	-1,2757	1
Gm9776	-1,2757	1
Gm43482	-1,2767	1

Maats1os	-1,2771	1
Tnk2	-1,2787	1
Gm23722	-1,2789	1
Tango6	-1,2814	1
Camk2n2	-1,2829	1
Mboat1	-1,283	1
Gm45828	-1,2836	1
Sfxn2	-1,2848	1
Fastkd1	-1,2851	1
Ribc1	-1,2854	1
Gm43813	-1,2889	1
Mrps28	-1,2892	1
Gm29438	-1,2896	1
Gm45890	-1,2897	1
Med24	-1,2909	1
Tesk2	-1,291	1
1700007L15Rik	-1,291	1
Fam19a3	-1,2946	1
Wdr35	-1,2955	1
Mylpf	-1,2973	1
Gm42967	-1,298	1
Gm16201	-1,2982	0,98935
Tctex1d4	-1,3002	1
Gm43445	-1,3005	1
Kntc1	-1,3007	1
Gm43793	-1,3023	1
Crebl2	-1,3043	1
Fkbp14	-1,3051	1
Gm37084	-1,3096	0,72131
Ddx59	-1,3111	1
Gm45809	-1,3138	1
Gm43462	-1,3158	1
9930014A18Rik	-1,3205	1
Gm15506	-1,3206	1
Gm37569	-1,3223	1
Cd5l	-1,3232	1
Tmem198b	-1,3246	1
Gm11451	-1,326	1
B3gntl1	-1,3295	1
Rabl2	-1,3301	1
D930016D06Rik	-1,3307	1
Gm15644	-1,3322	1
Agbl3	-1,3331	1
Kcnq1ot1	-1,3333	0,12842
Slc22a15	-1,336	1
RP23-104D6.2	-1,3376	1
Tfec	-1,3403	1
Tmigd3	-1,3425	1
Carmil1	-1,3449	1
Ganc	-1,3488	1
AI464131	-1,3565	1
Asah2	-1,3576	1

Gm44953	-1,3587	1
Tmem241	-1,3613	1
Lmln	-1,3628	1
Hmga2	-1,364	0,0049431
Gm37423	-1,3669	1
Spn	-1,3702	1
Zfhx4	-1,3733	0,44034
Senp8	-1,3754	1
RP24-175C20.18	-1,3756	1
Pde4d	-1,3772	1
Catsper2	-1,3776	1
Gm45084	-1,3785	1
Tmem67	-1,3786	1
Carf	-1,3789	1
Gm42481	-1,3823	1
RP24-323H7.5	-1,3828	1
Gm43696	-1,3845	1
Plxnb3	-1,3846	1
Pfas	-1,3849	0,98935
Ing4	-1,3884	1
Gm25636	-1,3922	1
Echdc3	-1,3962	1
Gm10698	-1,401	1
Upk1a	-1,4025	1
Gm15696	-1,4134	1
Lgals4	-1,4136	1
Gls2	-1,4196	1
Cnbd2	-1,4205	1
Mob3b	-1,424	1
Catsperg1	-1,424	1
Gm15327	-1,4251	1
Lrp8os3	-1,4299	1
3110070M22Rik	-1,4356	1
Fzd2	-1,4364	1
Gm44509	-1,4368	1
Gm15859	-1,4405	1
Spink5	-1,4422	0,0031561
Tctn2	-1,4454	1
Gm43112	-1,4454	1
Gm37105	-1,4461	1
Sla	-1,4489	0,39257
Cstad	-1,4515	1
Gm37642	-1,4516	1
Pou5f2	-1,4543	1
B230354K17Rik	-1,4574	1
B230216N24Rik	-1,4576	1
Exoc3l	-1,4586	1
Fam208a	-1,4603	1
Gm24927	-1,4627	1
Gm44694	-1,4629	1
Cspg5	-1,4662	1
Bcl2l15	-1,4671	1

Ankrd34a	-1,4736	1
Gm6245	-1,4757	1
Lrrc1	-1,4805	1
Gm8228	-1,4811	1
L3hypdh	-1,4832	0,91083
Prkdc	-1,4862	1
Magi2	-1,4879	1
Pxdn	-1,4879	1
Gm26930	-1,4896	1
Col15a1	-1,4957	1
Katnb1	-1,496	1
Ankrd16	-1,4977	1
Zscan22	-1,4978	1
Gm26497	-1,4986	1
Itgb3	-1,5052	1
Cc2d2a	-1,5056	1
Serinc2	-1,5082	0,45248
Scarna17	-1,5095	1
Gm25517	-1,5109	1
Gm37606	-1,5116	1
Gm43544	-1,5135	1
Tmem116	-1,5139	1
Gm14239	-1,5148	1
Amt	-1,5154	1
Gm43071	-1,5169	1
Il1rl1	-1,5173	1
Plch2	-1,5199	1
Mical2	-1,5207	0,79467
Lekr1	-1,5222	1
Zfp933	-1,5293	1
Col20a1	-1,5297	0,72131
Itgax	-1,5303	0,54405
Gm43728	-1,5327	1
C530043K16Rik	-1,533	0,7043
Tnfrsf4	-1,5334	1
Gm37728	-1,5374	1
Gm45728	-1,5386	1
Gm44510	-1,5427	1
Gm43628	-1,5542	1
Gm37531	-1,5555	1
Fads2	-1,5563	1
Gm43336	-1,5596	0,47425
Sec14l2	-1,5666	1
Shprh	-1,5699	0,38316
Gm43627	-1,5721	1
RP23-36H21.3	-1,573	1
Prss53	-1,574	1
Ano7	-1,5753	1
Gm26935	-1,5806	1
Hspbap1	-1,5835	1
Airn	-1,5852	0,7424
Iqce	-1,5865	1

Tpm2	-1,5893	0,9607
Gm37906	-1,5977	1
Gm43715	-1,5984	1
Actg1	-1,6011	1
Msantd3	-1,6016	0,73784
Mypopos	-1,6075	1
Gm13423	-1,611	1
Gm37978	-1,616	1
Zfp942	-1,6178	1
C130089K02Rik	-1,6204	0,39509
Src	-1,6215	0,98935
Dsn1	-1,6239	1
Gm29488	-1,6255	1
Mir763	-1,6262	1
Treml1	-1,6301	0,043237
Masp2	-1,6312	1
Pctp	-1,6329	0,98935
Hck	-1,6347	0,9823
Nudt5	-1,6379	0,84458
Ccdc171	-1,6382	1
Gm38062	-1,6397	1
Gper1	-1,6678	1
Extl1	-1,6688	1
Kdm4d	-1,6843	1
Gm37472	-1,6846	1
Gm37390	-1,6984	1
AA386476	-1,7098	1
Gm30238	-1,7111	1
Tmtc4	-1,7138	1
Gm11716	-1,7162	1
Gng8	-1,7254	1
Gm10575	-1,7254	1
B930086L07Rik	-1,7271	0,47425
Elfn2	-1,7294	1
Rnf152	-1,7362	1
BC055308	-1,7444	0,53484
Bbs1	-1,7462	1
RP24-282C4.4	-1,7509	1
Gm27010	-1,7518	1
Wnk2	-1,7529	0,98935
Pdpn	-1,7619	1
Gm37902	-1,7637	1
Gm42633	-1,7686	1
Gm12522	-1,7701	1
Etv4	-1,7761	1
Gm38082	-1,7769	0,28334
Vaultrc5	-1,7788	0,032545
Gm42639	-1,7819	0,7027
4930509H03Rik	-1,7838	1
Gm45358	-1,7846	1
Tmem91	-1,7914	1
Gm7856	-1,7971	1

Txk	-1,8015	1
BC024978	-1,8034	1
Gm5837	-1,8163	1
RP24-460E12.3	-1,8222	1
Gm44639	-1,824	1
Zfp862-ps	-1,8242	1
Cep72	-1,8285	1
Gm37106	-1,8309	1
Gm45289	-1,8358	1
Il20rb	-1,837	1
Pde4c	-1,8407	1
RP23-402A24.3	-1,8448	1
Arhgap19	-1,8492	1
Gm45342	-1,8506	0,98935
Gm37080	-1,8529	1
Rhbdd2	-1,857	1
Gm12444	-1,8626	1
Gm22973	-1,8674	1
RP23-243B24.1	-1,8682	1
RP23-38L16.4	-1,8689	1
Gm37519	-1,8699	1
Gm43727	-1,887	1
AI661453	-1,8892	1
Gm10478	-1,8902	1
Gm37289	-1,8982	1
Gnrh1	-1,903	1
Exd1	-1,9105	1
Ctsk	-1,9226	0,23643
Anxa9	-1,9265	1
A430110C17Rik	-1,9347	1
Gm44432	-1,936	1
Gm15937	-1,9381	1
2810021J22Rik	-1,9386	1
Cradd	-1,9399	0,7043
Gm37718	-1,9553	1
Emp2	-1,9559	0,38822
Gm15496	-1,9584	1
Ttc9	-1,9952	1
Gm17249	-2,0097	1
Gm37788	-2,012	1
Gm43609	-2,0289	1
Gm42482	-2,0298	0,92663
Bok	-2,0371	0,98935
B3glct	-2,0438	0,33191
Krcc1	-2,0469	1
Ptpn5	-2,0488	1
Gm37490	-2,084	0,98935
4833412K13Rik	-2,0856	0,082885
Mras	-2,1168	1
Gm42908	-2,1187	1
Ankdd1a	-2,1198	1
Gm38020	-2,1329	0,98935

Stc1	-2,134	1
Gm38220	-2,143	1
Gm42484	-2,1486	0,90552
Acsbg1	-2,1517	1
Ccdc122	-2,1567	1
Gm45343	-2,1636	0,28525
RP23-40D21.1	-2,1647	0,53484
Gm37297	-2,1782	0,98935
Gm45203	-2,1817	0,72131
Gm38036	-2,1834	1
Phxr4	-2,188	1
Tti1	-2,1896	1
Gm37060	-2,2064	0,081815
Nt5e	-2,218	0,68789
Gm37333	-2,2222	1
Gm26132	-2,2326	0,98935
Gm45698	-2,2369	0,94714
Gm12258	-2,2528	1
Enpp5	-2,2591	0,054353
Zfp169	-2,2739	0,98935
Slc12a5	-2,2798	0,47425
Gm23344	-2,2809	0,9823
Gm42486	-2,2809	1
Gm37699	-2,3391	0,63189
Gm25514	-2,3534	0,98935
Gm45206	-2,3869	1
Gm43111	-2,3906	0,84458
Gm44699	-2,4364	0,84042
Stc2	-2,4441	0,87175
Gm42480	-2,4551	0,15127
Slc35d2	-2,4569	0,52583
Gm26601	-2,4788	0,84458
Olfr933	-2,5427	0,28334
Gm38399	-2,5644	0,44451
Gm44951	-2,5681	0,85058
Rgs16	-2,5923	0,054353
Gm37219	-2,642	0,98935
Mmp9	-2,7185	0,29544
Gm45534	-2,7198	0,95171
Gm44270	-2,7257	0,82117
Gm42640	-2,7872	0,3804
Il34	-2,8665	0,90552
Ccpg1os	-2,8796	0,39257
Gm36963	-3,1092	0,22229
Gm11205	-3,8507	0,027043
