# Supplementary material for: Bone, dentin and cementum differentially influence the differentiation of osteoclast-like cells
Source: Sci Rep. 2025 Jun 5;15:19857. doi: 10.1038/s41598-025-04874-9 (PMC12141432; doi:10.1038/s41598-025-04874-9)
Supplement: Supplementary file 5 — Supplementary Information 5. [file 41598_2025_4874_MOESM5_ESM.pdf]

Tab. S4:
Transcripts induced in murine macrophage cells stimulated on cementum (n=6), fold of negative control

gene name	regulation of expression	adj.P.Val
Gm29358	111,4381961	2,80E-06
4921507G05Rik	47,69611983	1,92E-05
4930578M07Rik	44,191592	3,50E-06
Hspa1b	43,88633838	0,0010808
RP23-451J19.1	33,7566539	1,85E-05
Slc16a5	27,01146531	0,00031078
mt-Ti	25,33043592	2,24E-06
RP23-440L7.5	24,18934836	0,00010405
AY074887	22,53975611	6,38E-06
1500004A13Rik	21,26486808	1,28E-06
Zp1	21,13848387	0,0011315
mt-Ta	20,84168477	0,002093
Tsix	20,66618235	2,06E-05
1700030M09Rik	19,23817445	0,00025871
Rpl30-ps2	19,13709578	5,38E-08
Gm44652	18,62798861	4,72E-06
Gm18709	18,02715716	7,41E-06
Rn7sk	17,66228385	0,00011858
mt-Ts2	17,63659317	0,00015422
Gm23037	17,15788712	0,0020706
mt-Tm	16,79197478	1,89E-06
Fzd7	16,36906486	2,56E-07
RP24-174I4.1	16,10347345	2,90E-05
Adm	14,43901077	1,08E-08
Lbp	14,22246869	0,0013158
Gm12469	14,06853306	0,000327
mt-Tc	13,93750097	0,0024601
RP24-295J1.1	13,82780252	0,00087559
Gm27248	13,37252504	0,0059334
Gm28373	13,2076327	0,0059334
Gm8649	12,8973253	9,04E-12
Thap8	12,426947	0,000309
Gm8885	12,41489367	0,0011315
Mcm8	12,35394579	2,80E-05
Gm8317	12,32144882	1,44E-05
Med16	12,05282979	2,06E-09
RP23-136K21.4	11,97787436	0,019581
Gapdh	11,21766112	3,38E-05
Hist1h2bg	11,15717595	0,020931
Gm8623	11,04177344	0,00019649
Gm43878	10,9003307	0,0053384
Rgcc	10,86412441	2,25E-06
Aloxe3	10,60814136	0,0033715
Gdf15	10,56191843	2,11E-08
Gm10827	10,53997836	1,27E-07
Gm37052	10,49696242	0,042873
Gm26870	10,29020629	0,64261
Gm9521	10,25958166	0,0059779

Gm26226	10,17883116	0,036581
Acoxl	10,09871622	0,02077
Gm43714	10,0540161	0,0059343
2310058D17Rik	9,714905754	0,00019461
Gm16181	9,694053201	0,020667
Gm42670	9,662523381	0,0035897
Hes7	9,59311931	0,014239
Gm26656	9,563243432	0,0027026
Hba-ps4	9,550657125	0,081599
RP24-175C20.10	9,528836055	9,48E-07
4632415L05Rik	9,397649753	2,55E-07
mt-Tl1	9,382679594	7,65E-08
Hspa1a	9,368382626	0,068974
Gm26810	9,292713955	0,0038361
Gm10636	9,270839598	0,0042873
1700054M17Rik	9,199784071	0,010969
Gm14279	9,112203711	3,43E-06
Car7	8,978659071	1,79E-05
Rpl30-ps1	8,828081815	0,00011502
RP23-350F7.3	8,747070974	0,00010506
Ankrd37	8,719829947	1,72E-06
4930542C12Rik	8,61589101	0,035356
RP24-93F20.12	8,564090255	0,0056622
D430001F17Rik	8,546300233	0,00022005
Gm4607	8,466114497	0,00055583
Gm7351	8,282693483	0,022688
Rnf122	8,211808591	2,71E-05
Hist1h2be	8,181129286	2,32E-05
Gm45833	8,139837537	0,00013568
Gm43182	8,133633586	0,046427
Trf	8,100999958	0,006639
Rasd1	8,089777368	0,02235
Snord82	8,027217564	0,015401
Gm26730	7,896426968	0,02863
Gm45251	7,840251859	0,02235
Adamtsl1	7,823423156	0,26035
Gm14326	7,775847904	0,065819
Gm26983	7,757544137	0,0012231
Gm10800	7,732312795	1
Gad2	7,707697756	0,041582
Gm26772	7,648622792	0,016412
Gm11810	7,525565523	0,0003191
Hist2h4	7,522957813	0,049501
Gm24631	7,521914981	0,0047847
Mafb	7,482394333	1,52E-10
Kcnd1	7,4044881	0,041069
Mast4	7,390643579	0,094281
Lrrc2	7,37171349	0,01371
Bc1-ps1	7,315213646	0,021411
Id1	7,286873774	1,08E-08
Hlx	7,269217134	0,001025
Olfr286	7,248085631	0,055108

Teddm2	7,049397705	0,050097
A830008E24Rik	7,033291441	0,035187
Gm13383	6,983740729	0,033777
Gm25008	6,969233537	0,0033581
2810433D01Rik	6,95138283	0,11079
Hist1h4a	6,940309497	0,097107
Hba-a1	6,851405626	0,1329
4930578M01Rik	6,797951175	0,093169
Rhoh	6,785711058	0,21214
Gadd45g	6,779129357	3,43E-06
S1pr1	6,770676549	1,17E-07
Gm15877	6,76692313	0,023304
Gm43684	6,76410943	0,1586
Atp1b4	6,745381312	0,12227
Gm6450	6,712266515	0,074616
Cbx2	6,643760193	1,87E-05
Rybp	6,643299698	0,026915
Egln3	6,639156685	0,010254
Hbb-bh3	6,608395354	0,15586
Gm29228	6,605647571	0,0097988
Gm5112	6,568664133	0,002011
Gm8818	6,558655054	0,024293
Gm6564	6,520126703	0,0083375
Gm42432	6,491716512	0,0097932
Notch1	6,481375413	0,049375
5330426L24Rik	6,445087745	0,057934
Gm14034	6,323397882	0,067298
Hist1h1b	6,319892418	0,039342
Gm26664	6,309825029	0,0089775
Gm28731	6,285815818	0,057026
Efna3	6,235477315	0,013415
Plk2	6,14664856	6,15E-05
Xaf1	6,142815277	0,011835
Dynlt1b	6,130054923	0,13194
Gfod2	6,124958207	5,12E-06
Gm28041	6,119441546	0,078766
Lrrc17	6,042309149	1,03E-05
Arc	6,029339638	0,00083307
Mxd3	6,022239149	0,16691
4933437G19Rik	5,957883857	0,04164
Sez6	5,94798084	2,50E-05
Gm12280	5,906485714	0,0051774
Socs2	5,902393069	0,03478
Gm9381	5,896259417	0,074417
Ighd	5,878711586	0,082118
Stamos	5,839318485	0,013454
5930420M18Rik	5,837295082	0,1033
Gm12604	5,827592504	0,0010735
Clec3b	5,805820627	0,13181
RP24-316F13.7	5,794161913	0,038725
Rflnb	5,787739543	0,051872
Gm17541	5,787338382	0,13766

Wwc1	5,786135064	1,44E-07
A830073O21Rik	5,763319499	0,2036
Gm10382	5,751347465	0,0016261
RP23-226H21.3	5,74218575	0,037753
mt-Tv	5,725890179	0,0021547
Gm7099	5,695805966	0,0025378
Gm26397	5,670987593	0,061064
Lgals7	5,665879817	0,031133
Gm42731	5,664309119	0,13389
Gfap	5,659599638	0,10565
Gm6969	5,655678062	0,087548
Tspan15	5,653326421	0,091693
Snord89	5,647843059	5,47E-05
Gm45051	5,643147269	0,038307
Gm16045	5,634939028	8,23E-05
Gm42851	5,631034539	0,038809
Gm11343	5,614276043	0,033777
Gm12778	5,605721224	0,0043699
Esco2	5,600672232	0,037423
Wfdc17	5,587488667	4,81E-05
Sep 01	5,566613817	0,1997
Gm36936	5,517061261	0,1498
Fcrl5	5,504456036	0,18432
Gm8210	5,46643398	0,11026
Gm22513	5,452054561	0,088844
Fam72a	5,446766421	0,01464
Tmcc3	5,439974919	0,14581
Olfr912	5,434698495	0,51229
Dtd2	5,432062203	8,37E-06
Jup	5,427545819	0,036112
Dvl3	5,421529815	0,069511
Gm8203	5,411392924	0,19147
Aif1	5,411017848	0,00021092
Ciart	5,404270921	4,02E-06
Gm15542	5,404270921	0,021732
Rhov	5,363222104	0,0018058
Selenop	5,338372585	0,00058196
Txnip	5,33356439	3,50E-06
Gm43566	5,328391177	0,00011345
RP23-366E4.9	5,323591973	0,10169
1700031P21Rik	5,29525441	0,015937
Gm26759	5,285719988	0,093043
Gm42793	5,273277797	0,24251
Rhob	5,2634181	0,00019649
Rpl32-ps	5,254669403	0,056896
Atr	5,253941002	2,94E-05
Atf3	5,243390521	1,61E-07
Gm10801	5,240120544	0,7247
Ang	5,235763747	1,85E-05
Atp5l-ps1	5,212227301	0,0002975
C730034F03Rik	5,183404563	8,96E-07
Gm44884	5,17765917	0,21541

H2-Q10	5,169769651	0,076879
Otud1	5,143676819	0,0050181
Exo1	5,12872423	0,14692
Cytip	5,119844524	0,18931
Gm14057	5,094709901	0,16088
Gm6341	5,092238532	2,07E-07
Ndrg1	5,083421996	2,06E-09
Tstd1	5,054260417	0,0005591
Gm6520	5,053209522	0,0712
Gm5312	5,040965218	0,011523
Arhgap26	5,024917813	0,00024874
RP24-547N4.7	5,015522487	0,076127
Nsl1	4,999209546	0,00015392
Tnfrsf12a	4,968464304	2,56E-07
2900060B14Rik	4,937223654	0,093533
Gm44013	4,936197094	0,077939
Gm42522	4,934144614	0,057112
Gm28555	4,931067494	5,88E-07
Arhgap39	4,915710657	0,00021298
Hist1h2ae	4,90413944	0,087548
5430421F17Rik	4,90210029	0,13766
9330162G02Rik	4,900741328	0,45958
Pou6f2	4,893952165	0,49471
Ier5l	4,892934602	0,00010196
A330069E16Rik	4,880740337	0,0075145
Zfp36l1	4,850723683	1,27E-07
Ccng2	4,849378963	1,70E-05
Zfp36l2	4,846354706	2,31E-06
Ccdc36	4,846354706	0,00025107
Gm11759	4,844675379	0,034754
Gm27003	4,819555182	0,19285
Slc25a2	4,818219103	0,042237
Gm15728	4,816549525	0,00060768
Carnmt1	4,79922017	0,072822
Sit1	4,78361075	0,1824
Gm14585	4,778308497	0,070097
Olfr95	4,769374268	0,092553
Gm26710	4,767391153	0,09504
9530085L11Rik	4,741685576	0,025137
C3ar1	4,740042518	2,52E-05
4930589L23Rik	4,713177452	0,079896
Gm42856	4,69426732	0,23953
Gm29170	4,685165465	0,00056312
Crkl	4,678026389	0,027441
Gm8330	4,678026389	0,063715
Taco1os	4,676081257	0,088844
mt-Nd6	4,673812958	1,70E-05
Gm37486	4,663457598	0,091205
Cks2	4,656674365	0,19285
Gm42786	4,640885166	0,2264
Gm5100	4,630923741	0,11637
Gm44777	4,584295366	0,14961

RP24-511J14.2	4,562106102	0,0038745
RP24-324J2.1	4,55736525	0,096215
E130201H02Rik	4,532163789	0,1311
Gm44957	4,520868644	0,31206
Adgb	4,516483693	0,18922
Mir3091	4,490574415	0,22757
Gm19566	4,476899711	0,006836
Hist1h4d	4,463885432	0,14812
Hoxa3	4,437047507	0,084754
Gm16755	4,433051123	0,30733
Gm13226	4,413123129	0,079896
Snord7	4,413123129	0,20505
Gm14094	4,405787767	0,013881
Ccl6	4,391762385	0,14961
Gm12164	4,356893958	0,16691
Gm16585	4,348144845	0,025118
Polr2k	4,34693945	0,15721
Ppp1r18os	4,339112526	0,22349
Gm6682	4,294231594	0,36121
Rpl27a-ps1	4,287688209	0,028248
0610039K10Rik	4,27700235	0,040982
Depdc1b	4,273742542	0,12173
0610005C13Rik	4,271077274	0,16148
4932422M17Rik	4,259251735	0,0054849
Gm6919	4,25836614	0,051951
Gm9722	4,243045061	0,082118
Gm10343	4,241868802	0,0041431
Gm28578	4,220751816	0,020931
Rpl19-ps11	4,220751816	0,2789
Phlda1	4,21519683	0,00029118
Gm43011	4,202943318	0,21353
Gadd45b	4,199157788	8,40E-06
Gm9442	4,194503354	0,32023
Gm45167	4,188402239	0,00025874
Gm27043	4,169574021	0,29169
Bloc1s6os	4,169285018	0,072091
Cfh	4,159759154	3,99E-06
Rasl2-9	4,156588705	0,10039
Gm14130	4,141920894	0,12258
Gm4832	4,139911709	0,34499
Klf10	4,139050928	1,86E-06
Kif18b	4,11074534	0,25283
Mcm10	4,107042852	0,011218
Tspan33	4,104766055	0,23452
Gm28424	4,104481544	0,24977
Rpl35a-ps5	4,099647876	0,0008589
5033430I15Rik	4,098795466	0,18287
Gm42508	4,094252277	0,065758
Rpl31-ps22	4,093684732	0,17833
Klf11	4,089714122	0,00035847
Errfi1	4,084048503	0,00019649
Hist2h3c2	4,069072541	0,020748

Gm26530	4,067380613	0,15721
mt-Tq	4,06597121	0,067045
Polr2l	4,064562296	3,95E-05
Gm11631	4,053027601	0,2565
Igf1	4,052465771	1,56E-08
Gm5900	4,014721745	0,23197
Gm3617	4,008048594	0,20737
4930404I05Rik	4,00360599	0,40625
Gm29019	4,001663899	0,0053627
Rasgef1b	3,99916831	5,12E-06
Gtse1	3,996674277	0,00016061
Gm44283	3,996120259	0,33172
Gm7895	3,991967569	0,23154
Alpk2	3,987819194	0,16189
Insig1	3,986990036	2,38E-05
Pin4	3,976226665	0,24487
Gm29666	3,972920707	0,19775
Gm13140	3,959724342	0,076439
Kctd6	3,958626626	5,75E-05
B3gnt6	3,951498882	0,32988
Ppp2cb	3,949855842	0,064552
Gm20302	3,939738844	0,21208
Wdr62	3,920669412	0,11327
C78859	3,918224334	0,2126
Kif20b	3,909001125	8,23E-05
Gm26830	3,90115143	0,39993
Gm16439	3,899529324	0,22053
Ddit4	3,897097429	8,12E-06
Rps8-ps4	3,857590492	0,051872
Adgre5	3,852780512	0,088111
2900093K20Rik	3,847709819	3,73E-05
Gm14650	3,831209718	0,23331
Mafk	3,826432632	5,76E-06
Pmaip1	3,824841595	8,40E-06
Fth-ps3	3,819013448	0,00017026
Gmnn	3,818748743	0,00018624
RP24-282C4.3	3,818484057	0,23418
Gm37733	3,813722839	0,038345
Sap30	3,806592141	3,95E-05
RP24-418P10.4	3,806064472	0,21475
Tma7	3,80131874	0,0078754
Gm15131	3,790268365	0,18143
Adamts1	3,789480282	0,0075533
Gm18860	3,780298088	0,16494
Hist1h3d	3,76069681	0,22356
Gm28659	3,760175503	0,16186
Crip1	3,748984862	1,88E-08
Gm14439	3,746906559	0,46778
Map3k12	3,745348589	0,00029612
Gm10388	3,727995194	0,01535
Gm8423	3,726703393	0,092376
Gm7132	3,725670275	0,31886

Gm37785	3,723346805	0,17327
Gm43331	3,722056615	0,25462
Gm24991	3,706609161	0,39606
Hist1h1a	3,69762777	0,5168
Cped1	3,695577938	0,031296
Rps6-ps3	3,693785268	0,11278
Tnfsf9	3,685090372	0,038731
Ect2	3,682536947	0,0059334
Gm14537	3,680240377	0,20824
Gm13992	3,67922014	0,29334
Cpne9	3,675906319	0,17998
Enho	3,675396766	0,2138
Gm2076	3,670305114	0,29827
Gm12034	3,66725351	0,16892
Gm38299	3,665728659	0,085569
Rny1	3,664204442	0,0031138
Zic2	3,65659286	0,0062459
Gm17430	3,656339413	0,21183
Gm6177	3,651021084	0,17136
Gm12380	3,646721436	0,19425
Cenpu	3,642174387	0,028432
Gm11604	3,640155296	0,20031
4930579G24Rik	3,639902989	0,077691
Gm13567	3,62907029	0,064582
Gm9409	3,628064237	0,20782
Gm14013	3,620527728	0,12242
Spdl1	3,619524044	0,00072719
Slc25a25	3,61150458	0,00014115
D830025C05Rik	3,60825175	0,035505
Gm8326	3,606751433	0,38727
Gapdh-ps14	3,606251465	0,24306
Gm44913	3,601505232	0,0012641
Gm11363	3,590786808	0,049375
Gm10717	3,584569837	0,76273
D2hgdh	3,564994868	0,19997
Gm45133	3,561043354	0,14853
Gm15007	3,55906924	0,30128
Rad51ap1	3,558082593	0,0047927
Bvht	3,552660923	0,037661
Gm23301	3,5474934	0,36748
Rpl30-ps3	3,541351385	0,00058446
Itga6	3,540124259	0,00048787
Gm44851	3,537916503	0,28571
Kif21a	3,527631834	0,38296
Gm37696	3,52689836	0,338
Gm12090	3,521768309	0,34149
Rn7s6	3,521768309	0,49599
Nfil3	3,521524207	0,00058196
Mkln1os	3,520547969	0,23953
Gm15950	3,519815968	0,016938
E230029C05Rik	3,513965434	0,063715
Gm26737	3,508610988	0,042873

Rpl29	3,508124625	0,30723
Ntrk3	3,50375039	0,64926
Cep55	3,500594613	0,011371
Ezr	3,499866761	4,50E-07
Pigb	3,494049386	0,053403
Plscr4	3,493807206	0,55472
Tbc1d30	3,490176509	0,37071
Gm7634	3,489934597	0,20518
Gm7327	3,483892238	0,010581
Tmod1	3,481960894	0,0019782
Crtc2	3,478342508	0,39836
Gm5921	3,467269555	0,30017
Atp5g1	3,466068099	0,00025107
1500015A07Rik	3,454315841	0,0028718
Csrnp1	3,449291365	4,02E-06
Gm7266	3,448335151	0,070549
Ttc30a1	3,444512945	0,33143
Gm4890	3,441410522	0,1999
4930558J18Rik	3,439979578	0,42103
A330035P11Rik	3,439025945	0,34795
Gzmm	3,436404818	0,26405
Hmgb1-ps5	3,433547685	0,19425
Gm26594	3,432595836	0,40042
Gm6905	3,423091841	0,24625
BC024386	3,420008712	0,1576
Gm18943	3,420008712	0,50009
Il1b	3,414560746	0,38868
Unc13a	3,411721775	0,002421
Gm8925	3,408412625	0,26801
Rps19-ps11	3,407467744	0,053853
Gm26782	3,405578766	0,0044087
Fn1	3,405342718	0,0023462
2810013P06Rik	3,402275575	0,0016838
Rgmb	3,399446818	0,19206
Gm7363	3,398504422	0,30997
B930036N10Rik	3,393325911	0,19698
Asb10	3,391679859	0,0046205
Gm13433	3,389094817	0,27756
Rps19-ps6	3,388390148	0,11279
H2-Q5	3,386042309	0,40667
Gm9920	3,380882787	0,23714
Pbk	3,376199136	0,0008003
March4	3,365917929	0,20457
RP23-246F14.1	3,36568463	0,35302
Ccl4	3,362420133	5,88E-07
Gm6768	3,3603232	0,29656
Btbd6	3,356831218	0,10271
Lsm7	3,343362996	0,053066
B430305J03Rik	3,325105234	0,43665
2610528A11Rik	3,324413869	0,04095
Brip1	3,322570934	0,024627
Pea15a	3,300764019	3,69E-05

Dnd1	3,297562492	0,5216
Ier5	3,293222527	3,43E-06
Itga11	3,292537792	0,11651
Id2	3,289116251	1,57E-05
C230096K16Rik	3,283876793	0,060248
Gm13736	3,274557581	0,43409
Gm12940	3,268208443	0,49855
Hist1h1c	3,266849516	6,15E-05
2810001G20Rik	3,26345467	0,087338
Ppfia4	3,258707811	0,0016611
Iqgap3	3,25825609	0,0016895
Sdc3	3,256224118	5,11E-07
Gm5577	3,255321426	0,36756
Zfp101	3,253291284	0,02034
H2-T10	3,251037055	0,55203
Gm8894	3,24788376	0,31072
D130051D11Rik	3,242709983	0,013771
Gm2467	3,241811038	0,43529
Gm12466	3,240013895	0,24499
Rpl28-ps3	3,236646935	0,14621
Pmp22	3,235525393	7,12E-07
Gm8129	3,234180056	0,49035
RP23-88C11.5	3,232163099	0,34126
Zwilch	3,231267077	0,027011
Ankrd55	3,22410784	0,17554
Pole	3,223214052	0,21873
Gm5869	3,22075741	0,19066
Myc	3,21986455	0,3118
4932416K20Rik	3,216295586	0,13766
Slc2a1	3,215403963	2,12E-05
Gm14584	3,214958244	0,12438
Tnfrsf17	3,211172129	0,031361
Gadd45a	3,209614436	0,00506
Rgs9bp	3,20916952	0,45998
Gm26225	3,204723749	0,30959
Mapkapk5	3,204057414	0,44067
Gm44130	3,203613268	0,46314
Gm26847	3,191423354	0,59592
Pgf	3,189875241	0,03544
Atad5	3,182366512	0,042873
Rps11-ps3	3,181704826	0,22757
Gpr19	3,176636471	0,24808
Rps3a3	3,173775321	0,056363
Gm6946	3,170477196	0,38868
Gm13215	3,168719597	0,021605
Tgif2	3,167621593	0,0002975
Yjefn3	3,164110535	0,066535
Gm8930	3,157537789	0,40667
Gm43920	3,144433227	0,48013
Gm37482	3,131817185	0,36091
Gm24276	3,131383053	0,00016697
Gm11491	3,129647127	0,071824

Gm12583	3,1272618	0,39606
Gm43447	3,122496598	0,82891
RP24-122E11.4	3,120332998	0,62153
Nuf2	3,11752256	0,028523
Cenpk	3,115578354	0,019993
Btf3	3,10975301	0,058324
Gm12312	3,100927939	0,3087
Gm8250	3,100283186	0,49288
Hist1h2al	3,099638567	0,067445
Rpl30-ps5	3,099638567	0,25957
Polq	3,098779284	0,14812
Gm37653	3,0985645	0,23637
Fos	3,086988268	0,053872
Gm6913	3,086988268	0,40463
Sgk1	3,084421651	0,015937
Gm12643	3,082711758	0,39282
Jun	3,080148697	0,00030996
Hmgb1-ps8	3,078867965	0,37144
Ptrf	3,077161152	0,43529
Ckap2	3,07332428	0,0055238
Ska1	3,065027462	5,75E-05
Ormdl3	3,063328321	0,00019649
Gm43010	3,058660538	0,38868
Rpl9-ps7	3,056329315	0,32825
5830432E09Rik	3,055058488	0,04583
Creb5	3,051037686	0,25443
Gm7701	3,051037686	0,29963
Gm19028	3,048712273	0,40667
Gm7867	3,045966342	0,26163
Dnali1	3,043222884	0,36876
Ybx1-ps2	3,043011951	0,338
Gm38248	3,041114214	0,50001
Gm44419	3,031853408	0,13712
Sertad1	3,026604156	0,00084416
Nadk2	3,026394375	0,0024461
Gm37584	3,020945171	0,62619
Rps12-ps26	3,020735782	0,11594
Mgst3	3,016132901	0,042478
H2-T23	3,013416314	0,087548
Basp1	3,012789757	0,0004514
Gm4963	3,011537033	0,58142
RP23-356P21.1	3,010910867	0,047098
Gm26533	3,010910867	0,25443
Itgb3bp	3,010702174	0,22055
Gm12017	3,007990478	0,49502
Kifc5b	3,007573511	0,089593
Adam9	3,006531346	0,11279
Cd300lf	3,002990685	0,47494
Hmgb2	3,000701892	0,0061673
Dusp1	3,000285935	0,0012167
Gm11826	2,989076872	0,053066
Gm11989	2,98721277	0,36091

Trappc2	2,985142909	0,38555
4930532G15Rik	2,983901681	0,40199
Gm44198	2,983488053	0,20152
1700003G18Rik	2,983488053	0,60056
Rbmx	2,977703279	0,2414
Sec24a	2,974608908	0,00014115
Sep 02	2,974608908	0,011043
Gm11346	2,972341746	0,42108
Lin54	2,972135727	0,0015639
E2f7	2,97172373	0,18548
Hpse	2,962674232	0,010389
Gm14005	2,961237082	0,068682
Tob1	2,959800629	0,014866
Mpc1	2,957544754	0,4558
Pim1	2,953652292	1,87E-05
Sgol1	2,953447567	0,022796
Rps13-ps1	2,953447567	0,36756
Klf4	2,951605686	3,73E-05
Aurkb	2,949969421	0,0044033
Proscos	2,948538435	0,14493
Spsb2	2,948334065	0,003966
Sik1	2,945678543	0,067308
Arid5a	2,945270213	0,13523
Gm11895	2,939355793	0,47884
Gm12758	2,93915206	0,30723
Gm7079	2,938744635	0,3478
Adh5	2,938133604	3,18E-05
1700052K11Rik	2,934063308	0,32247
Kifc1	2,932640036	0,0075145
Tiparp	2,929795565	0,00032952
Gm26826	2,926548119	0,01233
RP23-168F21.4	2,92573682	0,41057
Rep15	2,924114897	0,15092
Rps15a-ps6	2,922696452	0,014537
4930430E12Rik	2,921076214	0,032818
Gm15610	2,91763619	0,6292
Gm34121	2,910163077	0,39571
Lockd	2,899089756	0,0040445
Zfp326	2,895474919	2,76E-05
AV099323	2,894471598	0,60488
Ltb	2,891263305	0,0034714
Kif11	2,889259929	0,0054353
Frat2	2,889059667	0,00047491
Gm3724	2,888258761	0,40686
Mcm3	2,887057818	0,00042368
Cks1brt	2,886057413	0,50794
Gm44258	2,885457337	0,08236
Rad51	2,883258124	0,040955
Maff	2,876271774	0,0025378
Gm10916	2,875474413	0,58458
Bbs5	2,869899069	0,16399
Gsg1	2,867711715	0,046708

Lonrf3	2,865724657	0,00033209
Mtfr2	2,858978954	0,20738
Gm3940	2,858780792	0,33179
Gm26698	2,853831192	0,20282
Gm42559	2,852842301	0,46919
RP23-320D23.6	2,851458429	0,15919
Ccrl2	2,851260788	0,028079
Rpl36-ps4	2,850865546	0,17374
Smarca5-ps	2,850272787	0,49159
Lilrb4a	2,846916146	0,01733
Gm38297	2,846718819	0,41952
Nemp1	2,845929651	0,0008968
Gm7964	2,840805385	0,091333
Gm45856	2,840805385	0,14913
Gm20594	2,836280071	0,68348
BC028528	2,835297262	0,011916
Gm29736	2,83215456	0,037494
Gm12005	2,826271379	0,41099
2010111I01Rik	2,82392153	0,00014731
Rps4x	2,823725797	0,25512
Gm12165	2,817860125	0,36019
Crip2	2,81590761	0,033835
Gm37234	2,81551727	0,43798
Gm10616	2,815126983	0,064249
Rps16	2,814541655	0,16903
C130013H08Rik	2,811811731	0,58875
Gm11470	2,811421958	0,080424
2410080I02Rik	2,806748896	0,070694
Gm10237	2,805192936	0,57614
Fcor	2,804998501	0,33575
Psmc3ip	2,801695177	0,15022
Gm26520	2,801500985	0,0032787
Gm25007	2,79820178	0,39577
Gm45184	2,796456716	0,010254
Gm42895	2,796456716	0,47877
Krtcap3	2,795293944	0,33911
6430511E19Rik	2,792776263	0,41361
RP23-325K4.10	2,791615022	0,0012641
AV356131	2,789487333	0,0004855
S100a3	2,782728196	0,33512
Gins1	2,782535319	0,12902
A730071L15Rik	2,782149605	0,54085
Gm5828	2,780607281	0,33014
Tnfrsf14	2,779258449	0,54659
Pard6b	2,776562748	0,073254
Fau	2,775600631	0,035187
Iscu	2,775023521	0,0038745
Gm43511	2,769642945	0,16148
Rpl7	2,765997783	0,070748
1600029O15Rik	2,763698047	0,49013
Ost4	2,761208824	0,062379
Gm5257	2,760634707	0,61862

A430035B10Rik	2,760443361	0,63656
Gm42728	2,757383628	0,48384
Socs1	2,756810306	0,10636
Depdc1a	2,756619225	0,65241
Rps19-ps5	2,756428157	0,10985
Mad2l1	2,756237103	0,02725
H2-Q6	2,756237103	0,37954
Gm10177	2,755473018	0,076879
1700120C14Rik	2,752228018	0,16761
Rassf7	2,751655768	0,064582
Gm15216	2,747843806	0,41057
Spc24	2,747082047	0,013881
Fam83d	2,746701247	0,20721
Gm12459	2,745559164	0,43858
Gm7504	2,744798038	0,070129
Gm8927	2,743656746	0,48055
Nr4a3	2,743276421	0,10039
Dlgap5	2,742706032	0,15747
Cfap126	2,740045785	0,58642
4930579K19Rik	2,738526802	0,56695
Isg15	2,735112169	0,23714
Gm31274	2,732080514	0,18143
Gm2986	2,729808978	0,32104
Gm15720	2,728863062	0,44933
Gm7990	2,72829567	0,20782
Sdc4	2,727350278	8,01E-05
1810041H14Rik	2,724516069	0,5989
Cox7c	2,723571988	0,26795
Ttk	2,721873465	0,0087195
Fam161b	2,715654606	0,50855
Cdc7	2,714337281	0,11499
Gm45749	2,710013428	0,3087
Hmmr	2,707947933	0,0041248
Rpl36-ps2	2,704946388	0,14042
Gm42466	2,704946388	0,59592
Gm17034	2,704571429	0,67232
Sirpa	2,704383968	2,57E-05
Hnrnph2	2,696896209	0,25684
Rbm48	2,696709281	0,0032743
Nsa2-ps2	2,696522366	0,72967
Col4a6	2,696335463	0,46041
Zscan21	2,695401147	0,073836
Gm43501	2,693720192	0,66934
Snord71	2,692040286	0,67874
Gm7965	2,687006851	0,46122
Gm37334	2,684586703	0,62656
Snord49b	2,681982828	0,0437
Tubb2b	2,681796933	0,49527
Gm7287	2,680124463	0,19206
Gm15798	2,675113308	0,40371
Gm37522	2,672889136	0,56101
Cox20	2,66881629	0,0014964

Gm9506	2,668631308	0,68966
Hist1h4h	2,667336794	0,3323
Gm15564	2,666042908	0,23953
Haus3	2,665303827	0,0045466
Gm19777	2,665119089	0,3297
Rpl39-ps	2,66474965	0,008987
Dusp5	2,662903226	0,013395
Gm5277	2,661242539	0,17949
Ly86	2,661058082	0,00063088
Gm14541	2,66013599	0,70362
Dnah8	2,656450813	0,61923
Rpl7a	2,655898477	0,077691
Zmynd10	2,655162208	0,38864
Rmi2	2,654978172	0,28571
Gm8909	2,654978172	0,37144
Rhox5	2,654058186	0,54659
Gm20091	2,650014034	0,56438
Gpat3	2,649830355	0,38868
Rdh13	2,64964669	0,00027494
Snord35a	2,649463036	0,30959
Gm45855	2,648177821	0,69386
Rnf19a	2,646709766	0,0019944
Ypel2	2,645609259	0,017274
Pdgfb	2,643592852	0,056966
Cox20-ps	2,641211806	0,0023684
Rps6-ps4	2,639015822	0,32695
Cbfa2t3	2,635177241	0,33265
H60b	2,634264116	7,35E-05
5031425F14Rik	2,63170905	0,6476
Gm12074	2,63152664	0,29548
Gm6472	2,627152595	0,049623
D930030I03Rik	2,625150254	0,60488
Gm26549	2,623513111	0,64731
Cdc6	2,620968473	0,020671
Ddah2	2,619333938	0,20782
Gm3531	2,619152386	0,7913
Gm11281	2,618063337	0,56003
Gm29438	2,617518983	0,36025
RP23-2N7.4	2,616974742	0,14965
Gm16238	2,614980159	0,41099
Gm6794	2,614074033	0,59093
Gm10358	2,610452667	0,3907
Gm36989	2,610452667	0,46706
Vdr	2,609186375	0,33179
Gm17827	2,609005526	0,64921
Snord87	2,606294299	0,20737
Gm8172	2,606294299	0,40667
Rpl34-ps1	2,605210597	0,55976
Gm6649	2,599979053	0,55583
AA474408	2,599078125	0,33932
Wdfy2	2,597637289	0,16804
Gm45223	2,593499335	0,010248

Troap	2,591163408	0,0822
Suco	2,590624647	8,40E-06
Gm44953	2,587394431	0,70267
Rc3h1	2,586856453	0,00086178
Rpl35a-ps4	2,585780834	0,33932
Gm13777	2,58434737	0,18473
Gm20223	2,581303897	0,70884
Gm45716	2,580588307	0,2865
Hsh2d	2,580230586	0,45161
Gm6134	2,57790661	0,13766
Rpl23a-ps14	2,577191962	0,39497
Gm15796	2,575406207	0,63586
Rin1	2,574692252	0,23154
Klf2	2,570590849	0,050698
Adrb2	2,566495979	0,12243
Gm4617	2,564717636	0,069327
Ube2t	2,562230025	0,013897
4930522L14Rik	2,560809617	0,30997
Snord15a	2,560277167	0,37702
Cenpw	2,555312969	0,0012475
Rpl6l	2,552126783	0,18193
Unc5b	2,550358396	0,35591
Arrdc2	2,549297952	0,018948
Gm8624	2,547531526	0,32583
Gm12176	2,546295756	0,38165
Cldn11	2,545766324	0,0021547
Tmem107	2,545237001	0,052222
Gm15013	2,544707789	0,27687
5730405O15Rik	2,541887182	0,66723
Scel	2,537310363	0,085512
Jund	2,536958641	0,00013848
Paqr5	2,536782799	0,64644
Gm8304	2,535376498	0,17998
Hcfc1r1	2,529583741	1,70E-05
Gm5786	2,529583741	0,31812
Rps6-ps1	2,527830975	0,044487
Gm23127	2,527655765	0,69765
Gm5580	2,526779898	0,39214
Gm5566	2,525029075	0,65434
Suv39h2	2,52380422	0,11578
Gm18969	2,52310457	0,51323
Phf13	2,520482609	0,0061288
Ccdc117	2,518736149	0,00086499
Gm7887	2,518736149	0,47563
Dedd2	2,518037904	0,00044681
Gm42551	2,517688854	0,63761
H2-Ob	2,514723882	0,0080381
RP23-182J19.2	2,514549581	0,66212
Aunip	2,514375291	0,17656
Gm12174	2,514201014	0,34884
Hist1h2bp	2,513678255	0,75972
Gm12844	2,513329809	0,065268

Ropn1l	2,513329809	0,21956
A430010J10Rik	2,513329809	0,7247
Lsp1	2,51124015	0,001122
3110062M04Rik	2,51106609	0,00084416
Gm28727	2,510718006	0,12586
Zfp36	2,509152227	0,041064
Uhrf1	2,508630518	0,20068
Shcbp1	2,504460747	0,26305
Gm38262	2,504460747	0,75203
Gm11516	2,50185816	0,79857
Pcna-ps2	2,501511353	0,32825
Gm4968	2,498738626	0,74916
Bub1	2,498392251	0,025195
Gm29759	2,496488048	0,73744
Gm13416	2,496141986	0,48207
Gm7832	2,495450004	0,41906
Gm16380	2,493202389	0,6387
Rassf3	2,493029579	0,0090827
Gm13827	2,487161173	0,061513
4931440P22Rik	2,487161173	0,10985
Rpa3	2,486644036	0,52636
Cd300c2	2,486471681	0,0016386
Map3k8	2,485437801	0,0066681
Gm13776	2,485437801	0,57135
Gm6210	2,484059964	0,40883
Usp50	2,483887788	0,35826
Zfp503	2,483715624	0,12405
Gm2011	2,483027087	0,43012
Gm19272	2,482166685	0,78966
Osgin1	2,481822607	0,073037
G2e3	2,478899874	0,0040879
Gm15446	2,477353938	0,20685
Rpl17-ps8	2,47683884	0,066017
Gm8394	2,473579041	0,38029
Rbm19	2,473407591	0,081599
Rbm3	2,473236153	0,45871
Gm11263	2,472721912	0,67999
Prdm10	2,472550522	0,073492
Mynn	2,472379144	0,0065216
Snord104	2,470837274	0,0053014
RP24-550H10.4	2,469125214	0,64797
Zdhhc18	2,468269628	0,0043591
Gm13349	2,46775642	0,68416
Gm7434	2,465191975	0,43031
Gm42632	2,464850251	0,64097
Gm5801	2,464679406	0,44619
RP23-440I21.3	2,464679406	0,73741
Gm12341	2,463313077	0,1409
Gm19726	2,462800898	0,77224
Tsc22d3	2,46160623	0,022688
Snrpf	2,459048177	0,22918
Svil	2,457855329	0,6119

Gm9530	2,455641574	0,2556
Pfkfb3	2,45496082	0,010014
Snx13	2,454450378	0,013656
Rps12-ps10	2,453940043	0,060635
Gm8599	2,452749672	0,1053
Fam26f	2,452749672	0,49942
Gm16288	2,452749672	0,71059
Necap1	2,449521577	0,0008589
Rps12-ps19	2,449012266	0,63299
Rplp1-ps1	2,448163651	0,65786
Gm4754	2,444602674	0,62338
Trim59	2,443416833	0,082272
Hist3h2a	2,443247474	0,0019255
Insig2	2,443078127	0,00207
Gm6285	2,442231567	0,053872
Gm5764	2,442231567	0,6292
Tmem171	2,441385301	0,04994
Gm12726	2,440708499	0,68659
Asf1b	2,440539328	0,088264
Tmem170	2,440370168	0,63299
Fzd5	2,439017315	0,021246
Gm3362	2,438003167	0,36906
RP23-134M7.3	2,435638461	0,62133
Gins3	2,435469642	0,0023217
Tmem44	2,434288231	0,57372
C030034I22Rik	2,433782088	0,00872
Rpl30-ps11	2,433782088	0,27824
Zfp367	2,43327605	0,01404
Ap1s3	2,430747437	0,004643
Ticrr	2,428894791	0,67801
Gm9769	2,427211793	0,71735
Rpl21-ps14	2,424689481	0,47491
Lhx5	2,4235133	0,60488
Gm6322	2,422337689	0,70541
Tma7-ps	2,421666168	0,16691
Rangrf	2,420155925	0,61862
Dusp8	2,419485008	0,44427
Gm4217	2,419485008	0,66843
Kif15	2,419317308	0,057934
Gm23849	2,418646624	0,71362
RP23-149L23.1	2,416300693	0,55429
Gm17108	2,414961183	0,50623
Rell1	2,414626421	0,0014066
Rps7	2,414291706	0,12996
Rps15a-ps1	2,412786063	0,40667
Cit	2,409610564	0,13746
Ccdc62	2,408274762	0,63586
Il10ra	2,407607139	0,0020906
Lin52	2,407273397	0,32293
Mogat1	2,40094108	0,47311
RP24-351I17.3	2,400109122	0,72289
Gm4859	2,398446069	0,1204

Ube2n	2,398279828	0,073075
Higd1a	2,397614975	0,073111
Tex30	2,395953651	0,027011
Gm44168	2,395621524	0,6119
H3f3a	2,395455478	0,015514
Zfp563	2,394293478	0,42396
Gm13092	2,392634455	0,39772
Tbc1d10a	2,389485479	0,0041518
Gm15032	2,388491926	0,25296
Pex5	2,385183067	0,21803
Gm11625	2,381713699	0,73873
Hsf2bp	2,381053439	0,16486
Fam174a	2,378579095	0,0016604
Gm11652	2,375119332	0,66731
Dpf1	2,373802651	0,41906
Gm12981	2,372157827	0,80186
Bbc3	2,371500216	0,0042038
H2-DMb2	2,371335842	0,49708
Gm43096	2,370678459	0,31942
Nr4a1	2,370349836	0,30128
Mxd1	2,3690358	0,0091449
RP23-43M12.2	2,367886615	0,079435
Nkapl	2,366409911	0,58209
Dnm3	2,365098059	0,56443
Gm6543	2,36427852	0,44412
Kif18a	2,363950784	0,049333
3110056K07Rik	2,361657905	0,47388
Gm6451	2,360185085	0,21358
Gm13360	2,359040194	0,80186
Gm44126	2,355445579	0,79914
Gm5445	2,354629385	0,26405
1110006O24Rik	2,353650326	0,86987
Smc2	2,353160949	0,0013763
Tnfaip3	2,352671674	0,20068
E230032D23Rik	2,352345547	0,16589
Gm26610	2,349086763	0,59502
Gm14138	2,348110008	0,32853
Rps19-ps12	2,347621783	0,27655
Fbxo5	2,345182181	0,17374
Gm8919	2,344857092	0,73923
Hist1h1e	2,344369543	0,010254
Gm10762	2,344369543	0,37071
Gm19898	2,341121809	0,20782
Clec12a	2,340797283	0,00082093
Gm7799	2,339499629	0,27923
Gm13453	2,338851072	0,43033
Cox4i2	2,33528722	0,20685
Ndufs5	2,33528722	0,40667
Carhsp1	2,334478012	0,00071672
Etv5	2,333183861	0,10985
Gm8825	2,332537055	0,77995
Gm28686	2,332213719	0,73923

Hist1h4i	2,330274645	0,0069063
Arsb	2,327530382	0,045123
Dennd4c	2,32720774	0,037472
Ptgs2os	2,327046436	0,705
Zfp747	2,325434009	0,18959
Zfand2a	2,324144872	0,0031138
C330011M18Rik	2,323178489	0,85316
Plaur	2,323017464	0,17543
Fam103a1	2,322051549	0,18287
Ppwd1	2,321407829	0,010014
Gm43421	2,320764287	0,92474
Hist1h2bc	2,319477739	0,0054353
Gm45718	2,319477739	0,64651
Ndfip1	2,317227995	0,072917
Cdca7	2,313536724	0,10297
Epha2	2,311773413	0,10266
Gm5525	2,311452955	0,21397
Gm37420	2,310331704	0,69322
Fem1c	2,310171569	0,002011
Smagp	2,309210996	0,0019088
Gng5	2,307930852	0,083823
Gm11686	2,306171813	0,32214
Nup205	2,305532495	0,089409
Dqx1	2,304733597	0,025837
Sowahc	2,304414115	0,020931
Fbxl3	2,304094678	0,14493
Gm45250	2,300743257	0,63415
Rnf144b	2,299946018	0,11126
AA465934	2,298989696	0,13766
Gm8806	2,298989696	0,33259
Gm20517	2,296441448	0,57389
Arvcf	2,295804828	0,31303
Hsd3b7	2,295327478	0,79857
Gm10863	2,292306568	0,38868
Gm10036	2,291988809	0,14089
Pank4	2,291194604	0,59733
4921524J17Rik	2,290559438	0,033303
Rpl10a-ps2	2,290083179	0,12516
Pclaf	2,289765728	0,01233
Ddx20	2,289765728	0,014906
Bsdc1	2,287544803	0,22787
Gm10736	2,285959748	0,30352
RP23-115A18.3	2,28247649	0,40553
Gm16754	2,281369294	0,040955
P4ha1	2,279472493	0,00059042
Gm16020	2,276630249	0,39606
Rpl13-ps3	2,276630249	0,41099
Gm44545	2,273633946	0,7885
Rps3a1	2,27253104	0,032484
Med22	2,271271231	0,018631
Oaz1	2,269068244	0,13104
Tspan13	2,267810354	0,00014134

Snhg11	2,267495991	0,81942
Amd2	2,266238973	0,4921
Ptger4	2,264982651	0,0036584
Gm15159	2,26482566	0,49492
Ddias	2,262942614	0,1999
Nsa2	2,262785765	0,17136
1110035H17Rik	2,26168812	0,43802
Cbx4	2,260277646	0,020931
Zeb2os	2,260120981	0,1289
D6Ertd527e	2,259807683	0,70529
Gm5873	2,258868051	0,40589
Gm3550	2,25652068	0,23953
Etv3	2,255113428	0,01181
Wdhd1	2,252769958	0,093043
Gm7600	2,252613813	0,65194
Gm30074	2,251365045	0,8255
Tmem267	2,250740921	0,64797
Gm10240	2,250428923	0,19147
RP24-82M14.1	2,249493191	0,75762
Cxcl10	2,249493191	0,79383
Tap2	2,248401995	0,54242
Fam19a2	2,247934501	0,60313
Thap6	2,247778692	0,39606
Rps4x-ps	2,247622893	0,31476
Mcm6	2,244664768	0,01233
Samd9l	2,24326491	0,75948
Ranbp9	2,242953949	0,011917
Ccna2	2,242643031	0,03246
Eno1	2,242021324	0,12586
Pgk1	2,239846709	0,11357
Rpl26-ps2	2,23969146	0,52336
Spty2d1	2,239225777	0,0023179
Fsbp	2,239070571	0,66988
Dnajb6	2,237674202	0,0014964
Pask	2,237519104	0,72595
Mki67	2,237053873	0,038809
Prc1	2,233799966	0,005061
Rgs2	2,233645136	0,00090905
Osm	2,233025924	0,44839
Rps13-ps5	2,232406884	0,44433
Rps19-ps7	2,231478645	0,219
2700099C18Rik	2,231169317	0,70887
Gm13094	2,230241593	0,64126
Gm7984	2,229932437	0,75203
Gm8292	2,229777876	0,054383
Gm45546	2,229468784	0,80295
Gm20620	2,229159736	0,16545
Rpl19-ps1	2,228541767	0,14042
Gm13680	2,226688889	0,55025
Gm5787	2,226688889	0,82568
RP24-454N4.2	2,226534552	0,044487
Rpl21-ps1	2,225608755	0,94291

Vsig8	2,223758315	0,81662
Ambp	2,223450058	0,67829
Mkrn2	2,22206343	0,041582
Anln	2,220369837	0,11342
Pcif1	2,219292768	0,0053384
Ywhah	2,21898513	0,076879
Ank3	2,216218307	0,9873
Gm11737	2,215296799	0,46634
Btg2	2,214836189	0,064249
RP24-84C23.4	2,212381219	0,054872
Gm26202	2,211614599	0,73518
Cd83	2,209316332	0,0015993
Gm37009	2,209316332	0,5372
Cenpn	2,209163199	0,47073
Kdm3a	2,208550774	0,053255
Lbr	2,208397694	0,0007815
Tgfb1	2,204726961	0,02235
Tor1aip2	2,204421342	0,0015939
Gm17251	2,203962994	0,57826
Gm14513	2,201825292	0,35724
Hus1b	2,200757219	0,86549
Bub1b	2,200147124	0,01032
Col11a2	2,199994627	0,37622
Rpl38-ps1	2,19984214	0,054433
Tpt1-ps3	2,197099185	0,60056
Gm37747	2,197099185	0,80902
RP23-159E10.1	2,195728991	0,86836
Rcbtb2	2,19542462	0,030849
Tmem29	2,19512029	0,24605
Ccdc163	2,194968141	0,066535
Tslp	2,194207555	0,54763
Gm15694	2,1932952	0,49446
BC030867	2,193143177	0,83832
Gm11964	2,192991165	0,53695
Gm8574	2,192839164	0,82289
Arl4c	2,192687173	0,0037131
Mcm7	2,191927377	0,038024
Gm12577	2,19177545	0,27756
Rac3	2,191471626	0,7673
Sag	2,190104942	0,21956
D830050J10Rik	2,188587403	0,29309
Gm6378	2,188284021	0,38995
n-R5s151	2,188284021	0,72967
Snord110	2,185858482	0,82814
Slc22a13b-ps	2,185101052	0,86386
D130020L05Rik	2,184041091	0,48143
Supt7l	2,181317839	0,046816
Stil	2,180561983	0,32988
Gm8618	2,179353158	0,11499
9230116N13Rik	2,177994031	0,6077
Per1	2,177541177	0,020485
Mcts2	2,176484883	0,27329

Dyrk3	2,176334026	0,016938
4930412F12Rik	2,172415407	0,82359
Cog3	2,1713616	0,61387
RP24-365N15.9	2,171060606	0,86911
RP23-70B19.5	2,170910125	0,39606
Bloc1s1	2,16970665	0,72234
Eno2	2,169105163	0,016412
Raf1	2,16790269	0,18337
Rps23-ps2	2,167151482	0,20245
4931428F04Rik	2,166400535	0,54966
Rpl30-ps9	2,162349913	0,69765
Gm1862	2,162050169	0,57845
Gm12902	2,160851606	0,38995
Mical1	2,160102841	0,75972
Gm37795	2,158456473	0,47
Haus6	2,158306865	0,028845
Gon7	2,157409437	0,012268
Hs3st3b1	2,156661866	0,66145
Lpl	2,15636291	0,0021218
RP24-550H10.3	2,153823456	0,40208
1700001G11Rik	2,153524893	0,74871
Gm38235	2,153375627	0,93593
Abcg4	2,152331056	0,77224
Gm19287	2,151734386	0,73213
Rgs1	2,151137881	0,034721
Ccdc58	2,150988781	0,059463
Gm4866	2,146966976	0,48688
Nrxn3	2,145479327	0,64126
Bnip3l	2,145181921	0,068046
Rrm1	2,144141325	0,022046
Slc25a30	2,143546925	0,73565
Gm44791	2,142804157	0,71083
Sertad3	2,141913175	0,040104
Meg3	2,140874164	0,77965
Plau	2,139094176	0,029994
Gm43581	2,13894591	0,29228
Rpl3-ps2	2,137760156	0,43031
B3gat3	2,137315667	0,037753
Cdkn2c	2,136426967	0,23074
Rccd1	2,135538637	0,31256
Birc5	2,134798644	0,0066964
Racgap1	2,133910991	0,033608
Cdc42ep3	2,133023707	0,32366
Calm1	2,131693472	0,00018233
Smc4	2,130954811	0,0055923
Csrp1	2,130068757	0,038217
Gm4468	2,128445279	0,3087
Selenbp1	2,126086066	0,10805
Gm45311	2,125644005	0,71083
Ppp1r2	2,125496672	0,038549
RP23-447C2.2	2,125496672	0,37542
Gm5822	2,125349349	0,80794

Gm17786	2,125202036	0,73046
Gm15393	2,124907441	0,77182
Ppp1r15a	2,124612886	0,033777
Spc25	2,124612886	0,075012
Gm43524	2,124465625	0,69705
Gm7514	2,124465625	0,86237
Spry2	2,124318373	0,47334
Fgd2	2,123287897	0,45998
Rpl18	2,122993568	0,024928
Gm15207	2,122699278	0,75393
Fanci	2,122552149	0,82039
Rps19-ps8	2,121963734	0,84095
Gm12504	2,120934401	0,62497
Tmem64	2,119317886	0,003115
Gm10335	2,119170991	0,47599
Gm2223	2,118730367	0,24461
9330020H09Rik	2,11755582	0,3263
RP23-312A24.1	2,11755582	0,5083
L1cam	2,117409047	0,54242
Prkd2	2,115795218	0,070244
Zfp932	2,115208677	0,19066
Gm42850	2,114036081	0,69584
RP24-240E7.1	2,113303539	0,33721
Cdc42ep4	2,112717688	0,076879
Mb21d1	2,112571251	0,0021547
Gm14006	2,112132	1
Gm12543	2,111546473	0,5494
Klf6	2,110522193	0,026327
Fam13b	2,109644634	0,062323
Gm12038	2,109644634	0,31557
Mybl2	2,10949841	0,44211
Gm4342	2,108475123	0,65434
Ndc80	2,107452333	0,25512
Tpi1	2,106138045	0,0031892
Gm9762	2,105846093	0,11474
Rpl36	2,104241078	0,1462
Sfn	2,103657739	0,52335
Gm6548	2,10336613	0,48384
Plekhf2	2,102637285	0,042873
Gm36189	2,102491547	0,31156
Gm5881	2,102345818	0,16777
Ifi213	2,100597863	0,088503
Tsg101-ps	2,099578894	0,80295
Taf5	2,098705885	0,22349
Gm5276	2,098414963	0,64784
D030056L22Rik	2,097687834	0,018374
Ska3	2,094781836	0,37542
Rps10-ps2	2,094491457	0,21809
Klhl15	2,094055965	0,13711
Rps8-ps3	2,090430385	0,48384
Gm4525	2,089995738	0,39344
Gm3695	2,089850875	0,83477

Rdh12	2,089271526	0,92368
Gm17150	2,088981911	0,80366
Ccne2	2,087534441	0,7264
RP24-131G14.13	2,085798801	0,30149
Rpl36-ps8	2,085509668	0,85316
Rassf2	2,085076043	0,0090827
Mapre2	2,08478701	0,0077624
Gm10263	2,083486858	0,87413
Tmem120b	2,083053654	0,42774
Gm6136	2,082909273	0,35633
Blm	2,08262054	0,099064
Rps12-ps23	2,08262054	0,83477
Dpep2	2,081610292	0,13224
Eno1b	2,080456322	0,47261
Gm5576	2,079014759	0,49059
Gm13204	2,077862228	0,70884
Npm3	2,077286202	0,15798
Gm20673	2,077286202	0,5616
Gm8254	2,077142221	0,66723
Gm44164	2,075990728	0,89813
St3gal6	2,075559082	0,0090827
Gm9385	2,075415221	0,0078868
Lpar6	2,073689657	0,040925
Gm12912	2,073689657	0,63415
9930022D16Rik	2,072252782	0,43529
Gm7312	2,071391136	0,50795
Rassf1	2,070960447	0,013854
Rpl13a-ps1	2,070386334	0,074417
Rpl36-ps10	2,069812381	0,070748
Papd5	2,069668918	0,11347
Traip	2,069238587	0,37702
Dis3	2,067518158	0,23031
Eif1b	2,066515235	0,003983
Gm8973	2,066228775	0,82612
mt-Tp	2,06608556	0,31812
Fam187b	2,064653955	0,85319
2810454H06Rik	2,063795468	0,65336
Plin2	2,063652422	0,019581
Pcgf5	2,063509385	0,55374
Ccdc50-ps	2,063080335	0,24277
Rbm4b	2,062079565	0,19066
Gm6023	2,061650812	0,11327
C630043F03Rik	2,059651144	0,77606
Gm13935	2,059651144	0,87651
Gm29994	2,059080167	0,79188
Gm13461	2,058652038	0,5806
Rpl21-ps6	2,058366668	0,25073
Gm11675	2,058223998	0,38345
Gm15464	2,05679784	0,07641
Erf	2,05537267	0,016341
Gm12762	2,055230207	0,57229
Arf2	2,054518042	0,004368

Asb11	2,052383027	0,61923
Rpl35	2,051956291	0,56732
Gm43309	2,051387446	0,14692
Gm10923	2,051387446	0,54033
Dhrs3	2,051103083	0,20411
Gm15484	2,050960917	0,73565
Ptpru	2,049255685	0,9327
Usp1	2,048829598	0,015401
Gm5830	2,04854559	0,74046
Ccsap	2,048261621	0,38345
Gm5578	2,048261621	0,80509
Gm12481	2,04740995	0,077691
Rpl36a	2,046984247	0,15022
Gm43273	2,046842366	0,99829
Dnajb4	2,046558633	0,014239
Gm9294	2,046558633	0,55583
Gm6987	2,046133107	0,37976
Gm8995	2,043865131	0,28964
Kif24	2,043581811	0,68103
Ubb	2,043440165	0,024302
Kpna4	2,042590499	0,027011
Gm14427	2,042448923	0,41952
Bend3	2,041741186	0,70518
Anxa2	2,040892227	0,028817
Ncapg2	2,040892227	0,16021
Ccdc18	2,040892227	0,50908
Rpl12	2,03990222	0,23053
Gm10941	2,039336717	0,72155
H60c	2,037923646	0,75315
Icam4	2,036511555	0,35282
Gm9711	2,0363704	0,8107
Eif4a2	2,035241509	0,20782
Gm10051	2,035241509	0,7884
Rpl23a-ps5	2,034818336	0,50598
Gm43721	2,034677298	0,83477
Rpl18a	2,03453627	0,36121
Gm12230	2,034113244	0,58811
Mir124-2hg	2,033690306	0,79942
Tnfsf12	2,032844694	0,14626
Gm21057	2,031295318	0,75393
Brca1	2,030732202	0,46778
Khk	2,029325093	0,023674
Llph	2,028621905	0,056966
Cd274	2,02749731	0,41099
H2afv	2,026654272	0,037
Arl4d	2,026654272	0,66934
Vps8	2,026232885	0,27502
Coprs	2,026092442	0,066666
Rpl18a-ps1	2,025109615	0,32749
Gm2367	2,024688549	0,83487
Kbtbd8	2,02426757	0,7885
Gm4374	2,023986967	0,44737

Ptchd1	2,02384668	0,04164
Rps29	2,023706402	0,26405
Gm12943	2,02300516	0,43878
Gm5599	2,022584532	0,51724
H2-K2	2,022304162	0,32672
Hspa8	2,02202383	0,69
Rpl27a-ps2	2,021883679	0,57229
Gm38022	2,02076282	0,79489
Gm13464	2,020342657	0,8538
Plekhh3	2,020202623	0,17374
2410022M11Rik	2,019782577	0,21183
Gm13445	2,019362619	0,81588
Gm6640	2,01880281	0,65832
Bach1	2,018662882	0,076879
Snapc1	2,017403968	0,012041
3110031N09Rik	2,016984504	0,73923
Gatsl2	2,015586921	0,025137
Adcy6	2,015307521	0,3907
Mfsd2a	2,0139111	0,3478
Gm12267	2,013771511	0,62338
C920021L13Rik	2,012934181	0,10554
Gm3511	2,012655149	0,21208
Gm15727	2,011957737	0,35003
Gm12468	2,011260566	0,87651
Sumo1	2,01042428	0,046966
Gm5835	2,009727641	0,44664
H3f3b	2,009309773	0,1158
Gm16630	2,009309773	0,90187
Rpl23a	2,008891992	0,37954
Gins2	2,006943497	0,33083
Ezh2	2,006804391	0,022796
Sap18	2,005552872	0,26875
Spag5	2,004996892	0,35302
Gm7224	2,003607615	0,86083
Gm9320	2,003052174	0,1999
Gm5139	2,001941754	0,92567
Spink10	2,001386775	0,11499
Gm8444	2,001109343	0,51095
Dab2	2,000970642	0,022013
Nr2c2ap	2,000138634	0,079056
Gm42548	1,997367774	0,81437
Gm15703	1,994324262	0,92225
Gm6394	1,993633202	0,2036
Gm9354	1,99321868	0,90186
Vezt	1,992942381	0,13712
Dok2	1,992389896	0,020483
Gm7658	1,991561457	0,058355
Gm7308	1,989905611	0,30959
2410004B18Rik	1,988526792	0,13037
RP23-288C18.3	1,98783774	0,40667
Gm11942	1,986735755	0,25913
Gm12182	1,985359132	0,69765

A930007I19Rik	1,984671178	0,40667
Gm10126	1,983295985	0,324
Gm17039	1,983295985	0,94277
Mad2l2	1,983021061	0,22757
Cenpe	1,982471327	0,024001
Rpl9	1,982333917	0,12996
Rps27a	1,981234983	0,3813
6820402A03Rik	1,981097659	0,95084
Gm14636	1,980823039	0,36383
Slfn3	1,980823039	0,74156
Gm45733	1,980411182	0,87703
A430018G15Rik	1,979313313	0,5494
RP23-162P10.8	1,978353177	0,25462
Gm5910	1,976571303	0,84332
5830454E08Rik	1,976434302	0,48336
Gm7638	1,976160329	0,36025
Gm14620	1,976023357	0,047714
Cnksr3	1,975475563	0,3697
Tnfrsf1b	1,974654157	0,12586
Tia1	1,973696281	0,21873
Cd9	1,971918601	0,55156
Gm14336	1,971918601	0,63654
Rps15a-ps4	1,971918601	0,7574
Aplf	1,970415661	0,49364
Eif3m	1,970279087	0,3332
Syap1	1,969869422	0,24581
Mef2c	1,969323334	0,01404
RP24-496O17.7	1,969186836	0,84553
Rpl31-ps13	1,968095189	0,32646
Gm14140	1,968095189	0,91371
Ly6g6d	1,967413218	0,7765
Gm5124	1,96686781	0,90293
Pcgf1	1,965913712	0,55363
Klhl28	1,964687693	0,29193
Gm12501	1,963054192	0,33259
Cdkal1	1,962646028	0,31265
Gm43637	1,962646028	0,70887
Hist2h2ac	1,961422048	0,96401
Ftl1	1,961286097	0,69074
Arhgap11a	1,961150156	0,14498
Glt1d1	1,961014224	0,73621
Zfp87	1,960878302	0,13955
Gm43800	1,959791261	0,88461
Ptgs2	1,959655424	0,17998
Gm5644	1,959519595	0,57229
Prr18	1,959247967	0,36756
Xirp1	1,959247967	0,82021
Ckap2l	1,95789039	0,21783
Gm11464	1,956669376	0,97078
Hoxb6	1,95626254	0,73425
Srfbp1	1,955584669	0,11718
Thap1	1,955449122	0,17962

Eef1a1	1,954500563	0,54033
Fmr1	1,95422963	0,04862
Matr3-ps2	1,953146276	0,70877
Gm5070	1,952604824	0,80794
Ulbp1	1,952198833	0,079056
A130014A01Rik	1,951116604	0,5168
Gm7847	1,950575715	0,74156
Klhl25	1,949494386	0,25899
Gm8722	1,948143567	0,64412
Gm42535	1,947198552	0,77182
Gm17511	1,947063587	0,55976
Chmp1b	1,946928631	0,021035
Gm13758	1,946928631	0,74452
Ncapg	1,945984205	0,29387
Gm14593	1,945849324	0,66723
Nfkbib	1,945444738	0,26739
Cox19	1,945175061	0,11174
Gm13392	1,944501031	0,79188
Sdhd	1,944096726	0,74806
Rps17	1,943961976	0,40199
Lsm3	1,943692504	0,25481
Senp3	1,943557782	0,29382
Bnip3	1,942480344	0,044168
Vps37b	1,942480344	0,067045
Cxcr4	1,940596264	0,036869
Gm527	1,939923821	0,84297
Gmfg	1,939520467	0,77995
6330403N20Rik	1,937236378	0,90171
Mmaa	1,936699337	0,68573
Bcl6b	1,936028244	0,99943
B4galt3	1,93522324	0,039563
Lzic	1,934418571	0,37271
Gm13456	1,931872658	0,55114
Calr-ps	1,931337103	0,032023
Ncapd3	1,930935535	0,21956
Trim13	1,930667869	0,32081
Gm10269	1,929463831	0,22146
9330175E14Rik	1,929330096	0,67815
Gm9009	1,928795246	0,88078
Tsc22d2	1,928260544	0,040468
Gm32340	1,928126892	0,75624
Tmem176a	1,927725991	0,15834
Gm13340	1,927325173	0,82186
Fbxo33	1,927058008	0,16189
Cdkn2d	1,926523788	0,04164
Dram1	1,926256734	0,20824
Ppp1r35	1,925989716	0,70884
Nuak2	1,925722736	0,1169
Hyal1	1,924655184	0,23952
Ercc1	1,924255005	0,32058
Gm8662	1,924255005	0,41953
Cd40	1,924121631	0,18432

Fanca	1,923321577	0,6119
Skp2	1,922788393	0,22055
Irx2	1,920657135	0,36648
Dkkl1	1,920390894	1
Rnf185	1,919991601	0,27336
Gm8508	1,918794222	0,85212
Gm1840	1,917996383	0,37071
Tubb2a	1,917331772	0,073111
Nudt8	1,91666739	0,83798
Riiad1	1,91666739	0,93125
Dgke	1,916136051	0,18931
Gm6198	1,915737643	0,92681
Prim1	1,915073814	0,50058
Rab20	1,914808347	0,33265
Papd4	1,914808347	0,63992
Gm7784	1,914410215	0,99411
Lat	1,913879501	0,8686
Spata2	1,913746846	0,71007
Rnf25	1,9136142	0,6681
Litaf	1,912685934	0,0044649
Rhoc	1,912420797	0,0088266
Gm18889	1,911625609	0,34988
Gm44292	1,910565873	0,79427
Atl2	1,909109694	0,052338
Pam16	1,909109694	0,70529
Snx18	1,908977369	0,11279
Zfp949	1,908845054	0,48343
Rdh10	1,908051353	0,32402
Rpl5-ps1	1,907390187	0,68836
Eif3s6-ps1	1,906993598	0,11499
Zfand4	1,906861419	0,84215
A930029G22Rik	1,905672228	0,9671
Gm11531	1,905011887	0,35455
Hmgb1-rs16	1,904879846	0,91371
Gm11450	1,904087794	0,66723
Manbal	1,902768438	0,11327
Gm45153	1,901977263	0,71502
Ska2	1,90065937	0,54085
RP23-187B11.16	1,90065937	0,817
Gm8268	1,90026418	0,67874
Gm16372	1,899869072	0,86096
Metrnl	1,899605713	0,035356
Socs4	1,89934239	0,23946
Rpl36a-ps2	1,898947474	0,2142
Rbm47	1,898815853	0,071361
Rpl28	1,898289463	0,50822
Gm10784	1,898289463	0,9306
Cnih1	1,898026322	0,078894
Dennd4a	1,896974125	0,066894
Ip6k2	1,896711166	0,14167
Gm5121	1,896711166	0,4674
Gm8667	1,896316797	0,84095

Rps23	1,896185359	0,36091
Dusp2	1,8957911	0,35703
Ppp1r13l	1,893821031	0,37542
Tpx2	1,893558509	0,11042
Gm11605	1,893164796	0,97112
Gm37383	1,893033576	1
Gm37510	1,892508789	0,9723
Nusap1	1,89159076	0,15363
Gm6493	1,89145965	0,70884
Anp32-ps	1,89145965	0,89073
Gm15541	1,891066373	1
Rbl1	1,890935299	0,38763
Rpl18-ps2	1,889494082	0,31065
Il27	1,889363117	0,8255
Ssfa2	1,889232161	0,16777
Rps28	1,887792242	0,39772
Gm14830	1,887661395	0,72558
Gm26912	1,887530557	0,93159
Itgam	1,885046354	0,37238
Tk1	1,884785049	0,67493
Cbwd1	1,88452378	0,42801
Hikeshi	1,88452378	0,46706
Med6	1,884262548	0,18143
Gm20689	1,884001352	0,73923
Casz1	1,88321798	0,40891
Rpph1	1,882956929	0,84945
Luc7l3	1,879957438	0,14884
Cdc45	1,879957438	0,69227
Txlng	1,879827134	0,50794
A930015D03Rik	1,879827134	0,76894
Smad7	1,879306008	0,2862
8030453O22Rik	1,878654803	0,82802
Crebrf	1,878524589	0,070176
Zfp655	1,878394384	0,10622
Ppp1r16a	1,878134001	0,49364
Chic2	1,877613344	0,068046
Xxylt1	1,877483202	0,69787
Gm11560	1,877222946	0,23426
RP23-114G13.1	1,876182281	0,94291
Igfbp4	1,876052238	0,032402
Gm20492	1,87579218	0,93967
Sema4c	1,875402161	0,25479
Gm11970	1,875142193	0,23952
Gm6745	1,874492431	0,99103
Gm43578	1,874362505	0,93017
Gm4784	1,872155151	0,68497
Gm45445	1,871895633	0,84215
Hbegf	1,870987604	0,38297
Gm12732	1,870857921	0,47615
Btbd7	1,870857921	0,56608
Hip1r	1,870598583	0,77204
Dalrd3	1,870339281	0,37811

Gm37065	1,869950396	0,94673
Fancd2	1,869561591	0,6119
Gm15289	1,869561591	0,91492
Rpl41	1,869432007	0,11253
Gm37352	1,86904331	0,78788
RP23-213P10.2	1,868525173	0,91469
Gm2735	1,868266158	0,7885
Nup98	1,867877704	0,5027
Arid3b	1,867748237	0,60982
Atxn7l2	1,86748933	0,71083
Fam64a	1,867230459	0,36548
Nedd4l	1,866971624	0,38318
Dmrt2	1,866195333	0,73535
Napsa	1,864772973	0,023304
Spsb3	1,864385245	0,70612
Gm24951	1,86373921	0,14016
2610203C20Rik	1,86361003	0,5494
Alox8	1,863351696	0,96196
Gm19196	1,862964264	0,57614
Upf2	1,86270602	0,11253
Tmsb10	1,86270602	0,62272
Clu	1,862318721	0,15022
Gm27605	1,861802448	0,62836
Lilr4b	1,861415338	0,29191
Hmgb3	1,861286319	0,49809
Pole2	1,860512393	0,78016
RP23-324E2.11	1,860512393	0,93898
Dusp10	1,86012555	0,66726
Gm5905	1,859352106	0,54402
Rpl5	1,858965505	0,24425
Mbip	1,858707816	0,46201
Taf6	1,857806184	0,13844
Chrnb1	1,857419904	0,73142
Cldnd1	1,856132885	0,36746
Pdcd7	1,855746953	0,20245
Usp53	1,855746953	0,21224
Tpt1	1,855103911	0,19294
Rpl23a-ps3	1,855103911	0,51095
Dleu2	1,854332556	0,5057
Pnp	1,852662385	0,40381
H2-T22	1,85240557	0,50719
Tmed7	1,851122025	0,35546
Ppard	1,850480586	0,37288
RP24-378K7.3	1,850352325	0,92593
Atp11a	1,848301356	0,33528
Gm8731	1,848045145	0,51095
Nxt1	1,847660895	0,16421
Rrm2	1,847660895	0,35087
Gm7783	1,847404772	0,706
Rpl36-ps3	1,847148685	0,54745
Gm13436	1,847148685	0,56366
Gm14633	1,846764621	0,87906

Tmem240	1,846508623	0,81389
Gm5239	1,845868783	0,82256
Gm16106	1,845740842	0,91371
Tusc3	1,845101267	0,16589
Dusp4	1,844589768	0,11499
Gm8318	1,843950592	0,87854
Slfn2	1,843567193	0,096215
Gm15198	1,843183874	0,28876
Cpeb2	1,842162079	0,32081
Tmx2	1,842162079	0,49003
Wdr20	1,842034395	0,36548
Gm5586	1,842034395	0,72236
Neil3	1,841651394	0,81414
Hexim1	1,841396105	0,31564
Gm13890	1,840502871	1
N4bp3	1,839482562	0,71502
1110003F10Rik	1,838845157	0,81662
Rps12-ps4	1,838590256	0,39065
Zfp280b	1,837443642	0,40086
Ints8	1,836679629	0,44658
Gm44567	1,835915934	0,78473
Sp140	1,834516652	0,34494
Etfdh	1,833499662	0,34274
Dennd5a	1,833372578	0,1038
Gm38375	1,832991378	0,95169
Gm10260	1,832610258	0,57046
Creb3	1,832483236	0,08238
Id3	1,832102221	0,39606
Gm20274	1,831848255	0,77524
Gm14173	1,831594325	0,717
Gm27039	1,83134043	0,99796
Hist4h4	1,83134043	0,99974
Gpam	1,830832745	0,92368
Gemin6	1,829564148	0,77961
Rnu11	1,82918374	0,84315
Polg2	1,828803412	0,42108
Uba3	1,828169706	0,3006
Pmf1	1,827029591	0,23248
Gm45185	1,826776328	0,84801
Cebpb	1,826269908	0,70374
Gm6204	1,825763629	0,23328
Gorasp1	1,825510542	0,39025
Gm8116	1,825510542	0,82814
Bicd2	1,82525749	0,15267
Gm38365	1,824877978	0,79857
2310022A10Rik	1,823739915	0,073075
Gm14126	1,823487108	1
Rpl10-ps2	1,822223601	0,78048
Zfp773	1,821971005	0,61924
Adgre1	1,82184472	0,077539
Rnf2	1,821592176	0,10452
Fam83a	1,821465917	0,74554

Gm37677	1,821465917	0,86549
Gm44254	1,821465917	0,95244
Rpl27-ps3	1,821213426	0,68729
Gm43137	1,820456162	0,92681
Gm14286	1,819951494	0,68641
Esco1	1,819573085	0,59592
2010015M23Rik	1,819446967	0,56087
1810026B05Rik	1,818186259	0,34795
Gm11808	1,817430254	0,11295
Sft2d1	1,81680049	1
Gm6807	1,815038311	0,49003
Magoh	1,814912506	0,077934
Tra2a	1,814912506	0,11912
Rab10os	1,814912506	0,13121
Gm43655	1,814912506	0,93967
Cd9-ps	1,814660923	0,98408
Arhgap25	1,814409375	0,21543
Cadm1	1,814283614	0,026474
2700038G22Rik	1,814283614	0,91912
Tob2	1,813152158	0,12412
Pth1r	1,813026485	0,94574
Rpl27	1,812398246	0,64499
Gm11334	1,812021408	0,84095
Gm7407	1,811519078	0,46719
Tcf20	1,810138391	0,16889
Gm2058	1,809260319	0,57229
Cdkn2aip	1,808884133	0,34739
Zfand5	1,808508025	0,1343
Stk17b	1,80800667	0,21103
Gm42743	1,807630745	0,85316
Gm14706	1,807380171	0,47599
Zbtb11	1,807254897	0,18287
Tmem170b	1,807129632	0,43003
Cnr2	1,807129632	0,56675
Stxbp3	1,805501977	0,7264
Gm9761	1,805501977	0,95266
Plekhh1	1,804250932	0,98755
Gm6159	1,804125875	0,31831
D8Ertd738e	1,804000827	0,10946
Gm15421	1,803875788	0,8602
4930427A07Rik	1,803500721	0,92355
N4bp2	1,80237599	0,4681
Slc19a2	1,80237599	0,69765
Gm7785	1,80237599	0,74266
Coq6	1,801876334	0,59333
Gm24916	1,801876334	0,60005
2700097O09Rik	1,80100227	0,69344
Prps1l3	1,800128631	0,79857
Gm12854	1,799879097	0,95793
Cycs	1,799255415	0,45627
Gm5614	1,799006002	0,71657
Anxa3	1,798756624	0,11253

Gm5384	1,798507281	0,80794
Rab26os	1,798257972	0,34795
Gm2214	1,79713651	0,73923
Zc3h7a	1,796389258	0,17751
Ddx23	1,795642316	0,21956
Ralb	1,795020101	0,1498
Gm4987	1,794273729	0,72236
Pdcd2l	1,794149363	0,35136
Asf1a	1,793154752	0,21929
Ptpn2	1,792906185	0,52049
Rusc2	1,792284918	0,5989
Fam134c	1,792160691	0,47388
Rpl6	1,791912262	0,37622
Gm8019	1,79178806	1
Atp7a	1,791415507	0,27756
Gm5841	1,791167182	1
Lmnb1	1,790670633	0,25531
Gm10080	1,790546518	0,79653
Snrpg	1,789429865	0,20782
Gm13532	1,789181815	0,76273
Pim3	1,789057802	0,085263
Arhgef17	1,788685817	0,90611
Leng9	1,78843787	0,7825
Gm37082	1,78843787	0,97003
Arrdc3	1,788066013	0,5168
Gsk3a	1,78682705	0,21508
Sp4	1,786331705	0,54627
Gm13039	1,785712717	0,55241
Rnf7	1,785217681	0,24306
Ak4	1,784599078	0,15432
Gm9645	1,784351698	0,67382
Nop10	1,78422802	0,087548
Oaz1-ps	1,78422802	0,89574
Whamm	1,783733396	0,35455
Piga	1,783486135	0,30294
1700084E18Rik	1,783238908	0,69705
Gm9434	1,783238908	0,95195
Gm13573	1,782620992	0,91958
Gm9378	1,782250345	0,85316
Gm6612	1,782003289	1
Rpl19	1,780645098	0,42774
Cdc20	1,780521677	0,30959
RP23-356D13.11	1,78002808	1
Snhg8	1,779781333	0,50503
Gm18737	1,779781333	0,84095
Ddx47	1,777931819	0,088156
Gm2962	1,77743894	0,59589
Kif14	1,777315741	0,49839
Samd8	1,776946197	0,13701
Gm12944	1,77657673	0,87354
E130102H24Rik	1,775838026	0,78361
Rpl36a-ps3	1,774976593	0,8764

Phf20l1	1,77436154	0,26766
Gm6501	1,774238555	0,92008
Gm12924	1,773623757	1
Map3k1	1,773500823	0,19987
Slc16a1	1,773132073	0,2138
Bcl6	1,772886281	0,23953
Tdp2	1,772886281	0,30959
Man1a	1,772763398	0,50469
Osgepl1	1,772394801	0,8094
Kpna3	1,772149112	0,14407
Gm9169	1,771166696	0,6627
Gm11249	1,770798431	0,84175
Abt1	1,770430242	0,64028
Coro1a	1,769203498	0,41952
Snrpert	1,768222715	0,48789
Rps12-ps9	1,768222715	0,53972
RP23-38L16.3	1,767855062	0,717
Gm9625	1,766630103	0,33883
Rpl21	1,766385213	0,46706
Cenpp	1,766385213	0,72236
Gm5321	1,766262781	0,82252
Uhrf2	1,765528365	0,066456
BC022687	1,765528365	0,63899
4933404O12Rik	1,765405993	0,54383
Gm13886	1,765405993	0,70529
Uimc1	1,764305018	0,23154
C330027C09Rik	1,764060451	0,51997
Gm13443	1,76393818	0,89206
5730508B09Rik	1,763693663	0,58036
Grcc10	1,763082519	0,91332
Trp53rkb	1,76210513	0,57229
Rubcnl	1,76210513	0,62619
Lnpep	1,761860867	0,50634
Gm17994	1,761860867	0,91946
Rpl26-ps4	1,761494536	0,95583
Ss18l1	1,760884155	0,46556
Rps27rt	1,760396002	0,33143
Rps15-ps2	1,759664026	0,85948
Gm9435	1,759542059	1
RP23-308G10.5	1,759542059	1
Nck1	1,758688531	0,34501
Cks1b	1,758444742	0,1046
Hsp90aa1	1,758079122	0,27608
Gm7027	1,757469924	0,6935
Snord65	1,757469924	0,84032
Gm7899	1,757348109	0,75193
Cnot3	1,756982717	0,26035
Gm22009	1,756982717	0,82359
9130230L23Rik	1,756008709	0,94347
Gm13022	1,754548709	0,88234
Klkb1	1,754305494	0,90805
Hnrnpa1	1,754062313	1

Gm5865	1,753819165	0,58209
H2-Q7	1,752725417	0,41433
Rps12-ps24	1,752725417	0,71717
Kif23	1,752360985	0,14542
Gm10076	1,752360985	0,28253
Bard1	1,752360985	0,80235
Tpst2	1,75199663	0,37455
Gm14794	1,751632351	1
Numb	1,751146762	0,50169
Golga7	1,750782659	0,58937
1500011K16Rik	1,750418632	0,50832
Gm12430	1,749690805	1
Wdr70	1,748357241	0,46141
Gm6142	1,748236058	0,3097
Gm44044	1,74787256	1
Arl1	1,746782521	0,29132
C920009B18Rik	1,746661447	0,5168
Top2a	1,745935182	0,21895
Gm6525	1,745088255	0,89188
Tecpr1	1,744967299	0,14414
Slc8b1	1,744846351	0,53411
Inpp5f	1,744362644	0,57432
Cygb	1,744362644	0,85316
Slc36a3os	1,743033141	1
Cdk9	1,742308384	0,50808
Rwdd1	1,742308384	0,73923
Gm10689	1,741463216	0,40088
Brms1l	1,740739112	0,71204
Cmtm6	1,740256544	0,17554
Gm12834	1,739412371	1
Polr2d	1,739291808	0,65969
Gm42549	1,739291808	1
Gm21781	1,738568608	0,83103
9930120I10Rik	1,738568608	0,89188
Arhgap17	1,738327608	0,22349
Zfp595	1,737725253	0,78428
Gm8430	1,737363941	0,39791
Gm44093	1,736039772	1
Prr3	1,735919443	0,78256
Gm16046	1,735438211	0,90781
Gna12	1,735197645	0,49327
Gm5451	1,734716613	0,49035
BC030336	1,734476146	0,31072
Mex3c	1,734355926	0,48485
Hmga1-rs1	1,733394461	0,74156
Gm2830	1,732673713	0,68673
Hax1	1,732313451	0,9327
Tacc3	1,731833218	0,57636
Timm22	1,730873152	0,41489
Zfp637	1,730873152	0,62249
Gm6305	1,730753181	0,92205
Gm10657	1,730753181	1

Pttg1	1,730633219	0,3697
Gm11539	1,730273381	0,26081
Nab2	1,730153452	0,4191
Gm9835	1,729913618	0,86788
Gm42666	1,72931418	0,92205
Fam20c	1,728475315	0,20953
Vamp2	1,728235714	0,55203
Ifnar1	1,727876375	0,47299
Gm4875	1,727636857	1
Plp2	1,727397372	0,5168
Gm15772	1,726679117	0,47877
Dnajb1	1,726679117	0,48343
Gm9701	1,726679117	0,53891
Hoxa5	1,726439765	0,79653
Anp32a	1,725961161	0,14087
Pkn2	1,725363092	0,16889
Ube2f	1,72500435	0,19254
RP23-354J5.3	1,72500435	0,70884
Rps15a-ps5	1,72500435	0,87509
Dbr1	1,724645683	0,33083
Mcm2	1,723809082	0,45998
Dnajc19	1,72273405	0,92368
Ccl3	1,722495245	0,31807
Gm10086	1,721898377	0,31812
Gm5069	1,721421031	1
Atg12	1,720228247	0,077691
Cdca5	1,718797996	0,71386
Slc16a10	1,71832151	0,32293
Rps15a-ps7	1,71832151	0,89188
Klf3	1,718202409	0,45409
Dut	1,717726088	0,30119
Gm13050	1,717607029	0,84544
Speer9-ps1	1,717130873	1
Daglb	1,716416887	0,26962
Zfyve26	1,716060005	0,53297
Vhl	1,715941061	0,33659
Rnaseh1	1,715941061	1
Cd200r3	1,715346464	1
Gm43817	1,714157888	0,8686
Hn1l	1,713801476	0,71502
Ftsj1	1,713682689	0,58865
Pax3	1,713682689	0,59357
Siva1	1,713088875	0,4518
Gm26244	1,710715675	0,74381
Ptp4a1	1,710478535	0,38995
Bloc1s4	1,710122888	0,37455
Tmod3	1,709648807	0,1997
Gpr137b	1,709411816	0,3342
Cdca8	1,708819482	0,19518
Hat1	1,70870104	0,19489
Chka	1,708227353	0,46376
Gm15779	1,708108951	0,90984

Gm6743	1,707872174	0,99218
Rab4a	1,707635429	0,85354
Iffo1	1,706925391	0,67074
Gm15163	1,706925391	0,99628
Efhd2	1,706333918	0,13712
3110009E18Rik	1,706333918	0,6327
Ss18l2	1,70574265	0,46244
Syne3	1,705624421	0,39606
Btaf1	1,705151587	0,6119
Dclre1a	1,705151587	0,84945
Gm14813	1,70491522	1
RP23-413G8.2	1,704678884	0,89188
Hspe1	1,703733872	0,33083
Clspn	1,703733872	0,68836
Gm11687	1,703615782	0,77995
Xlr	1,703379627	0,63586
Gm26799	1,703261562	0,83657
Ccdc84	1,702317335	0,73761
Gng12	1,701845418	0,13766
Erbin	1,701609508	0,25479
Gm14769	1,701609508	0,72153
Enc1	1,701255705	0,29228
Zbtb43	1,700430451	0,34031
Terf1	1,699369998	0,7112
Prrg4	1,699134432	0,64784
Nhlrc1	1,698427929	1
Gpsm1	1,698310207	0,59017
Med18	1,698192493	0,75203
Nrap	1,698192493	1
Rapgef5	1,69795709	0,71251
Gm38380	1,697721719	0,85682
Gm7353	1,697721719	1
2810408I11Rik	1,697368725	0,96495
Prpf39	1,696780563	0,4399
Naa20	1,696662956	0,38847
Abcd2	1,696545356	0,86531
Gm19353	1,694782339	0,42096
Tet2	1,694782339	0,51323
Mob2	1,694077646	0,24644
1700012D14Rik	1,693842813	0,93599
Gm17060	1,693373245	0,91204
Gm42671	1,693373245	1
Dpys	1,692786468	0,93362
Abca1	1,692669137	0,72289
Gm12990	1,692551814	0,87896
RP24-401G4.1	1,6924345	0,69803
Gm20072	1,691496275	0,69155
Nhlrc3	1,691379033	0,40685
Ncoa4	1,690792948	1
Rps11-ps4	1,690675755	0,94627
Hist1h2an	1,690207065	1
Uchl5	1,690089913	0,26488

Wbp4	1,689972769	0,25147
Coa6	1,689972769	0,86649
Gm28187	1,689504274	0,9306
Mettl16	1,689270075	0,40625
Gm13611	1,689152988	0,85132
Gm38115	1,689152988	0,97453
Mok	1,688918838	0,92613
Phactr4	1,688333604	0,38752
Ppia	1,688333604	0,85527
Tsr2	1,688099568	0,52268
Rhebl1	1,687631592	0,30907
Utp23	1,687397653	0,38573
Rpl9-ps6	1,687397653	0,47266
Gramd4	1,687280696	0,69827
Tmx4	1,686929872	0,20045
Plekha7	1,68669603	1
AC133103.1	1,686462221	0,5672
Gm5883	1,686345328	1
Btbd1	1,685760986	0,40284
Gm20900	1,685644142	0,69842
Cdk1	1,685527307	0,21896
Gm7666	1,685527307	0,9873
Rpl14-ps1	1,684359393	0,35136
Ccar1	1,683659033	0,22035
Nmrk1	1,683659033	0,78361
Rps12-ps1	1,683192288	1
Zfp574	1,683075622	0,58209
Gm5093	1,682492414	0,89221
Pgam1	1,682492414	1
Gm43868	1,682492414	1
Gla	1,681792831	0,41754
Stam	1,681792831	0,54454
Mphosph10	1,681443148	0,32388
Ift80	1,681326603	0,65361
Gm6382	1,681326603	1
Fbxl14	1,681210067	0,99198
Acrbp	1,680860505	0,84032
Uqcrh-ps1	1,680744001	0,88342
Gm10658	1,680744001	0,90682
Cdkn1a	1,679928696	0,18193
Cenpa	1,679812257	0,18922
Dvl2	1,679812257	0,38434
Hnrnph1	1,679346578	0,4152
Snn	1,679346578	0,95038
Wee1	1,677950318	0,54687
Snhg15	1,677834015	0,44469
Tdrd7	1,677717721	0,93245
6430710M23Rik	1,677485156	1
Gm7117	1,677020123	0,84929
Prkx	1,676555218	0,56592
E2f6	1,676439012	0,42801
mt-Co1	1,676206625	0,32988

Gm6085	1,676206625	0,33512
Atf1	1,675974269	0,38297
Gm8451	1,675509655	0,68994
RP24-547N4.5	1,675393522	0,92613
Gm26461	1,675393522	1
Pcna	1,675277396	0,58141
Zc3h12c	1,674464744	0,65592
Cd74	1,674116585	0,38868
Gm9392	1,673884519	0,82814
Incenp	1,673652485	0,17374
Slc25a38	1,673536481	0,65797
Dhx8	1,672608732	0,42926
Med25	1,671681498	0,56003
Apmap	1,671681498	0,59259
Calm3	1,671333918	0,34502
Vcpkmt	1,671218074	0,73923
Inafm1	1,670291611	0,45998
Epb41	1,669828572	0,41099
Spin1	1,669828572	0,72448
Hsd17b10	1,6695971	0,47599
Tec	1,6695971	0,57883
Acadm	1,669134254	0,40348
Fam214a	1,668787203	0,49364
Tmed8	1,668787203	0,72748
BC029214	1,668671536	0,71717
Hnrnpa3	1,668555876	0,64808
Gm14325	1,667977699	1
Ppp2r2d	1,667515302	0,31613
Arf6	1,667399722	0,15493
Cdca2	1,667053032	0,67385
Psme1	1,666937485	0,37702
Kdm2b	1,666128878	0,7313
Gm45033	1,665089817	1
RP24-75M13.2	1,664512841	1
Cnnm4	1,663936064	0,83057
Patl1	1,663590095	0,53555
Zfp53	1,662783111	0,79798
Gbe1	1,662437381	0,58937
Gm12967	1,661170319	0,76059
Gm13840	1,660709809	0,99796
Gtf2h5	1,660479602	0,22287
Diaph3	1,659789171	0,82802
Topors	1,658754064	0,37702
Lsm12	1,658294223	0,55472
Plekhf1	1,658294223	0,99218
Higd2a	1,65783451	0,23953
Rpl10a	1,657145179	0,86324
Pcnp	1,656685785	0,64171
Eif2s3x	1,656341323	0,87354
Tgoln1	1,655996932	0,15184
Gm7589	1,655767378	0,56438
Gm8357	1,655193632	0,93898

Klhl18	1,65496419	0,77546
Zfp69	1,65496419	0,95654
Clk3	1,654620085	0,24272
Ncaph	1,654046737	0,72234
Nup50	1,653932091	0,40863
Gm10031	1,653702823	0,37983
Gm8805	1,653473587	0,62495
Gm42918	1,653473587	0,99218
Vat1	1,653358981	0,25607
Gm4034	1,652671512	0,92434
Spns1	1,651984328	0,575
Gm5045	1,651640843	1
Prelid1	1,65129743	0,39089
Ift172	1,651068527	0,63586
Rfc4	1,650610817	0,6699
Gm4604	1,650267618	0,76714
Gm27046	1,650267618	0,98724
Kif20a	1,649695778	0,78256
Hdac5	1,649467097	0,4681
Pfdn2	1,649124136	0,5057
Fgfbp3	1,648781246	0,82911
Fbxo34	1,648438427	0,6077
Smg5	1,647981446	0,61393
9930104L06Rik	1,647638794	0,99974
Smarce1	1,647410398	0,69765
Sesn2	1,647296212	0,60448
Yars2	1,647296212	0,72379
Fam171a2	1,647296212	0,76685
Themis2	1,646953702	0,67809
RP23-58B7.2	1,646040691	0,72463
Pnrc1	1,6459266	0,37238
Oas1g	1,6459266	1
Gm26384	1,645698442	0,52268
Irf2bpl	1,645470315	0,30149
Apol11b	1,644900137	1
Mxi1	1,644672122	0,34795
Rpsa-ps12	1,64410222	0,85126
Gm43660	1,64410222	0,9763
Tbc1d31	1,643190789	0,66723
Gm16062	1,643190789	0,95266
Atp9a	1,642963011	0,54055
Nr1d1	1,642963011	0,60101
Rps24-ps3	1,642849133	0,48013
Uevld	1,642735263	0,75046
Hmga1	1,642735263	0,90187
Qk	1,642507548	0,38573
Mir703	1,642279863	0,3495
Rnps1	1,642052211	0,73923
Slc41a1	1,64182459	0,6731
Gm44836	1,64182459	1
Dtymk	1,641710791	0,54949
Rps27a-ps1	1,641710791	0,63168

Pafah1b1-ps2	1,641710791	0,95752
Rnf145	1,641597001	0,26577
Ctdp1	1,641483218	0,65117
D230017M19Rik	1,641483218	1
RP23-316F10.2	1,641369443	0,97779
Gm36378	1,640232126	0,89221
Gltp	1,640118438	0,21873
Gm15730	1,640118438	0,64261
Rps19bp1	1,640004758	0,36746
Fam162a	1,638868387	0,3783
Tnfrsf26	1,638527629	0,35136
Cyp2c55	1,63727879	0,83653
Map3k9	1,63727879	0,87049
2310022B05Rik	1,637165307	0,40503
E330011M16Rik	1,636938363	1
Cmtm4	1,636257721	0,63878
Fiz1	1,635577362	0,77355
Entpd1	1,635350639	0,88202
Banp	1,635237289	0,55376
Cds1	1,634897286	0,6292
Mknk2	1,634783968	0,40667
Clec10a	1,634330772	0,70745
Gm2991	1,633877702	0,83057
Arhgef39	1,633764454	0,67828
Hps5	1,633424758	0,71502
Gm12428	1,632745577	1
Gm45212	1,632519246	0,9873
Gm7561	1,632406092	0,97531
Stk25	1,632292946	0,87651
Ginm1	1,632179808	0,6455
Selenow	1,631953556	0,55376
Ptgs1	1,631727335	1
Nxt2	1,631274987	0,61923
Gm9008	1,631274987	0,96337
Mier1	1,630822764	0,37622
Gm45413	1,630822764	1
Oard1	1,629692755	0,71984
Gm8730	1,629353906	0,8136
Rgs12	1,629240971	0,99042
B330016D10Rik	1,629128045	0,92575
Atg14	1,629015126	0,4681
Sec61g	1,628676417	0,8416
Slc35f5	1,62856353	0,79282
Kbtbd7	1,627999211	0,82264
Gm14680	1,627547896	0,81662
Spata5	1,627322285	0,73565
Smyd5	1,627209492	0,66723
Ensa	1,626645642	0,70884
Tfeb	1,626420157	0,4625
Gm12696	1,626194702	0,48055
Mllt10	1,626194702	0,55583
8030462N17Rik	1,626194702	0,88843

Arpp19	1,625743888	0,32023
Lgr5	1,625631204	1
Arntl	1,625293199	0,68325
Serf2	1,625067901	0,70884
Gm2383	1,624279604	0,95867
Gm12704	1,624167022	0,96101
Sort1	1,623716769	0,66919
BC030499	1,623716769	1
Ing2	1,623604226	0,42469
Gm14017	1,623379162	0,92498
Gm6433	1,623266642	0,75762
Fchsd1	1,623266642	0,79588
RP23-65M10.2	1,621579775	0,9873
Bcas2	1,620456171	0,68724
Cebpa	1,619782382	0,66723
Lin9	1,619557848	0,57605
2810002D19Rik	1,619445593	1
Rpl31-ps8	1,619108874	0,76676
Trp53	1,618772224	0,76273
Syne2	1,618660024	0,9873
4833421G17Rik	1,618211298	1
Pcsk4	1,617874835	0,84332
Caprin2	1,617762697	1
Cd302	1,617426327	0,76993
Gm6155	1,61720212	0,94605
Gm43178	1,617090028	1
Coq10b	1,616753799	0,66282
Gm6808	1,616529684	0,86068
Tprn	1,616417639	0,57229
P4ha2	1,61540958	0,84945
Suv39h1	1,615185652	0,75972
Nr3c1	1,614514055	0,37144
Zc3h12a	1,614514055	0,7931
Gm9790	1,614402149	0,65584
Aldh9a1	1,613842737	0,38123
Rpl18-ps1	1,613730878	0,64797
Rpl7-ps7	1,613171698	0,5057
9930111J21Rik2	1,613059885	1
Syt11	1,61294808	0,6119
Apoo	1,61294808	1
Saraf	1,612500938	0,30959
B130021K23Rik	1,612389172	1
Gm5251	1,612277414	0,96227
Tusc2	1,612165663	0,5738
A930005H10Rik	1,611942185	0,74927
Rpl31	1,611495322	0,49364
Gm1976	1,611160255	0,90187
Gm43343	1,611160255	0,94468
Rpsa-ps9	1,611160255	0,95244
Sf3b1	1,611048582	0,41099
Mettl23	1,610825259	0,85132
Osgin2	1,610490332	0,55228

Setdb2	1,610155475	0,96423
Zfp143	1,609932275	0,52636
RP23-114G13.7	1,609709107	0,85316
Junos	1,609485969	0,97602
Cdc34b	1,609485969	0,9873
Gm5262	1,608816742	1
Camta1	1,608593728	0,74747
Zfp532	1,608482233	1
Gm5735	1,608370745	1
Slc31a2	1,607924872	0,72289
Nabp2	1,607479122	0,69765
Rtn4	1,607367704	0,35003
Gm2796	1,607256294	0,6121
Taf7	1,606922109	0,68497
Gm9727	1,606922109	1
Gm12254	1,606365289	0,7758
Snrpc	1,606365289	0,89288
Zbtb18	1,606253948	0,81662
Ctsc	1,606031289	0,66723
Hook2	1,606031289	0,7885
Pag1	1,605808661	0,72731
Gm12166	1,605808661	0,77995
Pla2g15	1,605697359	0,40136
Gm13487	1,605697359	0,73923
Eif1a	1,605029707	0,39606
Cdh23	1,604695985	1
Fam234b	1,604473542	0,85948
Tpra1	1,604251131	0,63433
Gm7488	1,603361792	1
Rpl12-ps1	1,602139755	0,98714
Kctd5	1,601806634	0,90171
Bhlhe41	1,601362581	0,67816
Sgol2a	1,600585783	0,73437
Pts	1,60036391	0,814
Bri3	1,599920257	0,84945
Pcdhgc4	1,599920257	1
Pim2	1,599587597	0,89405
Kif2c	1,599587597	0,90801
Gm15417	1,599476726	0,69165
Kctd10	1,599365863	0,6838
Dcp2	1,599255007	0,51387
Rpl13	1,599255007	0,64644
Smim11	1,599144159	0,55472
Gm37963	1,598811661	1
Gm9517	1,598590034	0,99218
Cenph	1,597925337	0,7247
Ilf3	1,597925337	0,7462
Mdm1	1,597482359	0,85823
Kdm4c	1,595711677	0,93362
Gm7299	1,595601074	1
Gm5944	1,595269313	1
Aarsd1	1,59416394	1

Abca5	1,593832477	1
Gm19620	1,593832477	1
Ccnl1	1,593722005	0,59502
Vamp3	1,593501083	0,54659
Ccl9	1,593390634	0,54673
Tatdn2	1,593280193	0,65434
Rbmx2	1,593059332	0,78558
RP23-205H11.3	1,592728099	1
Gm5362	1,592617704	1
Gigyf1	1,592507316	0,78016
4930447F24Rik	1,592286563	1
RP24-282C4.13	1,591514168	0,99796
Gm14539	1,591072968	0,79513
Dgat2	1,590962687	1
Gm37305	1,590742148	1
Tomm40l	1,590521639	0,85132
Rpl37a	1,590190934	0,41106
Rnf130	1,590080714	0,97111
RP24-275P22.2	1,589970502	0,92928
Lmtk2	1,5897501	0,96196
Rps6ka5	1,589639911	0,89823
Gm12751	1,58952973	1
Lig1	1,589309389	0,50855
Card9	1,589309389	1
Katnbl1	1,589199231	0,50169
Lamb2	1,589199231	1
Gm15975	1,589089079	0,9873
Tra2b	1,588648551	0,54661
Tmem256	1,588428333	0,61923
Rpl10-ps3	1,588428333	0,87069
Egln1	1,588208146	0,56695
Erfe	1,587987988	1
Mettl7a1	1,587547766	0,92016
Hyls1	1,587217679	1
Snhg9	1,586667686	0,73923
Dhx58	1,586447743	0,84032
Gm7206	1,585568273	1
Ifitm1	1,585568273	1
H2afz	1,585458374	0,84673
Mad2l1bp	1,585018852	0,59333
Gm15596	1,584908991	1
Sc5d	1,584140175	0,55472
Spire2	1,583701019	0,9044
Sf3a2	1,583042513	0,76059
Gm44152	1,583042513	1
Gm6123	1,582384281	0,96486
Rab27a	1,582274602	0,82377
Cwf19l1	1,582055268	0,84945
Rpl31-ps17	1,58161669	0,85316
Pwp1	1,581507064	0,65434
Atp8b2	1,581507064	0,78885
Gltscr1	1,581507064	0,91675

Tmub1	1,581178233	0,67153
Ugcg	1,580959051	0,80448
Gm13413	1,580630334	1
Cdca3	1,579425622	0,83114
Crls1	1,57822183	0,60488
E130317F20Rik	1,577893682	0,97447
Gm7863	1,577784314	1
Alkbh4	1,577456257	0,8814
Gm7776	1,577018954	0,82814
Wdcp	1,577018954	0,86447
Polr3g	1,576909647	0,7884
A330023F24Rik	1,576691056	0,92936
Zfp940	1,576363227	1
Polr2j	1,576144712	0,75393
Mettl17	1,575707772	0,88936
Fyttd1	1,575489348	0,50795
Kif4	1,575489348	0,95266
Pfdn5	1,57505259	0,4681
Rps26-ps1	1,574834256	0,61625
Gm8822	1,574834256	0,85424
Gm5867	1,574506813	0,9069
Gm42798	1,574506813	1
H2-K1	1,574070327	0,60147
Mien1	1,573633963	0,62094
Tmem168	1,572870616	0,70514
Gm12396	1,572870616	0,91554
Smpd4	1,572761597	0,79841
Midn	1,572543581	0,61862
Pramef8	1,572434584	0,75972
Gm12693	1,572434584	0,88216
Gm10704	1,571671819	0,82814
Terf2ip	1,571127214	0,71705
Gm4285	1,571018315	1
Vgll4	1,570800541	0,62458
Gm10602	1,570256237	0,98329
Arl13b	1,56982093	0,90518
Gm7336	1,569712122	1
Naa16	1,569385744	0,77577
Gm6576	1,569385744	1
Pfdn6	1,569276966	0,77237
Rad54b	1,569276966	1
Pot1b	1,569168196	1
4930503L19Rik	1,569059433	0,7587
Neurl3	1,56884193	0,78256
Rplp2	1,568515733	0,71898
Pfkp	1,568189603	0,64651
Krr1	1,568189603	0,73621
Apba3	1,568189603	0,94724
Icosl	1,567754869	0,99974
Psat1	1,567102993	0,51039
Gm17745	1,566994374	0,9327
Clic4	1,566234248	0,66258

Rars	1,565908593	0,52625
Rmnd5a	1,565474491	0,91554
Mtmr12	1,565365984	0,82891
Fgfr1op	1,565257485	0,66988
Stom	1,564823563	0,77204
Hbp1	1,564606648	0,53028
Mbd4	1,563522521	0,84553
Ppp1r12c	1,562980739	0,50009
Rmrp	1,562980739	1
Tnrc18	1,562330849	0,57219
Gm16580	1,562330849	0,73744
Bag3	1,56222256	0,68105
Tnni2	1,561681229	0,80307
Gpn2	1,561681229	1
Prpf38a	1,561031879	0,728
Wbp11	1,560707305	0,67964
Ric8b	1,56049096	0,80041
Blvrb	1,559842105	0,55534
Gm10110	1,559842105	0,94463
Nup35	1,559409685	0,88461
Leng1	1,559301599	0,8255
Fth-ps2	1,559301599	1
Gm5050	1,559085449	0,96764
Trim47	1,55876128	0,9785
Triap1	1,558329159	0,6627
Gm7424	1,558113144	0,99796
Ap3s1	1,557465278	1
Nup153	1,557141446	0,84095
Rpl31-ps11	1,556817681	1
Gm38305	1,556709774	0,95972
Tubb4b	1,556493983	0,84322
Zfp263	1,556386099	0,66843
Gm12338	1,556062491	0,91509
Sap30l	1,555954637	0,81437
Rpl17	1,555738951	0,84945
Nudt16l1	1,555631119	0,67801
Cib2	1,555523295	0,95583
Gm6733	1,555523295	0,97193
Wwp1	1,555415478	0,70541
Foxm1	1,555307668	0,82182
B230219D22Rik	1,554984285	0,72282
Rora	1,554984285	0,74617
Rpl22-ps1	1,554984285	0,90267
Rpl28-ps1	1,554984285	0,90325
Ccdc25	1,554876505	0,7955
Gm4613	1,554768733	1
Eif4e	1,554445462	0,49779
Gltscr2	1,55379912	0,53869
Gm16200	1,553691423	0,95402
Gm9825	1,553583733	0,88967
Mettl3	1,553583733	0,95752
Rfxap	1,553368375	0,83798

Gm10327	1,553368375	1
Gm9800	1,553045395	0,90844
Dynll2	1,55293775	0,54242
Rasgrp4	1,552614859	0,98976
Gm6977	1,552399636	0,952
Gas2l3	1,552292036	0,76587
Cracr2b	1,552076857	0,95752
Fbxl15	1,551861709	0,97628
Wasl	1,551754145	0,75762
Mrpl9	1,551539042	0,75365
Rpsa	1,551108923	0,70565
Pde1b	1,550786413	0,86622
Snord66	1,550356503	1
Azin1	1,550034149	0,69568
Rpl11	1,549926712	0,95583
Gm6419	1,549926712	1
Exosc3	1,549497041	1
Eif1-ps1	1,548745403	0,90801
Camk2b	1,548316059	1
RP24-232D3.1	1,548316059	1
E230016M11Rik	1,54735047	1
Sprydz7	1,547135977	0,70911
Yrdc	1,547028741	0,81183
Chchd6	1,547028741	0,85126
Dusp16	1,546814292	0,86802
Hmgb1-ps6	1,546814292	1
Rps15a	1,546599873	0,6528
Gm13712	1,5462783	1
Gpr157	1,546171124	0,92072
Dact3	1,546171124	0,95381
Rpl21-ps12	1,545956794	1
Parp6	1,545528223	0,85527
Gm24507	1,545421099	1
Cenpi	1,544564373	0,99901
Zcchc10	1,544457316	0,71138
Rrh	1,544243224	1
Rpl39	1,543815128	0,8819
Rnf26	1,543601125	0,93599
Dnajb5	1,543494134	1
Gyg	1,543387151	0,67874
Ggps1	1,543280175	0,90844
Cbx1	1,543066246	0,56003
Mrip-ps	1,542852346	1
D130017N08Rik	1,541890165	1
Gm5745	1,541783293	0,98976
Prr7	1,541676428	0,94799
2610021A01Rik	1,541569571	0,9873
Gm38067	1,540928584	0,99972
Mob1b	1,540714981	0,75393
Gpr35	1,539967602	1
E2f5	1,539860864	0,92891
Pde2a	1,539860864	1

Fancb	1,539433983	1
Ggct	1,539327281	0,73046
Gm8606	1,538900548	0,99218
Gm45477	1,538580576	1
BC055324	1,53815405	0,84945
Arhgap27os2	1,53815405	0,89173
Uprt	1,53815405	1
Sav1	1,538047437	0,8366
Usmg5	1,538047437	0,93259
Dhcr7	1,537940831	0,8338
Lgals3	1,537514483	0,61393
Ube2q2	1,537407914	0,85316
Polh	1,537301353	1
Tax1bp3	1,53655563	1
Rps11-ps1	1,536342633	0,94673
Gm5449	1,536236146	1
Rpl26	1,53581027	0,89021
Serpinb6b	1,535597376	0,86961
Wdr54	1,535597376	1
Nrbf2	1,535278091	0,92575
Atg16l1	1,534958872	0,70612
Gm45454	1,53485248	1
Nectin1	1,534639719	0,77507
Neurl1b	1,53453335	0,77488
Ccnyl1	1,534426988	1
Kif22	1,534214286	0,90645
Prdx4	1,534107946	0,77234
Prpf3	1,533895288	0,85767
Bcl2	1,533895288	0,85823
Iqsec2	1,533576357	0,79383
Pdzk1ip1	1,533470061	1
Gm32175	1,533470061	1
Mrpl34	1,533363772	0,68278
Casp6	1,533363772	0,92225
Gm4204	1,532513731	0,5494
Akirin1	1,532513731	0,84509
Card19	1,532195087	0,61923
AW554918	1,532195087	0,92498
Cby1	1,531557997	0,92474
0610040B10Rik	1,530284613	0,95239
Kdm6a	1,529966432	0,82131
Ybx1	1,529860386	0,69765
Gm43411	1,528906308	1
Rpl37	1,528694371	0,80535
Sp3	1,528588413	0,77384
Rnf103	1,528588413	0,90284
Pltp	1,528270585	0,78966
Rab1a	1,528270585	0,83548
Lsm14a	1,528164657	0,7885
Pigl	1,528164657	1
Gm9568	1,528164657	1
Tapbpl	1,528058737	0,96268

Gm9173	1,527211636	1
Gm11930	1,526682437	1
Gm13368	1,526576619	1
Ecm1	1,526470808	0,7955
Rpl36al	1,526470808	0,87651
Gm15575	1,526470808	1
Gm13005	1,52615342	1
Runx1	1,525730339	0,69835
Cic	1,525624588	0,64797
Oaz2	1,525413106	0,75972
Ccnj	1,525413106	1
Stard5	1,525201653	0,91326
Rrp12	1,52499023	0,86068
Clic1	1,52467315	0,74308
Rps24	1,524356136	0,78602
Gm12020	1,52425048	0,90171
Mcm4	1,52414483	0,88342
Tgds	1,523722306	1
Ccdc9	1,523511088	0,78788
Rab3il1	1,52308874	0,92898
Tex2	1,522772055	0,94291
Gpr155	1,522772055	0,95752
Gm33080	1,522560969	1
Aacs	1,522455437	0,98989
Csrp2	1,522138884	0,96387
Usf1	1,522033381	1
Mturn	1,521716916	1
Zbtb24	1,521400517	0,89206
Gm13015	1,521400517	0,97911
Gm2225	1,521400517	1
Gpr137b-ps	1,520978753	0,89206
3110045C21Rik	1,520978753	1
Mrps23	1,520767915	0,76595
Srsf1	1,520557107	0,8814
Fam118a	1,520557107	0,96217
Fbxo21	1,520240949	0,81862
Rps13	1,51960883	0,92509
Dot1l	1,51834538	0,72448
Slc30a5	1,51834538	0,89461
Dok4	1,518029682	1
Nectin4	1,517608853	1
Gm2000	1,517398482	0,91371
Tbpl1	1,517188141	0,72967
Eif3f	1,516977829	0,76514
Gm7332	1,516872683	1
E2f4	1,516662415	0,93128
Gm45729	1,516452175	1
Golga3	1,516241965	0,81862
Snx8	1,515611509	0,78042
Tial1	1,515611509	0,83408
Kbtbd4	1,514876308	0,93974
Tiprl	1,514666316	0,7885

Gm8276	1,514036515	0,74965
Sik2	1,514036515	0,95244
Rgs3	1,513931573	1
Star	1,513721713	0,99829
G6pc3	1,513616793	0,90844
S100a8	1,513302078	1
Kif9	1,512987428	1
Gas8	1,512777698	1
Dgkh	1,512567997	1
Plekha1	1,5122535	0,80902
Ccdc22	1,5122535	0,87651
Hcn2	1,511834273	1
Eif4b	1,511519928	0,61625
Taf8	1,511415161	0,96078
Grpel2	1,511205649	0,94096
Bzw1	1,511100904	0,80453
Mybl1	1,511100904	0,95266
Bbs10	1,510263204	1
Nceh1	1,509949186	0,76273
Srsf4	1,509739877	0,9873
Ankrd9	1,509635234	0,9834
Zfp874a	1,509425969	0,95752
Gm10443	1,509321347	0,82295
Pofut2	1,509321347	0,90325
Junb	1,509007525	0,92681
Brwd3	1,508693768	0,88042
Clk4	1,508589197	0,85316
Gm26881	1,508484633	0,97169
Snhg12	1,508380077	0,80535
Cir1	1,508275527	0,87651
Gm14323	1,508275527	1
Zfp553	1,507857403	0,99057
1810037I17Rik	1,50733491	0,96196
Gm10169	1,507125963	0,88936
Gm8213	1,506917046	0,92509
Sesn1	1,506186062	0,99042
Gm14681	1,506081665	0,91251
Zfp689	1,505977275	1
Aph1a	1,505872892	0,86531
5330406M23Rik	1,505768517	1
Fuca2	1,505664148	0,94228
Yipf4	1,505664148	1
Dnajb9	1,505559787	0,92517
Gm6028	1,505559787	1
Stard3nl	1,505246747	0,95859
Gm14048	1,505246747	1
Gm12589	1,504516574	1
Napg	1,503995239	0,73923
Ccdc15	1,503474084	0,98976
RP23-123D6.12	1,503474084	0,99974
Nt5dc3	1,50295311	0,87354
BC002163	1,502744771	0,99767

Gnpda1	1,502536461	1
Rps2-ps11	1,50222405	1
Polr1d	1,502015812	0,74927
Slc25a32	1,501599422	1
Swt1	1,501287206	0,87528
Vps29	1,501079098	0,88629
Dtl	1,500975054	0,98346
Gm10575	1,500975054	1
Iffo2	1,500871018	0,95381
Trmt10c	1,500766989	0,98921
Susd6	1,500662968	0,91326
Gm11353	1,500558953	1
Pik3ip1	1,500350946	0,81662
Fam198b	1,500142968	0,9956
Gm38190	1,500142968	1
Eda2r	1,500038989	0,92115
Bud13	1,499727097	0,90818
Prdm15	1,499727097	0,98685
Kctd18	1,499623148	0,95216
Mob3a	1,499207422	0,95583
Traf4	1,498999602	0,92644
Grasp	1,498687926	0,94347
Acer3	1,498584048	0,94627
Cdk5r1	1,498376315	0,92509
Lmnb2	1,498376315	0,99042
Pdlim7	1,498376315	1
Ndufb4	1,498272459	0,98755
Gm5523	1,498272459	1
Svep1	1,49816861	1
Prr11	1,497960934	0,99383
Leo1	1,497857107	0,96958
Vprbp	1,497441871	0,96642
Yipf7	1,497026749	1
Cox14	1,496300563	0,77994
K230015D01Rik	1,495885758	1
Rnf11	1,495782075	0,84032
Ctnnbip1	1,495782075	0,85319
Crnkl1	1,49557473	0,92205
Rps11	1,495471068	0,89827
Cgrrf1	1,495471068	0,94565
Gm10059	1,495367414	1
Ern1	1,494952867	0,92368
Trim33	1,494227688	0,8981
Rnf146	1,494124119	0,91711
Caap1	1,493917004	0,97324
Fbxl12os	1,493813457	1
Gm14040	1,493813457	1
1190002N15Rik	1,493709917	0,91299
Zbtb7b	1,49350286	0,80361
Gm6368	1,493295831	0,99767
Supt4a	1,492778383	0,99042
Aldh2	1,492157683	0,81086

Cbr2	1,492157683	0,9873
Gm12848	1,491537241	1
Gstcd	1,491330485	0,94574
Map3k2	1,491227117	0,93966
Hspb6	1,491227117	1
Gm4742	1,491227117	1
Rfc3	1,491020404	0,9222
Gm14253	1,490400435	1
Rps19-ps9	1,490400435	1
Dynlrb1	1,490193835	0,85126
Rps2	1,490193835	0,99961
Bod1	1,489058052	0,95595
RP23-184H3.5	1,48885164	0,92936
Psd2	1,48885164	1
Gm6654	1,488645255	1
Cdca4	1,488542074	0,90356
Casp8ap2	1,488438899	1
Aaed1	1,488335732	1
Pif1	1,488026274	1
C130026I21Rik	1,487304454	0,95216
Abhd17a	1,487201365	0,93423
Gm23935	1,487098284	0,99042
Rsrp1	1,486582984	0,95867
Gm27219	1,486582984	1
Nek2	1,486582984	1
Tgfbr1	1,486479946	0,96173
Gm9938	1,486479946	1
Car11	1,48627389	1
4732440D04Rik	1,485861865	1
Gpcpd1	1,485655895	0,98697
1600012H06Rik	1,484832301	0,98408
Mif	1,484729384	0,95867
Efnb1	1,484729384	1
Gm15753	1,48380345	1
Zyx	1,483597765	1
Adnp2	1,483392109	0,99662
Dda1	1,483083679	0,90812
Rpl15	1,482878094	0,96078
Gm13602	1,482878094	1
Borcs8	1,482775312	0,97098
Nexn	1,482775312	1
Gm7730	1,482775312	1
Snu13	1,482672538	0,97381
Rpl35a	1,48246701	1
Lrsam1	1,482158772	1
Ptrh1	1,48205604	0,95793
Upf1	1,481542487	0,95724
Dcstamp	1,481542487	1
Mrs2	1,481542487	1
Lars2	1,481131773	0,99579
Nlgn2	1,481029113	1
Mzt1	1,480618541	0,94929

Lemd3	1,480105487	0,98914
Gm12346	1,480002897	0,94228
St3gal4	1,480002897	0,99042
Gm15427	1,47959261	0,94972
Sdccag3	1,479490056	0,99315
Cep44	1,479284969	1
Tmem184b	1,478772377	0,91838
Gm11878	1,47856739	1
Kpna6	1,478259963	0,94205
Gm5619	1,478259963	1
Myo1f	1,478157501	0,95716
Shpk	1,477747725	1
Emilin2	1,477645299	0,86531
Pip4k2b	1,477440468	0,91492
Zfp516	1,477440468	0,96401
Tor1aip1	1,477235666	0,87714
Rpl30	1,477235666	1
A530041M06Rik	1,477133275	1
Qars	1,477030892	0,87651
Rbm18	1,477030892	0,94799
Gm13862	1,476621429	1
C030015A19Rik	1,476519081	1
Rpsa-ps1	1,475802843	0,98644
H2-DMb1	1,475802843	1
2610203C22Rik	1,475598267	1
4931406P16Rik	1,47549599	0,93898
Zfp280d	1,47549599	0,99042
Rps13-ps2	1,47539372	0,95244
Zbtb42	1,474984711	0,99904
Hmgcs1	1,474473609	0,96306
Mcm5	1,474473609	1
5430416N02Rik	1,473656213	1
Gm16177	1,473554071	1
Rpl31-ps1	1,473451935	1
Esrp2	1,473451935	1
Fam46a	1,473349807	0,99757
Lgmn	1,473247686	0,99796
Lfng	1,473043464	1
Lrrfip1	1,472839271	0,96019
RP24-389J11.1	1,472839271	0,9873
Tcn2	1,472737186	0,96306
Gm28404	1,472635107	1
Gm13612	1,472635107	1
Ctdspl2	1,472124819	0,99657
Praf2	1,471716716	1
Cpeb4	1,471614708	0,9873
Stk24	1,471410713	0,94673
Gm11478	1,470696953	0,95673
Gng2	1,470493085	0,96324
Elac1	1,470085434	1
Gm8659	1,469983539	1
Pdcl	1,469677897	0,952

Gm37760	1,469168633	1
Coil	1,46876135	1
Gm11474	1,468659546	1
Rpusd2	1,468252404	1
Sub1	1,467845374	0,88998
Rbm38	1,467641902	0,98597
Nfe2l2	1,467540176	0,8814
Gm15459	1,467540176	1
Gm5879	1,467336746	1
Erh	1,466828294	1
2700029L08Rik	1,466726625	1
Hoxb8	1,466320018	1
Hs1bp3	1,466320018	1
Mrps31	1,465811918	1
Maml1	1,465507143	1
Pggt1b	1,465507143	1
Gm16373	1,465405565	1
Bbof1	1,465303995	1
Rnf24	1,465100875	1
Hic2	1,464999325	1
Arl5b	1,464897783	1
Cryz	1,464390176	0,96324
Gm12716	1,464288675	1
Gm13815	1,464085696	1
Glul	1,463984217	0,99767
Gorab	1,463984217	1
Clp1	1,463679821	1
mt-Rnr2	1,463375489	1
Hmgcr	1,463172637	0,96306
Gm42547	1,463172637	1
4930426I24Rik	1,463172637	1
Trit1	1,462969812	0,9956
Tmem50b	1,462767015	0,95661
Lrrc41	1,461854778	1
Psmd9	1,461550826	0,98755
Angptl4	1,461550826	1
Morf4l2	1,46094311	1
2900055J20Rik	1,46094311	1
Gm13422	1,460740594	1
Bdp1	1,460335647	0,9873
Hdac3	1,460234428	1
Gm43149	1,459930812	1
Tspyl4	1,459930812	1
Ubfd1	1,45962726	1
Swap70	1,45952609	1
Ankzf1	1,459323771	1
Mb21d2	1,459323771	1
Lrig2	1,458919216	1
Gtf2f1	1,458818095	0,99004
Slc15a4	1,458312595	0,93878
Gm14303	1,457908321	0,98976
RP23-371B13.3	1,457908321	1

Ctss	1,457605189	0,9744
Ephx1	1,45730212	0,97894
Gm10275	1,456999114	1
Zswim3	1,456999114	1
Dpysl2	1,456696171	1
Dnajb2	1,456494244	1
Gm4994	1,456292345	1
Gm1947	1,455989549	1
Mss51	1,45578772	1
Ptprc	1,455686816	0,97072
Tcte2	1,45528327	1
Rcn2	1,454980684	0,98863
Rbm14	1,454879836	0,99796
Snord83b	1,454879836	1
St6galnac6	1,454778994	1
Zbtb2	1,454778994	1
Rita1	1,454577333	1
Eif4g2	1,4543757	0,9873
2900076A07Rik	1,454174095	1
Gm4880	1,454174095	1
Gm11977	1,453267217	1
Rpl38-ps2	1,45296505	1
Itpripl1	1,45276364	1
Rhoa	1,452662945	0,99042
Emc7	1,452461578	0,9963
Ndel1	1,452260238	0,96227
Paip2	1,452159578	0,9873
Igf2bp2	1,452159578	1
Foxn2	1,452159578	1
Fkbp7	1,451857641	1
Gm11517	1,45175701	1
Pdp1	1,451455157	1
Prosc	1,451354553	1
Zfr	1,450851639	0,9873
Gm6598	1,450851639	1
Zbtb34	1,450650522	1
Snhg20	1,450449433	1
Gm5845	1,450449433	1
Nbas	1,450348899	1
Zbtb17	1,450248372	1
Mrpl23	1,450248372	1
Zmym2	1,450047339	1
Gm3555	1,450047339	1
Gm12791	1,449946833	1
Tcf7l2	1,449745841	1
Serpinb9	1,449243485	1
Gm16740	1,449243485	1
F2	1,449143035	1
Rheb	1,448942155	1
Stx7	1,448440076	0,98131
RP23-349H12.3	1,448339682	1
Mfsd14a	1,448239294	1

Rsrc2	1,448138913	0,99829
Abl1	1,448138913	1
Cdc42se1	1,447938172	0,97324
Gm9531	1,447837812	1
Gm6565	1,447837812	1
Hilpda	1,447737459	1
Stt3a	1,447536774	1
2410131K14Rik	1,447436442	1
Fam117b	1,447135488	1
Pabpc4	1,447035183	1
Lysmd3	1,447035183	1
Ppp4r3a	1,446934886	1
Atp6v1g1	1,446834595	1
Gnai3	1,446433503	1
Crry-ps	1,446433503	1
Sema6c	1,446433503	1
Snx3	1,446232999	1
BC055308	1,446232999	1
Btg1	1,445932295	1
Ube2cbp	1,44573186	1
Itpkc	1,445230894	1
E330034L11Rik	1,445230894	1
Cdk17	1,445030556	1
Ube2g1	1,44442971	1
Gm16433	1,444329593	1
Tpd52-ps	1,444329593	1
Rcc1l	1,444229483	1
Hivep3	1,44412938	1
AA914427	1,44412938	1
Zfp639	1,444029285	1
Caml	1,443729038	1
Rpl23	1,44362897	1
Hnrnpf	1,44362897	1
Car12	1,443528909	1
Glipr2	1,443128733	1
Gm15846	1,442728668	1
Stx3	1,442528678	1
Smap2	1,442428693	1
Fads3	1,442028822	1
Tmem71	1,441828928	1
Foxo3	1,441728992	1
Rnf215	1,441728992	1
Anapc15	1,441629062	1
Gm10161	1,441129518	1
Pold3	1,44102963	1
Rpsa-ps11	1,44102963	1
Mis18a	1,440530294	1
Ubr7	1,440430447	1
Sclt1	1,440430447	1
Map3k14	1,439332592	1
Entpd6	1,439232829	1
Ap2b1	1,43843497	1

Orc1	1,438235574	1
Elf1	1,437537907	1
Akirin2	1,436940177	1
Gm17690	1,436840579	1
3110043O21Rik	1,436740988	1
Tmco3	1,436641404	1
Crcp	1,436442257	1
Gm9703	1,435844982	1
Retn	1,43574546	1
Fam133b	1,435347442	1
Rab11fip4os1	1,435148475	1
Tmsb4x	1,435049001	1
Gm37303	1,435049001	1
RP23-48A24.3	1,434949535	1
Ghdc	1,434850075	1
Tfb1m	1,434850075	1
Rab3a	1,434750622	1
Arrdc4	1,434551737	1
Gm9143	1,434452305	1
Irf1	1,43435288	1
Cep350	1,43415405	1
Mrps11	1,434054646	1
Slc7a6os	1,433955248	1
Gnas	1,433756473	1
Ruvbl2	1,433557726	1
Gm6166	1,433458363	1
Lyst	1,43296165	1
Cyth3	1,432862328	1
Txn-ps1	1,432763013	1
Gm11772	1,432663705	1
Kbtbd2	1,431968741	1
Fbl	1,431770242	1
Top1	1,43157177	1
Arhgdia	1,431373326	1
Slc39a10	1,430778158	1
Gm5302	1,430678988	1
Psmd8	1,430480667	1
Acot8	1,430381517	1
Gm24920	1,430381517	1
Pcnx4	1,429984986	1
Anapc7	1,42968766	1
Rictor	1,429489477	1
Med4	1,429192254	1
Commd1	1,429192254	1
Pak4	1,429192254	1
Ugp2	1,429093193	1
Amn1	1,429093193	1
Ube2s	1,42869702	1
Cebpg	1,428201957	1
Pcf11	1,428102965	1
Gm5687	1,427905001	1
Cox7a2l	1,42780603	1

Pop5	1,42780603	1
Mapk6	1,427608108	1
Rps9	1,427608108	1
Phlda3	1,427212346	1
Cox16	1,426816693	1
Pold1	1,426717797	1
Cdkn1b	1,426520026	1
Nrf1	1,426520026	1
Cd300ld	1,42642115	1
Melk	1,426124565	1
Ubxn4	1,425926876	1
Ccnb1	1,425828042	1
Spg21	1,425432773	1
Vbp1	1,425432773	1
Rps2-ps5	1,425432773	1
Chek1	1,425333973	1
Mrpl54	1,425037614	1
Clpx	1,424543819	1
Hspa9-ps1	1,424346349	1
Ero1l	1,424050196	1
Elavl1	1,423951491	1
Gm10175	1,42365542	1
Nat6	1,423458073	1
Ube2g2	1,42335941	1
Rap1a	1,422964825	1
Relt	1,422964825	1
4930520O04Rik	1,422866196	1
Ptcd3	1,422668959	1
Kras	1,42257035	1
Sirt1	1,422373154	1
Gm12184	1,422373154	1
Gm7846	1,422373154	1
Ranbp6	1,421880282	1
Gm15829	1,421781729	1
Gm15500	1,421486108	1
Tbcb	1,421486108	1
Arhgap27os1	1,421486108	1
Stard4	1,421190549	1
Wdr37	1,421190549	1
Dhx9	1,421092043	1
Tcof1	1,420993544	1
Gm8186	1,420599616	1
Gprasp1	1,420205796	1
Mgat1	1,419910504	1
Ogfrl1	1,419713676	1
Pdxk	1,419123356	1
Lcmt2	1,41843496	1
Sun2	1,418238337	1
Sccpdh	1,418238337	1
Arhgap15	1,417648631	1
Usp33	1,41755037	1
Dse	1,41755037	1

Psmd11	1,417452117	1
Ikzf5	1,41735387	1
Nfatc2	1,41735387	1
Gm7114	1,417157397	1
Hdac2	1,41696095	1
Sart1	1,416862738	1
Ccnb2	1,41656814	1
Cd300a	1,41656814	1
Cxcl16	1,416469954	1
Prcc	1,416469954	1
Snora17	1,416371775	1
Triobp	1,41607728	1
Srp9	1,415979128	1
Anxa1	1,415979128	1
Qrich1	1,415488472	1
S100a13	1,415390361	1
Nbr1	1,415292258	1
Msantd2	1,413919516	1
Zbtb21	1,413919516	1
Gm8181	1,413723518	1
Nufip1	1,413135689	1
Gm11599	1,413135689	1
Harbi1	1,413037742	1
Gm14277	1,412939801	1
Hcar2	1,412548105	1
Atpaf2	1,412254404	1
Trmt112	1,412156518	1
Gm42783	1,412058638	1
Zfp703	1,411960765	1
Gm9844	1,411667186	1
Zbtb8a	1,41156934	1
Secisbp2l	1,411275843	1
Exoc6	1,411275843	1
Gm25541	1,411275843	1
Chst12	1,411080213	1
Diaph1	1,410982407	1
Ifi27l2a	1,410591254	1
Denr	1,410493483	1
Zic5	1,41029796	1
Ighm	1,410102465	1
Larp4	1,410004728	1
Pabpn1	1,409711556	1
Gm4032	1,409516142	1
5430402O13Rik	1,409516142	1
Gm16124	1,409418445	1
Med10	1,409418445	1
Raly	1,409320755	1
Zkscan14	1,409320755	1
Tmem33	1,409223072	1
Gm7860	1,409027726	1
Xpa	1,408930063	1
Cul3	1,408930063	1

Capza2	1,408734757	1
Igbp1	1,408637114	1
Fignl1	1,408539478	1
Ift57	1,408441849	1
Sptssa	1,408344227	1
Znrf3	1,407856217	1
Ccnd3	1,40766106	1
Baz2a	1,407368375	1
Mars2	1,406978224	1
Gtf2e2	1,406783189	1
Fcf1	1,406685682	1
Mrpl42	1,405905866	1
Abcg1	1,405808419	1
Adap1	1,405808419	1
Gm9833	1,405710979	1
Spata5l1	1,40551612	1
Cep192	1,405029089	1
Bahd1	1,404931704	1
Gm4258	1,404931704	1
Mrps21	1,404542228	1
2310034G01Rik	1,404444876	1
Hirip3	1,40434753	1
H2-Q4	1,40415286	1
Rap2a	1,40415286	1
Rdm1	1,403569011	1
Tnf	1,402985404	1
Lnpk	1,40288816	1
Gm45762	1,40288816	1
Jag1	1,402790922	1
Guk1	1,402499251	1
Fis1	1,402304836	1
Gm37125	1,401916089	1
Snrpb2	1,401624599	1
Nfkbid	1,401430306	1
Gm5601	1,401041801	1
Naa10	1,401041801	1
Ppp6c	1,400944691	1
Gas5	1,400944691	1
Snx2	1,400556321	1
Gm37598	1,400556321	1
Gm6457	1,400168058	1
Zfp91	1,399973967	1
Efcab2	1,399973967	1
AK157302	1,399876932	1
Fez2	1,399779903	1
Fign	1,39929486	1
Traf3	1,399197872	1
Vezf1	1,399003915	1
Ifi207	1,398713031	1
Rbm34	1,398713031	1
Mmd	1,398422207	1
Atp6v0a1	1,398422207	1

4933439C10Rik	1,398228358	1
G3bp2	1,398131443	1
Tcp11l1	1,397937635	1
Rpl37rt	1,397937635	1
Dip2b	1,39784074	1
Vps13d	1,39784074	1
Aktip	1,397646972	1
Pabpc1l	1,397550098	1
Prcp	1,397550098	1
Cdc42	1,397550098	1
Elovl1	1,397259516	1
Ssna1	1,396388132	1
Mettl21b	1,396291345	1
Gm38387	1,396194564	1
Armcx5	1,396001024	1
Dctpp1	1,396001024	1
Bloc1s3	1,395807511	1
Ckb	1,395710764	1
Bmpr1a	1,395614024	1
Cnot4	1,395614024	1
Nrm	1,395614024	1
5830487J09Rik	1,395614024	1
Srpr	1,394937032	1
1810013L24Rik	1,394937032	1
Syf2	1,394550327	1
Cxxc5	1,394550327	1
Gm5611	1,394163729	1
Cdt1	1,394163729	1
Sdf2l1	1,394067097	1
Gm6851	1,393970471	1
Rhbdf2	1,393584034	1
Akr1b10	1,393487441	1
Tjp3	1,393487441	1
Exosc2	1,393390855	1
Mvp	1,393197704	1
Tmem261	1,393101138	1
Itga5	1,392328853	1
Rps11-ps2	1,392135848	1
Jmjd6	1,391846392	1
Edrf1	1,391653454	1
Arpc5l	1,391460544	1
Dock1	1,39126766	1
Npat	1,39126766	1
Gm14292	1,391171228	1
Enoph1	1,390978384	1
Mrpl50	1,390689168	1
Ddx43	1,390400012	1
Dpy30	1,390207275	1
Gm29650	1,390207275	1
Eif3e	1,390110917	1
Jtb	1,389436594	1
Mir6236	1,389243991	1

Map2k1	1,389051414	1
Ppp1r37	1,388858864	1
Ppp2r1a	1,388473844	1
Metap1d	1,387896514	1
Gm14137	1,387800316	1
Nup43	1,387704124	1
Gm15782	1,387704124	1
Taf4	1,387607939	1
Dohh	1,387607939	1
Rps27l	1,387415589	1
Gm13743	1,386742574	1
Pnn	1,386358141	1
Oat	1,386165964	1
Med19	1,386069886	1
Cxcl14	1,385109468	1
Gm44552	1,385109468	1
Dpy19l3	1,385109468	1
Acot9	1,384629509	1
Cdk2	1,383957845	1
Rps18-ps1	1,38386192	1
Gm11764	1,38386192	1
Gm23639	1,38386192	1
Cep135	1,383670089	1
Anp32b-ps1	1,383670089	1
Gm20768	1,383478285	1
Tmem109	1,383382393	1
Wdr83	1,383286508	1
A630072M18Rik	1,383094757	1
Mitd1	1,383094757	1
Cebpzos	1,382615496	1
Snrpe	1,382423838	1
Metrn	1,382136401	1
Lgals1	1,381944809	1
Dnajc4	1,381657472	1
Apoo-ps	1,381465947	1
Agtpbp1	1,381178709	1
Stk35	1,38098725	1
Foxj3	1,380795818	1
Zfpm1	1,380604412	1
Dhx38	1,380604412	1
Tfip11	1,380508719	1
Dym	1,380413033	1
Gm45109	1,38022168	1
Dennd2c	1,380030354	1
9330151L19Rik	1,378978535	1
Zfp948	1,378596254	1
Zfp955a	1,3785007	1
Gm5075	1,378405153	1
Psmb1	1,378309613	1
Smpdl3a	1,378118551	1
Trmt112-ps2	1,377736509	1
Tchp	1,377641015	1

Rnf181	1,377641015	1
Il1rn	1,377545527	1
2210406H18Rik	1,377545527	1
Sin3a	1,377545527	1
Tmem63a	1,377354572	1
Pld4	1,377259104	1
Mrpl23-ps1	1,376972741	1
Slc9a3r1	1,376400193	1
Gm15800	1,376304792	1
Klhl12	1,375732522	1
Rab6a	1,375732522	1
Cops9	1,375446476	1
Gm7936	1,375446476	1
Rpf1	1,375446476	1
Ddx51	1,375351141	1
Gm12355	1,375255812	1
Tjp2	1,37516049	1
Tmem41a	1,375065174	1
Vma21	1,375065174	1
Pbdc1	1,374779267	1
Gm8724	1,37449342	1
Kpnb1	1,37439815	1
Mpp3	1,37439815	1
Rps15	1,374302888	1
Irak1bp1	1,374207631	1
Cystm1	1,374017139	1
Rcsd1	1,374017139	1
Rps12	1,373826673	1
Ube2h	1,373636233	1
Gpr162	1,37344582	1
Adck2	1,373350624	1
Eef1d	1,373350624	1
Aspm	1,373255433	1
Calr	1,373255433	1
C330006A16Rik	1,373065073	1
Gm37738	1,373065073	1
Gm35106	1,372779582	1
Cuta	1,372684431	1
Gm28791	1,372589287	1
Csnk1e	1,37249415	1
Dek	1,372399019	1
Gm44075	1,372208778	1
9130019O22Rik	1,372113667	1
Gstz1	1,372113667	1
Birc2	1,371733289	1
Gm16379	1,371543139	1
Lpxn	1,371162919	1
Paip2b	1,370972849	1
Cd200r1	1,370972849	1
Mnat1	1,370687793	1
Gm10131	1,370592787	1
Atg101	1,370497788	1

Lztr1	1,37030781	1
Mkln1	1,37030781	1
Slc31a1	1,370117858	1
Gm12240	1,370022892	1
Plk4	1,369927933	1
Mnt	1,369738034	1
Taf1d	1,369738034	1
Lmbrd1	1,369358314	1
Gm6181	1,369358314	1
6720427I07Rik	1,369263401	1
Zfp281	1,369073594	1
H2afx	1,369073594	1
Nacc1	1,368883813	1
Gm15453	1,368788933	1
Cdyl2	1,368788933	1
Tspan32	1,368788933	1
Szrd1	1,368694059	1
Cct5	1,368694059	1
Apitd1	1,368599191	1
Gm13268	1,368504331	1
Gm13009	1,368030125	1
Stk40	1,368030125	1
Fbxo30	1,368030125	1
Fam107b	1,367556084	1
Fbxw11	1,367082208	1
Nup107	1,366892703	1
Cd52	1,366892703	1
Unc13b	1,366703224	1
Rpl27a	1,366513772	1
Alox5ap	1,366324346	1
Gm20667	1,366229643	1
Tmem242	1,365756226	1
Med26	1,365661562	1
Cbx8	1,365377611	1
Pgm1	1,365377611	1
Rbx1	1,365377611	1
Lonrf1	1,364715286	1
Ing1	1,364526109	1
Gm20186	1,364431531	1
Fcnaos	1,364431531	1
Bcl7a	1,364147835	1
Gm45113	1,364053283	1
March5	1,363958737	1
Smad3	1,363864198	1
Pmm1	1,363675139	1
Rpl10a-ps1	1,363675139	1
Gm15785	1,3633916	1
Asb6	1,362919167	1
Gm45809	1,3628247	1
Gmps	1,362635785	1
Rps27	1,362541338	1
Ryr1	1,362163613	1

Tmem11	1,362163613	1
Wdr44	1,361785994	1
Crbn	1,361785994	1
Cacybp	1,361597223	1
Lyz1	1,361597223	1
Eloc	1,361125411	1
Slc35g1	1,361031069	1
Hadhb	1,360465149	1
Ncaph2	1,360370852	1
Rnf38	1,359710956	1
Dck	1,359616712	1
Rpl22l1	1,359334017	1
Nup160	1,359239798	1
Hotairm1	1,359051381	1
1810062G17Rik	1,358957182	1
Ogfr	1,358768803	1
Setd4	1,358392125	1
Ctbp2	1,358203825	1
Narf	1,358203825	1
Pik3r3	1,358015551	1
Aida	1,358015551	1
Zfp174	1,358015551	1
Lta4h	1,357827303	1
Msl2	1,357827303	1
Slc35e4	1,357827303	1
Aph1b	1,357733189	1
Chmp2b	1,357450885	1
Gm12231	1,356792406	1
Slbp	1,356228249	1
Blcap	1,356134246	1
D730045B01Rik	1,355664327	1
Pank2	1,355476405	1
Dynlt1-ps1	1,355194571	1
Dyrk1a	1,355194571	1
Eif4e3	1,354912795	1
Mylip	1,354818883	1
Gm7972	1,354818883	1
Tomm7	1,354537186	1
Itgb1bp1	1,3544433	1
Mecp2	1,3544433	1
4933433G15Rik	1,35406782	1
Cenpf	1,35406782	1
Chchd7	1,353973967	1
Lrrc57	1,353692445	1
Pradc1	1,353598617	1
Zfp800	1,353504796	1
Rnd1	1,353410982	1
Gm13408	1,353410982	1
Trmt1l	1,353223372	1
Cep120	1,353035789	1
Usp22	1,353035789	1
RP24-325P4.5	1,352942007	1

C1qtnf6	1,352848231	1
Tomm20	1,352660699	1
RP23-269H21.1	1,352566943	1
Ppp1cc	1,352191984	1
Golm1	1,351723431	1
Lncpint	1,351442378	1
Get4	1,351442378	1
Hexb	1,351442378	1
Gemin2	1,351255041	1
BC051226	1,350974085	1
Gna11	1,350974085	1
1700056N10Rik	1,350974085	1
Ube2d1	1,350599567	1
Gm9013	1,350505954	1
Slc4a8	1,350225153	1
Ywhaq	1,350225153	1
Utp14a	1,349570179	1
Gm10501	1,349383101	1
Serpinf1	1,349289573	1
Mttp	1,349009025	1
Acbd5	1,348915522	1
Zbtb11os1	1,348915522	1
2610020C07Rik	1,348915522	1
Gm561	1,348822026	1
E2f8	1,348822026	1
Foxj2	1,348728536	1
1110046J04Rik	1,348167732	1
2210013O21Rik	1,347980849	1
Arsk	1,347420356	1
Zfand1	1,347420356	1
Pinx1	1,347420356	1
Rpl3	1,347326963	1
Cfl2	1,347326963	1
Gm10180	1,347046824	1
Ubald2	1,347046824	1
Gm9497	1,346860096	1
Ccdc34	1,346580054	1
Rangap1	1,346393391	1
Rpl7a-ps5	1,346206754	1
Fam20b	1,346206754	1
Cdc42se2	1,346113445	1
Sugt1	1,345740275	1
Tapbp	1,345273958	1
RP23-403D16.3	1,345180714	1
Pex26	1,344434994	1
Mtmr3	1,344341808	1
Ncapd2	1,344248629	1
Gm43859	1,344062289	1
Cdc26	1,343875976	1
Gm7380	1,343596554	1
Med11	1,34331719	1
Fam53c	1,343224081	1

Gm12582	1,343224081	1
Pex7	1,342758636	1
Pold4	1,342479446	1
Emc4	1,342200314	1
Gtf2f2	1,342200314	1
Mastl	1,341921241	1
Ttc25	1,341921241	1
Gm16061	1,341828229	1
Zfp619	1,341456247	1
Ppp2r5e	1,341456247	1
Sh3bp1	1,341177328	1
Ccdc59	1,341177328	1
Gm20432	1,340991414	1
Ovgp1	1,340805526	1
Zcchc14	1,340805526	1
Adpgk	1,340526742	1
Msh3	1,340433827	1
Itpk1	1,340340919	1
Xpo1	1,340248017	1
Cage1	1,340062232	1
Gm10250	1,340062232	1
Zmym1	1,340062232	1
Arhgap23	1,339969349	1
4921531C22Rik	1,339969349	1
Lamtor5	1,339969349	1
Pdcd5	1,339876472	1
Nfatc2ip	1,339783602	1
Gm7722	1,339319349	1
Celf5	1,339319349	1
Mef2d	1,338948063	1
Map4k2	1,338855257	1
4933427D14Rik	1,338855257	1
Cst3	1,338484099	1
Zfyve27	1,33783482	1
Cxx1a	1,33783482	1
Gm16973	1,337649369	1
Mrps30	1,337463945	1
Gm15832	1,337185856	1
Ets2	1,337185856	1
Nudt19	1,337000495	1
Usp2	1,336907825	1
Cysltr1	1,336537207	1
Dffb	1,336351936	1
Gtf3c5	1,335981472	1
Aen	1,335888872	1
Selenok	1,335888872	1
Ino80d	1,335518536	1
Nop56	1,335333407	1
Sqle	1,335240852	1
Gm4997	1,335240852	1
Desi2	1,335055761	1
Ints6l	1,334963225	1

Eps8	1,334963225	1
1110008P14Rik	1,334685656	1
Dimt1	1,334408145	1
Gm43275	1,33422317	1
2610001J05Rik	1,33422317	1
Eif2ak3	1,33422317	1
Naip5	1,334130692	1
Meis2	1,334130692	1
Gm4366	1,334130692	1
Tbc1d16	1,33403822	1
Pikfyve	1,333853296	1
Rnf115	1,333760843	1
Bsn	1,333575958	1
Slc8a1	1,333298677	1
Cmpk1	1,333298677	1
Oxr1	1,333206263	1
Mrpl32	1,333206263	1
Uck1	1,333021454	1
Kin	1,332744289	1
Gm6222	1,332467181	1
Scrn3	1,332282475	1
Pacsin2	1,332097794	1
Gm13622	1,331820821	1
Vps72	1,331820821	1
Gm15210	1,33172851	1
Sec62	1,331451613	1
Nfkbil1	1,331359327	1
Med7	1,331082508	1
Strip1	1,331082508	1
Aip	1,330805746	1
Bloc1s2	1,330713504	1
Irgq	1,33062127	1
Als2cl	1,330436819	1
Cpsf7	1,330160191	1
Kctd20	1,330160191	1
Gm13186	1,330067995	1
Gatad1	1,329791444	1
Alyref	1,329699273	1
Timm23	1,329607108	1
Tuba1b	1,32951495	1
Slc7a7	1,329238514	1
2810403A07Rik	1,329238514	1
Usp28	1,328777915	1
Zranb3	1,328685814	1
Urm1	1,32859372	1
Pirb	1,32840955	1
Gm37851	1,328041288	1
Usp42	1,327489086	1
Uspl1	1,327121079	1
Mrpl51	1,326385371	1
Hdac11	1,325833857	1
Rnf139	1,325833857	1

Ppt2	1,325741961	1
Siah1b	1,32565007	1
Arl3	1,325282573	1
Glipr1	1,325282573	1
Cbr1	1,325098863	1
Tmem176b	1,325098863	1
Rps16-ps2	1,325098863	1
Rps21	1,324639698	1
RP23-162P10.2	1,324456077	1
Rara	1,324364276	1
Ppp5c	1,324364276	1
0610037L13Rik	1,324364276	1
Cactin	1,324272481	1
Sem1	1,32408891	1
9130024F11Rik	1,323813602	1
Rnf34	1,323813602	1
Gm12857	1,323813602	1
Vps37c	1,323721846	1
Nucb1	1,323721846	1
Alkbh5	1,323630096	1
Wdr74	1,323538352	1
Pcnt	1,323538352	1
Pde12	1,323446614	1
H2-M3	1,32280463	1
Topbp1	1,32280463	1
Rpl38	1,322712943	1
Gm5436	1,322621263	1
Mknk1	1,322529589	1
Rps10-ps4	1,322254605	1
Depdc7	1,322162957	1
Abhd18	1,322071314	1
Aoc2	1,321979679	1
Unc119b	1,321979679	1
C87436	1,321246821	1
Lamc1	1,321246821	1
Pik3c2a	1,321155242	1
Frs2	1,32106367	1
Trappc6b	1,320605903	1
Hscb	1,320514369	1
Fam110a	1,320056793	1
2900009J06Rik	1,319873807	1
Ndufa6	1,319873807	1
Ccdc124	1,319782323	1
Cnst	1,319142116	1
Mettl9	1,319142116	1
Cfap20	1,319050683	1
Gm43328	1,319050683	1
Strn	1,318593614	1
Atp5l	1,318228073	1
Mgea5	1,318228073	1
Ripk3	1,318136704	1
Hist1h4n	1,317862634	1

Rttn	1,31777129	1
Gm13641	1,317679952	1
Actr1a	1,317132058	1
Ppif	1,316949477	1
Gm43756	1,316584392	1
Tspan14	1,316584392	1
Stk19	1,316584392	1
Gm45698	1,316493137	1
Gm19739	1,316128178	1
Dpp7	1,316036954	1
4930509H03Rik	1,315672122	1
Gm15501	1,315398564	1
Riox1	1,315398564	1
Uba6	1,315398564	1
Gm6863	1,31530739	1
Cwc15	1,315216223	1
Eef1g	1,315125063	1
Usp12	1,31494276	1
Gm10288	1,314851618	1
Pnpt1	1,314578231	1
Zxdc	1,314578231	1
Edem3	1,314578231	1
Nkiras1	1,314213803	1
Inip	1,31375841	1
Npm3-ps1	1,313667351	1
Mfng	1,313667351	1
Zfp868	1,31339421	1
Znhit3	1,313212147	1
Gm13771	1,31303011	1
Gm43133	1,312666111	1
Mea1	1,312575127	1
Zfp472	1,312211255	1
Gm26606	1,312211255	1
Tmem208	1,312211255	1
Ciao1	1,312029356	1
Top3a	1,311847483	1
Zfyve1	1,31139291	1
Nkap	1,31139291	1
Socs5	1,311302014	1
Rpsa-ps4	1,311120242	1
Pate2	1,311120242	1
Ptch1	1,311120242	1
Prdx3	1,31084763	1
Kdm3b	1,310665921	1
Treml4	1,310575076	1
Trmt13	1,310575076	1
Rngtt	1,310575076	1
Ceacam16	1,310484237	1
Foxn3	1,310484237	1
Gm11448	1,310393404	1
Mtmr14	1,310120944	1
Gm44024	1,309757751	1

Hacd2	1,309666969	1
Cbll1	1,309666969	1
Thyn1	1,309666969	1
Kcnk13	1,309303902	1
Smg8	1,309303902	1
Atp11b	1,309031669	1
Tollip	1,308578071	1
Rnf126	1,308487371	1
Rpa2	1,308396677	1
Cyth1	1,308215307	1
Ccdc92b	1,308124631	1
Gm35931	1,307761993	1
Psmc4	1,307671349	1
Rab5a	1,307580711	1
Patz1	1,307580711	1
Vma21-ps	1,307490079	1
Runx2os1	1,307399454	1
Crybb3	1,307399454	1
Seh1l	1,307399454	1
Gtf3c4	1,307308835	1
Got1	1,307218223	1
Nars2	1,306946423	1
Raph1	1,306765254	1
Mgarp	1,306765254	1
Lsm11	1,30658411	1
Cenpl	1,306493548	1
Ccnt2	1,306402992	1
Gm13578	1,306312442	1
Brcc3	1,306312442	1
Gm7496	1,306221898	1
Pole4	1,306131361	1
Rpia	1,305950306	1
AB124611	1,305859787	1
Rny3	1,305769275	1
Clip2	1,305769275	1
Eloa	1,305769275	1
Ascc3	1,305588269	1
Gm12186	1,305045403	1
Socs3	1,304593188	1
Erlin1	1,304502763	1
Hint3	1,304050735	1
Ypel5	1,303869968	1
Gm8762	1,303779594	1
Ubap1	1,303689226	1
Pwwp2b	1,303598864	1
Gm4895	1,303327816	1
Gdpd3	1,303327816	1
Gm14165	1,303056825	1
Pias4	1,302966507	1
Mta2	1,30278589	1
Eif3k	1,302695591	1
Mrpl47	1,302424731	1

Fkbp5	1,302424731	1
Abi1	1,302334457	1
Gm5297	1,302334457	1
Secisbp2	1,301973424	1
Gm44090	1,301792944	1
9530078K11Rik	1,301792944	1
Zfp984	1,301702714	1
Helb	1,30161249	1
Gm15445	1,301341855	1
Gm15441	1,301251656	1
Smox	1,301161464	1
Utp15	1,300620438	1
Rnf32	1,300530289	1
Tcea3	1,300440147	1
Ripk2	1,300440147	1
Fam71f2	1,30025988	1
Mapk1ip1	1,30025988	1
2200002J24Rik	1,300079638	1
Gm45266	1,300079638	1
Mgat4a	1,299899421	1
Tns1	1,299629143	1
2310015A10Rik	1,299629143	1
Dlgap4	1,299539062	1
Chd3os	1,298908675	1
Fam220a	1,298908675	1
Nsmce4a	1,298728621	1
Rp9	1,298728621	1
Rps25	1,298098626	1
Psmb2	1,297918684	1
Sirt2	1,297918684	1
Armc7	1,297828722	1
Trmt61b	1,297828722	1
Foxd2	1,297648818	1
Txndc17	1,297648818	1
Micu3	1,297558875	1
Sart3	1,297468938	1
Atg4c	1,297019347	1
Zcchc24	1,296839555	1
Fkbp1a	1,296839555	1
Atp5e	1,296839555	1
Dhrs11	1,296749668	1
Fbxo45	1,296480044	1
C5ar1	1,296390182	1
Arid5b	1,296300326	1
Vps37d	1,296210477	1
Strn4	1,296210477	1
Sh2b1	1,296210477	1
Gm45407	1,295761322	1
Pigg	1,29567151	1
Gm14857	1,295312323	1
Aplp2	1,294953236	1
Fxr1	1,294594249	1

Gm7808	1,294414792	1
Rpl23a-ps2	1,294325074	1
1110004F10Rik	1,294235361	1
Atf2	1,293517883	1
Macrod2	1,293428227	1
Rpp38	1,293428227	1
Fem1b	1,293428227	1
Fas	1,293069662	1
Ier2	1,292980036	1
Anapc16	1,292890417	1
Itga7	1,292532001	1
Dcun1d3	1,292263255	1
Hoxb7	1,291905013	1
Srcap	1,291725929	1
Orc4	1,291636397	1
Trim8	1,29145735	1
Ndufb2	1,291367836	1
Acat1	1,291188827	1
Pfn1	1,290830883	1
Supt20	1,290473039	1
Leng8	1,29020472	1
Gm1943	1,290115293	1
2510039O18Rik	1,289757647	1
Gm43088	1,289578861	1
Sar1b	1,289489477	1
Mpp6	1,289310728	1
Tfpt	1,289042652	1
Rpp25l	1,288953305	1
Pfkfb2	1,288863965	1
Ipo5	1,28877463	1
Aasdhppt	1,28806018	1
Sac3d1	1,287970902	1
Ythdc1	1,287524602	1
4933417C20Rik	1,287346126	1
Rnh1	1,287346126	1
Cntd1	1,287256897	1
2610037D02Rik	1,287256897	1
Trp53bp2	1,287167674	1
Gabarapl1	1,286632468	1
Zc3h3	1,286543288	1
Tnnt1	1,286008341	1
Ccdc137	1,286008341	1
Cdc27	1,285919205	1
Gm12183	1,285740951	1
Pcdhb17	1,285651833	1
Mrpl18	1,285651833	1
Iah1	1,285562722	1
Mrpl35	1,285473616	1
4930431P19Rik	1,285117257	1
Ssh2	1,285117257	1
Fam175a	1,285028182	1
Gosr1	1,284939114	1

Arl8b	1,284850052	1
F11r	1,284671946	1
Hltf	1,284671946	1
Cmc1	1,284671946	1
Fam126b	1,284671946	1
Gli1	1,284493865	1
Kiz	1,284404834	1
Gm7432	1,284315809	1
Pdss1	1,284137777	1
Gm12989	1,28395977	1
Atp5j2	1,283603831	1
BC031181	1,283514861	1
Syce2	1,283514861	1
Zfp622	1,283514861	1
Rab22a	1,28333694	1
B230217C12Rik	1,283247989	1
Jrkl	1,283247989	1
Rps8	1,283247989	1
Rev1	1,283159044	1
Tmem60	1,283159044	1
Furin	1,282892247	1
Als2	1,282803326	1
Ttc5	1,282625505	1
Ier3	1,282625505	1
Pafah1b3	1,282447707	1
Me2	1,282269935	1
Ptma	1,282181057	1
Mre11a	1,28155909	1
Cops2	1,281470262	1
Polr2h	1,281381441	1
Ube2b	1,281203816	1
Rpl31-ps16	1,281026216	1
Gm11362	1,28084864	1
Cep76	1,28084864	1
Chaf1a	1,28084864	1
Gm8770	1,280759861	1
Gnal	1,280671089	1
Rps10	1,280671089	1
Mier3	1,279961131	1
Polr2i	1,279872414	1
Tmem106a	1,279872414	1
Bbs2	1,279783703	1
Flt3l	1,279694998	1
Spred3	1,2796063	1
4933412L11Rik	1,2796063	1
Hnrnpd	1,279340241	1
Acyp1	1,278187957	1
Mtor	1,278010775	1
Akap1	1,277833618	1
RP23-13B8.12	1,277833618	1
Fam105a	1,277745048	1
Leprot	1,277656484	1

Vps26a		1,277567927	1
Copz2		1,277390831	1
Brix1		1,277302292	1
2900052L18Rik		1,277213759	1
Card14		1,277213759	1
Phip		1,277125233	1
Atp5c1		1,277036712	1
Dph3		1,276948198	1
Papd7		1,276859689	1
Elmo2		1,276771187	1
Ahdc1		1,276240304	1
Abi2		1,276151844	1
Etohd2		1,276063391	1
Zfp444		1,275974944	1
Ccdc174		1,275886504	1
Orc6		1,27570964	1
St14		1,27570964	1
Lamtor3		1,275532802	1
Gm16399		1,275444392	1
Mipep		1,275355988	1
Slc37a1		1,27526759	1
Klhl6		1,27526759	1
A430005L14Rik		1,275179198	1
Acsl3		1,275179198	1
Sgcb		1,275090812	1
Gm1848		1,27491406	1
Pabpc1		1,274560627	1
Dynll1		1,274560627	1
Trp53rka		1,274295617	1
Mterf3		1,274030663	1
Stac3		1,273942357	1
Stk16		1,273854057	1
E030030I06Rik		1,273589194	1
Slc29a2		1,273589194	1
Rhobtb2		1,272971394	1
	Sep 08	1,272971394	1
Mta3		1,272794935	1
Hinfp		1,272706715	1
9130230N09Rik		1,272530293	1
Pnrc2		1,272442091	1
Ndufs4		1,272265705	1
Mospd1		1,272177521	1
Ppig		1,272177521	1
Palld		1,271913007	1
Naa30		1,271913007	1
Gm15824		1,271736694	1
Gm12906		1,271736694	1
Dnaja2		1,271736694	1
Camk2n1		1,271560406	1
Klhl7		1,27129602	1
Tmem143		1,271119794	1
Rad9a		1,27059126	1

Mrpl16	1,27041513	1
Gm29257	1,270327075	1
Stac2	1,270150983	1
Tmem38a	1,26988689	1
Ttc7b	1,269798871	1
Gdap10	1,269710858	1
Ube2c	1,269534851	1
Pmvk	1,269446857	1
Tcea1-ps1	1,26909494	1
Ccser2	1,26909494	1
C330013E15Rik	1,269006976	1
Ube2v1	1,268743121	1
Gt(ROSA)26Sor	1,268655181	1
RP23-193N1.2	1,268303484	1
Rbm43	1,268303484	1
Bola2	1,268215575	1
6430573P05Rik	1,267864	1
Gm11956	1,267688249	1
Galnt10	1,267688249	1
Comp	1,267600382	1
Phospho2	1,267600382	1
Snhg6	1,267073312	1
Rpsa-ps10	1,267073312	1
Gpi1	1,26689767	1
Gm13196	1,266809859	1
Gm36964	1,266634254	1
Hnrnpr	1,266195348	1
Asnsd1	1,266195348	1
Yy1	1,266019828	1
Vkorc1	1,265932077	1
Gosr2	1,265932077	1
Man1c1	1,265668861	1
Xylt2	1,265317992	1
Tsc22d1	1,265317992	1
Aimp2	1,26523029	1
Ndufa1	1,265142594	1
Eif2a	1,265054904	1
Zgrf1	1,264879542	1
Gpr137	1,264879542	1
Creld2	1,264528892	1
Minos1	1,264528892	1
Cep57	1,264265968	1
Hace1	1,264178339	1
Slc25a5	1,264090716	1
Taf1a	1,263915487	1
Zfp422	1,263915487	1
Phf8	1,263827883	1
Gm10132	1,263652691	1
Nbn	1,263302381	1
B4galt7	1,263127262	1
Ppid	1,263127262	1
Phc2	1,263039712	1

Bcl2l2	1,262952167	1
Crk	1,262952167	1
Tmem59	1,262689571	1
Cript	1,262339528	1
M6pr	1,262077059	1
Zbed3	1,261639732	1
Gngt2	1,261464844	1
Mocs2	1,260852926	1
Map1lc3a	1,260852926	1
Slfn9	1,260765533	1
Mrpl30	1,260678147	1
Sptbn4	1,260503392	1
Glrx3	1,260328661	1
Chchd5	1,260153954	1
Gm9484	1,26006661	1
Bcl10	1,26006661	1
Cdc42bpb	1,259979272	1
Gm8869	1,25989194	1
Gm9256	1,25962998	1
Gm12816	1,259542672	1
Naga	1,259542672	1
Ndufaf1	1,259280785	1
Copb1	1,259106223	1
Gm5812	1,259018952	1
RP23-426K2.3	1,258931686	1
Vps51	1,258582685	1
Pcbp4	1,25849545	1
Gm3571	1,25849545	1
Gabpa	1,25849545	1
Lsm2	1,25840822	1
L3mbtl2	1,258320997	1
Sh3bgrl2	1,25823378	1
Jam2	1,257972165	1
Gm5871	1,257623429	1
Ints12	1,257623429	1
Ttc21b	1,25753626	1
Efcab11	1,257449098	1
Cd47	1,257361941	1
Rpl17-ps4	1,257187646	1
Arl4a	1,257100507	1
Dhrs4	1,257013375	1
Gm14673	1,257013375	1
Adipor1	1,257013375	1
Recql5	1,256926248	1
Slc5a6	1,256490706	1
Tmem183a	1,256490706	1
Rassf8	1,256403616	1
Rab33b	1,256403616	1
Arl5a	1,256316532	1
Gm5054	1,256229453	1
Ptms	1,256229453	1
2700060E02Rik	1,256142381	1

4930524J08Rik	1,255794153	1
4833439L19Rik	1,255707111	1
Snhg4	1,255271991	1
Erap1	1,255097986	1
Dr1	1,255097986	1
Itpkb	1,254924004	1
Elmsan1	1,254576114	1
Ilf2	1,254489156	1
Eif3l	1,254489156	1
Spata24	1,254054459	1
Ccdc115	1,253967537	1
Xk	1,253793712	1
Gm43430	1,253272383	1
Gm17971	1,253185515	1
Cep83	1,253185515	1
Naxd	1,253098654	1
Ubl5	1,253098654	1
Zfp39	1,253011799	1
Gabpb1	1,252838107	1
Tdpx-ps1	1,25275127	1
Gm38355	1,25275127	1
Wdr18	1,252664439	1
Pum1	1,252664439	1
Akt3	1,252577614	1
Spp1	1,252230373	1
Hsf2	1,252056789	1
Sdhaf2	1,251622935	1
Fbxo11	1,251536182	1
Tmem9b	1,251449435	1
Mettl13	1,251362694	1
Cyth2	1,25092908	1
Gm11221	1,250842375	1
Gm45902	1,250755677	1
Rbm8a2	1,250755677	1
Fig4	1,250668984	1
Lmf1	1,250668984	1
Dag1	1,250495616	1
Tbkbp1	1,250322273	1
Rnase4	1,249542524	1
Cdan1	1,249455916	1
Etfbkmt	1,249369313	1
Plppr2	1,249282716	1
Gm20568	1,249196126	1
Egfl8	1,249109541	1
Hspd1-ps3	1,249022962	1
Scyl2	1,248849823	1
Ndrg3	1,248503616	1
Etnk1	1,248330549	1
Mocs1	1,248244024	1
Deaf1	1,248244024	1
Layn	1,248244024	1
0610038B21Rik	1,248157506	1

Timm17a	1,248157506	1
Lonp2	1,247897985	1
Syncrip	1,247897985	1
Chst10	1,247811491	1
Jsrp1	1,247725002	1
Gm43961	1,247725002	1
Gm43223	1,247725002	1
Myl12a	1,247725002	1
Ccdc159	1,247552043	1
Nenf	1,247465572	1
Prmt2	1,247292648	1
Kif21b	1,247033308	1
Slc20a1	1,247033308	1
Rps18	1,246687605	1
Lats2	1,246601194	1
Gtpbp10	1,246341998	1
Rad54l	1,245996486	1
Rnf138	1,245737416	1
Fbxo2	1,245305751	1
Snx1	1,245219436	1
Tuba1a	1,245133127	1
Rabgef1	1,244874235	1
Ranbp10	1,244701671	1
Sec22c	1,24444287	1
Tmem251	1,24444287	1
Gamt	1,244356614	1
Rnf166	1,243925428	1
Fbxo44	1,243839209	1
Arpc1a	1,243839209	1
Mpst	1,243839209	1
Ntpcr	1,243752995	1
Mboat7	1,243666788	1
Nasp	1,243494391	1
Ube2l6	1,243408201	1
Mrps18b	1,243408201	1
Ric1	1,242977344	1
Eif3j1	1,24289119	1
Ctnnbl1	1,242805042	1
D330045A20Rik	1,242718901	1
Nol7	1,242632765	1
1700109H08Rik	1,242374394	1
Gm23458	1,242116076	1
Uqcrh-ps2	1,241943894	1
Unc119	1,241943894	1
Gatm	1,241857812	1
Cnih4	1,241857812	1
Gm14494	1,241427492	1
Amhr2	1,241341445	1
Renbp	1,241255405	1
Piezo1	1,241255405	1
Abhd10	1,24099732	1
Trib1	1,24099732	1

Mtpn	1,240911304	1
Rrn3	1,240739289	1
Hoxc6	1,240567298	1
Cul1	1,240481311	1
Mthfsl	1,240137424	1
Celf1	1,240137424	1
Ell	1,240051468	1
Nsmce2	1,239879572	1
Pgrmc1	1,2397077	1
Vps53	1,239278124	1
Sf3b6	1,239278124	1
Arpc3	1,239192227	1
Kif13b	1,239106336	1
Gm24924	1,239106336	1
Gm11688	1,239020451	1
Cdk19	1,238848698	1
Rraga	1,23876283	1
Zc3hc1	1,23841942	1
Hsd17b4	1,238333582	1
U2af2	1,23824775	1
Tomm22	1,237904483	1
Gm37274	1,237647095	1
Dcun1d4	1,23756131	1
Rmdn3	1,23756131	1
Hps3	1,237475532	1
Cd164	1,237475532	1
Tmf1	1,23738976	1
Rpl34	1,237218233	1
Med14	1,23704673	1
Taf11	1,236703796	1
Bst2	1,236618077	1
Prkd3	1,236446657	1
Rnaset2a	1,236275261	1
Cenpm	1,236189572	1
Fundc1	1,236103889	1
Gm12013	1,23593254	1
Calcrl	1,235846875	1
Cyp51	1,235589914	1
Pcsk7	1,235504273	1
Gm11694	1,235418637	1
Prpf18	1,235418637	1
Gm4540	1,235247384	1
Mob4	1,235076154	1
Gm45220	1,234990548	1
Ado	1,234733765	1
Rpl32	1,234648183	1
Zcchc9	1,234562607	1
Gm37780	1,234305914	1
Mtpap	1,234305914	1
Nsmce3	1,234220361	1
Fpgs	1,234049273	1
Abhd6	1,233792687	1

Gm44609	1,233621659	1
Cdyl	1,233621659	1
Skp1a	1,233450654	1
Sepsecs	1,233194192	1
Naa15	1,233108717	1
Slc26a11	1,232852325	1
Fcgr3	1,232595988	1
Brk1	1,232425125	1
Rai1	1,232083472	1
Rubcn	1,232083472	1
Pgm3	1,231998073	1
Psmb6-ps2	1,231827294	1
Gm7535	1,231571169	1
Pla2g4a	1,231485806	1
Gfpt1	1,231315098	1
Scp2-ps2	1,231144413	1
Rpl31-ps10	1,231144413	1
Plcg2	1,23105908	1
Fkbp1b	1,230973752	1
Med31	1,230717805	1
Rad51d	1,230547203	1
Nucks1	1,230547203	1
C130071C03Rik	1,230461911	1
Dazap2	1,230120802	1
Rps26	1,229694548	1
Tmem159	1,229609315	1
Zcchc11	1,229609315	1
Hmgn5	1,229353652	1
Ssbp3	1,228927664	1
1110038B12Rik	1,228757311	1
Gm7123	1,22858698	1
Dirc2	1,22858698	1
Dnajc19-ps	1,228501824	1
Ppme1	1,228501824	1
Klhdc4	1,228501824	1
Psma4	1,228246391	1
Gm43059	1,228161258	1
Smc3	1,228161258	1
Znrd1	1,227905896	1
Cebpd	1,227820787	1
Hsph1	1,227820787	1
Ndufv3	1,227735684	1
Guca1a	1,227650587	1
Gm43201	1,22748041	1
Gm43712	1,227310257	1
Dazap1	1,227225189	1
Rnf5	1,227055071	1
Zc3h10	1,227055071	1
Dhx40	1,226970021	1
Slc36a1	1,226714907	1
Aldh6a1	1,22662988	1
Ddit3	1,22654486	1

Gm13803	1,226204836	1
Actr6	1,22603486	1
Aurka	1,22603486	1
Dpf2	1,225949881	1
Lamtor4	1,225694978	1
Sap18b	1,225610022	1
AW549877	1,225610022	1
Crlf3	1,225610022	1
Mex3d	1,225440129	1
Ndufb6	1,22535519	1
Wdr47	1,225270258	1
4933421O10Rik	1,225270258	1
Cdc5l	1,225015497	1
Klc2	1,224760789	1
Nnt	1,224760789	1
Calm2	1,224675898	1
Shb	1,224591012	1
5031425E22Rik	1,224591012	1
Havcr2	1,224336392	1
Rnd2	1,224251531	1
Bmt2	1,224166675	1
Slc2a8	1,223996981	1
Hadha	1,223827311	1
Map1lc3b	1,223148865	1
Slirp	1,222979313	1
Rras2	1,222894545	1
Rer1	1,222894545	1
Dnmbp	1,222809783	1
Nipa2	1,222809783	1
Ddhd1	1,222555534	1
Gm8121	1,222470796	1
Gm9796	1,222470796	1
Snrnp48	1,222470796	1
Socs6	1,222216616	1
Ppp1r14b	1,222131902	1
Csnk1g3	1,222047193	1
Hccs	1,22196249	1
Slc35a2	1,221539063	1
Rplp0	1,221369734	1
Glo1	1,221115784	1
Gpbp1l1	1,221031145	1
Gch1	1,221031145	1
Gm23751	1,220946513	1
Hdac6	1,220946513	1
Gm12222	1,220269664	1
Eif4e2	1,220269664	1
Kti12	1,220015942	1
Otub1	1,220015942	1
Trappc8	1,21993138	1
Ndufa12	1,21993138	1
Fndc3a	1,219846824	1
Arl6ip1	1,219846824	1

Surf1	1,219762273	1
Tmem30a	1,219677729	1
Npepps	1,219677729	1
Naa60	1,219508657	1
Gm38247	1,219255094	1
RP24-550H10.6	1,219255094	1
Usp39	1,219170585	1
Gm6272	1,219086081	1
Gm45422	1,219001583	1
Gm13328	1,219001583	1
Pi16	1,219001583	1
Gm6140	1,218579183	1
Tardbp	1,21849472	1
Mrm2	1,218410264	1
Cdc34	1,218410264	1
Mrpl11	1,218325813	1
Psmd12	1,218156929	1
Zbtb37	1,218072495	1
Aga	1,217650417	1
Gm6206	1,217312859	1
Xkr8	1,216975395	1
Pdcl3	1,216975395	1
Mfsd14b	1,216806698	1
Slc17a7	1,216469375	1
Nostrin	1,216385058	1
Zfp382	1,216132144	1
RP23-128C4.4	1,216047852	1
Paqr4	1,215710738	1
Atp5h	1,215626474	1
Zfp654	1,215626474	1
Tnnt3	1,215457965	1
Phc1	1,215205243	1
Snrnp27	1,215121015	1
1810058I24Rik	1,214952575	1
Gnpnat1	1,214699959	1
Ube2k	1,214615765	1
Hsbp1	1,214615765	1
Chtf18	1,214447395	1
Psmb6	1,214279049	1
Gm9165	1,214194884	1
Usp32	1,214194884	1
RP23-331E5.10	1,213942426	1
Tspan31	1,213858285	1
Tmem158	1,213774149	1
2700062C07Rik	1,21369002	1
9430015G10Rik	1,21369002	1
5430427O19Rik	1,213605896	1
Mrps16	1,213521778	1
Pcdhb15	1,213185365	1
Fbxo18	1,213101277	1
Rdh11	1,213101277	1
Lrp5	1,21276498	1

Zfp746	1,212596867	1
Pibf1	1,212512819	1
Smarca5	1,212344741	1
Itgb1	1,212176686	1
Ttf1	1,211840646	1
Med13	1,211840646	1
Fam96a	1,21175665	1
R3hdm4	1,21175665	1
Gm15148	1,211168845	1
Trmt1	1,210917015	1
Suz12	1,210833084	1
Tsc1	1,210749158	1
Zfp511	1,210581324	1
Taf13	1,210581324	1
Naa50	1,210581324	1
Sh2d5	1,210161842	1
March2	1,210161842	1
Rchy1	1,210161842	1
Rad21	1,210161842	1
Rab1b	1,210077963	1
Anxa5	1,210077963	1
Nudcd2	1,209910222	1
Tprgl	1,20957481	1
Tmtc3	1,209490971	1
Tesk1	1,209490971	1
Mthfs	1,209239491	1
Pias2	1,209155676	1
Gm16104	1,209071866	1
Trappc1	1,208988063	1
Dtx2	1,208904265	1
Pycrl	1,208904265	1
Hspa5	1,208820473	1
Pkmyt1	1,208652907	1
Abhd8	1,208485363	1
Fbxo42	1,208234092	1
Slc25a23	1,208234092	1
2410006H16Rik	1,208234092	1
Ippk	1,208150347	1
Plekhg2	1,208066607	1
Pja2	1,208066607	1
A430046D13Rik	1,207982873	1
Sos1	1,207982873	1
Emp1	1,207982873	1
Ankle2	1,207899145	1
Dnajc3	1,207647995	1
Zfp646	1,207564291	1
Dnttip2	1,207396898	1
Abhd13	1,207313211	1
Snx30	1,207313211	1
Plcl2	1,207229529	1
Tmem132a	1,207145853	1
Taldo1	1,207145853	1

Adi1	1,207062183	1
Bola1	1,207062183	1
Api5	1,20689486	1
1110059G10Rik	1,206811208	1
Smad5	1,206727561	1
Tor3a	1,20664392	1
Eef1e1	1,206476655	1
Tm6sf1	1,206309413	1
Dgkd	1,206225801	1
Tcaim	1,206142195	1
Kdm1a	1,205975	1
Abcb1b	1,205975	1
Stam2	1,205891411	1
Borcs6	1,205807828	1
Ctr9	1,205640679	1
Eif4h	1,205557113	1
Ssb	1,205473553	1
Miip	1,205389999	1
Psmg4	1,205139371	1
Ndst1	1,20505584	1
Kdm4b	1,204972315	1
Abl2	1,204972315	1
Arfip1	1,204888796	1
Tle4	1,204721774	1
B230317F23Rik	1,204638272	1
Taf5l	1,204638272	1
Edf1	1,204638272	1
Ppp2r2a	1,204638272	1
Txnl1	1,204554776	1
Gm45286	1,204387801	1
Ambra1	1,204304322	1
Zfp607a	1,20405392	1
Myadm	1,20405392	1
Vti1b	1,203970464	1
D10Wsu102e	1,20380357	1
Tma16	1,203553272	1
Pigx	1,203469851	1
6030400A10Rik	1,203219622	1
Mrc1	1,203219622	1
Gm38192	1,203136224	1
Morc3	1,203052832	1
Cops8	1,202385903	1
Gm26670	1,20196926	1
Fam78a	1,201802643	1
1700096K18Rik	1,20163605	1
Myd88	1,20163605	1
0610012G03Rik	1,20163605	1
Tmem104	1,201386202	1
Drg1	1,201386202	1
Gm9774	1,201219666	1
Eif4enif1	1,201219666	1
Snrpb	1,201219666	1

Hdac9	1,201053154	1
Gm12882	1,200803427	1
Otud3	1,200803427	1
Wdr53	1,200636972	1
Gtf2a2	1,200387333	1
Rsbn1	1,200387333	1
Zbtb41	1,200304131	1
Gatad2b	1,200220935	1
B2m	1,200137745	1
Gm12770	1,19988821	1
Mapk8ip1	1,199805043	1
Pafah1b1-ps1	1,199805043	1
Ddx3x	1,199805043	1
Gm7128	1,199721881	1
Socs7	1,199721881	1
Tuba4a	1,199638726	1
Rabif	1,199555576	1
Gss	1,199555576	1
Ndufaf6	1,199306161	1
Nr1h3	1,199223035	1
Mtrf1	1,199223035	1
4930430F08Rik	1,199223035	1
Ufm1	1,199139914	1
Gm9843	1,198973689	1
Gm8185	1,198807488	1
Kctd13	1,198724396	1
Nop14	1,198641309	1
Rnf4	1,198475154	1
Mrpl49	1,198059866	1
Cmtr2	1,197893791	1
Hspa14	1,197478705	1
Gm8337	1,19731271	1
Ptrhd1	1,19731271	1
Commd2	1,19731271	1
P3h3	1,197229722	1
Gmcl1	1,197229722	1
Smim7	1,197229722	1
Hdac1	1,197146739	1
S100a11	1,197063762	1
Gm7094	1,196897825	1
Scp2	1,196897825	1
Rps19	1,196897825	1
Mfsd13a	1,196648963	1
Tmem131	1,196648963	1
Nicn1	1,196483084	1
Ldha	1,196483084	1
Rpl31-ps14	1,196317228	1
Zfp384	1,196317228	1
Gm11945	1,196317228	1
Ptdss2	1,196317228	1
Mat2b	1,196234308	1
1110004E09Rik	1,196068486	1

Atp5j	1,195654032	1
Asrgl1	1,195654032	1
Lum	1,195322573	1
Gm6444	1,195239722	1
Ccnt1	1,194991205	1
Gtf2e1	1,194908377	1
Zfp787	1,194908377	1
Pdcd4	1,194825555	1
Ctc1	1,194659929	1
Spire1	1,194659929	1
Acaa1b	1,194494325	1
Pot1a	1,194494325	1
Ppox	1,194411532	1
Gm3355	1,194328745	1
Rps13-ps7	1,194328745	1
2900026A02Rik	1,194328745	1
Snhg18	1,194245963	1
Calr3	1,194163187	1
Xrcc2	1,193997652	1
Slc25a3	1,193997652	1
Rnf113a1	1,193832141	1
Wdsub1	1,193666652	1
Mfsd8	1,193666652	1
Rbl2	1,193418462	1
Tax1bp1	1,193335743	1
Sparc	1,19325303	1
Slmap	1,19325303	1
Gm14270	1,193087622	1
Ccm2	1,192839552	1
Anp32e	1,192839552	1
Isca1	1,192756873	1
H2-DMa	1,192674201	1
Tmem248	1,192674201	1
Imp3	1,192674201	1
Gm13498	1,192591534	1
Gabarap	1,192591534	1
Pigf	1,192508872	1
Gm4978	1,192343567	1
Itch	1,192260923	1
Lck	1,192178284	1
Nsun6	1,192178284	1
Med21	1,192013025	1
Otud4	1,191930404	1
Prelid2	1,191765179	1
Ssr4	1,191682575	1
3110002H16Rik	1,191517384	1
Pdxdc1	1,191517384	1
Armc10	1,191517384	1
Dnajc21	1,191517384	1
Nhp2	1,191517384	1
Ahctf1	1,19126964	1
Cacnb1	1,191021949	1

Dusp6	1,191021949	1
Fgf13	1,190939396	1
Cotl1	1,190856849	1
Ccdc50	1,190691773	1
Bex3	1,190691773	1
Pomp	1,190609243	1
Cspg4	1,19052672	1
Nupl1	1,190196681	1
Ppih	1,190031696	1
Rnf13	1,190031696	1
Dad1	1,189949212	1
Fam53b	1,189866734	1
Snrnp35	1,189701795	1
Acadvl	1,189701795	1
R3hdm1	1,189619334	1
Dhrs9	1,189536879	1
Thumpd3	1,189454429	1
Mir99ahg	1,189371986	1
Mpc2	1,189207115	1
Cab39	1,189207115	1
Eif1	1,189124688	1
Mrpl55	1,188959852	1
Gtpbp1	1,188877442	1
Fpgt	1,188630248	1
Hexim2	1,18846548	1
Gm7565	1,18846548	1
Btbd10	1,18846548	1
Nudt4	1,188053661	1
Hspa13	1,187559666	1
Pald1	1,187395047	1
Cnpy4	1,187312746	1
Pfdn1	1,187312746	1
Ndufaf4	1,18723045	1
Abcc3	1,187148161	1
A430027C01Rik	1,187148161	1
Adarb1	1,186736798	1
Top1mt	1,186736798	1
Rmnd5b	1,186736798	1
Gpn3	1,186654542	1
Rasa4	1,186654542	1
Tmed5	1,186490048	1
Gm24339	1,186161129	1
Xpc	1,186161129	1
Txndc5	1,185914499	1
Ndufb10	1,185914499	1
Tmem115	1,185750108	1
Gde1	1,185667921	1
Ahsa1	1,185503564	1
Scoc	1,185503564	1
Tle3	1,185503564	1
Malt1	1,185421394	1
Ppie	1,185421394	1

Selenom	1,185257071	1
U2af1	1,185174918	1
Gm4332	1,185174918	1
Pfdn4	1,185092771	1
Lysmd4	1,184928494	1
Frg1	1,184928494	1
Ada	1,184846363	1
Etaa1	1,184271612	1
Capza1	1,184189527	1
1110065P20Rik	1,184189527	1
Ring1	1,183861246	1
Gm44916	1,183861246	1
Hps4	1,183697139	1
Zfp593	1,183286972	1
Zfand6	1,183204956	1
H1f0	1,18304094	1
Sestd1	1,182958941	1
Tpt1-ps5	1,182794959	1
Gm16523	1,182549029	1
Cry1	1,182467064	1
Pdgfa	1,182467064	1
Rbm12b2	1,182385105	1
Hnrnpk	1,182385105	1
Baat	1,18213926	1
Irf2	1,182057323	1
Mt1	1,181975392	1
Mapk1ip1l	1,181893467	1
Pigyl	1,181565822	1
Fam76b	1,181402033	1
Tmem144	1,181156393	1
Epb41l4aos	1,180992661	1
Zfp638	1,180910804	1
Trove2	1,180828952	1
Selenoh	1,180747106	1
8430429K09Rik	1,180665266	1
Snx5	1,180665266	1
Zfp707	1,180501603	1
Hmces	1,180501603	1
Mrpl1	1,180337962	1
P2ry2	1,18025615	1
Gm12350	1,180174343	1
Slc25a22	1,180010748	1
Gm22714	1,179928958	1
Phf12	1,179928958	1
Gopc	1,179928958	1
Cnot9	1,179683625	1
Mcee	1,179683625	1
Shoc2	1,179683625	1
Gm4149	1,179438342	1
Gm42986	1,179111377	1
Srek1ip1	1,178947929	1
Dusp11	1,178866214	1

Nbeal1	1,178784504	1
Prkab1	1,178621101	1
Dnajc17	1,178539408	1
Gtpbp2	1,178212693	1
Phf5a	1,178212693	1
Snupn	1,178049369	1
Ptbp1	1,177886068	1
Smarcal1	1,177559534	1
Gm37399	1,177477914	1
Zfp296	1,177396301	1
2410015M20Rik	1,177151494	1
Lgals9	1,176743595	1
Psmb8	1,176662032	1
Stat2	1,176580475	1
Hras	1,176498923	1
Pura	1,176498923	1
Slc35b3	1,176335837	1
Psen1	1,176172774	1
Eif2s3y	1,17609125	1
Fhod1	1,176009733	1
Pde7a	1,176009733	1
Atxn1l	1,175928221	1
Bak1	1,175928221	1
Slx4ip	1,175846714	1
Ripk1	1,175439268	1
Ints14	1,175439268	1
Kcnj2	1,175357795	1
Zfp146	1,175357795	1
Fam168b	1,175276328	1
a	1,175194867	1
Mutyh	1,175194867	1
Ube2j2	1,174950518	1
Gm15834	1,174787646	1
Nabp1	1,174787646	1
Ugdh	1,174624798	1
Car5b	1,174299167	1
Ptafr	1,174136386	1
Cln8	1,17356683	1
1110012L19Rik	1,173485487	1
Ifitm6	1,172753657	1
Nampt	1,17259109	1
Ankrd35	1,172509815	1
Meiob	1,172509815	1
Lacc1	1,172428546	1
Gm16556	1,172428546	1
Gm10073	1,172266024	1
Daxx	1,171941048	1
RP23-403E19.1	1,171859818	1
Pmpcb	1,171778594	1
Elob	1,171778594	1
Usp37	1,171697375	1
Ecsit	1,171534955	1

Lrpap1	1,171534955	1
Elovl5	1,171453753	1
Slc13a2	1,171291366	1
Wdr45b	1,171291366	1
Nap1l1	1,171291366	1
Arl6ip6	1,171210181	1
Gm7618	1,17096666	1
Ak6	1,170885498	1
Mrps25	1,17072319	1
Surf6	1,17072319	1
Rsl24d1	1,170642044	1
Nolc1	1,170560904	1
Phf1	1,170398641	1
Pcm1	1,170317518	1
Gm37675	1,170155289	1
Naca	1,169911988	1
Pi4kb	1,169587664	1
Gtpbp4	1,169506597	1
Wipi2	1,169182386	1
Pcgf2	1,169101347	1
Tle1	1,169101347	1
Asap1	1,169020314	1
Clcn6	1,168939287	1
Gm42876	1,168939287	1
Nxf1	1,168858265	1
Fgr	1,168696238	1
Cbx5	1,168696238	1
Sec14l1	1,168615233	1
Wdr34	1,168534233	1
Mcfd2	1,168534233	1
Il15	1,168291269	1
Fnbp4	1,16812932	1
Vamp8	1,16812932	1
Maea	1,167967395	1
Zyg11b	1,16788644	1
Lemd2	1,16788644	1
Hps6	1,167724548	1
Gm9246	1,16764361	1
Plek	1,16764361	1
Acadl	1,167481752	1
Ifi35	1,167400831	1
Tmem219	1,167400831	1
Cox4i1	1,167400831	1
Nde1	1,167239006	1
Cebpe	1,167158102	1
Actn4	1,167158102	1
Fam109a	1,166996311	1
Mdm2	1,166996311	1
Pex13	1,166834542	1
Eif2d	1,166834542	1
Gm16072	1,166511071	1
Gm11889	1,166430218	1

Ola1	1,166430218	1
Osbpl8	1,165945214	1
BC005624	1,1658644	1
Dzip1	1,165702788	1
Zfp65	1,16562199	1
Mllt11	1,165298856	1
Ctcf	1,165298856	1
Gnb1	1,165218086	1
Nap1l4	1,165056564	1
Spryd4	1,164975811	1
Gm10146	1,164733586	1
Zscan2	1,164733586	1
Adam8	1,164652856	1
Gm38257	1,164572131	1
Ckap5	1,164572131	1
Zcwpw1	1,164491412	1
Cfl1	1,164329991	1
Rhot1	1,164329991	1
Lactb2	1,164249288	1
Tox2	1,164007214	1
Gm37706	1,163765191	1
Rft1	1,163523218	1
Pwp2	1,163523218	1
Mettl5	1,163442572	1
Taf15	1,163442572	1
Urb2	1,163361931	1
Timm10b	1,163361931	1
Cxcl2	1,163200666	1
Phtf1os	1,163039423	1
Ywhaz	1,16295881	1
Nprl2	1,162717005	1
Kpna1	1,162717005	1
Smpd1	1,162555829	1
AI662270	1,162555829	1
Dolk	1,162314108	1
Vps35	1,162314108	1
Creb1	1,162233545	1
4833417C18Rik	1,162152988	1
Txnl4a	1,162152988	1
Sertad2	1,162152988	1
Prpsap2	1,16191135	1
Tet3	1,161830815	1
Gm10499	1,161669762	1
Tes	1,161669762	1
Cstf3	1,161428225	1
Hnrnpa2b1	1,160623463	1
Uqcrb	1,160543018	1
Rilpl1	1,160301715	1
Siah2	1,160221292	1
Fam210a	1,160060462	1
Snrpd1	1,159980055	1
Phf11d	1,159899655	1

Gm22581	1,159899655	1
Dip2c	1,159819259	1
Sf3b4	1,159738869	1
Pak1	1,159738869	1
Dpm3	1,159578107	1
St13	1,159578107	1
Pxmp2	1,159497734	1
Cox8a	1,159497734	1
Noct	1,159417366	1
RP23-23P9.3	1,159256648	1
Pdcd10	1,159256648	1
2610301B20Rik	1,159256648	1
Zfp131	1,159095952	1
Arf5	1,159095952	1
Gm37465	1,159015612	1
Traf3ip1	1,159015612	1
Gm44639	1,158935278	1
Kdm5c	1,15885495	1
Isg20l2	1,158774627	1
Ggh	1,158774627	1
Cap1	1,158774627	1
Gm14586	1,158694309	1
Lmo2	1,158613998	1
Gm25291	1,158453391	1
Dcaf5	1,158292806	1
Nelfb	1,158212522	1
Clcnkb	1,158132244	1
Psd3	1,158132244	1
F10	1,157891442	1
Snhg3	1,157811186	1
Wsb1	1,157811186	1
Mtm1	1,157730935	1
Icmt	1,157730935	1
Tm2d3	1,157490217	1
Hist2h2be	1,157490217	1
Mrpl48-ps	1,157249549	1
Zfp335	1,157249549	1
Snrpa	1,157169337	1
RP23-356D13.9	1,15700893	1
Ints2	1,15700893	1
Gm9396	1,156768362	1
Fkbp3	1,156287376	1
Etf1	1,156127091	1
Hnrnpc	1,155966829	1
Tpd52l2	1,155886707	1
Slc25a16	1,155806589	1
Fam160a2	1,155806589	1
Trim3	1,155726478	1
Mycn	1,155486176	1
Irf8	1,155406087	1
D330023K18Rik	1,155326003	1
Tpgs2	1,155245925	1

Eif3i	1,155245925	1
Rnf114	1,155005723	1
Stbd1	1,154765571	1
Clec4d	1,154765571	1
Tmem189	1,154765571	1
Fam98c	1,154605498	1
Mtss1	1,154605498	1
Proser1	1,154605498	1
Bud31	1,154605498	1
Psenen	1,154445447	1
Rfwd2	1,154445447	1
Sbds	1,154285418	1
Lmf2	1,154285418	1
Lrrc40	1,154285418	1
Ndufa3	1,154285418	1
Gnpda2	1,154125411	1
Rexo4	1,153965426	1
Gm11224	1,153885442	1
Fam76a	1,153725491	1
Fnip1	1,153725491	1
Scrn2	1,153645523	1
Gm15151	1,153245769	1
Snhg5	1,153165835	1
Gm37140	1,152766248	1
Prkcd	1,152766248	1
Ttc9c	1,152686347	1
Ccnc	1,152526562	1
Tnip3	1,152526562	1
Eif2s1	1,152526562	1
Anks3	1,152446677	1
Wdr82	1,152446677	1
Gm7809	1,152366799	1
Ltv1	1,152127196	1
Mpdu1	1,152047339	1
Yif1b	1,151887642	1
Ilkap	1,151807802	1
Orai1	1,151727968	1
D1Ertd622e	1,151648139	1
Mapk8	1,151488498	1
Atp1b3	1,151408685	1
Scaf1	1,151009707	1
Gm45806	1,150850154	1
Polr2e	1,150850154	1
Trim23	1,150291893	1
Pdhb	1,150052722	1
Tspyl1	1,150052722	1
Gm43351	1,149893302	1
Mfsd9	1,149654213	1
Fopnl	1,149574528	1
Gm38319	1,149494848	1
Fam122a	1,149335505	1
Zranb1	1,149255842	1

Ldlrap1	1,149096533	1
Epop	1,148937245	1
Fam214b	1,148857609	1
Gnb2	1,148698355	1
Ifitm2	1,148459515	1
Med12	1,148141138	1
Nfix	1,148141138	1
Mettl6	1,148061558	1
Ufsp1	1,147902414	1
Rps18-ps3	1,147902414	1
Gpbp1	1,147902414	1
Gm13181	1,147743292	1
Ppp4r3b	1,14750465	1
Trappc6a	1,147425114	1
Sla2	1,147345583	1
Ubl7	1,147107024	1
Gm11633	1,147107024	1
Ajuba	1,147027516	1
Pex1	1,146868515	1
Isoc1	1,146868515	1
Toe1	1,146709536	1
Parp9	1,146709536	1
Trappc10	1,146709536	1
Mkrn1	1,146709536	1
Serpinf2	1,146630055	1
Mrpl33	1,146471109	1
Rpl14	1,146471109	1
Pet100	1,146312186	1
Snip1	1,146232732	1
Tmem134	1,146232732	1
Crnde	1,146073842	1
Repin1	1,145517898	1
Gm12669	1,145438499	1
RP24-282K24.4	1,145200337	1
Epsti1	1,144882864	1
Aff1	1,144882864	1
Il13ra1	1,14480351	1
Sco1	1,144644817	1
1810032O08Rik	1,144486147	1
Mr1	1,144327498	1
Mink1	1,144248182	1
Chmp4b	1,144248182	1
Tbrg4	1,144248182	1
Kat7	1,144168872	1
Magohb	1,143930973	1
Tmem39b	1,143930973	1
Lpin1	1,143851685	1
Mrpl22	1,143772402	1
Tsta3	1,143772402	1
Ccdc77	1,143772402	1
Cndp2	1,143613852	1
Cep170	1,143376069	1

Ppil4	1,143217574	1
Trim36	1,143138335	1
Golph3l	1,143138335	1
Nus1	1,143059102	1
Ddx11	1,142900651	1
Osbpl3	1,142900651	1
Txndc15	1,142742223	1
Cuedc2	1,142742223	1
Chmp7	1,142742223	1
Tipin	1,142663016	1
Ccdc138	1,142583816	1
Slc25a17	1,142583816	1
Kitl	1,142425431	1
Cbx6	1,142425431	1
Tarbp2	1,142029565	1
Scaf8	1,142029565	1
Aprt	1,141950408	1
Rps6ka4	1,141871257	1
Ahcy	1,141792111	1
Trip10	1,141792111	1
Acbd3	1,141712971	1
Taf3	1,141633836	1
Dnaaf3	1,141554707	1
Dnmt3l	1,141475583	1
1700066M21Rik	1,141396465	1
Ccnh	1,141396465	1
Gm13835	1,141000956	1
Cfp	1,14092187	1
Pdpk1	1,140684647	1
Csnk1g2	1,140684647	1
Fxr2	1,140605583	1
Phlpp1	1,140210348	1
Smad4	1,140210348	1
Zfp706	1,139973273	1
Hmbs	1,139894259	1
4833445I07Rik	1,139894259	1
Osbp	1,139894259	1
Pdia6	1,139894259	1
Bicdl1	1,13981525	1
Ubl3	1,139420288	1
Csgalnact2	1,139183377	1
Ift22	1,139104418	1
Anapc2	1,139104418	1
A230028O05Rik	1,139025464	1
Usp35	1,138867572	1
Zfp30	1,138867572	1
RP24-84O13.9	1,138788635	1
Lpp	1,138788635	1
Ercc6	1,138630776	1
Cd14	1,138630776	1
Rbm12b1	1,138551855	1
Gm6030	1,138394029	1

Nelfe	1,138236225	1
AI846148	1,137920683	1
Gm9794	1,137920683	1
Pafah1b1	1,137841811	1
Ggta1	1,137762944	1
Trnau1ap	1,137684083	1
Atp5k	1,137684083	1
Cdadc1	1,137605228	1
Slc35a4	1,137526378	1
Rbm39	1,137368694	1
Gm20604	1,13728986	1
Tmem234	1,137211032	1
Eepd1	1,13697458	1
Isyna1	1,136423051	1
Cav2	1,136344283	1
Fam195a	1,136186763	1
M1ap	1,136186763	1
E4f1	1,136186763	1
Dhps	1,135950524	1
Hmgb1	1,135635615	1
Rest	1,135399491	1
Prune1	1,135399491	1
Dnm1l	1,135399491	1
Thumpd2	1,135242102	1
RP23-442M18.5	1,135084735	1
Gm37531	1,135084735	1
Klrg2	1,135084735	1
Plod3	1,135084735	1
Gm11598	1,13500606	1
Mat2a	1,13500606	1
Gm43672	1,13492739	1
Hoxb4	1,13492739	1
Myg1	1,134848725	1
Cpped1	1,134612765	1
Gm7670	1,134455485	1
Svbp	1,134455485	1
Usp36	1,134298227	1
Gm38043	1,134219606	1
Arnt	1,134219606	1
Atf6	1,133905177	1
Hprt	1,133826584	1
Zfp341	1,133512264	1
Necap2	1,133512264	1
2310009B15Rik	1,133433697	1
Nepro	1,133198031	1
Pias1	1,133198031	1
Rap2c	1,133198031	1
Zfp748	1,132805362	1
Gm9803	1,132648333	1
Hnrnpu	1,132648333	1
Clk1	1,132569827	1
Zfp275	1,132491326	1

Mrfap1	1,132491326	1
Ikbkap	1,132177376	1
Wdr91	1,132177376	1
Maip1	1,132020434	1
Sbk2	1,131941971	1
Nub1	1,131941971	1
0610030E20Rik	1,131863513	1
Cnot7	1,131706615	1
Usp21	1,131549738	1
Wtap	1,131549738	1
Slc30a1	1,131314463	1
Pcbd2	1,131236049	1
Mcub	1,13115764	1
Reep4	1,131079237	1
B3gnt2	1,130765679	1
Rabl3	1,130765679	1
Rbm22	1,130765679	1
Rras	1,130373853	1
Ltbp4	1,130217161	1
Fdps	1,130217161	1
Guf1	1,130217161	1
Thap2	1,130217161	1
Gm13998	1,130138823	1
Cbfa2t2	1,130138823	1
Ddx6	1,129668909	1
Lsm1	1,129434026	1
1500002F19Rik	1,129355742	1
Gm36266	1,129355742	1
Mcmbp	1,129355742	1
Thbs3	1,129120923	1
Ergic3	1,129120923	1
Papss1	1,129042661	1
Gm36930	1,128886154	1
Gm42890	1,128807908	1
Cnot8	1,128651433	1
Prdx6	1,128651433	1
Egr1	1,128573203	1
Rap2b	1,128416761	1
Stk11	1,12826034	1
Rbm7	1,128182137	1
Rps6kc1	1,128025749	1
Chac2	1,127947563	1
Abcb7	1,127869382	1
Abhd14b	1,127634873	1
Tshz1	1,127244132	1
Ccdc71l	1,127166	1
Ube2w	1,127166	1
Camkmt	1,127087874	1
Ptar1	1,126853527	1
Sufu	1,126775422	1
Gm5244	1,126697322	1
Rab5c	1,126697322	1

Erich1	1,12654114	1
Atp5s	1,126463057	1
Ube2a	1,126463057	1
Fcer1g	1,126306907	1
Letm2	1,126150778	1
Gm10320	1,126150778	1
Slc27a3	1,125760552	1
Alkbh1	1,125682523	1
Tgif1	1,125526481	1
Bsg	1,125448468	1
Ing3	1,12537046	1
Camk2g	1,125292458	1
Gm12421	1,125214462	1
Usp26	1,125214462	1
S100a1	1,125058485	1
Ctps2	1,12482456	1
Elof1	1,12482456	1
Utp18	1,124746595	1
Rbm6-ps1	1,124668637	1
Fyn	1,124434793	1
Mgme1	1,124278924	1
Mrnip	1,124123076	1
Tctn1	1,123967251	1
Wdr48	1,123655664	1
Gm6257	1,123577781	1
Eif3h	1,123577781	1
Abca7	1,123422031	1
Gspt1	1,123344164	1
Snord55	1,123266302	1
Casc3	1,123266302	1
Rad17	1,123188446	1
Cmss1	1,123110595	1
Gm15625	1,122877075	1
Usp47	1,122799246	1
Gm6265	1,122643604	1
Katna1	1,122565791	1
Tbca	1,122565791	1
Moap1	1,122410181	1
Bahcc1	1,122176806	1
Tjap1	1,122099026	1
Gm6542	1,121943481	1
St3gal5	1,121943481	1
Fam63b	1,121710203	1
Rab8b	1,121632455	1
Fbxo9	1,121399242	1
Ppm1a	1,121399242	1
Gm37914	1,121321515	1
Sgms1	1,121088367	1
Smad2	1,121010662	1
Aff4	1,120932962	1
Eif2b2	1,120855268	1
Lsg1	1,120622217	1

Nit2	1,120544544	1
Khnyn	1,120466876	1
Tpd52	1,120466876	1
Immp1l	1,119923354	1
Bet1	1,119690497	1
Pisd	1,119690497	1
Ndufb11	1,119690497	1
Rad23a	1,119612889	1
Lcor	1,119535286	1
Atad2	1,119535286	1
Gm17491	1,119302509	1
Igsf8	1,119302509	1
Msn	1,119302509	1
Sptbn1	1,119224928	1
Atp11c	1,119147352	1
Mettl2	1,119147352	1
Fam46c	1,118992216	1
Casp7	1,118914656	1
5430405H02Rik	1,118837101	1
Cers5	1,118759552	1
Wdr90	1,118682008	1
Mrm3	1,11860447	1
Llph-ps1	1,118526937	1
Adk	1,118526937	1
Akt1s1	1,118449409	1
Ndufaf2	1,118371887	1
Cdc25a	1,118371887	1
Yae1d1	1,11829437	1
Mkks	1,11829437	1
Akap8	1,117674427	1
Chd1	1,117519496	1
Tex264	1,117364585	1
Tmem201	1,117287138	1
Nova2	1,11713226	1
Arih2	1,117054829	1
Tube1	1,116977403	1
Sapcd1	1,116822568	1
Dpm1	1,116745158	1
Gm5380	1,116358191	1
Akt2	1,116280814	1
Hyal2	1,116203442	1
Clpb	1,116203442	1
Wrb	1,116048714	1
Nob1	1,115971358	1
Clip1	1,115971358	1
Rsad1	1,115739322	1
Sult6b1	1,115584659	1
Micall2	1,115584659	1
Rbm8a	1,115584659	1
Aste1	1,115430017	1
Timm29	1,115430017	1
Rtcb	1,115352704	1

Ccng1	1,115198094	1
Fam212a	1,114888938	1
Hsp90b1	1,114888938	1
Gm43138	1,114811662	1
Prkag1	1,114811662	1
Fut11	1,114657127	1
Urb1	1,114579868	1
Gm16253	1,114502614	1
Pelo	1,114502614	1
Ypel1	1,114425365	1
Gm45836	1,114425365	1
Gm5575	1,114348122	1
Uqcc2	1,114039201	1
Atg13	1,113961985	1
Gm37962	1,113807568	1
Gm22516	1,113498797	1
Simc1	1,113421618	1
Daam1	1,113421618	1
Siah1a	1,113267276	1
Prmt7	1,113267276	1
Hjurp	1,113267276	1
Uap1	1,113190113	1
Gm11222	1,112727246	1
Gins4	1,112650121	1
Brf2	1,112650121	1
Prpf4	1,112650121	1
Gm42819	1,112495885	1
Camsap2	1,112495885	1
Sh2d3c	1,112341671	1
Gm28438	1,112341671	1
Ik	1,112341671	1
Trim24	1,112264572	1
Trem2	1,112264572	1
Mrpl52	1,112264572	1
Rbm15	1,112187479	1
Asl	1,11211039	1
Gin1	1,111802091	1
Thoc5	1,111725029	1
Bckdha	1,111725029	1
2610044O15Rik8	1,111570922	1
7330423F06Rik	1,111570922	1
Tbc1d12	1,111493876	1
E2f2	1,111416836	1
Nemf	1,111416836	1
Bccip	1,110800707	1
Dnaja1	1,110800707	1
Hmg20b	1,110800707	1
Morn2	1,110646728	1
Baz1a	1,11049277	1
Mrpl20	1,110415799	1
Srsf2	1,110415799	1
Rock2	1,110338834	1

Alkbh6	1,110261873	1
Smg1	1,110261873	1
Snx12	1,110261873	1
Phf6	1,110184919	1
Myl12b	1,110184919	1
9530068E07Rik	1,110031025	1
Faim	1,109954086	1
Gm6560	1,109877153	1
2810428I15Rik	1,109877153	1
Snx14	1,109646384	1
Snora30	1,109569472	1
Tomm5	1,109569472	1
Gm6290	1,109492565	1
Gm15503	1,109415664	1
Gm5617	1,109261877	1
Dusp3	1,109261877	1
Tbccd1	1,109184991	1
Lpcat2	1,109108111	1
Plekhh2	1,108954367	1
Trappc2l	1,108954367	1
Tcp1	1,108800644	1
Gm11298	1,108493261	1
Ndufs2	1,108493261	1
Traf1	1,108416429	1
Stra8	1,108185964	1
Copg2	1,108185964	1
Zbtb3	1,108109153	1
Gm38335	1,107955548	1
Gm7384	1,107955548	1
Slfn4	1,107955548	1
St6galnac4	1,107955548	1
Gltscr1l	1,107571626	1
Dcp1a	1,107418095	1
Dbf4	1,107418095	1
Tmie	1,107264584	1
Slc35b2	1,107264584	1
Ifitm3	1,107264584	1
Ppp1cb	1,107187837	1
Glrx	1,107034359	1
Lyar	1,106957628	1
Lzts3	1,106880902	1
Plaa	1,106727467	1
RP24-225A16.3	1,106420659	1
Emc2	1,106420659	1
Gm37893	1,106343971	1
Fitm2	1,106343971	1
Mrps7	1,106190609	1
Tubb5	1,106190609	1
Paxbp1	1,106113937	1
Rps24-ps2	1,106113937	1
Homez	1,105960607	1
Kmt5a	1,105883951	1

Mapk8ip3	1,105500747	1
Park7	1,105424122	1
Bap1	1,105347503	1
Tm2d1	1,105270888	1
Actr3	1,105270888	1
RP23-47A1.1	1,10519428	1
Armt1	1,105041078	1
Spcs1	1,105041078	1
Gm4430	1,104887897	1
Tbx6	1,104811315	1
Slfn10-ps	1,104658166	1
Vcpip1	1,104658166	1
Zc3h11a	1,1045816	1
Ero1lb	1,1045816	1
4732491K20Rik	1,104505039	1
Rabggtb	1,104428483	1
Slc2a3	1,104351932	1
Gm7733	1,104275387	1
S100a6	1,104198847	1
Lsr	1,104045783	1
Ppp1r12a	1,104045783	1
Knstrn	1,103969259	1
Ftx	1,103892741	1
Arhgef40	1,103586719	1
Ogdh	1,103586719	1
Scaper	1,103510227	1
Clint1	1,103510227	1
Itgax	1,103280782	1
Steap3	1,103280782	1
Txn1	1,103280782	1
Gm38377	1,103051385	1
Hn1	1,102822036	1
Dgat1	1,102669163	1
Rwdd2a	1,102592735	1
Bloc1s6	1,102592735	1
Ubald1	1,102516311	1
Serinc1	1,102516311	1
Ctsb	1,102516311	1
Fubp1	1,102439893	1
Gm9575	1,102057883	1
Pygm	1,101981497	1
Gm45884	1,10182874	1
Ddx31	1,10175237	1
Zwint	1,101676005	1
C8g	1,101523291	1
Nod2	1,101370598	1
Insl6	1,101217926	1
Ppib	1,101217926	1
Hcst	1,101141598	1
Myl6b	1,101065275	1
Arpc4	1,101065275	1
Gm21399	1,100988958	1

Gpat4	1,100912646	1
Zfp277	1,100454884	1
Tor1a	1,100454884	1
Mesdc1	1,100226075	1
Gm43294	1,100149816	1
Pdia4	1,100149816	1
Rce1	1,100073562	1
Rps20	1,099997313	1
Grhl1	1,09992107	1
Knl1	1,099844832	1
Gm2950	1,099768599	1
Pcnx	1,099768599	1
Snrpd2	1,099692372	1
Pdap1	1,099692372	1
Gm6563	1,099616149	1
Vps18	1,099616149	1
Gm17494	1,099463721	1
Sp2	1,099311313	1
Ascc1	1,099311313	1
Pqbp1	1,099311313	1
Ifi204	1,099235117	1
Pola2	1,099235117	1
Mrrf	1,099235117	1
Ptgs2os2	1,099158927	1
Ccdc61	1,099006562	1
Cnep1r1	1,098854218	1
Zxdb	1,098778053	1
Ppa1	1,098778053	1
Armc8	1,09847345	1
Mark4	1,098245052	1
Mvk	1,09816893	1
Snrnp25	1,09816893	1
Mff	1,09816893	1
Pdia3	1,098092814	1
Plk1	1,098016702	1
Mis18bp1	1,097940596	1
Atp5a1	1,097940596	1
Zfp84	1,097864496	1
Pou2f1	1,097864496	1
Pcdhb16	1,097636225	1
RP23-40D21.1	1,097560145	1
Tmem86a	1,097408001	1
Irf2bp2	1,097255879	1
9130011E15Rik	1,097103778	1
Zfyve16	1,097027735	1
Zfp652	1,096951697	1
Kxd1	1,096951697	1
4933434E20Rik	1,096875665	1
Gm9332	1,096799638	1
Eif5a	1,096723616	1
Gm43588	1,0966476	1
Gm5898	1,096571589	1

Decr2	1,096419582	1
Cln6	1,096191612	1
Reep5	1,096191612	1
Zrsr2	1,095963689	1
Nkiras2	1,095887725	1
Cs	1,095887725	1
Luzp1	1,095735813	1
Plekhj1	1,095583922	1
A730011C13Rik	1,095280204	1
Tbp	1,095204288	1
Gxylt1	1,095204288	1
Rhog	1,09467302	1
Gm14776	1,094597146	1
Dus4l	1,094597146	1
Bag4	1,094597146	1
Mbnl2	1,094597146	1
Ethe1	1,094521277	1
Stip1	1,094445413	1
Sgpl1	1,094369555	1
Gm12276	1,094293701	1
Galnt3	1,094066173	1
Tfe3	1,093914514	1
Cpne3	1,093838692	1
Rida	1,093762875	1
Nectin3	1,093383872	1
Slc44a1	1,093383872	1
Gm2788	1,093232307	1
Max	1,093080763	1
Eapp	1,093004999	1
Rnf217	1,092777739	1
Brd2	1,092777739	1
Gm14248	1,092626258	1
Cdipt	1,092474799	1
Rpl8	1,092474799	1
Gpsm2	1,092399077	1
Efr3b	1,09232336	1
Myo1g	1,09232336	1
Idh3b	1,09232336	1
Ndufv2	1,092247649	1
Gm37339	1,092171942	1
Slc13a3	1,091944855	1
Tmbim6	1,091944855	1
Dcakd	1,09186917	1
Slc45a3	1,09179349	1
Htt	1,091642146	1
Tmem263	1,091642146	1
Thnsl1	1,09133952	1
Zfp873	1,091112606	1
Dusp14	1,091112606	1
Cd36	1,091036979	1
Fbxl5	1,091036979	1
Gm12618	1,090885739	1

Mycbp	1,090734521	1
Marf1	1,09065892	1
1700029J07Rik	1,090507733	1
Exosc8	1,090507733	1
Rrs1	1,090129857	1
Sec31b	1,090054298	1
RP24-370M23.1	1,090054298	1
Lrp1	1,090054298	1
Psmd13	1,089752112	1
Alg12	1,089601051	1
Zfp346	1,089601051	1
Scarna2	1,089450011	1
Edc4	1,089450011	1
Eid1	1,089450011	1
Ptpmt1	1,089374498	1
Rps2-ps10	1,089298991	1
Trmt10a	1,089223489	1
Nab1	1,089072501	1
Mbd2	1,088997015	1
Usp14	1,088997015	1
Uap1l1	1,088921534	1
Parpbp	1,088846059	1
Phf2	1,088695123	1
Polr1c	1,088619663	1
Wrnip1	1,08809159	1
2610318N02Rik	1,088016172	1
Phkg1	1,088016172	1
Edc3	1,087865351	1
Ppm1m	1,087789948	1
Gm29593	1,087639159	1
Tcp11l2	1,087488391	1
Stt3b	1,087413015	1
Skiv2l	1,087186917	1
Zfp729b	1,087186917	1
Sat2	1,087036211	1
AA986860	1,087036211	1
Rps15a-ps8	1,08658422	1
Ppp2r5a	1,08658422	1
C1d	1,086508906	1
1190005I06Rik	1,086358294	1
Gm14240	1,086207703	1
Uqcrfs1	1,085831318	1
Ncoa3	1,085756056	1
Gatc	1,0856808	1
Ptprj	1,085304597	1
Fcho2	1,085304597	1
Donson	1,085078938	1
BC037032	1,084778132	1
Rab9	1,084552582	1
Tpst1	1,084477409	1
Mfsd11	1,084251922	1
Slc9a4	1,084026481	1

Snora31	1,084026481	1
Nol8	1,083725967	1
Safb	1,083725967	1
Rbm27	1,083650851	1
Gm45853	1,083575741	1
Tcea1	1,083575741	1
Usf2	1,083350441	1
Mib2	1,083200267	1
Rnf44	1,083125188	1
Gm37206	1,082975046	1
Inppl1	1,082674823	1
Fcna	1,08259978	1
Xpnpep1	1,082524743	1
Gm45110	1,08244971	1
Atp5g2	1,082299661	1
Fuca1	1,082299661	1
Nr4a2	1,082149633	1
Med9	1,082149633	1
Psmb7	1,082074627	1
Mir17hg	1,08192463	1
Mex3a	1,081849639	1
Etv4	1,081774654	1
Sssca1	1,081624698	1
Ptp4a3	1,081624698	1
Pip5k1a	1,081474763	1
Zfp598	1,08132485	1
Ssr3	1,08132485	1
Zfp397	1,081174957	1
Nudc	1,080950156	1
Blmh	1,080950156	1
Tmem185b	1,080875233	1
Pprc1	1,080875233	1
Dbi	1,080875233	1
Dusp7	1,080800315	1
Nedd8	1,080800315	1
Utp14b	1,080725402	1
Acvr2a	1,080650494	1
Ythdf1	1,080650494	1
Gm13391	1,080575592	1
Lrig3	1,080425803	1
Iqcb1	1,080425803	1
Yme1l1	1,080425803	1
Dubr	1,080350916	1
Rrnad1	1,080126287	1
Smg7	1,080126287	1
Gm17018	1,080051421	1
Mirlet7b	1,079677168	1
Hoxb5	1,079452679	1
Psmc1	1,079303045	1
RP23-255F14.4	1,079228237	1
Dpp9	1,079078634	1
Zfp790	1,079003841	1

Ttc30b	1,078854269	1
March11	1,078779491	1
Cdc40	1,078704719	1
Smu1	1,078704719	1
Gm4737	1,078480432	1
Ptk2b	1,078181455	1
Hsdl1	1,078106724	1
Gpr132	1,078031998	1
Psmd14	1,078031998	1
Exd2	1,077957277	1
Atmin	1,077882561	1
Slc15a3	1,07780785	1
Spata6	1,07780785	1
Gcsh	1,07758375	1
Slc7a8	1,07758375	1
Syngr1	1,07750906	1
Gm42724	1,077285022	1
Noc3l	1,077285022	1
Arl2bp	1,077135689	1
Prkcsh	1,077135689	1
Rps3a2	1,076911728	1
Dtx3	1,076911728	1
Gnai2	1,076911728	1
Osbpl1a	1,076837085	1
Khsrp	1,076837085	1
Tox4	1,076837085	1
Dstn	1,076837085	1
Gm43466	1,076762446	1
Gm12529	1,076463946	1
Mark3	1,076165528	1
Acin1	1,076165528	1
Cerkl	1,075792622	1
Megf9	1,075718056	1
Oas1b	1,075643496	1
Map3k3	1,075643496	1
Rpl13a	1,075643496	1
Ski	1,075643496	1
Psmd7	1,075643496	1
Pdcd5-ps	1,07549439	1
Apobec1	1,07549439	1
Acbd6	1,075419845	1
Elf2	1,075270771	1
Rab18	1,075196241	1
Haus8	1,075047198	1
Xndc1	1,074972684	1
Polr2c	1,074972684	1
Rdh5	1,07467468	1
B230307C23Rik	1,07445123	1
RP24-323H7.5	1,07445123	1
1700123O20Rik	1,07445123	1
Gpank1	1,07445123	1
Tigd2	1,07445123	1

Gm42479	1,074376758	1
Timm50	1,074376758	1
Gm12497	1,07430229	1
Atp5sl	1,074153371	1
Mfsd7b	1,074153371	1
Zfp64	1,074078919	1
9430038I01Rik	1,07393003	1
Zfp142	1,073855593	1
Adamts10	1,073781162	1
Eif4a3	1,073781162	1
Hspbp1	1,073706736	1
Phf23	1,073706736	1
Ctsd	1,073706736	1
Gm5547	1,073632315	1
Csnk2a1	1,073557899	1
Tnfaip1	1,073409082	1
Cct2	1,073334682	1
Sash1	1,073185896	1
Nono	1,073185896	1
Pcmt1	1,073111511	1
Snapc2	1,073037131	1
Cdc73	1,072962757	1
Ak2	1,072962757	1
Nup54	1,072814023	1
Pcdhb22	1,072739664	1
Srebf1	1,072739664	1
Pan3	1,072665309	1
Uggt2	1,072516617	1
Aplp1	1,072516617	1
Gm23100	1,072516617	1
Vcp-rs	1,072293616	1
Krit1	1,072293616	1
Zfp248	1,071922052	1
Pcid2	1,071847755	1
Psmg3	1,071847755	1
Arhgap22	1,071773463	1
Morf4l1	1,071773463	1
Eif2b1	1,071699175	1
Ap3d1	1,071624893	1
Mrps18c	1,071402079	1
4930589O11Rik	1,071105064	1
Txn2	1,070956588	1
Nrp1	1,070882357	1
Gm45380	1,070585487	1
Rarg	1,070585487	1
Gm8242	1,070511282	1
Bcs1l	1,070437082	1
Snapc3	1,070288698	1
Tmem161a	1,070288698	1
Arl2	1,070140335	1
Rragc	1,070140335	1
Gm11427	1,069917829	1

Gm10167	1,069769517	1
Dpm2	1,069695369	1
Echs1	1,069695369	1
Gm44890	1,069547088	1
Chchd3	1,069472955	1
Ldlr	1,069472955	1
C1ra	1,069324705	1
H2afj	1,069324705	1
Hspb11	1,069250588	1
Trnt1	1,069176475	1
Klf13	1,068954169	1
Ifrd1	1,068583762	1
RP24-183O8.6	1,068509696	1
Tacc1	1,068509696	1
Zcchc8	1,068435635	1
Dhrs7	1,068287529	1
Cdkl3	1,068213484	1
Gm42633	1,068139443	1
Mcl1	1,067991378	1
Gm5963	1,067991378	1
Gnl2	1,067917353	1
Snhg17	1,067695309	1
4930526A20Rik	1,067695309	1
Rpa1	1,067621304	1
Anp32b	1,067547305	1
Napepld	1,067399322	1
Psme2	1,067177386	1
Zfp54	1,067103417	1
Gm14567	1,066955495	1
A630081D01Rik	1,066881542	1
Aimp1	1,066881542	1
Gm37452	1,066807594	1
G430095P16Rik	1,066807594	1
Ncbp2	1,066659713	1
Kansl3	1,066659713	1
Fbxo22	1,066585781	1
Tmem81	1,066290101	1
Fes	1,066216194	1
Tmpo	1,066142293	1
Nckap1	1,065994504	1
Ppp4r1l-ps	1,065994504	1
Qtrt1	1,065846736	1
Gm6162	1,06577286	1
Gm20442	1,06577286	1
Lhpp	1,065698989	1
Gm42511	1,065698989	1
Hectd3	1,065698989	1
Tyw5	1,065625123	1
Inpp5e	1,065625123	1
Fgd6	1,065625123	1
Dstyk	1,065625123	1
Pip4k2a	1,065625123	1

Tmx1	1,065625123	1
Commd8	1,065625123	1
Oraov1	1,065551262	1
Amz1	1,065551262	1
Gm6344	1,065477406	1
1810022K09Rik	1,065477406	1
Zfp651	1,065182034	1
Selenot	1,065182034	1
Dbndd2	1,065108203	1
Sat1	1,064960558	1
H2-D1	1,064886743	1
Rxra	1,064812934	1
Ddx49	1,064665329	1
Chd6	1,064296408	1
Emc6	1,064001364	1
Lpin2	1,063927615	1
Alms1	1,063706401	1
Mrpl14	1,063485232	1
Fam134b	1,063485232	1
Rcor3	1,063337812	1
Ptges3l	1,063337812	1
Gm15773	1,06326411	1
Peg12	1,063043033	1
Rnpep	1,062969351	1
Rbm41	1,062895674	1
Ppfia1	1,062895674	1
Srsf10	1,062748336	1
Zfpl1	1,062674674	1
Srp19	1,062674674	1
Golph3	1,062601018	1
5330438D12Rik	1,062527367	1
Rnf31	1,062527367	1
Gm6297	1,06245372	1
Rrp36	1,06245372	1
Tmem101	1,06245372	1
Mmgt1	1,062306443	1
Gm27010	1,062159186	1
Egr2	1,062159186	1
Chmp2a	1,062085566	1
Sgf29	1,06193834	1
Eef1b2	1,061864734	1
Smim3	1,061717539	1
Hpcal1	1,061717539	1
Heca	1,061643949	1
Pvr	1,061570364	1
Pop7	1,061496784	1
Serpine1	1,061349639	1
Zfp644	1,061349639	1
Eif4a1	1,061349639	1
Atxn7l1	1,061276075	1
Nmt1	1,061276075	1
Gnl1	1,061128961	1

Fcrl1	1,061055411	1
Pds5a	1,061055411	1
Mdh1	1,061055411	1
Qsox1	1,061055411	1
Tsacc	1,060981867	1
D17Wsu92e	1,060981867	1
Amz2	1,060761265	1
Cd44	1,060761265	1
Gm11520	1,060687741	1
Smad6	1,060614223	1
Rdh14	1,060540709	1
Tmed10	1,060540709	1
Tcaf1	1,060393697	1
Cenpc1	1,060320199	1
Gpr146	1,060320199	1
Ift46	1,060246705	1
Angel1	1,060173217	1
Ing5	1,060099734	1
E2f3	1,060026256	1
Cryga	1,059732395	1
Washc1	1,059658943	1
Psmf1	1,059585495	1
Aven	1,059438616	1
Rbmxl1	1,059365184	1
Vdac2	1,059365184	1
Arhgap12	1,058924698	1
Prox2	1,05877791	1
Asah1	1,05877791	1
Gm4673	1,058704523	1
Trp53i13	1,058704523	1
Gatad2a	1,058631142	1
Cyb561d1	1,058411029	1
Alkbh7	1,058337668	1
N4bp2l2	1,058337668	1
Tnnc1	1,058190961	1
Arl15	1,058044275	1
3110040N11Rik	1,058044275	1
Lrrc8c	1,057897609	1
Cxxc1	1,057677648	1
Ei24	1,057604338	1
Gm4734	1,057457733	1
Ap2s1	1,057311149	1
Slc9a5	1,057164585	1
Pkig	1,05709131	1
Ythdf3	1,057018041	1
Cecr5	1,056798263	1
Tmem106b	1,056578531	1
Gm15634	1,056505297	1
Wdr89	1,056505297	1
Hyal3	1,056285625	1
Ebna1bp2	1,056212412	1
Ndc1	1,056139203	1

Fcgr2b	1,056066	1
Gpatch2l	1,056066	1
Rnf19b	1,055992801	1
Satb2	1,055773237	1
Eaf1	1,055553718	1
Nudt2	1,055261097	1
1700061G19Rik	1,055187954	1
Gm23346	1,054895434	1
Kansl2	1,054895434	1
Wbp2	1,054822317	1
Ist1	1,054822317	1
Ino80	1,054749205	1
Txndc12	1,054749205	1
Slc37a3	1,054529899	1
Wdr83os	1,054529899	1
Abhd17c	1,054456807	1
Brd9	1,054456807	1
Prmt9	1,05438372	1
Tssc4	1,05438372	1
Gm43668	1,054310638	1
Cd99l2	1,054310638	1
Gm5422	1,054237562	1
Lrp6	1,054237562	1
Emc8	1,054237562	1
Ywhag	1,054237562	1
4933408B17Rik	1,05416449	1
Ifi30	1,054018362	1
Pik3cb	1,053507073	1
Agk	1,053434052	1
2310035C23Rik	1,053434052	1
Spopl	1,053434052	1
Bag1	1,053434052	1
Vps13b	1,053361036	1
Gk	1,053361036	1
Rel	1,053361036	1
Zfp688	1,053288025	1
Slc25a19	1,053215019	1
Msmo1	1,052339344	1
Tceal9	1,052266404	1
Zfp652os	1,052047614	1
Rad52	1,051901779	1
Lca5	1,051828869	1
Myl6	1,051828869	1
Fabp5l2	1,051683065	1
Dnajc18	1,051464397	1
Tmem218	1,051245773	1
D230025D16Rik	1,05110005	1
Sema4d	1,05110005	1
Afg3l2	1,051027196	1
Fbxw2	1,051027196	1
Ap2a1	1,050954347	1
Ddx19a	1,050954347	1

Caprin1	1,050954347	1
Sbf1	1,050881502	1
Ubl4a	1,050808663	1
Urgcp	1,050735829	1
Gm9828	1,050444544	1
Plcb3	1,050226133	1
Kremen1	1,050153339	1
Traf7	1,050153339	1
Agps	1,050153339	1
Pgrmc2	1,050153339	1
Usp18	1,050007767	1
C2cd5	1,049934989	1
Blzf1	1,049862215	1
Tmem185a	1,049571172	1
Zg16	1,049498424	1
Rrp1	1,049498424	1
Gpkow	1,049425681	1
Arel1	1,049425681	1
Zfas1	1,049425681	1
Tmem135	1,049280209	1
Rsf1	1,048989328	1
Ctnnd1	1,04891662	1
Brap	1,04891662	1
Myo10	1,048771219	1
Rplp1	1,048698526	1
Champ1	1,048625839	1
Gm6395	1,048553156	1
Nln	1,048553156	1
Ogfod2	1,048553156	1
Ift52	1,048480478	1
Sra1	1,048480478	1
Rpp30	1,048335138	1
Zmym5	1,048335138	1
Chml	1,048262476	1
Jmjd1c	1,048189818	1
Gm7285	1,048044518	1
Slc16a3	1,048044518	1
Strap	1,048044518	1
Uba2	1,047971876	1
Ano6	1,047971876	1
Lsm6	1,047899238	1
Fbxo8	1,047826606	1
Cnot6l	1,047826606	1
Plpp7	1,047681357	1
Flna	1,047681357	1
Elk4	1,047536127	1
Mlf2	1,047536127	1
Smndc1	1,04746352	1
Sgta	1,047318321	1
Tcta	1,047173142	1
Pole3	1,04710056	1
Rc3h2	1,046882844	1

Idnk	1,046810282	1
Dcps	1,046810282	1
Cep152	1,046737725	1
Cnppd1	1,046592627	1
Nup62	1,046592627	1
Tmem79	1,046375016	1
Anxa7	1,046375016	1
Eif1ax	1,046229968	1
Med23	1,04608494	1
Rab11a	1,045939932	1
Hk1	1,045939932	1
Psmb10	1,045867435	1
Zfp414	1,045722457	1
AI413582	1,045722457	1
Ubap2	1,045649976	1
Sike1	1,045505028	1
Kif5b	1,045505028	1
Kcnk6	1,044925438	1
Psma5	1,044853011	1
Lmbrd2	1,044708174	1
Nudt21	1,044635763	1
Pno1	1,044635763	1
Smg6	1,044563357	1
Immp2l	1,044201401	1
Xrcc1	1,043984288	1
Tnks1bp1	1,043911927	1
RP23-454I20.1	1,043911927	1
Gna13	1,043911927	1
Gm38345	1,043694875	1
Nfu1	1,043694875	1
Rnf10	1,043694875	1
Nfkb1	1,043405541	1
Fam135a	1,043260904	1
Gchfr	1,042971691	1
Cox6c	1,042971691	1
Gm16418	1,042827115	1
Etfa	1,042827115	1
Pank3	1,042754834	1
Zfp383	1,042682558	1
Ercc6l	1,042610287	1
Tbcc	1,042538022	1
Nme1	1,042538022	1
Rpl3l	1,042393505	1
Gm29462	1,042321254	1
Lrfn4	1,042176768	1
Cep85	1,042104532	1
Sys1	1,041887855	1
Mlf1	1,041743429	1
Trmt61a	1,041743429	1
Ttc12	1,041382451	1
Nfrkb	1,041382451	1
Sbno1	1,041382451	1

Mospd3	1,04131027	1
Ctdnep1	1,04131027	1
Brd4	1,041093758	1
Gm27477	1,041021598	1
Bag5	1,040877291	1
Adrm1	1,040588739	1
Zc3h18	1,040516613	1
B9d2	1,040300267	1
Gm23442	1,04015606	1
Nptn	1,04015606	1
Sdhaf1	1,040083965	1
Tom1l1	1,040083965	1
Snrnp70	1,040083965	1
Marc2	1,040083965	1
BC029722	1,040011874	1
Prpf8	1,039867708	1
Sep 10	1,039795632	1
Ppcs	1,039651496	1
Mrpl4	1,039651496	1
Ifnar2	1,039651496	1
Zfp867	1,039579435	1
Aqr	1,039435329	1
Ubc	1,039363283	1
Psmc6	1,039363283	1
Fam111a	1,039219207	1
Fosl1	1,03907515	1
Pym1	1,038931114	1
Hypk	1,038787098	1
Fam98b	1,038787098	1
Fen1	1,038499125	1
Med15	1,038499125	1
Sdc1	1,038283197	1
Atg5	1,038067315	1
Zfp58	1,037923418	1
Rogdi	1,037851477	1
4930440I19Rik	1,037419937	1
Sfr1	1,037419937	1
RP24-310D17.9	1,037348031	1
Tspo	1,037204234	1
Cr1l	1,037132343	1
Ebi3	1,037132343	1
Dexi	1,036988575	1
Rps10-ps1	1,036988575	1
Bpgm	1,036916699	1
Setd2	1,036844828	1
Rcor1	1,036844828	1
Gm7436	1,036772962	1
4932441J04Rik	1,036772962	1
Tbce	1,036485547	1
Adora2b	1,036270039	1
Lrch3	1,036198213	1
Sars	1,036126391	1

Mafg	1,035982764	1
Nup214	1,035767359	1
Pink1	1,035695568	1
Jade1	1,035695568	1
Sptlc2	1,035552	1
Atxn2	1,035552	1
Hmgn1	1,035552	1
Mrps22	1,035480224	1
Pla2g6	1,035480224	1
Prpf4b	1,035480224	1
5031439G07Rik	1,035480224	1
Hint1	1,035408452	1
Esf1	1,035193167	1
Gtf3c6	1,035121416	1
Prkra	1,035121416	1
Parp10	1,035049669	1
Gm28417	1,034977927	1
Samd4b	1,034619293	1
Rapgef2	1,034547582	1
March7	1,034547582	1
Mrps5	1,034475875	1
Trappc4	1,034475875	1
Sgk3	1,034260784	1
Bend4	1,034260784	1
Eefsec	1,034189097	1
Chek2	1,033974066	1
Lxn	1,033902398	1
Gba2	1,033830736	1
St5	1,033759079	1
Plekhm2	1,033759079	1
Hnrnpll	1,033687427	1
Nrde2	1,033544137	1
Pnpla8	1,0334725	1
Rpn2	1,033400868	1
Abhd16a	1,033186	1
Hcfc1	1,033186	1
Fam204a	1,032971178	1
Adprhl2	1,032827987	1
Gm37702	1,032684817	1
Gm29539	1,032470098	1
Sprtn	1,032326978	1
Dld	1,032326978	1
Srgn	1,032183877	1
Ermp1	1,032040795	1
Gm7236	1,031969262	1
Washc3	1,031826211	1
Ufl1	1,031754693	1
Zfp874b	1,031683179	1
Ifngr1	1,031611671	1
Nudt9	1,031540168	1
Naa38	1,031540168	1
Gpsm3	1,031325687	1

Vps50	1,031254204	1
Fam229b	1,031182725	1
Neu1	1,031182725	1
Gm5697	1,031111251	1
Smchd1	1,031111251	1
Gm12115	1,031039783	1
Rbpsuh-rs3	1,030968319	1
Fam53a	1,03089686	1
Selenoi	1,030825406	1
Cntrob	1,03053964	1
Yod1	1,03053964	1
Pebp1	1,030468211	1
Tmem129	1,030396787	1
Kdm5a	1,030396787	1
C530043K16Rik	1,030325368	1
Dnajc9	1,030253954	1
Zfp81	1,030253954	1
Gm12097	1,030182544	1
Atxn10	1,030182544	1
Agap3	1,03011114	1
Cog6	1,030039741	1
Kdm7a	1,029968346	1
Dusp12	1,029896957	1
Mrps10	1,029825572	1
Tyrobp	1,029682818	1
1810044D09Rik	1,029611448	1
Prrg2	1,029540083	1
Pop4	1,029326019	1
Fbxw8	1,029326019	1
Telo2	1,028826708	1
Adat1	1,028684092	1
Gm11868	1,028684092	1
Napb	1,028684092	1
Zfp772	1,028612792	1
Gm45420	1,028612792	1
Pgs1	1,028541496	1
Sh3bp2	1,028541496	1
A930004J17Rik	1,028470206	1
Klhl24	1,028470206	1
Tes3-ps	1,02839892	1
Eme1	1,028185093	1
Pik3r5	1,028185093	1
Clec4n	1,028113827	1
Ddx27	1,028113827	1
Cct4	1,028042566	1
Dnm1	1,02797131	1
Gm2272	1,027900059	1
Mief2	1,027900059	1
Pold2	1,027828812	1
Phka2	1,027757571	1
Mettl14	1,027686335	1
Gm10313	1,027472656	1

Parl	1,027472656	1
Chst1	1,027330228	1
Abce1	1,027330228	1
Dlx1	1,027259021	1
Ilk	1,027259021	1
Pom121	1,027187819	1
Gramd1a	1,026974244	1
Tti2	1,026831885	1
Il6ra	1,026618383	1
Emc10	1,026476074	1
Pa2g4	1,026262646	1
Matr3	1,026262646	1
Dync1i2	1,026262646	1
Cdk2ap2	1,026191514	1
Mrto4	1,026191514	1
Gm5909	1,026120386	1
Oxt	1,025907032	1
1700001P01Rik	1,025693723	1
Hexdc	1,025551542	1
Mir22hg	1,025551542	1
Anxa4	1,025551542	1
Baiap2	1,025480458	1
2300009A05Rik	1,02540938	1
Stap1	1,025267238	1
Arid4b	1,025267238	1
Cox7b	1,025267238	1
Cfap74	1,025125116	1
Lcmt1	1,025125116	1
Rsrc1	1,025054062	1
Cd300lb	1,024983013	1
Alas1	1,02484093	1
Tubg1	1,02484093	1
Cdkn2aipnl	1,02484093	1
Gpr65	1,024769896	1
Eed	1,024769896	1
Rps25-ps1	1,024769896	1
Greb1	1,024556823	1
Itgb5	1,024556823	1
Sh3gl1	1,024556823	1
Ndufa5	1,024414799	1
Pam	1,024414799	1
Rad18	1,024059826	1
Mtf2	1,0238469	1
Zfp623	1,023775935	1
Snrpa1	1,02363402	1
Tmem38b	1,023563069	1
Rfc1	1,023563069	1
Zfp90	1,023421183	1
Arhgef10l	1,023421183	1
St3gal1	1,023421183	1
Smim14	1,023279317	1
D330041H03Rik	1,023208391	1

Vmac	1,023208391	1
Il6st	1,022924736	1
Tcerg1	1,022924736	1
Ybey	1,022853835	1
Scrib	1,022782939	1
Arf4	1,022782939	1
Gmeb1	1,022712047	1
Med29	1,022641161	1
Bscl2	1,022641161	1
Aldh3a2	1,022499402	1
Ankle1	1,022357664	1
Gm45222	1,022215945	1
AI480526	1,022074245	1
Efcab7	1,022003403	1
Srsf11	1,021932566	1
Cul4b	1,021790905	1
Ipo13	1,021720083	1
Rpap3	1,021578452	1
Rps5	1,021578452	1
Stx8	1,021366043	1
Glrx2	1,02129525	1
Cacna1s	1,021224461	1
Gm16437	1,021153678	1
Pak1ip1	1,021153678	1
Atp23	1,021012126	1
Usp30	1,020941357	1
Ppp1r13b	1,020870593	1
Cacna1a	1,020870593	1
P4hb	1,020870593	1
Map2k7	1,02072908	1
Evi5	1,02072908	1
Thop1	1,020658331	1
Ercc2	1,020658331	1
Zmat3	1,020516848	1
Sdcbp	1,020516848	1
Cited2	1,020446113	1
Rprd1a	1,020304659	1
9430060I03Rik	1,02023394	1
Gps1	1,020163225	1
Paqr3	1,02002181	1
Atp9b	1,02002181	1
Dna2	1,01995111	1
Gm17259	1,019880415	1
Sp1	1,019880415	1
Prps1	1,019880415	1
Gcnt1	1,019739039	1
Fam49b	1,019739039	1
Kcnb1	1,019527012	1
Kat2b	1,019456347	1
Rgs10	1,019244379	1
Ctu2	1,019173732	1
Pik3r6	1,019103091	1

Dhrs7b	1,019103091	1
Fbxl12	1,018961823	1
Pak2	1,018820575	1
Nemp2	1,018749958	1
Atox1	1,018679346	1
Selenof	1,018608739	1
Rapgef6	1,018538137	1
Zkscan5	1,01846754	1
Pola1	1,01846754	1
Nup133	1,018255777	1
Gm12606	1,018114627	1
Metap1	1,018114627	1
Ago3	1,018044059	1
Gga1	1,017973496	1
Kansl1	1,017902938	1
Bag6	1,017902938	1
AI597479	1,017902938	1
Gm37726	1,017832385	1
Rpp14	1,017832385	1
Magee1	1,017761836	1
Klhl9	1,017691293	1
Zswim6	1,017550221	1
Gm45630	1,017338649	1
Gm28530	1,017197626	1
Gm13822	1,017197626	1
Ap1ar	1,017127122	1
Bax	1,016774673	1
Gm15644	1,016422347	1
Chchd10	1,016422347	1
Pwwp2a	1,016351897	1
Col4a5	1,016351897	1
Pdha1	1,016140574	1
Gm2756	1,015999717	1
Slc7a5	1,015999717	1
Gmeb2	1,015929296	1
Tsen34	1,015788468	1
Rabggta	1,015788468	1
Ddt	1,015788468	1
Kif1b	1,015788468	1
C130083A15Rik	1,015718061	1
Polrmt	1,015718061	1
Rpl3-ps1	1,015647659	1
Use1	1,015577262	1
Suclg2	1,01550687	1
Adprm	1,015436483	1
Smim13	1,015366101	1
Otud6b	1,015295723	1
Fuom	1,015225351	1
Vcl	1,015225351	1
Zfp692	1,015154983	1
Atp2c1	1,015154983	1
Trpm7	1,01494391	1

Rev3l	1,01473288	1
Mrpl43	1,014662547	1
Psmb4	1,014451575	1
Tnfrsf10b	1,014381261	1
Setd3	1,014381261	1
Snrnp40	1,014310952	1
2210408I21Rik	1,014029765	1
Actr10	1,014029765	1
Tmed4	1,0138892	1
Zfp607b	1,013818925	1
Rock1	1,013748655	1
Ndufab1-ps	1,013608129	1
Cbl	1,013537874	1
Atxn2l	1,013467623	1
Tnfrsf11a	1,013327136	1
C1galt1c1	1,0132569	1
Exoc3	1,013186669	1
Ece1	1,012976005	1
Zfp24	1,012976005	1
Bckdhb	1,012905793	1
Papola	1,012905793	1
Cox5a	1,012905793	1
Rpl22	1,012835586	1
Ndufab1	1,012695187	1
Idua	1,012484625	1
Nubp1	1,012203943	1
Gm37108	1,012133785	1
Mrpl48	1,012133785	1
Eef2	1,012063632	1
Ifrd2	1,011993483	1
Set	1,011993483	1
Fgf11	1,011853201	1
Rnf167	1,011853201	1
Dcun1d5	1,011853201	1
Npm1	1,011853201	1
Mrpl27	1,011783067	1
Rbm28	1,011783067	1
Phkg2	1,011642814	1
Lst1	1,011572695	1
6230400D17Rik	1,01150258	1
Flot2	1,011362366	1
Slc38a2	1,011222171	1
Mrps15	1,011152081	1
Anapc1	1,011081996	1
Polb	1,011081996	1
Nhlrc2	1,011011915	1
Vdac3-ps1	1,01094184	1
Cox11	1,01094184	1
Bnip1	1,01094184	1
Wdr78	1,010871769	1
Mthfd2l	1,010871769	1
Adat2	1,010801703	1

Spag9	1,010801703	1
Rnf14	1,010731642	1
Irf7	1,010661586	1
Edem1	1,010451446	1
Smc1a	1,010311378	1
Zfp148	1,010241351	1
Eps15l1	1,010101311	1
Ndufb3	1,010101311	1
Tsr1	1,010101311	1
Cmip	1,010031299	1
Sgpp1	1,009961291	1
Gm24601	1,009891289	1
S1pr2	1,009891289	1
Snx6	1,009821291	1
L2hgdh	1,009681309	1
Thap4	1,009401405	1
Phldb1	1,009331441	1
Rb1	1,009261482	1
Zfp428	1,009191528	1
Sdf2	1,009191528	1
Lcorl	1,009121578	1
Gm13864	1,008981694	1
Slc25a28	1,008981694	1
Akap10	1,008841829	1
Gm45137	1,008632068	1
Gm14822	1,008562158	1
1700007K09Rik	1,008562158	1
Ddx19b	1,008562158	1
Apoa1bp	1,008422351	1
Chuk	1,008352455	1
Ttc37	1,008282564	1
Zfp938	1,008142796	1
Mettl18	1,008072919	1
Oxsr1	1,007723608	1
Paics	1,00751408	1
Ndufa7	1,007234776	1
Maml3	1,007164962	1
Zfp420	1,007095153	1
Thap3	1,00695555	1
Gtf2a1	1,006885756	1
Wdr55	1,006746181	1
Rabac1	1,006606626	1
Uqcr10	1,006536856	1
Gm37660	1,006467091	1
Rfesd	1,006188077	1
Aldoart1	1,006118336	1
Trmt44	1,0060486	1
Ddx42	1,0060486	1
Fdx1	1,00583942	1
Utp11	1,00583942	1
Ccdc86	1,00569999	1
Plekhg3	1,00521214	1

Grk4	1,005142466	1
Nqo2	1,005142466	1
Lrrc28	1,005003134	1
Aar2	1,005003134	1
Mbd3	1,005003134	1
Gfm2	1,00486382	1
Ltc4s	1,004654887	1
Ubap2l	1,004654887	1
B4galt6	1,004585252	1
Tsr3	1,004585252	1
Ier3ip1	1,004585252	1
Zfyve21	1,004237149	1
Mbtd1	1,004167543	1
Mrpl45	1,003958754	1
Zfp592	1,003889167	1
Tagln2	1,003889167	1
Pex19	1,003819586	1
Ccdc69	1,003750009	1
Ranbp3	1,003680436	1
Rilpl2	1,003402196	1
Tpp2	1,003263104	1
Tmco6	1,003193566	1
Snx16	1,003124032	1
Esyt1	1,003124032	1
Hmgcl	1,003124032	1
Gm4707	1,00291546	1
Gse1	1,00291546	1
Cipc	1,002845945	1
Bhlhe40	1,002567936	1
Cfdp1	1,00242896	1
Ipmk	1,002220533	1
Ercc4	1,002151066	1
Tmem167b	1,002081605	1
Rab19	1,002012148	1
Mipol1	1,002012148	1
Gm6293	1,00187325	1
Git1	1,001803808	1
Chchd2	1,001664938	1
Exog	1,00159551	1
Tmem126a	1,001387256	1
Gm25857	1,001248444	1
Kcnc3	1,001179045	1
Nr2f6	1,001040261	1
Snord118	1,000901497	1
Bcat2	1,000901497	1
Ccdc82	1,000762753	1
Commd6	1,000762753	1
Gm6921	1,000624027	1
Cggbp1	1,000485321	1
Zc3h13	1,000277297	1
Csrnp2	1,000207966	1
Trib3	1,000207966	1

Mrpl24	1,000207966	1
Aes	1,000138639	1
Gm42418	1	1
Sap130	-0,00010235	1
Tmem123	-0,00020918	1
Axin1	-0,00051061	1
Pef1	-0,00066609	1
Casp3	-0,00066679	1
Zfp597	-0,00079728	1
RP23-359K10.8	-0,0008459	1
Ago4	-0,001019	1
Tnip2	-0,0011151	1
Apex1	-0,001271	1
Gm14328	-0,0014232	1
Pacs1	-0,0013717	1
Rpl4	-0,0017324	1
Sec22b	-0,0016879	1
Kif3a	-0,0017825	1
Mtmr1	-0,0020055	1
Spast	-0,0021877	1
Faf1	-0,0024129	1
Arhgap5	-0,0024002	1
1110051M20Rik	-0,0025935	1
Gm43329	-0,0027388	1
Gm11966	-0,0029064	1
1700088E04Rik	-0,0030476	1
Fmnl1	-0,0030892	1
Ccdc47	-0,0031547	1
Irak3	-0,0033305	1
Nt5c	-0,0034019	1
Ap2a2	-0,0036071	1
Cept1	-0,0036207	1
Stoml2	-0,0037317	1
Ogg1	-0,0039739	1
Nfxl1	-0,004084	1
Sf3b5	-0,0040833	1
Gm11110	-0,0042192	1
Bmp2k	-0,0045484	1
Eny2	-0,004505	1
Stambpl1	-0,0047955	1
Zfp35	-0,0049442	1
Zdhhc24	-0,0050513	1
Dhcr24	-0,0053006	1
Sf3a3	-0,0052567	1
mt-Tt	-0,0054627	1
2010016I18Rik	-0,0059284	1
Ldb1	-0,0059761	1
Tfap4	-0,0059669	1
Gm37472	-0,0062388	1
Mid1ip1	-0,0062845	1
Baz1b	-0,0062964	1
2610002M06Rik	-0,0063689	1

Gid4	-0,0065499	1
Pcbp1	-0,0064591	1
Rabep1	-0,0065063	1
mt-Nd5	-0,0069622	1
Akr7a5	-0,007082	1
Stim1	-0,0071042	1
Trappc5	-0,0073087	1
Hdgfrp2	-0,0074155	1
Slfn8	-0,0075257	1
Gm16310	-0,007515	1
Asna1	-0,0075615	1
Ddost	-0,0076926	1
Ankrd17	-0,0077263	1
Cox7a2	-0,0077463	1
Ints13	-0,0078305	1
Snw1	-0,0078006	1
Slc39a3	-0,0082194	1
Wdr5	-0,0084416	1
Zeb2	-0,0084408	1
Gm37199	-0,0084729	1
Exoc2	-0,0085083	1
Pdrg1	-0,0085653	1
Nae1	-0,0087926	1
Ctso	-0,0092473	1
Dtx3l	-0,0093197	1
Acvr2b	-0,0094012	1
Msh6	-0,0094431	1
Larp7	-0,0094644	1
Tbc1d8b	-0,0096075	1
Gpnmb	-0,0097835	1
Rffl	-0,0099203	1
Ptges2	-0,010018	1
Cdip1	-0,010131	1
Ndufb7	-0,010209	1
Gfer	-0,010208	1
Por	-0,010155	1
Prmt6	-0,010388	1
Abtb1	-0,010496	1
Ssr2	-0,011032	1
Cltb	-0,011245	1
1700037H04Rik	-0,011404	1
Sltm	-0,011391	1
Ppp4r2	-0,011645	1
Rab3gap1	-0,01161	1
Gm45568	-0,011731	1
Atg3	-0,011688	1
Ccny	-0,011754	1
Dhx37	-0,011996	1
Tmem222	-0,012228	1
Psmc5	-0,012338	1
Tnpo1	-0,012323	1
Llgl2	-0,012705	1

Sde2	-0,012679	1
Cep170b	-0,012935	1
Mrpl39	-0,013067	1
Aebp2	-0,013182	1
Cpsf2	-0,01325	1
2610306M01Rik	-0,013266	1
Avpi1	-0,01327	1
Tbc1d22b	-0,013399	1
Cisd3	-0,01353	1
2500002B13Rik	-0,013495	1
Atp1a3	-0,013626	1
Snapc5	-0,013667	1
Pnpo	-0,013718	1
Plxnc1	-0,013721	1
Kntc1	-0,013834	1
Gpx4	-0,013753	1
Mrps28	-0,013798	1
Ppm1d	-0,013822	1
Mustn1	-0,013934	1
Il13ra2	-0,01402	1
Tpm3	-0,014153	1
Sil1	-0,014303	1
Tmub2	-0,014427	1
Casp9	-0,014389	1
4930518I15Rik	-0,014526	1
Dkc1	-0,014633	1
Asph	-0,014553	1
Zc3h14	-0,015	1
Pih1d2	-0,015077	1
Tap1	-0,015276	1
Gm42748	-0,01541	1
Clock	-0,015793	1
Ift88	-0,016054	1
Smc6	-0,016078	1
Ndufs3	-0,016177	1
Ctbs	-0,016188	1
Uqcrc1	-0,016332	1
Sirt3	-0,016529	1
Tmem128	-0,016765	1
Abhd2	-0,017033	1
Uba52	-0,017402	1
Wipf2	-0,017535	1
Cebpz	-0,017687	1
Prkar2a	-0,017998	1
Zbtb5	-0,018259	1
Syngap1	-0,018459	1
Ubtd2	-0,018559	1
Vdac3	-0,018554	1
5830408C22Rik	-0,018709	1
Lactb	-0,018661	1
Phrf1	-0,018939	1
E230020A03Rik	-0,018974	1

Cnnm2	-0,019108	1
Snrpd3	-0,019051	1
Emp3	-0,019516	1
Slpi	-0,019658	1
Sumf1	-0,019895	1
Ywhae	-0,020075	1
Tmem39a	-0,020177	1
Tmem165	-0,020186	1
Actr2	-0,020151	1
Mthfd2	-0,020189	1
Ugt1a7c	-0,020445	1
Pcbp2	-0,020373	1
Selenon	-0,020907	1
Srsf7	-0,021024	1
Cmc2	-0,02123	1
Arf3	-0,021303	1
Slc22a17	-0,021353	1
Zc3h15	-0,021489	1
Gadd45gip1	-0,021662	1
Fam43a	-0,021758	1
Ndufa10	-0,021778	1
Git2	-0,022031	1
Slc35b1	-0,021956	1
Pigu	-0,022059	1
Epc1	-0,022105	1
Fh1	-0,022181	1
Rpl17-ps10	-0,02274	1
Ahnak	-0,022707	1
Znrf1	-0,022848	1
Slc39a2	-0,023004	1
Tsen15	-0,023318	1
2310043L19Rik	-0,023358	1
Rpf2	-0,0237	1
Nckap1l	-0,023684	1
D530018E20Rik	-0,023771	1
Gm11273	-0,023856	1
A930006K02Rik	-0,023945	1
Gm23502	-0,024044	1
Washc4	-0,024371	1
Cbx3	-0,02438	1
Ubqln1	-0,024463	1
Uchl4	-0,024567	1
Rnf141	-0,024664	1
Ndufb5	-0,024735	1
Arl10	-0,024792	1
Hipk3	-0,024806	1
Phtf1	-0,02495	1
Ppat	-0,025002	1
Actr1b	-0,025042	1
Macrod1	-0,025287	1
Polk	-0,025564	1
Ralgds	-0,02566	1

Eci2	-0,025843	1
Gm12096	-0,025897	1
Gm42571	-0,025852	1
Trappc3	-0,025991	1
Ndufc2	-0,026096	1
Fzr1	-0,026319	1
Rbm12	-0,026331	1
Zfp961	-0,026451	1
Rps19-ps3	-0,026587	1
Ncoa5	-0,026665	1
Hist1h2aa	-0,026863	1
Tlcd2	-0,026954	1
Ppp6r2	-0,027083	1
Atn1	-0,027094	1
Mtch1	-0,027078	1
Cct8	-0,027136	1
Nos1	-0,027237	1
Ddx3y	-0,027202	1
Zfp580	-0,02743	1
Pdik1l	-0,027462	1
P2rx7	-0,027719	1
4833420G17Rik	-0,02802	1
Gpt	-0,02809	1
Tmx3	-0,028231	1
Capn15	-0,028251	1
Mfsd12	-0,02863	1
Appbp2	-0,028785	1
Dhx30	-0,028801	1
Fip1l1	-0,028792	1
Tmem160	-0,028989	1
Lrrc42	-0,029828	1
Ngdn	-0,029916	1
Mtcl1	-0,030157	1
C2cd2l	-0,030328	1
Mcoln2	-0,030455	1
Lats1	-0,030586	1
Mtx2	-0,030947	1
Thoc1	-0,031017	1
Mapk7	-0,031076	1
Zfp526	-0,031175	1
Map4k5	-0,031343	1
Luc7l	-0,03129	1
St6gal1	-0,031529	1
AC168977.1	-0,031624	1
9230111E07Rik	-0,031924	1
Tssc1	-0,031877	1
Irf5	-0,031966	1
Man2c1os	-0,032053	1
Minpp1	-0,032119	1
Timm9	-0,032115	1
Ints4	-0,032293	1
Cd200r4	-0,032324	1

Ifi202b	-0,032403	1
Bora	-0,032426	1
Hgs	-0,032417	1
Rbms1	-0,032376	1
Asxl1	-0,032513	1
Gkap1	-0,032572	1
Ppp1r8	-0,032785	1
Ccdc167	-0,03289	1
Sord	-0,033013	1
Fam104a	-0,033119	1
Gm3375	-0,033202	1
Irak2	-0,033526	1
Rsbn1l	-0,033452	1
C030037D09Rik	-0,033581	1
Ntn5	-0,033747	1
Csnk1g1	-0,033744	1
Xrn2	-0,033756	1
Mtfmt	-0,033888	1
Zbtb40	-0,033981	1
Maz	-0,034093	1
Gdap2	-0,034064	1
Gnb5	-0,034342	1
Scarb2	-0,034423	1
Nek1	-0,034601	1
Lrp10	-0,03469	1
Mybbp1a	-0,034668	1
Gm42829	-0,034875	1
Tbc1d15	-0,035034	1
Abcb8	-0,035148	1
Wdr43	-0,035163	1
Gm8168	-0,035251	1
Hnrnpa0	-0,035418	1
Purb	-0,035494	1
Ulk4	-0,035592	1
Hsd17b12	-0,035742	1
Ptpn18	-0,035704	1
Arf1	-0,035767	1
Rab3d	-0,035947	1
Bzw2	-0,035885	1
Pdp2	-0,035985	1
Scaf4	-0,036514	1
Rin2	-0,036495	1
Ndufa2	-0,03674	1
Crem	-0,036776	1
Ttyh2	-0,036968	1
Clk2	-0,037154	1
Grwd1	-0,037328	1
Man2a2	-0,037572	1
Tmem258	-0,037792	1
Naa25	-0,037922	1
Lsm8	-0,038048	1
Tnpo3	-0,038057	1

Gm43387	-0,038388	1
Zmym3	-0,038403	1
Ube2v2	-0,038493	1
Ipp	-0,038593	1
Gm16286	-0,038704	1
Yaf2	-0,038912	1
Ube3c	-0,039257	1
Gm28809	-0,039479	1
Kctd9	-0,03965	1
E330020D12Rik	-0,039678	1
Zfp212	-0,039666	1
Jade3	-0,039801	1
Zfp740	-0,04004	1
Pigm	-0,040197	1
Alg10b	-0,040283	1
Nedd1	-0,040411	1
Crlf2	-0,040609	1
Mad1l1	-0,04065	1
Rpl10	-0,040704	1
Smdt1	-0,040668	1
Atp13a3	-0,040779	1
6030442K20Rik	-0,04094	1
Gm3145	-0,041094	1
Fdxacb1	-0,04134	1
Ube2e1	-0,041549	1
4930563E22Rik	-0,041559	1
Klhl42	-0,041629	1
1110008L16Rik	-0,041596	1
Gm9403	-0,041664	1
Dhrs13	-0,041661	1
Gps2	-0,041858	1
Mapk3	-0,042199	1
Srsf3	-0,042382	1
Uqcrh	-0,042378	1
Epb41l2	-0,042529	1
Adss	-0,042477	1
Zfp568	-0,04284	1
Ebag9	-0,04292	1
Rap1b	-0,042979	1
Irf9	-0,043076	1
Erc1	-0,043248	1
Bcl7b	-0,043381	1
Aggf1	-0,043462	1
Lpcat1	-0,044043	1
Inpp4a	-0,044239	1
Mbnl1	-0,044208	1
Trpm2	-0,044312	1
Rbbp6	-0,044416	1
Unc45a	-0,044559	1
Llph-ps2	-0,044594	1
Sep 06	-0,044574	1
Morc2a	-0,044889	1

Smim15	-0,04508	1
Dntt	-0,045188	1
Cd81	-0,04517	1
Atad3a	-0,045198	1
Ppm1j	-0,045164	1
Hint2	-0,045256	1
Hk2	-0,045392	1
Gnl3	-0,045462	1
Nek7	-0,045721	1
Flii	-0,045702	1
Nsd3	-0,045848	1
Mrpl44	-0,046022	1
Vamp7	-0,046074	1
Fosb	-0,046143	1
Gtf3c2	-0,046108	1
Zfp995	-0,046298	1
Plekha2	-0,046411	1
Odc1	-0,046725	1
Sms	-0,046715	1
Marcksl1	-0,047008	1
Mtrr	-0,047134	1
Chd7	-0,04714	1
Nf1	-0,047315	1
Chd2	-0,047411	1
Cbr3	-0,04746	1
Dclre1b	-0,047631	1
Cd320	-0,047788	1
Acd	-0,047773	1
Unc50	-0,047759	1
Atf4	-0,047816	1
Lmbr1l	-0,048279	1
Ndufa13	-0,048534	1
Tmem229b	-0,048624	1
Cops4	-0,048627	1
Epn1	-0,048903	1
Tuba1c	-0,049018	1
Tmem9	-0,049236	1
Tspyl3	-0,049316	1
Slc19a1	-0,049344	1
Gm15946	-0,049373	1
Lyrm9	-0,04945	1
Gm45053	-0,049687	1
Mir5128	-0,049657	1
Gm2810	-0,049695	1
Aup1	-0,049693	1
Nfyb	-0,049836	1
Nupr1	-0,049998	1
Mphosph6	-0,050097	1
Bst1	-0,050169	1
Smn1	-0,050224	1
Scyl1	-0,050348	1
Malsu1	-0,050429	1

Dcaf4	-0,050467	1
Engase	-0,050616	1
Zfp385a	-0,050864	1
Rprd1b	-0,05088	1
Exosc10	-0,050906	1
Nle1	-0,051015	1
Ppp2ca	-0,050963	1
Gemin4	-0,051057	1
Mri1	-0,051073	1
Peg13	-0,051175	1
Nrbp2	-0,051289	1
Itfg1	-0,051275	1
RP24-366E11.4	-0,05135	1
Capns1	-0,051409	1
Gale	-0,051609	1
Plxna1	-0,051606	1
Gar1	-0,051651	1
Csnk2b	-0,05195	1
Impad1	-0,051853	1
Gm15964	-0,051976	1
Hdgf	-0,051951	1
Med20	-0,05206	1
Zfp551	-0,052254	1
Tubgcp4	-0,052347	1
Zfp954	-0,05232	1
Atp6v1h	-0,052618	1
Gem	-0,052813	1
Acad8	-0,052859	1
Opa1	-0,052923	1
Thrap3	-0,052998	1
Cep57l1	-0,053616	1
Casp8	-0,053623	1
2210008F06Rik	-0,053664	1
Fam188b	-0,053801	1
Gm9134	-0,05378	1
Atl3	-0,053837	1
Timm8a1	-0,054016	1
Rps14	-0,05398	1
Erp44	-0,054016	1
Ganab	-0,054222	1
Mcf2l	-0,05434	1
Upf3b	-0,054377	1
Tmed9	-0,054764	1
Zfp830	-0,054931	1
Gm10138	-0,055173	1
Gm14121	-0,055434	1
Specc1	-0,055521	1
Mfsd4b4	-0,055608	1
Gm12151	-0,055584	1
Cisd1	-0,055836	1
4933440N22Rik	-0,055911	1
Chpf2	-0,055873	1

Cspp1	-0,05597	1
Sppl3	-0,056036	1
Slc9a1	-0,056098	1
Gm2a	-0,056139	1
Gm43794	-0,056499	1
Gfod1	-0,056599	1
Zfat	-0,056809	1
Fam208b	-0,056757	1
Ufd1l	-0,056796	1
Ubqln4	-0,056895	1
Atp6v0e2	-0,056859	1
F8a	-0,057181	1
Acot10	-0,0573	1
Alkbh8	-0,057261	1
Tnks	-0,0575	1
Phc3	-0,057715	1
Toporsos	-0,057984	1
Taf2	-0,058013	1
Fbxw4	-0,05797	1
Sae1	-0,058183	1
Rsl1d1	-0,058419	1
Mrps14	-0,058731	1
Tmco1	-0,058843	1
Nek8	-0,058939	1
Tmem216	-0,058854	1
Ddx28	-0,058935	1
Flad1	-0,059068	1
Brd8	-0,059062	1
Gm43848	-0,059302	1
Naa35	-0,059287	1
Lyrm4	-0,059378	1
Gdpd5	-0,059381	1
Tmem97	-0,059403	1
Zkscan6	-0,059449	1
Klhl21	-0,059446	1
Rbm25	-0,059406	1
Gm9333	-0,059633	1
Dennd6b	-0,059667	1
Gm45206	-0,059745	1
Otulin	-0,05977	1
Rufy2	-0,059879	1
Psmd3	-0,059889	1
Gm28557	-0,060103	1
Mpp5	-0,060112	1
Gm6526	-0,060181	1
Ndrg2	-0,060189	1
Glod4	-0,060218	1
Tmem8b	-0,060389	1
Got2-ps1	-0,060457	1
Ccdc181	-0,060748	1
Mtl5	-0,061048	1
Agtrap	-0,060975	1

Pxn	-0,061136	1
Gm11914	-0,06119	1
Rpl21-ps5	-0,061156	1
Fdft1	-0,061207	1
Slc12a6	-0,061323	1
Adam17	-0,061434	1
Lrrc51	-0,061502	1
Cxx1b	-0,061696	1
Prss36	-0,062078	1
Pygl	-0,062088	1
Pex12	-0,062093	1
Polr2g	-0,062079	1
Gm16537	-0,062331	1
Kat5	-0,062369	1
Gtpbp6	-0,062472	1
Atad2b	-0,06273	1
Snap29	-0,062732	1
Wasf2	-0,063051	1
Mterf4	-0,063241	1
Galnt6	-0,063185	1
Cdk2ap1	-0,063444	1
Milr1	-0,063418	1
Papolg	-0,063501	1
Srp14	-0,063544	1
Got2	-0,06367	1
Zbtb44	-0,063984	1
Fbrs	-0,06412	1
Nifk	-0,064188	1
Tceanc2	-0,064868	1
Zbtb1	-0,065174	1
Igip	-0,065177	1
Tmem259	-0,065247	1
Rab40c	-0,065241	1
Gtf3c1	-0,065268	1
Tmem70	-0,065735	1
Gm43775	-0,065841	1
Rps6ka1	-0,065912	1
Cops7a	-0,065954	1
Coq5	-0,066046	1
Lrrc8a	-0,066164	1
Cflar	-0,066196	1
Slc25a45	-0,066353	1
Slc25a26	-0,066951	1
Tmem230	-0,066984	1
Dnaaf2	-0,067232	1
Zrsr1	-0,067544	1
Ptcd1	-0,067579	1
Plrg1	-0,067601	1
Cfap43	-0,06787	1
Hmbox1	-0,067955	1
Nsd2	-0,068005	1
Grsf1	-0,067966	1

Ap1s2	-0,068157	1
Gm20703	-0,068407	1
Fbxl20	-0,068428	1
Cltc	-0,068372	1
Gm4950	-0,068517	1
Gm45050	-0,068682	1
Adcy9	-0,068831	1
Ccdc186	-0,069079	1
Anapc11	-0,069064	1
Sugp1	-0,069222	1
Slc25a4	-0,069164	1
Slc48a1	-0,069303	1
Hpf1	-0,069443	1
Mfsd5	-0,069426	1
Srrd	-0,069524	1
Olfr460	-0,069508	1
C330007P06Rik	-0,069846	1
Ap3s2	-0,069783	1
2310074N15Rik	-0,070023	1
Pgap1	-0,070465	1
Atg4b	-0,070631	1
Ube2z	-0,070896	1
Dagla	-0,071139	1
Gm43110	-0,071344	1
Plcd3	-0,071311	1
Zmat2	-0,07145	1
Zfp850	-0,07161	1
Ankrd24	-0,071718	1
Slc30a6	-0,071655	1
Vps54	-0,071769	1
Ewsr1	-0,071791	1
Cers6	-0,071752	1
Bola3	-0,071846	1
Arglu1	-0,07202	1
Rhoq	-0,071968	1
Thap12	-0,072202	1
E130309D02Rik	-0,07235	1
Asb13	-0,072678	1
Tatdn1	-0,072856	1
Camk2d	-0,072926	1
Gm9840	-0,073298	1
Ice1	-0,073402	1
Mrpl21	-0,073419	1
Scand1	-0,0735	1
Tm9sf2	-0,073523	1
Gm37470	-0,073599	1
Wbp1	-0,073578	1
Mast2	-0,073812	1
Gnl3l	-0,073934	1
Sh3bgrl3	-0,074143	1
Htatsf1	-0,074424	1
Ube2d3	-0,074447	1

Gdf9	-0,074623	1
Mapre1	-0,074689	1
Dfna5	-0,074811	1
Nfkbiz	-0,074759	1
Fam50a	-0,074933	1
Usp11	-0,075017	1
Evl	-0,075214	1
Il17ra	-0,075214	1
Gm4705	-0,075283	1
Tmem191c	-0,075318	1
Aamp	-0,075473	1
Gm43482	-0,07561	1
Nfic	-0,075704	1
BC085271	-0,075911	1
Slc26a6	-0,076268	1
March6	-0,076648	1
D830044I16Rik	-0,076828	1
Egln2	-0,076845	1
Msl1	-0,077023	1
Tada1	-0,077083	1
Ankdd1a	-0,077181	1
Uqcrq	-0,077239	1
Ddx52	-0,077328	1
Lancl2	-0,077398	1
Spg20	-0,07776	1
Cab39l	-0,077896	1
Usp7	-0,077918	1
Chtf8	-0,078031	1
Fahd1	-0,078076	1
Arl8a	-0,078196	1
Ctns	-0,078251	1
Ip6k1	-0,078623	1
Fnta	-0,078701	1
Tstd2	-0,078692	1
Bpnt1	-0,079225	1
Hhex	-0,0794	1
Itgb2	-0,079395	1
Gm3283	-0,079622	1
Tmem120a	-0,079619	1
Mpp1	-0,079565	1
Mrps17	-0,07956	1
Gm26569	-0,079813	1
Tbc1d2	-0,079763	1
Prim2	-0,079992	1
Stau1	-0,080126	1
Appl2	-0,080138	1
D5Ertd605e	-0,080165	1
Fkbp4	-0,080414	1
Mrps9	-0,08046	1
Timm17b	-0,081019	1
Hells	-0,08104	1
Iba57	-0,081069	1

Odf2	-0,08116	1
Tmc6	-0,08128	1
Stard10	-0,081252	1
Dnajb14	-0,081489	1
Far1	-0,081649	1
Uqcc3	-0,08181	1
Gm13450	-0,081902	1
Trip13	-0,081948	1
Dnajc15	-0,081984	1
Rhod	-0,082073	1
Kcnn4	-0,082264	1
B3gnt3	-0,082405	1
Dtnbp1	-0,082351	1
Timm13	-0,082643	1
Usp5	-0,082715	1
Fn3k	-0,082922	1
Elp5	-0,082959	1
Sec23b	-0,083087	1
Nop58	-0,083086	1
Cpox	-0,083322	1
Larp1	-0,083269	1
Klf7	-0,083277	1
Cdc37	-0,08337	1
Nmt2	-0,083471	1
Ergic2	-0,083704	1
Twistnb	-0,083786	1
Pstpip2	-0,083915	1
Erp27	-0,084022	1
Tatdn3	-0,084003	1
Capn5	-0,084077	1
Dcaf7	-0,084498	1
Pvt1	-0,084739	1
1700124L16Rik	-0,084792	1
Pkp4	-0,084888	1
Jmjd7	-0,085063	1
Ext1	-0,085148	1
Snx29	-0,085216	1
Atg16l2	-0,085165	1
Rps19-ps4	-0,085323	1
Zfyve28	-0,085415	1
Mvb12a	-0,085531	1
Zfp335os	-0,085745	1
Oxnad1	-0,085728	1
Slc25a44	-0,085751	1
Lysmd1	-0,085869	1
Epc2	-0,08633	1
Peli1	-0,086347	1
Rab35	-0,086273	1
Mrpl46	-0,086406	1
Fam175b	-0,086381	1
Dennd3	-0,086471	1
Bcl9	-0,086488	1

Fam192a	-0,086483	1
Mtf1	-0,086786	1
Plekha3	-0,086852	1
Acp6	-0,08704	1
Cd2bp2	-0,087057	1
Utp6	-0,08714	1
Ccdc127	-0,087052	1
Cyb5r3	-0,087306	1
Uhmk1	-0,087494	1
Cbr4	-0,087739	1
Ddx50	-0,087684	1
Trim32	-0,088009	1
Ankra2	-0,088052	1
Psmc2	-0,088362	1
Nosip	-0,088501	1
Brca2	-0,088635	1
Cacul1	-0,088833	1
Prkce	-0,088975	1
Mdp1	-0,089012	1
Dennd6a	-0,089164	1
BC025920	-0,089391	1
Timm8b	-0,08943	1
Fkbp2	-0,089622	1
Gm45840	-0,089737	1
Ccnk	-0,089909	1
Kif3c	-0,089921	1
Ddx39	-0,09026	1
Gm7856	-0,090762	1
Ptges3	-0,09077	1
Gm15265	-0,090869	1
0610009O20Rik	-0,090992	1
Mrps12	-0,091119	1
Eml4	-0,091137	1
Klhl26	-0,091236	1
R3hcc1l	-0,091253	1
Tnfrsf22	-0,091387	1
Gtf3a	-0,091425	1
Emsy	-0,091494	1
2700049A03Rik	-0,091564	1
Vwa5a	-0,091806	1
Sin3b	-0,091785	1
2810405F17Rik	-0,091892	1
Mrps26	-0,091905	1
Pex14	-0,09197	1
Tmem203	-0,092009	1
Nt5m	-0,092081	1
Zfp944	-0,092179	1
Tmem231	-0,092237	1
Lamtor1	-0,092405	1
Reep3	-0,092355	1
Mrpl41	-0,092816	1
Scnm1	-0,092905	1

Vps36	-0,093111	1
Gm15787	-0,093474	1
Tifa	-0,093743	1
Cep295	-0,093833	1
Sh3glb1	-0,093778	1
Polr2m	-0,09394	1
Usb1	-0,094016	1
9330104G04Rik	-0,094148	1
Rab3gap2	-0,094091	1
Phldb3	-0,0942	1
Gm10012	-0,094206	1
Mcoln1	-0,094178	1
Nfya	-0,094536	1
Tspan17	-0,094484	1
Zfp712	-0,0946	1
Oxa1l	-0,095396	1
Znhit1	-0,095594	1
Akip1	-0,095702	1
Nr6a1	-0,095786	1
Usp19	-0,095781	1
Rbm17	-0,095974	1
Rbbp7	-0,09605	1
2310001H17Rik	-0,096206	1
Mydgf	-0,096314	1
Impdh2	-0,096406	1
Bin1	-0,096706	1
Rab21	-0,09674	1
Helq	-0,0968	1
Uqcr11	-0,097027	1
Med28	-0,096966	1
Tinf2	-0,097218	1
Pdcd2	-0,097215	1
Galc	-0,097494	1
Ankrd52	-0,097621	1
2010107E04Rik	-0,09767	1
Dctn3	-0,097805	1
Gm42635	-0,097855	1
Vta1	-0,097955	1
Rbm33	-0,097988	1
Znrd1as	-0,098444	1
Zfp821	-0,098725	1
Atp5d	-0,098895	1
Copb2	-0,098927	1
Dnlz	-0,099058	1
1600002K03Rik	-0,09927	1
Atpif1	-0,099435	1
Fam114a2	-0,099403	1
Mrps36-ps2	-0,099532	1
Hnrnpdl	-0,099611	1
Dlg1	-0,099742	1
Rae1	-0,099738	1
Kdsr	-0,10013	1

Haus1	-0,10047	1
Dcaf12	-0,10051	1
4931406C07Rik	-0,10062	1
Nek6	-0,10087	1
Cramp1l	-0,10092	1
Gm18867	-0,10119	1
Klhl11	-0,10147	1
Ddi2	-0,10151	1
Hus1	-0,10159	1
Tbk1	-0,10173	1
Mob3c	-0,10193	1
Tmem43	-0,10198	1
Tmem41b	-0,10207	1
Usp27x	-0,10244	1
Atp6ap1	-0,10263	1
Agpat5	-0,10274	1
C77080	-0,10339	1
Vrk2	-0,10336	1
Gm43379	-0,10357	1
Serf1	-0,1037	1
Mapk11	-0,10393	1
Noxo1	-0,10394	1
Camk1	-0,10389	1
Gatsl3	-0,10396	1
Prdx2	-0,10409	1
Klc3	-0,10464	1
Rab5b	-0,1046	1
Foxo1	-0,10465	1
D630023F18Rik	-0,10465	1
Pstk	-0,10487	1
Dusp19	-0,10493	1
Gm10268	-0,10503	1
Pgpep1	-0,10501	1
Rps27-ps1	-0,10507	1
Chmp1a	-0,10511	1
9330160F10Rik	-0,10549	1
Nmral1	-0,10559	1
Ulk1	-0,10561	1
Ncoa1	-0,10595	1
Rala	-0,10595	1
Gm32856	-0,10635	1
Gm43788	-0,10656	1
Anpep	-0,10673	1
Thumpd1	-0,10667	1
Lzts2	-0,10681	1
Gm7160	-0,10714	1
Mrpl15	-0,10708	1
1600020E01Rik	-0,1074	1
Icam1	-0,10739	1
Maoa	-0,10766	1
Sf1	-0,10774	1
Mycbp2	-0,10776	1

Gnpat	-0,10787	1
Impdh1	-0,10804	1
Zfp606	-0,10833	1
Med30	-0,10843	1
Lztfl1	-0,10846	1
Mapre3	-0,10865	1
Mpzl1	-0,10867	1
Tstd3	-0,10874	1
Dnaaf5	-0,10868	1
Psrc1	-0,1089	1
Zfa-ps	-0,109	1
Zfp788	-0,10909	1
Sod2	-0,10915	1
Tmem50a	-0,10917	1
Sdhaf3	-0,10933	1
Trp53inp1	-0,10934	1
Ap1g1	-0,10941	1
Supv3l1	-0,10962	1
Ube2e3	-0,10958	1
Cops3	-0,10991	1
Ppm1k	-0,11022	1
Ppp1r15b	-0,11026	1
Eif3g	-0,11029	1
Pkd1	-0,1104	1
Rabgap1	-0,1104	1
Cog5	-0,11048	1
Psip1	-0,11081	1
Ino80e	-0,11079	1
Brd3	-0,11082	1
Uvrag	-0,11089	1
Galt	-0,11115	1
Prpf19	-0,11109	1
A630033H20Rik	-0,1114	1
Gm10074	-0,11152	1
Ssbp1	-0,11158	1
Gpd1l	-0,11165	1
Apopt1	-0,11174	1
Fbxo38	-0,11173	1
Ppp1r21	-0,11181	1
Dynlt3	-0,11182	1
Znhit2	-0,11185	1
Prdx5	-0,11201	1
Ctxn1	-0,1122	1
Ercc3	-0,11223	1
Cln5	-0,11231	1
Ankrd13a	-0,1123	1
Gpr179	-0,11242	1
5430403G16Rik	-0,11274	1
Smim20	-0,1127	1
Cinp	-0,11278	1
Ccdc126	-0,11286	1
Arap3	-0,11295	1

Gm18284	-0,11302	1
Cstb	-0,11302	1
Qtrtd1	-0,1131	1
Reps1	-0,11307	1
Lias	-0,11313	1
Gm12743	-0,11318	1
9930021J03Rik	-0,11317	1
Dusp28	-0,11342	1
Dnpep	-0,11359	1
Ttc39b	-0,11371	1
Xiap	-0,11397	1
Snap47	-0,11406	1
Herc6	-0,11421	1
Pop1	-0,11416	1
A830080D01Rik	-0,11425	1
Kdm6b	-0,11444	1
Psmd5	-0,11437	1
Stxbp1	-0,1144	1
B130034C11Rik	-0,11459	1
Gm5776	-0,11462	1
Vapa	-0,11473	1
Cep97	-0,11485	1
Ehbp1	-0,11483	1
Psmd4	-0,11475	1
Manf	-0,11477	1
Tmed2	-0,11488	1
Khdrbs1	-0,11491	1
Vdac1	-0,11494	1
Gm7676	-0,1151	1
Zfp784	-0,11523	1
Dicer1	-0,11519	1
Iws1	-0,11544	1
Cops7b	-0,11567	1
Ythdf2	-0,11566	1
Vps13a	-0,11581	1
Jpx	-0,11592	1
Ralbp1	-0,1161	1
Ormdl2	-0,11624	1
C1galt1	-0,11628	1
Ndfip2	-0,11641	1
Evi5l	-0,11646	1
Cwc22	-0,11647	1
Hars	-0,11652	1
Bad	-0,11656	1
Ran	-0,11673	1
A130010J15Rik	-0,11675	1
Rlf	-0,11714	1
Xdh	-0,11748	1
Dph7	-0,11749	1
Bcas3	-0,11772	1
Stx16	-0,11767	1
Usp3	-0,1179	1

Dars	-0,11796	1
Hoxc4	-0,11807	1
Kdelr2	-0,11809	1
Sqstm1	-0,11805	1
Nudcd3	-0,11816	1
B630019K06Rik	-0,11826	1
Phyh	-0,11832	1
Npl	-0,11847	1
Ahcyl1	-0,11846	1
Cdnf	-0,11884	1
Abhd17b	-0,11882	1
Pard6a	-0,11921	1
Lin7b	-0,11925	1
Gabarapl2	-0,11938	1
Srrt	-0,11948	1
Psma1	-0,11986	1
Vrk1	-0,12005	1
Slc35f6	-0,11998	1
AU020206	-0,12008	1
Dock4	-0,12026	1
Aamdc	-0,12043	1
Gm9712	-0,12054	1
Atf7	-0,12063	1
Ap4e1	-0,12067	1
Asb3	-0,12097	1
Slc35c2	-0,12104	1
Pitrm1	-0,12119	1
Supt16	-0,12161	1
Slamf9	-0,12186	1
1700001C19Rik	-0,12208	1
Memo1	-0,12208	1
Ifngr2	-0,12207	1
Eif2b4	-0,12222	1
Hadh	-0,12219	1
1110008F13Rik	-0,12226	1
Acot1	-0,12238	1
Gm14780	-0,12244	1
Gm12444	-0,12246	1
Stag1	-0,12253	1
Eif2ak1	-0,12249	1
Zfp449	-0,12268	1
Snord59a	-0,12273	1
Nfe2l1	-0,12278	1
Mcrs1	-0,12334	1
Cklf	-0,12344	1
Brat1	-0,12355	1
Aifm1	-0,12363	1
Hmgxb3	-0,12367	1
Mphosph8	-0,12389	1
Timm10	-0,12401	1
Snx33	-0,12417	1
Ddx18	-0,12418	1

Ten1	-0,12419	1
Grina	-0,1242	1
Rnaseh2c	-0,12429	1
Gm42600	-0,12438	1
Gm43560	-0,12458	1
Efna2	-0,12498	1
Gm37670	-0,12502	1
Zfp866	-0,12511	1
Gm20342	-0,12512	1
Mpeg1	-0,12522	1
Map4k3	-0,12547	1
Dnmt1	-0,12557	1
Gm7967	-0,12568	1
Zw10	-0,12584	1
Gm5446	-0,12589	1
Narfl	-0,12587	1
Kctd3	-0,126	1
Zfp1	-0,12603	1
Gm11488	-0,12612	1
Zfp560	-0,12621	1
Foxk1	-0,12625	1
4930402H24Rik	-0,12659	1
Sntb2	-0,1267	1
Nip7	-0,12685	1
Zfp319	-0,127	1
Mak16	-0,12756	1
Trim14	-0,12776	1
Rhof	-0,12782	1
Stk3	-0,12788	1
Snx21	-0,12819	1
Clcn7	-0,12819	1
Zfp120	-0,12843	1
Rap1gds1	-0,12838	1
1810043G02Rik	-0,12849	1
Cnot11	-0,12848	1
Atp2b1	-0,12894	1
Mtx1	-0,12901	1
Nol11	-0,1291	1
Arfgef1	-0,12909	1
Mrps24	-0,12932	1
Gm43162	-0,12935	1
Pds5b	-0,12945	1
Tsen54	-0,12958	1
Ddx54	-0,12965	1
Mrps36-ps1	-0,12979	1
Snai2	-0,12985	1
Glmn	-0,12999	1
Ndufaf8	-0,13023	1
Neurl2	-0,1303	1
Agpat4	-0,13057	1
Clcn2	-0,13067	1
Tut1	-0,13068	1

Amfr	-0,13075	1
Gmds	-0,13104	1
Lamtor2	-0,13116	1
Rps6	-0,13129	1
Riok1	-0,13132	1
Bbx	-0,13128	1
Nav2	-0,13137	1
Sep 07	-0,13164	1
Ccnd1	-0,13164	1
Add3	-0,13171	1
Phb	-0,1318	1
St7l	-0,13177	1
Mnd1	-0,13224	1
Chsy1	-0,13218	1
RP23-6C18.6	-0,13234	1
Taf10	-0,13233	1
Abhd14a	-0,13262	1
Prpf6	-0,13275	1
Ptpre	-0,13306	1
Hnrnpab	-0,1336	1
Serpinb6a	-0,13362	1
Tmppe	-0,13383	1
Gm44027	-0,13386	1
Gm18913	-0,13402	1
9330159M07Rik	-0,13413	1
Gm37558	-0,13425	1
Ldah	-0,13428	1
Tmem37	-0,13438	1
Tmem243	-0,13453	1
Cnbp	-0,13445	1
Wrn	-0,1347	1
Naif1	-0,13509	1
Cdk13	-0,13544	1
Tmem87b	-0,13539	1
Gm45248	-0,13545	1
Zfp189	-0,13552	1
Zfp358	-0,13564	1
Lrrc59	-0,13563	1
Rnft1	-0,13565	1
Gm43213	-0,13571	1
Man1a2	-0,13566	1
Il11ra1	-0,13588	1
Zfp770	-0,13594	1
Spata33	-0,13605	1
Dolpp1	-0,13611	1
Gm11423	-0,1363	1
Pik3r1	-0,13629	1
9430034N14Rik	-0,13663	1
Gm5939	-0,13671	1
Pes1	-0,13682	1
Rhno1	-0,13701	1
Fam63a	-0,13715	1

Knop1	-0,13743	1
Cyld	-0,1379	1
Fastkd1	-0,13804	1
Gpn1	-0,13802	1
Rrp15	-0,13817	1
Rac1	-0,13826	1
RP23-307F3.6	-0,13837	1
Terf2	-0,13835	1
Tmem110	-0,13847	1
Eif5	-0,13849	1
Ngrn	-0,13861	1
Ptgr1	-0,13879	1
Bbs4	-0,1392	1
Emd	-0,13935	1
Rcor2	-0,13951	1
Zfp266	-0,13977	1
Spag7	-0,13999	1
Zbtb14	-0,14	1
Tbc1d8	-0,14051	1
Cblb	-0,14055	1
Tmed3	-0,14078	1
Poldip3	-0,14097	1
Sun1	-0,1411	1
Erlec1	-0,14136	1
4930455G09Rik	-0,14151	1
Ammecr1l	-0,14173	1
Slu7	-0,1417	1
Rpp21	-0,14179	1
Acads	-0,14203	1
Fancl	-0,14223	1
Naf1	-0,14229	1
Eif4ebp2	-0,14234	1
Scpep1	-0,1423	1
Atp5f1	-0,14233	1
Atp6v0c	-0,14241	1
Cul7	-0,14254	1
Ppp2r5b	-0,14248	1
Tnpo2	-0,1426	1
Vps11	-0,14279	1
Nthl1	-0,14294	1
Mta1	-0,14296	1
Bcorl1	-0,14335	1
Grb2	-0,1434	1
Coq8a	-0,14374	1
Tbc1d20	-0,14394	1
Bri3bp	-0,14391	1
Slc30a9	-0,14401	1
Uqcrc2	-0,14401	1
Mon1a	-0,14412	1
Usp31	-0,14409	1
Ppp3r1	-0,1442	1
Ciz1	-0,14431	1

Enkd1	-0,14455	1
Arsa	-0,14474	1
Tep1	-0,14472	1
Dmtf1	-0,14467	1
Plxna3	-0,14485	1
Ggnbp2	-0,14489	1
Plagl2	-0,14499	1
Psmb3	-0,14529	1
Clta	-0,14536	1
Zfx	-0,14547	1
Ctsa	-0,14553	1
Zfp7	-0,14573	1
Gtf2h1	-0,14569	1
Ptprs	-0,14602	1
Ttc4	-0,14616	1
Hist1h1d	-0,1463	1
Sharpin	-0,14631	1
Jak2	-0,14633	1
Rfc5	-0,14639	1
Phax	-0,14654	1
Plekho2	-0,14663	1
Dctn2	-0,14659	1
Arid3a	-0,1467	1
D3Ertd254e	-0,14685	1
Ythdc2	-0,14699	1
Washc2	-0,14718	1
Rlim	-0,14755	1
Pxmp4	-0,14774	1
Gm2308	-0,14792	1
A530072M11Rik	-0,14794	1
Taok1	-0,1479	1
Gm43024	-0,14804	1
Tubgcp5	-0,14803	1
Snapc4	-0,14796	1
Lrrc73	-0,14809	1
mt-Rnr1	-0,14808	1
Mzf1	-0,14832	1
Cdk18	-0,14861	1
Fto	-0,14856	1
Otub2	-0,14866	1
Gm4879	-0,14877	1
Abracl	-0,14908	1
Gm43737	-0,14951	1
Arl6ip5	-0,14956	1
Pick1	-0,14966	1
Phb2	-0,14987	1
Eif3j2	-0,14997	1
Phf20-ps	-0,15006	1
Pmpca	-0,15019	1
Dnajc13	-0,15026	1
Gsg2	-0,15036	1
Aldh1l1	-0,15043	1

Sarnp	-0,15055	1
Rabepk	-0,15072	1
5730455P16Rik	-0,15106	1
Lin7c	-0,15134	1
Fhad1	-0,15137	1
Ccdc88a	-0,15137	1
Man2c1	-0,15152	1
Tmed1	-0,15158	1
Rcc1	-0,15159	1
Ppp2r3c	-0,1517	1
Rnf152	-0,15187	1
BC003965	-0,15185	1
Gm38200	-0,15215	1
Tmem14c	-0,15211	1
Esd	-0,15208	1
Rnf40	-0,1522	1
Otud7b	-0,15217	1
Cdk8	-0,1523	1
Arhgef2	-0,15233	1
Arid1a	-0,15325	1
Gm14056	-0,15341	1
Itm2c	-0,15344	1
Grpel1	-0,15351	1
Tfdp1	-0,15361	1
Cd151	-0,15362	1
Spop	-0,15364	1
Npepl1	-0,15372	1
Hdac10	-0,15372	1
Psma6	-0,15375	1
Tbl1x	-0,15403	1
Acot7	-0,15407	1
Fam126a	-0,15458	1
Tcf12	-0,15471	1
Map2k3	-0,15475	1
Gm12251	-0,15484	1
Ctsz	-0,1548	1
Gtf3c3	-0,15498	1
Cmtm7	-0,15534	1
Nup88	-0,15526	1
Fance	-0,15535	1
Gm5544	-0,15566	1
Gm13038	-0,15604	1
Tradd	-0,15614	1
Gripap1	-0,15621	1
Alg13	-0,15628	1
Dnal4	-0,15641	1
Ddx5	-0,15663	1
Eml2	-0,15663	1
Bbip1	-0,15673	1
Hspd1	-0,1567	1
Hira	-0,1568	1
Tbc1d23	-0,15678	1

Kmt2c	-0,15679	1
Isy1	-0,15677	1
Ninj1	-0,15684	1
Shq1	-0,15701	1
Gm43795	-0,15708	1
Dhx57	-0,15714	1
Mmadhc	-0,15724	1
RP23-359K10.9	-0,1575	1
Eya3	-0,15754	1
Dctn1	-0,15807	1
Uba7	-0,1581	1
Cry2	-0,15826	1
Adipor2	-0,1583	1
Rpl7l1-ps1	-0,15845	1
Gtf2ird1	-0,15837	1
Nbeal2	-0,15853	1
Galk2	-0,15848	1
Brd1	-0,15859	1
Pdlim5	-0,15873	1
Cdk11b	-0,15901	1
Slc35a5	-0,15923	1
Eftud2	-0,15932	1
E130311K13Rik	-0,15937	1
Gm13477	-0,15976	1
Rpap2	-0,15985	1
Fam219b	-0,15977	1
Nckipsd	-0,15991	1
Bysl	-0,15993	1
Ndufa8	-0,16001	1
Unc13d	-0,16015	1
Ndufaf5	-0,16019	1
Fmc1	-0,16042	1
Irx5	-0,16057	1
Dhx15	-0,16063	1
Kazald1	-0,16084	1
Gsr	-0,16078	1
Gm26652	-0,16114	1
Pphln1	-0,16117	1
Mblac1	-0,16136	1
Snrk	-0,16163	1
Arhgap10	-0,1618	1
Gm2531	-0,16193	1
Ice2	-0,16205	1
Wbscr22	-0,16201	1
Gm27029	-0,16221	1
Ldlrad3	-0,16215	1
Uxs1	-0,16218	1
Hdhd2	-0,1623	1
Gm37959	-0,16241	1
Slx1b	-0,16236	1
Soat1	-0,1624	1
Camsap1	-0,16282	1

Fastkd3	-0,16294	1
Mtmr6	-0,16293	1
Gm4799	-0,16332	1
Chd4	-0,16331	1
Flcn	-0,16339	1
AU019823	-0,16367	1
Cd68	-0,16367	1
Hmox1	-0,16389	1
Qdpr	-0,16394	1
Rfng	-0,16405	1
Gys1	-0,16408	1
Egfl7	-0,16416	1
Hspa4	-0,16451	1
Kyat3	-0,16471	1
9330111N05Rik	-0,16473	1
Nol9	-0,16483	1
Gm7292	-0,16495	1
Syngr2	-0,16498	1
Ifitm5	-0,16521	1
Ostf1	-0,16518	1
Tor2a	-0,16527	1
Ssx2ip	-0,1658	1
Tcf3	-0,16577	1
Mdfic	-0,16603	1
Clcc1	-0,16605	1
Ccdc93	-0,16628	1
Fnbp1l	-0,16628	1
Sgsm3	-0,16678	1
Gm15903	-0,16697	1
Vegfa	-0,16715	1
2810403D21Rik	-0,16742	1
Carm1	-0,16736	1
Emg1	-0,16737	1
Slc4a7	-0,1675	1
Colgalt1	-0,1675	1
Sh3pxd2b	-0,16775	1
Unk	-0,16781	1
Map3k10	-0,16779	1
Tmem138	-0,1679	1
5430434F05Rik	-0,1679	1
6330562C20Rik	-0,16823	1
Ipo11	-0,16827	1
Ccdc33	-0,16848	1
Fam207a	-0,16864	1
Wdyhv1	-0,16865	1
Fam134a	-0,16868	1
Zfp729a	-0,16896	1
Gm12454	-0,16902	1
1810014B01Rik	-0,16907	1
Gm13889	-0,1691	1
Gm37254	-0,16912	1
Pex10	-0,16931	1

Rab13	-0,16937	1
Ciapin1	-0,1694	1
Acap2	-0,16954	1
Fam117a	-0,16955	1
Mrpl57	-0,16956	1
Ndrg4	-0,16983	1
Alyref2	-0,16989	1
Zzz3	-0,16998	1
Rasip1	-0,17025	1
Casp2	-0,17052	1
Slc6a8	-0,17064	1
Fam178a	-0,17073	1
Dnajc7	-0,17073	1
Lyrm2	-0,1708	1
Fam160b1	-0,1711	1
Anapc5	-0,17114	1
Ap2m1	-0,17135	1
Polr2b	-0,17139	1
Cog2	-0,17156	1
Tgs1	-0,17161	1
Gm15920	-0,17172	1
Cox6a1	-0,17178	1
Zfp668	-0,17195	1
Exo5	-0,17214	1
Rps12-ps5	-0,17217	1
Pithd1	-0,17264	1
Mepce	-0,17276	1
Pex11a	-0,17295	1
Acap3	-0,17309	1
Zcchc17	-0,17332	1
Cenpo	-0,1734	1
Rexo1	-0,17336	1
Nomo1	-0,17353	1
Aph1c	-0,17356	1
Polr1e	-0,17369	1
Exosc9	-0,17403	1
Dus3l	-0,1741	1
Plgrkt	-0,17422	1
2310057M21Rik	-0,17426	1
Smpd2	-0,1744	1
Tmem181a	-0,17453	1
Amd1	-0,1745	1
Hivep2	-0,17446	1
Tyms	-0,17457	1
Kantr	-0,17483	1
Rbm6	-0,1749	1
Arhgap21	-0,17504	1
Cox7a1	-0,17508	1
Map4	-0,17507	1
Skiv2l2	-0,17525	1
2010204K13Rik	-0,17551	1
Pyroxd1	-0,17555	1

Sbno2	-0,17562	1
Hcfc2	-0,17557	1
Fam173a	-0,17595	1
Sar1a	-0,17597	1
Gm20430	-0,17613	1
Armc5	-0,17609	1
Hnrnpul2	-0,17641	1
Rab11b	-0,17649	1
Sephs2	-0,17657	1
Hibadh	-0,17669	1
Emc3	-0,17703	1
Vcp	-0,17709	1
Cdc14a	-0,17727	1
Myef2	-0,17736	1
Eif5b	-0,1774	1
Fundc2	-0,17741	1
Mcur1	-0,17746	1
Eif6	-0,17764	1
Smyd4	-0,17772	1
Usp45	-0,17771	1
Agfg1	-0,17773	1
Bcl2l12	-0,17786	1
Rbfa	-0,17789	1
Agpat2	-0,17799	1
Ggnbp1	-0,17798	1
Ctps	-0,17799	1
Eral1	-0,17812	1
Lipe	-0,17828	1
Ccdc112	-0,17829	1
Bcl7c	-0,17879	1
Rrp7a	-0,17884	1
Vars	-0,17893	1
Thra	-0,17906	1
Irgm1	-0,17923	1
Gm38120	-0,17917	1
Klhdc3	-0,17937	1
Setd7	-0,17943	1
Fam8a1	-0,17949	1
Cd101	-0,18007	1
Mnd1-ps	-0,18005	1
Tsku	-0,18041	1
Cyhr1	-0,18039	1
Tmbim4	-0,18038	1
Ube2m	-0,18045	1
Ntmt1	-0,18046	1
Ppdpf	-0,18058	1
Rab31	-0,18056	1
Utp3	-0,18056	1
Xab2	-0,18066	1
Myo1e	-0,18075	1
Txnrd1	-0,18083	1
Stag2	-0,18082	1

Cstf1	-0,18113	1
Notch4	-0,18122	1
Mlec	-0,18124	1
Arrdc1	-0,18125	1
Cd72	-0,18127	1
Manba	-0,18156	1
Gm24876	-0,1816	1
Gpalpp1	-0,18181	1
Cask	-0,18195	1
Ubxn1	-0,18207	1
Wdr19	-0,18223	1
Ghitm	-0,18234	1
Gng7	-0,18236	1
Fuk	-0,18241	1
Ndufs8	-0,18243	1
Btf3l4	-0,18269	1
Sgsm2	-0,18296	1
Mast3	-0,1831	1
Orc2	-0,18399	1
Sh2b2	-0,18425	1
Traf2	-0,18433	1
Timeless	-0,18442	1
Gtf2h4	-0,18448	1
Gm14239	-0,18456	1
Jmjd4	-0,18462	1
Btbd3	-0,18462	1
Smc5	-0,18457	1
Asb1	-0,18466	1
Prpf38b	-0,18473	1
Stk38	-0,18483	1
Nup188	-0,18492	1
Cdc16	-0,18512	1
Acvr1b	-0,18519	1
Gm10060	-0,18552	1
Rbms2	-0,18553	1
Mief1	-0,18564	1
P2rx4	-0,18558	1
RP23-380K24.3	-0,18571	1
Trmt10b	-0,18585	1
Lrrc58	-0,18609	1
Ift27	-0,18625	1
Rab28	-0,18636	1
Slc25a40	-0,18682	1
Mettl4	-0,18706	1
Gm42979	-0,18721	1
Elmo1	-0,1874	1
Cct6a	-0,18746	1
Gm42611	-0,18762	1
Fut10	-0,18758	1
Gm45203	-0,18758	1
Foxp4	-0,18764	1
Tlk1	-0,18784	1

Tldc1	-0,18786	1
Gm19503	-0,18794	1
Nat9	-0,18788	1
Dync1li1	-0,18787	1
Zfp667	-0,18797	1
Tpr	-0,18807	1
Gm44178	-0,18821	1
Zfp369	-0,18815	1
Twf1	-0,18861	1
Zfp958	-0,18873	1
R3hdm2	-0,1887	1
Srek1	-0,18896	1
Parvb	-0,18895	1
Zbed5	-0,18911	1
Gba	-0,18935	1
Riok3	-0,18972	1
Drap1	-0,18966	1
Sik3	-0,18968	1
Uhrf1bp1	-0,18976	1
Mrpl40	-0,19	1
Spcs3	-0,19022	1
Ppp2r5d	-0,19024	1
Mfsd13b	-0,19041	1
Dis3l2	-0,19036	1
Cnot6	-0,19042	1
Gpatch2	-0,1907	1
Zdhhc7	-0,19088	1
Dhx36	-0,19099	1
Ssh3	-0,19107	1
Hmg20a	-0,19148	1
Mtmr10	-0,1916	1
Usp10	-0,19168	1
Commd9	-0,1919	1
Epg5	-0,192	1
Sf3a1	-0,19195	1
Htatip2	-0,19206	1
Il12rb1	-0,19218	1
Nsun2	-0,19252	1
Ints9	-0,19256	1
Mir5136	-0,19262	1
Yipf6	-0,19283	1
Osbpl10	-0,19328	1
Chchd1	-0,19333	1
Asb8	-0,19361	1
Psmd1	-0,19365	1
2310036O22Rik	-0,19371	1
Cyb5b	-0,19365	1
Zfp119a	-0,19386	1
4632404H12Rik	-0,1939	1
Ddx10	-0,19415	1
Banf1	-0,19431	1
Wsb2	-0,1944	1

Rbbp5	-0,19451	1
Pik3ap1	-0,19455	1
Pkp2	-0,19456	1
Atf7ip	-0,19468	1
Rpl15-ps5	-0,19483	1
Zfp141	-0,19475	1
Kat2a	-0,19513	1
Aatk	-0,19528	1
Hnrnpl	-0,19549	1
Sugp2	-0,19563	1
Gigyf2	-0,19568	1
Recql	-0,19584	1
Ndufa11	-0,19582	1
Lmtk3	-0,19586	1
Supt6	-0,19586	1
Psme2b	-0,1961	1
Ranbp2	-0,19608	1
Pdcd6	-0,19614	1
Lsm5	-0,1966	1
Ppp4r1	-0,19663	1
Map3k4	-0,19662	1
BC060293	-0,19668	1
Clasp1	-0,19711	1
Slc25a51	-0,1972	1
2010320M18Rik	-0,19732	1
Diexf	-0,19729	1
Sec24b	-0,1974	1
Tpm4	-0,19743	1
Zfand2b	-0,19746	1
Gm38009	-0,19758	1
RP23-453B15.7	-0,19783	1
Gm43200	-0,19806	1
Mlh1	-0,19877	1
Bend6	-0,19884	1
Zfp524	-0,19881	1
Skap2	-0,19912	1
Rps6kb1	-0,19944	1
Bcap31	-0,1994	1
Traf3ip2	-0,19947	1
Prpf40a	-0,1996	1
Tmem161b	-0,1999	1
Tnfrsf23	-0,20014	1
Prr13	-0,20024	1
Sephs1	-0,20032	1
Zfp160	-0,20048	1
Dpy19l1	-0,20055	1
Gm10463	-0,20085	1
Trp53bp1	-0,20078	1
Comtd1	-0,20112	1
Ceacam1	-0,20143	1
Lonp1	-0,20138	1
Shc4	-0,2017	1

Rmnd1	-0,20181	1
Gm18916	-0,20184	1
Gm43774	-0,20193	1
Ccdc32	-0,20191	1
Ccpg1	-0,20192	1
Uba1	-0,20195	1
Zfp777	-0,20208	1
Mrpl38	-0,2021	1
Mapk1	-0,20211	1
Pigk	-0,20219	1
Rnf41	-0,2025	1
Chd9	-0,20266	1
Fbxo6	-0,20324	1
Ubxn7	-0,20325	1
Tirap	-0,20334	1
Atraid	-0,20347	1
Zc3hav1l	-0,20357	1
Susd3	-0,20361	1
C430049E01Rik	-0,20375	1
Ibtk	-0,20388	1
Mdm4-ps	-0,20403	1
Tceanc	-0,20402	1
Lrwd1	-0,20411	1
Isl2	-0,20419	1
Srebf2	-0,20417	1
Gm43360	-0,2044	1
Nrbp1	-0,20443	1
Wiz	-0,20435	1
Kcmf1	-0,20445	1
Brdt	-0,20508	1
Josd2	-0,20513	1
Fchsd2	-0,20551	1
Gm43106	-0,2058	1
Cpd	-0,20579	1
Tm2d2	-0,20593	1
Tbcel	-0,20591	1
Stk10	-0,20601	1
Fam84b	-0,20624	1
Mto1	-0,20634	1
Msra	-0,20635	1
Gm31166	-0,20703	1
Dnph1	-0,20712	1
Osgep	-0,20719	1
Scamp3	-0,20728	1
Prr14	-0,20748	1
Cpt2	-0,20761	1
Ube2o	-0,20765	1
C730045M19Rik	-0,2079	1
Thoc6	-0,20805	1
Stn1	-0,20841	1
Cbx7	-0,20843	1
Meis3	-0,20852	1

Myo5a	-0,20895	1
Fabp5	-0,20903	1
Slc44a2	-0,20896	1
Rnf6	-0,20906	1
1110037F02Rik	-0,20916	1
Ipo9	-0,20933	1
Mapkap1	-0,20985	1
Pigq	-0,20987	1
Col18a1	-0,21001	1
Pten	-0,20997	1
Gas6	-0,21004	1
Ppp2r1b	-0,21012	1
Ube2j1	-0,21008	1
Prune2	-0,21049	1
Nsun3	-0,21065	1
Gm20633	-0,21068	1
Pxk	-0,21067	1
Atp5b	-0,21073	1
RP23-390D8.2	-0,21094	1
Zmym4	-0,21085	1
Acot2	-0,21101	1
Pigo	-0,21095	1
Rfx1	-0,21113	1
Scarna9	-0,2112	1
Golga5	-0,21131	1
Trip12	-0,21164	1
Tubgcp2	-0,21174	1
Purg	-0,212	1
Saal1	-0,21242	1
Fbxw5	-0,21244	1
Slc3a2	-0,21261	1
Tbc1d22a	-0,21265	1
Ubr2	-0,21297	1
Arv1	-0,2131	1
Kdm2a	-0,21326	1
Tceal8	-0,21364	1
Pacs2	-0,2136	1
Dtwd1	-0,21373	1
Dlg4	-0,21381	1
Slc47a2	-0,21389	1
Fam71e1	-0,21401	1
Pepd	-0,21412	1
Ctif	-0,21451	1
Azi2	-0,21467	1
Mtif3	-0,21476	1
Haus2	-0,21494	1
Trip6	-0,21501	1
Rasgef1a	-0,21507	1
Copz1	-0,21524	1
Coasy	-0,21533	1
Slc4a1ap	-0,21552	1
Cntrl	-0,21548	1

Gm37255	-0,21568	1
Fam193b	-0,21584	1
Atp6v0e	-0,21581	1
Mecr	-0,21602	1
Zfp51	-0,21605	1
Chkb	-0,21617	1
Senp5	-0,2162	1
Klc1	-0,21624	1
Sapcd2	-0,21697	1
Man1b1	-0,21721	1
Fam195b	-0,21723	1
Sec61b	-0,21722	1
Reps2	-0,21727	1
Gspt2	-0,2178	1
Tmem206	-0,21781	1
Tfam	-0,21782	1
Eif2b5	-0,21798	1
Vegfb	-0,21806	1
Cela1	-0,21819	1
Ndufv1	-0,21845	1
Fxn	-0,21848	1
Zbtb26	-0,21846	1
Nkrf	-0,21876	1
Fam219a	-0,219	1
Ech1	-0,21911	1
Slc25a36	-0,21914	1
Nif3l1	-0,21936	1
Smcr8	-0,21955	1
Gipc2	-0,21961	1
Msantd4	-0,2196	1
Zfp952	-0,21971	1
Gna15	-0,21968	1
Creld1	-0,21979	1
Rdx	-0,21978	1
Ncor1	-0,21994	1
Vkorc1l1	-0,21999	1
Foxd2os	-0,22009	1
Vps25	-0,22013	1
Cnot2	-0,22057	1
Fam149b	-0,22078	1
Fam193a	-0,22076	1
Acp1	-0,22109	1
Ndufs6	-0,22112	1
Impact	-0,2212	1
Rnf216	-0,22128	1
Zfp626	-0,22147	1
Ptp4a2	-0,22175	1
Chrac1	-0,22181	1
Epb41l5	-0,22223	1
Akr1a1	-0,22224	1
Med27	-0,22272	1
Cetn2	-0,22308	1

Ccs	-0,22321	1
Lyn	-0,22321	1
Gm5131	-0,22336	1
Akr1c13	-0,22374	1
1700021F05Rik	-0,22372	1
Dusp22	-0,22394	1
Bcl2l13	-0,22392	1
Bcor	-0,22388	1
1110020A21Rik	-0,22403	1
Gm37183	-0,22409	1
Cryzl1	-0,22414	1
Ube3a	-0,22409	1
Msrb1	-0,22408	1
Recql4	-0,22434	1
Nlrc5	-0,22465	1
Slc35e1	-0,22497	1
Zmiz2	-0,2251	1
N4bp2l1	-0,22516	1
Ybx3	-0,2253	1
Gm11131	-0,22544	1
Gm28071	-0,22548	1
Rfk	-0,22555	1
Tarbp1	-0,2258	1
Gm16096	-0,22613	1
Gskip	-0,22619	1
Paox	-0,22633	1
Zswim1	-0,22641	1
Gm42893	-0,2265	1
Pnkd	-0,22667	1
Fastkd2	-0,22757	1
Lrrfip2	-0,22774	1
Taf1	-0,22781	1
Gm21975	-0,22786	1
Mpg	-0,22806	1
Cox6b1	-0,22807	1
Alox5	-0,22873	1
Srgap2	-0,22883	1
Pik3cd	-0,22894	1
Lig4	-0,22911	1
Mmachc	-0,22944	1
Scd1	-0,22962	1
D630029K05Rik	-0,22984	1
Slc39a6	-0,23028	1
Mtx3	-0,23056	1
Rnf149	-0,23061	1
Copg1	-0,23094	1
Sap30bp	-0,23126	1
Mtfp1	-0,23143	1
Gm16053	-0,2315	1
Gpaa1	-0,23158	1
Klf8	-0,2317	1
Sbf2	-0,23196	1

Eldr	-0,23219	1
Zfp609	-0,23249	1
Rnaseh2b	-0,23271	1
Tnrc6a	-0,23268	1
Xpot	-0,23277	1
Ppan	-0,23294	1
Mcts1	-0,23303	1
Zswim8	-0,23325	1
Fbxw9	-0,23322	1
Anxa6	-0,2333	1
Scarb1	-0,23339	1
Prokr1	-0,23386	1
Wdr3	-0,23389	1
Rnf169	-0,23396	1
Fbxl18	-0,23411	1
Taf6l	-0,23414	1
Qpctl	-0,23427	1
Ccne1	-0,23428	1
Rps15a-ps3	-0,23513	1
Gm9892	-0,23511	1
Shisa5	-0,2352	1
Amacr	-0,23604	1
Prkci	-0,23598	1
Prdm2	-0,23618	1
Nudc-ps1	-0,23627	1
Pcgf3	-0,23658	1
Fam129b	-0,23741	1
Bcdin3d	-0,23748	1
Cd200r2	-0,23761	1
Laptm4b	-0,2379	1
Akap8l	-0,23787	1
Mrps33	-0,23797	1
Sec13	-0,23801	1
Gm3608	-0,23821	1
H1fx	-0,23831	1
Rbbp8	-0,23844	1
Mysm1	-0,23845	1
Twsg1	-0,23851	1
Nop2	-0,23856	1
Bub3	-0,23856	1
Chmp5	-0,2387	1
Trim41	-0,23886	1
Cmbl	-0,23914	1
Pik3c3	-0,23932	1
Dera	-0,23932	1
2510016D11Rik	-0,23954	1
Hnrnpul1	-0,23978	1
Agpat3	-0,24004	1
Ankib1	-0,24005	1
Sfxn5	-0,24045	1
Fubp3	-0,24127	1
Glyr1	-0,24133	1

Otud5	-0,24155	1
Fam57a	-0,24174	1
Fam216a	-0,24204	1
Lyz2	-0,24228	1
Zfp512	-0,2424	1
Golga4	-0,24236	1
Map3k11	-0,24264	1
Ticam1	-0,24259	1
9130604C24Rik	-0,24285	1
Cables2	-0,24292	1
Rpap1	-0,24286	1
Zfp217	-0,24288	1
Tssk6	-0,24305	1
Afdn	-0,24309	1
Icam5	-0,2433	1
9330162012Rik	-0,24325	1
Ppp1ca	-0,24342	1
Pum2	-0,2435	1
Rack1	-0,24356	1
Ankrd27	-0,24366	1
Trpm1	-0,24389	1
Siglec1	-0,24406	1
Cdc42bpg	-0,24452	1
Thtpa	-0,2446	1
Apbb2	-0,2446	1
Hexa	-0,24467	1
Rrp1b	-0,24478	1
Usp4	-0,24481	1
Dap3	-0,24522	1
Grhpr	-0,24547	1
Vmp1	-0,24553	1
Gpx1	-0,24551	1
Zfp207	-0,24577	1
Mpv17l2	-0,24597	1
Mxd4	-0,24612	1
Arfip2	-0,24621	1
Dhx35	-0,24639	1
Vapb	-0,24645	1
Gemin7	-0,24674	1
Tab2	-0,24673	1
Trim35	-0,24668	1
Supt3	-0,24687	1
Pde6d	-0,24701	1
Lss	-0,24697	1
Fam206a	-0,24703	1
Aco1	-0,24713	1
A430105J06Rik	-0,24725	1
Hgsnat	-0,24717	1
Itsn2	-0,24725	1
Gm22299	-0,2477	1
RP23-139H6.1	-0,24769	1
Ccdc71	-0,24779	1

Gm43484	-0,2479	1
Inafm2	-0,24786	1
Gm16223	-0,24798	1
Coq9	-0,24818	1
Xrcc6	-0,24833	1
Cwc25	-0,24832	1
Ncbp3	-0,24868	1
Pgp	-0,24876	1
Cbfb	-0,24877	1
Leprotl1	-0,24897	1
Nol6	-0,24943	1
Farsa	-0,24964	1
B4galt5	-0,24967	1
Stambp	-0,24978	1
Wrap53	-0,25016	1
Cenpx	-0,25029	1
Capn2	-0,25032	1
Pbx2	-0,25031	1
Oas3	-0,2509	1
4930539J05Rik	-0,25101	1
Fam217b	-0,25124	1
Prkacb	-0,25126	1
Gm5778	-0,25155	1
Gm44901	-0,25155	1
Sft2d3	-0,25156	1
Arpc5	-0,25169	1
Zmynd19	-0,25188	1
Mllt3	-0,25198	1
Trak1	-0,25211	1
Magi1	-0,25221	1
Zbtb6	-0,25223	1
Rpe	-0,25233	1
Ap4m1	-0,25235	1
Vamp5	-0,2524	1
Pi4k2b	-0,25237	1
Mocos	-0,2525	1
Zfp455	-0,2527	1
Map4k1	-0,25268	1
Polr3c	-0,25271	1
Tspan4	-0,25273	1
Myo19	-0,25278	1
Emc1	-0,25282	1
Kif2a	-0,25292	1
Spata2l	-0,25297	1
Rptor	-0,25303	1
Atp5o	-0,253	1
Zdhhc3	-0,25307	1
Brox	-0,25309	1
Cryl1	-0,25315	1
Tbc1d1	-0,25342	1
Lmna	-0,25353	1
Fbxl4	-0,25362	1

Tnfaip8l1	-0,25356	1
Bmyc	-0,25358	1
Cstf2	-0,25359	1
Nmnat1	-0,2537	1
Sptan1	-0,25371	1
Lage3	-0,25392	1
Chp1	-0,25414	1
Prmt5	-0,25419	1
Zfc3h1	-0,25429	1
Heatr5b	-0,2545	1
Efemp2	-0,25486	1
Lamp2	-0,25506	1
Lman2l	-0,25524	1
Gm6377	-0,25527	1
Pkm	-0,25534	1
Slc12a9	-0,25536	1
Herpud2	-0,25551	1
Cops5	-0,25563	1
Gtf2h3	-0,25569	1
Tanc1	-0,25576	1
2010315B03Rik	-0,25585	1
Gm6418	-0,25609	1
Fbxo7	-0,25619	1
Fndc3b	-0,25618	1
Ndufa4	-0,25635	1
Heatr6	-0,25649	1
Tram2	-0,25657	1
Trrap	-0,25687	1
Zdhhc13	-0,25698	1
Ankrd50	-0,25714	1
Ncln	-0,25727	1
Samhd1	-0,25754	1
Dnajb11	-0,25776	1
Fxyd5	-0,25778	1
Rps3	-0,25795	1
Ap1b1	-0,25791	1
Dus2	-0,25795	1
Mgat2	-0,25804	1
Arid4a	-0,25796	1
Ttc1	-0,25814	1
Arl14ep	-0,25821	1
Ppp1r7	-0,25854	1
Gle1	-0,25847	1
Fam199x	-0,25855	1
Dcbld2	-0,25871	1
Plekhm1	-0,25875	1
Zbtb46	-0,25896	1
Tagap	-0,25906	1
Yeats2	-0,25918	1
Pcmtd2	-0,25942	1
Itm2b	-0,25935	1
Dvl1	-0,25962	1

Hipk1	-0,25963	1
Tfcp2l1	-0,25966	1
Clec4e	-0,25976	1
Gm37390	-0,25996	1
Zbtb49	-0,26024	1
Endov	-0,26053	1
Itgal	-0,26054	1
RP24-365A12.2	-0,2605	1
Jak1	-0,2605	1
2810402E24Rik	-0,26059	1
Cmtm3	-0,26055	1
Wdr12	-0,26083	1
Coa5	-0,26083	1
E130208F15Rik	-0,26087	1
Zkscan8	-0,26145	1
Tnfsf8	-0,26166	1
Atxn3	-0,26176	1
Tab3	-0,26222	1
Mbd1	-0,26364	1
Ptpn4	-0,26355	1
Ctnna1	-0,26365	1
Ntan1	-0,2638	1
Tcf19	-0,26383	1
Lipa	-0,26396	1
Mios	-0,26417	1
Oxct1	-0,26429	1
Adal	-0,26467	1
Auh	-0,2649	1
Tsfm	-0,26536	1
Mettl10	-0,26571	1
Nfam1	-0,26566	1
Sp100	-0,26594	1
Ostm1	-0,26597	1
Mon2	-0,26605	1
Shmt2	-0,26631	1
Scmh1	-0,26633	1
Nudcd1	-0,26638	1
Wdr36	-0,2667	1
9530082P21Rik	-0,26704	1
Mfge8	-0,2671	1
Inpp5a	-0,26734	1
Gm26982	-0,26742	1
Eif3c	-0,26769	1
Eid2b	-0,26783	1
Saa3	-0,26786	1
Skil	-0,2679	1
Trmt5	-0,26804	1
Mdm4	-0,26812	1
Gm14853	-0,26824	1
Trpt1	-0,26844	1
Gm6415	-0,26839	1
Ppt1	-0,26845	1

AI314180	-0,26853	1
Spag4	-0,26872	1
Gm13350	-0,26874	1
Atg2b	-0,26876	1
Pitpnb	-0,26883	1
Ap3m1	-0,26893	1
Chfr	-0,26899	1
Kcnab2	-0,2693	1
Ddb1	-0,26928	1
Acox1	-0,26942	1
Vwa8	-0,2695	1
Man2b1	-0,26954	1
Iqgap1	-0,2695	1
Slx4	-0,26958	1
Ckap4	-0,26973	1
Tulp3	-0,26988	1
Mvd	-0,26994	1
Snf8	-0,27012	1
Clcn3	-0,27008	1
Nt5c3	-0,2703	1
RP23-304C21.3	-0,27041	1
Ubqln2	-0,27041	1
Med8	-0,27069	1
Lipt1	-0,27082	1
Cpeb3	-0,27083	1
Isca2	-0,27092	1
Actl6a	-0,27091	1
Ehbp1l1	-0,27095	1
Gm29155	-0,27111	1
Pax6	-0,27137	1
Rrp8	-0,27153	1
Csnk1a1	-0,2719	1
Tank	-0,27215	1
Psme4	-0,27218	1
Rab7	-0,27231	1
Nipbl	-0,27243	1
Lmo4	-0,27254	1
Nectin2	-0,27268	1
2310039H08Rik	-0,27272	1
Spaca6	-0,27305	1
Ccdc107	-0,27328	1
Tubb6	-0,27331	1
Fam96b	-0,27328	1
Peak1	-0,27328	1
Dgkg	-0,27345	1
Dhx34	-0,27342	1
Gm8719	-0,27373	1
Rab11fip1	-0,27397	1
Txnrd2	-0,27413	1
Fasn	-0,2741	1
Slc6a6	-0,27421	1
Cetn3	-0,27467	1

Rpl19-ps9	-0,27481	1
Prkrip1	-0,27482	1
Pigc	-0,27481	1
Tnfrsf1a	-0,2749	1
Mrm1	-0,27497	1
Cox17	-0,27497	1
Cmtr1	-0,27516	1
Parp2	-0,27531	1
Ino80b	-0,27531	1
Poldip2	-0,27541	1
Rnf111	-0,27537	1
Hes6	-0,27538	1
Nsrp1	-0,27554	1
Lap3	-0,27556	1
Znfx1	-0,27566	1
Prep	-0,27576	1
Apeh	-0,27611	1
Tpgs1	-0,2763	1
B3galnt2	-0,27633	1
Sult2b1	-0,2763	1
Aspscr1	-0,27641	1
Slc35a3	-0,27635	1
Sec23ip	-0,27653	1
Spats1	-0,2766	1
Map1s	-0,2766	1
Clec16a	-0,27715	1
Fgd3	-0,27752	1
Lrrc45	-0,27775	1
Usp34	-0,27771	1
Stoml1	-0,27779	1
2410089E03Rik	-0,27788	1
Nit1	-0,27809	1
Shcbp1l	-0,2781	1
Canx	-0,27825	1
Elac2	-0,27828	1
Npy	-0,27844	1
Ubtd1	-0,27839	1
Synj1	-0,27862	1
Smtn	-0,27875	1
Cyp4v3	-0,27889	1
BC017643	-0,27897	1
CAAA01180111.2	-0,27926	1
Siae	-0,27926	1
Myo9a	-0,27943	1
Cchcr1	-0,27954	1
Traf5	-0,27947	1
Hsd17b11	-0,27996	1
Ppm1b	-0,28034	1
G3bp1	-0,28032	1
Mcm9	-0,2804	1
Tab1	-0,28042	1
Map3k5	-0,28039	1

Rac2	-0,28075	1
Mthfd1l	-0,28078	1
Ehd4	-0,28102	1
U2surp	-0,28151	1
Pgls	-0,28173	1
Parp14	-0,28206	1
Xkr5	-0,2822	1
Eif2ak4	-0,2823	1
Commd3	-0,28252	1
Ppp2r5c	-0,28263	1
Katnal1	-0,28283	1
Snx17	-0,2828	1
Gm6304	-0,28301	1
Fam13c	-0,28301	1
Snhg1	-0,28327	1
Man2b2	-0,28354	1
Alcam	-0,28355	1
Ndufs1	-0,28346	1
Lace1	-0,28357	1
Eif2s2	-0,28362	1
Snx25	-0,28381	1
Gm43773	-0,28414	1
Actr3b	-0,28452	1
Rgl3	-0,28474	1
E130307A14Rik	-0,28492	1
Ccdc166	-0,28492	1
Vsir	-0,28509	1
Cep70	-0,28526	1
Rtca	-0,28529	1
Cast	-0,28526	1
Rundc1	-0,28599	1
Dhx32	-0,28618	1
Mrap	-0,28638	1
Pde4b	-0,28653	1
Hdc	-0,2866	1
Txndc11	-0,28664	1
Trappc9	-0,28692	1
Slc5a3	-0,28695	1
Ube2r2	-0,28695	1
Arfgap2	-0,28735	1
Ncs1	-0,28736	1
Runx3	-0,28753	1
Rasal1	-0,28762	1
Gm6524	-0,28766	1
Extl3	-0,28784	1
Paxip1	-0,2879	1
Mfn2	-0,28805	1
Mroh1	-0,28819	1
Tlk2	-0,28824	1
Vps4a	-0,28826	1
Rab2a	-0,28837	1
A530013C23Rik	-0,28877	1

Fam92a	-0,28908	1
Fam32a	-0,28911	1
Stat5a	-0,28924	1
Izumo4	-0,28963	1
Selenoo	-0,28983	1
Gm12059	-0,28995	1
Hspa9	-0,29006	1
Oip5	-0,29032	1
Ccr10	-0,29054	1
Ankrd28	-0,29073	1
RP23-110E20.5	-0,29081	1
Pex6	-0,29101	1
Hgh1	-0,2911	1
Plekhg4	-0,29143	1
Inpp1	-0,29142	1
Phpt1	-0,29162	1
Ubr3	-0,29155	1
Arpc1b	-0,29164	1
Bmpr2	-0,29181	1
2510046G10Rik	-0,2921	1
Shkbp1	-0,29209	1
Stard3	-0,29209	1
Utrn	-0,2924	1
Eea1	-0,29238	1
B9d1	-0,29252	1
Cyb5a	-0,29255	1
Npc1	-0,29274	1
Mettl22	-0,29289	1
Aco2	-0,29292	1
Gstp1	-0,29374	1
RP24-497N7.2	-0,29421	1
Zfp715	-0,2943	1
Nploc4	-0,29429	1
Lrrc75a	-0,29463	1
Grin1	-0,29472	1
Slc38a7	-0,2949	1
Kctd2	-0,29492	1
Ankrd40	-0,29518	1
Ppil1	-0,29529	1
Zc3h8	-0,2953	1
Cd37	-0,29528	1
Fnbp1	-0,29544	1
Fancm	-0,29562	1
Rpl36a-ps1	-0,29569	1
Mia2	-0,29573	1
Rnf168	-0,29582	1
Llgl1	-0,29583	1
Zkscan3	-0,29589	1
Cyp27a1	-0,29641	1
1700025G04Rik	-0,29636	1
Arhgap19	-0,2972	1
Gm37354	-0,2973	1

Bin2	-0,2973	1
Dcaf13	-0,2973	1
Dnajc5	-0,29737	1
Tmem199	-0,29752	1
Srp68	-0,2975	1
Dctn5	-0,29768	1
Clmp	-0,29789	1
Map7d1	-0,29809	1
Gm26129	-0,29838	1
Ap1m1	-0,2984	1
Dock2	-0,2986	1
Gcn1l1	-0,29861	1
Ndst2	-0,29869	1
Tln1	-0,299	1
Ifih1	-0,2991	1
Ireb2	-0,29926	1
Lasp1	-0,2994	1
Aftph	-0,29939	1
Tufm	-0,29962	1
Mrpl12	-0,29982	1
Hagh	-0,29976	1
Rasa1	-0,29987	1
Mrps36	-0,29999	1
Psmb5	-0,30011	1
Hsp90ab1	-0,30007	1
Vps33a	-0,30022	1
Slc25a39	-0,30023	1
Usp15	-0,3003	1
Ywhab	-0,30043	1
Dpy19l4	-0,30051	1
Ighmbp2	-0,30047	1
Mzt2	-0,30085	1
Nudt18	-0,3011	1
Gclm	-0,30121	1
Pld3	-0,30163	1
Pik3ca	-0,30202	1
Spred2	-0,30215	1
Gm43533	-0,3022	1
Ctsh	-0,30242	1
Gm8494	-0,30254	1
Smurf2	-0,30251	1
Lsm4	-0,30261	1
Bid	-0,30257	1
Epn2	-0,30263	1
Hipk2	-0,30269	1
Gm42659	-0,30286	1
Srsf9	-0,30299	1
Gm8522	-0,3031	1
Cdv3	-0,30307	1
Pi4k2a	-0,30316	1
Gm16425	-0,30373	1
Rbsn	-0,30379	1

Pdzd8	-0,30375	1
Pex3	-0,30388	1
Pik3r4	-0,30407	1
Mtbp	-0,30432	1
Snx4	-0,30431	1
Rspry1	-0,30475	1
Mov10	-0,30478	1
Pign	-0,3048	1
Plekhm3	-0,30494	1
Arhgef11	-0,30499	1
Mfap1b	-0,30498	1
Ccz1	-0,30502	1
Batf2	-0,30508	1
Snx27	-0,3051	1
Fam65a	-0,3053	1
Coq7	-0,30568	1
Mob1a	-0,30597	1
Borcs5	-0,30638	1
Tyw1	-0,30666	1
Cdc25c	-0,307	1
Lpgat1	-0,30729	1
Pdk2	-0,30756	1
Scaf11	-0,30783	1
Homer1	-0,3079	1
Dip2a	-0,30803	1
Dnttip1	-0,30808	1
Srpk1	-0,30813	1
Hspa4l	-0,30824	1
Adgrl1	-0,30819	1
Espl1	-0,30829	1
Ndufb9	-0,3085	1
Tor1b	-0,30855	1
Rdh1	-0,30859	1
Gm43148	-0,30892	1
Rwdd3	-0,30893	1
Fam234a	-0,3089	1
Krtcap2	-0,30888	1
Yeats4	-0,30914	1
Rnf113a2	-0,30938	1
Bfar	-0,30957	1
Ptrh2	-0,31039	1
Sucla2	-0,31054	1
Sf3b2	-0,31049	1
Tysnd1	-0,31064	1
Coq8b	-0,31067	1
Ttbk2	-0,31096	1
Laptm4a	-0,31105	1
Setdb1	-0,31124	1
Slc16a13	-0,31138	1
Suds3	-0,31145	1
Mrpl36	-0,31149	1
Atp6v1f	-0,31146	1

Romo1	-0,31193	1
Wnt6	-0,31206	1
2610008E11Rik	-0,31241	1
Glce	-0,31258	1
Gm43513	-0,31291	1
Ndufs7	-0,31304	1
Casp4	-0,31312	1
Mbtps2	-0,31322	1
Efr3a	-0,31322	1
Aasdh	-0,31364	1
Nsfl1c	-0,31377	1
Slc25a20	-0,31388	1
Chd8	-0,31425	1
Degs1	-0,31463	1
Dnaja3	-0,31491	1
Adar	-0,3154	1
Ankrd13c	-0,31553	1
Glrx5	-0,31555	1
Akna	-0,31577	1
Med17	-0,31587	1
Atg7	-0,31598	1
Gm4943	-0,31624	1
Tada2b	-0,31634	1
Dgcr8	-0,31695	1
Taok2	-0,31703	1
Pml	-0,31717	1
Slc25a13	-0,31723	1
2310033P09Rik	-0,31734	1
Jak3	-0,31759	1
Fkbpl	-0,31768	1
Limk2	-0,31779	1
Prelid3b	-0,31842	1
Vps39	-0,31846	1
Enpp4	-0,31866	1
Ccdc51	-0,31884	1
Wdr1	-0,31902	1
Fam58b	-0,31907	1
Zfp513	-0,31924	1
Cep89	-0,31929	1
Gnb4	-0,31937	1
Myh11	-0,31947	1
Paf1	-0,31956	1
Pyroxd2	-0,3198	1
Commd7	-0,32002	1
Klf9	-0,32036	1
Herc3	-0,32054	1
Card11	-0,32051	1
Mrpl28	-0,32047	1
Aagab	-0,32051	1
Il7r	-0,32058	1
Trim16	-0,32079	1
B230118H07Rik	-0,32109	1

E430021H15Rik	-0,32118	1
Gm5590	-0,32142	1
Tfg	-0,32142	1
Fgd4	-0,32154	1
Ccdc43	-0,32184	1
Acsl1	-0,32184	1
A130050O07Rik	-0,32192	1
Mkl1	-0,32215	1
Abca3	-0,32219	1
Fra10ac1	-0,32216	1
Palm	-0,3223	1
Nxf7	-0,32229	1
Pygo2	-0,32247	1
Zfp943	-0,32258	1
Ttll4	-0,32263	1
Gm4017	-0,32267	1
Gm10425	-0,3227	1
Cnnm3	-0,32314	1
Stx18	-0,3232	1
Cdk4	-0,32321	1
Gm43571	-0,32329	1
Gm2199	-0,32336	1
Fxyd2	-0,32361	1
Cdkn3	-0,32374	1
Pomc	-0,32378	1
Gm10557	-0,32383	1
Mir142hg	-0,3238	1
Galm	-0,32384	1
Atp6v0d1	-0,32376	1
Endod1	-0,32392	1
Malat1	-0,32392	1
Brwd1	-0,32444	1
Usp38	-0,32482	1
Mppe1	-0,32491	1
9130008F23Rik	-0,32525	1
Pus3	-0,32521	1
Sdad1	-0,32518	1
Agrn	-0,32543	1
Ttc14	-0,32553	1
D930015E06Rik	-0,32587	1
Rab3ip	-0,32607	1
Mgst2	-0,32608	1
4921511C10Rik	-0,32615	1
Fam188a	-0,32661	1
Mis12	-0,32673	1
Sympk	-0,32699	1
Rrm2b	-0,32729	1
Ehmt1	-0,32749	1
Acot13	-0,32757	1
Gm8463	-0,32765	1
Ankmy2	-0,32783	1
Ccdc6	-0,32777	1

Atp13a1	-0,32802	1
Stxbp2	-0,32798	1
Ddx24	-0,32834	1
Tef	-0,32829	1
Ift81	-0,3284	1
Zfp68	-0,32838	1
Ppp1r11	-0,32893	1
C230037L18Rik	-0,32903	1
Tamm41	-0,32931	1
Mical3	-0,32973	1
Arap1	-0,32976	1
Prorsd1	-0,33012	1
Exoc5	-0,3302	1
Ppil2	-0,33069	1
Cers2	-0,33078	1
Mlxip	-0,33089	1
Gnptg	-0,331	1
Pitpnc1	-0,33123	1
Gm4117	-0,33122	1
Psmb9	-0,33149	1
Fer	-0,33178	1
Nkpd1	-0,33176	1
Cdk12	-0,33187	1
Mtmr4	-0,33198	1
Xpo7	-0,33199	1
Ptbp3	-0,33204	1
Wac	-0,33217	1
Atp6v1a	-0,33233	1
Faap20	-0,33252	1
Zcrb1	-0,33247	1
Gm15420	-0,33282	1
Zbtb7a	-0,33278	1
Def8	-0,33296	1
Snora73b	-0,3338	1
Eif3d	-0,33377	1
Fnip2	-0,33383	1
Zfp317	-0,33398	1
Tomm34	-0,33428	1
Spice1	-0,33447	1
Pyurf	-0,33464	1
2310068J16Rik	-0,33472	1
Scai	-0,3348	1
Eci1	-0,33496	1
Ptov1	-0,33496	1
Celf2	-0,33512	1
Ttc17	-0,33518	1
Dgcr6	-0,33564	1
Rnpc3	-0,33581	1
9630010A21Rik	-0,33587	1
Dnajc1	-0,33595	1
Scfd1	-0,33616	1
Apobr	-0,33631	1

Rbbp4	-0,33625	1
Nelfcd	-0,33662	1
Nrros	-0,3367	1
Gm5805	-0,33689	1
Clns1a	-0,33699	1
Exoc6b	-0,33716	1
Kank2	-0,33723	1
Tacc2	-0,3373	1
Fam212b	-0,33737	1
D16Ertd472e	-0,3374	1
RP23-151L20.5	-0,33744	1
Plod1	-0,33745	1
Mrps34	-0,33792	1
Brpf1	-0,3379	1
2810474O19Rik	-0,33789	1
Fam179b	-0,33831	1
Tmco4	-0,33849	1
Rtf1	-0,33863	1
Hemk1	-0,33877	1
Gm43378	-0,33877	1
Cpq	-0,33888	1
Syvn1	-0,33886	1
Btbd9	-0,33923	1
Uqcc1	-0,3393	1
5530601H04Rik	-0,33951	1
Nagk	-0,3395	1
Commd4	-0,33951	1
Tmem238	-0,33972	1
Rab24	-0,33985	1
Gm24890	-0,33999	1
Picalm	-0,34007	1
Akr1b7	-0,34055	1
Dock9	-0,34066	1
Pcmtd1	-0,34068	1
Men1	-0,34092	1
Ece2	-0,34096	1
Senp2	-0,341	1
Gm43350	-0,34122	1
Gm5453	-0,34129	1
Myo9b	-0,34134	1
Zfp865	-0,34142	1
Gm37503	-0,34148	1
Svip	-0,34163	1
Tomm40	-0,34164	1
Foxk2	-0,34161	1
Pex11b	-0,3416	1
Puf60	-0,34166	1
Slc6a13	-0,34191	1
Arfgap3	-0,34203	1
Josd1	-0,34196	1
Trim12c	-0,34211	1
Mapk14	-0,3421	1

Baz2b	-0,34215	1
Xpo5	-0,34224	1
1700017B05Rik	-0,34221	1
Kif1c	-0,34223	1
Alg6	-0,34245	1
Trps1	-0,34255	1
Fbrsl1	-0,3428	1
Nmd3	-0,34282	1
Antxr2	-0,34301	1
Snord72	-0,34334	1
Jkamp	-0,34335	1
Sel1l	-0,34337	1
Prkaca	-0,34348	1
Nova1	-0,34384	1
Lnx2	-0,344	1
Cptp	-0,34412	1
Dner	-0,34418	1
Psmd2	-0,34417	1
Nme4	-0,34466	1
Matn1	-0,34465	1
Efna1	-0,34475	1
Acss2	-0,34526	1
Dmxl2	-0,34534	1
Olfml3	-0,34536	1
Haghl	-0,34539	1
Trappc13	-0,34549	1
Vps16	-0,34561	1
Kmt5c	-0,34579	1
Wdtc1	-0,34604	1
Dctn6	-0,34625	1
Saysd1	-0,34637	1
Pbx3	-0,34647	1
Gpd2	-0,34667	1
Pdcd6ip	-0,3471	1
Lman1	-0,34738	1
Akr1e1	-0,34773	1
Micu1	-0,34865	1
Acbd4	-0,34865	1
Sec11a	-0,34877	1
Ssrp1	-0,34883	1
Sf3b3	-0,34894	1
Homer3	-0,34899	1
Fabp3	-0,34918	1
Hnrnph3	-0,34922	1
Akap13	-0,34928	1
Sfswap	-0,34946	1
Tnks2	-0,34962	1
Sumo3	-0,34964	1
Pqlc2	-0,35013	1
Las1l	-0,35012	1
Pip4k2c	-0,35041	1
Capzb	-0,35073	1

Tefm	-0,35093	1
Chtop	-0,35087	1
Umad1	-0,35095	1
Iqcf1	-0,35103	1
Rufy3	-0,35102	1
Gak	-0,35104	1
Eps15	-0,35119	1
Tgfbr2	-0,35138	1
Prps2	-0,35147	1
Pcgf6	-0,35182	1
Acad11	-0,35185	1
Pus10	-0,3519	1
Rbpj	-0,35198	1
Gls	-0,35231	1
Rbm15b	-0,35247	1
BC049715	-0,35256	1
Gm43247	-0,35264	1
Uggt1	-0,35263	1
Tbc1d7	-0,35292	1
Rwdd4a	-0,35295	1
Gm13378	-0,35309	1
Primpol	-0,35312	1
Comt	-0,35314	1
Senp6	-0,3533	1
B230322F03Rik	-0,3535	1
Cpsf4	-0,35353	1
Golgb1	-0,35351	1
Derl2	-0,35359	1
Gm33142	-0,35411	1
Ap3m2	-0,35412	1
4930448A20Rik	-0,35432	1
Tagap1	-0,35433	1
Gm37621	-0,3547	1
Cd63-ps	-0,35494	1
Tex261	-0,35498	1
Pnisr	-0,35503	1
Fam131a	-0,35514	1
Atxn7	-0,35536	1
Tbl3	-0,35553	1
Sypl	-0,35555	1
Dguok	-0,35557	1
Ift74	-0,3559	1
Spg7	-0,35591	1
Zhx1	-0,35608	1
Gm29284	-0,35626	1
Rgs19	-0,35628	1
Aurkaip1	-0,3563	1
Fbf1	-0,35644	1
Kctd21	-0,35651	1
Ptdss1	-0,35655	1
Fam171b	-0,35669	1
Gm7452	-0,35671	1

Mmp2	-0,35686	1
Pdzd11	-0,35691	1
Strn3	-0,3569	1
Lif	-0,35709	1
Usp9x	-0,35706	1
Zfp398	-0,35723	1
Agmo	-0,35737	1
Epm2a	-0,35755	1
Stard8	-0,35772	1
Ubxn6	-0,35768	1
Arpin	-0,35783	1
Sec61a2	-0,35786	1
Gm35315	-0,35805	1
Acadsb	-0,35797	1
Tigar	-0,35834	1
Tmem55b	-0,35836	1
Klf16	-0,3585	1
Utp4	-0,35849	1
1110025M09Rik	-0,35861	1
Adsl	-0,35859	1
Ercc5	-0,35861	1
Ube2e2	-0,35883	1
Adam15	-0,35882	1
Uba5	-0,35905	1
A530017D24Rik	-0,35938	1
Nom1	-0,35943	1
Nsmaf	-0,35945	1
Esrra	-0,35968	1
Nfkb2	-0,35993	1
Cc2d1a	-0,36024	1
Smug1	-0,36049	1
Uty	-0,36075	1
Tmem57	-0,36085	1
Plxnb2	-0,36088	1
Mbtps1	-0,36105	1
Wars	-0,36163	1
Zfp709	-0,36158	1
Tsn	-0,36167	1
Aim1l	-0,3618	1
Wdr76	-0,36195	1
Lsm14b	-0,36196	1
Pus1	-0,36199	1
Kat6b	-0,36223	1
Cdc14b	-0,36219	1
Det1	-0,36255	1
Gm43006	-0,36271	1
Lrp8	-0,36282	1
Gm12248	-0,36318	1
Tbc1d17	-0,36321	1
Slc38a6	-0,36336	1
Zik1	-0,36352	1
Klhdc10	-0,36358	1

Gmfb	-0,36395	1
Gm42908	-0,364	1
Cnot1	-0,36401	1
Ppp4c	-0,36423	1
Gm44423	-0,36417	1
Dedd	-0,36473	1
Chchd4	-0,36466	1
Med13l	-0,36496	1
2610507B11Rik	-0,36497	1
Supt5	-0,36517	1
Tecpr2	-0,3653	1
Trmt12	-0,36555	1
Alpk1	-0,36621	1
Trim21	-0,36666	1
3830406C13Rik	-0,36671	1
Ndufc1	-0,36697	1
Fndc10	-0,36715	1
Sfi1	-0,36724	1
Gtpbp3	-0,36718	1
Ahsa2	-0,36729	1
Gm29487	-0,36727	1
Osbpl9	-0,36763	1
Shc1	-0,36797	1
Arfrp1	-0,3682	1
Slc39a14	-0,36837	1
Chd3	-0,36851	1
RP23-261O3.5	-0,3688	1
Wdr81	-0,36901	1
Zfhx2	-0,36913	1
Aim2	-0,36909	1
Strada	-0,36949	1
Zfp235	-0,36959	1
Oxld1	-0,36963	1
Plpp1	-0,36957	1
Ptk2	-0,36985	1
Padi2	-0,37047	1
Exoc1	-0,37072	1
Cep95	-0,37072	1
Gm16540	-0,37097	1
Cyb5d2	-0,37108	1
Srrm1	-0,37134	1
Rhbdd3	-0,37137	1
Ccnf	-0,37142	1
Gm42576	-0,3716	1
Gars	-0,37159	1
Nr1d2	-0,37157	1
Gm44190	-0,3719	1
Tbl2	-0,3721	1
Gm42566	-0,37206	1
Zfp287	-0,37227	1
F830208F22Rik	-0,37262	1
Synj2	-0,373	1

Nras	-0,37297	1
Hyi	-0,37307	1
Gas2	-0,37318	1
Nol10	-0,3734	1
Zfp386	-0,37347	1
Fcgrt	-0,37361	1
Zfp202	-0,37415	1
Cln3	-0,37427	1
Gm10093	-0,37468	1
4933421A08Rik	-0,37503	1
Pgm2l1	-0,37511	1
Slc7a6	-0,37531	1
Tpm3-rs7	-0,37535	1
Ptpn9	-0,37568	1
Ttf2	-0,37577	1
Ep300	-0,3758	1
Stx6	-0,3762	1
Sppl2a	-0,37633	1
Prkar1a	-0,37638	1
Arhgdib	-0,37649	1
Mdh2	-0,37677	1
Glg1	-0,37697	1
Hdlbp	-0,37712	1
Riox2	-0,37741	1
Dock10	-0,3774	1
Tbl1xr1	-0,37759	1
Sep 11	-0,37782	1
Sep 09	-0,37835	1
Gm13373	-0,37836	1
Heatr1	-0,37838	1
9130401M01Rik	-0,37838	1
Ccr2	-0,37857	1
Ddhd2	-0,37865	1
1700047K16Rik	-0,37898	1
Arid1b	-0,37906	1
Plpp2	-0,37924	1
Ankrd13b	-0,37928	1
Bbs12	-0,37994	1
Polr2a	-0,37987	1
Tmem141	-0,37989	1
Ung	-0,38025	1
Ubac1	-0,38041	1
Nudt13	-0,3804	1
Atf6b	-0,38042	1
Wdfy3	-0,38052	1
Rad23b	-0,38058	1
Tfr2	-0,38073	1
Cx3cr1	-0,38066	1
Nlrp10	-0,38109	1
Arhgef6	-0,38123	1
Tmem167	-0,38134	1
B230216N24Rik	-0,38144	1

Dynlt1f	-0,38145	1
Xpo6	-0,38159	1
Tm7sf2	-0,382	1
Gm6266	-0,38215	1
Ipo4	-0,38205	1
Pdpr	-0,38222	1
Cdk7	-0,38242	1
Ptgr2	-0,38243	1
Plekhb2	-0,38276	1
Trmt2a	-0,38286	1
Jarid2	-0,38299	1
Ppfia3	-0,38306	1
Dmxl1	-0,38313	1
Trim39	-0,38339	1
Clptm1	-0,38385	1
Ppargc1b	-0,38387	1
Pcdh7	-0,38387	1
Tmem26	-0,38409	1
Mfsd10	-0,38414	1
Smco4	-0,38415	1
Kdelr1	-0,38412	1
Sec11c	-0,38419	1
Zbtb39	-0,38433	1
Armc1	-0,38439	1
Wfs1	-0,38463	1
Atp1a1	-0,38474	1
Mms22l	-0,38487	1
Ercc8	-0,38517	1
Kars	-0,3854	1
Nol12	-0,38551	1
Tnni3	-0,38593	1
Adcy7	-0,38589	1
Firre	-0,38612	1
Ltn1	-0,38617	1
Itpr1	-0,38646	1
D17H6S53E	-0,38702	1
Lrrc27	-0,3871	1
Zfp62	-0,38708	1
Rbm5	-0,38726	1
Sh2b3	-0,38764	1
5031434O11Rik	-0,38769	1
Ptpn6	-0,38772	1
Fbxo31	-0,38785	1
Kansl1l	-0,38809	1
Inhbe	-0,38835	1
Chst14	-0,38873	1
Apaf1	-0,38901	1
Zfp110	-0,38908	1
Sh3bgrl	-0,38924	1
Ctsf	-0,38918	1
Wdr4	-0,38934	1
Larp1b	-0,3894	1

Hectd1	-0,38997	1
Gm25596	-0,39017	1
Ankfy1	-0,39125	1
Hyou1	-0,3912	1
Zfp759	-0,39148	1
Mrpl3	-0,39147	1
Ddx56	-0,39171	1
Hs6st1	-0,39179	1
Cope	-0,39194	1
Flnc	-0,39188	1
Npr1	-0,39199	1
Slc2a4rg-ps	-0,39232	1
Mbd6	-0,3923	1
Ids	-0,39227	1
Gm13397	-0,39245	1
Cdpf1	-0,39256	1
Hars2	-0,39286	1
Rab11fip5	-0,39297	1
Ngly1	-0,39309	1
Polr3b	-0,39346	1
Galnt11	-0,39355	1
4932438A13Rik	-0,39355	1
Nfx1	-0,39374	1
Clec4a2	-0,39376	1
Slc25a46	-0,39403	1
D730003I15Rik	-0,39422	1
Cep19	-0,39435	1
Dnajc2	-0,39447	1
Prmt1	-0,39476	1
Nvl	-0,3949	1
Ndor1	-0,39501	1
Gdi1	-0,39524	1
Lamp1	-0,39531	1
Hpgds	-0,39548	1
Zc3h6	-0,39555	1
Smpdl3b	-0,39562	1
Slc33a1	-0,39569	1
F730043M19Rik	-0,3957	1
Tlr6	-0,3958	1
Itpa	-0,39606	1
RP23-164P21.3	-0,39611	1
Dennd1b	-0,3961	1
R3hcc1	-0,39651	1
Safb2	-0,39648	1
Pptc7	-0,39655	1
Wnk1	-0,39651	1
Slc29a1	-0,39662	1
Ypel3	-0,39665	1
2310011J03Rik	-0,39671	1
Smarca4	-0,39703	1
Gpr89	-0,39707	1
Pttg1ip	-0,3977	1

Phf11c	-0,39785	1
Dopey2	-0,39783	1
Dhdh	-0,39819	1
Ahcyl2	-0,39834	1
Arrb2	-0,3984	1
Ankrd11	-0,39838	1
Abhd4	-0,39884	1
Ccdc28b	-0,39893	1
Fhit	-0,39902	1
Fntb	-0,39909	1
Smyd3	-0,39912	1
St3gal3	-0,39924	1
Hapln3	-0,39952	1
Dnajc30	-0,39967	1
Thoc2	-0,39974	1
Gm8093	-0,39975	1
Sfxn1	-0,40012	1
Nol4l	-0,4002	1
Cd180	-0,40018	1
Cda	-0,40026	1
Pcyt2	-0,40042	1
Aldoa	-0,40055	1
Man2a1	-0,40075	1
Synj2bp	-0,40076	1
Plpp6	-0,4009	1
Hoxa7	-0,40104	1
Ezh1	-0,40122	1
C1rl	-0,40129	1
Nat10	-0,40137	1
Dnajc16	-0,40143	1
Taok3	-0,40138	1
Gm5117	-0,40146	1
Brd7	-0,40179	1
Zdhhc14	-0,40193	1
Fat1	-0,40242	1
Dgcr2	-0,4025	1
Nlrc3	-0,40265	1
Mgam	-0,4027	1
Btrc	-0,40317	1
5830444B04Rik	-0,4033	1
Ccdc90b	-0,40363	1
Snx20	-0,40373	1
Wdr61	-0,40365	1
Zfp810	-0,40376	1
Letm1	-0,40382	1
Smim8	-0,40388	1
Abhd5	-0,40388	1
Tapt1	-0,40391	1
Rbpms	-0,40397	1
A930001C03Rik	-0,40406	1
Arl5c	-0,40425	1
Grk2	-0,40426	1

Fzd9	-0,40481	1
Sigmar1	-0,40554	1
Ebp	-0,40565	1
Fam91a1	-0,40569	1
Zfp760	-0,40592	1
Pogz	-0,40594	1
Zdhhc16	-0,40598	1
Usp40	-0,40605	1
Rhot2	-0,40605	1
Kctd7	-0,40634	1
Ano7	-0,40629	1
Elp6	-0,40679	1
Cct7	-0,40722	1
Tnfaip2	-0,40734	1
Klra2	-0,40785	1
Gm37105	-0,40806	1
S100a10	-0,40814	1
1110038F14Rik	-0,4082	1
Snai1	-0,4083	1
Ctla2b	-0,40828	1
Ccdc114	-0,40837	1
Yipf1	-0,40843	1
Mrpl10	-0,40853	1
Cdo1	-0,40867	1
Sft2d2	-0,40878	1
Gm10039	-0,40904	1
Gm38376	-0,40944	1
Fam118b	-0,40945	1
Stat3	-0,40961	1
Pnpla2	-0,41021	1
Ick	-0,41026	1
Gm19552	-0,41035	1
Hspb7	-0,41053	1
Rab14	-0,41079	1
Ttc32	-0,41089	1
Zfp236	-0,41117	1
Dcaf8	-0,41131	1
Zswim7	-0,41156	1
Thap11	-0,41164	1
Vav2	-0,41168	1
Fam129a	-0,41169	1
Ubac2	-0,41207	1
Tmem177	-0,41236	1
Pxylp1	-0,41251	1
Thoc3	-0,4125	1
Agfg2	-0,41291	1
Eri3	-0,413	1
Ddb2	-0,4133	1
Pou6f1	-0,41329	1
Gm11952	-0,41338	1
Gm38104	-0,41335	1
Myo1c	-0,41377	1

9930012K11Rik	-0,4139	1
Cars2	-0,41404	1
Dctn4	-0,41402	1
Gorasp2	-0,41402	1
Gm42941	-0,41412	1
Cenpj	-0,41417	1
Cep250	-0,41463	1
Fkbp8	-0,41456	1
Dhfr	-0,41476	1
Desi1	-0,41491	1
Parp4	-0,41503	1
Sec24c	-0,41496	1
Tle6	-0,41513	1
Rab32	-0,41505	1
Rasa2	-0,41521	1
Ssbp4	-0,41533	1
Pbrm1	-0,41533	1
Ncl	-0,41532	1
Idi1	-0,41566	1
N6amt1	-0,41582	1
Gon4l	-0,41598	1
Slc25a24	-0,41596	1
Kmt2d	-0,41604	1
Prnp	-0,41604	1
Traf6	-0,41627	1
Tmem184c	-0,41632	1
Zdhhc5	-0,41631	1
Hdac4	-0,41637	1
1500011B03Rik	-0,41685	1
Pcx	-0,41705	1
Ccndbp1	-0,41697	1
4930529C04Rik	-0,41742	1
Fbxw17	-0,41746	1
Slc7a1	-0,41773	1
Gm38340	-0,41778	1
Zfp9	-0,41823	1
Bclaf1	-0,41825	1
Extl2	-0,41883	1
Rnf187	-0,4188	1
Rabl6	-0,4188	1
9530053A07Rik	-0,41911	1
Dffa	-0,4192	1
Ncor2	-0,41935	1
Poli	-0,41945	1
Ubxn11	-0,42007	1
Tonsl	-0,42033	1
Prag1	-0,42046	1
Rcbtb1	-0,42061	1
2210016L21Rik	-0,42073	1
Etfb	-0,42092	1
BC003331	-0,42124	1
Igsf6	-0,42122	1

Pls3	-0,42132	1
Tubgcp3	-0,42138	1
Trpm4	-0,42156	1
Pan2	-0,4216	1
Pgap2	-0,42156	1
Focad	-0,4219	1
Thg1l	-0,42208	1
Ankrd54	-0,42224	1
Coro2a	-0,42269	1
Srprb	-0,42326	1
Dnajc14	-0,42334	1
Zfp451	-0,42332	1
Tprkb	-0,42339	1
Gm12389	-0,42351	1
Bet1l	-0,42369	1
Dopey1	-0,42383	1
Stim2	-0,42375	1
Tango2	-0,42411	1
Ikzf1	-0,42415	1
Ncstn	-0,42426	1
Mcat	-0,42439	1
Nop9	-0,42449	1
Afp	-0,42473	1
Zfp799	-0,42481	1
Derl1	-0,42483	1
0610009B22Rik	-0,42488	1
Rpp40	-0,42493	1
Cul2	-0,42493	1
Rfc2	-0,42487	1
Larp4b	-0,42493	1
Psma2	-0,42529	1
Prkcg	-0,42563	1
Rfx7	-0,42559	1
AI467606	-0,42565	1
Ppm1g	-0,4257	1
Fbxl6	-0,42607	1
Sars2	-0,4263	1
Ninl	-0,42632	1
Os9	-0,42676	1
Prrc2b	-0,42724	1
Cdc37l1	-0,42739	1
Gm13270	-0,42749	1
Ralgapa1	-0,4276	1
Pnkp	-0,42767	1
Usp6nl	-0,42788	1
Tbrg1	-0,42846	1
Pgghg	-0,42858	1
Gstm1	-0,42877	1
Stk4	-0,42878	1
Gm15535	-0,42889	1
Gcc1	-0,42903	1
Dpagt1	-0,42898	1

Prrc1	-0,42924	1
Psmc3	-0,42937	1
N4bp1	-0,42964	1
Armc2	-0,43002	1
Zfp251	-0,43011	1
Plec	-0,43008	1
S100pbp	-0,43069	1
Ufc1	-0,43082	1
Rin3	-0,43105	1
Psme3	-0,4312	1
Pdlim2	-0,43153	1
Pex2	-0,43176	1
Smarcad1	-0,43206	1
Nsmf	-0,43219	1
Ube2l3	-0,43224	1
Ccnl2	-0,43217	1
Capg	-0,4326	1
Stk38l	-0,43272	1
Cd48	-0,4329	1
Cdk10	-0,433	1
Ehmt2	-0,43296	1
Cyb5r1	-0,43347	1
Xrcc4	-0,43374	1
Trip4	-0,43369	1
Yipf5	-0,43381	1
Efl1	-0,43404	1
Fam185a	-0,43397	1
Mndal	-0,43425	1
Dgkz	-0,43424	1
Nisch	-0,43474	1
Vaultrc5	-0,43538	1
Dcaf15	-0,4358	1
Gm42515	-0,43593	1
Gm11722	-0,43627	1
Avl9	-0,43646	1
Mrpl2	-0,43674	1
Tcf25	-0,43669	1
Tada3	-0,43694	1
Gm5391	-0,43696	1
Wdr75	-0,43728	1
Tac4	-0,43755	1
Snx11	-0,43782	1
Tmem51	-0,43851	1
Top2b	-0,43927	1
Mrpl13	-0,43936	1
Fbxo28	-0,43969	1
Phkb	-0,43982	1
Cldn12	-0,43995	1
Gm44116	-0,43986	1
Angptl6	-0,43996	1
Dhx33	-0,4401	1
Rad54l2	-0,44022	1

Tmem151a	-0,44042	1
St3gal2	-0,44052	1
Gldc	-0,44048	1
Nhej1	-0,44062	1
Prrc2c	-0,44119	1
Ddrgk1	-0,44137	1
Gm7102	-0,44141	1
Upp2	-0,44152	1
Rp2	-0,44155	1
Zmynd11	-0,44145	1
Ctu1	-0,44166	1
Capn7	-0,4419	1
Akt1	-0,44215	1
Jrk	-0,44221	1
Rnf225	-0,44217	1
AI837181	-0,44248	1
A130048G24Rik	-0,44249	1
Elmod2	-0,44273	1
Zbtb33	-0,44277	1
Zufsp	-0,4428	1
Net1	-0,44297	1
Mrpl58	-0,44314	1
Ndufa4l2	-0,4431	1
Nubpl	-0,44355	1
Rnf20	-0,44346	1
Crybg3	-0,44363	1
D630024D03Rik	-0,44395	1
Gm28151	-0,44395	1
Paip1	-0,44404	1
Cep290	-0,44423	1
Noa1	-0,44441	1
Gm12309	-0,44439	1
Ptpn22	-0,44443	1
Zc3hav1	-0,44454	1
Celsr3	-0,44464	1
Fam102b	-0,44471	1
Tnfaip8l2	-0,44491	1
Rassf5	-0,44488	1
Panx1	-0,44514	1
Adat3	-0,44514	1
Alg11	-0,44551	1
Pomt2	-0,44562	1
Dnajc25	-0,44562	1
Cog4	-0,44573	1
Dyrk2	-0,44574	1
Ssr1	-0,44574	1
Rnpepl1	-0,44635	1
Tgfbrap1	-0,44662	1
Cherp	-0,44674	1
Fbxo4	-0,44749	1
Kctd11	-0,44769	1
Tmlhe	-0,44789	1

Them4	-0,44796	1
Arcn1	-0,44801	1
Fyb	-0,44804	1
Mmgt2	-0,44856	1
Ccdc88b	-0,44879	1
Golt1b	-0,44896	1
Slc45a4	-0,44914	1
Klhdc1	-0,44941	1
AC149090.1	-0,44939	1
Gm44103	-0,44954	1
Ermap	-0,44974	1
Faf2	-0,44996	1
Dok1	-0,45023	1
Ccdc180	-0,45045	1
Sco2	-0,45074	1
Slain2	-0,45071	1
Dph5	-0,4509	1
Tkt	-0,45121	1
Cse1l	-0,45132	1
Wfikkn1	-0,45146	1
G6pd2	-0,45154	1
Sirt4	-0,45231	1
Csnk1d	-0,45229	1
Ogfod3	-0,45263	1
Zfp410	-0,45285	1
Galnt4	-0,45294	1
Ipo7	-0,45286	1
Meaf6	-0,45326	1
Nup210	-0,45344	1
Txlna	-0,45352	1
4930581F22Rik	-0,45365	1
Exosc4	-0,4538	1
Gm15050	-0,45401	1
Ptpn11	-0,45431	1
Srsf6	-0,45434	1
Slco4a1	-0,45468	1
Pianp	-0,45484	1
Cox18	-0,45519	1
Brpf3	-0,45518	1
Gm45221	-0,45555	1
Snx10	-0,45572	1
Idh3a	-0,45602	1
Gm15892	-0,45603	1
Ube2i	-0,45693	1
Fgfr1op2	-0,45703	1
Cd53	-0,45716	1
Lhfpl2	-0,4572	1
Tbc1d19	-0,45829	1
Cfap97	-0,45836	1
Slc30a4	-0,45844	1
Nans	-0,45845	1
Gm45501	-0,45862	1

Ccdc97	-0,45868	1
Ncbp1	-0,45886	1
Vps33b	-0,45905	1
Gm43457	-0,45902	1
Tmeff1	-0,45917	1
Arpc2	-0,45924	1
Csad	-0,45938	1
Gm28875	-0,45954	1
Btbd2	-0,45963	1
Tug1	-0,4597	1
Abhd12	-0,45992	1
Gabpb2	-0,45991	1
Mga	-0,45986	1
Ttl	-0,45999	1
Ykt6	-0,46001	1
Sema4g	-0,46009	1
Zfp599	-0,46032	1
Acvrl1	-0,46044	1
Vav3	-0,46061	1
Pcyox1l	-0,46083	1
Kdelc1	-0,46092	1
Nelfa	-0,46095	1
Phf3	-0,46106	1
Mvb12b	-0,46126	1
Trim11	-0,46132	1
Tmbim1	-0,46131	1
Gm42715	-0,46149	1
Snx9	-0,46163	1
Poc1a	-0,4616	1
Ap4b1	-0,46171	1
Cdk5rap3	-0,46184	1
Tpmt	-0,46181	1
Apool	-0,46197	1
Gnb1l	-0,46276	1
Plekha8	-0,46302	1
Prickle3	-0,46317	1
Ruvbl1	-0,46342	1
Osbpl2	-0,46354	1
Dtx4	-0,46354	1
Vash2	-0,46357	1
Tspan5	-0,46376	1
Tfpi	-0,46407	1
Ift140	-0,46422	1
Gm37566	-0,46428	1
Bcl2a1d	-0,46459	1
Mtrf1l	-0,46468	1
Slfn5	-0,46494	1
RP23-63H11.3	-0,46491	1
Trim26	-0,46511	1
Gm44291	-0,46531	1
Agbl5	-0,46537	1
Hmox2	-0,46548	1

Fbxo25	-0,46557	1
Foxc1	-0,46566	1
Klhdc2	-0,46584	1
Smco3	-0,46577	1
Esr1	-0,46583	1
Sqrdl	-0,46599	1
Zfp964	-0,46598	1
Dscr3	-0,4669	1
Rab11fip2	-0,46696	1
Kat8	-0,46707	1
Rapgef1	-0,46723	1
Gm44269	-0,46743	1
Flrt2	-0,46775	1
Gm45358	-0,4677	1
Mia3	-0,46781	1
Cad	-0,46823	1
Exosc1	-0,46833	1
D5Ertd579e	-0,46828	1
Trim56	-0,46832	1
Uchl3	-0,46839	1
Trmt2b	-0,46858	1
Txnl4b	-0,46886	1
Tmem147	-0,46889	1
Zfp956	-0,46903	1
Slc11a2	-0,46908	1
Lgals2	-0,46906	1
Ctsl	-0,46915	1
Usp16	-0,4695	1
Trim65	-0,46971	1
Ccdc116	-0,46984	1
Prdm4	-0,46997	1
Hmx3	-0,4701	1
BC052040	-0,47024	1
Nt5dc2	-0,47018	1
Tfb2m	-0,47074	1
Slk	-0,47072	1
Usp48	-0,47071	1
9030617O03Rik	-0,47077	1
Synpo	-0,47095	1
Cc2d1b	-0,47116	1
Them6	-0,47132	1
Zbtb4	-0,47156	1
Ints1	-0,47197	1
Map2k4	-0,47214	1
Myo18a	-0,47217	1
Bcl2l14	-0,47238	1
Psma8	-0,47262	1
Nop16	-0,47294	1
Tomm70a	-0,47349	1
Mtfr1l	-0,4735	1
Aaas	-0,47355	1
Herc4	-0,47363	1

Kbtbd3	-0,47378	1
Aatf	-0,47408	1
Srf	-0,47413	1
Pip5k1c	-0,47442	1
Cybb	-0,47454	1
Elk1	-0,47457	1
Wdr92	-0,47478	1
Swi5	-0,47484	1
Idh3g	-0,47485	1
Mthfd1	-0,47506	1
Eif4ebp1	-0,47523	1
Ncf2	-0,47546	1
Nup155	-0,47561	1
Farsb	-0,47616	1
Zmat5	-0,47626	1
Ggcx	-0,47636	1
Des	-0,47648	1
Il2rg	-0,47696	1
Cd2ap	-0,47699	1
Sfpq	-0,47702	1
Psmd6	-0,47731	1
Polr1b	-0,47739	1
Ddx1	-0,4775	1
Ap5m1	-0,47764	1
Ltbr	-0,47768	1
Lrrc25	-0,47803	1
Nat2	-0,47858	1
Sorl1	-0,47883	1
Aptx	-0,47905	1
Clasrp	-0,47919	1
Klhl22	-0,47943	1
1110002L01Rik	-0,47941	1
3830403N18Rik	-0,47973	1
Runx2	-0,47995	1
Gucy2g	-0,4804	1
Mogs	-0,48074	1
G6pdx	-0,48087	1
Pon2	-0,48087	1
Tsc22d4	-0,48122	1
Cutc	-0,48151	1
Atxn7l3b	-0,48194	1
Adamts6	-0,48194	1
Mum1	-0,48211	1
1110059E24Rik	-0,48244	1
Gm16638	-0,48243	1
Arhgap1	-0,4827	1
Psmg1	-0,48284	1
Ube2d-ps	-0,4829	1
Ubn1	-0,48288	1
Tnnc2	-0,48306	1
Ears2	-0,48337	1
Irf2bp1	-0,48339	1

Tigd5	-0,48381	1
B4gat1	-0,48393	1
Slc10a7	-0,48408	1
Gm42636	-0,48454	1
Abcc5	-0,48451	1
Murc	-0,48463	1
Braf	-0,48481	1
Mcu	-0,48482	1
Caly	-0,48488	1
Pla2g12a	-0,48503	1
Gm17807	-0,48502	1
Lins1	-0,48529	1
Mesdc2	-0,4853	1
Slc25a1	-0,48558	1
Ly9	-0,48566	1
3110001I22Rik	-0,48584	1
Gm20045	-0,48598	1
Dnm2	-0,48613	1
Pigs	-0,48633	1
Ptgir	-0,48663	1
Ppp6r1	-0,48658	1
Crygn	-0,48662	1
Ppp3cc	-0,48692	1
Map2k5	-0,48699	1
Il10rb	-0,48706	1
Atp6v1c1	-0,48725	1
Angpt2	-0,48736	1
Arhgdig	-0,48749	1
Rab34	-0,48746	1
Ttc33	-0,4879	1
Pelp1	-0,4883	1
Mau2	-0,48835	1
Orc3	-0,48856	1
Zdhhc17	-0,48921	1
Six1	-0,4894	1
Mtch2	-0,48938	1
Sdf4	-0,48938	1
Fam120aos	-0,48949	1
Spcs2	-0,48959	1
Gstp-ps	-0,48969	1
Ly6e	-0,48985	1
Nanos1	-0,48984	1
Dcxr	-0,48985	1
Clcn4	-0,48996	1
Ddx55	-0,49011	1
Rragb	-0,49018	1
Zfp219	-0,49025	1
Gm5914	-0,49032	1
H13	-0,49053	1
Zkscan1	-0,49093	1
St8sia4	-0,4915	1
Gm26890	-0,49168	1

Tars2	-0,49185	1
Ubn2	-0,49197	1
1600014C10Rik	-0,49211	1
Adam10	-0,49216	1
Slc41a3	-0,49294	1
Arhgef7	-0,49307	1
Clpp	-0,49326	1
Phlpp2	-0,49381	1
Blnk	-0,49376	1
Samd1	-0,49405	1
Ddx46	-0,49409	1
Wdr46	-0,49439	1
Gstt1	-0,49486	1
Ncdn	-0,49504	1
Atp5k-ps2	-0,49507	1
Pdk1	-0,4954	1
Ankrd10	-0,49539	1
Zfp811	-0,49537	1
Anapc13	-0,4955	1
Psma3	-0,49559	1
Psmd10	-0,49581	1
Tmem35b	-0,49594	1
Zfp839	-0,49625	1
Samsn1	-0,49629	1
Fmnl3	-0,49647	1
Atic	-0,49695	1
Atrx	-0,49702	1
Mms19	-0,49716	1
Lgals4	-0,49746	1
Cds2	-0,49763	1
Ube3b	-0,49804	1
Map3k20	-0,4981	1
Pros1	-0,49824	1
March8	-0,49822	1
Dnmt3a	-0,49817	1
Tmem164	-0,49835	1
Osbpl11	-0,49833	1
Slc4a11	-0,49835	1
Polr3f	-0,49863	1
Mrps35	-0,49862	1
Hddc2	-0,49901	1
Acad10	-0,49905	1
Dmap1	-0,49933	1
Gtf2h2	-0,49935	1
Rab8a	-0,49954	1
Specc1l	-0,49959	1
Exosc7	-0,49975	1
Serp1	-0,49997	1
Preb	-0,50017	1
Eml3	-0,50031	1
Gpatch3	-0,50077	1
Pih1d1	-0,50081	1

Dctd	-0,50124	1
Rsl1	-0,50122	1
Fam122b	-0,50127	1
Pfkl	-0,50222	1
Rtn3	-0,5023	1
Mfn1	-0,50232	1
Rpusd4	-0,50233	1
Gm22980	-0,50247	1
Porcn	-0,50268	1
Nek4	-0,50276	1
Abcf2	-0,50286	1
Gm37906	-0,50303	1
Ebpl	-0,50351	1
Casp1	-0,50359	1
Tcf4	-0,504	1
Smim4	-0,50416	1
Ogt	-0,50451	1
Tifab	-0,5045	1
Strbp	-0,50462	1
Zfp292	-0,50461	1
Lsm10	-0,50472	1
Wdr73	-0,50466	1
Ralgapb	-0,50518	1
Zfp950	-0,50544	1
Parg	-0,50558	1
Cenpq	-0,50567	1
2510002D24Rik	-0,50586	1
Gm44153	-0,50603	1
Zfp931	-0,50607	1
Gstt2	-0,50616	1
Mllt6	-0,50644	1
Prpsap1	-0,50647	1
Nup37	-0,50712	1
Cwf19l2	-0,50723	1
Xylt1	-0,5075	1
Klhl20	-0,50796	1
Tshz3	-0,50812	1
Rab10	-0,50815	1
AI506816	-0,50893	1
Arhgap45	-0,50915	1
Rnf219	-0,50909	1
Gipr	-0,50926	1
Kidins220	-0,5095	1
Trim44	-0,50955	1
Rmi1	-0,50949	1
Gm996	-0,50961	1
Gm43144	-0,50964	1
Otx1	-0,5098	1
Ticam2	-0,5099	1
Rxrb	-0,51049	1
AI839979	-0,51117	1
Lims1	-0,51143	1

Actr5	-0,51177	1
Lix1l	-0,51197	1
Gm4602	-0,51197	1
Dxo	-0,51206	1
Tmem192	-0,51231	1
Trp53inp2	-0,51262	1
Zfyve9	-0,51264	1
Neurl4	-0,51267	1
Nme7	-0,51325	1
Bloc1s5	-0,51341	1
Gm20628	-0,51345	1
Sdhb	-0,51361	1
Akap11	-0,5138	1
Nlrx1	-0,51379	1
Ogfod1	-0,51421	1
Kdm1b	-0,51421	1
Wdr33	-0,51443	1
Srm	-0,5144	1
Fbxo46	-0,51444	1
Zfp930	-0,51495	1
Znrf2	-0,51539	1
Srbd1	-0,51594	1
Polr2f	-0,51633	1
Ankrd44	-0,51625	1
Bcar3	-0,51665	1
Gm15472	-0,51671	1
Dcaf17	-0,51692	1
3300002I08Rik	-0,51693	1
4632427E13Rik	-0,51703	1
Stat6	-0,51747	1
Ormdl1	-0,51752	1
Gm37494	-0,51754	1
Hcls1	-0,51767	1
Hoxa1	-0,51784	1
Nipal3	-0,51789	1
Zdhhc8	-0,51788	1
Rit1	-0,51796	1
Tctex1d2	-0,51877	1
Tbc1d9b	-0,51886	1
Ubxn2b	-0,51904	1
Eif4g1	-0,51916	1
1190007I07Rik	-0,51944	1
Hacl1	-0,51975	1
Slf1	-0,52007	1
Dbp	-0,52028	1
Neu3	-0,52051	1
Cdon	-0,52077	1
Rab2b	-0,52096	1
Rusc1	-0,52116	1
Zcchc2	-0,52147	1
Sh2d2a	-0,52152	1
Mapkbp1	-0,52161	1

Hibch	-0,52164	1
Gm45629	-0,52199	1
Nr1h2	-0,52209	1
Hs2st1	-0,5225	1
Gm38213	-0,52249	1
Zmpste24	-0,52267	1
Faap100	-0,52268	1
Dlat	-0,52303	1
Bmi1	-0,52317	1
Tvp23b	-0,52331	1
Arhgap27	-0,52343	1
Foxred1	-0,52355	1
Rnaseh2a	-0,52359	1
Rbak	-0,52394	1
Ccdc66	-0,52385	1
Snapin	-0,52408	1
Ccdc57	-0,52441	1
Setd1a	-0,52472	1
Scamp2	-0,52496	1
Gfm1	-0,52504	1
Cirbp	-0,52502	1
Hddc3	-0,52505	1
Rnmt	-0,52521	1
Vav1	-0,52555	1
4930453N24Rik	-0,52551	1
BC005561	-0,52555	1
Numa1	-0,52573	1
Cers4	-0,52581	1
Hpdl	-0,52593	1
Parp1	-0,52596	1
Ulk2	-0,52631	1
C430042M11Rik	-0,52643	1
Rad51c	-0,52653	1
Plpp5	-0,52676	1
Ilvbl	-0,52698	1
Cep78	-0,52754	1
Gm43300	-0,52781	1
Cep104	-0,52808	1
Klhl2	-0,5282	1
Stx4a	-0,52835	1
Ubiad1	-0,52833	1
Wdr26	-0,52893	1
Mul1	-0,52888	1
Golga1	-0,52893	1
Amdhd2	-0,52946	1
Rrbp1	-0,52959	1
Taf12	-0,52957	1
Mzb1	-0,52957	1
Olfr920	-0,53003	1
Gm37084	-0,5303	1
Srl	-0,53041	1
Cnot10	-0,53052	1

BC037034	-0,53068	1
Tsg101	-0,53076	1
Alad	-0,53094	1
Luc7l2	-0,53139	1
Gng8	-0,53174	1
B230369F24Rik	-0,53187	1
Sdhc	-0,53214	0,98811
Ocel1	-0,53234	1
Hbs1l	-0,53272	0,99796
Wapl	-0,53294	0,99042
Heatr5a	-0,53296	1
Atf5	-0,53314	1
Arhgef1	-0,53329	1
Camkk2	-0,53369	1
Tmem19	-0,53373	1
Phka1	-0,53385	1
Hebp1	-0,53428	1
Pgam5	-0,53443	1
Babam1	-0,53437	1
Dcaf10	-0,53455	1
Gga3	-0,53463	1
Ssh1	-0,53459	1
1700030K09Rik	-0,53476	1
Nr0b2	-0,53534	1
Pkib	-0,53538	1
Rad50	-0,53566	1
Ankrd13d	-0,53574	1
Msl3	-0,53598	1
Stxbp5	-0,53627	1
Ap4s1	-0,5363	1
Efcab14	-0,53642	1
Mlycd	-0,53638	1
Coq3	-0,53641	1
Mcph1	-0,53648	1
Cpne8	-0,53657	1
Zfp523	-0,53657	1
Fam3a	-0,53658	1
CAAA01194877.2	-0,53672	1
1700022N22Rik	-0,53666	1
Birc6	-0,53694	1
Polr3d	-0,5379	1
C130036L24Rik	-0,53785	1
Isg20	-0,53856	1
Gm44771	-0,53866	1
Oprl1	-0,53891	1
Selplg	-0,53933	1
Acacb	-0,53951	1
Urod	-0,53963	1
Fli1	-0,53965	1
Lin37	-0,5399	1
Ints5	-0,54014	1
Zmym6	-0,54041	1

Top3b	-0,54046	1
Usp25	-0,54068	1
Cdc42ep2	-0,54107	1
Il15ra	-0,54229	1
Plekho1	-0,54259	0,92434
Gm26497	-0,54261	1
Zfp408	-0,54276	1
Appl1	-0,54366	0,96324
Tuft1	-0,5439	1
Tarsl2	-0,54386	1
Anapc10	-0,54401	1
Zbtb25	-0,54431	1
Serbp1	-0,54441	0,94291
Map4k4	-0,54441	0,96337
Acox3	-0,54441	0,98714
Atp5g3	-0,54447	0,95583
RP23-55A6.4	-0,54446	1
Slc20a2	-0,54459	1
Mocs3	-0,54479	1
Zfp628	-0,54494	1
Cabin1	-0,54553	0,99579
Gm43364	-0,54581	1
Sec63	-0,54598	0,95528
Mbd5	-0,54611	1
Serpinc1	-0,54624	1
Gab3	-0,54646	1
Smarcd2	-0,54694	0,9306
Vps41	-0,54691	1
Wdr11	-0,54689	1
Col7a1	-0,54687	1
Vps13c	-0,54698	0,95661
Kmt2a	-0,54753	0,95867
Upf3a	-0,54749	0,99442
Gm37407	-0,54755	1
Zfp119b	-0,54763	1
Gm10842	-0,54769	1
Ptpro	-0,54781	1
Pafah1b2	-0,54803	0,93245
Gdi2	-0,54796	0,94311
Gm17586	-0,54812	1
Ash1l	-0,54834	0,94773
Borcs7	-0,5486	1
Ptpra	-0,54872	0,97779
Slc37a4	-0,54865	1
Sgtb	-0,54874	1
Plxnd1	-0,54878	0,99218
Smg9	-0,5489	0,97894
Rpusd3	-0,54893	1
Pid1	-0,54907	1
Gnmt	-0,54905	1
Gm11918	-0,54971	1
Mrps18a	-0,54986	0,9873

Acaa1a	-0,5502	0,93159
Kdm5b	-0,55018	0,95585
Kmt2e	-0,55049	0,97111
Rnf128	-0,55056	0,96306
Msh2	-0,55058	1
Mon1b	-0,5508	1
Zswim4	-0,55101	1
H2-Oa	-0,5512	1
Itgav	-0,55149	0,9873
Abhd3	-0,55149	1
Gm2895	-0,55158	1
Ubxn2a	-0,55173	1
Pmm2	-0,55193	1
Acly	-0,55255	0,96324
Pum3	-0,55266	0,95583
Vac14	-0,55274	1
Atp2a2	-0,55293	0,94311
Taco1	-0,55291	1
Coq10a	-0,55326	0,98796
Twf2	-0,55344	0,99042
Anks1	-0,55346	0,98591
E2f1	-0,55366	1
Prpf31	-0,5538	1
Zfp180	-0,55395	0,99467
Spidr	-0,55398	1
Rnft2	-0,5541	1
Tram1	-0,55467	0,93259
Mtif2	-0,55483	1
Rnf183	-0,55514	1
Pcnx3	-0,55527	0,94574
Fbxo3	-0,55562	0,93966
Wwp2	-0,5556	0,99218
Phf20	-0,55623	0,95244
Usp24	-0,55617	0,99218
Tmem25	-0,55629	1
Tal1	-0,55637	1
Washc5	-0,55637	1
Snord92	-0,55667	1
Napa	-0,55677	0,93599
Atg4d	-0,55689	1
Tubd1	-0,55713	1
Ccdc12	-0,55716	1
Pla2g2e	-0,5572	1
Actb	-0,55749	0,95867
D11Wsu47e	-0,55746	1
Setd5	-0,55756	0,92681
Polr3k	-0,55768	1
Morc4	-0,55826	0,95239
Rfwd3	-0,55843	1
Abcb10	-0,5585	1
Slc35a1	-0,55861	1
Gm8738	-0,55892	1

Gm37900	-0,55905	1
Spr	-0,55918	1
Arl16	-0,55932	1
Gm19967	-0,55934	1
Cdc123	-0,55961	0,99057
Ccdc94	-0,55969	1
Usp46	-0,55983	0,99042
Micu2	-0,56014	0,98346
Itpka	-0,56016	1
Ptpn23	-0,56063	1
Ints6	-0,56057	1
Dst	-0,56138	0,99495
Gm43792	-0,56152	1
Dph2	-0,56159	1
Zfp608	-0,56157	1
Slc2a9	-0,56173	1
Gcfc2	-0,56187	1
Tmem87a	-0,56202	0,96587
Pidd1	-0,56195	1
Rnf214	-0,56238	0,98921
Psen2	-0,56304	0,95583
Vps45	-0,56321	0,95867
Sh3bp5	-0,56325	0,89311
Snora21	-0,56334	1
Ddx39b	-0,5634	0,93409
Epm2aip1	-0,56341	0,98162
Polg	-0,56342	1
Lgals8	-0,56347	0,92936
Fars2	-0,56358	0,97849
Arhgef15	-0,56373	1
Dis3l	-0,5638	1
Mettl25	-0,56376	1
Dnal1	-0,564	1
Scyl3	-0,56424	1
Sfxn3	-0,56443	0,9327
Gm44250	-0,56454	1
Erp29	-0,56499	0,84945
C1qbp	-0,56545	0,83407
Pms2	-0,56553	1
Stard7	-0,56564	0,92593
Ctdsp2	-0,56556	0,97058
Senp1	-0,56556	0,97078
Pdhx	-0,56579	1
Calu	-0,56601	0,97078
Gm20257	-0,56607	1
Spa17	-0,56635	1
Tnip1	-0,56677	0,91263
Spata1	-0,56683	1
Ptbp2	-0,56687	0,9873
Csrp2bp	-0,56688	1
Hp1bp3	-0,56703	0,83548
Mfap3	-0,56731	0,99495

Limd1	-0,56742	0,91711
Cyb561d2	-0,56746	1
Ddx41	-0,56758	0,98892
Cttnbp2nl	-0,56767	0,94495
Bcl2a1b	-0,56792	1
Fcgr4	-0,56801	1
Cd63	-0,56808	0,84553
Lrmp	-0,56837	0,93245
Nrip1	-0,56849	0,9873
1810024B03Rik	-0,56878	1
Hsf1	-0,56889	1
Mpi	-0,5691	1
Mars	-0,56915	0,95216
Zfp316	-0,56956	1
Atp6v1e1	-0,56977	0,84673
Tecr	-0,56993	0,95654
Tcam1	-0,56995	1
Atpaf1	-0,57015	1
Ctnnb1	-0,57052	0,7913
Cyp20a1	-0,57055	0,96764
Rab43	-0,57057	1
Helz2	-0,57059	1
Dock6	-0,5714	1
2310010J17Rik	-0,57178	1
Atp10d	-0,57193	1
Exoc4	-0,57213	0,85126
Dnmt3b	-0,57243	1
Creb3l4	-0,57252	1
Magt1	-0,57258	0,93119
Gm42967	-0,57274	1
Rnf220	-0,57304	0,92008
Snx32	-0,57336	1
Srpk2	-0,57353	0,78676
Eif2b3	-0,57349	0,92205
Fam167b	-0,57381	0,89461
Yipf2	-0,57396	0,95583
Tulp4	-0,57428	0,90113
Srsf5	-0,57468	0,89221
Rad9b	-0,57488	1
Gbf1	-0,5751	0,89546
Maged1	-0,57544	0,95931
Rftn1	-0,57542	0,97628
Gm44187	-0,57556	1
Fhl3	-0,57624	0,94574
Eprs	-0,57657	0,79653
Kiss1r	-0,57691	1
Ydjc	-0,577	0,91299
Exoc3l2	-0,57703	1
Mfsd1	-0,57707	0,7884
Poc5	-0,57741	0,99042
Tmem8	-0,57758	0,9873
Cacna1d	-0,57761	1

Yars	-0,57765	0,94428
Coro1b	-0,57779	0,94428
Camk1d	-0,57794	0,95819
Vps4b	-0,57803	0,90611
Uso1	-0,57856	0,92613
Apobec3	-0,57864	0,99218
Sipa1l3	-0,57876	1
Vasp	-0,57898	0,81164
Ppp3cb	-0,57901	0,86961
Gpatch8	-0,57936	0,93128
Vipas39	-0,57944	0,97482
Rbm4	-0,57948	1
Trpc4ap	-0,57965	0,85126
Plcg1	-0,57971	0,95195
Gm43628	-0,57969	1
Zfp764	-0,57982	1
Gm43681	-0,58041	1
Acp2	-0,58067	1
Prelid3a	-0,58068	1
CH25-309J2.1	-0,58069	1
Trem3	-0,58163	1
Sdr42e1	-0,58188	1
Mef2a	-0,582	0,8335
Zc2hc1a	-0,58199	0,9873
Zfp605	-0,58206	1
Kat6a	-0,58271	0,90157
Mtmr2	-0,58289	0,81451
Snd1	-0,58312	0,85126
Hip1	-0,58355	0,98976
Tpp1	-0,58377	0,84667
Ncoa2	-0,58378	0,93898
Tbc1d25	-0,5838	1
Cep131	-0,58385	1
Mtdh	-0,58397	0,69827
Dlst	-0,58412	0,8228
Nubp2	-0,58425	0,99796
Gm12966	-0,58434	1
Mprip	-0,58446	0,87267
Chmp6	-0,58453	0,98976
Parp12	-0,58466	0,98976
Zfp329	-0,5847	1
Zfp974	-0,58508	1
Trim37	-0,58534	0,87773
Cbarp	-0,58541	1
Rnf170	-0,58559	1
Zranb2	-0,58586	0,85527
Pdk3	-0,58613	0,93599
Syt8	-0,58625	1
Tnfrsf9	-0,5868	0,95395
Zfp959	-0,58693	1
Dennd1a	-0,5873	0,97894
P3h1	-0,5874	0,95673

Cfap36	-0,58747	0,99767
Snap23	-0,58779	0,8205
Thap7	-0,58786	0,94908
Cyfip1	-0,58801	0,91958
Cadps	-0,58796	0,99042
Mthfr	-0,58831	0,97531
Gm43420	-0,58829	1
Mmp19	-0,58838	0,95427
March9	-0,58882	0,9269
Ccdc28a	-0,58887	1
Abcf1	-0,58913	0,7931
Crot	-0,58911	0,97111
Serhl	-0,58939	0,9306
Sdcbp2	-0,58937	0,98629
Fam172a	-0,58937	1
Plag1	-0,58959	1
Snta1	-0,58968	0,82407
2510009E07Rik	-0,58969	0,85316
RP23-402A24.3	-0,58968	1
Trio	-0,59007	0,95244
Chrnb2	-0,59006	1
Atm	-0,59026	0,80581
Dgcr14	-0,59085	1
Atg4a-ps	-0,59082	1
Anapc4	-0,59089	0,95867
Aldh16a1	-0,59093	0,96686
Drg2	-0,59086	1
Usp54	-0,59093	1
Timmdc1	-0,59095	1
Herc2	-0,59132	0,81713
Cep128	-0,59162	1
Lrrk2	-0,59168	0,95032
Twnk	-0,59193	0,93159
Ociad1	-0,59198	0,78966
Samm50	-0,59195	0,81258
Stx2	-0,59204	0,90372
Mbp	-0,59243	0,77224
Trmu	-0,59252	1
Gm37145	-0,59254	1
Nfatc3	-0,59267	0,79857
Slc25a53	-0,59296	0,98606
Gm43323	-0,59319	1
Gm43499	-0,59325	1
Gas2l1	-0,59338	0,95585
Gm11944	-0,59367	1
Fsd1l	-0,59378	1
Cxcr3	-0,59403	1
Gm10642	-0,59398	1
Kdelc2	-0,59409	0,95247
Mrps27	-0,59412	1
Gaa	-0,59415	0,84729
Atrn	-0,59435	0,95427

Tor4a	-0,5943	0,99218
Fam45a	-0,59446	0,99796
1700020I14Rik	-0,59464	0,911
Prdx1	-0,59478	0,71386
Ppp1r18	-0,595	0,82377
Orc5	-0,59513	0,97059
Aqp11	-0,59532	1
Zscan12	-0,59542	0,9306
Lmntd2	-0,59549	1
Glud1	-0,59592	0,73923
Haus4	-0,59606	1
Zfp541	-0,5963	1
Sh3bp5l	-0,59666	0,90171
Prex1	-0,5969	0,86531
Relb	-0,59688	0,90518
Ampd2	-0,5972	0,75582
Gm37106	-0,59743	1
Cdk5	-0,59754	0,97193
Aim1	-0,59776	0,92517
Ak1	-0,59787	0,83798
Actr8	-0,5983	0,85423
Vps37a	-0,59854	0,84945
Nup93	-0,59885	0,8132
Nxpe3	-0,59912	0,89221
Gm45828	-0,59908	1
D130019J16Rik	-0,59919	1
Gpr108	-0,59926	0,86033
Rgp1	-0,5995	0,91684
Pomt1	-0,59958	0,9956
Gm15690	-0,59982	1
Col4a3bp	-0,60081	0,96173
Parp11	-0,60087	1
2410002F23Rik	-0,60119	0,83487
Slc9a6	-0,60124	0,85823
Wdr60	-0,60121	0,86096
Stx12	-0,60167	0,78676
Mlx	-0,60194	0,9154
Gm42872	-0,60243	1
Dnajc8	-0,60272	0,77224
Suclg1	-0,60314	0,83234
1110034G24Rik	-0,60306	1
Peak1os	-0,6033	1
Gm16199	-0,60367	1
Abcd4	-0,60406	0,94291
Crtap	-0,60426	0,99042
Spsb1	-0,60427	0,99463
Nme6	-0,60495	1
Tmem268	-0,60578	0,86117
Irf3	-0,60607	0,95583
Atxn1	-0,60615	0,86531
Ralgapa2	-0,60628	0,99767
Klhl35	-0,60632	1

Dync1h1	-0,60649	0,62375
Zfp871	-0,60734	0,86068
mt-Nd2	-0,6075	0,84729
Zfp827	-0,60791	0,89408
Nufip2	-0,60829	0,77935
Dnase1l1	-0,60836	0,89971
Hif1a	-0,60853	0,7639
Megf8	-0,60847	0,92936
Ddx58	-0,60856	0,95174
Gm37968	-0,60898	1
Acap1	-0,60895	1
Gm44935	-0,60928	1
Commd10	-0,60982	0,89112
Osbpl7	-0,61006	0,89408
Psap	-0,61058	0,8132
Cntnap1	-0,61062	1
Umps	-0,61075	0,90187
Zgpat	-0,61076	0,99796
Rbm26	-0,61145	0,84479
Zfp276	-0,61165	0,99921
Polm	-0,61158	1
Snx15	-0,61181	0,99218
Ube2q1	-0,61185	0,68348
Gm15268	-0,612	1
Fastk	-0,61213	0,76714
Mgmt	-0,61248	0,90187
Mxra8	-0,61325	0,98589
Echdc1	-0,61347	0,87386
Ube2d2a	-0,61346	0,92936
Rtn4ip1	-0,61361	0,92668
Itpripl2	-0,61394	0,73923
Coa4	-0,61399	0,97003
Ppm1f	-0,61417	0,87906
Ttpal	-0,61442	0,97392
Rpgrip1l	-0,61436	1
Ndufaf3	-0,61452	0,90187
Tsc2	-0,6148	1
Cluh	-0,6149	0,70884
Herc1	-0,61505	0,75374
Lrch1	-0,61509	0,95583
Fam168a	-0,61515	0,69386
Ndufa9	-0,6152	0,84036
Tmem63b	-0,61533	0,81183
Fam210b	-0,61539	0,90356
Tnfrsf13b	-0,61543	1
Neurl1a	-0,61551	1
Rhd	-0,61607	0,79857
Nipsnap1	-0,61617	0,88255
Nfkbia	-0,61709	0,56247
Srp72	-0,61717	0,60056
Dhx16	-0,61765	0,9306
Pddc1	-0,61774	0,9956

Trim46	-0,61788	1
BC065397	-0,61801	1
Yif1a	-0,61817	0,90187
Myo7a	-0,61855	0,94564
Slc30a7	-0,61873	0,85316
Ube4b	-0,61877	0,72289
Gata3	-0,61905	1
Grn	-0,61916	0,71679
Tns3	-0,61976	0,71423
Nrd1	-0,61998	0,76273
Trabd	-0,6203	0,62746
Sipa1	-0,62044	0,85567
Nfyc	-0,62039	1
Rab29	-0,62082	0,87712
Whrn	-0,62082	1
Trub1	-0,62102	0,86754
Immt	-0,62119	0,73923
Mrgbp	-0,62137	0,9327
Kptn	-0,62179	1
Ascl2	-0,6218	1
Ell2	-0,62197	0,70884
Mpv17l	-0,62195	0,9842
Zbtb22	-0,62236	0,84032
Fsd2	-0,62324	0,93327
Nars	-0,62357	0,64353
Gcc2	-0,62406	0,80041
Slc35c1	-0,62416	0,82199
Trip11	-0,62428	0,87413
Rpl7l1	-0,62445	0,79588
Exoc3l4	-0,62439	0,95247
D130007C19Rik	-0,62479	0,98261
Tkfc	-0,6249	0,92355
Miga1	-0,62501	0,86115
Ubp1	-0,62509	0,75046
Tmem245	-0,62544	0,75186
Wrap73	-0,62551	0,95673
Lrrc8d	-0,62601	0,77727
Rela	-0,62619	0,76126
Nsun5	-0,62621	0,99767
Gm43061	-0,62641	1
Tsen2	-0,62695	0,94096
Nin	-0,627	0,93119
Sec23a	-0,62724	0,73923
Mcm3ap	-0,62719	0,82814
Crtc3	-0,62757	0,91442
F7	-0,6277	1
Pdcd11	-0,62798	0,70905
Ss18	-0,628	0,80532
Herpud1	-0,62808	0,61923
Limd2	-0,62846	0,8484
Pias3	-0,6286	0,76673
Mdc1	-0,6288	0,90372

Lrp12	-0,62894	0,85028
Kirrel3	-0,62903	1
Tm9sf3	-0,6291	0,66723
Nadk	-0,62916	0,69827
9230112E08Rik	-0,62946	1
Nfia	-0,62971	0,85536
Sdha	-0,63043	0,59668
Kcnab3	-0,63102	0,99057
Gm37423	-0,63128	1
Kifc3	-0,63177	0,72155
Angel2	-0,63177	0,79925
Tln2	-0,63177	0,97236
Ikbip	-0,63205	0,86331
Rabep2	-0,63204	0,96227
Ccl25	-0,63257	0,91804
Rb1cc1	-0,63274	0,81549
Itgb7	-0,6327	0,86353
Agl	-0,63299	0,80909
Gclc	-0,63347	0,7457
Zfp418	-0,63383	1
Gpatch1	-0,63398	0,87773
6030460B20Rik	-0,63442	1
Abcf3	-0,63449	0,86447
Ulk3	-0,63453	0,89173
Trim25	-0,63472	0,69765
1810011H11Rik	-0,63491	0,95528
Tmem94	-0,63509	0,92898
Platr3	-0,63556	1
Gapvd1	-0,63585	0,62458
Crat	-0,63604	0,93159
Intu	-0,63611	1
Exoc3l	-0,63613	1
Alg5	-0,6364	0,79857
Scarna17	-0,63646	1
Gm12479	-0,63652	1
Akr1b8	-0,63662	0,6838
Ate1	-0,63698	0,61255
Gm38055	-0,63699	1
Pigt	-0,6372	0,88461
Rhbdd1	-0,63761	0,84215
Zfr2	-0,63764	0,95216
Rab11fip3	-0,63766	1
Gm37116	-0,6377	1
Trmo	-0,63775	0,99657
B3galt6	-0,63878	1
Lman2	-0,63889	0,6292
Ptpa	-0,63891	0,72289
Irf4	-0,63906	1
Rcan3	-0,6392	0,79653
Hsdl2	-0,63938	0,96217
Ttll13	-0,63964	1
Gm20056	-0,63987	1

Cstf2t	-0,64007	0,81183
Slc25a43	-0,64009	1
Gnaq	-0,6402	0,73565
Ankhd1	-0,64037	0,65638
Cdc25b	-0,64046	0,59094
Fus	-0,6408	0,849
Kmt5b	-0,64127	0,8255
Snrnp200	-0,64151	0,64126
Gpatch11	-0,64171	0,72479
1110032A03Rik	-0,64169	0,93224
Car6	-0,64201	1
Alg1	-0,6421	0,77995
Ksr1	-0,6428	1
Tgm2	-0,64289	0,61923
Rexo2	-0,64349	0,68659
Pja1	-0,64349	0,88484
Klhl36	-0,64357	1
Uhrf1bp1l	-0,6439	0,53839
Adssl1	-0,64395	0,67801
Spryd3	-0,64397	0,85527
Bmf	-0,64424	0,92368
Csde1	-0,64438	0,44113
Gm42970	-0,64465	1
Cisd2	-0,64501	0,82802
6330408A02Rik	-0,64498	0,99042
Fibp	-0,64534	0,86353
Trdmt1	-0,64609	1
Cmc4	-0,64636	1
Klf1	-0,64639	1
Glmp	-0,64667	0,70884
Gm4262	-0,64666	1
Pde6g	-0,64681	1
Tnrc6b	-0,6469	0,66723
2700046G09Rik	-0,64695	1
Chst3	-0,6471	0,94673
Huwe1	-0,64722	0,64797
Brms1	-0,64719	0,99796
H2afy	-0,64741	0,64445
Slc39a9	-0,64742	0,76059
Cd276	-0,64773	0,88255
Dcaf6	-0,64791	0,64921
Notch2	-0,64798	0,69413
Dsn1	-0,64827	0,99943
Gm37204	-0,64859	1
Cand1	-0,64884	0,67153
Cdc23	-0,64913	0,70884
Adamtsl4	-0,64914	0,93967
Gm8566	-0,64908	0,95867
Psma7	-0,64918	0,66723
Gm43062	-0,64915	1
Ptcd2	-0,64945	0,56054
Zfp566	-0,64954	1

Phf21a	-0,64956	0,57269
Gm45890	-0,64992	1
0610009L18Rik	-0,65066	1
E430018J23Rik	-0,65077	1
Naaa	-0,65114	0,95673
Pgm5	-0,65114	1
Atp6v1b2	-0,65162	0,35046
Sec22a	-0,65167	0,90267
Mettl26	-0,65181	0,78026
Pik3cg	-0,65194	0,89461
Tmem126b	-0,65215	0,90171
Hacd1	-0,65243	0,88461
Pqlc3	-0,65254	0,8594
Nradd	-0,65263	0,80361
Zfp579	-0,65315	0,96226
Gm37519	-0,65322	1
Pigw	-0,65381	0,92936
Dus1l	-0,65391	0,84215
Fermt3	-0,65404	0,75393
Prpf40b	-0,65396	0,77995
Chrna1os	-0,65417	1
Nrtn	-0,6542	1
Mfap3l	-0,65426	0,78982
Bcl9l	-0,65446	0,80186
Mospd2	-0,6545	0,88332
Dnajc24	-0,65451	0,93259
Soat2	-0,65459	1
Tas1r1	-0,65487	1
Opa3	-0,65502	0,74927
4921536K21Rik	-0,65502	1
Uck2	-0,65528	0,46634
Zcchc6	-0,65549	0,7053
Ranbp1	-0,65571	0,80741
Bcap29	-0,65594	0,72282
Cpsf6	-0,65606	0,60737
Sema5a	-0,65609	0,80361
Vps26b	-0,65646	0,57953
Pycr2	-0,65659	0,64353
Ifi211	-0,65671	1
Znhit6	-0,65708	0,82814
Bag2	-0,65708	0,84215
Thoc7	-0,65731	0,63586
Zcchc7	-0,65737	0,66843
Spata7	-0,65744	0,90171
Rnf121	-0,65753	0,89311
Ap1s1	-0,65772	0,5434
Fyco1	-0,65849	0,90293
Zfp28	-0,65858	1
Gmip	-0,65889	0,75762
Tm9sf4	-0,65902	0,36906
Gtdc1	-0,65904	0,89778
Nudt7	-0,6591	0,91711

Kifap3	-0,65932	0,7457
Galns	-0,65958	0,83477
1700112E06Rik	-0,65958	0,92498
Gart	-0,65999	0,73923
Nupl2	-0,66054	0,97531
Pex11g	-0,66092	1
Gm37949	-0,66089	1
Nfat5	-0,66155	0,68497
A230050P20Rik	-0,66146	0,93125
Rbmx2-ps	-0,66197	1
Gm37728	-0,66206	1
D430013B06Rik	-0,66222	1
Il18	-0,66229	0,69155
Slc22a21	-0,66229	1
2810414N06Rik	-0,66226	1
Add1	-0,66267	0,76059
Pi4ka	-0,66286	0,54006
Mir7078	-0,66348	1
Coa7	-0,66356	0,95528
Scd2	-0,66373	0,5929
Timp1	-0,66373	1
Mroh2a	-0,66376	1
Mrgpre	-0,66385	1
Sh3bgr	-0,66423	1
Cog1	-0,66441	0,97391
Pear1	-0,66451	1
Madd	-0,66469	0,78901
Dpp3	-0,66474	0,90187
Jagn1	-0,66491	0,75186
Pcyox1	-0,66538	0,69765
Gsdmd	-0,66626	0,79857
Gm13341	-0,66646	1
Fdxr	-0,66656	0,73611
Mut	-0,66674	0,73923
Ofd1	-0,66675	0,94162
Polr3h	-0,66679	0,69272
Kctd12	-0,66722	0,95585
Rab7b	-0,66748	0,71502
Pccb	-0,6679	0,90171
Ryk	-0,6681	0,50777
Trpv2	-0,66807	0,54771
Sacm1l	-0,66829	0,64973
Actg1	-0,66854	1
Tmem5	-0,66878	0,88406
Prkaa1	-0,6689	0,74108
Tlr13	-0,66933	0,89477
Speg	-0,6696	0,83481
Wdr41	-0,66991	0,87651
Rgs11	-0,67019	0,9785
Cpsf1	-0,67027	0,72704
C1rb	-0,6704	1
Scamp5	-0,67067	0,7247

Txndc9	-0,67083	0,62249
Itpr3	-0,67105	0,70617
C130023A14Rik	-0,67102	0,96196
Gm9025	-0,67136	0,97779
Ccdc173	-0,67142	0,97994
Timm44	-0,67152	0,69765
Trim28	-0,67231	0,64651
Slc25a11	-0,67242	0,61177
Gzf1	-0,67277	0,78646
E330037G11Rik	-0,67301	1
Smad1	-0,67349	0,84801
Prkab2	-0,67379	0,77995
Phf7	-0,67427	0,90171
Mgat4b	-0,67459	0,49364
Zc3h7b	-0,67493	0,89188
Gm43813	-0,67486	1
Fam120a	-0,67536	0,58726
Pkn1	-0,67603	0,57647
Lrrcc1	-0,6761	0,80794
Gm13398	-0,67607	1
Rab23	-0,67626	0,93218
Igtp	-0,67628	1
0610007P14Rik	-0,67643	0,6455
Tmem156	-0,67656	0,87787
Coa3	-0,67716	0,72967
Spef1	-0,67721	1
1600002H07Rik	-0,67773	0,82875
Ttc3	-0,67794	0,56223
Nsdhl	-0,67798	0,73621
Ap5s1	-0,67833	0,74126
AI606181	-0,67845	0,84891
Fth1	-0,67949	0,3087
Hacd4	-0,68029	0,63415
Gm13675	-0,68027	1
Ttc13	-0,68055	0,85316
Mpv17	-0,68088	0,75217
Dram2	-0,68092	0,81183
Dcun1d1	-0,68112	0,60005
Dnajc22	-0,68142	1
Mrps2	-0,68146	0,69584
Hdhd3	-0,68164	0,94428
Pus7	-0,68189	0,77021
A130071D04Rik	-0,68192	0,80662
Atg9b	-0,68189	0,84315
Cog8	-0,68203	0,79252
Clasp2	-0,68209	0,73573
Il16	-0,68209	0,86083
Gm43544	-0,68209	1
1700020D05Rik	-0,68219	1
Trim45	-0,68293	0,92053
Tnfaip8	-0,68471	0,42295
Kri1	-0,68482	0,78256

Gm43727	-0,68482	1
Rrad	-0,68479	1
Wdr24	-0,68546	0,83354
Zfp738	-0,68551	0,96078
Trim30a	-0,68545	0,97779
Tex9	-0,68549	0,98606
Colec12	-0,68556	0,87906
Car2	-0,68584	0,72153
Msi2	-0,68589	0,77965
Gm43707	-0,68593	1
Clec7a	-0,68597	0,7616
Atp8a1	-0,68614	0,73565
Dapk3	-0,68606	0,8686
Bivm	-0,68642	0,89813
Ep400	-0,68646	0,56661
Gm42820	-0,68664	0,97994
Plin3	-0,68686	0,66723
Sdccag8	-0,68714	0,79571
Vamp1	-0,68719	0,79798
Ttc27	-0,68724	0,8335
Zfp61	-0,68724	0,85057
Hif1an	-0,68733	0,73765
Trap1	-0,68739	0,66586
C330018D20Rik	-0,68752	0,89419
Rnf213	-0,68799	0,78463
Mt2	-0,68858	0,4191
Dsel	-0,68914	0,90627
Pigv	-0,68928	0,84945
Tbcd	-0,68943	0,69835
Kif16b	-0,69034	0,78966
Atxn7l3	-0,69076	0,55635
Coq4	-0,69077	0,93878
Gm43153	-0,69119	1
Cerk	-0,69184	0,44555
Uckl1	-0,69186	0,77827
Uros	-0,69186	1
Bcl2l1	-0,69286	0,37542
Cdk5rap2	-0,69294	0,79857
Rbm45	-0,69292	0,90187
Retsat	-0,69321	0,79914
Gm8013	-0,69334	0,97671
2610016A17Rik	-0,69333	0,98976
Impa1	-0,69339	0,45871
Ppa2	-0,69339	0,69765
Gm15440	-0,69341	0,90781
Fhod3	-0,69345	0,91299
Gm43696	-0,6935	0,98131
Psd	-0,69394	1
Tsga10	-0,69408	0,92936
Pknox1	-0,69463	0,79282
Manea	-0,69472	0,89514
Apex2	-0,69467	0,9873

Nfs1	-0,69497	0,55736
Mrpl19	-0,69509	0,54169
Tmem186	-0,69512	0,91554
Ppil3	-0,6956	0,77929
1700123M08Rik	-0,69584	1
Tspoap1	-0,69595	0,90157
Rcc2	-0,69614	0,28874
Coro1c	-0,69606	0,39892
Zfp11	-0,69607	1
Clcn5	-0,6962	0,79857
Arl6	-0,6962	0,97779
Slc25a33	-0,69646	0,5806
Mypop	-0,69689	0,93224
Commd5	-0,69716	0,79293
Nipsnap3b	-0,69765	0,74546
Zmynd8	-0,69789	0,42103
Defb25	-0,69828	0,90171
Frat1	-0,69923	0,84801
Gm17066	-0,69958	0,59502
Isoc2a	-0,69963	0,76273
Rnf8	-0,69991	0,77224
Hivep1	-0,69994	0,91371
Spata13	-0,70006	0,48857
Sirt7	-0,7001	0,6709
Sri	-0,70022	0,5616
Slc50a1	-0,70016	0,58458
Slc17a9	-0,70044	0,81258
Kmt2b	-0,70064	0,75972
Creb3l1	-0,7009	0,99218
Eid3	-0,70118	0,97159
Ifi47	-0,70121	1
As3mt	-0,70162	0,9327
Gm26542	-0,70176	0,93283
Gm37349	-0,70188	0,88255
Casd1	-0,70242	0,73565
Ocrl	-0,70282	0,60448
Dhdds	-0,70318	0,53297
Lrtm2	-0,70392	1
Tm9sf1	-0,704	0,95163
Ahrr	-0,70522	0,99257
Rprd2	-0,70571	0,49492
Dtd1	-0,70584	0,35136
Ikbkb	-0,7058	0,64097
2810004N23Rik	-0,70619	0,46665
Smarca2	-0,70634	0,35518
Atp6v0b	-0,70654	0,54173
Prr12	-0,7065	0,82802
Mier2	-0,70662	0,33617
Gm37902	-0,70658	1
Tk2	-0,70703	0,44555
Spen	-0,70714	0,62574
Tpk1	-0,70729	0,81401

Wdfy1	-0,70766	0,4399
Klk8	-0,70767	0,96306
Atp6v1d	-0,70796	0,30128
Ncoa6	-0,7085	0,52636
Igf2r	-0,70892	0,65434
Osbp2	-0,70908	1
Foxp1	-0,70915	0,32104
Spout1	-0,70926	0,61408
C630004M23Rik	-0,70942	1
Mapk12	-0,71045	0,94574
Tanc2	-0,71095	0,8644
Rhpn2	-0,71096	1
Gm24009	-0,71103	1
Slc38a10	-0,71119	0,36784
Bcl2l11	-0,71139	0,55466
Ppic	-0,71135	0,57367
Gsap	-0,71138	0,61923
4831440E17Rik	-0,71145	1
App	-0,71169	0,44066
Nup85	-0,71172	0,88796
Dnajc27	-0,71165	0,91675
Impa2	-0,71183	0,49553
Acsl5	-0,71246	0,33683
Galk1	-0,71264	0,58458
Il4ra	-0,71282	0,78966
6330418K02Rik	-0,71292	0,94291
Tom1l2	-0,7131	0,63826
Rtfdc1	-0,71383	0,52712
Elovl6	-0,7138	0,64126
Haus7	-0,71414	0,95096
Serpini1	-0,71419	0,80213
Gm43362	-0,71476	0,85682
Pdia5	-0,71498	0,77995
Alkbh2	-0,71504	0,78256
Was	-0,71514	0,77995
Kank3	-0,71536	0,8132
Apc	-0,71571	0,5799
Cd3eap	-0,7157	0,66586
Smarcb1	-0,71596	0,69173
Fignl2	-0,71663	0,97059
Surf4	-0,71667	0,23952
Gm13868	-0,71712	1
Zfp60	-0,71717	0,91728
Blvra	-0,7178	0,46919
Tlr2	-0,71885	0,55374
Elp3	-0,71894	0,64261
Mrpl37	-0,71896	0,39924
Abcd3	-0,71911	0,46651
Sipa1l2	-0,71946	0,87665
Ggact	-0,71954	0,90627
Gtf2b	-0,7196	0,71502
Slc26a9	-0,71957	1

Trim7	-0,71972	0,73535
Tbck	-0,71977	0,7354
Tfdp2	-0,72008	0,78966
Iqcg	-0,72009	0,8764
Nsmce1	-0,7203	0,76714
RP23-225D5.4	-0,72064	1
Synrg	-0,72074	0,6455
9230114K14Rik	-0,7207	0,71245
Zbed4	-0,72073	0,85316
Gm14403	-0,72083	1
Cdkl2	-0,72122	0,7055
Dgkq	-0,72133	0,71679
Ppip5k1	-0,72165	0,7053
Gm37124	-0,72172	0,9844
Ergic1	-0,72195	0,33179
Mtfr1	-0,72215	0,66689
Msto1	-0,72233	0,62656
Crybg3	-0,72234	1
Mfsd7a	-0,72243	1
Suox	-0,72295	0,84032
Zmiz1	-0,72324	0,50832
Bptf	-0,7233	0,36454
Gm44829	-0,72374	0,94428
Cacna1b	-0,72392	1
Fahd2a	-0,72405	0,91669
Vsig10	-0,72417	0,88843
Xpr1	-0,72442	0,25462
Trappc11	-0,72444	0,71465
Nt5c2	-0,72441	0,91311
Gm7815	-0,72532	1
Gm43359	-0,72552	0,84332
Fam69b	-0,72572	1
1810010D01Rik	-0,72633	0,94627
Zfp330	-0,72645	0,46236
Insr	-0,72636	0,72967
Ing4	-0,72672	1
Rreb1	-0,72691	0,69
Ccdc191	-0,72701	0,75948
Zfp768	-0,7271	0,55068
Orai2	-0,72723	0,55976
Zfp85	-0,7273	1
Gm6209	-0,72751	0,96078
Afmid	-0,72755	1
Plcb2	-0,72778	0,58299
Asns	-0,72787	0,73923
Tmem202	-0,72801	0,99218
Zdhhc21	-0,72912	0,40839
Gm26740	-0,72925	1
Stx1a	-0,72943	1
Capn3	-0,72936	1
Chpt1	-0,72986	0,75739
Gm8539	-0,73004	0,9873

Gm38062	-0,72995	1
Pnpla6	-0,73042	0,79727
Myh9	-0,73059	0,37425
Trafd1	-0,73088	0,40667
Usf3	-0,73095	0,6077
Ppp1r9b	-0,73113	0,56032
Lrp4	-0,73119	1
Zfp710	-0,73143	0,6077
Ylpm1	-0,73242	0,46141
Tmcc2	-0,73242	0,77042
Gm3699	-0,73239	1
Frmd4b	-0,73312	0,44054
Procr	-0,73308	0,77995
Gm45873	-0,73334	1
Gab2	-0,73363	0,31703
Sidt2	-0,73434	0,43447
Cyb5r4	-0,73432	0,67874
Phf14	-0,73435	0,65584
Ccdc134	-0,73454	0,84775
Pqlc1	-0,73469	0,36827
Mplkip	-0,73535	0,49942
Arhgap35	-0,73527	0,86178
9630013D21Rik	-0,73547	1
Maf1	-0,7358	0,40746
Taz	-0,73615	0,70617
Mtap	-0,73702	0,63586
Gm13421	-0,73701	1
Npc2	-0,73712	0,24977
Ppip5k2	-0,7377	0,49045
Lpar2	-0,73785	0,97822
Gtf2i	-0,73829	0,39282
Ahi1	-0,73886	0,55736
Mtg1	-0,73895	0,67874
Wars2	-0,73888	0,90844
Mlst8	-0,73909	0,52636
Rpn1	-0,73928	0,17273
Il23a	-0,7395	0,97392
Phykpl	-0,73961	0,81944
Limk1	-0,73972	0,87603
Ostc	-0,73999	0,35087
Lancl1	-0,74016	0,55182
Tlr4	-0,74022	0,72155
Fry	-0,74032	0,99796
Zfp12	-0,74055	0,79287
Zfp786	-0,74112	1
Gm44510	-0,74126	1
Faah	-0,7414	1
Fam114a1	-0,74173	0,61408
Utp20	-0,7417	0,6292
Gm45456	-0,74169	1
Gm5837	-0,74168	1
RP24-282C4.4	-0,74173	1

Pou4f1	-0,74177	1
Kif1bp	-0,74187	0,67801
Eif3b	-0,74196	0,28262
Churc1	-0,74242	0,92368
Stxbp4	-0,7427	0,92217
Dhx29	-0,74298	0,3783
Zfand3	-0,74313	0,32023
Zscan26	-0,74347	0,90844
Zfp664	-0,74375	0,64126
Ttll1	-0,74377	0,88629
Poll	-0,74393	0,47599
Slc17a5	-0,74398	0,39025
Gm23969	-0,74403	1
B4galt1	-0,74422	0,1738
Prob1	-0,74441	1
1700086O06Rik	-0,74445	1
Smim1	-0,74472	0,9154
4930568A12Rik	-0,74533	1
Vamp4	-0,74546	0,18287
Gm12933	-0,74562	1
Jmy	-0,74644	0,50808
Oma1	-0,7467	0,72155
Gm45084	-0,74693	0,98454
Cct3	-0,74745	0,19425
Fbxl17	-0,74769	0,82182
Gm12655	-0,74784	0,92613
Ankrd49	-0,74839	0,65226
Cep164	-0,7484	0,78885
Spats2	-0,74911	0,56032
Ncf1	-0,74917	0,17374
Alkbh3	-0,74986	0,50808
Gm43569	-0,75089	0,9044
Becn1	-0,75106	0,26577
Szt2	-0,7511	0,86068
Csf2rb2	-0,75118	0,85926
Mettl1	-0,75147	0,57485
Itsn1	-0,7516	0,41195
Rps6kb2	-0,75166	0,72967
Ddx21	-0,75185	0,21809
Naa40	-0,75222	0,60488
Gm25636	-0,75216	1
Ftsj3	-0,75254	0,33466
Emc9	-0,75265	0,90325
Vti1a	-0,75302	0,74927
Zfp512b	-0,75304	0,79327
B3galt4	-0,75341	1
Nek9	-0,75383	0,37015
Slc46a3	-0,7538	0,96495
Ncf4	-0,754	0,47299
Gm38162	-0,75404	0,91371
Gm6245	-0,75429	1
Yipf3	-0,75448	0,60147

Cops6	-0,75473	0,46661
Carf	-0,75469	0,84215
Clec4a3	-0,7548	0,95238
Slc22a5	-0,75517	0,80104
Pgm2	-0,75526	0,51599
Zfp771	-0,75564	0,48341
Rmdn1	-0,75583	0,61923
Irak1	-0,75589	0,2812
Cenpb	-0,75595	0,62458
Disp1	-0,75588	0,80535
Zfp445	-0,75635	0,4069
Scfd2	-0,75626	0,84893
Timm21	-0,75639	0,78966
Gm12089	-0,75636	1
Slc12a2	-0,75662	0,63654
Dand5	-0,75698	0,91234
Cdca7l	-0,75719	0,38954
Ubxn8	-0,75722	0,79857
Ercc6l2	-0,75762	0,77995
Ypel4	-0,75782	0,94724
Agpat1	-0,7579	0,42298
Wdr45	-0,75809	0,82576
Doc2g	-0,75811	0,94311
Fam98a	-0,75823	0,74244
Ubtf	-0,75847	0,39089
Pygb	-0,75893	0,36644
Map2k2	-0,75927	0,30128
Asxl2	-0,75968	0,39606
Wdr59	-0,75988	0,7457
Adprh	-0,76021	0,30354
Stradb	-0,76023	0,43254
Ikbkg	-0,76024	0,5216
Tm7sf3	-0,76056	0,45871
Gm2885	-0,76073	0,99042
Chid1	-0,76091	0,57367
Gm42690	-0,76104	1
Prrc2a	-0,76208	0,6119
6430531B16Rik	-0,76229	0,68673
Gm14843	-0,76248	0,9956
AA386476	-0,76375	1
Slc12a7	-0,76421	0,36383
Edil3	-0,76482	0,6292
Phf10	-0,76489	0,24461
Serpinb8	-0,76513	0,40835
Tmem237	-0,76536	0,73892
S100a4	-0,76563	0,19425
Helz	-0,76561	0,3655
Cwc27	-0,76632	0,50719
Crebzf	-0,76651	0,911
Mir155hg	-0,76649	0,95244
Hmcn2	-0,76723	0,95585
Zdhhc6	-0,76774	0,74607

Ankrd26	-0,76779	0,72967
D430042O09Rik	-0,76788	0,65434
Csk	-0,76851	0,39166
Rcl1	-0,76867	0,33818
Ifi27	-0,76904	0,39892
Gm19705	-0,76925	0,83512
Gga2	-0,76952	0,40201
Ints11	-0,7695	0,55068
Alg14	-0,76953	0,70529
Eri1	-0,76971	0,45076
Wdpcp	-0,76997	0,94565
Zfp780b	-0,77034	0,79571
Htr2b	-0,77034	1
Odf2l	-0,7708	0,77929
Tubgcp6	-0,77093	0,72289
Slc4a2	-0,77125	0,47121
Cdkl4	-0,77119	0,84315
Fmn1	-0,77131	0,33156
Nos3	-0,77157	0,67874
Chordc1	-0,77171	0,19116
Brip1os	-0,77217	0,66789
Rnf123	-0,77224	0,87651
Enpp1	-0,77235	0,77995
Galnt7	-0,77242	0,20685
Xrn1	-0,77282	0,36891
RP23-36H21.3	-0,77332	1
RP23-277D1.1	-0,77337	1
Acad12	-0,77366	0,902
Serinc3	-0,77401	0,35455
Pcca	-0,774	0,72448
Tex10	-0,77452	0,57229
Phtf2	-0,77448	0,58134
Zfp446	-0,77465	0,85316
Zbtb8os	-0,77486	0,679
Rcn1	-0,77505	0,47078
D10Jhu81e	-0,77527	0,49288
Wipf1	-0,77561	0,41099
Rftn2	-0,77607	0,84893
Gm20707	-0,77638	0,90518
Accs	-0,77682	0,84032
Smurf1	-0,77693	0,71059
Tmem205	-0,77704	0,38868
Tdrkh	-0,77701	0,53965
Coq2	-0,77743	0,76108
Zfp846	-0,77738	0,85693
Gm43761	-0,77776	0,87651
Mccc2	-0,77825	1
Gm20699	-0,7786	0,9306
Zkscan7	-0,77915	0,99218
Gm24959	-0,77909	1
Bop1	-0,77936	0,5949
Nupr1l	-0,7803	0,67014

Car13	-0,78043	0,81164
Gm26601	-0,78037	1
Golga2	-0,78081	0,079896
Smarcc1	-0,78111	0,21541
Zfp282	-0,78107	0,70887
Nkain1	-0,78129	1
Clec11a	-0,78143	0,78558
B130006D01Rik	-0,78154	0,86447
Ucp2	-0,78165	0,37071
Stk11ip	-0,78174	0,78059
Pms1	-0,78176	0,83979
Plcd1	-0,78176	1
Setmar	-0,78192	0,95752
Lrif1	-0,78245	0,7053
Ptpn12	-0,78272	0,24272
Gm37238	-0,78272	0,89173
Ankrd33b	-0,78291	0,97628
Bod1l	-0,78302	0,49942
Farp2	-0,78425	0,97324
Oas1c	-0,78466	0,79653
Tmem127	-0,78486	0,41099
Ago2	-0,78498	0,21853
Tspan3	-0,78523	0,21353
Nudt3	-0,78573	0,13712
Dapp1	-0,78583	0,66145
Ccdc120	-0,78581	0,97894
Lcp1	-0,78654	0,1974
Apip	-0,78658	0,84519
Dap	-0,78714	0,13712
Pla2g16	-0,78714	0,36759
Flt1	-0,78737	0,72155
9130221H12Rik	-0,7875	0,74965
Wwc2	-0,78758	0,92355
Creb3l3	-0,7878	1
Atg2a	-0,78806	0,22362
Gm44950	-0,78832	0,99004
Gm45292	-0,78831	1
Clstn1	-0,78928	0,41409
Hmx2	-0,78933	0,80794
Ankrd12	-0,78988	0,44211
Snx24	-0,79017	0,66639
Fbxo36	-0,79033	0,89188
Lmln	-0,79125	0,84945
Eif3a	-0,79146	0,11749
Gm45640	-0,79149	0,94724
Garnl3	-0,79159	0,98714
Pskh1	-0,79167	0,57826
Filip1l	-0,79174	0,70877
Jaml	-0,79179	0,35858
5430420F09Rik	-0,79179	1
Gpr183	-0,79202	0,44427
Acad9	-0,79236	0,84332

Lrrc24	-0,79246	0,7161
Ttc19	-0,793	0,60124
Mertk	-0,79341	0,71066
Parn	-0,79364	0,46312
Klc4	-0,79369	0,24499
Imp4	-0,79377	0,36558
Noc4l	-0,79381	0,59017
Arhgap30	-0,79469	0,38686
Dlg3	-0,79472	0,8538
Gm37033	-0,79477	0,92593
Acat2	-0,79493	0,43425
Msr1	-0,79498	0,17543
Mllt1	-0,79516	0,79857
Tpcn2	-0,7952	1
Prkch	-0,79566	0,34795
Usp49	-0,79601	0,77995
Wdr7	-0,79652	0,62338
Fbxl19	-0,79725	0,8397
Gusb	-0,79749	0,087548
Map3k7	-0,79755	0,1612
Trmt6	-0,79764	0,41753
Wipi1	-0,79823	0,43858
Lin28b	-0,79821	1
Lrrk1	-0,79841	0,60005
Gm23300	-0,79941	1
Smyd2	-0,79955	0,38763
Nedd9	-0,79962	0,90237
Tctn3	-0,79986	0,78966
Zfp775	-0,80021	0,69439
Zfp157	-0,8002	0,80267
Zfp945	-0,80056	0,892
Cdk6	-0,8013	0,15363
AU022252	-0,80169	0,47078
Vps9d1	-0,80202	0,47966
5730480H06Rik	-0,80287	0,77929
Per2	-0,80308	0,39778
Zfp322a	-0,80309	0,59592
Bcat1	-0,80342	0,4639
Gm15506	-0,80364	1
Aifm2	-0,80415	0,19066
Hsd17b7	-0,80451	0,42396
Ide	-0,80456	0,5083
Pitpnm1	-0,80484	0,5057
Edem2	-0,80556	0,40001
Piwil2	-0,80589	0,92265
Chmp3	-0,8065	0,1423
Trp53cor1	-0,80719	0,73611
Pced1b	-0,80777	0,97003
Ecel1	-0,80844	0,87651
Fam120b	-0,80863	0,7107
Drosha	-0,80878	0,40667
Prepl	-0,80944	0,85567

Srgap3	-0,80955	0,24461
1110019D14Rik	-0,80982	0,90293
Ndufb8	-0,8101	0,30733
Iqsec1	-0,81055	0,2538
Car9	-0,81051	0,54033
Ttll5	-0,81055	0,62886
Ctnnal1	-0,8111	0,85536
Ovca2	-0,81163	0,47388
Slc29a3	-0,8117	0,40667
Dido1	-0,8117	0,49059
Abhd11	-0,81193	0,29953
Gm12517	-0,812	0,84242
Ralgps1	-0,81208	0,83481
Acaca	-0,81218	0,45839
Gen1	-0,81222	1
C3	-0,81225	1
Wwox	-0,8129	0,57817
H2-Ab1	-0,81291	0,98921
Ano8	-0,81356	0,55999
Zfp518a	-0,8142	0,60326
Gm26947	-0,81424	0,81934
P2ry6	-0,81426	0,40667
Tbc1d14	-0,81671	0,5027
Tbc1d5	-0,81763	0,6077
A630001G21Rik	-0,81806	0,87703
Taf4b	-0,81834	0,77224
Wdr46-ps	-0,81827	0,94972
Entpd7	-0,81839	0,69584
Pgd	-0,81924	0,074616
Plk3	-0,81921	0,54834
2310047D07Rik	-0,81927	0,85316
Dnase2a	-0,81945	0,095067
Fbxw7	-0,81964	0,46236
Lyrm1	-0,82078	0,66759
Nktr	-0,82099	0,21956
Fan1	-0,82098	0,94673
Bco2	-0,82148	0,94627
Usp8	-0,82172	0,31646
Slc41a2	-0,82191	0,28219
Gsto1	-0,82245	0,3655
Zfp46	-0,82259	0,6292
E330009J07Rik	-0,82288	0,56011
Ptpn21	-0,82295	0,79282
Tmem173	-0,82288	0,84139
Zfp407	-0,82322	0,73403
BC004004	-0,82365	0,43881
Entpd5	-0,82403	0,73923
Arl6ip4	-0,8245	0,33617
Fn3krp	-0,82463	0,76181
Gm12689	-0,82502	0,92898
Tasp1	-0,82525	0,87296
Gm26532	-0,82535	0,7476

Selenos	-0,82551	0,15257
n-R5-8s1	-0,82558	0,85316
2700033N17Rik	-0,82613	0,79427
Gcdh	-0,82614	0,85126
Sass6	-0,82649	0,70042
Gm42483	-0,8265	0,97453
2810030D12Rik	-0,82686	0,8227
Tmem209	-0,82718	0,75972
Idh1	-0,82732	0,040955
Zdhhc4	-0,82928	0,52118
Deptor	-0,8296	0,27778
2210417A02Rik	-0,8298	1
Cars	-0,83009	0,21809
Psmg2	-0,8303	0,35302
Rgs14	-0,83029	0,7574
Olfr921	-0,8309	0,94748
Ppp3ca	-0,83101	0,13766
Cd82	-0,8311	0,26801
AI987944	-0,8311	0,55472
RP23-278O17.1	-0,83126	0,9873
Gdpd1	-0,83241	0,3342
Cox5b	-0,83244	0,71245
Rasgrp3	-0,83279	0,18922
Arfgef2	-0,83283	0,4188
Gtf2ird2	-0,83325	0,71502
Abca2	-0,83335	0,5929
Poglut1	-0,83361	0,20031
Cul5	-0,83379	0,13766
Laptm5	-0,83475	0,057264
Gm20696	-0,83489	0,91204
Mif4gd	-0,83501	0,58811
Keap1	-0,83513	0,23144
Prtn3	-0,83523	1
Rassf4	-0,83552	0,14151
Phf11b	-0,83621	0,94495
Ttyh3	-0,83658	0,25489
Ms4a6b	-0,83709	1
Gm13604	-0,83746	1
Zfp260	-0,83843	0,49381
Cyc1	-0,83848	0,32247
Myoz1	-0,83847	1
Sorbs1	-0,83884	1
B4galnt1	-0,83939	0,44507
Tbc1d10b	-0,8397	0,15022
Lig3	-0,8397	0,39606
Gpr107	-0,84002	0,092376
Camta2	-0,84005	0,2369
Mgrn1	-0,84044	0,29213
Gm9207	-0,84047	1
Brf1	-0,84063	0,44191
Nt5dc1	-0,84058	0,75948
Gm29340	-0,84175	0,54085

Tnfrsf18	-0,84177	0,87651
3110083C13Rik	-0,84241	1
Lgi4	-0,84278	1
Gm6088	-0,84295	1
Zfp318	-0,84332	0,38759
Tpcn1	-0,84405	0,19783
Dock8	-0,84398	0,44412
Zfp687	-0,84401	0,6077
Ubr5	-0,84468	0,1999
Slc6a12	-0,84469	0,3432
Ankrd46	-0,84539	0,64028
Atp13a2	-0,84568	0,24315
Gm15937	-0,84566	1
Gm24927	-0,8463	0,99796
Ndufaf7	-0,8467	0,3432
Rgl2	-0,84775	0,43713
Rad1	-0,84982	0,55068
Qrsl1	-0,85026	0,32134
Arsg	-0,85073	0,86549
Srd5a1	-0,85078	0,94428
Gm42595	-0,85109	0,849
C230035I16Rik	-0,85204	1
Ppp2r3d	-0,85291	0,50085
Etv1	-0,85318	0,40675
2010008C14Rik	-0,85326	0,85132
Ric8a	-0,85338	0,57229
Capn10	-0,85339	0,7485
Gm43692	-0,8536	0,7616
Ston1	-0,85381	0,85682
Gm10069	-0,85436	0,94574
Atp2a3	-0,85488	0,25588
Aak1	-0,855	0,1738
Arhgef4	-0,85518	0,73923
Dbt	-0,85544	0,46919
Samd10	-0,85631	0,86359
B930086L07Rik	-0,85677	0,706
Sirt5	-0,85713	0,6119
Dtwd2	-0,85805	0,90984
Dcun1d2	-0,85826	0,8059
Gm3650	-0,8584	0,77237
Gm11613	-0,85848	0,44979
Lgr4	-0,85926	0,70877
Zfp52	-0,8596	0,86096
Slc12a4	-0,8601	0,42157
Ikbke	-0,86065	0,73684
Gm43547	-0,86066	1
Ktn1	-0,86126	0,054383
Aig1	-0,8613	0,14582
Gm26930	-0,86179	1
Xpnpep3	-0,86187	0,62584
Spaca9	-0,86208	0,90356
Qser1	-0,86265	0,29334

Nt5c3b	-0,86272	0,73941
Rtkn	-0,86324	0,65839
Ptpn1	-0,86355	0,070991
Gm26917	-0,86368	0,048461
Crocc	-0,86366	0,92072
Tcirg1	-0,86384	0,070748
Setd6	-0,86378	0,31219
Crebbp	-0,86445	0,23248
Gm42747	-0,86448	0,77211
Gan	-0,86457	0,24271
Atp6v0a2	-0,86474	0,42917
Mettl15	-0,8657	0,56438
Vwf	-0,8661	0,85251
Ubr1	-0,86661	0,47658
Hook3	-0,86668	0,10554
Gm12799	-0,8672	0,90818
Smim12	-0,86825	0,26271
Stat5b	-0,86844	0,4852
Abcc1	-0,8685	0,087338
Dok3	-0,86858	0,19452
Lypla2	-0,86907	0,19425
Fktn	-0,86914	0,40667
Mks1	-0,86923	0,41099
Cyp4f13	-0,86978	0,57229
Clec1a	-0,86978	0,77513
Lrrc49	-0,86986	0,85527
Gemin8	-0,87022	0,96376
Ifit2	-0,87024	0,97628
Mdrl	-0,87033	0,9873
Grk6	-0,87053	0,4191
Ubr4	-0,87108	0,049282
Gm37589	-0,87108	0,96078
Gm26800	-0,87169	0,58384
Gm15513	-0,87167	0,82782
Sh3glb2	-0,87186	0,3642
Cul4a	-0,87218	0,3758
Gmppa	-0,87229	0,37773
Itfg2	-0,8723	0,54169
Elf4	-0,87323	0,59502
Ascc2	-0,87359	0,3815
Fkbp11	-0,87368	0,51323
Ccp110	-0,87531	0,78558
Cldn15	-0,87532	0,99916
6430590A07Rik	-0,8758	0,80267
Kdm5d	-0,87592	0,48595
Gm38125	-0,87615	0,9873
B230208H11Rik	-0,87669	0,94627
Glyctk	-0,87745	0,97078
A430105I19Rik	-0,87764	0,80352
Fam35a	-0,87915	0,66723
Afg3l1	-0,87993	0,1997
Rgl1	-0,88006	0,041069

Slc9a9	-0,88041	0,77488
Cep162	-0,88076	0,62153
Zzef1	-0,88099	0,32247
Surf2	-0,88118	0,46665
2610020H08Rik	-0,88134	0,77684
Cuedc1	-0,88178	0,21809
St7	-0,88243	0,55376
Ccdc14	-0,8836	0,56695
Dync2li1	-0,88376	0,88766
Arhgap18	-0,88401	0,15771
Zfp74	-0,88402	0,74617
Cmas	-0,8842	0,073521
Spi1	-0,88443	0,14778
Rbm10	-0,88444	0,25696
Pitpna	-0,88472	0,066573
Pnpla7	-0,88498	0,087548
Naglu	-0,88538	0,20031
Abcc10	-0,8859	0,97779
Mrpl17	-0,88631	0,15023
Zkscan17	-0,88641	0,068682
Eef2kmt	-0,88704	0,5168
Kcnn1	-0,88733	0,83798
Gm25517	-0,88739	0,88695
Zdhhc20	-0,8878	0,033161
D7Bwg0826e	-0,88814	0,91332
Tdp1	-0,88821	0,20737
Dapk1	-0,8884	0,056966
Gm15859	-0,88921	0,82891
Wdfy4	-0,88944	0,068974
Slc16a6	-0,88956	0,2132
Bin3	-0,89064	0,5288
Wdr5b	-0,8907	0,85316
B230377A18Rik	-0,8907	0,91677
Evi2a	-0,8923	0,31064
Snx19	-0,89256	0,58458
Ptpn14	-0,89258	0,65241
Gm29488	-0,8929	0,94574
Pou2f2	-0,89441	0,11127
Trmt11	-0,89498	0,68163
Hdac8	-0,89531	0,58209
Atp6v1g2	-0,89552	0,91311
A430033K04Rik	-0,89572	0,74871
Pip5k1b	-0,8959	0,20261
Hnrnpm	-0,89604	0,036953
Tmem68	-0,89607	0,49629
Invs	-0,89634	0,50169
Setx	-0,89647	0,41185
Pcyt1a	-0,89691	0,01908
Capn1	-0,89765	0,36264
A330074K22Rik	-0,89764	0,95691
Pogk	-0,89945	0,16124
Slc25a12	-0,89941	0,17554

Gm5624	-0,89949	0,70887
Celf4	-0,89973	0,75762
Txndc16	-0,89979	0,37238
Rere	-0,90012	0,010254
Tmem69	-0,9003	0,53028
Ano10	-0,90039	0,1176
Gm42463	-0,90102	0,80794
Uri1	-0,90146	0,17137
Arid2	-0,90177	0,33125
Gm44694	-0,90208	0,67145
D030028A08Rik	-0,90212	0,76636
Zcchc4	-0,90284	0,067298
Atg4a	-0,90337	0,33098
Fads1	-0,90352	0,061561
Gm8550	-0,90352	0,92613
Gm23344	-0,90407	0,93409
Zdhhc9	-0,90424	0,39259
Gid8	-0,90486	0,15022
Polr3a	-0,90518	0,52636
Prmt3	-0,90564	0,23616
Clgn	-0,90612	0,93159
Gm36445	-0,90652	1
Cst7	-0,90718	0,38868
Prss44	-0,90721	0,9327
Tpm1	-0,90829	0,0076907
Polr1a	-0,90887	0,19417
1300002E11Rik	-0,90909	0,77524
Cdk16	-0,90921	0,49345
Ppp1r10	-0,90964	0,12063
Abr	-0,90973	0,011835
Cnp	-0,90991	0,72967
Dnajb12	-0,9112	0,35302
Akap7	-0,9114	0,40166
Fam160b2	-0,91193	0,25481
Arhgap9	-0,91243	0,27294
Ext2	-0,91244	0,3907
Sh2d6	-0,91264	0,93382
Dennd4b	-0,91292	0,50638
Xbp1	-0,91301	0,070549
Fam173b	-0,91364	0,62495
Rpusd1	-0,91377	0,70742
Heatr3	-0,91389	0,57269
Gpr180	-0,91406	0,70887
C130089K02Rik	-0,91448	0,5494
Pbx1	-0,91455	1
Scarf1	-0,91481	0,52304
Cant1	-0,91532	0,046708
Rif1	-0,91541	0,15444
Cul9	-0,91566	0,33259
Ube4a	-0,91581	0,65241
Zc4h2	-0,91709	0,76685
Grtp1	-0,91773	0,57268

Bms1	-0,91775	0,055108
Zfp59	-0,91783	0,9327
Rsu1	-0,91796	0,37622
Gm4459	-0,9184	0,9873
Fam3c	-0,91845	0,17547
Gramd1b	-0,91921	0,056966
Fam136a	-0,91953	0,30128
Tyw3	-0,91962	0,88796
Mtus2	-0,92021	0,24306
Frg2f1	-0,9207	0,83867
Stub1	-0,92086	0,024905
Plekhg5	-0,92144	0,36019
Smim10l1	-0,92175	0,54217
Dhrs1	-0,92182	0,17797
Sergef	-0,92175	0,43036
Gemin5	-0,92191	0,53643
Txlnb	-0,92298	0,89112
Copa	-0,92329	0,061761
Micall1	-0,92353	0,1999
Nr2c2	-0,92381	0,25489
Ccdc91	-0,92455	0,39158
Atat1	-0,92474	0,57219
Ccdc106	-0,92492	0,71066
Gm10029	-0,92497	0,92567
Kdm4a	-0,92515	0,03478
Zfp672	-0,92554	0,49763
2210016F16Rik	-0,92564	0,44427
C2cd3	-0,92687	0,62701
RP24-460E12.3	-0,92732	0,91909
Hacd3	-0,92855	0,22287
Plekhn1	-0,92925	0,35677
Morn1	-0,92963	0,9873
Mdk	-0,93074	0,61862
Pex16	-0,93076	0,25889
Gm23054	-0,93174	0,99961
Htra2	-0,93268	0,1498
Eya4	-0,933	0,30844
Mavs	-0,93313	0,43858
Gm37490	-0,93324	0,86549
Cdr2	-0,93359	0,46236
Dennd1c	-0,93431	0,90984
Opn3	-0,93448	0,60114
Calcoco1	-0,93474	0,46244
Tubb4a	-0,93497	0,73923
Nfkbie	-0,93522	0,61923
Pon3	-0,93533	0,28236
Fancg	-0,93545	0,70745
Vim	-0,93561	0,021499
Wdr13	-0,93635	0,079875
Fam132a	-0,93642	0,83271
Hoxaas3	-0,93667	0,73923
Sec61a1	-0,93713	0,01361

Unc93b1	-0,93719	0,088034
D6Wsu163e	-0,93763	0,51037
Zfp280c	-0,93759	0,67195
Nlrp3	-0,93823	0,32711
Smarcd1	-0,93837	0,40874
Zadh2	-0,93849	0,30404
Gcat	-0,93919	0,54383
Tnrc6c	-0,93959	0,42271
Sacs	-0,94047	0,43311
Polr3gl	-0,94072	0,071361
Kyat1	-0,94078	0,56156
Tlcd1	-0,9413	0,88298
Proser3	-0,94158	0,89514
Tsga10ip	-0,94249	0,96587
Gm10399	-0,9426	0,91711
Zfp459	-0,94285	0,77907
Jmjd8	-0,94297	0,45811
Psph	-0,94312	0,60488
1600010M07Rik	-0,94376	0,66029
Gmpr2	-0,94443	0,50908
Cpeb1	-0,94441	0,97059
Gsk3b	-0,94459	0,045137
Slc38a9	-0,94506	0,13747
Pde8b	-0,9458	0,022139
Ccdc63	-0,94615	0,89772
Abcd1	-0,94648	0,55171
Mccc1	-0,94671	0,44054
Sh3pxd2a	-0,94834	0,13224
Tars	-0,94889	0,11408
Gm38021	-0,94933	0,88161
Pank1	-0,94979	0,54508
Tmem260	-0,94984	0,66988
Creg1	-0,94998	0,029994
Slc23a2	-0,95072	0,43226
Vars2	-0,95086	0,38209
Zfp72	-0,95089	0,9844
Odf3l1	-0,95106	0,69835
Cnpy2	-0,95117	0,38243
Hoga1	-0,95193	0,84553
Sdhaf4	-0,95212	0,3478
Fcgr1	-0,95271	0,43691
Pin1	-0,95314	0,27074
Nudt6	-0,95355	0,64126
Ccdc136	-0,95363	0,66348
B3gntl1	-0,95389	0,6528
Gda	-0,95478	0,15022
Neo1	-0,95516	0,20948
Fadd	-0,95519	0,95867
Serac1	-0,95555	0,82999
Pfkm	-0,95572	0,21873
Gm9732	-0,95613	0,77995
Inpp5k	-0,95652	0,24496

Cux1	-0,95689	0,015128
Kdelr3	-0,95701	0,94574
Tmem18	-0,95757	0,33617
Lgalsl	-0,95818	0,29382
Igf2bp1	-0,95868	0,89514
Ssu72	-0,95913	0,037066
Kifc2	-0,96031	0,97797
Dennd5b	-0,96048	0,84201
Ccdc85b	-0,96068	0,88091
Gm38111	-0,96128	0,94291
AW209491	-0,9617	0,34362
Cpt1a	-0,96203	0,19775
B230312C02Rik	-0,96229	0,44447
Gm5637	-0,96265	0,90682
Gm43336	-0,9631	0,46778
Trappc12	-0,96354	0,22764
Tcea2	-0,96352	0,89112
Kif3b	-0,96387	0,077691
Zfp809	-0,96419	0,02235
Mlh3	-0,96428	0,42995
Zfp870	-0,96472	0,51124
Csnk2a2	-0,96535	0,010389
Cib1	-0,9654	0,066894
Gm9347	-0,96741	0,66869
Vwa1	-0,96773	0,9956
Smarcc2	-0,96803	0,12536
Tns2	-0,96799	0,80794
Cep68	-0,9681	0,22121
Zfyve19	-0,96826	0,71297
Nrg4	-0,96829	0,96324
RP23-444K20.4	-0,96892	0,34522
Soga1	-0,96973	0,25531
Irgm2	-0,97037	0,77224
Slc9a8	-0,97072	0,11278
Flywch1	-0,97072	0,32596
Lypla1	-0,97097	0,12065
Itga4	-0,97152	0,079896
Cep63	-0,97154	0,65336
3110080O07Rik	-0,97301	0,91371
Fastkd5	-0,97311	0,60005
BC002059	-0,97325	0,15798
Clptm1l	-0,97363	0,02235
Gm12988	-0,97393	0,83103
Slc10a3	-0,97458	0,49553
Ufsp2	-0,97485	0,058355
Syk	-0,97539	0,066893
Stab1	-0,97542	0,52335
Bcar1	-0,97551	0,54158
Ubash3b	-0,97634	0,066666
Slc2a6	-0,97634	0,57229
9230102O04Rik	-0,97666	0,86018
Gnrh1	-0,97667	0,87069

Chil6	-0,97752	0,99112
Gm37642	-0,97803	0,62375
Tmem106c	-0,97848	0,40835
Creb3l2	-0,97878	0,20782
Amt	-0,97896	0,67787
Cdk5rap1	-0,97909	0,46706
Stat1	-0,97965	0,62458
Cd109	-0,97967	0,020671
Nr2c1	-0,9797	0,59705
Chpf	-0,98007	0,3445
mt-Nd1	-0,98084	0,063715
Gm23722	-0,98127	0,91425
Gm42835	-0,98185	0,77995
Gm22716	-0,98275	0,92205
Arhgef18	-0,98342	0,69344
Adck5	-0,98448	0,50238
RP23-138K22.2	-0,98513	0,91251
Mib1	-0,98535	0,051285
Zfp456	-0,98549	0,68573
Pced1a	-0,98558	0,27802
Nxn	-0,98631	0,6476
Ints10	-0,98669	0,55707
Eif1ad	-0,98687	0,15108
Ublcp1	-0,98693	0,86083
Tmem55a	-0,98704	0,10648
Map3k15	-0,98703	0,85823
Mpnd	-0,98711	0,09843
Slc36a4	-0,98753	0,1268
Gm16536	-0,98752	0,49763
Slc7a4	-0,98782	0,36774
Ap3b1	-0,98834	0,12819
Lrba	-0,98827	0,2507
Prr36	-0,98884	0,91711
Srrm2	-0,98895	0,011988
Gm37678	-0,98898	0,9327
Itpr2	-0,98933	0,019581
1810055G02Rik	-0,98954	0,66843
Ccdc92	-0,99016	0,69048
Frmd8os	-0,99151	0,89447
Dync1li2	-0,99161	0,02725
Zdhhc2	-0,9921	0,070217
Rhbdf1	-0,99211	0,55855
Gm10136	-0,9924	0,84032
Inpp5d	-0,99268	0,074005
Ccdc130	-0,9936	0,23248
Nmi	-0,99509	0,71465
Mettl21a	-0,99538	0,14893
Cog7	-0,99632	0,23953
6430571L13Rik	-0,9963	0,91299
Ankrd39	-0,99688	0,45972
Mgat5	-0,99706	0,082118
Tepsin	-0,99726	0,22651

Dennd2d	-0,99851	0,31557
Lair1	-0,99872	0,043884
Tada2a	-0,99913	0,079896
Ly96	-1,0001	0,56096
Tm4sf19	-1,0001	0,84095
Dock11	-1,001	0,37116
Rasal3	-1,0015	0,7628
Ccni	-1,0018	0,0097071
Slc26a2	-1,0025	0,058058
Pkd2	-1,0041	0,62733
Atad1	-1,0044	0,1999
1700037C18Rik	-1,0044	0,78205
Exosc5	-1,0047	0,14498
H6pd	-1,0052	0,25462
Gm45360	-1,0058	0,68641
Gpr85	-1,0058	0,86549
Rnasel	-1,0059	0,46561
Srd5a3	-1,0064	0,53483
Rpain	-1,0066	0,63004
Pou5f2	-1,0068	0,90187
Uaca	-1,0069	0,06791
Mapkapk2	-1,007	0,0076631
Rilp	-1,0072	0,94318
Rps6ka3	-1,0079	0,16353
BC048403	-1,0082	0,70612
Etv6	-1,0087	0,21685
Zfp106	-1,0105	0,0071908
Gm38082	-1,0108	0,44555
Mettl8	-1,011	0,33617
Pdss2	-1,0111	0,49553
Cntln	-1,0114	0,60056
2310061I04Rik	-1,0116	0,061698
Ifi203	-1,0119	0,55859
F830115B05Rik	-1,0129	0,92575
Gm26514	-1,0132	0,81886
Xrra1	-1,0133	0,90171
R74862	-1,0135	0,71645
Ap1g2	-1,0139	0,56003
Asb7	-1,0148	0,24176
D2Bwg1423e	-1,015	0,6838
Gm43793	-1,0154	0,77204
Cyb561	-1,0156	0,73621
Senp7	-1,0161	0,14921
Gphn	-1,0172	0,11033
Gm30329	-1,0179	0,78599
Phactr1	-1,0183	0,79857
Gm43715	-1,0186	0,99042
Srr	-1,0191	0,1999
Csf2ra	-1,0194	0,59094
Slc25a14	-1,0196	0,56721
Ubox5	-1,0196	0,75972
Ift20	-1,0208	0,16346

Gm43290	-1,0212	0,51387
E130308A19Rik	-1,0217	0,52977
Wbp1l	-1,0233	0,061064
Gm14698	-1,0233	0,74927
Phf19	-1,0234	0,63186
Zfp820	-1,0234	0,92593
Akap9	-1,0236	0,013395
Tlr1	-1,0236	0,51972
Ddn	-1,0239	0,90435
Dmwd	-1,0244	0,26345
Kbtbd11	-1,0256	0,0051574
2810029C07Rik	-1,0259	0,80186
Fam69a	-1,026	0,7053
Gm37569	-1,0263	0,66145
Ppp6r3	-1,0277	0,013486
Myh7b	-1,0281	0,84801
Gm43445	-1,0292	0,84531
1810030O07Rik	-1,0298	0,11167
4833412K13Rik	-1,0301	0,43098
Ccar2	-1,031	0,66988
Wls	-1,0312	0,054012
Ecd	-1,0323	0,19775
Dock7	-1,033	0,32247
Gm22973	-1,033	0,72748
Son	-1,0337	0,018374
Slc9a3r2	-1,034	0,32988
Slc27a1	-1,0345	0,32502
Alg3	-1,0348	0,45409
Wdr77	-1,035	0,17554
Mmab	-1,035	0,50238
Azin2	-1,0354	0,40675
1700003F12Rik	-1,038	0,36594
Galnt1	-1,0386	0,0083205
Rab12	-1,0393	0,046708
Mybpc3	-1,0393	0,41906
Kif13a	-1,0394	0,51387
Arl11	-1,0395	0,5509
Gm15157	-1,0397	0,86324
3110082I17Rik	-1,0399	0,34149
Slc39a8	-1,0404	0,90459
Thada	-1,0412	0,47033
Kcne3	-1,0416	0,89206
Tbc1d24	-1,0421	0,19412
Gm45343	-1,0429	0,58875
Zmat1	-1,0429	0,95585
Dhodh	-1,043	0,33639
Parvg	-1,0432	0,17686
Mtg2	-1,0432	0,48214
Smim19	-1,0433	0,18669
Sppl2b	-1,0438	0,28042
Zfp429	-1,0441	0,49532
Parp16	-1,0445	0,18793

Tmcc1	-1,0452	0,27797
Btd	-1,0453	0,48064
Zfp94	-1,0453	0,59524
Rrp9	-1,0454	0,19783
Arih1	-1,0456	0,0031892
Clybl	-1,0467	0,11466
Rpsa-ps2	-1,048	0,89288
D630045J12Rik	-1,0481	0,84471
Prtg	-1,0483	0,30589
Iars	-1,0487	0,086724
RP24-233B16.6	-1,0497	0,717
Eif4g3	-1,0498	0,039283
Stau2	-1,0499	0,28811
Klhl41	-1,0499	0,60821
Pde4a	-1,05	0,12074
Gm42467	-1,0504	0,4518
Diaph2	-1,051	0,38185
Fem1a	-1,0511	0,025195
Lyrm7	-1,0523	0,51527
Faap24	-1,0524	0,61387
Stx5a	-1,0525	0,20405
Trak2	-1,0533	0,081599
Poln	-1,0536	0,81389
Acsl4	-1,0541	0,0021102
Tm4sf5	-1,0543	0,74617
2810025M15Rik	-1,0544	0,0071908
Lat2	-1,0546	0,016791
Ppcdc	-1,0552	0,064249
Chaf1b	-1,0552	0,37791
Pbxip1	-1,0564	0,003983
Rbfox2	-1,0566	0,068692
Cenpv	-1,0572	0,19749
Slc1a5	-1,0576	0,004643
Tmem42	-1,0577	0,75762
Plxna2	-1,0579	0,0538
Tiam1	-1,0582	0,28065
Gm17100	-1,0585	0,83866
G730013B05Rik	-1,0611	0,055336
Gm42937	-1,0612	0,80432
Sp3os	-1,0619	0,33512
Prkag2	-1,0622	0,014681
Eml5	-1,0625	0,35003
Tbc1d32	-1,0625	0,36548
Macf1	-1,0628	0,011418
Fam151b	-1,063	0,93128
Tbc1d13	-1,0631	0,0674
Ppp1r12b	-1,0637	0,0055938
Mthfsd	-1,0643	0,49026
Slc6a9	-1,0648	0,64028
Pkd1l2	-1,0649	0,6387
Aldoc	-1,0654	0,0041248
Caskin2	-1,0662	0,71502

Scamp1	-1,0664	0,028911
Fkrp	-1,0664	0,40381
Scly	-1,0666	0,28925
Tmem62	-1,0671	0,74953
Paqr7	-1,0678	0,1746
Klhl8	-1,0682	0,3697
Pomk	-1,0683	0,37365
Hps1	-1,0688	0,17374
A430110C17Rik	-1,0694	0,7955
Gm42478	-1,0697	0,84215
RP24-499N24.6	-1,0699	0,49915
Ms4a6d	-1,0709	0,52377
Lrpprc	-1,0714	0,15284
Sp110	-1,0714	0,46122
Tspyl2	-1,0716	0,60789
Lrrc14	-1,072	0,30128
Gm15185	-1,0725	0,86549
Gm44667	-1,0741	0,70529
Slc35e2	-1,0747	0,44211
Arfgef3	-1,0748	0,11578
Grk5	-1,0751	0,43918
Ppm1h	-1,0752	0,24791
Arhgef25	-1,0754	0,25936
Snora57	-1,0761	0,50794
Als2cr12	-1,0764	0,94096
3300005D01Rik	-1,0775	0,11422
Mfsd6	-1,0776	0,058719
Elmod3	-1,0777	0,579
Dusp18	-1,0782	0,24387
Gm45495	-1,0783	0,57229
AA414768	-1,0796	0,71866
Mitf	-1,08	0,032541
Glb1l	-1,0803	0,65141
Gm38077	-1,0807	0,60831
Nipa1	-1,0809	0,6476
Gm42567	-1,0813	0,87348
Slc39a1	-1,0816	0,056896
Fndc7	-1,0816	0,79293
Itga2b	-1,0819	0,7068
Fbxl8	-1,0821	0,36756
RP24-282C4.10	-1,0823	0,30997
Sos2	-1,0826	0,10462
Enox2	-1,0826	0,12212
Fech	-1,0826	0,32366
Elp2	-1,083	0,07085
Gas7	-1,0832	0,1065
Csf2rb	-1,0834	0,1204
Gm5857	-1,0862	0,88817
Letmd1	-1,0865	0,29581
Rwdd2b	-1,0873	0,64016
Parp3	-1,0878	0,10022
Gk5	-1,0881	0,47306

B230398E01Rik	-1,0882	0,57389
Hoxa4	-1,0884	0,4681
Hspbap1	-1,0885	0,94311
Adck1	-1,0899	0,27593
Plekha5	-1,0903	0,30733
Mark2	-1,0907	0,081193
RP24-282C4.9	-1,0912	0,50398
D330050G23Rik	-1,0915	0,80794
Slc35d1	-1,0916	0,081849
Dgka	-1,0917	0,63586
Mphosph9	-1,092	0,52071
Eno3	-1,0927	0,12242
Ccr1	-1,093	0,80532
Ints7	-1,0946	0,11811
Cd33	-1,0952	0,0069063
Lcp2	-1,0967	0,018856
Gm38157	-1,0973	0,84945
Ttc39a	-1,0976	0,74953
Lrrc47	-1,0982	0,0046878
Trub2	-1,0985	0,197
Atp2b4	-1,0986	0,029797
Vps52	-1,0986	0,43226
Hk3	-1,0991	0,3783
Lmbr1	-1,0991	0,4681
Gbas	-1,0999	0,25571
9330102E08Rik	-1,1013	0,82568
Esyt2	-1,1023	0,01908
Abcc4	-1,1026	0,27776
Iqcc	-1,1026	0,53061
Gm13223	-1,103	0,8602
BC005537	-1,1033	0,015304
Zfp467	-1,1033	0,24583
Vamp7-ps	-1,1036	0,79857
Hal	-1,1039	0,74927
Etfrf1	-1,1049	0,20539
Gm42972	-1,105	0,8686
Shmt1	-1,1052	0,064249
6430548M08Rik	-1,1054	0,077713
Per3	-1,1056	0,15955
Cpt1c	-1,1065	0,37655
Vil1	-1,1074	0,71251
Foxo4	-1,1076	0,18852
Nyap1	-1,1085	0,42269
Mtr	-1,1086	0,061056
Gm28192	-1,1086	0,81069
Stard9	-1,1093	0,066285
Iars2	-1,1096	0,049501
Shprh	-1,1097	0,25874
Tmem246	-1,1098	0,55376
Ncoa7	-1,1102	0,18193
Ttll3	-1,1103	0,27698
Cish	-1,1112	0,8686

Slc27a4	-1,1129	0,39925
Gm37776	-1,1133	0,82484
Cdk14	-1,1151	0,14884
Glt8d1	-1,1159	0,09504
Tyk2	-1,1163	0,007415
Lpcat3	-1,1163	0,27958
Ficd	-1,1167	0,60056
Il18rap	-1,1174	0,71984
Slc38a1	-1,1178	0,0023462
Flot1	-1,1182	0,3097
Rinl	-1,1183	0,22336
Rabl2	-1,1186	0,49779
Tsnax	-1,1194	0,29656
Ap5z1	-1,1197	0,14498
Mus81	-1,1199	0,47599
Zfp26	-1,1207	0,1999
Idh2	-1,1209	0,018374
Traf3ip3	-1,1217	0,5443
Inpp5b	-1,1223	0,25511
Sod1	-1,1225	0,0013006
Zbtb12	-1,1225	0,20782
Nudt16	-1,1227	0,050698
Adgrl2	-1,1227	0,72595
Zfp40	-1,1228	0,54242
Depdc5	-1,1232	0,40675
Dmpk	-1,1242	0,42346
Cacfd1	-1,1251	0,0054353
Araf	-1,1252	0,1053
Fam222b	-1,1266	0,18473
Ttc38	-1,1273	0,60488
4930590J08Rik	-1,1278	0,81934
Smap1	-1,1281	0,02988
Gm37357	-1,1292	0,84531
Gm45802	-1,1302	0,51966
Islr2	-1,1304	0,81934
Nlk	-1,1315	0,19268
Gm11410	-1,1319	0,73923
Hdac7	-1,1324	0,54553
Diablo	-1,134	0,11499
Nphp1	-1,1343	0,71423
Med1	-1,1344	0,0031344
Gm37474	-1,1353	0,22679
Gm44834	-1,1353	0,86444
3010003L21Rik	-1,1356	0,78235
Akr1b3	-1,1363	0,40874
Metap2	-1,1365	0,014252
3830408C21Rik	-1,1372	0,80267
Pspc1	-1,1385	0,20634
C2	-1,1387	0,71617
Gm43462	-1,139	0,78651
Ttc26	-1,1396	0,54006
Catsperg1	-1,1399	0,79832

Dmrta2	-1,1399	0,79841
Zbtb48	-1,1403	0,70541
Dnajc10	-1,1406	0,0055938
Sfmbt1	-1,141	0,11279
Coro7	-1,1418	0,23633
Gfi1	-1,1432	0,76644
Btk	-1,1435	0,16077
Poc1b	-1,1436	0,081824
Mapk9	-1,1442	0,15917
Polr3e	-1,1443	0,16232
Zfp691	-1,1446	0,39418
Gm16201	-1,1454	0,24425
Wdr25	-1,1454	0,51966
Ifi44	-1,1454	0,79257
Zer1	-1,1457	0,23331
Aldh4a1	-1,147	0,30884
Fgfrl1	-1,1474	0,26801
Psca	-1,1479	0,67232
Fryl	-1,1492	0,038809
Tbxas1	-1,1493	0,82377
Gm19325	-1,1495	0,48064
Pemt	-1,1502	0,672
Plscr3	-1,1508	0,087548
Pfn2	-1,1513	0,55203
Slc25a37	-1,1516	0,0010126
Gucd1	-1,1542	0,50756
Pstpip1	-1,1543	0,00075927
Habp4	-1,1543	0,4681
Pck2	-1,1545	0,20737
Rfx5	-1,156	0,0054996
Ppm1l	-1,1561	0,0078977
4930432K21Rik	-1,1576	0,2156
Arfgap1	-1,1589	0,15287
Gns	-1,1591	0,0011621
Uxt	-1,1593	0,77995
Sec31a	-1,1594	0,0070753
Lrrc61	-1,1595	0,1261
Rufy1	-1,1595	0,211
Mmp9	-1,1597	0,66843
Slc40a1	-1,1601	0,35079
Oasl1	-1,1601	0,37965
C2cd2	-1,1602	0,35674
Inca1	-1,1614	0,78016
Ctdsp1	-1,1622	0,37702
RP24-286J14.3	-1,1622	0,41952
Tgfb1i1	-1,1622	0,64985
Sema6b	-1,1629	0,61064
Rpgrip1	-1,1632	0,44433
Cyth4	-1,1634	0,0044649
Spcs2-ps	-1,1645	0,77204
Npc1l1	-1,1659	0,75494
Nacc2	-1,1699	0,11253

Atg10	-1,1713	0,24791
Clec2l	-1,1715	0,69155
RP24-91J7.1	-1,172	0,7175
Nptxr	-1,1723	0,13455
Lclat1	-1,1725	0,45811
Vrk3	-1,1737	0,055303
A730062M13Rik	-1,1738	0,82466
Erlin2	-1,1741	0,17312
F9	-1,175	0,88461
Orai3	-1,1772	0,10297
Sh3tc1	-1,1776	0,20073
Nckap5l	-1,1778	0,30128
Tfcp2	-1,1779	0,3118
Cyba	-1,1783	0,002563
Srxn1	-1,179	0,043655
Ptpn7	-1,1798	0,12536
Dtnb	-1,1807	0,33683
Ints3	-1,1809	0,0010808
Swsap1	-1,1812	0,55338
Birc3	-1,1814	0,028845
Aldh1b1	-1,1822	0,25361
Pla2g2d	-1,1823	0,33624
Slc37a2	-1,1839	0,0024601
Syne1	-1,1841	0,022909
Tmem175	-1,1845	0,21809
Gm9726	-1,1845	0,61791
Zfp239	-1,1845	0,62886
Mob3b	-1,1845	0,7639
Fbxo32	-1,1849	0,33721
Tbc1d4	-1,1852	0,057934
Sema4a	-1,186	0,0059334
Endog	-1,186	0,34739
Atp6ap2	-1,1861	0,00013848
Taf1c	-1,1873	0,25895
Zbtb38	-1,1878	0,0059334
Kcnq1ot1	-1,1879	0,022568
Fut7	-1,1884	0,58721
Noc2l	-1,1886	0,001064
Oit3	-1,1895	0,32988
Adamts4	-1,1913	0,71679
9030624J02Rik	-1,1923	0,078598
Gm15708	-1,1926	0,8107
Dars2	-1,1929	0,23952
Tmem181b-ps	-1,193	0,41057
Iqsec3	-1,1935	0,70612
Zscan20	-1,1948	0,34935
Tmem14a	-1,1952	0,73565
Gm44822	-1,1957	0,71996
Eogt	-1,1965	0,21475
Ttc28	-1,1967	0,50923
Olfm1	-1,1974	0,0016261
Hoxb3	-1,1977	0,42677

Armc6	-1,1983	0,26415
RP23-243B24.1	-1,1983	0,6342
Aldh5a1	-1,1984	0,18598
Arrb1	-1,2003	0,022954
Slc12a5	-1,2005	0,55401
Ehd1	-1,2006	0,00061333
Gm45871	-1,2008	0,43867
Tfrc	-1,201	0,014752
Prr14l	-1,201	0,033835
Zfp951	-1,201	0,54242
Def6	-1,2024	0,26558
Eri2	-1,2033	0,34053
Trim27	-1,2034	0,0074952
Ffar4	-1,2034	0,54161
Snx7	-1,204	0,32502
Park2	-1,204	0,77306
1700034H15Rik	-1,2042	0,5669
Rbbp9	-1,2066	0,57229
Gm43128	-1,2076	0,40835
Hspa2	-1,2097	0,70807
Sphk2	-1,2104	0,0024805
2310009A05Rik	-1,2112	0,11212
Rtel1	-1,2115	0,63512
Erbb3	-1,2119	0,40667
Cep83os	-1,2123	0,26415
5730409E04Rik	-1,2126	0,17421
Gm12522	-1,2126	0,5956
Gm6329	-1,2142	0,046689
Dclre1c	-1,2143	0,32753
Pycr1	-1,2146	0,36548
Cnpy3	-1,2149	0,01662
Bcl3	-1,215	0,092376
AI464131	-1,2158	0,73565
Catsper2	-1,2165	0,55068
Abtb2	-1,2166	0,25894
Ipo8	-1,2168	0,065268
Gm4924	-1,217	0,36756
Msi1	-1,2171	0,26716
Tmem220	-1,2174	0,15022
Akap17b	-1,2174	0,30149
Pla1a	-1,2175	0,57435
Adamtsl5	-1,218	0,25936
Chm	-1,2197	0,44113
Sipa1l1	-1,2206	0,43878
Gm11451	-1,2211	0,717
Golim4	-1,2212	0,014711
Gca	-1,2221	0,41952
Ptpdc1	-1,2242	0,35052
Zc3h4	-1,2253	0,0050428
Zfp473	-1,2254	0,62938
1700086P04Rik	-1,2267	0,66723
RP24-226A8.2	-1,2287	0,80186

Gm37063	-1,2302	0,78599
Fads6	-1,2306	0,20594
Hmgxb4	-1,2311	0,11939
Gm26620	-1,2314	0,70689
Slc35b4	-1,2326	0,2838
Ccdc17	-1,2334	0,72371
9530062K07Rik	-1,234	0,23953
Nek3	-1,234	0,6862
Klhl40	-1,2345	0,77929
AU040320	-1,2347	0,024697
Pecr	-1,236	0,037423
Gm10605	-1,2366	0,58875
Abcb4	-1,2369	0,00057688
Inf2	-1,2382	0,00025207
Zfp994	-1,2382	0,61208
Pfkfb4	-1,2388	0,01597
Prdm9	-1,2391	0,48688
Sptlc1	-1,2408	0,037494
Dpp8	-1,2414	0,013395
Cd59a	-1,2416	0,1999
Scap	-1,242	0,059803
Sh3kbp1	-1,243	0,063369
Ccdc88c	-1,2452	0,70302
Zfp975	-1,2452	0,73057
Lgals3bp	-1,2455	0,13037
Rars2	-1,2462	0,12774
Cyp4f16	-1,2463	0,50638
Eif2ak2	-1,2465	0,30119
Foxred2	-1,2468	0,0025172
Plbd2	-1,2472	0,00022148
Vwa7	-1,2484	0,43184
Ift43	-1,249	0,31265
Zpr1	-1,2501	0,00057787
6720475M21Rik	-1,2506	0,73648
Gm5113	-1,2511	0,57636
Ppfibp1	-1,2515	0,0012475
Gm340	-1,252	0,28184
Gm44053	-1,2523	0,67801
Mrps6	-1,2525	0,0016369
Tlr7	-1,2529	0,025186
Frmd8	-1,2549	0,00207
Slc16a12	-1,2578	0,40667
Gm26935	-1,2581	0,60737
Arhgef12	-1,2613	0,0019255
Acta2	-1,2616	0,69803
Lamc2	-1,2627	0,71297
Il1rl1	-1,2628	0,8602
Flnb	-1,2634	0,0028707
Ribc1	-1,2645	0,72357
Csf1r	-1,2649	0,00019084
Exoc7	-1,2657	0,11615
Chd1l	-1,2659	0,20394

Trim68	-1,266	0,14498
Abcb9	-1,2679	0,61828
Rasa3	-1,2682	0,0066321
Gm26631	-1,2693	0,81437
Arhgef3	-1,2726	0,087548
Acy3	-1,2731	0,62495
Wdr6	-1,2736	0,11022
Cmklr1	-1,2738	0,63826
Gm30238	-1,2746	0,71576
Aars	-1,2774	0,081599
Bre	-1,278	0,0055238
Prss53	-1,2782	0,60101
Eef2k	-1,2783	0,40675
Armc9	-1,2798	0,23144
I830077J02Rik	-1,2803	0,49741
4930550C14Rik	-1,2816	0,58937
Ago1	-1,2825	0,063715
Phf21b	-1,2834	0,68966
Gm45224	-1,2843	0,69344
Frrs1	-1,285	0,00053542
Stx17	-1,2852	0,065262
Alg8	-1,2855	0,28876
Nav1	-1,2863	0,0028709
Plxdc1	-1,2863	0,024752
Map2k3os	-1,2879	0,64445
Nudt22	-1,2887	0,036035
Gm43728	-1,2887	0,46778
Gm17249	-1,2887	0,71984
Slc2a4	-1,2917	0,45803
Rbck1	-1,2919	0,0055938
Alg9	-1,2929	0,2138
Gm10033	-1,2941	0,41099
Gm43627	-1,2943	0,64364
Mtmr9	-1,2945	0,57787
Zfp763	-1,2947	0,33659
Txnrd3	-1,2948	0,30404
Ctbp1	-1,295	0,0019088
AW046200	-1,2961	0,41099
Cluap1	-1,2987	0,079875
Tesk2	-1,2992	0,40675
Fuz	-1,2993	0,46665
Zfp362	-1,2994	0,089425
Senp8	-1,3011	0,50778
Gdpgp1	-1,3012	0,4681
Tubg2	-1,3013	0,59017
Gm17530	-1,3018	0,63887
Nmb	-1,3019	0,46661
Mkl2	-1,3024	0,20506
Reep6	-1,3027	0,13044
D230022J07Rik	-1,3027	0,65361
RP23-3F1.8	-1,3043	0,056048
Zdhhc1	-1,3052	0,4583

Zfp942	-1,3066	0,41792
4833418N02Rik	-1,3093	0,69227
Gm42869	-1,3101	0,57229
Fkbp14	-1,3106	0,39651
Apbb1ip	-1,3124	9,03E-05
Calhm2	-1,3124	0,1999
Gm44237	-1,3143	0,55376
Echdc3	-1,3161	0,20909
Xpo4	-1,3162	0,12173
Neil1	-1,317	0,048715
Fam161a	-1,3183	0,050565
Lppos	-1,3186	0,60046
Uvssa	-1,3192	0,049542
Gm7769	-1,3206	0,69803
Zdhhc12	-1,3212	0,18131
Gm15327	-1,3215	0,63887
Ivns1abp	-1,3225	7,02E-06
C530005A16Rik	-1,3245	0,60982
Gm37060	-1,3252	0,19737
2610524H06Rik	-1,3256	0,37965
Lrrc1	-1,3266	0,47388
Zfp14	-1,3277	0,58771
Cd5l	-1,3278	0,45718
Gnptab	-1,3284	0,00042122
Msh5	-1,3298	0,19486
Oxsm	-1,3312	0,39158
Gpatch4	-1,332	0,015343
Jade2	-1,3321	0,13321
Crtc1	-1,334	0,33274
Cspg5	-1,335	0,65433
Egf	-1,3353	0,55156
4931414P19Rik	-1,3355	0,25899
3110070M22Rik	-1,3357	0,55068
Myo6	-1,3359	0,088123
Gm37101	-1,3367	0,59592
Pomgnt1	-1,3369	0,10297
Abi3	-1,3374	0,34577
Tst	-1,3385	0,48055
Ganc	-1,3391	0,21698
Ica1	-1,3394	0,073836
Zfp953	-1,3394	0,60448
Rnf157	-1,3404	0,0090015
Gm15530	-1,3418	0,52118
Gramd1c	-1,3431	0,52636
Eva1b	-1,3433	0,28426
Eef1akmt1	-1,3442	0,2582
Cyp26b1	-1,3444	0,60488
Hk1os	-1,3452	0,66723
Actn1	-1,3463	0,00020139
mt-Nd4	-1,3466	0,014224
Zbtb32	-1,3472	0,34421
Setd1b	-1,3473	0,013395

Pla2g5	-1,3482	0,0059612
Sbk1	-1,3489	0,48763
Sh3rf1	-1,3505	0,093043
Slc16a9	-1,3505	0,54281
Slc25a42	-1,3512	0,58875
Gm21816	-1,3519	0,53028
Nudt1	-1,3521	0,36264
Dnajc11	-1,3524	0,055108
mt-Cytb	-1,3529	0,0028087
Slc24a5	-1,3537	0,66843
Il17rc	-1,3539	0,41057
Gnat2	-1,3545	0,63717
Nat8f1	-1,3559	0,19066
Xylb	-1,357	0,6119
1700084J12Rik	-1,3576	0,73648
Hap1	-1,3594	0,10271
4930556M19Rik	-1,3606	0,58209
Gm7909	-1,3612	0,56721
Rbks	-1,3615	0,6917
Irak4	-1,363	0,057112
1700007L15Rik	-1,3649	0,78983
Zfp653	-1,3656	0,22679
Pigh	-1,3684	0,40675
Cd80	-1,3687	0,30723
Slc11a1	-1,3705	0,0043699
Sigirr	-1,3713	0,51323
Palb2	-1,3717	0,49303
Gabrd	-1,3722	0,49819
Gm12833	-1,3727	0,75762
Matk	-1,3731	0,61923
Tmem51os1	-1,3733	0,61923
Dbnl	-1,3753	0,022232
Pde8a	-1,3762	0,085569
A930024E05Rik	-1,3763	0,42917
A930018M24Rik	-1,377	0,71483
Tmem140	-1,3781	0,20068
Myof	-1,3783	0,00022785
Pmepa1	-1,3791	0,0048026
Tmem2	-1,3791	0,0075533
Nphp3	-1,3808	0,25571
Clec5a	-1,3809	0,017121
Gm44557	-1,3841	0,6838
1600014C23Rik	-1,3862	0,5956
Ermard	-1,3864	0,31236
B3galnt1	-1,3866	0,46122
Frmd4a	-1,3876	0,0085952
Prkdc	-1,3878	0,23314
Ino80c	-1,3879	0,00083369
Gtpbp8	-1,3884	0,18287
Ankrd16	-1,3887	0,36383
Bbs7	-1,391	0,31206
AW146154	-1,391	0,58937

Tdrd3	-1,3927	0,286
Nagpa	-1,3943	0,011043
Ssc5d	-1,3958	0,70989
Cat	-1,3974	2,64E-05
Sumf2	-1,4006	0,2816
Dph6	-1,4034	0,033512
Nudt12	-1,4042	0,28097
Cpne2	-1,4047	0,01181
Tmem65	-1,405	0,0027231
Gm20522	-1,4053	0,77237
Gm43071	-1,4062	0,55156
Nlrc4	-1,4064	0,54242
Amotl1	-1,4078	5,86E-05
Gm20554	-1,4084	0,5806
Arhgap4	-1,4091	0,49553
Ppp1r26	-1,4102	0,44447
Qsox2	-1,4117	0,38901
Six4	-1,4123	0,088973
Fam102a	-1,4129	1,17E-05
2210408F21Rik	-1,4133	0,092891
Taf1b	-1,415	0,056603
Gm45534	-1,4175	0,70261
Lipt2	-1,4225	0,48055
Alg2	-1,4228	0,37702
Gmppb	-1,4257	0,06901
Tmem17	-1,4277	0,55859
Cox15	-1,4282	0,13786
Zfp41	-1,4306	0,26758
DHRSX	-1,4313	0,11463
Fah	-1,4314	0,18931
Cyfip2	-1,4365	0,0008148
Zbtb20	-1,4368	0,00082524
Cdk20	-1,4372	0,24035
Cyb5rl	-1,4374	0,21237
Gm26132	-1,4383	0,52384
2900005J15Rik	-1,4391	0,26992
Gm44434	-1,44	0,63004
9430092D12Rik	-1,4406	0,60789
Lrrc8b	-1,4411	0,051425
Rab42	-1,4421	0,58903
Ddr1	-1,444	0,57195
Ahnak2	-1,4465	0,0010555
Dzip3	-1,4481	0,15257
Gm5609	-1,4484	0,25111
Ash2l	-1,4485	0,010054
Bnip2	-1,4507	0,00025107
Gm20712	-1,4507	0,25007
Gmpr	-1,4509	0,0041504
Ctdspl	-1,4509	0,3464
Gm15853	-1,4511	0,58875
Cass4	-1,4534	0,2474
Cstad	-1,4545	0,64028

Slc7a11	-1,455	0,013839
Adap2	-1,4551	0,32013
Maml2	-1,4557	0,1999
Nf2	-1,4571	0,00027494
Fbxo15	-1,4591	0,55641
Frmd6	-1,4595	0,37622
Zfp128	-1,4597	0,51095
Kif7	-1,46	0,34884
Lims2	-1,4601	0,20031
Pofut1	-1,4611	0,056966
Tmigd3	-1,4623	0,59586
C130050O18Rik	-1,4629	0,10745
Miga2	-1,4634	0,32793
Gm44775	-1,4642	0,41959
Bicd1	-1,4644	0,14115
Mboat1	-1,4647	0,19042
Pfas	-1,4672	0,085512
Zfp629	-1,4689	0,21393
Gpr68	-1,4708	0,032402
Gatb	-1,4715	0,12729
Gm10478	-1,4715	0,44382
Zfp617	-1,4727	0,39791
Crebl2	-1,4737	0,21873
Aldh3b1	-1,4741	0,013296
Pus7l	-1,4766	0,27923
Cox10	-1,4778	0,00014412
Zfp426	-1,4778	0,14498
Natd1	-1,4782	0,083619
Gm15696	-1,4792	0,38901
Lars	-1,4801	2,32E-05
Zfp27	-1,4818	0,30136
Asap3	-1,4825	0,56608
Loxl3	-1,4835	0,22757
Rcan1	-1,484	0,0004406
Lekr1	-1,4853	0,4248
Slc39a11	-1,4869	4,19E-05
Atad3aos	-1,4869	0,46041
Cep72	-1,487	0,46665
Il1rap	-1,4878	0,24977
Kif5a	-1,4884	0,11127
A930016O22Rik	-1,4898	0,5474
Spg11	-1,4907	0,47078
Rai14	-1,4932	0,001278
1700008J07Rik	-1,495	0,64357
Fancf	-1,4952	0,51323
Slc43a2	-1,496	0,00019649
Zfp719	-1,4972	0,19212
Naip6	-1,4976	0,17728
Zfp983	-1,4996	0,19066
Gm37699	-1,5	0,38901
Grik5	-1,5034	0,4321
Mfhas1	-1,5069	0,21929

Ttll12	-1,5096	0,00029324
Pld2	-1,5099	0,29309
Gm44623	-1,512	0,42886
Zfp882	-1,5139	0,49708
Fam213b	-1,5149	0,41168
Sirt6	-1,5154	0,31842
Rabgap1l	-1,5155	0,12763
Tctn2	-1,5155	0,42917
Acot6	-1,5164	0,24393
Lhx1	-1,5204	0,4417
Mdn1	-1,521	0,00035079
Acsf3	-1,521	0,42013
Acvr1	-1,5219	0,022781
Rint1	-1,5221	0,14057
Pkn3	-1,5255	0,013395
Zfp324	-1,5274	0,24472
Spink5	-1,5279	2,57E-05
Fam19a3	-1,5286	0,45718
Wbscr27	-1,5307	0,030849
8430408G22Rik	-1,5345	0,52115
Cpsf3	-1,5348	0,005265
Trpv4	-1,5354	0,11499
RP23-228B2.5	-1,5371	0,58744
Gipc1	-1,5399	0,10871
Sbk3	-1,5411	0,42043
Mfsd3	-1,5415	0,40299
Pigp	-1,5416	0,104
2810006K23Rik	-1,5454	0,3445
Slc22a4	-1,5464	0,24644
RP24-325N9.5	-1,5465	0,4498
Fam13a	-1,5492	0,66348
Rapgef3	-1,5511	0,44469
Eng	-1,5515	0,038487
Fkbp15	-1,5517	0,00015135
Ccl5	-1,5517	0,37074
Hsd17b14	-1,5536	0,48789
Ammecr1	-1,5571	0,0049148
Cnksr1	-1,5604	0,4994
Tnk2	-1,5609	0,073836
Fam213a	-1,5613	0,02235
Mst1	-1,563	0,11532
BC037039	-1,5643	0,4017
Zfp3	-1,5647	0,12461
Tmem67	-1,5648	0,3948
Nucb2	-1,5654	0,00038972
Oas2	-1,5661	0,4713
Neat1	-1,5672	0,0005591
Lyl1	-1,5676	0,0069063
Zhx3	-1,5676	0,044391
Arhgap31	-1,5678	0,070748
Slc43a3	-1,5679	0,03544
Slc52a2	-1,5684	0,12173

Ccdc80	-1,5687	0,46634
Lrrc20	-1,5689	0,017125
Prickle2	-1,5694	0,079896
Bphl	-1,5694	0,20721
RP23-476G10.1	-1,5706	0,46641
Tbx15	-1,5708	0,25285
Gm37080	-1,5712	0,34795
RP23-104D6.2	-1,5744	0,52556
Xrcc5	-1,5749	0,089223
L3hypdh	-1,5752	0,070097
Klhl23	-1,5764	0,55376
Glb1	-1,5783	2,24E-05
Numbl	-1,5783	0,038487
Gm37578	-1,5784	0,55877
1810021B22Rik	-1,5786	0,19997
Elp4	-1,5787	0,47576
Gsto2	-1,5792	0,39651
Catip	-1,5797	0,25512
Col20a1	-1,5813	0,055108
Ank2	-1,5824	0,26081
Ift122	-1,5829	0,10036
Cnrip1	-1,5832	0,14884
Tmem80	-1,5836	0,23114
Gm43924	-1,5838	0,37702
Gm22767	-1,584	0,40744
Gstt3	-1,5842	0,028845
Gm38366	-1,5875	0,4172
Gm44432	-1,5883	0,47884
6030458C11Rik	-1,5894	0,0065275
Dnajc12	-1,5901	0,36383
Igsf3	-1,5923	0,057934
Prkar2b	-1,5936	0,17745
Gm38036	-1,5977	0,48181
Apoe	-1,5992	0,36264
Rbfox1	-1,6001	0,41099
Acaa2	-1,6006	0,00872
Atp8b3	-1,6017	0,4065
Gm12663	-1,6035	0,46561
D3Ertd751e	-1,605	0,25283
Snord13	-1,6052	0,00065522
Katnb1	-1,6066	0,40667
Aldh18a1	-1,6068	0,070097
Tnfsf13b	-1,6071	0,22394
Ppp2r3a	-1,6131	0,18926
Cox6a2	-1,6135	0,18488
L3mbtl3	-1,6143	0,21208
Rnf135	-1,6146	0,34149
Gm8228	-1,6146	0,42013
Gm10698	-1,6161	0,28042
Gm45342	-1,6173	0,22663
Nsun4	-1,6176	0,053501
Agbl3	-1,618	0,087338

B230354K17Rik	-1,6197	0,22506
Btbd19	-1,6242	0,010014
Xrcc3	-1,625	0,33617
RP23-38L16.4	-1,6253	0,2816
Apbb1	-1,6256	0,30443
Sema4b	-1,6267	0,081599
Gm37121	-1,6275	0,099064
Slc22a15	-1,6299	0,31809
Mtmr11	-1,6306	0,39772
Stap2	-1,6317	0,19812
Gm37219	-1,6327	0,55799
Zfp229	-1,6333	0,46778
Gm28535	-1,6336	0,42043
Zfp113	-1,6343	0,040104
Ampd3	-1,6347	0,017202
Ak3	-1,6349	0,077168
Spn	-1,6378	0,17998
Fam65c	-1,641	0,01181
Tmem214	-1,6448	0,030505
Gm20632	-1,6462	0,097258
Marveld1	-1,6472	0,00016697
Tnfrsf4	-1,6502	0,26987
Gm42481	-1,6506	0,3783
Mfap1a	-1,6525	0,37791
Maats1os	-1,653	0,43633
Zkscan4	-1,6534	0,43546
Gm43742	-1,6541	0,069294
Camk2n2	-1,6542	0,14961
Zfp78	-1,6546	0,40199
Bckdk	-1,6568	0,0019043
Pld1	-1,657	0,1132
Gne	-1,658	0,038487
Zfp182	-1,6586	0,27756
Exoc8	-1,663	0,097107
Nudt5	-1,6631	0,077691
Fzd2	-1,6646	0,41099
Nadsyn1	-1,6655	0,4006
Tmem198b	-1,6663	0,14842
Plch2	-1,6672	0,44469
Rfxank	-1,6686	0,32023
Gm37297	-1,6703	0,35412
Mir763	-1,6708	0,17061
Nfatc1	-1,6719	2,37E-05
Gm37289	-1,6735	0,48
Dync2h1	-1,6758	0,00011347
Cetn4	-1,6777	0,18931
Med24	-1,6799	0,28103
Amer1	-1,6801	0,16019
Gm38220	-1,6818	0,46661
Dcp1b	-1,6832	0,35136
Ddx59	-1,6834	0,31131
Zfp365	-1,6837	0,16012

Zfp111	-1,6846	0,12109
Gm24336	-1,6858	0,40381
Ptpn5	-1,6863	0,38098
Dusp9	-1,6877	0,21956
Nsf	-1,689	0,00035847
Gm21967	-1,694	0,46349
Fam208a	-1,6942	0,087548
Rasal2	-1,6943	8,01E-05
Sgsm1	-1,6966	1,45E-06
Kdm4d	-1,6985	0,4399
Gm37606	-1,7003	0,29548
Card6	-1,7004	0,23248
Gm42639	-1,7006	0,085512
Gm37978	-1,7008	0,57662
Usp20	-1,7019	6,15E-05
Fancc	-1,7071	0,17068
Stx11	-1,7083	0,19997
6330403L08Rik	-1,7109	0,18922
Spred1	-1,7128	4,80E-05
Gm45289	-1,7134	0,43446
Nrp2	-1,7146	1,88E-08
Fam129c	-1,7152	0,38868
Masp2	-1,7154	0,36383
Gm43147	-1,7178	0,27315
Dennd2a	-1,7182	0,0060763
Gm16845	-1,7203	0,03608
Tns4	-1,7207	0,079896
Aldh7a1	-1,7214	0,30843
0610010F05Rik	-1,7232	0,041069
Elk3	-1,7272	0,0056581
Prss50	-1,7298	0,048486
Fabp7	-1,7314	0,40667
Mblac2	-1,7335	0,23346
Ccnd2	-1,743	1,85E-05
Ophn1	-1,7441	0,30058
Magi2	-1,7453	0,15798
Slc25a15	-1,7506	0,12405
Gpt2	-1,7507	0,0010442
D930016D06Rik	-1,7516	0,20953
Gm17455	-1,7523	0,31802
Dcaf11	-1,7529	0,0075533
Adora2a	-1,7532	0,35136
Cracr2a	-1,754	0,36859
9130023H24Rik	-1,7557	0,29953
Tmem241	-1,756	0,041064
Rsph1	-1,7562	0,24217
Zscan22	-1,7574	0,33179
Gm12258	-1,7612	0,44898
Pik3r2	-1,7621	0,0037445
Nmnat3	-1,7654	0,079056
Upk1a	-1,7665	0,18931
Sec24d	-1,7666	0,0097071

Pde4d	-1,7692	0,086489
Gm44270	-1,7692	0,42271
Plcb4	-1,7725	8,56E-07
Fut8	-1,7748	0,22335
Ttc7	-1,7833	0,0023179
Gls2	-1,7872	0,3781
Tmem150a	-1,7879	0,24969
Tmem91	-1,7908	0,29169
Amigo1	-1,7913	0,30997
Mypopos	-1,7919	0,41099
Fads2	-1,792	0,073142
Gm10676	-1,7996	0,44382
C030014I23Rik	-1,8014	0,28912
Slc24a3	-1,802	0,12173
4930556M19Rik	-1,8034	0,37015
Lima1	-1,8038	6,42E-05
Carmil1	-1,8111	0,4681
Hmga2	-1,8124	3,95E-07
Matn4	-1,8126	0,37613
Adcy2	-1,8139	0,070748
Cenpt	-1,8219	0,23045
Ldb3	-1,8224	0,19116
Slc30a2	-1,8232	0,33014
AI661453	-1,8276	0,16589
Ivd	-1,8291	0,027377
Pde4dip	-1,8301	0,0034711
Slc39a13	-1,8312	0,00015569
Timp2	-1,8333	0,00011858
Mtus1	-1,8338	0,35302
Bbs9	-1,8365	0,061076
Amigo3	-1,8387	0,2507
Bank1	-1,8392	0,37461
Rhobtb1	-1,8403	0,18931
Trim2	-1,8426	0,065262
2810428J06Rik	-1,8451	0,24461
Gm38399	-1,8484	0,2123
Sdsl	-1,8507	0,096215
Galnt15	-1,8507	0,20073
Prss35	-1,8509	0,145
Crtam	-1,8573	0,27213
Zfp658	-1,8585	0,20073
C030013C21Rik	-1,8692	0,12434
Tango6	-1,8706	0,38185
Apba1	-1,8715	0,30776
Ank	-1,8754	1,88E-08
Arhgef19	-1,8776	0,32023
Ltbp2	-1,8791	0,25443
Tmem98	-1,8808	0,20324
Agap1	-1,8831	0,0022146
Gm9951	-1,8857	0,18931
Glrp1	-1,8943	0,21873
Gm44951	-1,8985	0,31564

Abhd1	-1,8988	0,19042
Grap	-1,9015	0,0066681
Maged2	-1,9042	0,19315
Pgap3	-1,9046	0,17136
Gstm4	-1,9084	0,1768
Tlr3	-1,9094	0,17957
Airn	-1,9102	0,015094
Nudt14	-1,9118	0,022139
Sec16a	-1,9144	0,0066681
Nat14	-1,9145	0,25607
BC017158	-1,9155	0,16189
Cdhr4	-1,917	0,28571
Gm29243	-1,9186	0,23953
Dock5	-1,9188	0,00471
Mkx	-1,9245	0,23553
Treml1	-1,9263	9,34E-05
6720464F23Rik	-1,9314	0,38345
Hck	-1,9319	0,038487
Gm43112	-1,9341	0,28426
AW047730	-1,9346	0,23953
Fmo5	-1,9362	0,24272
Fblim1	-1,9371	0,00029922
Gm13423	-1,9439	0,25913
4930461G14Rik	-1,9441	0,19672
Slc25a10	-1,9449	0,014752
Gm37788	-1,9486	0,32074
Tmtc4	-1,9498	0,18143
Shox2	-1,9505	0,057934
Skor1	-1,9507	0,2271
Stc2	-1,9542	0,2507
Cnbd2	-1,9556	0,20922
Gm20156	-1,9622	0,34276
Gm22	-1,9682	0,082118
Lpin3	-1,971	0,003115
Klhl5	-1,9715	0,00010617
Ddx17	-1,9764	1,66E-06
Arl14epl	-1,9823	0,23154
Pafah2	-1,9834	0,13569
Zfp661	-1,9859	0,1169
Gm42482	-1,9908	0,12065
Gm37333	-1,9936	0,25283
Arhgef10	-1,9937	0,0288
Oscp1	-1,9951	0,29228
Prss42	-1,9976	0,14884
Ncmap	-2,0002	0,19518
Bdh2	-2,0007	2,57E-05
Gm33370	-2,001	0,27183
RP24-131G14.10	-2,0029	0,23418
Atp8b4	-2,0066	0,058355
Nprl3	-2,0077	0,028911
Pter	-2,0083	0,025137
Kif5c	-2,0096	0,34868

Zfhx4	-2,0118	0,00027494
Cep41	-2,0124	0,14853
Zscan29	-2,0147	0,050565
Ms4a6c	-2,0147	0,13766
Pde4c	-2,0159	0,22234
Gm44509	-2,0174	0,27045
Plxnb3	-2,021	0,1586
Gm43609	-2,0256	0,43184
Tbc1d2b	-2,0274	7,65E-08
Fosl2	-2,0286	5,38E-08
Sec16b	-2,0373	0,00018624
Hebp2	-2,0422	0,021246
Myom1	-2,0469	0,028845
Sfxn2	-2,05	0,01378
Zfp93	-2,0508	0,28925
Gm44699	-2,0647	0,18883
Tmem204	-2,0726	0,0089083
Naip2	-2,0806	0,00084416
Pctp	-2,0841	0,021393
Hlcs	-2,0852	0,10572
Slc16a7	-2,09	0,022796
Asah2	-2,093	0,14055
Gm37010	-2,1008	0,20836
Cyp2u1	-2,1012	0,020671
Tfec	-2,1016	0,061575
Rpgr	-2,1041	0,075012
Elfn2	-2,1049	0,14884
Zfp933	-2,1084	0,17222
Tpm2	-2,115	0,01233
Ceacam10	-2,1158	0,14498
Stc1	-2,118	0,219
Gm13657	-2,1252	0,15771
Lrp8os3	-2,1256	0,13766
Gm13205	-2,1256	0,17523
Klhl30	-2,1413	0,056363
Pitpnm2	-2,1448	0,014239
Ifi203-ps	-2,1475	0,33172
Gsn	-2,1524	1,88E-08
Lpar1	-2,1552	0,12536
Tctex1d4	-2,1568	0,2138
Shtn1	-2,1601	7,02E-06
Gm42480	-2,1666	0,022781
Slc46a1	-2,1679	0,10248
Gm37718	-2,173	0,13766
Bcl2l15	-2,1832	0,11312
Bbs1	-2,1868	0,20721
Me1	-2,1953	3,36E-07
Prkar1b	-2,1963	0,0232
Serinc2	-2,2004	0,00038972
Gm20219	-2,2016	0,068692
9030407P20Rik	-2,2076	0,096371
Nfatc4	-2,2086	0,20721

Tti1	-2,209	0,17222
Particl	-2,2108	0,062379
Fam83h	-2,2147	0,17962
Gm16712	-2,2151	0,11127
Nod1	-2,2155	0,011746
Rhou	-2,2228	0,19066
Fendrr	-2,2253	0,17797
Gm22748	-2,2274	0,030743
Gm11205	-2,2365	0,098526
Oscar	-2,2395	0,079056
Mylpf	-2,251	0,38905
Ttc9	-2,2525	0,082647
Celf6	-2,2574	0,089409
Gramd2	-2,2594	0,1017
Mical2	-2,2617	0,0016611
Frk	-2,2639	0,11491
Hfe	-2,2659	0,03478
Gm42484	-2,272	0,073142
Pdcd1	-2,2847	0,10964
Rgs20	-2,2871	0,064552
Aldh1l2	-2,2904	0,0089922
Gm9776	-2,2937	0,06901
Slc9b1	-2,2991	0,096885
Exd1	-2,3031	0,082118
RP23-268C22.3	-2,3269	0,10022
Angptl2	-2,3279	2,09E-06
Zbtb45	-2,3284	0,061513
Mlkl	-2,3328	0,13953
Celf3	-2,3391	0,13747
Zfp462	-2,344	0,01233
Rorc	-2,3451	0,10842
Ap5b1	-2,3472	0,022812
Gm45728	-2,36	0,14581
Fbxo10	-2,3619	0,14407
Lctl	-2,362	0,047996
Hrc	-2,3668	0,16258
Myo1d	-2,3669	1,27E-07
Arg1	-2,3677	0,14884
Rab11fip4	-2,3698	0,12461
Acot11	-2,3713	0,16494
Olfr933	-2,3784	0,03141
Tspan10	-2,3785	0,0031138
Bdh1	-2,382	0,038487
Gm43111	-2,3873	0,086149
Atp6v0d2	-2,3925	7,65E-08
Cd93	-2,393	0,17447
Ankrd34a	-2,3958	0,096698
Slc39a4	-2,3981	0,055108
Ppm1e	-2,4075	0,087548
B3glct	-2,408	0,0042237
Cradd	-2,4093	0,011418
Plekhs1	-2,4142	0,071389

Prkca	-2,4143	0,003391
Celsr1	-2,4157	0,10985
Prss46	-2,4291	0,10271
Chd5	-2,4389	0,10579
Wdr35	-2,4474	0,038095
Sec14l2	-2,4554	0,028911
Tmem116	-2,456	0,011636
Emp2	-2,4613	0,0025649
Jdp2	-2,4801	2,15E-05
Sgsh	-2,483	0,00021775
Lta	-2,4876	0,080498
St18	-2,5028	0,00016116
Serinc5	-2,5077	0,037494
Ptges	-2,5109	0,012717
Gm42486	-2,5168	0,17656
Acy1	-2,5225	0,020979
Sla	-2,5387	1,53E-05
Itgb3	-2,5484	0,084419
Gm5532	-2,5562	0,065918
2810021J22Rik	-2,5666	0,070097
Six5	-2,572	0,077934
Txk	-2,5775	0,021212
RP24-175C20.18	-2,5852	0,038809
Ip6k3	-2,5865	0,00079658
Krcc1	-2,5947	0,077934
Camk2a	-2,6016	0,00057286
Cc2d2a	-2,6033	0,12518
Calml4	-2,6316	0,0015064
F630040K05Rik	-2,6372	0,029711
Gm38020	-2,6459	0,032725
Slc6a4	-2,6522	0,015343
Phxr4	-2,6581	0,052481
Gm11716	-2,6693	0,01517
Iqce	-2,6953	0,018948
Oas1d	-2,7136	0,056966
Ehd2	-2,7205	0,026915
Ccdc171	-2,7292	0,14193
Zfp862-ps	-2,7425	0,010014
Anxa9	-2,7432	0,047345
Shisa3	-2,7908	0,096456
Col15a1	-2,7996	0,087012
Ccpg1os	-2,8014	0,034878
9930014A18Rik	-2,8132	0,015465
Ociad2	-2,8318	0,18287
Adh7	-2,8801	0,049407
Ccdc122	-2,8928	0,023784
Rap1gap	-2,8989	0,013177
Gm36963	-2,9115	0,021499
Zfp169	-2,9202	0,023862
Msantd3	-2,9227	8,18E-05
Epb41l1	-2,9247	0,0061416
Gper1	-2,9488	0,036501

Gm42640	-2,949	0,016175
Tiam2	-2,9494	0,02951
Extl1	-2,9607	0,014224
Enpp5	-2,9655	3,79E-05
Accsl	-2,98	0,0049208
Gja1	-2,9918	0,016887
Il20rb	-3,0491	0,0028709
Plat	-3,0557	0,032733
Pars2	-3,0809	0,075012
BC024978	-3,1411	0,0075533
Chac1	-3,1819	0,023391
Gm43154	-3,2037	0,019169
Rgs8	-3,2078	0,024752
Slc35d2	-3,2347	0,0036221
Scn11a	-3,24	0,0021218
Ddr2	-3,2435	0,0078754
Vegfc	-3,2453	0,022464
Src	-3,2891	9,79E-05
Dixdc1	-3,2985	0,003983
Rgs16	-3,3146	7,38E-05
Rhbdd2	-3,3251	0,0059334
Robo3	-3,3342	0,0017713
Wnk2	-3,3518	0,00022874
Olr1	-3,3591	0,002629
Gm25514	-3,5178	0,0070753
Pdpn	-3,58	0,00075927
Ablim1	-3,6681	0,0010808
Bok	-3,6765	0,00075108
Gm19026	-3,6822	0,0059779
Gm15496	-3,6882	0,0085253
Wisp1	-3,7563	0,0088431
Adamts7	-3,8387	0,0004291
Nt5e	-3,8745	0,00019084
Acsbg1	-3,9445	0,0013411
Met	-3,9691	0,00039274
Pxdn	-4,0517	0,00040273
Col27a1	-4,1156	0,00058604
Slc1a4	-4,1251	0,0027026
Rab15	-4,244	0,00084416
Acp5	-4,3574	5,32E-10
Il34	-4,4836	0,0034816
Mras	-4,4869	0,00034979
Acod1	-4,6299	0,00012991
Ctsk	-5,424	1,53E-09
Slc9b2	-6,1215	2,70E-06
