# Supplementary material for: Bone, dentin and cementum differentially influence the differentiation of osteoclast-like cells
Source: Sci Rep. 2025 Jun 5;15:19857. doi: 10.1038/s41598-025-04874-9 (PMC12141432; doi:10.1038/s41598-025-04874-9)
Supplement: Supplementary file 6 — Supplementary Information 6. [file 41598_2025_4874_MOESM6_ESM.pdf]

Tab. S5:
Transcripts induced in murine macrophage cells stimulated on bone (n=6), fold of stimulation control

gene name	regulation of expression	adj.P.Val
mt-Tc	47,01339626	4,55E-05
Hspa1b	33,25962506	1,10E-05
Hspa1a	26,32205096	0,00056436
Snord66	26,11487694	0,0020185
mt-Tm	25,22530836	1,21E-07
Snord83b	21,06097031	4,55E-05
Rasd1	20,80560016	0,00042982
Gm26225	17,84935454	0,0022741
mt-Tq	17,64026098	5,21E-05
Gm24631	16,20424615	0,00027129
Snord59a	15,84328945	0,0012494
Hist1h2an	14,8028186	0,0050484
Crtc2	13,81151812	0,0021124
mt-Tl1	13,79142858	8,84E-08
mt-Ta	13,12003806	0,0034301
Gm42793	12,29670602	0,011988
Lgr5	11,6778275	0,036865
Gm42908	11,23945363	0,012768
Rn7sk	10,90486496	1,10E-05
Gm23037	10,62359391	0,00019041
Gm8210	10,49769004	0,039416
Aloxe3	10,22550366	0,00067545
mt-Ti	10,18588903	0,00015586
Gm22513	9,886089723	0,0024583
Gm36989	9,864185975	0,004075
Gm26461	9,538748536	0,015874
Gm43800	9,466295776	0,012661
Gm24991	9,440740342	0,012445
Gm20186	9,380078523	0,0090065
Hspa8	9,213185112	4,92E-05
Gm26202	9,049261122	0,0021314
Gm18709	8,894416725	4,95E-05
Gm44292	8,837265328	0,0003632
4930589O11Rik	8,366939481	0,02083
Hist1h1a	8,347822935	0,05479
mt-Tp	8,183397891	2,05E-05
Gdf15	8,148305096	1,26E-06
Kif18b	7,774770017	0,048572
1700054M17Rik	7,766152301	0,0034301
Gm22980	7,761309034	0,0039193
mt-Ts2	7,643323007	0,02078
Snord7	7,638556341	0,054908
Rpl7	7,60105672	5,95E-06
Rnft2	7,57370905	0,086478
Tap2	7,567412035	0,0058505
Gm23969	7,502128633	0,0058505
Gm26810	7,445145275	0,0039193
Gm28404	7,398844611	0,0034301

Gm26594	7,322823381	0,022148
Gm37851	7,251603278	0,087197
Depdc1a	7,193031889	0,14686
RP24-282K24.4	7,141365707	0,030492
Mcm8	7,114685575	0,00019121
Hist2h2ac	7,021114229	0,068901
Hist1h4n	6,988583167	0,03058
Gm45220	6,963439102	0,012661
Gm25008	6,944640438	0,0022233
Gm12940	6,88663875	0,14393
Snord87	6,835277927	0,00065785
5930420M18Rik	6,830068274	0,055518
Gm37696	6,799836228	0,055346
Gm45698	6,767861289	0,0027161
Gm8337	6,725772592	0,036865
Hist2h4	6,621233462	0,0133
Gm10069	6,508837968	0,11167
Tnfsf9	6,495767519	0,0020861
Rbfox1	6,462534154	0,028725
Gm8203	6,430807926	0,13847
RP23-320D23.6	6,401011941	0,00018781
Snord71	6,378866122	0,14126
Gm5830	6,370470815	0,026566
Lgals7	6,351951954	0,025321
4930589L23Rik	6,316826731	0,024686
Gm23301	6,281895745	0,07374
Gm42743	6,234612954	0,00081241
Gm37204	6,005148823	0,085855
Gm23344	5,994336194	0,051496
Gm43714	5,97483967	0,03985
Snord110	5,951692541	0,15422
Gm42632	5,925759192	0,12956
Gm45185	5,880341737	0,017912
Rpl7a	5,804613466	5,08E-05
Gm18284	5,782526611	0,16387
Gm26772	5,738604705	0,031382
Gm9920	5,717165286	0,099971
Ccdc33	5,65842288	0,08606
4930578M01Rik	5,647843059	0,11736
Gm43059	5,561600049	0,19779
Adam9	5,552740596	0,0056723
Gm42576	5,510182109	0,017908
Gm29358	5,486933154	0,00043436
Snord15a	5,480091569	0,028214
Amd2	5,46643398	0,017751
Rbm3	5,438466847	0,0085734
Gm20594	5,431685694	0,14186
mt-Tv	5,408018174	0,0056051
Hist1h1d	5,376994515	0,0077135
Sdhd	5,35579225	0,01157
Gm24009	5,346890011	0,16377
Gm28151	5,338372585	0,046439

Hist4h4	5,300762859	0,076808
Snord89	5,293052633	0,00020982
Gm43006	5,271815936	0,072697
Gm43148	5,253212701	0,10845
Gm11491	5,229597817	0,00061649
Gm44270	5,128368746	0,034412
Kcnj2	5,092238532	0,0058096
St5	5,088357374	0,29903
Dynlt1b	5,051808669	0,1244
Gm42670	5,047958326	0,061312
Gm29170	4,992283971	4,56E-05
4930578M07Rik	4,980187294	0,0015948
Gm44090	4,97122017	0,0066021
RP23-356D13.9	4,953334329	0,10505
2310058D17Rik	4,925260392	0,0080064
Gm42522	4,901081033	0,14442
Fzd7	4,88784996	0,00012011
Hist1h2bp	4,880063769	0,23421
Rps2-ps10	4,867901581	0,066073
Gm37106	4,862842953	0,14784
Gm3550	4,856779538	0,0015948
1110035H17Rik	4,853414241	0,049107
C3	4,829252843	0,28826
Med12	4,826910243	0,14615
Cxcl10	4,774335664	0,18732
Ost4	4,739385454	0,0003708
4833421G17Rik	4,715791717	0,048987
Icosl	4,705343348	0,011332
4921507G05Rik	4,705343348	0,045418
Hist1h2be	4,691339797	0,00011336
RP23-205H11.3	4,688739087	0,012862
Adora2a	4,686139819	0,15841
Bard1	4,667338167	0,016145
Gfap	4,653125182	0,31775
Snord82	4,641850312	0,085327
Gm38365	4,629639955	0,012661
Rpl27-ps3	4,622265085	0,0079281
Gm29759	4,604996309	0,29134
Gm43011	4,602124459	0,094153
Slc16a5	4,590654956	0,043814
Gm43566	4,585884437	0,00025606
RP24-75M13.2	4,571602605	0,16356
Mkln1os	4,569068273	0,029705
Trf	4,567485029	0,067222
Hsd3b7	4,555154539	0,29246
Gm3531	4,539080266	0,2386
Hmgb1	4,517736102	0,0064443
Atp1b4	4,517109854	0,17402
Gm14137	4,510539491	0,1461
Gm12770	4,502105923	0,18459
Gm26226	4,494622656	0,19679
Myc	4,48901837	0,074728

Snord49b	4,487773922	0,00061515
Gadd45g	4,466361422	2,50E-05
Socs1	4,439816345	0,021936
Gm37383	4,436739965	0,34035
Gm28373	4,429365348	0,079106
Gm44552	4,41648925	0,083017
Gm42895	4,380209883	0,13969
Gm5100	4,37414183	0,23837
Vamp7-ps	4,368384966	0,31002
Gm43200	4,352969767	0,10236
Hist1h4d	4,352366359	0,02342
Gm44901	4,352064686	0,38357
Dnd1	4,350858205	0,34648
Gm42728	4,337909635	0,24199
Hist1h3d	4,334603405	0,067222
Rpl35a-ps4	4,329198645	0,0345
Gm43331	4,328898578	0,16559
Gm44652	4,328298507	0,00043436
Rpl23a-ps2	4,298102833	0,16287
4632415L05Rik	4,286796702	0,00033584
9330151L19Rik	4,285014244	0,02057
Pask	4,275520311	0,20864
Gm23849	4,249814882	0,18497
Mir124-2hg	4,240986822	0,18331
Gdap10	4,239811134	0,0058505
L1cam	4,234231064	0,022012
Mapkapk5	4,2321771	0,19779
Gm38319	4,226607078	0,25424
A530017D24Rik	4,209940957	0,095966
2700029L08Rik	4,199157788	0,1618
Gm17034	4,188402239	0,38974
D430001F17Rik	4,186370506	0,0044358
Gm45358	4,174490135	0,23945
Ptgs1	4,14852937	0,095966
Mrps28	4,138190326	0,098735
Klkb1	4,104766055	0,18756
A130014A01Rik	4,090848189	0,0058229
RP23-440L7.5	4,085180999	0,098969
Hist1h2bg	4,083765428	0,08606
Samd9l	4,058650186	0,40692
AV099323	4,029218379	0,12989
1700020D05Rik	4,011661839	0,16559
Gm12428	4,011661839	0,18497
Gm43149	3,994181799	0,066315
Psd2	3,987819194	0,21995
Gm12184	3,986437361	0,10415
Camk2b	3,985332239	0,15841
Snord72	3,982846834	0,090015
Gm4607	3,978707938	0,01444
Mrps18b	3,952594622	0,15383
Gm42666	3,947392562	0,075745
Pam16	3,946845375	0,11386

Hnrnpa1	3,946298264	0,25313
Gm23127	3,945204269	0,49972
4930529C04Rik	3,937827734	0,0065559
Gm37255	3,916866616	0,059337
Tob1	3,911440448	0,0027161
Rac3	3,903315287	0,27152
Rybp	3,879309818	0,1153
Rgmb	3,835726885	0,12042
Gm37052	3,827228399	0,19779
Gm37738	3,827228399	0,36798
Snora17	3,810551992	0,2602
Fam72a	3,808439559	0,13939
Hacd2	3,800791802	0,10349
Gm9008	3,777155032	0,14413
Gm6140	3,777155032	0,24113
Gm24924	3,775061113	0,1618
Nup98	3,762000394	0,0059512
Slc12a5	3,750284386	0,0484
Gm7336	3,747426027	0,056488
Gm15634	3,74508899	0,30143
Hyal1	3,741715842	5,94E-05
Cd274	3,731614619	0,0070719
Gm43628	3,731614619	0,13847
4933421A08Rik	3,728253608	0,30236
Gm25517	3,724895624	0,12767
Gm4285	3,714839808	0,45113
Gm7099	3,710722203	0,017946
4933437G19Rik	3,710465004	0,079589
Gm43061	3,705067946	0,23541
D530018E20Rik	3,70224405	0,31516
4833445I07Rik	3,697371479	0,28644
Gm43328	3,692249385	0,043814
Gm15421	3,678965124	0,18256
Eno1b	3,674887283	0,010209
Gm24920	3,67208639	0,15716
Gm26912	3,657353306	0,30998
Gm37334	3,653552656	0,4865
Gm37653	3,652033502	0,01917
RP23-182J19.2	3,652033502	0,33697
1700001P01Rik	3,646215928	0,25026
Ncoa4	3,637128759	0,34213
Gm30074	3,635616434	0,39457
Mpc1	3,630831553	0,53455
A730011C13Rik	3,628818751	0,475
Gm26947	3,628315724	0,056247
Fanci	3,625047753	0,2984
RP23-451J19.1	3,622787036	0,056488
Mrc1	3,599758194	0,51086
Ceacam16	3,594522165	0,16104
Olfr286	3,59352569	0,34146
Gm45084	3,593276615	0,20057
Pabpn1	3,580596625	0,0066296

Abcc10	3,580596625	0,263
Gm9938	3,571177889	0,25313
Gm15785	3,566725034	0,41346
Ier5l	3,55611012	0,0010853
C730045M19Rik	3,5474934	0,12573
Gm12981	3,541351385	0,48369
n-R5s151	3,536200312	0,25313
5430434F05Rik	3,53179107	0,38357
Gm15268	3,531301494	0,34342
Gm45248	3,530811985	0,14569
Gm45729	3,504721971	0,39645
Gm13890	3,501322617	0,45055
Erfe	3,499866761	0,45472
Gm26244	3,485583043	0,011988
Gm10131	3,47256091	0,087219
Gm7846	3,467990629	0,2677
Snord55	3,465107234	0,38357
Rpl10-ps3	3,460306904	0,0080064
Atp6v0c	3,459107861	0,2547
RP24-225A16.3	3,456711021	0,016249
Gm8019	3,449052287	0,45472
Gm38257	3,448335151	0,23953
Gm13456	3,446662415	0,03058
Gm28727	3,444990489	0,0035031
Hba-ps4	3,441410522	0,61034
Pank4	3,440456493	0,23207
1810026B05Rik	3,440218027	4,55E-05
Abcg4	3,42926644	0,32028
Gm16046	3,417165211	0,2617
Gm15937	3,412904392	0,24042
Gm22767	3,409121458	0,50342
Cit	3,407231564	0,0064844
Mterf3	3,40487067	0,048782
D830025C05Rik	3,40416272	0,045087
Gm43178	3,397797796	0,31007
Gm14057	3,393796359	0,32917
4930522L14Rik	3,389799633	0,086478
Rnf225	3,379008548	0,46711
C430049E01Rik	3,358925972	0,30648
Esco2	3,356365895	0,099334
Gm12902	3,355435442	0,0484
Nat6	3,351483892	0,00043436
Gm43868	3,347072961	0,40259
Slc25a2	3,331333986	0,20204
Zfp773	3,329025681	0,0063649
Gm43637	3,326949575	0,13199
Ckap2	3,320268701	0,0034115
Gm37678	3,318888127	0,34171
Apoo-ps	3,315898853	0,54888
Insig1	3,311534756	0,00032407
Gm26759	3,308552105	0,45113
2700038G22Rik	3,308093474	0,22674

5330426L24Rik	3,307405647	0,35775
Gm43010	3,303968655	0,3741
6330403N20Rik	3,303968655	0,38061
RP23-226H21.3	3,292766021	0,32188
Gm24916	3,287520749	0,0080135
Hist1h2aa	3,285698266	0,23766
Gm7351	3,282966435	0,47245
Wwc1	3,280009515	8,64E-05
Acoxl	3,276373885	0,3373
Cerkl	3,272288617	0,26592
Rps13-ps1	3,272061807	0,42917
Vwa1	3,267981916	0,32842
Gm43133	3,25825609	0,27265
Gm45153	3,25644983	0,08383
4930509H03Rik	3,25644983	0,1691
Got2-ps1	3,245408316	0,57841
Gm42478	3,244733522	0,46613
Syt8	3,240687707	0,34213
Zscan21	3,236646935	0,0074115
Ube2s	3,222097164	0,11924
3110056K07Rik	3,21651853	0,11948
Snord65	3,212285229	0,19244
Gm26129	3,211839943	0,43089
Gm15503	3,209614436	0,044617
Proscos	3,208947085	0,06333
Gm42856	3,208057499	0,45535
Btf3	3,207612799	0,043814
2900060B14Rik	3,207390472	0,29937
Gpr19	3,201393462	0,49649
Gm45630	3,200727821	0,44328
Gm8909	3,200284136	0,36104
Id1	3,194743269	0,00025558
4930412F12Rik	3,191644574	0,3745
Manba	3,16630449	0,080869
Rnf25	3,165426726	0,23337
Usp26	3,163671927	0,52103
Dnali1	3,159289185	0,27852
H2-Q4	3,15775666	0,053651
Gm16540	3,156224879	0,20261
Mtfr2	3,153163545	0,16559
RP23-40D21.1	3,152726454	0,067953
Gm2011	3,149886839	0,10845
Hapln3	3,143561526	0,44939
Gm45266	3,142254428	0,43726
Gm43420	3,139859491	0,43484
Hus1b	3,129213296	0,65204
Gm43445	3,128996403	0,46805
Gm23935	3,128345814	0,0053748
Ldb1	3,125311521	0,10576
Rpl12	3,11752256	0,0013263
Cks2	3,116442296	0,52103
Gm42819	3,108029075	0,061226

Morf4l1	3,107598241	0,0021292
Rnf130	3,103078083	0,15218
Ifitm5	3,102863002	0,38233
Gm20492	3,102863002	0,43103
C230096K16Rik	3,095773668	0,027795
Gmnn	3,085490815	0,0026753
Cog3	3,071407638	0,26258
Pin4	3,069279438	0,49166
Gm15289	3,067365319	0,37172
4930532G15Rik	3,064602588	0,46302
Rps12-ps9	3,061205719	0,0077888
RP24-295J1.1	3,059720773	0,30552
Npl	3,057176827	0,77597
Rpl28-ps1	3,055693835	0,0080064
Gm10382	3,05273001	0,054928
Hist1h4h	3,050403307	0,42762
Gm6136	3,04998046	0,032682
Hnrnpa3	3,049769059	0,0021091
Rps6	3,049769059	0,57344
Gm7638	3,044277768	0,018897
Gm11759	3,040692655	0,28846
Txlnb	3,040271155	0,56328
Lbp	3,038375126	0,36348
Gm45477	3,035217706	0,42462
5330406M23Rik	3,033955657	0,075358
Tnfaip3	3,03080283	0,06152
Gm22714	3,028492839	0,46655
Ltb	3,019689057	0,0018327
Pgam1	3,017387536	0,34704
Gm12924	3,015296767	0,68592
Msantd2	3,014251926	0,010209
Gm23100	3,005281225	0,65435
BC055308	3,001742036	0,03058
Gm42511	3,001117907	0,57162
Gm28187	3,000701892	0,33653
Gm16253	3,000285935	0,10236
Crkl	2,998830541	0,095966
Ncapd3	2,99675263	0,00043902
9130604C24Rik	2,995506575	0,58919
RP23-58B7.2	2,994261038	0,0077693
Rps12-ps10	2,994053498	0,0123
Gm24339	2,991356798	0,095966
Gm19272	2,99114946	0,48741
Gm37519	2,990942137	0,65779
Znrf3	2,990734827	0,087219
Gm10175	2,983901681	0,0034301
Gm6290	2,983901681	0,58177
Hist1h2ae	2,97997453	0,44082
Mafk	2,979767981	0,00033584
Gm37893	2,965345075	0,61999
Rasal1	2,964317543	0,42508
Ftl1	2,963701195	0,15005

Gm5841	2,961852918	0,6475
Ppia	2,961647625	0,16957
Rpl19	2,958159822	0,0021292
Gm44836	2,958159822	0,6583
Izumo4	2,957544754	0,20307
Snx32	2,95713478	0,48741
Gm19967	2,956929814	0,083126
Tsc22d3	2,943229415	0,0027602
Gm12882	2,942413492	0,06372
Mfsd7a	2,942005615	0,3741
Bst2	2,931014286	0,0011128
Gm42467	2,929186395	0,06152
Dennd4c	2,925939624	0,0031028
1110020A21Rik	2,91925452	0,55144
Atn1	2,91460424	0,42833
H2afz	2,911979098	0,010209
Ppp2cb	2,911979098	0,36899
Eno1	2,90552728	0,0031555
Gm5362	2,900496744	0,41057
Fcrl5	2,899290713	0,7345
Gm38043	2,89768345	0,11522
Itgam	2,897482605	0,0080064
Gem	2,895073549	0,51884
Gm42483	2,894672234	0,66249
2010016I18Rik	2,890662146	0,14272
Man2b2	2,88885942	0,039416
Gm25541	2,887858391	0,39944
Zfp335os	2,887858391	0,70261
Gtpbp2	2,885657348	0,064928
Gm42671	2,884857385	0,43242
AY074887	2,883657856	0,071373
Eme1	2,883058279	0,72592
Gm16439	2,881659749	0,49739
Tmem240	2,878066645	0,20954
3110045C21Rik	2,877468231	0,69174
Nek2	2,872287177	0,11653
H2-T10	2,872088092	0,72749
Gm26664	2,866718014	0,33075
Gm13383	2,86493022	0,46959
Rpl13a-ps1	2,862746652	0,0010853
Gadd45b	2,861159648	0,0023695
Gm42731	2,860961335	0,55153
Gm24876	2,857394039	0,52103
Hace1	2,848890162	0,24693
Gm43817	2,845929651	0,10514
5330438D12Rik	2,845535149	0,28682
Gm37949	2,843957686	0,6624
Hsd17b14	2,843366363	0,73615
Gm11516	2,841790103	0,59128
Gm37357	2,838640204	0,66905
Gm28659	2,835100741	0,37014
Rpl31-ps13	2,834904233	0,023181

Gm45251	2,83411834	0,49014
Trip10	2,830780723	0,16673
Sep 01	2,830584515	0,61375
Gm5112	2,828035049	0,10376
Snrpc	2,827643027	0,10505
Gm23639	2,813761406	0,65907
Rpl39-ps	2,80616531	0,0061956
Gm9320	2,805192936	0,0064443
Map3k15	2,80247208	0,53501
Gm25291	2,802083602	0,70368
Abhd18	2,801500985	0,11346
Nsl1	2,80091849	0,0081476
Ly86	2,795681481	0,00040412
Mmp2	2,795487705	0,62574
Gm8623	2,793357065	0,10992
Rsrp1	2,79296985	0,0020261
Tk1	2,788134193	0,12042
RP24-282C4.3	2,782728196	0,30711
Efcab11	2,772716281	0,70383
Gm5822	2,761591636	0,63513
Gm42635	2,761208824	0,054823
Gm12355	2,761208824	0,25313
2900055J20Rik	2,75910431	0,20204
Plekhh1	2,758721844	0,75096
Gm15542	2,757383628	0,39925
Gm37407	2,756810306	0,19294
Gm5131	2,756237103	0,62516
Rps12-ps4	2,754136378	0,011047
Col4a6	2,752800387	0,57841
Khnyn	2,738716629	0,30782
6820402A03Rik	2,729241389	0,75921
Wdhd1	2,725649399	0,016145
0610039K10Rik	2,719798931	0,28931
AI480526	2,717537606	0,23945
B430305J03Rik	2,715842847	0,55751
Maff	2,71396102	0,0065631
Sp4	2,713584812	0,02078
Lsm7	2,710764907	0,064418
Gm12276	2,710013428	0,73514
Mdm4-ps	2,709825591	0,055119
Gm14673	2,709825591	0,76773
Aunip	2,70738489	0,14768
Gm21781	2,705884013	0,20261
Zfp873	2,705508924	0,55144
Mxd3	2,701386373	0,73083
Ptpru	2,698953274	0,8099
Nectin4	2,696335463	0,7343
Gm19028	2,692413508	0,72076
Bub1	2,69148055	0,0071014
G2e3	2,691107458	0,0010071
Rnf122	2,689056373	0,06333
Lppos	2,686820609	0,52663

Gm11631	2,684772791	0,74819
Arl4d	2,682354655	0,35771
Gm37558	2,675113308	0,20702
2810029C07Rik	2,673259703	0,5309
Gm2531	2,672148157	0,089616
Gm37399	2,672148157	0,36758
Prkcg	2,670296607	0,27819
Isg20l2	2,668631308	0,13057
Gm27003	2,660873638	0,65416
Rps19-ps11	2,65571439	0,19092
Ect2	2,653874227	0,06505
Trmt112	2,651667715	0,0076742
Gm26520	2,651483921	0,010436
Fam71f2	2,648912153	0,31545
Gapdh	2,648728551	0,036327
Raf1	2,647994269	0,048097
A330069E16Rik	2,646893229	0,24442
Gm29650	2,646526317	0,37857
Rps28	2,64469252	0,016189
Rab5a	2,641211806	0,1106
Gm23300	2,638650002	0,78402
Kcnd1	2,633898955	0,53274
Dnm3	2,633898955	0,53469
Gm7488	2,630979487	0,46868
Gm10240	2,629338708	0,076186
Dpf1	2,628063254	0,12329
Cntrob	2,627698953	0,14186
Polq	2,626788419	0,2011
Adcy6	2,62314944	0,046274
Gm43138	2,62314944	0,29134
Cmtm7	2,619333938	0,0057965
Sft2d1	2,619152386	0,84466
Slc25a30	2,616793353	0,71586
RP23-390D8.2	2,615342697	0,43276
Gm29438	2,615161422	0,24422
Senp3	2,612262722	0,020114
Ppp1cc	2,609186375	0,68985
B230317F23Rik	2,608643866	0,5655
Zc3h12c	2,608101471	0,016637
Gm22009	2,603405428	0,28588
Rpl17-ps8	2,602864121	0,03058
Gm7285	2,602503313	0,57344
Slbp	2,600880293	0,16004
Klhl35	2,596737173	0,81191
Rps17	2,593139825	0,025141
RP24-174I4.1	2,589906473	0,27334
Rn7s6	2,589367973	0,71962
Gm38248	2,586497864	0,65345
D430013B06Rik	2,585422393	0,4374
Gm37914	2,584168243	0,50342
Tnfrsf13b	2,582377654	0,72076
Snhg20	2,578085303	0,0023779

Pigt	2,577727929	0,0345
Sez6	2,577727929	0,08976
Gm45809	2,577549261	0,60864
Gm11464	2,577191962	0,68912
Park2	2,57701333	0,76648
Idi1	2,576834712	0,75103
5730405O15Rik	2,576656105	0,90295
Gm7308	2,574513794	0,066315
Gm8228	2,569521992	0,46676
Tcf19	2,569165805	0,69174
Rccd1	2,565251009	0,088324
Rbmx	2,563828926	0,28644
Gm44187	2,562407632	0,5156
Gm38299	2,561519723	0,32393
Stom	2,561164645	0,0052592
Gm10358	2,561164645	0,4374
Nr4a1	2,560632121	0,35769
Gm14286	2,560277167	0,16524
Fsd1l	2,559567406	0,36303
Stc1	2,557971163	0,7793
Cep55	2,557084791	0,087127
Iqgap3	2,556198726	0,02083
Gm44639	2,554958752	0,47619
Gm16199	2,554250465	0,70113
Gm37902	2,551065604	0,55243
Gm38297	2,550358396	0,57352
Zwilch	2,550004866	0,10825
Gm26610	2,544178687	0,41012
Zfp36l2	2,542239587	0,011971
Timm8a1	2,539773779	0,77561
Gm15779	2,539245703	0,38169
Umad1	2,535376498	0,18732
RP23-23P9.3	2,534322283	0,57215
Usp35	2,533444106	0,090162
Slc17a7	2,53221517	0,77545
Scn11a	2,530986829	0,70841
Lrrc17	2,52940841	0,082157
Sema6c	2,527305382	0,68117
RP24-496O17.7	2,525729258	0,70867
Gm8093	2,525554194	0,6624
3010003L21Rik	2,525204102	0,65247
Efna3	2,524329085	0,32842
8030453O22Rik	2,522055458	0,46271
Galnt11	2,518037904	0,51257
Gm37531	2,513678255	0,58012
Npm3-ps1	2,512981412	0,68294
8030462N17Rik	2,51211063	0,1234
Rps6ka5	2,507761244	0,14767
Cox7c	2,507239823	0,39887
H3f3a	2,50689227	0,0057965
Gm23346	2,504460747	0,32866
2810428J06Rik	2,502378461	0,69144

Sh3bgrl2	2,501164594	0,0015076
C030015A19Rik	2,499951316	0,23354
Lncpint	2,498911831	0,23369
Anln	2,498565433	0,03658
Gm37470	2,498565433	0,70815
Gm13196	2,498219082	0,61005
Pate2	2,495968972	0,40278
Gm7353	2,492856781	0,6961
1700030M09Rik	2,492511222	0,54748
Pde2a	2,49233846	0,67455
Gm37670	2,491992973	0,64421
N4bp2	2,491647533	0,047424
Kif18a	2,489403342	0,055037
Col11a2	2,489403342	0,2547
4930430E12Rik	2,488713229	0,23865
Alox8	2,486816403	0,72396
Tnfrsf12a	2,485954687	0,0063006
AW554918	2,483027087	0,026566
Kif11	2,481822607	0,0345
Gm24959	2,480962622	0,75823
Tbc1d31	2,478384454	0,011988
Gm4832	2,475122621	0,90588
Hnrnph2	2,47477952	0,43416
B930086L07Rik	2,47409346	0,080869
Tarbp2	2,473750502	0,40416
Gm4784	2,473579041	0,32083
Spire2	2,473064728	0,24315
Rnf24	2,472550522	0,028185
Gm15501	2,470494767	0,024954
Cpne9	2,469981096	0,497
Gm10863	2,46741434	0,40231
Lzic	2,467072307	0,045087
Gm8430	2,466730322	0,010702
Satb2	2,458707304	0,095236
Gm15445	2,458536885	0,86192
0610005C13Rik	2,457173961	0,67469
Notch1	2,457173961	0,70746
Hax1	2,455471368	0,58816
C130083A15Rik	2,454280255	0,36253
Gm43360	2,454110143	0,29712
Gm23442	2,454110143	0,81712
Tcea3	2,453429813	0,70548
Tra2a	2,453089719	0,000844
Gm5914	2,451899761	0,73149
Gm34121	2,447315329	0,59139
1700124L16Rik	2,444094386	0,23945
Gm9009	2,440877682	0,65779
Papd4	2,440370168	0,41346
Gm26656	2,439524547	0,49457
2410080I02Rik	2,438848261	0,21623
Rny1	2,436144991	0,10236
RP23-213P10.2	2,434119505	0,92089

Magohb	2,432938749	0,38974
Wdfy2	2,432264289	0,11949
Hist2h3c2	2,430747437	0,22767
Gm12240	2,42738004	0,74754
Hmx3	2,425698091	0,71724
Bicdl1	2,424521421	0,70746
Gt(ROSA)26Sor	2,424185334	0,011988
RP24-131G14.10	2,424017308	0,8874
Suv39h2	2,423849294	0,10071
Gm11343	2,423849294	0,69765
Rnaseh1	2,423681291	0,76648
Gm12834	2,422841453	0,74754
Hsh2d	2,420659235	0,73384
9530085L11Rik	2,419820443	0,294
Snrpf	2,417305813	0,19244
1500004A13Rik	2,416970727	0,18083
Tia1	2,414626421	0,030239
Gm43153	2,41295331	0,54748
Gm44432	2,411949999	0,69824
Dlgap5	2,405605381	0,26887
Gm6987	2,404771806	0,13998
Cln6	2,403438687	0,0016282
Dhx9	2,403105523	0,068853
2210417A02Rik	2,402938958	0,95652
Gm44283	2,401273945	0,85936
A430035B10Rik	2,40094108	0,95933
Stamos	2,399277452	0,45055
Cox4i2	2,398113597	0,23061
2210408I21Rik	2,397282618	0,72946
Gm22516	2,396784169	0,95103
Nup205	2,396618043	0,065203
Rgs11	2,396285824	0,19559
9930104L06Rik	2,395621524	0,58327
Pole2	2,394957409	0,43416
Gm16380	2,394791409	0,70465
Tgfb1i1	2,393629731	0,77195
Selenbp1	2,392468616	0,019172
Scarna17	2,390976582	0,576
4932416K20Rik	2,389154248	0,38313
Gm9769	2,388326374	0,84589
Gm16223	2,387002372	0,71648
Rps27rt	2,385017745	0,011098
RP23-325K4.10	2,382869594	0,016688
Fancd2	2,382209014	0,11844
Tnnt1	2,381383546	0,71943
Ift80	2,379898425	0,048095
Gm43482	2,379733469	0,17189
Luc7l3	2,375283969	0,0065559
Pabpc1	2,372651155	0,021282
Gm6969	2,372157827	0,76471
4933439C10Rik	2,371664602	0,12329
Hoxb6	2,368871596	0,57721

Basp1	2,367886615	0,021282
Ogt	2,365589918	0,038317
Gm24507	2,364770209	0,90858
Clcn2	2,362149049	0,89841
Smarca5-ps	2,357242202	0,70744
Gm6807	2,354955829	0,084065
Prdm10	2,353650326	0,099334
Mogat1	2,353324064	0,54063
Rpl23a-ps14	2,353160949	0,5852
Pi16	2,351856441	0,046274
Diaph3	2,350226824	0,22614
Gm45289	2,347296357	0,77552
RP23-454I20.1	2,346645638	0,37978
Kat2b	2,346157718	0,11386
Uevld	2,345832494	0,11653
Rab11fip4os1	2,343882096	0,84217
Rps13-ps5	2,343719636	0,51884
Eid3	2,34339475	0,45472
Gzmm	2,341121809	0,54278
Gm43088	2,339337473	0,55144
RP24-550H10.3	2,339013194	0,24555
Sit1	2,338364772	0,4782
Pacs1	2,337392476	0,056488
Ppp1r37	2,336258641	0,25087
Ccdc36	2,33577288	0,1041
Camk2n1	2,334963503	0,68431
Gm12165	2,334154407	0,67883
Asf1b	2,333345591	0,15693
Fbxo33	2,332860436	0,028214
Tmed8	2,332860436	0,12736
Gm37677	2,331728799	0,67469
Prss42	2,326240083	0,83116
Hist1h1b	2,322373476	0,49322
Rps13	2,321246927	0,053398
Uba52	2,319638518	0,11653
Rpl12-ps1	2,316746191	0,49014
St3gal4	2,315301381	0,048782
1500015A07Rik	2,312895365	0,12749
Top2a	2,312254183	0,0030739
Cdkn2c	2,308090831	0,082235
RP23-151L20.5	2,307291046	0,99022
Cbx5	2,307131123	0,0031555
Gm2308	2,307131123	0,91128
Kif24	2,30697121	0,65185
Gm45546	2,306651418	0,65255
Blm	2,305852132	0,033133
Gm15453	2,305852132	0,2617
B130034C11Rik	2,305372694	0,66226
Ang	2,303296278	0,051494
4932422M17Rik	2,303136631	0,1265
Tpt1	2,302019413	0,013485
5033430I15Rik	2,301859855	0,67103

Rpl31-ps8	2,300902737	0,10349
Gm37906	2,295327478	0,3373
C230037L18Rik	2,295327478	0,66212
Pvr	2,295168384	0,27819
Pigb	2,294850228	0,43105
Helq	2,294373077	0,99022
Rbm4b	2,293101159	0,10516
Gm45137	2,292942219	0,80847
Syne2	2,288813639	0,56141
Lysmd3	2,288654996	0,18083
Ints4	2,288337743	0,19244
Gm21816	2,283584223	0,78503
Gm8550	2,283109414	0,72291
Gm43696	2,282001911	0,3581
Gm7363	2,280420696	0,74731
Gm37598	2,277893031	0,7343
Bco2	2,27599912	0,53469
Gm14427	2,274579721	0,23668
Gm43727	2,274579721	0,85692
Dram1	2,270956387	0,054908
Rad54b	2,269540133	0,68215
Gm42724	2,267810354	0,30239
Spred3	2,267338826	0,34704
Fam103a1	2,267024527	0,20297
Gm4987	2,263099475	0,30181
Vamp2	2,262628926	0,045087
A930029G22Rik	2,260591009	0,82084
Trpm2	2,259024629	0,79239
Dtl	2,258398382	0,076937
Akap10	2,25652068	0,1471
D930030I03Rik	2,255895126	0,81943
Arc	2,255582414	0,38974
Gm45133	2,255269746	0,72781
Gm5124	2,254019505	0,87562
Vps13b	2,251989343	0,10503
Lnpep	2,251989343	0,11009
Gm42535	2,251989343	0,72745
H2-Q10	2,251833252	0,78214
Marf1	2,251365045	0,054505
Gm27010	2,251208998	0,78418
Grin1	2,250428923	0,99075
Pcdhb17	2,249181367	0,67103
Gm13416	2,249181367	0,78628
Gm5601	2,248246153	0,22357
Ifi213	2,247934501	0,035977
Gm42798	2,246376892	0,55084
Gm42820	2,244664768	0,49649
Mxd1	2,244353613	0,027709
9630013D21Rik	2,244042501	0,83749
Gm37206	2,244042501	0,90617
Car11	2,243731432	0,23108
Cd48	2,243731432	0,65204

Pclaf	2,24031252	0,012514
Snhg6	2,240157239	0,58044
Ppp1r18os	2,239846709	0,7868
Gpn2	2,239536221	0,56002
9630010A21Rik	2,239380994	0,37069
Ier3	2,237519104	0,0018553
RP23-162P10.2	2,231323976	0,71106
Spcs2-ps	2,229932437	0,78628
Gm14537	2,229623325	0,66128
Gm9207	2,229159736	1
Sptbn4	2,227460734	0,38725
Kdm4b	2,227306344	0,19038
Pmf1	2,226688889	0,01989
Cep135	2,22622591	0,31351
Gm8268	2,225763027	0,46655
Capza1	2,225300241	0,31482
Itgal	2,220831598	0,1563
Anapc5	2,21898513	0,0034301
Gm44567	2,217908733	0,65725
Gm14636	2,217140198	0,10845
Ccdc18	2,214682674	0,23354
Pcgf5	2,214682674	0,46188
Gm7312	2,214375675	0,43726
Gtse1	2,213608364	0,12668
Cenpn	2,212841318	0,55118
mt-Rnr1	2,211921215	0,02078
Gm6640	2,211308025	0,60864
Socs4	2,211154754	0,064928
Fam76b	2,207632455	0,040576
Rpl10a-ps2	2,205949861	0,1618
Gm13567	2,203810232	0,5549
Zp1	2,201214901	0,81805
Comp	2,200604679	0,70815
Gm2000	2,20045215	0,077557
Exoc6	2,199689664	0,49739
Gm9143	2,199384743	0,87417
Gm37522	2,1955768	0,93746
Itga11	2,194968141	0,58468
Ntrk3	2,194968141	0,99495
Nemp1	2,194816003	0,045875
Kif15	2,193447232	0,1136
Gm45343	2,190256753	0,3464
Lekr1	2,190104942	0,65889
Ncapd2	2,1894978	0,063228
mt-Nd6	2,1894978	0,091712
Ahcyl2	2,1894978	0,24422
H2-T22	2,189346041	0,19244
Nt5c2	2,189194292	0,53911
Gm28791	2,188739109	0,44814
Kctd13	2,187829027	0,052801
Arf2	2,186464614	0,0014205
Psenen	2,186464614	0,36819

Rps6-ps4	2,185706975	0,58294
Tslp	2,184949598	0,61921
Itgb3bp	2,184949598	0,65955
Gm13864	2,18464672	0,51086
Tsc22d2	2,183889711	0,004355
Spdl1	2,182073957	0,18751
Rplp1-ps1	2,180713133	0,78099
Ncmap	2,180410843	0,95807
Gm42432	2,179202102	0,57344
Gm37472	2,179202102	0,67196
RP23-413G8.2	2,178295986	0,53677
Gm4459	2,177692118	0,9711
Tmem259	2,177239326	0,02078
Gm23502	2,175278318	0,96489
Dgat2	2,174675287	0,87562
Gm10501	2,173921732	0,61258
Rpl36-ps2	2,172565992	0,46035
Oaz1	2,172415407	0,13847
RP23-288C18.3	2,171963713	0,20979
4933417C20Rik	2,1713616	0,87029
Med16	2,170458744	0,077557
Gm12034	2,170007456	0,76307
Gm29228	2,169556263	0,64449
Gm5277	2,169405886	0,50953
H2-T23	2,169105163	0,38974
Gm43721	2,168353539	0,89508
Sap30	2,167301703	0,056308
Bloc1s3	2,166851072	0,72789
C920009B18Rik	2,166400535	0,15684
Ankrd52	2,164299266	0,58044
Rgs14	2,163849259	0,2549
Cchcr1	2,163699278	0,67848
Mrip-ps	2,162499801	0,68512
Arpin	2,161900312	0,44082
Usp28	2,161450804	0,31351
RP24-240E7.1	2,161151184	0,38357
Cdca2	2,159354337	0,08383
Rps12-ps26	2,159354337	0,53469
Gm15216	2,157409437	0,70113
Ncapg2	2,15546629	0,095236
Gm13204	2,15546629	0,77501
Gpkow	2,154271377	0,49649
Metap1d	2,153077127	0,08606
Selenow	2,151137881	0,016724
4930503L19Rik	2,150988781	0,05037
C330011M18Rik	2,150988781	0,98519
Epha2	2,150392483	0,2445
Nup107	2,149945368	0,65889
Nup210	2,149200383	0,20702
Riiad1	2,149051417	0,86744
Nme7	2,145925513	0,5903
Gbe1	2,143546925	0,045087

Gm12166	2,142507122	0,12558
Rnf152	2,14235862	0,65185
C030037D09Rik	2,139687341	0,30231
Gm7114	2,139390738	0,60421
Meiob	2,139242452	0,89894
Slc22a13b-ps	2,139242452	0,98527
B230312C02Rik	2,13864941	0,25557
Acrbp	2,138352951	0,36581
Gpat3	2,137760156	0,73483
Klf1	2,135834706	0,90858
Tmem170b	2,135686666	0,14413
Gm11956	2,134058907	0,64013
BC049715	2,133910991	0,60809
Smg6	2,133171562	0,032513
Rgs12	2,132875862	0,75823
Scarna9	2,132728027	0,31863
Zbtb49	2,12918307	0,95933
Aoc2	2,128887923	0,30364
Nfyc	2,128445279	1
Shcbp1	2,128150235	0,58443
D2hgdh	2,127855231	0,94645
Kif4	2,126380825	0,40675
Gm11952	2,1240239	0,67176
Gm13038	2,122993568	0,57721
Gm11531	2,120052513	0,14356
Gm10167	2,119758631	1
2810013P06Rik	2,118583513	0,17107
Dock6	2,117262284	0,3741
Gm7117	2,116381923	0,53819
C030034I22Rik	2,115501927	0,052408
Lif	2,113743034	0,98026
P4ha2	2,112132	0,13398
Med30	2,11096111	0,57352
Sgol2a	2,108621276	0,079685
4732440D04Rik	2,107452333	0,83174
Grtp1	2,106430038	0,39374
Rpl30	2,106284037	0,37395
Wdcp	2,10540824	0,23061
Tmem107	2,103074562	0,1691
Cnep1r1	2,102637285	0,18439
Ccng2	2,102491547	0,12601
Piga	2,101908693	0,039915
Dennd6b	2,101617327	0,73384
Gm15575	2,100452266	0,79916
Nrap	2,099142344	0,89378
Jmjd7	2,098560419	0,32842
Gm17541	2,094636641	0,95226
March4	2,094636641	0,96775
Man1a	2,094055965	0,19474
Neurl1b	2,093330347	0,028214
Gm45222	2,093185253	0,35931
Cd74	2,092024866	0,023466

Gm15796	2,091879863	0,87067
Pkmyt1	2,091444914	0,77501
Tpcn2	2,091444914	0,93353
Ip6k2	2,091010056	0,036865
C78859	2,09014061	0,94645
Numb	2,089706023	0,11161
Nxt2	2,088981911	0,07374
Hoxaas3	2,087534441	0,65779
Bsdc1	2,086232575	0,41192
Sparc	2,085509668	1
Gm37510	2,085076043	1
Cytip	2,084498017	0,95103
Gm4968	2,084498017	0,96142
C230035I16Rik	2,084209065	1
Hsp90aa1	2,083920152	0,036249
Gm12230	2,083486858	0,58468
Prr7	2,083198045	0,3887
Srfbp1	2,082476189	0,067222
Dnm1	2,082476189	0,40853
Ciart	2,081898884	0,1529
Zfp36l1	2,081033227	0,055167
Donson	2,080744754	0,69358
B3galnt2	2,080312121	0,19294
Rdh13	2,079591264	0,03658
Wdr20	2,075990728	0,19378
Pfkfb3	2,075846836	0,098206
Rab2b	2,075127527	0,27622
Zswim8	2,073689657	0,14579
Gm11625	2,072971095	1
Rpl21-ps6	2,072827413	0,27031
RP23-43M12.2	2,072396425	0,219
RP23-162P10.8	2,071247563	0,14377
Atr	2,070673371	0,2386
6430710M23Rik	2,070099338	0,84615
Rmnd5a	2,069955854	0,36798
Arsb	2,068808345	0,14677
Whrn	2,068664952	0,78214
Pabpc4	2,068234829	0,037502
Lmf2	2,068234829	0,12042
Pbk	2,068091475	0,10504
Vdac3-ps1	2,066801735	0,7343
Gm13886	2,064510849	0,45382
Thap8	2,064510849	0,57352
Zfyve26	2,063652422	0,12739
9330175E14Rik	2,063080335	0,65779
Gm7327	2,062794351	0,29043
Mif	2,062651374	0,095236
Gm43848	2,06236545	0,46098
mt-Rnr2	2,06179372	0,10514
Rnf5	2,060793574	0,43726
Coa6	2,060793574	0,61885
Lilrb4a	2,060079481	0,24442

Ddit4	2,058794738	0,095966
Pik3r3	2,058081337	0,71586
Mturn	2,057938687	0,37921
Gm20091	2,057510795	0,96683
Rad51c	2,057082992	0,46302
Gm13641	2,052383027	0,71255
Rps19-ps4	2,050392348	0,50342
Pigg	2,048971617	0,74754
Gm12943	2,04854559	0,40232
Hmgb1-ps5	2,0484036	0,73483
2900076A07Rik	2,04755187	0,33
Rps15a-ps6	2,045282322	0,28202
Gm8624	2,045140558	0,71692
Rpl19-ps11	2,045140558	0,95431
Gli1	2,044857061	1
Ift22	2,044573603	0,3758
Egfl7	2,043581811	0,45587
Gm21975	2,043015288	0,64195
Acad11	2,041175174	0,61921
Car12	2,040609318	0,11736
Crip2	2,04018503	0,31074
Trappc2	2,039478078	0,94571
C330006A16Rik	2,038630059	0,064486
Klf11	2,037499916	0,25087
Gm43737	2,037358692	0,45458
Dnmt3l	2,0363704	0,28588
Gm28438	2,034959384	1
Golga7	2,034254243	0,19779
RP24-460E12.3	2,034113244	0,90588
Gm44164	2,033690306	0,88589
H2-Q5	2,033549347	0,9725
Gm45762	2,032985605	0,79062
Ovgp1	2,032562902	0,65112
RP23-403D16.3	2,030169241	0,62329
Gm42748	2,02988782	0,56332
Gm29736	2,028903151	0,40675
9130024F11Rik	2,028200108	0,58012
RP24-84C23.4	2,026935246	0,19334
Apba3	2,026935246	0,56265
Trim59	2,026232885	0,36172
mt-Co1	2,021743538	0,028725
Ap2s1	2,021042976	0,059337
Ncapg	2,021042976	0,17933
Rab3gap2	2,019922583	0,27819
Mir155hg	2,019922583	0,86744
Gm15832	2,017543808	0,34213
Gm6565	2,016285591	0,56974
Mcm5	2,015167835	0,17233
Gas2	2,015167835	0,91028
Prpf8	2,014748836	0,094611
Gm4963	2,014748836	1
Cdan1	2,014329925	0,65255

Gm43773	2,013213253	0,69765
Gm32175	2,013213253	0,89378
Gm4017	2,011678839	0,99022
Nova1	2,010981765	0,59107
Gm29019	2,010703003	0,45113
Sik2	2,00861352	0,2373
RP23-2N7.4	2,007778335	0,53402
Gm12604	2,007082613	0,54629
Gm43813	2,004718959	0,6668
Gm15441	2,003607615	0,93474
Raly	2,002913338	0,19361
S1pr1	2,002913338	0,24236
Bend4	2,002774511	0,099534
9530078K11Rik	2,002080523	0,88531
Rad51ap1	2,001525505	0,35931
Gm17971	1,999306973	0,66013
Ypel2	1,999168396	0,22767
Gm15728	1,999029829	0,48128
Zfp326	1,998614186	0,03967
Pcsk7	1,998614186	0,24422
Gm30238	1,998060129	0,96489
Slc19a2	1,997783159	0,59094
Pou6f1	1,996814063	0,48741
Espl1	1,996260506	0,46138
Aldh6a1	1,995430457	0,49313
Mfsd14a	1,994600753	0,30561
Mcts2	1,993080526	0,50342
Gm19726	1,992942381	1
RP23-70B19.5	1,992389896	0,58565
Gm43329	1,991699506	0,6317
Frat2	1,991285387	0,073529
Crebzf	1,989629771	0,7168
Kpna4	1,989491865	0,060516
Zfp367	1,988802479	0,16154
Zic2	1,988802479	0,4326
Gm2367	1,988388962	0,79147
Hoxb8	1,987975532	0,12525
Dmpk	1,987699959	0,49577
Rnu11	1,987562187	0,62551
Abcc3	1,987424424	0,98417
Gm7887	1,987286671	0,89508
G6pd2	1,986598049	0,9229
Gm28555	1,985083921	0,13683
RP24-366E11.4	1,984808749	0,63203
Zdhhc18	1,982608746	0,095236
Ceacam1	1,982059127	0,99336
Igfbp4	1,978764607	0,011773
B230219D22Rik	1,977667651	0,062955
Atp11b	1,977667651	0,081673
Tmx2	1,976160329	0,32894
Ttc21b	1,975749441	0,47245
Gm15829	1,975201723	0,87562

Rfwd2	1,974791034	0,23354
Lamc1	1,973559479	0,1197
Pole	1,973559479	0,83292
Ints6l	1,973422687	0,29381
Card14	1,973149132	0,6624
Tmed7	1,973012368	0,25509
RP24-310D17.9	1,973012368	0,42567
Gm45733	1,972875614	1
Recql	1,972602135	0,54615
RP23-307F3.6	1,972602135	0,71781
Gm6501	1,970142523	0,96875
Gm42559	1,969596359	1
Gm44258	1,969459842	0,34566
Gm4613	1,969323334	1
Dnajb4	1,969050347	0,03985
4930542C12Rik	1,967822372	0,96041
Lmnb1	1,967276852	0,10825
Hoxb5	1,967004148	0,52103
Teddm2	1,966186264	0,96791
Gm42611	1,96536872	0,68431
Gnpda1	1,964551516	0,99368
Phkb	1,964143042	0,31919
Ntn5	1,963870773	0,81715
Med14	1,963190265	0,44029
Nrbp2	1,962782073	0,5157
RP23-149L23.1	1,962782073	0,82104
Gm28424	1,962782073	0,96775
Ap2b1	1,962646028	0,10825
Gm14813	1,962646028	0,94952
Med22	1,962509993	0,13449
Cdc7	1,962373967	0,56157
Gatm	1,962101943	0,34231
Itpka	1,960878302	1
Arhgap15	1,960742389	0,24273
Gm26549	1,959791261	0,98778
Chek1	1,958433308	0,48128
Ccdc117	1,95789039	0,075168
Gm29488	1,956533754	0,99768
Lockd	1,95626254	0,21339
Zfp280d	1,955991363	0,12525
Lyst	1,955991363	0,18256
Ccdc163	1,955991363	0,25509
Plch2	1,955855789	0,90356
Gpcpd1	1,955449122	0,2545
Uhrf1	1,954365092	0,54278
Snord35a	1,953010898	0,86838
Stxbp3	1,952604824	0,6475
Slc27a3	1,952469484	0,5407
Gm14633	1,95152237	0,88693
Gm10033	1,951387105	0,86027
Lonrf3	1,95125185	0,11754
Gm44957	1,950575715	1

2410089E03Rik	1,950170146	0,70113
Tsga10	1,949764662	0,67395
Gm7565	1,948953946	0,84089
Gm44013	1,948953946	0,97103
Gm10237	1,94881886	1
Atad5	1,948413656	0,61921
Tmem151a	1,948413656	1
Gm16580	1,948008537	0,1195
Gm13349	1,946928631	1
9330020H09Rik	1,946793685	0,42917
Srebf2	1,945849324	0,16273
Gm43343	1,945849324	0,71196
Dgkh	1,945040236	0,52792
Ubxn11	1,944770615	1
Gm43774	1,943153673	0,69824
Gm6649	1,940865306	0,94009
Tbc1d8b	1,938445266	0,88126
Gnb2	1,937639257	0,19094
Gm28041	1,937102104	1
Rps19-ps6	1,936833583	0,77411
Txnip	1,936699337	0,32511
Itga6	1,936430872	0,17627
Ece1	1,936430872	0,96142
B3gnt6	1,936430872	0,98167
Fam13a	1,934820864	1
Masp2	1,933748269	0,98026
Prkx	1,932408361	0,23297
Syngap1	1,931872658	0,51823
Epm2a	1,930667869	0,49212
E130208F15Rik	1,929998866	1
Atp6ap1	1,929597576	0,028237
Tgfb1	1,928928944	0,11524
Jun	1,928661557	0,2304
Plekhh3	1,928661557	0,3417
Creld2	1,927859615	0,2756
Myef2	1,926390256	0,19294
Leng8	1,926256734	0,53469
Lig1	1,92612322	0,031498
Ift88	1,925722736	0,61258
4930520O04Rik	1,925455793	0,48027
Parp6	1,925322335	0,17237
Zfp619	1,924922017	1
Ezr	1,924788596	0,0496
Atp5g2	1,923988265	0,67848
4921536K21Rik	1,923054967	0,8816
Gm20673	1,92265512	0,83048
C2	1,92145608	0,92089
Gm8394	1,920923413	0,8278
Gm43581	1,920657135	0,40168
Rai1	1,920390894	0,45113
H2-Ob	1,920257787	0,19378
Tmem63b	1,919858522	0,11721

Tmem176a	1,919725452	0,16377
Gys1	1,91945934	0,063228
Oaz2	1,918528239	0,045087
D730045B01Rik	1,918129333	0,7345
Snora30	1,917198877	0,97632
Gm14650	1,916800248	1
Cbr2	1,914542916	0,41057
Timeless	1,914542916	0,81943
Gm42851	1,913083707	0,9386
4932441J04Rik	1,912685934	0,67467
Nuf2	1,912023162	0,59934
Aurkb	1,91149311	0,35855
Gm14620	1,910698308	0,11026
Orc1	1,910698308	0,78503
Gm17249	1,910433447	0,9391
Gm37065	1,910433447	0,97009
Dynlt1f	1,908183613	1
Gm10036	1,907125785	0,35663
Gm12522	1,907125785	0,78378
Slc25a25	1,906993598	0,23865
Zbtb34	1,904747815	0,4965
5830408C22Rik	1,904615792	0,58498
Zbed3	1,904351775	0,6421
Gm14279	1,903691891	0,53501
Agbl5	1,903428002	0,52858
Arrdc2	1,903296071	0,26478
Neil3	1,902900332	0,78073
Fdps	1,901845433	0,30711
Gm27029	1,901713612	0,91327
2810403A07Rik	1,90065937	0,20111
Gm45413	1,899869072	1
Pdia3	1,89934239	0,051251
Atf5	1,898947474	0,12329
Pitrm1	1,898684242	0,5652
Coq10b	1,898421047	0,24446
Cdk16	1,898289463	0,51983
Siah1b	1,896448245	0,49617
Gm44168	1,8957911	1
Mapk8ip3	1,894346182	0,10445
3110083C13Rik	1,894083588	1
Gm4430	1,893952305	0,93965
Srsf1	1,892771164	0,23354
Prss35	1,892115293	1
Pnpt1	1,890935299	0,5425
2900005J15Rik	1,88975604	0,74726
Gm43484	1,889625056	0,6159
Rpl6	1,889363117	0,24422
Melk	1,889363117	0,67202
Rab26os	1,889101214	0,22687
Gm45051	1,888708427	0,90982
Otub1	1,887923098	0,18751
Rrad	1,886745718	1

Rpl26	1,886484178	0,28644
Slx4ip	1,886222674	0,67296
Ttk	1,88517702	0,22977
Pnrc1	1,884393159	0,08383
Rps19-ps12	1,884001352	0,69433
Arhgap26	1,882695914	0,56577
Palm	1,882173992	0,57062
Snn	1,882173992	0,84147
Gm12504	1,881782646	0,92852
3110031N09Rik	1,881521793	0,89508
Bank1	1,881391381	1
Gm10177	1,881260977	0,67418
Nnt	1,880087751	0,15222
Nucb1	1,879566553	0,054928
Cldnd1	1,879306008	0,38974
Inpp5e	1,878915257	1
Zfc3h1	1,878524589	0,18751
Mettl17	1,876962725	0,43484
Kifc1	1,875402161	0,38418
Rflnb	1,875142193	1
Gm12017	1,875012222	1
Gm45203	1,874752309	0,76758
Slc31a1	1,874622365	0,033365
Trp53	1,874362505	0,43103
Arsa	1,874102682	0,38974
Rpl7a-ps5	1,872674294	0,85692
Cd300lf	1,872414704	1
Epsti1	1,872155151	0,4652
Ndrg1	1,869950396	0,089375
Jpx	1,86904331	0,61672
Dnajb1	1,868913762	0,32688
Atf6	1,866712824	0,1126
Usp50	1,866583438	0,76621
Gm42941	1,864902234	0,94083
Tnf	1,864772973	0,29769
Abca1	1,864643721	0,57708
4933404O12Rik	1,86425602	0,46438
Ssh3	1,86361003	0,40645
Eri3	1,863480859	0,42285
Mef2c	1,863093399	0,064418
Rpl32	1,860512393	0,19921
Notch4	1,860512393	0,94731
RP24-325P4.5	1,85999662	1
Gm36964	1,859738788	0,73615
Cldn11	1,859609885	0,21937
Capn3	1,859609885	1
Gm15265	1,85922323	1
Rpl31	1,859094363	0,094759
Timm23	1,856390217	1
Traip	1,856132885	0,67196
Tpx2	1,856004232	0,13612
Skp2	1,855618327	0,34528

Cdip1	1,85548971	0,23586
Gm42986	1,855361101	0,59128
4930579G24Rik	1,855232502	0,78835
Gm13611	1,854718194	0,68622
Svip	1,853047676	0,92599
Gm42566	1,852919237	1
Rpl27a-ps2	1,852662385	0,82601
Prim1	1,851378663	0,60864
B9d1	1,851378663	0,91028
Gm37140	1,851378663	1
Naa20	1,850480586	0,2113
Troap	1,850352325	0,65503
Naa60	1,850224073	0,20111
Atp1a3	1,850224073	0,24555
Snora31	1,84894204	1
1700084E18Rik	1,848301356	0,6007
Lzts3	1,847532829	1
Gm37706	1,845612909	1
Fam234b	1,845484985	0,64015
Gm5883	1,845229164	1
Elavl1	1,844589768	0,13036
Ccnl1	1,844589768	0,16559
Intu	1,843694984	0,86192
Kif20b	1,842672906	0,31324
3110062M04Rik	1,842545186	0,16448
Gm17108	1,842034395	0,78014
Nsdhl	1,841396105	0,33761
Trp53rkb	1,841268474	0,49313
Nup153	1,840885632	0,36186
Aif1	1,839992646	0,59735
Usp53	1,839865112	0,24042
Gm42633	1,838717702	1
Gm45053	1,837571008	0,78378
Zfp87	1,837443642	0,30143
Msi2	1,836425029	0,39374
Gm3940	1,836170464	0,92188
Nfix	1,835915934	0,27504
Hap1	1,835915934	0,54483
Plekha7	1,835406981	1
Gm43182	1,835152557	1
Tmem64	1,835025358	0,084888
Pea15a	1,834389497	0,23766
Anxa2	1,833118436	0,17627
Lrp1	1,832864329	0,10651
Trdmt1	1,832864329	1
Mcm7	1,832483236	0,25601
Serpinf1	1,832356222	1
Psma5	1,832102221	0,23865
Gm45568	1,831721286	0,5016
F730043M19Rik	1,831467373	0,36585
Ptcd3	1,831086569	0,68407
Ptprj	1,830959653	0,56002

Gm2788	1,830832745	0,99287
Acat1	1,830452073	0,62862
Nfkbid	1,82918374	0,36172
Gm5735	1,82918374	1
Gm23751	1,829056955	0,81943
Gm37349	1,828549903	0,63312
Slc25a22	1,828423162	0,74261
Rragb	1,828423162	0,97437
2310074N15Rik	1,828042992	1
Fam53b	1,8276629	0,36513
Btaf1	1,827156235	0,48969
Scel	1,826902955	0,6915
A830080D01Rik	1,82664971	0,73083
Zbtb21	1,82664971	0,74778
Gm15859	1,826143325	0,89334
2810454H06Rik	1,825890186	0,80849
Gm5070	1,825637081	1
Maz	1,82525749	0,57968
Adm	1,824751491	0,54783
RP23-426K2.3	1,824498544	0,95288
Hes7	1,824372084	1
Lrrc2	1,823866331	0,87562
Hoxa5	1,823234337	0,8202
Mospd3	1,8229816	0,31616
Cep128	1,822349912	0,84823
Tfr2	1,822223601	1
Ifnar1	1,821971005	0,38313
Klc2	1,821087193	0,52103
B4galt3	1,820456162	0,13465
Tapbp	1,819825349	0,21937
Gm16523	1,819194755	0,98854
Gm28578	1,81856438	0,81947
Olfr921	1,81856438	0,99277
Pde1b	1,818186259	0,38357
Gm44834	1,818060236	1
Mospd2	1,817682221	0,53274
Gm13815	1,817430254	0,80108
Upf2	1,815415776	0,19244
Gm7701	1,815164124	0,98347
Cenpf	1,815038311	0,26492
Trmt1l	1,814535145	0,43335
Map3k8	1,813780658	0,28644
Ctdspl2	1,812900819	0,3464
Gm9435	1,812900819	1
Tubg1	1,811519078	0,30174
Acadsb	1,810263864	0,3994
Hdac1	1,810012926	0,85676
9930120I10Rik	1,808884133	1
Lnpk	1,808758755	0,52858
Prosc	1,808131996	0,2128
Gm10499	1,807254897	1
Gm11539	1,80625302	0,17653

Snord92	1,80625302	1
Gm44777	1,805126572	1
Sdc4	1,804876346	0,18279
A530041M06Rik	1,804876346	0,85112
Tle6	1,804751246	0,65841
Gm42551	1,804501072	1
Cmpk1	1,804125875	0,27537
Cpsf7	1,804125875	0,41672
Gm14005	1,803875788	0,68874
Agtpbp1	1,802625869	0,10761
Mocs1	1,80237599	0,68117
Cops7a	1,801876334	0,56002
Tmtc3	1,801127111	0,77679
Tpt1-ps3	1,801127111	0,90091
Fam120aos	1,800877439	1
Gm13092	1,80000386	0,88073
Klhl7	1,799255415	0,43726
9430092D12Rik	1,798756624	1
Gm14843	1,798507281	1
E2f8	1,798382622	1
Hba-a1	1,797634851	1
Rpl17-ps10	1,797510253	0,94099
Rpl13	1,79713651	0,30197
Cep192	1,796887392	0,72002
Fbl	1,796638307	0,40018
Cad	1,796389258	0,47115
Atf7	1,796264746	0,36104
Gm26132	1,795891262	1
Mcm2	1,795642316	0,43416
Srsf2	1,795144527	0,23865
Cmtm6	1,794646876	0,1481
Fam174a	1,794398103	0,22207
Gm10275	1,793651989	0,32197
Gadd45a	1,793651989	0,57567
Pear1	1,793403353	0,98026
AW046200	1,793403353	0,98527
Pdzk1ip1	1,793154752	1
Gm38082	1,792657652	0,63016
Vkorc1	1,792160691	0,30161
Gabpb1	1,792160691	0,65889
Ccr10	1,789926069	1
Smg7	1,789553903	0,33052
Gm26397	1,789057802	1
Tctn1	1,788313909	0,65262
Dpy19l3	1,788313909	1
Tcf20	1,788066013	0,24199
Dis3l2	1,788066013	0,57062
Rasa4	1,787446424	0,23297
Wdr74	1,787198649	0,90859
Gm43660	1,787198649	1
Pim1	1,786950907	0,21241
Gm12090	1,786703201	1

4930581F22Rik	1,786331705	0,82752
Zgrf1	1,785712717	0,67196
Adgre1	1,785465181	0,12736
Gla	1,784722782	0,31301
Gabpb2	1,784475384	0,33174
Hspbap1	1,784351698	1
Tmem33	1,783238908	0,12329
Ccdc58	1,782126813	0,37395
Arhgef11	1,781138866	0,67196
Gm10762	1,780768527	0,90946
Ccnj	1,77953462	0,84102
Cracr2a	1,77953462	1
Gm12762	1,778794686	0,98452
Ddah2	1,77743894	0,77438
Mxi1	1,77657673	0,14784
Nfkbiz	1,775714939	0,50751
Lix1l	1,774853566	0,33482
F11r	1,77436154	0,68215
Exd2	1,773377898	0,89381
Gm6305	1,773377898	1
Kifc5b	1,773009173	0,79062
Tiparp	1,772517658	0,3767
Gm6612	1,772271952	1
Acss2	1,77202628	0,60272
Pfn1	1,771412249	0,63335
Sf3a2	1,771166696	0,46438
Mad2l1	1,770798431	0,61827
Gm10051	1,770798431	1
Zmynd10	1,770552963	0,9624
Rnf38	1,770184825	0,4865
Pfkp	1,769939442	0,21331
Exosc3	1,769939442	0,97277
Matr3-ps2	1,769448779	0,98227
Ddx11	1,765773137	1
Arl14epl	1,765528365	1
Rpl39	1,764794256	0,57517
Gm12097	1,764305018	1
Ddr1	1,763693663	1
Rhov	1,763204731	0,7343
4833417C18Rik	1,762838121	0,97814
Casp7	1,762715935	0,76491
Gm37219	1,762471587	1
Ppp1r15a	1,76210513	0,40675
Rps4x	1,761982994	0,94074
Fmr1	1,761250358	0,26967
Rap1gds1	1,760273984	0,31516
Lsr	1,760273984	1
Gm38190	1,75832286	1
Vamp1	1,758079122	0,51531
Lamtor2	1,757957265	0,24693
RP24-454N4.2	1,757591746	0,42555
Kdm3a	1,757104506	0,4965

Gm19353	1,756860936	0,33067
Pcyt2	1,756617401	0,47619
Nbeal1	1,756008709	0,21937
Atp11c	1,755886996	0,61584
Gm21967	1,755156895	1
Triobp	1,75467033	0,72551
Cntnap1	1,754183899	1
Rgcc	1,754062313	0,59743
Prr11	1,753819165	0,62225
Cox19	1,753697603	0,41059
Itpr3	1,75333297	0,37984
Foxj3	1,75333297	0,50719
Ggh	1,753089923	0,58619
Plekha1	1,75284691	0,21001
Dvl2	1,752725417	0,27334
Plxna3	1,75199663	1
2900052L18Rik	1,751632351	0,77561
9330104G04Rik	1,751510941	1
D230022J07Rik	1,751025386	1
Tpp2	1,750904019	0,36441
Papss1	1,750782659	0,32136
Mrpl42	1,750418632	0,24315
Dkkl1	1,749812088	1
Rsrc2	1,749690805	0,16954
Osbpl3	1,749327005	0,3896
Rpl13-ps3	1,749205755	0,94449
Gm16437	1,749205755	1
Slc38a2	1,74896328	0,30927
Gm12643	1,748720839	1
Celf1	1,748478432	0,34481
Serpinb9	1,748357241	0,99228
Dnajc7	1,748236058	0,23421
Dr1	1,74787256	0,47619
Map3k12	1,746782521	0,58816
Dpep2	1,746661447	0,43089
Arfgef3	1,746540382	0,49739
Pidd1	1,746540382	1
Clec4n	1,746056205	1
Matk	1,745451173	1
Pik3c2a	1,744846351	0,31516
R3hdm4	1,743879071	0,2452
Gm13413	1,743637335	1
Pan2	1,743516479	0,60024
Abhd10	1,743274793	0,69356
Fbxo5	1,743153963	0,69356
U2af2	1,742791522	0,61426
RP23-168F21.4	1,742791522	1
Rpl23a-ps3	1,742429156	0,69467
Ska1	1,742308384	0,33174
Epb41	1,742187621	0,35811
Selenop	1,742187621	0,81623
Dpm1	1,741463216	0,54501

Gm6576	1,741221815	1
Kmt2d	1,740377174	0,2934
2810433D01Rik	1,739774109	1
Gm44130	1,739171254	1
RP24-418P10.4	1,739050708	1
Pif1	1,73820712	0,63651
Rell1	1,73748437	0,36104
Zfp52	1,737123108	1
Pyroxd2	1,735919443	0,7343
Fam151b	1,735558507	1
Smg1	1,735317924	0,44932
Gm15787	1,735197645	1
Rhoa	1,734957112	0,23354
Hmmr	1,734836858	0,48271
Ube2t	1,734836858	0,56992
Kbtbd8	1,734476146	1
Tpra1	1,73411551	0,39457
0610009O20Rik	1,733995314	0,58327
Tcn2	1,733514615	0,36343
Mettl21b	1,732553617	0,70398
Gm10110	1,73243353	0,77862
Pdia6	1,732313451	0,1563
Gigyf1	1,731713181	0,58816
Pbdc1	1,731233115	0,33174
Gm5778	1,729793714	1
Arhgef1	1,72931418	0,57967
Gm8599	1,729194317	0,79294
Hist2h2be	1,728595128	0,40449
Gm42547	1,72835551	0,89972
Gm5121	1,728235714	0,75096
Tacc3	1,727277642	0,58919
Llgl2	1,727277642	1
Kiz	1,727157921	0,32573
Poc1a	1,727157921	0,9391
Ptms	1,726798806	0,25087
Snord104	1,726798806	0,43103
Recql4	1,726320102	1
Rad52	1,725123922	0,53911
A430110C17Rik	1,723689601	1
Atp11a	1,72273405	0,6475
Strn4	1,722614643	0,43687
Gm4525	1,722137099	0,88693
Epn2	1,721659687	0,2547
E330034L11Rik	1,721659687	0,64668
4932438A13Rik	1,720228247	0,39944
Cebpb	1,719512972	0,92477
Vps51	1,719393789	0,52633
RP23-36H21.3	1,719393789	1
Ubc	1,718917138	0,65435
Spns1	1,718440619	0,497
Nsa2-ps2	1,718440619	1
Plcb3	1,715584278	0,47773

Yae1d1	1,715584278	0,81972
Cnot3	1,714989805	0,45124
Rc3h1	1,714514375	0,43089
Nadk2	1,714157888	0,5794
Cbwd1	1,713801476	0,76223
Ccl5	1,712495266	1
Lmbrd2	1,7121392	0,68496
Lmtk3	1,712020527	1
Gm44044	1,712020527	1
Dennd4a	1,711308666	0,34648
Dubr	1,711308666	0,69086
6330562C20Rik	1,711190051	0,89005
Creb3l3	1,710952846	1
Fam122b	1,710241429	0,70113
Gm527	1,710122888	1
Spata1	1,710004356	1
Tcp11l1	1,709411816	0,50502
Stard4	1,709056391	0,36253
Tgif2	1,708937932	0,54682
Tsg101-ps	1,708345762	1
Rarg	1,707635429	0,67226
Tma7-ps	1,707517069	0,79581
Arhgap27os1	1,707280373	1
Rps24	1,70704371	0,34979
Syap1	1,706925391	0,70791
Zw10	1,706452196	0,19294
Csrp2	1,705387988	0,80108
Cacna1s	1,70408819	0,88154
Platr3	1,70385197	1
C2cd5	1,703615782	0,61921
Gm13477	1,703025456	1
Gm43878	1,702907415	1
2210406H18Rik	1,702671359	1
Gm37390	1,702317335	1
Rusc1	1,701963385	0,41886
Dock1	1,701963385	0,83669
Stxbp4	1,701845418	0,95493
Lrrc49	1,701255705	1
Glt1d1	1,700784083	1
Pdcl3	1,699369998	0,89635
Eif4g2	1,698545659	0,30368
Rusc2	1,698545659	0,828
Ubald2	1,698192493	0,64698
Srebf1	1,69795709	0,73553
Gm37621	1,697721719	1
Rps16	1,697604046	0,97437
Mettl25	1,697015803	0,99768
Tmem109	1,696310181	0,28609
Gpr137b-ps	1,69595748	0,57062
Gm7299	1,695839929	1
Cep44	1,695722387	0,68215
Slc4a8	1,695722387	1

Rrm1	1,695369808	0,45321
Acad10	1,695369808	0,98127
Anp32a	1,695017302	0,24422
Ptgs2	1,69466487	0,60705
Dync2li1	1,69466487	1
Gm9506	1,694429956	1
Upf1	1,693960225	0,52991
Rps27	1,693608013	0,68407
Hist1h4a	1,693608013	1
Kremen1	1,693255873	0,7428
Dip2a	1,69313851	0,81943
4931428F04Rik	1,69313851	0,93563
Copz2	1,69313851	1
1110006O24Rik	1,693021155	1
Rbx1	1,692786468	0,2549
Primpol	1,691496275	0,78861
RP23-324E2.11	1,691027358	1
1700001G11Rik	1,690910149	1
Pikfyve	1,690792948	0,45472
Apex2	1,690675755	1
Naga	1,68868472	0,73483
Dnajb6	1,688450635	0,32794
Rpl21	1,688099568	0,62225
Slfn9	1,687982562	1
4933433G15Rik	1,687865564	1
4632404H12Rik	1,687280696	0,6755
Rpl15-ps5	1,687280696	1
Gm43421	1,686812947	1
Glipr2	1,68669603	0,87562
RP24-389J11.1	1,686579121	0,55308
Ssbp4	1,686228443	0,46297
Olfr933	1,685877839	0,98026
Zc3h11a	1,685527307	0,99287
Map3k3	1,685410479	0,75823
Tet2	1,684826461	0,6184
Gpr179	1,684476148	0,79818
Bnip3l	1,684359393	0,5903
Mcoln1	1,683308962	0,74768
Taf2	1,683075622	0,92044
2700046G09Rik	1,682375797	1
Gm11945	1,682259187	1
E230032D23Rik	1,682142586	0,82388
a	1,682025993	1
Chfr	1,681210067	0,51059
Ltbp4	1,680860505	1
Asb10	1,6801616	0,83129
Gm11249	1,678997404	1
Gm37084	1,678648303	0,74819
Traf3ip3	1,678531952	1
Btg1	1,677252623	0,58986
Svep1	1,677252623	1
Tial1	1,675161279	0,43806

Map3k2	1,675161279	0,56031
Knstrn	1,675045169	0,36448
Slc12a6	1,675045169	0,58565
Fendrr	1,674812975	1
Zcwpw1	1,67469689	0,99022
Kantr	1,674116585	0,86787
Serpinb6b	1,674000548	0,68407
Gtf2ird1	1,674000548	0,82933
Trpt1	1,673768498	1
Gm6344	1,673768498	1
Mapre2	1,673536481	0,37042
Gm6525	1,673072542	1
Lsm12	1,672724673	0,60581
RP23-366E4.9	1,6724928	1
Taz	1,672376875	0,69335
Gm5075	1,672376875	0,89378
Gm43544	1,671681498	1
Cry1	1,67144977	0,64195
Uba7	1,67098641	0,81193
2700097O09Rik	1,67098641	0,89438
Gm4737	1,669712832	0,81972
Eya3	1,669249953	0,54278
Npr1	1,669018562	1
Gm18943	1,668440225	1
Rab27a	1,668324581	0,71766
Camk2g	1,667746484	0,60057
Gm15210	1,667168588	0,49212
St6galnac6	1,666590891	0,48741
Papd5	1,666475376	0,70841
Ralgapa1	1,666475376	0,79916
Nlgn2	1,666359868	0,76551
Asb3	1,665551542	0,84896
Adam15	1,665320663	0,56141
Nup88	1,665320663	0,61463
Rapgef3	1,665089817	1
Phf8	1,664282106	0,56509
Supt20	1,664051404	0,58816
Slc16a10	1,663474787	0,50342
Asph	1,662322154	0,27852
Gipr	1,662322154	0,87356
Isyna1	1,661861324	0,61794
Gm37105	1,661630957	1
Gm16045	1,661285467	0,81943
Gm13005	1,660824925	1
Aldh1l2	1,66036451	0,88157
B3gnt2	1,659789171	1
Gm45534	1,659099028	1
Gm12280	1,658294223	1
Tubgcp2	1,657489809	0,88825
Aaed1	1,657260048	1
Gm13935	1,657145179	1
Trmt13	1,656915466	0,64705

Dock4	1,656915466	1
Vps8	1,656456135	0,86444
Oxct1	1,653702823	0,36448
Gm12816	1,653702823	0,90391
Gm9833	1,653702823	1
Gm44890	1,653702823	1
Vwa8	1,652556961	0,75618
Phip	1,651640843	0,36513
Gm37785	1,651640843	1
Gm13215	1,651411893	0,89943
Plin2	1,650610817	0,49332
Txlng	1,649581434	0,9362
Ube2h	1,648781246	0,39122
Pcna	1,648438427	0,70643
Racgap1	1,648209921	0,59094
Psma3	1,647524592	0,87417
Pigs	1,647296212	0,87067
H2-DMb1	1,647182035	0,87562
Kmt5c	1,646839548	0,58077
Gm15975	1,646383011	1
Gm12038	1,646268896	0,92044
Elk4	1,645812517	0,67555
Dynlt1-ps1	1,645014157	1
Unc13a	1,644216185	0,81794
Clic1	1,64410222	0,43832
Cybb	1,643532517	0,57315
Fam20b	1,6434186	0,576
Gm8318	1,6434186	1
Tpi1	1,643304691	0,3954
Tnpo2	1,643190789	0,3373
Hs3st3b1	1,643190789	1
Gm13408	1,643076896	0,98167
Col4a5	1,642735263	0,74397
Pde7a	1,642621402	0,68592
Gm38376	1,642507548	1
Asap3	1,642393702	1
Krit1	1,642279863	0,50974
Chd5	1,642166033	1
Rp2	1,642052211	0,70744
Zfp101	1,642052211	0,93338
Luc7l2	1,64182459	0,41705
Bbs5	1,641369443	1
Gm12481	1,641141916	0,65416
Gm43290	1,639891085	0,99336
Gm27248	1,63977742	1
Susd6	1,639663764	0,67196
Gm5045	1,639322841	1
Ugcg	1,638981988	0,76773
Prim2	1,638868387	0,99228
Lman2l	1,638754793	0,81623
Rpl15	1,638641207	0,65951
Rbm19	1,638073396	0,95685

Etfdh	1,637959857	0,76804
Gm11686	1,637959857	1
Lypla1	1,63727879	0,63763
Klf6	1,636938363	0,63194
Hipk2	1,636711451	0,60809
Adap1	1,636598007	0,38785
Parpbp	1,636598007	1
Sec22c	1,63648457	0,88693
Cebpg	1,636371142	0,51001
AW549877	1,636257721	0,62409
Nmrk1	1,636257721	0,90755
Cystm1	1,635804117	0,95368
Uimc1	1,635690736	0,58468
RP23-269H21.1	1,635463997	0,54278
Fnip1	1,635350639	0,5415
Fam135a	1,635350639	0,74754
Tomm40l	1,635350639	0,78022
Jund	1,635123947	0,50342
Smc2	1,635010613	0,50369
Fam64a	1,635010613	0,75608
Tsix	1,634783968	1
Spata2	1,634783968	1
Casp2	1,634444059	0,89141
Mapk6	1,634217493	0,53911
Nop56	1,633877702	0,59517
Gm14513	1,633198333	0,96791
Gm37962	1,633198333	1
Gm10657	1,633085132	1
Swi5	1,632858754	0,57352
Gm6418	1,632858754	1
Gm12089	1,632292946	1
Rbms2	1,632066678	0,57081
Hdhd2	1,631840441	0,92852
Gm20689	1,631274987	1
Phf1	1,63104886	0,52346
Mthfs	1,6305967	0,9163
Rpl34-ps1	1,6305967	1
Gm6450	1,6305967	1
Rbpms	1,630370667	0,94347
Zc3h3	1,630370667	1
Gatad2b	1,630257662	0,58548
Gm10138	1,629918694	0,78281
Gm43668	1,629128045	1
Gm26620	1,628224915	1
Fanca	1,62788637	1
Sf3b1	1,627773538	0,45113
B230217C12Rik	1,627547896	0,92672
Rpl10-ps2	1,627547896	1
Stap2	1,627209492	1
Dgkz	1,626758396	0,6421
Rhog	1,626645642	0,71898
Pwwp2a	1,626532895	0,77545

Bax	1,626194702	0,55118
Dclre1a	1,625743888	1
Atf3	1,625631204	0,72007
Dntt	1,625631204	1
Topbp1	1,625518528	0,68431
Tbc1d5	1,624054447	0,874
Gtpbp10	1,62394188	1
Hmox1	1,623716769	0,73548
2410004B18Rik	1,623604226	0,76041
Cenpk	1,623379162	0,89111
Bub1b	1,623041625	0,62574
Nyap1	1,622591685	1
Scai	1,622141869	0,90617
Hdac8	1,621354992	0,95607
Gm11868	1,621242612	1
5730508B09Rik	1,62079317	0,87369
Rnf217	1,62079317	0,90753
Zfp207	1,620680829	0,53795
Pak4	1,620006947	0,99228
Erf	1,619894661	0,57234
Nptn	1,619894661	0,61426
Oat	1,619445593	0,75795
Bcat2	1,619445593	0,8343
Yod1	1,619333345	0,79124
Tmem29	1,619221106	0,94731
Ggcx	1,619221106	1
Ccdc134	1,61899665	0,98242
Gm26737	1,618884433	1
Eif3m	1,618547831	0,90753
Lbr	1,618211298	0,46281
Kdm6b	1,617538443	0,76152
Rad54l	1,617426327	0,95283
Dut	1,61731422	0,58565
Ccdc84	1,616753799	0,98189
Ocrl	1,616641738	0,70261
Cd36	1,616641738	0,75688
Fam43a	1,616529684	1
Gm9844	1,616081549	1
Gna12	1,615969535	0,79239
Pola1	1,614849818	0,89841
2610037D02Rik	1,614849818	1
Trappc9	1,614625968	0,72551
R3hdm2	1,614402149	0,53199
Atp5g1	1,613842737	0,78503
Ccdc9	1,613395347	0,59435
Ugp2	1,613395347	0,65009
Tmem38b	1,612389172	0,67418
Atg4c	1,612389172	1
Usp22	1,612277414	0,60421
Uros	1,612277414	1
Cdk5	1,611830457	0,88168
RP23-63H11.3	1,611718738	1

Pex1	1,611495322	0,84589
Luzp1	1,611383625	0,581
Tbc1d22b	1,610267086	0,94645
Plxnd1	1,609932275	0,70867
Suv39h1	1,609597534	0,84971
Jak3	1,609597534	1
Zfp414	1,609485969	0,81712
Hspa9-ps1	1,609262862	0,94817
Tbcd	1,609039787	0,75088
Bbs4	1,609039787	0,90127
Fam83a	1,609039787	1
Nhlrc3	1,60892826	0,7087
Lpin2	1,608816742	0,61005
RP23-6C18.6	1,608816742	1
Gm6341	1,608705231	0,71586
Zfp81	1,608593728	0,90982
Ankzf1	1,608482233	0,82728
Sp1	1,608370745	0,6175
Cdkal1	1,608370745	0,89005
Usp37	1,608147793	0,71622
Gm5312	1,608147793	1
Capns1	1,607701981	0,61205
Ddit3	1,607256294	0,72076
Gm10698	1,607033496	1
Sgcb	1,606587994	1
Bcl2l2	1,606587994	1
Mcm3	1,606476637	0,73615
Slc45a4	1,606476637	1
Ints1	1,606142614	1
Mob1b	1,605586064	0,63651
Sf3b3	1,605252227	0,75506
C330027C09Rik	1,605252227	0,84645
Rdh5	1,605252227	1
Gm7783	1,605029707	1
Kdm6a	1,60402875	0,71124
Rara	1,603695236	0,85472
Nfatc2	1,603028416	0,79294
Chmp4b	1,602695111	0,60421
Lacc1	1,602695111	0,99061
Qk	1,602584024	0,59142
Hist1h1e	1,602250811	0,71766
Kansl1	1,602028707	0,80099
Smc4	1,601695609	0,60251
Jsrp1	1,600252985	1
Phka2	1,600142068	0,87448
Gm45380	1,599920257	1
Gm5786	1,599920257	1
Zfp652	1,599033318	0,6159
Lmtk2	1,598479232	1
Sep 11	1,598257651	0,71869
Gm6142	1,597925337	0,7345
Zfp511	1,597593092	0,87298

Gm28417	1,597593092	1
Dnaaf5	1,597260917	0,69919
Sh2b1	1,597150207	0,65684
Zdhhc13	1,596596773	1
Bmt2	1,596375453	0,57841
Raph1	1,595601074	0,95368
Ccdc69	1,595601074	1
Spidr	1,595269313	0,99768
Gm1976	1,595158741	1
Ctps2	1,594716531	0,95355
Sympk	1,594384953	0,78224
Rnaset2a	1,593942957	1
Arhgap19	1,593832477	1
Gm8649	1,592507316	0,71389
Insig2	1,592176198	0,71869
Ccna2	1,59206584	0,77336
Pmaip1	1,591734814	0,7941
Pycr1	1,591734814	1
Lum	1,591293552	1
Ndc80	1,590962687	0,95844
Sdc3	1,590190934	0,58498
Gm15690	1,590190934	1
Braf	1,590080714	0,83014
9230112E08Rik	1,589970502	1
Gm9354	1,589970502	1
Gm38022	1,589639911	1
Mtbp	1,588758672	1
Ptchd1	1,588538439	0,69043
Dck	1,588318236	0,82074
Eda2r	1,58765781	0,77882
Psma8	1,587217679	0,89413
Cep70	1,587217679	0,97437
Tbc1d10a	1,586997659	0,79147
Kdm5c	1,586447743	0,78281
Gm43323	1,58622783	1
Gm42659	1,585128721	0,99851
Snx13	1,584799137	0,88531
Arhgef40	1,584689291	0,84445
Cmtm4	1,584579453	0,83559
Slc25a36	1,584249983	0,68147
Lrig2	1,584030374	0,81193
AV356131	1,583591249	0,74754
Efcab7	1,583481487	1
Txndc5	1,583371732	0,79062
Rpa3	1,583042513	1
Gramd4	1,582932789	0,94571
Pgap3	1,582932789	1
Fbxo21	1,582823072	0,73095
Megf8	1,582823072	0,87567
Prepl	1,582713363	1
Gm8770	1,582713363	1
Tmem80	1,582274602	1

Ctsh	1,582274602	1
1700086P04Rik	1,582274602	1
Ahdc1	1,582164931	0,90035
Rps23	1,582164931	0,93338
Gm14292	1,582164931	1
Mkrn2	1,582055268	0,88589
Ddx43	1,581945612	0,87972
Lamtor3	1,581507064	0,89334
Dtd2	1,581507064	0,94571
Atp5l-ps1	1,581397446	0,97437
Gm45853	1,580630334	1
Ermp1	1,579973105	0,84225
RP23-55A6.4	1,579973105	1
Gm42715	1,579206683	1
Gm37733	1,578768894	1
Rps6-ps3	1,577565602	1
Gm18889	1,57734692	0,94431
BC055324	1,577128269	0,82905
Clgn	1,576800348	1
Pla2g6	1,576581772	0,98668
Gm43273	1,576035465	1
Palb2	1,575707772	1
Hspe1	1,575380147	0,71586
Hjurp	1,575161768	0,71653
Gm43466	1,574943419	1
Fam126b	1,574615953	0,73095
Lgmn	1,574615953	0,92089
Ttc9c	1,574506813	0,87806
Gm43560	1,57439768	1
Tnfrsf17	1,574070327	1
Rrnad1	1,573633963	0,92461
Iqcb1	1,573088677	0,98533
Ly9	1,573088677	1
RP23-128C4.4	1,572979643	1
Nfrkb	1,572761597	0,86444
R3hdm1	1,572434584	0,71586
Vma21-ps	1,572434584	1
Nfil3	1,57210764	0,93503
Ppp5c	1,571998673	0,81191
Vcl	1,571998673	0,92852
Wdr92	1,571998673	1
A330023F24Rik	1,571780763	1
Rad21	1,571345033	0,76773
Pcbp4	1,571127214	0,96739
Atp2a3	1,571018315	0,768
Gm26652	1,570800541	1
Gsk3a	1,570691665	0,79916
Pam	1,570582797	0,65608
Gm43309	1,570582797	0,92827
Gm17259	1,570365083	1
Gm12164	1,570147399	1
Elac1	1,570038569	0,96875

Laptm4b	1,569929746	1
Arfgap2	1,56982093	1
Kcnc3	1,56982093	1
Rpl10a	1,569603322	1
Rnf185	1,569385744	0,95493
Gm12248	1,569385744	1
Arf5	1,569168196	0,67196
Clec12a	1,568950678	0,69027
Per3	1,568950678	0,90982
Eldr	1,568950678	1
Nr4a3	1,568298305	1
Gm43924	1,568298305	1
Gnas	1,567972221	0,79545
Fsbp	1,567972221	1
Rps20	1,567320255	0,74768
7330423F06Rik	1,567211621	1
Gm16062	1,566777157	1
Rhoh	1,566777157	1
Gm24276	1,566451389	0,85558
Fyco1	1,566234248	1
Cdkn3	1,565474491	1
Ccdc94	1,565148993	1
Gm45223	1,564823563	0,95182
C530043K16Rik	1,564823563	1
Lin52	1,564823563	1
Phf20l1	1,564715102	0,8343
Mfsd13a	1,564281331	0,99356
Psat1	1,564172907	0,61405
Rhou	1,56384768	1
Fubp1	1,563522521	0,64076
Ahcy	1,56341415	1
Tbcel	1,563305786	0,77882
Diaph2	1,563197429	1
Rnf145	1,562980739	0,61794
Lsp1	1,562980739	0,79062
Ropn1l	1,562764079	1
Iqsec2	1,562330849	0,80099
Hoxc6	1,561897739	0,94645
Traf3ip1	1,561897739	0,9613
Fam122a	1,56092368	1
Rps12-ps19	1,56092368	1
Tdrd7	1,560707305	1
Atxn2l	1,560599129	0,77438
Ctxn1	1,560599129	1
RP24-183O8.6	1,559950229	1
Samd1	1,559842105	0,85933
Slc30a2	1,559733989	1
Tmem191c	1,559409685	0,76758
Gm16372	1,559301599	1
Pabpc1l	1,558869329	0,87088
Zbtb26	1,55876128	1
Pex13	1,558653238	1

Camta1	1,558545205	0,95174
Crebrf	1,558221148	0,75068
Snapc3	1,558113144	0,94009
Arpc3	1,557465278	0,68661
Tubb5	1,557357326	0,658
Flna	1,556925595	0,86411
Pnp	1,556493983	1
Snrpd3	1,555954637	0,7469
Iffo1	1,555199866	1
Stab1	1,554553211	1
Gm6919	1,554337719	1
Uck1	1,553691423	0,95095
Med31	1,553583733	1
Errfi1	1,553260708	0,98131
Gm7863	1,553260708	1
Nfat5	1,553153048	0,87521
2610318N02Rik	1,553153048	1
Pnn	1,553045395	0,74225
F830208F22Rik	1,553045395	1
Prelid3a	1,55293775	1
Zfat	1,552722482	1
Ppwd1	1,552292036	0,88679
Gm26533	1,552076857	1
Esr1	1,552076857	1
Eif2s3y	1,551754145	0,86846
Utrn	1,551539042	0,8667
Rps15a-ps4	1,551539042	1
Rnf32	1,551431501	1
Ubb	1,551216442	0,81588
3830408C21Rik	1,550893909	1
Tarbp1	1,550786413	1
Plcl2	1,550463969	0,99431
Gltscr1	1,550463969	1
Ric1	1,549819283	0,89508
Maats1os	1,549711862	1
Cacna1d	1,54928225	1
Gm25636	1,548530716	1
Gtdc1	1,548423384	1
1810022K09Rik	1,547564994	0,95723
Rnd1	1,545956794	1
Arl5b	1,545635355	0,94567
Cul7	1,545635355	1
Inca1	1,545635355	1
Dis3	1,545421099	1
Nckap5l	1,545421099	1
Gm43275	1,545421099	1
Lims1	1,545206873	0,94731
Cnnm2	1,545206873	1
Hsf2bp	1,545206873	1
Xrcc2	1,545206873	1
Snx25	1,543922141	1
Dnaaf3	1,543815128	1

Usp18	1,543708123	1
Gna13	1,543387151	0,74225
Ern1	1,543280175	0,90858
Gm8181	1,543280175	1
Birc2	1,543066246	0,95961
9230111E07Rik	1,542959292	1
Pdpk1	1,542852346	0,90533
Dtx3	1,542103931	0,95685
Mmd	1,541783293	0,94645
Slc25a5	1,541355879	1
Eloc	1,541249044	1
Rps15a	1,540714981	0,77761
Zc3h7a	1,54060819	0,87618
1810037I17Rik	1,540394631	1
B230322F03Rik	1,539540692	1
Zscan26	1,539433983	1
Gm7634	1,539220587	1
Gm8213	1,538793883	0,95368
1810010D01Rik	1,538793883	1
Calcrl	1,538687226	1
Scarna2	1,538580576	1
Spink10	1,537407914	0,95288
Clu	1,537194799	0,88825
8430408G22Rik	1,537194799	1
Safb	1,536981713	0,875
Chp1	1,536875181	0,82933
4931440P22Rik	1,536768657	1
Mnd1	1,53655563	0,99228
Ddx19b	1,53581027	1
Mknk1	1,535703819	1
Adi1	1,535278091	0,96425
C77080	1,535278091	1
Mtx3	1,535171677	1
Hpse	1,535065271	1
Gm11694	1,535065271	1
Lpar6	1,534958872	0,97535
Ndufb4	1,534746096	1
Fbxl4	1,534746096	1
A930004J17Rik	1,534214286	1
Kat2a	1,533895288	0,93353
Rps18-ps3	1,53368266	1
Ahrr	1,533576357	1
Ppdpf	1,533257491	1
Ubxn7	1,533044952	0,89721
Ccdc22	1,533044952	0,92606
Gm38335	1,532938693	1
Ppfia3	1,532832441	1
Cd164	1,53261996	0,93309
H2-Q7	1,531982694	0,96895
RP23-354J5.3	1,531557997	1
Gm12174	1,531557997	1
Rps13-ps2	1,530815061	0,92029

Atxn7l2	1,530178545	1
Copg2	1,530072485	0,94449
Gm7236	1,529860386	1
Amhr2	1,529648317	0,99228
Chst1	1,528906308	1
Nampt	1,528800335	0,81095
Phc1	1,528800335	1
Cfap20	1,528694371	1
Fam53c	1,528270585	1
Mphosph9	1,528164657	1
E030030I06Rik	1,528058737	1
Ankrd13a	1,527846918	0,93503
Immp1l	1,527211636	1
Csnk1g2	1,526999934	0,80331
Gm45890	1,526999934	1
Gm13803	1,526576619	1
Adck2	1,526576619	1
Lrp5	1,526576619	1
Cda	1,525941865	1
Hn1l	1,525730339	1
Nf2	1,525518843	0,94725
Dusp4	1,525307376	0,90982
Tmx4	1,525095938	0,78046
Gm15464	1,525095938	0,98528
Gm43499	1,52499023	1
Ift172	1,524778836	0,99756
5031434O11Rik	1,524778836	1
Trappc11	1,5244618	1
Slx1b	1,523933554	1
Rpl27	1,523722306	1
Gpsm1	1,523616694	1
Patl1	1,52340549	0,99768
Gm43430	1,522772055	1
Fam193b	1,522349912	0,97004
Xaf1	1,522349912	1
Apmap	1,521822397	1
Dhcr7	1,521611442	0,97405
Col18a1	1,521505976	0,94472
Mir99ahg	1,521400517	1
Gm10916	1,521295065	1
Cox20-ps	1,52118962	0,95538
Arhgap11a	1,520978753	0,98778
Mta3	1,520978753	1
Camkmt	1,520978753	1
Dstn	1,520873331	0,82074
Kpnb1	1,520873331	0,98242
Zdhhc17	1,520873331	1
Zfp931	1,520873331	1
BC025920	1,520451714	1
Rbm39	1,520346327	0,83441
Pja2	1,520240949	0,90569
Gm9134	1,520240949	1

Fem1c	1,519924856	0,93503
Zfp182	1,519924856	1
Gm12912	1,519292869	1
Tom1l1	1,518661144	0,97437
Ppp1r2	1,518555882	0,99228
Id3	1,51824014	1
Slc6a9	1,518134907	1
Rfwd3	1,517924464	1
Pygl	1,517398482	1
Atg4a-ps	1,517293308	1
Mier1	1,517082981	0,875
M6pr	1,516347066	1
Ehmt2	1,516241965	0,96142
Rab10os	1,516031784	0,92763
Mis18a	1,516031784	1
Gm10335	1,515926704	1
Gon7	1,515821632	0,9534
Gnai3	1,515716567	1
Tmem41b	1,515716567	1
Gm4997	1,515506458	0,90982
Ctns	1,515506458	1
Cpsf1	1,51519135	1
Ptbp1	1,514561331	0,88753
C330007P06Rik	1,514561331	1
Brpf3	1,514561331	1
Kdm4c	1,514141463	1
Pdlim7	1,513931573	1
Pik3r6	1,513616793	1
Slc7a7	1,512043872	1
Nod2	1,511834273	1
Arglu1	1,511519928	0,91347
Prc1	1,511519928	0,94645
Hdgfrp2	1,511519928	0,97525
Lamb2	1,511310402	1
N4bp1	1,510891436	0,87088
Lipe	1,510786712	1
2610528A11Rik	1,510786712	1
Sema4g	1,510681996	0,99022
Pcnp	1,510577287	1
Gm11613	1,510577287	1
Gm7899	1,510263204	1
Hcar2	1,510263204	1
Rps15a-ps7	1,510158524	1
Stt3a	1,509321347	1
Mybl2	1,509321347	1
Gm43756	1,509112125	1
Chd6	1,508902932	0,97107
Pmpcb	1,508902932	1
Gm38220	1,508902932	1
Prkar2a	1,508693768	0,96683
Smg5	1,508589197	1
Taf15	1,508380077	0,96489

Cd47	1,508170985	0,89243
Foxo3	1,508170985	0,9208
Hmgb2	1,507961923	1
Rpsa	1,507857403	0,9534
Adh7	1,506812598	1
Ccne2	1,506708157	1
Vrk1	1,506603723	1
Gm6457	1,506290467	1
Gm7856	1,506186062	1
Scpep1	1,505977275	0,95549
Jade3	1,505977275	1
9330160F10Rik	1,505768517	1
Gm26514	1,505559787	1
Jak2	1,505142415	0,99768
Fgf11	1,505142415	1
Klf10	1,50503809	1
Gm38200	1,504933773	1
Stac2	1,504829462	1
Inpp5f	1,504829462	1
Iqcf1	1,504203751	1
Gemin4	1,503786755	1
Gm12833	1,503786755	1
Zcchc11	1,503578301	0,97103
Ilf2	1,50295311	1
Als2cl	1,502848937	1
Caprin1	1,502744771	0,89508
Cdc42ep4	1,502640612	1
Slc35a1	1,502640612	1
Meis3	1,502640612	1
Gm11989	1,502640612	1
Selenof	1,502015812	1
Rngtt	1,501807602	1
Spcs3	1,501599422	1
Osbpl1a	1,501391271	1
Cep83os	1,501391271	1
A630001G21Rik	1,501079098	1
Eno2	1,500975054	1
Zfp72	1,500975054	1
Rev1	1,500246953	1
Zkscan8	1,500142968	1
Gramd1a	1,499831054	0,93563
Ccdc50	1,499623148	0,95685
Dhx40	1,499623148	1
Gm42979	1,499519205	1
Hmcn2	1,499207422	1
Bbc3	1,498791811	1
Gls	1,498376315	0,94571
Gm10941	1,498272459	1
Smarcc2	1,497960934	1
Gm5687	1,49733808	0,9968
Wee1	1,497234296	1
Tmem168	1,496300563	1

Rapgef5	1,496093147	1
Gm5445	1,495263766	1
Gpi1	1,494538436	1
Cyth3	1,494434846	1
Edrf1	1,494227688	1
Gm2214	1,494227688	1
Pyurf	1,493813457	1
Gm43571	1,493606385	1
Cdc6	1,493399341	1
Sntb2	1,493295831	1
Tkt	1,493192327	1
Gm8121	1,492571455	1
Fam229b	1,491950841	1
Uxs1	1,49164063	1
2310047D07Rik	1,49164063	1
Cpeb2	1,491330485	1
Gm6564	1,491330485	1
Rassf1	1,491227117	1
Dusp10	1,491227117	1
Ttc39b	1,491123757	1
Gm14541	1,490503745	1
Slc2a4	1,490193835	1
Snhg4	1,489987265	1
Cox16	1,489987265	1
Pcgf6	1,489883991	1
Kirrel3	1,489780723	1
Ccdc50-ps	1,489677463	1
Zdhhc8	1,489677463	1
Kctd6	1,489367726	1
Rfc4	1,489367726	1
Gm10093	1,489264494	1
Clk1	1,489058052	0,98778
Rnpep	1,489058052	0,99336
2900093K20Rik	1,489058052	1
Scd1	1,488748444	0,99707
Gm9442	1,488748444	1
Spata5	1,488232572	1
Gm42869	1,487820004	1
Tpmt	1,487820004	1
Gm44126	1,48771688	1
Ccdc15	1,48740755	1
Bend3	1,48740755	1
9930111J21Rik2	1,48740755	1
Ift57	1,48699521	1
Tbx6	1,48699521	1
Gm4540	1,486376915	1
Lrrc41	1,486376915	1
2310035C23Rik	1,486067863	1
Rbm48	1,48596486	1
Gm14239	1,485758876	1
Dcp2	1,485655895	1
Nusap1	1,485655895	1

Gm45749	1,485655895	1
Ftsj1	1,485449954	1
Mospd1	1,485346994	1
Slc30a5	1,484935225	1
A930007I19Rik	1,484935225	1
B2m	1,484626474	1
Zfp54	1,484626474	1
Gm44027	1,483906303	1
Rpl5	1,483597765	1
Abca7	1,483494934	1
Pdxk	1,483186482	1
Gm10923	1,483186482	1
Gm10784	1,483186482	1
RP23-356P21.1	1,483083679	1
Txndc11	1,482878094	1
Etfa	1,482775312	1
Nup93	1,482672538	1
Slc7a8	1,48256977	1
Noc3l	1,482364257	1
Efcab2	1,48205604	1
Gm37060	1,481645183	1
Gm13050	1,481542487	1
Zfp609	1,481234441	1
Gm26497	1,481029113	1
A330035P11Rik	1,481029113	1
Rpl30-ps5	1,480721173	1
Rpl22l1	1,480618541	0,96651
Tmem134	1,480618541	0,98026
Zfand1	1,480310687	1
Hnrnpd	1,478464907	0,99697
Alg6	1,478464907	1
Spire1	1,478259963	1
Mrps36	1,477952599	1
Ino80d	1,477747725	1
Fam172a	1,477747725	1
Khdrbs1	1,47754288	1
Lgals9	1,47754288	1
Hist1h2bc	1,476212079	1
Rnf144b	1,476212079	1
Gm4374	1,476212079	1
Cysltr1	1,476212079	1
H2-Ab1	1,475905141	1
Fancb	1,475700551	1
Depdc1b	1,475598267	1
Cbx4	1,47549599	1
RP24-275P22.2	1,47539372	1
Gm6266	1,475291457	1
Kpna3	1,475189201	1
Abhd8	1,475189201	1
Fam46a	1,475086952	1
Pcx	1,475086952	1
Smim7	1,474780249	1

2610203C22Rik	1,474780249	1
Wfdc17	1,474575815	1
Dusp11	1,474473609	0,99356
Clic4	1,474371409	1
Fgfr1op	1,473554071	1
Mettl10	1,473247686	1
Mir7078	1,473043464	1
Rpl18a-ps1	1,472737186	1
Hnrnpdl	1,472635107	1
Vcpkmt	1,472328913	1
Gm43300	1,472226862	1
Glrp1	1,471920753	1
1810041H14Rik	1,471716716	1
Gm16630	1,471308726	1
Gm37303	1,471308726	1
1700020I14Rik	1,471206746	1
Skiv2l2	1,471206746	1
Gm44237	1,471206746	1
Crcp	1,470900849	1
Ldlrad3	1,470900849	1
B930036N10Rik	1,470900849	1
Prune2	1,470595015	1
Gm4673	1,470595015	1
Bcl6b	1,470595015	1
Sgk1	1,470493085	1
Mlkl	1,470493085	1
Fkbp7	1,470085434	1
Mcm4	1,470085434	1
Gm15720	1,46977977	1
Vsig8	1,46977977	1
C030013C21Rik	1,469677897	1
Cpeb4	1,46957603	1
Incenp	1,46947417	1
Sugp2	1,469270472	1
Osbp	1,468964977	1
Cfh	1,46886316	1
Ccl3	1,46876135	1
Setd1a	1,468659546	1
Olfr920	1,468659546	1
Zfp948	1,468659546	1
Per1	1,467743635	1
Sh3bp1	1,467641902	1
Lrwd1	1,467540176	1
Ap2a2	1,467336746	1
Srsf6	1,467336746	1
Tex30	1,467336746	1
Pex2	1,467133344	1
Gm14769	1,467031654	1
Gm42466	1,466726625	1
C630004M23Rik	1,466624963	1
Plk4	1,466218384	1
Ints8	1,466015137	1

2610020H08Rik	1,465913524	1
Gm16973	1,465811918	1
Galnt10	1,465303995	1
Mpp3	1,464796247	1
Rdm1	1,464694719	1
Vrk2	1,464390176	1
Trem3	1,464288675	1
Pfdn5	1,464187182	1
Paics	1,464085696	1
Gm9835	1,463882745	1
Armc2	1,463375489	1
Htt	1,46327406	1
Rpe	1,463172637	1
Borcs7	1,462969812	1
Mtpn	1,462767015	1
Naip5	1,462767015	1
Gm38111	1,462665627	1
Ccnl2	1,462158794	1
Gm6159	1,462057448	1
Atxn1l	1,462057448	1
Tm2d1	1,462057448	1
Cbx2	1,461854778	1
0610030E20Rik	1,461550826	1
Snrpa	1,461348226	1
Gm5525	1,461246936	1
AI846148	1,461145654	1
Ercc6l	1,460538107	1
Gm16754	1,460234428	1
Kif14	1,460234428	1
A730071L15Rik	1,460032011	1
Wwp1	1,459829621	1
Fam117b	1,459829621	1
Ube2n	1,45962726	1
H2-K2	1,459323771	1
Zfp385a	1,459222621	1
Ppid	1,459121479	1
Ube2c	1,459020344	1
Gm42484	1,458919216	1
Mki67	1,458514774	1
Arhgap23	1,458413681	1
A830008E24Rik	1,458312595	1
BC028528	1,458110444	1
Actr1a	1,458009379	1
Akt3	1,457908321	1
Poc5	1,457908321	1
Hps3	1,457605189	1
Kntc1	1,457605189	1
Rpl21-ps12	1,45730212	1
A530013C23Rik	1,457201111	1
Gm26799	1,456898126	1
Tmem183a	1,456292345	1
Ormdl3	1,456191406	1

Macrod2	1,455585919	1
Aldh2	1,455485029	1
Nr3c1	1,455485029	1
Pkd1l2	1,455384146	1
Zfp712	1,45528327	1
Gm13758	1,454879836	1
Hist1h2al	1,45467816	1
Helz2	1,453871739	1
Gm12758	1,453770968	1
Actr2	1,453569446	1
Lin54	1,453468696	1
Prpf3	1,453267217	1
Ascc3	1,453267217	1
Ypel5	1,453166487	1
Zfp652os	1,453166487	1
Ets2	1,453065765	1
Tle4	1,45276364	1
Apaf1	1,45276364	1
Tmem81	1,452461578	1
Ccdc85b	1,452461578	1
Glrx3	1,452159578	1
Rpl14	1,451857641	1
Ppfia4	1,450952208	1
Gng5	1,450751077	1
Dus4l	1,450248372	1
Gm14094	1,450248372	1
Tfe3	1,450147852	1
Zfp212	1,450147852	1
Rps19-ps3	1,449343942	1
Zfp174	1,449243485	1
Hsp90ab1	1,448741302	1
Cd37	1,448640887	1
Fbxl19	1,448640887	1
Dcp1a	1,448540478	1
Rcbtb1	1,448339682	1
Zfp932	1,448239294	1
Gm12346	1,447938172	1
Arhgap5	1,447737459	1
Atf1	1,447737459	1
Rasgef1b	1,446634036	1
Gnpnat1	1,445631653	1
Btk	1,445531453	1
Pim3	1,445331073	1
Arntl	1,444730102	1
BC051226	1,444730102	1
Gm16020	1,444629964	1
Arfip1	1,444229483	1
Cdc42	1,44412938	1
4933427D14Rik	1,44412938	1
Swap70	1,444029285	1
Pcna-ps2	1,443929196	1
Supt3	1,443829113	1

Xiap	1,443829113	1
Ethe1	1,443829113	1
Bloc1s1	1,44362897	1
9330111N05Rik	1,443228767	1
Cygb	1,442928687	1
Slc31a2	1,442428693	1
Abcg1	1,441928871	1
Gins3	1,441728992	1
Gsg1	1,441728992	1
Trim3	1,441728992	1
Syt11	1,441529139	1
Fastkd1	1,441429224	1
Hspbp1	1,441229413	1
Slc25a38	1,440929749	1
Dna2	1,440730007	1
Lcorl	1,440630147	1
Slc39a14	1,440630147	1
Klhl41	1,440630147	1
Adamts6	1,440630147	1
Cep164	1,440330608	1
Gatsl2	1,440230775	1
Gm15950	1,440230775	1
Sh3bgrl	1,44003113	1
Ap1ar	1,44003113	1
Gm37589	1,439931319	1
Rnf19a	1,439831514	1
Smagp	1,439432363	1
Gm45407	1,439332592	1
Grina	1,439133072	1
Uhrf2	1,439033323	1
Cnot4	1,43893358	1
Rpl36-ps8	1,438734115	1
Sf3b4	1,438634393	1
Fbxl20	1,438534678	1
Arl6ip6	1,438534678	1
Gm15800	1,438534678	1
Fbxo8	1,437936533	1
Neat1	1,437836866	1
Pttg1	1,437637553	1
Gm6493	1,437438268	1
Mettl7a1	1,437139393	1
Mthfd2	1,437039781	1
Mir142hg	1,437039781	1
Phlda1	1,436940177	1
Eif2ak3	1,436442257	1
Armc8	1,436044046	1
Dnal1	1,435944511	1
Terf2ip	1,43574546	1
Letm2	1,43574546	1
Ctnnd1	1,43574546	1
Ptpn9	1,435645945	1
Gm43213	1,435546437	1

Higd2a	1,435446936	1
Snx12	1,435446936	1
Bcl6	1,435446936	1
Tjp3	1,435446936	1
Abt1	1,435347442	1
Thg1l	1,435247955	1
Lactb2	1,435049001	1
Dmxl1	1,434750622	1
P4ha1	1,434551737	1
Ckap5	1,434452305	1
Snx2	1,434452305	1
2310009B15Rik	1,434253462	1
Gm27477	1,434253462	1
Adpgk	1,43415405	1
Gm3724	1,43415405	1
Spc24	1,433955248	1
Glg1	1,433855857	1
Gm10012	1,433557726	1
Zfpm1	1,433359007	1
Utp14b	1,433259657	1
Polg2	1,433060979	1
Gm24336	1,43296165	1
Eml2	1,432564404	1
Vezt	1,432365822	1
Gm10263	1,432266542	1
Abi2	1,432167268	1
Scand1	1,431770242	1
Ifitm2	1,431671003	1
Toporsos	1,43157177	1
Gm6382	1,431373326	1
Tbc1d19	1,431274114	1
Rpl29	1,43097652	1
Pmp22	1,430778158	1
Gm14439	1,430678988	1
Jmjd1c	1,430183238	1
Gna11	1,430084109	1
Slc25a23	1,429588565	1
mt-Nd5	1,429192254	1
Gm17827	1,429192254	1
Traf4	1,428498974	1
E130317F20Rik	1,428498974	1
Galnt6	1,428399961	1
Snx27	1,428399961	1
Pgls	1,428201957	1
Ybx1	1,428201957	1
Gm8618	1,428102965	1
Nup35	1,42800398	1
Rhoq	1,427905001	1
Rpl10	1,42780603	1
Anxa9	1,426915596	1
Ccdc25	1,426816693	1
Fkbp14	1,426816693	1

Gm7206	1,426816693	1
Rheb	1,426618908	1
Plcg2	1,42622342	1
Arrdc1	1,426124565	1
Pgm3	1,426124565	1
Nipsnap3b	1,425926876	1
Bach1	1,425828042	1
Rpl37	1,425729214	1
Gas6	1,425136394	1
Cdca5	1,425136394	1
Fam98c	1,424741317	1
Cyth1	1,424642565	1
Fer	1,424543819	1
Psca	1,424543819	1
Tlcd1	1,424148907	1
Efhd2	1,423951491	1
Fn1	1,423951491	1
Hacd4	1,423852794	1
Ndrg3	1,423852794	1
Gm10689	1,423458073	1
Nlrc3	1,423458073	1
Hdac5	1,42335941	1
Cox6c	1,422866196	1
Gabpa	1,42257035	1
Ascc1	1,422077411	1
Gm7799	1,421683182	1
Acin1	1,421584642	1
Igf2r	1,421486108	1
Rbm33	1,421289062	1
Aatk	1,421289062	1
Mbtd1	1,421092043	1
Gm4617	1,420107359	1
Anpep	1,420008928	1
Hsph1	1,419713676	1
Unc93b1	1,419713676	1
Stip1	1,419615272	1
Cep120	1,419418485	1
Fam134c	1,419418485	1
Prpf40b	1,419221726	1
Zfand5	1,419123356	1
Seh1l	1,419024993	1
Rab28	1,418926638	1
Dock2	1,418631611	1
Zbtb37	1,418631611	1
Lrrc28	1,41843496	1
Nrm	1,41755037	1
Manf	1,417452117	1
RP23-268C22.3	1,417452117	1
Ccdc62	1,41725563	1
Prkcsh	1,417157397	1
Irak3	1,41705917	1
Mark4	1,416862738	1

Fto	1,416666332	1
Zgpat	1,416469954	1
C87436	1,416175438	1
Itgb1	1,415979128	1
Stbd1	1,415880983	1
Hsf1	1,415782845	1
Pitpnc1	1,41558659	1
Cnst	1,41558659	1
Ogfr	1,415488472	1
Cenpu	1,415488472	1
Dmxl2	1,415292258	1
Etv1	1,41519416	1
Hprt	1,414997986	1
Nacc1	1,414997986	1
Adat1	1,41489991	1
Cbx6	1,414703776	1
Gm9165	1,41450767	1
Ctdp1	1,41362553	1
Cdkn1b	1,413331605	1
Brms1l	1,413331605	1
Anapc16	1,413233644	1
Tarsl2	1,413135689	1
Dedd2	1,412939801	1
Atpaf2	1,412743939	1
Lpcat2	1,412548105	1
Lpcat1	1,412352298	1
Pcm1	1,412254404	1
Shprh	1,412058638	1
Foxk1	1,412058638	1
Rnf13	1,412058638	1
Gm10636	1,412058638	1
Mad2l2	1,411471501	1
Osgepl1	1,411373669	1
Pcmtd1	1,411275843	1
Mcm6	1,411080213	1
Thop1	1,410884609	1
Yme1l1	1,410689032	1
Spag5	1,410591254	1
Lpp	1,410493483	1
Stil	1,41029796	1
Ckb	1,410004728	1
Eps15l1	1,409613846	1
Gm45902	1,409516142	1
Mta1	1,409418445	1
Smc5	1,409320755	1
Atp7a	1,409027726	1
Pla2g15	1,409027726	1
Gm7561	1,408734757	1
Prox2	1,408734757	1
Cnot6l	1,408539478	1
Hnrnpu	1,408539478	1
Fam92a	1,408441849	1

Casp4	1,408344227	1
Snap47	1,408246611	1
Ccnt2	1,407953805	1
Fzd2	1,407563492	1
Ctsa	1,40746593	1
E4f1	1,407368375	1
Vamp7	1,407270827	1
Accsl	1,407270827	1
Gm12799	1,407270827	1
Ak4	1,407173286	1
Oxr1	1,406880703	1
Ankrd13d	1,406685682	1
Rps27l	1,406588181	1
Gm12577	1,406100779	1
Poln	1,406100779	1
Pnpla6	1,405905866	1
Gm13573	1,405613546	1
1700034H15Rik	1,40551612	1
Rtkn	1,405223881	1
Eaf1	1,405223881	1
Desi2	1,405223881	1
Edc3	1,404639587	1
Adamts10	1,404542228	1
4930427A07Rik	1,404542228	1
Gm12583	1,404444876	1
Mrps33	1,404250192	1
Rbm47	1,404250192	1
Atf4	1,403958217	1
Gm37788	1,403958217	1
Klhl15	1,403569011	1
Mtmr11	1,403277177	1
Caap1	1,402985404	1
Sestd1	1,40288816	1
Ccnb2	1,402499251	1
Huwe1	1,402499251	1
Rnf26	1,40240204	1
Btf3l4	1,402110449	1
Rictor	1,402110449	1
Fzd5	1,401916089	1
Slc20a1	1,401916089	1
Ano7	1,401818919	1
Fxyd2	1,401430306	1
Gm26826	1,401138917	1
Gm6946	1,400944691	1
Polb	1,400750493	1
Gm37490	1,400750493	1
Foxm1	1,400653403	1
Zfp692	1,400556321	1
Cd40	1,400265114	1
Chd3os	1,399973967	1
Tnrc18	1,399973967	1
Dst	1,399876932	1

Tet3	1,399779903	1
Rps25	1,399488857	1
Emc10	1,39929486	1
Helb	1,39910089	1
Gm12454	1,398906947	1
Mb21d1	1,397259516	1
Zbtb33	1,396581725	1
Uba6	1,396194564	1
Polh	1,395710764	1
Sf3a1	1,395614024	1
Slc17a9	1,394743666	1
Micu3	1,394550327	1
Secisbp2	1,394453668	1
Ppp2r3d	1,394453668	1
Lin9	1,394357015	1
Atf6b	1,394163729	1
Med20	1,393970471	1
Sesn1	1,393777239	1
Ptma	1,393777239	1
Acad9	1,393584034	1
Rbfox2	1,393584034	1
Chuk	1,392714942	1
Rpl31-ps1	1,392521884	1
Ssfa2	1,392521884	1
Ncaph	1,392425365	1
Ifitm3	1,392232347	1
Zyx	1,391460544	1
Gtf2h4	1,391074802	1
Prkacb	1,390978384	1
Snx18	1,390689168	1
Slc9a9	1,390592776	1
Nfkb1	1,390110917	1
Cir1	1,390014565	1
Gm561	1,389147699	1
Phpt1	1,389147699	1
Kpna1	1,389051414	1
Rcbtb2	1,388858864	1
1700056N10Rik	1,388858864	1
Mrpl4	1,388570089	1
Slc25a17	1,388570089	1
Nmral1	1,388185149	1
Gm38036	1,388185149	1
Trrap	1,387896514	1
Gm7984	1,387896514	1
Yipf4	1,387319424	1
Slc25a3	1,387319424	1
Fam179b	1,387030969	1
Acot9	1,386742574	1
Plxnb3	1,386742574	1
Timm10b	1,386646456	1
Gm43920	1,386165964	1
Gm42508	1,385973814	1

Moap1	1,385589594	1
Mbnl2	1,385109468	1
Smarcal1	1,385013463	1
Clasp1	1,385013463	1
Abhd2	1,385013463	1
Zfp397	1,384821473	1
Dnajb9	1,384341613	1
2810403D21Rik	1,384245661	1
Vil1	1,383957845	1
Agrn	1,383766001	1
Igsf6	1,383670089	1
9330162G02Rik	1,383574184	1
Klhl24	1,383286508	1
Tmem165	1,382998891	1
Fam131a	1,382615496	1
9230102O04Rik	1,382423838	1
Hnrnpa2b1	1,381944809	1
Golga1	1,381849024	1
Prcp	1,381849024	1
Atp6v1h	1,381753244	1
Iscu	1,381657472	1
Zfp949	1,381465947	1
Smtn	1,381274448	1
Got1	1,381274448	1
Gpr137	1,381082976	1
Isca1	1,380795818	1
Got2	1,380604412	1
Mgarp	1,380413033	1
Plxnc1	1,38022168	1
Gm6768	1,38022168	1
Gm15834	1,380126014	1
Calr	1,380030354	1
Irf1	1,379360922	1
Dhdh	1,379074122	1
Nono	1,378882955	1
Tsga10ip	1,378787381	1
RP24-370M23.1	1,378691814	1
Pou2f1	1,3785007	1
Kctd10	1,378309613	1
Xrcc4	1,378214079	1
Pts	1,378118551	1
Tmem243	1,377927517	1
Lsm11	1,377927517	1
Scrib	1,37783201	1
Rnf2	1,37783201	1
Cdk2ap2	1,377641015	1
Rock2	1,377545527	1
Abhd4	1,377259104	1
Ilf3	1,377163643	1
4933421O10Rik	1,377068189	1
Rpl37a	1,377068189	1
Gm13889	1,376972741	1

Map3k9	1,3768773	1
Paip1	1,3768773	1
Ifitm6	1,3768773	1
Agmo	1,376686438	1
Il6ra	1,376591016	1
Mettl16	1,376591016	1
Eif4a2	1,376400193	1
Flcn	1,376018627	1
Gltp	1,376018627	1
Mcf2l	1,375255812	1
Rpl35a	1,375065174	1
Ift52	1,374969865	1
Gm13340	1,374874563	1
Prpf39	1,374779267	1
Erlin1	1,374683978	1
Spin1	1,374588696	1
Kdm3b	1,374588696	1
Lsm14a	1,37449342	1
Capn15	1,374112382	1
Nr1d1	1,374112382	1
Rmnd1	1,373826673	1
Rrh	1,37344582	1
Grn	1,37316025	1
Map2k3os	1,373065073	1
Cdkl3	1,372969903	1
Dcaf7	1,372969903	1
Prdm15	1,372874739	1
Igf1	1,372874739	1
Dnpep	1,372874739	1
Hmces	1,372303895	1
Stag1	1,372208778	1
Gtf2h5	1,372018562	1
Gm8116	1,371733289	1
Gm5835	1,371162919	1
Cdo1	1,370972849	1
Hinfp	1,370877824	1
Pcif1	1,370782805	1
Prickle3	1,370022892	1
Pfkfb2	1,370022892	1
Churc1	1,370022892	1
Gm43362	1,369263401	1
Ano8	1,3689787	1
Usp32	1,368883813	1
Pdlim5	1,368788933	1
Serp1	1,368504331	1
1190002N15Rik	1,368219788	1
Dtymk	1,368124953	1
Agps	1,368124953	1
Il16	1,367840489	1
Crocc	1,367840489	1
Zscan2	1,367840489	1
Rps7	1,367745681	1

Map2k7	1,367745681	1
Cry2	1,367650879	1
Sh3bp2	1,367650879	1
Otud3	1,367556084	1
Akap8l	1,367556084	1
Acta2	1,367556084	1
Arv1	1,367366514	1
H2-Q6	1,367271739	1
Vezf1	1,36717697	1
Cracr2b	1,366987452	1
Chek2	1,366608495	1
Frk	1,366608495	1
1700031P21Rik	1,366513772	1
Bivm	1,365850896	1
Idh3a	1,365850896	1
Ube2cbp	1,365850896	1
Gm45884	1,365566905	1
Wdr82	1,365282973	1
Nisch	1,365188342	1
Tceal9	1,364715286	1
Ncor2	1,364526109	1
Arhgap39	1,364431531	1
Cbl	1,364431531	1
Arhgdia	1,364053283	1
Maged1	1,363769665	1
Rnf6	1,363769665	1
9930022D16Rik	1,363675139	1
Gm6543	1,363675139	1
Tecr	1,363486107	1
Pag1	1,3633916	1
Gga1	1,3633916	1
Ppp2r1a	1,363108121	1
Gng8	1,36301364	1
Rbm12b2	1,362919167	1
Nbas	1,3628247	1
RP23-402A24.3	1,3628247	1
Tm6sf1	1,362446897	1
Mis18bp1	1,362258035	1
Stard10	1,362163613	1
Rpsa-ps11	1,361785994	1
Mgat4a	1,361691605	1
Tmem50b	1,361691605	1
Spa17	1,361597223	1
Ap3d1	1,361597223	1
Knl1	1,361219761	1
Tube1	1,361219761	1
Vegfb	1,361219761	1
Atg4a	1,361031069	1
Crls1	1,361031069	1
Qpctl	1,360936732	1
Gm17690	1,360936732	1
Smpd4	1,360936732	1

Thbs3	1,360842403	1
Wdr37	1,360842403	1
Pcnt	1,36074808	1
Arhgef25	1,360653763	1
Ctsf	1,360653763	1
Fam26f	1,360465149	1
Rps11-ps4	1,360465149	1
Ppa1	1,360465149	1
Eif2d	1,360276562	1
Gm8276	1,360088001	1
Zfp382	1,359805208	1
Nufip1	1,359522474	1
Polr1e	1,359428242	1
Sun2	1,359239798	1
Mpzl1	1,359145586	1
Aurka	1,359145586	1
Coq2	1,358674624	1
Cenpc1	1,358015551	1
9230116N13Rik	1,358015551	1
Fbxl5	1,357827303	1
Qtrt1	1,357733189	1
Pex5	1,357450885	1
Dpm3	1,357356797	1
Gm9531	1,357074572	1
Zfp871	1,35698051	1
Klhl28	1,356792406	1
Znrf1	1,356228249	1
Drap1	1,356228249	1
Atxn2	1,356134246	1
Gm20517	1,355946259	1
Phc3	1,355946259	1
Trnt1	1,355664327	1
Nomo1	1,355664327	1
Pop4	1,355570363	1
Ttc39a	1,355288509	1
Tgoln1	1,355194571	1
Eif4h	1,354724977	1
Myl12a	1,354537186	1
Ero1l	1,354537186	1
Cops2	1,354537186	1
RP23-134M7.3	1,354255547	1
Arl5a	1,353692445	1
Trpm7	1,353504796	1
Chrnb1	1,353410982	1
Bhlhe41	1,353410982	1
Trappc8	1,353317174	1
Lss	1,353223372	1
4930402H24Rik	1,352754462	1
Gpbp1l1	1,352754462	1
Cep76	1,352566943	1
Paqr3	1,352191984	1
Bbs2	1,351723431	1

Dock7	1,351723431	1
Mipol1	1,35162974	1
Noxo1	1,35162974	1
AI597479	1,35162974	1
Ypel1	1,35162974	1
Alox5ap	1,35162974	1
Mfge8	1,35162974	1
Borcs8	1,351536056	1
Pkn2	1,351442378	1
Ap1s2	1,351161382	1
Exoc6b	1,35106773	1
Asah1	1,350225153	1
Gm5257	1,350131566	1
Lamp2	1,349944411	1
Gm11687	1,349850843	1
Tmem216	1,349757282	1
Dnajc15	1,349663727	1
Rnd2	1,349570179	1
Rnf126	1,34919605	1
Hdac11	1,349009025	1
Nop10	1,349009025	1
Jmjd8	1,348822026	1
Srcap	1,348728536	1
Rel	1,348635052	1
Eif1b	1,348635052	1
Sash1	1,348167732	1
Ttc5	1,347513755	1
Tmem248	1,347513755	1
Mettl23	1,347420356	1
Rpl9	1,347326963	1
Rpl17	1,347046824	1
Xpo1	1,346860096	1
Crnkl1	1,346673395	1
Cd200r4	1,346580054	1
Gfpt1	1,346486719	1
Cacybp	1,346300069	1
Rpl11	1,346020143	1
Ankrd16	1,345740275	1
Car6	1,345740275	1
Peak1os	1,345646999	1
Gm11977	1,345553729	1
Msrb1	1,345553729	1
Gm10343	1,345460466	1
Slc7a5	1,345367209	1
Stk11	1,344807802	1
Samd4b	1,344714591	1
Strbp	1,344621385	1
Gm43578	1,344528187	1
Slc25a26	1,344155456	1
Etv3	1,344155456	1
Adk	1,344062289	1
Ext2	1,343969129	1

Sbf2	1,343969129	1
Arl2	1,343969129	1
Nudt19	1,343782828	1
Gm10658	1,343689688	1
Zfyve16	1,343503426	1
Gm20257	1,34331719	1
Mtmr1	1,343130979	1
Prkcd	1,343037884	1
Mboat7	1,342851712	1
Cyp2c55	1,342014259	1
Cmip	1,342014259	1
Ogfod3	1,341921241	1
Spty2d1	1,341921241	1
Gm19503	1,341921241	1
Fam63b	1,341921241	1
Foxj2	1,341828229	1
Cs	1,341735224	1
Rps4x-ps	1,341642225	1
Arl6ip5	1,341084368	1
Pias4	1,340991414	1
Klhl6	1,340898467	1
Aph1b	1,340805526	1
Gm43147	1,340433827	1
Mrpl58	1,340340919	1
Med15	1,340155121	1
Ppp1r13l	1,339876472	1
Dip2b	1,339690739	1
Rras	1,339690739	1
Pitpnm1	1,339597882	1
Bscl2	1,339597882	1
Gm11604	1,339505031	1
Stard9	1,339226518	1
Pgap2	1,339226518	1
Acadm	1,339040875	1
Mynn	1,338948063	1
Wdr54	1,338855257	1
Vwa5a	1,338855257	1
Iqcg	1,338762458	1
Oas2	1,338669665	1
Gm6472	1,338576879	1
Tmem150a	1,338576879	1
Pros1	1,338576879	1
2210408F21Rik	1,338391326	1
Hmgb1-rs16	1,338391326	1
Spc25	1,338205798	1
Agl	1,338113044	1
Dusp3	1,337927555	1
Gm5093	1,33783482	1
Acd	1,33783482	1
Ccser2	1,33783482	1
Gm15753	1,337742091	1
Gm20667	1,337742091	1

Ints9	1,337649369	1
Tcte2	1,337371242	1
Rpph1	1,337371242	1
Gm15159	1,336907825	1
Gm16104	1,336722503	1
Plekhg4	1,336629852	1
Cnksr3	1,336537207	1
Ndufc2	1,336537207	1
Gm15564	1,336351936	1
B330016D10Rik	1,336351936	1
Gm20072	1,336351936	1
Gm16425	1,33625931	1
Bcl10	1,336166691	1
Cdk2	1,336166691	1
1500011K16Rik	1,336074078	1
Nup43	1,335796278	1
Kat8	1,335796278	1
Plekhn1	1,335796278	1
Bmpr1a	1,335703691	1
E230020A03Rik	1,335703691	1
Mgst3	1,335611111	1
St13	1,335518536	1
Fnbp1l	1,335425968	1
Stam	1,335055761	1
Abcb7	1,335055761	1
Tubgcp4	1,334778173	1
Rpl30-ps1	1,334593146	1
Pold1	1,334593146	1
Rangrf	1,33422317	1
Gm1862	1,334130692	1
Tmem128	1,334130692	1
Stx7	1,334130692	1
Arhgap27os2	1,333945755	1
Anxa5	1,333945755	1
Rpl3	1,333853296	1
Slc16a1	1,333575958	1
Gm43411	1,333298677	1
Gm13680	1,333206263	1
Tsc1	1,333113855	1
Gm5845	1,333021454	1
Pianp	1,332929059	1
Pds5a	1,332744289	1
Gm16379	1,332559544	1
Dleu2	1,332374825	1
6030400A10Rik	1,332005464	1
Gm17251	1,331636204	1
Grasp	1,331636204	1
Slc22a5	1,331543906	1
Gm43533	1,331543906	1
E2f2	1,331543906	1
Selenoi	1,330805746	1
Gm2383	1,330713504	1

Bak1	1,330713504	1
Cox7a2l	1,330529041	1
2310022B05Rik	1,330067995	1
Adh5	1,329975805	1
Slc37a1	1,329699273	1
Prrg4	1,329607108	1
Capn1	1,329146381	1
Gm5787	1,329146381	1
Mgea5	1,329146381	1
Cox20	1,328962135	1
Necap1	1,328870022	1
Bckdha	1,328777915	1
Tecpr1	1,32859372	1
Atp13a1	1,328501632	1
Pqbp1	1,328317475	1
Bet1	1,328041288	1
Scarb1	1,32730507	1
Adat2	1,327213071	1
Ctc1	1,327121079	1
Brcc3	1,326753174	1
Slc2a8	1,32656926	1
Tmed10	1,326477312	1
Mllt3	1,326477312	1
Mbd2	1,326385371	1
Usp45	1,326201507	1
Cstf2	1,326109585	1
St7	1,32601767	1
P3h3	1,325833857	1
Atp6v0a1	1,325833857	1
H1fx	1,32565007	1
Ttc12	1,32565007	1
Wdr48	1,32565007	1
Pfdn4	1,325466309	1
Atp8b2	1,325282573	1
Aldh3a2	1,325098863	1
Gmds	1,325098863	1
Tollip	1,325098863	1
Rmi1	1,325007017	1
Chchd6	1,324823345	1
Anxa1	1,324731518	1
Abca3	1,324731518	1
Papolg	1,324547884	1
Clspn	1,323721846	1
Aldh1l1	1,323721846	1
Sp100	1,323446614	1
Usp15	1,323354883	1
Sigirr	1,323079728	1
4933408B17Rik	1,322988022	1
Nck1	1,322896323	1
Ncstn	1,32234626	1
Casp6	1,322254605	1
Gm36266	1,322162957	1

Ice2	1,322162957	1
Sqle	1,322162957	1
Bcas3	1,321979679	1
Fahd1	1,321979679	1
Itgav	1,321704809	1
Ift27	1,321521595	1
Atl3	1,321429997	1
St3gal1	1,321429997	1
Naa15	1,321429997	1
Crlf3	1,321338406	1
Gca	1,321246821	1
Gm37578	1,321246821	1
Gm35106	1,321155242	1
Mycn	1,321155242	1
Dact3	1,32106367	1
Dctn1	1,320880544	1
Ubn2	1,320880544	1
Cstf3	1,320697444	1
Gm17491	1,320422841	1
Paip2	1,320422841	1
Bicd2	1,320331319	1
Ola1	1,320239804	1
Emc2	1,319965297	1
Gm9385	1,319965297	1
Pigu	1,319690846	1
Pcf11	1,319599375	1
Egln1	1,319142116	1
RP24-282C4.9	1,318867837	1
Aco1	1,318867837	1
Arl16	1,318593614	1
Hk1os	1,318410831	1
Rps27a	1,318319449	1
Vps37d	1,318319449	1
Ddx20	1,318228073	1
Aldoa	1,318228073	1
Gchfr	1,318136704	1
Anxa3	1,318045341	1
Gins2	1,317862634	1
Psd3	1,317862634	1
Erh	1,31777129	1
Gm43462	1,31777129	1
Gm37566	1,317679952	1
Rps18-ps1	1,31758862	1
Gm26983	1,317223358	1
Nfxl1	1,317223358	1
Rpsa-ps12	1,317040765	1
Wdr34	1,316858197	1
Msl1	1,316858197	1
Lrrc57	1,316766922	1
Taf5	1,316675654	1
Ndufs1	1,316584392	1
Ebi3	1,316584392	1

Gm6378	1,316493137	1
Chil6	1,316401887	1
Ctdnep1	1,316401887	1
Snhg11	1,316036954	1
Hdac2	1,315945736	1
Hoxb7	1,315854525	1
Etv5	1,31576332	1
Oas1b	1,315489743	1
Rps11-ps3	1,315398564	1
Plod1	1,31530739	1
Lrrc51	1,315125063	1
Cd93	1,31494276	1
Slc35a5	1,314851618	1
Nek1	1,314851618	1
Fcho2	1,314760483	1
Gm44178	1,314487115	1
Chd1l	1,314396005	1
Ptprc	1,314304901	1
Tstd1	1,314122712	1
Plek	1,314031627	1
Gm38375	1,313849476	1
Arl6ip1	1,31375841	1
Etnk1	1,313576297	1
Ndufaf4	1,313212147	1
Usp33	1,313121125	1
Eif2a	1,313121125	1
Lpxn	1,31303011	1
Crry-ps	1,312757101	1
Cd82	1,312666111	1
Nt5dc3	1,312211255	1
Alpk2	1,311847483	1
Adarb1	1,311756556	1
Deptor	1,311756556	1
Fam210a	1,311665635	1
Pip4k2b	1,311483812	1
Lctl	1,311120242	1
Heca	1,311120242	1
Gm8326	1,310938494	1
Phf2	1,310938494	1
Mrps16	1,31084763	1
Gm5577	1,310665921	1
Nrf1	1,310665921	1
Gabarapl2	1,310665921	1
Abcb9	1,310575076	1
Patz1	1,310575076	1
9430034N14Rik	1,310484237	1
B230216N24Rik	1,310120944	1
Csnk1e	1,310120944	1
1190005I06Rik	1,309939335	1
2300009A05Rik	1,309939335	1
Lxn	1,30984854	1
Pgs1	1,30984854	1

Rnf181	1,309666969	1
Fbxo2	1,309485423	1
Mnt	1,308940937	1
Blcap	1,308940937	1
Pla2g4a	1,308940937	1
Tmod3	1,308759491	1
Atg16l1	1,308668778	1
Mpst	1,308487371	1
Gm996	1,308396677	1
Runx1	1,308396677	1
Mt1	1,308396677	1
Svbp	1,308124631	1
Tmem238	1,308033962	1
Ubl5	1,308033962	1
Orc4	1,307943299	1
Fgd2	1,307852643	1
Soat2	1,307671349	1
3830403N18Rik	1,307399454	1
Gm11353	1,307218223	1
Lsm5	1,307127616	1
BC030867	1,30658411	1
Phkg2	1,30658411	1
Gm24927	1,306402992	1
Zranb3	1,306312442	1
Urb2	1,306131361	1
Sccpdh	1,306131361	1
Dexi	1,306131361	1
Inppl1	1,30604083	1
Slc41a3	1,305859787	1
Snu13	1,305769275	1
Slc6a8	1,305678769	1
Gm44024	1,305407289	1
Kank2	1,305407289	1
Vmp1	1,305226334	1
Slc44a1	1,305135865	1
Dhx8	1,304683618	1
Lta4h	1,304593188	1
Dhrs3	1,304412345	1
Scrn2	1,304412345	1
Foxn3	1,304412345	1
Tbc1d12	1,304321933	1
Hspa5	1,304321933	1
Slc30a7	1,304050735	1
Neurl4	1,303869968	1
Cacna1a	1,303869968	1
Ppp2r3a	1,303779594	1
Prkd3	1,303779594	1
G6pc3	1,303598864	1
Ldlr	1,303598864	1
Gm6210	1,303418159	1
Zfp516	1,30323748	1
Rbm14	1,303147149	1

Sap18	1,302966507	1
Vps13c	1,302876196	1
Pi4kb	1,302695591	1
Mroh1	1,302605298	1
Gabarap	1,302605298	1
Gripap1	1,302515012	1
Hmgcr	1,302334457	1
Gnal	1,30224419	1
Gm4875	1,302153928	1
Rpl18	1,302153928	1
Adgrl1	1,302153928	1
1700120C14Rik	1,302063673	1
Aco2	1,301973424	1
2510039O18Rik	1,301883181	1
Scyl2	1,301792944	1
P4hb	1,301522272	1
Dhx34	1,301341855	1
Prps1l3	1,301251656	1
Spg7	1,300981097	1
Abcb1b	1,300890923	1
Anapc1	1,300710594	1
Lrch3	1,300710594	1
H60b	1,300710594	1
Spice1	1,300620438	1
Tmod1	1,300620438	1
Spaca9	1,300530289	1
Gps1	1,30025988	1
Nprl2	1,30025988	1
Cyb5r4	1,299989526	1
9130230L23Rik	1,299899421	1
Gm15446	1,299629143	1
Enoph1	1,299629143	1
Eef1a1	1,299539062	1
Me2	1,299539062	1
Wdr83os	1,299358921	1
Spop	1,299088755	1
Zfp995	1,298818645	1
Zmat3	1,298638603	1
Fam105a	1,298368586	1
2810402E24Rik	1,297558875	1
S100a11	1,297468938	1
Vbp1	1,297379007	1
Jak1	1,296839555	1
Polr2h	1,296749668	1
Tusc3	1,296749668	1
Zmym2	1,296480044	1
Mdm1	1,296390182	1
Mast3	1,296210477	1
Gm44269	1,296120633	1
Kmt2c	1,296120633	1
Cdk8	1,296030796	1
Pmpca	1,296030796	1

Stt3b	1,295761322	1
Gda	1,295402111	1
Ubl7	1,295222542	1
Cul1	1,295222542	1
Amn1	1,294504518	1
Rab6a	1,294504518	1
Banp	1,294414792	1
Gm37718	1,294235361	1
Eif4ebp2	1,294145654	1
Gm16181	1,293697215	1
Mvd	1,293697215	1
Slfn4	1,293517883	1
Uba3	1,293338576	1
Dnmbp	1,293069662	1
Mss51	1,293069662	1
Plau	1,293069662	1
Ptpn5	1,292800804	1
Ogdh	1,292800804	1
Bsg	1,292800804	1
Mxd4	1,292621596	1
Pdk3	1,292442413	1
Utp14a	1,292442413	1
Irgq	1,292352831	1
Ranbp9	1,291994564	1
Pank2	1,291994564	1
Sbno1	1,291994564	1
1110051M20Rik	1,291905013	1
Elf1	1,291815468	1
Ube2f	1,291815468	1
Bloc1s2	1,29154687	1
Atp9a	1,29154687	1
Oxa1l	1,29145735	1
Hnrnpr	1,291367836	1
E2f5	1,291009843	1
Secisbp2l	1,291009843	1
Gm9409	1,290830883	1
Gm37199	1,290830883	1
Ids	1,290830883	1
Aga	1,290741413	1
Ifitm1	1,290651949	1
Ipo5	1,290651949	1
Gm5619	1,290383593	1
Chmp1b	1,290383593	1
Fyttd1	1,290383593	1
Ahctf1	1,290294153	1
Rnf146	1,290294153	1
Nab2	1,290115293	1
Cenph	1,289936458	1
Kif23	1,289847049	1
Csnk2a1	1,289847049	1
D16Ertd472e	1,289578861	1
Hist1h4i	1,289578861	1

Lrrk2	1,289489477	1
Gm43588	1,2894001	1
Fbxo30	1,2894001	1
AI606181	1,289310728	1
Tmem132a	1,289221363	1
Fam234a	1,289221363	1
Aldh9a1	1,289042652	1
Sarnp	1,288953305	1
Chaf1a	1,288953305	1
Prpf38b	1,28877463	1
Cnot7	1,288506665	1
Myo6	1,288328053	1
Asna1	1,288328053	1
Gm13341	1,28806018	1
Itpkb	1,28788163	1
Gm27605	1,287524602	1
Exoc2	1,287524602	1
Vps52	1,287167674	1
Dzip3	1,286989247	1
Ubap2l	1,286989247	1
Mrps24	1,286900043	1
Pgghg	1,286810845	1
Fat1	1,286721653	1
4930447F24Rik	1,286543288	1
Psmc3ip	1,286454115	1
Uap1	1,286275787	1
Rprd1a	1,286186632	1
Grik5	1,286097483	1
Hltf	1,286008341	1
Pwp1	1,285740951	1
Zfp219	1,285651833	1
Tcam1	1,285562722	1
Gm43775	1,285473616	1
Gm19566	1,285295424	1
2200002J24Rik	1,285295424	1
Mastl	1,285295424	1
Nxf7	1,285295424	1
Carm1	1,285295424	1
Cadps	1,285028182	1
Ppp3cc	1,284939114	1
Edil3	1,284671946	1
Dcaf6	1,284582903	1
Thrap3	1,284493865	1
Itgb2	1,284404834	1
Zfp787	1,28422679	1
Dynll2	1,28422679	1
Gm26542	1,283781788	1
Dennd3	1,283692806	1
Rnf10	1,283692806	1
Ube2j2	1,283603831	1
Gm42783	1,283514861	1
Far1	1,283159044	1

Usp24	1,283070106	1
Prdx2	1,282981173	1
Elof1	1,282803326	1
Rad9a	1,282269935	1
Rcsd1	1,282269935	1
Tmem97	1,282181057	1
Ddx23	1,282003322	1
Hip1r	1,281914463	1
Rpl14-ps1	1,281736764	1
Phc2	1,281736764	1
Lats2	1,28155909	1
Gm11298	1,281203816	1
Creb1	1,281203816	1
Gm28809	1,281115013	1
D10Wsu102e	1,280937425	1
Cpd	1,28084864	1
Asf1a	1,280582323	1
Sep 06	1,280316061	1
Hspa4l	1,280316061	1
Rbm25	1,280227319	1
Larp4	1,280138584	1
Ska3	1,279961131	1
Pml	1,279783703	1
Gm7504	1,2796063	1
Klf4	1,2796063	1
Plaur	1,279340241	1
Irak1	1,279251567	1
Fuom	1,279162899	1
Aldh16a1	1,279162899	1
Fh1	1,279162899	1
Inafm1	1,279162899	1
Gm19287	1,279074237	1
Dmwd	1,279074237	1
Phkg1	1,278985581	1
Lrrcc1	1,278985581	1
Msh3	1,278719651	1
Dnajc5	1,278719651	1
Tardbp	1,278542395	1
Gm5069	1,278453777	1
Cul4b	1,278453777	1
Trim46	1,277833618	1
Wdr70	1,277833618	1
Acadvl	1,277745048	1
Htr2b	1,277656484	1
Gas8	1,277567927	1
Paxbp1	1,277567927	1
Socs5	1,277479376	1
Mbd5	1,277390831	1
4833420G17Rik	1,277302292	1
Gnrh1	1,277213759	1
Cdc20	1,277213759	1
Rnpc3	1,277125233	1

C430042M11Rik	1,276859689	1
Bud13	1,276771187	1
Nars2	1,276682691	1
Scd2	1,276682691	1
Fgfbp3	1,276505718	1
Klhl21	1,27641724	1
Fam78a	1,276240304	1
B230307C23Rik	1,275974944	1
Map3k1	1,275974944	1
Nudt2	1,275886504	1
Kctd20	1,275798069	1
Arl1	1,275621218	1
Siglec1	1,275532802	1
Dnajc13	1,275444392	1
Prdx4	1,275355988	1
Pcmtd2	1,275179198	1
Tmem176b	1,275179198	1
Dpp7	1,275090812	1
RP23-356D13.11	1,275002433	1
Akt1s1	1,27491406	1
Traf7	1,274737331	1
Golga5	1,274560627	1
Zdhhc9	1,274472285	1
Cdc25c	1,274295617	1
Chtf8	1,274295617	1
Edem1	1,274207293	1
Rnase4	1,274207293	1
Cks1brt	1,274030663	1
Wasf2	1,273854057	1
Gm7432	1,273589194	1
Plxna1	1,273589194	1
Rps6ka1	1,273500918	1
Smad6	1,273412649	1
Dennd1c	1,273412649	1
9330102E08Rik	1,273412649	1
Pole4	1,273324386	1
Napsa	1,273324386	1
4831440E17Rik	1,273236129	1
Colgalt1	1,272883162	1
Snhg17	1,272794935	1
Il17rc	1,272442091	1
A230050P20Rik	1,272001172	1
Ranbp10	1,272001172	1
Srrt	1,271913007	1
Gm12469	1,271736694	1
Atad2	1,271736694	1
Tfeb	1,271560406	1
Tmem106b	1,271560406	1
Gpatch2l	1,27059126	1
Szt2	1,270503192	1
Rtel1	1,27041513	1
Lfng	1,27041513	1

Camk1d	1,270150983	1
Sharpin	1,270150983	1
Men1	1,270062945	1
Celf4	1,269798871	1
Rpl30-ps2	1,269798871	1
Calr-ps	1,269534851	1
0610037L13Rik	1,269446857	1
RP24-175C20.10	1,268391399	1
Fam13b	1,268127672	1
Rab31	1,268039775	1
Chka	1,267688249	1
Scp2-ps2	1,267600382	1
Mccc2	1,266985488	1
Trove2	1,26689767	1
Klk8	1,26654646	1
Asap1	1,26654646	1
Add3	1,266458673	1
Glrx	1,266370892	1
Dnajc22	1,266195348	1
Gm7832	1,266107585	1
Ctbp2	1,266107585	1
Pigo	1,266019828	1
Gm11605	1,266019828	1
Bcl2	1,265844333	1
Pcnx3	1,265844333	1
Il27	1,2654057	1
Rps3a1	1,2654057	1
AA474408	1,265317992	1
Sidt2	1,265054904	1
Cenpx	1,264791871	1
Bahd1	1,264791871	1
Aph1a	1,264704205	1
Ptk2b	1,264616545	1
Batf2	1,264265968	1
Tas1r1	1,264003098	1
Cox6a1	1,263915487	1
Nabp1	1,263827883	1
Sema4a	1,263565104	1
Guf1	1,263389949	1
Meaf6	1,263389949	1
Gm37702	1,263302381	1
Fuca2	1,263302381	1
D130019J16Rik	1,263214818	1
Plcb2	1,263214818	1
Mthfd1l	1,263127262	1
Fbxw4	1,263127262	1
Dopey1	1,262864629	1
Rcn2	1,262864629	1
Gpn3	1,262689571	1
Metrn	1,262602051	1
Numa1	1,262602051	1
Rbmx2-ps	1,262514537	1

Ube2g1	1,262514537	1
Atrx	1,262514537	1
Tdp1	1,26242703	1
Clock	1,262339528	1
Hadha	1,262252032	1
Hsp90b1	1,262252032	1
Atxn10	1,26190211	1
Kdm5a	1,261814645	1
Slc26a11	1,261727185	1
Hibch	1,261727185	1
Btbd9	1,261639732	1
Csrp1	1,261464844	1
Zbtb46	1,26128998	1
Strn	1,261202557	1
Icam5	1,261202557	1
Mtf2	1,26111514	1
Anp32-ps	1,260852926	1
D130051D11Rik	1,260852926	1
Mrpl38	1,260765533	1
Slc5a3	1,260678147	1
Ggps1	1,260416023	1
Gm6560	1,260328661	1
Hdlbp	1,260241305	1
Gm5384	1,260153954	1
Hikeshi	1,260153954	1
Kti12	1,260153954	1
Pnrc2	1,25989194	1
Kptn	1,259717294	1
Nr4a2	1,259717294	1
Zbtb43	1,259717294	1
Megf9	1,25962998	1
Gm4032	1,25945537	1
Gm38062	1,25945537	1
Cmc1	1,259368074	1
Cds1	1,259280785	1
Zzz3	1,259280785	1
Fabp5l2	1,259193501	1
Trmt1	1,258931686	1
Mdh1	1,258582685	1
Dalrd3	1,25840822	1
Ntan1	1,258320997	1
Gm26601	1,258059364	1
Dffb	1,257972165	1
Rpl32-ps	1,257884972	1
Carhsp1	1,257623429	1
5530601H04Rik	1,257449098	1
Uhrf1bp1	1,257449098	1
Commd10	1,257449098	1
Flt1	1,257361941	1
Mre11a	1,257361941	1
Tpd52-ps	1,25727479	1
Usp14	1,25727479	1

Sqstm1	1,257100507	1
Tep1	1,257013375	1
Dixdc1	1,256926248	1
Gm15903	1,256839128	1
Rbl2	1,256752013	1
Phf12	1,256664905	1
Tcp11l2	1,256664905	1
Gm5302	1,256316532	1
Aaas	1,256316532	1
Hal	1,256229453	1
Gorasp1	1,256142381	1
Klhdc10	1,255707111	1
2510002D24Rik	1,255620075	1
Gm15530	1,255533045	1
Fasn	1,255359003	1
Klhl9	1,255184985	1
Kbtbd11	1,255010992	1
Gpr157	1,254924004	1
Id2	1,254924004	1
Commd1	1,254576114	1
Narf	1,254576114	1
Brd9	1,254489156	1
Gm4705	1,25422832	1
Rpl22-ps1	1,254054459	1
B4galnt1	1,253967537	1
Edem3	1,253967537	1
Ncaph2	1,253967537	1
Plekho2	1,253880622	1
Pus10	1,253793712	1
Fam178a	1,253793712	1
Jag1	1,253793712	1
Ywhah	1,253619912	1
Them6	1,25353302	1
Psme1	1,25353302	1
Gm7909	1,253272383	1
Plekha2	1,253272383	1
4930579K19Rik	1,253185515	1
Gm30329	1,253098654	1
Gps2	1,25292495	1
Gm11810	1,252838107	1
Gm8304	1,252838107	1
Gm42479	1,252838107	1
Cpne3	1,252838107	1
Zfp280b	1,252664439	1
Npepps	1,252664439	1
Kif7	1,252577614	1
Gm36936	1,252577614	1
Tex10	1,252490794	1
Ercc1	1,252403981	1
Sh3glb1	1,252403981	1
Zfp277	1,252317174	1
Gm5944	1,252230373	1

Gm7407	1,252230373	1
Gm45828	1,252143578	1
Pkib	1,25188323	1
Ptpn4	1,251709694	1
Rpl21-ps14	1,251362694	1
Hmbox1	1,251362694	1
Gm8667	1,251275959	1
Ss18l2	1,251102508	1
Rchy1	1,251102508	1
Gm9396	1,25092908	1
Gm11722	1,250755677	1
Gtf3a	1,250668984	1
Ino80	1,250322273	1
Msn	1,250322273	1
Fam168b	1,250062303	1
Vps29	1,249889019	1
Gm44190	1,249629139	1
Slc33a1	1,249629139	1
Ipo4	1,249629139	1
Wrn	1,249542524	1
Gm9790	1,249542524	1
Gm12933	1,249022962	1
Gnl3l	1,249022962	1
Synrg	1,24893639	1
Pcdh7	1,24893639	1
Chd3	1,24893639	1
Tmed2	1,248763262	1
2010111I01Rik	1,248763262	1
Gm10827	1,248676708	1
Irak1bp1	1,248590159	1
Ccnc	1,24841708	1
Yars2	1,24841708	1
Lrrc45	1,24841708	1
Npc2	1,248244024	1
1110003F10Rik	1,248070993	1
Cd80	1,247897985	1
Defb25	1,247638519	1
Vps37b	1,247465572	1
Xdh	1,247033308	1
Cln5	1,247033308	1
Scrn3	1,246860445	1
Ankrd50	1,246687605	1
Fgd4	1,246601194	1
Tspan31	1,246601194	1
Ugdh	1,246514789	1
Ppm1k	1,246514789	1
Pcbp2	1,246428391	1
Ikbke	1,246341998	1
Gm12005	1,245737416	1
Coprs	1,245737416	1
Qsox1	1,245564732	1
Tufm	1,245392072	1

Hypk	1,24478795	1
Gm45873	1,244615398	1
RP23-359K10.8	1,244615398	1
1600012H06Rik	1,244615398	1
A830073O21Rik	1,244529131	1
Nav2	1,24444287	1
4930448A20Rik	1,244356614	1
Mlst8	1,244270365	1
Nfatc4	1,244184122	1
Taf10	1,244097885	1
E330037G11Rik	1,244011653	1
Cfap74	1,244011653	1
Nckipsd	1,243925428	1
Abi1	1,243839209	1
Fam133b	1,243752995	1
Slc25a42	1,243580586	1
Abca2	1,243494391	1
Fkbp3	1,243408201	1
Ppp1r12c	1,243408201	1
Cenpq	1,243322018	1
Asns	1,243149669	1
Anxa6	1,243149669	1
Shc1	1,243063503	1
Rcor1	1,242977344	1
Sfi1	1,242805042	1
Mmgt1	1,242718901	1
Rangap1	1,242374394	1
Ier5	1,242202176	1
Ndufab1	1,242116076	1
Gyg	1,242116076	1
Gm10443	1,242029982	1
Napepld	1,241943894	1
Gm9521	1,241857812	1
Hdac7	1,241599602	1
Ppt2	1,241599602	1
Ppme1	1,241513544	1
Ankrd10	1,241255405	1
Snx5	1,240567298	1
Nelfcd	1,240481311	1
Ripk3	1,24039533	1
Mlxip	1,24039533	1
Sipa1l3	1,240309356	1
Il2rg	1,240223387	1
Pias2	1,239879572	1
Rac2	1,239793633	1
Rps8-ps3	1,2397077	1
Myoz1	1,239535852	1
Ppp4r3b	1,239535852	1
Srsf5	1,239278124	1
Ctnnal1	1,239192227	1
Nup54	1,239106336	1
Eif2ak4	1,239106336	1

Hook2	1,238934571	1
Dtx3l	1,238934571	1
Gm5586	1,238848698	1
Prdx3	1,238848698	1
Gm14776	1,23876283	1
Ppp4c	1,238505264	1
1110008P14Rik	1,238333582	1
Vgll4	1,238333582	1
Tmigd3	1,237904483	1
Arhgap12	1,237904483	1
Med25	1,237818681	1
Nicn1	1,237818681	1
Stk17b	1,237732885	1
Ghdc	1,23756131	1
Rpl9-ps6	1,23756131	1
Ttyh3	1,237475532	1
Lmln	1,23738976	1
Klhl42	1,23704673	1
Gm13397	1,236875251	1
Rgp1	1,236875251	1
Med18	1,23678952	1
Ybx3	1,236703796	1
Gm14494	1,236618077	1
Gm17066	1,236446657	1
Cblb	1,236275261	1
Tspyl3	1,236018211	1
Zfp473	1,236018211	1
Aplp2	1,235761215	1
Pign	1,235675562	1
Txndc16	1,235589914	1
Hnrnpul1	1,235589914	1
2610020C07Rik	1,235418637	1
Dnajc4	1,235247384	1
Cdkn2d	1,235247384	1
Dnajc10	1,235247384	1
Irf2	1,234819354	1
Mrpl50	1,234562607	1
Tomm20	1,234477037	1
B3gnt3	1,234391472	1
Firre	1,234305914	1
Aqr	1,234305914	1
Gm9392	1,234220361	1
Ciz1	1,234049273	1
Lsm4	1,234049273	1
Lmo2	1,234049273	1
Zc3h18	1,233963739	1
Pak2	1,23387821	1
Csrnp1	1,233536154	1
Gm16585	1,233450654	1
Vav3	1,233194192	1
Commd4	1,233108717	1
Mlec	1,232852325	1

Rsf1	1,232852325	1
Ctso	1,232766874	1
Dap3	1,232766874	1
Isg20	1,232681428	1
Akap1	1,232595988	1
Tprn	1,232510554	1
Smchd1	1,232510554	1
Zdhhc1	1,232254287	1
Evi5	1,231998073	1
Myo5a	1,231998073	1
Kras	1,231998073	1
Orai2	1,231912681	1
Prpf4b	1,231827294	1
Gm20628	1,231741913	1
Trappc6b	1,231656538	1
Hbb-bh3	1,231571169	1
A630081D01Rik	1,231485806	1
Rps2-ps5	1,231400449	1
Gcn1l1	1,231400449	1
1700061G19Rik	1,231315098	1
Mtpap	1,231229753	1
Fuca1	1,231229753	1
Sppl2a	1,231144413	1
Mtl5	1,23105908	1
Ensa	1,230973752	1
RP24-316F13.7	1,230888431	1
Hadhb	1,230888431	1
Nf1	1,230461911	1
Clasp2	1,230461911	1
Peak1	1,230291345	1
2310036O22Rik	1,23020607	1
Pcgf1	1,230120802	1
Agfg2	1,230035539	1
Slc9a5	1,229950283	1
Tpm3	1,229950283	1
Gm11346	1,229865032	1
Cfp	1,229694548	1
Cspp1	1,229694548	1
1700025G04Rik	1,229694548	1
Eif4b	1,229268443	1
Csf2ra	1,228927664	1
Gm9169	1,228842484	1
Fis1	1,228842484	1
Slc35a3	1,228672143	1
Otud4	1,228672143	1
Lgals1	1,22858698	1
Esco1	1,228246391	1
Golga3	1,227905896	1
Sort1	1,227735684	1
2610301B20Rik	1,227650587	1
Ttc14	1,22748041	1
Pltp	1,22748041	1

Guca1a	1,22739533	1
Runx2	1,227310257	1
Cnpy4	1,227310257	1
Gm10288	1,227225189	1
Gm43336	1,227140127	1
Dctd	1,226884977	1
Eps15	1,226799939	1
Wdr90	1,226714907	1
Akr7a5	1,226714907	1
Mndal	1,22662988	1
Pigh	1,22654486	1
Spaca6	1,22654486	1
Brwd3	1,226459845	1
Hddc2	1,226374836	1
Hat1	1,226289833	1
Sem1	1,226289833	1
BC085271	1,226204836	1
Gm16536	1,22603486	1
Nek9	1,22603486	1
Tmem63a	1,225949881	1
Zmiz2	1,22577994	1
Mapk3	1,22577994	1
Zfp36	1,225440129	1
Myadm	1,225440129	1
Irx2	1,225270258	1
4921524J17Rik	1,225185332	1
Syne3	1,224930588	1
Usp19	1,224930588	1
Hcfc1	1,224760789	1
Nfya	1,224675898	1
Polr1d	1,224336392	1
Macrod1	1,224166675	1
Gm43247	1,223996981	1
Cog5	1,223742485	1
RP23-350F7.3	1,22357285	1
A430027C01Rik	1,223488041	1
Fktn	1,223403238	1
Stk35	1,223148865	1
Plgrkt	1,222725028	1
Pdk1	1,222555534	1
Tmem263	1,222555534	1
Sep 08	1,222470796	1
Ddx19a	1,222386063	1
Gltscr2	1,222386063	1
Dynlt3	1,222386063	1
Pgpep1	1,222301337	1
Dnajc3	1,222301337	1
Accs	1,222216616	1
Klhl12	1,222131902	1
Flot1	1,222047193	1
Aplp1	1,22196249	1
Dennd6a	1,22196249	1

Serinc1	1,22196249	1
Cnih1	1,221708416	1
Arid5b	1,221623737	1
Gak	1,221623737	1
Prelid1	1,221623737	1
Lca5	1,221539063	1
Sbf1	1,221454396	1
Higd1a	1,221369734	1
Nktr	1,221285078	1
Kif5a	1,221200428	1
Gnb1	1,221115784	1
Arid3b	1,220946513	1
DHRSX	1,220777265	1
AU020206	1,220777265	1
Ddhd1	1,22069265	1
Ctdsp2	1,22069265	1
RP24-323H7.5	1,220523438	1
Ssr3	1,220523438	1
Ewsr1	1,22010051	1
Smg9	1,219846824	1
Hnrnpll	1,21959319	1
Zbed4	1,219339609	1
Man2b1	1,219086081	1
Rbbp7	1,219086081	1
Gm3650	1,219001583	1
Xk	1,218917092	1
Sub1	1,218832606	1
Robo3	1,218663651	1
Gm13840	1,218663651	1
Peli1	1,218579183	1
Snap23	1,218579183	1
Wdr45b	1,218579183	1
Lztfl1	1,21849472	1
Dars	1,21849472	1
Ankmy2	1,21849472	1
Itfg1	1,218325813	1
Gpsm3	1,218072495	1
Wdr83	1,218072495	1
Car5b	1,217903646	1
Cops4	1,217734821	1
Gm37080	1,217566019	1
Rdh1	1,217481626	1
Pou2f2	1,217481626	1
Fam49b	1,217481626	1
Vps13a	1,21739724	1
Gm45501	1,217312859	1
Memo1	1,217312859	1
Car13	1,217228484	1
Tor1aip2	1,217144116	1
Gm14303	1,216722359	1
Pygb	1,216469375	1
Zfyve27	1,216385058	1

Vamp8	1,216385058	1
Gm42571	1,216216443	1
Adipor2	1,216216443	1
Sin3a	1,216216443	1
Phf11c	1,215879283	1
Chchd2	1,215879283	1
Rad51	1,215795008	1
Dhfr	1,215542217	1
Actr3	1,215542217	1
Inpp4a	1,215289478	1
Chd4	1,215205243	1
Atrn	1,215121015	1
Rhod	1,215121015	1
Gm13350	1,215036792	1
Gm44153	1,214868364	1
L2hgdh	1,214784158	1
Tmed4	1,214531577	1
Rplp1	1,214531577	1
Cdk12	1,214279049	1
Gm26847	1,214194884	1
Gm45167	1,214194884	1
Luc7l	1,214194884	1
Rps2-ps11	1,214026573	1
Gprasp1	1,213942426	1
Agk	1,213774149	1
Tbc1d30	1,213437666	1
Polr2d	1,213437666	1
Zmym5	1,213437666	1
Wls	1,21335356	1
Cdc37	1,21326946	1
E130307A14Rik	1,213185365	1
Naa16	1,213185365	1
Sptan1	1,213101277	1
Tnks	1,213101277	1
Igsf8	1,213017194	1
Gm26982	1,21276498	1
Heatr5b	1,21276498	1
Rbm4	1,212680921	1
Cuta	1,212680921	1
Cenpe	1,212680921	1
Harbi1	1,212512819	1
Cct2	1,212512819	1
Prmt7	1,212344741	1
Usp21	1,21226071	1
Rpl30-ps3	1,21226071	1
Nab1	1,21226071	1
Yy1	1,212176686	1
Ing5	1,212092667	1
Gm12013	1,211840646	1
Baat	1,21167266	1
Hist3h2a	1,211588677	1
Mrpl1	1,211588677	1

Hk2	1,211588677	1
Vdac3	1,211504699	1
Ppp2r2d	1,211252799	1
Srek1	1,211168845	1
Oprl1	1,211000953	1
Wdr1	1,211000953	1
Tbrg4	1,210497416	1
Snrpg	1,210413514	1
Ltbp2	1,210329617	1
C1galt1	1,210245727	1
Sar1b	1,210245727	1
Fzr1	1,209994089	1
Arpc2	1,209994089	1
Bmi1	1,20957481	1
Kif1c	1,20957481	1
Bcas2	1,209490971	1
Stx3	1,209407139	1
Gm15459	1,209323312	1
Dusp22	1,209239491	1
Lmf1	1,209155676	1
Dpf2	1,209071866	1
Minos1	1,209071866	1
Ctnnbip1	1,208988063	1
Ubl3	1,208904265	1
Catsperg1	1,208736687	1
Spag9	1,208736687	1
Cyb5b	1,208736687	1
Abhd14b	1,208569132	1
Sgol1	1,208485363	1
H2-M3	1,208401601	1
Cenpl	1,208401601	1
Copb1	1,208234092	1
Gnb4	1,208066607	1
Herc1	1,208066607	1
Kansl3	1,208066607	1
Rcor3	1,207982873	1
Bckdhb	1,207731706	1
Stim1	1,207731706	1
Lgr4	1,207647995	1
Gm12479	1,207564291	1
Gm15163	1,207480591	1
Zxdc	1,207480591	1
Isoc2a	1,207480591	1
Acvr2b	1,207480591	1
Gins1	1,207313211	1
Rilpl2	1,207313211	1
Mex3d	1,207145853	1
Khsrp	1,207062183	1
Sae1	1,207062183	1
Gm18916	1,206978519	1
Gm13181	1,20689486	1
Slc35g1	1,20689486	1

Lsm2	1,206811208	1
Tmem120b	1,20664392	1
Usf1	1,206560284	1
Armc10	1,206309413	1
Ndufa4l2	1,206058594	1
2510016D11Rik	1,205891411	1
Akr1b10	1,205640679	1
Rasal3	1,205473553	1
Wdr47	1,205222908	1
Rassf7	1,205139371	1
Ube2j1	1,205139371	1
Txndc17	1,204972315	1
Zfp760	1,204805282	1
Icam4	1,204805282	1
Stat2	1,204805282	1
Evl	1,204721774	1
Pdcd10	1,204638272	1
Gm11930	1,204471285	1
Zcchc9	1,204387801	1
St14	1,204387801	1
Maea	1,204304322	1
Nectin3	1,204220849	1
Isoc1	1,204137381	1
Rlim	1,20405392	1
Loxl3	1,20380357	1
Gm10086	1,203553272	1
AU022252	1,203469851	1
Parp11	1,203303026	1
Yif1b	1,203303026	1
Alkbh1	1,203303026	1
Dazap1	1,203219622	1
Nectin1	1,203136224	1
Hmgcs1	1,203052832	1
Gdi1	1,202886065	1
Crbn	1,202886065	1
Vps41	1,20280269	1
Sgsm2	1,20280269	1
Cep83	1,202469249	1
Map4k2	1,202302563	1
Fgf13	1,202302563	1
Smox	1,202302563	1
Pgm1	1,202052577	1
Gm11716	1,20196926	1
1500002F19Rik	1,20196926	1
Xkr8	1,201885949	1
Banf1	1,20163605	1
Reep4	1,201302931	1
Plod3	1,201302931	1
Gm42890	1,201219666	1
Ddx6	1,201136407	1
Phactr1	1,200886664	1
Ptpn18	1,200886664	1

1110034G24Rik	1,200803427	1
Polk	1,200803427	1
Ddx47	1,200636972	1
Cep170	1,20047054	1
Pura	1,20047054	1
Gcat	1,200220935	1
Lpar2	1,200137745	1
Tbpl1	1,199971382	1
Stra8	1,199805043	1
Gpr137b	1,199638726	1
Top1	1,199555576	1
Dsn1	1,199472432	1
Esyt1	1,199472432	1
Pum1	1,199389294	1
Gm35931	1,199139914	1
Tmppe	1,199139914	1
Gm1848	1,199056799	1
Bcs1l	1,199056799	1
Mdm4	1,199056799	1
Mia2	1,198890586	1
Zfp266	1,198807488	1
Gm44694	1,198724396	1
Mbd4	1,198641309	1
Gopc	1,198558229	1
Yaf2	1,198309021	1
Ripk2	1,198309021	1
Fam188b	1,198225964	1
Tsta3	1,198142912	1
Cdc23	1,198142912	1
RP24-324J2.1	1,198059866	1
Slc39a3	1,197976826	1
Plpp5	1,197893791	1
Mpi	1,197727739	1
Gm11110	1,197644722	1
Rbm38	1,19756171	1
Zer1	1,19731271	1
Tbc1d7	1,197229722	1
Gm43201	1,197229722	1
Icmt	1,197229722	1
Hcn2	1,196897825	1
Rbl1	1,196814866	1
Dym	1,196814866	1
Slc25a40	1,196566021	1
Hsd17b12	1,196400153	1
RP23-278O17.1	1,196317228	1
Samd8	1,196068486	1
Pmvk	1,196068486	1
Pstpip2	1,195736912	1
Vhl	1,195736912	1
Gmfb	1,195736912	1
Tbca	1,195488291	1
2310022A10Rik	1,195405429	1

Dpysl2	1,195322573	1
As3mt	1,195239722	1
C730034F03Rik	1,195156877	1
Pcid2	1,194659929	1
Eif4g3	1,194577124	1
Slc36a1	1,194577124	1
Smim13	1,194494325	1
Brd8	1,194494325	1
Eif3s6-ps1	1,194163187	1
Szrd1	1,193914894	1
Lrrfip2	1,193583916	1
AI662270	1,193583916	1
Auh	1,193501186	1
Gpaa1	1,193501186	1
Litaf	1,193501186	1
Smarcad1	1,193087622	1
Celsr3	1,193004926	1
Tmem185a	1,192922236	1
Tbc1d9b	1,192922236	1
Swt1	1,192922236	1
Bag4	1,192756873	1
Znhit1	1,192756873	1
Cpsf4	1,192508872	1
Hexim1	1,192426217	1
Pdia4	1,192343567	1
Zbtb44	1,192343567	1
Cdc42se1	1,192343567	1
Gfod1	1,192095652	1
Mphosph6	1,192013025	1
Polr3g	1,191682575	1
Gm13223	1,191599976	1
Tmem9	1,191599976	1
Arcn1	1,191517384	1
Hectd3	1,191434797	1
Clip2	1,19126964	1
Rragc	1,19126964	1
Rgs9bp	1,191021949	1
Arl5c	1,191021949	1
Lsm8	1,191021949	1
Mat2a	1,190939396	1
BC030336	1,190774308	1
Gm20342	1,190444201	1
Fas	1,190444201	1
Tbl2	1,190444201	1
Ube2i	1,190361689	1
Tmlhe	1,190279182	1
Prps2	1,190279182	1
Tank	1,190196681	1
Fcer1g	1,190196681	1
Vegfa	1,190196681	1
Lancl2	1,190031696	1
Kdm7a	1,190031696	1

Eif4e3		1,189784262	1
Gm15013		1,189701795	1
Smad5		1,189042267	1
Pex7		1,188959852	1
Ddx50		1,188959852	1
Gm7266		1,188877442	1
Tmem123		1,18871264	1
Chmp1a		1,188630248	1
Myh9		1,188547861	1
Mob2		1,18846548	1
Zfr2		1,188383105	1
Pgk1		1,188300735	1
Srsf4		1,188136013	1
Josd2		1,188136013	1
Qars		1,188136013	1
Malat1		1,188053661	1
Clcn6		1,187971314	1
Cox14		1,187971314	1
Sirt1		1,187888973	1
Epb41l5		1,187724308	1
	Sep 09	1,187724308	1
Eif4e		1,187724308	1
Baiap2		1,187559666	1
Arhgap17		1,187477354	1
Khk		1,187477354	1
Gm6085		1,187312746	1
Pola2		1,18723045	1
Gm29340		1,18723045	1
Ercc6		1,18723045	1
4931406P16Rik		1,18723045	1
Rpia		1,186736798	1
Mipep		1,186490048	1
Trip12		1,186490048	1
Fxr2		1,186325577	1
Rap1a		1,186325577	1
Bag6		1,18624335	1
Jam2		1,186078913	1
Sbno2		1,186078913	1
Dek		1,185996704	1
2700033N17Rik		1,185832301	1
Hif1a		1,185832301	1
Epg5		1,185750108	1
Anp32e		1,185750108	1
Supt7l		1,18558574	1
Dnajc24		1,185257071	1
Zfp143		1,185174918	1
Dcakd		1,185092771	1
Ppp4r1l-ps		1,185092771	1
Gm37584		1,185010629	1
Trp53cor1		1,184846363	1
Ppp1r35		1,184764239	1
E130309D02Rik		1,184764239	1

Uba1	1,18468212	1
Eef1b2	1,18468212	1
Man2c1	1,184600007	1
0610040B10Rik	1,184353702	1
1600014C10Rik	1,184107448	1
Ccm2	1,183861246	1
Gm5523	1,18377919	1
Tmco3	1,183533055	1
Birc5	1,183286972	1
Pik3ip1	1,183286972	1
Vprbp	1,183204956	1
Cep295	1,183204956	1
Rnf115	1,183122945	1
Igip	1,182958941	1
RP23-114G13.7	1,182794959	1
Gm6433	1,182712977	1
Vps35	1,182631	1
Sart1	1,182549029	1
Tnpo1	1,182549029	1
Lrrc42	1,182549029	1
Slc25a39	1,182549029	1
Ccnyl1	1,182221203	1
Gm8522	1,182057323	1
Celf5	1,181975392	1
Ube2d3	1,181975392	1
Mien1	1,181893467	1
Gm37145	1,181647724	1
Gm13368	1,181565822	1
Prpf38a	1,181483925	1
Ubqln4	1,181402033	1
Katna1	1,181402033	1
Sec14l1	1,181320148	1
Tctn3	1,181074524	1
Gm2225	1,180992661	1
Slc7a6os	1,180992661	1
Sec22b	1,180992661	1
Hspb11	1,180910804	1
Gm26530	1,180828952	1
Gnai2	1,180828952	1
Psmd14	1,180747106	1
Klc1	1,180665266	1
Ube2e2	1,180501603	1
Wdr62	1,180337962	1
Chd9	1,180337962	1
Pofut1	1,180092543	1
Ago3	1,180092543	1
Smurf2	1,179928958	1
Golph3l	1,179928958	1
Gm43609	1,179847175	1
Dda1	1,179847175	1
Slc25a1	1,179438342	1
Rc3h2	1,179274848	1

Coq4	1,178947929	1
Gm29593	1,178947929	1
Uba2	1,178947929	1
Cic	1,178866214	1
Wdsub1	1,178784504	1
Brat1	1,178784504	1
Smc3	1,178784504	1
Pan3	1,178702799	1
Rbm22	1,178702799	1
Tmem39a	1,178539408	1
Nelfe	1,178539408	1
Rsl1d1	1,178539408	1
Strip1	1,178457721	1
Apool	1,178049369	1
Mms22l	1,177886068	1
Atm	1,177804426	1
Nlk	1,177641159	1
Anxa4	1,177641159	1
Gm23722	1,177477914	1
Col27a1	1,177396301	1
Fkbp1a	1,177396301	1
Rps8	1,177314693	1
Mok	1,176988317	1
RP23-65M10.2	1,176906737	1
Eloa	1,176825163	1
Il13ra1	1,176743595	1
Rpl7-ps7	1,176662032	1
Ogfod1	1,176580475	1
Cars2	1,176335837	1
Galm	1,176335837	1
Ggnbp1	1,176335837	1
Magoh	1,176335837	1
Slpi	1,176254302	1
Kif9	1,17609125	1
4930430F08Rik	1,176009733	1
March7	1,176009733	1
Fnbp4	1,175846714	1
Dhx35	1,175765214	1
Gosr1	1,175683719	1
Emc1	1,175683719	1
9330159M07Rik	1,175439268	1
4930524J08Rik	1,175357795	1
Gm11363	1,175276328	1
Creb3l1	1,175276328	1
Cmklr1	1,175276328	1
Sat1	1,175276328	1
Gm16399	1,175276328	1
Srgn	1,175194867	1
Polr3b	1,175113412	1
Tbk1	1,174950518	1
Rbms1	1,174950518	1
Gse1	1,174869079	1

Bmpr2	1,174706219	1
Gstcd	1,174624798	1
Hibadh	1,174624798	1
Gxylt1	1,174380567	1
Gm12906	1,174299167	1
Tulp4	1,174299167	1
Flii	1,174299167	1
Stk24	1,174136386	1
Mknk2	1,173892257	1
Gnb1l	1,173648178	1
Fam117a	1,173648178	1
Eral1	1,173485487	1
Pias3	1,17340415	1
Eif2s1	1,173322819	1
Ttll4	1,173241493	1
C2cd2l	1,173241493	1
Rab1b	1,173160173	1
Eva1b	1,173078859	1
Fbxw11	1,173078859	1
Rnf111	1,17299755	1
Birc6	1,172834949	1
Gm5867	1,172753657	1
Bcl7a	1,172753657	1
Znrd1	1,172428546	1
4921531C22Rik	1,172266024	1
Rab7	1,172266024	1
Ipo7	1,172266024	1
Gm15694	1,172103525	1
Dennd1a	1,172103525	1
Srpk1	1,172103525	1
Stn1	1,172022284	1
Pebp1	1,171941048	1
Cpne2	1,171859818	1
Fam107b	1,171778594	1
Aste1	1,171616162	1
Cers5	1,171534955	1
Clec4e	1,171534955	1
Ptprs	1,171453753	1
Wdr44	1,171372557	1
Smim14	1,171291366	1
Cdk6	1,171291366	1
Slc35a4	1,171210181	1
Hsd17b4	1,171210181	1
Dennd5a	1,171210181	1
5830432E09Rik	1,171129002	1
Mut	1,170804341	1
Nucks1	1,17047977	1
Sec62	1,17047977	1
Rps26	1,170398641	1
Ube2k	1,170398641	1
Snx29	1,170236401	1
Ddt	1,170074183	1

Eif3e	1,170074183	1
Gm5609	1,169993082	1
Gm3511	1,169993082	1
Gtf3c2	1,169993082	1
Cln3	1,169911988	1
Ahi1	1,169911988	1
Stradb	1,169668737	1
Atg12	1,169668737	1
Bvht	1,169587664	1
Tipin	1,169506597	1
Trp53inp1	1,169425536	1
Prdx6	1,16926343	1
Zbtb18	1,169020314	1
Mzt1	1,168939287	1
Pxn	1,168696238	1
Cfl2	1,168534233	1
Abtb1	1,16845324	1
Pcyox1l	1,16845324	1
Gm15420	1,168291269	1
Epm2aip1	1,168210292	1
Gm37289	1,167967395	1
Tnfrsf26	1,167805491	1
Mir5136	1,167481752	1
Cnih4	1,167481752	1
Cdca7	1,167400831	1
Ndufb2	1,167400831	1
Tmbim6	1,167400831	1
P2rx7	1,167319916	1
Unc119	1,167158102	1
Cd302	1,166996311	1
Foxk2	1,166996311	1
Cactin	1,166834542	1
Amd1	1,166591931	1
Nfic	1,166591931	1
Dnajb5	1,166511071	1
Smarce1	1,166511071	1
Rab5b	1,166511071	1
Rpl36-ps4	1,166430218	1
Ythdc2	1,166187691	1
Rnf216	1,166187691	1
Pick1	1,166187691	1
Cyth2	1,165783591	1
Nkain1	1,165702788	1
Selenom	1,165218086	1
Rny3	1,165137322	1
Arhgef10l	1,165137322	1
Ada	1,164975811	1
Gm14706	1,164895064	1
Mrpl32	1,164814322	1
Prss53	1,164572131	1
Gm10463	1,164249288	1
Tcf7l2	1,164168591	1

Rpl9-ps7	1,164007214	1
Armc9	1,164007214	1
Lypla2	1,16384586	1
Pdk2	1,163765191	1
Fbxl18	1,163765191	1
Slc25a28	1,163765191	1
Actn4	1,163684528	1
Zg16	1,163523218	1
Casp3	1,163442572	1
Blnk	1,163120042	1
Gm12844	1,16295881	1
Suz12	1,162878203	1
Dot1l	1,162797601	1
Nbn	1,162636414	1
Nat10	1,162314108	1
Clpx	1,162314108	1
H60c	1,162233545	1
Rbbp5	1,162233545	1
Ctsl	1,162233545	1
Gm2885	1,162072436	1
Dad1	1,16191135	1
Gpr155	1,161830815	1
Gm11944	1,161750286	1
Rnf7	1,161669762	1
Erp44	1,161589244	1
Map1lc3a	1,161347724	1
Mrpl34	1,161347724	1
Akr1b7	1,161186738	1
Ppp3cb	1,161186738	1
Rab14	1,161186738	1
Ogfrl1	1,161106253	1
Chst10	1,161106253	1
Snd1	1,161025774	1
Scaf1	1,160864833	1
Cd68	1,160703914	1
Timm22	1,160623463	1
B9d2	1,160543018	1
Slc19a1	1,160543018	1
A930005H10Rik	1,160462578	1
Npm3	1,160462578	1
Cotl1	1,160221292	1
Fam206a	1,160140874	1
Gm13433	1,160060462	1
BC029214	1,159899655	1
Itsn1	1,159819259	1
Ube2e3	1,159819259	1
Cntrl	1,159578107	1
Amigo3	1,159256648	1
Usp34	1,159176297	1
Fbxo42	1,158935278	1
Cdc42bpb	1,158935278	1
Anapc15	1,15885495	1

Fabp3	1,15885495	1
Fam76a	1,158694309	1
Taf1	1,158613998	1
Mllt6	1,158453391	1
Setdb1	1,158292806	1
Armcx5	1,158292806	1
Tmem9b	1,158212522	1
Gm26782	1,158132244	1
Calm1	1,158132244	1
Mlh1	1,158051971	1
Rfx7	1,158051971	1
Mbnl1	1,158051971	1
Snhg8	1,157891442	1
Rassf2	1,157891442	1
Sumo1	1,157811186	1
Trim41	1,157811186	1
Bbip1	1,157730935	1
Gm13009	1,157409989	1
Ccsap	1,157409989	1
Fam120a	1,157409989	1
Mapk7	1,157249549	1
Mmp9	1,157089131	1
Uggt1	1,157089131	1
D930016D06Rik	1,15700893	1
Kcne3	1,15700893	1
Sptbn1	1,156928735	1
Hnrnph3	1,156848546	1
Rbm18	1,156688184	1
Nmt2	1,156688184	1
Clec10a	1,156367526	1
Pld2	1,156207231	1
Gm37238	1,156127091	1
Ube2a	1,156127091	1
Ubfd1	1,155966829	1
Slc2a1	1,155966829	1
Tspan13	1,155966829	1
Gm7964	1,155646372	1
Zeb2os	1,155406087	1
Ech1	1,155406087	1
1810032O08Rik	1,155165852	1
Cdca8	1,155085785	1
Bzw2	1,155085785	1
Gm13328	1,154845616	1
Abhd12	1,154525469	1
Mfn1	1,154525469	1
Rpl18-ps1	1,154445447	1
Kars	1,154365429	1
Gm4742	1,154205411	1
Med21	1,154205411	1
Ppm1m	1,154205411	1
Rhof	1,154125411	1
Hrc	1,153965426	1

Erbin	1,153885442	1
Gm38021	1,153725491	1
Snx6	1,153645523	1
RP24-282C4.4	1,153485605	1
Bbs10	1,153405654	1
Gm5745	1,153245769	1
Mecp2	1,153245769	1
Insr	1,153085907	1
Phldb1	1,153005984	1
Pot1b	1,152846154	1
Med13	1,152846154	1
Gm8172	1,152766248	1
Polr2l	1,152686347	1
Coq5	1,152606451	1
Lamp1	1,152606451	1
Gm42972	1,152366799	1
Neu3	1,152286925	1
Golm1	1,152286925	1
Trim37	1,152286925	1
Gm11808	1,152286925	1
Anapc2	1,152127196	1
Mpp5	1,152127196	1
Ptpdc1	1,152047339	1
Ska2	1,151967488	1
Mapk8ip1	1,151887642	1
Mtm1	1,151807802	1
Trmt2b	1,151328879	1
Myo1g	1,151249077	1
Srsf3	1,151169282	1
Dgke	1,151089491	1
Upp2	1,150929928	1
Nxn	1,150690623	1
2810414N06Rik	1,150531115	1
Gm43364	1,150371628	1
Tspyl4	1,150291893	1
Cep170b	1,150291893	1
Fchsd1	1,150052722	1
Dlg1	1,149893302	1
Rpl28-ps3	1,149733904	1
Fam98b	1,149733904	1
Brwd1	1,149733904	1
Farsa	1,149733904	1
Psmb3	1,149415174	1
Ltc4s	1,149335505	1
Washc2	1,149255842	1
Mtmr14	1,148857609	1
Lilr4b	1,148777979	1
Ssx2ip	1,148618736	1
Pdxdc1	1,148459515	1
G3bp2	1,148459515	1
Repin1	1,148379912	1
Gm2962	1,148300315	1

Snrpb	1,148220724	1
Snx3	1,148141138	1
Map2k1	1,148061558	1
Ltn1	1,147981983	1
Zfp786	1,147743292	1
Irf2bp2	1,147743292	1
Scoc	1,147584192	1
Gm5921	1,14750465	1
Plppr2	1,147425114	1
Cd300c2	1,147266058	1
Cped1	1,147186538	1
Brip1	1,147107024	1
Ticrr	1,146789023	1
Zfp827	1,146789023	1
Dusp28	1,146550579	1
Gm2058	1,146550579	1
Pcbd2	1,146550579	1
Rnf215	1,146471109	1
Limd2	1,146391645	1
Ctsd	1,146391645	1
Kctd9	1,146073842	1
Akirin2	1,145359106	1
Gm13498	1,145200337	1
Rsrc1	1,145200337	1
Setd7	1,145120961	1
Gpr35	1,144962224	1
Psip1	1,144962224	1
Lonp1	1,144962224	1
Gm12693	1,144882864	1
Vps13d	1,144882864	1
Fndc3a	1,14480351	1
Atad2b	1,144644817	1
Hdac6	1,144565479	1
Fkbp1b	1,144486147	1
Sp3	1,144486147	1
Ctnna1	1,14440682	1
Cenpi	1,144248182	1
RP24-351I17.3	1,144248182	1
Gm37101	1,144248182	1
Gm9825	1,144168872	1
Paip2b	1,144168872	1
Tcf25	1,144010267	1
Fundc1	1,143930973	1
Phf5a	1,143851685	1
Steap3	1,143772402	1
Phf6	1,143772402	1
Arhgap10	1,143376069	1
Gm43672	1,143217574	1
Rps29	1,142979874	1
Gnpda2	1,142742223	1
Nit1	1,142663016	1
Smarcb1	1,142663016	1

Ezh1	1,142504621	1
Rps19-ps5	1,142425431	1
Coro7	1,142425431	1
Ctr9	1,142267068	1
Dpy19l4	1,142187895	1
Slc4a7	1,142187895	1
Cib2	1,142108727	1
Nup188	1,141712971	1
Cbfb	1,141712971	1
Matr3	1,141712971	1
Gstt2	1,141633836	1
Spp1	1,141633836	1
Gm10132	1,141554707	1
Dvl1	1,141554707	1
Zmym1	1,141396465	1
Kif21b	1,141317352	1
Gm11826	1,141000956	1
Plekhh2	1,14084279	1
Gng12	1,140605583	1
Nap1l1	1,140605583	1
Ifi30	1,140447473	1
Rps19-ps9	1,140289384	1
Gm3555	1,140289384	1
Sik3	1,140289384	1
Acap3	1,140131318	1
Efr3b	1,140052292	1
Vars	1,140052292	1
Tanc2	1,139973273	1
Smad4	1,139973273	1
AC168977.1	1,139736247	1
Cenpm	1,139262342	1
Ppif	1,139183377	1
Larp1	1,138867572	1
Tmpo	1,138788635	1
Usp1	1,138709703	1
Napg	1,138630776	1
Gm37728	1,138472939	1
Tmsb10	1,138472939	1
Taf4	1,138394029	1
Cabin1	1,138315124	1
Ankrd37	1,138157331	1
Ints12	1,13799956	1
Nexn	1,137920683	1
Gm11221	1,137841811	1
Zfr	1,137841811	1
Tfb1m	1,137762944	1
Gm11889	1,137762944	1
Dohh	1,137762944	1
Itm2c	1,137762944	1
Hnrnph1	1,137684083	1
Vcp	1,137684083	1
Stxbp1	1,137684083	1

Slc6a6	1,137526378	1
Gigyf2	1,137526378	1
Ccng1	1,137447533	1
Slc37a2	1,137211032	1
Tchp	1,13713221	1
Rfxap	1,137053392	1
A930006K02Rik	1,13697458	1
Gm12716	1,136738178	1
Rnf4	1,136738178	1
Mkln1	1,136423051	1
Tnfrsf10b	1,136344283	1
Skp1a	1,136029265	1
Cdc42se2	1,136029265	1
Ndufc1	1,135793059	1
Tnrc6c	1,135556902	1
Clk4	1,135556902	1
Celf2	1,135556902	1
Acbd5	1,135478194	1
Gnpat	1,135242102	1
Sptssa	1,135163416	1
Slc35e4	1,135084735	1
Myh7b	1,134770066	1
Tgds	1,134691413	1
Slc40a1	1,134612765	1
Ifit2	1,134376853	1
Fbxo3	1,134376853	1
Alg9	1,134219606	1
Bloc1s6os	1,134062381	1
Stk40	1,133826584	1
Sdf2	1,133590836	1
Zfp60	1,133512264	1
Rpl3-ps2	1,133433697	1
Tpk1	1,133355136	1
Ier3ip1	1,133355136	1
Nadk	1,133276581	1
Snhg9	1,133198031	1
Itpkc	1,133119486	1
Zfp598	1,133040947	1
Hyou1	1,132962414	1
Bbx	1,132883885	1
Uqcrh	1,132805362	1
Blmh	1,132805362	1
Trim8	1,132726845	1
Rpl38-ps1	1,132648333	1
Pdcl	1,13241283	1
Gm15730	1,132177376	1
Armt1	1,132098902	1
Rras2	1,131863513	1
Ythdf3	1,131863513	1
Ift122	1,131628173	1
Selenon	1,131392882	1
RP24-365N15.9	1,13115764	1

Rps10-ps2	1,131079237	1
Xpo6	1,131079237	1
Rab9	1,131079237	1
Tmem94	1,131000839	1
Mtr	1,130922447	1
Hmgn5	1,130922447	1
Rab22a	1,13084406	1
Ik	1,130765679	1
Uspl1	1,130687303	1
St3gal2	1,130608932	1
Ip6k1	1,130608932	1
Inafm2	1,130452207	1
Opa1	1,130373853	1
Cwf19l1	1,130138823	1
Bin1	1,13006049	1
Copz1	1,129825525	1
Ndel1	1,129825525	1
Napb	1,129668909	1
Zdhhc3	1,129590609	1
Gm44623	1,129277464	1
RP23-453B15.7	1,129199191	1
Kcnab3	1,129042661	1
Fam102b	1,128886154	1
Lrrc59	1,128807908	1
BC030499	1,128651433	1
Bdp1	1,128573203	1
Arsg	1,128416761	1
Hccs	1,128416761	1
Gm7860	1,128338548	1
Rab5c	1,127947563	1
Nme1	1,127869382	1
Fam188a	1,127322269	1
Ctsc	1,127322269	1
Syncrip	1,127322269	1
Zfp563	1,127244132	1
Efna1	1,127244132	1
Shoc2	1,127244132	1
Rgs3	1,127087874	1
Fcgr3	1,127087874	1
Pdpr	1,127009753	1
Git2	1,127009753	1
Plekhj1	1,127009753	1
Srsf11	1,127009753	1
Vps50	1,126853527	1
Gm24951	1,126775422	1
Tab2	1,126775422	1
Cryzl1	1,126697322	1
Synj1	1,126697322	1
Lrrc24	1,126463057	1
AC133103.1	1,126384979	1
Flt3l	1,126384979	1
Psmd4	1,12622884	1

Tpp1	1,126072722	1
Mms19	1,126072722	1
Tcf12	1,125994671	1
Gm14326	1,125916626	1
Greb1	1,125604499	1
Gm4859	1,125526481	1
Slc8b1	1,125526481	1
Cxcl16	1,125526481	1
Fau	1,125448468	1
Dnm1l	1,125448468	1
Rbbp9	1,12537046	1
Atp2c1	1,12537046	1
Bbof1	1,125292458	1
Nhlrc2	1,125214462	1
Siae	1,125136471	1
Itgb7	1,125058485	1
Elob	1,124980504	1
Rogdi	1,12482456	1
RP24-497N7.2	1,12482456	1
Zfp449	1,124434793	1
Fign	1,124356856	1
Akt1	1,124278924	1
Ywhaq	1,124278924	1
Trp53bp2	1,124200997	1
Ubp1	1,124045161	1
Herpud1	1,124045161	1
Stk38	1,123967251	1
Ptp4a1	1,123889346	1
Btbd1	1,123733553	1
Rufy3	1,123577781	1
Galk2	1,123499903	1
Erlec1	1,123266302	1
S100a3	1,123188446	1
Gm6548	1,123188446	1
5430402O13Rik	1,123110595	1
Rfc5	1,123110595	1
Sowahc	1,122877075	1
Upf3b	1,122799246	1
Vma21	1,122721422	1
9030407P20Rik	1,122643604	1
Snora57	1,122565791	1
Gm38055	1,122487983	1
Yipf6	1,122487983	1
Gm43742	1,122332384	1
Haghl	1,122332384	1
Bclaf1	1,122254592	1
Ndufa3	1,122176806	1
Atxn7l1	1,122099026	1
Idh3g	1,122099026	1
Tnrc6b	1,121943481	1
Atg14	1,121865716	1
Gm20568	1,121787957	1

Cdca3	1,121710203	1
Baz2a	1,121710203	1
Rabl3	1,121399242	1
Rhbdf2	1,121243794	1
C1qtnf6	1,121010662	1
Gm37009	1,120932962	1
Rrbp1	1,120932962	1
Gm5451	1,120855268	1
Bc1-ps1	1,120777579	1
Hbp1	1,120777579	1
AI314180	1,120699895	1
Dap	1,120699895	1
Ncbp2	1,120544544	1
Zswim6	1,120544544	1
Ywhaz	1,120466876	1
Gm44291	1,120389214	1
Zfp784	1,120311557	1
Itch	1,120311557	1
Xpo7	1,120078619	1
Impact	1,120000984	1
Gm12115	1,11984573	1
Ino80b	1,11984573	1
C130071C03Rik	1,119768111	1
Stambpl1	1,119768111	1
Cdk5rap3	1,119690497	1
Lpgat1	1,119690497	1
Ikzf5	1,119690497	1
Rps2	1,119612889	1
Tmem229b	1,119612889	1
Gm13226	1,119302509	1
Apobr	1,119224928	1
Ubr7	1,119224928	1
Zbtb7b	1,119224928	1
Ctss	1,119147352	1
Nedd8	1,119147352	1
Dazap2	1,119147352	1
Rasgrp4	1,119069781	1
Zfp78	1,119069781	1
Cops7b	1,118992216	1
Nxf1	1,118914656	1
Tacc1	1,118914656	1
Slc2a3	1,118837101	1
Cpox	1,118837101	1
Rmi2	1,118759552	1
Nsd3	1,118759552	1
Efr3a	1,11860447	1
Yipf3	1,118526937	1
Afdn	1,117984356	1
Sh2d6	1,117906865	1
Noct	1,117442038	1
Acox1	1,117442038	1
0610009B22Rik	1,117364585	1

Brms1	1,117287138	1
Blvrb	1,11713226	1
3830406C13Rik	1,117054829	1
Gm45360	1,116745158	1
Alkbh5	1,116745158	1
Por	1,116667754	1
Ctsz	1,116512962	1
Scamp3	1,116358191	1
Phf11d	1,116280814	1
Lcor	1,116280814	1
Bmp2k	1,116280814	1
Mprip	1,116280814	1
Spg21	1,116203442	1
Zfp874b	1,116126075	1
4930461G14Rik	1,116048714	1
Mybl1	1,116048714	1
Ndfip1	1,116048714	1
Rab3gap1	1,116048714	1
Clstn1	1,115971358	1
Ppp4r3a	1,115816662	1
Snapc1	1,115584659	1
Gm8508	1,115507335	1
Ccdc136	1,115275396	1
Dgkg	1,115198094	1
Adamtsl4	1,115120797	1
Abracl	1,114734392	1
E230029C05Rik	1,114657127	1
Nufip2	1,114348122	1
Itm2b	1,114270884	1
Tns2	1,114193651	1
Ptpn2	1,114193651	1
Fignl1	1,114116423	1
Srp72	1,114116423	1
Srrm1	1,113961985	1
Zfp532	1,113884774	1
Gm6654	1,113884774	1
1810013L24Rik	1,113884774	1
Gng2	1,113653172	1
Med23	1,113421618	1
Gm11427	1,113344444	1
Nqo2	1,113344444	1
Faf1	1,113112955	1
Atg2a	1,113112955	1
Polr2i	1,113035803	1
Mdh2	1,113035803	1
Dync1h1	1,113035803	1
Relt	1,112958655	1
Ndufa1	1,112804377	1
Msh6	1,112804377	1
Grhpr	1,112727246	1
D930015E06Rik	1,112727246	1
Rnf11	1,112650121	1

Entpd5	1,112573	1
Rap2c	1,112418776	1
Sprydd7	1,112341671	1
Hnrnpa0	1,112341671	1
Hip1	1,112187479	1
Fam210b	1,112187479	1
Ptbp2	1,112187479	1
Atl2	1,112187479	1
Wdr91	1,112033307	1
Iqgap1	1,11195623	1
Gm5611	1,111725029	1
Ap3s1	1,111647973	1
Ppp1r16a	1,111493876	1
Nfia	1,111185748	1
Arnt	1,111185748	1
Nae1	1,110954707	1
Trip13	1,110723714	1
Gm13270	1,110723714	1
Mrpl9	1,110723714	1
Pigq	1,110723714	1
Tmtc4	1,110646728	1
Rrm2	1,110646728	1
Rabggtb	1,110569746	1
Chic2	1,110569746	1
1700066M21Rik	1,11049277	1
Rin1	1,110415799	1
S100pbp	1,110415799	1
Gm8242	1,110261873	1
4930431P19Rik	1,110107969	1
Cnot1	1,110107969	1
Mars2	1,109954086	1
Gm14539	1,109954086	1
Glyctk	1,109877153	1
Clasrp	1,109877153	1
Xylt1	1,109723302	1
Tmc6	1,109723302	1
Hsf2	1,109723302	1
Trim56	1,109723302	1
Ptrh1	1,109569472	1
Rps15a-ps8	1,109492565	1
Clk2	1,109415664	1
Cyp26b1	1,109261877	1
Morc3	1,109261877	1
Gm11737	1,108800644	1
Cdc14b	1,108800644	1
Wasl	1,108800644	1
1110065P20Rik	1,108493261	1
Pdgfb	1,108416429	1
Ak2	1,108339602	1
Mycbp2	1,108262781	1
Ube2e1	1,108185964	1
Cenpw	1,108185964	1

Gm13464	1,108109153	1
Mrps11	1,108109153	1
Scfd2	1,108032348	1
Taf11	1,108032348	1
Trio	1,107955548	1
Stag2	1,107955548	1
Cltc	1,107955548	1
Nde1	1,107878753	1
Arhgef6	1,107801963	1
Eif3l	1,107801963	1
Spata6	1,107725179	1
Ei24	1,107725179	1
Mkx	1,1076484	1
AB124611	1,1076484	1
Ambp	1,107418095	1
Mecr	1,107418095	1
Nkapl	1,107187837	1
Mbd3	1,107111096	1
Nfatc2ip	1,107034359	1
Elovl1	1,107034359	1
Rita1	1,106804182	1
Klhl30	1,106727467	1
Fam160a2	1,106727467	1
Ddx39b	1,106650757	1
Rb1	1,106650757	1
D17Wsu92e	1,106650757	1
Srp9	1,106574052	1
Arpp19	1,106574052	1
Ppt1	1,106574052	1
Gm7514	1,106497353	1
Npc1	1,106497353	1
Gm5578	1,106343971	1
Arfrp1	1,106343971	1
Sh3bgrl3	1,106190609	1
St6galnac4	1,105730653	1
Dock11	1,105654013	1
Vps11	1,105654013	1
Nat2	1,105500747	1
Cnot10	1,105424122	1
Unc5b	1,105347503	1
Pdcd5	1,105117676	1
Cul3	1,105041078	1
Dcaf4	1,104964485	1
Rps6kb2	1,104887897	1
Lsm1	1,104887897	1
Rnf213	1,104887897	1
Tmem39b	1,104658166	1
Dennd1b	1,104428483	1
Scaper	1,104428483	1
Gm17994	1,104275387	1
Dtwd2	1,104122313	1
Kitl	1,104045783	1

Ighm	1,104045783	1
Zdhhc21	1,103739719	1
Asb13	1,103510227	1
Gm2986	1,10343374	1
Lonp2	1,103357258	1
Rhoc	1,103357258	1
Ppp2r5e	1,103280782	1
Tcea2	1,102898481	1
E2f6	1,102669163	1
Kif1b	1,102439893	1
4930404I05Rik	1,102363481	1
Tnfrsf22	1,102363481	1
Crk	1,102210671	1
Krtcap3	1,102134274	1
Invs	1,102134274	1
Gm16072	1,102057883	1
Slc25a32	1,10182874	1
Camk2d	1,10182874	1
Tspan10	1,10175237	1
Ankrd55	1,101676005	1
D8Ertd738e	1,101676005	1
Myo7a	1,101370598	1
Stard5	1,101370598	1
Mrps27	1,101217926	1
Csnk1a1	1,101217926	1
Rbm8a2	1,100912646	1
Cgrrf1	1,100531165	1
Zfp287	1,100226075	1
Isg15	1,09992107	1
Psmd9	1,099844832	1
Lrrk1	1,099768599	1
Hspa13	1,099768599	1
Atp5e	1,099692372	1
Rab8b	1,099616149	1
S100a8	1,099539932	1
Tmem171	1,099387514	1
Gm45184	1,099387514	1
Exosc10	1,099387514	1
Aacs	1,099311313	1
Babam1	1,099311313	1
Cd9-ps	1,099158927	1
Kctd2	1,099158927	1
Tmem167b	1,099158927	1
Mgst2	1,098778053	1
Rora	1,098701895	1
A930018M24Rik	1,098625741	1
Ap5m1	1,098549593	1
Mpp6	1,098549593	1
Traf3	1,09847345	1
Zc3hav1l	1,09832118	1
Sptlc2	1,09832118	1
Ckap2l	1,09832118	1

Washc4	1,09832118	1
Bnip3	1,098245052	1
Supt4a	1,098016702	1
Nfkb2	1,097864496	1
Gm6285	1,097864496	1
Zfp746	1,0977884	1
Marcksl1	1,09771231	1
Wbp1	1,097636225	1
Lpin1	1,097636225	1
Rps25-ps1	1,097636225	1
Atp5b	1,097560145	1
Txn2	1,097255879	1
Tor1aip1	1,097255879	1
Gm44198	1,097103778	1
Rps19bp1	1,097027735	1
Chml	1,096875665	1
Erap1	1,096723616	1
Tox2	1,0966476	1
Cxxc5	1,0966476	1
Piezo1	1,0966476	1
Atf7ip	1,096115632	1
Snx21	1,095583922	1
Fndc10	1,095507985	1
Klf3	1,095204288	1
Chaf1b	1,094900675	1
2210013O21Rik	1,094900675	1
Glul	1,094824785	1
Iqcc	1,094521277	1
Rnf114	1,094521277	1
Pno1	1,094369555	1
Gas5	1,094369555	1
Cpeb3	1,094293701	1
Pank3	1,094066173	1
Coro2a	1,093914514	1
Prdm2	1,093914514	1
Gm12309	1,093838692	1
Otx1	1,093838692	1
Timp1	1,093459662	1
Rpa1	1,093459662	1
Rab11a	1,093459662	1
Cby1	1,093383872	1
Oscar	1,093232307	1
Gm5909	1,092929241	1
Kat7	1,092929241	1
Tma7	1,092777739	1
Fxr1	1,092777739	1
Rwdd2b	1,092474799	1
Gm44116	1,092474799	1
Btbd7	1,09232336	1
Gm16373	1,092247649	1
Ccl4	1,092171942	1
Ly6e	1,092096241	1

Specc1	1,091944855	1
Msmo1	1,091944855	1
Rsl1	1,09186917	1
Zfp131	1,09186917	1
Ttc38	1,09179349	1
Hells	1,091642146	1
Tef	1,091490823	1
Proser3	1,091112606	1
Gab3	1,091112606	1
Hexdc	1,091036979	1
Gm22716	1,091036979	1
Nfx1	1,090885739	1
Yars	1,090810128	1
Rnf138	1,090734521	1
Gm4707	1,09065892	1
Ppp6c	1,090583324	1
Tmem164	1,090356567	1
Tagln2	1,090356567	1
Purb	1,090356567	1
Gm17745	1,090280992	1
Slc38a9	1,090280992	1
Map1lc3b	1,090280992	1
Gm11599	1,090205422	1
E2f7	1,090129857	1
Ap1b1	1,090129857	1
Snupn	1,090054298	1
Mmachc	1,089676579	1
Ganc	1,089676579	1
Zfp280c	1,089298991	1
Pym1	1,089298991	1
Gm38235	1,089223489	1
Gm45728	1,089072501	1
Chchd5	1,089072501	1
Mapkbp1	1,088997015	1
Aasdh	1,088921534	1
Il18rap	1,088544208	1
Tomm22	1,088544208	1
RP24-226A8.2	1,088393315	1
Pak1	1,088393315	1
Gm4204	1,088242442	1
Shox2	1,088167013	1
St3gal6	1,08809159	1
Bcl2l13	1,087940759	1
Hsbp1	1,087940759	1
Homer1	1,087865351	1
Imp3	1,087865351	1
Gm6913	1,087789948	1
Cyp27a1	1,087639159	1
Tfrc	1,087563772	1
Prkd2	1,087488391	1
1700037H04Rik	1,087413015	1
Gpr132	1,087337643	1

Zfyve28	1,087262278	1
Fes	1,087111561	1
Kcnq1ot1	1,087036211	1
Parvb	1,087036211	1
Arf1	1,086885526	1
Crot	1,086810192	1
Aspm	1,086734863	1
Slc9a8	1,086659538	1
Pax3	1,086508906	1
Vcpip1	1,086508906	1
Tomm34	1,086433597	1
Kyat3	1,086207703	1
Chd7	1,086207703	1
Gm42481	1,086132416	1
Gm15727	1,086132416	1
Zbtb8a	1,086057133	1
Spcs2	1,086057133	1
Clpb	1,085981856	1
Saa3	1,085906584	1
Lhx1	1,085906584	1
Pigl	1,085831318	1
Malt1	1,085831318	1
Pqlc3	1,0856808	1
Fam126a	1,0856808	1
A130050O07Rik	1,085605549	1
Ikzf1	1,085530303	1
Capza2	1,085530303	1
Rps3a2	1,085455062	1
Gamt	1,085455062	1
Tmem184b	1,085455062	1
Sh3gl1	1,085304597	1
Tmcc3	1,085154152	1
Sesn2	1,085078938	1
Rp9	1,085078938	1
Rev3l	1,085003728	1
Reep5	1,085003728	1
Iqsec1	1,084928524	1
Acap1	1,084853325	1
Cpped1	1,084853325	1
Csnk2b	1,084702943	1
Stx4a	1,084702943	1
Txndc12	1,084552582	1
Hyls1	1,084477409	1
Pld4	1,084327079	1
Gm12778	1,083876214	1
Kif2c	1,083876214	1
Gm11470	1,083876214	1
Atp6v1a	1,083725967	1
Ube3b	1,083650851	1
Nckap1l	1,083650851	1
Gid4	1,083575741	1
Alkbh8	1,083500636	1

Bphl	1,083350441	1
Acsl1	1,083200267	1
Nfkbil1	1,083125188	1
Nr2c2ap	1,083050114	1
Ubxn2a	1,082975046	1
1810021B22Rik	1,082899982	1
Zfp956	1,082899982	1
Cycs	1,082824924	1
Nup133	1,08259978	1
Ttf2	1,08259978	1
Acads	1,08259978	1
Zic5	1,082524743	1
Trappc2l	1,082524743	1
Rnf34	1,082299661	1
Tjp2	1,082149633	1
Clcn3	1,082074627	1
2310010J17Rik	1,081999626	1
Pex26	1,08192463	1
Arhgdib	1,08192463	1
Ncoa3	1,081849639	1
Cnnm4	1,081774654	1
Trim7	1,081774654	1
Csrnp2	1,081699673	1
Acyp1	1,081699673	1
Pbx1	1,081624698	1
2410002F23Rik	1,081399804	1
Gfm1	1,08132485	1
Ap3m2	1,081249901	1
Csnk1g3	1,081174957	1
Glipr1	1,081174957	1
Plat	1,081100018	1
Atp1a1	1,081100018	1
Galnt3	1,081025084	1
Lrba	1,081025084	1
Soat1	1,080800315	1
Zc3hav1	1,080800315	1
Ugt1a7c	1,080575592	1
Gm42600	1,080575592	1
D030056L22Rik	1,080575592	1
Cln8	1,080575592	1
Pes1	1,080425803	1
Slc46a3	1,080276034	1
Ephx1	1,079826854	1
Fsd2	1,079752008	1
2410022M11Rik	1,079602333	1
Myl12b	1,079452679	1
Mettl9	1,07937786	1
Cdyl	1,07937786	1
Mrpl16	1,07937786	1
Fpgs	1,079228237	1
Phtf1	1,079228237	1
2610002M06Rik	1,079153433	1

Gm15625	1,079003841	1
B4galt1	1,078929052	1
Mvp	1,078704719	1
Rab34	1,078704719	1
Abhd13	1,078629951	1
Calm3	1,078629951	1
Tatdn2	1,078480432	1
Caly	1,078181455	1
Kif16b	1,078106724	1
Rbm15	1,078106724	1
Sec31a	1,078031998	1
0610012G03Rik	1,078031998	1
Gm37354	1,077957277	1
Vdac1	1,077882561	1
Fubp3	1,077733145	1
Tnpo3	1,077733145	1
Gm45716	1,077658445	1
Klf8	1,077658445	1
Hist1h1c	1,07750906	1
Cenpp	1,077434375	1
Gan	1,077434375	1
Amfr	1,077285022	1
Gm6394	1,07706103	1
Polr2j	1,076986376	1
Arid1a	1,076986376	1
Bzw1	1,076986376	1
Gm31166	1,076837085	1
Serpinb6a	1,076613186	1
Gm5117	1,076538563	1
Dnaja1	1,076538563	1
Ubxn4	1,076538563	1
Rpsa-ps9	1,076389334	1
Hmgb1-ps8	1,076389334	1
Unc13b	1,076090937	1
Fchsd2	1,076090937	1
Pdcd7	1,07601635	1
Cab39l	1,075941769	1
Kctd5	1,075867193	1
Nfam1	1,075643496	1
Rexo1	1,075643496	1
Tssc4	1,075643496	1
Sdhaf3	1,075568941	1
Jmjd6	1,075121717	1
Ints6	1,075047198	1
Ncoa5	1,075047198	1
2310034G01Rik	1,074749173	1
Tln2	1,074749173	1
Sdhaf2	1,074525708	1
Fcgr4	1,074376758	1
Wipf2	1,07430229	1
Rbm26	1,074153371	1
Ccnt1	1,074153371	1

Slmap	1,073855593	1
Atox1	1,073781162	1
Psmg1	1,073632315	1
Kif3a	1,073632315	1
Flywch1	1,073557899	1
Stx16	1,073557899	1
Bend6	1,073185896	1
Rsu1	1,073111511	1
Nabp2	1,072962757	1
Rnps1	1,072739664	1
Zfand2a	1,072739664	1
Gm15535	1,072665309	1
Mccc1	1,072665309	1
Gas2l3	1,072590961	1
Ttc3	1,072516617	1
Cxx1b	1,072293616	1
H2afv	1,072293616	1
Zfp771	1,072144975	1
Gucy2g	1,072070662	1
Milr1	1,071996355	1
Lman1	1,071699175	1
Samd10	1,071624893	1
Syngr1	1,071402079	1
Fam65a	1,071327817	1
Ssr1	1,071253561	1
4833439L19Rik	1,071105064	1
Slc15a4	1,071105064	1
Cntd1	1,070956588	1
Gm8013	1,070956588	1
Fig4	1,070882357	1
Gm4866	1,070808132	1
Cct8	1,070659697	1
Arel1	1,070511282	1
Ghitm	1,070511282	1
Ifi204	1,070066161	1
Exoc3l	1,070066161	1
Zbtb11	1,069991993	1
Acap2	1,069917829	1
Tigd2	1,069917829	1
Prps1	1,069917829	1
Man2a2	1,069917829	1
C130013H08Rik	1,06984367	1
Gm19620	1,06984367	1
Rap2a	1,069621226	1
Rnf167	1,069621226	1
Tm4sf19	1,069398827	1
Idnk	1,069324705	1
Brk1	1,069250588	1
Rrp12	1,069176475	1
Myo9a	1,069176475	1
Dld	1,069028266	1
Arf6	1,06873191	1

2810001G20Rik	1,068657834	1
Pla2g2d	1,068583762	1
Uqcrh-ps2	1,068435635	1
Slc9a1	1,06836158	1
Ccdc28a	1,068139443	1
Bri3bp	1,068139443	1
Gm43359	1,068065408	1
Taf6l	1,067991378	1
Cbfa2t2	1,067917353	1
Eif3h	1,067843333	1
Mcu	1,067621304	1
Lsm3	1,067547305	1
Gm17786	1,067325338	1
Syce2	1,067325338	1
Chmp2b	1,067251359	1
Rnf19b	1,067177386	1
Pik3ap1	1,067103417	1
4930426I24Rik	1,067029454	1
1110008L16Rik	1,066733651	1
Gatc	1,066585781	1
Cr1l	1,066437931	1
Tmem87b	1,066364014	1
Trp53i13	1,066142293	1
1700007K09Rik	1,066142293	1
Serpini1	1,066068396	1
Tdp2	1,066068396	1
Dusp1	1,065994504	1
Morf4l2	1,065920617	1
Gm11878	1,06577286	1
Galc	1,065698989	1
Plp2	1,065698989	1
Gm12618	1,065551262	1
Olfr95	1,065477406	1
Sh2d3c	1,065403555	1
Gm29666	1,065329709	1
RP24-232D3.1	1,065255869	1
Sertad3	1,065255869	1
Gm26384	1,065182034	1
Gm45033	1,065108203	1
3110043O21Rik	1,065108203	1
Snrpe	1,065108203	1
Gm45836	1,065034378	1
Gm29257	1,064960558	1
Ndufs6	1,064960558	1
Zfp512b	1,064665329	1
Fam193a	1,064665329	1
Sipa1l2	1,064591535	1
C920021L13Rik	1,064591535	1
Tanc1	1,064591535	1
Thoc2	1,064517746	1
Terf1	1,064443962	1
Cyfip1	1,064443962	1

Alg12	1,064370182	1
Rapgef2	1,064370182	1
Rasl2-9	1,064296408	1
Mga	1,064296408	1
Gm5828	1,06422264	1
Golgb1	1,06422264	1
1700012D14Rik	1,064075117	1
Rps5	1,064075117	1
March5	1,063927615	1
Lrrc58	1,063780134	1
Npm1	1,063780134	1
Cask	1,063706401	1
Rpf1	1,063632673	1
Phtf1os	1,063485232	1
Klhdc4	1,06341152	1
Gm7809	1,06326411	1
Gm35315	1,063043033	1
Sep 10	1,063043033	1
Capn7	1,063043033	1
Nmt1	1,062969351	1
Clcn5	1,062674674	1
Shcbp1l	1,062527367	1
Trim33	1,062527367	1
Sec24a	1,062085566	1
Wbp2	1,06201195	1
Cdc42bpg	1,061791134	1
Mr1	1,061791134	1
Gm43681	1,061717539	1
Rpl23a-ps5	1,061643949	1
Acaca	1,061570364	1
Micu1	1,061496784	1
H2-K1	1,061496784	1
Rps8-ps4	1,061423209	1
Bpnt1	1,061055411	1
Phldb3	1,061055411	1
Gm29243	1,061055411	1
Plxnb2	1,061055411	1
Mrpl30	1,061055411	1
Dnajc18	1,060981867	1
Fmnl1	1,060908328	1
Tmem135	1,060908328	1
Clec3b	1,060834794	1
Arid2	1,060761265	1
Calcoco1	1,060540709	1
Ddx3y	1,060540709	1
Ctps	1,060393697	1
Trim24	1,060320199	1
Snrnp200	1,060320199	1
Zcchc14	1,060173217	1
Gm8463	1,060099734	1
Gm13578	1,060026256	1
Slc6a12	1,060026256	1

Zmym3	1,059952783	1
Suco	1,059952783	1
Gm43387	1,059879316	1
Usp8	1,059879316	1
Lmo4	1,059805853	1
Gm10575	1,059732395	1
Cnppd1	1,059658943	1
Mthfsd	1,059585495	1
Map3k14	1,059585495	1
Iws1	1,059438616	1
Prkci	1,059365184	1
Npat	1,059291757	1
Uty	1,059291757	1
Eef2	1,059144918	1
Ppp1r18	1,058998099	1
Fam199x	1,058851301	1
Diaph1	1,058704523	1
Zfp954	1,058557766	1
4930440I19Rik	1,058484395	1
Bbs7	1,058337668	1
Nacc2	1,058190961	1
Parl	1,058117616	1
Ddx51	1,058044275	1
Entpd1	1,058044275	1
Slc12a7	1,058044275	1
Yipf1	1,058044275	1
Ubtd2	1,05797094	1
Snapc4	1,057750964	1
Aifm2	1,057750964	1
Rps19-ps7	1,057604338	1
Myo1f	1,057604338	1
B3gat3	1,057531033	1
Tifab	1,057384439	1
Gm45221	1,057384439	1
Gm44609	1,057384439	1
Atp5sl	1,057384439	1
Rpl22	1,057384439	1
Krr1	1,057311149	1
Gdf9	1,057164585	1
Prkca	1,056871517	1
Parvg	1,056725014	1
Haus6	1,056725014	1
Ankfy1	1,056725014	1
Hsd17b7	1,056651769	1
Slc12a9	1,056578531	1
Gm11652	1,056505297	1
Fermt3	1,056505297	1
Rps13-ps7	1,056432068	1
Mpv17l2	1,056432068	1
Tbl1x	1,056432068	1
Gm44950	1,056285625	1
Ttc19	1,056285625	1

Card19	1,056139203	1
Setd5	1,056066	1
Gm6088	1,055919608	1
Fam214a	1,055919608	1
Kcnab2	1,055919608	1
Arhgef39	1,055700059	1
Inip	1,055700059	1
Ipo11	1,055626886	1
Max	1,055626886	1
Ncoa1	1,055480555	1
Gm45250	1,055407397	1
Parp9	1,055334244	1
Anapc7	1,055261097	1
Ablim1	1,055187954	1
Wac	1,055114816	1
Rpn2	1,055041684	1
Dynlrb1	1,054968557	1
Ric8b	1,054822317	1
Gphn	1,054822317	1
Ddb1	1,054822317	1
Gm13736	1,054529899	1
Dtnb	1,054529899	1
Gm14593	1,054456807	1
Ttpal	1,054310638	1
Sirt2	1,054310638	1
Gm10320	1,054237562	1
Antxr2	1,05416449	1
Ulk1	1,05416449	1
Tmem60	1,05416449	1
E130308A19Rik	1,054091423	1
Mbtps2	1,054018362	1
Med6	1,054018362	1
Zc3h14	1,053872254	1
Gm12989	1,053799208	1
Chchd7	1,053726166	1
Dab2	1,053507073	1
Myl6b	1,053361036	1
Cdkn2aip	1,053361036	1
Wdtc1	1,053288025	1
Tmem14a	1,053142018	1
D830050J10Rik	1,052996032	1
Sos1	1,052996032	1
Alg10b	1,052923046	1
Park7	1,05277709	1
Mef2d	1,05270412	1
Eif2b2	1,052558194	1
Pus7	1,052485239	1
Usp2	1,052485239	1
Baz1b	1,052485239	1
Mtx2	1,052412289	1
Zcchc8	1,052412289	1
Papola	1,052412289	1

Mob1a	1,052339344	1
Mrfap1	1,052266404	1
Trp53bp1	1,052193469	1
Rest	1,052047614	1
Fam118a	1,051974694	1
1700007L15Rik	1,051974694	1
Vamp4	1,051901779	1
C330013E15Rik	1,051828869	1
Brd4	1,051683065	1
Exoc4	1,051683065	1
Ptar1	1,051464397	1
Gtf3c1	1,051391517	1
Pgm2l1	1,051245773	1
Gm7658	1,051245773	1
Plekhb2	1,051172909	1
Selenok	1,051172909	1
Ccar1	1,051172909	1
Pfkm	1,051027196	1
Fam168a	1,051027196	1
Cks1b	1,050954347	1
N4bp2l1	1,050808663	1
Pnpla2	1,050735829	1
RP24-122E11.4	1,050663001	1
Trim14	1,050663001	1
Rbbp8	1,050663001	1
Man1a2	1,050371735	1
Csnk1g1	1,050371735	1
Cc2d1b	1,050298932	1
Pip4k2c	1,050226133	1
Dand5	1,050153339	1
Sep 02	1,050007767	1
Spcs1	1,050007767	1
Safb2	1,049862215	1
Skap2	1,049862215	1
Rab3a	1,049643925	1
Yrdc	1,049571172	1
Dock10	1,049571172	1
Pex6	1,049425681	1
RP23-331E5.10	1,049207481	1
Dnm2	1,049207481	1
Prrc2a	1,048989328	1
Rgs1	1,048989328	1
Dhx15	1,048989328	1
Ccdc167	1,048843917	1
Sass6	1,048843917	1
Emsy	1,048553156	1
Tbc1d24	1,048480478	1
Gm13776	1,048407806	1
Anp32b	1,048407806	1
Tmem35b	1,048189818	1
Saraf	1,048189818	1
Hcfc2	1,048117166	1

Cct5	1,047899238	1
H2-DMb2	1,047826606	1
Ccdc124	1,047681357	1
Zfp607a	1,047536127	1
Cers6	1,04746352	1
Bhlhe40	1,04746352	1
Cdc5l	1,047245729	1
2810408I11Rik	1,04710056	1
Rpl31-ps11	1,04710056	1
Fancl	1,04710056	1
Trim23	1,047027983	1
Mpp1	1,046955411	1
Eml5	1,046882844	1
Clec4a3	1,046737725	1
Rpl18a	1,046737725	1
Fitm2	1,046665173	1
Tmx3	1,046592627	1
Gmcl1	1,04630249	1
Pold4	1,046157451	1
Atp8b3	1,046012433	1
Ergic2	1,045939932	1
Cd72	1,045939932	1
Zbtb17	1,045649976	1
Atp6v0e	1,045505028	1
Cdc40	1,045432562	1
Rps24-ps3	1,045287644	1
Macf1	1,045070305	1
Stx8	1,044925438	1
Gltscr1l	1,044708174	1
Ptger4	1,044708174	1
Abhd14a	1,044635763	1
Gatsl3	1,044635763	1
Phactr4	1,044563357	1
Git1	1,044563357	1
4930539J05Rik	1,04441856	1
Ccdc93	1,044273782	1
Tmem199	1,044201401	1
Lrp6	1,044056654	1
Map4k4	1,043694875	1
Ddx54	1,043622534	1
Ppp2r1b	1,043477867	1
Med27	1,04333322	1
Odf3l1	1,043260904	1
Cuedc1	1,043260904	1
Aven	1,043043987	1
Dok2	1,043043987	1
Gm37900	1,0428994	1
Xlr	1,042827115	1
9530082P21Rik	1,042754834	1
0610009L18Rik	1,042465761	1
Smc1a	1,042393505	1
Kansl2	1,042321254	1

Gtpbp1	1,042321254	1
Pde6d	1,042249009	1
Gm26935	1,042176768	1
Pds5b	1,042032302	1
Zbtb41	1,041960076	1
Tfap4	1,041960076	1
Pphln1	1,041887855	1
Cbx1	1,041887855	1
Ccne1	1,04181564	1
2010107E04Rik	1,041743429	1
Ndst2	1,041671223	1
Tap1	1,041526827	1
Dusp19	1,041382451	1
Ubr2	1,041382451	1
Gpd1l	1,04131027	1
Mrps17	1,041093758	1
Atp5f1	1,041093758	1
Suclg2	1,041021598	1
Zfp994	1,040949442	1
Scmh1	1,040949442	1
Aen	1,040877291	1
Senp2	1,040733005	1
Tob2	1,040444493	1
Pfkl	1,040372377	1
Pkm	1,040372377	1
Gm45109	1,040300267	1
Map2k5	1,040228161	1
Cep89	1,04015606	1
Rpp21	1,040011874	1
Crlf2	1,039939788	1
Cox7b	1,039939788	1
Zfp638	1,039723561	1
Mllt11	1,039723561	1
Bcor	1,039651496	1
Mff	1,039435329	1
Cdyl2	1,039147176	1
Sgf29	1,039147176	1
Lyrm9	1,03907515	1
Tecpr2	1,038787098	1
Trim35	1,038787098	1
Wwp2	1,038499125	1
Llph	1,038499125	1
Gm12380	1,038427144	1
Zfp275	1,038211231	1
Rnf128	1,038211231	1
Sart3	1,038139271	1
Nrp1	1,038067315	1
Gm17455	1,038067315	1
Nthl1	1,038067315	1
Emc6	1,037995364	1
Cd52	1,037995364	1
Gm6419	1,037851477	1

Cdk11b	1,037851477	1
Ints13	1,03770761	1
Tmem230	1,03770761	1
Dctn4	1,037635684	1
Mapre1	1,037563763	1
Gm9294	1,037491848	1
Ago1	1,037491848	1
Rpl36	1,037491848	1
Coq8a	1,037419937	1
Vps9d1	1,037348031	1
Hk1	1,037348031	1
Pcca	1,03727613	1
Ssc5d	1,037204234	1
Mtmr12	1,037204234	1
Ift46	1,037132343	1
Rpa2	1,037060457	1
Fbxw8	1,037060457	1
Ilvbl	1,036988575	1
1810058I24Rik	1,036988575	1
Nrg4	1,036844828	1
Pcgf3	1,036844828	1
Trappc10	1,036772962	1
Slc29a1	1,036772962	1
Cisd2	1,036701101	1
Zufsp	1,036557394	1
Gng7	1,036485547	1
Crat	1,036485547	1
Zfp160	1,036485547	1
Fam173a	1,036413706	1
Pycrl	1,03634187	1
Scaf8	1,03634187	1
9930021J03Rik	1,036270039	1
Tfdp1	1,036198213	1
Pop1	1,036126391	1
Hdac3	1,036054575	1
Rpl23a	1,035767359	1
Mrps14	1,035767359	1
Pard6b	1,035695568	1
Abl1	1,035623782	1
Gnaq	1,035623782	1
Oas3	1,035552	1
Zbtb11os1	1,035480224	1
Usp40	1,035408452	1
Top2b	1,035336685	1
Cog6	1,035264924	1
Srpr	1,035193167	1
Arvcf	1,034906191	1
Pold3	1,034906191	1
Nudt16l1	1,034906191	1
Cox5b	1,034834459	1
Sdccag3	1,034834459	1
Brd7	1,034834459	1

Mfsd7b	1,034762732	1
Dgcr14	1,03469101	1
Flot2	1,03469101	1
Fxn	1,034619293	1
Mrps31	1,034475875	1
Tmf1	1,034475875	1
Snrnp70	1,034475875	1
Mrpl35	1,034475875	1
Il15	1,034404173	1
Trim21	1,034332476	1
Cdk5rap2	1,034332476	1
Shisa5	1,034332476	1
Phf11b	1,034260784	1
Spast	1,034260784	1
Rpsa-ps2	1,034189097	1
Taok2	1,034189097	1
Llph-ps1	1,034117415	1
Gm15207	1,034117415	1
Impdh1	1,034045738	1
Sh3pxd2b	1,033974066	1
Zyg11b	1,033902398	1
Pim2	1,033830736	1
Mapk14	1,03361578	1
Ninj1	1,03361578	1
Alcam	1,0334725	1
Calm2	1,0334725	1
Zfp384	1,033400868	1
Glyr1	1,033400868	1
Tapt1	1,03332924	1
2310068J16Rik	1,033257618	1
Zfp541	1,033257618	1
Cdhr4	1,033114388	1
Ormdl2	1,033114388	1
Arl8b	1,03304278	1
Ppp2r5b	1,03289958	1
Adnp2	1,032827987	1
Eci2	1,032827987	1
Piwil2	1,0327564	1
Rufy2	1,0327564	1
Dph3	1,032684817	1
Nek3	1,032613239	1
Usf2	1,032613239	1
Nemf	1,032398535	1
Gnptg	1,032326978	1
1700022N22Rik	1,032255425	1
Apoa1bp	1,032255425	1
Tfdp2	1,032183877	1
Atp13a2	1,032183877	1
Aip	1,032112334	1
Dlgap4	1,031969262	1
Mlx	1,031826211	1
Grk4	1,031468669	1

Gm9800	1,031468669	1
Slc35e1	1,031468669	1
Nfe2l2	1,031468669	1
Pcyox1	1,031397176	1
Ccdc82	1,031325687	1
Impdh2	1,031254204	1
Tbck	1,031254204	1
Pxk	1,031254204	1
Ccdc47	1,031182725	1
Cnot8	1,030968319	1
Ttll3	1,03089686	1
Acbd6	1,030753957	1
Taok1	1,030682513	1
Samhd1	1,030611074	1
Fyb	1,030468211	1
Heatr5a	1,030325368	1
Tsr2	1,030039741	1
Atg5	1,030039741	1
Gm5865	1,029968346	1
Nudcd2	1,029968346	1
Fam84b	1,029896957	1
Haus3	1,029896957	1
Adcy7	1,029825572	1
Prpf40a	1,029825572	1
Ppp2ca	1,029825572	1
Cyb5r1	1,029754193	1
Aprt	1,029754193	1
Tpt1-ps5	1,029611448	1
2810030D12Rik	1,029611448	1
Gale	1,029540083	1
Exoc1	1,029111999	1
Gas7	1,028898023	1
Atp6v0d1	1,028826708	1
Lst1	1,028684092	1
Polr1b	1,028612792	1
Brd3	1,028612792	1
Tbc1d23	1,028327639	1
Gm44953	1,028256363	1
Cope	1,028185093	1
Yipf7	1,028113827	1
Grpel2	1,028042566	1
Vps39	1,028042566	1
Spen	1,02797131	1
Cep57	1,027900059	1
Hmg20b	1,027900059	1
Acer3	1,027828812	1
Osm	1,027757571	1
Gm13450	1,027615104	1
Gm9625	1,027472656	1
Rab1a	1,027472656	1
Zc3hc1	1,027472656	1
Arpc1b	1,027472656	1

Mgat4b	1,027401439	1
Mlf1	1,027330228	1
Phf21a	1,027330228	1
Tmem242	1,027259021	1
Cd81	1,027187819	1
Rps24-ps2	1,027045431	1
Traf1	1,026831885	1
Ap3s2	1,026831885	1
Hcfc1r1	1,026831885	1
Yeats2	1,026689546	1
Micall1	1,026618383	1
Arap1	1,026618383	1
Gm5898	1,026476074	1
Ccdc106	1,026476074	1
Gm37339	1,026476074	1
Inpp5k	1,026476074	1
Hacl1	1,026404926	1
Spata33	1,026333784	1
Gm8995	1,026333784	1
Snhg3	1,026333784	1
Twsg1	1,026191514	1
Gm5963	1,026191514	1
Gm45802	1,026120386	1
Mef2a	1,025978145	1
B230398E01Rik	1,025907032	1
Tmem143	1,025907032	1
Bag3	1,025907032	1
Mtfr1l	1,025835924	1
Nbr1	1,02562263	1
March2	1,025551542	1
Xylb	1,025338306	1
Gm20620	1,025125116	1
Cbr1	1,024983013	1
Bbs12	1,024911969	1
Ide	1,024911969	1
Gm10388	1,024769896	1
Fmnl3	1,0242018	1
2010015M23Rik	1,024059826	1
Rpl30-ps11	1,023775935	1
Gm43788	1,023775935	1
Atf2	1,023704975	1
Nup62	1,02363402	1
Gpd2	1,023492124	1
Crem	1,023208391	1
Rplp0	1,023066554	1
Zfp580	1,022995643	1
Nupr1	1,022853835	1
Atp10d	1,022782939	1
Tnfrsf11a	1,022782939	1
Fam45a	1,022712047	1
Col4a3bp	1,022641161	1
BC065397	1,022428531	1

Tkfc	1,022428531	1
Setd2	1,022215945	1
Scamp2	1,022145093	1
Sgsm3	1,022074245	1
mt-Nd4	1,022003403	1
Nedd1	1,021932566	1
Ccdc88a	1,021932566	1
2610021A01Rik	1,021861733	1
Fgr	1,021861733	1
Ndc1	1,021720083	1
Gm11970	1,021720083	1
Alkbh6	1,021649265	1
Rfc1	1,021649265	1
Tle3	1,021578452	1
Mink1	1,021436841	1
Thap4	1,021436841	1
Vcp-rs	1,021153678	1
Carmil1	1,021012126	1
Tmem30a	1,021012126	1
Cklf	1,020941357	1
Ccpg1	1,020941357	1
Rhbdd1	1,020870593	1
Ciao1	1,020870593	1
Mycbp	1,020799834	1
Sec61b	1,020587587	1
Gtf2e2	1,020446113	1
Mcub	1,020375384	1
Lgals3	1,020163225	1
Rnf123	1,020092515	1
Ambra1	1,020092515	1
Gm3699	1,02002181	1
Hmx2	1,02002181	1
D330045A20Rik	1,019809724	1
Top3b	1,019739039	1
Nek7	1,019739039	1
Clec16a	1,019385686	1
Cdipt	1,019385686	1
Gm7160	1,01931503	1
Zfp655	1,01931503	1
Rassf3	1,019103091	1
Ppp1cb	1,019103091	1
Zfp608	1,018961823	1
Ppil2	1,018961823	1
Cflar	1,018961823	1
Slc5a6	1,018820575	1
Rpap3	1,018820575	1
Gm45286	1,018749958	1
Ppp1r21	1,018749958	1
Rabggta	1,018679346	1
B230369F24Rik	1,018608739	1
Gm32856	1,018608739	1
Ndufv3	1,018608739	1

Vaultrc5	1,018538137	1
Srbd1	1,018538137	1
Ccdc34	1,018538137	1
Anapc10	1,01846754	1
Gm37660	1,01832636	1
Snrpb2	1,01832636	1
Azin2	1,018255777	1
Smim11	1,018255777	1
Sh2d5	1,0181852	1
2610016A17Rik	1,018114627	1
Rps15a-ps5	1,018114627	1
Gm14584	1,018044059	1
Gm13712	1,018044059	1
Hpgds	1,017973496	1
Snrpert	1,017902938	1
Brdt	1,017902938	1
Zfp410	1,017832385	1
Gdpd3	1,017761836	1
Uhmk1	1,017691293	1
Lmntd2	1,017620755	1
Gm12857	1,017479692	1
Fam96a	1,017409168	1
Gm4978	1,017268135	1
Gm38120	1,017197626	1
E330011M16Rik	1,017197626	1
Anks1	1,017197626	1
Ran	1,017197626	1
Gm37183	1,016986128	1
Tbcb	1,016986128	1
Ppih	1,016915638	1
Cmc4	1,016915638	1
Nrd1	1,016915638	1
Eid1	1,016845153	1
Xndc1	1,016774673	1
Cds2	1,016704198	1
Epb41l4aos	1,016633728	1
Cdadc1	1,016563263	1
Cggbp1	1,016563263	1
Ehd2	1,016492803	1
Trem2	1,016422347	1
Ahcyl1	1,016422347	1
Gmppa	1,016351897	1
Ppp1r9b	1,016070143	1
Cep97	1,015999717	1
Xrn1	1,015999717	1
Dpy19l1	1,015999717	1
Borcs6	1,015999717	1
2610001J05Rik	1,015929296	1
Gm26670	1,015858879	1
Myof	1,015858879	1
Gm11362	1,015647659	1
Slc25a13	1,015577262	1

Tec	1,015295723	1
Trim39	1,015225351	1
Plekha5	1,015154983	1
Ago4	1,015084621	1
Brca1	1,015014263	1
Nkiras1	1,015014263	1
Ciapin1	1,015014263	1
Gcdh	1,01494391	1
Gm43096	1,01494391	1
Zfp142	1,01494391	1
Ddx39	1,014803218	1
Thada	1,01473288	1
Cd320	1,014521894	1
Gm43294	1,014451575	1
Gm14857	1,014451575	1
G730013B05Rik	1,014310952	1
Mtor	1,014310952	1
Chm	1,014240648	1
Ncor1	1,014100054	1
Coq10a	1,01395948	1
Emilin2	1,01395948	1
Wdr41	1,0138892	1
Kmt2a	1,0138892	1
Sf3b2	1,0138892	1
D330041H03Rik	1,013818925	1
Gm43111	1,013748655	1
Sil1	1,013608129	1
Mrrf	1,013537874	1
Pdcd6ip	1,013537874	1
Oraov1	1,013467623	1
Gm13862	1,013467623	1
Fam114a2	1,013467623	1
Kcmf1	1,013467623	1
Rpl36-ps10	1,013397377	1
Tmem70	1,013397377	1
Gm44791	1,013327136	1
Sacm1l	1,013186669	1
Herc6	1,012905793	1
Gm8606	1,012905793	1
Aebp2	1,012765384	1
Gm8254	1,012695187	1
Saal1	1,012554807	1
Gm38157	1,012274106	1
Nfkbib	1,012133785	1
Lrrc40	1,012063632	1
Dscr3	1,011993483	1
Tnnc1	1,01192334	1
Mkl2	1,011853201	1
Kdm5d	1,011783067	1
Nrtn	1,011642814	1
Sec23b	1,011572695	1
Tm9sf3	1,01150258	1

Fundc2	1,01150258	1
Glce	1,011432471	1
Ccdc92	1,011292266	1
Psd	1,011292266	1
Wdr26	1,011152081	1
Gm6524	1,011081996	1
Stxbp2	1,011081996	1
Lats1	1,010871769	1
Utp4	1,010871769	1
Frg1	1,010801703	1
Chrnb2	1,010661586	1
Gk	1,010521488	1
Ncbp3	1,01038141	1
Zfp317	1,01038141	1
Prcc	1,010101311	1
Btg2	1,010101311	1
Eed	1,010101311	1
Dennd2c	1,010031299	1
Psmg4	1,010031299	1
Rdx	1,009751298	1
Maoa	1,009471374	1
Nenf	1,009471374	1
Gm11675	1,009331441	1
Usp39	1,009331441	1
Aes	1,009331441	1
Trappc6a	1,009261482	1
Rida	1,009191528	1
Mtus1	1,009191528	1
Dynll1	1,009191528	1
Pip4k2a	1,008981694	1
D130017N08Rik	1,008911759	1
Nmd3	1,008911759	1
Rab21	1,008841829	1
Fastk	1,008771904	1
Fam208a	1,008562158	1
4930518I15Rik	1,008072919	1
Ccdc112	1,008072919	1
Map4	1,00765376	1
Haus8	1,00751408	1
Nr2c2	1,007444246	1
Mrto4	1,007444246	1
Pon3	1,007374418	1
Tor2a	1,007374418	1
Cpq	1,007304595	1
Naa38	1,007304595	1
Abl2	1,007164962	1
Vapa	1,007164962	1
Tmco6	1,007025349	1
Ptpn23	1,00695555	1
Ercc4	1,006885756	1
Edf1	1,006815966	1
Prkab1	1,006746181	1

Actb	1,006676401	1
Klhdc2	1,006676401	1
Desi1	1,006467091	1
Tle1	1,006467091	1
Prr14	1,00639733	1
Pcnx	1,00639733	1
Rrn3	1,00639733	1
Extl1	1,006257823	1
Kdm2b	1,006257823	1
Cept1	1,006188077	1
Spred2	1,006118336	1
Prr36	1,005909142	1
Slc30a9	1,005909142	1
Neil1	1,00583942	1
Stx6	1,005769703	1
Pigf	1,005769703	1
Ccny	1,005769703	1
Gm2756	1,00569999	1
Gm12231	1,00569999	1
Ctcf	1,00569999	1
Gm15484	1,00556058	1
Cdk4	1,005490883	1
Ssbp3	1,00542119	1
Snhg12	1,005281818	1
Echdc1	1,00521214	1
Tnfrsf23	1,005142466	1
Cacfd1	1,005003134	1
Gm23458	1,00486382	1
Scp2	1,00486382	1
Fosb	1,004724526	1
Lyar	1,004585252	1
Vps26b	1,004585252	1
Ube2w	1,004445996	1
Gm5239	1,004376376	1
Gtf3c5	1,004376376	1
Prkra	1,004237149	1
Gm20430	1,004097942	1
Rasip1	1,003819586	1
Gm11474	1,003819586	1
Kdm1a	1,003680436	1
Sapcd1	1,003680436	1
Cpsf6	1,003541306	1
Itga4	1,003054503	1
Epb41l2	1,00291546	1
Serf2	1,002845945	1
Gm44152	1,002706931	1
Ldah	1,002706931	1
Pcgf2	1,002706931	1
Bola3	1,002290004	1
Akap13	1,002290004	1
Zxdb	1,002151066	1
Gm9645	1,002151066	1

Cetn3	1,002151066	1
Pomc	1,002012148	1
Taf8	1,002012148	1
Gm11942	1,001942697	1
Tstd2	1,001942697	1
Mttp	1,00187325	1
Pvt1	1,001803808	1
1700086O06Rik	1,00159551	1
Nsun6	1,001526087	1
Atp5s	1,001456669	1
Tcp1	1,001456669	1
Gm6023	1,001109651	1
Atg3	1,001040261	1
Nus1	1,001040261	1
Dusp7	1,000970877	1
Eif3d	1,000970877	1
Atic	1,000762753	1
Appbp2	1,000624027	1
Slc39a4	1,000415975	1
Ulbp1	1,000415975	1
Mirlet7b	1,000346634	1
Pdha1	1,000346634	1
Dcun1d3	1,000069317	1
Gm42418	1	1
Fancc	-0,00014003	1
D630023F18Rik	-5,03E-05	1
Itgb5	-0,00019423	1
Timm21	-0,0009068	1
Kmt5a	-0,00094839	1
Get4	-0,0010802	1
Atp13a3	-0,001123	1
C130026I21Rik	-0,001566	1
Slc39a10	-0,0016194	1
Ctsb	-0,001696	1
Eif2s2	-0,0020061	1
Ezh2	-0,0020697	1
Rac1	-0,0021355	1
BC037039	-0,0021855	1
D230025D16Rik	-0,0024096	1
Leo1	-0,0025237	1
Gatad1	-0,0025078	1
Ubr4	-0,0024506	1
Cxxc1	-0,0025743	1
Mrpl33	-0,0026033	1
Gm38366	-0,0027784	1
Gmip	-0,0028447	1
Gm38355	-0,002852	1
Plcg1	-0,0028799	1
Kdm2a	-0,0030169	1
Sipa1l1	-0,0033434	1
Slc35c2	-0,0034225	1
Snapc2	-0,0034554	1

Dph6	-0,0035896	1
Ptch1	-0,0040321	1
Sft2d2	-0,0039822	1
Gm20274	-0,0041451	1
L3mbtl2	-0,0041864	1
Sdha	-0,0046428	1
Plekhm3	-0,004737	1
Csgalnact2	-0,0049024	1
Naa30	-0,0049445	1
Dis3l	-0,0049982	1
Zranb1	-0,0051378	1
Atp2a2	-0,0052854	1
Akap8	-0,0055224	1
Kpna6	-0,0057415	1
Rhot1	-0,0057482	1
Bora	-0,0058134	1
Gm5617	-0,0058497	1
Ubqln1	-0,0059	1
Arf3	-0,0060914	1
Bloc1s6	-0,0063002	1
Wsb1	-0,0064735	1
Asb1	-0,0066065	1
Tex264	-0,006674	1
Cetn2	-0,0066789	1
Fopnl	-0,0068419	1
Fbxl12os	-0,0068866	1
Rpl34	-0,007198	1
Gm43062	-0,0072559	1
Myg1	-0,0072838	1
Ddx41	-0,0073736	1
Ubr3	-0,007373	1
H2afx	-0,0075406	1
Pld3	-0,0075488	1
Stat3	-0,0075032	1
St3gal3	-0,0076326	1
Sap130	-0,0076591	1
Gm7132	-0,0079294	1
Spg20	-0,0080181	1
Map2k4	-0,0080816	1
Eny2	-0,0083301	1
Tnip2	-0,0084325	1
Dkc1	-0,0085195	1
Gtf2a1	-0,008751	1
Cadm1	-0,0088293	1
Gm6377	-0,0091452	1
Dhx16	-0,0090782	1
Prex1	-0,0090895	1
Canx	-0,0090918	1
Pax6	-0,0092648	1
Fam129b	-0,0093414	1
Gm1943	-0,0094447	1
Gm8822	-0,0094582	1

Hpf1	-0,0096128	1
Slc25a37	-0,0095771	1
Gm43795	-0,0097377	1
Cdt1	-0,0096868	1
Nit2	-0,0097912	1
Gmps	-0,0098804	1
Atp2b1	-0,010019	1
Zfp358	-0,010075	1
Mcur1	-0,010279	1
Vps53	-0,010331	1
Pdcd6	-0,010315	1
Rps12-ps1	-0,010633	1
Apobec3	-0,010652	1
Tfg	-0,010742	1
Hdgf	-0,010726	1
Sec63	-0,011031	1
Ipo9	-0,01154	1
Wipi1	-0,011452	1
Foxp4	-0,011863	1
Arl3	-0,011913	1
Tsnax	-0,012193	1
Thap1	-0,01258	1
Acly	-0,01261	1
Spsb2	-0,012836	1
Smyd5	-0,01285	1
Nsd2	-0,012924	1
Ap3m1	-0,012986	1
Gm5869	-0,013123	1
RP23-246F14.1	-0,0131	1
Itpr1	-0,013214	1
Hnrnpm	-0,013158	1
Glo1	-0,013166	1
Crip1	-0,013289	1
Irak2	-0,01348	1
E130201H02Rik	-0,013713	1
Angel1	-0,013871	1
Trmt10c	-0,013856	1
Phrf1	-0,013932	1
Tmed9	-0,013921	1
Lamtor5	-0,013969	1
Nphp3	-0,014125	1
Rabgap1	-0,014127	1
E2f1	-0,014157	1
Ero1lb	-0,014584	1
Txndc15	-0,01472	1
Plag1	-0,015094	1
Hus1	-0,015148	1
Gdpd5	-0,01519	1
Hira	-0,015294	1
Pald1	-0,015391	1
Ttc25	-0,015686	1
Camsap1	-0,015694	1

Gm13094	-0,015805	1
Zfp667	-0,01599	1
Agap1	-0,016116	1
Spats2	-0,016347	1
Pip5k1a	-0,016253	1
Lin7c	-0,016319	1
Ccdc180	-0,016398	1
Fdxr	-0,016363	1
Oaz1-ps	-0,016481	1
Sbds	-0,016782	1
Gm9484	-0,017119	1
Neurl2	-0,017068	1
Rdh11	-0,017096	1
Gm22581	-0,017312	1
Pigp	-0,017382	1
Tmem159	-0,017394	1
Amz2	-0,017464	1
Gm44509	-0,017659	1
Pot1a	-0,017699	1
Zfx	-0,017687	1
Hmgcl	-0,017731	1
Gm9726	-0,017847	1
Gm6905	-0,017877	1
Cdon	-0,017895	1
Dolpp1	-0,01795	1
Slc45a3	-0,018036	1
Pex10	-0,01813	1
Phf19	-0,018274	1
Cryz	-0,018321	1
Creb3l2	-0,01842	1
Mgrn1	-0,01841	1
Pgm2	-0,018412	1
Rxra	-0,018505	1
Hnrnpc	-0,018453	1
Efemp2	-0,018601	1
Hint3	-0,01863	1
Peg13	-0,018906	1
Skiv2l	-0,019096	1
Cryl1	-0,019145	1
Ccdc14	-0,019081	1
Nsmaf	-0,019083	1
Gm8423	-0,019216	1
Slc9a6	-0,019501	1
Ing1	-0,019518	1
Galnt4	-0,019597	1
Snrnp48	-0,019624	1
Hmgb1-ps6	-0,019654	1
Tyk2	-0,019841	1
Ecm1	-0,019907	1
Rhbdd2	-0,019952	1
Mbd6	-0,020082	1
Ccdc181	-0,020111	1

Mfng	-0,020079	1
Gm2a	-0,020203	1
Arap3	-0,02035	1
Pbrm1	-0,020369	1
Dnaja3	-0,020607	1
Sdcbp	-0,020588	1
Eif2b5	-0,020652	1
Ccdc159	-0,020926	1
Prelid3b	-0,020863	1
Txnl1	-0,020916	1
Gm9703	-0,02108	1
Odf2	-0,021054	1
9430015G10Rik	-0,021153	1
Mthfd1	-0,021445	1
Wwox	-0,021732	1
Atg13	-0,021658	1
Vat1	-0,021914	1
Ube3a	-0,021957	1
Fkbp5	-0,022142	1
Nln	-0,022302	1
Agpat3	-0,022324	1
Ash1l	-0,022483	1
Rab19	-0,022778	1
Uckl1	-0,02278	1
Pi4ka	-0,022774	1
Zfp800	-0,022976	1
Map3k7	-0,023023	1
Ttll5	-0,023395	1
Fabp5	-0,023593	1
Pdp1	-0,023638	1
Gm13827	-0,023732	1
Trp53inp2	-0,023713	1
Tmem59	-0,02368	1
Stx5a	-0,023981	1
Anapc11	-0,024143	1
Nudt9	-0,024305	1
Gm15596	-0,024633	1
Gm15644	-0,024607	1
Heatr6	-0,024579	1
Gm16200	-0,024689	1
Snx30	-0,024882	1
F830115B05Rik	-0,025018	1
Thap2	-0,025028	1
Rnf141	-0,02499	1
Ascl2	-0,025072	1
Ralb	-0,025223	1
B4galt7	-0,025306	1
Tbce	-0,025288	1
Srrm2	-0,025293	1
Ndfip2	-0,025585	1
Mafg	-0,025732	1
Mob3b	-0,026028	1

Pgrmc2	-0,025986	1
Dnajc14	-0,026231	1
Cdca7l	-0,02619	1
Stx18	-0,026437	1
Mapk8	-0,026484	1
Tbrg1	-0,026522	1
C130036L24Rik	-0,027025	1
Ppp2r2a	-0,027211	1
Vdac2	-0,027308	1
Rad51d	-0,027365	1
Ggta1	-0,027515	1
Pi4k2a	-0,027522	1
Mvk	-0,027902	1
Sfxn3	-0,027931	1
Plbd2	-0,028149	1
Hipk1	-0,028221	1
Uvrag	-0,028541	1
Csde1	-0,028511	1
Msl3	-0,028601	1
Gm15417	-0,028571	1
Eif5a	-0,028655	1
Tex2	-0,028861	1
Slc35b3	-0,028998	1
Slc25a10	-0,029075	1
Fam185a	-0,029106	1
Kdelc2	-0,029169	1
Haus1	-0,029188	1
Trip4	-0,029315	1
Gm16053	-0,029501	1
Mcmbp	-0,029493	1
Gm8662	-0,03013	1
Osbpl10	-0,030179	1
Actr6	-0,03017	1
Gm37963	-0,030326	1
Ankle2	-0,030312	1
Mak16	-0,030322	1
Rsbn1l	-0,030508	1
Kif5b	-0,03046	1
Kif5c	-0,030603	1
Gm28557	-0,030743	1
Fam217b	-0,030839	1
Hic2	-0,03079	1
Cxcr4	-0,030785	1
Pibf1	-0,03089	1
Erc1	-0,031107	1
Pold2	-0,031412	1
Stat6	-0,031548	1
Dip2c	-0,031551	1
Lsm6	-0,031656	1
Nupl1	-0,031808	1
Fyn	-0,031842	1
Dusp2	-0,031931	1

Mpc2	-0,031866	1
Gm7776	-0,03204	1
Ube3c	-0,032028	1
Abcb8	-0,032363	1
Tubgcp5	-0,032443	1
Uchl5	-0,03243	1
Gm12663	-0,032918	1
Mrps9	-0,033164	1
Trim44	-0,033394	1
Ccdc137	-0,033558	1
Tex9	-0,033747	1
Ywhag	-0,03377	1
Hcst	-0,033964	1
Tuba1b	-0,033994	1
Rbpsuh-rs3	-0,034034	1
Mcrs1	-0,034055	1
Gm11517	-0,034087	1
Map1s	-0,034214	1
Entpd6	-0,034377	1
Tubb4a	-0,034529	1
Gm42482	-0,034516	1
Coq8b	-0,034501	1
Vwa7	-0,034646	1
Pik3cg	-0,03469	1
Zfp369	-0,034722	1
Rbbp6	-0,034663	1
Polr3k	-0,034828	1
Hmg20a	-0,034805	1
Gm14056	-0,034906	1
Ano10	-0,035122	1
Gm15920	-0,0352	1
Wdpcp	-0,035263	1
9130011E15Rik	-0,035803	1
D730003I15Rik	-0,035856	1
Enho	-0,03601	1
RP24-282C4.10	-0,036027	1
Zfp710	-0,036149	1
Sgpl1	-0,036094	1
Emp3	-0,036194	1
Zfp59	-0,036365	1
Lrpprc	-0,036578	1
Ptk2	-0,036555	1
Gm20900	-0,036683	1
Prrc2b	-0,036831	1
Rpgrip1l	-0,03687	1
Atxn3	-0,03711	1
Fam63a	-0,037142	1
Phlpp1	-0,037155	1
Gm8885	-0,037269	1
Fiz1	-0,037383	1
Tspan17	-0,037402	1
Slc29a2	-0,037622	1

Adamtsl5	-0,038041	1
Cxcr3	-0,038203	1
Eif4a3	-0,038244	1
Surf6	-0,038521	1
Usp49	-0,038712	1
Maged2	-0,038916	1
Cdkn1a	-0,038945	1
Dgkd	-0,039095	1
Usf3	-0,039166	1
Gcc2	-0,039166	1
Hes6	-0,039217	1
Camk2n2	-0,039339	1
Mrpl47	-0,039263	1
Socs7	-0,039486	1
Arpc5	-0,039701	1
Gm7380	-0,039882	1
Smurf1	-0,039947	1
Gtf2ird2	-0,040015	1
Bst1	-0,040054	1
Abce1	-0,040146	1
Fbrsl1	-0,040424	1
Pcdhb15	-0,04041	1
Nol7	-0,040587	1
Gm8719	-0,040718	1
4930558J18Rik	-0,040724	1
Epn1	-0,040928	1
Gm10169	-0,040978	1
Hirip3	-0,041147	1
2410131K14Rik	-0,04106	1
Rif1	-0,041211	1
Dyrk1a	-0,04131	1
Gm15782	-0,041437	1
Rptor	-0,041397	1
Ikbkap	-0,041439	1
Ucp2	-0,041518	1
Eif3i	-0,041505	1
Prmt1	-0,041606	1
Fgfr1op2	-0,041718	1
Ppip5k1	-0,041901	1
C1qbp	-0,042083	1
Mllt10	-0,042153	1
Ctbs	-0,042437	1
Nubp1	-0,042372	1
4933434E20Rik	-0,042389	1
Gm38192	-0,042711	1
Dcps	-0,04268	1
Prpf4	-0,042778	1
Nradd	-0,043095	1
Arpc4	-0,043097	1
Casp8	-0,043342	1
Lrrfip1	-0,043367	1
Rhebl1	-0,043383	1

Tm7sf2	-0,043466	1
Gstz1	-0,043516	1
Smyd2	-0,043688	1
Ccdc174	-0,04384	1
Itga7	-0,043921	1
Cdc37l1	-0,043853	1
Ccnd3	-0,04394	1
9530068E07Rik	-0,044058	1
Npepl1	-0,04434	1
Mcfd2	-0,044319	1
D5Ertd605e	-0,044381	1
Cers2	-0,044453	1
Grsf1	-0,04462	1
Urm1	-0,044686	1
Gm5837	-0,044825	1
Nudt4	-0,044837	1
Mbtps1	-0,044755	1
AW047730	-0,044899	1
Gm20633	-0,045336	1
Orc3	-0,045294	1
Amt	-0,045407	1
Sc5d	-0,045387	1
Grk2	-0,045505	1
Sumo3	-0,045587	1
Sos2	-0,045739	1
Spopl	-0,046129	1
Fam132a	-0,046243	1
Gm7102	-0,046176	1
Sugp1	-0,046184	1
RP23-187B11.16	-0,046387	1
Cpt1c	-0,046379	1
Meis2	-0,04669	1
Hlx	-0,046831	1
Smad1	-0,046764	1
Odc1	-0,046816	1
Larp7	-0,046807	1
Gm13532	-0,046918	1
Rpl36a	-0,046917	1
Pum2	-0,046878	1
Nceh1	-0,046926	1
Gm9530	-0,047186	1
Galt	-0,047203	1
Reps1	-0,047304	1
Ercc3	-0,047301	1
Timm29	-0,047333	1
Kansl1l	-0,047383	1
Farp2	-0,047437	1
Tspan32	-0,047487	1
4833412K13Rik	-0,047482	1
Srsf10	-0,047647	1
Gm38104	-0,047739	1
Nphp1	-0,047702	1

Dctpp1	-0,047837	1
Gm24601	-0,047878	1
Cd83	-0,047904	1
Ufm1	-0,048016	1
Hyal2	-0,048251	1
Rgl3	-0,048372	1
Ptp4a3	-0,048366	1
Rps6ka4	-0,04854	1
Man2a1	-0,048493	1
Stk3	-0,048505	1
Plekhf2	-0,048526	1
Serpinb8	-0,048715	1
Tapbpl	-0,048844	1
Gm9761	-0,048964	1
Tbc1d16	-0,049336	1
Rabl2	-0,049478	1
Mapk9	-0,049622	1
Hdac9	-0,049769	1
Rpl8	-0,049761	1
Gm2272	-0,049879	1
Rps10	-0,049989	1
Dcaf8	-0,049989	1
1700029J07Rik	-0,050078	1
Klra2	-0,050094	1
Timm17a	-0,050147	1
Miip	-0,050161	1
5031425E22Rik	-0,050184	1
Tmem261	-0,050301	1
Samsn1	-0,050439	1
Aplf	-0,050485	1
Kcnb1	-0,050849	1
Spry2	-0,051065	1
Arrdc3	-0,051318	1
Nagk	-0,051319	1
Tpr	-0,051287	1
Man1c1	-0,051373	1
H2afj	-0,051607	1
Orc5	-0,051662	1
Slc6a13	-0,052529	1
Meg3	-0,052587	1
Pxmp2	-0,052798	1
Cd2ap	-0,052937	1
Dusp8	-0,053118	1
Agfg1	-0,053117	1
Mettl15	-0,053253	1
Ggct	-0,053338	1
Wbp11	-0,053443	1
Paf1	-0,053502	1
Mrs2	-0,053626	1
Pcdhb16	-0,053572	1
Adipor1	-0,053847	1
Rab33b	-0,053935	1

Bex3	-0,053868	1
Srsf7	-0,054141	1
Ppib	-0,054084	1
Star	-0,054212	1
Eif4a1	-0,054167	1
Trap1	-0,054339	1
Smpdl3a	-0,054374	1
Psmd5	-0,05448	1
Ddx49	-0,054465	1
Zfp574	-0,054585	1
Snx17	-0,05481	1
Dnajc9	-0,054943	1
Washc3	-0,054943	1
Bnip1	-0,054899	1
Eif3j1	-0,055146	1
RP23-3F1.8	-0,05506	1
Rfng	-0,055068	1
Acsl3	-0,055212	1
Rsbn1	-0,055244	1
Gm44699	-0,055393	1
Cox7a2	-0,055354	1
Kif20a	-0,055584	1
Psen1	-0,05564	1
Btbd3	-0,055656	1
Cxx1a	-0,055727	1
Gm6563	-0,055742	1
Nova2	-0,055975	1
A930024E05Rik	-0,056092	1
Sec24d	-0,056223	1
Gmeb1	-0,05622	1
Slc7a1	-0,056205	1
Phka1	-0,056295	1
Rnf44	-0,056449	1
Brip1os	-0,056597	1
Tmem208	-0,056748	1
Tbl1xr1	-0,056791	1
N4bp2l2	-0,056994	1
Lcp2	-0,057618	1
Gm10076	-0,057746	1
Wdr76	-0,057754	1
Serinc3	-0,057813	1
Agpat5	-0,057964	1
Pex19	-0,058094	1
Pdp2	-0,058097	1
Fndc7	-0,058207	1
Pten	-0,058261	1
Acp1	-0,058417	1
Serf1	-0,05845	1
Psmd11	-0,058539	1
Sumf1	-0,058616	1
4930556M19Rik	-0,058678	1
Ndufa2	-0,058776	1

Rbm28	-0,059024	1
Pla2g12a	-0,059211	1
Gfi1	-0,059315	1
Unc45a	-0,059304	1
Jtb	-0,059325	1
Tst	-0,059859	1
Gm28192	-0,059943	1
Srsf9	-0,05997	1
Ndufaf1	-0,060066	1
Gm14130	-0,060335	1
Lyn	-0,060342	1
Casp8ap2	-0,060439	1
Dus3l	-0,060474	1
Selenot	-0,060724	1
Slc3a2	-0,060825	1
Zfand6	-0,060778	1
Gpt	-0,060895	1
Flnc	-0,060859	1
Slc22a17	-0,060962	1
Gtf2a2	-0,060985	1
Zmym4	-0,061052	1
Tmub1	-0,061212	1
Cramp1l	-0,061224	1
Fabp7	-0,061392	1
Lemd3	-0,061417	1
Fndc3b	-0,061427	1
Ngly1	-0,061536	1
Mast2	-0,061509	1
Slc41a1	-0,061495	1
Dhcr24	-0,061473	1
Mdp1	-0,061467	1
Hdac10	-0,061677	1
C8g	-0,061833	1
A430046D13Rik	-0,061929	1
Rps26-ps1	-0,06185	1
Adgre5	-0,062195	1
Ccndbp1	-0,062221	1
Rps6kb1	-0,062321	1
Smad7	-0,062368	1
Ski	-0,062373	1
Ddx5	-0,062662	1
Gpatch8	-0,06295	1
Fos	-0,06302	1
Tcerg1	-0,062964	1
Gga3	-0,063075	1
Ctif	-0,063189	1
D2Bwg1423e	-0,063417	1
Havcr2	-0,063491	1
Map3k20	-0,063527	1
Nr6a1	-0,063565	1
Ddx3x	-0,063641	1
Prkaca	-0,063672	1

Triap1	-0,063985	1
Adcy9	-0,064708	1
Tspyl1	-0,064881	1
Zfp706	-0,064875	1
Gm6368	-0,065512	1
Phyh	-0,065527	1
Was	-0,065607	1
Ireb2	-0,06559	1
Zfp7	-0,065845	1
Urod	-0,065883	1
Ist1	-0,066088	1
Bub3	-0,066232	1
6720427I07Rik	-0,066715	1
March8	-0,066683	1
Nelfb	-0,067085	1
Pon2	-0,067115	1
Ube2b	-0,067371	1
2900026A02Rik	-0,067507	1
Tpst1	-0,067497	1
Camk2a	-0,067749	1
Ppp2r3c	-0,067726	1
Tpgs2	-0,068104	1
Nt5dc1	-0,068244	1
Tmem245	-0,068212	1
Shkbp1	-0,068394	1
Dok3	-0,068424	1
Hivep3	-0,068545	1
Gm7287	-0,068635	1
Gm13373	-0,068676	1
Kctd18	-0,068688	1
Mxra8	-0,068855	1
Plcd3	-0,069013	1
Ncs1	-0,069034	1
Mnd1-ps	-0,069107	1
Rttn	-0,069227	1
Ifi202b	-0,069343	1
Nuak2	-0,06941	1
Mtch1	-0,069405	1
Dnttip1	-0,069625	1
Akirin1	-0,069638	1
Ganab	-0,069683	1
Strap	-0,070002	1
Rcc2	-0,070003	1
Slc13a3	-0,070264	1
Prmt5	-0,07049	1
Inpp5a	-0,070513	1
Gm7847	-0,070643	1
Rasa1	-0,070551	1
Hscb	-0,070627	1
Agap3	-0,070694	1
Fcna	-0,071045	1
D130020L05Rik	-0,071318	1

Gm13868	-0,071326	1
Skil	-0,071273	1
Hddc3	-0,071646	1
Gm44884	-0,072181	1
1110012L19Rik	-0,072438	1
Cuedc2	-0,072684	1
Vps25	-0,072788	1
Aim1	-0,073064	1
Tprgl	-0,07315	1
Kdelr1	-0,073238	1
Mfap1a	-0,073255	1
Fads6	-0,073411	1
D1Ertd622e	-0,0735	1
Adgb	-0,073566	1
Dlg4	-0,073637	1
Lair1	-0,073576	1
Tsn	-0,073652	1
Pbx3	-0,073769	1
Cenpa	-0,073824	1
Gnmt	-0,0739	1
Rps21	-0,07398	1
Rsad1	-0,074101	1
Gm12176	-0,074075	1
Leprot	-0,074222	1
Dnajb2	-0,074273	1
Tnip3	-0,074718	1
Tyrobp	-0,074736	1
Il23a	-0,074779	1
Sco1	-0,075051	1
Gm6297	-0,075273	1
Tax1bp3	-0,075392	1
Gpr68	-0,075635	1
Rab18	-0,075619	1
Il12rb1	-0,075723	1
Msl2	-0,075986	1
Rab40c	-0,07616	1
Gm17039	-0,076341	1
Gna15	-0,076322	1
Junos	-0,076445	1
Asnsd1	-0,076352	1
Rpl35	-0,076525	1
Qdpr	-0,076655	1
Morc4	-0,0769	1
Yipf5	-0,076934	1
Gm16845	-0,077047	1
Ergic3	-0,077045	1
Fdft1	-0,077129	1
Irf2bpl	-0,077095	1
Mrpl23-ps1	-0,07774	1
Tbc1d22a	-0,077661	1
Arrb2	-0,077774	1
Uqcr10	-0,077993	1

Nubpl	-0,078108	1
Nup50	-0,078219	1
Aff4	-0,078271	1
Ap1g1	-0,078306	1
RP24-93F20.12	-0,078436	1
Cct4	-0,078522	1
Otud7b	-0,078641	1
Adam8	-0,078566	1
Stx12	-0,078689	1
Med19	-0,078837	1
Gpsm2	-0,07906	1
Ago2	-0,079345	1
Atg16l2	-0,079257	1
Eif4e2	-0,079444	1
Tubgcp6	-0,079583	1
Dph7	-0,079827	1
Zfp560	-0,079753	1
Pink1	-0,07998	1
Gm5881	-0,080137	1
Snta1	-0,080148	1
Kifc2	-0,08019	1
Atxn1	-0,080243	1
Sdc1	-0,080491	1
Gm16177	-0,080628	1
Gm15007	-0,08064	1
Fads2	-0,080712	1
Fam213a	-0,080661	1
Gm42876	-0,081014	1
Gstm1	-0,081227	1
Sephs1	-0,081328	1
2410006H16Rik	-0,081602	1
Nsmce3	-0,081713	1
Proser1	-0,081695	1
Copg1	-0,081845	1
Lipa	-0,081755	1
Bin2	-0,081934	1
Bcap31	-0,081871	1
Gm11488	-0,081977	1
Ubr5	-0,082147	1
Ppil4	-0,082251	1
Ndufaf2	-0,082414	1
Atp6v0e2	-0,082407	1
Cdk19	-0,082671	1
Elfn2	-0,082814	1
Smc6	-0,082868	1
Eif1	-0,082951	1
Irf3	-0,083214	1
Pafah1b1-ps1	-0,083327	1
Ythdc1	-0,083407	1
Zcchc4	-0,083535	1
Rab24	-0,08362	1
Add1	-0,08404	1

Abcc4	-0,084355	1
Ptdss1	-0,084741	1
Col15a1	-0,084985	1
Zfp422	-0,084993	1
Slc4a1ap	-0,085212	1
Vps18	-0,085413	1
Ppm1j	-0,085632	1
Immp2l	-0,086029	1
Cst3	-0,085987	1
Mapkapk2	-0,086222	1
Rubcn	-0,086471	1
Zfp157	-0,086735	1
Mrpl15	-0,08671	1
Slc38a6	-0,086788	1
Dnal4	-0,086886	1
E330009J07Rik	-0,087089	1
Klf7	-0,087106	1
Deaf1	-0,087248	1
Dgat1	-0,087152	1
Ddhd2	-0,087406	1
Icam1	-0,087439	1
Lcp1	-0,087395	1
Rpp30	-0,087641	1
Cndp2	-0,087683	1
Zrsr2	-0,088114	1
2610524H06Rik	-0,088268	1
Alg13	-0,08847	1
Gm6272	-0,0887	1
Mrm1	-0,088771	1
Prpsap2	-0,088895	1
Cd14	-0,089192	1
Gm37795	-0,089425	1
Thumpd2	-0,089546	1
Bcl9l	-0,089538	1
Cdc27	-0,08948	1
AK157302	-0,08963	1
Mettl4	-0,089768	1
Shmt2	-0,089949	1
Srm	-0,090193	1
Rab11b	-0,090211	1
Rpusd1	-0,09027	1
Cdk17	-0,090255	1
Gm18737	-0,090452	1
Smco4	-0,090495	1
Dvl3	-0,091388	1
Arih2	-0,091359	1
Pbx2	-0,091579	1
Hagh	-0,091616	1
Srd5a3	-0,091723	1
Tctex1d2	-0,091696	1
Pbxip1	-0,091768	1
Alyref	-0,092091	1

Engase	-0,092518	1
Atp6v1g1	-0,092489	1
Pcmt1	-0,092637	1
Pmm2	-0,092863	1
Frmd6	-0,092984	1
Anp32b-ps1	-0,092973	1
Tex261	-0,092962	1
Mertk	-0,0932	1
Dcbld2	-0,093172	1
Inhbe	-0,093439	1
Sh3bgr	-0,093589	1
Nol8	-0,093845	1
Eif4enif1	-0,093869	1
Dmtf1	-0,093954	1
Eri1	-0,094094	1
Ubald1	-0,09429	1
Hps1	-0,094321	1
Rab11fip2	-0,094255	1
Ap4e1	-0,094448	1
Cul9	-0,094399	1
Os9	-0,094447	1
Olfr460	-0,094553	1
Mrpl23	-0,094964	1
Tmem68	-0,094964	1
Thap3	-0,095036	1
Nrxn3	-0,095106	1
Tsc22d1	-0,095071	1
Agpat1	-0,09515	1
Bcl2a1d	-0,095394	1
Smpdl3b	-0,095374	1
Med24	-0,095491	1
Thoc5	-0,09605	1
Glrx2	-0,096117	1
Ift140	-0,096263	1
Tpst2	-0,096372	1
Tuft1	-0,096509	1
Gm7027	-0,096695	1
Alpk1	-0,096742	1
Aarsd1	-0,097045	1
Plpp2	-0,097005	1
1810043G02Rik	-0,097128	1
Nipbl	-0,097474	1
Mettl14	-0,097787	1
Slc35b2	-0,097804	1
E330020D12Rik	-0,097876	1
Tmem256	-0,097918	1
Gm8805	-0,098062	1
Fbxo45	-0,098101	1
Atp5j2	-0,098214	1
Ldha	-0,098181	1
Dpm2	-0,098532	1
Cstb	-0,098498	1

Ndufs4	-0,098652	1
Vrk3	-0,09907	1
Ssb	-0,099116	1
Sapcd2	-0,099216	1
Gm27039	-0,099175	1
Atp9b	-0,099239	1
Sh3glb2	-0,099438	1
Cep350	-0,099468	1
Zfas1	-0,099553	1
Ing3	-0,099683	1
Ubap2	-0,099728	1
Ndufs3	-0,099789	1
Stau2	-0,10013	1
Uqcrb	-0,10012	1
Gm14165	-0,10023	1
Psmb1	-0,10018	1
Cfap97	-0,10026	1
Hadh	-0,10035	1
Commd8	-0,10043	1
Psmd2	-0,10058	1
Araf	-0,10079	1
Fam207a	-0,10109	1
Myom1	-0,10117	1
Fbxl3	-0,10116	1
Prpsap1	-0,10123	1
Morc2a	-0,10141	1
Ank3	-0,10148	1
Exoc3	-0,10174	1
Tmem258	-0,10178	1
Zfp53	-0,10213	1
Gm37780	-0,10206	1
Mrpl21	-0,10208	1
RP24-511J14.2	-0,1025	1
Rps6-ps1	-0,10256	1
Cpt1a	-0,10256	1
Pomt1	-0,10269	1
Rorc	-0,1027	1
Ddx52	-0,10269	1
Them4	-0,10324	1
Map3k10	-0,10321	1
Gm6134	-0,10327	1
Pigw	-0,10354	1
Echdc3	-0,10347	1
Appl2	-0,10359	1
Cyb5r3	-0,10368	1
Gtf2f1	-0,10383	1
Ssna1	-0,10397	1
Rps15	-0,1041	1
Nav1	-0,10457	1
Ctnnb1	-0,10459	1
Pggt1b	-0,10468	1
Kat5	-0,10465	1

Eif3f	-0,10503	1
Arfgef2	-0,10513	1
Ginm1	-0,10529	1
Vamp3	-0,10533	1
Nipsnap1	-0,10541	1
Ddost	-0,1054	1
Btbd6	-0,10549	1
Lyrm4	-0,10554	1
Siah2	-0,10552	1
Tmed5	-0,10552	1
Ptpa	-0,10553	1
Azi2	-0,10582	1
Strada	-0,10588	1
Synpo	-0,10588	1
BC037034	-0,10587	1
Il6st	-0,10591	1
Mri1	-0,10608	1
4931406C07Rik	-0,10609	1
Slc25a16	-0,10618	1
Dnajc21	-0,10627	1
Unc13d	-0,10642	1
Snw1	-0,10642	1
Prmt9	-0,10647	1
Cbx7	-0,10665	1
Rpl13a	-0,10673	1
Fads3	-0,10681	1
Abhd3	-0,10712	1
Tsen15	-0,10733	1
Psmd3	-0,10732	1
Plec	-0,10754	1
App	-0,10763	1
Ypel3	-0,10761	1
Dpagt1	-0,1079	1
1190007I07Rik	-0,10798	1
1110025M09Rik	-0,10821	1
Nol11	-0,10843	1
Ttc37	-0,10861	1
Lgals2	-0,1087	1
Zfp383	-0,10872	1
Nsun2	-0,10868	1
Herc4	-0,10889	1
9430060I03Rik	-0,10902	1
Gm15198	-0,10898	1
Mau2	-0,10914	1
Ano6	-0,10908	1
Mrpl48-ps	-0,1092	1
Tmem11	-0,10933	1
Gm16556	-0,10936	1
Sacs	-0,10952	1
2010204K13Rik	-0,10994	1
Tgfbr1	-0,10989	1
Gm26830	-0,11011	1

Gm28530	-0,11078	1
Ubtf	-0,11092	1
Ccpg1os	-0,11135	1
Ywhab	-0,11132	1
Lrpap1	-0,11131	1
Angel2	-0,11144	1
Ralgds	-0,11154	1
Tnfaip1	-0,1115	1
Gm20696	-0,11165	1
Rgs19	-0,11157	1
Plekho1	-0,11175	1
Rbm7	-0,11184	1
Elac2	-0,1119	1
Mtmr6	-0,11187	1
Mark2	-0,11199	1
Bcat1	-0,11203	1
9030617O03Rik	-0,11198	1
Cox5a	-0,11201	1
Ints7	-0,1124	1
Thumpd3	-0,11238	1
Ppp3r1	-0,11254	1
Ptgs2os	-0,11261	1
Gnat2	-0,11272	1
Ptges3l	-0,11315	1
Tmem57	-0,11317	1
Psmc6	-0,11333	1
Gm45629	-0,11336	1
Wnk1	-0,11344	1
Zfp622	-0,11391	1
Gm12944	-0,1143	1
Vps33b	-0,11431	1
Mrps30	-0,11447	1
Pth1r	-0,11465	1
Abhd17a	-0,11487	1
Sertad1	-0,11503	1
Trpc4ap	-0,11509	1
Speg	-0,11529	1
Dhx57	-0,11544	1
Ndufs2	-0,11539	1
Gm14253	-0,11551	1
Cnot9	-0,11559	1
Ofd1	-0,11571	1
Slc48a1	-0,11569	1
Sema4c	-0,11578	1
Letmd1	-0,11604	1
Aktip	-0,11612	1
Nipa2	-0,11619	1
Rpl10a-ps1	-0,11623	1
Tax1bp1	-0,1162	1
Stxbp5	-0,11655	1
Lmnb2	-0,11668	1
Xpnpep1	-0,1169	1

Bcorl1	-0,11685	1
Cep57l1	-0,11719	1
Mrpl3	-0,11745	1
Gm3145	-0,11764	1
Osbpl8	-0,11757	1
Golph3	-0,11772	1
Gm43110	-0,11784	1
Tatdn3	-0,11779	1
Tnrc6a	-0,11791	1
Nfyb	-0,11793	1
Psma6	-0,1182	1
Mtcl1	-0,11828	1
Fbxo22	-0,11831	1
Rnf166	-0,11838	1
Cdkl2	-0,11854	1
Smug1	-0,11864	1
Foxred2	-0,11867	1
Gosr2	-0,11887	1
Txlna	-0,11906	1
Rtcb	-0,11907	1
Gm37352	-0,1194	1
Taf4b	-0,11944	1
Rwdd4a	-0,11954	1
Gspt1	-0,11967	1
Gapvd1	-0,11969	1
2810428I15Rik	-0,12003	1
Wdr19	-0,12044	1
Phtf2	-0,12042	1
Kbtbd2	-0,12041	1
Gm8317	-0,12063	1
Gemin8	-0,12059	1
Tmx1	-0,12062	1
Cspg5	-0,12073	1
Qrsl1	-0,1209	1
Acsl5	-0,12134	1
Irf4	-0,12141	1
RP23-444K20.4	-0,12147	1
Tpd52	-0,12149	1
Skor1	-0,12157	1
Etf1	-0,12162	1
Gm6444	-0,12181	1
Rnf14	-0,12177	1
Vps72	-0,12185	1
Rnf220	-0,12192	1
Gm7618	-0,12216	1
Mettl5	-0,12223	1
Lztr1	-0,12236	1
Tram2	-0,12249	1
Bad	-0,12247	1
Haus7	-0,12255	1
Pik3r5	-0,12259	1
Dpp8	-0,12302	1

Ttc4	-0,12295	1
Rer1	-0,12353	1
Zdhhc2	-0,12394	1
Setx	-0,12395	1
Echs1	-0,12396	1
Gm9843	-0,12415	1
Cops3	-0,12451	1
Sav1	-0,12466	1
Aak1	-0,12492	1
Fahd2a	-0,12501	1
Fam46c	-0,12497	1
Gm25596	-0,12511	1
Psme4	-0,12514	1
Gnb5	-0,12523	1
Fancm	-0,12532	1
Amigo1	-0,12529	1
Acp6	-0,12544	1
Rapgef6	-0,12541	1
Plscr3	-0,12565	1
Asxl1	-0,12565	1
Hs2st1	-0,12567	1
Cntln	-0,12579	1
Ube2d2a	-0,1259	1
Sec61a2	-0,12605	1
5430416N02Rik	-0,12617	1
Rbm8a	-0,12619	1
Daam1	-0,12625	1
Tcea1	-0,12634	1
Plekha8	-0,12642	1
Aldh5a1	-0,12653	1
S100a6	-0,12655	1
Nxt1	-0,12681	1
Nploc4	-0,12702	1
Atp5k	-0,12695	1
Sp140	-0,12736	1
Ifrd2	-0,12751	1
Tesk1	-0,12745	1
Nr1h3	-0,12756	1
Taf1d	-0,12773	1
Nol12	-0,12793	1
Paox	-0,12803	1
Vps26a	-0,12796	1
Gm7967	-0,12825	1
1700109H08Rik	-0,12818	1
Hexb	-0,12846	1
Mapk1	-0,12868	1
Slc2a6	-0,12876	1
Cyhr1	-0,12911	1
Fbxw9	-0,12934	1
Washc5	-0,12936	1
Cib1	-0,12937	1
Xpo4	-0,12946	1

Ttc26	-0,12973	1
Gm20442	-0,1303	1
Akr1b8	-0,13029	1
Tnfrsf1b	-0,13039	1
Gm10074	-0,13047	1
Mfsd12	-0,13049	1
Sik1	-0,13062	1
Ube2l6	-0,13091	1
Prpf31	-0,13092	1
Akr1e1	-0,13094	1
Tor1a	-0,13132	1
Bop1	-0,13149	1
Rwdd1	-0,13168	1
Mrpl45	-0,13172	1
Zfp597	-0,13187	1
3300002I08Rik	-0,13218	1
Neo1	-0,13218	1
Nvl	-0,13224	1
Timmdc1	-0,13228	1
Orc6	-0,13229	1
Ccnd2	-0,13232	1
Gin1	-0,1327	1
Sap30bp	-0,13267	1
Dync1li2	-0,13266	1
Ilk	-0,13281	1
Plpp6	-0,1329	1
Mcm10	-0,13305	1
Cdc73	-0,13308	1
Gm6745	-0,13327	1
Rpl18-ps2	-0,13326	1
Lmbrd1	-0,13351	1
Gm6520	-0,13356	1
Tcea1-ps1	-0,1336	1
2700049A03Rik	-0,13374	1
Akap11	-0,13372	1
1700003G18Rik	-0,13406	1
Erp27	-0,13414	1
Hspd1-ps3	-0,13405	1
Zfp592	-0,13409	1
Rpsa-ps4	-0,13434	1
Sfpq	-0,13431	1
Rps23-ps2	-0,13439	1
Foxo4	-0,13465	1
Zfp282	-0,13481	1
Snx8	-0,13482	1
Mon2	-0,13501	1
Zfp346	-0,13512	1
Tspo	-0,13507	1
Gm17511	-0,13521	1
Nemp2	-0,13515	1
Plxna2	-0,13549	1
Pmm1	-0,13546	1

Ttf1	-0,13558	1
Trim36	-0,1357	1
Cyp4v3	-0,13593	1
Adprm	-0,13592	1
Capn2	-0,1362	1
Usp3	-0,1362	1
M1ap	-0,13645	1
Arl15	-0,13662	1
Asrgl1	-0,13658	1
Gm37423	-0,13665	1
Utp18	-0,13673	1
Ncf1	-0,13682	1
Phf20-ps	-0,13686	1
Ascc2	-0,13695	1
Polr2e	-0,13692	1
Gm37474	-0,13702	1
Slc1a4	-0,1372	1
Aurkaip1	-0,13721	1
Rpl19-ps9	-0,13737	1
Cops5	-0,13744	1
Sbk3	-0,13763	1
Vasp	-0,13758	1
Mcoln2	-0,13777	1
Txndc9	-0,13777	1
Parn	-0,13793	1
Gm5637	-0,13802	1
Polrmt	-0,13801	1
Magi1	-0,13798	1
Ppm1b	-0,13808	1
Cdk1	-0,13814	1
Rnf103	-0,13821	1
Natd1	-0,13834	1
Ndufv1	-0,13842	1
Tcf4	-0,13853	1
Ssbp1	-0,13861	1
Denr	-0,13866	1
Ttc13	-0,13926	1
Ifi27l2a	-0,13944	1
Fam114a1	-0,13949	1
Phlpp2	-0,13956	1
Celf3	-0,13956	1
Rpl28	-0,13967	1
Hsd17b10	-0,13973	1
Pcsk4	-0,14005	1
Fam220a	-0,14003	1
Rad18	-0,13997	1
RP24-378K7.3	-0,14006	1
Coa4	-0,14012	1
H13	-0,14012	1
Mob3a	-0,14016	1
Glt8d1	-0,14016	1
Rhobtb2	-0,14026	1

Mylpf	-0,14031	1
Ppp1r11	-0,14041	1
Pom121	-0,14065	1
Ubxn1	-0,14067	1
Ipp	-0,14101	1
Mzb1	-0,14104	1
Rexo2	-0,14098	1
Creb3	-0,14103	1
Ifnar2	-0,14103	1
Dhx37	-0,14107	1
Zfp617	-0,14107	1
Tti1	-0,14108	1
Btbd2	-0,14144	1
Mkrn1	-0,14162	1
Bud31	-0,14169	1
Ppargc1b	-0,14191	1
Mib1	-0,14188	1
Ranbp2	-0,14213	1
5430420F09Rik	-0,14229	1
Sirpa	-0,14234	1
Pkp2	-0,14237	1
Pigk	-0,14236	1
Hpcal1	-0,14261	1
Zfp865	-0,14327	1
mt-Cytb	-0,14328	1
E2f3	-0,14336	1
Lmbr1l	-0,1438	1
Clec2l	-0,14404	1
Fra10ac1	-0,144	1
Gm10736	-0,14412	1
Gm43128	-0,14435	1
Mfsd14b	-0,14459	1
Ube4b	-0,14472	1
AI506816	-0,14506	1
Thoc1	-0,14515	1
Gpnmb	-0,14521	1
Necap2	-0,1455	1
Smarca2	-0,14554	1
Spata2l	-0,14555	1
Pigx	-0,14564	1
Mcph1	-0,14567	1
Ep300	-0,14578	1
Sord	-0,14577	1
Smim15	-0,14631	1
Tomm7	-0,14662	1
A430005L14Rik	-0,14722	1
Zfp148	-0,14746	1
Nudt5	-0,14767	1
Naxd	-0,14793	1
4930526A20Rik	-0,14791	1
Acbd3	-0,14786	1
Mterf4	-0,14809	1

Sec23ip	-0,1481	1
Smim8	-0,14829	1
Brd2	-0,14834	1
Cox17	-0,14828	1
Cnot6	-0,14832	1
Lamc2	-0,14838	1
Ubxn2b	-0,1484	1
Npy	-0,14855	1
Apeh	-0,1486	1
Gm17018	-0,14887	1
Zfp687	-0,14915	1
Rbm17	-0,14943	1
Cox7a1	-0,14949	1
Arrdc4	-0,14945	1
Treml4	-0,14972	1
Clta	-0,14997	1
Gm11914	-0,15022	1
Vps4b	-0,15024	1
Rpl31-ps10	-0,15122	1
Gbf1	-0,15123	1
Klf2	-0,15141	1
Scarb2	-0,1514	1
Fam149b	-0,15147	1
Fam162a	-0,1516	1
Pigm	-0,15177	1
Gm12396	-0,15186	1
1110046J04Rik	-0,15198	1
2900009J06Rik	-0,15235	1
Ints14	-0,15238	1
Cdc45	-0,15252	1
Pip5k1b	-0,15248	1
Gm15853	-0,15263	1
Klhdc1	-0,15258	1
Nudcd3	-0,15268	1
H2-DMa	-0,15283	1
Lace1	-0,15291	1
Fam187b	-0,15295	1
Stk19	-0,15329	1
Ube2g2	-0,15327	1
Nfe2l1	-0,15339	1
Nrbp1	-0,15362	1
Wapl	-0,1537	1
Tln1	-0,15401	1
Ice1	-0,15411	1
Tmem50a	-0,15414	1
Ccdc57	-0,15439	1
Ston1	-0,1544	1
Rrm2b	-0,15441	1
Ttc7b	-0,15445	1
Lpl	-0,15436	1
Tmem69	-0,15471	1
Msh5	-0,15485	1

4930455G09Rik	-0,15483	1
Kdsr	-0,15498	1
Gm32340	-0,15515	1
Psmd1	-0,15513	1
2610008E11Rik	-0,15518	1
H1f0	-0,15519	1
Trappc3	-0,15534	1
U2af1	-0,15547	1
Txn-ps1	-0,15573	1
Tulp3	-0,15582	1
Gm26698	-0,1559	1
Rffl	-0,15591	1
Ctnnbl1	-0,15589	1
Efcab14	-0,156	1
Kdm5b	-0,15629	1
Znrf2	-0,15645	1
Anks3	-0,15655	1
Katnbl1	-0,15672	1
Lrp4	-0,15678	1
Specc1l	-0,15699	1
Atp8b4	-0,15712	1
Ndrg2	-0,15707	1
Zbtb42	-0,15718	1
Gm5697	-0,15728	1
Amer1	-0,15748	1
Wrap53	-0,15754	1
Slc25a12	-0,15752	1
Tbccd1	-0,15757	1
Dnajc19	-0,15773	1
Depdc7	-0,15786	1
Wbp4	-0,15785	1
Mt2	-0,15811	1
Cnot11	-0,15821	1
Serpinf2	-0,15829	1
Pced1b	-0,15834	1
RP23-228B2.5	-0,15876	1
Qser1	-0,15881	1
Fam171b	-0,15891	1
Sec11a	-0,15895	1
Gm9828	-0,15899	1
Tinf2	-0,15903	1
Hn1	-0,15896	1
Nedd4l	-0,1592	1
Baz2b	-0,15929	1
Sms	-0,1595	1
Mrps15	-0,15953	1
Gaa	-0,15957	1
1700096K18Rik	-0,15974	1
Mitf	-0,15971	1
Slc2a9	-0,15983	1
Rnf121	-0,16023	1
Tcaim	-0,16015	1

Thyn1	-0,16022	1
Cenpj	-0,16038	1
Pgap1	-0,16047	1
Rps3a3	-0,16061	1
Lamtor4	-0,16074	1
Golt1b	-0,16066	1
Atp5a1	-0,16067	1
Ap3b1	-0,16078	1
Plekhg3	-0,16116	1
Zfp445	-0,16124	1
Stau1	-0,1612	1
Ostf1	-0,16143	1
Rad23b	-0,16137	1
Cd99l2	-0,16145	1
Aamp	-0,16146	1
Gm45495	-0,16177	1
Eif4g1	-0,1619	1
Zbtb40	-0,1623	1
Gemin5	-0,16237	1
Arrb1	-0,16258	1
Gm45833	-0,16266	1
Gcfc2	-0,16297	1
Sirt3	-0,1631	1
Naf1	-0,16306	1
Fip1l1	-0,16324	1
Gm36378	-0,16339	1
Tnip1	-0,16336	1
Hoxc4	-0,16356	1
Rock1	-0,16385	1
Psmd8	-0,16393	1
Tcof1	-0,16388	1
Relb	-0,164	1
Pogk	-0,16398	1
AI839979	-0,16406	1
Stambp	-0,16426	1
Stk10	-0,16445	1
Gm6245	-0,16458	1
5730455P16Rik	-0,16458	1
Smu1	-0,16483	1
Zfyve1	-0,16492	1
Slc35b1	-0,16492	1
Camk1	-0,1655	1
Mical3	-0,16564	1
Gm10269	-0,16573	1
Trappc13	-0,1659	1
Gm31274	-0,16613	1
Asb11	-0,16628	1
Snrnp40	-0,16637	1
Cfdp1	-0,1664	1
Celsr1	-0,16651	1
Mir5128	-0,16663	1
Trabd	-0,16677	1

Ptbp3	-0,16697	1
Fhit	-0,16708	1
Smarca5	-0,1676	1
Spag4	-0,16776	1
Rhd	-0,16777	1
Cd59a	-0,16792	1
Slc12a4	-0,16798	1
Sec23a	-0,16804	1
Mybbp1a	-0,16807	1
Ccdc59	-0,16847	1
Nkap	-0,16876	1
Csnk2a2	-0,16897	1
Slc29a3	-0,16916	1
Tpm4	-0,16938	1
Tes	-0,16936	1
Prnp	-0,16947	1
Pacs2	-0,16992	1
Cd63	-0,1699	1
Slc4a2	-0,17015	1
Ddi2	-0,17032	1
Snhg1	-0,17038	1
Exoc5	-0,17064	1
Coa5	-0,17077	1
Zbtb20	-0,1709	1
Fam102a	-0,171	1
Cyp51	-0,17107	1
Chst3	-0,17131	1
Mrpl28	-0,17129	1
Ormdl1	-0,17156	1
Ptrhd1	-0,17164	1
Chtop	-0,17166	1
Chmp5	-0,17223	1
Cript	-0,17274	1
2310061I04Rik	-0,17285	1
Gen1	-0,17289	1
Cpsf2	-0,17302	1
Eef2kmt	-0,17314	1
Unk	-0,17309	1
Stac3	-0,17319	1
Usp54	-0,17339	1
Arpc5l	-0,17338	1
Mrpl52	-0,17337	1
Hps5	-0,1735	1
Ccnd1	-0,17353	1
Lars2	-0,17369	1
Zfp69	-0,1738	1
Ccdc88b	-0,17384	1
Taf3	-0,17378	1
Srgap3	-0,17392	1
2210016L21Rik	-0,17403	1
Ywhae	-0,17426	1
Ak6	-0,17436	1

Nfatc1	-0,17487	1
4732491K20Rik	-0,17507	1
Pigv	-0,17524	1
Glod4	-0,1756	1
Dbt	-0,17571	1
Ccrl2	-0,17592	1
Gm12466	-0,17597	1
Clcn4	-0,176	1
Gfm2	-0,17602	1
Dnase2a	-0,17603	1
Zcchc24	-0,17611	1
Cipc	-0,17614	1
Ptdss2	-0,17619	1
Plk3	-0,17626	1
Gm13998	-0,17654	1
Ccl6	-0,1766	1
Tmed1	-0,17659	1
Rpl35a-ps5	-0,17681	1
Ing2	-0,17704	1
Pafah1b3	-0,17714	1
Kif22	-0,17727	1
Casp9	-0,17758	1
Sf3b5	-0,17762	1
Cep290	-0,17768	1
Eif5	-0,17768	1
3110002H16Rik	-0,17779	1
Zfp91	-0,17779	1
Mcts1	-0,17813	1
Dbi	-0,1781	1
Wsb2	-0,17821	1
Eea1	-0,17823	1
Porcn	-0,17829	1
Ndufb6	-0,17836	1
Usp48	-0,17836	1
Dstyk	-0,17851	1
Zfp607b	-0,17859	1
Gata3	-0,17867	1
Sod2	-0,17884	1
Slc25a24	-0,17894	1
Cnp	-0,17897	1
Ccdc171	-0,17934	1
BC003331	-0,17944	1
Gm26631	-0,17953	1
Atad3a	-0,17965	1
Ppfia1	-0,17997	1
Fbrs	-0,18005	1
Dgka	-0,18025	1
Gm13822	-0,18018	1
Snrnp25	-0,18017	1
Gm10717	-0,18036	1
Drg1	-0,18035	1
Mcm9	-0,18051	1

Snapin	-0,18061	1
Tdpx-ps1	-0,18057	1
Mrpl41	-0,18064	1
Helz	-0,18109	1
Setdb2	-0,18124	1
Prr3	-0,18117	1
Polr2b	-0,18147	1
Idh3b	-0,18165	1
Ccdc126	-0,18193	1
8430429K09Rik	-0,18206	1
Esrra	-0,18231	1
Apoe	-0,1824	1
Slc25a45	-0,18244	1
Gm13392	-0,18258	1
Trim2	-0,18288	1
Utp15	-0,18286	1
Ccni	-0,18301	1
Paxip1	-0,18307	1
Tbc1d8	-0,18317	1
Tspan5	-0,18331	1
Mgat1	-0,18331	1
Mfsd10	-0,18342	1
Magt1	-0,18341	1
Eif2b1	-0,18357	1
Gm13015	-0,18368	1
Actr1b	-0,18374	1
Rilp	-0,18404	1
Zdhhc6	-0,18396	1
Hnrnpl	-0,18414	1
Nop16	-0,1842	1
Fam3a	-0,18446	1
Ggact	-0,18473	1
Cd33	-0,18502	1
Krtcap2	-0,18505	1
Shpk	-0,18529	1
Hdac4	-0,18528	1
Gm12988	-0,18551	1
Fam13c	-0,1855	1
Crebbp	-0,18552	1
Klf13	-0,18548	1
Dync1li1	-0,18554	1
Furin	-0,18551	1
Ifi207	-0,1856	1
Aftph	-0,18563	1
Oxsr1	-0,18573	1
Setd3	-0,18605	1
Snhg15	-0,18634	1
Grcc10	-0,18654	1
Tnfrsf9	-0,1867	1
Acadl	-0,18678	1
Ddrgk1	-0,18693	1
Plrg1	-0,18695	1

Syne1	-0,1871	1
Aspscr1	-0,18736	1
Ube2q2	-0,18765	1
Rab13	-0,1879	1
Tmem110	-0,18794	1
Gm17060	-0,18797	1
1110038B12Rik	-0,18819	1
Fpgt	-0,18827	1
Ndufa6	-0,18829	1
Senp1	-0,18871	1
Ece2	-0,18868	1
Ralbp1	-0,18894	1
Gm6209	-0,18904	1
Nsf	-0,189	1
Six4	-0,18913	1
Mcl1	-0,18909	1
Lclat1	-0,18924	1
Acvr2a	-0,18916	1
Thra	-0,18917	1
Nol10	-0,18943	1
Elp5	-0,18956	1
BC017643	-0,18992	1
Herc2	-0,18988	1
Maml3	-0,19012	1
Sh2b2	-0,19008	1
Larp1b	-0,19009	1
Zfp9	-0,19035	1
Trmt61b	-0,19044	1
Med9	-0,19044	1
Gars	-0,19064	1
Itpripl1	-0,19072	1
Dcxr	-0,19078	1
Dsel	-0,19092	1
Gm5380	-0,19105	1
Gm2796	-0,1911	1
Gm37333	-0,19147	1
Eif1ax	-0,19185	1
Gm13360	-0,19195	1
Tmem86a	-0,19192	1
Gm43627	-0,19214	1
Gm7589	-0,19228	1
Psrc1	-0,19237	1
Mitd1	-0,19259	1
Adss	-0,19264	1
Mir703	-0,19272	1
Ptges2	-0,19281	1
Mat2b	-0,19284	1
Vipas39	-0,19287	1
Tcf3	-0,19303	1
Whamm	-0,19322	1
Upf3a	-0,19325	1
Gm10073	-0,19331	1

Ralgps1	-0,19347	1
Ydjc	-0,19395	1
Uso1	-0,19418	1
Synj2bp	-0,19438	1
Dbp	-0,19445	1
Picalm	-0,19447	1
Creb3l4	-0,19468	1
Gm44250	-0,19469	1
Smpd1	-0,19465	1
Akt2	-0,19479	1
Tada1	-0,19478	1
Clptm1	-0,19508	1
Rrp15	-0,19517	1
Xrn2	-0,19528	1
Gm6265	-0,196	1
Ufc1	-0,19609	1
Cwc15	-0,19646	1
Wdr13	-0,19656	1
Pdcd4	-0,19692	1
Gm43513	-0,19698	1
Osgep	-0,19759	1
Lap3	-0,19788	1
Gm9840	-0,19805	1
Mcee	-0,1981	1
Cd151	-0,19835	1
Frmd4b	-0,19826	1
Rps16-ps2	-0,1983	1
Llgl1	-0,19845	1
Naaa	-0,19875	1
Rwdd2a	-0,19878	1
Unc119b	-0,19884	1
Gm10642	-0,199	1
Cenpt	-0,19913	1
Tmem218	-0,19913	1
Fntb	-0,1992	1
Zfp644	-0,1993	1
Ndufaf5	-0,19945	1
Cmc2	-0,19943	1
Isca2	-0,19946	1
2700060E02Rik	-0,1996	1
Emd	-0,19958	1
Aup1	-0,19982	1
Mfhas1	-0,19992	1
Nat14	-0,19989	1
Ccdc61	-0,20015	1
Zkscan3	-0,20017	1
Trps1	-0,20023	1
Rbm27	-0,20024	1
Gnl1	-0,2005	1
Arhgap22	-0,20072	1
Senp7	-0,20067	1
Leprotl1	-0,20074	1

Rabgap1l	-0,20083	1
Eps8	-0,20081	1
5430405H02Rik	-0,20094	1
Taf6	-0,20104	1
Nol4l	-0,20113	1
Dhrs1	-0,20112	1
Sppl3	-0,20106	1
Myd88	-0,20114	1
Tmem237	-0,20123	1
Apitd1	-0,20152	1
Bin3	-0,20155	1
Oard1	-0,20146	1
Atp5d	-0,20161	1
Sra1	-0,20168	1
Vti1b	-0,20171	1
Rps11-ps1	-0,20178	1
D6Ertd527e	-0,20192	1
Gm37254	-0,20193	1
Chsy1	-0,20205	1
Senp5	-0,2022	1
Gm12704	-0,20243	1
Med13l	-0,20296	1
Brd1	-0,20306	1
Smarcc1	-0,20314	1
Ppp1r14b	-0,20329	1
Vps33a	-0,20339	1
Sec24c	-0,2034	1
Chkb	-0,20359	1
Dcaf15	-0,20357	1
Casc3	-0,20363	1
Zkscan7	-0,20373	1
Gk5	-0,20375	1
Acacb	-0,20411	1
Nup155	-0,20432	1
Srprb	-0,20426	1
Mapk1ip1l	-0,20425	1
Zik1	-0,20452	1
Clp1	-0,20462	1
Hotairm1	-0,20484	1
Fam8a1	-0,20503	1
Med17	-0,20515	1
Zfp335	-0,20513	1
Wdr33	-0,20519	1
Ankib1	-0,20532	1
Ppp2r5d	-0,20536	1
Med28	-0,20541	1
Chmp6	-0,20557	1
Ankhd1	-0,20565	1
Gm5446	-0,20574	1
Zmpste24	-0,2059	1
Set	-0,20594	1
Gngt2	-0,20596	1

Ampd2	-0,20598	1
Itsn2	-0,20596	1
Sergef	-0,20654	1
Aida	-0,20654	1
AA465934	-0,20655	1
Cd53	-0,20662	1
Fam69a	-0,20696	1
Tada3	-0,20696	1
Kri1	-0,20715	1
Tnks2	-0,20724	1
Tra2b	-0,2074	1
Socs2	-0,2077	1
Gpr89	-0,20772	1
Cox4i1	-0,20781	1
Cse1l	-0,20802	1
Tspan33	-0,20817	1
Ifi203	-0,20833	1
Fxyd5	-0,20829	1
Manea	-0,20848	1
Gtf2e1	-0,20863	1
Nudc	-0,20881	1
Pprc1	-0,20889	1
Zfp664	-0,20888	1
Rmdn3	-0,20886	1
Ndrg4	-0,20889	1
Ppie	-0,20896	1
Tmem71	-0,20933	1
Sf1	-0,20929	1
Mfsd8	-0,20936	1
Tmem131	-0,20949	1
Mical2	-0,20952	1
Ube2r2	-0,20947	1
Zmym6	-0,2096	1
Osbpl9	-0,20967	1
C2cd3	-0,20977	1
Osgin2	-0,20982	1
Fbxo18	-0,20986	1
Cd2bp2	-0,20991	1
Tmco1	-0,20986	1
Gm12606	-0,21	1
Plekhg2	-0,21006	1
Myo18a	-0,21013	1
Ttyh2	-0,21036	1
Hgsnat	-0,21064	1
Cnpy3	-0,2107	1
Kif2a	-0,21067	1
Gm13622	-0,21092	1
Gm3608	-0,21098	1
Rps12-ps23	-0,21107	1
Aldh7a1	-0,21107	1
Ten1	-0,21113	1
Cap1	-0,21107	1

Xrcc6	-0,21127	1
Trim28	-0,21154	1
Bag1	-0,2117	1
Slc38a10	-0,2117	1
Dpp9	-0,21223	1
Senp8	-0,21229	1
Ankrd35	-0,21227	1
Cdc42ep3	-0,21238	1
Ptpn6	-0,21274	1
Rpl6l	-0,21284	1
Gbas	-0,2137	1
Ap1m1	-0,21371	1
Gm2991	-0,21375	1
Dync2h1	-0,21398	1
Mafb	-0,21411	1
Snx14	-0,21437	1
Gm20707	-0,21473	1
Rufy1	-0,21466	1
Pdcd2l	-0,21476	1
Actl6a	-0,21489	1
Ergic1	-0,21485	1
Snx33	-0,21508	1
Tmem234	-0,21523	1
2510046G10Rik	-0,21529	1
Gdpd1	-0,21553	1
Zfp451	-0,21561	1
Pnpla8	-0,21572	1
Extl3	-0,21601	1
Naa35	-0,21604	1
Metrnl	-0,21614	1
Plekhm2	-0,21611	1
Acot1	-0,21661	1
Tmem42	-0,21704	1
Mapk1ip1	-0,21696	1
Bcl7c	-0,21715	1
Api5	-0,21708	1
Coq7	-0,21742	1
Pfdn1	-0,21737	1
Nr1d2	-0,21737	1
Nudcd1	-0,2175	1
Cnot2	-0,21751	1
Htatip2	-0,21757	1
Purg	-0,21818	1
Tuba1a	-0,21842	1
Hspd1	-0,2187	1
Slc35f5	-0,21881	1
Lsg1	-0,21885	1
Uqcrc2	-0,2188	1
Homer3	-0,219	1
Carnmt1	-0,21911	1
Vps45	-0,21911	1
Cfl1	-0,21929	1

Ddn	-0,21944	1
Frs2	-0,21952	1
Ncl	-0,21965	1
Naa50	-0,21967	1
Def8	-0,21995	1
Gm10602	-0,22003	1
Rpl41	-0,22014	1
Prpf18	-0,22019	1
Tmem120a	-0,22038	1
Foxn2	-0,22049	1
Prkaa1	-0,22057	1
Gm13436	-0,22068	1
Ddx17	-0,22065	1
Ndufa7	-0,22071	1
Copa	-0,22078	1
Gtf3c3	-0,22092	1
Ppp2r5c	-0,22093	1
Tfcp2	-0,22119	1
Azin1	-0,22121	1
Zdhhc20	-0,22117	1
Trib3	-0,22134	1
Xpa	-0,22133	1
Ogg1	-0,22144	1
Isy1	-0,22176	1
Tmem106c	-0,22203	1
Ptpn14	-0,22214	1
Sag	-0,22221	1
Arpc1a	-0,22262	1
Ptpmt1	-0,22281	1
Fam134a	-0,22284	1
Gm7079	-0,22291	1
Clec4a2	-0,22292	1
Ncln	-0,22287	1
Eif3k	-0,22301	1
Wdr81	-0,22322	1
Ap5z1	-0,22335	1
Pgf	-0,22345	1
Gm44913	-0,22384	1
Zfp950	-0,22385	1
Arfgef1	-0,22388	1
Ssrp1	-0,22388	1
Vkorc1l1	-0,22398	1
Rbm41	-0,22415	1
Enkd1	-0,22429	1
Aff1	-0,22431	1
Oscp1	-0,22448	1
Ube2q1	-0,22474	1
Mettl26	-0,2248	1
Ociad1	-0,22479	1
Stk11ip	-0,22494	1
Ppp1r15b	-0,22485	1
Dirc2	-0,22497	1

Gpbp1	-0,22505	1
Impa1	-0,22561	1
Sys1	-0,22579	1
Zdhhc5	-0,22602	1
Fam195b	-0,22603	1
Prpf6	-0,22604	1
Fnbp1	-0,22599	1
Qrich1	-0,22633	1
Grb2	-0,2264	1
Actr10	-0,22651	1
Ndufb10	-0,2266	1
Nup214	-0,22678	1
Smap2	-0,22692	1
Doc2g	-0,22698	1
Mrpl51	-0,227	1
Gabarapl1	-0,2272	1
Gm2223	-0,22731	1
Smyd3	-0,22743	1
Gm14822	-0,22747	1
D630024D03Rik	-0,22752	1
Rpl21-ps1	-0,22747	1
Alms1	-0,22763	1
Slc35d1	-0,22763	1
Taf5l	-0,22773	1
Pnkp	-0,22789	1
Gm7332	-0,22798	1
Fhod1	-0,22818	1
Phf13	-0,22862	1
Hcls1	-0,22866	1
Asl	-0,22895	1
Chst12	-0,2289	1
Gclm	-0,22887	1
Smco3	-0,2291	1
Dcaf12	-0,22931	1
Slc16a3	-0,22944	1
Mrps23	-0,2295	1
Klhl20	-0,22958	1
Arfgap1	-0,22982	1
Btbd10	-0,22983	1
Gtpbp4	-0,22977	1
Focad	-0,22993	1
3300005D01Rik	-0,22985	1
Eif1-ps1	-0,23002	1
Gm20703	-0,23019	1
Tes3-ps	-0,2303	1
Sap18b	-0,23028	1
Pdhb	-0,23029	1
Osgin1	-0,23045	1
Cep152	-0,23036	1
Mrpl19	-0,23047	1
Galnt7	-0,23052	1
Hgs	-0,23059	1

Cars	-0,23059	1
Ppp1r12a	-0,23058	1
Gm13743	-0,23069	1
Sec24b	-0,23073	1
B4galt5	-0,23073	1
Tram1	-0,23069	1
Abcf1	-0,23071	1
Abcd1	-0,23084	1
Plk1	-0,23079	1
Ccdc88c	-0,23086	1
Ndufaf3	-0,23086	1
Acot7	-0,23089	1
Slco4a1	-0,23109	1
Wtap	-0,23119	1
Ube2v1	-0,2317	1
Elk1	-0,23172	1
Zfp811	-0,23181	1
Inpp5d	-0,23179	1
Abhd11	-0,23177	1
Sugt1	-0,23186	1
1500011B03Rik	-0,23196	1
Nat9	-0,23198	1
Gm43569	-0,23214	1
Zswim1	-0,23234	1
Pitpna	-0,23234	1
B230354K17Rik	-0,23271	1
BC029722	-0,23271	1
Srpk2	-0,23269	1
Phf21b	-0,23281	1
Emc7	-0,23297	1
Zmynd11	-0,23296	1
Aldh4a1	-0,23348	1
Eml4	-0,23356	1
Dennd5b	-0,23399	1
Zbed5	-0,23397	1
Cab39	-0,23421	1
Esyt2	-0,23426	1
Zfp362	-0,23441	1
Ccdc127	-0,23439	1
Surf1	-0,23443	1
Rprd1b	-0,23487	1
Zfp646	-0,23505	1
Gm10161	-0,23524	1
Trit1	-0,23531	1
Jade1	-0,23571	1
Tonsl	-0,23566	1
Kdm4d	-0,2359	1
Ubtd1	-0,23595	1
Uxt	-0,23611	1
Zfp654	-0,23616	1
Kank3	-0,2363	1
Rps10-ps1	-0,23631	1

Eef1d	-0,23638	1
Polr3gl	-0,23647	1
Sfswap	-0,23671	1
Clip1	-0,23673	1
Phlda3	-0,23682	1
Slc50a1	-0,23679	1
Cyld	-0,23687	1
3110040N11Rik	-0,23722	1
Gm9701	-0,23733	1
Clk3	-0,23734	1
Hoxa4	-0,23751	1
Mthfsl	-0,23772	1
Arl2bp	-0,23774	1
Eif3c	-0,23785	1
Gm38162	-0,23795	1
Nod1	-0,23803	1
Afg3l1	-0,23797	1
Kxd1	-0,23807	1
Tmem91	-0,2382	1
Cep85	-0,2382	1
Lamtor1	-0,23819	1
Gm7808	-0,23829	1
Papd7	-0,23831	1
Man1b1	-0,23854	1
Gm45113	-0,23868	1
Tmem5	-0,23874	1
Gm6204	-0,23874	1
Otud1	-0,23902	1
Lgals4	-0,23909	1
Lhpp	-0,23918	1
Zfp770	-0,23926	1
Gm19196	-0,23955	1
Rps6ka3	-0,23948	1
Gm12517	-0,23961	1
Camta2	-0,23958	1
Dcun1d1	-0,23968	1
Glmp	-0,23979	1
Polm	-0,23987	1
Fgd6	-0,23996	1
Angptl6	-0,24069	1
Ep400	-0,2408	1
Svil	-0,24098	1
B3glct	-0,24104	1
Gm15513	-0,24108	1
Socs6	-0,24107	1
Slc9a3r2	-0,24128	1
Gm5900	-0,24136	1
Kif21a	-0,2414	1
Polr2a	-0,24144	1
Smyd4	-0,24146	1
Pef1	-0,24149	1
Exd1	-0,24156	1

Tmeff1	-0,24175	1
C130089K02Rik	-0,24187	1
Lage3	-0,24192	1
Sri	-0,24202	1
Gm2467	-0,24212	1
Mpg	-0,24213	1
Kctd12	-0,24217	1
Slc26a2	-0,24256	1
Dnajb14	-0,24258	1
Ttc17	-0,24275	1
AI413582	-0,24313	1
Dock8	-0,24367	1
Pole3	-0,24366	1
Phf23	-0,24372	1
Arid4a	-0,24381	1
Ndufa13	-0,24377	1
Lgalsl	-0,24385	1
Acat2	-0,24389	1
Zfp110	-0,24397	1
Gm7292	-0,24431	1
Ubac1	-0,24428	1
Fam171a2	-0,24447	1
Mphosph10	-0,24455	1
Cables2	-0,24463	1
2310009A05Rik	-0,2446	1
Rpl30-ps9	-0,24471	1
Creld1	-0,24493	1
Ankrd11	-0,24494	1
Tspyl2	-0,24501	1
Ptcd1	-0,24497	1
Tmem189	-0,24524	1
Smarca4	-0,24525	1
Ppp6r2	-0,24538	1
Wdr75	-0,24536	1
Osbpl7	-0,24569	1
Zc2hc1a	-0,24579	1
Mid1ip1	-0,24577	1
Pnisr	-0,24602	1
Crybg3	-0,24614	1
Cwf19l2	-0,24651	1
Ssr2	-0,24648	1
Grk5	-0,24663	1
Med26	-0,24663	1
Ruvbl1	-0,24669	1
Stat1	-0,24683	1
Fam160b1	-0,24705	1
Gm38077	-0,24712	1
Gm45840	-0,2473	1
Bptf	-0,2473	1
Fbxo11	-0,24737	1
Gfer	-0,24739	1
Slk	-0,2478	1

Smad2	-0,24811	1
Mta2	-0,24806	1
Xpr1	-0,24812	1
Slc39a6	-0,24847	1
Tmem147	-0,24857	1
Selenoh	-0,2488	1
Sclt1	-0,24893	1
Itpr2	-0,24918	1
Arhgef4	-0,24936	1
Gm26569	-0,24942	1
Dusp12	-0,24974	1
Dync1i2	-0,24973	1
Gm7123	-0,24975	1
Map3k4	-0,24981	1
S100a10	-0,24976	1
Gm12791	-0,25061	1
Fam214b	-0,25059	1
Gm12696	-0,25066	1
Aim1l	-0,2509	1
Kctd3	-0,25095	1
Arhgef7	-0,25102	1
Sfmbt1	-0,25131	1
RP23-371B13.3	-0,25146	1
Sufu	-0,25158	1
Mettl6	-0,25177	1
BC037032	-0,25185	1
Tbkbp1	-0,25191	1
Chpf2	-0,25205	1
Odf2l	-0,25223	1
Zmynd8	-0,25218	1
Scaf11	-0,25222	1
Arf4	-0,25221	1
Sh2d2a	-0,25243	1
Ibtk	-0,25243	1
Prkag2	-0,25245	1
Itfg2	-0,25277	1
Apopt1	-0,2528	1
Akip1	-0,25281	1
Gm7867	-0,25305	1
Rpp40	-0,25313	1
Capg	-0,25319	1
Neurl3	-0,25326	1
Sorbs1	-0,25352	1
Mettl1	-0,25347	1
Cast	-0,25361	1
Gm45855	-0,2537	1
Mtss1	-0,25374	1
Tmem55b	-0,25383	1
Iah1	-0,25391	1
L3mbtl3	-0,25414	1
C030014I23Rik	-0,25418	1
Igbp1	-0,25449	1

Chchd1	-0,2545	1
Cbx3	-0,25462	1
Aldoc	-0,25475	1
Nhp2	-0,25465	1
Gpx4	-0,25484	1
Msto1	-0,25476	1
Zc3h13	-0,25475	1
Murc	-0,25486	1
Klhl8	-0,25495	1
Pofut2	-0,25488	1
Terf2	-0,25488	1
Cdh23	-0,25495	1
Nlrc5	-0,25521	1
Ino80e	-0,25515	1
Yipf2	-0,25551	1
Gm12751	-0,25565	1
Selenoo	-0,25571	1
Rps19-ps8	-0,25605	1
Alox5	-0,25612	1
Arid4b	-0,25632	1
Mgmt	-0,25663	1
Ccdc116	-0,25669	1
Rpl17-ps4	-0,257	1
Prrc2c	-0,2571	1
Ppp2r5a	-0,25715	1
Alg1	-0,25742	1
Pradc1	-0,25747	1
Galnt1	-0,25746	1
Retn	-0,25772	1
Uprt	-0,25775	1
Dctn3	-0,25776	1
Gm45456	-0,25801	1
Eprs	-0,25803	1
Ptges	-0,25807	1
Mnat1	-0,25825	1
Gm36189	-0,25849	1
Slc36a4	-0,25884	1
Pde4d	-0,25888	1
Med7	-0,25893	1
Pkd1	-0,25924	1
Cdc26	-0,25939	1
Mfap3l	-0,25948	1
A430105J06Rik	-0,25963	1
Dgcr2	-0,26016	1
Cyp4f13	-0,26047	1
Rtn3	-0,26098	1
Slc35a2	-0,26119	1
Gm5547	-0,26134	1
Abcb4	-0,26162	1
Rabac1	-0,26163	1
Dzip1	-0,26167	1
Dctn5	-0,26167	1

Rmdn1	-0,26195	1
Fbxl6	-0,26193	1
Gcsh	-0,262	1
Tmem173	-0,26213	1
Gramd1b	-0,26217	1
Rbmx2	-0,2623	1
Car7	-0,26235	1
Tsc22d4	-0,26269	1
Sgtb	-0,26289	1
Fkbp8	-0,26289	1
Tyw1	-0,26322	1
Heatr1	-0,26326	1
Dnmt3a	-0,26338	1
Tbc1d20	-0,26354	1
Uqcrh-ps1	-0,26373	1
Ankra2	-0,26373	1
Usp5	-0,26367	1
Dqx1	-0,26392	1
Tjap1	-0,26386	1
Enpp5	-0,26406	1
Trappc1	-0,26409	1
Rgl2	-0,26452	1
Cyp20a1	-0,26449	1
Psmd13	-0,2645	1
Rnf113a1	-0,26467	1
Mtch2	-0,26467	1
Map4k5	-0,26478	1
Apc	-0,26489	1
Tyms	-0,26503	1
Rad50	-0,26499	1
Hspa9	-0,265	1
Arhgef12	-0,26539	1
Kbtbd7	-0,26547	1
Gm42567	-0,26624	1
Glud1	-0,26627	1
Wdfy1	-0,26662	1
Zeb2	-0,26662	1
Rack1	-0,2667	1
Zfyve9	-0,26685	1
Tmem186	-0,26694	1
Rps12-ps5	-0,2669	1
Rnf187	-0,26693	1
Sap30l	-0,26698	1
Mvb12a	-0,26702	1
Mtrr	-0,26713	1
Cd44	-0,26708	1
Pkn1	-0,26734	1
Net1	-0,26783	1
Ubqln2	-0,26794	1
Gm9762	-0,26791	1
Cops8	-0,26805	1
A430010J10Rik	-0,26812	1

Snrnp27	-0,26832	1
Zmat2	-0,26854	1
Tab1	-0,26859	1
Irf9	-0,26887	1
Gm12096	-0,26922	1
Fam69b	-0,26936	1
Cnbd2	-0,26983	1
Psmf1	-0,26983	1
Gtf3c4	-0,2698	1
Snx9	-0,27004	1
Atg7	-0,26997	1
Gm15877	-0,27006	1
Gm13039	-0,27012	1
Xpo5	-0,27021	1
Nin	-0,27015	1
Commd6	-0,27032	1
Zfp788	-0,27048	1
Gm1947	-0,27062	1
Dpys	-0,27059	1
Mrps7	-0,27058	1
RP23-159E10.1	-0,27073	1
Atraid	-0,27078	1
Synj2	-0,27099	1
Rtn4	-0,27102	1
Rasgef1a	-0,27125	1
Mfap3	-0,27128	1
Ncoa7	-0,27143	1
Prss44	-0,27149	1
Gm12444	-0,27159	1
Rps19	-0,27156	1
Dpy30	-0,27177	1
Gm19777	-0,27186	1
Cluap1	-0,27192	1
Ndufb11	-0,2719	1
Kidins220	-0,27205	1
Ufsp1	-0,27209	1
Aamdc	-0,27211	1
Rnf40	-0,27209	1
Rps11	-0,27223	1
Mdfic	-0,27227	1
Zfyve21	-0,27264	1
Mrps18c	-0,27258	1
Rfx1	-0,27262	1
Ccp110	-0,27276	1
Ap4b1	-0,2728	1
Mier3	-0,273	1
Smad3	-0,27311	1
Fam134b	-0,2733	1
Srp19	-0,27334	1
Zfp292	-0,27361	1
Lonrf1	-0,27369	1
Gm19898	-0,27377	1

Dcun1d4	-0,27421	1
Ccnf	-0,2743	1
Stk38l	-0,27429	1
Stk4	-0,27453	1
Sltm	-0,27468	1
Pip5k1c	-0,27484	1
1600002K03Rik	-0,27491	1
Tfpi	-0,27487	1
Ppp3ca	-0,27502	1
Mrpl57	-0,27534	1
Nhej1	-0,27541	1
Gm37465	-0,27557	1
Zfp780b	-0,27568	1
Xpnpep3	-0,27571	1
E430021H15Rik	-0,27577	1
Ccdc77	-0,27586	1
Wdfy3	-0,27608	1
Taldo1	-0,27624	1
Mob4	-0,27621	1
Apobec1	-0,27626	1
Afp	-0,27657	1
Gm26800	-0,27689	1
Clybl	-0,2769	1
Eef1e1	-0,27707	1
Uqcc2	-0,27719	1
Tac4	-0,27733	1
Tpgs1	-0,27738	1
Gm9892	-0,27763	1
Mrps5	-0,27766	1
Gm4880	-0,27803	1
Renbp	-0,27805	1
Gm20522	-0,27818	1
Pank1	-0,27828	1
RP23-442M18.5	-0,27854	1
Fam98a	-0,27855	1
Ptpn22	-0,27859	1
Cct6a	-0,27877	1
Tmem222	-0,27904	1
Usp36	-0,279	1
Nob1	-0,2794	1
Eefsec	-0,27978	1
Rpap2	-0,27982	1
Ulk4	-0,27988	1
Rpap1	-0,28001	1
Rbmxl1	-0,28035	1
Rps18	-0,28033	1
Zfp715	-0,28053	1
Ppp1r12b	-0,28084	1
Tubb2a	-0,28133	1
Slc11a2	-0,2813	1
Gm43106	-0,28138	1
Ecel1	-0,28145	1

Cebpzos	-0,28143	1
Vdr	-0,28155	1
Kmt2b	-0,28153	1
Dnaja2	-0,28154	1
Gba	-0,28173	1
Pgam5	-0,2818	1
Gm15427	-0,28185	1
Celf6	-0,28223	1
Fam20c	-0,28221	1
Mios	-0,28229	1
Hk3	-0,28233	1
6430531B16Rik	-0,28245	1
Cep104	-0,28251	1
Hexa	-0,28247	1
Aldoart1	-0,28256	1
Gm42937	-0,28279	1
Fbxo34	-0,28285	1
RP24-91J7.1	-0,28296	1
Rps12	-0,28312	1
Syngr2	-0,28319	1
Spsb1	-0,28343	1
D430042O09Rik	-0,28357	1
Mia3	-0,28369	1
Sfr1	-0,28367	1
Daglb	-0,28401	1
Foxd2	-0,28434	1
Rps11-ps2	-0,28435	1
Gpx1	-0,28448	1
Arl6	-0,28471	1
1600029O15Rik	-0,28478	1
Nepro	-0,2849	1
Serpinc1	-0,28512	1
Wdr11	-0,28514	1
A230028O05Rik	-0,28515	1
Rnf113a2	-0,28517	1
Tbc1d1	-0,28524	1
Ppard	-0,28544	1
Fam198b	-0,28554	1
Lck	-0,28551	1
Gm42872	-0,28573	1
Hspa14	-0,28582	1
Dhx36	-0,28581	1
Gm15708	-0,28593	1
Tlr2	-0,28611	1
Gm4149	-0,28652	1
Dner	-0,28657	1
Hnrnpul2	-0,28666	1
Rab35	-0,28691	1
Gm6329	-0,28698	1
Chchd3	-0,28696	1
Ecsit	-0,28715	1
Coq9	-0,28722	1

Gm4890	-0,28732	1
Elmo1	-0,28739	1
Fbxw2	-0,28735	1
Slc35e2	-0,28753	1
Poldip3	-0,28747	1
Zzef1	-0,28787	1
Parg	-0,28811	1
Ipo8	-0,28822	1
Ptpre	-0,28815	1
Bola2	-0,2884	1
Gm43684	-0,28858	1
Baz1a	-0,28869	1
Gga2	-0,28887	1
Uqcrfs1	-0,2891	1
Vps16	-0,28922	1
Rbm6	-0,28932	1
Nop2	-0,28953	1
Enpp4	-0,28974	1
Tm7sf3	-0,28973	1
Msh2	-0,28976	1
Tbc1d15	-0,28989	1
Ndufa12	-0,29013	1
Gch1	-0,29016	1
Arl14ep	-0,29032	1
Gm13422	-0,29049	1
Dhx58	-0,29069	1
Mtmr3	-0,29072	1
Zbtb48	-0,29081	1
Nmnat1	-0,29091	1
Tsacc	-0,29141	1
Zfp68	-0,29192	1
Uqcr11	-0,29187	1
Dtd1	-0,29236	1
Ralgapa2	-0,2925	1
Psmd12	-0,29284	1
Gkap1	-0,29303	1
Lrp8	-0,29303	1
Cluh	-0,29296	1
Pik3cd	-0,29313	1
Zfp429	-0,29319	1
Fbxo7	-0,29324	1
Cdc14a	-0,29335	1
Mtfr1	-0,29337	1
Ccdc17	-0,29347	1
Glmn	-0,29348	1
Mcm3ap	-0,29352	1
Tmem167	-0,2937	1
Gm6198	-0,29376	1
Ntpcr	-0,2939	1
Tmem126a	-0,29402	1
Hipk3	-0,29413	1
Usp47	-0,29457	1

Gm45445	-0,29501	1
Pcdhb22	-0,29505	1
Il18	-0,29505	1
Chac2	-0,29509	1
Sfxn1	-0,29518	1
Snf8	-0,29529	1
Gm5624	-0,29545	1
Gm44075	-0,29539	1
Zcchc6	-0,29543	1
Esd	-0,2954	1
C530005A16Rik	-0,29552	1
Nsfl1c	-0,29567	1
Aim2	-0,29566	1
Lrtm2	-0,29576	1
Pls3	-0,2958	1
H2-D1	-0,29581	1
Crnde	-0,29589	1
Spag7	-0,29595	1
Rps15-ps2	-0,29604	1
Mtmr2	-0,29604	1
Atp8a1	-0,29607	1
Tvp23b	-0,29608	1
Eif3a	-0,29631	1
Ddx21	-0,29639	1
Rassf5	-0,29655	1
Cdk14	-0,29696	1
Smim19	-0,29699	1
Parp2	-0,29755	1
Ppig	-0,29765	1
Gm5614	-0,29772	1
Parp14	-0,29779	1
Chd8	-0,29785	1
Pepd	-0,2979	1
1810062G17Rik	-0,29809	1
Serpine1	-0,29809	1
Rbm12	-0,29823	1
Zfp626	-0,29849	1
Dcaf5	-0,29852	1
Hfe	-0,29863	1
Gon4l	-0,29857	1
Zfp959	-0,29884	1
Zfp672	-0,29898	1
Map2k2	-0,29897	1
Ikbip	-0,29912	1
Pramef8	-0,29923	1
Zfp938	-0,29929	1
Map4k3	-0,29951	1
Mapk12	-0,30003	1
Scnm1	-0,29995	1
Mysm1	-0,30009	1
Poll	-0,30009	1
Gm10031	-0,30032	1

Smdt1	-0,30037	1
Fam129c	-0,30053	1
2010320M18Rik	-0,30053	1
Akr1a1	-0,30063	1
Uchl3	-0,30074	1
Tyw5	-0,30079	1
Twf2	-0,30078	1
Ankrd44	-0,30111	1
Mrpl18	-0,30111	1
Gsdmd	-0,30131	1
Ndufb5	-0,30135	1
P3h1	-0,30138	1
Ccdc63	-0,30147	1
Bcl2l1	-0,30145	1
Recql5	-0,30255	1
Cep250	-0,30258	1
Praf2	-0,30277	1
Mum1	-0,30277	1
Atp6v1f	-0,30282	1
Elp4	-0,30314	1
Oma1	-0,30316	1
Gm14048	-0,30334	1
Clcn7	-0,30371	1
Sec61a1	-0,30379	1
Ipmk	-0,30421	1
Dxo	-0,30453	1
Gclc	-0,3045	1
Tns3	-0,30464	1
Amacr	-0,3048	1
Siva1	-0,3049	1
Phb2	-0,30495	1
Rpl31-ps17	-0,30541	1
Herpud2	-0,30544	1
Myo1e	-0,30544	1
Elovl5	-0,30551	1
Gm12020	-0,30569	1
Wdr89	-0,30587	1
Tbc1d4	-0,30608	1
Twistnb	-0,3062	1
Nek8	-0,30662	1
Cdkn2aipnl	-0,30674	1
Aifm1	-0,30673	1
Psmd7	-0,30675	1
Oxsm	-0,30693	1
Hmox2	-0,30692	1
Hnrnpab	-0,30693	1
Cdc25a	-0,30701	1
Psme3	-0,3071	1
Xxylt1	-0,30732	1
Tmem87a	-0,30729	1
Acot13	-0,3073	1
Gm16238	-0,30785	1

Tbc1d17	-0,30792	1
Hacd1	-0,30798	1
Cirbp	-0,30812	1
Gm2810	-0,30816	1
Rad54l2	-0,30819	1
Chd1	-0,30819	1
Arl8a	-0,30837	1
Telo2	-0,30888	1
Nras	-0,30901	1
Ksr1	-0,30906	1
Yeats4	-0,30906	1
Spg11	-0,3092	1
mt-Nd1	-0,30947	1
Enc1	-0,3097	1
Ppp1r8	-0,30975	1
Tmem144	-0,30994	1
Ubap1	-0,31009	1
1700003F12Rik	-0,3103	1
Gm9013	-0,31038	1
Pik3c3	-0,31044	1
Fam129a	-0,31043	1
Ufd1l	-0,31042	1
Mrpl39	-0,31051	1
Mrpl24	-0,31049	1
Metap1	-0,31064	1
Gm5776	-0,311	1
Uqcrq	-0,311	1
Ifngr2	-0,31112	1
Smarcd2	-0,31109	1
Arhgef10	-0,31138	1
Aldh18a1	-0,31169	1
Ndufa8	-0,31183	1
Atxn7	-0,3122	1
Mtf1	-0,31219	1
Ttc33	-0,3124	1
Fbxl12	-0,31239	1
1700123O20Rik	-0,31253	1
E2f4	-0,31246	1
Usp31	-0,31255	1
Hivep2	-0,31275	1
Rcl1	-0,31297	1
Itga5	-0,31306	1
Smcr8	-0,31324	1
Fam104a	-0,31323	1
2510009E07Rik	-0,31359	1
Gm8494	-0,31391	1
Tnnt3	-0,31387	1
Rabep1	-0,31412	1
1700030K09Rik	-0,31421	1
Gm23054	-0,31419	1
Fam91a1	-0,31417	1
Abhd17b	-0,31424	1

Zfp365	-0,31427	1
2810474O19Rik	-0,31429	1
Gm13777	-0,31449	1
Cacnb1	-0,31505	1
Nt5c3	-0,31559	1
Gm6451	-0,31565	1
Vim	-0,31567	1
Asxl2	-0,31576	1
Ahnak	-0,31576	1
Slfn3	-0,31592	1
Fcgr2b	-0,31599	1
Sp3os	-0,31599	1
6430573P05Rik	-0,31613	1
Gemin2	-0,31659	1
Vash2	-0,31659	1
Impad1	-0,31659	1
Vta1	-0,31678	1
Ylpm1	-0,31692	1
Slc30a4	-0,31713	1
Nectin2	-0,31722	1
Idua	-0,31718	1
Gm12389	-0,31735	1
Gm8825	-0,3177	1
AC149090.1	-0,31768	1
Tor3a	-0,31776	1
Rhob	-0,31811	1
Gm21399	-0,31818	1
Ankrd24	-0,31821	1
Zdhhc4	-0,31821	1
Gm15050	-0,31834	1
Dnase1l1	-0,31843	1
Foxo1	-0,31846	1
Psap	-0,31853	1
Trip6	-0,31872	1
Fut10	-0,3188	1
Sptlc1	-0,31881	1
Xrcc1	-0,31877	1
Ggnbp2	-0,31889	1
Fkbp4	-0,31899	1
Zfp322a	-0,31963	1
Psmb2	-0,31972	1
Nbeal2	-0,32034	1
Lrmp	-0,32026	1
Tsen54	-0,32044	1
Rmrp	-0,32053	1
G430095P16Rik	-0,3206	1
Cux1	-0,32063	1
Rab3ip	-0,32087	1
Pi4k2b	-0,32085	1
Gm12848	-0,32121	1
Zfp459	-0,32122	1
Trmt112-ps2	-0,32187	1

Exosc7	-0,32197	1
Gm38247	-0,32222	1
Gm8806	-0,32226	1
Gsap	-0,32232	1
Gm12341	-0,32273	1
Fuk	-0,32276	1
Tsg101	-0,32283	1
Ltv1	-0,32291	1
Pyroxd1	-0,32298	1
Neu1	-0,323	1
Fam50a	-0,32304	1
B130006D01Rik	-0,32308	1
Ncf2	-0,32312	1
Afg3l2	-0,32322	1
Rab29	-0,32343	1
Ly6g6d	-0,3237	1
Ankrd40	-0,32402	1
Calhm2	-0,32411	1
Srek1ip1	-0,32456	1
Rxrb	-0,32462	1
Smim20	-0,32466	1
Fn3krp	-0,32484	1
Gm7128	-0,32493	1
RP24-282C4.13	-0,32487	1
Dmap1	-0,32508	1
Slc36a3os	-0,32537	1
9230114K14Rik	-0,32564	1
Slc27a1	-0,32597	1
Armc1	-0,32603	1
Top1mt	-0,32608	1
Serinc2	-0,32627	1
Brap	-0,32639	1
Atp6ap2	-0,32637	1
Wnt6	-0,32652	1
Gm10136	-0,32656	1
Gss	-0,32687	1
Rab10	-0,32688	1
Gm29462	-0,32702	1
Guk1	-0,32716	1
Hnrnpk	-0,32729	1
5730480H06Rik	-0,32753	1
Sgk3	-0,32749	1
Tnk2	-0,32768	1
Faim	-0,32767	1
Gm7424	-0,32785	1
Ppm1f	-0,3279	1
Phax	-0,32789	1
Gm5139	-0,32832	1
Wdfy4	-0,32845	1
Plk2	-0,3288	1
Gm8818	-0,32883	1
Mfsd1	-0,32953	1

Usp30	-0,32958	1
Napa	-0,32961	1
Ube2v2	-0,32989	1
Ttc32	-0,33006	1
Limk2	-0,33018	1
Ifi211	-0,33028	1
Rcor2	-0,33049	1
Zbtb14	-0,3306	1
Cnnm3	-0,3307	1
A630033H20Rik	-0,33086	1
Farsb	-0,3314	1
Mb21d2	-0,33158	1
2810002D19Rik	-0,33168	1
Gm14240	-0,33191	1
Mtmr10	-0,33221	1
Ndst1	-0,33244	1
Dock9	-0,33242	1
Tmub2	-0,33251	1
Gm14794	-0,33249	1
March6	-0,33247	1
Trpm1	-0,33266	1
Crybg3	-0,33269	1
Mettl3	-0,3329	1
Gm44775	-0,33302	1
Ado	-0,33312	1
Gm12183	-0,33369	1
Morn2	-0,33391	1
Ahsa2	-0,33387	1
Il10ra	-0,33388	1
Ptges3	-0,33402	1
Slc10a7	-0,33444	1
Gmeb2	-0,33444	1
Zfp593	-0,33443	1
Ick	-0,33462	1
Mapkap1	-0,33459	1
Gorasp2	-0,33482	1
Zfp503	-0,33486	1
Mfn2	-0,33495	1
Gm15846	-0,33521	1
Ikbkg	-0,33529	1
Cxcl14	-0,33551	1
Ms4a6c	-0,33572	1
Aars	-0,3361	1
1700123M08Rik	-0,33638	1
Dguok	-0,33643	1
Nt5dc2	-0,33654	1
Vps37a	-0,33657	1
Gins4	-0,33673	1
Rnh1	-0,33678	1
Gm10250	-0,33693	1
Golga4	-0,33714	1
Il1b	-0,33717	1

Mbd1	-0,3374	1
Mrpl22	-0,33791	1
Gpr162	-0,3379	1
Zfp777	-0,33806	1
Idh1	-0,33818	1
Lims2	-0,33843	1
Tmbim4	-0,3385	1
Gtf2h1	-0,33861	1
Trim65	-0,33874	1
Mzt2	-0,33866	1
Rpl27a	-0,33875	1
Clec1a	-0,33935	1
Tatdn1	-0,33952	1
Sike1	-0,33946	1
Ufl1	-0,33963	1
Arhgef15	-0,33974	1
Laptm5	-0,33966	1
Dcun1d5	-0,33976	1
Pnpla7	-0,34014	1
Tbc1d2	-0,34031	1
Lzts2	-0,34035	1
Golga2	-0,34052	1
Mmadhc	-0,34056	1
Irf8	-0,34081	1
Copb2	-0,34076	1
Txk	-0,34101	1
Casd1	-0,34109	1
Dag1	-0,3412	1
Supt5	-0,34123	1
Fam204a	-0,34163	1
4930550C14Rik	-0,34168	1
Tbc1d10b	-0,34184	1
Lrch1	-0,34188	1
Mfsd11	-0,34192	1
Cav2	-0,3422	1
Zfp566	-0,34241	1
Uap1l1	-0,34242	1
Aagab	-0,34264	1
Ilkap	-0,34323	1
AI837181	-0,34326	1
Ank2	-0,34374	1
Fryl	-0,3437	1
Ndufs8	-0,34366	1
Tpcn1	-0,34366	1
Ccz1	-0,34382	1
Cct7	-0,34388	1
Map2k3	-0,3439	1
Gm6123	-0,344	1
Rbm15b	-0,34395	1
Micall2	-0,34433	1
Ryr1	-0,34441	1
Slc52a2	-0,34449	1

Gm44093	-0,34447	1
Sars2	-0,34456	1
Flad1	-0,34466	1
Atg4b	-0,34536	1
Apex1	-0,34541	1
Zfp236	-0,34558	1
Rfc2	-0,34573	1
Ap1s3	-0,34591	1
Esf1	-0,34609	1
Supv3l1	-0,34624	1
Insl6	-0,34644	1
Tox4	-0,34639	1
Rnft1	-0,34647	1
Lgi4	-0,34646	1
Pih1d1	-0,34657	1
Acp2	-0,34667	1
Cyb5d2	-0,34669	1
Klhl26	-0,3469	1
G3bp1	-0,34694	1
Rbpj	-0,34689	1
Gm38067	-0,3472	1
Fth-ps3	-0,34737	1
Nkiras2	-0,34774	1
Fut11	-0,34787	1
S100a4	-0,34803	1
Gm15773	-0,34821	1
RP23-243B24.1	-0,34816	1
Ankrd46	-0,3483	1
Chd2	-0,34836	1
Acot10	-0,34853	1
Cisd3	-0,34847	1
Mrpl14	-0,34864	1
Mocos	-0,3489	1
Sde2	-0,34903	1
Cd9	-0,34919	1
C1d	-0,34934	1
1110059G10Rik	-0,34938	1
Arhgef2	-0,3494	1
Tmem25	-0,34961	1
Dgkq	-0,34959	1
Nudt8	-0,34984	1
Gm43379	-0,34982	1
Pdss1	-0,34997	1
Pdgfa	-0,35064	1
Stk16	-0,35098	1
Pdzd8	-0,3514	1
Prkab2	-0,35136	1
Fars2	-0,35164	1
Gtf2i	-0,35171	1
Ptgr2	-0,35193	1
Abcd4	-0,35198	1
Sdf2l1	-0,3521	1

Gm4604	-0,35219	1
Dnajc27	-0,35228	1
Gm7436	-0,35231	1
Rpsa-ps1	-0,3523	1
Smarcd1	-0,35237	1
Hyal3	-0,35255	1
Topors	-0,35275	1
Letm1	-0,35265	1
Pccb	-0,35284	1
Wrap73	-0,35337	1
Mdn1	-0,35339	1
Gm7434	-0,35354	1
Zfand3	-0,35355	1
Rpl21-ps5	-0,35369	1
Rars	-0,35379	1
Zfp595	-0,35397	1
Supt6	-0,354	1
Cst7	-0,35408	1
Ap2a1	-0,35423	1
Hacd3	-0,35429	1
Cd180	-0,35436	1
Aasdhppt	-0,3545	1
0610038B21Rik	-0,35465	1
Zmat1	-0,35475	1
Slain2	-0,3547	1
Nub1	-0,35469	1
Cdc16	-0,35529	1
Atp5g3	-0,35551	1
Zdhhc24	-0,35558	1
Scamp5	-0,35566	1
Zfp329	-0,3558	1
Mrps25	-0,35623	1
Ly96	-0,35629	1
Cmtm3	-0,35633	1
Lrrc1	-0,35644	1
Ext1	-0,35651	1
Gm16310	-0,35665	1
Txnrd3	-0,35691	1
Ndufb3	-0,35689	1
Ercc5	-0,3569	1
Nxpe3	-0,35699	1
Psmb10	-0,35715	1
Shmt1	-0,35768	1
Arl13b	-0,35778	1
Tars2	-0,35798	1
Atp5c1	-0,35804	1
Slc17a5	-0,35801	1
Pafah1b2	-0,35808	1
Zfp296	-0,35859	1
Mvb12b	-0,35859	1
Rbm45	-0,3587	1
B630019K06Rik	-0,35884	1

Aph1c	-0,35893	1
Tsr3	-0,36024	1
Itpk1	-0,36037	1
Slc2a4rg-ps	-0,36079	1
Ccnh	-0,36078	1
Ptrh2	-0,36116	1
Ccl9	-0,36118	1
Ndufa11	-0,36148	1
Fbxo4	-0,36167	1
Gm14270	-0,36189	1
Mfsd2a	-0,36218	1
Myo1d	-0,36215	1
Mpeg1	-0,36227	1
Snai1	-0,36248	1
Sdhaf1	-0,36247	1
Cox6a2	-0,36257	1
Atp6v0a2	-0,3627	1
Rrp7a	-0,36287	1
Itga2b	-0,36291	1
Palld	-0,36289	1
Ssr4	-0,36289	1
Tmem41a	-0,36297	1
Trmt10a	-0,36305	1
A430018G15Rik	-0,36349	1
Gm42595	-0,36408	1
Gm44935	-0,36492	1
Gpatch11	-0,36508	1
Nap1l4	-0,36508	1
Dusp18	-0,36521	1
Blzf1	-0,36518	1
Ints2	-0,36532	1
Rgs10	-0,36528	1
Nsun5	-0,36545	1
Ttbk2	-0,36545	1
Usmg5	-0,36555	1
Gne	-0,36561	1
Ammecr1	-0,36571	1
1110004F10Rik	-0,36584	1
Klrg2	-0,36613	1
Zfp943	-0,36615	1
Sdccag8	-0,36617	1
Ate1	-0,36651	1
Cnbp	-0,36673	1
Ktn1	-0,36745	1
Six1	-0,36761	1
Kiss1r	-0,36757	1
Dbndd2	-0,36786	1
Crtam	-0,36793	1
Sirt6	-0,36796	1
Mplkip	-0,36804	1
Gm7224	-0,36813	1
Abcd3	-0,36841	1

Rpgrip1	-0,3685	1
Tnfrsf14	-0,3686	1
Rpl38-ps2	-0,36871	1
Snx11	-0,36912	1
Camkk2	-0,36914	1
Cdc123	-0,36935	1
Zfp512	-0,36972	1
Mocs2	-0,36986	1
Ccdc43	-0,37011	1
Pex11a	-0,3701	1
Parp1	-0,37054	1
Epc2	-0,37065	1
Mogs	-0,37084	1
Rab2a	-0,37102	1
Midn	-0,37161	1
2610507B11Rik	-0,37159	1
Rnf183	-0,37187	1
Clint1	-0,37214	1
Msr1	-0,37239	1
CAAA01194877.2	-0,37275	1
Inpp1	-0,37279	1
Poldip2	-0,37276	1
Cwc27	-0,37292	1
Wiz	-0,3733	1
Cox18	-0,37344	1
Lyrm7	-0,37351	1
Prob1	-0,37345	1
Ap1g2	-0,3736	1
Nol9	-0,37376	1
Zfp24	-0,37386	1
Rpl23	-0,37389	1
Fcor	-0,37421	1
Atpaf1	-0,37455	1
Ptp4a2	-0,3747	1
Ccdc90b	-0,37487	1
Zmiz1	-0,37503	1
Rmnd5b	-0,37513	1
Fcgrt	-0,37523	1
Zcrb1	-0,37524	1
Stap1	-0,37517	1
Actr3b	-0,37533	1
Rps6kc1	-0,37555	1
Mon1b	-0,37595	1
Pstk	-0,37596	1
4930563E22Rik	-0,37603	1
Fam53a	-0,37639	1
Gm42480	-0,37659	1
Coro1b	-0,37668	1
Usp9x	-0,37671	1
Nudt6	-0,37679	1
Asb6	-0,37685	1
Timm8b	-0,37713	1

Gm6166	-0,37733	1
Ppp1ca	-0,3773	1
Sbk2	-0,37788	1
Pex3	-0,37853	1
Uhrf1bp1l	-0,37853	1
Zfp281	-0,37859	1
Slc25a4	-0,37873	1
Gdap2	-0,37875	1
Ddx46	-0,37895	1
Ninl	-0,37911	1
Traf3ip2	-0,3793	1
Dhx29	-0,37952	1
Atp1b3	-0,37965	1
Gm11520	-0,37976	1
Txnrd2	-0,37978	1
Gm15892	-0,38013	1
Cecr5	-0,38015	1
Arhgap21	-0,38035	1
Hlcs	-0,38035	1
Psmc4	-0,3805	1
Ms4a6d	-0,38073	1
Erp29	-0,38165	1
St3gal5	-0,3818	1
Gtpbp3	-0,38189	1
Gm10039	-0,38217	1
Tssc1	-0,38235	1
Polr2m	-0,38236	1
Ebpl	-0,38269	1
Tmem161b	-0,38276	1
Acsl4	-0,38276	1
Gm10059	-0,38308	1
Sf3b6	-0,38315	1
Zfp668	-0,38334	1
Slc11a1	-0,38333	1
Lgals3bp	-0,3835	1
Vps54	-0,38353	1
Polr3c	-0,38429	1
Rab3d	-0,38443	1
Edem2	-0,38461	1
Ndufv2	-0,38465	1
Rbm10	-0,38473	1
Tm9sf4	-0,3848	1
Armc6	-0,38505	1
Rrp36	-0,38532	1
Apba1	-0,38583	1
Gm11273	-0,38604	1
Gm33370	-0,38601	1
Ap2m1	-0,38597	1
Rabif	-0,38626	1
Bod1	-0,38627	1
Emp1	-0,38639	1
Siah1a	-0,38674	1

Gm5764	-0,38673	1
Hmgb3	-0,38708	1
Otud5	-0,38732	1
Prdx5	-0,38743	1
Edc4	-0,38754	1
Gm15157	-0,3875	1
Gm43071	-0,388	1
Gm12501	-0,38837	1
Rnpepl1	-0,38849	1
Zcchc7	-0,38852	1
Gde1	-0,38849	1
Cyp4f16	-0,38863	1
Slc25a44	-0,3886	1
Pias1	-0,3889	1
Ankrd13c	-0,38903	1
Rpl36a-ps2	-0,38905	1
Tada2a	-0,38919	1
Tmem205	-0,38929	1
Usp16	-0,38933	1
Dhx38	-0,39004	1
Lig3	-0,39021	1
1110004E09Rik	-0,39027	1
Myh11	-0,39038	1
Agpat4	-0,39041	1
Slc9b1	-0,39083	1
Ngdn	-0,39075	1
Pms1	-0,39101	1
Gm6222	-0,39106	1
Ostm1	-0,39111	1
Ppip5k2	-0,39106	1
Pfdn2	-0,39138	1
Snx15	-0,39174	1
2410015M20Rik	-0,39168	1
Cyb561	-0,39193	1
Gm42918	-0,3919	1
Pla2g16	-0,39186	1
Gpr85	-0,39204	1
Bre	-0,39201	1
5031439G07Rik	-0,39212	1
Cd109	-0,39222	1
Gm5262	-0,39231	1
Zfp444	-0,3925	1
Gm10616	-0,39255	1
Zswim7	-0,39273	1
Nhlrc1	-0,39281	1
Slc30a1	-0,39284	1
Tbc1d14	-0,39306	1
1110059E24Rik	-0,39317	1
Dctn6	-0,39318	1
Car2	-0,39332	1
Bri3	-0,39379	1
Fbxw5	-0,39382	1

Faap20	-0,39425	1
Tmem141	-0,39441	1
Slc43a3	-0,39456	1
Mthfd2l	-0,3948	1
Gm4799	-0,39478	1
Elmsan1	-0,39476	1
Lgals8	-0,39479	1
Hmga1-rs1	-0,39502	1
Car9	-0,39512	1
Hspb6	-0,39527	1
Adrb2	-0,39556	1
Snrnp35	-0,39562	1
Mgme1	-0,39576	1
Arl4c	-0,3961	1
Slc25a53	-0,39621	1
Camsap2	-0,39633	1
Rfc3	-0,3964	1
Tmem62	-0,39656	1
Xpc	-0,39683	1
Sep 07	-0,39675	1
Pdrg1	-0,39676	1
Las1l	-0,39692	1
Gm16638	-0,39696	1
Vav2	-0,39725	1
Gm8250	-0,39719	1
Gm12689	-0,39758	1
Slc44a2	-0,39771	1
Trim13	-0,39782	1
Ccnk	-0,39794	1
Dctn2	-0,39801	1
Rhobtb1	-0,39817	1
S100a1	-0,39819	1
Limd1	-0,3983	1
Serhl	-0,39854	1
Xrcc3	-0,39875	1
Rps27a-ps1	-0,39894	1
Abcc1	-0,39889	1
Oas1g	-0,39903	1
Immt	-0,39922	1
Fus	-0,39943	1
Ccdc91	-0,39945	1
Gm7496	-0,40025	1
Foxp1	-0,40024	1
Gab2	-0,40053	1
Phf3	-0,4007	1
Myo19	-0,40127	1
Gle1	-0,40143	1
Gapdh-ps14	-0,40158	1
Pnkd	-0,40184	1
Gm15772	-0,40198	1
Sco2	-0,40207	1
Gm16124	-0,40223	1

5830487J09Rik	-0,40223	1
Drosha	-0,40285	1
Hmgxb3	-0,40303	1
Slc4a11	-0,40307	1
Noa1	-0,40315	1
Gm5939	-0,40307	1
Als2	-0,40349	1
Rpl27a-ps1	-0,40369	1
CAAA01180111.2	-0,40376	1
Abcc5	-0,40414	1
Ppp6r3	-0,4041	1
Vapb	-0,40425	1
Ppp4r2	-0,40432	1
Hmga1	-0,40442	1
Kifc3	-0,40485	1
Gm8869	-0,40499	1
Ankrd28	-0,40535	1
Dram2	-0,40538	1
Wdr3	-0,4054	1
Uqcrc1	-0,40548	1
Zfp867	-0,40563	1
Fbxo38	-0,40556	1
Appl1	-0,40567	1
Slc16a7	-0,40591	1
Rce1	-0,40586	1
Amz1	-0,40605	1
2700099C18Rik	-0,40628	1
Gm6395	-0,4064	1
Rasa2	-0,40702	1
Cdnf	-0,40714	1
Rfk	-0,40724	1
Wdr18	-0,40727	1
Rassf4	-0,40737	1
Adam10	-0,40774	1
Nolc1	-0,40774	1
Pja1	-0,40805	1
Itgb1bp1	-0,40843	1
Fignl2	-0,40849	1
Rbm43	-0,40852	1
Gm11224	-0,40866	1
Bod1l	-0,40866	1
Nrde2	-0,40883	1
Zc3h6	-0,40889	1
Otulin	-0,40909	1
Taf13	-0,40925	1
Fech	-0,40936	1
3110009E18Rik	-0,40982	1
Rps15a-ps1	-0,40982	1
Usp25	-0,40978	1
Tceal8	-0,40988	1
Vps37c	-0,40986	1
Trappc4	-0,40986	1

Cldn12	-0,41	1
Gm12854	-0,41007	1
Uck2	-0,41008	1
Nostrin	-0,41034	1
Gm11478	-0,41046	1
Ubr1	-0,41063	1
Zc3h15	-0,41058	1
Iars	-0,41101	1
Psmb5	-0,41105	1
Wdr7	-0,41115	1
Comtd1	-0,4112	1
Cbx8	-0,41132	1
Usp6nl	-0,41134	1
9330162012Rik	-0,41135	1
Stoml2	-0,41154	1
Ighd	-0,41145	1
Gatad2a	-0,41157	1
Zkscan17	-0,41179	1
Chpt1	-0,41202	1
Bcl2l15	-0,412	1
Slc7a4	-0,41235	1
Alas1	-0,41242	1
Lias	-0,41315	1
Fbxl14	-0,41329	1
Dcaf17	-0,41361	1
Col20a1	-0,41412	1
Ica1	-0,41434	1
Timm50	-0,41427	1
Gm37082	-0,41442	1
Mief1	-0,41457	1
Adam17	-0,41457	1
Pkig	-0,41468	1
Pla2g2e	-0,41485	1
Wars	-0,41517	1
Sypl	-0,41522	1
Emg1	-0,41526	1
Zfp260	-0,41582	1
Gemin7	-0,41583	1
Spryd4	-0,41661	1
Gm15703	-0,41659	1
Dera	-0,41679	1
Ddx1	-0,41676	1
Serbp1	-0,41676	1
Gm11560	-0,41691	1
Poc1b	-0,41692	1
Zfhx4	-0,41705	1
Ivns1abp	-0,4172	1
Lancl1	-0,41732	1
Cog4	-0,41754	1
Tmem160	-0,41778	1
Gm10029	-0,41801	1
Kcnk6	-0,41823	1

Cd5l	-0,41851	1
Crtc3	-0,41852	1
Smap1	-0,41847	1
Ndufa10	-0,41862	1
Rbbp4	-0,41871	1
Hspa4	-0,41874	1
Mtap	-0,41891	1
Ncf4	-0,41889	1
Gm13445	-0,41885	1
Gm27043	-0,41922	1
Gm4332	-0,41918	1
Clcnkb	-0,41944	1
Trim25	-0,41959	1
Tiprl	-0,41965	1
Wisp1	-0,41963	1
Gm12338	-0,41976	1
Gm14681	-0,41983	1
Slc25a51	-0,42003	1
Rnf20	-0,42005	1
Eif5b	-0,4201	1
Prkag1	-0,42019	1
Sar1a	-0,4202	1
Mob3c	-0,42034	1
Pqlc1	-0,4204	1
Rpn1	-0,4204	1
Slc24a3	-0,4205	1
Lrrc27	-0,42051	1
Abhd16a	-0,42049	1
Foxred1	-0,42054	1
Stx2	-0,4205	1
Reep3	-0,4206	1
Pik3ca	-0,4208	1
1700037C18Rik	-0,42128	1
Gm14138	-0,42142	1
Gart	-0,42158	1
Rae1	-0,42192	1
Cand1	-0,42204	1
Cep72	-0,42209	1
Gm5599	-0,42231	1
Herc3	-0,42239	1
Rpsa-ps10	-0,42248	1
Pdlim2	-0,42249	1
Rps27-ps1	-0,42263	1
Atp5o	-0,42257	1
Zfp637	-0,42276	1
Leng1	-0,4229	1
Cops9	-0,42305	1
Mmaa	-0,42306	1
C5ar1	-0,42306	1
Klf16	-0,42305	1
1810011H11Rik	-0,42387	1
Taco1os	-0,42413	1

Rpl36-ps3	-0,42423	1
Mypop	-0,42417	1
Cherp	-0,42423	1
Gm14586	-0,42424	1
Gm13391	-0,42434	1
Gm38115	-0,42436	1
Alyref2	-0,42461	1
Gm6851	-0,4247	1
Stim2	-0,42498	1
Tmco4	-0,42553	1
Tesk2	-0,42548	1
D330050G23Rik	-0,42545	1
Wdr25	-0,42582	1
Zfp467	-0,42592	1
Fth-ps2	-0,42585	1
Nop58	-0,42592	1
Tnfsf13b	-0,42604	1
Tubg2	-0,42603	1
Gm15393	-0,42608	1
Hivep1	-0,42625	1
Rtn4ip1	-0,42627	1
Manbal	-0,42639	1
Gm12074	-0,42676	1
Anapc13	-0,4268	1
Zfp740	-0,42704	1
Nle1	-0,42731	1
Tmem206	-0,42806	1
Gpam	-0,4281	1
Tm9sf2	-0,42827	1
Faah	-0,42838	1
Hook3	-0,42863	1
Gm5297	-0,42881	1
Dnajc12	-0,42908	1
Zfand2b	-0,42923	1
Apip	-0,42932	1
Hbs1l	-0,42952	1
Slc37a4	-0,42978	1
Erich1	-0,42997	1
Rsl24d1	-0,43026	1
Gm12967	-0,43077	1
Aimp2	-0,43118	1
Josd1	-0,4313	1
Tlk1	-0,43153	1
Junb	-0,43195	1
Stub1	-0,43205	1
Sft2d3	-0,43215	1
Stat5a	-0,4321	1
Dcun1d2	-0,43251	1
Faap24	-0,43253	1
Gm7670	-0,43253	1
Txn1	-0,43246	1
Gusb	-0,43248	1

Nudt21	-0,43269	1
Pet100	-0,43265	1
Fads1	-0,43281	1
Rrp1	-0,43289	1
Psma1	-0,43288	1
Gm5910	-0,43298	1
Gm8451	-0,43316	1
2010315B03Rik	-0,43384	1
Apbb2	-0,43382	1
Mtfmt	-0,43407	1
Cdk18	-0,43416	1
Fam110a	-0,43439	1
Cox8a	-0,43446	1
Cutc	-0,43461	1
Rtca	-0,43461	1
Fmn1	-0,43471	1
Fbxl15	-0,435	1
Fam219b	-0,435	1
Nelfa	-0,43509	1
Mif4gd	-0,43536	1
Ap4m1	-0,43547	1
Abhd5	-0,43614	1
Socs3	-0,43634	1
Rexo4	-0,43638	1
Eef1g	-0,4364	1
Mib2	-0,43671	1
Dnph1	-0,43675	1
Srp14	-0,43684	1
Snx10	-0,43694	1
Nfkbia	-0,43685	1
Atp5l	-0,43721	1
Atg2b	-0,43724	1
Hif1an	-0,43745	1
Rcc1	-0,43737	1
Gstp-ps	-0,43746	1
Nudt14	-0,43765	1
Pecr	-0,43774	1
Gpatch1	-0,43807	1
Tcaf1	-0,43851	1
RP23-447C2.2	-0,43869	1
Polr2f	-0,4389	1
Gm42850	-0,43927	1
RP23-47A1.1	-0,43973	1
Utp6	-0,44002	1
Fam111a	-0,43999	1
Mrpl27	-0,44021	1
Ebag9	-0,44026	1
Rbm6-ps1	-0,44042	1
Ifi27	-0,4404	1
Eif3g	-0,44047	1
Gm6794	-0,44083	1
Tbp	-0,44138	1

Igf2bp2	-0,44181	1
Arfip2	-0,44203	1
Suds3	-0,4422	1
Surf4	-0,44217	1
Miga2	-0,44272	1
Ndor1	-0,44267	1
Lrp10	-0,44268	1
Nup160	-0,44282	1
Prdx1	-0,44276	1
B230118H07Rik	-0,44304	1
Nek6	-0,44325	1
Ap4s1	-0,44347	1
Calu	-0,44384	1
Cacna1b	-0,44386	1
Gm8730	-0,444	1
Coro1a	-0,44402	1
Lactb	-0,44412	1
Rps12-ps24	-0,44418	1
Gm10704	-0,44424	1
Gm4943	-0,44441	1
Tmem219	-0,44436	1
Matn4	-0,44443	1
Gm1840	-0,44465	1
Fkbp2	-0,44506	1
Gm43524	-0,44541	1
Csk	-0,4455	1
Senp6	-0,44557	1
Mkks	-0,44566	1
Rpl31-ps22	-0,44575	1
Dcaf10	-0,44579	1
Zfp113	-0,44587	1
Pkp4	-0,44612	1
Ddias	-0,44625	1
Gm10126	-0,44657	1
Cct3	-0,44668	1
Bmyc	-0,44683	1
Dnajc16	-0,44678	1
Arhgap45	-0,44692	1
Ddr2	-0,44697	1
Slc7a6	-0,44698	1
Jmjd4	-0,44712	1
Ptgs2os2	-0,44727	1
Gm42747	-0,44744	1
Kif3c	-0,44769	1
Ulk2	-0,44802	1
Gfod2	-0,44854	1
Rreb1	-0,44846	1
Ddx55	-0,44852	1
Gm43162	-0,44856	1
Rpain	-0,44876	1
BC031181	-0,44878	1
Eftud2	-0,44915	1

Hint1	-0,44908	1
Dse	-0,44964	1
Sp2	-0,44963	1
Gm21057	-0,44966	1
Kifap3	-0,44979	1
Rprd2	-0,44983	1
Aimp1	-0,4502	1
Mks1	-0,4503	1
Orc2	-0,45044	1
Mrpl11	-0,45078	1
Ranbp3	-0,45092	1
Etv4	-0,45103	1
Exoc3l2	-0,45124	1
Zfp428	-0,45147	1
Lrrc8b	-0,45221	1
Jarid2	-0,45233	1
Ldb3	-0,45251	1
Arhgap1	-0,45278	1
Commd7	-0,45312	1
Bcl2l14	-0,45341	1
Zfp524	-0,4536	1
Mark3	-0,45367	1
Cd300lb	-0,45377	1
Gm10425	-0,45406	1
Eri2	-0,45418	1
Gorab	-0,45419	1
Nlrx1	-0,4543	1
Gm7600	-0,45471	1
Gm37116	-0,45471	1
Acox3	-0,45479	1
Gm12497	-0,45496	1
Pced1a	-0,45508	1
Lrrc25	-0,45535	1
Endod1	-0,45527	1
Srgap2	-0,45583	1
Sepsecs	-0,45598	1
Klhl2	-0,45611	1
Mto1	-0,45609	1
AA986860	-0,45606	1
Wdr6	-0,45628	1
Kdelr2	-0,45665	1
Tmem126b	-0,45682	1
Snrpd2	-0,45721	1
Cep78	-0,45811	1
Timm13	-0,45815	1
Gm15946	-0,45833	1
Gm15472	-0,45864	1
Gm12059	-0,45871	1
Gm4366	-0,45882	1
Tnfaip2	-0,45878	1
Cyb5a	-0,45884	1
Abhd6	-0,45888	1

Nsun3	-0,45898	1
Ankrd17	-0,45907	1
Nsmce1	-0,45993	1
Ndufs7	-0,45995	1
Ppox	-0,46004	1
Ube2m	-0,45997	1
Nos1	-0,46043	1
Rraga	-0,4606	1
Timm10	-0,46085	1
Rai14	-0,46086	1
Prmt6	-0,46112	1
A930016O22Rik	-0,46108	1
Gm6030	-0,46158	1
Nsmce4a	-0,46168	1
Cacul1	-0,46191	1
Larp4b	-0,46196	1
Elmod2	-0,46225	1
Ttc27	-0,46228	1
Ehmt1	-0,46229	1
Dicer1	-0,46229	1
Ncoa2	-0,46242	1
Mtus2	-0,46273	1
Lmbr1	-0,46345	1
Dclre1c	-0,46356	1
Dock5	-0,46359	1
Cpne8	-0,46386	1
Etohd2	-0,46397	1
Sh2b3	-0,46407	1
Nsa2	-0,46428	1
Runx3	-0,46426	1
Abcf2	-0,46453	1
Gns	-0,46454	1
Gm14680	-0,46471	1
Gm5453	-0,46494	1
Gm44851	-0,46495	1
Bcl2l11	-0,46505	1
1700047K16Rik	-0,46524	1
Qtrtd1	-0,46533	1
Tomm40	-0,46583	1
Gm11450	-0,46591	1
Cbll1	-0,46615	1
Gstt1	-0,46616	1
Fcrl1	-0,46631	1
Mrps18a	-0,46626	1
Tmem267	-0,46671	1
Mrpl40	-0,46688	1
Fbxo10	-0,46719	1
Pcbp1	-0,46738	1
Cdk13	-0,46812	1
Gid8	-0,46815	1
Pdap1	-0,46841	1
Fbxl17	-0,46849	1

Clns1a	-0,46899	1
Katnal1	-0,46914	1
Zfp866	-0,46906	1
Slc39a9	-0,46922	1
Fbxo44	-0,46963	1
Gm16712	-0,46967	1
Gm10327	-0,46992	1
RP24-131G14.13	-0,47001	1
Commd9	-0,47011	1
D3Ertd254e	-0,47008	1
Gm16288	-0,47007	1
Osbpl11	-0,47042	1
Zswim4	-0,47038	1
Pa2g4	-0,47058	1
Cox15	-0,47076	1
Ndufaf8	-0,47107	1
Ruvbl2	-0,47127	1
Csad	-0,47144	1
Degs1	-0,47146	1
Atp2b4	-0,4716	1
Naip6	-0,4717	1
Pomp	-0,47172	1
Rsph1	-0,47182	1
1700008J07Rik	-0,4721	1
Atg101	-0,47233	1
Rpl37rt	-0,47229	1
Ccdc186	-0,47259	1
Mbp	-0,47264	1
Spout1	-0,47293	1
Tal1	-0,47331	1
Ccdc71	-0,47351	1
Zfp955a	-0,47364	1
Gm5575	-0,47386	1
Crtc1	-0,4742	1
Adar	-0,47438	1
Zfp407	-0,47446	1
Rep15	-0,47446	1
Pak1ip1	-0,47447	1
Alg8	-0,47465	1
Pck2	-0,47476	1
Lcmt1	-0,47491	1
Prkar1a	-0,47489	1
Gm14853	-0,47515	1
Gm13140	-0,47524	1
Pafah1b1	-0,4757	1
Ccdc6	-0,47582	1
RP23-304C21.3	-0,47594	1
Naa25	-0,47599	1
Trim12c	-0,4762	1
Rhbdd3	-0,47639	1
Smndc1	-0,47651	1
Evi5l	-0,4767	1

Pde4dip	-0,47695	1
Tlk2	-0,47702	1
Cers4	-0,47705	1
Csrp2bp	-0,47721	1
Clec11a	-0,47753	1
Rin3	-0,47746	1
Gm17150	-0,47781	1
Uggt2	-0,47776	1
Gm12582	-0,47815	1
Osbpl2	-0,4782	1
Snrpd1	-0,4782	1
Plekhg5	-0,47838	1
1810014B01Rik	-0,47849	1
Tfec	-0,47846	1
Sumf2	-0,47886	1
Map3k5	-0,47892	1
Atp5j	-0,47958	1
9130023H24Rik	-0,47958	1
Glb1	-0,47983	1
Ppm1a	-0,47989	1
Zkscan14	-0,48005	1
Ryk	-0,47996	1
Plpp1	-0,48017	1
Lrig3	-0,48026	1
Gm16096	-0,48064	1
Rdh12	-0,48055	1
Plin3	-0,48078	1
Mad2l1bp	-0,48083	1
Sh3kbp1	-0,48088	1
Cc2d2a	-0,48105	1
Gm4950	-0,48124	1
Gm6733	-0,48135	1
Pinx1	-0,48153	1
Wdr60	-0,4815	1
Pdzd11	-0,48161	1
Prtn3	-0,48157	1
Dcaf13	-0,48173	1
Thoc6	-0,48178	1
Gm9332	-0,48212	1
Pfas	-0,48231	1
RP23-123D6.12	-0,48257	1
Chmp7	-0,48259	1
Cd200r1	-0,48284	1
Cryga	-0,48285	1
Ndufa9	-0,48295	1
Themis2	-0,48326	1
Zfp341	-0,48352	1
Dusp16	-0,48349	1
Pgrmc1	-0,48352	1
Snhg18	-0,48352	1
Ssh2	-0,48359	1
Bcap29	-0,48382	1

Gm9803	-0,48404	1
Tfpt	-0,48415	1
AA386476	-0,48426	1
Snora73b	-0,48444	1
Bpgm	-0,48448	1
Nr2f6	-0,48466	1
AW146154	-0,48472	1
Gm8731	-0,48487	1
Capn5	-0,48495	1
Amotl1	-0,4851	1
Mrps26	-0,48509	1
Slc20a2	-0,48573	1
Zbtb7a	-0,48601	1
Clec4d	-0,48595	1
Enpp1	-0,48633	1
Atp6v1b2	-0,48636	1
Ube2l3	-0,48694	1
Commd3	-0,48696	1
Il13ra2	-0,48713	1
Zfp169	-0,4873	1
Leng9	-0,48736	1
Btrc	-0,48748	1
Daxx	-0,48764	1
Cnpy2	-0,48766	1
Gm8722	-0,48768	1
Tmcc1	-0,48786	1
Zfp639	-0,4879	1
Gm4994	-0,48792	1
Mmgt2	-0,48801	1
Znrd1as	-0,48822	1
Arid1b	-0,48827	1
Tmbim1	-0,48843	1
Ppm1g	-0,48871	1
Akr1b3	-0,48875	1
Dnmt1	-0,48883	1
Gm8930	-0,48914	1
Gm14121	-0,48918	1
Abcd2	-0,49	1
Lemd2	-0,49006	1
Clmp	-0,49022	1
Gm6155	-0,49036	1
Timm17b	-0,4909	1
Derl1	-0,49099	1
Nifk	-0,4911	1
Gm8330	-0,49106	1
Eif2s3x	-0,49127	1
Mon1a	-0,49164	1
Lrif1	-0,4918	1
Eef2k	-0,49207	1
Exosc9	-0,49251	1
Psmg3	-0,49261	1
Samm50	-0,49263	1

Islr2	-0,49291	1
Cldn15	-0,49291	1
Zfp330	-0,49297	1
Parp10	-0,49314	1
Prickle2	-0,49324	1
Slc25a11	-0,49319	1
Klhdc3	-0,49331	1
Gm37968	-0,49337	1
Acvrl1	-0,49347	1
Otub2	-0,4937	1
Son	-0,49407	1
Ankdd1a	-0,49435	1
RP23-48A24.3	-0,49441	1
Ythdf1	-0,4951	1
Rpgr	-0,4951	1
Slc15a3	-0,49514	1
Becn1	-0,4953	1
Gm22748	-0,49529	1
Notch2	-0,49553	1
Galns	-0,49568	1
Kdm4a	-0,4958	1
Uchl4	-0,49595	1
6030458C11Rik	-0,49599	1
Gm14140	-0,49597	1
Vamp5	-0,49622	1
Ap1s1	-0,49655	1
Spata24	-0,49674	1
Pex16	-0,49721	1
Xrcc5	-0,49715	1
Prelid2	-0,49735	1
Hilpda	-0,49734	1
Strn3	-0,49727	1
Cdca4	-0,49757	1
Sema4d	-0,49787	1
Ppa2	-0,49798	1
Nr1h2	-0,49804	1
Phf7	-0,49812	1
Urgcp	-0,49819	1
Nars	-0,49835	1
Arhgap27	-0,49854	1
Atat1	-0,49854	1
Ptov1	-0,49878	1
Akap9	-0,49905	1
Abtb2	-0,49909	1
Pcdhgc4	-0,49913	1
Acaa1a	-0,49942	1
Rps15a-ps3	-0,4997	1
Cpt2	-0,49994	1
Ube2d1	-0,50003	1
Etaa1	-0,50017	1
Ttc1	-0,50044	1
Capzb	-0,50051	1

Ccdc115	-0,50052	1
Tgm2	-0,50069	1
Pirb	-0,50072	1
Coq6	-0,5008	1
Slc7a11	-0,50088	1
Rab3il1	-0,50117	1
Hsdl2	-0,50124	1
Ankrd12	-0,50126	1
Arl6ip4	-0,50144	1
Gm5244	-0,50164	1
Dbr1	-0,50192	1
Slc25a14	-0,50189	1
Mrpl48	-0,50217	1
Znhit3	-0,50243	1
Polr3f	-0,50263	1
RP24-499N24.6	-0,50267	1
Gm37063	-0,50284	1
Bcl7b	-0,50311	1
Dlst	-0,50355	1
Rspry1	-0,50358	1
Srr	-0,50376	1
Gm9774	-0,50378	1
Rbm34	-0,50465	1
Ybx1-ps2	-0,50477	1
Tab3	-0,50512	1
Tmem251	-0,50516	1
Angpt2	-0,50524	1
Tspan15	-0,50544	1
Adcy2	-0,50548	1
Trafd1	-0,50558	1
Gm15131	-0,50561	1
Rtf1	-0,50602	1
Eif1a	-0,5061	1
Setmar	-0,50626	1
Cinp	-0,50663	1
Rpl3l	-0,50673	1
Rbck1	-0,50718	1
Pddc1	-0,50725	1
Gls2	-0,50723	1
Lasp1	-0,50744	1
Pelo	-0,50746	1
Dnlz	-0,50746	1
Gm37978	-0,50751	1
Laptm4a	-0,50767	1
Zc3h7b	-0,50775	1
Xirp1	-0,50772	1
Riok3	-0,50776	1
Syk	-0,50797	1
Hspa2	-0,50888	1
Zdhhc14	-0,50916	1
Zfp975	-0,50926	1
Rrp1b	-0,50962	1

Ptcd2	-0,50994	1
Gskip	-0,50988	1
Gm4879	-0,50999	1
Sirt7	-0,51025	1
Psmd10	-0,51033	1
Lpcat3	-0,5105	1
Mettl13	-0,51105	1
Pdcd5-ps	-0,51112	1
Sirt5	-0,5111	1
Mrps34	-0,5113	1
Ankrd13b	-0,51129	1
Faap100	-0,51161	1
Ublcp1	-0,51158	1
Imp4	-0,51182	1
Tsc2	-0,51187	1
Adrm1	-0,51203	1
Fcf1	-0,51253	1
Commd2	-0,51252	1
Gm5050	-0,51257	1
AA414768	-0,51315	1
Emc3	-0,51327	1
Gm45420	-0,51358	1
Rcc1l	-0,51378	1
Trappc5	-0,51396	1
Mphosph8	-0,51427	1
Csnk1d	-0,51436	1
Rnf169	-0,51442	1
Tmem161a	-0,51441	1
Ube2z	-0,5146	1
Tmem241	-0,51457	1
Mars	-0,51479	1
Gm16201	-0,51478	1
Scyl1	-0,51504	1
Ripk1	-0,51511	1
Reep6	-0,51554	1
Gpalpp1	-0,51606	1
Agpat2	-0,51618	1
Gm5113	-0,51616	1
Dhdds	-0,51631	1
Sorl1	-0,5163	1
Wdr36	-0,51638	1
Atmin	-0,5165	1
Padi2	-0,51649	1
Ppcdc	-0,51664	1
Gm12186	-0,51658	1
Gm3617	-0,51663	1
Gm43547	-0,51669	1
Zfp983	-0,5169	1
Zwint	-0,5171	1
Ifi35	-0,51743	1
Wdyhv1	-0,51752	1
Pqlc2	-0,51771	1

Fam118b	-0,51818	1
Rpl5-ps1	-0,51914	1
Zfp26	-0,51915	1
Abcb10	-0,51923	1
Gm3355	-0,51925	1
9430038I01Rik	-0,51952	1
Gm11410	-0,51968	1
Mrgbp	-0,51999	1
Dnajc1	-0,52016	1
Tom1l2	-0,5203	1
Zfp513	-0,52052	1
Cdr2	-0,52067	1
Vmac	-0,52079	1
Sin3b	-0,5209	1
Uvssa	-0,52091	1
Ehbp1l1	-0,52119	1
Ubxn6	-0,52123	1
Olfml3	-0,52165	1
Mrpl20	-0,5218	1
L3hypdh	-0,52205	1
Zfp146	-0,52206	1
Miga1	-0,52248	1
Gm37452	-0,52253	1
C1galt1c1	-0,52271	1
Nup37	-0,52348	1
Wdr5	-0,52403	1
Cdk20	-0,52413	1
Rere	-0,52419	1
Gm43859	-0,52418	1
Cdv3	-0,52426	1
Nupr1l	-0,52434	1
Ube2o	-0,52441	1
Plekha3	-0,52483	1
Ttc9	-0,52514	1
Eml3	-0,52522	1
D830044I16Rik	-0,52564	1
Wnk2	-0,52573	1
Btd	-0,5265	1
Rpl26-ps4	-0,52663	1
Mpdu1	-0,52695	1
mt-Tt	-0,52692	1
Ccar2	-0,52709	1
Tmem26	-0,52719	1
Pex11b	-0,52748	1
Ak1	-0,52782	1
Nup85	-0,52777	1
Ppic	-0,52808	1
Atp5h	-0,52884	1
Iars2	-0,52887	1
Mical1	-0,52889	1
Madd	-0,52916	1
Cited2	-0,52932	1

Egln3	-0,52934	1
Kmt5b	-0,5294	1
Wdr45	-0,52972	1
Hoxb4	-0,52984	1
Ipo13	-0,52981	1
Prkce	-0,53012	1
Psmc5	-0,53043	1
Map4k1	-0,53047	1
Ulk3	-0,53069	1
6230400D17Rik	-0,53067	1
Gm5422	-0,53078	1
Ccdc122	-0,53083	1
Txnrd1	-0,53098	1
Vps36	-0,53129	1
Rcan3	-0,53164	1
Gm14780	-0,53172	1
Mpv17	-0,5318	1
Tmem268	-0,53209	1
Plekhm1	-0,53233	1
Egln2	-0,53276	1
Psmc2	-0,53342	1
Tmsb4x	-0,53349	1
Tusc2	-0,53381	1
Gm19739	-0,53393	1
Mapre3	-0,53402	1
Dusp5	-0,53429	1
Nsmf	-0,53484	1
Gdi2	-0,535	1
Zfp248	-0,53529	1
Rap2b	-0,53543	1
Fblim1	-0,53558	1
Cyfip2	-0,5357	1
Gm27046	-0,53568	1
2500002B13Rik	-0,5358	1
Ralgapb	-0,53605	1
Pisd	-0,53641	1
Fbxo28	-0,53638	1
Clptm1l	-0,53728	1
Rhbdf1	-0,53751	1
Vars2	-0,53757	1
Wrb	-0,53762	1
Gm11423	-0,53765	1
Tmem8b	-0,53777	1
Dopey2	-0,53814	1
Ptpn1	-0,53837	1
Sh3pxd2a	-0,53844	1
Cd300ld	-0,53837	1
Polg	-0,53855	1
Gm8444	-0,53877	1
Gm7722	-0,539	1
Tfcp2l1	-0,53922	1
A130010J15Rik	-0,53945	1

Gm7094	-0,53979	1
Cdk2ap1	-0,53994	1
Sf3a3	-0,54006	1
Gm43501	-0,54015	1
Prr18	-0,54046	1
A530072M11Rik	-0,54049	1
Slc8a1	-0,54077	1
Etfrf1	-0,54115	1
Bola1	-0,54116	1
Preb	-0,54139	1
RP23-138K22.2	-0,54152	1
Csf1r	-0,54185	1
Gm38377	-0,54205	1
St7l	-0,54216	1
F2	-0,54219	1
Slc35b4	-0,54273	1
Coasy	-0,54284	1
Mad1l1	-0,54288	1
Ehbp1	-0,54304	1
Usp7	-0,54344	1
RP23-225D5.4	-0,54367	1
Adat3	-0,54405	1
Ddx24	-0,54437	1
Gcnt1	-0,54455	1
Gm12267	-0,54482	1
Fam96b	-0,54512	1
Trmt10b	-0,54526	1
Gm7785	-0,54527	1
Eif2ak2	-0,54561	1
Trip11	-0,54573	1
Acaa1b	-0,54581	1
Tmem101	-0,54594	1
Rnaseh2b	-0,54595	1
Tmem43	-0,5466	1
Elovl6	-0,54675	1
Myo9b	-0,54675	1
Tctex1d4	-0,54666	1
Rpl36al	-0,54746	1
U2surp	-0,54758	1
Gm8927	-0,54807	1
Med11	-0,54828	1
D7Bwg0826e	-0,54828	1
Cbr4	-0,54849	1
Gm6177	-0,5486	1
Ahnak2	-0,54878	1
RP23-184H3.5	-0,54891	1
Gm26881	-0,54889	1
Pafah1b1-ps2	-0,54935	1
RP23-255F14.4	-0,54939	1
Pex14	-0,54953	1
Puf60	-0,5501	1
Gm43712	-0,55038	1

Gm37606	-0,55086	1
Gsk3b	-0,55098	1
Efl1	-0,55101	1
Tnfrsf18	-0,55166	1
Fam216a	-0,55178	1
Ppp4r1	-0,55184	1
Gm43511	-0,55186	1
Entpd7	-0,55207	1
Ash2l	-0,55216	1
Galnt15	-0,55219	1
Dyrk3	-0,55242	1
Ikbkb	-0,5527	1
Matn1	-0,55268	1
Cisd1	-0,55279	1
Tpd52l2	-0,5529	1
Gm9434	-0,55331	1
Avl9	-0,55344	1
9030624J02Rik	-0,55351	1
Klhl22	-0,55369	1
Sdf4	-0,55419	1
Gm14567	-0,55435	1
Per2	-0,55438	1
Snora21	-0,55455	1
Utp11	-0,55471	1
Opa3	-0,55485	1
Gm13602	-0,55524	1
Gm5391	-0,55544	1
Cela1	-0,55551	1
Zfp263	-0,55582	1
Ccdc86	-0,55584	1
Ahsa1	-0,556	1
Thnsl1	-0,55616	1
Dbf4	-0,5563	1
Atxn7l3	-0,55632	1
Cat	-0,5564	1
Dhx30	-0,55649	1
Fli1	-0,55691	1
Alg3	-0,55703	1
Tsen2	-0,55707	1
Zfp930	-0,55766	1
Tspan14	-0,55772	1
Cenpo	-0,55769	1
Prr13	-0,5579	1
Gspt2	-0,55831	1
Slc16a9	-0,55863	1
Emc4	-0,55858	1
Heatr3	-0,55897	1
Osbp2	-0,55909	1
Ankrd33b	-0,55961	1
Decr2	-0,55975	1
Jkamp	-0,5601	1
Hmgn1	-0,56022	1

Gm8186	-0,56037	1
Hsd17b11	-0,56064	1
Lmna	-0,56065	0,98227
Ell2	-0,56072	1
Colec12	-0,56074	1
Gm5576	-0,56087	1
Thap6	-0,56095	1
Sec61g	-0,56106	1
Gm38305	-0,56161	1
Mrpl10	-0,56189	1
Use1	-0,56201	1
Kif13b	-0,56199	1
Sdhaf4	-0,56224	1
Med10	-0,56234	1
Dgcr8	-0,56243	1
Pfdn6	-0,56263	1
Emc8	-0,56259	1
Gm11964	-0,56316	1
Cmtr1	-0,56349	1
Treml1	-0,56379	1
Dars2	-0,56389	1
BC002059	-0,56399	1
Rabepk	-0,56468	1
Dhrs7	-0,56493	1
Fam71e1	-0,56499	1
Eif4ebp1	-0,5652	1
Parp4	-0,56536	1
Adck5	-0,56579	1
Sdhc	-0,56594	0,96791
Zdhhc16	-0,56614	1
Tcta	-0,5662	1
Dph2	-0,5665	1
Gm20302	-0,56667	1
Maml1	-0,5668	1
Zfand4	-0,56701	1
Cdc25b	-0,56711	0,99768
Thap11	-0,56781	1
Gm5812	-0,5678	1
Myo1c	-0,56801	1
Bloc1s4	-0,56795	1
Fhl3	-0,56831	1
Mrps10	-0,56874	1
Gm8925	-0,56867	1
Gm15148	-0,56903	1
Ttll1	-0,56908	1
Sars	-0,56931	1
Rpusd3	-0,56927	1
Bahcc1	-0,56982	1
RP23-13B8.12	-0,56996	1
R3hcc1	-0,57015	1
Mief2	-0,57035	1
Tceanc	-0,57025	1

Oxnad1	-0,57038	1
Ddx18	-0,57085	1
Usp46	-0,57109	1
Mfsd13b	-0,5711	1
Ncbp1	-0,57117	1
Thap12	-0,57116	1
Grk6	-0,5713	1
Klc4	-0,57169	1
Exosc1	-0,57223	1
Disp1	-0,57238	1
Nudt3	-0,57266	0,96967
Tprkb	-0,57299	1
Neurl1a	-0,57303	1
Rbks	-0,57308	1
Nucb2	-0,57354	1
Tdrkh	-0,57439	1
Des	-0,57444	1
Gm37959	-0,57487	1
Ddb2	-0,57526	1
Ing4	-0,57533	1
Naglu	-0,57608	1
Vti1a	-0,57615	1
Jade2	-0,57613	1
Dimt1	-0,57635	1
Mdc1	-0,57669	1
Gm17530	-0,57703	1
Gm14403	-0,57696	1
Cenpv	-0,5773	1
Snap29	-0,57744	0,98842
Agtrap	-0,57736	1
Lrrc8c	-0,57783	1
Gm26710	-0,57782	1
Rint1	-0,57793	1
Tmem14c	-0,57797	0,98026
R3hcc1l	-0,57811	1
Pdia5	-0,57834	1
Avpi1	-0,57864	1
Fbxl8	-0,5789	1
Tmem127	-0,57902	1
Ercc2	-0,579	1
Gm16537	-0,57913	1
Rnf168	-0,57935	1
Tgfbrap1	-0,57933	1
Exoc7	-0,57943	1
Gm27219	-0,57938	1
Unc50	-0,57964	1
Carf	-0,57956	1
Il11ra1	-0,57969	1
Nkpd1	-0,57979	1
Tpm1	-0,58024	0,87562
Atp6v1d	-0,58048	0,94472
Mrps21	-0,58047	1

Aatf	-0,58098	1
Gm25007	-0,58108	1
Nfs1	-0,58157	1
Tti2	-0,58156	1
Bfar	-0,5819	0,96489
Dus1l	-0,58218	1
Tcirg1	-0,58242	0,95954
Garnl3	-0,58249	1
Kat6a	-0,58259	1
Rps3	-0,58312	0,97405
Ercc8	-0,58318	1
Prdm9	-0,58361	1
Ammecr1l	-0,58393	1
Eif3b	-0,5841	0,95933
Mrpl49	-0,58568	1
Adck1	-0,58567	1
Gm5801	-0,58582	1
Cfap36	-0,58588	1
Pacsin2	-0,58617	1
Mepce	-0,58677	1
Hbegf	-0,58682	1
Med1	-0,58769	0,96667
Elp3	-0,58766	1
Zdhhc7	-0,58772	1
Polr2k	-0,58783	1
Zfa-ps	-0,58799	1
Tnni3	-0,58807	1
Tfam	-0,58833	1
RP24-401G4.1	-0,58826	1
Tagap1	-0,58853	1
Nans	-0,58866	0,97556
Dnajb11	-0,58893	1
Gm2076	-0,58907	1
Tmem104	-0,58923	1
Gm43137	-0,58926	1
Sun1	-0,58941	1
Tgs1	-0,58991	1
Snai2	-0,59	1
Hpdl	-0,59048	1
Snx1	-0,5907	0,94725
Dnajb12	-0,59081	1
Fam19a2	-0,59122	1
Ifngr1	-0,59149	1
Tceanc2	-0,59175	1
Ddx31	-0,59197	1
Wdr43	-0,59221	0,87088
Sod1	-0,59227	0,90858
Gtf2f2	-0,5923	0,96723
Exoc3l4	-0,59254	1
Rfesd	-0,59267	1
Ppp1r13b	-0,59286	1
Syvn1	-0,5934	1

Usp4	-0,59392	0,90858
Mettl8	-0,59412	1
Rpl26-ps2	-0,59421	1
Stard3	-0,59454	1
Gm45311	-0,59455	1
Gm6682	-0,59463	1
Romo1	-0,59482	0,98189
Rabep2	-0,59475	1
Gadd45gip1	-0,59499	1
Hmgxb4	-0,59548	1
Rubcnl	-0,59546	1
Dhps	-0,59569	1
Gmfg	-0,59569	1
Supt16	-0,59599	0,85293
Trnau1ap	-0,5962	1
Scarf1	-0,59632	1
Ogfod2	-0,59642	1
Rilpl1	-0,59675	0,98802
Brix1	-0,59678	0,90982
Psmb6-ps2	-0,59706	1
Plcb4	-0,59806	0,92477
Ddx59	-0,59848	1
Hectd1	-0,59935	0,84421
Pcyt1a	-0,59955	0,83048
Gm42829	-0,59962	1
Dclre1b	-0,59966	1
Gm6977	-0,59982	1
Ccdc66	-0,60009	1
Ube2d-ps	-0,60027	1
Mrpl54	-0,60056	1
1700088E04Rik	-0,60071	1
Usp11	-0,60112	1
Slc47a2	-0,60133	1
RP23-476G10.1	-0,60162	1
Micu2	-0,60177	0,99336
Spink5	-0,60193	0,90982
Abi3	-0,60197	1
Gm11633	-0,60208	0,94449
Exosc8	-0,60207	1
Ocel1	-0,60222	1
Ndufa4	-0,60244	1
Ankrd26	-0,60265	1
P2rx4	-0,6031	1
Rasgrp3	-0,60346	0,94606
Thtpa	-0,60351	1
Gm8894	-0,6035	1
Rplp2	-0,60359	0,97259
Sssca1	-0,60363	1
Trpm4	-0,60357	1
Klc3	-0,60378	1
Dnajc19-ps	-0,60387	1
Atad1	-0,604	1

Tamm41	-0,60601	1
Zfp398	-0,60613	1
Tmem214	-0,60688	1
Rnf214	-0,60697	1
Prune1	-0,60714	0,89041
Eya4	-0,60723	1
Acvr1	-0,60732	1
Npc1l1	-0,60737	1
Tsfm	-0,60773	1
Msra	-0,60772	1
Ppat	-0,60795	0,97678
Gm13398	-0,6081	1
Lysmd1	-0,6083	1
1700001C19Rik	-0,60834	1
Coq3	-0,60836	1
Gm11263	-0,60844	1
Pik3r1	-0,60846	0,92477
Fam167b	-0,60863	0,88753
Diexf	-0,60874	1
Pde12	-0,6088	0,98937
Gpatch2	-0,60972	1
Apbb1ip	-0,61023	0,84159
Gm38125	-0,61037	1
Nom1	-0,61055	0,98456
Tspan3	-0,61091	0,86429
Tor1b	-0,61126	0,94216
Dtwd1	-0,6115	1
Ebna1bp2	-0,6122	0,96489
Aqp11	-0,61224	1
Bccip	-0,61233	1
Def6	-0,61243	1
Tmed3	-0,61287	0,85558
Stk25	-0,61305	1
Atg4d	-0,61307	1
Arhgap30	-0,61321	0,99228
Tnfsf12	-0,61328	0,99495
Zfp446	-0,61344	1
Utp3	-0,61356	0,67455
Rasal2	-0,61356	0,94567
Mlf2	-0,61367	0,96876
RP23-115A18.3	-0,61404	1
Prrc1	-0,61462	0,78657
BC005561	-0,61519	0,97986
Axin1	-0,61561	0,99368
1600010M07Rik	-0,61558	1
Rpl36a-ps3	-0,61556	1
Creg1	-0,61577	0,84312
Tug1	-0,61578	1
Traf5	-0,61586	0,98047
Gm42636	-0,61641	1
Acaa2	-0,61646	1
Pde8b	-0,61658	0,78073

D5Ertd579e	-0,61666	0,94645
Map7d1	-0,61679	0,75375
Pdcd2	-0,61709	1
Ankrd27	-0,61719	0,92557
Rala	-0,61754	0,81191
Gm5879	-0,61795	1
Fdx1	-0,6181	0,95785
Pigyl	-0,61814	0,98937
Nsmce2	-0,61811	1
Gm43792	-0,61808	1
Mdrl	-0,6182	1
Akr1c13	-0,6186	0,9774
Tacc2	-0,61861	1
Arsk	-0,61928	1
Mrpl44	-0,61987	0,9391
Eapp	-0,62031	0,80099
Psmb9	-0,62031	0,94645
Cltb	-0,62055	1
Iqce	-0,62099	1
Plekhs1	-0,62113	1
Tlr3	-0,62127	1
Rft1	-0,62165	1
Wipi2	-0,62191	0,91128
Ifih1	-0,62224	1
Fkbp15	-0,62259	0,88753
Tbxas1	-0,623	1
Zbtb4	-0,62335	0,85558
Elp6	-0,62343	1
Gm3571	-0,62359	1
Slc38a1	-0,62433	0,81403
Psen2	-0,62473	0,90858
Gm14006	-0,62483	1
Layn	-0,62493	0,81403
Gm14173	-0,62488	1
Snhg5	-0,62529	0,93503
Gm11966	-0,62527	1
Gemin6	-0,6254	1
BC002163	-0,62567	1
Phf14	-0,6258	1
Chtf18	-0,62601	1
Rpl31-ps14	-0,62608	1
Srp68	-0,62616	0,80445
Elk3	-0,62625	1
Washc1	-0,62637	0,97368
Ift81	-0,62635	1
Kdelr3	-0,6266	1
Fmc1	-0,62668	0,9646
Shc4	-0,62687	1
Zfp553	-0,62708	1
Minpp1	-0,62733	1
Gm10146	-0,62761	1
Gm15151	-0,62757	1

Pde8a	-0,62783	1
Gm18867	-0,62783	1
Gm6162	-0,62814	1
Mrpl13	-0,62828	0,94645
Gsn	-0,62865	0,81943
Enox2	-0,6288	0,98231
Atp6v1c1	-0,62905	0,6488
Pemt	-0,6293	1
Arfgap3	-0,62939	0,94009
Cul2	-0,62954	0,94396
Gm18860	-0,62965	1
Atp6v0b	-0,62978	0,88825
1600020E01Rik	-0,62991	1
Lrrc61	-0,63004	1
Dnajc25	-0,63035	0,91093
Arl4a	-0,63029	0,96531
Dmrta2	-0,63055	1
Cant1	-0,63097	0,7682
Timm44	-0,63099	0,92378
Gm12966	-0,63118	1
A430105I19Rik	-0,63122	1
Gm5873	-0,63195	1
Dtnbp1	-0,63205	0,98417
Hs1bp3	-0,63225	1
Nptxr	-0,63236	1
Rab11fip1	-0,63255	0,90449
Gm5805	-0,6326	0,90982
Gm10676	-0,63269	1
Tomm70a	-0,63294	0,67555
Riox2	-0,63303	1
Atxn7l3b	-0,6333	0,70744
Mfsd5	-0,63339	0,82728
RP24-550H10.4	-0,63342	1
Ykt6	-0,63364	0,79964
Prdm4	-0,63365	1
Pygo2	-0,63374	0,82089
Pogz	-0,63383	0,87652
Gm43715	-0,63381	1
Cfap43	-0,63393	1
RP23-104D6.2	-0,634	1
Ubl4a	-0,63444	0,98776
Eno3	-0,63445	0,99016
Noc4l	-0,63446	1
Il10rb	-0,63509	0,89378
Cul4a	-0,63518	0,99449
Psma4	-0,63569	0,77134
Pgp	-0,63623	0,875
Gm15496	-0,63633	1
Thoc7	-0,63661	0,80048
Sec16b	-0,63684	0,96755
Gm26730	-0,63676	1
Cd300a	-0,6372	1

Wdr46	-0,63718	1
Gm5544	-0,63716	1
Akna	-0,63757	1
Aar2	-0,63773	0,96489
Cep131	-0,63789	1
Zscan29	-0,63844	1
Ints11	-0,63901	0,95577
Gm5321	-0,63917	1
6430548M08Rik	-0,63936	0,93918
Nrp2	-0,6397	0,61034
Noc2l	-0,63986	0,74754
P2ry2	-0,63998	1
Atg9b	-0,64006	1
Psmb4	-0,64023	0,81193
Rad23a	-0,64019	0,93746
Uqcc3	-0,64033	0,79149
Tshz1	-0,64077	0,90884
Ppm1e	-0,64121	1
Zcchc17	-0,64158	0,79959
Htatsf1	-0,64191	0,87088
5830454E08Rik	-0,64195	1
C630043F03Rik	-0,64201	1
Ehd1	-0,64263	0,71586
Mrpl43	-0,64301	0,6377
Stx17	-0,64297	1
St18	-0,64317	1
Gm45206	-0,64354	1
H2afy	-0,64394	0,77561
Jmy	-0,6439	0,89334
G6pdx	-0,64413	0,92852
Smim10l1	-0,64424	1
Pum3	-0,64482	0,75082
Tubgcp3	-0,64508	0,94571
Pus1	-0,64512	0,95103
Cstf1	-0,64525	0,8343
Gm13604	-0,64532	1
Mrnip	-0,64569	1
B230377A18Rik	-0,64569	1
Tpm3-rs7	-0,64584	0,78427
E130102H24Rik	-0,64584	1
Lhfpl2	-0,64592	0,57793
Rfxank	-0,64649	1
Nlrc4	-0,64664	1
Abr	-0,64668	0,57351
Psmc1	-0,64677	0,72076
Gm9794	-0,64708	0,65203
Gm13186	-0,64719	1
Rbfa	-0,6473	0,79239
Atpif1	-0,64775	0,70192
Ddx42	-0,64792	0,93746
Nrbf2	-0,64808	1
Nop14	-0,64859	0,71638

Bckdk	-0,64892	0,93955
Tmem55a	-0,64947	0,89243
Ptpra	-0,6498	0,77134
Gm3362	-0,64986	1
Zc3h8	-0,6505	1
Txnl4a	-0,65103	0,98227
Fry	-0,65128	1
Gm9025	-0,65136	1
Mavs	-0,65157	1
Rabl6	-0,65172	0,66678
Gm3283	-0,65171	1
1810044D09Rik	-0,65178	1
Zfp462	-0,6518	1
Gsg2	-0,65186	1
Rbm5	-0,65212	0,78427
Pop5	-0,65231	0,98527
Ldlrap1	-0,65248	0,61999
Soga1	-0,6527	0,99191
Gm11688	-0,65302	1
Zfp141	-0,65385	1
Oxt	-0,6539	1
Fam161b	-0,65496	1
Klf9	-0,65522	0,57566
BC005537	-0,65518	0,79555
Gpr107	-0,65541	0,65881
Hebp1	-0,6558	0,99765
Gm4895	-0,65599	0,94472
Prkrip1	-0,65628	0,85558
Actr5	-0,65702	0,88693
Cwc22	-0,65717	0,92557
Zcchc2	-0,65726	0,7793
Spata7	-0,65734	1
Nosip	-0,65743	0,91428
Gm45050	-0,65743	1
Tmem106a	-0,65794	0,72016
Cmss1	-0,65788	1
Fcnaos	-0,65793	1
Zbtb3	-0,65789	1
Slc26a6	-0,65842	1
Mrpl36	-0,65969	0,69824
Mgat5	-0,65966	0,84188
Pop7	-0,65991	0,93474
Gm4342	-0,66013	1
Gm9517	-0,66045	1
Tmem18	-0,66101	0,93746
Habp4	-0,66123	1
Tomm5	-0,66189	0,98937
Sult2b1	-0,66216	1
Apoo	-0,66269	1
Pxylp1	-0,66289	1
Sec11c	-0,66298	0,60474
A130071D04Rik	-0,663	0,9229

Slc24a5	-0,66349	1
Pdss2	-0,66356	1
Wdr61	-0,66376	0,63581
Timm9	-0,66383	0,78504
Polr1c	-0,66387	0,78492
Gm13487	-0,66393	0,91016
Spi1	-0,66425	0,78046
Pus3	-0,66472	0,93094
Vps4a	-0,66478	0,83559
Impa2	-0,66531	0,72341
Xpot	-0,66554	0,79062
Pxmp4	-0,66547	0,96489
Sgpp1	-0,66561	0,54278
Tiam2	-0,66626	1
Pou5f2	-0,66628	1
Zranb2	-0,66643	0,67555
Akap7	-0,6669	0,96489
Zfp964	-0,66698	1
Kif3b	-0,66707	0,74225
Zfp629	-0,66715	1
1700112E06Rik	-0,66716	1
Actn1	-0,66734	0,67103
Gm20554	-0,66726	1
Rab11fip5	-0,66762	0,6834
Rpl38	-0,6676	0,81202
Arhgap9	-0,66784	0,89141
Tmem156	-0,66807	1
Mea1	-0,66825	1
Pitpnm2	-0,6689	1
Mkl1	-0,66896	0,76758
Parp16	-0,66896	0,9391
Ppp6r1	-0,66916	0,74754
Fcgr1	-0,66921	0,98509
Jrkl	-0,66917	0,98527
Zfp235	-0,66943	1
Map3k11	-0,67001	0,96791
Zhx1	-0,67015	0,8915
Pdcd11	-0,67109	0,71622
Tnni2	-0,67134	0,84147
Scaf4	-0,67248	0,88753
Gm19705	-0,67257	1
Eif6	-0,67303	0,6679
Gm36963	-0,67311	1
Chmp3	-0,67323	0,58468
Zfp106	-0,67329	0,57564
Psmd6	-0,67327	0,67883
Fnip2	-0,67336	0,53274
Gm9575	-0,67377	1
Rnf219	-0,6739	0,92852
Gm45640	-0,67385	1
Gas2l1	-0,6741	0,92873
Adssl1	-0,67422	0,6852

Vsir	-0,67479	0,47608
H6pd	-0,67484	0,92469
Parp3	-0,67514	0,86139
Gstp1	-0,67518	1
Ints3	-0,67536	0,61027
Rabgef1	-0,67571	0,81943
Tiam1	-0,67586	0,93214
Lrp8os3	-0,67629	1
Spata13	-0,67642	0,68482
Pcnx4	-0,67635	0,99768
Sult6b1	-0,67653	1
Flnb	-0,67661	0,66936
Sigmar1	-0,67662	0,90572
Gar1	-0,67682	0,9386
RP23-349H12.3	-0,67693	1
Nudt7	-0,67739	1
Usp10	-0,67769	0,74748
4930453N24Rik	-0,67784	0,99336
Tmem170	-0,67834	1
AU040320	-0,67847	0,87029
Kdelc1	-0,67845	1
Arih1	-0,67861	0,53911
Ppm1d	-0,67862	0,79561
Clcc1	-0,67895	0,75096
Taok3	-0,6794	0,5453
Gsto1	-0,67938	0,8121
Mus81	-0,67952	1
Gm38380	-0,67974	1
Exog	-0,67982	1
F7	-0,67983	1
Uaca	-0,6801	0,79062
Gm6598	-0,68042	1
Cdk10	-0,68049	1
Mrps12	-0,68063	0,81513
Aggf1	-0,68069	0,66013
Tmem192	-0,68115	0,79545
Dbnl	-0,68122	0,90718
Hp1bp3	-0,68125	0,4183
Mex3a	-0,68212	0,96425
1700052K11Rik	-0,6827	1
1110008F13Rik	-0,68318	0,53199
Gm43154	-0,68338	1
Hars	-0,68386	0,6159
Sipa1	-0,68388	0,80678
Hoxa3	-0,68394	1
Acy3	-0,6842	1
Rab32	-0,68449	0,77552
Ric8a	-0,68457	0,95288
Pitpnb	-0,68506	0,67218
Ybey	-0,68511	1
Mrpl55	-0,68528	0,92226
Stam2	-0,68553	0,75096

Zc4h2	-0,68579	1
Zfp526	-0,68613	1
Gm9712	-0,68641	1
Gm37486	-0,68665	1
Procr	-0,68692	0,93503
Gm13461	-0,68715	1
Tmem138	-0,68749	1
Polr1a	-0,68791	0,79462
Hmbs	-0,68798	1
Rpl31-ps16	-0,68811	1
Ss18l1	-0,68833	0,85067
Hint2	-0,68842	0,88589
Nat8f1	-0,68841	0,98776
Rnf139	-0,68858	0,75158
Orai1	-0,68862	0,86838
Taf12	-0,68892	0,83438
Tspan4	-0,68968	0,599
Setd6	-0,68986	0,75096
Htra2	-0,69008	0,74819
Wipf1	-0,69019	0,77416
Ubn1	-0,69027	0,6049
Cmbl	-0,69087	1
Nagpa	-0,69118	0,81712
F8a	-0,6914	0,93503
6720475M21Rik	-0,69136	1
mt-Nd2	-0,69185	0,73618
Nasp	-0,69199	0,6571
Gm8659	-0,69206	1
Stard8	-0,69222	0,99287
Zfp661	-0,69234	1
Mroh2a	-0,69263	1
Kyat1	-0,69282	0,99768
Dhrs7b	-0,69289	0,85923
Hs6st1	-0,69324	0,58468
2310015A10Rik	-0,69325	1
Etfbkmt	-0,69369	1
Ccdc97	-0,69397	0,73615
Rfx5	-0,69492	0,65255
Ifrd1	-0,69497	0,7343
9130008F23Rik	-0,69514	1
D130007C19Rik	-0,69517	1
Smpd2	-0,69548	0,8816
Fgd3	-0,69577	0,68844
Zfp651	-0,69603	0,83048
RP23-139H6.1	-0,69611	0,88069
Fam208b	-0,69623	1
Fam160b2	-0,69627	0,77761
Zkscan1	-0,69644	0,75795
Rab42	-0,69647	1
Trim26	-0,69658	0,89219
Cox6b1	-0,69683	0,61794
Ring1	-0,69681	0,99321

Gm5436	-0,69685	1
Cdc34	-0,69694	0,75094
Itpa	-0,6972	1
Tmem115	-0,69731	0,86832
Rpl4	-0,69751	0,68431
Mblac2	-0,69751	1
Cnksr1	-0,69758	1
Zcchc10	-0,69773	0,6915
Ssh1	-0,69767	0,9229
Foxd2os	-0,69787	1
Alad	-0,6987	0,86013
Atp23	-0,69959	0,86838
Mtg1	-0,69974	0,84188
Cox10	-0,6998	0,63763
Znfx1	-0,70035	0,64015
Thumpd1	-0,70036	0,73483
Glrx5	-0,7008	0,45975
Zbtb32	-0,70109	0,99061
Hmga2	-0,70117	0,52346
Brca2	-0,7012	1
Dnajc2	-0,70135	0,59142
1110037F02Rik	-0,70143	0,6838
Mtx1	-0,70153	0,80108
Gm42548	-0,70177	1
Zmynd19	-0,70187	0,77724
Gm37420	-0,70222	1
Ccdc28b	-0,70266	0,96667
Snord118	-0,70281	1
Tubd1	-0,70303	1
Gm44822	-0,70351	1
Gm6028	-0,70359	1
Prpf19	-0,7042	0,7343
Polr2g	-0,70436	0,60705
BC005624	-0,70448	0,86553
Alg2	-0,70472	1
Dusp9	-0,70501	1
Dffa	-0,7053	1
Slirp	-0,70587	0,55104
Phb	-0,70632	0,98456
Tmem181a	-0,70628	1
Ndufb7	-0,707	0,74225
Shtn1	-0,7078	0,70815
Alkbh3	-0,7078	0,74754
Gm26870	-0,70828	1
A130048G24Rik	-0,7084	1
Gm14126	-0,70847	1
Hars2	-0,70858	0,72396
R74862	-0,70857	1
Jaml	-0,70926	0,63763
Snx16	-0,70957	0,90449
Gm45806	-0,71003	0,9899
Gm9347	-0,70998	1

Zfp51	-0,71034	0,96967
Zfpl1	-0,71076	0,59128
Lins1	-0,71077	1
Gm16286	-0,71093	0,68407
Irak4	-0,71102	0,89635
Tmem38a	-0,71098	0,96607
Gm13675	-0,71095	1
Gm45871	-0,71112	1
RP23-440I21.3	-0,71114	1
Stard3nl	-0,71131	0,82388
Ehd4	-0,71181	0,34213
Ceacam10	-0,71206	1
Simc1	-0,71222	0,92044
Mrm3	-0,71226	0,91327
Gm42486	-0,71257	1
Zfp809	-0,71291	0,45689
1810030O07Rik	-0,71305	0,77438
Rpl19-ps1	-0,7134	0,89013
Gm7676	-0,71358	1
Gm7784	-0,71374	1
BC022687	-0,71381	0,9729
Slc23a2	-0,71397	0,94571
Cops6	-0,71447	0,6377
Klhl11	-0,71481	1
Rrp8	-0,7149	0,6183
Zbtb8os	-0,71485	0,94571
Nudt1	-0,71488	1
Gm29539	-0,71523	1
Prkar1b	-0,71564	1
Eif2b3	-0,71609	0,58294
Trmt11	-0,71613	1
Gm6808	-0,71616	0,93503
Lyz2	-0,71634	0,44888
Gm340	-0,7167	0,96489
Caml	-0,71709	0,86192
Rpl3-ps1	-0,71752	0,74748
Lsm14b	-0,71753	0,81943
Rad9b	-0,71767	1
Fam175b	-0,71844	0,42588
Arhgap18	-0,71853	0,61426
Rps9	-0,71871	0,60272
Wdr59	-0,71877	0,93002
Cyc1	-0,71911	0,67395
Gm16418	-0,71937	0,96425
Gm37108	-0,71939	1
B3gntl1	-0,71958	1
Adamts1	-0,71969	1
Plaa	-0,72009	0,71622
Hsdl1	-0,72046	0,92599
Bcl3	-0,72047	0,96378
Morn1	-0,72058	1
Cerk	-0,721	0,45975

Efnb1	-0,72099	1
Trpv2	-0,72126	0,44124
Gmpr2	-0,72139	0,89956
Ddx10	-0,72174	0,62862
Rad17	-0,72168	0,66785
Cdk7	-0,72208	0,85676
Atp6v1e1	-0,72237	0,32467
Slc26a9	-0,72235	1
Arl10	-0,7225	0,88791
Blvra	-0,72294	0,61005
Msantd4	-0,72373	0,53917
Mesdc2	-0,72369	0,62892
Fan1	-0,72498	1
Rnf31	-0,72567	0,96952
Zfp120	-0,72595	0,72315
Flrt2	-0,726	0,74768
Gm5276	-0,72609	1
D630045J12Rik	-0,72677	1
Zmat5	-0,72752	0,87806
H3f3b	-0,72782	0,70468
Slc30a6	-0,72778	0,71898
Stx1a	-0,72779	1
Gm14328	-0,72794	1
Tgif1	-0,72813	0,53199
Eif2ak1	-0,72825	0,63174
Bsn	-0,72838	1
Mrpl12	-0,72845	0,66226
Tbc1d2b	-0,72863	0,46098
Gm37726	-0,72869	1
Atg10	-0,72903	0,93002
Haus4	-0,72902	1
Slc25a19	-0,7291	0,65908
March11	-0,72916	0,9729
Yjefn3	-0,72944	1
Sucla2	-0,72985	0,57708
Fam212b	-0,72996	1
Mmp19	-0,73015	0,81943
Pfn2	-0,73039	1
Gm12543	-0,73037	1
Nubp2	-0,73061	0,88791
D330023K18Rik	-0,73071	0,93369
Tfb2m	-0,73075	1
Marc2	-0,73084	0,41886
Arid5a	-0,7311	1
Pnpo	-0,73123	0,81191
Sdad1	-0,73146	0,46868
Ankrd39	-0,7315	0,9386
Cebpz	-0,73194	0,52908
Snx24	-0,73199	0,87618
Kazald1	-0,73196	0,98189
Dagla	-0,73196	1
Psmb6	-0,73207	0,37782

Dlat	-0,73211	0,42323
Sgta	-0,73262	0,58816
Phf10	-0,73266	0,42664
Zfp523	-0,73284	0,85692
Gm9381	-0,73289	1
Pgd	-0,73322	0,2927
Tbc1d32	-0,73318	0,91825
Gpr108	-0,73366	0,55308
Comt	-0,73396	0,74225
Fem1b	-0,73436	0,36798
Gm12254	-0,73447	0,7793
Dok1	-0,7347	0,40059
Trmt61a	-0,73488	0,83485
Cyb5rl	-0,73494	1
Gm2895	-0,73521	0,88353
Trak1	-0,73537	0,65889
Gm44771	-0,73571	1
Rab23	-0,73611	0,98802
Urb1	-0,73669	0,86744
Zfp568	-0,73671	1
Fbf1	-0,73681	0,99228
Psmg2	-0,73706	0,67003
RP23-38L16.3	-0,73743	0,94472
Abcf3	-0,73793	0,75096
A930001C03Rik	-0,73804	1
Ippk	-0,73829	0,96489
Zfp606	-0,7393	0,99022
Zfp11	-0,73975	1
Gm6304	-0,73984	1
Ccdc107	-0,74004	0,9044
Tmem209	-0,74025	0,96967
Tirap	-0,74059	0,73514
Uba5	-0,74068	0,52991
Alkbh4	-0,74149	0,94571
Scyl3	-0,74232	0,90315
Lrp12	-0,74285	0,77351
Rapgef1	-0,74334	0,45472
Opn3	-0,7438	0,93494
Haus2	-0,7441	0,5152
Arhgef19	-0,74412	1
Sqrdl	-0,74441	0,82728
Pycr2	-0,74467	0,46304
Acvr1b	-0,74466	1
Rnaseh2a	-0,74484	0,94731
Lman2	-0,745	0,36561
Rpl7l1	-0,74533	0,59142
Sel1l	-0,74576	0,24442
Rnaseh2c	-0,74605	0,73466
Gpat4	-0,74695	0,40735
Rgs16	-0,74696	0,72403
Gm20768	-0,7473	0,55615
Brox	-0,74736	0,46266

Acbd4	-0,74744	0,6014
Smg8	-0,74769	0,62488
St6gal1	-0,74804	0,9391
Hdc	-0,7481	1
Rap1b	-0,74859	0,24422
Plcd1	-0,74867	1
Nt5m	-0,74876	0,87014
Dfna5	-0,74875	0,93002
Calml4	-0,74931	0,94645
Elp2	-0,74941	0,66714
Arhgap4	-0,74939	1
Nudt13	-0,74954	0,52792
Exosc5	-0,75009	0,62692
Casp1	-0,75042	0,7102
Umps	-0,75055	0,67455
Cnrip1	-0,75055	0,94731
Elf2	-0,7507	0,77862
Fah	-0,75076	0,96214
Xkr5	-0,75124	1
Mir6236	-0,75137	1
Tmem8	-0,75198	0,93503
2310033P09Rik	-0,75246	0,67395
Gucd1	-0,75253	1
Pptc7	-0,75264	0,45113
Gm42549	-0,75297	1
Zbtb25	-0,75312	0,667
Eif3j2	-0,75367	0,32532
Psmb7	-0,75391	0,73466
Gm15032	-0,75412	0,94645
BC004004	-0,75461	0,6915
Gm16061	-0,75461	1
Gm43112	-0,75484	1
Esrp2	-0,75484	1
Zfp30	-0,75519	0,63909
Snx7	-0,7554	0,94571
Gm14698	-0,75551	1
Gm2830	-0,75569	0,77299
Gm37494	-0,75614	0,96667
Magee1	-0,75606	0,98417
Rrs1	-0,75639	0,4232
Gm6415	-0,75663	0,828
Dyrk2	-0,75677	0,68215
Zfp952	-0,75701	0,84342
Nanos1	-0,75704	1
Slc25a46	-0,75748	0,58454
Kif1bp	-0,75746	0,79124
Sh3bp5	-0,75814	0,27504
Tasp1	-0,75812	1
Gm15500	-0,75817	0,42323
Wrnip1	-0,75827	0,97004
Syf2	-0,75863	0,42216
Pde6g	-0,75866	1

Ccdc71l	-0,75985	0,98748
Gm12589	-0,75987	1
Hnrnpf	-0,76021	0,84963
Dnajc17	-0,76075	0,94645
Trappc12	-0,7615	0,73514
Pstpip1	-0,76159	0,25807
Anxa7	-0,76208	0,45975
Maip1	-0,76228	0,92461
Fam3c	-0,76282	0,59128
Gldc	-0,76284	1
Myl6	-0,76365	0,73652
Tmcc2	-0,76389	0,84312
Klhl23	-0,76414	1
Dtx2	-0,76444	0,88753
Nsun4	-0,7644	0,90884
Pwwp2b	-0,7645	0,71586
Lin28b	-0,76471	1
Ndufb8	-0,76476	0,49926
Runx2os1	-0,76488	1
Gm43223	-0,7649	1
Iffo2	-0,76501	0,80445
Mier2	-0,76512	0,26642
Gmpr	-0,76537	0,62574
D630029K05Rik	-0,7666	1
Lars	-0,76701	0,37116
Ccdc12	-0,76699	0,82482
Ptpn11	-0,76764	0,60967
Ppil3	-0,76801	0,82457
Fez2	-0,7683	0,57566
Gm37503	-0,76837	1
Prrg2	-0,76902	0,58919
Scap	-0,76906	0,75096
Nme4	-0,76935	0,93792
Myo10	-0,76966	0,4346
Vav1	-0,76967	0,46271
Ebp	-0,76975	0,65435
Tm2d2	-0,76991	0,57352
Tshz3	-0,77001	0,85558
Rab8a	-0,77115	0,45321
Gm15964	-0,77116	1
Mbip	-0,7714	0,90982
Inf2	-0,7715	0,25825
Fam65c	-0,7717	0,76758
Gm26740	-0,7718	1
Tm4sf5	-0,7719	1
Nrip1	-0,77228	0,70472
Gm13205	-0,77264	1
Cdc34b	-0,77268	0,9968
Rasa3	-0,77289	0,51086
Lyz1	-0,77299	0,24315
Tpm2	-0,77312	0,82388
Rassf8	-0,77385	0,67164

Rhox5	-0,7742	1
Rhno1	-0,7744	0,4346
Oas1d	-0,77443	1
K230015D01Rik	-0,77448	1
Selplg	-0,77532	0,35558
Mettl2	-0,7756	0,85001
Mrps36-ps2	-0,77617	1
Sgms1	-0,77637	0,68072
Ifi203-ps	-0,77658	1
Timp2	-0,7767	0,56157
Psmb8	-0,77699	0,4964
Bcar1	-0,77759	0,95355
Gm43793	-0,77763	1
Wwc2	-0,77805	1
Gm45856	-0,77814	0,98937
Slc37a3	-0,77826	0,84044
Gm4924	-0,77834	0,94571
Foxc1	-0,7789	1
Pik3cb	-0,77903	0,25026
2310057M21Rik	-0,77895	0,76621
Trmu	-0,77943	0,92571
Lrfn4	-0,7796	0,98463
Ttll12	-0,7797	0,42462
Gm11598	-0,77979	1
Ndufb9	-0,78042	0,4881
Gm2735	-0,78075	0,98776
Rnf8	-0,7808	0,74768
Dhrs11	-0,78086	1
Ank	-0,78111	0,20935
Gm42639	-0,78224	0,87562
Smim12	-0,78228	0,53402
Glb1l	-0,78231	0,98167
Gm7384	-0,7826	1
Me1	-0,78383	0,3994
9130401M01Rik	-0,78403	0,53199
Ftsj3	-0,78485	0,37782
Ccdc191	-0,78485	0,71586
Sp110	-0,78534	0,93557
Pdik1l	-0,78547	0,64449
Trub1	-0,78652	0,58012
Rnf170	-0,78646	1
Zbtb1	-0,78684	0,93366
Fam192a	-0,78687	0,43089
I830077J02Rik	-0,78729	1
Cdk5r1	-0,78891	0,61265
Gm16106	-0,78921	1
Polr2c	-0,78997	0,50922
Uri1	-0,79019	0,51912
Adsl	-0,79056	0,69919
Smim3	-0,79086	0,45047
BC017158	-0,79109	1
Ythdf2	-0,7912	0,33478

Mettl21a	-0,79183	0,57841
Ndufaf6	-0,79188	0,72745
Otud6b	-0,79214	0,49586
Slu7	-0,79221	0,23766
Smim4	-0,79228	0,52285
Rpp25l	-0,79271	0,29713
Dapk1	-0,79267	0,2984
Prtg	-0,79271	0,80273
Tigar	-0,79291	0,39457
Usb1	-0,7939	0,30197
Adamts7	-0,79416	1
9530062K07Rik	-0,79453	0,77029
Gstt3	-0,79494	0,78099
Gm5871	-0,79502	0,93474
Cep95	-0,79532	0,61921
Gm42970	-0,79551	1
Slc22a15	-0,79579	1
Rb1cc1	-0,79634	0,50342
Gm12732	-0,79637	0,75795
Bloc1s5	-0,79693	0,61794
Chpf	-0,79694	0,65255
Gm7452	-0,79697	1
Ubac2	-0,79736	0,60676
Nr2c1	-0,79737	0,97678
5430427O19Rik	-0,79772	0,83651
Phf20	-0,79778	0,32842
9930012K11Rik	-0,79823	0,77882
Tmem201	-0,79836	0,88157
Rad1	-0,79877	0,72255
Zbtb5	-0,79939	0,75088
Gm10399	-0,79994	1
Gm45292	-0,80072	1
Ndufa5	-0,80078	0,41705
Prep	-0,80105	0,61862
Pik3r4	-0,80122	0,53677
Suclg1	-0,80163	0,34481
Slfn5	-0,8018	0,67883
Ndufaf7	-0,80216	0,54864
Egr1	-0,80217	0,80445
Ndufab1-ps	-0,80237	1
Ptpn12	-0,80311	0,28644
Nfatc3	-0,80341	0,24719
Eif2b4	-0,80353	0,3464
Tmem79	-0,80354	1
Adprh	-0,80359	0,28638
N6amt1	-0,80369	0,82261
Polr3d	-0,80378	0,57793
Psma2	-0,80402	0,17107
Gm37274	-0,80431	0,92029
Tns1	-0,8043	1
Trim32	-0,80462	0,67218
Ears2	-0,80572	0,79545

Ccr2	-0,80597	1
Bbs1	-0,80619	1
Cog7	-0,80627	0,618
Itgb3	-0,80763	1
Gm8292	-0,80767	0,54629
Retsat	-0,80802	0,68938
Dennd4b	-0,80809	0,81943
Fgfrl1	-0,80864	0,84342
Mybpc3	-0,80861	0,98167
Xrra1	-0,80862	1
Ngrn	-0,8088	0,28878
Naa10	-0,80909	0,66776
Gm7733	-0,80911	1
Scfd1	-0,80984	0,47571
4632427E13Rik	-0,80977	0,56509
Pik3r2	-0,80977	0,6769
Psmc3	-0,81031	0,38024
Nme6	-0,81062	0,99813
Lrrc73	-0,81084	1
Dhrs4	-0,81161	0,89841
Fth1	-0,81177	0,073529
Ranbp6	-0,81251	0,70867
Cetn4	-0,813	0,96775
Itpripl2	-0,81344	0,24422
D10Jhu81e	-0,81347	0,45472
Gm12251	-0,81392	0,80847
Mtif2	-0,81421	0,56031
Ccdc114	-0,81428	0,97568
Gtpbp6	-0,81449	0,72396
Lin7b	-0,81462	0,96489
Gm38020	-0,8146	1
Ap5s1	-0,81544	0,59735
Lrsam1	-0,81541	0,87446
Aig1	-0,81594	0,2736
RP23-136K21.4	-0,81608	1
Slc39a2	-0,81618	0,96811
Gm45342	-0,81634	0,95103
Exosc2	-0,81663	0,79965
9130221H12Rik	-0,81689	0,80362
Gm5580	-0,817	1
Ctu2	-0,81803	0,58565
Gm15185	-0,81829	1
Tango2	-0,81867	0,61386
Pde4b	-0,81866	0,875
Met	-0,81883	1
RP24-286J14.3	-0,81888	0,88978
Gm44510	-0,81895	1
Mtrf1	-0,81973	0,8707
Brf2	-0,81984	0,53199
Zfp318	-0,81992	0,58651
Trmt2a	-0,82103	0,44
Rdh10	-0,82107	0,75413

Diablo	-0,82237	0,60085
Ccnb1	-0,82236	0,70841
Mzf1	-0,82238	1
Rgl1	-0,82256	0,12989
Slc16a6	-0,82442	0,43089
Zfp64	-0,82477	0,581
Dhodh	-0,82481	0,79996
Gm9403	-0,82476	0,98227
Gm45224	-0,82508	1
Wbscr27	-0,82535	0,74225
Zfp62	-0,82549	0,78014
Gm15541	-0,82639	1
Sec13	-0,82649	0,38758
Borcs5	-0,82662	0,65518
Mpnd	-0,8267	0,3487
Gm43794	-0,8272	0,90858
Utp23	-0,8279	0,39887
Prr14l	-0,82871	0,56489
Btbd19	-0,82871	0,6488
Fnta	-0,82877	0,3581
Anapc4	-0,82884	0,61005
Rab11fip4	-0,82896	1
Sema5a	-0,82923	0,46868
Lyrm2	-0,82963	0,65169
Zrsr1	-0,82984	0,42616
Fam83d	-0,82992	1
Bms1	-0,83034	0,23837
Pttg1ip	-0,83032	0,32511
Rab11fip3	-0,83032	0,98937
Ccdc32	-0,83055	0,63763
Narfl	-0,83096	0,49586
Cep63	-0,83099	0,94934
Gm43707	-0,83123	1
Twnk	-0,8313	0,59094
Cog2	-0,83143	0,71313
Ankrd34a	-0,83218	1
Alg11	-0,83252	0,69358
Mtdh	-0,83275	0,03058
Cpeb1	-0,83315	1
Wdr77	-0,83328	0,6175
Sfxn2	-0,83329	0,8467
Tars	-0,83371	0,3746
Sbk1	-0,83384	1
BC052040	-0,83395	0,85558
Ccdc120	-0,83392	1
Med29	-0,83412	0,92029
Rpp14	-0,83447	0,2411
Acot6	-0,83462	1
Prorsd1	-0,83514	0,18279
Dnttip2	-0,83517	0,37042
Fbxo31	-0,83561	0,70468
Zbtb24	-0,83635	0,57841

Prkar2b	-0,83687	0,90858
RP23-88C11.5	-0,83686	1
Gm4217	-0,83689	1
Gm12459	-0,83706	1
Sertad2	-0,83734	0,30774
Slf1	-0,83728	0,92852
Mtmr4	-0,83737	0,40645
Tmem175	-0,83811	0,79124
Gm44053	-0,83851	1
Wdr55	-0,83861	0,75096
Gm28875	-0,8388	0,18937
Cdk9	-0,83891	0,57147
Gm4034	-0,83886	0,91028
Grap	-0,83904	0,70548
Gm11451	-0,83901	1
Fbxo25	-0,83927	0,51767
Sfxn5	-0,83944	0,93503
Gm4734	-0,84005	1
Taco1	-0,8403	0,79124
Smn1	-0,84073	0,19361
Ypel4	-0,84094	0,91295
Gm14040	-0,84109	1
Gm8762	-0,84202	1
S1pr2	-0,84226	0,18294
Gm8539	-0,8423	1
Slc41a2	-0,8434	0,36598
Calr3	-0,84368	1
Alg14	-0,84453	0,67418
Polr3h	-0,84477	0,30534
AU019823	-0,84484	0,47717
Rab4a	-0,84476	0,86846
Derl2	-0,84515	0,33682
Cc2d1a	-0,84541	0,83879
Chordc1	-0,84665	0,11405
Lta	-0,84703	1
Fam213b	-0,84736	1
Bnip2	-0,84768	0,26492
Gm11281	-0,84773	1
Mtfp1	-0,84805	0,79124
Plxdc1	-0,84816	0,47571
Chchd10	-0,8486	1
Trak2	-0,84877	0,46868
Tdrd3	-0,84932	0,9391
Wdr12	-0,85006	0,3373
Gm13423	-0,85012	1
Ss18	-0,85048	0,35257
Nipal3	-0,85073	0,58919
Pithd1	-0,8509	0,32157
Brpf1	-0,85126	0,24779
Ak3	-0,85128	0,77862
Gm14325	-0,85186	1
Zfp472	-0,85199	0,53402

Mlycd	-0,85255	0,51912
Med4	-0,85266	0,3464
Gm12222	-0,85321	0,76353
Arid3a	-0,8533	0,70192
Rbsn	-0,85332	0,71032
Setd4	-0,85337	0,65416
Mir3091	-0,85346	1
Dnaaf2	-0,85363	0,45689
Ostc	-0,85389	0,17247
2610044O15Rik8	-0,85435	1
5031425F14Rik	-0,85484	1
Taf1a	-0,85494	0,73553
Chrac1	-0,856	0,53199
Upk1a	-0,85712	1
Cul5	-0,85733	0,17189
Pelp1	-0,85792	0,60705
Riok1	-0,85815	0,2617
Klhl25	-0,85821	0,58919
Faf2	-0,8584	0,32842
Cwc25	-0,85845	0,3562
Nprl3	-0,85893	0,90982
B230208H11Rik	-0,85909	1
Cox11	-0,85915	0,58919
Pygm	-0,85923	1
Gm38262	-0,85923	1
Ddx58	-0,85971	0,70368
F9	-0,85993	1
Wfikkn1	-0,86149	0,81734
Lrrc8d	-0,86173	0,30998
Gm13453	-0,86204	0,9711
Tubb2b	-0,86196	1
Taf1c	-0,86226	0,76491
Acad8	-0,86288	0,37984
Gm9497	-0,86331	0,99356
Gm9732	-0,86337	1
Gm37305	-0,86369	0,99851
Zbtb12	-0,86389	0,57216
Plagl2	-0,86403	0,57708
Zfp386	-0,86442	0,70867
Poglut1	-0,86463	0,24442
Ube4a	-0,86467	0,82469
Uqcc1	-0,86511	0,30998
Gm11334	-0,8651	0,93338
Ermard	-0,86523	0,96167
Tmem231	-0,86534	0,56672
Gm2950	-0,86548	1
Mex3c	-0,8662	0,57162
Hspb7	-0,86623	0,71622
RP23-312A24.1	-0,86643	0,92226
Srd5a1	-0,86664	1
Gm42786	-0,86757	1
Prmt2	-0,86775	0,75088

Wdr4	-0,86772	0,75381
Usp20	-0,86779	0,26492
Gm43457	-0,86812	1
Plekhf1	-0,86853	1
Cfap126	-0,86859	1
Naa40	-0,86884	0,53469
Grhl1	-0,86916	0,99431
Slc1a5	-0,86927	0,10793
Ddx56	-0,86932	0,50454
Xylt2	-0,86939	0,43794
Gm6863	-0,86953	0,30795
Wbp1l	-0,87034	0,32532
1110019D14Rik	-0,87036	0,96489
Gpank1	-0,8705	0,47245
Irx5	-0,87221	1
Rbm12b1	-0,87299	1
Reps2	-0,87358	1
Sla2	-0,87367	1
RP23-277D1.1	-0,87513	1
Mrpl37	-0,87773	0,13211
Ppfibp1	-0,87859	0,18256
Gm37010	-0,87861	1
Tut1	-0,87915	0,58619
Gm9256	-0,88011	1
Tyw3	-0,88017	1
Prmt3	-0,8804	0,3464
Tnfaip8	-0,88133	0,072697
Tmem17	-0,88144	1
Tubb4b	-0,8815	0,41012
Ercc6l2	-0,8816	0,75096
Gm7965	-0,88251	1
Nip7	-0,88286	0,13038
Cd276	-0,88315	0,51912
Eci1	-0,88338	0,68091
Ccl25	-0,88355	0,57656
Gm8129	-0,88388	1
Gm6206	-0,88423	0,80445
Amdhd2	-0,88458	0,34146
Cd101	-0,88473	0,91898
Fut8	-0,88481	1
Slfn8	-0,88483	1
Rps14	-0,88536	0,33278
Rap1gap	-0,88554	1
Bysl	-0,88685	0,24519
Dnajc30	-0,88721	0,80409
Gm11772	-0,88716	1
Paqr4	-0,88758	0,78693
Naca	-0,88794	0,28249
Acad12	-0,88805	0,91093
Gm9246	-0,88805	1
Nol6	-0,88894	0,73483
Gm44103	-0,88914	1

Alkbh7	-0,88933	0,64352
4930568A12Rik	-0,88943	1
Gja1	-0,88954	0,94934
Pld1	-0,88959	0,76621
Etfb	-0,89026	0,30927
Sdcbp2	-0,89081	0,59862
Tango6	-0,89085	1
Snapc5	-0,89102	0,83443
Pms2	-0,89112	0,67196
Rnf41	-0,89181	0,7832
March9	-0,89213	0,3741
Zfp945	-0,89257	0,9743
Gm5532	-0,89256	1
Mgam	-0,89334	1
Gm37297	-0,89359	0,97444
Adap2	-0,89425	0,89484
Polr3a	-0,89489	0,73236
Caskin2	-0,89494	1
Zfp940	-0,89498	1
Snrk	-0,89534	0,49288
Gtf2b	-0,89556	0,58044
Slc38a7	-0,89578	0,38974
Gm14830	-0,89584	0,80442
Gm14034	-0,89599	1
Eepd1	-0,8961	0,20057
Gtf2h2	-0,89619	0,57352
Fam32a	-0,89667	0,067953
Gm43761	-0,897	0,92378
Ticam2	-0,89739	0,62574
Gm8357	-0,89753	0,86744
Prr12	-0,89772	0,66905
Ankrd9	-0,8981	0,75908
Fbxo6	-0,89821	0,45809
Atp6v1g2	-0,89881	1
Fhod3	-0,89907	0,75088
Lrrc75a	-0,89937	0,20935
Gm13378	-0,89959	0,94449
Zswim3	-0,90005	0,72624
Kcnn1	-0,9002	0,92852
Tsr1	-0,90133	0,15946
Riox1	-0,90143	0,14722
Gm9727	-0,90147	1
C1rb	-0,90153	1
Gm10557	-0,90188	0,99336
Ckap4	-0,90247	0,049575
Mrps22	-0,90367	0,82434
Trim27	-0,90445	0,20153
Cdpf1	-0,90467	0,71375
Eng	-0,90503	0,71898
Gm17100	-0,90539	1
Gm14323	-0,90633	0,98842
Ccs	-0,90653	0,25674

Gm44667	-0,90653	0,99768
Mydgf	-0,90659	0,19294
Zfp984	-0,90656	0,73352
Gm9722	-0,90679	0,95283
Trim16	-0,90713	0,99321
Lipt1	-0,9075	0,667
Tssk6	-0,90788	1
Dus2	-0,90824	0,5508
Irf5	-0,90837	0,065707
Gm13835	-0,90879	0,43242
Tlr7	-0,91013	0,43726
Ms4a6b	-0,91012	1
Pla1a	-0,9105	0,93503
Tma16	-0,91122	0,22595
Tsen34	-0,91172	0,73615
Rtfdc1	-0,91223	0,2332
Gm43655	-0,91239	0,97339
Nudt16	-0,91248	0,28172
Tmem129	-0,91256	0,72238
Mpv17l	-0,91263	0,79916
Cx3cr1	-0,9137	0,27939
Snx4	-0,91383	0,22076
Dolk	-0,91391	0,77501
RP23-193N1.2	-0,91396	0,88126
Parp12	-0,9145	0,57967
Airn	-0,91458	0,6488
Gm20432	-0,91618	0,73083
Cstf2t	-0,91774	0,44706
Usp12	-0,91777	0,92852
Slfn2	-0,91838	0,13465
Kmt2e	-0,91849	0,13401
Brf1	-0,91855	0,38656
Hyi	-0,919	0,24442
Lipt2	-0,92011	0,99768
Gm38213	-0,92025	0,63651
Zfp821	-0,92028	0,52633
Ppm1h	-0,92128	0,56974
Drg2	-0,92144	0,82434
Slc27a4	-0,92168	0,80445
Ftx	-0,92222	0,82052
Tbc1d13	-0,92241	0,24866
1700017B05Rik	-0,92317	0,18248
Xab2	-0,9241	0,42791
Lrrc20	-0,92406	0,43105
Adprhl2	-0,92433	0,32354
Dido1	-0,92514	0,53906
RP23-316F10.2	-0,9252	0,91128
3110001I22Rik	-0,92536	0,70468
Gpr65	-0,92614	0,37545
Ctbp1	-0,92619	0,18497
Chmp2a	-0,92682	0,19977
Nos3	-0,92697	0,46953

Fibp	-0,92717	0,46232
Pknox1	-0,92734	0,55144
Ift74	-0,92803	0,78771
Shb	-0,92904	0,48741
Aptx	-0,92907	0,36825
Mis12	-0,92916	0,40753
C3ar1	-0,92923	0,59107
Gm5857	-0,92927	1
Gm6542	-0,9295	0,50639
Psme2	-0,92961	0,72076
Tmem203	-0,92996	0,23286
Ell	-0,93048	0,13401
Pkn3	-0,93085	0,46232
Gm7730	-0,93145	0,89635
Dapk3	-0,93189	0,61921
Gm5644	-0,93207	0,70155
Sdhb	-0,93221	0,12779
Ranbp1	-0,93311	0,39374
Kin	-0,93381	0,28588
Slc35c1	-0,93399	0,2549
Slc16a12	-0,93404	0,88353
Rlf	-0,93499	0,30711
Ufsp2	-0,93562	0,1244
Ecd	-0,93656	0,52633
Idh2	-0,93732	0,11985
Arg1	-0,93809	1
S100a13	-0,9389	0,32783
Spsb3	-0,93962	0,93347
Fn3k	-0,93986	0,99697
Gm29284	-0,94018	0,84445
Zfhx2	-0,94064	0,7832
Gm37569	-0,94069	0,87501
Nek4	-0,94218	0,86765
Klhl5	-0,94246	0,3276
Gm13612	-0,94251	1
Pard6a	-0,94259	0,56184
Asb7	-0,94294	0,43357
Ncoa6	-0,94363	0,16287
Slc39a13	-0,94379	0,2549
Ints10	-0,94388	0,77416
St8sia4	-0,94453	0,17466
Ccdc138	-0,94476	0,97226
4933412L11Rik	-0,94587	1
Zkscan5	-0,94646	0,82005
Klhl18	-0,94663	0,65435
Zfp119a	-0,94662	1
Trub2	-0,94702	0,46271
Exo5	-0,94725	0,67395
Gm25857	-0,94723	1
Frmd8os	-0,9477	1
Kdm1b	-0,94821	0,97977
Fbxw17	-0,94863	0,58919

Fkbp11	-0,9487	0,48741
Usp38	-0,94916	0,56002
2700062C07Rik	-0,94918	0,74225
Clec5a	-0,94954	0,32842
9530053A07Rik	-0,94961	0,95706
Hras	-0,9511	0,29022
Gatb	-0,95197	0,64992
RP23-261O3.5	-0,95251	0,82753
Zfp688	-0,95427	0,26642
Gm9333	-0,95453	0,58919
Coil	-0,95475	0,45055
Mrpl2	-0,95515	0,16799
Prokr1	-0,95735	1
Gm45212	-0,95786	0,91016
Gm44419	-0,95827	0,83468
Bid	-0,95854	0,094611
Dgcr6	-0,95893	0,39645
Cmas	-0,95921	0,048849
Gsr	-0,95943	0,44649
Fen1	-0,95939	0,56595
Stat5b	-0,95946	0,52346
6430511E19Rik	-0,95962	1
Bdh2	-0,9604	0,19094
Elf4	-0,96056	0,69815
Gm12655	-0,9618	0,93002
Agbl3	-0,96211	0,60975
Wdr78	-0,963	0,79147
Etv6	-0,96347	0,39457
Rin2	-0,96384	0,10349
Acot8	-0,96422	0,44814
Ddx27	-0,96433	0,077329
Pdhx	-0,96445	0,36186
Trpv4	-0,96624	0,73514
Det1	-0,96647	0,71769
Abhd17c	-0,96744	0,30998
Crtap	-0,96761	0,60202
Cyba	-0,9677	0,060788
Ssu72	-0,968	0,05924
Zfp599	-0,96798	0,86846
Cxcl2	-0,96913	0,61626
Dapp1	-0,96919	0,43103
Fance	-0,96939	0,25488
Ppm1l	-0,96957	0,067222
Olfr912	-0,96967	1
Gnl3	-0,96989	0,075293
1300002E11Rik	-0,97092	0,86744
Gm26606	-0,97189	1
F10	-0,97223	0,20204
Pspc1	-0,97232	0,60272
2810025M15Rik	-0,97244	0,036865
Alg5	-0,97416	0,18035
5830444B04Rik	-0,97425	0,24042

Marveld1	-0,97438	0,14686
Dhrs13	-0,97442	0,75056
Oas1c	-0,97454	0,71622
Zfp703	-0,9753	0,42123
Gm13421	-0,97549	0,91397
C130050O18Rik	-0,97574	0,54227
Gm29994	-0,97568	1
Gm8185	-0,97597	0,65319
Prss46	-0,97604	1
Sec16a	-0,97651	0,56157
Zbtb2	-0,97685	0,37193
Prkdc	-0,97709	0,72283
Gipc2	-0,97715	1
Klhl36	-0,97727	0,9229
2810004N23Rik	-0,97775	0,067222
2610203C20Rik	-0,97776	0,69358
Cep162	-0,97862	0,69467
Gm5449	-0,97857	1
Cep68	-0,97935	0,25674
Wars2	-0,98016	0,77438
Gpatch4	-0,9805	0,22687
Zfp958	-0,98131	0,24284
Dusp14	-0,98254	0,3009
Gm15610	-0,98262	0,94645
Zfp868	-0,98324	0,81943
D030028A08Rik	-0,98383	0,90295
Zfp579	-0,98413	0,76771
Gm2199	-0,98434	0,57934
Acot2	-0,98437	0,84589
Pde4a	-0,98453	0,23061
Pmepa1	-0,98476	0,18937
Gm20223	-0,98482	1
Sephs2	-0,98564	0,19679
Dennd2d	-0,98568	0,40856
B4gat1	-0,98611	0,46953
Panx1	-0,98635	0,52103
Gm44434	-0,98631	1
Rcn1	-0,98661	0,14361
Slc13a2	-0,98737	1
Ptrf	-0,98761	1
Rela	-0,98794	0,1133
Chchd4	-0,98811	0,043814
Stard7	-0,98971	0,056488
Mfap1b	-0,99053	0,6317
Fastkd3	-0,99203	0,19784
Actr8	-0,99293	0,088251
Fam35a	-0,99288	0,71898
Zfp217	-0,993	0,095966
Scly	-0,99304	0,60809
Zfp74	-0,99296	0,78281
Wbscr22	-0,99358	0,25674
Ncdn	-0,99384	0,46841

Snrpa1	-0,99426	0,09386
6720464F23Rik	-0,99481	1
Gpt2	-0,99487	0,29995
Gm10080	-0,99485	0,75096
Gm7535	-0,99521	0,79916
Ermap	-0,99601	0,75381
Thoc3	-0,99633	0,10505
1700021F05Rik	-0,99633	0,19094
Arhgap25	-0,99743	0,14443
Akap17b	-0,99748	0,62518
Dnah8	-0,99753	1
Gm4468	-0,99771	0,55178
Kcnk13	-0,9983	0,34042
Fam175a	-0,99873	0,62574
Malsu1	-0,99884	0,11167
Nudt22	-0,99953	0,27712
Asb8	-1,0012	0,27411
Inpp5b	-1,0012	0,69919
Gm20156	-1,0015	1
Kbtbd3	-1,0018	0,23586
Rab12	-1,002	0,1153
Twf1	-1,0021	0,37984
Serinc5	-1,0029	0,86744
RP23-380K24.3	-1,003	0,96791
Pex12	-1,0036	0,52607
Elmo2	-1,004	0,497
Nr0b2	-1,0044	1
Zfp729a	-1,0047	0,667
Tmem184c	-1,0052	0,54278
Metap2	-1,0067	0,090162
RP23-403E19.1	-1,0075	0,99228
Zfp874a	-1,0078	0,54615
Il1rap	-1,0081	0,81636
Rps10-ps4	-1,0093	0,89754
Cep41	-1,0094	0,92852
Gm17494	-1,0097	0,69358
Hoxa1	-1,0099	0,84044
Tldc1	-1,01	0,53786
Gm11764	-1,0104	1
Ung	-1,0108	0,41705
A330074K22Rik	-1,0111	1
Gm5566	-1,0115	1
Rhpn2	-1,0126	0,96142
Nudt18	-1,0144	0,25126
Gm4602	-1,0146	0,97678
Gm28535	-1,0147	0,90982
Tefm	-1,0153	0,74768
6030460B20Rik	-1,0153	1
Tbc1d25	-1,0167	0,71648
Dtx4	-1,0173	0,028214
Ttc28	-1,0174	0,875
Xbp1	-1,0185	0,042549

Galk1	-1,0206	0,10962
Fadd	-1,0214	1
Rars2	-1,0218	0,46266
Chid1	-1,0225	0,18947
Pde4c	-1,0225	0,96875
Gm12430	-1,0229	1
5430421F17Rik	-1,023	0,99214
RP24-233B16.6	-1,0235	0,89228
Sirt4	-1,0238	1
Jdp2	-1,024	0,34648
Ivd	-1,024	0,55178
Ndufs5	-1,024	0,74778
Snx20	-1,0241	0,17189
Zfp882	-1,0244	0,9729
Gpn1	-1,0248	0,31516
Tlr1	-1,0251	0,79282
Gm4258	-1,0265	0,81193
Ticam1	-1,0268	0,36065
D230017M19Rik	-1,0279	0,94883
Gm26917	-1,0284	0,0068458
Rpf2	-1,029	0,050938
Slc25a20	-1,029	0,41345
Cbarp	-1,0292	0,89854
Rpusd4	-1,0299	0,10386
Ociad2	-1,0299	1
Gm16755	-1,0301	1
Gm29155	-1,0307	0,86192
Tns4	-1,0308	0,77765
n-R5-8s1	-1,0309	0,75096
Sec31b	-1,0322	0,99768
Mylip	-1,0327	0,35598
Catsper2	-1,0328	0,78281
Oip5	-1,0333	0,71622
Cebpe	-1,0335	0,60659
Fam57a	-1,0335	0,89508
Dok4	-1,0337	0,91016
Zfp128	-1,0338	0,98227
0610007P14Rik	-1,0356	0,08377
Ttc30b	-1,036	1
1110038F14Rik	-1,0364	0,082157
Gm15798	-1,0371	0,86192
Gm38399	-1,0381	0,80445
Traf6	-1,0388	0,67067
RP24-550H10.6	-1,0388	0,92953
Gm14336	-1,0394	0,63121
0610010F05Rik	-1,0395	0,56677
Gm37675	-1,0397	1
Mst1	-1,0398	0,60975
Cdk5rap1	-1,0398	0,6488
Rgs20	-1,0399	0,94731
Clpp	-1,0404	0,26533
Psph	-1,0405	0,56372

Mrps2	-1,0406	0,20122
Gm13771	-1,0406	0,67103
Rpl36a-ps1	-1,0407	1
Zfp85	-1,0409	1
Gm18913	-1,0411	0,40416
Tuba4a	-1,0416	0,064547
Nt5c	-1,0421	0,45472
Ovca2	-1,0436	0,2982
Setd1b	-1,0441	0,24042
Ppan	-1,0445	0,028922
Nfu1	-1,0463	0,48815
Gm4754	-1,0464	0,97226
Slc25a43	-1,0466	0,7793
C2cd2	-1,0471	0,54615
Gm20045	-1,0477	0,5596
Psme2b	-1,049	0,13449
Mustn1	-1,0494	0,24789
Gm10605	-1,0497	0,93528
Tmem19	-1,0498	0,42462
Nudt12	-1,05	0,74608
Ficd	-1,05	0,81972
Tifa	-1,0507	0,11283
Trim68	-1,051	0,38633
Emp2	-1,0513	0,39374
Taf7	-1,0516	0,2527
Gm42967	-1,0516	0,98417
Itgax	-1,0517	0,14615
Mrps6	-1,0524	0,033308
Trim11	-1,0533	0,017806
Rnasel	-1,0533	0,58177
Prss50	-1,0549	0,5366
Gm44254	-1,0549	0,96575
Gm45422	-1,0558	0,95601
Cog8	-1,056	0,34235
Zfp456	-1,0571	0,75109
Gm7990	-1,0574	0,6624
Coro1c	-1,0579	0,0098944
Slc25a15	-1,0582	0,72396
Irf7	-1,0584	0,67883
Rhot2	-1,0585	0,60809
Dph5	-1,0592	0,21807
Naif1	-1,0594	0,39925
2310039H08Rik	-1,0595	0,16387
Slc12a2	-1,0599	0,27265
Mfsd6	-1,0602	0,11754
Gm8574	-1,0604	0,99431
Bcl9	-1,0605	0,31444
1110032A03Rik	-1,0606	0,36454
Gm38345	-1,0612	1
Gm37124	-1,0624	0,92852
Erbb3	-1,0638	0,88004
Taf1b	-1,0639	0,36677

Tstd3	-1,0642	0,09807
Gm10268	-1,0647	0,94009
Fuz	-1,0648	0,89508
Dusp6	-1,0659	0,075293
Trmt12	-1,066	0,87088
Gm12312	-1,0664	0,89605
Fam19a3	-1,0665	0,88353
BC048403	-1,0673	0,81712
Gm42893	-1,0689	0,98347
Gm8738	-1,069	0,75158
Gm6181	-1,0691	0,93002
Gm5590	-1,0693	0,98026
Ctdsp1	-1,0694	0,52044
Utp20	-1,0696	0,25227
Als2cr12	-1,0697	1
RP23-164P21.3	-1,0699	0,75789
Znhit6	-1,0712	0,24714
Hps4	-1,0717	0,099334
Gm43144	-1,0717	1
Plscr4	-1,0719	0,94571
Ppp1r7	-1,073	0,27852
Gm38340	-1,0731	0,45458
Gm13992	-1,074	0,87567
Il17ra	-1,0743	0,1244
Mov10	-1,0745	0,70746
Coa3	-1,0746	0,10888
1110002L01Rik	-1,0758	0,63312
Lrrc47	-1,076	0,01548
Zfp316	-1,0766	0,39645
Il34	-1,0775	0,70037
RP23-114G13.1	-1,0811	1
Fam109a	-1,0822	0,22687
Acy1	-1,0823	0,69919
Zfp239	-1,0824	0,90982
Rgs2	-1,0841	0,0066296
Nif3l1	-1,0844	0,21192
Mmab	-1,0852	0,52103
Gm12669	-1,0854	0,36448
Ppil1	-1,0861	0,50044
Fam161a	-1,0863	0,27679
Aldh3b1	-1,0875	0,18256
Ift43	-1,0875	0,44932
Ier2	-1,0877	0,36585
Tnfrsf1a	-1,088	0,011705
Kctd21	-1,0887	1
Gm37482	-1,0889	0,828
Fhad1	-1,089	0,58327
Fmo5	-1,0903	0,97161
AW209491	-1,0919	0,30998
Bcl2a1b	-1,0919	0,46959
Gm45110	-1,0929	0,96967
Slamf9	-1,0932	0,88531

Spef1	-1,0933	0,84829
Bdh1	-1,0935	0,79818
Arhgdig	-1,0936	0,71778
Msi1	-1,095	0,56092
6330418K02Rik	-1,0957	0,67196
Gm7972	-1,0959	0,97368
Pwp2	-1,0962	0,3464
Tmem198b	-1,0963	0,65283
Llph-ps2	-1,0964	0,098296
Gm15440	-1,0965	0,46271
Mrpl17	-1,0967	0,031498
Sh3bp5l	-1,0975	0,1041
Frg2f1	-1,0986	0,88069
Ptgr1	-1,0988	0,48741
Orai3	-1,0991	0,19882
Dhx33	-1,1004	0,32157
Zfp707	-1,1005	0,52792
Tmem65	-1,1006	0,086736
Zfp653	-1,1008	0,63206
Spata5l1	-1,1013	0,2458
Zbtb38	-1,1018	0,047134
Man2c1os	-1,1023	0,6769
Phykpl	-1,1024	0,55089
Gm8724	-1,1025	0,59157
Epc1	-1,1037	0,28644
Zscan20	-1,1049	0,57216
RP24-82M14.1	-1,1049	0,85112
Jagn1	-1,1057	0,1557
Wdr53	-1,1059	0,2527
Zfp961	-1,1065	0,49739
Zc3h12a	-1,1066	0,50435
Rpusd2	-1,1088	0,65501
C1rl	-1,1092	0,85558
Tmem220	-1,1095	0,31863
Grpel1	-1,1104	0,028072
Atad3aos	-1,1107	0,88825
Golim4	-1,1109	0,067222
Ttl	-1,1113	0,10322
Fam195a	-1,1114	0,40449
1810024B03Rik	-1,1119	0,80156
Phospho2	-1,112	0,093504
Grwd1	-1,1128	0,38278
Sh3tc1	-1,1145	0,43103
Apol11b	-1,1145	1
Efna2	-1,1148	0,93002
Toe1	-1,1153	0,39163
E230016M11Rik	-1,1155	0,94472
Tmem51os1	-1,1159	0,95538
Mypopos	-1,117	0,84309
Trmt5	-1,1176	0,56476
Pars2	-1,1179	0,96489
4921511C10Rik	-1,118	0,59094

Crebl2	-1,1196	0,56002
Wdr46-ps	-1,1199	0,9229
H2-Oa	-1,1201	0,88195
AI987944	-1,1202	0,34356
Dennd2a	-1,1207	0,20215
Crybb3	-1,1215	0,79147
RP24-547N4.5	-1,1229	0,61921
4933440N22Rik	-1,123	0,83237
Dcaf11	-1,1231	0,23354
Frrs1	-1,1238	0,011813
Rab43	-1,1252	0,53469
Adamtsl1	-1,1259	1
Rnf149	-1,127	0,22035
Bcar3	-1,1271	0,048097
Gm14248	-1,1276	0,9968
Zfp839	-1,1277	0,23502
Acot11	-1,1278	0,98757
Sppl2b	-1,1279	0,22165
Bicd1	-1,1287	0,39819
Top3a	-1,129	0,3197
Pid1	-1,1297	0,085855
Ptpn21	-1,1308	0,63758
Scamp1	-1,1316	0,040576
Mthfr	-1,1318	0,40645
Tysnd1	-1,1327	0,17488
Spryd3	-1,1329	0,17137
Gm43692	-1,1339	0,60809
Slc25a33	-1,1342	0,046423
Bap1	-1,1345	0,10804
A630072M18Rik	-1,1363	0,69708
Hoga1	-1,1375	0,92378
Egr2	-1,1382	0,2547
Slc35d2	-1,1387	0,6488
Trmt6	-1,1397	0,086478
Sprtn	-1,1412	0,48704
Zfyve19	-1,1412	0,5652
Sdsl	-1,1414	0,63194
Zfp1	-1,1426	0,38758
Cebpd	-1,1429	0,1041
Rnmt	-1,1438	0,011904
Bag5	-1,1442	0,067222
Csf2rb	-1,1449	0,23023
Rit1	-1,145	0,089447
Gm37033	-1,1451	0,77552
Crygn	-1,1454	0,52908
B3galt4	-1,1454	0,96967
Nt5c3b	-1,1461	0,50454
Ift20	-1,1463	0,10576
Chst14	-1,1477	0,57984
Trmt44	-1,1491	0,59139
Gtf3c6	-1,1504	0,15458
Bbs9	-1,1504	0,42589

Phxr4	-1,1518	0,85558
Gm12258	-1,1521	0,93312
Armc5	-1,1526	0,52103
Gm12726	-1,1526	0,95034
Usp42	-1,1533	0,10516
Cd63-ps	-1,1535	0,023271
Cdkl4	-1,1538	0,6087
Gm10478	-1,1547	0,71622
6430590A07Rik	-1,1563	0,70867
Atp5k-ps2	-1,1571	0,70368
Gm5905	-1,1574	0,28507
RP23-359K10.9	-1,1586	0,75795
Frmd8	-1,1602	0,014398
Dnajc11	-1,162	0,24442
Nckap1	-1,1621	0,6887
Cish	-1,1623	1
Eid2b	-1,1626	0,33346
Tmem116	-1,1632	0,48332
2210016F16Rik	-1,1664	0,2756
Tfip11	-1,167	0,13727
Tk2	-1,1673	0,01445
Snord13	-1,1683	0,08377
Tmem67	-1,1695	0,88753
Pex11g	-1,1698	0,83749
Mtif3	-1,1699	0,16296
Mppe1	-1,17	0,27097
Wdr5b	-1,1725	0,71769
Fam173b	-1,1729	0,43416
Cradd	-1,173	0,41425
Kcnn4	-1,1737	0,0024583
Ttc7	-1,1743	0,1697
Gm11448	-1,1748	0,85625
Lrrc8a	-1,1749	0,27819
Zfp180	-1,175	0,063712
Gm26930	-1,1751	0,88195
Hebp2	-1,1758	0,51086
Dhx32	-1,1761	0,38916
Tubb6	-1,1766	0,024866
Gnl2	-1,177	0,06505
Gm12743	-1,1778	0,93687
Rrp9	-1,1807	0,12427
Arhgap31	-1,1815	0,30711
Sphk2	-1,1823	0,01247
Ampd3	-1,1825	0,30711
Zfp942	-1,1827	0,58548
BC003965	-1,1847	0,0484
Tm2d3	-1,1849	0,055037
Gm45718	-1,1852	0,84147
Med8	-1,1859	0,093272
Ctdspl	-1,1862	0,618
4930590J08Rik	-1,1872	0,93442
Gm16433	-1,1873	0,61426

B4galt6	-1,1885	0,10349
Tbcc	-1,1889	0,14953
Surf2	-1,1889	0,22687
Gm10260	-1,1892	0,24422
Acp5	-1,1899	0,061934
Prag1	-1,1904	0,26351
Gm28731	-1,1913	0,78046
Dedd	-1,1927	0,24442
Ptgir	-1,1943	0,049107
Gm15506	-1,1943	0,81947
Igtp	-1,1949	0,7339
Creb5	-1,1949	0,77416
Lnx2	-1,1952	0,07296
Naip2	-1,1965	0,20558
Bag2	-1,1967	0,19779
Bok	-1,1971	0,37026
AA914427	-1,1977	0,99336
Zfp944	-1,198	0,80427
Kat6b	-1,1988	0,25904
Gm44951	-1,1995	0,84255
Gm6526	-1,2012	0,7793
Dnajc8	-1,2017	0,0024583
Yif1a	-1,2021	0,10641
Oxld1	-1,2025	0,29134
Gnptab	-1,2031	0,0086175
Gm33080	-1,2033	0,69919
Adgrl2	-1,2038	0,80847
Gm9776	-1,204	0,64861
Gm36445	-1,204	0,93002
Cdc42ep2	-1,2046	0,29725
Gm10842	-1,2052	0,90617
Irgm1	-1,2054	0,72315
Gm44545	-1,2054	0,94781
Gm19325	-1,2066	0,57708
Arhgef3	-1,2074	0,24714
Pdpn	-1,2074	0,65255
Mtrf1l	-1,2096	0,27508
Eogt	-1,2099	0,3767
Zfp420	-1,212	0,40841
Slc39a8	-1,2121	0,9498
Gm14013	-1,2123	0,71911
Fam58b	-1,2124	0,17237
Il1rn	-1,2124	0,45113
Maf1	-1,2139	0,010854
Paqr5	-1,2139	0,89508
Fkrp	-1,2143	0,39002
Adal	-1,2158	0,48128
Psma7	-1,2169	0,004957
Coa7	-1,2194	0,46953
Zfp119b	-1,2197	0,80182
2810405F17Rik	-1,2197	0,84589
Zfp605	-1,2203	0,68091

Gpr183	-1,2206	0,11283
Gm12468	-1,2206	0,72002
Nfkbie	-1,2216	0,50953
Fosl2	-1,222	0,0040886
Gzf1	-1,2235	0,11754
Pla2g5	-1,2236	0,034412
Ankrd54	-1,2259	0,21984
Dhrs9	-1,2265	0,45472
Gm17807	-1,2281	0,86139
Gstm4	-1,2285	0,76482
Iba57	-1,231	0,48271
Cebpa	-1,2327	0,060236
Actg1	-1,2334	0,86013
Bet1l	-1,2336	0,016819
CH25-309J2.1	-1,2337	0,90946
RP24-84O13.9	-1,2342	0,6488
Acsf3	-1,2349	0,82074
Zbtb6	-1,2351	0,080869
Tmem158	-1,2353	0,13447
Gm15327	-1,2354	0,80445
Jup	-1,236	0,69919
Casz1	-1,2365	0,28249
D3Ertd751e	-1,2383	0,60705
Tnfaip8l2	-1,2387	0,16273
Gm10060	-1,2397	0,65951
Tbx15	-1,24	0,52103
Hexim2	-1,2416	0,73483
Arhgef18	-1,2426	0,72076
Zfp830	-1,2432	0,066315
RP24-547N4.7	-1,2436	0,71032
Zfp14	-1,2443	0,7713
Prss36	-1,2445	0,67296
Cpsf3	-1,2458	0,068901
Stx11	-1,2475	0,64449
Rnf157	-1,2477	0,02892
Chac1	-1,2493	0,92408
Pin1	-1,2499	0,070692
Tm9sf1	-1,2545	0,57062
Gm19552	-1,2551	0,58468
Gm22299	-1,2554	0,86846
Gm14277	-1,2558	0,36798
Zfp846	-1,2559	0,57216
Mfsd4b4	-1,2564	0,88157
Mir17hg	-1,2567	0,29273
Fbxo9	-1,2572	0,2756
Gm9951	-1,2581	0,74748
Gtpbp8	-1,2598	0,45321
Erlin2	-1,2619	0,20323
Catip	-1,2625	0,65725
Gm12350	-1,2646	0,25867
Champ1	-1,2654	0,0038989
Gm3375	-1,2655	0,46232

Hgh1	-1,2659	0,2617
Mllt1	-1,2663	0,41886
Shisa3	-1,2663	0,9614
Traf2	-1,269	0,27824
Nsrp1	-1,2694	0,017566
Rundc1	-1,2695	0,12783
Nlrp3	-1,2697	0,14443
Fem1a	-1,2703	0,0070719
Igf2bp1	-1,2703	0,81943
Exosc4	-1,2706	0,039838
Zfp747	-1,271	0,49371
Fam136a	-1,2723	0,080478
Zadh2	-1,2734	0,079896
Knop1	-1,2735	0,0034074
Selenos	-1,2736	0,0023795
Src	-1,2741	0,36172
Slc6a4	-1,2744	0,73505
Zfp820	-1,2761	0,93002
Thap7	-1,2772	0,061226
Zfp426	-1,2774	0,32076
Mrap	-1,2775	0,84832
Cyth4	-1,2778	0,0030739
Ntmt1	-1,2781	0,037009
Kctd11	-1,2784	0,36513
Snip1	-1,2789	0,14818
Tlr6	-1,2807	0,16226
Tnfaip8l1	-1,2826	0,61613
Tlr13	-1,283	0,41705
Ptpro	-1,2844	0,10825
Serac1	-1,2849	0,71898
Rpp38	-1,2851	0,27712
Gm28686	-1,2853	0,8118
Sgsm1	-1,2855	0,0008647
Zfp46	-1,2871	0,3009
Pter	-1,2884	0,37891
Kctd7	-1,2888	0,36766
Gm6322	-1,2918	0,77882
Sat2	-1,2953	0,53786
Fbxw7	-1,2965	0,095966
6330408A02Rik	-1,2986	0,59646
Gm9378	-1,3001	0,54273
Slc43a2	-1,3002	0,0076818
Trim47	-1,3009	0,51086
Stoml1	-1,3032	0,08383
Sgsh	-1,3042	0,16273
Kbtbd4	-1,306	0,19538
Il4ra	-1,3066	0,19779
Gm11895	-1,3071	0,76758
Mapk11	-1,3075	0,69919
Rab20	-1,3108	0,24236
6330403L08Rik	-1,3109	0,50953
Ophn1	-1,3115	0,66322

Apbb1	-1,3115	0,68407
Mgat2	-1,3139	0,041522
Extl2	-1,3147	0,25674
Exoc8	-1,3169	0,43726
4833418N02Rik	-1,3173	0,78214
Vac14	-1,3174	0,28487
Zfp748	-1,3184	0,39311
Bcl2l12	-1,3195	0,84971
Eef1akmt1	-1,32	0,37042
Gm43351	-1,3212	0,47825
Gm20712	-1,3217	0,59377
Gad2	-1,322	0,61921
Lig4	-1,3222	0,2549
Trmo	-1,3237	0,60705
Gm4117	-1,3252	0,9464
Rdh14	-1,3303	0,086299
Kif13a	-1,3313	0,41867
Gm44829	-1,3318	0,62574
Poli	-1,333	0,16559
Dnmt3b	-1,333	0,61671
Trim30a	-1,3344	0,57344
Gm43024	-1,335	0,61643
Lat2	-1,3377	0,001439
Srrd	-1,3377	0,40753
Msantd3	-1,3391	0,079886
Mrps36-ps1	-1,3395	0,39457
Zfp799	-1,3402	0,44815
Wfs1	-1,3411	0,1265
Gm22973	-1,3419	0,35261
Slc39a11	-1,342	0,0008647
Rnf135	-1,3423	0,58565
Gm20604	-1,3438	0,17488
Gm13022	-1,3439	0,51086
Ubash3b	-1,344	0,0026606
Zfp84	-1,3456	0,087063
Cyb561d1	-1,3464	0,38734
Ankle1	-1,3472	0,7345
Gm37699	-1,3475	0,45055
Il15ra	-1,3483	0,4881
Acsbg1	-1,3487	0,45472
Gm12529	-1,3487	0,76947
3110080O07Rik	-1,3489	0,74518
Nmb	-1,3492	0,707
Armc7	-1,3494	0,14864
Peg12	-1,3494	0,91327
Ighmbp2	-1,3505	0,13401
Pigc	-1,3529	0,010696
C330018D20Rik	-1,3529	0,32842
Suox	-1,3542	0,33
Zfp551	-1,3546	0,30774
F630040K05Rik	-1,3556	0,61921
Zbtb22	-1,3563	0,024424

Zfp628	-1,3563	0,36433
Nt5e	-1,357	0,23535
Pdcd1	-1,3576	0,46098
Nipa1	-1,3579	0,56509
6430571L13Rik	-1,359	0,81636
Arhgef17	-1,3591	0,73767
Pfkfb4	-1,3617	0,022031
Mettl22	-1,3635	0,35067
Tradd	-1,3655	0,26492
Gm11131	-1,3683	0,36598
Il1rl1	-1,3706	0,76758
Lsm10	-1,3723	0,10236
Mutyh	-1,373	0,76758
Gm12182	-1,3733	0,38583
Cyp2u1	-1,3744	0,31516
Zc3h10	-1,3757	0,020515
1700084J12Rik	-1,3764	0,82074
Abca5	-1,3767	0,61128
Zscan22	-1,3771	0,69433
Tmem2	-1,3773	0,051784
Zfp709	-1,3774	0,875
Sema4b	-1,3785	0,24866
Zfp455	-1,3798	0,74768
Tbl3	-1,3805	0,062736
Fkbpl	-1,3807	0,1083
Arl11	-1,3807	0,44814
Dlx1	-1,3823	0,13254
Ltbr	-1,3833	0,015369
Ajuba	-1,3846	0,74768
Zpr1	-1,3862	0,00033584
2810006K23Rik	-1,3866	0,50342
Arhgap35	-1,3868	0,38974
Fancg	-1,3893	0,42567
5430403G16Rik	-1,3897	0,45472
Wdr24	-1,3913	0,16954
Isl2	-1,393	0,30774
Pih1d2	-1,3932	0,78046
Tmie	-1,3934	0,21937
Evi2a	-1,3949	0,053931
Slc35f6	-1,3961	0,0052592
Acod1	-1,3963	0,75088
Mesdc1	-1,3966	0,17627
Gm8919	-1,3974	0,64411
Fzd9	-1,3977	0,72781
Gm14585	-1,3987	0,6184
Ccdc51	-1,3992	0,2368
Zkscan6	-1,4002	0,031152
Lyl1	-1,4045	0,049107
2010008C14Rik	-1,406	0,73048
5730409E04Rik	-1,4098	0,10825
Mdm2	-1,4101	0,0008647
Spr	-1,4127	0,10566

Tor4a	-1,4136	0,15715
Depdc5	-1,4144	0,39857
Zfp658	-1,4149	0,67775
Gm13657	-1,4162	0,67848
Six5	-1,4163	0,72283
Gm26532	-1,4169	0,38758
Ribc1	-1,4169	0,78476
Srf	-1,417	0,0019979
Slc22a21	-1,418	0,51912
Gm25514	-1,4184	0,54278
Gba2	-1,4185	0,46138
Fbxo15	-1,4188	0,6488
Ttc30a1	-1,4193	0,70199
Maml2	-1,4227	0,45809
Fdxacb1	-1,4243	0,17019
Keap1	-1,4247	0,0018553
Hhex	-1,4255	0,077898
Pus7l	-1,4259	0,66721
Ccdc173	-1,4276	0,41192
Gm37121	-1,4277	0,44685
Slc16a13	-1,4277	0,618
Gm5054	-1,4302	0,73638
Fbxo46	-1,4318	0,16004
Cbr3	-1,4346	0,0023695
Gm44557	-1,4347	0,66212
Gm15696	-1,4363	0,53501
Tgfbr2	-1,4365	0,0059926
Lyrm1	-1,4381	0,12929
Abhd1	-1,4382	0,60705
Cenpb	-1,4393	0,046274
D11Wsu47e	-1,4396	0,14767
Lhx5	-1,4423	0,67771
Paqr7	-1,4447	0,044687
Gm6257	-1,4475	0,65885
Rftn2	-1,4476	0,3103
Il7r	-1,4478	0,81623
Ptafr	-1,449	0,41705
Susd3	-1,4496	0,0018553
Sec14l2	-1,4502	0,2617
Tmem246	-1,4512	0,32866
Mrgpre	-1,4516	0,52908
Gm29487	-1,4516	0,79818
Zscan12	-1,4525	0,02342
Spn	-1,4525	0,30711
Zdhhc12	-1,4535	0,19784
Gm37760	-1,4544	0,15383
Lin37	-1,4546	0,40841
Frmd4a	-1,4571	0,021936
Iqsec3	-1,4585	0,62947
Zfp319	-1,4588	0,27265
Gm10313	-1,459	0,73483
Cd3eap	-1,4601	0,028725

Katnb1	-1,4601	0,67395
Nudc-ps1	-1,461	0,19679
AI661453	-1,4616	0,30998
Tmem140	-1,4631	0,2445
Gm18969	-1,4642	0,59107
Oit3	-1,4656	0,24444
Cep19	-1,4675	0,052059
RP24-365A12.2	-1,4676	0,6364
Zfp775	-1,4693	0,13408
Saysd1	-1,4701	0,084928
2810021J22Rik	-1,4705	0,52103
Gpr146	-1,4712	0,055969
Gabrd	-1,4723	0,6184
Gsto2	-1,4738	0,59628
Pafah2	-1,4751	0,51086
Zfp763	-1,4763	0,38725
Magi2	-1,4777	0,35316
Gm17430	-1,4785	0,62574
Smim1	-1,479	0,36819
Gm12151	-1,4839	0,70744
Rgs8	-1,4922	0,71586
Zkscan4	-1,4926	0,66614
Gm38009	-1,4946	0,45472
Zfp418	-1,4947	0,67196
Prkch	-1,4959	0,0027161
Gm12990	-1,4971	0,27644
Gm37125	-1,4974	0,70716
Ip6k3	-1,5	0,23364
Fancf	-1,5039	0,69383
Slfn10-ps	-1,5053	0,38758
1600002H07Rik	-1,5074	0,066206
Stc2	-1,5076	0,43687
Rpl7l1-ps1	-1,5077	0,58548
Mfsd3	-1,5078	0,58294
Gm22	-1,5101	0,60421
Gm8566	-1,5109	0,11524
Zfp759	-1,5141	0,6915
Frat1	-1,5149	0,12749
Nlrp10	-1,5167	0,35692
Zhx3	-1,5183	0,12042
Mtmr9	-1,5195	0,57967
Vsig10	-1,5242	0,24442
Chrna1os	-1,525	0,46138
Zfp518a	-1,5256	0,14199
Cage1	-1,5285	0,43412
Lima1	-1,5305	0,0050027
Gm43447	-1,5313	0,86203
Mrpl46	-1,5353	0,060507
Tada2b	-1,5368	0,095966
Zbtb39	-1,5402	0,47766
C130023A14Rik	-1,5403	0,32689
Ubxn8	-1,5407	0,30711

2310011J03Rik	-1,5412	0,019015
2610306M01Rik	-1,5425	0,18279
Gm13268	-1,5433	0,53274
B130021K23Rik	-1,5441	0,56031
Gm19026	-1,5461	0,64086
Slx4	-1,5463	0,35561
2210008F06Rik	-1,5467	0,59094
Mrps35	-1,5482	0,0048132
Pkd2	-1,5489	0,36766
Gm43350	-1,5493	0,5652
Gm42690	-1,5504	0,55069
Gm13443	-1,5509	0,4346
Pxdn	-1,554	0,38974
Gtf2h3	-1,5559	0,44328
Ankrd49	-1,557	0,045087
Rab15	-1,5586	0,63763
Gm9711	-1,5588	0,61566
Zfp951	-1,5636	0,59712
AI464131	-1,5678	0,63957
Gm14017	-1,5682	0,24284
Zfp623	-1,5696	0,19094
Gm8973	-1,571	0,616
Fbxo36	-1,5733	0,3103
Klhl40	-1,5742	0,69919
Shq1	-1,5778	0,16547
Polr3e	-1,58	0,048782
Tmem185b	-1,5817	0,064928
Gm9796	-1,5822	0,63203
Plpp7	-1,5831	0,16954
Slc22a4	-1,5861	0,34171
Egf	-1,5871	0,49529
Capn10	-1,5895	0,27819
Eif1ad	-1,5896	0,0024583
Cog1	-1,5898	0,41564
Zc3h4	-1,591	0,0023328
Gm43961	-1,5934	0,60142
Gramd2	-1,5967	0,46232
Birc3	-1,5996	0,0077888
Tmem177	-1,6013	0,080478
Lat	-1,6019	0,48516
Tsku	-1,6055	0,61128
Fam219a	-1,606	0,12329
Afmid	-1,6083	0,64195
Gm12421	-1,6085	0,59789
Gm42515	-1,6102	0,55078
Zfp933	-1,6106	0,44127
Cd200r3	-1,6108	0,66411
Ttll13	-1,6132	0,73503
Gm38387	-1,6171	0,61405
Srl	-1,6196	0,46438
Gm45454	-1,6244	0,27504
Mlh3	-1,6248	0,14186

Ccdc92b	-1,6274	0,54457
Gm7936	-1,6314	0,39678
Tnks1bp1	-1,6334	0,52103
Exo1	-1,6348	0,58077
Zfp93	-1,6358	0,64058
Ccr1	-1,636	0,61794
Cnr2	-1,6415	0,14176
Gm20699	-1,6444	0,45182
Gm6293	-1,6457	0,20864
Sac3d1	-1,6489	0,016819
Alkbh2	-1,6489	0,049107
Bcdin3d	-1,6519	0,12697
BC024978	-1,6521	0,43105
RP23-38L16.4	-1,6533	0,41346
E130311K13Rik	-1,6552	0,096629
Tnnc2	-1,6565	0,58632
Qsox2	-1,6567	0,39594
Gm16740	-1,6581	0,58201
Pomt2	-1,661	0,32532
Ddx28	-1,6615	0,01752
Cptp	-1,6682	0,24261
Slc39a1	-1,6687	0,0019979
Fbxo32	-1,6713	0,14615
A930015D03Rik	-1,6747	0,45472
Igsf3	-1,6773	0,049508
Olfm1	-1,6775	2,27E-05
Cmtr2	-1,6775	0,00085025
Ints5	-1,6804	0,02057
Mdk	-1,6817	0,23286
Clec7a	-1,6855	0,0055685
Fastkd2	-1,6885	0,066315
Nkrf	-1,6889	0,24442
Fosl1	-1,6896	0,021714
Zfp850	-1,6901	0,23369
BC060293	-1,6901	0,54636
A730062M13Rik	-1,6927	0,58619
Asah2	-1,6928	0,3741
4930556M19Rik	-1,6941	0,47687
Ifi47	-1,6985	0,73023
2310043L19Rik	-1,7016	0,59128
Gm26890	-1,7019	0,23369
Angptl4	-1,7061	0,28644
Tmem181b-ps	-1,7072	0,16356
4930432K21Rik	-1,7095	0,049329
B3galnt1	-1,71	0,33174
Gm6743	-1,7104	0,55144
Pskh1	-1,7127	0,033215
A430033K04Rik	-1,7132	0,28644
Ino80c	-1,7137	0,0001722
Dcp1b	-1,715	0,57793
Zfp772	-1,7157	0,21061
Gm7666	-1,7183	0,48073

Hck	-1,7231	0,07858
Pomk	-1,7239	0,082235
P2ry6	-1,7248	0,0023695
Epop	-1,7252	0,27023
Cd200r2	-1,7273	0,023271
Tagap	-1,7285	0,060507
Zfp65	-1,7289	0,066932
Fam212a	-1,7301	0,22259
RP23-110E20.5	-1,732	0,62698
Rab7b	-1,7323	0,00035579
D6Wsu163e	-1,7323	0,14953
Gm44916	-1,7342	0,4782
Gper1	-1,7361	0,52792
Dmrt2	-1,7365	0,46232
Cyb561d2	-1,737	0,42798
Mboat1	-1,741	0,1691
N4bp3	-1,7413	0,32502
Fam120b	-1,7477	0,086511
4931414P19Rik	-1,7529	0,10963
Rcan1	-1,754	0,00025558
Col7a1	-1,7597	0,45113
9130230N09Rik	-1,7612	0,37984
Mast4	-1,7654	0,5407
Nmi	-1,7656	0,29204
Rwdd3	-1,7673	0,046274
Zfp764	-1,768	0,099531
Gm42640	-1,7685	0,23346
Atp6v0d2	-1,7719	3,77E-05
Pou6f2	-1,7741	0,75795
Nmnat3	-1,7759	0,13449
Gm36930	-1,7769	0,3973
Mocs3	-1,7816	0,079106
Tlr4	-1,783	0,038791
Adora2b	-1,7863	0,090652
Dpp3	-1,7874	0,049107
Nupl2	-1,7886	0,31169
6030442K20Rik	-1,7886	0,49739
Gm11918	-1,7929	0,22595
Sdr42e1	-1,7935	0,10289
2310001H17Rik	-1,7957	0,54615
BC024386	-1,796	0,43105
Zfp324	-1,7973	0,22656
Gcc1	-1,7974	0,00062809
Bmf	-1,8004	0,058583
Srxn1	-1,8011	0,0042504
Nop9	-1,8035	0,020571
Gm7769	-1,8073	0,50953
Gm20219	-1,8079	0,45482
Irf2bp1	-1,8085	0,00059348
Pgm5	-1,8091	0,16862
Gm20056	-1,8127	0,43416
Rftn1	-1,8165	0,0065631

Lpin3	-1,8198	0,034412
Ifi44	-1,8217	0,52858
Gm11222	-1,8278	0,45482
Zfp689	-1,8312	0,22595
Gm6921	-1,8369	0,45382
Gm8168	-1,8421	0,39887
Zfp251	-1,8499	0,059337
Gm28071	-1,8579	0,49322
Caprin2	-1,8626	0,57352
Zfp202	-1,8635	0,16338
Gm3695	-1,8651	0,30236
Tepsin	-1,8671	0,0073675
Slc9a3r1	-1,8715	0,19655
Zfp729b	-1,8731	0,061166
Gm9173	-1,8733	0,16004
Tnfrsf4	-1,8736	0,26492
Mcat	-1,8789	0,021282
Endov	-1,8846	0,13998
Zfp276	-1,8922	0,10825
Pctp	-1,8949	0,055053
Card9	-1,8974	0,31444
Egfl8	-1,8995	0,45975
Tspoap1	-1,9012	0,19294
Card6	-1,9052	0,27852
Zfp862-ps	-1,9065	0,18279
Aldh1b1	-1,9074	0,080478
Zfp39	-1,9102	0,41022
Mir22hg	-1,911	0,00024244
Zfp810	-1,9143	0,066397
Speer9-ps1	-1,9159	0,63659
Zfp974	-1,9161	0,20532
Olr1	-1,9247	0,24686
Gm37747	-1,9256	0,41672
Tmem260	-1,9329	0,16296
Znhit2	-1,9365	0,028214
Gpr180	-1,9385	0,19779
Hps6	-1,9393	0,004355
Tmem98	-1,9419	0,25904
Zfp12	-1,942	0,066802
Wdr73	-1,9427	0,011556
Ubiad1	-1,9437	0,032749
Nadsyn1	-1,9482	0,33314
Endog	-1,9503	0,093834
Sema6b	-1,9551	0,29134
Ubox5	-1,9571	0,35771
Emc9	-1,9624	0,12042
Csf2rb2	-1,9642	0,27508
Lpar1	-1,9653	0,31301
Gm17586	-1,9673	0,25026
Pomgnt1	-1,9708	0,014484
RP24-175C20.18	-1,9711	0,28249
Nrros	-1,9721	4,95E-05

Zfp953	-1,9754	0,36474
Gm42463	-1,9789	0,32083
Tmem202	-1,9806	0,17279
Zfp229	-1,9891	0,39457
Hemk1	-1,9987	0,043814
Zfp189	-2,004	0,076757
Cstad	-2,004	0,52285
Ctla2b	-2,0052	0,30263
Gm37776	-2,0068	0,43416
Ccdc130	-2,0086	0,0095361
Angptl2	-2,0123	0,00012879
C1ra	-2,0136	0,42917
Gm44423	-2,0142	0,37384
Lcmt2	-2,0164	0,027225
Slc46a1	-2,0165	0,21937
Txnl4b	-2,0179	0,095966
Ptpn7	-2,0183	0,0065559
Ccdc166	-2,0222	0,043814
Zbtb45	-2,0339	0,34932
Sh3rf1	-2,0343	0,025321
Mul1	-2,0372	0,013993
Tctn2	-2,0392	0,33278
Lysmd4	-2,0494	0,000844
Gm7895	-2,0518	0,43103
Fam222b	-2,0524	0,026498
Cspg4	-2,0526	0,17824
Mtg2	-2,0551	0,073472
Limk1	-2,0578	0,14375
Slc9a4	-2,0651	0,3581
Card11	-2,0661	0,29388
Gm5251	-2,0684	0,23953
Rinl	-2,0842	0,029757
Trim45	-2,0842	0,042372
Zfp768	-2,0848	0,00025558
Gm9568	-2,0848	0,2756
Ppcs	-2,094	0,018371
Tuba1c	-2,0941	0,00014419
Ctu1	-2,0948	0,0063649
Tlcd2	-2,1032	0,24132
Gm43728	-2,1232	0,1166
Mrm2	-2,1235	0,016637
Nedd9	-2,1244	0,30339
Snx19	-2,1371	0,043993
Tmem37	-2,1371	0,067953
Cttnbp2nl	-2,1378	3,12E-05
Gdpgp1	-2,1446	0,30774
Oasl1	-2,15	0,15044
Vwf	-2,1578	0,18405
Gm15824	-2,1592	0,26048
Krcc1	-2,1621	0,42306
Adamts4	-2,1644	0,32783
Mblac1	-2,1701	0,07296

Wdr35	-2,1723	0,19679
Fam83h	-2,1763	0,2756
Dcstamp	-2,1825	0,000844
Spats1	-2,1828	0,24333
Zfp94	-2,1841	0,14048
Spred1	-2,1887	1,10E-05
Gm37234	-2,1897	0,25313
Gm10800	-2,1918	1
Ffar4	-2,1998	0,098427
Numbl	-2,2014	0,018509
Trib1	-2,2034	0,00031075
Gramd1c	-2,2061	0,2778
Gpatch3	-2,2111	0,043814
Gmppb	-2,2135	0,0065055
Ppp1r10	-2,2165	4,55E-05
Tnfsf8	-2,2405	0,2756
Gm43378	-2,2461	0,27819
Gm7815	-2,2857	0,3103
Swsap1	-2,2859	0,1461
1810055G02Rik	-2,2872	0,095236
B3galt6	-2,2884	0,13415
RP24-325N9.5	-2,2887	0,19977
1600014C23Rik	-2,2933	0,16356
Gm10180	-2,294	0,1406
Irgm2	-2,3088	0,22082
Lrrc14	-2,3303	0,016145
AI467606	-2,3307	0,00074579
Cass4	-2,3355	0,08377
Homez	-2,3416	0,19606
Gm37642	-2,3426	0,082235
Jrk	-2,3477	0,16957
Zfp790	-2,361	0,13449
Zfp61	-2,3668	0,0065631
Gm4262	-2,3819	0,1373
3110070M22Rik	-2,3827	0,11985
Zfp58	-2,3862	0,1905
Hoxa7	-2,3959	0,052459
Zfp738	-2,4033	0,086736
Trp53rka	-2,4046	0,0027076
Zfp35	-2,4142	0,017726
Fastkd5	-2,4158	0,094611
Mettl18	-2,4172	0,032685
Cbfa2t3	-2,4248	0,15398
Hoxb3	-2,451	0,10505
E430018J23Rik	-2,4642	0,067222
Sla	-2,4732	0,00033584
Gm42835	-2,4845	0,13199
Gm33142	-2,4949	0,14615
Il20rb	-2,5016	0,0091506
Zfp40	-2,504	0,078714
Gm24890	-2,5084	0,24264
Slc10a3	-2,5136	0,024686

Tigd5	-2,5394	0,10804
Sec22a	-2,5606	0,0051
Gipc1	-2,5698	0,0073819
Ap5b1	-2,5754	0,073002
Hdhd3	-2,5821	0,051251
9130019O22Rik	-2,5904	0,043814
9930014A18Rik	-2,6098	0,07374
Zfp870	-2,6197	0,010436
Zfp90	-2,6246	0,01052
Tmem44	-2,6369	0,16766
Ccdc80	-2,6426	0,12752
Dlg3	-2,6432	0,011332
Zfp691	-2,6502	0,043894
Tmem204	-2,6533	0,043814
Sfn	-2,6647	0,10576
Zfp3	-2,6863	0,011813
Mfsd9	-2,688	0,016819
Elmod3	-2,6926	0,052059
Zfp27	-2,705	0,072697
RP23-308G10.5	-2,7224	0,57183
Tmem51	-2,7229	0,0018327
Fut7	-2,7236	0,081154
Pou4f1	-2,7659	0,14811
Gm20632	-2,7926	0,039833
Zfp28	-2,7957	0,10504
3110082I17Rik	-2,8143	0,0023795
Particl	-2,8485	0,0345
Zfp719	-2,8552	0,037406
D17H6S53E	-2,8942	0,021936
Mir763	-2,9288	0,042142
Commd5	-2,9542	0,00034541
Mras	-2,9716	0,043082
Gm10801	-2,9802	0,57393
Zfp408	-2,999	0,0014727
Zfp111	-3,0033	0,027065
Epb41l1	-3,0093	0,028185
Zfp41	-3,1422	0,033199
Filip1l	-3,1725	0,0093122
Rbak	-3,2428	0,010357
Ctsk	-3,2534	1,26E-06
Vegfc	-3,2592	0,066315
Ppp1r26	-3,4726	0,056488
Gm11205	-3,5408	0,0020051
Usp27x	-3,9797	0,042549
Slc9b2	-4,0916	0,0073819
