# Supplementary material for: Bone, dentin and cementum differentially influence the differentiation of osteoclast-like cells
Source: Sci Rep. 2025 Jun 5;15:19857. doi: 10.1038/s41598-025-04874-9 (PMC12141432; doi:10.1038/s41598-025-04874-9)
Supplement: Supplementary file 7 — Supplementary Information 7. [file 41598_2025_4874_MOESM7_ESM.pdf]

Tab. S6:
Transcripts induced in murine macrophage cells stimulated on dentin (n=6), fold of stimulation control

gene name	regulation of expression	adj.P.Val
mt-Tc	30,1293992	0,00047184
Gm26225	17,10919528	0,011176
mt-Ta	13,91819287	0,016728
mt-Ti	12,96275083	0,00049497
Gm24991	10,94651703	0,034639
Ccdc33	10,88296682	0,031686
Hist1h2bg	10,83404438	0,029431
4930589O11Rik	10,71009966	0,029431
Rasd1	10,51006728	0,01782
mt-Tq	10,34742581	0,00061947
mt-Tm	10,16402554	4,63E-05
Snord59a	9,65114425	0,022907
Snord83b	8,963113714	0,00049497
Gm23969	8,492562909	0,018926
mt-Tl1	8,218641822	2,36E-06
Snord66	8,013319502	0,081742
Hist1h2be	7,751094276	5,04E-05
1700054M17Rik	7,731240943	0,019133
Hist1h3d	7,43070966	0,054011
Hist1h2an	7,349265136	0,069887
4933437G19Rik	7,076323483	0,033185
Arhgap27os1	6,857582151	0,35368
Hist4h4	6,768799579	0,1367
Gm24631	6,553656236	0,010145
Gm20594	6,509289142	0,24968
Hist1h1a	6,477782372	0,23719
Trf	6,474640094	0,084574
Gm26202	6,338757165	0,023307
Gm29759	6,304141862	0,35954
Rn7sk	6,182969976	0,00049497
mt-Tp	6,078435319	0,00032
Gm23037	5,969458218	0,0047342
Hist1h1d	5,95045505	0,022907
Dnali1	5,939329203	0,16327
Rrh	5,82315088	0,31655
Hspa8	5,818712641	0,0012033
Il13ra2	5,710036628	0,29607
Gm11464	5,60183698	0,46083
Gm44187	5,51820862	0,22544
RP23-134M7.3	5,453566402	0,35368
Gm7132	5,278397501	0,37299
Hist1h4n	5,263782945	0,18873
Gm43006	5,202842374	0,20584
Gm24507	5,190595271	0,64385
Mapkapk5	5,099656238	0,32546
mt-Tv	5,098595905	0,029431
Gm25008	5,068293247	0,025215
Slc25a2	5,004756922	0,17693

AV099323	4,955738285	0,22544
Gm6290	4,951274723	0,50956
St5	4,947158081	0,60655
Rpl7a	4,880402041	0,00061947
RP24-282C4.3	4,774666607	0,17793
Gm43878	4,757487942	0,38315
Gm43714	4,750567937	0,21056
Gm14137	4,66507411	0,33592
Gm42793	4,632207884	0,27558
Olfr95	4,629639955	0,37975
Gm37696	4,621304011	0,34592
Gm26870	4,615221854	1
Vwa1	4,608189356	0,36872
Gm20492	4,607869952	0,42808
Snord71	4,604677126	0,53211
Atp1b4	4,602443465	0,38153
Gm22513	4,56558586	0,091354
F830208F22Rik	4,533420546	0,57229
Gm26461	4,528081735	0,27558
mt-Ts2	4,486840813	0,27536
Snord87	4,480935624	0,017151
H2-DMb2	4,477210036	0,19433
C3	4,463885432	0,63673
Gm26759	4,445359205	0,56097
Gm45546	4,436739965	0,35349
4930412F12Rik	4,413429034	0,42717
Notch4	4,387198552	0,36801
Rn7s6	4,382943253	0,63893
Hspa1a	4,378995599	0,10992
Gm5778	4,366871261	0,49984
Hist1h4d	4,362333294	0,083208
Gm18284	4,35025509	0,57115
Gm8550	4,344830814	0,43168
Pgam1	4,28382635	0,35368
Rybp	4,256595504	0,22557
Snord15a	4,250993344	0,17728
Pgap3	4,229537754	0,36241
RP23-451J19.1	4,155724456	0,10919
Gm8181	4,135609594	0,6409
Rhoh	4,125302748	0,8795
2310074N15Rik	4,105050585	0,62369
Gm37470	4,101068952	0,63335
Hist2h2ac	4,074152548	0,46965
Gm8203	4,070483018	0,63655
Gm13935	4,056119058	0,61873
Gm38248	4,055837919	0,60655
5930420M18Rik	4,035926777	0,41738
Crtc2	4,026147424	0,17055
Gm7799	4,019455282	0,084574
4930589L23Rik	4,011939916	0,26236
Hist1h2ae	4,007770787	0,49984
Psca	3,969342355	0,46965

Med12	3,967141895	0,47244
B430305J03Rik	3,93073744	0,55815
Ncoa4	3,914152592	0,60655
Gm5830	3,911711578	0,28486
4921507G05Rik	3,888193452	0,22594
Adora2a	3,885499295	0,51429
Tnfsf9	3,87500991	0,069887
Gpat3	3,829882152	0,44923
Aloxe3	3,821661504	0,10747
Gm11631	3,810551992	0,85568
Gm4742	3,807119883	0,60655
RP24-295J1.1	3,785017577	0,41023
Gm43149	3,770876757	0,21708
Gm28373	3,762782761	0,3418
Gm26912	3,751584361	0,59911
Snord72	3,746906559	0,28259
RP23-454I20.1	3,735237558	0,19364
4930578M01Rik	3,725670275	0,60655
Slc4a8	3,724121134	0,48645
Gdf15	3,719993241	0,0013043
Gm12981	3,718704213	0,82561
H2-Ab1	3,70455435	0,70844
Park2	3,69993519	0,82683
Gm15453	3,699422306	0,084574
Gm37851	3,683047491	0,66153
Mkln1os	3,681771265	0,20975
Gm23639	3,66725351	0,80932
Gfap	3,651527258	0,82683
Gm3699	3,642931834	0,5164
Rpl7	3,638137325	0,0041586
n-R5s151	3,628567229	0,49119
Rps13-ps5	3,61826983	0,3539
Myc	3,60500185	0,35349
Gm42666	3,604751979	0,26995
Hspa1b	3,602503919	0,031023
Gadd45g	3,601255603	0,00059897
4833445I07Rik	3,592031496	0,6246
Gm28659	3,583824524	0,42808
Gm19272	3,580844822	0,68264
Gm12466	3,578611671	0,52341
Gm23751	3,571177889	0,27582
Gm5822	3,562524658	0,81122
Zfp54	3,550691456	0,54169
Rps2-ps10	3,536690567	0,37975
Dynlt1b	3,536445431	0,60157
Apoo-ps	3,521524207	0,85777
Dntt	3,517377062	0,71147
4930578M07Rik	3,511043815	0,039732
4930430E12Rik	3,491144326	0,16961
Gm14057	3,489692702	0,60655
D430001F17Rik	3,479789411	0,051064
Fzd7	3,458148925	0,0064051

Gm7099	3,444274198	0,091666
Gm42670	3,441887636	0,41424
C230037L18Rik	3,439025945	0,6003
Gm7351	3,42332912	0,79146
Camk2b	3,421431349	0,51361
Rab11fip4os1	3,397326794	0,91122
Mcm8	3,393796359	0,045372
Gm10069	3,391679859	0,76817
Snord89	3,391444773	0,024472
Gm45733	3,37854015	0,91999
Plscr4	3,371521973	1
Timm8a1	3,369652922	0,94449
Gm8019	3,355668031	0,83621
Gm7285	3,355435442	0,66153
Rac3	3,351483892	0,71596
RP24-370M23.1	3,34962595	0,81268
Rps13-ps7	3,34313126	0,80138
Rbfox1	3,332257756	0,46182
Gm13890	3,330410472	0,85948
Gemin4	3,327872129	0,3815
Gm22980	3,32533572	0,22594
Gm42743	3,30969896	0,070546
Snord7	3,307176403	0,65259
Slc16a5	3,304426714	0,34837
Gm16380	3,303052728	0,76857
Mturn	3,29984898	0,10435
Acoxl	3,285015095	0,64421
8030453O22Rik	3,278872947	0,49984
Gm26226	3,273196014	0,71547
Insig1	3,269114708	0,0012033
Hist1h1b	3,268661544	0,40484
Gm18709	3,255321426	0,035021
Snord82	3,247433539	0,48941
Hba-ps4	3,243609178	1
RP23-413G8.2	3,239789322	0,34837
2700046G09Rik	3,22075741	0,63673
Gm8623	3,211839943	0,17156
Gm11516	3,20939197	0,87686
RP23-440L7.5	3,20916952	0,45475
Hyal1	3,19186581	0,0012033
Rnf122	3,183469628	0,074444
AY074887	3,172675565	0,14439
Soat2	3,162575672	0,96062
Gm44836	3,143779429	0,99584
Hist2h4	3,139424245	0,37311
Gm15216	3,094700941	0,63335
Hist1h4a	3,093843026	0,79146
Gm24920	3,079081384	0,55258
Tnnt1	3,072685266	0,85568
Dnm3	3,070343354	0,73316
Xk	3,067790576	1
Cerkl	3,067152713	0,62369

Rpl19-ps11	3,063965388	0,94943
Gm9920	3,056329315	0,73697
Gm5131	3,045755219	0,93527
Gm43273	3,03290435	0,90649
Gm7327	3,026184609	0,083435
Snord65	3,001533979	0,52341
Gm7114	2,984729109	0,51181
RP24-282K24.4	2,982040807	0,62016
Gm45248	2,964317543	0,53425
Rbm3	2,96390663	0,30032
Proscos	2,963495774	0,24972
Gm15542	2,963084974	0,66031
Gm12902	2,962263547	0,23724
Gm26810	2,955085761	0,3594
Lgi4	2,949560497	1
1700034H15Rik	2,946291143	0,56381
Gm4034	2,945474371	0,71707
Gm42731	2,945474371	0,91485
Gm29462	2,945474371	1
Eno1b	2,941801698	0,13344
Gm10358	2,937319093	0,61788
Riiad1	2,923101651	0,95166
Gm45629	2,919456874	1
Lgr5	2,91925452	0,93777
Gm16062	2,916018758	0,59566
Snx32	2,911777262	0,88097
Umad1	2,910970058	0,27261
Kyat3	2,90331277	1
Adamtsl1	2,892465998	1
Gm45698	2,891864589	0,30466
Rpl23a-ps14	2,890261443	0,70124
Tmem151a	2,88725794	1
Gm44552	2,885857374	0,60497
Gm22767	2,885257339	1
Hsh2d	2,878266145	0,96902
0610039K10Rik	2,875673732	0,54169
Gm12017	2,874876537	1
Inhbe	2,874677272	1
Icosl	2,873681158	0,28069
Gm10717	2,873681158	1
Snn	2,872088092	0,60711
Gem	2,870694885	0,91451
Gm44292	2,85620593	0,17812
Rps3a2	2,853633386	0,92192
Efemp2	2,843760565	0,64736
Gm44652	2,836869921	0,054791
Lbp	2,835297262	0,81122
Gapdh	2,83215456	0,071493
Gm6525	2,831565691	0,72767
Gm16104	2,829995973	0,91485
Plekhh1	2,82666321	1
4921536K21Rik	2,824900396	0,76156

Lum	2,824117276	1
Arhgap26	2,8108374	0,21008
Lamb2	2,810252964	0,95746
Gm43800	2,807916433	0,67619
RP24-174I4.1	2,80772181	0,44187
Lppos	2,802666339	0,84142
A930029G22Rik	2,796650558	0,99967
Gm43721	2,796262887	1
Ier5l	2,795681481	0,034639
1810026B05Rik	2,792195582	0,0021695
Rpsa-ps9	2,781378336	0,61306
Gm8210	2,778680579	0,86759
Depdc1a	2,775408248	1
Gm12583	2,773100688	1
Gm29170	2,768875144	0,025215
Sult2b1	2,764656038	1
Gm9009	2,763506489	0,9007
Fam72a	2,762931893	0,67424
Gm6649	2,754900093	0,93273
Wwc1	2,747082047	0,0033953
Gm12454	2,745749478	0,96868
Palld	2,743276421	1
9330151L19Rik	2,740425662	0,35349
Gm14292	2,739476067	0,69629
Hist1h4h	2,739096322	0,91485
4831440E17Rik	2,736629257	1
Rps12-ps19	2,73606025	1
Clec4n	2,732459287	1
Rps17	2,724893794	0,062095
Ccdc18	2,722816958	0,20584
Gm45729	2,722628233	1
Rpl35a-ps4	2,718668034	0,44187
Gm43578	2,713396727	0,89904
Comp	2,71170455	0,85168
Gm14005	2,707760239	0,29874
C430049E01Rik	2,699140357	0,88097
Gm43010	2,698205069	0,93777
Amigo1	2,697644051	0,94611
Hist1h2al	2,696335463	0,3815
Gm15937	2,695961698	0,80245
Gm3940	2,682354655	0,78396
RP23-2N7.4	2,682168735	0,36928
4930522L14Rik	2,681053484	0,48941
Galnt10	2,680310242	0,71596
Rpl10a-ps2	2,679752945	0,12847
Poln	2,679010062	1
Klhl35	2,676968198	1
Gm12428	2,67585511	0,85739
Rpl29	2,673630321	0,97786
Ntrk3	2,66881629	1
Mir124-2hg	2,667336794	0,88803
Gm15575	2,666042908	0,91425

Maff	2,664010927	0,031602
Pla2g2d	2,662718654	0,90824
Snord49b	2,660873638	0,094325
Gm30074	2,660689207	1
Gm10382	2,660504788	0,29874
Gm11346	2,659767242	0,99433
C230096K16Rik	2,658477028	0,21056
Klf1	2,658477028	1
Unc13b	2,652586872	1
Gm16439	2,649830355	0,96508
Bco2	2,648361385	0,71029
Bcl2a1d	2,647994269	1
Pank4	2,646526317	0,81122
Rps15a-ps6	2,645792645	0,15496
RP23-324E2.11	2,645792645	1
Gm19726	2,635725267	1
Nrap	2,63499459	1
Wdfy2	2,633533844	0,21236
Rny1	2,633168784	0,18778
Orc1	2,632256355	0,70475
Gm5117	2,63152664	1
Rgs12	2,620060272	0,89431
Gm29358	2,617881873	0,17055
Rpl17-ps8	2,615886599	0,093267
Hax1	2,614255233	0,89904
A730011C13Rik	2,614074033	1
Gm37670	2,613892845	0,96965
Meg3	2,612624884	1
Itgam	2,611176539	0,073347
Gm6987	2,609367236	0,23897
Izumo4	2,605030024	0,61306
Hoxaas3	2,60322498	0,70637
Esco2	2,599979053	0,55741
Gm37204	2,596197253	0,95202
Hist2h3c2	2,594218506	0,39896
Gapdh-ps14	2,593499335	1
5033430I15Rik	2,591881931	0,92006
Gm26610	2,587215093	0,7445
Hapln3	2,581482826	1
Gm12577	2,580588307	0,27211
Gm17108	2,57790661	0,59757
Gas2	2,571303666	1
Ost4	2,569521992	0,1367
Cacna1s	2,569521992	0,46086
Ang	2,566495979	0,064127
Hba-a1	2,560454638	1
Gm43153	2,559744828	0,85568
Gm13226	2,557793864	1
RP23-320D23.6	2,555135854	0,1753
Zfp532	2,554958752	1
Gm12230	2,554427518	0,6239
Tk1	2,553542375	0,42247

3830408C21Rik	2,551773007	1
A430035B10Rik	2,551419281	1
Tmcc3	2,549297952	1
Itgal	2,548414586	0,18277
Gm29243	2,54823795	1
Plat	2,547531526	1
Gnpda1	2,547001837	1
Upp2	2,544178687	0,35974
Hsd3b7	2,541182519	1
Gm13641	2,540830261	0,7966
Gm19566	2,538893713	0,60655
RP24-310D17.9	2,538541772	0,28069
RP23-151L20.5	2,536782799	1
Efna3	2,534497955	0,6409
Gm15163	2,534146624	1
Ltb	2,532917346	0,043218
Rpl12	2,53221517	0,045372
Gm23301	2,530811401	0,91425
Gm13477	2,53046058	1
Gm14056	2,52993444	0,85817
Dpf1	2,527830975	0,37214
Fanci	2,524854059	1
Rab42	2,522929688	1
RP23-288C18.3	2,521880649	0,22544
Gm44090	2,521880649	0,41454
Pla1a	2,518736149	1
Gm43328	2,514026749	0,51804
Gm38257	2,512458907	0,89196
Gm9435	2,512284762	1
Tcea3	2,507935074	1
Inca1	2,504287157	1
Nsa2-ps2	2,50289887	1
Gm19028	2,502378461	1
Ccl6	2,501164594	1
Kcnj2	2,50047122	0,40844
Adgb	2,49943152	1
Rhov	2,498738626	0,37975
4930427A07Rik	2,498565433	1
Gm5112	2,492511222	0,43515
9530085L11Rik	2,491474831	0,57954
Gm13408	2,483027087	0,59244
Slc47a2	2,483027087	1
D830025C05Rik	2,480618712	0,43681
Gm10110	2,477697396	0,3815
Gm6457	2,475637362	0,60655
9330162G02Rik	2,472036423	1
Gm42728	2,471522431	1
Olr1	2,468098547	1
Nup98	2,461776861	0,25366
Gm3531	2,46075325	1
5330438D12Rik	2,459559575	0,80245
Gm12834	2,456663059	1

0610009O20Rik	2,455471368	0,1367
Bard1	2,452579667	0,53211
Ube2s	2,447315329	0,64736
Gm43566	2,444094386	0,13727
1700001G11Rik	2,443586203	1
Gm15564	2,441554531	0,62728
Nectin4	2,437834183	1
Nsl1	2,433613397	0,10571
Gm6210	2,43024203	0,91485
Saa3	2,42990515	1
Gm7701	2,425866234	1
Gm36936	2,424689481	1
Aarsd1	2,424353371	1
Gm38299	2,423849294	0,74329
Gm12882	2,422001905	0,40689
Gmnn	2,419820443	0,10406
Gm42856	2,41914962	1
Zfp786	2,41914962	1
Pabpn1	2,418646624	0,28069
H2-Q5	2,417305813	1
Pde2a	2,416803201	1
Ccdc63	2,415965746	1
Hoxb8	2,412618827	0,056103
Macrod2	2,411448501	0,56131
Gm5523	2,409276544	1
Mafk	2,407607139	0,022907
Gm29019	2,407273397	0,43515
Adm	2,404771806	0,21056
Nat6	2,4042718	0,069887
Gm10175	2,399111152	0,10197
Btf3	2,398612323	0,43681
Tsc22d3	2,397448791	0,084574
Gm43011	2,396618043	0,91485
Gm5362	2,395455478	0,99433
Cdkn2c	2,395123421	0,17812
B3gnt2	2,393463823	1
Gm2011	2,391971168	0,65083
Fabp7	2,390148076	1
Rpa3	2,38650606	0,99433
Tnfrsf14	2,38650606	1
Gm16372	2,386175243	1
mt-Nd6	2,383860808	0,12847
Gm44957	2,383034767	1
Rps12-ps26	2,382704432	0,74954
Gadd45b	2,380228372	0,069837
Rps15a-ps4	2,375777946	0,99967
Gm13383	2,372980097	1
8030462N17Rik	2,3690358	0,40689
C030034I22Rik	2,368871596	0,045314
Gm29650	2,365425954	0,89647
1700001P01Rik	2,362312786	1
Gm11694	2,360839558	1

4932422M17Rik	2,359203716	0,28486
Gm13889	2,355772136	0,64469
Gm12940	2,354466181	1
Ttc39a	2,353487189	1
C730045M19Rik	2,352345547	0,82683
Crip2	2,351204459	0,34837
Ntn5	2,35071559	0,96965
Hacd2	2,347947256	0,8202
RP23-43M12.2	2,346808301	0,22544
2900055J20Rik	2,343719636	0,71596
Whrn	2,342420362	1
RP23-356D13.9	2,339823975	1
Crkl	2,335125356	0,60655
Ccdc85b	2,333992621	1
H3f3a	2,331728799	0,05401
Gm19967	2,328014429	0,55812
Gm15484	2,328014429	1
Rps19-ps9	2,327046436	0,90649
Gm4673	2,326401331	0,59383
Cytip	2,323500572	1
3110056K07Rik	2,322534456	0,76524
B230377A18Rik	2,320120924	1
RP23-55A6.4	2,315622372	1
Gm24959	2,314017861	1
Gm10237	2,313216022	1
Gm8909	2,312735053	1
Abhd18	2,310331704	0,6003
Aldh1l2	2,308890893	0,49119
Gm4459	2,306811309	1
Mettl21b	2,306011967	0,37399
3010003L21Rik	2,306011967	1
Dixdc1	2,305212903	0,92275
Rps19-ps3	2,300424328	0,85598
Tmco4	2,299946018	1
Dkkl1	2,2996272	1
Zcwpw1	2,299467807	0,85568
9130024F11Rik	2,299308426	0,72319
Hist1h2bp	2,297715219	1
Murc	2,296759825	1
Gm29736	2,294691166	0,48444
Socs1	2,293101159	0,69156
P4ha2	2,291671094	0,19144
Sorl1	2,291671094	1
Gm13416	2,291353423	1
Gm15459	2,291035796	1
Mfsd7a	2,290876999	1
Gm23300	2,288496364	1
Gm17034	2,287861946	1
Gm24009	2,287544803	1
2700038G22Rik	2,286435149	0,96965
Gm6136	2,285326033	0,42717
Rps19-ps4	2,285326033	0,66153

Npr1	2,285167632	1
Npm3-ps1	2,283267673	1
Gm9409	2,283109414	1
Gm10863	2,279472493	0,88097
Zfa-ps	2,277261552	1
Rasal1	2,275368167	1
Mrps18b	2,274106785	1
Cd274	2,272846102	0,42781
Manba	2,27205853	0,67424
Tob1	2,271271231	0,33592
Pdzk1ip1	2,271113803	1
Gm27029	2,27016947	1
Tma7-ps	2,268753706	0,52341
Gm37893	2,268439212	1
Gm13611	2,268124761	0,67424
Rnft2	2,266710273	1
4833417C18Rik	2,266396062	1
Lgals7	2,264982651	0,95166
Gm8172	2,260277646	1
Gm42432	2,257459335	0,8944
Hnrnpa1	2,256833521	1
Gm12643	2,256833521	1
Lsm7	2,256677095	0,43681
Arhgef15	2,256207881	1
4932441J04Rik	2,254332	0,75201
Gm7308	2,254019505	0,37399
Proser3	2,252301556	1
Satb2	2,251677172	0,39269
Gm26530	2,250428923	1
Zp1	2,248869585	1
Ifi213	2,248713711	0,10507
1700084E18Rik	2,248401995	0,5164
Gcat	2,246999806	0,68866
9330020H09Rik	2,246532604	0,43354
Eme1	2,245598492	1
Gm24339	2,241555158	0,6844
Uba7	2,241244434	0,43681
Gm6344	2,24031252	1
Kif18b	2,239225777	1
Jsrp1	2,237519104	0,68875
Ifitm5	2,237364016	1
Gm8121	2,235658761	1
Gm8228	2,233335509	1
Rpl17-ps10	2,232716382	1
Gm7783	2,231942716	0,76444
Rpl39-ps	2,231633324	0,17987
Gm4607	2,231478645	0,59566
4632415L05Rik	2,23101467	0,31655
Acap1	2,23101467	1
Gm42548	2,230860033	1
Gm45051	2,230550792	1
Zscan21	2,23008701	0,34592

Gm14427	2,229623325	0,54222
Gm9381	2,229623325	1
Ccne2	2,229314255	1
Gzmm	2,229005227	0,99433
Zfand1	2,228232847	0,50956
Gm45185	2,224220781	0,87607
Pate2	2,223295946	0,95144
Rps28	2,221139492	0,22594
Gm26594	2,220677667	1
Mdm4-ps	2,217908733	0,43681
Rnaset2a	2,217755005	0,88998
C030015A19Rik	2,213915256	0,72386
Kif18a	2,213301514	0,33977
Cygb	2,212074539	1
5330426L24Rik	2,211001494	1
Gm9392	2,210695005	0,77149
Gm7336	2,210388559	0,79146
Gm10036	2,208856965	0,31655
4933417C20Rik	2,208856965	1
Pigb	2,208244625	0,85708
Arl4d	2,207479439	0,9797
Adh7	2,206408623	1
Ceacam10	2,206255691	1
Rorc	2,206255691	1
Gm5735	2,204115766	1
Gm15421	2,202130551	1
Gpr19	2,200604679	1
Gm5883	2,20045215	1
Gm16253	2,196033405	0,73186
Gm9506	2,194511758	1
Gm24336	2,193903394	1
Rps12-ps10	2,192687173	0,35368
Gm4784	2,191471626	0,84142
Slc7a7	2,191471626	0,99168
Usp18	2,19101597	0,84957
Gm12758	2,188435706	1
C030037D09Rik	2,188132346	0,57246
Pin4	2,186616174	1
Mmp2	2,186464614	1
Rab5a	2,186161527	0,60561
Gm12184	2,184041091	0,98389
Gm10657	2,182073957	1
Gm28187	2,181317839	1
Gm7899	2,181015465	0,78876
Nme7	2,179202102	0,96902
Snora17	2,178597983	1
Oaz1	2,177692118	0,33977
Stamos	2,177088417	0,9717
Gm13758	2,176484883	1
Gm26129	2,1713616	1
Sh3bgrl2	2,167451934	0,045629
Hist1h2aa	2,165349645	1

Gm7436	2,16489942	0,94611
Cox4i2	2,164449289	0,69779
Ptgs1	2,164449289	1
Rpl34-ps1	2,16160063	1
Gm4285	2,16160063	1
Gm8927	2,158755719	1
Rps12-ps9	2,158306865	0,36699
Gm10762	2,158306865	0,99425
Gm4737	2,157409437	0,54455
Slc9b1	2,1551675	1
Traf3ip3	2,154271377	0,99675
Gm9320	2,153823456	0,23946
4930447F24Rik	2,153226372	1
Gm4032	2,151734386	1
Gm12933	2,149945368	1
H2-T23	2,147264629	0,76524
Olfr912	2,142655634	1
Lockd	2,14235862	0,2336
Abcc10	2,14235862	1
Sap30	2,142061646	0,1753
mt-Rnr1	2,141913175	0,090638
Gm43138	2,141616263	0,94463
Sit1	2,137167525	0,99433
Gm6768	2,137167525	1
Rpl30-ps5	2,134502718	1
D530018E20Rik	2,130511738	1
Mast4	2,130364067	1
Got2-ps1	2,12918307	1
Selenop	2,129035491	0,80013
Gm28151	2,128445279	1
Man2b2	2,126233441	0,55343
Slc25a25	2,125496672	0,22544
2310058D17Rik	2,123729469	0,85655
Mfsd13a	2,123582268	0,55258
Pkmyt1	2,12240503	1
Cxxc5	2,122257921	1
Gm14240	2,122257921	1
Pomc	2,121669588	0,99433
Dubr	2,121375483	0,41454
Gm29228	2,120934401	1
Gm36989	2,117702602	0,97201
Rpl26-ps4	2,117409047	1
Rpl23a-ps3	2,115355297	0,56381
Gm44775	2,114915467	1
RP23-278O17.1	2,114915467	1
Gm10131	2,114475728	0,90649
RP24-183O8.6	2,112424823	1
Gm11652	2,111400117	1
Gm13204	2,110375908	1
Gm14813	2,110083368	1
Gm3550	2,10832898	0,56801
Lysmd3	2,107598415	0,59566

Coq2	2,107160198	0,59383
RP23-23P9.3	2,105992064	1
Usp50	2,104678688	0,95746
Gm16199	2,104241078	1
Selenow	2,104095228	0,075999
Gm20517	2,104095228	1
Gm37108	2,10336613	1
Alox8	2,100015534	1
Gm8337	2,099578894	1
Eno1	2,098705885	0,26973
Gm13360	2,098705885	1
Car11	2,098269517	0,64385
Gm4968	2,098269517	1
Gm15829	2,097833239	1
Fign	2,096379636	1
Cd300lf	2,095653212	1
Gm13622	2,095217479	1
Trappc2	2,095072254	1
Gm7160	2,093330347	0,90189
Gm43868	2,09304017	1
Zfp36l1	2,09216988	0,14883
Tsg101-ps	2,089850875	1
Rassf7	2,087534441	0,7168
Gm7846	2,086232575	1
Gm37738	2,085509668	1
4933412L11Rik	2,084498017	1
Gm26569	2,084498017	1
Gm42972	2,083920152	1
Gm44791	2,083342447	1
Creb3l3	2,083198045	1
Rnf152	2,082764902	1
RP24-316F13.7	2,082187517	1
Ubb	2,080888986	0,093267
Gm8885	2,079879577	1
Gm15268	2,078150301	1
Sparc	2,078150301	1
Mthfs	2,077718207	0,69637
Gm25541	2,077142221	1
Rpsa-ps12	2,076854288	0,85781
Polq	2,076566394	0,8913
Gm10177	2,075127527	0,88097
Bcat2	2,074552259	0,43681
4930556M19Rik	2,074120913	1
Dpep2	2,073833399	0,23385
Rpl36-ps2	2,071678312	0,92161
RP23-403D16.3	2,071678312	0,98253
Amd2	2,071391136	0,94952
6330403N20Rik	2,071104	1
Gm10658	2,067804797	1
Gm10029	2,066372	1
Fhit	2,066372	1
Gm42535	2,065512799	1

Ceacam16	2,064081591	1
Zfp773	2,062937338	0,49984
Gm43059	2,06236545	1
Gm45456	2,061365027	1
Slc39a4	2,061079281	1
Gm6807	2,060936422	0,48038
Zgpat	2,060936422	0,99967
Gm5525	2,06022228	0,75133
Pbx1	2,06022228	1
Gm26244	2,058794738	0,64593
Zc3h12c	2,058652038	0,36438
Rpl28-ps1	2,057653416	0,46182
Sdhd	2,057225583	0,96965
Slc25a30	2,05679784	1
Gm7847	2,055087755	1
Gm17060	2,054945312	1
Gm37383	2,054945312	1
Gps1	2,054660456	1
Clgn	2,05124526	1
B9d1	2,04854559	1
Gm2383	2,047693801	1
Slc1a4	2,047693801	1
AK157302	2,045849473	1
Sp4	2,045424095	0,44761
Tmem29	2,044998805	0,76857
Snord110	2,044857061	1
3110083C13Rik	2,043723466	1
Gm16585	2,043156904	1
Asap3	2,043156904	1
RP24-232D3.1	2,042732086	1
Insig2	2,042590499	0,14977
Cd74	2,042024251	0,096644
Dhrs11	2,041599669	1
4933433G15Rik	2,041316662	1
Selenbp1	2,040892227	0,27582
Ift22	2,040892227	0,7298
Rplp1-ps1	2,039195366	1
H2afz	2,037782393	0,4473
A530017D24Rik	2,037358692	1
Cdkal1	2,03665272	0,56381
C2	2,03566477	1
Prim1	2,03453627	0,76505
Acta2	2,02749731	1
Arf2	2,0265138	0,022907
Gm12013	2,0265138	1
Rpl19	2,025390374	0,32546
Gm11249	2,024548213	1
Gm37052	2,023706402	1
Igfbp4	2,023566134	0,024428
2010016I18Rik	2,021883679	0,94676
Gm15420	2,021883679	1
Ypel1	2,021883679	1

Rpl13a-ps1	2,021183069	0,23441
Tnfrsf17	2,019642581	0,79315
Khnyn	2,018662882	1
Gm15753	2,017543808	0,99433
Rps12-ps4	2,016285591	0,42808
8430408G22Rik	2,015726636	1
Nkain1	2,015447216	1
Zfp36l2	2,015167835	0,34592
4921524J17Rik	2,015028159	0,36928
H2-DMb1	2,014469552	0,75133
Rpl10-ps2	2,013771511	1
Gm7863	2,013631932	1
Gm8254	2,011121161	1
Llph-ps1	2,010284933	1
Ftl1	2,009866949	0,99476
Gm12816	2,009588342	0,82597
Olfml3	2,009170503	1
Vamp2	2,008195884	0,35349
Gpr137	2,007639171	0,72026
Fam103a1	2,007360873	0,74954
Rpph1	2,007221738	1
E130317F20Rik	2,005413862	1
Gm13215	2,004996892	0,82325
Gm10636	2,004996892	1
Gm17786	2,004302133	1
mt-Rnr2	2,003052174	0,32762
Rell1	2,002913338	0,20584
RP23-354J5.3	2,002913338	0,71713
1500004A13Rik	2,002080523	0,82338
RP24-75M13.2	1,998475657	1
Gm20302	1,998337138	1
Gm8242	1,997229332	1
Aif1	1,996675659	0,78941
L1cam	1,995568774	0,93596
Ticrr	1,995568774	1
Gm28424	1,994047809	1
Car12	1,991975634	0,3539
Gm25291	1,990733362	1
Pi16	1,99059538	0,43681
Gm10916	1,988251143	1
2900093K20Rik	1,987699959	0,35136
Gm10051	1,987424424	1
D930030I03Rik	1,986460354	1
Tubb2b	1,98563438	1
Serpinb9	1,984946331	1
Atg4a-ps	1,98425852	1
Rps27rt	1,984120986	0,27558
Gm29593	1,983983462	1
Gm6969	1,983708443	1
Cd48	1,982059127	1
Osbpl1a	1,981921745	0,82683
Gm18889	1,981097659	0,61873

Alpk2	1,979587723	1
Shox2	1,978764607	1
Gm11491	1,976023357	0,67164
Gm9294	1,975886394	1
Tmem267	1,974517289	1
Zfp931	1,972328693	1
Cep128	1,970142523	1
RP23-63H11.3	1,970005968	1
1110006O24Rik	1,969732886	1
Itga6	1,969323334	0,36575
Qpctl	1,967549593	1
4930529C04Rik	1,966595164	0,82508
Ip6k2	1,966186264	0,22544
Gm13433	1,965913712	1
Slc13a3	1,96482388	1
C130083A15Rik	1,963870773	1
Matr3-ps2	1,963870773	1
Gm26520	1,963326347	0,44187
RP23-187B11.16	1,962509993	1
Lrrc2	1,961965945	1
Gm43148	1,960742389	1
Mxd3	1,959791261	1
Cmtm7	1,959247967	0,35794
Ly86	1,958976376	0,20316
Samd9l	1,958704823	1
C330013E15Rik	1,957754684	1
Fdps	1,956533754	0,52341
Gadd45a	1,956533754	0,69637
Prdm10	1,954500563	0,6771
Gm15975	1,953146276	1
Ccdc36	1,953010898	0,69002
Dennd4c	1,952875531	0,42247
H2-Ob	1,951792927	0,38056
Gm12912	1,951792927	1
Hist2h2be	1,951387105	0,28608
Emc10	1,950575715	0,29124
1700123M08Rik	1,949764662	1
Maz	1,948683782	0,78374
Slbp	1,948683782	0,95022
Gm17690	1,948008537	1
Gm7312	1,945175061	1
Nrbp2	1,944231485	0,91485
Lzic	1,944096726	0,60655
Rps18-ps1	1,942884313	1
Rbmx	1,942076459	1
3110031N09Rik	1,942076459	1
Gm7638	1,940596264	0,73186
Gm45250	1,939520467	1
Gm13456	1,939251611	0,92045
Gm10240	1,938176559	0,80245
Gm14130	1,937504955	1
RP24-389J11.1	1,937236378	0,37333

Morf4l1	1,936833583	0,47563
Gm10138	1,936430872	0,59566
Stap2	1,935894054	1
Gm23935	1,933078199	0,6409
Dennd5b	1,932944213	1
2810013P06Rik	1,932274421	0,64593
Atn1	1,932274421	1
Plekha7	1,932274421	1
Gm14843	1,931203238	1
5430402O13Rik	1,929998866	1
Gm23442	1,928795246	1
Ttk	1,92745877	0,41225
Ncapd3	1,92612322	0,31655
Gm7722	1,925856221	1
Gm34121	1,92558926	1
2810029C07Rik	1,925055447	1
Serpinf1	1,924655184	1
Gm7867	1,924255005	1
Akr1b7	1,923721562	1
Gm14048	1,923588225	1
Rpl26	1,923321577	0,52341
Gm11343	1,923321577	1
Adat2	1,922388602	0,80245
Hmgb1	1,921855677	0,91485
Adam9	1,92145608	0,98426
Gm12355	1,920923413	1
Golga7	1,920390894	0,61306
Gm5093	1,917065991	1
Gm12770	1,917065991	1
Slc30a2	1,915870436	1
Gm43182	1,914012166	1
Gm5764	1,911625609	1
Mettl7a1	1,91149311	0,90082
Snhg20	1,910830752	0,33592
Gm10263	1,910830752	1
Gm8318	1,909242028	1
Rpl10-ps3	1,908183613	0,85568
Mrps28	1,906861419	1
Klf11	1,90672925	0,73386
Cenpx	1,90646494	0,60691
Gm42511	1,906332798	1
Tbc1d30	1,905936429	1
Slc2a4	1,905804324	1
Gm15720	1,905672228	1
4932416K20Rik	1,904087794	1
Spdl1	1,901977263	0,79693
Rccd1	1,901845433	0,86385
H2-T10	1,901449996	1
Kbtbd8	1,901054642	1
Tmem71	1,90065937	1
Cks2	1,899737388	1
A930006K02Rik	1,899474047	1

Gm8093	1,899210742	1
Gtpbp10	1,898421047	1
Rps27a	1,89763168	0,88097
Gm14513	1,89750015	1
Trim3	1,895659698	0,9797
Fam71f2	1,895528305	1
Txlnb	1,894346182	1
Gm15596	1,893821031	1
E2f8	1,892771164	1
H2-Q4	1,89159076	0,98253
Pacs1	1,890804234	0,63765
Ctdspl2	1,890542131	0,52137
Gm15950	1,890280064	1
Upf2	1,889101214	0,28194
B930036N10Rik	1,888577516	1
Gm5100	1,888446614	1
Rpl21	1,887007295	0,60602
Hdac11	1,885961206	0,79832
Tnfaip3	1,885046354	0,98024
Il18rap	1,884131945	1
Apol11b	1,88308745	1
Tmem240	1,882826417	1
Arhgap15	1,882304459	0,64736
Gm20673	1,882173992	1
Msantd2	1,881521793	0,73953
Jmjd7	1,879827134	0,95166
RP23-366E4.9	1,879566553	1
Gm7909	1,879566553	1
Gm9169	1,878915257	1
Cln6	1,877092831	0,2324
Gm33370	1,876182281	1
RP23-58B7.2	1,875922205	0,75366
Gm7363	1,875532158	1
Pcgf5	1,875272172	1
Ints4	1,875012222	0,9154
Aunip	1,874622365	1
Rpl30	1,874362505	1
Cul7	1,873972783	1
RP24-454N4.2	1,873842894	0,5312
Lsm12	1,87345328	0,52706
Rgs11	1,87345328	0,99293
Greb1	1,87345328	1
1700020D05Rik	1,87293392	1
Ldb1	1,872025387	1
1500015A07Rik	1,870857921	0,84517
Kdm4b	1,870598583	0,88075
Nlrc3	1,870080015	1
Gpkow	1,869820785	1
Gm10941	1,869820785	1
Ypel2	1,868654694	0,70475
Tbc1d10a	1,86800718	0,50176
Gm13822	1,867748237	1

Zwilch	1,86684222	0,95144
Gm10335	1,866583438	1
Gm10501	1,865936642	1
Mterf3	1,863997597	1
Rsrp1	1,86270602	0,51959
Hmgb1-ps5	1,862576911	1
Gm26800	1,86218964	1
Gm9332	1,861544366	1
Gm12924	1,861544366	1
Rps19-ps8	1,860383436	1
Nmrk1	1,8598677	0,97786
Gm6564	1,859480991	1
4930579G24Rik	1,859094363	1
Gm24916	1,858965505	0,83381
Snrpf	1,858965505	1
9330104G04Rik	1,858192544	1
Gm45568	1,858063748	0,82763
Atp2a3	1,857419904	0,37082
Rps6	1,857419904	1
Fam107b	1,856904989	1
E230020A03Rik	1,856261547	1
Gm12240	1,855618327	1
RP23-149L23.1	1,85548971	1
Slc17a7	1,855103911	1
Ssc5d	1,854075509	1
Clcn2	1,853818497	1
Gm5069	1,853561521	1
Epsti1	1,853304581	0,87195
Tnfrsf12a	1,853047676	0,46842
3110062M04Rik	1,852277175	0,35662
Efcab2	1,850608856	1
Ciart	1,850352325	0,71713
Rnf225	1,849582945	1
Gm8304	1,849070203	1
Gm8649	1,848045145	0,37975
Rpl31-ps11	1,848045145	1
Zfp541	1,847404772	1
Gm14094	1,847148685	1
Hspa9-ps1	1,847020655	0,99293
Rpl11	1,846252661	0,96857
Rab26os	1,845996733	0,5677
Gm11810	1,845996733	1
Gm7488	1,845101267	1
Rpl13-ps3	1,844973379	1
Gm17827	1,844589768	1
Gm9835	1,841140851	1
Gm26947	1,841013237	1
1190005I06Rik	1,840502871	1
Gtf2ird1	1,838845157	0,98087
Gm12943	1,838462819	1
Rpl31-ps13	1,836934265	0,85568
Gm7079	1,836806942	1

Gm28727	1,836170464	0,82683
Abhd3	1,836170464	1
Gm11952	1,835025358	1
Calr-ps	1,833880967	0,24972
Hsd17b14	1,833372578	1
Clec3b	1,832737289	1
D2hgdh	1,832610258	1
Ccr10	1,831975234	1
Rps27	1,831467373	0,81145
Gm24876	1,83134043	1
Arrdc2	1,830832745	0,69156
Dtx3	1,829817797	0,69002
Rpl17	1,82918374	0,72319
Rab27a	1,82918374	0,80292
9330160F10Rik	1,828803412	1
Akap10	1,82829643	0,91485
Gm43637	1,828169706	1
Gm5075	1,82740955	1
Gm5121	1,826902955	1
Fbxo33	1,823992756	0,63673
Gm2214	1,823866331	1
Lrrc49	1,823613507	1
Gm7565	1,822602561	1
Sgk1	1,822476232	1
Gm42671	1,822223601	1
Gm15441	1,82184472	1
Gm23100	1,82184472	1
Tslp	1,820203811	1
Fam229b	1,820077648	1
Faah	1,819951494	1
Gm1848	1,819446967	1
Matk	1,819320857	1
Cit	1,819194755	0,90082
Ccdc50-ps	1,817430254	0,88743
Frat2	1,817052369	0,46478
Gm5577	1,816422736	1
B230317F23Rik	1,816296835	1
Tap2	1,815919186	1
Hnrnpa3	1,815793321	0,69156
Gm2788	1,815793321	1
Rpsa-ps2	1,815793321	1
Ccng2	1,815541616	0,75896
Gm8719	1,814409375	1
Rps23-ps2	1,80900952	1
Csrp2	1,808758755	1
Gm14494	1,808633386	1
Gm5909	1,807505454	0,99168
Myh11	1,806628659	1
Gm12097	1,806378225	1
Gm45380	1,806002638	1
E130201H02Rik	1,805501977	1
Snord55	1,805251699	1

Gm38297	1,804751246	1
RP24-91J7.1	1,804626154	1
Cenpp	1,803125733	1
Gm6088	1,80237599	1
Gsg1	1,802001235	0,97786
Gm14537	1,800378199	1
Gm13567	1,799380134	1
Gm12751	1,799130704	1
Tcte2	1,798507281	1
Diaph3	1,798257972	1
Kif11	1,798008698	0,82508
Gm11956	1,795393404	1
Rilp	1,795393404	1
Piga	1,795268961	0,53708
Ahcyl2	1,793154752	1
Bub1	1,792409154	0,73611
Ccdc69	1,792036472	1
Gm11759	1,791912262	1
Ctps2	1,791415507	1
Spata2	1,79091889	1
Gm44270	1,790794758	1
Rpl21-ps6	1,789926069	0,98276
Gm8494	1,789926069	1
Gm13270	1,789429865	1
Rps11-ps4	1,788809804	1
Sep 01	1,788561839	1
Nbas	1,788313909	0,82763
Teddm2	1,787570325	1
Uhrf1	1,787198649	1
Mtfr2	1,787198649	1
Srfbp1	1,78620789	0,64448
Gm6159	1,786084084	0,76857
Capn1	1,785465181	0,66992
Gm15853	1,783980691	1
2700033N17Rik	1,783733396	1
RP23-426K2.3	1,783486135	1
RP24-275P22.2	1,783362517	1
Myh7b	1,782373885	1
Ect2	1,781879775	1
Slc36a3os	1,781509282	1
Gm13092	1,781262329	1
Setdb2	1,781262329	1
Gm37653	1,781015411	1
Sik2	1,780274861	0,89904
Gm44884	1,779287941	1
A130014A01Rik	1,778301568	1
Ahcy	1,77743894	1
Gm26656	1,77743894	1
Runx2os1	1,776330462	1
4933421A08Rik	1,77559186	1
Rps15a-ps5	1,77546879	1
Gm42908	1,775345728	1

Treml4	1,775345728	1
Rnu11	1,77436154	1
Pfkfb3	1,772886281	0,75819
9330102E08Rik	1,772886281	1
Lig1	1,771903457	0,31655
Gm15501	1,771289468	0,85568
2410080I02Rik	1,771166696	1
Rps4x-ps	1,769816764	1
4931428F04Rik	1,769448779	1
Vps51	1,768958252	0,78876
Gm15530	1,768835641	1
Ift80	1,767977605	0,87916
Gon7	1,766752561	0,58493
Sft2d1	1,766752561	1
Gm15289	1,766385213	1
Socs2	1,766385213	1
Ccdc134	1,765283628	1
Atp5g2	1,765038925	1
Dis3l2	1,763693663	0,99433
Tle6	1,763204731	1
Rpsa-ps4	1,762715935	1
Depdc1b	1,76210513	1
Med22	1,761494536	0,71713
Cfp	1,759542059	1
Tbc1d31	1,75832286	0,7687
Cenpk	1,758079122	1
Dock6	1,758079122	1
E230032D23Rik	1,757469924	1
G6pc3	1,757348109	0,75896
Gm45749	1,755400228	1
Eno2	1,755035241	0,61999
Fsd1l	1,755035241	1
Rpl31-ps1	1,754062313	1
Adarb1	1,753940734	0,97386
Gm12469	1,752725417	1
Cdc6	1,752482454	1
Bloc1s1	1,752118073	1
RP24-418P10.4	1,751510941	1
Aim1l	1,751268147	1
Platr3	1,751146762	1
Nucb1	1,750539966	0,37975
Tsc22d2	1,750297307	0,41207
Col11a2	1,74896328	1
Gm12479	1,74896328	1
Gm5944	1,747751411	1
Atf7	1,747630271	0,80292
Arl14epl	1,747509138	1
Kif20b	1,747388014	0,8302
Pygl	1,746782521	1
Chchd10	1,746540382	1
Gm38319	1,746298277	1
Gm5239	1,745693161	1

Med16	1,743758199	0,87445
Gm12604	1,743516479	1
Mtl5	1,743274793	1
Gm14794	1,743153963	1
Zfyve28	1,742791522	1
A230028O05Rik	1,742670725	1
Oas1d	1,742549936	1
Cnnm2	1,742066866	1
Ccdc117	1,741704651	0,60655
Gm37678	1,739894705	1
Rpl36	1,739412371	1
Capza1	1,738809641	1
Gstt2	1,738086641	1
Ift88	1,737725253	1
C330006A16Rik	1,737363941	0,70844
Zfp511	1,737363941	0,97665
C430042M11Rik	1,736761921	1
Nudt19	1,736039772	0,91425
Gm11989	1,735438211	1
Rpl27-ps3	1,734716613	1
Vdac3-ps1	1,734716613	1
Zeb2os	1,734476146	1
Gm20689	1,734235714	1
BC055324	1,73411551	0,82683
Gm42567	1,733514615	1
Hcn2	1,733514615	1
5031425F14Rik	1,733394461	1
Gm17150	1,731953264	1
Pnpt1	1,731833218	1
Uggt2	1,731833218	1
Gm20223	1,731833218	1
Mad2l1	1,731593152	1
Atf5	1,730633219	0,67619
Tmsb10	1,730153452	1
Gm10012	1,729793714	1
Gm38043	1,729673818	1
Nadk2	1,727996146	0,93871
Eri3	1,727996146	1
C78859	1,727277642	1
Kifc2	1,727157921	1
Pltp	1,726798806	0,57246
Trmt112	1,726798806	0,88097
Uba52	1,726798806	1
Pmaip1	1,726679117	0,8302
Gm13998	1,726679117	1
Ccnj	1,726559437	1
Rpl30-ps2	1,726200446	1
Ppfia3	1,725961161	0,99433
Gm26737	1,723809082	1
Edil3	1,723689601	0,89904
Tgif2	1,722972888	0,88644
Icam4	1,721182408	1

Gm13498	1,720824536	1
1110035H17Rik	1,720228247	1
Pxylp1	1,717964232	1
Bsdc1	1,717249899	1
Mif	1,715584278	0,87959
Pabpc4	1,715227569	0,7038
Prr11	1,714395538	1
Hsf2bp	1,714395538	1
Cox5b	1,714157888	1
Gna12	1,713801476	0,98276
Gm11531	1,71356391	1
Ube2v1	1,713445138	1
Bnip3l	1,712376569	0,90176
Epm2a	1,71225788	1
Gm7504	1,71225788	1
AV356131	1,711071445	0,76524
5730508B09Rik	1,711071445	1
Zfp326	1,710715675	0,62415
Cbx5	1,710597101	0,60655
Fancb	1,710597101	1
Gm28557	1,710597101	1
B230219D22Rik	1,710359978	0,69628
Ndufa4l2	1,710004356	1
8430429K09Rik	1,709767315	1
RP24-84C23.4	1,709648807	1
Lnpep	1,709648807	1
C87436	1,709648807	1
Tia1	1,709174858	0,96965
Gm43569	1,709056391	1
Hnrnph2	1,709056391	1
Mogat1	1,709056391	1
Itgb3bp	1,709056391	1
Oas1b	1,708345762	1
Spsb2	1,708227353	1
Ccdc17	1,707517069	1
Fbxo5	1,706215648	1
Serpini1	1,705269784	1
9230116N13Rik	1,704442582	1
Gm23849	1,704324443	1
Snrpc	1,704206313	1
Gm8430	1,703970076	0,91009
Ppp2cb	1,703379627	1
Ywhah	1,702435335	1
Gm14650	1,702435335	1
2610318N02Rik	1,701609508	1
Foxj3	1,70031259	0,99433
Hip1r	1,699605597	1
Nfatc2	1,699487794	0,96902
Kcnd1	1,698074787	1
RP23-325K4.10	1,69795709	0,91122
Nfkb2	1,697015803	1
Pyroxd2	1,696898179	1

Tra2a	1,696310181	0,63408
Gm11868	1,696310181	1
Gm42576	1,695252298	1
B3gnt6	1,695134796	1
Atxn2l	1,695017302	0,71713
Plxna3	1,694899817	1
Rps6-ps4	1,694312511	1
Gm13712	1,694312511	1
Rps3a1	1,692903807	1
Htr2b	1,692786468	1
1700061G19Rik	1,6924345	0,96965
Gm12280	1,6924345	1
Ahdc1	1,692199895	1
Hist1h2bc	1,692082604	0,82508
Rps15a-ps7	1,692082604	1
Gm45630	1,691379033	1
RP24-351I17.3	1,691379033	1
Rpl32-ps	1,690792948	1
Rasgef1b	1,690441394	0,9085
Coa6	1,690089913	1
RP23-205H11.3	1,689270075	1
Gm11539	1,688918838	0,71713
Abcg4	1,687982562	1
Arsa	1,687748574	1
Flt1	1,687397653	1
Vwa8	1,687280696	1
Espl1	1,686812947	1
Prr3	1,686228443	1
Gm6640	1,685877839	1
Tmem176a	1,685644142	0,91122
Prelid3a	1,684592911	1
Siglec1	1,684476148	1
Tma7	1,684359393	1
Gm13038	1,684009177	1
Gm5601	1,683659033	1
AA474408	1,683659033	1
Zdhhc18	1,683192288	0,87195
Gbe1	1,683192288	0,90649
Ankzf1	1,683192288	1
2900076A07Rik	1,682492414	1
Gm11889	1,682259187	1
Rps10-ps2	1,681676262	1
Gt(ROSA)26Sor	1,681559701	0,93527
Ncapg	1,681559701	1
Gm12164	1,681443148	1
Otub1	1,681093538	0,91122
Cenpn	1,6801616	1
Gm9008	1,6801616	1
Atp6v0c	1,679928696	1
Trp53rkb	1,679346578	1
Jund	1,678531952	0,70124
Gys1	1,678415609	0,709

Map3k8	1,677717721	0,93621
Rps19-ps12	1,677717721	1
Cdhr4	1,677717721	1
Gm14769	1,677601434	1
Gm14279	1,676903885	1
Gm12090	1,676439012	1
Smco3	1,676322815	1
Gm9521	1,676206625	1
Rpl31-ps8	1,675741946	1
Igf1	1,675625797	0,76982
Cdkl3	1,67469689	1
Gm9143	1,674580813	1
Kremen1	1,674116585	1
Pyurf	1,674116585	1
Tmx2	1,673188515	1
1700003G18Rik	1,672956578	1
Fancd2	1,672608732	1
Klkb1	1,670754778	1
Gm2986	1,670638974	1
Blm	1,67040739	1
Gm23127	1,67040739	1
Nek2	1,670175839	1
Id1	1,669249953	0,96965
Rpl30-ps1	1,668555876	1
mt-Co1	1,668093318	0,75366
Gm42851	1,667515302	1
Naa60	1,667168588	0,91122
Gm12089	1,667168588	1
Hsp90aa1	1,666937485	0,86224
Rps6-ps3	1,666706414	1
Tsga10ip	1,666706414	1
Fam134c	1,666590891	1
Parp6	1,666359868	0,97884
Atp5l-ps1	1,666013395	1
Rps7	1,665897919	1
Tgfb1	1,665666993	0,89904
Batf2	1,665205236	1
Anp32-ps	1,664859002	1
RP23-114G13.7	1,664743607	1
Slx1b	1,664282106	1
Gm44258	1,663820733	1
Chaf1b	1,66370541	1
Gm24601	1,663474787	1
Gm44291	1,663474787	1
Gm45220	1,663359488	1
Map3k12	1,663013638	1
Arsb	1,662783111	1
Cir1	1,661515786	0,96868
9130604C24Rik	1,660249426	1
Nxn	1,659444064	1
Gm13368	1,659444064	1
Rdh13	1,658064351	0,88803

Parpbp	1,658064351	1
Gm26397	1,65783451	1
Gm37558	1,657489809	1
Cox7a1	1,657489809	1
Gm5745	1,657145179	1
Morn2	1,657145179	1
Lonrf3	1,656456135	0,96062
Coq10b	1,655882151	1
BC051226	1,655537856	1
Raly	1,655308365	1
Clec4a2	1,6545054	1
Hist1h1e	1,654161391	0,96868
Lamtor3	1,653817453	1
Aldh6a1	1,653473587	1
Gm13803	1,653015211	1
Pnp	1,652900636	1
Ccdc15	1,652671512	1
Rpl19-ps9	1,65175533	1
2310047D07Rik	1,651640843	1
Zfp78	1,651182974	1
Metap1d	1,650954088	1
Spire1	1,650496409	0,98276
Gm13450	1,650267618	1
Car13	1,650153234	1
Prss35	1,649352769	1
Accsl	1,649352769	1
Klhl40	1,649352769	1
Lin28b	1,649352769	1
Robo3	1,649009831	1
H2-Q7	1,648209921	1
Gm16045	1,648209921	1
Anapc5	1,646953702	0,76857
Acad10	1,646839548	1
Sowahc	1,646497133	1
Tpt1-ps3	1,6459266	1
Ankrd35	1,645014157	1
Gdap10	1,644900137	1
Gm5446	1,644672122	1
4933404O12Rik	1,644558126	1
Mocs1	1,644330157	1
Gm6272	1,64410222	1
Hipk2	1,643646442	0,96965
Slc25a38	1,643532517	1
Pea15a	1,643304691	0,99433
Rpl13	1,643304691	1
Pclaf	1,642963011	0,94611
Gm8268	1,642279863	1
Cnep1r1	1,641597001	1
Ksr1	1,641483218	1
Aldh1l1	1,640914421	1
Gm17100	1,640914421	1
Wdcp	1,640800686	1

Thap8	1,640686958	1
Mroh2a	1,640573238	1
2610203C22Rik	1,640459526	1
Rarg	1,640232126	1
Gm12504	1,640118438	1
Rpl21-ps12	1,640118438	1
Gm8894	1,640004758	1
Gm43061	1,63977742	1
Supt20	1,639095598	1
Nsdhl	1,638981988	1
Gm22009	1,638527629	1
Ms4a6b	1,638300497	1
Luc7l3	1,638186942	0,98403
Rpl31	1,637959857	0,83381
Rps19-ps11	1,637619288	1
Ank3	1,636824903	1
Grtp1	1,636711451	1
Nacc1	1,636144308	1
Kifc1	1,636144308	1
RP23-162P10.8	1,636030903	1
Rps13	1,635917506	1
Acvr2a	1,635463997	1
Lgr4	1,635350639	1
Gm8599	1,635237289	1
1810010D01Rik	1,635237289	1
Gngt2	1,635010613	1
Slfn9	1,634897286	1
Gm4617	1,634783968	1
Dock2	1,634557354	1
Gm8394	1,634330772	1
Gm21057	1,634330772	1
Kat2b	1,634217493	1
2010015M23Rik	1,634104221	1
2610016A17Rik	1,633877702	1
Spcs2-ps	1,633877702	1
Hmmr	1,633764454	1
Metrn	1,633424758	1
Gm5873	1,633198333	1
Svep1	1,632066678	1
6430710M23Rik	1,632066678	1
Klf6	1,631840441	1
Rpl12-ps1	1,631388062	1
Gng5	1,630370667	1
Ubald2	1,629128045	1
Cdh23	1,628902215	1
1700022N22Rik	1,628902215	1
Gm45251	1,628112059	1
Prcc	1,627435087	1
Prrg4	1,627322285	1
Lif	1,627322285	1
Gm4987	1,626871158	1
Gm17249	1,626758396	1

Dhrs3	1,626532895	1
Arhgap27os2	1,626420157	1
Stard4	1,625067901	0,96062
Asb10	1,624955264	1
Rusc1	1,624504793	0,99967
Cdca2	1,624392195	1
Atl3	1,623379162	1
Gm8463	1,622929128	1
Nr4a3	1,622704158	1
Ifi211	1,622591685	1
Gm6394	1,622254311	1
Fam13a	1,621917008	1
Gm8317	1,621804589	1
Atr	1,621692178	1
Gnb1l	1,621692178	1
Gm45153	1,621579775	1
Gm6418	1,621467379	1
Creld2	1,621354992	1
Gm15210	1,621242612	0,99476
Iqcc	1,621017875	1
Vcpkmt	1,62079317	1
Gm11298	1,620680829	1
Gm43110	1,620568496	1
Ap3s1	1,620456171	1
Fndc7	1,620456171	1
Rpl9-ps7	1,619782382	1
Numb	1,619445593	1
Toporsos	1,619333345	1
Gm6543	1,619108874	1
Esr1	1,619108874	1
Dusp28	1,618660024	1
Spc24	1,618435645	1
Gm37334	1,618435645	1
Rnf32	1,617538443	1
Gm42522	1,617426327	1
Rpl9	1,616977944	1
Gm7887	1,616193571	1
Fam71e1	1,616081549	1
Iqgap3	1,615857528	1
Rpsa	1,615185652	0,96868
Gm44126	1,614849818	1
Klc2	1,614402149	1
Rdm1	1,614066478	1
Plekhh2	1,614066478	1
Slc35a2	1,613730878	1
Lsm5	1,613507183	1
Gm23502	1,613507183	1
2900060B14Rik	1,613283518	1
Sigirr	1,613171698	1
Ift57	1,613059885	0,99293
Brms1l	1,61205392	1
Sdc4	1,611942185	0,98276

Etfdh	1,611718738	1
Socs4	1,611607026	1
Tmem35b	1,610601967	1
Gm5869	1,610490332	1
Gm6493	1,610378705	1
Crebrf	1,609374412	0,96259
Speg	1,609374412	1
Gm15013	1,60892826	1
Tpt1	1,608816742	1
AI839979	1,608482233	1
Rps18-ps3	1,607813423	1
Timeless	1,607813423	1
Cmpk1	1,607144891	1
Med14	1,606810729	1
Gm2058	1,606365289	1
Pvr	1,606031289	1
Rnd2	1,605363498	1
Il16	1,605363498	1
Gm18969	1,605029707	1
Kif5c	1,604807218	1
Gm26631	1,604807218	1
Pim1	1,604695985	1
Cox20	1,604473542	1
Vamp1	1,604362333	1
Sec22c	1,604251131	1
Borcs8	1,604139936	1
Cyp2c55	1,60402875	1
Fam172a	1,602917307	1
Mef2c	1,602695111	0,90649
Gm2467	1,602584024	1
Stxbp3	1,602361874	1
Psd2	1,601473583	1
B130034C11Rik	1,601362581	1
Gm45873	1,601251587	1
RP23-213P10.2	1,60091865	1
Smtn	1,600696731	1
Tnf	1,600585783	1
Cox16	1,600585783	1
Nfil3	1,599809362	1
Id3	1,598257651	1
Gm13578	1,598146872	1
Rasgrp4	1,597925337	1
Hist1h4i	1,597039505	1
Mxd1	1,596486109	1
Zfp81	1,596264804	1
Fabp3	1,59604353	1
Cacnb1	1,595822287	1
Gm20072	1,595601074	1
Acadsb	1,595379892	1
Gm9517	1,594716531	1
Supt4a	1,59416394	1
Tsix	1,59416394	1

Gm2000	1,593942957	1
Gm18943	1,593501083	1
Pxmp2	1,592948914	1
Slc31a2	1,592176198	1
Carnmt1	1,59206584	1
Gm43096	1,591845148	1
Dnajb6	1,591734814	0,99168
Gm1862	1,591734814	1
AI480526	1,590962687	1
Papd4	1,59063189	1
Gm43331	1,589860297	1
Crry-ps	1,589199231	1
Rpl27a-ps2	1,589089079	1
Phlda1	1,588978936	1
Dpp7	1,587987988	1
RP24-324J2.1	1,5873277	1
Lilrb4a	1,587217679	1
Haus7	1,587107665	1
Eif4a2	1,58688766	1
Gm26826	1,586667686	1
Gm4374	1,586337782	1
Pcyt2	1,585128721	1
AW554918	1,584908991	1
Nfix	1,583920582	1
Kpna4	1,583371732	1
Zfp87	1,583261985	1
Gm37677	1,583152245	1
Mboat7	1,582932789	1
4933408B17Rik	1,582603661	1
Gm10642	1,582164931	1
Rps8	1,582055268	0,84226
C630004M23Rik	1,58161669	1
Ccl5	1,581287836	1
Zic2	1,581178233	1
Adgre1	1,580959051	0,99433
Gm43200	1,580849471	1
Pcgf1	1,580411227	1
2700029L08Rik	1,580192151	1
Adamts10	1,579973105	1
4933421O10Rik	1,579973105	1
Olfr286	1,579973105	1
2900009J06Rik	1,579425622	1
Gm12309	1,579425622	1
4833421G17Rik	1,578987773	1
Zfp882	1,578659466	1
Ppp1r37	1,578550046	1
4632404H12Rik	1,578440633	1
Gm8250	1,578440633	1
Gm28578	1,578112439	1
Tbx6	1,577674954	1
Fcor	1,577565602	1
Ccdc159	1,57734692	1

Gm16238	1,577237591	1
Snora31	1,576909647	1
Rsph1	1,576472496	1
Rps23	1,576144712	1
Dusp4	1,575816996	1
Pbk	1,575380147	1
Gm8522	1,575270954	1
Gm6565	1,574943419	1
Cebpb	1,574943419	1
Yae1d1	1,574725101	1
Smarce1	1,574615953	1
Scp2-ps2	1,574615953	1
Smagp	1,57439768	1
Gm16379	1,57439768	1
Gm15625	1,574288555	1
Sfi1	1,574179437	1
Dnaaf3	1,574179437	1
Gtpbp2	1,57385213	1
Olfr921	1,57385213	1
2610002M06Rik	1,572761597	1
Papss1	1,572434584	1
Eva1b	1,572216614	1
Rpl5	1,57210764	1
Vamp7-ps	1,571889714	1
Gm17541	1,571889714	1
Gm43466	1,571780763	1
Plaur	1,571562883	1
Cox7c	1,571453954	1
Ropn1l	1,57123612	1
Gm12791	1,57123612	1
Flt3l	1,571127214	1
Pcdhgc4	1,570691665	1
Ercc6l	1,570147399	1
Ypel5	1,570038569	0,99584
Asf1b	1,570038569	1
Nbn	1,570038569	1
Shcbp1	1,56982093	1
Zfp9	1,569276966	1
Abi2	1,569168196	1
Dennd6b	1,569059433	1
Gm12844	1,568950678	1
Dlgap5	1,56884193	1
Errfi1	1,567211621	1
Mapk8ip3	1,566777157	1
Ormdl3	1,566234248	1
Zfp932	1,566234248	1
Mnd1	1,565908593	1
Ogfod3	1,565908593	1
Crem	1,565474491	1
Rnf19a	1,565365984	1
Atad5	1,565365984	1
Tmed7	1,565148993	1

Galnt6	1,564932033	1
Gm6142	1,564932033	1
1600014C10Rik	1,564932033	1
Gm16425	1,564932033	1
Rpl23a-ps2	1,564932033	1
Rnf181	1,564823563	1
Svip	1,564823563	1
4930542C12Rik	1,564715102	1
Pdia3	1,564498201	1
Abhd8	1,564498201	1
Gm12833	1,564498201	1
Chtf18	1,563956082	1
Gm43420	1,56384768	1
Scn11a	1,563522521	1
Gm10463	1,563522521	1
Slc16a10	1,56341415	1
Dgkh	1,563089081	1
Tmem107	1,562872405	1
Rpl3	1,562547449	1
Gm22716	1,562547449	1
Gm10736	1,56222256	1
Hspe1	1,561897739	1
Gm12038	1,561897739	1
Pabpc1l	1,56178948	1
Bend3	1,561681229	1
Hap1	1,561572985	1
Pnrc1	1,561248299	1
Cystm1	1,55962588	1
Cpt1c	1,559085449	1
Gm6341	1,558977385	1
Col4a6	1,558869329	1
Hist3h2a	1,558545205	1
Itga11	1,558437178	1
Aplf	1,556709774	1
Gpr179	1,556386099	1
Plch2	1,556386099	1
Cep70	1,554660968	1
Tcf19	1,554660968	1
Crcp	1,554337719	1
Ptgs2	1,554337719	1
Gm5262	1,554122257	1
Jpx	1,553906825	1
Gm20186	1,55379912	1
H2-K2	1,553691423	1
Gm10343	1,553583733	1
Pafah1b1-ps2	1,553260708	1
Tpx2	1,553153048	1
Gm5384	1,553045395	1
Ppp1r18os	1,552722482	1
Gm28555	1,551431501	1
St3gal4	1,550893909	1
Arfgap2	1,550786413	1

Rpl36-ps10	1,550571443	1
Rpl7a-ps5	1,550463969	1
Gm5614	1,550356503	1
Smg6	1,550141593	1
Gm43133	1,550141593	1
Ablim1	1,550141593	1
Gm14620	1,549819283	1
Gipr	1,549711862	1
Rragb	1,549604448	1
Exosc3	1,549497041	1
RP23-390D8.2	1,549174866	1
Hjurp	1,549067489	1
Rpl6	1,548745403	1
Nlgn2	1,548638056	1
Lsm11	1,548101432	1
Rbx1	1,547457728	1
Pabpc1	1,547457728	1
Gm12165	1,54735047	1
Gm16177	1,54724322	1
Gm5445	1,546063956	1
Ppwd1	1,545742494	1
Rap1gds1	1,545206873	1
Ncapg2	1,54488559	1
1700031P21Rik	1,54477851	1
Ppia	1,544564373	1
Atp6ap1	1,544350266	1
Gm44777	1,544350266	1
A530013C23Rik	1,544243224	1
Slc35a1	1,544029161	1
Il27	1,544029161	1
Trappc9	1,543708123	1
Tuba1a	1,543387151	1
Gm13094	1,543387151	1
Cfh	1,543173207	1
Cbr2	1,542959292	1
Aldh5a1	1,542852346	1
Ubfd1	1,542531552	1
RP23-246F14.1	1,542424636	1
Gm6285	1,541783293	1
Nnt	1,541462722	1
Hbb-bh3	1,541462722	1
Hpse	1,541355879	1
Rps2-ps5	1,541355879	1
A830073O21Rik	1,541355879	1
RP24-550H10.3	1,540714981	1
Ccdc9	1,540287863	1
Gm26983	1,540181102	1
Gm42478	1,540074348	1
Gm16124	1,539860864	1
Nck1	1,539647408	1
Mex3d	1,5391139	1
Rab2b	1,53900722	1

Ctsh	1,53900722	1
4930509H03Rik	1,538367298	1
Zmym1	1,53826067	1
Rps8-ps3	1,538047437	1
Dmpk	1,537727642	1
Palb2	1,537621059	1
Igsf6	1,537407914	1
Gm2367	1,536875181	1
A930024E05Rik	1,536875181	1
Macrod1	1,536449128	1
Plcl2	1,536449128	1
Scel	1,536449128	1
Gm27003	1,536449128	1
Gm32175	1,536023193	1
Tomm40l	1,535916728	1
Gm5881	1,53581027	1
2210408I21Rik	1,53549094	1
Pmf1	1,535171677	1
Cep44	1,534426988	1
Fcnaos	1,53378897	1
Nkapl	1,533576357	1
Vps8	1,533470061	1
Nuf2	1,533257491	1
Ndfip1	1,533044952	1
Fis1	1,53261996	1
Rab1b	1,532301294	1
Psat1	1,531770331	1
Syt11	1,531451841	1
Vhl	1,530815061	1
Gm15634	1,530602859	1
Gm16754	1,530072485	1
Rps19-ps7	1,529860386	1
Ndufb4	1,529648317	1
Icam5	1,528588413	1
Gm38375	1,528588413	1
Pcnp	1,528482463	1
Slc31a1	1,527952824	1
Pigt	1,527952824	1
Lmo2	1,527846918	1
Ift27	1,527846918	1
Gm6140	1,527741019	1
Dedd2	1,527635128	1
Gm6548	1,527529244	1
Gm4705	1,527529244	1
B230322F03Rik	1,527529244	1
Crlf2	1,526788262	1
Ezr	1,526576619	1
Maged1	1,52615342	1
Gm13675	1,52615342	1
Oaz2	1,525941865	1
Gm11605	1,525518843	1
Gm11353	1,525413106	1

Gm12481	1,525307376	1
H2-T22	1,525307376	1
Usp53	1,525201653	1
Ckap2	1,52499023	1
Notch1	1,524778836	1
Exd2	1,5244618	1
Ptrh1	1,524356136	1
Rgmb	1,524356136	1
Gm13886	1,52414483	1
Chchd6	1,523722306	1
St6galnac6	1,523616694	1
Gkap1	1,522772055	1
Senp3	1,522455437	1
Aen	1,522349912	1
Klhl6	1,522138884	1
RP24-82M14.1	1,522138884	1
Fxn	1,521927885	1
Gm24924	1,521927885	1
Ggps1	1,521822397	1
Cd36	1,521611442	1
Tufm	1,521611442	1
Pou6f1	1,520873331	1
Fam53b	1,520767915	1
H2-Q6	1,520030213	1
Hacd4	1,519714165	1
Ndrg3	1,519714165	1
Gm43737	1,519714165	1
Higd1a	1,519503502	1
Tpcn2	1,519503502	1
2210408F21Rik	1,519398182	1
Gm19353	1,519187563	1
Trip10	1,518766413	1
Ccdc167	1,518661144	1
Clec12a	1,518450628	1
Rps6-ps1	1,518450628	1
Gm11930	1,517924464	1
Stab1	1,517398482	1
Gm8806	1,517398482	1
Bscl2	1,517293308	1
Arl5a	1,517293308	1
Snord104	1,516977829	1
Gm37339	1,516662415	1
Snx12	1,516241965	1
Usp26	1,515926704	1
Cldn11	1,515506458	1
Dynlt1f	1,514981315	1
Cdc34b	1,514771308	1
C920021L13Rik	1,514666316	1
Gm1976	1,514666316	1
Cox6a2	1,513616793	1
Dennd5a	1,513197187	1
Vkorc1	1,512672844	1

Ifitm2	1,512567997	1
4933439C10Rik	1,512463158	1
Ghdc	1,511834273	1
Ccna2	1,510786712	1
Hexdc	1,510786712	1
Cdk5	1,510577287	1
Rsrc2	1,510472586	1
2410004B18Rik	1,510367891	1
Urm1	1,510367891	1
Gins2	1,509949186	1
Abca7	1,509844528	1
Oxct1	1,509739877	1
Lfng	1,509321347	1
Stom	1,509216732	1
Pex5	1,509112125	1
C920009B18Rik	1,508798346	1
Cblb	1,508798346	1
Bend4	1,508693768	1
Trpm2	1,508589197	1
Tmx4	1,508170985	1
Pcif1	1,50806645	1
Triobp	1,507752889	1
Tmed8	1,507439394	1
Prelid1	1,50733491	1
Rbm48	1,507230433	1
Top2a	1,507021501	1
Aurkb	1,506708157	1
Wfdc17	1,506290467	1
Rpl35	1,505768517	1
Ndrg1	1,505664148	1
Cep135	1,505455433	1
Crnkl1	1,505351087	1
Cdk2ap1	1,505351087	1
Rnf185	1,505142415	1
D630029K05Rik	1,504620863	1
Rps6ka5	1,504516574	1
Fgfbp3	1,504412293	1
Isoc2a	1,503995239	1
Tcn2	1,503682524	1
Jun	1,503474084	1
Foxd2os	1,503474084	1
Tmem259	1,50305729	1
Gm43178	1,502536461	1
Inafm1	1,502328179	1
Lacc1	1,502119927	1
G2e3	1,501807602	1
Tcea1-ps1	1,501599422	1
Phkg1	1,501495343	1
Gm12854	1,501495343	1
Sptbn4	1,501391271	1
Gm37914	1,500662968	1
Polg2	1,500350946	1

Mipep	1,500142968	1
Fancc	1,499935018	1
Nfkbid	1,499831054	1
Rad54b	1,499727097	1
Zfand5	1,499103508	1
Pcbp4	1,498999602	1
Clu	1,498895703	1
4930461G14Rik	1,498791811	1
Gm11110	1,498480178	1
Rrm1	1,497960934	1
Fam46a	1,497649475	1
Gm5914	1,497649475	1
Snd1	1,497234296	1
Coa4	1,497234296	1
Gm12848	1,497026749	1
Ptpru	1,496922987	1
Dock1	1,496508009	1
Gm14138	1,496404283	1
Gm13868	1,496404283	1
Ada	1,496093147	1
Fkbp14	1,495056493	1
Amhr2	1,494952867	1
Gm5841	1,494745637	1
Calm3	1,494642033	1
Mob1b	1,494538436	1
Zfp873	1,494538436	1
Rpl18a-ps1	1,494434846	1
Isyna1	1,494020558	1
Rapgef5	1,493813457	1
Gm19196	1,493192327	1
Dpy19l3	1,492778383	1
Ccdc25	1,492571455	1
RP23-350F7.3	1,492571455	1
Ap2s1	1,492364555	1
Bicd2	1,492364555	1
Mir5128	1,492364555	1
Eda2r	1,492261115	1
Bet1	1,492157683	1
Gm15159	1,491950841	1
Gm37962	1,491950841	1
Gabarapl2	1,491744027	1
Gm10076	1,49164063	1
Gm13005	1,49164063	1
Arsg	1,491227117	1
Plpp5	1,490917057	1
Rcbtb2	1,490710387	1
Megf8	1,490710387	1
Hdac2	1,490503745	1
Haghl	1,490297131	1
9130230L23Rik	1,490297131	1
4930532G15Rik	1,489987265	1
Atf1	1,489883991	1

Rps24	1,489677463	1
Nipsnap3b	1,48957421	1
Mgst3	1,489367726	1
Gm37733	1,489367726	1
Sort1	1,489264494	1
Serpinf2	1,489058052	1
Gm7117	1,488232572	1
Mastl	1,487820004	1
Pnrc2	1,48771688	1
Zfp787	1,487201365	1
Gm23344	1,487201365	1
Gm6085	1,48699521	1
Patz1	1,48668603	1
Pgk1	1,486582984	1
Gm6368	1,486170873	1
E130208F15Rik	1,486170873	1
Ntan1	1,486067863	1
Tstd1	1,48596486	1
Kif24	1,48596486	1
Eif1b	1,485655895	1
Dnal1	1,485655895	1
Gm5544	1,485141095	1
Ccdc58	1,485038156	1
Kpnb1	1,484729384	1
Osbpl3	1,484523571	1
Spata6	1,484214905	1
BC030499	1,484214905	1
Lrwd1	1,48411203	1
Rpl15	1,48411203	1
Snord35a	1,483906303	1
Gm16106	1,483700604	1
Jtb	1,483597765	1
Gm7380	1,483597765	1
Bst2	1,483494934	1
Pnn	1,483083679	1
1810037I17Rik	1,483083679	1
Myg1	1,482775312	1
Meiob	1,482672538	1
Lxn	1,48205604	1
Lpp	1,481850597	1
Polr3g	1,481747887	1
Dhcr7	1,481542487	1
Ddah2	1,481542487	1
Gnas	1,481234441	1
6230400D17Rik	1,481234441	1
Rps2-ps11	1,481029113	1
Gm43533	1,480926459	1
C330027C09Rik	1,480823813	1
Recql	1,480823813	1
Pde1b	1,480721173	1
Zfp212	1,480515916	1
Taco1os	1,480515916	1

Gm5865	1,480413298	1
Fam187b	1,480413298	1
Rbm4b	1,480002897	1
Tjp3	1,480002897	1
Cep120	1,479900315	1
Erap1	1,47959261	1
Gm5921	1,479490056	1
Pkd1l2	1,479182437	1
Gins1	1,478977393	1
Pdlim5	1,47866988	1
Gm10698	1,47856739	1
Tas1r1	1,478464907	1
Gm9354	1,478259963	1
Reep5	1,478055046	1
Anln	1,477645299	1
Mipol1	1,477645299	1
D16Ertd472e	1,477440468	1
Ier3	1,477235666	1
Celf6	1,477235666	1
Rpl37a	1,477030892	1
Mcoln1	1,476928515	1
Stn1	1,476826146	1
Mapre2	1,476519081	1
Hn1l	1,476519081	1
Klra2	1,474780249	1
Zfp280d	1,474371409	1
Gm3695	1,474371409	1
Rai1	1,474269217	1
Gm13573	1,474064854	1
Sdf2l1	1,473962683	1
Mycbp	1,47386052	1
Rnf121	1,47386052	1
Gm11942	1,473758363	1
Nr2c2ap	1,473656213	1
Crebzf	1,473656213	1
Tnfrsf9	1,473247686	1
D730045B01Rik	1,473043464	1
Krtcap3	1,473043464	1
2810408I11Rik	1,472941364	1
Erf	1,472839271	1
Ero1l	1,472737186	1
4931440P22Rik	1,472737186	1
Dvl2	1,472022782	1
Tapbpl	1,471818731	1
Plekhh3	1,471410713	1
Gm6419	1,471410713	1
Usp28	1,471308726	1
Gm13862	1,471308726	1
1110020A21Rik	1,471308726	1
Atp11b	1,471104773	1
Gm15877	1,470798897	1
Gm44639	1,469983539	1

Rpl35a-ps5	1,469881651	1
Mcts2	1,46957603	1
Nup205	1,469372317	1
Basp1	1,46876135	1
Fem1c	1,468455961	1
Dnajc15	1,468455961	1
Raf1	1,468455961	1
Gm3724	1,468354179	1
Rps15a	1,468252404	1
Dtl	1,468252404	1
Rab10os	1,468150636	1
Pole2	1,468150636	1
Tcf20	1,468048875	1
Med30	1,467947121	1
Gm5045	1,467743635	1
Bcl2	1,467540176	1
Map3k9	1,467336746	1
Dram1	1,467235042	1
Gm9575	1,467031654	1
Trp53	1,46692997	1
Wdr83os	1,466523307	1
Kif4	1,466523307	1
Gm15393	1,466218384	1
Nos1	1,465913524	1
Eif1-ps1	1,46571032	1
Chp1	1,465405565	1
Selenom	1,465100875	1
Spred3	1,464796247	1
Prim2	1,464796247	1
Rprd1a	1,464593197	1
Clspn	1,464288675	1
Acot9	1,464187182	1
Oscp1	1,463984217	1
Taz	1,46357837	1
Arid5b	1,46357837	1
Aacs	1,46357837	1
Acd	1,463476926	1
Cnst	1,463172637	1
Meis3	1,46286841	1
Gm10275	1,462767015	1
Ttc21b	1,462361506	1
4930404I05Rik	1,462158794	1
Mmgt1	1,461449522	1
Exoc6	1,460841849	1
Capn15	1,460234428	1
Aph1a	1,458818095	1
Gm43309	1,458818095	1
Pmvk	1,458413681	1
Alg10b	1,458413681	1
C030014I23Rik	1,458211516	1
Pcsk7	1,457908321	1
Csrnp2	1,457908321	1

Cib2	1,457706226	1
Dusp11	1,457605189	1
Fahd1	1,457605189	1
Fam234b	1,45730212	1
Gm13827	1,457201111	1
2610528A11Rik	1,457100109	1
Pam16	1,457100109	1
Pdk3	1,456595204	1
Ppp1r15a	1,455989549	1
Nono	1,455182401	1
Smarcal1	1,455182401	1
Wdr83	1,455081539	1
Smim13	1,455081539	1
Hace1	1,455081539	1
Tmem50b	1,454174095	1
RP23-269H21.1	1,453670204	1
Gm15446	1,453670204	1
Mettl23	1,453367953	1
Rassf1	1,452562258	1
Rpl27a-ps1	1,452461578	1
Papd5	1,452058925	1
Gm9385	1,45175701	1
Ddit4	1,450650522	1
Rpl27	1,450248372	1
Camk2g	1,450047339	1
Gm3608	1,449846334	1
Gm7776	1,449745841	1
Ankrd37	1,449645356	1
Gm5867	1,449645356	1
Calml4	1,449243485	1
Cadps	1,449143035	1
Fam98c	1,448540478	1
Ap2b1	1,448239294	1
Slc5a6	1,448239294	1
Gm9800	1,448138913	1
Fcgr2b	1,448138913	1
Peak1os	1,447938172	1
B230217C12Rik	1,447336117	1
Dalrd3	1,446834595	1
Cgrrf1	1,446232999	1
Bicdl1	1,446232999	1
Rhox5	1,446232999	1
Noxo1	1,445832074	1
Nup43	1,445631653	1
2310036O22Rik	1,445531453	1
Il15	1,445431259	1
Txndc11	1,445431259	1
Klk8	1,445331073	1
Gm15832	1,445230894	1
Gm5312	1,445130722	1
Carhsp1	1,445030556	1
Rpl35a	1,444930398	1

Aplp1	1,444830246	1
Cox20-ps	1,444629964	1
AB124611	1,444529834	1
Prune2	1,443729038	1
Arf5	1,44362897	1
Rpl15-ps5	1,44362897	1
2900052L18Rik	1,443528909	1
Mir7078	1,442928687	1
6820402A03Rik	1,442828674	1
Gm16373	1,441828928	1
C130013H08Rik	1,441828928	1
Bcl2l14	1,441728992	1
Pbdc1	1,441529139	1
Gm7266	1,441229413	1
Nup210	1,441129518	1
Gm16020	1,441129518	1
Atf6	1,440929749	1
Il1b	1,440929749	1
Spire2	1,440829875	1
Gm13009	1,440630147	1
Lnpk	1,440230775	1
Tbcel	1,439631924	1
Abcb9	1,439631924	1
Rpl14-ps1	1,43843497	1
Elac1	1,43843497	1
Kdm3a	1,438235574	1
Gm15610	1,438036206	1
Znrf1	1,437836866	1
Phf20l1	1,437737206	1
Oxr1	1,437737206	1
Svbp	1,437537907	1
Gm32340	1,437239011	1
Fer	1,437039781	1
A430105J06Rik	1,437039781	1
Ccdc22	1,436840579	1
Coq8a	1,436641404	1
Prdx4	1,436442257	1
Calcrl	1,436342694	1
Sugp2	1,436243138	1
Zc3h11a	1,435944511	1
AU022252	1,43574546	1
Gm11687	1,435546437	1
Cpne9	1,435247955	1
Gm2223	1,435148475	1
Gm13464	1,435049001	1
Ripk3	1,434949535	1
1700124L16Rik	1,434850075	1
Syap1	1,434551737	1
Rgs14	1,434551737	1
Tmem191c	1,434452305	1
Sep 11	1,434452305	1
Abhd14b	1,434253462	1

Polh	1,43415405	1
Rnf7	1,433855857	1
Prkcg	1,433756473	1
Tmem243	1,433359007	1
Atf3	1,433259657	1
Gpx4	1,43296165	1
Nenf	1,432862328	1
RP24-366E11.4	1,432862328	1
Anapc16	1,432763013	1
Suv39h1	1,432068001	1
Knstrn	1,431968741	1
Rad51ap1	1,431968741	1
Rmnd1	1,431671003	1
Necap1	1,431373326	1
Rnf26	1,430678988	1
Cyp26b1	1,430579824	1
D8Ertd738e	1,430480667	1
Gm43813	1,430480667	1
Fam174a	1,429984986	1
Gm42724	1,429588565	1
Gm2076	1,429390396	1
Rad52	1,429291322	1
Map3k10	1,429192254	1
Fam83a	1,42899414	1
Gm13461	1,428895093	1
Ankrd13a	1,428796053	1
Poc1a	1,42869702	1
Cuta	1,428498974	1
Gm8276	1,428399961	1
Plp2	1,428399961	1
Zfp101	1,428399961	1
S100a3	1,428201957	1
Lin52	1,427707065	1
Gm14253	1,427707065	1
Calr3	1,427608108	1
Prdx2	1,427509157	1
Gm17745	1,427509157	1
Cpq	1,427410213	1
Gps2	1,427212346	1
Gm3511	1,427113422	1
Kiz	1,426915596	1
Rpl36-ps8	1,426816693	1
Gm5257	1,426520026	1
Gm12166	1,426322282	1
Gm45223	1,426322282	1
H1fx	1,425828042	1
Traip	1,425828042	1
Calr	1,425630394	1
Hcar2	1,425333973	1
Car6	1,425333973	1
Mycn	1,424938841	1
Cdc7	1,424938841	1

Isoc1	1,424741317	1
Gm5687	1,424543819	1
Jmjd8	1,424346349	1
Pitpnc1	1,424148907	1
Als2cl	1,424148907	1
Mkrn2	1,423458073	1
BC049715	1,42335941	1
Gm10086	1,423260753	1
Cdca5	1,423162104	1
Slc2a9	1,423063461	1
5031434O11Rik	1,422964825	1
Map2k7	1,422668959	1
Tmem134	1,42257035	1
Gm45222	1,422175985	1
Gm45113	1,422175985	1
Gm42895	1,422077411	1
Timm10b	1,421683182	1
Vps13b	1,421486108	1
Man1a	1,421486108	1
Lamc1	1,421092043	1
Pask	1,420796566	1
Gm45053	1,420698087	1
Gm15728	1,420599616	1
Lyst	1,420402692	1
Fgd2	1,420402692	1
Sep 02	1,420008928	1
Gm16418	1,420008928	1
Rnf217	1,419713676	1
Get4	1,419713676	1
Lmf2	1,419615272	1
Sapcd2	1,419615272	1
Sdhaf2	1,419221726	1
Aven	1,419221726	1
Gm12059	1,419221726	1
Zfp563	1,419123356	1
Smim7	1,419024993	1
Zfp746	1,418828288	1
Ascc1	1,418336645	1
Fam120aos	1,418336645	1
Map4k2	1,418238337	1
Inpp5a	1,418041741	1
Vmac	1,418041741	1
Stk35	1,417943453	1
Apex2	1,417845172	1
Mnd1-ps	1,417845172	1
1700086P04Rik	1,417746898	1
Fiz1	1,41755037	1
Arl4c	1,41705917	1
Caprin1	1,416862738	1
Ndc80	1,416862738	1
Gm8013	1,416764532	1
Gm9769	1,416469954	1

Agtpbp1	1,416175438	1
Tpi1	1,415880983	1
Itgb7	1,415684714	1
Anp32a	1,41558659	1
Suv39h2	1,415390361	1
Lmntd2	1,415292258	1
Nfkbiz	1,41519416	1
Osbpl10	1,41519416	1
Gla	1,41489991	1
4930402H24Rik	1,414703776	1
Mylpf	1,414703776	1
4930431P19Rik	1,41450767	1
Cd5l	1,414311592	1
Celf4	1,414213562	1
Snx21	1,414017524	1
Gm8213	1,414017524	1
Gm4832	1,414017524	1
Gm15772	1,413919516	1
Vsig8	1,413919516	1
Hoxc6	1,413723518	1
Cmtm4	1,41362553	1
Kctd13	1,413527548	1
Snrpa	1,413331605	1
Gm12389	1,413331605	1
Rps15a-ps3	1,413331605	1
Steap3	1,413233644	1
Ubtd2	1,413037742	1
Rpl18-ps1	1,412743939	1
Gm13196	1,412743939	1
Slc25a22	1,412646019	1
2610524H06Rik	1,412352298	1
Tsga10	1,412058638	1
Vat1	1,411862899	1
Psd3	1,411862899	1
Rps4x	1,411765039	1
Ddx11	1,411765039	1
Mthfd2	1,41156934	1
2200002J24Rik	1,411275843	1
Cep83	1,411080213	1
Nop56	1,410493483	1
Syne2	1,410493483	1
Rhou	1,410493483	1
Dnd1	1,410200209	1
Bloc1s2	1,409516142	1
D130051D11Rik	1,409418445	1
Gm11878	1,409320755	1
Bcs1l	1,408930063	1
Raph1	1,408832406	1
Gm8508	1,408344227	1
Gm42571	1,408344227	1
Capns1	1,408149002	1
R3hdm4	1,407953805	1

Ctsf	1,407953805	1
Rnf24	1,407758635	1
Gm7128	1,40766106	1
Gm11895	1,406783189	1
Mrpl42	1,406685682	1
Psmc3ip	1,406490687	1
Naga	1,4063932	1
RP23-70B19.5	1,4063932	1
Ogt	1,40629572	1
Mpc1	1,405808419	1
Gpr157	1,405613546	1
Cox19	1,40551612	1
Pik3r6	1,405223881	1
Klhl7	1,405126482	1
Ndufb2	1,404931704	1
Pam	1,404736952	1
AI662270	1,404639587	1
Idi1	1,404542228	1
Polr2i	1,404444876	1
Rnf126	1,404250192	1
Rgcc	1,40415286	1
Asns	1,4037636	1
Coro1a	1,4037636	1
Bcl2l2	1,403666302	1
D730003I15Rik	1,402790922	1
Tmem97	1,402499251	1
Hes7	1,402499251	1
Lrig2	1,402013266	1
Osgepl1	1,402013266	1
Me2	1,401916089	1
Diaph2	1,401721756	1
Nicn1	1,401624599	1
B4galt3	1,40123604	1
Gyg	1,401138917	1
Mcm7	1,401041801	1
Ahi1	1,400847589	1
Adk	1,400847589	1
Gm14857	1,400750493	1
Rhob	1,400653403	1
Gm27039	1,400168058	1
Kif15	1,400071009	1
Nomo1	1,399876932	1
Pstpip2	1,399779903	1
Gm16580	1,399779903	1
Tarbp2	1,399682881	1
Eif2s3y	1,399585866	1
Dcakd	1,399391855	1
Cdip1	1,399197872	1
Crk	1,399197872	1
AI597479	1,399003915	1
Ube2c	1,399003915	1
Tacc3	1,398519141	1

Birc2	1,398519141	1
C630043F03Rik	1,398519141	1
E230029C05Rik	1,398131443	1
Flot1	1,39784074	1
Gm8818	1,39745323	1
Aoc2	1,39735637	1
Cd47	1,397259516	1
Tmem33	1,397162668	1
1810044D09Rik	1,397065828	1
Armcx5	1,396872167	1
Nup153	1,396872167	1
Cacybp	1,396775346	1
Evl	1,396581725	1
Selenoh	1,396581725	1
Rps19-ps5	1,396388132	1
Arhgap39	1,396097791	1
Sh2b1	1,396097791	1
Lncpint	1,396001024	1
DHRSX	1,396001024	1
RP23-226H21.3	1,396001024	1
E030030I06Rik	1,394937032	1
Cpeb2	1,394743666	1
Adap1	1,394646993	1
Unc13d	1,394357015	1
1110008P14Rik	1,393873851	1
Txlng	1,393680633	1
Dut	1,393584034	1
Emc1	1,393584034	1
Gm5786	1,393004579	1
C130036L24Rik	1,392811481	1
Cenph	1,392714942	1
Bbs4	1,392521884	1
Abhd14a	1,392328853	1
Naa20	1,392328853	1
Ubxn11	1,392328853	1
Pi4kb	1,392232347	1
Leng8	1,392135848	1
Fau	1,391846392	1
Pla2g15	1,391846392	1
Zbtb18	1,39174992	1
Pigq	1,390978384	1
F2	1,390881972	1
Dhcr24	1,390785566	1
Gm6472	1,390592776	1
4921531C22Rik	1,39030364	1
Rpl5-ps1	1,39030364	1
Dclre1a	1,390207275	1
Supt3	1,390207275	1
Gm12267	1,390110917	1
Kcne3	1,390014565	1
Ppp1cc	1,38991822	1
Rhog	1,389821881	1

RP24-225A16.3	1,38972555	1
Mcm3	1,389147699	1
Gm29257	1,388762599	1
Trpm1	1,388473844	1
Iqcg	1,388185149	1
Nxt2	1,388185149	1
Gm12020	1,388088931	1
Slc35a4	1,387896514	1
Gm14673	1,387511761	1
Ap3s2	1,387415589	1
0610037L13Rik	1,387415589	1
Rras2	1,387127114	1
Fmnl3	1,387030969	1
Rpl22l1	1,386934831	1
Gm5871	1,386838699	1
Ccdc94	1,386742574	1
Harbi1	1,386550344	1
Brcc3	1,386454239	1
Dnm1	1,386358141	1
Smc4	1,386069886	1
Acad11	1,385589594	1
Gm26664	1,385301498	1
Ube2cbp	1,385301498	1
Gpsm1	1,38520548	1
RP23-182J19.2	1,385109468	1
Gm13864	1,385013463	1
Gm11826	1,384917464	1
Dnajc7	1,384917464	1
Sun2	1,384725487	1
Gm12693	1,383957845	1
Mta3	1,383766001	1
Pced1b	1,383766001	1
Spns1	1,383190629	1
Prcp	1,383190629	1
Gm44027	1,383190629	1
Zmym6	1,383094757	1
Gm14584	1,382998891	1
Rbl1	1,382711335	1
Gm28417	1,382519664	1
Map3k2	1,382423838	1
Chd3os	1,382136401	1
Tatdn2	1,381944809	1
Plin2	1,381944809	1
Actr1a	1,381657472	1
Gm15903	1,381561706	1
Tcp11l1	1,381561706	1
Decr2	1,381082976	1
Cda	1,381082976	1
Etohd2	1,380700112	1
Kif21a	1,380604412	1
Pfdn4	1,380413033	1
A930007I19Rik	1,379265315	1

4930448A20Rik	1,379074122	1
Fbxl4	1,378978535	1
Gamt	1,378596254	1
Gm5586	1,378596254	1
Gm26698	1,3785007	1
Ddr1	1,378405153	1
Nexn	1,378309613	1
Rpl36-ps4	1,378309613	1
Gm11599	1,378309613	1
Gtf2h5	1,378309613	1
Nudt6	1,378118551	1
Gm12944	1,37783201	1
Vgll4	1,37783201	1
Defb25	1,377736509	1
Gm2756	1,377450046	1
Ints6	1,377163643	1
Slx4ip	1,377068189	1
Cdkn1b	1,377068189	1
Lsm3	1,3768773	1
Ogdh	1,3768773	1
Tmem170b	1,376304792	1
Gm9790	1,376304792	1
Gorasp1	1,376209397	1
Strn4	1,376209397	1
Gm26847	1,376209397	1
Gm10499	1,376209397	1
Tpt1-ps5	1,376114009	1
Ilf3	1,375827884	1
Yif1b	1,375732522	1
E4f1	1,375637167	1
Kctd6	1,375446476	1
Bsg	1,375446476	1
Ovgp1	1,374969865	1
Vwa7	1,374588696	1
Gm12182	1,37439815	1
Hoxb7	1,37439815	1
Csrp1	1,374302888	1
Ubl7	1,374017139	1
Mpst	1,373921903	1
Lsm14a	1,37373145	1
Plekhj1	1,373541024	1
Srsf2	1,372779582	1
Chfr	1,372684431	1
Mcm5	1,372589287	1
Pebp1	1,372399019	1
Lgals9	1,372303895	1
Prkx	1,371923464	1
Sdc3	1,371257964	1
Stxbp4	1,371162919	1
C330011M18Rik	1,370877824	1
Mmd	1,370782805	1
Copz2	1,370687793	1

Gm12704	1,370687793	1
Gm9165	1,370497788	1
Babam1	1,370212831	1
Btaf1	1,370117858	1
Ugp2	1,370117858	1
D130017N08Rik	1,369927933	1
Gm2810	1,36983298	1
Tiam2	1,369738034	1
Arfip1	1,369453234	1
Foxo3	1,369263401	1
Gins3	1,368788933	1
Cyb5r4	1,368694059	1
Gm6501	1,368599191	1
Tfr2	1,368409476	1
D330045A20Rik	1,368314629	1
Sf3b1	1,368124953	1
BC029214	1,368030125	1
9330111N05Rik	1,367935304	1
Sympk	1,367935304	1
Palm	1,367745681	1
Scarb1	1,367745681	1
Gm5845	1,367366514	1
Nptn	1,367366514	1
Hoxb6	1,367271739	1
Gm10800	1,367082208	1
Ltbp4	1,366892703	1
Rpl31-ps10	1,366892703	1
Eldr	1,36679796	1
Zmynd10	1,366703224	1
Gm7634	1,366703224	1
Gm13815	1,366608495	1
Usp35	1,366324346	1
Zfp874b	1,366134947	1
Gm16540	1,366134947	1
Gm27046	1,365945573	1
Upf1	1,365850896	1
Chek2	1,365661562	1
Lpar6	1,365566905	1
Auh	1,365282973	1
Ppp1r21	1,365093718	1
Cenpl	1,3649991	1
Hdac7	1,364809884	1
Arl2	1,364620694	1
Lrrc28	1,364526109	1
Rhod	1,364053283	1
Snap47	1,363864198	1
Rfxap	1,363769665	1
Lmtk2	1,363675139	1
Pdlim7	1,363486107	1
Ifitm1	1,363202607	1
Gm11944	1,363202607	1
Hat1	1,36301364	1

2700097O09Rik	1,3628247	1
Acad12	1,362635785	1
Ncbp2	1,362258035	1
Spink10	1,362258035	1
2810428J06Rik	1,362258035	1
Tbc1d8b	1,362163613	1
Stac2	1,362163613	1
Pop4	1,361880389	1
Ppfia4	1,361597223	1
2010111I01Rik	1,361408479	1
N4bp2	1,360936732	1
Inpp5f	1,360653763	1
Vav3	1,360559453	1
Gm4430	1,360559453	1
Mrpl9	1,360559453	1
Gm37407	1,360465149	1
Mllt10	1,360465149	1
Swap70	1,360370852	1
Bcl6b	1,360370852	1
Gpr68	1,360088001	1
Ctsc	1,359710956	1
Gm27605	1,359239798	1
Intu	1,359239798	1
Hddc2	1,359239798	1
Ccser2	1,359239798	1
Cep55	1,359145586	1
H2-M3	1,358768803	1
Rheb	1,358486285	1
Rnf130	1,358392125	1
Msi2	1,358392125	1
Spata1	1,358392125	1
Sgol1	1,357827303	1
Skp2	1,357827303	1
Bbc3	1,357450885	1
2410022M11Rik	1,35698051	1
Oprl1	1,356886455	1
Eef1a1	1,356792406	1
Gm12778	1,356792406	1
D030056L22Rik	1,356698363	1
Rras	1,356698363	1
Gm16712	1,356604327	1
Slc25a17	1,356510298	1
Psmb6-ps2	1,355852275	1
Tmod1	1,355852275	1
Ipo5	1,355758298	1
Ints8	1,355664327	1
Cbx4	1,355664327	1
mt-Nd5	1,355476405	1
St3gal2	1,355288509	1
Sez6	1,355194571	1
Ptms	1,355194571	1
Rabl3	1,355100639	1

Gm2272	1,354912795	1
Rnf5	1,354724977	1
Hyal3	1,354724977	1
Tspan33	1,354724977	1
Tbcd	1,35434942	1
A230050P20Rik	1,35416168	1
Heca	1,35406782	1
Ift122	1,35388012	1
Aspscr1	1,35388012	1
Gm11474	1,353786279	1
Gm21781	1,353692445	1
Uimc1	1,353410982	1
Gm42635	1,353317174	1
Tbcb	1,353223372	1
Pop1	1,352942007	1
Srp9	1,352754462	1
Fam76b	1,352660699	1
Bcl7a	1,352566943	1
Rbm39	1,352473194	1
Zfp949	1,352285714	1
Wdr48	1,352191984	1
Tef	1,352004544	1
Atg12	1,351910833	1
Gm9938	1,351817129	1
1810022K09Rik	1,351817129	1
Mrpl4	1,351723431	1
Gm12696	1,351723431	1
Ap1s2	1,35162974	1
2310068J16Rik	1,351536056	1
Hk1os	1,351442378	1
Pld2	1,351348706	1
Rpl22-ps1	1,351348706	1
Tmem171	1,351255041	1
Dhx40	1,351255041	1
Ube2h	1,350974085	1
Wdsub1	1,350786813	1
Gm43696	1,350693187	1
Pla2g6	1,350599567	1
2310009B15Rik	1,350505954	1
Ptchd1	1,350225153	1
Stra8	1,350225153	1
Neurl2	1,350131566	1
Mospd3	1,350037985	1
Gtse1	1,349944411	1
Gm16973	1,349757282	1
Wdr20	1,349570179	1
Uqcc2	1,349476637	1
Aldh9a1	1,349383101	1
Sertad1	1,34919605	1
Lctl	1,34919605	1
Prosc	1,34919605	1
RP24-365A12.2	1,34919605	1

Myl12b	1,34919605	1
Dtnb	1,349009025	1
Immp1l	1,348915522	1
Fam151b	1,348822026	1
Tmem183a	1,348822026	1
Iqcb1	1,348541575	1
Psma8	1,348541575	1
Fbxo30	1,348541575	1
Sep 06	1,348448104	1
Rtkn	1,34835464	1
Fbl	1,348261183	1
Acss2	1,348167732	1
Csrnp1	1,348167732	1
Lmf1	1,347980849	1
Prpf3	1,347980849	1
Rpl18	1,347980849	1
Lss	1,347607161	1
Tmem64	1,347513755	1
Gm5619	1,347420356	1
Rpl36-ps3	1,347233577	1
Pole4	1,347233577	1
Gm27477	1,347140197	1
Fam122b	1,347140197	1
Prss42	1,347140197	1
Ubr7	1,346766742	1
Arpin	1,346673395	1
Rps13-ps1	1,346673395	1
Cops2	1,346580054	1
Chic2	1,346486719	1
S1pr1	1,346113445	1
Fundc1	1,345740275	1
Tbc1d5	1,345646999	1
Atp9a	1,345460466	1
Pdia4	1,345460466	1
Gm42820	1,345367209	1
Mars2	1,344528187	1
Rab13	1,344434994	1
Coprs	1,344434994	1
Drap1	1,344434994	1
Gm11362	1,343969129	1
Gm6520	1,343969129	1
Rpe	1,343969129	1
Luzp1	1,343875976	1
Gm45109	1,343782828	1
Eral1	1,343689688	1
Rps29	1,343689688	1
Ambp	1,343689688	1
Ccdc88c	1,343596554	1
Litaf	1,343503426	1
Napsa	1,343410304	1
Ndufs3	1,343224081	1
Pola1	1,343130979	1

Ggh	1,343037884	1
4930539J05Rik	1,343037884	1
Abca1	1,342944795	1
Psma5	1,342758636	1
Gm13341	1,342479446	1
Znrf3	1,342386396	1
B3gat3	1,342386396	1
Fcgr4	1,342200314	1
Prc1	1,342200314	1
Gm6023	1,341828229	1
Snx18	1,341735224	1
Actr2	1,341549233	1
Sema4g	1,341456247	1
Clstn1	1,341363267	1
Tpra1	1,341363267	1
Pold4	1,341084368	1
Nek3	1,340898467	1
Gm45809	1,340526742	1
Sipa1l2	1,339969349	1
Itpkc	1,339969349	1
Ifitm6	1,339969349	1
Gm5609	1,339690739	1
Gm16200	1,339597882	1
Ssfa2	1,339412187	1
B9d2	1,339133693	1
Ska1	1,339133693	1
Atp11c	1,338948063	1
Gm13604	1,338948063	1
Kcnab3	1,338855257	1
Gm12231	1,338762458	1
C2cd5	1,338762458	1
Gm7809	1,338669665	1
Gnpnat1	1,338484099	1
Znhit1	1,338391326	1
Gm45802	1,338205798	1
Gm11613	1,338205798	1
Gm6378	1,337927555	1
B330016D10Rik	1,337649369	1
Ifi27l2a	1,337649369	1
Gm13736	1,337649369	1
Eif3l	1,337556654	1
Per1	1,337371242	1
Gm37472	1,337185856	1
Adcy6	1,337185856	1
Pik3ap1	1,337093172	1
Commd1	1,336907825	1
Ccdc163	1,336815161	1
Rnf38	1,336815161	1
Ak2	1,336537207	1
Ubl5	1,336537207	1
Zfyve27	1,336351936	1
Ppid	1,33625931	1

Gm28404	1,336166691	1
Gm5828	1,335333407	1
Casp7	1,335148303	1
0610005C13Rik	1,335055761	1
Rpl39	1,335055761	1
Pwwp2a	1,334963225	1
Paics	1,334870696	1
Gm36266	1,334593146	1
Khk	1,334593146	1
Wdhd1	1,334408145	1
Wdr34	1,334315654	1
Cad	1,33422317	1
Stbd1	1,33403822	1
Rpl9-ps6	1,33403822	1
Adi1	1,333760843	1
Cd82	1,333668397	1
Gm13340	1,332374825	1
Zfp938	1,332097794	1
Fkbp7	1,332097794	1
Tmem263	1,332097794	1
Mrps11	1,331636204	1
Gna11	1,331636204	1
Fam76a	1,331451613	1
Cox7a2l	1,331451613	1
Them6	1,330990247	1
Gm37785	1,330897993	1
Gm21975	1,330805746	1
Gm27219	1,330805746	1
R3hdm2	1,33062127	1
Mrps18c	1,330436819	1
Repin1	1,330344603	1
Cbx6	1,330160191	1
Nemp1	1,329975805	1
Pafah1b1-ps1	1,329791444	1
Dnajb2	1,32951495	1
Timm22	1,329422798	1
Aldh2	1,329238514	1
Ddr2	1,329054255	1
Gm11410	1,329054255	1
Primpol	1,328962135	1
Tdp2	1,328962135	1
Pros1	1,328870022	1
Sil1	1,328685814	1
Rps25	1,328501632	1
Nrxn3	1,328133344	1
Sar1b	1,327857195	1
RP24-401G4.1	1,327765158	1
Rpl10	1,327581104	1
Gm15464	1,327581104	1
Nrf1	1,327489086	1
Glrx3	1,327213071	1
Rpn2	1,327213071	1

Atf4	1,326845141	1
A930005H10Rik	1,326661214	1
Eif3m	1,326477312	1
Bbs2	1,326109585	1
Spag5	1,326109585	1
Cldnd1	1,326109585	1
Sart1	1,32601767	1
Mndal	1,325741961	1
Tmem132a	1,325558187	1
RP23-268C22.3	1,325374438	1
Pgs1	1,325190715	1
Smco4	1,325098863	1
Gm44901	1,325007017	1
Pex13	1,324915178	1
Zfyve26	1,324823345	1
Gm11273	1,324731518	1
Mxd4	1,324639698	1
Cebpzos	1,324547884	1
Gm14541	1,324547884	1
Gm9844	1,324364276	1
Myo6	1,324364276	1
Stil	1,324272481	1
Hmgb2	1,324272481	1
Tkt	1,324180692	1
Pycrl	1,32408891	1
Lonp2	1,32408891	1
Vars	1,32408891	1
Eif2a	1,323997135	1
Gm11686	1,323813602	1
Pja2	1,323813602	1
Stk40	1,322988022	1
Rbms2	1,322988022	1
Anxa2	1,32234626	1
Ppme1	1,322254605	1
Arc	1,322162957	1
Gabpb1	1,322162957	1
Bahd1	1,322162957	1
Trem3	1,321888049	1
Plau	1,321888049	1
Isg20l2	1,321796426	1
Dusp3	1,321704809	1
H60b	1,321521595	1
Cracr2b	1,321429997	1
Commd10	1,321429997	1
Pla2g12a	1,321429997	1
Gm43360	1,321246821	1
Gm37065	1,321246821	1
Iscu	1,321246821	1
Acrbp	1,320972104	1
Taf10	1,320972104	1
Slc25a23	1,320880544	1
Kif14	1,320788991	1

Sh3bgr	1,320697444	1
Cd164	1,320697444	1
Unc13a	1,320605903	1
Bub1b	1,320514369	1
1700037C18Rik	1,320422841	1
Mlec	1,319965297	1
Pigs	1,319599375	1
Mgarp	1,319507911	1
Serpinb6b	1,319050683	1
Ccnl1	1,318959257	1
Gm12346	1,318867837	1
Gm13445	1,318776423	1
Cdk2ap2	1,318776423	1
Kansl3	1,318776423	1
Polr2j	1,318593614	1
Clcn6	1,31850222	1
Wdr62	1,31850222	1
Samd1	1,31850222	1
Fhod1	1,318319449	1
Lmnb1	1,318319449	1
Alox5ap	1,318136704	1
Cox14	1,317862634	1
Gng7	1,317405976	1
Gm5575	1,317314664	1
Isg20	1,317223358	1
Kcnab2	1,317132058	1
Plod3	1,317040765	1
Nup35	1,316858197	1
Pcna	1,316858197	1
Snx2	1,316766922	1
Hrc	1,316675654	1
Gm43447	1,316675654	1
Lrrc17	1,316584392	1
Ucp2	1,316584392	1
Gm45133	1,316310644	1
Gm7965	1,316310644	1
Phf8	1,316310644	1
Rps19-ps6	1,316128178	1
Cenpu	1,316128178	1
Narf	1,316128178	1
Borcs7	1,315945736	1
Gm14248	1,315945736	1
2510046G10Rik	1,315854525	1
Plcb3	1,31576332	1
2610021A01Rik	1,315672122	1
Mrip-ps	1,315672122	1
Ankrd55	1,315672122	1
Nxf7	1,315489743	1
Impdh2	1,315125063	1
Vegfb	1,314851618	1
Donson	1,314760483	1
Fuca1	1,314760483	1

Crbn	1,314760483	1
F11r	1,314396005	1
Fgfr1op	1,314396005	1
Ndufv3	1,314304901	1
Gm10689	1,314213803	1
Enho	1,313849476	1
Alkbh6	1,31375841	1
Fgf11	1,312757101	1
Efna1	1,312757101	1
Mt1	1,31248415	1
Tspan5	1,312302213	1
Rnf25	1,312211255	1
Mrrf	1,312211255	1
Atg4c	1,312120302	1
Gm15779	1,312120302	1
B3galnt2	1,312029356	1
Gm10602	1,311938416	1
Gltscr2	1,311938416	1
Scaf1	1,311847483	1
Mrpl48-ps	1,311847483	1
Cnih1	1,311756556	1
Kras	1,311665635	1
Caap1	1,31157472	1
Foxn3	1,311483812	1
Klf8	1,311120242	1
1110034G24Rik	1,311120242	1
Dnajb1	1,310938494	1
3300002I08Rik	1,310665921	1
Gramd4	1,310665921	1
Apmap	1,310665921	1
Hikeshi	1,310575076	1
4930520O04Rik	1,310575076	1
Hmgcs1	1,310575076	1
2210013O21Rik	1,310484237	1
Uhrf2	1,310393404	1
Gas8	1,310302577	1
Gm43684	1,310302577	1
Gm14681	1,310120944	1
Sntb2	1,309939335	1
Rnf13	1,309939335	1
Il13ra1	1,309757751	1
Fcgr3	1,309576193	1
Gm5050	1,30939466	1
Spry2	1,309213152	1
Scd1	1,309213152	1
Rfwd2	1,308668778	1
Gltscr1	1,308578071	1
Gm15503	1,308215307	1
Dnmt3l	1,308215307	1
Ap2a1	1,307761993	1
Dusp2	1,307671349	1
Gm12096	1,307580711	1

Coro2a	1,307580711	1
Gm5611	1,307580711	1
Glipr2	1,307218223	1
Gm11808	1,307218223	1
Gm22516	1,306855835	1
Cfl1	1,306674679	1
Mad2l2	1,30658411	1
Uba3	1,30658411	1
Akt3	1,30658411	1
Elof1	1,306493548	1
Fam210b	1,306312442	1
Gm10250	1,305859787	1
Rpsa-ps11	1,305769275	1
Clic1	1,305588269	1
Hsph1	1,305497776	1
Scaper	1,305497776	1
Kif3a	1,305407289	1
Hoxa5	1,305226334	1
Eif2d	1,305226334	1
Guf1	1,305226334	1
Gm6450	1,305135865	1
Ermp1	1,305045403	1
Lamtor2	1,305045403	1
Nfatc4	1,304954948	1
Itfg1	1,304683618	1
N4bp1	1,304593188	1
Tnpo2	1,304593188	1
Zranb3	1,304502763	1
Mdrl	1,304502763	1
Dpysl2	1,304412345	1
Dnajb4	1,304412345	1
Gm11675	1,304321933	1
Tomm20	1,304141128	1
Ss18l2	1,303960348	1
Prdx3	1,303869968	1
Napepld	1,303689226	1
Pfkp	1,303689226	1
Rbm19	1,303508509	1
Kifc5b	1,303418159	1
Ube2e2	1,303327816	1
Gm9625	1,30323748	1
D5Ertd605e	1,303147149	1
Cxx1b	1,302966507	1
Marcksl1	1,30278589	1
Sdc1	1,302695591	1
Rnf2	1,302695591	1
Egfl7	1,302605298	1
Laptm4b	1,302515012	1
Tec	1,30224419	1
Uqcr11	1,30224419	1
Deptor	1,301883181	1
Gm15417	1,30161249	1

Gng2	1,30161249	1
Creb3l2	1,301432061	1
Rchy1	1,301432061	1
Gchfr	1,301161464	1
Ccdc180	1,301071277	1
Psme1	1,301071277	1
Mthfd1l	1,300710594	1
Ube2f	1,300620438	1
Mzb1	1,300530289	1
Otud3	1,300440147	1
Vezt	1,30035001	1
Nckipsd	1,300169756	1
Arpp19	1,299629143	1
Dpm1	1,299358921	1
Fam20b	1,298998712	1
Gm6305	1,298908675	1
Rmnd5a	1,298908675	1
9330162012Rik	1,298818645	1
Tiparp	1,298638603	1
Bvht	1,298548591	1
Ssh3	1,298548591	1
Pdk2	1,298458586	1
Pdia6	1,298278594	1
Dcaf4	1,298098626	1
Mrpl58	1,298098626	1
Acad9	1,297828722	1
Nr3c1	1,297648818	1
Erlec1	1,297558875	1
Tusc3	1,297558875	1
Kpna3	1,297558875	1
Lrrc51	1,297468938	1
Dnajc4	1,297379007	1
Islr2	1,297289083	1
Ugcg	1,297109253	1
Mthfsl	1,297019347	1
Cdan1	1,296839555	1
Sp140	1,296839555	1
Gm8919	1,296839555	1
Pdxk	1,296569912	1
Sep 10	1,296300326	1
Mki67	1,296300326	1
5430434F05Rik	1,296120633	1
Rad51c	1,296030796	1
Fam234a	1,296030796	1
Asf1a	1,296030796	1
Cped1	1,295491904	1
Gcdh	1,295402111	1
Prpf8	1,295402111	1
Kdm6a	1,295402111	1
Mlst8	1,295312323	1
A430018G15Rik	1,294953236	1
Atp5g1	1,294953236	1

Mrc1	1,29486348	1
Tgoln1	1,294683986	1
Chd1l	1,294594249	1
Higd2a	1,294414792	1
Minos1	1,294414792	1
Pan2	1,294325074	1
D130020L05Rik	1,29396626	1
Fabp5l2	1,293876572	1
Mapk1ip1	1,293517883	1
1810032O08Rik	1,293517883	1
Cxcl14	1,293338576	1
Aldh16a1	1,293338576	1
Snrpg	1,293338576	1
Ube2m	1,293069662	1
Anapc15	1,292800804	1
Rpia	1,292800804	1
Ifi203	1,292711197	1
Tmlhe	1,292711197	1
Zfp609	1,292711197	1
Gm6155	1,292442413	1
Rpl32	1,292442413	1
Ccdc92b	1,292352831	1
Gm43379	1,292084121	1
Arl1	1,292084121	1
Ppp2r2d	1,292084121	1
Gm15785	1,291994564	1
Gm7407	1,291994564	1
Prkcsh	1,291994564	1
Naip5	1,291815468	1
Gm45413	1,291725929	1
Npm3	1,291636397	1
Isg15	1,29145735	1
Dis3	1,291367836	1
Ifnar1	1,291367836	1
Gpn3	1,291278329	1
Ddt	1,291188827	1
Pgm3	1,291009843	1
Ppm1e	1,29092036	1
Ap3d1	1,29092036	1
Gm12989	1,290830883	1
Atxn10	1,290830883	1
Gm7102	1,29056249	1
2810402E24Rik	1,290473039	1
Ap2a2	1,290473039	1
Rcbtb1	1,290383593	1
Got1	1,29020472	1
Tubg1	1,289847049	1
Msrb1	1,289847049	1
Cog3	1,289578861	1
Dip2a	1,2894001	1
4930440I19Rik	1,289310728	1
Bckdhb	1,289310728	1

Gm4204	1,289310728	1
A930004J17Rik	1,289042652	1
Zfp784	1,288953305	1
Nubp1	1,288863965	1
Gpsm3	1,288685303	1
Gm27248	1,287970902	1
Cd200r4	1,287970902	1
Rad51d	1,287970902	1
Phkb	1,28788163	1
Ezh2	1,28788163	1
Enoph1	1,287792363	1
Rps24-ps3	1,287792363	1
Tmed1	1,28761385	1
Gm9726	1,287524602	1
Plekha1	1,287524602	1
Ankrd13d	1,287346126	1
Gm11427	1,287256897	1
Reps2	1,287256897	1
Foxk2	1,287256897	1
Cd40	1,287256897	1
Hcst	1,287256897	1
Tmem216	1,287167674	1
Selenon	1,287167674	1
Ddx47	1,286810845	1
Zfp607b	1,286721653	1
Nfkbib	1,286364948	1
Topbp1	1,286364948	1
Zfp788	1,286186632	1
Gm44237	1,286097483	1
Tapbp	1,285740951	1
Klf10	1,285384517	1
Eif4e3	1,285384517	1
Paip2b	1,285117257	1
Kat8	1,284939114	1
Cbwd1	1,284939114	1
Ndufs2	1,284850052	1
Mrps24	1,284760996	1
Ptges3l	1,284493865	1
Gpr162	1,284404834	1
Gm9246	1,284404834	1
Swi5	1,284404834	1
Ifitm3	1,284404834	1
Ppp4r1l-ps	1,283692806	1
Tbc1d22a	1,283692806	1
Abcd2	1,283070106	1
Gm3555	1,282981173	1
Gm24951	1,282981173	1
Ebi3	1,282892247	1
Gm12174	1,282803326	1
Ing5	1,282803326	1
G6pd2	1,282625505	1
Tex30	1,282625505	1

Mynn	1,282625505	1
Usf2	1,282625505	1
Itpka	1,282447707	1
Dad1	1,282358818	1
Glrp1	1,282181057	1
Mettl17	1,281914463	1
Gm24276	1,28155909	1
Irak1bp1	1,281381441	1
Mtor	1,281292625	1
Rnf146	1,281203816	1
Hdac5	1,28084864	1
Tmub1	1,280759861	1
Nabp1	1,280671089	1
Pikfyve	1,280671089	1
Cic	1,280582323	1
Cmc2	1,280404809	1
Spata5	1,280227319	1
Galk2	1,280227319	1
Acadvl	1,280227319	1
Stx8	1,280138584	1
Gm15445	1,279961131	1
BC030336	1,279961131	1
Dpm3	1,279961131	1
Fry	1,279783703	1
Kitl	1,279783703	1
Itgav	1,279783703	1
Kantr	1,2796063	1
Rpl18a	1,279428921	1
Zfc3h1	1,279340241	1
Lsm2	1,279340241	1
Bax	1,279340241	1
Gm4525	1,279251567	1
Anapc2	1,279251567	1
Gm6451	1,279162899	1
RP24-323H7.5	1,279162899	1
Tmem9	1,278985581	1
Bbs5	1,278896932	1
Lamtor4	1,278896932	1
1500011B03Rik	1,278896932	1
2310034G01Rik	1,278808289	1
Atp6v1h	1,278808289	1
2210406H18Rik	1,278187957	1
Gm37349	1,278099363	1
S100a1	1,278099363	1
Gm6322	1,278010775	1
Gm4978	1,277922193	1
Polr1e	1,277656484	1
Apool	1,277656484	1
Ceacam1	1,277656484	1
Txn2	1,277567927	1
Lrig3	1,277479376	1
Rab3a	1,277479376	1

Stag1	1,277036712	1
Gm37399	1,276859689	1
Dexi	1,276682691	1
Dym	1,276682691	1
Gm14165	1,276594202	1
Gm13680	1,276505718	1
Wdr70	1,276505718	1
Mrps16	1,276240304	1
Cuedc2	1,276151844	1
Fmr1	1,276151844	1
Uqcrh-ps2	1,276063391	1
Rrnad1	1,276063391	1
Zfp207	1,275886504	1
Zfp367	1,27570964	1
Sp100	1,275532802	1
Slc24a5	1,275002433	1
Cd302	1,27491406	1
Sugp1	1,274825692	1
Usp11	1,274737331	1
Cbr1	1,274648976	1
Pink1	1,274472285	1
Gm14439	1,274383948	1
Smox	1,274295617	1
Aqr	1,274207293	1
Gm17491	1,274030663	1
Fuca2	1,274030663	1
Gm9531	1,274030663	1
Gm8624	1,273942357	1
Tbc1d12	1,273942357	1
Tbpl1	1,273412649	1
Pgm2l1	1,273324386	1
Stim1	1,273324386	1
Smarca5-ps	1,273236129	1
Gpatch2l	1,273236129	1
RP23-47A1.1	1,273147878	1
Gm15730	1,273059633	1
Utp18	1,272971394	1
Edc3	1,272971394	1
Gm20628	1,272883162	1
Rfxank	1,272706715	1
Mr1	1,272706715	1
Pdxdc1	1,272618501	1
Mri1	1,272618501	1
Chchd5	1,272618501	1
Rad9a	1,272442091	1
Trappc6a	1,272442091	1
Slc52a2	1,272353895	1
Ncaph	1,272265705	1
Gm42970	1,272177521	1
Zgrf1	1,272089344	1
Trim7	1,272001172	1
Mier1	1,271736694	1

Clic4	1,271648547	1
Gatsl3	1,271560406	1
Nmral1	1,271472272	1
Nupr1	1,27129602	1
Cry1	1,27129602	1
Mss51	1,271031689	1
Txndc15	1,271031689	1
Bckdha	1,271031689	1
Klhl15	1,270855499	1
Qars	1,270855499	1
Gm37598	1,27059126	1
A730071L15Rik	1,27059126	1
Col18a1	1,27041513	1
P4hb	1,27041513	1
Il6ra	1,270150983	1
H2afv	1,269974914	1
Khdrbs1	1,26988689	1
Cd59a	1,269622851	1
Tep1	1,269622851	1
Pik3r3	1,269534851	1
Cep83os	1,269446857	1
Vamp8	1,269270886	1
Rps12-ps1	1,26918291	1
Rab6a	1,269006976	1
Gm43088	1,268567248	1
Traf3ip1	1,268303484	1
Nbeal1	1,268303484	1
Map2k3os	1,268215575	1
Asah1	1,268215575	1
Gstcd	1,268127672	1
Gm5898	1,268039775	1
Slc41a3	1,268039775	1
Noc3l	1,267864	1
Rpl13a	1,267864	1
Vbp1	1,267776121	1
Lztr1	1,267424668	1
Sf3a2	1,26733682	1
Ssna1	1,267073312	1
Stt3b	1,266985488	1
Itgb1	1,26689767	1
Zfat	1,266809859	1
Mdh1	1,266809859	1
Svil	1,266722053	1
Slc40a1	1,266458673	1
Dld	1,266283117	1
Erh	1,266195348	1
Haus3	1,266195348	1
Zfp692	1,266195348	1
Slc6a8	1,266195348	1
Epn2	1,266195348	1
Osbp	1,266107585	1
Gm2531	1,265932077	1

Nfam1	1,265932077	1
Gm14633	1,265668861	1
Cryga	1,26523029	1
Ndufv1	1,265142594	1
Gm30329	1,26496722	1
Cs	1,264791871	1
Txnrd3	1,264528892	1
Rc3h1	1,264528892	1
Chchd7	1,264353603	1
Nfxl1	1,264178339	1
Arl16	1,264090716	1
Sdhaf3	1,264090716	1
Mettl16	1,263915487	1
Prdm2	1,263827883	1
Dmwd	1,263565104	1
K230015D01Rik	1,262952167	1
Tln2	1,262864629	1
Mrpl34	1,262864629	1
RP23-136K21.4	1,262777097	1
Atp6v0a1	1,262777097	1
Tnnc1	1,262602051	1
Card14	1,26242703	1
Commd9	1,262252032	1
Anp32b-ps1	1,262252032	1
Bach1	1,262164543	1
Ddx43	1,262077059	1
Foxp4	1,261814645	1
Zfp60	1,261727185	1
Ttc12	1,261727185	1
Helq	1,261552285	1
Utrn	1,261202557	1
Gm26549	1,26111514	1
Cdon	1,261027729	1
Wwp1	1,261027729	1
Psmb5	1,260940324	1
Zfp277	1,260940324	1
Nhlrc2	1,260852926	1
Brdt	1,260765533	1
Sh3bgrl	1,260678147	1
Desi2	1,260590766	1
4930524J08Rik	1,260416023	1
Lamc2	1,260416023	1
Gm15800	1,260328661	1
Mrps36	1,260328661	1
Ptpn18	1,259979272	1
Gtf3a	1,25989194	1
Plod1	1,25989194	1
Ttc38	1,259804614	1
Mcoln2	1,259804614	1
Xrcc6	1,259804614	1
Ube2g1	1,259804614	1
Rps15-ps2	1,259717294	1

Cds1	1,259717294	1
Cdk16	1,25962998	1
Pfdn5	1,25962998	1
Apip	1,25945537	1
Camkmt	1,259106223	1
Slc25a3	1,259106223	1
Zfp948	1,258931686	1
Hscb	1,258931686	1
Fabp5	1,258844427	1
Tmem218	1,258844427	1
Acadl	1,258757174	1
Gatad2b	1,258669926	1
Lix1l	1,258582685	1
Aip	1,25849545	1
Nprl2	1,25849545	1
Hdac6	1,25840822	1
Cybb	1,257797785	1
Gm4707	1,257449098	1
Rasa4	1,257449098	1
Grn	1,25727479	1
Rnf31	1,257187646	1
Ralb	1,257187646	1
Ppdpf	1,257100507	1
AW549877	1,257013375	1
Cchcr1	1,256752013	1
Slc39a3	1,256752013	1
Hyal2	1,256664905	1
Gm8770	1,256577802	1
Chrnb2	1,256577802	1
Mir6236	1,256490706	1
Ppp2r1a	1,256403616	1
Gm21399	1,256142381	1
Ndufaf5	1,255968255	1
Fam193b	1,255968255	1
Atp11a	1,255794153	1
Pip4k2b	1,255794153	1
Casp2	1,255707111	1
Jak2	1,255620075	1
Snhg6	1,255184985	1
Ifi202b	1,255184985	1
Tubb5	1,254837023	1
Hexb	1,254663077	1
Ybx1	1,254489156	1
2510002D24Rik	1,254402205	1
Tmem42	1,254315259	1
Plekhg3	1,254315259	1
Dhx9	1,254315259	1
Arhgef11	1,25422832	1
B4galt7	1,254054459	1
Fam214a	1,253967537	1
Cdkn3	1,253793712	1
Mettl26	1,253793712	1

Cdk5rap3	1,253706809	1
Txndc12	1,253706809	1
Zfp59	1,25353302	1
Gm5599	1,253446135	1
Zfp652	1,253446135	1
Dusp7	1,253359256	1
Grcc10	1,253272383	1
Atp6v1g1	1,253098654	1
Ift52	1,252664439	1
Cebpg	1,252490794	1
Gna15	1,252403981	1
Ndufc1	1,252230373	1
Pcx	1,252056789	1
Gm8618	1,251970007	1
Chchd3	1,251796459	1
Sav1	1,251709694	1
Mzt1	1,251709694	1
Gm6560	1,251622935	1
Cyth1	1,251536182	1
Srpr	1,251536182	1
Lpcat1	1,251536182	1
Cd80	1,251449435	1
Zg16	1,251449435	1
Rpl14	1,251449435	1
Gm37357	1,251362694	1
Spty2d1	1,251362694	1
Prkd3	1,251275959	1
Rhoa	1,251275959	1
Lgals1	1,251275959	1
1700120C14Rik	1,251189231	1
1500011K16Rik	1,251189231	1
BC025920	1,251102508	1
Gm43924	1,25092908	1
Tm7sf2	1,250322273	1
Mb21d2	1,250322273	1
Nudt2	1,25023561	1
Polr2k	1,250148953	1
Itgb2	1,250062303	1
Aplp2	1,250062303	1
Surf6	1,24971576	1
9230112E08Rik	1,249629139	1
Mrpl32	1,249629139	1
Epha2	1,249282716	1
Fam43a	1,249196126	1
Guca1a	1,249109541	1
Eif4e	1,248849823	1
Gm36378	1,248676708	1
Csnk2b	1,248676708	1
Apopt1	1,248590159	1
Alg9	1,24841708	1
B3gnt3	1,248244024	1
Med20	1,248244024	1

Saraf	1,248157506	1
Tmco3	1,247984486	1
Trmt13	1,247984486	1
Nt5m	1,247897985	1
Ptbp1	1,247552043	1
Ethe1	1,247379107	1
Zfyve16	1,247379107	1
Pld4	1,247379107	1
Phc1	1,247292648	1
Tbck	1,247206196	1
Pitrm1	1,247119749	1
Pck2	1,247033308	1
Mvk	1,246946873	1
Gm4540	1,246860445	1
Cnih4	1,246860445	1
AC168977.1	1,246601194	1
Grina	1,246428391	1
Tdpx-ps1	1,246255611	1
Braf	1,24616923	1
Cxcl16	1,245996486	1
Gm9843	1,245910123	1
Tifab	1,245823766	1
Selenok	1,245823766	1
5430416N02Rik	1,245737416	1
Fbxo2	1,245651071	1
Phkg2	1,245564732	1
Smc2	1,245392072	1
Pmp22	1,245392072	1
Aldh3a2	1,245219436	1
Ywhaz	1,245219436	1
Casp6	1,245133127	1
Zfp580	1,245133127	1
Sf3a1	1,244874235	1
Nelfcd	1,244529131	1
Idnk	1,244356614	1
Gm6204	1,244184122	1
Polr1d	1,244097885	1
Lekr1	1,243925428	1
Pgghg	1,243752995	1
9530082P21Rik	1,243752995	1
Micu1	1,243752995	1
Mrpl33	1,243580586	1
Brip1	1,243494391	1
Elovl1	1,243494391	1
Rps2	1,243322018	1
Gramd1a	1,24323584	1
Mvd	1,24323584	1
Ccdc124	1,243063503	1
Gm4997	1,243063503	1
Pofut2	1,242977344	1
Ube2j2	1,24289119	1
Lamtor5	1,242632765	1

Fkbp3	1,242632765	1
Zfp414	1,242546635	1
Kdm4c	1,242460511	1
Mfsd13b	1,242460511	1
Hnrnpul1	1,242460511	1
U2af2	1,242374394	1
Rnf215	1,242374394	1
Leo1	1,242288282	1
Sbno1	1,242288282	1
Trim14	1,242202176	1
Dnajc24	1,241943894	1
Wdr37	1,241857812	1
Eif4b	1,241771736	1
Hspbp1	1,241685666	1
Gm44130	1,241599602	1
Zcchc9	1,241427492	1
Ubl3	1,241255405	1
Tmod3	1,241169371	1
Id2	1,241083342	1
Qk	1,241083342	1
Wipi1	1,240739289	1
Tial1	1,240567298	1
Spg7	1,24039533	1
Gm27043	1,240309356	1
Ptpn4	1,240051468	1
Mknk1	1,240051468	1
Rpl6l	1,239965517	1
Fn1	1,239965517	1
Ppp4r3a	1,239879572	1
Ski	1,239621773	1
Cstf2	1,239449937	1
Sep 08	1,239364028	1
Trp53cor1	1,239278124	1
Mrps33	1,239192227	1
Racgap1	1,239192227	1
Polm	1,239106336	1
Gm42986	1,239106336	1
Crnde	1,238934571	1
Gm15032	1,238848698	1
Utp14b	1,23876283	1
Timp1	1,238676969	1
E330020D12Rik	1,238591113	1
Nek1	1,238505264	1
Gpbp1l1	1,23824775	1
Ppt2	1,23824775	1
Gm14822	1,237990291	1
9430092D12Rik	1,237990291	1
Scpep1	1,237990291	1
Tmem81	1,237475532	1
Mcm6	1,237475532	1
Rnps1	1,237303994	1
Polr2l	1,237303994	1

Lmtk3	1,237218233	1
Gm44024	1,237132479	1
Tarsl2	1,23704673	1
Sumf1	1,23704673	1
C1d	1,236960988	1
Lgmn	1,23678952	1
Engase	1,236618077	1
Plcg1	1,236618077	1
Rilpl2	1,236618077	1
Flii	1,236618077	1
Wdr91	1,236532364	1
Chd6	1,236446657	1
Gm16523	1,236360956	1
Aste1	1,236275261	1
Hlx	1,236275261	1
Zc3h3	1,236189572	1
Gm44609	1,236189572	1
Arhgef10l	1,236018211	1
Slamf9	1,23593254	1
Adcy2	1,235846875	1
Psenen	1,235846875	1
Haus8	1,235846875	1
Xlr	1,235675562	1
Blvrb	1,235161766	1
F730043M19Rik	1,235076154	1
Chka	1,235076154	1
Ptprc	1,234819354	1
Rac2	1,234819354	1
Atp5e	1,234819354	1
Gm7658	1,234733765	1
Sh3bp1	1,234562607	1
Gm43609	1,234477037	1
Gm8822	1,234391472	1
Gm7589	1,234220361	1
Cmtm6	1,234220361	1
Stk11	1,234049273	1
2300009A05Rik	1,234049273	1
Rrp7a	1,233963739	1
Car5b	1,233963739	1
4930430F08Rik	1,233792687	1
Cox6c	1,233792687	1
Smg5	1,23370717	1
Rbmx2-ps	1,233621659	1
Cby1	1,233621659	1
AC133103.1	1,233621659	1
Arglu1	1,233536154	1
Bcat1	1,233450654	1
Tom1l1	1,233450654	1
Rest	1,233450654	1
Gm14593	1,233279674	1
Dctpp1	1,233279674	1
Gm5277	1,233023247	1

Trmt2b	1,232937783	1
Elf1	1,232937783	1
Zkscan8	1,232766874	1
RP23-65M10.2	1,232254287	1
Nrm	1,232254287	1
Gm26830	1,232168876	1
Fndc10	1,231912681	1
Sestd1	1,231912681	1
Mark4	1,231912681	1
Cul4b	1,231912681	1
Cct5	1,231912681	1
Tpd52-ps	1,231827294	1
Cnot4	1,231741913	1
Mtpn	1,231656538	1
Hsp90b1	1,231656538	1
Stard10	1,231485806	1
Irgq	1,231400449	1
Rpl41	1,231315098	1
Rnf11	1,231229753	1
Zbtb42	1,231144413	1
Cers5	1,231144413	1
Vdac1	1,23105908	1
Mtch1	1,230888431	1
Gm8606	1,230803115	1
Smg9	1,230803115	1
Chmp1b	1,230803115	1
9630013D21Rik	1,230717805	1
Gm6851	1,230632501	1
Ulk1	1,230632501	1
Wrap53	1,230376625	1
Gm45477	1,230376625	1
Lsm4	1,23020607	1
mt-Tt	1,230120802	1
Ap1b1	1,230120802	1
Ociad2	1,229865032	1
Kdm5c	1,229865032	1
Fnbp1l	1,229438867	1
Coq4	1,229353652	1
Galnt3	1,229268443	1
Adh5	1,229183239	1
Smc1a	1,229183239	1
Rpl10a-ps1	1,229098042	1
Smpd4	1,22901285	1
S100a11	1,22901285	1
Zbtb32	1,228927664	1
Gmds	1,228927664	1
Dcp2	1,228927664	1
Nhlrc3	1,228757311	1
Pkn2	1,22858698	1
Eif2s3x	1,22833153	1
Polr3b	1,22833153	1
Nelfe	1,227905896	1

Ptges	1,227735684	1
Ppp1r13l	1,227650587	1
Mospd1	1,22748041	1
Gja1	1,22748041	1
Cenpe	1,22748041	1
Tceal9	1,22748041	1
5330406M23Rik	1,227310257	1
Gm43137	1,227310257	1
Utp14a	1,227310257	1
Gm43581	1,227140127	1
Lsp1	1,227055071	1
E2f7	1,226970021	1
RP23-228B2.5	1,226799939	1
Rps12-ps24	1,226714907	1
Ndufaf2	1,22654486	1
Ttc9c	1,226459845	1
Hmces	1,226459845	1
Lancl2	1,226204836	1
Gm12468	1,225949881	1
Plxna1	1,225949881	1
RP24-547N4.5	1,225525073	1
Tbl2	1,22535519	1
Hpcal1	1,225270258	1
Spa17	1,225100411	1
Gss	1,225015497	1
Ppp1r35	1,224930588	1
Nectin3	1,224930588	1
Max	1,224930588	1
Gm13777	1,224845685	1
March4	1,224760789	1
Tmem176b	1,224591012	1
Zfp382	1,224506133	1
Gm11478	1,22442126	1
Prepl	1,224166675	1
Plek	1,224166675	1
RP23-40D21.1	1,224081825	1
Slc25a39	1,224081825	1
Pcbd2	1,223912143	1
Zbtb7b	1,223657664	1
Dnal4	1,22357285	1
Mgea5	1,22323365	1
Cd37	1,222894545	1
Chmp4b	1,222894545	1
Slc44a1	1,222894545	1
Cep76	1,222809783	1
Eps15	1,222809783	1
Trim2	1,222725028	1
Ip6k1	1,222640278	1
Gm9484	1,222301337	1
Ggnbp1	1,222216616	1
Rps13-ps2	1,222216616	1
Samd8	1,222216616	1

3110045C21Rik	1,222047193	1
Leng9	1,22196249	1
Gm31274	1,221708416	1
Nt5dc3	1,221708416	1
Dstn	1,221708416	1
Camta1	1,221539063	1
Plekhf2	1,221539063	1
Mrpl1	1,221539063	1
Vcl	1,221454396	1
Lpcat2	1,221200428	1
Cog5	1,221115784	1
Rpl37	1,221031145	1
Kmt2d	1,220861886	1
Stard5	1,220608041	1
Ctsa	1,220608041	1
Eif4h	1,220608041	1
Eef1d	1,220523438	1
Ints12	1,220438841	1
Bak1	1,220269664	1
Marf1	1,22010051	1
Cenpw	1,22010051	1
5830487J09Rik	1,220015942	1
Sec24a	1,220015942	1
Nop10	1,220015942	1
Gm37902	1,21993138	1
Tmem38b	1,219846824	1
Zfand6	1,219846824	1
Ak4	1,219762273	1
Zfpm1	1,21942413	1
Nit1	1,219339609	1
Gdf9	1,219339609	1
Dph6	1,219339609	1
Sccpdh	1,219339609	1
Pddc1	1,219255094	1
Zdhhc17	1,219255094	1
Pgm1	1,219001583	1
Dtymk	1,218748126	1
Mgst2	1,218579183	1
Sesn2	1,218410264	1
Cycs	1,218072495	1
Kif7	1,217903646	1
A330035P11Rik	1,217819231	1
Mtus1	1,217819231	1
Ccdc62	1,217819231	1
Pdha1	1,217650417	1
Mre11a	1,217481626	1
Pak4	1,21739724	1
Mapk6	1,217312859	1
Fam132a	1,216806698	1
Uevld	1,216806698	1
Kdm1a	1,216722359	1
Gm37255	1,216385058	1

Gm44168	1,216216443	1
Gm6166	1,216216443	1
Wdr74	1,216132144	1
Hotairm1	1,216132144	1
Heatr5b	1,216132144	1
Cd9	1,216047852	1
Gatad1	1,216047852	1
Slc25a5	1,215963564	1
Chd3	1,215963564	1
Ecm1	1,215879283	1
Dis3l	1,215795008	1
Gm14539	1,215710738	1
Ssr3	1,215626474	1
Gm7964	1,215373718	1
Sarnp	1,215373718	1
Psmg4	1,215373718	1
Nod1	1,215121015	1
Slc37a2	1,215036792	1
Hnrnpd	1,215036792	1
E330011M16Rik	1,214952575	1
Aldoa	1,214868364	1
Rbl2	1,214615765	1
Rpl18-ps2	1,214615765	1
Actr10	1,214447395	1
Abhd17a	1,214279049	1
Snrnp48	1,214279049	1
BC037032	1,214194884	1
Atp5b	1,214110726	1
Selenoi	1,21369002	1
Wdr54	1,213605896	1
2810403A07Rik	1,213605896	1
Abhd10	1,213521778	1
Aco2	1,213521778	1
Gm1947	1,21335356	1
Snx6	1,21335356	1
Tbc1d22b	1,21326946	1
0610009L18Rik	1,213017194	1
Ccl3	1,212933117	1
Sqle	1,212933117	1
Casp4	1,21276498	1
Banp	1,21276498	1
Rangap1	1,21276498	1
Srebf1	1,212428777	1
Anxa5	1,21226071	1
Spag4	1,212008654	1
Smarcc2	1,212008654	1
Leng1	1,211924647	1
Hes6	1,211924647	1
Arrdc1	1,211840646	1
Gm7730	1,21175665	1
Zyx	1,21175665	1
Txnl1	1,21167266	1

Ppp1r2	1,211588677	1
Atpaf2	1,211504699	1
Slc8a1	1,211168845	1
Mbd3	1,211084896	1
Fdxr	1,211000953	1
Hmg20b	1,210917015	1
Gm8326	1,210833084	1
Cpsf4	1,210833084	1
Klf4	1,210749158	1
Hacl1	1,210665238	1
2410131K14Rik	1,210665238	1
Atox1	1,210665238	1
Otx1	1,210581324	1
AA465934	1,210581324	1
Gm10161	1,210497416	1
Srebf2	1,210329617	1
Eid1	1,210161842	1
Got2	1,210077963	1
Tex264	1,209658654	1
Gm10784	1,20957481	1
Dvl3	1,209490971	1
Gm10327	1,209490971	1
Gm36964	1,209490971	1
Mien1	1,209323312	1
Paip2	1,209155676	1
Rnaseh1	1,208904265	1
Dip2c	1,208652907	1
Ccnb2	1,208485363	1
Cndp2	1,208485363	1
Tcp1	1,208401601	1
Ddit3	1,208066607	1
Rpl10a	1,207899145	1
Abtb1	1,207899145	1
Tcf7l2	1,207899145	1
Rps16	1,207647995	1
Gm12857	1,207647995	1
1110051M20Rik	1,207564291	1
Gm26881	1,207396898	1
Ncaph2	1,207313211	1
Hspd1-ps3	1,207229529	1
Ankrd52	1,207145853	1
Anapc7	1,207062183	1
Fendrr	1,206727561	1
Pitpnm1	1,20664392	1
Tor1aip2	1,20664392	1
Gabarap	1,20664392	1
Gm44434	1,206309413	1
Hist1h1c	1,206309413	1
Fam171b	1,206225801	1
Pgap2	1,206142195	1
Dlgap4	1,206142195	1
Gm43773	1,206058594	1

Pola2	1,205975	1
Scrn3	1,205975	1
Tst	1,205807828	1
Dcaf6	1,20572425	1
Inafm2	1,205473553	1
Atp1a3	1,205389999	1
Klhl24	1,205306451	1
Dnpep	1,205306451	1
Hspa5	1,205222908	1
Foxm1	1,205139371	1
Pcmtd1	1,205139371	1
Armc8	1,20505584	1
Ascc3	1,204888796	1
Gm42466	1,204721774	1
2310022B05Rik	1,204638272	1
Gm7236	1,204554776	1
Ric1	1,204554776	1
1500002F19Rik	1,204471285	1
Rel	1,204471285	1
Fam105a	1,204304322	1
Zbtb34	1,204220849	1
Ptprj	1,204220849	1
Abi1	1,204220849	1
Secisbp2	1,20405392	1
Ftsj1	1,203887014	1
Tfe3	1,20380357	1
Bcas3	1,203636699	1
Cyth3	1,203553272	1
Prkra	1,203553272	1
Abcc3	1,203469851	1
Acap3	1,203386436	1
Scarna9	1,203219622	1
Tspan13	1,203136224	1
Lman2l	1,202886065	1
Cd93	1,202719321	1
Kctd10	1,202719321	1
Eif3e	1,202719321	1
Ogfr	1,202635958	1
Itga5	1,202469249	1
Cdc42ep4	1,202385903	1
Gm10059	1,202219228	1
Gm16630	1,202219228	1
St13	1,202219228	1
Rab11fip4	1,2021359	1
Haus1	1,2021359	1
Faf1	1,202052577	1
Hprt	1,202052577	1
Tbrg4	1,202052577	1
Ckb	1,202052577	1
Ube2a	1,20196926	1
Hook2	1,201885949	1
Aamdc	1,201386202	1

Phyh	1,201386202	1
Tmem238	1,201219666	1
St14	1,201219666	1
Ube2l6	1,201136407	1
Slc8b1	1,201136407	1
Anxa3	1,201136407	1
Polb	1,201136407	1
Gm16845	1,200969906	1
Rgl3	1,200886664	1
Sys1	1,200803427	1
Rflnb	1,200720197	1
BC002163	1,200636972	1
Ctbp2	1,200553753	1
Tmem168	1,20047054	1
Dck	1,200387333	1
Phf5a	1,200387333	1
Rasal3	1,200220935	1
Mta1	1,200220935	1
Tmem30a	1,200220935	1
Aldoart1	1,199555576	1
Ddx23	1,199555576	1
Kazald1	1,199472432	1
Gm6123	1,199472432	1
Gm13328	1,199223035	1
Cd81	1,199056799	1
Usf1	1,198890586	1
Cep164	1,198890586	1
Lta	1,198807488	1
Met	1,198558229	1
Osbp2	1,198558229	1
Rpl36a	1,198309021	1
Bcl6	1,198225964	1
Pdcl	1,198225964	1
Unc119	1,198225964	1
Slc41a1	1,198142912	1
Uqcrh-ps1	1,197893791	1
Gm6733	1,197893791	1
Stk25	1,197893791	1
Mknk2	1,197893791	1
Slc35e4	1,197727739	1
Tubb4a	1,197644722	1
Fam13b	1,19756171	1
Ppm1k	1,19756171	1
Sfxn3	1,19756171	1
Ola1	1,19756171	1
Tmem106c	1,197478705	1
Mcm9	1,197395705	1
4933427D14Rik	1,197395705	1
Tmem219	1,197395705	1
Sema6c	1,19731271	1
Snhg12	1,197146739	1
1700030M09Rik	1,197063762	1

Lrp5	1,196897825	1
Ctnnbip1	1,196897825	1
Gm4859	1,196731911	1
Btf3l4	1,196731911	1
Usp32	1,196731911	1
Ehmt2	1,196731911	1
Micu3	1,196648963	1
Hinfp	1,196648963	1
Phip	1,196566021	1
Nmt1	1,196566021	1
Cd83	1,196483084	1
Ccdc50	1,196483084	1
Gm9703	1,196400153	1
Uck1	1,196400153	1
Ints9	1,196317228	1
Ten1	1,196317228	1
Trmt1	1,196234308	1
Arhgdia	1,196151394	1
Asph	1,195902688	1
Stip1	1,195819797	1
Cnksr3	1,195736912	1
Acads	1,195654032	1
Cxcl10	1,195571159	1
Zbtb26	1,195571159	1
Zfp428	1,195488291	1
Uros	1,195488291	1
Gm12396	1,195488291	1
Adam15	1,195488291	1
Gm37522	1,195405429	1
Gm6433	1,195405429	1
Ift172	1,195322573	1
Ccdc126	1,195156877	1
Dr1	1,195156877	1
RP23-349H12.3	1,194991205	1
Lipe	1,194908377	1
Fam114a1	1,194908377	1
Brd9	1,194908377	1
Fcrl5	1,194742739	1
Smc3	1,194742739	1
Spsb1	1,194659929	1
4930579K19Rik	1,194494325	1
Usp45	1,194494325	1
Neurl1b	1,194494325	1
Gm11977	1,194411532	1
Angptl6	1,194411532	1
Rnf34	1,194245963	1
Eloc	1,194080417	1
Fam64a	1,193997652	1
Arl5b	1,193997652	1
Bmt2	1,193997652	1
Gm11221	1,193914894	1
Kirrel3	1,193832141	1

Pgls	1,193749393	1
Ric8b	1,193583916	1
Mpv17l2	1,193583916	1
2810403D21Rik	1,193501186	1
Samd4b	1,193501186	1
Efhd2	1,193335743	1
Mvp	1,19325303	1
Usp1	1,193087622	1
Bbof1	1,193004926	1
Traf4	1,192756873	1
Arhgap11a	1,192756873	1
Cab39l	1,192756873	1
Fam168a	1,192674201	1
Gna13	1,192591534	1
Hdac3	1,192508872	1
Slmap	1,192508872	1
Tmed4	1,192508872	1
Slc37a1	1,192343567	1
Cxcr4	1,192343567	1
Cops7a	1,192178284	1
Galt	1,192178284	1
Snx8	1,192178284	1
2810414N06Rik	1,192095652	1
Tfdp2	1,192095652	1
Sirt2	1,192095652	1
Gmppa	1,192013025	1
Agpat3	1,192013025	1
Maged2	1,191930404	1
Rbm22	1,191930404	1
Ppp5c	1,191847788	1
Gm4604	1,191765179	1
Rbm4	1,191682575	1
Chek1	1,191434797	1
Vprbp	1,191434797	1
Incenp	1,191187071	1
Fam126b	1,191021949	1
2900005J15Rik	1,190939396	1
Cdc25c	1,190856849	1
Hdgfrp2	1,190691773	1
Eif4g2	1,190609243	1
E2f6	1,19052672	1
Kpna1	1,19052672	1
Gm17994	1,190114186	1
Gm4994	1,190114186	1
Tac4	1,190031696	1
Nusap1	1,190031696	1
March2	1,189866734	1
Snx13	1,189701795	1
Mettl5	1,189619334	1
Nucks1	1,189536879	1
Mpzl1	1,189454429	1
Bbs12	1,189371986	1

Nudt9	1,189207115	1
Pdgfa	1,189207115	1
Pfn1	1,189124688	1
Fgf13	1,189042267	1
Polr2d	1,188959852	1
Cbx1	1,188959852	1
Ttc25	1,188877442	1
Ppp6r2	1,188795039	1
Pgpep1	1,188630248	1
Bank1	1,188383105	1
Thop1	1,188383105	1
RP24-550H10.4	1,188218372	1
Bcas2	1,188136013	1
Fyttd1	1,188136013	1
Apobec3	1,188053661	1
Slc12a6	1,188053661	1
Fh1	1,188053661	1
Szrd1	1,188053661	1
Hoxa4	1,187971314	1
Dmxl2	1,187888973	1
Stradb	1,187641984	1
Zbtb43	1,187312746	1
Pak1	1,187312746	1
Ap4m1	1,18723045	1
5830432E09Rik	1,187065877	1
Tuba1b	1,186901326	1
Ptma	1,186572292	1
Larp7	1,18640781	1
Cyth2	1,186325577	1
Chmp1a	1,18624335	1
Ikzf5	1,18624335	1
Smdt1	1,186161129	1
Zbtb8a	1,186078913	1
Glo1	1,186078913	1
2810454H06Rik	1,185996704	1
Mrpl50	1,185996704	1
Exoc2	1,185914499	1
Cnot3	1,185832301	1
Vezf1	1,185750108	1
Cdipt	1,185503564	1
Gm25517	1,185421394	1
Abca3	1,185421394	1
Myadm	1,18533923	1
Rpl23a-ps5	1,185257071	1
Gm5302	1,185174918	1
Mob2	1,185174918	1
Vps35	1,185174918	1
Gm43756	1,185092771	1
Prr7	1,185092771	1
Hoxb5	1,18468212	1
Ndufa3	1,18468212	1
Rptor	1,184600007	1

H2afj	1,184600007	1
Tssc4	1,1845179	1
Gm4963	1,184435798	1
Crip1	1,184353702	1
Acadm	1,184271612	1
2310010J17Rik	1,184189527	1
Tmem109	1,184107448	1
Clpb	1,184025375	1
Kdm3b	1,183943308	1
Morf4l2	1,183615094	1
Wwox	1,183451022	1
Flna	1,183451022	1
RP24-122E11.4	1,183204956	1
Dusp19	1,183204956	1
Jak3	1,182958941	1
Ndufab1	1,182958941	1
Mrpl52	1,182958941	1
Gm13453	1,182876947	1
Gm45050	1,182876947	1
Gm5547	1,182794959	1
Yeats2	1,182712977	1
Lrrc45	1,182712977	1
Lypla1	1,182631	1
Slc2a1	1,182549029	1
0610012G03Rik	1,182549029	1
2310061I04Rik	1,182467064	1
Gtf2f1	1,182385105	1
Serf2	1,182303151	1
Ogfrl1	1,182303151	1
Spidr	1,182221203	1
Acaa1b	1,18213926	1
Cdkn2d	1,181975392	1
Pik3ip1	1,181975392	1
Pdpk1	1,181811547	1
Uchl5	1,181811547	1
Oxa1l	1,181811547	1
Ppil4	1,181647724	1
Ppm1m	1,181074524	1
Mecp2	1,181074524	1
Gatsl2	1,180910804	1
Hif1a	1,180828952	1
Lsm1	1,180747106	1
Timp2	1,180501603	1
Ddost	1,180501603	1
Mxi1	1,180501603	1
Gm13050	1,180337962	1
Gpi1	1,180337962	1
Gas5	1,180010748	1
Entpd1	1,179847175	1
Slc27a3	1,179847175	1
Tmed10	1,179847175	1
Ubap2	1,179765397	1

Arhgap22	1,179683625	1
Sgol2a	1,179601858	1
Arrdc4	1,179601858	1
Commd4	1,179601858	1
Akirin1	1,179520097	1
Gm43655	1,179111377	1
Plcg2	1,179111377	1
Pank2	1,179111377	1
Dus4l	1,179029651	1
Adgrl1	1,178947929	1
Rufy3	1,178376039	1
Hsp90ab1	1,178376039	1
Asrgl1	1,178294363	1
Ccdc84	1,178212693	1
Itga7	1,177967716	1
Gm16223	1,177886068	1
Nsun6	1,177886068	1
Acbd6	1,177559534	1
Ska2	1,177477914	1
Hsf1	1,177396301	1
Colgalt1	1,177314693	1
Gid4	1,17723309	1
Hnrnpa2b1	1,17723309	1
Fdft1	1,177151494	1
Crls1	1,176335837	1
Acot1	1,176254302	1
Mbd2	1,176172774	1
D330023K18Rik	1,175928221	1
Pmm1	1,175928221	1
Galnt11	1,175846714	1
Cyb5r1	1,175683719	1
Pigo	1,175602229	1
Armc6	1,175602229	1
4932438A13Rik	1,175602229	1
Gmcl1	1,175602229	1
Gm38335	1,175276328	1
Sapcd1	1,175276328	1
Ncapd2	1,175113412	1
Isca1	1,174950518	1
Idh3a	1,174869079	1
Hnrnpdl	1,174869079	1
Tsta3	1,174787646	1
Ddx51	1,174706219	1
Brpf3	1,174706219	1
Serpinb8	1,174543382	1
Mrpl47	1,174217774	1
Eif3s6-ps1	1,174136386	1
Tmed3	1,173973628	1
Hcfc1r1	1,173648178	1
Mybl2	1,17356683	1
Vps52	1,17356683	1
Zfp827	1,17356683	1

Snhg4	1,173485487	1
Cops9	1,173485487	1
Ndufs1	1,17340415	1
Zfp397	1,173322819	1
Ccnl2	1,173322819	1
Frk	1,173241493	1
Spag7	1,173241493	1
Ints6l	1,173241493	1
Rer1	1,173160173	1
Fam96a	1,173078859	1
Gm26384	1,17299755	1
Reep4	1,172834949	1
Gm11970	1,172834949	1
Sptssa	1,172753657	1
Gm5637	1,17259109	1
Galc	1,17259109	1
Gm15796	1,172428546	1
Gm15727	1,172428546	1
Gatm	1,172428546	1
9930104L06Rik	1,172103525	1
Msh3	1,171941048	1
Gstz1	1,171859818	1
Ankle2	1,171697375	1
Sptlc2	1,171534955	1
Gca	1,171453753	1
Stt3a	1,171453753	1
Taf1d	1,171453753	1
Stam	1,171291366	1
Crat	1,171210181	1
Hmox1	1,171210181	1
Nemf	1,171210181	1
Dennd4a	1,171210181	1
Gm13743	1,171047828	1
Gm20522	1,171047828	1
Fam210a	1,171047828	1
Med19	1,170804341	1
Sat1	1,170804341	1
Hadha	1,170804341	1
Exoc6b	1,17047977	1
Gm15782	1,170398641	1
Tspan17	1,170317518	1
Cacna1d	1,170236401	1
Trim24	1,170236401	1
Ssbp4	1,170236401	1
Gltp	1,170236401	1
Meaf6	1,170074183	1
Per3	1,169911988	1
Plxnc1	1,169911988	1
Trem2	1,169830898	1
Trmt1l	1,169668737	1
Ggct	1,169668737	1
Fam53c	1,169587664	1

Anp32b	1,169506597	1
Brk1	1,169506597	1
Tmem164	1,16934448	1
Lcorl	1,169182386	1
Matr3	1,169182386	1
Tprgl	1,169020314	1
Cldn15	1,168858265	1
Bst1	1,168858265	1
Tnfsf8	1,168858265	1
Wisp1	1,168858265	1
Vps29	1,168858265	1
Rps15	1,168777249	1
Stx18	1,16845324	1
Kdm2b	1,168372251	1
Gm42890	1,168291269	1
Gm37949	1,168048355	1
Gm561	1,168048355	1
C330007P06Rik	1,167967395	1
Uxs1	1,167967395	1
Gpr137b-ps	1,16788644	1
Fasn	1,16788644	1
Csnk1g2	1,16788644	1
Manf	1,16788644	1
Chrna1os	1,167562678	1
Gsk3a	1,167400831	1
Nt5c2	1,167158102	1
BC029722	1,167158102	1
P4ha1	1,167158102	1
Map3k15	1,166996311	1
Gm44935	1,166996311	1
Cd200r1	1,166915423	1
Tctn1	1,166915423	1
Btbd7	1,166753666	1
Pigx	1,166753666	1
Pgrmc2	1,166672795	1
Dnajb9	1,166591931	1
Ccdc137	1,166591931	1
As3mt	1,166511071	1
Tpk1	1,166511071	1
Cdyl2	1,166268527	1
Grasp	1,166106859	1
Zcchc11	1,166106859	1
Psmb2	1,166026034	1
Gm13422	1,166026034	1
Rbm14	1,166026034	1
RP24-499N24.6	1,1658644	1
Il2rg	1,165379631	1
Agpat4	1,165379631	1
Insl6	1,165218086	1
Rps20	1,165137322	1
Mllt3	1,165056564	1
Pcna-ps2	1,164975811	1

Gpr155	1,164975811	1
Golga3	1,164975811	1
Acvr2b	1,164895064	1
Vamp7	1,164652856	1
Tfrc	1,164652856	1
Mvb12a	1,164652856	1
Serp1	1,164652856	1
Socs5	1,164572131	1
Mrpl21	1,164491412	1
Mcfd2	1,164329991	1
Sprysd4	1,164249288	1
Melk	1,164168591	1
Sumo1	1,164168591	1
Cdca3	1,1640879	1
Ltc4s	1,1640879	1
Lin54	1,164007214	1
Gm26772	1,163926534	1
Irf2bp2	1,163684528	1
Fcer1g	1,16360387	1
Ext2	1,163200666	1
Gm8662	1,163120042	1
Zfp383	1,163120042	1
Anapc13	1,163039423	1
Yy1	1,163039423	1
Lzts3	1,16295881	1
Vps41	1,16295881	1
Swt1	1,16295881	1
Atxn2	1,16295881	1
Urod	1,162878203	1
Zfp182	1,162717005	1
Pcca	1,162636414	1
Tmem41b	1,162636414	1
Pcm1	1,162636414	1
Snora30	1,162555829	1
Adck2	1,162555829	1
Dynll2	1,16247525	1
Kank2	1,162394676	1
Zdhhc4	1,162072436	1
Ube2t	1,162072436	1
Ctdp1	1,161830815	1
Snrpert	1,161750286	1
Snhg18	1,161750286	1
Mgat4a	1,161750286	1
Msn	1,161750286	1
D630023F18Rik	1,161669762	1
A830080D01Rik	1,161347724	1
Hmgcr	1,161347724	1
Manbal	1,161267228	1
Fam49b	1,161267228	1
E130307A14Rik	1,161025774	1
Zfp346	1,161025774	1
Dnajc18	1,161025774	1

Gm6682	1,160864833	1
Gm13015	1,160703914	1
Cope	1,160623463	1
Cln8	1,160623463	1
Nme1	1,160543018	1
1110025M09Rik	1,160382144	1
Aph1b	1,160382144	1
Blmh	1,160221292	1
Gm14173	1,160140874	1
Xpa	1,159899655	1
Arhgap23	1,159819259	1
Leprot	1,159819259	1
Gm5835	1,159738869	1
Pop5	1,159738869	1
Naa16	1,159738869	1
Dgka	1,159658485	1
Pdcd1	1,159578107	1
Fam63b	1,159497734	1
Ipo4	1,159337004	1
Smurf1	1,159176297	1
Gm12341	1,159095952	1
Slc15a4	1,159015612	1
Gm4217	1,158935278	1
Sec62	1,158935278	1
Elavl1	1,15885495	1
Skp1a	1,158774627	1
Tmem242	1,158694309	1
Jmjd6	1,158613998	1
Tarbp1	1,158533691	1
Ube2k	1,158533691	1
Park7	1,158533691	1
Cdadc1	1,158373096	1
Dynlt3	1,158373096	1
Tnfrsf18	1,158292806	1
Ndufa2	1,158292806	1
Ypel3	1,158212522	1
Sap18b	1,158132244	1
Tulp4	1,158132244	1
Calm1	1,158051971	1
Gnpda2	1,157891442	1
Ccsap	1,157811186	1
Fcgrt	1,157811186	1
Gm4924	1,157730935	1
Tcam1	1,15765069	1
Bmpr2	1,157570451	1
Cpeb4	1,157409989	1
Cdc42	1,157409989	1
Gm42819	1,157249549	1
Slfn4	1,156688184	1
Abhd4	1,156688184	1
Josd2	1,156608011	1
Vrk1	1,156608011	1

Rap2a	1,156527844	1
Selenof	1,156207231	1
Atp7a	1,156207231	1
Smpd1	1,156207231	1
Bzw1	1,156127091	1
Msl3	1,156046958	1
Acot8	1,155966829	1
Yme1l1	1,155886707	1
Rsad1	1,155646372	1
Gm6444	1,155646372	1
Rpap3	1,155486176	1
Syngr1	1,155486176	1
Gm26620	1,155326003	1
Agbl5	1,155326003	1
Gm44567	1,155245925	1
Rps3a3	1,155245925	1
Ddx19b	1,155165852	1
Apeh	1,155005723	1
Arl5c	1,154925667	1
Mtpap	1,154765571	1
Tubgcp2	1,154685532	1
Gm11716	1,154445447	1
Cd320	1,154445447	1
Trp53inp2	1,154445447	1
Mnt	1,154445447	1
Abracl	1,154285418	1
Stambpl1	1,154285418	1
Scyl2	1,154205411	1
Mrps9	1,154125411	1
Plpp2	1,153885442	1
Atxn1l	1,153805464	1
Lbr	1,153725491	1
Srd5a1	1,153485605	1
St6galnac4	1,153485605	1
Arfrp1	1,153405654	1
Cep57	1,153165835	1
Gm6905	1,153085907	1
Ei24	1,153005984	1
Arhgap10	1,153005984	1
Zfp459	1,152766248	1
Gm43628	1,152606451	1
Abcb7	1,152606451	1
5830408C22Rik	1,152526562	1
Gm19503	1,152526562	1
Gm15427	1,152526562	1
Tesk1	1,152446677	1
Zfp429	1,152366799	1
Pfdn1	1,152286925	1
Rfc4	1,152207058	1
Tmem120b	1,152127196	1
Gm43445	1,152127196	1
D630024D03Rik	1,152047339	1

Pnkd	1,152047339	1
Pigu	1,152047339	1
Orc4	1,152047339	1
Itpa	1,151967488	1
Sec61g	1,151967488	1
Capza2	1,151967488	1
Rab3gap1	1,151967488	1
Eid3	1,151887642	1
Tkfc	1,151887642	1
Pcdhb17	1,151727968	1
Rps19bp1	1,151727968	1
Lpxn	1,151648139	1
Mrpl23	1,151328879	1
Aktip	1,151328879	1
Cntln	1,151249077	1
Gm9803	1,151169282	1
Zbtb21	1,151089491	1
Gm31166	1,151089491	1
Nova1	1,151009707	1
Akr1b10	1,151009707	1
Gm42547	1,150929928	1
Sdccag3	1,150850154	1
Epb41	1,150850154	1
Gm38376	1,150770386	1
Gm6654	1,150770386	1
Ogfod1	1,150531115	1
Cenpf	1,150451369	1
Gm6377	1,150291893	1
Ctnna1	1,150291893	1
Aco1	1,150212164	1
Mis18bp1	1,150212164	1
Atad2	1,150212164	1
Shc1	1,150212164	1
Aes	1,15013244	1
Gm26542	1,150052722	1
1700056N10Rik	1,150052722	1
Pdcd10	1,1498136	1
Gm14303	1,1498136	1
Gm37531	1,149733904	1
Gm5449	1,149654213	1
Cryz	1,149574528	1
Pex10	1,149415174	1
Tecpr1	1,149415174	1
Gm13436	1,149335505	1
Gm38111	1,149176185	1
Fam171a2	1,149096533	1
Ptar1	1,148937245	1
Drg1	1,148937245	1
Baz2a	1,148937245	1
Rps12	1,148698355	1
Vps37b	1,148698355	1
Gm8731	1,148618736	1

Btbd9	1,148618736	1
Rhd	1,148539123	1
Gm15131	1,148459515	1
Irf2bpl	1,148300315	1
Arhgef25	1,148141138	1
2810030D12Rik	1,148061558	1
Cspp1	1,148061558	1
Fanca	1,147981983	1
Zfp667	1,14782285	1
Camk2n2	1,147663739	1
Ubap2l	1,147663739	1
Klhl28	1,147584192	1
Arfgef3	1,147584192	1
Ifi30	1,14750465	1
Atp5sl	1,147345583	1
Zfp637	1,147186538	1
Sugt1	1,147186538	1
Eif2s2	1,147027516	1
Gm11560	1,146948012	1
Sqstm1	1,146630055	1
Fuom	1,146471109	1
Tprn	1,146471109	1
4930503L19Rik	1,146391645	1
Tmem63b	1,146312186	1
Mtbp	1,146153284	1
D830050J10Rik	1,146153284	1
2610020H08Rik	1,146073842	1
Dzip3	1,146073842	1
Katna1	1,146073842	1
Top1	1,145914973	1
Scand1	1,145835547	1
1700025G04Rik	1,145676711	1
Rps11-ps3	1,145517898	1
Hdhd2	1,145517898	1
Gm43817	1,145200337	1
Gm16638	1,145120961	1
Pick1	1,14504159	1
Eif1	1,14504159	1
Parvg	1,144962224	1
Anapc11	1,144882864	1
Vps13c	1,144882864	1
A430046D13Rik	1,144724161	1
Wdr82	1,144644817	1
Six1	1,144565479	1
Lrrcc1	1,144565479	1
Rab9	1,144565479	1
Gm12606	1,144565479	1
Ckap5	1,144486147	1
Nrg4	1,14440682	1
Wdr45b	1,14440682	1
Amn1	1,144327498	1
Usp33	1,144248182	1

Sf3b3	1,144089567	1
Rpl21-ps1	1,143930973	1
Men1	1,143930973	1
Napg	1,143930973	1
Ptgs2os	1,143772402	1
Dek	1,143772402	1
Prickle3	1,143693124	1
Cc2d1b	1,143613852	1
Phf6	1,143613852	1
Arf6	1,143534586	1
Fam133b	1,143455325	1
Rp9	1,143217574	1
Qtrt1	1,143138335	1
Dph3	1,143059102	1
Skiv2l2	1,142979874	1
Ccnd3	1,142821434	1
Wdyhv1	1,142583816	1
Rapgef3	1,142583816	1
Nudt16l1	1,142504621	1
Fbxl19	1,142267068	1
Sgcb	1,141950408	1
Slc13a2	1,141950408	1
Ripk2	1,141792111	1
Pih1d1	1,141712971	1
Ciao1	1,141712971	1
Ring1	1,141633836	1
Gm9701	1,141475583	1
Ranbp9	1,141475583	1
Zfp771	1,141475583	1
Ganab	1,141475583	1
Tchp	1,141317352	1
Usp37	1,141159143	1
Lpin2	1,14092187	1
Mettl25	1,14084279	1
Cdyl	1,14084279	1
Irx2	1,140763716	1
Ankrd50	1,140684647	1
Urb2	1,140605583	1
Arpc1b	1,140605583	1
Serpinc1	1,140368426	1
Fyb	1,140368426	1
Imp3	1,140289384	1
Gm9396	1,140210348	1
Gm20430	1,140210348	1
Gpsm2	1,140210348	1
Gosr1	1,140131318	1
Gm37352	1,139973273	1
Prmt7	1,139973273	1
Mcee	1,139973273	1
St3gal1	1,139973273	1
Rgs9bp	1,139894259	1
Runx3	1,13981525	1

Phf11d	1,139736247	1
Zfp143	1,139736247	1
Amfr	1,139657249	1
Srsf5	1,139657249	1
Sh3glb1	1,139657249	1
Lims1	1,13949927	1
Pphln1	1,139420288	1
Zfp622	1,139420288	1
Iqcf1	1,139341313	1
Ensa	1,139183377	1
Tmem256	1,139183377	1
Gm7670	1,138946515	1
Epb41l5	1,138867572	1
Arl6ip1	1,138709703	1
Ino80b	1,138630776	1
Nabp2	1,138630776	1
Gm8825	1,138551855	1
Dact3	1,138394029	1
Gm5644	1,138315124	1
Gm44190	1,138315124	1
Cr1l	1,138315124	1
Zdhhc1	1,138236225	1
Yaf2	1,13799956	1
Cfl2	1,13799956	1
Tbc1d16	1,137920683	1
Gm3145	1,137762944	1
E2f5	1,137684083	1
Clasrp	1,137684083	1
Ncor2	1,137684083	1
Cdc42se2	1,137684083	1
Fastk	1,137605228	1
Mlkl	1,137526378	1
Tnfrsf10b	1,137447533	1
Gm43154	1,137211032	1
Zfyve1	1,13697458	1
Copz1	1,13697458	1
Asnsd1	1,13697458	1
5430421F17Rik	1,136895774	1
Klhl41	1,136816973	1
Adat1	1,136738178	1
Syne3	1,136738178	1
Memo1	1,136659388	1
Gm28686	1,136659388	1
Mfap1a	1,136501824	1
Cers6	1,136501824	1
Spcs1	1,136501824	1
Foxd2	1,136423051	1
Gabpa	1,136423051	1
Shoc2	1,136423051	1
Dctn1	1,136344283	1
Rbm33	1,13626552	1
Med21	1,136186763	1

Stxbp2	1,136186763	1
Gm42551	1,136108011	1
Gm4149	1,136108011	1
Ccl9	1,136029265	1
Txndc17	1,135950524	1
Ptp4a1	1,135871789	1
P3h3	1,135793059	1
Aaas	1,135556902	1
Gm9840	1,135556902	1
Fbxl5	1,135556902	1
Dnajc3	1,135399491	1
Wdr44	1,135320794	1
Ppox	1,135242102	1
Cstf3	1,135242102	1
Npm1	1,135242102	1
Zfp811	1,135163416	1
Itpkb	1,135084735	1
Mospd2	1,13500606	1
Hnrnph1	1,13500606	1
Abcg1	1,13500606	1
St7	1,13492739	1
Gm44834	1,134848725	1
Parl	1,134848725	1
Zfp598	1,134848725	1
Irf2	1,134848725	1
Fam188b	1,134770066	1
Galnt15	1,134691413	1
Gm8973	1,134691413	1
Rpl31-ps17	1,134691413	1
Gm22581	1,134691413	1
Gm12430	1,134691413	1
Hsbp1	1,134691413	1
Bbs10	1,134612765	1
Stat2	1,134612765	1
Kif1c	1,134612765	1
Ndufa13	1,134534122	1
Chchd2	1,134455485	1
Glg1	1,134298227	1
Micall1	1,134219606	1
Ero1lb	1,134140991	1
Pcmtd2	1,134140991	1
Yipf4	1,134062381	1
Pcf11	1,134062381	1
RP23-128C4.4	1,133905177	1
Fnip1	1,133905177	1
Mcur1	1,133826584	1
Ctns	1,133826584	1
Vma21	1,133826584	1
Pigl	1,133747995	1
Gm26782	1,133512264	1
Siva1	1,133433697	1
Gga1	1,133433697	1

Pex2	1,133276581	1
Mrpl20	1,133276581	1
Galm	1,133198031	1
Rwdd1	1,133119486	1
Asap1	1,133119486	1
Fbxl20	1,133119486	1
Plk4	1,133040947	1
Aup1	1,133040947	1
Tspan10	1,132883885	1
Fignl2	1,132805362	1
Surf1	1,132726845	1
Rbmx2	1,132569827	1
Anxa6	1,132569827	1
Dpf2	1,132569827	1
Cep290	1,132491326	1
Gm44044	1,13241283	1
Naa10	1,13241283	1
Gng12	1,13241283	1
Ccdc174	1,132255855	1
Tcf25	1,132255855	1
Myef2	1,132098902	1
Irf1	1,131863513	1
Gm10923	1,131785061	1
C77080	1,131785061	1
Txndc5	1,131785061	1
Cotl1	1,131785061	1
Ssbp3	1,131706615	1
Terf2ip	1,131549738	1
Gm37906	1,131314463	1
Arl6ip5	1,13115764	1
Ndufaf4	1,13115764	1
Tfcp2	1,130922447	1
Spin1	1,130922447	1
Dda1	1,130765679	1
RP24-175C20.10	1,130608932	1
Gm4943	1,130530567	1
RP24-240E7.1	1,130530567	1
Snrnp35	1,130452207	1
Lrch3	1,130295504	1
Ccdc34	1,130217161	1
Uqcr10	1,130138823	1
Hdac1	1,13006049	1
Eif3k	1,13006049	1
1700037H04Rik	1,129825525	1
Gm12669	1,129668909	1
Rab3gap2	1,129668909	1
Bola3	1,129668909	1
Spg11	1,129512315	1
Fubp1	1,129512315	1
Fchsd2	1,129434026	1
Cryzl1	1,129434026	1
Kif22	1,129199191	1

Ddn	1,129120923	1
Dctd	1,129120923	1
Lrpap1	1,129120923	1
Phf12	1,129042661	1
Taf4	1,128886154	1
Gm9761	1,128807908	1
R3hdm1	1,128807908	1
Cdc37	1,128729668	1
Pqbp1	1,128573203	1
Gm11964	1,128338548	1
Gm37589	1,128338548	1
Vps26b	1,128338548	1
Zfp954	1,128338548	1
Snhg17	1,128103941	1
RP24-325P4.5	1,128025749	1
Asb3	1,127947563	1
Tsc22d1	1,127947563	1
E2f2	1,127947563	1
Wasf2	1,127947563	1
Hadh	1,127947563	1
Syncrip	1,127869382	1
Fahd2a	1,127791207	1
Rpl36a-ps2	1,127791207	1
Gm45184	1,127713037	1
Rock2	1,127634873	1
Sub1	1,127634873	1
Mrpl54	1,12747856	1
Rnase4	1,127400412	1
Gm13413	1,127322269	1
2310022A10Rik	1,127322269	1
Commd6	1,127244132	1
Ckap2l	1,127009753	1
Arap3	1,126931637	1
Csnk1e	1,126931637	1
Gm13391	1,126853527	1
Cdk12	1,126853527	1
Pkib	1,126697322	1
Scp2	1,126619228	1
Odc1	1,126463057	1
Rad51	1,126384979	1
Pwp1	1,126306907	1
Dvl1	1,126306907	1
Rpl8	1,126306907	1
Gm23346	1,12622884	1
Pex26	1,12622884	1
Idh3b	1,126072722	1
Itch	1,125916626	1
Fam118a	1,125838586	1
Pdk1	1,125760552	1
Herpud1	1,125760552	1
Strbp	1,125604499	1
Arpc3	1,125604499	1

Rpl21-ps14	1,125448468	1
Cpd	1,125448468	1
Brd8	1,125214462	1
Tmem248	1,125136471	1
Flcn	1,125058485	1
Gm9530	1,124746595	1
Srsf1	1,124746595	1
Gm10031	1,124746595	1
Hsd17b10	1,124668637	1
Gpr137b	1,124668637	1
Vps25	1,124590683	1
Tmed2	1,124590683	1
Clec4e	1,124590683	1
Ccdc93	1,124512735	1
Myoz1	1,124356856	1
Ddx41	1,124356856	1
Phpt1	1,124356856	1
D10Wsu102e	1,124356856	1
Taf8	1,124278924	1
Gm10169	1,124278924	1
Fes	1,124123076	1
Cript	1,124123076	1
Snx25	1,124045161	1
Sin3a	1,123967251	1
Ccni	1,123967251	1
Lrp1	1,123811447	1
5530601H04Rik	1,123733553	1
Mzf1	1,123733553	1
Pold1	1,123655664	1
Prkar2a	1,123655664	1
Thap4	1,123577781	1
Mob3a	1,123499903	1
Hmgn5	1,123499903	1
Psmb3	1,123422031	1
Patl1	1,123422031	1
Fgd4	1,123110595	1
Clasp2	1,122721422	1
Tmem184b	1,122721422	1
Plekhn1	1,122643604	1
Esyt1	1,122643604	1
Fzd5	1,122565791	1
Cmip	1,122565791	1
Rps27l	1,122565791	1
Nup133	1,122254592	1
2010107E04Rik	1,122254592	1
Psmb1	1,122176806	1
Gm7123	1,122099026	1
Hdlbp	1,12202125	1
Hbp1	1,12202125	1
Tube1	1,121865716	1
Rps18	1,121787957	1
Qdpr	1,121787957	1

Gm9025	1,121632455	1
Tmem165	1,121632455	1
Gm12517	1,121476974	1
Ern1	1,121399242	1
Chst10	1,121321515	1
Rc3h2	1,121321515	1
Kdelc2	1,121243794	1
Gm17259	1,121088367	1
Nt5dc2	1,121088367	1
Kars	1,121088367	1
Mertk	1,121010662	1
Sap30l	1,121010662	1
Ctnnbl1	1,121010662	1
Arhgap12	1,121010662	1
Stk11ip	1,120932962	1
Lims2	1,120932962	1
Mrpl28	1,120855268	1
Usp22	1,120855268	1
Copb1	1,120855268	1
Cysltr1	1,120777579	1
1700007L15Rik	1,120777579	1
RP24-550H10.6	1,120699895	1
Gm10269	1,120699895	1
Gm8129	1,120622217	1
Tex10	1,120544544	1
Ssx2ip	1,120466876	1
Mphosph9	1,120466876	1
Npl	1,120389214	1
Zscan2	1,120311557	1
Usp15	1,120311557	1
Rcn2	1,120233906	1
Ccar1	1,120233906	1
Efcab7	1,12015626	1
Uhmk1	1,12015626	1
RP23-168F21.4	1,120078619	1
Creb1	1,120078619	1
Txnrd2	1,119923354	1
Gm38247	1,11984573	1
Slc16a1	1,119768111	1
Klhdc4	1,119690497	1
Gm15773	1,119535286	1
Cpsf7	1,119457688	1
Scamp3	1,119457688	1
Bzw2	1,119457688	1
Man1a2	1,119302509	1
BC031181	1,119224928	1
Atp5h	1,119147352	1
Gm12276	1,118992216	1
Pvt1	1,118992216	1
Sf3b4	1,118837101	1
2210417A02Rik	1,118759552	1
9530053A07Rik	1,118682008	1

Celf3	1,118682008	1
Mfge8	1,118449409	1
Ppm1j	1,11829437	1
Tmem80	1,118061851	1
Gm5451	1,117829381	1
Prkaa1	1,117829381	1
Glrx	1,117829381	1
Pet100	1,117751901	1
Med9	1,117751901	1
Gm18737	1,117596959	1
E2f1	1,117442038	1
Slc35b1	1,117442038	1
Hspa13	1,11713226	1
Rcsd1	1,116899983	1
1110003F10Rik	1,116822568	1
Tor2a	1,116822568	1
Atf2	1,116822568	1
Gm42798	1,116745158	1
Cyhr1	1,116745158	1
Zbtb37	1,116358191	1
Fto	1,116358191	1
Hal	1,116280814	1
Ergic3	1,116203442	1
Tcta	1,115661988	1
Tmem161a	1,115661988	1
Iqsec2	1,115584659	1
Cd200r3	1,115507335	1
Ctxn1	1,115430017	1
Lta4h	1,115352704	1
Rabggtb	1,115198094	1
Tyw5	1,115120797	1
Dap3	1,115120797	1
Dtd2	1,115043505	1
Zfas1	1,115043505	1
1110065P20Rik	1,114966219	1
Ssr4	1,114888938	1
Cfap20	1,114734392	1
Paqr3	1,114734392	1
Rrp36	1,114734392	1
Sars	1,114734392	1
Nkap	1,114657127	1
Edf1	1,114657127	1
Gm35931	1,114502614	1
Aprt	1,114502614	1
Gm43128	1,114270884	1
Rhbdd1	1,114193651	1
Adipor1	1,114193651	1
Smim11	1,114116423	1
Bdh2	1,113961985	1
Gigyf1	1,113961985	1
Gm10443	1,113961985	1
Cnpy2	1,113884774	1

Etfa	1,113884774	1
Cnot10	1,113807568	1
Agps	1,113807568	1
Nfe2l1	1,113807568	1
Ggact	1,113730367	1
Oard1	1,113730367	1
Ubxn4	1,113498797	1
Cds2	1,113421618	1
Slc19a2	1,113344444	1
Abca5	1,113344444	1
Ctsz	1,113267276	1
E330034L11Rik	1,113190113	1
Rngtt	1,113190113	1
Mir5136	1,113112955	1
Dst	1,112958655	1
Stx3	1,112958655	1
Rnf10	1,112804377	1
Etnk1	1,112804377	1
Mms22l	1,112650121	1
2410089E03Rik	1,112573	1
Ly9	1,112573	1
Arid3b	1,112495885	1
Gm10827	1,112341671	1
Itpr3	1,112341671	1
Gm16399	1,112341671	1
Nars2	1,112264572	1
Acy3	1,112187479	1
Gm13039	1,112187479	1
Ptges2	1,112187479	1
Echdc1	1,112187479	1
Slc45a3	1,112187479	1
Psmd14	1,112187479	1
Acat1	1,111879158	1
H2-K1	1,111879158	1
Acox1	1,111725029	1
Pdp1	1,111647973	1
Nampt	1,111647973	1
Trappc2l	1,111570922	1
Jade3	1,111493876	1
Gins4	1,111493876	1
Camk2n1	1,111339801	1
Ms4a6d	1,111339801	1
Rpl3-ps2	1,111262772	1
Trappc11	1,111262772	1
Cst7	1,111185748	1
Psmd9	1,111185748	1
Spryd7	1,110646728	1
Megf9	1,110338834	1
Mrto4	1,110261873	1
Tmem208	1,110184919	1
Uqcrq	1,110184919	1
Mttp	1,110107969	1

Fam173a	1,110107969	1
Cops4	1,110107969	1
Gm14706	1,110031025	1
Znrd1	1,110031025	1
Gnb4	1,109877153	1
Oat	1,109800225	1
Amd1	1,109723302	1
Ndufb11	1,109723302	1
Pim3	1,109723302	1
Rps6kb2	1,109569472	1
Copg2	1,109415664	1
Zfand2a	1,109415664	1
Fhod3	1,109338768	1
Gm42467	1,109338768	1
Tspan31	1,109031236	1
Dennd6a	1,109031236	1
Cd2bp2	1,109031236	1
Fbxl3	1,108800644	1
Gm7434	1,108646942	1
Dus3l	1,108646942	1
Gm42747	1,108570099	1
Nr1d1	1,108570099	1
Eloa	1,108570099	1
Emilin2	1,108570099	1
Ap3m2	1,108493261	1
Ptcd3	1,108339602	1
Pld3	1,108339602	1
Def8	1,108262781	1
Naa15	1,108262781	1
Smg7	1,108185964	1
Traf3	1,108032348	1
Kxd1	1,108032348	1
Gm11914	1,107878753	1
Bhlhe40	1,107878753	1
Gm11945	1,107801963	1
Dnajc5	1,107801963	1
Gm45360	1,1076484	1
Icam1	1,107341337	1
Gm12176	1,107187837	1
Kcnc3	1,107111096	1
1700086O06Rik	1,107111096	1
Ssbp1	1,107111096	1
Mis18a	1,106957628	1
M6pr	1,106957628	1
Eif2s1	1,106957628	1
Nat2	1,106727467	1
Rrm2	1,106650757	1
Birc5	1,106650757	1
Cdca8	1,106650757	1
Trip13	1,106420659	1
Mgmt	1,106343971	1
Dgkg	1,106267287	1

Fars2	1,106267287	1
Zbtb49	1,106113937	1
Rxra	1,106113937	1
Unc5b	1,106037269	1
Ext1	1,106037269	1
Rhoq	1,105960607	1
Fam65a	1,105883951	1
Prpf4	1,105883951	1
Havcr2	1,105730653	1
Cdk6	1,105730653	1
Gm28438	1,105654013	1
Gxylt1	1,105577377	1
Gm9134	1,105500747	1
Smad7	1,105500747	1
Cnppd1	1,105500747	1
Clip2	1,105424122	1
Mbnl2	1,105347503	1
Gm13532	1,105270888	1
Tceal8	1,105117676	1
Sorbs1	1,105117676	1
Epm2aip1	1,104887897	1
Sharpin	1,104811315	1
G3bp2	1,104734738	1
Fam213a	1,104658166	1
Gm10288	1,104428483	1
1810058I24Rik	1,104275387	1
Cwf19l1	1,103969259	1
Ddx49	1,103969259	1
Ctcf	1,103663216	1
Sgf29	1,10343374	1
Fxyd2	1,10343374	1
Tardbp	1,10343374	1
Bola2	1,103357258	1
Kdm5d	1,103204311	1
Atf6b	1,103204311	1
Pdcd5	1,103051385	1
Xpnpep1	1,10297493	1
Tpp2	1,102898481	1
Slc7a8	1,102822036	1
Lpl	1,102822036	1
Eef2kmt	1,102516311	1
Map2k1	1,102516311	1
Rpl30-ps3	1,102439893	1
Tle1	1,102439893	1
Ubqln4	1,102363481	1
Prpf4b	1,102363481	1
Fcna	1,102210671	1
Slc35c2	1,101981497	1
Hlcs	1,101905116	1
Snx27	1,10182874	1
Pura	1,10182874	1
Gm43362	1,10175237	1

Wbp1	1,10175237	1
Tmtc3	1,101676005	1
Atxn7l1	1,101676005	1
Cox6a1	1,101599645	1
Cntd1	1,101523291	1
Os9	1,101523291	1
Prpf38b	1,101523291	1
Gm10146	1,101370598	1
Med25	1,101370598	1
Gm5617	1,101370598	1
Sord	1,101294259	1
Tmem261	1,101217926	1
Hip1	1,101141598	1
Tmco6	1,100988958	1
Slc22a13b-ps	1,100988958	1
Rps24-ps2	1,100988958	1
Rp2	1,100912646	1
Gcn1l1	1,100836339	1
Tcp11l2	1,100760038	1
Galnt4	1,10060745	1
RP23-356P21.1	1,10060745	1
Gm43742	1,100454884	1
Sptan1	1,100378609	1
Tmem106b	1,100378609	1
Abcb8	1,10030234	1
Fam126a	1,10030234	1
Eif3i	1,100149816	1
Timmdc1	1,099997313	1
Mbd4	1,099997313	1
Klhdc1	1,099997313	1
Map1lc3a	1,09992107	1
Igsf8	1,09992107	1
H6pd	1,099616149	1
Jam2	1,099616149	1
Gm8730	1,099463721	1
Zfp524	1,099387514	1
Immp2l	1,099311313	1
Mpp5	1,099235117	1
Gm8805	1,098854218	1
Ddx6	1,098778053	1
Tmc6	1,098625741	1
Tusc2	1,098625741	1
Zmym2	1,098549593	1
Gabpb2	1,09832118	1
Bloc1s6	1,09832118	1
Ppp2r5d	1,098245052	1
Bdp1	1,098245052	1
Mettl4	1,098092814	1
Inppl1	1,098092814	1
Cep295	1,098016702	1
Tomm22	1,097864496	1
Kansl1l	1,0977884	1

Padi2	1,09771231	1
Nsa2	1,09771231	1
B2m	1,09771231	1
Fopnl	1,09771231	1
Tmem206	1,097484071	1
Rgs1	1,097408001	1
Rnf187	1,097408001	1
Pla2g4a	1,097408001	1
Gm19898	1,097255879	1
Gm15198	1,097255879	1
Ranbp10	1,097255879	1
Srp72	1,097179826	1
Ndufb10	1,097103778	1
Tor3a	1,097027735	1
Ppa1	1,097027735	1
Zfp444	1,096951697	1
Pianp	1,096951697	1
Fchsd1	1,096875665	1
Cmc1	1,096799638	1
Rpl38-ps1	1,096723616	1
B230312C02Rik	1,0966476	1
Ryr1	1,096571589	1
Fam179b	1,096571589	1
Fam135a	1,096495583	1
Gm36189	1,096419582	1
Syce2	1,096419582	1
Gm9892	1,096419582	1
Kctd2	1,096343587	1
Inip	1,096343587	1
Phf1	1,096343587	1
Gm44913	1,096191612	1
Wee1	1,096191612	1
Arel1	1,096191612	1
Ywhaq	1,096191612	1
Rab5c	1,096191612	1
Rplp1	1,096191612	1
Ttc4	1,096115632	1
Vps11	1,096039658	1
Taf2	1,095887725	1
Armc10	1,095887725	1
Pak2	1,095887725	1
Pex7	1,095811766	1
Slc9a5	1,095735813	1
A330069E16Rik	1,095432053	1
Gm17971	1,095432053	1
Cbx2	1,095432053	1
Vrk2	1,095432053	1
Creld1	1,095356126	1
Kcmf1	1,094900675	1
Pcdh7	1,094824785	1
Clk1	1,094824785	1
Chmp2b	1,094824785	1

Gm43430	1,0947489	1
Yipf3	1,0947489	1
Msto1	1,0947489	1
Alkbh1	1,094597146	1
1810043G02Rik	1,094521277	1
Hsd17b12	1,094521277	1
Cyb5b	1,094445413	1
Gm5453	1,094369555	1
Fkbp1a	1,094369555	1
Atp6v0e	1,094293701	1
Mapk3	1,094293701	1
Parn	1,094217853	1
Git1	1,094217853	1
Neil3	1,09414201	1
Ptger4	1,094066173	1
Fam92a	1,093990341	1
E2f3	1,093687064	1
Dhx8	1,093687064	1
Mcm2	1,093611258	1
Yod1	1,093535457	1
Zw10	1,093459662	1
Mphosph10	1,093308087	1
Vma21-ps	1,093156533	1
Gm9497	1,093156533	1
Prox2	1,093156533	1
Trnt1	1,093156533	1
Cntrob	1,093080763	1
Eif4enif1	1,093080763	1
Celf1	1,092929241	1
1700112E06Rik	1,092626258	1
Ugdh	1,092626258	1
Gucy2g	1,092474799	1
Mief2	1,092474799	1
Igbp1	1,092474799	1
Gpr35	1,09232336	1
Relt	1,09232336	1
Lsm6	1,092171942	1
Gm11625	1,092096241	1
Ndufa4	1,092096241	1
Nmt2	1,092096241	1
Spata2l	1,092020546	1
Pald1	1,092020546	1
Map3k3	1,092020546	1
Plbd2	1,092020546	1
Cnpy4	1,09186917	1
Psen1	1,09186917	1
Arl3	1,09186917	1
Rsrc1	1,09186917	1
Nap1l1	1,09186917	1
Erfe	1,091566482	1
Lmbrd2	1,091415169	1
2510039O18Rik	1,091415169	1

Myo1g	1,09133952	1
Fkbp5	1,09133952	1
Hyou1	1,09133952	1
Atp5k	1,09133952	1
Ap4s1	1,091263877	1
Gm44950	1,091263877	1
Tubgcp5	1,091263877	1
Fbxo21	1,091112606	1
Arfip2	1,091036979	1
Zfp275	1,091036979	1
Rrn3	1,090734521	1
Gprasp1	1,090583324	1
Fbxl17	1,090507733	1
Ctso	1,090507733	1
Coro1b	1,090507733	1
Nfrkb	1,090507733	1
Ldha	1,090507733	1
Rps27-ps1	1,090280992	1
Edem1	1,090280992	1
Pmpcb	1,090205422	1
Dnph1	1,090054298	1
Pradc1	1,089827651	1
Pcdhb15	1,089827651	1
Cdc5l	1,089827651	1
Rps26	1,089827651	1
Sh2d5	1,089676579	1
Syngap1	1,089525528	1
Wrn	1,089450011	1
Por	1,089450011	1
Llph	1,089298991	1
Smg1	1,089147993	1
Mpc2	1,089072501	1
Nfic	1,088997015	1
Mms19	1,088921534	1
A330074K22Rik	1,088846059	1
Zbtb11	1,088846059	1
Serinc1	1,088695123	1
Nedd8	1,088695123	1
Gpcpd1	1,088619663	1
Atp5c1	1,088619663	1
Btbd1	1,088619663	1
Bnip1	1,088242442	1
Pax3	1,088167013	1
Bad	1,088167013	1
Lonp1	1,088167013	1
Pqlc2	1,088016172	1
Ppp3cb	1,088016172	1
Gm16437	1,087940759	1
Gm7292	1,087940759	1
Baiap2	1,087940759	1
Agk	1,087789948	1
Hmgb3	1,087639159	1

Snhg8	1,087639159	1
Cry2	1,087639159	1
Prob1	1,087563772	1
BC028528	1,087488391	1
Serpinb6a	1,087413015	1
Trim8	1,087337643	1
Blcap	1,087337643	1
Tnni3	1,087262278	1
Phf2	1,087186917	1
Zfp280b	1,086960866	1
Kctd9	1,086960866	1
Rbm38	1,086885526	1
Aga	1,086885526	1
Gm45716	1,086810192	1
2810433D01Rik	1,086810192	1
Lrrc41	1,086810192	1
Rpl36al	1,086659538	1
Gm37486	1,086433597	1
Sart3	1,086433597	1
Procr	1,086358294	1
Gm12501	1,086358294	1
Rps26-ps1	1,086282996	1
Bcl7c	1,086207703	1
Cnot8	1,086207703	1
Xrcc2	1,086132416	1
Abcb1b	1,086132416	1
Gm4875	1,086057133	1
Scoc	1,086057133	1
Qsox1	1,086057133	1
Pole3	1,085831318	1
Grhpr	1,085605549	1
Vcp-rs	1,085605549	1
Ece2	1,085530303	1
Banf1	1,085530303	1
Mettl10	1,085379827	1
Specc1	1,085379827	1
Zc3hc1	1,085379827	1
Tet3	1,085304597	1
Ppp1r12c	1,085304597	1
Sae1	1,085229372	1
Cers4	1,085154152	1
4833439L19Rik	1,085003728	1
Bag1	1,084928524	1
Rsf1	1,084702943	1
Pdcl3	1,084477409	1
Acsl3	1,084477409	1
Rabgap1l	1,084402241	1
Bcl2l13	1,084402241	1
Idh3g	1,084402241	1
C030013C21Rik	1,084251922	1
Larp1	1,084026481	1
Eef1b2	1,083876214	1

Pear1	1,083801088	1
Gm15694	1,083500636	1
Slc9a3r2	1,083200267	1
Mrpl51	1,083200267	1
Pde7a	1,083200267	1
Maea	1,083200267	1
Ankmy2	1,083125188	1
Guk1	1,083125188	1
Gls	1,083125188	1
Nxt1	1,082899982	1
Prnp	1,082899982	1
Ier3ip1	1,082899982	1
Rab14	1,082899982	1
Mbd5	1,082824924	1
Trap1	1,082824924	1
0610030E20Rik	1,082824924	1
Upf3b	1,082824924	1
Icmt	1,082749871	1
Ipo7	1,082674823	1
Cd99l2	1,08259978	1
Rhbdf2	1,08244971	1
Golt1b	1,08244971	1
Dmrta2	1,082374683	1
Ilf2	1,082299661	1
Prkacb	1,082299661	1
RP23-277D1.1	1,082224645	1
Lrrc59	1,082224645	1
Tap1	1,082224645	1
Kti12	1,082149633	1
Cul1	1,082149633	1
Trim59	1,082074627	1
Gnai2	1,081999626	1
Cenpc1	1,08192463	1
2410006H16Rik	1,08192463	1
Bbx	1,081774654	1
N4bp2l1	1,081774654	1
Gm43482	1,081624698	1
Erlin1	1,081399804	1
Golm1	1,081399804	1
Agl	1,081399804	1
Gm37219	1,081249901	1
C2cd2l	1,081249901	1
Arhgef40	1,081100018	1
Kptn	1,081025084	1
Tomm7	1,080950156	1
Tecr	1,080875233	1
Tsen15	1,080800315	1
Map4	1,080800315	1
Dync1h1	1,080800315	1
Ube2w	1,080725402	1
Tle4	1,080725402	1
Hagh	1,080650494	1

Sidt2	1,080350916	1
Zfr	1,080350916	1
Pth1r	1,080276034	1
B930086L07Rik	1,080201158	1
Saal1	1,080201158	1
Aimp1	1,080201158	1
Fbxl8	1,080051421	1
Atp5j	1,080051421	1
Zfp131	1,080051421	1
Smad5	1,080051421	1
Wsb2	1,07997656	1
Grpel2	1,079826854	1
Nckap1l	1,079826854	1
Gm12380	1,079752008	1
Mpp6	1,079602333	1
Gm13022	1,079527504	1
Rpl34	1,07937786	1
Sec23b	1,079303045	1
Foxn2	1,079228237	1
Ice2	1,079153433	1
Rpl3l	1,079078634	1
Cdc20	1,078929052	1
Tm6sf1	1,078929052	1
Vamp4	1,078854269	1
Fig4	1,078704719	1
H2-Q10	1,078704719	1
Tspan15	1,078629951	1
Tjp2	1,078480432	1
Snu13	1,07840568	1
Psmb7	1,078256191	1
Siae	1,078256191	1
Elp5	1,078181455	1
Snrpe	1,078181455	1
Skor1	1,078031998	1
Tm2d1	1,078031998	1
Gm11520	1,077957277	1
Ydjc	1,077957277	1
Eif4ebp2	1,07780785	1
Pcbp2	1,077733145	1
Dcps	1,077658445	1
Stk19	1,07758375	1
Cox7b	1,07758375	1
AI846148	1,07750906	1
Trmt10b	1,07750906	1
Gm42559	1,077434375	1
Snhg9	1,077434375	1
Zfp655	1,077434375	1
Ncoa1	1,077359696	1
Zc3h14	1,077285022	1
Trit1	1,077285022	1
Gm43364	1,077210353	1
Map3k1	1,077210353	1

Agap3	1,07706103	1
Itm2c	1,07706103	1
Tbl1xr1	1,076986376	1
Ccdc59	1,076986376	1
Rragc	1,076986376	1
Zfp385a	1,076837085	1
Snx3	1,076837085	1
Gm5578	1,076762446	1
Rab3ip	1,076687814	1
Nostrin	1,076687814	1
3110043O21Rik	1,076687814	1
Slc2a8	1,076538563	1
Ly6g6d	1,076389334	1
Dhx34	1,076240125	1
Stag2	1,076240125	1
Dok2	1,076240125	1
Dennd3	1,076165528	1
Poc5	1,076165528	1
Arhgap5	1,076165528	1
Gm35106	1,076090937	1
Gtdc1	1,07601635	1
1110008L16Rik	1,07601635	1
Ralgapa1	1,075941769	1
Lpgat1	1,075941769	1
Psmc5	1,075867193	1
Hmcn2	1,075718056	1
Phldb1	1,075718056	1
Xndc1	1,075643496	1
Txnl4a	1,075568941	1
Sp1	1,07549439	1
Sh3bgrl3	1,07549439	1
9930021J03Rik	1,075419845	1
Actr3	1,075419845	1
Dip2b	1,075345306	1
Gm8722	1,075196241	1
Hibch	1,075196241	1
Dnajc17	1,075121717	1
Atg16l1	1,075121717	1
E330037G11Rik	1,075047198	1
Ptdss2	1,075047198	1
Zfp566	1,074972684	1
Odf3l1	1,074972684	1
Rubcn	1,074749173	1
Itm2b	1,074749173	1
Ndufs8	1,074376758	1
Lin9	1,074376758	1
Fpgt	1,074227828	1
2010320M18Rik	1,074227828	1
Lst1	1,074227828	1
Rps27a-ps1	1,074078919	1
Pygb	1,074078919	1
Gm38365	1,074004472	1

Tmem185a	1,074004472	1
Pif1	1,07393003	1
Spop	1,07393003	1
Chaf1a	1,073855593	1
Uap1	1,073855593	1
Mir155hg	1,073781162	1
Lpin1	1,073781162	1
Luc7l2	1,073706736	1
Zfp608	1,073632315	1
Nt5c3b	1,073334682	1
Smap2	1,073260286	1
1190007I07Rik	1,073185896	1
Gm10388	1,073037131	1
Fxr2	1,072888387	1
Tpgs2	1,072590961	1
Rad54l	1,072590961	1
C130026I21Rik	1,072516617	1
Gm38220	1,072293616	1
Brwd1	1,072219293	1
Tnrc6c	1,072144975	1
Denr	1,072144975	1
Clec1a	1,072070662	1
Anxa4	1,072070662	1
Mical1	1,071996355	1
Clec10a	1,071922052	1
Hspb11	1,071847755	1
Cdnf	1,071773463	1
Fbxo34	1,071773463	1
Tbc1d20	1,071773463	1
Ttll5	1,071624893	1
Pdcd2l	1,071624893	1
Dand5	1,071476345	1
Gm9347	1,071476345	1
Rnf114	1,071476345	1
Tars2	1,071402079	1
Glb1	1,071402079	1
Pkm	1,071402079	1
Hdac8	1,071327817	1
Vcpip1	1,071327817	1
Hint3	1,071253561	1
Ndufa1	1,071105064	1
Dtx3l	1,071105064	1
Paip1	1,071030823	1
Msl1	1,070882357	1
Mrpl18	1,070808132	1
Pias2	1,070733912	1
Rpl23	1,070733912	1
Ulk4	1,070437082	1
Lats2	1,070362888	1
Rhbdd2	1,070288698	1
Gnptg	1,070288698	1
Ptpn2	1,070288698	1

Arl6ip6	1,070214514	1
Nat9	1,070214514	1
Nedd4l	1,070140335	1
Slc25a1	1,070140335	1
Psip1	1,070066161	1
Fgr	1,069991993	1
Ttc5	1,069991993	1
Msmo1	1,069991993	1
Man1b1	1,069917829	1
Hspa4l	1,069917829	1
Fam78a	1,06984367	1
Phf21b	1,06984367	1
Cd9-ps	1,06984367	1
Runx2	1,06984367	1
Necap2	1,069769517	1
Rps21	1,069769517	1
Bhlhe41	1,069695369	1
Ndufs4	1,069472955	1
Gtf3c5	1,069398827	1
Phf13	1,069324705	1
Pitpnm2	1,069250588	1
Pfdn2	1,069250588	1
Atg101	1,069176475	1
Gm8116	1,069102368	1
Cklf	1,069028266	1
Mtcl1	1,06873191	1
Gm8659	1,068657834	1
Tmem234	1,068657834	1
Agrn	1,068583762	1
Mir142hg	1,068509696	1
Gm2962	1,06836158	1
Eif2ak4	1,06836158	1
Oraov1	1,068213484	1
Tet2	1,068213484	1
Zbtb14	1,068065408	1
Rap1a	1,067917353	1
Usp30	1,067843333	1
Papolg	1,067695309	1
Fndc3a	1,067695309	1
4930550C14Rik	1,067621304	1
Atp5j2	1,067621304	1
Gm22714	1,067473311	1
BC055308	1,067473311	1
Mtmr14	1,067473311	1
Rpsa-ps1	1,067473311	1
Gm12967	1,067399322	1
Gm12522	1,067251359	1
Gm7984	1,067251359	1
Cdca7	1,067177386	1
Pfdn6	1,067177386	1
Emc2	1,067103417	1
Hdac10	1,067029454	1

RP23-138K22.2	1,066955495	1
Dnaaf5	1,066955495	1
Fbxl12os	1,066881542	1
Ndel1	1,066881542	1
Als2cr12	1,066733651	1
Nup50	1,066659713	1
Gm42850	1,066585781	1
Fbxw4	1,066585781	1
Alg12	1,066364014	1
Nemp2	1,066290101	1
Esrra	1,066216194	1
Rpl31-ps14	1,066068396	1
Atg5	1,066068396	1
Arpc5l	1,065920617	1
Bmpr1a	1,065846736	1
Zfp14	1,06577286	1
Casp3	1,06577286	1
Ttll4	1,065698989	1
Slc38a2	1,065625123	1
Efr3b	1,065551262	1
Emc6	1,065477406	1
Gnb1	1,065403555	1
Gripap1	1,065329709	1
Vwa5a	1,065255869	1
Gm14336	1,065182034	1
Gm42715	1,065108203	1
Bag3	1,065108203	1
Ppp4r3b	1,065108203	1
Slc12a5	1,065034378	1
Slc25a4	1,065034378	1
Retn	1,064960558	1
Prpf38a	1,064960558	1
Mcph1	1,064812934	1
Mrpl19	1,064812934	1
Rnf4	1,064739129	1
Creb3	1,064739129	1
Mrpl16	1,064739129	1
Arhgef1	1,064591535	1
Klhl21	1,064591535	1
Triap1	1,064591535	1
Supt7l	1,064517746	1
Cln3	1,064517746	1
Tmem70	1,064517746	1
Phf11b	1,064443962	1
Mdp1	1,064443962	1
Prdm15	1,064370182	1
Lmnb2	1,064370182	1
Dstyk	1,064370182	1
Zfp553	1,064296408	1
Siah1b	1,064296408	1
Txndc16	1,064296408	1
Ppp2r5e	1,064296408	1

Cmc4	1,06422264	1
Il12rb1	1,06422264	1
Slc39a10	1,064148876	1
Rps12-ps5	1,064075117	1
Tgm2	1,064001364	1
Dhfr	1,063927615	1
Arhgdib	1,063780134	1
Gnai3	1,063706401	1
Pdlim2	1,063706401	1
Rpl28	1,06355895	1
St3gal5	1,06355895	1
Fgd6	1,063485232	1
Cd2ap	1,063485232	1
Acbd5	1,06341152	1
Nek6	1,063337812	1
A430010J10Rik	1,06326411	1
Ephx1	1,06326411	1
Aifm2	1,06326411	1
Mfsd14a	1,06311672	1
Esco1	1,062822003	1
Cox17	1,062601018	1
Agfg2	1,062601018	1
Gm17430	1,062527367	1
RP23-164P21.3	1,06245372	1
Fut11	1,062380079	1
Slc25a16	1,062380079	1
Klhdc10	1,062380079	1
Synrg	1,062085566	1
Cdk4	1,062085566	1
Heatr1	1,06201195	1
Wdr92	1,06193834	1
Rpl27a	1,06193834	1
Ube2g2	1,061864734	1
Tmem167b	1,061791134	1
Slc26a11	1,061717539	1
Ccdc112	1,061717539	1
Usp34	1,061643949	1
Fsd2	1,061570364	1
Gm20620	1,061349639	1
Mtmr1	1,061349639	1
Rbm18	1,061349639	1
Huwe1	1,061349639	1
RP23-261O3.5	1,061128961	1
Cnbd2	1,060908328	1
D130019J16Rik	1,060908328	1
Nacc2	1,060908328	1
Mtfp1	1,060834794	1
Lztfl1	1,060761265	1
Pias4	1,060761265	1
Ccnt2	1,060761265	1
Gabarapl1	1,060687741	1
Kif20a	1,060540709	1

Ppip5k1	1,060540709	1
Ccdc43	1,0604672	1
Abca2	1,060320199	1
Ubxn6	1,060320199	1
Bin1	1,060246705	1
Cenpt	1,060173217	1
Rps11-ps2	1,060173217	1
Mrps23	1,060173217	1
Ino80	1,060099734	1
2010204K13Rik	1,060026256	1
Vps13a	1,059952783	1
Gm12689	1,059879316	1
Npepl1	1,059879316	1
Slc30a5	1,059879316	1
Mtmr11	1,059805853	1
Cnot6l	1,059805853	1
Casp8ap2	1,059732395	1
Tfap4	1,059732395	1
Lrpprc	1,059658943	1
Slc35g1	1,059658943	1
Gm6794	1,059512053	1
Med31	1,059512053	1
Tnfrsf22	1,059512053	1
Ifngr2	1,059438616	1
Gm25007	1,059365184	1
Zfp871	1,059365184	1
Gm43524	1,059218335	1
Gm7332	1,059071506	1
Golph3	1,059071506	1
Sik3	1,058998099	1
Clk2	1,058924698	1
Gm10320	1,058851301	1
Gm6563	1,058851301	1
Ddx19a	1,058851301	1
Eny2	1,058851301	1
Atm	1,05877791	1
Cep192	1,058704523	1
Ptgs2os2	1,058631142	1
Lmln	1,058557766	1
Ciz1	1,058557766	1
Mrfap1	1,058557766	1
Zc3h7a	1,058484395	1
Iffo1	1,058411029	1
Lrrc40	1,058411029	1
Rab24	1,058337668	1
Gm5900	1,058264312	1
Rfc2	1,058264312	1
Snx5	1,058264312	1
Gm28530	1,058190961	1
Rgp1	1,058190961	1
Eif3h	1,05797094	1
Gm4342	1,057897609	1

RP23-184H3.5	1,057824284	1
Hk2	1,057824284	1
Ccng1	1,057824284	1
Diaph1	1,057677648	1
Prdx6	1,057677648	1
Cct8	1,057677648	1
Mrps27	1,057604338	1
Pbx3	1,057531033	1
Lyrm4	1,057384439	1
Rpp40	1,057311149	1
Nudt22	1,057237864	1
Eps15l1	1,057237864	1
Eif4e2	1,057237864	1
Npc2	1,05709131	1
Cdc26	1,057018041	1
Cenpq	1,057018041	1
Acvrl1	1,057018041	1
Kdelr3	1,056944776	1
Gm8724	1,056871517	1
Rtn4ip1	1,056651769	1
Rab22a	1,056651769	1
Pot1a	1,056578531	1
Gm17018	1,056505297	1
Trp53bp2	1,056358844	1
Asna1	1,056358844	1
Akr1b8	1,056358844	1
A930018M24Rik	1,056212412	1
Edrf1	1,056139203	1
Slc17a9	1,056066	1
Gm43571	1,055992801	1
Gm2a	1,055992801	1
Pfkm	1,055700059	1
Mff	1,055553718	1
Parp9	1,055480555	1
Cenpj	1,055334244	1
Ifi27	1,055334244	1
Desi1	1,055187954	1
Pafah1b3	1,055187954	1
Pcyox1	1,055187954	1
Mettl3	1,055041684	1
Rpa1	1,055041684	1
Vegfa	1,055041684	1
Gm7287	1,054895434	1
Mettl6	1,054895434	1
Tmem41a	1,054895434	1
Mapk1ip1l	1,054895434	1
Letm2	1,054822317	1
Helb	1,054822317	1
Ldlr	1,054822317	1
Bud31	1,054822317	1
Ndufab1-ps	1,054676098	1
Trappc8	1,054602996	1

Rai14	1,054602996	1
Gphn	1,054456807	1
Ywhae	1,054456807	1
Cct2	1,05438372	1
Cela1	1,054237562	1
Sgpl1	1,054237562	1
Vdac3	1,05416449	1
Xdh	1,054018362	1
Gfer	1,054018362	1
Limd2	1,053945305	1
Gnl1	1,053726166	1
Gm8869	1,053580099	1
Magoh	1,053288025	1
Zdhhc8	1,053215019	1
Churc1	1,053142018	1
Rpl7-ps7	1,053069023	1
Gm26533	1,052996032	1
2700060E02Rik	1,052996032	1
Gars	1,052850066	1
Flot2	1,05277709	1
Srsf6	1,05277709	1
Snapc3	1,052631155	1
4931406P16Rik	1,052631155	1
Rfc1	1,052485239	1
Rnf166	1,052485239	1
Tmem57	1,052412289	1
Gm7808	1,052339344	1
Rabggta	1,052266404	1
Lrrk2	1,052266404	1
Hpf1	1,052266404	1
Gm14636	1,052047614	1
Arf1	1,052047614	1
Cwc15	1,051901779	1
Rrm2b	1,051828869	1
Ptprs	1,051828869	1
Ppp4c	1,051755965	1
Spcs3	1,051610171	1
Rps19	1,051610171	1
Tsc1	1,051537281	1
Gdi1	1,051464397	1
C1rb	1,051391517	1
Dpagt1	1,051245773	1
Rmdn3	1,051027196	1
Clec4a3	1,050954347	1
Rps8-ps4	1,050808663	1
Rfwd3	1,050808663	1
Krr1	1,050735829	1
Actr3b	1,050735829	1
Cln5	1,050735829	1
Prps1	1,050735829	1
Agmo	1,050663001	1
Gtf2a2	1,050663001	1

Fnbp4	1,050663001	1
Map1lc3b	1,050663001	1
Dnajc21	1,050590177	1
Cyp51	1,050590177	1
mt-Nd4	1,050153339	1
Fra10ac1	1,050153339	1
Clk3	1,050153339	1
Gm7432	1,050080551	1
Sec63	1,050080551	1
Acyp1	1,050007767	1
Ldlrad3	1,050007767	1
Emp3	1,050007767	1
Wdr41	1,049934989	1
Ddb2	1,049716684	1
Rdh12	1,049716684	1
Fam162a	1,049716684	1
Uba6	1,049571172	1
Trim46	1,049352943	1
Flad1	1,049134758	1
Ostf1	1,049134758	1
Gm6266	1,048989328	1
Fkbp2	1,048771219	1
Sec61b	1,048771219	1
Mettl9	1,048698526	1
Prr36	1,048625839	1
Rplp2	1,048625839	1
Morc4	1,048625839	1
Xrcc1	1,048553156	1
Eif3f	1,048553156	1
Nme4	1,048480478	1
Suz12	1,048480478	1
Zc2hc1a	1,048407806	1
Uba1	1,048407806	1
Idua	1,048335138	1
Mrpl11	1,048262476	1
Gm12762	1,047899238	1
AU020206	1,047826606	1
Pbx2	1,047826606	1
Gm37621	1,047753979	1
Naa38	1,047753979	1
Smim20	1,047681357	1
Mfsd2a	1,047536127	1
Tsr3	1,047536127	1
Snhg3	1,04710056	1
Lca5	1,047027983	1
Prkab1	1,046955411	1
Taf3	1,046955411	1
Cog4	1,046882844	1
Cacna1a	1,046882844	1
RP23-447C2.2	1,046810282	1
Wipf2	1,046810282	1
Rnf145	1,046810282	1

Trim33	1,046737725	1
Ppp6c	1,046737725	1
Rpap2	1,046665173	1
Glt1d1	1,046592627	1
Cyp4f13	1,046592627	1
Golph3l	1,046592627	1
Yipf1	1,046447548	1
Hypk	1,046375016	1
Gm15535	1,046229968	1
Rassf3	1,046229968	1
Zfp516	1,046229968	1
Mafg	1,046229968	1
E2f4	1,046157451	1
Tmed9	1,046157451	1
Myo1d	1,046012433	1
Wasl	1,045939932	1
Ubxn7	1,045939932	1
Sra1	1,045939932	1
Gm1943	1,045867435	1
Ighd	1,045794944	1
Ntpcr	1,045794944	1
Grk4	1,045649976	1
Scarna2	1,045649976	1
AA414768	1,045577499	1
Mb21d1	1,045577499	1
Klhl8	1,045505028	1
Prpf40a	1,045505028	1
Trim21	1,045432562	1
Fads3	1,045287644	1
Vti1b	1,045287644	1
Gsdmd	1,045215193	1
Polr3c	1,045215193	1
Arpc5	1,045215193	1
Ik	1,045215193	1
Taf4b	1,045142746	1
Osgep	1,045070305	1
Gale	1,044997869	1
Strip1	1,044997869	1
BC003331	1,044925438	1
Snx30	1,044925438	1
Gm5070	1,044490956	1
Gm7424	1,044346168	1
Tspyl4	1,044201401	1
Gm45884	1,044201401	1
Btk	1,044201401	1
Myo5a	1,044129025	1
Smad2	1,044056654	1
Mfap3l	1,043911927	1
Bok	1,043839571	1
Fitm2	1,043839571	1
Snapc4	1,043767221	1
Seh1l	1,043767221	1

Gm9013	1,043694875	1
Mlf1	1,043694875	1
Gm20156	1,043694875	1
Enpp1	1,043622534	1
Slc19a1	1,043550198	1
Tmem63a	1,043405541	1
Thap1	1,04333322	1
Taf6	1,04333322	1
Fam168b	1,04333322	1
Wdtc1	1,043260904	1
Bop1	1,043188594	1
Ccdc107	1,043116288	1
Tspo	1,042971691	1
Lrrfip1	1,042754834	1
Blnk	1,042538022	1
Ube2q2	1,042465761	1
9130230N09Rik	1,042393505	1
Tubgcp4	1,042393505	1
Dazap2	1,042393505	1
Otud4	1,042249009	1
Gmfg	1,042032302	1
Wdpcp	1,041960076	1
Rogdi	1,041960076	1
Clpx	1,041960076	1
Bri3bp	1,041960076	1
Ndufaf1	1,041743429	1
Sec14l1	1,041671223	1
Loxl3	1,041599023	1
Slc37a4	1,041454636	1
Zfp654	1,04131027	1
Man2c1	1,041238095	1
Shkbp1	1,041238095	1
Ppif	1,041165924	1
Usp14	1,041093758	1
Kif9	1,041021598	1
Usp39	1,041021598	1
Ppp3cc	1,040949442	1
Mrpl39	1,040949442	1
Gm37106	1,040877291	1
Fxr1	1,040877291	1
Noct	1,040805146	1
D830044I16Rik	1,04066087	1
1810024B03Rik	1,040516613	1
Arl8b	1,040444493	1
Fip1l1	1,040228161	1
BC017643	1,040011874	1
Gm8357	1,039939788	1
Mcm4	1,039795632	1
Erbin	1,039795632	1
Pigf	1,039651496	1
Ctr9	1,039579435	1
Pla2g16	1,03950738	1

Safb	1,039363283	1
Elob	1,039363283	1
Tcerg1	1,039363283	1
Abcf2	1,039291243	1
Dgke	1,039291243	1
Gmfb	1,039291243	1
Ybx3	1,039291243	1
Rab1a	1,039219207	1
Slc6a13	1,039219207	1
Xiap	1,039147176	1
Tmem60	1,03907515	1
Gm9833	1,03900313	1
Mocs2	1,038859103	1
Rad18	1,038787098	1
Tnrc6b	1,038787098	1
Pts	1,038427144	1
Wtap	1,038283197	1
Rbfox2	1,038211231	1
Ddx54	1,038211231	1
RP24-497N7.2	1,038067315	1
Mfn1	1,037995364	1
Lrp4	1,037851477	1
E230016M11Rik	1,037851477	1
Zdhhc3	1,037635684	1
Erich1	1,037563763	1
Plk2	1,037563763	1
Prss44	1,037419937	1
Pym1	1,037419937	1
Cbl	1,03727613	1
Rusc2	1,037060457	1
Neo1	1,037060457	1
Dohh	1,036916699	1
Psmd4	1,036844828	1
Tubb2a	1,036772962	1
Dcaf7	1,036772962	1
Plxnd1	1,036557394	1
Rab34	1,036557394	1
Rps6ka1	1,036485547	1
Eif2b5	1,036485547	1
Cmss1	1,036198213	1
Gm13421	1,036198213	1
Carm1	1,036126391	1
Def6	1,036054575	1
Runx1	1,036054575	1
Zfp995	1,035982764	1
Rpl19-ps1	1,035982764	1
Srek1	1,035982764	1
Gm12034	1,035839156	1
Pi4k2a	1,035767359	1
D430013B06Rik	1,035695568	1
Rab21	1,035695568	1
Pgf	1,035623782	1

Trappc1	1,035552	1
Gm44164	1,035480224	1
Clptm1	1,035408452	1
Srsf11	1,035408452	1
Mnat1	1,035336685	1
Rida	1,035193167	1
Thap3	1,035193167	1
Cdc45	1,035049669	1
Acly	1,035049669	1
Ufm1	1,035049669	1
Rbm8a2	1,034977927	1
Arl6ip4	1,034834459	1
Bbip1	1,034762732	1
Gaa	1,03469101	1
Ddx52	1,034547582	1
Alkbh5	1,034260784	1
Rab28	1,034189097	1
Glmp	1,034189097	1
Pcnt	1,034045738	1
March5	1,034045738	1
Cox4i1	1,034045738	1
Fn3krp	1,033974066	1
Fbxo8	1,033830736	1
Ndufa6	1,033830736	1
Spag9	1,033687427	1
Rhobtb1	1,03361578	1
Gm16046	1,033544137	1
Gm13443	1,033544137	1
3110040N11Rik	1,033544137	1
1810013L24Rik	1,0334725	1
Glrx2	1,0334725	1
Jak1	1,03332924	1
Sec22b	1,03332924	1
Nfx1	1,033257618	1
Apba3	1,033186	1
Hadhb	1,033186	1
Nfatc1	1,033186	1
Wdr1	1,033114388	1
Ttyh3	1,03304278	1
Hspb7	1,03289958	1
Cpsf1	1,03289958	1
Nup62	1,03289958	1
Gm38366	1,032827987	1
Alg6	1,032684817	1
Aurka	1,032684817	1
Cd52	1,032613239	1
Rnf103	1,032541666	1
Mrpl23-ps1	1,032541666	1
Slpi	1,032541666	1
Tmem59	1,032541666	1
Apoa1bp	1,032470098	1
Mrps21	1,032326978	1

Paf1	1,032326978	1
Acot7	1,032255425	1
Rala	1,032183877	1
Gtf2i	1,032112334	1
Rnf19b	1,031969262	1
Tyrobp	1,031897734	1
Ap1ar	1,031826211	1
Dera	1,031540168	1
Ocrl	1,031540168	1
Gm9762	1,031468669	1
Plcb2	1,031397176	1
Irak2	1,031397176	1
Egln1	1,031397176	1
Cdc42se1	1,031397176	1
Brca1	1,031325687	1
Mrps25	1,031254204	1
Sh3glb2	1,031182725	1
Ambra1	1,031182725	1
Furin	1,031111251	1
Rdh1	1,030753957	1
Lactb2	1,030753957	1
Afdn	1,030753957	1
Lrrc42	1,030611074	1
Rara	1,03053964	1
Rabac1	1,030468211	1
1110012L19Rik	1,030325368	1
Rbbp5	1,030325368	1
Cpped1	1,030325368	1
Atg14	1,030325368	1
Cks1brt	1,030039741	1
Pus10	1,030039741	1
Xpo5	1,029968346	1
Whamm	1,029968346	1
Ap1g2	1,029825572	1
Tmem126b	1,029611448	1
Fcf1	1,029540083	1
Rnf115	1,029540083	1
Cadm1	1,029540083	1
Fam117b	1,029468724	1
Gm23458	1,029397369	1
Borcs6	1,029326019	1
Gm12248	1,029254674	1
Vps50	1,029254674	1
Cdc16	1,029254674	1
Tbc1d9b	1,029183334	1
Fbxl6	1,029111999	1
Zfp706	1,029111999	1
Cks1b	1,028969343	1
Mkln1	1,028826708	1
Trim41	1,028684092	1
Sipa1l1	1,028612792	1
Kat2a	1,028612792	1

Gm20568	1,028612792	1
Fam134a	1,028612792	1
Igf2bp1	1,028541496	1
Uqcrc2	1,028327639	1
Ccpg1os	1,028185093	1
Gm7832	1,028185093	1
Camk2d	1,028113827	1
Etv5	1,028042566	1
Nudcd2	1,027900059	1
Fosb	1,027828812	1
Eif4g3	1,027615104	1
Ing2	1,027615104	1
Xpo1	1,027330228	1
6330562C20Rik	1,027259021	1
Rbpsuh-rs3	1,027259021	1
Nr4a1	1,027045431	1
Doc2g	1,026974244	1
Klhl12	1,026760713	1
Tigd2	1,026760713	1
Med13	1,026689546	1
Stau2	1,026476074	1
Gnal	1,026404926	1
Dusp1	1,026404926	1
Herc6	1,026333784	1
Mrps15	1,026333784	1
Chmp6	1,026262646	1
Cyp27a1	1,026191514	1
Ecsit	1,026120386	1
Thoc5	1,025978145	1
Dusp22	1,025907032	1
Ctsl	1,025907032	1
Ndufc2	1,025835924	1
Rabep1	1,025835924	1
Akt1s1	1,025764821	1
Gm4366	1,025693723	1
Dhx16	1,025551542	1
Fkbp1b	1,025551542	1
2310035C23Rik	1,025551542	1
Gdpd3	1,025551542	1
Ifi203-ps	1,025480458	1
Gm37199	1,025480458	1
Srgn	1,02540938	1
Fbxw8	1,025267238	1
Pex1	1,025125116	1
Ctdnep1	1,025125116	1
Pigm	1,025054062	1
Rad21	1,024983013	1
Rmi1	1,024698867	1
Abhd12	1,024627842	1
Fbxo22	1,024627842	1
Arvcf	1,024556823	1
Zfp595	1,024556823	1

Col4a5	1,024485809	1
Dnase1l1	1,024343795	1
Map2k2	1,024343795	1
Ebp	1,0242018	1
Ythdc2	1,023988846	1
Sec61a2	1,023917871	1
Spast	1,023917871	1
Rnaseh2b	1,023775935	1
Rhot1	1,023704975	1
Pttg1	1,02363402	1
Gm9333	1,023563069	1
Phf7	1,023563069	1
Sap18	1,023563069	1
Gm8995	1,023421183	1
C230035I16Rik	1,023350247	1
Psma3	1,023279317	1
Sdf2	1,023279317	1
Gm43106	1,023066554	1
Rps15a-ps8	1,022995643	1
Gm5905	1,022995643	1
mt-Cytb	1,022995643	1
Pfkl	1,022782939	1
Exoc4	1,022782939	1
Rab7	1,022712047	1
Gopc	1,022641161	1
H2-DMa	1,022570279	1
9130023H24Rik	1,022428531	1
Ube2e3	1,022357664	1
Fam193a	1,022215945	1
Dnm1l	1,022215945	1
Ythdc1	1,022215945	1
Gm12254	1,021649265	1
Noa1	1,021578452	1
Kidins220	1,021578452	1
Kbtbd11	1,021507644	1
Rbm25	1,021507644	1
Gm43774	1,021436841	1
Tbl1x	1,021436841	1
Cisd2	1,021224461	1
Tmem39a	1,021224461	1
Gm26652	1,021153678	1
Traf7	1,021153678	1
Gnpat	1,021082899	1
Bex3	1,021082899	1
Sec24d	1,021012126	1
Ino80d	1,021012126	1
Nrd1	1,020941357	1
Trove2	1,020941357	1
Sult6b1	1,020870593	1
Coq10a	1,020799834	1
Smim15	1,020658331	1
Scarna17	1,020516848	1

Fbxl18	1,020446113	1
Gmps	1,020375384	1
Nlk	1,020375384	1
Pqlc1	1,020375384	1
Stxbp1	1,02023394	1
Slc2a6	1,020163225	1
Gm3571	1,020163225	1
Cenpm	1,019809724	1
Gm12183	1,019809724	1
Arhgef4	1,019668359	1
Sep 07	1,019668359	1
Tcof1	1,019597683	1
Slc4a1ap	1,019527012	1
Slc6a9	1,019456347	1
Casp8	1,019456347	1
Nae1	1,019456347	1
Rps10	1,019456347	1
Psmd11	1,019456347	1
Ctsb	1,019385686	1
Pign	1,019103091	1
Lair1	1,019032455	1
A530072M11Rik	1,018961823	1
Skap2	1,018891197	1
Tfdp1	1,018820575	1
Zfp384	1,018820575	1
5830454E08Rik	1,018749958	1
Itgax	1,018749958	1
Rnpep	1,018749958	1
Tmbim6	1,018749958	1
RP24-496O17.7	1,018608739	1
Stard3nl	1,01846754	1
Skiv2l	1,018396947	1
1700001C19Rik	1,018396947	1
Tmem9b	1,018396947	1
Evi5	1,01832636	1
Clp1	1,018255777	1
Snap23	1,018255777	1
Vrk3	1,0181852	1
Zbtb33	1,017902938	1
Ercc5	1,017832385	1
Zrsr2	1,017620755	1
Angel1	1,017479692	1
Pml	1,017268135	1
Acat2	1,017268135	1
Tbc1d2	1,017197626	1
Pomp	1,017197626	1
Gm42611	1,017056622	1
Med15	1,017056622	1
Copg1	1,016986128	1
Ccdc88a	1,016986128	1
Ppic	1,016986128	1
Nat10	1,016774673	1

Stoml2	1,016704198	1
Atrx	1,016704198	1
Slc22a17	1,016492803	1
Irf3	1,016492803	1
Nup188	1,016492803	1
3830406C13Rik	1,016351897	1
Canx	1,016351897	1
Ncoa7	1,01621101	1
Psmc4	1,01621101	1
Prkd2	1,016140574	1
Rps5	1,016070143	1
Abhd2	1,015999717	1
Nfe2l2	1,015999717	1
Mast3	1,015858879	1
Erc1	1,015858879	1
Brip1os	1,015788468	1
Sp3	1,015788468	1
Rbm47	1,015788468	1
Ttc13	1,015718061	1
E330009J07Rik	1,015718061	1
Dnaja1	1,015718061	1
Etv3	1,015718061	1
Sdhaf1	1,015577262	1
Fam178a	1,015577262	1
Ppib	1,015577262	1
Pes1	1,01550687	1
Haus6	1,015436483	1
Hk1	1,015436483	1
Gm26799	1,015366101	1
Nr1d2	1,015295723	1
Galns	1,015225351	1
Tnfrsf26	1,015154983	1
Gm43343	1,015014263	1
Tgfbr1	1,015014263	1
Tnfsf12	1,015014263	1
Med18	1,014873562	1
Hectd3	1,014873562	1
Hbegf	1,014873562	1
Ubn2	1,014873562	1
RP23-123D6.12	1,014662547	1
Sptbn1	1,014592218	1
Rpl30-ps11	1,014451575	1
Spc25	1,014240648	1
Eif2b1	1,014100054	1
Lrrc27	1,014029765	1
Rora	1,0138892	1
Dazap1	1,013818925	1
Chd5	1,013748655	1
Smpdl3a	1,013748655	1
Acot10	1,013678389	1
Gm527	1,013678389	1
Usp5	1,013678389	1

Fancl	1,013608129	1
Crot	1,013608129	1
Tes	1,013537874	1
Ilvbl	1,013397377	1
Snhg5	1,013397377	1
Psmd8	1,013397377	1
Nr2f6	1,013116443	1
Pag1	1,013116443	1
Cirbp	1,013046221	1
2810002D19Rik	1,012976005	1
Grsf1	1,012976005	1
Samhd1	1,012905793	1
Pim2	1,012835586	1
Ska3	1,012835586	1
Baz1a	1,012835586	1
BC085271	1,012765384	1
Mcf2l	1,012765384	1
Tfg	1,012765384	1
Ccdc181	1,012695187	1
Rexo1	1,012624995	1
Mrpl45	1,012624995	1
9530078K11Rik	1,012554807	1
Cd68	1,012484625	1
Gm43201	1,012414447	1
Nyap1	1,012414447	1
Mtfr1	1,012414447	1
Adpgk	1,012344274	1
Gm13487	1,012203943	1
Nufip1	1,012203943	1
Mrpl12	1,012133785	1
Usp54	1,011993483	1
Zyg11b	1,011993483	1
Paxbp1	1,011993483	1
Slc25a36	1,011993483	1
Polrmt	1,01192334	1
Xpnpep3	1,011853201	1
Smim3	1,011712938	1
Brd3	1,011712938	1
Mea1	1,011572695	1
Gm37566	1,01150258	1
Gm11604	1,01150258	1
Specc1l	1,01150258	1
Nubpl	1,011432471	1
2410015M20Rik	1,011362366	1
Deaf1	1,011292266	1
Frg1	1,011292266	1
Dxo	1,011222171	1
Hnrnpr	1,011152081	1
Tnrc18	1,011152081	1
Nf2	1,011011915	1
Ctsd	1,011011915	1
Slc12a9	1,01094184	1

Snrnp27	1,01094184	1
Polr1c	1,010801703	1
Rpl22	1,010801703	1
Zfp593	1,010731642	1
S100a8	1,010661586	1
Cactin	1,010661586	1
Ankfy1	1,010661586	1
Iah1	1,010591535	1
Gm38104	1,010521488	1
Cacfd1	1,010521488	1
Clta	1,010521488	1
Nudt4	1,010451446	1
Myh9	1,010451446	1
Gm14680	1,01038141	1
Fbxw11	1,01038141	1
Atp13a1	1,010311378	1
Mkrn1	1,010171329	1
Ano10	1,010101311	1
Dnajc12	1,009821291	1
Spef1	1,009821291	1
Tes3-ps	1,009681309	1
Cluap1	1,009611326	1
Tmem11	1,009541348	1
Fam204a	1,009471374	1
Ptpmt1	1,009331441	1
Xylt1	1,009261482	1
Setd1a	1,009261482	1
Rgs19	1,009191528	1
Gm15050	1,009191528	1
Dynlrb1	1,009191528	1
Ran	1,009191528	1
Prpf40b	1,009121578	1
Ddx50	1,009051634	1
Kif2a	1,008981694	1
Opa1	1,008981694	1
Milr1	1,008911759	1
Ndc1	1,008841829	1
Ikbke	1,008771904	1
Neurl3	1,008771904	1
Gosr2	1,008771904	1
Coa5	1,008771904	1
Crlf3	1,008701984	1
Tspan32	1,008562158	1
Rpsa-ps10	1,008562158	1
Gm4895	1,008492252	1
Scarb2	1,008422351	1
Sep 09	1,008282564	1
Zfp760	1,008212677	1
Adipor2	1,008072919	1
Tbca	1,008072919	1
Trim36	1,008003047	1
Vps53	1,00793318	1

1110038B12Rik	1,007793461	1
Ttc39b	1,007723608	1
Pbxip1	1,00765376	1
Atg7	1,007374418	1
Luc7l	1,007304595	1
Psmf1	1,007234776	1
Chm	1,007095153	1
Ing1	1,006815966	1
Gata3	1,006746181	1
Ppig	1,006676401	1
Fam98a	1,006606626	1
Capn2	1,006606626	1
Lamtor1	1,006536856	1
Kdelr2	1,006536856	1
Gas6	1,006467091	1
Pef1	1,00639733	1
Kdm5a	1,006257823	1
Purb	1,006257823	1
Cops7b	1,006188077	1
Ak1	1,006188077	1
Gm45358	1,006118336	1
Gm32856	1,0060486	1
Rita1	1,0060486	1
Cul3	1,0060486	1
H60c	1,005909142	1
Ggcx	1,005909142	1
Klhl9	1,00583942	1
Dscr3	1,00583942	1
Tmem170	1,005630283	1
Sap30bp	1,00556058	1
Atg13	1,00556058	1
Gm45833	1,005490883	1
Pde6d	1,00542119	1
Tpm3	1,00542119	1
Zmat3	1,00542119	1
Hras	1,005142466	1
Nxf1	1,005142466	1
RP24-365N15.9	1,005072798	1
Ddx20	1,005072798	1
Gtf2e2	1,005072798	1
Rpl37rt	1,00486382	1
Polk	1,00486382	1
Dhdh	1,004794171	1
Gm42508	1,004515622	1
Pex19	1,004515622	1
Kif5b	1,004515622	1
Rpf1	1,004445996	1
Enpp4	1,00430676	1
Fam26f	1,004237149	1
Gm43544	1,004237149	1
Gm11488	1,004237149	1
Cfdp1	1,004237149	1

Ssr1	1,004237149	1
Bahcc1	1,004167543	1
Atg4a	1,004167543	1
Pold3	1,004167543	1
Cap1	1,004167543	1
Zfp369	1,004028346	1
Gm8185	1,003958754	1
Csnk2a1	1,003958754	1
Rfng	1,003819586	1
Mrpl48	1,003819586	1
Clk4	1,003750009	1
Gtf2f2	1,003680436	1
Uspl1	1,003541306	1
Emc7	1,003471749	1
Cdkn2aip	1,003402196	1
Jmjd1c	1,003332647	1
Brap	1,003263104	1
Tpst2	1,003193566	1
Mrpl35	1,003054503	1
Cetn2	1,003054503	1
Lcor	1,002984979	1
Glyr1	1,002984979	1
Mrpl30	1,002845945	1
Fundc2	1,002845945	1
Porcn	1,002776436	1
Gm7353	1,002706931	1
Hnrnpl	1,002706931	1
Fam195b	1,002706931	1
2610001J05Rik	1,002567936	1
Sdccag8	1,002498446	1
Thnsl1	1,002498446	1
Tspyl1	1,002498446	1
Rnaseh2c	1,00242896	1
Ago3	1,00242896	1
Aurkaip1	1,00242896	1
Gstm1	1,00235948	1
Bloc1s6os	1,002220533	1
Zranb1	1,002081605	1
Ywhag	1,002081605	1
Dbi	1,002081605	1
Gm6265	1,001942697	1
Rpain	1,001803808	1
Plcd3	1,001803808	1
Wbp11	1,001803808	1
Larp4	1,001803808	1
Tax1bp1	1,00173437	1
Scai	1,00159551	1
Rpa2	1,00159551	1
Mia2	1,00159551	1
Sgsm2	1,00159551	1
Mthfsd	1,001526087	1
Gm4799	1,001526087	1

Hexim1	1,001526087	1
Col15a1	1,001456669	1
Mrps31	1,001456669	1
Arpc1a	1,001387256	1
Stk16	1,001317847	1
Zfp687	1,001248444	1
Ube2d3	1,001248444	1
Rev1	1,001109651	1
Ulbp1	1,001109651	1
Ndufa7	1,001109651	1
Ccne1	1,000832123	1
Elk4	1,000832123	1
Arpc4	1,000832123	1
Mpi	1,000762753	1
Mrps7	1,000762753	1
Phtf2	1,000485321	1
Kntc1	1,000415975	1
Gm11423	1,000415975	1
Ube2e1	1,000277297	1
Akap1	1,000207966	1
Zzz3	1,000138639	1
Rps9	1,000138639	1
Ssr2	1,000138639	1
Gm42418	1	1
Lrrc58	-0,00032806	1
Wsb1	-0,00026781	1
Prokr1	-0,00053856	1
Uqcrh	-0,00051671	1
Ttc7b	-0,0005818	1
Parvb	-0,00064334	1
Golga1	-0,00071344	1
Gpt	-0,00067801	1
2610301B20Rik	-0,0006934	1
Tmem160	-0,00077584	1
Afp	-0,00083079	1
Unc93b1	-0,00083565	1
Kif1b	-0,00083296	1
D230022J07Rik	-0,00094269	1
Chtf8	-0,00086304	1
Gm28791	-0,0013757	1
Synj1	-0,0013781	1
Epn1	-0,0014974	1
Srsf9	-0,0018704	1
S100a13	-0,0020599	1
Gba	-0,002115	1
Sephs1	-0,0022027	1
Slc33a1	-0,0023122	1
Gm5297	-0,0023866	1
Zfp53	-0,0025412	1
Nlrc4	-0,0026371	1
1700020I14Rik	-0,0026178	1
Leprotl1	-0,0025777	1

Exosc10	-0,0027873	1
Ddx39	-0,0027919	1
Ap3b1	-0,0028887	1
Etfbkmt	-0,0030359	1
Gm13771	-0,0032343	1
Nde1	-0,0033413	1
Rb1	-0,0032802	1
Gne	-0,0033543	1
Yars	-0,0035017	1
Traf3ip2	-0,0035588	1
Synj2bp	-0,0036134	1
Nat8f1	-0,0037075	1
Arl4a	-0,0036927	1
Anxa1	-0,0039781	1
Tnni2	-0,0039566	1
Gm6808	-0,0041831	1
Dennd1b	-0,0042043	1
Ubac1	-0,0043119	1
Gm37009	-0,0044024	1
Pdcd5-ps	-0,0045431	1
Mllt11	-0,0044666	1
Taf5	-0,0045656	1
Bora	-0,0046116	1
Stk24	-0,0046425	1
Dner	-0,0047005	1
Tlcd1	-0,0049329	1
Nepro	-0,0051476	1
Erp29	-0,005293	1
Ppp1r14b	-0,0055885	1
Naa30	-0,0055556	1
Cfap74	-0,0056865	1
Gstt1	-0,0057287	1
Susd6	-0,0057715	1
Dok3	-0,0057785	1
Ccm2	-0,0058667	1
Serhl	-0,0059637	1
Prkar1b	-0,0060506	1
Exoc3l4	-0,0061272	1
Eef2	-0,0060967	1
Rhoc	-0,0062731	1
Gm45266	-0,0066094	1
Chrnb1	-0,006791	1
Pds5b	-0,0067666	1
Fbxl15	-0,0069565	1
Hnrnpu	-0,007036	1
Mkks	-0,0074219	1
Camk1	-0,007359	1
Zfp68	-0,0074941	1
Lgals3	-0,0074895	1
Pycr1	-0,0075708	1
Avpi1	-0,0076492	1
Pmpca	-0,0076712	1

Slc9a6	-0,0082353	1
Tnpo1	-0,0082393	1
RP23-304C21.3	-0,0084514	1
Gm11224	-0,0085095	1
Lman1	-0,008473	1
Nup107	-0,0088373	1
Nmnat1	-0,0089202	1
Chuk	-0,0088506	1
Htt	-0,0089209	1
Amz2	-0,0091162	1
Apitd1	-0,0091556	1
Rpp21	-0,0092147	1
Hnrnpc	-0,0091765	1
Cd300a	-0,0093052	1
B4galnt1	-0,0093537	1
Heatr6	-0,0093914	1
Trp53inp1	-0,009439	1
Stk38	-0,0094391	1
Nus1	-0,0094201	1
Qtrtd1	-0,0095794	1
Gm14326	-0,0095698	1
Dnaja2	-0,0098089	1
Gpaa1	-0,0099209	1
Acaa2	-0,010018	1
Mrps14	-0,010193	1
Otud1	-0,010434	1
Gm35315	-0,010397	1
Exoc1	-0,010422	1
Cdc34	-0,010529	1
Rasl2-9	-0,010589	1
Junos	-0,010739	1
Herc1	-0,010727	1
Erp44	-0,01083	1
Camk2a	-0,011115	1
Hsf2	-0,011135	1
Hccs	-0,011488	1
Spg20	-0,011819	1
Srp19	-0,011813	1
Nhp2	-0,012214	1
Aatk	-0,01235	1
Med28	-0,012369	1
Rps25-ps1	-0,012459	1
Magohb	-0,012591	1
Mapre1	-0,012601	1
Setd7	-0,012685	1
Coq5	-0,012682	1
Slc25a26	-0,012767	1
D17Wsu92e	-0,012799	1
Fam213b	-0,012892	1
Gin1	-0,012923	1
Ttc19	-0,012889	1
Snhg15	-0,01308	1

Ift46	-0,013136	1
Ascc2	-0,01322	1
Csde1	-0,013428	1
Fcrl1	-0,013615	1
Prkca	-0,013707	1
Ptrhd1	-0,013697	1
Cnot7	-0,013718	1
Fam199x	-0,013731	1
Rfc5	-0,013919	1
Krit1	-0,013901	1
Gm5566	-0,01401	1
Pcnx4	-0,014335	1
Kpna6	-0,014472	1
Xpo7	-0,014478	1
Tpp1	-0,014487	1
Smc6	-0,014481	1
Sh2d2a	-0,014666	1
Slc7a6os	-0,014657	1
Ddb1	-0,014737	1
Rpl30-ps9	-0,014834	1
Brwd3	-0,014793	1
Lats1	-0,014962	1
Smad4	-0,014957	1
Man2b1	-0,015058	1
Tatdn3	-0,015221	1
Gm9645	-0,015304	1
Tmem128	-0,015345	1
L3mbtl2	-0,015271	1
Spata5l1	-0,015522	1
Rnf6	-0,015656	1
Ufc1	-0,015659	1
Arap1	-0,015694	1
Myo1f	-0,015843	1
Gm20442	-0,015918	1
Sirt1	-0,015992	1
Gm43329	-0,016056	1
Tnks2	-0,016083	1
Gm42786	-0,016162	1
Kdm6b	-0,01626	1
Gm6946	-0,016741	1
Pik3c2a	-0,016705	1
Phc2	-0,016737	1
5730455P16Rik	-0,016785	1
Mcts1	-0,016759	1
Zfp512	-0,017077	1
Zfx	-0,017077	1
Kat5	-0,01732	1
Gnb2	-0,017469	1
Cd44	-0,01754	1
Coq8b	-0,01766	1
Renbp	-0,017693	1
Kat7	-0,017715	1

Ptpa	-0,017907	1
Ccnh	-0,017988	1
Ubtd1	-0,018071	1
Ndufv2	-0,018176	1
Gm13612	-0,018403	1
Lyz1	-0,01837	1
Tnnc2	-0,01848	1
Kmt5a	-0,018721	1
St3gal3	-0,018794	1
Fam206a	-0,019004	1
Myo18a	-0,019027	1
Zfp91	-0,019017	1
Eif3c	-0,019246	1
Armc2	-0,019477	1
Stk17b	-0,01949	1
Usp24	-0,019614	1
Btg1	-0,019587	1
Tex2	-0,019698	1
Dnajb14	-0,01982	1
C1galt1	-0,019882	1
Usp3	-0,019898	1
Phldb3	-0,019986	1
Psmd1	-0,019973	1
Cox7a2	-0,020064	1
Rufy1	-0,020343	1
Kmt5c	-0,020254	1
AI314180	-0,020268	1
Amacr	-0,020401	1
Gm9711	-0,02041	1
Cdk18	-0,02037	1
Chd9	-0,0204	1
Ndufb5	-0,020644	1
Ewsr1	-0,020673	1
Snrnp25	-0,020716	1
Hnrnpll	-0,020833	1
Mcl1	-0,020871	1
Tpd52l2	-0,021037	1
Gpn2	-0,021137	1
Nme6	-0,021177	1
Mccc1	-0,021212	1
Efcab11	-0,02136	1
Anapc1	-0,021663	1
Tmem192	-0,021843	1
Gm42941	-0,022126	1
Gm12799	-0,02207	1
Slc25a11	-0,022176	1
Usp48	-0,022199	1
Adamtsl4	-0,022449	1
Fgfr1op2	-0,022393	1
Asb6	-0,022477	1
Gm12312	-0,022577	1
Gm8444	-0,02271	1

Gm6134	-0,022759	1
Natd1	-0,022844	1
Rffl	-0,022831	1
Lage3	-0,023105	1
Fbxo18	-0,023222	1
Mrpl41	-0,023349	1
Tmem14a	-0,023446	1
Gm12529	-0,023399	1
Slc29a1	-0,023592	1
Echs1	-0,023705	1
Miip	-0,023743	1
Ndufs6	-0,023726	1
Fbxo7	-0,023827	1
Mrpl24	-0,02379	1
Rgs10	-0,023973	1
Tmem159	-0,024079	1
Rab11a	-0,024053	1
Tmem126a	-0,024234	1
Tacc1	-0,024204	1
Gm6162	-0,024294	1
Bod1	-0,024253	1
B4galt1	-0,024301	1
Tdp1	-0,024593	1
Timm29	-0,024597	1
Slc35a5	-0,024676	1
Rnf113a1	-0,024751	1
Polr2h	-0,024924	1
Fam207a	-0,025095	1
Eif5a	-0,025131	1
Golgb1	-0,025335	1
Gstp1	-0,025445	1
Phka2	-0,025396	1
Kmt2c	-0,0255	1
Prpf39	-0,025572	1
Eci2	-0,02565	1
Gm11737	-0,025675	1
Them4	-0,02577	1
Lcmt1	-0,02584	1
Zfp335os	-0,026003	1
Mrs2	-0,026251	1
Gm44152	-0,026308	1
Gse1	-0,026308	1
Gpr85	-0,026389	1
Dars	-0,026438	1
Brd4	-0,026373	1
Gm1840	-0,026516	1
Mrps17	-0,02651	1
Safb2	-0,026557	1
Map2k5	-0,026649	1
Rpusd3	-0,026686	1
Cenpa	-0,026772	1
Gm2796	-0,026911	1

Atg2a	-0,026867	1
Zdhhc6	-0,026997	1
Mrps5	-0,027132	1
Bri3	-0,027212	1
Gm10704	-0,027269	1
Acin1	-0,027388	1
Mid1ip1	-0,02738	1
Ppp2r3a	-0,02784	1
Slc50a1	-0,027944	1
Polr2b	-0,028197	1
Sdha	-0,028247	1
Rnf14	-0,028642	1
Exoc3	-0,028716	1
Anpep	-0,028808	1
Akt2	-0,028814	1
Tmem135	-0,028839	1
Mgat1	-0,029202	1
Tob2	-0,029401	1
Atp5f1	-0,029439	1
Klhl42	-0,029693	1
Gigyf2	-0,029725	1
Gm42566	-0,029797	1
Gda	-0,029886	1
Ier5	-0,030036	1
Sem1	-0,030102	1
Ppie	-0,030162	1
Acaca	-0,03028	1
Pole	-0,030439	1
Lamp2	-0,030609	1
Mga	-0,030687	1
Ccnt1	-0,030728	1
Gm13373	-0,030784	1
Nol7	-0,030787	1
Isca2	-0,031002	1
Pcsk4	-0,031202	1
Gm6198	-0,031213	1
Ccpg1	-0,031245	1
Foxj2	-0,031167	1
Armc9	-0,031316	1
Gm14040	-0,031566	1
Rlim	-0,031554	1
Mrpl40	-0,031663	1
Pkig	-0,03179	1
Ano6	-0,031841	1
Sf3b2	-0,031758	1
Papola	-0,032024	1
Usmg5	-0,032178	1
Zswim6	-0,032642	1
Xpo6	-0,03274	1
Morc3	-0,032688	1
Ptpn9	-0,032917	1
Prmt9	-0,032911	1

Uvrag	-0,03307	1
Nod2	-0,033256	1
Irak1	-0,033317	1
Atrn	-0,033405	1
Epb41l4aos	-0,033414	1
Abhd6	-0,033529	1
Ginm1	-0,033816	1
D10Jhu81e	-0,033907	1
Trpm4	-0,034084	1
Yars2	-0,03415	1
Snw1	-0,034155	1
Pon2	-0,034362	1
Fmnl1	-0,034467	1
Wnt6	-0,034629	1
Hint2	-0,034575	1
Yipf2	-0,034583	1
Stx7	-0,034605	1
Gm43547	-0,034967	1
Pmepa1	-0,034978	1
Ubald1	-0,035173	1
Slc35b2	-0,035297	1
Hgs	-0,03552	1
Tbc1d17	-0,035647	1
Prpf6	-0,035643	1
Uqcrfs1	-0,035808	1
Creb3l4	-0,035909	1
Map3k14	-0,035903	1
Fbrs	-0,035965	1
Efr3a	-0,036068	1
Lrrc57	-0,036634	1
Cnnm3	-0,03657	1
Zfp263	-0,036613	1
Timm17a	-0,036606	1
Pik3c3	-0,036813	1
Cdc40	-0,036767	1
RP23-48A24.3	-0,036983	1
Hps3	-0,037027	1
Gm5963	-0,036957	1
Vps33a	-0,037142	1
Cast	-0,037235	1
Tpr	-0,037189	1
Lars2	-0,037353	1
Sfxn1	-0,037546	1
0610038B21Rik	-0,037583	1
Fam160a2	-0,037844	1
Gm14140	-0,037949	1
Rbm45	-0,03804	1
2310009A05Rik	-0,037981	1
Ndrg2	-0,038143	1
2210016L21Rik	-0,038242	1
Thyn1	-0,038249	1
Rpl31-ps16	-0,038269	1

Layn	-0,038332	1
Xrcc4	-0,038447	1
Ctdsp2	-0,038696	1
Tollip	-0,03866	1
Atp5a1	-0,038678	1
Psrc1	-0,038846	1
Snx9	-0,038868	1
Eif2ak3	-0,038859	1
Srprb	-0,03888	1
Pxk	-0,038852	1
Pnisr	-0,039399	1
Gan	-0,039677	1
Zswim7	-0,040233	1
Gm43290	-0,040298	1
Itgb5	-0,040318	1
Itgb1bp1	-0,040379	1
Rabgap1	-0,040484	1
Ctps	-0,040532	1
Prdx5	-0,040718	1
Gpd2	-0,040696	1
Npat	-0,040829	1
Myo19	-0,040936	1
Slc25a37	-0,041233	1
Xrcc3	-0,041351	1
Armt1	-0,041352	1
Dlg1	-0,041483	1
Gm16556	-0,041626	1
Rhof	-0,041573	1
Nbr1	-0,041654	1
Synpo	-0,041914	1
Fbxo45	-0,04187	1
Srsf3	-0,041865	1
Sirpa	-0,042023	1
Slc48a1	-0,041958	1
Agpat5	-0,041992	1
Ube2n	-0,042138	1
Ctc1	-0,042058	1
Snupn	-0,04208	1
Ercc6	-0,042274	1
Pogk	-0,04248	1
Lmo4	-0,04262	1
Gm9403	-0,042653	1
Gm44075	-0,042681	1
RP23-88C11.5	-0,042826	1
Rnf216	-0,042833	1
Nploc4	-0,042815	1
Ogg1	-0,04287	1
Hacd1	-0,043023	1
RP23-115A18.3	-0,043129	1
9930022D16Rik	-0,043096	1
Nrp1	-0,043052	1
Gm6415	-0,043204	1

Gm9207	-0,043409	1
Zc3hav1	-0,043361	1
Kctd12	-0,043525	1
Tmem38a	-0,043622	1
Tmeff1	-0,043571	1
Pds5a	-0,043594	1
Rpl38-ps2	-0,043716	1
Wbp2	-0,043732	1
Wls	-0,043808	1
Tmed5	-0,043883	1
Yrdc	-0,044112	1
Mir703	-0,044112	1
Gm12186	-0,044325	1
Dpy19l4	-0,044263	1
Ptp4a3	-0,04445	1
Hddc3	-0,044475	1
Lyrm9	-0,044619	1
Clec2l	-0,04467	1
Mlxip	-0,044869	1
Akap8l	-0,044888	1
Sergef	-0,044978	1
Nphp1	-0,04506	1
Fkbp8	-0,045211	1
Zfp644	-0,045231	1
Washc2	-0,045255	1
Clasp1	-0,045253	1
Fcho2	-0,045276	1
Psmd3	-0,045382	1
Cryl1	-0,045456	1
Coq6	-0,045647	1
Nab1	-0,045573	1
Sec31a	-0,045677	1
Mocos	-0,04579	1
2810428I15Rik	-0,046	1
Tor1a	-0,045977	1
D230025D16Rik	-0,046073	1
Dhrs4	-0,046241	1
Zbtb17	-0,046168	1
Tmem123	-0,04625	1
Psmb10	-0,046424	1
Olfr920	-0,046639	1
Gm15920	-0,046667	1
Azi2	-0,046855	1
Ctss	-0,047207	1
Zfp560	-0,047336	1
Bcorl1	-0,047281	1
Dot1l	-0,047396	1
Pip4k2a	-0,047502	1
Zer1	-0,047588	1
Nkiras1	-0,047586	1
Hnrnph3	-0,047786	1
Pa2g4	-0,047918	1

Osbpl9	-0,048163	1
Usp2	-0,048332	1
Lyn	-0,048321	1
Cpne3	-0,048348	1
Csf2ra	-0,048588	1
Zfp512b	-0,048627	1
Txnip	-0,048596	1
Gm10033	-0,048802	1
Ptpn23	-0,048849	1
Ap1s3	-0,048799	1
Myl12a	-0,048763	1
Mcu	-0,048822	1
Ccny	-0,048991	1
Kif23	-0,048954	1
Fam19a2	-0,049242	1
Trim13	-0,049244	1
Trappc3	-0,049375	1
Rcor3	-0,049423	1
Rps16-ps2	-0,049351	1
Snhg1	-0,049505	1
1190002N15Rik	-0,049574	1
Zfp652os	-0,049653	1
Cdt1	-0,049686	1
Epc2	-0,049845	1
Gm13776	-0,050122	1
4933434E20Rik	-0,050059	1
Phax	-0,050129	1
Ndufs7	-0,050215	1
Rplp0	-0,050248	1
Insr	-0,05025	1
Brat1	-0,050395	1
3830403N18Rik	-0,050433	1
1110046J04Rik	-0,05053	1
Mcub	-0,050681	1
Rab31	-0,050751	1
Senp2	-0,050919	1
Ptpn1	-0,050903	1
Rps12-ps23	-0,051248	1
Syf2	-0,051189	1
Plekha2	-0,051424	1
Epb41l2	-0,05153	1
Srrt	-0,051842	1
Akt1	-0,051755	1
Tmem258	-0,052189	1
Cdkn1a	-0,052283	1
Gtpbp1	-0,052395	1
Gm10313	-0,052483	1
Ndufb6	-0,052507	1
Ccdc28b	-0,05255	1
Lsm8	-0,052617	1
Extl3	-0,052609	1
Ltbp2	-0,052689	1

Zswim8	-0,052717	1
Ergic1	-0,052775	1
Eif2b2	-0,052811	1
Gtpbp4	-0,052897	1
Ppt1	-0,052971	1
Stk38l	-0,053143	1
Ptges3	-0,05335	1
Plaa	-0,053374	1
Spaca9	-0,05359	1
Zcchc14	-0,053684	1
Scrn2	-0,053795	1
Cpeb1	-0,053885	1
Twsg1	-0,053947	1
Sh3bp2	-0,053943	1
Ndufa10	-0,053876	1
Ywhab	-0,054223	1
Cdk2	-0,054385	1
Trmt112-ps2	-0,054537	1
Cipc	-0,054676	1
Man2a2	-0,054776	1
Srpk1	-0,054869	1
Plppr2	-0,055118	1
Zfp287	-0,055296	1
Col4a3bp	-0,05527	1
Gm8566	-0,055351	1
Ets2	-0,055354	1
Msl2	-0,055353	1
Coq7	-0,055647	1
Tbrg1	-0,05567	1
Pigk	-0,055673	1
Npepps	-0,055669	1
Gm44283	-0,055785	1
Iqsec1	-0,05588	1
Atg9b	-0,056188	1
Sf3b5	-0,056399	1
Spice1	-0,056639	1
Prkag1	-0,056561	1
Ercc1	-0,056762	1
Daam1	-0,056832	1
Dpm2	-0,056784	1
Hsd17b4	-0,057186	1
Zfp36	-0,057368	1
Dnttip1	-0,05737	1
Pgm2	-0,057369	1
Slc9a1	-0,057385	1
BC037034	-0,057516	1
Upf3a	-0,057507	1
Tnfaip1	-0,057532	1
Bag4	-0,057642	1
Ruvbl2	-0,057797	1
Pcnx	-0,057947	1
Troap	-0,058162	1

Ifi35	-0,058173	1
Prps2	-0,0582	1
Nceh1	-0,058233	1
A830008E24Rik	-0,058441	1
Gm10260	-0,058351	1
Sbk2	-0,058685	1
Slc25a28	-0,058704	1
Sppl2a	-0,058759	1
Zfand2b	-0,058863	1
Lrrc24	-0,058994	1
Dmxl1	-0,059066	1
Snrpb2	-0,059087	1
Far1	-0,059255	1
Inpp4a	-0,059396	1
Rap2c	-0,059775	1
Nf1	-0,059915	1
Serf1	-0,059894	1
Dnase2a	-0,060141	1
Gm12222	-0,060305	1
Ppp1r16a	-0,060307	1
Bclaf1	-0,060262	1
Ctif	-0,060372	1
Prpf31	-0,060437	1
Cab39	-0,060415	1
Rps11	-0,060738	1
Mettl1	-0,060797	1
Xrn2	-0,060772	1
Tbc1d7	-0,060913	1
Akr7a5	-0,060856	1
Gfpt1	-0,060947	1
Reps1	-0,06138	1
Ift81	-0,061495	1
Cpox	-0,061466	1
Aldh7a1	-0,061679	1
Tnfsf13b	-0,061846	1
Ttf1	-0,061831	1
Atp2a2	-0,061807	1
Cracr2a	-0,061871	1
Cyp20a1	-0,061934	1
Scamp5	-0,061903	1
Gm38022	-0,062061	1
Six4	-0,06221	1
St3gal6	-0,062183	1
Nek9	-0,062355	1
Dolpp1	-0,062497	1
Gm44771	-0,062587	1
Pou2f1	-0,062845	1
Klhl22	-0,062977	1
Hltf	-0,063033	1
Sash1	-0,063148	1
Klhdc2	-0,063098	1
Fam69a	-0,063184	1

Rab40c	-0,06317	1
Arfgap3	-0,063306	1
Gpd1l	-0,063447	1
Ubqln1	-0,063401	1
Slc46a3	-0,063594	1
Gm8451	-0,063598	1
Trip6	-0,063593	1
Kctd20	-0,063723	1
Nol8	-0,063798	1
Atp2c1	-0,063851	1
Nckap5l	-0,063969	1
Dennd2c	-0,064	1
Bud13	-0,063963	1
Ppp2r3c	-0,064225	1
Trim56	-0,064265	1
RP23-371B13.3	-0,064799	1
Alcam	-0,064843	1
Dkc1	-0,064852	1
Gm5787	-0,065005	1
Psmc6	-0,064963	1
Atf7ip	-0,065498	1
Smyd2	-0,065487	1
Tagln2	-0,065453	1
Unc119b	-0,065606	1
Gm12338	-0,065791	1
Secisbp2l	-0,065959	1
Elovl5	-0,066005	1
Gm8423	-0,066124	1
Rnf138	-0,066063	1
F9	-0,066242	1
Selenoo	-0,066171	1
Dhrs7	-0,066184	1
Foxo4	-0,0663	1
Net1	-0,066586	1
6720475M21Rik	-0,066552	1
A630001G21Rik	-0,066673	1
Nab2	-0,066688	1
Mfng	-0,066724	1
Agpat1	-0,066851	1
Gm17511	-0,067036	1
Gm15846	-0,067126	1
Mmp9	-0,067074	1
Fyco1	-0,067201	1
Sos2	-0,067203	1
Ahctf1	-0,067329	1
Bnip3	-0,067727	1
Atp6v1a	-0,067804	1
Gm37780	-0,068211	1
Bcl7b	-0,068166	1
Myo7a	-0,068308	1
Ids	-0,068256	1
Mprip	-0,068254	1

Atl2	-0,068398	1
Alyref2	-0,06851	1
Klf2	-0,068493	1
Gm996	-0,068645	1
Sgsm3	-0,068586	1
Ncstn	-0,068661	1
Cnot9	-0,068823	1
Csgalnact2	-0,068776	1
Tex9	-0,06894	1
Eya3	-0,068923	1
Mrpl38	-0,06895	1
Nr4a2	-0,069073	1
Mrps30	-0,069348	1
Agtrap	-0,069428	1
Mydgf	-0,069405	1
Kif5a	-0,069565	1
Gm11450	-0,069714	1
Trappc10	-0,069749	1
Nr1h3	-0,070258	1
4833420G17Rik	-0,070279	1
Tcf12	-0,070427	1
Ghitm	-0,070367	1
Sesn1	-0,070498	1
RP23-331E5.10	-0,070632	1
Dhrs7b	-0,070584	1
Nek7	-0,070616	1
Phlpp1	-0,070849	1
Mkx	-0,070873	1
Maml3	-0,070898	1
Trrap	-0,070886	1
Elp3	-0,070989	1
Mterf4	-0,071119	1
Clybl	-0,071164	1
Chkb	-0,071271	1
Bag6	-0,071394	1
Gcsh	-0,071498	1
Nisch	-0,071728	1
Gm20900	-0,071849	1
Dusp8	-0,071913	1
Gm16288	-0,071991	1
Nup93	-0,071969	1
Rfc3	-0,072112	1
Ndufa11	-0,072128	1
Gm8330	-0,072314	1
Ndufa12	-0,072412	1
F8a	-0,072703	1
Jag1	-0,072726	1
Mpg	-0,072815	1
Cenpi	-0,073047	1
Faap20	-0,073	1
Gm11281	-0,073097	1
Abl2	-0,073124	1

Lrtm2	-0,073174	1
Wdr13	-0,073225	1
Tsku	-0,073686	1
Cd109	-0,073689	1
Smad1	-0,073781	1
Slc25a12	-0,073941	1
Rpl26-ps2	-0,073995	1
Tmem229b	-0,073984	1
Slc30a7	-0,073957	1
Nfkbil1	-0,07412	1
March7	-0,074225	1
Dock4	-0,074257	1
Ift140	-0,074382	1
G730013B05Rik	-0,074478	1
Cdca7l	-0,074506	1
Mon1b	-0,07462	1
Hcfc2	-0,074589	1
Cbfb	-0,074834	1
Wdr18	-0,075077	1
Tpst1	-0,07525	1
Mfsd12	-0,075249	1
Tfb1m	-0,075456	1
Tra2b	-0,075514	1
Gm5124	-0,075757	1
Gm45084	-0,07585	1
Cisd3	-0,075852	1
Pip4k2c	-0,075946	1
Acot13	-0,075867	1
Hint1	-0,075919	1
Tfcp2l1	-0,076348	1
Snx10	-0,07627	1
9530068E07Rik	-0,076321	1
Selenot	-0,076501	1
Napb	-0,07683	1
Rbm43	-0,077085	1
Zxdc	-0,077053	1
Timm13	-0,07719	1
Nob1	-0,077176	1
Kctd5	-0,07735	1
Fbxo3	-0,077331	1
Vps72	-0,077338	1
Mtmr3	-0,077355	1
Rcor1	-0,07745	1
Invs	-0,077472	1
Larp1b	-0,077527	1
Gm7785	-0,077733	1
Dab2	-0,077695	1
Tctn3	-0,077779	1
Fxyd5	-0,077813	1
Ptbp2	-0,077918	1
4930581F22Rik	-0,077988	1
Cyp4v3	-0,077985	1

Slc30a9	-0,078122	1
Csnk1g3	-0,078379	1
Rbm7	-0,078392	1
Rnf40	-0,078539	1
Osbpl7	-0,078788	1
Stard3	-0,07877	1
Cdc123	-0,079036	1
Thap2	-0,079042	1
Daglb	-0,079024	1
Zcrb1	-0,079113	1
Fam109a	-0,079215	1
4930455G09Rik	-0,079243	1
Trim37	-0,079192	1
Mapk8ip1	-0,07929	1
Nle1	-0,079388	1
Rictor	-0,079563	1
Adamtsl5	-0,07977	1
Gm43213	-0,080136	1
Slc35a3	-0,080081	1
Arrb2	-0,080124	1
Pxn	-0,080166	1
Pls3	-0,080185	1
Gm10399	-0,080348	1
Top1mt	-0,080315	1
RP24-511J14.2	-0,08038	1
Gm4468	-0,080619	1
Washc5	-0,080586	1
Ubxn1	-0,080612	1
Abl1	-0,080924	1
Suco	-0,080997	1
Mapk14	-0,081236	1
Elac2	-0,081822	1
Arpc2	-0,081812	1
Thoc2	-0,081758	1
Mrpl15	-0,081835	1
Krtcap2	-0,081856	1
Sdf4	-0,082025	1
Dap	-0,081954	1
RP23-476G10.1	-0,082367	1
Arid5a	-0,082558	1
Emc8	-0,082658	1
Arf3	-0,082719	1
Abce1	-0,082678	1
Wbp4	-0,082759	1
Sumo3	-0,082941	1
Rac1	-0,082935	1
Neu1	-0,083032	1
Rbpms	-0,083143	1
Scaf8	-0,083223	1
Hmx3	-0,083305	1
Yipf6	-0,083267	1
Mtf1	-0,08334	1

Uggt1	-0,083307	1
Gm28731	-0,083388	1
Smim14	-0,083821	1
Mzt2	-0,08394	1
Gm4332	-0,083946	1
Arcn1	-0,083963	1
Ndufa9	-0,084048	1
Mier3	-0,084117	1
Snapc2	-0,084392	1
Atp8b2	-0,084425	1
Cog6	-0,084468	1
Phf23	-0,084558	1
Scmh1	-0,084688	1
Fam167b	-0,08466	1
Coro7	-0,085017	1
Ech1	-0,084971	1
Gorasp2	-0,084974	1
Mrpl49	-0,085234	1
Hs2st1	-0,085229	1
Gm6297	-0,085396	1
Gak	-0,085373	1
Pan3	-0,085451	1
Taf11	-0,085545	1
Zkscan14	-0,085844	1
Ttc3	-0,085842	1
Mat2b	-0,085885	1
Rbbp8	-0,086119	1
Zdhhc9	-0,086068	1
Cnnm4	-0,086211	1
Rnf135	-0,086207	1
Ipmk	-0,086208	1
Soat1	-0,086315	1
Exoc3l	-0,086536	1
2610044O15Rik8	-0,086552	1
Lap3	-0,086688	1
Nfkb1	-0,086758	1
Slc38a10	-0,086897	1
Rnpepl1	-0,087035	1
Anks1	-0,087007	1
Dhx35	-0,087145	1
Atp8b3	-0,087158	1
Polr2m	-0,087242	1
Ddx31	-0,087296	1
1110004F10Rik	-0,087326	1
Anp32e	-0,0873	1
Mrpl57	-0,087441	1
Baz1b	-0,087399	1
Lhpp	-0,087566	1
Tpmt	-0,087666	1
Ints1	-0,087849	1
Mrps22	-0,087889	1
Pold2	-0,087879	1

Trp53bp1	-0,08787	1
Akirin2	-0,088012	1
Mir99ahg	-0,088185	1
Peli1	-0,088212	1
Gorab	-0,088543	1
Tuft1	-0,088503	1
Gm16181	-0,088631	1
Alad	-0,088621	1
Meis2	-0,088706	1
Zfp72	-0,088769	1
Suclg2	-0,088838	1
Snrpd3	-0,088753	1
Sh3kbp1	-0,088902	1
Timm50	-0,088968	1
Slc39a14	-0,089021	1
Nipa2	-0,089017	1
Myd88	-0,089369	1
Zc3hav1l	-0,089508	1
Fcgr1	-0,08947	1
Snx17	-0,089533	1
Cdkn2aipnl	-0,089648	1
Lamp1	-0,089671	1
Tnfrsf1b	-0,090001	1
Acp1	-0,090184	1
Pidd1	-0,090162	1
Scnm1	-0,090242	1
Sgtb	-0,090296	1
Hmg20a	-0,09034	1
Slc4a2	-0,090424	1
Snapc1	-0,090403	1
Gm37702	-0,090494	1
Nhlrc1	-0,090614	1
Nfyc	-0,090888	1
Ubl4a	-0,09089	1
Tsr2	-0,090891	1
Dpy19l1	-0,090897	1
Ptgr2	-0,091091	1
AW046200	-0,091205	1
Entpd5	-0,091308	1
Ppil2	-0,091276	1
Cdk8	-0,09135	1
Adcy7	-0,091472	1
Fzr1	-0,091589	1
Tmem268	-0,091682	1
Abhd11	-0,091684	1
Tmem87b	-0,09208	1
4930558J18Rik	-0,092246	1
Cetn3	-0,092197	1
Tpd52	-0,092235	1
Aldh18a1	-0,092512	1
Gcc2	-0,09263	1
Vti1a	-0,092789	1

Gm36445	-0,092939	1
Tbc1d24	-0,092934	1
Rrp1	-0,092907	1
Emd	-0,092878	1
Ormdl2	-0,093113	1
Akap8	-0,093079	1
Gnb5	-0,093302	1
Akip1	-0,09345	1
Itsn1	-0,093434	1
Frmd6	-0,093479	1
Rab11b	-0,093461	1
a	-0,093666	1
Btrc	-0,093693	1
Orai2	-0,093737	1
Vmp1	-0,093677	1
1600010M07Rik	-0,093826	1
Pno1	-0,093806	1
Caly	-0,093869	1
Nthl1	-0,093902	1
Trim23	-0,094113	1
Slc35f5	-0,094054	1
Ptpn6	-0,09425	1
Parp11	-0,094445	1
Ahsa2	-0,094416	1
Zfp119b	-0,094478	1
Tle3	-0,0945	1
Limk2	-0,094477	1
Ly6e	-0,094517	1
Trpt1	-0,094591	1
Sar1a	-0,094907	1
Arl10	-0,095123	1
Pkd1	-0,095282	1
Dnajc14	-0,095252	1
Nudcd1	-0,095432	1
Fubp3	-0,095417	1
Cyb5a	-0,095378	1
Glipr1	-0,095502	1
Xrra1	-0,09563	1
Med26	-0,095633	1
Nradd	-0,095617	1
Smchd1	-0,095575	1
Pgam5	-0,095647	1
Gfi1	-0,095743	1
Spsb3	-0,095871	1
Rdx	-0,095872	1
Tmem150a	-0,095979	1
Gm44269	-0,095999	1
Dctn5	-0,09597	1
Tmem199	-0,096062	1
Hdgf	-0,096428	1
Pex6	-0,096535	1
Psap	-0,096699	1

Ppp2r5c	-0,096743	1
Tnip2	-0,096836	1
Thumpd3	-0,096773	1
Strap	-0,096769	1
Mak16	-0,096814	1
Gm10180	-0,096929	1
Stau1	-0,096893	1
Vipas39	-0,097027	1
Hcfc1	-0,096994	1
Dctn4	-0,097001	1
Gm12115	-0,097093	1
Gpbp1	-0,09708	1
Rufy2	-0,097367	1
Chd4	-0,0974	1
Nup88	-0,097455	1
Dnaja3	-0,097704	1
Apobr	-0,097745	1
Gm11334	-0,097833	1
Ubxn2a	-0,097792	1
Nova2	-0,097899	1
Metap1	-0,098134	1
Gm16072	-0,098194	1
Tomm34	-0,098288	1
Dcxr	-0,09829	1
Mdm4	-0,098582	1
Dhx15	-0,098641	1
Rnh1	-0,098573	1
Ndufb3	-0,098765	1
Psma4	-0,098857	1
Nop14	-0,098921	1
Ppp2r2a	-0,098958	1
Clec16a	-0,099099	1
Ehmt1	-0,09912	1
Arnt	-0,099326	1
Casc3	-0,099543	1
Depdc7	-0,09973	1
Tbc1d19	-0,099793	1
Pprc1	-0,099814	1
Mdh2	-0,1001	1
Pdrg1	-0,10022	1
Hic2	-0,10042	1
Arhgap4	-0,10047	1
Ccdc106	-0,10065	1
Smyd4	-0,10082	1
Calm2	-0,10092	1
Ipo8	-0,10127	1
AI413582	-0,10133	1
Mapk7	-0,10143	1
Pdp2	-0,10152	1
C730034F03Rik	-0,10157	1
Atp5o	-0,1016	1
Mrps18a	-0,10171	1

Pigv	-0,10183	1
Bmp2k	-0,10193	1
Ing3	-0,1021	1
Zfp956	-0,10222	1
Wrb	-0,10224	1
Adnp2	-0,10234	1
Rack1	-0,10252	1
Gm28809	-0,103	1
Trappc6b	-0,10315	1
Eaf1	-0,10333	1
Pias3	-0,1034	1
Acsl5	-0,10342	1
Atp6v0b	-0,10363	1
Tmbim4	-0,10375	1
Ide	-0,10381	1
Apaf1	-0,10379	1
Tmpo	-0,10377	1
Gcnt1	-0,10388	1
M1ap	-0,10395	1
Phlda3	-0,10396	1
Abcc4	-0,10421	1
Pnkp	-0,10417	1
Cltc	-0,10425	1
Hnrnpa0	-0,10426	1
Yipf7	-0,10443	1
Gm43681	-0,10455	1
Btg2	-0,10454	1
Tssc1	-0,10449	1
Pcbp1	-0,10448	1
A130010J15Rik	-0,10459	1
Uchl3	-0,1046	1
Thg1l	-0,1051	1
Gtf3c2	-0,1051	1
Gm7561	-0,1056	1
Dpy30	-0,10594	1
Antxr2	-0,10597	1
Zfp219	-0,10611	1
Atp2b4	-0,10608	1
Snrnp70	-0,10609	1
Tcea1	-0,10632	1
Tmem143	-0,10654	1
Drosha	-0,10703	1
Oip5	-0,10734	1
S100a10	-0,10744	1
Dennd1a	-0,10758	1
Lmbr1l	-0,1076	1
Itga2b	-0,10779	1
Metrnl	-0,10791	1
Lgalsl	-0,10809	1
Dnajc13	-0,10813	1
Sin3b	-0,10815	1
Hexa	-0,10826	1

Gm15207	-0,1084	1
Fermt3	-0,10836	1
Etv1	-0,10847	1
Gm12005	-0,10858	1
Vps26a	-0,10863	1
Ate1	-0,10859	1
Gm37145	-0,10877	1
Atp5l	-0,10878	1
Rab8b	-0,10884	1
Ctnnal1	-0,10893	1
Kbtbd2	-0,10892	1
Khsrp	-0,10891	1
Zfp503	-0,10902	1
Exoc3l2	-0,10898	1
Txndc9	-0,10912	1
Pdcd4	-0,10912	1
N4bp2l2	-0,10912	1
Mtfr1l	-0,10919	1
Med7	-0,10931	1
Nsmaf	-0,10979	1
Ergic2	-0,10981	1
Snord92	-0,10987	1
Rnf213	-0,11008	1
Eri1	-0,11009	1
Ccdc77	-0,11013	1
Rhobtb2	-0,11016	1
Itpripl1	-0,11022	1
Mcmbp	-0,11022	1
Etf1	-0,11027	1
Lace1	-0,11038	1
Syngr2	-0,11044	1
Rraga	-0,11046	1
Mcrs1	-0,1106	1
Lrrfip2	-0,11057	1
Il11ra1	-0,11081	1
Gm14006	-0,11088	1
Gm5939	-0,11102	1
Alyref	-0,11097	1
Mrps34	-0,11114	1
Spg21	-0,11111	1
Med23	-0,11127	1
Rars	-0,11134	1
Rassf2	-0,11155	1
Prelid2	-0,1116	1
Ankrd10	-0,11163	1
Gm42659	-0,1117	1
Dctn3	-0,11169	1
Sik1	-0,11194	1
Stx16	-0,11189	1
Nudcd3	-0,11191	1
Mlh1	-0,11203	1
Dusp10	-0,11253	1

Mad2l1bp	-0,11255	1
Ahcyl1	-0,11245	1
Capn3	-0,11257	1
Rab5b	-0,11258	1
Jade1	-0,11266	1
Gm12497	-0,11299	1
Fan1	-0,11312	1
Pcyox1l	-0,11311	1
Neurl4	-0,11321	1
Cbx7	-0,11319	1
Nup54	-0,11332	1
Sfpq	-0,11329	1
Kif21b	-0,11339	1
Abhd13	-0,11344	1
Fam50a	-0,11336	1
Gm11222	-0,11349	1
Gm43660	-0,11371	1
Chst3	-0,11388	1
9330159M07Rik	-0,114	1
Gm7618	-0,11403	1
Ccdc91	-0,11415	1
Dsn1	-0,11427	1
Slc6a12	-0,11433	1
Cdk19	-0,1143	1
Ggnbp2	-0,11433	1
Rubcnl	-0,11451	1
Gm12074	-0,11465	1
Cep170	-0,11472	1
Cwf19l2	-0,11466	1
Sco1	-0,11479	1
Tceanc2	-0,11501	1
RP23-13B8.12	-0,11512	1
Actn4	-0,11511	1
Ppp2ca	-0,11514	1
Eif3j1	-0,11516	1
Mbtps2	-0,11528	1
Rbbp4	-0,11533	1
Srek1ip1	-0,11537	1
Mef2a	-0,11551	1
Plgrkt	-0,11559	1
Epg5	-0,11573	1
Phactr4	-0,11577	1
Pcgf6	-0,116	1
Qrsl1	-0,11632	1
Crocc	-0,11643	1
Thbs3	-0,11685	1
Agap1	-0,11685	1
Lck	-0,11695	1
Cdk5rap2	-0,11697	1
Kctd18	-0,117	1
Sos1	-0,11724	1
Bcap31	-0,11726	1

Comtd1	-0,11754	1
Uhrf1bp1	-0,11752	1
Nsd2	-0,11762	1
Zbtb44	-0,11763	1
Gm10073	-0,11774	1
Scd2	-0,1177	1
Rab3d	-0,11784	1
Scaf11	-0,11784	1
Nans	-0,11813	1
Gm7600	-0,11838	1
Commd7	-0,11847	1
Mdm1	-0,11859	1
Tmf1	-0,11857	1
Qser1	-0,11873	1
Sgta	-0,11865	1
2810001G20Rik	-0,11882	1
Nub1	-0,11881	1
Akap13	-0,11929	1
Brd7	-0,11932	1
Acbd3	-0,11928	1
Dbp	-0,11941	1
Gm6329	-0,11964	1
Ddrgk1	-0,11981	1
Slc30a1	-0,11978	1
Rsbn1l	-0,12014	1
Irf9	-0,12027	1
Rbm41	-0,12044	1
Snf8	-0,12076	1
Btbd2	-0,12094	1
Ifi204	-0,12096	1
Coq9	-0,12098	1
Cst3	-0,12096	1
Rbbp9	-0,12126	1
Me1	-0,12152	1
Trmt10a	-0,12147	1
Pum1	-0,12152	1
Clip1	-0,12155	1
Hspb6	-0,12168	1
Fam122a	-0,12171	1
CAAA01180111.2	-0,12206	1
F10	-0,12214	1
Gm20707	-0,12223	1
Pibf1	-0,12222	1
Gm43775	-0,12216	1
Dnm2	-0,12222	1
Gm10080	-0,12271	1
Mthfd1	-0,12273	1
Hspd1	-0,12269	1
Rpl36a-ps3	-0,12277	1
Adrb2	-0,12282	1
Slc25a32	-0,12302	1
H1f0	-0,12302	1

Wwc2	-0,12308	1
Rapgef2	-0,12312	1
Zfp157	-0,12318	1
Baat	-0,12315	1
Mroh1	-0,12319	1
Rnf144b	-0,12327	1
Phf20-ps	-0,1234	1
Rnf169	-0,12341	1
Snta1	-0,12359	1
Gm2735	-0,12373	1
Slc20a1	-0,12384	1
Ubc	-0,12386	1
Syt8	-0,12402	1
Atp13a2	-0,12397	1
Eml2	-0,12407	1
Prss53	-0,12421	1
Gm7784	-0,12424	1
Firre	-0,12444	1
Stk3	-0,12442	1
Acap2	-0,1246	1
Dcun1d5	-0,1246	1
Tnpo3	-0,12466	1
Gm5812	-0,12476	1
Eef1g	-0,12478	1
Baz2b	-0,12495	1
Pkp2	-0,12503	1
Rgs3	-0,12512	1
Slc5a3	-0,12514	1
Nfya	-0,12543	1
Zfp467	-0,12548	1
Sacm1l	-0,12554	1
Inpp1	-0,12561	1
Kdm2a	-0,12565	1
Gm6913	-0,12575	1
Ezh1	-0,12568	1
Tank	-0,12624	1
Rpl17-ps4	-0,12628	1
Cd72	-0,12637	1
Gm15185	-0,12654	1
Tmx3	-0,1265	1
Kansl1	-0,12657	1
Nap1l4	-0,12703	1
9030617O03Rik	-0,12722	1
Gm20554	-0,12733	1
Dok4	-0,1274	1
Eif3d	-0,12743	1
Arhgap17	-0,12764	1
Lactb	-0,12771	1
Fam102a	-0,12774	1
Ptk2b	-0,12784	1
Topors	-0,12792	1
Sfr1	-0,12789	1

Gm7027	-0,12815	1
Ddhd1	-0,12825	1
Cox8a	-0,12825	1
Rin1	-0,12833	1
Atp6v0d1	-0,12837	1
Cdc23	-0,12841	1
Psmd12	-0,12843	1
Dnajc22	-0,12846	1
Zbed3	-0,12847	1
Uap1l1	-0,12854	1
Mtf2	-0,12861	1
Anapc10	-0,1287	1
Parp1	-0,12868	1
Zmym3	-0,12915	1
Tbce	-0,12939	1
Smap1	-0,12946	1
Rexo2	-0,12975	1
Nsmce4a	-0,12981	1
Cyb5d2	-0,1299	1
Rcan3	-0,12999	1
Naa50	-0,13014	1
Eml4	-0,1302	1
Rbbp7	-0,13025	1
Golga4	-0,1303	1
1600002K03Rik	-0,13042	1
Neil1	-0,13037	1
Camk1d	-0,13041	1
Lrp6	-0,13045	1
Gm5113	-0,13056	1
Fam104a	-0,13064	1
Gm44432	-0,13082	1
Apex1	-0,13091	1
Plrg1	-0,13087	1
Psmb8	-0,131	1
Cacul1	-0,13121	1
Tipin	-0,13133	1
Mfhas1	-0,13143	1
Slc35d1	-0,13139	1
Nipsnap1	-0,1314	1
Tnks	-0,13136	1
Parg	-0,13145	1
Arl13b	-0,13156	1
Setd3	-0,13172	1
Mpdu1	-0,13185	1
Pdhx	-0,13211	1
Zfp24	-0,13211	1
Taf13	-0,13206	1
Lypla2	-0,13208	1
Pdcd6	-0,13211	1
Senp8	-0,13241	1
Mphosph6	-0,13236	1
Gdpd5	-0,13254	1

Nipbl	-0,13247	1
RP23-159E10.1	-0,13262	1
Ube3b	-0,13256	1
Tnfrsf23	-0,1327	1
Gm26606	-0,13284	1
Srsf4	-0,1329	1
Sap130	-0,1331	1
Taf15	-0,13327	1
Spink5	-0,13348	1
Use1	-0,13356	1
Rbfa	-0,13367	1
Il18	-0,1338	1
Kmt2a	-0,13383	1
Nt5c3	-0,13399	1
Hfe	-0,13432	1
Ttc33	-0,13435	1
Map2k4	-0,13457	1
Zbed4	-0,13469	1
Wwp2	-0,13471	1
Aebp2	-0,1351	1
Gm6863	-0,13516	1
Gm43501	-0,13551	1
Gnl3l	-0,1355	1
Rbm8a	-0,13564	1
Cbr4	-0,13579	1
Dcaf12	-0,13577	1
Prep	-0,13588	1
Dusp16	-0,13599	1
Ncf2	-0,13605	1
Adrm1	-0,13618	1
Msh6	-0,13624	1
Psma6	-0,1362	1
Aaed1	-0,13629	1
Gpatch1	-0,13634	1
Gm14286	-0,1365	1
Ppp1ca	-0,13653	1
Mto1	-0,13677	1
Crebbp	-0,13684	1
Trip12	-0,13679	1
Fas	-0,13695	1
Bcl10	-0,13697	1
Ubap1	-0,1371	1
Zfp800	-0,13718	1
Hspbap1	-0,13731	1
Plpp1	-0,13733	1
Hibadh	-0,13749	1
Spata24	-0,13769	1
Ccnc	-0,13768	1
Etaa1	-0,13778	1
Mpp1	-0,13791	1
Shmt2	-0,13795	1
Aldh4a1	-0,13806	1

Aldoc	-0,13809	1
Trim35	-0,13816	1
Zfyve9	-0,13833	1
9430015G10Rik	-0,1384	1
Vcp	-0,13843	1
Ado	-0,13841	1
Tmem167	-0,13854	1
Gemin2	-0,1388	1
Nsun3	-0,13929	1
Trpm7	-0,13933	1
Impact	-0,13964	1
Tox2	-0,13972	1
Siah2	-0,13996	1
Sc5d	-0,14014	1
Dopey1	-0,14018	1
Znrd1as	-0,1403	1
Fam214b	-0,14047	1
Zfp592	-0,14051	1
Washc1	-0,14066	1
Snrpb	-0,14074	1
Cd200r2	-0,14085	1
Acer3	-0,14092	1
Rasgef1a	-0,14125	1
Scrib	-0,14126	1
Mapk8	-0,14127	1
Exosc8	-0,14145	1
Zmiz2	-0,14137	1
Smad6	-0,1416	1
Vav2	-0,14168	1
Ube2j1	-0,14172	1
Atp6ap2	-0,1418	1
Eif2ak2	-0,14218	1
Fam117a	-0,14218	1
Flnc	-0,1422	1
Gm43323	-0,14235	1
Tonsl	-0,14226	1
Gle1	-0,14262	1
Clint1	-0,14277	1
Zfp142	-0,14299	1
Nqo2	-0,14317	1
Ttc1	-0,14318	1
Tomm5	-0,14333	1
Gm15787	-0,14343	1
Tlr2	-0,14339	1
Pdhb	-0,14363	1
Sumf2	-0,14372	1
Usp49	-0,1438	1
Phka1	-0,14384	1
Clcnkb	-0,14395	1
Ddx1	-0,14394	1
Tmem138	-0,14415	1
Rfx7	-0,14407	1

Fntb	-0,1442	1
Ccdc127	-0,1442	1
Ppa2	-0,14429	1
Dlg4	-0,14433	1
Gm45836	-0,14452	1
Edem2	-0,14453	1
Cggbp1	-0,14451	1
Uqcrc1	-0,14466	1
Mitd1	-0,1448	1
9430038I01Rik	-0,145	1
Rwdd4a	-0,14502	1
Maoa	-0,14514	1
Ugt1a7c	-0,14527	1
Ncbp3	-0,14535	1
Capg	-0,14548	1
1700096K18Rik	-0,14566	1
Gm2199	-0,14577	1
Rdh11	-0,14592	1
Gm8186	-0,14599	1
Eea1	-0,14603	1
Ptk2	-0,14612	1
Kdm7a	-0,14634	1
Zfp950	-0,14649	1
Tmem144	-0,14649	1
Elk1	-0,14658	1
Rapgef6	-0,14665	1
Ago1	-0,1468	1
Asl	-0,14712	1
Zfp626	-0,14763	1
Mrpl14	-0,14764	1
Gm9442	-0,14774	1
Snai2	-0,14771	1
Cpne8	-0,1477	1
Bivm	-0,14779	1
Tgds	-0,14787	1
Cept1	-0,1479	1
Exosc9	-0,14804	1
Nectin1	-0,14798	1
Ap5m1	-0,14821	1
Gm44153	-0,14829	1
Ybx1-ps2	-0,14844	1
Thoc1	-0,14844	1
Shisa5	-0,14842	1
Fpgs	-0,14847	1
Pdzd11	-0,14847	1
Poldip2	-0,14878	1
Mecr	-0,14903	1
Rpl31-ps22	-0,14937	1
Gm7936	-0,14938	1
Smyd5	-0,14942	1
Yeats4	-0,1494	1
Gng8	-0,14957	1

Arhgef39	-0,14956	1
Zkscan17	-0,14962	1
Rfk	-0,14983	1
Tmem50a	-0,14975	1
Fam219b	-0,14986	1
Stx12	-0,15021	1
1110004E09Rik	-0,1503	1
Tal1	-0,15049	1
Rsu1	-0,15053	1
Psmd7	-0,15073	1
Tspan3	-0,15083	1
Ufd1l	-0,15086	1
Ifrd2	-0,15123	1
Gtf3c4	-0,15121	1
Chsy1	-0,15127	1
Gm7452	-0,1514	1
Taf5l	-0,15143	1
Pisd	-0,15137	1
Atg3	-0,15151	1
Wdr89	-0,1517	1
Ndufb9	-0,15218	1
Stat6	-0,1524	1
Hmgn1	-0,15261	1
Gm5879	-0,15275	1
Cish	-0,15276	1
Ccdc88b	-0,15314	1
Gm38021	-0,15324	1
Ankrd16	-0,15329	1
Pepd	-0,15331	1
RP23-6C18.6	-0,15339	1
Pgp	-0,15342	1
Vamp3	-0,15348	1
Mrps26	-0,1535	1
Myo1e	-0,15358	1
Zfp85	-0,15391	1
Vps16	-0,1539	1
Tmem68	-0,15405	1
Gemin7	-0,15403	1
Tfeb	-0,15395	1
Sri	-0,15411	1
Hmx2	-0,15427	1
Ralbp1	-0,15429	1
Nrbf2	-0,15464	1
Gclm	-0,15475	1
Alkbh8	-0,15466	1
Ptdss1	-0,15507	1
Fam131a	-0,15515	1
Med11	-0,15527	1
AI606181	-0,1554	1
2610306M01Rik	-0,15551	1
Prelid3b	-0,15551	1
Nvl	-0,15575	1

Lrba	-0,15597	1
Atp5s	-0,15611	1
Snrpd2	-0,1561	1
Psmb4	-0,15613	1
Polr2e	-0,15625	1
Tfpt	-0,15643	1
Xkr5	-0,15653	1
Exoc5	-0,1565	1
Rangrf	-0,15656	1
Dctn2	-0,15661	1
Mical3	-0,15672	1
Fam129b	-0,15665	1
Skil	-0,15672	1
St7l	-0,15677	1
Ist1	-0,15678	1
Tmem55b	-0,15685	1
Npc1	-0,15697	1
Psmd13	-0,15764	1
Gm42479	-0,15772	1
Ube2r2	-0,15778	1
1600012H06Rik	-0,15804	1
Ubr4	-0,1582	1
Rpusd1	-0,15836	1
Clec4d	-0,1584	1
Gm13186	-0,15861	1
Taf1	-0,15886	1
Uxt	-0,15899	1
Anks3	-0,15935	1
Disp1	-0,15936	1
Esf1	-0,15942	1
Gm15859	-0,15948	1
Gfm2	-0,15963	1
Rpl38	-0,15972	1
S100a4	-0,15971	1
Zdhhc21	-0,15989	1
Mon2	-0,16	1
Prkag2	-0,16005	1
Tsacc	-0,16045	1
Usf3	-0,16052	1
Ece1	-0,16061	1
Eif1ax	-0,16056	1
Ndufb7	-0,16084	1
Fam98b	-0,16075	1
Preb	-0,16085	1
Wdr78	-0,16089	1
Rcc1l	-0,16091	1
Bmyc	-0,16095	1
Aagab	-0,16098	1
Pard6b	-0,16114	1
Nfatc2ip	-0,16117	1
Polr2f	-0,16123	1
Atp8a1	-0,16132	1

Sms	-0,16133	1
Gtf2a1	-0,16148	1
Pdap1	-0,16162	1
9430034N14Rik	-0,16182	1
Xaf1	-0,16177	1
Tlk1	-0,16183	1
Fyn	-0,16193	1
Fnta	-0,16191	1
Zc3h15	-0,16185	1
Bphl	-0,16202	1
Prkcd	-0,16199	1
Tex261	-0,16197	1
Vdac2	-0,16199	1
Rce1	-0,16206	1
Arhgef6	-0,16209	1
Tmem5	-0,1622	1
Mars	-0,16226	1
Amdhd2	-0,16255	1
Tmem222	-0,16263	1
Vps39	-0,16262	1
Dcbld2	-0,16266	1
Arrb1	-0,16317	1
Ubr2	-0,16333	1
Trip4	-0,16354	1
Ebpl	-0,16371	1
Ankrd46	-0,16384	1
Neu3	-0,16379	1
Acp6	-0,16379	1
Coq3	-0,16396	1
Gm38213	-0,1642	1
Fdx1	-0,16451	1
Slc44a2	-0,16449	1
Fbxo42	-0,16475	1
Txlna	-0,16484	1
Cpt1a	-0,16496	1
Gm20768	-0,16498	1
Csnk1a1	-0,16503	1
Mapk1	-0,16515	1
Tax1bp3	-0,16542	1
Map1s	-0,1656	1
Gm21967	-0,16575	1
Nol12	-0,16567	1
Il1rl1	-0,16584	1
Nipal3	-0,1658	1
9130008F23Rik	-0,16585	1
Dnajc1	-0,16588	1
Abt1	-0,16598	1
Gm45762	-0,16609	1
Scarf1	-0,16605	1
Sirt6	-0,16617	1
Rnf139	-0,16635	1
Smarca2	-0,16653	1

Rab18	-0,16648	1
Ciapin1	-0,16661	1
P3h1	-0,16702	1
Akr1e1	-0,16697	1
Rps10-ps4	-0,16715	1
Pou2f2	-0,16713	1
Rpn1	-0,1672	1
Gm45203	-0,16731	1
Rab32	-0,16736	1
Fnbp1	-0,1674	1
Cul9	-0,16748	1
Ncoa5	-0,16754	1
Ranbp3	-0,16748	1
Tmx1	-0,16748	1
Glod4	-0,16754	1
Tprkb	-0,16769	1
4930526A20Rik	-0,16781	1
Klf7	-0,16815	1
Ssb	-0,16854	1
Rpl23a	-0,16861	1
Camta2	-0,16864	1
Fkbp4	-0,16868	1
Ahnak	-0,16886	1
Gadd45gip1	-0,16905	1
Gm15690	-0,1691	1
Pdcd7	-0,16923	1
Dync1i2	-0,16923	1
Smarca4	-0,1693	1
Gmpr2	-0,16941	1
Dyrk1a	-0,16937	1
Slc16a7	-0,16952	1
Shcbp1l	-0,16954	1
Nup85	-0,16969	1
Zdhhc2	-0,16979	1
1700003F12Rik	-0,16987	1
Cars2	-0,17004	1
Cox5a	-0,17023	1
Xkr8	-0,17031	1
Sh2d3c	-0,1703	1
Pdcd2	-0,17035	1
Mbtd1	-0,17027	1
Gdi2	-0,17034	1
Api5	-0,17037	1
Aff4	-0,17047	1
Tpm4	-0,17065	1
Dnlz	-0,17094	1
Ccdc28a	-0,17121	1
Syne1	-0,17122	1
Ccdc82	-0,1713	1
Cops5	-0,17126	1
Smpd2	-0,17152	1
Dnajc19-ps	-0,1716	1

Trim44	-0,17172	1
Kiss1r	-0,17184	1
Tiprl	-0,17185	1
Ick	-0,17201	1
Mrpl55	-0,17197	1
Dgkd	-0,17196	1
Gm43247	-0,17236	1
Zmym5	-0,17254	1
Gm11633	-0,17245	1
Lyar	-0,17254	1
Akr1a1	-0,17272	1
Cc2d2a	-0,17277	1
Atp5d	-0,17278	1
Gm12350	-0,17301	1
Rbms1	-0,17296	1
RP23-308G10.5	-0,17319	1
Ctbs	-0,17327	1
Vasp	-0,17329	1
Nadk	-0,17348	1
Aftph	-0,1736	1
Gm6576	-0,17365	1
Zfp975	-0,17382	1
Mitf	-0,17375	1
Cep72	-0,17388	1
Psmb6	-0,17386	1
Hmga1-rs1	-0,17421	1
Cib1	-0,1742	1
Gas2l3	-0,17442	1
Uqcrb	-0,17445	1
Gm45420	-0,17464	1
Zswim1	-0,17463	1
Gm6177	-0,1747	1
Spp1	-0,17471	1
Slc2a3	-0,1747	1
AC149090.1	-0,17473	1
Usp6nl	-0,17481	1
Chmp5	-0,17475	1
Egln2	-0,17486	1
Aifm1	-0,17488	1
Dock7	-0,17516	1
7330423F06Rik	-0,17539	1
Traf1	-0,17542	1
Thrap3	-0,17557	1
AI837181	-0,17571	1
A430005L14Rik	-0,17572	1
Gspt2	-0,17584	1
Map4k4	-0,17579	1
Rab2a	-0,17589	1
Oas3	-0,17604	1
Ptch1	-0,17596	1
Il6st	-0,17601	1
Gm14830	-0,17623	1

Ppp1r11	-0,17618	1
Klhl25	-0,17657	1
Tmem110	-0,1766	1
Tmco1	-0,17657	1
D930015E06Rik	-0,17674	1
Ccdc6	-0,17667	1
Tinf2	-0,1768	1
Xpr1	-0,17679	1
Malt1	-0,17685	1
Gm42918	-0,17703	1
Atp10d	-0,17712	1
Akap11	-0,17744	1
Birc6	-0,17752	1
Eif3g	-0,17754	1
Gm6542	-0,17755	1
Lrrk1	-0,17767	1
Impa2	-0,17783	1
Edem3	-0,17787	1
Wrap73	-0,17795	1
Zrsr1	-0,17806	1
Pars2	-0,17817	1
Rbm34	-0,17855	1
Gm12988	-0,17861	1
Zfp322a	-0,17855	1
Tcf3	-0,17864	1
Cwc22	-0,17872	1
Mtch2	-0,17877	1
Timm8b	-0,17881	1
Tmbim1	-0,17905	1
Shmt1	-0,17907	1
Tbc1d8	-0,17934	1
D1Ertd622e	-0,17927	1
Cdc42bpg	-0,17936	1
Gm2950	-0,17951	1
Ss18l1	-0,17949	1
Gm2225	-0,1798	1
Adprm	-0,18007	1
Klc1	-0,18005	1
Tmem106a	-0,18029	1
Cebpz	-0,18039	1
Gm42632	-0,18045	1
Zfyve19	-0,18066	1
Igf2r	-0,18079	1
Tnfrsf11a	-0,18088	1
2610020C07Rik	-0,1811	1
Ndufs5	-0,18118	1
Vim	-0,18129	1
Sh3gl1	-0,18149	1
Pax6	-0,18176	1
Gm13398	-0,18176	1
Ulk3	-0,18181	1
Aamp	-0,182	1

Stk4	-0,18205	1
Gm45856	-0,18229	1
Cyfip1	-0,18243	1
Ccdc114	-0,18251	1
Gm11966	-0,18249	1
Ercc4	-0,1831	1
Asxl1	-0,18346	1
Pik3r5	-0,18371	1
Vps45	-0,18383	1
Gm26670	-0,18387	1
Gm25636	-0,18393	1
Gm12732	-0,18386	1
Ube3c	-0,18388	1
Usp19	-0,18389	1
Zmat2	-0,18388	1
Osgin2	-0,18398	1
Gm45806	-0,18409	1
Slc7a4	-0,18413	1
Gm26982	-0,18408	1
Gstp-ps	-0,18423	1
Vps18	-0,1844	1
Mvb12b	-0,18449	1
Mcm10	-0,18462	1
Adcy9	-0,18459	1
Papd7	-0,18471	1
Smndc1	-0,1848	1
Snrnp200	-0,18491	1
Naxd	-0,18505	1
Paqr4	-0,1851	1
Pex14	-0,18509	1
Mta2	-0,18506	1
Gm10126	-0,18537	1
Psmg1	-0,18537	1
Aimp2	-0,18548	1
Stxbp5	-0,18557	1
Fam65c	-0,18573	1
Gm13349	-0,18594	1
Nit2	-0,18613	1
Ppargc1b	-0,18622	1
Sbno2	-0,1863	1
Amigo3	-0,18643	1
Vps37c	-0,18639	1
Cfap97	-0,18648	1
Orc6	-0,18666	1
Panx1	-0,18704	1
Vps9d1	-0,18703	1
Gm5321	-0,18706	1
2810474O19Rik	-0,18715	1
Gm19026	-0,18729	1
Cd300c2	-0,18726	1
Ubr3	-0,18742	1
Numa1	-0,18755	1

Otud7b	-0,18756	1
Malat1	-0,18769	1
4930426I24Rik	-0,18808	1
Ago4	-0,18823	1
Tm7sf3	-0,18819	1
Slc36a1	-0,18824	1
Cmbl	-0,18834	1
Rspry1	-0,18829	1
Mob4	-0,18829	1
Cpeb3	-0,18835	1
Rpl4	-0,1884	1
Smu1	-0,18835	1
Tanc2	-0,18851	1
Fam217b	-0,1886	1
Itpr1	-0,1887	1
Rnf44	-0,18881	1
Picalm	-0,18883	1
Tmem127	-0,18894	1
Gde1	-0,18894	1
0610009B22Rik	-0,18901	1
Cops3	-0,18914	1
RP23-162P10.2	-0,18919	1
Gm2895	-0,18932	1
Cdc25a	-0,18927	1
Zufsp	-0,18951	1
Nsun2	-0,18957	1
Gm7990	-0,1897	1
Pon3	-0,18984	1
Fbxw9	-0,18975	1
Gm10136	-0,19032	1
Scamp2	-0,19026	1
Tbk1	-0,19035	1
Cct4	-0,19061	1
Prps1l3	-0,19068	1
Pofut1	-0,19078	1
Mut	-0,19083	1
Gpat4	-0,19076	1
Lipa	-0,19115	1
Trappc5	-0,19125	1
Tbc1d2b	-0,19133	1
Gemin5	-0,19144	1
Rev3l	-0,19148	1
Glul	-0,19166	1
Hira	-0,19183	1
Ly96	-0,1919	1
Acaa1a	-0,19192	1
Csf1r	-0,19202	1
Sod2	-0,19248	1
Vamp5	-0,19259	1
Trpv2	-0,19264	1
Aim2	-0,19259	1
Gapvd1	-0,1927	1

Mtmr6	-0,19271	1
Slc24a3	-0,19291	1
Rps10-ps1	-0,19301	1
Arl15	-0,19308	1
Atp9b	-0,19315	1
Fuk	-0,19331	1
Hmgcl	-0,19331	1
Map4k3	-0,19349	1
U2af1	-0,19348	1
Trio	-0,19362	1
Ncln	-0,19402	1
Gpr132	-0,19413	1
Prr14	-0,19407	1
Plekhb2	-0,19411	1
Dnajc10	-0,19407	1
Zfp410	-0,19438	1
Nucb2	-0,19455	1
Gm14586	-0,19457	1
Dync2li1	-0,1948	1
Gm37303	-0,19478	1
Ube2d-ps	-0,19494	1
Bloc1s5	-0,19504	1
Mrap	-0,19511	1
Ttyh2	-0,19516	1
Gm5624	-0,19531	1
Ttc37	-0,19534	1
Gm6222	-0,19526	1
Usp40	-0,19535	1
Cnp	-0,1954	1
Fbxo11	-0,19546	1
Zfand3	-0,1955	1
Gm20703	-0,19586	1
Arntl	-0,19587	1
Naca	-0,19612	1
Ak6	-0,19613	1
Cenpo	-0,19623	1
Ppih	-0,19632	1
Pnpla2	-0,19637	1
Rtn3	-0,19639	1
Nudc	-0,19658	1
Ppp1r12a	-0,19661	1
Hmga1	-0,19674	1
Ppard	-0,19674	1
Gm5139	-0,19702	1
Fam3a	-0,19711	1
Hgsnat	-0,19734	1
Trmt10c	-0,19738	1
Ppp2r5b	-0,19742	1
Gm6745	-0,19747	1
Gm11764	-0,19751	1
Dgat2	-0,19769	1
Add3	-0,19775	1

Gm26710	-0,19768	1
Maats1os	-0,1979	1
Prkab2	-0,1981	1
Actr1b	-0,19815	1
Wdfy1	-0,19815	1
Glrx5	-0,19824	1
Tmem101	-0,19827	1
Cox6b1	-0,19828	1
Foxk1	-0,19845	1
Ilk	-0,19881	1
Rbm26	-0,19888	1
Gpatch11	-0,19905	1
Cep350	-0,19906	1
Grk2	-0,19906	1
Pggt1b	-0,19923	1
Hmbox1	-0,1992	1
Card19	-0,19933	1
March11	-0,19947	1
Sertad3	-0,1995	1
Zwint	-0,19954	1
Gm6030	-0,19948	1
Sec61a1	-0,1995	1
Snrnp40	-0,19958	1
Dclre1c	-0,19971	1
Armc1	-0,19973	1
Zc3h18	-0,19986	1
Ptrh2	-0,20005	1
Hmgb1-rs16	-0,20046	1
2900026A02Rik	-0,20057	1
Pnpla6	-0,20086	1
Pla2g2e	-0,201	1
Pcmt1	-0,20099	1
Arid4b	-0,201	1
RP23-316F10.2	-0,20126	1
Gm45454	-0,2013	1
Mmachc	-0,2014	1
Ino80e	-0,20152	1
Brms1	-0,20161	1
Dcun1d1	-0,20159	1
Mtrf1	-0,20167	1
Nsmce1	-0,2017	1
Rilpl1	-0,20178	1
Setd2	-0,20181	1
Rbbp6	-0,20182	1
Gm5590	-0,20194	1
Dleu2	-0,20191	1
Lin7c	-0,20198	1
Nuak2	-0,20205	1
Ninj1	-0,20224	1
Plekhg2	-0,2028	1
Snx29	-0,20303	1
Cxxc1	-0,20328	1

Bre	-0,20335	1
Gm42869	-0,20347	1
Arl2bp	-0,20352	1
Gm6028	-0,20363	1
Exd1	-0,20357	1
Fth-ps3	-0,20358	1
Ankhd1	-0,20423	1
Nars	-0,2044	1
Tstd2	-0,20445	1
Wdr60	-0,20453	1
B4galt5	-0,20473	1
Sirt3	-0,20488	1
Gm12716	-0,20503	1
Commd8	-0,20505	1
Socs7	-0,20517	1
Chmp2a	-0,20582	1
Ndufaf3	-0,20602	1
Nptxr	-0,20607	1
Odf2	-0,2063	1
Dmap1	-0,20645	1
Rpgr	-0,20653	1
Cct7	-0,20647	1
Trib3	-0,20656	1
Uba2	-0,20661	1
Cyb5r3	-0,20677	1
Cdk11b	-0,20676	1
Abi3	-0,20699	1
BC004004	-0,20695	1
Rtf1	-0,20705	1
D230017M19Rik	-0,20732	1
Stap1	-0,20745	1
Ltn1	-0,20749	1
Brd1	-0,20754	1
Umps	-0,20757	1
Dnajc19	-0,20771	1
Appl2	-0,20799	1
Mafb	-0,20813	1
Pitpna	-0,20832	1
Vps13d	-0,2085	1
Pigh	-0,20889	1
Nr0b2	-0,20904	1
Irx5	-0,20904	1
Uqcc3	-0,20913	1
Pip5k1a	-0,20915	1
Mycbp2	-0,20945	1
Dock11	-0,20963	1
Gm42633	-0,20982	1
Rttn	-0,21044	1
Gm43712	-0,21064	1
Prpsap2	-0,21071	1
Zfp282	-0,2107	1
Gm5436	-0,21081	1

Cep170b	-0,21077	1
P2ry2	-0,21086	1
Sbk3	-0,21102	1
Stx5a	-0,21104	1
Gm6293	-0,21111	1
Ints13	-0,21111	1
Atad2b	-0,21107	1
Rtcb	-0,21114	1
Gatc	-0,21137	1
Rft1	-0,21197	1
Acp5	-0,21197	1
Jup	-0,21214	1
Pdia5	-0,21219	1
Ankrd13c	-0,21224	1
Bin3	-0,21232	1
Ercc6l2	-0,21237	1
Eprs	-0,21261	1
Iqgap1	-0,21271	1
Mpnd	-0,21283	1
Arid1a	-0,21285	1
Irf4	-0,21292	1
Nkiras2	-0,21287	1
Zxdb	-0,2132	1
Taldo1	-0,21319	1
Vkorc1l1	-0,21326	1
Ykt6	-0,21334	1
Ccndbp1	-0,21332	1
Pafah1b1	-0,21332	1
Btbd6	-0,21384	1
Bmi1	-0,2139	1
Ap1g1	-0,21399	1
Naa35	-0,21411	1
Moap1	-0,21432	1
Serinc2	-0,21439	1
Atp6v1b2	-0,21441	1
Nupl1	-0,2145	1
Ppp2r5a	-0,2145	1
Dnajc9	-0,21474	1
Srd5a3	-0,21491	1
Srp14	-0,21502	1
2700062C07Rik	-0,21509	1
Klf3	-0,21511	1
Mybl1	-0,21532	1
Atp1b3	-0,21541	1
Pigyl	-0,21552	1
Aida	-0,21558	1
Limd1	-0,21558	1
Emsy	-0,21575	1
Wdr90	-0,21577	1
Dcp1a	-0,2158	1
Ahrr	-0,21584	1
Ncl	-0,21583	1

Clcn4	-0,2159	1
Camkk2	-0,21589	1
Gm5776	-0,21608	1
Pex11b	-0,2162	1
Prr13	-0,2162	1
3300005D01Rik	-0,21658	1
Plxnb2	-0,21661	1
Spcs2	-0,21685	1
Osbpl11	-0,21693	1
Taco1	-0,21696	1
Gm2991	-0,21701	1
Sod1	-0,21703	1
Laptm4a	-0,21699	1
Srcap	-0,21728	1
Mrm1	-0,21731	1
Aff1	-0,21727	1
Szt2	-0,21784	1
Ppp1r9b	-0,21789	1
Odf2l	-0,21817	1
Gad2	-0,2186	1
Gm44890	-0,21909	1
Gm26730	-0,21916	1
Tspyl3	-0,21935	1
Mib2	-0,21926	1
Chtop	-0,21925	1
Rasip1	-0,21945	1
Zfp148	-0,21935	1
Asb1	-0,21951	1
Gm9434	-0,21962	1
Add1	-0,21969	1
C530043K16Rik	-0,22002	1
Tab1	-0,2201	1
Fam20c	-0,22008	1
Wdr5b	-0,22038	1
Zfp715	-0,22044	1
Mus81	-0,22084	1
Zmat1	-0,22111	1
Cflar	-0,22111	1
Wapl	-0,22108	1
Fads1	-0,22115	1
Bub3	-0,22122	1
Psmd2	-0,22123	1
Abhd17c	-0,22134	1
Klf16	-0,22137	1
Smyd3	-0,22145	1
Ktn1	-0,22146	1
Tmem189	-0,22169	1
Katnal1	-0,22191	1
H13	-0,22213	1
Hs3st3b1	-0,22225	1
Pkp4	-0,22225	1
Inpp5k	-0,22244	1

Ppp1cb	-0,22239	1
Mettl14	-0,22236	1
Itpk1	-0,22249	1
Tmem120a	-0,22255	1
Gm29487	-0,22282	1
Cisd1	-0,22279	1
Xpc	-0,22297	1
Rock1	-0,22296	1
Cpsf2	-0,22307	1
Mapkbp1	-0,22324	1
Sp110	-0,22331	1
Trappc4	-0,22334	1
Csnk1g1	-0,22334	1
Zbtb41	-0,22346	1
Phrf1	-0,22362	1
Htatip2	-0,22365	1
Rrp12	-0,2242	1
Utp15	-0,22426	1
Rsl24d1	-0,22438	1
Plec	-0,22439	1
Smc5	-0,22459	1
Srsf10	-0,22463	1
Gm12618	-0,22479	1
Gm20667	-0,2249	1
Tmcc2	-0,22498	1
Rps6kb1	-0,22498	1
Atp1a1	-0,22498	1
Phc3	-0,22505	1
Nek8	-0,22518	1
Hsdl2	-0,22519	1
Gm14780	-0,22527	1
Was	-0,22532	1
Rgl2	-0,22527	1
Stambp	-0,22538	1
Myof	-0,22543	1
Ndst2	-0,22547	1
Tctex1d2	-0,22553	1
Gfod1	-0,22561	1
Hn1	-0,22574	1
Gm10093	-0,22614	1
Tmem147	-0,22607	1
Mtx2	-0,22654	1
Lrsam1	-0,22685	1
Homer3	-0,22684	1
Micu2	-0,22684	1
Nsmce3	-0,227	1
Synj2	-0,22708	1
Gm44093	-0,22738	1
Camsap1	-0,22746	1
Ubp1	-0,22747	1
Hmox2	-0,22763	1
Wdr4	-0,2278	1

Pyroxd1	-0,22797	1
Gm19705	-0,2282	1
Cog7	-0,2282	1
Tmem230	-0,22833	1
Ppp3ca	-0,2283	1
Dcaf8	-0,22834	1
Lsr	-0,22836	1
Pdpr	-0,22843	1
Gspt1	-0,22842	1
Gm42829	-0,22863	1
Smcr8	-0,22857	1
Cbfa2t2	-0,22873	1
Dcun1d2	-0,22877	1
Fam134b	-0,22887	1
Stx4a	-0,22894	1
Zfp51	-0,22907	1
Atraid	-0,22914	1
Dtnbp1	-0,22919	1
9130011E15Rik	-0,22927	1
Mllt6	-0,2293	1
Rnf113a2	-0,22943	1
Acp2	-0,2295	1
Ttf2	-0,22953	1
Foxo1	-0,22954	1
Ppm1b	-0,22963	1
Rhebl1	-0,22965	1
Cdk9	-0,22975	1
Gm42783	-0,22991	1
Dnmbp	-0,22988	1
Wdr36	-0,23013	1
Slc25a19	-0,23017	1
Neat1	-0,23017	1
Stard9	-0,23021	1
Tm4sf19	-0,23029	1
Pigp	-0,23035	1
5730405O15Rik	-0,23044	1
Washc4	-0,23035	1
Tspan4	-0,23041	1
Sclt1	-0,23049	1
Sh3pxd2b	-0,23063	1
Ndufa8	-0,23061	1
Zfp110	-0,23072	1
B230307C23Rik	-0,23094	1
Smim4	-0,23102	1
Zfp607a	-0,23137	1
Pqlc3	-0,23147	1
Poldip3	-0,23161	1
Gm44013	-0,23176	1
Ccdc90b	-0,23178	1
Sec11a	-0,23189	1
Setd5	-0,23189	1
Ddx18	-0,23221	1

1810021B22Rik	-0,23234	1
Rbm15	-0,23233	1
Poc1b	-0,23247	1
Hivep2	-0,23245	1
Smarcd2	-0,23247	1
mt-Nd1	-0,23275	1
Dennd1c	-0,23277	1
Ankib1	-0,23306	1
Abcf1	-0,23324	1
Elmo1	-0,23342	1
Ofd1	-0,23351	1
Rab11fip2	-0,23345	1
Psmd5	-0,23375	1
Mfn2	-0,23386	1
Ccl4	-0,23404	1
Hoxc4	-0,23414	1
Gbf1	-0,23405	1
S100a6	-0,23429	1
Glmn	-0,23453	1
Cnot1	-0,23449	1
Dyrk3	-0,23461	1
Mmab	-0,23476	1
Magt1	-0,23486	1
Commd3	-0,23494	1
Mmadhc	-0,23489	1
Ap4e1	-0,23499	1
Dph7	-0,23524	1
Pbrm1	-0,23532	1
Timm10	-0,23538	1
Klrg2	-0,23556	1
Capn7	-0,23569	1
Letm1	-0,23572	1
Trabd	-0,23566	1
Sft2d2	-0,23588	1
Adap2	-0,23615	1
Zfp712	-0,2363	1
Tmem141	-0,23627	1
Nav1	-0,23655	1
Smarcc1	-0,23662	1
Smarcb1	-0,23683	1
Setd4	-0,23691	1
Grap	-0,23704	1
Nagk	-0,23698	1
Fmn1	-0,23708	1
Vsir	-0,23718	1
Rbks	-0,23734	1
March6	-0,23735	1
Uso1	-0,23741	1
Gdap2	-0,23749	1
Pcdhb22	-0,23783	1
Gtf2e1	-0,23791	1
Smpdl3b	-0,2379	1

Nt5dc1	-0,238	1
Mtrr	-0,23799	1
Riox2	-0,23854	1
Gm16286	-0,23849	1
Supt6	-0,23868	1
Slc7a1	-0,23866	1
Thra	-0,2388	1
C1qtnf6	-0,23891	1
Bola1	-0,23892	1
Zfp629	-0,23906	1
Snx15	-0,2393	1
Ptp4a2	-0,23939	1
Gm5837	-0,23965	1
Cdr2	-0,23973	1
Dnmt3a	-0,23983	1
Ccdc136	-0,23994	1
Man1c1	-0,24018	1
Cdc37l1	-0,24024	1
Ddx3x	-0,24044	1
Afg3l1	-0,24071	1
Gm19739	-0,24067	1
1110019D14Rik	-0,24083	1
Usp21	-0,24103	1
Eed	-0,241	1
RP23-453B15.7	-0,24108	1
Ptcd2	-0,24111	1
S100pbp	-0,24122	1
Exosc2	-0,24129	1
Plcd1	-0,24128	1
Gldc	-0,24128	1
Gm10039	-0,24172	1
Rgs16	-0,24205	1
Mfsd7b	-0,24219	1
Azin1	-0,24216	1
Fam53a	-0,24233	1
Elmod2	-0,24258	1
Utp11	-0,24266	1
Dgkz	-0,24287	1
Gm2830	-0,24299	1
Fktn	-0,2432	1
Zmym4	-0,24324	1
Cdc73	-0,24318	1
Serinc5	-0,24334	1
Gm9796	-0,24351	1
Ikbkg	-0,24351	1
Btbd3	-0,24374	1
Nudt14	-0,24365	1
Gm16061	-0,24366	1
Srpk2	-0,24379	1
Aasdhppt	-0,24412	1
Qrich1	-0,24421	1
Glce	-0,2444	1

Chd1	-0,24459	1
Rrp8	-0,24469	1
Smim12	-0,24478	1
H2afx	-0,24494	1
Zcchc17	-0,24494	1
Rps3	-0,24512	1
Fam198b	-0,24522	1
1700030K09Rik	-0,24531	1
Abhd5	-0,24532	1
Chpt1	-0,24536	1
Gm43848	-0,24553	1
Gm4117	-0,24566	1
Gm9712	-0,2459	1
Nsmce2	-0,24588	1
Mllt1	-0,24624	1
Atp6v1f	-0,24626	1
Prkaca	-0,2464	1
Cyld	-0,24682	1
Mlx	-0,24692	1
Nrtn	-0,24722	1
Ube3a	-0,24732	1
Spopl	-0,24775	1
Pop7	-0,24778	1
Cars	-0,24781	1
Zfp386	-0,24785	1
Gm43484	-0,24789	1
Paox	-0,248	1
Gm5380	-0,24838	1
Zfp358	-0,24843	1
Gm45890	-0,2484	1
Tyms	-0,24862	1
Orc3	-0,24862	1
Rnf141	-0,24859	1
D3Ertd751e	-0,24879	1
Gm10167	-0,24908	1
Zcchc8	-0,2491	1
P2rx7	-0,24917	1
Atp6v0e2	-0,24922	1
Pigg	-0,24939	1
Slc35e1	-0,24952	1
Tox4	-0,24969	1
Gnmt	-0,24986	1
Naf1	-0,24992	1
Traf2	-0,25006	1
Cd180	-0,25023	1
Arhgap21	-0,25026	1
Coasy	-0,25042	1
Zfp710	-0,25059	1
Ddx55	-0,25078	1
Nmd3	-0,25077	1
Ndufa5	-0,2508	1
Kdelr1	-0,25077	1

Il23a	-0,25163	1
Nfat5	-0,25175	1
Snhg11	-0,25191	1
Pias1	-0,25211	1
Lsg1	-0,25206	1
Isy1	-0,25214	1
RP23-3F1.8	-0,25224	1
Cwc27	-0,25223	1
Eif3a	-0,25219	1
Nsd3	-0,25234	1
Rbmxl1	-0,25238	1
Itpr2	-0,25238	1
Ube2b	-0,25246	1
Rtel1	-0,2526	1
Ap5z1	-0,2527	1
mt-Nd2	-0,25269	1
Ccdc47	-0,25285	1
Ptpdc1	-0,25302	1
L2hgdh	-0,25333	1
Riok3	-0,25329	1
Cnot6	-0,25329	1
Snrpd1	-0,25335	1
Map4k1	-0,2539	1
Snx14	-0,2539	1
Rad50	-0,25402	1
Tnip1	-0,25403	1
Rbm28	-0,25402	1
Fbxw2	-0,25403	1
Cdk1	-0,25407	1
Il7r	-0,25443	1
Pdgfb	-0,25448	1
Cntrl	-0,25452	1
Cdc42bpb	-0,25457	1
Znrf2	-0,25466	1
Gm14239	-0,25482	1
3110009E18Rik	-0,25484	1
Lsm14b	-0,25501	1
Chd2	-0,25503	1
Trim65	-0,25528	1
Ankrd13b	-0,25527	1
Slc25a40	-0,25529	1
Rab11fip1	-0,25531	1
Bfar	-0,25529	1
Zfp646	-0,25541	1
Dgcr14	-0,25538	1
Tomm40	-0,25544	1
Map3k4	-0,25535	1
Bin2	-0,25549	1
Plekha5	-0,25558	1
Rad23b	-0,2559	1
Dirc2	-0,25596	1
Acox3	-0,25597	1

Gm6919	-0,25613	1
Uckl1	-0,25607	1
Mettl15	-0,25634	1
Kif16b	-0,25673	1
Mrpl43	-0,25695	1
Gm26935	-0,25757	1
Ica1	-0,2576	1
Srsf7	-0,25776	1
Mogs	-0,25796	1
Gm9828	-0,25822	1
Peg13	-0,25817	1
Ncf1	-0,25827	1
Tpgs1	-0,25864	1
Ipp	-0,25873	1
Zfp462	-0,25865	1
Gm37519	-0,2588	1
Bbs7	-0,25886	1
Psmc3	-0,25889	1
Mrgbp	-0,25901	1
Ddx39b	-0,25923	1
Spats2	-0,25947	1
Plk3	-0,25972	1
Prrc2c	-0,25971	1
Cdca4	-0,25985	1
Sf3b6	-0,25988	1
Tbc1d23	-0,26007	1
RP23-255F14.4	-0,26015	1
Orc5	-0,26029	1
Atpif1	-0,26034	1
Kdm5b	-0,26034	1
Dnajc16	-0,26054	1
Mapkap1	-0,26046	1
Ociad1	-0,26068	1
Grik5	-0,26087	1
Timm9	-0,26105	1
Fads2	-0,26108	1
Tab2	-0,2612	1
Klhl26	-0,26128	1
Ankrd24	-0,2615	1
Gm20432	-0,26164	1
Eif4a1	-0,2617	1
Derl1	-0,26172	1
Cspg5	-0,26207	1
Flnb	-0,26212	1
Tubb4b	-0,26255	1
Gm13392	-0,2628	1
Gm28192	-0,26295	1
Ddx3y	-0,26318	1
Rmi2	-0,26329	1
Ogfod2	-0,26331	1
Eif6	-0,26334	1
Tram2	-0,26336	1

Gm29155	-0,2634	1
1600014C23Rik	-0,26343	1
Zc3h6	-0,2634	1
Pkn1	-0,26364	1
Psmd10	-0,26383	1
H2-D1	-0,26379	1
Gga2	-0,26403	1
Nfia	-0,26426	1
Bptf	-0,26433	1
Usp47	-0,26434	1
Acbd4	-0,26475	1
Mmgt2	-0,26467	1
Gm7535	-0,26483	1
Dpp9	-0,26491	1
1110059E24Rik	-0,26502	1
Nupr1l	-0,26535	1
Glud1	-0,26536	1
Gm17066	-0,26545	1
Dcun1d3	-0,26574	1
Ttc14	-0,26569	1
Utp23	-0,26579	1
Zdhhc13	-0,26611	1
Thtpa	-0,26614	1
Rnd1	-0,26629	1
Rpgrip1l	-0,26652	1
Gm7496	-0,26657	1
Ints14	-0,26664	1
Ireb2	-0,26659	1
Alpk1	-0,26671	1
Apc	-0,26681	1
Prmt5	-0,26683	1
1110008F13Rik	-0,26684	1
Fam45a	-0,26689	1
Ppp1r18	-0,26691	1
Ttll1	-0,2671	1
Mtmr10	-0,26708	1
Malsu1	-0,26741	1
Gm7733	-0,26778	1
Slc11a2	-0,26778	1
Dgcr6	-0,26821	1
Zfp317	-0,26828	1
Sec24c	-0,26852	1
Rbm10	-0,26864	1
Gm15946	-0,26873	1
Sacs	-0,26871	1
Pirb	-0,26876	1
Fbxo6	-0,26904	1
Vapb	-0,26904	1
Smug1	-0,26912	1
Srbd1	-0,26907	1
Immt	-0,26914	1
Slc9a9	-0,26922	1

Gm20257	-0,2692	1
Ostm1	-0,26931	1
Rasa1	-0,26932	1
Cep97	-0,26958	1
Rmdn1	-0,26972	1
Tatdn1	-0,26987	1
Chml	-0,27003	1
Utp6	-0,27028	1
Iws1	-0,2703	1
Tsn	-0,27028	1
Samsn1	-0,27039	1
2810025M15Rik	-0,27048	1
Stk10	-0,2708	1
Zfp664	-0,27088	1
Sdcbp	-0,27125	1
Polr3gl	-0,27127	1
Rsbn1	-0,27126	1
Acsl1	-0,27138	1
Tdrd7	-0,27155	1
Rprd1b	-0,27156	1
Slc12a4	-0,27163	1
Zfp638	-0,27172	1
BC030867	-0,2719	1
Ifnar2	-0,27186	1
Mink1	-0,27199	1
Edc4	-0,2721	1
Dusp12	-0,27224	1
Sec23ip	-0,27232	1
Ash2l	-0,27294	1
Atp6v0a2	-0,27317	1
Nlrx1	-0,27318	1
Gm15500	-0,27324	1
Fadd	-0,27327	1
Zmpste24	-0,27329	1
Tmsb4x	-0,27328	1
Slc25a13	-0,27329	1
Gm8930	-0,27352	1
Fbxl12	-0,2735	1
Praf2	-0,27361	1
Sbf2	-0,27374	1
Brix1	-0,27375	1
Strada	-0,27409	1
Mia3	-0,27411	1
A130048G24Rik	-0,27419	1
Cltb	-0,27427	1
Entpd6	-0,27448	1
1810011H11Rik	-0,27468	1
Smim8	-0,27484	1
Piezo1	-0,27482	1
Prickle2	-0,27489	1
Pip5k1c	-0,27497	1
Adss	-0,27497	1

Nelfb	-0,27515	1
Sec11c	-0,27515	1
6430573P05Rik	-0,27519	1
RP24-378K7.3	-0,27573	1
Gm16201	-0,27608	1
Fam114a2	-0,27627	1
Pced1a	-0,27635	1
Rab33b	-0,27643	1
Slc7a5	-0,27637	1
Rabgef1	-0,2768	1
Ppm1f	-0,27676	1
Ccnk	-0,27696	1
Gm45311	-0,2773	1
Trmt61b	-0,27791	1
2700099C18Rik	-0,278	1
Tsg101	-0,27802	1
Cdc25b	-0,27806	1
Relb	-0,27829	1
Zfp597	-0,27838	1
Arhgap19	-0,27846	1
Gm10575	-0,27851	1
Rwdd2a	-0,27868	1
Tyk2	-0,27873	1
Eef1e1	-0,27886	1
Ahsa1	-0,27901	1
Senp5	-0,279	1
Map4k5	-0,27915	1
Fbxo38	-0,27917	1
1810041H14Rik	-0,27926	1
Zfp120	-0,27933	1
Nosip	-0,27973	1
Gm44623	-0,27988	1
Tmem245	-0,28003	1
Ostc	-0,27999	1
Cbx3	-0,27998	1
Eml5	-0,28007	1
Aars	-0,28005	1
Selplg	-0,28007	1
Ankrd40	-0,28027	1
B3glct	-0,28047	1
Nup155	-0,2806	1
Tanc1	-0,28071	1
Tango6	-0,28076	1
Rif1	-0,28083	1
Rbm17	-0,28092	1
Atg16l2	-0,28119	1
Ifi207	-0,28146	1
Zscan26	-0,2816	1
Ppcdc	-0,28171	1
Lyrm7	-0,28178	1
Hells	-0,28184	1
Top2b	-0,28177	1

Chchd1	-0,28179	1
Rpp30	-0,28192	1
Akna	-0,28209	1
Dnajb12	-0,28213	1
Abcc5	-0,28214	1
Lemd2	-0,2823	1
Nsmf	-0,28236	1
Dync2h1	-0,28244	1
Alox5	-0,28277	1
Eri2	-0,28286	1
Dhx30	-0,28308	1
Letmd1	-0,28324	1
Ccnyl1	-0,28344	1
Adamts6	-0,28337	1
Rnf20	-0,28354	1
Eif1a	-0,28391	1
Ube2d2a	-0,28413	1
Rbm27	-0,28414	1
Wdr26	-0,28416	1
Gm29340	-0,28427	1
Farsa	-0,28462	1
Romo1	-0,28491	1
Ap2m1	-0,28513	1
Ppm1g	-0,28514	1
Mast2	-0,28551	1
Echdc3	-0,28549	1
Ndufaf8	-0,28554	1
Zscan29	-0,2856	1
Trappc13	-0,28558	1
Otud5	-0,28563	1
Kifap3	-0,2857	1
Gm9794	-0,28576	1
Csad	-0,28604	1
Slirp	-0,2861	1
Ssrp1	-0,28611	1
Thumpd2	-0,28652	1
Ninl	-0,28653	1
Pdcd6ip	-0,28648	1
Slc12a7	-0,28662	1
Ibtk	-0,28683	1
Serbp1	-0,28717	1
Lilr4b	-0,28741	1
Zfp341	-0,28777	1
Neurl1a	-0,28787	1
Las1l	-0,28799	1
Ighm	-0,28844	1
Sec23a	-0,28838	1
Chd7	-0,28855	1
Jmjd4	-0,28869	1
4931406C07Rik	-0,28866	1
Fam220a	-0,28876	1
Orai3	-0,28908	1

Appbp2	-0,28916	1
Terf1	-0,28934	1
Fblim1	-0,28934	1
Cep250	-0,28939	1
Copa	-0,28952	1
Lyz2	-0,28961	1
Uty	-0,28967	1
Eef2k	-0,29018	1
Tapt1	-0,29016	1
Vps54	-0,29043	1
Aim1	-0,29041	1
Pten	-0,29044	1
9630010A21Rik	-0,29055	1
Gm20633	-0,29061	1
Gm5857	-0,29107	1
Cdc14b	-0,29137	1
Spout1	-0,29176	1
Gm6181	-0,29195	1
Cluh	-0,29205	1
Gm11517	-0,29206	1
Eps8	-0,29212	1
Lasp1	-0,29212	1
Tyw3	-0,29217	1
Nop58	-0,29219	1
Tmem86a	-0,29246	1
Adprhl2	-0,29258	1
Psme3	-0,29271	1
Mob1a	-0,29269	1
Tm4sf5	-0,29282	1
Xab2	-0,29295	1
Hps5	-0,29308	1
Hus1b	-0,29322	1
Plekhs1	-0,29323	1
Pdzd8	-0,29343	1
Hps1	-0,29335	1
Prrg2	-0,29343	1
Fam110a	-0,29378	1
Slc35b3	-0,29397	1
Setmar	-0,2941	1
Herc2	-0,29443	1
Mbtps1	-0,2946	1
Rabep2	-0,29524	1
Gli1	-0,29534	1
Ppp2r1b	-0,29531	1
Srgap3	-0,29525	1
Nin	-0,29536	1
Tulp3	-0,2954	1
Rnf183	-0,29556	1
Ap3m1	-0,29562	1
Lcp1	-0,29565	1
Trpc4ap	-0,29568	1
Midn	-0,29584	1

Tstd3	-0,29594	1
Atp5g3	-0,29608	1
Gm37082	-0,29627	1
Ube2o	-0,29647	1
RP23-359K10.8	-0,29672	1
Colec12	-0,29713	1
Cask	-0,29731	1
Kbtbd3	-0,29741	1
Bcl2a1b	-0,29766	1
Agfg1	-0,29773	1
Focad	-0,2979	1
Phf21a	-0,29803	1
Mef2d	-0,29797	1
Ldah	-0,29834	1
Ccdc115	-0,29825	1
Fam102b	-0,29828	1
Gpr89	-0,29846	1
Stat3	-0,29932	1
Tmem131	-0,29948	1
Zfp994	-0,29959	1
Polr3k	-0,29957	1
9530062K07Rik	-0,29985	1
Gm43588	-0,29997	1
Rps11-ps1	-0,30007	1
Asb13	-0,30018	1
Eif5b	-0,30023	1
Btd	-0,30033	1
Wdr33	-0,30027	1
Impdh1	-0,30026	1
Gm18860	-0,30051	1
Lemd3	-0,30046	1
Dusp18	-0,30069	1
Gm14567	-0,30105	1
Irak3	-0,30099	1
Fignl1	-0,30113	1
Slc4a7	-0,30114	1
B230369F24Rik	-0,30127	1
Sfmbt1	-0,30145	1
Mybbp1a	-0,30159	1
Gm45343	-0,30177	1
Mfsd14b	-0,30192	1
Gm15265	-0,30206	1
Slc25a44	-0,30206	1
Tbp	-0,30223	1
Zbtb40	-0,30237	1
Pank3	-0,30243	1
Zfp984	-0,3025	1
Gen1	-0,30256	1
1700066M21Rik	-0,30277	1
Ccdc122	-0,30288	1
Gm45167	-0,30292	1
Lgals8	-0,30288	1

Oxt	-0,30326	1
Gm15148	-0,30365	1
Copb2	-0,30357	1
Arid4a	-0,30385	1
Lmbrd1	-0,30418	1
Tln1	-0,30428	1
Grb2	-0,30442	1
G430095P16Rik	-0,30451	1
5031425E22Rik	-0,30451	1
Gm8667	-0,30459	1
Ttc17	-0,30455	1
Pstpip1	-0,30489	1
Mtx3	-0,30497	1
Tssk6	-0,30577	1
5430405H02Rik	-0,30599	1
Timm44	-0,30597	1
Map3k20	-0,30596	1
Golga5	-0,30611	1
Suds3	-0,3061	1
Tmem237	-0,30621	1
Abcd4	-0,30619	1
Slc27a1	-0,3065	1
Gclc	-0,30655	1
Rap2b	-0,30654	1
Prrc1	-0,30666	1
Prpsap1	-0,30682	1
Ankrd11	-0,30682	1
Stim2	-0,30711	1
Scfd2	-0,30725	1
Msh5	-0,30718	1
Cstb	-0,30715	1
Twf2	-0,30733	1
Hook3	-0,30732	1
Trmt61a	-0,30774	1
Trnau1ap	-0,30778	1
Gm45718	-0,30785	1
Gm6395	-0,30775	1
Psmg3	-0,30814	1
RP23-193N1.2	-0,30822	1
Slc38a9	-0,30816	1
Rnf167	-0,30825	1
Angpt2	-0,30832	1
Ppfia1	-0,30832	1
Cdk10	-0,30854	1
Tada3	-0,30859	1
Sh2b2	-0,30873	1
Gm44419	-0,30899	1
Idh2	-0,30901	1
Eftud2	-0,30921	1
Inpp5d	-0,30916	1
Smim19	-0,30931	1
Dpys	-0,30938	1

Arv1	-0,30965	1
Zfp777	-0,30975	1
Cant1	-0,30974	1
Supt5	-0,30983	1
Vdr	-0,30986	1
Zfp574	-0,30998	1
Macf1	-0,31027	1
Scyl1	-0,31051	1
Nlrc5	-0,31064	1
Puf60	-0,31056	1
Tada1	-0,31073	1
Gemin6	-0,31083	1
Nfyb	-0,31093	1
Ulk2	-0,31104	1
Arl14ep	-0,31096	1
Gusb	-0,31099	1
Pelo	-0,31115	1
Gm5244	-0,31137	1
Usp16	-0,31138	1
Fam188a	-0,31164	1
Ap1m1	-0,31158	1
Homer1	-0,31172	1
Ruvbl1	-0,31181	1
Csrp2bp	-0,31182	1
Tmem251	-0,31178	1
Zfp599	-0,312	1
Rad23a	-0,31222	1
Mrpl13	-0,31234	1
Pi4ka	-0,31234	1
Gm14270	-0,31243	1
Vapa	-0,31241	1
Gm7094	-0,31259	1
Ncoa3	-0,31261	1
Kif2c	-0,31267	1
Taf6l	-0,31267	1
Zfp52	-0,31325	1
Dnajc25	-0,31342	1
Gm29438	-0,31368	1
Dzip1	-0,31368	1
Gm17530	-0,31373	1
Zkscan1	-0,31371	1
Ap1s1	-0,31367	1
Rnpc3	-0,3138	1
Commd2	-0,31386	1
Sppl3	-0,31415	1
Aasdh	-0,31469	1
Lias	-0,31467	1
Araf	-0,31505	1
Cnpy3	-0,31544	1
Clcn7	-0,31554	1
Akr1b3	-0,31565	1
Rab11fip5	-0,31571	1

Rabl2	-0,3161	1
Mical2	-0,31617	1
Slc9b2	-0,31634	1
Polg	-0,31699	1
Gtf3c1	-0,31699	1
Sco2	-0,31707	1
Hexim2	-0,31754	1
Ash1l	-0,31798	1
Surf4	-0,31799	1
Ube2i	-0,31808	1
Gatad2a	-0,31827	1
Habp4	-0,31843	1
Wac	-0,31843	1
Gm13602	-0,31848	1
Tns2	-0,31848	1
Msh2	-0,3185	1
Med10	-0,31853	1
Hilpda	-0,31858	1
Trim39	-0,31888	1
Rpl21-ps5	-0,31913	1
Arih2	-0,31918	1
Gm11722	-0,31943	1
Srm	-0,31943	1
Ncor1	-0,31935	1
Ptbp3	-0,31962	1
Cops8	-0,31971	1
Gm38062	-0,31983	1
Rassf4	-0,32002	1
Rmnd5b	-0,32017	1
Wdr53	-0,32065	1
Sirt5	-0,32084	1
Ubac2	-0,32078	1
Samm50	-0,32086	1
Hspa4	-0,32089	1
Atp13a3	-0,32101	1
Taf7	-0,32123	1
Cbll1	-0,32116	1
Pccb	-0,32129	1
Tmem214	-0,32134	1
Slc6a6	-0,3213	1
Tbccd1	-0,32163	1
Sbf1	-0,3216	1
Eno3	-0,32178	1
Ddhd2	-0,32193	1
Gm37254	-0,32226	1
Zfp513	-0,32229	1
Hsd17b11	-0,3228	1
Myo9a	-0,32333	1
Senp7	-0,32335	1
Ufl1	-0,32348	1
Capzb	-0,3235	1
Mrpl44	-0,3236	1

Sema4d	-0,32359	1
Casp9	-0,32365	1
Wdr81	-0,32385	1
Hspa9	-0,32386	1
Ttc9	-0,32428	1
Fancm	-0,32437	1
Cdk20	-0,32454	1
Slain2	-0,32446	1
Arhgap1	-0,32462	1
Tubgcp6	-0,32457	1
Mphosph8	-0,32459	1
Mtus2	-0,32473	1
Hbs1l	-0,32488	1
Cct6a	-0,32489	1
Wdr47	-0,325	1
4930556M19Rik	-0,32496	1
Telo2	-0,32498	1
Enox2	-0,32511	1
Gm7860	-0,32523	1
BC022687	-0,32532	1
Sgk3	-0,32539	1
Pwwp2b	-0,32563	1
Gm12589	-0,32568	1
Ammecr1l	-0,32617	1
Usp8	-0,32617	1
Rdh5	-0,32634	1
Sltm	-0,32631	1
Gm45286	-0,32644	1
Ndst1	-0,32674	1
Pank1	-0,3269	1
Hoxb4	-0,32693	1
Dus2	-0,32696	1
Ddx5	-0,32696	1
Hoxa3	-0,32746	1
Rny3	-0,32792	1
Rcn1	-0,32796	1
Gm45289	-0,32813	1
Trak1	-0,32822	1
Gm43300	-0,32846	1
Jaml	-0,32876	1
Dync1li2	-0,32905	1
Rab4a	-0,32907	1
Peak1	-0,32912	1
Ell2	-0,32915	1
Borcs5	-0,32932	1
Sla2	-0,32932	1
Ppil1	-0,32936	1
Nktr	-0,32944	1
Rps15a-ps1	-0,32945	1
Tom1l2	-0,3296	1
Gm8292	-0,32975	1
Slc3a2	-0,32973	1

Gch1	-0,32969	1
Zfp422	-0,33012	1
Ccdc171	-0,33021	1
Unc45a	-0,33062	1
Psma1	-0,33064	1
Slc25a46	-0,33086	1
Cdkl2	-0,3313	1
Zcchc7	-0,33135	1
Igip	-0,33149	1
Acot11	-0,33183	1
Tbc1d15	-0,3318	1
Napa	-0,33192	1
Dna2	-0,33197	1
Plxdc1	-0,33202	1
Agpat2	-0,33211	1
Spen	-0,33226	1
Mrpl3	-0,33226	1
Pcgf3	-0,33242	1
Cramp1l	-0,33245	1
Mettl8	-0,3326	1
Washc3	-0,33272	1
Gm11688	-0,33278	1
Tns1	-0,33294	1
Mapre3	-0,33329	1
Eif3j2	-0,33328	1
Azin2	-0,33339	1
Klc4	-0,33343	1
Llgl1	-0,33374	1
Thap11	-0,33372	1
Rsl1d1	-0,33382	1
Nectin2	-0,33386	1
Prpf19	-0,33394	1
Arfgef1	-0,33388	1
Phb2	-0,33404	1
Spata33	-0,33423	1
Ivns1abp	-0,33432	1
Eif5	-0,33439	1
H2-Oa	-0,33452	1
Pomt1	-0,3346	1
Mat2a	-0,33459	1
Cdk17	-0,33465	1
Lancl1	-0,33481	1
Tbc1d10b	-0,33481	1
Utp4	-0,33504	1
Zfp329	-0,33515	1
Gm45033	-0,3351	1
Vars2	-0,33515	1
Gm6977	-0,33539	1
Ndfip2	-0,33544	1
Ipo11	-0,3356	1
Katnbl1	-0,33571	1
Tor1aip1	-0,33568	1

Atic	-0,33586	1
2610008E11Rik	-0,33601	1
RP23-139H6.1	-0,33611	1
Oscar	-0,33632	1
Tmem205	-0,33668	1
Camsap2	-0,33674	1
Ndrg4	-0,3367	1
D7Bwg0826e	-0,3369	1
Gm16536	-0,33704	1
Slc25a45	-0,33701	1
Nsf	-0,33702	1
Sbds	-0,33709	1
Oas1g	-0,33707	1
Gtf2h4	-0,33744	1
Hyi	-0,3374	1
1700123O20Rik	-0,33754	1
Gm37010	-0,33754	1
Tmem14c	-0,33751	1
Atp2b1	-0,3375	1
Elfn2	-0,33771	1
Dqx1	-0,33796	1
Tuba4a	-0,33802	1
Setx	-0,33854	1
Atp6v1g2	-0,33882	1
Slc26a2	-0,33878	1
Ube4b	-0,33882	1
Slc43a3	-0,33916	1
Gm3650	-0,33929	1
Gm11598	-0,33936	1
Arg1	-0,33943	1
Sertad2	-0,33961	1
Gm37706	-0,34003	1
Nsfl1c	-0,33999	1
Atxn3	-0,34005	1
Lrmp	-0,34021	1
Plxna2	-0,34078	1
Plekho1	-0,34079	1
Orc2	-0,34088	1
Cxx1a	-0,34099	1
Dgcr2	-0,34123	1
Grin1	-0,34134	1
Opa3	-0,34133	1
Psen2	-0,34139	1
Tmem231	-0,3418	1
Alkbh7	-0,3421	1
Rabif	-0,34237	1
Gmeb1	-0,34238	1
Mdfic	-0,34263	1
Gm43147	-0,34303	1
Tcaim	-0,34305	1
Gm4866	-0,34309	1
Zbtb46	-0,34321	1

Xxylt1	-0,3433	1
Gm8762	-0,34356	1
Tmem104	-0,34368	1
Zfp952	-0,34399	1
RP23-356D13.11	-0,34403	1
Gm4613	-0,34402	1
Polr2g	-0,34408	1
Col27a1	-0,34436	1
Zfyve21	-0,34475	1
Lzts2	-0,3447	1
Pik3cd	-0,34487	1
9130401M01Rik	-0,34525	1
March8	-0,34531	1
Fbxo31	-0,34558	1
Clec11a	-0,34577	1
Rrbp1	-0,34597	1
Cd33	-0,34624	1
Ppp1r12b	-0,34619	1
Ttc26	-0,34642	1
Atg4b	-0,34647	1
Tjap1	-0,34684	1
Suclg1	-0,34685	1
Mok	-0,34723	1
Otulin	-0,34724	1
D430042O09Rik	-0,34758	1
Gm45640	-0,3478	1
Mark3	-0,34803	1
Clock	-0,34799	1
RP24-282C4.13	-0,34819	1
Cnbp	-0,34818	1
Gm13840	-0,34834	1
RP23-307F3.6	-0,34836	1
Mlf2	-0,34859	1
Mtfmt	-0,34861	1
Tspyl2	-0,3489	1
Gtpbp3	-0,34893	1
Gm15703	-0,34886	1
Gm4950	-0,3492	1
Yjefn3	-0,34932	1
Otud6b	-0,34979	1
Yipf5	-0,35001	1
Lpar1	-0,35031	1
Ccnd2	-0,35041	1
Glt8d1	-0,35049	1
Eml3	-0,35061	1
Prr18	-0,35085	1
Pde8b	-0,35079	1
Psmc2	-0,35087	1
Ubr5	-0,35102	1
E130309D02Rik	-0,35112	1
Gpatch8	-0,3512	1
Wars	-0,35139	1

Gm33080	-0,35135	1
Ikbkb	-0,35136	1
1700109H08Rik	-0,35152	1
Nln	-0,35167	1
Ppp2r3d	-0,35179	1
Slc39a6	-0,35183	1
Mrpl22	-0,35199	1
Dbr1	-0,35202	1
Wfikkn1	-0,35209	1
Mkl2	-0,35206	1
Tmem161b	-0,35237	1
Cd63	-0,35237	1
Gm13181	-0,35248	1
Dynll1	-0,35254	1
Gon4l	-0,3526	1
Zranb2	-0,35263	1
Cd53	-0,35266	1
Gpr108	-0,35271	1
Fmc1	-0,35286	1
Gm19287	-0,35317	1
Dlst	-0,35328	1
Psme4	-0,35328	1
Unk	-0,35378	1
Sufu	-0,354	1
9330175E14Rik	-0,35455	1
B3galt4	-0,35456	1
Tram1	-0,35478	1
Mcm3ap	-0,35524	1
Rwdd2b	-0,3553	1
Tyw1	-0,35535	1
Cdc27	-0,35551	1
Ankra2	-0,35554	1
Sema4c	-0,35581	1
Ggta1	-0,35584	1
Nfkbia	-0,3562	1
Cep104	-0,35657	1
Mustn1	-0,35687	1
Gm7676	-0,35699	1
Zfp365	-0,35719	1
D2Bwg1423e	-0,35724	1
Gab2	-0,35753	1
Ikbkap	-0,35759	1
Rnf168	-0,35772	1
Kif3c	-0,35772	1
Mib1	-0,35788	1
Dnajc2	-0,35793	1
Blzf1	-0,35791	1
Poli	-0,35824	1
Xrcc5	-0,35844	1
Gm45212	-0,35846	1
Ncf4	-0,35851	1
Olfr460	-0,3586	1

Hdac4	-0,3586	1
Chst12	-0,35886	1
Oaz1-ps	-0,35896	1
Sp2	-0,35901	1
Clcn3	-0,35895	1
Fos	-0,35922	1
Cyfip2	-0,35923	1
Nop16	-0,35931	1
Lrp10	-0,35949	1
Pnpla8	-0,35962	1
Tmem69	-0,36004	1
Kansl2	-0,36002	1
Bloc1s4	-0,36012	1
Dtd1	-0,36037	1
Rbm15b	-0,36053	1
Scyl3	-0,36061	1
Pot1b	-0,36074	1
BC005624	-0,36147	1
Amotl1	-0,36154	1
Exosc7	-0,36155	1
Zcchc4	-0,36175	1
Anxa9	-0,36187	1
Arhgef19	-0,36205	1
Foxred1	-0,362	1
Dag1	-0,36225	1
Gm11363	-0,36238	1
Cd151	-0,36255	1
Alg1	-0,36259	1
Gm38190	-0,36267	1
Slc36a4	-0,36284	1
Gm43672	-0,36288	1
Hipk1	-0,36287	1
Ddx21	-0,36297	1
Herpud2	-0,36309	1
Hsd17b7	-0,36325	1
Zeb2	-0,36334	1
Pak1ip1	-0,36354	1
Rtn4	-0,36366	1
Fbxo44	-0,36388	1
Gm28041	-0,36398	1
Snx1	-0,36398	1
Rgl1	-0,36404	1
Pnpo	-0,36428	1
Ctu2	-0,36427	1
Lrrc61	-0,36444	1
Plekhm3	-0,36439	1
Josd1	-0,36435	1
Gm43162	-0,36494	1
Rab29	-0,36497	1
Dock8	-0,36498	1
Lmna	-0,36503	1
Fsbp	-0,36505	1

Ube2q1	-0,36538	1
Pafah1b2	-0,36574	1
Atg10	-0,36576	1
Txn-ps1	-0,36596	1
Sdhc	-0,36598	1
Tsen54	-0,3661	1
Ndufaf6	-0,36613	1
Gm11470	-0,36619	1
Gm5910	-0,3662	1
Fam63a	-0,36698	1
Zfp146	-0,36707	1
1810014B01Rik	-0,36728	1
Gmip	-0,36726	1
Lman2	-0,3673	1
Galnt7	-0,36735	1
Tshz1	-0,36755	1
Utp3	-0,36759	1
Lin7b	-0,36776	1
Gm7206	-0,36782	1
Prex1	-0,36795	1
Heatr5a	-0,36798	1
Rfesd	-0,36808	1
Map3k5	-0,36812	1
Dpp8	-0,36844	1
Lrp8	-0,3684	1
Car9	-0,36853	1
Prmt1	-0,36851	1
Stx6	-0,36856	1
Atp23	-0,36936	1
Rpp25l	-0,36935	1
Fbxo4	-0,36982	1
Gtf2ird2	-0,3707	1
Stard8	-0,37086	1
Atg4d	-0,37086	1
Fads6	-0,37102	1
Scaf4	-0,37098	1
Ralgps1	-0,37121	1
Degs1	-0,37117	1
9230102O04Rik	-0,37128	1
Cnot2	-0,37146	1
Gm16053	-0,3716	1
Ptpn22	-0,37175	1
Zmynd11	-0,37166	1
Srrm1	-0,37169	1
Mad1l1	-0,37211	1
Phlpp2	-0,3722	1
Fhl3	-0,37225	1
Iqsec3	-0,37243	1
Zcchc24	-0,37247	1
Crtap	-0,37271	1
Tm9sf3	-0,37269	1
Masp2	-0,37278	1

Apobec1	-0,37275	1
Sfswap	-0,37286	1
Sec16b	-0,37297	1
Gm44178	-0,37302	1
Dok1	-0,37308	1
Cyc1	-0,3732	1
Vav1	-0,37331	1
Git2	-0,37337	1
Smarcd1	-0,37364	1
Mtg1	-0,37357	1
Bcor	-0,374	1
Bcl2l11	-0,37403	1
Eapp	-0,37405	1
Reep3	-0,37408	1
Dhrs1	-0,37432	1
Tsfm	-0,37438	1
Gm17039	-0,37443	1
Sppl2b	-0,37481	1
Zfp266	-0,37505	1
Mxra8	-0,37508	1
Fam91a1	-0,37513	1
Gm37503	-0,37516	1
Strn	-0,37524	1
Ccrl2	-0,37537	1
Ypel4	-0,37543	1
Timm17b	-0,37539	1
Pde12	-0,3756	1
Nup214	-0,37579	1
Gm15496	-0,37588	1
Wdr45	-0,37606	1
Parp2	-0,37662	1
Gm7856	-0,37672	1
Smarcad1	-0,37687	1
Gatb	-0,37722	1
Cyba	-0,37732	1
Nedd1	-0,37742	1
Fndc3b	-0,37741	1
Gm14034	-0,37843	1
Fth-ps2	-0,37883	1
Zfp451	-0,37884	1
Spaca6	-0,37965	1
Endod1	-0,37971	1
Stx1a	-0,37968	1
Map3k7	-0,37973	1
Slc29a2	-0,37983	1
Gm10132	-0,38002	1
Mfsd11	-0,38016	1
Tvp23b	-0,38052	1
Naa25	-0,38066	1
Magi1	-0,38075	1
Med13l	-0,381	1
Eci1	-0,38124	1

Rxrb	-0,38123	1
Mfsd1	-0,38119	1
Chst1	-0,38137	1
Myl6	-0,38146	1
Gm12990	-0,3816	1
Tldc1	-0,38252	1
Abcd1	-0,38257	1
Faim	-0,38258	1
Ikzf1	-0,3826	1
Apoo	-0,38269	1
Cstf1	-0,38272	1
Hivep3	-0,38283	1
Esrp2	-0,38293	1
Dhx37	-0,38298	1
Traf5	-0,38319	1
Slc38a6	-0,38341	1
Mbnl1	-0,38337	1
Pacs2	-0,38381	1
Dnajb11	-0,38389	1
Stat5a	-0,38418	1
Gpx1	-0,38469	1
Ttc32	-0,38477	1
Naaa	-0,38485	1
Emg1	-0,38516	1
Sike1	-0,38535	1
Prkar1a	-0,38539	1
Gm37060	-0,38546	1
Shprh	-0,38557	1
Aph1c	-0,38599	1
4930453N24Rik	-0,3861	1
Ubtf	-0,38631	1
Set	-0,38669	1
Gm37423	-0,38681	1
Gm8574	-0,38706	1
5430403G16Rik	-0,38733	1
Usp31	-0,38735	1
Arfgef2	-0,3875	1
Cabin1	-0,3876	1
Arid2	-0,3879	1
Ube2v2	-0,38797	1
Mtr	-0,38796	1
Gm15151	-0,38798	1
Nol11	-0,38808	1
Nufip2	-0,38808	1
Ythdf1	-0,3881	1
Adam10	-0,38817	1
D930016D06Rik	-0,38837	1
Ms4a6c	-0,38863	1
Helz	-0,38874	1
Zfp335	-0,38869	1
Xpo4	-0,38887	1
Zmynd19	-0,38915	1

Atad3a	-0,3891	1
Smad3	-0,38913	1
Gpam	-0,38947	1
Hyls1	-0,38982	1
Marc2	-0,38978	1
Dusp9	-0,38995	1
Gm38157	-0,39002	1
Tbc1d1	-0,39	1
Rpl3-ps1	-0,3901	1
Ranbp2	-0,39028	1
RP23-110E20.5	-0,39072	1
Amz1	-0,39073	1
Mettl13	-0,39091	1
Fam160b1	-0,39093	1
Shpk	-0,39106	1
Slc25a24	-0,39107	1
Sag	-0,39143	1
Hars2	-0,3914	1
Fam8a1	-0,39154	1
Star	-0,39163	1
Aig1	-0,39192	1
Fam96b	-0,39221	1
1110037F02Rik	-0,39238	1
Pxmp4	-0,39286	1
Sdhb	-0,39296	1
Nrbp1	-0,39321	1
Rasa3	-0,3934	1
Dicer1	-0,39348	1
Dock10	-0,39356	1
Sipa1	-0,39374	1
Zfpl1	-0,39374	1
Clmp	-0,39404	1
Snap29	-0,39399	1
Lrrc75a	-0,39421	1
Cdk14	-0,3943	1
Lrfn4	-0,39461	1
Mfap3	-0,39456	1
Gm43920	-0,39471	1
Knl1	-0,39484	1
Smurf2	-0,39497	1
Ccdc61	-0,39506	1
Chd8	-0,39544	1
Cebpe	-0,39575	1
Gm45840	-0,39569	1
Tnip3	-0,39573	1
9130221H12Rik	-0,39581	1
Zcchc6	-0,39583	1
Alg13	-0,39616	1
Gm3355	-0,39626	1
Rcc2	-0,39661	1
Akap7	-0,39673	1
Slfn2	-0,39712	1

Gm43668	-0,39718	1
Frs2	-0,39717	1
Comt	-0,39742	1
Nav2	-0,39739	1
Cnot11	-0,39753	1
Trp53i13	-0,3976	1
Ndufb8	-0,39764	1
Tbxas1	-0,39758	1
Rnf214	-0,39798	1
Nop2	-0,39827	1
Clcn5	-0,39841	1
Cacna1b	-0,39856	1
Nol10	-0,39913	1
Ufsp1	-0,39917	1
Lig3	-0,39933	1
Dhx38	-0,39928	1
Rbm6-ps1	-0,39944	1
Cct3	-0,39944	1
Kmt2b	-0,39962	1
Arhgap9	-0,3998	1
Plekho2	-0,3998	1
Slc45a4	-0,40011	1
Ankrd12	-0,4001	1
Bpgm	-0,40056	1
Angel2	-0,40073	1
Slc11a1	-0,40091	1
Arhgef2	-0,40106	1
Hmgxb3	-0,40138	1
C1qbp	-0,40141	1
Gns	-0,40158	1
Atp6v1e1	-0,40188	1
Ccdc138	-0,40215	1
Nudt3	-0,40219	1
Rab35	-0,40231	1
Med27	-0,40236	1
Exosc5	-0,40254	1
Gas7	-0,40259	1
Slc25a10	-0,40267	1
Gm20274	-0,40285	1
Gm23054	-0,40287	1
Fah	-0,40303	1
1700012D14Rik	-0,40297	1
Med6	-0,40318	1
Sypl	-0,40343	1
Plin3	-0,40368	1
Fam120a	-0,40372	1
Gm3362	-0,40395	1
Slc15a3	-0,40402	1
Zbtb20	-0,40414	1
Emc3	-0,40433	1
Nolc1	-0,40436	1
Gm45902	-0,40449	1

Zfp7	-0,40463	1
Trps1	-0,4046	1
Eif4ebp1	-0,40456	1
Wnk1	-0,40477	1
Ddx46	-0,40512	1
Ppp3r1	-0,40513	1
Tpm2	-0,4052	1
Tmem26	-0,40528	1
Ngdn	-0,40528	1
Sec24b	-0,40542	1
Elp4	-0,40548	1
Hcls1	-0,40563	1
Ptpre	-0,40582	1
Mt2	-0,40581	1
Gm45407	-0,40592	1
Tmem62	-0,40626	1
Rgs20	-0,40626	1
Trafd1	-0,40634	1
Uck2	-0,40627	1
Serpine1	-0,40651	1
Gm44116	-0,4067	1
Fez2	-0,40705	1
Helz2	-0,40725	1
Ptafr	-0,40732	1
Ilkap	-0,40737	1
Tfam	-0,40776	1
Pttg1ip	-0,40777	1
Etfb	-0,4079	1
Dcaf5	-0,40823	1
Osbpl8	-0,4082	1
1810062G17Rik	-0,40863	1
Mplkip	-0,40856	1
Eif4g1	-0,40863	1
Gm13657	-0,4087	1
Atxn7l2	-0,40891	1
Ehbp1l1	-0,40942	1
Gm4017	-0,40963	1
Nanos1	-0,40969	1
Wipi2	-0,40971	1
Prpf18	-0,40982	1
Farsb	-0,40979	1
Taf12	-0,41017	1
Hnrnpk	-0,41029	1
Gm43499	-0,41042	1
Fam129a	-0,41044	1
Zfp296	-0,41065	1
Chil6	-0,4106	1
C8g	-0,41083	1
Miga2	-0,41094	1
Rps14	-0,41099	1
Ecel1	-0,41125	1
Twistnb	-0,41172	1

Bod1l	-0,41193	1
Sipa1l3	-0,41196	1
Gm14776	-0,41218	1
Rbpj	-0,41252	1
Nubp2	-0,4126	1
Pinx1	-0,41268	1
Arl8a	-0,41294	1
Cnrip1	-0,41322	1
Ehd1	-0,41318	1
Golga2	-0,41352	1
Zfp959	-0,41358	1
Htatsf1	-0,41371	1
Tomm70a	-0,41368	1
Rcor2	-0,41377	1
2410002F23Rik	-0,41377	1
Atxn1	-0,41422	1
Nrde2	-0,4144	1
Pemt	-0,4145	1
Morc2a	-0,41462	1
Gm9825	-0,41466	1
Klf13	-0,41481	1
Rb1cc1	-0,41511	1
Gm5697	-0,41517	1
Thap7	-0,41519	1
Thoc7	-0,41554	1
Cutc	-0,41599	1
Pstk	-0,41618	1
Fam192a	-0,41635	1
Socs6	-0,41654	1
Med17	-0,41652	1
A930016O22Rik	-0,41718	1
Fkbp11	-0,41736	1
Siah1a	-0,41742	1
Txn1	-0,41741	1
Zfp707	-0,41764	1
Gm4880	-0,41781	1
Lrrc25	-0,41793	1
Cops6	-0,41788	1
Oas2	-0,41791	1
Ston1	-0,41811	1
Gm26497	-0,41841	1
Gm38305	-0,41847	1
Abhd16a	-0,41853	1
Becn1	-0,41847	1
Gm37578	-0,4187	1
Ctnnb1	-0,41865	1
Map7d1	-0,41885	1
Tdrkh	-0,41902	1
A530041M06Rik	-0,4192	1
Klhl2	-0,4192	1
Gm5422	-0,41927	1
Gcfc2	-0,41934	1

Lrch1	-0,41949	1
Hdac9	-0,41946	1
Wiz	-0,41948	1
6720427I07Rik	-0,41969	1
Alas1	-0,41988	1
Ddi2	-0,41999	1
Supv3l1	-0,4202	1
Rpgrip1	-0,42032	1
Pcid2	-0,42036	1
Pcyt1a	-0,42047	1
Smim10l1	-0,42091	1
Zkscan3	-0,42096	1
Gm10425	-0,42105	1
Ccr1	-0,42133	1
Ikbip	-0,42163	1
Zfp407	-0,42203	1
Pgap1	-0,42199	1
Mccc2	-0,42196	1
Dnajc30	-0,42222	1
Gm42748	-0,42222	1
Ercc3	-0,42229	1
Gm45110	-0,42242	1
Gm37101	-0,42252	1
Sssca1	-0,42305	1
Zfp84	-0,42315	1
RP23-312A24.1	-0,42321	1
Il17rc	-0,42334	1
Gab3	-0,42343	1
Tbc1d4	-0,42339	1
Hpgds	-0,42364	1
Adck1	-0,42385	1
Fem1b	-0,42429	1
Hnrnpf	-0,42462	1
Ccnd1	-0,42488	1
Axin1	-0,42542	1
Gm37084	-0,4255	1
Csnk2a2	-0,42592	1
Itsn2	-0,42616	1
Dmtf1	-0,42631	1
Ralgds	-0,42633	1
Arhgef12	-0,42625	1
Impad1	-0,42637	1
Dcun1d4	-0,4265	1
2810405F17Rik	-0,42666	1
Myom1	-0,42694	1
Bcl9l	-0,42691	1
Foxred2	-0,4269	1
Gsn	-0,4269	1
Abcb10	-0,42734	1
Ttll12	-0,42749	1
Zdhhc5	-0,42768	1
Rexo4	-0,42772	1

Poglut1	-0,42826	1
Fam84b	-0,42848	1
Ap4b1	-0,42856	1
6430571L13Rik	-0,42881	1
Chmp3	-0,42902	1
Orai1	-0,4291	1
Gm15472	-0,42919	1
Rbm12b2	-0,42915	1
Mgam	-0,4292	1
Dhx36	-0,42926	1
Rnaseh2a	-0,42937	1
Terf2	-0,4295	1
Wdr12	-0,42972	1
Senp1	-0,4298	1
Anxa7	-0,43003	1
Fam149b	-0,43012	1
Calu	-0,43063	1
Cerk	-0,43074	1
Herc4	-0,43093	1
Ercc8	-0,43106	1
Mgrn1	-0,4312	1
Adam8	-0,43134	1
Cc2d1a	-0,43163	1
Tmem158	-0,43156	1
Zzef1	-0,43159	1
Pus7	-0,4317	1
Rnf111	-0,43182	1
Ppp1r8	-0,43187	1
Dhrs13	-0,43204	1
Gltscr1l	-0,43202	1
Tigar	-0,43196	1
Psme2	-0,43209	1
Adam17	-0,43231	1
Dgat1	-0,43282	1
Usp36	-0,43322	1
Msr1	-0,43364	1
Actb	-0,43401	1
Slc35b4	-0,43411	1
Gm5580	-0,43409	1
Plekhm1	-0,43408	1
A130071D04Rik	-0,43424	1
Gm4890	-0,43428	1
A430110C17Rik	-0,43441	1
Plekha8	-0,43451	1
Usp46	-0,43448	1
Rmrp	-0,43522	1
Aspm	-0,43591	1
Cfap36	-0,43625	1
E130308A19Rik	-0,43716	1
Abr	-0,43717	1
Elp6	-0,43743	1
Celf2	-0,43744	1

Mgat4b	-0,43749	1
Prkrip1	-0,43757	1
Pgd	-0,43757	1
6430531B16Rik	-0,43774	1
Dync1li1	-0,43765	1
Rpp14	-0,43827	1
Mapkapk2	-0,43832	1
Mrm3	-0,43879	1
G3bp1	-0,43887	1
Erp27	-0,43915	1
Tsr1	-0,43961	1
Gm42515	-0,43967	1
Pex16	-0,43978	1
Gas2l1	-0,43997	1
Cse1l	-0,44002	1
Gm37354	-0,44028	1
Gm44254	-0,44064	1
Mios	-0,44094	1
Fech	-0,44125	1
Ankrd44	-0,44141	1
1300002E11Rik	-0,4414	1
Slc7a6	-0,44144	1
Rnf128	-0,44136	1
Arhgef7	-0,44194	1
Mrpl36	-0,44192	1
Car7	-0,44233	1
Slc25a51	-0,44231	1
Recql5	-0,44237	1
Phtf1os	-0,44243	1
Ormdl1	-0,44249	1
Rgs8	-0,44279	1
Pip5k1b	-0,44292	1
Snx33	-0,443	1
Fbf1	-0,44303	1
Pramef8	-0,4433	1
Tbx15	-0,44336	1
Hdc	-0,4434	1
B630019K06Rik	-0,44366	1
Cbx8	-0,44418	1
Llph-ps2	-0,44456	1
Ttll3	-0,44489	1
Fryl	-0,44496	1
Gstm4	-0,44524	1
Zfp449	-0,44526	1
Flrt2	-0,44532	1
Eif4a3	-0,44535	1
Irgm1	-0,44543	1
Myo1c	-0,44551	1
Kcnk6	-0,44559	1
Abhd17b	-0,44582	1
Prss46	-0,44605	1
Madd	-0,44621	1

Bcap29	-0,44631	1
Ampd2	-0,44664	1
Ncmap	-0,44679	1
Fbrsl1	-0,44697	1
P2rx4	-0,44716	1
Lmbr1	-0,44742	1
Eif2b3	-0,44737	1
Zfp160	-0,44748	1
Ganc	-0,44771	1
Gpnmb	-0,44774	1
Gm14325	-0,44782	1
Ccdc71	-0,44805	1
Ttc27	-0,44808	1
Elmsan1	-0,44809	1
Smarca5	-0,44816	1
RP24-282C4.10	-0,44828	1
RP24-282C4.4	-0,44897	1
Usp4	-0,44922	1
Ythdf3	-0,44932	1
Trim28	-0,44939	1
Ccp110	-0,44965	1
Gpank1	-0,44978	1
Urgcp	-0,45014	1
Zbtb12	-0,45079	1
Klhl11	-0,45082	1
Rabepk	-0,45119	1
Ptpn14	-0,45136	1
Tbc1d14	-0,4515	1
2500002B13Rik	-0,45162	1
Asb11	-0,45156	1
Oxld1	-0,4517	1
Zfp688	-0,45204	1
Gnaq	-0,45216	1
Ipo13	-0,45241	1
Gm21816	-0,45237	1
L3mbtl3	-0,45257	1
Ddx10	-0,45275	1
Gm3283	-0,45374	1
Plpp6	-0,45381	1
Tmem94	-0,45378	1
Gm14121	-0,45419	1
Cuedc1	-0,45451	1
Zbtb4	-0,45453	1
Esd	-0,45473	1
Ptov1	-0,45485	1
Ccdc186	-0,455	1
Zfp606	-0,45624	1
Crybg3	-0,45622	1
Fbxw5	-0,45633	1
Rrp15	-0,45642	1
Fastkd1	-0,45664	1
Ssh2	-0,45668	1

Pex3	-0,45691	1
Jrkl	-0,45711	1
Dctn6	-0,45711	1
Mepce	-0,45749	1
Txnrd1	-0,45765	1
Usp9x	-0,45799	1
Gm30238	-0,45796	1
Cog2	-0,45811	1
Bcar1	-0,45815	1
Ss18	-0,45844	1
Srp68	-0,45854	1
Rps6ka4	-0,45858	1
Klhl30	-0,4593	1
Rab8a	-0,45951	1
Atp6v1c1	-0,45954	1
Gm29666	-0,45956	1
Mtss1	-0,45958	1
Ube2z	-0,45976	1
Akr1c13	-0,45987	1
Gm37206	-0,45993	1
6030400A10Rik	-0,46013	1
Slc16a9	-0,46016	1
Prorsd1	-0,46029	1
Tnfrsf13b	-0,46039	1
Wdr55	-0,46046	1
Prss50	-0,46095	1
D5Ertd579e	-0,46104	1
Gm23722	-0,46098	1
AW047730	-0,46097	1
Znhit3	-0,46147	1
Extl1	-0,46172	1
Sp3os	-0,46198	1
Hnrnpul2	-0,46219	1
Slc20a2	-0,46229	1
5031439G07Rik	-0,46241	1
Dhx57	-0,46258	1
Gm13140	-0,46283	1
Slc29a3	-0,46301	1
Pygo2	-0,4632	1
Gm16537	-0,46324	1
Gm14277	-0,46333	1
Rbck1	-0,46334	1
Srr	-0,46337	1
Cat	-0,46353	1
Gm45855	-0,46382	1
Tpm3-rs7	-0,46404	1
Junb	-0,46413	1
Rcc1	-0,46413	1
Mesdc2	-0,46422	1
Gm17807	-0,46426	1
Polr3h	-0,46473	1
Fam185a	-0,4648	1

Taok1	-0,46485	1
Phtf1	-0,46518	1
A130050O07Rik	-0,4655	1
R3hcc1	-0,46556	1
Tnrc6a	-0,46556	1
Ccdc97	-0,46622	1
Zc3h13	-0,46626	1
Pcgf2	-0,46637	1
Mtap	-0,46655	1
Egln3	-0,4667	1
Rfx1	-0,46687	1
Cinp	-0,46754	1
Tmem39b	-0,46767	1
Rnf220	-0,46806	1
Ybey	-0,46819	1
Pecr	-0,46823	1
Apbb2	-0,4684	1
Gk5	-0,46847	1
Dlat	-0,46854	1
Nr2c2	-0,46867	1
Cldn12	-0,46879	1
Apbb1	-0,46902	1
Ep400	-0,46929	1
Zbtb24	-0,46959	1
Kifc3	-0,46969	1
Ak3	-0,46982	1
Tfpi	-0,46979	1
Zfp330	-0,46975	1
Map2k3	-0,46992	1
Trmt12	-0,47014	1
Mrps36-ps1	-0,47015	1
Snx7	-0,4705	1
Trmt2a	-0,47049	1
Tmem18	-0,47074	1
Ankrd28	-0,47068	1
Il34	-0,47069	1
Pik3r4	-0,47077	1
Gm38082	-0,47106	1
Pum3	-0,47115	1
Abcb4	-0,47116	1
F7	-0,47126	1
Ints2	-0,47143	1
Myo9b	-0,47154	1
Gsto1	-0,4716	1
Cyb561	-0,47163	1
Mtif2	-0,47177	1
Nudt7	-0,47194	1
Timm21	-0,47199	1
Prdm9	-0,47212	1
Pik3cb	-0,47209	1
Tmem25	-0,47234	1
Adamts7	-0,47279	1

Elovl6	-0,4729	1
Rasal2	-0,47293	1
Psmc1	-0,47306	1
Gm44557	-0,47368	1
Nxpe3	-0,47384	1
Ptcd1	-0,47388	1
Cyp4f16	-0,47401	1
Frmd4b	-0,47425	1
Rep15	-0,47418	1
Gm38377	-0,47419	1
Xrn1	-0,47473	1
Wdfy3	-0,47491	1
Txk	-0,47504	1
Mbd6	-0,4753	1
Pex11a	-0,47534	1
Nol9	-0,47543	1
Actr6	-0,47562	1
Gm15964	-0,47556	1
Cenpv	-0,47576	1
9230114K14Rik	-0,47601	1
Gm9774	-0,47624	1
Opn3	-0,47644	1
Pld1	-0,4764	1
Gm44198	-0,47639	1
Tm2d2	-0,47652	1
Ube2d1	-0,47679	1
Afg3l2	-0,47752	1
Ppp1r15b	-0,47793	1
Pmm2	-0,47827	1
Foxp1	-0,47831	1
Tnfaip8	-0,4785	1
Mtdh	-0,47851	1
Pacsin2	-0,47863	1
Diablo	-0,47888	1
Vps36	-0,47935	1
Tasp1	-0,47968	1
Wdr59	-0,47983	1
Ranbp6	-0,47999	1
BC005561	-0,48002	1
Parp10	-0,4801	1
Proser1	-0,48045	1
Ammecr1	-0,48101	1
Prmt6	-0,48162	1
Gm12726	-0,48161	1
Polr2c	-0,4817	1
Btbd10	-0,48182	1
Akap9	-0,48201	1
Zdhhc20	-0,48205	1
Gm5576	-0,48245	1
Tcf4	-0,48278	1
Ptpn5	-0,48306	1
Tnfaip8l2	-0,48352	1

Ipo9	-0,48346	1
Sf3a3	-0,48382	1
Ppm1d	-0,48398	1
Capn5	-0,48439	1
Ank2	-0,48446	1
Plekhg5	-0,48481	1
1110059G10Rik	-0,48503	1
Ebag9	-0,48561	1
Stam2	-0,48569	1
Iars2	-0,48567	1
Hspa14	-0,48582	1
Gart	-0,48605	1
Rae1	-0,48619	1
Pik3cg	-0,48661	1
Card9	-0,48679	1
Zdhhc24	-0,48707	1
Zfp809	-0,48745	1
L3hypdh	-0,48745	1
Ints7	-0,48774	1
Setd6	-0,48768	1
Irf7	-0,48785	1
Kank3	-0,48804	1
Ccz1	-0,48821	1
Exosc1	-0,48842	1
Minpp1	-0,48852	1
2010315B03Rik	-0,48861	1
Senp6	-0,48879	1
Amer1	-0,4889	1
Ylpm1	-0,48903	1
Il4ra	-0,48925	1
Cd276	-0,48951	1
Hacd3	-0,4895	1
Gm14328	-0,48959	1
B230118H07Rik	-0,48982	1
Dguok	-0,48994	1
Ubxn2b	-0,49047	1
Med4	-0,4905	1
Brox	-0,49059	1
Narfl	-0,49073	1
Laptm5	-0,49071	1
Usp10	-0,49082	1
Nhej1	-0,49077	1
Gm44851	-0,49095	1
Pdss1	-0,49135	1
Crybg3	-0,49172	1
Clns1a	-0,49178	1
Arhgap45	-0,49192	1
RP24-282C4.9	-0,49197	1
Oxsr1	-0,49208	1
Lipt2	-0,49242	1
Srrm2	-0,49252	1
Kdelc1	-0,49253	1

Cpne2	-0,49257	1
Arhgap27	-0,4928	1
Tmem79	-0,4928	1
Dtwd2	-0,49289	1
Gm6209	-0,49331	1
Wdr3	-0,49399	1
Ift43	-0,49421	1
Arid1b	-0,4942	1
F830115B05Rik	-0,49489	1
Msra	-0,49564	1
Mtmr12	-0,49578	1
Atmin	-0,49577	1
Oma1	-0,49604	1
Cd63-ps	-0,496	1
Slc16a3	-0,49606	1
Tifa	-0,49683	1
0610040B10Rik	-0,49705	1
Atp6v1d	-0,49746	1
Rnf219	-0,49759	1
Pik3ca	-0,49772	1
RP23-36H21.3	-0,4977	1
Cep95	-0,49792	1
Pptc7	-0,49786	1
Slk	-0,49788	1
Tma16	-0,49796	1
Bcl2l1	-0,49803	1
Gm7384	-0,49813	1
Serinc3	-0,49828	1
Carmil1	-0,49832	1
Nudc-ps1	-0,49914	1
Sirt7	-0,49918	1
Ankrd54	-0,49939	1
Cand1	-0,49946	1
9030407P20Rik	-0,49949	1
Snrpa1	-0,50015	1
2700049A03Rik	-0,50011	1
Brf2	-0,50023	1
Slc26a6	-0,50074	1
Mrpl37	-0,50087	1
Gsap	-0,50092	1
Lrrc73	-0,50095	1
Cul4a	-0,50114	1
Tmppe	-0,50166	1
Dnmt1	-0,5019	1
Cspg4	-0,50187	1
Idh1	-0,50216	1
Pi4k2b	-0,50234	1
Ocel1	-0,50228	1
Ccr2	-0,50227	1
Gm4879	-0,5024	1
Lysmd1	-0,50285	1
N6amt1	-0,50289	1

Gm44103	-0,50293	1
R3hcc1l	-0,50298	1
Gm12543	-0,503	1
Zfp958	-0,5031	1
Ung	-0,50306	1
Zbtb25	-0,50327	1
Zfp639	-0,50336	1
Gm5276	-0,5034	1
Zfp940	-0,50338	1
2610507B11Rik	-0,50354	1
Uba5	-0,50356	1
Reep6	-0,50374	1
Gm3617	-0,50396	1
Trim25	-0,50434	1
Phf3	-0,50451	1
Gm5805	-0,50445	1
1700047K16Rik	-0,5047	1
Mgat5	-0,50503	1
Cox18	-0,50499	1
Calcoco1	-0,50512	1
Nt5e	-0,5051	1
Rpl28-ps3	-0,50573	1
Zfhx2	-0,50576	1
Lyrm2	-0,50618	1
Gm9727	-0,50622	1
Cul2	-0,50628	1
Purg	-0,50628	1
Gm37510	-0,50634	1
Snapc5	-0,50676	1
Gtpbp6	-0,50697	1
Tm9sf4	-0,50719	1
Slc25a43	-0,50742	1
Pdik1l	-0,50756	1
Zc3h8	-0,50799	1
Med24	-0,50797	1
Maml1	-0,50808	1
Gm20342	-0,50854	1
Stub1	-0,50885	1
Ano8	-0,50902	1
Naip6	-0,5096	1
A630033H20Rik	-0,51001	1
Dynlt1-ps1	-0,51001	1
Noc4l	-0,51034	1
Ncs1	-0,51039	1
Hipk3	-0,51039	1
Dolk	-0,51039	1
Ints11	-0,51091	1
Lhx1	-0,51109	1
Thoc6	-0,51117	1
Gm43560	-0,51119	1
Sh2d6	-0,51159	1
Bcl2l12	-0,51159	1

Ccnb1	-0,51184	1
Gfm1	-0,512	1
Fam111a	-0,51218	1
Fkbp15	-0,51228	1
Zfp141	-0,51244	1
Efcab14	-0,51261	1
Zfp780b	-0,51261	1
Wdr25	-0,51296	1
Ankrd27	-0,51315	1
Ift20	-0,51324	1
Rad9b	-0,51368	1
Tnfrsf1a	-0,51394	1
Rbm12	-0,51401	1
Dcaf15	-0,51418	1
Mau2	-0,51461	1
Ppat	-0,51474	1
Nudt13	-0,51498	1
Mob3b	-0,51504	1
Rhno1	-0,51511	1
Gucd1	-0,5153	1
Impa1	-0,51568	1
Spata7	-0,51593	1
Itfg2	-0,51588	1
Hnrnpab	-0,51602	1
Vps33b	-0,51609	1
Mgme1	-0,51618	1
Rnf149	-0,51625	1
Cdv3	-0,51642	1
Wdr46	-0,51652	1
R74862	-0,51683	1
Accs	-0,51688	1
Adat3	-0,51723	1
Lcp2	-0,51754	1
Derl2	-0,51763	1
Pgrmc1	-0,51779	1
Pum2	-0,51801	1
Dhdds	-0,51827	1
Grk6	-0,51834	1
Gm12966	-0,51842	1
Gm20091	-0,51859	1
Itgb3	-0,51881	1
Hs6st1	-0,51887	1
Apoe	-0,51891	1
Vps37a	-0,51915	1
Gm15007	-0,51964	1
Gm13223	-0,51961	1
Syk	-0,52003	1
Abcc1	-0,52006	1
Parp12	-0,52008	1
Morn1	-0,5201	1
Rasa2	-0,52053	1
Phf10	-0,52081	1

Mtmr2	-0,52131	1
Ddx24	-0,5221	1
Zfp316	-0,52224	1
Clcc1	-0,52231	1
Acad8	-0,52243	1
Gm6304	-0,52245	1
Gm12251	-0,52247	1
Prkce	-0,52276	1
Cox10	-0,52296	1
Prrc2a	-0,52295	1
Dcaf10	-0,52329	1
Dgkq	-0,52348	1
Tmub2	-0,52348	1
Ccdc191	-0,52381	1
Zbtb7a	-0,52419	1
Bbs9	-0,52424	1
Rabl6	-0,52427	1
Nudt21	-0,52435	1
G6pdx	-0,52442	1
9430060I03Rik	-0,52446	1
Tcirg1	-0,52464	1
Zmat5	-0,52464	1
Lrrc20	-0,52474	1
Alms1	-0,52466	1
Dffa	-0,52499	1
Gtf3c3	-0,52546	1
Gnrh1	-0,52547	1
Abcd3	-0,52561	1
Plekha3	-0,52568	1
Gnl2	-0,52568	1
Zkscan7	-0,52575	1
Gbas	-0,52579	1
Eepd1	-0,52594	1
Cep162	-0,52603	1
Ebna1bp2	-0,52623	1
Gpt2	-0,52616	1
Cep152	-0,52616	1
Zfp867	-0,52619	1
Larp4b	-0,52632	1
Lhfpl2	-0,52636	1
Gsr	-0,52641	1
Ncbp1	-0,52645	1
Glb1l	-0,52654	1
Rprd2	-0,52701	1
Mpv17	-0,52792	1
Wdr43	-0,52823	1
Miga1	-0,52828	1
Parp14	-0,52829	1
Eif2b4	-0,52845	1
Taf1a	-0,52873	1
Sass6	-0,52874	1
Ctnnd1	-0,52869	1

Rcl1	-0,52898	1
Wdr5	-0,52917	1
Tmem186	-0,5294	1
Osbpl2	-0,52978	1
Gm19777	-0,52981	1
Gm12743	-0,52975	1
Prrc2b	-0,53039	1
Tctex1d4	-0,5304	1
Cox11	-0,5305	1
Acsl4	-0,53086	1
Slc17a5	-0,53109	1
Rpusd4	-0,5313	1
Hus1	-0,53226	1
Gm5801	-0,53235	1
Rab10	-0,53311	1
Chid1	-0,53309	1
Zfp964	-0,53313	1
Gm17251	-0,53317	1
Fn3k	-0,53345	1
Cd14	-0,53403	1
Ngrn	-0,53397	1
Eif3b	-0,53408	1
Vps4b	-0,53416	1
1600020E01Rik	-0,53421	1
Gm42876	-0,53431	1
Imp4	-0,53462	1
Tbkbp1	-0,53508	1
Kyat1	-0,53511	1
Mark2	-0,53542	1
B4gat1	-0,53553	1
Gm12151	-0,53576	1
Kif3b	-0,53617	1
Cmklr1	-0,53642	1
Pygm	-0,53642	1
Zfp30	-0,53683	1
Gm43387	-0,5369	1
Creg1	-0,53751	1
Sigmar1	-0,53752	1
Gm7299	-0,53753	1
Galnt1	-0,53809	1
1700021F05Rik	-0,53814	1
Ppp6r1	-0,53838	1
Nras	-0,53854	1
Tceanc	-0,53848	1
Kcnq1ot1	-0,53879	1
Fam216a	-0,53875	1
Wdr77	-0,53888	1
Per2	-0,53928	1
Vps4a	-0,53929	1
B130006D01Rik	-0,53936	1
Gm38077	-0,53978	1
Trak2	-0,53993	1

Arsk	-0,54016	1
Tada2a	-0,54055	1
Tspan14	-0,54064	1
Sdhaf4	-0,54072	1
Gm12906	-0,5408	1
S1pr2	-0,54122	1
Gm8539	-0,54127	1
Plk1	-0,54148	1
Rtca	-0,54167	1
Mthfd2l	-0,5417	1
Zfp292	-0,54189	1
Fbxo28	-0,54237	1
D130007C19Rik	-0,54246	1
Ankrd17	-0,54274	1
Cd300ld	-0,54302	1
Csk	-0,54322	1
Usp12	-0,54345	1
Mfsd5	-0,54364	1
Zfp280c	-0,54403	1
Lrp8os3	-0,54433	1
Ep300	-0,54506	1
Ankrd34a	-0,54514	1
Plscr3	-0,54577	1
Zfp930	-0,54611	1
Poll	-0,5463	1
Gk	-0,54643	1
Mbd1	-0,54672	1
Tm2d3	-0,54693	1
Socs3	-0,54706	1
Zfp651	-0,54718	1
Mmaa	-0,54737	1
Stx17	-0,54761	1
Prtn3	-0,54775	1
Il10ra	-0,54829	1
Mrps12	-0,54844	1
Snai1	-0,54854	1
Ago2	-0,54866	1
Tagap1	-0,54894	1
Hps4	-0,54895	1
Hnrnpm	-0,5492	1
Trim12c	-0,5492	1
Elk3	-0,54979	1
Cpsf6	-0,54988	1
Rad17	-0,54999	1
Rassf8	-0,55021	1
Klhl20	-0,55021	1
Msantd3	-0,55027	1
Kdsr	-0,55067	1
Gm42484	-0,55069	1
Ddx42	-0,55123	1
Zfr2	-0,55136	1
RP24-131G14.10	-0,55154	1

Rad54l2	-0,55177	1
Zdhhc16	-0,55179	1
Zfp260	-0,55191	1
Gm16096	-0,55212	1
Atxn7	-0,55224	1
Csnk1d	-0,5527	1
Nasp	-0,55279	1
Bcar3	-0,5528	1
Crtc3	-0,553	1
BC017158	-0,55308	1
Psd	-0,55307	1
Gm13423	-0,55342	1
Tmem201	-0,55348	1
Gm37140	-0,55369	1
Snora21	-0,55384	1
Rgs2	-0,55421	1
Kcnn1	-0,55508	1
Efl1	-0,55523	1
Ercc2	-0,55515	1
Ccdc12	-0,55521	1
Tpm1	-0,5555	1
Fam195a	-0,55612	1
Fbxl14	-0,55629	1
Pnpla7	-0,55643	1
Sh2b3	-0,55715	1
Gm14323	-0,55724	1
Ltv1	-0,55743	1
Trim26	-0,5575	1
Aar2	-0,55792	1
Cecr5	-0,55798	1
Rere	-0,5585	1
Gga3	-0,55851	1
Snapin	-0,55881	1
Ccnf	-0,55884	1
Mtm1	-0,55878	1
Ehbp1	-0,55876	1
Aak1	-0,55887	1
Daxx	-0,55985	1
Zfp568	-0,55981	1
Klhl18	-0,55992	1
2310039H08Rik	-0,56009	1
Zfp672	-0,56025	1
Spata13	-0,56036	1
Supt16	-0,56061	1
Vta1	-0,56088	1
Dnajc27	-0,56095	1
Rps6kc1	-0,56102	1
Lonrf1	-0,56111	1
Cmas	-0,56123	1
Kctd3	-0,56151	1
Gpatch2	-0,56158	1
Top3a	-0,56169	1

Gba2	-0,56165	1
Gm45828	-0,56204	1
Dtwd1	-0,56213	1
Gm37474	-0,56212	1
Gm3375	-0,56216	1
Eefsec	-0,56249	1
Pkn3	-0,56256	1
Mrps10	-0,56262	1
2310043L19Rik	-0,56291	1
Mtx1	-0,56296	1
Rreb1	-0,56322	1
Nt5c	-0,56316	1
Nudt1	-0,56323	1
Prkci	-0,56383	1
Gm28535	-0,56415	1
Ddx27	-0,56421	1
Bloc1s3	-0,56472	1
Slu7	-0,56565	1
Polr3d	-0,56583	1
Gm37105	-0,56595	1
Usb1	-0,56613	1
Mrpl2	-0,56646	1
Manea	-0,56704	1
Mbp	-0,56716	1
Psma2	-0,5672	1
Bckdk	-0,56732	1
1110032A03Rik	-0,56731	1
Rbm5	-0,56746	1
Pithd1	-0,5676	1
Thumpd1	-0,56782	1
Snx11	-0,56809	1
Gpr65	-0,56822	1
Gm11263	-0,56877	1
Dagla	-0,56915	1
Gm43462	-0,56947	1
Ripk1	-0,56962	1
Cables2	-0,56986	1
Appl1	-0,57024	1
Gpr107	-0,57035	1
Ptpn11	-0,57074	1
Dbt	-0,57083	1
Zfp740	-0,57118	1
Arrdc3	-0,57134	1
Sf1	-0,57234	1
Pus1	-0,57258	1
Pcnx3	-0,57266	1
Mfsd10	-0,57284	1
Gfod2	-0,57292	1
Inf2	-0,57323	1
Nup160	-0,57333	1
Caml	-0,57402	1
Tsnax	-0,57399	1

Tcaf1	-0,57398	1
Magee1	-0,57403	1
Xylb	-0,57414	1
Atg2b	-0,57435	1
Prkar2b	-0,57449	1
Slc30a4	-0,57468	1
Gm27010	-0,57493	1
Gm43859	-0,57495	1
Uprt	-0,57521	1
Aggf1	-0,57525	1
Gm10616	-0,57568	1
Pfkfb2	-0,57575	1
Nifk	-0,57607	1
Zfp236	-0,57617	1
Plekhm2	-0,5763	1
Esyt2	-0,57644	1
Zbtb3	-0,57657	1
Cetn4	-0,57678	1
Mis12	-0,57687	1
Hivep1	-0,57691	1
Enpp5	-0,57707	1
Fut10	-0,57746	1
1700084J12Rik	-0,5777	1
1700008J07Rik	-0,57805	1
Mief1	-0,57834	1
Gm43351	-0,57846	1
Tmem91	-0,57863	1
Arhgap25	-0,57872	1
Mpp3	-0,57873	1
Cep57l1	-0,5789	1
Spred2	-0,57907	1
Mtmr4	-0,5793	1
Coa3	-0,57955	1
Cmtm3	-0,57996	1
Gm10676	-0,58009	1
Gm43336	-0,58042	1
Ccs	-0,581	1
Dapk3	-0,58114	1
Diexf	-0,58119	1
Hebp1	-0,5813	1
Anapc4	-0,58148	1
Abtb2	-0,58159	1
Pik3r1	-0,58184	1
Uaca	-0,58236	1
Gm43511	-0,58238	1
Jkamp	-0,58252	1
Tmem55a	-0,58248	1
Fam118b	-0,58267	1
Kat6a	-0,5829	1
Celf5	-0,58322	1
Lins1	-0,58336	1
Thap12	-0,58371	1

Gm17455	-0,58368	1
Pigw	-0,58389	1
Psmb9	-0,58446	1
Snx4	-0,58467	1
Dcaf13	-0,58492	1
Itpripl2	-0,58501	1
Rassf5	-0,58523	1
Ube2l3	-0,58576	1
Jdp2	-0,58617	1
Atxn7l3b	-0,58655	1
Gm340	-0,58666	1
Naglu	-0,58684	1
Actl6a	-0,58689	1
Chpf	-0,58764	1
Etfrf1	-0,58759	1
Prune1	-0,58811	1
Mrpl27	-0,58823	1
Pja1	-0,58848	1
Gid8	-0,58937	1
Zcchc2	-0,58963	1
Notch2	-0,58962	1
Slfn3	-0,58986	1
Mob3c	-0,5903	1
Asb8	-0,59062	1
Dbf4	-0,59084	1
Gm14017	-0,59093	1
Hars	-0,59103	1
Brf1	-0,59101	1
Nol4l	-0,59114	1
Wdr75	-0,59158	1
Ptpn12	-0,5917	1
Il17ra	-0,5921	1
Slc26a9	-0,59206	1
BC065397	-0,5921	1
Gm37289	-0,59236	1
C2cd2	-0,5926	1
Ldb3	-0,5928	1
Gm16755	-0,59293	1
Unc50	-0,59305	1
Atp8b4	-0,59324	1
Usp7	-0,59332	1
Crtam	-0,59326	1
Tns3	-0,59354	1
Slc37a3	-0,59363	1
Ldlrap1	-0,59378	1
Dffb	-0,5939	1
Kin	-0,59398	1
Oxnad1	-0,59455	1
Chmp7	-0,5957	1
Znfx1	-0,59596	1
Cdk7	-0,59599	1
Wdfy4	-0,59622	1

Tmem173	-0,59647	1
Dhx29	-0,59692	1
Sqrdl	-0,59714	1
Sh3pxd2a	-0,59736	1
Irf8	-0,5975	1
Dapp1	-0,59786	1
Prdm4	-0,59802	1
Vil1	-0,59857	1
Ddias	-0,59884	1
Zmynd8	-0,59913	1
Tamm41	-0,59922	1
Brd2	-0,59933	1
Creb3l1	-0,59929	1
Fam175b	-0,59954	1
Wdr19	-0,59958	1
Mrpl10	-0,60002	1
Il1rn	-0,59998	1
Cux1	-0,60011	1
Dnttip2	-0,60011	1
Gm15440	-0,60008	1
Abcf3	-0,60024	1
Tlk2	-0,60028	1
Fat1	-0,6005	1
Recql4	-0,60078	1
Mpeg1	-0,60104	1
Prdx1	-0,60134	1
Tsen2	-0,60155	1
Ccdc92	-0,60152	1
Ufsp2	-0,60171	1
Grk5	-0,60205	1
A330023F24Rik	-0,60223	1
Rpl7l1	-0,60228	1
Mapk9	-0,60268	1
Ric8a	-0,60265	1
Mif4gd	-0,60379	1
Hif1an	-0,60425	1
Kcnk13	-0,60432	1
Fibp	-0,60429	1
Zfp668	-0,60452	1
Usp25	-0,60464	1
Taok2	-0,60458	1
Nudt18	-0,60494	1
Tor4a	-0,60493	1
Gskip	-0,60516	1
Dram2	-0,60549	1
Nos3	-0,6055	1
Arf4	-0,60604	1
Lat	-0,60651	1
Dennd4b	-0,60682	1
Iba57	-0,60715	1
Gm42937	-0,60743	1
Uhrf1bp1l	-0,60757	1

Atp6v0d2	-0,6076	1
Ubox5	-0,6081	1
Iars	-0,60832	1
Wdr76	-0,60861	1
Cdpf1	-0,60874	1
Slc10a7	-0,60901	1
Adar	-0,60944	1
Mapk12	-0,60941	1
Sgpp1	-0,60974	1
Fbxo15	-0,60984	1
Pus3	-0,61013	1
Grpel1	-0,61072	1
Dusp6	-0,61123	1
3110002H16Rik	-0,61141	1
Yif1a	-0,61152	1
Snora57	-0,61236	1
Phf19	-0,61251	1
2510009E07Rik	-0,61278	1
Phactr1	-0,61283	1
Cdk13	-0,61288	1
AA386476	-0,61299	1
Coil	-0,61319	1
Dtx2	-0,61323	1
Plcb4	-0,61429	1
Wdr7	-0,61439	1
RP23-225D5.4	-0,61588	1
Zfp318	-0,61636	1
Fam161a	-0,61637	1
Man2c1os	-0,61636	1
Gm13350	-0,61645	1
Rrp1b	-0,6166	1
Chpf2	-0,61717	1
Bsn	-0,6174	1
Zswim4	-0,61802	1
Sec14l2	-0,61816	1
Emp1	-0,61852	1
Zfp820	-0,61898	1
Prmt2	-0,61913	1
Ddx56	-0,61934	1
Sucla2	-0,61936	1
Nudt16	-0,61971	1
Gm18913	-0,61994	1
Ehd4	-0,62012	1
Mfsd8	-0,62037	1
Taok3	-0,62114	1
Ubr1	-0,62164	1
Slc25a53	-0,62173	1
Hmbs	-0,62181	1
Setdb1	-0,62218	1
Clpp	-0,62227	1
Nagpa	-0,62258	1
Exog	-0,62279	1

Phf14	-0,62317	1
6030458C11Rik	-0,62325	1
Ncoa6	-0,62343	1
D330050G23Rik	-0,6234	1
Apbb1ip	-0,62345	1
Tgif1	-0,62347	1
Atat1	-0,62348	1
Exoc7	-0,62359	1
Trmu	-0,62372	1
Zfp174	-0,62369	1
Enkd1	-0,62386	1
Nbeal2	-0,62387	1
Cav2	-0,62413	1
Slc12a2	-0,6242	1
Pom121	-0,62438	1
Srgap2	-0,62449	1
Wdr61	-0,62455	1
Als2	-0,62477	1
Rint1	-0,62485	1
Ermap	-0,62493	1
2610037D02Rik	-0,62504	1
Scfd1	-0,62576	1
Klhdc3	-0,62654	1
Cdc42ep3	-0,62652	1
Ppm1a	-0,62656	1
Pitpnb	-0,62704	1
Cherp	-0,62723	1
Ddx58	-0,6275	1
Sema5a	-0,62763	1
C3ar1	-0,62785	1
Cntnap1	-0,62929	1
Dph5	-0,62971	1
Rnf123	-0,63011	1
Chrac1	-0,63042	1
Map3k11	-0,63045	1
Hs1bp3	-0,63065	1
5730480H06Rik	-0,63084	1
Gm38055	-0,63085	1
Gm15157	-0,63116	1
App	-0,63145	1
1110038F14Rik	-0,63232	1
C2cd3	-0,63238	1
Zfp106	-0,63248	1
Gm6382	-0,63256	1
Wbp1l	-0,63283	1
Gm5391	-0,63285	1
Npy	-0,63305	1
Haus2	-0,63332	1
Gmeb2	-0,63343	1
Sec13	-0,6334	1
Shtn1	-0,63342	1
Sptlc1	-0,63353	1

Blvra	-0,63367	1
Ppp6r3	-0,63433	1
Eif2ak1	-0,63446	1
Cox15	-0,63447	1
9230111E07Rik	-0,63517	1
Gm7967	-0,63528	1
Lgals4	-0,63531	1
Il10rb	-0,63556	1
Nlrp10	-0,63559	1
Ap5s1	-0,63589	1
RP23-380K24.3	-0,63641	1
Slc9a8	-0,63661	1
Cpt2	-0,63662	1
Mir3091	-0,637	1
Sel1l	-0,63706	1
Rhbdd3	-0,63735	1
Gm7224	-0,63795	1
Tmem209	-0,63884	1
Rpusd2	-0,63919	1
Man2a1	-0,63929	1
Gm43294	-0,63948	1
Ubn1	-0,63962	1
Psmd6	-0,63963	1
Ccdc32	-0,64001	1
Scap	-0,64042	1
Hk3	-0,64042	1
Rnft1	-0,64044	1
Gm11918	-0,64042	1
Tfec	-0,64064	1
Ubqln2	-0,64072	1
Dock9	-0,64091	1
Lpar2	-0,64133	1
Tecpr2	-0,64138	1
Zdhhc14	-0,64165	1
Dhx58	-0,64195	1
Mblac2	-0,64238	1
Gm9378	-0,64236	1
4732491K20Rik	-0,6437	1
Ppp4r2	-0,64396	1
Ptpra	-0,64414	1
Ccdc86	-0,64421	1
Gm45501	-0,64448	1
Irak4	-0,64461	1
Mirlet7b	-0,6451	1
RP24-460E12.3	-0,64526	1
Zcchc10	-0,6456	1
Xylt2	-0,64582	1
Dbnl	-0,64578	1
Ank	-0,64615	1
Polr2a	-0,6462	1
Zc3h7b	-0,6465	1
Gm37125	-0,64647	1

Eya4	-0,64662	1
Shb	-0,64662	1
4933440N22Rik	-0,6469	1
Iqce	-0,64759	1
Atp5k-ps2	-0,64762	1
Rab15	-0,64787	1
Gm43359	-0,64814	1
Stx2	-0,64855	1
Lpcat3	-0,6486	1
Pik3r2	-0,64928	1
Avl9	-0,64965	1
Hoxa1	-0,64963	1
BC052040	-0,65147	1
Gm37728	-0,6519	1
Tor1b	-0,65198	1
Bap1	-0,65196	1
Gm12459	-0,6521	1
Tpcn1	-0,65263	1
Zbed5	-0,6526	1
Thada	-0,65275	1
Ngly1	-0,65347	1
Ppp1r13b	-0,65353	1
Trub2	-0,65379	1
C130050O18Rik	-0,65393	1
Vash2	-0,65401	1
Psma7	-0,65412	1
Lrrc8c	-0,65409	1
Naif1	-0,65431	1
Slc25a42	-0,65467	1
Tars	-0,65528	1
C5ar1	-0,65569	1
Alkbh3	-0,65587	1
Itga4	-0,65636	1
Slc30a6	-0,65639	1
Rab3il1	-0,6564	1
Cfap43	-0,65711	1
Son	-0,65732	1
Calhm2	-0,65788	1
Slc16a12	-0,65803	1
Klc3	-0,65816	1
Pou6f2	-0,65849	1
Tmem181a	-0,65873	1
Actr8	-0,65904	1
Nrp2	-0,65922	0,88097
Sema4a	-0,65935	1
Adora2b	-0,65932	1
Hp1bp3	-0,65941	0,86543
Gdpd1	-0,65937	1
Sun1	-0,66027	1
Dfna5	-0,66044	1
Stc1	-0,66043	1
Mex3c	-0,66078	1

Kcnb1	-0,6609	1
Trip11	-0,66134	1
Asxl2	-0,66137	1
St18	-0,66156	1
Cers2	-0,66228	0,94611
Tmem115	-0,66236	1
Prmt3	-0,66247	1
Adamts1	-0,66251	1
Tmtc4	-0,66258	1
Gtf2h1	-0,66304	1
Fam32a	-0,66308	0,99584
C130071C03Rik	-0,66314	1
Hectd1	-0,66315	0,93061
Riox1	-0,66335	1
Ctdsp1	-0,66342	1
Gm14853	-0,66367	1
Gm26514	-0,66373	1
Stac3	-0,6637	1
Stard7	-0,66392	1
Zfp661	-0,66422	1
Osgin1	-0,66426	1
Tnfaip2	-0,66463	1
Mrpl17	-0,66482	1
Gm37718	-0,66587	1
Parp3	-0,66627	1
Bpnt1	-0,66668	1
Fzd9	-0,66693	1
Gm37606	-0,66706	1
Gm37305	-0,66715	1
Hmgb1-ps6	-0,66737	1
Nol6	-0,66768	1
Zfp759	-0,66766	1
Sft2d3	-0,6679	1
Slc41a2	-0,66833	1
Dock5	-0,66861	1
Pard6a	-0,66893	1
Pycr2	-0,66914	1
Zfp445	-0,66926	1
Rps6ka3	-0,66963	1
Sephs2	-0,6706	1
Usp20	-0,67104	1
Nsun4	-0,67132	1
Kbtbd7	-0,6713	1
Catsperg1	-0,67133	1
Ttbk2	-0,67191	1
Ttpal	-0,67234	1
Zbtb48	-0,6723	1
Ehd2	-0,67243	1
Cpsf3	-0,67255	1
Uqcc1	-0,67281	1
Mypopos	-0,67288	1
Wdr6	-0,6732	1

Llgl2	-0,67315	1
Gm5532	-0,67344	1
Dennd2d	-0,67357	1
Parp4	-0,67361	1
Epc1	-0,67372	1
Zfp955a	-0,67513	1
Ppm1l	-0,67526	1
Thoc3	-0,67558	1
Lin37	-0,67613	1
Gramd1b	-0,6765	1
Gm11131	-0,67647	1
E130102H24Rik	-0,67649	1
Rsl1	-0,67653	1
Psme2b	-0,67677	1
RP24-84O13.9	-0,67689	1
Gm28071	-0,67694	1
Gm42893	-0,67743	1
Frmd8os	-0,67769	1
Zfp472	-0,67785	1
Ckap4	-0,67823	0,88998
Flywch1	-0,67839	1
Gm8168	-0,67858	1
Zfp473	-0,67907	1
Bcl2l15	-0,67942	1
Alg3	-0,68072	1
9030624J02Rik	-0,68131	1
Ier2	-0,68235	1
RP24-131G14.13	-0,6825	1
Hsdl1	-0,6825	1
Plekhg4	-0,68247	1
Gm38235	-0,68321	1
Dus1l	-0,68341	1
Cyb5rl	-0,68381	1
Zfp579	-0,68391	1
Slco4a1	-0,68401	0,79924
Nup37	-0,6841	1
Cdc14a	-0,68432	1
Gm45137	-0,68432	1
1110002L01Rik	-0,68442	1
6430548M08Rik	-0,68481	1
Gm38120	-0,68549	1
A630081D01Rik	-0,68554	1
Zc4h2	-0,68607	1
Rap1b	-0,6862	0,79589
Mkl1	-0,68619	1
Sdad1	-0,68629	0,99653
Mrps35	-0,68649	1
Tmigd3	-0,68664	1
Alg5	-0,68693	1
Rpap1	-0,68709	1
Gm45495	-0,68733	1
Ints3	-0,68737	0,94463

Dhps	-0,68739	1
Cdk5r1	-0,68802	1
Btbd19	-0,68822	1
Akap17b	-0,68847	1
Mbip	-0,68886	1
Fance	-0,689	1
Tmem129	-0,68916	1
Gm9732	-0,68949	1
Stat1	-0,68967	1
Hspa2	-0,68999	1
Dnaaf2	-0,69005	1
Jarid2	-0,69127	0,99716
Slfn5	-0,69234	1
Gm28875	-0,69294	0,96252
Slc7a11	-0,69315	1
Slc25a33	-0,69326	1
Rhbdf1	-0,69345	1
9930012K11Rik	-0,69443	1
Gabrd	-0,69442	1
Dyrk2	-0,69451	1
D630045J12Rik	-0,69468	1
Tacc2	-0,69481	1
Bc1-ps1	-0,69489	1
Zbtb5	-0,69513	1
Ints10	-0,6953	1
Ssu72	-0,69539	0,96965
Tgfb1i1	-0,69548	1
Tmem19	-0,69625	1
Iffo2	-0,69656	1
Tmem220	-0,69676	1
Arl11	-0,6976	1
Gm37675	-0,69791	1
Crygn	-0,69834	1
Zfp865	-0,6986	1
Rbm6	-0,69932	1
Phospho2	-0,69937	1
Chchd4	-0,69981	0,94611
Ccdc120	-0,70003	1
Pxdn	-0,70023	1
Tsc22d4	-0,70046	1
Gm38200	-0,70133	1
Themis2	-0,70157	1
Casd1	-0,70171	1
Zfp235	-0,7021	1
Gm38262	-0,70229	1
Slc35e2	-0,70238	1
Tmem8b	-0,70249	1
Rab19	-0,70284	1
Pex11g	-0,70343	1
Myo10	-0,70395	1
RP23-402A24.3	-0,70446	1
Ntmt1	-0,70491	1

Cd300lb	-0,70516	0,81122
Noc2l	-0,70551	0,86336
Nsun5	-0,7058	1
Riok1	-0,70616	1
Mettl21a	-0,70671	1
Adprh	-0,70677	0,94053
Chordc1	-0,70709	0,7966
Fbxo25	-0,7082	1
Asb7	-0,70824	1
Aatf	-0,70861	1
Pex12	-0,70856	1
Ice1	-0,70902	1
5430427O19Rik	-0,70925	1
Fam46c	-0,70928	1
Nphp3	-0,70937	1
Mysm1	-0,70978	1
Cebpd	-0,70989	1
Fam208b	-0,71061	1
Dbndd2	-0,71066	1
U2surp	-0,71096	0,77955
Adck5	-0,71125	1
Fam160b2	-0,71158	1
E430021H15Rik	-0,71303	1
Hoga1	-0,71306	1
Elf2	-0,71366	1
Cd101	-0,71384	1
Cdo1	-0,71393	1
Gm44509	-0,71432	1
Gm19620	-0,7143	1
Parp16	-0,71544	1
Gm42481	-0,71551	1
Slc1a5	-0,7156	0,80245
Coro1c	-0,71628	0,79011
Gm44699	-0,71642	1
Hirip3	-0,7167	1
Coa7	-0,71705	1
Nfs1	-0,71811	1
Tlr7	-0,71809	1
Dennd2a	-0,71851	1
Tdrd3	-0,7187	1
Mst1	-0,71893	1
Actn1	-0,71923	0,84226
Slc22a5	-0,71935	1
0610007P14Rik	-0,7195	1
Ythdf2	-0,7216	0,90082
Rpf2	-0,72245	0,95318
Gm8925	-0,72265	1
Enc1	-0,72302	0,88097
Gm43707	-0,72311	1
Adssl1	-0,72339	0,88743
Zdhhc7	-0,72356	1
Dnajb5	-0,72387	1

Gm37900	-0,72408	1
Myl6b	-0,72435	1
Sgms1	-0,72447	1
Zfp113	-0,72461	1
0610010F05Rik	-0,7246	1
Pelp1	-0,72493	1
Car2	-0,72547	1
Gmpr	-0,72631	1
Kif13b	-0,72741	1
Nip7	-0,72753	0,8302
BC005537	-0,72749	0,95022
Gm22748	-0,72754	1
Slc38a7	-0,72805	1
Fkbpl	-0,72907	1
Wbscr27	-0,7296	1
Rnasel	-0,72997	1
Cwc25	-0,7301	1
Faf2	-0,73045	1
Rtfdc1	-0,73064	1
Wrnip1	-0,73109	1
Tmem43	-0,7315	0,59911
Rdh14	-0,73149	1
Gsg2	-0,73149	1
Catip	-0,732	1
Igf2bp2	-0,73284	0,86543
B230216N24Rik	-0,73294	1
Zfp446	-0,733	1
Pde8a	-0,73307	1
Gnl3	-0,73318	0,86543
Cmtr1	-0,7333	1
Rapgef1	-0,73362	0,83621
Egr2	-0,73379	1
Gm13835	-0,73464	1
Cul5	-0,73488	0,81122
Gm38380	-0,73509	1
Pde4a	-0,73539	1
Acsf3	-0,7355	1
Clptm1l	-0,73604	0,74095
9930111J21Rik2	-0,73659	1
Gm43788	-0,73717	1
Retsat	-0,73768	1
Slc2a4rg-ps	-0,73772	1
Med8	-0,73785	1
Ascl2	-0,73787	1
Gm43411	-0,73786	1
Klf9	-0,73862	0,52341
Adsl	-0,73869	1
Arhgap31	-0,73909	1
Arfgap1	-0,73922	1
Jmy	-0,73931	0,99623
Mdn1	-0,74002	0,88803
Il1rap	-0,74038	1

Slc39a9	-0,74202	1
Snx16	-0,74247	1
Kri1	-0,74412	1
Aptx	-0,74429	1
Ifngr1	-0,74458	1
Slc39a2	-0,74579	1
Ccar2	-0,74582	1
Zfp821	-0,74642	1
Gm14585	-0,74684	1
Rpl36a-ps1	-0,74687	1
Gnat2	-0,74699	1
Slc38a1	-0,74715	0,70124
Paxip1	-0,74755	1
Maip1	-0,7483	1
Cep78	-0,74835	1
Gm38162	-0,74832	1
Ppfibp1	-0,74867	0,82763
Phf20	-0,74889	0,81122
6330403L08Rik	-0,749	1
Snord118	-0,74911	1
Zfp983	-0,74934	1
Prtg	-0,74937	1
1810030O07Rik	-0,74993	1
Mrnip	-0,75011	1
Rasgrp3	-0,75021	0,80292
Zbtb8os	-0,75078	1
Rrad	-0,75175	1
Lgals2	-0,75179	1
Lrrc8b	-0,75195	1
Hmgxb4	-0,75234	1
RP23-403E19.1	-0,75342	1
Zpr1	-0,7538	0,72537
Rrp9	-0,75528	1
Ralgapb	-0,75564	0,85541
5830444B04Rik	-0,75629	1
Eid2b	-0,75632	1
Fam129c	-0,75645	1
Ccdc57	-0,75703	1
Tsc2	-0,75747	1
Nelfa	-0,75786	1
Gtf2h2	-0,75868	1
Rfx5	-0,7588	0,80732
Spryd3	-0,75935	1
Gm14403	-0,75968	1
Tm9sf2	-0,76183	0,60655
Emp2	-0,762	1
D3Ertd254e	-0,76234	1
Vac14	-0,76247	1
Inpp5e	-0,76267	1
Eef1akmt1	-0,763	1
Ccdc80	-0,76351	1
Psmg2	-0,7639	0,98024

Mlycd	-0,7639	1
Faap24	-0,76431	1
Tbc1d32	-0,7647	1
Rcan1	-0,76483	0,98329
Tti2	-0,7655	1
Micall2	-0,76595	1
Uchl4	-0,76634	1
Gm37660	-0,76701	1
6720464F23Rik	-0,76701	1
Tubb6	-0,76789	0,89904
Hebp2	-0,76857	1
Gm22973	-0,76859	1
Ndufaf7	-0,76884	1
Gm6245	-0,76889	1
Phb	-0,76919	1
Tiam1	-0,76945	1
Gm45871	-0,76972	1
Tmem37	-0,77	1
Otub2	-0,77048	1
Gm44250	-0,77067	1
Gm37238	-0,77071	1
Wars2	-0,77079	1
Ift74	-0,77089	1
Ranbp1	-0,77099	1
2810004N23Rik	-0,77179	0,72319
Clec5a	-0,77209	1
Tnk2	-0,77275	1
Tsen34	-0,77275	1
Med1	-0,7728	0,61306
Zbtb39	-0,77289	1
Ddx17	-0,774	0,79924
Gm6206	-0,7744	1
Dsel	-0,77481	1
Fam13c	-0,77508	1
Exosc4	-0,77528	1
Smg8	-0,77605	0,92647
Acsbg1	-0,77601	1
Mmp19	-0,77614	1
Osm	-0,77623	1
AW146154	-0,77629	1
Zfp943	-0,77663	1
Ifit2	-0,77695	1
Gm44053	-0,77699	1
Ifrd1	-0,77733	0,86769
A730062M13Rik	-0,77744	1
Creb5	-0,77815	1
Acvr1	-0,77836	1
Tmem87a	-0,77885	0,83917
Zfp362	-0,77969	1
Fam35a	-0,77979	1
Pou5f2	-0,77981	1
Gm10557	-0,78006	1

Gm2308	-0,78014	1
Zfp69	-0,78014	1
Ttc7	-0,78084	1
Shc4	-0,78107	1
Alg2	-0,78198	1
Ltbr	-0,78217	1
Pdcd11	-0,7829	0,70124
Fus	-0,78295	1
Samd10	-0,78289	1
Gm29284	-0,78329	1
Ophn1	-0,78335	1
Dtx4	-0,78405	0,59383
Htra2	-0,78422	0,85568
Alkbh4	-0,78417	1
Zfp281	-0,78448	1
Wdr11	-0,78513	1
Trdmt1	-0,78528	1
Zfp839	-0,78551	1
Zfp217	-0,78563	0,79146
Tmem203	-0,78632	0,88998
Nr6a1	-0,7867	1
Pogz	-0,78701	0,75133
Wipf1	-0,78799	0,88182
AA986860	-0,78823	1
Syvn1	-0,78974	0,83635
Gm42979	-0,78986	1
H2afy	-0,79044	0,60655
Ccdc66	-0,79045	1
Adgre5	-0,79129	1
Gm4734	-0,79145	1
Dopey2	-0,79172	0,94611
Gm43111	-0,7917	1
Smn1	-0,7918	0,58493
Atpaf1	-0,79193	1
Ptpn21	-0,7919	1
Tmem65	-0,79235	0,93061
Pdss2	-0,79272	1
Slc25a20	-0,79276	1
Fth1	-0,79323	0,22865
Gtf3c6	-0,79326	1
Gm10074	-0,79329	1
Mpv17l	-0,79335	1
Gm43792	-0,79344	1
Ncdn	-0,79533	1
B230354K17Rik	-0,7953	1
Zfhx4	-0,79575	0,97786
AU040320	-0,79738	0,96252
2510016D11Rik	-0,79753	1
Rrs1	-0,79757	0,60655
Plxnb3	-0,79762	1
Ttl	-0,79815	0,96062
Gm45853	-0,79835	1

Stoml1	-0,79881	1
Timm23	-0,79992	1
Gm13397	-0,80017	1
Ahnak2	-0,80062	0,79693
Gm43223	-0,80066	1
Rdh10	-0,80115	1
Rnf41	-0,80112	1
Rnf157	-0,80174	0,90569
Top3b	-0,80188	1
Mrps6	-0,80206	0,61068
2810006K23Rik	-0,80217	1
Rhot2	-0,80333	1
Trim32	-0,80461	1
Pla2g5	-0,80563	0,88776
4930590J08Rik	-0,8069	1
Tubd1	-0,80696	1
Rela	-0,80752	0,74329
RP24-93F20.12	-0,80766	1
Wnk2	-0,80878	1
Entpd7	-0,80893	1
Ell	-0,809	0,64497
4930563E22Rik	-0,809	1
Galk1	-0,80953	0,79364
Strn3	-0,8099	0,2049
Aldh3b1	-0,80987	1
Pid1	-0,81013	0,89904
Gm12444	-0,81015	1
2310033P09Rik	-0,81018	0,91169
Kmt5b	-0,81063	1
Ubash3b	-0,81083	0,69648
Gm5251	-0,81091	1
Trim27	-0,81111	0,70829
Mettl2	-0,81224	1
Gm20045	-0,81248	1
Sdcbp2	-0,81259	1
Snx24	-0,81287	1
Lrrc1	-0,81293	1
Pou4f1	-0,81293	1
Gm10268	-0,81298	1
RP23-38L16.3	-0,81346	1
Gm12663	-0,81356	1
Nsrp1	-0,81387	0,82683
Cep68	-0,81457	0,94611
Cep89	-0,81581	1
Ippk	-0,81577	1
Sh3bp5	-0,81603	0,34592
Sfxn5	-0,81628	1
Cxcr3	-0,81722	1
Fam219a	-0,81771	1
Gm43795	-0,81865	1
Taf1c	-0,8199	1
Dcaf11	-0,82011	1

Bnip2	-0,8212	0,6117
RP23-359K10.9	-0,82142	1
Sde2	-0,82222	0,27536
Cep63	-0,82296	1
Gm24927	-0,82298	1
Nudt5	-0,82315	1
Med29	-0,8243	1
Acy1	-0,82442	1
Arhgap30	-0,82453	0,79686
Gm10801	-0,82496	1
Gm45445	-0,82522	1
Slc39a13	-0,82602	0,83917
Dusp14	-0,82609	0,96868
1700029J07Rik	-0,8262	1
Atxn7l3	-0,82634	0,64385
Kctd11	-0,82655	1
Herc3	-0,82656	1
D330041H03Rik	-0,82658	1
Caskin2	-0,82692	1
Gm42636	-0,82703	1
Ptgr1	-0,82723	1
Col20a1	-0,82778	1
D11Wsu47e	-0,82828	1
Mavs	-0,82853	1
Tgfbrap1	-0,82895	1
Exo5	-0,83128	1
Lgals3bp	-0,83152	1
Mrps36-ps2	-0,83161	1
Foxc1	-0,83183	1
Golim4	-0,83214	0,78374
Gm4602	-0,83373	1
Wdr46-ps	-0,83367	1
Nom1	-0,83383	0,71596
Dcaf17	-0,83378	1
Zmiz1	-0,8341	0,63986
Gpalpp1	-0,83516	0,96868
Gpn1	-0,83554	1
Rnmt	-0,83622	0,50956
Tango2	-0,83649	0,96062
Gm15834	-0,83719	1
Zfp93	-0,83717	1
Kbtbd4	-0,83747	1
Slc6a4	-0,83753	1
Cep41	-0,8392	1
Eng	-0,84006	1
Alg14	-0,84052	1
Zfp770	-0,84135	1
Kdm4a	-0,8419	0,35974
Sgsm1	-0,84264	0,3815
Gm38036	-0,84266	1
Fnip2	-0,84349	0,22544
Ears2	-0,84351	1

Gm16433	-0,84454	1
Fam3c	-0,84498	0,72553
Zadh2	-0,84588	0,98099
Nat14	-0,84649	1
Saysd1	-0,84662	1
Gm18916	-0,84697	1
Gm37963	-0,84715	1
Zik1	-0,84717	1
Thap6	-0,84724	1
Ctla2b	-0,84727	1
Grwd1	-0,84791	1
4930518I15Rik	-0,8481	1
Gm37452	-0,84834	1
BC060293	-0,84865	1
Npc1l1	-0,84882	1
AU019823	-0,85052	0,8202
4732440D04Rik	-0,85091	1
Casz1	-0,851	1
Tug1	-0,85118	1
Sac3d1	-0,85148	1
Glyctk	-0,85211	1
Ryk	-0,85273	0,31655
Cep85	-0,85271	0,75896
Vaultrc5	-0,85298	0,65532
Tgs1	-0,85328	0,65428
Toe1	-0,85335	1
Tbc1d13	-0,85345	0,70124
Dedd	-0,8535	1
Zfp62	-0,85357	1
Zbtb2	-0,85383	0,96995
Oxsm	-0,85391	1
1600029O15Rik	-0,85423	1
Rnf8	-0,85483	0,99293
Ppp4r1	-0,85539	1
Gm42872	-0,85651	1
Tmem2	-0,85675	0,98309
Ctbp1	-0,85713	0,59542
Mier2	-0,85756	0,24468
Fhad1	-0,85777	1
Zfp64	-0,85794	0,88075
Stat5b	-0,85829	1
Slc9a3r1	-0,85957	1
Casp1	-0,86006	0,82508
Tirap	-0,86035	0,82825
Ptgir	-0,86046	0,75896
Sgsh	-0,86062	1
Rin3	-0,86128	0,88097
Gsk3b	-0,86151	0,36693
Ttc28	-0,86146	1
Mtrf1l	-0,86206	1
Gm37274	-0,86283	1
Gstt3	-0,86337	1

Bysl	-0,86362	0,57345
Dpp3	-0,8637	1
Gm45728	-0,86405	1
Acot2	-0,86444	1
Twnk	-0,86532	0,8913
Mum1	-0,86559	0,92717
Sars2	-0,86564	1
Klhl23	-0,86559	1
Emc4	-0,86566	1
Trub1	-0,86638	0,73186
Tigd5	-0,86686	1
Irf5	-0,86792	0,24968
Zfp418	-0,86835	1
Adgrl2	-0,86867	1
Gm7972	-0,8688	1
Metap2	-0,86908	0,52341
6330418K02Rik	-0,86917	1
Elmo2	-0,8701	1
Gm42483	-0,87059	1
Tmem17	-0,87068	1
Ccdc116	-0,87068	1
Slc4a11	-0,87131	1
Msantd4	-0,8715	0,38327
Zbtb1	-0,8716	1
Ddx59	-0,87166	1
Gm7514	-0,87194	1
Fgd3	-0,87292	0,49119
Gm13205	-0,87306	1
Elf4	-0,87366	1
Gm37333	-0,87371	1
Nkpd1	-0,87375	1
Zfp455	-0,87412	1
2310057M21Rik	-0,87419	0,97084
Tcea2	-0,87423	1
Msi1	-0,87433	1
Nckap1	-0,87463	1
Uvssa	-0,87489	0,8537
Rit1	-0,87608	0,76524
Gm6598	-0,87614	1
Ftsj3	-0,8763	0,39955
Plekhf1	-0,87646	1
Mon1a	-0,87681	1
Pde4d	-0,87716	1
Lrp12	-0,8778	0,85777
Dse	-0,87785	1
Ankrd26	-0,87788	1
Gm37795	-0,87827	1
Arhgap18	-0,87838	0,46182
Gpatch4	-0,87982	0,71976
Lrrc8d	-0,88019	0,55125
Zfp229	-0,88022	1
Zfand4	-0,88045	1

Zfp775	-0,88057	1
Tmem184c	-0,88111	1
Tnfaip8l1	-0,88124	1
Tmem140	-0,88126	1
Mutyh	-0,88141	1
Zfp961	-0,88157	1
Dapk1	-0,88174	0,31655
Bag5	-0,88227	0,69156
Elp2	-0,88249	0,69002
Pms1	-0,88367	1
Piwil2	-0,88385	1
Ralgapa2	-0,88418	1
Psph	-0,88426	1
Atad1	-0,88448	0,81145
Pfkfb4	-0,88515	0,79146
Bccip	-0,8865	1
Arl6	-0,88666	1
Zfp398	-0,88679	1
Gm13992	-0,88704	1
Olfr933	-0,88759	1
B4galt6	-0,88793	0,83385
Zfp119a	-0,88798	1
Mybpc3	-0,88845	1
Pfn2	-0,88925	1
Cited2	-0,88935	0,55815
Ankrd33b	-0,8902	1
Sh3bp5l	-0,89032	0,69156
Usp38	-0,89055	1
Arih1	-0,89061	0,16529
Mex3a	-0,8909	0,95746
Lipt1	-0,8909	1
Mrpl46	-0,89131	1
Ppil3	-0,89148	0,99293
Dnajc8	-0,89186	0,25211
Actr5	-0,89211	0,60691
Gm29488	-0,89213	1
Gm45292	-0,89264	1
Nif3l1	-0,89285	0,85781
Zscan12	-0,89333	0,86543
Gm12582	-0,89488	1
Ankdd1a	-0,89595	1
Gm37482	-0,8976	1
Wbscr22	-0,89856	0,75745
BC037039	-0,89869	1
Zfp26	-0,89995	0,97386
Ifih1	-0,89988	1
Kif13a	-0,90056	1
1700088E04Rik	-0,90098	1
Dusp5	-0,90112	0,97386
Bicd1	-0,90112	1
Oas1c	-0,90146	1
Ccl25	-0,90159	0,91451

Ppcs	-0,90183	1
Zfp526	-0,90207	1
Aqp11	-0,90352	1
AW209491	-0,90389	0,97084
Usp42	-0,90421	0,75366
H3f3b	-0,90566	0,6003
Fam208a	-0,90566	1
Tut1	-0,9058	0,9122
Plagl2	-0,90608	0,85568
Ncoa2	-0,90729	0,60441
Des	-0,90785	1
Lclat1	-0,90819	1
Tmem246	-0,90826	1
Zhx1	-0,90837	0,66031
Ticam1	-0,90864	0,92006
Heatr3	-0,91106	1
Pms2	-0,91133	1
Nek4	-0,91164	1
Brpf1	-0,91181	0,35325
Gm37183	-0,91182	1
Ppm1h	-0,91217	0,96293
Bcl3	-0,9128	1
Spi1	-0,91311	0,37975
Farp2	-0,91382	1
Dgcr8	-0,91394	0,97786
Hpdl	-0,91404	1
Slc39a8	-0,91463	1
Trmt11	-0,9148	1
Fastkd3	-0,91536	0,60655
Dars2	-0,91567	1
Chac1	-0,91595	1
Gm14013	-0,91729	1
Peg12	-0,91746	1
Tmem156	-0,91781	1
Kcnn4	-0,91879	0,1753
Zkscan6	-0,9201	0,7966
Nfu1	-0,92045	1
Polr1a	-0,92081	0,53333
Slc25a15	-0,92078	1
Sdsl	-0,92135	1
Arhgef10	-0,92138	1
Mov10	-0,92194	1
Gm9776	-0,9237	1
Six5	-0,92431	1
Arhgap35	-0,92504	1
Zfp830	-0,92512	0,72473
Gm15541	-0,92522	1
Gm14126	-0,92752	1
Rpp38	-0,92762	1
Dhodh	-0,9282	1
Ssh1	-0,92952	0,83381
Trmt6	-0,93039	0,60655

Tfip11	-0,93123	0,78251
Gls2	-0,93115	1
Scamp1	-0,93192	0,41424
Fam19a3	-0,93265	1
Pin1	-0,93288	0,72319
Gm5054	-0,93301	1
Cyb561d2	-0,93383	1
Gm12421	-0,9338	1
Gm19325	-0,93419	1
Naa40	-0,93451	0,74963
Gm43062	-0,93512	1
Gm6524	-0,93518	1
Cage1	-0,93565	1
Gm11451	-0,93582	1
Gzf1	-0,93682	0,80684
Tysnd1	-0,93729	0,78876
Kmt2e	-0,9392	0,26995
March9	-0,9397	0,58135
Gm37584	-0,93978	1
Ptrf	-0,93987	1
Fosl2	-0,94012	0,22594
Arhgef3	-0,94009	0,97786
Pde4dip	-0,94082	0,85623
Lat2	-0,94168	0,24606
Kctd7	-0,94234	1
Mtif3	-0,9432	0,80245
Hmgb1-ps8	-0,94412	1
Ighmbp2	-0,94481	0,96965
Gm42600	-0,94493	1
Tnnt3	-0,94506	1
Tubgcp3	-0,94517	0,55806
Polr3f	-0,94534	0,75115
Trim68	-0,94641	0,91485
AI987944	-0,94644	0,96868
Snx20	-0,94658	0,55258
Trmt5	-0,94714	1
Fuz	-0,94833	1
Frrs1	-0,94923	0,20689
Fam58b	-0,94928	0,85972
Tab3	-0,94927	1
Zfp128	-0,95088	1
Acacb	-0,9509	1
Zfp523	-0,95128	0,80292
Ube4a	-0,95137	1
Zbtb22	-0,9518	0,61999
Slc22a15	-0,95181	1
3110001I22Rik	-0,95186	1
Alg11	-0,95198	0,85085
Rbsn	-0,95204	0,92647
Slc27a4	-0,95233	1
Cebpa	-0,95361	0,60655
Gm13378	-0,95361	1

Vps37d	-0,9538	1
RP23-114G13.1	-0,95425	1
Lrrc47	-0,95475	0,17055
Spr	-0,95486	0,96062
Ctdspl	-0,95495	1
Gm45422	-0,95507	1
Klhl36	-0,95536	1
C1galt1c1	-0,95605	0,95746
Gm38340	-0,95605	0,99716
Bend6	-0,95611	1
Fdxacb1	-0,95625	1
Zfp703	-0,95747	0,79693
Rad1	-0,95774	0,79146
Ivd	-0,96081	1
Fli1	-0,96096	0,42947
Tshz3	-0,96116	0,9122
Nfatc3	-0,96148	0,14064
Tmem116	-0,96158	1
Egr1	-0,96233	0,92161
Efnb1	-0,96227	1
Arid3a	-0,96313	0,8913
Mylip	-0,96322	0,81268
Trappc12	-0,96352	0,65894
Gm42690	-0,96498	1
5730409E04Rik	-0,96594	0,94463
Cyp2u1	-0,96587	1
Csf2rb	-0,96651	0,81145
Zscan20	-0,96743	1
Fen1	-0,9679	0,92192
Mypop	-0,96819	1
Plag1	-0,96873	1
Bid	-0,96915	0,21236
Rab11fip3	-0,96921	1
Gm37116	-0,96989	1
Gm26132	-0,97021	1
Nmi	-0,97087	1
Ndor1	-0,9717	1
Rab23	-0,97192	1
Dhx32	-0,97246	1
Epop	-0,97422	1
Gm20056	-0,97467	1
Gm45534	-0,97608	1
CH25-309J2.1	-0,97824	1
Nr1h2	-0,97865	0,80056
Prr14l	-0,97905	0,56801
Gm42639	-0,98005	0,95298
Ppip5k2	-0,98013	0,35349
Nfkbie	-0,98126	1
Ppan	-0,98155	0,15558
Sec16a	-0,98171	0,91999
Snora73b	-0,98456	0,96062
Zbtb38	-0,98471	0,29124

Mdc1	-0,98501	0,63359
Zbtb11os1	-0,98509	1
Lyrm1	-0,98515	0,96965
Zfp426	-0,98574	1
Shq1	-0,98678	1
Paqr7	-0,98688	0,74954
Kif1bp	-0,98769	0,70124
Sepsecs	-0,98831	0,92565
Ankrd39	-0,98839	0,90031
Slc16a6	-0,98908	0,34837
Ublcp1	-0,99006	1
Dnah8	-0,99054	1
Gm38067	-0,99108	1
Fancg	-0,99209	1
Ip6k3	-0,99325	1
Cfap126	-0,99408	1
Gm44822	-0,99551	1
Mppe1	-0,99601	0,85623
Ticam2	-0,99705	0,82683
Polr3a	-0,99968	0,96965
Zhx3	-1,0001	0,99433
BC003965	-1,0002	0,38538
Nprl3	-1,001	1
4930568A12Rik	-1,0024	1
Slc25a14	-1,003	1
Gm37760	-1,0033	0,99168
Sec31b	-1,0038	1
Sbk1	-1,0039	1
Lnx2	-1,0044	0,48645
Knop1	-1,005	0,17055
Uri1	-1,0056	0,32422
Ficd	-1,0062	1
Gm42482	-1,0067	1
Tmem202	-1,0076	1
Zfp319	-1,008	1
Gm20219	-1,0102	1
Trim11	-1,011	0,084574
6430511E19Rik	-1,0112	1
Ppp1r7	-1,0115	0,6844
Zfp180	-1,0117	0,40844
Zfp623	-1,0118	1
Gm42480	-1,0127	0,80245
6430590A07Rik	-1,0127	1
Mocs3	-1,013	1
Rin2	-1,015	0,17098
6030460B20Rik	-1,015	1
Gm42595	-1,0152	1
Simc1	-1,0158	0,71803
Pter	-1,0166	1
Eif1ad	-1,0173	0,41207
Ovca2	-1,0175	0,63986
Ankrd9	-1,018	0,98099

Gm44694	-1,0183	0,87686
2210008F06Rik	-1,0185	1
BC002059	-1,0189	0,38534
Cradd	-1,0198	0,98283
Cep131	-1,0204	0,98546
Gm14698	-1,0227	1
Bet1l	-1,0244	0,24972
Gm43275	-1,0245	1
Celsr3	-1,0248	1
RP24-286J14.3	-1,0251	0,99168
Ccdc14	-1,0254	0,7298
Fam212b	-1,0258	1
Fam175a	-1,0264	0,97201
Utp20	-1,0265	0,60655
Tradd	-1,0269	1
Zfp1	-1,0273	0,90649
Twf1	-1,0275	0,64736
Gm15708	-1,0297	1
C130089K02Rik	-1,0308	0,60655
Mfsd6	-1,0313	0,34168
Pigc	-1,0318	0,33592
Cyb561d1	-1,0326	1
Rab20	-1,0341	0,92192
Sprtn	-1,0347	0,99476
Lars	-1,0356	0,081432
Cbr3	-1,0359	0,23719
Fgfrl1	-1,0365	0,87694
Faap100	-1,0369	0,57276
D6Ertd527e	-1,0382	1
Zfp772	-1,0383	1
Snrk	-1,0393	0,53333
4833418N02Rik	-1,0397	1
Zfp874a	-1,0404	0,85781
Amt	-1,0407	1
Pgm5	-1,0408	1
Fem1a	-1,0411	0,1782
B3galnt1	-1,0421	1
Kdm4d	-1,0423	1
Mettl22	-1,0435	1
Agbl3	-1,0437	0,85781
1600002H07Rik	-1,0442	0,79693
Gm15798	-1,0445	1
Gm37494	-1,046	0,96062
Gm6612	-1,0471	1
Gm37490	-1,0486	1
Gm15824	-1,049	1
Gm9256	-1,0494	1
Zkscan5	-1,0516	1
Gm37959	-1,0517	1
Garnl3	-1,0517	1
AI661453	-1,0525	1
Nudt8	-1,0537	1

Susd3	-1,0557	0,18873
Cog8	-1,0559	0,64593
Gramd2	-1,0564	1
Tmcc1	-1,0576	0,58493
Polr1b	-1,058	1
B3gntl1	-1,0582	0,96062
n-R5-8s1	-1,0583	1
Xpot	-1,0584	0,15785
Prss36	-1,0609	1
Nrip1	-1,0622	0,42808
Zfp74	-1,0628	1
Dhx33	-1,0635	0,69156
Sdr42e1	-1,0635	1
St6gal1	-1,0639	0,82683
Hmga2	-1,064	0,035021
Lrrc8a	-1,064	0,75366
Selenos	-1,065	0,07913
Prr12	-1,065	0,76524
Trpv4	-1,0658	0,99967
B230208H11Rik	-1,0682	1
Gm38345	-1,0721	1
Mcat	-1,073	0,85623
4921511C10Rik	-1,0731	1
Gm15513	-1,0735	1
Slc35c1	-1,0736	0,24798
Slc39a11	-1,0752	0,065042
Fam120b	-1,0752	0,99107
Gm10060	-1,0765	1
Zdhhc12	-1,078	0,94449
Zfp551	-1,0783	0,98087
Rab12	-1,0795	0,16961
Maf1	-1,0798	0,12499
Matn4	-1,0811	1
Rftn2	-1,0821	1
Angptl2	-1,0831	0,34837
Tbl3	-1,0833	0,56097
Tagap	-1,0837	0,88998
Mgat2	-1,085	0,37923
Fbxo36	-1,0853	1
St8sia4	-1,0854	0,15496
Jagn1	-1,086	0,37975
Gm38192	-1,0869	1
Gm43457	-1,0872	1
Pspc1	-1,0888	0,7966
Etv6	-1,0899	0,46182
Tmem241	-1,0899	0,82338
Mesdc1	-1,0905	0,82683
Gm37776	-1,0907	1
Armc5	-1,0919	0,96965
Sat2	-1,0921	1
Nadsyn1	-1,0952	1
Gm26930	-1,0963	1

Cdk5rap1	-1,0964	0,96508
Mapk11	-1,097	1
Ddx28	-1,0972	0,60655
Gm9173	-1,1002	1
Ino80c	-1,1009	0,16145
Gtpbp8	-1,1031	1
Evi5l	-1,1041	1
Cbarp	-1,1042	1
Gm16310	-1,1051	1
Znhit6	-1,1057	0,43681
Fbxo32	-1,1067	0,99623
Slfn10-ps	-1,1069	1
Gm37978	-1,1074	1
Hhex	-1,1082	0,61306
Taf1b	-1,1094	0,60655
Zfp944	-1,1101	1
Brca2	-1,1107	0,80017
Gm15506	-1,1112	1
Tlr6	-1,1113	0,62369
Smim1	-1,1119	1
Fam136a	-1,1123	0,41424
Mthfr	-1,1145	0,76982
Alkbh2	-1,116	0,74329
Zfp605	-1,116	1
Afmid	-1,1168	1
Slc22a4	-1,117	1
Fut8	-1,1172	1
Tbcc	-1,1173	0,44187
Gm26917	-1,1177	0,0059982
Tmem175	-1,1179	0,7298
Polr3e	-1,1183	0,66309
Gm9722	-1,1183	1
Celsr1	-1,119	1
Urb1	-1,1192	0,50462
Mrps2	-1,1203	0,29874
Ccdc71l	-1,1214	0,91122
RP23-104D6.2	-1,1214	1
Fbxo9	-1,1216	0,76524
Dnajc11	-1,1231	0,57276
Cstf2t	-1,124	0,36928
3110080O07Rik	-1,125	1
Sphk2	-1,1258	0,069887
Gm44423	-1,1261	1
2210016F16Rik	-1,127	0,61766
Armc7	-1,1287	0,66153
Tk2	-1,1291	0,069528
Sh3tc1	-1,1302	0,75133
Frat1	-1,1305	0,8093
Klhl5	-1,1306	0,27187
Lrif1	-1,131	0,7298
Ecd	-1,1311	0,51429
Zfp729a	-1,1312	0,88097

Tesk2	-1,1316	0,99293
Isl2	-1,133	0,95202
Cenpb	-1,1347	0,46478
Alg8	-1,1349	0,89491
Gm26532	-1,1349	1
Tefm	-1,1358	0,99741
4833412K13Rik	-1,136	0,3815
Sema4b	-1,1395	0,85299
Haus4	-1,1396	0,95166
Tmem8	-1,1402	0,70637
Prkch	-1,1409	0,17055
Clec7a	-1,1446	0,37333
Igsf3	-1,1447	0,72452
Pfas	-1,1457	0,59383
Zc3h10	-1,1481	0,25064
Zfp868	-1,1484	1
Mfsd3	-1,1485	1
Oit3	-1,1487	0,91519
Mks1	-1,1491	0,35349
RP24-547N4.7	-1,1495	1
Catsper2	-1,1503	1
Nr2c1	-1,1506	0,94987
Soga1	-1,1507	0,36801
Tlr3	-1,1513	1
Scly	-1,1516	0,73934
Bms1	-1,1548	0,024472
Crebl2	-1,1555	0,88743
Chac2	-1,156	1
Cep19	-1,1569	0,49167
Gm37788	-1,1572	1
Gm7815	-1,1574	1
Acvr1b	-1,1579	1
Mfap1b	-1,1589	0,75896
Ermard	-1,159	1
Wfs1	-1,1591	0,56024
Slc35f6	-1,1615	0,12504
Gm10478	-1,1616	1
Keap1	-1,1617	0,07286
Champ1	-1,1635	0,038501
Zfp420	-1,1637	0,81854
Fkrp	-1,1649	0,79693
Gm15892	-1,1659	1
E130311K13Rik	-1,1661	0,81646
Gm19552	-1,1664	1
Matn1	-1,1664	1
Gm26601	-1,1667	1
Sfxn2	-1,1679	0,74954
Slc16a13	-1,1686	1
Mir17hg	-1,1719	0,71596
Ifi44	-1,1722	1
Tns4	-1,1723	1
Gm38115	-1,1731	1

F630040K05Rik	-1,1751	1
Gm7895	-1,1756	1
Zbtb6	-1,1763	0,27582
Trim30a	-1,1782	1
Gm22299	-1,1799	1
Gm45221	-1,1801	1
Rlf	-1,1826	0,17156
Pomt2	-1,1826	1
Fbxw7	-1,1829	0,37975
Mrm2	-1,1829	0,82508
Serac1	-1,1837	1
Xbp1	-1,1851	0,020261
B230398E01Rik	-1,1862	0,93729
Gm45224	-1,1866	1
Gm37747	-1,187	1
Slf1	-1,1878	0,88378
Gm43793	-1,1882	1
Cptp	-1,1897	1
Rhpn2	-1,1909	1
Mtmr9	-1,1919	1
Mdm2	-1,1931	0,029431
Zfp11	-1,1932	1
Hps6	-1,1951	0,47974
Fosl1	-1,1955	0,49984
Gar1	-1,1958	0,27696
AI506816	-1,1964	0,3815
Srf	-1,1978	0,054011
Tmie	-1,1983	0,72553
Treml1	-1,1994	0,094325
Lsm10	-1,2015	0,46067
2310011J03Rik	-1,2017	0,34024
Ttll13	-1,2018	1
Dlx1	-1,2024	0,55159
Suox	-1,2031	0,84226
Rundc1	-1,2061	0,3815
Jade2	-1,2062	0,66031
Zc3h12a	-1,2068	0,71713
Marveld1	-1,2069	0,063408
Zfp709	-1,211	1
Slc23a2	-1,2117	0,45959
Zfp945	-1,2118	1
Gm45342	-1,2137	0,85299
A430027C01Rik	-1,214	1
Fam57a	-1,2143	1
Gtf2b	-1,2156	0,36265
4931414P19Rik	-1,2164	0,85781
Zfp456	-1,2166	0,97786
Rars2	-1,2175	0,47515
Acod1	-1,2186	1
Bdh1	-1,2188	1
Tnks1bp1	-1,2191	1
Etv4	-1,2205	1

Zfp763	-1,221	0,99653
N4bp3	-1,2245	1
Gm37080	-1,2297	0,99293
Gm7769	-1,2303	1
Csf2rb2	-1,2312	1
A430033K04Rik	-1,2333	1
Zfp850	-1,2338	0,97786
Gpatch3	-1,2349	0,93596
Det1	-1,2372	0,73386
Ccdc166	-1,2372	0,8302
Irf2bp1	-1,2373	0,14977
6330408A02Rik	-1,2379	1
Zswim3	-1,238	0,59911
Fbxw17	-1,2381	0,46965
Tm9sf1	-1,2399	0,96703
RP23-243B24.1	-1,2416	0,99476
Gm7666	-1,2442	1
Pcdhb16	-1,2444	1
Frmd8	-1,248	0,019679
Tada2b	-1,248	0,58124
Zfp46	-1,2482	0,6409
Zfp764	-1,2485	0,80245
Ttc30b	-1,2493	1
Gm2885	-1,2494	1
Gm43513	-1,2508	1
Xirp1	-1,251	1
Gm4754	-1,2516	1
Cmtr2	-1,2537	0,095423
Lcmt2	-1,2546	0,71713
Cdc42ep2	-1,2554	0,50956
Ccdc173	-1,2562	0,95166
Apba1	-1,2566	1
Extl2	-1,2583	0,60655
Rbm12b1	-1,2583	1
Paqr5	-1,2589	1
Phf11c	-1,2595	1
Igtp	-1,2623	1
Gm18867	-1,2633	1
Gm8738	-1,2653	0,94463
Gm20696	-1,2655	1
Gnptab	-1,2659	0,016928
Zfp846	-1,2659	0,93471
Airn	-1,2668	0,43681
Drg2	-1,2675	0,78876
Gm25857	-1,2677	1
Gpr146	-1,2732	0,34837
Spn	-1,2735	0,82597
Gm11772	-1,2747	1
Gm43627	-1,2751	1
Setd1b	-1,2754	0,17055
Kdm1b	-1,2756	1
Naip2	-1,2764	0,33943

Gm12655	-1,2766	1
Fam173b	-1,2776	0,62415
Zfp748	-1,2782	0,78374
Trim47	-1,2786	0,90082
Spats1	-1,2786	1
Gm20604	-1,2822	0,46086
Zfp747	-1,2834	0,83917
Zfp689	-1,2841	1
Snip1	-1,2849	0,34406
Tmem181b-ps	-1,2862	0,82763
Src	-1,2868	0,65434
Zfp617	-1,2869	1
Ankrd49	-1,29	0,35974
Dclre1b	-1,2908	0,47571
4632427E13Rik	-1,2929	0,083435
Cd3eap	-1,293	0,20833
Dhrs9	-1,2937	0,71713
Inpp5b	-1,2959	0,69628
E430018J23Rik	-1,2984	1
Zfp653	-1,2985	0,76731
Nop9	-1,2989	0,43168
Gm37465	-1,3004	0,96703
RP23-444K20.4	-1,3007	0,24972
Dimt1	-1,3019	1
Atad3aos	-1,304	1
Lpin3	-1,3046	0,53708
Gm13268	-1,3073	1
Shisa3	-1,3083	1
Il20rb	-1,309	0,79255
RP24-226A8.2	-1,3097	1
Pknox1	-1,3111	0,27558
Dph2	-1,3115	0,6488
Bag2	-1,3128	0,27558
1700017B05Rik	-1,315	0,017151
9930120I10Rik	-1,3151	1
Zfp790	-1,3184	1
Mrgpre	-1,3187	1
Ctsk	-1,3188	0,27696
Evi2a	-1,3198	0,20975
Tmem177	-1,3203	0,49016
Gm37297	-1,3207	0,95022
Gpr183	-1,3211	0,17098
Tuba1c	-1,3213	0,11302
Slc39a1	-1,3214	0,084574
Tmem198b	-1,3222	0,75899
A430105I19Rik	-1,3224	0,96965
Tlcd2	-1,3224	1
CAAA01194877.2	-1,323	0,70124
Gm10605	-1,323	1
Wdr73	-1,3251	0,42894
2610203C20Rik	-1,3268	0,6003
2810021J22Rik	-1,331	1

Slc43a2	-1,3321	0,019679
Dcp1b	-1,333	1
Frmd4a	-1,334	0,14439
Asah2	-1,334	1
Fam161b	-1,3355	1
Wdr24	-1,3381	0,43354
Zc3h4	-1,3394	0,061179
Lig4	-1,3403	0,49984
Il15ra	-1,3428	0,85568
Pwp2	-1,3429	0,29874
Kat6b	-1,3435	0,34115
Frg2f1	-1,3439	1
Gm20712	-1,3501	0,95144
Fam83h	-1,3506	1
Sirt4	-1,3509	1
Srrd	-1,3514	0,72913
Zfp942	-1,3517	0,75819
Slc22a21	-1,3526	0,95166
Gemin8	-1,354	1
RP23-440I21.3	-1,3552	1
Eogt	-1,3568	0,49167
Gm15327	-1,3569	1
Fam212a	-1,3577	0,85299
Gm25514	-1,3578	0,96965
Zfp169	-1,3598	0,95746
Zfp933	-1,3599	1
Gm25596	-1,3619	1
Jrk	-1,3633	1
Cnksr1	-1,3644	1
Gm38125	-1,3661	1
1700052K11Rik	-1,3671	1
Rpl7l1-ps1	-1,3694	1
Fzd2	-1,3698	1
Zfp189	-1,3706	0,76731
2310001H17Rik	-1,3706	1
Nedd9	-1,3707	1
C530005A16Rik	-1,3732	1
Pafah2	-1,3752	0,96965
Gm43961	-1,3763	1
Abhd1	-1,3774	1
Homez	-1,3775	1
Slc46a1	-1,3779	1
BC024978	-1,3792	1
Bcl9	-1,3812	0,1753
Stx11	-1,3845	0,88998
Phykpl	-1,3858	0,52341
Gm44510	-1,3875	1
Rnf170	-1,3895	0,89904
A930001C03Rik	-1,39	0,83381
Gm37726	-1,39	1
Gm37968	-1,3905	0,96062
Lyl1	-1,3919	0,14813

Lhx5	-1,3929	1
Gm37234	-1,3936	1
Trim16	-1,3955	0,94023
Zfp619	-1,3961	0,99396
Hgh1	-1,3973	0,36699
Gm9568	-1,4003	1
Pde4b	-1,4027	0,41207
Gm9951	-1,4032	1
Zfp658	-1,4049	1
Egf	-1,4052	1
Gm42549	-1,4052	1
Cyth4	-1,4061	0,0021695
Arhgdig	-1,4096	0,75896
Gm17494	-1,4133	0,55258
Fam83d	-1,4149	0,79146
Emc9	-1,4159	0,80056
Nudt12	-1,4162	0,72319
Ftx	-1,4184	0,59383
Ampd3	-1,4185	0,29124
Rab7b	-1,4195	0,019679
Tlr4	-1,4195	0,37975
Trim45	-1,4203	0,62603
Ffar4	-1,4219	0,8913
Pde6g	-1,4237	1
Gm10842	-1,4242	1
C1rl	-1,4245	0,96965
Zfp799	-1,4246	0,69923
Plpp7	-1,4266	0,54774
Olfm1	-1,4273	0,00082689
Mdk	-1,4276	0,75133
Gm37390	-1,4276	0,96703
Mblac1	-1,4314	0,80138
Zfp202	-1,4323	0,79146
Grhl1	-1,4346	0,82508
Mul1	-1,4347	0,38538
Znhit2	-1,4363	0,41707
Gm43071	-1,437	0,99629
AA914427	-1,4374	1
Pskh1	-1,4433	0,27558
Dcstamp	-1,4443	0,18245
Carf	-1,4443	0,35662
Zkscan4	-1,4443	1
Zfp738	-1,4446	0,96062
Zfp251	-1,446	0,49011
Gm6921	-1,447	1
Card6	-1,4495	0,97665
Fastkd2	-1,4506	0,37082
Fbxo10	-1,4523	1
Arhgef17	-1,4537	1
Sfn	-1,4542	1
Upk1a	-1,4548	0,80292
Pdpn	-1,4595	0,77332

Erlin2	-1,4599	0,21817
Gm24890	-1,4601	1
2310015A10Rik	-1,4625	1
Erbb3	-1,463	0,90082
Pomk	-1,4692	0,42717
Gcc1	-1,4712	0,029431
Gm43350	-1,4731	0,99584
Surf2	-1,4735	0,16145
Tlr13	-1,4741	0,5164
Gsto2	-1,4742	0,98024
Gm43794	-1,4786	0,42808
Trp53rka	-1,4801	0,36282
Zfp248	-1,4804	0,94376
D030028A08Rik	-1,4822	0,79693
Hemk1	-1,4828	0,50051
Traf6	-1,4844	0,48158
C130023A14Rik	-1,4881	0,69156
Rftn1	-1,4885	0,1367
Prag1	-1,4888	0,18873
Cnr2	-1,4898	0,47244
Ptpro	-1,49	0,096644
Crtc1	-1,4922	0,64792
Magi2	-1,4934	0,64385
Ccdc51	-1,4937	0,37975
Hck	-1,4958	0,38327
Bbs1	-1,4959	1
Ptpn7	-1,4964	0,22884
Trmt44	-1,4992	0,54516
Gm43378	-1,4998	1
Gm37420	-1,5002	0,96965
Maml2	-1,5013	0,72553
Tfb2m	-1,5036	0,85781
Ints5	-1,5075	0,14883
Prkdc	-1,5077	0,41707
Slc35d2	-1,5136	0,60493
Zfp974	-1,5145	0,80732
Gm44667	-1,5147	0,82508
Snord13	-1,515	0,02024
Cx3cr1	-1,5189	0,0070612
Zbtb45	-1,5256	1
Adamts4	-1,5323	1
Ttc30a1	-1,5326	1
Zic5	-1,5334	0,96965
Zfp628	-1,535	0,46716
Fbxo46	-1,5358	0,26236
Bcdin3d	-1,5358	0,40689
Nkrf	-1,5368	0,65083
Adal	-1,5384	0,42947
Gm44916	-1,5452	0,99293
Gipc2	-1,5457	1
Tubg2	-1,5485	0,96543
Speer9-ps1	-1,549	1

Nipa1	-1,5507	0,73798
Ankle1	-1,5507	0,96965
Phxr4	-1,5528	0,90649
Tmem51os1	-1,5541	1
Gm43421	-1,5565	1
Tgfbr2	-1,5598	0,0066269
Gm29539	-1,5623	0,88075
Tmem98	-1,5663	0,86158
Stc2	-1,5682	0,72386
Zfp276	-1,5683	0,5454
Gm37033	-1,5704	0,76524
Nmb	-1,5718	0,90649
Dido1	-1,5723	0,07009
Gmppb	-1,5767	0,27261
Srl	-1,5779	0,85781
Gm4258	-1,579	0,61011
Zfp810	-1,5795	0,41424
Gm38355	-1,5806	0,91006
Birc3	-1,5808	0,029431
Mfsd4b4	-1,5831	1
Tmem185b	-1,5845	0,17098
Ubxn8	-1,5865	0,55258
C330018D20Rik	-1,5871	0,36872
P2ry6	-1,5918	0,022056
Ctu1	-1,5941	0,18873
Cdkl4	-1,5959	0,46486
Zfp12	-1,596	0,42247
Gm37699	-1,5971	0,52137
Zfp324	-1,5984	0,64736
Gm6257	-1,5988	0,92192
Tbc1d25	-1,6018	0,39035
Chst14	-1,6046	0,39905
Exoc8	-1,6068	0,44187
Slfn8	-1,6103	0,82561
Vsig10	-1,6113	0,41116
Rab43	-1,6143	0,30732
RP24-233B16.6	-1,6234	0,70895
Capn10	-1,6246	0,52375
Sh3rf1	-1,627	0,29989
Kctd21	-1,6316	1
Txnl4b	-1,6321	0,54037
Sema6b	-1,638	0,8537
Gm44953	-1,6382	0,89431
BC048403	-1,6392	0,62569
Gm44829	-1,6403	0,71596
Gm6743	-1,6403	0,9797
A930015D03Rik	-1,6449	0,82765
Gm43112	-1,6488	0,95415
Ajuba	-1,6522	0,94023
Tepsin	-1,653	0,079326
Pctp	-1,6549	0,30779
Gm37569	-1,6568	0,46182

Gm42835	-1,6572	0,90649
Exo1	-1,66	0,94053
BC024386	-1,6608	0,88998
Zfp239	-1,662	0,80292
Angptl4	-1,6636	0,60711
Nmnat3	-1,6668	0,40689
Zfp953	-1,6675	0,93527
Slc9a4	-1,6689	0,97842
Gm43727	-1,6708	0,71713
Gm36963	-1,6713	0,42032
Gm15696	-1,6723	0,66547
Cxcl2	-1,6736	0,14145
Hoxa7	-1,6801	0,60655
Gm15644	-1,6823	0,60196
Pomgnt1	-1,6839	0,16145
Zfp768	-1,6864	0,016929
Lima1	-1,6868	0,0046683
Egfl8	-1,6875	0,97665
Mras	-1,6926	0,86033
Aldh1b1	-1,6961	0,35662
Gm22	-1,6961	0,82508
Pde4c	-1,6977	0,7966
4930432K21Rik	-1,6981	0,14746
Gm17586	-1,6986	0,75133
Zfp518a	-1,7021	0,18873
Mtg2	-1,7032	0,42717
Endog	-1,7064	0,40844
Crybb3	-1,709	0,61306
Caprin2	-1,7092	1
Zfp61	-1,7117	0,24968
Rap1gap	-1,7139	0,82338
Cass4	-1,7193	0,63876
2010008C14Rik	-1,7198	0,87445
Gm11448	-1,7276	0,78345
Tmem260	-1,7338	0,53425
Gm12258	-1,7356	0,86033
Rwdd3	-1,7423	0,14439
Card11	-1,7424	0,83917
6030442K20Rik	-1,744	0,89904
Nupl2	-1,7443	0,64736
Slx4	-1,7478	0,46674
Gm38020	-1,7478	0,60655
Ing4	-1,7485	0,63408
Zscan22	-1,7497	0,76817
Gm44545	-1,751	0,96252
Gm43692	-1,7513	0,30941
Srxn1	-1,7583	0,019679
Gpr180	-1,7685	0,57246
Bmf	-1,7709	0,17504
1810055G02Rik	-1,7772	0,604
Gm43715	-1,7786	1
Depdc5	-1,7798	0,36064

Tlr1	-1,7805	0,37975
Gm6526	-1,7838	0,65259
Numbl	-1,7849	0,22594
Acot6	-1,7854	0,6003
Endov	-1,7884	0,39436
Zfp39	-1,7904	0,83917
Actg1	-1,7922	0,81122
Zfp65	-1,7926	0,14701
Gm43144	-1,7962	0,96868
Gm45206	-1,7999	0,76982
Vwf	-1,8071	0,67302
Mlh3	-1,8178	0,18873
Zfp729b	-1,818	0,19178
Gtf2h3	-1,8199	0,54455
Pih1d2	-1,8232	0,85541
Sec22a	-1,8249	0,22594
Gm43761	-1,8266	0,33592
Gm38387	-1,8297	0,83918
Nrros	-1,8298	0,00049497
RP23-442M18.5	-1,8338	0,71713
Fancf	-1,8375	0,82338
I830077J02Rik	-1,8376	0,64385
Gipc1	-1,8406	0,26995
Zfp90	-1,8463	0,34205
A630072M18Rik	-1,8497	0,35662
Dlg3	-1,8502	0,3472
Ubiad1	-1,8529	0,1367
Tmem44	-1,8574	0,88803
Zfp35	-1,8606	0,27582
Gm44951	-1,8612	0,6771
Snx19	-1,864	0,2609
Gm38399	-1,8723	0,35625
Zfp94	-1,8754	0,55125
C1ra	-1,8757	0,86385
Qsox2	-1,8791	0,5312
Trib1	-1,884	0,0081259
B130021K23Rik	-1,8841	0,6246
Ccdc130	-1,8877	0,056103
Fmo5	-1,895	0,73904
Lysmd4	-1,9048	0,0075008
B3galt6	-1,9073	0,58493
Swsap1	-1,9088	0,60655
5430420F09Rik	-1,9149	0,80292
AI464131	-1,9232	0,75133
Katnb1	-1,9268	0,67667
D6Wsu163e	-1,9345	0,20671
Gm37063	-1,9379	0,85781
Ano7	-1,9383	0,49984
Cog1	-1,9414	0,43168
Mettl18	-1,9447	0,31507
Fam69b	-1,9448	0,6003
Gper1	-1,9474	0,72694

Zfp866	-1,9482	0,62415
Ribc1	-1,9552	0,7966
Tmem204	-1,9581	0,47563
Gm33142	-1,9594	0,69648
Trmo	-1,9677	0,3815
Gm26740	-1,9683	0,73699
Cttnbp2nl	-1,971	0,00034365
Slc10a3	-1,9712	0,30466
9930014A18Rik	-1,9854	0,55258
Tmem67	-1,9886	0,60655
Zfp862-ps	-1,9904	0,34837
Tspoap1	-1,9919	0,35349
Particl	-1,9922	0,50956
RP24-175C20.18	-1,9986	0,54169
Gm43728	-2,015	0,35349
Gm42967	-2,0196	0,60655
3110082I17Rik	-2,0215	0,14813
Mboat1	-2,0231	0,18873
Epb41l1	-2,0245	0,52706
Commd5	-2,0298	0,070546
1700007K09Rik	-2,0318	0,79364
Tnfrsf4	-2,0326	0,40844
RP24-325N9.5	-2,0347	0,61011
Pkd2	-2,0382	0,27582
Lrrc14	-2,0429	0,13727
Zfp951	-2,0458	0,59566
Zfp28	-2,0491	0,69923
Efna2	-2,0638	0,54169
9130019O22Rik	-2,0674	0,36282
AI467606	-2,0712	0,011531
Gm36930	-2,0732	0,49011
Wdr35	-2,0763	0,48769
Zfp40	-2,0792	0,43354
D17H6S53E	-2,0799	0,3815
Limk1	-2,0933	0,31655
Tti1	-2,0984	0,52907
Nlrp3	-2,1025	0,0041506
Zfp27	-2,1067	0,50462
Gm26890	-2,1099	0,19178
Arhgef18	-2,1099	0,35443
Zfp3	-2,1214	0,20411
Ap5b1	-2,1221	0,41707
Hdhd3	-2,1234	0,35662
Zfp870	-2,1236	0,16812
Gm42486	-2,1256	0,75896
Rinl	-2,1305	0,073347
Ppp1r26	-2,1352	0,79686
Fam222b	-2,1361	0,056103
Gm20699	-2,1384	0,39955
Irgm2	-2,1404	0,57877
Mir22hg	-2,1475	0,00010988
Gm42640	-2,1548	0,21246

Gramd1c	-2,1549	0,60423
Tmem51	-2,1631	0,060406
Gm37121	-2,1771	0,17812
Ifi47	-2,186	0,80371
Spred1	-2,1953	4,34E-05
Fut7	-2,2038	0,46965
Col7a1	-2,2044	0,44187
Sla	-2,2083	0,0052152
Dnmt3b	-2,2222	0,24972
Pus7l	-2,2265	0,40488
Mfsd9	-2,2436	0,1782
Ppp1r10	-2,2742	9,21E-05
3110070M22Rik	-2,2893	0,34834
Vegfc	-2,3213	0,6246
Gm37124	-2,3387	0,28869
Cbfa2t3	-2,3604	0,37975
Gm20632	-2,3697	0,27212
Cstad	-2,3739	0,63483
Zfp408	-2,3763	0,054011
Dmrt2	-2,4234	0,32947
Gm42463	-2,4268	0,33592
Hoxb3	-2,4316	0,26973
Gdpgp1	-2,4326	0,40844
RP23-38L16.4	-2,4433	0,19726
Oasl1	-2,4532	0,18873
Elmod3	-2,4585	0,22594
Gm29994	-2,4599	0,44923
Usp27x	-2,5164	0,67357
Gm43024	-2,5386	0,12847
Gm4262	-2,5726	0,22544
Gm38009	-2,6014	0,084574
Fastkd5	-2,6293	0,15423
Zfp719	-2,6367	0,17098
Tctn2	-2,6817	0,25211
Filip1l	-2,6897	0,10853
Zfp111	-2,894	0,099549
Zfp691	-2,9025	0,065042
Gm16740	-2,909	0,17812
Zfp41	-2,982	0,1367
Gm37642	-2,9964	0,039732
Zfp58	-3,0354	0,1431
Mir763	-3,0736	0,083208
Rbak	-3,1235	0,045372
Krcc1	-3,1821	0,22594
Gm11205	-3,5714	0,0057795
