# Supplementary material for: Bone, dentin and cementum differentially influence the differentiation of osteoclast-like cells
Source: Sci Rep. 2025 Jun 5;15:19857. doi: 10.1038/s41598-025-04874-9 (PMC12141432; doi:10.1038/s41598-025-04874-9)
Supplement: Supplementary file 8 — Supplementary Information 8. [file 41598_2025_4874_MOESM8_ESM.pdf]

Tab. S7:
Transcripts induced in murine macrophage cells stimulated on cementum (n=6), fold of stimulation control

gene name	regulation of expression	adj.P.Val
Hspa1b	226,9609053	3,02E-06
Gm29358	125,7574449	8,63E-07
Rn7sk	72,41416366	5,14E-08
mt-Tm	71,88406054	2,90E-10
Hspa1a	63,71229937	9,56E-06
Gm23037	60,94348092	2,50E-06
4930578M07Rik	58,14567581	4,37E-07
mt-Tc	42,42668343	2,58E-06
Gm44652	36,63879117	4,23E-08
4921507G05Rik	35,69870908	2,76E-05
mt-Ta	35,30987968	7,67E-05
Slc16a5	33,76835509	4,13E-05
RP23-451J19.1	31,45461939	1,12E-05
Aloxe3	29,24463342	3,18E-06
Hist1h2bg	26,47575646	0,00013994
Gm18709	26,29287505	3,04E-07
mt-Ti	25,11191247	9,58E-07
Gm37052	25,06322223	0,0005292
Gapdh	23,84310137	6,86E-08
1700054M17Rik	23,06766386	2,19E-05
Hist1h2an	22,4866992	0,00022689
Hist2h4	21,47222814	9,63E-05
Gm42793	21,0376259	0,00049873
Sit1	20,84746412	7,48E-05
Mcm8	20,49499879	4,01E-07
Gm28373	20,23386473	0,00024645
Snord83b	20,16386087	6,78E-07
Hist1h4d	19,89177345	1,73E-05
Rasd1	19,85595712	7,37E-05
AY074887	19,79274768	5,62E-06
Fzd7	19,63832529	3,47E-08
Adm	19,2595221	5,01E-10
Gm26870	19,02467628	0,241
RP23-440L7.5	18,52626307	0,00015941
1500004A13Rik	18,1387106	1,54E-06
Gm45698	17,94611928	1,03E-05
Gm22513	17,92622743	4,81E-05
Crtc2	17,66350815	0,00013527
Gm26810	17,19002812	2,80E-05
Gadd45g	16,91346096	2,90E-10
Hist1h1a	16,83626246	0,002006
Gm24991	16,57802226	0,00037868
mt-Tq	16,44754096	1,97E-06
Gm26226	16,39972821	0,0021342
Hist1h2be	16,10570602	2,33E-08
Gm24631	15,87076758	1,03E-05
Gm10800	14,76797387	0,65818
mt-Tl1	14,26788908	7,21E-10

4933437G19Rik	14,21655496	9,92E-05
Gm8623	13,84410612	1,32E-05
Atp1b4	13,67624246	0,0035661
Dynlt1b	13,62137087	0,0021494
4632415L05Rik	13,547925	6,98E-09
AV099323	13,3985038	0,00097343
2310058D17Rik	13,25532373	1,08E-05
D430001F17Rik	13,13186574	2,45E-06
Hspa8	13,09006177	5,95E-07
Rgcc	13,05472353	1,90E-07
Acoxl	12,89911338	0,0028378
Rpl7	12,8812438	1,10E-08
Snord66	12,8741029	0,0010652
Thap8	12,83845774	8,85E-05
Gm26772	12,82778346	0,00036245
Zp1	12,79226655	0,0054145
Gm43714	12,51945626	0,00070892
Gm26594	12,47787191	0,00076807
Gm26202	12,45713156	6,38E-05
Rpl30-ps2	12,23125046	6,98E-07
Gm4607	12,15939885	1,20E-05
Gm27248	11,96708607	0,0035196
Gm25008	11,88936699	2,66E-05
Hist1h1d	11,79824199	4,36E-05
Hist1h4a	11,59314428	0,0056036
Id1	11,39240156	7,38E-11
Hist1h1b	11,32626276	0,00058185
1700030M09Rik	11,30116815	0,0021079
Gdf15	11,09547865	7,85E-09
RP24-174I4.1	11,03259297	0,000149
Gm37653	11,0134915	1,64E-05
Ankrd37	10,91318268	6,86E-08
4930578M01Rik	10,86487748	0,0066833
Esco2	10,84305963	0,00020846
Rybp	10,83179173	0,00065964
Gm23969	10,76293767	0,00016273
5930420M18Rik	10,42156473	0,0032805
Snord82	10,38910882	0,0014466
Mkln1os	10,380471	0,00022808
RP23-320D23.6	10,35173008	9,20E-07
Gm29170	10,20638455	4,42E-08
Med16	10,15768686	5,42E-09
Gm12469	9,995647326	0,0010072
Gm8649	9,992876328	7,38E-11
Lrrc2	9,949334303	0,00084152
mt-Ts2	9,895002008	0,0017262
Gm20594	9,892944614	0,0086979
Gm43011	9,884719315	0,0018068
Gm43273	9,729057156	0,01767
Gm26225	9,634434574	0,0002857
RP23-366E4.9	9,501794466	0,0026041
Gm42670	9,432237204	0,0014419

Gm44292	9,374878545	1,34E-05
RP24-295J1.1	9,365785519	0,0029655
Hist1h3d	9,335323387	0,00096638
Gm42743	9,255429884	9,05E-06
Kcnd1	9,167955236	0,0062396
Trf	9,100841823	0,0012493
Gm5112	9,067469579	4,98E-05
Gm19272	8,997349094	0,013413
RP24-282C4.3	8,963113714	0,0014875
Snord89	8,775613578	4,52E-07
Kif18b	8,743433926	0,0059789
Efna3	8,672211849	0,00048523
Gm26656	8,590844746	0,0021653
Hist4h4	8,545707869	0,0039379
Gm14057	8,454972135	0,0096861
Hba-a1	8,426888288	0,033302
Nsl1	8,378546566	1,55E-07
Wwc1	8,236891586	7,21E-10
Hist1h4n	8,224340526	0,0028672
Rnf122	8,210670271	9,41E-06
Gm23301	8,210670271	0,0063144
4932422M17Rik	8,188504553	3,10E-06
Gm26461	8,165832642	0,003761
Lbp	8,160740119	0,0091332
Crkl	8,074091856	0,00013265
Tsix	8,071853553	0,0029616
Gm7099	7,994456744	3,88E-05
Gm3531	7,911218948	0,027329
Olfr286	7,896974326	0,017212
Gm11491	7,823423156	4,11E-06
Lgals7	7,795275438	0,0020277
4930589L23Rik	7,768305834	0,0018853
Gm26847	7,747334333	0,025663
Gm43566	7,711438462	5,47E-07
Ier5l	7,650743734	2,32E-07
Sep 01	7,646502437	0,035261
Gm42856	7,631676404	0,022407
Rbm3	7,61160133	0,00022803
4930542C12Rik	7,562168522	0,025163
Rhoh	7,477209729	0,090178
Gm8203	7,458057913	0,031472
Gm44130	7,425560869	0,014291
Hist2h2ac	7,39987038	0,012597
Gm8210	7,39987038	0,015199
9530085L11Rik	7,369159091	0,00034143
Gm26983	7,355380626	0,00066456
Gm42432	7,354361025	0,001626
Gm37696	7,34722777	0,010189
2810433D01Rik	7,344681856	0,050328
Gm43878	7,339592674	0,015673
Gm45251	7,314706612	0,014411
Gm36989	7,302548324	0,0011767

Gm10382	7,275770315	6,24E-05
Snord7	7,262166464	0,01336
Gm5577	7,252608633	0,0060478
B430305J03Rik	7,180578108	0,018351
Gzmm	7,141860726	0,003852
Hba-ps4	7,137901535	0,092088
Myc	7,017221976	0,002916
4930509H03Rik	6,969233537	0,0018666
Dpf1	6,957649484	9,37E-05
Gm8317	6,928293272	0,0004627
Mapkapk5	6,876621788	0,015295
5330426L24Rik	6,849981064	0,019062
Gm11516	6,742109226	0,043974
Gm42671	6,695538076	0,014271
Snord59a	6,664053579	0,0026474
RP24-282K24.4	6,652054553	0,0062396
Gm13383	6,641918407	0,018351
Mxd3	6,641918407	0,070054
mt-Tv	6,640537402	0,00017251
Gm45546	6,639156685	0,012624
2900060B14Rik	6,61939792	0,010797
Lsm7	6,611602545	3,04E-05
C230096K16Rik	6,596039325	2,66E-05
Gm42731	6,590098368	0,036154
RP23-350F7.3	6,586901608	0,00038116
Rpl32-ps	6,580968881	0,0073198
Gm43800	6,564567665	0,0060478
Dvl3	6,537323088	0,011278
Rpl12	6,515157249	6,88E-08
Rpl7a	6,488567476	9,58E-07
Snord87	6,476435495	3,26E-05
Gm44258	6,445087745	9,91E-06
Rn7s6	6,406782437	0,059931
RP23-205H11.3	6,39923745	0,0003536
B3gnt6	6,363409653	0,039562
Gm10657	6,336999932	0,01894
Rhov	6,326905291	0,00011525
Gm10827	6,316388897	8,47E-06
Ang	6,29715422	5,76E-07
Hes7	6,293227076	0,03559
Ccng2	6,263200223	3,07E-07
Ost4	6,252356322	3,32E-06
Rnf152	6,245426081	0,0066444
Ndrg1	6,228565782	7,79E-11
Sdhd	6,224249965	0,00084368
Gm23849	6,217781846	0,019642
Gm2011	6,189401881	0,00090837
Gm17108	6,133029966	0,0018082
Tnfsf9	6,110116975	0,0001793
Gm44639	6,031847692	0,0093486
RP23-40D21.1	6,02432666	0,00038116
Gm11810	6,014313203	0,00076539

Gadd45b	5,996414032	5,14E-08
Gm42908	5,991013149	0,011641
Txnip	5,970285818	5,75E-07
Stamos	5,955406558	0,0045057
Gm29228	5,912630004	0,0073306
Hbb-bh3	5,889723881	0,11072
Gm10717	5,848635176	0,27333
Dnali1	5,827996456	0,011256
Gm43182	5,823958196	0,070649
Gmnn	5,790147097	5,15E-07
Gm37785	5,78413009	0,0081271
Gm42895	5,75015163	0,016703
Gm15937	5,748956044	0,014288
Tap2	5,746963953	0,0028611
Hist1h2bp	5,727477953	0,059773
Gm14279	5,701335893	0,00011434
Gm45729	5,697780324	0,05183
Ntrk3	5,692253845	0,16088
Insig1	5,672166964	7,58E-08
Gm17541	5,66745095	0,079626
Gm10636	5,656854249	0,025427
Ccdc36	5,641191842	1,43E-05
Gm43331	5,629473501	0,02387
Ppp1r18os	5,592913431	0,049944
Sap30	5,589038062	9,51E-08
4930589O11Rik	5,578587974	0,014983
Gm9521	5,569315411	0,045255
Hist2h3c2	5,520504056	0,00044972
Clec3b	5,516296486	0,071661
Gm43920	5,515531818	0,049769
Tmcc3	5,490737725	0,07162
Snord71	5,470224337	0,060625
5033430I15Rik	5,467570812	0,025617
Gm14537	5,457347836	0,017111
Tsc22d3	5,448654454	3,75E-07
RP24-75M13.2	5,440729112	0,025721
Gm28727	5,429050863	9,73E-06
Eno1	5,426041192	5,97E-07
Rbmx	5,410642798	0,0024132
Gm27003	5,410642798	0,065836
Atp5l-ps1	5,399029127	7,26E-05
Gm2367	5,398654908	0,015272
Teddm2	5,359877395	0,066744
4833421G17Rik	5,35913441	0,0047073
Itga6	5,297089923	4,91E-07
Dkkl1	5,281691364	0,094492
A330069E16Rik	5,268893431	0,0019055
Polq	5,26305328	0,001205
2700029L08Rik	5,261958973	0,020717
4930412F12Rik	5,251392392	0,038293
Rpl30-ps1	5,238304771	0,0028968
Gm12940	5,22778569	0,082824

Ciart	5,20644996	2,44E-06
Gm16045	5,173712907	6,76E-05
Nsa2-ps2	5,173712907	0,11151
Gm7351	5,172995728	0,07162
BC055308	5,172637175	8,33E-05
Cep55	5,156170606	7,37E-05
Slc25a2	5,148670697	0,011854
Gm29438	5,148313831	0,0027466
Cxcl10	5,137975461	0,049158
Zscan21	5,135483105	1,97E-05
Mafk	5,12446005	3,80E-08
Wdfy2	5,121619234	9,37E-05
Atr	5,101070359	1,59E-05
1810026B05Rik	5,084126757	3,17E-08
Proscos	5,06232454	0,0008372
Tstd1	5,051458517	0,00016955
Rgmb	5,033981798	0,011048
Eno1b	5,026311209	0,00013527
RP23-356D13.9	5,010310454	0,027291
Rpl35a-ps4	5,004410031	0,0025259
Dnd1	4,998516556	0,10824
Hnrnpa1	4,996438164	0,043222
Pabpn1	4,977426458	7,26E-05
Gm12280	4,954021054	0,0066924
Cpne9	4,950931539	0,0191
D830025C05Rik	4,937908146	0,00073383
Gm7132	4,931751133	0,070199
Gm28659	4,930042214	0,020892
mt-Nd6	4,912985564	3,02E-06
Gm12604	4,902779913	0,0015936
Gm45833	4,893273766	0,0036024
Gm26664	4,892934602	0,019403
mt-Tp	4,875330423	7,62E-06
Gm7327	4,871276923	8,08E-05
Gm29759	4,869926505	0,092814
Gm43328	4,868576462	0,0015624
Gm28424	4,858799835	0,079626
Gm26610	4,857452877	0,013351
Ccl6	4,854087114	0,048061
RP24-122E11.4	4,849042841	0,1613
Arc	4,847698588	0,0021313
Cks2	4,837293248	0,09077
Vsig8	4,82456878	0,071827
Gm38299	4,821894212	0,0061551
Gm14439	4,817217287	0,17392
Rpl19-ps11	4,812544897	0,10231
Ccdc18	4,804878665	0,00019661
Ect2	4,785268911	0,00010499
Gad2	4,769374268	0,12048
Pbk	4,759466937	1,48E-06
Gm45051	4,743329204	0,03559
RP24-175C20.10	4,742014256	0,00028187

Adam9	4,737086447	0,0011814
Lockd	4,730524001	1,77E-06
Gm38248	4,718734499	0,079769
Gm5830	4,717426369	0,013527
Gm16439	4,715791717	0,057785
Pgam1	4,704691094	0,029837
0610039K10Rik	4,70175707	0,009164
Kif20b	4,698824876	1,81E-06
RP23-226H21.3	4,695569028	0,042263
Snrpf	4,693291276	0,0005292
Klf10	4,689714184	1,24E-07
Gm12090	4,668308814	0,072107
Hmgb1-ps5	4,661518524	0,021394
Gm6520	4,658288525	0,045297
Olfr912	4,648289745	0,43887
Gm44044	4,640563496	0,061582
Gm12758	4,637990934	0,018062
Gm38365	4,621624347	0,0016664
Cytip	4,620983698	0,13356
Gm45220	4,615861704	0,0073263
Mrps28	4,615541768	0,017744
Gm5362	4,60371971	0,039903
Phlda1	4,603081543	3,13E-05
Rnu11	4,601167573	0,004636
2700038G22Rik	4,598935614	0,022243
B930036N10Rik	4,595111918	0,020505
Gm16181	4,584295366	0,1604
Tnfrsf12a	4,577309993	3,47E-07
Gm15610	4,574772497	0,1451
Pfkfb3	4,564636572	6,78E-07
Hmgb2	4,546952711	1,10E-05
Gm45809	4,541597964	0,073597
Zfp36l2	4,541283175	1,91E-06
3110056K07Rik	4,540968409	0,0061032
Fam72a	4,539394902	0,018503
Gm45477	4,52933736	0,062167
4932416K20Rik	4,521182017	0,0063839
Gm28041	4,504603113	0,12107
1700031P21Rik	4,480003937	0,018712
Snord15a	4,477830752	0,0083162
2900093K20Rik	4,461720066	1,48E-06
Sez6	4,448749914	0,00019848
Notch1	4,441355335	0,09787
RP23-136K21.4	4,429365348	0,22801
Hist1h2ae	4,429058338	0,065739
Aif1	4,42875135	0,00050512
Gm43868	4,423535803	0,082841
Gm6969	4,420164311	0,10736
Gm44164	4,419857939	0,080193
Wfdc17	4,419551589	0,00020226
Gm13890	4,415877037	0,12207
Mrps18b	4,415570963	0,03267

Ttk	4,409759573	1,72E-06
Gm15796	4,409453923	0,087468
Olfr95	4,406093163	0,064258
Gm12643	4,405177037	0,065116
Rnaseh1	4,402124659	0,12704
Ltb	4,398769486	2,55E-06
4930579G24Rik	4,392675722	0,010189
Rpl29	4,391762385	0,079609
Gm6341	4,386590399	6,54E-07
Rpl9-ps7	4,377478218	0,035441
Slc25a25	4,372626131	2,85E-06
Gm7336	4,368082183	0,004179
Errfi1	4,365358081	2,98E-05
Gm22980	4,361426267	0,003957
Hyal1	4,344830814	6,75E-07
Gm15877	4,341519308	0,082339
Gm11759	4,327398556	0,027755
Depdc1a	4,325299399	0,148
L1cam	4,322302368	0,0025259
Pin4	4,319307415	0,099022
Gm37383	4,319008033	0,14562
Snord49b	4,312426894	4,49E-05
n-R5s151	4,310633779	0,046503
Gm14636	4,282638786	2,83E-05
Gm37472	4,279671316	0,03624
Lrrc17	4,274631334	0,00016041
Rplp1-ps1	4,272853934	0,076099
Fanci	4,270485218	0,071282
P4ha2	4,266638854	1,67E-05
Zwilch	4,258070983	0,00049873
Gm9442	4,25748073	0,17294
Frat2	4,255120536	2,39E-07
Gm42551	4,240986822	0,081
Amd2	4,237167027	0,0069134
Btf3	4,234231064	0,0012251
Gm36936	4,218119595	0,17579
Gm30074	4,217827227	0,12519
Gm26830	4,212276091	0,20594
Zfp773	4,20673226	7,86E-05
Gm45185	4,20352601	0,011897
Gm2467	4,200031069	0,12978
Icosl	4,199157788	0,0025545
Gm8885	4,188111931	0,11347
9330162G02Rik	4,185500065	0,38467
RP23-356P21.1	4,183469741	0,0005767
RP23-182J19.2	4,183179775	0,098566
A330035P11Rik	4,179122361	0,10241
4930522L14Rik	4,177384674	0,0066447
Smarca5-ps	4,15716497	0,088992
Gm11464	4,156300602	0,13851
Rps19-ps11	4,152557082	0,0039814
Dtd2	4,151693672	7,76E-05

Iqgap3	4,146516979	1,59E-05
Gm14130	4,142782272	0,060414
Klf11	4,125016814	8,86E-05
Gm43010	4,12415913	0,082107
Gfap	4,111600234	0,16562
Ncapd3	4,110175509	6,62E-07
RP23-23P9.3	4,100784697	0,067203
Kcnj2	4,096523242	0,0019055
Zfp36l1	4,094536078	6,54E-07
Gm15542	4,094536078	0,042102
Gm10575	4,09198257	0,24038
Gsg1	4,07330544	0,00028844
Suv39h2	4,070765172	0,00026291
Ckap2	4,066253052	5,36E-05
Fancd2	4,06597121	0,00028612
Gm43578	4,064280572	0,076359
Rps17	4,06315387	4,03E-05
Adamtsl1	4,052184885	0,48639
Alpk2	4,051342344	0,078728
Gm9381	4,049377098	0,14835
Gm43149	4,037885496	0,012604
Adcy6	4,03760562	0,00015386
Arhgap26	4,035926777	0,0010081
Cit	4,034248631	0,00015476
Gm28555	4,015000034	4,11E-06
Rad51ap1	4,015000034	0,00038121
Tmem240	4,008048594	0,013184
Gm42666	3,995843278	0,013937
H2-DMb2	3,991967569	0,026629
Gm29019	3,98367513	0,0018388
Tspan15	3,982570774	0,16413
Rps19-ps8	3,979259542	0,1191
Snord110	3,977053584	0,12737
2810454H06Rik	3,971544037	0,014231
Abcg4	3,957803538	0,079769
Gm38319	3,950951126	0,1194
Tnfrsf17	3,945477739	0,0013254
Atf3	3,941650881	3,64E-06
Gm12981	3,941377676	0,18342
Gm43817	3,940011935	0,0031888
Gm3699	3,935917552	0,066781
4931428F04Rik	3,930464992	0,010459
Rny1	3,918767553	0,00039928
Gm17034	3,913067509	0,18884
Cenpk	3,912796285	0,0004325
Cd274	3,909001125	0,00066456
Gm15216	3,904127043	0,055207
C730045M19Rik	3,903585854	0,023522
Gm45153	3,894937019	0,0063448
Gm38297	3,894937019	0,080031
H2-T23	3,890350122	0,0043451
Vwa1	3,888732507	0,071661

Fcrl5	3,886576734	0,26484
Gm3550	3,884422155	0,0005022
Egln3	3,879847641	0,076699
Rpl17-ps8	3,87608444	8,36E-05
D130051D11Rik	3,870983076	0,00061167
Ddah2	3,870983076	0,0051158
Gm23344	3,862406476	0,051002
Gm19726	3,862138763	0,19659
Bc1-ps1	3,858392738	0,15238
G2e3	3,855184752	5,95E-07
Ddit4	3,85491754	4,13E-06
Crip1	3,852513467	6,47E-09
Cdkn2c	3,848510011	9,50E-05
Gm42851	3,843444937	0,10345
Gm13935	3,839717044	0,14152
Gm43684	3,838120483	0,34351
Bub1	3,837854454	1,40E-05
Gm15441	3,837056477	0,1383
Gm37531	3,835992767	0,082781
Gm5100	3,834929351	0,11996
Cfh	3,823516235	4,22E-06
Gm15564	3,819278171	0,011024
Gm26759	3,813722839	0,14992
Pask	3,81292988	0,090178
A130014A01Rik	3,810287874	0,00084152
B230317F23Rik	3,809759693	0,10068
Tmem107	3,806064472	0,00010147
Plscr4	3,80052836	0,31015
Selenbp1	3,796315776	7,97E-06
1700001P01Rik	3,788954985	0,079671
Gm26912	3,782394912	0,11485
5330406M23Rik	3,778726234	0,0042473
Gm34121	3,773753004	0,08695
Gm43581	3,762521954	0,00037789
Gm9506	3,762000394	0,21295
Gm7634	3,761218189	0,070835
2810013P06Rik	3,754706139	0,00013841
Ezr	3,749764522	5,32E-08
Arrdc2	3,742493991	2,46E-05
Gm4968	3,731873283	0,21733
Basp1	3,730063006	9,07E-06
Usp50	3,729804467	0,017211
Kifc5b	3,725928527	0,0064826
Gm14005	3,724379279	0,0030261
H3f3a	3,723863007	3,18E-06
Ank3	3,716385088	0,36245
Gm12428	3,716127497	0,080794
Gm26549	3,71535483	0,17484
0610005C13Rik	3,711236654	0,1394
Gm15728	3,708151017	0,0032507
Rac3	3,703270672	0,12207
Gm44090	3,699422306	0,0042691

Gm24920	3,69685895	0,044022
Gm28578	3,695834105	0,019678
Hsd3b7	3,694553449	0,18582
Gm8337	3,692761275	0,059541
Gm15950	3,684324159	0,0039912
Dlgap5	3,684324159	0,0063839
Gm20492	3,681771265	0,11802
Gm5312	3,680495481	0,039235
Gm10036	3,67922014	0,00017535
Selenop	3,667761934	0,0069435
Gm16380	3,665474579	0,14041
Slc2a1	3,664458434	9,58E-07
Rpl35a-ps5	3,657606823	0,0011432
Ptgs2os	3,657353306	0,12667
Gm3940	3,654565776	0,064229
Gm7114	3,654059181	0,031472
H2-T10	3,651527258	0,28066
RP23-149L23.1	3,65051498	0,068887
Oaz1	3,642426852	0,00016058
RP24-324J2.1	3,640155296	0,12119
Tob1	3,639398426	0,00040604
Gm6649	3,638137325	0,1265
Ly86	3,637380874	7,16E-07
Zic2	3,631083232	0,0020826
Gm44126	3,626807064	0,23903
Arf2	3,620527728	7,88E-09
S1pr1	3,612506041	0,00015403
Gm6136	3,61150458	0,0014018
Ifitm5	3,607251469	0,10296
Nek2	3,602254222	0,0071734
Gm42522	3,598760268	0,11297
Nadk2	3,59352569	5,36E-05
Gm10358	3,585066798	0,05507
RP24-323H7.5	3,584072944	0,25149
Rasgef1b	3,580100284	9,32E-06
Gm7784	3,57885973	0,22961
Gm15596	3,57315872	0,059946
Rpl39-ps	3,570930363	4,65E-05
H2-Q5	3,569692987	0,22484
H2-Q10	3,566725034	0,15586
Ftl1	3,563265542	0,017321
Gm25541	3,562030822	0,088573
Pmaip1	3,561537054	1,03E-05
Gm13092	3,56079653	0,031963
Rpl13a-ps1	3,556356619	6,61E-06
Rpl36-ps8	3,553399755	0,1714
Gm45203	3,5474934	0,025602
Gtse1	3,544543904	0,0002623
Rflnb	3,543806914	0,17185
Ifi213	3,541596861	1,40E-05
Gm6946	3,54036965	0,16754
D930030I03Rik	3,535220005	0,18821

Snhg20	3,533260206	3,02E-06
C730034F03Rik	3,533015307	5,84E-05
Gm13413	3,52689836	0,31031
Fancb	3,526409462	0,1409
Mtfr2	3,521524207	0,027587
1500015A07Rik	3,517133265	0,00070418
Hist1h2al	3,516889484	0,0097619
Tspan33	3,515670834	0,20675
Ptgs1	3,515427155	0,053486
Gm10263	3,515427155	0,19254
Dpep2	3,513965434	1,74E-05
Mir124-2hg	3,512504321	0,11059
Gm7363	3,510800456	0,14539
6330403N20Rik	3,509827191	0,14291
Cerkl	3,50375039	0,081149
Gm15503	3,499866761	0,0043856
Gm44777	3,491386322	0,19677
Gm13567	3,487999903	0,035466
Ccdc117	3,485824654	1,05E-06
Uhrf1	3,485583043	0,0057994
C330011M18Rik	3,47737824	0,2952
Gm44270	3,475450507	0,034998
Tk1	3,471598246	0,0072795
Gm37065	3,469433225	0,16089
Rps28	3,46486706	0,00011147
Gm9409	3,461746304	0,13948
Maff	3,459347636	4,25E-05
Atp5g1	3,457190256	7,95E-05
Luc7l3	3,445468099	3,17E-06
Ifitm1	3,442842063	0,12459
Gm15289	3,441649071	0,11099
Pank4	3,440933475	0,081751
Tpt1-ps3	3,438549228	0,053941
Gm14094	3,437357724	0,033302
Pde2a	3,437357724	0,16596
Gm11346	3,435452176	0,16515
Cd300lf	3,434975955	0,21057
Pea15a	3,434737869	6,83E-06
Rpl23a-ps2	3,434499799	0,10169
Pclaf	3,430455139	2,52E-06
Cbx2	3,429504147	0,0072601
Gm16585	3,424990532	0,049116
Tnnt1	3,424278399	0,17347
Gm5921	3,421431349	0,17388
Cdh23	3,416691524	0,1932
Gm43637	3,416217903	0,032523
Rps8-ps4	3,414560746	0,045393
Kifc1	3,41361416	0,0002928
Pigb	3,412431296	0,025582
Gm12240	3,409357769	0,22038
Sdc3	3,408885164	8,91E-08
Cox20-ps	3,407940152	7,85E-06

Map3k12	3,397797796	0,00033984
4833445I07Rik	3,396149574	0,14319
Gm12466	3,395443438	0,1113
4930447F24Rik	3,392620362	0,14888
AV356131	3,391209704	4,63E-06
Eno2	3,388390148	3,18E-06
Prdm10	3,381117141	0,00071671
Unc13a	3,37854015	0,00076779
Gm13408	3,373625894	0,01894
Rpa3	3,369652922	0,10994
Gm38043	3,367318066	0,01127
Gm42466	3,362420133	0,22285
Lzic	3,361022033	0,00030291
Cox7c	3,357296606	0,052065
Nfil3	3,356365895	0,0003536
Gm42798	3,350090339	0,054383
Gm6564	3,345681242	0,13228
Gm9920	3,344290102	0,12978
Igfbp4	3,339194201	3,58E-08
Crip2	3,336417892	0,0015705
RP24-550H10.3	3,33387497	0,0081661
C030034I22Rik	3,332719737	1,45E-05
Gadd45a	3,331564904	0,0010081
Tgif2	3,324183446	4,98E-05
Zmynd10	3,32211036	0,081751
RP23-43M12.2	3,313141912	0,00054519
A530017D24Rik	3,312912271	0,068642
Ypel2	3,311305226	0,0002623
Gm37733	3,304655768	0,039595
Gm10343	3,30351066	0,013527
Arsb	3,302137054	0,00015176
Kif11	3,301908175	0,0002228
Anln	3,29984898	0,00028748
Itgam	3,29984898	0,0002946
Rsrp1	3,297791069	3,05E-05
Rab5a	3,297105384	0,0048394
Rccd1	3,295734442	0,0025379
Gm5257	3,292081381	0,27565
RP23-325K4.10	3,2868372	3,27E-05
Gm26730	3,285926021	0,31886
Rps6-ps4	3,281373916	0,057592
RP23-288C18.3	3,280919053	0,0015953
Rpl19	3,278872947	6,55E-05
Vamp2	3,272742284	7,37E-05
9330020H09Rik	3,270927993	0,0024725
Cog3	3,265943878	0,078503
Gbe1	3,265717508	1,79E-05
A930029G22Rik	3,262323838	0,22894
RP24-351I17.3	3,259159595	0,23885
Hmgb1	3,257804431	0,0057573
9330151L19Rik	3,257352834	0,012512
6430710M23Rik	3,255547075	0,16851

RP23-454I20.1	3,253967857	0,030384
Ube2s	3,253967857	0,030542
Gm44283	3,253967857	0,32026
Gm26737	3,249910527	0,027369
Car7	3,249685268	0,042615
Itgax	3,246533284	0,0010457
Spsb2	3,244733522	0,0004225
Fbxo5	3,244733522	0,0024132
Gm24916	3,241361659	0,0011207
Gm12164	3,240912342	0,23666
Gm13215	3,23687129	0,0062631
Rps19-ps6	3,236422596	0,066751
Kif15	3,234852655	0,00040242
2900055J20Rik	3,23462844	0,028272
Psd2	3,232163099	0,14769
Hmmr	3,221203933	7,37E-05
Taco1os	3,217410462	0,20356
Lilrb4a	3,216295586	0,0010793
Lonrf3	3,212062578	1,59E-05
Gm44957	3,20916952	0,41181
Gm42728	3,208724665	0,19674
1700001G11Rik	3,20561241	0,1616
Gm9009	3,202725161	0,17192
Gm19566	3,202059243	0,034958
Zfp326	3,200284136	1,61E-06
Gm7266	3,198731725	0,05173
Gm6987	3,194743269	0,0037915
4930532G15Rik	3,190317482	0,19755
Gm18284	3,188548885	0,22429
Ska1	3,186118665	8,93E-06
RP23-134M7.3	3,185897827	0,19071
Gm44013	3,183028336	0,22801
Rps12-ps10	3,181925373	0,00084441
Tiparp	3,181704826	3,79E-05
Rps15a-ps6	3,179059459	0,0015913
Gm13456	3,174875458	0,0090767
Gm11604	3,173335373	0,1676
Gm14427	3,168060749	0,0060396
Gm12902	3,163014128	0,012903
Pole	3,159070207	0,12687
Mad2l1	3,158413366	0,0019205
Gm9320	3,151415544	0,0001993
Rpl27-ps3	3,151197112	0,0096321
E2f8	3,151197112	0,18794
Ccne2	3,150323536	0,11365
Fn1	3,149886839	0,0020777
Anxa2	3,14792245	5,09E-06
Gdap10	3,14792245	0,004603
Gm42633	3,146613538	0,30988
Rpl27a-ps1	3,14530517	0,080107
Senp3	3,13964186	0,00041401
Tmod1	3,13964186	0,0021653

Gm24276	3,138118866	5,13E-05
Map3k8	3,137683861	3,88E-05
Gm37334	3,134205987	0,30882
Nrxn3	3,13268563	0,095077
Gm12034	3,132034274	0,1614
Gm13622	3,130948981	0,18794
Rps11-ps3	3,130297986	0,14115
Gm26520	3,129647127	0,0001993
Gm8330	3,129430204	0,17185
Gm16754	3,128345814	0,00010068
Snord104	3,126178161	3,09E-05
Gm15453	3,123795478	0,011546
Zdhhc18	3,113635362	2,27E-05
Spc24	3,112556445	0,00080763
Rps4x	3,112556445	0,084586
Ccdc58	3,110184144	7,37E-05
Gm7701	3,10609079	0,15942
S100a3	3,101787817	0,11145
Msantd2	3,099638567	0,00096638
RP23-2N7.4	3,097920239	0,01785
Fth-ps3	3,09448644	0,0010076
Aarsd1	3,093414158	0,28224
Itga11	3,09127071	0,075896
Hist1h1e	3,089985354	2,76E-05
Dennd4c	3,089342876	0,0001458
Fam103a1	3,087630256	0,0054669
Gm37738	3,086774302	0,25818
8030462N17Rik	3,08656035	0,0063016
Nemp1	3,085704693	7,34E-05
Gm527	3,080362204	0,16094
9530078K11Rik	3,071620539	0,18596
Golga7	3,068428571	0,00061665
Rdh12	3,068003226	0,35377
Sgol1	3,06523992	0,0056463
Tma7-ps	3,064390173	0,0085532
Ormdl3	3,062691386	7,37E-05
H2-Q4	3,06099354	0,011851
Gm5445	3,057176827	0,020343
4930558J18Rik	3,054846734	0,36006
Pcgf5	3,052095278	0,044178
Ncapg	3,049769059	0,00037826
Gm6290	3,040692655	0,29577
Tma7	3,039849713	0,021801
E130201H02Rik	3,036059364	0,27204
Gm42819	3,035848928	0,014288
Gm42632	3,030172658	0,2567
Cldn11	3,026604156	4,69E-05
Rassf7	3,025974857	0,0097619
Lpl	3,023458968	6,98E-07
Rhob	3,022830323	0,020683
A830073O21Rik	3,022830323	0,49072
Mogat1	3,021782871	0,10613

Gm10175	3,016132901	0,00038273
Gm15785	3,014460865	0,26605
Hlx	3,013416314	0,14391
Gm4832	3,010702174	0,43467
Mcm7	3,010076181	6,43E-05
Neil3	3,009867545	0,076502
Gm26244	3,009450318	0,0056981
Gm12577	3,008407503	0,0074164
C130013H08Rik	3,00673975	0,33898
Wdhd1	3,005281225	0,00076539
Rps2-ps10	3,003615205	0,08855
8030453O22Rik	3,003407017	0,11575
Gm26129	2,998207016	0,23024
Gm12184	2,997999204	0,084139
Gm44884	2,996129538	0,45853
Bnip3l	2,995506575	0,00016495
Gm24339	2,995506575	0,023206
Gm12778	2,995091338	0,11151
Mki67	2,992393703	0,00012501
Riiad1	2,989905735	0,26896
Cdc7	2,989284066	0,021556
Tomm40l	2,988869692	0,00095157
Mafb	2,983074483	3,01E-05
H2-Ob	2,981214124	0,00014391
Itgb3bp	2,979561447	0,12323
Gm1862	2,978941929	0,092745
Il13ra2	2,977703279	0,27204
Nup205	2,976671465	0,0014574
Cox20	2,975640008	6,81E-05
Rpl10a-ps2	2,974608908	0,0025143
A730071L15Rik	2,972341746	0,30996
RP23-162P10.8	2,970693987	0,00054555
Tgfb1	2,969458767	3,05E-05
Gm10240	2,968635572	0,0056897
RP24-418P10.4	2,966989868	0,27721
4931440P22Rik	2,965345075	0,0077781
Sdc4	2,963701195	7,60E-06
Gm11343	2,963701195	0,24371
Tia1	2,962674232	0,0004225
Gm27043	2,962263547	0,41573
Gm12583	2,961031832	0,28848
Zfp101	2,958159822	0,01702
Hsp90aa1	2,956929814	2,08E-05
Chrnb1	2,953242857	0,047733
Jsrp1	2,953038161	0,02573
2410080I02Rik	2,948742819	0,019216
Gm15268	2,948538435	0,22931
Gm12882	2,943841506	0,012597
Rps12-ps26	2,940170869	0,060299
Lgr5	2,935690749	0,27666
Csrnp1	2,927562559	3,05E-05
Wdr62	2,925939624	0,20009

Rpl36-ps4	2,92553403	0,077469
Zfp367	2,924723013	0,00029442
1700084E18Rik	2,923506907	0,011158
Ptchd1	2,922493873	1,34E-05
Cdca2	2,920873747	0,00040682
Aunip	2,919861625	0,025721
Ccna2	2,91925452	0,00012861
Sp4	2,91258469	0,0014149
Tnfaip3	2,910163077	0,015912
Gm10916	2,909356321	0,40218
Gm11531	2,907541936	0,001103
Gm15829	2,906332975	0,20369
Gm7799	2,904721808	0,027898
Gm14813	2,903514019	0,26636
Mturn	2,898085182	0,01268
Gm8019	2,896679363	0,3089
4930404I05Rik	2,894471598	0,49401
Gm28404	2,892065045	0,042715
Arhgap39	2,891864589	0,021842
Rpl23a-ps14	2,889259929	0,14892
Morf4l1	2,886457533	0,00046394
Tmsb10	2,883457983	0,019547
Alox8	2,88205926	0,29782
Gm12943	2,880860894	0,014701
Gm14034	2,878066645	0,39875
Gm14326	2,87686994	0,5025
Gm6576	2,876471149	0,35149
RP23-356D13.11	2,875873066	0,34224
4933433G15Rik	2,869899069	0,15114
Mgst3	2,868506923	0,026494
Gm43447	2,866916726	0,68222
Gm5525	2,865128808	0,015685
Gm12230	2,864334536	0,06789
Gm29666	2,864136002	0,30767
Hoxb8	2,860961335	0,00018814
Pole2	2,859573524	0,093675
Snord72	2,855414131	0,095302
Bard1	2,854820426	0,035725
Higd1a	2,852249131	0,0039121
Gm16630	2,850865546	0,32429
Spdl1	2,848692699	0,0050344
E230032D23Rik	2,846916146	0,011851
Gm43059	2,846718819	0,32707
Fam174a	2,844746308	2,42E-05
Rps6-ps3	2,842578124	0,19699
Numb	2,841790103	0,00053285
RP23-58B7.2	2,839821007	0,002916
Hnrnpa3	2,838246712	0,00047419
H2-Q6	2,836673291	0,20489
Gm8254	2,832939908	0,1366
Gm43501	2,832939908	0,43206
Nup98	2,830976945	0,0062637

Rps12-ps4	2,829603679	0,0012493
Rpl28-ps1	2,829603679	0,0026716
Gm11631	2,827447036	0,38595
Hus1b	2,827055096	0,42574
3110031N09Rik	2,826467288	0,16782
Dgkh	2,821964815	0,01988
Ticrr	2,821378065	0,31975
Gm18943	2,82020493	0,47563
Tpi1	2,820009456	1,91E-06
Atad5	2,817860125	0,04299
Dtl	2,817664813	0,0013125
Ttc25	2,816493223	0,25294
Ube2t	2,813566378	0,0012975
Acrbp	2,812591439	0,024296
Kif21a	2,812591439	0,40471
Mmp9	2,812201558	0,043356
RP24-454N4.2	2,8108374	0,0003536
Pif1	2,810447763	0,003766
Krtcap3	2,810058179	0,19606
Gm11826	2,809863407	0,035261
Gm7846	2,80830572	0,19794
Gm16104	2,8075272	0,26683
Smc2	2,806943452	1,08E-05
Gm15720	2,806748896	0,25606
Gm26397	2,806748896	0,35255
Gm8304	2,806359825	0,038637
Gm42508	2,80577632	0,19139
1700003G18Rik	2,797038283	0,45959
Blm	2,796069071	0,0004244
Nat6	2,795487705	0,00066456
Gm14286	2,794131655	0,025944
Rpl30	2,793357065	0,022586
Rps12-ps23	2,79296985	0,32861
Gm5302	2,791228048	0,29891
Rps12-ps9	2,791034582	0,0039039
Polr2l	2,790067451	0,0028347
Lsp1	2,788907335	4,98E-05
BC028528	2,788520738	0,0059077
Gm21781	2,788134193	0,052468
Gm5045	2,784464694	0,27067
Raf1	2,781378336	0,0063448
Insig2	2,779643763	7,17E-05
Gm12834	2,777525198	0,3232
Gm7638	2,775408248	0,0063016
Gm4987	2,774061938	0,028933
H2-T22	2,771947628	0,0069832
Mettl7a1	2,771563381	0,015868
Tra2a	2,769067074	1,20E-05
Rps27rt	2,764464413	0,00024409
Gm5075	2,763314944	0,029217
Smtn	2,762931893	0,14769
Ppp2cb	2,762548896	0,18367

Tpt1	2,762357418	0,00014509
3110062M04Rik	2,761208824	4,81E-05
Gm7783	2,761208824	0,052793
Snhg11	2,759678109	0,4215
AW554918	2,75891307	0,0011794
Xaf1	2,758148243	0,26727
Gm7312	2,755664019	0,06216
Selenow	2,755473018	3,61E-05
Meg3	2,753372875	0,30532
Gm15459	2,751655768	0,28205
RP24-310D17.9	2,75032098	0,014049
RP23-413G8.2	2,75032098	0,093332
Hnrnph2	2,747653346	0,12107
Tbc1d31	2,7463205	0,0004244
Gm2076	2,745368863	0,38378
Tsc22d2	2,744798038	6,83E-06
Plk2	2,742706032	0,05507
Adgb	2,741945698	0,41042
Gm19967	2,740425662	0,032373
Gm29736	2,740235717	0,020117
Rc3h1	2,737388117	0,00013542
Rpl36-ps2	2,736629257	0,063796
Nuf2	2,734733028	0,033302
Gm13611	2,73321699	0,043536
Gm15779	2,73321699	0,12323
Fau	2,732080514	0,014586
RP24-183O8.6	2,731512452	0,3448
Gm43133	2,729052219	0,19032
Rbl1	2,726783201	0,0035333
RP24-511J14.2	2,726783201	0,088726
Asb10	2,725838333	0,019234
Gm14513	2,725082675	0,044093
Rps16	2,724138397	0,11121
Rpl10-ps2	2,723949581	0,088877
Gm12017	2,723949581	0,41618
Ccdc50-ps	2,722062137	0,0049109
Pmf1	2,721118906	0,00013634
Gm4617	2,720741705	0,01438
Rps12-ps19	2,718291172	0,33657
Tpx2	2,717160902	4,49E-05
Bloc1s1	2,716407649	0,28506
Gm12380	2,716031101	0,29917
Ip6k2	2,715842847	8,89E-05
Hist1h2bc	2,713020596	0,00013994
Mcm3	2,712080498	0,00038273
Gm15832	2,71170455	0,01091
A430010J10Rik	2,708886599	0,45791
Supt20	2,70869884	0,0003442
Aurkb	2,708323361	0,0042412
Gm37470	2,706446744	0,34351
9930104L06Rik	2,704571429	0,19802
Rpl31-ps13	2,703821666	0,005539

Arl4d	2,703072111	0,14178
Fam64a	2,690547916	0,0021658
RP23-426K2.3	2,688310912	0,21885
Col11a2	2,687751951	0,061033
Med22	2,686634379	0,00039646
Gatsl2	2,685889589	1,54E-05
Mxd1	2,684586703	0,0003536
Gm17249	2,681425183	0,2768
Gm8430	2,681239327	0,00072199
Kif4	2,680310242	0,040775
Clec12a	2,679010062	1,47E-05
Cenpw	2,67566964	0,00018814
Dnm3	2,675484183	0,25971
Slc13a3	2,67492789	0,22626
Socs1	2,672889136	0,061768
Zfp280d	2,671592557	0,00031595
Gm13464	2,66881629	0,34518
2010016I18Rik	2,66807644	0,06216
Car12	2,667336794	0,00086777
Gm26826	2,666782195	0,012624
Gm16238	2,665858119	0,24484
Ncapg2	2,665303827	0,0016633
Rassf1	2,663826278	3,31E-05
Gm6919	2,65718744	0,23214
Parp6	2,657003264	0,00051938
Rpl9	2,656450813	0,00049873
Piga	2,655162208	0,0001641
Top2a	2,653322427	3,88E-05
Pcdhgc4	2,651300141	0,33564
Gm6745	2,650014034	0,44012
Slbp	2,648912153	0,042485
Gm6472	2,648361385	0,018528
Gm26530	2,648177821	0,34836
4933439C10Rik	2,645425886	0,013401
Gm23751	2,645059178	0,11232
Pim1	2,641944207	6,46E-05
Gm6807	2,640113586	0,0060171
Gm5277	2,639015822	0,097505
Gm13360	2,635359903	0,47408
H2afz	2,633351308	0,0041079
2610528A11Rik	2,632438816	0,091374
Kctd6	2,63152664	0,006225
Depdc1b	2,631344243	0,36101
Zc3h12c	2,630250125	0,0024132
Mast4	2,630250125	0,60168
Tmx2	2,628245424	0,0087083
Gm16755	2,628063254	0,55324
RP23-70B19.5	2,624786357	0,071498
Bend3	2,624422509	0,19915
Tacc3	2,623331269	0,0035181
Pi16	2,62314944	0,0024165
RP24-93F20.12	2,618607805	0,39164

Gm6210	2,618244814	0,19755
A830008E24Rik	2,617881873	0,47437
Gm43178	2,617700422	0,26987
Rab26os	2,616067925	0,00085078
2310074N15Rik	2,612443797	0,36208
Abcc10	2,61153855	0,26223
Bsdc1	2,611357538	0,043459
Gm28791	2,609909895	0,079609
Gm45749	2,608643866	0,20078
Lin54	2,607559187	0,0035709
Ankrd55	2,607378451	0,22484
Slc22a13b-ps	2,606655633	0,48985
Gm28731	2,603405428	0,45807
Gm15421	2,60322498	0,19937
Gm42724	2,60070002	0,047733
Dedd2	2,597997423	7,86E-05
Gm14633	2,597637289	0,26737
Pcna-ps2	2,597457241	0,15326
Ldb1	2,596917171	0,079853
RP23-354J5.3	2,596737173	0,0193
Gpat3	2,595477534	0,25334
Rps19-ps12	2,594578166	0,079484
RP24-275P22.2	2,592960088	0,037541
Racgap1	2,592780364	0,00035607
Rpl11	2,590983809	0,022003
Gm8927	2,590983809	0,36208
Gm4963	2,590265535	0,52084
Plin2	2,58972696	8,00E-05
H2-K2	2,58972696	0,016454
Ift80	2,586677152	0,0035333
Trim59	2,585601607	0,022003
Cd74	2,583810026	0,00010699
E2f7	2,583451858	0,18521
Gm5822	2,581124981	0,39742
Gm29650	2,580588307	0,17829
Rps13-ps1	2,57790661	0,34248
Tslp	2,57629893	0,18392
Gm9844	2,575941804	0,26422
Gfod2	2,572729895	0,030999
Gm9769	2,572729895	0,45695
Snn	2,57166015	0,17724
Palld	2,571303666	0,45911
Nkapl	2,570412675	0,31528
Gstt2	2,569521992	0,085516
Svep1	2,568987731	0,30055
AK157302	2,567385612	0,36403
Cox4i2	2,567207661	0,047664
Brip1	2,566140211	0,0827
Rpl23a-ps3	2,564717636	0,018961
Gm10388	2,560632121	0,11625
Gm42576	2,560277167	0,1022
C530043K16Rik	2,559922262	0,023291

Rpl6	2,557262041	0,0015374
Rpl10-ps3	2,556553115	0,02704
C78859	2,554427518	0,43395
Zfp932	2,552834481	0,010762
Rpl12-ps1	2,551773007	0,15577
Gm17827	2,551596138	0,46836
Rps18-ps1	2,550888783	0,13587
Apoo-ps	2,548767896	0,44175
Dnajb6	2,547178388	3,85E-05
Wdr54	2,546295756	0,096515
Bvht	2,543649695	0,13801
Hsf2bp	2,542944543	0,04689
Hist1h1c	2,542415808	0,001483
mt-Co1	2,540830261	7,86E-05
Gm37902	2,53818988	0,27443
Gm12165	2,533268507	0,3114
Ndc80	2,531864155	0,023206
Gm37893	2,530986829	0,45281
Bloc1s6os	2,52993444	0,30642
Iscu	2,528707206	0,0053018
Rpl21-ps12	2,528181431	0,34611
Car11	2,527130209	0,030478
Socs4	2,526779898	0,0030261
Gm38022	2,525379142	0,33532
Slc25a38	2,525029075	0,0042533
Rpl21-ps6	2,524154118	0,01739
Smc4	2,522754818	7,06E-05
2700097O09Rik	2,522405114	0,055276
Gm8318	2,51925996	0,27984
Gm8268	2,518910741	0,12342
Spag5	2,51559557	0,02584
4921524J17Rik	2,515246858	0,0029136
Gm8394	2,515072521	0,21885
Tmem176a	2,514898195	0,00097281
Gm7856	2,513329809	0,46756
Gm5735	2,510718006	0,38216
Gm43096	2,508804409	0,12882
Cdc6	2,507935074	0,012167
Gm45358	2,506544765	0,34587
Cd9-ps	2,5058499	0,45013
Gm5841	2,503940013	0,46408
Prim1	2,503419387	0,041036
Gm26698	2,502725388	0,20279
Zeb2os	2,501337968	0,021083
Arhgap27os1	2,497180318	0,41343
Rnf24	2,496834159	0,0050434
Rps4x-ps	2,496315011	0,085694
Asf1b	2,493548044	0,026629
Mettl17	2,490611501	0,014349
Rdh13	2,490266253	0,00034115
Fam71f2	2,489921053	0,15586
Gm10501	2,48854073	0,19677

Camk2b	2,487333576	0,25754
Gm43482	2,486816403	0,037272
Cenpn	2,486644036	0,16844
Plau	2,486471681	0,0013066
Gm44552	2,484404352	0,19677
2810001G20Rik	2,48251081	0,18288
Eif4a2	2,482338742	0,013174
Gins2	2,480962622	0,024956
Gm11539	2,479587266	0,00017289
Rasl2-9	2,477525661	0,36353
Ska2	2,476667164	0,054203
Khk	2,475980582	0,00016273
Ppfia4	2,474436466	0,018351
Gm2383	2,473921975	0,16808
Zfyve26	2,473236153	0,0038928
Rpl27	2,472207777	0,050553
Rab11fip4os1	2,47169375	0,47053
Apol11b	2,470837274	0,7041
Calr-ps	2,470494767	7,08E-05
Gm45266	2,469981096	0,35591
Ppp1r37	2,469638707	0,0608
Cchcr1	2,468440722	0,25174
Pmp22	2,468098547	8,00E-05
Ier5	2,468098547	0,00031718
Gm10237	2,465191975	0,5126
BC051226	2,462630196	0,0088865
C130083A15Rik	2,462630196	0,14772
Birc5	2,461947505	0,00012193
Rpl21	2,461947505	0,0047073
Rps18-ps3	2,46160623	0,03596
9130230L23Rik	2,457855329	0,33236
Psmc3ip	2,456492782	0,16158
Gm22009	2,455982022	0,14324
Jun	2,455471368	0,0043637
C330027C09Rik	2,455130991	0,0084119
Sowahc	2,453599878	0,0026736
Rpl30-ps5	2,453089719	0,34672
Slc27a3	2,452239691	0,062167
Ercc6l	2,451899761	0,18341
Timm8a1	2,450880257	0,49225
Ywhah	2,450370664	0,0077246
Gramd4	2,450200823	0,019475
Ptgs2	2,44833335	0,0046811
Gm2531	2,44833335	0,043506
Gm37106	2,44748497	0,34351
Rnf19a	2,446806477	0,0023019
Gm11878	2,446467301	0,061768
Rnf130	2,44392498	0,15059
Mef2c	2,443416833	4,86E-05
Syne2	2,442570156	0,22597
Gm11470	2,44206229	0,10008
Gm38257	2,44206229	0,27691

S100a8	2,441893025	0,29367
Kntc1	2,440877682	0,28205
Gm16540	2,439693648	0,19674
Gm8624	2,439355458	0,2295
Snhg6	2,437665211	0,23871
Gm43153	2,437327303	0,26169
Gm42635	2,436820527	0,032028
D530018E20Rik	2,436313857	0,36386
Mettl21b	2,434963253	0,028705
Rpl30-ps9	2,433782088	0,34715
Rps27a	2,42990515	0,037023
Eldr	2,42990515	0,35111
B3gat3	2,428221451	0,001761
Gm13641	2,426875333	0,23184
Gm37851	2,42267352	0,4002
Rpph1	2,419988178	0,32026
Col4a6	2,418311352	0,40262
Arhgap19	2,417473374	0,52709
4930426I24Rik	2,416635687	0,58255
Gm12844	2,416300693	0,037864
Gm37906	2,416133214	0,10096
Bbc3	2,413455122	0,0013868
Erf	2,412618827	0,00024431
Abhd18	2,411782822	0,082824
Hcar2	2,411448501	0,19794
Gm37914	2,411281358	0,29899
Plxna3	2,408441697	0,34584
Gm45133	2,407607139	0,35997
Shcbp1	2,407106544	0,17451
Mdm4-ps	2,406439245	0,030918
C030037D09Rik	2,406439245	0,049116
Dpy19l3	2,405938892	0,41353
Nmrk1	2,405605381	0,050235
Sft2d1	2,405105201	0,5833
Plaur	2,404938498	0,061725
Rps6	2,403771897	0,45968
1810041H14Rik	2,403605286	0,51934
Gm27010	2,401440394	0,40506
Gm42511	2,401440394	0,44806
B330016D10Rik	2,401273945	0,13077
Klf4	2,399443763	0,00076863
Rps19-ps4	2,395953651	0,10348
Hist1h4i	2,395621524	0,0018403
Cped1	2,394459444	0,1999
Rpl6l	2,391308065	0,1356
Gm4673	2,389319858	0,092614
Gm37584	2,389319858	0,63848
Diaph3	2,389154248	0,065206
H2-Q7	2,387498787	0,0043629
Gm24924	2,386175243	0,26302
Rpl17-ps10	2,386009852	0,26981
Dnajb4	2,385679104	0,00018359

Hist3h2a	2,385183067	0,0011814
Gm6640	2,384687134	0,24371
Tmed7	2,384521846	0,013571
Pde1b	2,384191304	0,0067037
Gm7565	2,382704432	0,29375
Gm12770	2,380063393	0,36127
Gm7308	2,376601471	0,030814
Smagp	2,375777946	0,00035506
Gm10736	2,375283969	0,13016
Gm37352	2,375119332	0,25608
Gm7984	2,374790094	0,466
Arhgap15	2,373638118	0,0097923
Cebpb	2,372157827	0,1676
Rps7	2,371171479	0,068642
Gm23100	2,371171479	0,52948
Gm14138	2,370349836	0,17354
Gm12481	2,369856987	0,0028968
Ropn1l	2,369856987	0,15158
2700099C18Rik	2,369856987	0,43864
Pltp	2,367722491	0,00030874
Gm12924	2,366737988	0,56709
Plekha7	2,366573944	0,426
Stxbp3	2,365262	0,1459
Gapdh-ps14	2,365098059	0,4704
Sgol2a	2,362640295	0,004049
Dclre1a	2,362149049	0,15414
1110035H17Rik	2,360348687	0,21993
Rrm1	2,359040194	0,0012669
P4ha1	2,356915441	0,00013088
Kdm4b	2,356752078	0,036238
Gm13573	2,356588726	0,36006
Gm13038	2,355608852	0,18943
Rpl36-ps10	2,354139805	0,0042739
N4bp2	2,353487189	0,018183
Troap	2,353487189	0,084152
Rpl30-ps3	2,353324064	0,039447
Gm10051	2,353160949	0,40594
Rpl31-ps8	2,351856441	0,029653
Dram1	2,350878535	0,0098813
Adh5	2,350389735	0,0010081
Sep 02	2,349575293	0,052231
Rps23	2,347784514	0,023087
Lig1	2,347296357	8,39E-05
Eef1a1	2,343557188	0,1538
Gm5900	2,343069909	0,54073
Prr11	2,342258004	0,027291
Cdkal1	2,340797283	0,02747
Gm4784	2,340472802	0,15903
Carhsp1	2,339337473	0,00022245
Ppwd1	2,337878573	0,0031071
Zg16	2,336096709	0,45484
Gm15753	2,335610982	0,15586

Kdm3a	2,334801662	0,009777
Kif18a	2,334639831	0,021842
Eif1b	2,334154407	0,00010125
Ppp1r2	2,333345591	0,004636
Bub1b	2,332537055	0,0013245
Gm43309	2,332537055	0,015265
Rpl31	2,33124398	0,00030585
Crebrf	2,330920824	0,00036494
Prc1	2,330597712	0,00065964
Usp53	2,330597712	0,0025259
Clu	2,330274645	0,001294
Ncoa4	2,329144263	0,42905
Jund	2,328498576	0,00037066
Srfbp1	2,328337183	0,0031772
Ptpru	2,328337183	0,59869
Trmt112	2,327853069	0,0063004
5730508B09Rik	2,327369056	0,032028
Pgk1	2,32720774	0,033482
Rgs11	2,326401331	0,079716
Kpna4	2,323017464	0,0010793
Npm3-ps1	2,322051549	0,45484
Gm13992	2,321729667	0,54879
Hoxb6	2,321407829	0,30498
Rdm1	2,320442583	0,0032179
Hpse	2,319960111	0,05507
Rnft2	2,318674008	0,45484
Fign	2,318352594	0,52801
Trim3	2,314017861	0,087712
Tmem29	2,312574752	0,079716
Gm12166	2,312254183	0,016572
Bcl6b	2,309531142	0,6194
Gm43138	2,309050939	0,19677
Gm24876	2,305372694	0,42117
Cks1brt	2,304414115	0,56418
Nxt2	2,301859855	0,0051661
Gm13498	2,301700308	0,19677
2900052L18Rik	2,301062229	0,099239
Cystm1	2,299467807	0,11548
Gm6023	2,296759825	0,013413
Gm8618	2,2956457	0,02584
Fgd2	2,2956457	0,18865
Gm18889	2,294532116	0,029275
Trp53rkb	2,294373077	0,033251
Spink10	2,29326011	0,0071875
Dvl2	2,291512253	0,0010205
Rpl13-ps3	2,291512253	0,25334
Ift22	2,290718213	0,068697
Ttc21b	2,289765728	0,085984
Gm12038	2,289289634	0,091513
6820402A03Rik	2,289130958	0,59601
Gm5069	2,288972293	0,55324
Rps23-ps2	2,288654996	0,060072

Manba	2,288337743	0,1366
Stom	2,286435149	0,0050784
Mcm5	2,286435149	0,011897
Tmem64	2,284217455	0,00020846
RP23-159E10.1	2,283425942	0,62997
Parpbp	2,282634704	0,30478
Klf6	2,278840577	0,0037216
Tarbp2	2,278524684	0,23916
A530041M06Rik	2,277735145	0,22255
Gm16062	2,27710371	0,27809
Hspe1	2,27599912	0,00086777
Zfp87	2,275368167	0,0074164
Psenen	2,274422065	0,12352
Gm11989	2,274106785	0,47491
Gm23127	2,272688565	0,58635
Gm13340	2,271901048	0,41208
Mapre2	2,271586117	0,00042395
Gm3617	2,270956387	0,56937
Nr2c2ap	2,270012119	0,0043929
Gm4737	2,267181671	0,062278
RP23-246F14.1	2,267181671	0,57341
Gm13050	2,266553162	0,18943
Rpl18a-ps1	2,265767771	0,072401
Gm43628	2,26482566	0,29054
RP24-240E7.1	2,264197804	0,12342
Gm2000	2,263256347	0,018307
1110006O24Rik	2,261374606	0,67948
Per1	2,261061134	0,0050784
Gm11625	2,260277646	0,59844
Gm19353	2,259964327	0,0029179
Gm5881	2,259181218	0,03559
2010111I01Rik	2,258554927	0,0036399
Hacd2	2,256677095	0,2358
Trappc2	2,255269746	0,50864
Gm8894	2,254957121	0,49966
Gm7887	2,254488264	0,42012
Pold1	2,253707053	0,046964
Uba52	2,252926113	0,04587
Nrbp2	2,252769958	0,10402
Tpt1-ps5	2,252301556	0,44224
E230029C05Rik	2,251989343	0,31313
Gm10941	2,251521103	0,38458
Mcts2	2,251208998	0,11477
Gm9008	2,251052961	0,30018
Slc12a5	2,250584917	0,16235
Oaz2	2,250116969	0,0003595
RP24-370M23.1	2,249805059	0,48948
Arhgap11a	2,248869585	0,0083369
Gm10177	2,248557848	0,15167
Gm37204	2,247311328	0,41306
Gm6548	2,246844061	0,22723
Comp	2,245909819	0,38259

Ceacam16	2,243420406	0,30366
Hcfc1r1	2,242487588	0,00013499
Etv3	2,241710536	0,0054405
Gm16253	2,241710536	0,14511
Dut	2,240467812	0,0012155
Rpl36	2,240157239	0,034679
Pcif1	2,239846709	0,0017686
Rpl5	2,239380994	0,0077945
Icam4	2,239380994	0,092745
Rbm4b	2,238139562	0,036605
Ezh2	2,237829311	0,00080789
B230219D22Rik	2,236898818	0,0019958
Gm12174	2,235813731	0,33099
Wdr70	2,235038991	0,013696
Ccdc163	2,235038991	0,024848
Gm6382	2,234109658	0,63242
Bcat2	2,233954806	0,032602
4930520O04Rik	2,232716382	0,078433
Akr1b7	2,232716382	0,25104
1700120C14Rik	2,232097428	0,26058
Gm15501	2,231788015	0,024001
Gm23935	2,230396187	0,040084
Gm12479	2,228078403	0,48164
Gm37255	2,226380226	0,21002
Polg2	2,225917311	0,033205
Eif3m	2,223758315	0,066534
Gm7867	2,223604181	0,45133
E330034L11Rik	2,221909414	0,036238
Gm5586	2,221293455	0,22863
B4galt3	2,220677667	0,00096339
Samd9l	2,218369982	0,56867
Gm2830	2,217293884	0,095157
AA474408	2,217293884	0,35963
Gm15159	2,216371929	0,3449
Rps3a1	2,216218307	0,018939
Ankrd52	2,214222191	0,27016
Gm12933	2,214222191	0,4574
Ankdd1a	2,214068718	0,53708
Gm37486	2,212687941	0,54622
Tusc3	2,212534575	0,0037357
Sirpa	2,211614599	0,00090393
Rgs9bp	2,210082156	0,63914
Cfp	2,209316332	0,066744
Cdkn2d	2,209010077	0,0012892
Gm14040	2,208244625	0,67532
Gm5121	2,208091567	0,090178
Cd9	2,207326433	0,2398
Tex30	2,207020454	0,032041
Gm10762	2,206561564	0,29397
Orc1	2,204574147	0,29464
Gm28151	2,203199291	0,30783
Cox16	2,20106233	0,42051

Mcm6	2,200909769	0,0063448
4930427A07Rik	2,20045215	0,45246
RP24-232D3.1	2,198775029	0,48217
Gm6142	2,198470235	0,0063967
Gm43200	2,197860774	0,34294
Rasal1	2,197403789	0,43014
Metap1d	2,197099185	0,0182
Swap70	2,196946899	0,0035284
Gm10863	2,196946899	0,27483
F11r	2,196642359	0,090371
2610020C07Rik	2,196490105	0,61019
Vcpkmt	2,19527245	0,068938
Rpl13	2,194055469	0,0079885
Eme1	2,193903394	0,62867
Traip	2,193599276	0,14892
Necap1	2,192839164	0,0031812
Ndufa4l2	2,192687173	0,55272
Gm6285	2,192383223	0,066566
Gm23346	2,192383223	0,23842
A730011C13Rik	2,192231264	0,57323
Epha2	2,19177545	0,079484
Atp11b	2,190256753	0,0039912
C030015A19Rik	2,18995314	0,16897
Gm43813	2,189346041	0,25471
AI480526	2,188587403	0,23914
Gm44913	2,187525751	0,11047
Gm7899	2,186161527	0,18063
Sik2	2,182527754	0,035574
2900076A07Rik	2,182225212	0,079716
Dhx9	2,181922713	0,051332
Tmem170b	2,180713133	0,034993
Gm43696	2,180561983	0,18571
1500011K16Rik	2,180259714	0,049944
Gm45184	2,180259714	0,071079
Kdm4c	2,179957487	0,18287
H2afv	2,179051056	0,0050531
Nucb1	2,178748997	0,0005292
Rps27	2,178446979	0,04168
Mocs1	2,178446979	0,11858
Hip1r	2,178446979	0,34196
Hist2h2be	2,177994031	0,00655
Cenpx	2,177541177	0,028702
Pex5	2,177390246	0,20418
Calm3	2,176786629	0,0013254
Gm37219	2,176484883	0,50559
Polh	2,174524555	0,44737
Ppp1r35	2,173469725	0,33197
Kat2b	2,173319077	0,065206
Gm5124	2,172264832	0,57287
AC133103.1	2,170609194	0,021596
Gm12912	2,169857048	0,37187
Gtpbp2	2,169556263	0,12793

Pttg1	2,16925552	0,0086124
Rps8-ps3	2,16925552	0,25822
Dleu2	2,169105163	0,098994
Zfp949	2,168804482	0,11824
Gm12005	2,168804482	0,52619
Ndfip1	2,168654158	0,066326
Cenpf	2,16790269	0,0076834
Ero1l	2,167301703	0,0024819
Mb21d1	2,167001272	0,0004225
Csrp1	2,166550703	0,011849
Raly	2,165349645	0,027039
Adgre1	2,164299266	0,00062306
Gm8599	2,164299266	0,13618
1700020D05Rik	2,164299266	0,39434
Usp26	2,164299266	0,54338
Znrf3	2,164149253	0,15791
Gins3	2,163999251	0,0072601
Gm12816	2,163849259	0,12978
Slc16a10	2,162649699	0,0043065
Pfkp	2,162499801	0,0020277
Uimc1	2,161450804	0,0047297
Gon7	2,160252574	0,004704
9330175E14Rik	2,15995312	0,30532
Upf2	2,159803408	0,0036456
Pygl	2,158456473	0,085783
Ckap2l	2,157409437	0,034993
Gm9790	2,156512383	0,01179
Gm12944	2,1551675	0,36975
Rps15a-ps4	2,154420705	0,42597
Fam26f	2,153524893	0,46681
Gm20186	2,152927892	0,34171
Man1a	2,15248025	0,04929
Gm20091	2,1520327	0,59209
4921531C22Rik	2,151734386	0,36182
Gm9354	2,151436113	0,6043
Wdcp	2,150690611	0,068642
Pate2	2,150690611	0,31259
Map3k9	2,149200383	0,16897
Lmnb1	2,14860458	0,0086606
Gm10110	2,148306741	0,13312
Osbpl3	2,147115797	0,010189
Trmt13	2,146966976	0,017869
Izumo4	2,146371792	0,27691
Gm10076	2,145479327	0,00771
E130317F20Rik	2,144884556	0,27016
Gm42986	2,143844104	0,12025
Gemin4	2,14295269	0,32799
Snx13	2,141616263	0,035672
Lum	2,141467823	0,63602
Rpl26	2,141319393	0,02542
Rapgef5	2,141319393	0,093675
Gm7504	2,139094176	0,20021

Fcor	2,138352951	0,4823
Gm37399	2,137908339	0,34072
Ubb	2,13746382	0,004049
C3	2,13746382	0,57811
Fzd5	2,135982756	0,047664
Gm45248	2,135686666	0,33357
Gm13886	2,135390618	0,16518
Lbr	2,135242609	0,00066456
Hax1	2,134206834	0,44027
Glt1d1	2,133910991	0,41343
Gm2788	2,133171562	0,48101
Timeless	2,132875862	0,36101
Gm44953	2,131693472	0,68982
Emc10	2,130954811	0,010153
Gm11930	2,130659419	0,26925
Gm13204	2,130659419	0,4704
Umad1	2,129330659	0,17458
RP23-114G13.7	2,128445279	0,10928
Gm8508	2,128445279	0,51404
Mif	2,128002728	0,021747
Oas1b	2,127855231	0,45309
Tmed8	2,126823039	0,081884
Ska3	2,126675624	0,19618
Rab10os	2,126233441	0,0033485
Gm7353	2,125938702	0,53886
Dusp4	2,125791349	0,0046974
Gm15420	2,125202036	0,57008
Prelid1	2,124907441	0,0023588
Gm6457	2,124171132	0,2388
Gm11942	2,123876679	0,070564
Chchd6	2,122993568	0,023637
Cldnd1	2,122110823	0,055468
Skp2	2,121816656	0,03266
Zcwpw1	2,120934401	0,30204
Hist1h2aa	2,120346435	0,39317
Gm43061	2,119170991	0,44554
4930430E12Rik	2,116381923	0,20279
Scp2-ps2	2,115795218	0,38481
Dnajb1	2,115501927	0,03768
Rad51	2,113010593	0,21081
Snord65	2,112864136	0,34351
Pcsk7	2,112424823	0,051543
Rps13-ps5	2,112132	0,36082
Gm45223	2,111839216	0,057222
Gm8818	2,111107435	0,62854
Fbxo33	2,110083368	0,024283
H60b	2,108475123	0,0034633
Gm14239	2,10730626	0,20437
Mapk6	2,107160198	0,0042139
Gm15013	2,10540824	0,38176
Man2b2	2,104095228	0,10508
Cdc20	2,103949388	0,016454

Plekha1	2,103803558	0,0023594
Tmx4	2,103657739	0,00049873
Rps2-ps5	2,101180353	0,41747
Rpl3	2,100597863	0,13423
Rps6ka5	2,098560419	0,14682
Gm15007	2,098414963	0,64773
Gng5	2,097542438	0,099202
Rpsa-ps2	2,097251677	0,50853
Gm37678	2,09347545	0,48059
Rps12-ps1	2,09347545	0,56989
Gm13827	2,092750033	0,1366
Gm44836	2,092459936	0,64224
Gm8909	2,091010056	0,49072
Vezt	2,090285493	0,03266
Rasgrp4	2,09014061	0,27372
Mynn	2,089995738	0,031478
Gem	2,088837119	0,53258
Srsf1	2,087679143	0,028418
Gm44791	2,087534441	0,52999
Gm43148	2,087389749	0,47658
Rpl17	2,08666644	0,040534
Rpl27a-ps2	2,08666644	0,34351
Rpsa	2,086377187	0,0049735
Gm4374	2,086087974	0,2373
Gm8292	2,084931522	0,06172
Atp9a	2,08478701	0,01291
Gm5239	2,084209065	0,44806
Scel	2,084064603	0,188
G6pc3	2,083631279	0,021129
Arhgap27os2	2,083486858	0,046587
Grcc10	2,083342447	0,46533
Sgk1	2,082187517	0,19937
Gm7847	2,080744754	0,44377
RP24-84C23.4	2,079735416	0,048447
Pcna	2,07915887	0,038355
Lin52	2,078438414	0,36266
Fam76b	2,078294352	0,02172
Gm12267	2,078006259	0,36294
Leng8	2,075415221	0,16413
Gm37558	2,074264685	0,25674
AA465934	2,072827413	0,15595
Gm9835	2,070673371	0,37735
Akap10	2,069955854	0,097969
Itgal	2,068808345	0,091401
Ak4	2,067518158	0,0059086
Gm6525	2,067518158	0,44171
St6galnac6	2,067374854	0,008649
Spry2	2,06608556	0,33957
Gm10784	2,06608556	0,61019
Aoc2	2,064940197	0,14717
Rps13	2,064797071	0,050553
Cdca5	2,063652422	0,15114

Ccdc33	2,063652422	0,48059
Gm27605	2,063223342	0,25585
Gng8	2,062079565	0,65191
Pcgf1	2,06179372	0,29054
Nrap	2,060936422	0,57811
Tcf19	2,060936422	0,59404
5330438D12Rik	2,059365636	0,37084
Gt(ROSA)26Sor	2,058794738	0,021556
Psat1	2,058081337	0,00084757
Syt11	2,057796046	0,01268
Rpl18	2,057796046	0,018712
Gm37407	2,056227653	0,27555
B9d1	2,055800117	0,44326
Gm13815	2,054945312	0,29569
C330006A16Rik	2,054518042	0,014558
Calm1	2,054233245	0,00024409
Gm6085	2,053948488	0,0050344
9130024F11Rik	2,053094452	0,27939
C920021L13Rik	2,052525293	0,035083
Gm16580	2,051956291	0,020505
Cenpe	2,05124526	0,0042989
Rps24	2,050676613	0,0075506
Rab27a	2,050108123	0,06858
Rps3a2	2,049539792	0,55857
Gm5835	2,048971617	0,26751
Gm9294	2,048829598	0,36463
Gm26533	2,048687589	0,52909
Inafm1	2,047977691	0,015968
Gm8326	2,04740995	0,74017
Chka	2,047126138	0,033482
Esr1	2,044998805	0,64683
Gys1	2,044715327	0,0053968
Bco2	2,043581811	0,39113
Clcn2	2,043440165	0,68705
Cbx5	2,043015288	0,0051344
Pnn	2,041599669	0,003761
Gm6394	2,041599669	0,080303
Cd48	2,041458161	0,46245
Gm42786	2,041458161	0,74833
Coprs	2,040750768	0,028418
Lysmd3	2,04032645	0,14115
Vps51	2,039760829	0,038355
Gm7079	2,039054025	0,57941
2810408I11Rik	2,038771371	0,52372
Slc19a2	2,037923646	0,29096
Gm38375	2,037076274	0,61119
Tbc1d30	2,037076274	0,71308
Gm8228	2,035946992	0,44631
Gm6768	2,035241509	0,63672
Gpr137b-ps	2,034677298	0,039162
Gm5093	2,034395252	0,36754
Gm4859	2,034113244	0,2057

Tbx6	2,033972255	0,44292
St5	2,033690306	0,66877
Klkb1	2,032844694	0,50282
Gm19028	2,032703793	0,65447
Rmnd5a	2,032562902	0,19008
Cdc25c	2,032562902	0,53371
Gm44187	2,03242202	0,47351
B230217C12Rik	2,032281148	0,17573
Lnpep	2,032140286	0,084139
Gm43420	2,031999434	0,55544
Gm24009	2,028903151	0,57341
Rpl21-ps14	2,028621905	0,53069
Gm20072	2,028059529	0,12455
Gm11363	2,026794754	0,44292
Gm42559	2,025249989	0,65813
Gm15464	2,024828894	0,041837
Cenpp	2,024688549	0,25944
Gm43343	2,024688549	0,34742
Snrpc	2,02300516	0,22449
Gna12	2,022724732	0,060888
Gm10138	2,022163991	0,072107
Coa6	2,022163991	0,34948
Ulbp1	2,021743538	0,020419
Gm11970	2,021042976	0,05507
Gm1976	2,020902893	0,26507
St3gal4	2,020482702	0,055468
Gm17786	2,020062598	0,57854
Rps19-ps9	2,019502595	0,41474
Nexn	2,019082695	0,26128
Snx32	2,018103268	0,56638
Gm37962	2,016984504	0,63519
Klhl28	2,014748836	0,12186
Gm11249	2,014190307	0,44377
C630004M23Rik	2,012515647	0,69092
D030056L22Rik	2,0120972	0,016091
Gas2	2,0120972	0,57613
1700124L16Rik	2,011539405	0,25149
Med30	2,011539405	0,35255
Ctdspl2	2,010284933	0,041965
Dock1	2,010006268	0,21654
Mastl	2,009309773	0,23492
Sec24a	2,00861352	0,038746
4933404O12Rik	2,008474298	0,11767
Papd5	2,007500017	0,076359
Gm35931	2,007360873	0,41818
Raph1	2,006109006	0,16264
Ccdc15	2,005413862	0,14916
Rusc2	2,005413862	0,22014
Gm37303	2,004857921	0,63015
Cnnm2	2,004441065	0,18633
Tor1aip2	2,004024297	0,0057994
Nnt	2,002358089	0,018528

Rell1	2,000693267	0,019642
Kif24	2,000138634	0,5061
Sh3bgrl2	1,999861375	0,01247
Gm10335	1,999029829	0,39624
Rrh	1,998337138	0,61818
Ppia	1,996398881	0,34584
Gm14620	1,996260506	0,016702
Gm5384	1,99612214	0,45078
Pcbp4	1,995707101	0,12882
Suv39h1	1,995292149	0,092993
Rps15a-ps7	1,995153851	0,45485
Rpl34-ps1	1,994877283	0,66146
Egln1	1,994739013	0,0065599
Dis3	1,994600753	0,15903
Tcf20	1,994462503	0,016821
Gm19898	1,993633202	0,29647
Gm37482	1,99321868	0,65946
RP24-365N15.9	1,992804245	0,7377
Cnih1	1,991975634	0,016322
Kif14	1,99018149	0,11471
1810037I17Rik	1,989905611	0,12569
Gm14650	1,989767686	0,69949
Gm43006	1,989491865	0,48775
Ints8	1,988664631	0,14152
Ddx20	1,98783774	0,052658
Lnpk	1,986460354	0,11878
Gm5869	1,984808749	0,55903
Ap1s3	1,984396063	0,047543
5430434F05Rik	1,983295985	0,6043
Gm15210	1,982883613	0,01958
Nusap1	1,981097659	0,037288
Otud1	1,980273915	0,51836
Gm9938	1,979587723	0,5126
Snhg8	1,979313313	0,16409
Gm45873	1,977667651	0,65594
Gm11652	1,977256449	0,67532
Rassf3	1,976982362	0,087769
Ints4	1,976982362	0,18225
BC055324	1,975886394	0,045186
B930086L07Rik	1,974654157	0,14632
Rbm48	1,973559479	0,09547
Sertad1	1,973422687	0,10763
Gm10923	1,972465409	0,41224
Tmco3	1,972191986	0,11706
Cmtm4	1,972055289	0,060168
Gm26782	1,972055289	0,27806
Prkx	1,971918601	0,067129
Gm5944	1,971781923	0,66175
Dubr	1,971508595	0,14263
Gm13680	1,970962052	0,52102
Gm6451	1,970688838	0,33099
Gm10275	1,969323334	0,046793

Gm10131	1,968095189	0,34491
Gm43721	1,967549593	0,65429
Gpn2	1,967140495	0,44131
Nup153	1,967004148	0,076699
Rsrc2	1,96686781	0,0058497
Rps10-ps2	1,96577745	0,19157
Ifnar1	1,96536872	0,076142
3110045C21Rik	1,96482388	0,7085
Rpl17-ps4	1,964551516	0,57613
Rps6-ps1	1,964415349	0,19417
4930503L19Rik	1,964279191	0,043504
Btaf1	1,96346244	0,13008
2610203C22Rik	1,963054192	0,1394
Tial1	1,962918128	0,017954
Rnps1	1,962373967	0,14428
Gm43088	1,962373967	0,46821
Hdac1	1,961558008	0,40582
Rpl15	1,961422048	0,048648
Gm6450	1,961150156	0,79919
Gm43329	1,959927108	0,36403
Pth1r	1,959791261	0,63954
Ift172	1,959655424	0,075175
RP24-82M14.1	1,959655424	0,70525
RP24-225A16.3	1,959247967	0,19741
Metrn	1,958704823	0,079484
Gm38235	1,958433308	0,81031
Fam134c	1,958297565	0,1394
Asf1a	1,95626254	0,031127
Cd300c2	1,955584669	0,043459
Gm9385	1,95504254	0,01251
Gm7964	1,954365092	0,39862
Rbfox1	1,954094178	0,47708
Ptrh1	1,953281662	0,037804
Etv5	1,953281662	0,22909
Gm37598	1,952875531	0,58909
Gm6378	1,952604824	0,41153
Gm24507	1,952604824	0,73216
Sh2d5	1,951657644	0,20872
Pam16	1,951657644	0,46681
RP24-547N4.7	1,950170146	0,69934
Dtymk	1,949629519	0,036655
Spc25	1,949494386	0,09547
Gm8825	1,949494386	0,7377
Nudt19	1,948413656	0,082824
Nr4a1	1,948143567	0,42876
Sf3b1	1,947468509	0,0072795
Gm45380	1,946119095	0,38267
Rps15a	1,943153673	0,0096098
Fmr1	1,942884313	0,022363
0610009O20Rik	1,942614991	0,10844
Lamc1	1,942480344	0,050226
Gm7285	1,942480344	0,57722

Gm37519	1,942480344	0,71681
Gm45716	1,942076459	0,50125
C630043F03Rik	1,942076459	0,64141
Lncpint	1,938445266	0,31793
Rpl31-ps22	1,938176559	0,70847
Cbx4	1,937639257	0,077766
Zbtb34	1,937370662	0,21078
Wdr20	1,937102104	0,12597
Coro1a	1,936430872	0,11767
Kif20a	1,936296653	0,21411
4930579K19Rik	1,935894054	0,73638
Arntl	1,935759872	0,089069
Gm11605	1,934150422	0,73519
RP24-389J11.1	1,932810236	0,059337
Gm6419	1,93254231	0,75269
Gm44168	1,932274421	0,66877
Fam13b	1,931738755	0,08793
Syne3	1,931203238	0,044146
Id2	1,929330096	0,062183
Fam162a	1,928527877	0,013463
RP24-496O17.7	1,928527877	0,66186
1110008P14Rik	1,927993249	0,05995
Gm24951	1,927325173	0,041999
Gm8213	1,927191586	0,079318
Ankzf1	1,926390256	0,11517
Pdlim7	1,925722736	0,26193
Hn1l	1,92558926	0,267
Tmem132a	1,925188886	0,26183
Dock2	1,924388389	0,1127
Gm10086	1,924255005	0,033351
Tdp2	1,924255005	0,057143
Etfdh	1,923188268	0,12107
Gnas	1,922521857	0,041802
Mkrn2	1,922521857	0,11023
Fam83a	1,922255357	0,44494
RP24-316F13.7	1,922122121	0,72161
Spp1	1,921855677	0,19667
Osbpl1a	1,921189728	0,25515
Unc5b	1,921056566	0,56943
2410004B18Rik	1,920790269	0,096102
Epsti1	1,920257787	0,17947
A330023F24Rik	1,919725452	0,30622
Ncapd2	1,918794222	0,086213
Anp32a	1,918661226	0,0076625
RP23-390D8.2	1,918262293	0,50282
Dcp2	1,917996383	0,013465
RP23-115A18.3	1,917996383	0,49459
Cln6	1,917331772	0,021046
Rpl18-ps1	1,916800248	0,065836
Gm26384	1,91666739	0,05214
Sptbn4	1,91666739	0,3512
Tbc1d10a	1,916534542	0,064161

Serpinf1	1,916003239	0,72296
Gm5614	1,915870436	0,39862
Gm43511	1,915870436	0,47556
Snora17	1,915737643	0,58126
Rasa4	1,915206561	0,03175
Pgf	1,915206561	0,4404
Pcnp	1,913348935	0,11497
Fis1	1,912818515	0,075193
Gm23639	1,912685934	0,70493
Polr2d	1,911890635	0,25975
Gm16046	1,911890635	0,55478
Abca1	1,911625609	0,25047
Mfsd7a	1,910565873	0,55857
Gm10689	1,910168623	0,081751
Gm15575	1,910168623	0,59113
Hs3st3b1	1,910168623	0,61019
Fdps	1,910036225	0,12394
RP24-366E11.4	1,909771456	0,40182
Gm36964	1,908977369	0,39434
Mpc1	1,908315883	0,73379
Tpra1	1,908183613	0,05162
Rgs14	1,90646494	0,23685
Rpl21-ps1	1,906332798	0,86949
Gm6266	1,905672228	0,69011
Gm44093	1,905408064	0,84036
Gm12444	1,905275996	0,63554
Rpl14-ps1	1,904615792	0,03734
Gm21057	1,904615792	0,62417
Gm12504	1,904087794	0,57808
Gm9165	1,904087794	0,61019
Nfatc2	1,903559942	0,096295
Rad52	1,902768438	0,10241
Gm12468	1,902636553	0,72663
Fgfr1op	1,901449996	0,025892
Lmf2	1,901449996	0,096469
Fem1c	1,901318202	0,036739
Rnf7	1,900791118	0,059873
Npm3	1,900791118	0,1762
Rpl9-ps6	1,90026418	0,089487
Gm561	1,899869072	0,25897
Cox19	1,899737388	0,072427
Intu	1,899079103	0,49236
Gigyf1	1,898947474	0,1394
Espl1	1,898157888	0,28292
Ccnj	1,898157888	0,41475
Entpd1	1,897894766	0,38146
Rpsa-ps12	1,896974125	0,35434
Gm12693	1,896842641	0,23068
Plekhh3	1,896711166	0,16218
Gm15446	1,896316797	0,44171
A430035B10Rik	1,896185359	0,82231
Gm8667	1,895134182	0,62867

Clec10a	1,894608812	0,14812
Spns1	1,894214881	0,080765
Gm17690	1,893033576	0,50189
Gm43775	1,892771164	0,35527
Tspan32	1,892639972	0,82297
Gm12248	1,892508789	0,73628
Triobp	1,892246449	0,27691
Gm10327	1,892246449	0,56795
Pfdn5	1,892115293	0,0070043
Gm19196	1,892115293	0,35037
Pou6f2	1,892115293	0,95632
Gins1	1,89172188	0,47197
Fiz1	1,89145965	0,22051
Ddx43	1,890804234	0,123
2810403A07Rik	1,890673177	0,089069
Plk4	1,890673177	0,27428
Mxi1	1,890542131	0,016244
Cmtm7	1,888708427	0,080965
Uevld	1,887661395	0,20391
Rpl39	1,887138097	0,18858
Nectin4	1,886745718	0,73767
Fam126b	1,885961206	0,037457
Mbd4	1,885830485	0,14906
Gm2214	1,885699774	0,45092
Gm5745	1,885438379	0,41198
Snord55	1,885438379	0,65106
Gm6159	1,884785049	0,1048
Klf2	1,884785049	0,30366
Ssfa2	1,884131945	0,082558
Gm11686	1,884131945	0,46421
Clic4	1,883479068	0,041213
Pabpc1l	1,883479068	0,075781
Zfand5	1,882826417	0,036739
Nck1	1,882304459	0,089348
Sp140	1,882043534	0,15362
Gm6305	1,881652215	0,59869
Pkmyt1	1,881521793	0,58126
Epm2a	1,880218074	0,27333
Creld2	1,879957438	0,13777
H2-DMb1	1,879696839	0,26925
Nop56	1,879436276	0,049904
Mettl23	1,879436276	0,28872
Gm17150	1,879045498	0,71151
Socs2	1,878394384	0,73803
Nop10	1,878134001	0,016327
Matr3-ps2	1,876312332	0,56748
C920009B18Rik	1,876182281	0,18797
Tet2	1,875662165	0,1224
Cenpu	1,875142193	0,51987
Dennd4a	1,875012222	0,037381
Gm8172	1,87345328	0,64254
Copz2	1,873193582	0,52477

Gm14292	1,872544495	0,53557
Pabpc4	1,872284923	0,054164
Gm37677	1,872155151	0,61162
Fbl	1,871636151	0,11755
Igf1	1,871506424	0,036433
Adamts10	1,870857921	0,16413
Gm11675	1,870209644	0,38176
Scarna17	1,86904331	0,56419
Tnfrsf14	1,868654694	0,77396
Rpl23a-ps5	1,868136664	0,4695
Topbp1	1,866971624	0,084152
Gpcpd1	1,86684222	0,15342
D8Ertd738e	1,866454061	0,029228
Gm7561	1,866324693	0,57613
Gm12276	1,866195333	0,75791
Ccsap	1,866065983	0,39498
Rpl31-ps1	1,865548671	0,35434
Mcm10	1,864643721	0,53678
4833417C18Rik	1,863351696	0,56748
Gm23502	1,863222543	0,76744
Tcte2	1,863093399	0,56877
Gm7600	1,863093399	0,6881
Kpna3	1,862964264	0,030892
Dalrd3	1,861673403	0,22723
Gm6204	1,861286319	0,11234
Ahdc1	1,861286319	0,1648
Amn1	1,86012555	0,18571
Lsm12	1,859738788	0,10817
Cep44	1,85922323	0,18762
Sec22c	1,858836656	0,27417
Ubald2	1,858707816	0,16689
Gm4525	1,858063748	0,41976
Cntrob	1,857162429	0,35149
Lyst	1,857033705	0,11744
Ugcg	1,856904989	0,16841
9130604C24Rik	1,856776283	0,72696
Gm37522	1,856261547	0,76364
Btbd1	1,856132885	0,065555
Nsa2	1,854075509	0,354
Rbx1	1,853946998	0,017665
Gtf2h5	1,853818497	0,012019
Gm15772	1,853690005	0,15059
Usp1	1,853433046	0,04299
Bcl6	1,85240557	0,076632
Dpysl2	1,851635336	0,10374
Cbwd1	1,850737135	0,29226
RP23-55A6.4	1,850737135	0,73411
Cd47	1,850095829	0,019139
Stard4	1,84983937	0,053638
Vkorc1	1,849070203	0,083512
Kpnb1	1,84894204	0,11052
Rnf145	1,848557603	0,012831

Efr3b	1,847276724	0,19802
Gm6134	1,847276724	0,47164
Snrpg	1,846764621	0,069568
Rpl35a	1,846764621	0,34224
Rhoc	1,846124693	0,01093
Vps8	1,846124693	0,30544
2810002D19Rik	1,845612909	0,77859
Nacc1	1,845229164	0,10919
Ilf2	1,844717629	0,090178
Taf5	1,84407841	0,31516
Map3k2	1,843183874	0,096216
Cops7a	1,843183874	0,25173
Ift57	1,843056119	0,047531
Cep128	1,843056119	0,50603
Gm9484	1,842928372	0,67701
Qk	1,842417475	0,037999
Snord35a	1,841651394	0,61179
Gm8250	1,841396105	0,82115
Gm4997	1,840758036	0,039916
Rps24-ps3	1,840120189	0,079196
Snx25	1,840120189	0,52801
Gm43524	1,840120189	0,66832
Camta1	1,839865112	0,18712
Macrod2	1,839355063	0,40722
Gm43466	1,839227573	0,82231
Stil	1,838462819	0,45527
Prdx4	1,836934265	0,054281
Cmpk1	1,836934265	0,092533
Clspn	1,836425029	0,29801
Ovgp1	1,836043195	0,50178
Lsm14a	1,835915934	0,061641
Mrpl42	1,835534206	0,042745
Neurl1b	1,833753857	0,070212
Gm7117	1,833118436	0,46492
Ilf3	1,832356222	0,19674
Anxa1	1,831467373	0,50806
Capza1	1,830578955	0,37341
5430421F17Rik	1,829690968	0,82752
Gm28187	1,829564148	0,59557
Arrdc4	1,829310534	0,043869
Papd4	1,829310534	0,51898
Gm16177	1,828930179	0,71425
Fam46a	1,828676653	0,15844
Gm9392	1,828423162	0,43874
Zfand2a	1,827536221	0,089069
Rad9a	1,827536221	0,28416
Clic1	1,827282888	0,038326
Gm17994	1,827282888	0,6744
Phip	1,826902955	0,025257
Rnf26	1,826776328	0,32026
Gm11281	1,826523101	0,7647
Gm13009	1,826269908	0,50402

Gm25517	1,826269908	0,53579
Kif22	1,826143325	0,211
Rps3a3	1,825890186	0,54038
Atf6	1,825637081	0,057143
5031425F14Rik	1,825004473	0,8287
Haus3	1,824877978	0,22051
Prpf39	1,823739915	0,12346
Nr3c1	1,8229816	0,01894
Bst2	1,822349912	0,14514
Syap1	1,82184472	0,26169
Tspan13	1,821339667	0,018307
D830050J10Rik	1,820834755	0,4433
Sapcd1	1,820582351	0,061857
Gnpda1	1,820203811	0,73749
Pdgfb	1,819068663	0,41259
Tmem81	1,818816504	0,54282
Gm45407	1,818060236	0,59133
H2-M3	1,817934222	0,38595
Eda2r	1,817682221	0,11572
Apmap	1,817682221	0,18496
Trmt1l	1,817052369	0,20421
2610318N02Rik	1,81680049	0,33231
Pyroxd2	1,81680049	0,33598
Gm9169	1,81680049	0,40268
Dsn1	1,81680049	0,45013
Cds1	1,815415776	0,15586
Hace1	1,815415776	0,52656
Rpsa-ps11	1,815289946	0,5172
Phf20l1	1,814912506	0,10844
Vhl	1,814535145	0,094197
Sun2	1,814283614	0,017261
Gm45733	1,814283614	0,76491
Kctd13	1,813654941	0,14396
Cnksr3	1,813026485	0,37545
Mfsd13a	1,812900819	0,30726
Gm9143	1,812900819	0,74131
Lgmn	1,812775163	0,22952
1500002F19Rik	1,812398246	0,49225
Bcl2	1,812272625	0,14769
Zfp931	1,811644648	0,49635
Gm11944	1,811393518	0,75225
Cks1b	1,811142423	0,029653
Ube2c	1,811016888	0,10189
Gm9531	1,811016888	0,19677
Letm2	1,810389347	0,34351
Hoxb7	1,809511153	0,056933
Gltscr1	1,809260319	0,4077
4921536K21Rik	1,809260319	0,6332
4930529C04Rik	1,809134915	0,38043
Gm5619	1,808508025	0,56011
Gm13416	1,80825733	0,70779
Pak4	1,806753889	0,44535

Cdca8	1,80625302	0,037603
Ggps1	1,806002638	0,27545
Gm3511	1,80587746	0,28573
Gm11956	1,805627129	0,56955
Rps8	1,805376833	0,014417
5830454E08Rik	1,805251699	0,47468
Mir3091	1,805001455	0,85956
C130026I21Rik	1,804876346	0,1451
Rcbtb2	1,804000827	0,17616
Gm6433	1,804000827	0,27514
Gm13005	1,804000827	0,59322
Napsa	1,803375716	0,02419
Kif23	1,803125733	0,042296
Upf1	1,802625869	0,14316
Nme7	1,801876334	0,5543
Cnep1r1	1,800752616	0,24774
Enoph1	1,799130704	0,20779
Gm43773	1,798008698	0,55857
Ralb	1,797634851	0,081383
Ap2b1	1,796638307	0,11858
Lsm3	1,795766784	0,28262
Ccnl1	1,795144527	0,10016
Prrg4	1,795144527	0,31578
Gm7832	1,795020101	0,68306
Cmtm6	1,794895684	0,056262
Polr3g	1,794895684	0,2003
Cebpg	1,794646876	0,064918
Bend4	1,794522485	0,1351
Plp2	1,794149363	0,24226
Supt4a	1,793900659	0,29961
Gm7488	1,793900659	0,63203
Mob1b	1,793527666	0,079882
Pomc	1,792781914	0,55833
Cdkn1b	1,792657652	0,11011
Prr3	1,792533399	0,51284
Cfap20	1,792036472	0,52477
Gm37621	1,79178806	0,66755
Nlgn2	1,789926069	0,28079
Gm1840	1,789802005	0,35981
Ddx47	1,78967795	0,039266
Gm5599	1,78967795	0,53693
Trp53	1,788809804	0,28449
Rhoa	1,788561839	0,060072
Rpl37a	1,787942078	0,027329
Fgf13	1,787694234	0,45701
Gm16288	1,787198649	0,84262
Gm8919	1,787074774	0,8259
Gm43544	1,786950907	0,81622
BC049715	1,78657936	0,56867
Cox7a2l	1,786455528	0,039562
Eloc	1,786455528	0,29569
Usp28	1,786084084	0,39434

Dok2	1,785588945	0,072107
Ppdpf	1,784599078	0,57323
Dhcr7	1,784351698	0,14831
Phf13	1,783733396	0,23496
Gm5837	1,783733396	0,67741
Smox	1,783238908	0,068891
Tcp11l1	1,782373885	0,15903
Trip10	1,781756268	0,49243
Bbs5	1,781756268	0,62867
Gm12346	1,781632771	0,096613
Fsd1l	1,781509282	0,56913
Gm26652	1,781385801	0,40181
Anapc16	1,780891965	0,21152
Agtpbp1	1,780521677	0,04587
Cep135	1,779657972	0,42597
Maz	1,779287941	0,34948
Rps19-ps7	1,779287941	0,46689
Ybx1	1,778424835	0,047995
Crcp	1,77805506	0,14772
Gm26670	1,777931819	0,67704
Gm5687	1,777562147	0,10143
Apba3	1,776946197	0,49072
Chic2	1,776699878	0,089069
Foxj3	1,776699878	0,22014
Srsf2	1,776453592	0,1191
Fam229b	1,774607536	0,38481
Gm43774	1,77399261	0,54427
Gm15207	1,77399261	0,76939
Gm12341	1,773869651	0,49225
Snhg12	1,7737467	0,060627
Gm8550	1,773377898	0,70264
Chek1	1,773254981	0,40035
Gm14137	1,772763398	0,67017
Tapbp	1,772640524	0,1316
Gm15730	1,772640524	0,21993
Zfp511	1,772640524	0,26507
Vat1	1,771903457	0,038637
Usp35	1,771289468	0,35377
Fam20b	1,770675693	0,12704
Taf6	1,769939442	0,14263
Gm45855	1,769816764	0,87832
Gorasp1	1,769571432	0,29801
Jup	1,768958252	0,7895
Higd2a	1,768835641	0,040084
Rps19-ps5	1,768835641	0,57127
Ube2n	1,768100156	0,41573
Arl1	1,767977605	0,14247
Gm14541	1,767977605	0,88765
Cdc42ep4	1,766140357	0,26302
Gm5451	1,766140357	0,27424
Mgarp	1,765895535	0,34013
Ubfd1	1,765283628	0,31551

Aph1a	1,765161272	0,11094
R3hdm4	1,765038925	0,085039
Fam234b	1,764671934	0,42905
Ier3	1,764305018	0,059765
Gm45343	1,764060451	0,45602
Gm9800	1,76344918	0,37831
RP23-403D16.3	1,76344918	0,54724
Aplf	1,762227274	0,52581
Dtx3	1,76210513	0,22378
Mis18bp1	1,76210513	0,24542
Rnf185	1,761982994	0,30942
Hdac5	1,761616638	0,14576
Nup43	1,760029976	0,50901
Gm9517	1,759420101	0,65716
Uhrf2	1,759298152	0,037127
Gstcd	1,759298152	0,1858
Commd1	1,759298152	0,40218
Cd40	1,758932354	0,2505
Rnf2	1,758810438	0,097505
Oaz1-ps	1,757713578	0,69831
Gm9722	1,757226303	0,75412
Tmod3	1,756495645	0,068697
Rab1b	1,756373899	0,077598
Gm11808	1,756373899	0,099168
Gabpb1	1,756373899	0,41227
9230116N13Rik	1,75613043	0,70701
Efcab2	1,756008709	0,83459
Tmem171	1,755886996	0,40773
Gm14769	1,755521907	0,44156
Bnip3	1,755400228	0,11514
Gm10012	1,754913595	0,82817
Tnfsf12	1,754548709	0,30445
Rpl22-ps1	1,754183899	0,38706
Gm7434	1,753819165	0,71308
Lpp	1,753089923	0,27679
Rnd2	1,752968413	0,38595
Rgs12	1,75284691	0,68446
Tmem191c	1,751875195	0,1048
Calr	1,751753769	0,016454
Prr7	1,751753769	0,44375
Cyp2c55	1,751632351	0,47052
Gm6418	1,751632351	0,71527
Mis18a	1,751268147	0,38523
BC030336	1,751025386	0,15697
Blvrb	1,750904019	0,05162
Lgals1	1,74956953	0,024848
Gm43201	1,748599631	0,56464
Ccdc22	1,748236058	0,16432
RP23-65M10.2	1,74787256	0,67646
Cic	1,747509138	0,032721
Gm8662	1,747145792	0,45649
Adarb1	1,746056205	0,31615

Ifi202b	1,745572163	0,56435
Ccl4	1,744967299	0,1163
Psme1	1,744846351	0,11873
Prcc	1,744846351	0,51368
Gm8606	1,744483558	0,55374
Meis3	1,743637335	0,57069
Hinfp	1,743516479	0,21792
Gm5778	1,742791522	0,74808
Tmem267	1,742670725	0,77109
Med31	1,742308384	0,49554
Ccl3	1,741825381	0,17381
Exosc3	1,741101127	0,66175
Ybx1-ps2	1,740135923	0,7895
Fam133b	1,739894705	0,092814
RP23-269H21.1	1,739774109	0,12667
Sf3a2	1,739774109	0,27067
Sort1	1,739532942	0,25233
D130017N08Rik	1,739532942	0,83711
Gm15445	1,73893017	0,83732
Cyth3	1,738809641	0,27802
Slc25a22	1,738809641	0,52964
4932441J04Rik	1,73868912	0,54008
Klhl7	1,738568608	0,27832
Dck	1,73820712	0,23596
Gm10658	1,73820712	0,64182
Atg12	1,736641542	0,032078
Rpl41	1,736521171	0,16897
Gm7589	1,735678811	0,25243
Haus6	1,735197645	0,23526
Wdr34	1,735197645	0,49966
Gm45311	1,734476146	0,763
Gm5523	1,73411551	0,76373
Efhd2	1,733875127	0,055438
Gprasp1	1,733634778	0,35566
Naa20	1,733514615	0,18288
Rpsa-ps9	1,733394461	0,62646
St3gal6	1,733274316	0,12287
Stam	1,733274316	0,27796
Ccnb2	1,733154179	0,26661
Ptms	1,732313451	0,1067
Ndufb2	1,732073318	0,19848
Klhl15	1,731233115	0,37162
Pot1b	1,731233115	0,93062
Mcm2	1,730993131	0,27809
Timm10b	1,730633219	0,14263
F830208F22Rik	1,730513265	0,78282
Gm38190	1,729793714	0,76366
Rad54b	1,72955393	0,70618
Mien1	1,729434051	0,12587
R3hdm1	1,729074463	0,12665
Fam98c	1,729074463	0,18751
Iqsec2	1,728834779	0,16174

Kremen1	1,728834779	0,38176
Gm13532	1,727636857	0,61395
Ascc3	1,727397372	0,31638
Id3	1,727397372	0,38467
Tdrd7	1,727397372	0,68038
Gm8181	1,727397372	0,79674
Crry-ps	1,726559437	0,41668
Hsph1	1,726080799	0,13423
Ctnnbip1	1,725961161	0,10844
Pfn1	1,725721908	0,42024
Rnf181	1,725243503	0,12548
Glipr2	1,725123922	0,49957
Gm11263	1,72500435	0,87125
Cdca3	1,724645683	0,36131
D130020L05Rik	1,724526144	0,66529
Lsm5	1,72428709	0,68244
Acad11	1,723928572	0,59408
Gm6543	1,723689601	0,71367
Napepld	1,723450664	0,39907
Gm42478	1,722853465	0,77832
Gm14240	1,722853465	0,85956
Hcn2	1,722853465	0,86601
Ensa	1,72273405	0,34363
Gm13803	1,722256473	0,43186
Snx18	1,722137099	0,21885
Kiz	1,721659687	0,14524
Gm16020	1,721659687	0,65714
Smim11	1,721540355	0,14163
Rnf126	1,721540355	0,16488
4930448A20Rik	1,721182408	0,66473
Gm8121	1,721063109	0,68222
Arl5a	1,720943818	0,26644
Tcn2	1,720705261	0,17947
Rheb	1,720705261	0,24754
Cpeb2	1,720585995	0,34161
mt-Rnr2	1,720228247	0,29211
Gm7776	1,719989789	0,36101
Tmem256	1,718678862	0,18878
Gm9396	1,718678862	0,67279
E2f6	1,718083316	0,1984
Efemp2	1,718083316	0,66529
RP23-168F21.4	1,717726088	0,82464
Fignl1	1,717487977	0,60806
Coq10b	1,717130873	0,27372
Tmem216	1,716654849	0,40262
Gm42535	1,716535864	0,73377
Gm14494	1,715822125	0,75225
Gm8423	1,715703197	0,74834
Tmem50b	1,715108683	0,10402
Abhd8	1,714989805	0,34584
Dpm1	1,714870935	0,31358
Tmem33	1,714395538	0,095812

Gm20620	1,714395538	0,49966
Ube2f	1,713801476	0,1169
Rps15a-ps5	1,713682689	0,67145
Pts	1,713445138	0,41844
Atp11a	1,712970136	0,36618
Ripk3	1,712851407	0,48964
4933417C20Rik	1,712732685	0,78812
Zc3h7a	1,7121392	0,18858
Cracr2b	1,711190051	0,55478
RP23-88C11.5	1,710004356	0,8445
Gm14539	1,709885831	0,37759
Dpp7	1,708937932	0,25676
Samd8	1,70870104	0,14169
Zbtb37	1,70870104	0,45501
Cpeb4	1,70846418	0,28224
Tmem259	1,707517069	0,20357
Zfp938	1,70680708	0,57584
Gm37670	1,706688777	0,74589
Ptpn2	1,706452196	0,45812
Rpl26-ps4	1,706333918	0,78364
Klc2	1,705860887	0,40203
Arhgef39	1,70574265	0,34044
Mier1	1,705624421	0,11369
Spred3	1,705269784	0,55443
Ncaph2	1,705151587	0,17338
Nrp1	1,704560729	0,60168
Spty2d1	1,704324443	0,17073
Gsk3a	1,703970076	0,21824
Gm27477	1,703615782	0,62867
Mthfs	1,703497701	0,48016
Gm45222	1,703497701	0,49103
Gm8276	1,703261562	0,099202
Ogt	1,702199343	0,32939
Ckb	1,701963385	0,054203
Gm13461	1,701845418	0,67704
Hmgn5	1,701373632	0,18686
Tbcb	1,701255705	0,42004
Elavl1	1,699723409	0,17198
Tatdn2	1,699605597	0,26272
Gm43421	1,699487794	0,97254
Bicd2	1,699369998	0,2095
Me2	1,699369998	0,4497
Urm1	1,699016661	0,35963
Gm16379	1,699016661	0,37187
Fanca	1,699016661	0,62812
Chmp1b	1,698545659	0,138
Nhlrc3	1,698427929	0,2484
Sdf2l1	1,698192493	0,50461
Prelid3a	1,698074787	0,49864
Gm15393	1,697721719	0,83518
Gm13862	1,697015803	0,54879
Rpl31-ps11	1,697015803	0,76334

Gm14006	1,696662956	0,99511
Bcs1l	1,695604852	0,33956
Gm7424	1,695252298	0,65387
4933427D14Rik	1,694547409	0,29366
Gm20673	1,694429956	0,69136
Mad2l2	1,694077646	0,43618
Rpl18-ps2	1,693490625	0,42215
2210408I21Rik	1,693490625	0,78585
Gm37949	1,692903807	0,83962
Ahcy	1,692786468	0,59869
Gpsm1	1,69055857	0,40801
Zfp873	1,689855633	0,76018
Camk2g	1,689621385	0,28557
Knstrn	1,68938717	0,14574
Gpi1	1,688801775	0,2183
Aldh6a1	1,688450635	0,53876
Spata2	1,687631592	0,7041
Dnmt3l	1,687514619	0,44224
Dennd5a	1,68669603	0,19197
Rad54l	1,686579121	0,52522
Tmem168	1,685877839	0,25149
Bach1	1,685176847	0,32709
Pradc1	1,684476148	0,69225
Mnd1	1,684242646	0,37735
Ctps2	1,683425645	0,48775
Gm7332	1,682725673	0,62812
E230020A03Rik	1,682492414	0,84797
Patz1	1,682142586	0,25173
Kras	1,681909408	0,14151
Hbegf	1,681443148	0,47566
Smg5	1,681326603	0,35996
Dntt	1,681326603	0,7895
Mapk8ip3	1,680394536	0,21419
Gm4978	1,680278064	0,69151
Gm37009	1,679462986	0,74587
Banp	1,679113788	0,2995
Itpkb	1,678881029	0,34161
Atp8b2	1,677950318	0,39107
Lin9	1,677834015	0,26957
Prpf3	1,677601434	0,34285
Zbtb18	1,677485156	0,48578
Borcs8	1,677252623	0,34044
Ccr10	1,676903885	0,88635
Acadm	1,676671432	0,24752
Gm13777	1,675858104	0,67891
Vbp1	1,675741946	0,24287
Selenom	1,675277396	0,42125
Acat1	1,674580813	0,55478
Gm45568	1,674464744	0,45049
Pop5	1,674464744	0,52342
Jmjd7	1,674348683	0,52225
Flt3l	1,67423263	0,30018

Cbx6	1,674116585	0,089513
Ugdh	1,674116585	0,45078
Aldh2	1,673884519	0,11382
Gm24601	1,672956578	0,86613
Nenf	1,672376875	0,2138
Mmd	1,672029151	0,34298
Flna	1,671913258	0,29499
Cd302	1,671333918	0,46408
Rpl22l1	1,668902879	0,098285
Pnrc2	1,668902879	0,22925
Gm7380	1,668787203	0,74604
Rpl36a	1,668555876	0,45602
Cygb	1,668555876	0,7146
Klra2	1,668093318	0,61559
Llph	1,667746484	0,32991
Hikeshi	1,667284151	0,56919
Syce2	1,667168588	0,19095
Atf1	1,667168588	0,2609
Ss18l2	1,667053032	0,3645
Gm4742	1,666821946	0,79239
S100a11	1,666706414	0,25294
Pikfyve	1,666244369	0,26879
Fubp1	1,666013395	0,13777
Exoc3l	1,665551542	0,87704
2010015M23Rik	1,665436099	0,56967
Pmpcb	1,665205236	0,6203
Rpl31-ps10	1,66462822	0,75234
Gm10161	1,663936064	0,84447
Adap1	1,663474787	0,13914
Pnpt1	1,663359488	0,53555
Gm31274	1,663359488	0,71527
Gm5867	1,663244197	0,56943
C230037L18Rik	1,66266786	0,75415
Naa60	1,661861324	0,2884
Akt3	1,661400622	0,20507
Cdip1	1,661400622	0,32366
Hsd17b10	1,660709809	0,33171
Pbdc1	1,66036451	0,25294
Gm44567	1,660249426	0,73602
Abi2	1,659904223	0,33729
RP23-187B11.16	1,659674127	0,78774
Fam171a2	1,659444064	0,54007
Gm11110	1,659214032	0,42352
Zc3h3	1,659099028	0,81693
Mfsd14a	1,658869044	0,49103
Pkn2	1,658754064	0,17752
Pam	1,658639092	0,17185
Kdm6a	1,658409171	0,30214
Naga	1,658409171	0,47888
Rbm19	1,658409171	0,59932
Arid3b	1,658294223	0,64628
Toporsos	1,658179283	0,54073

Lmnb2	1,658064351	0,52253
Tufm	1,657949426	0,56206
Plekhf2	1,65783451	0,37262
2310034G01Rik	1,657604701	0,59376
Prcp	1,657489809	0,25515
Snhg9	1,656915466	0,40801
Gm5883	1,656341323	0,87032
Gm43360	1,655996932	0,6343
Ggct	1,655767378	0,2304
Arpp19	1,655078907	0,14514
Jpx	1,655078907	0,57613
Serpinb9	1,655078907	0,73411
Hoxa5	1,65496419	0,66858
Trmt112-ps2	1,654734779	0,72552
Zfp652os	1,654734779	0,73053
Atp7a	1,654620085	0,36294
Gm13196	1,654620085	0,76813
Pmm1	1,654046737	0,30375
Rnf32	1,654046737	0,73092
Gm12096	1,653932091	0,69085
0610037L13Rik	1,653702823	0,30149
Galnt10	1,653702823	0,71073
RP24-131G14.13	1,653588201	0,60295
Rps29	1,653358981	0,5616
Incenp	1,653015211	0,1237
Rnf144b	1,653015211	0,57722
Cbr2	1,652671512	0,49809
Ltbp4	1,652556961	0,7265
Acot1	1,652213357	0,73467
Tmem243	1,651068527	0,62438
Eif2s3x	1,650725233	0,67827
Actr1a	1,650153234	0,29464
Mrip-ps	1,650153234	0,73637
Tpd52-ps	1,650038858	0,73078
Csrp2	1,64981013	0,56958
Wdr83	1,649238448	0,35852
Sapcd2	1,649009831	0,65219
Polr2k	1,648781246	0,90893
5430416N02Rik	1,64832417	0,53932
RP23-324E2.11	1,648209921	0,86608
Crlf2	1,648095679	0,60743
Osgepl1	1,647638794	0,71812
Slc25a40	1,647524592	0,40725
Cox14	1,646040691	0,1344
Ern1	1,645812517	0,34351
Alox5ap	1,645470315	0,12587
Rpl38-ps1	1,645470315	0,47197
Fbxl12os	1,645128185	0,69679
Gla	1,645014157	0,33517
Brms1l	1,644558126	0,63071
Cadm1	1,644444137	0,13241
Ap3s1	1,644330157	0,76018

Gpr19	1,643874315	0,83883
Cnot4	1,643532517	0,32613
Hist1h4h	1,643532517	0,79661
B3galnt2	1,642963011	0,47292
Ppid	1,642279863	0,20988
Narf	1,641938397	0,30544
Rfxap	1,64182459	0,4387
Gm42547	1,64182459	0,66186
Rpl37	1,641710791	0,33956
Gm15975	1,641028165	0,73219
Gm4285	1,640914421	0,86554
Ypel5	1,640800686	0,17611
Acaa1b	1,640800686	0,7799
Pim3	1,640118438	0,2152
Gm11889	1,639095598	0,64353
Bsg	1,638981988	0,10924
Prkd2	1,638981988	0,46609
Rbm47	1,638868387	0,30733
Exoc6	1,638641207	0,68027
Slc25a30	1,638414059	0,85958
Ccdc9	1,637619288	0,2741
Rcn2	1,637165307	0,25294
Atxn2l	1,637165307	0,28715
Akr1b10	1,636711451	0,50517
Nup107	1,636711451	0,74145
Ascc1	1,63648457	0,304
Timm22	1,636371142	0,42676
Anapc5	1,636257721	0,23229
Gltscr2	1,636144308	0,16137
Pold4	1,635350639	0,43286
RP23-128C4.4	1,635237289	0,64109
5830432E09Rik	1,635010613	0,70144
Tnrc18	1,634330772	0,21055
Gm43484	1,634330772	0,62105
Pdia3	1,633990958	0,23775
Bloc1s2	1,633877702	0,56265
Sap18	1,633651214	0,58055
Gm15727	1,633311541	0,63027
Rpl18a	1,632858754	0,63954
Gm11353	1,632858754	0,64611
Melk	1,632632408	0,64773
Caap1	1,632519246	0,49983
Tuba1b	1,632292946	0,27473
Smg1	1,632066678	0,39742
Arf5	1,631953556	0,23869
Dusp1	1,631953556	0,50864
Gm6493	1,631953556	0,74535
Smg6	1,631614236	0,33885
Spin1	1,631388062	0,57935
Myadm	1,631161919	0,26661
Rpl36-ps3	1,63104886	0,63306
Gm1947	1,630935808	0,73078

Ptma	1,630370667	0,27111
Nectin3	1,629692755	0,35737
Gm6682	1,629579798	0,99899
Cenpl	1,629240971	0,31015
Gm36378	1,629240971	0,69949
Ift88	1,628902215	0,64045
Plekhj1	1,62856353	0,2775
Med14	1,628224915	0,57713
Txlng	1,628112059	0,63205
Ccnd3	1,627547896	0,28646
Gm2058	1,627322285	0,61713
Rassf2	1,626081987	0,30942
Hdac2	1,62596928	0,34742
3110083C13Rik	1,625405859	0,85641
Mllt10	1,625180546	0,3794
1700056N10Rik	1,625067901	0,94163
Atf7	1,624730013	0,41801
Sh3bp1	1,624392195	0,29801
Iffo1	1,624392195	0,59464
Atp11c	1,624279604	0,53206
Nrm	1,624054447	0,32991
Zgrf1	1,624054447	0,59234
1600029O15Rik	1,624054447	0,90441
Stac2	1,62394188	0,49031
Gm13349	1,623266642	0,92387
Arsa	1,623154129	0,50703
Arhgdia	1,622704158	0,15094
Ccl9	1,621804589	0,30612
Ints6l	1,620343853	0,53416
Vps29	1,620119241	0,36118
Exo1	1,620119241	0,95136
Erfe	1,620006947	0,86613
Tbpl1	1,619557848	0,22655
Gm10029	1,619557848	0,83099
Birc2	1,619108874	0,47769
Saraf	1,61899665	0,17669
Gm9645	1,618547831	0,67532
Primpol	1,618323468	0,58682
Notch4	1,618323468	0,79791
Got2-ps1	1,617986982	0,89646
Zscan2	1,61720212	0,88558
Cryz	1,616641738	0,30724
Lgals3	1,616081549	0,21055
Htr2b	1,616081549	0,86608
Gm5845	1,615633539	0,72661
Lsm2	1,615521555	0,32613
Lmf1	1,615185652	0,57443
Papss1	1,614849818	0,36067
Ppp1r15a	1,614849818	0,45449
H2-K1	1,613842737	0,32991
Fastkd1	1,613842737	0,62544
Gm37510	1,613619026	0,93325

Gng12	1,613507183	0,21068
Pola1	1,613507183	0,57323
Gm7658	1,613171698	0,41995
Bhlhe41	1,612724494	0,47241
Nabp1	1,612612712	0,64045
Ugp2	1,612389172	0,37265
Hdac11	1,612277414	0,56011
Klhl6	1,611607026	0,30783
mt-Nd5	1,611607026	0,40801
Sh2b1	1,611383625	0,33494
Nbeal1	1,611160255	0,30942
Spire1	1,611160255	0,41455
Ube2e2	1,611048582	0,57584
Rnf25	1,611048582	0,74726
Hsh2d	1,611048582	0,85575
Paip2	1,609374412	0,27453
Gm6913	1,609262862	0,92132
Hat1	1,607479122	0,28429
Gm3695	1,607144891	0,91725
Wee1	1,606699358	0,5019
Szrd1	1,606142614	0,27684
Gm9625	1,606142614	0,47617
Calcrl	1,605586064	0,59725
Isg20l2	1,605474777	0,6587
Gm26710	1,605140963	0,91895
1700061G19Rik	1,604918459	0,4721
Arf6	1,604251131	0,19576
Sqle	1,604139936	0,34395
Gm13758	1,604139936	0,81487
Lztr1	1,603917571	0,3798
Cacybp	1,603361792	0,35206
Ttc12	1,603250659	0,45507
Dhrs3	1,602917307	0,60818
Map3k1	1,602806205	0,39515
Hmgcs1	1,602584024	0,44748
Gm12857	1,602139755	0,46391
Gm12618	1,602139755	0,85341
Gm10443	1,602028707	0,36118
Eif4g2	1,601917667	0,32655
Rps13-ps2	1,601917667	0,42004
Gm5446	1,601917667	0,81596
Eif3s6-ps1	1,601806634	0,45527
Cbx1	1,601584592	0,21443
Ap2s1	1,601473583	0,42022
Gkap1	1,601473583	0,73628
Cenpi	1,601362581	0,74169
Sptssa	1,600031159	0,55993
Rrm2	1,600031159	0,57956
Gm6654	1,600031159	0,66473
Rps12-ps5	1,600031159	0,89936
5830408C22Rik	1,599476726	0,65768
Fkbp7	1,599033318	0,47403

Hnrnpd	1,598479232	0,26313
Abhd14b	1,597703833	0,63554
Lsm11	1,597150207	0,41688
Slc31a1	1,596818123	0,2549
Suco	1,596486109	0,29211
Hoxc6	1,596375453	0,54797
Anxa3	1,595158741	0,35833
Elac1	1,595048177	0,59316
Gm43848	1,594605997	0,6789
Srp9	1,594384953	0,47316
Nr4a3	1,594384953	0,74833
Gm5611	1,593942957	0,56706
Tgoln1	1,593722005	0,19823
Prkcg	1,593059332	0,73177
Nfix	1,592948914	0,47385
Gm11560	1,592948914	0,55439
Exd2	1,592396935	0,74208
Otub1	1,592176198	0,45517
Gm15417	1,592176198	0,50729
Gm15032	1,591734814	0,75478
Gm5070	1,591514168	0,85958
Camkmt	1,591514168	0,85982
Dgke	1,591293552	0,54658
Yipf4	1,591183256	0,72552
Slc16a1	1,590852413	0,44219
Gm36189	1,590852413	0,70365
Mrpl9	1,590742148	0,45484
Crnkl1	1,59063189	0,46689
Mpst	1,590521639	0,40119
Gm9701	1,590411396	0,59527
Eif2s3y	1,590301161	0,45302
Arl5b	1,590301161	0,5126
Etv4	1,590080714	0,9413
Terf2ip	1,589860297	0,47038
Gm13864	1,589860297	0,73628
Plekhh1	1,589860297	0,92317
Enho	1,589639911	0,92013
Pnp	1,589419556	0,63264
Glrx3	1,589309389	0,4433
Atp5g2	1,589309389	0,72452
Chd3os	1,588758672	0,64045
Pde7a	1,588318236	0,51154
Gm5865	1,588208146	0,64202
Ambp	1,58765781	0,88725
Cenph	1,587107665	0,53932
Satb2	1,586557711	0,61818
C330013E15Rik	1,586337782	0,83325
Gatm	1,58567818	0,61019
Selenoi	1,584908991	0,52619
Arfip1	1,584249983	0,46895
Pmvk	1,583701019	0,33219
Aldh9a1	1,583481487	0,32206

Fbxo21	1,583371732	0,43942
Pip4k2b	1,582932789	0,36316
Fam117b	1,582932789	0,5597
Slc8b1	1,582932789	0,62484
Lamtor3	1,581945612	0,56988
Gm20900	1,581945612	0,65946
Nbn	1,58161669	0,64737
Rrnad1	1,581507064	0,57814
Rpl3-ps2	1,580411227	0,7799
Gng2	1,579973105	0,44976
Polr1d	1,579754089	0,28176
Bcas2	1,579535104	0,58494
Sfi1	1,579097224	0,64773
Prdm15	1,57887833	0,65342
Litaf	1,578659466	0,25606
Gm5786	1,578440633	0,82394
Hoxb5	1,578003057	0,69192
Asph	1,577893682	0,3114
Ccser2	1,577893682	0,47351
Pik3ip1	1,577784314	0,36074
Cdk2ap2	1,577565602	0,4726
Ghdc	1,57734692	0,56218
Nfkbid	1,577128269	0,55094
Noc3l	1,577018954	0,6358
Fbxl3	1,576909647	0,70937
Rfwd2	1,576253965	0,57374
Rps13-ps7	1,575816996	0,87977
Stx3	1,575707772	0,37603
Hnrnph1	1,575270954	0,50005
Ahcyl2	1,575270954	0,66175
Ptger4	1,574070327	0,48726
Lmln	1,573197719	0,72844
Gm45884	1,572434584	0,69225
Gm26881	1,572216614	0,66453
Paip2b	1,571998673	0,62292
Fuca1	1,571889714	0,43547
Ccdc84	1,571562883	0,71308
Slc31a2	1,571453954	0,59408
Nrf1	1,571345033	0,50603
Pabpc1	1,571345033	0,57573
Flt1	1,57123612	0,62148
Tuba1a	1,570800541	0,55993
Scrn3	1,570691665	0,55857
Gm12522	1,570691665	0,77834
Tctn1	1,570038569	0,67023
4933421O10Rik	1,56982093	0,65481
Trmt1	1,569276966	0,40079
Gas5	1,569168196	0,29028
Ranbp9	1,56884193	0,55374
Tmem134	1,568298305	0,33326
Bmt2	1,568189603	0,40218
Gm23458	1,567972221	0,90073

Zfc3h1	1,567754869	0,51155
Rpl15-ps5	1,567754869	0,94437
Serf2	1,567646204	0,6248
Tle6	1,567102993	0,69011
Atpaf2	1,566777157	0,56419
Gm5776	1,566451389	0,82492
Fam53b	1,565908593	0,58256
Gm7287	1,565474491	0,80902
Erlin1	1,565257485	0,65716
Ppp2r2d	1,564498201	0,43376
Prr18	1,564498201	0,71305
Irx2	1,564281331	0,69504
Pacs1	1,563956082	0,60303
P3h3	1,563956082	0,79674
Acadvl	1,563522521	0,41343
Slc15a4	1,563089081	0,32429
Ccdc124	1,562980739	0,52142
Gm15427	1,562980739	0,52964
Gnpnat1	1,562980739	0,61395
Pla2g15	1,562439145	0,38327
Gm14056	1,562439145	0,81559
Tmem38b	1,562330849	0,52709
Rnf5	1,562006005	0,71926
Lacc1	1,56092368	0,72161
Tmem261	1,560599129	0,53678
Msh3	1,560599129	0,66754
Mipep	1,56049096	0,5679
Pfkfb2	1,56005836	0,80334
RP24-378K7.3	1,558977385	0,91732
Adi1	1,558869329	0,57884
Zfp607b	1,558869329	0,91155
Gm43655	1,558869329	0,91358
Eif2a	1,55876128	0,39547
Eri3	1,55876128	0,64098
Inpp5f	1,55876128	0,68766
Trappc6a	1,558545205	0,44803
Xk	1,558005148	0,94212
Smim7	1,557573237	0,37786
Nabp2	1,556925595	0,60532
Gm2735	1,556386099	0,84262
Rpl32	1,556170353	0,56108
Gm4032	1,55584679	0,8426
Pex13	1,555738951	0,69016
Aplp1	1,555631119	0,71689
1810010D01Rik	1,555631119	0,78254
2210013O21Rik	1,555415478	0,40802
Mboat7	1,555415478	0,5832
Gm14830	1,555415478	0,82521
Gm14048	1,555307668	0,76914
Rarg	1,555199866	0,64801
Ndrg3	1,554984285	0,54622
Mospd3	1,554876505	0,57621

Gm15198	1,554660968	0,63479
Ssna1	1,55379912	0,60806
Zfp655	1,553368375	0,54759
Pax3	1,553153048	0,68027
Kctd10	1,552830112	0,59316
Gm6368	1,552830112	0,71026
Gm43571	1,552399636	0,79117
Rpl36a-ps3	1,552292036	0,85958
Nptn	1,552076857	0,52709
Gm16199	1,55164659	0,9075
Usf1	1,551539042	0,78252
Pvr	1,551323967	0,73244
Gm16223	1,550893909	0,88685
Pdcd7	1,550249044	0,61045
Rpl30-ps11	1,550249044	0,81794
Gm12355	1,550141593	0,78391
Gm12176	1,550034149	0,87615
Eif4e	1,549926712	0,3601
Mybl2	1,549926712	0,8002
Adrb2	1,549604448	0,78196
Fam107b	1,54928225	0,77022
Yae1d1	1,549067489	0,73249
Btg1	1,548745403	0,60818
Anapc7	1,548530716	0,46278
Fam210a	1,547994129	0,5416
Slc17a7	1,547994129	0,93396
Slx4ip	1,547457728	0,75694
Rpl7-ps7	1,547135977	0,52801
Pole4	1,546921513	0,5635
Gm7407	1,546921513	0,70906
Gm22714	1,546599873	0,88264
Gtpbp10	1,546492675	0,83069
Rnf38	1,546063956	0,63451
Il27	1,54584964	0,87977
Atxn1l	1,545742494	0,69666
Jmjd6	1,545635355	0,45867
Erh	1,545635355	0,86248
Tubb2b	1,545635355	0,93728
Ccdc34	1,545421099	0,56054
Gltp	1,545313983	0,37755
Tmub1	1,545313983	0,56958
Eif4e3	1,544457316	0,56088
Fbxo2	1,544457316	0,68625
BC029214	1,544350266	0,71966
Gm11474	1,544243224	0,73515
Emilin2	1,543815128	0,41259
Gpkow	1,541997044	0,7796
Pxmp2	1,541676428	0,77549
Rps15-ps2	1,541249044	0,85971
Pcyt2	1,541035397	0,63203
Gm2796	1,540928584	0,59603
Lfng	1,54060819	0,72176

Gm21399	1,540287863	0,98351
Pcdhb17	1,540181102	0,85249
4930402H24Rik	1,539860864	0,64446
Atg16l1	1,539754132	0,49652
Gm6272	1,539540692	0,8521
Dym	1,53900722	0,57659
A430018G15Rik	1,538900548	0,80902
Gm4204	1,538580576	0,36208
Gm11964	1,538047437	0,86056
Gm17971	1,537621059	0,79973
E2f5	1,537514483	0,72844
Reep5	1,537088252	0,54166
Uqcrh-ps1	1,536981713	0,82359
Snrpa	1,53655563	0,61574
Gpr155	1,536449128	0,73497
Desi2	1,536342633	0,59162
Gnai3	1,536342633	0,64156
Mettl16	1,535703819	0,60743
Gm25007	1,535703819	0,9232
Dis3l2	1,535597376	0,69949
Ecm1	1,535384512	0,56943
Naa16	1,535065271	0,64925
Pdia6	1,534426988	0,46774
Casp6	1,534320633	0,71966
Daglb	1,534214286	0,56913
Crls1	1,534001613	0,55478
Zbtb7b	1,533895288	0,45507
Stt3a	1,533895288	0,72216
Yipf7	1,533895288	0,80254
Celf1	1,53378897	0,6009
Rpl28-ps3	1,533576357	0,88085
Tardbp	1,533257491	0,49966
Gngt2	1,533151218	0,71868
Rngtt	1,532938693	0,59209
Tsc1	1,532832441	0,64446
Arl2	1,532195087	0,58441
Gm8730	1,531557997	0,75809
1110020A21Rik	1,531345693	0,90743
Eif4b	1,530921172	0,36006
Gipr	1,530921172	0,74695
4933408B17Rik	1,530708956	0,70928
Rras	1,53049677	0,5268
Bbs4	1,53049677	0,71689
Mdm1	1,53049677	0,75809
Gm16973	1,530390688	0,81559
Peak1os	1,530072485	0,86903
Tmtc3	1,529118274	0,78391
Seh1l	1,528694371	0,62095
Rpl36a-ps2	1,528694371	0,69092
Prosc	1,528164657	0,59408
RP23-308G10.5	1,528164657	1
Fcna	1,527952824	0,70415

Gm12396	1,527952824	0,77332
Gm6565	1,527423367	0,78663
Gm5601	1,526894094	0,74546
Pnrc1	1,526576619	0,56387
Txndc15	1,526470808	0,69028
Arl3	1,526259209	0,58126
Anxa5	1,52615342	0,49764
Ppp5c	1,52615342	0,62867
Gm9134	1,526047639	0,94169
Ube2g1	1,525941865	0,65646
Gatad1	1,525730339	0,56376
Spire2	1,525730339	0,78395
Rpl5-ps1	1,525730339	0,84851
Zfp787	1,525624588	0,63441
Cnot3	1,525518843	0,62867
Polr2j	1,525095938	0,67279
Nono	1,52467315	0,59662
Serpinb6b	1,524567472	0,69085
Chst1	1,524567472	0,79993
Gm10602	1,52425048	0,83455
Hps3	1,52414483	0,7215
Gtf2ird1	1,52414483	0,74982
Atn1	1,523827926	0,87704
Rictor	1,523511088	0,59544
Il13ra1	1,523194316	0,50657
Gm31166	1,523194316	0,94555
Gm7027	1,522666508	0,79487
Gm44152	1,521822397	0,97012
Gm13477	1,521505976	0,98415
AI662270	1,521084183	0,53734
Dexi	1,520873331	0,66529
Mrpl34	1,520557107	0,5361
Tspyl4	1,520346327	0,98403
Dnajc19	1,520240949	0,89425
Chchd5	1,519924856	0,59603
Tm6sf1	1,519924856	0,64773
Epb41	1,519819507	0,63954
Gm18737	1,519714165	0,87756
Gm13039	1,51960883	0,76775
Rangap1	1,519292869	0,48322
Ptcd3	1,518976974	0,78235
Dab2	1,518555882	0,61043
Ubl5	1,518134907	0,43395
Ogfrl1	1,518134907	0,61039
Rnf217	1,518029682	0,75215
Gm29257	1,517608853	0,80757
Cycs	1,517398482	0,75265
Snx3	1,517082981	0,62438
Anpep	1,517082981	0,89697
Gm14681	1,516767545	0,69174
Vwa8	1,516767545	0,72074
Vps37b	1,516662415	0,65169

Caprin1	1,516347066	0,52964
Gm5787	1,516031784	0,99638
Slfn3	1,515086329	0,90043
Nfkbib	1,514561331	0,75506
Med12	1,514351383	0,88435
Gm9435	1,514246419	0,9755
U2af2	1,513721713	0,7398
Hspa9-ps1	1,513616793	0,77264
Yars2	1,513406976	0,75883
Slc36a3os	1,513406976	0,96766
Nab2	1,513302078	0,7063
Fahd1	1,512463158	0,57613
Fgfbp3	1,512463158	0,80603
Isyna1	1,512358325	0,68306
Plcb3	1,512148683	0,67279
Tmem183a	1,512043872	0,52964
Pank2	1,512043872	0,66091
Xrcc2	1,511415161	0,82464
Vezf1	1,510891436	0,61221
Rnf146	1,510786712	0,67017
Baz2a	1,510681996	0,6274
Gm14303	1,510681996	0,64446
Magoh	1,510053852	0,60532
Otud3	1,509844528	0,71844
Hmga1	1,509321347	0,84835
Gm37140	1,509112125	0,96227
Gm12854	1,508693768	0,95074
Pwp1	1,50806645	0,66644
Gm11427	1,50806645	0,7398
Mrps33	1,507752889	0,83273
Jtb	1,507021501	0,67601
Arid5b	1,506812598	0,76299
Mzt1	1,506499297	0,68222
Mgea5	1,506290467	0,50039
Cd83	1,506081665	0,62943
Ppp4r3a	1,506081665	0,66091
Myl12b	1,505872892	0,60916
Rap1gds1	1,505768517	0,66832
Mterf3	1,505768517	0,80634
Gm38062	1,505142415	0,95074
Stk25	1,50503809	0,83069
Relt	1,504725159	0,80688
Gm20689	1,504620863	0,87775
Ccnt2	1,504308018	0,6445
Aacs	1,503682524	0,82296
Tmem170	1,503474084	0,96978
Dusp3	1,503161478	0,58599
Chchd7	1,50295311	0,68138
Cdkn2aip	1,502744771	0,74924
Oard1	1,502640612	0,76491
Jam2	1,502536461	0,70761
Minos1	1,50222405	0,52964

A830080D01Rik	1,502119927	0,81901
Wdr74	1,501911703	0,85559
Morf4l2	1,501391271	0,7326
Tnni2	1,501391271	0,74651
Mss51	1,501079098	0,95049
Ppt2	1,500871018	0,67906
Immp1l	1,500871018	0,73981
Ino80d	1,500454946	0,72739
Nprl2	1,500350946	0,6744
Chd6	1,500350946	0,67692
Manbal	1,500246953	0,71151
Cacna1a	1,500246953	0,71951
Lamb2	1,499519205	0,92198
Dennd6b	1,498895703	0,87876
1810032O08Rik	1,498791811	0,72758
Amhr2	1,498584048	0,7398
Cuta	1,49816861	0,67657
Gm7808	1,497130519	0,88751
Adpgk	1,497026749	0,75638
Mir703	1,496715484	0,64856
Ttc5	1,496611743	0,82803
Slc25a23	1,496404283	0,66859
Mcoln1	1,496404283	0,77907
Gm32340	1,496300563	0,91004
Gm6905	1,496300563	0,96711
Foxo3	1,495989449	0,64751
Zfp692	1,495989449	0,75986
Snhg4	1,495885758	0,76539
Lamtor4	1,495367414	0,73638
Gm12454	1,495367414	0,92526
Cfl2	1,495263766	0,69048
Polr2i	1,494124119	0,7297
Lman2l	1,493917004	0,77859
Ric8b	1,49350286	0,75269
Itga5	1,493399341	0,83302
Rgs1	1,49308883	0,74943
Hdac3	1,492778383	0,78829
Zfp36	1,492468001	0,80987
Rab3a	1,492261115	0,75377
B2m	1,492054258	0,69993
Gm20430	1,492054258	0,87339
Eif3f	1,490917057	0,67017
Gm14336	1,490917057	0,88264
Gm4217	1,490917057	0,98568
Noxo1	1,490813719	0,93522
Uchl5	1,490607062	0,68038
Pcf11	1,490607062	0,71226
Pdzk1ip1	1,490090547	0,92063
4933421A08Rik	1,489883991	0,92141
Rita1	1,489470964	0,785
Psma5	1,489367726	0,73365
Rai1	1,489367726	0,8025

Kitl	1,489367726	0,81165
Fkbp14	1,489367726	0,84087
Iqcb1	1,48916127	0,80476
Gm5644	1,488748444	0,86737
Pex1	1,488542074	0,77999
Ctss	1,488026274	0,68244
Efcab11	1,487613762	0,97133
9330104G04Rik	1,487098284	0,90583
Irf2bpl	1,486892143	0,66644
Cmc1	1,486479946	0,72074
Helq	1,48627389	1
Gm12696	1,486170873	0,71951
RP23-6C18.6	1,486170873	0,91953
Dctpp1	1,486067863	0,77683
Mtpn	1,48596486	0,69798
Gm4604	1,485758876	0,82492
Dusp11	1,485449954	0,63212
Map3k14	1,485038156	0,78502
Gm42820	1,484935225	0,86286
Slc12a6	1,484832301	0,75225
Triap1	1,484317786	0,71609
Pard6b	1,484317786	0,8655
Pbx1	1,484317786	0,99909
Rnf11	1,484214905	0,68426
Ppp1r12c	1,48411203	0,64773
Gm12989	1,48380345	0,85958
Gm6177	1,483700604	0,96741
Rps25	1,483289292	0,66923
Ifitm2	1,483289292	0,73183
Crbn	1,483186482	0,71136
Gm8731	1,483083679	0,83962
Gm14673	1,483083679	0,98712
Ctxn1	1,482878094	0,91055
Eif3e	1,482672538	0,66832
Pwwp2a	1,482158772	0,78257
Slc25a5	1,482158772	0,85956
Atp5e	1,481645183	0,6264
Ube2h	1,481645183	0,69079
Kif9	1,481645183	0,87217
Rps15a-ps8	1,481131773	0,87775
E030030I06Rik	1,480926459	0,87033
5430402O13Rik	1,480823813	0,94476
Cir1	1,480721173	0,75225
Guf1	1,480413298	0,78698
Ttc9c	1,480105487	0,77925
Stk11	1,480002897	0,71689
Zfp174	1,479900315	0,94971
Gm7990	1,47959261	0,91041
Edil3	1,479182437	0,77659
Ssh3	1,479182437	0,80085
Tmlhe	1,479079911	0,94137
Napg	1,478977393	0,65768

Tubb5	1,478362431	0,68455
Gm11478	1,478362431	0,74667
Eif2d	1,478259963	0,81259
1190002N15Rik	1,478157501	0,75591
Ndufb4	1,478055046	0,83268
Gm2986	1,477747725	0,93399
Cacna1s	1,477645299	0,85292
Rps15a-ps3	1,47754288	0,90996
Itpkc	1,477440468	0,83062
Rps19bp1	1,477235666	0,71977
Dnaaf3	1,477030892	0,84323
D730045B01Rik	1,476723784	0,86956
Prdx2	1,476621429	0,70525
D330045A20Rik	1,476621429	0,79015
Gm38220	1,476212079	0,9341
Gm7432	1,476109759	0,92132
Snrpert	1,476007447	0,82673
Gm5262	1,475905141	0,99624
Cdkl3	1,475802843	0,86903
Sclt1	1,475700551	0,85971
Gm45762	1,475189201	0,9075
Zfp532	1,474882476	1
Ifi27l2a	1,474780249	0,81448
Zyx	1,474780249	0,84957
RP24-282C4.4	1,474473609	0,982
Ppp2r1a	1,47386052	0,71241
Miip	1,47386052	0,78671
Rps11-ps2	1,473554071	0,83236
Gm7809	1,473145572	0,80084
Sertad3	1,473043464	0,80334
Gm14706	1,472839271	0,83933
Zcchc24	1,472635107	0,84373
Gm43737	1,472533035	0,85115
Ptprc	1,47243097	0,72088
D10Wsu102e	1,472226862	0,73183
Cby1	1,472226862	0,83396
A430046D13Rik	1,471920753	0,8324
Prdx3	1,471818731	0,77012
Srebf1	1,471818731	0,83242
Tmem109	1,471512707	0,73238
Dhx8	1,471512707	0,7799
Uba3	1,471512707	0,81693
1110065P20Rik	1,471410713	0,7698
Slc7a6os	1,471206746	0,79859
Clpx	1,470900849	0,75113
Vps13b	1,470493085	0,82196
Vprbp	1,470493085	0,82912
Zfand1	1,470391162	0,8445
Ankrd35	1,470187336	0,8981
Mrps16	1,469983539	0,74017
Gm45084	1,469881651	0,90694
Pdcl3	1,46977977	0,86396

Hotairm1	1,469677897	0,81559
Gm10269	1,469677897	0,82982
Gm36266	1,46957603	0,91972
Smarce1	1,469372317	0,83518
Gm10288	1,469168633	0,78252
Gm5764	1,469168633	0,99114
Btf3l4	1,469066802	0,85076
Slc25a17	1,468964977	0,77832
Btbd7	1,468964977	0,87033
Hnrnpr	1,46886316	0,74664
Vgll4	1,46886316	0,76213
Itgb1	1,468354179	0,71305
1810022K09Rik	1,468252404	0,81559
C2cd5	1,468150636	0,81693
Gyg	1,467743635	0,75269
Gpr137b	1,467540176	0,78671
Dr1	1,466523307	0,81136
Nup50	1,466421659	0,78078
Gpr137	1,466421659	0,84936
Snx2	1,466320018	0,80505
Hmgcr	1,466218384	0,76675
Capns1	1,465811918	0,77219
Uba7	1,46571032	0,84851
Cfl1	1,465608728	0,78061
Gm27029	1,465303995	0,92234
Tet3	1,465202431	0,82464
Ddx23	1,465100875	0,80334
RP23-402A24.3	1,464694719	0,96337
Cdyl2	1,464390176	0,85094
Gm24959	1,464390176	0,96864
Gm15131	1,464187182	0,98388
Thap1	1,463375489	0,83871
Lxn	1,463172637	0,88129
Nucks1	1,463071221	0,72552
Atf5	1,462665627	0,7895
Anp32b-ps1	1,462564247	0,83268
Rpl23a	1,462564247	0,87399
Gm5117	1,462564247	0,96265
Tmem41b	1,462361506	0,8271
Zfp382	1,462158794	0,90666
Creb1	1,46195611	0,78252
Krit1	1,461854778	0,78391
Susd6	1,461246936	0,82282
Ppp4r1l-ps	1,461145654	0,86038
Zfyve27	1,460841849	0,80367
C87436	1,460841849	0,86396
Magohb	1,460133216	0,89134
Gm12501	1,459728437	0,86613
Utrn	1,45962726	0,8092
Eef1d	1,459424927	0,81449
Kantr	1,458919216	0,86613
Gm8093	1,458919216	0,95179

Slc30a5	1,458818095	0,83551
Fnbp1l	1,458514774	0,79993
Gm13433	1,458514774	0,99774
Kirrel3	1,458413681	0,91093
Ywhaq	1,458211516	0,7575
Tmem63a	1,458009379	0,77608
Fyttd1	1,457908321	0,7734
Cenpm	1,45780727	0,87329
Actr2	1,457605189	0,77608
Ggh	1,457504159	0,84957
Cdc42	1,456898126	0,76272
Lmtk2	1,456494244	0,90893
Apip	1,456393291	0,87977
Rnf13	1,455989549	0,82296
Ldha	1,455888631	0,76031
Psmd9	1,45578772	0,81527
Kbtbd8	1,455686816	0,98012
Napb	1,455485029	0,83236
Gm14165	1,455384146	0,87675
Ccdc25	1,45528327	0,84262
Cd36	1,455182401	0,84066
Uba6	1,455081539	0,80448
AB124611	1,454980684	0,78391
Sin3a	1,454980684	0,79064
Spcs2-ps	1,454879836	0,95379
Whamm	1,454577333	0,85132
Capn1	1,4543757	0,83225
Gps1	1,454174095	0,91148
4632404H12Rik	1,453972517	0,85286
Hcst	1,453871739	0,84836
Gm8805	1,453770968	0,85563
Snhg18	1,453770968	0,86193
Gm15163	1,453569446	0,97606
Ruvbl2	1,453367953	0,88184
Cd52	1,453166487	0,7398
Erap1	1,453065765	0,85958
Aldh1l1	1,452864341	0,84653
Snu13	1,452662945	0,85249
Gm44024	1,452461578	0,92938
Gm11694	1,452461578	0,93325
Mipol1	1,452260238	0,86228
Dhx40	1,452058925	0,84481
RP24-325P4.5	1,452058925	1
9330111N05Rik	1,451857641	0,84851
Gm26497	1,451555767	0,94257
Hmga1-rs1	1,450952208	0,89799
Gm45630	1,450952208	0,95518
Gm14173	1,450851639	0,91372
Patl1	1,450751077	0,85286
Lta4h	1,450248372	0,82743
Swt1	1,450047339	0,83416
Ggnbp1	1,449946833	0,84938

E330037G11Rik	1,449143035	0,91377
Qars	1,449042591	0,79942
Golga3	1,448942155	0,83455
Lamtor5	1,448741302	0,83347
Mrpl50	1,448440076	0,82724
Nr1d1	1,448339682	0,85996
Nicn1	1,448239294	0,84653
Gm15694	1,448038539	0,93648
Lactb2	1,447737459	0,85688
Ncaph	1,447637113	0,87992
Slc35a2	1,447637113	0,88683
Rnase4	1,447536774	0,81001
Prkcsh	1,447436442	0,80769
Cenpa	1,446734312	0,81165
Uck1	1,446634036	0,86608
Bcl7a	1,446232999	0,86895
Dusp28	1,44573186	0,89181
Stk40	1,445631653	0,86608
Gm26601	1,445631653	0,96929
Gm37706	1,445431259	0,98567
Adk	1,445331073	0,85465
Mta3	1,445230894	0,87855
Gm13736	1,445130722	1
Etfa	1,444529834	0,87945
Cdk2	1,444029285	0,86608
Fkbp3	1,443528909	0,83933
Gm10499	1,443528909	1
Arl6ip6	1,443328807	0,87137
Cers5	1,443128733	0,83944
Cnst	1,442928687	0,88758
Gm11687	1,442728668	0,9072
Mroh2a	1,442728668	1
Guca1a	1,442628669	0,82401
Cops2	1,442428693	0,8468
2510039O18Rik	1,442028822	0,8224
Tubg1	1,441928871	0,86353
Arpc3	1,441529139	0,83856
Gm37354	1,441529139	0,99951
RP23-331E5.10	1,441229413	0,90088
Gm45206	1,441129518	1
Capza2	1,440730007	0,85111
Abi1	1,440530294	0,87027
Rin1	1,440130949	0,93325
Rps11-ps4	1,44003113	0,95719
Gm37589	1,439931319	0,9633
Pdxk	1,439831514	0,85249
Kcnab2	1,439731715	0,86934
Ola1	1,438734115	0,86286
Rpl19-ps1	1,438534678	0,90327
2310022A10Rik	1,438235574	0,85858
Spryd7	1,438235574	0,86286
2310009B15Rik	1,437737206	0,88058

Selenoh	1,437637553	0,86737
E2f2	1,437338636	0,87622
Rab6a	1,437039781	0,85335
Rock2	1,436740988	0,86981
Ap1s2	1,436143589	0,87676
Ppme1	1,436044046	0,88093
Sdccag3	1,436044046	0,89425
Ulk4	1,436044046	0,91359
Adamts1	1,435247955	0,93067
Gm8806	1,435148475	0,93132
Gm12020	1,435049001	0,89409
Coq2	1,435049001	0,90179
Gm4707	1,434651176	0,90195
Aplp2	1,434452305	0,85641
Sumo1	1,434452305	0,88373
Hbp1	1,433955248	0,86248
Zbtb8a	1,433855857	0,9224
Arhgef11	1,433756473	0,90612
Cd300a	1,433756473	0,90743
Rpl35	1,433160315	0,93885
Dynll2	1,432862328	0,86266
Stbd1	1,43246511	0,94241
Usp37	1,432266542	0,88769
Diaph1	1,432167268	0,87395
Gpn3	1,43157177	0,91041
Mettl10	1,43157177	0,92279
Klhl35	1,43157177	1
Xxylt1	1,431373326	0,93885
Gm7206	1,430877336	0,9609
Ccdc137	1,429984986	0,89768
Phka2	1,429786762	0,9063
Rmrp	1,429390396	0,94919
Bcl2l2	1,42899414	0,93348
Gm12967	1,428895093	0,91732
Pdpk1	1,428201957	0,90126
Lrp1	1,42780603	0,8928
Snx8	1,427410213	0,89425
Gm14323	1,427212346	0,99759
Il15	1,427113422	0,90583
Tmem208	1,427014506	0,90282
Mb21d2	1,426816693	0,91989
Gatad2b	1,426520026	0,90243
Gm10616	1,426520026	0,93321
Cdc34b	1,42642115	0,95297
Rara	1,425828042	0,9127
Lmbrd2	1,425729214	0,91498
Gm14584	1,42553158	0,9633
BC085271	1,425333973	0,95518
Zfp81	1,425136394	0,91808
Map2k1	1,424642565	0,90831
Rpl7a-ps5	1,424642565	0,941
Hypk	1,424445081	0,92155

Gm7722	1,424445081	0,94404
Gm44190	1,424346349	0,9938
0610030E20Rik	1,424148907	0,90903
Tcea1-ps1	1,423260753	0,92013
Zfat	1,423063461	0,92526
Bbof1	1,42257035	0,92155
Utp14a	1,422471749	0,90893
Tapbpl	1,422274566	0,92526
Sccpdh	1,421683182	0,92155
Stip1	1,420993544	0,91372
Dhx58	1,420993544	0,92451
Rbm39	1,420599616	0,91377
Wdr37	1,420599616	0,92013
Txndc17	1,420402692	0,91849
Tfb1m	1,420304241	0,93745
Gm13436	1,420008928	0,93132
Tnfrsf26	1,419713676	0,9191
Dek	1,419615272	0,92032
Repin1	1,419516875	0,92132
Pfdn2	1,419516875	0,9224
Adgre5	1,419516875	0,98999
Mrpl33	1,419418485	0,92132
Acd	1,419418485	0,9232
Slc35a4	1,419418485	0,92526
AC168977.1	1,419221726	0,92181
Snapc1	1,419123356	0,92132
Sap30l	1,419123356	0,925
Dnaaf5	1,418926638	0,9224
Sep 11	1,418041741	0,92507
Nbas	1,418041741	0,92639
Zcchc11	1,417943453	0,92451
Fbxo30	1,416175438	0,93067
Gpr179	1,41607728	0,93234
Gm9530	1,415782845	0,95111
Cct5	1,41558659	0,92912
Dnm1	1,415390361	0,93396
Arhgap17	1,414997986	0,93321
BC030499	1,41460572	1
Mdh1	1,413821513	0,93605
Rbm14	1,413527548	0,93661
Runx1	1,413135689	0,93809
Atp1a3	1,412450198	0,9387
Ndufv3	1,411373669	0,94376
Elof1	1,411275843	0,94373
Hnrnpa2b1	1,410689032	0,945
Lamtor2	1,410591254	0,94404
Tsg101-ps	1,410591254	1
Hprt	1,410395718	0,94476
Lrig2	1,41029796	0,94314
Slfn9	1,410200209	1
Gm37060	1,410102465	0,94163
Stk19	1,409809273	0,94396

Cd5l	1,409809273	0,94476
Lmtk3	1,409613846	0,9633
Ccar1	1,409516142	0,94729
E4f1	1,409320755	0,94399
Pafah1b1-ps2	1,409125395	0,9692
Pafah1b3	1,408930063	0,94476
Gm15484	1,408734757	0,99732
Slc9a5	1,408539478	0,94404
Rpl28	1,408246611	0,94435
Scarna2	1,4080514	0,94466
Foxm1	1,4080514	0,94476
Oxr1	1,407563492	0,94476
Gm7863	1,407173286	0,99327
2610021A01Rik	1,407075752	0,94756
Pdcd2l	1,406783189	0,94651
Gm32175	1,406783189	0,98533
Mob2	1,40629572	0,95518
Col18a1	1,406003319	0,95375
Ppp1r16a	1,405905866	0,94538
Rhox5	1,405710979	1
Gm9762	1,405613546	0,94689
Rbm18	1,4054187	0,95433
Plch2	1,405321287	0,97759
Gm43560	1,405321287	0,98088
Atl3	1,405223881	0,95326
Gm44609	1,405126482	0,94919
Ctdp1	1,405126482	0,94971
Vaultrc5	1,404834325	0,95123
Aurka	1,404639587	0,95411
Cxcl16	1,404639587	0,95786
Cuedc2	1,403860905	0,95518
Lrwd1	1,403666302	0,95738
Sugp2	1,403179913	0,95793
Mex3d	1,403082655	0,95305
Lrrc45	1,402985404	0,95315
Gm17491	1,40288816	0,95179
Ripk2	1,402790922	0,9633
B3gnt2	1,402499251	0,9964
Oat	1,402110449	0,95786
Zmym6	1,401624599	0,95793
Gm42748	1,401527449	0,95111
Gm10260	1,40123604	0,95136
Ipo5	1,40123604	0,98127
Aip	1,400944691	0,9633
Gna11	1,400847589	0,96227
Gpr157	1,400653403	0,95297
Dnajb2	1,400653403	0,95433
Ccdc50	1,400265114	0,97345
Gm6501	1,400265114	1
Xlr	1,399779903	0,95433
Acyp1	1,399682881	0,95326
RP23-307F3.6	1,399682881	0,95672

Sntb2	1,399682881	0,96741
Ftsj1	1,399585866	0,95593
Sparc	1,39929486	1
RP24-550H10.4	1,399197872	1
Lars2	1,398906947	0,95835
Mrpl23	1,398906947	0,96337
Zbtb11	1,398519141	0,96929
Hsp90b1	1,398228358	0,98629
Rest	1,39784074	0,97554
B230307C23Rik	1,397646972	0,96698
Rev1	1,39745323	0,96373
Ssr3	1,39735637	0,98533
D2hgdh	1,397259516	1
Spaca6	1,397162668	0,95653
Mycn	1,396968994	0,97501
Fkbp1a	1,396872167	0,9938
Gm7160	1,396678532	0,95642
Tmem120b	1,396484925	0,96149
Slc8a1	1,396097791	0,99018
Prpf8	1,395807511	0,97419
Pja2	1,395807511	0,98406
Mrpl51	1,395130425	0,97312
Rad51d	1,395033725	0,97312
Ap2a2	1,395033725	0,98406
Unc119	1,394840346	0,99751
Zfp143	1,394646993	0,98313
Etnk1	1,394646993	0,99414
Ndufaf2	1,393390855	0,96872
A630081D01Rik	1,393294276	1
Ssbp3	1,393004579	0,96929
Traf4	1,392521884	0,97759
Eif3k	1,392425365	0,99759
Lsr	1,392232347	1
Gm26799	1,392135848	0,96766
Icam5	1,391942871	0,96995
Clpb	1,391942871	0,98673
Vcl	1,391846392	0,97443
Marf1	1,39174992	0,9755
Sult2b1	1,391653454	1
Anp32-ps	1,391074802	1
Zbtb21	1,390978384	0,96337
Zmym2	1,390881972	0,99414
5730405O15Rik	1,390881972	1
Cebpzos	1,390592776	0,97278
Gm43445	1,390592776	1
Abcb1b	1,390207275	1
Abca7	1,390110917	0,97988
Snx12	1,38972555	0,99481
Gm16523	1,389340289	0,99114
Med21	1,389243991	0,98376
Evl	1,389051414	0,98762
Cpsf7	1,388570089	0,98406

Gm7436	1,388088931	0,96853
Gm13578	1,387896514	0,96741
Rps15	1,387896514	1
Ppfia3	1,387800316	0,98281
Gm26947	1,387607939	0,96766
Orc4	1,387223266	1
Gm37105	1,387127114	0,99018
RP23-123D6.12	1,387030969	0,96864
Eps15l1	1,386742574	0,98627
Gm5909	1,386646456	0,97584
Ctbp2	1,386550344	0,99739
Cs	1,386550344	1
Hspb11	1,386069886	0,9938
Pebp1	1,386069886	1
Ikzf5	1,385973814	1
Cep164	1,38578169	0,97759
Stxbp4	1,385589594	0,96978
Stag1	1,385301498	0,98762
Gm4540	1,384917464	0,98085
Gm15541	1,384917464	1
Gm10704	1,384629509	0,98251
Strn4	1,384341613	1
Metrnl	1,384053777	1
Cry1	1,38386192	0,99533
Xpa	1,383766001	0,99327
Akirin2	1,383574184	0,98999
Nfam1	1,383478285	0,98088
Cwf19l1	1,383286508	0,9783
Cd164	1,382998891	1
Gm43756	1,382998891	1
Sdhaf2	1,38280718	1
Fgd4	1,382711335	0,98762
Gm12074	1,382711335	1
Smc3	1,382711335	1
Atp6ap1	1,382519664	1
Gm8995	1,382423838	0,97443
Aen	1,382040602	1
Ubr7	1,381753244	1
Fcgr3	1,381753244	1
Gm10031	1,381465947	1
Ube2g2	1,381082976	1
Zfp652	1,38098725	1
Ifitm6	1,380700112	1
Stk24	1,38022168	1
Katna1	1,38022168	1
Hjurp	1,379934701	1
Supt7l	1,379839055	0,99247
Galnt6	1,379456535	1
Xpo1	1,379265315	1
Inhbe	1,379074122	1
Tfe3	1,378787381	1
Pcm1	1,378787381	1

P4hb	1,3785007	1
Gm14822	1,377450046	0,98388
Rap2a	1,377450046	1
Agps	1,377354572	1
1110003F10Rik	1,377163643	1
Gxylt1	1,376972741	1
Mkln1	1,376591016	1
Bloc1s3	1,376304792	0,99414
Phf6	1,376209397	1
Sem1	1,376114009	1
Rps18	1,376018627	0,99055
Cda	1,375732522	0,99624
Psmb1	1,375637167	1
Cdc42se1	1,375255812	1
Hapln3	1,375065174	1
Plcl2	1,374874563	0,99909
Siva1	1,374779267	1
Lppos	1,374683978	1
Tusc2	1,374683978	1
Creb3	1,37439815	1
Rgs3	1,374207631	0,98673
Fam214a	1,374207631	1
Nt5dc3	1,374207631	1
Pgm3	1,373921903	0,9938
Zc3h11a	1,373826673	0,98406
Myo1f	1,37344582	1
Gabarap	1,37344582	1
Vdac3-ps1	1,373350624	0,99018
Mapk1ip1	1,37316025	1
Sgcb	1,372969903	0,98406
Acot9	1,372874739	1
Armc8	1,372684431	1
Star	1,372589287	0,98454
1110046J04Rik	1,372589287	0,98713
Gm11450	1,372589287	0,98999
Nectin1	1,37249415	1
D16Ertd472e	1,372399019	0,99476
1810058I24Rik	1,372303895	1
Gm45856	1,372208778	0,99759
Gm25636	1,371923464	1
Dpm3	1,371923464	1
Gm17039	1,371257964	1
Gnb2	1,371067881	1
2310022B05Rik	1,370592787	1
Nxt1	1,370402796	1
Bscl2	1,37030781	1
Prpf38a	1,369927933	1
Pop1	1,369738034	0,99476
Tomm20	1,369738034	1
Plekhh2	1,369548161	1
Gm12751	1,369358314	1
Cnih4	1,369168494	1

Gm25291	1,3689787	1
Bax	1,368883813	1
Bckdhb	1,368504331	1
Rps26-ps1	1,368409476	1
Eif3l	1,368314629	1
Tprn	1,368219788	1
Gm11334	1,368124953	1
Med18	1,367745681	0,99055
Rhbdf2	1,367745681	1
Skp1a	1,367271739	1
Gm45109	1,366703224	0,99414
Rer1	1,366608495	1
Gm3283	1,366513772	1
Csnk1g2	1,366229643	1
Snx21	1,366134947	1
Akirin1	1,365945573	1
Irgq	1,365661562	1
Dna2	1,365377611	1
Sar1b	1,365377611	1
Rps27a-ps1	1,364904489	1
Glrx	1,364904489	1
Hexb	1,364809884	1
Abhd2	1,364620694	1
Pik3c2a	1,364431531	1
Inip	1,364336959	1
Top1	1,364147835	1
Acadl	1,364053283	1
Tcf7l2	1,363958737	1
Gm43247	1,363958737	1
Gm11952	1,363675139	0,99624
Casp7	1,363108121	1
5031434O11Rik	1,363108121	1
Ergic3	1,363108121	1
Tchp	1,36301364	1
Kansl3	1,36301364	1
Rpl10a-ps1	1,362919167	1
2410131K14Rik	1,362730239	1
Klhl24	1,362730239	1
Noct	1,362635785	0,9964
Zbtb43	1,362635785	1
Elovl1	1,362635785	1
Sub1	1,362446897	1
E330020D12Rik	1,362163613	1
Scp2	1,362163613	1
Gm13140	1,36197479	1
Usp32	1,361880389	1
Gm43275	1,361691605	1
Casp8ap2	1,361219761	1
Bahd1	1,361219761	1
2200002J24Rik	1,361125411	1
Itpka	1,361125411	1
Gm5905	1,360842403	0,99785

Mars2	1,36074808	1
Wbp1	1,360653763	1
Terf1	1,360276562	1
Pigl	1,359805208	0,99941
Tpp2	1,359522474	1
Pag1	1,359334017	1
Adck2	1,359145586	1
Cgrrf1	1,358957182	1
Htt	1,358862989	1
Prmt7	1,358674624	1
RP23-38L16.3	1,358580451	0,99797
Luzp1	1,358580451	1
Gm12182	1,357921423	1
Pcbd2	1,357921423	1
Engase	1,357827303	1
Sep 10	1,357356797	1
2410006H16Rik	1,357074572	1
Kazald1	1,356886455	1
Poc1a	1,356792406	0,99951
Slc7a7	1,356416275	1
Cstf3	1,356416275	1
Paxbp1	1,356416275	1
Mre11a	1,356228249	1
a	1,356040249	1
Smyd5	1,356040249	1
Gm43533	1,355758298	1
B130034C11Rik	1,355570363	1
Pld4	1,355194571	1
Atp6v1g1	1,355100639	1
Cd200r3	1,354724977	1
Upp2	1,3544433	1
1700034H15Rik	1,354255547	1
Mtor	1,354255547	1
Mnt	1,35406782	1
Gm13015	1,353692445	1
Rhog	1,353692445	1
Car5b	1,353598617	1
Bzw1	1,353598617	1
Serpine1	1,353317174	1
Gtf2f1	1,353317174	1
Rpl23	1,352942007	1
Sirt2	1,352942007	1
Yjefn3	1,352848231	1
BC030867	1,352566943	1
Ift52	1,352191984	1
Gli1	1,352191984	1
Zfp444	1,352004544	1
Arrdc3	1,351910833	1
Tmem30a	1,351817129	1
Mknk1	1,351536056	1
Wdr44	1,351161382	1
Als2cl	1,351161382	1

Cib2	1,350225153	1
Cyth2	1,350131566	1
Il6ra	1,350131566	1
Mlf1	1,349757282	1
Jag1	1,349570179	1
Sympk	1,349476637	1
Hps5	1,349289573	1
Bak1	1,348728536	1
Gm4866	1,348635052	1
Gchfr	1,348541575	1
Foxn3	1,348448104	1
Nudt16l1	1,348261183	1
A430110C17Rik	1,348167732	1
Taf10	1,347980849	1
Phf1	1,347980849	1
Zfp414	1,347887417	1
Tubb2a	1,347887417	1
Ppp6c	1,347700573	1
Ptprj	1,347420356	1
Nudt2	1,347233577	1
Eif4h	1,347046824	1
Prpf4b	1,346300069	1
Aaed1	1,345553729	1
Pik3r6	1,345553729	1
Tmem242	1,345460466	1
Sgsm2	1,345367209	1
Zfp609	1,345180714	1
Snrpe	1,344994245	1
Stk35	1,344901021	1
Grasp	1,344901021	1
Cops9	1,344901021	1
Max	1,344901021	1
Hsbp1	1,344621385	1
Gm11221	1,344528187	1
Dusp7	1,344434994	1
Hmces	1,344341808	1
Rps20	1,344341808	1
Ebi3	1,343969129	1
Gm6794	1,343875976	1
D430013B06Rik	1,343782828	1
Pigg	1,343503426	1
Gm9843	1,343503426	1
Casp4	1,34331719	1
Gm42783	1,343130979	1
Npepps	1,343037884	1
9430015G10Rik	1,342758636	1
Gm42548	1,342479446	1
Fkbp1b	1,342386396	1
Rhebl1	1,342014259	1
Stx7	1,341735224	1
Sharpin	1,341642225	1
Btbd6	1,341084368	1

Srsf4	1,340991414	1
Pi4kb	1,340805526	1
Lrig3	1,340619664	1
Rab4a	1,340526742	1
M6pr	1,340526742	1
Aga	1,340526742	1
Taf8	1,340433827	1
Stard10	1,340155121	1
Gm11488	1,339876472	1
Sart1	1,339690739	1
Gm12338	1,339505031	1
Aspm	1,339226518	1
Gm2225	1,339226518	1
Pdp1	1,339226518	1
Rpl14	1,339226518	1
9330160F10Rik	1,338669665	1
Blcap	1,338484099	1
Nufip1	1,338391326	1
Wasl	1,338298559	1
Ppp1r13l	1,338205798	1
Edc3	1,338205798	1
Nomo1	1,338205798	1
Gm11599	1,338113044	1
Angptl6	1,338020296	1
Nmral1	1,338020296	1
Rad21	1,337927555	1
A930005H10Rik	1,337556654	1
Tomm7	1,337463945	1
Dmpk	1,337371242	1
Gpx4	1,337371242	1
Trem2	1,337185856	1
Zbtb26	1,336907825	1
Tecpr1	1,336907825	1
Gm5801	1,336815161	1
S100a13	1,336722503	1
Gm15625	1,336444568	1
Rps10	1,336351936	1
Rap1a	1,334870696	1
Leo1	1,334778173	1
Arpc5l	1,334778173	1
Murc	1,334500642	1
Ccdc85b	1,33422317	1
Sestd1	1,334130692	1
Gm13602	1,333391098	1
G3bp2	1,332651913	1
Lpin2	1,332559544	1
Ndufa1	1,332467181	1
Maged1	1,332190132	1
Gripap1	1,331820821	1
Gm16418	1,331082508	1
Gm2756	1,331082508	1
Gtf2e2	1,331082508	1

Copg2	1,330897993	1
Cox6c	1,330805746	1
Hsf2	1,330713504	1
D630029K05Rik	1,33062127	1
Gm16399	1,330436819	1
Srpr	1,330344603	1
Cotl1	1,330344603	1
Hscb	1,330252394	1
Map3k3	1,329975805	1
mt-Rnr1	1,329791444	1
Gna13	1,329791444	1
Cdk1	1,329699273	1
Card14	1,32951495	1
Gm2223	1,32951495	1
Plod3	1,32951495	1
Znrf1	1,329054255	1
Sil1	1,329054255	1
Mtl5	1,32840955	1
Gm8451	1,328225406	1
Phkg2	1,328133344	1
Fbxl5	1,328133344	1
Txn2	1,328133344	1
Bet1	1,327949238	1
Yy1	1,327949238	1
Ercc1	1,327857195	1
Calr3	1,327581104	1
Cep76	1,327489086	1
Pym1	1,327397075	1
Txndc5	1,327029093	1
Mgat4a	1,327029093	1
Dohh	1,326845141	1
Macrod1	1,326661214	1
Lrrfip1	1,326293436	1
Gm4430	1,326201507	1
Cbr1	1,326201507	1
Ppm1m	1,326201507	1
Hmg20b	1,326201507	1
Galnt11	1,326109585	1
Itgb7	1,325833857	1
Gm16200	1,325374438	1
Aktip	1,325374438	1
Gm37349	1,325282573	1
Rab2b	1,324180692	1
Rpl27a	1,32408891	1
Rbpms	1,323997135	1
Mrpl32	1,323905365	1
Akr7a5	1,323813602	1
Dusp19	1,323263158	1
Bmpr1a	1,323079728	1
Sag	1,322988022	1
Gm45902	1,322896323	1
Gm7123	1,322712943	1

Casp2	1,322712943	1
Ss18l1	1,322621263	1
Ywhaz	1,322529589	1
Gfpt1	1,322437921	1
Utp14b	1,32234626	1
Elf1	1,32234626	1
Vamp1	1,321979679	1
Glo1	1,321888049	1
Bdp1	1,321796426	1
Cep192	1,321613199	1
Rbm25	1,321613199	1
Selenof	1,321521595	1
Gm15800	1,321429997	1
Rpl19-ps9	1,320972104	1
Klhdc4	1,320880544	1
Cdc42se2	1,320788991	1
Ak2	1,320605903	1
Scaf1	1,320514369	1
Brwd3	1,320514369	1
Znhit1	1,320514369	1
Gnrh1	1,320239804	1
Gramd1a	1,320239804	1
Gm1943	1,319965297	1
Gm3724	1,319873807	1
Osbp	1,319782323	1
Zfyve16	1,319782323	1
Hmgb3	1,319690846	1
Gabpa	1,319690846	1
Gm45413	1,319599375	1
St13	1,319325001	1
Lmo2	1,319142116	1
Cd320	1,318776423	1
Cdk2ap1	1,318593614	1
RP23-184H3.5	1,318593614	1
Zfp280b	1,318136704	1
Acer3	1,318136704	1
Myl12a	1,317953984	1
Tbc1d12	1,317862634	1
Cep120	1,31777129	1
Slc35e4	1,317679952	1
Gm12762	1,31758862	1
Nuak2	1,317497295	1
Tmem263	1,317497295	1
Rpl34	1,317497295	1
Tom1l1	1,317040765	1
Yod1	1,316858197	1
Fam187b	1,316766922	1
Tnpo2	1,316766922	1
Arvcf	1,316675654	1
Palb2	1,316675654	1
Hic2	1,316310644	1
Gm12115	1,31576332	1

Thbs3	1,31576332	1
Tpcn2	1,31576332	1
Usmg5	1,315580929	1
Dda1	1,31530739	1
Mbnl2	1,31530739	1
Dnajc4	1,314669354	1
Mvp	1,314304901	1
Brca1	1,314122712	1
Gm6140	1,314122712	1
Tmem218	1,314122712	1
Ddit3	1,314122712	1
Tsta3	1,314031627	1
Med25	1,313940549	1
Mpp6	1,313667351	1
Snx30	1,313576297	1
Ndufa3	1,313576297	1
Arhgef40	1,31339421	1
Gm4613	1,313303175	1
Pla2g4a	1,313212147	1
Rny3	1,312939101	1
Lrrc49	1,312666111	1
Crlf3	1,312575127	1
Arl4c	1,312393178	1
Gm15859	1,312029356	1
Gm45890	1,311665635	1
Elk4	1,311665635	1
Ckap5	1,31157472	1
Trim8	1,31139291	1
Gm20442	1,311302014	1
Msn	1,311211125	1
Tubgcp2	1,310484237	1
Rab3gap2	1,310484237	1
Slc38a2	1,310484237	1
Slc39a10	1,310484237	1
Rab1a	1,310393404	1
Clec4n	1,310120944	1
Rragb	1,310030136	1
Llph-ps1	1,309757751	1
Gm12732	1,309666969	1
Xndc1	1,309576193	1
Tjp3	1,30939466	1
Rnf115	1,309213152	1
Kcnc3	1,309122407	1
Isg15	1,309122407	1
Prkd3	1,308668778	1
Mrps23	1,308396677	1
Gm6808	1,308215307	1
Kdm2b	1,308215307	1
Idnk	1,308124631	1
Slc7a8	1,307852643	1
Myo1g	1,307671349	1
Polb	1,307580711	1

Naa10	1,307308835	1
Snrnp48	1,307127616	1
Tmem11	1,306946423	1
Zmym1	1,306765254	1
Ntan1	1,30658411	1
Scarb1	1,306493548	1
G430095P16Rik	1,306221898	1
Clcn6	1,306221898	1
Smpd4	1,30604083	1
Vdr	1,305950306	1
Mtch1	1,305588269	1
Pycrl	1,305316808	1
Pfdn4	1,305045403	1
Tube1	1,304864498	1
Fam172a	1,304864498	1
Gm28557	1,304774055	1
Lyz1	1,304321933	1
Syngap1	1,304141128	1
Gm8444	1,304050735	1
Gm6322	1,304050735	1
Rps27l	1,304050735	1
2900009J06Rik	1,303147149	1
Pold3	1,303147149	1
Tprgl	1,303147149	1
Gm7299	1,303056825	1
Ptbp1	1,302876196	1
Scrn2	1,30278589	1
Pura	1,30224419	1
Ube2m	1,302063673	1
Cox7a1	1,301883181	1
Gm3571	1,30161249	1
Gm45033	1,301522272	1
8430429K09Rik	1,301432061	1
Gm10250	1,301341855	1
Hadha	1,301251656	1
Brk1	1,301251656	1
Arrdc1	1,300981097	1
Itgb2	1,300981097	1
RP23-447C2.2	1,300890923	1
Tcof1	1,300620438	1
Pim2	1,300530289	1
Mknk2	1,300169756	1
Lpar6	1,299989526	1
Gm20568	1,299809322	1
Sec62	1,299629143	1
Cul7	1,299539062	1
Ncbp2	1,299539062	1
Psmd11	1,299178804	1
Clip2	1,298908675	1
Pqbp1	1,298728621	1
Armc10	1,298548591	1
Tbcel	1,298368586	1

Gm9703	1,298188607	1
Cspp1	1,298188607	1
Prickle3	1,297918684	1
Rp9	1,297918684	1
Chp1	1,297828722	1
Atp5j2	1,297738767	1
Mgat1	1,297648818	1
Fam53c	1,297558875	1
Stk16	1,297468938	1
Dnpep	1,297468938	1
Ndel1	1,297468938	1
Helb	1,297379007	1
Gm10801	1,297379007	1
Cep83	1,297289083	1
Sugt1	1,297289083	1
Srcap	1,296120633	1
Rpa2	1,295940965	1
Mcm4	1,29567151	1
Strip1	1,295491904	1
Mark4	1,295312323	1
Gm5617	1,295312323	1
Esco1	1,295042999	1
Ppox	1,294953236	1
Trove2	1,294414792	1
Arl6ip1	1,294325074	1
Gm21975	1,294235361	1
Park7	1,294235361	1
Fam13a	1,294145654	1
Clk3	1,29396626	1
Gm5050	1,293786891	1
Irak1bp1	1,293786891	1
Usp22	1,293607546	1
Crk	1,293428227	1
Fundc1	1,293248932	1
Gabarapl2	1,293159294	1
Ppm1j	1,293159294	1
Fads3	1,293069662	1
Mthfd2	1,292980036	1
Rps12	1,292442413	1
Gm37795	1,292352831	1
Tec	1,292263255	1
Ephx1	1,292173685	1
Tesk1	1,291994564	1
Chmp4b	1,291815468	1
Gm6198	1,291636397	1
Taf1d	1,29154687	1
Slc25a26	1,291278329	1
Heca	1,291278329	1
Eid3	1,291188827	1
Npr1	1,290830883	1
Zfp746	1,290830883	1
Cnot6l	1,29056249	1

Ipp	1,290473039	1
Ift27	1,290473039	1
Fh1	1,290025872	1
Nkap	1,289757647	1
Mrpl1	1,289578861	1
Rplp2	1,289489477	1
Kdm5c	1,289489477	1
A930007I19Rik	1,288863965	1
Ptpn18	1,288595981	1
Kctd5	1,288238756	1
Dot1l	1,287970902	1
Uqcc2	1,287792363	1
Gm16425	1,287703104	1
Ppif	1,287435361	1
Ddx19b	1,287346126	1
Setdb2	1,287078458	1
Golph3l	1,286900043	1
Gm13368	1,286275787	1
Tmed10	1,286097483	1
Gm13450	1,286008341	1
Cmc2	1,285830075	1
Ganab	1,285830075	1
Atxn7l2	1,285740951	1
Lpxn	1,285562722	1
Myef2	1,285473616	1
Mrpl30	1,285473616	1
Gm35106	1,285384517	1
Adipor1	1,285206337	1
Pgm1	1,285117257	1
Ing1	1,284582903	1
Fam193b	1,284493865	1
Zfp948	1,284404834	1
Wwp1	1,284404834	1
Pex26	1,28422679	1
Npl	1,28422679	1
Zfr	1,283870776	1
Igbp1	1,283781788	1
Zfp53	1,283425898	1
Tsr2	1,283425898	1
Fbxl14	1,28333694	1
Zfp580	1,283247989	1
Sdc1	1,283247989	1
Phc2	1,283159044	1
Gm11423	1,282981173	1
Gnb1	1,282981173	1
Serpini1	1,282892247	1
Mcub	1,282892247	1
Recql	1,282625505	1
Gm12848	1,282358818	1
Pdlim5	1,282269935	1
C330007P06Rik	1,282092186	1
Tln2	1,282092186	1

Sf3b3	1,282092186	1
Ubl3	1,282092186	1
Rpl31-ps17	1,282003322	1
4930539J05Rik	1,281914463	1
Aamdc	1,281736764	1
Tfap4	1,281470262	1
Card19	1,281381441	1
Gm43294	1,281292625	1
Hspbap1	1,281292625	1
Zbtb17	1,281292625	1
Mcfd2	1,281292625	1
Phf5a	1,281026216	1
Phkg1	1,280671089	1
Gm4705	1,280582323	1
B3gnt3	1,279961131	1
Vma21-ps	1,279872414	1
Acvr2b	1,279872414	1
Kpna6	1,279783703	1
Sep 06	1,279428921	1
Fam96a	1,279428921	1
Got1	1,279162899	1
Gm20628	1,278985581	1
Rras2	1,278808289	1
Alg10b	1,278542395	1
Tmem159	1,278187957	1
Gm8129	1,278187957	1
Pex7	1,278010775	1
Gm37084	1,277922193	1
Arfgap2	1,277656484	1
Adgrl1	1,277479376	1
Gpam	1,277213759	1
Gm6612	1,277125233	1
Nup35	1,276859689	1
Aph1b	1,276328769	1
Lekr1	1,276151844	1
Plcg2	1,275974944	1
Sc5d	1,275974944	1
Unc13b	1,275886504	1
Smarcal1	1,275886504	1
Slc35g1	1,275798069	1
Prelid2	1,27570964	1
Nampt	1,27491406	1
Pgghg	1,274737331	1
Cdipt	1,274560627	1
Yif1b	1,274295617	1
Hnrnpu	1,274207293	1
Hdac6	1,274118975	1
Gm20302	1,274030663	1
Pdcd5	1,273942357	1
Gm10698	1,273765763	1
Irak3	1,273765763	1
Rangrf	1,273589194	1

Gm37423	1,273500918	1
Slc4a8	1,272883162	1
Bckdha	1,272353895	1
Vamp8	1,272353895	1
Atp5k	1,272353895	1
Hdhd2	1,272265705	1
Ppp4r3b	1,272001172	1
Sep 08	1,271560406	1
Polr2h	1,271472272	1
Drap1	1,27129602	1
Gm17511	1,270943591	1
Gm28686	1,270943591	1
Lsm1	1,270679333	1
Gm12231	1,27059126	1
Gm17251	1,27041513	1
Slc2a8	1,270327075	1
Rbms2	1,270327075	1
1110038B12Rik	1,270327075	1
Mir99ahg	1,270239026	1
Rbm33	1,269974914	1
Cdc45	1,26988689	1
Megf8	1,26988689	1
Gm7514	1,269622851	1
Sp3	1,269622851	1
1700012D14Rik	1,269006976	1
Zkscan8	1,268919018	1
Mlec	1,268919018	1
Kmt2d	1,268831066	1
B230216N24Rik	1,268567248	1
Abt1	1,268391399	1
AW549877	1,268215575	1
Ogfr	1,268127672	1
Gm14585	1,267951885	1
Gm45167	1,267864	1
Gpsm3	1,267688249	1
Ube2k	1,267424668	1
1810013L24Rik	1,26733682	1
Tnf	1,267073312	1
Nap1l1	1,26689767	1
Rnf225	1,266370892	1
Tnfrsf22	1,266283117	1
Vma21	1,266283117	1
Pdcd10	1,266107585	1
Sp100	1,265844333	1
Gm2991	1,265756594	1
Nme1	1,265668861	1
Sbno1	1,265668861	1
Slc44a1	1,265668861	1
Mybl1	1,265317992	1
Kdm3b	1,265142594	1
Edf1	1,26496722	1
Ndufab1	1,264791871	1

S100a1	1,264791871	1
Cdc26	1,264704205	1
Zfp207	1,264704205	1
Zfpm1	1,264528892	1
Rora	1,264528892	1
Gpsm2	1,264441244	1
Uqcr11	1,264441244	1
Tor3a	1,264178339	1
Add3	1,264003098	1
Rpl13a	1,263302381	1
Rpn2	1,263302381	1
Asah1	1,263127262	1
Lrrc41	1,263039712	1
Clasp1	1,263039712	1
Gm13181	1,262864629	1
Gm12089	1,262689571	1
Gm37199	1,262689571	1
Ccdc62	1,262339528	1
Mrpl4	1,262339528	1
Atp5c1	1,262252032	1
Gm2962	1,262077059	1
Glul	1,261814645	1
Scarna9	1,261552285	1
Gm44027	1,261552285	1
Gm17745	1,26111514	1
Cep295	1,260852926	1
Stk17b	1,260590766	1
Abhd17a	1,260328661	1
Gm13422	1,26006661	1
Gm14857	1,259979272	1
Nova1	1,25989194	1
AA386476	1,259717294	1
Ermp1	1,25962998	1
Sik1	1,259542672	1
Adat2	1,25945537	1
Rps26	1,259280785	1
Klf3	1,259193501	1
Nedd8	1,259018952	1
Gm5566	1,258931686	1
Atp5l	1,258931686	1
Gm11895	1,258844427	1
Guk1	1,258844427	1
F730043M19Rik	1,258757174	1
Tfeb	1,258757174	1
Rrp12	1,258669926	1
Sdf2	1,258669926	1
Dstn	1,258669926	1
Mpc2	1,258582685	1
Fnip1	1,258146569	1
Tarbp1	1,258059364	1
Gm16124	1,258059364	1
RP23-151L20.5	1,258059364	1

Gm5139	1,257972165	1
Pfdn1	1,257884972	1
Pds5a	1,257710604	1
Rfc4	1,257361941	1
Ciao1	1,256926248	1
Tbl2	1,256664905	1
Rbm38	1,256577802	1
Edrf1	1,256316532	1
Samd4b	1,256316532	1
Trappc9	1,256055315	1
Scaper	1,255794153	1
Plod1	1,255794153	1
Rab5c	1,255010992	1
Cdyl	1,254663077	1
Mrpl54	1,254576114	1
Fgf11	1,254576114	1
A930004J17Rik	1,25422832	1
Palm	1,25422832	1
Vwa5a	1,254054459	1
Plek	1,253880622	1
Gm45534	1,253793712	1
Ndufa6	1,253619912	1
Wdr91	1,253359256	1
Tmem184b	1,253098654	1
Hltf	1,253011799	1
Pgls	1,25292495	1
Ptp4a1	1,252838107	1
AU020206	1,25275127	1
Pak1	1,252490794	1
Micu3	1,252143578	1
Tmed4	1,252056789	1
Mical1	1,251970007	1
Armt1	1,251709694	1
Fnbp4	1,251622935	1
Tmem219	1,251189231	1
Tmpo	1,251189231	1
Gm15535	1,251102508	1
Arhgef1	1,251015791	1
Tssc4	1,250842375	1
Get4	1,250755677	1
Denr	1,250582297	1
2610001J05Rik	1,250408942	1
4930431P19Rik	1,250322273	1
Ccdc174	1,25023561	1
Rpe	1,24971576	1
Gm8357	1,249542524	1
Abcg1	1,249542524	1
Ppp1cc	1,249455916	1
Rnf103	1,249282716	1
Tpst2	1,249282716	1
Gm8770	1,249109541	1
Mettl5	1,248676708	1

Pfdn6	1,248676708	1
RP24-401G4.1	1,248503616	1
Pias4	1,248503616	1
Ube2j2	1,247984486	1
Rabggtb	1,247811491	1
Rc3h2	1,247811491	1
Ubl7	1,247552043	1
Khdrbs1	1,247379107	1
Uros	1,247292648	1
Gm12013	1,247206196	1
Pigx	1,247206196	1
Rps9	1,247119749	1
Tcp11l2	1,246946873	1
Gnb4	1,246687605	1
Map3k10	1,246687605	1
Aven	1,246601194	1
Alms1	1,24616923	1
Cr1l	1,245823766	1
Gnai2	1,245737416	1
Mmgt1	1,245564732	1
Nek1	1,245478399	1
Ing2	1,245392072	1
Lck	1,245305751	1
Nudt4	1,244960526	1
Ginm1	1,244874235	1
Stt3b	1,24478795	1
Myg1	1,244615398	1
Imp3	1,24444287	1
Ap2a1	1,244356614	1
Itfg1	1,244356614	1
Otud4	1,244356614	1
Gnptg	1,244270365	1
Anapc2	1,244270365	1
Slc39a14	1,244184122	1
Ube2a	1,244097885	1
Pdxdc1	1,244011653	1
Zfp277	1,243925428	1
Tacc1	1,243752995	1
Phc1	1,243666788	1
Gmds	1,243408201	1
Rpia	1,243322018	1
Trem3	1,24323584	1
Asb3	1,243063503	1
Swi5	1,24289119	1
Eif3j1	1,242632765	1
Ercc6	1,242460511	1
Dus4l	1,242288282	1
Vps53	1,242202176	1
Arhgef10l	1,241943894	1
Mrpl52	1,241943894	1
Snx29	1,241685666	1
Dnajc7	1,241685666	1

Nfxl1	1,241599602	1
Cdt1	1,241513544	1
Hadhb	1,241427492	1
Prkra	1,241427492	1
Gm44153	1,240911304	1
Ubxn7	1,240911304	1
Cnnm4	1,240825293	1
Klhl42	1,24065329	1
Smc1a	1,24065329	1
Mrpl23-ps1	1,240567298	1
Ptges3l	1,240481311	1
Tmem128	1,24039533	1
Mt1	1,240223387	1
2610020H08Rik	1,240137424	1
Dlgap4	1,240137424	1
Gm27039	1,240051468	1
Chek2	1,239879572	1
Mrpl35	1,239879572	1
Eaf1	1,239793633	1
Zfp397	1,239793633	1
Gm12716	1,2397077	1
Katnbl1	1,239621773	1
Atl2	1,239535852	1
Psd3	1,239449937	1
Tjp2	1,239278124	1
Cd37	1,239020451	1
Kpna1	1,238848698	1
Rpsa-ps1	1,238676969	1
Snhg15	1,23841942	1
Eid1	1,23824775	1
B9d2	1,238076105	1
Sys1	1,237904483	1
Cul3	1,237647095	1
A530072M11Rik	1,23738976	1
Txn-ps1	1,237303994	1
Gm43609	1,237303994	1
Fkbp5	1,23704673	1
Alyref	1,236960988	1
Acap1	1,236875251	1
1110051M20Rik	1,236703796	1
Zfp786	1,236532364	1
Dnajc3	1,236532364	1
Golm1	1,236446657	1
Ankrd13a	1,236360956	1
Cxcr4	1,236275261	1
Mrps31	1,236103889	1
Cadps	1,23593254	1
Zfand6	1,235846875	1
Mphosph10	1,235675562	1
Ndufs4	1,235589914	1
Blmh	1,235589914	1
Fuca2	1,235504273	1

Cstf2	1,235333007	1
Col4a5	1,235076154	1
Agrn	1,235076154	1
Snx5	1,235076154	1
Fcrl1	1,234904948	1
Map4k2	1,234819354	1
Cdca7	1,234819354	1
Tra2b	1,234819354	1
Ndufs5	1,234733765	1
Mrps18c	1,234733765	1
Naa15	1,234733765	1
Dnajc22	1,234648183	1
Paics	1,234648183	1
Rps11	1,234562607	1
Ogdh	1,234562607	1
Gm45137	1,234391472	1
Mbd2	1,234305914	1
Phf8	1,234305914	1
RP24-547N4.5	1,234134814	1
Syncrip	1,234134814	1
Ssr4	1,233792687	1
Map2k7	1,23370717	1
Arhgap23	1,233536154	1
Eral1	1,233450654	1
Dad1	1,233279674	1
Ppa1	1,233023247	1
Lztfl1	1,232937783	1
Tollip	1,232937783	1
RP23-36H21.3	1,232766874	1
Gm4890	1,232681428	1
Mr1	1,232254287	1
Brcc3	1,231912681	1
Taf4	1,231144413	1
Gm6297	1,23105908	1
Phpt1	1,230973752	1
Hnrnpdl	1,230973752	1
Fbxo44	1,230803115	1
Taz	1,230717805	1
Pitrm1	1,230632501	1
Aes	1,230632501	1
Acad10	1,230547203	1
Nxn	1,230547203	1
Elob	1,230461911	1
Spg21	1,229865032	1
Pan2	1,229779787	1
Trim33	1,229779787	1
Brd8	1,229438867	1
Mir5128	1,229353652	1
Irf2	1,229268443	1
Rsf1	1,229098042	1
Kdm1a	1,22901285	1
Scoc	1,22901285	1

Zfp622	1,228757311	1
Gm15798	1,228672143	1
B4galt7	1,22858698	1
Rps2	1,228161258	1
Smg7	1,228161258	1
Mbtd1	1,227565495	1
Nova2	1,22739533	1
Eif1-ps1	1,22739533	1
Gm11737	1,227140127	1
Gmfg	1,226970021	1
Gm45806	1,226884977	1
Gm38082	1,226884977	1
Meaf6	1,226884977	1
Lrpap1	1,226799939	1
Hspb7	1,22654486	1
Gm37357	1,22654486	1
Rabl3	1,226459845	1
Tmed1	1,226459845	1
Lrch3	1,226289833	1
Mrrf	1,226119845	1
Chchd3	1,226119845	1
Sae1	1,225610022	1
Gm27046	1,225440129	1
Zw10	1,225440129	1
Crebzf	1,22535519	1
Atxn2	1,22535519	1
Fam105a	1,225185332	1
Tle4	1,225100411	1
Adora2a	1,225015497	1
Mfng	1,224930588	1
Bola2	1,224675898	1
Ogfod3	1,224591012	1
Drg1	1,224251531	1
Psmb6-ps2	1,223912143	1
Gm8494	1,223827311	1
Zfp503	1,223657664	1
Capn15	1,22357285	1
Dock6	1,223148865	1
Iah1	1,223064086	1
Rps19-ps3	1,222894545	1
Dip2a	1,222809783	1
Acbd5	1,222725028	1
Oxct1	1,222725028	1
Rbm22	1,222725028	1
Ndufa2	1,222640278	1
Eif3i	1,222386063	1
Grina	1,222216616	1
Fam49b	1,222216616	1
Pigt	1,222131902	1
Cep57l1	1,221793102	1
Dnajc15	1,221793102	1
Emc1	1,221623737	1

Ranbp10	1,221200428	1
Bst1	1,221115784	1
Sav1	1,221115784	1
Tmem165	1,220523438	1
Phkb	1,220185084	1
Gm44935	1,22010051	1
Dnal1	1,21993138	1
Spata1	1,219846824	1
Cdkn1a	1,219762273	1
Gm7128	1,21959319	1
Harbi1	1,219508657	1
Pou2f1	1,219508657	1
Cwc15	1,21942413	1
Gm11977	1,219339609	1
Tdpx-ps1	1,219339609	1
Leprot	1,219339609	1
Eif3h	1,219255094	1
Dnaja1	1,218663651	1
Sf3b4	1,218579183	1
Mrpl47	1,218579183	1
Prkcd	1,218410264	1
Dact3	1,218072495	1
Ccnt1	1,217903646	1
Rps21	1,217903646	1
Lonp2	1,217734821	1
Cnot7	1,217734821	1
3010003L21Rik	1,217566019	1
Hook2	1,217481626	1
Yrdc	1,217481626	1
Ctsa	1,21739724	1
Gm8930	1,217228484	1
Kctd9	1,217144116	1
Tfcp2l1	1,216891044	1
Tnnc1	1,216891044	1
Slc37a1	1,216891044	1
Rps19	1,216806698	1
Ubxn4	1,216553697	1
Gm43137	1,216385058	1
Tle1	1,216216443	1
Abcb8	1,215963564	1
Bud13	1,215879283	1
Pdcl	1,215879283	1
Svil	1,215795008	1
Atp6v0a1	1,215795008	1
Gng7	1,215710738	1
Taf15	1,215542217	1
Edem3	1,215036792	1
Lgals4	1,214868364	1
Igsf6	1,214868364	1
Gm23300	1,214784158	1
Yme1l1	1,214784158	1
Arl13b	1,214531577	1

Wdr48	1,214531577	1
Fbxo8	1,214447395	1
Alkbh5	1,21369002	1
Armcx5	1,213605896	1
Med19	1,213605896	1
C430049E01Rik	1,213437666	1
Gmcl1	1,21335356	1
Krr1	1,212933117	1
Trit1	1,212849046	1
Fcer1g	1,212849046	1
Gm8722	1,21276498	1
Cdnf	1,21276498	1
Hexdc	1,212512819	1
Gm37702	1,212428777	1
Irf1	1,21226071	1
Morn2	1,212176686	1
Rusc1	1,212176686	1
Rnf34	1,212092667	1
Gm10169	1,212092667	1
Txnl1	1,212092667	1
Cyth1	1,211924647	1
Vps35	1,211924647	1
Ctnnbl1	1,21175665	1
Gpbp1l1	1,21167266	1
Brd9	1,211588677	1
Stim1	1,211588677	1
Mettl9	1,211504699	1
H2afj	1,21133676	1
Urb2	1,211252799	1
Fam76a	1,211168845	1
Gm45113	1,211084896	1
Stn1	1,211084896	1
Snrpb2	1,210917015	1
Sumf1	1,210749158	1
F10	1,210413514	1
Fam135a	1,210413514	1
Dhcr24	1,210413514	1
2700060E02Rik	1,210413514	1
Smad7	1,210329617	1
Wdr83os	1,20982636	1
Psmd14	1,20982636	1
Cep170	1,209742504	1
Gm15782	1,20957481	1
Ric1	1,209407139	1
Ppp4c	1,209239491	1
Cry2	1,209071866	1
Ufm1	1,208988063	1
Tspan17	1,208820473	1
Mocs2	1,208736687	1
Cntd1	1,208485363	1
Naip5	1,208485363	1
Rufy3	1,208485363	1

2810403D21Rik	1,208317843	1
Cdk19	1,207982873	1
Fam161b	1,207899145	1
Sec61g	1,207815423	1
St3gal1	1,207815423	1
Tmem143	1,207647995	1
Rp2	1,207647995	1
Gm6977	1,207313211	1
Nfkb1	1,207145853	1
Dip2b	1,207145853	1
Camk2n1	1,206811208	1
Chmp1a	1,20664392	1
Asb11	1,206560284	1
Pcnt	1,206560284	1
Auh	1,206476655	1
Cdk5	1,206393031	1
Tagln2	1,206393031	1
Atg14	1,206309413	1
Gm13226	1,206142195	1
Lcor	1,206058594	1
Exoc6b	1,205975	1
Gm14253	1,205640679	1
Gm12312	1,205557113	1
Gm28809	1,205306451	1
Lss	1,205306451	1
Mrpl18	1,205306451	1
Ankrd13d	1,20505584	1
Ube2l6	1,204888796	1
Prim2	1,204638272	1
Smpdl3a	1,204638272	1
Psip1	1,204471285	1
Mvb12a	1,204304322	1
Gm6851	1,204137381	1
Gm10059	1,20405392	1
4833439L19Rik	1,20405392	1
BC002163	1,203887014	1
Socs5	1,20380357	1
Txndc11	1,20380357	1
Vamp3	1,203553272	1
4930581F22Rik	1,203469851	1
Ndufa12	1,203469851	1
Rps12-ps24	1,203219622	1
Anp32b	1,202969446	1
Tmem59	1,202969446	1
Mrps30	1,202719321	1
Sesn1	1,202552601	1
Ak6	1,202552601	1
Gm45053	1,202302563	1
Tmem176b	1,202302563	1
Gm28417	1,2021359	1
Med11	1,2021359	1
Safb	1,20196926	1

Mycbp	1,201885949	1
Pomp	1,201885949	1
Snapc3	1,201802643	1
Fuom	1,201719343	1
Clk1	1,201302931	1
Ndufaf4	1,201136407	1
Fos	1,200969906	1
Zfp369	1,200886664	1
Atxn10	1,200886664	1
Alkbh1	1,200553753	1
Gm6344	1,200137745	1
Atox1	1,199971382	1
Rpl36al	1,19988821	1
Anxa6	1,19988821	1
Snrnp25	1,199805043	1
Gm20517	1,199638726	1
Atp5h	1,199638726	1
Itpripl1	1,199638726	1
Slc25a3	1,199555576	1
Tmed2	1,199472432	1
Tnnt3	1,199389294	1
0610009L18Rik	1,199223035	1
Fndc7	1,198973689	1
Gm13392	1,198641309	1
Cnpy4	1,198475154	1
Fam173a	1,198475154	1
Gm12309	1,198392085	1
Tbca	1,198392085	1
Srrt	1,198225964	1
Pdha1	1,198225964	1
Sirt1	1,197976826	1
Psmd8	1,197810762	1
Pet100	1,197727739	1
Tmco6	1,19756171	1
Sptbn1	1,19756171	1
1700022N22Rik	1,197395705	1
Mecp2	1,197395705	1
Cript	1,19731271	1
Eif2s1	1,19731271	1
Mrpl16	1,196897825	1
Mapk3	1,196731911	1
Gm45836	1,196566021	1
Ube2v1	1,196483084	1
Tmem97	1,196483084	1
Selenok	1,196400153	1
Fto	1,196317228	1
Pofut2	1,196068486	1
Gm8242	1,195985584	1
Rcsd1	1,195985584	1
Atp5sl	1,195985584	1
Whrn	1,195654032	1
Stard5	1,195654032	1

Zcchc9	1,195488291	1
Spata5	1,195322573	1
Csnk1e	1,195156877	1
Nrtn	1,194659929	1
Mpzl1	1,194245963	1
Tbrg4	1,193583916	1
Aida	1,193335743	1
Zbtb42	1,19325303	1
Gm6444	1,193087622	1
Rsrc1	1,192839552	1
Nudcd2	1,192756873	1
Olfr921	1,192591534	1
Pigq	1,192591534	1
Coro2a	1,192508872	1
Tmem9b	1,192260923	1
Erlec1	1,192013025	1
Isoc1	1,191599976	1
Nemf	1,191517384	1
Hmbox1	1,191104507	1
Bloc1s6	1,191104507	1
Epb41l4aos	1,191104507	1
Gm15834	1,190774308	1
Apool	1,190774308	1
Arhgap5	1,190774308	1
2610037D02Rik	1,190691773	1
Gm13094	1,190609243	1
Gamt	1,19052672	1
Fam63b	1,19052672	1
2810029C07Rik	1,190444201	1
Gosr2	1,190444201	1
Eif2ak3	1,190279182	1
4930524J08Rik	1,190031696	1
Mir142hg	1,189866734	1
Clec4a2	1,189784262	1
Vti1b	1,189701795	1
AI597479	1,189207115	1
Nol7	1,189207115	1
RP24-460E12.3	1,188877442	1
Svip	1,188630248	1
Spcs3	1,188630248	1
Mob3a	1,188547861	1
Smap2	1,18846548	1
Gcn1l1	1,188383105	1
Erbin	1,188053661	1
Fndc3a	1,187971314	1
Gm4342	1,187806638	1
Gm44419	1,187724308	1
Rpl31-ps14	1,187724308	1
Wrn	1,187559666	1
Smc5	1,187559666	1
Anp32e	1,187477354	1
Creb3l4	1,187148161	1

Itch	1,187148161	1
Snupn	1,186983598	1
Fam168b	1,186983598	1
Ccnc	1,186819059	1
Dnm1l	1,186819059	1
Haus8	1,186572292	1
Manf	1,186572292	1
Ptdss2	1,18640781	1
9930022D16Rik	1,18624335	1
Pcsk4	1,186078913	1
Ddx11	1,185914499	1
Ppie	1,185914499	1
Sgf29	1,185750108	1
Kxd1	1,18558574	1
Tmem9	1,185257071	1
Anxa4	1,185257071	1
Uggt2	1,185010629	1
Saal1	1,184846363	1
Ero1lb	1,184435798	1
Mndal	1,184435798	1
Mir5136	1,184353702	1
Ddost	1,184353702	1
Lpcat1	1,184353702	1
Gm42611	1,184271612	1
Gm8013	1,184025375	1
2010204K13Rik	1,183943308	1
Ech1	1,183368994	1
Gtf3c5	1,183286972	1
Gm7730	1,183204956	1
Ythdc1	1,183122945	1
Rps24-ps2	1,18304094	1
Inppl1	1,182712977	1
Gm16373	1,182631	1
Zbtb49	1,182385105	1
Hadh	1,182221203	1
Aprt	1,182221203	1
Wbp11	1,18213926	1
Ap1ar	1,181975392	1
Ap3d1	1,181811547	1
Mrpl11	1,181565822	1
Gm8659	1,181402033	1
Psma8	1,181320148	1
Rprd1a	1,181074524	1
Gm9761	1,181074524	1
Apopt1	1,180828952	1
Acbd6	1,180828952	1
Ufsp1	1,180583431	1
Mcf2l	1,180583431	1
Ppm1k	1,180583431	1
Vrk1	1,180583431	1
Tgds	1,180501603	1
Mospd2	1,180337962	1

Rbmx2	1,18025615	1
Nup54	1,18025615	1
Fopnl	1,180092543	1
Dhrs11	1,179356592	1
Cul1	1,17919311	1
Tspan31	1,178866214	1
Hipk2	1,178457721	1
Ifi30	1,178376039	1
Fxr1	1,178212693	1
Zfp954	1,178131028	1
Ptpn4	1,178049369	1
Pgs1	1,17772279	1
Larp4	1,17772279	1
BC029722	1,177477914	1
Dazap1	1,177396301	1
Nup210	1,17723309	1
Tnfrsf23	1,17723309	1
Map3k15	1,177151494	1
Gdpd3	1,177069902	1
Prdx6	1,177069902	1
Trip13	1,176988317	1
Gm17018	1,176743595	1
Zfp212	1,176580475	1
Cd44	1,176498923	1
Mrpl20	1,176417377	1
Rnf215	1,176335837	1
Srsf11	1,176335837	1
Sesn2	1,17609125	1
Usp33	1,176009733	1
Uap1	1,176009733	1
9630010A21Rik	1,175846714	1
Tomm22	1,175683719	1
Cpq	1,175602229	1
Dynlt1f	1,175520746	1
Lats2	1,175439268	1
Fbxl4	1,175357795	1
Supt3	1,175357795	1
Atp6v1h	1,175357795	1
Bola3	1,175276328	1
Rnf114	1,175276328	1
Zfas1	1,175194867	1
Rpl10a	1,175113412	1
Pde6d	1,174706219	1
Anapc15	1,174706219	1
Nmt1	1,174706219	1
Gm8116	1,174624798	1
Rcor3	1,174461971	1
Slc45a3	1,174380567	1
Ryr1	1,174299167	1
Nupr1	1,174299167	1
Pitpnc1	1,174217774	1
Tor1aip1	1,174136386	1

Thop1	1,173973628	1
Mllt11	1,173973628	1
Abhd14a	1,173810892	1
Ddt	1,173648178	1
Gm14593	1,17340415	1
Grpel2	1,173322819	1
Spaca9	1,173160173	1
D330023K18Rik	1,173078859	1
Arpin	1,173078859	1
Nubp1	1,17299755	1
Prpf38b	1,172834949	1
Tmem234	1,172509815	1
Cops4	1,172509815	1
Pcbp2	1,172347282	1
Carnmt1	1,172022284	1
Slmap	1,171941048	1
Gm42979	1,171534955	1
Asnsd1	1,171534955	1
Coq4	1,171291366	1
Hspa5	1,17072319	1
Dqx1	1,170560904	1
Secisbp2l	1,170236401	1
Hspd1-ps3	1,170236401	1
Copb1	1,170236401	1
Lcorl	1,170236401	1
Lilr4b	1,170155289	1
Hmx2	1,169911988	1
Tceal9	1,169749815	1
Vdac3	1,169668737	1
Rnf166	1,169425536	1
Fam109a	1,169182386	1
Mrfap1	1,169101347	1
H60c	1,169020314	1
Ssbp1	1,168939287	1
Csnk2b	1,168615233	1
Scyl2	1,16845324	1
Tnpo1	1,16845324	1
A530013C23Rik	1,168372251	1
Stard3nl	1,168372251	1
Rpl37rt	1,168372251	1
Gm9833	1,16812932	1
Gpatch2l	1,168048355	1
Zfa-ps	1,167805491	1
Uxs1	1,167805491	1
Sf3a1	1,167805491	1
Mir6236	1,16764361	1
Thap2	1,16764361	1
Arglu1	1,16764361	1
Gm37728	1,167562678	1
Mapk8ip1	1,167319916	1
Chaf1a	1,167319916	1
Emc7	1,167319916	1

Tm2d1	1,167158102	1
Tmem60	1,167158102	1
Bbs2	1,166268527	1
Neurl2	1,166106859	1
Gm12254	1,165945214	1
Gm44851	1,165702788	1
Nelfe	1,165702788	1
Cep57	1,165702788	1
Qtrt1	1,165460412	1
Bag3	1,165460412	1
Ndufb10	1,165379631	1
Gm12517	1,165218086	1
Epb41l5	1,165218086	1
Atad2	1,165218086	1
Rfc3	1,165137322	1
Rel	1,165137322	1
Aatk	1,164895064	1
2810414N06Rik	1,164814322	1
Aim1l	1,164814322	1
Nqo2	1,164814322	1
Ly6g6d	1,164733586	1
Calm2	1,164652856	1
Nit1	1,164491412	1
Mettl6	1,164329991	1
Cysltr1	1,164249288	1
Apitd1	1,16360387	1
Abhd10	1,16360387	1
Gm12704	1,163361931	1
Gm20223	1,163361931	1
Slc39a3	1,162878203	1
Dld	1,162636414	1
Utp18	1,16247525	1
Rwdd1	1,162394676	1
Ptch1	1,162314108	1
Gm44432	1,162233545	1
Gm3555	1,162152988	1
Wdr92	1,162152988	1
Hspbp1	1,162072436	1
Cactin	1,16199189	1
Gm27219	1,16191135	1
Usp21	1,16191135	1
Gm16106	1,161830815	1
Surf1	1,161669762	1
Gm5828	1,161589244	1
Sart3	1,161186738	1
Zfp91	1,161186738	1
Asrgl1	1,161106253	1
Hdgfrp2	1,160945301	1
Reep4	1,160864833	1
Ntpcr	1,160543018	1
Ethe1	1,160462578	1
RP23-371B13.3	1,160301715	1

Phactr4	1,160301715	1
Ppib	1,160301715	1
Rplp0	1,160301715	1
Kbtbd2	1,160060462	1
Fgr	1,159980055	1
Rpl10	1,159899655	1
Aldoart1	1,159899655	1
Arl8b	1,159899655	1
1700029J07Rik	1,159658485	1
Zdhhc17	1,159658485	1
Slc17a9	1,159578107	1
Rep15	1,159497734	1
Olfr920	1,159337004	1
L3mbtl2	1,159176297	1
Ado	1,159015612	1
Eloa	1,158935278	1
Atp2c1	1,158935278	1
Ets2	1,15885495	1
Mrps25	1,158613998	1
Kif2c	1,158533691	1
Gm8574	1,158453391	1
Rnpep	1,158453391	1
Gm7618	1,158373096	1
Smdt1	1,158051971	1
Amd1	1,157971704	1
Pign	1,157971704	1
Gm9013	1,157891442	1
Gm37963	1,157811186	1
Kif1b	1,157811186	1
Wdr45b	1,157730935	1
Mrps11	1,157490217	1
Dynll1	1,157490217	1
Map1lc3a	1,157249549	1
Gm7094	1,157089131	1
Gm42467	1,156928735	1
Ago3	1,156928735	1
Pcnx4	1,156848546	1
Cbl	1,156768362	1
C2	1,156608011	1
Snhg3	1,156608011	1
Pex2	1,156527844	1
C130036L24Rik	1,156207231	1
Dgat2	1,156127091	1
Tmed5	1,156127091	1
Upf3b	1,156046958	1
March5	1,156046958	1
Slx1b	1,155806589	1
Pdk1	1,155726478	1
Tmf1	1,155646372	1
Wbp4	1,155486176	1
Gm4468	1,155406087	1
Ttc37	1,155165852	1

Gm4034	1,155005723	1
2900026A02Rik	1,154925667	1
Pcmtd2	1,154845616	1
Tbc1d19	1,154685532	1
Snora31	1,154525469	1
Sbds	1,154285418	1
Gpt	1,154125411	1
2410015M20Rik	1,154125411	1
Txndc12	1,153965426	1
Ddx50	1,153965426	1
Stx8	1,153725491	1
Neurl3	1,153725491	1
Atp2a3	1,153725491	1
Spidr	1,153325709	1
Lrrc28	1,153325709	1
Ctcf	1,153325709	1
Dip2c	1,153245769	1
Sash1	1,153245769	1
Shc1	1,153165835	1
Tob2	1,153085907	1
Tsc22d1	1,152846154	1
Med13	1,152766248	1
Smchd1	1,152766248	1
Zfp940	1,152606451	1
Chmp2b	1,152606451	1
Mmaa	1,152526562	1
Mttp	1,152526562	1
Atg4c	1,152366799	1
Tubgcp4	1,152047339	1
Actn4	1,152047339	1
Slc44a2	1,151967488	1
Gid4	1,151967488	1
Cap1	1,151967488	1
1190005I06Rik	1,151887642	1
Cd81	1,151807802	1
Dhdh	1,151807802	1
Defb25	1,151727968	1
Acads	1,151568316	1
Osgin2	1,151488498	1
Lrrc40	1,151488498	1
Rab22a	1,151488498	1
4930563E22Rik	1,151328879	1
Jmjd1c	1,151249077	1
0610012G03Rik	1,151249077	1
Rbm8a2	1,151249077	1
Galm	1,151169282	1
Zfp383	1,151089491	1
Alg12	1,150929928	1
Dph3	1,150929928	1
Prkar2a	1,150929928	1
Kptn	1,150770386	1
Igsf8	1,150531115	1

Cpped1	1,150451369	1
Gm7965	1,150371628	1
Gtf3a	1,150291893	1
Brwd1	1,150291893	1
Tsen15	1,150291893	1
Fxr2	1,150212164	1
RP23-359K10.8	1,15013244	1
Cdc5l	1,15013244	1
Isca1	1,149973009	1
Syngr1	1,149973009	1
Nhlrc2	1,149733904	1
Ddx6	1,149574528	1
Lims1	1,149335505	1
Mxd4	1,149255842	1
Psmg4	1,149176185	1
Nde1	1,148777979	1
Gm14121	1,148539123	1
2610524H06Rik	1,148141138	1
Vdac1	1,147981983	1
Ppp1r14b	1,147902414	1
Mthfd1l	1,14782285	1
6330562C20Rik	1,147743292	1
Srgn	1,147743292	1
Abtb1	1,147584192	1
Nus1	1,147584192	1
Gm6733	1,147425114	1
Ino80	1,147425114	1
Ndufs2	1,147425114	1
Dbi	1,147345583	1
Haus1	1,147266058	1
Timm29	1,146391645	1
Trappc2l	1,146153284	1
Nemp2	1,145597302	1
Nfrkb	1,145597302	1
Ifitm3	1,145200337	1
Jak3	1,14504159	1
Irf2bp2	1,144962224	1
Gm37420	1,144724161	1
Emc2	1,144724161	1
Gm22516	1,144644817	1
Shoc2	1,144486147	1
Zdhhc8	1,14440682	1
Vars	1,14440682	1
Asb6	1,144010267	1
Zbtb33	1,143613852	1
Bnip1	1,143613852	1
RP23-255F14.4	1,143534586	1
Cdan1	1,143376069	1
Sema4g	1,143376069	1
Acad9	1,143296819	1
7330423F06Rik	1,143138335	1
Kansl1	1,142979874	1

Zcchc14	1,142742223	1
Nav2	1,142742223	1
Fam122a	1,142663016	1
Sp1	1,142504621	1
Eif5a	1,142187895	1
Ctc1	1,141950408	1
0610038B21Rik	1,141871257	1
Atg101	1,140684647	1
Nxf1	1,140605583	1
Rpl38-ps2	1,140526525	1
Clk4	1,140526525	1
Fbxl18	1,140447473	1
2410022M11Rik	1,140368426	1
March4	1,140289384	1
Nae1	1,140052292	1
R3hdm2	1,139894259	1
RP24-282C4.13	1,13981525	1
Gm16556	1,139657249	1
Kif21b	1,139657249	1
Zfp598	1,139578257	1
Ctsf	1,139578257	1
Klf1	1,13949927	1
Rpsa-ps10	1,139341313	1
Mrc1	1,139262342	1
Spata6	1,139104418	1
Sdhaf3	1,139104418	1
Mdp1	1,139104418	1
Maea	1,138867572	1
Gm6560	1,138551855	1
Gm20768	1,138078443	1
Dhfr	1,137920683	1
Fam20c	1,137920683	1
Rmdn3	1,137841811	1
Uqcr10	1,137841811	1
Zfp595	1,137684083	1
Phf12	1,137684083	1
Ddx49	1,137368694	1
Rab28	1,13697458	1
Gm4366	1,136816973	1
Tpm3	1,136816973	1
Npat	1,135950524	1
Agk	1,135871789	1
Plekha2	1,135871789	1
Zic5	1,135793059	1
Entpd6	1,135793059	1
Med6	1,135242102	1
Dcakd	1,135084735	1
Rpl8	1,135084735	1
Vegfb	1,134612765	1
Baat	1,134534122	1
Msl1	1,134534122	1
Dynlt3	1,134298227	1

Serinc1	1,134140991	1
Rbm12b2	1,134062381	1
Kat8	1,133905177	1
Rab9	1,133905177	1
Ppp2r5e	1,133905177	1
Flii	1,133826584	1
Kat2a	1,133669413	1
Commd6	1,133433697	1
Rnf4	1,133433697	1
Gm1848	1,133276581	1
Csnk2a1	1,132648333	1
Cln8	1,132491326	1
Phf2	1,13241283	1
Smad5	1,132020434	1
Clk2	1,131863513	1
Pgrmc2	1,131863513	1
Isoc2a	1,131785061	1
Nr2f6	1,131236049	1
Idh3b	1,131079237	1
Oxa1l	1,131000839	1
Gtf2a2	1,130922447	1
Vamp7	1,130687303	1
Pdia4	1,130608932	1
Zfp516	1,130373853	1
Glrx2	1,130373853	1
Itgb1bp1	1,129668909	1
Tubb4b	1,129512315	1
Trnt1	1,129512315	1
Snap47	1,129434026	1
Mff	1,129120923	1
2810402E24Rik	1,129042661	1
Cst3	1,128573203	1
Papolg	1,128103941	1
Slc41a1	1,128025749	1
Borcs6	1,128025749	1
Tcp1	1,127791207	1
Colgalt1	1,127634873	1
Gm13487	1,127322269	1
Phyh	1,127087874	1
Zfp9	1,126931637	1
Gm44198	1,126619228	1
Prss53	1,126306907	1
Qpctl	1,126150778	1
Sipa1l2	1,126072722	1
Pex19	1,125994671	1
Zc3hc1	1,125994671	1
C3ar1	1,125526481	1
Bag4	1,12537046	1
Spa17	1,125136471	1
Cpne3	1,124980504	1
Mnat1	1,124590683	1
Gm12833	1,124512735	1

Nfkbil1	1,124512735	1
Fchsd1	1,124200997	1
Bex3	1,124123076	1
Gm2272	1,123811447	1
S100a6	1,123811447	1
Fam43a	1,123655664	1
Agmo	1,123655664	1
Traf3	1,123577781	1
Scpep1	1,123499903	1
Cox7b	1,123344164	1
Zfp541	1,123266302	1
Cep72	1,123188446	1
Mbd3	1,12303275	1
Cdc37	1,12295491	1
Snx6	1,122877075	1
Tep1	1,122643604	1
Pigf	1,122643604	1
Cryzl1	1,122410181	1
Gm6181	1,122176806	1
Gm19620	1,122176806	1
Gstz1	1,12202125	1
Arhgap12	1,12202125	1
Parl	1,121476974	1
Memo1	1,121166078	1
Msi2	1,121166078	1
Icmt	1,121166078	1
Fam198b	1,121088367	1
Topors	1,121088367	1
Zbed3	1,120932962	1
Gabarapl1	1,120855268	1
Ift46	1,120777579	1
Wdsub1	1,120622217	1
Gm8186	1,120622217	1
Ubap2l	1,120544544	1
Ccnyl1	1,120466876	1
Anapc11	1,120466876	1
F2	1,120389214	1
BC031181	1,120389214	1
Ahctf1	1,120389214	1
Gm6265	1,120233906	1
Rpf1	1,120233906	1
Kif3a	1,12015626	1
D130019J16Rik	1,11984573	1
Alcam	1,11984573	1
Zfp263	1,119768111	1
Nhp2	1,119768111	1
Gm19739	1,119612889	1
Apex2	1,119535286	1
Nob1	1,119302509	1
Bcl2l14	1,118992216	1
Ctsc	1,118759552	1
Ei24	1,118682008	1

Ddx19a	1,118682008	1
Fam19a2	1,118526937	1
Kmt2c	1,118449409	1
Gm43795	1,118371887	1
Galk2	1,118216858	1
Gm8822	1,118139352	1
Leng1	1,118061851	1
Dyrk3	1,117984356	1
N4bp1	1,117906865	1
Acot8	1,117829381	1
Rplp1	1,117596959	1
Gnpda2	1,117519496	1
Baiap2	1,117442038	1
Gtf3c4	1,117209696	1
Uqcrh-ps2	1,11713226	1
Selenon	1,116822568	1
1810043G02Rik	1,116667754	1
Gm8925	1,116435574	1
Gm10126	1,116126075	1
Mcee	1,116048714	1
Gm9332	1,115894007	1
4930430F08Rik	1,115894007	1
Dynlrb1	1,115894007	1
Cdk5rap3	1,115739322	1
Atf6b	1,115661988	1
Gorab	1,115584659	1
Lipe	1,115584659	1
Nfe2l2	1,115584659	1
Rsbn1	1,115352704	1
Fbxw11	1,115275396	1
Samd1	1,115198094	1
Gm5879	1,114966219	1
Rad51c	1,114966219	1
Fhod1	1,114966219	1
Slc35a1	1,114811662	1
Acin1	1,114811662	1
Myoz1	1,114657127	1
Cdadc1	1,114657127	1
Cox6a1	1,114270884	1
Dtnb	1,114116423	1
Gm2a	1,114039201	1
Khnyn	1,113961985	1
Gnal	1,113884774	1
Cmip	1,113884774	1
2310036O22Rik	1,113807568	1
Agfg2	1,113807568	1
Ttc39b	1,113653172	1
Plxna1	1,113575982	1
Rchy1	1,113575982	1
2410089E03Rik	1,113344444	1
Gm12606	1,113344444	1
Zdhhc1	1,113112955	1

Aimp1	1,112958655	1
1600012H06Rik	1,112881514	1
Smim13	1,112341671	1
Zswim6	1,112187479	1
Dnajc13	1,112187479	1
Rps16-ps2	1,112187479	1
Bzw2	1,11195623	1
Fam78a	1,111879158	1
2810405F17Rik	1,111879158	1
Mrpl58	1,111802091	1
Larp1	1,111802091	1
4931406P16Rik	1,111725029	1
Cd99l2	1,111647973	1
Rubcn	1,111493876	1
Tarsl2	1,111185748	1
Dnajc21	1,111108729	1
Kctd18	1,110800707	1
Cab39l	1,110569746	1
Tsr3	1,110338834	1
Arap3	1,110184919	1
Atg5	1,110184919	1
Smpd1	1,110031025	1
Chchd2	1,109954086	1
Lrrc57	1,109800225	1
2610301B20Rik	1,109800225	1
Lpin1	1,109800225	1
Slc35b1	1,109800225	1
Ints9	1,109646384	1
Hpf1	1,109415664	1
Smad6	1,109338768	1
Rida	1,109338768	1
Snd1	1,109338768	1
Zfp275	1,109261877	1
Hyal2	1,109184991	1
Unc119b	1,109108111	1
Aco2	1,109108111	1
Pola2	1,109031236	1
Trmt10a	1,108877502	1
Psmb7	1,108646942	1
Snhg17	1,108646942	1
Lsm6	1,108570099	1
Cdc42bpb	1,108493261	1
Pole3	1,108493261	1
Gopc	1,108416429	1
Khsrp	1,108416429	1
Nxf7	1,108339602	1
Rtkn	1,108185964	1
Hsf1	1,107801963	1
Rnf10	1,107801963	1
Sh3bgrl3	1,107725179	1
A930006K02Rik	1,107494858	1
Lst1	1,107264584	1

Xdh	1,107187837	1
Rab13	1,107111096	1
Gm42659	1,106957628	1
Banf1	1,106804182	1
Agap3	1,106727467	1
Gm15634	1,106650757	1
Nthl1	1,106574052	1
Tmbim6	1,106574052	1
Hddc2	1,106497353	1
Rpl26-ps2	1,106037269	1
Msl2	1,105960607	1
Pcmtd1	1,105960607	1
Pla2g6	1,105807299	1
Mrpl38	1,105730653	1
Lca5	1,105654013	1
Emc6	1,105424122	1
Abl2	1,105270888	1
Sgsm3	1,104734738	1
Adam15	1,104658166	1
Ccdc180	1,1045816	1
Atp5b	1,104428483	1
Dazap2	1,104351932	1
Surf6	1,104275387	1
Zfp593	1,104198847	1
Steap3	1,104122313	1
3110009E18Rik	1,103892741	1
Crem	1,103739719	1
Pigs	1,103739719	1
Lpgat1	1,103739719	1
2300009A05Rik	1,103586719	1
Aldoa	1,103127846	1
Frg1	1,102745597	1
RP23-316F10.2	1,102669163	1
Rfc1	1,102669163	1
Snora30	1,102592735	1
Gm43162	1,102592735	1
Acsl3	1,102516311	1
Gfer	1,102516311	1
Mrps21	1,102439893	1
Sptlc2	1,102439893	1
Skiv2l2	1,101981497	1
Rab31	1,10175237	1
Gm8724	1,101676005	1
Cblb	1,101523291	1
Peak1	1,101523291	1
Cdca4	1,101523291	1
Lrrc42	1,101446942	1
Igip	1,101294259	1
Zbtb14	1,101217926	1
Ctsd	1,101217926	1
Rrn3	1,101141598	1
Tm7sf2	1,100988958	1

Tmc6	1,100912646	1
Vps13d	1,100912646	1
Gm38120	1,10060745	1
Rilp	1,100454884	1
Diaph2	1,100454884	1
Spata33	1,100149816	1
Mief2	1,099844832	1
Dusp10	1,099768599	1
Ube2q2	1,099616149	1
Tcea3	1,099539932	1
Kdm7a	1,099539932	1
Tes	1,099463721	1
Chfr	1,099387514	1
Gm15472	1,099311313	1
Rnf121	1,099158927	1
Cyb5b	1,099006562	1
Stambpl1	1,098930387	1
Zfp54	1,098625741	1
Odf3l1	1,098625741	1
Lix1l	1,09847345	1
Osbpl10	1,09816893	1
Adat1	1,098092814	1
Fpgt	1,097940596	1
Plxnc1	1,097864496	1
Tmem248	1,0977884	1
2310047D07Rik	1,09771231	1
Ccdc159	1,097560145	1
Ap3m2	1,097560145	1
Hnrnpc	1,097560145	1
Vrk2	1,097408001	1
Luc7l	1,097331938	1
Clmp	1,097179826	1
Hes6	1,097027735	1
Mrps36	1,096951697	1
Mta1	1,096951697	1
Fam131a	1,096875665	1
Gucy2g	1,096875665	1
Faf1	1,096799638	1
Tmem123	1,096799638	1
Megf9	1,096723616	1
Gm4332	1,096343587	1
Kdm5a	1,096343587	1
Cct2	1,096039658	1
Nkiras1	1,095887725	1
Abhd13	1,095659865	1
Ecsit	1,095204288	1
Ormdl2	1,095128377	1
Svbp	1,095052471	1
Ccdc114	1,09497657	1
Pum1	1,09497657	1
2510002D24Rik	1,094597146	1
Yaf2	1,094293701	1

Got2	1,094293701	1
Layn	1,09414201	1
Polr2e	1,093838692	1
Ppp2r2a	1,093611258	1
Sos1	1,093459662	1
Psmb10	1,093232307	1
Hsd17b12	1,093004999	1
Tmem79	1,092777739	1
Prepl	1,092701996	1
Rpl22	1,092701996	1
Snrpd3	1,092626258	1
Prps1	1,092399077	1
G6pd2	1,09232336	1
Havcr2	1,092247649	1
Snrpb	1,092096241	1
Apoa1bp	1,09186917	1
1700037H04Rik	1,09179349	1
Nmt2	1,09179349	1
Sigirr	1,091717815	1
Gm3362	1,091566482	1
Luc7l2	1,091566482	1
Gm14843	1,091490823	1
Gm13612	1,091415169	1
Rnaset2a	1,091112606	1
Kat7	1,091112606	1
Lsm8	1,090961356	1
Kansl1l	1,090734521	1
Srebf2	1,090734521	1
Rps11-ps1	1,09065892	1
Traf3ip1	1,090583324	1
Arl6ip5	1,090583324	1
6030400A10Rik	1,090432147	1
Ctso	1,090205422	1
Serpinb6a	1,090205422	1
Leng9	1,090129857	1
Anapc13	1,090129857	1
Cab39	1,090129857	1
Rpa1	1,089978743	1
9530068E07Rik	1,089978743	1
Akap1	1,089903194	1
Pfkl	1,089827651	1
Vps13a	1,089752112	1
Nostrin	1,089601051	1
Necap2	1,089601051	1
Slc36a1	1,089525528	1
Mier3	1,089450011	1
Eef1b2	1,089450011	1
Speg	1,089374498	1
Suz12	1,089374498	1
Cd300ld	1,089223489	1
Cep350	1,088997015	1
Flot2	1,088846059	1

Mrps24	1,088770588	1
Gm45828	1,088619663	1
Tkt	1,088544208	1
Ndrg2	1,088544208	1
A230050P20Rik	1,087940759	1
Gm15500	1,087940759	1
Aldh3a2	1,087865351	1
2310035C23Rik	1,087789948	1
Ndufb6	1,087714551	1
Med9	1,087186917	1
Wdr82	1,087036211	1
BC025920	1,08658422	1
Lrrc51	1,086508906	1
H2-Ab1	1,086282996	1
Gm43411	1,086282996	1
Acap3	1,086207703	1
Kctd20	1,086207703	1
RP23-349H12.3	1,085906584	1
Mettl26	1,085605549	1
Cdkn3	1,085530303	1
Ccpg1os	1,085455062	1
Fbxo22	1,085304597	1
RP23-453B15.7	1,085154152	1
AI846148	1,085078938	1
Ppt1	1,085078938	1
Slc35f5	1,084552582	1
Cox4i1	1,084402241	1
Ing5	1,084327079	1
Bag6	1,084327079	1
1110004F10Rik	1,084327079	1
Thap4	1,084327079	1
Wdr90	1,084251922	1
D730003I15Rik	1,084251922	1
Urod	1,084026481	1
Josd2	1,083951345	1
Il10ra	1,083876214	1
Man2a2	1,083801088	1
Cenpc1	1,083725967	1
Spg7	1,083575741	1
Eif4e2	1,083575741	1
Fcho2	1,083425536	1
Ccdc134	1,083350441	1
Ndufa13	1,083200267	1
Acss2	1,082824924	1
Vps50	1,082824924	1
2700046G09Rik	1,082749871	1
Psen1	1,082524743	1
Cdc42ep3	1,08244971	1
Esyt1	1,08244971	1
Rcbtb1	1,082374683	1
Fam178a	1,082224645	1
Ppig	1,082224645	1

Gm4017	1,082149633	1
Cnot8	1,082149633	1
Bora	1,082074627	1
Tiprl	1,081999626	1
Lemd3	1,081774654	1
Hpcal1	1,081699673	1
Gm19287	1,081474763	1
1700096K18Rik	1,081399804	1
Psmb2	1,08132485	1
Pick1	1,08132485	1
Purb	1,081249901	1
Gm9256	1,081174957	1
Csnk1g3	1,081174957	1
Aimp2	1,081100018	1
Rhobtb2	1,080875233	1
Gm14013	1,080800315	1
Gm13712	1,080800315	1
Gm43323	1,080725402	1
Rtn4	1,080425803	1
Man1c1	1,080276034	1
Ttll4	1,080201158	1
Celf4	1,080126287	1
Zfp563	1,079901704	1
Ccdc59	1,079826854	1
Meis2	1,079452679	1
Bag1	1,079452679	1
Tmem42	1,07937786	1
Atp5j	1,079228237	1
Wnt6	1,079153433	1
Siah1b	1,078929052	1
0610040B10Rik	1,078854269	1
Pyurf	1,078854269	1
Dpf2	1,078779491	1
Alkbh6	1,078629951	1
3110040N11Rik	1,078629951	1
Scrib	1,078555189	1
Foxj2	1,078480432	1
Zfp574	1,078330933	1
Abcb9	1,078256191	1
Malt1	1,078256191	1
Ppil4	1,078256191	1
Ubxn11	1,078031998	1
Erc1	1,077957277	1
Gm3145	1,077882561	1
Samhd1	1,077882561	1
Kansl2	1,077658445	1
Ndufc2	1,07758375	1
Atf2	1,077210353	1
Psmd13	1,076911728	1
Idua	1,076837085	1
Sh3bgrl	1,076613186	1
Gm6030	1,076463946	1

Usp14	1,076389334	1
Fer	1,076240125	1
Mtf2	1,07601635	1
Rbbp8	1,075867193	1
Rbm4	1,075718056	1
Lonp1	1,075718056	1
Pnkd	1,075643496	1
Brd3	1,075643496	1
Rab11a	1,075643496	1
Asna1	1,075568941	1
1700020I14Rik	1,075568941	1
Hccs	1,07549439	1
Fcf1	1,075345306	1
Ccdc112	1,075196241	1
Timm23	1,075121717	1
Nsun6	1,075121717	1
Trpt1	1,074972684	1
Insl6	1,074972684	1
Golgb1	1,074972684	1
Soat2	1,074898175	1
Fbxo34	1,074823671	1
Phc3	1,074823671	1
Tmem70	1,074823671	1
Stat2	1,074749173	1
Ppic	1,074749173	1
Dvl1	1,074749173	1
Pkd1l2	1,07467468	1
Epn2	1,07467468	1
Tgfbr1	1,074600191	1
Hsd17b4	1,074376758	1
Aqr	1,07430229	1
E130208F15Rik	1,074227828	1
Etv1	1,074227828	1
Rgs2	1,074153371	1
Ptk2b	1,074078919	1
4932438A13Rik	1,074078919	1
Gm5297	1,074078919	1
Rnh1	1,074078919	1
Trappc8	1,073855593	1
Bloc1s4	1,073855593	1
Gas8	1,073781162	1
Zfp637	1,073557899	1
Ttc7b	1,073557899	1
Wdr18	1,073557899	1
Gm37490	1,073409082	1
Gss	1,073409082	1
Usp15	1,073334682	1
Gm4459	1,073260286	1
Uspl1	1,073260286	1
Rnf138	1,073185896	1
Slc19a1	1,073111511	1
Zzz3	1,073037131	1

Hyal3	1,072962757	1
Slc25a36	1,072962757	1
Inafm2	1,072814023	1
Gm45629	1,072665309	1
Pmpca	1,072590961	1
Renbp	1,072590961	1
Prkag1	1,072219293	1
Atg13	1,071996355	1
Gm10313	1,071699175	1
5830487J09Rik	1,071624893	1
Gm11517	1,071550617	1
Ggcx	1,071327817	1
Knl1	1,071253561	1
Psma3	1,071253561	1
Cers6	1,071253561	1
Dyrk1a	1,07117931	1
Znrd1	1,071105064	1
Actr3	1,070956588	1
Brpf3	1,070808132	1
Nudc	1,070808132	1
Gls	1,070659697	1
Ski	1,070585487	1
Spcs1	1,070585487	1
Scai	1,070437082	1
Pdk3	1,070437082	1
1600014C10Rik	1,070362888	1
Coq8a	1,069769517	1
Ndufs6	1,069695369	1
Msmo1	1,069695369	1
Slc25a28	1,069472955	1
Gm9727	1,069324705	1
Pdgfa	1,069250588	1
Anapc1	1,069176475	1
Ndufc1	1,069176475	1
Dars	1,069028266	1
Klf7	1,068954169	1
Pdcd4	1,068657834	1
Vmac	1,068435635	1
Ttc30a1	1,068213484	1
Mrto4	1,068213484	1
Osgep	1,068065408	1
Trim36	1,067843333	1
Usp2	1,067769318	1
Gm44771	1,067695309	1
Ewsr1	1,067547305	1
Ap3s2	1,067547305	1
H2-DMa	1,067473311	1
Agbl5	1,067325338	1
Ndufv2	1,067325338	1
Sap18b	1,067177386	1
Scand1	1,067177386	1
Gin1	1,067103417	1

Ifi35	1,067103417	1
Sarnp	1,067103417	1
Hk1	1,067103417	1
Rnf167	1,067029454	1
Phtf1os	1,066955495	1
Ldlr	1,066881542	1
Gm17430	1,066807594	1
C2cd2l	1,066807594	1
Gm4117	1,066585781	1
Gga1	1,066511853	1
Ier3ip1	1,066364014	1
Gm20667	1,066290101	1
Tmsb4x	1,066290101	1
Pros1	1,066068396	1
Pcmt1	1,066068396	1
Emsy	1,065994504	1
Nup133	1,065846736	1
Pdrg1	1,065698989	1
Paqr5	1,065625123	1
Pyroxd1	1,065034378	1
Pianp	1,065034378	1
Ogg1	1,064960558	1
3110043O21Rik	1,064886743	1
1700007L15Rik	1,064812934	1
Ifi211	1,064739129	1
Ctdnep1	1,064739129	1
Lrrc58	1,064665329	1
Med20	1,064591535	1
Maml3	1,064591535	1
Dbr1	1,064443962	1
Cep70	1,06422264	1
Adam8	1,06422264	1
Trappc6b	1,064148876	1
Gm13453	1,064001364	1
Retn	1,063927615	1
Scd1	1,063780134	1
Gm12726	1,06355895	1
Ighm	1,06355895	1
Aup1	1,06341152	1
Il1rl1	1,063337812	1
Usf2	1,06326411	1
Tcta	1,06311672	1
Gm12988	1,063043033	1
Cfap74	1,062969351	1
Bicdl1	1,062969351	1
Med7	1,062674674	1
Tnfrsf10b	1,06245372	1
Dstyk	1,062306443	1
Ddias	1,062232812	1
Uqcrq	1,062232812	1
Rcc1l	1,06201195	1
Tmem229b	1,061791134	1

Tnni3	1,061717539	1
Rabac1	1,061349639	1
Rttn	1,061202515	1
Gm14794	1,061202515	1
Eya3	1,061202515	1
Atxn7l1	1,061128961	1
Fam234a	1,060908328	1
Hmgb1-rs16	1,060761265	1
Mbd5	1,060173217	1
Ccdc167	1,060026256	1
Zfp146	1,060026256	1
Tfpt	1,059952783	1
Mrpl24	1,059879316	1
Sugp1	1,059291757	1
Gatsl3	1,059071506	1
Ehmt2	1,059071506	1
Nudt8	1,05877791	1
Mtmr14	1,058704523	1
Fasn	1,058557766	1
Per3	1,058484395	1
Adipor2	1,058484395	1
Gm13328	1,058337668	1
Ube2e3	1,058264312	1
Man1b1	1,058044275	1
Map1lc3b	1,058044275	1
Vps26a	1,057897609	1
Rrp36	1,057677648	1
Ccdc63	1,057604338	1
Sema4c	1,057457733	1
Nkain1	1,057384439	1
Gm7236	1,057311149	1
Hint3	1,057311149	1
Mdm4	1,05709131	1
Mvk	1,056871517	1
Chtf8	1,056871517	1
Azin1	1,056871517	1
B230312C02Rik	1,056798263	1
Sat1	1,056651769	1
Nit2	1,056651769	1
Gm5873	1,056432068	1
Abracl	1,056432068	1
Mta2	1,056432068	1
Wsb1	1,056358844	1
Arhgap22	1,056212412	1
Scaf8	1,055992801	1
Gm4994	1,05584642	1
Angel1	1,05584642	1
Gm43387	1,055700059	1
Cdc34	1,055700059	1
Psmf1	1,055626886	1
Mpv17l2	1,055334244	1
Mms19	1,054822317	1

Bcas3	1,054602996	1
Dgkd	1,054602996	1
Trmt2b	1,05416449	1
Emp3	1,054018362	1
St6galnac4	1,054018362	1
Arfrp1	1,053872254	1
Npm1	1,05365313	1
Them6	1,053507073	1
Gm37339	1,053361036	1
Shprh	1,053142018	1
Galnt3	1,052850066	1
Snrpd2	1,05277709	1
Edem1	1,05277709	1
Slc25a4	1,05270412	1
Tmem106b	1,052558194	1
Nfatc2ip	1,052412289	1
Proser3	1,052339344	1
Avpi1	1,052339344	1
Secisbp2	1,052193469	1
Asap1	1,052120539	1
Sec14l1	1,052120539	1
Sord	1,052047614	1
Ccnl2	1,051974694	1
Rpl38	1,051683065	1
Furin	1,051318643	1
Eef1g	1,051172909	1
Ndufb11	1,05110005	1
Mcmbp	1,05110005	1
Tmem135	1,050954347	1
Srsf10	1,050881502	1
Gnl1	1,050735829	1
Haus7	1,050663001	1
Ambra1	1,050590177	1
2810428I15Rik	1,050371735	1
Ifi207	1,050298932	1
Fam193a	1,050298932	1
Gm4149	1,050226133	1
Ik	1,050226133	1
Dnajc18	1,050153339	1
Naxd	1,050007767	1
4833420G17Rik	1,050007767	1
Bad	1,050007767	1
Sars	1,050007767	1
Thrap3	1,049934989	1
Tspo	1,049934989	1
Doc2g	1,049352943	1
Matr3	1,049352943	1
Mkrn1	1,049352943	1
Itpr3	1,049207481	1
Cct8	1,048843917	1
Dnal4	1,048771219	1
Usp39	1,048698526	1

Uhrf1bp1	1,048553156	1
Zfp131	1,048553156	1
Gm13776	1,048407806	1
A430027C01Rik	1,048189818	1
Tap1	1,048189818	1
Ptges2	1,048117166	1
Sbf1	1,048117166	1
Mrps15	1,047971876	1
Fdft1	1,047899238	1
Zyg11b	1,047826606	1
Pik3r3	1,047753979	1
Ceacam1	1,047681357	1
Lyar	1,047681357	1
Vps18	1,047681357	1
Thyn1	1,047536127	1
Ube2b	1,047536127	1
Hspa13	1,04746352	1
Gm9840	1,047390918	1
9930111J21Rik2	1,047173142	1
Exoc2	1,04710056	1
Use1	1,046955411	1
Lmntd2	1,046882844	1
Csgalnact2	1,046810282	1
Gm4875	1,046737725	1
Hk2	1,046737725	1
Gm9825	1,046665173	1
Gm22973	1,046592627	1
Snx27	1,046447548	1
Hoxaas3	1,046375016	1
Mri1	1,04630249	1
Rbl2	1,046157451	1
Gm43110	1,04608494	1
Ten1	1,04608494	1
Gm26935	1,046012433	1
Tmem63b	1,045794944	1
Tmem167b	1,045722457	1
Npepl1	1,045432562	1
BC017643	1,045287644	1
Gm5380	1,045070305	1
Dcaf7	1,044997869	1
Gm44834	1,044925438	1
Dcps	1,044925438	1
Bud31	1,044925438	1
Serpinf2	1,044853011	1
Rcor1	1,044853011	1
Ccdc106	1,04478059	1
Tmem206	1,044635763	1
Ahnak	1,044635763	1
Glg1	1,044563357	1
Ptprs	1,044490956	1
Btg2	1,044201401	1
Mgst2	1,044129025	1

Fbxw4	1,044129025	1
Eny2	1,043550198	1
Fitm2	1,043260904	1
Gas2l3	1,043188594	1
Haghl	1,0428994	1
Pafah1b1-ps1	1,042827115	1
Gm43213	1,042754834	1
Bahcc1	1,042393505	1
Polr1c	1,042249009	1
Fxn	1,042104532	1
Fam45a	1,042104532	1
Gins4	1,042104532	1
Jak2	1,041887855	1
Pigu	1,04181564	1
A430005L14Rik	1,041743429	1
Slc35b2	1,041743429	1
Hnrnpll	1,041526827	1
Gch1	1,041526827	1
Rnf44	1,041454636	1
Gnb1l	1,041382451	1
Ankle2	1,041382451	1
Afdn	1,041382451	1
Gm13743	1,04131027	1
Pak2	1,041093758	1
Otx1	1,041021598	1
Pot1a	1,040949442	1
Ddhd1	1,040733005	1
Stx16	1,040516613	1
Tmco4	1,040444493	1
Zfp874b	1,040444493	1
C77080	1,040372377	1
Gabpb2	1,040228161	1
Taf11	1,040083965	1
Zfp60	1,040011874	1
Mpp5	1,039723561	1
Qsox1	1,039723561	1
Atp5a1	1,039651496	1
Gm37959	1,039579435	1
Rab8b	1,039579435	1
Mafg	1,039579435	1
Hdac10	1,03950738	1
Smco4	1,03950738	1
Cyp51	1,039435329	1
Dcp1a	1,039363283	1
Rps2-ps11	1,039219207	1
Cdk17	1,038931114	1
Rogdi	1,038787098	1
Laptm4b	1,038643101	1
Atp13a3	1,03857111	1
Chd7	1,038211231	1
Gm43788	1,037851477	1
Ncoa3	1,037851477	1

Taf2	1,037491848	1
Acot7	1,037419937	1
C1galt1	1,037348031	1
Wrap53	1,03727613	1
Fam220a	1,037204234	1
Med28	1,036916699	1
Smg9	1,036772962	1
E130307A14Rik	1,036629245	1
Nup88	1,036629245	1
Galt	1,036485547	1
Nipsnap3b	1,036270039	1
Gm37183	1,036198213	1
Gfod1	1,035982764	1
Med15	1,035910957	1
Nars2	1,035839156	1
Map4	1,035552	1
Gm8719	1,035408452	1
Nbr1	1,035336685	1
Fyco1	1,034834459	1
Oraov1	1,034619293	1
Vps72	1,034547582	1
Pik3ap1	1,034404173	1
Parp9	1,034332476	1
Xrcc1	1,034332476	1
Fbxl20	1,034332476	1
Tor1a	1,034189097	1
Erp44	1,034189097	1
Fam92a	1,034117415	1
Foxk2	1,034045738	1
Srek1ip1	1,033974066	1
Snrnp70	1,033974066	1
Mitd1	1,033902398	1
RP23-312A24.1	1,033759079	1
Snhg5	1,033687427	1
Gm6863	1,03361578	1
Tyrobp	1,03361578	1
Gm42747	1,0334725	1
Man2c1	1,03332924	1
Cope	1,03332924	1
Sep 09	1,033186	1
Tigd2	1,033186	1
Trappc11	1,032971178	1
Rhot1	1,032971178	1
H2afx	1,032827987	1
Dis3l	1,032613239	1
Slc22a17	1,032613239	1
Ndufa7	1,032470098	1
Cdc14b	1,032040795	1
Timm17a	1,031826211	1
Mrs2	1,031754693	1
Zfp638	1,031611671	1
Eif4enif1	1,031611671	1

Bbx	1,031540168	1
Amz2	1,031468669	1
Ltc4s	1,031397176	1
Akap8	1,031325687	1
Ipo4	1,031325687	1
Cox17	1,031182725	1
Slc5a3	1,031182725	1
Rgl3	1,031111251	1
Fam110a	1,030968319	1
Arhgap10	1,030968319	1
2700033N17Rik	1,030825406	1
Vmp1	1,030825406	1
Msh6	1,030825406	1
Hsp90ab1	1,030753957	1
Slc20a1	1,030611074	1
Tstd2	1,03053964	1
Rbbp7	1,030468211	1
Gm6028	1,030396787	1
Myl6b	1,030396787	1
Tcaim	1,030325368	1
Gm12222	1,030253954	1
Timm13	1,030253954	1
Fzr1	1,030039741	1
Chd3	1,029682818	1
Gm11914	1,029611448	1
Gm12097	1,029611448	1
Ptar1	1,029611448	1
Dhrs4	1,029540083	1
Thoc5	1,029254674	1
Stk38	1,029183334	1
Greb1	1,028684092	1
Nisch	1,028470206	1
Arpc4	1,028470206	1
Zmat3	1,028256363	1
Rps10-ps1	1,028113827	1
Dbndd2	1,028042566	1
Tmem189	1,027900059	1
Gm10320	1,027828812	1
Mrpl48-ps	1,027757571	1
Gm42549	1,027686335	1
Braf	1,027686335	1
Nsmce4a	1,027543877	1
Dync2li1	1,027330228	1
Agpat3	1,027330228	1
Snrpd1	1,027330228	1
Atp6v0e2	1,027259021	1
Usp45	1,027187819	1
Idh3a	1,026689546	1
Gm15773	1,026262646	1
Cad	1,026191514	1
Zfp346	1,026191514	1
Polrmt	1,026120386	1

Zfand4	1,025907032	1
Mfsd12	1,025764821	1
RP23-162P10.2	1,025693723	1
Polk	1,025693723	1
Ptpmt1	1,025551542	1
6230400D17Rik	1,025196174	1
Abl1	1,025125116	1
Baz1b	1,025125116	1
Srek1	1,025054062	1
2010107E04Rik	1,025054062	1
Tef	1,024911969	1
Tmem120a	1,02484093	1
March2	1,024769896	1
Hint2	1,024769896	1
Ttc39a	1,024556823	1
Tipin	1,024556823	1
Chst12	1,024343795	1
1110008L16Rik	1,024343795	1
Rbpsuh-rs3	1,0242018	1
Acadsb	1,023988846	1
Commd10	1,023917871	1
Gnb5	1,0238469	1
Zc3h14	1,023775935	1
Gm14776	1,023704975	1
Fcgr2b	1,023492124	1
Zbtb44	1,023492124	1
Bcl10	1,023350247	1
Spata5l1	1,023208391	1
Capn2	1,02313747	1
Thap3	1,022995643	1
Pou6f1	1,022924736	1
Heatr5b	1,022924736	1
Gcsh	1,022641161	1
Ano6	1,022641161	1
Gm5321	1,022570279	1
Prps1l3	1,022570279	1
Ago4	1,022499402	1
Lage3	1,022286802	1
Nap1l4	1,022003403	1
AI413582	1,021932566	1
Hnrnpul1	1,021649265	1
Pcx	1,021507644	1
Ostf1	1,021507644	1
Cd2bp2	1,021436841	1
Mettl3	1,021224461	1
Tmem160	1,021082899	1
Eif2b2	1,021082899	1
Gm11520	1,020587587	1
Abca3	1,020587587	1
Rab3gap1	1,020587587	1
Gm37747	1,020516848	1
Cd200r1	1,020516848	1

Actr10	1,020516848	1
Ptpn9	1,020446113	1
Odc1	1,020446113	1
Slc35a5	1,020163225	1
Cdk9	1,020163225	1
Fam195b	1,020163225	1
Mettl25	1,02002181	1
Mtm1	1,019880415	1
Pkm	1,019668359	1
Tsga10	1,019597683	1
Zkscan14	1,019456347	1
Sult6b1	1,019244379	1
Ccdc107	1,019103091	1
Mfge8	1,019032455	1
Dnmbp	1,018820575	1
Polr1e	1,018749958	1
St14	1,018749958	1
Ece1	1,018538137	1
Gm5436	1,018396947	1
Gm8869	1,018255777	1
Naa38	1,018044059	1
Ctsb	1,017973496	1
Tex2	1,017902938	1
Ndufs1	1,017832385	1
Eml2	1,017761836	1
Hyou1	1,017620755	1
Gm5812	1,017409168	1
Extl3	1,017338649	1
Tax1bp3	1,017268135	1
Tbc1d22a	1,017197626	1
Mtmr3	1,017056622	1
Dnajb9	1,017056622	1
Rnf14	1,016986128	1
Tmem39a	1,016774673	1
Mad2l1bp	1,016774673	1
Nudt6	1,016633728	1
Mlh1	1,016633728	1
Klhl25	1,016563263	1
Spag9	1,016563263	1
Bri3	1,016492803	1
Pkd1	1,016492803	1
Tbcd	1,016492803	1
Scamp3	1,016492803	1
Dtx3l	1,016492803	1
AI839979	1,016351897	1
Ctnna1	1,016281451	1
Coa4	1,01621101	1
Rbms1	1,016140574	1
Tomm5	1,015788468	1
Prune2	1,015647659	1
2610002M06Rik	1,015577262	1
Selenot	1,015366101	1

Slfn4	1,015225351	1
Wdr1	1,015084621	1
Arpc1b	1,015014263	1
Dnlz	1,014803218	1
Myd88	1,014451575	1
Gm45110	1,014170349	1
Sipa1l1	1,014100054	1
Cklf	1,013748655	1
Rpgrip1l	1,013467623	1
Ccny	1,013397377	1
Junos	1,013327136	1
Zfp646	1,012905793	1
Gm10080	1,012554807	1
Sra1	1,012484625	1
Eif1	1,012414447	1
Efcab7	1,012274106	1
Ndufv1	1,012203943	1
Smad4	1,012133785	1
Hoxa3	1,01192334	1
Pgap2	1,011783067	1
H1fx	1,011712938	1
D630023F18Rik	1,01150258	1
Fbxo42	1,011432471	1
Snord118	1,011292266	1
Gm43336	1,011222171	1
Vcp-rs	1,011152081	1
Lrrfip2	1,011081996	1
Padi2	1,011011915	1
Lyrm9	1,011011915	1
Gpaa1	1,01094184	1
Trim23	1,010871769	1
Lpcat2	1,010801703	1
Ptp4a3	1,010661586	1
Ankrd10	1,010451446	1
Ube2d1	1,01038141	1
Atg3	1,01038141	1
Tpgs2	1,010311378	1
Paip1	1,010241351	1
Ssbp4	1,010171329	1
Trim24	1,010031299	1
Psmc6	1,010031299	1
Lmbr1l	1,009681309	1
Ube2w	1,009681309	1
Dzip1	1,009611326	1
Ilk	1,009611326	1
Fam160a2	1,009541348	1
Nat2	1,009401405	1
Xiap	1,009401405	1
RP24-550H10.6	1,009121578	1
Smarcad1	1,009121578	1
Zfp384	1,008981694	1
Aco1	1,008841829	1

Gemin6	1,008701984	1
Gm5453	1,008701984	1
Akt1s1	1,008701984	1
Gm44901	1,008632068	1
Papd7	1,008492252	1
Bod1	1,008422351	1
Eps15	1,008352455	1
Kcnb1	1,008212677	1
Immp2l	1,008003047	1
Mphosph9	1,00765376	1
Gm37390	1,007583918	1
Psmb8	1,00751408	1
Zxdc	1,007374418	1
Kcnab3	1,007164962	1
Tmed9	1,007095153	1
M1ap	1,00639733	1
Hdac9	1,00639733	1
Dpy19l1	1,006327574	1
Prkab1	1,006257823	1
Slc25a32	1,006188077	1
Nipa2	1,006188077	1
Hectd3	1,006118336	1
1810044D09Rik	1,005978868	1
Tnks	1,005909142	1
Prpf40b	1,00583942	1
Gm6155	1,005769703	1
Abhd12	1,00569999	1
RP23-164P21.3	1,00556058	1
Ybx3	1,00556058	1
Dctd	1,005351502	1
Odf2	1,005351502	1
Huwe1	1,005142466	1
D17Wsu92e	1,005003134	1
C130071C03Rik	1,00486382	1
Fam118a	1,00486382	1
Gm12183	1,004794171	1
Trappc1	1,004794171	1
9430060I03Rik	1,004724526	1
Rps6ka4	1,004724526	1
Gm9828	1,004724526	1
Uap1l1	1,004724526	1
Tmem41a	1,004585252	1
Ccm2	1,004585252	1
E2f4	1,004515622	1
Rufy2	1,004376376	1
Rhof	1,004376376	1
Tnfrsf11a	1,00430676	1
Gtpbp1	1,004237149	1
Plk1	1,004167543	1
Tspyl3	1,004097942	1
C1d	1,004097942	1
Cyb5r4	1,004028346	1

Gm2308	1,003889167	1
Mid1ip1	1,003889167	1
Sf3b5	1,003889167	1
Gm11613	1,003819586	1
Vps11	1,003541306	1
Gm22716	1,003471749	1
Mapk1ip1l	1,003471749	1
Ip6k1	1,003402196	1
Prdx5	1,003193566	1
Hspa4l	1,003193566	1
Cnppd1	1,003193566	1
Xirp1	1,00291546	1
Bin1	1,00291546	1
Zfp871	1,00291546	1
Cdc37l1	1,002845945	1
Dync1h1	1,002776436	1
Nfya	1,002706931	1
Srsf3	1,002706931	1
Il11ra1	1,002498446	1
Ddx39	1,002290004	1
Hmgcl	1,002290004	1
Dpm2	1,002220533	1
Fam120aos	1,002081605	1
Mef2d	1,002012148	1
Tmx3	1,001942697	1
Kctd2	1,00187325	1
Cox7a2	1,00187325	1
Gm12990	1,00159551	1
Hivep3	1,001526087	1
Taldo1	1,001456669	1
Washc3	1,001179045	1
Uba1	1,001179045	1
Fbxw8	1,001109651	1
Dap3	1,001040261	1
Pcgf2	1,000970877	1
Cpox	1,000901497	1
Mea1	1,000832123	1
Tbc1d22b	1,000832123	1
Chst10	1,000762753	1
Gm15846	1,000693387	1
Trp53bp2	1,000624027	1
Lyrm4	1,000554672	1
Hdac7	1,000485321	1
Sik3	1	1
Gm42418	1	1
Anks3	-7,86E-05	1
Golt1b	-0,00016591	1
Spag4	-0,00027248	1
Dpy30	-0,00033309	1
Pno1	-0,00026543	1
Dennd6a	-0,00040095	1
Mgmt	-0,0007683	1

1700025G04Rik	-0,00078672	1
Dcaf4	-0,00089469	1
Klhl21	-0,00089058	1
Tspan5	-0,0014495	1
Mrps9	-0,0013968	1
Mrps14	-0,0015657	1
RP23-13B8.12	-0,0017812	1
Tmem258	-0,0018243	1
Gm18860	-0,0022204	1
Ttf1	-0,0022433	1
Snx9	-0,0024124	1
Cinp	-0,0025996	1
Spop	-0,0027799	1
Cdk8	-0,0033479	1
Gm20274	-0,0034964	1
Wasf2	-0,0034664	1
Vdac2	-0,0035647	1
Psmb3	-0,0036564	1
Gna15	-0,0038426	1
Gdi1	-0,0037981	1
Ccdc88a	-0,0039626	1
Naa30	-0,0044144	1
Plcd3	-0,0045286	1
Fchsd2	-0,0044847	1
Slc25a39	-0,0044624	1
Atp13a1	-0,0046026	1
Aebp2	-0,0046838	1
Gm9497	-0,0048336	1
Gm6222	-0,0050359	1
Ncoa1	-0,0050853	1
Fmnl1	-0,005179	1
Eef2	-0,005225	1
Gm16437	-0,0053565	1
Ttc14	-0,0055349	1
Gm11688	-0,0056219	1
Gm12669	-0,0057143	1
Ube2e1	-0,0058758	1
Ccdc181	-0,006269	1
Ciz1	-0,0065593	1
Gm25857	-0,0068193	1
Rragc	-0,0072276	1
Gm10146	-0,0078788	1
Fbxo45	-0,0079579	1
Dgkg	-0,0081206	1
Commd9	-0,0083957	1
Sh3bp2	-0,0084815	1
Fip1l1	-0,0087826	1
Pold2	-0,0089974	1
Csnk1g1	-0,0089975	1
Apex1	-0,009318	1
Dlg4	-0,0094482	1
Gm11868	-0,0095278	1

Eci2	-0,009482	1
Slc13a2	-0,010038	1
Casp9	-0,010123	1
Mcoln2	-0,010293	1
Rb1	-0,010284	1
Fgd6	-0,010384	1
Copz1	-0,010501	1
Hcfc2	-0,010569	1
Echs1	-0,0109	1
Tbc1d5	-0,011035	1
Clock	-0,011041	1
Lamtor1	-0,011024	1
Tmem106a	-0,010988	1
Cxx1b	-0,011319	1
Ubap1	-0,01125	1
Dnajc24	-0,011399	1
Gm10073	-0,011527	1
Sgtb	-0,011564	1
Coq6	-0,011635	1
Tmem238	-0,011882	1
Rhoq	-0,01207	1
Nckap1l	-0,012443	1
Batf2	-0,012654	1
Fut11	-0,012682	1
Cndp2	-0,012732	1
Zranb1	-0,012704	1
Lcmt1	-0,012754	1
Grhpr	-0,013055	1
Rgp1	-0,013248	1
Agl	-0,013394	1
Ywhae	-0,013398	1
Gm37108	-0,013455	1
Gm37566	-0,013579	1
Fam199x	-0,013626	1
Sec22b	-0,013597	1
Tmed3	-0,013983	1
Skap2	-0,013995	1
Gm9378	-0,014275	1
Bcap31	-0,014401	1
Mrpl28	-0,014511	1
Ifrd2	-0,014604	1
Clta	-0,015045	1
Rps25-ps1	-0,015359	1
Gm6563	-0,015509	1
E2f1	-0,0156	1
Gm6598	-0,015695	1
Gm43430	-0,015756	1
Gm4799	-0,015827	1
Foxd2os	-0,016062	1
Dnajb14	-0,016189	1
Plppr2	-0,016367	1
Nudt9	-0,016489	1

Wsb2	-0,016747	1
Fbxl12	-0,016652	1
Icam1	-0,016776	1
Rilpl2	-0,01679	1
Jade1	-0,016925	1
Akap8l	-0,01712	1
Tpd52	-0,017128	1
Tle3	-0,017293	1
D1Ertd622e	-0,01733	1
Gm11945	-0,017444	1
Elp5	-0,017472	1
2500002B13Rik	-0,018114	1
Cmss1	-0,018338	1
Dnaja2	-0,018288	1
3300002I08Rik	-0,018471	1
Camk2d	-0,019113	1
Alkbh8	-0,019353	1
Snrnp27	-0,019466	1
Pdhb	-0,019864	1
9430092D12Rik	-0,020223	1
Fkbp8	-0,020224	1
Nr1h3	-0,020303	1
Kmt5a	-0,020417	1
Tbc1d8b	-0,020468	1
Nsd2	-0,02048	1
Fam122b	-0,020586	1
Mkks	-0,020601	1
Adss	-0,020584	1
Tmem164	-0,020691	1
Ptrf	-0,020828	1
Dync1i2	-0,020794	1
Myl6	-0,020942	1
Ppp2r5d	-0,020891	1
Rac2	-0,021017	1
Hmg20a	-0,021137	1
1700109H08Rik	-0,021191	1
Ints13	-0,021309	1
Gm30238	-0,021427	1
Hmgb1-ps8	-0,021394	1
Prkacb	-0,021485	1
Olfr460	-0,021795	1
Impdh2	-0,022138	1
Tkfc	-0,022265	1
Abcb7	-0,022294	1
Iqcg	-0,02244	1
Zfp410	-0,0224	1
Pdk2	-0,02273	1
Fmnl3	-0,02287	1
Nr4a2	-0,023069	1
Atad2b	-0,023078	1
Far1	-0,023056	1
Igf2bp2	-0,023238	1

Syt8	-0,023446	1
Gm4950	-0,023411	1
Mettl4	-0,024162	1
Mpp3	-0,024209	1
Cog5	-0,024221	1
Ints1	-0,0244	1
Lat	-0,024564	1
6720427I07Rik	-0,02459	1
Bhlhe40	-0,024611	1
Dhx34	-0,024739	1
Psmd4	-0,025048	1
Clgn	-0,025159	1
Rps5	-0,025368	1
Hmox1	-0,025566	1
Mfsd2a	-0,025708	1
Rps6ka1	-0,025861	1
Trmt61b	-0,026043	1
Xpnpep1	-0,026117	1
Mphosph6	-0,026137	1
Rhod	-0,026171	1
Deaf1	-0,026744	1
Chml	-0,026997	1
Mvd	-0,027068	1
Nod2	-0,027412	1
Ubl4a	-0,027553	1
Snrnp35	-0,027813	1
5530601H04Rik	-0,027949	1
Cd68	-0,027936	1
Pcnx	-0,028034	1
Rnf6	-0,028177	1
Taf3	-0,028157	1
Pex10	-0,02828	1
Kdm6b	-0,028415	1
Mat2b	-0,028522	1
Rab19	-0,028728	1
Snapc2	-0,028683	1
Golph3	-0,028669	1
Nfkbiz	-0,028944	1
Ikbkap	-0,028924	1
Ssb	-0,028929	1
Hint1	-0,028983	1
Kti12	-0,02933	1
Iqcc	-0,029417	1
Fam65a	-0,030098	1
Ccdc47	-0,030407	1
Gps2	-0,030406	1
Fam126a	-0,030374	1
Stxbp1	-0,030466	1
Chd4	-0,030595	1
Gm44890	-0,030736	1
Cnot10	-0,030737	1
Plgrkt	-0,030815	1

Apaf1	-0,030944	1
Fam219b	-0,030989	1
Dpagt1	-0,031079	1
Itgb5	-0,031154	1
Rpsa-ps4	-0,03155	1
Arpc1a	-0,031722	1
Atp6v0c	-0,031825	1
St3gal2	-0,032216	1
Zmym3	-0,032317	1
Kars	-0,032283	1
RP24-497N7.2	-0,032403	1
Rab24	-0,032397	1
Gtf2f2	-0,032845	1
Myo5a	-0,032858	1
Phldb1	-0,033135	1
Smim14	-0,033334	1
Tnpo3	-0,033381	1
Gm5963	-0,033381	1
Ralgapa1	-0,033455	1
Ndufb3	-0,033734	1
Faim	-0,033896	1
Rptor	-0,033944	1
Cetn3	-0,033883	1
Ubqln1	-0,03392	1
Git1	-0,033997	1
Gcnt1	-0,034503	1
Zranb3	-0,03458	1
Tnip2	-0,034606	1
Slc35b3	-0,035433	1
Ap1b1	-0,035399	1
Mtpap	-0,03581	1
Agpat4	-0,03599	1
Ubn2	-0,036355	1
Ndrg4	-0,036406	1
Opa1	-0,036769	1
Slc35a3	-0,036969	1
Gm6123	-0,037127	1
Chd9	-0,037209	1
Ccdc77	-0,03733	1
9930012K11Rik	-0,037415	1
Ccdc115	-0,037568	1
Nup62	-0,037607	1
Hibch	-0,037847	1
Cpd	-0,037803	1
Zfp654	-0,037932	1
Glod4	-0,037898	1
Trpm7	-0,038302	1
Neu1	-0,038359	1
Pibf1	-0,038469	1
Mecr	-0,038495	1
Dhrs7b	-0,038458	1
Tbl1x	-0,038549	1

Nedd4l	-0,03856	1
Mast3	-0,038707	1
Tcea1	-0,038692	1
Gm37206	-0,039126	1
Smim8	-0,039176	1
Rnf19b	-0,03919	1
Gse1	-0,039442	1
Bri3bp	-0,039603	1
Plaa	-0,039686	1
Lsm4	-0,039668	1
Naa35	-0,039995	1
Gm15690	-0,040179	1
Dctn2	-0,040194	1
Zfp706	-0,040226	1
Zfp644	-0,040318	1
Arel1	-0,04044	1
Fxyd5	-0,040416	1
D630024D03Rik	-0,041325	1
Trmt10c	-0,041305	1
Gm14586	-0,041716	1
Cfdp1	-0,041717	1
9230112E08Rik	-0,041952	1
Kmt5c	-0,042074	1
Man1a2	-0,042217	1
Slc41a3	-0,042385	1
Ube2cbp	-0,042486	1
Prox2	-0,04263	1
Pias2	-0,042948	1
Ube2d3	-0,042929	1
Papola	-0,043059	1
Hn1	-0,043228	1
Dcaf6	-0,043656	1
Ccng1	-0,043747	1
Ssr2	-0,043714	1
Naa50	-0,043735	1
Nt5c2	-0,043757	1
Arf1	-0,043844	1
Rtcb	-0,043777	1
Gm11362	-0,043969	1
Dus3l	-0,043954	1
1700037C18Rik	-0,044077	1
Ankrd16	-0,044246	1
Btbd9	-0,044216	1
Bola1	-0,044498	1
Klhl9	-0,044684	1
Gigyf2	-0,044995	1
Zfp955a	-0,045294	1
Ctbs	-0,045267	1
Clcnkb	-0,045393	1
Rab40c	-0,045642	1
Acox1	-0,045733	1
Hmgn1	-0,045697	1

Idi1	-0,045839	1
Mthfd2l	-0,045907	1
Recql4	-0,045904	1
Zfp428	-0,046018	1
Morc3	-0,04631	1
Gm38262	-0,046487	1
BC037032	-0,046641	1
Cenpq	-0,046735	1
Bcl2a1d	-0,046777	1
Pkp2	-0,047128	1
Xrcc4	-0,047279	1
Dynlt1-ps1	-0,047425	1
Mospd1	-0,047444	1
Gm6245	-0,047461	1
Ppp1cb	-0,047725	1
Hexim1	-0,047932	1
H3f3b	-0,047929	1
Rbm15	-0,04797	1
Wdr47	-0,048119	1
Gm14680	-0,048238	1
Ube2i	-0,048159	1
Cggbp1	-0,048411	1
Rad23a	-0,048456	1
Pla2g12a	-0,048539	1
Nelfcd	-0,049127	1
Gnpat	-0,049266	1
Rars	-0,049389	1
Gnl3l	-0,049622	1
Zc3h18	-0,050034	1
4930518I15Rik	-0,050325	1
Rpap3	-0,050291	1
Mapre1	-0,050379	1
Tfdp1	-0,050477	1
Gm15903	-0,050607	1
Plekhg2	-0,051139	1
Adnp2	-0,051146	1
Lrp6	-0,051127	1
Nupl1	-0,051072	1
Asb1	-0,051327	1
Cetn2	-0,052284	1
Gm45718	-0,052426	1
Gm38355	-0,052356	1
Cyp4v3	-0,052365	1
Mapk8	-0,052565	1
Ankrd24	-0,052807	1
Tatdn1	-0,053094	1
Gm4895	-0,053086	1
Epb41l2	-0,053724	1
Fbxo11	-0,054129	1
Brd4	-0,05435	1
Efna1	-0,054525	1
Mrpl45	-0,054923	1

Gm4754	-0,05501	1
Ctns	-0,054954	1
Mrpl39	-0,055133	1
Pvt1	-0,055171	1
Nle1	-0,055552	1
Mrps5	-0,05557	1
Rsl24d1	-0,055807	1
Nat9	-0,055842	1
U2af1	-0,056062	1
Uggt1	-0,056109	1
Rps15a-ps1	-0,05632	1
Osm	-0,056384	1
Nfe2l1	-0,056562	1
Mga	-0,056641	1
Dag1	-0,056658	1
Gm5576	-0,056935	1
Pan3	-0,057026	1
Gm38067	-0,057232	1
Setd2	-0,057328	1
Aste1	-0,057475	1
Zbtb32	-0,057817	1
Hibadh	-0,05789	1
Senp8	-0,058043	1
Slamf9	-0,058072	1
2810030D12Rik	-0,058231	1
Zfp385a	-0,058239	1
Selenoo	-0,058247	1
Smad3	-0,058431	1
Bbip1	-0,058485	1
Casp3	-0,05846	1
Rala	-0,058996	1
March7	-0,059057	1
Enc1	-0,059212	1
Gtpbp4	-0,059414	1
Arhgef4	-0,059457	1
Gm5276	-0,059943	1
Zfp553	-0,060241	1
Thumpd3	-0,060208	1
Gm29593	-0,060327	1
Tmx1	-0,06031	1
Mrpl21	-0,060397	1
Gm38104	-0,060473	1
Nckipsd	-0,060742	1
Tmem185a	-0,060803	1
Gm16310	-0,061024	1
Bbs12	-0,060981	1
Rassf8	-0,061136	1
Pi4k2a	-0,061215	1
Slc2a9	-0,061313	1
Vcpip1	-0,061301	1
Nup188	-0,061592	1
Wdr89	-0,061654	1

Setd3	-0,06203	1
Wipf2	-0,062084	1
Chuk	-0,062628	1
Kmt2a	-0,062796	1
Gen1	-0,063402	1
Zfp422	-0,063511	1
Appl2	-0,063451	1
Rnaseh2c	-0,063563	1
Sorbs1	-0,063749	1
Hnrnpl	-0,063672	1
Tmem144	-0,064182	1
Ubqln4	-0,06435	1
Gm14567	-0,064619	1
RP23-47A1.1	-0,064719	1
Ctnnd1	-0,065151	1
Nup85	-0,065283	1
Gm45853	-0,065523	1
Cxcl14	-0,065627	1
Akt2	-0,065595	1
Hirip3	-0,065731	1
Lgr4	-0,066013	1
Tcerg1	-0,066033	1
Fam132a	-0,066159	1
Glce	-0,066334	1
Atrx	-0,06637	1
Gm45360	-0,06648	1
Gm37254	-0,066708	1
Slc6a8	-0,067228	1
Men1	-0,0672	1
Gm7670	-0,067269	1
Klhl12	-0,067287	1
Nagk	-0,067353	1
Eif1ax	-0,067431	1
Aaas	-0,067741	1
Pds5b	-0,068103	1
Micu1	-0,068068	1
4930526A20Rik	-0,068768	1
Nek6	-0,069324	1
Cdc40	-0,069478	1
Atp5s	-0,069563	1
Soat1	-0,069777	1
Ciapin1	-0,069961	1
Gemin2	-0,07006	1
Dmwd	-0,070076	1
1110012L19Rik	-0,070399	1
Clp1	-0,07059	1
Rubcnl	-0,071072	1
Emc8	-0,071536	1
Slc2a3	-0,071476	1
Cdk12	-0,071487	1
Gm15050	-0,071649	1
Npy	-0,071693	1

Socs7	-0,071722	1
Sgpl1	-0,071697	1
Gmps	-0,071777	1
Ddx54	-0,071842	1
Cnot9	-0,072049	1
Arl5c	-0,072097	1
Zfp560	-0,072217	1
Scnm1	-0,072341	1
4933434E20Rik	-0,072426	1
Ctsz	-0,072677	1
Atp6v0e	-0,072906	1
Man2b1	-0,073207	1
Siae	-0,073227	1
1110004E09Rik	-0,073339	1
Setd7	-0,073357	1
Rfng	-0,073516	1
Nsd3	-0,073556	1
Rnf187	-0,073735	1
Pip4k2a	-0,073866	1
Zmym5	-0,074139	1
Uqcrfs1	-0,074112	1
Gm2810	-0,074819	1
Dcun1d3	-0,074774	1
Evi5	-0,074824	1
Chaf1b	-0,074951	1
Milr1	-0,07498	1
Ptrhd1	-0,075361	1
Herc1	-0,075393	1
Prrg2	-0,07552	1
Grtp1	-0,075908	1
Gm15920	-0,075902	1
4831440E17Rik	-0,075965	1
Emp1	-0,076439	1
Rxra	-0,076648	1
Gm26631	-0,07674	1
Hilpda	-0,076829	1
Clasrp	-0,076967	1
Inpp5a	-0,076963	1
Cd72	-0,077186	1
Fkbp2	-0,077336	1
Apeh	-0,077277	1
Git2	-0,077517	1
4930440I19Rik	-0,077901	1
Lrfn4	-0,07802	1
Gtdc1	-0,078623	1
Ece2	-0,078562	1
Mnd1-ps	-0,078709	1
Ypel1	-0,078827	1
Rbm7	-0,078909	1
Unc13d	-0,079649	1
Kif3c	-0,080244	1
Tmem230	-0,080265	1

4833412K13Rik	-0,080471	1
Pef1	-0,080545	1
Ccdc126	-0,080815	1
BC022687	-0,080845	1
Commd4	-0,080861	1
Mrps17	-0,081049	1
Zfyve28	-0,081888	1
Gosr1	-0,081994	1
Plekhm3	-0,081958	1
Fam98b	-0,082059	1
1700123M08Rik	-0,08217	1
Foxn2	-0,082252	1
Atf7ip	-0,082439	1
Ndufab1-ps	-0,082543	1
B230322F03Rik	-0,082583	1
Pdlim2	-0,082676	1
Ubap2	-0,082856	1
Sh3glb1	-0,082873	1
9630013D21Rik	-0,082974	1
Phf23	-0,083048	1
Prpf4	-0,083192	1
Tmem161a	-0,083478	1
Qdpr	-0,08353	1
Rprd1b	-0,083567	1
Klhdc10	-0,083649	1
Donson	-0,083897	1
Nfkb2	-0,083983	1
Gm43362	-0,084007	1
Ncor2	-0,084126	1
Ran	-0,084115	1
Suclg2	-0,084186	1
Cc2d1b	-0,084243	1
Rnf113a1	-0,084323	1
Psmd12	-0,084321	1
Ankrd50	-0,084465	1
Cdk4	-0,084662	1
Poc5	-0,08483	1
Gm5131	-0,08492	1
Rfc5	-0,084976	1
Dnajc5	-0,085027	1
Vav3	-0,085308	1
Rdh11	-0,085259	1
Piezo1	-0,085448	1
Setd4	-0,085527	1
Stoml2	-0,085576	1
Gm10093	-0,08613	1
Tifab	-0,086235	1
Uba2	-0,086171	1
Fcgrt	-0,086288	1
RP23-63H11.3	-0,086373	1
AI314180	-0,08677	1
Kcmf1	-0,086811	1

Tssc1	-0,086941	1
Slc9a1	-0,086985	1
Traf1	-0,087313	1
Syf2	-0,087319	1
Gm15148	-0,087329	1
Mllt3	-0,087357	1
Tns1	-0,087861	1
Zrsr2	-0,08795	1
Rnf141	-0,087933	1
Ttc13	-0,088113	1
Tcf25	-0,08816	1
Cybb	-0,088251	1
Vegfa	-0,088332	1
Gm45420	-0,088366	1
Thumpd2	-0,088526	1
Zdhhc21	-0,08887	1
Pax6	-0,089292	1
Mycbp2	-0,089315	1
Etaa1	-0,089363	1
Gm9803	-0,089405	1
Cramp1l	-0,089533	1
Trappc10	-0,089797	1
Dusp16	-0,09017	1
Usp34	-0,090424	1
Gm5914	-0,090479	1
Midn	-0,090477	1
Fyn	-0,090562	1
Mob4	-0,090561	1
Dusp22	-0,090654	1
Dctn1	-0,091222	1
Brdt	-0,091434	1
Gde1	-0,091535	1
Jmjd8	-0,091579	1
Gm13373	-0,091655	1
Rpl31-ps16	-0,091791	1
Tnfaip1	-0,091774	1
Trappc4	-0,091834	1
Fam179b	-0,092172	1
Serf1	-0,092509	1
Mrpl27	-0,092635	1
Washc4	-0,092695	1
Ppil2	-0,092806	1
Gm13022	-0,092886	1
Acvr2a	-0,093049	1
Dopey1	-0,09299	1
Rmi2	-0,093205	1
Slc30a9	-0,093213	1
Strbp	-0,093382	1
Csf2ra	-0,09352	1
Kif5b	-0,093494	1
Gm43569	-0,093607	1
Gm38192	-0,093693	1

Ext2	-0,093674	1
Lhx5	-0,093884	1
Tor2a	-0,093897	1
Pon2	-0,09413	1
Phf21b	-0,094218	1
Foxk1	-0,094212	1
Zfp142	-0,094282	1
Hnrnpf	-0,094364	1
Trrap	-0,094391	1
Stap2	-0,094574	1
Smpdl3b	-0,094658	1
Hcfc1	-0,094837	1
Synj2bp	-0,095313	1
Fabp5l2	-0,095465	1
Inpp5e	-0,09574	1
Pxn	-0,095714	1
Gm45250	-0,095936	1
Eef1e1	-0,096032	1
Dennd1b	-0,096054	1
Bivm	-0,09629	1
Pphln1	-0,096265	1
Eif4a1	-0,096291	1
Pepd	-0,096365	1
Ints6	-0,096494	1
Rpap2	-0,097016	1
Dpys	-0,097102	1
Pigv	-0,097205	1
Mrpl49	-0,097327	1
Smc6	-0,097295	1
Tmem50a	-0,097324	1
Gm42941	-0,097498	1
Gm10069	-0,097558	1
Dcaf5	-0,097596	1
Slc35c2	-0,097639	1
Mzt2	-0,097659	1
Spopl	-0,097715	1
Arhgdib	-0,097743	1
Mink1	-0,09795	1
Abce1	-0,098014	1
Yipf1	-0,098314	1
Agpat5	-0,098478	1
Ndfip2	-0,098765	1
Usp3	-0,098773	1
Gm37465	-0,099114	1
Trp53i13	-0,099098	1
Cox8a	-0,099056	1
Invs	-0,099253	1
Dmxl1	-0,099336	1
Ttf2	-0,099412	1
Rap2c	-0,09947	1
Gm3650	-0,099626	1
Sike1	-0,099685	1

Gm28438	-0,099766	1
Faap20	-0,09975	1
Gm5547	-0,099898	1
Fes	-0,10032	1
Rpp21	-0,10029	1
Arhgap25	-0,10046	1
Usp19	-0,10054	1
Grsf1	-0,10051	1
Mrps27	-0,10065	1
Wbp2	-0,10075	1
Smad2	-0,10089	1
Chmp6	-0,10104	1
Psmc4	-0,10111	1
Psmg3	-0,10106	1
Cep97	-0,10122	1
Jade3	-0,10128	1
Bmpr2	-0,10143	1
Plec	-0,10154	1
Pes1	-0,10145	1
Fpgs	-0,10167	1
Sh3gl1	-0,10182	1
Tspyl1	-0,10223	1
Tes3-ps	-0,10235	1
Larp7	-0,1024	1
Gm45286	-0,1026	1
Porcn	-0,1026	1
Gpd2	-0,10321	1
Ndufa10	-0,10318	1
Nfic	-0,10333	1
Gm19503	-0,10349	1
Ilkap	-0,10345	1
Dhx15	-0,10355	1
Lzts3	-0,10374	1
Ccnh	-0,10379	1
Pop4	-0,10397	1
Ndufaf5	-0,10415	1
Yipf6	-0,10421	1
Ywhab	-0,10424	1
Gm6524	-0,10439	1
Rbbp5	-0,1044	1
Acbd3	-0,10443	1
Psmd3	-0,10441	1
Rabl2	-0,10456	1
Cnp	-0,10472	1
Clip1	-0,10471	1
Cyp27a1	-0,10501	1
Caprin2	-0,10525	1
Praf2	-0,10532	1
Mroh1	-0,10526	1
4732440D04Rik	-0,10564	1
Exosc10	-0,10567	1
Adcy7	-0,10595	1

Scarb2	-0,10604	1
Tex264	-0,10672	1
Ggta1	-0,10678	1
Prmt2	-0,10703	1
Pbx2	-0,10708	1
Aasdhppt	-0,10721	1
Kif7	-0,10746	1
As3mt	-0,10748	1
Gm42890	-0,10791	1
Aldh16a1	-0,10791	1
Rps27-ps1	-0,10799	1
Xpo7	-0,10813	1
Hk1os	-0,10821	1
Gm37780	-0,10821	1
Tax1bp1	-0,1083	1
Gm43712	-0,10861	1
Iws1	-0,10868	1
Dnttip1	-0,10879	1
Lrsam1	-0,10906	1
Apoo	-0,10938	1
Sptan1	-0,10953	1
Gm20342	-0,10969	1
Kank2	-0,10989	1
Tbce	-0,10999	1
Slc48a1	-0,1102	1
Crat	-0,11026	1
Traf7	-0,11051	1
Zfp995	-0,11089	1
Tmem222	-0,11097	1
Cul4b	-0,11141	1
Smim20	-0,11149	1
Plekhg4	-0,11164	1
Dhx35	-0,11168	1
Cdkn2aipnl	-0,11171	1
Pcid2	-0,1118	1
Gm8522	-0,11198	1
Thnsl1	-0,11209	1
Bmp2k	-0,11244	1
Trip6	-0,11242	1
Lamp2	-0,1127	1
Rab7	-0,11284	1
Pwwp2b	-0,11318	1
Wdpcp	-0,1134	1
Jak1	-0,11352	1
Mpg	-0,1138	1
Epn1	-0,11381	1
Rab11b	-0,11388	1
Gm43499	-0,11429	1
Eml4	-0,11438	1
Ogfod1	-0,11453	1
Fam69a	-0,11471	1
Mthfsl	-0,1148	1

Gm5580	-0,11495	1
Gm2950	-0,11519	1
Wrb	-0,11574	1
Gm44545	-0,11585	1
Ndufs8	-0,11639	1
Treml4	-0,11647	1
Pdcd5-ps	-0,11682	1
Gm13445	-0,11692	1
Metap1	-0,11701	1
Alox5	-0,11703	1
Uqcrb	-0,11703	1
Fndc10	-0,11757	1
Smndc1	-0,11782	1
Gm13604	-0,1179	1
Rab34	-0,11793	1
Twsg1	-0,1182	1
Dnajb5	-0,11834	1
Zfyve1	-0,11827	1
Ctsh	-0,11839	1
CAAA01180111.2	-0,11846	1
Hyls1	-0,11871	1
Lrrk2	-0,11865	1
Clstn1	-0,11877	1
Malat1	-0,1188	1
Sbf2	-0,11893	1
Psrc1	-0,11904	1
Psmc5	-0,11939	1
Cct4	-0,11957	1
RP23-193N1.2	-0,11984	1
Kdelc2	-0,11977	1
Uqcrh	-0,11982	1
Stap1	-0,11993	1
Rab3d	-0,12002	1
Fxyd2	-0,12001	1
Gm10074	-0,12032	1
Ctr9	-0,12029	1
Skiv2l	-0,12045	1
Ptpn5	-0,12052	1
Ints12	-0,12052	1
Ndufb5	-0,12048	1
Phlpp1	-0,12077	1
Ppp1ca	-0,12082	1
Smu1	-0,12077	1
Itm2c	-0,12077	1
Fubp3	-0,12104	1
Ints14	-0,12117	1
Stag2	-0,12122	1
Rps10-ps4	-0,12154	1
Ddx51	-0,12157	1
Itgav	-0,12193	1
Clasp2	-0,12215	1
Ndufb7	-0,12243	1

Arid4b	-0,12242	1
Slc25a16	-0,12272	1
Shisa5	-0,12312	1
Gm7860	-0,1232	1
Gm16201	-0,12331	1
Zfp157	-0,1234	1
2210016L21Rik	-0,12423	1
AU022252	-0,12439	1
Gm44254	-0,12491	1
Casc3	-0,12487	1
Por	-0,12546	1
Gm18969	-0,12561	1
Zfp449	-0,12583	1
Pkig	-0,1258	1
2900005J15Rik	-0,12586	1
Creld1	-0,12604	1
Hdlbp	-0,12614	1
Fam210b	-0,12624	1
Gm6166	-0,12628	1
Hacd4	-0,12631	1
Arih2	-0,12633	1
Tpm4	-0,12711	1
Cpsf4	-0,12717	1
Coq5	-0,1275	1
Taf1	-0,12758	1
Trpm4	-0,12777	1
Hspb6	-0,12792	1
N4bp2l2	-0,12796	1
Emc4	-0,12814	1
Xylt1	-0,12809	1
Prps2	-0,12823	1
Arnt	-0,12824	1
5730455P16Rik	-0,12827	1
Armc6	-0,1284	1
Pa2g4	-0,12848	1
Ralbp1	-0,12848	1
Hnrnpa0	-0,12858	1
Pigk	-0,12871	1
Specc1	-0,12875	1
Gmfb	-0,1294	1
Vps13c	-0,1294	1
Gm45212	-0,12962	1
Nab1	-0,12957	1
Gm43300	-0,12973	1
Orc6	-0,12995	1
Pnpla8	-0,13032	1
Fra10ac1	-0,13029	1
Zbtb40	-0,13041	1
9930021J03Rik	-0,13042	1
Ppp2ca	-0,13055	1
Gm5637	-0,1309	1
Cspg5	-0,13093	1

Glipr1	-0,13103	1
Atp5d	-0,13099	1
Casp8	-0,13117	1
Usp18	-0,13149	1
Mlst8	-0,13149	1
Exosc8	-0,13158	1
Cacna1d	-0,13181	1
Gm35315	-0,13192	1
Gtf3c2	-0,13196	1
Pdap1	-0,13211	1
Etf1	-0,13245	1
Prpf18	-0,13276	1
Nfyb	-0,13281	1
Tex10	-0,13319	1
Plpp2	-0,13322	1
Hoxc4	-0,1333	1
Cox5a	-0,13344	1
Vps52	-0,13347	1
Zfp148	-0,13357	1
Shkbp1	-0,1338	1
Strap	-0,13429	1
Mrpl40	-0,13464	1
Firre	-0,13466	1
Hdac8	-0,13491	1
Flrt2	-0,13512	1
Rad18	-0,13524	1
Nsdhl	-0,13521	1
Strada	-0,13532	1
Uqcc3	-0,13532	1
Tpd52l2	-0,1353	1
Cbx7	-0,13537	1
Nub1	-0,13553	1
Spast	-0,13621	1
Fgfr1op2	-0,13619	1
Mtbp	-0,13632	1
Gm37675	-0,1363	1
Cln5	-0,13716	1
Smim15	-0,13715	1
L2hgdh	-0,13746	1
Usp30	-0,13758	1
Apobr	-0,13763	1
Rapgef6	-0,13769	1
Dnah8	-0,13784	1
Ctps	-0,13777	1
2210417A02Rik	-0,13789	1
Gatc	-0,13788	1
1500011B03Rik	-0,13808	1
Sfr1	-0,13805	1
Aasdh	-0,13822	1
Cltc	-0,13825	1
Dirc2	-0,13844	1
Timm17b	-0,13839	1

Slc5a6	-0,13853	1
Zfp651	-0,13849	1
Spice1	-0,13855	1
Ddx41	-0,13863	1
Gba	-0,13859	1
Hdgf	-0,13869	1
Gm14325	-0,1389	1
Gm5449	-0,13886	1
Exoc1	-0,13885	1
Zmym4	-0,13903	1
Cyhr1	-0,13921	1
Etfbkmt	-0,13931	1
Washc1	-0,13932	1
Tox2	-0,13958	1
Pstk	-0,13959	1
Fsd2	-0,1397	1
Usp5	-0,13976	1
Mybbp1a	-0,13979	1
Bcl2l13	-0,13995	1
Ptges3	-0,14026	1
Rack1	-0,14027	1
Dennd2c	-0,14037	1
Aff1	-0,14064	1
Mrps26	-0,14067	1
3830406C13Rik	-0,14088	1
Ino80e	-0,14182	1
Gm42567	-0,14198	1
Sf3b6	-0,14196	1
Xpc	-0,14206	1
Cept1	-0,14211	1
Wwp2	-0,1424	1
Safb2	-0,14246	1
Kif1c	-0,14274	1
Psca	-0,14289	1
Plpp5	-0,14285	1
Gm29462	-0,14346	1
Ring1	-0,14354	1
Mfsd14b	-0,14346	1
Gm33080	-0,14357	1
6430531B16Rik	-0,14382	1
Dock7	-0,14395	1
Ndufaf1	-0,14387	1
Lrp5	-0,14401	1
Rfwd3	-0,14406	1
Gdpd5	-0,14413	1
Ptpn6	-0,14418	1
Frs2	-0,14428	1
Epm2aip1	-0,14431	1
Slfn2	-0,14444	1
Psma4	-0,14437	1
Uqcrc2	-0,14455	1
Mrpl48	-0,14474	1

Ifi203	-0,14482	1
Rnd1	-0,14522	1
Ccdc94	-0,14543	1
N4bp2l1	-0,14538	1
Sod2	-0,14561	1
Strn	-0,14572	1
Ppp2r3c	-0,14566	1
Srsf9	-0,14571	1
Dlg1	-0,14577	1
Xrn2	-0,14584	1
Rlim	-0,14586	1
Eif4ebp2	-0,14609	1
Zcchc8	-0,14619	1
Stxbp2	-0,1466	1
Zbtb11os1	-0,14695	1
Arcn1	-0,14689	1
Mthfsd	-0,14702	1
Galc	-0,14707	1
Ywhag	-0,14706	1
Mfn1	-0,14737	1
Arfgef3	-0,14745	1
Rexo4	-0,14755	1
Psma6	-0,14747	1
Gm9892	-0,14767	1
Gm26620	-0,14827	1
Gm11633	-0,14832	1
Ndufa8	-0,14826	1
Gm37082	-0,14839	1
Cars2	-0,14865	1
2210408F21Rik	-0,1488	1
Tmem151a	-0,149	1
Rbm34	-0,14902	1
Traf3ip3	-0,14915	1
Commd8	-0,14911	1
Clint1	-0,14922	1
Hip1	-0,14936	1
Prpf40a	-0,14942	1
Acvrl1	-0,14966	1
Slc29a2	-0,15003	1
Pcdh7	-0,14998	1
Eif2s2	-0,15006	1
Irf9	-0,15043	1
Nfia	-0,1506	1
Nipsnap1	-0,15099	1
Chd2	-0,15121	1
Yeats2	-0,15128	1
Pdp2	-0,15133	1
Snw1	-0,15133	1
Mtx2	-0,15185	1
Gpbp1	-0,152	1
Car2	-0,1522	1
Cdc25a	-0,15232	1

Camk1d	-0,15238	1
Tpr	-0,15239	1
Lrrc27	-0,15255	1
Thg1l	-0,15245	1
Ubald1	-0,15273	1
Mettl14	-0,15267	1
H2-D1	-0,15273	1
Cflar	-0,15341	1
Txnl4a	-0,15353	1
Cisd3	-0,15405	1
Rabep1	-0,15407	1
Gm13822	-0,15419	1
Slc33a1	-0,15423	1
Pgpep1	-0,15421	1
Azin2	-0,1544	1
Nol8	-0,15469	1
Slc16a3	-0,1549	1
Ndufaf3	-0,15513	1
Nat10	-0,15522	1
Irak2	-0,15531	1
Ctif	-0,15536	1
Mlxip	-0,15545	1
Clec16a	-0,15605	1
Cbll1	-0,15609	1
Leprotl1	-0,1561	1
Ddrgk1	-0,15622	1
Rnf111	-0,15615	1
Sppl2a	-0,15651	1
Eif1a	-0,15649	1
Chst3	-0,15669	1
Sltm	-0,15673	1
Sqstm1	-0,15678	1
Pitpnm1	-0,15694	1
Zmiz2	-0,15718	1
Slc4a7	-0,15721	1
Cdk11b	-0,15717	1
Dctn3	-0,15734	1
Katnal1	-0,15775	1
Moap1	-0,15828	1
Mcur1	-0,15837	1
Itpk1	-0,15903	1
Cops7b	-0,15898	1
Eps8	-0,15912	1
Ghitm	-0,15937	1
Gm29488	-0,15953	1
Pkp4	-0,1595	1
Zfp800	-0,15949	1
Rexo1	-0,15969	1
Gm10642	-0,1598	1
Mapk7	-0,16002	1
Thra	-0,15995	1
Rhbdd1	-0,16011	1

Acp6	-0,16012	1
Gpd1l	-0,16011	1
Zfp335	-0,16012	1
Akr1e1	-0,16036	1
Arap1	-0,16061	1
Peli1	-0,16069	1
Rbbp4	-0,16074	1
Pih1d1	-0,16077	1
Eif2ak4	-0,16091	1
Park2	-0,16095	1
Ubr3	-0,16121	1
Itga7	-0,16136	1
Sgta	-0,16143	1
Hspa14	-0,16162	1
Vamp5	-0,16174	1
Chsy1	-0,16175	1
Ndufa4	-0,1618	1
Elovl5	-0,16179	1
Ocrl	-0,16209	1
Klc1	-0,16222	1
Mical3	-0,16228	1
Ncoa5	-0,16248	1
Pdcd6	-0,16247	1
Sdhaf1	-0,16269	1
Aff4	-0,16283	1
Gm37452	-0,16301	1
Cdk6	-0,16298	1
Gm23722	-0,16331	1
Atm	-0,1633	1
Commd2	-0,16349	1
Dst	-0,16361	1
Mia2	-0,16379	1
9430034N14Rik	-0,16424	1
Srp19	-0,16441	1
Msrb1	-0,16438	1
Srpk1	-0,16457	1
Ube3c	-0,16565	1
Gm43106	-0,16568	1
Plekhf1	-0,16614	1
Hap1	-0,16617	1
Sh3pxd2b	-0,16624	1
Slc26a11	-0,16718	1
Nsmce3	-0,16745	1
Tmem110	-0,16736	1
Tmem126b	-0,16748	1
Cdk18	-0,16769	1
Lin7b	-0,16784	1
Hras	-0,16779	1
Pdcd2	-0,16794	1
Pgm2l1	-0,16793	1
Prmt9	-0,16799	1
Ctsl	-0,16816	1

Abhd4	-0,16854	1
Bsn	-0,16873	1
Ctu2	-0,16878	1
9430038I01Rik	-0,16881	1
Atxn3	-0,1688	1
Gm43588	-0,16911	1
Fam117a	-0,16909	1
Serp1	-0,16932	1
Tbrg1	-0,16947	1
S100a10	-0,16971	1
Taf13	-0,17049	1
Serhl	-0,17064	1
Fsbp	-0,17103	1
Bcorl1	-0,17116	1
Zfp597	-0,17129	1
Bclaf1	-0,1716	1
Rce1	-0,17176	1
Wdtc1	-0,17182	1
Snapc4	-0,17186	1
Zdhhc3	-0,17225	1
Kifc2	-0,17246	1
Gm6415	-0,17249	1
Rac1	-0,17249	1
Camk2n2	-0,17257	1
Gale	-0,17256	1
Efr3a	-0,17274	1
Mcts1	-0,17275	1
Gm11598	-0,17288	1
Gm38305	-0,17289	1
Atf4	-0,17287	1
Uhmk1	-0,17304	1
Mtmr12	-0,17319	1
Rnf139	-0,17404	1
Xrcc6	-0,17414	1
Ebpl	-0,17414	1
Gm38376	-0,1742	1
Lmbrd1	-0,17443	1
Pank3	-0,1744	1
Gm13771	-0,17472	1
K230015D01Rik	-0,17529	1
Lancl2	-0,17529	1
Chkb	-0,17528	1
BC003331	-0,17549	1
Esrra	-0,17548	1
Gm26132	-0,17594	1
Mrpl55	-0,1761	1
Ndufaf8	-0,17606	1
2610016A17Rik	-0,17616	1
Gm45454	-0,17626	1
Dock4	-0,17637	1
Ints2	-0,17644	1
Ids	-0,17639	1

Sms	-0,17639	1
Ccpg1	-0,1772	1
Naca	-0,17733	1
Abcd2	-0,17735	1
Spg20	-0,17751	1
Polr3b	-0,17762	1
Gtf2a1	-0,17786	1
Mrpl14	-0,17799	1
Ptbp2	-0,1781	1
Chmp2a	-0,17832	1
Ice2	-0,17843	1
Neurl4	-0,17865	1
Mrpl12	-0,1788	1
Zfp512	-0,17881	1
Ddx31	-0,17886	1
Mapk14	-0,17899	1
Utp15	-0,17898	1
Mccc1	-0,17907	1
Sap30bp	-0,17921	1
5031425E22Rik	-0,17922	1
Rgs10	-0,17923	1
Atp5f1	-0,18044	1
Tmem87b	-0,18045	1
Iqgap1	-0,18077	1
Fam168a	-0,18077	1
Gm12906	-0,18089	1
Siah1a	-0,18096	1
Mrm1	-0,18102	1
Ipo7	-0,18148	1
Bin2	-0,18171	1
Isca2	-0,18175	1
Pex6	-0,18197	1
Ddx3y	-0,18205	1
Cdc73	-0,18221	1
Fbxw2	-0,18219	1
Asap3	-0,18234	1
Zfp266	-0,18229	1
Gm13270	-0,18245	1
Api5	-0,18243	1
Fam84b	-0,18258	1
Rab21	-0,18277	1
Tnfrsf9	-0,18304	1
Gm38335	-0,18309	1
Asl	-0,18335	1
Dusp2	-0,18382	1
Ldlrad3	-0,18411	1
Golga1	-0,1845	1
Ppp2r1b	-0,18471	1
Cfap126	-0,18486	1
Synpo	-0,1849	1
Brap	-0,18499	1
A430105J06Rik	-0,18549	1

Lman1	-0,18549	1
Gstp1	-0,18564	1
Pnpla6	-0,18636	1
Parvb	-0,1866	1
Lmo4	-0,18682	1
Ranbp2	-0,18704	1
Tnfrsf1b	-0,18771	1
Gm12389	-0,18833	1
Cd80	-0,18845	1
Gan	-0,18841	1
Eea1	-0,18843	1
Gm8185	-0,18855	1
Brat1	-0,18858	1
Znrd1as	-0,18935	1
Fhit	-0,18936	1
Hacd1	-0,18948	1
Ulk1	-0,18984	1
Pkib	-0,18994	1
Gda	-0,19012	1
Mapk1	-0,19008	1
Pink1	-0,19041	1
Dock10	-0,19043	1
Gmppa	-0,1907	1
Gm15151	-0,19124	1
Zswim8	-0,19128	1
Thoc2	-0,19154	1
Dusp8	-0,1926	1
Clec4d	-0,19284	1
Atp9b	-0,19309	1
Cep170b	-0,19308	1
Stx6	-0,19336	1
Mms22l	-0,19348	1
Rbm28	-0,19362	1
Usp47	-0,19407	1
1190007I07Rik	-0,1942	1
Fbrs	-0,19427	1
Pgm2	-0,19449	1
St3gal5	-0,19456	1
Fam206a	-0,19476	1
Ndc1	-0,19491	1
Irak1	-0,195	1
Gm19777	-0,1952	1
Crnde	-0,19543	1
Brip1os	-0,19543	1
Ankfy1	-0,19542	1
Lrrcc1	-0,19557	1
Eed	-0,1956	1
Arl16	-0,19572	1
Dcbld2	-0,19578	1
Gsdmd	-0,19616	1
Cbx3	-0,19622	1
Tmem26	-0,19641	1

Ppard	-0,19663	1
Psmd7	-0,19658	1
Cbfb	-0,19699	1
Eefsec	-0,19713	1
Daam1	-0,1971	1
Atp1b3	-0,1971	1
Eif2b5	-0,19776	1
Emd	-0,19789	1
Clcn3	-0,19813	1
Trim13	-0,19859	1
Tecr	-0,19862	1
Cntrl	-0,19867	1
Ndufa11	-0,19921	1
Rab14	-0,19933	1
Ppih	-0,19995	1
Creb5	-0,2001	1
Shcbp1l	-0,20018	1
Slc2a4rg-ps	-0,20029	1
Zfp219	-0,20035	1
Klhdc2	-0,20038	1
Atpif1	-0,20047	1
Snx1	-0,20055	1
Ap1g1	-0,20063	1
Rbbp9	-0,20074	1
Gm9207	-0,20074	1
Btbd10	-0,2008	1
Arpc5	-0,20082	1
Mmp2	-0,20134	1
Rab18	-0,2013	1
Vamp7-ps	-0,20138	1
Plekhn1	-0,20143	1
Uqcrc1	-0,2015	1
Xpo6	-0,20191	1
Pigyl	-0,20188	1
Dffb	-0,20224	1
Lats1	-0,20229	1
Rabgap1	-0,20231	1
Klf8	-0,20262	1
Ube3b	-0,20259	1
Dolpp1	-0,20265	1
Gm20432	-0,20281	1
Heatr6	-0,20284	1
Depdc7	-0,20293	1
Sirt3	-0,20291	1
Usp48	-0,20289	1
Copg1	-0,20315	1
Nelfb	-0,20317	1
Fdxr	-0,20331	1
Ythdf3	-0,20343	1
Stx18	-0,20346	1
Dcstamp	-0,20371	1
Pcyox1	-0,20367	1

Gm7936	-0,20382	1
RP23-48A24.3	-0,20396	1
Trp53bp1	-0,20417	1
Prr14	-0,20442	1
Elmo1	-0,20448	1
Fig4	-0,20473	1
Hacl1	-0,20495	1
Cln3	-0,20502	1
Med26	-0,20514	1
Ddx3x	-0,20515	1
Dcun1d5	-0,20535	1
Gm13186	-0,20543	1
Echdc3	-0,20544	1
Hagh	-0,20535	1
Tcaf1	-0,20546	1
Rrm2b	-0,2055	1
Paf1	-0,20567	1
Gm45289	-0,20582	1
Fam188a	-0,20577	1
Pald1	-0,20608	1
Spred2	-0,20621	1
Os9	-0,20633	1
Pgrmc1	-0,20646	1
Gm24927	-0,20657	1
Nek7	-0,20662	1
Mdh2	-0,20657	1
Pygb	-0,20698	1
Gm6209	-0,20725	1
Slc25a45	-0,20749	1
Slc4a1ap	-0,20751	1
Washc2	-0,20751	1
Gm20703	-0,20758	1
Rasal3	-0,20766	1
Vcp	-0,20818	1
Cryga	-0,20834	1
Slc47a2	-0,20846	1
Flcn	-0,20848	1
Rrp1	-0,20848	1
Enkd1	-0,20867	1
Rdh5	-0,20893	1
Gm2895	-0,20896	1
Gfm2	-0,20915	1
Ccdc82	-0,20937	1
Gm17066	-0,20943	1
Zfp72	-0,20948	1
Nop14	-0,20954	1
Pggt1b	-0,2096	1
Usp6nl	-0,2097	1
Camk1	-0,21012	1
Cdc27	-0,21029	1
Snx14	-0,21055	1
Akap13	-0,21051	1

Fundc2	-0,21064	1
Cxxc1	-0,21087	1
Ahcyl1	-0,21094	1
Ddhd2	-0,21164	1
Rdx	-0,2117	1
Gak	-0,21183	1
Ap4e1	-0,21188	1
Vps54	-0,21207	1
Sco1	-0,21211	1
Rbm8a	-0,21229	1
Ubxn6	-0,21289	1
Mrpl41	-0,21289	1
2310068J16Rik	-0,213	1
Pdzd11	-0,21337	1
Fam134a	-0,21383	1
Zfand2b	-0,21423	1
Cbr4	-0,21437	1
Trim56	-0,21482	1
Phrf1	-0,2153	1
Krtcap2	-0,21543	1
Trim41	-0,21555	1
Unc45a	-0,21563	1
Itm2b	-0,21573	1
Phf20-ps	-0,21592	1
Gm29340	-0,21598	1
Timm50	-0,2163	1
Mfsd7b	-0,21651	1
Sssca1	-0,21669	1
Gm9794	-0,21674	1
Gm13889	-0,21715	1
Dner	-0,21711	1
Rsbn1l	-0,21731	1
Fam102b	-0,21743	1
Sema6c	-0,2181	1
Ydjc	-0,21829	1
Hdc	-0,21845	1
0610009B22Rik	-0,21853	1
Fam204a	-0,21883	1
Aamp	-0,21895	1
Gm38377	-0,21898	1
Desi1	-0,21921	1
Spink5	-0,21917	1
Parg	-0,2194	1
Tpk1	-0,21968	1
Appbp2	-0,21966	1
Nudt21	-0,21983	1
Afg3l2	-0,21978	1
Timmdc1	-0,21987	1
Fam104a	-0,21989	1
Ppp2r5b	-0,22	1
Ppp2r5a	-0,2202	1
Jrkl	-0,22065	1

Sumo3	-0,22069	1
Lrrc59	-0,22101	1
Rnaseh2b	-0,22123	1
Fbxl19	-0,22166	1
Fkbp4	-0,22195	1
Zwint	-0,222	1
Psmd1	-0,22279	1
Ufd1l	-0,22291	1
Pbx3	-0,22303	1
Ddb1	-0,22303	1
Nktr	-0,2231	1
Rbm6-ps1	-0,22344	1
Nepro	-0,22341	1
Farsa	-0,22346	1
Sf3b2	-0,22355	1
Bmi1	-0,22358	1
Nsun2	-0,22366	1
Alg6	-0,22391	1
Nlrc3	-0,224	1
Sep 07	-0,22472	1
Gm996	-0,22486	1
Usp24	-0,22524	1
Tbc1d16	-0,22529	1
Amfr	-0,2255	1
Setd1a	-0,22617	1
Ttc4	-0,22636	1
Zfp287	-0,22702	1
Slc25a1	-0,22717	1
Zfx	-0,22715	1
Syngr2	-0,2274	1
Atg16l2	-0,22745	1
Pirb	-0,22774	1
Zfp956	-0,22785	1
Mrpl15	-0,22791	1
RP23-213P10.2	-0,228	1
Trim46	-0,22808	1
Tmem39b	-0,22814	1
Ahi1	-0,22814	1
Tnrc6b	-0,22807	1
Deptor	-0,22816	1
Rbm26	-0,2283	1
5430405H02Rik	-0,22848	1
3110002H16Rik	-0,22873	1
Osbpl8	-0,2287	1
Tada1	-0,22886	1
Klf16	-0,22901	1
Canx	-0,22901	1
Mlx	-0,2291	1
Mcl1	-0,22908	1
Dennd3	-0,2292	1
Ddx52	-0,2295	1
Cdk16	-0,2302	1

Abhd6	-0,2304	1
1700066M21Rik	-0,23053	1
Hells	-0,23049	1
Arl10	-0,23064	1
Tmem8b	-0,23091	1
Mrpl57	-0,23087	1
Lactb	-0,23119	1
Slc25a19	-0,23137	1
Cpeb3	-0,2315	1
Tmem101	-0,2317	1
Nceh1	-0,23171	1
Cask	-0,23249	1
Plxnd1	-0,2328	1
Ifi204	-0,23305	1
Tbkbp1	-0,23308	1
Pear1	-0,23335	1
D930015E06Rik	-0,23343	1
Dnaja3	-0,23365	1
Tbccd1	-0,23405	1
Znhit3	-0,23412	1
Pcyox1l	-0,23434	1
Zfp592	-0,23429	1
Ada	-0,23447	1
Gm12350	-0,23461	1
Rpl4	-0,2347	1
Rhd	-0,23469	1
Dhx38	-0,23492	1
Tcf12	-0,23491	1
Ccdc28a	-0,23513	1
Tcam1	-0,23517	1
Tpst1	-0,23537	1
Ubxn1	-0,23535	1
Psmg1	-0,23563	1
Rmi1	-0,23587	1
Actr6	-0,23619	1
Stx4a	-0,23628	1
Arf3	-0,23648	1
Dnph1	-0,23684	1
Cep83os	-0,23696	1
Stk3	-0,23725	1
Gm13840	-0,23767	1
Smarcc2	-0,23776	1
Snrnp40	-0,23794	1
Apobec3	-0,23818	1
Gm7452	-0,23826	1
Set	-0,23833	1
Ogfod2	-0,23843	1
Trip12	-0,23842	1
Insr	-0,23863	1
Atad3a	-0,23884	1
Slc7a5	-0,23884	1
2010315B03Rik	-0,23902	1

Heatr5a	-0,23946	1
Map1s	-0,23949	1
Stard9	-0,23962	1
Gm9568	-0,2398	1
Ipmk	-0,2398	1
Tbc1d17	-0,23986	1
Dgcr14	-0,24039	1
Acot13	-0,24112	1
Glyr1	-0,2415	1
Maoa	-0,24215	1
Cog6	-0,24231	1
Nol12	-0,24255	1
Srsf7	-0,24257	1
Nrbf2	-0,24293	1
Pafah1b1	-0,24287	1
Vps41	-0,2433	1
Fam217b	-0,24341	1
Acly	-0,24337	1
Mtmr1	-0,24355	1
Nfat5	-0,24355	1
Gtf2h4	-0,24411	1
Cnot1	-0,24406	1
Polr3c	-0,24441	1
Mettl1	-0,24465	1
Fancl	-0,24473	1
Rapgef2	-0,24477	1
Lin7c	-0,24493	1
Tspan4	-0,24502	1
Ifnar2	-0,24517	1
Fktn	-0,24532	1
Herc6	-0,24536	1
Sephs1	-0,24544	1
Actr1b	-0,24562	1
Birc6	-0,24556	1
Pxk	-0,24575	1
Rbm17	-0,24592	1
Orai2	-0,24595	1
Txn1	-0,24592	1
A630001G21Rik	-0,24613	1
Trappc3	-0,24613	1
Vps37c	-0,24648	1
Htatip2	-0,24742	1
Kidins220	-0,24736	1
Lsg1	-0,24784	1
Ube2j1	-0,24792	1
Endod1	-0,2481	1
Phb2	-0,24827	1
Marcksl1	-0,24851	1
Ttll5	-0,24855	1
Pip5k1a	-0,24893	1
Atp5o	-0,24916	1
Usp54	-0,24948	1

1600002K03Rik	-0,25026	1
Chd1l	-0,25033	1
Exoc3	-0,25031	1
Sec63	-0,25036	1
Taok1	-0,25045	1
Rabggta	-0,25059	1
Stk10	-0,25058	1
Mau2	-0,25102	1
Ing3	-0,25116	1
Pten	-0,25157	1
Gm11224	-0,25184	1
Ntn5	-0,25188	1
Zmat2	-0,25191	1
Gm15946	-0,25228	1
Saa3	-0,25301	1
Cd2ap	-0,25304	1
Sgk3	-0,25336	1
Gm43128	-0,25345	1
Siah2	-0,25364	1
Prdm2	-0,2539	1
Zfp608	-0,25405	1
Dse	-0,25424	1
Tbck	-0,25419	1
Eif2b1	-0,25417	1
Zbtb46	-0,25434	1
Atrn	-0,25455	1
Trnau1ap	-0,25459	1
Gm42715	-0,25507	1
Mtss1	-0,25506	1
Fam207a	-0,25535	1
Gm45050	-0,25554	1
Zfp524	-0,25549	1
Ap4m1	-0,25556	1
Eif3c	-0,25563	1
Arid1a	-0,25588	1
Fdx1	-0,25634	1
Sec61b	-0,25661	1
Carm1	-0,25672	1
Smurf2	-0,25685	1
Gm38036	-0,25697	1
Acaa1a	-0,25703	1
Csnk1a1	-0,25705	1
Yipf3	-0,25741	1
Ccdc28b	-0,25762	1
Mcu	-0,25779	1
Myo9a	-0,25788	1
Aifm2	-0,25819	1
Lemd2	-0,25822	1
Gtf2e1	-0,25854	1
Cast	-0,25858	1
Mpi	-0,25867	1
Hspd1	-0,25869	1

Il6st	-0,25882	1
Aspscr1	-0,25914	1
Dnajc14	-0,25917	1
Kdm2a	-0,25926	1
Erich1	-0,25937	1
Coasy	-0,2596	1
Rps14	-0,2598	1
Vkorc1l1	-0,26011	1
Tatdn3	-0,2603	1
Hnrnph3	-0,26034	1
Zswim7	-0,26039	1
Gm5910	-0,26044	1
Peg13	-0,2604	1
Impad1	-0,26041	1
Aldh1l2	-0,26064	1
Dnajc1	-0,26057	1
Mpp1	-0,26103	1
Ly6e	-0,26161	1
Phlda3	-0,26189	1
Slc12a9	-0,26188	1
Ankmy2	-0,26197	1
4931406C07Rik	-0,26206	1
Klrg2	-0,26218	1
Gm23442	-0,26239	1
Slirp	-0,26239	1
Slc30a1	-0,26257	1
Gm17807	-0,26271	1
Homer1	-0,26315	1
Idh3g	-0,26355	1
Sdccag8	-0,26362	1
Mlf2	-0,26375	1
Hddc3	-0,26381	1
Scmh1	-0,26443	1
Nup93	-0,26442	1
DHRSX	-0,26458	1
Sipa1l3	-0,26489	1
Fam53a	-0,26491	1
3830408C21Rik	-0,26512	1
Golga4	-0,26515	1
Arfip2	-0,26532	1
Zfp335os	-0,26539	1
Psmb6	-0,26537	1
Fam114a2	-0,26548	1
Rwdd4a	-0,26661	1
Cyb5r1	-0,26703	1
Zfp24	-0,26697	1
Gm4602	-0,26706	1
Ypel4	-0,26731	1
Map4k1	-0,26727	1
Gadd45gip1	-0,2674	1
Ucp2	-0,26741	1
Mut	-0,2675	1

Capn5	-0,26769	1
Bmyc	-0,26795	1
Ppp1r12a	-0,26792	1
Recql5	-0,26809	1
Rab42	-0,2683	1
Cpsf1	-0,26828	1
Eef2kmt	-0,26842	1
Rbm43	-0,26848	1
Ezh1	-0,26883	1
Mtfmt	-0,26895	1
Stradb	-0,26926	1
Churc1	-0,26991	1
Pacs2	-0,26993	1
Coq9	-0,2701	1
Cyb5r3	-0,27042	1
Usp12	-0,27066	1
4930455G09Rik	-0,27084	1
2610008E11Rik	-0,27093	1
Tbc1d2	-0,27116	1
Rmnd1	-0,27127	1
Rasip1	-0,2716	1
Tinf2	-0,27191	1
Anxa7	-0,27255	1
Rasa1	-0,27267	1
Il1rn	-0,27313	1
Brms1	-0,2732	1
Plcb2	-0,27316	1
Mprip	-0,2732	1
Uty	-0,27347	1
Fastk	-0,27353	1
Lrrc24	-0,27402	1
Mpdu1	-0,27402	1
March11	-0,27405	1
Nf1	-0,27421	1
Arpc2	-0,27417	1
Pik3r5	-0,27423	1
H1f0	-0,27455	1
Car13	-0,27457	1
Rsl1d1	-0,27493	1
Rev3l	-0,27503	1
Mob3b	-0,27555	1
Nsmce2	-0,2757	1
Gtf3c1	-0,27579	1
Gm15265	-0,27599	1
Fabp3	-0,27601	1
Slc25a13	-0,27617	1
Ergic2	-0,27619	1
Aurkaip1	-0,27632	1
Ighd	-0,27637	1
Il1b	-0,27659	1
Gcc2	-0,27665	1
Rab3il1	-0,27679	1

Fam171b	-0,27697	1
Col4a3bp	-0,27761	1
Zfp771	-0,27761	1
Zcrb1	-0,27765	1
Ist1	-0,27756	1
Ranbp3	-0,27772	1
Gm20522	-0,27803	1
Fam8a1	-0,2784	1
Trp53inp1	-0,27852	1
Pstpip2	-0,27891	1
Sfxn3	-0,27895	1
Zc3hav1l	-0,27902	1
Snx17	-0,27902	1
Dennd5b	-0,27905	1
Rnf31	-0,27905	1
Tmbim4	-0,2791	1
Sh3bgr	-0,27915	1
Azi2	-0,27936	1
Dnase1l1	-0,27964	1
Pcbp1	-0,27962	1
Ino80b	-0,27976	1
Tshz1	-0,27992	1
Polr2m	-0,27985	1
Nlk	-0,28022	1
1700086O06Rik	-0,28051	1
Timm9	-0,28052	1
Slc35e1	-0,28058	1
Ccdc93	-0,28065	1
Capg	-0,28141	1
Spryd4	-0,28166	1
Lypla1	-0,28172	1
Cav2	-0,2821	1
Tank	-0,28222	1
Dpy19l4	-0,28236	1
Dera	-0,28255	1
Plekho2	-0,28263	1
Gm17259	-0,28293	1
Card9	-0,28289	1
Slc30a7	-0,28288	1
Psmb5	-0,28308	1
Plrg1	-0,28318	1
Map3k5	-0,28331	1
Mydgf	-0,28326	1
Rbbp6	-0,28362	1
Dus2	-0,28391	1
Dhrs7	-0,28442	1
Gemin7	-0,28436	1
Flad1	-0,28462	1
Cacul1	-0,28476	1
Brix1	-0,28514	1
S100pbp	-0,28543	1
Als2	-0,28564	1

Med10	-0,28582	1
Crot	-0,28594	1
Borcs7	-0,28597	1
Rbfox2	-0,28612	1
Rmnd5b	-0,2862	1
Cnot6	-0,28643	1
Nhlrc1	-0,28678	1
Dcaf12	-0,28686	1
RP23-261O3.5	-0,28711	1
1700030K09Rik	-0,28715	1
Spef1	-0,2874	1
Sufu	-0,28781	1
Tex261	-0,28785	1
Runx3	-0,28814	1
Grk4	-0,28865	1
Uvrag	-0,28887	1
Rab5b	-0,28895	1
Coro1b	-0,28895	1
Cnnm3	-0,28906	1
Pigm	-0,28922	1
Prpsap2	-0,28934	1
Psmd5	-0,28939	1
Usp40	-0,29009	1
Gm7676	-0,29036	1
Pnisr	-0,29045	1
Hexa	-0,29047	1
Dgcr6	-0,29057	1
2010320M18Rik	-0,29073	1
Psmb9	-0,29109	1
Proser1	-0,29111	1
Cep89	-0,29152	1
Mir155hg	-0,29161	1
Mtfp1	-0,29158	1
Chchd10	-0,29156	1
Cops3	-0,29156	1
Thoc1	-0,29194	1
Acsl1	-0,29219	1
Stau1	-0,2925	1
Dfna5	-0,29259	1
Gm10136	-0,29258	1
Uprt	-0,29283	1
Sbno2	-0,29289	1
Plekhg3	-0,29341	1
Atp2b1	-0,29346	1
Ccne1	-0,29373	1
Vps39	-0,2938	1
Ssr1	-0,29441	1
Ankrd11	-0,29454	1
Zbtb41	-0,2948	1
Nfx1	-0,29515	1
Siglec1	-0,29525	1
Ankib1	-0,29521	1

Gm45802	-0,29531	1
Gle1	-0,29533	1
Abhd16a	-0,29551	1
Ggnbp2	-0,29565	1
Sh3glb2	-0,29578	1
Cstb	-0,29601	1
Ccnd1	-0,29599	1
Nipbl	-0,29656	1
Mocos	-0,29681	1
Fam129b	-0,29689	1
Baz1a	-0,29692	1
Ppp3cb	-0,2969	1
Zfp784	-0,29699	1
Fas	-0,29719	1
Pias3	-0,29735	1
Btbd2	-0,29763	1
Ubc	-0,29778	1
AC149090.1	-0,29775	1
Irf7	-0,29796	1
Armc2	-0,29799	1
Btbd3	-0,29796	1
Runx2	-0,29817	1
Actr3b	-0,29817	1
Cisd2	-0,29832	1
Smpd2	-0,29843	1
Six1	-0,29859	1
Tmem5	-0,29894	1
Bpgm	-0,29932	1
9530082P21Rik	-0,29929	1
Lap3	-0,29929	1
Shpk	-0,29958	1
Slc52a2	-0,30045	1
Gltscr1l	-0,30069	1
Srp14	-0,30071	1
Mtx3	-0,30123	1
Polr2c	-0,30123	1
Zfp7	-0,30128	1
Ncstn	-0,30134	1
Mark3	-0,30127	1
Rock1	-0,30143	1
E2f3	-0,3015	1
Nudcd3	-0,30153	1
Mtfr1l	-0,30158	1
Nt5dc1	-0,30201	1
Dkc1	-0,3022	1
1810014B01Rik	-0,30239	1
Exosc2	-0,30243	1
Paqr3	-0,3024	1
Telo2	-0,30281	1
Elac2	-0,30294	1
Il2rg	-0,30314	1
Msto1	-0,30312	1

Sertad2	-0,30338	1
Acap2	-0,30345	1
Kif13b	-0,30374	1
Tox4	-0,30375	1
Nln	-0,30397	1
Gm13397	-0,30446	1
Tmem131	-0,30445	1
Cpeb1	-0,30468	1
Fcgr4	-0,30468	1
Car6	-0,30475	1
Mrpl19	-0,30481	1
Gm42872	-0,30504	1
Pgam5	-0,30516	1
Nup155	-0,30529	1
Ncor1	-0,30533	1
Fosb	-0,30538	1
Nkiras2	-0,30543	1
Tmem126a	-0,30558	1
Rbfa	-0,30564	1
Cdc42bpg	-0,3058	1
2700062C07Rik	-0,30589	1
Isg20	-0,30589	1
Gm38115	-0,30601	1
Kdm5b	-0,30612	1
Vti1a	-0,30616	1
Mrm3	-0,30677	1
Dgat1	-0,30686	1
Ncln	-0,30702	1
Prkci	-0,30707	1
2700049A03Rik	-0,30719	1
Caly	-0,30731	1
Smyd3	-0,30726	1
Cep104	-0,30729	1
Kyat3	-0,30764	1
Atraid	-0,30757	1
Qrich1	-0,308	1
Exoc5	-0,30797	1
Cryl1	-0,30805	1
Mettl13	-0,30824	1
Eif6	-0,30861	1
Nans	-0,30866	1
Amz1	-0,30873	1
Ankrd40	-0,30961	1
Dusp5	-0,30981	1
Trim35	-0,31001	1
Trp53cor1	-0,3101	1
2310010J17Rik	-0,31013	1
Mgme1	-0,3101	1
Zfp639	-0,31052	1
Pddc1	-0,31073	1
Gm12543	-0,31082	1
Timm8b	-0,31089	1

Pus7	-0,31101	1
Ifngr2	-0,3112	1
Ptpa	-0,31119	1
Numa1	-0,31118	1
Akr1b8	-0,31131	1
Slc6a13	-0,31141	1
Oas3	-0,31158	1
Cds2	-0,31195	1
Ddx39b	-0,31228	1
Cstf1	-0,31288	1
Paqr4	-0,313	1
Arid5a	-0,313	1
Isy1	-0,31298	1
Cops8	-0,31313	1
Spata24	-0,31342	1
Asb13	-0,31336	1
Fntb	-0,31337	1
Gm9025	-0,31364	1
Elmsan1	-0,31376	1
Gtf2i	-0,31389	1
Aifm1	-0,31395	1
Chrnb2	-0,31408	1
Dhx37	-0,31411	1
Abcc3	-0,3143	1
Senp2	-0,31425	1
Cdc23	-0,31428	1
AI837181	-0,31443	1
Slc25a11	-0,31454	1
Ireb2	-0,31491	1
Med23	-0,31497	1
Ldah	-0,31497	1
Chmp5	-0,31497	1
Cnbp	-0,31502	1
Arhgef15	-0,31511	1
Gm45501	-0,31519	1
Dscr3	-0,31544	1
Gm20045	-0,31569	1
Mak16	-0,31565	1
Coa5	-0,31567	1
Bub3	-0,31622	1
Bpnt1	-0,31634	1
Gm16372	-0,3163	1
Snhg1	-0,31634	1
Zfyve21	-0,31649	1
Ncs1	-0,31659	1
Capn7	-0,31655	1
Plxnb2	-0,3166	1
Stx12	-0,31679	1
Vamp4	-0,31719	1
Coq8b	-0,31737	1
Armc9	-0,31749	1
Prpf31	-0,31774	1

Cela1	-0,31777	1
Coq10a	-0,31787	1
Grn	-0,31802	1
Parp14	-0,31812	1
Coro7	-0,31814	1
Cyfip1	-0,31809	1
Ttc19	-0,31815	1
Tdp1	-0,31852	1
Tcf3	-0,31858	1
Cxx1a	-0,31872	1
Zc3hav1	-0,31883	1
Egfl7	-0,31899	1
Gm37145	-0,31914	1
Eif3d	-0,31915	1
Kdelr2	-0,31964	1
Map2k3os	-0,31994	1
Tmem86a	-0,32011	1
Fam134b	-0,32006	1
Heatr1	-0,32021	1
Clcn7	-0,32019	1
Slc25a24	-0,32055	1
Pisd	-0,32077	1
Pprc1	-0,32117	1
Ndufs3	-0,32127	1
Gspt1	-0,32148	1
Agfg1	-0,32148	1
Rbm27	-0,32161	1
Nploc4	-0,32184	1
Apobec1	-0,32207	1
Lims2	-0,32233	1
Sft2d2	-0,3224	1
Ccdc61	-0,32249	1
Rps3	-0,32268	1
Gm7785	-0,32292	1
H13	-0,32289	1
Mrpl43	-0,32309	1
Cox5b	-0,32323	1
Gm18916	-0,32319	1
Tbc1d9b	-0,32327	1
Gm28530	-0,32347	1
1110008F13Rik	-0,32351	1
Gm7102	-0,3236	1
Prr36	-0,32357	1
Meiob	-0,32384	1
Stk38l	-0,32397	1
Epg5	-0,32427	1
Msl3	-0,32457	1
Gpat4	-0,32456	1
Kdsr	-0,32466	1
Gpnmb	-0,32476	1
C130089K02Rik	-0,32487	1
Rab33b	-0,32487	1

Rgs19	-0,32487	1
Gstm1	-0,32523	1
Fbxl15	-0,32591	1
Baz2b	-0,32601	1
Zdhhc13	-0,32605	1
Gm7535	-0,32618	1
Gm15644	-0,32658	1
Fbxo7	-0,32768	1
Minpp1	-0,3279	1
Reep3	-0,32822	1
Tsen54	-0,32866	1
Magi1	-0,32874	1
Utp6	-0,32871	1
Fam50a	-0,32901	1
Hnrnpk	-0,32898	1
Rwdd2a	-0,32912	1
Gm5575	-0,3292	1
Fabp5	-0,32928	1
Kif2a	-0,32951	1
Alyref2	-0,33005	1
Morc2a	-0,3301	1
Cog4	-0,33008	1
Foxp4	-0,33033	1
Zfp827	-0,33035	1
Fam149b	-0,33067	1
Hoxa4	-0,33073	1
Limd2	-0,33068	1
Polr2g	-0,33076	1
Trappc5	-0,33194	1
Babam1	-0,33219	1
Pou5f2	-0,33226	1
Creb3l3	-0,33258	1
Rtn4ip1	-0,33276	1
Tfdp2	-0,33287	1
Srsf5	-0,33304	1
Gpr132	-0,33359	1
Npc1	-0,33431	1
Ccdc127	-0,33461	1
Ltn1	-0,33472	1
Amigo1	-0,33499	1
Gnaq	-0,33505	1
Nt5c	-0,33513	1
Sar1a	-0,33508	1
Ly9	-0,33542	1
Mterf4	-0,33553	1
Rft1	-0,33695	1
Atg7	-0,33691	1
Cdc16	-0,33695	1
Fez2	-0,33741	1
Mtfr1	-0,33761	1
Vps16	-0,33762	1
Smim3	-0,33782	1

Prkaca	-0,33807	1
Hoxb4	-0,33822	1
Chchd1	-0,33827	1
Fam83d	-0,33844	1
Lzts2	-0,3386	1
Rfx7	-0,33891	1
Top1mt	-0,33896	1
Polr2b	-0,33899	1
Ube3a	-0,33914	1
Gm26800	-0,33982	1
Mbnl1	-0,33999	1
Chtf18	-0,34011	1
Smarca5	-0,34036	1
Tmem35b	-0,34089	1
Prmt1	-0,34093	1
Pigo	-0,3413	1
Mto1	-0,34154	1
Xkr8	-0,3418	1
Vps26b	-0,34194	1
Orc2	-0,34211	1
Tmem80	-0,34224	1
Gm12430	-0,34253	1
P2ry2	-0,34286	1
Ndufa5	-0,34292	1
Arl4a	-0,34317	1
Srsf6	-0,34329	1
Magt1	-0,3434	1
Alg13	-0,34365	1
Gm7224	-0,34372	1
Otud6b	-0,34375	1
Arrb2	-0,34401	1
Sec61a2	-0,34444	1
Gm44699	-0,34469	1
Ercc3	-0,3448	1
Sla2	-0,34487	1
Zxdb	-0,3451	1
Psmb4	-0,34538	1
Psma1	-0,34539	1
Prmt5	-0,34641	1
Gm8973	-0,34673	1
Pgp	-0,34692	1
Gm12459	-0,34699	1
Pinx1	-0,34698	1
Fnta	-0,34733	1
Map2k5	-0,34732	1
Kcnk13	-0,34744	1
Poldip3	-0,34797	1
Fars2	-0,34826	1
Stard8	-0,34878	1
Utp23	-0,34897	1
Wars	-0,3491	1
Mat2a	-0,34914	1

2810474O19Rik	-0,34916	1
Pnpla2	-0,34964	1
Ano10	-0,34993	1
Ninj1	-0,35015	1
Hipk1	-0,35022	1
B4galnt1	-0,35025	1
Tab2	-0,35047	1
Ext1	-0,35075	1
Rfx1	-0,35071	1
Lias	-0,3508	1
Fignl2	-0,35103	1
Smyd4	-0,35097	1
Nrd1	-0,35096	1
Gm5244	-0,35137	1
Usp36	-0,35156	1
Cisd1	-0,35275	1
Daxx	-0,35284	1
Exoc3l2	-0,3529	1
Sec23b	-0,35333	1
Cops5	-0,35335	1
Rnf213	-0,35349	1
Phf7	-0,35363	1
Pnpo	-0,35356	1
Wdr55	-0,35379	1
Cd200r4	-0,35421	1
Rps6kb1	-0,35443	1
Ypel3	-0,35452	1
Lsm14b	-0,35465	1
Fam111a	-0,35456	1
Themis2	-0,35472	1
Pld3	-0,35468	1
Gm14780	-0,3549	1
Rps6kc1	-0,35508	1
Tbl1xr1	-0,35524	1
Fmc1	-0,35533	1
Cdca7l	-0,35526	1
Chd1	-0,35526	1
Bbs10	-0,35557	1
Ikzf1	-0,35579	1
Ibtk	-0,35609	1
Ikbip	-0,35617	1
Ufc1	-0,35624	1
Slc39a6	-0,35639	1
Myh9	-0,35639	1
Grk2	-0,3564	1
Eif4a3	-0,3566	1
Prmt6	-0,35665	1
Nek8	-0,35686	1
Phf21a	-0,35695	1
Hebp1	-0,35723	1
Cwf19l2	-0,35729	1
Gm14277	-0,35752	1

St3gal3	-0,35766	1
Purg	-0,3581	1
Fahd2a	-0,35858	1
Map2k4	-0,35859	1
Taf6l	-0,35859	1
Abhd17c	-0,35883	1
Sidt2	-0,35889	1
Osgin1	-0,35921	1
Zfp715	-0,35923	1
2510046G10Rik	-0,35962	1
Tanc1	-0,35987	1
Klhl26	-0,36017	1
Zfp68	-0,3606	1
Tma16	-0,36062	1
Gm11273	-0,36069	1
D230022J07Rik	-0,36071	1
Mtrr	-0,36071	1
Bcat1	-0,36083	1
Tmem55b	-0,36101	1
Fam167b	-0,36117	1
Arfgef1	-0,36121	1
Zfp317	-0,36137	1
Ppp1r18	-0,36147	1
Zfp292	-0,36172	1
Mphosph8	-0,36182	1
Ccdc88b	-0,36199	1
Gm13391	-0,36231	1
Mfsd10	-0,36228	1
Atp6v1f	-0,36243	1
Tspan14	-0,36267	1
Tonsl	-0,36285	1
Dusp12	-0,36312	1
Gpr89	-0,36332	1
Cipc	-0,36343	1
Ankrd44	-0,36358	1
Parp11	-0,36366	1
Dhx16	-0,36385	1
Rbm12	-0,36377	1
Rcor2	-0,36455	1
Gm38200	-0,36469	1
Gm22581	-0,36475	1
Cdc123	-0,36473	1
Golga5	-0,36469	1
Ubp1	-0,36467	1
D830044I16Rik	-0,36482	1
Cyb5d2	-0,36513	1
Tmem71	-0,36564	1
Ppp1r21	-0,36579	1
Myo1e	-0,36581	1
Helz2	-0,36643	1
Zfp160	-0,36649	1
Chil6	-0,3666	1

1110034G24Rik	-0,36693	1
Dph7	-0,36703	1
Mfn2	-0,36708	1
Inca1	-0,36716	1
Sbk2	-0,36751	1
Cabin1	-0,36754	1
Gm37289	-0,36765	1
Myo7a	-0,36863	1
Cirbp	-0,36874	1
Ccrl2	-0,36894	1
Arv1	-0,36903	1
Ptdss1	-0,3692	1
Dhps	-0,36932	1
Chmp7	-0,36949	1
Tulp4	-0,36955	1
Rpgrip1	-0,36991	1
Tmem106c	-0,36993	1
Ncbp3	-0,37003	1
Tprkb	-0,3702	1
Utp11	-0,37024	1
Acp1	-0,37045	1
Aagab	-0,37049	1
Otulin	-0,37061	1
Ano8	-0,37067	1
Fat1	-0,37084	1
Sdha	-0,37076	1
Mrps34	-0,37091	1
Taf5l	-0,37114	1
Irf3	-0,37139	1
B230118H07Rik	-0,37143	1
Cep152	-0,3716	1
Zgpat	-0,3717	1
Tmco1	-0,3717	1
Atp1a1	-0,37182	1
Pls3	-0,3724	1
Tmem231	-0,37301	1
Eif3g	-0,37398	1
Smyd2	-0,37443	1
Ahsa1	-0,37491	1
Camsap2	-0,37509	1
Zkscan3	-0,37517	1
Taf4b	-0,37532	1
Bcl7b	-0,37533	1
Upf3a	-0,3753	1
Ap5m1	-0,37587	1
Ppp1r11	-0,37649	1
Cpsf2	-0,37648	1
Ggact	-0,37662	1
Gm5697	-0,37666	1
Trappc13	-0,37673	1
Ankra2	-0,37684	1
Herc4	-0,37687	1

Med4	-0,37703	1
Fbxw9	-0,37703	1
Lyn	-0,37714	1
Arid4a	-0,37739	1
Mon2	-0,37781	1
Gm14270	-0,3779	1
Map3k20	-0,37791	1
Tmeff1	-0,37813	1
Dctn5	-0,37817	1
Igf2r	-0,37826	1
Pik3cd	-0,3783	1
Dbf4	-0,37867	1
Coq7	-0,3791	1
Tbc1d1	-0,37944	1
Il23a	-0,37959	1
Rabgap1l	-0,37978	1
Atpaf1	-0,38002	1
Pgap1	-0,38003	1
Cdon	-0,38018	1
Naf1	-0,38061	1
Ssh2	-0,38093	1
Ppp2r5c	-0,38092	1
Gm43223	-0,38102	1
Akr1a1	-0,3813	1
Ccnb1	-0,38163	1
Dap	-0,38203	1
Gcat	-0,38206	1
Ctdsp2	-0,38206	1
Ubtd1	-0,38238	1
Ndufs7	-0,38274	1
Adcy9	-0,38304	1
Dctn4	-0,38306	1
Esd	-0,38306	1
Txlnb	-0,38332	1
Setd5	-0,38332	1
Abca5	-0,38342	1
Pcgf3	-0,38342	1
Impdh1	-0,38353	1
Trim37	-0,38355	1
Pidd1	-0,38427	1
Gm12186	-0,38443	1
RP23-304C21.3	-0,38436	1
Zeb2	-0,38456	1
Chd8	-0,38534	1
Wtap	-0,38543	1
Snf8	-0,3856	1
Zfp667	-0,38576	1
Ssrp1	-0,38577	1
Rasgef1a	-0,38611	1
Il18rap	-0,3865	1
Rdh10	-0,38663	1
Synj1	-0,38661	1

Anapc10	-0,38665	1
Gm42571	-0,38683	1
Zc3h15	-0,38695	1
Catsperg1	-0,38729	1
Tmem141	-0,38735	1
Camkk2	-0,38743	1
Il18	-0,38764	1
Snap23	-0,38756	1
Gca	-0,38793	1
Atxn7	-0,38791	1
Fndc3b	-0,38847	1
Gm10399	-0,38867	1
Abhd5	-0,38867	1
Dhx30	-0,38867	1
Ap2m1	-0,38878	1
Ergic1	-0,38884	1
Cep95	-0,38894	1
Rfk	-0,38909	1
Hivep2	-0,38919	1
Adam17	-0,38945	1
Plcg1	-0,38951	1
Ahsa2	-0,38973	1
9030617O03Rik	-0,38965	1
Manea	-0,38981	1
Mtf1	-0,39002	1
Tbc1d20	-0,39008	1
Zscan26	-0,39017	1
Gas6	-0,39054	1
Gm5422	-0,3906	1
Ttbk2	-0,39055	1
Gm12689	-0,39059	1
Gpatch2	-0,39069	1
Tars2	-0,39087	1
Nolc1	-0,39087	1
Coil	-0,3912	1
Agtrap	-0,39119	1
Ccndbp1	-0,39117	1
Limk2	-0,39154	1
Glb1l	-0,39181	1
Atp23	-0,39186	1
Riox1	-0,39196	1
Tyw5	-0,39211	1
Otud7b	-0,39219	1
Pofut1	-0,39251	1
Nvl	-0,39247	1
Osbpl9	-0,39247	1
Rrbp1	-0,39257	1
Gm43462	-0,39275	1
Ubxn2a	-0,39286	1
Gpr162	-0,39292	1
Ddi2	-0,39302	1
Map4k5	-0,39309	1

Fam114a1	-0,39313	1
Pqlc3	-0,39324	1
Lace1	-0,39334	1
Kcnq1ot1	-0,39343	1
Picalm	-0,39346	1
Rnf40	-0,39373	1
Comtd1	-0,39398	1
Gpx1	-0,39409	1
Bank1	-0,39428	1
Bop1	-0,39434	1
Zfp777	-0,39442	1
Cbx8	-0,39455	1
Tmem167	-0,3945	1
Fry	-0,39463	1
Rcc1	-0,39465	1
Cd180	-0,39501	1
Abcd4	-0,39508	1
Atg4a	-0,39513	1
Ptpn22	-0,39513	1
Cd59a	-0,39531	1
Pus10	-0,39574	1
Rbmx2-ps	-0,39595	1
Them4	-0,39599	1
Tbk1	-0,39627	1
Gm37503	-0,39648	1
Gm5939	-0,39648	1
Srp72	-0,39653	1
Net1	-0,39693	1
Cers4	-0,39696	1
Brd7	-0,39695	1
Gm9246	-0,39713	1
Prpf6	-0,39776	1
Adprm	-0,39799	1
Nmd3	-0,39809	1
Wdr19	-0,39824	1
Dhx36	-0,39836	1
Fbxo18	-0,39851	1
Ttc1	-0,39862	1
Myo18a	-0,39856	1
Gm44269	-0,39868	1
Gm38055	-0,39897	1
Arl14ep	-0,3992	1
Phf11b	-0,39932	1
Tmem199	-0,39983	1
Fnbp1	-0,39979	1
Snord92	-0,40004	1
Slc37a4	-0,40014	1
Fn3krp	-0,40069	1
Phf11d	-0,40133	1
Itsn2	-0,40126	1
Gm7972	-0,40144	1
Ebna1bp2	-0,40152	1

Dcxr	-0,40179	1
Mbd1	-0,40187	1
A130071D04Rik	-0,40191	1
Gm17060	-0,40277	1
Gm14853	-0,40291	1
Nop58	-0,40293	1
Sec11c	-0,40313	1
Gm9575	-0,40368	1
Zcchc17	-0,40368	1
Kcnk6	-0,40381	1
Med27	-0,40406	1
Junb	-0,40421	1
Srrm1	-0,4044	1
Abca2	-0,4045	1
Fam151b	-0,40453	1
Prnp	-0,40507	1
Pex14	-0,40541	1
Mmadhc	-0,40536	1
Fam214b	-0,4055	1
Tomm34	-0,40555	1
Homer3	-0,406	1
Gm37125	-0,40605	1
Gm7909	-0,40625	1
Gm7384	-0,40639	1
Klhdc1	-0,40652	1
Mmachc	-0,40677	1
P2rx7	-0,40707	1
Tm2d3	-0,40729	1
1700047K16Rik	-0,40733	1
Slc29a1	-0,40732	1
Trpm2	-0,40747	1
Gmeb1	-0,40783	1
Anks1	-0,40814	1
E130102H24Rik	-0,40825	1
Gm42972	-0,40821	1
Ms4a6d	-0,40845	1
Phtf1	-0,40844	1
Gm15853	-0,40864	1
Egln2	-0,40874	1
Cd14	-0,40868	1
Hyi	-0,40917	1
Galns	-0,40936	1
Rrp7a	-0,40962	1
Tmem245	-0,40957	1
Txnrd2	-0,4097	1
Fem1b	-0,41002	1
Gm17494	-0,41007	1
Vps45	-0,41012	1
Mthfd1	-0,41033	1
Ube2d-ps	-0,41084	1
Mfsd8	-0,41103	1
St7l	-0,41112	1

Nt5c3	-0,41123	1
Cox6b1	-0,41128	1
Trio	-0,41139	1
Trim39	-0,41147	1
E130309D02Rik	-0,41154	1
Wnk1	-0,41197	1
Mfap3l	-0,41206	1
Fbxo3	-0,41213	1
Spag7	-0,41221	1
Pcca	-0,41219	1
Ruvbl1	-0,41232	1
Arhgap45	-0,41239	1
Dxo	-0,41257	1
Tmem57	-0,41259	1
Lipa	-0,41269	1
Stat3	-0,41292	1
Rad23b	-0,41306	1
Mef2a	-0,41347	1
Arl6ip4	-0,41357	1
Procr	-0,41372	1
Il16	-0,41401	1
Rffl	-0,41402	1
Tjap1	-0,41402	1
Tmem147	-0,41397	1
Pdik1l	-0,41423	1
Alkbh7	-0,41442	1
Ndufb9	-0,41438	1
Itga2b	-0,41456	1
Ndufa9	-0,4149	1
Ptpre	-0,4149	1
Nr1d2	-0,41518	1
Hnrnpul2	-0,41561	1
Trim7	-0,41578	1
9230111E07Rik	-0,41594	1
Tsn	-0,41593	1
Brd1	-0,41605	1
Exoc4	-0,41611	1
Paox	-0,41624	1
Mob1a	-0,41616	1
Urgcp	-0,41628	1
Ipo9	-0,41658	1
Il12rb1	-0,41666	1
Oxsr1	-0,41673	1
Pde12	-0,41676	1
Cd300lb	-0,41684	1
Pias1	-0,41718	1
Ppp1r15b	-0,41724	1
Copb2	-0,41721	1
Wwc2	-0,41729	1
A230028O05Rik	-0,41743	1
Mrps18a	-0,41746	1
Mex3c	-0,41757	1

Apbb2	-0,41774	1
Mrps7	-0,41788	1
D230025D16Rik	-0,41798	1
Acsl5	-0,41851	1
Nudcd1	-0,41861	1
Napa	-0,4186	1
Rab11fip2	-0,41934	1
Scaf11	-0,41926	1
Ndst2	-0,41937	1
Camsap1	-0,41977	1
Cyld	-0,42003	1
Gm43290	-0,42013	1
Dok4	-0,42013	1
Pdpr	-0,42011	1
Frmd6	-0,4202	1
Sec11a	-0,42025	1
1110025M09Rik	-0,42072	1
9130011E15Rik	-0,42079	1
Pcgf6	-0,42142	1
Tsg101	-0,42171	1
Dnm2	-0,42181	1
Mrpl44	-0,42223	1
C030014I23Rik	-0,42235	1
Sec24b	-0,42234	1
Trip4	-0,42234	1
Rif1	-0,42234	1
Morc4	-0,42227	1
Plekhb2	-0,42242	1
Gm4734	-0,42251	1
Larp1b	-0,42265	1
Msh5	-0,42318	1
Fermt3	-0,42327	1
Lrp10	-0,42346	1
Ptp4a2	-0,42352	1
Txndc16	-0,42381	1
Fyb	-0,42378	1
Fbxo6	-0,42394	1
Gm37305	-0,42388	1
Wdr5	-0,42398	1
Clec4e	-0,42403	1
Zer1	-0,4241	1
Gm28192	-0,42438	1
Wipi1	-0,42493	1
Marc2	-0,42499	1
Sirt5	-0,4251	1
Nek9	-0,42531	1
Antxr2	-0,42557	1
Ptbp3	-0,42577	1
Mcph1	-0,42628	1
Usp31	-0,42648	1
Shmt2	-0,42651	1
Inpp4a	-0,42658	1

Mast2	-0,42723	1
Wwox	-0,42715	1
Tomm40	-0,42732	1
Ercc4	-0,42736	1
Las1l	-0,42758	1
Arfgap3	-0,42779	1
Srbd1	-0,42782	1
C230035I16Rik	-0,42783	1
Dnajc17	-0,42791	1
Dand5	-0,42789	1
Dgkz	-0,42867	1
Xpr1	-0,42927	1
Prrc2b	-0,42955	1
Vps9d1	-0,42964	1
Ptpdc1	-0,42972	1
Ranbp6	-0,42967	1
Pik3cb	-0,42977	1
Fam185a	-0,42999	1
Gm14140	-0,43035	1
Gpatch8	-0,43044	1
Timm10	-0,43036	1
Tapt1	-0,43075	1
Rpp25l	-0,43104	1
Gm43794	-0,43117	1
Gm5898	-0,43146	1
Gm12589	-0,4317	1
Ebp	-0,43182	1
Pml	-0,43211	1
Aldh5a1	-0,43273	1
Herc2	-0,43293	1
Ybey	-0,43319	1
Ssx2ip	-0,43352	1
Dmap1	-0,43348	1
AA914427	-0,4341	1
Gphn	-0,43423	1
Trap1	-0,43454	1
Pbrm1	-0,43456	1
Gm13868	-0,43468	1
Abhd17b	-0,4347	1
March6	-0,43486	1
Caml	-0,43528	1
Rbm6	-0,43533	1
Reps1	-0,43554	1
Tmem181a	-0,43593	1
Tpp1	-0,43599	1
Sin3b	-0,43603	1
Lonrf1	-0,43652	1
Rpl21-ps5	-0,43658	1
Enpp4	-0,4368	1
Rtel1	-0,43696	1
Nasp	-0,43719	1
Eapp	-0,43729	1

Ufl1	-0,43732	1
Ap4s1	-0,43791	1
Mtmr10	-0,43791	1
Gm42566	-0,43793	1
Ganc	-0,43802	1
Mtmr6	-0,43834	1
Myo6	-0,43836	1
Mllt6	-0,43858	1
Ppp3r1	-0,43858	1
Wac	-0,43902	1
Pxmp4	-0,43924	1
Zc3h13	-0,43958	1
Cct6a	-0,43958	1
Emg1	-0,43966	1
Nrde2	-0,43988	1
Ndst1	-0,44003	1
Smurf1	-0,44023	1
Rtn3	-0,44072	1
Cep290	-0,44077	1
Nt5m	-0,44097	1
Dguok	-0,44097	1
Itsn1	-0,44109	1
Grb2	-0,4416	1
Brd2	-0,44169	1
Wdr41	-0,44184	1
Gm5871	-0,44193	0,99951
Sdcbp	-0,44189	1
Gm4943	-0,44196	0,99819
Gm13998	-0,44204	1
Cct7	-0,44225	1
Psmd2	-0,44217	1
Rilpl1	-0,44231	1
Arsk	-0,44252	1
Parp10	-0,44259	1
Trpm1	-0,44343	1
Zfp710	-0,44379	1
Ppp6r2	-0,44444	1
Phax	-0,44438	1
Ube2r2	-0,44474	1
Sap130	-0,44484	1
Gars	-0,44494	1
Ppm1b	-0,44545	1
Chtop	-0,44564	1
Prr13	-0,44592	1
Ppfia1	-0,44596	1
Irf8	-0,44679	1
Tmem14c	-0,44686	1
Pak1ip1	-0,44695	1
Kdelr1	-0,44741	1
1700123O20Rik	-0,44748	0,9974
Ptrh2	-0,44784	1
Was	-0,44794	1

Uckl1	-0,44842	1
Trpc4ap	-0,44851	1
Pomt1	-0,44857	0,99624
Usp8	-0,44892	1
Nipal3	-0,44909	1
C5ar1	-0,44909	1
Cst7	-0,44928	1
Yipf2	-0,44927	1
Flot1	-0,44943	0,99951
Lamp1	-0,44962	1
Cnbd2	-0,44975	1
Prelid3b	-0,44981	1
Gm16072	-0,45004	0,99211
Rnpepl1	-0,45003	1
D430042O09Rik	-0,45031	1
Acaca	-0,45027	1
Cenpj	-0,45036	0,99055
Gm4880	-0,45067	1
Tbc1d15	-0,45074	1
Pip4k2c	-0,45086	1
Foxo1	-0,45112	1
Gm22299	-0,4511	1
Rpp30	-0,45125	0,99486
Lair1	-0,45134	1
Gm8762	-0,45125	1
Stim2	-0,45142	1
Senp5	-0,45153	1
Mapk12	-0,45168	0,99327
Hipk3	-0,45172	1
Rabepk	-0,45174	1
Llgl1	-0,45179	1
Ttc3	-0,45193	1
Rab3ip	-0,45204	1
Zfyve9	-0,45225	1
Sfxn1	-0,4523	1
Vsir	-0,45239	1
Sh2b2	-0,45253	1
Gmpr2	-0,45255	0,99414
Mylpf	-0,45324	1
Gm26982	-0,45328	0,99878
Zfp512b	-0,45354	0,99055
Slc6a6	-0,45369	1
Snrnp200	-0,45407	1
C1rb	-0,45416	1
Gm16845	-0,45433	0,99774
E330011M16Rik	-0,45432	1
Dolk	-0,45437	0,98999
Sema4d	-0,45487	1
Ankrd27	-0,45501	1
Tctn3	-0,45532	0,98533
Tln1	-0,4553	1
Add1	-0,45575	1

Psme4	-0,45664	1
Nhej1	-0,45673	1
Gm6542	-0,4569	0,98999
Wdr36	-0,45693	1
Rab2a	-0,45691	1
Masp2	-0,457	1
Dpp9	-0,45727	0,99414
Ercc5	-0,45726	1
Ubr2	-0,45731	1
Gm44694	-0,45745	0,98403
Map2k2	-0,45834	1
Ngdn	-0,45839	1
Gm7967	-0,45851	0,98875
Clptm1	-0,45846	1
Gm15703	-0,45865	1
Atg4a-ps	-0,45923	1
Zfp740	-0,45917	1
Tnrc6c	-0,45928	0,99299
Tmppe	-0,45945	1
Runx2os1	-0,4597	0,99476
Eif2ak2	-0,46001	0,98306
Tfpi	-0,46005	0,99486
Eif5b	-0,46034	1
Dtnbp1	-0,46039	0,99732
Celf2	-0,46053	1
Nanos1	-0,46069	0,9964
Wdr46	-0,46079	0,98544
Orai1	-0,46165	0,99414
Eif4g3	-0,46195	1
Commd7	-0,46242	1
Esf1	-0,46237	1
Gm43660	-0,46253	1
Ptk2	-0,46262	0,99601
Gatad2a	-0,46261	1
Ccdc6	-0,46295	1
Ptpn23	-0,46315	0,98426
Kat5	-0,46323	0,9964
R3hcc1	-0,4633	0,97756
Sumf2	-0,46342	0,97759
Rfxank	-0,46351	0,9938
RP24-365A12.2	-0,4639	1
Usp49	-0,46398	0,97638
Adamtsl5	-0,46412	0,98085
Fam63a	-0,46429	1
Zfp788	-0,4646	0,97832
Wipi2	-0,4647	1
Fam71e1	-0,46492	1
Usf3	-0,46501	1
Zbed5	-0,46515	0,97759
Tnfsf8	-0,46506	1
Gk	-0,46518	0,99501
Trim21	-0,46515	1

Gm32856	-0,46515	1
Rbmxl1	-0,46529	0,98484
Sfswap	-0,46551	1
Prrc2c	-0,4656	1
Slc25a20	-0,46607	0,9755
Ncf2	-0,46634	1
Akt1	-0,46635	1
Gm14126	-0,46625	1
Ston1	-0,46641	0,98992
Ube2z	-0,46646	0,99951
Zfp51	-0,46661	0,97418
Psmc1	-0,46673	1
Slc45a4	-0,46688	0,99057
Tmem161b	-0,46727	1
Gm12497	-0,46771	0,982
Slc37a3	-0,46832	0,97254
Mib2	-0,46849	0,97759
Gm26930	-0,46854	1
Gm43727	-0,46858	1
Zdhhc6	-0,46882	0,97449
Focad	-0,46891	0,98913
Colec12	-0,46926	0,97254
Dbp	-0,46937	1
Capzb	-0,46961	1
Vta1	-0,46992	0,99909
Rae1	-0,4703	1
Nrg4	-0,47028	1
Dcaf8	-0,47044	1
Dgcr8	-0,47047	0,96978
Lhpp	-0,4706	0,97481
Gm17100	-0,47062	1
Snta1	-0,47091	1
Inpp1	-0,47125	0,98309
Nyap1	-0,47132	0,96872
AI506816	-0,47132	0,97013
Prep	-0,47133	0,97759
Skil	-0,47176	1
Noa1	-0,47195	0,97584
Rbm41	-0,47195	0,99018
Tmem251	-0,47216	0,98406
Agpat1	-0,47245	0,99486
Cwc22	-0,4731	0,97759
Ptov1	-0,4731	0,99669
Snrpa1	-0,47343	0,98999
Tyw1	-0,47371	0,97418
Twistnb	-0,47389	0,99055
Tbc1d23	-0,47414	0,98426
Spcs2	-0,47476	0,98406
Dzip3	-0,47553	0,9653
Acot10	-0,47559	0,9719
Kbtbd11	-0,47562	1
Cdkl2	-0,47614	0,97443

Entpd5	-0,47629	0,9633
Decr2	-0,47635	0,96355
Uso1	-0,47674	0,98406
Csrp2bp	-0,47682	0,96929
Ppm1f	-0,4769	0,97759
Edem2	-0,47693	0,98305
Ehbp1l1	-0,47689	1
Sp3os	-0,47703	0,96711
Klhl18	-0,47719	0,9633
Prkrip1	-0,47716	0,97759
Otud5	-0,47732	0,99018
Ubac1	-0,47754	0,97638
Slc50a1	-0,47774	0,98426
Tada3	-0,47777	0,96813
Itpa	-0,4779	0,9633
Cbfa2t2	-0,47805	0,96396
Ap3m1	-0,47804	0,97988
Plekha3	-0,47822	0,99819
Elk1	-0,47826	0,96978
Ralgds	-0,47881	0,98533
Arhgef2	-0,47899	0,99569
Zmat1	-0,47899	1
Hsdl2	-0,47935	0,96006
Nsmaf	-0,47939	0,98762
Hus1	-0,47962	0,95986
Qtrtd1	-0,47967	0,96929
Ccdc69	-0,47974	1
Dcun1d4	-0,47978	0,96924
Edc4	-0,48019	0,96853
Ppp1r9b	-0,48019	0,97479
Terf2	-0,48059	0,97974
Plpp1	-0,48097	0,96978
Hgs	-0,48114	0,97013
Afp	-0,48106	0,97449
S100a4	-0,48117	0,98913
Mbtps1	-0,48174	0,98475
9230102O04Rik	-0,4819	1
Mrpl13	-0,48218	0,96766
Zfp329	-0,48236	0,95614
Tmem43	-0,48288	0,98673
Wls	-0,4831	0,97418
Mvb12b	-0,48321	0,97755
Gm3355	-0,48323	0,98947
Zc2hc1a	-0,48331	0,95848
Zfp664	-0,48334	0,9633
Rab11fip1	-0,48333	0,96835
Gclm	-0,4834	0,97449
Rfc2	-0,48351	0,96864
Mirlet7b	-0,48364	0,99018
Hk3	-0,48368	0,95442
Mbtps2	-0,48373	0,96337
Specc1l	-0,48408	0,96741

Tubgcp5	-0,48425	0,95738
Rrp15	-0,4844	0,96112
Efcab14	-0,48512	0,96853
Akip1	-0,48509	0,96929
Cuedc1	-0,48535	0,96741
Coq3	-0,48544	0,96106
Drosha	-0,48546	0,96337
Gm38162	-0,48548	0,96396
Srprb	-0,48585	0,9633
Fam216a	-0,48598	0,96227
Gm9403	-0,4865	0,97759
Srgap3	-0,48693	0,96741
Fam96b	-0,48706	0,96183
Arhgef6	-0,48734	0,96413
Thtpa	-0,48744	0,95205
Tmbim1	-0,48742	0,97449
Alg9	-0,4877	0,95029
Csad	-0,48777	0,95179
Zc3h6	-0,48799	0,95828
Pik3r1	-0,48798	0,9633
Stard3	-0,48827	0,95614
Gm9347	-0,48838	0,95433
Mrpl22	-0,48917	0,94898
Nup214	-0,48935	0,95379
Rnf168	-0,48943	0,95475
Ptgs2os2	-0,48939	0,96766
Itpr1	-0,48964	0,95523
Ppip5k1	-0,48986	0,95084
Creb3l2	-0,49043	0,95165
Olfml3	-0,49039	0,97449
Zfr2	-0,4905	0,94651
Trp53inp2	-0,49122	0,952
Cd151	-0,49137	0,94538
Gfm1	-0,49156	0,94971
Rnf169	-0,49159	0,95433
Hspa9	-0,49169	0,96741
Trmt61a	-0,49178	0,94499
Ccdc88c	-0,49185	1
Rpl3-ps1	-0,49191	0,94729
Vapa	-0,4919	0,96413
Cep85	-0,49206	0,94729
Supt16	-0,49231	0,95786
March8	-0,49236	0,94699
Mrps36-ps2	-0,49237	0,97781
Lrrc8c	-0,49255	0,94476
Dcaf15	-0,49245	0,95074
Trim44	-0,49271	0,9493
Nradd	-0,49283	0,94646
Nedd1	-0,49293	0,94476
Gm9726	-0,49316	0,97347
Smad1	-0,49375	0,94376
Mbip	-0,49371	0,94539

Top2b	-0,49366	0,95297
Osbpl7	-0,49401	0,94476
Glmn	-0,4941	0,9445
Gm38380	-0,49409	0,96929
Tmem38a	-0,49452	0,94376
Ppp1r8	-0,49453	0,94409
Ttyh2	-0,49502	0,94373
Slc25a51	-0,49502	0,94729
Tceanc2	-0,49526	0,94435
B4galt5	-0,49559	0,94476
Tal1	-0,49569	0,94201
Tctex1d2	-0,49584	0,94919
Ccdc43	-0,49591	0,94203
Epc2	-0,4961	0,94212
Ppp2r3d	-0,49622	0,94125
Mertk	-0,49646	0,94065
Asxl1	-0,49659	0,94296
Fads2	-0,49678	0,9413
Gm45456	-0,49678	1
Cdc25b	-0,49702	0,94373
Ash1l	-0,49695	0,94376
Gm9434	-0,49698	1
2610507B11Rik	-0,4973	0,94376
Ilvbl	-0,49741	0,93905
Tpmt	-0,49745	0,9964
Kdm5d	-0,49753	0,9387
Mex3a	-0,4976	0,9387
Tuba4a	-0,49793	0,939
Dgka	-0,49795	0,95786
Gm16096	-0,49823	0,938
Setdb1	-0,49841	0,93735
Tstd3	-0,49843	0,93757
Srpk2	-0,49839	0,94125
Ankrd28	-0,49851	0,93735
Nckap5l	-0,49847	0,938
Dph6	-0,49872	0,93728
Echdc1	-0,49866	0,93735
Ocel1	-0,4989	0,93734
Senp6	-0,49886	0,93804
Sec24c	-0,49897	0,93728
Cd200r2	-0,49916	0,939
Pgap3	-0,49918	0,952
Vapb	-0,49954	0,93652
Sh2b3	-0,49955	0,93557
Mtch2	-0,49992	0,93446
Parvg	-0,50004	0,9341
Nras	-0,50001	0,9341
Ift81	-0,49999	0,94476
C430042M11Rik	-0,50013	0,94814
Ttc17	-0,50021	0,93396
Psmc2	-0,50058	0,93325
Spout1	-0,50077	0,93325

Htatsf1	-0,50093	0,93321
Hs2st1	-0,50106	0,93234
Klhdc3	-0,50118	0,93225
Sec23a	-0,50127	0,93172
Nupr1l	-0,50142	0,93325
Rpusd1	-0,50142	0,94373
Stat6	-0,5015	0,93181
Arl2bp	-0,50162	0,93132
Gm42893	-0,50161	1
Spg11	-0,5017	0,96929
Maml1	-0,502	0,94212
Iqsec1	-0,50223	0,92917
Nsfl1c	-0,50218	0,93199
Esrp2	-0,50225	1
Gm12582	-0,50239	1
Macf1	-0,50261	0,92704
Adprhl2	-0,50257	0,93067
Armc1	-0,50279	0,92731
Tceal8	-0,50286	0,93396
Mir7078	-0,50307	0,9633
Tnrc6a	-0,50335	0,92422
Gm45840	-0,50348	0,94583
Rpp14	-0,50371	0,92517
Zfp182	-0,50372	0,95433
Fbf1	-0,50389	0,94188
Sacm1l	-0,50398	0,925
Gm16286	-0,50409	0,92558
P3h1	-0,50421	0,92678
Amt	-0,50428	0,94404
Ddx18	-0,50439	0,92569
Nubpl	-0,50443	0,93036
Sec23ip	-0,50478	0,9232
Pfkm	-0,50477	0,925
Sp2	-0,5049	0,93067
Abcf2	-0,5053	0,92507
Tfg	-0,50581	0,9224
Ankrd13b	-0,50582	0,9258
Gm36445	-0,506	1
Naaa	-0,50606	0,93325
Ddx5	-0,50628	0,91702
Dmxl2	-0,50645	0,9224
Atp6v1a	-0,50653	0,91091
Ipo11	-0,50649	0,9224
Ccdc90b	-0,50675	0,92132
Tab1	-0,50684	0,9224
Rap2b	-0,50705	0,91483
Znrf2	-0,50707	0,92132
Actl6a	-0,50739	0,91546
Nol11	-0,5074	0,92049
Fcnaos	-0,50753	1
Fuk	-0,50762	0,92279
Nadk	-0,50791	0,91224

Suds3	-0,50838	0,90784
Impact	-0,50884	0,92132
Dennd1a	-0,50888	0,92132
Pex11a	-0,50901	0,91822
Sema5a	-0,50909	0,91895
Gm38111	-0,50934	1
Hdac4	-0,50942	0,91224
Mogs	-0,50961	0,90126
Stam2	-0,50985	0,91377
Zfp322a	-0,50981	0,92004
Dnajc9	-0,50987	0,93067
Klhl11	-0,50999	0,96953
Emc3	-0,51011	0,90604
Rabgef1	-0,51012	0,91505
Zdhhc9	-0,51014	0,91889
Fabp7	-0,5101	0,98127
Rufy1	-0,51022	0,92013
Pum2	-0,51167	0,90482
Zswim1	-0,51175	0,92181
Dtx2	-0,51192	0,92449
Scd2	-0,51286	0,9005
Blzf1	-0,51323	0,89968
Ascc2	-0,51325	0,9095
Slc9a6	-0,51396	0,90123
Iqcf1	-0,5141	0,96029
Nosip	-0,51445	0,90903
Hira	-0,51435	0,91224
Ehmt1	-0,51494	0,90126
Clec1a	-0,51494	0,93355
Fam129a	-0,5151	0,90279
Wapl	-0,51534	0,87715
Arhgef7	-0,51598	0,89134
Fbxw5	-0,51608	0,90526
Mtmr11	-0,51637	0,97756
Cpt1a	-0,51684	0,89931
Gm2199	-0,51718	0,92132
Taok2	-0,51766	0,9035
Acox3	-0,51779	0,88093
Ccp110	-0,51801	0,93132
Setx	-0,51815	0,90482
Gga2	-0,51821	0,88751
Zfp687	-0,51869	0,90903
Rpusd3	-0,51887	0,92569
Zfp619	-0,51891	0,99731
Tulp3	-0,51914	0,87855
Eml3	-0,51906	0,91077
Ctnnb1	-0,51917	0,85241
Dnmt1	-0,51934	0,86613
Ndufaf6	-0,51988	0,90743
Mob3c	-0,51986	0,91792
Hspa4	-0,52003	0,86056
Gm11966	-0,52132	0,89813

Gm20633	-0,52127	1
Glrx5	-0,52144	0,86248
Tmem115	-0,52136	0,90027
2410002F23Rik	-0,52172	0,87339
Serpinb8	-0,52228	0,87704
Wdr76	-0,52235	0,91786
Grk5	-0,52241	0,91093
Shox2	-0,52236	0,91377
Wdr25	-0,52266	0,92558
Aftph	-0,52284	0,85983
Adam10	-0,52293	0,89134
Wrap73	-0,52297	0,90739
Med17	-0,52345	0,86296
Rrp1b	-0,52382	0,87033
Zfp688	-0,52376	0,88927
Slc6a9	-0,52378	0,93325
E330009J07Rik	-0,52402	0,89453
Alpk1	-0,52427	0,88223
Clcn5	-0,52428	0,89425
Lrp4	-0,5245	0,96227
Prpf19	-0,52458	0,88115
Phf19	-0,52501	0,92451
Slc38a9	-0,52519	0,86903
Dnajc19-ps	-0,52516	0,90126
Mapkap1	-0,52537	0,87109
Aim2	-0,52557	0,85109
Ak1	-0,52556	0,86193
Nsmf	-0,5256	0,89646
Zufsp	-0,52591	0,87872
Cyb5a	-0,52605	0,85559
Hmgxb3	-0,52612	0,87946
Mtx1	-0,52624	0,88392
Gorasp2	-0,52634	0,84314
Sergef	-0,52633	0,89134
Phtf2	-0,52689	0,8809
Laptm4a	-0,52723	0,83069
Cecr5	-0,52735	0,90658
Lrrk1	-0,52742	0,88683
Rnaseh2a	-0,52778	0,91069
Lgals8	-0,52829	0,8502
Asxl2	-0,52849	0,85858
Gm10039	-0,52859	0,87766
Rfesd	-0,52863	0,90126
Ikbke	-0,5286	0,90915
Btrc	-0,52871	0,89286
Gm11722	-0,52929	0,90985
Tagap1	-0,52965	0,86608
Hmbs	-0,52982	0,91077
Efnb1	-0,53015	0,94199
Csde1	-0,53023	0,80417
Med13l	-0,53017	0,83711
Gm13443	-0,53021	0,97793

BC037034	-0,53045	0,86903
Rnf216	-0,53093	0,87704
Fbxl17	-0,53182	0,89839
Zfp984	-0,53191	0,90976
Pxylp1	-0,53188	1
BC005624	-0,53196	0,88062
Stau2	-0,53206	0,87977
Tmem158	-0,53221	0,88773
Ap1m1	-0,53234	0,85958
Gm12791	-0,53229	0,94979
Ppat	-0,53244	0,85938
Btk	-0,53267	0,87704
Gbf1	-0,53302	0,83501
Gpr35	-0,53302	0,99751
Oxnad1	-0,53321	0,85956
Nos1	-0,53317	0,98762
Tm2d2	-0,53345	0,86248
Dmtf1	-0,53353	0,83933
Cdk5rap2	-0,53352	0,87704
Ppp3cc	-0,53352	0,95074
Ralgapa2	-0,53379	0,89832
Smarca4	-0,53386	0,80902
Mrpl3	-0,5339	0,85274
Nufip2	-0,53406	0,83371
Gm5805	-0,53424	0,85575
Ninl	-0,53442	0,85996
Psme2	-0,53494	0,90975
Arf4	-0,53497	0,80317
Plekhm2	-0,53563	0,81347
Cmtm3	-0,536	0,87832
Msh2	-0,53627	0,86608
Pdcd6ip	-0,53642	0,80224
Pi4ka	-0,53637	0,8092
Smim4	-0,53641	0,85835
Dnajc16	-0,53646	0,85559
Prkab2	-0,53654	0,86091
Crybg3	-0,53676	0,85956
Zmat5	-0,53684	0,88085
Nol10	-0,53733	0,83617
Ppargc1b	-0,53747	0,85956
Atxn1	-0,53764	0,84481
Ift122	-0,53766	0,8848
Psmc3	-0,53781	0,84312
Rnpc3	-0,53786	0,87255
Gm11131	-0,53801	0,9268
Ptgr2	-0,53808	0,84911
Commd3	-0,53845	0,81767
Stxbp5	-0,53891	0,82682
Zbed4	-0,53914	0,88758
9330159M07Rik	-0,53943	0,93396
Unc50	-0,53973	0,83069
Alkbh4	-0,54009	0,89333

Trim2	-0,5402	0,88373
Arhgef25	-0,54038	0,86477
Lmna	-0,54049	0,75975
Klf13	-0,54053	0,81794
Alad	-0,54063	0,86266
Gm42479	-0,54059	1
Atp2a2	-0,54091	0,79487
Gm13350	-0,54089	1
Glmp	-0,54101	0,82151
Usp11	-0,54118	0,92938
Prkar1a	-0,54154	0,78257
Zcchc4	-0,54161	0,79239
Trib3	-0,54156	0,87756
Prkce	-0,54168	0,89618
Yars	-0,54175	0,83518
Phb	-0,542	0,88758
Six4	-0,54218	0,86608
Exosc9	-0,54231	0,83518
Synrg	-0,54231	0,84312
Riok3	-0,54257	0,818
Cwc27	-0,54258	0,83711
Tram2	-0,54313	0,9191
Cacnb1	-0,54327	1
Ythdc2	-0,54339	0,86613
Ascl2	-0,54355	0,94929
Ccni	-0,54391	0,7702
Fam3a	-0,54438	0,86613
Lin37	-0,5452	0,94373
9330162012Rik	-0,54522	1
Pip5k1c	-0,54529	0,8321
Scamp2	-0,54561	0,76466
St6gal1	-0,54604	0,88537
Cxxc5	-0,54604	0,95379
Neil1	-0,54647	0,83875
Ice1	-0,54646	0,84341
Tbp	-0,54673	0,87775
Sfpq	-0,54689	0,78918
Fth-ps2	-0,54719	0,99896
Cfap97	-0,54749	0,85956
Gm3608	-0,54756	0,96201
Tmem94	-0,54807	0,86194
Gsr	-0,54857	0,86248
Zfp429	-0,54863	0,87832
RP23-440I21.3	-0,54882	1
Ankhd1	-0,54886	0,77332
Rab32	-0,54896	0,82464
Cyp4f13	-0,54895	0,85996
Snora57	-0,54957	0,88373
Cacfd1	-0,54965	0,76108
Unk	-0,54971	0,85076
Pdia5	-0,55003	0,85417
Klk8	-0,55012	0,91358

Thoc6	-0,55051	0,82282
Wdr26	-0,55068	0,7459
Nsmce1	-0,55066	0,84911
Hsd17b11	-0,55126	0,8252
Atg4b	-0,55145	0,80634
E130308A19Rik	-0,55144	0,87796
A130010J15Rik	-0,55169	0,84957
Vasp	-0,55184	0,73238
6430511E19Rik	-0,55185	0,99951
Sdf4	-0,55213	0,74563
Mettl15	-0,55219	0,85465
Cebpz	-0,55322	0,78585
Erp29	-0,55395	0,70967
Gga3	-0,55404	0,80063
Micu2	-0,55423	0,8029
St7	-0,55453	0,85111
Polr3k	-0,55458	0,84373
Ate1	-0,55471	0,73404
Mfhas1	-0,55481	0,88341
Rhno1	-0,55613	0,78391
RP23-442M18.5	-0,5562	0,99785
Scyl1	-0,55653	0,82342
Atg2a	-0,55658	0,73967
Fam160b1	-0,55661	0,79132
Gm37238	-0,5567	0,90123
Zfp120	-0,55686	0,81817
Zfp358	-0,55729	0,85574
Mcrs1	-0,55733	0,88093
Tfam	-0,55745	0,81839
Herpud1	-0,55781	0,7146
Npc2	-0,55785	0,72323
Gm7496	-0,55821	1
Sgpp1	-0,55852	0,70656
Zdhhc16	-0,55867	0,85973
Supt6	-0,55918	0,74018
Fam98a	-0,55917	0,84371
Mrgbp	-0,5599	0,83754
Rsu1	-0,56014	0,81794
Tnip3	-0,56031	0,7895
Inpp5k	-0,56056	0,80474
Hcls1	-0,56074	0,73591
Dicer1	-0,56094	0,76886
Galnt4	-0,56086	0,85575
Pex11b	-0,56135	0,77401
Gpr68	-0,56169	0,81559
Atmin	-0,56179	0,78038
Cul9	-0,56194	0,80769
G3bp1	-0,56262	0,73716
Dync1li2	-0,56291	0,71531
Romo1	-0,56295	0,76005
Gm29284	-0,56303	0,91093
Ckap4	-0,56309	0,70268

Ankrd17	-0,56321	0,72696
Mfsd11	-0,56368	0,7799
Yeats4	-0,56396	0,7071
Gcdh	-0,56398	0,89813
Nectin2	-0,56405	0,83371
Gm13421	-0,56428	0,94404
Preb	-0,56444	0,76299
Psme3	-0,56441	0,76497
Lyrm7	-0,56439	0,86396
Bod1l	-0,56447	0,79239
Poldip2	-0,56503	0,78395
Pnkp	-0,56584	0,8092
Zzef1	-0,56612	0,78215
Ifi27	-0,56644	0,75818
Slc40a1	-0,56653	0,85559
Gdap2	-0,56703	0,75809
Aim1	-0,56705	0,7895
Odf2l	-0,56721	0,84481
Fam192a	-0,56735	0,75804
Rtca	-0,56743	0,75816
Spen	-0,56766	0,7702
Bfar	-0,56812	0,69945
Crygn	-0,56819	0,88373
Klhl2	-0,56834	0,80445
Med29	-0,5683	0,88671
Socs6	-0,56862	0,75412
Synj2	-0,56855	0,79306
Trabd	-0,5687	0,65714
Pycr1	-0,5688	0,85942
Rad50	-0,56891	0,78671
Tmem104	-0,56897	0,88758
Stambp	-0,56937	0,73078
Adrm1	-0,5697	0,86524
Tecpr2	-0,5698	0,82986
Ubr4	-0,56988	0,6681
3300005D01Rik	-0,56989	0,77596
Arrb1	-0,5703	0,75786
Gm43668	-0,57055	0,8635
Dock11	-0,57056	0,81559
Slc9a3r2	-0,57059	0,81794
Gm42937	-0,57064	0,93905
Smug1	-0,57072	0,80254
Eif3j2	-0,57085	0,69092
Gm38213	-0,57094	0,84943
Xab2	-0,57102	0,81297
Ep400	-0,57111	0,72088
Cnpy2	-0,57111	0,80996
RP23-243B24.1	-0,57166	0,92556
Gm9774	-0,57198	0,82232
Snx11	-0,57208	0,72344
Ankrd13c	-0,57218	0,75975
Atg2b	-0,57228	0,77261

Pik3c3	-0,57228	0,84121
Akr1c13	-0,57241	0,7702
Tmem192	-0,57264	0,7668
Pqlc1	-0,57282	0,69766
Twf2	-0,57278	0,74707
Gm6293	-0,57285	0,90543
Gdi2	-0,57325	0,64995
Maats1os	-0,57388	0,93661
Ldlrap1	-0,57405	0,65478
Fam195a	-0,57408	0,85111
Tmem237	-0,57545	0,81693
Xpnpep3	-0,5755	0,82958
Ddx10	-0,57569	0,74208
Trps1	-0,57576	0,71527
Gm43513	-0,5759	0,94104
Snap29	-0,57601	0,67692
Myo10	-0,576	0,72422
Trmt10b	-0,57615	0,83062
Prpsap1	-0,57616	0,73365
Thoc7	-0,57645	0,70264
Zfp296	-0,57638	0,82461
Ccz1	-0,57661	0,67675
Gm4879	-0,57673	0,78585
4933412L11Rik	-0,57673	0,98876
Gm10268	-0,57694	0,97501
9130401M01Rik	-0,57699	0,75665
Ap1s1	-0,5771	0,66025
D330050G23Rik	-0,57741	0,94699
Gm5624	-0,578	0,85956
Tmem91	-0,57795	0,91077
Ankrd12	-0,57809	0,74664
Rdh1	-0,57818	1
Clns1a	-0,5789	0,66091
Rad17	-0,5789	0,74589
Gm14017	-0,57912	0,89604
Ykt6	-0,57928	0,68557
L3hypdh	-0,57952	0,81053
Clcc1	-0,57975	0,73177
Gm42690	-0,58001	0,97312
Bloc1s5	-0,58027	0,78203
Zfp451	-0,58036	0,63255
Gpank1	-0,58067	0,78289
Rpl3l	-0,581	0,9127
Uchl3	-0,58112	0,70146
Cers2	-0,5816	0,62049
Ppil1	-0,5816	0,85249
Ttc9	-0,58158	0,86264
2310009A05Rik	-0,58166	0,77321
Ociad1	-0,58186	0,6332
G730013B05Rik	-0,58188	0,7146
Gm21816	-0,58186	0,92013
Sh3kbp1	-0,58205	0,75225

Man2c1os	-0,58217	0,8976
Zbtb24	-0,58241	0,78662
Cox18	-0,58273	0,7382
Chpf	-0,58304	0,78391
Cpne8	-0,58321	0,65594
Fbxl6	-0,58324	0,76813
Vim	-0,58334	0,6268
Arhgap21	-0,58349	0,75786
Pramef8	-0,58356	0,78078
Rsad1	-0,58366	0,88765
Map3k4	-0,58385	0,73126
Naa25	-0,58407	0,66172
Tsfm	-0,58426	0,86613
Apc	-0,58437	0,71966
Clybl	-0,58487	0,73078
Zmynd11	-0,58525	0,62324
Gm9796	-0,5854	1
1110059G10Rik	-0,58554	0,75225
Utp3	-0,58556	0,53106
Dnajc10	-0,58555	0,6358
Dimt1	-0,58567	0,99018
Myo19	-0,58581	0,84851
Gm44510	-0,58601	1
Fam188b	-0,58626	0,96929
Ncl	-0,5868	0,57644
Gm9333	-0,58709	0,82817
Tsnax	-0,58758	0,79908
Vps4a	-0,58775	0,72665
Slc25a43	-0,58767	0,90694
Wdr12	-0,58815	0,71992
Oprl1	-0,58822	0,97759
Abhd11	-0,58865	0,67704
Gpatch11	-0,58884	0,67458
Wdfy3	-0,58912	0,74833
Sppl3	-0,58942	0,69035
Glt8d1	-0,58988	0,7245
Prkaa1	-0,59009	0,7202
Cdk5r1	-0,59045	0,75249
Snx10	-0,59083	0,63554
Slc12a4	-0,59092	0,74604
Trak1	-0,59095	0,72623
Grik5	-0,59099	0,89765
Pigh	-0,59119	0,8624
Dnmt3a	-0,59146	0,58008
Slc7a1	-0,59168	0,58874
Klhl20	-0,59166	0,79977
Zfp950	-0,5918	0,80254
Zcchc7	-0,59224	0,65818
Setd6	-0,59232	0,71222
Rab35	-0,59269	0,67906
Gm44291	-0,59274	0,98533
Tnks2	-0,59325	0,61426

Nop16	-0,59332	0,58008
Eif4g1	-0,59351	0,55526
Immt	-0,59366	0,62818
Man2a1	-0,59367	0,79989
Poc1b	-0,59427	0,71689
Gm15506	-0,59427	0,96074
Farsb	-0,59441	0,60692
Lrp8	-0,59442	0,63211
Maip1	-0,59497	0,82803
Exd1	-0,59616	0,87704
Rapgef3	-0,59616	0,92013
Polr2f	-0,5964	0,64978
Ell2	-0,5965	0,60264
Tceanc	-0,59662	0,81297
Ltv1	-0,59693	0,71966
Zfp281	-0,59704	0,76539
5031439G07Rik	-0,59726	0,58126
Gaa	-0,59751	0,63306
Tmem18	-0,59752	0,74664
Gm13341	-0,59756	0,91093
Josd1	-0,59772	0,59468
Ammecr1l	-0,59768	0,68244
Relb	-0,59796	0,69342
Slc7a6	-0,59801	0,69788
Ep300	-0,5982	0,6661
Ddx55	-0,59834	0,74664
Vipas39	-0,5988	0,73689
Fancc	-0,59937	0,82997
Alas1	-0,59945	0,73377
Gm26542	-0,59967	0,83627
2610306M01Rik	-0,59985	0,85373
Ddx1	-0,60031	0,64773
Gm38340	-0,6003	0,81596
Lrmp	-0,60063	0,6343
Ccs	-0,60058	0,69798
Gm38366	-0,60066	0,9035
Kifap3	-0,60082	0,68222
Eprs	-0,60089	0,50127
Chpf2	-0,60108	0,70014
Lrba	-0,60121	0,72161
Pip5k1b	-0,60132	0,65429
AI606181	-0,60136	0,76775
Rcc2	-0,60196	0,49713
Rpp40	-0,60232	0,7811
Cacna1b	-0,60239	1
Gm10557	-0,60246	0,92259
Ercc8	-0,60289	0,72588
Zfp668	-0,60296	0,76808
Smarcc1	-0,60361	0,56369
Acbd4	-0,60358	0,68417
Amdhd2	-0,60375	0,70656
Clcn4	-0,60377	0,73251

Fmn1	-0,60388	0,6102
Map2k3	-0,60485	0,45897
Gart	-0,60479	0,67416
Tuft1	-0,60504	0,65106
Tgif1	-0,60531	0,62581
Ccdc91	-0,60541	0,73078
Glud1	-0,60579	0,51645
Ppm1g	-0,60597	0,51038
Tom1l2	-0,6061	0,68222
Nudt13	-0,60699	0,64561
1700001C19Rik	-0,60698	0,96149
Samm50	-0,60706	0,56407
Zfp282	-0,60724	0,7665
Vav2	-0,60727	0,83459
Aar2	-0,60735	0,71367
Supt5	-0,60751	0,59408
Pi4k2b	-0,60746	0,6744
Zfp110	-0,60751	0,68115
Pafah1b2	-0,60756	0,49225
Zdhhc4	-0,6076	0,72663
Stat1	-0,60765	0,82488
Wdr81	-0,60794	0,68244
Gtpbp6	-0,60804	0,77109
Cd276	-0,60811	0,74604
Sdhc	-0,60835	0,4679
Taf12	-0,60863	0,7082
Tpgs1	-0,60881	0,79291
Cenpo	-0,60875	0,82752
Thap11	-0,60897	0,53706
Vps37a	-0,60907	0,61758
Klhl22	-0,60914	0,73249
Gm38247	-0,6091	0,94163
Dennd1c	-0,60925	0,9341
Smarcd2	-0,60926	0,46835
Dcun1d2	-0,6099	0,83416
Fbxo38	-0,61019	0,61559
Exosc7	-0,61032	0,64228
Jarid2	-0,61064	0,59527
Def8	-0,61064	0,74604
Clec11a	-0,6109	0,79064
Rnf20	-0,61104	0,50402
2610203C20Rik	-0,61098	0,8321
Cdo1	-0,61106	0,93225
Traf3ip2	-0,61159	0,73628
Kiss1r	-0,6117	0,96005
Wdr78	-0,61189	0,85574
Dnajb11	-0,61232	0,61758
Dhrs13	-0,6125	0,84741
Spsb1	-0,61259	0,78881
Zfyve19	-0,61274	0,84696
Pitpna	-0,61295	0,52581
Gpr108	-0,61299	0,62076

Sun1	-0,61327	0,66529
Abcf1	-0,61353	0,50559
Ubxn2b	-0,61387	0,75696
Flnc	-0,61407	0,58504
Ube2d2a	-0,61406	0,7203
BC005561	-0,61455	0,66149
Utp4	-0,61512	0,68115
Prune1	-0,61562	0,55478
Tsc22d4	-0,61565	0,61404
Hnrnpab	-0,61609	0,47617
Slc3a2	-0,61608	0,55848
Mmgt2	-0,61611	0,58702
Nubp2	-0,61618	0,75809
Cluap1	-0,61626	0,70336
Pdss1	-0,6166	0,90583
Mum1	-0,61666	0,73238
Oas1g	-0,61665	0,99951
Szt2	-0,61692	0,8107
Gfi1	-0,61688	0,92639
Smarca2	-0,61724	0,49232
Tsr1	-0,61745	0,59848
Srgap2	-0,6182	0,52261
Ddb2	-0,61871	0,79451
Wdr3	-0,6188	0,43286
Pck2	-0,61879	0,72551
Wiz	-0,61916	0,62564
Mrps10	-0,61927	0,74007
Gtpbp3	-0,61977	0,76381
Plekha5	-0,62022	0,73638
Myh7b	-0,62047	0,91895
Disp1	-0,62111	0,77659
Lasp1	-0,62132	0,59873
Mepce	-0,62192	0,62922
Stat5a	-0,62191	0,67675
Arl15	-0,62193	0,83518
Stk4	-0,62202	0,57073
Psap	-0,62234	0,59162
Tm9sf3	-0,62241	0,50714
Pogk	-0,62238	0,58504
Eif5	-0,62251	0,3671
Cyp20a1	-0,62269	0,64449
Slc25a46	-0,62276	0,63941
Klhl8	-0,62277	0,74432
D10Jhu81e	-0,62285	0,64446
Lypla2	-0,62301	0,59306
Vars2	-0,62313	0,71151
Zfp398	-0,62308	0,75562
Gon4l	-0,62347	0,63954
Rbm45	-0,62365	0,78358
Jkamp	-0,62384	0,6274
Acat2	-0,62423	0,63071
Nbeal2	-0,6244	0,7799

Vps36	-0,62475	0,59268
Ccdc138	-0,62473	0,91358
Cd82	-0,62497	0,57659
Eftud2	-0,62514	0,6343
Timp1	-0,62517	0,93172
Enpp1	-0,62569	0,76539
Dgcr2	-0,6259	0,66394
Cdk20	-0,62633	0,78918
Pik3ca	-0,62714	0,61611
9230114K14Rik	-0,6278	0,68358
Malsu1	-0,62853	0,61045
Unc93b1	-0,62857	0,56218
Hif1a	-0,62867	0,50833
Gskip	-0,62866	0,68244
Syne1	-0,62893	0,57591
Cntln	-0,62897	0,80063
Cebpa	-0,62908	0,68222
Gmip	-0,6294	0,62031
Usp9x	-0,62974	0,56967
Zbtb2	-0,62985	0,71308
Pla2g16	-0,63014	0,57535
Mrps12	-0,63008	0,6343
1700052K11Rik	-0,63036	0,941
Ccdc191	-0,63053	0,70856
Nrbp1	-0,63065	0,60818
Top3a	-0,63058	0,75591
Wdr13	-0,63069	0,51404
Tubd1	-0,63091	0,84938
Pcdhb15	-0,63105	0,98309
Gata3	-0,6313	0,7963
Gm30329	-0,63135	0,87821
Slc9a9	-0,63136	0,8137
5430403G16Rik	-0,63153	0,86608
Pou2f2	-0,6317	0,5061
Pkn1	-0,63179	0,5181
Ehbp1	-0,63194	0,73592
Map4k3	-0,63212	0,74664
Lancl1	-0,63217	0,60557
Tiam1	-0,63253	0,69371
Slc38a6	-0,63299	0,59869
Tlcd1	-0,6331	0,9072
Ide	-0,63325	0,64856
Ttc26	-0,63333	0,81914
Pcnx3	-0,6335	0,48522
Usp10	-0,6338	0,57613
Kri1	-0,63454	0,67027
Polr3h	-0,63484	0,57647
Plekha8	-0,63492	0,67347
Zrsr1	-0,63516	0,61713
Psme2b	-0,63572	0,63954
Gm6206	-0,63591	0,79859
Eri1	-0,63603	0,57807

Stab1	-0,636	0,74833
Nxpe3	-0,63708	0,58599
Socs3	-0,63731	0,6982
Ippk	-0,63726	0,77401
Atp6v0b	-0,63739	0,56059
Sec31a	-0,63758	0,49966
Bcor	-0,63782	0,63954
Zmpste24	-0,63798	0,65646
Lgi4	-0,63796	0,96758
Vrk3	-0,63833	0,59869
Psmd10	-0,63844	0,51265
Taf1a	-0,63848	0,75269
Grin1	-0,63859	0,94539
Ubtd2	-0,63873	0,94203
Rbm15b	-0,63898	0,65594
Wdr43	-0,63923	0,38133
Elmo2	-0,63931	0,74612
Rcan3	-0,63951	0,58656
Vps33a	-0,63977	0,5597
Pithd1	-0,64054	0,57621
Gm5590	-0,64073	0,95342
Zfp84	-0,64101	0,71951
Slc6a12	-0,64116	0,58315
Tifa	-0,64116	0,6133
Pmm2	-0,64122	0,67502
Impa2	-0,64125	0,5126
Rrp8	-0,64218	0,54267
Nphp1	-0,64231	0,87625
Axin1	-0,64241	0,6165
Rspry1	-0,64241	0,64446
Zfp770	-0,64252	0,70212
Degs1	-0,64313	0,3389
Parp1	-0,64312	0,3454
Gm5609	-0,64328	0,77834
Csrnp2	-0,6433	0,92558
Cse1l	-0,64335	0,38869
Ythdf1	-0,64369	0,44119
Cops6	-0,64372	0,55196
Nol9	-0,6443	0,49882
Gab3	-0,64429	0,63954
Gstp-ps	-0,64449	0,7913
Sh2d3c	-0,64451	0,84187
Stc1	-0,64451	0,90831
B3glct	-0,64464	0,69934
Gm6395	-0,64476	0,94296
Usp4	-0,64502	0,41105
Gm37333	-0,6451	0,8558
Dok3	-0,64529	0,52065
D230017M19Rik	-0,64569	0,92132
Txlna	-0,64612	0,50147
Mars	-0,64614	0,55037
Bphl	-0,64607	0,76775

Lyz2	-0,64628	0,43206
Cnot11	-0,64643	0,57613
Gm43707	-0,64637	0,85519
Tacc2	-0,64664	0,62949
Scaf4	-0,64679	0,62925
Lysmd1	-0,64683	0,67517
Eif4ebp1	-0,64693	0,47708
Sf1	-0,64712	0,36074
Limd1	-0,64755	0,46346
Txnrd3	-0,64765	0,75415
Atic	-0,64814	0,49209
R3hcc1l	-0,64817	0,55286
Ppm1d	-0,64835	0,57435
Sos2	-0,64854	0,5811
Rraga	-0,64875	0,63437
Ppm1a	-0,64893	0,48147
Nr6a1	-0,64896	0,7264
Casz1	-0,64907	0,75405
RP24-282C4.9	-0,64937	0,75252
Mrps22	-0,64946	0,80099
Sypl	-0,64965	0,39719
Llph-ps2	-0,6497	0,60866
Sqrdl	-0,64966	0,68847
Gm6377	-0,64968	0,7799
Narfl	-0,65015	0,6136
Zfp780b	-0,65012	0,73207
Tmem138	-0,65022	0,75282
Gm16053	-0,65047	0,92558
Gm12529	-0,65061	0,95136
Ifngr1	-0,65069	0,64563
Gm13835	-0,65067	0,65342
Cited2	-0,65094	0,57174
Ddx46	-0,65097	0,44739
Gm14403	-0,65207	0,95433
Asns	-0,65269	0,65761
Usp7	-0,65318	0,34092
Ccdc92	-0,65368	0,79859
Zranb2	-0,65386	0,44221
Celf5	-0,65389	0,93731
Trmt2a	-0,65428	0,56913
Mplkip	-0,655	0,50864
Psd	-0,65505	0,88373
Prrc1	-0,6555	0,35255
Derl1	-0,65578	0,28955
Neat1	-0,65674	0,56435
Fam118b	-0,65689	0,55857
F8a	-0,65744	0,67347
Cluh	-0,65791	0,36074
Map4k4	-0,65893	0,36101
Lrrc1	-0,65902	0,81622
Chpt1	-0,66037	0,66041
Zik1	-0,66069	0,75506

Rmdn1	-0,66174	0,59854
Ppp4r2	-0,66242	0,48492
Aph1c	-0,66274	0,45864
Hars	-0,66285	0,41181
Borcs5	-0,66279	0,65818
A630033H20Rik	-0,66287	0,67052
Zbtb4	-0,66317	0,4297
Rpl36a-ps1	-0,66322	0,96741
Tcf4	-0,66337	0,34351
Dync1li1	-0,66346	0,34351
Nup37	-0,66347	0,51645
1810011H11Rik	-0,66356	0,70761
Ppp3ca	-0,66369	0,38578
Atp6v0d1	-0,66385	0,50272
Stx2	-0,66407	0,50864
Mok	-0,66433	0,87468
Snai2	-0,66432	0,89661
Zfpl1	-0,66453	0,45507
Senp1	-0,66464	0,5232
Rhbdd3	-0,6646	0,71308
Gm13268	-0,66514	0,9075
Ermap	-0,66516	0,80063
Reps2	-0,66549	0,92586
Phlpp2	-0,66559	0,69017
Gm42829	-0,66557	0,92912
Tubb4a	-0,66567	0,78698
Simc1	-0,66646	0,67502
Zfp280c	-0,66663	0,75477
Txnrd1	-0,66684	0,4102
Brf2	-0,66678	0,57956
Lrrc75a	-0,66706	0,49041
Rhbdf1	-0,6671	0,73219
B230369F24Rik	-0,66726	0,86
Snx15	-0,66755	0,73078
Tmem68	-0,66763	0,64281
Qser1	-0,66774	0,50852
Arl6	-0,66819	0,8092
Zdhhc5	-0,6685	0,51391
Gm5857	-0,6688	0,93396
Wdr4	-0,66898	0,72696
Zfp964	-0,66938	0,95379
Gm340	-0,66956	0,71192
Spats2	-0,67037	0,5361
RP23-114G13.1	-0,67072	0,9633
Xkr5	-0,67091	0,938
Foxred1	-0,67104	0,52709
H2-Oa	-0,67157	0,90743
Gm10167	-0,67158	0,94476
Mapkbp1	-0,67182	0,44493
Kbtbd7	-0,67191	0,73705
Cnot2	-0,67225	0,40262
Pacsin2	-0,67232	0,6165

Acad8	-0,67249	0,54122
Tbc1d24	-0,67284	0,56877
Nop2	-0,67312	0,51942
Dnase2a	-0,67336	0,27984
Zmynd19	-0,67336	0,54797
Crtc3	-0,67353	0,62523
Lins1	-0,67381	0,73238
Gm42484	-0,67411	0,76813
Ttc33	-0,67426	0,39515
Cdhr4	-0,67439	0,8558
Ebag9	-0,67485	0,59844
Phospho2	-0,67538	0,55763
Cd63	-0,67563	0,25294
Cltb	-0,67593	0,58698
Ttyh3	-0,67628	0,44748
Gm9712	-0,67632	0,91106
Matk	-0,67636	0,89221
Etfb	-0,67646	0,52342
Herc3	-0,67653	0,72552
Pex3	-0,67728	0,55631
Dtd1	-0,67765	0,28205
Srm	-0,67807	0,49966
Slc2a6	-0,67808	0,70818
Gm11298	-0,6782	0,90583
Prtn3	-0,67863	0,94399
Micall1	-0,67866	0,47679
Gm44116	-0,67926	0,94971
Ccnk	-0,67963	0,41747
Zfp958	-0,67961	0,56059
Plekhm1	-0,67971	0,32026
Ncf1	-0,67982	0,23701
Pttg1ip	-0,67978	0,45073
Osbpl2	-0,67978	0,50517
1110032A03Rik	-0,68017	0,6681
Txndc9	-0,68036	0,40119
Pycr2	-0,6809	0,39862
Eepd1	-0,68087	0,44748
Tst	-0,6814	0,79487
Rad54l2	-0,6816	0,44414
Slc12a7	-0,68174	0,40196
Nckap1	-0,68188	0,82885
Fam120a	-0,68204	0,40237
Rbpj	-0,68216	0,26113
B3gntl1	-0,6826	0,74017
Selplg	-0,68339	0,37003
Zc3h8	-0,68402	0,65851
Gm8566	-0,68413	0,71813
Gm28875	-0,68425	0,34919
Ppp2r3a	-0,68443	0,72623
Mrnip	-0,68484	0,77668
Aatf	-0,68487	0,51154
Nudt3	-0,68495	0,25809

Trpv2	-0,68578	0,31313
Mpeg1	-0,68578	0,43206
Cdk7	-0,68633	0,61019
A130048G24Rik	-0,68653	0,86773
Cd101	-0,68656	0,79306
Rnf214	-0,68666	0,50864
Xylt2	-0,68756	0,54622
Dctn6	-0,68778	0,36504
Dclre1b	-0,68783	0,70268
Pbxip1	-0,68832	0,2592
Tram1	-0,68826	0,27691
Ccdc186	-0,68835	0,37935
Gm38021	-0,68859	0,86241
Helz	-0,68933	0,37514
Slc25a37	-0,68996	0,25585
Tvp23b	-0,69	0,47236
Nin	-0,69011	0,62173
Vps33b	-0,69011	0,65927
Letm1	-0,69032	0,3114
Nav1	-0,69039	0,36082
Ttll3	-0,69035	0,61019
Foxo4	-0,69087	0,55993
Ap4b1	-0,69111	0,52084
Ube2v2	-0,69113	0,74589
Klc3	-0,69141	0,72422
Cox11	-0,69153	0,6046
Gm45292	-0,69162	0,88537
Nsun3	-0,69179	0,48239
Rxrb	-0,69247	0,25674
Ccnf	-0,6925	0,62867
Puf60	-0,6927	0,36006
Gtf2h1	-0,69293	0,52344
Wfikkn1	-0,69307	0,72088
Rpn1	-0,69315	0,18582
Zfp760	-0,69343	0,70011
RP23-277D1.1	-0,69345	0,88673
Rpap1	-0,69372	0,5348
Gm44950	-0,6944	0,85996
Atp10d	-0,69464	0,74449
Scfd2	-0,69571	0,71305
Atp6v1b2	-0,69575	0,11563
Nmnat1	-0,69613	0,67789
Umps	-0,69682	0,50895
P2rx4	-0,69706	0,46278
Ncoa7	-0,69734	0,55651
S1pr2	-0,69782	0,31528
Scamp5	-0,69778	0,45484
Gm44557	-0,69841	0,90264
Osbpl11	-0,69866	0,64846
Fan1	-0,69873	0,83627
Cd53	-0,69881	0,40033
Herpud2	-0,69881	0,50864

Map3k11	-0,69915	0,59527
Gk5	-0,69921	0,70269
C1qbp	-0,69944	0,15316
Timm21	-0,69952	0,66172
Atp2b4	-0,69963	0,35591
Pdhx	-0,6996	0,57082
Prex1	-0,70009	0,39313
Arfgef2	-0,7004	0,48964
App	-0,70046	0,32939
Llgl2	-0,70048	0,83724
Nifk	-0,7008	0,23526
Cfap43	-0,70076	0,74502
Camta2	-0,70095	0,35696
Mon1b	-0,70113	0,66172
Orc3	-0,7012	0,45484
Crocc	-0,70129	0,83871
Plekhg5	-0,70223	0,52716
Hs6st1	-0,70244	0,30351
Ttc27	-0,70254	0,59468
Tsen2	-0,70253	0,62093
Abtb2	-0,70299	0,64523
Dnajc25	-0,70318	0,40597
Dnaaf2	-0,70345	0,50282
Usb1	-0,70362	0,31477
Mfap3	-0,70407	0,53557
Prrc2a	-0,70408	0,53749
Map7d1	-0,70418	0,19267
Mkl1	-0,7042	0,38634
Kcnn4	-0,70484	0,27044
Pdzd8	-0,70506	0,50559
Cdv3	-0,70528	0,20138
Snai1	-0,70533	0,70175
Atp5g3	-0,70545	0,20749
Ugt1a7c	-0,7055	0,83627
Exosc5	-0,70564	0,45847
Ercc2	-0,70576	0,51804
Abcb10	-0,70652	0,69783
Tango2	-0,70663	0,54797
Parp2	-0,7071	0,53057
Clec4a3	-0,70723	0,78554
Bptf	-0,70748	0,26128
Nlrc5	-0,7077	0,59209
Cpsf6	-0,70795	0,27666
Sri	-0,70796	0,35268
Tsen34	-0,70803	0,69934
Gsg2	-0,70806	0,77332
Pygo2	-0,70825	0,31623
Dagla	-0,7085	0,9341
Tomm70a	-0,70904	0,17935
Mdfic	-0,70905	0,31974
Zdhhc2	-0,70914	0,33225
Cmbl	-0,70909	0,75809

Tm9sf2	-0,70928	0,2549
Sgms1	-0,70964	0,53057
9530062K07Rik	-0,70981	0,61019
Gm5578	-0,71015	0,92387
Tcea2	-0,7107	0,85575
Rrad	-0,71079	0,87832
Tbc1d8	-0,71195	0,92132
Cpt1c	-0,71211	0,63909
Bcl7c	-0,71208	0,78252
Zfp407	-0,71238	0,66091
Zfp445	-0,71248	0,34351
Zcchc10	-0,71259	0,37339
Dcaf17	-0,71264	0,65636
Mutyh	-0,71279	0,92132
1700112E06Rik	-0,71309	0,63899
Elfn2	-0,71323	0,75809
Thumpd1	-0,71349	0,41823
Rexo2	-0,71445	0,29401
Miga1	-0,71436	0,45484
Ube4b	-0,71448	0,25268
Tlk1	-0,71447	0,30116
Dcaf10	-0,71528	0,36082
Cib1	-0,71555	0,30613
Ampd2	-0,71561	0,1892
G6pdx	-0,71565	0,44455
Prdx1	-0,71596	0,14215
Oma1	-0,71614	0,55857
Srp68	-0,71683	0,25149
Rnf128	-0,71688	0,26785
Sacs	-0,71686	0,57174
Polg	-0,71711	0,56943
1700086P04Rik	-0,71706	0,83062
Gm43672	-0,71721	0,91837
Nacc2	-0,71744	0,48081
Gm37726	-0,71784	0,96396
Dcaf13	-0,71823	0,15326
Smarcb1	-0,71858	0,47708
Tmem203	-0,71887	0,41343
Dffa	-0,71912	0,79458
Gclc	-0,71922	0,31998
RP23-139H6.1	-0,71919	0,52225
Fhod3	-0,72017	0,6789
Mcm3ap	-0,72041	0,40237
Cnrip1	-0,72065	0,66625
Bin3	-0,72088	0,58576
Tfcp2	-0,72134	0,61758
Pon3	-0,72137	0,44292
Slc25a44	-0,72165	0,43697
Rab10	-0,72269	0,15362
Olfr933	-0,72335	0,64603
Ppa2	-0,72359	0,41795
Gm37760	-0,72366	0,67334

Calu	-0,7238	0,41153
Gm33370	-0,72401	0,85249
Tti2	-0,72412	0,61818
Gm42480	-0,72427	0,60826
1110037F02Rik	-0,72485	0,34948
Slco4a1	-0,7253	0,14438
Wdr60	-0,7253	0,33543
Gapvd1	-0,72535	0,19101
Tex9	-0,72552	0,74664
Thap6	-0,72553	0,78786
Mtdh	-0,72564	0,07162
Mad1l1	-0,72555	0,68115
D5Ertd579e	-0,72609	0,34707
Rps6kb2	-0,72606	0,56003
Cul2	-0,72616	0,39064
Mrpl10	-0,72634	0,31703
Wdr75	-0,72643	0,27796
2510009E07Rik	-0,7265	0,27204
Ngly1	-0,7265	0,7041
Mtg1	-0,72669	0,4823
Ap5z1	-0,72754	0,46278
Ylpm1	-0,72774	0,30094
Kif16b	-0,72892	0,5062
Fbxl8	-0,7289	0,59554
Gm12059	-0,72898	0,7202
Tsacc	-0,72948	0,80277
Gm11448	-0,72946	0,87089
Plekho1	-0,72957	0,07612
Sigmar1	-0,72955	0,48239
Nme4	-0,72962	0,66727
Phf3	-0,72969	0,15462
Lgalsl	-0,72981	0,45562
Hs1bp3	-0,72989	0,6351
Slain2	-0,73018	0,22285
Mdc1	-0,7306	0,51172
Gm43859	-0,73076	0,94539
Rcn1	-0,73114	0,37755
Phldb3	-0,73139	0,62417
Tsga10ip	-0,73143	0,90128
Zfp821	-0,73169	0,56035
Ubr5	-0,73283	0,27153
Ofd1	-0,73312	0,66529
Dlat	-0,73362	0,20804
Slc30a4	-0,73373	0,19032
Dyrk2	-0,73391	0,44739
B4galt1	-0,73452	0,11586
Snx16	-0,73471	0,54221
Chac2	-0,73481	0,7895
Hmcn2	-0,73487	0,78196
Dgkq	-0,73525	0,48921
Gm17530	-0,73525	0,81222
Kmt2b	-0,73543	0,47816

Zmynd8	-0,73552	0,18794
Gm43681	-0,73573	0,90583
Fads1	-0,7359	0,19071
Jaml	-0,73586	0,30733
Mief1	-0,73617	0,3925
Iffo2	-0,73625	0,55478
Xpo4	-0,73693	0,52179
Lyrm2	-0,73694	0,52619
Mpnd	-0,73702	0,32696
Pelo	-0,73746	0,26896
Opn3	-0,7376	0,6203
Fam208b	-0,73758	0,63071
Ift140	-0,73772	0,57374
3830403N18Rik	-0,73788	0,87796
Cdk10	-0,73821	0,64523
Slc38a10	-0,73842	0,1676
Rnf220	-0,73839	0,27305
Slc4a2	-0,7384	0,35255
D330041H03Rik	-0,73864	0,74861
Zfp513	-0,73881	0,36082
Zfp865	-0,73894	0,61538
Mdrl	-0,73929	0,87117
Comt	-0,73952	0,44631
Cldn12	-0,73979	0,62303
Tmem14a	-0,73997	0,85292
Eif2b3	-0,74024	0,27046
Tubb6	-0,74039	0,3421
Dhdds	-0,7417	0,26272
C2cd3	-0,74194	0,62982
Nfu1	-0,74213	0,62224
Fbxo4	-0,74223	0,50039
Senp7	-0,74284	0,37262
Pom121	-0,74284	0,38688
Gm10478	-0,74298	0,78252
Hpgds	-0,74321	0,25471
Neu3	-0,74335	0,88184
Mios	-0,74373	0,57338
Tmie	-0,74376	0,65946
Hgsnat	-0,74425	0,45642
Wrnip1	-0,74441	0,66473
Tspan3	-0,74481	0,18296
Fam91a1	-0,74481	0,28679
Hps1	-0,74477	0,4165
Slk	-0,74503	0,21792
Elp3	-0,74517	0,38259
Dusp14	-0,74542	0,4642
Abcc5	-0,74558	0,24106
6430573P05Rik	-0,74556	0,83302
Traf5	-0,74565	0,35434
Gm11716	-0,74596	0,62269
Riox2	-0,74624	0,53786
Aldh4a1	-0,74632	0,56494

Tyms	-0,74644	0,70289
Nat8f1	-0,74653	0,57811
A930024E05Rik	-0,74657	0,7297
B630019K06Rik	-0,74707	0,56867
Hars2	-0,74782	0,35386
Rabif	-0,74841	0,60826
Rpain	-0,74846	0,67079
Gm42869	-0,74857	0,77301
Bap1	-0,74872	0,43729
Ift74	-0,74873	0,68859
Gm16638	-0,749	0,87151
Slc25a12	-0,74912	0,27511
Gm45221	-0,74952	0,80334
Gm6329	-0,74984	0,34877
Gm37660	-0,74978	0,73238
Impa1	-0,74992	0,17303
Spata7	-0,75023	0,54888
Ms4a6b	-0,7503	0,87756
Zbtb7a	-0,75071	0,14717
Znfx1	-0,75083	0,25674
Nsun5	-0,75121	0,66923
Gm7666	-0,75115	0,87151
Zkscan17	-0,75162	0,15651
Tbc1d7	-0,75158	0,67906
Mtcl1	-0,75177	0,94929
Sdad1	-0,75198	0,2012
Ubac2	-0,752	0,42352
Dlst	-0,75252	0,14291
Pccb	-0,75274	0,56988
Gm9173	-0,75282	0,76039
Qrsl1	-0,75322	0,32651
Zfp30	-0,75351	0,36754
Thap12	-0,75476	0,38378
Top3b	-0,75498	0,56088
Sipa1	-0,75564	0,35255
Orc5	-0,75569	0,49839
Yipf5	-0,75584	0,17579
Ago2	-0,75577	0,1758
Gm26606	-0,75575	0,91849
Atg9b	-0,75603	0,51581
Gtf3c3	-0,75697	0,48985
Gm36930	-0,75723	0,85111
Flnb	-0,75768	0,20369
BC024386	-0,75791	0,86608
Gm37699	-0,75804	0,73078
Adamtsl4	-0,75813	0,58256
Nars	-0,75907	0,1159
Mt2	-0,7593	0,13603
Gas2l1	-0,75954	0,44737
Eif3a	-0,75976	0,10169
Fryl	-0,75984	0,29024
Phka1	-0,75983	0,48081

Arid2	-0,75988	0,37445
Kin	-0,75999	0,37315
RP24-282C4.10	-0,76034	0,49966
Mesdc2	-0,76055	0,28147
Dsel	-0,76103	0,59794
Ric8a	-0,76145	0,50203
Ntmt1	-0,76175	0,40218
Zfp472	-0,76176	0,43299
Crybg3	-0,76193	0,80417
Ostm1	-0,76201	0,34366
Nfkbia	-0,76223	0,054073
Smn1	-0,76297	0,17792
Dock9	-0,76311	0,71308
Tlk2	-0,76394	0,17579
Xpo5	-0,76414	0,63602
Pptc7	-0,76418	0,20964
Lrpprc	-0,76424	0,41286
Snapc5	-0,76531	0,6678
Hmgb1-ps6	-0,7658	0,80334
Cdk13	-0,7661	0,46408
Actb	-0,76622	0,22162
Zfp930	-0,76633	0,29226
Smim19	-0,76699	0,38327
Trim12c	-0,76722	0,64659
Ccdc17	-0,76751	0,83285
1110059E24Rik	-0,76783	0,28429
Ankrd46	-0,76845	0,5348
Dnajc12	-0,76853	0,73078
Ano7	-0,76925	0,81165
Mrpl36	-0,76953	0,15844
Cspg4	-0,76973	0,80319
Gm44250	-0,76984	0,52619
Gm7733	-0,7702	0,85996
Rabl6	-0,77031	0,11891
Rnf41	-0,77116	0,63039
Opa3	-0,77137	0,28857
Tmem209	-0,77175	0,60826
Vav1	-0,77189	0,23769
Psen2	-0,7721	0,2309
Mcm9	-0,77214	0,61757
Zbtb5	-0,77306	0,50054
Gm43062	-0,77355	0,85956
Uba5	-0,77382	0,21885
Tm7sf3	-0,77392	0,2592
Plagl2	-0,77392	0,45527
Mesdc1	-0,77387	0,59987
Araf	-0,77405	0,35833
Pld2	-0,77398	0,66172
Dcun1d1	-0,77406	0,20737
Pde8b	-0,77417	0,093748
Natd1	-0,77448	0,49031
Smcr8	-0,77464	0,42883

Ccdc97	-0,77482	0,27806
Kctd3	-0,77489	0,58635
Gbas	-0,77499	0,46681
6030442K20Rik	-0,77521	0,88115
Galnt7	-0,77586	0,1184
Cc2d1a	-0,77585	0,61559
Mfsd5	-0,77604	0,17957
Gsap	-0,77612	0,2821
Larp4b	-0,77646	0,095633
Fen1	-0,77668	0,54622
Ap3b1	-0,77691	0,26661
Gm10033	-0,77691	0,6653
Gm42595	-0,77686	0,697
Hmox2	-0,77707	0,19072
Tfrc	-0,77743	0,21596
Msra	-0,77744	0,71218
Gcfc2	-0,77756	0,49225
Spsb3	-0,77756	0,75215
Slc11a2	-0,77784	0,06637
Rgl2	-0,77805	0,37262
Ubtf	-0,77831	0,19976
Sepsecs	-0,77834	0,56361
Vash2	-0,77833	0,91106
Hmx3	-0,77842	0,73519
Ptcd2	-0,77889	0,10266
Mir17hg	-0,77897	0,59662
Ap1g2	-0,77927	0,61838
1700003F12Rik	-0,77968	0,48831
Usp16	-0,78003	0,16593
Nos3	-0,78001	0,45527
Slu7	-0,7801	0,11802
C1qtnf6	-0,78036	0,7702
Per2	-0,78054	0,2708
D5Ertd605e	-0,78052	0,83455
Ttc38	-0,78149	0,68914
Wdfy1	-0,78164	0,14233
Slc37a2	-0,78167	0,12551
Zfp606	-0,78171	0,61658
Zfp141	-0,78195	0,49394
Tmem201	-0,78231	0,5833
Tbc1d14	-0,78312	0,36395
Fam175b	-0,78349	0,10351
Fam32a	-0,78379	0,1027
Gm15185	-0,78408	0,84704
Ehd4	-0,78454	0,06216
Ddx27	-0,78464	0,17198
Lig3	-0,78482	0,30783
Cebpe	-0,78486	0,61714
Eif2b4	-0,78565	0,19032
Tubgcp6	-0,78582	0,49446
Ccdc14	-0,78617	0,49222
1810021B22Rik	-0,78627	0,59601

Poln	-0,78715	0,79859
Ppp1r12b	-0,78728	0,09143
Zfp952	-0,78772	0,48831
Zfp64	-0,78796	0,37862
Grpel1	-0,7884	0,20479
Gm43111	-0,78839	0,68986
Tnip1	-0,78857	0,13086
Grwd1	-0,78882	0,54888
Zfp260	-0,78943	0,39435
Hlcs	-0,78942	0,63255
Cyb561	-0,78944	0,71132
Sec31b	-0,78982	0,84957
Dram2	-0,79012	0,41943
Zfp626	-0,79018	0,56193
Morn1	-0,79022	0,87704
Vps4b	-0,79035	0,17947
Serbp1	-0,79094	0,051002
Tnfaip8	-0,79086	0,083869
Dusp6	-0,7913	0,25567
Akap11	-0,79142	0,22434
Ksr1	-0,79148	0,73467
Cct3	-0,7918	0,060713
Polm	-0,79187	0,7202
Tgs1	-0,79352	0,25471
Pced1a	-0,79359	0,36474
Slc35d1	-0,79369	0,26625
Dnajc2	-0,79414	0,1502
Slc35e2	-0,79424	0,54267
Rrs1	-0,79444	0,15104
Gm26917	-0,79461	0,068014
Tmem268	-0,79511	0,22267
Dnttip2	-0,79522	0,24201
Ikbkg	-0,7952	0,28079
Ulk2	-0,79557	0,35555
Gm11772	-0,79573	0,79474
Ngrn	-0,79584	0,14982
Gm42876	-0,79611	0,88435
Tpm3-rs7	-0,79633	0,15408
Slc2a4	-0,79646	0,66819
Mia3	-0,79657	0,19682
Gm15440	-0,79675	0,56992
Bcap29	-0,79679	0,22051
Xrra1	-0,79711	0,85826
Zc3h7b	-0,79725	0,49809
Hsd17b7	-0,79738	0,27305
Appl1	-0,79764	0,076632
Il17rc	-0,7978	0,64449
Cyp4f16	-0,79798	0,6774
Chm	-0,79822	0,62417
Diexf	-0,79853	0,31365
Adar	-0,79862	0,13502
Lrrc25	-0,79856	0,45867

Gnl2	-0,79875	0,31866
1600020E01Rik	-0,79883	0,64043
Klc4	-0,79944	0,13331
Cand1	-0,79946	0,14832
Mitf	-0,79954	0,19073
Aldoc	-0,79959	0,081053
Timm44	-0,79958	0,2295
Dhrs1	-0,80051	0,21826
Rnf149	-0,80116	0,43808
Ttc32	-0,80151	0,54008
Mustn1	-0,80185	0,39292
Ptcd1	-0,80221	0,30282
Stx5a	-0,80239	0,343
Gm10060	-0,80243	0,73967
Wdr11	-0,80275	0,50864
Arl8a	-0,8034	0,22903
Cdk14	-0,80356	0,3504
Golga2	-0,80398	0,027503
Suclg1	-0,80396	0,16106
Gm43792	-0,80461	0,63264
Gm16061	-0,80471	0,89375
Srrm2	-0,80477	0,076525
Tdrkh	-0,80544	0,29176
Ly96	-0,80538	0,56595
Gm38387	-0,8056	0,86259
Uhrf1bp1l	-0,80569	0,052335
Adsl	-0,80566	0,39099
Acvr1b	-0,80584	0,70656
Zfp236	-0,80588	0,5172
Cdpf1	-0,80593	0,55289
Zfp52	-0,80591	0,69342
Parn	-0,80635	0,27044
Pik3cg	-0,80637	0,43208
Tmigd3	-0,80648	0,80334
Mtrf1	-0,80671	0,57323
Pum3	-0,80729	0,10844
Brox	-0,80808	0,1409
1110019D14Rik	-0,80848	0,69825
Gm21967	-0,80863	0,78395
Mzf1	-0,80878	0,86491
Rabep2	-0,80892	0,52769
Acaa2	-0,80905	0,28752
Nr2c2	-0,80921	0,26854
D930016D06Rik	-0,80928	0,64156
Atp13a2	-0,80938	0,16841
Dok1	-0,80963	0,089513
Rasa2	-0,80975	0,25149
Ppp1r13b	-0,81017	0,6103
Gm11222	-0,81021	0,85111
Gpalpp1	-0,81127	0,36074
Gm7895	-0,81169	0,90027
Mapre3	-0,81184	0,65169

Bre	-0,81234	0,13812
Supv3l1	-0,81233	0,29362
Cntnap1	-0,81226	0,71689
1700021F05Rik	-0,81255	0,27555
Nt5dc2	-0,81249	0,46681
Crebbp	-0,81267	0,1815
Gm37297	-0,81287	0,72363
Ift43	-0,81297	0,53424
Sdhb	-0,81309	0,15586
Rtf1	-0,81432	0,049328
Riok1	-0,81428	0,16857
Afg3l1	-0,81442	0,16754
Slc38a7	-0,81449	0,30366
Rnf113a2	-0,81453	0,34083
Trim32	-0,81477	0,37339
Snapin	-0,81477	0,37831
Cdc14a	-0,81497	0,67017
Atp6v1e1	-0,81546	0,042539
Cdr2	-0,81557	0,42579
Zkscan1	-0,81615	0,23004
Rb1cc1	-0,81669	0,23007
Kif5c	-0,8171	0,83371
Stub1	-0,81727	0,05249
Plpp6	-0,81847	0,41439
Gm16433	-0,81853	0,67626
Plxna2	-0,81863	0,15799
Zcchc6	-0,81863	0,16392
Cep250	-0,81913	0,29367
Exog	-0,81944	0,67741
Myo1c	-0,8201	0,094088
Carf	-0,82008	0,5503
Sec61a1	-0,82035	0,041551
Trub1	-0,82054	0,26183
Zfp709	-0,82077	0,89931
Polr1b	-0,82135	0,62729
Mtif2	-0,82154	0,2884
Tmem205	-0,8216	0,16897
Angel2	-0,82171	0,18984
Gmeb2	-0,82197	0,13656
Stoml1	-0,82212	0,39759
Mtap	-0,82221	0,28857
Zbtb12	-0,82238	0,36895
Pja1	-0,82238	0,37412
Fcgr1	-0,82264	0,41286
Dtwd1	-0,82335	0,29688
Hsdl1	-0,82342	0,44926
Traf2	-0,82421	0,53876
Slc25a42	-0,82426	0,73638
Alg1	-0,82493	0,19848
Ccdc86	-0,82491	0,29782
RP23-403E19.1	-0,82501	0,80334
Cyc1	-0,82558	0,19827

Ube2o	-0,82562	0,3006
Ddx24	-0,82566	0,14433
1600010M07Rik	-0,82639	0,57621
Poll	-0,82696	0,16596
Wdr33	-0,82715	0,20779
Akap9	-0,82769	0,064735
Rnf183	-0,82768	0,66091
Taf7	-0,82782	0,35645
Tmem87a	-0,82802	0,17163
Abcc4	-0,82831	0,39515
Slc30a6	-0,82836	0,25076
Ifrd1	-0,82904	0,1944
Nudt1	-0,82909	0,58599
Slc16a9	-0,82949	0,70993
Fam13c	-0,82959	0,3454
Dph5	-0,82968	0,33225
Abr	-0,82976	0,022575
1110038F14Rik	-0,83003	0,17669
Sfmbt1	-0,83002	0,28049
Gm43961	-0,83039	0,83416
Casd1	-0,8306	0,29349
Hp1bp3	-0,83065	0,022152
Garnl3	-0,83066	0,75225
Ralgapb	-0,83076	0,14002
Gm11451	-0,83098	0,75791
Ipo13	-0,83129	0,50152
Ikbkb	-0,83148	0,20872
Tigar	-0,83173	0,14063
2310039H08Rik	-0,83175	0,28578
Atp5k-ps2	-0,83176	0,69766
Samsn1	-0,83243	0,45055
Smg8	-0,83288	0,20509
Lcp1	-0,83362	0,067569
Mgat4b	-0,834	0,064705
Gm37900	-0,83417	0,85443
Prkag2	-0,83446	0,073063
Kcne3	-0,83498	0,81156
Parp4	-0,83512	0,44224
A130050O07Rik	-0,83515	0,78812
Polr2a	-0,8356	0,14152
Rassf4	-0,83567	0,077371
Sass6	-0,83569	0,4829
Gm10132	-0,83621	0,79694
Paxip1	-0,83636	0,38523
Pdcd1	-0,83645	0,66175
Aqp11	-0,83662	0,62544
Ripk1	-0,83673	0,4579
2310061I04Rik	-0,83689	0,12168
Rap1b	-0,83698	0,029375
Cd63-ps	-0,83732	0,15362
Sec13	-0,83727	0,17443
Gpatch1	-0,8373	0,30942

Etohd2	-0,83768	0,75797
Gm43742	-0,83786	0,43376
Lman2	-0,83824	0,062457
D3Ertd254e	-0,83823	0,30642
Shb	-0,83867	0,37315
Esyt2	-0,83906	0,099202
Ints11	-0,83951	0,23947
Polr3gl	-0,83985	0,096451
Rprd2	-0,84034	0,10169
Zdhhc20	-0,84069	0,027898
Abhd3	-0,84146	0,77785
Fgd3	-0,84204	0,1376
Mtus2	-0,84218	0,21006
Mfsd1	-0,84255	0,018307
Wdr53	-0,84298	0,38056
2810428J06Rik	-0,84323	0,68496
Kyat1	-0,84351	0,47173
Ccdc12	-0,84397	0,40582
Zbtb25	-0,84413	0,23004
Xrn1	-0,84431	0,12025
Gm37274	-0,84427	0,55355
Gm12421	-0,84448	0,82886
Gm10425	-0,84473	0,86396
Usp46	-0,84511	0,21666
Ccl25	-0,84511	0,36463
Zfp943	-0,84512	0,74651
Pemt	-0,84551	0,69389
Rhpn2	-0,84566	0,76675
Derl2	-0,84582	0,15679
Ralgps1	-0,84603	0,57647
Retsat	-0,84647	0,34742
Rpf2	-0,84659	0,11052
Gm42483	-0,84695	0,76491
Atp8a1	-0,8476	0,23615
Chmp3	-0,84807	0,044541
Slc24a3	-0,84834	0,52854
Nudt5	-0,84836	0,45159
Agpat2	-0,84882	0,4704
Pus1	-0,84916	0,24692
Zfp59	-0,85017	0,78395
Sf3a3	-0,8503	0,15128
Fbxo28	-0,85044	0,14574
Zfp607a	-0,85057	0,63662
Rapgef1	-0,85074	0,082107
Tm9sf4	-0,85077	0,0098131
Gm16536	-0,85099	0,4536
Trim28	-0,85139	0,12587
Acta2	-0,8517	0,78698
Gm37116	-0,85183	0,84094
Amacr	-0,85191	0,68473
Pop7	-0,85213	0,2609
Rab11fip5	-0,85266	0,071867

Atp6v1c1	-0,85281	0,014201
Actr5	-0,85343	0,18868
Zfp867	-0,85348	0,73497
Prickle2	-0,85383	0,40109
Wdfy4	-0,85426	0,043917
Ndufb8	-0,85434	0,13316
Uck2	-0,85443	0,021596
Chchd4	-0,85437	0,067876
Itga4	-0,85476	0,12699
Dhx29	-0,85486	0,079716
Sft2d3	-0,85498	0,2775
Vwa7	-0,85499	0,58399
Pex16	-0,8554	0,21821
Gm45445	-0,85556	0,76914
Gm37606	-0,85577	0,63154
BC065397	-0,85653	0,77219
Fam175a	-0,85666	0,51393
Ddx42	-0,85734	0,21792
Mypopos	-0,85727	0,73628
Elovl6	-0,85751	0,19269
Entpd7	-0,85749	0,43317
Zfp961	-0,85826	0,51917
Eif2ak1	-0,85846	0,14137
Cherp	-0,85851	0,14566
Ube2q1	-0,85878	0,02685
Ell	-0,85913	0,1048
Sod1	-0,85949	0,034478
Actg1	-0,85956	0,80413
1700084J12Rik	-0,85956	0,84957
Jmy	-0,86032	0,14892
C1galt1c1	-0,86025	0,43448
mt-Tt	-0,86041	0,56455
Tor1b	-0,86085	0,10262
Tmem127	-0,86087	0,14717
Capn3	-0,86118	0,78257
Gm43924	-0,86264	0,65804
Slc10a7	-0,86271	0,34351
Def6	-0,86276	0,39875
Zfp341	-0,86304	0,42475
Cog2	-0,86401	0,37819
Adamts6	-0,86414	0,72124
Tirap	-0,86433	0,23773
Otub2	-0,86523	0,57078
5830444B04Rik	-0,8656	0,20889
Ticam2	-0,86594	0,39719
Snx24	-0,86658	0,35758
Micall2	-0,8668	0,2975
Mrpl2	-0,86706	0,14148
Mif4gd	-0,86731	0,34083
Ccdc71	-0,86762	0,2884
Copa	-0,86788	0,051002
Ankrd54	-0,8684	0,41112

Psma2	-0,86855	0,02848
Pank1	-0,86959	0,44542
Tango6	-0,8699	0,74214
B4gat1	-0,87	0,38176
Gpr65	-0,87007	0,24994
Rcl1	-0,87038	0,079769
Uchl4	-0,87067	0,62818
Ncoa2	-0,87134	0,1764
AU019823	-0,87227	0,20745
Actr8	-0,87237	0,10169
Fut10	-0,87244	0,55341
Trim65	-0,87259	0,61166
Surf4	-0,87312	0,013279
2210406H18Rik	-0,87308	0,78711
Gusb	-0,87349	0,013955
Ung	-0,87373	0,36474
Frmd4b	-0,87414	0,081689
Abcf3	-0,87412	0,23459
Ttpal	-0,87471	0,41198
Dhrs9	-0,87495	0,55478
AA414768	-0,87509	0,67052
Ick	-0,87586	0,49798
Chid1	-0,87655	0,20683
Coa3	-0,87786	0,17957
Usp42	-0,87795	0,23643
Il17ra	-0,87806	0,19506
Cnpy3	-0,87842	0,10345
Col20a1	-0,87933	0,32199
Ccdc57	-0,87994	0,50461
Exoc3l4	-0,88022	0,39759
Ulk3	-0,88053	0,27447
Irf5	-0,8821	0,031102
Psma7	-0,88223	0,067666
Atp6v0a2	-0,88252	0,2295
Tamm41	-0,88249	0,28932
Idh2	-0,88263	0,079769
Ppcdc	-0,88257	0,11023
Gm3375	-0,88337	0,57374
Adssl1	-0,88346	0,062457
Jmjd4	-0,88363	0,56147
Gm37718	-0,88356	0,61404
Btd	-0,88379	0,46368
Dhx57	-0,88398	0,44457
Ipo8	-0,88406	0,18733
Gm12251	-0,88411	0,41618
Mysm1	-0,88418	0,14363
Wdr59	-0,88429	0,36074
Shc4	-0,88442	0,6043
9130221H12Rik	-0,88458	0,41353
Kifc3	-0,88486	0,062457
Inpp5d	-0,88498	0,084632
Tfip11	-0,88504	0,27212

Slc36a4	-0,88511	0,13112
Ube4a	-0,88589	0,49834
Asb8	-0,88633	0,23903
Gm13423	-0,88631	0,6661
Hivep1	-0,88638	0,48469
Mpv17	-0,88658	0,20279
Hook3	-0,88672	0,036655
Tmub2	-0,88676	0,36463
Wdr45	-0,88686	0,46822
AA986860	-0,88719	0,83062
Ube2l3	-0,88749	0,04974
Bcl2l1	-0,88772	0,01958
H6pd	-0,88807	0,2398
Ormdl1	-0,88823	0,39862
Fbrsl1	-0,88872	0,31727
Tbc1d32	-0,8891	0,37262
Gm37101	-0,88923	0,68914
Sde2	-0,88934	0,015625
Zfand3	-0,88944	0,037864
Bcl9l	-0,88969	0,19495
Gm45422	-0,89028	0,75225
Exosc1	-0,89044	0,16563
Dync2h1	-0,8906	0,060414
Susd3	-0,89062	0,11586
8430408G22Rik	-0,89062	0,69949
Pecr	-0,89066	0,15427
Csnk2a2	-0,89078	0,014201
Ankrd9	-0,8909	0,47236
Zfp994	-0,89091	0,6522
Atxn7l3b	-0,89127	0,017159
Gm6088	-0,8914	0,83069
Zbtb20	-0,89159	0,071145
Dock8	-0,89164	0,21295
Sucla2	-0,89234	0,081149
Cfap36	-0,89228	0,38643
Itpr2	-0,89264	0,027691
Tas1r1	-0,89299	0,7085
Nfyc	-0,89328	0,80224
Aggf1	-0,8934	0,051651
Gtf3c6	-0,89359	0,26643
Fam58b	-0,89393	0,32987
Tanc2	-0,8941	0,43491
Gstt1	-0,89456	0,6358
Plbd2	-0,89485	0,016576
Gm44075	-0,89531	0,83711
Ddx21	-0,89541	0,018712
Elf2	-0,89561	0,2592
Maged2	-0,89562	0,56943
F830115B05Rik	-0,89588	0,79306
Psmd6	-0,89622	0,046793
Slc20a2	-0,89637	0,56898
Dnajc27	-0,89654	0,50585

Ktn1	-0,89683	0,014745
Med24	-0,89698	0,57621
Zfp526	-0,89735	0,68358
Zfp747	-0,89755	0,57613
Lif	-0,89753	0,70847
Tmtc4	-0,89854	0,57082
Hnrnpm	-0,89865	0,01702
Gm42639	-0,89878	0,40801
Pigw	-0,89879	0,42369
Gm37234	-0,89877	0,78358
Zdhhc24	-0,89889	0,52297
Tada2a	-0,8993	0,079609
Iars2	-0,90027	0,096216
Rbm10	-0,90114	0,11858
Gm43793	-0,90136	0,64045
Msr1	-0,90155	0,023896
Tyw3	-0,90148	0,69504
Rai14	-0,90165	0,069928
Erp27	-0,90167	0,76619
Sdcbp2	-0,90183	0,3161
Gm44916	-0,90194	0,76619
RP23-228B2.5	-0,90193	0,78842
9130008F23Rik	-0,90305	0,47351
Hectd1	-0,90326	0,011696
Rad1	-0,90332	0,2821
Mib1	-0,90353	0,048272
Xrcc3	-0,90379	0,61019
4930556M19Rik	-0,90471	0,73638
Pitpnb	-0,90575	0,052877
Cep78	-0,90586	0,49031
Madd	-0,90602	0,17214
Serinc2	-0,90657	0,14103
Cd109	-0,90733	0,021649
Fam213a	-0,90734	0,2075
Nudt14	-0,90731	0,31313
Cpt2	-0,90785	0,24893
Foxp1	-0,90819	0,013527
Rab8a	-0,90858	0,06812
Ostc	-0,90873	0,037063
Mbd6	-0,90918	0,27773
Plin3	-0,90967	0,10924
Fam46c	-0,90974	0,81884
Anapc4	-0,91006	0,23149
Gar1	-0,91012	0,24212
B230377A18Rik	-0,91042	0,69504
Zfp386	-0,91058	0,36006
Mylip	-0,91089	0,29464
Sh3bp5	-0,9112	0,015353
Zfp707	-0,91297	0,46767
Elp6	-0,91301	0,50037
Lpar2	-0,91302	0,64683
Eci1	-0,91314	0,36145

RP24-286J14.3	-0,91324	0,45847
CAAA01194877.2	-0,91386	0,52253
Tpm1	-0,91488	0,003094
Gtf2ird2	-0,91644	0,40609
Sirt6	-0,91675	0,54181
Rdh14	-0,91709	0,28561
Calcoco1	-0,9174	0,29119
Cutc	-0,918	0,44737
Cars	-0,91864	0,044234
Myo9b	-0,91892	0,18865
Sfxn5	-0,91995	0,53106
Nt5c3b	-0,92045	0,48137
Letmd1	-0,92054	0,28848
D6Ertd527e	-0,92061	0,80318
Pard6a	-0,92156	0,32843
Stk11ip	-0,92172	0,40892
2810025M15Rik	-0,9227	0,021649
Wdr61	-0,92305	0,017869
Kcnn1	-0,92383	0,57814
Pced1b	-0,92386	0,67121
Bysl	-0,92406	0,076336
Rsph1	-0,92443	0,56867
Zdhhc7	-0,92459	0,21811
Lhfpl2	-0,9247	0,0040801
Hif1an	-0,92565	0,15997
Fam160b2	-0,92572	0,11273
Nom1	-0,92589	0,090386
Il7r	-0,92591	0,83268
Cux1	-0,92662	0,011641
Ptpra	-0,92678	0,039038
Mis12	-0,92698	0,19895
Ptpn11	-0,92731	0,11946
Ccdc92b	-0,92734	0,73981
Tmem186	-0,92742	0,45159
Gramd1b	-0,92783	0,025018
Efl1	-0,92789	0,079769
Sirt7	-0,928	0,12704
Ago1	-0,92818	0,16844
Serinc3	-0,92838	0,059844
Trim14	-0,92844	0,73177
Rin3	-0,92861	0,183
Sorl1	-0,92891	0,71689
Blnk	-0,92895	0,1394
Dpp8	-0,92978	0,066326
Cebpd	-0,93025	0,16333
Egr2	-0,93042	0,29603
Alg14	-0,93057	0,27984
Mrpl37	-0,93086	0,025864
Tpm2	-0,93088	0,30011
BC060293	-0,93095	0,76347
Uqcc1	-0,93096	0,080899
Tyk2	-0,93111	0,024033

Strn3	-0,93159	0,0028935
Lgals9	-0,93187	0,61818
Gm37474	-0,9321	0,25173
Tasp1	-0,93223	0,56988
Fance	-0,93231	0,14319
Tbxas1	-0,93308	0,77596
Eva1b	-0,93317	0,44156
Galk1	-0,93355	0,088478
Wdr7	-0,93392	0,24758
Toe1	-0,93391	0,36504
Usp25	-0,93474	0,046804
Slc22a5	-0,93479	0,36895
Acad12	-0,93551	0,54203
Zfp566	-0,93562	0,58118
U2surp	-0,93584	0,011851
Hps4	-0,93647	0,10598
Slc25a53	-0,93646	0,29041
Cmtr1	-0,93648	0,48656
Gemin5	-0,93701	0,33043
Arhgap27	-0,93759	0,030384
Taok3	-0,93778	0,010536
Arhgef12	-0,93781	0,025721
Mtr	-0,93822	0,087683
Mapk11	-0,93834	0,68625
Rps6ka3	-0,93877	0,11252
Fhl3	-0,93892	0,095157
Eml5	-0,93895	0,3006
Oxld1	-0,93922	0,36074
Sel1l	-0,93936	0,004843
Gas7	-0,93965	0,13423
Zfp316	-0,93981	0,32991
Snx33	-0,93996	0,31578
Tnfrsf13b	-0,94027	0,63211
Becn1	-0,94051	0,014701
Slf1	-0,94047	0,49772
Tut1	-0,94065	0,2459
Celsr3	-0,94091	0,58008
Pex12	-0,94119	0,34428
Ptgr1	-0,94133	0,40931
Trmu	-0,94136	0,40816
Eif3b	-0,94155	0,013798
Csk	-0,94155	0,055929
Mon1a	-0,94218	0,32738
Pik3r4	-0,94246	0,1092
Cenpv	-0,94256	0,17579
Apoe	-0,94263	0,60814
Lgals2	-0,94275	0,73177
Ncbp1	-0,94286	0,019449
Abcd3	-0,94336	0,038355
Mkl2	-0,94487	0,32228
Foxc1	-0,9455	0,53706
Rab20	-0,94557	0,38894

Ddn	-0,94568	0,74852
Tbc1d10b	-0,94616	0,02178
Tmem202	-0,94645	0,63211
R74862	-0,94679	0,56435
Hrc	-0,94782	0,62818
Alg5	-0,94785	0,088399
Gm37080	-0,94795	0,57374
Trim47	-0,94828	0,55774
Agap1	-0,94865	0,12525
4930568A12Rik	-0,94874	0,74219
Nudt18	-0,9495	0,1621
Cant1	-0,94961	0,012919
Rassf5	-0,94974	0,07641
Il10rb	-0,95083	0,054939
Tmem129	-0,95081	0,38803
Gm38077	-0,95089	0,5172
AW046200	-0,95192	0,48948
Slpi	-0,95253	0,079716
Slc26a9	-0,95253	0,61713
Csnk1d	-0,95288	0,011757
Tgm2	-0,95287	0,014701
Eri2	-0,95295	0,37022
Tnfrsf1a	-0,9533	0,018521
Zfhx2	-0,95346	0,4704
Msantd4	-0,95508	0,028059
Ppp6r1	-0,95547	0,037541
Ltbp2	-0,95553	0,59073
Adck1	-0,95582	0,24509
RP24-91J7.1	-0,9558	0,69092
Gm4258	-0,95593	0,57038
Pstpip1	-0,95629	0,006927
Naglu	-0,95643	0,05614
Pms1	-0,95639	0,44325
Cmc4	-0,95642	0,51111
Plscr3	-0,95668	0,11824
Kif3b	-0,95713	0,033736
Cox15	-0,95714	0,3114
Tlr2	-0,95733	0,064432
Dopey2	-0,95742	0,10119
Slc29a3	-0,95762	0,093748
B230354K17Rik	-0,95758	0,45621
Ubqln2	-0,95791	0,16623
Dapp1	-0,95788	0,22499
Pigp	-0,95857	0,30933
Trim26	-0,95931	0,17392
Ccdc136	-0,95944	0,44979
Ppm1l	-0,96118	0,024044
Imp4	-0,96161	0,055281
Washc5	-0,96157	0,31846
Mtmr2	-0,96199	0,0071097
Ankrd33b	-0,96204	0,64446
Fbxo31	-0,96262	0,25694

Zcchc2	-0,96295	0,033371
Glyctk	-0,9633	0,71689
Enox2	-0,96367	0,10807
Kif5a	-0,96423	0,28578
Rgs16	-0,96432	0,13408
Carmil1	-0,96453	0,73177
Acp2	-0,96544	0,30532
Cep131	-0,96572	0,39434
Pwp2	-0,96607	0,28224
Nup160	-0,96607	0,53057
1700088E04Rik	-0,96654	0,60255
Thoc3	-0,96665	0,050653
Dapk3	-0,96673	0,3114
Ankrd39	-0,96713	0,30366
Rnf219	-0,9673	0,15269
Fancm	-0,96749	0,5126
Cwc25	-0,96763	0,069148
Pgm5	-0,96755	0,55162
Gm42482	-0,96769	0,46547
Mrps36-ps1	-0,96788	0,48877
Rpusd2	-0,96837	0,5025
Tpcn1	-0,9688	0,028977
Tysnd1	-0,96928	0,18051
Avl9	-0,97003	0,059342
Taco1	-0,97014	0,33848
Zfp217	-0,97022	0,041663
Myof	-0,97032	0,015625
Bccip	-0,97039	0,27814
Noc4l	-0,97047	0,17255
Ss18	-0,97088	0,062962
Scyl3	-0,97154	0,2752
Gab2	-0,97162	0,010492
Gm5391	-0,9725	0,74882
Gm6526	-0,97256	0,65576
Rpusd4	-0,97269	0,062457
Cerk	-0,97278	0,011329
Psph	-0,97304	0,37162
Rwdd2b	-0,9732	0,52619
Mccc2	-0,97391	0,72696
Crtap	-0,97413	0,32608
Mettl2	-0,97417	0,27806
E230016M11Rik	-0,97418	0,72124
Gm20156	-0,97537	0,70518
Adat3	-0,9757	0,094492
Syvn1	-0,97622	0,056482
Galnt1	-0,97686	0,0068016
Casp1	-0,97707	0,11514
Bcl2l12	-0,97711	0,763
Prorsd1	-0,97725	0,011702
Idh1	-0,9788	0,0011639
Aig1	-0,97892	0,019654
Shmt1	-0,97941	0,062167

6430548M08Rik	-0,97954	0,076796
Arid1b	-0,9804	0,016454
Gm45342	-0,98038	0,45602
H2afy	-0,98064	0,01615
Abcc1	-0,98076	0,010232
Sdhaf4	-0,98109	0,17579
Sephs2	-0,98146	0,084434
Brpf1	-0,98199	0,02704
Bid	-0,98215	0,025201
Rad9b	-0,98261	0,48562
Fkbpl	-0,98267	0,27162
Gm6162	-0,98347	0,69943
Zfp568	-0,98379	0,4159
Gm42481	-0,98383	0,6046
Ecel1	-0,98395	0,50729
Dmrta2	-0,98466	0,71772
Mark2	-0,98503	0,076153
Zkscan7	-0,98564	0,63264
Nol6	-0,98668	0,33225
Nf2	-0,98687	0,024925
Ppp4r1	-0,98702	0,32991
Pelp1	-0,98714	0,18191
Gsto1	-0,98749	0,064014
Rnft1	-0,98771	0,53706
Mbp	-0,98881	0,0042059
Gm18913	-0,98915	0,24689
Catsper2	-0,98925	0,52477
Notch2	-0,98939	0,026249
Brf1	-0,98993	0,11858
Gm8463	-0,99002	0,48831
Sars2	-0,99022	0,51391
E430021H15Rik	-0,99085	0,40582
AI661453	-0,99085	0,47827
Miga2	-0,99106	0,45867
Gm37578	-0,99139	0,73238
Slc9a8	-0,99183	0,038175
Pgd	-0,99211	0,0022665
Hbs1l	-0,99212	0,0044088
Gm43547	-0,99217	0,75006
Chordc1	-0,99301	0,0046974
Smap1	-0,99298	0,038355
Mgrn1	-0,99303	0,046609
Gm9711	-0,99308	0,71308
Tcirg1	-0,99353	0,0057512
Ccdc71l	-0,99365	0,40773
Haus2	-0,99391	0,024099
Spata13	-0,99466	0,01707
BC004004	-0,99539	0,09559
Map3k7	-0,99546	0,0057568
Gm37494	-0,99581	0,36463
Mxra8	-0,99599	0,3114
Gm43627	-0,99733	0,6343

Ythdf2	-0,99748	0,016134
Pld1	-0,99782	0,34919
Frmd8os	-0,99796	0,66535
Rab23	-0,99854	0,35696
A630072M18Rik	-0,99847	0,52801
2310033P09Rik	-0,99964	0,079853
Tfr2	-0,99976	0,61119
Tmem69	-1,0002	0,23261
Gm29155	-1,0003	0,56929
Naip6	-1,0008	0,30996
BC052040	-1,0008	0,35186
Smim10l1	-1,0011	0,2742
Kctd11	-1,0015	0,39624
6720475M21Rik	-1,002	0,7146
Nudc-ps1	-1,0032	0,37953
Ank2	-1,0032	0,42823
Akna	-1,0034	0,32707
Foxred2	-1,0038	0,011851
Zfp330	-1,0046	0,023755
Gm15964	-1,0053	0,57346
Gne	-1,0058	0,19853
Zfp809	-1,0068	0,0052513
Tldc1	-1,0074	0,28768
Pnpla7	-1,0076	0,0079758
Fhad1	-1,0076	0,39837
2310057M21Rik	-1,0084	0,17198
Cenpt	-1,0086	0,49966
Atp6ap2	-1,0091	0,0013357
Zmiz1	-1,0093	0,032462
Accs	-1,0101	0,38514
Tmcc1	-1,0102	0,16664
Farp2	-1,0108	0,60806
Wdyhv1	-1,011	0,30381
Osbp2	-1,0111	0,75377
Parp3	-1,0113	0,067891
Kbtbd3	-1,012	0,091374
Slc7a4	-1,012	0,18661
Nlrx1	-1,0122	0,082522
Mmab	-1,013	0,343
Tnfsf13b	-1,0132	0,39909
Serpinc1	-1,0134	0,51599
Sprtn	-1,0135	0,36182
Neo1	-1,0144	0,065149
Gnl3	-1,0145	0,014735
Ash2l	-1,0145	0,063085
RP23-110E20.5	-1,0149	0,7702
Arhgap9	-1,0155	0,073545
Zfp882	-1,0158	0,65621
Mtus1	-1,016	0,62985
Scfd1	-1,017	0,048378
Dus1l	-1,0172	0,12647
Angpt2	-1,0173	0,067123

Eya4	-1,0176	0,099769
Ttll1	-1,0181	0,40703
Lrch1	-1,0187	0,23669
Zdhhc14	-1,0188	0,086478
Crybb3	-1,0188	0,57374
Fth1	-1,019	0,00044509
Bbs7	-1,0196	0,39543
Ddr1	-1,0198	0,67091
Rreb1	-1,02	0,11506
Urb1	-1,02	0,17478
Psmg2	-1,0202	0,052231
Gm44822	-1,0217	0,63205
9930120I10Rik	-1,0224	0,61818
Tmem25	-1,0229	0,37205
RP24-499N24.6	-1,0247	0,36395
Phf20	-1,0248	0,012042
Tmem156	-1,0248	0,24324
6030460B20Rik	-1,0251	0,70901
Sat2	-1,026	0,49704
Arid3a	-1,0264	0,21389
RP23-3F1.8	-1,0268	0,096367
Zfp874a	-1,0272	0,27067
Sco2	-1,0276	0,28433
Dclre1c	-1,0277	0,30389
Mrpl17	-1,0286	0,019642
Gm14248	-1,0286	0,73767
Gm26569	-1,0288	0,71966
Gm13675	-1,0291	0,56088
Gm42515	-1,0293	0,66126
Smim12	-1,0295	0,040783
Ankle1	-1,0296	0,66066
Gm5054	-1,0302	0,70701
Thada	-1,0305	0,29119
Nme6	-1,0311	0,46361
B4galt6	-1,0313	0,10687
Srd5a3	-1,0316	0,30783
Mtmr4	-1,0317	0,041036
Nip7	-1,032	0,0069682
Trim25	-1,0323	0,0096155
Fn3k	-1,0328	0,59933
6030458C11Rik	-1,0331	0,064616
Armc7	-1,0333	0,24283
Ttl	-1,0342	0,066733
Gucd1	-1,0348	0,41259
Kdelr3	-1,0351	0,67593
Grhl1	-1,0355	0,52396
F9	-1,0356	0,75442
Kdelc1	-1,0358	0,35177
Tnfaip2	-1,0361	0,0059077
Gm44053	-1,0361	0,64546
Wars2	-1,0365	0,42448
9030624J02Rik	-1,0371	0,072764

Phf14	-1,0372	0,092624
Gm44178	-1,0372	0,65818
Prob1	-1,0376	0,65818
Nol4l	-1,038	0,12548
4933440N22Rik	-1,038	0,58635
Clpp	-1,0381	0,11858
Zswim3	-1,0383	0,2876
Ccdc122	-1,0389	0,42291
Rin2	-1,0393	0,016273
Zfp942	-1,0406	0,44167
C2cd2	-1,0408	0,29499
Gm4924	-1,0408	0,32669
D2Bwg1423e	-1,0413	0,45159
Klf9	-1,0416	0,00076777
Trafd1	-1,0421	0,010119
Tnfrsf18	-1,0423	0,48817
Atat1	-1,0427	0,256
Creg1	-1,0428	0,0040542
Smarcd1	-1,0436	0,14263
Gm43351	-1,0437	0,46053
Snora21	-1,0444	0,41286
Ccdc32	-1,0445	0,12222
Hal	-1,0445	0,58682
Gm26514	-1,0448	0,59869
Zfp467	-1,045	0,16458
C030013C21Rik	-1,0451	0,38805
Itfg2	-1,0458	0,17425
Stac3	-1,0459	0,67948
Mus81	-1,0468	0,36266
Bend6	-1,0471	0,64228
Gm28535	-1,0474	0,56088
Vps37d	-1,0484	0,70289
1700007K09Rik	-1,0491	0,74304
Slc17a5	-1,0497	0,011557
Gm29487	-1,0498	0,74471
Gm44237	-1,0499	0,52477
Zfp672	-1,0505	0,18805
Gm43359	-1,0506	0,27698
Arfgap1	-1,0516	0,1105
Gm8738	-1,0531	0,47094
Prdm9	-1,0538	0,44002
Tnk2	-1,0549	0,18464
Faf2	-1,055	0,025516
St8sia4	-1,0555	0,024925
Gpr107	-1,0561	0,0019433
Kdm4d	-1,0565	0,59209
Sppl2b	-1,0567	0,1356
Mical2	-1,0572	0,089719
Fadd	-1,0573	0,6977
Myh11	-1,0577	0,70415
Fkbp11	-1,0579	0,14709
Oip5	-1,0595	0,40301

Lipt1	-1,06	0,22285
Fech	-1,0608	0,17155
Dars2	-1,0609	0,19748
Nsf	-1,0615	0,023014
Gm38157	-1,0617	0,65576
Rgl1	-1,0619	0,0011231
Pus3	-1,0619	0,069158
Tbc1d4	-1,0625	0,050328
Mapk9	-1,0629	0,10844
Uxt	-1,063	0,62949
Tmem214	-1,0644	0,13596
Ifit2	-1,0647	0,63792
Tug1	-1,0651	0,21283
Bcl2l11	-1,0653	0,021048
Pfn2	-1,0653	0,43016
Cep68	-1,0654	0,060321
Arhgdig	-1,0672	0,4536
Zfp78	-1,0676	0,60806
Tns3	-1,0681	0,0034847
BC003965	-1,0681	0,039886
Glb1	-1,0688	0,0023874
Adprh	-1,0694	0,0051165
Fbxo15	-1,0694	0,6084
Dusp18	-1,0698	0,12882
Sac3d1	-1,0698	0,15253
Ubr1	-1,0704	0,1138
Tgfb1i1	-1,071	0,52801
Atg4d	-1,0718	0,18751
Pla1a	-1,0719	0,46321
Fastkd3	-1,0726	0,04063
Akr1b3	-1,0726	0,27832
Ddx56	-1,0728	0,076502
Rnf8	-1,0728	0,11432
Nptxr	-1,0734	0,096343
Mmp19	-1,0738	0,090178
L3mbtl3	-1,0741	0,37275
2810004N23Rik	-1,075	0,0062914
Gm8168	-1,0754	0,6358
Ptpn1	-1,0758	0,0015936
Gm6743	-1,0763	0,67733
Lrrc73	-1,0772	0,46246
Exoc7	-1,0773	0,11287
Habp4	-1,0774	0,3332
Dtwd2	-1,0776	0,5597
Stc2	-1,0783	0,50729
Poli	-1,0784	0,20675
Cldn15	-1,0787	0,68759
Tspyl2	-1,0789	0,4083
Gm44623	-1,08	0,51596
Rab29	-1,0808	0,064432
Chrac1	-1,081	0,07162
Uvssa	-1,0811	0,071867

RP23-359K10.9	-1,0813	0,52089
Loxl3	-1,0815	0,32994
Tmem150a	-1,0817	0,45878
Tmem19	-1,0819	0,183
Plat	-1,0824	0,5025
Prmt3	-1,0836	0,030918
Phf10	-1,0842	0,0023445
Prr12	-1,0856	0,18858
Brca2	-1,0857	0,24497
Ncf4	-1,0862	0,022069
Slfn5	-1,0865	0,087184
Pcdhb16	-1,0865	0,60168
Itpripl2	-1,0866	0,0031772
Ing4	-1,0869	0,54613
Snx4	-1,0878	0,016572
Nucb2	-1,0883	0,0083157
Sec24d	-1,089	0,079609
Alg3	-1,0893	0,22709
Rere	-1,0896	0,00012236
Cmas	-1,0899	0,0022936
Usp38	-1,0904	0,16484
Slc39a9	-1,0906	0,026249
Ctnnal1	-1,0915	0,39742
Tmcc2	-1,0916	0,1459
Fibp	-1,0918	0,093675
Gpn1	-1,0924	0,098194
Nrip1	-1,093	0,066326
4930550C14Rik	-1,093	0,52619
Gm37788	-1,0937	0,53173
2310015A10Rik	-1,0938	0,69949
Wbscr22	-1,0939	0,054
Amer1	-1,0942	0,32055
Atg10	-1,0946	0,16623
Pogz	-1,0947	0,0086365
Gm20707	-1,095	0,43206
Gm22767	-1,0955	0,54797
Wdr5b	-1,0961	0,48985
Zbtb48	-1,0963	0,52801
Fam219a	-1,097	0,29846
Rbsn	-1,0985	0,15844
Lrrc61	-1,0989	0,065286
0610007P14Rik	-1,0993	0,014271
1810062G17Rik	-1,0993	0,57174
Ftx	-1,0997	0,34351
Mdn1	-1,0998	0,0066849
Gm14698	-1,101	0,47094
A330074K22Rik	-1,101	0,63314
n-R5-8s1	-1,1017	0,39719
Gm11410	-1,1022	0,53984
Ears2	-1,1024	0,1616
Acy3	-1,1024	0,5348
4930453N24Rik	-1,1026	0,14345

Ryk	-1,103	0,0014452
Kctd7	-1,103	0,30962
Alkbh3	-1,1041	0,02419
Nagpa	-1,1044	0,033409
Gm13378	-1,1045	0,43562
Slc26a6	-1,1047	0,30532
Abcb4	-1,1048	0,0014856
Gm7292	-1,1054	0,66186
Nfatc4	-1,1055	0,57367
Akap7	-1,1057	0,081083
RP23-278O17.1	-1,1064	0,60818
Cog7	-1,1065	0,055207
Gm20696	-1,1067	0,48964
Gm42918	-1,1079	0,58859
Rasgrp3	-1,1084	0,0046328
Ndufaf7	-1,1084	0,0296
Parp12	-1,1084	0,13153
B130021K23Rik	-1,1085	0,58099
Snrk	-1,1086	0,072401
Gm14328	-1,1094	0,32669
Zfp69	-1,1097	0,57014
Oxsm	-1,11	0,37091
Gm43147	-1,111	0,45159
Zfp14	-1,1115	0,56943
Pde4a	-1,1135	0,035181
Kat6a	-1,1141	0,0096321
Matn1	-1,1149	0,50729
Gm12743	-1,1164	0,64773
Fbxo25	-1,117	0,039916
Slc39a2	-1,1173	0,35565
Mtif3	-1,1178	0,086625
Mdm2	-1,118	0,0059453
Elmod2	-1,118	0,18117
Mlycd	-1,1181	0,048525
Amotl1	-1,1189	0,00098233
3110080O07Rik	-1,1192	0,60978
Mettl8	-1,1196	0,11191
Trdmt1	-1,1199	0,56435
Trmt5	-1,1201	0,2995
Cables2	-1,1204	0,27057
Hspa2	-1,1205	0,56049
Gm19705	-1,1206	0,28872
Aptx	-1,1208	0,047116
Mfsd13b	-1,1212	0,71425
Htra2	-1,1216	0,01336
Zc3h10	-1,1225	0,043295
Arhgef17	-1,1229	0,6069
Kank3	-1,1239	0,15942
Tab3	-1,124	0,2075
Gpr146	-1,1247	0,12623
Grk6	-1,125	0,059254
Tgfbrap1	-1,1251	0,13423

Panx1	-1,1256	0,15192
Dbt	-1,126	0,078861
Mavs	-1,1264	0,11858
Dnajb12	-1,1268	0,061175
Zfp319	-1,1274	0,32384
Angptl4	-1,1283	0,46278
Zbtb1	-1,1292	0,2508
Stard7	-1,1308	0,0029036
Rbm5	-1,131	0,0037554
Parp16	-1,131	0,05183
Lclat1	-1,1312	0,30942
Mrps35	-1,1322	0,038355
Atp6v1g2	-1,1328	0,52769
Tubgcp3	-1,1329	0,027148
Coa7	-1,1346	0,30619
Laptm5	-1,1361	0,00016771
Tnfaip8l2	-1,1368	0,11088
Ptpn12	-1,137	0,0019615
1110002L01Rik	-1,1372	0,30733
Ccr2	-1,1378	0,56218
Gnat2	-1,1382	0,60204
4732491K20Rik	-1,1389	0,46402
Arhgap18	-1,139	0,0058601
Fam208a	-1,1396	0,19795
Slc26a2	-1,1397	0,0050344
Tesk2	-1,1399	0,33225
B230208H11Rik	-1,1399	0,57341
Xpot	-1,1402	0,0069486
Slc25a10	-1,1405	0,10262
Bag5	-1,1412	0,0219
Rnf123	-1,1421	0,34832
Ppan	-1,1429	0,0021313
Zfp959	-1,1429	0,41747
Gspt2	-1,143	0,58374
Fbxw17	-1,1437	0,14605
Gm37968	-1,1447	0,48823
Rpl7l1	-1,1451	0,009777
Zfp703	-1,1454	0,084152
Aak1	-1,1465	0,0037915
Csf1r	-1,1474	0,00042504
Als2cr12	-1,148	0,70026
Zfp523	-1,1518	0,076502
Nlrp10	-1,1518	0,39742
Rint1	-1,1527	0,20042
Chrna1os	-1,1527	0,47689
Zfp729a	-1,1528	0,25149
Klhl41	-1,1528	0,34196
Fads6	-1,1529	0,12168
Zfp62	-1,1531	0,14091
Eno3	-1,1532	0,035962
Kbtbd4	-1,1534	0,15586
Zfp1	-1,1535	0,17787

March9	-1,1536	0,023896
Sptlc1	-1,1537	0,023502
Mrpl46	-1,154	0,14041
Hacd3	-1,1549	0,021046
Trmt11	-1,1551	0,25173
Ctdsp1	-1,1558	0,19823
Eef2k	-1,156	0,3114
Gm38399	-1,1562	0,40218
Pdss2	-1,1567	0,20904
6330418K02Rik	-1,1572	0,33901
Faap100	-1,1577	0,059719
Slc35b4	-1,1578	0,17865
Arhgap1	-1,1583	0,11099
Ssh1	-1,1585	0,06789
Dhx32	-1,1588	0,19677
Tmem246	-1,1592	0,33885
Ptges	-1,1595	0,16484
Eid2b	-1,1596	0,15598
Mtrf1l	-1,1601	0,14862
Adck5	-1,1608	0,18861
Ccdc66	-1,1611	0,15687
D630045J12Rik	-1,1611	0,56946
Nfs1	-1,1641	0,004625
Zfp868	-1,1651	0,35255
Gm11918	-1,1655	0,42125
Trmt12	-1,1655	0,47563
Cyba	-1,1661	0,0013432
Fam19a3	-1,1666	0,48916
Snx7	-1,1673	0,18037
Mier2	-1,1675	0,00026419
Spats1	-1,169	0,58256
Cetn4	-1,1705	0,30126
Gm44103	-1,1711	0,62607
Lrrc20	-1,1713	0,04531
Naif1	-1,1722	0,11443
Gm44434	-1,1724	0,57579
mt-Nd1	-1,1728	0,0038394
Fzd9	-1,1732	0,58126
Plk3	-1,1738	0,064797
Ncoa6	-1,1739	0,0054125
Fam213b	-1,1739	0,41365
RP23-380K24.3	-1,1739	0,51391
Tm4sf5	-1,1745	0,46557
Mettl21a	-1,1753	0,01767
Zswim4	-1,176	0,14006
Bicd1	-1,176	0,15731
Gid8	-1,1774	0,004704
Ammecr1	-1,1783	0,016585
Anxa9	-1,1786	0,35963
Reep6	-1,1791	0,085694
Ssc5d	-1,1796	0,65429
C130050O18Rik	-1,1807	0,1194

Zfp599	-1,181	0,36463
Twf1	-1,1837	0,064705
Mfap1a	-1,1841	0,48177
Clptm1l	-1,186	0,00045593
Zfp830	-1,1862	0,032462
Sema4a	-1,1864	0,0021492
Fkbp15	-1,1881	0,0016172
Des	-1,1885	0,40203
Aldh18a1	-1,1889	0,1274
Ufsp2	-1,1899	0,0023588
Nphp3	-1,1906	0,23092
Islr2	-1,191	0,57295
Kat6b	-1,1914	0,11614
Gm44951	-1,1917	0,53473
Dixdc1	-1,1918	0,17947
Gm37978	-1,1922	0,66453
Zfp106	-1,1926	0,00017251
Samd10	-1,1928	0,39377
Rbm12b1	-1,1928	0,56246
A730062M13Rik	-1,1943	0,61115
B230398E01Rik	-1,1947	0,29375
Gm44423	-1,1949	0,60307
Kif1bp	-1,195	0,052714
Faah	-1,1982	0,53119
Slc24a5	-1,1984	0,58702
Rpp38	-1,1985	0,1701
Gm43715	-1,1987	0,69225
Gm29539	-1,1992	0,49459
Scarf1	-1,1996	0,11296
Slc46a3	-1,2003	0,43467
Nudt16	-1,2005	0,0098312
Trub2	-1,2006	0,05867
Bcl9	-1,2019	0,064034
Fbxo36	-1,2021	0,35792
Yif1a	-1,2026	0,038076
Tbcc	-1,2028	0,053608
Prkar2b	-1,2035	0,23529
Exosc4	-1,2045	0,019642
Gtf2h2	-1,2045	0,062457
Nif3l1	-1,2076	0,037603
5730480H06Rik	-1,2079	0,20478
Coro1c	-1,208	0,0002279
Zc4h2	-1,2082	0,34351
Atxn7l3	-1,2083	0,0026934
Zfp180	-1,2091	0,014161
Son	-1,2098	0,00078781
Mks1	-1,2098	0,037541
Exo5	-1,2108	0,15813
Ap5s1	-1,2121	0,024017
Hoxa1	-1,2127	0,36182
Cage1	-1,2127	0,41181
Knop1	-1,2134	0,0014574

Ddx58	-1,2138	0,092716
Rftn2	-1,2149	0,27864
Smim1	-1,2152	0,3389
Dennd2d	-1,2155	0,05614
Mapkapk2	-1,2159	0,00013604
Cnr2	-1,2161	0,23835
Rab11fip3	-1,2167	0,32119
Spryd3	-1,2169	0,038355
Nr1h2	-1,2172	0,063138
Spr	-1,2181	0,10348
Alg11	-1,2183	0,062183
AW146154	-1,2194	0,50889
Slc27a1	-1,2195	0,070619
Cul4a	-1,2196	0,023291
Gm18867	-1,2197	0,44292
Bcar3	-1,2202	0,0050784
A930018M24Rik	-1,2204	0,60702
Trip11	-1,2224	0,025792
Diablo	-1,2229	0,025357
Rbck1	-1,223	0,0032179
Lcp2	-1,2241	0,0019639
Ehd1	-1,2248	0,00015955
Hmgxb4	-1,2261	0,057018
Gm20257	-1,2278	0,40218
Cxcr3	-1,2278	0,54453
Ier2	-1,2279	0,094298
Oxt	-1,2279	0,5275
Pfas	-1,228	0,087306
Xrcc5	-1,2282	0,13077
Tmem173	-1,2295	0,30366
Ubn1	-1,2299	0,00043129
Sh3pxd2a	-1,2306	0,0047151
Dnajc8	-1,2315	0,00030933
Ranbp1	-1,2331	0,02538
Snora73b	-1,2339	0,1192
Slc16a6	-1,2352	0,0054043
Polr1a	-1,2354	0,0063115
4833418N02Rik	-1,2355	0,53494
Znhit6	-1,2367	0,036655
Tor4a	-1,2372	0,13326
D11Wsu47e	-1,2373	0,13851
Prdm4	-1,2378	0,099202
Mblac2	-1,2378	0,30793
Adap2	-1,238	0,27293
Ppip5k2	-1,2385	0,0042739
Wipf1	-1,2385	0,0052898
D130007C19Rik	-1,2385	0,18821
Ppcs	-1,2393	0,19157
Lrp8os3	-1,24	0,41795
Tmem44	-1,24	0,61182
Srr	-1,2404	0,023124
Lrrc8a	-1,2409	0,088743

A930016O22Rik	-1,2411	0,53557
Hexim2	-1,2418	0,4433
Lgals3bp	-1,2423	0,051002
Arsg	-1,2428	0,35255
Tbc1d13	-1,2432	0,0052898
Ints7	-1,2432	0,018393
Syk	-1,2438	0,0015193
Ddx59	-1,2439	0,36082
Gm12258	-1,244	0,54226
Slc22a15	-1,2457	0,35206
1810024B03Rik	-1,246	0,39759
Slc35c1	-1,2471	0,0067471
Irgm1	-1,2479	0,40182
Rnmt	-1,248	0,00072199
Rtfdc1	-1,2481	0,0032082
Cbr3	-1,2483	0,0035426
Pin1	-1,2488	0,023206
Ctdspl	-1,2495	0,30018
Gm6304	-1,2503	0,64087
Txk	-1,251	0,18934
Srrd	-1,2516	0,2543
Pigc	-1,2523	0,006756
Gm15327	-1,2534	0,49031
Dnajc30	-1,2546	0,16789
Haus4	-1,2558	0,22282
Slc4a11	-1,2559	0,17572
Mzb1	-1,258	0,2593
Inf2	-1,2581	7,48E-05
Sh3bp5l	-1,2583	0,0082578
Akap17b	-1,2593	0,14279
Fdxacb1	-1,2599	0,13618
Med1	-1,2601	0,0001971
Tnfaip8l1	-1,2604	0,36006
Mgat5	-1,2628	0,0026934
Wdr6	-1,2631	0,047649
Tbx15	-1,2633	0,25894
Calhm2	-1,264	0,10096
Gdpd1	-1,2648	0,0057579
Ltbr	-1,2649	0,011263
Car9	-1,2651	0,031039
Lpcat3	-1,2654	0,078912
Fendrr	-1,2656	0,40218
Atad1	-1,2658	0,01905
Blvra	-1,2662	0,0013254
Tmem55a	-1,2669	0,0037031
Gm5113	-1,2671	0,3798
Emp2	-1,2674	0,063511
Rasa3	-1,2682	0,0021664
Faap24	-1,2683	0,26388
Ercc6l2	-1,2686	0,11669
Tmem184c	-1,269	0,095249
Pde4d	-1,2691	0,13812

Rrp9	-1,2692	0,024283
Pcdhb22	-1,2697	0,54275
Ppm1h	-1,2703	0,054203
Cc2d2a	-1,2705	0,42733
Gsk3b	-1,2709	0,0002857
Arhgap30	-1,2711	0,0045236
Kctd12	-1,2727	0,21596
Zfp362	-1,2729	0,039235
Peg12	-1,2736	0,62544
Gm15530	-1,2742	0,40123
Fah	-1,2748	0,1309
Slc43a3	-1,2749	0,038761
Zbtb8os	-1,275	0,064201
Gm15787	-1,2756	0,43265
Zc3h12a	-1,276	0,14376
Cul5	-1,2762	0,00023473
Stra8	-1,2763	0,46278
Fnip2	-1,2765	2,31E-05
5430427O19Rik	-1,2773	0,082981
Lamc2	-1,2779	0,49966
Mblac1	-1,2784	0,31949
Zfp944	-1,2787	0,44414
Stx17	-1,2801	0,022846
Thap7	-1,2803	0,018301
Lyrm1	-1,2811	0,10121
9530053A07Rik	-1,2814	0,37315
Abcd1	-1,2819	0,12997
Zfp420	-1,2826	0,1539
Kmt2e	-1,2831	0,0005556
Enpp5	-1,2835	0,011005
Gns	-1,2845	7,95E-05
Gm10180	-1,285	0,44806
Ssu72	-1,2853	0,00022245
RP23-444K20.4	-1,2856	0,03838
1300002E11Rik	-1,2859	0,29226
Tradd	-1,2866	0,15506
Zfp365	-1,2867	0,20096
Polr3f	-1,287	0,021842
Xylb	-1,2882	0,46689
Zbtb3	-1,2902	0,52906
Noc2l	-1,2907	9,37E-05
Elp4	-1,2908	0,43729
Pdcd11	-1,2917	0,00038391
Ak3	-1,2925	0,096013
mt-Nd4	-1,294	0,010829
Gldc	-1,2942	0,56748
Gm42967	-1,2944	0,49041
Zfp712	-1,2948	0,49041
Gm15708	-1,2949	0,53741
Gm44509	-1,295	0,45206
Adcy2	-1,2964	0,13071
Mrps6	-1,2968	0,00038116

Lnx2	-1,2971	0,009777
Pde6g	-1,2972	0,46149
mt-Cytb	-1,2973	0,0023325
Gipc2	-1,2979	0,53259
Zscan29	-1,2986	0,15554
Gls2	-1,2987	0,46928
Dtx4	-1,2991	0,0001324
Mfap1b	-1,2999	0,1191
Pqlc2	-1,3002	0,46533
Asb7	-1,3004	0,022166
Gpatch4	-1,3006	0,0057573
Atp6v1d	-1,3009	1,78E-05
Zfp748	-1,3016	0,19823
Cep63	-1,3017	0,20958
Acsl4	-1,3018	1,64E-05
Col15a1	-1,3018	0,36069
Pla2g2e	-1,3024	0,18123
Slc12a2	-1,303	0,024424
Fam173b	-1,303	0,13417
Prkca	-1,3034	0,04063
Dennd4b	-1,3046	0,071498
Ftsj3	-1,3057	0,00036245
Med8	-1,3058	0,012019
Lig4	-1,3059	0,1191
Fli1	-1,3061	0,0031071
Mpv17l	-1,3063	0,16413
Gm25596	-1,3072	0,45602
Fam102a	-1,3081	1,60E-05
Gm44775	-1,3089	0,38216
Sirt4	-1,3089	0,48757
Zfp26	-1,3097	0,041003
Nsrp1	-1,31	0,0028637
2810006K23Rik	-1,3106	0,3089
Ubash3b	-1,311	0,0007513
Ptpro	-1,312	0,032133
Sp110	-1,3123	0,13239
Gm15892	-1,3123	0,33171
Slc22a21	-1,3127	0,33976
Gm8539	-1,3127	0,36546
Zfp446	-1,3131	0,2141
Ift20	-1,3133	0,0099012
Trim11	-1,3139	0,00010067
2510016D11Rik	-1,3139	0,47563
Gm43379	-1,3146	0,41823
Magee1	-1,3152	0,1394
Oas1c	-1,3152	0,15292
Ivns1abp	-1,3154	3,02E-06
9330102E08Rik	-1,3154	0,49966
Rundc1	-1,3166	0,035181
Tnnc2	-1,3166	0,51562
Gm15513	-1,3168	0,29112
Prtg	-1,3171	0,048031

Npc1l1	-1,3182	0,46308
Zfp629	-1,3197	0,16305
Saysd1	-1,3205	0,063805
Slc16a7	-1,3208	0,098194
Heatr3	-1,3215	0,10764
Cyb5rl	-1,3216	0,15023
RP23-476G10.1	-1,322	0,46311
Poglut1	-1,3222	0,00050507
Cmtr2	-1,3222	0,004603
Gm19552	-1,3225	0,27529
Actn1	-1,3235	9,37E-05
Znhit2	-1,324	0,12621
Nelfa	-1,3241	0,010221
1600014C23Rik	-1,3244	0,44554
Crebl2	-1,3249	0,15527
Gm43071	-1,3263	0,41343
Zbtb6	-1,3264	0,012792
Cd33	-1,3272	0,00013527
Arih1	-1,3285	1,62E-05
Ncdn	-1,3285	0,042679
Agbl3	-1,3286	0,076308
Acacb	-1,3305	0,57323
Orai3	-1,3306	0,015968
Epc1	-1,3311	0,035466
Fam3c	-1,3317	0,0020958
Fbxo9	-1,3326	0,087683
Twnk	-1,333	0,011297
Mppe1	-1,333	0,050959
Fam57a	-1,3331	0,34948
Crtam	-1,3333	0,33064
Hfe	-1,3337	0,15249
Hsd17b14	-1,3341	0,41259
Gm42636	-1,3343	0,026409
Slc25a14	-1,3354	0,18081
Bcar1	-1,3368	0,12891
Gm43364	-1,3368	0,50471
Abi3	-1,3369	0,19506
Ppp1r7	-1,3376	0,023448
Tsku	-1,3383	0,49966
Slc35f6	-1,3387	0,0019864
A930001C03Rik	-1,3396	0,27385
Zfp983	-1,34	0,14215
Gnmt	-1,34	0,45527
Ppp6r3	-1,341	7,76E-05
Ddx28	-1,3416	0,036238
Tars	-1,3418	0,0011712
Rlf	-1,3422	0,0049064
Zbtb39	-1,3429	0,35996
Dedd	-1,3437	0,046587
Ldb3	-1,3439	0,25062
Ints3	-1,345	3,41E-05
Ttll12	-1,3467	0,00032975

Neurl1a	-1,3468	0,49966
Polr3d	-1,3482	0,0045723
Gm38125	-1,349	0,49957
Atp8b4	-1,3496	0,12135
Klhl36	-1,3506	0,31846
N4bp3	-1,3518	0,34475
Pcyt1a	-1,3519	3,97E-06
Slc25a33	-1,352	0,0012167
Wfs1	-1,353	0,042558
9130230N09Rik	-1,353	0,39592
Slc41a2	-1,3532	0,0010463
Lrrc8d	-1,3541	0,00089359
Ccdc173	-1,3544	0,24443
Gm43761	-1,3546	0,22566
Tshz3	-1,3553	0,041445
Ppil3	-1,3559	0,031127
Plxdc1	-1,3564	0,0040324
Zfp318	-1,3567	0,0066094
Rasal2	-1,358	0,00036444
RP23-104D6.2	-1,3582	0,49225
Pms2	-1,3588	0,049042
Gm12966	-1,3602	0,14695
Zfp235	-1,3602	0,14835
Clec7a	-1,3607	0,014828
Gm37569	-1,3608	0,22204
Gatb	-1,3615	0,076487
Slc23a2	-1,3618	0,045094
Mfsd4b4	-1,3629	0,49232
Trim68	-1,3636	0,035083
Gm11764	-1,364	0,48964
Frk	-1,3652	0,34806
Gzf1	-1,366	0,015228
Clec5a	-1,3664	0,0061879
Bckdk	-1,3665	0,0034846
Igtp	-1,3674	0,31941
Irak4	-1,3679	0,017568
Gm42850	-1,3681	0,46464
Nfatc3	-1,3687	4,25E-05
Tac4	-1,3695	0,53807
Slc1a5	-1,3697	2,01E-05
Cox6a2	-1,3699	0,13777
Cep19	-1,3701	0,028428
Rhbdd2	-1,3702	0,11858
Slc11a1	-1,371	0,0012161
1810030O07Rik	-1,3712	0,0032015
Pid1	-1,3722	0,0033629
Zfp839	-1,3722	0,020591
Zfp689	-1,3727	0,29041
BC005537	-1,3736	0,00028612
Vps25	-1,374	0,43097
Gm42970	-1,3746	0,50703
Elp2	-1,3748	0,0023109

Etfrf1	-1,3755	0,024848
Pex11g	-1,3755	0,38943
Hps6	-1,376	0,035901
Gm13223	-1,3781	0,50511
Rhot2	-1,3787	0,11868
Platr3	-1,3789	0,35792
Kdm4a	-1,3792	2,59E-05
Cracr2a	-1,3795	0,35738
Lmbr1	-1,381	0,14041
2210016F16Rik	-1,3811	0,038355
Nfatc1	-1,3813	0,0001641
Bet1l	-1,382	0,00083994
Cat	-1,3833	1,02E-05
Gm5544	-1,3837	0,4875
Mllt1	-1,384	0,1394
Atp8b3	-1,3855	0,39325
Phactr1	-1,3858	0,36018
Tbl3	-1,3865	0,018321
Tctex1d4	-1,387	0,36754
Tm4sf19	-1,3889	0,39903
Maf1	-1,3891	0,00029254
Slc16a13	-1,3896	0,37003
Trim27	-1,3922	0,00034125
Apbb1ip	-1,3926	1,03E-05
Gm13398	-1,393	0,45762
Cyb561d1	-1,3935	0,15903
Spi1	-1,3949	0,00028736
Bcl2l15	-1,3955	0,26585
Ica1	-1,3968	0,018301
Naa40	-1,3971	0,0081005
Trappc12	-1,3982	0,0068305
Zhx1	-1,3988	0,0025675
Mov10	-1,3998	0,18342
Aldh3b1	-1,3999	0,0063448
BC002059	-1,4006	0,0023184
2210008F06Rik	-1,4015	0,40868
Tfec	-1,4019	0,14091
Hhex	-1,402	0,028702
Prss44	-1,4023	0,41806
Gm15157	-1,4027	0,44591
4632427E13Rik	-1,403	0,0027045
Rit1	-1,4031	0,0030769
Gm6921	-1,4041	0,45206
Lsm10	-1,4043	0,028776
Smco3	-1,4053	0,49778
Pknox1	-1,4054	0,021596
Taf1c	-1,4055	0,054939
Adora2b	-1,4061	0,13417
Gm37010	-1,4061	0,30271
Sh2d6	-1,4077	0,42229
Zfp799	-1,4089	0,18169
Prkdc	-1,4094	0,11443

Zfp74	-1,4115	0,16754
Zadh2	-1,4116	0,0090214
Zfp975	-1,4118	0,45078
Plcd1	-1,4155	0,4631
Flywch1	-1,4171	0,018636
Bcl2a1b	-1,4183	0,062871
F7	-1,4187	0,42831
Rela	-1,4201	0,0003129
Creb3l1	-1,4201	0,30206
Dbnl	-1,4209	0,0048116
Lrp12	-1,4209	0,0053647
Ccdc171	-1,4213	0,38992
Zfp456	-1,4214	0,19283
Rnf157	-1,4248	0,0013245
Cog8	-1,4252	0,018381
Gdf9	-1,4264	0,42416
Tmem62	-1,4276	0,3208
Zfp759	-1,4276	0,45507
Tm9sf1	-1,4282	0,1962
Speer9-ps1	-1,4289	0,60946
Arhgap31	-1,4297	0,040534
Sbk1	-1,4305	0,25505
Pde8a	-1,4311	0,020601
Gm45871	-1,4315	0,14888
Tsc2	-1,4323	0,2028
Gm45495	-1,433	0,1616
Rsl1	-1,4331	0,46895
Plxnb3	-1,434	0,30011
Zfhx4	-1,4342	0,0023313
Uri1	-1,4351	0,00056528
Trmt44	-1,4351	0,14319
Nudt22	-1,4356	0,0041901
Bag2	-1,4356	0,018961
Mfsd6	-1,4375	0,0010886
Oscp1	-1,4375	0,41181
Rpgr	-1,4384	0,16007
Bbs9	-1,4392	0,066168
Gm15824	-1,4397	0,41488
Gemin8	-1,4422	0,41198
Fam212b	-1,4427	0,10342
Alg8	-1,4432	0,096295
Utp20	-1,4434	0,0077762
Etv6	-1,444	0,0075223
Ifih1	-1,4444	0,15064
Nek4	-1,4448	0,16675
Vsig10	-1,4449	0,13675
Gm9732	-1,4449	0,25149
Zkscan5	-1,4458	0,13851
Slc15a3	-1,4472	0,18288
Eif1ad	-1,448	0,0018622
Zfp945	-1,4491	0,22484
Ifi203-ps	-1,4506	0,43856

Camk2a	-1,4511	0,011851
Kmt5b	-1,4519	0,0047073
Metap2	-1,4527	0,00018232
Glrp1	-1,4545	0,23769
Tefm	-1,4572	0,14148
Fblim1	-1,4575	0,0014574
Afmid	-1,4585	0,45078
Fam212a	-1,4616	0,19417
Ptpn21	-1,4617	0,14628
Tgfbr2	-1,462	0,0010229
Pou4f1	-1,4634	0,49704
Ankrd34a	-1,4674	0,22367
C530005A16Rik	-1,4682	0,34351
Slc38a1	-1,469	7,70E-06
Mgam	-1,4702	0,4165
Zscan12	-1,4703	0,0051914
Ccdc51	-1,4716	0,071867
Iqsec3	-1,472	0,34351
Cd93	-1,4723	0,2952
Gm20056	-1,4723	0,38438
Iars	-1,4724	0,0011814
Rhobtb1	-1,4734	0,18582
Gm36963	-1,4736	0,14859
Ficd	-1,4742	0,21479
Plag1	-1,4744	0,24398
Rfx5	-1,4748	4,41E-05
Zkscan6	-1,4754	0,0044995
1700017B05Rik	-1,4756	0,00015476
Gmpr	-1,4774	0,00077364
Ahnak2	-1,4783	0,00023722
Fam65c	-1,4789	0,0068899
Dhodh	-1,479	0,032077
Cx3cr1	-1,4791	0,00062139
AU040320	-1,4795	0,0012135
Gtf2b	-1,4796	0,011546
Lrrc8b	-1,4803	0,015272
C1rl	-1,4817	0,2895
Fgfrl1	-1,4827	0,035261
Gm43457	-1,483	0,36674
2610044O15Rik8	-1,4831	0,40582
Evi5l	-1,4832	0,16675
Stat5b	-1,4835	0,016375
Gm44667	-1,4841	0,2543
Trp53rka	-1,4842	0,06216
Zfp551	-1,4845	0,084879
Tmem241	-1,4846	0,041208
Champ1	-1,4853	4,69E-05
Ankrd26	-1,4856	0,037063
Gm42600	-1,4863	0,42686
Timp2	-1,4871	0,00041078
Dennd2a	-1,4873	0,0059856
Trmt6	-1,4877	0,0011806

Mypop	-1,4877	0,13423
A930015D03Rik	-1,4886	0,32544
Wbp1l	-1,4889	0,00028612
Aars	-1,4889	0,010189
Cradd	-1,4893	0,05614
AW209491	-1,4902	0,012936
Zfp426	-1,4913	0,060713
Gm43692	-1,4914	0,1197
Pih1d2	-1,4922	0,41823
Mettl22	-1,4931	0,10638
Ttll13	-1,4934	0,5125
Zfp623	-1,4939	0,10008
Sec16b	-1,4949	0,0010166
Treml1	-1,4956	0,0004193
Cyfip2	-1,4957	0,00012342
Zfp605	-1,4957	0,22051
Tk2	-1,4971	5,59E-05
Golim4	-1,4975	0,0003676
Jagn1	-1,498	0,0024581
Rnf135	-1,4981	0,23008
Celf6	-1,4986	0,20412
Cyp26b1	-1,4989	0,33564
Gm29994	-1,4994	0,45013
RP24-233B16.6	-1,5013	0,23769
Gpr85	-1,5027	0,36182
Soga1	-1,5029	0,004875
Gstt3	-1,5029	0,012919
Wdr77	-1,5038	0,0041976
RP24-226A8.2	-1,5038	0,45957
Xbp1	-1,504	1,96E-05
Fkrp	-1,5043	0,059674
RP24-131G14.10	-1,5049	0,33827
Zfp775	-1,5059	0,041445
Sec14l2	-1,5069	0,092624
mt-Nd2	-1,5089	0,0001793
B130006D01Rik	-1,5093	0,15129
Zfp113	-1,5095	0,022161
Sh2d2a	-1,5102	0,16716
Hmga2	-1,5123	3,89E-06
Ighmbp2	-1,5124	0,019199
Lrif1	-1,5128	0,032384
Gm19325	-1,5134	0,13423
Cep162	-1,5139	0,066711
1700008J07Rik	-1,5152	0,44634
Prss36	-1,5168	0,21792
Setmar	-1,5176	0,2775
Zfp579	-1,5184	0,10546
Lin28b	-1,5198	0,49839
Slc7a11	-1,5199	0,0020777
Fam136a	-1,52	0,0039305
Acot2	-1,5203	0,15615
Btbd19	-1,5221	0,0043995

Fus	-1,5226	0,0023082
Gm7769	-1,524	0,40262
Zbtb22	-1,5244	0,0013573
Dlx1	-1,5257	0,021747
Nudt7	-1,5263	0,054
Zfp850	-1,5264	0,15566
Lipt2	-1,5266	0,24505
Surf2	-1,5271	0,011851
5730409E04Rik	-1,5286	0,019526
Rab12	-1,5291	0,00013499
Cbarp	-1,5292	0,23184
Pmepa1	-1,5307	0,0003536
Dlg3	-1,5317	0,15023
Fam35a	-1,5327	0,08046
Phykpl	-1,5346	0,06188
Keap1	-1,535	9,13E-05
Usp20	-1,5353	6,64E-05
Zscan20	-1,5377	0,068014
Myo1d	-1,5385	1,21E-05
Tada2b	-1,5386	0,032235
2310011J03Rik	-1,5388	0,0048285
Gm16537	-1,5407	0,40202
Spn	-1,5411	0,10262
Lrrc47	-1,5413	6,10E-06
Plpp7	-1,5422	0,077428
Nsun4	-1,5424	0,023849
Tmem220	-1,5427	0,012208
Vac14	-1,5437	0,047733
Zfp251	-1,5447	0,06789
Cdc42ep2	-1,5449	0,024283
Srd5a1	-1,5458	0,32361
Ccar2	-1,5469	0,17128
Mocs3	-1,549	0,068697
4921511C10Rik	-1,5492	0,080965
Ajuba	-1,5503	0,35461
Epop	-1,5513	0,18537
Trim16	-1,5532	0,21369
Polr3a	-1,5553	0,029986
Serac1	-1,5553	0,267
Gm37033	-1,5579	0,20907
Uaca	-1,5603	0,00013265
Trak2	-1,561	0,00055718
Ndor1	-1,5613	0,13917
Itgb3	-1,562	0,18392
4930556M19Rik	-1,564	0,30771
Gm12151	-1,564	0,36787
Nr2c1	-1,5642	0,082148
Dpp3	-1,5651	0,042876
Piwil2	-1,5651	0,2335
Adgrl2	-1,5654	0,27806
Gm12799	-1,5664	0,24856
3110001I22Rik	-1,5669	0,031356

Mcat	-1,5675	0,029653
Prkch	-1,5679	0,00024761
Bnip2	-1,5708	2,36E-05
Nop9	-1,5711	0,020429
N6amt1	-1,5714	0,015912
Fosl1	-1,5721	0,011784
Shq1	-1,5745	0,065238
Nek3	-1,5745	0,30724
Scap	-1,5747	0,0024888
Drg2	-1,5748	0,076359
Ints10	-1,5755	0,064705
Frmd8	-1,5773	1,89E-05
Bok	-1,5774	0,033205
Zfp811	-1,5787	0,31535
Ddx17	-1,58	3,00E-05
Gm20604	-1,5819	0,0196
Snip1	-1,5832	0,0085532
RP23-225D5.4	-1,5844	0,34307
Ahrr	-1,5856	0,21885
Gm38345	-1,5874	0,46744
Ccl5	-1,588	0,20988
Slfn10-ps	-1,5908	0,14654
Zfp455	-1,5908	0,33225
Cenpb	-1,5912	0,0043856
Plcb4	-1,5913	1,61E-06
Zpr1	-1,5913	3,10E-06
Airn	-1,5917	0,013824
Slc27a4	-1,5918	0,065449
Cd3eap	-1,594	0,0026066
Ccdc120	-1,5944	0,28449
Gba2	-1,5945	0,14628
Ttc30b	-1,5946	0,40229
Myom1	-1,5956	0,042326
Fzd2	-1,598	0,26867
Pitpnm2	-1,5982	0,02584
Foxd2	-1,5993	0,27488
Tmem175	-1,5995	0,018049
Mgat2	-1,6003	0,00094881
Tmem177	-1,6027	0,025675
Ppfibp1	-1,6034	7,34E-06
Cox10	-1,6038	1,00E-05
Ublcp1	-1,6038	0,2995
Pla2g5	-1,6042	0,0001993
D3Ertd751e	-1,6048	0,13431
Eef1akmt1	-1,6049	0,063805
Cyth4	-1,605	1,02E-05
Bms1	-1,6056	4,13E-06
Nod1	-1,6062	0,02747
Ovca2	-1,6101	0,0023985
Adal	-1,6111	0,062774
Tssk6	-1,6121	0,3114
Lhx1	-1,6122	0,24856

Mrps2	-1,6133	0,00061557
Dhx33	-1,6137	0,0068583
Marveld1	-1,6141	5,46E-05
AI987944	-1,6141	0,011024
Fam129c	-1,6149	0,2775
Ccdc116	-1,6195	0,31078
RP24-84O13.9	-1,6197	0,148
Taf1b	-1,6211	0,0063448
Acvr1	-1,6213	0,0044655
Ppm1e	-1,6229	0,22134
Dph2	-1,6235	0,046433
Rnf170	-1,6239	0,15651
BC017158	-1,6243	0,14115
Ccnd2	-1,6261	1,05E-05
Tdrd3	-1,6262	0,080031
RP23-138K22.2	-1,6269	0,34351
BC048403	-1,6278	0,15162
Erlin2	-1,6283	0,010326
Tmem17	-1,6311	0,29451
Il15ra	-1,6333	0,11744
Iba57	-1,6337	0,06433
Zfp846	-1,6338	0,11206
Lrtm2	-1,6361	0,33523
Gm24890	-1,6381	0,41153
6430590A07Rik	-1,6383	0,13539
Paqr7	-1,6386	0,0032015
Frat1	-1,6386	0,025126
Pkn3	-1,639	0,0018433
Rnasel	-1,6406	0,030478
Ank	-1,6417	1,68E-07
Arhgef10	-1,6424	0,031127
Emc9	-1,6428	0,12107
Irf2bp1	-1,6429	0,00041609
Fem1a	-1,6433	1,99E-05
Wbscr27	-1,6451	0,0058265
Zfp772	-1,6458	0,10759
Grap	-1,6471	0,0047414
Rab43	-1,6477	0,039369
Klhl30	-1,6478	0,059765
Dcaf11	-1,6481	0,002515
Ophn1	-1,6487	0,19056
Nipa1	-1,6511	0,14899
6330403L08Rik	-1,6513	0,1007
Nrp2	-1,6517	2,42E-08
Mst1	-1,652	0,032462
Gm23054	-1,6534	0,41206
Pik3r2	-1,6545	0,0013549
Fam69b	-1,6565	0,24754
Fancg	-1,6569	0,093048
Matn4	-1,6574	0,31906
Dock5	-1,6576	0,0032395
Il4ra	-1,6586	0,012222

Det1	-1,6594	0,036811
1600002H07Rik	-1,6604	0,0075763
Zfp119a	-1,6607	0,36101
Fastkd2	-1,6621	0,023671
Zfp248	-1,6623	0,20918
Hgh1	-1,6639	0,015968
Tmem198b	-1,664	0,065459
Aldh7a1	-1,6661	0,17572
Dapk1	-1,6668	1,16E-06
Ddr2	-1,6677	0,12639
C130023A14Rik	-1,6695	0,1041
Cdkl4	-1,6704	0,071357
Ptgir	-1,6724	0,00012759
Rars2	-1,6754	0,0062637
Zfp85	-1,6763	0,32729
Elf4	-1,6771	0,026866
Atad3aos	-1,6779	0,21821
Gm26740	-1,6787	0,31764
Snx20	-1,6795	0,00013841
Igf2bp1	-1,6803	0,26843
Isl2	-1,682	0,047096
Cpsf3	-1,6828	0,00053285
Frrs1	-1,6835	1,84E-06
Gm24336	-1,6836	0,26271
Zfp202	-1,6838	0,10994
Gm10463	-1,6841	0,23023
Gm45728	-1,6854	0,26282
Fut8	-1,6856	0,14391
4930590J08Rik	-1,69	0,3114
Alkbh2	-1,6902	0,011192
Ccdc166	-1,6921	0,049641
Lat2	-1,6937	3,03E-06
Lcmt2	-1,6941	0,032721
Nmi	-1,6952	0,15586
6430571L13Rik	-1,6955	0,31473
Card11	-1,696	0,26867
Prr14l	-1,6977	0,00026988
Ermard	-1,6997	0,086376
Nkpd1	-1,7008	0,31769
Kif13a	-1,7021	0,061768
Rbks	-1,7021	0,33225
Cpne2	-1,7049	0,00044509
Gm26890	-1,7068	0,093897
Shtn1	-1,7069	2,01E-05
Mrap	-1,7081	0,28484
Slc39a13	-1,7082	7,08E-05
E130311K13Rik	-1,7082	0,025201
Fam161a	-1,7086	0,0018433
Lysmd4	-1,7096	0,0017644
Slc16a12	-1,7137	0,076359
Fbxw7	-1,7138	0,0015913
Tlr6	-1,7143	0,0041344

6720464F23Rik	-1,7152	0,34672
C330018D20Rik	-1,7162	0,03714
Cbfa2t3	-1,7171	0,26193
Oit3	-1,7182	0,038091
Bcdin3d	-1,7186	0,034993
Egfl8	-1,7192	0,31313
Zfp820	-1,7226	0,34395
Stx1a	-1,725	0,2821
BC037039	-1,7267	0,19034
Cstf2t	-1,729	0,00051975
Srf	-1,7305	1,03E-05
Mlkl	-1,7306	0,16364
Msi1	-1,7313	0,025094
Ttc28	-1,7314	0,12246
Gm10676	-1,7314	0,31432
Ffar4	-1,7329	0,13085
Elk3	-1,733	0,0013814
Bdh2	-1,7332	2,32E-05
Tmem65	-1,7339	4,81E-05
B3galnt1	-1,7355	0,14178
Spata2l	-1,7355	0,29801
Trim45	-1,7359	0,049332
Irf4	-1,7367	0,34283
Gm15696	-1,7381	0,14033
Zfp128	-1,7392	0,22484
Dnajc11	-1,7414	0,0026934
Ptafr	-1,742	0,089513
Crtc1	-1,7432	0,074063
Zfp239	-1,7447	0,189
Prss50	-1,7458	0,013527
Selenos	-1,748	1,11E-06
Prss42	-1,749	0,15326
Gm45640	-1,749	0,20274
Magi2	-1,7508	0,068049
Extl1	-1,7536	0,06208
Zfp866	-1,7555	0,21885
Iqce	-1,7564	0,047323
Tiam2	-1,7568	0,16316
Tmem51os1	-1,757	0,25451
Egf	-1,7578	0,17669
Slc39a8	-1,762	0,3114
Ms4a6c	-1,7632	0,096632
Phf11c	-1,7632	0,21826
Slc39a11	-1,7646	6,98E-07
Zfp459	-1,7655	0,14562
Slc39a4	-1,7689	0,076699
A430105I19Rik	-1,7695	0,10924
Gm43728	-1,771	0,11858
Zfp462	-1,772	0,021386
Scamp1	-1,7724	1,00E-05
Rhou	-1,7726	0,19375
Jade2	-1,7756	0,010969

Nfkbie	-1,7757	0,039595
Cish	-1,7788	0,33803
Tns2	-1,7791	0,16413
Tuba1c	-1,7807	0,0002501
Pde4b	-1,7824	0,011851
AI464131	-1,7825	0,25705
5430420F09Rik	-1,7839	0,28429
Tlr3	-1,7873	0,12323
Zfp46	-1,7878	0,013932
Tmem181b-ps	-1,7894	0,046384
Gm12655	-1,7915	0,10325
Ctbp1	-1,7916	4,76E-06
Pde4dip	-1,7918	0,0011785
Hck	-1,7931	0,018228
2310043L19Rik	-1,7977	0,2821
Gm5251	-1,7984	0,18203
Extl2	-1,7991	0,011052
Mtmr9	-1,8001	0,1815
Traf6	-1,8005	0,028059
Gsn	-1,8016	2,71E-07
Igsf3	-1,8019	0,0069726
Nudt12	-1,8043	0,053214
Zfp653	-1,8058	0,035083
9030407P20Rik	-1,8081	0,11517
Wdr24	-1,8082	0,0069832
Me1	-1,8088	1,06E-06
Sdsl	-1,8117	0,047418
Sh3tc1	-1,8132	0,0067421
Trpv4	-1,8147	0,022194
9130023H24Rik	-1,8149	0,15342
Prag1	-1,8154	0,0028968
Gm43378	-1,8159	0,26896
D030028A08Rik	-1,8174	0,092012
Upk1a	-1,8187	0,085013
Tspan10	-1,8211	0,0041913
4931414P19Rik	-1,8229	0,028657
Ivd	-1,8235	0,0062804
0610010F05Rik	-1,8243	0,0086606
Zfp661	-1,8297	0,079512
P2ry6	-1,8322	0,00017183
Trim30a	-1,8334	0,085605
Arhgap35	-1,8342	0,041883
Cdk5rap1	-1,8359	0,02094
Capn10	-1,8362	0,057268
Fam120b	-1,8389	0,019131
Ticam1	-1,8419	0,00066456
Kdm1b	-1,8426	0,1194
Wdr46-ps	-1,8436	0,18757
Sema4b	-1,8437	0,012882
Tlr7	-1,8447	0,00013542
Mrgpre	-1,8449	0,1104
Ncmap	-1,845	0,16244

Wdr73	-1,8455	0,0048341
Gm38009	-1,8459	0,093423
Slc25a15	-1,846	0,043356
Slx4	-1,8498	0,06789
Mir22hg	-1,8521	5,36E-05
Frg2f1	-1,8531	0,13597
Zfp764	-1,8593	0,022554
Hoga1	-1,8641	0,18958
Tmem140	-1,8653	0,020455
Chst14	-1,8677	0,025721
Oas2	-1,8681	0,19667
Klhl40	-1,8683	0,26854
Zdhhc12	-1,8694	0,01194
Msantd3	-1,8714	0,00048583
Cyb561d2	-1,8719	0,15903
Irx5	-1,8723	0,20064
Pde4c	-1,8729	0,15269
Efna2	-1,8791	0,17094
Fbxo32	-1,8829	0,02303
Rab7b	-1,8844	1,30E-05
Slc9a3r1	-1,8866	0,075668
Gpt2	-1,8867	0,00010067
Gsto2	-1,887	0,14267
Scly	-1,8871	0,0056131
Hemk1	-1,8872	0,020421
Sphk2	-1,8877	6,98E-07
Cptp	-1,8903	0,049204
Nat14	-1,8914	0,15326
Txnl4b	-1,8919	0,051344
Sgsm1	-1,8934	5,32E-08
Nadsyn1	-1,8938	0,17163
Zkscan4	-1,8938	0,19053
Bmf	-1,8943	0,011069
Arhgef3	-1,895	0,0017234
Zfp418	-1,8975	0,19677
Ecd	-1,8992	0,00061417
Zbtb38	-1,8993	1,95E-06
Ceacam10	-1,8996	0,13678
Setd1b	-1,9035	9,93E-05
Tmem116	-1,9037	0,011897
Il1rap	-1,9048	0,044541
B3galt4	-1,9051	0,22488
Dido1	-1,9055	0,00047157
Tnks1bp1	-1,9081	0,15816
Gm2885	-1,9104	0,13538
Hoxa7	-1,911	0,075175
Tbc1d25	-1,9115	0,020461
Pomk	-1,9128	0,011069
Mfsd3	-1,9141	0,14115
Rftn1	-1,9147	0,00072327
Klhl5	-1,9153	3,90E-05
Ino80c	-1,9166	2,58E-06

Gm20699	-1,9189	0,12704
Tbc1d2b	-1,9217	4,48E-08
Pygm	-1,922	0,18879
AI467606	-1,9238	0,0016867
Suox	-1,9245	0,014917
C8g	-1,928	0,12568
Robo3	-1,9288	0,0079853
Eogt	-1,929	0,0062631
Gm37063	-1,929	0,26295
Lars	-1,9292	6,86E-08
Lta	-1,93	0,13553
Gja1	-1,9308	0,043099
Sfxn2	-1,933	0,0050344
Ribc1	-1,9343	0,22769
Bbs1	-1,9365	0,15199
Gcc1	-1,9388	2,79E-05
Zfp933	-1,9391	0,10374
Pfkfb4	-1,9414	2,66E-05
Gm9776	-1,9417	0,050043
Ccr1	-1,9448	0,20421
Ptpn14	-1,9468	0,022756
Prkar1b	-1,9476	0,012955
Mthfr	-1,9494	0,0028612
Stx11	-1,9501	0,060627
Polr3e	-1,9506	0,0013104
Cep41	-1,9537	0,088387
Sec16a	-1,9543	0,0014609
Gm11205	-1,9572	0,077421
2810021J22Rik	-1,9591	0,082711
4930432K21Rik	-1,9626	0,0034716
Ankrd49	-1,9696	0,00083819
Pspc1	-1,9727	0,0022481
Cmklr1	-1,9729	0,1394
Gm44829	-1,9732	0,079484
Zfp953	-1,9732	0,17185
Zfp276	-1,9756	0,02788
Zfp65	-1,9768	0,0057579
Tmem37	-1,9812	0,038355
Zfp628	-1,9817	0,015526
Gm45224	-1,9835	0,1776
Slc22a4	-1,9844	0,049931
Gm37124	-1,9858	0,10763
Gtpbp8	-1,9867	0,015161
Zfp473	-1,9867	0,13469
Gm10605	-1,9884	0,10842
Gm20554	-1,9892	0,1614
Gnptab	-1,992	2,01E-07
Gm26532	-1,993	0,027452
Ttc7	-1,9936	0,00014862
Slc39a1	-1,9939	1,87E-05
Tfb2m	-2,0004	0,076308
Lyl1	-2,0016	0,0001534

Arl14epl	-2,0028	0,12838
Hpdl	-2,0036	0,18081
Zfp169	-2,0061	0,06073
Zscan22	-2,0093	0,11829
Rwdd3	-2,0101	0,0035029
Armc5	-2,0113	0,0082547
Gm29243	-2,0126	0,11532
Mkx	-2,0172	0,11188
Catip	-2,0173	0,058125
Tmem8	-2,0209	0,0015061
Phxr4	-2,023	0,10339
Mrm2	-2,034	0,0062631
Katnb1	-2,0374	0,13609
D7Bwg0826e	-2,0378	0,11755
Arl11	-2,0387	0,027133
Evi2a	-2,0393	0,00012236
2310001H17Rik	-2,0397	0,19016
Apbb1	-2,0406	0,081258
Tubg2	-2,0428	0,11858
Adh7	-2,0497	0,088726
Galnt15	-2,0541	0,075781
Prss35	-2,0543	0,047813
Zhx3	-2,0548	0,0025196
Calml4	-2,0567	0,0016172
Ctu1	-2,0583	0,0017156
Pomt2	-2,0591	0,049274
6330408A02Rik	-2,0611	0,041668
Caskin2	-2,0644	0,098717
Zfp324	-2,0647	0,039562
Fbxo46	-2,0662	0,0020991
Pskh1	-2,067	0,0010229
Eng	-2,0682	0,0017409
Serinc5	-2,0685	0,03596
Asah2	-2,0694	0,069991
Sdr42e1	-2,0696	0,010872
Olfm1	-2,0713	3,00E-08
Tlcd2	-2,072	0,10844
Tlr4	-2,0732	0,0020756
Zfp189	-2,0744	0,018521
Apba1	-2,0749	0,13609
Gm37776	-2,0776	0,18759
Tmem185b	-2,0798	0,0010793
Homez	-2,0803	0,13757
Pla2g2d	-2,0816	0,011954
Gm33142	-2,0816	0,1394
Csf2rb	-2,0846	0,00013298
Pomgnt1	-2,0863	0,0018068
Slfn8	-2,0865	0,079402
Il34	-2,0877	0,016458
Tlr13	-2,0894	0,0083787
Snord13	-2,0975	6,97E-06
E430018J23Rik	-2,0998	0,063484

Gm15496	-2,1056	0,035902
Pctp	-2,1061	0,0060925
Zfp35	-2,1065	0,016218
A430033K04Rik	-2,1086	0,038761
Zfp617	-2,1086	0,074813
Nrros	-2,1104	1,78E-06
Ampd3	-2,1113	0,00066834
Gm17455	-2,1116	0,094678
Pter	-2,1154	0,0052513
Frmd4a	-2,1161	2,32E-05
Zfp11	-2,1162	0,15798
Tti1	-2,1178	0,10819
Zfp768	-2,1188	2,62E-05
Trmo	-2,1192	0,044307
Gramd2	-2,1199	0,065941
Ints5	-2,1242	0,00024645
Bcl3	-2,1278	0,0005292
Rorc	-2,1288	0,099197
Qsox2	-2,1292	0,060055
Rcan1	-2,133	8,78E-07
Cyp2u1	-2,1345	0,0057994
Zfp229	-2,1354	0,14514
Gm43112	-2,1375	0,12498
Egr1	-2,1378	0,00049174
Gm43144	-2,1418	0,18333
Fuz	-2,1439	0,060627
St18	-2,1445	7,37E-05
Arhgef19	-2,1446	0,11802
CH25-309J2.1	-2,1457	0,14391
Cxcl2	-2,1467	0,00090622
Tnfrsf4	-2,1494	0,05507
Alg2	-2,1496	0,051743
Acot11	-2,1551	0,14407
Nt5e	-2,1615	0,0015483
Pkd2	-2,162	0,027306
Zfp39	-2,1632	0,12102
Ptpn7	-2,1657	0,00056973
Acy1	-2,1658	0,012562
Tmem67	-2,1748	0,093236
Gpr183	-2,1782	2,32E-05
Mul1	-2,1786	0,001598
3110070M22Rik	-2,1894	0,0714
Tepsin	-2,1924	0,00016884
Zfp810	-2,1928	0,0061032
Zfp729b	-2,1945	0,0039647
Nprl3	-2,1948	0,0055092
Prokr1	-2,1963	0,079196
Gtf2h3	-2,1979	0,040679
RP23-38L16.4	-2,1997	0,050481
Zfp61	-2,2028	0,003349
Mboat1	-2,2048	0,011204
Acsf3	-2,2051	0,081807

Zfp518a	-2,2053	0,0016508
Amigo3	-2,2063	0,069991
Vwf	-2,2083	0,063596
Zfp763	-2,2084	0,016327
Atp6v0d2	-2,2104	4,27E-08
Arg1	-2,2125	0,11858
Cnksr1	-2,218	0,12048
4930461G14Rik	-2,2193	0,062435
Naip2	-2,2251	7,76E-05
Exoc8	-2,2262	0,0072795
Nkrf	-2,2263	0,016682
9130019O22Rik	-2,2276	0,03873
Abhd1	-2,228	0,057268
Gm16740	-2,2298	0,10763
Tagap	-2,2302	0,0013184
Nr0b2	-2,2323	0,076191
Pafah2	-2,2341	0,036739
Gm6257	-2,2412	0,073816
Celf3	-2,2435	0,075781
Sfn	-2,2463	0,097846
Birc3	-2,2471	9,06E-06
Depdc5	-2,2476	0,010616
Trib1	-2,2527	3,05E-05
Gpatch3	-2,2561	0,01054
Vil1	-2,2571	0,058126
Gmppb	-2,2584	0,00111
Mettl18	-2,2604	0,016129
Gm38020	-2,2608	0,03266
Gabrd	-2,267	0,065238
Gstm4	-2,2694	0,049332
B3galt6	-2,2713	0,05249
Zfp119b	-2,2722	0,057785
Nmnat3	-2,2724	0,0063415
Gm42835	-2,2739	0,081149
Tmem260	-2,2755	0,020455
Sema6b	-2,2761	0,05911
Clec2l	-2,2795	0,082981
1810055G02Rik	-2,2888	0,032545
Endov	-2,2895	0,011897
Gpr180	-2,2905	0,028428
Slc35d2	-2,2914	0,0074164
Cog1	-2,2955	0,028776
Mybpc3	-2,2994	0,0050942
Chd5	-2,2994	0,066326
Ifi44	-2,3033	0,11583
Ccdc80	-2,3069	0,0998
Slc9a4	-2,3132	0,10503
Nlrp3	-2,3151	5,00E-05
Adamts4	-2,3156	0,11087
Hebp2	-2,3158	0,0024132
Slc43a2	-2,3164	5,32E-08
Gm42640	-2,3166	0,015968

Slc9b1	-2,3193	0,046587
Zfp93	-2,3204	0,11354
Zfp90	-2,3254	0,0084119
Gm13657	-2,3286	0,059517
Gm10842	-2,3316	0,084152
Nlrc4	-2,3335	0,09143
Kctd21	-2,3362	0,21654
Endog	-2,3389	0,0066466
Cttnbp2nl	-2,3397	5,49E-07
Arhgef18	-2,3457	0,029254
Zfp12	-2,3458	0,0035045
Mtg2	-2,3473	0,0072795
Mdk	-2,3492	0,0084119
Gm43024	-2,3566	0,023291
Gm7815	-2,3575	0,12346
Klhl23	-2,3576	0,14495
Lima1	-2,3584	6,62E-07
Gm42486	-2,3616	0,13719
Tmem98	-2,3643	0,036646
Mlh3	-2,372	0,0015624
Inpp5b	-2,3727	0,0014609
Arhgap4	-2,3764	0,060993
Gm17586	-2,3768	0,036757
Cstad	-2,3769	0,15128
Zc3h4	-2,3771	3,75E-07
Tmem2	-2,3826	7,16E-06
Tlr1	-2,3858	0,0066201
Zfp974	-2,3927	0,01894
Dusp9	-2,3935	0,027995
Gm16712	-2,3992	0,032658
I830077J02Rik	-2,4036	0,037063
Wnk2	-2,4076	0,00023678
C1ra	-2,4152	0,097409
Six5	-2,4167	0,062391
Gm28071	-2,4175	0,08885
Snx19	-2,4208	0,003761
Ubiad1	-2,4214	0,00068792
Mfsd9	-2,4223	0,011466
Slc30a2	-2,4381	0,072401
Oscar	-2,4429	0,02542
Tns4	-2,4461	0,0054043
Srxn1	-2,4483	7,62E-06
Srl	-2,4566	0,02848
Zfp790	-2,4566	0,038293
Sgsh	-2,4619	4,13E-05
Sbk3	-2,4628	0,042296
Slc46a1	-2,466	0,025224
Pars2	-2,4672	0,062963
RP24-325N9.5	-2,4682	0,05173
Zfp738	-2,4684	0,023755
Commd5	-2,4709	0,00063186
Tigd5	-2,4768	0,044996

Prss46	-2,4784	0,045094
Ociad2	-2,4842	0,14291
Skor1	-2,4845	0,045178
Jrk	-2,49	0,04587
Ubxn8	-2,4923	0,0040593
Bdh1	-2,4929	0,012012
Fam83h	-2,4942	0,059765
Jdp2	-2,4946	4,63E-06
Ehd2	-2,5048	0,016043
Gm20712	-2,5055	0,010801
Oas1d	-2,5069	0,028418
Fancf	-2,5139	0,059052
Il20rb	-2,5211	0,0019866
Gm25514	-2,5221	0,013413
Gm37642	-2,5228	0,015868
Nupl2	-2,5248	0,01785
Gm12663	-2,5252	0,062216
Ccdc130	-2,5253	9,92E-05
Swsap1	-2,5278	0,028001
RP23-268C22.3	-2,5303	0,035141
Card6	-2,5397	0,022085
Gipc1	-2,549	0,0018427
D6Wsu163e	-2,5546	0,0018082
Zfp58	-2,5591	0,050553
Nmb	-2,5598	0,02728
Aldh1b1	-2,5611	0,0014466
Limk1	-2,5634	0,010512
Fbxo10	-2,5653	0,055468
Lctl	-2,5654	0,014021
Sh3rf1	-2,5684	0,00040682
Acsbg1	-2,5688	0,0028612
Dmrt2	-2,5692	0,033351
Gm42463	-2,5772	0,036083
Tmem51	-2,5834	0,00061583
Acot6	-2,5862	0,012903
Sec22a	-2,5872	0,0009108
Gm22748	-2,6058	0,0036873
Gm9951	-2,6073	0,016702
Angptl2	-2,6088	1,39E-07
Zfp870	-2,613	0,0025309
Plekhs1	-2,6176	0,023484
Fosl2	-2,6362	1,60E-10
Maml2	-2,6418	0,0040324
3110082I17Rik	-2,648	0,0010047
Cass4	-2,6513	0,010104
Tspoap1	-2,6539	0,0063448
Dcp1b	-2,6562	0,05214
Irgm2	-2,6667	0,039595
Gm43350	-2,6684	0,021084
Lpin3	-2,6687	7,49E-05
Rpl7l1-ps1	-2,6754	0,019335
F630040K05Rik	-2,6796	0,0099732

Zfp658	-2,6877	0,024017
Erbb3	-2,6923	0,0095732
Gm4262	-2,6944	0,020701
Col7a1	-2,7072	0,024426
BC024978	-2,7169	0,011765
Fut7	-2,7192	0,02685
Numbl	-2,7217	0,00036795
Lrrc14	-2,7345	0,00061557
Gm13205	-2,7404	0,028476
Fam222b	-2,7452	0,00020766
Hdhd3	-2,7464	0,0089466
Tctn2	-2,7517	0,029837
Zfp3	-2,7657	0,0020277
Dnmt3b	-2,7753	0,0052739
Zfp94	-2,7794	0,0079885
Elmod3	-2,7805	0,011818
Ctla2b	-2,7846	0,01924
AW047730	-2,7884	0,022161
Zfp951	-2,8024	0,020898
Ubox5	-2,8091	0,020898
Rab11fip4	-2,818	0,026249
Fmo5	-2,8287	0,021338
Rinl	-2,8296	0,00020244
D17H6S53E	-2,8335	0,0069832
Gm37121	-2,8919	0,0013122
Zfp862-ps	-2,9087	0,0022986
Zfp28	-2,9164	0,025944
Ifi47	-2,9366	0,06188
2010008C14Rik	-2,9422	0,016068
Lpar1	-2,9474	0,011069
Src	-2,9544	5,48E-05
Zfp408	-2,9662	0,00028674
Gm5532	-2,9679	0,015519
Particl	-2,9807	0,0063448
Rgs8	-2,9916	0,021375
Fastkd5	-2,9951	0,0055366
Spred1	-2,9987	1,77E-09
Rgs20	-3,0008	0,0063448
Scn11a	-3,0238	0,0021653
Slc10a3	-3,0338	0,00079056
Csf2rb2	-3,0442	0,0061809
Gm43154	-3,0485	0,014649
Celsr1	-3,0495	0,014071
Ppp1r10	-3,0832	1,08E-08
Mir763	-3,1182	0,0069682
Nedd9	-3,1558	0,011298
Accsl	-3,1834	0,0012413
Hoxb3	-3,204	0,0037915
RP24-175C20.18	-3,2082	0,003761
Ip6k3	-3,2123	3,51E-05
Pus7l	-3,2276	0,004387
Wdr35	-3,2282	0,0040542

Gper1	-3,2283	0,0093926
Adamts7	-3,2386	0,00022106
Zfp27	-3,2427	0,0047919
Gm19026	-3,258	0,0075205
Gramd1c	-3,2605	0,0092359
Pxdn	-3,264	0,00049873
Pdpn	-3,2776	0,00031149
Sla	-3,2982	4,44E-07
Zfp40	-3,3149	0,0017008
Zfp719	-3,3481	0,0022584
Oasl1	-3,364	0,0011139
Gdpgp1	-3,4111	0,0063448
Zfp111	-3,424	0,0019607
Shisa3	-3,4246	0,015066
Ablim1	-3,4519	0,0010457
Zfp691	-3,463	0,00076539
9930014A18Rik	-3,4781	0,0015855
Olr1	-3,4821	0,0007818
Gm22	-3,4873	0,0020443
Acp5	-3,5701	1,18E-10
Slc6a4	-3,594	0,00077723
Rbak	-3,6639	0,00061557
Ap5b1	-3,6657	0,00076539
Slc1a4	-3,7009	0,0032062
Ppp1r26	-3,7031	0,010659
Filip1l	-3,7136	0,0003536
Krcc1	-3,7299	0,0097336
Zfp41	-3,7487	0,0015913
Met	-3,7528	0,00038059
Rap1gap	-3,7924	0,00083436
Gm20219	-3,8091	0,0013254
Gm20632	-3,871	0,00032617
Epb41l1	-3,9032	0,0004244
Usp27x	-3,9127	0,014332
Wisp1	-3,9597	0,0028002
Chac1	-3,9741	0,0022118
Mras	-4,0627	0,00043777
Zbtb45	-4,1862	0,00054555
Col27a1	-4,4785	0,00010125
Tmem204	-4,5627	1,77E-05
Rab15	-4,607	0,00015403
Vegfc	-4,6292	0,00068267
Ctsk	-4,8202	7,38E-11
Acod1	-5,3988	2,31E-05
Slc9b2	-7,7212	2,59E-07
