# Supplementary material for: Bone, dentin and cementum differentially influence the differentiation of osteoclast-like cells
Source: Sci Rep. 2025 Jun 5;15:19857. doi: 10.1038/s41598-025-04874-9 (PMC12141432; doi:10.1038/s41598-025-04874-9)
Supplement: Supplementary file 9 — Supplementary Information 9. [file 41598_2025_4874_MOESM9_ESM.pdf]

Tab. S8:
Transcripts induced in murine macrophage cells stimulated on dentin (n=6), fold of bone

gene name	regulation of expression	adj.P.Val
Slc9b2	13,69236736	0,14278
Olr1	9,370330929	0,30584
Il13ra2	8,003327798	0,57294
Gm26870	7,540708078	1
Mast4	7,242561349	1
Plscr4	7,087122569	1
Adamtsl1	6,312449758	1
Gm10800	6,245858996	1
RP23-308G10.5	5,853096238	1
Tnfsf8	5,523566123	1
Gm3695	5,371406848	0,97422
Gm7132	5,307380562	1
Gm4034	5,268163058	0,57294
Klhl40	4,911283148	1
Pla1a	4,734460377	1
Gm10180	4,585566579	1
Gm10801	4,454612687	1
Gm18969	4,428444383	1
Sult2b1	4,375051502	1
Olfr95	4,345131985	1
H2-DMb2	4,272557773	0,97113
Rrh	4,239811134	1
Olfr912	4,19595731	1
Rab42	4,088296981	1
1600014C23Rik	4,083765428	1
Apol11b	4,077542743	1
Gm12466	4,042926568	1
RP23-134M7.3	4,026984725	1
Arhgap27os1	4,016948597	1
4933412L11Rik	4,01555667	1
Ccdc92b	3,992521011	1
Pou4f1	3,871519745	1
Ctsk	3,82272124	9,65E-05
Gm43447	3,805800665	1
Slc47a2	3,766958136	1
Lgi4	3,750284386	1
RP24-365A12.2	3,731355972	1
Gm9381	3,705581613	1
Gm29462	3,694809544	1
Ccdc80	3,678455147	1
B230377A18Rik	3,629573421	1
Gm42548	3,628315724	1
Ceacam10	3,614008752	1
Tubb2b	3,609002143	1
Gm3699	3,571425433	1
Gm12182	3,560549723	1
9130230N09Rik	3,533260206	1
Palld	3,527876359	1

Dhrs11	3,507881469	1
Gm11895	3,480995623	1
Gm37108	3,463186304	1
Gm20223	3,427365378	1
Gapdh-ps14	3,425940273	1
Zfa-ps	3,423091841	1
Gm8919	3,416217903	1
Cd200r3	3,406995401	1
Gm8973	3,371288285	1
Chrna1os	3,360090289	1
Gm4742	3,298476897	1
Gm11222	3,281373916	1
Sorl1	3,277736773	1
RP24-82M14.1	3,273876726	1
Gm10717	3,25644983	1
Zfp882	3,211172129	1
Tigd5	3,187664954	1
Rps13-ps7	3,164549205	1
Gm45629	3,158194449	1
Gm8927	3,156443659	1
Chchd10	3,145087162	1
5031425F14Rik	3,134857794	1
Gm6322	3,128996403	1
Tnnc2	3,112556445	1
Tmco4	3,089128747	1
Tnfrsf14	3,08121638	1
Ms4a6b	3,078654562	1
Gm17100	3,073537314	1
Inhbe	3,066940122	1
Lpar1	3,063115994	1
Rpl26-ps4	3,050191876	1
Gm13443	3,028282927	1
Runx2os1	3,01822425	1
Cd200r2	3,003198844	0,30584
Oas1d	2,980800869	1
Ccdc63	2,97729051	1
Pdcd1	2,971517753	1
Gm17430	2,960826596	1
Gm20302	2,959800629	1
Amigo1	2,942413492	1
Cspg4	2,929592494	1
F830208F22Rik	2,91905218	1
Gm9711	2,904721808	1
Tsku	2,891463719	1
Abca5	2,891062905	1
Olfml3	2,884457487	1
Efemp2	2,880661215	1
Gm14248	2,875474413	1
Gm12468	2,856997948	1
Arhgef15	2,855414131	1
Gm15610	2,841593132	1
Bcl2a1d	2,829015341	1

Gm7799	2,827447036	1
Ccl6	2,826859146	1
Slc9b1	2,825879602	1
Lin28b	2,802277834	1
Gm7722	2,79820178	1
Gm43878	2,793744333	1
Gm7936	2,793550692	1
Psca	2,786395407	1
Gm28686	2,77041096	1
Gm7436	2,763889619	1
Gm10029	2,760826066	1
Usp27x	2,757383628	1
Gm8566	2,742706032	1
Murc	2,74061562	1
Gm13022	2,740425662	1
Tmem267	2,728673918	1
Pxylp1	2,71998746	1
Meg3	2,709637766	1
Gm6293	2,703072111	1
Gm45454	2,681611052	1
Gm8181	2,679567205	1
Kyat3	2,672703872	1
Pgap3	2,671777744	1
Gm14240	2,671222221	1
RP24-547N4.5	2,669001285	1
Gm44775	2,664010927	1
Card9	2,658477028	1
Hist1h2bg	2,652954624	1
Mroh2a	2,651667715	1
Gm10313	2,651116373	1
Slamf9	2,636821663	1
Rhoh	2,632986273	1
Adgb	2,630250125	1
Rps3a2	2,628792009	1
2610306M01Rik	2,615342697	1
Gm9332	2,600339511	1
Aarsd1	2,593139825	1
Gm14056	2,592061593	1
Cdc34b	2,587753145	1
Rpsa-ps9	2,583989128	1
4930556M19Rik	2,583272793	1
Tmem37	2,579515293	1
Gm19026	2,564717636	1
Gm16106	2,564184373	1
Calr3	2,561874849	1
D630029K05Rik	2,559744828	1
Ubox5	2,547354951	1
RP23-110E20.5	2,533795341	1
Gm5873	2,530811401	1
Gm9796	2,529233091	1
Ppp1r26	2,527130209	1
Zfp14	2,524679056	1

Gm5778	2,524504064	1
Acad12	2,521880649	1
Gm9517	2,520657322	1
Xk	2,516816441	1
Ociad2	2,51124015	1
H2-Ab1	2,509848008	1
Gm12529	2,5058499	1
Gm5764	2,499258278	1
Gm13675	2,498045925	1
Fabp7	2,494066616	1
Pla2g2d	2,491820247	1
Gm8894	2,491820247	1
Igf2bp1	2,480962622	1
Gm26569	2,477869142	1
Rhox5	2,473407591	1
Gm45456	2,465021107	1
Unc13b	2,464850251	1
1700123M08Rik	2,461776861	1
Gm33370	2,451729815	1
Faah	2,449012266	1
Gm13935	2,447654622	1
Gm5117	2,444433233	1
Foxd2os	2,438848261	1
Calml4	2,436313857	1
RP24-370M23.1	2,429568317	1
Gm7965	2,426875333	1
Mras	2,42670712	1
Gm13622	2,425193734	1
Fcnaos	2,419988178	1
Soat2	2,418478982	1
Chtf18	2,413789721	1
Gm17150	2,411949999	1
Uggt2	2,411782822	1
Gm5871	2,406272449	1
Gm29243	2,401606855	1
Gm13360	2,397448791	1
Gm12669	2,397116457	1
Bok	2,393297926	1
Zfp54	2,391639594	1
Gm5251	2,390810858	1
Fam71e1	2,390810858	1
Gm44434	2,389816754	1
Rnf135	2,388491926	1
Gm9246	2,377095723	1
Nt5c3b	2,375448616	1
Gm14048	2,373802651	1
Rorc	2,3690358	1
Myh11	2,36805075	1
Reps2	2,358549697	1
Notch4	2,358059301	1
Plat	2,356425385	1
Tmcc3	2,349412438	1

Gm29487	2,343719636	1
Gm17060	2,34095954	1
Gm9575	2,340310578	1
Gm16418	2,338040628	1
Ccr1	2,320764287	1
Fhit	2,320120924	1
Gm15484	2,315140902	1
Sfn	2,314178262	1
Gm7730	2,310972352	1
5430421F17Rik	2,310331704	1
Gm5544	2,309851334	1
Gm12430	2,305692308	1
Il1rl1	2,304893355	1
RP24-550H10.6	2,302498155	1
Gm13461	2,300743257	1
Zfp532	2,293578045	1
Gm7867	2,293101159	1
Il7r	2,286910649	1
Il20rb	2,285642868	1
Gm13226	2,285167632	1
Ppcs	2,285009241	1
Gm5905	2,281843741	1
Gja1	2,274106785	1
Dennd5b	2,273318776	1
Pafah1b1-ps2	2,273003649	1
Gm8724	2,269382826	1
Gm28071	2,267338826	1
Gm5449	2,26545369	1
Slc13a2	2,264040867	1
Gm26800	2,256207881	1
Acot8	2,255269746	1
Slc1a4	2,251989343	1
Gm42515	2,251052961	1
1810024B03Rik	2,248869585	1
E230016M11Rik	2,248713711	1
2310074N15Rik	2,245598492	1
E430018J23Rik	2,243575914	1
Atp6v0d2	2,241244434	0,092566
Gm8168	2,240001968	1
Als2cr12	2,239070571	1
Saa3	2,237674202	1
Slc36a3os	2,23225215	1
Gm21057	2,232097428	1
Ctla2b	2,231478645	1
RP23-164P21.3	2,230396187	1
Gm8494	2,22499177	1
RP23-136K21.4	2,223295946	1
Gm11918	2,222987753	1
Rps19-ps8	2,221601413	1
Pcdhgc4	2,219908172	1
Gm43655	2,219292768	1
Gemin4	2,212994706	1

Upp2	2,210541777	1
Gm42970	2,208244625	1
2310043L19Rik	2,201825292	1
Tmem71	2,197556107	1
Slc4a8	2,196185628	1
RP24-91J7.1	2,195728991	1
1810044D09Rik	2,194968141	1
Gm14794	2,194968141	1
Gm24507	2,194968141	1
A330074K22Rik	2,194359651	1
Gm10260	2,18980135	1
Gm14336	2,189346041	1
Cdk2ap1	2,188739109	1
Rsph1	2,186313065	1
Gm7815	2,186009999	1
Adora2b	2,184041091	1
C630043F03Rik	2,182376478	1
Zfp119b	2,181469042	1
AK157302	2,177088417	1
Tiam2	2,173771053	1
Gm11464	2,173620384	1
Gm5644	2,171963713	1
Bdh2	2,167602175	0,57294
Pou6f2	2,166851072	1
Gm12990	2,166700882	1
Dntt	2,163699278	1
Gm11346	2,162649699	1
9530053A07Rik	2,160552069	1
K230015D01Rik	2,160402316	1
Gm36445	2,160102841	1
4930556M19Rik	2,160102841	1
Gm15824	2,158755719	1
Gm7847	2,158157268	1
Zfp229	2,156811359	1
Lta	2,15636291	1
Gm28731	2,15531689	1
Spef1	2,154570043	1
Gm44187	2,153524893	1
Gm2076	2,150243434	1
Gm13453	2,149945368	1
4831440E17Rik	2,149200383	1
Gad2	2,14860458	1
Gm12751	2,14786006	1
Gm7079	2,14369551	1
6230400D17Rik	2,139835658	1
Rab11fip4	2,135390618	1
Gm8885	2,134354771	1
Gm12350	2,131102523	1
A230028O05Rik	2,123582268	1
Iqsec3	2,122846418	1
Spata5l1	2,122552149	1
Mrap	2,11755582	1

Pomc	2,117262284	1
Gm16104	2,117262284	1
Gm13822	2,11608855	1
Mir6236	2,115062067	1
Met	2,11418262	1
Nudt22	2,113743034	1
Gm4117	2,113450027	1
Itgax	2,11169284	0,97113
Zfp786	2,107744508	1
Srd5a1	2,10336613	1
Gm15163	2,098705885	1
Nt5m	2,096815611	1
1700034H15Rik	2,096234331	1
Gm13604	2,094346283	1
Ccdc17	2,092750033	1
Gm20156	2,08956118	1
Gm15032	2,089416348	1
Morn2	2,088692337	1
Gm42567	2,08478701	1
Aim1l	2,083920152	1
Rnf31	2,078870658	1
Gm8250	2,078726566	1
Gm4217	2,069955854	1
Rgs8	2,069668918	1
Fhod3	2,068808345	1
Gm24890	2,068091475	1
Gm8129	2,067948131	1
Proser3	2,064224667	1
Slc13a3	2,062937338	1
Gm12312	2,06179372	1
Slc39a4	2,060079481	1
Zfp790	2,059936692	1
Ptafr	2,058652038	1
Acap1	2,056512727	1
Gm44791	2,055942619	1
Gm7160	2,05366377	1
RP23-261O3.5	2,053521426	1
Gm13771	2,052525293	1
Psmb6-ps2	2,05081876	1
Fcor	2,044715327	1
Rps19-ps9	2,040609318	1
Ifi211	2,04004362	1
Socs2	2,03990222	1
Rilp	2,039760829	1
Gm5262	2,039760829	1
Gm13421	2,037499916	1
Decr2	2,03566477	1
Gm5523	2,035100442	1
Gm16124	2,034959384	1
Vmac	2,03453627	1
Ksr1	2,033549347	1
Jup	2,033267456	1

Mllt1	2,02791896	1
Gm12267	2,02791896	1
RP23-187B11.16	2,026654272	1
Traf2	2,026373338	1
F2	2,025390374	1
Timp2	2,022444342	1
Gm14130	2,020202623	1
Setdb2	2,019642581	1
Slc24a5	2,019502595	1
Gm43578	2,018103268	1
Nos1	2,017124315	1
Slc9a3r1	2,016565127	1
Ppm1e	2,013352803	1
Cish	2,013352803	1
Ube2v1	2,011957737	1
Gm13657	2,01042428	1
Fry	2,010006268	1
Gm43569	2,007360873	1
Fam109a	2,00416321	1
Gm14138	2,004024297	1
5430403G16Rik	2,00319102	1
Mdrl	2,002496887	1
Gm11652	1,998475657	1
Rps15a-ps3	1,998337138	1
Zbtb32	1,997921639	1
Gm5566	1,996537265	1
RP24-401G4.1	1,99612214	1
Gm12854	1,995153851	1
Lat	1,993633202	1
Rpl5-ps1	1,992389896	1
Kazald1	1,992251799	1
Rfxank	1,992251799	1
Gm9497	1,988664631	1
Gm27219	1,988526792	1
Mzf1	1,987148928	1
Taco1os	1,986598049	1
Gm8254	1,985909666	1
Gm4924	1,98563438	1
Rps23-ps2	1,985496751	1
D3Ertd751e	1,985496751	1
Cacnb1	1,985221522	1
RP23-277D1.1	1,985083921	1
Rcan1	1,984946331	0,86146
Naa10	1,984120986	1
Gm9333	1,983570947	1
Gm15853	1,983021061	1
Gm27046	1,980136657	1
Epb41l1	1,978901769	1
Jrk	1,978490311	1
Abhd3	1,977667651	1
Gm19566	1,975338638	1
Gm8185	1,974654157	1

Gm12583	1,974517289	1
Bcl2l14	1,974106742	1
Gm15393	1,970005968	1
Acp5	1,969596359	1
Treml4	1,969459842	1
Gm14017	1,968777397	1
Gpatch3	1,967413218	1
Poli	1,96536872	1
Smco3	1,96482388	1
C1rb	1,964006902	1
Tmem202	1,962782073	1
Gm12454	1,962782073	1
Gm8172	1,960742389	1
Gm16238	1,952469484	1
Gm23751	1,952469484	1
D330023K18Rik	1,951387105	1
Gm13998	1,951387105	1
Homez	1,950981368	1
Gm12059	1,950440516	1
Nudc-ps1	1,947873516	1
Gm44557	1,946658748	1
Zfyve19	1,946119095	1
Cox6a2	1,946119095	1
Llph-ps1	1,943961976	1
Cdh23	1,943827235	1
Zfp738	1,94342307	1
Hras	1,943288367	1
Prokr1	1,940999841	1
8430429K09Rik	1,939654909	1
Gm8357	1,937236378	1
Gm36936	1,935759872	1
Cxxc5	1,935357384	1
Rpsa-ps4	1,934820864	1
Wdr5b	1,934686757	1
Slc35a2	1,934016362	1
Usp11	1,933614236	1
Pmepa1	1,931604862	1
Clec4a2	1,930935535	1
Gm12151	1,929330096	1
Gm15596	1,926523788	1
Gm43273	1,924521782	1
Gm45546	1,923454897	1
Ccdc33	1,923321577	1
Rpl27a-ps1	1,92145608	1
Mrm2	1,91945934	1
Pars2	1,918129333	1
RP24-232D3.1	1,917597589	1
Vegfc	1,915737643	1
2700046G09Rik	1,914410215	1
S100a13	1,914277523	1
Prr3	1,911890635	1
RP23-349H12.3	1,910565873	1

Coro1a	1,909639085	1
Mtfp1	1,909639085	1
4933437G19Rik	1,907125785	1
Gm5614	1,905804324	1
Ankrd35	1,905672228	1
Gm26530	1,905672228	1
6430571L13Rik	1,905540142	1
Gm11410	1,905408064	1
Poln	1,905275996	1
Pbx1	1,904747815	1
Gm9294	1,904483779	1
Etohd2	1,904483779	1
Angptl2	1,90421978	1
Gm8550	1,903032236	1
Oip5	1,900000766	1
Trp53rka	1,898026322	1
Commd5	1,897894766	1
Gm13612	1,89750015	1
Hexim2	1,89736863	1
Gm8806	1,897237119	1
Dpp3	1,896974125	1
Gm5446	1,896711166	1
Gm2467	1,895396922	1
Zfp84	1,895265548	1
Gm15459	1,894346182	1
Cd5l	1,890280064	1
Gm4468	1,888315722	1
Gm37776	1,887138097	1
Gngt2	1,885830485	1
A730062M13Rik	1,885830485	1
Hspb7	1,882826417	1
Sdf2l1	1,881391381	1
Rab15	1,879957438	1
Dnali1	1,879957438	1
Polr2k	1,878915257	1
Gm16712	1,878524589	1
Gm17786	1,877873654	1
Dok4	1,874232589	1
Gcat	1,872155151	1
Spats1	1,871506424	1
Gm44884	1,870598583	1
Gm12848	1,870339281	1
Gm12791	1,869302432	1
Rpl29	1,868395661	1
Gm15772	1,868136664	1
Gm5050	1,867877704	1
Itpa	1,867748237	1
Gm8719	1,866324693	1
Gm45050	1,865807309	1
Gkap1	1,865677986	1
Arl4c	1,864772973	1
Gm6210	1,864643721	1

Acvr2a	1,864514478	1
Fign	1,864514478	1
C330013E15Rik	1,861286319	1
Gm16062	1,861157309	1
Lamb2	1,859480991	1
Hist1h2al	1,853561521	1
Carnmt1	1,853176124	1
BC002163	1,852533973	1
Gm44423	1,850737135	1
Ring1	1,850480586	1
Zfp85	1,849326556	1
Gm43137	1,846380637	1
Mfsd13b	1,845868783	1
RP24-550H10.4	1,843183874	1
Nlrp10	1,841779052	1
Ndufab1-ps	1,839355063	1
Cox7a1	1,838462819	1
Gm45250	1,837698383	1
Gm45718	1,837061596	1
1700003G18Rik	1,835788683	1
Gm7895	1,835534206	1
Gm12577	1,835152557	1
Dixdc1	1,833880967	1
Stk25	1,832229217	1
Gm10080	1,8303252	1
Gm5575	1,829437336	1
Galnt10	1,82918374	1
Gm15564	1,827029591	1
Mrps36-ps1	1,826776328	1
Hist1h4a	1,826776328	1
Gm43154	1,826143325	1
Kifc2	1,825890186	1
Islr2	1,825637081	1
Gm7990	1,824751491	1
Gm5590	1,824372084	1
Pop5	1,822855245	1
E130201H02Rik	1,822728899	1
Dnajc17	1,821718444	1
Il1b	1,820329982	1
Shox2	1,81856438	1
Psmb7	1,81831229	1
Thap7	1,817556233	1
Gm26631	1,817430254	1
Mrps35	1,817178322	1
Rpl19-ps9	1,81680049	1
Gm10642	1,816170944	1
Gm6155	1,815541616	1
Ddr2	1,811770226	1
Gba2	1,811142423	1
Particl	1,810389347	1
Gm42972	1,808382673	1
Rpl36-ps3	1,807756044	1

Pxdn	1,807129632	1
Nadsyn1	1,806378225	1
Gm33080	1,805001455	1
Nt5e	1,804876346	1
Gm42786	1,804250932	1
Gm37125	1,803625735	1
Mmab	1,803125733	1
Fcgr2b	1,802750822	1
F10	1,802625869	1
AI839979	1,802251063	1
Abcd2	1,802001235	1
Gm8242	1,798881309	1
Zfp759	1,798008698	1
Celf6	1,796389258	1
Tuba1a	1,795766784	1
Tmed3	1,795393404	1
Spsb3	1,794646876	1
Ndufs5	1,793651989	1
Apbb1	1,793154752	1
Rps10-ps4	1,792781914	1
BC060293	1,79178806	1
Gpat3	1,791539683	1
Gm9392	1,791167182	1
Zfp9	1,790546518	1
Nudt6	1,789429865	1
Gm14173	1,789057802	1
Gm26606	1,788933799	1
Gm2199	1,788313909	1
Zfp541	1,787818152	1
Gm20056	1,787570325	1
Ssc5d	1,787570325	1
Nme4	1,787074774	1
Acy3	1,787074774	1
Mydgf	1,78657936	1
9330162G02Rik	1,78657936	1
Fam213b	1,783238908	1
Lrig3	1,782003289	1
Pdhx	1,780521677	1
Sec14l2	1,780151467	1
4930427A07Rik	1,779041297	1
Ube2m	1,778671394	1
Lum	1,774853566	1
Gm42835	1,77436154	1
Gm15877	1,773500823	1
mt-Tt	1,772517658	1
Fam83h	1,772394801	1
Gm9409	1,768590445	1
Cryga	1,768100156	1
1700037C18Rik	1,768100156	1
RP23-278O17.1	1,767855062	1
Gm26881	1,766385213	1
D230017M19Rik	1,766140357	1

Osbp2	1,765895535	1
Rpl31-ps11	1,764916586	1
Gm7128	1,763326951	1
Gm8317	1,763204731	1
Gm13445	1,762960316	1
Gm19196	1,762838121	1
Il4ra	1,762227274	1
Slc8a1	1,761982994	1
Gm9025	1,761738748	1
Gm12389	1,761128282	1
Mrps22	1,760273984	1
Gm43110	1,758566632	1
D17H6S53E	1,758444742	1
Gm11764	1,756739164	1
Ifi203-ps	1,756739164	1
Ank3	1,75613043	1
Rpsa-ps2	1,755765291	1
H6pd	1,755521907	1
2900009J06Rik	1,755278557	1
Gm8818	1,755156895	1
Adcy2	1,754427097	1
Orai3	1,753211443	1
Gm6682	1,753089923	1
Rps12-ps19	1,75284691	1
Gm14681	1,752725417	1
Gm9347	1,752725417	1
Gm14040	1,752603931	1
Fbxo15	1,751875195	1
Tor4a	1,751510941	1
Bcl2l12	1,750661309	1
Smim3	1,750418632	1
Procr	1,748842056	1
Gm37486	1,748720839	1
Commd9	1,748478432	1
Trpm1	1,748478432	1
Gm6272	1,748357241	1
Mcat	1,748236058	1
Gm28557	1,747509138	1
Ebp	1,746298277	1
Rhob	1,746177237	1
Tmem158	1,745572163	1
Cyb561d2	1,744967299	1
Rps15a-ps5	1,743999952	1
Wdr78	1,743637335	1
Speg	1,743274793	1
Ccdc107	1,742187621	1
Panx1	1,740256544	1
Ticrr	1,74001531	1
Zfp93	1,739532942	1
1700084J12Rik	1,739532942	1
C030014I23Rik	1,739171254	1
Gm20219	1,738327608	1

Gm5239	1,738086641	1
Sac3d1	1,73796617	1
Tbx15	1,737123108	1
Ttc39a	1,736521171	1
Gm37234	1,736400809	1
Acot11	1,73616011	1
Nr0b2	1,735558507	1
1700112E06Rik	1,734957112	1
Nmi	1,734716613	1
Bcl2a1b	1,73411551	1
RP23-88C11.5	1,733875127	1
F9	1,733514615	1
Pck2	1,73303405	1
Pddc1	1,73303405	1
Dlg3	1,732673713	1
2810405F17Rik	1,732673713	1
Gm12222	1,732553617	1
3110082I17Rik	1,73243353	1
Rassf7	1,732073318	1
Gm14277	1,732073318	1
Gm14292	1,731473131	1
Gm11273	1,731113119	1
Il18rap	1,730873152	1
Gm15420	1,730633219	1
B3galt4	1,730033531	1
Hyal3	1,729673818	1
Nkain1	1,728954616	1
Nme6	1,72835551	1
Msantd3	1,727756612	1
Haus7	1,727756612	1
Clec3b	1,727636857	1
9330162012Rik	1,727277642	1
Fam219a	1,727038207	1
RP23-47A1.1	1,726798806	1
Eif2s3x	1,726559437	1
Gm8659	1,726439765	1
Gm10736	1,726320102	1
Acaa1b	1,725721908	1
H2-Oa	1,723809082	1
Spsb2	1,723570128	1
Wdr53	1,723331207	1
Ccdc166	1,723092319	1
Gm12933	1,721301716	1
Gstm4	1,720943818	1
A430105J06Rik	1,720347488	1
Gm9403	1,719632164	1
Gm24601	1,719632164	1
Eif1-ps1	1,719036288	1
5430402O13Rik	1,71832151	1
Tlcd2	1,718083316	1
D10Jhu81e	1,716654849	1
Tmem44	1,716654849	1

Cpeb1	1,716297918	1
RP24-282C4.3	1,715822125	1
Gm12020	1,715703197	1
Lin37	1,715346464	1
Zfp90	1,715227569	1
Gabrd	1,71463322	1
Ffar4	1,714514375	1
Hist1h3d	1,714276709	1
Gm6181	1,713801476	1
Leng9	1,712970136	1
Gm45733	1,712495266	1
Gm13889	1,710834256	1
Gm11281	1,710715675	1
Oscp1	1,710478535	1
Gpx4	1,709767315	1
Mrpl54	1,709530308	1
Gm9173	1,708937932	1
Tuba1c	1,708582606	1
Gm4032	1,708582606	1
Gm6088	1,706925391	1
Mocs3	1,703497701	1
Tstd3	1,703261562	1
Pgm5	1,703261562	1
2610044O15Rik8	1,702671359	1
Gm11334	1,70208136	1
Zbtb39	1,70208136	1
Gm29155	1,70208136	1
Gm10146	1,701609508	1
Pitpnm2	1,699959057	1
Dhrs4	1,699841229	1
Gm12254	1,699723409	1
Sec61g	1,699487794	1
Rpl19-ps1	1,698663397	1
Gm17807	1,698074787	1
Tnfrsf18	1,6978394	1
Apip	1,696075039	1
Gm2810	1,69595748	1
Lcmt2	1,695722387	1
Gm13433	1,69466487	1
Irgm1	1,693490625	1
Serinc5	1,693021155	1
Rn7s6	1,692551814	1
Gm6525	1,6924345	1
RP24-316F13.7	1,691613525	1
Ppil1	1,689621385	1
Txnl4a	1,688918838	1
Gm38213	1,688801775	1
Tnfaip8l2	1,687865564	1
Lyz1	1,687163746	1
Fndc7	1,687163746	1
Nedd9	1,686111567	1
Sppl2b	1,685410479	1

S100a1	1,684359393	1
Aldh5a1	1,684359393	1
Gm6542	1,684009177	1
Epop	1,682958965	1
Gm29593	1,682958965	1
Oxld1	1,682725673	1
Rnaseh2c	1,681210067	1
Gm2223	1,6801616	1
Gm5599	1,679695825	1
Fadd	1,679579401	1
Gm45113	1,678066629	1
Gm3608	1,678066629	1
Zfp28	1,677834015	1
Gm43378	1,677485156	1
Abhd17c	1,677252623	1
Mrpl12	1,677020123	1
Tnfrsf9	1,676787654	1
Rpl36	1,676555218	1
Psmb5	1,676555218	1
Dmrta2	1,675741946	1
Gm10327	1,675277396	1
Mir5128	1,675161279	1
Hps6	1,675045169	1
4930447F24Rik	1,673768498	1
Cenpp	1,673652485	1
Gm43351	1,673304495	1
Gm6733	1,672376875	1
Gm12013	1,672260959	1
Ap2a1	1,671681498	1
Gm4342	1,671681498	1
A930006K02Rik	1,670523178	1
Rps12-ps24	1,669018562	1
Gm4994	1,669018562	1
Mblac1	1,668671536	1
Gm37747	1,668555876	1
Dcstamp	1,668093318	1
Stard3nl	1,667515302	1
Gm11964	1,667053032	1
A430018G15Rik	1,666013395	1
Sec22a	1,665205236	1
Greb1	1,664397469	1
Gm13712	1,664282106	1
Galnt15	1,663820733	1
Gm6863	1,66370541	1
2700062C07Rik	1,663359488	1
Csf2rb2	1,662091723	1
Serpinf2	1,661746137	1
Rpl35a-ps5	1,661630957	1
Malsu1	1,660249426	1
Fzd9	1,659559091	1
Selenoh	1,659444064	1
Gm6290	1,659329044	1

Gm7535	1,659099028	1
Taf7	1,658984032	1
Sdr42e1	1,658639092	1
Gm27043	1,658524128	1
Ndufa4	1,658064351	1
Gipc1	1,657719602	1
Macrod2	1,656685785	1
Akr1b7	1,656685785	1
Cox5b	1,656456135	1
Gm16585	1,656456135	1
Ankrd54	1,654620085	1
Sars	1,654046737	1
Gm5909	1,653817453	1
Pxmp2	1,652327884	1
Hist1h2be	1,652213357	1
Tcta	1,651869825	1
Gm6851	1,651869825	1
RP24-499N24.6	1,65175533	1
RP23-316F10.2	1,651640843	1
Coa4	1,650038858	1
Amdhd2	1,649467097	1
Gm10250	1,649352769	1
Chmp2a	1,64832417	1
Tcea1-ps1	1,647296212	1
Rnf121	1,647067865	1
Gm14830	1,646725402	1
Gm10399	1,646725402	1
Gm43379	1,646611264	1
Fam187b	1,646040691	1
Rps9	1,6459266	1
Rpl31-ps14	1,645356264	1
3830408C21Rik	1,645356264	1
Cldn15	1,644900137	1
4921524J17Rik	1,644786126	1
Zfp553	1,643646442	1
Gm6457	1,643532517	1
Hoxa7	1,642393702	1
Gm37470	1,641369443	1
Gm6415	1,639663764	1
Zfp938	1,639209215	1
Xrra1	1,639209215	1
Kif5c	1,639209215	1
Gm6808	1,638073396	1
Gm16177	1,636257721	1
Wdyhv1	1,635690736	1
Uqcc2	1,635350639	1
Prkar1b	1,635350639	1
Cmss1	1,634897286	1
Zfp428	1,634670657	1
Tnni3	1,634444059	1
Syf2	1,632858754	1
Cxcl14	1,632066678	1

Kif21a	1,632066678	1
Kdelr3	1,631840441	1
Sult6b1	1,631614236	1
Cd200r1	1,630709728	1
Gm15131	1,630483679	1
Rps6-ps1	1,630370667	1
Kbtbd3	1,629466848	1
Tmem219	1,629240971	1
Tuba4a	1,62856353	1
Gm3375	1,628224915	1
Gm11478	1,627322285	1
Paqr4	1,627322285	1
Mrpl20	1,627096706	1
2810025M15Rik	1,626758396	1
Gm5869	1,625293199	1
Leng1	1,624730013	1
Snhg18	1,624279604	1
Cdhr4	1,62394188	1
Gm5881	1,62349169	1
Gpr162	1,623379162	1
Mief2	1,622254311	1
Inca1	1,620231543	1
Txnrd3	1,619445593	1
Tmem204	1,619108874	1
Mustn1	1,616081549	1
Slc52a2	1,615521555	1
Naca	1,615297612	1
Gm11131	1,615297612	1
Gm15185	1,615297612	1
Atp5h	1,614625968	1
Dgcr6	1,614066478	1
Etfbkmt	1,614066478	1
Fbxl8	1,613283518	1
5830487J09Rik	1,612277414	1
Aplf	1,61205392	1
Prcc	1,611160255	1
Gm13270	1,611048582	1
Gm9803	1,610155475	1
Cpt1c	1,609932275	1
Cebpzos	1,609820687	1
Tmem170	1,609374412	1
Cdc34	1,609262862	1
Gm2950	1,608816742	1
Gm12696	1,608259265	1
Nipal3	1,607590548	1
Gm8731	1,607479122	1
Mea1	1,607479122	1
Nat8f1	1,607367704	1
Mrps18c	1,607144891	1
Gm9568	1,607144891	1
Ccdc85b	1,606922109	1
Gm13450	1,605919971	1

Arl11	1,605586064	1
Ap4m1	1,605474777	1
Zfp707	1,605252227	1
2610016A17Rik	1,604918459	1
Gm15453	1,604473542	1
Klhl25	1,603917571	1
Gm13487	1,603695236	1
Mturn	1,60358408	1
Zfyve28	1,602917307	1
Polr1c	1,601473583	1
Gm32340	1,600474843	1
Ccdc159	1,60036391	1
Gm43684	1,60036391	1
Serpini1	1,599476726	1
Zmym6	1,599365863	1
Zfp772	1,599255007	1
A930024E05Rik	1,597814581	1
Gm12421	1,596264804	1
2010015M23Rik	1,595711677	1
Gm8574	1,594827072	1
Gm44771	1,594495471	1
Uqcr11	1,594274443	1
Fam120b	1,593832477	1
Tmem161a	1,59361154	1
Rplp2	1,593390634	1
Gm6451	1,59206584	1
Gm12726	1,59206584	1
RP23-454I20.1	1,591845148	1
Gm45110	1,591624487	1
Tusc2	1,590521639	1
Ercc6l2	1,590190934	1
Slc2a9	1,5897501	1
5830454E08Rik	1,5897501	1
Gm4895	1,589089079	1
B3galnt1	1,588758672	1
Tnni2	1,588208146	1
Tmem38a	1,588208146	1
Rpl36al	1,588098063	1
Rpl35	1,587767862	1
Fnta	1,58765781	1
Plaa	1,587437729	1
Rps19-ps3	1,587217679	1
Rrp7a	1,58688766	1
Prep	1,585788095	1
Gm25007	1,584799137	1
Fam107b	1,584689291	1
Trim45	1,584249983	1
Def6	1,583920582	1
Rala	1,583591249	1
Irx5	1,583591249	1
Me1	1,582603661	1
Pcnx4	1,582274602	1

Gm4673	1,581835964	1
Gps1	1,580192151	1
Gm6166	1,579754089	1
Tmem192	1,579316149	1
Rcn1	1,578659466	1
Adap2	1,578003057	1
1700022N22Rik	1,578003057	1
Gm9378	1,577674954	1
Cd63-ps	1,57734692	1
Gm12704	1,577128269	1
Rpsa-ps12	1,576909647	1
Zfp820	1,576909647	1
Gm2735	1,576800348	1
Ccdc88c	1,576800348	1
Pfdn6	1,576144712	1
C1d	1,575926227	1
Gm12096	1,575816996	1
Zfp599	1,575707772	1
Zfp61	1,574725101	1
Gmfg	1,574725101	1
Wwc2	1,574615953	1
Gm20432	1,574070327	1
Mb21d2	1,573415826	1
Cops9	1,573306769	1
Hint2	1,573306769	1
Runx3	1,572434584	1
Trmt10b	1,572434584	1
Gm13868	1,572216614	1
Gm3571	1,571780763	1
Zrsr1	1,571127214	1
Llph-ps2	1,571127214	1
Gm14585	1,571127214	1
Fkbpl	1,571018315	1
Myh7b	1,570691665	1
Tbc1d30	1,570691665	1
Gnl2	1,570582797	1
Padi2	1,570256237	1
Ccdc28b	1,569276966	1
Rpl31-ps16	1,568950678	1
Gstp1	1,56884193	1
Ifi44	1,56873319	1
Gm43096	1,568407015	1
Gm44419	1,568407015	1
Gm38248	1,568080908	1
Arhgap4	1,567972221	1
Gm10059	1,567863541	1
Arid5a	1,567537547	1
Gm12743	1,567102993	1
Gm21399	1,566125689	1
Tspan33	1,565040509	1
Clec4n	1,564932033	1
Psmb8	1,564823563	1

RP23-324E2.11	1,564606648	1
Gm7452	1,564389762	1
Ddb2	1,564064491	1
Anapc13	1,56341415	1
Tagap	1,56341415	1
Wisp1	1,56341415	1
Snhg5	1,563197429	1
Mrpl46	1,56265576	1
Nlrc4	1,56265576	1
Cnpy2	1,561897739	1
Ppox	1,561681229	1
Manbal	1,560599129	1
Gm13094	1,560382799	1
Mylpf	1,55919352	1
Wdr4	1,558221148	1
Urm1	1,557897159	1
Pqlc2	1,557789177	1
Slc46a1	1,556817681	1
RP23-246F14.1	1,556493983	1
Gm5857	1,556386099	1
Tm2d3	1,556170353	1
Cebpe	1,555954637	1
Gm6368	1,555199866	1
Gmppb	1,554876505	1
Spryd4	1,554014538	1
Psmc5	1,553906825	1
RP23-138K22.2	1,55293775	1
Tnfsf12	1,552614859	1
1700001C19Rik	1,552614859	1
Gm10076	1,552507244	1
Gm20517	1,551754145	1
Rgs20	1,551431501	1
Zfp788	1,551323967	1
Zfp189	1,551216442	1
Ift20	1,550893909	1
Coq2	1,550893909	1
Zpr1	1,550141593	1
Adamts4	1,549819283	1
Cela1	1,549497041	1
Ccdc114	1,549497041	1
Adprhl2	1,549389642	1
Cd9	1,548960119	1
Xab2	1,548745403	1
Gm6768	1,548423384	1
RP23-184H3.5	1,547564994	1
1110019D14Rik	1,547028741	1
Fbxo6	1,546707079	1
Gm14253	1,546707079	1
Mical1	1,546707079	1
Rps15-ps2	1,546599873	1
Jtb	1,54584964	1
Idh2	1,545742494	1

Nfkb2	1,545742494	1
Ndufb9	1,545635355	1
Cd300a	1,545313983	1
Tldc1	1,54488559	1
Arl10	1,54477851	1
Fhod1	1,544243224	1
Pced1b	1,544243224	1
Arl4a	1,543922141	1
Mrps21	1,543708123	1
Rps13-ps5	1,543708123	1
Zfp418	1,543708123	1
Bbs9	1,543387151	1
6720475M21Rik	1,541997044	1
Aspscr1	1,541676428	1
9930014A18Rik	1,541569571	1
Tma7	1,541462722	1
Iba57	1,540928584	1
Saysd1	1,54060819	1
Taco1	1,540394631	1
Zfp408	1,539754132	1
Dhrs7b	1,539327281	1
Gm10658	1,538900548	1
Gm2383	1,538793883	1
Gm4943	1,538367298	1
Gm11694	1,537940831	1
Zfp637	1,537834233	1
Gm7670	1,537088252	1
Gss	1,53655563	1
Tmem35b	1,53655563	1
Gm26698	1,53581027	1
Tubb4b	1,535703819	1
F8a	1,53549094	1
Map3k10	1,535171677	1
Armc6	1,535171677	1
Mtrf1	1,53485248	1
Wdr73	1,534320633	1
Zfp386	1,533257491	1
Gm12017	1,533257491	1
Setd4	1,533151218	1
Rpl3l	1,533151218	1
Cygb	1,533044952	1
Cass4	1,532832441	1
Rab13	1,531345693	1
Gm11560	1,531239552	1
Rps15a-ps4	1,531133418	1
Tspan15	1,531133418	1
Gm24336	1,531027292	1
Gm8463	1,530921172	1
Gm44254	1,530708956	1
Sgta	1,53049677	1
Mpnd	1,530390688	1
Gm16372	1,530284613	1

Rpl36-ps10	1,530072485	1
Crem	1,529966432	1
Mthfsl	1,529330269	1
Ino80c	1,529224268	1
Gm9169	1,529012287	1
Gm12732	1,528906308	1
Il1rn	1,528906308	1
Fan1	1,528270585	1
Gm10602	1,528164657	1
D11Wsu47e	1,527635128	1
2510046G10Rik	1,527635128	1
Agpat4	1,527529244	1
BC031181	1,527529244	1
Chchd3	1,527211636	1
Rnf149	1,526999934	1
Klra2	1,526894094	1
Alad	1,526365005	1
Tango6	1,526365005	1
Snn	1,526047639	1
Cdk9	1,525413106	1
Gm7380	1,525201653	1
Hbegf	1,524356136	1
Gm13777	1,523194316	1
Il34	1,522877609	1
Tapbpl	1,522560969	1
Gstt2	1,522560969	1
Acaa2	1,522455437	1
Gm6123	1,522455437	1
Pnkd	1,522033381	1
Ubxn6	1,521716916	1
RP23-193N1.2	1,521716916	1
Gm4604	1,521295065	1
Cfl1	1,521084183	1
Crnde	1,520978753	1
Sapcd2	1,520662507	1
Zfp984	1,520030213	1
Tmsb10	1,519714165	1
Gm6162	1,519714165	1
Chaf1b	1,519503502	1
Mul1	1,518450628	1
Rpl31-ps10	1,517924464	1
Tssk6	1,517924464	1
Grap	1,517819253	1
Bloc1s5	1,517714049	1
Gm1848	1,517398482	1
Ltbr	1,516872683	1
Pdgfa	1,516347066	1
Gm45212	1,515086329	1
Rdh14	1,514456353	1
Nsa2	1,514456353	1
Arg1	1,514351383	1
Zfp27	1,513931573	1

Gm2986	1,513931573	1
Gtf2f2	1,513197187	1
Gm13391	1,5122535	1
Gm42747	1,511729484	1
Gm45856	1,511415161	1
Trmt12	1,511310402	1
Gm13408	1,511205649	1
Gm5453	1,510472586	1
Cers4	1,510367891	1
Crtap	1,510367891	1
Xkr5	1,510158524	1
Prss50	1,509425969	1
Alpk2	1,509112125	1
Fbxl17	1,508902932	1
Pet100	1,508589197	1
Ift43	1,508589197	1
Gm15753	1,508170985	1
Cage1	1,508170985	1
Cacna1s	1,507857403	1
Gm16638	1,507857403	1
Gm13578	1,507648384	1
Gm8722	1,507648384	1
Rpa3	1,507439394	1
Cenpx	1,50733491	1
Shq1	1,506290467	1
Gm6394	1,506186062	1
Atp5j	1,505977275	1
Yif1a	1,505872892	1
Cyba	1,505664148	1
Zfp524	1,505559787	1
Tgm2	1,505455433	1
Gm12758	1,505351087	1
Zfand1	1,505246747	1
Gm7784	1,504725159	1
2700033N17Rik	1,504203751	1
Zfp775	1,503890994	1
Lrsam1	1,503786755	1
Fignl2	1,503578301	1
Mapk1ip1	1,503474084	1
Tyw3	1,503161478	1
Gm44935	1,502848937	1
Gm13498	1,502744771	1
Slc25a2	1,502328179	1
1500011B03Rik	1,502015812	1
Layn	1,501703509	1
Fcgrt	1,501703509	1
Exoc3l4	1,501495343	1
2610524H06Rik	1,501391271	1
Zeb2os	1,501183148	1
Unc13d	1,501183148	1
Fam65c	1,501079098	1
Gm14005	1,501079098	1

Rpph1	1,500871018	1
Gm42690	1,500454946	1
Fancc	1,500038989	1
Gm27039	1,499831054	1
Gm7909	1,499623148	1
2810006K23Rik	1,49941527	1
Atg9b	1,498895703	1
C230037L18Rik	1,498272459	1
D830044I16Rik	1,49816861	1
Rpl19-ps11	1,49816861	1
Gm8121	1,497857107	1
Gm20768	1,497234296	1
Svil	1,497026749	1
Tmem140	1,496715484	1
Hyi	1,496508009	1
Dus2	1,496196851	1
Gm13758	1,495989449	1
Ypel1	1,495885758	1
Gm8730	1,495678399	1
Rnaseh2b	1,494745637	1
Fcgr1	1,494538436	1
Glb1	1,494227688	1
Itga5	1,493917004	1
Ifi203	1,493606385	1
Arhgap26	1,492985341	1
Cog7	1,492881858	1
AA414768	1,492157683	1
Gm7769	1,491744027	1
Gm12944	1,491433859	1
Bahcc1	1,490607062	1
Myg1	1,490297131	1
Tspan5	1,490090547	1
Exosc2	1,489987265	1
Gatb	1,489470964	1
Inpp5a	1,489058052	1
Bcar3	1,488954842	1
Tnfrsf1a	1,488645255	1
Ostc	1,488542074	1
Acvrl1	1,488129419	1
Gm22973	1,487923135	1
Ntmt1	1,487820004	1
Cpsf3	1,487820004	1
Apopt1	1,48771688	1
Mnd1-ps	1,48740755	1
Acsbg1	1,487304454	1
RP23-413G8.2	1,487304454	1
Snrnp35	1,487201365	1
Eif1ad	1,48699521	1
Rps10-ps2	1,486789083	1
Gm13477	1,48596486	1
Irf2bp1	1,485758876	1
Avpi1	1,485552921	1

RP23-115A18.3	1,485449954	1
Irf7	1,485141095	1
Gm20492	1,485141095	1
Gm44291	1,484626474	1
Trpm4	1,484009163	1
Ap4s1	1,484009163	1
Atg101	1,483289292	1
Pofut2	1,483186482	1
Acta2	1,48256977	1
Gm7776	1,48226151	1
Arfip2	1,482158772	1
A530072M11Rik	1,48205604	1
Tmem151a	1,481850597	1
Top3a	1,481747887	1
Insl6	1,481439798	1
Gm13827	1,481337116	1
Iqcc	1,481029113	1
Arfgap3	1,480515916	1
Ccne2	1,47959261	1
Zfp3	1,479284969	1
Ift81	1,479284969	1
Fbxo32	1,478977393	1
Ddx28	1,47866988	1
Gm11889	1,47856739	1
RP24-351I17.3	1,478157501	1
Gm37339	1,47754288	1
Psma7	1,477133275	1
Rpl36a-ps2	1,476826146	1
Six1	1,476723784	1
Tmem206	1,476519081	1
Utp23	1,47641674	1
Sp110	1,476314406	1
6330403L08Rik	1,476212079	1
Gm8304	1,475905141	1
Aimp1	1,475802843	1
Smim4	1,475598267	1
Dnph1	1,47549599	1
Zfp78	1,47539372	1
Rps18-ps1	1,474575815	1
Gm6520	1,474371409	1
Atp6v1g2	1,474269217	1
Thnsl1	1,474064854	1
Tmem51	1,474064854	1
Zscan12	1,473451935	1
Ifi27l2a	1,473349807	1
Gm11810	1,473349807	1
Psmb6	1,47243097	1
Zfp623	1,472124819	1
RP24-84O13.9	1,471512707	1
Eef1akmt1	1,471206746	1
Fxn	1,471104773	1
Gm11942	1,471002807	1

Gpat4	1,470391162	1
Cmc2	1,470187336	1
Fam195a	1,46947417	1
Ablim1	1,469168633	1
Vac14	1,469066802	1
4921536K21Rik	1,468964977	1
Fcf1	1,468659546	1
Zfp316	1,468455961	1
Tceal8	1,468252404	1
Ptges	1,468252404	1
Tifa	1,468048875	1
Gm6204	1,468048875	1
Crlf2	1,468048875	1
Zfp35	1,467743635	1
Gm7327	1,467031654	1
Ppic	1,466421659	1
Sowahc	1,466320018	1
Gmpr2	1,466116757	1
Cebpz	1,465608728	1
RP23-55A6.4	1,465608728	1
Psmc3	1,465507143	1
Mettl26	1,465202431	1
Arl6ip4	1,464999325	1
Rdh12	1,464694719	1
Adh7	1,464390176	1
Ndufa5	1,464085696	1
Nop14	1,46378128	1
Gm1947	1,463679821	1
Polm	1,463172637	1
Aen	1,462564247	1
Gm14494	1,462564247	1
Enpp1	1,462057448	1
BC004004	1,461753454	1
Rps27-ps1	1,461348226	1
Sertad1	1,461246936	1
Alkbh7	1,461246936	1
Zfp689	1,461145654	1
Gm3940	1,460740594	1
Smdt1	1,460639347	1
Emc9	1,460538107	1
Ndufb7	1,460234428	1
Gm2895	1,459930812	1
Ccl9	1,459222621	1
Aldoart1	1,459121479	1
Gm15625	1,459020344	1
Xrcc6	1,458514774	1
Sla2	1,458413681	1
Tmem42	1,457908321	1
Ap3s1	1,457706226	1
Ubl4a	1,457504159	1
2610002M06Rik	1,457403136	1
Tmem138	1,45730212	1

Txnrd2	1,457201111	1
Umps	1,456999114	1
Tmem246	1,456999114	1
Rpusd3	1,456595204	1
Ccdc138	1,456595204	1
Nsa2-ps2	1,456494244	1
Slc10a3	1,456393291	1
Dhrs13	1,456393291	1
Zfp607b	1,456292345	1
Rrp36	1,455989549	1
Fam19a2	1,455989549	1
Psmb2	1,455384146	1
Tmed1	1,45528327	1
Gm7733	1,45528327	1
Gm37010	1,455081539	1
Zgpat	1,454980684	1
Clec7a	1,454879836	1
Fiz1	1,45467816	1
Gramd2	1,4543757	1
Spsb1	1,453972517	1
Gm20554	1,453871739	1
Gpr137	1,453670204	1
Sdhb	1,453166487	1
Slc25a43	1,453166487	1
Tmcc2	1,452864341	1
Gm11423	1,452260238	1
Supt4a	1,451857641	1
Gm13862	1,45175701	1
Pih1d1	1,451656385	1
Rai14	1,451555767	1
Smim12	1,451455157	1
Dhcr24	1,451354553	1
Tbc1d2b	1,451253956	1
Psma4	1,450751077	1
Aamdc	1,450751077	1
Ak1	1,450650522	1
4931414P19Rik	1,450449433	1
Gm5069	1,449846334	1
Gm12341	1,449645356	1
Gm14822	1,449444407	1
Gm33142	1,449444407	1
Rab11fip4os1	1,449444407	1
Slc7a7	1,449343942	1
Zdhhc4	1,448841725	1
Adat2	1,448440076	1
Gm12479	1,448339682	1
Hcn2	1,448339682	1
Ccdc167	1,447938172	1
B430305J03Rik	1,447336117	1
Ubash3b	1,447135488	1
Alkbh2	1,446934886	1
Ms4a6d	1,446934886	1

Rps19-ps7	1,446533766	1
Igsf3	1,446433503	1
Gm42850	1,446132757	1
Tpd52l2	1,445832074	1
Nek6	1,445832074	1
Qpctl	1,44573186	1
BC037032	1,44573186	1
Phf7	1,445631653	1
Fkbp11	1,445230894	1
Tmem218	1,445130722	1
Osbpl10	1,445130722	1
1700001G11Rik	1,445130722	1
Calr-ps	1,444629964	1
Catip	1,44442971	1
Gm7899	1,44412938	1
Gm12309	1,443929196	1
Prss46	1,443929196	1
Rgs2	1,443829113	1
Mycbp	1,443729038	1
March11	1,44362897	1
Ptgs2os2	1,443428855	1
1300002E11Rik	1,443428855	1
9130008F23Rik	1,443228767	1
Trpv2	1,442528678	1
Gm43524	1,442328714	1
Tac4	1,442328714	1
Ndufaf5	1,442128779	1
Nxn	1,442128779	1
2210008F06Rik	1,442128779	1
B3gnt2	1,442128779	1
Ophn1	1,442028822	1
Rpl30-ps5	1,441529139	1
Spag7	1,440430447	1
Zfp459	1,440230775	1
Atp6v0b	1,44003113	1
Zfp444	1,439931319	1
Gm45806	1,439831514	1
Rps27a	1,439432363	1
Ndufs3	1,439432363	1
Gm12967	1,438833844	1
D730003I15Rik	1,438135887	1
Uqcrh-ps1	1,438135887	1
Gm6794	1,438135887	1
Fam167b	1,437936533	1
Smpd2	1,437936533	1
Eef1d	1,437836866	1
Tmem231	1,437438268	1
Gm5865	1,437338636	1
Rpl6l	1,437039781	1
9130019O22Rik	1,436940177	1
Gm5745	1,436940177	1
Hps4	1,436740988	1

3300002I08Rik	1,436442257	1
Rnf139	1,436143589	1
Ptpn7	1,435844982	1
1600010M07Rik	1,43574546	1
Park2	1,43574546	1
Gldc	1,435546437	1
Slfn2	1,435247955	1
Tmem2	1,434452305	1
Gm15216	1,434452305	1
Rpl41	1,43435288	1
Hlcs	1,43435288	1
Abcf2	1,43415405	1
Rpl26-ps2	1,43415405	1
Mzb1	1,43415405	1
Fut7	1,433855857	1
Ssr4	1,433657096	1
Zfp764	1,433557726	1
Vat1	1,433458363	1
Gpr68	1,433359007	1
RP23-476G10.1	1,433259657	1
Zfp263	1,433160315	1
Nod1	1,433060979	1
Gm5093	1,43296165	1
Acadl	1,432763013	1
Mrpl11	1,432663705	1
Tomm5	1,432564404	1
Zhx3	1,432167268	1
Ifi27	1,432068001	1
Gm22716	1,432068001	1
Gm14034	1,43157177	1
Galns	1,431472545	1
Gm17690	1,431373326	1
Rab32	1,431075711	1
Nostrin	1,430877336	1
Cfp	1,430877336	1
N4bp3	1,430778158	1
Elp3	1,430678988	1
Gm10110	1,430282374	1
Plxdc1	1,430084109	1
Gm8444	1,430084109	1
Hemk1	1,429885871	1
Stard3	1,429786762	1
Lamtor4	1,42968766	1
Rab4a	1,42968766	1
Lpin3	1,429192254	1
Icam4	1,428597993	1
Rasgrp4	1,427905001	1
Gm14006	1,427905001	1
Fkbp2	1,42780603	1
Rbfa	1,427608108	1
Mrpl55	1,427311276	1
Gm2830	1,426717797	1

Cd59a	1,426322282	1
Grcc10	1,42622342	1
Gm13422	1,426124565	1
Pdlim2	1,425630394	1
9130023H24Rik	1,425630394	1
Gm15773	1,425136394	1
Gm10161	1,424938841	1
Gm16061	1,424938841	1
Hba-a1	1,424346349	1
1810055G02Rik	1,424050196	1
Atp5k-ps2	1,423556743	1
Wfikkn1	1,423458073	1
Rpl4	1,423162104	1
Ss18l1	1,422866196	1
C920021L13Rik	1,422668959	1
Hmgb3	1,422373154	1
Zbtb45	1,422373154	1
Rpl38	1,421978843	1
Agtrap	1,421978843	1
Gm12501	1,421978843	1
Ung	1,421880282	1
Mrpl48	1,421683182	1
Slc5a6	1,421486108	1
Plcd1	1,421486108	1
Cdnf	1,421289062	1
Actr10	1,420895052	1
Ip6k3	1,420895052	1
Get4	1,420796566	1
Pdcd5-ps	1,420698087	1
Cst7	1,420304241	1
Gm20522	1,420107359	1
Rtn4ip1	1,419910504	1
Gm10636	1,419910504	1
Zfp455	1,419812086	1
Pgam1	1,419713676	1
Gm6649	1,419418485	1
Gm11631	1,419418485	1
Nenf	1,419320102	1
Lrrc75a	1,419320102	1
Usp18	1,419320102	1
C130036L24Rik	1,419123356	1
Rgs16	1,419024993	1
Gm8825	1,418926638	1
Ddx31	1,418828288	1
RP23-447C2.2	1,418828288	1
Nop9	1,418729946	1
Nrxn3	1,418631611	1
Stoml1	1,418533282	1
Ndufa4l2	1,417943453	1
Gm5547	1,417746898	1
Gm6419	1,417746898	1
RP23-123D6.12	1,417648631	1

Nr2f6	1,41755037	1
Trf	1,41755037	1
Lims2	1,41725563	1
Rhov	1,41705917	1
Cpq	1,41696095	1
Glrx5	1,416764532	1
Cmbl	1,416666332	1
Sp140	1,41656814	1
Gm7434	1,41656814	1
Coa7	1,416469954	1
Zfp688	1,416371775	1
Eci1	1,416273603	1
Rps27a-ps1	1,416175438	1
0610009O20Rik	1,416175438	1
Ext1	1,416175438	1
Mtl5	1,416175438	1
Coro1b	1,415880983	1
Nt5dc2	1,415684714	1
Mrpl48-ps	1,414997986	1
Ctu1	1,41489991	1
Plekhh2	1,41480184	1
Mrpl49	1,41460572	1
Znhit2	1,414409628	1
Gm6028	1,414213562	1
Grpel1	1,413919516	1
2310039H08Rik	1,41362553	1
Acsf3	1,41362553	1
Tmem126b	1,413135689	1
Slc25a19	1,413135689	1
Dhrs7	1,413037742	1
Rpusd4	1,412841867	1
Cox6b1	1,412743939	1
Gm8762	1,412743939	1
Tdpx-ps1	1,412352298	1
Zfp429	1,412058638	1
Sertad2	1,411960765	1
Dusp28	1,411862899	1
Psme2	1,411765039	1
Fars2	1,411667186	1
Borcs5	1,41156934	1
Gm5054	1,411471501	1
Angptl6	1,411275843	1
Smco4	1,410884609	1
Gm15440	1,410689032	1
Pomp	1,410689032	1
Zfp870	1,410395718	1
Gm7589	1,410200209	1
Vwa1	1,410102465	1
Gm5525	1,409906997	1
Gm4366	1,409809273	1
Exosc4	1,409613846	1
Selplg	1,409613846	1

Coa3	1,409418445	1
Ppp6r2	1,409223072	1
Sh3bgr	1,409223072	1
9530062K07Rik	1,409027726	1
Prob1	1,408930063	1
Pafah1b1-ps1	1,408832406	1
Gm15530	1,408734757	1
Fosl1	1,408441849	1
Snrpa1	1,408441849	1
Vwa7	1,407953805	1
Nrbf2	1,407758635	1
1110032A03Rik	1,40766106	1
RP23-114G13.7	1,40746593	1
Hist1h1b	1,40746593	1
Rpl36a-ps3	1,407173286	1
Pltp	1,406783189	1
2810408I11Rik	1,406685682	1
Six5	1,4063932	1
Mutyh	1,405905866	1
Afmid	1,405905866	1
Klhl22	1,405126482	1
1190005I06Rik	1,405029089	1
Fbxo31	1,404444876	1
Gm13368	1,404444876	1
Gm6285	1,40434753	1
Sys1	1,40415286	1
Gm9800	1,403958217	1
E130317F20Rik	1,403860905	1
Chid1	1,403860905	1
Gins4	1,403666302	1
E130311K13Rik	1,403569011	1
Gm13186	1,403082655	1
Slc37a4	1,402790922	1
4930430E12Rik	1,402790922	1
Gm2272	1,402596468	1
Plekhg3	1,402596468	1
Fbxo36	1,402499251	1
Gm11520	1,402499251	1
Pfdn2	1,402499251	1
Rpl31-ps17	1,402207639	1
Impa2	1,402013266	1
Ptpn1	1,402013266	1
Hotairm1	1,401624599	1
Mvb12a	1,401430306	1
Gm15427	1,401138917	1
Gm2058	1,400944691	1
Mllt10	1,400750493	1
Hoxa4	1,400556321	1
Gm5436	1,400556321	1
Man2c1os	1,400459245	1
Washc1	1,400265114	1
Siva1	1,400168058	1

Gm38247	1,400071009	1
Dnajb2	1,399682881	1
Utp18	1,399585866	1
Gm15500	1,399488857	1
Spg11	1,399488857	1
Gm17108	1,399391855	1
Psmb4	1,398713031	1
Gm36378	1,398422207	1
Jsrp1	1,398228358	1
Cog4	1,398228358	1
Hdac11	1,398034536	1
Vti1a	1,398034536	1
Erich1	1,39784074	1
Rps12	1,39784074	1
Filip1l	1,39745323	1
Pgp	1,39745323	1
Il15	1,39735637	1
Acbd4	1,39735637	1
Bap1	1,397162668	1
Etfb	1,397065828	1
Lamc2	1,396968994	1
AA465934	1,396872167	1
Il17ra	1,396872167	1
Tmem106c	1,396678532	1
Gm43547	1,396388132	1
Gm12689	1,396194564	1
Zfp51	1,396001024	1
Rubcnl	1,395710764	1
Gm5944	1,395710764	1
Atp5c1	1,395227131	1
Gm7114	1,395130425	1
Gm8869	1,394937032	1
Zkscan6	1,394840346	1
Flt3l	1,394840346	1
Emc8	1,394646993	1
A430033K04Rik	1,394550327	1
Gm14680	1,394357015	1
Rpl37rt	1,394067097	1
Emc10	1,393970471	1
Tm4sf5	1,393873851	1
Nt5c	1,393777239	1
Cptp	1,393294276	1
Cwc22	1,393294276	1
Pomt2	1,393197704	1
Zfp958	1,393004579	1
Gm8292	1,392714942	1
Ndufv1	1,392618409	1
Tmem106a	1,392425365	1
Ryr1	1,392232347	1
9330102E08Rik	1,392232347	1
Ndufs2	1,391846392	1
Rpl9-ps7	1,391653454	1

Tceanc2	1,391556996	1
Rnaset2a	1,391460544	1
Ubac2	1,391460544	1
Pfkfb4	1,391364098	1
4933440N22Rik	1,390978384	1
Ighd	1,390881972	1
Lmntd2	1,390785566	1
Aldh1l2	1,390592776	1
Gm7783	1,390592776	1
Prrg2	1,390496391	1
E230020A03Rik	1,389629225	1
Serpinc1	1,389532906	1
Rps14	1,389340289	1
Ptges3l	1,389340289	1
Gm7666	1,389051414	1
Trim2	1,387992719	1
Mettl18	1,387511761	1
Rpl23	1,387511761	1
Gm16286	1,387415589	1
Gm12186	1,387319424	1
Sdf4	1,387223266	1
Tns1	1,386454239	1
Slc25a11	1,386069886	1
Mcoln2	1,385973814	1
Gm5577	1,385877749	1
St3gal5	1,38578169	1
Gm9435	1,38578169	1
Gm4149	1,385685639	1
Nans	1,385589594	1
Calm3	1,385589594	1
Rrp8	1,385301498	1
Alg10b	1,385109468	1
Fam161a	1,385109468	1
Nkapl	1,385109468	1
Gm10031	1,385013463	1
Ten1	1,384917464	1
Coq8a	1,384821473	1
Rpusd2	1,384725487	1
Slc25a4	1,384725487	1
Hexdc	1,384629509	1
Slc22a4	1,384245661	1
Il11ra1	1,384053777	1
Foxd2	1,383957845	1
Gpt2	1,38386192	1
Kbtbd4	1,383670089	1
Spink5	1,383670089	1
Fdxacb1	1,383286508	1
Gm7785	1,382711335	1
RP23-304C21.3	1,382711335	1
4930412F12Rik	1,382711335	1
Gnb1l	1,381753244	1
Pex11g	1,381657472	1

Kdelr2	1,381274448	1
Ypel4	1,380795818	1
Gm14857	1,380795818	1
Gm5879	1,380508719	1
Dnajc30	1,380317353	1
Lztr1	1,379552155	1
Mgam	1,379456535	1
Gm16380	1,379265315	1
1600002H07Rik	1,378596254	1
Tfcp2l1	1,378309613	1
Gm12469	1,378214079	1
Lsm14b	1,377927517	1
Orc1	1,377641015	1
Sdc1	1,377450046	1
Arhgap35	1,377259104	1
Fabp3	1,377259104	1
Uqcrq	1,377259104	1
Polr3e	1,377163643	1
Tsr1	1,377163643	1
Dera	1,377068189	1
Pla2g12a	1,376781866	1
Exosc8	1,376114009	1
Gstt1	1,375923252	1
Tspan3	1,375637167	1
Ifi35	1,374874563	1
Tspan4	1,374874563	1
1700061G19Rik	1,37449342	1
Mus81	1,374302888	1
Hdhd3	1,374302888	1
Rgl1	1,374112382	1
Gm13743	1,374017139	1
1700021F05Rik	1,373826673	1
Ptrh1	1,373826673	1
Tyw5	1,373636233	1
Ndfip1	1,373636233	1
Gm14140	1,373636233	1
Spr	1,37344582	1
Paqr7	1,37344582	1
Zcwpw1	1,373065073	1
Tars2	1,373065073	1
RP23-48A24.3	1,373065073	1
Zbtb42	1,372874739	1
Pstpip1	1,372399019	1
Wrap53	1,372303895	1
Zfp850	1,372018562	1
Qtrtd1	1,371543139	1
Card6	1,371543139	1
Rpl11	1,371543139	1
Hexb	1,371543139	1
BC029722	1,371448074	1
Nsrp1	1,371257964	1
Coq6	1,370972849	1

Gm31274	1,370782805	1
Ccdc43	1,370592787	1
RP23-13B8.12	1,370592787	1
Suclg1	1,370592787	1
Dusp6	1,370497788	1
C2cd2	1,37030781	1
Mri1	1,369738034	1
Gm2756	1,369643094	1
Ctu2	1,369643094	1
RP23-228B2.5	1,369548161	1
Fdx1	1,369453234	1
Npr1	1,369263401	1
Ap5b1	1,369168494	1
Gm29759	1,3689787	1
Lyrm1	1,368883813	1
Fcrl1	1,368599191	1
Cep162	1,368504331	1
Gm14165	1,368409476	1
Gm5321	1,368030125	1
Kctd11	1,367745681	1
Rpain	1,367366514	1
Tor3a	1,367366514	1
Gm37760	1,367082208	1
Zfp319	1,366892703	1
Acot1	1,36679796	1
B4gat1	1,366608495	1
Gm28535	1,366513772	1
Neurl2	1,366229643	1
Pqlc1	1,365566905	1
Rpl13a	1,365282973	1
Lcmt1	1,365188342	1
Sav1	1,364715286	1
Med8	1,364242394	1
Polr3c	1,364242394	1
Pla2g16	1,363864198	1
Ndufs8	1,3633916	1
Crygn	1,363297101	1
Lin7b	1,363108121	1
Zfp566	1,36301364	1
Actr3b	1,36301364	1
Pdcd2	1,362919167	1
Zfp120	1,3628247	1
Gm16200	1,362730239	1
Rpsa-ps1	1,362730239	1
Rps18	1,362352462	1
Reep5	1,362352462	1
Rhobtb1	1,362163613	1
Rnf157	1,362163613	1
Ssna1	1,361785994	1
Spag4	1,361502848	1
Fam171a2	1,361314116	1
Mfsd9	1,360842403	1

Gm19705	1,36074808	1
Coq3	1,36074808	1
Aven	1,360653763	1
Nr2c2ap	1,360653763	1
Mypopos	1,360559453	1
5730409E04Rik	1,360276562	1
Riiad1	1,360182278	1
C430042M11Rik	1,360088001	1
Gm6548	1,35999373	1
Sgsh	1,35999373	1
RP23-312A24.1	1,359616712	1
Dnajc12	1,359616712	1
D130020L05Rik	1,359522474	1
5430416N02Rik	1,359522474	1
Commd6	1,359522474	1
Gm5812	1,359428242	1
Rpl30-ps2	1,359334017	1
Sgsm1	1,359334017	1
Gm11966	1,359239798	1
Arhgap31	1,358862989	1
Guk1	1,358862989	1
Snai2	1,358768803	1
Otud6b	1,358768803	1
Gm14584	1,358580451	1
Sap18b	1,358486285	1
Rbmx2	1,358392125	1
Ywhah	1,358015551	1
Rpl17	1,357921423	1
D130017N08Rik	1,357827303	1
Gm9843	1,357827303	1
Mfsd13a	1,35754498	1
Slc25a33	1,35754498	1
Gcnt1	1,357262716	1
Lrrc27	1,357168641	1
Gm9712	1,357074572	1
Mcm9	1,35698051	1
Gm11598	1,35698051	1
Tmem268	1,35698051	1
Tmem41a	1,356698363	1
Spry2	1,356416275	1
Clec1a	1,356322259	1
mt-Nd2	1,355852275	1
Ccdc126	1,355758298	1
Arhgap22	1,355758298	1
Car13	1,355664327	1
Pwwp2b	1,355570363	1
9130401M01Rik	1,355476405	1
Ucp2	1,355006714	1
Mkks	1,354912795	1
Jdp2	1,354631078	1
Tbc1d22a	1,354631078	1
Vsir	1,35434942	1

Lgr4	1,354255547	1
Fmnl3	1,354255547	1
RP24-183O8.6	1,35406782	1
Gm16755	1,353973967	1
Slc6a4	1,353692445	1
Poglut1	1,353223372	1
Robo3	1,353035789	1
4930550C14Rik	1,352942007	1
Slc12a2	1,352566943	1
Hpcal1	1,352566943	1
Stoml2	1,352379451	1
Gm15013	1,352285714	1
Sep 02	1,352285714	1
Wrb	1,352285714	1
Xpa	1,352191984	1
Mrpl40	1,352098261	1
Fut11	1,352098261	1
Zfhx2	1,351817129	1
A130010J15Rik	1,351817129	1
Bcl7b	1,351817129	1
Rpsa-ps10	1,351723431	1
Gm12589	1,351161382	1
A130048G24Rik	1,351161382	1
Ap3s2	1,351161382	1
Noa1	1,350974085	1
Nbn	1,350412347	1
Gsr	1,350037985	1
Cebpd	1,350037985	1
Smarce1	1,349850843	1
Gm9521	1,349663727	1
Dnajc19-ps	1,349383101	1
Gm12844	1,349102534	1
Erap1	1,349102534	1
Peg12	1,349102534	1
Mrpl17	1,349009025	1
D630024D03Rik	1,348822026	1
Sap30l	1,348822026	1
Gm11474	1,348635052	1
Zfp202	1,348448104	1
Uqcc3	1,34835464	1
G6pc3	1,348074287	1
Kif13a	1,347980849	1
Zmym1	1,347700573	1
C130013H08Rik	1,347700573	1
Csrnp2	1,347700573	1
Scarf1	1,347513755	1
Bola2	1,347513755	1
Epc1	1,347233577	1
Ly6g6d	1,347140197	1
Pink1	1,347140197	1
Sugt1	1,347140197	1
Enho	1,347046824	1

Gm11633	1,346860096	1
Dph5	1,346766742	1
Fam171b	1,346673395	1
Mfsd2a	1,346486719	1
P2ry2	1,346393391	1
Anp32b-ps1	1,346300069	1
Use1	1,345740275	1
Gm9701	1,345553729	1
Zfp629	1,345460466	1
Fbxl15	1,345367209	1
Lipt2	1,345087476	1
Zadh2	1,344901021	1
Rab3ip	1,344901021	1
Rfc2	1,344901021	1
Tcte2	1,344807802	1
Hist1h2ae	1,344807802	1
Tsr3	1,344714591	1
Trmt61a	1,344528187	1
Myo1d	1,344528187	1
Rpl32-ps	1,344155456	1
Lyz2	1,344155456	1
Slc25a46	1,344062289	1
D5Ertd605e	1,343875976	1
Gm5297	1,343875976	1
Nptxr	1,343782828	1
Gm13398	1,343782828	1
Rps8	1,343782828	1
Gm26759	1,343596554	1
Gadd45gip1	1,343410304	1
Ndufa13	1,343410304	1
Capn1	1,34331719	1
Ndufaf6	1,343224081	1
Zbtb14	1,343130979	1
Rpl27a	1,343037884	1
Rrp9	1,342944795	1
Abhd6	1,342944795	1
4930461G14Rik	1,342851712	1
Gm10463	1,342851712	1
2310001H17Rik	1,342665566	1
Mocs2	1,342479446	1
Zfp40	1,342386396	1
Sep 07	1,342386396	1
Flt1	1,342107283	1
Hoga1	1,342014259	1
Cuedc2	1,342014259	1
Usp30	1,341921241	1
D330045A20Rik	1,341828229	1
Edil3	1,341735224	1
Aig1	1,341642225	1
Ubb	1,341456247	1
Cmtr2	1,341456247	1
Uba7	1,341270294	1

Gm13039	1,341177328	1
Rpp25l	1,341084368	1
Gm20632	1,340712592	1
Pa2g4	1,340433827	1
Plp2	1,340340919	1
Gm6023	1,340340919	1
Eva1b	1,340248017	1
Pttg1ip	1,340248017	1
Spata6	1,339876472	1
Ppp1r21	1,339876472	1
Ndufb11	1,339876472	1
Pfdn1	1,339690739	1
Gm13436	1,339319349	1
Rpl21-ps1	1,339319349	1
9130221H12Rik	1,338948063	1
Gm18913	1,338948063	1
Tmem181b-ps	1,338855257	1
Gpank1	1,338576879	1
Gm6344	1,338484099	1
Eif3j2	1,338298559	1
Wdr12	1,338205798	1
Ykt6	1,338205798	1
Gm7504	1,338113044	1
Rps3a1	1,33783482	1
Slirp	1,337742091	1
Adarb1	1,337093172	1
Dusp2	1,336907825	1
Gm8330	1,336815161	1
Arhgap25	1,336722503	1
Krtcap3	1,336537207	1
Tpt1-ps5	1,336537207	1
Gm7701	1,336444568	1
Tmub1	1,33625931	1
Gm10657	1,336166691	1
Gm1840	1,336166691	1
Clpp	1,336166691	1
Atp6v1g1	1,336074078	1
Pla2g5	1,336074078	1
RP23-2N7.4	1,335888872	1
Gm26730	1,335703691	1
Ubtd2	1,335611111	1
Gm10704	1,335148303	1
Tmem160	1,335148303	1
Creb5	1,334870696	1
2900093K20Rik	1,334870696	1
Numbl	1,334685656	1
Ubfd1	1,334500642	1
1110008F13Rik	1,334500642	1
Gm7123	1,33422317	1
Ap1g2	1,33422317	1
Gm10263	1,334130692	1
Mrpl52	1,33403822	1

Bcat1	1,333113855	1
Zfp811	1,333021454	1
2310061I04Rik	1,333021454	1
Abhd14a	1,332836671	1
Flnb	1,332836671	1
Tnks1bp1	1,332744289	1
Gm8186	1,332744289	1
Gm8522	1,332651913	1
Gm7102	1,332559544	1
Surf1	1,332559544	1
Flad1	1,332282475	1
Rpl10a-ps1	1,332282475	1
Cc2d1a	1,332190132	1
Rpl18-ps2	1,332190132	1
Cdk18	1,332190132	1
Ruvbl2	1,331913139	1
Tma16	1,33172851	1
Pld1	1,331636204	1
Zbtb12	1,331543906	1
Disp1	1,331451613	1
Zfp952	1,331451613	1
Aacs	1,331359327	1
Sin3b	1,331267048	1
Gm12231	1,331174774	1
Tmem220	1,331174774	1
Cbr4	1,331174774	1
Phospho2	1,331082508	1
Mettl21b	1,330990247	1
AA474408	1,330713504	1
Pop7	1,33062127	1
Tbcb	1,330529041	1
Mettl7a1	1,329975805	1
Ttll13	1,329975805	1
Gm26830	1,329883621	1
Asb8	1,329146381	1
Gm9892	1,329146381	1
Mettl3	1,328870022	1
Tma7-ps	1,328685814	1
Rft1	1,32840955	1
Ndufs7	1,32840955	1
Eif6	1,32840955	1
Gm12396	1,328133344	1
Nudt18	1,328133344	1
AI661453	1,327857195	1
Haghl	1,327857195	1
Gm15417	1,327673128	1
Tubb6	1,327489086	1
Cyp2u1	1,32730507	1
Nubp1	1,327213071	1
Timm8a1	1,326753174	1
2410015M20Rik	1,326753174	1
Gm19898	1,32656926	1

Hmgn1	1,326477312	1
4930404I05Rik	1,326385371	1
Sh3rf1	1,326201507	1
Hebp2	1,326109585	1
Trak1	1,32601767	1
Hip1r	1,325833857	1
Rpl34-ps1	1,32565007	1
E330020D12Rik	1,325558187	1
Fn3k	1,325374438	1
Eid2b	1,325282573	1
Ctdsp1	1,325007017	1
RP24-324J2.1	1,324915178	1
Htr2b	1,324915178	1
Hspd1-ps3	1,324823345	1
Ighmbp2	1,324731518	1
Ier2	1,324456077	1
Ube2d-ps	1,324364276	1
Tprkb	1,324364276	1
Naif1	1,324180692	1
Gm12517	1,32408891	1
Gm12606	1,323905365	1
Gm43501	1,323721846	1
Zbtb22	1,323630096	1
Egr2	1,323538352	1
Gm11362	1,32317144	1
Gm14325	1,32317144	1
1110038F14Rik	1,32317144	1
Smpd1	1,32317144	1
Zfp251	1,323079728	1
2010320M18Rik	1,322988022	1
Gm8013	1,322896323	1
Psmc4	1,322896323	1
Dolk	1,322712943	1
Eif2ak2	1,322621263	1
Gm19726	1,322529589	1
Gm11878	1,32234626	1
Dtnbp1	1,322162957	1
Hscb	1,322162957	1
Timm9	1,322071314	1
Tnfaip8	1,322071314	1
Pigyl	1,321888049	1
Mgmt	1,321704809	1
1600014C10Rik	1,321613199	1
Arsg	1,321521595	1
Dennd2a	1,321521595	1
Rcan3	1,32106367	1
Zfp974	1,320972104	1
Sdhaf2	1,320788991	1
Tnfaip8l1	1,320697444	1
Siglec1	1,320605903	1
Gna15	1,320422841	1
Rnf187	1,320422841	1

Anp32-ps	1,320331319	1
Gm16373	1,320056793	1
Osbpl1a	1,320056793	1
Mrps25	1,320056793	1
Gm11224	1,319690846	1
Mrps34	1,319690846	1
Ddx27	1,319599375	1
Mrpl18	1,319325001	1
Rps4x-ps	1,319142116	1
Eif3k	1,318959257	1
Lrrc20	1,318867837	1
Engase	1,31850222	1
Rabgef1	1,31850222	1
Ndufa9	1,31850222	1
Tfcp2	1,318319449	1
Gm13015	1,318319449	1
Cbr3	1,318319449	1
Creb3l2	1,318136704	1
Slfn10-ps	1,318045341	1
Zfp768	1,318045341	1
Hcst	1,317953984	1
Adm	1,317862634	1
Gm16288	1,31777129	1
Cmas	1,317679952	1
Sorbs1	1,317405976	1
Batf2	1,317223358	1
Gtf2i	1,317040765	1
Fancg	1,316949477	1
Ifi202b	1,316949477	1
Gm4799	1,316675654	1
Cdc16	1,316675654	1
Zcchc17	1,316401887	1
Sugp1	1,316310644	1
Fam114a1	1,316219408	1
Vav1	1,316219408	1
Gm11450	1,316036954	1
Slc9a4	1,316036954	1
Rpp14	1,316036954	1
Ccdc180	1,315945736	1
Lat2	1,315854525	1
Ube2l6	1,315216223	1
Gm9727	1,315125063	1
BC030499	1,315033908	1
Mpdu1	1,315033908	1
Abi3	1,31494276	1
Abhd17a	1,31494276	1
Rilpl1	1,31494276	1
Trim3	1,314851618	1
Rps11-ps4	1,314760483	1
Ddn	1,314578231	1
Riox2	1,314487115	1
Parp12	1,314396005	1

Ifngr2	1,314396005	1
Dkkl1	1,314213803	1
Zkscan14	1,314213803	1
Cgrrf1	1,314122712	1
Dgka	1,314031627	1
Kcnk13	1,314031627	1
Btrc	1,313849476	1
Susd3	1,313849476	1
Nlrc3	1,31375841	1
Cd276	1,313667351	1
Ttc7	1,31348525	1
Gm37503	1,313303175	1
Zfp462	1,313212147	1
Med11	1,313121125	1
Serhl	1,312757101	1
Rad51d	1,312575127	1
Gm15950	1,31248415	1
Nbas	1,312211255	1
Sh3kbp1	1,312211255	1
Ss18	1,312211255	1
Smim20	1,312120302	1
Zfp940	1,311847483	1
Gm42893	1,311756556	1
1110002L01Rik	1,311665635	1
Gm36189	1,31157472	1
8430408G22Rik	1,311302014	1
Rnf169	1,311302014	1
Sil1	1,31084763	1
Gm7424	1,310756772	1
Tmem127	1,310484237	1
Gm6921	1,310211757	1
Stk11ip	1,310030136	1
Zfp595	1,309485423	1
Gm17745	1,309303902	1
Mrpl2	1,309213152	1
BC022687	1,309031669	1
Snx8	1,308940937	1
Sec11c	1,308396677	1
Arrdc4	1,308305989	1
2310068J16Rik	1,308033962	1
Atpif1	1,308033962	1
Xrcc1	1,307852643	1
Atg4a-ps	1,307761993	1
Mcee	1,307761993	1
RP23-354J5.3	1,307671349	1
Klhl18	1,307399454	1
Gm10916	1,306946423	1
Gm44103	1,306946423	1
Kxd1	1,306855835	1
Mrpl43	1,306855835	1
Prelid2	1,30658411	1
Txnl4b	1,306493548	1

Gm5113	1,306402992	1
Casz1	1,306402992	1
St7l	1,306221898	1
Msto1	1,306221898	1
Rab1b	1,306131361	1
Idua	1,30604083	1
Lrfn4	1,305859787	1
Pisd	1,305859787	1
Pop1	1,305769275	1
Sdhaf1	1,305678769	1
Frat1	1,305316808	1
Snapc5	1,305226334	1
Gm45802	1,304141128	1
AB124611	1,304141128	1
Gm29257	1,304050735	1
Gm5580	1,303960348	1
Fbxo34	1,303960348	1
Pkig	1,303960348	1
Zkscan1	1,303779594	1
Gm9726	1,303598864	1
Gspt2	1,303598864	1
Ercc5	1,303508509	1
Gm23639	1,303327816	1
Myl6	1,303327816	1
Arhgef19	1,30323748	1
Igbp1	1,30323748	1
Dcp1b	1,303147149	1
Plk2	1,303147149	1
Scyl3	1,302876196	1
Pradc1	1,302695591	1
Ak3	1,302695591	1
Gm9840	1,302695591	1
Prkaa1	1,302424731	1
Rb1cc1	1,302424731	1
B3galt6	1,302334457	1
Timm13	1,30224419	1
Gpr108	1,30224419	1
Drg1	1,301973424	1
Preb	1,301792944	1
Jaml	1,301792944	1
4930590J08Rik	1,30161249	1
Yjefn3	1,301432061	1
Gm26710	1,301432061	1
Polr3h	1,301341855	1
Aup1	1,301341855	1
Clgn	1,300890923	1
Rasa3	1,300890923	1
Nucb2	1,300440147	1
8030453O22Rik	1,299989526	1
Wdr55	1,299719229	1
Ulk4	1,299629143	1
Dnal4	1,299539062	1

Hyal2	1,299448988	1
Nosip	1,299268859	1
Lman2	1,299268859	1
E2f4	1,299088755	1
Rab11fip1	1,298818645	1
Swsap1	1,298728621	1
Oas1b	1,298728621	1
Ndufaf2	1,298638603	1
Nek3	1,298548591	1
D8Ertd738e	1,298548591	1
Mrpl37	1,298548591	1
Erp29	1,298098626	1
Creld1	1,298008652	1
Adrm1	1,297558875	1
Traf3ip2	1,297468938	1
Tmem98	1,297379007	1
Zdhhc12	1,297289083	1
Sod1	1,297019347	1
Zfp931	1,296749668	1
Micu2	1,296749668	1
Prorsd1	1,296659787	1
Gm15720	1,296569912	1
Gnpda1	1,296480044	1
Gm7863	1,296390182	1
9430038I01Rik	1,296390182	1
Usp12	1,296210477	1
Gm6177	1,295851141	1
Spryd3	1,295581704	1
Cog2	1,295312323	1
Asrgl1	1,295312323	1
Mphosph10	1,295222542	1
Hint1	1,295222542	1
Gm9506	1,295132768	1
Gm13464	1,295042999	1
Fn3krp	1,295042999	1
Mettl5	1,29486348	1
Mrrf	1,294683986	1
Fam212a	1,294414792	1
Ski	1,294414792	1
Nsmce2	1,294325074	1
Psme2b	1,294325074	1
Gm9013	1,294235361	1
Cdk10	1,294055954	1
Esrp2	1,294055954	1
Metrn	1,293697215	1
Rps15a-ps6	1,293517883	1
Dapp1	1,293517883	1
Ermap	1,293338576	1
Gm42829	1,293248932	1
Fam192a	1,292800804	1
Zfand2b	1,292711197	1
Prpf19	1,292621596	1

Eepd1	1,292532001	1
Zfp746	1,292442413	1
Gm11675	1,292263255	1
Gm4737	1,292084121	1
Sdccag8	1,292084121	1
Gm8451	1,291994564	1
Dennd5a	1,291994564	1
Nudt19	1,291815468	1
Gm45109	1,291725929	1
Pmm1	1,291725929	1
Mtch1	1,29154687	1
Rps3a3	1,291278329	1
Rps11-ps2	1,291188827	1
Mrpl39	1,29092036	1
Def8	1,290830883	1
Pkn3	1,290830883	1
Mad2l1bp	1,290830883	1
Acot10	1,290741413	1
Pigx	1,290741413	1
Gm17827	1,290651949	1
Snx21	1,290651949	1
Gm12643	1,29056249	1
Itgb1bp1	1,29056249	1
Tpm2	1,290473039	1
Gm5637	1,290294153	1
Tnnt1	1,290294153	1
Ndufv3	1,290294153	1
Rhd	1,29020472	1
Gm5822	1,290025872	1
Lsm3	1,289757647	1
Ndufb8	1,289757647	1
Ezh2	1,289757647	1
Smim1	1,289668251	1
Cyp26b1	1,289668251	1
Pnpo	1,289668251	1
Speer9-ps1	1,289578861	1
Gm43721	1,289578861	1
Snd1	1,289578861	1
Zbtb24	1,289489477	1
Gm5921	1,2894001	1
RP23-380K24.3	1,289310728	1
Gm7285	1,289310728	1
Bri3	1,289310728	1
Ndufv2	1,289221363	1
Pdia5	1,288953305	1
Gm7292	1,288685303	1
Dvl3	1,288595981	1
Tm7sf2	1,288595981	1
Gorab	1,288506665	1
Gpr85	1,288417356	1
Rftn2	1,288328053	1
Sumf1	1,288328053	1

Atp2b4	1,288328053	1
Gsdmd	1,287970902	1
Tbc1d2	1,287792363	1
Gdi2	1,28761385	1
Ndufa10	1,28761385	1
2610021A01Rik	1,287524602	1
Mrps18a	1,287524602	1
Pex14	1,287346126	1
Tnfsf13b	1,287167674	1
RP24-310D17.9	1,286632468	1
Prkag1	1,286632468	1
Gm45873	1,286543288	1
Tlr4	1,286543288	1
Tbc1d20	1,286543288	1
Cyb5a	1,286543288	1
Atg10	1,286364948	1
Hist3h2a	1,286364948	1
Ehmt1	1,286275787	1
Pcbp1	1,286008341	1
4930539J05Rik	1,285919205	1
Alkbh6	1,285919205	1
4833417C18Rik	1,285651833	1
Pole3	1,285651833	1
Etaa1	1,285562722	1
Zfp59	1,285562722	1
Gne	1,285473616	1
Med4	1,285384517	1
Bod1	1,285206337	1
Gm12183	1,285117257	1
Dok1	1,284850052	1
Pcgf1	1,284760996	1
Cenpo	1,284760996	1
Gm12912	1,284671946	1
Fez2	1,284582903	1
Gm9794	1,284582903	1
Ada	1,28422679	1
Tigar	1,28422679	1
Cndp2	1,28422679	1
Txndc15	1,284048771	1
Snx10	1,284048771	1
B930036N10Rik	1,28395977	1
Hoxa1	1,283692806	1
Myo19	1,283692806	1
Polr2g	1,283692806	1
Ndufb10	1,283692806	1
Surf6	1,283514861	1
Traf3ip3	1,283425898	1
RP23-139H6.1	1,283425898	1
Tnfrsf17	1,283070106	1
Insig2	1,282892247	1
Mfsd3	1,282714412	1
Cd2bp2	1,282714412	1

Spata24	1,282625505	1
Tec	1,282536603	1
Asah2	1,282447707	1
Ntpcr	1,282092186	1
Pold4	1,281914463	1
Nup85	1,281736764	1
Gpr65	1,28155909	1
Egln2	1,28155909	1
Tatdn2	1,281381441	1
Rpp38	1,281203816	1
Xrcc3	1,281115013	1
Bcat2	1,281115013	1
Ints6	1,281026216	1
Ttc1	1,281026216	1
Cep290	1,280937425	1
Gtf3c6	1,280937425	1
Oard1	1,280582323	1
Gm7676	1,280404809	1
A430010J10Rik	1,280404809	1
Slc9a3r2	1,280404809	1
Rps12-ps5	1,280316061	1
Hoxa3	1,280316061	1
Apeh	1,280316061	1
Gm44913	1,280227319	1
Vhl	1,280227319	1
Ybx1-ps2	1,280138584	1
4930589O11Rik	1,280049854	1
Alyref2	1,279961131	1
Taf1d	1,279961131	1
Fabp5	1,2796063	1
Akna	1,279428921	1
Ralb	1,279428921	1
Dtnb	1,279251567	1
Cnbd2	1,279162899	1
Fam204a	1,279162899	1
Prkch	1,279074237	1
Slc2a4	1,278896932	1
Rps12-ps1	1,278542395	1
Asb6	1,278453777	1
Dnajc1	1,278365164	1
Mtdh	1,278276558	1
Gm12338	1,278099363	1
Rer1	1,278010775	1
Ppa2	1,277833618	1
Pycrl	1,277745048	1
Tmem19	1,277656484	1
Dnase1l1	1,277302292	1
Idh3b	1,277213759	1
Stk16	1,277125233	1
Rcc1l	1,277125233	1
Hlx	1,277036712	1
Zscan29	1,277036712	1

Hist4h4	1,276948198	1
Zfp512	1,276859689	1
Cdon	1,276771187	1
Zswim7	1,276771187	1
Kif22	1,276771187	1
Mars	1,276771187	1
Mis12	1,276594202	1
Ttll12	1,276505718	1
Gcdh	1,27641724	1
Cib2	1,276328769	1
B4galt7	1,276328769	1
Mtg2	1,276240304	1
Rab11fip5	1,276240304	1
Gas2	1,276063391	1
Ccni	1,275974944	1
Med9	1,275532802	1
Rabep1	1,275444392	1
Dapk3	1,27526759	1
Vwf	1,275179198	1
Impdh2	1,275179198	1
Oxt	1,275090812	1
Mex3d	1,275002433	1
Zfp346	1,275002433	1
Trmt2a	1,275002433	1
2810002D19Rik	1,274825692	1
Dctpp1	1,274825692	1
Zfyve1	1,274648976	1
Heatr1	1,274648976	1
Rraga	1,274648976	1
Zfp593	1,274383948	1
Csf1r	1,274383948	1
4930431P19Rik	1,274295617	1
Mthfs	1,274207293	1
Gm14323	1,273765763	1
Ankrd37	1,273677475	1
Gm14094	1,273589194	1
Ulk3	1,273589194	1
Trim13	1,273324386	1
Retn	1,273236129	1
Stx1a	1,272883162	1
Znrd1as	1,272706715	1
Nle1	1,272706715	1
mt-Ti	1,272618501	1
Fah	1,272530293	1
Tmem101	1,272530293	1
Exosc5	1,272442091	1
Mtrf1l	1,272353895	1
Dusp16	1,272353895	1
Zfand6	1,272353895	1
Nanos1	1,272265705	1
Foxp4	1,272265705	1
Slc39a1	1,272177521	1

S100a3	1,271648547	1
Prdx5	1,271648547	1
Kctd7	1,271384143	1
Mvk	1,27129602	1
Cltb	1,27129602	1
Ptges2	1,271207904	1
Gng7	1,271031689	1
Zfp12	1,271031689	1
Nfkbib	1,270943591	1
Cyc1	1,270943591	1
Rpap2	1,270767413	1
Gtf2f1	1,27059126	1
Kcnn1	1,270239026	1
Gamt	1,270062945	1
Ddost	1,269974914	1
Exosc9	1,269710858	1
Crk	1,269446857	1
Rps19	1,269446857	1
B3gat3	1,269358868	1
Gm15575	1,269270886	1
Diablo	1,268831066	1
Dnajc2	1,268743121	1
Gm13736	1,268479321	1
BC005624	1,268391399	1
Gm9625	1,268391399	1
Habp4	1,268215575	1
Ccnh	1,268215575	1
Plpp1	1,268215575	1
Gm16433	1,268127672	1
Trit1	1,268127672	1
Mtif2	1,267864	1
Exoc3l2	1,267776121	1
Mrpl51	1,267776121	1
Zfp839	1,267688249	1
Gm12497	1,267512522	1
Gm37675	1,26733682	1
Prr13	1,267248978	1
Coil	1,267161142	1
Cript	1,267161142	1
Coro1c	1,267161142	1
Gm16845	1,26689767	1
Rgs10	1,26689767	1
Marc2	1,266722053	1
Pdzk1ip1	1,26654646	1
Sfi1	1,26654646	1
Cdkal1	1,266283117	1
Ppp2r5d	1,266283117	1
Acad8	1,266195348	1
Ggact	1,265932077	1
Narfl	1,265932077	1
Rrp1	1,265756594	1
Kin	1,265581135	1

Gm43712	1,265493415	1
2310011J03Rik	1,265317992	1
Cdc26	1,26523029	1
Dedd	1,265054904	1
Gm11298	1,26496722	1
Ptpn21	1,264791871	1
Gm45420	1,264791871	1
Tradd	1,264616545	1
Ddx18	1,264528892	1
Gm11599	1,264353603	1
Mipep	1,264265968	1
Ubap2	1,264178339	1
Gm28659	1,264090716	1
Rps3	1,264003098	1
Thtpa	1,263477524	1
Cnrip1	1,263389949	1
Plekhf2	1,263389949	1
RP23-359K10.9	1,263214818	1
Mtor	1,263127262	1
Tmem29	1,262952167	1
Dnlz	1,262689571	1
Comt	1,262689571	1
Bcl7c	1,262602051	1
Gm7600	1,262514537	1
Gm8508	1,262514537	1
Man1b1	1,262339528	1
Mrpl28	1,262164543	1
Rbks	1,261989581	1
Hnrnpf	1,26190211	1
Polr2i	1,261814645	1
Zfp975	1,261727185	1
Adgrl2	1,261552285	1
Gm4332	1,261552285	1
Sumf2	1,261464844	1
Zfp810	1,261202557	1
Commd7	1,26111514	1
Trappc6a	1,260765533	1
Ufsp2	1,260416023	1
Actr8	1,260416023	1
Ptgir	1,260328661	1
Gm11930	1,260241305	1
Gm9434	1,260241305	1
Map2k2	1,260241305	1
Rpp40	1,26006661	1
Stn1	1,26006661	1
Chac1	1,259979272	1
Usmg5	1,259979272	1
Tubb2a	1,259979272	1
Pdzd11	1,259717294	1
Ogfod2	1,259717294	1
Rpl31-ps1	1,25962998	1
Ranbp6	1,259193501	1

Gm7809	1,259106223	1
Zfp563	1,258931686	1
Wdr18	1,258931686	1
Tesk1	1,258931686	1
Sipa1l2	1,258757174	1
Anxa7	1,258757174	1
Nsmce4a	1,258669926	1
Mrps26	1,25840822	1
Itgb7	1,258320997	1
Gm15050	1,258320997	1
Rbm43	1,25823378	1
Brap	1,257972165	1
Rpn2	1,257972165	1
Gm9835	1,257710604	1
Cacul1	1,257623429	1
Foxn2	1,257449098	1
Gm6444	1,257449098	1
Pik3r4	1,257449098	1
Selenom	1,257361941	1
Phf21b	1,257187646	1
Gm10269	1,257187646	1
Atp23	1,257187646	1
Phyh	1,257187646	1
Cisd1	1,257013375	1
Tst	1,256926248	1
2410022M11Rik	1,256926248	1
Nat9	1,256926248	1
Fam132a	1,256490706	1
Bag1	1,256403616	1
Snx4	1,256316532	1
Gm4978	1,256229453	1
Gm18860	1,256229453	1
Rps15	1,256229453	1
Cntln	1,256142381	1
Smap2	1,256142381	1
Mettl6	1,256055315	1
Gm18889	1,255968255	1
Fgd6	1,255881201	1
Cox11	1,255794153	1
Atp5l	1,255620075	1
D030056L22Rik	1,255533045	1
Rftn1	1,255359003	1
Nrap	1,255271991	1
Rad23a	1,255271991	1
Plekhs1	1,255184985	1
Macrod1	1,255097986	1
Mtmr9	1,255010992	1
9230116N13Rik	1,255010992	1
Tfpt	1,255010992	1
Clec4d	1,254924004	1
Brf1	1,254837023	1
Derl2	1,254837023	1

Gm5828	1,254750047	1
Rpl3-ps1	1,254750047	1
Lamtor5	1,254750047	1
E230029C05Rik	1,254315259	1
Cirbp	1,25422832	1
Zfp383	1,254141386	1
Bfar	1,254054459	1
Taf1a	1,253706809	1
Cyp4f13	1,253706809	1
Mtg1	1,253706809	1
Gcc1	1,253706809	1
Gm45640	1,253619912	1
Rbm34	1,253619912	1
Stard7	1,253359256	1
Ccs	1,253098654	1
Pik3ap1	1,253011799	1
Timm50	1,25292495	1
Phf13	1,25292495	1
Nkap	1,25292495	1
Sar1a	1,25292495	1
Slc35b1	1,25275127	1
Rpl38-ps2	1,252664439	1
Timm44	1,252664439	1
Gm6085	1,252403981	1
Pigq	1,252317174	1
Prss44	1,252230373	1
Dusp7	1,252143578	1
Ecsit	1,252143578	1
Rfc3	1,252056789	1
Gm12778	1,251796459	1
Rps2-ps5	1,251709694	1
Zfp276	1,251709694	1
Hbb-bh3	1,251622935	1
Card11	1,251536182	1
Rnasel	1,251275959	1
Lyrm2	1,251275959	1
Gfer	1,251189231	1
Gm11110	1,251102508	1
Gm5900	1,251015791	1
Pgk1	1,251015791	1
Ppil4	1,25092908	1
Brix1	1,25092908	1
Fibp	1,250842375	1
1110025M09Rik	1,250755677	1
Pygm	1,250755677	1
Tal1	1,250755677	1
Trappc5	1,250668984	1
Wipi1	1,250668984	1
Slc41a1	1,250322273	1
Rpl30-ps1	1,25023561	1
Myl12b	1,249975658	1
Gsg1	1,249889019	1

C130050O18Rik	1,249889019	1
Mrpl21	1,249889019	1
Nos3	1,249629139	1
Adamts7	1,249542524	1
Stard8	1,249542524	1
Mfap3l	1,249542524	1
Faap20	1,249455916	1
Cant1	1,249369313	1
Fcgr4	1,249282716	1
Pigc	1,249282716	1
Mrpl19	1,249282716	1
Atp5o	1,249196126	1
Mst1	1,249109541	1
Epc2	1,249022962	1
Dxo	1,24893639	1
Rbm45	1,24893639	1
Ctnnbl1	1,24893639	1
Thoc3	1,24893639	1
Atp6v1e1	1,248763262	1
Efcab2	1,248676708	1
Gm11688	1,248503616	1
Ncoa6	1,248503616	1
Mettl22	1,248330549	1
Nsun3	1,248070993	1
Gm10941	1,247984486	1
Pid1	1,247984486	1
Brf2	1,247984486	1
Ypel3	1,247897985	1
Bcar1	1,247811491	1
Tnnc1	1,247811491	1
Trib1	1,247811491	1
Aktip	1,247811491	1
Mto1	1,247725002	1
Sbk2	1,247638519	1
Klhl8	1,247552043	1
Kcnab2	1,247379107	1
Fis1	1,247206196	1
Hoxaas3	1,247033308	1
Cd109	1,247033308	1
Gm6564	1,246946873	1
Dhrs3	1,246946873	1
Nubp2	1,246601194	1
Zfp830	1,246601194	1
Tdp2	1,246601194	1
Babam1	1,246514789	1
Dyrk3	1,246428391	1
Rabgap1l	1,246341998	1
Zwint	1,246255611	1
Hhex	1,245996486	1
Gm45311	1,245910123	1
Hars2	1,245910123	1
Sema6b	1,245823766	1

0610038B21Rik	1,245823766	1
Mrpl33	1,245823766	1
Pin1	1,245737416	1
Zfpl1	1,245737416	1
Oit3	1,245651071	1
2410131K14Rik	1,245651071	1
Dnajc25	1,245651071	1
Isoc2a	1,245564732	1
Dpp7	1,245392072	1
Hes6	1,245305751	1
Lactb	1,245219436	1
Cpne8	1,245046824	1
0610007P14Rik	1,244960526	1
Mrpl13	1,24478795	1
Gm13009	1,244701671	1
Klf1	1,244615398	1
Plpp5	1,244615398	1
Leo1	1,244529131	1
Rnaseh2a	1,24444287	1
Rspry1	1,244270365	1
Dusp9	1,244097885	1
Higd1a	1,244097885	1
Asap3	1,244011653	1
Sephs2	1,244011653	1
0610010F05Rik	1,243925428	1
Gemin6	1,243666788	1
Pdcd2l	1,243666788	1
Cbr1	1,243580586	1
Trappc1	1,243580586	1
Chrnb2	1,243408201	1
Polr3g	1,243408201	1
Gm43106	1,243322018	1
Zc2hc1a	1,243149669	1
Cyb561d1	1,243063503	1
Eef2kmt	1,243063503	1
Zranb2	1,242977344	1
Mrps23	1,242977344	1
Nupr1	1,24289119	1
Gm8822	1,242460511	1
Ttl	1,242374394	1
Scp2-ps2	1,242202176	1
Rpl17-ps10	1,242202176	1
Rab7b	1,242202176	1
Xpo5	1,242116076	1
Usf2	1,242116076	1
Eno3	1,242029982	1
Zmynd19	1,242029982	1
Gm9762	1,241943894	1
Fdft1	1,241771736	1
Rgl3	1,241771736	1
Gabarapl1	1,241599602	1
Gm12074	1,241513544	1

Dennd2d	1,241513544	1
Utp11	1,241513544	1
Rwdd1	1,241427492	1
Gm7808	1,241341445	1
Rab8a	1,241083342	1
Eef1g	1,241083342	1
Pex11b	1,240825293	1
Mtfr1	1,240739289	1
Rps8-ps3	1,24065329	1
Tmem234	1,240567298	1
Gm7332	1,24039533	1
Tmem79	1,240309356	1
Psmb10	1,240309356	1
Cep19	1,240223387	1
Ccnb1	1,240137424	1
Smad2	1,239965517	1
Fem1b	1,239793633	1
Lrrc73	1,239621773	1
Dubr	1,239621773	1
Aldh1l1	1,239621773	1
Romo1	1,239621773	1
Dnajc8	1,239535852	1
Sgk1	1,239364028	1
Tmbim1	1,239192227	1
Plcg1	1,239106336	1
Hsd17b10	1,239020451	1
Akap17b	1,238848698	1
1110037F02Rik	1,238848698	1
Nfe2l1	1,23876283	1
Trak2	1,238676969	1
Tef	1,238676969	1
Zfp94	1,238591113	1
Brdt	1,238591113	1
Dnajb12	1,238591113	1
Hist1h4i	1,238505264	1
A830073O21Rik	1,23841942	1
Gm37352	1,238333582	1
Cc2d2a	1,23824775	1
Polr2c	1,23824775	1
Tmem65	1,23824775	1
Mnat1	1,23824775	1
Lmo2	1,238161925	1
Mob3a	1,238076105	1
Gm13641	1,237990291	1
Clk3	1,237990291	1
Prrc1	1,237990291	1
Zfp953	1,237904483	1
Rpl36a	1,237904483	1
Akirin1	1,237904483	1
Rnmt	1,237647095	1
Gtpbp6	1,23756131	1
Acaa1a	1,23756131	1

Ppig	1,23738976	1
Rpl10-ps2	1,237218233	1
Ece2	1,237218233	1
Baz1a	1,237218233	1
A930029G22Rik	1,237132479	1
RP24-295J1.1	1,23704673	1
Pik3cb	1,23704673	1
Pdap1	1,236960988	1
Gm9484	1,236875251	1
Cd83	1,236875251	1
Rpf2	1,23678952	1
Fau	1,236703796	1
Ptgs2os	1,236618077	1
Gm14780	1,236618077	1
Med19	1,236618077	1
Cox8a	1,236446657	1
Mesdc1	1,236360956	1
Gm5867	1,236103889	1
Steap3	1,235589914	1
Dip2c	1,235418637	1
Gm5898	1,235333007	1
Rabac1	1,235333007	1
Hdc	1,235161766	1
Cenpb	1,235076154	1
Lsm5	1,234477037	1
Gm3145	1,234477037	1
Ccr2	1,234305914	1
Enox2	1,234305914	1
Rnf7	1,234305914	1
Vamp5	1,234220361	1
Plpp2	1,234134814	1
Fbxl6	1,234049273	1
Rbbp4	1,234049273	1
Prelid1	1,23387821	1
Mocos	1,233792687	1
Dcaf11	1,23370717	1
Kidins220	1,233536154	1
Ydjc	1,233108717	1
Nhlrc1	1,233023247	1
Bola1	1,233023247	1
Zfp622	1,233023247	1
Ttll1	1,232852325	1
Ecm1	1,232766874	1
Gpatch1	1,232595988	1
Scaf4	1,232425125	1
Rnpepl1	1,232425125	1
Gm5384	1,232339703	1
Celf3	1,232339703	1
C3ar1	1,232339703	1
Comp	1,232254287	1
Mon1b	1,232254287	1
Snrpd2	1,232083472	1

Gm8539	1,231998073	1
Rps24-ps3	1,231998073	1
S1pr2	1,231998073	1
Galt	1,231912681	1
N6amt1	1,231827294	1
B4galt6	1,231656538	1
Gm43128	1,231485806	1
Zfp467	1,231485806	1
Gm10335	1,231400449	1
Zfp579	1,231315098	1
Snrnp48	1,230888431	1
Rabep2	1,230717805	1
Ccdc6	1,230376625	1
Rtf1	1,230291345	1
AV099323	1,229950283	1
Fahd2a	1,229865032	1
Tprgl	1,229865032	1
Mrpl23	1,229694548	1
Elp5	1,229609315	1
Gm11914	1,229438867	1
Haus3	1,229438867	1
Ccdc18	1,229353652	1
Txnl1	1,229353652	1
Tmem171	1,229183239	1
Slc39a8	1,22901285	1
Cep95	1,228927664	1
Tbl3	1,228757311	1
Ap1s3	1,228672143	1
Atl3	1,22858698	1
Rgs12	1,228501824	1
Plaur	1,228416674	1
Gm13423	1,22833153	1
Slc30a2	1,22833153	1
Rpl23a-ps14	1,228246391	1
Cops6	1,228246391	1
Pdrg1	1,228161258	1
Rpl31-ps22	1,228076132	1
Mrpl44	1,227991011	1
Zcrb1	1,227820787	1
H2afj	1,227735684	1
Cdpf1	1,227650587	1
Enpp4	1,227650587	1
Drosha	1,227565495	1
Pgd	1,22748041	1
Gm15964	1,22739533	1
Lpl	1,227310257	1
Polr2m	1,226970021	1
Gm14013	1,226884977	1
Pex12	1,226884977	1
Haus1	1,226714907	1
Tubb4a	1,22662988	1
Ncoa7	1,22662988	1

Haus8	1,22662988	1
Zfp654	1,226459845	1
Wdr77	1,226374836	1
Ppm1l	1,226289833	1
Gm12230	1,226119845	1
Cnnm3	1,226119845	1
Nars	1,22603486	1
Fbf1	1,225864907	1
Rpl36a-ps1	1,225864907	1
Scamp5	1,225864907	1
Comtd1	1,22577994	1
Rras2	1,225525073	1
Tm2d2	1,225525073	1
Larp7	1,225525073	1
Gm19739	1,225440129	1
Trub2	1,22535519	1
Gins1	1,225015497	1
Gm12989	1,225015497	1
Stx8	1,225015497	1
Nudt16	1,225015497	1
Xpnpep3	1,224930588	1
Mrpl9	1,224930588	1
Fdxr	1,224845685	1
Coasy	1,224675898	1
Maged2	1,224506133	1
Gm5939	1,22442126	1
Polr2j	1,224336392	1
Wnt6	1,224251531	1
Zfp426	1,224081825	1
4930453N24Rik	1,224081825	1
Smndc1	1,224081825	1
Zfp874b	1,223996981	1
Gm12251	1,223912143	1
Fpgt	1,223912143	1
Prrg4	1,223912143	1
Rpl18-ps1	1,223742485	1
Tsg101-ps	1,223318441	1
Laptm4a	1,22323365	1
H2-DMb1	1,222979313	1
Gm13611	1,222894545	1
Cdc123	1,222894545	1
Cerk	1,222894545	1
Sipa1	1,222725028	1
Klhdc1	1,222640278	1
Srp9	1,222470796	1
Dph6	1,222386063	1
Rps19-ps5	1,222301337	1
RP23-63H11.3	1,222301337	1
Rpl18a	1,222216616	1
Ndufa11	1,222131902	1
Selenop	1,222047193	1
Emp2	1,222047193	1

Wsb2	1,222047193	1
RP23-255F14.4	1,22196249	1
Smap1	1,221793102	1
Cdc25b	1,221793102	1
Tufm	1,221708416	1
Gm44957	1,221708416	1
Itgb3	1,221623737	1
Tada2b	1,221623737	1
Gm6159	1,221623737	1
Ghdc	1,221623737	1
Rpgr	1,221454396	1
Trnau1ap	1,221285078	1
Cyhr1	1,221285078	1
Glmp	1,221200428	1
Chchd4	1,221200428	1
Fam98a	1,221031145	1
Leprot	1,221031145	1
Sfxn3	1,220946513	1
Gm2214	1,220608041	1
Igf1	1,220523438	1
Nckap1	1,220438841	1
Cblb	1,220438841	1
Lrrc14	1,220438841	1
Alg5	1,220269664	1
Ankrd34a	1,22010051	1
Il27	1,22010051	1
Pomgnt1	1,22010051	1
Dis3l	1,220015942	1
Ccdc50-ps	1,220015942	1
Rps2-ps11	1,219846824	1
RP23-350F7.3	1,219846824	1
Fam229b	1,219846824	1
Mrpl24	1,219846824	1
Cdkl3	1,219762273	1
Gm3511	1,219762273	1
Gzf1	1,219762273	1
Lrpap1	1,219677729	1
Wdr46-ps	1,21942413	1
Sep 10	1,21942413	1
Aip	1,21942413	1
Wdr36	1,21942413	1
Arhgap27os2	1,219339609	1
Pmvk	1,219339609	1
Sigirr	1,219255094	1
Bmyc	1,219170585	1
Tmem256	1,219170585	1
Rptor	1,219086081	1
Cluap1	1,219001583	1
Tmem214	1,218832606	1
Ssbp1	1,218748126	1
Wtap	1,218748126	1
Plekhj1	1,218748126	1

Ccdc91	1,218663651	1
Neurl1a	1,218579183	1
Fam219b	1,21849472	1
Sord	1,218410264	1
Snx7	1,218325813	1
3110040N11Rik	1,218241368	1
Smim13	1,218156929	1
Gatad1	1,218156929	1
4930440I19Rik	1,218072495	1
Cxcr4	1,218072495	1
Gm7384	1,217988068	1
Rps7	1,217988068	1
Amacr	1,217903646	1
Gm4705	1,217903646	1
Zfp68	1,217903646	1
Prnp	1,217903646	1
Creb3l3	1,217566019	1
Snrnp27	1,217566019	1
Urod	1,217228484	1
Gatsl3	1,217228484	1
Pdha1	1,217228484	1
Cenpt	1,217144116	1
Zfp580	1,217144116	1
Rab3a	1,217059752	1
Ranbp3	1,217059752	1
Pithd1	1,216975395	1
Usp5	1,216975395	1
Psen2	1,216975395	1
Zfp606	1,216806698	1
Klf8	1,216638025	1
Rsad1	1,216553697	1
1700084E18Rik	1,216469375	1
Shb	1,216216443	1
Gm26532	1,215879283	1
Nmnat1	1,215879283	1
Ntrk3	1,215879283	1
Kif3a	1,215795008	1
Dner	1,215795008	1
Ncbp2	1,215710738	1
Fosl2	1,215710738	1
CAAA01180111.2	1,215626474	1
Mapk1ip1l	1,215373718	1
Gm12816	1,215289478	1
Tex264	1,215289478	1
Tuba1b	1,215205243	1
Gm8606	1,215121015	1
Cxx1b	1,215121015	1
Tmem177	1,215036792	1
Cdr2	1,214952575	1
Serpinb8	1,214868364	1
Rpl10a-ps2	1,214784158	1
Flrt2	1,214784158	1

Rmdn3	1,214784158	1
Ptges3	1,214784158	1
Ndufb5	1,214784158	1
Atg7	1,214699959	1
Ptdss2	1,214699959	1
9330160F10Rik	1,214531577	1
Fance	1,214531577	1
Gm9776	1,214447395	1
Psmf1	1,214447395	1
Itga7	1,214363219	1
Apoo	1,214194884	1
Gm13215	1,214110726	1
Gm9703	1,214026573	1
Nepro	1,214026573	1
Rpl23a-ps3	1,213942426	1
Gba	1,213858285	1
Bloc1s1	1,21369002	1
Gm11826	1,21369002	1
Ggct	1,21369002	1
Hoxb8	1,213521778	1
Ash2l	1,213521778	1
Cebpa	1,213437666	1
Ambp	1,21335356	1
Taf12	1,213185365	1
Sort1	1,213101277	1
Golim4	1,213101277	1
1110004F10Rik	1,212933117	1
Tasp1	1,212849046	1
Eif1	1,21276498	1
Cul7	1,212428777	1
Pdik1l	1,212428777	1
Tssc1	1,212428777	1
Chic2	1,212428777	1
Phax	1,212344741	1
4833418N02Rik	1,21226071	1
Gm7266	1,21226071	1
Kdm1a	1,21226071	1
Polr2f	1,21226071	1
Aldh3b1	1,212176686	1
Arhgef4	1,212092667	1
Gm10762	1,212008654	1
Icam5	1,212008654	1
Taf13	1,211840646	1
Ahsa1	1,21167266	1
Wdr60	1,21167266	1
Rab20	1,211420726	1
Acot7	1,211420726	1
Mars2	1,21133676	1
Map4k1	1,21133676	1
Irf2bpl	1,21133676	1
Nexn	1,211252799	1
Pgm2l1	1,211252799	1

Zfp551	1,211168845	1
Hsdl2	1,210917015	1
Slc16a12	1,210833084	1
Gnl1	1,210833084	1
Cd99l2	1,210749158	1
Crry-ps	1,210581324	1
Afp	1,210581324	1
Agpat3	1,210581324	1
Clcnkb	1,210413514	1
Ccdc59	1,210413514	1
Eif2b4	1,210077963	1
Golt1b	1,209994089	1
Esrra	1,20982636	1
Ngrn	1,20982636	1
4933433G15Rik	1,209490971	1
Tbck	1,209407139	1
Nudt9	1,209407139	1
2700060E02Rik	1,209239491	1
Pgf	1,209155676	1
Hadh	1,209155676	1
Srpr	1,208988063	1
Fmc1	1,208988063	1
Tsga10ip	1,208904265	1
Pard6a	1,208904265	1
Gm12693	1,208904265	1
Hspb6	1,208820473	1
Pik3c3	1,208820473	1
RP24-275P22.2	1,208736687	1
Mrm3	1,208736687	1
Dhx30	1,208652907	1
Uchl5	1,208652907	1
Tshz1	1,208485363	1
Rabl3	1,208401601	1
Slc30a1	1,208401601	1
Snx19	1,208317843	1
BC024978	1,208234092	1
Ddx1	1,208150347	1
Adrb2	1,208066607	1
Cisd3	1,207982873	1
Spata2l	1,207982873	1
Pxmp4	1,207982873	1
E2f3	1,207982873	1
Ssu72	1,207982873	1
Gm19325	1,207815423	1
Tbc1d17	1,207731706	1
Gstz1	1,207731706	1
Eif3l	1,207480591	1
Atp6v1b2	1,207480591	1
Ggps1	1,207396898	1
Pter	1,207313211	1
C87436	1,207229529	1
Mrps7	1,207229529	1

Cox20	1,207229529	1
Dnaja2	1,207229529	1
Ptgr1	1,207145853	1
Mtus1	1,206727561	1
Tcp1	1,206727561	1
Gm10012	1,20664392	1
Mrpl47	1,20664392	1
Ndufa2	1,206476655	1
Ctsc	1,206225801	1
Armcx5	1,205975	1
Akap7	1,205975	1
Ak2	1,205891411	1
Gm11249	1,205807828	1
Frmd8os	1,205807828	1
Timmdc1	1,205640679	1
Tmem126a	1,205640679	1
Tlk1	1,205557113	1
Cwc15	1,205389999	1
Pskh1	1,205306451	1
Fbxo7	1,205306451	1
Iah1	1,20505584	1
Gm11605	1,204972315	1
Mapkapk5	1,204972315	1
Ddx10	1,204972315	1
Gm5735	1,204888796	1
Rit1	1,204888796	1
Ptcd2	1,204805282	1
Tsc22d1	1,204721774	1
Eif2b3	1,204721774	1
Gm17586	1,204638272	1
Acat2	1,204638272	1
Dstyk	1,204554776	1
Knop1	1,204554776	1
Gm15782	1,204471285	1
Gpsm2	1,204471285	1
Gm15846	1,204220849	1
Arhgap9	1,204137381	1
Parn	1,20405392	1
Il1rap	1,203887014	1
Opn3	1,203636699	1
Trmt112-ps2	1,203553272	1
Arhgef3	1,203553272	1
Ndufb2	1,203469851	1
Ankrd49	1,203303026	1
Psmg4	1,203303026	1
Ubr7	1,203303026	1
Slc39a11	1,203052832	1
Rab3d	1,202969446	1
Sra1	1,202886065	1
Psmb1	1,202886065	1
Neurl3	1,202385903	1
Pde8b	1,202302563	1

Vti1b	1,202219228	1
Lrrc61	1,2021359	1
Arpc5l	1,202052577	1
Doc2g	1,20196926	1
Clstn1	1,20196926	1
Gm15148	1,20196926	1
Ttc25	1,201885949	1
Bst1	1,201802643	1
Mrps11	1,201719343	1
Mlf2	1,201719343	1
Hacd1	1,20163605	1
Grwd1	1,201552761	1
Sla	1,201552761	1
Gars	1,201552761	1
Kcne3	1,201386202	1
Wwox	1,201386202	1
Gm6977	1,201136407	1
Tspan17	1,201053154	1
Klhl11	1,200803427	1
Sec16b	1,200720197	1
Dclre1c	1,200636972	1
Mcfd2	1,200636972	1
Colec12	1,20047054	1
Dlat	1,20047054	1
Fam134a	1,20047054	1
Gm17530	1,200220935	1
Rpl9	1,200220935	1
Fam58b	1,200054561	1
Lonp2	1,200054561	1
Keap1	1,200054561	1
Chchd7	1,19988821	1
Eif3g	1,19988821	1
Gm6198	1,199638726	1
Cstf1	1,199555576	1
Prkar2b	1,199472432	1
Pafah1b1	1,199472432	1
Rfxap	1,199389294	1
Taf6	1,199389294	1
Zkscan17	1,199306161	1
Gm6030	1,199223035	1
Toe1	1,199139914	1
Bag5	1,199056799	1
Gemin7	1,198973689	1
St3gal2	1,198807488	1
Mettl8	1,198724396	1
Srp19	1,198724396	1
Asnsd1	1,198724396	1
Mr1	1,198558229	1
Gm10126	1,198475154	1
Osgep	1,198475154	1
Prkra	1,198475154	1
Stk19	1,198392085	1

Gm20594	1,198309021	1
Mrgbp	1,198309021	1
Ccdc191	1,198309021	1
Ptgr2	1,198142912	1
Uqcrc1	1,198142912	1
Arid5b	1,198059866	1
Ganab	1,197976826	1
Gm15496	1,197810762	1
Renbp	1,197810762	1
Tmsb4x	1,197644722	1
Isl2	1,197478705	1
Ahi1	1,197395705	1
Gm29019	1,197146739	1
Gm19272	1,197146739	1
AI467606	1,197146739	1
Orai1	1,197063762	1
Mesdc2	1,197063762	1
Ntn5	1,196897825	1
Rhno1	1,196897825	1
2210013O21Rik	1,196897825	1
Glo1	1,196897825	1
Slc16a13	1,196814866	1
Apobec3	1,196814866	1
Uqcrc2	1,196731911	1
Nupr1l	1,196648963	1
Ankle2	1,196648963	1
Tomm7	1,196566021	1
Gle1	1,196483084	1
Ift122	1,196400153	1
4933408B17Rik	1,196234308	1
Xpnpep1	1,196068486	1
Nsmce1	1,195985584	1
Mtmr4	1,195902688	1
Ndufb3	1,195902688	1
Mertk	1,195819797	1
Mfap1a	1,195736912	1
Ate1	1,195736912	1
Acy1	1,195654032	1
Ammecr1l	1,195654032	1
Sfxn1	1,195488291	1
Coro2a	1,195322573	1
Rfk	1,195322573	1
Cyp2c55	1,195239722	1
Cir1	1,195239722	1
Crip1	1,195239722	1
Nedd4l	1,194991205	1
A430046D13Rik	1,194908377	1
Brox	1,194825555	1
Rnf181	1,194825555	1
Zfas1	1,194742739	1
Wdr83	1,194577124	1
E330011M16Rik	1,194411532	1

Uqcrh-ps2	1,194328745	1
Ankrd55	1,194245963	1
Depdc1b	1,194245963	1
Cox4i1	1,194245963	1
Ankrd13b	1,194163187	1
Ccdc137	1,194080417	1
Glb1l	1,193997652	1
Gm20072	1,193997652	1
Vav2	1,193832141	1
Gm27029	1,193749393	1
CH25-309J2.1	1,193666652	1
Ttc4	1,193666652	1
Zfp763	1,193583916	1
Rhot2	1,193501186	1
Itga2b	1,193418462	1
Trip6	1,193418462	1
Kcnn4	1,19325303	1
Pptc7	1,193170323	1
Gm1862	1,193087622	1
Pomk	1,193087622	1
Mzt2	1,193087622	1
Pafah1b3	1,193087622	1
AU022252	1,193004926	1
Riok3	1,192922236	1
Uqcr10	1,192922236	1
9530082P21Rik	1,192839552	1
RP23-403E19.1	1,192591534	1
Mdk	1,192591534	1
RP24-325N9.5	1,192508872	1
Gm5883	1,192426217	1
Osbpl11	1,192095652	1
Gm14567	1,191930404	1
Uqcrfs1	1,191930404	1
Gstp-ps	1,191847788	1
Rpn1	1,191847788	1
Atp5k	1,191765179	1
Smurf1	1,191682575	1
Gm44075	1,191434797	1
Nsmf	1,19126964	1
Commd3	1,190856849	1
Snhg12	1,190856849	1
Saraf	1,190774308	1
Gm10268	1,190609243	1
Zbtb18	1,19052672	1
Zc3h4	1,19052672	1
Eid1	1,190114186	1
Stx18	1,190031696	1
Gm18737	1,189949212	1
2310034G01Rik	1,189866734	1
Ywhae	1,189866734	1
Zfp933	1,189784262	1
Pef1	1,189784262	1

Stap2	1,189701795	1
Rnd2	1,189619334	1
Mrps5	1,189619334	1
Mrps6	1,189454429	1
Olfm1	1,189454429	1
Fbxl3	1,189289547	1
Ddx56	1,189207115	1
Hsh2d	1,189042267	1
Zbtb25	1,189042267	1
Gorasp2	1,189042267	1
Cd44	1,188795039	1
H2-Q5	1,18871264	1
Pde4a	1,18846548	1
Usp42	1,18846548	1
Zc3h15	1,188136013	1
Ints10	1,188053661	1
Prmt2	1,188053661	1
Lfng	1,188053661	1
Gm10138	1,187971314	1
Pgrmc2	1,187888973	1
Cep72	1,187724308	1
Gm8662	1,187641984	1
Fam214a	1,187559666	1
Tiprl	1,187395047	1
Gm6543	1,18723045	1
Dpep2	1,18723045	1
Ube2q2	1,18723045	1
Lamtor1	1,18723045	1
Anapc4	1,187065877	1
Gm10237	1,186983598	1
Inpp1	1,186901326	1
Borcs8	1,186901326	1
Katnal1	1,186901326	1
Gm12857	1,186901326	1
Marcksl1	1,186736798	1
Abhd14b	1,186736798	1
Ppm1j	1,186654542	1
Dtx3	1,186572292	1
Ccdc124	1,186490048	1
Ssr2	1,186490048	1
Gm15975	1,186325577	1
BC017643	1,186325577	1
Psmd10	1,186325577	1
Eapp	1,186161129	1
Fblim1	1,186078913	1
4921531C22Rik	1,185996704	1
Utp3	1,185914499	1
Gm3362	1,185832301	1
Kcnab3	1,185750108	1
Top1mt	1,185750108	1
Aimp2	1,185667921	1
Tmem104	1,185503564	1

Clptm1	1,18533923	1
Emc1	1,18533923	1
Selenok	1,185174918	1
Tmem201	1,185010629	1
Slc35a4	1,185010629	1
Xlr	1,184928494	1
Gm4204	1,184764239	1
Bad	1,184600007	1
Dld	1,1845179	1
Tes3-ps	1,184435798	1
Chmp3	1,184435798	1
H2afv	1,184353702	1
Endog	1,184189527	1
Rce1	1,184107448	1
Med10	1,184107448	1
Rnf122	1,183861246	1
Chmp6	1,183451022	1
BC003331	1,183286972	1
Gm15198	1,183286972	1
Necap2	1,183286972	1
Isoc1	1,183204956	1
Qdpr	1,182958941	1
Rars	1,182958941	1
Nhp2	1,182958941	1
Cyp51	1,182876947	1
Vps25	1,182794959	1
Atg4d	1,182794959	1
Itpkc	1,182549029	1
Parp1	1,182549029	1
Aplp1	1,182467064	1
Gm10086	1,182467064	1
Trim14	1,182385105	1
Zfp24	1,182385105	1
Nxt1	1,182385105	1
Atp2a3	1,182303151	1
Trmt10a	1,182303151	1
Dctn2	1,18213926	1
Scaper	1,181975392	1
Rpl36-ps4	1,181647724	1
Ppp1ca	1,181647724	1
Gm45749	1,181565822	1
Gm16379	1,181402033	1
Tmem14c	1,181402033	1
Nfkbie	1,181320148	1
Edem2	1,181074524	1
Gm45051	1,180992661	1
Ntan1	1,180992661	1
Pctp	1,180992661	1
Ubac1	1,180992661	1
1110004E09Rik	1,180992661	1
Neu1	1,180992661	1
Taf3	1,180992661	1

Mrps9	1,180992661	1
Rnf113a1	1,180910804	1
Ube2g2	1,180910804	1
Dcakd	1,180828952	1
Pnrc2	1,180828952	1
Gm15151	1,180665266	1
Ahsa2	1,180583431	1
Puf60	1,180583431	1
Elovl5	1,180583431	1
Nr1d2	1,180419779	1
Denr	1,180337962	1
Sema4b	1,180174343	1
Wdr59	1,180092543	1
Gm16201	1,179928958	1
Gm14328	1,179683625	1
Map4k2	1,179601858	1
Klc4	1,179601858	1
Hacl1	1,179520097	1
Rnh1	1,179520097	1
Cenpj	1,179438342	1
Riox1	1,179438342	1
Nap1l4	1,179438342	1
BC017158	1,179356592	1
Camk2n2	1,179356592	1
BC029214	1,179356592	1
Polr3d	1,179356592	1
Emc7	1,179356592	1
Fastkd2	1,179274848	1
Sh2d2a	1,17919311	1
Msl3	1,17919311	1
Hsd17b11	1,17919311	1
Smc1a	1,17919311	1
Cdca4	1,179111377	1
Serf2	1,178947929	1
Wbp1l	1,178947929	1
Mboat7	1,178784504	1
Gucd1	1,178702799	1
4932441J04Rik	1,178621101	1
Uba5	1,178621101	1
Fgfbp3	1,178539408	1
Gm13602	1,178376039	1
Psma2	1,178376039	1
Gnl3	1,178294363	1
Tifab	1,178212693	1
Ccnd3	1,178212693	1
Usp6nl	1,178131028	1
Slc16a7	1,178049369	1
Hmg20b	1,178049369	1
Slc25a20	1,177967716	1
Cuta	1,177967716	1
Ndufa6	1,177967716	1
Cetn4	1,177886068	1

Mcph1	1,177886068	1
Ptpmt1	1,177886068	1
Gm13268	1,177641159	1
Cox17	1,177641159	1
Nub1	1,177641159	1
Tfip11	1,177559534	1
Whamm	1,177559534	1
Tbc1d16	1,177477914	1
Ergic3	1,177477914	1
Limk2	1,177477914	1
Gm20045	1,177069902	1
Tbc1d10a	1,177069902	1
Dnttip2	1,176988317	1
Nfam1	1,176906737	1
Ppcdc	1,176825163	1
AI662270	1,176825163	1
Tmbim4	1,176743595	1
Asb7	1,176662032	1
Slc35f6	1,176580475	1
Nudt13	1,176580475	1
Icam1	1,176580475	1
Fundc1	1,176417377	1
Elmod3	1,176254302	1
Gm5611	1,176172774	1
Gas2l1	1,176172774	1
Thap11	1,176172774	1
Nsun6	1,17609125	1
Gm8805	1,17609125	1
Os9	1,17609125	1
Mmgt1	1,176009733	1
Trim7	1,175846714	1
Fam32a	1,175765214	1
Rps29	1,175602229	1
Tmem30a	1,175602229	1
Cct5	1,175602229	1
Tsfm	1,175520746	1
Dtx4	1,175520746	1
Pde12	1,175439268	1
Slc44a2	1,175439268	1
Sdhaf3	1,175276328	1
Tln2	1,175031962	1
Bcs1l	1,175031962	1
Traf5	1,175031962	1
2310036O22Rik	1,175031962	1
Dcaf4	1,174787646	1
Ddx58	1,174624798	1
Gm7658	1,174624798	1
Fam210b	1,174543382	1
Pirb	1,174461971	1
Inafm1	1,174461971	1
Arpp19	1,174461971	1
Isg15	1,174217774	1

Fam134c	1,174136386	1
Creb3	1,174136386	1
Ell2	1,174136386	1
Phkg1	1,173973628	1
Acot13	1,173973628	1
Ctdspl	1,173810892	1
Fhad1	1,173810892	1
Snx9	1,173810892	1
Il16	1,173648178	1
Trp53inp2	1,17356683	1
Cdc6	1,173485487	1
Clp1	1,173485487	1
Cep83	1,173485487	1
Gm11944	1,17340415	1
Ostf1	1,17340415	1
Skor1	1,172834949	1
Gmppa	1,172834949	1
Rpl30-ps9	1,172753657	1
Fam195b	1,172753657	1
Gm14586	1,17259109	1
Furin	1,17259109	1
Rest	1,172428546	1
Ehd1	1,172428546	1
Clint1	1,172347282	1
Derl1	1,172266024	1
Fem1a	1,172184772	1
Accsl	1,172022284	1
Mgat2	1,171941048	1
Rap2b	1,171941048	1
A130071D04Rik	1,171859818	1
Mtf1	1,171859818	1
Micu1	1,171778594	1
Otud1	1,171697375	1
Rpl28	1,171697375	1
Itpripl2	1,171534955	1
Rpl3	1,171453753	1
Slc7a4	1,171372557	1
Htatsf1	1,171372557	1
Gm17018	1,171372557	1
Gm16425	1,171129002	1
Ccdc122	1,171129002	1
Ube2o	1,171129002	1
6720464F23Rik	1,171047828	1
Ccdc97	1,171047828	1
Usb1	1,171047828	1
Nemp2	1,17096666	1
Sapcd1	1,17096666	1
Pebp1	1,17096666	1
Slc24a3	1,170885498	1
Bicd1	1,170885498	1
Zc3h10	1,170885498	1
Gm12543	1,17072319	1

1810043G02Rik	1,17072319	1
Pstpip2	1,170642044	1
Rrm2b	1,170642044	1
Hck	1,170642044	1
Gm7094	1,170560904	1
Gm7972	1,17047977	1
Mtif3	1,170155289	1
Snx1	1,170155289	1
Slu7	1,170074183	1
6330418K02Rik	1,169993082	1
Tas1r1	1,169749815	1
Btd	1,169749815	1
Arl13b	1,169749815	1
Gm14593	1,169587664	1
Pth1r	1,169587664	1
Zfp503	1,169425536	1
Gm26983	1,16926343	1
Chrac1	1,16926343	1
Eno2	1,16926343	1
Timm10	1,169182386	1
Zfp809	1,169182386	1
Tbxas1	1,169101347	1
Gm44609	1,169101347	1
Gtpbp10	1,169020314	1
Gng2	1,168858265	1
Pld2	1,168777249	1
Zfp961	1,168777249	1
Dlgap4	1,168777249	1
Snrpd1	1,168696238	1
Topors	1,168615233	1
Mettl4	1,168534233	1
Rasgef1b	1,168534233	1
Chchd5	1,168534233	1
Rfesd	1,16845324	1
Arpc1a	1,16845324	1
Fam213a	1,16812932	1
Ckap4	1,16812932	1
3110080O07Rik	1,167967395	1
Poldip2	1,167967395	1
Pkp4	1,16788644	1
Gm9761	1,167724548	1
Rassf8	1,16764361	1
Xylt2	1,16764361	1
Vps37c	1,167562678	1
Kif2a	1,167562678	1
Gm6905	1,167481752	1
Tmem129	1,167481752	1
Smim10l1	1,167400831	1
Ulk1	1,167400831	1
Mmgt2	1,167400831	1
Cd81	1,167319916	1
Ldha	1,167319916	1

Ccdc174	1,167158102	1
Gm11353	1,166996311	1
Dnajb14	1,166996311	1
Gm5276	1,166915423	1
Ogfod3	1,166915423	1
Lrtm2	1,166834542	1
Immp2l	1,166834542	1
Stk40	1,166834542	1
Ndufa7	1,166591931	1
Eif4enif1	1,166511071	1
Eif1a	1,166511071	1
Nfatc1	1,166268527	1
Psap	1,166187691	1
Srp14	1,166187691	1
Polg	1,166026034	1
Rnf168	1,166026034	1
Fam162a	1,166026034	1
Rpl8	1,1658644	1
Dnm3	1,165783591	1
Nol6	1,165783591	1
Thoc7	1,16562199	1
Stk38l	1,16562199	1
Gm12176	1,165541198	1
Gon7	1,165541198	1
Yipf2	1,165460412	1
Hmga1-rs1	1,165379631	1
Armc7	1,165298856	1
Msi1	1,165218086	1
Fip1l1	1,164895064	1
Sdsl	1,164814322	1
Efna1	1,164572131	1
Elmod2	1,164491412	1
Txn2	1,164329991	1
Tsen15	1,164249288	1
Cryz	1,164249288	1
Anapc11	1,164168591	1
Tomm70a	1,164168591	1
Aldh18a1	1,1640879	1
Srf	1,1640879	1
Gm5532	1,164007214	1
Pex10	1,163926534	1
Ebpl	1,163926534	1
Nck1	1,16384586	1
Lage3	1,163765191	1
Idnk	1,163684528	1
Odc1	1,163684528	1
Prkrip1	1,163684528	1
Bud31	1,163684528	1
Bco2	1,16360387	1
Eif3c	1,16360387	1
Zfp719	1,163523218	1
0610009L18Rik	1,163523218	1

Gm38375	1,163442572	1
1110006O24Rik	1,163442572	1
Zfp30	1,163442572	1
Mid1ip1	1,163442572	1
Prickle2	1,163361931	1
Ipmk	1,163120042	1
5830444B04Rik	1,163120042	1
Prmt3	1,163039423	1
Ubtd1	1,163039423	1
Mrpl36	1,16295881	1
Cdipt	1,16295881	1
Ndufa12	1,162878203	1
Fkbp8	1,162878203	1
Gm6304	1,162636414	1
Mib2	1,162636414	1
Gm43961	1,162394676	1
Cdkn2aipnl	1,162394676	1
Cap1	1,162394676	1
Mdm2	1,162314108	1
Ybey	1,162233545	1
Gm9530	1,162152988	1
Cyb5d2	1,162152988	1
Fcgr3	1,16199189	1
Mir3091	1,16191135	1
Gm14513	1,161830815	1
Cast	1,161830815	1
Gm8318	1,161750286	1
D630023F18Rik	1,161750286	1
Ing2	1,161750286	1
Vars	1,161428225	1
Gm8930	1,161186738	1
Pls3	1,161186738	1
RP23-288C18.3	1,161106253	1
Kdm2b	1,161106253	1
Lasp1	1,160945301	1
Dcun1d5	1,160864833	1
Gm42918	1,160623463	1
Zfp667	1,160623463	1
Pemt	1,160543018	1
Bbs12	1,160462578	1
Gm8649	1,160382144	1
Max	1,160382144	1
Fra10ac1	1,160382144	1
Apex1	1,160301715	1
March2	1,160221292	1
Mtcl1	1,160060462	1
Tepsin	1,159980055	1
Pla2g2e	1,159819259	1
Uap1l1	1,159819259	1
Gm24991	1,159497734	1
Opa3	1,159497734	1
Mtmr3	1,159417366	1

Stk35	1,159256648	1
Bcl2	1,159256648	1
Evl	1,159256648	1
Fancb	1,159176297	1
Fam35a	1,159176297	1
Kitl	1,159176297	1
Smtn	1,158774627	1
Metap1	1,158694309	1
Repin1	1,158533691	1
Setmar	1,158453391	1
Wipi2	1,158453391	1
Jrkl	1,158373096	1
Egfl8	1,158292806	1
Gm10036	1,158212522	1
Ccdc12	1,158132244	1
Phf10	1,158132244	1
Zfp787	1,158051971	1
Fads1	1,158051971	1
Gm42571	1,157971704	1
Fcna	1,157891442	1
Rps11	1,157891442	1
D7Bwg0826e	1,157811186	1
Ptpn12	1,157811186	1
Aldh1b1	1,157730935	1
Gm6377	1,15765069	1
Mastl	1,157570451	1
Galk1	1,157570451	1
Mapk11	1,157089131	1
Lrp4	1,15700893	1
Tomm40	1,15700893	1
Ripk3	1,156848546	1
1190007I07Rik	1,156608011	1
Gm10169	1,156608011	1
Adipor1	1,156527844	1
Scnm1	1,156447682	1
Fam50a	1,156447682	1
Abr	1,156287376	1
Wars2	1,156207231	1
Ift27	1,156207231	1
Gm11427	1,156207231	1
Nacc1	1,156207231	1
Akr1b8	1,156207231	1
Chpf	1,156127091	1
Trmt6	1,156127091	1
Dus3l	1,156046958	1
Srek1ip1	1,156046958	1
Txndc12	1,155966829	1
Bet1l	1,155966829	1
Rnf214	1,155886707	1
Slc50a1	1,155806589	1
Atg12	1,155806589	1
Gaa	1,155726478	1

F7	1,155566271	1
Havcr2	1,155486176	1
Selenos	1,155486176	1
Rbl1	1,155326003	1
Prdm2	1,155326003	1
Erlec1	1,155165852	1
Gm11989	1,154925667	1
Tfdp2	1,154925667	1
Gsto1	1,154925667	1
Bloc1s4	1,154925667	1
Lemd2	1,154925667	1
Hmga1	1,154845616	1
Gm10343	1,154685532	1
Tmem208	1,154685532	1
Zfp217	1,154605498	1
Smad7	1,154365429	1
Sh3bp5l	1,154365429	1
Golga4	1,154125411	1
Lmnb2	1,154045416	1
9330020H09Rik	1,153965426	1
2310009A05Rik	1,153965426	1
Pnisr	1,153965426	1
Ttc38	1,153885442	1
B9d2	1,153885442	1
Rnps1	1,153485605	1
Aph1a	1,153485605	1
2810004N23Rik	1,153485605	1
Gdf9	1,153405654	1
Rsl24d1	1,153405654	1
Bcl7a	1,153325709	1
Clybl	1,153245769	1
Nudt7	1,153085907	1
Fam96a	1,153085907	1
Eif4a2	1,153005984	1
Mex3c	1,153005984	1
Gm4707	1,152926066	1
Rell1	1,152766248	1
Dnajb11	1,152686347	1
Daglb	1,152686347	1
Crip2	1,152366799	1
Ggnbp2	1,152366799	1
Scyl1	1,152286925	1
Dock2	1,152207058	1
Yeats2	1,152047339	1
Rad9b	1,151887642	1
Eif2b1	1,151727968	1
Dcun1d2	1,151648139	1
Dhx32	1,151568316	1
Zfp318	1,151568316	1
Ppm1g	1,151568316	1
Hagh	1,151568316	1
Sat2	1,151249077	1

Gm4617	1,151169282	1
Csnk2b	1,151169282	1
Pygl	1,151089491	1
Zmat5	1,151009707	1
Hdac3	1,151009707	1
Hoxb4	1,151009707	1
Trem2	1,151009707	1
Cyp20a1	1,150770386	1
Vars2	1,150610866	1
Zfp784	1,150531115	1
Ccdc115	1,150531115	1
Cdc45	1,150451369	1
Setd6	1,150451369	1
Nob1	1,150451369	1
Pld4	1,150371628	1
Eef2k	1,150212164	1
Sirt6	1,15013244	1
Chchd6	1,15013244	1
Klf16	1,150052722	1
Gsn	1,150052722	1
Tmem116	1,149973009	1
Sema5a	1,149973009	1
Dalrd3	1,1498136	1
Gm8623	1,149733904	1
Dennd4b	1,149733904	1
Ikbkb	1,149733904	1
Net1	1,149654213	1
Mrpl14	1,149494848	1
Ttc9	1,149415174	1
Mapre3	1,149255842	1
Morn1	1,149096533	1
Fam76a	1,149096533	1
Golph3	1,149096533	1
Carhsp1	1,149016886	1
Sec24a	1,148698355	1
Sdhc	1,148698355	1
RP24-389J11.1	1,148618736	1
Eif2s2	1,148618736	1
Stam2	1,148539123	1
Scamp1	1,148459515	1
Gm22581	1,148379912	1
Cdk20	1,148379912	1
Gde1	1,148300315	1
1110038B12Rik	1,148220724	1
Umad1	1,148141138	1
Tmem260	1,147981983	1
Gm11970	1,147902414	1
Zfp324	1,14782285	1
Hspa9-ps1	1,14782285	1
Gm6265	1,147743292	1
Acox3	1,147743292	1
Commd2	1,147663739	1

Kctd12	1,147584192	1
Ncdn	1,14750465	1
Itgal	1,147425114	1
4933421O10Rik	1,147345583	1
Inf2	1,147345583	1
Timm8b	1,147345583	1
Tmeff1	1,147266058	1
Zfp513	1,147266058	1
Leprotl1	1,147266058	1
Gm3283	1,147107024	1
Gpr146	1,147107024	1
RP23-451J19.1	1,147027516	1
Map7d1	1,147027516	1
Gm10358	1,146868515	1
Ddx55	1,146868515	1
Ppp1r14b	1,146868515	1
Fmn1	1,146789023	1
Ddx49	1,146471109	1
9130230L23Rik	1,146391645	1
Mrps15	1,146312186	1
Angpt2	1,146232732	1
Ptpn11	1,146232732	1
Hist1h2bc	1,146153284	1
Usp20	1,146073842	1
Gm37305	1,145914973	1
Wdsub1	1,145914973	1
BC051226	1,145914973	1
Pelo	1,145756126	1
D16Ertd472e	1,145676711	1
Gins2	1,145676711	1
Ero1lb	1,145676711	1
Fhl3	1,145597302	1
Uchl3	1,145597302	1
Timm22	1,145438499	1
Zfp341	1,145359106	1
Gm6222	1,145359106	1
Kiss1r	1,145279719	1
Tspo	1,145279719	1
Lmna	1,145200337	1
Ndufs4	1,145120961	1
Eral1	1,14504159	1
Tysnd1	1,14504159	1
Gm5609	1,144962224	1
2410006H16Rik	1,144882864	1
Tmie	1,14480351	1
Hgs	1,14480351	1
Rab2a	1,14480351	1
Rad18	1,144644817	1
Ado	1,144486147	1
Ppm1d	1,14440682	1
Rufy1	1,144168872	1
Kdelc2	1,144168872	1

Nudt14	1,143930973	1
2010204K13Rik	1,143930973	1
Auh	1,143930973	1
Flot1	1,143851685	1
Anapc7	1,143772402	1
Rnf40	1,143613852	1
Gm14769	1,143534586	1
DHRSX	1,143534586	1
Slc25a16	1,143534586	1
Harbi1	1,143534586	1
Tmem251	1,143455325	1
Yif1b	1,143296819	1
A930005H10Rik	1,143217574	1
Sec13	1,143217574	1
Med28	1,143138335	1
Arap3	1,142979874	1
Hcfc1r1	1,142979874	1
Mettl1	1,142900651	1
Atp5j2	1,142821434	1
Bop1	1,142742223	1
Gm28530	1,142663016	1
Apitd1	1,142663016	1
Uqcc1	1,142583816	1
Rnf166	1,142504621	1
Acbd6	1,142504621	1
Tlr7	1,142346247	1
Tcof1	1,142187895	1
R74862	1,142108727	1
Vdac1	1,142108727	1
Nif3l1	1,141950408	1
Mcur1	1,141950408	1
Gm37482	1,141792111	1
Gm13532	1,141792111	1
Tnks2	1,141712971	1
Cyb5r1	1,141633836	1
Myo18a	1,141633836	1
Abhd5	1,141396465	1
Synj2bp	1,141396465	1
Snrpert	1,141317352	1
Lnx2	1,141317352	1
Tom1l2	1,141317352	1
Klk8	1,141238245	1
Usp54	1,141238245	1
Ccdc32	1,141159143	1
Rubcn	1,141159143	1
Gcsh	1,141159143	1
Cbx1	1,141159143	1
Prpf6	1,141080047	1
Hrc	1,141000956	1
Sirt5	1,141000956	1
Gm5244	1,141000956	1
Tmem18	1,141000956	1

Axin1	1,14092187	1
Slc25a38	1,140605583	1
Brms1l	1,140526525	1
Mphosph8	1,140526525	1
Gm15946	1,140447473	1
Prr18	1,140447473	1
Selenoo	1,140368426	1
Tmem261	1,140289384	1
Gpn1	1,140131318	1
Gm44916	1,139973273	1
Mier3	1,139894259	1
Tes	1,13981525	1
Bre	1,139736247	1
Galk2	1,13949927	1
Cdadc1	1,13949927	1
Aes	1,13949927	1
Gata3	1,139420288	1
S100a4	1,139420288	1
AW209491	1,139183377	1
Hnrnpl	1,139183377	1
Prpf4	1,139183377	1
Gps2	1,139104418	1
Mrpl57	1,139104418	1
Pigm	1,138788635	1
Rprd1a	1,138709703	1
Gm24951	1,138630776	1
Ncf2	1,138472939	1
Gm13992	1,138394029	1
Ipo8	1,138315124	1
Usp10	1,138315124	1
Gabarapl2	1,138236225	1
Tmem57	1,138236225	1
RP23-371B13.3	1,138157331	1
Esf1	1,138157331	1
Fbxo18	1,138078443	1
Cd320	1,13799956	1
Trappc4	1,13799956	1
Dnajc16	1,137762944	1
Pgam5	1,137762944	1
Lyrm4	1,137684083	1
Selenon	1,137684083	1
Elp6	1,137605228	1
Kdelc1	1,137526378	1
Ndufaf8	1,137211032	1
Galnt3	1,13713221	1
Csad	1,13713221	1
Ahcy	1,13697458	1
H2-DMa	1,136895774	1
Gm12459	1,136816973	1
Nmrk1	1,136659388	1
Aptx	1,136659388	1
Neo1	1,136580603	1

Slc37a3	1,136501824	1
Atp6ap2	1,136501824	1
Ankrd46	1,136423051	1
Poc1b	1,136423051	1
Psmg3	1,136423051	1
Rnf103	1,136344283	1
BC003965	1,136344283	1
Dusp19	1,13626552	1
Agpat2	1,136108011	1
Scfd1	1,136108011	1
Prss36	1,135714334	1
Snap29	1,135635615	1
Gm20430	1,135556902	1
Coa5	1,135478194	1
Serpinb9	1,135399491	1
Taf4b	1,135399491	1
Psmd8	1,135320794	1
2210408F21Rik	1,135242102	1
Klhl6	1,135242102	1
Ap1s1	1,135163416	1
Limd1	1,13500606	1
Fam104a	1,13492739	1
BC052040	1,134848725	1
Psmc2	1,134848725	1
Psen1	1,134770066	1
Fam168a	1,134770066	1
Porcn	1,134691413	1
Nemf	1,134534122	1
Kansl1l	1,134455485	1
Magee1	1,134455485	1
Wfs1	1,134455485	1
Egf	1,134376853	1
Patz1	1,134376853	1
Gm11808	1,134376853	1
Tpm3-rs7	1,134298227	1
Aagab	1,134219606	1
Rpap3	1,134140991	1
Rtfdc1	1,134140991	1
Nt5c3	1,134140991	1
Spout1	1,133826584	1
Rps26-ps1	1,133826584	1
9130024F11Rik	1,133747995	1
Nlrx1	1,133747995	1
Dbi	1,133747995	1
Gm4602	1,133590836	1
Gm26847	1,133433697	1
Tfam	1,133355136	1
Sssca1	1,133355136	1
F630040K05Rik	1,133198031	1
Rela	1,133198031	1
Ufc1	1,133198031	1
Dffa	1,133119486	1

Dpagt1	1,132883885	1
Timp1	1,132805362	1
Dlx1	1,132805362	1
Bscl2	1,132726845	1
Hdac2	1,132648333	1
Ap5s1	1,132491326	1
RP23-43M12.2	1,13241283	1
Gm26826	1,13241283	1
Nicn1	1,13241283	1
Whrn	1,13233434	1
Eif3i	1,132255855	1
Gm9009	1,132177376	1
Pcyt1a	1,132177376	1
Spn	1,132098902	1
Gm23969	1,132020434	1
Ppie	1,131941971	1
Hilpda	1,131941971	1
Pced1a	1,131863513	1
Ogg1	1,131785061	1
Shmt1	1,131785061	1
Csf2rb	1,131628173	1
Tars	1,131628173	1
Ric8b	1,131549738	1
Lxn	1,131471307	1
Ttc33	1,131314463	1
Toporsos	1,13115764	1
Gemin2	1,13115764	1
Agap3	1,13115764	1
Acod1	1,131079237	1
Gm29736	1,131000839	1
Tstd1	1,13084406	1
Clec5a	1,13084406	1
Dnajc21	1,13084406	1
Sirt2	1,130687303	1
1700031P21Rik	1,130530567	1
Capzb	1,130530567	1
Zfp568	1,130452207	1
Bnip1	1,130452207	1
Gtpbp4	1,130452207	1
Ankrd13c	1,130373853	1
Cox10	1,130373853	1
Bicd2	1,130295504	1
Ddx41	1,130138823	1
Cyfip2	1,130138823	1
Pdp1	1,13006049	1
Scaf1	1,129982163	1
Crat	1,129982163	1
Rnf219	1,129982163	1
Mpv17l2	1,129903842	1
mt-Cytb	1,129825525	1
Cenpl	1,129590609	1
Smim15	1,129590609	1

Gm11516	1,129355742	1
Cped1	1,129277464	1
Wrap73	1,129277464	1
Asns	1,129199191	1
Slc41a2	1,129042661	1
Hist2h2be	1,128886154	1
Gm45380	1,128807908	1
Cope	1,128807908	1
Spc24	1,128729668	1
Eml3	1,128651433	1
Wdr83os	1,128651433	1
Frrs1	1,128573203	1
Snf8	1,128573203	1
Hs6st1	1,128494979	1
E2f1	1,128416761	1
Sema4d	1,128416761	1
Pdia4	1,128416761	1
Gm13835	1,128338548	1
Ap1b1	1,128338548	1
Hexa	1,128338548	1
Cby1	1,12826034	1
Zfp472	1,12826034	1
Mvb12b	1,12826034	1
Mppe1	1,128182137	1
Gm9732	1,128103941	1
Zfp821	1,128103941	1
Xpc	1,128103941	1
AI987944	1,128025749	1
Cab39	1,128025749	1
Psmc1	1,127947563	1
Gyg	1,127947563	1
Tmem242	1,127947563	1
Pum3	1,127947563	1
Specc1l	1,127713037	1
Fastk	1,127713037	1
Eif3f	1,127713037	1
Cadps	1,127634873	1
Snhg15	1,127634873	1
Usp50	1,127556714	1
Akr1b3	1,12747856	1
Atox1	1,12747856	1
Cnnm2	1,127400412	1
Ubap1	1,127400412	1
Ints5	1,127322269	1
Syngr2	1,127244132	1
Sgtb	1,127087874	1
Bola3	1,127087874	1
Pdk3	1,127009753	1
Cfdp1	1,127009753	1
Acsl3	1,126775422	1
Tkfc	1,126619228	1
Samm50	1,126463057	1

Rad17	1,126384979	1
Prdx4	1,126306907	1
Slc45a3	1,12622884	1
Ccdc173	1,126150778	1
RP24-122E11.4	1,126150778	1
Eri2	1,126072722	1
Ppfia3	1,126072722	1
Nup50	1,126072722	1
Rps21	1,126072722	1
5033430I15Rik	1,125994671	1
Cd101	1,125760552	1
Mad1l1	1,125682523	1
Lsm10	1,125682523	1
Nmt1	1,125682523	1
Fads3	1,125604499	1
Ssbp3	1,125604499	1
1110008P14Rik	1,125604499	1
Litaf	1,125604499	1
Rpl21-ps12	1,125448468	1
E2f7	1,125448468	1
Trmt5	1,125448468	1
Gm45833	1,125448468	1
Pygo2	1,125448468	1
Nudt3	1,125448468	1
Stra8	1,125292458	1
Abcb8	1,125292458	1
Cep41	1,125214462	1
Gm3555	1,125136471	1
Gpr180	1,125136471	1
Cycs	1,124902529	1
Adamts10	1,124902529	1
Utp6	1,12482456	1
Atp6v1c1	1,124668637	1
Tlr6	1,124590683	1
Mpg	1,124512735	1
Fndc10	1,124512735	1
Tpgs2	1,124434793	1
Gm15730	1,124434793	1
Trap1	1,124434793	1
H2-M3	1,124356856	1
Zbtb4	1,124123076	1
Lck	1,123967251	1
Gng5	1,123889346	1
Isca2	1,123889346	1
Khk	1,123889346	1
Irgm2	1,123811447	1
RP23-366E4.9	1,123733553	1
Stxbp2	1,123733553	1
Mcl1	1,123655664	1
Sgol1	1,123577781	1
Gmcl1	1,123577781	1
Bet1	1,123499903	1

Lsm11	1,123499903	1
Ctps2	1,123344164	1
Paip2b	1,123266302	1
Slc38a7	1,123266302	1
Stap1	1,123266302	1
Elovl1	1,123266302	1
AI837181	1,123188446	1
Srp68	1,123188446	1
Vps26b	1,123188446	1
Ccdc69	1,123110595	1
Gm6329	1,12295491	1
Sesn2	1,122877075	1
Cd3eap	1,122799246	1
Atp5e	1,122799246	1
Osbpl7	1,122643604	1
Cttnbp2nl	1,122643604	1
Rhod	1,122565791	1
Chpt1	1,122487983	1
Gm10051	1,122332384	1
Zfp709	1,122332384	1
Cdyl2	1,122332384	1
Rpl14-ps1	1,122332384	1
Vps33a	1,122099026	1
Coq9	1,122099026	1
Znhit1	1,122099026	1
Taf8	1,12202125	1
Akt2	1,121943481	1
Gpatch11	1,121943481	1
Dpy30	1,121787957	1
Cep57	1,121787957	1
Dctn5	1,121710203	1
Ccdc77	1,121710203	1
Il12rb1	1,121554712	1
Csrp2bp	1,121476974	1
Srprb	1,121476974	1
Rab24	1,121476974	1
Sart1	1,121321515	1
Clta	1,121243794	1
Scp2	1,121088367	1
Cep68	1,121010662	1
Pcca	1,120855268	1
Arl5a	1,120855268	1
Pcdhb15	1,120777579	1
Ly96	1,120699895	1
Pyurf	1,120699895	1
Psmd12	1,120699895	1
Hfe	1,120622217	1
Gm2796	1,120544544	1
Nhlrc2	1,120544544	1
Atp1b3	1,120544544	1
Rack1	1,120544544	1
Rps15a-ps7	1,120466876	1

Oprl1	1,120466876	1
Kifap3	1,120466876	1
Pkd1	1,120389214	1
Rxra	1,120389214	1
Gm6493	1,120311557	1
Tbl1xr1	1,120233906	1
Gpr157	1,120078619	1
Dnaaf2	1,120078619	1
Lsm12	1,120000984	1
Scarb2	1,120000984	1
Tmem144	1,119923354	1
Extl3	1,119923354	1
1810032O08Rik	1,11984573	1
Zfp180	1,11984573	1
Gm10060	1,119768111	1
C030034I22Rik	1,119768111	1
Cdk5rap3	1,119690497	1
Hist1h1c	1,119535286	1
Dagla	1,119457688	1
Ick	1,119302509	1
Cd300lf	1,119224928	1
Maged1	1,119069781	1
Ranbp1	1,118914656	1
Ergic1	1,118914656	1
Adck1	1,118682008	1
Ttc13	1,118682008	1
Coq7	1,118682008	1
Gm15446	1,118526937	1
Sergef	1,118526937	1
Osbpl9	1,118449409	1
Ciao1	1,118371887	1
Mcts1	1,118371887	1
Il18	1,11829437	1
Ptrh2	1,118139352	1
D130051D11Rik	1,117829381	1
Rpl21	1,117829381	1
Crnkl1	1,117829381	1
Eif4ebp1	1,117751901	1
Pik3r2	1,117674427	1
Zfp41	1,117442038	1
Sec61a2	1,117364585	1
Fam136a	1,117287138	1
Rps12-ps23	1,11713226	1
Eif4e2	1,11713226	1
Wdr62	1,117054829	1
Yeats4	1,117054829	1
Pvt1	1,116977403	1
Ufd1l	1,116977403	1
Snrnp25	1,116899983	1
Akip1	1,116822568	1
Phf23	1,116667754	1
Spire1	1,116512962	1

Pbx2	1,116512962	1
Rpl23a-ps5	1,116435574	1
Lsm6	1,116435574	1
Creb3l4	1,116358191	1
Zfp91	1,116358191	1
Akr1c13	1,116280814	1
Slc40a1	1,116203442	1
Jmjd4	1,116048714	1
Tubgcp5	1,116048714	1
Gm15159	1,115971358	1
Camk1	1,115816662	1
Tmem141	1,115816662	1
Pdk2	1,115739322	1
Zfand3	1,115739322	1
Galnt4	1,115661988	1
Anapc15	1,115661988	1
Ptrhd1	1,115661988	1
Ap3b1	1,115661988	1
Slc26a6	1,115507335	1
Kyat1	1,115507335	1
Anp32b	1,115507335	1
Slc2a6	1,115352704	1
Mrpl41	1,115198094	1
Mgst3	1,115120797	1
2810030D12Rik	1,115043505	1
Ift74	1,115043505	1
Entpd1	1,115043505	1
Zdhhc6	1,114966219	1
Gm29284	1,114888938	1
Gm38262	1,114888938	1
Lmf1	1,114811662	1
Cytip	1,114734392	1
Gtpbp8	1,114734392	1
Ica1	1,114734392	1
Parg	1,114734392	1
Clcc1	1,114657127	1
Rps19-ps4	1,114579868	1
Plpp7	1,114579868	1
Slc25a25	1,114579868	1
Dusp14	1,114579868	1
Psph	1,114425365	1
Trim30a	1,114348122	1
Usp48	1,114348122	1
Mkrn1	1,114348122	1
Ang	1,114270884	1
Ttc28	1,114193651	1
Psmd7	1,114193651	1
P2rx4	1,114116423	1
Pld3	1,114116423	1
Sfr1	1,114039201	1
Trmu	1,113961985	1
D5Ertd579e	1,113884774	1

Celf4	1,113730367	1
Tdrkh	1,113730367	1
Hdac10	1,113653172	1
Nip7	1,113653172	1
Tmem167	1,113575982	1
Polrmt	1,113498797	1
Plbd2	1,113498797	1
Zfp146	1,113421618	1
3110009E18Rik	1,113421618	1
Mpc2	1,113421618	1
Zfp60	1,113112955	1
Mgat5	1,113112955	1
Npm3	1,113035803	1
Pbx3	1,113035803	1
Il13ra1	1,113035803	1
Triap1	1,112881514	1
Wdr89	1,112804377	1
Gm5586	1,112727246	1
Zfp644	1,112727246	1
Mgat1	1,112727246	1
Prdx2	1,112650121	1
Ttc7b	1,112573	1
Vgll4	1,112573	1
Wdr45	1,112418776	1
Slc1a5	1,112418776	1
2610203C22Rik	1,112341671	1
Psrc1	1,112264572	1
Gm43533	1,112187479	1
Caprin2	1,112187479	1
Fbxo22	1,112187479	1
Sdccag3	1,11211039	1
Prkab2	1,11211039	1
Polr2b	1,11211039	1
Camkk2	1,11211039	1
Cradd	1,112033307	1
Wdr91	1,11195623	1
H2-Q6	1,111802091	1
Pex5	1,111802091	1
Fam96b	1,111802091	1
D130007C19Rik	1,111647973	1
Mov10	1,111493876	1
Hat1	1,111493876	1
Snx15	1,111416836	1
Ywhaz	1,111339801	1
Nkrf	1,111262772	1
Ercc8	1,111185748	1
Ppp4r3a	1,111185748	1
Ccdc15	1,111108729	1
Igsf6	1,111108729	1
Riok1	1,111108729	1
Myo1e	1,111031715	1
Nudt1	1,110877704	1

Cnr2	1,110877704	1
Ddx52	1,110877704	1
Lhpp	1,110800707	1
Ccpg1os	1,110723714	1
Mdp1	1,110723714	1
Rps2	1,11049277	1
Dtx2	1,11049277	1
Suox	1,110415799	1
Morf4l2	1,110415799	1
Slmap	1,110415799	1
Med26	1,110338834	1
Ddx51	1,110261873	1
Zfp626	1,110261873	1
Zfp687	1,110261873	1
Anapc2	1,110261873	1
Bvht	1,110184919	1
Arpc1b	1,110107969	1
Dcps	1,110031025	1
Matr3-ps2	1,109877153	1
Clcn6	1,109877153	1
Fkbp4	1,109800225	1
Dlst	1,109800225	1
Ccdc34	1,109723302	1
Hsf2bp	1,109492565	1
Dbr1	1,109492565	1
Mrpl45	1,109415664	1
Cox14	1,109338768	1
Cfap36	1,109261877	1
Med7	1,109261877	1
Aldh7a1	1,109108111	1
Mgst2	1,109108111	1
1810011H11Rik	1,108954367	1
Rps16-ps2	1,10872379	1
Zfp651	1,108646942	1
Tor2a	1,108570099	1
Cab39l	1,108570099	1
Irf9	1,108493261	1
Mir703	1,108493261	1
Hddc2	1,108339602	1
4930448A20Rik	1,108262781	1
Stard5	1,108262781	1
Fkbp5	1,108262781	1
Pdxdc1	1,108109153	1
Mttp	1,108032348	1
5730455P16Rik	1,107878753	1
Sqrdl	1,107494858	1
Trim36	1,107418095	1
2310047D07Rik	1,107264584	1
Ahnak	1,107187837	1
Edf1	1,107111096	1
Cxcl16	1,107034359	1
Dph3	1,106880902	1

Otx1	1,106727467	1
Hist1h1d	1,106650757	1
Fyb	1,106650757	1
Rnf11	1,106574052	1
Dctn3	1,106574052	1
B9d1	1,106497353	1
Ktn1	1,106497353	1
Echdc1	1,106420659	1
Gm28875	1,106420659	1
Gm7287	1,106343971	1
Capn2	1,106267287	1
Clspn	1,106190609	1
Mvp	1,106190609	1
Atp5b	1,106190609	1
Zc3h6	1,106113937	1
Morc4	1,106037269	1
Phlda1	1,105883951	1
Gm5617	1,105807299	1
Cbll1	1,105730653	1
Thap3	1,105730653	1
Blvrb	1,105654013	1
Usp4	1,105500747	1
Lysmd4	1,105424122	1
Pepd	1,105424122	1
1700086O06Rik	1,105347503	1
1600002K03Rik	1,105347503	1
Casp8ap2	1,105117676	1
Gm5131	1,105041078	1
Fbrs	1,105041078	1
Fam69a	1,104811315	1
Fth-ps3	1,104811315	1
Sh3glb2	1,104734738	1
Micall1	1,104734738	1
6430590A07Rik	1,104658166	1
Slc35c2	1,104658166	1
Tmem203	1,104658166	1
Glrx2	1,104658166	1
Acads	1,104428483	1
Gm38305	1,104275387	1
Defb25	1,104275387	1
Caml	1,104275387	1
Ccdc90b	1,104275387	1
Rpl7l1	1,104275387	1
AC149090.1	1,104198847	1
Ube2e2	1,104045783	1
Tlr2	1,103969259	1
Nudt16l1	1,103969259	1
Zc3h8	1,103816227	1
Slc31a2	1,103816227	1
Nrg4	1,103739719	1
Pald1	1,103739719	1
Trim24	1,103739719	1

Nrros	1,103663216	1
Dad1	1,103663216	1
S100a10	1,103663216	1
Ddt	1,103510227	1
Rps12-ps26	1,10343374	1
Rnf34	1,10343374	1
Vav3	1,103280782	1
Cd2ap	1,103280782	1
AI413582	1,103280782	1
Gm10177	1,103127846	1
Bloc1s6	1,103127846	1
Foxk2	1,103051385	1
Spata7	1,10297493	1
Zfp141	1,10297493	1
Fopnl	1,10297493	1
Gdap2	1,102898481	1
Zfp322a	1,102745597	1
Fahd1	1,102592735	1
Sap30bp	1,102439893	1
Aurkaip1	1,102439893	1
Ndufc1	1,102439893	1
Smyd4	1,102363481	1
Fam110a	1,102363481	1
Rasal2	1,102363481	1
Btbd19	1,102287073	1
Gm10039	1,102287073	1
1700003F12Rik	1,102210671	1
Kif20a	1,102210671	1
Mrto4	1,102057883	1
Abhd11	1,101981497	1
Rdm1	1,101905116	1
Eftud2	1,10182874	1
Eif1b	1,101599645	1
Chordc1	1,101599645	1
2810021J22Rik	1,101523291	1
Gm9790	1,101446942	1
Tbp	1,101294259	1
Gm26384	1,101294259	1
Nasp	1,101294259	1
Trim26	1,101217926	1
Sigmar1	1,101217926	1
Tssc4	1,101217926	1
Tsen34	1,101141598	1
Cenpw	1,101065275	1
Minpp1	1,100988958	1
Arl3	1,100988958	1
Xrcc5	1,100912646	1
Vcpkmt	1,100836339	1
Mtx1	1,100836339	1
Rpl7l1-ps1	1,100683741	1
Alg9	1,100683741	1
Uhmk1	1,100683741	1

Gm6563	1,10060745	1
RP23-159E10.1	1,100454884	1
Galc	1,100378609	1
Mtus2	1,100378609	1
Park7	1,100378609	1
C1ra	1,10030234	1
Stau2	1,10030234	1
Itpk1	1,10030234	1
AY074887	1,100226075	1
Abcf3	1,100149816	1
Pcif1	1,100149816	1
Metap2	1,100073562	1
Gtf2h2	1,099997313	1
Thap4	1,099997313	1
Slc25a15	1,09992107	1
Zscan20	1,09992107	1
Cct7	1,09992107	1
Gm9385	1,099768599	1
Ulk2	1,099616149	1
BC055324	1,099539932	1
RP23-151L20.5	1,099463721	1
Hdac6	1,099463721	1
Pias1	1,099463721	1
Usp46	1,099311313	1
Gm15903	1,099235117	1
Hdac7	1,099235117	1
Rnf20	1,099235117	1
Lars2	1,099082742	1
Gm10136	1,099006562	1
Prim1	1,098930387	1
Svbp	1,098930387	1
Suds3	1,098930387	1
Lgalsl	1,098701895	1
2210016L21Rik	1,098701895	1
Zfyve27	1,098625741	1
Upf3a	1,098625741	1
Fbxo9	1,098549593	1
Abcb9	1,09847345	1
Gtf2ird1	1,09847345	1
Vps16	1,09832118	1
Ptp4a2	1,09832118	1
BC024386	1,098245052	1
BC005561	1,098245052	1
Psmd1	1,098245052	1
Vapb	1,098245052	1
Ank	1,098092814	1
2200002J24Rik	1,098016702	1
Nkiras2	1,098016702	1
Atp8a1	1,097864496	1
Xrn2	1,09771231	1
P3h1	1,097636225	1
Vim	1,097636225	1

Scaf11	1,097636225	1
Ctcf	1,097484071	1
Fam53a	1,097408001	1
Cul4a	1,097331938	1
Maf1	1,097331938	1
Fam19a3	1,097255879	1
Rybp	1,097255879	1
Ccdc120	1,097255879	1
Elmo2	1,097255879	1
Zfp964	1,097179826	1
Sar1b	1,097179826	1
Gm7496	1,097103778	1
Nop58	1,097103778	1
Zmat1	1,097027735	1
0610012G03Rik	1,096951697	1
Unc119b	1,096723616	1
Rffl	1,0966476	1
Pelp1	1,096571589	1
P2ry6	1,096571589	1
Thoc5	1,096571589	1
Mrgpre	1,096495583	1
Phlda3	1,096495583	1
Rab27a	1,096419582	1
Plod3	1,096343587	1
Thumpd1	1,096191612	1
Kbtbd8	1,096039658	1
Chkb	1,096039658	1
Gtf2a2	1,096039658	1
Mrps12	1,095963689	1
Lrp8os3	1,095811766	1
Gm4950	1,095811766	1
Tra2b	1,095735813	1
Clec11a	1,095659865	1
2610318N02Rik	1,095659865	1
Abi2	1,095659865	1
Cwf19l2	1,095659865	1
Aifm1	1,095659865	1
Gorasp1	1,095583922	1
Galnt6	1,095583922	1
Spata2	1,095507985	1
Gosr2	1,095432053	1
Tgfbr1	1,095356126	1
Tle1	1,095356126	1
Gm5139	1,095280204	1
Gm14539	1,095280204	1
AC133103.1	1,095128377	1
Fbxo25	1,095128377	1
Cds2	1,095128377	1
Tsacc	1,095052471	1
Lockd	1,095052471	1
Kif3b	1,09497657	1
Ppp6r1	1,094900675	1

Rpl17-ps4	1,094824785	1
Tdrd3	1,0947489	1
Klrg2	1,0947489	1
Fxyd5	1,09467302	1
Slc26a9	1,094521277	1
2310010J17Rik	1,094445413	1
Arhgap21	1,094369555	1
Immt	1,094369555	1
Dnajc18	1,094369555	1
Gm340	1,094293701	1
Rasgef1a	1,094293701	1
Ppfibp1	1,094217853	1
Smim11	1,09414201	1
Serbp1	1,093990341	1
Acd	1,093914514	1
Mbd3	1,093914514	1
Fuk	1,093838692	1
Tmem97	1,093838692	1
Mepce	1,093762875	1
AC168977.1	1,093762875	1
Smagp	1,093762875	1
Brk1	1,093762875	1
Pphln1	1,093611258	1
Psmc3ip	1,093308087	1
Tmco6	1,093308087	1
Zbtb7b	1,093308087	1
Emilin2	1,093308087	1
Scap	1,093232307	1
Mipol1	1,093156533	1
Zfyve9	1,093156533	1
2700099C18Rik	1,093004999	1
Npepl1	1,093004999	1
Cwc25	1,093004999	1
Crbn	1,093004999	1
Arhgap1	1,092929241	1
Csrnp1	1,092929241	1
Gm5805	1,092853487	1
Ints11	1,092853487	1
1110059E24Rik	1,092853487	1
0610037L13Rik	1,092853487	1
Faf2	1,092777739	1
Gpbp1	1,092777739	1
Ptar1	1,092701996	1
Akr1a1	1,092701996	1
Gm5075	1,092626258	1
Cd302	1,092474799	1
Rabl6	1,092399077	1
Lins1	1,09232336	1
Zbtb8a	1,092171942	1
Slx1b	1,092096241	1
2810474O19Rik	1,092096241	1
Six4	1,091944855	1

RP23-114G13.1	1,09186917	1
1110034G24Rik	1,09186917	1
Ppip5k1	1,09179349	1
Shkbp1	1,09179349	1
Kansl3	1,091642146	1
Fbxo38	1,091566482	1
Armc1	1,091490823	1
Rhbdd1	1,091415169	1
Bloc1s2	1,09133952	1
Cdk7	1,09133952	1
Dpysl2	1,091263877	1
Nfu1	1,091188239	1
Ndufb6	1,091188239	1
Atf2	1,091036979	1
Gadd45a	1,090885739	1
Vps4a	1,090885739	1
Pak1	1,090885739	1
Vrk3	1,090507733	1
Ccdc134	1,090356567	1
Slc35f5	1,090356567	1
Kdm5d	1,090356567	1
Slc38a10	1,090356567	1
Rgs19	1,090280992	1
2010111I01Rik	1,090280992	1
Clpb	1,090280992	1
Ppp2r5c	1,089903194	1
Surf4	1,089903194	1
Tmem184c	1,089827651	1
Noc4l	1,089827651	1
Mrpl23-ps1	1,089676579	1
Tmem263	1,089601051	1
Pnkp	1,089525528	1
Ascc2	1,089525528	1
Csrp1	1,089450011	1
Amotl1	1,089450011	1
Rpia	1,089374498	1
Hmgcs1	1,089374498	1
Cul2	1,089223489	1
Wdr13	1,089223489	1
Sf3b6	1,089223489	1
Ttll5	1,089147993	1
Frmd4a	1,089072501	1
Tmem11	1,089072501	1
Gm3355	1,088997015	1
Zbtb2	1,088997015	1
Jade1	1,088997015	1
Cc2d1b	1,088846059	1
mt-Nd6	1,088770588	1
Myo1c	1,088619663	1
Cul5	1,088619663	1
D830050J10Rik	1,088393315	1
Nudcd1	1,088317876	1

Abcc5	1,088242442	1
Thyn1	1,088242442	1
Atp5a1	1,088242442	1
Tmem26	1,088167013	1
Gm10557	1,08809159	1
Ostm1	1,08809159	1
Dnajc15	1,088016172	1
Pogk	1,087865351	1
Cipc	1,087865351	1
Ell	1,087865351	1
Gusb	1,087865351	1
Lrrc47	1,087714551	1
Hook3	1,087714551	1
Sf3b5	1,087639159	1
Phtf2	1,087563772	1
Ccnk	1,087488391	1
Mrpl34	1,087488391	1
Zfp511	1,087413015	1
Ccdc130	1,087413015	1
Snhg1	1,087413015	1
Wdr46	1,087262278	1
Cwc27	1,087262278	1
Ero1l	1,087262278	1
Rnf19a	1,087186917	1
Lrpprc	1,086885526	1
Mettl13	1,086810192	1
Tyrobp	1,086734863	1
Acot2	1,086659538	1
Eif5b	1,086659538	1
Gin1	1,08658422	1
Zfp39	1,086508906	1
G6pdx	1,086508906	1
Gid4	1,086433597	1
Lhfpl2	1,086433597	1
Cic	1,086282996	1
Mpv17l	1,086207703	1
Rbak	1,086132416	1
Maml3	1,086132416	1
Atxn2l	1,086132416	1
Tmlhe	1,086057133	1
Fgd2	1,086057133	1
Ndufaf1	1,085981856	1
Fam175b	1,085906584	1
Ndrg2	1,085906584	1
Tax1bp1	1,085756056	1
Tcerg1	1,085756056	1
Dmap1	1,0856808	1
Gtf3c4	1,0856808	1
Ptpn6	1,085605549	1
Gm44126	1,085455062	1
Ticam1	1,085379827	1
Gm37082	1,085304597	1

Rhbdd2	1,085229372	1
Aif1	1,085154152	1
Atp5sl	1,085154152	1
Stim2	1,085154152	1
Hint3	1,085154152	1
Gm6987	1,085078938	1
Slc39a13	1,085078938	1
P4ha2	1,085003728	1
Cers6	1,085003728	1
Cdc25a	1,085003728	1
Prelid3a	1,084853325	1
Rpl34	1,084778132	1
Fam172a	1,084702943	1
Pmaip1	1,084702943	1
Ift57	1,084702943	1
Acp2	1,08462776	1
Gm44093	1,084552582	1
Gm38340	1,084552582	1
Apool	1,084552582	1
Zbtb38	1,084552582	1
Letm1	1,084402241	1
Aldoc	1,084251922	1
Fuz	1,084101623	1
RP24-174I4.1	1,084101623	1
Brip1	1,084026481	1
Tmem222	1,084026481	1
Usp3	1,083951345	1
Bcdin3d	1,083801088	1
Rbm10	1,083801088	1
Spata13	1,083801088	1
Exo5	1,083725967	1
Rrn3	1,083725967	1
Gm16537	1,083650851	1
Rnf215	1,083650851	1
Dnase2a	1,083650851	1
Parvg	1,083575741	1
1110012L19Rik	1,083350441	1
Natd1	1,083350441	1
Timm17b	1,083350441	1
Edc4	1,083275352	1
Ppp4r1l-ps	1,083200267	1
Zfp1	1,083200267	1
Supt6	1,083200267	1
Clip1	1,083125188	1
Ythdc1	1,083050114	1
Cenpk	1,082975046	1
Wdfy2	1,082749871	1
Gm13803	1,082749871	1
Gm20442	1,082524743	1
Aprt	1,082374683	1
Spcs1	1,082299661	1
Nup43	1,082149633	1

Slc15a4	1,082074627	1
Fbxw7	1,081849639	1
Rabepk	1,081849639	1
Zrsr2	1,081699673	1
Zfp932	1,081549728	1
Slc4a1ap	1,081549728	1
Ip6k1	1,081474763	1
Rps27	1,081399804	1
Mogs	1,081399804	1
Gm12966	1,08132485	1
Dedd2	1,081249901	1
Rap2a	1,081249901	1
Larp1b	1,081174957	1
Cep128	1,081100018	1
Sgpl1	1,080950156	1
Rny1	1,080875233	1
Msra	1,080800315	1
Mndal	1,080800315	1
Serinc2	1,080650494	1
Myd88	1,080575592	1
Ehbp1l1	1,080575592	1
AV356131	1,080500695	1
Ypel5	1,080425803	1
Prdm9	1,080350916	1
Gm5422	1,080350916	1
Kcmf1	1,080350916	1
Eif4e3	1,080276034	1
Pdlim5	1,080276034	1
1810014B01Rik	1,080126287	1
Lap3	1,080126287	1
Bsn	1,07997656	1
Lrrc8a	1,07997656	1
Faf1	1,07997656	1
Pak1ip1	1,079901704	1
Map3k5	1,079826854	1
Haus2	1,079826854	1
Cenph	1,079677168	1
E2f6	1,079677168	1
Gstcd	1,079602333	1
Sucla2	1,079602333	1
Aasdhppt	1,079527504	1
Gm15727	1,079452679	1
Fkbp15	1,079452679	1
Fam214b	1,079303045	1
Coprs	1,079228237	1
Gm6206	1,079078634	1
3110083C13Rik	1,079003841	1
E330009J07Rik	1,078929052	1
Orc2	1,078929052	1
Slfn5	1,078854269	1
Zfp111	1,078779491	1
Me2	1,078779491	1

Mettl23	1,078629951	1
Nmnat3	1,078555189	1
Pprc1	1,078555189	1
Mycn	1,078555189	1
Syngr1	1,078480432	1
Las1l	1,07840568	1
Emc3	1,07840568	1
C630004M23Rik	1,078330933	1
Pcsk4	1,078330933	1
Sel1l	1,078256191	1
Gas5	1,078256191	1
Slc35b4	1,078181455	1
Rpl9-ps6	1,077957277	1
Gm43728	1,077882561	1
Metrnl	1,077882561	1
Gm43707	1,07780785	1
Slc6a13	1,07780785	1
Gm17994	1,077733145	1
Jmjd6	1,077658445	1
Rpl22-ps1	1,07758375	1
Capg	1,07750906	1
Pacsin2	1,077359696	1
2010107E04Rik	1,077285022	1
Necap1	1,077135689	1
Mat2b	1,076986376	1
Ythdf1	1,076986376	1
Psmd13	1,076911728	1
Irf3	1,076837085	1
Ccdc93	1,076762446	1
Emd	1,076762446	1
Emp3	1,076687814	1
Sprtn	1,076613186	1
Zfp706	1,076463946	1
Copg1	1,076314727	1
Map4k3	1,076240125	1
Trappc3	1,076240125	1
Ncoa4	1,076165528	1
Peak1os	1,07601635	1
Fbxo44	1,07601635	1
Ube2w	1,07601635	1
Mmadhc	1,07601635	1
Mark3	1,075941769	1
Lysmd1	1,075792622	1
H2-Q7	1,075792622	1
Aggf1	1,075792622	1
Lrrc2	1,075718056	1
Zg16	1,075568941	1
H1fx	1,075568941	1
Ormdl3	1,075568941	1
Tgif1	1,075270771	1
R3hcc1	1,075196241	1
Trim68	1,075196241	1

Hbs1l	1,075196241	1
Gm14813	1,075121717	1
Zfp605	1,075047198	1
Ercc6l	1,075047198	1
Znfx1	1,075047198	1
Zbtb5	1,074972684	1
Sec61a1	1,074972684	1
BC030336	1,074898175	1
Cd180	1,074823671	1
Casc3	1,074823671	1
Ankrd27	1,074749173	1
Gm15542	1,074600191	1
Gm4374	1,074600191	1
9930012K11Rik	1,074600191	1
Lrrfip1	1,074600191	1
Rbm41	1,074525708	1
Arpc5	1,074376758	1
Deaf1	1,07430229	1
Eml4	1,07430229	1
Cox16	1,074227828	1
Krtcap2	1,074153371	1
Cln8	1,074153371	1
Aim2	1,074078919	1
Pbxip1	1,073855593	1
Gm14843	1,073781162	1
Tatdn3	1,073706736	1
Cnih1	1,073706736	1
C130026I21Rik	1,073632315	1
Bzw1	1,073557899	1
Polr2l	1,073409082	1
Psma1	1,073409082	1
Tmem9	1,073334682	1
Rcbtb2	1,073334682	1
Champ1	1,073260286	1
Gltscr2	1,073260286	1
Otud5	1,073037131	1
1110046J04Rik	1,072888387	1
Cenpv	1,072888387	1
Tm7sf3	1,072888387	1
Tmem209	1,072814023	1
Mrpl32	1,072739664	1
Serac1	1,072665309	1
Stat5b	1,072665309	1
Ankrd12	1,072665309	1
Rpl37a	1,072665309	1
Mfn2	1,072590961	1
Map4	1,072590961	1
Tpst2	1,072516617	1
Parl	1,072516617	1
4930524J08Rik	1,072442278	1
Ints12	1,072442278	1
Tmem164	1,072442278	1

Cdk5r1	1,072442278	1
Ap2m1	1,072367945	1
Lair1	1,072367945	1
Gm45422	1,072293616	1
Gpatch4	1,072293616	1
Lrrc49	1,071996355	1
Fbxl12os	1,071996355	1
Echs1	1,071996355	1
Rps24-ps2	1,071996355	1
Sirpa	1,071996355	1
Degs1	1,071996355	1
Gm5624	1,071847755	1
Ddx47	1,071773463	1
Pafah2	1,071699175	1
Ocel1	1,071699175	1
Zfp53	1,071476345	1
Clec2l	1,071327817	1
Psd3	1,071327817	1
Paf1	1,071327817	1
Rpsa	1,07117931	1
Trafd1	1,07117931	1
Fam173a	1,07117931	1
Gm2a	1,070956588	1
Gm12164	1,070882357	1
Trmt2b	1,070882357	1
Itfg1	1,070882357	1
Tfrc	1,070882357	1
Mzt1	1,070808132	1
Gm6395	1,070733912	1
Mlec	1,070733912	1
Slc16a9	1,070659697	1
Lias	1,070659697	1
Minos1	1,070585487	1
Plekhm1	1,070511282	1
Sh2d5	1,070214514	1
Fam118a	1,070140335	1
Hspa4	1,070140335	1
Uaca	1,070066161	1
Ufm1	1,070066161	1
Arf6	1,069991993	1
Noxo1	1,069695369	1
Pot1a	1,069621226	1
Qars	1,069621226	1
Ahdc1	1,069547088	1
Tmem55b	1,069547088	1
Adprh	1,069398827	1
Ddx42	1,069324705	1
Tox4	1,069324705	1
Gapdh	1,069250588	1
Gm31166	1,068954169	1
Clmp	1,068954169	1
Endov	1,068954169	1

Slc35d1	1,068954169	1
Wdr35	1,068805991	1
Hspd1	1,068805991	1
Pi4kb	1,06873191	1
Lsm1	1,06873191	1
Wbscr27	1,068657834	1
Vipas39	1,068657834	1
1600020E01Rik	1,068583762	1
Isg20	1,068583762	1
Snrpg	1,068509696	1
Slc37a2	1,068435635	1
Aldh4a1	1,06836158	1
Stx17	1,06836158	1
Naa25	1,068287529	1
March6	1,068139443	1
Btbd7	1,068065408	1
Pyroxd1	1,068065408	1
Wbscr22	1,068065408	1
Gm5776	1,067991378	1
Cnih4	1,067991378	1
Tmem50b	1,067917353	1
Fuca1	1,067843333	1
Wdpcp	1,067695309	1
Maz	1,067695309	1
Nubpl	1,067695309	1
Bckdhb	1,067473311	1
Pcbd2	1,067473311	1
Cr1l	1,067473311	1
RP24-547N4.7	1,067325338	1
Ndrg3	1,067325338	1
Hist2h3c2	1,067251359	1
Hacd4	1,067251359	1
Nit1	1,067177386	1
Slc8b1	1,067177386	1
Eif2ak1	1,067177386	1
Npm1	1,067177386	1
Sec23b	1,066955495	1
Dcaf12	1,066955495	1
3110070M22Rik	1,066881542	1
Tonsl	1,066881542	1
Saal1	1,066807594	1
Gatad2a	1,066807594	1
Trim27	1,066807594	1
Chd2	1,066807594	1
Dym	1,066733651	1
Ppat	1,066733651	1
Ppp2r2d	1,066733651	1
Nsun4	1,066659713	1
Vps54	1,066659713	1
Rnf14	1,066659713	1
Sptlc2	1,066585781	1
Cat	1,066511853	1

Hars	1,066437931	1
Inafm2	1,066364014	1
Ninl	1,066290101	1
Gm20707	1,066216194	1
Stk4	1,066216194	1
Dcxr	1,066216194	1
RP24-454N4.2	1,066142293	1
Prkag2	1,066142293	1
Zfp954	1,065920617	1
Abcb10	1,06577286	1
Lgals8	1,06577286	1
Map3k9	1,065698989	1
Ikbkg	1,065698989	1
Ccar1	1,065698989	1
Lyrm7	1,065625123	1
Commd1	1,065625123	1
Man1a2	1,065625123	1
Ehd4	1,065625123	1
1700007L15Rik	1,065403555	1
Slc39a10	1,065329709	1
Slc15a3	1,065182034	1
Bcl2l11	1,065108203	1
Ogdh	1,065108203	1
Ppme1	1,065034378	1
Mafg	1,065034378	1
Ubiad1	1,064960558	1
Eny2	1,064960558	1
Gm26737	1,064886743	1
Rnf41	1,064886743	1
Sp2	1,064812934	1
1810058I24Rik	1,064812934	1
Gtf3c5	1,064739129	1
Hax1	1,064665329	1
H2-K2	1,064665329	1
Zfp771	1,064665329	1
Kras	1,064665329	1
A230050P20Rik	1,064591535	1
Ccdc127	1,064517746	1
Kif3c	1,064370182	1
Mfsd5	1,06422264	1
Atp6v0a2	1,064001364	1
Prkar1a	1,064001364	1
Naa50	1,064001364	1
Bsg	1,063927615	1
Blvra	1,063853872	1
Foxo1	1,06355895	1
Gm11687	1,063485232	1
Gm19552	1,06341152	1
Ppard	1,06341152	1
Mlycd	1,06341152	1
Gm4859	1,06326411	1
5430427O19Rik	1,06326411	1

Gm20689	1,06311672	1
Dfna5	1,06311672	1
Vcp-rs	1,06311672	1
Snx30	1,06311672	1
Gm11488	1,062969351	1
Zfp944	1,062895674	1
Sri	1,062822003	1
Mob4	1,062822003	1
Gm44836	1,062748336	1
Pdcd4	1,062748336	1
1810010D01Rik	1,062674674	1
A430035B10Rik	1,062674674	1
Capn15	1,062601018	1
Ptpa	1,062601018	1
Rassf4	1,06245372	1
Kat5	1,062380079	1
Syt11	1,062380079	1
Col15a1	1,062159186	1
Gm15829	1,062085566	1
Elf4	1,062085566	1
Apoo-ps	1,06201195	1
Pnp	1,06193834	1
Pdhb	1,06193834	1
Pigk	1,06193834	1
Sgf29	1,061864734	1
Taf10	1,061864734	1
Them6	1,061791134	1
Slc25a44	1,061791134	1
Psmd11	1,061717539	1
Sec24d	1,061570364	1
Ebna1bp2	1,061423209	1
Mtch2	1,061349639	1
Nabp2	1,061202515	1
Capza2	1,061202515	1
Tbpl1	1,061202515	1
Klhl26	1,061128961	1
Mtfmt	1,061055411	1
Tbx6	1,060981867	1
9330104G04Rik	1,060908328	1
Tor1a	1,060908328	1
Gm43153	1,060834794	1
Ubl7	1,060834794	1
mt-Ta	1,060834794	1
Arhgef10l	1,060834794	1
Elk1	1,060761265	1
Mettl21a	1,060761265	1
Gpr107	1,060761265	1
Sco2	1,060687741	1
Csrp2	1,060614223	1
Abhd8	1,060540709	1
Gna12	1,060540709	1
Mir17hg	1,0604672	1

Smcr8	1,0604672	1
Rbm5	1,0604672	1
Zmat2	1,0604672	1
Slc22a17	1,060393697	1
Gstm1	1,060393697	1
Bbof1	1,060173217	1
Nfatc2	1,060173217	1
Znrf1	1,060173217	1
2500002B13Rik	1,060099734	1
Nploc4	1,060099734	1
Lsm2	1,060099734	1
Bex3	1,059952783	1
Bpgm	1,059879316	1
Smox	1,059879316	1
Exosc1	1,059805853	1
A530013C23Rik	1,059732395	1
Gm43162	1,059658943	1
Rpl5	1,059658943	1
Slc25a12	1,059658943	1
Trim65	1,059585495	1
Ikzf5	1,059512053	1
C2	1,059438616	1
Hpf1	1,059365184	1
Lrp10	1,059365184	1
Gm15506	1,059291757	1
Gm4459	1,059291757	1
Zfp128	1,059218335	1
Madd	1,059218335	1
Atp6v1d	1,059218335	1
Car6	1,059144918	1
Klf2	1,059144918	1
Ivns1abp	1,059144918	1
Mbip	1,058851301	1
Lancl1	1,058851301	1
Ric8a	1,058411029	1
Asl	1,058337668	1
Ing5	1,058337668	1
Bckdk	1,058190961	1
Pdcl	1,058117616	1
Sec63	1,058117616	1
Nek8	1,058044275	1
Pole4	1,058044275	1
Zbtb3	1,05797094	1
Gpsm3	1,05797094	1
Xndc1	1,057897609	1
Amfr	1,057897609	1
Ndufa8	1,057897609	1
Bhlhe40	1,057677648	1
Nfkbia	1,057531033	1
Gm6134	1,057384439	1
Itpripl1	1,057384439	1
Dync1i2	1,057384439	1

Rps15a-ps1	1,057311149	1
0610039K10Rik	1,057237864	1
Hivep2	1,057237864	1
Gm5121	1,05709131	1
Senp8	1,056944776	1
Cdyl	1,056944776	1
Zfp655	1,056944776	1
Aar2	1,056871517	1
Slc4a2	1,056798263	1
Crcp	1,056798263	1
Gm5257	1,056578531	1
Ifitm2	1,056578531	1
Rps6-ps3	1,056505297	1
Hmox2	1,056505297	1
Brip1os	1,056432068	1
Tomm20	1,056432068	1
Arsk	1,056358844	1
Sephs1	1,056358844	1
Zfand5	1,056358844	1
Zc3hc1	1,056358844	1
Cdk4	1,056285625	1
Snx12	1,056285625	1
Tmed9	1,056285625	1
Ifitm1	1,056212412	1
Gm26930	1,056139203	1
Jmjd8	1,055992801	1
Smarcd2	1,055992801	1
Gm5576	1,05584642	1
Acot9	1,05584642	1
Creg1	1,055773237	1
Pi4k2a	1,055773237	1
Gm27605	1,055700059	1
5730508B09Rik	1,055700059	1
Sdcbp2	1,055700059	1
Ndufa3	1,055700059	1
Usp16	1,055553718	1
Acap3	1,055480555	1
Nono	1,055334244	1
Rps10	1,055334244	1
Aste1	1,055187954	1
Them4	1,055187954	1
Snx20	1,055187954	1
Slc35e4	1,055187954	1
Ipo13	1,055114816	1
Zfp950	1,055114816	1
Napsa	1,055041684	1
Ano6	1,054968557	1
Uxt	1,054895434	1
Plin3	1,054895434	1
Mterf4	1,054822317	1
Rpl13-ps3	1,054676098	1
Becn1	1,054676098	1

mt-Nd1	1,054602996	1
Fastkd3	1,054602996	1
Tmco3	1,054529899	1
Tmem5	1,054456807	1
Elk3	1,054456807	1
Rnf183	1,054310638	1
Taf5l	1,054310638	1
Stim1	1,054310638	1
Ppib	1,054310638	1
Gm43350	1,054237562	1
Oraov1	1,054091423	1
Fam207a	1,054091423	1
Smarca4	1,054091423	1
Midn	1,053945305	1
Gm22299	1,053799208	1
Endod1	1,053799208	1
4930455G09Rik	1,053799208	1
Gm10073	1,053799208	1
Pycr2	1,053726166	1
Zfp608	1,05365313	1
Atp5l-ps1	1,053507073	1
Myo9b	1,053507073	1
Cnot8	1,053507073	1
Rp9	1,053507073	1
Evi2a	1,053434052	1
R3hcc1l	1,053434052	1
Wbp4	1,053434052	1
Dbp	1,053361036	1
Ccdc112	1,053215019	1
Sep 06	1,053215019	1
Flii	1,053069023	1
Hebp1	1,052996032	1
Shtn1	1,052923046	1
Klhdc4	1,052923046	1
Cyb5r4	1,052850066	1
Plekha7	1,05277709	1
Id3	1,05270412	1
Gm1943	1,05270412	1
Mapkap1	1,05270412	1
Gclm	1,05270412	1
Smpdl3a	1,05270412	1
2810428I15Rik	1,052631155	1
Elovl6	1,052558194	1
E2f8	1,052485239	1
Thap1	1,052485239	1
Ttf1	1,052412289	1
Prdx3	1,052412289	1
Slc48a1	1,052412289	1
Prmt9	1,052339344	1
Pigv	1,052193469	1
Txnrd1	1,052120539	1
Oas1c	1,051974694	1

Exoc3	1,051901779	1
Snx6	1,051828869	1
Rbpsuh-rs3	1,051828869	1
Gm7964	1,051683065	1
Pus1	1,051537281	1
Eif4e	1,051537281	1
Eif2b5	1,051464397	1
Snw1	1,051391517	1
Plcd3	1,050881502	1
Tbcc	1,050881502	1
Commd10	1,050881502	1
Zfp275	1,050881502	1
Cers5	1,050881502	1
Rpl35a	1,050808663	1
Rps17	1,050808663	1
Ginm1	1,050808663	1
Slc30a6	1,050735829	1
Smarcal1	1,050663001	1
Xpr1	1,050663001	1
Cfh	1,050590177	1
Casp8	1,050517358	1
Lpp	1,050517358	1
Tmem70	1,050444544	1
Serpinf1	1,050371735	1
Grpel2	1,050371735	1
Camta2	1,050371735	1
Ipo5	1,050371735	1
Dnajc4	1,050298932	1
Gm8276	1,050226133	1
Gm12981	1,050080551	1
Prpf31	1,050080551	1
Slc39a2	1,050007767	1
Mpst	1,050007767	1
Bend3	1,049934989	1
Retsat	1,049934989	1
Gm6743	1,049789447	1
Napepld	1,049789447	1
Rps19bp1	1,049789447	1
Tmem59	1,049643925	1
Fchsd2	1,049571172	1
Phf5a	1,049498424	1
Tatdn1	1,049425681	1
Bcorl1	1,049425681	1
Ythdf2	1,049425681	1
Mylip	1,049280209	1
Daam1	1,049280209	1
Gm11221	1,049207481	1
Ptpn18	1,049207481	1
4930430F08Rik	1,049134758	1
Slc39a3	1,04906204	1
Ctbp1	1,04906204	1
Tbc1d13	1,048989328	1

Narf	1,048989328	1
Gm37670	1,04891662	1
1700030K09Rik	1,04891662	1
Pinx1	1,04891662	1
Llph	1,04891662	1
Nfatc4	1,048843917	1
Dicer1	1,048843917	1
Rufy3	1,048771219	1
Nagpa	1,048698526	1
Gm13573	1,048625839	1
Mfhas1	1,048625839	1
Iffo2	1,048625839	1
Wdfy1	1,048625839	1
Tspyl1	1,048625839	1
Smc6	1,048553156	1
Foxo4	1,048480478	1
Desi1	1,048480478	1
Gm17511	1,048407806	1
Rps18-ps3	1,048335138	1
Caskin2	1,048262476	1
Imp3	1,048262476	1
Gm38377	1,048189818	1
RP24-323H7.5	1,048044518	1
Gm15703	1,048044518	1
Gm16556	1,048044518	1
Zcchc2	1,047971876	1
Dnajc24	1,047826606	1
Fkbp14	1,047753979	1
Gm16754	1,047753979	1
Usp1	1,047753979	1
Ddrgk1	1,047608739	1
Tmem55a	1,047536127	1
Git1	1,047536127	1
Josd1	1,047536127	1
H2-K1	1,04746352	1
Gm5910	1,047390918	1
Ptger4	1,047173142	1
Gm10388	1,04710056	1
Fgr	1,04710056	1
Irak4	1,04710056	1
RP23-65M10.2	1,047027983	1
Cklf	1,047027983	1
Hmbs	1,046955411	1
Polg2	1,046955411	1
Aldh9a1	1,046810282	1
Atp6v0e	1,046665173	1
Junos	1,046592627	1
Myo10	1,046592627	1
Cmc4	1,046520085	1
Sart3	1,046520085	1
Ankzf1	1,046447548	1
Slc22a21	1,046375016	1

Gm43609	1,04630249	1
Gm11868	1,04630249	1
Mtmr10	1,046157451	1
Rgs1	1,046157451	1
Eng	1,04608494	1
Ccdc25	1,04608494	1
Trp53cor1	1,045939932	1
Fbxo30	1,045867435	1
4930402H24Rik	1,045794944	1
Lrrc40	1,045794944	1
Pex19	1,045794944	1
Rtn3	1,045794944	1
Dusp18	1,045722457	1
Lamtor3	1,045722457	1
Cbfa2t3	1,045649976	1
Stau1	1,045577499	1
Synpo	1,0453601	1
Bahd1	1,0453601	1
Wdr43	1,0453601	1
Polr3b	1,045287644	1
Nckipsd	1,045215193	1
Wars	1,045215193	1
4931428F04Rik	1,045070305	1
Chd1	1,045070305	1
Brcc3	1,044997869	1
Rundc1	1,044925438	1
Grsf1	1,044853011	1
E230032D23Rik	1,04478059	1
Ivd	1,04478059	1
Nipsnap3b	1,044635763	1
Gns	1,044635763	1
Mta2	1,044635763	1
Taldo1	1,044635763	1
Ppan	1,044563357	1
Gm38104	1,044490956	1
Hoxb7	1,044490956	1
Egln3	1,044346168	1
Dok3	1,044346168	1
Lst1	1,044346168	1
Slc35b2	1,044273782	1
Gm5619	1,044201401	1
Pigl	1,044201401	1
Rap1b	1,044201401	1
Dyrk2	1,044129025	1
Acly	1,044129025	1
Eef1a1	1,044056654	1
Fam20c	1,043984288	1
Oas1g	1,043911927	1
Psmd3	1,043839571	1
Otulin	1,043767221	1
Gm20604	1,043622534	1
Tmed5	1,043622534	1

Gm44053	1,043550198	1
Ppp1r7	1,043477867	1
Vamp8	1,043477867	1
Cnppd1	1,043260904	1
Esr1	1,043188594	1
St6galnac4	1,043188594	1
Lppos	1,043116288	1
Abhd1	1,043043987	1
Gm43182	1,042971691	1
Armc5	1,042971691	1
Hsbp1	1,042971691	1
6330408A02Rik	1,0428994	1
Gm25514	1,0428994	1
Ctdspl2	1,042827115	1
Rfng	1,042827115	1
Gm7351	1,042754834	1
Coq4	1,042754834	1
Ncf4	1,042754834	1
Rnf32	1,042610287	1
Gpt	1,042610287	1
Cep120	1,042610287	1
Zfp715	1,042538022	1
Arfrp1	1,042538022	1
Ubxn1	1,042538022	1
Tmod1	1,042465761	1
Prr36	1,042393505	1
Tspan32	1,042321254	1
Matk	1,042321254	1
Nudt4	1,042321254	1
Ss18l2	1,042249009	1
Adk	1,042249009	1
Gm13341	1,042176768	1
Camsap2	1,042176768	1
Rmnd1	1,042032302	1
Atp5g3	1,042032302	1
Nelfe	1,041887855	1
Ubald1	1,04181564	1
Azi2	1,041743429	1
Glmn	1,041743429	1
Atp9a	1,041743429	1
Zbtb6	1,041599023	1
Maml1	1,041526827	1
Ldlrap1	1,041526827	1
Zfp119a	1,041454636	1
Usp38	1,041454636	1
Pfdn4	1,041454636	1
Fnbp1	1,041454636	1
Foxred1	1,041382451	1
Taok3	1,041238095	1
Depdc7	1,041093758	1
Srsf9	1,041093758	1
Pdzd8	1,041021598	1

Mlst8	1,041021598	1
Irak2	1,041021598	1
Pitpnb	1,041021598	1
Cyp4v3	1,040949442	1
Pex26	1,040949442	1
Ei24	1,040877291	1
Tcirg1	1,040877291	1
Zswim1	1,040805146	1
Rnf126	1,040805146	1
Tspan13	1,040805146	1
Cdc5l	1,04066087	1
Chst3	1,040588739	1
Pex16	1,040588739	1
Upf2	1,040588739	1
Nr1h3	1,040516613	1
Pcdhb22	1,040444493	1
Adamts1	1,040444493	1
Zcchc7	1,040444493	1
Gm12833	1,040372377	1
Ywhab	1,040372377	1
Exog	1,040300267	1
Rchy1	1,040228161	1
Tmem243	1,04015606	1
Map2k7	1,04015606	1
Ankrd24	1,040083965	1
Naa38	1,040083965	1
Camk2a	1,040011874	1
Atp6v1f	1,040011874	1
Hikeshi	1,039939788	1
Prkab1	1,039939788	1
Sphk2	1,039939788	1
Extl2	1,039867708	1
Gm12380	1,039795632	1
Fbxo45	1,039723561	1
Sf3a3	1,039723561	1
Aars	1,039651496	1
Wbp11	1,039651496	1
Ube2a	1,039651496	1
Plrg1	1,039651496	1
Poldip3	1,03950738	1
Sgpp1	1,03950738	1
Rnf113a2	1,039363283	1
Slc43a3	1,039147176	1
Tap1	1,03907515	1
1700037H04Rik	1,03907515	1
Arid4b	1,03907515	1
Mfsd12	1,03900313	1
Mblac2	1,038931114	1
Zfp729b	1,038931114	1
Ggnbp1	1,038931114	1
Gm6472	1,038859103	1
Fntb	1,038859103	1

Rmnd5b	1,038859103	1
Ube2z	1,038715097	1
Pus3	1,03857111	1
Inip	1,038499125	1
Gm13328	1,038355168	1
Setd3	1,038355168	1
Bag3	1,038283197	1
Elmo1	1,038139271	1
Tnfaip1	1,038139271	1
Erc1	1,037995364	1
Psme1	1,037923418	1
Papd7	1,037851477	1
Ino80e	1,037851477	1
Gm5801	1,037779541	1
Cdca3	1,037779541	1
9930021J03Rik	1,037779541	1
Cdkn2c	1,03770761	1
Wdr24	1,037635684	1
Tcf7l2	1,037563763	1
Cacybp	1,037491848	1
Grk6	1,037419937	1
Gm10676	1,037132343	1
Tmem205	1,037132343	1
Zfp407	1,036988575	1
Iqcg	1,036916699	1
C130023A14Rik	1,036844828	1
Gnptg	1,036772962	1
Zcchc10	1,036772962	1
Nup133	1,036629245	1
Sgms1	1,036629245	1
Adsl	1,036629245	1
Alkbh3	1,036629245	1
Vps36	1,036629245	1
Fbxo11	1,036629245	1
Ube2r2	1,036485547	1
2810414N06Rik	1,036198213	1
Cox7b	1,036198213	1
Gramd1c	1,036126391	1
Cyb5rl	1,036054575	1
Ppm1f	1,036054575	1
Gm42432	1,035910957	1
Chsy1	1,035839156	1
Cadm1	1,035839156	1
Ubn1	1,035695568	1
Wdr41	1,035552	1
Abracl	1,035552	1
Gm20620	1,035336685	1
Eif3b	1,035264924	1
Rpl18	1,035193167	1
Slc25a39	1,035121416	1
AI597479	1,035049669	1
Ano10	1,034977927	1

Atg5	1,034977927	1
Tmem258	1,034977927	1
Gm14270	1,034906191	1
Lhx5	1,034834459	1
Gm38366	1,034834459	1
Fkrp	1,034834459	1
Dtwd1	1,034834459	1
Ctsf	1,034762732	1
Nr4a3	1,03469101	1
Dnal1	1,034619293	1
Phf20	1,034475875	1
Smn1	1,034475875	1
Washc5	1,034404173	1
Crebbp	1,034332476	1
Ubxn4	1,034332476	1
Gm4734	1,034260784	1
Gm14137	1,034260784	1
Tmem184b	1,034260784	1
Tab1	1,034189097	1
Zfand2a	1,034189097	1
Psmc6	1,034117415	1
Zkscan4	1,034045738	1
Zfp420	1,034045738	1
Fes	1,034045738	1
BC037034	1,034045738	1
Gm5445	1,033974066	1
Ppp1r35	1,033902398	1
Urgcp	1,033902398	1
Gpatch2	1,033902398	1
Stat5a	1,033759079	1
Abtb1	1,033759079	1
Mitd1	1,033687427	1
Pigf	1,033687427	1
Ptrf	1,03361578	1
Cnst	1,03361578	1
Tmem181a	1,033544137	1
Gm20628	1,03332924	1
Cct3	1,033257618	1
Cenpa	1,033186	1
Gm44950	1,033114388	1
Fth-ps2	1,033114388	1
Gm6654	1,033114388	1
Sptssa	1,033114388	1
Guf1	1,033114388	1
Dusp4	1,033114388	1
Calr	1,03304278	1
Rabggta	1,032971178	1
Cnot9	1,032971178	1
Atxn7l3b	1,032971178	1
Ppp3ca	1,03289958	1
Api5	1,03289958	1
Crebrf	1,032827987	1

Reep3	1,0327564	1
Heca	1,0327564	1
Nomo1	1,032613239	1
Hist1h1e	1,032398535	1
Aqr	1,032326978	1
Abcd4	1,032255425	1
Fam65a	1,032255425	1
3110031N09Rik	1,032183877	1
Rab3gap1	1,032183877	1
Dhx16	1,032040795	1
Skiv2l	1,031969262	1
Eprs	1,031969262	1
Cacna1b	1,031897734	1
Gm7407	1,031826211	1
Dok2	1,031826211	1
Sdad1	1,031826211	1
Mrps30	1,031754693	1
4921511C10Rik	1,031611671	1
Tsg101	1,031540168	1
6030442K20Rik	1,031468669	1
Ncbp1	1,031468669	1
Usp39	1,031397176	1
Vps72	1,031325687	1
Fam134b	1,031254204	1
Nupl2	1,031182725	1
Arpc4	1,031182725	1
Tjp3	1,031039783	1
Eif3a	1,031039783	1
Sipa1l1	1,030968319	1
A930016O22Rik	1,03089686	1
Klhl35	1,03089686	1
Rabif	1,03089686	1
Synj2	1,03089686	1
Ptov1	1,03089686	1
Ficd	1,030825406	1
Rbck1	1,030825406	1
Stambpl1	1,030825406	1
Ankrd40	1,030825406	1
Zcchc9	1,030682513	1
Odf3l1	1,030468211	1
Prtg	1,030468211	1
Scarb1	1,030468211	1
Fam102a	1,030468211	1
Rps10-ps1	1,030468211	1
Lancl2	1,030396787	1
Utp20	1,030396787	1
Iars2	1,030396787	1
Gab2	1,030253954	1
Rfc1	1,030182544	1
Srxn1	1,030039741	1
Ppp2r5a	1,029968346	1
Angptl4	1,029896957	1

Lace1	1,029896957	1
Pkm	1,029896957	1
Taf1c	1,029825572	1
Mthfd1l	1,029754193	1
Trim11	1,029754193	1
9530085L11Rik	1,029611448	1
Gm42731	1,029468724	1
N4bp2l1	1,029468724	1
Srl	1,029326019	1
Tbl2	1,029326019	1
Dpm2	1,029326019	1
Tpk1	1,029254674	1
Atraid	1,029254674	1
Phf11b	1,029183334	1
Atf1	1,029111999	1
Lctl	1,029040669	1
Nme1	1,028969343	1
Vps51	1,028898023	1
Rbm6-ps1	1,028826708	1
Znhit3	1,028826708	1
6430573P05Rik	1,028755398	1
Fdps	1,028755398	1
Nudcd3	1,028684092	1
Zfp106	1,028684092	1
Cntd1	1,028541496	1
Zfp369	1,028470206	1
Tnfrsf1b	1,02839892	1
Srr	1,02839892	1
Hs2st1	1,02839892	1
Irf5	1,02839892	1
Fam198b	1,028327639	1
Gm14057	1,028256363	1
Tspan10	1,028256363	1
Zfp748	1,028256363	1
Qser1	1,028185093	1
Tmem132a	1,028113827	1
Myo6	1,02797131	1
Gm11263	1,027900059	1
Eif3e	1,027900059	1
Map3k11	1,027828812	1
Tagap1	1,027828812	1
Platr3	1,027757571	1
Rhot1	1,027757571	1
2210016F16Rik	1,027686335	1
Epn1	1,027686335	1
Rgl2	1,027615104	1
Sec61b	1,027615104	1
Polh	1,027543877	1
Slc35a1	1,027543877	1
Fabp5l2	1,027543877	1
mt-Nd4	1,027543877	1
Wdr61	1,027543877	1

Cinp	1,027472656	1
Gmpr	1,027472656	1
Gmps	1,027401439	1
Trappc2	1,027330228	1
Angel1	1,027330228	1
Zfp46	1,027330228	1
Dnajc11	1,027330228	1
Fbxl12	1,027330228	1
Syce2	1,027259021	1
Gpam	1,027116623	1
Ccdc181	1,026903062	1
Tk2	1,026831885	1
Trappc2l	1,026831885	1
Dcun1d1	1,026760713	1
Ubl3	1,026760713	1
Exd1	1,026689546	1
Hsdl1	1,026689546	1
Gm13373	1,026618383	1
Rassf3	1,026618383	1
Rab21	1,026618383	1
Unc119	1,026618383	1
Jund	1,026547226	1
Emc6	1,026476074	1
Bcap29	1,026333784	1
Lrrc25	1,026262646	1
Irak1bp1	1,026262646	1
Ckap2l	1,026120386	1
Copb2	1,026120386	1
n-R5s151	1,026049263	1
Elf2	1,025978145	1
Kif5b	1,025978145	1
Dhx33	1,025907032	1
Klhl2	1,025907032	1
Chmp2b	1,025835924	1
Ywhag	1,025835924	1
Gm5845	1,025764821	1
Gm26912	1,025764821	1
Rida	1,025764821	1
Gm45184	1,025764821	1
Gas8	1,02562263	1
Vamp4	1,025551542	1
Lmbr1l	1,02540938	1
Ap3m2	1,025267238	1
Timm10b	1,025267238	1
Psmb9	1,025125116	1
Hk1os	1,025054062	1
Clasrp	1,025054062	1
Prdm4	1,024983013	1
Dbnl	1,02484093	1
Supt16	1,02484093	1
Vps45	1,024769896	1
Mxd4	1,024769896	1

Cox7a2	1,024769896	1
Plekhh1	1,024627842	1
Gm14121	1,024556823	1
Tmem115	1,024556823	1
Gm23458	1,024414799	1
4932422M17Rik	1,024343795	1
Rpl21-ps5	1,024272795	1
L3hypdh	1,024272795	1
Baz2a	1,024272795	1
Ralbp1	1,024272795	1
Rilpl2	1,0242018	1
Blmh	1,0242018	1
Mecp2	1,02413081	1
Gm20703	1,024059826	1
Matr3	1,024059826	1
Baz2b	1,024059826	1
Arl2bp	1,023988846	1
Odf2l	1,023917871	1
Arel1	1,023917871	1
Gm3724	1,0238469	1
Gm12097	1,0238469	1
Ppp1r12a	1,0238469	1
Gm37780	1,023775935	1
Psmd6	1,023563069	1
Nxf7	1,023492124	1
Ccr10	1,023421183	1
Zfp827	1,023421183	1
Rbm7	1,023421183	1
Ndufaf7	1,023350247	1
Tmco1	1,023350247	1
Fbxw2	1,023350247	1
Ppm1m	1,023279317	1
Mien1	1,023279317	1
Arhgap39	1,023208391	1
Gm10425	1,02313747	1
Gtpbp3	1,02313747	1
Fosb	1,023066554	1
Pcyox1	1,023066554	1
Slc2a1	1,022995643	1
Pon2	1,022995643	1
Atxn10	1,022924736	1
M1ap	1,022782939	1
Canx	1,022782939	1
Gpn3	1,022712047	1
Zfp560	1,022712047	1
C030037D09Rik	1,022641161	1
Igfbp4	1,022641161	1
H1f0	1,022570279	1
Eea1	1,022570279	1
D630045J12Rik	1,022499402	1
Ttk	1,022428531	1
H2-D1	1,022428531	1

Gnb5	1,022357664	1
Miga2	1,022286802	1
Tcea3	1,022286802	1
Tceanc	1,022286802	1
Plau	1,022286802	1
Zc3h14	1,022215945	1
Ska2	1,022145093	1
Pccb	1,022074245	1
Supt5	1,022003403	1
Oxr1	1,021932566	1
Eif1ax	1,021932566	1
Samd8	1,021861733	1
Ubl5	1,021790905	1
Zp1	1,021578452	1
Wfdc17	1,021507644	1
Nek4	1,021366043	1
Gm12089	1,021366043	1
Tmem161b	1,02129525	1
Slain2	1,021153678	1
Usp47	1,021153678	1
Rhobtb2	1,021082899	1
Elac2	1,021082899	1
Emg1	1,021082899	1
Phf11d	1,021012126	1
Vwa8	1,021012126	1
Nol8	1,021012126	1
Zfp212	1,020941357	1
A930015D03Rik	1,020870593	1
Atg13	1,020799834	1
Ncoa1	1,02072908	1
Gm15472	1,020658331	1
Bmf	1,020658331	1
Gm12280	1,020587587	1
Ctsb	1,020587587	1
Ing1	1,020516848	1
Tpd52	1,020516848	1
RP23-403D16.3	1,020446113	1
Praf2	1,020446113	1
Dand5	1,020375384	1
Mtrr	1,020375384	1
Tfg	1,020375384	1
Mknk2	1,020375384	1
Mfsd6	1,02023394	1
Snhg3	1,02023394	1
Ncln	1,02023394	1
Rnf26	1,020163225	1
Atp5d	1,020163225	1
Txndc9	1,020092515	1
Wdr48	1,02002181	1
Skp1a	1,02002181	1
Acbd3	1,02002181	1
Sec31b	1,019880415	1

Zfp777	1,019809724	1
Agpat1	1,019809724	1
Dnaaf3	1,019739039	1
Zfp661	1,019668359	1
Rbmxl1	1,019597683	1
Rpl26	1,019527012	1
Diexf	1,01931503	1
Gm9774	1,019244379	1
Mrs2	1,019173732	1
Mnd1	1,019173732	1
Wrnip1	1,019032455	1
Nipa2	1,019032455	1
Hddc3	1,019032455	1
Gucy2g	1,018961823	1
Atp1b4	1,018891197	1
Gm13416	1,018820575	1
Ssh2	1,018820575	1
Adat3	1,018749958	1
Sun2	1,018749958	1
Car9	1,018608739	1
Pik3r1	1,018608739	1
Bnip2	1,018538137	1
Miip	1,01846754	1
Elof1	1,01846754	1
Lgals1	1,018396947	1
Ovca2	1,01832636	1
Ogfrl1	1,01832636	1
B4galt5	1,0181852	1
Osgin2	1,018044059	1
Ccdc71	1,017832385	1
Abca7	1,017761836	1
Guca1a	1,017691293	1
Nfil3	1,017620755	1
Cyth2	1,017620755	1
Zbtb11	1,017620755	1
Rwdd3	1,017479692	1
Flot2	1,017409168	1
4933417C20Rik	1,017338649	1
Ndufaf3	1,017338649	1
Gga2	1,017338649	1
Tpm1	1,017268135	1
Nectin3	1,017197626	1
Tfap4	1,017127122	1
Itga6	1,016986128	1
Gm26610	1,016915638	1
Ino80b	1,016774673	1
Pitpna	1,016774673	1
Fam98c	1,016704198	1
Bnip3l	1,016633728	1
Ercc2	1,016633728	1
Dnmt3a	1,016492803	1
H2-Ob	1,016422347	1

Tmem189	1,016422347	1
Pkd1l2	1,016351897	1
Slc20a2	1,016351897	1
Nucks1	1,016351897	1
Gm6378	1,016281451	1
Sfxn5	1,01621101	1
Ube2d1	1,01621101	1
Zfp330	1,01621101	1
Bysl	1,01621101	1
Polr1d	1,016140574	1
Brca1	1,016070143	1
Cd82	1,016070143	1
Gm28424	1,015999717	1
Ccser2	1,015999717	1
H60b	1,015999717	1
Mlf1	1,015929296	1
Pola2	1,015858879	1
Timm29	1,015858879	1
Hspa13	1,015788468	1
Tmem147	1,015718061	1
Trim47	1,015577262	1
Ddx24	1,015577262	1
Nme7	1,01550687	1
Mff	1,01550687	1
RP24-511J14.2	1,015436483	1
Arl2	1,015436483	1
Stradb	1,015366101	1
Klhl36	1,015295723	1
Ift140	1,015295723	1
Fam46a	1,015295723	1
Cpsf4	1,015295723	1
Vps53	1,015225351	1
Prpf40a	1,015225351	1
Hp1bp3	1,015225351	1
Sdhaf4	1,015014263	1
Gale	1,01494391	1
Pkmyt1	1,014803218	1
Rpl18a-ps1	1,014662547	1
Sfpq	1,014662547	1
Zfp329	1,014451575	1
Ccdc88b	1,014451575	1
Atmin	1,014451575	1
Gm45568	1,014381261	1
Msl2	1,014381261	1
Btbd2	1,014310952	1
Rbm48	1,014240648	1
Ube2cbp	1,014170349	1
Pgm1	1,014100054	1
Gm12834	1,01395948	1
Tcea1	1,01395948	1
Irf2bp2	1,01395948	1
Rdh10	1,0138892	1

Anapc16	1,0138892	1
Sep 08	1,013818925	1
Grk4	1,013748655	1
Jagn1	1,013748655	1
Adam10	1,013678389	1
Hoxb3	1,013608129	1
5830432E09Rik	1,013608129	1
Slc27a1	1,013608129	1
Smc3	1,013608129	1
Fgfr1op2	1,013467623	1
Vps26a	1,013467623	1
Nabp1	1,013397377	1
Fam126a	1,013397377	1
Herc6	1,0132569	1
Wdr34	1,0132569	1
Gnpda2	1,0132569	1
Prune1	1,0132569	1
Car5b	1,013186669	1
Parpbp	1,013116443	1
Borcs6	1,013116443	1
Sik1	1,013046221	1
Tpgs1	1,013046221	1
4833439L19Rik	1,013046221	1
Birc3	1,013046221	1
Cutc	1,012976005	1
Cnbp	1,012976005	1
Arl1	1,012905793	1
Fth1	1,012905793	1
Sclt1	1,012835586	1
Ltc4s	1,012835586	1
Nrf1	1,012765384	1
Rras	1,012695187	1
Acsl5	1,012484625	1
Zfp703	1,012414447	1
Ccdc186	1,012274106	1
Snrpe	1,012274106	1
Cenpe	1,012203943	1
Mthfr	1,012133785	1
Pkp2	1,012063632	1
Asf1a	1,012063632	1
Tspan14	1,01192334	1
Rab9	1,01192334	1
Akap9	1,011853201	1
Pcnp	1,011783067	1
Coq8b	1,011712938	1
Pgs1	1,011712938	1
Lipt1	1,011572695	1
Aplp2	1,011572695	1
Tyms	1,011432471	1
Rab1a	1,011432471	1
Gfm2	1,011432471	1
Tctex1d4	1,011362366	1

Lrwd1	1,011292266	1
Ufl1	1,011292266	1
B3gnt3	1,011222171	1
G430095P16Rik	1,011222171	1
Rbm22	1,011222171	1
Lmbr1	1,011152081	1
Agpat5	1,011152081	1
Aldh16a1	1,011081996	1
Syne1	1,011081996	1
Thumpd3	1,010871769	1
Ambra1	1,010871769	1
3110043O21Rik	1,010801703	1
Trim21	1,010731642	1
Gm6433	1,010731642	1
Ptp4a1	1,010731642	1
Amz1	1,010661586	1
Ttyh2	1,010591535	1
Heatr6	1,010591535	1
Per2	1,010521488	1
Txn1	1,010521488	1
Csde1	1,010521488	1
Trib3	1,010311378	1
Uba3	1,010241351	1
Psmd9	1,010241351	1
Tm9sf1	1,010171329	1
Eif4b	1,010171329	1
Zdhhc16	1,010031299	1
Tcf3	1,010031299	1
Tep1	1,010031299	1
Psme3	1,010031299	1
Serpinb6a	1,010031299	1
Ttc27	1,009891289	1
Vma21	1,009891289	1
1700096K18Rik	1,009821291	1
Exoc5	1,009821291	1
Tpr	1,009821291	1
Maip1	1,009751298	1
Gm30329	1,009471374	1
Gm36266	1,009401405	1
Phf20-ps	1,009401405	1
Timm17a	1,009401405	1
Slc11a2	1,009401405	1
Hgsnat	1,009261482	1
3300005D01Rik	1,009261482	1
Cep250	1,009191528	1
Calu	1,009191528	1
Zfp110	1,009191528	1
Parp10	1,009051634	1
Fam216a	1,009051634	1
Rab40c	1,009051634	1
Nup62	1,009051634	1
Errfi1	1,008981694	1

Smg9	1,008911759	1
Gm3617	1,008841829	1
Fer	1,008771904	1
Lyl1	1,008771904	1
1110008L16Rik	1,008701984	1
Traf3	1,008701984	1
Gm13140	1,008632068	1
Tomm22	1,008632068	1
Dnttip1	1,008562158	1
Gm45716	1,008492252	1
Fuca2	1,008492252	1
Nudc	1,008492252	1
Arhgap10	1,008422351	1
Aftph	1,008352455	1
Lyn	1,008352455	1
Eif2a	1,008352455	1
Reep6	1,008212677	1
Tgif2	1,008212677	1
Mrpl1	1,008212677	1
Sypl	1,008212677	1
Shoc2	1,008142796	1
Nadk2	1,008072919	1
Kbtbd2	1,008003047	1
Zfp930	1,008003047	1
Etf1	1,00793318	1
4930432K21Rik	1,007863318	1
Slc9a6	1,007863318	1
Tmem110	1,007863318	1
Rexo2	1,007793461	1
Sirt7	1,007723608	1
Cetn2	1,007723608	1
Rad50	1,00765376	1
Gdpd3	1,00765376	1
Cnot10	1,007583918	1
Tmem17	1,00751408	1
Vkorc1l1	1,007444246	1
Gtf3a	1,007374418	1
Fig4	1,007304595	1
RP24-418P10.4	1,007164962	1
Arl8b	1,007164962	1
Zfp658	1,007025349	1
Vprbp	1,00695555	1
Zfp277	1,006885756	1
Med29	1,006815966	1
Ube2f	1,006815966	1
Nol7	1,006815966	1
Rapgef1	1,006746181	1
Gm4968	1,006676401	1
Mia3	1,006676401	1
Coq10a	1,006676401	1
Mdh1	1,006606626	1
Hnrnpc	1,006467091	1

Tex2	1,00639733	1
Ppm1h	1,006327574	1
Copz1	1,006327574	1
Parp3	1,006188077	1
Mical3	1,006188077	1
Ifi30	1,006188077	1
Pitpnc1	1,0060486	1
Rexo4	1,0060486	1
Mrfap1	1,005978868	1
1110065P20Rik	1,005909142	1
Amz2	1,005769703	1
Gm7618	1,005630283	1
Glod4	1,005630283	1
Zranb1	1,005630283	1
Cep63	1,00556058	1
Sumo1	1,00556058	1
Tuft1	1,00556058	1
Hnrnpul1	1,00556058	1
Cacfd1	1,005490883	1
Ropn1l	1,00542119	1
Cdv3	1,00542119	1
Zfp36l1	1,005351502	1
Foxn3	1,005351502	1
Gm4879	1,005281818	1
Fam234a	1,005281818	1
3110062M04Rik	1,005281818	1
Prkce	1,005142466	1
Ctnna1	1,005142466	1
Aifm2	1,005142466	1
Plekhg2	1,005072798	1
Rpl17-ps8	1,005072798	1
Blzf1	1,005072798	1
Itch	1,004933475	1
Dnah8	1,00486382	1
Aff1	1,00486382	1
Tbc1d10b	1,00486382	1
Gm12988	1,004794171	1
Pop4	1,004724526	1
Tamm41	1,004724526	1
Napg	1,004654887	1
Gtf2h5	1,004585252	1
Gm10320	1,004445996	1
Pfkm	1,004445996	1
Hipk2	1,00430676	1
Wsb1	1,00430676	1
Rps6kb2	1,004237149	1
Mrps10	1,004237149	1
Smyd3	1,004167543	1
Mafb	1,004167543	1
Rabggtb	1,004167543	1
Hpse	1,004097942	1
Efna3	1,004028346	1

Clec4a3	1,003958754	1
Ndst1	1,003958754	1
Ptpra	1,003958754	1
Arhgap27	1,003958754	1
Nrd1	1,003889167	1
Picalm	1,003889167	1
Il15ra	1,003819586	1
Slc7a6	1,003819586	1
Wbp1	1,003750009	1
Tchp	1,003610869	1
Mrpl15	1,003402196	1
Nelfcd	1,003263104	1
Qrsl1	1,003193566	1
Mt1	1,003193566	1
Tmem50a	1,003054503	1
Cd80	1,00291546	1
Alg14	1,002776436	1
Heatr5b	1,002776436	1
Gpr155	1,002706931	1
Mpv17	1,002706931	1
Scamp3	1,002706931	1
Ptp4a3	1,002706931	1
Acoxl	1,002637431	1
Tbc1d8	1,002637431	1
Ncl	1,002637431	1
Uck2	1,002637431	1
Nacc2	1,002567936	1
Pkn1	1,002567936	1
Oaz1	1,00242896	1
Cryzl1	1,00242896	1
Ppp2r3c	1,00242896	1
Eif5a	1,00242896	1
Nolc1	1,00235948	1
Hist1h4d	1,002290004	1
4930579G24Rik	1,002151066	1
1500002F19Rik	1,002081605	1
Gpatch2l	1,002081605	1
Vps35	1,002081605	1
Alg12	1,001942697	1
Ctif	1,001942697	1
Pabpc1l	1,001942697	1
Acadvl	1,001942697	1
Nphp1	1,001803808	1
Phf14	1,001803808	1
Zfp598	1,001664938	1
Pax3	1,00159551	1
Herpud1	1,001526087	1
Cdc42se2	1,001526087	1
Sgk3	1,001456669	1
Abhd16a	1,001387256	1
Naa35	1,001317847	1
Hmgxb3	1,001179045	1

Hbp1	1,001179045	1
Nat2	1,001109651	1
Hs1bp3	1,001109651	1
B330016D10Rik	1,000970877	1
Tnfrsf10b	1,000970877	1
Prcp	1,000970877	1
Ankhd1	1,000970877	1
Bora	1,000832123	1
Cox7a2l	1,000762753	1
Ulbp1	1,000693387	1
Unc5b	1,000624027	1
Tusc3	1,000624027	1
Cluh	1,000624027	1
Glud1	1,000624027	1
Snora21	1,000485321	1
Syncrip	1,000485321	1
Frg1	1,000485321	1
Capn5	1,000415975	1
Zfx	1,000415975	1
Ccsap	1,000346634	1
Ifi213	1,000346634	1
Cds1	1,000346634	1
Ap1s2	1,000346634	1
6030460B20Rik	1,000207966	1
Rep15	1,000207966	1
Apoa1bp	1,000207966	1
Parp9	1,000138639	1
Cog8	1,000069317	1
Trim32	1,000005141	1
Calm1	1	1
Gm42418	1	1
Pip5k1c	-0,00012217	1
Phf6	-0,0001898	1
Zc4h2	-0,00027679	1
Kat6a	-0,00031472	1
Gsto2	-0,00044876	1
Frmd6	-0,00049536	1
Gphn	-0,00053965	1
Il10rb	-0,00047407	1
Arrb1	-0,00059236	1
Naa30	-0,0006111	1
Lsm14a	-0,00076035	1
Fkbp3	-0,00081648	1
Plekha3	-0,00084875	1
Chek2	-0,00094627	1
Atg4b	-0,0011092	1
Tpm4	-0,0012773	1
Ilf3	-0,0014205	1
2510002D24Rik	-0,0014198	1
Serf1	-0,0014437	1
Diaph1	-0,0013744	1
Wdr70	-0,0015353	1

Pik3ip1	-0,0016031	1
Eef1e1	-0,001794	1
Sms	-0,0018346	1
Apc	-0,001926	1
Zranb3	-0,0019887	1
Cdca5	-0,0019768	1
Gclc	-0,0020448	1
Dhdds	-0,0019612	1
Spryd7	-0,0021414	1
Rapgef5	-0,0022006	1
Tmem120a	-0,0021664	1
Napa	-0,0023113	1
Ing3	-0,0024154	1
Arrb2	-0,0023505	1
Mrps24	-0,0024249	1
Chmp5	-0,0025249	1
Abcf1	-0,0025256	1
Ift22	-0,0026353	1
Prrc2c	-0,002605	1
Pdcd5	-0,0026983	1
Afg3l1	-0,0027384	1
Tmem185b	-0,0028275	1
Anks3	-0,0027953	1
Gm43466	-0,0028535	1
Chmp1a	-0,0029082	1
Nudcd2	-0,0029824	1
Ube2g1	-0,0030835	1
Cog5	-0,0030747	1
Itpr2	-0,0032073	1
Gm2991	-0,003252	1
Thoc1	-0,0032839	1
Bod1l	-0,0032673	1
Rab11b	-0,0032508	1
Gm16020	-0,0034491	1
Hnrnph1	-0,0034715	1
Kctd2	-0,0036964	1
Fitm2	-0,0038337	1
Ascc1	-0,0037848	1
St14	-0,003774	1
Spg20	-0,0038008	1
Gm5451	-0,0038975	1
Ppt2	-0,0039107	1
Mob2	-0,0040484	1
Gm8995	-0,004054	1
Efl1	-0,0042141	1
Lzts2	-0,0043488	1
Ctsz	-0,0042502	1
Lsm4	-0,004549	1
Uvrag	-0,004529	1
Brd1	-0,0044775	1
Bcl2l13	-0,0046498	1
Cd300ld	-0,0046463	1

Klf6	-0,0045938	1
Ndufb4	-0,004797	1
Tjp2	-0,0048367	1
Eps15	-0,0047941	1
Sparc	-0,0050644	1
Wdr5	-0,0051366	1
Nckap1l	-0,0051299	1
Sec16a	-0,0052009	1
Rrm2	-0,0052105	1
Rps23	-0,0054702	1
Map3k4	-0,0055449	1
Sat1	-0,0054782	1
Trip13	-0,0055808	1
Nrde2	-0,005572	1
AU019823	-0,0056818	1
Miga1	-0,0057993	1
Ssr3	-0,0058085	1
Rac2	-0,0058357	1
Arid1b	-0,0059326	1
Lss	-0,0059927	1
Fbxo28	-0,0059843	1
Snip1	-0,0060737	1
Ppwd1	-0,0061491	1
Hif1a	-0,0061061	1
Htatip2	-0,0060846	1
Rasal3	-0,0062918	1
Plekhg5	-0,0064334	1
Zfp592	-0,0064234	1
1700054M17Rik	-0,0064546	1
Cbx7	-0,0065451	1
Spred1	-0,0066369	1
Twf2	-0,006558	1
Tmem159	-0,0066844	1
Gm10478	-0,0069303	1
Ccdc88a	-0,0069845	1
Szrd1	-0,007091	1
Gm15798	-0,0073784	1
Acox1	-0,0073788	1
Alas1	-0,0074565	1
Cd52	-0,0075383	1
Ddx54	-0,0075549	1
4933434E20Rik	-0,0076699	1
Pafah1b2	-0,0076527	1
Rmdn1	-0,0077696	1
Homer3	-0,0078347	1
Tpst1	-0,0077521	1
Plekhf1	-0,007933	1
2810402E24Rik	-0,0079396	1
Fbxo4	-0,00815	1
Fen1	-0,0085172	1
Cops2	-0,0084731	1
Kpna6	-0,00873	1

Specc1	-0,0087281	1
Krr1	-0,0089021	1
Dscr3	-0,0088655	1
Aida	-0,0090448	1
Lig3	-0,0091212	1
Usf1	-0,0092107	1
Dhx38	-0,009238	1
Rbm8a	-0,0094425	1
Hmgn5	-0,0094537	1
Osgepl1	-0,0096342	1
Tmem62	-0,0097062	1
Gm6560	-0,010033	1
Zfp846	-0,010026	1
Tbrg4	-0,010032	1
Cyld	-0,0099568	1
Gm6418	-0,010107	1
Gm6297	-0,010123	1
Chm	-0,010179	1
Rnf146	-0,010237	1
Gng12	-0,010412	1
Pros1	-0,010457	1
Bid	-0,010613	1
Bin3	-0,010773	1
Naglu	-0,010759	1
Nus1	-0,010923	1
Gm38345	-0,010981	1
Deptor	-0,010981	1
Rad9a	-0,011145	1
L3mbtl2	-0,011084	1
Srpk2	-0,011097	1
Zfp81	-0,011158	1
St3gal3	-0,011161	1
B230322F03Rik	-0,011312	1
Nceh1	-0,011307	1
Gm16045	-0,011366	1
Ran	-0,011361	1
Dgkg	-0,011538	1
Gm15920	-0,011467	1
Gm45360	-0,011809	1
Clcn7	-0,011822	1
Gm2788	-0,011958	1
Rpl36-ps8	-0,012039	1
Ints3	-0,012015	1
Taf4	-0,012071	1
Syk	-0,012058	1
Gem	-0,012198	1
Smim19	-0,012321	1
Zfp747	-0,012427	1
Hspe1	-0,01237	1
Fundc2	-0,012365	1
Tor1aip2	-0,012521	1
Fam3a	-0,012642	1

Thap12	-0,01255	1
Vps11	-0,012614	1
Drap1	-0,012633	1
Src	-0,012755	1
Plcl2	-0,013094	1
Dnmt1	-0,013076	1
Rpl30-ps11	-0,013228	1
Enoph1	-0,013169	1
Apbb1ip	-0,013225	1
Cdkn1a	-0,013338	1
Eif3h	-0,013346	1
Unc50	-0,01341	1
Ptpn4	-0,013481	1
Sod2	-0,013639	1
Nrbp2	-0,013679	1
Srrd	-0,013694	1
Wdtc1	-0,013788	1
Mitf	-0,01404	1
Mrpl22	-0,014078	1
Reps1	-0,014077	1
Sec62	-0,014326	1
Arvcf	-0,014422	1
Fam208b	-0,014376	1
Hip1	-0,014401	1
Map4k5	-0,014371	1
Tmc6	-0,014439	1
Actr1b	-0,014411	1
Rps8-ps4	-0,014516	1
Spast	-0,014499	1
Ctnnbip1	-0,014534	1
Ngdn	-0,014532	1
H2-T23	-0,014631	1
Epsti1	-0,014586	1
Ube2k	-0,014661	1
Arl14epl	-0,014827	1
Tcf25	-0,014835	1
Gm13092	-0,015141	1
9030617O03Rik	-0,015239	1
Fam160b2	-0,015306	1
Zfp639	-0,015452	1
Sh3tc1	-0,015639	1
Magi2	-0,01568	1
Ehbp1	-0,015716	1
Zfp131	-0,015692	1
Selenot	-0,015777	1
Fgf13	-0,01594	1
Yipf1	-0,015894	1
Eri1	-0,015994	1
Telo2	-0,016105	1
Id2	-0,016063	1
Tmx4	-0,016117	1
Plcb4	-0,01623	1

Kremen1	-0,016317	1
Cnp	-0,01643	1
Fbxw8	-0,01653	1
Nectin2	-0,016639	1
Rbm15b	-0,01658	1
Dusp11	-0,016556	1
Sap30	-0,016969	1
Bptf	-0,017032	1
Mkl1	-0,017229	1
Cars	-0,017223	1
Dusp3	-0,017546	1
Arfgap2	-0,017632	1
Dmwd	-0,017578	1
Slc11a1	-0,017583	1
Pla2g15	-0,017636	1
Cct8	-0,01758	1
Adam17	-0,017742	1
Tinf2	-0,017776	1
Tango2	-0,017829	1
Kmt5a	-0,017773	1
Qrich1	-0,01788	1
Gm32856	-0,017952	1
Lig4	-0,018042	1
Ccl25	-0,018048	1
Psma6	-0,017999	1
Ndc1	-0,018316	1
Gm9645	-0,018372	1
St18	-0,018383	1
Chd3os	-0,018493	1
Lrrc8d	-0,018459	1
Insig1	-0,018676	1
Gchfr	-0,018668	1
Dusp8	-0,018795	1
Alyref	-0,018881	1
Gm43006	-0,019008	1
Snapc4	-0,019188	1
2610001J05Rik	-0,019168	1
Dvl1	-0,019324	1
Polr2e	-0,019331	1
Lrrc51	-0,019515	1
Nrp2	-0,019519	1
Mrpl16	-0,019658	1
Dis3l2	-0,019844	1
Abcc4	-0,019856	1
4930526A20Rik	-0,019901	1
Tulp4	-0,019954	1
Lrmp	-0,019954	1
Pms2	-0,02021	1
Vcpip1	-0,020256	1
Zfp646	-0,020368	1
Zfp384	-0,020494	1
Prmt6	-0,020499	1

Aamp	-0,020535	1
Arl14ep	-0,020642	1
Kmt2e	-0,020707	1
Tmem60	-0,02079	1
Azin1	-0,020946	1
Dag1	-0,021046	1
Smarca2	-0,020987	1
Bmpr2	-0,021206	1
Rapgef6	-0,02124	1
Smarcd1	-0,02127	1
Sos2	-0,021464	1
Trim33	-0,021624	1
Cisd2	-0,021725	1
Supt20	-0,021817	1
Smyd2	-0,021799	1
Ak6	-0,021763	1
Nfe2l2	-0,021777	1
Chd1l	-0,021949	1
Gtf2e2	-0,021872	1
Srp72	-0,022136	1
Snx32	-0,022242	1
Ephx1	-0,022254	1
Slc25a51	-0,022275	1
Jkamp	-0,022415	1
Dusp12	-0,022501	1
Med21	-0,022704	1
Imp4	-0,022801	1
Clns1a	-0,022785	1
Cfap43	-0,023176	1
RP24-282C4.13	-0,023327	1
Etfdh	-0,023389	1
Nvl	-0,023518	1
Smu1	-0,023523	1
Sdha	-0,023605	1
Bbs10	-0,023703	1
Ifrd2	-0,023722	1
Brd3	-0,023912	1
Ddx39	-0,02396	1
Hjurp	-0,024043	1
Oxnad1	-0,024168	1
Tfdp1	-0,024426	1
Eif3s6-ps1	-0,024376	1
Arhgdib	-0,024417	1
Fsd2	-0,024519	1
Pfkl	-0,02458	1
Mss51	-0,024787	1
1700123O20Rik	-0,025006	1
Samhd1	-0,024969	1
Mon2	-0,02499	1
Mpp6	-0,02512	1
Exo1	-0,025182	1
Rbm4	-0,025158	1

Nin	-0,025213	1
Twf1	-0,025421	1
Cbx3	-0,025353	1
Rwdd4a	-0,025488	1
Bcas2	-0,025708	1
Mthfd2	-0,025886	1
Gm44851	-0,025994	1
Nipsnap1	-0,025992	1
Prps1	-0,026037	1
Ddx46	-0,026178	1
Cryl1	-0,026312	1
Rabgap1	-0,026356	1
Tmed4	-0,02636	1
3110001I22Rik	-0,0265	1
Pick1	-0,026458	1
Atxn7l1	-0,026512	1
Iqce	-0,0266	1
Prune2	-0,026595	1
Tut1	-0,02665	1
Alox5	-0,02665	1
Maff	-0,026779	1
Psmg2	-0,026843	1
Rcc1	-0,026763	1
Strap	-0,026767	1
Relt	-0,026999	1
Smad1	-0,027018	1
Aph1c	-0,027061	1
Chchd1	-0,027289	1
n-R5-8s1	-0,027414	1
Kcnk6	-0,027363	1
RP24-175C20.18	-0,027474	1
Bbs2	-0,027567	1
Fam175a	-0,027707	1
Ankib1	-0,027744	1
Zfp62	-0,028082	1
Vdr	-0,028307	1
Gm43742	-0,028342	1
Tsta3	-0,028405	1
Smg8	-0,028357	1
Gm20712	-0,028475	1
Rpl3-ps2	-0,028541	1
A430005L14Rik	-0,028505	1
Sec22c	-0,028702	1
Msh2	-0,028732	1
Rab6a	-0,028718	1
Gm14427	-0,028781	1
Gemin5	-0,029075	1
Tbrg1	-0,029148	1
Phb2	-0,029085	1
Msmo1	-0,02921	1
Gtf2e1	-0,029272	1
Nudt2	-0,029309	1

Polr2d	-0,029417	1
Ap5z1	-0,029353	1
Thoc6	-0,029387	1
Tpd52-ps	-0,029542	1
Pim2	-0,029537	1
Mir7078	-0,029804	1
Cep70	-0,029895	1
Gm44152	-0,030236	1
Gm6142	-0,030168	1
Psat1	-0,030244	1
Isy1	-0,030383	1
Gm13204	-0,030478	1
Gm43511	-0,030523	1
Gm11205	-0,030527	1
Pecr	-0,030484	1
Cks1b	-0,030519	1
E130102H24Rik	-0,030646	1
Slc27a4	-0,03065	1
Lats1	-0,030611	1
Rnf114	-0,030622	1
Siah1a	-0,03068	1
Hk1	-0,030743	1
Gm20673	-0,030784	1
Zfp949	-0,030779	1
Fbxo5	-0,030893	1
Zyg11b	-0,030863	1
Dirc2	-0,03099	1
Yipf3	-0,030971	1
Gm26782	-0,031058	1
Gm7027	-0,031457	1
Tbc1d32	-0,03152	1
Cdk19	-0,031625	1
Slc25a37	-0,031656	1
Acp1	-0,031767	1
Lpin1	-0,031772	1
Fech	-0,03189	1
Selenow	-0,031922	1
Slc43a2	-0,031906	1
Junb	-0,032181	1
Mad2l1	-0,032312	1
Ogfod1	-0,032324	1
9530068E07Rik	-0,032264	1
Stt3b	-0,032414	1
Zfp874a	-0,032597	1
Fcer1g	-0,032562	1
Gm29228	-0,032653	1
Zfp235	-0,032667	1
Pyroxd2	-0,032715	1
Prr11	-0,032747	1
Sugp2	-0,032723	1
RP23-426K2.3	-0,032778	1
Cbx8	-0,032865	1

Snx16	-0,032896	1
E4f1	-0,032935	1
Dexi	-0,032925	1
Psmb3	-0,032997	1
Ilvbl	-0,033155	1
Amhr2	-0,033152	1
Zfp64	-0,033165	1
Snrnp40	-0,033203	1
Lsg1	-0,033213	1
Rab26os	-0,033278	1
Vbp1	-0,033287	1
Rbmx2-ps	-0,033369	1
Tle6	-0,033531	1
Smarcc1	-0,033486	1
Phlpp1	-0,033695	1
Cwf19l1	-0,033838	1
Slc25a17	-0,033789	1
Cops5	-0,033825	1
Tbc1d12	-0,03405	1
Twnk	-0,034022	1
Pgrmc1	-0,034264	1
Rere	-0,034309	1
Ibtk	-0,034399	1
Siah2	-0,034438	1
Znhit6	-0,034546	1
Dbf4	-0,034534	1
Apbb2	-0,034583	1
Reep4	-0,034666	1
Kpnb1	-0,034715	1
Snx17	-0,034723	1
Car12	-0,034835	1
Polr3gl	-0,034796	1
Rangap1	-0,034849	1
Cd74	-0,034914	1
P4hb	-0,034892	1
Stab1	-0,034956	1
Capn10	-0,035013	1
Cst3	-0,034972	1
Nipbl	-0,034992	1
As3mt	-0,035051	1
Tex9	-0,035193	1
Gm20900	-0,035165	1
Palb2	-0,035375	1
Bcap31	-0,03539	1
Hsp90b1	-0,035355	1
Limk1	-0,035483	1
Grhpr	-0,03559	1
Vasp	-0,035714	1
Por	-0,035667	1
Bri3bp	-0,035726	1
Gm43223	-0,03576	1
Ssx2ip	-0,035757	1

Crebl2	-0,035808	1
Ppp1r11	-0,035772	1
Cotl1	-0,035793	1
Zbtb8os	-0,035925	1
Ociad1	-0,035889	1
Xxylt1	-0,035981	1
Gm15535	-0,035998	1
Akr1e1	-0,036031	1
Alox5ap	-0,036197	1
Tsix	-0,036286	1
Lgals3	-0,036274	1
Gfi1	-0,036428	1
Zfp157	-0,036444	1
Clu	-0,036484	1
Mapk1	-0,036468	1
Ywhaq	-0,036522	1
Mettl2	-0,036636	1
Rprd1b	-0,036691	1
Swt1	-0,036736	1
Txndc11	-0,036905	1
1810030O07Rik	-0,03688	1
Naxd	-0,037116	1
Sumo3	-0,037354	1
Ebi3	-0,03735	1
Wipf2	-0,037404	1
Cdkn1b	-0,037505	1
Chka	-0,037612	1
Nol12	-0,03774	1
Ears2	-0,037789	1
Naip6	-0,037898	1
Rassf1	-0,03787	1
U2af1	-0,038007	1
Mrpl10	-0,038125	1
Mfsd11	-0,038239	1
Cfl2	-0,038151	1
Zbtb7a	-0,038183	1
Csnk1d	-0,038342	1
Acp6	-0,038351	1
Dhcr7	-0,038485	1
Mrps16	-0,038619	1
Chd3	-0,038606	1
Meis3	-0,038722	1
Dpys	-0,038786	1
Mrpl4	-0,038774	1
Josd2	-0,038817	1
Cd9-ps	-0,039034	1
Pgm2	-0,038957	1
Gm5578	-0,039054	1
Itgb2	-0,03913	1
Ifitm6	-0,039279	1
Bdp1	-0,039305	1
Svep1	-0,03941	1

B3glct	-0,039432	1
Eya4	-0,039393	1
1700047K16Rik	-0,03946	1
Snx14	-0,039531	1
Gch1	-0,039526	1
Gm8019	-0,039579	1
Atf7	-0,039603	1
Exosc7	-0,039589	1
Sco1	-0,039734	1
Clcn4	-0,039895	1
Nedd8	-0,039879	1
Ctsh	-0,039986	1
Cdt1	-0,039999	1
Gm42890	-0,040087	1
Itgb5	-0,040124	1
Exoc4	-0,040125	1
Pstk	-0,04022	1
Pgpep1	-0,04028	1
Gm15210	-0,040286	1
Mplkip	-0,040528	1
Kif7	-0,040555	1
St5	-0,040605	1
Sh3bgrl3	-0,040608	1
Rnf152	-0,040719	1
Tor1b	-0,040714	1
March5	-0,04108	1
Rrs1	-0,041177	1
Rab5c	-0,041173	1
Utp15	-0,041396	1
Rab29	-0,041542	1
4833445I07Rik	-0,041634	1
Adamtsl5	-0,041729	1
mt-Rnr2	-0,041681	1
Mettl9	-0,041658	1
Sirt3	-0,041782	1
Acad10	-0,041882	1
Nova2	-0,041924	1
Stag2	-0,041882	1
Tbc1d15	-0,041909	1
Shisa3	-0,042024	1
Meis2	-0,042016	1
Plagl2	-0,042057	1
Kars	-0,042256	1
Dync1h1	-0,04234	1
Slc41a3	-0,042412	1
Abce1	-0,042532	1
Alkbh4	-0,042675	1
Zfp365	-0,042921	1
Acyp1	-0,042916	1
Glyr1	-0,043104	1
Uso1	-0,043229	1
Senp6	-0,043218	1

Skap2	-0,043205	1
Pdp2	-0,043418	1
Snta1	-0,043438	1
Pold3	-0,043495	1
Ccnj	-0,043594	1
D230025D16Rik	-0,043664	1
Akap11	-0,043727	1
Hook2	-0,043831	1
Katna1	-0,043762	1
Exoc7	-0,044162	1
Foxj3	-0,044236	1
Adprm	-0,044152	1
Nsfl1c	-0,044313	1
Ppargc1b	-0,044307	1
Slc6a8	-0,044294	1
Pde2a	-0,044394	1
Gosr1	-0,044352	1
Arl5c	-0,044403	1
Tsen2	-0,044476	1
Dolpp1	-0,044547	1
Mrpl35	-0,044485	1
Cib1	-0,044829	1
Paqr5	-0,044966	1
Wdr44	-0,045039	1
Arhgap45	-0,045001	1
Bzw2	-0,045214	1
Gm13340	-0,045305	1
Ebag9	-0,045347	1
Gm6341	-0,045305	1
Taf1b	-0,045482	1
6430548M08Rik	-0,045451	1
Gm9165	-0,045634	1
Tsen54	-0,045658	1
Zdhhc2	-0,045844	1
Btbd1	-0,045758	1
Txlna	-0,045774	1
Ubxn8	-0,045899	1
Sike1	-0,045893	1
Mmp19	-0,04599	1
Slfn9	-0,046137	1
Cct6a	-0,046123	1
Mrpl58	-0,046215	1
Wiz	-0,046175	1
Snord118	-0,046298	1
Rinl	-0,046309	1
Snap47	-0,046279	1
Rab10os	-0,046282	1
Etfrf1	-0,046449	1
mt-Rnr1	-0,046426	1
Rpl38-ps1	-0,046519	1
Parp16	-0,046482	1
Itgav	-0,046497	1

Ube2j2	-0,046533	1
Mre11a	-0,04657	1
Mfng	-0,046645	1
Mrps31	-0,046744	1
Abi1	-0,046724	1
B230118H07Rik	-0,046775	1
Gm8423	-0,046908	1
Rint1	-0,046918	1
Fancl	-0,04691	1
Tmx1	-0,046858	1
Arf1	-0,046972	1
Nprl2	-0,047061	1
Hn1l	-0,047226	1
Clec12a	-0,047254	1
Mtap	-0,047636	1
March9	-0,047565	1
Chtop	-0,047589	1
Plxna3	-0,047786	1
Shmt2	-0,048003	1
Myadm	-0,048046	1
Scrn3	-0,048092	1
Vamp3	-0,048147	1
Asb10	-0,048156	1
Snrpa	-0,04818	1
Kctd18	-0,048312	1
Cdca7l	-0,048316	1
Rnf19b	-0,048381	1
Mms19	-0,048449	1
Vps41	-0,048518	1
Pank1	-0,048616	1
Tbl1x	-0,048601	1
Cracr2b	-0,048925	1
Pgghg	-0,049165	1
Rbl2	-0,049227	1
Adssl1	-0,049165	1
Idh3g	-0,04931	1
Thra	-0,049634	1
Fem1c	-0,049715	1
Tmem167b	-0,049915	1
Usp7	-0,049884	1
Dnajc19	-0,049975	1
Cpsf2	-0,050046	1
Vegfb	-0,050012	1
Sfmbt1	-0,050144	1
Rpa2	-0,050175	1
Chst10	-0,05024	1
Gm43859	-0,050765	1
Cdc42ep2	-0,05076	1
Rhbdf2	-0,050769	1
Rin2	-0,051148	1
Magt1	-0,051445	1
6430511E19Rik	-0,05154	1

4930558J18Rik	-0,051523	1
Mcm10	-0,051573	1
Osbpl2	-0,051579	1
Rpa1	-0,051604	1
Sltm	-0,051633	1
Sf3b2	-0,051622	1
Gm17491	-0,051683	1
Snx18	-0,051695	1
Arhgef2	-0,051667	1
Pik3cd	-0,051743	1
Mfsd1	-0,051663	1
Cops8	-0,051665	1
Rpgrip1	-0,051819	1
Exoc1	-0,051784	1
Acvr2b	-0,051823	1
Pde6d	-0,051914	1
Ppp2r5e	-0,051907	1
Actn1	-0,051892	1
Dzip1	-0,05201	1
Zfp296	-0,052063	1
Itm2b	-0,052055	1
N4bp2l2	-0,052124	1
2610528A11Rik	-0,052164	1
Dek	-0,052281	1
Upf3b	-0,052384	1
Nradd	-0,052522	1
Dpp9	-0,052678	1
Ms4a6c	-0,052911	1
Foxc1	-0,052927	1
Sp100	-0,05315	1
Elmsan1	-0,053333	1
Cul4b	-0,053504	1
Gcc2	-0,053465	1
Map1lc3b	-0,053457	1
Arap1	-0,053584	1
Galm	-0,053844	1
Gm13378	-0,054024	1
Gon4l	-0,054029	1
Thap2	-0,054014	1
Hyou1	-0,054016	1
Klc3	-0,054382	1
Orc6	-0,054372	1
Ripk1	-0,054508	1
1810037I17Rik	-0,05463	1
Dusp1	-0,054638	1
Supt3	-0,054558	1
Ttc32	-0,054713	1
Ncaph2	-0,054691	1
2010315B03Rik	-0,054772	1
Faim	-0,054909	1
Plxna1	-0,055005	1
Ndufa1	-0,055134	1

Mrps36-ps2	-0,055436	1
Gm38380	-0,055354	1
Nlrc5	-0,05543	1
Pigb	-0,055494	1
Hmg20a	-0,055535	1
Smim7	-0,055605	1
Epb41l2	-0,055713	1
Vsig8	-0,055917	1
Lrrc28	-0,055872	1
Kat8	-0,056017	1
G3bp2	-0,056011	1
Dpf1	-0,056082	1
Arrdc2	-0,056063	1
Ssfa2	-0,056085	1
Dkc1	-0,056332	1
Zfp143	-0,056376	1
Arl15	-0,056454	1
Pold2	-0,056467	1
Atp2a2	-0,056521	1
Sbno1	-0,056545	1
Cdk5rap1	-0,056594	1
Rxrb	-0,056605	1
A930018M24Rik	-0,056778	1
Tnfrsf22	-0,057215	1
Epb41l4aos	-0,057179	1
Gm5380	-0,057332	1
Ccny	-0,057269	1
Mvd	-0,057393	1
Erh	-0,057547	1
1700120C14Rik	-0,057535	1
Lpgat1	-0,0575	1
Rab34	-0,057483	1
Ubqln1	-0,057501	1
Herpud2	-0,057648	1
Haus6	-0,057552	1
2310033P09Rik	-0,057723	1
Ppm1k	-0,057725	1
Tmem134	-0,057703	1
Ccne1	-0,057817	1
Ppp1r10	-0,057766	1
Asxl1	-0,05781	1
Mrpl50	-0,057869	1
Sh3bp5	-0,057889	1
Ptpn2	-0,057964	1
Ube2v2	-0,058076	1
Rarg	-0,058086	1
Gm11716	-0,058202	1
Cox5a	-0,05822	1
Mrps27	-0,058297	1
Tbc1d14	-0,058438	1
Map2k1	-0,058377	1
Tmem176b	-0,058393	1

Gm37274	-0,058519	1
Gm45871	-0,0586	1
Fam193a	-0,058704	1
Cldn12	-0,058786	1
Ptpn23	-0,058841	1
Arhgap15	-0,05892	1
Sh3pxd2a	-0,058917	1
Trim8	-0,058925	1
Hspa9	-0,058858	1
Clec4e	-0,059024	1
Tmem68	-0,059082	1
Rock1	-0,059115	1
Ppp6c	-0,059232	1
Prkd3	-0,059279	1
Nfxl1	-0,059283	1
Tigd2	-0,059332	1
Rtkn	-0,059525	1
Eif2d	-0,059621	1
Yy1	-0,05968	1
Ubald2	-0,059913	1
Eif3j1	-0,060011	1
Arid4a	-0,060042	1
Rnf185	-0,060293	1
Fbxw9	-0,06041	1
Pip4k2a	-0,060362	1
Brpf1	-0,060543	1
Stc2	-0,060605	1
Rpsa-ps11	-0,060634	1
Epb41l5	-0,060591	1
Ube2c	-0,060611	1
Ppp1r13b	-0,060669	1
Mllt11	-0,060682	1
Lrrc59	-0,060817	1
Arl16	-0,060948	1
Mrps14	-0,060882	1
Fam91a1	-0,060954	1
Msrb1	-0,061008	1
Gm26225	-0,06113	1
Ccpg1	-0,061126	1
Stambp	-0,061123	1
Pik3r5	-0,061113	1
Msr1	-0,061248	1
4931440P22Rik	-0,061336	1
Dda1	-0,061293	1
Siae	-0,061377	1
Mcm3ap	-0,061719	1
Ttc17	-0,0618	1
Cdca8	-0,061838	1
Ankrd11	-0,061877	1
Zfp664	-0,062001	1
Mynn	-0,062015	1
Plod1	-0,062139	1

Ssrp1	-0,062235	1
Cpt1a	-0,062397	1
Fbxw5	-0,062512	1
Pip4k2b	-0,062636	1
Gm13680	-0,062753	1
Gnptab	-0,062767	1
Phb	-0,062868	1
Cdk2ap2	-0,063001	1
Nap1l1	-0,062979	1
Rgs9bp	-0,063257	1
E130307A14Rik	-0,063408	1
Cdkn2d	-0,063528	1
Zfp65	-0,063702	1
Slc12a9	-0,063714	1
Hectd1	-0,063802	1
Csgalnact2	-0,063874	1
Dctn6	-0,06393	1
Rfx5	-0,063879	1
D430042O09Rik	-0,064007	1
Dynlrb1	-0,064028	1
Commd4	-0,063963	1
Bckdha	-0,064079	1
Ilkap	-0,064141	1
Gm6745	-0,064194	1
Cd200r4	-0,064184	1
Ankfy1	-0,064287	1
Rae1	-0,06427	1
Larp4b	-0,064362	1
Iscu	-0,064483	1
Gart	-0,064477	1
Fam118b	-0,064486	1
Cops3	-0,064637	1
Trappc9	-0,064742	1
Kifc3	-0,064836	1
Rpl22	-0,065028	1
Ruvbl1	-0,065117	1
Mgarp	-0,06513	1
Naa16	-0,065069	1
Sec24c	-0,065128	1
Ankrd28	-0,065328	1
Ppp1r12b	-0,065348	1
Noc2l	-0,065643	1
Rbpj	-0,065632	1
Naa40	-0,065675	1
Ier3ip1	-0,065663	1
Rpp21	-0,065819	1
Ift46	-0,065754	1
Mapre1	-0,065752	1
Tgoln1	-0,06591	1
Ilk	-0,066002	1
Top1	-0,065972	1
Prtn3	-0,066185	1

Pds5b	-0,066171	1
Gm18943	-0,066322	1
Pnn	-0,066471	1
Mak16	-0,066492	1
Ddx21	-0,066586	1
Bbx	-0,066618	1
Rps5	-0,066628	1
Tmem120b	-0,066654	1
Hn1	-0,066779	1
Akr1b10	-0,066898	1
Ccdc9	-0,066877	1
Pcnx	-0,067143	1
Rusc1	-0,067113	1
Dhrs9	-0,067159	1
Pdcd10	-0,067182	1
Elf1	-0,067311	1
Clk2	-0,067251	1
Igtp	-0,067386	1
Zmpste24	-0,067386	1
Wapl	-0,067385	1
Pcbp4	-0,067792	1
Gpr35	-0,067897	1
Mpp5	-0,067889	1
Focad	-0,067962	1
Dtd1	-0,068005	1
Rpusd1	-0,068093	1
Chmp1b	-0,06818	1
Fam49b	-0,06823	1
Fchsd1	-0,068326	1
Gstt3	-0,068438	1
Rpl36-ps2	-0,068547	1
Tmem216	-0,068519	1
Dync2h1	-0,06846	1
Copa	-0,068741	1
Cystm1	-0,068824	1
Spg7	-0,068796	1
Mir5136	-0,068762	1
Chp1	-0,068775	1
Rsrc1	-0,068836	1
Trabd	-0,068889	1
Phkg2	-0,069026	1
Dnajc14	-0,069021	1
Stxbp5	-0,069019	1
Tex261	-0,069012	1
Nkiras1	-0,069087	1
4930579K19Rik	-0,069191	1
Ssb	-0,069423	1
Dcbld2	-0,069492	1
Rps15a	-0,069484	1
Gm14706	-0,069592	1
Hic2	-0,069634	1
Ripk2	-0,069631	1

Irgq	-0,069662	1
Zfp74	-0,06982	1
Cdkn2aip	-0,070077	1
Il6st	-0,070099	1
Brd9	-0,070236	1
Fxr1	-0,070164	1
Atp6v0a1	-0,070263	1
5031439G07Rik	-0,07029	1
Ptdss1	-0,070334	1
Ndufab1	-0,070403	1
Mdfic	-0,070361	1
Pym1	-0,070455	1
Hoxc4	-0,070581	1
Rheb	-0,070672	1
Dlg4	-0,070695	1
Gzmm	-0,070806	1
Map3k12	-0,070923	1
Sun1	-0,07086	1
Ttc12	-0,071241	1
Larp1	-0,071197	1
Rpl27	-0,071265	1
Gm16096	-0,071488	1
Stx12	-0,07152	1
Ppp2r2a	-0,071747	1
Gm5687	-0,071939	1
Zfp148	-0,071886	1
Fbxo2	-0,072027	1
Amd1	-0,072146	1
Zfyve21	-0,072106	1
RP23-225D5.4	-0,072209	1
Gm13890	-0,072161	1
Fam111a	-0,07219	1
Daxx	-0,072207	1
BC005537	-0,072305	1
Eci2	-0,072279	1
Otud3	-0,072548	1
Stard4	-0,072701	1
Rc3h2	-0,072683	1
Klhdc2	-0,072676	1
Snhg9	-0,072807	1
Sec11a	-0,072939	1
Trim12c	-0,073003	1
Gapvd1	-0,073005	1
Golga2	-0,073008	1
Cul9	-0,073082	1
Sbk3	-0,073396	1
Lonp1	-0,073448	1
Trmt1	-0,073684	1
Rnf8	-0,074024	1
Dqx1	-0,074041	1
Supv3l1	-0,073965	1
Cep104	-0,074053	1

Gpd2	-0,074224	1
Gm5963	-0,074241	1
Cpped1	-0,074348	1
Uqcrb	-0,074331	1
Yaf2	-0,074485	1
Cox20-ps	-0,074495	1
Utp14a	-0,074569	1
Gm1976	-0,074652	1
Usp31	-0,074794	1
Zfr	-0,074817	1
Gins3	-0,074879	1
Rps25	-0,07504	1
Cbarp	-0,075051	1
Lrp8	-0,075372	1
Ccna2	-0,07556	1
Slc29a1	-0,075712	1
Gm12038	-0,075916	1
Zfp282	-0,075888	1
Stk3	-0,075916	1
Golga3	-0,075924	1
RP23-38L16.3	-0,076031	1
Tacc2	-0,076194	1
Lrrc8c	-0,076266	1
Nup155	-0,076282	1
Kctd9	-0,076383	1
mt-Nd5	-0,076359	1
Tmod3	-0,07645	1
Mcrs1	-0,076547	1
Kif20b	-0,076591	1
Arf3	-0,076628	1
Arfip1	-0,076682	1
Ctbp2	-0,076662	1
Senp5	-0,076801	1
Ap3d1	-0,076848	1
Stub1	-0,076799	1
Pik3ca	-0,076916	1
Myo1g	-0,07707	1
Dnaja3	-0,077097	1
Rprd2	-0,077174	1
Alg2	-0,077255	1
9330111N05Rik	-0,077299	1
Gm5391	-0,077405	1
Inpp5d	-0,077371	1
Cand1	-0,077426	1
Gm44024	-0,077492	1
Ippk	-0,077478	1
Dcaf10	-0,077497	1
Lrch1	-0,077615	1
Als2cl	-0,077605	1
Rab12	-0,077552	1
Actr1a	-0,077553	1
Dynlt3	-0,077584	1

Lrrc45	-0,077962	1
Grb2	-0,07802	1
Nbr1	-0,078109	1
Foxp1	-0,078071	1
S100a11	-0,078244	1
Fam188b	-0,078442	1
Dennd2c	-0,078383	1
Pex3	-0,078379	1
Nfx1	-0,078379	1
Map1lc3a	-0,078353	1
Farsb	-0,078385	1
Maml2	-0,078551	1
Znrd1	-0,078915	1
Parp2	-0,079065	1
Itm2c	-0,07909	1
Gm16399	-0,07937	1
Scoc	-0,079534	1
Gsg2	-0,07963	1
Cyb561	-0,0797	1
Gm38299	-0,079664	1
Mrps2	-0,079663	1
Mcub	-0,079738	1
Mybpc3	-0,079835	1
Klf4	-0,079816	1
Rwdd2a	-0,079896	1
Trub1	-0,079856	1
Naip2	-0,079972	1
Gm13392	-0,080221	1
Epm2aip1	-0,080401	1
Gm8599	-0,080621	1
Mcmbp	-0,080724	1
Rnf2	-0,080839	1
Mtmr6	-0,080836	1
Rac1	-0,0808	1
Snx24	-0,080879	1
Snapc2	-0,080936	1
Nsun2	-0,080888	1
Klf7	-0,081044	1
Dgcr2	-0,081063	1
RP23-149L23.1	-0,081175	1
Mthfsd	-0,081246	1
Usp9x	-0,081278	1
Flnc	-0,08134	1
Mrpl30	-0,081387	1
Atf4	-0,081488	1
Agbl3	-0,08157	1
Gm14303	-0,081594	1
H13	-0,08201	1
Gnas	-0,082102	1
Mms22l	-0,082198	1
Fam3c	-0,082158	1
Adss	-0,082326	1

Ifrd1	-0,08237	1
Abtb2	-0,082497	1
Fam13b	-0,082552	1
Rpf1	-0,082609	1
Ndel1	-0,082646	1
Gbf1	-0,082819	1
Nmt2	-0,082844	1
Tnrc6c	-0,082923	1
Gm43149	-0,082986	1
Tjap1	-0,082981	1
Hnrnpk	-0,082994	1
Golga7	-0,083087	1
Pxk	-0,083241	1
Cdk6	-0,083155	1
Rplp0	-0,083169	1
Rcor2	-0,08328	1
Hcar2	-0,083447	1
Abl2	-0,083442	1
Klf9	-0,083399	1
Plk3	-0,083469	1
Map2k5	-0,08357	1
Fam222b	-0,083678	1
Ankrd17	-0,083672	1
Zfp862-ps	-0,083927	1
Cyp27a1	-0,083875	1
Dnaja1	-0,0839	1
Rbm27	-0,083901	1
Gm37733	-0,08403	1
Eif2s1	-0,084053	1
Gm5302	-0,084178	1
Wasl	-0,084168	1
Zfp799	-0,084434	1
Tvp23b	-0,084438	1
8030462N17Rik	-0,084615	1
2310022A10Rik	-0,084615	1
Mrps17	-0,084572	1
Trim25	-0,08475	1
Dpm3	-0,084738	1
Zbtb1	-0,084756	1
B630019K06Rik	-0,084815	1
mt-Tv	-0,084928	1
Sarnp	-0,084867	1
Caprin1	-0,084902	1
Nifk	-0,084969	1
Txndc17	-0,085051	1
Dnajb6	-0,085106	1
Snrpb2	-0,085237	1
Cs	-0,085274	1
Cyp4f16	-0,085374	1
Hba-ps4	-0,085354	1
Zbtb43	-0,085364	1
Skil	-0,085452	1

Hdgf	-0,085702	1
Stbd1	-0,085897	1
Swap70	-0,086077	1
2300009A05Rik	-0,086143	1
Cfap97	-0,086217	1
Eloa	-0,086208	1
Brd8	-0,08623	1
Nek1	-0,086288	1
Psmd14	-0,086254	1
Megf8	-0,086496	1
Stat2	-0,086559	1
Hmmr	-0,086609	1
Tob2	-0,086616	1
Slc30a9	-0,086592	1
Ube4a	-0,0867	1
Ttc37	-0,086737	1
Casp6	-0,086689	1
2310009B15Rik	-0,08682	1
Naf1	-0,086861	1
Wdr25	-0,087141	1
Gxylt1	-0,087134	1
Rexo1	-0,087068	1
Vsig10	-0,087156	1
Phka1	-0,087549	1
Cd36	-0,087465	1
Trmt61b	-0,087474	1
Milr1	-0,087493	1
Col4a3bp	-0,087589	1
Akap8	-0,087557	1
Atp5f1	-0,087563	1
Frmd8	-0,087774	1
Snord72	-0,088107	1
Ccdc86	-0,088371	1
Tnip2	-0,088403	1
Wdr3	-0,088589	1
Gm23054	-0,088682	1
Ip6k2	-0,088845	1
Prmt7	-0,088753	1
Cbx6	-0,088945	1
Eef2	-0,089027	1
Spag5	-0,089098	1
Rbm8a2	-0,089126	1
Dapk1	-0,089061	1
Gm26917	-0,089228	1
Eda2r	-0,089329	1
Pes1	-0,089425	1
Polr1e	-0,089516	1
Lrrc58	-0,089479	1
Slc16a10	-0,089515	1
Rsbn1l	-0,089636	1
Aldh3a2	-0,089726	1
Il2rg	-0,089724	1

Trp53bp2	-0,089805	1
Tax1bp3	-0,090024	1
Tmem131	-0,089996	1
Ptprc	-0,08997	1
Snhg8	-0,090272	1
Gm27477	-0,090398	1
Asah1	-0,090442	1
Tomm40l	-0,090521	1
Wdr45b	-0,090581	1
Tspoap1	-0,090745	1
Tnip1	-0,090663	1
Nsmaf	-0,090711	1
Ist1	-0,090689	1
Gpr89	-0,090742	1
Stil	-0,090813	1
Rab18	-0,090861	1
Gm9531	-0,091174	1
Pibf1	-0,091326	1
Eps8	-0,091313	1
Twsg1	-0,09128	1
Dcaf6	-0,091382	1
Irf4	-0,091508	1
Ppm1b	-0,091547	1
Ftsj3	-0,091451	1
Ppfia4	-0,091624	1
Golm1	-0,091601	1
Nudt21	-0,091658	1
Dhps	-0,091705	1
Ddb1	-0,091709	1
Ankra2	-0,091808	1
Gm45292	-0,091917	1
Gm12090	-0,091878	1
Ttc19	-0,091879	1
G3bp1	-0,091932	1
Bin1	-0,092006	1
Tyw1	-0,092129	1
Rtcb	-0,092061	1
Aff4	-0,092203	1
Kif1c	-0,092313	1
Mtpap	-0,092437	1
Mier2	-0,092444	1
Snx3	-0,092512	1
Rtn4	-0,092638	1
Sh2b3	-0,093078	1
Gpr137b	-0,093124	1
Ptpn22	-0,093162	1
Ssr1	-0,093231	1
Cerkl	-0,093373	1
Lacc1	-0,093502	1
Prkca	-0,09348	1
Cdk5	-0,093577	1
Tiam1	-0,093587	1

Ccdc22	-0,093582	1
Atp9b	-0,093911	1
Ppp3cb	-0,093926	1
Slc44a1	-0,093908	1
Banp	-0,094008	1
Htra2	-0,094138	1
Notch2	-0,094088	1
Dpf2	-0,094225	1
Rpl22l1	-0,094309	1
Rnf4	-0,09443	1
Pggt1b	-0,09455	1
Ccdc51	-0,094517	1
Mbp	-0,094519	1
Rad23b	-0,094527	1
Acbd5	-0,094638	1
Arhgef17	-0,09469	1
1500011K16Rik	-0,094658	1
Sc5d	-0,09475	1
Gm43364	-0,094787	1
G730013B05Rik	-0,094983	1
Wdr33	-0,095085	1
2310057M21Rik	-0,095238	1
Gskip	-0,09528	1
Cetn3	-0,095291	1
Jmy	-0,095412	1
Esyt1	-0,09553	1
Klf11	-0,09577	1
Ifi207	-0,095853	1
Ddx23	-0,095856	1
4632404H12Rik	-0,096249	1
Avl9	-0,096201	1
Slc26a2	-0,096227	1
Fam199x	-0,096192	1
Apobec1	-0,096493	1
Gm7565	-0,096641	1
Cpeb2	-0,096611	1
Myo1f	-0,096639	1
Birc5	-0,096571	1
Car11	-0,096727	1
Ankrd13a	-0,096673	1
Vps29	-0,096695	1
Zeb2	-0,09672	1
Gm11451	-0,096811	1
Safb2	-0,096781	1
Rplp1-ps1	-0,096886	1
RP23-268C22.3	-0,096911	1
Nrp1	-0,096955	1
Gm11539	-0,096973	1
Caap1	-0,097263	1
Cdk14	-0,097341	1
Ypel2	-0,097395	1
1110051M20Rik	-0,097405	1

Efr3b	-0,097502	1
Cstf2	-0,097471	1
Psip1	-0,097596	1
Chd8	-0,097589	1
Cdc25c	-0,097734	1
Csk	-0,097723	1
Wipf1	-0,097794	1
Snord65	-0,09789	1
Prkd2	-0,09794	1
Ap1m1	-0,097875	1
Rab22a	-0,09789	1
Tmem14a	-0,098172	1
Znrf2	-0,09821	1
Rps11-ps1	-0,098287	1
Got1	-0,098415	1
Gm35315	-0,098646	1
Rbsn	-0,098718	1
Gm15541	-0,098828	1
Nek7	-0,098777	1
Ube2l3	-0,098813	1
Memo1	-0,098883	1
Brd4	-0,099027	1
Vps18	-0,098986	1
Cspp1	-0,099111	1
Gm9828	-0,099229	1
Calcrl	-0,099309	1
Sccpdh	-0,099261	1
Nfic	-0,099296	1
Trpv4	-0,0996	1
Cep350	-0,099595	1
Ticam2	-0,099665	1
Lbp	-0,099752	1
5031434O11Rik	-0,099758	1
Lyrm9	-0,09987	1
Camk2d	-0,099928	1
Sgsm3	-0,10005	1
Ubqln4	-0,099951	1
Fam129a	-0,10001	1
Chchd2	-0,099953	1
Phldb1	-0,1001	1
Zc3h12a	-0,10015	1
Gpx1	-0,10021	1
Gm13205	-0,10042	1
Zcchc6	-0,1004	1
Rasl2-9	-0,10054	1
Gm24959	-0,10047	1
Tmem86a	-0,10054	1
Gpr183	-0,10049	1
Fndc3a	-0,10054	1
Crot	-0,10062	1
Wdr81	-0,10062	1
Brwd1	-0,10064	1

Agap1	-0,10074	1
Cdc40	-0,10084	1
Spty2d1	-0,10077	1
Rab14	-0,10076	1
Gipr	-0,10117	1
Dus1l	-0,10123	1
Fryl	-0,10126	1
Mthfd1	-0,10128	1
Klhl23	-0,10145	1
Gm44027	-0,10144	1
Aco2	-0,10151	1
Tada3	-0,10162	1
Nol9	-0,10166	1
1700025G04Rik	-0,10209	1
Plek	-0,10219	1
Elp4	-0,10234	1
Sufu	-0,10242	1
Noct	-0,10252	1
Dazap2	-0,10251	1
Nucb1	-0,10264	1
Acad9	-0,10273	1
Gna11	-0,10289	1
Rps26	-0,10296	1
Nt5dc3	-0,10314	1
Cyb5r3	-0,10309	1
Irx2	-0,10317	1
Dcaf13	-0,1032	1
Dhodh	-0,10339	1
Slc39a6	-0,10336	1
Mob1b	-0,1034	1
Stag1	-0,10367	1
Slc12a4	-0,10365	1
Phf3	-0,10381	1
Tspyl2	-0,10389	1
Tubd1	-0,10394	1
Fbxo46	-0,104	1
Slc36a4	-0,104	1
Kdm5b	-0,10405	1
Gm2962	-0,1041	1
Tbce	-0,1041	1
Fasn	-0,10417	1
Cdc37	-0,1042	1
Mrnip	-0,10442	1
2410004B18Rik	-0,10435	1
Tprn	-0,10444	1
Appl2	-0,10439	1
H3f3a	-0,10453	1
Arl8a	-0,10457	1
Herc2	-0,10456	1
Ccng1	-0,10463	1
Commd8	-0,10463	1
Fam173b	-0,10466	1

Cnpy3	-0,10475	1
Nsd2	-0,10469	1
Polr3a	-0,10479	1
Tbc1d1	-0,10477	1
Cenpu	-0,10498	1
Tmem237	-0,10498	1
Cnot6	-0,10498	1
Gm9354	-0,10508	1
Usp49	-0,10509	1
5430405H02Rik	-0,10505	1
Rpl10	-0,10505	1
Zkscan5	-0,10519	1
Pde8a	-0,10524	1
Pex11a	-0,10525	1
Alg1	-0,10517	1
Phldb3	-0,10552	1
Twistnb	-0,10553	1
Fkbp1a	-0,1055	1
Gm35931	-0,1056	1
Nlgn2	-0,10564	1
Gm12481	-0,10561	1
Gm19028	-0,10565	1
Papola	-0,10572	1
D1Ertd622e	-0,10577	1
1700008J07Rik	-0,10595	1
Mtmr14	-0,10595	1
Ints2	-0,1061	1
Msn	-0,10609	1
Stk10	-0,10635	1
Dnajc3	-0,10632	1
A930007I19Rik	-0,1065	1
Lgals9	-0,10657	1
Mical2	-0,10665	1
Gm43309	-0,10658	1
Fh1	-0,1066	1
Sik3	-0,10679	1
Pigo	-0,10688	1
Rrp1b	-0,10697	1
Zfyve16	-0,10713	1
Rtca	-0,10706	1
Pik3r6	-0,10717	1
Gm7099	-0,10749	1
S100a6	-0,10775	1
Cep295	-0,10777	1
Syne3	-0,10776	1
Cdk12	-0,10782	1
Clip2	-0,1079	1
1810013L24Rik	-0,10809	1
Ipo4	-0,10821	1
Parp4	-0,10825	1
Lman1	-0,10835	1
Btbd6	-0,10836	1

Rnf13	-0,10839	1
Serpine1	-0,10842	1
Copb1	-0,10836	1
Pcmt1	-0,10835	1
Fam105a	-0,10849	1
Prpf3	-0,10847	1
Senp2	-0,10853	1
Hadha	-0,10845	1
Gmds	-0,10866	1
Nop2	-0,10874	1
Gspt1	-0,10874	1
Eri3	-0,10894	1
Otud7b	-0,10892	1
Jam2	-0,10919	1
Clec10a	-0,10941	1
Lipa	-0,10939	1
Ptk2	-0,10956	1
Casp1	-0,10964	1
Arf2	-0,10958	1
Dcaf5	-0,10971	1
Arid3a	-0,10983	1
Impad1	-0,10978	1
Gm26397	-0,10991	1
Rdx	-0,10989	1
Arfgef1	-0,11	1
Cdc73	-0,1101	1
Rad51	-0,1102	1
Cd47	-0,11023	1
1810062G17Rik	-0,11054	1
Rogdi	-0,11046	1
Zbtb17	-0,11054	1
Gabarap	-0,11047	1
Tmem183a	-0,11059	1
Socs3	-0,11072	1
Meaf6	-0,1107	1
Alkbh5	-0,11067	1
Gnpnat1	-0,11106	1
Tnrc6b	-0,11113	1
Vps8	-0,11135	1
Mad2l2	-0,11136	1
Anks1	-0,11159	1
Atp6v1h	-0,11172	1
Ensa	-0,11178	1
Pdcd11	-0,11181	1
Shc1	-0,11205	1
Cct4	-0,11208	1
Gtpbp1	-0,11221	1
Sub1	-0,1122	1
Alcam	-0,11234	1
Bcl6b	-0,11242	1
Map3k8	-0,11251	1
Colgalt1	-0,11261	1

Ints8	-0,11282	1
4930568A12Rik	-0,11295	1
Sppl3	-0,11309	1
Chmp7	-0,11311	1
Ttc30a1	-0,1133	1
Gm15694	-0,11342	1
Pou5f2	-0,11353	1
C3	-0,11347	1
Rasa2	-0,11351	1
Ik	-0,1135	1
Adh5	-0,11363	1
Dpy19l1	-0,1138	1
Sin3a	-0,11383	1
Yrdc	-0,11395	1
Slc25a23	-0,11402	1
Hspa5	-0,11398	1
Gpr179	-0,11414	1
Tmem135	-0,11414	1
Gm10863	-0,11424	1
Zfp800	-0,11421	1
Ints14	-0,11426	1
Relb	-0,11429	1
Elob	-0,11428	1
Zdhhc1	-0,11448	1
Ndufs6	-0,11451	1
Gm8326	-0,11464	1
Zfp959	-0,11475	1
Map2k3os	-0,11466	1
Rmrp	-0,11469	1
Ipo7	-0,11468	1
Proscos	-0,11482	1
Kctd6	-0,11484	1
Rreb1	-0,11476	1
Mapk8	-0,11479	1
Ccl5	-0,11506	1
Golgb1	-0,11511	1
Gm10689	-0,11522	1
Ammecr1	-0,1153	1
Rab35	-0,1154	1
Trip11	-0,11561	1
Actr2	-0,11574	1
RP24-497N7.2	-0,11585	1
Purb	-0,11583	1
Rora	-0,1159	1
Cdk1	-0,11592	1
Tada1	-0,11595	1
Smad3	-0,11602	1
Bag2	-0,11613	1
Il6ra	-0,11606	1
Gm37606	-0,1162	1
Ifitm3	-0,11631	1
C330027C09Rik	-0,11637	1

Pspc1	-0,11648	1
Acaca	-0,11652	1
Nup35	-0,11687	1
Nxpe3	-0,11686	1
Plec	-0,11685	1
Rps15a-ps8	-0,11707	1
9430015G10Rik	-0,11725	1
Afdn	-0,11724	1
Tbc1d4	-0,11731	1
Gdpd5	-0,11735	1
Catsper2	-0,11746	1
Ppp2r1a	-0,11752	1
Ipp	-0,11772	1
Pex7	-0,11773	1
Ptpre	-0,11767	1
Ofd1	-0,1178	1
C2cd2l	-0,11785	1
Aldoa	-0,11778	1
Kmt2b	-0,1181	1
Gm13776	-0,1183	1
Ppil2	-0,11834	1
Exoc2	-0,11859	1
Sh2b2	-0,11866	1
Lysmd3	-0,11892	1
AU040320	-0,11891	1
Tbcel	-0,11893	1
Gm16973	-0,11902	1
Vps37b	-0,11899	1
Pdk1	-0,119	1
Btg2	-0,11906	1
Fyttd1	-0,11908	1
Fnbp1l	-0,11934	1
Psmd4	-0,11928	1
Map3k14	-0,11942	1
Alg11	-0,11947	1
Zfp446	-0,11957	1
Hps5	-0,11958	1
Trappc13	-0,11968	1
Dctd	-0,11977	1
Tirap	-0,11977	1
Fmnl1	-0,1198	1
Mgea5	-0,11981	1
Ankrd9	-0,11986	1
Adcy9	-0,11989	1
Stx3	-0,11986	1
Paox	-0,11997	1
Gm10698	-0,12022	1
Gm37254	-0,12033	1
Sec23a	-0,12034	1
Arf5	-0,1203	1
Mgme1	-0,12042	1
Lig1	-0,12035	1

Tefm	-0,12052	1
Tmem191c	-0,12053	1
Zfp867	-0,12056	1
Mga	-0,12063	1
Psmd2	-0,12065	1
Stat6	-0,12085	1
Srsf5	-0,12089	1
Gm16439	-0,12102	1
Hsph1	-0,12099	1
Parvb	-0,121	1
Gid8	-0,12122	1
Abcc1	-0,12116	1
Dock8	-0,12131	1
Limd2	-0,12132	1
Srgap3	-0,12133	1
Fam133b	-0,12126	1
Usf3	-0,12135	1
Gm15327	-0,12151	1
Cd40	-0,12148	1
Ncf1	-0,12145	1
S100a8	-0,12165	1
Prpsap2	-0,12181	1
Irf2	-0,12179	1
Pdcd6	-0,1218	1
Gpd1l	-0,12181	1
Trappc10	-0,12187	1
Gm8213	-0,12198	1
Ppp1r8	-0,12212	1
Phf20l1	-0,12213	1
Cbx4	-0,12219	1
Usp2	-0,12215	1
Gnb4	-0,12226	1
Srsf11	-0,12231	1
Mccc1	-0,12244	1
Tm9sf4	-0,12239	1
Ikbip	-0,12252	1
Zdhhc3	-0,1225	1
Akap13	-0,12255	1
Rpe	-0,12256	1
Kdm2a	-0,12263	1
Tmem25	-0,12273	1
Senp7	-0,12268	1
Atg14	-0,12276	1
Slc38a1	-0,12282	1
Tmem238	-0,1229	1
Srd5a3	-0,12319	1
Mettl16	-0,12318	1
Mfap3	-0,12329	1
Tgfbr2	-0,12328	1
Tlk2	-0,12326	1
Yae1d1	-0,12355	1
Ppil3	-0,12347	1

Snx2	-0,1235	1
Alg3	-0,12369	1
Pura	-0,12373	1
Mbd4	-0,12384	1
Npat	-0,1239	1
Nae1	-0,12394	1
Stk11	-0,12403	1
Tram1	-0,12409	1
Sec23ip	-0,12422	1
Rbm18	-0,12419	1
Mmaa	-0,1243	1
Atp5g2	-0,12439	1
Gfod2	-0,12438	1
Sqle	-0,12437	1
Mettl14	-0,12457	1
Gm42535	-0,12473	1
Ptgs2	-0,12469	1
Atat1	-0,12494	1
Bak1	-0,12502	1
Gm20568	-0,12511	1
Ctps	-0,12508	1
Bdh1	-0,1253	1
Tle3	-0,12525	1
Sap130	-0,12544	1
Cfap126	-0,12548	1
Msh3	-0,12581	1
Map2k3	-0,12602	1
Gnai2	-0,12609	1
Ppp1r13l	-0,1262	1
Elac1	-0,12631	1
Baiap2	-0,12633	1
Casp3	-0,12643	1
Zfp729a	-0,12653	1
Map2k4	-0,12649	1
Smim8	-0,12654	1
Etv6	-0,12648	1
Lmo4	-0,12647	1
Cebpb	-0,12667	1
Nmral1	-0,12667	1
Usp34	-0,12675	1
Eif2b2	-0,12671	1
Tk1	-0,12679	1
Pnpt1	-0,12678	1
Clec16a	-0,12678	1
Satb2	-0,12688	1
Ppid	-0,12685	1
Paip2	-0,12697	1
Ufsp1	-0,12708	1
Grn	-0,12714	1
6030458C11Rik	-0,12727	1
Aatf	-0,12763	1
Gpbp1l1	-0,12756	1

9030624J02Rik	-0,1278	1
Ndrg4	-0,1278	1
Ankrd13d	-0,12794	1
Oxa1l	-0,12799	1
Dyrk1a	-0,12806	1
Ankrd50	-0,12819	1
Cyth4	-0,12829	1
Hsd17b12	-0,12833	1
Eef1b2	-0,12829	1
Ppfia1	-0,12835	1
Mrm1	-0,12854	1
Ddx6	-0,12846	1
AA386476	-0,12873	1
Cd93	-0,12874	1
Rdh11	-0,12882	1
Eif3d	-0,1288	1
Yars	-0,12888	1
Mapk14	-0,12889	1
Uhrf1	-0,129	1
Rpl13	-0,12911	1
Pdss2	-0,12916	1
Cdca7	-0,1295	1
St6galnac6	-0,12945	1
Rbx1	-0,12952	1
Gen1	-0,12967	1
Sit1	-0,1298	1
Ecel1	-0,12981	1
Ppif	-0,1298	1
Glrx3	-0,12983	1
Tpt1-ps3	-0,12999	1
Chst12	-0,12996	1
Fyn	-0,13009	1
Ncaph	-0,13019	1
Nup160	-0,13051	1
Gm37962	-0,13051	1
Wbp2	-0,13051	1
Vps50	-0,13074	1
Ctbs	-0,13083	1
Ifnar2	-0,13083	1
Gm42666	-0,13094	1
Aldh2	-0,1309	1
3010003L21Rik	-0,13104	1
Hgh1	-0,1314	1
Abl1	-0,13142	1
Map1s	-0,13139	1
Oxct1	-0,13143	1
E030030I06Rik	-0,13153	1
Zdhhc24	-0,13148	1
Rbm17	-0,13149	1
Cox18	-0,13155	1
Abhd17b	-0,13158	1
Pank2	-0,13184	1

Wdr37	-0,13192	1
2610507B11Rik	-0,13195	1
Vamp1	-0,13198	1
Nav1	-0,13198	1
Frat2	-0,13209	1
Clasp2	-0,13219	1
Lin52	-0,13235	1
Mocs1	-0,13235	1
Rnf44	-0,13236	1
Zdhhc14	-0,13249	1
Nuak2	-0,13264	1
Sqstm1	-0,13265	1
Trp53rkb	-0,1328	1
2810013P06Rik	-0,13276	1
Dgkd	-0,13286	1
Selenoi	-0,13295	1
2900026A02Rik	-0,13306	1
Elp2	-0,13308	1
Klhdc3	-0,13323	1
Vps52	-0,13326	1
Paics	-0,13328	1
Prkcsh	-0,13339	1
Socs5	-0,13351	1
Mybbp1a	-0,13352	1
Cops4	-0,1335	1
Tsr2	-0,13358	1
Gm9396	-0,13367	1
Gm45223	-0,13375	1
Tctex1d2	-0,13383	1
Atf5	-0,13395	1
Adcy7	-0,13386	1
Dohh	-0,1339	1
Ccnt1	-0,13392	1
B4galt1	-0,13391	1
Txk	-0,13403	1
Anxa3	-0,13401	1
Arid3b	-0,13422	1
Trip4	-0,13423	1
Per1	-0,13425	1
Anxa6	-0,13444	1
Ift52	-0,1344	1
Crtc3	-0,13448	1
Cog6	-0,13448	1
Gm44190	-0,13464	1
Prelid3b	-0,13464	1
Gm12346	-0,13466	1
Vdac2	-0,13468	1
Scaf8	-0,13476	1
Lrp12	-0,13494	1
Adar	-0,13506	1
Hacd3	-0,13521	1
Uhrf2	-0,13516	1

Bcas3	-0,13532	1
Llgl1	-0,13529	1
Btbd9	-0,13538	1
Cul3	-0,13536	1
Tcaf1	-0,13546	1
Anxa4	-0,13547	1
1110059G10Rik	-0,13564	1
Add1	-0,13565	1
RP24-378K7.3	-0,13566	1
Ap1g1	-0,13568	1
Zdhhc7	-0,13585	1
Ahcyl1	-0,13592	1
Ctr9	-0,13596	1
Zfp260	-0,1361	1
Sfswap	-0,13615	1
Sh3glb1	-0,13612	1
Cln3	-0,1362	1
3830406C13Rik	-0,13631	1
Mta3	-0,1364	1
Nfs1	-0,13655	1
Alkbh1	-0,13652	1
Smyd5	-0,13656	1
Cpt2	-0,13668	1
Galnt7	-0,13683	1
Xylt1	-0,13686	1
Stx11	-0,137	1
Rpl30-ps3	-0,13703	1
Hmgb1-ps5	-0,13715	1
Atg2b	-0,13711	1
Gm15441	-0,13723	1
Erf	-0,13733	1
Teddm2	-0,13738	1
Gpsm1	-0,13741	1
Pkn2	-0,13752	1
Rita1	-0,13772	1
Zcchc14	-0,13802	1
Anxa5	-0,13799	1
Lpcat3	-0,1381	1
Npm3-ps1	-0,13826	1
Trim44	-0,13832	1
Ccdc106	-0,1384	1
Armc10	-0,13851	1
Ttll4	-0,13866	1
Smad5	-0,13872	1
Zmynd11	-0,13869	1
Zc3h7b	-0,13875	1
Neil1	-0,13879	1
Dnm1l	-0,13881	1
Rab11fip3	-0,13888	1
Zfp512b	-0,13906	1
Rab11fip2	-0,1392	1
Ide	-0,13934	1

Vezt	-0,13943	1
Foxo3	-0,13944	1
Tspyl4	-0,13953	1
Exosc10	-0,13949	1
Slc35c1	-0,13956	1
Tulp3	-0,13958	1
Gm4880	-0,13978	1
Recql5	-0,13982	1
Hspb11	-0,13981	1
RP23-331E5.10	-0,13994	1
Rbm38	-0,13991	1
Slc25a3	-0,13987	1
Dennd6a	-0,13995	1
Zbtb40	-0,14007	1
Tnfrsf23	-0,14014	1
Ormdl2	-0,14007	1
Stamos	-0,1402	1
Ldb3	-0,14029	1
Ankrd44	-0,1403	1
Slc25a1	-0,14027	1
Ap4e1	-0,14055	1
Ube2q1	-0,14064	1
Ank2	-0,14072	1
Myoz1	-0,1407	1
Sptan1	-0,14075	1
Ola1	-0,14072	1
Tram2	-0,14087	1
St8sia4	-0,14089	1
Rttn	-0,14121	1
Igsf8	-0,14124	1
Cspg5	-0,14133	1
Pdss1	-0,14138	1
Tns4	-0,14146	1
Kif1b	-0,14153	1
Wdr54	-0,14173	1
Birc2	-0,14187	1
Edc3	-0,14199	1
Nprl3	-0,1421	1
Fkbp7	-0,14225	1
Hcfc2	-0,14238	1
Smpdl3b	-0,14252	1
Scmh1	-0,14256	1
Rpl15	-0,1429	1
Bbc3	-0,14292	1
2310022B05Rik	-0,1429	1
Fbxl14	-0,143	1
Arhgap12	-0,14309	1
Cyb5b	-0,14324	1
Mcu	-0,14327	1
Klhl15	-0,14337	1
Tmem143	-0,14349	1
Dhx36	-0,14345	1

Adgrl1	-0,14345	1
Mkln1	-0,14347	1
Pgm3	-0,14362	1
Stac2	-0,14374	1
Zzef1	-0,14373	1
Dynll2	-0,14366	1
Hoxc6	-0,14378	1
Cda	-0,14394	1
Rufy2	-0,14391	1
Snrk	-0,14393	1
Fam160b1	-0,14388	1
Pnpla8	-0,1439	1
Pramef8	-0,14407	1
Zfp740	-0,14414	1
Cep44	-0,14417	1
Sacm1l	-0,14441	1
Ccz1	-0,14439	1
Yipf7	-0,14448	1
Kif14	-0,14474	1
Kat6b	-0,14466	1
Dap3	-0,14471	1
Prdx6	-0,14468	1
Mir763	-0,14482	1
Cdk13	-0,14476	1
Atad2	-0,14484	1
Nop10	-0,14493	1
Icmt	-0,14501	1
Fxr2	-0,14501	1
Gm9951	-0,14506	1
Nsmce3	-0,14529	1
Inpp5f	-0,14531	1
Nectin4	-0,1454	1
Adck5	-0,14546	1
Zfp692	-0,14552	1
Stard10	-0,14548	1
Rragc	-0,14551	1
Cep76	-0,14556	1
Aaas	-0,14578	1
Kat7	-0,14587	1
Sp3os	-0,14599	1
Cd53	-0,14604	1
Mtfr1l	-0,14596	1
Casp9	-0,14607	1
Zfp597	-0,14651	1
H2afy	-0,1465	1
Banf1	-0,14657	1
Pip4k2c	-0,14669	1
Rasgrp3	-0,14674	1
Ppm1a	-0,14667	1
Suclg2	-0,14681	1
Mthfd2l	-0,1469	1
Rps25-ps1	-0,14689	1

Gm4890	-0,14696	1
Eogt	-0,14696	1
Smpd4	-0,14709	1
Fam129b	-0,14731	1
Gan	-0,1473	1
Dgke	-0,14737	1
Dennd1b	-0,14754	1
Trem3	-0,14755	1
Zswim4	-0,14764	1
Sdf2	-0,14763	1
Ube2e1	-0,14772	1
Nthl1	-0,14779	1
Msantd4	-0,14777	1
Rplp1	-0,14783	1
Cox4i2	-0,14792	1
Mrpl27	-0,14801	1
Nsf	-0,14803	1
Acsl4	-0,14809	1
Ift88	-0,14824	1
Pcyt2	-0,14822	1
Rhoc	-0,14822	1
Gm45840	-0,14839	1
Calm2	-0,14841	1
Fam220a	-0,14873	1
Eif2s3y	-0,14882	1
Rhou	-0,14889	1
Adnp2	-0,14898	1
Nup214	-0,14901	1
Higd2a	-0,1492	1
Suz12	-0,14933	1
Rb1	-0,14952	1
Prpf38a	-0,14977	1
Itgam	-0,15011	1
Erbin	-0,15021	1
Dhx40	-0,1503	1
Prr14l	-0,15034	1
Naa60	-0,15029	1
Tln1	-0,15027	1
Dctn4	-0,15033	1
9230114K14Rik	-0,15038	1
Smug1	-0,15048	1
Eif4h	-0,15046	1
Fgd4	-0,15055	1
Hivep1	-0,15066	1
Pitpnm1	-0,1508	1
Ccdc171	-0,15087	1
Usp45	-0,15087	1
Pdlim7	-0,151	1
Men1	-0,15098	1
Pgap2	-0,151	1
Arsa	-0,15109	1
Nde1	-0,15109	1

Ccndbp1	-0,1511	1
Laptm5	-0,15106	1
Tcam1	-0,15118	1
Zfp382	-0,15121	1
Tmed2	-0,15118	1
Gm6257	-0,1513	1
Tmem185a	-0,15143	1
Tada2a	-0,15135	1
Abcd1	-0,15172	1
Rap1a	-0,15174	1
St13	-0,15168	1
Ube3c	-0,15185	1
Pkib	-0,15195	1
Atp8b3	-0,15206	1
Gm10288	-0,15206	1
Hdac5	-0,1522	1
Dcun1d4	-0,15229	1
Msh5	-0,15233	1
Man2a2	-0,15227	1
Spa17	-0,15239	1
Mkrn2	-0,1524	1
Mef2a	-0,15251	1
Ddias	-0,15259	1
Gtf2a1	-0,15273	1
Slc9a1	-0,15275	1
Ptbp3	-0,15265	1
Borcs7	-0,15282	1
Gnpat	-0,15287	1
Dab2	-0,15292	1
Tceal9	-0,15294	1
Rab11a	-0,15291	1
Rpl21-ps14	-0,15301	1
Atg3	-0,153	1
Maea	-0,15296	1
Ifngr1	-0,15308	1
Pogz	-0,15318	1
Cops7b	-0,15331	1
Aebp2	-0,15341	1
Slc19a1	-0,15335	1
Isyna1	-0,15351	1
Mfn1	-0,15351	1
Dguok	-0,1535	1
Sae1	-0,15349	1
Grk2	-0,15355	1
Atm	-0,15364	1
Kmt2a	-0,15374	1
Gm8925	-0,15397	1
Sec31a	-0,15403	1
Cnot2	-0,15395	1
Gm5312	-0,15423	1
Shc4	-0,15419	1
Usp36	-0,15422	1

Afg3l2	-0,15431	1
Fbxl4	-0,15445	1
Lcor	-0,15439	1
Gprasp1	-0,1546	1
Phf12	-0,15455	1
Slc25a45	-0,15457	1
Nxf1	-0,15471	1
Gm13005	-0,155	1
Papss1	-0,15498	1
Snrnp70	-0,155	1
Bub3	-0,15499	1
Anapc10	-0,15507	1
Rab3il1	-0,15523	1
Pim1	-0,15524	1
Hist1h4h	-0,15531	1
6430531B16Rik	-0,15529	1
Dph7	-0,15542	1
Supt7l	-0,15542	1
Ptprs	-0,15535	1
Zc3hav1	-0,15545	1
Ubr4	-0,15574	1
Ap4b1	-0,15575	1
Med22	-0,15582	1
Rhbdf1	-0,15594	1
Slc22a15	-0,15603	1
Sep 11	-0,15599	1
Gm12943	-0,1561	1
Lima1	-0,15633	1
Desi2	-0,15669	1
Eif5	-0,15671	1
Gm15207	-0,15682	1
Cept1	-0,15676	1
Hmgxb4	-0,15686	1
Gm10443	-0,15699	1
Diaph2	-0,15722	1
Abcd3	-0,1572	1
Stxbp1	-0,15722	1
Slc37a1	-0,15728	1
Mfsd14b	-0,15733	1
Rbbp8	-0,15743	1
Ppp2ca	-0,15752	1
Clpx	-0,15773	1
Fam217b	-0,15776	1
Blnk	-0,15793	1
Fuom	-0,15798	1
Agk	-0,15811	1
Akt3	-0,15813	1
Nfatc3	-0,15806	1
Ube2d2a	-0,15824	1
1700086P04Rik	-0,15834	1
Atp5s	-0,15825	1
Fkbp1b	-0,15832	1

Scly	-0,15854	1
Tmem199	-0,1585	1
Mios	-0,15865	1
Prdx1	-0,15858	1
Jmjd7	-0,15875	1
Maoa	-0,1587	1
Pea15a	-0,15875	1
Gm26542	-0,15878	1
Pfn2	-0,15886	1
Crybg3	-0,15903	1
Rad1	-0,15897	1
D17Wsu92e	-0,15904	1
Tnfrsf4	-0,15907	1
Sacs	-0,15919	1
Med20	-0,15928	1
Magoh	-0,15932	1
Esd	-0,15933	1
Gm5045	-0,15949	1
Zfp456	-0,15947	1
Cenpm	-0,15974	1
Was	-0,15971	1
Dnajc9	-0,1598	1
Arhgap18	-0,15986	1
Egr1	-0,16015	1
Lrrcc1	-0,16019	1
Prpf4b	-0,16017	1
Rpl7-ps7	-0,16016	1
1110003F10Rik	-0,1603	1
Abca3	-0,1603	1
Ncbp3	-0,16025	1
Trim23	-0,16043	1
Rbm14	-0,16045	1
Cnksr3	-0,16057	1
Asap1	-0,16056	1
Tex10	-0,16065	1
Nup37	-0,16061	1
Itgb1	-0,16058	1
Arhgef12	-0,16086	1
Rhbdd3	-0,16096	1
Pam	-0,161	1
Ints9	-0,16114	1
Tbc1d24	-0,16124	1
Cd33	-0,16122	1
Trp53bp1	-0,16125	1
Baz1b	-0,16116	1
Slfn4	-0,16135	1
Hsf2	-0,16129	1
Gcn1l1	-0,16171	1
Gm29650	-0,16199	1
Prim2	-0,16201	1
Bbip1	-0,162	1
Hmces	-0,16213	1

Tfec	-0,16218	1
5530601H04Rik	-0,16221	1
Fnbp4	-0,16247	1
Rps6kb1	-0,16266	1
Rel	-0,16309	1
Sdc4	-0,16306	1
Zfp451	-0,16324	1
Son	-0,16325	1
U2surp	-0,16338	1
Fam210a	-0,16361	1
Rnu11	-0,16369	1
Cox15	-0,16371	1
Mapk3	-0,16373	1
Mief1	-0,16377	1
Opa1	-0,16391	1
Idh1	-0,16398	1
Tbccd1	-0,16406	1
Cd151	-0,1642	1
Zfp142	-0,16435	1
Appl1	-0,16458	1
Tmem8b	-0,16472	1
Cmtm4	-0,16466	1
Slc16a6	-0,16467	1
Fgfr1op	-0,16484	1
Sf3a1	-0,16482	1
Suco	-0,16504	1
Zfp868	-0,16521	1
Hmgcr	-0,16534	1
Ubap2l	-0,1653	1
Kif13b	-0,16542	1
Cdk5rap2	-0,16563	1
Nr3c1	-0,16556	1
Socs7	-0,16568	1
RP24-175C20.10	-0,16592	1
Pex6	-0,16616	1
Ndufs1	-0,16616	1
Snupn	-0,16649	1
Rcbtb1	-0,16656	1
Serinc1	-0,16657	1
Slc9a5	-0,16667	1
Xbp1	-0,16665	1
Gm10827	-0,1668	1
Hif1an	-0,1668	1
Cmpk1	-0,16682	1
Dtymk	-0,1668	1
Atp6v0e2	-0,16681	1
Pate2	-0,16695	1
Gm14439	-0,16698	1
Letmd1	-0,1672	1
Ndc80	-0,16721	1
Med13	-0,16723	1
Rbbp6	-0,16715	1

Rps19-ps12	-0,16725	1
Dzip3	-0,1673	1
Prr12	-0,16725	1
Kank2	-0,16734	1
Otub1	-0,16736	1
Tmf1	-0,16745	1
Nup188	-0,16757	1
Hdlbp	-0,16762	1
Dhx15	-0,16766	1
Nsdhl	-0,16802	1
Strada	-0,1682	1
Cggbp1	-0,16824	1
Rpl30	-0,16829	1
Mfap1b	-0,16833	1
Mif4gd	-0,16844	1
Med25	-0,16852	1
Suv39h1	-0,16856	1
Tspan31	-0,16871	1
Cnpy4	-0,16867	1
Brd7	-0,16875	1
Srgap2	-0,16866	1
Ghitm	-0,1687	1
Lpar6	-0,16876	1
Ccdc117	-0,16878	1
Rbm39	-0,16881	1
Tmed10	-0,16895	1
Zfp942	-0,169	1
Gm37009	-0,16914	1
Pcmtd2	-0,16909	1
Sag	-0,16922	1
Gm9256	-0,16932	1
B230217C12Rik	-0,16935	1
Atp6v0d1	-0,16941	1
Kif18a	-0,1696	1
Mmd	-0,16961	1
Atp13a2	-0,16966	1
Cmtr1	-0,16982	1
Nt5dc1	-0,16976	1
Sbk1	-0,17003	1
Mob3c	-0,16995	1
Ankmy2	-0,16997	1
Alpk1	-0,16997	1
RP23-269H21.1	-0,16996	1
Ciart	-0,17014	1
Mki67	-0,17015	1
Fnip2	-0,17012	1
Gm14541	-0,17027	1
Hal	-0,17041	1
RP24-365N15.9	-0,17049	1
Trim41	-0,17064	1
Lmbrd1	-0,17067	1
Acvr1	-0,17103	1

Pak2	-0,17105	1
Gm14650	-0,17114	1
Fam83a	-0,17115	1
Prc1	-0,17134	1
Gm7634	-0,17144	1
Sema4g	-0,17137	1
Ptch1	-0,17193	1
Thoc2	-0,17193	1
Gm11977	-0,17199	1
Vamp2	-0,17208	1
Samd4b	-0,17207	1
Ylpm1	-0,17211	1
Uspl1	-0,17208	1
Sp3	-0,17216	1
Abhd12	-0,17227	1
Pax6	-0,17249	1
Golph3l	-0,17291	1
Rcn2	-0,1729	1
Slc17a5	-0,17308	1
Dhrs1	-0,1732	1
Epm2a	-0,17327	1
Hdac4	-0,17333	1
Mbd2	-0,17342	1
Iqsec1	-0,17343	1
Tkt	-0,17338	1
Strip1	-0,17339	1
Synj1	-0,17349	1
Bin2	-0,17355	1
Scyl2	-0,1736	1
Gca	-0,17367	1
4931406P16Rik	-0,17367	1
Klc2	-0,17382	1
Rbbp5	-0,17381	1
Vegfa	-0,17385	1
Dgkq	-0,17389	1
Sik2	-0,17408	1
Snhg17	-0,1741	1
Lpcat1	-0,17436	1
Ccdc94	-0,17455	1
Spink10	-0,17456	1
Terf2	-0,17462	1
Ergic2	-0,17461	1
Gm11737	-0,1747	1
Gm4784	-0,17473	1
Sec24b	-0,1747	1
Gm11613	-0,1748	1
Slc46a3	-0,175	1
Nop16	-0,17512	1
C030015A19Rik	-0,17535	1
Gm43792	-0,17536	1
Fam103a1	-0,1755	1
Itpr1	-0,17548	1

Socs6	-0,17547	1
Wasf2	-0,17549	1
Hnrnpul2	-0,17553	1
Adgre1	-0,17559	1
Hmgcl	-0,17558	1
Bach1	-0,17592	1
Kif9	-0,17596	1
Gnmt	-0,17596	1
Lrig2	-0,17615	1
Nit2	-0,17634	1
Uba1	-0,17632	1
Hira	-0,17654	1
Zfp518a	-0,17655	1
Rnase4	-0,17657	1
Actr3	-0,17673	1
Ddx3x	-0,17679	1
Il23a	-0,17685	1
Ccp110	-0,17689	1
Ctso	-0,1769	1
Mavs	-0,17696	1
Hcls1	-0,17697	1
Srsf10	-0,17699	1
Fgd3	-0,17715	1
Acadm	-0,17729	1
Kdelr1	-0,17753	1
Ralgps1	-0,17774	1
Frs2	-0,17765	1
Cecr5	-0,17783	1
H3f3b	-0,17784	1
Amn1	-0,17788	1
Clk4	-0,17798	1
Med13l	-0,17804	1
Stk38	-0,17802	1
Xpo7	-0,17808	1
Bloc1s6os	-0,17825	1
Rhpn2	-0,17833	1
Dcaf8	-0,17835	1
Gsap	-0,17861	1
Phactr4	-0,17865	1
Zfp628	-0,17869	1
Ranbp9	-0,17869	1
Rrm1	-0,17865	1
Cd48	-0,17893	1
Katnbl1	-0,17899	1
Immp1l	-0,17912	1
Lyar	-0,1791	1
A630033H20Rik	-0,17915	1
Naip5	-0,17922	1
Psmd5	-0,17927	1
Raph1	-0,17954	1
Pygb	-0,17953	1
Adamtsl4	-0,17965	1

Gm12902	-0,17983	1
Car7	-0,17998	1
Glrx	-0,18	1
Ctsl	-0,18001	1
Ubr3	-0,18004	1
Mrps33	-0,18041	1
Fads2	-0,18037	1
Pja1	-0,18043	1
Uqcrh	-0,18038	1
Acer3	-0,18054	1
Trpc4ap	-0,18059	1
Gm14326	-0,18068	1
Unk	-0,18069	1
Mapre2	-0,18072	1
Fam57a	-0,18077	1
2610008E11Rik	-0,18083	1
4933404O12Rik	-0,18082	1
Set	-0,18075	1
Hspa2	-0,18111	1
Mnt	-0,18116	1
Ctsd	-0,18125	1
Rfc5	-0,18144	1
Atg2a	-0,18144	1
Pi4k2b	-0,18149	1
Sec14l1	-0,1815	1
Atf3	-0,18173	1
Megf9	-0,18198	1
Echdc3	-0,18202	1
Antxr2	-0,18204	1
Ube2e3	-0,18203	1
Gm5112	-0,18214	1
Itpkb	-0,18224	1
Zmym4	-0,18219	1
Acadsb	-0,18229	1
Morc3	-0,18227	1
E2f5	-0,1824	1
Slc7a1	-0,18246	1
Cd63	-0,18246	1
Vps37a	-0,18258	1
4931406C07Rik	-0,18257	1
Rpl31	-0,18263	1
Gm14403	-0,18272	1
Nupl1	-0,1827	1
Prkaca	-0,18273	1
Kctd5	-0,18289	1
Hmx2	-0,18291	1
Tcaim	-0,18289	1
Izumo4	-0,18314	1
Gm14279	-0,18306	1
Gm10616	-0,18313	1
Orc4	-0,18306	1
D930030I03Rik	-0,18347	1

Trmt10c	-0,18352	1
Zfp335	-0,18356	1
Atp6v1a	-0,18383	1
Tti2	-0,18394	1
St3gal6	-0,18403	1
Fam160a2	-0,18416	1
Exoc3l	-0,18426	1
AI314180	-0,18466	1
Rsf1	-0,1847	1
Tmem87b	-0,1848	1
Tmem43	-0,18489	1
Kpna3	-0,18511	1
Med1	-0,1851	1
Ube2b	-0,18508	1
Odf2	-0,18524	1
Ap2a2	-0,18533	1
Scd1	-0,18541	1
Rps6kc1	-0,18546	1
Dock1	-0,18555	1
Tmem9b	-0,18565	1
Nagk	-0,18566	1
Hccs	-0,18575	1
Lin7c	-0,18566	1
Tpp1	-0,18581	1
Gm22	-0,18601	1
Snai1	-0,18606	1
Naaa	-0,1861	1
Man2c1	-0,18612	1
Rhebl1	-0,18627	1
Tacc1	-0,18634	1
Fam8a1	-0,18651	1
Vps13c	-0,18651	1
Cpd	-0,18654	1
Ttc3	-0,18685	1
Cyth1	-0,18688	1
Snord104	-0,18686	1
Gm7312	-0,18703	1
Stx5a	-0,18706	1
Ybx1	-0,1871	1
Mllt3	-0,18719	1
Hmgb2	-0,18735	1
Sestd1	-0,18747	1
Tmem176a	-0,18753	1
Fermt3	-0,18763	1
Ythdc2	-0,18766	1
Slc35g1	-0,18773	1
Dnpep	-0,18778	1
Rap1gds1	-0,18792	1
D10Wsu102e	-0,18804	1
Btbd3	-0,18809	1
Slpi	-0,18805	1
Klhl5	-0,18819	1

Ciapin1	-0,18816	1
Gm14853	-0,18851	1
Mcoln1	-0,18873	1
Stam	-0,18872	1
Man1c1	-0,18881	1
Sntb2	-0,18901	1
Wwp2	-0,18925	1
Lpxn	-0,18918	1
Nfkbil1	-0,18931	1
Mcm6	-0,1894	1
Sptbn1	-0,1894	1
Arhgdia	-0,18953	1
Fbxl18	-0,18962	1
Prpf18	-0,18963	1
Gm8618	-0,18987	1
Ugdh	-0,1901	1
Pcdh7	-0,19006	1
Tagln2	-0,19023	1
Got2	-0,19027	1
Ccdc66	-0,19036	1
Gm4262	-0,19072	1
Gm15800	-0,19078	1
Gm43681	-0,19093	1
Arhgef7	-0,19093	1
Tmub2	-0,19097	1
Pwp1	-0,19099	1
Csnk1g3	-0,19098	1
Tlr13	-0,19107	1
Rnf217	-0,19107	1
Mbtps2	-0,19113	1
Tcp11l2	-0,19113	1
Tshz3	-0,19115	1
Capns1	-0,19123	1
Cd72	-0,19118	1
Rnf138	-0,19137	1
Grasp	-0,19158	1
Dnm2	-0,19156	1
Sos1	-0,19174	1
Gm7308	-0,1918	1
Exosc3	-0,19186	1
B3gnt6	-0,19193	1
Sh3bgrl	-0,1919	1
Tarsl2	-0,19204	1
Dhfr	-0,19221	1
Tmx3	-0,19218	1
Bcl3	-0,19233	1
Slc7a11	-0,19228	1
Gm6807	-0,19243	1
Taz	-0,19237	1
Mpzl1	-0,1924	1
Lrp6	-0,19266	1
Sec22b	-0,1927	1

Nipa1	-0,1928	1
Oma1	-0,19288	1
Pip5k1a	-0,1929	1
Ercc4	-0,19295	1
Nat10	-0,19304	1
Trim35	-0,19303	1
Nfyb	-0,19301	1
Zfp287	-0,19312	1
Mlh3	-0,19306	1
Atad3aos	-0,19322	1
9130011E15Rik	-0,19347	1
Gys1	-0,19357	1
Usp32	-0,19395	1
Creb1	-0,19387	1
Gm44237	-0,19399	1
Tex30	-0,19404	1
Gdi1	-0,19408	1
Sh2b1	-0,19408	1
Pdxk	-0,19405	1
Ube4b	-0,1941	1
Rfx1	-0,19425	1
Rpp30	-0,19428	1
Ecd	-0,1945	1
Bcl2l2	-0,19473	1
Gm5837	-0,19482	1
Ccdc61	-0,19491	1
Tfpi	-0,19492	1
Usp25	-0,19485	1
1810022K09Rik	-0,19501	1
Rbm28	-0,195	1
Urb2	-0,19552	1
Gm6598	-0,19572	1
Rars2	-0,19566	1
Hk2	-0,19576	1
Pigu	-0,19596	1
Hinfp	-0,19609	1
Ppp1r12c	-0,19623	1
Prmt5	-0,19634	1
Syvn1	-0,19633	1
Gm8738	-0,19639	1
Zcchc24	-0,19636	1
Zfp609	-0,19637	1
Cdc37l1	-0,19638	1
Dync1li2	-0,19639	1
Tmbim6	-0,19651	1
Kctd10	-0,19656	1
Rps24	-0,19656	1
Bcl2l1	-0,19658	1
Tsn	-0,19662	1
Zswim6	-0,19681	1
Srgn	-0,19675	1
Gnb1	-0,19682	1

Kifc1	-0,19687	1
Rab7	-0,19691	1
Thumpd2	-0,19698	1
Shisa5	-0,19714	1
Cd68	-0,19712	1
RP23-3F1.8	-0,19718	1
Fgf11	-0,19732	1
Coq10b	-0,19726	1
Iars	-0,19731	1
Aco1	-0,19735	1
Qsox1	-0,19764	1
Zfp653	-0,19772	1
Wrn	-0,19777	1
Gm42639	-0,19781	1
Efr3a	-0,19779	1
Atf7ip	-0,19787	1
D330050G23Rik	-0,19795	1
Erlin2	-0,19798	1
Prss35	-0,19812	1
Runx2	-0,19806	1
Spdl1	-0,19824	1
Gm12504	-0,19835	1
Gm42851	-0,19827	1
Cox6a1	-0,19829	1
L3mbtl3	-0,19842	1
Slc6a12	-0,19841	1
Soat1	-0,19841	1
Lrrc42	-0,19842	1
Gm10382	-0,19845	1
Usp33	-0,19856	1
Trmt11	-0,19867	1
Papd5	-0,19871	1
Klhl24	-0,19869	1
Wdfy3	-0,19882	1
Rasa1	-0,19876	1
Clptm1l	-0,19875	1
Nars2	-0,19896	1
Hps1	-0,19903	1
Snx11	-0,19896	1
Fancm	-0,19905	1
Eldr	-0,19906	1
Glrp1	-0,19911	1
Ppt1	-0,19909	1
Rcsd1	-0,19922	1
Cmc1	-0,19934	1
Ccrl2	-0,19945	1
Zyx	-0,1995	1
Rps11-ps3	-0,19958	1
Cnnm4	-0,19963	1
Fn1	-0,19962	1
Pja2	-0,19961	1
Gm5786	-0,19978	1

Vps13a	-0,19978	1
Ago1	-0,19987	1
Lztfl1	-0,20001	1
Hspa14	-0,2	1
Dram2	-0,20011	1
Gm15464	-0,20011	1
Pmp22	-0,20021	1
A730071L15Rik	-0,20044	1
Pon3	-0,20044	1
Rnf115	-0,20062	1
Vdac3	-0,20074	1
Cxxc1	-0,2007	1
Ireb2	-0,201	1
Zfp101	-0,20116	1
Sharpin	-0,20121	1
Slx4	-0,20146	1
Zfp955a	-0,20149	1
Spopl	-0,20162	1
Cct2	-0,20159	1
Camk2g	-0,20172	1
Zdhhc5	-0,20166	1
Mark4	-0,20175	1
Atg16l2	-0,20193	1
Trappc12	-0,20203	1
Nrm	-0,20214	1
Tnfrsf26	-0,20212	1
D6Wsu163e	-0,20221	1
Gm8228	-0,20227	1
Gmfb	-0,20235	1
Caly	-0,20248	1
Vps39	-0,20248	1
Lrch3	-0,20259	1
Nsl1	-0,2028	1
Bnip3	-0,20289	1
Gm28192	-0,20301	1
Adi1	-0,203	1
Cherp	-0,203	1
Gm12604	-0,20313	1
Mettl15	-0,20308	1
Wdr7	-0,20324	1
Orc3	-0,20333	1
Prr14	-0,20331	1
Fubp3	-0,20345	1
Ankle1	-0,20347	1
Srsf7	-0,20362	1
Smad4	-0,204	1
Mpp1	-0,20411	1
Gpkow	-0,20425	1
Trps1	-0,20437	1
Gm28578	-0,20458	1
Zfp280b	-0,20472	1
Dock5	-0,20502	1

Gm4754	-0,2052	1
Tcn2	-0,20518	1
Tmem69	-0,20533	1
Plxna2	-0,20528	1
Gm23300	-0,20539	1
Ctsa	-0,20545	1
Vezf1	-0,20548	1
Ptpro	-0,20561	1
Prpsap1	-0,20559	1
Cox6c	-0,20569	1
Shpk	-0,20577	1
Tnfaip2	-0,20585	1
Mtss1	-0,20584	1
Rnf10	-0,20611	1
RP24-286J14.3	-0,20625	1
Cstf2t	-0,20621	1
Ak4	-0,20618	1
Rab10	-0,20622	1
Arl6ip1	-0,20632	1
Gm26670	-0,20656	1
Sh3bgrl2	-0,20665	1
Tmem39a	-0,20669	1
Nlk	-0,2068	1
Dnajc5	-0,207	1
Exoc6b	-0,20706	1
Ddx19a	-0,20718	1
Tstd2	-0,20721	1
Cactin	-0,20737	1
Grina	-0,20741	1
Eif4a1	-0,20753	1
Myo7a	-0,20759	1
Helz	-0,20765	1
Nelfb	-0,20806	1
Glipr1	-0,20812	1
Setd7	-0,20815	1
Lcp1	-0,20825	1
H60c	-0,20844	1
Ext2	-0,20841	1
Parp6	-0,20838	1
Bbs4	-0,20854	1
Aph1b	-0,20846	1
Gm28417	-0,20856	1
Olfr921	-0,20855	1
Orc5	-0,20863	1
Tmem106b	-0,20859	1
Dnajc27	-0,20867	1
Esco1	-0,20873	1
Smc4	-0,20866	1
Cstb	-0,20865	1
Loxl3	-0,20881	1
Mex3a	-0,20878	1
Tecpr1	-0,20896	1

Hnrnpab	-0,20909	1
B230208H11Rik	-0,20916	1
Cdc42se1	-0,20916	1
Mbd1	-0,20932	1
Atad3a	-0,20945	1
B230219D22Rik	-0,20952	1
Abcb4	-0,20954	1
Atp10d	-0,20964	1
Ctss	-0,20961	1
Mcm3	-0,20967	1
Gm19353	-0,20969	1
Phrf1	-0,20969	1
Nol10	-0,2097	1
Ago4	-0,20983	1
Pcbp2	-0,20975	1
Lpcat2	-0,21005	1
Dtx3l	-0,20999	1
Gm45855	-0,21012	1
Crybg3	-0,21007	1
Txn-ps1	-0,21024	1
5330438D12Rik	-0,21027	1
Utp14b	-0,21034	1
Glt8d1	-0,21033	1
Xpo6	-0,21044	1
Crls1	-0,2104	1
E330037G11Rik	-0,21062	1
Gm42636	-0,21062	1
Fam63b	-0,21076	1
Gm11952	-0,21101	1
Ubr1	-0,21101	1
Ethe1	-0,21101	1
Agfg2	-0,2111	1
Wwp1	-0,2112	1
Gper1	-0,21131	1
Arhgap30	-0,21133	1
Med17	-0,21137	1
Gm9722	-0,21148	1
Zc3h13	-0,21151	1
Pias2	-0,21163	1
Pard6b	-0,21171	1
Rpl21-ps6	-0,21172	1
Ccng2	-0,21175	1
Tgfb1	-0,21168	1
Camsap1	-0,21176	1
Tmem245	-0,21182	1
N4bp1	-0,2118	1
Secisbp2	-0,21185	1
Abcb7	-0,212	1
Arih1	-0,21201	1
Slc25a24	-0,21213	1
Nop56	-0,21208	1
Rsrc2	-0,21206	1

RP24-131G14.13	-0,21249	1
Tardbp	-0,21248	1
Gm38387	-0,21256	1
Ppp1r16a	-0,21279	1
Ubxn2a	-0,21277	1
Cdkl2	-0,21276	1
Asb1	-0,2129	1
Nfix	-0,21294	1
Tbc1d9b	-0,21293	1
Pigp	-0,21297	1
Rapgef2	-0,21313	1
Zmym3	-0,21312	1
St3gal1	-0,21316	1
Ranbp10	-0,21319	1
Tnfrsf11a	-0,21334	1
Ttc30b	-0,21336	1
Clic4	-0,21341	1
Fastkd5	-0,21354	1
Rap2c	-0,21345	1
Traf1	-0,21367	1
Sdc3	-0,21366	1
Eif2ak4	-0,21385	1
2810403D21Rik	-0,21392	1
Pacs2	-0,21389	1
Araf	-0,21425	1
Stx16	-0,21432	1
Rpl14	-0,21435	1
Osbp	-0,21437	1
Zfp710	-0,21445	1
Il10ra	-0,2144	1
Trim56	-0,21445	1
Setx	-0,21459	1
2810001G20Rik	-0,21466	1
Dact3	-0,2147	1
Zfp87	-0,21483	1
Ccnb2	-0,21483	1
Cd164	-0,2148	1
Mrpl3	-0,21482	1
Dut	-0,21476	1
Tpi1	-0,21492	1
Hspbp1	-0,215	1
Pbrm1	-0,21495	1
Txndc16	-0,21521	1
Ndufaf4	-0,21524	1
Nhej1	-0,21535	1
Kdm3b	-0,21545	1
D2hgdh	-0,21547	1
Apmap	-0,21555	1
Uri1	-0,21546	1
Fbxw11	-0,21547	1
Casp4	-0,21566	1
Gigyf2	-0,21565	1

Ccdc82	-0,21578	1
Rbm12	-0,21578	1
Zfp526	-0,21594	1
Rcl1	-0,21601	1
Mib1	-0,216	1
Rsbn1	-0,21602	1
B130006D01Rik	-0,21628	1
Zxdb	-0,21628	1
Hipk3	-0,21625	1
Pnpla7	-0,21629	1
Ttc26	-0,21668	1
Wdr6	-0,21692	1
Foxm1	-0,21696	1
Dctn1	-0,21713	1
Zfp956	-0,21709	1
Pfdn5	-0,21708	1
Spata5	-0,21725	1
Eif4g3	-0,21722	1
Mef2c	-0,21722	1
Csnk1e	-0,21722	1
Aim1	-0,21735	1
Idh3a	-0,21737	1
Dhx29	-0,21741	1
Coq5	-0,2176	1
Invs	-0,21778	1
Tomm34	-0,21789	1
Ccnd2	-0,21809	1
Exd2	-0,21824	1
Mkx	-0,21834	1
Themis2	-0,2183	1
Cstf3	-0,2183	1
Zfp523	-0,21844	1
Khdrbs1	-0,21847	1
Pcf11	-0,2186	1
Ccar2	-0,21872	1
Ifi204	-0,21868	1
Ccdc62	-0,21876	1
Triobp	-0,21884	1
Ppp4r3b	-0,21883	1
Gm10842	-0,21892	1
Gm14126	-0,21905	1
Gm43457	-0,21911	1
Rpl23a	-0,21934	1
Gm19503	-0,21947	1
Kti12	-0,21962	1
Ralgapb	-0,21959	1
C230096K16Rik	-0,21974	1
Zfp410	-0,21993	1
Rac3	-0,21995	1
Samsn1	-0,21995	1
Camk2b	-0,22007	1
Frk	-0,22011	1

Itsn2	-0,2202	1
Armt1	-0,22032	1
Gm10501	-0,22036	1
AU020206	-0,22036	1
Tnf	-0,22046	1
Ppih	-0,22057	1
Kptn	-0,22063	1
Akt1	-0,22075	1
Micu3	-0,22076	1
Zkscan3	-0,22079	1
Sympk	-0,22102	1
Zfp668	-0,22118	1
Als2	-0,22128	1
Ciz1	-0,22126	1
Srfbp1	-0,22145	1
Rpl27a-ps2	-0,22149	1
Ly6e	-0,22158	1
Atpaf2	-0,22172	1
Atl2	-0,22177	1
Atxn2	-0,22177	1
Polb	-0,22189	1
Scamp2	-0,22188	1
Ubr2	-0,22187	1
Acap2	-0,22213	1
Lca5	-0,22235	1
Zfp948	-0,22234	1
Iqcb1	-0,22225	1
Qsox2	-0,22237	1
Rab33b	-0,2225	1
Kpna1	-0,22251	1
Nmb	-0,2226	1
Abhd13	-0,22265	1
Pla2g6	-0,22312	1
P3h3	-0,22317	1
1700109H08Rik	-0,22334	1
Nom1	-0,22328	1
Cpox	-0,22349	1
Cmtm3	-0,22364	1
Rpl37	-0,22359	1
Rnf141	-0,2236	1
Pno1	-0,2239	1
Mast2	-0,224	1
Cnot4	-0,22424	1
Zfp326	-0,22445	1
St7	-0,22446	1
Zc3hav1l	-0,22481	1
Edem1	-0,22486	1
Rasip1	-0,22495	1
Rpl15-ps5	-0,22496	1
Gm44777	-0,22517	1
Lmln	-0,22518	1
Rgp1	-0,22518	1

Crtam	-0,22533	1
Mtmr2	-0,22527	1
Srek1	-0,22535	1
Ube3a	-0,22537	1
Slc25a53	-0,22553	1
Noc3l	-0,22555	1
Spag9	-0,22571	1
Dclre1a	-0,2258	1
Tmem198b	-0,22599	1
Lta4h	-0,22616	1
Knstrn	-0,22619	1
Oas3	-0,22645	1
Prpf38b	-0,22652	1
Bclaf1	-0,22667	1
Mprip	-0,22695	1
Erp44	-0,22694	1
Rhog	-0,22699	1
Piga	-0,22754	1
Pfkfb3	-0,2276	1
Klf10	-0,22758	1
Arhgef39	-0,22773	1
Sidt2	-0,22769	1
Arih2	-0,22782	1
Pcmtd1	-0,22778	1
Snx33	-0,22792	1
Fgfrl1	-0,22788	1
Stx2	-0,22804	1
Psme4	-0,22814	1
Btf3l4	-0,22851	1
Ep400	-0,22849	1
Ptcd1	-0,22891	1
Uhrf1bp1l	-0,22904	1
Plekho1	-0,22904	1
Gopc	-0,22906	1
Mgat4a	-0,22913	1
Lmtk2	-0,22922	1
Ptpn14	-0,22922	1
Srm	-0,22924	1
Snx5	-0,22923	1
Hyal1	-0,22935	1
Selenbp1	-0,22931	1
Klf13	-0,22933	1
Wdr1	-0,22926	1
Nras	-0,22953	1
Snora31	-0,22964	1
Rpgrip1l	-0,22965	1
Mrps36	-0,22979	1
Abca2	-0,22998	1
Kif1bp	-0,23023	1
Ppp6r3	-0,23022	1
Ckb	-0,23024	1
Parp14	-0,23049	1

Zfp236	-0,23059	1
Lrrc24	-0,23078	1
C330006A16Rik	-0,23077	1
Adap1	-0,23079	1
Hibch	-0,23087	1
Tmem81	-0,23107	1
Zc3h11a	-0,23122	1
Setd1b	-0,23129	1
Ptbp2	-0,23129	1
Hnrnpa2b1	-0,23128	1
Hectd3	-0,23136	1
Ssh1	-0,23185	1
Mta1	-0,23182	1
Gm17249	-0,23195	1
Pomt1	-0,23191	1
Sft2d2	-0,2319	1
Cse1l	-0,23199	1
Emp1	-0,23213	1
Gm22748	-0,23225	1
Ptma	-0,23223	1
Pten	-0,23218	1
Fut8	-0,23237	1
Zfp983	-0,23243	1
Marveld1	-0,2325	1
Phlpp2	-0,23265	1
C5ar1	-0,23263	1
Tmem63a	-0,23259	1
Tnpo3	-0,23261	1
Serp1	-0,23265	1
Isca1	-0,23286	1
Polr1a	-0,2329	1
Gm8394	-0,23312	1
Zbtb20	-0,23325	1
Uckl1	-0,23329	1
Zfpm1	-0,23325	1
Fam206a	-0,2333	1
Hap1	-0,23343	1
Taf11	-0,23352	1
Setd2	-0,23353	1
Gltp	-0,23369	1
Synrg	-0,23383	1
C330018D20Rik	-0,23425	1
Cenpq	-0,23422	1
Atp2c1	-0,23421	1
Myh9	-0,23426	1
Pex13	-0,23438	1
Ltv1	-0,23452	1
Ddhd2	-0,23452	1
Ptchd1	-0,23456	1
Arrdc1	-0,23493	1
Psma8	-0,23504	1
Actr5	-0,23508	1

Pqbp1	-0,23511	1
Trp53inp1	-0,23521	1
Ogfr	-0,23517	1
Gm28373	-0,23527	1
Hprt	-0,2353	1
2900055J20Rik	-0,23541	1
Tug1	-0,2354	1
Gm45836	-0,23544	1
Ago3	-0,23542	1
Ugp2	-0,23572	1
Ppp4c	-0,23585	1
Rab23	-0,2358	1
Sft2d3	-0,23576	1
Gm15696	-0,23603	1
Ampd3	-0,23601	1
Dpy19l4	-0,23611	1
Zdhhc18	-0,23622	1
Stk24	-0,2362	1
Sep 09	-0,2363	1
Lonrf3	-0,23633	1
Pcx	-0,23641	1
Mir22hg	-0,23648	1
Ddx50	-0,23668	1
Osbpl3	-0,23676	1
Zfp780b	-0,23693	1
Tmem229b	-0,23695	1
Dusp22	-0,23728	1
H2afx	-0,2374	1
Otud4	-0,23742	1
Map4k4	-0,23748	1
Trim28	-0,23784	1
Ino80	-0,23811	1
Zhx1	-0,23822	1
Mbd5	-0,23834	1
Gm16072	-0,23842	1
Zfp358	-0,23836	1
Plxnc1	-0,23856	1
Rragb	-0,23868	1
Myo5a	-0,23867	1
Mpeg1	-0,23878	1
Jarid2	-0,23895	1
Lemd3	-0,23904	1
Zmym2	-0,23909	1
Zfp397	-0,23916	1
Etnk1	-0,23921	1
Peg13	-0,23926	1
Cnot11	-0,23932	1
E2f2	-0,2394	1
Ube2d3	-0,23944	1
Nrbp1	-0,23959	1
Rif1	-0,23961	1
Ppp4r2	-0,23964	1

AA914427	-0,23967	1
Npc2	-0,23981	1
Sema4c	-0,24004	1
Atxn7	-0,24005	1
Vps9d1	-0,23996	1
Pcgf5	-0,24006	1
Tmx2	-0,24007	1
Entpd6	-0,24011	1
Rdh1	-0,2402	1
Usp15	-0,24035	1
Pi16	-0,24057	1
Ampd2	-0,24066	1
Gm22767	-0,24077	1
Ppp1r9b	-0,24088	1
Senp1	-0,24109	1
Klhdc10	-0,24112	1
Efnb1	-0,24128	1
Zufsp	-0,24128	1
Npc1l1	-0,24145	1
Gm5835	-0,24157	1
Klhl28	-0,24163	1
Tcf12	-0,24162	1
Gm43201	-0,24188	1
Dhx37	-0,24191	1
Cep83os	-0,24216	1
Map3k20	-0,24244	1
Samd1	-0,24254	1
Xdh	-0,24256	1
Magi1	-0,24277	1
Espl1	-0,24302	1
Dlg1	-0,24298	1
Spats2	-0,24312	1
Txlng	-0,24323	1
Gm44258	-0,24324	1
Cdc20	-0,24343	1
Rad54l2	-0,24358	1
Gm15157	-0,24365	1
Svip	-0,24393	1
Alox8	-0,2439	1
Fbxw4	-0,244	1
Ppp2r5b	-0,24409	1
Mis18bp1	-0,24414	1
Vta1	-0,2441	1
Map3k1	-0,2443	1
Cdk17	-0,2444	1
Nptn	-0,24453	1
Cd37	-0,24446	1
Vkorc1	-0,24462	1
Agfg1	-0,24462	1
Gltscr1	-0,24469	1
Tubgcp6	-0,24499	1
Arhgef25	-0,24501	1

Srsf3	-0,24495	1
Zfp422	-0,24512	1
Entpd5	-0,24516	1
Frg2f1	-0,24534	1
Gm20633	-0,24527	1
Dpp8	-0,24542	1
Usp40	-0,24552	1
Tsc2	-0,2456	1
RP24-84C23.4	-0,24559	1
Arl6ip5	-0,24561	1
Rab8b	-0,2458	1
Washc2	-0,24591	1
Tbca	-0,24603	1
Poll	-0,24621	1
Dap	-0,24631	1
Eif4g1	-0,24673	1
Pwp2	-0,24679	1
Dnajb9	-0,24698	1
Dazap1	-0,24704	1
Cln5	-0,2471	1
Gm6913	-0,24716	1
Stip1	-0,24744	1
Rlf	-0,24761	1
Thop1	-0,24765	1
Mt2	-0,24769	1
Aga	-0,248	1
Pold1	-0,24815	1
Qtrt1	-0,24815	1
Myof	-0,24814	1
Mtpn	-0,2481	1
Snapc1	-0,24815	1
Cflar	-0,24819	1
Ranbp2	-0,24815	1
Rhof	-0,24842	1
Ube3b	-0,24842	1
Tbcd	-0,24862	1
Spi1	-0,24886	1
Slc26a11	-0,24899	1
Gm11956	-0,24932	1
Ccm2	-0,24939	1
Sh3bp1	-0,24951	1
Tgfbrap1	-0,24962	1
Gm37699	-0,24965	1
Scel	-0,24973	1
Tmem156	-0,24974	1
Ppp1cb	-0,24967	1
Ddi2	-0,24967	1
Creld2	-0,24984	1
Mbtps1	-0,24984	1
Yipf6	-0,24994	1
Ninj1	-0,24999	1
Gm561	-0,25008	1

Slk	-0,25008	1
Tnpo1	-0,25011	1
Gm37423	-0,25016	1
Rpl7a	-0,25023	1
Inppl1	-0,25025	1
Armc9	-0,25042	1
Hypk	-0,25051	1
Sf3b1	-0,25063	1
Syap1	-0,25075	1
Sdcbp	-0,25066	1
Plekhn1	-0,2508	1
Ybx3	-0,25096	1
A930004J17Rik	-0,25118	1
Ccnd1	-0,25134	1
Klhl12	-0,25137	1
Arnt	-0,25144	1
Cd300lb	-0,25139	1
Kank3	-0,25174	1
Hexim1	-0,25168	1
Abhd4	-0,25175	1
D130019J16Rik	-0,25179	1
Kdm4b	-0,25177	1
Ahnak2	-0,25185	1
Dvl2	-0,25182	1
Timeless	-0,25192	1
Rps28	-0,25188	1
Btbd10	-0,25199	1
Pdpn	-0,25214	1
Slc16a1	-0,25214	1
Dync1li1	-0,25211	1
Cltc	-0,25212	1
Gm45248	-0,2523	1
Zfp691	-0,25228	1
Rsu1	-0,25234	1
Hadhb	-0,25262	1
Dmxl2	-0,2527	1
Napb	-0,25271	1
Vdac3-ps1	-0,25277	1
Armc8	-0,25298	1
Ppp1r15b	-0,25308	1
Plppr2	-0,25347	1
Gm4540	-0,25349	1
Ltb	-0,2536	1
Dopey2	-0,25358	1
Apex2	-0,25393	1
Naa15	-0,25385	1
Rnf213	-0,25401	1
Gm24920	-0,25411	1
Wdr82	-0,25432	1
5031425E22Rik	-0,25432	1
Pla2g4a	-0,25433	1
Dennd3	-0,25444	1

2510039O18Rik	-0,25442	1
Fam13a	-0,25451	1
Dhx8	-0,25452	1
Mier1	-0,25449	1
Fam78a	-0,25458	1
Mdh2	-0,25458	1
Slc7a6os	-0,25467	1
Elfn2	-0,2549	1
Efhd2	-0,25488	1
Zfp574	-0,2554	1
Eed	-0,25546	1
Gm12174	-0,25571	1
Insr	-0,25575	1
Wwc1	-0,25586	1
Plin2	-0,25633	1
Gcfc2	-0,25637	1
Strbp	-0,25658	1
Irf8	-0,25669	1
Entpd7	-0,25686	1
Ankrd39	-0,25689	1
Csnk2a2	-0,25694	1
Ugt1a7c	-0,25704	1
Zfp760	-0,25699	1
Shcbp1l	-0,25702	1
Tpx2	-0,257	1
Polk	-0,25705	1
Cmip	-0,25755	1
Slc30a4	-0,25755	1
Hnrnpa0	-0,25782	1
Gm5697	-0,2579	1
Fbxl5	-0,25787	1
Gm19777	-0,25795	1
Gm6969	-0,25796	1
Gm11517	-0,25797	1
Hnrnph3	-0,258	1
Ccdc36	-0,25816	1
Fpgs	-0,25847	1
Fut10	-0,25866	1
Pgls	-0,25869	1
Gse1	-0,25882	1
Tyk2	-0,25889	1
Hspa4l	-0,25894	1
Mpi	-0,25912	1
Dis3	-0,25911	1
Snap23	-0,25909	1
Cul1	-0,25933	1
Fbxo3	-0,25926	1
Xpo4	-0,25941	1
Mia2	-0,25936	1
Chd9	-0,2596	1
Lats2	-0,2598	1
Anp32a	-0,2599	1

Inpp5k	-0,26017	1
Traf4	-0,26023	1
Col18a1	-0,26016	1
Tmem248	-0,26021	1
Apobr	-0,26027	1
Abcb1b	-0,26028	1
Cdk11b	-0,26033	1
Gm12115	-0,26044	1
Uap1	-0,26039	1
Prickle3	-0,26047	1
Ttyh3	-0,2605	1
Bcl6	-0,26062	1
Bmp2k	-0,26065	1
Dpy19l3	-0,26066	1
Glce	-0,26082	1
Fam168b	-0,26085	1
Zdhhc20	-0,26088	1
Zfp672	-0,26127	1
Rrp15	-0,26125	1
Jak2	-0,26146	1
Pgap1	-0,26153	1
Sh2d3c	-0,2617	1
Tfb1m	-0,26167	1
Gm4875	-0,26177	1
Klhl21	-0,26177	1
Luzp1	-0,2619	1
Ncor2	-0,26226	1
Tmem186	-0,26246	1
Ddah2	-0,26272	1
Slc25a32	-0,26296	1
Itfg2	-0,26311	1
Ccdc58	-0,26307	1
Chaf1a	-0,26344	1
Tgs1	-0,26337	1
Tslp	-0,26352	1
Nmd3	-0,26356	1
Ston1	-0,26371	1
Nrtn	-0,26393	1
Naga	-0,26389	1
Eif4ebp2	-0,26391	1
Olfr460	-0,26405	1
Gtf2h3	-0,26402	1
Pbdc1	-0,26415	1
Sgsm2	-0,26412	1
Ddx5	-0,2643	1
Sirt1	-0,26442	1
Lsm7	-0,26451	1
Ints13	-0,26451	1
Rnf216	-0,26464	1
Gk5	-0,26472	1
Cep164	-0,2647	1
Polr3k	-0,26474	1

Slc35b3	-0,26498	1
Dot1l	-0,26496	1
Gpaa1	-0,2651	1
Fam53b	-0,26521	1
Ncapg	-0,2653	1
Gadd45b	-0,26552	1
Rps27rt	-0,26549	1
Pola1	-0,26581	1
Cdc42bpg	-0,26584	1
Dcun1d3	-0,26589	1
Mif	-0,26585	1
Hsf1	-0,26599	1
Cdc27	-0,26603	1
Plekhb2	-0,26612	1
Cbfb	-0,26605	1
Med23	-0,26629	1
Ccdc28a	-0,26631	1
Jpx	-0,26644	1
Gga1	-0,26653	1
Rabl2	-0,26662	1
Slc16a3	-0,26662	1
D430001F17Rik	-0,26677	1
RP23-23P9.3	-0,26706	1
Gm14698	-0,26716	1
Bcl2l15	-0,26741	1
Cenpi	-0,26745	1
Ajuba	-0,26762	1
Blcap	-0,26766	1
Wdfy4	-0,26777	1
Pnpla2	-0,26781	1
Prdm10	-0,26805	1
Dock6	-0,26818	1
Zfp292	-0,26828	1
Lrba	-0,26836	1
Tfe3	-0,26863	1
Lars	-0,26863	1
Ubr5	-0,26887	1
Slc3a2	-0,2689	1
Laptm4b	-0,26937	1
Snrnp200	-0,26938	1
Luc7l	-0,26945	1
Spg21	-0,26973	1
Gm7224	-0,26982	1
Pabpc4	-0,27001	1
Atxn7l3	-0,27002	1
Phf2	-0,27004	1
Adora2a	-0,27029	1
4933427D14Rik	-0,27033	1
Uchl4	-0,27039	1
Alkbh8	-0,27036	1
Ascc3	-0,27047	1
Usp53	-0,27054	1

Det1	-0,27073	1
Gramd1a	-0,27071	1
Adipor2	-0,27076	1
Ormdl1	-0,27094	1
Aldh6a1	-0,27112	1
Cramp1l	-0,27122	1
Tbc1d5	-0,27129	1
Unc45a	-0,27132	1
Rad52	-0,27141	1
Pnrc1	-0,27142	1
Gm13383	-0,27173	1
1500004A13Rik	-0,2717	1
Ncoa5	-0,27192	1
1700056N10Rik	-0,27214	1
Usp14	-0,27219	1
Gramd4	-0,27226	1
Rpl7a-ps5	-0,27236	1
Camkmt	-0,27259	1
Cyfip1	-0,27258	1
Tox2	-0,2728	1
Rab5a	-0,27282	1
Slc39a9	-0,2728	1
Vamp7	-0,273	1
Yipf5	-0,27307	1
Ddx59	-0,27318	1
Gm10605	-0,27335	1
Uimc1	-0,27331	1
Mfge8	-0,27329	1
Poc1a	-0,2737	1
Dcp2	-0,27374	1
Slfn3	-0,27394	1
Mapk8ip3	-0,27389	1
Dhx34	-0,27398	1
Bccip	-0,27418	1
Plcg2	-0,27451	1
Slc10a7	-0,27458	1
Spop	-0,27455	1
Evi5	-0,27484	1
Raly	-0,27495	1
Mospd1	-0,27505	1
Pdcd7	-0,27497	1
4921507G05Rik	-0,27515	1
C78859	-0,27506	1
Ankrd26	-0,27522	1
Ppp1r15a	-0,27536	1
Ubtf	-0,27539	1
Gas2l3	-0,27555	1
Kiz	-0,27551	1
Ermp1	-0,27577	1
BC085271	-0,27586	1
Gm5362	-0,27597	1
Snu13	-0,27601	1

Frmd4b	-0,27599	1
Ccl3	-0,27616	1
Otub2	-0,27677	1
Coro7	-0,27713	1
Map3k2	-0,27709	1
Plekha5	-0,27728	1
mt-Co1	-0,27738	1
Bphl	-0,2775	1
Fam193b	-0,27751	1
Slc25a28	-0,27751	1
Ifih1	-0,27764	1
Mmp9	-0,27755	1
Washc3	-0,27778	1
Pign	-0,27799	1
Abhd18	-0,2781	1
Card19	-0,27815	1
Zcchc4	-0,27822	1
Gm7432	-0,2784	1
Fam149b	-0,27864	1
Paqr3	-0,27861	1
March8	-0,27863	1
Zfp113	-0,27874	1
Sh3pxd2b	-0,27878	1
R3hdm2	-0,27888	1
Tube1	-0,279	1
2900052L18Rik	-0,27907	1
Rin1	-0,27941	1
Manf	-0,27944	1
Cux1	-0,27948	1
Gm16199	-0,2796	1
Nol11	-0,27966	1
Tmem175	-0,27977	1
Ptbp1	-0,27982	1
Pdia3	-0,27987	1
Atad1	-0,28047	1
Smarca5	-0,28055	1
Pcm1	-0,28062	1
Galnt1	-0,28063	1
Gm12882	-0,28073	1
Zfp398	-0,28066	1
Bsdc1	-0,28076	1
Arl4d	-0,28112	1
Pianp	-0,28106	1
Kmt5b	-0,28124	1
BC025920	-0,28133	1
Spin1	-0,28156	1
4930426I24Rik	-0,28169	1
Tmem230	-0,28172	1
Cav2	-0,28193	1
Gm7363	-0,28197	1
Mboat1	-0,28212	1
A330035P11Rik	-0,2823	1

Arfgef2	-0,28238	1
Ap3m1	-0,28264	1
Eefsec	-0,28271	1
Zbtb41	-0,28279	1
Scpep1	-0,28276	1
Med12	-0,28298	1
Wnk2	-0,28305	1
Ctdp1	-0,28296	1
Hnrnpd	-0,2831	1
Zkscan8	-0,28325	1
Zfp317	-0,28317	1
Tbkbp1	-0,28316	1
Mapk8ip1	-0,28329	1
2900076A07Rik	-0,28335	1
Herc1	-0,28338	1
Phykpl	-0,28346	1
Pus10	-0,28356	1
Atg16l1	-0,28363	1
Trim37	-0,28373	1
Ap5m1	-0,28382	1
Akirin2	-0,28377	1
Gm42466	-0,28395	1
Srpk1	-0,28393	1
Trim46	-0,28421	1
Hapln3	-0,28422	1
Emsy	-0,28416	1
Ndst2	-0,28435	1
Ccnf	-0,28454	1
Tcf20	-0,28448	1
Surf2	-0,28457	1
Pwwp2a	-0,28502	1
Stxbp3	-0,28514	1
Hsd17b4	-0,28515	1
Asb11	-0,28528	1
Kdm4c	-0,28525	1
Cxx1a	-0,28526	1
Atp7a	-0,28529	1
Med18	-0,28536	1
Irf1	-0,28536	1
Gm4997	-0,28597	1
Coa6	-0,28609	1
Gmeb1	-0,28616	1
Thap6	-0,28629	1
Nfkbid	-0,28639	1
Asna1	-0,2864	1
Gatsl2	-0,28644	1
Ppdpf	-0,2865	1
Nnt	-0,2865	1
Rpl6	-0,28683	1
Eif3m	-0,28715	1
Gm9844	-0,28716	1
Ints4	-0,28741	1

Chd6	-0,28738	1
Lonrf1	-0,28742	1
Yme1l1	-0,28741	1
Ube2h	-0,28745	1
Brat1	-0,28767	1
Psmg1	-0,28787	1
Gdpgp1	-0,28804	1
Purg	-0,2881	1
Zscan2	-0,28807	1
Ahcyl2	-0,28813	1
Gpatch8	-0,28825	1
Rassf5	-0,28868	1
Rock2	-0,28883	1
Tns3	-0,2889	1
Mdm4-ps	-0,28899	1
Med14	-0,28899	1
Kdm3a	-0,28895	1
Angel2	-0,28929	1
Fbxw17	-0,28949	1
Gm24276	-0,28952	1
Pi4ka	-0,28956	1
Exoc8	-0,28984	1
Gm37494	-0,28982	1
Ercc6	-0,28988	1
Nrip1	-0,28987	1
Appbp2	-0,29003	1
Gripap1	-0,29003	1
Impa1	-0,29007	1
Cep78	-0,29024	1
Smg5	-0,29026	1
Pip5k1b	-0,29044	1
Msh6	-0,29044	1
Flcn	-0,29051	1
Osbpl8	-0,29063	1
Sertad3	-0,29074	1
Yipf4	-0,29078	1
Tpm3	-0,29082	1
Igf2bp2	-0,29103	1
Aqp11	-0,29128	1
Tmpo	-0,29132	1
Wnk1	-0,29133	1
Gm16630	-0,29139	1
Mfsd7b	-0,29152	1
AW146154	-0,29157	1
Rbbp9	-0,2917	1
Stat3	-0,29181	1
Gm44822	-0,292	1
Mlx	-0,2921	1
Gm43010	-0,29221	1
D3Ertd254e	-0,29226	1
Gm13567	-0,29254	1
Cep97	-0,29252	1

Plcb2	-0,2925	1
Chfr	-0,29254	1
Ppp3r1	-0,29259	1
Gm43138	-0,29265	1
Gm38077	-0,29266	1
Ice2	-0,29294	1
Arglu1	-0,29315	1
Ech1	-0,29333	1
Lrrk2	-0,29338	1
Zfat	-0,29361	1
Phc1	-0,29365	1
D2Bwg1423e	-0,29382	1
Ermard	-0,2938	1
Slc29a3	-0,29385	1
Uggt1	-0,29382	1
Rnft1	-0,29397	1
2700097O09Rik	-0,29404	1
Nkpd1	-0,29396	1
Gm10784	-0,29413	1
Slc35e1	-0,29419	1
Gm10275	-0,29416	1
Csnk1g1	-0,29422	1
Lamp1	-0,29459	1
Inpp5b	-0,29472	1
Slc31a1	-0,29493	1
Cldn11	-0,29512	1
C1galt1	-0,29516	1
Srbd1	-0,29553	1
Ash1l	-0,29549	1
Malt1	-0,29562	1
Slc25a13	-0,29561	1
Tmem123	-0,29561	1
Fam114a2	-0,29561	1
Fam212b	-0,29584	1
Cep152	-0,2958	1
Tsc22d3	-0,29597	1
Gm35106	-0,29599	1
Fos	-0,2962	1
Mecr	-0,29624	1
Pde1b	-0,29619	1
Gm36930	-0,29628	1
Slc38a6	-0,29662	1
2610301B20Rik	-0,29663	1
Agps	-0,29672	1
Kbtbd11	-0,29692	1
Alms1	-0,29704	1
Ccdc47	-0,2971	1
Rps6ka1	-0,29707	1
Atg4c	-0,29734	1
P4ha1	-0,29752	1
Fads6	-0,29761	1
Bub1b	-0,29759	1

Rgs3	-0,29772	1
Hsp90ab1	-0,29793	1
Gm17039	-0,29809	1
Nqo2	-0,29809	1
Gm7887	-0,29815	1
Zfp607a	-0,29834	1
Msl1	-0,29833	1
Ltbp4	-0,29837	1
Prag1	-0,29846	1
Dus4l	-0,29862	1
Utrn	-0,2989	1
Gmeb2	-0,29899	1
2510009E07Rik	-0,29919	1
Fam45a	-0,29926	1
Sh3gl1	-0,29955	1
Rlim	-0,29946	1
Lrrc8b	-0,29973	1
Rpl12	-0,30006	1
Tubgcp3	-0,30009	1
Tom1l1	-0,30008	1
Mtx2	-0,3002	1
Wdr26	-0,30019	1
Gm7832	-0,30029	1
1700088E04Rik	-0,30027	1
Tbc1d23	-0,30033	1
Traf3ip1	-0,30037	1
Man2b1	-0,30089	1
1810026B05Rik	-0,30109	1
Lcorl	-0,30116	1
Slc2a8	-0,30128	1
Akap1	-0,30139	1
Gm14620	-0,30199	1
Rbm26	-0,3021	1
Plxnb2	-0,30212	1
Ldah	-0,30219	1
Tmem165	-0,30221	1
Zdhhc21	-0,30229	1
Rrnad1	-0,3024	1
Gpnmb	-0,30253	1
Mink1	-0,30259	1
Rbms2	-0,30286	1
Atxn3	-0,30294	1
Incenp	-0,3029	1
Npc1	-0,30298	1
Oasl1	-0,30313	1
Impact	-0,30313	1
Ftsj1	-0,3032	1
5730480H06Rik	-0,30331	1
Nbeal2	-0,30353	1
Ppp4r1	-0,30355	1
Simc1	-0,30359	1
Ppp3cc	-0,30375	1

Akap10	-0,30368	1
Fam178a	-0,30396	1
Kif21b	-0,30412	1
Csnk1a1	-0,30411	1
Gm6209	-0,30427	1
Gatc	-0,30437	1
Gtf3c3	-0,30454	1
Dgcr14	-0,30457	1
Tacc3	-0,30469	1
Copz2	-0,30477	1
Lsm8	-0,30478	1
Erp27	-0,30501	1
Ikbke	-0,30514	1
Pxn	-0,30511	1
Phpt1	-0,30517	1
Ldlr	-0,30546	1
Smarcc2	-0,30556	1
Pacs1	-0,30589	1
Pdcd6ip	-0,30593	1
1500015A07Rik	-0,30606	1
Mxra8	-0,30623	1
Oxsr1	-0,30635	1
Ttbk2	-0,30646	1
Cks1brt	-0,30671	1
Emc2	-0,30689	1
Emc4	-0,30708	1
Gm11531	-0,30708	1
Tcp11l1	-0,30719	1
Gm11686	-0,30725	1
Agl	-0,30735	1
Hnrnpll	-0,30728	1
Itsn1	-0,30734	1
Zer1	-0,30743	1
Mafk	-0,30759	1
Alg13	-0,30769	1
Ewsr1	-0,3077	1
Nfatc2ip	-0,30787	1
Plekha8	-0,30809	1
March7	-0,30814	1
Kdm6a	-0,30822	1
Foxred2	-0,30823	1
Mknk1	-0,30844	1
Gm44829	-0,3085	1
Dock9	-0,30849	1
Gm14537	-0,30854	1
R3hdm4	-0,3087	1
Tpcn1	-0,30897	1
Rev3l	-0,3092	1
Brwd3	-0,30932	1
Mapk12	-0,30938	1
Prps2	-0,30954	1
Pcna-ps2	-0,30964	1

Nr1d1	-0,30978	1
Arhgef6	-0,30978	1
Ndfip2	-0,30986	1
Trnt1	-0,31053	1
Upf1	-0,31055	1
Gsk3b	-0,31053	1
Kctd3	-0,31056	1
Dcaf15	-0,31061	1
Ppa1	-0,31056	1
Setd5	-0,31064	1
Gadd45g	-0,31069	1
Plk1	-0,31069	1
Carm1	-0,31092	1
Papolg	-0,31099	1
Cbr2	-0,3113	1
Trove2	-0,3114	1
Gm37785	-0,31148	1
Mkln1os	-0,31149	1
Dars	-0,31159	1
Tubb5	-0,31159	1
Stxbp4	-0,3117	1
Smim14	-0,31197	1
Bag4	-0,31196	1
Gbas	-0,31208	1
Mapk7	-0,31216	1
Cyth3	-0,31235	1
Ndrg1	-0,31253	1
Atrx	-0,31249	1
Strn3	-0,31263	1
Enpp5	-0,31301	1
Spns1	-0,31313	1
Morc2a	-0,31321	1
Zfand4	-0,31343	1
Gm43362	-0,31365	1
Akt1s1	-0,31375	1
2010008C14Rik	-0,31379	1
Ctnnb1	-0,31406	1
Hivep3	-0,31429	1
Atrn	-0,31446	1
Sptlc1	-0,31472	1
Gm12655	-0,31479	1
Gm10175	-0,31476	1
Lrrk1	-0,31483	1
Ralgds	-0,31479	1
Gpr132	-0,31498	1
C1rl	-0,31525	1
Arsb	-0,3153	1
Mrpl42	-0,31535	1
Dgkh	-0,3154	1
C430049E01Rik	-0,31548	1
Med16	-0,31575	1
Adat1	-0,31584	1

Atad5	-0,31582	1
Arhgdig	-0,31592	1
St6gal1	-0,31586	1
Fndc3b	-0,31598	1
Dstn	-0,31596	1
Ikbkap	-0,31615	1
Unc13a	-0,31623	1
Gm12618	-0,31639	1
Myc	-0,31639	1
Cacna1a	-0,31677	1
Zfp516	-0,31689	1
Gm26935	-0,31719	1
Dip2b	-0,31716	1
Uprt	-0,31746	1
Ccnl2	-0,31749	1
Atp5g1	-0,31756	1
Bag6	-0,31782	1
Tmem168	-0,31776	1
Tctn3	-0,31791	1
Ezh1	-0,31787	1
Cpeb3	-0,3184	1
Nuf2	-0,31852	1
Zdhhc17	-0,31894	1
Gpalpp1	-0,3191	1
Zfp945	-0,3192	1
Cox19	-0,31926	1
Tsc22d2	-0,31932	1
Cdc42	-0,31932	1
Kirrel3	-0,31947	1
Klhl9	-0,31948	1
Rps4x	-0,31966	1
Arid1a	-0,31981	1
Atp13a3	-0,31989	1
Pigs	-0,32005	1
Gtf2b	-0,32006	1
Traf7	-0,32006	1
Nusap1	-0,32013	1
Cdkn3	-0,32028	1
Sbds	-0,32031	1
Gm25857	-0,32044	1
Lin54	-0,3204	1
Tbc1d7	-0,32062	1
Klhl20	-0,32063	1
Fzd5	-0,32063	1
Cfap74	-0,32072	1
Dhx35	-0,3207	1
Rrp12	-0,32071	1
Bcl9	-0,32078	1
Plpp6	-0,32091	1
Eno1b	-0,32105	1
Rgcc	-0,321	1
Tnip3	-0,32101	1

Gm6450	-0,32117	1
Atp6ap1	-0,32127	1
Scand1	-0,32142	1
C130083A15Rik	-0,32158	1
Cdc42ep4	-0,32159	1
Zkscan7	-0,32202	1
Rcor3	-0,32204	1
Herc4	-0,32203	1
Hsp90aa1	-0,32218	1
Glul	-0,32232	1
Adal	-0,32256	1
Ddx11	-0,32271	1
Pan3	-0,32268	1
Vapa	-0,32268	1
Nelfa	-0,32277	1
Wls	-0,32277	1
Zcchc8	-0,32282	1
Rbm33	-0,32288	1
Rassf2	-0,32301	1
Nab1	-0,32325	1
Cbfa2t2	-0,32349	1
Hpdl	-0,32356	1
Mlh1	-0,3237	1
Slc33a1	-0,32382	1
Strn4	-0,32394	1
Capn7	-0,3239	1
Pcna	-0,32399	1
Gabpa	-0,32407	1
Gtf2h1	-0,32443	1
Sem1	-0,32436	1
Vcp	-0,32454	1
Bms1	-0,32446	1
Gm42937	-0,32464	1
Rps13-ps2	-0,32484	1
Cables2	-0,32523	1
Mmachc	-0,32526	1
Mbnl2	-0,32547	1
Rps27l	-0,32547	1
Bmpr1a	-0,32557	1
Rbm25	-0,32571	1
Myom1	-0,32577	1
Gm37452	-0,32581	1
Ckap5	-0,32578	1
Ccdc50	-0,32582	1
Ddx43	-0,32588	1
Ppp1r2	-0,32587	1
Hnrnpdl	-0,3259	1
Zfp995	-0,32618	1
Gm43924	-0,3262	1
Ctdsp2	-0,3264	1
Kdm5a	-0,32648	1
Mfsd4b4	-0,32664	1

Rcc2	-0,32661	1
Rdh13	-0,32675	1
Prmt1	-0,3269	1
Sirt4	-0,32704	1
Tm4sf19	-0,32708	1
Stk17b	-0,32717	1
Nfkbiz	-0,32732	1
4932416K20Rik	-0,32738	1
Kdm1b	-0,32742	1
Fam174a	-0,32751	1
Atp2b1	-0,32748	1
Jak1	-0,32767	1
B230369F24Rik	-0,32791	1
Gm13050	-0,3279	1
Cenpc1	-0,32792	1
Zfp638	-0,32794	1
Ltbp2	-0,32805	1
Ggta1	-0,32833	1
Lnpk	-0,32871	1
Kpna4	-0,32932	1
Nln	-0,32937	1
Rbm19	-0,32963	1
Plch2	-0,32966	1
Fam63a	-0,32984	1
Vwa5a	-0,32981	1
Tgds	-0,33013	1
Oaz2	-0,33032	1
Arhgap23	-0,33045	1
Rpl39-ps	-0,33048	1
Cry2	-0,33049	1
Ankrd33b	-0,33059	1
Gtf2ird2	-0,33069	1
4930542C12Rik	-0,33066	1
Gm43714	-0,33081	1
Zic2	-0,33093	1
Dst	-0,33088	1
D930015E06Rik	-0,33089	1
Pml	-0,33123	1
Thap8	-0,33142	1
Amer1	-0,33142	1
Gtf3c2	-0,3316	1
Faap24	-0,33178	1
Top2b	-0,33183	1
Gm37199	-0,33203	1
Spata1	-0,33206	1
AA986860	-0,33217	1
Car2	-0,33215	1
Zzz3	-0,33237	1
Dmtf1	-0,33236	1
Clic1	-0,33263	1
Ttc5	-0,3327	1
Dynlt1f	-0,33284	1

Gm8770	-0,33288	1
Tnpo2	-0,33286	1
Anp32e	-0,33307	1
Des	-0,33341	1
Fancf	-0,33365	1
Tm9sf2	-0,33356	1
Gm11343	-0,33379	1
Calhm2	-0,33377	1
Actn4	-0,33376	1
Atxn1	-0,33397	1
Pias3	-0,33414	1
Gm13038	-0,33419	1
Tmed7	-0,33416	1
Tmem173	-0,33434	1
Ezr	-0,33435	1
Sfxn2	-0,33457	1
Hipk1	-0,33465	1
Rab5b	-0,33474	1
Gla	-0,33498	1
Tecr	-0,33513	1
Zfp36l2	-0,33523	1
B4galnt1	-0,33581	1
Spcs2	-0,33594	1
Dnajc7	-0,33605	1
Paxbp1	-0,3362	1
Apoe	-0,33652	1
Tm6sf1	-0,33651	1
Klhl42	-0,33655	1
Dennd1a	-0,33669	1
Slc2a3	-0,33667	1
Arcn1	-0,33676	1
Runx1	-0,33679	1
Atic	-0,33694	1
Phf21a	-0,33693	1
Star	-0,33742	1
Vash2	-0,33741	1
Bcl9l	-0,33737	1
Ugcg	-0,33745	1
Tapt1	-0,3375	1
Topbp1	-0,3375	1
Atp1a1	-0,33748	1
Pcyox1l	-0,33775	1
Atf6b	-0,3377	1
Ska3	-0,33779	1
Zswim3	-0,33796	1
Pias4	-0,33815	1
Glg1	-0,33817	1
4930522L14Rik	-0,33844	1
Lipe	-0,33844	1
Phf8	-0,33846	1
B3gntl1	-0,33865	1
Zfp7	-0,33879	1

Fam179b	-0,33902	1
Polq	-0,33913	1
Bhlhe41	-0,33934	1
Fam20b	-0,3393	1
Ncor1	-0,33952	1
Mtmr1	-0,33965	1
B130021K23Rik	-0,34008	1
Snrpb	-0,34014	1
Gm15937	-0,34018	1
RP23-162P10.8	-0,34031	1
Tmem91	-0,34043	1
Nsun5	-0,34036	1
Gpi1	-0,34041	1
Bbs7	-0,34062	1
Fam234b	-0,3406	1
Trim39	-0,34067	1
Inpp4a	-0,34069	1
Rbm15	-0,34084	1
Zc3h12c	-0,34127	1
Ehmt2	-0,34135	1
Trio	-0,34152	1
Atxn1l	-0,34163	1
Klhl41	-0,34169	1
Gm16053	-0,3421	1
Ubxn2b	-0,34207	1
Slc25a5	-0,34217	1
Slc29a2	-0,34221	1
Esyt2	-0,34218	1
Tsc1	-0,34235	1
Oaz1-ps	-0,34248	1
Fbxl19	-0,34282	1
Dbndd2	-0,3428	1
Ier5	-0,34295	1
6430710M23Rik	-0,34299	1
Aurkb	-0,34327	1
Gnal	-0,34341	1
Terf2ip	-0,34355	1
Lzic	-0,34367	1
Fto	-0,3437	1
Serpinb6b	-0,34378	1
Prss53	-0,34404	1
Ttf2	-0,34402	1
Hdgfrp2	-0,34419	1
Fbxl20	-0,34432	1
Tcf4	-0,34426	1
Fam122b	-0,34435	1
Fam120aos	-0,34452	1
Cpeb4	-0,3445	1
Snhg4	-0,34455	1
Gm7236	-0,34492	1
Fxyd2	-0,3449	1
Gm19967	-0,34498	1

Zfp207	-0,3451	1
Abcg1	-0,34535	1
Asxl2	-0,34561	1
Zbtb37	-0,3457	1
Dgat1	-0,34567	1
Gm34121	-0,34587	1
Csf2ra	-0,34595	1
Drg2	-0,34601	1
Rnf220	-0,34614	1
Bax	-0,3461	1
Kdm4a	-0,3461	1
Eml5	-0,34617	1
Erlin1	-0,34619	1
Mapkbp1	-0,34625	1
Wdr75	-0,34622	1
Klf3	-0,3463	1
Swi5	-0,34633	1
Stx4a	-0,34629	1
Snord13	-0,34666	1
Zmynd8	-0,34695	1
Dhx57	-0,34714	1
9330159M07Rik	-0,34716	1
1810021B22Rik	-0,34725	1
Ier5l	-0,34716	1
9230112E08Rik	-0,34744	1
Slc25a30	-0,34737	1
Gm23442	-0,34749	1
C2cd5	-0,34771	1
Tnrc6a	-0,34766	1
Primpol	-0,34795	1
Gm38111	-0,348	1
Ddx3y	-0,34798	1
RP23-440L7.5	-0,34812	1
Sap18	-0,34823	1
Agtpbp1	-0,34815	1
Lgmn	-0,34839	1
Gm27248	-0,34846	1
Impdh1	-0,34856	1
Gbe1	-0,34875	1
Gm43756	-0,34878	1
C8g	-0,34899	1
0610009B22Rik	-0,34915	1
2610203C20Rik	-0,34906	1
Zxdc	-0,34909	1
Lsp1	-0,3491	1
Gm37145	-0,34955	1
Clcn2	-0,34957	1
Ppp1r18	-0,34957	1
Ptms	-0,34957	1
Gfap	-0,34973	1
Trmt44	-0,35008	1
Pqlc3	-0,35012	1

Slf1	-0,35049	1
Gmnn	-0,35063	1
Utp4	-0,35063	1
Chtf8	-0,35055	1
Zfp36	-0,35066	1
Arhgap11a	-0,35076	1
Lrp5	-0,35091	1
Lypla2	-0,35097	1
Dhx58	-0,35126	1
Ift172	-0,35129	1
Zfp652	-0,35132	1
Dgcr8	-0,35151	1
Camta1	-0,35147	1
Braf	-0,35157	1
Cog1	-0,35168	1
Mast3	-0,35169	1
Gm37589	-0,35181	1
Dars2	-0,35179	1
Tmem33	-0,352	1
Heatr3	-0,35209	1
Mapkapk2	-0,3521	1
Airn	-0,3522	1
Atr	-0,35259	1
Adam8	-0,35278	1
Uxs1	-0,35291	1
Hnrnpr	-0,35289	1
6720427I07Rik	-0,35298	1
Nr2c1	-0,35319	1
Gm45286	-0,35323	1
Trp53	-0,3536	1
Iws1	-0,35358	1
Larp4	-0,35373	1
Arf4	-0,35383	1
Uvssa	-0,35398	1
Nadk	-0,35398	1
Yars2	-0,35427	1
Itgb3bp	-0,35437	1
Mmp2	-0,35447	1
Dock11	-0,3545	1
Fbxo33	-0,35501	1
Egln1	-0,355	1
2610020H08Rik	-0,35511	1
Rgs11	-0,35511	1
AI464131	-0,35537	1
Grk5	-0,35542	1
Akr7a5	-0,35564	1
Gm28555	-0,35564	1
Ddr1	-0,35565	1
Mlxip	-0,35571	1
Rfx7	-0,35578	1
Capza1	-0,35589	1
Etv5	-0,35599	1

Gm6565	-0,35634	1
Nek9	-0,35637	1
Cln6	-0,35659	1
Efcab14	-0,35661	1
Klhl7	-0,35665	1
Tubgcp4	-0,35673	1
Ppp2r1b	-0,35671	1
Map3k7	-0,3567	1
Arpc2	-0,35677	1
Cnep1r1	-0,35711	1
Zfp871	-0,35719	1
Tipin	-0,35725	1
Zfp994	-0,35751	1
Kif5a	-0,35784	1
Mycbp2	-0,35779	1
Hk3	-0,35809	1
Manea	-0,35856	1
C730034F03Rik	-0,3588	1
Sppl2a	-0,35878	1
Prex1	-0,35886	1
Nf1	-0,35916	1
Jun	-0,3593	1
C1qbp	-0,35932	1
Gm8116	-0,35954	1
Azin2	-0,35951	1
Gm38021	-0,35956	1
Wdcp	-0,35975	1
Plekhm3	-0,35966	1
Trappc8	-0,35982	1
Cited2	-0,36003	1
Bbs5	-0,36006	1
Plekhm2	-0,36019	1
Ttc9c	-0,36038	1
Eme1	-0,36049	1
Mdm4	-0,36048	1
Casd1	-0,36062	1
Mfsd7a	-0,36082	1
Tdp1	-0,36082	1
Ctdnep1	-0,36079	1
Crkl	-0,36089	1
Ccl4	-0,3612	1
Parp11	-0,3614	1
Ccdc71l	-0,36152	1
Epg5	-0,36151	1
Csnk2a1	-0,36147	1
Brms1	-0,36162	1
Gm45224	-0,36157	1
Fam98b	-0,36205	1
Prkacb	-0,36203	1
Gm10575	-0,36224	1
Paip1	-0,36242	1
Sf3b4	-0,36268	1

Hmga2	-0,36278	1
Mybl2	-0,36299	1
Sf1	-0,36305	1
Fam151b	-0,36367	1
Ric1	-0,36365	1
Grtp1	-0,36398	1
Vcl	-0,36403	1
Skiv2l2	-0,36423	1
Gm45729	-0,3643	1
Gmip	-0,36441	1
Fam92a	-0,36454	1
Chpf2	-0,36513	1
Kif16b	-0,36524	1
Depdc5	-0,36539	1
Stt3a	-0,36556	1
Dynll1	-0,36571	1
Washc4	-0,36569	1
Spp1	-0,36578	1
Prox2	-0,36586	1
Hist1h1a	-0,36588	1
Lrrfip2	-0,36586	1
Iqgap1	-0,36586	1
Nudt12	-0,36625	1
Mob1a	-0,36629	1
2700049A03Rik	-0,36636	1
Snord15a	-0,36646	1
Fzr1	-0,36659	1
Brpf3	-0,36665	1
Zcchc11	-0,36668	1
Dusp5	-0,36683	1
Tet3	-0,36712	1
Prdm15	-0,36726	1
Kdm5c	-0,36726	1
Rps6-ps4	-0,36742	1
Glipr2	-0,36767	1
Numb	-0,36774	1
Slc12a7	-0,36801	1
Eloc	-0,36816	1
Neu3	-0,36829	1
Esco2	-0,36841	1
Rhoq	-0,36866	1
Qk	-0,36881	1
Mkl2	-0,36906	1
Zmat3	-0,36922	1
Hibadh	-0,36965	1
Hdac9	-0,3697	1
Cstad	-0,36989	1
Dmpk	-0,37024	1
Rfc4	-0,37021	1
Mlkl	-0,37035	1
Tpra1	-0,37041	1
Prepl	-0,37057	1

Rmi1	-0,37076	1
Tanc1	-0,37098	1
Gm45053	-0,37124	1
Taf1	-0,37128	1
Zbtb44	-0,37138	1
Gm2367	-0,37161	1
Spata33	-0,37171	1
Plk4	-0,37187	1
Fam43a	-0,37196	1
Actl6a	-0,372	1
Gna13	-0,37196	1
Spen	-0,37206	1
Mef2d	-0,37212	1
Slc25a22	-0,37226	1
Arl5b	-0,37241	1
Pex2	-0,37254	1
Zscan22	-0,37257	1
Uty	-0,37274	1
Ubqln2	-0,37277	1
Ryk	-0,37276	1
Agrn	-0,37299	1
Zfp414	-0,37323	1
Fam117a	-0,37321	1
Gatad2b	-0,37318	1
Peli1	-0,37338	1
Zmynd10	-0,37347	1
Atf6	-0,3735	1
Slc25a10	-0,37359	1
Cpne3	-0,37357	1
Mybl1	-0,37368	1
Macf1	-0,37383	1
Gak	-0,37418	1
Snx27	-0,37451	1
Phc2	-0,37484	1
Slc35d2	-0,37486	1
C330007P06Rik	-0,37487	1
Ercc3	-0,37499	1
Ints7	-0,37533	1
Arhgap17	-0,37557	1
Lin9	-0,37604	1
Lilrb4a	-0,37619	1
Dsn1	-0,37666	1
Stx6	-0,37684	1
Slc2a4rg-ps	-0,37693	1
E130208F15Rik	-0,37693	1
Uck1	-0,37701	1
Slc35a3	-0,37714	1
Plekha2	-0,37709	1
Lif	-0,3773	1
Tank	-0,3774	1
Selenof	-0,3775	1
Fbxo42	-0,3775	1

Tanc2	-0,37756	1
B4galt3	-0,37767	1
Rpl10a	-0,37785	1
Snapin	-0,3782	1
Cep170	-0,3783	1
Pank4	-0,37852	1
Chd4	-0,37861	1
Zfhx4	-0,3787	1
Snrpf	-0,37893	1
Gm7984	-0,37905	1
Usp24	-0,37923	1
Hes7	-0,37939	1
Cnot7	-0,37944	1
Churc1	-0,37951	1
Terf1	-0,37947	1
Ska1	-0,37967	1
Ubn2	-0,38023	1
Casp2	-0,38034	1
AW549877	-0,38035	1
Traip	-0,38046	1
Khsrp	-0,38045	1
Npepps	-0,38071	1
Zfp26	-0,38079	1
D730045B01Rik	-0,38094	1
Gm9320	-0,38115	1
Pim3	-0,38125	1
Gm37566	-0,38142	1
Rai1	-0,38141	1
Zbtb48	-0,38149	1
Ercc1	-0,38147	1
Vrk1	-0,38146	1
Rasal1	-0,38163	1
Srsf4	-0,38161	1
Ndufc2	-0,38167	1
Pmpca	-0,38178	1
Cep131	-0,38255	1
Urb1	-0,38249	1
Tmem41b	-0,38253	1
Zbtb26	-0,38269	1
Plcb3	-0,38276	1
Gm38335	-0,38321	1
Metap1d	-0,38319	1
Fus	-0,38352	1
Trip12	-0,38352	1
Pknox1	-0,38378	1
Rin3	-0,38382	1
Vps4b	-0,38392	1
Pum1	-0,38386	1
Ggh	-0,38433	1
Pcgf3	-0,38463	1
Pdpk1	-0,38461	1
Mon1a	-0,38517	1

Cnot1	-0,38515	1
Rbm12b1	-0,38533	1
Pmm2	-0,38541	1
Cd300c2	-0,38544	1
Cebpg	-0,38575	1
Mphosph6	-0,3858	1
Fam53c	-0,38586	1
Cybb	-0,38594	1
Diaph3	-0,3862	1
Gm42640	-0,38633	1
Cask	-0,38639	1
Zfp182	-0,38648	1
Gm37893	-0,38654	1
Mdn1	-0,38663	1
Acsl1	-0,38668	1
Csnk1g2	-0,38685	1
Srsf2	-0,38696	1
Gm45884	-0,38712	1
Mcm7	-0,38726	1
Gm11772	-0,38754	1
Gm12240	-0,38748	1
Kantr	-0,38773	1
Tmem8	-0,38825	1
Fas	-0,38845	1
Irak1	-0,3886	1
Nhlrc3	-0,3889	1
Cdc23	-0,38925	1
Tm9sf3	-0,38917	1
Gtf3c1	-0,38926	1
Mfsd10	-0,38941	1
Hcfc1	-0,38949	1
Crlf3	-0,38959	1
Agmo	-0,38987	1
Gm12716	-0,38996	1
Nol4l	-0,39001	1
Gm10923	-0,39011	1
Gm32175	-0,39025	1
Chmp4b	-0,39017	1
Plekhh3	-0,39036	1
Xpo1	-0,39067	1
Cdc14a	-0,39097	1
Atp11c	-0,39103	1
Rcor1	-0,39122	1
Ddx20	-0,3913	1
Gm6640	-0,39135	1
1700017B05Rik	-0,39178	1
S100pbp	-0,39231	1
Pbk	-0,39254	1
Smc2	-0,39273	1
Pou6f1	-0,39283	1
Xpot	-0,39289	1
Dip2a	-0,39299	1

Adck2	-0,39301	1
Gm10499	-0,39319	1
Gm9143	-0,39332	1
Rab31	-0,39336	1
Acss2	-0,39443	1
Orai2	-0,39466	1
Cry1	-0,39477	1
Atp13a1	-0,39494	1
Dbt	-0,39513	1
Cbl	-0,39555	1
Kdsr	-0,39569	1
Flna	-0,39577	1
Wac	-0,39587	1
Pcnt	-0,39607	1
Lgals4	-0,39623	1
Ubp1	-0,39617	1
Snord35a	-0,39628	1
Aurka	-0,39631	1
Ube2s	-0,39688	1
Gm2011	-0,39713	1
Smchd1	-0,39722	1
Ccdc136	-0,3973	1
Cenpn	-0,3973	1
Gm45342	-0,39734	1
Kdm7a	-0,39735	1
Cysltr1	-0,39736	1
Lnpep	-0,39752	1
Med15	-0,39803	1
Nup153	-0,3982	1
Gm20274	-0,3987	1
Srrt	-0,39882	1
Proser1	-0,39876	1
Jag1	-0,39907	1
Erbb3	-0,39927	1
Zc3h3	-0,3993	1
Ep300	-0,39928	1
Ppp5c	-0,39946	1
Enkd1	-0,39957	1
Amigo3	-0,39961	1
Kmt2c	-0,39966	1
Polr2h	-0,39981	1
Spc25	-0,39996	1
Baat	-0,40012	1
Rbmx	-0,40071	1
Iqcf1	-0,40076	1
Pikfyve	-0,40077	1
Casp7	-0,40086	1
Pdpr	-0,40098	1
Anapc1	-0,40101	1
Phxr4	-0,40105	1
Slc17a9	-0,40134	1
Taf5	-0,40145	1

Ankdd1a	-0,4016	1
Vps33b	-0,40178	1
Huwe1	-0,40204	1
Nr4a2	-0,40222	1
Tmem38b	-0,40254	1
Rpl39	-0,40262	1
Fam126b	-0,40275	1
Pdgfb	-0,40297	1
Mrpl38	-0,40324	1
Gda	-0,40331	1
Chml	-0,40341	1
Gm17494	-0,40359	1
C1qtnf6	-0,40375	1
Vps13d	-0,40372	1
Trappc6b	-0,40378	1
Dck	-0,40399	1
Fli1	-0,40406	1
Herc3	-0,40417	1
Etv3	-0,4042	1
Cars2	-0,40436	1
Cacna1d	-0,40482	1
Polr2a	-0,40476	1
Mtmr11	-0,40506	1
Rnf38	-0,40509	1
Gm11604	-0,40519	1
Dcaf7	-0,40518	1
Rnf167	-0,40534	1
Ddx39b	-0,40545	1
Mau2	-0,40547	1
Rbms1	-0,40561	1
Plgrkt	-0,40565	1
Kbtbd7	-0,40584	1
Zfp281	-0,40589	1
Atad2b	-0,40599	1
Fbrsl1	-0,40655	1
Ltn1	-0,40658	1
Rpap1	-0,40709	1
Eif4a3	-0,40711	1
Gm42856	-0,40718	1
Cdip1	-0,40726	1
Taf6l	-0,40752	1
Helb	-0,4075	1
Birc6	-0,40756	1
Gm38157	-0,40766	1
Gm26890	-0,40793	1
Sp4	-0,40787	1
Zfp280d	-0,40785	1
Chd7	-0,40788	1
Piezo1	-0,40787	1
Pts	-0,40829	1
Mdc1	-0,40833	1
Hells	-0,4083	1

Tbc1d22b	-0,40849	1
Ccnt2	-0,40854	1
Nedd1	-0,40869	1
Bend4	-0,40871	1
Trmt13	-0,40882	1
Hist1h4n	-0,40885	1
Brca2	-0,40948	1
Anxa1	-0,40968	1
Mysm1	-0,40969	1
Rps6ka4	-0,41004	1
Rbm6	-0,41	1
Usp8	-0,4101	1
Tnks	-0,41011	1
Naa20	-0,41034	1
Cnot6l	-0,41035	1
Ppp2r3a	-0,41052	1
Zfp943	-0,41047	1
Gm43793	-0,41062	1
Haus4	-0,41058	1
Cdca2	-0,41073	1
Gm18284	-0,41069	1
Ddx19b	-0,41092	1
Ube2j1	-0,41088	1
Mfsd8	-0,411	1
Heatr5a	-0,41109	1
Gm8093	-0,41119	1
Gm13815	-0,4113	1
Racgap1	-0,41143	1
Uba6	-0,4117	1
Ddit3	-0,41187	1
Pak4	-0,41216	1
RP24-282C4.10	-0,41225	1
Med24	-0,41248	1
Etfa	-0,41268	1
Mgrn1	-0,41279	1
Cep170b	-0,4128	1
Tfr2	-0,41321	1
Acvr1b	-0,41327	1
Enc1	-0,41333	1
Col20a1	-0,41366	1
Snx13	-0,41371	1
Ipo11	-0,41366	1
2210406H18Rik	-0,41375	1
Recql	-0,41375	1
Cdc42ep3	-0,41414	1
Gm17541	-0,41417	1
Fbl	-0,41421	1
Nsd3	-0,41425	1
Gramd1b	-0,41433	1
Slc35e2	-0,41485	1
Nectin1	-0,41476	1
Eps15l1	-0,41497	1

Kctd20	-0,41513	1
Btaf1	-0,41523	1
9430060I03Rik	-0,41544	1
Cbwd1	-0,4155	1
Ddhd1	-0,41592	1
Rbbp7	-0,41604	1
AW047730	-0,41607	1
Pdia6	-0,41612	1
Kif23	-0,41618	1
Gm24339	-0,41633	1
Gm6136	-0,41631	1
Cbx2	-0,41629	1
Nfia	-0,41633	1
RP23-453B15.7	-0,41643	1
Slbp	-0,41648	1
Bud13	-0,41648	1
Gm12582	-0,41673	1
Gm5601	-0,41712	1
Atpaf1	-0,41738	1
Nfya	-0,41784	1
Ctnnal1	-0,41829	1
Mut	-0,41833	1
Mb21d1	-0,41827	1
Zbed5	-0,41864	1
Ctns	-0,41867	1
Vrk2	-0,41877	1
Cmtm7	-0,41899	1
Far1	-0,41899	1
Bmt2	-0,41901	1
Btf3	-0,41932	1
Slc35a5	-0,4196	1
Klc1	-0,41968	1
Gm2225	-0,41984	1
Kansl2	-0,41984	1
Myo9a	-0,4198	1
Plscr3	-0,42013	1
Dcaf17	-0,42017	1
Tarbp1	-0,42073	1
Zbed4	-0,42079	1
Dennd6b	-0,42162	1
Micall2	-0,42162	1
Dpm1	-0,42245	1
Prosc	-0,42241	1
C2cd3	-0,42261	1
Nup54	-0,42266	1
Gm43737	-0,42286	1
Tbk1	-0,42292	1
BC028528	-0,4231	1
Per3	-0,42343	1
Mark2	-0,42343	1
4833420G17Rik	-0,42339	1
Ankrd10	-0,42342	1

Tnfrsf12a	-0,42383	1
Gls2	-0,42392	1
Rbfox2	-0,42476	1
Mapk6	-0,42489	1
Prkar2a	-0,42504	1
Gm37474	-0,4251	1
Rmi2	-0,42517	1
Gm37033	-0,42532	1
Gm12005	-0,42555	1
Nufip1	-0,42562	1
Uba52	-0,42582	1
Hltf	-0,42594	1
Gm45033	-0,42605	1
Ncoa3	-0,42616	1
Arl6ip6	-0,42674	1
Tle4	-0,4268	1
Atp11b	-0,42695	1
Slc7a8	-0,42689	1
Gm37297	-0,4271	1
Mir142hg	-0,42752	1
Gm37702	-0,42767	1
Arhgap5	-0,42784	1
Rps20	-0,42783	1
Firre	-0,42812	1
Gsk3a	-0,4281	1
Dse	-0,42821	1
Tmem107	-0,4283	1
Pag1	-0,42845	1
Ift80	-0,42874	1
Gm4525	-0,42887	1
Kif2c	-0,42887	1
mt-Tp	-0,429	1
Gm44153	-0,42906	1
Cad	-0,42914	1
Smarcb1	-0,42922	1
Pofut1	-0,42963	1
Pan2	-0,42981	1
Lamtor2	-0,42975	1
Snx29	-0,42987	1
Pih1d2	-0,43001	1
Bcor	-0,43014	1
Rps6ka3	-0,43015	1
Anapc5	-0,43009	1
RP23-356P21.1	-0,4303	1
Gm23502	-0,43098	1
Tmem128	-0,43124	1
Rab2b	-0,43119	1
Ighm	-0,43124	1
Uros	-0,43145	1
Cbx5	-0,43164	1
Rccd1	-0,43169	1
Rngtt	-0,43197	1

Snhg20	-0,4321	1
Pank3	-0,43213	1
Spidr	-0,43222	1
Sbno2	-0,43247	1
0610030E20Rik	-0,43265	1
Spaca9	-0,43266	1
F11r	-0,43293	1
Slc38a9	-0,4329	1
4930563E22Rik	-0,43297	1
Crebzf	-0,43305	1
Homer1	-0,43323	1
C1galt1c1	-0,43333	1
Mospd3	-0,43333	1
Tial1	-0,43336	1
Tab2	-0,43336	1
Gm26520	-0,43346	1
Lrrc57	-0,43361	1
3110002H16Rik	-0,43362	1
Osgin1	-0,43381	1
Aak1	-0,43395	1
Ttc21b	-0,43416	1
Nab2	-0,43421	1
Secisbp2l	-0,43443	1
Msi2	-0,43493	1
Ahctf1	-0,43504	1
Zmiz2	-0,43508	1
Xkr8	-0,43561	1
B2m	-0,43558	1
Fam185a	-0,4357	1
Spice1	-0,43581	1
Zdhhc9	-0,43599	1
Atp8b4	-0,43613	1
Malat1	-0,43632	1
Ids	-0,43653	1
3830403N18Rik	-0,4371	1
Scrn2	-0,43721	1
E430021H15Rik	-0,43726	1
Ap2s1	-0,43752	1
Aasdh	-0,43756	1
Gm13886	-0,4378	1
Tsc22d4	-0,43777	1
Fam26f	-0,43797	1
Poc5	-0,43804	1
Jade3	-0,43818	1
Tmem51os1	-0,4382	1
Zfp385a	-0,43843	1
Ap2b1	-0,43843	1
Gm8268	-0,43862	1
Atg4a	-0,43865	1
C030013C21Rik	-0,43882	1
Gabpb1	-0,43879	1
Shcbp1	-0,43899	1

Gm15007	-0,439	1
Rc3h1	-0,43922	1
AI846148	-0,43938	1
Lims1	-0,43944	1
Patl1	-0,43944	1
Slc25a26	-0,43949	1
Mcts2	-0,4396	1
Tm2d1	-0,43966	1
Intu	-0,43981	1
C77080	-0,4399	1
Gm10240	-0,44004	1
Khnyn	-0,44011	1
Fcho2	-0,44011	1
Cdc14b	-0,44038	1
Serinc3	-0,44047	1
Mettl10	-0,44085	1
Pfkp	-0,44103	1
Mcf2l	-0,44145	1
Ncs1	-0,44136	1
Mllt6	-0,44155	1
Gm4866	-0,44182	1
Cdkl4	-0,44208	1
Man2b2	-0,44219	1
Asb13	-0,44224	1
Polr3f	-0,44271	1
Hsd17b7	-0,44272	1
Stat1	-0,44284	1
Seh1l	-0,44312	1
Chrnb1	-0,44335	1
Epn2	-0,44335	1
Actb	-0,44363	1
Gas7	-0,4437	1
Clk1	-0,44365	1
Gdpd1	-0,44384	1
Kifc5b	-0,44389	1
Tab3	-0,44415	1
Uba2	-0,4441	1
Jak3	-0,44432	1
Col7a1	-0,44464	1
Letm2	-0,44484	1
Slc36a1	-0,44477	1
Tollip	-0,44479	1
Cd14	-0,44484	1
Ncoa2	-0,44487	1
Tnk2	-0,44507	1
Plekha1	-0,44515	1
Actr6	-0,44545	1
Traf6	-0,44557	1
Tmem64	-0,44558	1
Lman2l	-0,44608	1
Abhd2	-0,44703	1
Pclaf	-0,44734	1

Mphosph9	-0,44763	1
Gm42463	-0,4479	1
Lgals3bp	-0,44802	1
Uhrf1bp1	-0,44806	1
Psma5	-0,44835	1
Spcs2-ps	-0,44868	1
Tiparp	-0,44878	1
Slc17a7	-0,4489	1
Gm6305	-0,44926	1
Hpgds	-0,44939	1
Rps12-ps10	-0,44947	1
Zwilch	-0,4499	1
Rps12-ps4	-0,44988	1
Wee1	-0,44988	1
Gm16253	-0,45021	1
Ctc1	-0,45037	1
Gm30074	-0,45045	1
Stx7	-0,45048	1
Skp2	-0,45059	1
Brd2	-0,45099	1
Pou2f2	-0,451	1
Lrrc41	-0,45173	1
Zmym5	-0,45168	1
Pik3cg	-0,45192	1
Slco4a1	-0,45292	1
Gm42876	-0,45329	1
Fam64a	-0,45352	1
Zfp11	-0,45345	1
Mis18a	-0,45374	1
Slx4ip	-0,45391	1
1700066M21Rik	-0,45399	1
M6pr	-0,45397	1
Gm25008	-0,45448	1
BC002059	-0,45492	1
Pcsk7	-0,4551	1
Scfd2	-0,45521	1
Mbd6	-0,45522	1
Gm44623	-0,45526	1
Foxj2	-0,45542	1
Cfap20	-0,45554	1
Fam129c	-0,45592	1
Trappc11	-0,45608	1
Atp11a	-0,45617	1
Zbtb33	-0,45631	1
Rab28	-0,45629	1
Socs4	-0,4563	1
Gnl3l	-0,45631	1
Lrrc1	-0,45649	1
Ccdc84	-0,45652	1
Gm996	-0,45647	1
D830025C05Rik	-0,45664	1
Gm26656	-0,45671	1

Dhdh	-0,45673	1
Gm38257	-0,4568	1
Chst14	-0,45692	1
Slc30a7	-0,45693	1
Fignl1	-0,45701	1
Ptpdc1	-0,45717	1
Rpl31-ps8	-0,45739	1
Gm26226	-0,45752	1
Ccnc	-0,45782	1
Snx25	-0,45787	1
Pds5a	-0,45798	1
Zmiz1	-0,45907	1
Dnajc13	-0,45914	1
Slc5a3	-0,45933	1
Gm9134	-0,45957	1
Nampt	-0,45976	1
Lcp2	-0,45992	1
Gm28187	-0,46011	1
Gm16181	-0,46009	1
Gm28809	-0,4604	1
Palm	-0,46064	1
Smg6	-0,46061	1
Ern1	-0,4607	1
Rps2-ps10	-0,46093	1
Tpcn2	-0,46095	1
2410080I02Rik	-0,46144	1
Mogat1	-0,46145	1
Lyst	-0,46159	1
Cep57l1	-0,46172	1
Cdc7	-0,46176	1
Gm26982	-0,46235	1
Fam72a	-0,46303	1
Mxd3	-0,46296	1
Rnf6	-0,46323	1
Mtf2	-0,46332	1
Dock10	-0,46341	1
Med27	-0,46355	1
Lamp2	-0,46348	1
Copg2	-0,46383	1
Pde4dip	-0,46387	1
Gm43247	-0,46392	1
Gm42508	-0,46445	1
Fmr1	-0,46474	1
Oscar	-0,46493	1
Kif11	-0,46497	1
Phip	-0,46496	1
Blm	-0,46514	1
Gm2000	-0,46529	1
Cdk8	-0,46542	1
Tsga10	-0,4655	1
Ptk2b	-0,46656	1
Katnb1	-0,4667	1

Rbm47	-0,46723	1
Srrm2	-0,46723	1
Golga1	-0,46734	1
Cdc42bpb	-0,46735	1
Cntrl	-0,46809	1
Gls	-0,46811	1
Slc4a11	-0,46823	1
Leng8	-0,46843	1
Arpc3	-0,4685	1
Lmtk3	-0,46861	1
4732491K20Rik	-0,46863	1
Zfp219	-0,4686	1
Gm8909	-0,46858	1
Pmpcb	-0,46889	1
Dnajc22	-0,46897	1
Fubp1	-0,46912	1
Btk	-0,46916	1
Garnl3	-0,46922	1
Eno1	-0,46929	1
Lypla1	-0,46934	1
Ago2	-0,46932	1
Nbeal1	-0,46935	1
Tnrc18	-0,46944	1
Tor1aip1	-0,46959	1
3110056K07Rik	-0,4698	1
F830115B05Rik	-0,46987	1
RP24-366E11.4	-0,47003	1
Scd2	-0,47012	1
Gm44269	-0,47023	1
Pcgf2	-0,47027	1
Snord71	-0,47027	1
Extl1	-0,47077	1
Fam84b	-0,47101	1
Timm21	-0,47108	1
Pum2	-0,47113	1
Mtbp	-0,47115	1
Anxa2	-0,47116	1
Hmox1	-0,47127	1
Rhoa	-0,47152	1
Tmem87a	-0,47156	1
Ifitm5	-0,47172	1
Ncstn	-0,47175	1
Ipo9	-0,47192	1
RP23-128C4.4	-0,47219	1
P2rx7	-0,47236	1
Cdk2	-0,47251	1
Clcn3	-0,47275	1
Fendrr	-0,47291	1
Arid2	-0,473	1
Fam102b	-0,47314	1
Sde2	-0,47319	1
A730011C13Rik	-0,47327	1

Abca1	-0,47347	1
1600012H06Rik	-0,47375	1
Ilf2	-0,47368	1
2810029C07Rik	-0,47372	1
Lamc1	-0,47378	1
Slc16a5	-0,47425	1
Xiap	-0,47451	1
Tns2	-0,47451	1
Asph	-0,47503	1
Camk1d	-0,47537	1
Gm25541	-0,47548	1
Gm5787	-0,47551	1
Fbxo8	-0,476	1
Gm4987	-0,47621	1
Gm43430	-0,47616	1
Mgat4b	-0,47653	1
Usp19	-0,47658	1
Gm16580	-0,47674	1
Gm37959	-0,47679	1
Dopey1	-0,4769	1
Lactb2	-0,47734	1
Gm4430	-0,47727	1
Vmp1	-0,47798	1
R3hdm1	-0,47814	1
Adam15	-0,4782	1
Ublcp1	-0,47848	1
Manba	-0,47875	1
Srsf6	-0,479	1
Hmcn2	-0,47897	1
Wdr19	-0,47914	1
Med6	-0,47913	1
Nat6	-0,47923	1
Gfod1	-0,47916	1
Nr2c2	-0,47935	1
Gm15501	-0,48007	1
Pigw	-0,48035	1
Ncapg2	-0,48047	1
Nr1h2	-0,48062	1
Ap1ar	-0,48091	1
Gm15832	-0,48122	1
Ubxn11	-0,48201	1
Hoxa5	-0,48222	1
Zfp951	-0,48216	1
Gm11470	-0,48236	1
Fam71f2	-0,48284	1
Sf3a2	-0,48294	1
Rwdd2b	-0,48294	1
Gm42479	-0,4829	1
4632427E13Rik	-0,48309	1
Rad51ap1	-0,48315	1
Pom121	-0,48373	1
Ccnl1	-0,4839	1

Txndc5	-0,48441	1
Gm43775	-0,48448	1
Fam188a	-0,48449	1
Ctxn1	-0,48452	1
Npy	-0,48449	1
Prpf39	-0,48472	1
Iqsec2	-0,48587	1
Farsa	-0,48589	1
Srebf1	-0,48598	1
Clcn5	-0,48612	1
2610020C07Rik	-0,48612	1
Gm44130	-0,48619	1
Ldlrad3	-0,4863	1
Gm17259	-0,48625	1
Slc30a5	-0,4865	1
Myl12a	-0,48652	1
Chuk	-0,48675	1
Gm43813	-0,48689	1
RP23-104D6.2	-0,48741	1
Lilr4b	-0,48752	1
Ifi47	-0,48749	1
Lbr	-0,48809	1
U2af2	-0,4883	1
Trim16	-0,48837	1
Bmi1	-0,48838	1
Sf3b3	-0,4886	1
Ttll3	-0,48878	1
RP23-325K4.10	-0,48891	1
Mob3b	-0,48902	1
Rab43	-0,48915	1
Pkd2	-0,4893	1
Zgrf1	-0,48925	1
Pin4	-0,48927	1
Pttg1	-0,48992	1
Trp53i13	-0,48999	1
Tubg1	-0,48997	1
Gfpt1	-0,49014	1
Smad6	-0,49031	1
Stac3	-0,49051	1
9930022D16Rik	-0,49059	1
Atp8b2	-0,49076	1
Rps16	-0,49132	1
Mxd1	-0,49141	1
Prpf40b	-0,49202	1
Gm6266	-0,49201	1
Sgcb	-0,49252	1
1700012D14Rik	-0,49262	1
Pms1	-0,49266	1
Gm15728	-0,49279	1
Gm38220	-0,49283	1
Slc4a7	-0,49291	1
Mdm1	-0,49313	1

Gm25291	-0,4932	1
Prrc2b	-0,49356	1
Itga11	-0,49403	1
Sash1	-0,49415	1
Gm20699	-0,49406	1
Prkx	-0,49427	1
Usp37	-0,49487	1
Gm26620	-0,49499	1
Gltscr1l	-0,49515	1
Tbc1d31	-0,4952	1
Gga3	-0,49544	1
Rnf128	-0,49547	1
Slc12a6	-0,49559	1
Lpin2	-0,49579	1
Neurl4	-0,496	1
Lix1l	-0,49596	1
Ftx	-0,49621	1
Akap8l	-0,49649	1
Ifnar1	-0,49658	1
Xrn1	-0,49759	1
Slc23a2	-0,49776	1
Soga1	-0,49805	1
D030028A08Rik	-0,49832	1
Tia1	-0,49852	1
Dnajc10	-0,4989	1
Gm38297	-0,49899	1
Zfp160	-0,49922	1
Fzd7	-0,49925	1
Gm7860	-0,49938	1
Efcab7	-0,49946	1
Mcm4	-0,49958	1
Ly9	-0,49964	1
Wdr11	-0,49999	1
Zdhhc8	-0,50013	1
Snora73b	-0,50012	1
Gm43571	-0,50016	1
Tfeb	-0,50058	1
Edrf1	-0,50061	1
Ikzf1	-0,50105	1
Tmem80	-0,50105	1
Slc25a14	-0,50108	1
Hnrnpu	-0,50119	1
Tapbp	-0,50116	1
Xrcc4	-0,50128	1
Gas6	-0,50174	1
1190002N15Rik	-0,5019	1
Rpl13a-ps1	-0,50214	1
RP23-70B19.5	-0,50243	1
Adpgk	-0,5025	1
Gnaq	-0,50268	1
Cldnd1	-0,50304	1
Gm6946	-0,50313	1

Zc3h18	-0,5032	1
Pigh	-0,50346	1
Fam117b	-0,50384	1
Kcnc3	-0,50379	1
Fanca	-0,50397	1
Dtd2	-0,50424	1
Rps12-ps9	-0,50418	1
Edem3	-0,50433	1
4930578M07Rik	-0,50434	1
Mroh1	-0,50459	1
Rps13	-0,50482	1
Ddit4	-0,5052	1
Pus7	-0,50549	1
Sh3bp2	-0,50562	1
Arhgef11	-0,50598	1
Rpl12-ps1	-0,50605	1
Unc93b1	-0,50648	1
Hyls1	-0,50681	1
Gm7967	-0,50703	1
Slc6a6	-0,50721	1
Rasa4	-0,50733	1
Zfp445	-0,50803	1
Gm7117	-0,50797	1
Taok1	-0,5085	1
Xrcc2	-0,50864	1
Tbc1d8b	-0,50899	1
Col11a2	-0,50927	1
Arfgap1	-0,5094	1
Fancd2	-0,51022	1
Gm38162	-0,51037	1
Hdhd2	-0,51053	1
Wdr90	-0,51054	1
Rrbp1	-0,5107	1
Gnai3	-0,51084	1
Kmt2d	-0,51144	1
Jmjd1c	-0,51144	1
Dnajb1	-0,51154	1
Gm7488	-0,51192	1
Eif4g2	-0,51259	1
Ly86	-0,51306	1
E130308A19Rik	-0,51319	1
Gm38376	-0,51327	1
Gm26202	-0,51368	1
Spcs3	-0,51397	1
Slc25a40	-0,51421	1
Dynlt1b	-0,51452	1
H2afz	-0,51499	1
Pvr	-0,51502	1
Tlcd1	-0,515	1
Gm12799	-0,51501	1
Clasp1	-0,51516	1
Gm37116	-0,51517	1

Snord82	-0,51541	1
Cuedc1	-0,5156	1
2010016I18Rik	-0,51564	1
Gm37349	-0,51673	1
Tmem150a	-0,51671	1
Tpt1	-0,51684	1
Hus1	-0,51711	1
Alg6	-0,51772	1
Tmem109	-0,5186	1
Usp21	-0,51869	1
Gar1	-0,51894	1
Aatk	-0,51956	1
Arrdc3	-0,52002	1
Nisch	-0,52083	1
Gm26799	-0,52096	1
Sars2	-0,52108	1
Ssh3	-0,52122	1
H2-T22	-0,52146	1
Tubgcp2	-0,5215	1
Kat2b	-0,52163	1
C920009B18Rik	-0,52192	1
Fanci	-0,52185	1
AI606181	-0,52204	1
B230317F23Rik	-0,52226	1
Abhd10	-0,52257	1
Hmbox1	-0,52269	1
Gm12355	-0,52349	1
App	-0,52382	1
Numa1	-0,52396	1
Med30	-0,52407	1
Usp22	-0,5245	1
Vma21-ps	-0,52453	1
Gtdc1	-0,52503	1
Ccnyl1	-0,52499	1
Tspyl3	-0,52508	1
Faap100	-0,52529	1
Rpl32	-0,52556	1
Gm45495	-0,52556	1
Gm6501	-0,52564	1
B130034C11Rik	-0,52573	1
Pou2f1	-0,52592	1
2410002F23Rik	-0,52662	1
Ssbp4	-0,5271	1
Srrm1	-0,52743	1
Gt(ROSA)26Sor	-0,52774	1
Fnip1	-0,52821	1
Nfrkb	-0,52835	1
Carmil1	-0,52831	1
Ppp1r18os	-0,5286	1
Meiob	-0,52889	1
Ndor1	-0,52903	1
Tlr3	-0,53001	1

Gm45445	-0,53021	1
Aunip	-0,53031	1
Rfwd3	-0,53053	1
Prkdc	-0,53064	1
Phtf1os	-0,53126	1
Gm29340	-0,53185	1
Gm37065	-0,53204	1
Tra2a	-0,53219	1
Sepsecs	-0,53233	1
Cnot3	-0,53237	1
2700038G22Rik	-0,53289	1
Ino80d	-0,53344	1
Gm8430	-0,53371	1
L2hgdh	-0,53399	1
Hnrnph2	-0,53404	1
Fktn	-0,53407	1
Gm44116	-0,53426	1
Tmem259	-0,53456	1
Anpep	-0,53475	1
Acin1	-0,53493	1
2310035C23Rik	-0,53511	1
Kif4	-0,53593	1
Hnrnpm	-0,53605	1
Luc7l3	-0,53603	1
Pmf1	-0,53646	1
Thrap3	-0,53674	1
Fbxo21	-0,53669	1
Kri1	-0,53697	1
Zscan21	-0,53743	1
Gpr137b-ps	-0,53825	1
Ribc1	-0,53828	1
Add3	-0,53859	1
Gm12444	-0,53855	1
9430092D12Rik	-0,53902	1
Cmtm6	-0,54015	1
Gpr19	-0,54086	1
Setd1a	-0,54121	1
Zc3h7a	-0,54144	1
Lmf2	-0,5429	1
Kctd21	-0,54293	1
Kntc1	-0,54309	1
Rapgef3	-0,54331	1
Trrap	-0,54379	1
Mypop	-0,54402	1
Nufip2	-0,54431	1
Fsd1l	-0,54441	1
Oat	-0,54522	1
Zfp362	-0,54529	1
Gm44545	-0,54558	1
Git2	-0,54585	1
Rpl19	-0,5465	1
Oxsm	-0,54698	1

Dennd4a	-0,54718	1
Rnpep	-0,54759	1
Dr1	-0,54837	1
Rny3	-0,54838	1
Mtmr12	-0,54845	1
Gm37354	-0,54859	1
Ccdc163	-0,54913	1
4932438A13Rik	-0,54922	1
Gab3	-0,54919	1
Sesn1	-0,54945	1
Mettl17	-0,55014	1
Wdr76	-0,55086	1
Gm29666	-0,55091	1
Ubxn7	-0,55161	1
Arhgef40	-0,55167	1
9430034N14Rik	-0,55192	1
Med31	-0,55219	1
Gm4258	-0,55252	1
Gm42670	-0,55253	1
Cpsf1	-0,55281	1
Gm11448	-0,55284	1
Mapk9	-0,55306	1
Ddx17	-0,55334	1
Mcm5	-0,55406	1
Zfc3h1	-0,55421	1
Gm43328	-0,55448	1
Ptpn9	-0,55465	1
Ice1	-0,55492	1
Usp28	-0,55487	1
Cpsf6	-0,55496	1
4930520O04Rik	-0,55496	1
Bcl10	-0,5551	1
Iffo1	-0,55527	1
Zfp865	-0,55533	1
Wdr74	-0,55539	1
Aspm	-0,55592	1
Gm43672	-0,55601	1
Gm45413	-0,55666	1
Gm16523	-0,55715	1
Scarna2	-0,55725	1
Gm37696	-0,55724	1
Rps19-ps6	-0,55742	1
Gm45222	-0,55761	1
Htt	-0,55809	1
Gm37677	-0,55856	1
Actg1	-0,55876	1
Man1a	-0,55893	1
Acad11	-0,55887	1
1700020I14Rik	-0,55962	1
Gm26533	-0,55971	1
Ftl1	-0,56031	1
Ttc14	-0,56135	1

Eif2ak3	-0,56138	1
Thbs3	-0,56139	1
Gk	-0,56153	1
Tsnax	-0,5618	1
Nfkb1	-0,56192	1
Asb3	-0,56233	1
B230354K17Rik	-0,56259	1
Mum1	-0,56283	1
Szt2	-0,56322	1
Ovgp1	-0,56387	1
Safb	-0,56441	1
Cep135	-0,56439	1
Paxip1	-0,56448	1
Gm26129	-0,56487	1
Ttc39b	-0,56523	1
Grhl1	-0,56545	1
Mtr	-0,56547	1
Ppp1r37	-0,56556	1
Snapc3	-0,56581	1
Wdr92	-0,56587	1
Pot1b	-0,5659	1
Pabpn1	-0,56604	1
Yod1	-0,56641	1
Rictor	-0,5672	1
Sft2d1	-0,56801	1
Gm42715	-0,56827	1
Crocc	-0,56835	1
F730043M19Rik	-0,56848	1
Gipc2	-0,56852	1
Gm44510	-0,56854	1
Armc2	-0,56875	1
Sptbn4	-0,56914	1
Psd	-0,56926	1
Ube2i	-0,5695	1
1600029O15Rik	-0,56945	1
Tmcc1	-0,56972	1
Rpl28-ps1	-0,57048	1
Prps1l3	-0,57057	1
Rsl1d1	-0,57079	1
Gm42872	-0,57079	1
Gm10132	-0,57101	1
Gm17066	-0,57165	1
Asf1b	-0,57158	1
Ganc	-0,57165	1
BC048403	-0,5719	1
Rangrf	-0,57259	1
Mettl25	-0,57286	1
Phf1	-0,57308	1
S1pr1	-0,57326	1
Igip	-0,5739	1
Cabin1	-0,57454	1
Slc6a9	-0,57451	1

Ect2	-0,57466	1
Pfn1	-0,57501	1
Phtf1	-0,57518	1
Tmem241	-0,57532	1
Trpm7	-0,57603	1
Gm44250	-0,57598	1
Kat2a	-0,57654	1
Nup210	-0,57658	1
Fam135a	-0,57674	1
Rp2	-0,57681	1
Lmnb1	-0,57749	1
Ube2t	-0,57802	1
St3gal4	-0,57804	1
Rnf145	-0,57829	1
Zfp239	-0,57957	1
Dmxl1	-0,57991	1
Col27a1	-0,57997	1
Sp1	-0,5806	1
Hps3	-0,58061	1
5330426L24Rik	-0,58098	1
Dennd1c	-0,58147	1
Ankrd16	-0,5817	1
Dnaaf5	-0,58202	1
Mok	-0,58234	1
Gm42986	-0,58239	1
Trpm2	-0,58248	1
B230307C23Rik	-0,58255	1
Gm6526	-0,58262	1
Gm45167	-0,58293	1
Dennd4c	-0,58328	1
Gm12258	-0,58351	1
Rev1	-0,58363	1
Dsel	-0,58388	1
A130050O07Rik	-0,58403	1
Pde4b	-0,58406	1
BC049715	-0,58418	1
Gm12428	-0,5842	1
Fam46c	-0,58431	1
Rsrp1	-0,58441	1
Fam83d	-0,58499	1
Tbc1d25	-0,58509	1
Bub1	-0,58649	1
Gm42547	-0,58651	1
Mat2a	-0,58667	1
Gm12166	-0,58701	1
Dnm1	-0,58699	1
Prkcd	-0,58745	1
Crybb3	-0,58752	1
Ube2n	-0,58773	1
Spred2	-0,58788	1
Zfp652os	-0,58886	1
Ppip5k2	-0,58908	1

Nxt2	-0,58964	1
Fam13c	-0,58958	1
Mxi1	-0,58969	1
Calcoco1	-0,58997	1
Gm42783	-0,59	1
Exoc6	-0,59051	1
Man2a1	-0,5908	1
Cdk16	-0,59171	1
Ralgapa2	-0,59168	1
Ngly1	-0,59193	1
Prrc2a	-0,59195	1
Rad54l	-0,59252	1
C730045M19Rik	-0,59272	1
Rgs14	-0,59296	1
Atn1	-0,59304	1
Gm12165	-0,59308	1
Nf2	-0,59344	1
Rfwd2	-0,59367	1
Dnajb4	-0,59414	1
Eaf1	-0,59412	1
Phf19	-0,59423	1
Wdr47	-0,59426	1
Ets2	-0,59443	1
Mbnl1	-0,59504	1
H2-T10	-0,59505	1
Gm11363	-0,59534	1
E130309D02Rik	-0,59571	1
Cops7a	-0,59589	1
Gm44044	-0,59636	1
Gng8	-0,59641	1
Bivm	-0,59761	1
Sass6	-0,59757	1
Upk1a	-0,59765	1
Rad54b	-0,59768	1
Hdac8	-0,59786	1
Rtel1	-0,59787	1
Epb41	-0,59814	1
D930016D06Rik	-0,59875	1
Ier3	-0,59895	1
Gm37978	-0,59988	1
Gm7561	-0,60001	1
RP24-233B16.6	-0,59995	1
4930578M01Rik	-0,60022	1
Pde7a	-0,60067	1
Dnmbp	-0,60069	1
Olfr920	-0,60109	1
Hist1h2aa	-0,60155	1
Gm43213	-0,60177	1
Arl6	-0,60196	1
Zfp770	-0,60209	1
RP24-550H10.3	-0,60226	1
Ccdc57	-0,60264	1

Gm11945	-0,60266	1
A830008E24Rik	-0,60276	1
Rnf170	-0,60306	1
Scrib	-0,60362	1
Gm6245	-0,60431	1
Ptprj	-0,60449	1
RP23-213P10.2	-0,60452	1
Cx3cr1	-0,6052	1
Rnf5	-0,60519	1
Klhl30	-0,60558	1
Arfgef3	-0,6059	1
Zfp69	-0,60634	1
Pitrm1	-0,60642	1
Aoc2	-0,60735	1
Got2-ps1	-0,60812	1
Gm44667	-0,60816	1
Gm12248	-0,6086	1
Phkb	-0,60896	1
Gm9825	-0,60899	1
Snord87	-0,6092	1
Etv1	-0,60943	1
Gm44652	-0,60954	1
Dock7	-0,61001	1
Arhgef10	-0,61001	1
Kcnb1	-0,61005	1
Gm44198	-0,6101	1
Sbf1	-0,61018	1
Xylb	-0,61021	1
Slc20a1	-0,61125	1
Tmem39b	-0,61124	1
Nup98	-0,61183	1
Tmem240	-0,61223	1
RP23-359K10.8	-0,6124	1
Itpka	-0,6126	1
Luc7l2	-0,61273	1
Matn1	-0,61367	1
Gm44552	-0,61391	1
Ccdc116	-0,61399	1
Zfp449	-0,61445	1
Cep85	-0,61451	1
Fam120a	-0,6146	1
Slc39a14	-0,61573	1
Dleu2	-0,6159	1
Tctn1	-0,61594	1
Rad21	-0,61648	1
Dlgap5	-0,61663	1
Pabpc1	-0,61665	1
Gm37101	-0,61694	1
Tbc1d19	-0,61711	1
Gm43692	-0,61739	1
Papd4	-0,61753	1
Top2a	-0,61763	1

Ccdc92	-0,6177	1
Cers2	-0,61782	1
Nktr	-0,6178	1
Atp1a3	-0,61828	1
Pde4d	-0,61828	1
Gm21975	-0,6184	1
Trmt112	-0,61882	1
Taf2	-0,61904	1
Gm36964	-0,62068	1
Gm43387	-0,62083	1
Celf2	-0,62089	1
Hmgb1-rs16	-0,621	1
Dtl	-0,62126	1
Wdr20	-0,62132	1
Gm26652	-0,62129	1
2810454H06Rik	-0,62248	1
Fam131a	-0,62253	1
Grik5	-0,62384	1
Gm13181	-0,62381	1
BC065397	-0,62415	1
Gm14633	-0,62473	1
Gfm1	-0,62483	1
Gm3650	-0,62497	1
Rpl31-ps13	-0,62604	1
Map3k3	-0,62612	1
1700001P01Rik	-0,6262	1
Thg1l	-0,62637	1
Evi5l	-0,62735	1
Ythdf3	-0,62799	1
Gm8667	-0,62803	1
Peak1	-0,62811	1
E330034L11Rik	-0,6291	1
Gm13413	-0,62916	1
Nod2	-0,62951	1
Lhx1	-0,62998	1
Tmed8	-0,63006	1
Jade2	-0,63008	1
Kctd13	-0,63018	1
Dram1	-0,63026	1
Spred3	-0,6303	1
Gm16536	-0,631	1
Ralgapa1	-0,63124	1
Tnnt3	-0,63119	1
G6pd2	-0,63124	1
Tmem94	-0,63135	1
Smarcad1	-0,63161	1
Rbm4b	-0,63171	1
Hsd17b14	-0,63307	1
Kcnd1	-0,63329	1
Trmt1l	-0,63344	1
Ceacam1	-0,63354	1
Gm13840	-0,63361	1

Smurf2	-0,63367	1
Tmem170b	-0,63397	1
Gnat2	-0,63427	1
Kif24	-0,63458	1
Plxnd1	-0,63526	1
Cdan1	-0,63537	1
Gm18867	-0,63552	1
Treml1	-0,63557	1
Dtwd2	-0,63583	1
Gm26549	-0,63607	1
Gm42480	-0,63616	1
Gigyf1	-0,63646	1
Zfp266	-0,6367	1
Matn4	-0,63666	1
Prpf8	-0,6372	1
Gm17455	-0,6376	1
Ncapd3	-0,63769	1
RP23-168F21.4	-0,63778	1
Rara	-0,63797	1
Dnmt3l	-0,63832	1
Lrif1	-0,63916	1
Zfyve26	-0,63941	1
Nup93	-0,64014	1
Gm44168	-0,6404	1
Zfp367	-0,64062	1
Gm24876	-0,64185	1
mt-Tc	-0,64193	1
Zw10	-0,64209	1
Snord89	-0,6422	1
Gm11722	-0,64223	1
Tctn2	-0,64244	1
Apaf1	-0,6426	1
Eml2	-0,64265	1
Zik1	-0,64266	1
2210408I21Rik	-0,64271	1
Lgals2	-0,64309	1
Rflnb	-0,64316	1
Gm38067	-0,64387	1
Trmo	-0,64397	1
RP23-440I21.3	-0,64409	1
Card14	-0,64432	1
Gm20667	-0,64468	1
9330151L19Rik	-0,64486	1
Mirlet7b	-0,64557	1
Gm43581	-0,64635	1
Slc25a36	-0,6466	1
Nat14	-0,6466	1
Prkci	-0,64704	1
Dido1	-0,64714	1
2810403A07Rik	-0,64722	1
AW554918	-0,64768	1
Hmgb1-ps6	-0,64772	1

Kif15	-0,6478	1
Pdcl3	-0,64802	1
Snhg11	-0,64811	1
Ocrl	-0,64823	1
Srcap	-0,64884	1
4933439C10Rik	-0,649	1
Zfp58	-0,64926	1
Lmbrd2	-0,64957	1
Gm7638	-0,64961	1
Egfl7	-0,64969	1
C330011M18Rik	-0,64992	1
Gm42595	-0,65109	1
Gm43794	-0,65142	1
Stard9	-0,65161	1
A930001C03Rik	-0,65198	1
Gm42549	-0,65218	1
Pex1	-0,65263	1
A630001G21Rik	-0,65264	1
Gm38062	-0,65258	1
Taok2	-0,65312	1
A830080D01Rik	-0,65338	1
Gm37642	-0,6538	1
Gm42511	-0,65467	1
4930589L23Rik	-0,65495	1
RP24-282C4.4	-0,65501	1
Golga5	-0,65615	1
Itpr3	-0,65657	1
Tet2	-0,65733	1
Gm13864	-0,65755	1
BC030867	-0,65768	1
Cenpf	-0,65784	1
Rnf123	-0,65877	1
Kcnq1ot1	-0,65924	1
Gm8203	-0,65977	1
Alg8	-0,66025	1
Gm43294	-0,66016	1
Itga4	-0,66075	1
Tpmt	-0,66082	1
Gm44699	-0,66102	1
Gm37726	-0,66132	1
Zbtb34	-0,66156	1
Gm44951	-0,66173	1
Iqgap3	-0,66176	1
Rnf111	-0,66204	1
Donson	-0,6623	1
Sep 01	-0,6623	1
Gm10074	-0,66282	1
Gm42671	-0,66281	1
Bank1	-0,66284	1
Hspa8	-0,66305	1
Pfas	-0,66335	1
Vps13b	-0,66378	1

Phc3	-0,66438	1
B3galnt2	-0,66499	1
Pde6g	-0,66499	1
Gm42724	-0,66573	1
2900005J15Rik	-0,66608	1
Zbtb21	-0,66621	1
9030407P20Rik	-0,66641	1
Rad51c	-0,66648	1
Gm43588	-0,66663	1
Tpp2	-0,66673	1
Rnpc3	-0,66673	1
Cnksr1	-0,66677	1
Zfp280c	-0,66745	1
Gm42551	-0,6675	1
Lzts3	-0,66777	1
Gm22009	-0,66807	1
Mir124-2hg	-0,669	1
Gm9833	-0,67054	1
Elavl1	-0,67055	1
Rpl35a-ps4	-0,6712	1
Smg1	-0,67198	1
Rmnd5a	-0,67226	1
Sh2d6	-0,67236	1
Thada	-0,67384	1
6330403N20Rik	-0,67376	1
Cox7c	-0,67398	1
Spaca6	-0,67423	1
RP23-58B7.2	-0,67457	1
Zfp72	-0,67471	1
Pde4c	-0,67521	1
Nudt5	-0,67548	1
Hirip3	-0,67555	1
Pcid2	-0,67698	1
Mbtd1	-0,67727	1
Gm43868	-0,67726	1
Celf1	-0,67787	1
Zbtb46	-0,67816	1
Pycr1	-0,67822	1
2810433D01Rik	-0,67888	1
Mospd2	-0,67938	1
Hdac1	-0,67961	1
Msantd2	-0,67992	1
Gm44834	-0,67985	1
Rab19	-0,68006	1
Nyap1	-0,68047	1
Fam161b	-0,68052	1
Col4a5	-0,68122	1
Morf4l1	-0,68202	1
Gm37333	-0,68224	1
Txlnb	-0,68257	1
Arpin	-0,68287	1
Gm8624	-0,6829	1

Clock	-0,68406	1
1700052K11Rik	-0,68443	1
Moap1	-0,68483	1
Srebf2	-0,68502	1
Tnfaip3	-0,68507	1
Gm16437	-0,68512	1
Rnf144b	-0,68518	1
Ggcx	-0,68677	1
Dmrt2	-0,68692	1
Psma3	-0,68704	1
Abt1	-0,68735	1
Igf2r	-0,68817	1
Notch1	-0,68842	1
Cpsf7	-0,68854	1
Basp1	-0,68905	1
Dusp10	-0,68908	1
Bbs1	-0,68967	1
Zfp773	-0,6904	1
Gm37558	-0,69063	1
Dclre1b	-0,6911	1
Pcgf6	-0,69121	1
A430105I19Rik	-0,69117	1
Smg7	-0,69144	1
Nckap5l	-0,69197	1
Pnpla6	-0,6924	1
Mir99ahg	-0,69362	1
Gm23935	-0,69453	1
RP23-356D13.11	-0,69452	1
Pif1	-0,69467	1
Acrbp	-0,69487	1
Hacd2	-0,6949	1
Scn11a	-0,69493	1
Gm20257	-0,69495	1
Nphp3	-0,69525	1
Scai	-0,69562	1
Chd5	-0,69585	1
Gm12924	-0,69588	1
Ehd2	-0,69603	1
Foxk1	-0,69624	1
Tecpr2	-0,6963	1
Gm37621	-0,69635	1
Gm23722	-0,69666	1
Gm44509	-0,69666	1
Pik3r3	-0,69691	1
Rps19-ps11	-0,69751	1
Syne2	-0,69839	1
Melk	-0,69857	1
Mks1	-0,69875	1
Arhgef1	-0,69985	1
Sbf2	-0,7002	1
Gabpb2	-0,70016	1
Glt1d1	-0,70044	1

Phka2	-0,70364	1
Gm5830	-0,70363	1
Nudt8	-0,70385	1
Slc7a5	-0,70437	1
Cxcl2	-0,70444	1
Gm37472	-0,70461	1
Gm37219	-0,70498	1
Lrp1	-0,70563	1
Nup205	-0,70577	1
Pole2	-0,70602	1
Tesk2	-0,70616	1
Fam76b	-0,70668	1
Rpl28-ps3	-0,707	1
Gm19287	-0,70824	1
Tmtc3	-0,70924	1
Kdm6b	-0,71011	1
Strn	-0,71007	1
Gm38120	-0,71013	1
Gm5914	-0,7112	1
Icosl	-0,71141	1
Rusc2	-0,71178	1
Ascl2	-0,7128	1
Nav2	-0,71285	1
A630072M18Rik	-0,71342	1
Gtse1	-0,71355	1
Cks2	-0,71407	1
Fyco1	-0,7145	1
Slc38a2	-0,71472	1
Snord59a	-0,71509	1
Cd274	-0,7153	1
Rps6	-0,71538	1
Mcm2	-0,71547	1
Gm10131	-0,71575	1
Gm37569	-0,71607	1
Mrip-ps	-0,71692	1
Chek1	-0,71698	1
Elk4	-0,71766	1
Lclat1	-0,71896	1
Susd6	-0,71917	1
Acat1	-0,71922	1
Smc5	-0,71957	1
Agbl5	-0,72028	1
Gm14776	-0,7211	1
Cpne2	-0,72132	1
RP24-325P4.5	-0,72143	1
Snora30	-0,7217	1
Gm9442	-0,72187	1
Gm23100	-0,7221	1
Sipa1l3	-0,72264	1
Nemp1	-0,72267	1
Nr6a1	-0,72313	1
Abcc3	-0,72368	1

Ptcd3	-0,72433	1
Gm43144	-0,72447	1
5830408C22Rik	-0,72474	1
Tcf19	-0,72475	1
Slfn8	-0,72543	1
Dimt1	-0,72552	1
Taf15	-0,72624	1
Plekho2	-0,72619	1
Snrpd3	-0,72653	1
Pear1	-0,72663	1
Arntl	-0,72667	1
Slc27a3	-0,72665	1
Adgre5	-0,72909	1
RP24-93F20.12	-0,72922	1
Krit1	-0,72962	1
Snrpc	-0,73052	1
Gm38022	-0,7308	1
Hoxb5	-0,73147	1
Gm7846	-0,73325	1
Gm43544	-0,73521	1
6330562C20Rik	-0,73615	1
Rps6ka5	-0,73711	1
Gm42820	-0,73844	1
H2-Q4	-0,73927	1
Gm43773	-0,73927	1
Ldb1	-0,73942	1
Kmt5c	-0,73993	1
Gm15421	-0,74038	1
Abcc10	-0,74097	1
0610040B10Rik	-0,74118	1
Gatm	-0,7429	1
Gm43359	-0,74314	1
Xirp1	-0,74326	1
Gm15708	-0,74381	1
Tmem63b	-0,74395	1
Gm38043	-0,74443	1
Slc9a9	-0,74491	1
Dph2	-0,74498	1
Bicdl1	-0,74534	1
Tnfsf9	-0,74535	1
mt-Tl1	-0,74682	1
Acacb	-0,74679	1
Hnrnpa3	-0,74814	1
Gm16310	-0,74842	1
Ttpal	-0,74865	1
Gm38115	-0,74879	1
Gm37121	-0,74935	1
Ogt	-0,75022	1
Ints6l	-0,75029	1
Dcp1a	-0,75042	1
Srsf1	-0,7509	1
Gm37303	-0,75187	1

Bpnt1	-0,75218	1
Gm9769	-0,75368	1
Gm43290	-0,75392	1
Neat1	-0,7541	1
Snord49b	-0,75407	1
Pigt	-0,7545	1
Gm527	-0,75448	1
Tlr1	-0,75537	1
RP23-390D8.2	-0,75552	1
Gm38125	-0,7557	1
Slc9a8	-0,75652	1
Gm43696	-0,75657	1
Anln	-0,7578	1
5930420M18Rik	-0,75903	1
Gm26244	-0,75959	1
Gm20342	-0,76007	1
Arv1	-0,76101	1
Gm7336	-0,76167	1
Mtm1	-0,76268	1
Stom	-0,76293	1
Gm11759	-0,76289	1
Cep192	-0,76316	1
Mccc2	-0,76334	1
Gm12762	-0,76344	1
Tmppe	-0,7637	1
Fam122a	-0,76414	1
Gm15268	-0,76495	1
Btg1	-0,76569	1
Gm43147	-0,76575	1
Gm4963	-0,76641	1
Myef2	-0,76691	1
Gm44178	-0,76749	1
Xaf1	-0,76804	1
1700124L16Rik	-0,76839	1
mt-Ts2	-0,76856	1
2310015A10Rik	-0,76923	1
Etv4	-0,76945	1
Cmklr1	-0,76939	1
mt-Tq	-0,76957	1
Arc	-0,77063	1
Il17rc	-0,77095	1
Gm43788	-0,77103	1
Ints1	-0,77145	1
AI480526	-0,77244	1
Gm13223	-0,77248	1
Gm12906	-0,77261	1
Tfb2m	-0,77281	1
Ppp2cb	-0,77353	1
March4	-0,77415	1
Suv39h2	-0,77604	1
1700029J07Rik	-0,77612	1
Gm42659	-0,7763	1

Gm42869	-0,77664	1
Gm45133	-0,7768	1
Zfp712	-0,77764	1
Senp3	-0,77894	1
Cxcr3	-0,77902	1
Snora57	-0,77914	1
Spire2	-0,7794	1
Gm12663	-0,78064	1
Flywch1	-0,78077	1
Gm4285	-0,78118	1
Nek2	-0,78223	1
Cpne9	-0,78319	1
Epha2	-0,78342	1
Cchcr1	-0,78377	1
Tob1	-0,78422	1
Gm37914	-0,78419	1
Gm37900	-0,78472	1
Hist2h2ac	-0,78515	1
Gm15892	-0,78574	1
Prss42	-0,78802	1
Rbpms	-0,78833	1
Gm37795	-0,78884	1
C130089K02Rik	-0,78889	1
RP23-38L16.4	-0,78998	1
Gm27003	-0,79226	1
Hoxb6	-0,79296	1
Gm37660	-0,79319	1
Gm15289	-0,7962	1
D530018E20Rik	-0,79722	1
Gm44639	-0,79741	1
Mrps18b	-0,79752	1
Gm38055	-0,79751	1
Gm37420	-0,79801	1
Neil3	-0,79845	1
Myl6b	-0,79931	1
Gm45251	-0,79972	1
Ceacam16	-0,80032	1
Zfr2	-0,80041	1
Pus7l	-0,80061	1
Gm10093	-0,80076	1
Dock4	-0,8028	1
Rsl1	-0,80332	1
Irak3	-0,80389	1
Fmo5	-0,80472	1
Nova1	-0,80505	1
Gm5070	-0,80568	1
Hnrnpa1	-0,80617	1
Kansl1	-0,80645	1
Accs	-0,80639	1
Kdm4d	-0,80641	1
Snora17	-0,80663	1
Gm38235	-0,80656	1

Chil6	-0,80723	1
Gm4832	-0,8077	1
Gm43323	-0,8079	1
Eya3	-0,80817	1
Gm43795	-0,80891	1
D230022J07Rik	-0,80915	1
Neurl1b	-0,80941	1
2410089E03Rik	-0,80972	1
Adamts6	-0,81007	1
Gm43111	-0,81136	1
Gm19620	-0,81174	1
Gm42728	-0,81166	1
Rnf24	-0,81265	1
Gtf2h4	-0,81365	1
Prr7	-0,81372	1
Tmtc4	-0,81398	1
Gm25636	-0,81486	1
Gm5277	-0,81507	1
Osm	-0,81572	1
Lekr1	-0,81617	1
Gm37289	-0,81632	1
Gm37963	-0,81682	1
Rn7sk	-0,81857	1
Mtfr2	-0,8191	1
Tmem67	-0,8191	1
Pik3c2a	-0,81981	1
Col4a6	-0,82037	1
Gm37578	-0,82056	1
Tarbp2	-0,82156	1
Gm13196	-0,82233	1
Gm24916	-0,82253	1
Psenen	-0,82305	1
7330423F06Rik	-0,82356	1
Celf5	-0,82445	1
Scarna9	-0,82584	1
Gm14239	-0,82597	1
Syngap1	-0,82625	1
C130071C03Rik	-0,82633	1
Rap1gap	-0,82832	1
Gm13456	-0,82969	1
Maats1os	-0,82991	1
Top3b	-0,83007	1
Ppp2r3d	-0,83147	1
Gm23037	-0,83153	1
Creb3l1	-0,83225	1
Gm15513	-0,83245	1
Nlrp3	-0,83278	1
Ahrr	-0,83276	1
Nup88	-0,8333	1
Siah1b	-0,83344	1
Gm38399	-0,83413	1
Setdb1	-0,83421	1

Gm4607	-0,83432	1
Gm42481	-0,83475	1
Gm15796	-0,83526	1
Snhg6	-0,83571	1
D6Ertd527e	-0,83631	1
1810041H14Rik	-0,83673	1
Gm12522	-0,83748	1
Trpt1	-0,83764	1
Sgol2a	-0,83804	1
Gm15690	-0,83834	1
Rab3gap2	-0,83844	1
9230102O04Rik	-0,83843	1
Oas2	-0,8387	1
Gm37728	-0,83898	1
RP23-6C18.6	-0,83935	1
Knl1	-0,8397	1
Lncpint	-0,84004	1
G2e3	-0,84152	1
Hsd3b7	-0,84202	1
Gm37598	-0,8422	1
Ankrd52	-0,84234	1
Slc19a2	-0,84342	1
a	-0,84389	1
Gpcpd1	-0,84495	1
D330041H03Rik	-0,8464	1
Gm29539	-0,84708	1
Gm29170	-0,85041	1
Ptpru	-0,85048	1
Raf1	-0,8506	1
9130604C24Rik	-0,85142	1
Ptpn5	-0,85353	1
Zfp873	-0,85621	1
Dna2	-0,85874	1
Rpl10-ps3	-0,85869	1
Gm45890	-0,8591	1
Bc1-ps1	-0,85937	1
4930518I15Rik	-0,85973	1
Chac2	-0,86089	1
Helq	-0,86289	1
Gm43637	-0,86381	1
Carf	-0,86476	1
Aaed1	-0,86507	1
Gm43920	-0,86584	1
C530043K16Rik	-0,86603	1
Farp2	-0,86638	1
9630013D21Rik	-0,8666	1
Arhgef18	-0,86724	1
Gm17971	-0,86806	1
Gm12184	-0,86808	1
Mterf3	-0,86917	1
Rnf225	-0,86937	1
Gm42559	-0,87026	1

Apba1	-0,87072	1
N4bp2	-0,87254	1
Zfp169	-0,87251	1
Gm17034	-0,87246	1
Cep89	-0,87265	1
6030400A10Rik	-0,8737	1
Gtpbp2	-0,87454	1
Anxa9	-0,87477	1
Gm22516	-0,87506	1
Idi1	-0,87554	1
Gm43336	-0,87567	1
Rbm3	-0,87572	1
Rbm12b2	-0,87586	1
Gm37738	-0,87586	1
Phactr1	-0,87697	1
Gm37522	-0,87703	1
2810428J06Rik	-0,87734	1
Gnrh1	-0,87851	1
Vaultrc5	-0,87946	1
0610005C13Rik	-0,88012	1
Gm28438	-0,88009	1
Gm7206	-0,88063	1
Samd10	-0,8827	1
Gm43088	-0,88284	1
Ost4	-0,88321	1
Gm3531	-0,88332	1
Shprh	-0,88338	1
Marf1	-0,88376	1
Gm43300	-0,88643	1
Gm45828	-0,88646	1
Nfat5	-0,88698	1
Smarca5-ps	-0,88861	1
Dnmt3b	-0,88922	1
Usp35	-0,89076	1
RP24-282C4.9	-0,89129	1
Gm6919	-0,89248	1
Gm43360	-0,89333	1
Cracr2a	-0,89333	1
RP23-243B24.1	-0,89342	1
Gm43112	-0,89391	1
Pidd1	-0,89462	1
Rnd1	-0,89475	1
BC037039	-0,8965	1
Gm37968	-0,89711	1
Prkcg	-0,89718	1
Gm13350	-0,89748	1
Ncapd2	-0,89777	1
Trip10	-0,89836	1
Gm45902	-0,89968	1
Gm9920	-0,90343	1
Zscan26	-0,90397	1
Lpar2	-0,90451	1

Trim59	-0,90509	1
Ubc	-0,90531	1
Cit	-0,90533	1
Nt5c2	-0,90737	1
Gm28727	-0,90774	1
Gm43566	-0,90785	1
4930503L19Rik	-0,90787	1
Mfsd14a	-0,90787	1
Ppp1cc	-0,90864	1
Mir155hg	-0,9116	1
Cep55	-0,91177	1
Pcnx3	-0,91272	0,89908
Gm42743	-0,91354	1
Tdrd7	-0,91374	1
4930581F22Rik	-0,91496	1
Fam208a	-0,918	1
Gm37052	-0,91931	1
6820402A03Rik	-0,9196	1
Gm42566	-0,91957	1
Gm45630	-0,92016	1
Gm42941	-0,92127	1
Mtx3	-0,92342	1
Gm11625	-0,92457	1
Dffb	-0,92497	1
Sez6	-0,92764	1
Gm43774	-0,92783	1
Gm43062	-0,92787	1
Gm45501	-0,92818	1
Bard1	-0,92835	1
Gm43761	-0,92964	1
Piwil2	-0,93032	1
Gm45407	-0,93132	1
Gm37678	-0,93168	1
Gm38020	-0,93323	1
AW046200	-0,93394	1
RP24-240E7.1	-0,93484	1
Gm37183	-0,93617	1
Id1	-0,93646	1
Dhx9	-0,93799	1
Ptgs1	-0,93856	1
Gm15787	-0,93857	1
Hs3st3b1	-0,93878	1
Gm43343	-0,93897	1
Ppia	-0,93915	1
Gm10069	-0,94039	1
Snord55	-0,94072	1
Gm44567	-0,94092	1
Zdhhc13	-0,9411	1
Gm43011	-0,94124	1
Gm15445	-0,94176	1
Lrrc17	-0,94207	1
4632415L05Rik	-0,94225	1

Gm45809	-0,94318	1
Zbtb49	-0,94471	1
Gm44890	-0,94479	1
Catsperg1	-0,94485	1
Dgkz	-0,94488	1
Zfp248	-0,94514	1
Troap	-0,94597	1
Dnajb5	-0,94609	1
Gm17251	-0,94637	1
Uevld	-0,94697	1
Helz2	-0,94715	1
Efna2	-0,94899	1
CAAA01194877.2	-0,95022	1
Gm42611	-0,95036	1
Acot6	-0,95083	1
Arhgap19	-0,95101	1
Gm6524	-0,9511	1
Gm43660	-0,95142	1
Celsr1	-0,95245	1
Gm15779	-0,95258	1
Gm37060	-0,9527	1
Socs1	-0,95324	1
Plag1	-0,95363	1
Rbfox1	-0,95561	1
Dync2li1	-0,95584	1
Slc22a13b-ps	-0,9583	1
Ifit2	-0,9589	1
Ano8	-0,96217	1
Snord92	-0,96284	1
B230312C02Rik	-0,9636	1
Fat1	-0,96422	1
Pcdhb17	-0,9656	1
Gm14673	-0,96568	1
Gm43462	-0,96757	1
Gm7856	-0,96766	1
Gm42967	-0,96799	1
Slc25a42	-0,96919	1
Hace1	-0,96933	1
Gnb2	-0,97175	1
Adcy6	-0,9721	1
Apba3	-0,97223	1
Gm42482	-0,97216	1
Hspbap1	-0,97267	1
Gm44164	-0,97373	1
Gm6576	-0,97379	1
Gm21967	-0,97731	1
Gm37851	-0,9774	1
Gm26947	-0,97879	1
Gm44090	-0,97915	1
Gm37238	-0,98006	1
Bst2	-0,98237	1
9530078K11Rik	-0,98351	1

Fastkd1	-0,9841	1
Zfp473	-0,98479	1
Gm26497	-0,98499	1
Fbxo10	-0,98508	1
Rasd1	-0,9852	1
A630081D01Rik	-0,98593	1
Gm5841	-0,98657	1
Gm45728	-0,98715	1
1110020A21Rik	-0,98841	1
2900060B14Rik	-0,99145	1
Chst1	-0,99389	1
Tmigd3	-0,99456	1
Amt	-0,99528	1
Sema4a	-0,99686	0,86146
Gm36963	-0,99822	1
Gm21781	-0,99914	1
4930529C04Rik	-1,0016	1
Txnip	-1,0022	1
Glyctk	-1,0025	1
Rnaseh1	-1,0035	1
Gm43420	-1,0056	1
Gm45153	-1,0059	1
Ccdc14	-1,0064	1
Capn3	-1,0076	1
Gm42798	-1,0083	1
Rdh5	-1,0092	1
Gm43329	-1,0101	1
Hist1h2an	-1,0102	1
Pole	-1,0112	1
Gm10033	-1,0133	1
Isg20l2	-1,0136	1
Fsbp	-1,014	1
Tcea2	-1,0155	1
Gm7514	-1,0179	1
Crtc1	-1,018	1
Gm37407	-1,0189	1
Gm16223	-1,019	1
Gm43499	-1,0192	1
Krcc1	-1,0201	1
Gm37906	-1,0207	1
Atxn7l2	-1,0226	1
Abcg4	-1,0226	1
Eid3	-1,0246	1
Gm37465	-1,0249	1
Kcnj2	-1,0261	1
C230035I16Rik	-1,0262	1
Gm15859	-1,0283	1
Gm42635	-1,0288	1
Pfkfb2	-1,03	1
Wdhd1	-1,0303	1
9930104L06Rik	-1,0313	1
Zbtb11os1	-1,0354	1

Gm37653	-1,036	1
Gm37718	-1,0379	1
4930532G15Rik	-1,0403	1
Atp6v0c	-1,042	1
Lsr	-1,0442	1
Gm38192	-1,0442	1
A530017D24Rik	-1,047	1
Gm43071	-1,049	1
Gm26664	-1,0492	1
I830077J02Rik	-1,0503	1
Hmgb1-ps8	-1,0503	1
Ctnnd1	-1,0505	1
Samd9l	-1,0511	1
AI506816	-1,0513	1
Gm43513	-1,0539	1
Gm42600	-1,0567	1
Zbed3	-1,0578	1
Bend6	-1,058	1
1700030M09Rik	-1,0581	1
Usp26	-1,0614	1
H2-Q10	-1,0618	1
Rpl7	-1,0631	1
Gm37902	-1,0643	1
Gm37399	-1,0655	1
Gm12034	-1,067	1
Vil1	-1,0674	1
Gm29358	-1,0676	1
2510016D11Rik	-1,0677	1
Mcm8	-1,0678	1
4933421A08Rik	-1,0702	1
Camk2n1	-1,0711	1
Gm43133	-1,0718	1
Gm45203	-1,074	1
Gm26461	-1,0748	1
Gm42478	-1,0751	1
Gm14636	-1,0755	1
Hist2h4	-1,0766	1
C530005A16Rik	-1,0777	1
Gm2531	-1,0778	1
Sema6c	-1,0778	1
4930509H03Rik	-1,0819	1
RP23-356D13.9	-1,082	1
Gm43627	-1,0829	1
Slc45a4	-1,084	1
L1cam	-1,0853	1
Hist1h2bp	-1,0867	1
4833412K13Rik	-1,0885	1
Gm42633	-1,0885	1
Gm42484	-1,0956	1
Galnt11	-1,0986	1
Polr1b	-1,0987	1
1700020D05Rik	-1,0989	1

Plekhg4	-1,101	1
Gm43668	-1,1013	1
2210417A02Rik	-1,1029	1
Zswim8	-1,105	1
Gm38009	-1,1068	1
Gm13397	-1,1069	1
Zfp52	-1,11	1
Gm37383	-1,1104	1
Nup107	-1,1131	1
Ece1	-1,114	1
Gm22513	-1,1145	1
Mrps28	-1,1178	1
Gm18916	-1,1184	1
Tubg2	-1,1224	1
Ckap2	-1,1225	1
B230216N24Rik	-1,1226	1
Gm37531	-1,1284	1
Mpp3	-1,1294	1
Gdf15	-1,1311	1
Gm28791	-1,1314	1
Slc22a5	-1,1325	1
Gm43482	-1,1376	1
Gm43715	-1,1448	1
Zfp617	-1,1458	1
Gm13349	-1,1471	1
Gm6382	-1,15	1
RP23-402A24.3	-1,1511	1
Gm43411	-1,1528	1
Gm23346	-1,1529	1
Gm20696	-1,1539	1
Znrf3	-1,1557	1
Gm45206	-1,1564	1
Zfp174	-1,159	1
Gm43484	-1,166	1
Gm43560	-1,166	1
Gm9008	-1,1687	1
Cdo1	-1,1692	1
Gm37084	-1,1728	0,96453
Ing4	-1,1732	1
Gm37334	-1,1748	1
Gm43061	-1,176	1
Gm38190	-1,1769	1
Gm43178	-1,1771	1
RP23-444K20.4	-1,1792	1
Nfyc	-1,1807	1
Gm37357	-1,1818	1
Gpn2	-1,1843	1
Gm37584	-1,1846	1
Olfr286	-1,1854	1
Gm45762	-1,1897	1
Pcdhb16	-1,1908	1
Rnf130	-1,1917	1

RP24-75M13.2	-1,1938	1
Gm44013	-1,1944	1
B930086L07Rik	-1,1956	1
Gm26740	-1,1965	1
Gm15265	-1,1967	1
Gm9207	-1,1999	1
A130014A01Rik	-1,2018	1
Gm43024	-1,2037	1
Gm3550	-1,2039	1
Snord7	-1,2077	1
Gm16540	-1,2081	1
Gm24927	-1,2085	1
Gm37204	-1,2098	1
Gm5100	-1,2119	1
2310058D17Rik	-1,2136	1
A430110C17Rik	-1,2199	1
Gm22980	-1,2228	1
B230398E01Rik	-1,2231	1
Gm37706	-1,2241	1
Gm24009	-1,2249	1
Gm45698	-1,2267	1
Dynlt1-ps1	-1,2281	1
Scarna17	-1,2283	1
Gm12770	-1,2317	1
Snord83b	-1,2324	1
Hmgb1	-1,2331	1
Gemin8	-1,2334	1
3110045C21Rik	-1,2355	1
Gm25596	-1,2368	1
RP23-226H21.3	-1,238	1
Gm23127	-1,2399	1
Gm5124	-1,2482	1
Gm16740	-1,2509	1
Cog3	-1,252	1
Gm26514	-1,254	1
A330023F24Rik	-1,2546	1
RP24-282K24.4	-1,2598	1
Gm45221	-1,2606	1
9230111E07Rik	-1,2608	1
Map3k15	-1,2639	1
Cntrob	-1,2654	1
Plxnb3	-1,2693	1
Rnf25	-1,2703	1
A530041M06Rik	-1,2711	1
A330069E16Rik	-1,2729	1
Gm38319	-1,2753	1
Gm12276	-1,276	1
Gm37124	-1,2763	1
Fcrl5	-1,2791	1
Celsr3	-1,2795	1
RP23-36H21.3	-1,2796	1
Gm44694	-1,2797	1

Rps13-ps1	-1,2808	1
Gm37949	-1,2838	1
Pigg	-1,2842	1
Gm43848	-1,2898	1
Gm15634	-1,2909	1
Gm38200	-1,291	1
Gm15503	-1,2948	1
Magohb	-1,2953	1
Klkb1	-1,2968	1
Gm37105	-1,2986	1
Gm7299	-1,2996	1
Gm15834	-1,302	1
Gm45477	-1,3027	1
5330406M23Rik	-1,3057	1
Gm6140	-1,3059	1
Gm24631	-1,306	1
9930111J21Rik2	-1,3094	1
RP24-496O17.7	-1,3101	1
Gm24924	-1,3105	1
mt-Tm	-1,3114	1
Gm23301	-1,3117	1
Gm38082	-1,3131	0,86146
Gm43817	-1,3133	1
Gm7353	-1,3139	1
RP24-225A16.3	-1,3146	1
Gm38036	-1,3159	1
Psd2	-1,3162	1
2610037D02Rik	-1,3164	1
Gm28041	-1,3179	1
Nr4a1	-1,318	1
Gm23849	-1,3183	1
Dgat2	-1,3185	1
Gm44283	-1,3196	1
D430013B06Rik	-1,3199	1
Gm4613	-1,3217	1
Masp2	-1,3242	1
RP23-320D23.6	-1,3249	1
Gm28151	-1,3266	1
Gli1	-1,3273	1
RP23-307F3.6	-1,3284	1
Rgmb	-1,3313	1
Gm10167	-1,3329	1
Gm26810	-1,3331	1
RP23-162P10.2	-1,347	1
Vps37d	-1,3525	1
Hmx3	-1,3617	1
RP23-40D21.1	-1,3649	1
Gdap10	-1,3661	1
Mpc1	-1,3688	1
Depdc1a	-1,3739	1
Gm42979	-1,3744	1
Sdhd	-1,3803	1

Recql4	-1,3885	1
RP23-182J19.2	-1,3987	1
9330175E14Rik	-1,3994	1
Amd2	-1,4	1
Gm42467	-1,4008	1
Gm44432	-1,401	1
Gm9938	-1,4015	1
Gm45185	-1,4026	1
Gm11491	-1,4041	1
Gm42793	-1,4085	1
2700029L08Rik	-1,41	1
Gm42486	-1,4131	1
Rpl27-ps3	-1,4139	1
Gm4017	-1,4181	1
Aloxe3	-1,42	1
Gm43148	-1,4218	1
Gm42819	-1,4252	1
Gm43059	-1,4312	1
RP24-226A8.2	-1,4319	1
Gm45343	-1,4328	0,96453
Gm37063	-1,4351	1
Pam16	-1,4376	1
Cntnap1	-1,4401	1
Gm43445	-1,4414	1
Gm37140	-1,4423	1
Gm42748	-1,4436	1
Gm43331	-1,4451	1
5430434F05Rik	-1,4462	1
Npl	-1,4482	1
Gm18709	-1,4502	1
9630010A21Rik	-1,4536	1
Gm30238	-1,4565	1
Rpl23a-ps2	-1,4576	1
Gm45853	-1,4589	1
Gm43200	-1,4613	1
Llgl2	-1,4617	1
Gm15785	-1,465	1
Gm2885	-1,466	1
RP23-205H11.3	-1,4729	1
Vamp7-ps	-1,4746	1
Mrc1	-1,4751	1
Zfp866	-1,4792	1
Gm29994	-1,4842	1
Lgals7	-1,4877	1
BC055308	-1,4916	0,74465
Efcab11	-1,4927	1
Gm14286	-1,4928	0,96453
1110035H17Rik	-1,4964	1
Gm26601	-1,498	1
Gm22714	-1,5044	1
A430027C01Rik	-1,5051	1
Grin1	-1,5116	1

Gm37080	-1,5137	1
Gm44270	-1,5178	1
Adam9	-1,531	1
Gm37490	-1,5348	1
Snord110	-1,5413	1
Phf11c	-1,5415	1
Gm12940	-1,5484	1
RP23-442M18.5	-1,5553	1
Zfp335os	-1,556	1
Gm45289	-1,5591	1
Gm20091	-1,5595	1
Gm37510	-1,5664	1
Ncmap	-1,5714	1
Gm38355	-1,5777	1
4833421G17Rik	-1,5785	1
Pask	-1,5894	1
5730405O15Rik	-1,596	1
Gm42522	-1,5994	1
Gm42895	-1,623	1
Dnd1	-1,6254	1
Gm37206	-1,626	1
Gm44292	-1,6295	1
Olfr933	-1,6411	0,86146
Gm21816	-1,6436	1
Gm37788	-1,6467	1
Zic5	-1,6478	1
Gm25517	-1,6519	1
Gm43275	-1,6525	1
Gm15644	-1,6577	1
Gm45266	-1,6585	1
Trdmt1	-1,6593	1
Rrad	-1,6677	1
RP24-460E12.3	-1,6697	1
Inpp5e	-1,6726	1
Fam69b	-1,6754	1
Gm44953	-1,6784	1
Gm8337	-1,6796	1
Bloc1s3	-1,6803	1
Erfe	-1,681	1
Gm37255	-1,6871	1
Timm23	-1,6925	1
Gm43628	-1,695	1
Gm29438	-1,7006	0,86146
Gm42576	-1,7006	1
Snord66	-1,7044	1
Gm45534	-1,7065	1
Gm44901	-1,7156	1
Gm26594	-1,7214	1
Gm16046	-1,7253	1
Rnft2	-1,7404	1
Gm27010	-1,7456	1
Gm43800	-1,7533	1

5430420F09Rik	-1,7726	1
Crtc2	-1,7785	1
Kif18b	-1,7958	1
Gm26132	-1,8149	1
Slc12a5	-1,816	0,86146
Syt8	-1,8203	1
RP24-131G14.10	-1,8289	1
Tnfrsf13b	-1,8291	1
Gm37519	-1,8394	1
Gm29488	-1,8604	1
Fzd2	-1,863	1
Gm6612	-1,8727	1
Gm45137	-1,8815	1
Gm8210	-1,9175	1
Gm45084	-1,9212	1
4732440D04Rik	-1,9264	1
Hus1b	-1,939	1
Tgfb1i1	-1,9547	1
Tti1	-1,9573	1
Gm2308	-1,9862	1
Cxcl10	-1,9976	1
Lgr5	-2	1
Gm23344	-2,011	1
Stc1	-2,0154	1
Gm45358	-2,0528	1
Tap2	-2,0591	1
Gm45220	-2,0656	1
Gm38365	-2,1079	0,33876
1700007K09Rik	-2,1242	1
9930120I10Rik	-2,1702	1
Gm37390	-2,1951	1
Gm36989	-2,2198	1
Gm37106	-2,2239	1
Gm26772	-2,3017	0,86146
Gm43421	-2,3108	1
Zfp619	-2,3409	1
Gm42483	-2,404	1
Ano7	-2,4255	0,96453
Gm28404	-2,4693	0,33876
Hspa1a	-2,5876	1
Gm20186	-2,5939	0,86146
Gm42908	-2,6624	1
Gm42632	-2,7475	0,97422
Gm43727	-2,8563	0,53165
Hspa1b	-3,2067	0,10852
