# Supplementary material for: Bone, dentin and cementum differentially influence the differentiation of osteoclast-like cells
Source: Sci Rep. 2025 Jun 5;15:19857. doi: 10.1038/s41598-025-04874-9 (PMC12141432; doi:10.1038/s41598-025-04874-9)
Supplement: Supplementary file 10 — Supplementary Information 10. [file 41598_2025_4874_MOESM10_ESM.pdf]

Tab. S9:
Transcripts induced in murine macrophage cells stimulated on cementum (n=6), fold of bone

gene name	regulation of expression	adj.P.Val
Gm10800	67,4712	0,57302
Gm26870	31,0840	0,27112
Gm29358	22,9194	0,0030048
Gad2	11,9249	0,0060419
4930578M07Rik	11,6754	0,0051452
Adm	10,5546	6,65E-08
Gm10801	10,2383	0,41572
RP23-308G10.5	10,0847	0,34513
Rpl30-ps2	9,6318	1,25E-05
Ankrd37	9,5885	3,30E-07
Olfr912	9,1034	0,26066
Gm15610	9,0405	0,025306
Gapdh	9,0017	0,00018124
Mast4	8,9420	0,054585
Sit1	8,9154	0,018962
Adamtsl1	8,8434	0,22706
RP23-451J19.1	8,6818	0,024694
Gm44652	8,4649	0,001163
Gm43447	8,2873	0,20665
Plscr4	7,9895	0,082657
Gm12469	7,8598	0,010843
RP23-136K21.4	7,7985	0,097382
4921507G05Rik	7,5868	0,08503
Gm8317	7,5329	0,00060105
1500004A13Rik	7,5047	0,0016428
Rgcc	7,4426	4,38E-05
Slc16a5	7,3559	0,09697
Gm27248	7,2980	0,075053
Dvl3	6,9649	0,019829
AY074887	6,8642	0,012386
Hist1h4a	6,8452	0,11229
Hspa1b	6,8239	0,35129
Gm9381	6,7300	0,040865
Itgax	6,7295	8,08E-08
Rn7sk	6,6405	0,032117
Gm10717	6,6277	0,41062
Gm37052	6,5487	0,20335
Hist1h2bg	6,4827	0,15526
Pou6f2	6,4715	0,35826
Gm26847	6,3806	0,12017
Gm8649	6,2745	6,08E-09
Thap8	6,2186	0,02178
Gm43273	6,1731	0,17458
Gm28731	5,9451	0,075012
Gm7784	5,8698	0,086389
Gm3695	5,8547	0,029977
Zp1	5,8115	0,20679
Gm23037	5,7370	0,18884

RP23-366E4.9	5,6812	0,080032
Tspan15	5,6537	0,099403
Egln3	5,5995	0,024774
Gm26983	5,5840	0,010482
Gm5577	5,5335	0,057826
Ccl6	5,4862	0,058736
Gm45833	5,4771	0,002723
Lrrc2	5,4551	0,058948
RP23-350F7.3	5,3837	0,0040633
Gm16755	5,3669	0,21318
Gm14034	5,3558	0,12804
Apol11b	5,3499	0,3466
Gm15877	5,2354	0,076329
Rpl32-ps	5,2318	0,056303
Clec3b	5,2000	0,19547
Gm6520	5,1103	0,060164
Gm26730	5,1092	0,15947
Tmcc3	5,0602	0,19856
Gm10827	5,0585	0,0001613
Exo1	5,0312	0,17296
Gm2467	4,9674	0,14603
Gm7132	4,9588	0,14369
Gm8623	4,9561	0,035734
Tsix	4,9376	0,08503
Gm13992	4,8878	0,12483
Hist1h1b	4,8770	0,11956
Gm11810	4,8006	0,0092825
Hbb-bh3	4,7823	0,39784
Rhoh	4,7724	0,52021
Rps19-ps8	4,7522	0,12762
Gm8919	4,7076	0,10361
Gm43684	4,6878	0,42399
Hba-a1	4,6874	0,42264
Med16	4,6803	3,05E-05
RP24-547N4.7	4,6178	0,10401
RP24-122E11.4	4,6152	0,37157
Hist1h4d	4,5703	0,14376
Gm28373	4,5681	0,31652
Gm26830	4,5463	0,31717
RP23-440L7.5	4,5350	0,25729
1700030M09Rik	4,5337	0,23969
Gm9521	4,4847	0,21989
Gm12468	4,4340	0,10899
Gm8330	4,3985	0,07573
Gm14130	4,3196	0,099804
Taco1os	4,3169	0,13188
Gm43878	4,3100	0,26826
Gm8885	4,2978	0,19867
Rdh12	4,2809	0,27193
Gm44130	4,2696	0,26826
RP24-174I4.1	4,2598	0,10899
Gm37482	4,2398	0,14593

2810433D01Rik	4,2216	0,46258
RP24-82M14.1	4,2149	0,17115
Il13ra2	4,1736	0,17376
Jup	4,1664	0,11442
Rpl27a-ps1	4,1609	0,027083
Cd200r3	4,1376	0,37147
Olfr95	4,1350	0,17182
Rpl35a-ps5	4,1345	0,00045887
Gm2076	4,1296	0,22589
Gm43501	4,1193	0,30943
Cdh23	4,0770	0,20966
Tspan33	4,0615	0,26965
Fzd7	4,0175	0,027583
Gm10636	4,0061	0,22478
Ank3	3,9873	0,58499
Gm43920	3,9790	0,32032
Gm27043	3,9614	0,3884
Gm14040	3,9559	0,41202
Ptgs2os	3,9542	0,17534
Dcstamp	3,9417	2,20E-05
Acoxl	3,9370	0,4839
Fth-ps3	3,9367	3,84E-05
Rpl30-ps1	3,9250	0,044421
Car7	3,8976	0,020335
Crip1	3,8882	4,63E-09
Gm10657	3,8804	0,27257
Gm37747	3,8769	0,25788
Gm10575	3,8613	0,52544
Gm3699	3,8584	0,14231
Tstd1	3,8440	0,0051479
4930542C12Rik	3,8429	0,4865
Gm12466	3,8360	0,12804
4933437G19Rik	3,8315	0,25094
H2-DMb2	3,8098	0,066176
Gm8927	3,7884	0,18319
Gadd45g	3,7866	0,001542
Rhob	3,7685	0,0043863
Rpl9-ps7	3,7607	0,15295
Pcdhgc4	3,7474	0,19036
Gm2830	3,7438	0,00050932
RP24-175C20.10	3,7386	0,0066208
Gm42786	3,7249	0,45015
5430421F17Rik	3,7182	0,35826
Gm8292	3,6493	9,23E-05
Gm26226	3,6487	0,66665
Gm15596	3,6346	0,10173
Gm13622	3,6238	0,20569
Gm9568	3,5925	0,26965
Rhov	3,5881	0,033707
Id1	3,5660	5,65E-05
Gm37486	3,5615	0,26066
4932422M17Rik	3,5554	0,01937

Gm16181	3,5436	0,58417
Gm26656	3,5218	0,31005
Gm12182	3,5179	0,045533
Gfod2	3,5110	0,0022557
Gm37785	3,5021	0,22678
Kcnd1	3,4808	0,52002
Mafb	3,4603	3,78E-06
Hes7	3,4495	0,54879
Plk2	3,4448	0,014629
Bc1-ps1	3,4426	0,41029
Efna3	3,4355	0,24455
Hist1h2be	3,4331	0,040518
Gm8894	3,4262	0,27698
Tnfsf8	3,4233	0,41634
Atp5l-ps1	3,4141	0,014055
Caprin2	3,3806	0,56971
Gm42432	3,3746	0,25661
Gm36936	3,3676	0,61792
Gm14439	3,3669	0,71544
Lpl	3,3648	9,96E-08
Nrxn3	3,3461	0,12673
Gm14585	3,3429	0,29855
Ndrg1	3,3309	9,74E-07
Gm17108	3,3295	0,17213
AV099323	3,3253	0,5744
Kif21a	3,3249	0,54617
Aarsd1	3,3088	0,4637
Palld	3,3067	0,4839
5031425F14Rik	3,3008	0,4886
Gm16238	3,3001	0,17261
Gm9722	3,2946	0,24826
Gm11281	3,2871	0,35554
B3gnt6	3,2862	0,62028
Vsig8	3,2825	0,51055
1700031P21Rik	3,2786	0,19596
Spsb2	3,2736	0,00084546
Gm44258	3,2727	0,02178
A430010J10Rik	3,2623	0,56798
Mir3091	3,2612	0,59191
Gm3617	3,2488	0,42428
Hist2h4	3,2429	0,64463
Rps12-ps23	3,2331	0,39153
Esco2	3,2306	0,36706
RP24-282C4.3	3,2210	0,44179
Rps8-ps4	3,2170	0,12804
Gm5112	3,2063	0,17213
Phlda1	3,2034	0,0047153
Gm10260	3,1952	0,016037
Gm6322	3,1928	0,4839
Gm12758	3,1903	0,23502
Gm14138	3,1744	0,038969
Gm43182	3,1736	0,7204

Slc2a1	3,1698	2,13E-05
4632415L05Rik	3,1604	0,01508
Gm44884	3,1499	0,76471
4930558J18Rik	3,1423	0,63005
2700099C18Rik	3,1407	0,34312
D430001F17Rik	3,1368	0,19234
B930036N10Rik	3,1242	0,27199
Gapdh-ps14	3,1242	0,43267
Ankdd1a	3,1188	0,37654
Klf10	3,1162	9,95E-05
Gm11895	3,1149	0,51327
Gm16045	3,1143	0,025892
Hlx	3,1128	0,2191
Gm28686	3,0977	0,50569
Alpk2	3,0883	0,43258
Txnip	3,0827	0,0031271
Gm7990	3,0793	0,089337
D130051D11Rik	3,0703	0,014055
Rpl29	3,0693	0,53382
1700003G18Rik	3,0693	0,69661
E130201H02Rik	3,0650	0,52028
Gm14336	3,0644	0,06718
Card9	3,0621	0,31994
RP24-295J1.1	3,0610	0,61058
Gm4607	3,0559	0,27698
RP23-88C11.5	3,0542	0,45015
Rnf122	3,0534	0,099403
Gzmm	3,0506	0,44446
9530085L11Rik	3,0455	0,24732
RP24-324J2.1	3,0384	0,45799
Gm5905	3,0354	0,030228
Gm12267	3,0314	0,080032
C630043F03Rik	3,0306	0,33495
Atp1b4	3,0277	0,84984
9330162G02Rik	3,0251	1
Gm43578	3,0228	0,44978
Dkkl1	3,0184	0,78141
Gm37653	3,0157	0,26233
Hmgb2	3,0153	0,0043498
Nsa2-ps2	3,0107	0,78141
Gm13360	3,0105	0,68186
Gm19272	3,0080	0,78791
Gm11205	2,9972	0,017401
Wfdc17	2,9972	0,027717
2900093K20Rik	2,9963	0,00088797
Gm14279	2,9949	0,078429
Nsl1	2,9914	0,025936
Gm12280	2,9874	0,25465
Lat	2,9841	0,3225
Gm5921	2,9816	0,53466
Ccng2	2,9789	0,0057048
Gm17430	2,9728	0,44451

1700054M17Rik	2,9703	0,66147
Gm18709	2,9561	0,40075
C730034F03Rik	2,9559	0,0016428
Gm10313	2,9465	0,60775
RP24-323H7.5	2,9367	0,73937
RP23-115A18.3	2,9355	0,098741
Gm43655	2,9339	0,35554
RP24-511J14.2	2,9276	0,099403
Rnf152	2,9152	0,4886
Gm6745	2,9065	0,67246
Gm14830	2,8941	0,10798
Adgb	2,8855	0,68272
Rpl30-ps9	2,8839	0,363
Mcm8	2,8809	0,33273
Gm45546	2,8783	0,64665
Aloxe3	2,8600	0,66623
Gm9442	2,8596	0,83639
Gm15564	2,8580	0,15409
Ttc30a1	2,8570	0,63802
Meg3	2,8556	0,52267
mt-Tm	2,8497	0,26414
RP24-351I17.3	2,8483	0,64906
Ttc25	2,8471	0,46443
Gm5644	2,8404	0,078429
Gm12990	2,8273	0,099685
Gsg1	2,8253	0,034283
A330035P11Rik	2,8218	0,66389
RP23-356P21.1	2,8206	0,059063
5830454E08Rik	2,8171	0,033554
Cd9	2,8118	0,15473
Errfi1	2,8104	0,014202
C130013H08Rik	2,8104	0,74005
Tubb2b	2,8091	0,52021
Gm43511	2,8085	0,12804
Slc13a3	2,8085	0,34641
Gm8254	2,7974	0,26066
Gm11346	2,7934	0,6082
Rybp	2,7924	0,66237
Gm26698	2,7883	0,21506
Tma7	2,7820	0,082657
Gm9517	2,7810	0,20569
Gm16585	2,7768	0,27199
Fcor	2,7716	0,38637
Rpl6l	2,7714	0,082657
Gm5900	2,7696	0,713
RP24-93F20.12	2,7648	0,65719
Gm12778	2,7635	0,29989
S100a3	2,7616	0,35229
RP23-159E10.1	2,7547	0,82722
Il1rl1	2,7496	0,69661
Gm13461	2,7400	0,15855
Itga6	2,7357	0,008444

Ang	2,7340	0,029087
AK157302	2,7319	0,58038
Gm45454	2,7285	0,19195
Gm6341	2,7268	0,00165
Teddm2	2,7260	0,83707
Gm29228	2,7253	0,55651
Gm2367	2,7151	0,58642
Gm44044	2,7104	0,70313
Gm17541	2,7057	0,92867
Sep 01	2,7012	0,98117
Dynlt1b	2,6963	0,94767
Crkl	2,6924	0,398
2310058D17Rik	2,6913	0,41572
mt-Ta	2,6913	1
Gm7266	2,6905	0,24981
Gm7936	2,6900	0,34157
Gm29666	2,6885	0,68186
RP24-547N4.5	2,6878	0,19594
Lbp	2,6859	0,83671
Gm15032	2,6846	0,111
Tnfsf12	2,6838	0,0016035
Gm9409	2,6818	0,61058
Gm14323	2,6749	0,5117
Gm2735	2,6738	0,24246
Gm4117	2,6725	0,94767
Ifitm1	2,6675	0,58924
Gm4217	2,6631	0,68073
Gm22973	2,6530	0,11198
Gm8818	2,6517	0,71189
Gm45698	2,6515	0,66389
Gm7867	2,6498	0,47127
Dpf1	2,6473	0,28676
B430305J03Rik	2,6440	0,86114
Rpl31-ps22	2,6397	0,72097
4930447F24Rik	2,6370	0,64629
Coro1a	2,6343	0,0050415
Tmem44	2,6335	0,57168
Pop5	2,6317	0,029977
Gm11263	2,6299	0,76488
4930404I05Rik	2,6257	0,99338
Dtd2	2,6252	0,036873
Gm10736	2,6248	0,10608
Gm10327	2,6208	0,30927
Gm45311	2,6190	0,52873
Gm17150	2,6168	0,65171
Gm14006	2,6162	1
Gm12380	2,6155	0,6132
Gm12090	2,6128	0,81106
Cfh	2,6030	0,0034038
Gm21057	2,6012	0,45852
Ntrk3	2,5935	1
2810405F17Rik	2,5895	0,72433

Gm45251	2,5809	0,87376
Gm37352	2,5801	0,30943
Sap30	2,5788	0,0097773
S100a13	2,5626	0,013691
Gm15950	2,5581	0,15706
Nsa2	2,5578	0,046282
Gm14326	2,5551	1
Gm44913	2,5546	0,045533
Gm7600	2,5534	0,56358
Kif20b	2,5498	0,019226
Krtcap3	2,5497	0,53233
Lhx5	2,5463	0,66623
Gm5566	2,5380	0,78983
Sgol1	2,5364	0,065045
Tmsb10	2,5327	0,10247
Gm18969	2,5291	0,61978
Gm6946	2,5271	0,8053
Hbegf	2,5256	0,01252
Gm12312	2,5249	0,65327
Ap1s3	2,5221	0,0013752
Gm15798	2,5214	0,57041
Gm12643	2,5191	0,7788
Rps23-ps2	2,5121	0,03505
Gm45051	2,5116	0,74346
Wwc1	2,5112	0,01508
Rassf7	2,5109	0,099403
Ndufs5	2,5109	0,3153
Rpl36-ps4	2,5081	0,31055
Tnfrsf17	2,5065	0,15474
Gm43524	2,5057	0,52956
Gm14013	2,5043	0,55892
Ciart	2,5008	0,045533
Gm10388	2,4987	0,22038
Ppp1r18os	2,4968	0,93133
Gm11334	2,4920	0,3884
Gm14057	2,4915	0,97845
Gm19566	2,4913	0,30943
Stamos	2,4822	0,64628
Wdr62	2,4789	0,64906
Polr2k	2,4780	0,83574
Fam109a	2,4755	0,02477
Gm16288	2,4755	0,88628
Gm28424	2,4755	0,94596
Rasgef1b	2,4748	0,0065657
Rn7s6	2,4743	1
Gm43096	2,4720	0,24246
Paqr5	2,4719	0,85401
Rpl36-ps8	2,4696	0,86367
Ybx1-ps2	2,4690	0,70313
Pou4f1	2,4666	0,74202
mt-Ti	2,4654	0,77601
Atr	2,4635	0,099403

Gm11826	2,4625	0,17666
Gm6682	2,4608	1
Ticrr	2,4601	0,83612
Mxd3	2,4589	1
Rpl26-ps4	2,4582	0,63448
Gm15541	2,4558	1
Gm10343	2,4553	0,24246
Gm10263	2,4545	0,89353
Gm6451	2,4526	0,14603
Gm15772	2,4494	0,0043863
Gm14537	2,4477	0,77583
Gm37234	2,4470	0,76101
Gm7634	2,4436	0,69317
Gm12604	2,4426	0,3948
Sirpa	2,4409	0,0002226
Lsm7	2,4390	0,3036
Gm5257	2,4385	0,95518
Gm9378	2,4383	0,37691
Gm44126	2,4377	0,98583
Cdc34b	2,4370	0,33654
Gm1840	2,4358	0,045533
Cspg4	2,4331	0,49262
Mmp9	2,4304	0,21011
Gm8825	2,4297	0,91369
A830073O21Rik	2,4289	1
Fau	2,4275	0,084574
Gm8250	2,4249	0,97534
Atf3	2,4247	0,013308
Polr2l	2,4205	0,034473
Hspa1a	2,4205	1
Gm44093	2,4193	1
Lockd	2,4180	0,033707
Hist1h2al	2,4176	0,27077
Gm8574	2,4160	0,91369
Ccdc36	2,4151	0,16872
Cenpw	2,4145	0,0023164
Tmod1	2,4140	0,07573
Tnfrsf14	2,4126	0,90671
Cenpk	2,4103	0,14369
Gm19898	2,4103	0,14458
Gm13464	2,4086	0,81088
Tmem267	2,4083	0,69398
Rab4a	2,4074	0,26752
Aif1	2,4069	0,24246
Tmem170	2,4059	0,81144
Rhox5	2,4041	0,84269
Gm5599	2,3983	0,22038
Gm26710	2,3958	0,87201
Acaa1b	2,3953	0,49366
AA465934	2,3918	0,082657
Tnni2	2,3911	0,017401
Fancb	2,3897	0,85532

Gm5881	2,3883	0,031862
Gm10382	2,3834	0,48191
Calr3	2,3825	0,64463
Gm13022	2,3801	0,45752
Rps11-ps3	2,3797	0,70799
Gm7079	2,3797	0,74346
Gm42856	2,3789	1
Rpl36a-ps3	2,3784	0,56697
5033430I15Rik	2,3753	0,90288
F10	2,3748	0,0050976
Csrnp1	2,3733	0,0033135
Gm11516	2,3725	1
Grcc10	2,3710	0,55726
Gm14094	2,3702	0,48528
E2f7	2,3699	0,51643
Gm11604	2,3690	0,80486
4930579G24Rik	2,3677	0,57309
Ankrd55	2,3669	0,6082
Gm8724	2,3656	0,2797
Gm12726	2,3644	0,92867
Adamts1	2,3636	0,26396
Gm7895	2,3621	0,95072
Gm7327	2,3615	0,21509
Gm44639	2,3608	0,84854
Hist1h1c	2,3595	0,013691
Rpl19-ps1	2,3587	0,06367
Gm8129	2,3587	1
Gm5614	2,3550	0,20335
Gm6181	2,3545	0,80552
Gm45856	2,3532	0,36821
Rpl19-ps11	2,3530	1
RP23-134M7.3	2,3523	0,84801
Gm42551	2,3502	0,91452
Rpl17-ps4	2,3476	0,64629
Cbx2	2,3460	0,27044
Cd200r2	2,3427	0,043091
Ttk	2,3390	0,035734
Otud1	2,3371	0,54617
Gm8973	2,3364	0,85586
Higd1a	2,3353	0,077526
Gm12944	2,3329	0,50572
Mroh2a	2,3317	1
Rasl2-9	2,3279	0,80852
Gm8357	2,3277	0,40266
Gm28041	2,3254	1
Rpl36-ps10	2,3230	0,010482
2810001G20Rik	2,3229	0,4839
2810002D19Rik	2,3227	0,93133
Nkapl	2,3217	0,76488
4931428F04Rik	2,3214	0,51464
Gm42671	2,3209	0,98557
Eif2s3x	2,3204	0,3036

Gm13383	2,3184	1
Gm13935	2,3171	0,97539
Rnu11	2,3151	0,57168
Gm26810	2,3089	0,94459
Gm44419	2,3078	0,49486
Gm4468	2,3071	0,15205
Gm42731	2,3035	1
Gm20223	2,3025	1
Stk25	2,3020	0,18818
Pbk	2,3015	0,066979
Gm26881	2,3001	0,049512
Gm12444	2,2999	0,74346
Gm25007	2,2974	0,80744
B3gat3	2,2961	0,0092825
Gm11878	2,2955	0,18244
Gm6023	2,2942	0,029087
Slc25a25	2,2928	0,052516
Gm42466	2,2923	0,99853
Naa10	2,2906	0,07944
RP24-131G14.13	2,2904	0,1229
Gm5312	2,2887	0,65182
RP23-246F14.1	2,2879	0,97597
Gm15393	2,2811	0,8742
Cd9-ps	2,2798	0,95916
Rbl1	2,2784	0,063969
Rgs2	2,2773	0,00075647
Hmgb1-ps5	2,2757	0,81842
Prr18	2,2755	0,11107
Gm15459	2,2752	0,87236
Gm12732	2,2744	0,10962
Gm11964	2,2725	0,53662
Mkln1os	2,2719	0,89674
Hist2h3c2	2,2711	0,54617
RP23-38L16.3	2,2651	0,22049
4930426I24Rik	2,2647	1
D630029K05Rik	2,2637	0,92867
Eno2	2,2575	0,010509
Gm7730	2,2567	0,57168
RP23-356D13.11	2,2556	1
Kazald1	2,2536	0,30201
Gm11470	2,2531	0,3036
Gm45206	2,2514	1
Gstt2	2,2509	0,34942
mt-Nd6	2,2439	0,11544
Gm6564	2,2434	0,87376
Yjefn3	2,2431	0,43867
Gm26530	2,2425	0,93133
Ezh2	2,2411	0,001265
Gm7434	2,2409	0,66237
Cox20-ps	2,2403	0,019184
Gm8304	2,2400	0,34141
Cox20	2,2391	0,017384

Snord82	2,2381	1
Pmaip1	2,2375	0,033554
Sep 02	2,2375	0,14603
Gm14173	2,2374	0,35554
Brip1	2,2371	0,35129
Pgf	2,2361	0,35554
Hspb7	2,2360	0,17376
Gm26772	2,2353	1
Gm19196	2,2338	0,2058
Gm10076	2,2330	0,010044
Gm1862	2,2329	0,65182
Rpl21-ps1	2,2319	1
Bloc1s6os	2,2310	0,81088
Cd300a	2,2299	0,082657
Lyz1	2,2289	0,00092086
Gm5302	2,2218	0,95992
S100a8	2,2208	0,73914
Gm9506	2,2202	1
Gm15007	2,2190	1
Gm12341	2,2185	0,25051
Cytip	2,2168	1
Fn1	2,2121	0,16826
Sult2b1	2,2021	0,93133
Pfkfb3	2,1989	0,055942
Olfr286	2,1977	1
Gm6204	2,1963	0,029252
Spata5l1	2,1954	0,079455
Hist1h1d	2,1942	0,91369
Gm45718	2,1928	0,92358
Gm16418	2,1915	0,25182
Rpl36-ps3	2,1886	0,20569
Tpt1-ps5	2,1875	0,87201
Rpl5-ps1	2,1866	0,50692
Sowahc	2,1851	0,030775
Gm7847	2,1851	0,69333
Hcfc1r1	2,1839	0,00067372
Gm42548	2,1836	0,91489
Chrnb1	2,1821	0,52873
Acot8	2,1810	0,12926
Ddah2	2,1778	0,52329
2810454H06Rik	2,1751	0,69661
Bvht	2,1748	0,56759
Gm9173	2,1742	0,45752
Etv4	2,1736	1
Gm44164	2,1735	1
Spc24	2,1706	0,13702
RP24-550H10.4	2,1705	1
Gm6177	2,1702	0,93133
Ccdc18	2,1696	0,43817
Socs2	2,1693	1
Gm14240	2,1685	1
Sdf2l1	2,1676	0,19195

Zeb2os	2,1649	0,17182
Gm10029	2,1638	0,82903
Gm45110	2,1632	1
Dlg3	2,1607	0,22706
Rpl36	2,1591	0,099403
Gm7099	2,1544	0,72197
Hist1h3d	2,1537	1
Ier5l	2,1513	0,3134
Ska2	2,1499	0,28867
Gm6808	2,1492	0,24246
Arc	2,1492	0,67042
Gm43011	2,1480	1
Mgst3	2,1477	0,41202
Gm3283	2,1468	0,83471
Sdc3	2,1437	0,0034038
Arhgap26	2,1437	0,42732
C3ar1	2,1434	0,21506
Zfp940	2,1434	1
Gm16754	2,1424	0,065045
Fam162a	2,1422	0,0032057
Atp5g1	2,1422	0,111
AV356131	2,1415	0,029977
Spry2	2,1404	0,53457
4930509H03Rik	2,1401	0,96561
Gm12577	2,1395	0,3036
Sertad1	2,1372	0,097964
Frat2	2,1369	0,042201
Nfil3	2,1349	0,17182
Ss18l1	2,1313	0,078908
C230096K16Rik	2,1307	0,59346
Gm37125	2,1307	1
Rpa3	2,1286	0,94542
Gm44545	2,1280	1
Gm7424	2,1277	0,53558
Gm13827	2,1276	0,19988
Gm11560	2,1267	0,07573
Pth1r	2,1218	0,98675
Gm7965	2,1208	0,97539
Gm11942	2,1199	0,12804
Arhgap39	2,1195	0,41202
Gm12669	2,1136	0,33273
RP23-164P21.3	2,1108	0,69661
Rbmx	2,1102	0,85474
Gm45855	2,1101	1
Rps6-ps1	2,1091	0,18884
Rps12-ps1	2,1089	1
Gm12583	2,1083	1
Gm15796	2,1079	1
Wdfy2	2,1057	0,52312
Selenop	2,1051	0,59609
Gm6293	2,1037	0,45045
Ormdl3	2,1031	0,063801

Kifc5b	2,1015	0,61792
Gm16104	2,1003	1
Zfp938	2,1002	0,37156
Gm13612	2,0975	1
Snhg11	2,0970	1
Nadk2	2,0964	0,15168
Nr2c2ap	2,0959	0,03505
Gadd45b	2,0958	0,1236
Gm43714	2,0954	1
RP23-316F10.2	2,0939	0,77583
Klf2	2,0933	0,30314
Gm37733	2,0932	0,7384
Ccne2	2,0909	0,90825
Phf13	2,0901	0,086946
Rpl12	2,0899	0,1945
Rps15a-ps3	2,0891	0,69297
Cped1	2,0872	0,67652
Khk	2,0849	0,013308
Gm33080	2,0845	0,79777
Gm13777	2,0841	0,5679
Gm8730	2,0835	0,25355
Ccl9	2,0832	0,0078764
Tubb4b	2,0809	0,072212
Birc5	2,0806	0,010482
Gm15131	2,0787	1
Gm12844	2,0777	0,26075
Gm8731	2,0754	0,26485
Gm5239	2,0751	0,8287
H2-Q6	2,0747	0,96561
Hba-ps4	2,0741	1
RP24-550H10.6	2,0733	1
Gm44953	2,0731	1
5330426L24Rik	2,0711	1
Gm7722	2,0695	0,6082
Trmt112-ps2	2,0684	0,66219
Gm4034	2,0659	0,64463
Kif22	2,0649	0,095116
Gm14005	2,0647	0,55726
RP24-232D3.1	2,0641	0,98555
Gm12164	2,0641	1
2900060B14Rik	2,0638	1
Pafah1b1-ps2	2,0621	0,69732
Fign	2,0618	1
Gm44791	2,0602	0,97597
Sp140	2,0558	0,092604
Unc13a	2,0548	0,30943
BC024386	2,0534	0,95992
Gm13408	2,0532	0,68186
4921524J17Rik	2,0530	0,099906
Rps9	2,0524	0,049748
Mki67	2,0517	0,095189
Slamf9	2,0493	0,9979

Tmem202	2,0478	0,64131
Mcm10	2,0449	0,71779
Gm5445	2,0446	0,58924
Gm29170	2,0444	0,54859
Gm7799	2,0432	0,5735
Gm12350	2,0420	0,35766
Rps3a3	2,0409	0,64787
Adrb2	2,0383	0,55409
A730071L15Rik	2,0359	1
Taf6	2,0347	0,035466
Gm27003	2,0334	1
Snhg18	2,0327	0,16818
Prr3	2,0324	0,53424
H2afv	2,0323	0,044652
Gm28578	2,0323	0,83707
Slc8a1	2,0310	0,84711
Gm14681	2,0290	0,08503
Arrdc4	2,0289	0,011032
Gm13443	2,0289	0,85715
Rpsa-ps2	2,0279	0,96052
Gm36930	2,0275	0,9873
Dhrs11	2,0262	1
Gm11222	2,0245	1
Klf11	2,0244	0,39655
Gm42508	2,0244	0,97217
Rpl41	2,0229	0,036873
Gm13413	2,0227	1
Gm28555	2,0226	0,17213
Ulbp1	2,0209	0,041243
E230029C05Rik	2,0205	0,81791
P4ha2	2,0202	0,3036
Spats1	2,0191	1
6030442K20Rik	2,0187	1
Calm3	2,0181	0,015453
Gm10080	2,0178	0,70799
Hist1h1a	2,0168	1
Cep55	2,0164	0,5855
Gm37584	2,0163	1
Manbal	2,0161	0,040495
Ruvbl2	2,0149	0,34141
Pradc1	2,0135	0,72556
Gm10180	2,0127	0,84961
Kif20a	2,0124	0,27199
Dpep2	2,0117	0,15203
Gm13498	2,0100	0,68272
Gm6210	2,0089	0,92741
Gm42851	2,0089	1
Gm16106	2,0078	1
Zg16	2,0076	1
Gm26737	2,0075	0,76488
Gm45806	2,0069	0,47911
Gm5801	2,0064	0,96052

Rad51ap1	2,0060	0,51767
Gm3571	2,0054	1
RP24-365A12.2	2,0051	1
Gm12854	2,0047	1
Cpne9	2,0044	1
Polq	2,0036	0,87793
Gm24276	2,0033	0,11956
Gm5869	2,0029	0,9873
Rpl36a-ps2	2,0018	0,15984
Gm6548	2,0004	0,6938
Smtn	2,0001	0,92266
Usp50	1,9981	0,86958
Hmga1	1,9978	0,68809
Gm9727	1,9975	1
Gm6285	1,9968	0,27257
Gm9796	1,9956	1
Gm12096	1,9932	0,66147
Creb5	1,9928	1
Trf	1,9925	1
A330069E16Rik	1,9906	0,88781
Abca5	1,9906	0,98391
Tusc2	1,9903	0,035734
Gm3940	1,9902	1
Gm9256	1,9899	1
Gm4754	1,9881	1
Ier5	1,9869	0,052038
Gm14017	1,9849	0,64706
Gm6134	1,9843	0,60852
Gm7589	1,9832	0,10361
Gm45184	1,9832	0,35013
Gm29019	1,9812	0,68186
A430018G15Rik	1,9799	0,65927
Gm6472	1,9785	0,43817
1600029O15Rik	1,9785	1
Gm13092	1,9782	0,91374
Gm11970	1,9781	0,14689
Gm9294	1,9748	0,7532
Rpl9	1,9718	0,11956
Gm7332	1,9708	0,63351
Slc13a2	1,9688	1
Hist3h2a	1,9685	0,07653
Gm29438	1,9685	0,97533
Zfp36l1	1,9675	0,14507
Gm1947	1,9674	0,76101
Arrdc2	1,9663	0,26826
Fam171a2	1,9659	0,29989
Mir6236	1,9656	0,96052
Gm11478	1,9650	0,054961
Gm2011	1,9648	0,98936
Ckap2l	1,9643	0,18986
Gm37472	1,9639	1
Gkap1	1,9622	0,66237

Apip	1,9612	0,41689
Gm5525	1,9607	0,57309
Gm13215	1,9601	0,61361
Rplp2	1,9594	0,099804
Gm42690	1,9594	1
Gm13602	1,9592	0,98929
Rplp1-ps1	1,9592	1
Gm43581	1,9590	0,51767
Gm10161	1,9586	1
Pgk1	1,9584	0,33273
1600014C23Rik	1,9573	0,96352
Tspan32	1,9560	1
Gm7666	1,9550	1
Ywhah	1,9546	0,23602
Ptrf	1,9545	1
Gm17807	1,9526	1
Ezr	1,9481	0,047667
2810408I11Rik	1,9471	1
D330023K18Rik	1,9467	0,49323
Calr-ps	1,9461	0,033554
Tgif2	1,9452	0,22217
Map3k12	1,9450	0,3948
Gm5776	1,9433	0,90083
Ndfip1	1,9432	0,33495
Snrpf	1,9414	0,76488
Fiz1	1,9411	0,3134
Rpl30-ps3	1,9411	0,39771
Rassf3	1,9399	0,24246
Gm12254	1,9399	0,28449
RP23-123D6.12	1,9380	0,44655
Gm19726	1,9379	1
Gm16630	1,9376	1
Gm5262	1,9371	1
Rps18-ps1	1,9360	0,85212
Gm44777	1,9342	1
Gm16177	1,9340	1
Gm13453	1,9339	0,92626
Gm14636	1,9316	0,47653
4931440P22Rik	1,9296	0,57168
RP23-184H3.5	1,9292	0,095824
Gm10036	1,9292	0,47645
Pltp	1,9289	0,049279
Hist1h1e	1,9285	0,1478
Mettl7a1	1,9285	0,57044
C920021L13Rik	1,9280	0,14603
AC133103.1	1,9271	0,1889
Gm12967	1,9260	0,39784
Ccdc114	1,9255	0,90352
Rps12-ps5	1,9252	1
Rpl11	1,9249	0,52687
4930578M01Rik	1,9239	1
Plau	1,9228	0,16818

Akr1b7	1,9228	0,84824
2610306M01Rik	1,9221	0,61792
Gm5764	1,9209	1
Gm21399	1,9204	1
Acot1	1,9200	0,87376
Gm14048	1,9192	0,61792
Gm25857	1,9191	1
Il1rn	1,9176	0,68415
Cdk2ap1	1,9172	0,30943
Sh2d5	1,9168	0,41893
Gm4617	1,9157	0,55726
Gm15441	1,9149	1
Stard3nl	1,9131	0,24981
BC028528	1,9124	0,48398
Gm10916	1,9124	1
RP23-40D21.1	1,9108	0,98408
Gm12501	1,9107	0,51767
Gm20768	1,9104	0,099505
Gm15720	1,9096	1
Psmc3ip	1,9095	0,8742
Gm26610	1,9092	1
Tpt1-ps3	1,9091	1
Gm8659	1,9086	1
Gm8508	1,9081	1
Hmga1-rs1	1,9079	0,67652
Tmem79	1,9074	1
Gm44254	1,9053	1
Gm26826	1,9033	0,52544
Gm36189	1,9030	0,58523
Mrpl54	1,9024	0,21057
Wdr54	1,9018	0,80567
Fam161b	1,9018	1
Gm15427	1,9001	0,11815
Gm4604	1,8966	0,41202
Slc36a3os	1,8963	1
Gm11450	1,8958	0,82709
1700001G11Rik	1,8958	1
Gm26549	1,8958	1
Gm6394	1,8955	0,30943
Gm8444	1,8945	0,76488
Gm13140	1,8933	1
Gm17786	1,8926	1
Trp53rka	1,8925	0,34035
4932416K20Rik	1,8924	1
Rps15-ps2	1,8922	0,95416
Gm45203	1,8922	0,99202
Sec24a	1,8911	0,19514
9130230L23Rik	1,8907	1
Rflnb	1,8899	1
Gm6863	1,8886	0,19332
Rps3a2	1,8882	1
Gm20302	1,8869	1

Slfn3	1,8860	1
RP23-312A24.1	1,8847	0,89436
Cd5l	1,8843	0,70799
Gm10704	1,8840	0,28676
Gins2	1,8824	0,53369
Gm38299	1,8824	1
Bloc1s1	1,8817	1
Plaur	1,8798	0,64463
Cenpp	1,8792	0,69297
Lin37	1,8783	0,87653
RP24-401G4.1	1,8771	0,48215
Gm4342	1,8771	1
Gmnn	1,8766	0,51263
Zmynd10	1,8763	1
Klf4	1,8751	0,13259
Gm9701	1,8748	0,33438
Gm6649	1,8745	1
Gm10616	1,8727	0,76024
Pea15a	1,8724	0,23493
Ddit4	1,8724	0,30068
Gm11652	1,8715	1
Gm37675	1,8705	1
Lrig3	1,8702	0,98427
Psmb7	1,8696	0,41266
Gm42670	1,8685	1
Daglb	1,8680	0,068948
Rasgrp4	1,8677	0,82903
Eno1	1,8675	0,52731
Gm31274	1,8663	0,94591
Gm9484	1,8649	1
Gatsl2	1,8648	0,077918
Gm14277	1,8639	0,68186
Gm6969	1,8635	1
Gm37420	1,8624	1
Gm11423	1,8623	0,6474
Arhgap25	1,8622	0,15168
D030056L22Rik	1,8621	0,11592
Gm6419	1,8621	1
Pfdn2	1,8619	0,1889
Fbxo5	1,8614	0,68648
Gm12222	1,8612	0,62546
Rps4x-ps	1,8608	0,81303
Carhsp1	1,8601	0,06718
RP23-149L23.1	1,8599	1
Gm2383	1,8590	0,97539
Icam4	1,8587	0,64353
Gm12074	1,8587	1
Hist1h4i	1,8577	0,21524
BC002163	1,8575	0,58499
Rpl18-ps2	1,8574	0,40078
Gadd45a	1,8574	0,63351
Hmmr	1,8568	0,3517

Rgs9bp	1,8556	1
Itga5	1,8554	0,6474
Mblac1	1,8552	0,79547
Gm15728	1,8551	0,85586
Gmfg	1,8542	1
Gm11675	1,8529	0,71535
Tsc22d3	1,8513	0,47653
Psmb6-ps2	1,8513	0,64706
Rps10-ps4	1,8504	1
Pcnx4	1,8487	0,64463
Rpl31-ps10	1,8487	1
Gm5446	1,8474	1
Gm5050	1,8457	0,78141
Uqcrh-ps1	1,8454	0,90083
Jsrp1	1,8452	0,85939
Gm12479	1,8452	1
Dnali1	1,8447	1
Pfdn6	1,8442	0,25031
Rps27a	1,8432	0,63802
Klhl25	1,8428	0,42264
Tnfrsf12a	1,8414	0,37883
Gm15500	1,8401	0,14603
Gm5837	1,8401	1
Ptchd1	1,8396	0,16818
Tmem256	1,8394	0,17534
Dedd2	1,8388	0,14229
Gm13268	1,8381	1
Hist1h2bc	1,8378	0,2262
4921531C22Rik	1,8354	1
Ppp1r35	1,8346	1
Cd300lf	1,8345	1
Ccna2	1,8336	0,30507
Rpl31-ps14	1,8331	0,65617
Gm13039	1,8325	0,5659
Ifi202b	1,8316	0,84074
Iscu	1,8302	0,45289
Gm43137	1,8301	1
Hsd17b10	1,8297	0,20751
Gm6977	1,8297	0,84074
Ralb	1,8293	0,14444
Rnps1	1,8293	0,47852
Ska1	1,8287	0,24516
Gm24890	1,8280	1
Tomm40l	1,8277	0,53566
Tuba1a	1,8275	0,2314
Ccdc50-ps	1,8273	0,58397
Zic2	1,8258	0,8621
Aplf	1,8250	0,82374
Cdk9	1,8248	0,51055
Gm36378	1,8246	0,85157
Gm38248	1,8244	1
Gm6794	1,8241	1

Gm13771	1,8225	0,90996
Gm20594	1,8212	1
Kifc1	1,8203	0,6223
Gm15420	1,8191	1
Ndufa4l2	1,8182	1
Pmm1	1,8168	0,19867
Rnaseh1	1,8163	1
Gm37065	1,8162	1
Dnah8	1,8147	1
Sapcd1	1,8138	0,15073
Gm22513	1,8134	1
Gm9497	1,8132	1
D130020L05Rik	1,8119	1
Xaf1	1,8118	1
Snord104	1,8105	0,33438
Gm15216	1,8098	1
Tmem107	1,8096	0,68186
Rpph1	1,8094	1
2010111I01Rik	1,8088	0,26752
Cks1brt	1,8088	1
Ppcs	1,8085	0,67042
Notch1	1,8075	1
Gm4859	1,8073	0,75127
Dhrs4	1,8070	0,87201
C130026I21Rik	1,8069	0,28613
Mical1	1,8064	1
Tdp2	1,8050	0,24711
S1pr1	1,8038	0,66842
Eif4a2	1,8035	0,60852
Eef1a1	1,8034	0,98408
Cops9	1,8033	0,21506
Ect2	1,8031	0,91944
Gm45716	1,8021	1
Rps6-ps3	1,8019	1
Zfp101	1,8015	0,90083
Gm527	1,8013	1
Prelid2	1,8009	0,24535
Rps27a-ps1	1,7998	0,45075
A530072M11Rik	1,7998	1
RP23-114G13.7	1,7995	0,67829
Gm26384	1,7994	0,25788
Vat1	1,7990	0,065045
5430416N02Rik	1,7990	0,61058
Pcna-ps2	1,7989	1
Gm38235	1,7980	1
Lmnb2	1,7978	0,57612
B330016D10Rik	1,7969	0,97217
Cfp	1,7966	0,65018
Mb21d2	1,7955	0,66623
Tiparp	1,7951	0,40631
A830008E24Rik	1,7951	1
Tma7-ps	1,7946	0,87376

Rps11-ps2	1,7945	0,54359
Gm8806	1,7944	0,98583
Lin54	1,7940	0,53755
Entpd1	1,7938	0,87771
H2-T23	1,7935	1
Gm8451	1,7934	0,60599
Gm5586	1,7930	0,98555
Pgm5	1,7920	0,97533
Gm12338	1,7919	0,78931
Ube2m	1,7910	0,39785
Gm5069	1,7904	1
Gm4968	1,7903	1
Gm5449	1,7897	1
Pomc	1,7892	0,98555
Cdkn2d	1,7884	0,18543
Zfp36l2	1,7863	0,61792
Rassf1	1,7862	0,19874
Gm13487	1,7862	0,51767
Gm17827	1,7855	1
Rmrp	1,7850	1
Gm13532	1,7847	0,98408
Lsp1	1,7844	0,2751
Gm8662	1,7840	0,73665
Mad2l1	1,7836	0,78141
Spag5	1,7834	0,75587
Uhrf1	1,7834	0,98117
Depdc1b	1,7834	1
Sec61g	1,7819	0,81947
Ppox	1,7813	0,79611
Ccdc117	1,7805	0,29393
Gm44916	1,7805	1
Bnip3l	1,7784	0,49514
Gm11131	1,7781	0,96352
Oaz1-ps	1,7779	1
Gm44771	1,7779	1
2610020C07Rik	1,7779	1
Gm27046	1,7765	1
Gm45212	1,7755	1
Gm10602	1,7753	0,99104
Zfp444	1,7747	0,44384
H2-K2	1,7746	0,76365
Nexn	1,7744	0,90083
Calm1	1,7737	0,045533
Gm12020	1,7737	0,45799
Gm8566	1,7737	0,86126
Gm12933	1,7728	1
2810013P06Rik	1,7724	0,76728
Gm7436	1,7720	0,92626
Haus3	1,7719	0,53755
Xxylt1	1,7712	1
Rps19-ps9	1,7709	1
Gm15013	1,7696	1

Gm11930	1,7690	1
Gngt2	1,7683	0,58795
Tfcp2l1	1,7683	1
Gm12696	1,7682	0,21506
Adh5	1,7672	0,29389
Kctd6	1,7669	0,68186
Rps4x	1,7665	1
Sapcd2	1,7664	0,9228
Gm7808	1,7660	1
Gm44423	1,7645	1
Snhg12	1,7644	0,14369
Bend3	1,7644	1
Zfp607b	1,7643	1
Smarca5-ps	1,7636	1
RP23-447C2.2	1,7632	0,94596
Zfp932	1,7628	0,7034
AA386476	1,7621	1
Gm45809	1,7621	1
RP24-365N15.9	1,7619	1
Ptrh1	1,7604	0,3134
Rps24-ps3	1,7604	0,31997
Rpl23a-ps5	1,7598	1
Gm2796	1,7592	0,32156
Gm7776	1,7587	0,54694
Med11	1,7580	0,85474
RP24-275P22.2	1,7575	0,9145
Gm38262	1,7565	1
Gm24991	1,7560	1
Gm45749	1,7558	1
Fgd2	1,7554	1
Zfa-ps	1,7554	1
H2-Q5	1,7554	1
Gm38387	1,7550	1
Rpl36al	1,7537	0,51263
Sclt1	1,7537	0,8742
Rpl23	1,7532	0,27503
Urm1	1,7525	0,55558
AA474408	1,7525	1
Slc25a38	1,7524	0,60093
E2f8	1,7524	1
Rps3a1	1,7514	0,54942
RP24-183O8.6	1,7509	1
Gm26670	1,7502	1
Rps8-ps3	1,7498	1
Wdr70	1,7491	0,51643
Spef1	1,7482	1
Gm22299	1,7463	1
Lztr1	1,7459	0,26066
Gm15753	1,7459	1
Insig2	1,7458	0,39072
Ccdc58	1,7452	0,55974
Ulk4	1,7435	0,91594

Gm3531	1,7429	1
2610528A11Rik	1,7424	1
Rps12-ps19	1,7415	1
Gm12005	1,7411	1
Efemp2	1,7404	1
Prelid1	1,7395	0,24739
Gm28659	1,7389	1
Gm8423	1,7387	1
Dhx58	1,7382	0,67829
Rad51	1,7380	0,97533
Unc5b	1,7380	1
Rps10-ps2	1,7378	0,79443
Gm27219	1,7362	0,79709
RP23-349H12.3	1,7360	1
Slc35a2	1,7350	0,84984
Rpl21-ps12	1,7347	1
RP23-193N1.2	1,7340	1
Rps7	1,7336	0,93197
Gm42549	1,7320	1
Atp5h	1,7308	0,54942
Eif1b	1,7306	0,20335
Gm6085	1,7299	0,26826
Map3k8	1,7299	0,5659
Gm24601	1,7294	1
D830050J10Rik	1,7291	0,97597
0610039K10Rik	1,7287	1
Oard1	1,7279	0,6507
Prcc	1,7274	0,9514
Arl4c	1,7270	0,26757
Klra2	1,7270	0,96561
Psmb8	1,7264	0,48837
Gm9790	1,7260	0,49875
Gm8185	1,7260	0,94459
Hmgb3	1,7258	0,86022
Sez6	1,7258	0,98583
Pmp22	1,7251	0,27199
Gm11363	1,7246	1
D130017N08Rik	1,7240	1
Rpl36a	1,7237	0,65887
Cks1b	1,7233	0,18739
BC055308	1,7232	1
Gm8606	1,7223	1
Cenpx	1,7217	0,65198
Hras	1,7211	0,55257
Gm7783	1,7203	1
Mafk	1,7198	0,61047
Eef1d	1,7192	0,44675
Gpam	1,7184	1
Ggct	1,7181	0,26414
RP24-378K7.3	1,7180	1
Csrp1	1,7175	0,52544
Anxa2	1,7174	0,4401

Smc2	1,7168	0,34157
Tpi1	1,7161	0,24739
Gm7380	1,7156	1
Gm15207	1,7155	1
Plekhf2	1,7146	0,52881
Tm2d3	1,7143	0,64463
1700120C14Rik	1,7143	1
RP23-187B11.16	1,7139	1
Insig1	1,7127	0,76488
Gm3362	1,7127	1
Gm25008	1,7120	1
Dok2	1,7119	0,27557
Cdc34	1,7114	0,60664
Gm42633	1,7114	1
Gm7701	1,7113	1
Gm5879	1,7111	1
Gm7984	1,7111	1
Mir703	1,7107	0,27193
Gm42793	1,7107	1
Gm24951	1,7105	0,44446
Slfn2	1,7099	0,32156
Gm9800	1,7096	0,85157
Gm6919	1,7095	1
Snhg8	1,7094	0,8911
Fbxl14	1,7091	1
Gm8930	1,7086	1
Selenoh	1,7082	0,37849
Gm7114	1,7079	1
Sap30l	1,7075	0,57309
Clec12a	1,7074	0,31892
Gm26664	1,7068	1
Fancd2	1,7067	1
Rps2-ps5	1,7063	1
Cfl1	1,7062	0,36821
Tusc3	1,7062	0,46328
Fcrl1	1,7061	0,64032
RP24-418P10.4	1,7061	1
Ppfia4	1,7055	0,87236
Cd300c2	1,7046	0,50782
Atp5l	1,7046	0,6488
BC051226	1,7046	0,77583
Fbxo44	1,7044	1
Gm26594	1,7040	1
Zfand2a	1,7036	0,4391
Ankrd35	1,7033	1
Setd4	1,7027	0,79345
Gm26782	1,7027	0,99338
Tmem219	1,7026	0,52544
Ccdc107	1,7022	0,91853
Gm8168	1,7014	1
Gm10031	1,7011	0,33478
Gm8722	1,7004	1

4933433G15Rik	1,7003	1
Rnf19a	1,6994	0,62989
Hip1r	1,6994	1
Gm5045	1,6985	1
Siva1	1,6983	0,68731
Gm12421	1,6982	1
AA914427	1,6977	1
Gm12396	1,6975	0,94315
Gm43961	1,6970	1
Dnajc19	1,6958	1
RP23-354J5.3	1,6955	0,96848
Usmg5	1,6949	0,96973
Rpl7	1,6947	1
9930012K11Rik	1,6944	0,87201
D8Ertd738e	1,6943	0,33273
Prmt2	1,6943	0,97196
Irf7	1,6941	1
Hotairm1	1,6938	0,63448
E230032D23Rik	1,6926	1
Gm9762	1,6924	0,77184
BC060293	1,6924	1
Fbxl3	1,6915	0,98705
Cenpe	1,6914	0,32215
5430403G16Rik	1,6913	1
Gm7964	1,6911	1
Smim11	1,6907	0,35875
Gm18943	1,6903	1
Cebpzos	1,6902	0,72587
Lrrc17	1,6900	1
Mrpl20	1,6890	0,69492
Pold1	1,6887	0,89006
Mcm3	1,6882	0,66147
Igfbp4	1,6875	0,2751
Layn	1,6873	0,28708
Fam19a2	1,6851	1
Gm15937	1,6845	1
Plp2	1,6835	0,74083
Spp1	1,6834	0,94315
Rps15a-ps5	1,6832	1
Rps19-ps7	1,6824	1
Lsm3	1,6821	0,8053
Gm7094	1,6821	1
Gm2950	1,6821	1
Gm43566	1,6816	1
9330020H09Rik	1,6802	1
Chmp2a	1,6800	0,60852
Suv39h2	1,6796	1
Cdc6	1,6793	0,90913
Gm4602	1,6789	1
Ercc6l	1,6788	1
Tbc1d30	1,6788	1
Gm6028	1,6781	1

Ccnd3	1,6780	0,40834
Gm15198	1,6767	0,71779
Oaz1	1,6767	0,97597
Gm13573	1,6766	1
Pcgf1	1,6761	1
Rpl27a	1,6746	0,48191
Gm13862	1,6745	1
Kntc1	1,6745	1
Gm35931	1,6740	1
Rpl10-ps2	1,6737	1
RP23-255F14.4	1,6735	1
Mllt10	1,6734	0,54859
Myl6	1,6733	0,83424
Rhoc	1,6731	0,24732
Tbcb	1,6728	0,84961
Gm20620	1,6724	0,98755
Lamtor4	1,6717	0,65198
Gm7504	1,6716	1
Tuba1b	1,6712	0,41634
Rps18	1,6711	0,87236
Rps19-ps6	1,6710	1
Rpl38	1,6705	0,70799
Ssna1	1,6699	0,66389
Zwilch	1,6698	1
Rpl26-ps2	1,6698	1
Scp2-ps2	1,6691	1
Gm7856	1,6687	1
Gm14513	1,6686	1
Etv3	1,6677	0,66237
Cdkn2c	1,6673	1
S100a1	1,6668	0,63448
Pomp	1,6667	0,59191
Tmco3	1,6665	0,85558
Gm32340	1,6662	1
Gm44557	1,6659	1
Serpine1	1,6639	0,52687
Zcchc24	1,6638	0,94311
Whamm	1,6630	0,76112
Gm16124	1,6626	1
Ska3	1,6614	1
Gm20045	1,6609	1
Rpl18-ps1	1,6604	0,67214
Gm37760	1,6596	0,9873
Snord89	1,6581	1
Gm15159	1,6578	1
Gm12693	1,6568	0,96052
Rpl30-ps5	1,6567	1
Nenf	1,6566	0,51204
Ypel2	1,6563	0,97533
Arf2	1,6559	0,38448
Gm8925	1,6559	1
Stk16	1,6548	0,85161

Gm17994	1,6547	1
Cadm1	1,6546	0,26485
Gm13436	1,6546	1
Fbxl12os	1,6530	1
Gm8186	1,6526	0,62028
Gm13340	1,6524	1
Gm5075	1,6522	1
Gm6576	1,6519	1
Btbd1	1,6518	0,60396
Gm4978	1,6518	1
Hint2	1,6514	0,84824
Polr1c	1,6512	0,69661
Gm5436	1,6507	1
Smagp	1,6505	0,56603
Necap1	1,6502	0,61792
Uggt2	1,6502	1
Gm10269	1,6486	0,93174
Mrps21	1,6486	0,94312
Cdc20	1,6472	0,72197
Ndufab1-ps	1,6470	1
Gm15824	1,6466	1
Tor1aip2	1,6465	0,49338
Lonrf3	1,6462	0,78662
Snord118	1,6462	1
Zfp263	1,6460	0,52873
Hsf2bp	1,6457	1
Fam64a	1,6456	0,93197
4930412F12Rik	1,6454	1
Gm44957	1,6452	1
RP23-110E20.5	1,6439	1
Gm10250	1,6438	1
Tns1	1,6431	1
P4ha1	1,6430	0,50603
Mcm7	1,6426	0,81653
Sdc4	1,6421	0,64353
Haus6	1,6421	0,73648
Nrp1	1,6421	1
Gm7287	1,6418	1
Pdgfb	1,6411	1
Dyrk3	1,6397	0,71189
Gm20432	1,6397	1
Gm14325	1,6392	1
Rassf8	1,6389	0,87771
Clec10a	1,6385	0,89436
Coprs	1,6383	0,78931
Tubb2a	1,6381	0,65018
Npm3	1,6378	0,96052
Cryz	1,6373	0,52994
Gm5873	1,6372	1
Gm6272	1,6372	1
Rps12-ps24	1,6371	1
Naca	1,6365	0,83802

Gm16439	1,6365	1
Rnf7	1,6363	0,73013
C530043K16Rik	1,6359	1
Crip2	1,6354	1
Gm5745	1,6349	1
Gm12751	1,6349	1
Pcif1	1,6340	0,65939
Supt4a	1,6338	0,97533
1810041H14Rik	1,6333	1
Mief2	1,6332	0,80744
Cbfa2t3	1,6332	1
Gm44013	1,6332	1
Mrpl51	1,6329	0,74005
Pdcd2l	1,6326	0,85435
Nt5c	1,6324	1
Gm26930	1,6320	1
1500011K16Rik	1,6318	1
RP24-370M23.1	1,6318	1
Lrsam1	1,6316	1
Gm12176	1,6316	1
F2	1,6315	1
G430095P16Rik	1,6313	1
Gm37108	1,6312	1
Cnih1	1,6305	0,66237
Enho	1,6299	1
Zfp84	1,6297	0,93133
Sult6b1	1,6291	1
Gm7027	1,6282	1
Dleu2	1,6280	1
Tmem158	1,6279	0,91613
Supt20	1,6278	0,85676
Usp1	1,6277	0,59876
Cldn11	1,6277	0,84961
Plekhf1	1,6272	1
Gm5580	1,6269	1
2010015M23Rik	1,6263	1
Lrfn4	1,6263	1
Gm45729	1,6259	1
Hoxa3	1,6256	1
Eif3k	1,6252	0,53181
Gm8172	1,6251	1
Gm6368	1,6250	1
Mapkapk5	1,6248	1
Gm4673	1,6246	1
Ccdc92b	1,6245	1
Ifi27l2a	1,6244	0,94459
Gm15417	1,6241	0,81653
Gm20900	1,6227	1
Rpl27	1,6225	1
Asb10	1,6224	1
Gm12529	1,6223	1
Gm36445	1,6223	1

Iqgap3	1,6221	1
Ube2t	1,6218	1
Gm19739	1,6211	0,76028
H60b	1,6210	0,64463
Rpl21-ps14	1,6210	1
Rpl37rt	1,6209	0,70313
Zmym6	1,6208	1
Rpl31-ps11	1,6207	1
Efr3b	1,6203	0,97533
Gm7972	1,6183	1
Gm18737	1,6181	1
RP23-23P9.3	1,6181	1
Atp5c1	1,6178	0,79094
Oas1b	1,6177	1
Prrg2	1,6172	0,87653
RP23-320D23.6	1,6172	1
Pif1	1,6170	1
Pet100	1,6165	0,94337
C330011M18Rik	1,6165	1
Gm6851	1,6163	1
Tmem261	1,6160	0,74233
Arhgef39	1,6159	0,92074
Gm9645	1,6151	1
Mrpl11	1,6150	0,88028
Chka	1,6148	0,89353
Atp9a	1,6142	0,8053
Xpa	1,6133	0,87236
Gm11464	1,6128	1
Gm14822	1,6127	1
Tmub1	1,6123	0,72433
Hist4h4	1,6123	1
Ccsap	1,6122	1
Gm14121	1,6122	1
Rep15	1,6110	1
Rpsa-ps9	1,6104	1
Bbc3	1,6103	0,87201
Gm44851	1,6089	1
Rny1	1,6086	1
Cdnf	1,6083	1
Metrnl	1,6077	0,62824
Gm8618	1,6075	1
Tomm5	1,6071	1
Cfap126	1,6063	1
Llph	1,6059	0,84961
Fcna	1,6051	0,97533
Trim3	1,6051	1
Rps18-ps3	1,6050	1
Rnaseh2c	1,6049	1
Rps16	1,6047	1
Foxd2os	1,6041	1
Pex5	1,6040	1
Eif3f	1,6035	0,66237

Plaa	1,6026	0,97533
Chchd6	1,6025	0,95278
Gm27605	1,6025	1
Gm12430	1,6025	1
Gm12848	1,6021	1
H3f3b	1,6020	1
Gm6733	1,6018	1
Zfp326	1,6013	0,80552
Gtse1	1,6013	1
Inafm1	1,6010	0,84824
Gm14056	1,6007	1
Pole	1,6007	1
Ero1l	1,6000	0,78287
Chic2	1,5998	0,74693
RP24-454N4.2	1,5993	0,99346
Pxmp2	1,5991	1
Gm10086	1,5988	0,80486
RP23-43M12.2	1,5987	1
Bnip3	1,5984	0,75197
G6pc3	1,5984	0,91853
Ccl4	1,5977	0,76324
Tmem171	1,5973	1
Hcar2	1,5968	1
Rc3h1	1,5966	0,91874
Arsb	1,5962	1
Mapk1ip1	1,5960	0,95518
Tnni3	1,5960	1
Gpx4	1,5957	1
Cdc25c	1,5950	1
Snhg5	1,5945	0,94124
Gm9844	1,5939	1
St3gal6	1,5929	0,74233
Pafah1b3	1,5929	0,90825
Syf2	1,5925	0,80852
Gm5321	1,5925	1
Murc	1,5924	1
Bloc1s4	1,5920	0,90288
Ndc80	1,5914	1
Ap2a1	1,5906	0,97533
Mea1	1,5905	1
Gm7535	1,5900	1
Dgcr6	1,5893	1
Gm45873	1,5891	1
Gm38022	1,5886	1
Vdr	1,5873	1
Gm5362	1,5872	1
Gm5276	1,5868	1
Ssr4	1,5867	0,97533
Selenbp1	1,5867	1
Zscan21	1,5867	1
Hnrnpf	1,5864	1
RP23-261O3.5	1,5860	1

Fam174a	1,5853	0,90043
Gm6598	1,5852	1
Crlf2	1,5848	1
Rpl31-ps17	1,5844	1
Lgals3	1,5841	0,63014
Rdm1	1,5841	0,97248
Gm5384	1,5839	1
H2-Q10	1,5838	1
Gm6198	1,5834	1
Fam26f	1,5830	1
Nr2f6	1,5828	0,9873
Gm13567	1,5827	1
Atg101	1,5825	0,96052
Cd300ld	1,5819	1
1700084E18Rik	1,5818	1
Neil3	1,5817	1
Rpsa-ps1	1,5813	1
Angptl6	1,5810	1
1810044D09Rik	1,5805	1
Rpl18	1,5803	0,96561
Mutyh	1,5803	1
Gm5139	1,5794	1
Guk1	1,5792	0,9514
Lars2	1,5779	1
Gm13009	1,5779	1
Proscos	1,5776	1
Syne3	1,5767	0,94767
Gm28727	1,5759	1
Tatdn2	1,5758	0,98755
Tspan13	1,5757	0,65182
Tor3a	1,5757	0,85308
Gm5451	1,5757	1
Gm7351	1,5757	1
Polr2d	1,5756	1
Ifi213	1,5755	1
C030034I22Rik	1,5754	1
Basp1	1,5752	1
Smc4	1,5751	0,85715
Rpl3	1,5748	1
Gm12857	1,5746	0,97845
Gm5944	1,5745	1
Tcta	1,5741	1
Dhrs7b	1,5740	1
Mbd4	1,5734	1
Racgap1	1,5731	0,9979
Rps12	1,5727	1
Gm7123	1,5727	1
6430710M23Rik	1,5727	1
Avpi1	1,5716	0,91853
Gm44935	1,5712	1
Zdhhc18	1,5706	1
Pwwp2b	1,5706	1

Bcl6b	1,5705	1
Jtb	1,5704	0,94737
Kcnk13	1,5701	1
Gm10059	1,5701	1
Zfp949	1,5699	1
Plin2	1,5688	0,93174
Gm26397	1,5688	1
Tmie	1,5687	1
Irf2bpl	1,5685	0,77601
Ctu2	1,5684	1
Stk19	1,5679	0,99897
Blvrb	1,5673	0,72997
Snx8	1,5673	0,83707
D930030I03Rik	1,5671	1
Rps17	1,5669	1
Gins1	1,5669	1
4930427A07Rik	1,5668	1
Gm11688	1,5663	1
Usp12	1,5660	1
Tmem106a	1,5659	0,83554
Gm15730	1,5657	1
Lin7b	1,5657	1
Cdca8	1,5637	0,81088
Rps19-ps11	1,5636	1
Gm9625	1,5633	1
Pold4	1,5632	1
Serf2	1,5632	1
Mastl	1,5632	1
Myc	1,5632	1
Gm4366	1,5625	1
Syce2	1,5620	0,85401
Mocs2	1,5620	1
Arhgap27os2	1,5619	1
Zbtb32	1,5619	1
2700062C07Rik	1,5619	1
Gm8326	1,5619	1
RP24-316F13.7	1,5616	1
Tcea1-ps1	1,5614	1
Lilrb4a	1,5612	1
Mynn	1,5609	1
Map3k9	1,5609	1
Gm6382	1,5608	1
Uqcc2	1,5605	0,97597
Gm23458	1,5604	1
Gm14165	1,5601	1
Rpl18a	1,5600	1
Mcm6	1,5598	1
Anp32b-ps1	1,5598	1
Pgam1	1,5593	1
Gm11944	1,5592	1
Gm9332	1,5588	1
Ldha	1,5584	0,70031

H2-Q7	1,5583	1
E2f6	1,5581	0,98408
Sars	1,5580	1
Polh	1,5579	1
Cmss1	1,5578	1
Cd83	1,5569	0,90494
1110008P14Rik	1,5569	1
Ecm1	1,5567	0,94767
Fis1	1,5566	1
Arl13b	1,5564	1
Gm2223	1,5564	1
Exoc3l	1,5564	1
Gm8805	1,5561	1
Gemin6	1,5561	1
Cdca4	1,5552	0,9336
Rps15a-ps6	1,5543	1
Polg2	1,5533	1
Acadl	1,5526	0,93133
H2-Ob	1,5525	1
Cks2	1,5522	1
Gm6743	1,5521	1
Triap1	1,5516	0,97597
Rpl28	1,5514	1
Metrn	1,5512	1
Mb21d1	1,5509	0,85583
BC022687	1,5508	1
Gm38375	1,5505	1
Ppic	1,5498	0,90288
Dpysl2	1,5491	1
Rpl17	1,5491	1
Eldr	1,5486	1
Gm37959	1,5485	1
Rps19-ps5	1,5483	1
Nup50	1,5482	0,99338
Uqcr11	1,5480	1
Gramd4	1,5479	1
Aamdc	1,5478	1
Bahcc1	1,5474	1
Use1	1,5456	1
Slc25a2	1,5455	1
Mmaa	1,5453	1
Gm11918	1,5448	1
Gm18860	1,5448	1
4930579K19Rik	1,5448	1
Saraf	1,5445	0,78662
Trappc6a	1,5441	0,96052
Tmsb4x	1,5434	1
Gm3511	1,5434	1
Zfp503	1,5434	1
Sag	1,5433	1
RP23-2N7.4	1,5430	1
Rps14	1,5429	1

2310034G01Rik	1,5423	1
Txndc15	1,5421	1
Gm5865	1,5420	1
Gm34121	1,5420	1
Prc1	1,5419	1
Fignl1	1,5416	1
Dus2	1,5415	1
Gm13422	1,5411	1
Clmp	1,5411	1
Per1	1,5405	1
Gm14539	1,5405	1
Mydgf	1,5404	1
Mesdc1	1,5398	1
Gm7363	1,5395	1
Tgfb1	1,5394	1
Adcy6	1,5392	1
Arl3	1,5390	1
Rpl18a-ps1	1,5385	1
Gm11474	1,5384	1
Cdca3	1,5376	1
Gm13015	1,5375	1
Id2	1,5373	1
Cbx1	1,5372	0,83219
Ppp1r2	1,5366	1
A430046D13Rik	1,5366	1
Gm10146	1,5366	1
Dctpp1	1,5362	1
Ube2g2	1,5359	1
Rpl9-ps6	1,5355	1
Gm7658	1,5345	1
9530078K11Rik	1,5342	1
Mrps23	1,5340	1
Fcf1	1,5340	1
Rfc3	1,5336	1
Rps8	1,5335	0,85586
Vmac	1,5329	1
Gm8318	1,5329	1
Bcl2l14	1,5322	1
Svep1	1,5317	1
Ptpn2	1,5316	1
Dlgap5	1,5316	1
Igbp1	1,5315	1
Pclaf	1,5312	1
Dusp1	1,5308	1
Gm16380	1,5306	1
Dok4	1,5300	1
Gm13050	1,5299	1
C330027C09Rik	1,5294	1
BC031181	1,5292	1
Znhit2	1,5288	1
Gatad1	1,5284	1
Gorab	1,5284	1

Leng9	1,5283	1
Morn2	1,5278	1
Gm7128	1,5277	1
Gm11605	1,5277	1
Asf1a	1,5276	1
Ifi35	1,5274	1
Ubfd1	1,5272	1
Gm38297	1,5272	1
Rpsa-ps10	1,5270	1
Gm37531	1,5262	1
Miip	1,5259	1
5930420M18Rik	1,5258	1
Snrpg	1,5257	1
Gm6433	1,5254	1
Clk3	1,5253	0,97217
1110046J04Rik	1,5251	1
Abhd17c	1,5248	1
Uchl5	1,5245	1
Gm11808	1,5243	1
Zfp655	1,5239	1
Fzd5	1,5236	1
Sugt1	1,5235	1
Gbe1	1,5235	1
Fam13b	1,5233	1
Cdc7	1,5233	1
Ubl4a	1,5230	1
Gm11598	1,5230	1
Emc10	1,5228	1
Cenpa	1,5227	0,98058
Emilin2	1,5226	0,96052
Zfp146	1,5222	1
Hps5	1,5217	1
Swap70	1,5214	1
Gm8494	1,5213	1
Gm10126	1,5211	1
1500015A07Rik	1,5207	1
Gm5093	1,5207	1
Aimp1	1,5206	1
H2-K1	1,5203	1
Zfand4	1,5198	1
Cebpa	1,5195	1
Hist1h2an	1,5191	1
Gm4895	1,5188	1
Tacc3	1,5187	1
Lck	1,5178	1
Slc44a2	1,5176	1
Gm20072	1,5176	1
Gm44152	1,5176	1
Vhl	1,5175	1
Ccdc34	1,5174	1
Arhgap19	1,5167	1
Rcc1l	1,5163	1

Ropn1l	1,5163	1
Clu	1,5159	1
Gm15506	1,5158	1
Creb3	1,5155	1
Gm17494	1,5153	1
Gm6905	1,5150	1
K230015D01Rik	1,5149	1
Dsn1	1,5147	1
Tmed3	1,5146	1
Gm8667	1,5145	1
Rpl30-ps11	1,5142	1
2410015M20Rik	1,5141	1
Cdc26	1,5138	1
Gm11989	1,5134	1
Gng8	1,5129	1
Egln1	1,5121	1
Rpl31-ps16	1,5118	1
Hpse	1,5113	1
Rpl35	1,5112	1
Gm15472	1,5108	1
RP23-288C18.3	1,5107	1
0610005C13Rik	1,5105	1
2700097O09Rik	1,5095	1
Rpl5	1,5094	1
Pttg1	1,5090	1
Ncapg	1,5090	1
Dnajb6	1,5086	1
Tmem218	1,5086	1
Golga7	1,5084	1
C330013E15Rik	1,5083	1
Gm5812	1,5081	1
Ddx20	1,5080	1
1500002F19Rik	1,5079	1
Dbr1	1,5074	1
Rad23a	1,5070	1
Prkd2	1,5070	1
Ptger4	1,5067	1
Suco	1,5062	1
Flrt2	1,5062	1
Ppwd1	1,5061	1
Polr3g	1,5061	1
Arpc5l	1,5052	1
Cep72	1,5049	1
Atp5j	1,5048	1
Gm37699	1,5046	1
Gm43162	1,5046	1
Hmgn5	1,5044	1
H2-M3	1,5044	1
Gm4890	1,5044	1
Tex30	1,5042	1
Gm15727	1,5038	1
Gm12618	1,5036	1

Gm10941	1,5027	1
Casz1	1,5026	1
3110031N09Rik	1,5022	1
Pot1b	1,5017	1
E130317F20Rik	1,5015	1
Rps6-ps4	1,5013	1
Arf6	1,5012	1
Gins3	1,5011	1
Ndufb7	1,4996	1
Itgb1bp1	1,4994	1
Gm6415	1,4991	1
Leng1	1,4989	1
3110062M04Rik	1,4986	1
Il11ra1	1,4983	1
Gm37009	1,4983	1
Gstcd	1,4978	1
Rab1b	1,4970	1
Nfkbib	1,4964	1
Nxt1	1,4963	1
Gm11491	1,4961	1
Chchd3	1,4960	1
Crebrf	1,4959	1
Gm42515	1,4959	1
Arpp19	1,4957	1
Bola2	1,4957	1
Gm5835	1,4943	1
4930563E22Rik	1,4942	1
Mrpl23	1,4941	1
Sac3d1	1,4939	1
Tmem132a	1,4932	1
Angptl4	1,4927	1
Gm45477	1,4923	1
Hexb	1,4920	1
Crygn	1,4920	1
Ccdc124	1,4918	1
Yipf7	1,4918	1
Rps15	1,4917	1
Spink10	1,4916	1
Rps13-ps7	1,4916	1
Mrps18c	1,4915	1
Gm42798	1,4914	1
Vcpkmt	1,4910	1
Rps11	1,4909	1
Ryr1	1,4909	1
Tnnt3	1,4908	1
Ddx47	1,4906	1
Erf	1,4894	1
Paqr4	1,4893	1
Rpl17-ps8	1,4892	1
Rer1	1,4887	1
Hcst	1,4885	1
Ypel4	1,4882	1

Fgf13	1,4870	1
Pigb	1,4870	1
Flt3l	1,4864	1
Hist1h2ae	1,4863	1
Rpl14-ps1	1,4861	1
Gm20430	1,4859	1
Sgta	1,4858	1
H3f3a	1,4854	1
Asb6	1,4854	1
Gm43309	1,4851	1
RP23-65M10.2	1,4851	1
Mrpl18	1,4850	1
Klhl28	1,4849	1
Gm15542	1,4849	1
Atg12	1,4847	1
Etv5	1,4845	1
Gm42743	1,4845	1
Tmem208	1,4843	1
Rps23	1,4839	1
Ndufb2	1,4837	1
Smox	1,4832	1
Gm6378	1,4832	1
Mrps25	1,4831	1
RP23-13B8.12	1,4827	1
Tfpt	1,4827	1
Cic	1,4824	1
Gm6030	1,4824	1
Adgre5	1,4820	1
Gm42893	1,4817	1
Gm9392	1,4814	1
Zfp553	1,4812	1
Gm9385	1,4810	1
6230400D17Rik	1,4810	1
Gm4994	1,4807	1
Tomm7	1,4805	1
Tstd3	1,4802	1
Gm9711	1,4801	1
Dalrd3	1,4794	1
Gm14813	1,4794	1
Gm6913	1,4794	1
Ap3s1	1,4792	1
Arhgap11a	1,4786	1
Pim1	1,4785	1
Gm9169	1,4784	1
Gm13680	1,4784	1
1810026B05Rik	1,4779	1
Coil	1,4779	1
Hps6	1,4776	1
Slc25a20	1,4773	1
Rpl10a-ps1	1,4772	1
Cox16	1,4772	1
Cmc2	1,4764	1

Cuedc2	1,4764	1
8430429K09Rik	1,4764	1
Thap1	1,4762	1
Ndufaf2	1,4754	1
Dnajb2	1,4747	1
Kif15	1,4747	1
Psmb1	1,4746	1
Gm6768	1,4746	1
Bsn	1,4740	1
Gtf2f2	1,4738	1
Mgat1	1,4736	1
Gm13611	1,4736	1
RP23-426K2.3	1,4735	1
Susd3	1,4732	1
Gm7452	1,4729	1
Rala	1,4727	1
Rpl38-ps2	1,4727	1
Gm43775	1,4723	1
Rpl23a-ps3	1,4719	1
Zfp651	1,4717	1
Ndufa12	1,4716	1
Gm15829	1,4714	1
Inhbe	1,4713	1
Tapbpl	1,4712	1
Chchd10	1,4712	1
Zfp709	1,4709	1
Mtfp1	1,4707	1
BC030336	1,4706	1
Zfp367	1,4706	1
Nmrk1	1,4702	1
Ak4	1,4693	1
Rps19	1,4688	1
Etfbkmt	1,4686	1
Gm2991	1,4679	1
Anapc7	1,4674	1
Ring1	1,4674	1
Apoo	1,4674	1
3110009E18Rik	1,4665	1
Gm4332	1,4660	1
Anapc13	1,4654	1
Gm5523	1,4649	1
Tma16	1,4647	1
Gm9843	1,4642	1
Fam187b	1,4641	1
Mphosph10	1,4640	1
Tpx2	1,4640	1
Mrpl12	1,4638	1
Ccdc137	1,4637	1
Mien1	1,4634	1
Cdk1	1,4633	1
Gm23849	1,4630	1
Gm9530	1,4629	1

Lamtor5	1,4628	1
Arhgap27os1	1,4627	1
Pfdn1	1,4624	1
Snhg9	1,4622	1
Gm43201	1,4604	1
B230317F23Rik	1,4604	1
Ado	1,4600	1
0610038B21Rik	1,4600	1
Gm12174	1,4598	1
Vezt	1,4594	1
Slc15a4	1,4593	1
Setdb2	1,4593	1
Alms1	1,4591	1
Gm15446	1,4590	1
Iah1	1,4584	1
Rpl21	1,4584	1
E330020D12Rik	1,4578	1
Aimp2	1,4577	1
Ly6g6d	1,4577	1
Tuba4a	1,4576	1
Tcof1	1,4571	1
Spc25	1,4567	1
Ltb	1,4567	1
Cdkal1	1,4554	1
Rrh	1,4549	1
Gm18889	1,4548	1
Gtf2f1	1,4543	1
Zfp595	1,4541	1
Gm16062	1,4534	1
Dclre1a	1,4530	1
Rpl19-ps9	1,4530	1
Rpl38-ps1	1,4528	1
Gm12017	1,4528	1
Fam195a	1,4513	1
Nabp2	1,4511	1
Dgkh	1,4509	1
Luc7l3	1,4507	1
D830025C05Rik	1,4505	1
Snrpert	1,4500	1
Nudt19	1,4498	1
Nkap	1,4497	1
Mvb12a	1,4491	1
2900009J06Rik	1,4482	1
Nostrin	1,4480	1
Cdca5	1,4480	1
Sertad2	1,4479	1
D330045A20Rik	1,4479	1
Engase	1,4478	1
Sys1	1,4476	1
Gm7899	1,4475	1
1110038B12Rik	1,4473	1
Tiprl	1,4473	1

Ddias	1,4473	1
Rps29	1,4465	1
Vamp2	1,4464	1
Padi2	1,4462	1
Atad5	1,4462	1
Gm23751	1,4462	1
Gng5	1,4459	1
Gm5297	1,4458	1
Gm17039	1,4458	1
Snrpd2	1,4453	1
Plekhj1	1,4450	1
Hspe1	1,4448	1
Atp6v1g1	1,4448	1
Ptgs2	1,4447	1
Gm4742	1,4441	1
Gm12481	1,4440	1
Rfxap	1,4440	1
Cox7a1	1,4440	1
Hdc	1,4435	1
Gm12034	1,4433	1
A930029G22Rik	1,4431	1
Btbd6	1,4428	1
Sla2	1,4427	1
Dnlz	1,4425	1
Zfyve19	1,4424	1
Cpeb1	1,4424	1
Cds1	1,4416	1
Dab2	1,4414	1
Gm13604	1,4413	1
Srp9	1,4408	1
Pdcd7	1,4407	1
Hdac3	1,4407	1
Rrm2	1,4406	1
Gm11889	1,4406	1
Prdx4	1,4403	1
Rpsa-ps12	1,4403	1
Dennd5a	1,4401	1
Gm2895	1,4401	1
Kdm4c	1,4397	1
Eif1-ps1	1,4395	1
Hcn2	1,4394	1
Hoxb8	1,4391	1
Zfp637	1,4390	1
Rubcnl	1,4383	1
Tnnt1	1,4379	1
Ergic3	1,4378	1
Selenom	1,4377	1
Jmjd6	1,4376	1
Bub1b	1,4371	1
Amn1	1,4370	1
Svil	1,4368	1
Prdm10	1,4365	1

Aktip	1,4364	1
Gm6654	1,4363	1
2410006H16Rik	1,4360	1
5730508B09Rik	1,4359	1
Gm9013	1,4358	1
Il7r	1,4358	1
Gm20442	1,4353	1
Pdcd1	1,4350	1
Zbtb18	1,4349	1
Prkag1	1,4348	1
Snrpa1	1,4348	1
Gm23969	1,4347	1
RP23-162P10.8	1,4343	1
Man2c1os	1,4341	1
Lgals4	1,4339	1
Gas5	1,4338	1
Gm5611	1,4338	1
Ambp	1,4337	1
Akirin1	1,4335	1
Pard6b	1,4332	1
Ggps1	1,4329	1
G2e3	1,4326	1
Mrpl9	1,4322	1
Cd302	1,4322	1
Topors	1,4317	1
2500002B13Rik	1,4317	1
Bcl2	1,4316	1
Rapgef5	1,4313	1
Snrpd1	1,4311	1
1700084J12Rik	1,4308	1
Ntmt1	1,4303	1
Nuf2	1,4303	1
Pax3	1,4295	1
2310074N15Rik	1,4291	1
Ube2d1	1,4290	1
Gm45113	1,4290	1
Samd8	1,4286	1
Hmgn1	1,4285	1
Rnf139	1,4285	1
Ndufa6	1,4284	1
Rps26-ps1	1,4284	1
Exosc2	1,4282	1
Gm12912	1,4282	1
Tmem29	1,4281	1
Gprasp1	1,4280	1
Trit1	1,4277	1
RP24-282C4.13	1,4277	1
Ctnnbip1	1,4276	1
Syt11	1,4275	1
Pofut2	1,4271	1
Cdkn2aip	1,4266	1
Chchd7	1,4263	1

Trmt10a	1,4262	1
Cd200r1	1,4262	1
Smdt1	1,4261	1
RP23-226H21.3	1,4260	1
Bub1	1,4259	1
Xirp1	1,4259	1
Tra2b	1,4256	1
Ufsp1	1,4256	1
RP24-550H10.3	1,4253	1
Tsr3	1,4252	1
Rad9a	1,4252	1
Gon7	1,4251	1
Star	1,4251	1
Gm29257	1,4250	1
Gemin4	1,4250	1
Dtymk	1,4249	1
Gcnt1	1,4241	1
Lgals1	1,4240	1
Jund	1,4240	1
Hyi	1,4239	1
Wdr18	1,4238	1
Riox1	1,4236	1
Rgs1	1,4234	1
Mrpl55	1,4233	1
Gm11759	1,4232	1
Fam219a	1,4231	1
Nck1	1,4230	1
Ipp	1,4230	1
Ntpcr	1,4228	1
Eef1g	1,4225	1
Pygl	1,4225	1
Mrpl33	1,4220	1
Zkscan14	1,4220	1
Rad51d	1,4218	1
Rab3a	1,4217	1
Pnkd	1,4212	1
Hspa8	1,4207	1
2210013O21Rik	1,4206	1
Casp8ap2	1,4194	1
Gm2058	1,4193	1
Uqcc3	1,4191	1
Mad2l1bp	1,4189	1
Hsp90aa1	1,4189	1
Gng2	1,4187	1
Ppil1	1,4186	1
Gm14239	1,4184	1
Gm5867	1,4182	1
Gm11488	1,4181	1
Cinp	1,4181	1
Gm5576	1,4180	1
Gm7832	1,4177	1
Sort1	1,4169	1

Aurkb	1,4169	1
Reep5	1,4167	1
Napsa	1,4163	1
5830487J09Rik	1,4163	1
Sgk1	1,4161	1
Polr2j	1,4160	1
Ric8b	1,4159	1
Cd44	1,4158	1
Rbmx2	1,4156	1
Timm13	1,4154	1
Nsmce4a	1,4151	1
Gng12	1,4147	1
Gm31166	1,4145	1
Gm9835	1,4144	1
Bcs1l	1,4142	1
Gm4204	1,4139	1
Gm4374	1,4131	1
Gm6155	1,4128	1
Top3a	1,4126	1
A730062M13Rik	1,4126	1
Rpl23a	1,4121	1
Slc25a19	1,4120	1
3110056K07Rik	1,4119	1
Txnl4a	1,4118	1
Cdc45	1,4115	1
Tesk1	1,4114	1
Taf1d	1,4111	1
Bola1	1,4110	1
Aldoart1	1,4109	1
Gm6457	1,4102	1
Timm22	1,4099	1
Tmem11	1,4099	1
Sptssa	1,4095	1
Gm42747	1,4093	1
Cdyl2	1,4092	1
Snhg15	1,4091	1
Gorasp1	1,4087	1
Morf4l2	1,4084	1
Mturn	1,4083	1
Itga11	1,4083	1
Gm12989	1,4081	1
Ing2	1,4081	1
Pin4	1,4073	1
Apopt1	1,4070	1
Tmem231	1,4067	1
Slc8b1	1,4064	1
Gm12943	1,4063	1
Arl10	1,4063	1
Nemp1	1,4059	1
Fam83d	1,4059	1
Cystm1	1,4057	1
Psma4	1,4057	1

Dqx1	1,4055	1
Mettl21b	1,4054	1
Tmem206	1,4054	1
Emc8	1,4054	1
Gm9246	1,4053	1
Ube2v1	1,4049	1
Gm12459	1,4045	1
Gm12240	1,4045	1
Rassf2	1,4043	1
Gm14567	1,4042	1
Gstp1	1,4040	1
Anxa7	1,4040	1
Coq6	1,4037	1
Gm17249	1,4036	1
Polr2c	1,4032	1
Pdrg1	1,4030	1
Mrpl49	1,4029	1
Rab10os	1,4025	1
Commd1	1,4023	1
Gm10268	1,4022	1
2410131K14Rik	1,4021	1
Washc1	1,4016	1
Gm5619	1,4015	1
Map3k14	1,4015	1
Speer9-ps1	1,4015	1
Cycs	1,4013	1
Gm15484	1,4009	1
Arrdc3	1,4008	1
Taf5	1,4006	1
Mrpl52	1,4006	1
Psmb10	1,4004	1
Gm14584	1,4003	1
Gm37423	1,4000	1
Cd52	1,4000	1
Gm10358	1,3998	1
Hoxa7	1,3996	1
Rbm3	1,3996	1
Cenpi	1,3995	1
Ensa	1,3995	1
Wdr55	1,3994	1
Fam133b	1,3989	1
Rpl22-ps1	1,3988	1
Kxd1	1,3982	1
Commd5	1,3980	1
Kif23	1,3979	1
Tmco4	1,3975	1
1110004F10Rik	1,3973	1
Cwc15	1,3973	1
Hscb	1,3971	1
Gm15148	1,3964	1
5830432E09Rik	1,3961	1
Fnta	1,3961	1

Gm17511	1,3958	1
Stoml1	1,3958	1
Aph1a	1,3957	1
Chchd5	1,3956	1
Myl12b	1,3951	1
Nckap1	1,3949	1
Mettl23	1,3948	1
Parpbp	1,3947	1
Rpl3-ps2	1,3944	1
RP24-310D17.9	1,3940	1
Dusp4	1,3938	1
Utp23	1,3937	1
Ndufv2	1,3935	1
Fam110a	1,3932	1
Tcte2	1,3931	1
Gm10784	1,3931	1
Gm14427	1,3929	1
Tmem240	1,3926	1
Mir17hg	1,3925	1
Fads3	1,3924	1
Gm5453	1,3923	1
Zfp593	1,3922	1
Nop10	1,3922	1
Klf6	1,3920	1
Psme1	1,3919	1
Rrm1	1,3915	1
Riiad1	1,3912	1
Gm17690	1,3910	1
Smap2	1,3909	1
Trpm4	1,3907	1
Gm12038	1,3906	1
Med4	1,3905	1
2700038G22Rik	1,3903	1
Pabpn1	1,3901	1
2700060E02Rik	1,3900	1
Alkbh7	1,3898	1
Atp5k	1,3894	1
Fbxo6	1,3892	1
Atp5j2	1,3891	1
Rps15a-ps4	1,3886	1
Clec4a2	1,3886	1
Gm38340	1,3878	1
Napepld	1,3877	1
Sdccag3	1,3877	1
Gm6542	1,3876	1
Gm14494	1,3874	1
Clspn	1,3873	1
Drg1	1,3873	1
RP23-454I20.1	1,3867	1
Mettl6	1,3863	1
Tmed1	1,3862	1
Aim1l	1,3861	1

Arl1	1,3860	1
AI661453	1,3859	1
Exosc8	1,3856	1
Ndufv3	1,3856	1
Cox14	1,3856	1
Ssbp3	1,3855	1
Gm7809	1,3855	1
Gm11110	1,3854	1
Dut	1,3853	1
Rab26os	1,3849	1
Zbtb14	1,3848	1
Klhl18	1,3845	1
Hnrnph1	1,3845	1
Gm43817	1,3844	1
Enoph1	1,3843	1
Kcnn4	1,3841	1
Fam20c	1,3838	1
Rpsa	1,3837	1
BC029722	1,3836	1
Rps10	1,3835	1
4930448A20Rik	1,3831	1
Rhebl1	1,3829	1
Sertad3	1,3827	1
Anxa1	1,3825	1
Gm2199	1,3824	1
Dgke	1,3824	1
Nuak2	1,3824	1
Ffar4	1,3820	1
Tdpx-ps1	1,3819	1
Psmb6	1,3819	1
Katnbl1	1,3819	1
Rrp36	1,3816	1
RP23-371B13.3	1,3813	1
Ripk3	1,3809	1
Parp6	1,3800	1
Mir5128	1,3798	1
Pigx	1,3797	1
Fam134c	1,3796	1
Il15	1,3796	1
Cebpb	1,3796	1
Bcat2	1,3796	1
RP23-325K4.10	1,3794	1
Tmx4	1,3794	1
Vaultrc5	1,3793	1
Pde2a	1,3792	1
Sik1	1,3789	1
Txn-ps1	1,3783	1
Ccdc115	1,3783	1
Rpl4	1,3782	1
Pcgf5	1,3781	1
Nup85	1,3779	1
Ubb	1,3778	1

Lcmt1	1,3776	1
Zfp53	1,3775	1
Rps19-ps12	1,3772	1
Commd9	1,3772	1
2610021A01Rik	1,3770	1
Aida	1,3770	1
Slc25a40	1,3769	1
Denr	1,3768	1
Gm26202	1,3766	1
Gm6142	1,3758	1
Gm12517	1,3757	1
Neurl3	1,3752	1
Hoxb7	1,3752	1
Dolk	1,3751	1
Gm37795	1,3750	1
Rnf103	1,3749	1
Gm12230	1,3748	1
Plxna3	1,3747	1
Slc25a43	1,3746	1
Zfp689	1,3740	1
Gm9396	1,3739	1
Paip2b	1,3739	1
Eif3m	1,3738	1
RP23-331E5.10	1,3736	1
Ndufa5	1,3735	1
Nudc-ps1	1,3734	1
Gm4950	1,3734	1
Gm11539	1,3728	1
Gm5735	1,3725	1
Pbx1	1,3722	1
C630004M23Rik	1,3722	1
Ppm1j	1,3722	1
Dfna5	1,3719	1
Emc7	1,3718	1
Ncapd3	1,3715	1
Gm11531	1,3714	1
March11	1,3709	1
Tprgl	1,3709	1
Ppie	1,3708	1
Zbtb7b	1,3706	1
Snhg20	1,3705	1
Gm13736	1,3704	1
Gm7160	1,3702	1
Hyal3	1,3700	1
Mtch1	1,3699	1
Gm11633	1,3696	1
Itpripl1	1,3692	1
Med22	1,3690	1
Smim3	1,3689	1
Slc25a4	1,3688	1
Smyd5	1,3682	1
Vti1b	1,3682	1

Tbx6	1,3678	1
Eno1b	1,3677	1
Aacs	1,3677	1
Gm561	1,3677	1
Commd6	1,3670	1
Insl6	1,3667	1
Lrrfip1	1,3667	1
Surf1	1,3667	1
Fam198b	1,3664	1
Snn	1,3663	1
Il10ra	1,3661	1
Zfp280d	1,3659	1
Pdcd2	1,3653	1
Kras	1,3652	1
RP23-205H11.3	1,3648	1
Abhd17a	1,3648	1
Mex3c	1,3647	1
H2-Oa	1,3646	1
Ube2e2	1,3646	1
Jrkl	1,3646	1
Otud6b	1,3645	1
Carnmt1	1,3642	1
Tmem160	1,3640	1
Itgb3bp	1,3637	1
C1rb	1,3636	1
Ndufb10	1,3636	1
Kcnab2	1,3635	1
Atpif1	1,3635	1
Pdgfa	1,3634	1
Igf1	1,3632	1
Kif14	1,3629	1
Llph-ps2	1,3629	1
Rnf31	1,3628	1
Gm37303	1,3626	1
9430015G10Rik	1,3626	1
Lzic	1,3624	1
Fam103a1	1,3620	1
Gdf15	1,3617	1
Rps12-ps26	1,3616	1
Gm11448	1,3616	1
Ccdc28b	1,3613	1
Pdp1	1,3613	1
Traf2	1,3611	1
Tmem234	1,3611	1
Tspan4	1,3610	1
Thnsl1	1,3605	1
Gm13392	1,3604	1
Nbn	1,3603	1
Rpl13a	1,3603	1
Numb	1,3600	1
Slirp	1,3599	1
Ncaph2	1,3598	1

Snrnp25	1,3593	1
Mettl5	1,3591	1
Creb3l4	1,3587	1
Gm5117	1,3586	1
Nob1	1,3585	1
Gm13186	1,3583	1
Gm43712	1,3583	1
Arid3b	1,3582	1
Gm4737	1,3578	1
Akr1b10	1,3575	1
Aip	1,3574	1
Mapre2	1,3574	1
Ccnb1	1,3573	1
Ndufa4	1,3572	1
Ak6	1,3571	1
Sdc1	1,3568	1
Dock2	1,3566	1
Cnksr3	1,3565	1
Ints8	1,3565	1
Rnh1	1,3565	1
Gm37305	1,3564	1
Nop14	1,3557	1
Scp2	1,3556	1
Gm44283	1,3551	1
Gm15453	1,3548	1
Gm11427	1,3545	1
Gm10060	1,3541	1
Asb11	1,3540	1
Wdr83	1,3540	1
Rpl6	1,3535	1
Phospho2	1,3534	1
Nectin3	1,3533	1
Wnt6	1,3532	1
Gm9354	1,3532	1
Gm15151	1,3531	1
Ssfa2	1,3530	1
Tubb6	1,3530	1
Psmd11	1,3529	1
Diaph1	1,3528	1
Cdca2	1,3527	1
B130021K23Rik	1,3525	1
Prim1	1,3522	1
Relt	1,3521	1
Gm17100	1,3517	1
Rdh10	1,3513	1
Snx13	1,3513	1
Gtf2h5	1,3512	1
Cct5	1,3509	1
Gm29736	1,3507	1
Prrg4	1,3501	1
Ctnnbl1	1,3500	1
Gm6921	1,3499	1

Ppp4r3a	1,3498	1
Cript	1,3497	1
Tbpl1	1,3497	1
Psmb2	1,3496	1
Gm45223	1,3495	1
Ganab	1,3495	1
Nudt8	1,3493	1
Kitl	1,3490	1
Rpl10a-ps2	1,3484	1
Gm8869	1,3483	1
Gm16200	1,3483	1
Ccdc15	1,3483	1
Cenpl	1,3483	1
Tmem161a	1,3481	1
Fez2	1,3481	1
Zfp688	1,3477	1
Emc4	1,3476	1
Gm9794	1,3475	1
Gm43378	1,3474	1
Atp5e	1,3472	1
Gm8995	1,3470	1
Gtf3c4	1,3470	1
Baz2a	1,3468	1
Stc2	1,3466	1
Cby1	1,3465	1
Rps19bp1	1,3465	1
Gss	1,3464	1
Gm9165	1,3461	1
Gm5590	1,3459	1
Lum	1,3457	1
Rita1	1,3457	1
Zfp955a	1,3456	1
Rab42	1,3456	1
Dpy19l3	1,3454	1
Szrd1	1,3453	1
Mnat1	1,3450	1
Btbd7	1,3447	1
Hic2	1,3447	1
Gm15832	1,3442	1
Dusp7	1,3431	1
Rpl35a	1,3430	1
Ugdh	1,3430	1
Cnr2	1,3429	1
Gm45420	1,3428	1
Mkks	1,3427	1
Cox7a2l	1,3427	1
Polr2i	1,3424	1
Xlr	1,3422	1
Tmod3	1,3422	1
Psmd8	1,3420	1
Gm10689	1,3419	1
Idua	1,3416	1

Ptges3l	1,3416	1
Eif3s6-ps1	1,3414	1
Gm7676	1,3409	1
Ccar1	1,3409	1
Fcrl5	1,3404	1
Ptdss2	1,3404	1
Dpp7	1,3402	1
Gpat4	1,3402	1
Arntl	1,3399	1
Rpl31-ps1	1,3397	1
Wrb	1,3397	1
Adprhl2	1,3396	1
Gm2986	1,3392	1
Map3k10	1,3391	1
Cox7c	1,3390	1
Ginm1	1,3390	1
Ifi211	1,3387	1
Lsm2	1,3387	1
Gm12704	1,3386	1
Gltscr2	1,3385	1
Hilpda	1,3384	1
Apitd1	1,3380	1
Hint1	1,3380	1
Taf8	1,3377	1
Gm12543	1,3375	1
Gm37354	1,3373	1
Leo1	1,3372	1
Igf2bp2	1,3366	1
Sfn	1,3364	1
Ecsit	1,3364	1
Snx30	1,3364	1
Gm14794	1,3363	1
Arid5a	1,3362	1
Lmf1	1,3359	1
Nhp2	1,3359	1
Gpsm2	1,3357	1
Lsm14a	1,3357	1
Tpd52l2	1,3357	1
Gm4734	1,3356	1
1110006O24Rik	1,3356	1
Bri3	1,3355	1
Prr11	1,3355	1
Tpst2	1,3355	1
Mtor	1,3351	1
Rps15a-ps8	1,3350	1
Gm14680	1,3347	1
Sun2	1,3347	1
Gm9531	1,3345	1
Pigyl	1,3345	1
Mrps35	1,3342	1
Adipor1	1,3341	1
Rpl28-ps3	1,3339	1

Rnf11	1,3339	1
Glo1	1,3339	1
Nusap1	1,3335	1
Cap1	1,3335	1
Pik3ip1	1,3334	1
Rpsa-ps11	1,3330	1
Gm28557	1,3328	1
Ss18l2	1,3325	1
Pyroxd1	1,3323	1
Hmgcs1	1,3321	1
Adamts10	1,3320	1
Gm33142	1,3317	1
Osgin2	1,3317	1
Rdh14	1,3316	1
Psme2b	1,3316	1
Ddx31	1,3315	1
BC029214	1,3314	1
Sav1	1,3313	1
Tufm	1,3312	1
Gm14633	1,3311	1
2610203C22Rik	1,3311	1
Adarb1	1,3311	1
Myadm	1,3310	1
Zbtb42	1,3306	1
Kif11	1,3304	1
Ilf3	1,3304	1
Gm10478	1,3303	1
Ppig	1,3302	1
Gm10923	1,3300	1
Tmx2	1,3300	1
Gm10399	1,3299	1
Gm13421	1,3298	1
Zfp622	1,3297	1
Psmc5	1,3296	1
Ctss	1,3296	1
Gsr	1,3295	1
Etaa1	1,3294	1
RP23-114G13.1	1,3291	1
Gm10051	1,3290	1
Gm44609	1,3290	1
Kdm3a	1,3288	1
Gm1943	1,3287	1
Rest	1,3287	1
Gm6987	1,3286	1
Gm27039	1,3283	1
Tifa	1,3283	1
Lin52	1,3282	1
Rbm48	1,3280	1
Gm11520	1,3279	1
Aen	1,3278	1
Gm15464	1,3277	1
Rpl17-ps10	1,3274	1

1110065P20Rik	1,3274	1
Capza2	1,3272	1
9130230N09Rik	1,3270	1
Mettl17	1,3269	1
Zfand5	1,3268	1
Dbndd2	1,3267	1
6430511E19Rik	1,3267	1
Ube2f	1,3267	1
Prpf39	1,3266	1
Rpl30	1,3262	1
AI467606	1,3258	1
Cep95	1,3254	1
Cep57l1	1,3252	1
Lrpap1	1,3252	1
Snrnp48	1,3250	1
Erap1	1,3249	1
Cenpu	1,3248	1
Gm7561	1,3248	1
Pnrc2	1,3246	1
Zfp428	1,3245	1
Mrm3	1,3245	1
Nrbf2	1,3243	1
Rpl34	1,3241	1
Pmvk	1,3241	1
Zfp532	1,3240	1
Tmem30a	1,3240	1
Psmd9	1,3236	1
Gm2756	1,3234	1
1810058I24Rik	1,3234	1
Fam219b	1,3232	1
Hikeshi	1,3231	1
Ndufs4	1,3231	1
Litaf	1,3227	1
Gpr155	1,3224	1
Fam107b	1,3222	1
Abhd14b	1,3221	1
Pop1	1,3220	1
Tspyl4	1,3217	1
Gm43868	1,3216	1
Ppp1r14b	1,3216	1
Uimc1	1,3214	1
Snx3	1,3213	1
Gm45050	1,3212	1
Gm29487	1,3212	1
Mcfd2	1,3212	1
Rps15a-ps7	1,3212	1
Nipal3	1,3210	1
Anln	1,3208	1
Zbtb8a	1,3202	1
Btf3	1,3201	1
Nudt6	1,3201	1
Gm26533	1,3200	1

Gm5054	1,3195	1
Lbr	1,3195	1
Ube2l6	1,3193	1
Ost4	1,3192	1
Lsm5	1,3191	1
Gm10335	1,3187	1
Gm43328	1,3186	1
5430402O13Rik	1,3184	1
Alyref	1,3184	1
Bod1	1,3180	1
Uqcrq	1,3178	1
Wdr34	1,3177	1
Fkbpl	1,3177	1
Nup43	1,3176	1
Esr1	1,3176	1
RP23-70B19.5	1,3175	1
Rnf181	1,3173	1
Gm9703	1,3172	1
Smpd2	1,3169	1
Sc5d	1,3168	1
Mier3	1,3164	1
Nap1l4	1,3162	1
Chmp1b	1,3162	1
Gins4	1,3160	1
Gm43794	1,3159	1
Psat1	1,3158	1
Trem2	1,3157	1
Acrbp	1,3154	1
Gm4707	1,3153	1
Crry-ps	1,3152	1
Mipep	1,3152	1
Rmdn3	1,3151	1
Rpl7-ps7	1,3148	1
Psme2	1,3147	1
Gm9803	1,3146	1
Pnn	1,3146	1
Mrpl24	1,3144	1
Pdcd5-ps	1,3143	1
Mlf1	1,3138	1
2900052L18Rik	1,3137	1
Gm45109	1,3137	1
AB124611	1,3135	1
Zfp91	1,3135	1
Dusp16	1,3134	1
Cbx4	1,3132	1
Slc47a2	1,3130	1
Gm42895	1,3127	1
Rgmb	1,3124	1
Pole3	1,3124	1
Tpd52-ps	1,3123	1
Prdx5	1,3122	1
Gm9009	1,3121	1

Gabarapl1	1,3120	1
Slc39a10	1,3119	1
Gm16201	1,3118	1
Psmg3	1,3118	1
Mef2c	1,3115	1
Pde1b	1,3113	1
Sil1	1,3113	1
Gm8762	1,3111	1
Glb1l	1,3108	1
Hat1	1,3108	1
Snx1	1,3105	1
Tmem176a	1,3101	1
Rpl13-ps3	1,3100	1
N4bp3	1,3100	1
Rps28	1,3100	1
Mob3a	1,3098	1
Tmem242	1,3098	1
Gm8394	1,3093	1
Mrpl34	1,3093	1
Gm38305	1,3092	1
Gm13578	1,3092	1
Mrpl23-ps1	1,3092	1
Gfer	1,3088	1
Narf	1,3087	1
Gm12816	1,3085	1
Me2	1,3077	1
Fbxo34	1,3076	1
Sssca1	1,3076	1
Ogfrl1	1,3075	1
Dvl2	1,3073	1
Gm23301	1,3070	1
Car12	1,3070	1
Zbtb11	1,3070	1
Pcsk4	1,3069	1
Gm6222	1,3068	1
Bap1	1,3066	1
Gm15773	1,3064	1
Gm14253	1,3061	1
Fhod1	1,3060	1
Bcas2	1,3060	1
Spdl1	1,3055	1
Cyp2c55	1,3052	1
Eif4e	1,3050	1
Gm43223	1,3049	1
Gm17018	1,3047	1
Gtf2e2	1,3044	1
Timm9	1,3043	1
Itpkc	1,3040	1
Nacc1	1,3040	1
Ranbp6	1,3039	1
Spink5	1,3039	1
Rtn4	1,3037	1

Stil	1,3036	1
Itpkb	1,3036	1
Gm14586	1,3036	1
Ccdc63	1,3034	1
1700061G19Rik	1,3034	1
D230017M19Rik	1,3033	1
Gm3375	1,3032	1
Stx3	1,3029	1
Nudt16l1	1,3028	1
0610037L13Rik	1,3027	1
Rnf26	1,3026	1
Mrpl46	1,3025	1
Ing1	1,3022	1
Mrps30	1,3020	1
1110032A03Rik	1,3018	1
Adora2b	1,3015	1
Fam214a	1,3014	1
Ly86	1,3011	1
Gm5617	1,3006	1
Renbp	1,3005	1
Gm43331	1,3004	1
Gm4799	1,3004	1
Ahnak	1,3003	1
Kdm2b	1,3001	1
Slc16a10	1,3001	1
Cd320	1,3000	1
Gnl2	1,2998	1
Pnpo	1,2993	1
Rpl36a-ps1	1,2991	1
Rcbtb2	1,2990	1
Napg	1,2990	1
Rnd2	1,2989	1
1700037C18Rik	1,2988	1
Gm29284	1,2988	1
Ip6k2	1,2988	1
Myo1f	1,2986	1
Rpl37a	1,2984	1
Stam	1,2983	1
Gm6444	1,2982	1
Eif4e3	1,2981	1
Dbi	1,2981	1
Gm7384	1,2979	1
1810032O08Rik	1,2976	1
Cnnm2	1,2973	1
Banp	1,2971	1
Ywhaq	1,2970	1
Rin1	1,2969	1
Gm6297	1,2969	1
RP23-47A1.1	1,2968	1
Zfp984	1,2965	1
Rsl24d1	1,2964	1
Vps29	1,2963	1

Rcn2	1,2963	1
Clcnkb	1,2959	1
Nek6	1,2959	1
Actg1	1,2958	1
Trmt13	1,2958	1
Polr2g	1,2956	1
Zfp747	1,2955	1
Srek1ip1	1,2949	1
Brca1	1,2947	1
Vamp3	1,2947	1
mt-Ts2	1,2946	1
Chrna1os	1,2944	1
Il13ra1	1,2944	1
Gm37503	1,2941	1
Acyp1	1,2940	1
Nle1	1,2940	1
Cbr1	1,2939	1
Mrps36-ps1	1,2938	1
Tmem159	1,2937	1
Psmd13	1,2936	1
Gm10658	1,2935	1
Mis18bp1	1,2935	1
Fbxo36	1,2934	1
Pdik1l	1,2934	1
9230116N13Rik	1,2932	1
Spryd7	1,2931	1
Gosr2	1,2927	1
Malsu1	1,2926	1
Card11	1,2924	1
Inip	1,2924	1
Pfdn5	1,2923	1
Tmem41a	1,2920	1
1810037I17Rik	1,2918	1
Gm6266	1,2917	1
Mapk11	1,2916	1
Vbp1	1,2916	1
Ppp2r2d	1,2916	1
4933412L11Rik	1,2915	1
Gm16433	1,2912	1
Eif3j1	1,2910	1
Dcp2	1,2910	1
Dis3	1,2907	1
E330034L11Rik	1,2906	1
Snrnp35	1,2904	1
Bcl6	1,2904	1
Fgfr1op	1,2904	1
Fkbp2	1,2903	1
Hmg20b	1,2903	1
Polr1d	1,2902	1
Gm9333	1,2900	1
Xrcc1	1,2900	1
Kif3c	1,2900	1

Gm10443	1,2898	1
2610203C20Rik	1,2895	1
Plk4	1,2895	1
Plekha7	1,2894	1
Mapk6	1,2894	1
Gm6159	1,2891	1
Wbp4	1,2890	1
Zfand6	1,2890	1
Ccdc174	1,2889	1
Ndufb9	1,2888	1
Mzt1	1,2888	1
Napb	1,2884	1
Gm7670	1,2881	1
Rab20	1,2881	1
Xndc1	1,2880	1
Gm12606	1,2879	1
Nlrp10	1,2878	1
Rap2a	1,2878	1
Elmo2	1,2875	1
E430018J23Rik	1,2873	1
Eif6	1,2873	1
Bicd2	1,2871	1
Sdhaf2	1,2869	1
Ssbp1	1,2868	1
Mpc2	1,2867	1
Gm19353	1,2864	1
Mettl3	1,2863	1
Rps27	1,2863	1
Nfam1	1,2862	1
Lsm14b	1,2860	1
Usf1	1,2859	1
9130019O22Rik	1,2859	1
Dym	1,2859	1
Zfp9	1,2858	1
Gm4963	1,2857	1
Ift172	1,2852	1
Dhrs13	1,2851	1
Srpr	1,2851	1
Fdx1	1,2850	1
Rpp25l	1,2849	1
Kpna6	1,2849	1
Rccd1	1,2847	1
S100a11	1,2846	1
Nudt4	1,2843	1
Hspd1-ps3	1,2842	1
Tshz1	1,2842	1
Mogat1	1,2841	1
Wwc2	1,2841	1
Magoh	1,2838	1
Leprot	1,2837	1
Patz1	1,2835	1
Gm6265	1,2832	1

Sirt2	1,2832	1
1110008F13Rik	1,2832	1
Gm37452	1,2830	1
Idh3b	1,2829	1
Ndufa13	1,2826	1
Acer3	1,2824	1
Smndc1	1,2823	1
Cab39	1,2823	1
Rps21	1,2820	1
Med19	1,2818	1
Clpb	1,2817	1
Ogfod2	1,2816	1
Dctn2	1,2815	1
Ankrd54	1,2812	1
Mrpl48	1,2811	1
Gm4149	1,2809	1
Selenow	1,2809	1
Mcee	1,2803	1
Cenpm	1,2797	1
Cfl2	1,2797	1
Osbpl1a	1,2796	1
Rbm34	1,2795	1
Hbp1	1,2795	1
Mgarp	1,2793	1
Cmtr2	1,2793	1
Cdk19	1,2793	1
C1d	1,2792	1
Crtc2	1,2788	1
Agpat4	1,2785	1
Xk	1,2783	1
RP24-282C4.4	1,2782	1
Utp18	1,2780	1
Terf1	1,2778	1
2010204K13Rik	1,2777	1
Rps15a-ps1	1,2777	1
Gm5121	1,2777	1
Clic4	1,2775	1
Gm5871	1,2773	1
Xab2	1,2773	1
Trim47	1,2769	1
Sep 10	1,2768	1
Timm17b	1,2768	1
Fuca1	1,2767	1
Irx2	1,2766	1
Gm43728	1,2765	1
Rnf2	1,2765	1
Asrgl1	1,2764	1
Trim59	1,2761	1
Nabp1	1,2761	1
Rps16-ps2	1,2761	1
Nova2	1,2760	1
Dhrs9	1,2760	1

Rnf126	1,2760	1
Wdr78	1,2755	1
Rab1a	1,2754	1
9430038I01Rik	1,2752	1
Gm16379	1,2750	1
Stk40	1,2750	1
Maff	1,2746	1
Ghdc	1,2746	1
Mlf2	1,2745	1
AI662270	1,2745	1
Eif1a	1,2742	1
Fcgr2b	1,2741	1
Ddost	1,2741	1
Fbxo15	1,2740	1
Max	1,2740	1
Gm38213	1,2739	1
Uap1l1	1,2739	1
Pik3cb	1,2739	1
Commd2	1,2737	1
Mthfd2l	1,2736	1
Gch1	1,2736	1
Rwdd1	1,2735	1
Ndufa2	1,2735	1
Jun	1,2731	1
Aplp1	1,2731	1
Trim45	1,2730	1
Unc119b	1,2730	1
Psmf1	1,2728	1
Tec	1,2728	1
Rpl39-ps	1,2725	1
Mrpl27	1,2724	1
Rabac1	1,2724	1
Arvcf	1,2723	1
Snapc1	1,2722	1
Pdlim7	1,2721	1
Retn	1,2720	1
Troap	1,2719	1
Zbtb2	1,2719	1
Tmem216	1,2718	1
Hinfp	1,2718	1
Med7	1,2716	1
Gpr146	1,2715	1
Lmln	1,2714	1
H2-T10	1,2714	1
3110040N11Rik	1,2714	1
Arl5a	1,2713	1
Dusp19	1,2707	1
Sfi1	1,2706	1
Smpd1	1,2703	1
Pcbp4	1,2702	1
Spty2d1	1,2701	1
Mrps26	1,2696	1

Rnf166	1,2694	1
Calr	1,2694	1
Acvrl1	1,2691	1
Ndufb11	1,2691	1
Stard8	1,2687	1
Gm5909	1,2687	1
Mettl26	1,2687	1
Cdkn1b	1,2684	1
Bcl2l12	1,2679	1
Nsmce2	1,2679	1
Bsg	1,2677	1
Pa2g4	1,2676	1
Hadh	1,2674	1
Rbfa	1,2672	1
Eif3e	1,2672	1
Trnau1ap	1,2672	1
Gm1976	1,2669	1
Jam2	1,2669	1
Usp53	1,2667	1
Pcnp	1,2666	1
Llph-ps1	1,2665	1
Zcwpw1	1,2665	1
Gm12183	1,2662	1
Gm20056	1,2661	1
Hnrnpa1	1,2661	1
H2-T22	1,2661	1
Atp8b2	1,2661	1
Tnnc2	1,2658	1
Pdlim2	1,2656	1
Lysmd4	1,2656	1
Clpx	1,2655	1
Naa16	1,2653	1
Ckap4	1,2652	1
Bloc1s2	1,2651	1
Gm10237	1,2650	1
Htr2b	1,2649	1
Ppp1r16a	1,2649	1
Gm14706	1,2644	1
Gm9403	1,2643	1
Bbof1	1,2642	1
Gm6543	1,2640	1
Ebp	1,2639	1
Gm9025	1,2637	1
Smad7	1,2637	1
Macrod2	1,2637	1
Zfp773	1,2637	1
Gm12231	1,2634	1
Piga	1,2632	1
Gm45407	1,2631	1
Dhcr24	1,2631	1
Gm43294	1,2630	1
Cxcr4	1,2630	1

Kpna3	1,2628	1
Snrpe	1,2628	1
Mfsd2a	1,2627	1
RP23-413G8.2	1,2626	1
Pdap1	1,2625	1
Gm15846	1,2624	1
Apex1	1,2623	1
Hn1l	1,2622	1
Slc35f5	1,2622	1
Gm2214	1,2621	1
A530041M06Rik	1,2620	1
Ccdc80	1,2619	1
Cox8a	1,2617	1
Fut11	1,2616	1
Pcna	1,2613	1
Gm13890	1,2612	1
Rps15a	1,2612	1
Rab13	1,2611	1
Kbtbd2	1,2610	1
Sumf1	1,2610	1
Psma7	1,2610	1
Dusp28	1,2609	1
Letm2	1,2609	1
Vamp5	1,2609	1
Gm7618	1,2608	1
Cbr4	1,2606	1
Pou5f2	1,2605	1
Kif4	1,2605	1
Anapc16	1,2602	1
Hist2h2be	1,2600	1
Kif9	1,2598	1
Scrn3	1,2597	1
Rpl36-ps2	1,2596	1
Smarce1	1,2596	1
Pycrl	1,2596	1
E230020A03Rik	1,2595	1
Gm4866	1,2595	1
Dnajc17	1,2595	1
Tmem50b	1,2595	1
Pold3	1,2592	1
Cstf1	1,2591	1
Zfas1	1,2591	1
Mrpl40	1,2589	1
Zfp319	1,2583	1
Nemp2	1,2581	1
Eif3i	1,2581	1
Pim2	1,2580	1
Ermap	1,2578	1
Tmem26	1,2577	1
Rpp14	1,2577	1
Fam96a	1,2576	1
Gm13433	1,2573	1

Raph1	1,2573	1
Mcub	1,2573	1
Ccdc166	1,2571	1
mt-Co1	1,2568	1
Tsc22d2	1,2568	1
Bzw1	1,2568	1
Prep	1,2568	1
Edf1	1,2564	1
Psmb9	1,2563	1
Fkbp5	1,2562	1
Map3k1	1,2562	1
Slc27a3	1,2561	1
Cd40	1,2561	1
Gm12589	1,2554	1
Bst1	1,2554	1
H2afj	1,2554	1
Gadd45gip1	1,2549	1
Glt1d1	1,2548	1
Mta2	1,2547	1
Tmem42	1,2546	1
Fos	1,2546	1
Rps11-ps1	1,2544	1
Zfp580	1,2544	1
Nek2	1,2541	1
Cdc42ep3	1,2541	1
Rpl31	1,2540	1
Sap18	1,2537	1
Stoml2	1,2535	1
Ythdc1	1,2535	1
BC030499	1,2534	1
Rnf149	1,2534	1
2700029L08Rik	1,2531	1
Cdkn1a	1,2531	1
Atp5k-ps2	1,2530	1
BC055324	1,2528	1
Gm8213	1,2525	1
Clec7a	1,2525	1
Nr3c1	1,2525	1
Rnaseh2b	1,2524	1
Ube2q2	1,2524	1
Cox4i1	1,2524	1
Pigl	1,2523	1
Gm8276	1,2523	1
Tmem81	1,2522	1
Mrpl47	1,2522	1
Acadm	1,2521	1
Fem1b	1,2521	1
Sap18b	1,2519	1
Bsdc1	1,2518	1
Cracr2b	1,2518	1
Grwd1	1,2518	1
Gna12	1,2517	1

Gm8599	1,2516	1
Get4	1,2516	1
Gm13450	1,2514	1
Vps53	1,2511	1
Ndufb3	1,2511	1
Gm38377	1,2510	1
Fpgt	1,2509	1
Myg1	1,2509	1
Fem1c	1,2509	1
Smpdl3a	1,2509	1
Lcmt2	1,2503	1
B4galt7	1,2503	1
Rilp	1,2502	1
Cdipt	1,2502	1
Grpel1	1,2501	1
Bag1	1,2501	1
Nucks1	1,2501	1
Cdt1	1,2499	1
Ufm1	1,2499	1
Pld4	1,2498	1
Flt1	1,2497	1
Rpa2	1,2496	1
Gm11353	1,2491	1
B230217C12Rik	1,2487	1
Sema5a	1,2484	1
Ccdc138	1,2483	1
Fndc7	1,2483	1
Rab5a	1,2483	1
Tfb1m	1,2483	1
Man1b1	1,2483	1
Gm43010	1,2482	1
Ddx28	1,2482	1
Mrpl43	1,2482	1
Timm10b	1,2481	1
Emc9	1,2480	1
Cxcl16	1,2480	1
Dtl	1,2476	1
E2f4	1,2474	1
Fam58b	1,2470	1
Gm10169	1,2470	1
Snx21	1,2469	1
Trappc4	1,2466	1
Trmt1	1,2465	1
Ankle1	1,2463	1
Trp53rkb	1,2462	1
Tsr2	1,2460	1
Slc7a6os	1,2457	1
1110004E09Rik	1,2457	1
5330406M23Rik	1,2455	1
Ttc37	1,2455	1
Eif4b	1,2455	1
AC168977.1	1,2452	1

Ybx1	1,2452	1
Tk1	1,2451	1
Cd63-ps	1,2450	1
Gm15782	1,2449	1
Armc7	1,2449	1
Tmem64	1,2448	1
Cage1	1,2447	1
Cmtm4	1,2445	1
Gm7312	1,2444	1
Ndufa10	1,2444	1
Slc35b1	1,2443	1
Mtmr3	1,2441	1
Tmed5	1,2438	1
Rps27-ps1	1,2437	1
Abcb8	1,2435	1
Pts	1,2433	1
Tmem101	1,2433	1
Cd99l2	1,2432	1
Ndufs2	1,2430	1
Vprbp	1,2428	1
Lpp	1,2428	1
Tuba1c	1,2426	1
Tmlhe	1,2425	1
Rpl13a-ps1	1,2424	1
Minos1	1,2424	1
Mfng	1,2421	1
Nup205	1,2420	1
Gm14857	1,2420	1
Klhdc4	1,2420	1
Abcd2	1,2419	1
Ogg1	1,2417	1
Gm14303	1,2416	1
Ube2c	1,2413	1
Zfp383	1,2412	1
Brix1	1,2411	1
Mcat	1,2410	1
Borcs8	1,2409	1
Katnal1	1,2409	1
Map2k1	1,2409	1
Med9	1,2407	1
Gm10138	1,2406	1
Tspan17	1,2406	1
St6galnac6	1,2405	1
Gm10110	1,2401	1
Cbll1	1,2398	1
Fam46a	1,2397	1
Emp1	1,2397	1
Slc37a3	1,2396	1
Suv39h1	1,2396	1
Wbp1	1,2396	1
Ift57	1,2394	1
Gm10762	1,2391	1

Tsen15	1,2391	1
Sumo1	1,2390	1
Gm15625	1,2386	1
Zfp87	1,2383	1
Snx18	1,2383	1
Ubxn6	1,2382	1
F11r	1,2380	1
Nt5m	1,2378	1
Ikzf5	1,2378	1
Zfp35	1,2377	1
Atp23	1,2377	1
Hdac5	1,2376	1
Nudc	1,2376	1
Tmem8b	1,2370	1
Gm6123	1,2370	1
Ncapg2	1,2365	1
Tes	1,2364	1
Ccs	1,2363	1
Marc2	1,2362	1
Prkra	1,2362	1
Repin1	1,2361	1
Hsbp1	1,2359	1
Gm6525	1,2358	1
Ppp6c	1,2358	1
Ccnb2	1,2357	1
Runx2os1	1,2356	1
Zfp251	1,2356	1
Hdac2	1,2356	1
Gm15694	1,2354	1
Gm7407	1,2354	1
Cuta	1,2354	1
Gm4032	1,2353	1
Asnsd1	1,2352	1
Eif3l	1,2352	1
Cd74	1,2351	1
Gm37962	1,2350	1
Gm37219	1,2349	1
Rars	1,2349	1
Cgrrf1	1,2348	1
Nfu1	1,2347	1
Ubr7	1,2346	1
Gm25541	1,2335	1
Klhl15	1,2335	1
Arid5b	1,2335	1
Bcl7a	1,2332	1
Crbn	1,2330	1
Ppp4r1l-ps	1,2329	1
Gm13445	1,2328	1
Ggnbp1	1,2326	1
Zfp958	1,2326	1
Aspm	1,2323	1
4930522L14Rik	1,2323	1

Higd2a	1,2323	1
Azin1	1,2320	1
Cers5	1,2318	1
Tsc22d1	1,2314	1
Ciao1	1,2312	1
Gm15440	1,2309	1
Ndufb6	1,2309	1
Elovl1	1,2309	1
2610001J05Rik	1,2308	1
Gm10557	1,2306	1
Minpp1	1,2306	1
Zfp90	1,2305	1
Gm12346	1,2305	1
Cenph	1,2304	1
Znrd1as	1,2302	1
Zfp369	1,2301	1
Ubap1	1,2301	1
Hebp1	1,2300	1
Tfap4	1,2300	1
4930589L23Rik	1,2298	1
Txnl1	1,2298	1
Gm16556	1,2294	1
Park7	1,2294	1
Dhrs3	1,2288	1
Rab27a	1,2288	1
8030462N17Rik	1,2288	1
P2ry2	1,2287	1
Gde1	1,2286	1
Aldh9a1	1,2284	1
Rnf121	1,2283	1
Doc2g	1,2282	1
Nanos1	1,2281	1
mt-Tv	1,2280	1
Rpl23a-ps14	1,2278	1
Osbpl3	1,2275	1
Fbxl12	1,2275	1
Gm11599	1,2274	1
Gm16310	1,2274	1
4930539J05Rik	1,2274	1
Ilf2	1,2274	1
Pkn2	1,2273	1
Tssc1	1,2272	1
Lgals7	1,2271	1
Tia1	1,2270	1
Zwint	1,2270	1
Ndufaf8	1,2269	1
Psmb4	1,2268	1
Wbp11	1,2268	1
S100a6	1,2268	1
Cd47	1,2267	1
Faap20	1,2265	1
Gcsh	1,2263	1

Gnas	1,2260	1
Mars2	1,2259	1
Fcgr3	1,2259	1
Gm4987	1,2259	1
Faim	1,2259	1
Zbtb17	1,2254	1
Clec4d	1,2253	1
Pdcd4	1,2249	1
Osgep	1,2248	1
Ckap2	1,2247	1
Kdm1a	1,2245	1
Tmem246	1,2244	1
Mdm2	1,2244	1
Gm10012	1,2241	1
Cwf19l1	1,2240	1
Acadvl	1,2237	1
Idnk	1,2234	1
Rpl34-ps1	1,2233	1
Gpr137b	1,2233	1
Nol7	1,2231	1
RP23-48A24.3	1,2230	1
Rangap1	1,2229	1
Tmem59	1,2229	1
Gm38067	1,2226	1
Gm11343	1,2226	1
Gm7565	1,2226	1
Gpank1	1,2225	1
Uhrf2	1,2225	1
C78859	1,2221	1
Gm45033	1,2220	1
Tmem126b	1,2220	1
Pmf1	1,2220	1
Pgp	1,2220	1
Gm9761	1,2219	1
Gm13743	1,2218	1
Pfkp	1,2218	1
Fcgrt	1,2217	1
Pkig	1,2217	1
Il27	1,2216	1
Rexo4	1,2216	1
A330023F24Rik	1,2214	1
Aup1	1,2214	1
Rpl13	1,2209	1
2610524H06Rik	1,2205	1
Msh3	1,2204	1
Arl4a	1,2202	1
Rhbdf2	1,2198	1
B4galt3	1,2198	1
Pkp4	1,2198	1
Tatdn1	1,2196	1
Qars	1,2196	1
Noct	1,2194	1

Rgs3	1,2193	1
Aes	1,2193	1
Chtf18	1,2192	1
n-R5s151	1,2189	1
Cox6b1	1,2188	1
Paip2	1,2188	1
CAAA01180111.2	1,2187	1
Lig1	1,2187	1
Pym1	1,2185	1
Mvp	1,2185	1
Slc22a13b-ps	1,2184	1
Tmem189	1,2184	1
Rft1	1,2182	1
Neu1	1,2181	1
Dag1	1,2180	1
Zfp36	1,2179	1
Rpl21-ps6	1,2177	1
Eid1	1,2177	1
Efhd2	1,2177	1
Tsr1	1,2175	1
Acot7	1,2175	1
Mrps36-ps2	1,2174	1
Alox5ap	1,2174	1
Gm7769	1,2170	1
Brk1	1,2170	1
Coasy	1,2169	1
Pkd1	1,2166	1
Gm5277	1,2166	1
Gm45380	1,2164	1
Gm4832	1,2164	1
Tfip11	1,2159	1
Vps37b	1,2159	1
Hsph1	1,2159	1
Caml	1,2157	1
Kpnb1	1,2157	1
Tnfrsf26	1,2157	1
Swt1	1,2155	1
Mpst	1,2155	1
Mkrn2	1,2152	1
Midn	1,2151	1
Rbm18	1,2150	1
Amdhd2	1,2149	1
Pole4	1,2149	1
Dhrs7	1,2146	1
Gm4997	1,2146	1
Maml3	1,2145	1
Ranbp9	1,2144	1
Alg10b	1,2143	1
Nans	1,2142	1
Gpat3	1,2141	1
Arhgap22	1,2139	1
Ndrg2	1,2138	1

Psmc4	1,2137	1
Fam98c	1,2136	1
Ccdc59	1,2136	1
Sqle	1,2133	1
Card19	1,2133	1
Taldo1	1,2128	1
Lfng	1,2127	1
Blm	1,2126	1
Mbip	1,2123	1
Yars2	1,2123	1
Adgre1	1,2121	1
Gm5845	1,2120	1
Gm43351	1,2120	1
Slc35a4	1,2120	1
Pgrmc1	1,2118	1
Kctd11	1,2115	1
Dnajb4	1,2115	1
Mrpl30	1,2115	1
Stxbp3	1,2114	1
Mkln1	1,2114	1
Ube2n	1,2113	1
Med10	1,2113	1
Rps10-ps1	1,2111	1
Ten1	1,2110	1
Arhgap15	1,2106	1
Txn2	1,2104	1
Dzip1	1,2103	1
Usp42	1,2103	1
2900026A02Rik	1,2102	1
Anxa3	1,2102	1
Cd276	1,2100	1
Mrrf	1,2098	1
Dusp6	1,2097	1
Cryga	1,2096	1
Ift43	1,2096	1
AI413582	1,2095	1
Ttc12	1,2094	1
2410080I02Rik	1,2091	1
Lage3	1,2090	1
Gm12988	1,2089	1
Crcp	1,2088	1
Ppt2	1,2087	1
Mocs1	1,2087	1
Ube2g1	1,2087	1
Tmed7	1,2085	1
Procr	1,2085	1
Tle1	1,2084	1
Pirb	1,2083	1
Pabpc1l	1,2082	1
Glrx2	1,2082	1
1700096K18Rik	1,2081	1
Akirin2	1,2081	1

Nubp1	1,2079	1
Psmc3	1,2079	1
Tbc1d10a	1,2076	1
Ndufaf6	1,2075	1
Gnl1	1,2074	1
Ckb	1,2071	1
Fmc1	1,2070	1
Dpy30	1,2070	1
Wasl	1,2070	1
Pip4k2b	1,2070	1
Trappc1	1,2066	1
Rheb	1,2061	1
Tmem181a	1,2061	1
Gm9575	1,2059	1
Gm12248	1,2059	1
Gm5251	1,2058	1
Vti1a	1,2058	1
Cxcl14	1,2057	1
Gnptg	1,2053	1
Sat2	1,2053	1
Necap2	1,2052	1
Gmpr2	1,2048	1
Papd5	1,2046	1
Ppib	1,2046	1
Fam212a	1,2045	1
Zbtb37	1,2045	1
Znhit3	1,2044	1
Pdzd11	1,2043	1
Lrrc27	1,2041	1
Gm15163	1,2039	1
Gpt	1,2039	1
Snx9	1,2038	1
Gm13094	1,2037	1
Hspb6	1,2036	1
Med21	1,2036	1
Skp1a	1,2036	1
Tsku	1,2034	1
Serpini1	1,2033	1
Lin9	1,2033	1
Phf6	1,2032	1
2310022A10Rik	1,2031	1
Ndufa7	1,2031	1
Cdk18	1,2029	1
Polr2e	1,2027	1
Gm6450	1,2026	1
Tnnc1	1,2026	1
Gm37082	1,2025	1
Ftl1	1,2023	1
Med29	1,2023	1
Mfsd9	1,2023	1
Dstyk	1,2022	1
Ankrd24	1,2020	1

Senp3	1,2019	1
Klhl6	1,2019	1
Dlgap4	1,2017	1
Pdhx	1,2016	1
Gm9840	1,2015	1
Rps24	1,2013	1
Rab3d	1,2011	1
Uqcr10	1,2011	1
Zfyve27	1,2010	1
Smim1	1,2006	1
1700029J07Rik	1,2006	1
Plekhh2	1,2005	1
Chst12	1,2005	1
Oxld1	1,2002	1
Mad2l2	1,2002	1
Plekha1	1,2002	1
Dnaja2	1,2002	1
Map4k1	1,2001	1
Tpt1	1,2000	1
Wdr53	1,1999	1
Gpr137b-ps	1,1997	1
Gm11249	1,1996	1
Prcp	1,1996	1
Tardbp	1,1992	1
Hsf2	1,1991	1
Wdr12	1,1991	1
Nrf1	1,1989	1
Homez	1,1986	1
Tchp	1,1986	1
Mrpl35	1,1986	1
Zfyve26	1,1984	1
A130071D04Rik	1,1984	1
Tmem243	1,1982	1
Ptges2	1,1980	1
Gm5857	1,1979	1
Cst3	1,1979	1
Rps3	1,1978	1
Pdha1	1,1978	1
Fam126b	1,1977	1
Ankzf1	1,1976	1
Rdh13	1,1975	1
Kctd5	1,1974	1
Ndufa1	1,1974	1
Sec11c	1,1974	1
Rpn2	1,1974	1
Mypopos	1,1973	1
Gm10288	1,1971	1
Dnajc1	1,1971	1
Rpl15	1,1970	1
Dek	1,1970	1
Ptpmt1	1,1968	1
Gm14769	1,1966	1

Ephx1	1,1966	1
Ero1lb	1,1965	1
Sf3b1	1,1964	1
Sin3a	1,1963	1
Bloc1s6	1,1963	1
Rp9	1,1962	1
Kif13b	1,1961	1
Mxd1	1,1961	1
Gm45153	1,1960	1
Dnajc21	1,1960	1
Gm13835	1,1959	1
Rbbp4	1,1958	1
Mpp6	1,1958	1
Furin	1,1956	1
Med28	1,1956	1
Gm10177	1,1953	1
Ddx43	1,1952	1
Aven	1,1952	1
Hdac11	1,1952	1
Gm38062	1,1951	1
Unc119	1,1951	1
Ccnh	1,1950	1
Mrps31	1,1949	1
Gtf3c6	1,1949	1
Ttc7b	1,1948	1
Fry	1,1947	1
Fam83a	1,1947	1
Armc6	1,1947	1
Ppp1ca	1,1946	1
Ifi207	1,1945	1
1810043G02Rik	1,1944	1
Apmap	1,1944	1
Cenpf	1,1944	1
Smim4	1,1941	1
Mgmt	1,1941	1
Hpcal1	1,1941	1
Pole2	1,1940	1
Tmem120a	1,1940	1
Actr10	1,1940	1
Rcn1	1,1937	1
Mrpl39	1,1937	1
Ppp1r12c	1,1936	1
Stap1	1,1935	1
Xpc	1,1932	1
Nasp	1,1932	1
Fam195b	1,1932	1
Gm5380	1,1930	1
Poli	1,1930	1
Tln2	1,1929	1
Slc16a1	1,1929	1
Gm8624	1,1928	1
1700123M08Rik	1,1927	1

Mrps22	1,1927	1
1700012D14Rik	1,1926	1
Gm28791	1,1925	1
Zbtb24	1,1924	1
BC017643	1,1923	1
Zfp646	1,1921	1
Fam72a	1,1918	1
Snx25	1,1918	1
Zc3h10	1,1918	1
Gm16020	1,1918	1
Bhlhe41	1,1916	1
Mpdu1	1,1916	1
Ak2	1,1916	1
Arhgap17	1,1915	1
Esrp2	1,1914	1
Dtx2	1,1913	1
Rhobtb2	1,1913	1
Zfp931	1,1912	1
Cacybp	1,1910	1
E2f5	1,1909	1
Selenoi	1,1909	1
8030453O22Rik	1,1909	1
Ybey	1,1908	1
Mid1ip1	1,1904	1
Sgtb	1,1903	1
Isg15	1,1902	1
Smc1a	1,1902	1
RP24-75M13.2	1,1901	1
Gm6166	1,1900	1
Vwa1	1,1899	1
Upf2	1,1898	1
Papd7	1,1896	1
Enc1	1,1896	1
Arhgdia	1,1896	1
Fam229b	1,1895	1
Gm15210	1,1893	1
Tmem63a	1,1893	1
Tmco6	1,1891	1
Slc45a3	1,1891	1
Snrpb2	1,1891	1
Mdp1	1,1886	1
Ascc3	1,1885	1
Snrnp27	1,1883	1
Cebpe	1,1881	1
Thap2	1,1881	1
Prdx3	1,1881	1
Bnip1	1,1880	1
Gm27477	1,1879	1
Zfp787	1,1879	1
Gm6206	1,1878	1
Zfp961	1,1878	1
Nfatc2	1,1875	1

Mustn1	1,1871	1
Gm5687	1,1871	1
Fam167b	1,1871	1
Eif2a	1,1871	1
Pex26	1,1870	1
Aasdhppt	1,1870	1
Spin1	1,1868	1
H2-DMa	1,1868	1
Gm20492	1,1866	1
Gm15050	1,1865	1
Vps51	1,1864	1
Fkbp1a	1,1864	1
Gemin2	1,1863	1
Gm14541	1,1862	1
Vgll4	1,1862	1
Ccl3	1,1858	1
Tmem144	1,1858	1
Fopnl	1,1857	1
Unc13b	1,1856	1
Fgfbp3	1,1849	1
Efcab2	1,1848	1
Timm29	1,1847	1
Wdr89	1,1844	1
Pcbd2	1,1844	1
Gm6136	1,1842	1
Cit	1,1840	1
Zfp51	1,1840	1
Trmt61a	1,1835	1
Gm14292	1,1835	1
Cbx6	1,1834	1
Mob2	1,1833	1
Fkbp8	1,1832	1
2410004B18Rik	1,1830	1
Sec31b	1,1830	1
Acaa1a	1,1830	1
Gm14126	1,1828	1
Pebp1	1,1827	1
Rnf145	1,1827	1
Gm13641	1,1825	1
Gm37963	1,1824	1
Zscan2	1,1823	1
Cib2	1,1822	1
Sf3b6	1,1820	1
Bach1	1,1819	1
Car2	1,1819	1
Traip	1,1818	1
Mzt2	1,1818	1
Gm11221	1,1816	1
Gyg	1,1816	1
Extl3	1,1816	1
Mitd1	1,1816	1
Cd2bp2	1,1814	1

Crnkl1	1,1812	1
Ddx49	1,1812	1
Dock1	1,1810	1
Nrtn	1,1809	1
Ilkap	1,1808	1
Sike1	1,1808	1
Rusc2	1,1807	1
Rsrp1	1,1807	1
Mdm1	1,1806	1
Camta1	1,1805	1
Gm26520	1,1803	1
Cmc1	1,1803	1
Ywhaz	1,1803	1
Rubcn	1,1802	1
Fam210a	1,1802	1
Speg	1,1800	1
Taf13	1,1800	1
Golga3	1,1800	1
Neurl2	1,1799	1
Gtf2a2	1,1798	1
Ccdc77	1,1798	1
P3h3	1,1796	1
Ccser2	1,1795	1
Fbxo2	1,1794	1
Phyh	1,1794	1
Zcchc17	1,1793	1
Tslp	1,1792	1
Cela1	1,1791	1
E130102H24Rik	1,1790	1
Txndc17	1,1789	1
Dip2c	1,1787	1
Dusp14	1,1786	1
Itgb7	1,1785	1
Plk1	1,1784	1
Gtf3c5	1,1781	1
C130036L24Rik	1,1781	1
Tm2d2	1,1781	1
Pstk	1,1780	1
Fbxo22	1,1780	1
Fanci	1,1780	1
Arhgef17	1,1779	1
Haus8	1,1777	1
Mycbp	1,1774	1
Rrp12	1,1772	1
Tmem143	1,1772	1
Slc41a1	1,1772	1
Hist1h4n	1,1768	1
Upp2	1,1768	1
Zfp143	1,1768	1
Atg16l1	1,1765	1
Ahdc1	1,1764	1
Rce1	1,1762	1

Tgoln1	1,1760	1
Zfp746	1,1759	1
Gm26917	1,1759	1
Stk24	1,1756	1
Gm2810	1,1755	1
Tcaf1	1,1754	1
Cdc42ep4	1,1754	1
Guca1a	1,1753	1
Gm44510	1,1752	1
Fbf1	1,1752	1
Pafah1b1	1,1751	1
Gm12115	1,1750	1
Rftn2	1,1750	1
Mocs3	1,1750	1
Rpl8	1,1750	1
Nop9	1,1748	1
Lrrc75a	1,1747	1
Atp7a	1,1743	1
Lemd2	1,1743	1
Etfdh	1,1741	1
Hspb11	1,1738	1
Hist1h2bp	1,1736	1
Crk	1,1735	1
4933427D14Rik	1,1734	1
Mrpl50	1,1732	1
Trim36	1,1732	1
Erh	1,1730	1
Fkbp1b	1,1730	1
Nab2	1,1730	1
Smc3	1,1730	1
Gng7	1,1729	1
Pwp1	1,1729	1
Oaz2	1,1728	1
Cnih4	1,1728	1
3110080O07Rik	1,1727	1
Dhcr7	1,1727	1
Bola3	1,1726	1
Ccpg1os	1,1725	1
Gxylt1	1,1725	1
Fgd6	1,1725	1
Dph5	1,1724	1
2900055J20Rik	1,1723	1
Cfap20	1,1723	1
Pex19	1,1723	1
Dync1i2	1,1719	1
Guf1	1,1718	1
Tial1	1,1718	1
Gm6526	1,1717	1
Tcf7l2	1,1716	1
Abhd6	1,1716	1
Epb41l4aos	1,1716	1
RP23-55A6.4	1,1714	1

Bdp1	1,1712	1
Tnfrsf23	1,1711	1
Serhl	1,1711	1
2310039H08Rik	1,1710	1
Scnm1	1,1709	1
Rnf146	1,1709	1
Haus1	1,1707	1
Ripk2	1,1707	1
Ndufs8	1,1706	1
Lamtor1	1,1706	1
Thyn1	1,1706	1
Ndufa3	1,1706	1
Mrps15	1,1705	1
Orai1	1,1704	1
Pank2	1,1704	1
Cyp51	1,1703	1
Saal1	1,1702	1
1700056N10Rik	1,1701	1
Nicn1	1,1700	1
Gin1	1,1699	1
Dhps	1,1699	1
Dnajb14	1,1699	1
2610318N02Rik	1,1697	1
Pxmp4	1,1697	1
Ptma	1,1697	1
Gm3145	1,1695	1
Nt5c3b	1,1693	1
Rpl3-ps1	1,1693	1
Rps19-ps4	1,1685	1
Fzd9	1,1685	1
Dusp5	1,1684	1
Katna1	1,1684	1
Rab3il1	1,1683	1
Rrp1	1,1683	1
Cr1l	1,1682	1
Gm43466	1,1678	1
Sbds	1,1678	1
Taf7	1,1678	1
Kpna4	1,1676	1
Osgepl1	1,1674	1
Tnrc18	1,1674	1
Rbm47	1,1671	1
Clint1	1,1671	1
Cpq	1,1670	1
Gltscr1	1,1669	1
Bex3	1,1669	1
Gm7785	1,1667	1
Cep350	1,1667	1
Vsir	1,1667	1
Dpp3	1,1665	1
Mrps5	1,1664	1
Gm15535	1,1663	1

Myd88	1,1662	1
Dohh	1,1662	1
Serpinf2	1,1660	1
Gm9347	1,1660	1
Msl2	1,1658	1
Gm7336	1,1656	1
Ptch1	1,1656	1
Gm2788	1,1651	1
Eif5a	1,1651	1
Kctd18	1,1650	1
E330037G11Rik	1,1649	1
Sra1	1,1644	1
Dexi	1,1643	1
Fam192a	1,1643	1
Itpa	1,1642	1
Nub1	1,1641	1
Ppdpf	1,1639	1
Gpatch2	1,1639	1
Sord	1,1639	1
Supt7l	1,1638	1
Jade1	1,1638	1
Brms1l	1,1635	1
Caap1	1,1635	1
Cyth3	1,1635	1
Naxd	1,1634	1
Gm2272	1,1634	1
Rhno1	1,1634	1
Entpd6	1,1632	1
Tssc4	1,1629	1
Cdc42se2	1,1626	1
Ifitm5	1,1626	1
Cpeb4	1,1626	1
Capn5	1,1626	1
L3mbtl2	1,1626	1
Hs3st3b1	1,1625	1
Abhd8	1,1625	1
BC085271	1,1624	1
Mex3d	1,1624	1
Rnaseh2a	1,1624	1
Cdyl	1,1624	1
Rbm6-ps1	1,1623	1
Agap3	1,1623	1
Sdhd	1,1622	1
Atp5a1	1,1622	1
Gm38043	1,1621	1
Bloc1s5	1,1621	1
Tmem38a	1,1619	1
Taf1a	1,1619	1
Gm26606	1,1616	1
Hyal1	1,1612	1
Pisd	1,1612	1
Fkbp3	1,1610	1

Mss51	1,1609	1
Eepd1	1,1609	1
Slc35e4	1,1609	1
Zfand2b	1,1607	1
Uqcrfs1	1,1607	1
Aplp2	1,1607	1
Ubl5	1,1606	1
Mr1	1,1605	1
Smad3	1,1605	1
Gm45629	1,1604	1
Meis3	1,1604	1
Casp6	1,1604	1
G3bp2	1,1604	1
Gm30074	1,1601	1
1700022N22Rik	1,1601	1
Yrdc	1,1600	1
Phf20l1	1,1599	1
Chpf	1,1598	1
Gm8203	1,1597	1
Ncbp2	1,1597	1
Etfb	1,1597	1
Tgfbr1	1,1597	1
Gmcl1	1,1597	1
4930520O04Rik	1,1596	1
Prpf38a	1,1595	1
Smim20	1,1593	1
Nucb1	1,1592	1
Eloc	1,1591	1
Rps27rt	1,1591	1
Lsm11	1,1591	1
Gm7432	1,1590	1
Nudt21	1,1590	1
Abi2	1,1590	1
Lactb	1,1590	1
Alox8	1,1589	1
Ttc21b	1,1589	1
Mfsd13a	1,1589	1
Rsbn1	1,1589	1
Cebpd	1,1589	1
Gm20568	1,1588	1
Mboat7	1,1584	1
Toporsos	1,1583	1
Abi1	1,1581	1
Cyth2	1,1581	1
Nudcd2	1,1581	1
Trim8	1,1578	1
Mkrn1	1,1577	1
Tmem203	1,1576	1
Trim33	1,1574	1
Tmem120b	1,1573	1
Gm42835	1,1572	1
Ebna1bp2	1,1572	1

Wrap53	1,1570	1
4933408B17Rik	1,1570	1
Pithd1	1,1570	1
Pdhb	1,1570	1
Ppme1	1,1568	1
Gm17745	1,1567	1
Polr3h	1,1566	1
Gdpd3	1,1565	1
Gstz1	1,1564	1
Cygb	1,1564	1
Fam173a	1,1564	1
Mapk1ip1l	1,1561	1
Rpl35a-ps4	1,1560	1
Vps26a	1,1560	1
Gm42986	1,1555	1
Ebpl	1,1555	1
Psmd12	1,1555	1
Reps2	1,1552	1
Mis18a	1,1552	1
Egr2	1,1549	1
Endod1	1,1544	1
Prpf3	1,1544	1
Tmem184b	1,1544	1
9130401M01Rik	1,1543	1
Nemf	1,1542	1
Abcg4	1,1541	1
Gm16286	1,1541	1
Nprl2	1,1539	1
Orc1	1,1538	1
Gtpbp6	1,1538	1
Cpeb2	1,1537	1
1110038F14Rik	1,1537	1
A430005L14Rik	1,1537	1
Trip6	1,1537	1
Gm15946	1,1536	1
Wdr44	1,1536	1
Cdc42se1	1,1535	1
Siah1a	1,1533	1
Gm8121	1,1532	1
Evl	1,1530	1
Tspo	1,1530	1
BC037032	1,1528	1
Pdcd5	1,1528	1
Bud31	1,1528	1
Gm26759	1,1526	1
A930005H10Rik	1,1526	1
Klhl11	1,1525	1
Glul	1,1525	1
Pgrmc2	1,1524	1
Dubr	1,1520	1
Rps24-ps2	1,1519	1
Uqcrc1	1,1519	1

Tsen34	1,1516	1
Atf1	1,1516	1
Gm45884	1,1515	1
Rpl37	1,1515	1
Rell1	1,1515	1
Edil3	1,1514	1
Lgmn	1,1512	1
Tcaim	1,1512	1
Rora	1,1510	1
Tprkb	1,1509	1
Mettl13	1,1509	1
Prdx2	1,1509	1
Ssr2	1,1509	1
Ascc1	1,1508	1
Pak1	1,1508	1
Cacna1a	1,1506	1
D11Wsu47e	1,1505	1
0610009L18Rik	1,1504	1
Mttp	1,1504	1
Nop56	1,1504	1
St6gal1	1,1503	1
Gm11686	1,1502	1
Lsm1	1,1501	1
Prdm15	1,1500	1
Arfgap3	1,1500	1
Usp6nl	1,1500	1
Aldh2	1,1500	1
Pepd	1,1499	1
Alkbh4	1,1498	1
Tsc1	1,1498	1
Klf3	1,1497	1
Itpk1	1,1497	1
Qk	1,1496	1
Dcaf5	1,1494	1
D10Wsu102e	1,1494	1
Gm26601	1,1491	1
Ppm1m	1,1490	1
Dnd1	1,1489	1
Tnfrsf22	1,1487	1
Cisd1	1,1487	1
Dnph1	1,1486	1
Sdhaf1	1,1485	1
Topbp1	1,1485	1
Ndel1	1,1484	1
Trim13	1,1481	1
Nupr1	1,1481	1
Aprt	1,1481	1
Nrbp2	1,1477	1
Wdr4	1,1477	1
Cdk5r1	1,1475	1
Top2a	1,1475	1
Gm7514	1,1474	1

Anp32b	1,1474	1
Cd101	1,1473	1
Zfp654	1,1473	1
Zfp524	1,1472	1
Krr1	1,1472	1
Timm50	1,1471	1
Cnnm4	1,1470	1
Yipf4	1,1469	1
Hmx2	1,1469	1
Dynll1	1,1469	1
Gm42724	1,1469	1
Hyal2	1,1469	1
Ctcf	1,1469	1
Mrpl44	1,1468	1
Praf2	1,1467	1
Selenoo	1,1467	1
Cotl1	1,1466	1
Zbtb39	1,1465	1
Gm12834	1,1464	1
Chd2	1,1464	1
Nus1	1,1464	1
Coa3	1,1461	1
RP24-389J11.1	1,1460	1
Pddc1	1,1459	1
Sema4c	1,1458	1
Il17ra	1,1457	1
Nrm	1,1456	1
Acbd6	1,1456	1
Acsl3	1,1455	1
RP23-182J19.2	1,1454	1
Tjp2	1,1453	1
Slc9a5	1,1452	1
Cdk2ap2	1,1452	1
Map3k5	1,1452	1
Dse	1,1450	1
Zmym1	1,1449	1
Eda2r	1,1449	1
Ssr3	1,1449	1
Eif4e2	1,1449	1
Gm10501	1,1447	1
Tspan14	1,1447	1
Cdkn2aipnl	1,1447	1
2010315B03Rik	1,1446	1
Zrsr1	1,1445	1
Hypk	1,1443	1
Spr	1,1443	1
Cisd3	1,1442	1
Fahd1	1,1441	1
Anxa5	1,1441	1
Klf16	1,1439	1
Imp3	1,1439	1
Haus7	1,1439	1

Ptges3	1,1438	1
Myo10	1,1437	1
Metap1	1,1436	1
Skp2	1,1435	1
3110070M22Rik	1,1434	1
Rnf187	1,1433	1
Fbxo11	1,1433	1
Bad	1,1430	1
Fam220a	1,1429	1
Ddx23	1,1428	1
Socs4	1,1427	1
Cox17	1,1427	1
Ccdc163	1,1427	1
Dtx3	1,1427	1
Gm11914	1,1426	1
Ostf1	1,1424	1
Pde12	1,1423	1
Usp30	1,1423	1
Rras	1,1423	1
Cnot4	1,1422	1
Hoxb4	1,1420	1
Eif3h	1,1418	1
2810428I15Rik	1,1416	1
Ppil4	1,1416	1
Arpc1a	1,1415	1
Zfp512	1,1415	1
Efnb1	1,1414	1
Havcr2	1,1414	1
Grpel2	1,1413	1
Zmat5	1,1413	1
D10Jhu81e	1,1412	1
H2-DMb1	1,1411	1
Acad8	1,1411	1
Sgf29	1,1411	1
Creb1	1,1411	1
Cchcr1	1,1409	1
Scel	1,1408	1
Nectin1	1,1408	1
Arhgef4	1,1407	1
Sorbs1	1,1406	1
Gm5787	1,1406	1
Ccdc22	1,1404	1
Ube2d-ps	1,1403	1
Acap1	1,1401	1
Arl2	1,1401	1
4933421O10Rik	1,1400	1
Ints2	1,1399	1
Gm44190	1,1397	1
Zfp821	1,1397	1
Akt3	1,1395	1
Anapc11	1,1394	1
Spaca6	1,1391	1

Pcbp1	1,1390	1
Itgam	1,1389	1
Zfp707	1,1388	1
Zfp652os	1,1387	1
AI839979	1,1387	1
Erlin1	1,1387	1
Pfkfb2	1,1386	1
St3gal5	1,1386	1
4930402H24Rik	1,1383	1
Selenok	1,1382	1
Txn1	1,1380	1
Id3	1,1378	1
Cbr3	1,1378	1
D630024D03Rik	1,1378	1
Agtrap	1,1378	1
Lrrc40	1,1378	1
1810013L24Rik	1,1378	1
Psmg4	1,1378	1
A930015D03Rik	1,1377	1
Uba3	1,1377	1
Rgs10	1,1377	1
Kctd7	1,1374	1
Pih1d1	1,1374	1
Scaf1	1,1374	1
Hnrnpr	1,1374	1
Gm38380	1,1373	1
Mob4	1,1373	1
Top1	1,1372	1
Peak1os	1,1371	1
Bpgm	1,1370	1
Scaper	1,1370	1
Gm43707	1,1367	1
Pcmt1	1,1367	1
Mrpl32	1,1366	1
4833421G17Rik	1,1364	1
Mex3a	1,1364	1
Ppp2r5d	1,1364	1
Utp11	1,1364	1
Cwc22	1,1361	1
Bag3	1,1360	1
Rnase4	1,1360	1
Sec22c	1,1359	1
Myo1g	1,1359	1
1700021F05Rik	1,1359	1
Gm29462	1,1357	1
Spata24	1,1355	1
Klf7	1,1355	1
Sf3b5	1,1355	1
Gm6493	1,1353	1
Rfxank	1,1352	1
Eapp	1,1352	1
Gm43110	1,1352	1

Fgr	1,1352	1
Rpl26	1,1351	1
Rpap2	1,1351	1
Eif3j2	1,1351	1
Fen1	1,1350	1
Chd3os	1,1349	1
RP23-403E19.1	1,1348	1
Atp1b3	1,1348	1
Rpf2	1,1348	1
Pim3	1,1348	1
Tm7sf2	1,1347	1
Urod	1,1347	1
Mcm5	1,1346	1
Gm7887	1,1345	1
Trappc5	1,1345	1
Tspan5	1,1344	1
Xylt2	1,1343	1
Gm10073	1,1343	1
RP23-277D1.1	1,1342	1
Surf6	1,1341	1
Rplp0	1,1341	1
Gm26652	1,1341	1
Cep57	1,1341	1
Gm2225	1,1340	1
Toe1	1,1340	1
Ccnt1	1,1338	1
Gpn3	1,1337	1
Mybl1	1,1337	1
Ahsa1	1,1337	1
Eef1e1	1,1337	1
Ndufb5	1,1336	1
Narfl	1,1335	1
Mgea5	1,1333	1
Egfl8	1,1332	1
Lsm6	1,1332	1
Rnf113a1	1,1331	1
Bckdhb	1,1330	1
Ugcg	1,1330	1
Psmc1	1,1329	1
Sart1	1,1328	1
Ap4m1	1,1328	1
Zfp202	1,1327	1
Tor1a	1,1327	1
Ddx27	1,1326	1
Prpf19	1,1326	1
Glrx5	1,1324	1
Ift74	1,1323	1
Prkrip1	1,1322	1
Fhod3	1,1320	1
Dnaja1	1,1320	1
Bmyc	1,1320	1
Gm16399	1,1320	1

Dnajb1	1,1319	1
Anp32a	1,1319	1
Actr1a	1,1319	1
Cebpz	1,1319	1
Slc25a11	1,1318	1
Gm8268	1,1316	1
A630081D01Rik	1,1314	1
Atp5sl	1,1312	1
Shcbp1	1,1311	1
B230219D22Rik	1,1311	1
Dbf4	1,1310	1
Zfp639	1,1308	1
Gm13815	1,1307	1
Cacul1	1,1307	1
Fundc1	1,1305	1
Arsk	1,1304	1
Gm14780	1,1304	1
Arfip2	1,1303	1
Gm37465	1,1301	1
Ppif	1,1301	1
Wdr46	1,1301	1
Ubxn4	1,1301	1
Tor4a	1,1300	1
Gpi1	1,1300	1
1810014B01Rik	1,1298	1
Runx3	1,1298	1
Nat9	1,1298	1
Fyttd1	1,1298	1
Tmem115	1,1297	1
Rras2	1,1297	1
Gm2a	1,1297	1
Naa35	1,1297	1
Naa50	1,1297	1
Pcf11	1,1296	1
Mcts2	1,1295	1
Stam2	1,1295	1
Cldnd1	1,1292	1
Mtx1	1,1292	1
Polrmt	1,1291	1
Ypel5	1,1291	1
Gm10240	1,1290	1
Mllt11	1,1290	1
9930104L06Rik	1,1290	1
Lemd3	1,1288	1
Tra2a	1,1288	1
Srsf4	1,1287	1
Galnt10	1,1285	1
Cntd1	1,1284	1
Zfr	1,1283	1
Kansl3	1,1283	1
Gm13803	1,1282	1
Sar1b	1,1282	1

Epop	1,1281	1
Kin	1,1280	1
Dhx8	1,1279	1
Vma21	1,1279	1
Dpys	1,1278	1
mt-Nd5	1,1277	1
Atp5o	1,1277	1
Ranbp3	1,1276	1
Hes6	1,1272	1
Kansl1l	1,1272	1
Pde6d	1,1272	1
Rpl22l1	1,1272	1
Crls1	1,1271	1
Wdr91	1,1271	1
Gm5910	1,1270	1
BC005624	1,1270	1
Ndrg4	1,1270	1
Rny3	1,1269	1
Zfp346	1,1269	1
Snhg3	1,1269	1
Calm2	1,1269	1
Elp5	1,1267	1
Tmem168	1,1267	1
Adss	1,1267	1
Kctd10	1,1266	1
Sep 07	1,1266	1
Dtnbp1	1,1264	1
Rpl27a-ps2	1,1263	1
Car11	1,1263	1
4930431P19Rik	1,1263	1
Vps54	1,1262	1
Mri1	1,1262	1
Tcp1	1,1262	1
Gm43795	1,1259	1
Arap3	1,1259	1
Hmgcr	1,1258	1
Vps72	1,1257	1
Mrpl14	1,1256	1
Erich1	1,1255	1
Ppid	1,1255	1
Gm8822	1,1254	1
Gtpbp4	1,1254	1
Rap1a	1,1252	1
Psen1	1,1251	1
Nedd8	1,1250	1
Incenp	1,1249	1
C030037D09Rik	1,1247	1
Kif18b	1,1246	1
Capn2	1,1244	1
Zfp120	1,1244	1
Mier1	1,1243	1
4833439L19Rik	1,1241	1

Rsrc2	1,1241	1
Wfikkn1	1,1238	1
Lrrc45	1,1238	1
9530068E07Rik	1,1238	1
Cenpn	1,1237	1
Abhd14a	1,1237	1
Adam8	1,1237	1
Rabggtb	1,1237	1
Tmem222	1,1236	1
Dusp3	1,1235	1
Gm9320	1,1234	1
Gm45836	1,1234	1
Tmem191c	1,1234	1
Fxyd5	1,1234	1
Mdh1	1,1233	1
Dhx30	1,1233	1
Cox11	1,1232	1
Spag4	1,1231	1
Casp9	1,1231	1
Gltp	1,1231	1
Maip1	1,1230	1
Mfsd12	1,1229	1
Arl8b	1,1228	1
Rnf185	1,1227	1
Rhpn2	1,1226	1
Trmt2a	1,1225	1
Mettl9	1,1224	1
Sem1	1,1223	1
3110082I17Rik	1,1221	1
Nme1	1,1221	1
Gm15289	1,1220	1
Gm9828	1,1218	1
Irf2bp1	1,1217	1
Rabgef1	1,1216	1
Gm45422	1,1216	1
Med31	1,1215	1
Cd81	1,1214	1
Osbpl10	1,1213	1
Mrps16	1,1213	1
Mtfmt	1,1212	1
Gm6807	1,1211	1
Fdxacb1	1,1207	1
Trmt61b	1,1207	1
Prmt7	1,1207	1
Phb	1,1206	1
Mat2b	1,1206	1
Sgol2a	1,1205	1
Ift22	1,1204	1
Zfp61	1,1204	1
0610009O20Rik	1,1203	1
Ptprc	1,1203	1
Borcs5	1,1202	1

Zfp850	1,1202	1
Cul3	1,1201	1
Ndufa8	1,1201	1
Vps37c	1,1199	1
Rnf34	1,1199	1
Tysnd1	1,1199	1
Phf5a	1,1199	1
Coa4	1,1198	1
Zfp574	1,1198	1
Rnf144b	1,1198	1
Fastkd1	1,1196	1
Man1c1	1,1195	1
Gm8181	1,1193	1
RP23-440I21.3	1,1191	1
Ndufa11	1,1191	1
Ankrd27	1,1190	1
Spsb3	1,1189	1
Sgsm2	1,1186	1
Sart3	1,1185	1
Wsb2	1,1184	1
Gm5547	1,1184	1
Gps1	1,1183	1
Srfbp1	1,1181	1
Pef1	1,1180	1
Ywhae	1,1180	1
D830044I16Rik	1,1179	1
Rpl7a	1,1178	1
Phf23	1,1178	1
Ski	1,1178	1
Sub1	1,1178	1
Slc2a4rg-ps	1,1177	1
Golt1b	1,1177	1
Atox1	1,1175	1
Rabep1	1,1174	1
Ccdc180	1,1172	1
Ms4a6b	1,1171	1
Mrps18b	1,1171	1
Tmf1	1,1171	1
Lxn	1,1171	1
Mob1b	1,1171	1
Mtfr2	1,1169	1
Hpf1	1,1168	1
Gnb5	1,1167	1
Zfp644	1,1165	1
Plekhg2	1,1164	1
Pigf	1,1162	1
Gpr157	1,1161	1
Hmbs	1,1159	1
Alad	1,1158	1
Dynll2	1,1157	1
Tm6sf1	1,1156	1
Mettl16	1,1156	1

Lmbr1l	1,1155	1
Tcf20	1,1154	1
Mrps28	1,1154	1
4933439C10Rik	1,1154	1
Ptgr1	1,1154	1
Pi16	1,1154	1
Pak4	1,1153	1
B4galt6	1,1151	1
Wipi2	1,1151	1
Lyrm1	1,1150	1
Meis2	1,1150	1
Dda1	1,1149	1
Mettl18	1,1148	1
Gm11224	1,1148	1
Mrpl28	1,1148	1
Slc35b2	1,1148	1
Ptp4a1	1,1147	1
Anks3	1,1146	1
Vps8	1,1145	1
Smg5	1,1145	1
Dph3	1,1145	1
Atl2	1,1145	1
Ppp2r2a	1,1144	1
Gm43247	1,1143	1
F830208F22Rik	1,1143	1
Tmem37	1,1141	1
Ndufv1	1,1141	1
Timeless	1,1140	1
Dnal4	1,1138	1
Ccdc159	1,1136	1
Dhx38	1,1135	1
Rttn	1,1133	1
Tet2	1,1133	1
Hdac6	1,1132	1
Cdc25a	1,1132	1
Gm28875	1,1131	1
Fip1l1	1,1130	1
Gm12981	1,1130	1
Rilpl1	1,1130	1
Zfp954	1,1130	1
Gm14593	1,1129	1
Ccdc191	1,1129	1
Senp8	1,1128	1
Rab5c	1,1127	1
Gm44024	1,1126	1
Snu13	1,1126	1
Brf2	1,1120	1
Lsm12	1,1118	1
Hnrnpc	1,1117	1
Kbtbd4	1,1116	1
Lta4h	1,1116	1
Vps18	1,1116	1

Gm36266	1,1115	1
D730003I15Rik	1,1115	1
Tacc1	1,1115	1
Car5b	1,1114	1
Mark3	1,1114	1
Zc3h7a	1,1114	1
Mphosph8	1,1114	1
Clic1	1,1114	1
Hexdc	1,1113	1
AW554918	1,1111	1
Mrfap1	1,1110	1
Phactr4	1,1108	1
Nap1l1	1,1107	1
Ttc5	1,1106	1
Rprd1b	1,1106	1
B9d1	1,1104	1
Hnrnph2	1,1103	1
Sdf2	1,1103	1
Borcs6	1,1103	1
Sec62	1,1103	1
Tprn	1,1101	1
Rab6a	1,1101	1
Pttg1ip	1,1100	1
Fcnaos	1,1099	1
Gm5244	1,1098	1
Dnaaf2	1,1097	1
Gm13038	1,1096	1
Gpsm1	1,1096	1
Zfp511	1,1096	1
Tomm20	1,1095	1
Afmid	1,1094	1
Sp3	1,1093	1
Saysd1	1,1093	1
Fgd4	1,1092	1
Atpaf2	1,1090	1
Rab11fip1	1,1090	1
Ctdspl2	1,1089	1
Mrpl16	1,1089	1
Pla2g15	1,1089	1
Mrpl48-ps	1,1086	1
Rpl19	1,1084	1
Tob2	1,1083	1
Tbc1d31	1,1081	1
Hsp90b1	1,1077	1
Dot1l	1,1076	1
2510039O18Rik	1,1076	1
Mknk2	1,1076	1
Atp11b	1,1075	1
Smad2	1,1074	1
Terf2ip	1,1073	1
Nos3	1,1073	1
Tmem60	1,1072	1

Znhit1	1,1071	1
Ilk	1,1070	1
Tdrd7	1,1068	1
Smco4	1,1068	1
Gm11737	1,1067	1
Sh3bp1	1,1067	1
Plod3	1,1067	1
Iqsec2	1,1067	1
Mrpl13	1,1066	1
Ddx10	1,1066	1
Rnf14	1,1066	1
Rnf115	1,1065	1
Chid1	1,1064	1
Tagln2	1,1064	1
Copz2	1,1063	1
M1ap	1,1063	1
Taf3	1,1062	1
Sfr1	1,1062	1
Phip	1,1061	1
Cdk17	1,1060	1
2210008F06Rik	1,1059	1
Trappc2	1,1059	1
Med18	1,1058	1
Nono	1,1057	1
Armt1	1,1057	1
Gm8013	1,1056	1
Gm5575	1,1055	1
Fdft1	1,1055	1
Phf7	1,1053	1
Trak1	1,1053	1
S1pr2	1,1053	1
Vezf1	1,1051	1
Nmt1	1,1051	1
Pex14	1,1050	1
Acbd4	1,1049	1
Pafah1b1-ps1	1,1048	1
Elovl5	1,1047	1
Gba	1,1043	1
H2-D1	1,1043	1
Nosip	1,1042	1
Stx8	1,1041	1
Otud3	1,1041	1
Rad17	1,1040	1
Nudt13	1,1039	1
Lonp2	1,1037	1
Icam5	1,1037	1
Zfp942	1,1035	1
Pmpcb	1,1035	1
Fam175a	1,1035	1
1110003F10Rik	1,1034	1
Anp32-ps	1,1032	1
Eif1ad	1,1031	1

Fmr1	1,1031	1
E230016M11Rik	1,1030	1
Brap	1,1030	1
Srsf1	1,1030	1
Ano6	1,1030	1
Noxo1	1,1029	1
Rad52	1,1029	1
Htatsf1	1,1027	1
Gm13758	1,1026	1
Wdhd1	1,1026	1
Dyrk1a	1,1023	1
Rpusd2	1,1022	1
Spns1	1,1022	1
Mrps34	1,1022	1
Gm5422	1,1021	1
Rbm43	1,1019	1
Nxt2	1,1018	1
Polr3c	1,1018	1
Spg21	1,1018	1
Tmem51	1,1015	1
Rpl12-ps1	1,1014	1
Erc1	1,1014	1
Alox5	1,1012	1
Eif4enif1	1,1009	1
Phf21b	1,1008	1
6430531B16Rik	1,1008	1
Gm43569	1,1008	1
Utp14a	1,1006	1
Foxn2	1,1005	1
Stk11	1,1005	1
C730045M19Rik	1,1004	1
Tpra1	1,1004	1
Map3k2	1,1003	1
A630072M18Rik	1,1002	1
Elof1	1,1001	1
Glod4	1,1001	1
Thoc5	1,1001	1
Ung	1,0997	1
R3hdm1	1,0997	1
Kdelr2	1,0996	1
Clcn6	1,0995	1
Stk35	1,0995	1
Zfp316	1,0995	1
Rida	1,0993	1
Strip1	1,0993	1
Gm2962	1,0991	1
Srp14	1,0989	1
Tmpo	1,0986	1
Rab32	1,0985	1
Fbxo30	1,0983	1
Hps4	1,0983	1
Lnpk	1,0982	1

Gm37254	1,0982	1
Cdc5l	1,0982	1
Ighd	1,0982	1
Spryd4	1,0980	1
Clta	1,0980	1
Gm10275	1,0979	1
Daxx	1,0979	1
Slc25a46	1,0979	1
Tnfrsf1a	1,0979	1
Pinx1	1,0978	1
Dcun1d5	1,0976	1
Aldh1l1	1,0976	1
Ube2b	1,0976	1
Dera	1,0975	1
Abl2	1,0974	1
Clp1	1,0973	1
Arid4b	1,0973	1
Gm11517	1,0971	1
Chchd4	1,0971	1
Rps2	1,0970	1
Arfip1	1,0970	1
Gamt	1,0968	1
Ttf1	1,0968	1
Tsta3	1,0967	1
Cebpg	1,0967	1
Gigyf1	1,0966	1
Cdadc1	1,0965	1
Zfp14	1,0964	1
Cep44	1,0964	1
Mnd1	1,0961	1
Zswim7	1,0960	1
Gm12186	1,0960	1
Zc3hc1	1,0959	1
Clip1	1,0958	1
Dennd4a	1,0957	1
Gm12309	1,0956	1
Yy1	1,0955	1
Gemin7	1,0954	1
B230307C23Rik	1,0954	1
Rbx1	1,0952	1
Ctbp2	1,0951	1
Tmem14c	1,0951	1
Pxylp1	1,0951	1
St7l	1,0951	1
Syncrip	1,0947	1
Ubald2	1,0945	1
Slc30a1	1,0945	1
4930556M19Rik	1,0944	1
Glrx3	1,0944	1
Pwp2	1,0944	1
Dck	1,0944	1
Gm9892	1,0943	1

Capn1	1,0942	1
Rrn3	1,0941	1
Sugp1	1,0938	1
Elob	1,0938	1
Rabl3	1,0937	1
Akt2	1,0937	1
Gm12389	1,0935	1
Prelid3a	1,0935	1
Desi2	1,0933	1
Themis2	1,0932	1
Mpg	1,0930	1
Prpf4b	1,0930	1
Psmb5	1,0928	1
Tes3-ps	1,0927	1
Thap3	1,0927	1
Bcl7b	1,0926	1
Sprtn	1,0926	1
Psmc6	1,0926	1
Mcoln2	1,0924	1
Lmnb1	1,0922	1
Gm43813	1,0921	1
Ndrg3	1,0921	1
Fnbp1l	1,0921	1
Ift81	1,0916	1
Rack1	1,0916	1
Gm5828	1,0915	1
Gm14286	1,0913	1
Slmap	1,0913	1
Txnl4b	1,0912	1
Snapc5	1,0910	1
Frg1	1,0910	1
Golph3l	1,0907	1
Blmh	1,0907	1
Gm12716	1,0906	1
Nipa2	1,0906	1
Abhd16a	1,0905	1
Gm15503	1,0904	1
Gm11625	1,0904	1
Clip2	1,0904	1
Cfdp1	1,0903	1
Coro2a	1,0901	1
Eif1ax	1,0901	1
Gm42567	1,0900	1
Spire1	1,0899	1
Hspa14	1,0899	1
Egln2	1,0898	1
Setd3	1,0898	1
Usp5	1,0897	1
Ola1	1,0897	1
Slc31a2	1,0895	1
Mthfsl	1,0889	1
Coq3	1,0889	1

Snupn	1,0889	1
Fam204a	1,0888	1
Tgif1	1,0888	1
Gm13368	1,0886	1
Tyrobp	1,0885	1
Sccpdh	1,0885	1
Atg13	1,0882	1
Snhg6	1,0882	1
Mylip	1,0881	1
Fkbp7	1,0876	1
Atp6v0e2	1,0876	1
Dld	1,0876	1
Gpr108	1,0873	1
Rnf41	1,0872	1
Nedd4l	1,0872	1
Actr8	1,0872	1
Hnrnpl	1,0871	1
Pik3r1	1,0871	1
Zfp541	1,0870	1
Gm8430	1,0870	1
Ift80	1,0869	1
Alkbh5	1,0868	1
Eif2d	1,0867	1
Ttll13	1,0867	1
Sik2	1,0866	1
Rictor	1,0866	1
March5	1,0866	1
Bora	1,0864	1
Mff	1,0863	1
Gm13005	1,0862	1
Srsf10	1,0862	1
Eml4	1,0861	1
Sdhb	1,0861	1
Creld1	1,0859	1
Ipo5	1,0857	1
Stard5	1,0856	1
Hacd1	1,0856	1
Bbs5	1,0855	1
Ltbr	1,0855	1
Irf9	1,0855	1
Gm38115	1,0855	1
Gm12165	1,0854	1
Sltm	1,0852	1
Als2	1,0851	1
Hdac10	1,0849	1
Fosl1	1,0849	1
Gsk3a	1,0848	1
Pura	1,0848	1
Rbm14	1,0847	1
Gm6245	1,0846	1
Mmd	1,0846	1
Fam117b	1,0843	1

BC003965	1,0841	1
Sesn2	1,0839	1
B4gat1	1,0838	1
Thtpa	1,0838	1
Hdac1	1,0837	1
Macrod1	1,0837	1
9430060I03Rik	1,0836	1
Alcam	1,0836	1
Dgkd	1,0836	1
Gm43106	1,0835	1
Taf10	1,0835	1
Hn1	1,0835	1
Cox19	1,0833	1
Gm37470	1,0832	1
Pdxdc1	1,0832	1
Rnf44	1,0831	1
Ube2k	1,0829	1
Asb8	1,0828	1
Gm16373	1,0827	1
Ahcy	1,0827	1
Mto1	1,0827	1
Nubp2	1,0825	1
Stard4	1,0824	1
3300002I08Rik	1,0820	1
Gm38120	1,0820	1
Zbtb43	1,0818	1
Ino80e	1,0817	1
Echs1	1,0815	1
Chmp7	1,0815	1
Nfe2l2	1,0815	1
Taf4	1,0815	1
Ifrd2	1,0814	1
Rabepk	1,0814	1
Fam32a	1,0814	1
Alg12	1,0813	1
Ppp2r1a	1,0812	1
Raly	1,0812	1
Gnpnat1	1,0812	1
Hnrnpd	1,0812	1
Sgsm3	1,0810	1
Emp3	1,0808	1
Tango2	1,0807	1
Cdk2	1,0807	1
Prr13	1,0807	1
Ift46	1,0807	1
Gm37696	1,0805	1
Lcor	1,0804	1
Hemk1	1,0804	1
1190002N15Rik	1,0804	1
Fam53a	1,0804	1
Utp6	1,0802	1
Cox7b	1,0802	1

Tomm22	1,0801	1
Chmp2b	1,0801	1
Cbwd1	1,0800	1
Runx1	1,0800	1
Anapc2	1,0799	1
Col11a2	1,0797	1
Nkiras1	1,0797	1
E2f2	1,0795	1
Psmd7	1,0794	1
Ece2	1,0793	1
Rfc1	1,0793	1
Gm12166	1,0792	1
Stac2	1,0792	1
Akr7a5	1,0792	1
Mtl5	1,0791	1
Gm4525	1,0789	1
Gen1	1,0789	1
Cep83	1,0789	1
Crem	1,0787	1
Ifnar1	1,0787	1
Mre11a	1,0786	1
Gm45292	1,0786	1
Fbxl15	1,0786	1
Smim8	1,0786	1
Aunip	1,0785	1
Gm6640	1,0784	1
Triobp	1,0784	1
Srp19	1,0784	1
5830444B04Rik	1,0783	1
Fxr1	1,0783	1
Tmem192	1,0781	1
Npepl1	1,0780	1
Orc2	1,0780	1
Glrx	1,0778	1
Zcchc14	1,0778	1
Atg5	1,0778	1
Gm13226	1,0776	1
Tmem263	1,0776	1
Mvk	1,0775	1
Leng8	1,0774	1
Fam20b	1,0774	1
Zfp786	1,0774	1
4933404O12Rik	1,0774	1
Csnk2b	1,0774	1
Seh1l	1,0773	1
Gm8242	1,0772	1
Gm9769	1,0772	1
Nr1h3	1,0772	1
Slc22a17	1,0772	1
Med6	1,0771	1
Sgpp1	1,0771	1
Mtdh	1,0771	1

1700109H08Rik	1,0770	1
R3hcc1	1,0769	1
Zfp24	1,0769	1
Acbd5	1,0768	1
Brwd3	1,0767	1
Lyrm4	1,0765	1
Stard3	1,0765	1
Gm15779	1,0764	1
Jag1	1,0764	1
Armc10	1,0764	1
Lst1	1,0764	1
Gle1	1,0763	1
Dcps	1,0763	1
Nhlrc1	1,0762	1
Bahd1	1,0762	1
Cxcl10	1,0761	1
Ube2a	1,0761	1
Rps26	1,0759	1
Slc22a21	1,0757	1
Fbxw2	1,0756	1
Rufy3	1,0756	1
Gsdmd	1,0756	1
Zfp382	1,0753	1
Adk	1,0753	1
Atg14	1,0753	1
Chmp6	1,0751	1
Prmt6	1,0751	1
Tpd52	1,0750	1
Cdkl3	1,0750	1
Pex7	1,0748	1
RP23-402A24.3	1,0747	1
Klrg2	1,0747	1
Gm11966	1,0747	1
Btaf1	1,0746	1
Supt16	1,0745	1
Mipol1	1,0745	1
Afg3l2	1,0743	1
Ocel1	1,0742	1
Nlgn2	1,0742	1
Rpsa-ps4	1,0739	1
Gm26631	1,0739	1
Flna	1,0739	1
Rnf114	1,0739	1
Ssh2	1,0738	1
Crybb3	1,0737	1
Mrps33	1,0737	1
Sdccag8	1,0737	1
Pdcl	1,0737	1
Pigq	1,0737	1
Polr2m	1,0736	1
Tbc1d22a	1,0735	1
Nsun6	1,0734	1

Sp4	1,0733	1
Syngr1	1,0733	1
Tnfaip8l2	1,0732	1
Ttc1	1,0731	1
Wee1	1,0730	1
Golm1	1,0730	1
Dpm2	1,0730	1
Ptpn18	1,0730	1
Zmym2	1,0728	1
Mcmbp	1,0728	1
Gm6418	1,0727	1
Ccdc126	1,0726	1
Ippk	1,0725	1
Npat	1,0724	1
Eif1	1,0724	1
Pigc	1,0722	1
Crnde	1,0721	1
Usp47	1,0721	1
Dctn3	1,0721	1
Npepps	1,0721	1
Spata33	1,0719	1
Fam71e1	1,0718	1
Tax1bp3	1,0718	1
Wdr5	1,0718	1
Tmem258	1,0718	1
Rasa4	1,0715	1
U2af1	1,0713	1
Clcc1	1,0712	1
Synj2bp	1,0711	1
Hint3	1,0711	1
Gm14248	1,0710	1
Galt	1,0710	1
Scoc	1,0710	1
Mrs2	1,0709	1
Cdpf1	1,0708	1
Cerkl	1,0707	1
Slc2a9	1,0707	1
Gm5805	1,0706	1
Cox4i2	1,0705	1
Exoc3l2	1,0705	1
Tor1aip1	1,0701	1
Zcrb1	1,0700	1
Immp2l	1,0699	1
Cep19	1,0699	1
Setd6	1,0699	1
Amz1	1,0698	1
Wdr73	1,0697	1
Fkbp4	1,0695	1
Nxn	1,0695	1
Nfe2l1	1,0694	1
Rpl39	1,0693	1
Tceanc2	1,0692	1

Pgm1	1,0691	1
Ptrhd1	1,0690	1
Gm12454	1,0690	1
Gm43544	1,0690	1
Lmo2	1,0690	1
Kif2c	1,0689	1
Acvr2a	1,0689	1
Gm11687	1,0687	1
Asf1b	1,0687	1
Abcb1b	1,0687	1
Nup153	1,0685	1
Junos	1,0684	1
Zfp275	1,0684	1
Ccnt2	1,0684	1
Anpep	1,0684	1
Timm17a	1,0684	1
Ndufaf5	1,0683	1
Pkp2	1,0682	1
Zfp692	1,0681	1
0610012G03Rik	1,0679	1
Sqrdl	1,0678	1
Alyref2	1,0678	1
Gm45133	1,0675	1
Syap1	1,0673	1
Syne2	1,0673	1
1500011B03Rik	1,0673	1
Rtn4ip1	1,0670	1
B9d2	1,0668	1
Fam104a	1,0668	1
Gm27010	1,0667	1
Ccnj	1,0667	1
Ccdc112	1,0666	1
Ern1	1,0664	1
Lyrm2	1,0664	1
Rgl3	1,0663	1
Rnf32	1,0661	1
Reep3	1,0661	1
Angel1	1,0661	1
Fam69a	1,0660	1
Nthl1	1,0660	1
Arhgef10l	1,0659	1
Dgcr8	1,0658	1
Selplg	1,0658	1
2900076A07Rik	1,0657	1
Cep295	1,0657	1
RP23-304C21.3	1,0656	1
Colec12	1,0655	1
Fubp1	1,0655	1
Gm11772	1,0654	1
Gys1	1,0653	1
Cir1	1,0652	1
Gm8719	1,0650	1

Nnt	1,0650	1
Vdac1	1,0650	1
Xpnpep1	1,0650	1
Emc6	1,0650	1
Cops2	1,0649	1
Tnfaip8	1,0647	1
Shb	1,0647	1
Usb1	1,0646	1
Zfp472	1,0645	1
Plagl2	1,0644	1
Fbxo45	1,0644	1
Mrpl1	1,0644	1
Fnbp4	1,0644	1
D16Ertd472e	1,0643	1
Dnajc4	1,0643	1
Rnf168	1,0643	1
Upf1	1,0642	1
Mpnd	1,0641	1
Mxi1	1,0641	1
Mrm2	1,0640	1
Txndc12	1,0640	1
Noc3l	1,0639	1
Nit2	1,0639	1
RP23-269H21.1	1,0638	1
Acads	1,0637	1
Golph3	1,0636	1
Rmnd5b	1,0636	1
Ankle2	1,0636	1
Ppard	1,0635	1
Atl3	1,0634	1
Cog3	1,0633	1
Gid4	1,0631	1
Mrpl2	1,0630	1
Ap1s2	1,0629	1
Arl6ip4	1,0628	1
Rps12-ps10	1,0627	1
Tcea1	1,0627	1
Coro1b	1,0627	1
Cog4	1,0625	1
Ufd1l	1,0625	1
Psma1	1,0625	1
Gm17491	1,0625	1
Prpf18	1,0625	1
6330403N20Rik	1,0624	1
Galk1	1,0622	1
Kctd9	1,0621	1
Rpp38	1,0619	1
Gpr137	1,0618	1
Dirc2	1,0618	1
Zfp759	1,0617	1
Paxbp1	1,0617	1
Etnk1	1,0617	1

Gm13712	1,0616	1
Galns	1,0616	1
Thap4	1,0616	1
Srrd	1,0615	1
Sirt5	1,0614	1
Med25	1,0614	1
Dad1	1,0614	1
Mgme1	1,0612	1
Eif2ak2	1,0611	1
Erlec1	1,0611	1
Acd	1,0611	1
Wsb1	1,0611	1
Gm6305	1,0610	1
Snx29	1,0610	1
Fam111a	1,0610	1
Pnpla8	1,0610	1
Lrrc8c	1,0609	1
Gm44292	1,0608	1
Sar1a	1,0608	1
4933440N22Rik	1,0607	1
Orc4	1,0606	1
Sin3b	1,0606	1
Tmem70	1,0606	1
Ubl3	1,0605	1
Coa7	1,0605	1
9530062K07Rik	1,0605	1
Cpped1	1,0605	1
Matr3-ps2	1,0604	1
Mrto4	1,0603	1
Ercc1	1,0602	1
Ormdl2	1,0600	1
Acvr2b	1,0600	1
Rps25	1,0600	1
Parl	1,0599	1
Zyx	1,0598	1
Trip13	1,0597	1
Aff1	1,0597	1
Cyp4v3	1,0597	1
Itch	1,0597	1
Arpc4	1,0597	1
Decr2	1,0595	1
Tmem134	1,0592	1
Tpgs2	1,0591	1
Elmo1	1,0591	1
P4hb	1,0591	1
Sdhaf3	1,0591	1
2200002J24Rik	1,0590	1
Eefsec	1,0590	1
Selenot	1,0590	1
Yif1b	1,0589	1
Trappc2l	1,0588	1
Rps11-ps4	1,0585	1

Urgcp	1,0584	1
4833418N02Rik	1,0583	1
Mafg	1,0583	1
Kdm4b	1,0581	1
Rc3h2	1,0581	1
Fhad1	1,0580	1
Slc38a7	1,0580	1
Slc25a17	1,0579	1
Cdca7	1,0578	1
Sipa1l2	1,0578	1
Elmsan1	1,0578	1
Dynlrb1	1,0578	1
Apoa1bp	1,0578	1
Gm37297	1,0575	1
Gm29759	1,0575	1
Mycn	1,0574	1
Xkr5	1,0572	1
Atxn1l	1,0572	1
Taf12	1,0572	1
4833417C18Rik	1,0571	1
Ppp1r37	1,0571	1
Slc35g1	1,0571	1
Cers4	1,0571	1
S100a10	1,0570	1
Pcsk7	1,0569	1
Irgq	1,0567	1
Vsig10	1,0565	1
Dtnb	1,0564	1
Sharpin	1,0564	1
4930526A20Rik	1,0564	1
2410022M11Rik	1,0563	1
Tradd	1,0562	1
Coq2	1,0562	1
Casc3	1,0561	1
Dnajc19-ps	1,0561	1
Pam	1,0561	1
Smim7	1,0561	1
Nudt2	1,0559	1
Dennd4c	1,0558	1
Ldlrap1	1,0558	1
Alkbh6	1,0558	1
Cspp1	1,0557	1
Ctps2	1,0556	1
Tbl2	1,0556	1
Pigv	1,0556	1
Nhlrc3	1,0556	1
Gm37621	1,0554	1
Smn1	1,0554	1
RP23-359K10.9	1,0550	1
Actr3b	1,0550	1
Ndufs7	1,0550	1
Gnai2	1,0550	1

Ttl	1,0548	1
Vps4a	1,0548	1
Npy	1,0547	1
Dpagt1	1,0547	1
Mrpl45	1,0547	1
Gm19777	1,0546	1
Rab11fip4os1	1,0545	1
Dnm1l	1,0545	1
Wdr5b	1,0544	1
Esco1	1,0544	1
Gabarap	1,0543	1
2810006K23Rik	1,0541	1
Odc1	1,0541	1
Nr1d1	1,0540	1
Peg12	1,0539	1
Hist2h2ac	1,0539	1
Zfp623	1,0539	1
Porcn	1,0539	1
Ndufaf3	1,0539	1
Pot1a	1,0538	1
Ppat	1,0537	1
Rtcb	1,0536	1
Frs2	1,0535	1
Gm16580	1,0534	1
Cndp2	1,0534	1
Rpf1	1,0532	1
Gm11631	1,0531	1
Odf3l1	1,0531	1
Slc16a3	1,0530	1
Cnp	1,0528	1
Uqcrc2	1,0528	1
A430105J06Rik	1,0527	1
Sgk3	1,0527	1
Gm37906	1,0526	1
Flot2	1,0523	1
Atf2	1,0523	1
Zfp280b	1,0522	1
Psrc1	1,0521	1
Ccdc173	1,0520	1
Psip1	1,0520	1
Gpbp1	1,0520	1
Olfr460	1,0518	1
Rrp8	1,0517	1
Gna15	1,0515	1
Snrpa	1,0515	1
Gm14853	1,0513	1
Mmab	1,0513	1
Tubd1	1,0512	1
Eral1	1,0512	1
Golga4	1,0512	1
Pdcd10	1,0510	1
B230118H07Rik	1,0509	1

Ell	1,0507	1
Gnb1	1,0507	1
Apeh	1,0507	1
Sppl2b	1,0506	1
Raf1	1,0504	1
Csgalnact2	1,0504	1
Hivep3	1,0503	1
Cops6	1,0503	1
Kif1b	1,0502	1
Icam1	1,0502	1
Gm4258	1,0502	1
Atp5d	1,0502	1
Nmi	1,0500	1
Ssb	1,0499	1
Lyz2	1,0498	1
Cdc25b	1,0498	1
Zfp772	1,0496	1
Cnot8	1,0496	1
Eny2	1,0496	1
Cc2d1a	1,0494	1
Rbm38	1,0494	1
Dner	1,0494	1
Birc2	1,0493	1
Zic5	1,0492	1
Tbc1d2	1,0491	1
Atxn2l	1,0491	1
Six1	1,0490	1
Gm19620	1,0488	1
Parg	1,0488	1
Gm43609	1,0487	1
Msn	1,0487	1
Ptpn6	1,0487	1
Mrpl42	1,0486	1
Rbpsuh-rs3	1,0486	1
Tbc1d17	1,0483	1
Mrpl17	1,0483	1
Ndufa9	1,0483	1
Cln8	1,0481	1
Psph	1,0479	1
Bcap31	1,0479	1
Btf3l4	1,0478	1
Armcx5	1,0478	1
RP24-240E7.1	1,0477	1
Rpia	1,0477	1
Pfkl	1,0476	1
Usp27x	1,0475	1
Cd274	1,0475	1
Sgms1	1,0473	1
Nt5dc3	1,0472	1
Ddx18	1,0472	1
Eif3g	1,0472	1
Acot13	1,0470	1

Timm8b	1,0470	1
9330175E14Rik	1,0469	1
Wars	1,0469	1
Exosc4	1,0469	1
Slc25a23	1,0468	1
Tet3	1,0467	1
Fam216a	1,0467	1
Pyroxd2	1,0466	1
Mettl4	1,0465	1
Gm14140	1,0465	1
Ercc6	1,0465	1
Gm42783	1,0464	1
Rps13-ps2	1,0464	1
Camkmt	1,0464	1
Gm13181	1,0464	1
Nqo2	1,0462	1
Nudt18	1,0460	1
Vamp8	1,0460	1
Gm20091	1,0459	1
Maml1	1,0459	1
Samhd1	1,0459	1
Plcd3	1,0457	1
Rbm8a2	1,0457	1
Brd3	1,0457	1
Serpinf1	1,0456	1
Hps3	1,0456	1
Ipmk	1,0456	1
Uqcrh-ps2	1,0456	1
Dlg4	1,0455	1
Rfesd	1,0454	1
Fam76a	1,0453	1
Pycr2	1,0452	1
Ptp4a3	1,0451	1
Tmem43	1,0451	1
Gm43482	1,0450	1
Ifi203	1,0450	1
Tmem18	1,0450	1
Gm14620	1,0448	1
Mthfs	1,0447	1
Mrps11	1,0446	1
Iffo1	1,0445	1
Kcnb1	1,0443	1
Tigd5	1,0443	1
Gm45167	1,0442	1
Pex12	1,0442	1
Lias	1,0442	1
Gm16973	1,0441	1
Amz2	1,0440	1
Gm13391	1,0439	1
Wdr37	1,0439	1
1110019D14Rik	1,0438	1
Fkbp14	1,0438	1

Adpgk	1,0438	1
Srsf11	1,0438	1
Fanca	1,0437	1
Nepro	1,0436	1
Gm12743	1,0435	1
Calcrl	1,0435	1
Agpat3	1,0434	1
Kif3a	1,0433	1
D17H6S53E	1,0430	1
Rock2	1,0430	1
Il1b	1,0429	1
Lyar	1,0429	1
Rad54l	1,0428	1
Deaf1	1,0428	1
Ralbp1	1,0428	1
Tcp11l1	1,0427	1
Tmx1	1,0427	1
Thoc7	1,0426	1
Cav2	1,0425	1
Flad1	1,0425	1
Knop1	1,0425	1
Usp11	1,0424	1
Uba6	1,0422	1
Wdr36	1,0420	1
Tmbim4	1,0420	1
Fbl	1,0417	1
Hdac9	1,0417	1
Tagap1	1,0417	1
Mir22hg	1,0417	1
Rsrc1	1,0416	1
Cbx3	1,0413	1
Fam96b	1,0411	1
Cox7a2	1,0411	1
4930532G15Rik	1,0410	1
1700047K16Rik	1,0409	1
Gpsm3	1,0407	1
Fignl2	1,0407	1
Nbas	1,0405	1
Slc35f6	1,0405	1
Gm43343	1,0404	1
Rpusd4	1,0404	1
Tgds	1,0404	1
Zfp830	1,0403	1
Psenen	1,0402	1
Sep 08	1,0402	1
Tmem50a	1,0402	1
Phb2	1,0401	1
Washc3	1,0400	1
Arf5	1,0400	1
Gm9434	1,0398	1
Rpgrip1l	1,0397	1
Impad1	1,0397	1

D1Ertd622e	1,0397	1
Ncaph	1,0397	1
Gpr65	1,0397	1
Ap3s2	1,0397	1
RP23-128C4.4	1,0396	1
Syngr2	1,0394	1
Gm43571	1,0394	1
Atp13a3	1,0394	1
1700001P01Rik	1,0391	1
Rps6ka4	1,0391	1
Usp39	1,0390	1
Emc1	1,0390	1
Idh2	1,0386	1
Aga	1,0386	1
Tex2	1,0385	1
Nadsyn1	1,0384	1
Sos1	1,0384	1
Ykt6	1,0384	1
Ntan1	1,0384	1
Gm20628	1,0383	1
Nfkbil1	1,0383	1
H2afx	1,0383	1
Mecp2	1,0383	1
Tmem183a	1,0383	1
Gm14650	1,0381	1
Umps	1,0379	1
Fndc3a	1,0377	1
Rbm22	1,0373	1
Rhot1	1,0371	1
1700052K11Rik	1,0369	1
B3gnt3	1,0369	1
Nde1	1,0369	1
Itgb1	1,0369	1
Thumpd3	1,0368	1
Mocos	1,0368	1
Mtif3	1,0368	1
Gm18913	1,0366	1
Cnbp	1,0365	1
Dis3l	1,0362	1
Irak1bp1	1,0361	1
Ifitm2	1,0361	1
Rab24	1,0361	1
Ttc13	1,0361	1
Inpp5f	1,0358	1
Parp10	1,0357	1
Mrps12	1,0357	1
Rpusd3	1,0356	1
BC030867	1,0353	1
Akap8	1,0353	1
Dram1	1,0352	1
2210016L21Rik	1,0351	1
Sptbn1	1,0351	1

2810025M15Rik	1,0350	1
Mtrf1l	1,0348	1
Xrn2	1,0348	1
mt-Tl1	1,0345	1
Mnt	1,0345	1
Mrps18a	1,0344	1
Gm13886	1,0343	1
Bcl2a1d	1,0343	1
Dntt	1,0342	1
Gm26912	1,0342	1
Kdm6a	1,0340	1
Kansl2	1,0339	1
Ube2z	1,0339	1
Cdc37l1	1,0338	1
Foxn3	1,0338	1
Gnrh1	1,0337	1
Btg2	1,0337	1
Abhd5	1,0335	1
Aurka	1,0335	1
Micu2	1,0335	1
Cs	1,0334	1
Rpl22	1,0334	1
Stn1	1,0333	1
Ifi30	1,0333	1
Gm340	1,0332	1
Zfyve1	1,0328	1
Plekha3	1,0328	1
Akr1c13	1,0325	1
Zfpl1	1,0325	1
Rbm25	1,0323	1
Spata2	1,0323	1
Simc1	1,0323	1
Cab39l	1,0322	1
Asl	1,0321	1
Gnb4	1,0320	1
Ubac2	1,0320	1
Scarb2	1,0320	1
Vkorc1	1,0318	1
Mif	1,0317	1
Zfp335	1,0317	1
C87436	1,0316	1
Leprotl1	1,0314	1
Exosc5	1,0313	1
Mrpl57	1,0313	1
Coq8a	1,0312	1
Ehbp1l1	1,0312	1
Rnf13	1,0310	1
Smu1	1,0310	1
Slc52a2	1,0310	1
Usp10	1,0309	1
Riok1	1,0309	1
Tmed4	1,0309	1

Hadha	1,0309	1
Rhoa	1,0308	1
Catsper2	1,0307	1
Cnot9	1,0306	1
Acbd3	1,0305	1
Tiam1	1,0305	1
2310022B05Rik	1,0305	1
Gm21781	1,0304	1
Sema4d	1,0303	1
Intu	1,0300	1
Rcc1	1,0300	1
Ambra1	1,0300	1
Tox4	1,0300	1
Atp2c1	1,0299	1
Nkiras2	1,0298	1
Tpm4	1,0298	1
Usf2	1,0297	1
6720427I07Rik	1,0296	1
Erbin	1,0296	1
Upf3b	1,0296	1
Eps8	1,0293	1
Mrpl21	1,0293	1
Tmem9b	1,0293	1
Pi4kb	1,0293	1
Cnnm3	1,0293	1
Brd8	1,0293	1
Mlec	1,0293	1
Gm12013	1,0292	1
Med26	1,0292	1
Zbtb12	1,0292	1
Arl5b	1,0289	1
Plpp7	1,0288	1
Lmntd2	1,0288	1
Hk1	1,0288	1
Tstd2	1,0285	1
Gm2000	1,0285	1
Spata6	1,0283	1
Msantd2	1,0283	1
Chsy1	1,0283	1
Gm42559	1,0283	1
Gm6563	1,0283	1
Appl2	1,0282	1
Dnttip2	1,0281	1
Unc50	1,0280	1
Nme4	1,0279	1
Man1a	1,0279	1
Ppp2r5e	1,0278	1
Map4	1,0277	1
Gm10642	1,0275	1
Rps12-ps4	1,0274	1
Gm7733	1,0273	1
Dnase1l1	1,0273	1

Map4k2	1,0270	1
Tomm40	1,0270	1
Cstf3	1,0270	1
Otulin	1,0270	1
Ccl25	1,0270	1
Gm44198	1,0269	1
Gm43637	1,0269	1
Mybl2	1,0269	1
Slc16a13	1,0268	1
Gtf2i	1,0265	1
Gm36964	1,0265	1
Usp3	1,0263	1
Tmem138	1,0262	1
AU020206	1,0262	1
Nae1	1,0262	1
Gm37339	1,0261	1
Oxnad1	1,0261	1
Ppp4r3b	1,0261	1
RP24-84C23.4	1,0260	1
Xrcc6	1,0260	1
Rnf215	1,0260	1
Fance	1,0260	1
B3gntl1	1,0260	1
Lins1	1,0260	1
Ppp2r5a	1,0260	1
Cd300lb	1,0259	1
Zfp64	1,0258	1
Epsti1	1,0257	1
Ctu1	1,0256	1
Gm37728	1,0256	1
5730455P16Rik	1,0255	1
Gm3355	1,0253	1
Abca1	1,0252	1
Cdk7	1,0251	1
Eif2s3y	1,0249	1
Trpv2	1,0249	1
Vps35	1,0248	1
Psmd14	1,0246	1
Api5	1,0243	1
Recql5	1,0242	1
Ech1	1,0242	1
Arhgap45	1,0242	1
Xpo1	1,0241	1
Gm15185	1,0240	1
Rbbp8	1,0240	1
Gm10136	1,0238	1
F8a	1,0238	1
Elf1	1,0236	1
Treml4	1,0234	1
Sod2	1,0233	1
Gucy2g	1,0231	1
Gchfr	1,0231	1

Tmem251	1,0231	1
Ubash3b	1,0231	1
Ankrd52	1,0231	1
Rbm7	1,0231	1
Zfp408	1,0230	1
Psmc2	1,0230	1
Cops7a	1,0229	1
Bscl2	1,0229	1
Traf3	1,0229	1
Glipr2	1,0228	1
Cers6	1,0228	1
Blcap	1,0226	1
Gripap1	1,0225	1
Zbtb40	1,0224	1
Romo1	1,0224	1
Mrps9	1,0223	1
Rpl31-ps8	1,0221	1
Snx2	1,0221	1
Hoxc6	1,0221	1
Ticam2	1,0220	1
Tlcd2	1,0219	1
Olfml3	1,0219	1
Ints14	1,0219	1
Nhlrc2	1,0218	1
Trove2	1,0217	1
Tfam	1,0216	1
Capzb	1,0216	1
Gm44153	1,0215	1
Wdcp	1,0215	1
Ddrgk1	1,0215	1
Prkcsh	1,0214	1
Itfg1	1,0214	1
Rab40c	1,0214	1
Timm10	1,0214	1
Gm29155	1,0213	1
Aar2	1,0213	1
Hoxc4	1,0212	1
Ppm1d	1,0212	1
Zfp174	1,0212	1
Pnp	1,0212	1
Tmem170b	1,0211	1
Gm4705	1,0210	1
Kremen1	1,0210	1
Prps1	1,0210	1
Cnrip1	1,0209	1
Oraov1	1,0209	1
Ebi3	1,0209	1
L1cam	1,0208	1
Thoc3	1,0208	1
Hnrnpa2b1	1,0208	1
Slc37a4	1,0207	1
Rngtt	1,0207	1

Comp	1,0206	1
Metap1d	1,0204	1
Prkx	1,0204	1
Eif2s1	1,0204	1
Thra	1,0204	1
Btbd10	1,0203	1
Clk2	1,0202	1
Slc19a2	1,0202	1
Iffo2	1,0202	1
AI837181	1,0202	1
Odf2	1,0202	1
Ccdc25	1,0200	1
Zmynd19	1,0200	1
Rap2b	1,0199	1
Pdia5	1,0198	1
Chkb	1,0198	1
Mybbp1a	1,0198	1
Spcs1	1,0196	1
Utp3	1,0196	1
Junb	1,0194	1
Nxf1	1,0194	1
Epha2	1,0193	1
Cnst	1,0193	1
Gm44168	1,0192	1
Pak1ip1	1,0192	1
Gm3555	1,0192	1
Ctsb	1,0192	1
Nit1	1,0191	1
Fcer1g	1,0191	1
Gm6290	1,0190	1
1700030K09Rik	1,0190	1
Fam45a	1,0190	1
Scaf8	1,0190	1
Gm37183	1,0189	1
Unc13d	1,0187	1
Lilr4b	1,0186	1
Arhgdig	1,0185	1
Fastkd2	1,0185	1
Stk17b	1,0185	1
Slbp	1,0184	1
Zbtb5	1,0184	1
Alg5	1,0184	1
Irf5	1,0184	1
Fan1	1,0183	1
Gm20156	1,0183	1
Ndufab1	1,0183	1
Gm43533	1,0182	1
Gm13822	1,0182	1
Rab22a	1,0182	1
Cmpk1	1,0181	1
Scaf4	1,0180	1
Ptov1	1,0180	1

Ccdc106	1,0178	1
Zc3h3	1,0177	1
Gm11273	1,0177	1
Adam17	1,0176	1
Kidins220	1,0173	1
Arhgef15	1,0172	1
1600002K03Rik	1,0172	1
Zfp706	1,0172	1
Gm7224	1,0171	1
Zbtb34	1,0171	1
Tmed9	1,0169	1
Impa2	1,0168	1
Nr4a3	1,0166	1
Emsy	1,0166	1
Diaph3	1,0166	1
Vdac2	1,0166	1
Sipa1l1	1,0165	1
Zc3h15	1,0165	1
Dagla	1,0164	1
Tsfm	1,0164	1
Hhex	1,0164	1
Phkg2	1,0164	1
Adap1	1,0164	1
Stt3a	1,0163	1
Ggnbp2	1,0162	1
Nop58	1,0161	1
Nupr1l	1,0160	1
Dyrk2	1,0160	1
Elac1	1,0159	1
Mtpn	1,0159	1
Zfp217	1,0159	1
Rnf169	1,0159	1
Dnm3	1,0158	1
Gm20703	1,0158	1
Abhd13	1,0158	1
Pold2	1,0156	1
Tnfaip8l1	1,0155	1
Adi1	1,0154	1
Sae1	1,0154	1
A130048G24Rik	1,0153	1
Hsdl2	1,0153	1
Ino80d	1,0153	1
Shoc2	1,0153	1
Chmp1a	1,0152	1
Ap3m2	1,0151	1
Ccdc9	1,0151	1
Zfp422	1,0150	1
Camsap2	1,0148	1
Hmgcl	1,0147	1
Pard6a	1,0147	1
Hars	1,0147	1
Tango6	1,0146	1

Gipc1	1,0145	1
Usp2	1,0145	1
Mir5136	1,0145	1
Mbd3	1,0145	1
Tmem110	1,0144	1
Rps6kc1	1,0143	1
Tmem104	1,0142	1
Lmna	1,0141	1
AC149090.1	1,0139	1
Gm43788	1,0138	1
Gatsl3	1,0138	1
Anapc15	1,0137	1
Tnfaip1	1,0137	1
Gnai3	1,0136	1
Bak1	1,0135	1
Zyg11b	1,0135	1
Mapk8ip1	1,0134	1
Lrp10	1,0134	1
Foxj3	1,0133	1
Camk2g	1,0132	1
Tbkbp1	1,0131	1
Man2a2	1,0130	1
Itgb2	1,0129	1
Cdc16	1,0128	1
Otud4	1,0128	1
Rfk	1,0127	1
Serpinb6a	1,0126	1
4930524J08Rik	1,0125	1
Eif2b4	1,0125	1
2810414N06Rik	1,0124	1
Rbm19	1,0124	1
Isca2	1,0123	1
Gm42666	1,0123	1
9130024F11Rik	1,0123	1
1700007L15Rik	1,0122	1
R3hdm4	1,0121	1
Dhx32	1,0120	1
Comtd1	1,0120	1
B3galt6	1,0119	1
Lztfl1	1,0119	1
Coq9	1,0119	1
Gm12504	1,0119	1
Stbd1	1,0118	1
Ubap2	1,0118	1
Nolc1	1,0118	1
Cbx8	1,0117	1
Zfp748	1,0116	1
Dnajc3	1,0116	1
D630023F18Rik	1,0116	1
Zmat2	1,0116	1
RP23-356D13.9	1,0115	1
Dus3l	1,0115	1

Cep128	1,0114	1
Lig4	1,0114	1
Lims2	1,0112	1
Apbb2	1,0112	1
Tmem201	1,0112	1
Lpin1	1,0111	1
Gm8738	1,0110	1
Gm43149	1,0109	1
1810010D01Rik	1,0109	1
Enkd1	1,0109	1
Apool	1,0109	1
Auh	1,0109	1
Gm10175	1,0108	1
Ccdc167	1,0107	1
Sumf2	1,0107	1
Dpm3	1,0107	1
Naa38	1,0107	1
Tbc1d12	1,0104	1
Chst3	1,0102	1
Gm43153	1,0101	1
Golgb1	1,0101	1
Kcnk6	1,0100	1
Ankrd40	1,0100	1
Grasp	1,0099	1
Ei24	1,0099	1
Ube2s	1,0098	1
Rnf24	1,0098	1
Wrnip1	1,0097	1
Zfp148	1,0097	1
Ccdc181	1,0096	1
Bfar	1,0096	1
Stag1	1,0095	1
Nbr1	1,0095	1
Fra10ac1	1,0095	1
Pigk	1,0095	1
Hmg20a	1,0095	1
Mzf1	1,0095	1
Atg3	1,0093	1
Mtrf1	1,0091	1
Ngrn	1,0091	1
Caprin1	1,0091	1
Ccdc6	1,0090	1
Lactb2	1,0089	1
Sh2b1	1,0089	1
Cdc42	1,0088	1
Zranb2	1,0088	1
Ikbkap	1,0087	1
Hadhb	1,0086	1
Knstrn	1,0086	1
Zc3h12c	1,0085	1
Sirt1	1,0085	1
Fh1	1,0085	1

Slu7	1,0084	1
Xrra1	1,0080	1
Dapp1	1,0079	1
Arl4d	1,0077	1
C330006A16Rik	1,0077	1
Cep170	1,0077	1
Gm5778	1,0076	1
Ccny	1,0076	1
Gm37726	1,0075	1
Gm43421	1,0075	1
Pla2g12a	1,0074	1
Dusp11	1,0074	1
Fxn	1,0073	1
Ago4	1,0073	1
Sbk2	1,0072	1
Gm10074	1,0071	1
Gm9712	1,0070	1
2010320M18Rik	1,0068	1
Gm24920	1,0067	1
Agps	1,0067	1
Prdx6	1,0067	1
Hsd17b11	1,0065	1
Anxa4	1,0065	1
Nsmf	1,0064	1
Tap1	1,0064	1
Smim15	1,0064	1
Atp5b	1,0063	1
Tecpr1	1,0063	1
Fam134a	1,0063	1
Mettl1	1,0061	1
9330111N05Rik	1,0060	1
Zfp882	1,0059	1
H60c	1,0058	1
Harbi1	1,0058	1
Ppm1l	1,0058	1
Zfp598	1,0058	1
Stx7	1,0057	1
Gm11451	1,0056	1
Tax1bp1	1,0055	1
Gm44951	1,0054	1
Nudt9	1,0054	1
Foxm1	1,0053	1
Ube2w	1,0053	1
Zdhhc16	1,0052	1
Zfp560	1,0052	1
Rptor	1,0052	1
Kat6b	1,0051	1
Ankrd9	1,0050	1
Rrnad1	1,0050	1
B2m	1,0050	1
Cope	1,0050	1
Gm5637	1,0049	1

Ywhab	1,0049	1
Tmem141	1,0049	1
Gm12689	1,0049	1
Esrra	1,0047	1
Zfp870	1,0047	1
Gm5939	1,0046	1
Ndufs6	1,0045	1
C2cd2	1,0044	1
Clk4	1,0044	1
Opn3	1,0043	1
Eml3	1,0043	1
Fdps	1,0043	1
Telo2	1,0042	1
Plec	1,0042	1
Ext1	1,0040	1
1700037H04Rik	1,0040	1
Gm9134	1,0039	1
Ap4s1	1,0039	1
Snhg4	1,0039	1
Fam49b	1,0039	1
Ankrd13b	1,0038	1
Slc48a1	1,0038	1
Sptlc2	1,0038	1
Prkd3	1,0038	1
Mcts1	1,0038	1
Vars	1,0038	1
Gm26935	1,0037	1
C130083A15Rik	1,0034	1
Phtf1os	1,0033	1
Arl6ip6	1,0033	1
Rad18	1,0033	1
Pla2g4a	1,0033	1
Cdc123	1,0032	1
Ptms	1,0032	1
Stim1	1,0032	1
Fut7	1,0031	1
Ddt	1,0031	1
Klhl42	1,0029	1
Sephs2	1,0029	1
Gm7206	1,0028	1
Ifitm6	1,0028	1
Actr2	1,0028	1
BC003331	1,0027	1
Utp15	1,0027	1
Snx14	1,0026	1
Tnfrsf9	1,0026	1
Rab9	1,0025	1
2310009B15Rik	1,0024	1
Tbca	1,0024	1
Fars2	1,0024	1
Mical3	1,0024	1
Mmgt1	1,0023	1

Shq1	1,0022	1
Emc3	1,0022	1
Eid2b	1,0021	1
Bag5	1,0020	1
Psmd3	1,0020	1
Tldc1	1,0018	1
2310011J03Rik	1,0017	1
Gm4943	1,0017	1
Orc6	1,0016	1
Clpp	1,0016	1
Ak1	1,0016	1
Zfp953	1,0015	1
Mis12	1,0015	1
Sdf4	1,0015	1
Anp32e	1,0015	1
1110012L19Rik	1,0014	1
Trmt1l	1,0014	1
E4f1	1,0014	1
Gm24339	1,0013	1
Phkg1	1,0013	1
Sh3bgrl3	1,0013	1
Lzts2	1,0012	1
Zfp30	1,0012	1
Emd	1,0012	1
Phc2	1,0011	1
1700001C19Rik	1,0010	1
Stip1	1,0010	1
Snap29	1,0010	1
Lrrc58	1,0009	1
Mfsd14b	1,0008	1
Thumpd2	1,0007	1
Pin1	1,0007	1
Tmbim1	1,0007	1
Gm12882	1,0006	1
Brwd1	1,0006	1
Oxr1	1,0005	1
Smpdl3b	1,0005	1
Gm20274	1,0004	1
BC005561	1,0004	1
Fbxo21	1,0003	1
Naa30	1,0003	1
Lamtor3	1,0003	1
Csrp2bp	1,0003	1
Pank4	1,0001	1
Gm20522	1,0001	1
Zrsr2	1,0001	1
Cmtm6	1,0001	1
Gm13776	1,0000	1
Csnk1g3	1,0000	1
Gm42418	1,0000	1
Gm37589	-6,47E-05	1
Ifngr2	-7,84E-05	1

Bet1	-0,00010709	1
Med13	-0,00017293	1
Hexim2	-0,00026294	1
3110043O21Rik	-0,00038073	1
Yif1a	-0,00047717	1
Prkab1	-0,00065581	1
Derl2	-0,00066294	1
Snrnp70	-0,00070128	1
Plpp1	-0,00080242	1
Ugp2	-0,00084603	1
Sep 06	-0,0010192	1
Pola1	-0,0011382	1
March2	-0,0011093	1
Mtss1	-0,0013233	1
Rpgrip1	-0,001411	1
Tmem177	-0,0013817	1
E2f1	-0,0014433	1
Mpv17l2	-0,0014408	1
Dlat	-0,0015089	1
1810055G02Rik	-0,0015574	1
Hnrnpk	-0,0016915	1
Tada2b	-0,0018093	1
Ppp1r13l	-0,001861	1
Scrn2	-0,0018539	1
Kif21b	-0,002085	1
Gas2	-0,002168	1
Vav1	-0,0022204	1
Suclg1	-0,0023316	1
Kat7	-0,0023189	1
Zfp964	-0,0024073	1
Gm38365	-0,0025	1
Trmt5	-0,0024826	1
Pcgf2	-0,0025068	1
Pex13	-0,002722	1
Ctbs	-0,0028297	1
Ap2m1	-0,0028155	1
Add3	-0,0028638	1
Tcerg1	-0,0030686	1
Thap7	-0,0031629	1
Alkbh1	-0,0032916	1
Rnf167	-0,003499	1
Irf2bp2	-0,0034924	1
Chst1	-0,0041403	1
Rnf4	-0,0042477	1
Pag1	-0,0043873	1
Rsf1	-0,0044049	1
Kiz	-0,0045736	1
Hprt	-0,0046392	1
Rpa1	-0,0046114	1
Gm15975	-0,004657	1
Fitm2	-0,0046696	1
Gm26890	-0,0048308	1

Cast	-0,004975	1
Cdk5rap3	-0,0051374	1
Map1lc3a	-0,0051339	1
Atpaf1	-0,0054735	1
Cryzl1	-0,0054517	1
Comt	-0,0055552	1
Gm43387	-0,0057673	1
Rab19	-0,0059498	1
Tbl3	-0,0059809	1
Cactin	-0,005977	1
Fam50a	-0,0059671	1
Gm37780	-0,0061446	1
Slc35b3	-0,006435	1
Irf2	-0,0064452	1
Snx5	-0,0063827	1
Crtap	-0,0065211	1
Inpp5a	-0,00645	1
Galk2	-0,0067617	1
Qdpr	-0,0068758	1
Gm37902	-0,0072422	1
Aifm1	-0,0072239	1
Mtif2	-0,0073262	1
Usp32	-0,007448	1
Pibf1	-0,0075787	1
2810403A07Rik	-0,007648	1
Atp6v0b	-0,0076106	1
Nsd2	-0,0075566	1
Zranb1	-0,0075664	1
Tmem9	-0,0077297	1
Klhdc3	-0,007868	1
Hexa	-0,0079942	1
Tsen54	-0,008218	1
Chd6	-0,0081955	1
Baz1a	-0,0082378	1
Atp11a	-0,008276	1
Eif4h	-0,0082635	1
Prune1	-0,0084785	1
Ctdp1	-0,0086817	1
Hs6st1	-0,0092045	1
Dimt1	-0,0093257	1
Commd7	-0,0092964	1
Coq4	-0,0093724	1
Mnd1-ps	-0,0096017	1
Crlf3	-0,0096039	1
Zfand1	-0,0096619	1
Zfp277	-0,0097066	1
6030460B20Rik	-0,0097689	1
Malt1	-0,010032	1
Trim32	-0,010147	1
Pex10	-0,01015	1
Cyhr1	-0,010092	1
Cspg5	-0,0102	1

Cklf	-0,010214	1
Kbtbd3	-0,01016	1
Eea1	-0,010194	1
Zcchc9	-0,010719	1
Tcn2	-0,010721	1
Amd1	-0,010774	1
Sec13	-0,010775	1
Zswim6	-0,010751	1
Etf1	-0,010831	1
Zfp1	-0,010873	1
Ctsc	-0,010943	1
Sdcbp2	-0,011019	1
Lamb2	-0,011245	1
Tcp11l2	-0,01119	1
Zfp638	-0,011329	1
Tmc6	-0,011527	1
Pptc7	-0,011541	1
Prkce	-0,01156	1
Tmem126a	-0,011553	1
Foxo3	-0,011706	1
Wfs1	-0,011949	1
Purb	-0,0121	1
A130010J15Rik	-0,012239	1
Rapgef6	-0,012276	1
Gm38104	-0,012734	1
Gm12497	-0,012749	1
Zfhx2	-0,012816	1
Top1mt	-0,012874	1
Thumpd1	-0,013132	1
Klhl26	-0,013271	1
Iqsec3	-0,013516	1
Pgghg	-0,013626	1
Bmpr1a	-0,013633	1
Tmem135	-0,013613	1
Npm1	-0,013744	1
Dffa	-0,013812	1
Ammecr1l	-0,013751	1
Dpy19l1	-0,013815	1
Tbcc	-0,01393	1
Vcp-rs	-0,014236	1
Rrp1b	-0,014198	1
Fbrs	-0,014221	1
Qtrtd1	-0,014335	1
Acot9	-0,01442	1
Smc6	-0,014427	1
Actn4	-0,014491	1
Tfeb	-0,014612	1
Abl1	-0,014732	1
Dnajc18	-0,014788	1
Zcchc10	-0,014859	1
Nfyb	-0,01488	1
Isoc1	-0,015039	1

Adsl	-0,0151	1
Srsf2	-0,015085	1
Slc9a3r1	-0,015152	1
Pbx2	-0,015504	1
Nelfe	-0,015802	1
Gm14270	-0,016017	1
Nagk	-0,016034	1
Csad	-0,016328	1
Slc25a16	-0,016535	1
Clcn7	-0,016481	1
Gm13204	-0,016657	1
Zfp398	-0,016946	1
Sms	-0,016898	1
Uqcrb	-0,016903	1
Tnpo1	-0,017217	1
Gm16096	-0,017596	1
St13	-0,017553	1
Arpc1b	-0,017604	1
Zscan12	-0,017843	1
Agtpbp1	-0,017805	1
Eif3c	-0,017782	1
Ddx39	-0,017851	1
Gm15327	-0,017972	1
Gm6209	-0,018215	1
Cmbl	-0,018218	1
Camkk2	-0,018284	1
Cyb5d2	-0,018441	1
Rfng	-0,018448	1
Selenon	-0,018687	1
Nomo1	-0,01876	1
Txlng	-0,018908	1
Dazap2	-0,019121	1
Gm42872	-0,019315	1
Nupl1	-0,019264	1
Gabarapl2	-0,019343	1
Zfp874a	-0,019423	1
Gm5786	-0,019469	1
Il20rb	-0,019471	1
Kmt5a	-0,019468	1
Laptm4a	-0,019568	1
Ercc8	-0,019705	1
Fam193a	-0,019653	1
Fam132a	-0,019916	1
Parp14	-0,020327	1
Rb1cc1	-0,020348	1
Tbrg4	-0,020278	1
Gm24916	-0,02043	1
Sdad1	-0,020518	1
Lpgat1	-0,02071	1
RP23-168F21.4	-0,020849	1
Pikfyve	-0,021078	1
Ube4a	-0,02122	1

Faf1	-0,021235	1
Tyw3	-0,021303	1
Cox5a	-0,021429	1
Abhd2	-0,021454	1
Arl6ip1	-0,021523	1
Klhl24	-0,021632	1
Heca	-0,021995	1
Eloa	-0,02214	1
Dpm1	-0,022159	1
Fhit	-0,022278	1
Pfdn4	-0,022338	1
Nup133	-0,02242	1
Tal1	-0,022382	1
Nufip1	-0,022574	1
Scarb1	-0,022773	1
Lamc1	-0,022921	1
Gm13373	-0,022979	1
Aebp2	-0,022994	1
Preb	-0,02305	1
RP23-139H6.1	-0,023077	1
Gm43329	-0,023223	1
Tbx15	-0,023297	1
2010107E04Rik	-0,023315	1
Dnajb11	-0,02339	1
1110037F02Rik	-0,023421	1
Rnf138	-0,023406	1
Pgam5	-0,023355	1
Eps15l1	-0,02354	1
Stard10	-0,023461	1
Gm38190	-0,023628	1
Kcnn1	-0,023625	1
Bid	-0,02361	1
Rab11a	-0,023631	1
Exosc3	-0,023696	1
Rab11b	-0,023666	1
Sun1	-0,023857	1
Plekhh3	-0,02407	1
Ift52	-0,024117	1
Eif2b3	-0,024147	1
Gm12582	-0,024245	1
Ydjc	-0,024339	1
Emg1	-0,024404	1
Ltbp4	-0,024526	1
Usp48	-0,024527	1
Hirip3	-0,024584	1
Cep104	-0,024777	1
Slc25a45	-0,025047	1
Ttbk2	-0,025106	1
Snx16	-0,02514	1
Immp1l	-0,02507	1
Phlda3	-0,025062	1
Hook2	-0,025225	1

Snapc2	-0,025227	1
B3galnt1	-0,025539	1
Tgfbr2	-0,02552	1
Slc30a5	-0,025604	1
Bmt2	-0,025685	1
Sf3a2	-0,025778	1
Zfyve16	-0,025758	1
C5ar1	-0,026023	1
Gfpt1	-0,026012	1
Naga	-0,026116	1
Abtb1	-0,026063	1
Mapk8	-0,026082	1
Oip5	-0,026209	1
Tnip2	-0,026174	1
Tnfrsf11a	-0,026227	1
Rmnd5a	-0,026266	1
Tceanc	-0,026365	1
Stim2	-0,026435	1
Nrap	-0,02649	1
Sec22a	-0,026587	1
Jaml	-0,026606	1
Axin1	-0,026803	1
Cep76	-0,027036	1
Mtg1	-0,026951	1
Pon2	-0,027015	1
Stambpl1	-0,027051	1
Coa6	-0,027231	1
Trmt10c	-0,027448	1
AU019823	-0,02743	1
5430405H02Rik	-0,027536	1
Epb41l5	-0,02762	1
Ptpro	-0,027617	1
Adgrl1	-0,027591	1
Ms4a6d	-0,027716	1
Spout1	-0,027842	1
Aagab	-0,027852	1
Tacc2	-0,028029	1
Il6ra	-0,028003	1
Ubqln1	-0,02802	1
Capg	-0,028223	1
Cramp1l	-0,028309	1
Pias1	-0,028282	1
Ppt1	-0,028294	1
Polr2h	-0,02839	1
Ago3	-0,028552	1
Cbx7	-0,02872	1
Gm26497	-0,029009	1
Parp9	-0,028964	1
Lsg1	-0,028993	1
Pkm	-0,02896	1
Gabpb1	-0,029057	1
Map3k11	-0,029143	1

Hoxb6	-0,029189	1
Fbxl18	-0,029168	1
Znrf1	-0,029261	1
Psma6	-0,029274	1
Strada	-0,029433	1
Akr1e1	-0,029425	1
Amhr2	-0,029582	1
Msmo1	-0,029627	1
Gna11	-0,029737	1
Hmces	-0,029747	1
Sbno1	-0,0297	1
Cct8	-0,029735	1
Eci1	-0,029754	1
Dnajc8	-0,029755	1
Txnrd2	-0,029913	1
Htatip2	-0,02985	1
Gm24631	-0,030033	1
4933434E20Rik	-0,030037	1
Ptar1	-0,030363	1
Krtcap2	-0,030374	1
Lpxn	-0,030524	1
Dnmt1	-0,030519	1
Kirrel3	-0,030667	1
Zfp952	-0,030705	1
Las1l	-0,030665	1
Trmt10b	-0,030889	1
Itgb5	-0,030959	1
Galm	-0,031142	1
Nrde2	-0,03105	1
Dazap1	-0,031275	1
Tmem209	-0,031503	1
Gm38220	-0,031652	1
Myl6b	-0,031769	1
Gm37199	-0,031825	1
Mib2	-0,031782	1
Xrcc2	-0,031901	1
Fbxl5	-0,031893	1
Lsm10	-0,03197	1
Tmem19	-0,032127	1
Fuca2	-0,03209	1
Atf6	-0,032118	1
Mfn2	-0,032131	1
Zfp740	-0,032127	1
Gnpda2	-0,032179	1
Mrpl41	-0,03225	1
Socs7	-0,032237	1
Set	-0,032398	1
Pqbp1	-0,032515	1
Cog2	-0,032583	1
Gm28535	-0,032695	1
Fchsd1	-0,032781	1
Tars2	-0,032887	1

4833412K13Rik	-0,032988	1
Uba5	-0,033137	1
Gfm2	-0,033133	1
Zc3h8	-0,033523	1
Pi4k2a	-0,033693	1
Gm42819	-0,033841	1
Maged1	-0,033828	1
Pex11b	-0,033868	1
Trub1	-0,034017	1
Zfp768	-0,034044	1
Zfp180	-0,034042	1
Serf1	-0,034059	1
Tada1	-0,034083	1
Rcor2	-0,034058	1
Srgn	-0,034078	1
Ift27	-0,034273	1
Cnpy4	-0,034268	1
Zfp384	-0,034427	1
Fbxo7	-0,034439	1
Ppp4c	-0,034539	1
Pcm1	-0,034562	1
Mapk3	-0,034635	1
Acp6	-0,034679	1
Larp1	-0,034712	1
Dapk3	-0,034848	1
Riok3	-0,03481	1
2810474O19Rik	-0,034878	1
Rbm4b	-0,035027	1
Arih2	-0,034966	1
Uprt	-0,03508	1
Ccdc88a	-0,035243	1
Mepce	-0,035153	1
Mkl1	-0,035248	1
Sh2b3	-0,035483	1
Sgpl1	-0,035603	1
Ell2	-0,035778	1
Fadd	-0,035899	1
Vkorc1l1	-0,036134	1
Gm13423	-0,036191	1
Sufu	-0,036232	1
Plpp2	-0,036217	1
Creld2	-0,036256	1
Dnajc22	-0,036366	1
Gm29650	-0,036373	1
Zfp775	-0,036597	1
Gm10320	-0,036571	1
Zfp157	-0,036667	1
Maz	-0,036815	1
Traf4	-0,036831	1
Atraid	-0,036795	1
RP23-324E2.11	-0,036945	1
Mesdc2	-0,036861	1

Cetn3	-0,036977	1
Pcnx	-0,03723	1
Mgat4a	-0,037186	1
Bysl	-0,037214	1
Ppp1r12a	-0,037344	1
0610030E20Rik	-0,037356	1
Tspyl1	-0,037353	1
Hltf	-0,037467	1
Pfn1	-0,037718	1
Etfa	-0,037739	1
Tapbp	-0,037841	1
A930006K02Rik	-0,037962	1
Rrs1	-0,038045	1
Baz1b	-0,037955	1
Galnt3	-0,038108	1
Lacc1	-0,038108	1
Epm2a	-0,038237	1
Gdi2	-0,038245	1
Tmem129	-0,03825	1
Retsat	-0,038448	1
Lysmd1	-0,03853	1
Gm37383	-0,038839	1
Tigar	-0,038823	1
Snx6	-0,038969	1
Mrnip	-0,039156	1
Dnttip1	-0,039162	1
Ccnl1	-0,039165	1
Hars2	-0,039249	1
Zfp597	-0,039428	1
Lyrm9	-0,039423	1
Myl12a	-0,039487	1
Gm35106	-0,039571	1
Sirt3	-0,039812	1
Zbtb4	-0,03982	1
Mrgbp	-0,039913	1
Gm37274	-0,039959	1
Hspd1	-0,039993	1
Mcl1	-0,039984	1
Gas2l3	-0,040086	1
Eif5b	-0,040244	1
Rufy2	-0,04028	1
Prpf4	-0,040414	1
Agpat5	-0,040514	1
Rcor3	-0,040573	1
Nsrp1	-0,040554	1
Cd180	-0,040645	1
Ube2j2	-0,040603	1
Gm15920	-0,040703	1
Abcf2	-0,040762	1
Ccdc84	-0,040902	1
Lmbrd1	-0,040927	1
Prrc1	-0,040887	1

Cct4	-0,041045	1
Btrc	-0,041227	1
Thap11	-0,041163	1
Alkbh2	-0,041257	1
Mettl10	-0,041431	1
Fam208b	-0,041354	1
Scyl1	-0,041487	1
Zc3h14	-0,041743	1
Ptgs2os2	-0,042123	1
Gm15703	-0,042057	1
Eif4a1	-0,042124	1
Uba52	-0,042168	1
Meaf6	-0,042291	1
Zfp606	-0,042406	1
Sdhc	-0,042414	1
Irgm1	-0,042498	1
Rgs11	-0,042718	1
Ppp5c	-0,042775	1
Pdxk	-0,042785	1
Slc39a3	-0,042917	1
Ddx50	-0,043047	1
Abcd4	-0,043095	1
Ddb2	-0,043448	1
Map1lc3b	-0,043363	1
Zfyve21	-0,043847	1
Eif2b2	-0,043774	1
Aoc2	-0,044015	1
Mtfr1	-0,044238	1
Slc44a1	-0,044311	1
Pnisr	-0,044436	1
Camk1	-0,044622	1
Tmed10	-0,044599	1
Gnl3	-0,04463	1
Fxr2	-0,044569	1
Asb1	-0,044721	1
H2-Q4	-0,044879	1
Pik3ap1	-0,044945	1
Gmppb	-0,044901	1
Ranbp2	-0,044917	1
Snw1	-0,044905	1
Gpatch3	-0,044999	1
Dennd5b	-0,045061	1
Depdc7	-0,045064	1
Stx16	-0,045148	1
Cops8	-0,045086	1
E030030I06Rik	-0,04519	1
Atmin	-0,045283	1
Edem3	-0,045507	1
Tmem38b	-0,045505	1
Nipsnap1	-0,045586	1
Cetn2	-0,045606	1
Apobec1	-0,045812	1

1700112E06Rik	-0,045924	1
Copb1	-0,046101	1
Zfp386	-0,046162	1
B230216N24Rik	-0,046446	1
Sap30bp	-0,046544	1
Dhx40	-0,046491	1
Snx12	-0,046642	1
Rrp7a	-0,046751	1
Fam134b	-0,04676	1
Cyb561d1	-0,047041	1
Wdr43	-0,047016	1
Rundc1	-0,047121	1
Chd1	-0,047069	1
Bicd1	-0,047297	1
Acad12	-0,047464	1
Csrp2	-0,047752	1
Zfp410	-0,047948	1
Tle3	-0,048044	1
Prss53	-0,048212	1
Msto1	-0,04836	1
Vps16	-0,048396	1
Rae1	-0,048383	1
Pde7a	-0,048492	1
Thbs3	-0,048677	1
Disp1	-0,048725	1
Commd8	-0,048687	1
Ptp4a2	-0,048817	1
Cdc73	-0,04913	1
Trib1	-0,049299	1
Gm38355	-0,049504	1
Reep4	-0,049483	1
Klhl7	-0,049467	1
Ankrd11	-0,0496	1
Stk38l	-0,049685	1
Exosc9	-0,049802	1
Gtf2e1	-0,049907	1
Gm16061	-0,0501	1
Nek8	-0,050236	1
Brd9	-0,050214	1
Galnt6	-0,050243	1
Frmd8os	-0,05026	1
Tigd2	-0,0504	1
Vwf	-0,05048	1
Mrps10	-0,050525	1
Mndal	-0,050463	1
Znfx1	-0,050487	1
Flii	-0,050554	1
Trim23	-0,050708	1
Vdac3	-0,05072	1
3110002H16Rik	-0,050944	1
Steap3	-0,050924	1
Fbxw8	-0,050923	1

Gm38192	-0,050982	1
Gatad2a	-0,051048	1
Cenpo	-0,051065	1
Rrm2b	-0,051092	1
Usp4	-0,051096	1
Anxa6	-0,051163	1
Gcn1l1	-0,051271	1
Timm8a1	-0,05136	1
Commd3	-0,05149	1
Spopl	-0,051586	1
Itga2b	-0,051654	1
Tmem41b	-0,051665	1
Proser3	-0,052186	1
Mrps14	-0,052255	1
Ehmt1	-0,052648	1
E130311K13Rik	-0,052938	1
Sigmar1	-0,052935	1
Mcm2	-0,052988	1
Nmral1	-0,053068	1
Mrpl37	-0,053126	1
Gm5124	-0,053239	1
Senp6	-0,053286	1
Gm16425	-0,053394	1
1700034H15Rik	-0,053569	1
Gtpbp1	-0,053724	1
Pgm3	-0,053784	1
Ighm	-0,053916	1
Ubl7	-0,054129	1
Ndufb4	-0,054291	1
Tkfc	-0,054283	1
Bcorl1	-0,054308	1
Cops4	-0,054535	1
Ostc	-0,054833	1
Mettl14	-0,054888	1
Bzw2	-0,054912	1
Gpr162	-0,055019	1
Rabl2	-0,055084	1
Sp2	-0,055273	1
Cltb	-0,05538	1
Klhl22	-0,055453	1
RP24-366E11.4	-0,05555	1
Larp7	-0,055589	1
Cep164	-0,055667	1
Ahsa2	-0,055864	1
Grsf1	-0,055889	1
Eci2	-0,056111	1
Gsg2	-0,056208	1
Wars2	-0,056326	1
Cadps	-0,05629	1
Ttc9	-0,056438	1
Zfp777	-0,056362	1
Vps13d	-0,056486	1

Slc25a5	-0,056574	1
Sec14l2	-0,056783	1
Tmem33	-0,056815	1
Heatr1	-0,056948	1
Ikbip	-0,057056	1
Srcap	-0,057325	1
Tnfrsf1b	-0,057321	1
Fmnl3	-0,057356	1
RP23-453B15.7	-0,057456	1
Snord49b	-0,057476	1
L3hypdh	-0,057469	1
Vps50	-0,057483	1
Dcaf12	-0,057548	1
Aamp	-0,057485	1
Adrm1	-0,057665	1
Cdk10	-0,057715	1
Pvt1	-0,057806	1
Slc25a26	-0,057873	1
Epb41l2	-0,057907	1
Abce1	-0,057869	1
Stt3b	-0,057886	1
Ctsd	-0,058037	1
Nat8f1	-0,058126	1
Tmed2	-0,05811	1
Rxra	-0,058143	1
Ubald1	-0,058438	1
Zfp131	-0,058358	1
Tnfaip3	-0,058537	1
1110008L16Rik	-0,058516	1
Fyn	-0,05872	1
Ranbp10	-0,058825	1
Ifngr1	-0,059191	1
Atad3a	-0,059189	1
4930568A12Rik	-0,059308	1
Gm37702	-0,059284	1
Smpd4	-0,059415	1
Atp6v1f	-0,059608	1
Ncoa3	-0,05991	1
Tmem5	-0,060198	1
Pbdc1	-0,060235	1
Myo1e	-0,060375	1
Poldip3	-0,060501	1
Cirbp	-0,060614	1
Brox	-0,060726	1
Nol8	-0,060844	1
Tube1	-0,061023	1
Acvr1b	-0,061179	1
Usp21	-0,061173	1
1110002L01Rik	-0,061448	1
Limk2	-0,061363	1
Mapk1	-0,0614	1
6330418K02Rik	-0,061491	1

Prmt9	-0,061517	1
Tut1	-0,061503	1
Rnpepl1	-0,061538	1
Nptn	-0,061751	1
Zfp563	-0,061893	1
Gmps	-0,061897	1
Actr1b	-0,061884	1
4930518I15Rik	-0,061959	1
Dcakd	-0,062189	1
Slc2a8	-0,062485	1
Ankrd44	-0,062467	1
Bckdha	-0,062611	1
Snord83b	-0,062754	1
Tmx3	-0,06283	1
Gm26800	-0,062923	1
Rnf141	-0,062943	1
1110051M20Rik	-0,063003	1
Acox3	-0,062994	1
Kif5b	-0,063034	1
Fbxl17	-0,063333	1
Ddx6	-0,063284	1
Ctdspl	-0,063384	1
Jkamp	-0,063734	1
0610007P14Rik	-0,063682	1
Dnaaf3	-0,063763	1
Primpol	-0,063817	1
Sp100	-0,064169	1
Lrwd1	-0,064177	1
Cdc42bpb	-0,064168	1
Strap	-0,064292	1
Mbd1	-0,064462	1
Psma2	-0,064534	1
Fam234b	-0,064555	1
Mmadhc	-0,0648	1
Fam175b	-0,065044	1
Armc8	-0,065073	1
Zfp738	-0,06515	1
Chd7	-0,065225	1
Shkbp1	-0,065404	1
Rbm12	-0,065541	1
Gm43696	-0,065565	1
Gm26799	-0,065665	1
Snd1	-0,065702	1
Uqcc1	-0,065848	1
Gm42829	-0,065956	1
Lrrc8a	-0,065954	1
Fam118a	-0,066127	1
Suds3	-0,066179	1
Gm11362	-0,066406	1
Sugp2	-0,066401	1
Ogdh	-0,066423	1
Impdh2	-0,066558	1

Prkar1a	-0,066649	1
Bcdin3d	-0,066709	1
Gm4879	-0,066737	1
Cfap43	-0,066822	1
Gpcpd1	-0,066887	1
Atg7	-0,066942	1
Rhd	-0,066922	1
Plod1	-0,066854	1
Rangrf	-0,067103	1
Cyb5a	-0,067205	1
Rasd1	-0,067392	1
Gm19552	-0,067385	1
Trp53	-0,06736	1
Gpn1	-0,067613	1
Plek	-0,067624	1
Ngdn	-0,067638	1
9130221H12Rik	-0,067687	1
Psmd1	-0,067661	1
Gm17251	-0,067866	1
Casp4	-0,068228	1
Gm9774	-0,0682	1
Rdh11	-0,068163	1
Rpl31-ps13	-0,06831	1
Zfp799	-0,068738	1
Zfp68	-0,068686	1
Thoc6	-0,068728	1
Noa1	-0,068804	1
St6galnac4	-0,069099	1
Hccs	-0,069223	1
Morn1	-0,069636	1
Med30	-0,069556	1
Acap3	-0,069945	1
Edc3	-0,069904	1
Hck	-0,069983	1
4931414P19Rik	-0,070058	1
R3hcc1l	-0,07006	1
Trim24	-0,07012	1
Gm12251	-0,070189	1
Arfrp1	-0,070177	1
Nup54	-0,070271	1
Hspa13	-0,070342	1
Zfp189	-0,070377	1
Gtpbp10	-0,070502	1
Bud13	-0,070524	1
Patl1	-0,070573	1
Eif2b1	-0,070601	1
Zfp420	-0,070673	1
Gm5963	-0,070665	1
Gm37776	-0,07077	1
n-R5-8s1	-0,07084	1
Daam1	-0,070851	1
Smad5	-0,070948	1

Aph1b	-0,07104	1
N4bp2l2	-0,070967	1
Hmbox1	-0,071229	1
Mef2d	-0,07127	1
Nup62	-0,071262	1
Otx1	-0,071354	1
Brf1	-0,071381	1
Atxn10	-0,071449	1
Gm37060	-0,071465	1
G6pdx	-0,071516	1
Snrnp40	-0,071564	1
Atp5s	-0,07171	1
Gm7815	-0,071795	1
Aspscr1	-0,071775	1
Sipa1	-0,071754	1
Tmem167b	-0,071896	1
Prkch	-0,071951	1
Cggbp1	-0,072137	1
Bbs4	-0,072204	1
Snrpb	-0,07225	1
Ccdc51	-0,072468	1
Sestd1	-0,072535	1
Slc25a22	-0,072564	1
Usp36	-0,072568	1
Nos1	-0,072742	1
Espl1	-0,072734	1
Ehd4	-0,072722	1
Dnajc25	-0,07283	1
Snord7	-0,072872	1
Tfe3	-0,072854	1
Epn1	-0,07288	1
Fbxw11	-0,072903	1
Ccnc	-0,073042	1
Nfatc2ip	-0,072982	1
Ap2a2	-0,072986	1
Wdsub1	-0,073099	1
Ndfip2	-0,07318	1
Wrn	-0,073367	1
Fam8a1	-0,073371	1
Mgst2	-0,073599	1
Ctxn1	-0,073687	1
Mta3	-0,07379	1
Wdr45b	-0,073856	1
Gm42547	-0,074086	1
Snai2	-0,074313	1
Rpl3l	-0,074278	1
Mrpl19	-0,074345	1
Pygo2	-0,074507	1
Mbnl2	-0,074645	1
Ccdc47	-0,07466	1
Ints13	-0,074703	1
Fam168b	-0,074687	1

Lyst	-0,07486	1
Dcun1d3	-0,074923	1
Dok1	-0,074931	1
Ppp1cb	-0,074998	1
Slc25a51	-0,074989	1
Gm14403	-0,075106	1
Tubb5	-0,075133	1
Zkscan6	-0,075169	1
Uspl1	-0,075189	1
Asph	-0,0752	1
Fbxo9	-0,075377	1
Rin2	-0,0755	1
Inafm2	-0,075574	1
Adat2	-0,075584	1
4930430F08Rik	-0,075703	1
Atxn7	-0,075713	1
Fn3krp	-0,075849	1
Rac3	-0,075885	1
1810022K09Rik	-0,075875	1
Ppp2cb	-0,076009	1
Usp54	-0,076087	1
Tomm70a	-0,076101	1
Igsf8	-0,076324	1
RP23-58B7.2	-0,076422	1
Tmem229b	-0,076433	1
Hivep2	-0,076435	1
Copb2	-0,076443	1
Tbccd1	-0,07648	1
Pls3	-0,076597	1
Pdia4	-0,076693	1
Ankrd12	-0,076828	1
Ric8a	-0,076874	1
Ccdc12	-0,076981	1
Mt1	-0,077129	1
Plekhm3	-0,077221	1
Rpap3	-0,077181	1
Tmbim6	-0,077234	1
Ccnyl1	-0,077396	1
Dna2	-0,077509	1
Abracl	-0,077491	1
Mtf1	-0,077834	1
Snord87	-0,077887	1
Prickle3	-0,077934	1
Ppp2r3c	-0,077934	1
Zmym4	-0,077981	1
Wdpcp	-0,078141	1
Rfx1	-0,078087	1
Nek1	-0,078209	1
Als2cr12	-0,078293	1
Ppm1k	-0,078375	1
Hcfc2	-0,07836	1
Zfp715	-0,078705	1

Serpinb9	-0,079031	1
Synpo	-0,079015	1
Phf20-ps	-0,079057	1
Zc3h6	-0,079103	1
Cnppd1	-0,079081	1
Iqcb1	-0,07916	1
Ggta1	-0,079264	1
Zfp3	-0,079464	1
Rnf214	-0,079686	1
Gm11694	-0,079809	1
Gm4784	-0,079849	1
Smyd3	-0,079837	1
Csnk1g1	-0,079882	1
Pax6	-0,080027	1
Gm12151	-0,080087	1
Sfxn5	-0,080514	1
Zfp944	-0,080625	1
Tspan31	-0,080552	1
Atxn7l1	-0,080613	1
Fastkd3	-0,080581	1
Ap1s1	-0,080551	1
Maea	-0,080665	1
Akr1a1	-0,080673	1
Ccdc97	-0,08085	1
Rab8b	-0,080968	1
Pdk1	-0,081094	1
Flnb	-0,081073	1
Anapc4	-0,081217	1
Gm1848	-0,081323	1
Cnot7	-0,081524	1
Dnpep	-0,081558	1
Qrich1	-0,081668	1
Eif4ebp1	-0,081727	1
Tmem181b-ps	-0,082256	1
N4bp2	-0,082346	1
Drosha	-0,082615	1
Kmt2a	-0,082706	1
Glce	-0,082754	1
Rpl21-ps5	-0,082889	1
C130089K02Rik	-0,082999	1
Ncoa1	-0,082978	1
Nmt2	-0,083251	1
Zfp276	-0,083415	1
Cnpy2	-0,083449	1
Phlpp1	-0,083615	1
Pds5a	-0,083593	1
Fam105a	-0,083718	1
Ndufaf1	-0,083804	1
Chchd1	-0,083772	1
Bag4	-0,083882	1
Spryd3	-0,083977	1
Gm22009	-0,084132	1

Ncln	-0,084152	1
Skap2	-0,084158	1
Eif4g2	-0,084499	1
Tmem161b	-0,08451	1
Zcchc11	-0,08462	1
Aff4	-0,08456	1
Snord35a	-0,08471	1
Tbce	-0,084705	1
Lrch3	-0,08494	1
Tmem176b	-0,084885	1
Gm12902	-0,085249	1
Smg8	-0,085185	1
Pik3cd	-0,085173	1
Nfxl1	-0,085349	1
Gas2l1	-0,085444	1
Slfn10-ps	-0,085528	1
Vars2	-0,085557	1
Nsmce3	-0,085737	1
Socs5	-0,085697	1
Srsf9	-0,085739	1
Tom1l2	-0,0858	1
Ftsj1	-0,085917	1
Rab2a	-0,085892	1
Alg14	-0,086048	1
Stk10	-0,086125	1
Cep68	-0,086059	1
Srrt	-0,086109	1
Rbm8a	-0,086096	1
1190007I07Rik	-0,086223	1
Pacsin2	-0,086151	1
Ctdsp1	-0,086344	1
Ptrh2	-0,086673	1
Pip4k2a	-0,086726	1
Dvl1	-0,086927	1
Fam76b	-0,087116	1
Ndufc1	-0,087205	1
Map7d1	-0,08739	1
Ptpn4	-0,087492	1
Chd8	-0,087494	1
Klc3	-0,087632	1
Timmdc1	-0,087586	1
2610002M06Rik	-0,087665	1
Esd	-0,087663	1
Casp8	-0,087832	1
Baiap2	-0,087787	1
Elmod3	-0,087887	1
Ier3ip1	-0,087901	1
4921536K21Rik	-0,087996	1
Gm28809	-0,087967	1
Utrn	-0,08809	1
Zfp292	-0,088114	1
Dclre1b	-0,088162	1

Eef2	-0,088155	1
Pcbp2	-0,088321	1
Tnfsf9	-0,088377	1
Hspa9-ps1	-0,088402	1
Smg1	-0,088449	1
Rap1b	-0,088393	1
Psd3	-0,088495	1
Rrp9	-0,088532	1
Spn	-0,08862	1
Ehbp1	-0,088892	1
Napa	-0,088985	1
Setd2	-0,089051	1
Ttc9c	-0,089191	1
Fam71f2	-0,089308	1
Ndufb8	-0,089584	1
Dnajc16	-0,089679	1
Ankib1	-0,089895	1
A330074K22Rik	-0,08997	1
Otud5	-0,09	1
Fuom	-0,090069	1
Lonp1	-0,090061	1
Snf8	-0,090315	1
Fmnl1	-0,090516	1
Kdelc2	-0,090606	1
Nsmce1	-0,090734	1
Srp68	-0,090673	1
Trappc3	-0,090786	1
Tef	-0,090799	1
Zbtb25	-0,091004	1
Fam120b	-0,091123	1
Zfp764	-0,091231	1
Zbtb6	-0,091231	1
Taf11	-0,091243	1
Isy1	-0,091224	1
Tyw5	-0,091314	1
Gm45902	-0,091434	1
Bhlhe40	-0,091493	1
Ciapin1	-0,09151	1
Gmds	-0,091735	1
Gm12258	-0,091916	1
Ctif	-0,092175	1
Kifc2	-0,092266	1
Mrm1	-0,092253	1
Edem2	-0,092313	1
Kif18a	-0,09257	1
Cdk4	-0,092556	1
Il18	-0,092588	1
Edc4	-0,092652	1
Dnajc2	-0,09279	1
Spata7	-0,09289	1
Fn3k	-0,092967	1
Tceal8	-0,092985	1

Bcar3	-0,093048	1
Atp6v1e1	-0,093095	1
Hoxa4	-0,093219	1
Ankrd28	-0,093158	1
Lman2	-0,093243	1
Pdp2	-0,093233	1
Drap1	-0,093336	1
Isoc2a	-0,093445	1
Sntb2	-0,093407	1
Vps36	-0,093461	1
Mtrr	-0,093584	1
Pign	-0,093726	1
Atp9b	-0,093848	1
Bmf	-0,093931	1
Abca7	-0,093881	1
P2rx4	-0,093963	1
Ccr10	-0,094178	1
Naa20	-0,094174	1
Slc35c2	-0,094217	1
Tbc1d1	-0,094201	1
Suz12	-0,094176	1
Pdlim5	-0,094179	1
Vwa5a	-0,094347	1
RP24-286J14.3	-0,094358	1
Klc2	-0,094373	1
Mms19	-0,094379	1
Rnf217	-0,094472	1
Fgfr1op2	-0,094468	1
Ubxn1	-0,094685	1
Baat	-0,094835	1
Icmt	-0,094773	1
4931406P16Rik	-0,094836	1
Tmem131	-0,094965	1
Dhfr	-0,095249	1
Cst7	-0,095202	1
Eef2kmt	-0,095281	1
Vapb	-0,095289	1
Zfp790	-0,095621	1
Nt5c3	-0,09564	1
Agk	-0,095705	1
Osm	-0,095875	1
2310043L19Rik	-0,096099	1
Fam199x	-0,096086	1
Asah1	-0,096189	1
Plrg1	-0,096233	1
Bbs12	-0,096482	1
Rbm17	-0,096493	1
Cox6c	-0,096457	1
Smchd1	-0,096585	1
Cul2	-0,09662	1
Wdr25	-0,096839	1
Git1	-0,09692	1

Tnfrsf10b	-0,097009	1
2810004N23Rik	-0,09728	1
Gm38319	-0,097353	1
Gm5822	-0,097486	1
Hs1bp3	-0,097639	1
Nol6	-0,09774	1
Ufl1	-0,097696	1
Adnp2	-0,097792	1
Naa15	-0,097881	1
Rbm39	-0,097885	1
Gm17034	-0,098102	1
Samd4b	-0,098093	1
Rftn1	-0,098176	1
Pola2	-0,098298	1
Ppan	-0,098358	1
Cct7	-0,098368	1
Inpp1	-0,098458	1
Dicer1	-0,098646	1
Tsg101	-0,098878	1
Pih1d2	-0,099021	1
Ccdc50	-0,098954	1
Gm26129	-0,099288	1
Polb	-0,099305	1
Trmt12	-0,09954	1
Mob3c	-0,099516	1
Bin2	-0,099775	1
Tmem97	-0,099797	1
Gm37914	-0,099926	1
Wdr20	-0,099894	1
Tex264	-0,10005	1
Pacs2	-0,10001	1
Fam19a3	-0,10007	1
Zfp592	-0,1002	1
Ccdc127	-0,10021	1
Wbscr22	-0,10034	1
2810030D12Rik	-0,10036	1
Ercc5	-0,10036	1
Rcsd1	-0,10055	1
Slc30a6	-0,10059	1
Tmem167	-0,1008	1
mt-Tq	-0,10104	1
Nup155	-0,10097	1
Tpr	-0,10111	1
Sh3kbp1	-0,10118	1
Zfp874b	-0,10126	1
Hspa4	-0,10129	1
Skiv2l	-0,10135	1
Lap3	-0,10142	1
Slc30a9	-0,10168	1
Tfdp1	-0,10176	1
Lnx2	-0,10189	1
Echdc3	-0,10196	1

Gm4540	-0,10204	1
Cdk20	-0,1022	1
Itgal	-0,10232	1
Cstf2	-0,10231	1
RP24-282K24.4	-0,1024	1
March6	-0,10239	1
Afdn	-0,10243	1
A130014A01Rik	-0,10248	1
Rev1	-0,10246	1
Lrrc42	-0,10252	1
Dcbld2	-0,1026	1
Smim13	-0,10282	1
Hsdl1	-0,10296	1
Wdr83os	-0,103	1
Uck1	-0,10302	1
Pias4	-0,1031	1
Slf1	-0,10318	1
Igip	-0,10321	1
Ttc4	-0,10341	1
Cdc14b	-0,10351	1
Mapre1	-0,10353	1
Hnrnpa3	-0,10373	1
Ibtk	-0,10366	1
Mbd2	-0,10385	1
Aatf	-0,10389	1
Tob1	-0,10395	1
Tollip	-0,10398	1
Ankrd17	-0,10414	1
Ifnar2	-0,10414	1
Rnmt	-0,10424	1
Tor2a	-0,10452	1
Dguok	-0,10453	1
Rad1	-0,10455	1
Pggt1b	-0,10492	1
Tmem128	-0,10507	1
Tbp	-0,10535	1
Mtmr10	-0,1057	1
Haghl	-0,10592	1
Rpl15-ps5	-0,10601	1
Irf8	-0,10598	1
Kcmf1	-0,10607	1
Mfsd4b4	-0,10651	1
Morf4l1	-0,10649	1
Cyc1	-0,10647	1
Ik	-0,1066	1
Htt	-0,10679	1
Brcc3	-0,10702	1
Cep120	-0,10721	1
Tmem55b	-0,10718	1
P2ry6	-0,10738	1
Rapgef1	-0,1074	1
Ndst1	-0,10759	1

Serinc1	-0,10759	1
Rcan3	-0,10787	1
Slc39a6	-0,10792	1
Dynlt3	-0,10787	1
Dxo	-0,10805	1
Naa25	-0,10808	1
Pld1	-0,10823	1
Gabpa	-0,10832	1
Dmap1	-0,1084	1
Shc1	-0,10836	1
Ran	-0,10869	1
Urb2	-0,1088	1
Helb	-0,1089	1
Arl14ep	-0,10887	1
Pick1	-0,10902	1
Gm43128	-0,1091	1
Fkbp11	-0,10924	1
Cwc25	-0,10918	1
Rps27l	-0,10919	1
D5Ertd579e	-0,10944	1
Smyd4	-0,10951	1
Gpx1	-0,1096	1
Gm19967	-0,10969	1
Usp7	-0,10974	1
Mrpl36	-0,10984	1
Clns1a	-0,1099	1
Gnpda1	-0,11012	1
Gm12428	-0,11033	1
Fchsd2	-0,11028	1
Keap1	-0,11025	1
Kat2b	-0,11039	1
Pink1	-0,11043	1
Msh6	-0,11038	1
Trpm1	-0,11078	1
Cwf19l2	-0,11078	1
Rpl28-ps1	-0,11092	1
Got1	-0,11088	1
St8sia4	-0,111	1
Osbpl8	-0,11113	1
Pdpk1	-0,11143	1
Arpc3	-0,11155	1
Cdc37	-0,11168	1
Os9	-0,11189	1
Cxx1b	-0,11204	1
Klhl2	-0,11224	1
Atp11c	-0,11233	1
Pprc1	-0,11228	1
Nudt3	-0,11229	1
Slc29a2	-0,1124	1
Dhx36	-0,11255	1
Naif1	-0,11279	1
Gopc	-0,11279	1

Tinf2	-0,11288	1
Slc19a1	-0,11304	1
Ankra2	-0,11311	1
Man1a2	-0,11309	1
Riox2	-0,11321	1
Lrp6	-0,11334	1
Ywhag	-0,11329	1
Dusp12	-0,11337	1
Tpm3	-0,11362	1
Hagh	-0,11374	1
RP23-359K10.8	-0,11386	1
Usp31	-0,11393	1
Lasp1	-0,11388	1
Col18a1	-0,1139	1
Npm3-ps1	-0,11396	1
Kdm7a	-0,11412	1
Nudt1	-0,11421	1
Fntb	-0,11417	1
RP23-6C18.6	-0,11432	1
Shpk	-0,11429	1
Samm50	-0,11444	1
Pex1	-0,11452	1
Nol12	-0,11463	1
Rogdi	-0,11481	1
Rasgef1a	-0,11486	1
Btg1	-0,11501	1
Nsun2	-0,11498	1
Rps5	-0,115	1
Adam10	-0,11518	1
Gm7308	-0,11543	1
Defb25	-0,1154	1
Pomgnt1	-0,11541	1
2010016I18Rik	-0,1156	1
Zfp142	-0,11564	1
2610008E11Rik	-0,11575	1
Atg9b	-0,11597	1
4930455G09Rik	-0,11601	1
Tmem123	-0,11602	1
Utp14b	-0,11616	1
Spag7	-0,11626	1
Zmym3	-0,11629	1
Esf1	-0,11628	1
Gm42639	-0,11654	1
Dctn5	-0,1165	1
Ptpn22	-0,11654	1
Rpl14	-0,11647	1
Phax	-0,11649	1
Gm20689	-0,11661	1
Papss1	-0,11663	1
Papola	-0,11676	1
Brdt	-0,11702	1
Dact3	-0,11716	1

Ppm1g	-0,11725	1
Ap4e1	-0,11743	1
Itga7	-0,11744	1
Gla	-0,11761	1
Mllt1	-0,11767	1
Tbl1x	-0,11774	1
Mtmr14	-0,11788	1
Elavl1	-0,11795	1
Zeb2	-0,11794	1
Gm38399	-0,11805	1
Fam171b	-0,11806	1
Smarcal1	-0,11841	1
Tarbp2	-0,11853	1
Gm13456	-0,11855	1
Rabl6	-0,11859	1
Banf1	-0,11858	1
Memo1	-0,11868	1
Gipr	-0,1188	1
5730409E04Rik	-0,11883	1
Gskip	-0,11878	1
Kif2a	-0,11884	1
Ttll4	-0,11913	1
Cpne8	-0,11935	1
H1f0	-0,11936	1
Zkscan1	-0,1197	1
Kdm3b	-0,11976	1
Gm3724	-0,11987	1
Med8	-0,11985	1
Ppil2	-0,11988	1
Rplp1	-0,11999	1
Gm6524	-0,12032	1
Larp4	-0,12034	1
Ss18	-0,1204	1
Abhd17b	-0,12046	1
Gtf3a	-0,12062	1
Zfp28	-0,12071	1
Gm22716	-0,12071	1
Cdc27	-0,1208	1
Gm45733	-0,12088	1
Eef1b2	-0,12088	1
Mdrl	-0,12109	1
Fth-ps2	-0,12134	1
Gpatch2l	-0,12134	1
Lmf2	-0,12129	1
Copg1	-0,1213	1
Rbm27	-0,12136	1
Cited2	-0,12161	1
Rnf40	-0,12164	1
Matr3	-0,12173	1
Slc25a28	-0,12186	1
Ccdc61	-0,12234	1
Fbxw5	-0,12225	1

Slc4a1ap	-0,1223	1
Ap1g1	-0,12232	1
Ccdc85b	-0,12249	1
Gm15800	-0,12253	1
Ccnd1	-0,12246	1
4833445I07Rik	-0,12262	1
Nav2	-0,12295	1
Rprd1a	-0,12296	1
Khsrp	-0,12304	1
Sft2d1	-0,12306	1
Hmgxb3	-0,12309	1
Nif3l1	-0,12319	1
Pja2	-0,12316	1
Gm43793	-0,12373	1
Frat1	-0,12373	1
Vwa8	-0,12368	1
Tirap	-0,12374	1
Ino80	-0,12386	1
Zmat1	-0,12425	1
Zfp60	-0,12422	1
Cep97	-0,12416	1
Ddhd2	-0,12424	1
Nbeal1	-0,12416	1
Hsd17b4	-0,12443	1
Akap10	-0,12459	1
Igsf3	-0,12463	1
Mvb12b	-0,12462	1
Lats2	-0,12469	1
Atp6v0a1	-0,12498	1
Tcf3	-0,12555	1
Epc2	-0,12545	1
Traf1	-0,12556	1
Rab18	-0,12568	1
2610507B11Rik	-0,12571	1
Zfat	-0,12584	1
Ccdc43	-0,12579	1
Gm43756	-0,12592	1
Ppp1r15a	-0,12594	1
Gm13328	-0,12599	1
Ifi27	-0,12603	1
Atad2	-0,12613	1
Gm2531	-0,12624	1
Cnot6l	-0,12619	1
Wdr48	-0,12628	1
Tbc1d20	-0,12654	1
Zfp329	-0,12656	1
Lsm8	-0,12659	1
Ercc2	-0,12676	1
Ddx52	-0,12681	1
Ptpre	-0,12675	1
Fbxo31	-0,127	1
Brd4	-0,127	1

Acot10	-0,12706	1
Stk38	-0,12707	1
Trmt2b	-0,12718	1
Tonsl	-0,12719	1
Hmgb1-ps8	-0,12755	1
Ap2b1	-0,12752	1
Pds5b	-0,12751	1
Slc37a1	-0,12785	1
Ddx19a	-0,12791	1
Scarna2	-0,12797	1
Hdgf	-0,12796	1
Zfp141	-0,12811	1
Psmd10	-0,12811	1
Mmgt2	-0,1281	1
Tmem86a	-0,12819	1
Map4k5	-0,12831	1
Nckap1l	-0,12839	1
Acat1	-0,12846	1
Mink1	-0,12855	1
Gm9825	-0,12854	1
Ndufaf4	-0,12868	1
Osgin1	-0,12877	1
Gdpd5	-0,12894	1
Hnrnpul2	-0,12895	1
Pde4a	-0,12897	1
5031425E22Rik	-0,12904	1
Rab3gap1	-0,12899	1
Net1	-0,12909	1
C130023A14Rik	-0,12918	1
Pelp1	-0,12922	1
Ckap5	-0,12925	1
Uap1	-0,12928	1
Gm45286	-0,1294	1
Mark4	-0,12939	1
Gm45568	-0,12955	1
Fam65a	-0,1295	1
Rhbdf1	-0,1296	1
Bag6	-0,12961	1
Arhgef2	-0,12959	1
Mettl22	-0,12968	1
St3gal1	-0,12974	1
Taco1	-0,12984	1
Traf5	-0,12979	1
Greb1	-0,12987	1
Zfp551	-0,12991	1
Fam173b	-0,13005	1
Eif2ak1	-0,13021	1
Rwdd2a	-0,13034	1
Znrd1	-0,1304	1
Pno1	-0,13036	1
Hsd17b12	-0,13039	1
Kptn	-0,1305	1

Cmas	-0,13072	1
Brpf1	-0,13073	1
Slc25a32	-0,13102	1
4930503L19Rik	-0,131	1
Yaf2	-0,13107	1
Nat2	-0,13124	1
Rab3ip	-0,13116	1
Ddx41	-0,13126	1
Stau1	-0,1313	1
Gm43774	-0,13144	1
Mars	-0,13135	1
Pum1	-0,13139	1
Chchd2	-0,13147	1
Atxn3	-0,13169	1
Ccdc90b	-0,13188	1
Ogfod3	-0,13204	1
Particl	-0,13223	1
Ube2e3	-0,13216	1
A230028O05Rik	-0,13228	1
Plekhg3	-0,13225	1
Csnk1e	-0,13245	1
Arrdc1	-0,13251	1
Josd2	-0,13248	1
Fam160a2	-0,13264	1
Tjp3	-0,13265	1
Foxo1	-0,13266	1
Rtca	-0,13282	1
Slc36a1	-0,13282	1
Rnf19b	-0,13297	1
Clasp1	-0,13305	1
S100a4	-0,13313	1
Camk2n2	-0,13323	1
Rps12-ps9	-0,13325	1
Gm7638	-0,13342	1
Tmed8	-0,13344	1
Clec11a	-0,13337	1
Capns1	-0,1334	1
Lman2l	-0,13348	1
Lgals8	-0,13349	1
Ddx54	-0,13349	1
Cdc40	-0,13356	1
Arid4a	-0,13359	1
Ube3c	-0,13362	1
Tmem230	-0,13365	1
Kctd2	-0,13371	1
9330162012Rik	-0,13387	1
Cd3eap	-0,13392	1
Tspan3	-0,1339	1
Pwwp2a	-0,13409	1
Ifrd1	-0,13407	1
1810024B03Rik	-0,13415	1
Lmtk2	-0,13424	1

Snx24	-0,13458	1
Rbm28	-0,13459	1
0610040B10Rik	-0,13467	1
Bri3bp	-0,13465	1
Cyb561d2	-0,13488	1
Rarg	-0,13491	1
Serpinb6b	-0,13489	1
1700123O20Rik	-0,13495	1
Atg2b	-0,13504	1
Alkbh8	-0,13505	1
Bbx	-0,13524	1
Gtf2ird1	-0,13532	1
Scamp3	-0,13526	1
Cul1	-0,13536	1
Txnrd1	-0,13586	1
Isyna1	-0,13596	1
Prpf38b	-0,13602	1
Nelfb	-0,13609	1
Abcf3	-0,13619	1
Cd48	-0,13629	1
Tmeff1	-0,13638	1
Zfp800	-0,13651	1
Gpd2	-0,13674	1
Zgrf1	-0,13689	1
Gm15575	-0,137	1
Sco1	-0,13706	1
C2cd2l	-0,13719	1
Atp6v0e	-0,13715	1
Arfgef1	-0,13733	1
Cyb5b	-0,13729	1
Exoc5	-0,13733	1
Pigm	-0,13745	1
Rab8a	-0,13743	1
Mrpl4	-0,13746	1
4932441J04Rik	-0,13756	1
Rock1	-0,13758	1
Rfc2	-0,13778	1
Mthfd1l	-0,13815	1
Cnot6	-0,13811	1
Zadh2	-0,13821	1
Zswim3	-0,13827	1
Nrros	-0,13832	1
Fam118b	-0,13871	1
Brip1os	-0,13884	1
Rspry1	-0,13882	1
Arel1	-0,13879	1
Mogs	-0,13877	1
Pex11a	-0,13891	1
Zfp609	-0,13896	1
Mrps17	-0,13911	1
Aurkaip1	-0,13911	1
D17Wsu92e	-0,13906	1

Panx1	-0,13923	1
Dhx9	-0,13932	1
Enpp1	-0,13937	1
Dusp8	-0,13948	1
Exog	-0,13962	1
Hoxa5	-0,13967	1
Vps11	-0,13979	1
Purg	-0,13992	1
Nup37	-0,13999	1
Ier2	-0,14015	1
Gm43721	-0,14024	1
Chek2	-0,14038	1
Prkcd	-0,14043	1
Nkain1	-0,14068	1
Stard7	-0,14112	1
Usp38	-0,14122	1
Cep152	-0,14125	1
Pik3r4	-0,14124	1
Mrpl15	-0,1412	1
Tnpo3	-0,14134	1
Mul1	-0,14142	1
Bcl9	-0,14141	1
Gcc1	-0,14143	1
Ddx3x	-0,14151	1
Slc25a24	-0,14162	1
Secisbp2l	-0,14168	1
Irak2	-0,14183	1
Arhgap12	-0,14181	1
Mau2	-0,14188	1
Trappc10	-0,14192	1
Plekha2	-0,14188	1
Ppa1	-0,14192	1
Cd72	-0,142	1
Prorsd1	-0,14212	1
Gm11977	-0,14217	1
Suclg2	-0,14215	1
Jrk	-0,14228	1
Tatdn3	-0,1425	1
Gzf1	-0,14251	1
Gm7863	-0,14256	1
Tti2	-0,14256	1
Puf60	-0,14261	1
Chmp5	-0,14275	1
Mfsd5	-0,14265	1
Tbrg1	-0,14294	1
Chaf1a	-0,14304	1
Pcmtd2	-0,14297	1
Inppl1	-0,14309	1
Ncf2	-0,14322	1
Agfg2	-0,14321	1
Chek1	-0,14325	1
Ppfia3	-0,14332	1

Dlx1	-0,14339	1
Taf5l	-0,14342	1
2310036O22Rik	-0,14343	1
Phf12	-0,14352	1
Dnajc15	-0,14357	1
Pabpc4	-0,14357	1
Lca5	-0,14376	1
Pck2	-0,14403	1
2510046G10Rik	-0,14433	1
Hnrnpu	-0,1446	1
Fbxo33	-0,14471	1
Coq10b	-0,14482	1
Atf7	-0,14482	1
Dcaf4	-0,14485	1
Elf2	-0,14492	1
Coa5	-0,14491	1
H2afz	-0,14504	1
Tsen2	-0,14546	1
Tmem164	-0,14548	1
1600014C10Rik	-0,14568	1
2610301B20Rik	-0,14568	1
Cct2	-0,14571	1
Luc7l	-0,14595	1
Rbm6	-0,14601	1
Snhg1	-0,14595	1
Zfp229	-0,14631	1
Fgd3	-0,14627	1
Gm10039	-0,14642	1
Ubtd1	-0,14643	1
Atxn2	-0,14638	1
Chordc1	-0,14637	1
Hus1b	-0,1465	1
Gm6560	-0,14662	1
Gm15501	-0,14662	1
Mga	-0,14658	1
Ovgp1	-0,14673	1
Zdhhc1	-0,14673	1
Thoc1	-0,14678	1
Enpp4	-0,14706	1
Rwdd4a	-0,14707	1
Yae1d1	-0,14724	1
Cecr5	-0,1472	1
Ptpn7	-0,14735	1
Mrps7	-0,1473	1
Psen2	-0,14737	1
Plekhm1	-0,14737	1
Tmem106c	-0,1479	1
mt-Tc	-0,14804	1
Zfp729a	-0,14805	1
Blzf1	-0,14805	1
Mcur1	-0,14809	1
Esyt1	-0,14813	1

Siah2	-0,14812	1
Mpp5	-0,14813	1
Eif2s2	-0,14805	1
Lnpep	-0,14824	1
Atg16l2	-0,14819	1
Hddc2	-0,14835	1
Cd164	-0,14829	1
Adcy7	-0,14834	1
Bet1l	-0,14849	1
Exoc3	-0,14857	1
Ythdf1	-0,14859	1
Spaca9	-0,14869	1
Map2k7	-0,14882	1
Nudcd3	-0,14885	1
Ppm1f	-0,149	1
Fam126a	-0,14902	1
Nip7	-0,14915	1
Arhgap10	-0,1493	1
Pbx3	-0,14927	1
Hp1bp3	-0,1494	1
Cda	-0,14952	1
Ddx55	-0,14983	1
Kdm5b	-0,14982	1
Rps25-ps1	-0,14979	1
Fbxl8	-0,15	1
Rps13-ps5	-0,1501	1
Coro1c	-0,1501	1
Tjap1	-0,15016	1
Dip2b	-0,15027	1
Dync1h1	-0,15044	1
Tpm3-rs7	-0,15049	1
Kyat1	-0,1507	1
Dedd	-0,15098	1
Slc7a6	-0,15103	1
Kifap3	-0,15102	1
Cept1	-0,15097	1
Adamts4	-0,15117	1
Mrpl22	-0,15126	1
Ing3	-0,15148	1
Mapk12	-0,15165	1
Pik3r5	-0,15164	1
Dusp2	-0,15189	1
Cd36	-0,15186	1
Cenpb	-0,15189	1
D330050G23Rik	-0,15196	1
Rab7b	-0,15212	1
Paf1	-0,15217	1
Pdcd6	-0,15215	1
Pqlc1	-0,15242	1
Mthfd2	-0,15242	1
Lipt1	-0,15254	1
Mfap3l	-0,15259	1

Pkmyt1	-0,15265	1
Il6st	-0,15291	1
1600002H07Rik	-0,15302	1
Vta1	-0,15314	1
Sh3bp5	-0,15306	1
Myoz1	-0,15322	1
Pgs1	-0,15334	1
Fcgr1	-0,15343	1
Als2cl	-0,15352	1
Sat1	-0,15346	1
Irak3	-0,15375	1
Ubr3	-0,15383	1
Mdc1	-0,15392	1
Snx10	-0,15389	1
Ube2e1	-0,15403	1
Micu1	-0,15413	1
Wtap	-0,15424	1
Ube2h	-0,15419	1
Fam207a	-0,15426	1
Osbp	-0,15454	1
Dennd2c	-0,15475	1
Naa60	-0,15483	1
Fam214b	-0,15491	1
Hyou1	-0,15493	1
Cc2d1b	-0,15507	1
Exd2	-0,15528	1
Micu3	-0,15534	1
Ninl	-0,1553	1
Bco2	-0,15542	1
Pphln1	-0,15544	1
Cpne3	-0,15537	1
Twsg1	-0,15553	1
Gpr89	-0,15559	1
Gm5883	-0,15581	1
Rnf135	-0,15578	1
Bod1l	-0,1558	1
Hspa5	-0,15583	1
Tbc1d32	-0,15592	1
Scyl2	-0,15592	1
4931406C07Rik	-0,15597	1
C130071C03Rik	-0,15617	1
Btbd2	-0,15618	1
Ltc4s	-0,15619	1
Tmem71	-0,15631	1
Unc45a	-0,15632	1
Rbm15	-0,15648	1
Rb1	-0,15653	1
Slc7a7	-0,15667	1
Rpp21	-0,1569	1
Sfxn1	-0,15712	1
Elp3	-0,1575	1
Polr2f	-0,1575	1

Polr2b	-0,15751	1
Hipk3	-0,15758	1
Pde8b	-0,15759	1
Tpm2	-0,15777	1
Gm7496	-0,15796	1
E2f3	-0,15813	1
Gm17530	-0,15822	1
Jarid2	-0,15831	1
Cacna1b	-0,15853	1
Bend4	-0,15848	1
Ogfr	-0,15868	1
Gpbp1l1	-0,15892	1
Camk2d	-0,15903	1
Usp33	-0,15912	1
Cdip1	-0,15942	1
Map2k2	-0,15937	1
Peak1	-0,15946	1
Iqcc	-0,15971	1
Zfp397	-0,15968	1
Ptpn11	-0,15967	1
Vps13a	-0,15976	1
4930440I19Rik	-0,15988	1
Ap1b1	-0,15994	1
Magt1	-0,15999	1
Ppp2r5c	-0,15999	1
Ufc1	-0,16015	1
Chml	-0,16038	1
Csnk1g2	-0,16048	1
Plcg2	-0,16057	1
Ing5	-0,16062	1
Cpox	-0,16074	1
Sh3bp5l	-0,16084	1
Cct6a	-0,16081	1
Tbc1d15	-0,16085	1
Sp3os	-0,16104	1
Arpc5	-0,16112	1
Pla1a	-0,16138	1
Nup35	-0,16142	1
1600012H06Rik	-0,16141	1
Coq7	-0,16168	1
5031434O11Rik	-0,16177	1
Tmco1	-0,16184	1
Trmu	-0,16193	1
Ighmbp2	-0,16188	1
Ssrp1	-0,16189	1
Crat	-0,16198	1
Phka2	-0,1624	1
Srgap2	-0,16237	1
Rbm33	-0,1624	1
Sucla2	-0,16248	1
Stxbp2	-0,16254	1
Pum3	-0,16247	1

Gm13477	-0,16257	1
Lonrf1	-0,16283	1
Mppe1	-0,16296	1
Aig1	-0,16298	1
Gm43773	-0,16313	1
Tep1	-0,1631	1
Stom	-0,16364	1
Gm43560	-0,16391	1
Exoc6b	-0,16393	1
Gm45342	-0,16404	1
Arf1	-0,16404	1
Icosl	-0,16413	1
Sephs1	-0,16411	1
Agrn	-0,16407	1
Hdhd3	-0,16431	1
Dpf2	-0,16443	1
Mrpl10	-0,16445	1
Thap6	-0,16459	1
Fibp	-0,16459	1
Stat2	-0,16473	1
Kcnab3	-0,1648	1
Derl1	-0,16479	1
Kpna1	-0,16507	1
Pask	-0,16526	1
Slc5a6	-0,1654	1
Dgkg	-0,16538	1
Tmem147	-0,1654	1
Wdr59	-0,16552	1
Znhit6	-0,16549	1
Ptprs	-0,1655	1
Wipf2	-0,16547	1
Ruvbl1	-0,16562	1
Ajuba	-0,16568	1
Lysmd3	-0,16572	1
Susd6	-0,16621	1
Grhl1	-0,16631	1
Josd1	-0,16642	1
Cblb	-0,16649	1
Foxc1	-0,1666	1
D430042O09Rik	-0,16674	1
Cyb5r3	-0,16674	1
Baz2b	-0,16671	1
Tifab	-0,16676	1
BC052040	-0,16684	1
Ift20	-0,16693	1
Ccdc62	-0,16701	1
Scaf11	-0,16704	1
Lss	-0,16709	1
Usp37	-0,16712	1
Sec14l1	-0,16713	1
Cops3	-0,16705	1
Grhpr	-0,16716	1

Gm10863	-0,16741	1
Capn15	-0,16745	1
Lrig2	-0,16757	1
Erp44	-0,16757	1
Adck2	-0,16766	1
Twistnb	-0,1677	1
Tpst1	-0,16787	1
Krit1	-0,16794	1
Trp53bp2	-0,16805	1
Zbtb22	-0,16807	1
Rchy1	-0,16806	1
C030014I23Rik	-0,16817	1
Timm44	-0,16858	1
Bcl2l2	-0,16891	1
1600020E01Rik	-0,16892	1
Rps13	-0,16888	1
Cnot3	-0,16891	1
Ppm1a	-0,16904	1
Gtf2a1	-0,16911	1
Fmn1	-0,16917	1
Zc3h12a	-0,16936	1
Spg20	-0,16949	1
Wrap73	-0,1696	1
Rara	-0,16961	1
Msra	-0,16972	1
9330160F10Rik	-0,16974	1
Kdsr	-0,16968	1
Cwc27	-0,16966	1
Mxd4	-0,16966	1
Pik3r6	-0,16983	1
Mcoln1	-0,16982	1
Zfp703	-0,17009	1
Rac1	-0,17035	1
Egf	-0,17062	1
Dnaaf5	-0,17076	1
RP23-380K24.3	-0,17091	1
Mdm4-ps	-0,17121	1
Ctnna1	-0,1713	1
Xdh	-0,17161	1
Smad4	-0,17162	1
Prpf6	-0,17172	1
Degs1	-0,17167	1
Shisa5	-0,17184	1
Ip6k1	-0,17221	1
Bin1	-0,17229	1
Erp29	-0,1723	1
Igtp	-0,17251	1
Gnb1l	-0,17254	1
Sep 11	-0,17253	1
Gm4880	-0,17264	1
Fam63b	-0,17263	1
Dhx15	-0,17258	1

Smarca5	-0,17276	1
Zfp58	-0,17295	1
Rbm4	-0,17286	1
Mapk8ip3	-0,17294	1
Ppp2ca	-0,17294	1
Dhx37	-0,17304	1
Arid3a	-0,17311	1
Plcl2	-0,17341	1
2700049A03Rik	-0,17344	1
Azi2	-0,17354	1
Pcnt	-0,17345	1
Tm9sf1	-0,17363	1
D130019J16Rik	-0,17376	1
Gm45250	-0,17378	1
Fnbp1	-0,1738	1
Slc12a6	-0,17389	1
Snx4	-0,17398	1
Ergic1	-0,17399	1
Tasp1	-0,17411	1
Phpt1	-0,17441	1
Foxk2	-0,17451	1
Gm25636	-0,17478	1
Zfp81	-0,17475	1
Chmp3	-0,17484	1
Colgalt1	-0,17482	1
Zkscan3	-0,17499	1
Pdia6	-0,17496	1
Sumo3	-0,1751	1
Maf1	-0,17524	1
Milr1	-0,1753	1
Jpx	-0,17543	1
Cnot10	-0,17536	1
Mtm1	-0,17552	1
Cbx5	-0,1755	1
Clec1a	-0,17559	1
Vcl	-0,1756	1
F9	-0,17566	1
Gba2	-0,17603	1
Tbc1d16	-0,17595	1
Eftud2	-0,176	1
Pgm2	-0,17608	1
Gm20517	-0,17668	1
Armc1	-0,17676	1
Pou2f1	-0,17677	1
Stx18	-0,17702	1
Rnf157	-0,17712	1
Eif2b5	-0,17711	1
Poc1b	-0,17735	1
Dcun1d2	-0,17739	1
Ctso	-0,17736	1
Ftx	-0,17748	1
Akap1	-0,17753	1

M6pr	-0,1778	1
Cry2	-0,17776	1
Wdr90	-0,17804	1
Abt1	-0,17832	1
Heatr6	-0,17826	1
Gfap	-0,17854	1
Polg	-0,17856	1
Mfsd10	-0,17886	1
Rabgap1l	-0,17895	1
Fam149b	-0,1792	1
RP24-325N9.5	-0,17954	1
Fam172a	-0,17957	1
Gpnmb	-0,17956	1
Cmtm3	-0,17968	1
Yipf1	-0,1797	1
Rtn3	-0,17974	1
Cnbd2	-0,17992	1
Gm37642	-0,18021	1
Exoc1	-0,18022	1
Gm42635	-0,18033	1
Tmem165	-0,18031	1
Azin2	-0,18052	1
Cflar	-0,18049	1
Eaf1	-0,18069	1
Zfpm1	-0,18086	1
Slc7a8	-0,18089	1
Akr1b8	-0,18102	1
Vcpip1	-0,18098	1
Cdv3	-0,18102	1
Xpr1	-0,18115	1
Nhej1	-0,18132	1
Manea	-0,18134	1
Twf1	-0,18154	1
Zfp868	-0,18184	1
9930021J03Rik	-0,18182	1
Mdm4	-0,18177	1
Cox6a1	-0,18177	1
Soat1	-0,18187	1
Upf3a	-0,18205	1
Plcb3	-0,18206	1
Mcm4	-0,18219	1
Gm20673	-0,18231	1
Slc9a1	-0,18235	1
Gm43275	-0,18258	1
Mad1l1	-0,18267	1
Rgs14	-0,18269	1
Actr3	-0,18276	1
Osbpl9	-0,18281	1
H13	-0,18277	1
Ankrd13c	-0,18314	1
Abr	-0,18308	1
Gart	-0,18321	1

Cyld	-0,18316	1
Ddx1	-0,18355	1
Thap12	-0,1836	1
Clec16a	-0,18373	1
Gm43138	-0,18403	1
Gramd1a	-0,18401	1
Mknk1	-0,18429	1
Myo19	-0,18454	1
Selenof	-0,1847	1
Dolpp1	-0,1847	1
AI597479	-0,18466	1
Wwp1	-0,18467	1
Spast	-0,18483	1
Ppp1r8	-0,18478	1
Zc3h13	-0,18483	1
Nceh1	-0,18478	1
Pnpt1	-0,18495	1
Rbmxl1	-0,18494	1
Fuk	-0,18487	1
Rab11fip5	-0,18505	1
Tfpi	-0,18518	1
Prkab2	-0,18518	1
1190005I06Rik	-0,18543	1
Zfyve9	-0,1854	1
Sh3bgr	-0,18556	1
Mecr	-0,1857	1
Gm44434	-0,18604	1
Ptgr2	-0,18615	1
Irf1	-0,18634	1
Gm43792	-0,18653	1
Mpzl1	-0,18655	1
Copz1	-0,18658	1
Prpf31	-0,18683	1
Rsbn1l	-0,1868	1
Col4a6	-0,18694	1
Alas1	-0,18703	1
Homer3	-0,187	1
Fdxr	-0,18695	1
Gpn2	-0,18706	1
Abcb10	-0,18729	1
Cipc	-0,18729	1
Mterf4	-0,18744	1
Csnk2a1	-0,18751	1
Wbp2	-0,18753	1
Igsf6	-0,18767	1
Tctn1	-0,18781	1
Prr12	-0,18785	1
Mlh1	-0,18788	1
Trmt112	-0,18794	1
Rraga	-0,18815	1
Ccdc88b	-0,18815	1
Rhbdd3	-0,18821	1

Rabgap1	-0,18818	1
Ate1	-0,1882	1
Gdap2	-0,18828	1
Myo18a	-0,18843	1
Srsf7	-0,18843	1
Fbxo18	-0,18865	1
Stk3	-0,18874	1
Pomk	-0,18888	1
Dars	-0,18886	1
Pcyt2	-0,1889	1
Celf1	-0,18894	1
Pgls	-0,18889	1
Uba7	-0,18911	1
Yeats2	-0,18926	1
Mrpl38	-0,18932	1
Mir763	-0,18943	1
Sik3	-0,18943	1
Stat5a	-0,18981	1
Diexf	-0,18978	1
Gm5100	-0,18989	1
Apba3	-0,18995	1
Rhbdd1	-0,18992	1
Zfp322a	-0,19018	1
Ncapd2	-0,19039	1
Pald1	-0,19069	1
Magi1	-0,19076	1
Mapkap1	-0,19077	1
Lyrm7	-0,19088	1
Tipin	-0,19092	1
Hjurp	-0,19089	1
Elac2	-0,19104	1
Neurl1b	-0,19103	1
Nras	-0,191	1
Vps45	-0,19101	1
Rnf20	-0,19099	1
C030015A19Rik	-0,19109	1
Oxa1l	-0,19137	1
Trp53i13	-0,19149	1
Gorasp2	-0,19152	1
Sec24b	-0,19161	1
Aptx	-0,19173	1
Prpf40a	-0,19182	1
Gm23346	-0,19194	1
Ggact	-0,19189	1
Miga1	-0,19188	1
Iws1	-0,19196	1
Pop7	-0,19222	1
Hddc3	-0,19216	1
Poldip2	-0,19226	1
Ppp1r15b	-0,19239	1
Spg11	-0,19251	1
Acaa2	-0,19259	1

Gatad2b	-0,19258	1
Ewsr1	-0,19268	1
Ccrl2	-0,19302	1
Pten	-0,19331	1
Zfp948	-0,19345	1
Sel1l	-0,19359	1
Plxna1	-0,19368	1
Yipf2	-0,19377	1
Parp12	-0,1939	1
Paqr7	-0,1939	1
Zfp788	-0,19412	1
Mtmr4	-0,19434	1
Immt	-0,19444	1
Pstpip1	-0,1947	1
Nploc4	-0,19482	1
Tex261	-0,19489	1
Fbxw17	-0,19505	1
Rnf38	-0,1953	1
Itsn2	-0,19531	1
Rab21	-0,19549	1
Morc3	-0,1959	1
2810403D21Rik	-0,19605	1
Fbxo42	-0,19632	1
Sh3glb2	-0,19634	1
St3gal4	-0,19642	1
Actr5	-0,19641	1
Psmd4	-0,1965	1
Faf2	-0,19664	1
Epb41	-0,19694	1
Wwp2	-0,19694	1
Prtn3	-0,19706	1
Zbtb11os1	-0,19723	1
Man2c1	-0,19715	1
Cstb	-0,19751	1
Gm44053	-0,19759	1
Dnajc13	-0,19758	1
Nr1d2	-0,19781	1
Gm5070	-0,19808	1
2510002D24Rik	-0,19806	1
Uhmk1	-0,19834	1
Cyba	-0,19836	1
Kantr	-0,19853	1
Arap1	-0,1985	1
Mettl2	-0,19857	1
Smad6	-0,19904	1
Fam78a	-0,19897	1
Picalm	-0,19899	1
Ppp6r2	-0,19906	1
Abhd12	-0,19911	1
Nipbl	-0,19909	1
Snhg17	-0,19924	1
Srsf3	-0,19922	1

Megf9	-0,19981	1
Ubap2l	-0,19977	1
Cd2ap	-0,2001	1
Aqr	-0,20032	1
Adipor2	-0,20031	1
Prpsap2	-0,20045	1
Rhof	-0,20051	1
Ints11	-0,2005	1
Glmn	-0,20062	1
2810402E24Rik	-0,20078	1
Ints6	-0,20089	1
Socs3	-0,20097	1
Sep 09	-0,20101	1
9930022D16Rik	-0,20109	1
Nudcd1	-0,20111	1
Notch4	-0,20125	1
Copg2	-0,2012	1
Hk3	-0,20134	1
Phrf1	-0,20136	1
Stx6	-0,20164	1
Osbpl2	-0,20158	1
Mfsd8	-0,20166	1
Cttnbp2nl	-0,20184	1
Gspt1	-0,20181	1
Gm45828	-0,20194	1
2610016A17Rik	-0,20205	1
2700033N17Rik	-0,20211	1
RP24-497N7.2	-0,20212	1
Rasa1	-0,20212	1
Hax1	-0,20226	1
Zfyve28	-0,20254	1
Gfod1	-0,20251	1
Hoxa1	-0,20284	1
P3h1	-0,20283	1
Rab31	-0,2028	1
Ino80c	-0,20286	1
Kdm5a	-0,20286	1
Ddx51	-0,20298	1
Snx11	-0,20296	1
Arhgap21	-0,20314	1
RP23-403D16.3	-0,20318	1
U2af2	-0,20335	1
Prmt3	-0,20325	1
Ddx56	-0,20347	1
Cpsf2	-0,20346	1
Nfkb1	-0,20357	1
Tgs1	-0,20361	1
Tle6	-0,20365	1
Ahctf1	-0,20374	1
Zcchc7	-0,20372	1
Abtb2	-0,2039	1
Pus1	-0,20404	1

Ap3d1	-0,20429	1
A930007I19Rik	-0,20436	1
Psmb3	-0,20454	1
Afp	-0,2045	1
Fbxo38	-0,20463	1
Nfix	-0,20474	1
Fam129a	-0,20467	1
Gm13378	-0,20489	1
C130050O18Rik	-0,20496	1
Tmem109	-0,2051	1
Zpr1	-0,20513	1
5031439G07Rik	-0,20514	1
Crebl2	-0,20524	1
Zfp516	-0,20521	1
Supt6	-0,20518	1
Map1s	-0,20528	1
Tom1l1	-0,20547	1
Emc2	-0,20554	1
Pex11g	-0,20564	1
Dcun1d4	-0,20556	1
Cacna1s	-0,20573	1
Chaf1b	-0,20573	1
Ptpa	-0,20566	1
Kif24	-0,20586	1
Acox1	-0,20589	1
Actr6	-0,20602	1
Ccdc91	-0,20595	1
Atg4b	-0,20609	1
Srebf1	-0,20623	1
Pik3ca	-0,20634	1
Ythdf2	-0,20629	1
Nsfl1c	-0,2065	1
Gm43859	-0,20658	1
Abcg1	-0,20665	1
Gstp-ps	-0,20703	1
Ccpg1	-0,20708	1
Scfd1	-0,20715	1
Fth1	-0,20724	1
Gm7117	-0,20727	1
Arsa	-0,20738	1
C920009B18Rik	-0,20749	1
9130008F23Rik	-0,20791	1
Oat	-0,20794	1
Cxxc1	-0,20829	1
Prrc2c	-0,2085	1
Zfp930	-0,20867	1
Clcn2	-0,20903	1
Adssl1	-0,20924	1
Cox18	-0,20929	1
St3gal2	-0,20929	1
Ctsa	-0,2093	1
Pdcl3	-0,2094	1

Gm43484	-0,20937	1
Ddx42	-0,20943	1
Proser1	-0,20941	1
Atp6v1b2	-0,20939	1
Zfx	-0,20947	1
Rbms1	-0,20957	1
Amigo1	-0,2097	1
Nifk	-0,2097	1
Slc25a3	-0,20976	1
Vav2	-0,21002	1
Klhdc2	-0,20996	1
Eed	-0,21007	1
Tyw1	-0,21048	1
Melk	-0,21065	1
Cdk11b	-0,21074	1
1600010M07Rik	-0,2108	1
Trappc13	-0,21083	1
Zfp455	-0,21095	1
Ints4	-0,21094	1
Trappc6b	-0,21098	1
Rel	-0,21101	1
Hk2	-0,21098	1
Dcxr	-0,21101	1
Zfp526	-0,21122	1
Pctp	-0,21121	1
Mbtd1	-0,2112	1
Phf2	-0,21121	1
Arhgdib	-0,21135	1
Slc39a14	-0,21149	1
Zfp652	-0,21152	1
Ist1	-0,21148	1
Fbxo32	-0,21157	1
Fpgs	-0,21166	1
Dtwd1	-0,21184	1
Gm26244	-0,21188	1
Cbr2	-0,21218	1
Fam178a	-0,2123	1
Tubgcp4	-0,21245	1
Fam217b	-0,21257	1
Rnf10	-0,21261	1
Gm28530	-0,21269	1
Kmt2c	-0,21269	1
Safb2	-0,21269	1
Asxl2	-0,21273	1
Aggf1	-0,2127	1
Bbs2	-0,21285	1
Gm15834	-0,21292	1
Sesn1	-0,2129	1
Zfp599	-0,21302	1
Tmem39a	-0,21299	1
Dnaja3	-0,21304	1
Brd1	-0,21299	1

Pes1	-0,21305	1
Ptpru	-0,21317	1
H6pd	-0,21323	1
Snrk	-0,21329	1
Rgs19	-0,2133	1
Wdr3	-0,21341	1
Opa1	-0,2136	1
Nxf7	-0,21371	1
Prr14	-0,21366	1
Zfp426	-0,21385	1
Akap13	-0,21377	1
Ttf2	-0,2139	1
Nfkbid	-0,21387	1
Ccdc32	-0,214	1
Gmppa	-0,21409	1
Rabep2	-0,21417	1
Chst10	-0,21441	1
Sh3pxd2b	-0,2144	1
Gale	-0,21459	1
AI464131	-0,21473	1
Plppr2	-0,21472	1
4632404H12Rik	-0,21469	1
C2cd5	-0,21465	1
Gm17060	-0,2148	1
Pate2	-0,21481	1
2210016F16Rik	-0,21475	1
Atf7ip	-0,21481	1
Lancl1	-0,21485	1
Gna13	-0,21489	1
Spred2	-0,21502	1
Msl1	-0,215	1
Grb2	-0,21521	1
G3bp1	-0,21569	1
Yme1l1	-0,21575	1
Ccdc186	-0,21577	1
Cops5	-0,21591	1
Emp2	-0,21603	1
Abhd18	-0,21606	1
Pemt	-0,21621	1
Jmy	-0,21643	1
Opa3	-0,21652	1
Stxbp1	-0,21661	1
Ctc1	-0,21684	1
Sptbn4	-0,21689	1
N4bp2l1	-0,21685	1
Pdia3	-0,2171	1
Ptbp1	-0,21722	1
Ppp1r13b	-0,21731	1
Rgs16	-0,21736	1
Oas1g	-0,21762	1
Naf1	-0,21755	1
Zfp296	-0,21779	1

Slc25a33	-0,21776	1
Lats1	-0,21794	1
Atad2b	-0,218	1
Zfp513	-0,21829	1
Sec61a2	-0,21838	1
Zfp281	-0,21845	1
Piezo1	-0,2185	1
Gpd1l	-0,21847	1
Nfkb2	-0,21865	1
Gm45890	-0,21934	1
Gm5697	-0,21938	1
Dis3l2	-0,21961	1
Zfp668	-0,21962	1
Pgap1	-0,21956	1
Dgat1	-0,2197	1
Sh3gl1	-0,21987	1
Zcchc8	-0,21991	1
Champ1	-0,21987	1
Canx	-0,21992	1
Gm35315	-0,22017	1
Qtrt1	-0,22024	1
Mcf2l	-0,22025	1
Zbtb44	-0,22024	1
Nme6	-0,22049	1
Appbp2	-0,22052	1
3110083C13Rik	-0,22065	1
Pitpnb	-0,22069	1
Fes	-0,22084	1
Sbf1	-0,22075	1
Gm37334	-0,22119	1
Rps20	-0,22144	1
Zbtb26	-0,22149	1
Ndufs3	-0,22148	1
Snord71	-0,22176	1
Mfsd11	-0,22176	1
Gm37598	-0,22205	1
Cptp	-0,22214	1
Ctps	-0,22233	1
C1galt1	-0,22239	1
Tceal9	-0,2224	1
Ddi2	-0,2227	1
Fam53b	-0,22302	1
Psmd6	-0,22295	1
A430027C01Rik	-0,22315	1
Ankrd13d	-0,2232	1
Dus4l	-0,2233	1
RP23-243B24.1	-0,22351	1
Gm23502	-0,22345	1
Cnep1r1	-0,2236	1
Gm8116	-0,22375	1
Gpatch11	-0,22376	1
Atp6v1c1	-0,22376	1

Plekhg5	-0,22385	1
Usp14	-0,22411	1
Ppih	-0,22419	1
Gm26461	-0,22421	1
Snx17	-0,22421	1
Rps2-ps11	-0,22431	1
Aqp11	-0,22438	1
Chrac1	-0,22499	1
Fam84b	-0,22511	1
Ttll5	-0,22516	1
Sash1	-0,22532	1
Rap1gds1	-0,22525	1
Gnal	-0,22543	1
Dhdds	-0,2254	1
A230050P20Rik	-0,22551	1
Mtx2	-0,22551	1
Cd37	-0,2255	1
Ppa2	-0,22561	1
Terf2	-0,22571	1
Rdx	-0,22572	1
Gm45360	-0,22579	1
Mtmr12	-0,22586	1
Ndc1	-0,2259	1
Ucp2	-0,22589	1
Cyp27a1	-0,22618	1
Per2	-0,22616	1
Vac14	-0,22637	1
Mapk14	-0,22664	1
Sgcb	-0,2267	1
Casp1	-0,22665	1
Hspa9	-0,22668	1
E130307A14Rik	-0,22682	1
Cfap74	-0,2269	1
Phf20	-0,22704	1
Fundc2	-0,22712	1
Clasrp	-0,22732	1
Rab34	-0,22726	1
Cd59a	-0,22739	1
Epc1	-0,22743	1
Efna1	-0,22736	1
Polk	-0,22737	1
Apobec3	-0,22753	1
Rpe	-0,22747	1
Hnrnpll	-0,22775	1
Klc4	-0,22775	1
Washc4	-0,22803	1
Atp2b4	-0,22803	1
Cacnb1	-0,22822	1
Osbpl11	-0,22823	1
Gm37893	-0,22848	1
Desi1	-0,22851	1
Lin7c	-0,22861	1

Morc2a	-0,22869	1
Sfswap	-0,2288	1
Mtf2	-0,22898	1
Fubp3	-0,22907	1
Adam9	-0,22918	1
Scyl3	-0,22922	1
Gga2	-0,22934	1
2310057M21Rik	-0,22941	1
Mospd3	-0,22945	1
Fam160b2	-0,22945	1
Ccng1	-0,22952	1
Gm7353	-0,22969	1
Dennd2d	-0,22981	1
Aim2	-0,22991	1
Pelo	-0,22999	1
Ddhd1	-0,23011	1
Slc26a9	-0,23018	1
Ncoa6	-0,23026	1
Arf3	-0,23039	1
Ppp1r26	-0,23049	1
Mthfsd	-0,23051	1
Acly	-0,23076	1
Gatc	-0,23088	1
Zzz3	-0,23086	1
Gigyf2	-0,23092	1
Oxsr1	-0,231	1
Tdrkh	-0,23105	1
Zbed5	-0,23118	1
Msantd4	-0,23134	1
Zdhhc21	-0,23127	1
Aco2	-0,23128	1
Lhpp	-0,23143	1
Slc31a1	-0,2314	1
Papolg	-0,23161	1
Orai3	-0,23156	1
Ctsz	-0,2317	1
Rad21	-0,23204	1
Kctd20	-0,23215	1
Gnpat	-0,23223	1
Traf3ip2	-0,23229	1
Fzr1	-0,23229	1
Akip1	-0,23228	1
Larp1b	-0,23256	1
Pip5k1a	-0,23268	1
Nsun3	-0,23282	1
Ccz1	-0,23279	1
Dnajc14	-0,23293	1
Cyth1	-0,23328	1
Gar1	-0,2333	1
Ubac1	-0,23326	1
Celf4	-0,23342	1
Slc2a3	-0,23344	1

Atp6v1h	-0,2335	1
Fahd2a	-0,23357	1
4930430E12Rik	-0,2338	1
Nt5dc1	-0,23377	1
Ccdc71l	-0,2338	1
Atp6v1g2	-0,234	1
Gm15964	-0,23413	1
Gm44027	-0,23417	1
Sypl	-0,23443	1
Ahcyl1	-0,2344	1
Ebag9	-0,23459	1
Smg9	-0,23467	1
Nek7	-0,23478	1
Lrrc41	-0,23488	1
Psmd5	-0,23491	1
Dnal1	-0,23521	1
Mtch2	-0,23525	1
Ube2r2	-0,23527	1
Zfp7	-0,23543	1
Nsd3	-0,23546	1
Ankrd39	-0,23563	1
Ptk2b	-0,23559	1
Prnp	-0,23559	1
Hyls1	-0,23571	1
Grina	-0,2357	1
Gt(ROSA)26Sor	-0,23576	1
9330104G04Rik	-0,23607	1
Trem3	-0,23608	1
Fam53c	-0,23606	1
Ppp1r11	-0,23608	1
Tmtc3	-0,23618	1
1110059G10Rik	-0,23616	1
Kat8	-0,23637	1
Dcaf13	-0,23651	1
Ccm2	-0,23688	1
Zc2hc1a	-0,23752	1
Gcc2	-0,23748	1
Mycbp2	-0,23766	1
Zfp407	-0,23792	1
Gtpbp3	-0,23788	1
Gm20604	-0,23808	1
Stx12	-0,2381	1
R74862	-0,23822	1
Cbl	-0,23819	1
Tab1	-0,23825	1
Pla2g16	-0,23828	1
Gm6395	-0,23836	1
Phldb1	-0,23855	1
Atp5f1	-0,23857	1
Gm45456	-0,23877	1
Galc	-0,23883	1
Ptgs1	-0,23892	1

Bag2	-0,23887	1
Focad	-0,23898	1
Sec63	-0,23933	1
Sf3b4	-0,23944	1
Dennd1b	-0,23939	1
Kars	-0,23939	1
Lrrfip2	-0,23938	1
Rgl1	-0,23935	1
Gpaa1	-0,23948	1
Invs	-0,23956	1
Gm37494	-0,23967	1
Mrps27	-0,23971	1
1810011H11Rik	-0,23968	1
Lpar6	-0,2397	1
Pgm2l1	-0,24002	1
Ttc27	-0,24026	1
C330007P06Rik	-0,24036	1
Lace1	-0,24043	1
Carf	-0,24053	1
Sympk	-0,2406	1
Srek1	-0,24068	1
BC004004	-0,24078	1
Aldh6a1	-0,24094	1
Slc50a1	-0,24095	1
Gm11956	-0,24112	1
Mrps24	-0,24121	1
2610020H08Rik	-0,2413	1
Ralgapa2	-0,24129	1
Ift88	-0,24148	1
Pdk2	-0,24152	1
Arhgap23	-0,24153	1
Peg13	-0,24149	1
Zkscan8	-0,2415	1
St14	-0,24147	1
Smarcad1	-0,24166	1
Swsap1	-0,24189	1
Gm19287	-0,24202	1
Gm37522	-0,24212	1
Btbd3	-0,2423	1
Gm5841	-0,24231	1
Exosc10	-0,24237	1
Hacl1	-0,24254	1
Pid1	-0,24246	1
Rbbp7	-0,24251	1
Gm42967	-0,24271	1
Gm15785	-0,24271	1
Rwdd3	-0,24274	1
Prosc	-0,24275	1
Uba1	-0,24281	1
Mon2	-0,2428	1
Cd68	-0,24291	1
B3gnt2	-0,24298	1

Herc2	-0,24306	1
Slc12a2	-0,24317	1
Siae	-0,24328	1
Sf3b2	-0,24341	1
Stx2	-0,24357	1
Glipr1	-0,24365	1
Acad11	-0,24372	1
Fbxo8	-0,24369	1
Wdr60	-0,2438	1
Hectd3	-0,24392	1
Fto	-0,24393	1
Chp1	-0,24389	1
2310001H17Rik	-0,24402	1
Gstm1	-0,244	1
Kiss1r	-0,24413	1
Rfc4	-0,24429	1
Khdrbs1	-0,24429	1
Mrps6	-0,24438	1
Zfp839	-0,24447	1
Dpp9	-0,24503	1
Pak2	-0,24502	1
Rps19-ps3	-0,24514	1
Rbsn	-0,24513	1
Fam193b	-0,24514	1
Ccp110	-0,24525	1
Heatr5b	-0,24568	1
Otub1	-0,24573	1
Wiz	-0,24585	1
Tle4	-0,24586	1
Fasn	-0,24594	1
Paics	-0,24594	1
Ppp2r1b	-0,2461	1
Luzp1	-0,2462	1
Slc25a39	-0,2464	1
Msh2	-0,24651	1
Elk1	-0,24654	1
Lrrc57	-0,24663	1
Capn10	-0,24665	1
Wdtc1	-0,2467	1
Nectin2	-0,24683	1
Ypel3	-0,24691	1
Smc5	-0,24698	1
Zfp568	-0,24708	1
Lrrc20	-0,24722	1
Smim12	-0,24721	1
2310033P09Rik	-0,24718	1
2300009A05Rik	-0,24738	1
Gm38162	-0,24753	1
Fam136a	-0,24762	1
Ncs1	-0,24755	1
Fbxw9	-0,24769	1
Rbm41	-0,2478	1

Zfp65	-0,24796	1
Slc9a4	-0,24807	1
Garnl3	-0,24818	1
Arhgap31	-0,24827	1
Chpt1	-0,24835	1
Osbpl7	-0,24832	1
Pcyox1	-0,24829	1
Bax	-0,24852	1
Bcat1	-0,2488	1
Dlst	-0,24898	1
Rbbp6	-0,24896	1
Fndc10	-0,24921	1
Limd1	-0,24925	1
Senp5	-0,24933	1
Ireb2	-0,24932	1
Mob3b	-0,24952	1
Pafah1b2	-0,24948	1
Tor1b	-0,24958	1
Haus2	-0,24981	1
Spag9	-0,24977	1
Prr7	-0,25	1
Git2	-0,25	1
Bub3	-0,24999	1
Cul7	-0,25025	1
Edrf1	-0,25025	1
Def6	-0,25033	1
Gpr179	-0,2504	1
Fsd2	-0,2504	1
Rcor1	-0,25043	1
Foxred1	-0,25051	1
Hgs	-0,25054	1
Gas8	-0,25078	1
Them6	-0,25082	1
Wdr74	-0,25091	1
Sfxn3	-0,25102	1
Atxn7l2	-0,2511	1
AI314180	-0,25116	1
Pex6	-0,25159	1
Rad23b	-0,25168	1
Umad1	-0,25183	1
Gm8019	-0,25181	1
Cerk	-0,25178	1
5830408C22Rik	-0,25193	1
Nme7	-0,2521	1
Myo1c	-0,25209	1
Rfc5	-0,2525	1
Fam168a	-0,25254	1
Oit3	-0,2526	1
Nup188	-0,25274	1
Snapc4	-0,25282	1
Kbtbd8	-0,25285	1
Zfp39	-0,25305	1

Usp9x	-0,25303	1
4930432K21Rik	-0,25315	1
Rap2c	-0,25315	1
Dr1	-0,25322	1
Llgl1	-0,25334	1
Gm43088	-0,25342	1
Trub2	-0,25361	1
Fam114a1	-0,25364	1
Sec22b	-0,25359	1
6820402A03Rik	-0,25377	1
Ccdc82	-0,25384	1
Cep85	-0,25386	1
Klhdc1	-0,25394	1
Lpcat1	-0,25391	1
Herc3	-0,25414	1
Ufsp2	-0,25425	1
Gars	-0,2543	1
Gclm	-0,25453	1
Ddx19b	-0,25461	1
Wdr1	-0,25462	1
Txndc5	-0,25484	1
Yeats4	-0,2549	1
Crtc3	-0,25501	1
Rmi2	-0,25508	1
Alg13	-0,25518	1
Zfp429	-0,25544	1
Specc1	-0,25565	1
Gm43133	-0,25573	1
Maoa	-0,25571	1
Ctsf	-0,25583	1
Taf4b	-0,25588	1
Zfp414	-0,25592	1
Gm15853	-0,256	1
Gm45840	-0,25618	1
Kdm2a	-0,25624	1
BC049715	-0,25636	1
Svbp	-0,25652	1
Manf	-0,25655	1
Dnajc24	-0,2566	1
Rps6ka5	-0,25701	1
Aldoa	-0,257	1
Sf3a1	-0,25708	1
Psme3	-0,25731	1
Shc4	-0,25754	1
Ttc17	-0,25745	1
Eri3	-0,25763	1
Rpl32	-0,25766	1
Ghitm	-0,2577	1
Atxn7l3b	-0,25796	1
Rffl	-0,25812	1
Ppp4r2	-0,2581	1
Rit1	-0,25819	1

Taok3	-0,25838	1
Hnrnpdl	-0,25857	1
Ptbp3	-0,2588	1
Slc6a13	-0,25889	1
Pgd	-0,25888	1
Palb2	-0,25911	1
Wdr61	-0,25929	1
Slfn9	-0,25943	1
Gm9435	-0,25965	1
Rragc	-0,25973	1
Dhdh	-0,25977	1
2310068J16Rik	-0,26021	1
Gm15268	-0,26022	1
Nvl	-0,26023	1
Mir99ahg	-0,26032	1
Dock10	-0,26027	1
Uvrag	-0,26033	1
Asna1	-0,26043	1
Gm37105	-0,26056	1
Trim11	-0,2606	1
Qsox1	-0,26061	1
Nars	-0,26072	1
Zfc3h1	-0,2609	1
Ccdc88c	-0,26098	1
Map2k3	-0,26095	1
Aasdh	-0,26109	1
mt-Rnr2	-0,26128	1
Sec11a	-0,2613	1
Zxdc	-0,26147	1
Bcl2l13	-0,2615	1
Smim14	-0,26148	1
March9	-0,26145	1
Nat6	-0,26171	1
Pik3c3	-0,26184	1
Akap17b	-0,26185	1
Kcnc3	-0,26204	1
Nampt	-0,26202	1
Uhrf1bp1	-0,2621	1
Adprm	-0,26207	1
Gm13349	-0,26228	1
Rab23	-0,26242	1
Gm4924	-0,26247	1
Sh2b2	-0,26245	1
Nup214	-0,26257	1
Trim26	-0,26273	1
Vegfb	-0,26274	1
Fgf11	-0,26275	1
Bop1	-0,26285	1
Cxx1a	-0,26299	1
Tmem268	-0,26302	1
Farsb	-0,26301	1
Cep290	-0,26309	1

Trnt1	-0,26326	1
Clptm1	-0,26338	1
Exo5	-0,26351	1
Tmem184c	-0,26374	1
Spg7	-0,26385	1
Herc6	-0,26387	1
Isca1	-0,2639	1
Arl2bp	-0,26387	1
Lmbrd2	-0,264	1
Ppp1r7	-0,26456	1
B230377A18Rik	-0,26473	1
Rbl2	-0,26466	1
Zbtb7a	-0,2647	1
Rad9b	-0,26494	1
Rbm45	-0,26495	1
Rhog	-0,26494	1
Rexo1	-0,26486	1
B4galt5	-0,26486	1
Arhgef7	-0,26496	1
Rbm12b2	-0,26518	1
Thop1	-0,26521	1
Gm13998	-0,2655	1
Mlycd	-0,26558	1
Srpk2	-0,26569	1
Mfsd7b	-0,26584	1
Adprh	-0,26581	1
Mfsd14a	-0,2659	1
Ulk1	-0,26599	1
Ppfia1	-0,266	1
Arrb2	-0,26623	1
Lrrc73	-0,26634	1
Supt5	-0,26628	1
Ggh	-0,2664	1
Slc17a9	-0,26647	1
Pdss1	-0,26664	1
Uggt1	-0,26663	1
Ppp2r5b	-0,26667	1
Ncoa5	-0,26686	1
Ddx3y	-0,26686	1
Pdzk1ip1	-0,26707	1
Ldb1	-0,26719	1
Sod1	-0,26722	1
Zfp324	-0,26742	1
Asap1	-0,2676	1
Fancg	-0,26766	1
Vim	-0,26767	1
Tbcel	-0,26791	1
Herc4	-0,26798	1
Msh5	-0,26833	1
Agfg1	-0,26836	1
Cmip	-0,26875	1
Setd7	-0,26882	1

Mbd5	-0,26888	1
Ptcd2	-0,26895	1
Ccdc86	-0,26907	1
Stag2	-0,26914	1
Dcaf10	-0,26949	1
Ptcd3	-0,26969	1
Pitpnc1	-0,26965	1
Ints9	-0,26965	1
Mrpl58	-0,26971	1
Parp4	-0,26976	1
Bbip1	-0,26982	1
Med14	-0,26994	1
Chtf8	-0,2699	1
Tnks	-0,27023	1
Gm12059	-0,27027	1
Gdi1	-0,27032	1
Serac1	-0,27037	1
Pip5k1c	-0,27045	1
Nol9	-0,27054	1
Kctd13	-0,27064	1
Lmo4	-0,27067	1
Tada3	-0,27081	1
Slc16a9	-0,27086	1
Arhgef40	-0,27089	1
Rab33b	-0,27093	1
Yipf6	-0,27088	1
Dnmt3l	-0,27101	1
Eif2ak3	-0,27123	1
Bmp2k	-0,27116	1
Xpo7	-0,27173	1
Lpin2	-0,27179	1
Mir124-2hg	-0,27188	1
Pdk3	-0,27193	1
Twf2	-0,27201	1
Gse1	-0,27195	1
Ddx46	-0,27203	1
Mir142hg	-0,2723	1
Prss36	-0,27237	1
Cry1	-0,2724	1
Sarnp	-0,27253	1
Vps37a	-0,27251	1
Parp1	-0,27257	1
Tox2	-0,27266	1
Tmem106b	-0,27272	1
Rpn1	-0,27275	1
Dstn	-0,27292	1
Pmpca	-0,27304	1
Magi2	-0,27312	1
Qpctl	-0,27319	1
Tmem87b	-0,27318	1
Itpripl2	-0,27321	1
Prdx1	-0,2732	1

Ltv1	-0,27401	1
Usp46	-0,27402	1
Chtop	-0,27398	1
Mlx	-0,27428	1
Hnrnpul1	-0,27431	1
Prkaca	-0,2744	1
Gm20699	-0,27451	1
Zfp664	-0,27445	1
Tnks1bp1	-0,27464	1
Fbxw4	-0,27472	1
Dbp	-0,27492	1
Ankfy1	-0,27502	1
Phf1	-0,27498	1
Gm14776	-0,27513	1
Spa17	-0,27517	1
Usp28	-0,27518	1
Zfp605	-0,27536	1
Naip5	-0,27542	1
Edem1	-0,27543	1
Lrrc51	-0,27554	1
Atp5g2	-0,2757	1
Tnfrsf4	-0,27583	1
Snx15	-0,27581	1
Grk5	-0,27577	1
Prmt5	-0,27592	1
Sdr42e1	-0,27613	1
Tsg101-ps	-0,27632	1
Gm24507	-0,27631	1
Chd9	-0,27641	1
Tamm41	-0,27648	1
Casp3	-0,27682	1
Ptpra	-0,27697	1
Ipo4	-0,27703	1
Rasip1	-0,27711	1
Clstn1	-0,2771	1
Sigirr	-0,27731	1
Rabggta	-0,27733	1
Gm7102	-0,27742	1
Trp53bp1	-0,27755	1
RP23-442M18.5	-0,27767	1
Fbxo25	-0,27768	1
9030617O03Rik	-0,27767	1
Mapre3	-0,27782	1
Wdr19	-0,2778	1
Xylt1	-0,2783	1
Zzef1	-0,27825	1
Zfp810	-0,2785	1
Chd3	-0,27849	1
Gm9143	-0,27883	1
Ikbkb	-0,27878	1
Lhfpl2	-0,27877	1
Gm43336	-0,27914	1

Fcho2	-0,2792	1
Zswim1	-0,27941	1
Fam210b	-0,27963	1
Mphosph6	-0,27957	1
Gm12522	-0,27991	1
Calu	-0,27996	1
Nxpe3	-0,28009	1
Mccc1	-0,28029	1
Lmtk3	-0,28041	1
Uchl3	-0,28037	1
Lrmp	-0,28037	1
Arnt	-0,28035	1
Mtmr9	-0,2806	1
Fam98a	-0,28062	1
Mcph1	-0,28061	1
B230208H11Rik	-0,28083	1
Tm9sf2	-0,28101	1
Zfp608	-0,28111	1
1700124L16Rik	-0,28105	1
Fam234a	-0,28118	1
Ddx24	-0,28129	1
Abcb9	-0,28149	1
Srprb	-0,28158	1
Gm12791	-0,28169	1
Capza1	-0,28169	1
Nln	-0,28167	1
Ccnk	-0,28169	1
Dtx4	-0,28174	1
Thoc2	-0,28171	1
Tmem127	-0,28185	1
Arhgap5	-0,28188	1
Ifitm3	-0,28188	1
Gm44103	-0,28201	1
Hnrnpa0	-0,28214	1
A830080D01Rik	-0,2822	1
Tcf25	-0,28223	1
Gucd1	-0,28229	1
Gm5624	-0,28256	1
Heatr5a	-0,28257	1
Uso1	-0,28256	1
4833420G17Rik	-0,28275	1
Soat2	-0,28281	1
Arf4	-0,28277	1
1700086O06Rik	-0,28285	1
Coq8b	-0,28287	1
Anapc1	-0,28285	1
Tlk1	-0,28295	1
Eps15	-0,28295	1
Rgs12	-0,2831	1
Psmg2	-0,28313	1
Urb1	-0,28333	1
Fam98b	-0,28335	1

Atp2b1	-0,28344	1
Snx19	-0,2837	1
Ube2d3	-0,28415	1
Pramef8	-0,28433	1
Ints6l	-0,28449	1
Ptdss1	-0,28446	1
Aldh3a2	-0,28457	1
Slfn5	-0,28466	1
Ttyh2	-0,28466	1
Por	-0,28469	1
Fam114a2	-0,28482	1
Eef1akmt1	-0,2849	1
Zdhhc6	-0,28485	1
Ap1ar	-0,28489	1
Sirt4	-0,28502	1
Il15ra	-0,28504	1
Mak16	-0,28533	1
Lman1	-0,28536	1
Dtx3l	-0,28545	1
Acad9	-0,28554	1
Slx4ip	-0,28563	1
Aste1	-0,28594	1
Sec61b	-0,28603	1
Trmt44	-0,28606	1
Cltc	-0,28612	1
Fastk	-0,28618	1
Ppp6r1	-0,28631	1
Mgat2	-0,28644	1
Pi4k2b	-0,28661	1
Gm38247	-0,28687	1
Tlk2	-0,28692	1
Aatk	-0,28698	1
Mplkip	-0,28696	1
Dph7	-0,2872	1
Dctd	-0,2873	1
Fam129b	-0,28755	1
Exoc3l4	-0,28768	1
Shcbp1l	-0,28766	1
Gm42937	-0,28785	1
Nudt16	-0,28798	1
Irf3	-0,28817	1
Paox	-0,28821	1
Zbtb33	-0,28828	1
Exosc7	-0,28835	1
Cyp4f13	-0,28848	1
Nfya	-0,28849	1
6030400A10Rik	-0,28864	1
Rilpl2	-0,28863	1
Smarca4	-0,2886	1
Cryl1	-0,28891	1
Bbs9	-0,28886	1
Dcaf15	-0,28888	1

Glyr1	-0,28885	1
Isl2	-0,28901	1
Ldlr	-0,28911	1
Rrp15	-0,28923	1
Gm44699	-0,28929	1
Zdhhc4	-0,28939	1
Zc3h7b	-0,2895	1
Elp6	-0,28957	1
Dgcr14	-0,28957	1
Dennd6a	-0,28963	1
Slc5a3	-0,28985	1
Cenpj	-0,28998	1
Fkrp	-0,28996	1
Hibadh	-0,29004	1
Pxk	-0,29014	1
Kif3b	-0,29006	1
Tlr4	-0,29025	1
Ddit3	-0,29047	1
Crybg3	-0,29062	1
Txnrd3	-0,29074	1
A930004J17Rik	-0,29077	1
Thrap3	-0,29084	1
Trim12c	-0,29102	1
Snord15a	-0,29144	1
Lgi4	-0,2915	1
Rnf8	-0,29195	1
Rps6kb1	-0,2921	1
Mtg2	-0,29224	1
Cog6	-0,29232	1
Actl6a	-0,29249	1
Them4	-0,29275	1
Ptafr	-0,29302	1
Cd151	-0,29302	1
Agmo	-0,29305	1
March7	-0,29297	1
Nipa1	-0,29318	1
Brd2	-0,29335	1
Rnf219	-0,2934	1
Dkc1	-0,29368	1
Zfp809	-0,29392	1
Klhl40	-0,29411	1
Taok1	-0,2941	1
Pan3	-0,29426	1
Dctn6	-0,2946	1
Ralgapb	-0,29471	1
Cdr2	-0,29491	1
Zfp449	-0,29502	1
Zc3h11a	-0,29503	1
Gm37140	-0,29498	1
Rbm15b	-0,29503	1
Gpalpp1	-0,29521	1
Gm43411	-0,29558	1

Rufy1	-0,29556	1
Sec24c	-0,29557	1
Tmem248	-0,29568	1
Msl3	-0,29597	1
Gan	-0,29603	1
Commd10	-0,29645	1
Stxbp4	-0,29661	1
Ears2	-0,2967	1
Ankrd10	-0,29682	1
Supt3	-0,29677	1
Vma21-ps	-0,29702	1
Arfgap2	-0,29717	1
Gm45222	-0,2972	1
Acsl5	-0,29716	1
Gm7860	-0,29735	1
Ercc3	-0,2975	1
Synj2	-0,29756	1
Gm29593	-0,29779	1
Phc1	-0,2978	1
Cdk13	-0,29798	1
Exoc2	-0,29819	1
Smarcd2	-0,29818	1
Yod1	-0,29832	1
Arl6ip5	-0,29835	1
Lrrc28	-0,2985	1
Tbl1xr1	-0,29845	1
Slc25a13	-0,29849	1
Wnk1	-0,29852	1
Ybx3	-0,29853	1
Tbck	-0,29861	1
Pex3	-0,29875	1
Psma5	-0,29883	1
Dcaf17	-0,29903	1
Kdm5c	-0,29898	1
1700025G04Rik	-0,29912	1
Prmt1	-0,29932	1
Tmem57	-0,29942	1
Fam57a	-0,29959	1
Ube2i	-0,29959	1
Rpain	-0,2997	1
Uqcrh	-0,29968	1
Tspyl3	-0,29979	1
Xpnpep3	-0,29978	1
Rpl10	-0,29985	1
Lcorl	-0,29985	1
Ranbp1	-0,3	1
Ddb1	-0,3	1
Apobr	-0,30015	1
Rbpms	-0,30024	1
Cog7	-0,30026	1
Dap3	-0,30039	1
Aaed1	-0,30057	1

3830406C13Rik	-0,30059	1
Txndc11	-0,30078	1
Slc39a2	-0,30107	1
Ube2o	-0,30121	1
Glmp	-0,30122	1
Ttc14	-0,3012	1
Tln1	-0,30129	1
Lrp8	-0,30139	1
Ipo13	-0,30148	1
Zfp212	-0,30168	1
Nars2	-0,30167	1
Gm15696	-0,30182	1
Tarbp1	-0,30177	1
Hexim1	-0,30179	1
Gm15644	-0,30198	1
Usp15	-0,30211	1
Hsd3b7	-0,30219	1
9230114K14Rik	-0,30216	1
Lrrc1	-0,30259	1
Hip1	-0,30273	1
Nfia	-0,30267	1
Fads1	-0,30309	1
Psd2	-0,30316	1
Fig4	-0,30354	1
Slx4	-0,30354	1
Usp34	-0,30349	1
Psap	-0,30381	1
Aph1c	-0,30381	1
Pnrc1	-0,3038	1
Rad50	-0,30392	1
Hectd1	-0,30391	1
Nbeal2	-0,30406	1
Pank3	-0,30409	1
Snip1	-0,30435	1
Gm7846	-0,30439	1
Il23a	-0,30481	1
Gm37606	-0,30491	1
Ubqln4	-0,30488	1
Ubxn7	-0,305	1
Casp2	-0,30527	1
Entpd7	-0,30542	1
Ankrd13a	-0,30542	1
Fabp5	-0,30569	1
Zcchc2	-0,30569	1
Rab35	-0,30578	1
Ctdnep1	-0,30603	1
Aldh5a1	-0,3062	1
Ndufaf7	-0,30626	1
Cfap36	-0,3064	1
Gm19325	-0,30684	1
Itm2c	-0,30692	1
Parvb	-0,30695	1

Ints12	-0,30705	1
Naaa	-0,30731	1
Gatm	-0,30725	1
Rhod	-0,30727	1
Ppm1b	-0,30736	1
Clybl	-0,30796	1
Gsto1	-0,30812	1
Gtf2h2	-0,3083	1
Rtf1	-0,3083	1
Ccr1	-0,30885	1
Hnrnpab	-0,30916	1
Cyb5r1	-0,30934	1
Mfap1b	-0,3094	1
Fam160b1	-0,30956	1
Gm24876	-0,30971	1
Tbc1d19	-0,30982	1
Clk1	-0,30979	1
Col4a3bp	-0,30993	1
Ciz1	-0,30994	1
Tbxas1	-0,31008	1
Gm37738	-0,31018	1
Sf3a3	-0,31024	1
Klf8	-0,31048	1
6720475M21Rik	-0,31064	1
Hspbp1	-0,31064	1
Ndufc2	-0,3107	1
a	-0,3108	1
Elovl6	-0,31077	1
Grk2	-0,31089	1
Chmp4b	-0,3111	1
Fancl	-0,31117	1
Taf15	-0,31133	1
Hdac7	-0,31158	1
Chd4	-0,31181	1
Clcn3	-0,31193	1
Acp1	-0,31203	1
Ston1	-0,31201	1
Gm45343	-0,31214	1
Fosb	-0,31214	1
Ctr9	-0,31222	1
AI480526	-0,31236	1
Aldh3b1	-0,31238	1
1110025M09Rik	-0,31251	1
Gm4262	-0,31257	1
Trim68	-0,3126	1
Mylpf	-0,31292	1
Arhgef11	-0,31303	1
Wdfy3	-0,31304	1
Bcap29	-0,31297	1
Jmjd1c	-0,313	1
Srgap3	-0,31301	1
Slc35c1	-0,31306	1

Uevld	-0,31354	1
Srf	-0,31355	1
Otud7b	-0,31355	1
Mum1	-0,3139	1
Kcnj2	-0,31398	1
Fbxo28	-0,31405	1
Map3k20	-0,31438	1
Larp4b	-0,3145	1
Gm13416	-0,31477	1
Ano10	-0,31481	1
1300002E11Rik	-0,31503	1
Gm4875	-0,31497	1
Odf2l	-0,31498	1
Ethe1	-0,31525	1
Tmem185a	-0,31526	1
Nom1	-0,31533	1
Foxj2	-0,3153	1
Gosr1	-0,31551	1
Strn4	-0,31546	1
Cog5	-0,31554	1
Vamp7	-0,31566	1
Il10rb	-0,31574	1
Cass4	-0,31587	1
Cradd	-0,31623	1
Spata5	-0,31624	1
Gm37677	-0,31665	1
Lyn	-0,31679	1
Ube3a	-0,31718	1
Ssu72	-0,31734	1
Wdr47	-0,31739	1
Lamtor2	-0,3176	1
Dgka	-0,3177	1
Usp22	-0,31769	1
Megf8	-0,31784	1
Fam83h	-0,31789	1
Hoxb5	-0,31787	1
Tcf19	-0,318	1
Gm13675	-0,31816	1
Gm44822	-0,31818	1
Exosc1	-0,31821	1
Spata13	-0,31824	1
Snap47	-0,31831	1
Adcy9	-0,31834	1
Med17	-0,31831	1
Ube3b	-0,31845	1
Eif4a3	-0,31835	1
Sft2d2	-0,31842	1
Foxp4	-0,31847	1
Ap1m1	-0,31863	1
Cant1	-0,31863	1
A530013C23Rik	-0,31875	1
Ldah	-0,31882	1

Apc	-0,31948	1
Cd14	-0,31948	1
Plekhm2	-0,31952	1
Tnpo2	-0,3195	1
Rab28	-0,31956	1
Rbm12b1	-0,31976	1
Sergef	-0,31979	1
Rgp1	-0,31997	1
Gpatch4	-0,32011	1
Rhot2	-0,32019	1
Trib3	-0,32022	1
Eif3d	-0,32052	1
Nrip1	-0,32075	1
Pxn	-0,32066	1
Gipc2	-0,32077	1
Tbc1d13	-0,32076	1
Plgrkt	-0,32087	1
Sema6b	-0,32098	1
Cops7b	-0,32119	1
Fbxl6	-0,32131	1
Rbbp5	-0,32134	1
Zfp729b	-0,32139	1
Gm42511	-0,32158	1
Ripk1	-0,32162	1
Cask	-0,32157	1
Mief1	-0,3216	1
Atf6b	-0,32156	1
Gm37399	-0,32187	1
Hipk1	-0,322	1
Scmh1	-0,3223	1
Gm3550	-0,32238	1
Sepsecs	-0,32235	1
Sh3bgrl2	-0,3227	1
Cep135	-0,32298	1
Mpeg1	-0,32352	1
Rpl23a-ps2	-0,3236	1
Uba2	-0,32366	1
Pianp	-0,32375	1
Hdac4	-0,32414	1
Gm10499	-0,3242	1
Adar	-0,32424	1
Arl5c	-0,3243	1
Sf3b3	-0,32431	1
Gon4l	-0,3249	1
Tm2d1	-0,32501	1
Rab11fip2	-0,32509	1
B930086L07Rik	-0,32522	1
Mon1b	-0,32519	1
Rfwd2	-0,32524	1
Slc35e1	-0,32525	1
Slc39a1	-0,32524	1
Mov10	-0,32528	1

Tepsin	-0,32527	1
Nfkbia	-0,32538	1
Ncor1	-0,32549	1
Cenpc1	-0,32556	1
Nfic	-0,32561	1
Jmjd7	-0,32578	1
Bcas3	-0,32598	1
Cln6	-0,326	1
Ppp3r1	-0,32605	1
AI506816	-0,32626	1
Bcl2a1b	-0,32641	1
Ets2	-0,32638	1
Mtmr6	-0,32646	1
Fam213b	-0,32658	1
Ermp1	-0,32689	1
Fndc3b	-0,32704	1
Specc1l	-0,32708	1
Gm20667	-0,32717	1
Frmd6	-0,32722	1
Mpp1	-0,32723	1
Cyth4	-0,32721	1
Med13l	-0,32721	1
Elk4	-0,32737	1
Zfp62	-0,32759	1
Socs6	-0,32755	1
Tmcc2	-0,32767	1
Cep131	-0,32783	1
Dnmt3a	-0,32808	1
Pros1	-0,32837	1
Zfp933	-0,32846	1
Akap9	-0,32864	1
Wdr82	-0,32881	1
Ccndbp1	-0,32894	1
Phf3	-0,32899	1
Cdca7l	-0,32907	1
Efcab14	-0,32912	1
Spsb1	-0,32916	1
Tubg1	-0,32921	1
Chrnb2	-0,32934	1
Slc9a3r2	-0,32931	1
Cldn12	-0,32979	1
Tk2	-0,32983	1
Ccdc28a	-0,33023	1
Gm7967	-0,33026	1
Plxnc1	-0,33026	1
Ep400	-0,33031	1
Smyd2	-0,33074	1
Ifi204	-0,33078	1
Ptpn21	-0,33095	1
Lipa	-0,33094	1
Rac2	-0,33115	1
Tpgs1	-0,33143	1

Ptbp2	-0,33146	1
Rbm26	-0,33153	1
Psme4	-0,3315	1
9530053A07Rik	-0,3318	1
Ccr2	-0,33181	1
A630033H20Rik	-0,33201	1
Prr36	-0,33212	1
Hcls1	-0,33208	1
Coq5	-0,33242	1
Gm45802	-0,33252	1
Agpat2	-0,33264	1
Map4k3	-0,33261	1
Dscr3	-0,33269	1
Amfr	-0,33288	1
Slc25a44	-0,33305	1
Sec23a	-0,33323	1
Ssh3	-0,3333	1
Grk4	-0,33337	1
Trpc4ap	-0,33342	1
mt-Tt	-0,33348	1
Trappc8	-0,33366	1
Bmpr2	-0,33374	1
Vps39	-0,33366	1
Myo5a	-0,33385	1
Iqgap1	-0,33392	1
Ptpn12	-0,33388	1
Fam131a	-0,33399	1
Gm13270	-0,33396	1
Map3k4	-0,33404	1
Slc35a3	-0,33402	1
Gm44187	-0,3343	1
Efr3a	-0,33447	1
Ncdn	-0,33462	1
Tpm1	-0,33464	1
Rapgef2	-0,33478	1
Pfn2	-0,33488	1
Enox2	-0,33488	1
Gm10698	-0,33533	1
Got2	-0,33534	1
Rbpj	-0,33527	1
Phc3	-0,33526	1
Hmgb1-rs16	-0,33548	1
Ap2s1	-0,33569	1
Gm8228	-0,33579	1
Rtfdc1	-0,33584	1
Mtpap	-0,33587	1
Cds2	-0,33587	1
Noc4l	-0,33601	1
Parp3	-0,33615	1
6430571L13Rik	-0,33642	1
Gm3608	-0,33658	1
Shmt2	-0,33656	1

H2afy	-0,3367	1
Zmat3	-0,33679	1
Zdhhc7	-0,33688	1
2310009A05Rik	-0,33706	1
Aftph	-0,33721	1
Ophn1	-0,33726	1
Spcs3	-0,33727	1
C1galt1c1	-0,33754	1
Letm1	-0,33767	1
Akt1s1	-0,3379	1
Pnkp	-0,33794	1
Coq10a	-0,33792	1
Gm33370	-0,338	1
Bclaf1	-0,338	1
Psmg1	-0,33813	1
Lgr4	-0,33819	1
Surf2	-0,33818	1
Lsr	-0,33838	1
Mtfr1l	-0,33836	1
Pced1a	-0,33851	1
Tubgcp2	-0,33898	1
Aifm2	-0,3392	1
Dnajc12	-0,33946	1
Vegfa	-0,33952	1
Glud1	-0,33952	1
Tmem91	-0,33975	1
Zkscan17	-0,33983	1
3300005D01Rik	-0,34004	1
Taf1	-0,34	1
6430548M08Rik	-0,34018	1
Cdc42ep2	-0,34032	1
Usp40	-0,34026	1
6330403L08Rik	-0,34037	1
A430035B10Rik	-0,3405	1
B3galnt2	-0,34047	1
Vmp1	-0,34053	1
Dnajb5	-0,34056	1
Oas1c	-0,34066	1
Il12rb1	-0,34094	1
Ergic2	-0,34099	1
Pigu	-0,34109	1
Olfr920	-0,34112	1
Slc12a9	-0,34133	1
Abcc5	-0,34145	1
Tmem245	-0,34136	1
Gm8770	-0,34155	1
Psmd2	-0,34159	1
Map3k3	-0,34168	1
Gm23100	-0,34188	1
Scamp5	-0,34212	1
Rab7	-0,3421	1
4933417C20Rik	-0,34233	1

Zbtb1	-0,34233	1
Tsn	-0,34228	1
Cysltr1	-0,34241	1
Tmem260	-0,34262	1
Rtkn	-0,3426	1
Zfp956	-0,34272	1
Snai1	-0,34285	1
Ier3	-0,34277	1
Eprs	-0,34286	1
Lsm4	-0,34308	1
Pex2	-0,34317	1
Lrrc25	-0,34321	1
Ccdc93	-0,34319	1
Ankhd1	-0,34321	1
Rps6	-0,34342	1
Mmp19	-0,34361	1
Adap2	-0,34372	1
Dnmbp	-0,34393	1
Rps13-ps1	-0,34397	1
Cluap1	-0,34434	1
Rnf130	-0,3445	1
Cct3	-0,34511	1
Dhx35	-0,34523	1
Morc4	-0,34537	1
Gm14698	-0,34553	1
Knl1	-0,34557	1
A530017D24Rik	-0,3457	1
Pomt1	-0,34588	1
Tarsl2	-0,34684	1
Clock	-0,34712	1
Pld3	-0,34713	1
Dlg1	-0,34726	1
Gm45266	-0,34738	1
Gm22581	-0,34744	1
Stk4	-0,34748	1
Ulk2	-0,34755	1
Prps1l3	-0,3477	1
Gm42480	-0,34768	1
Arhgap9	-0,34767	1
Nol10	-0,34789	1
Trmt6	-0,34803	1
Herc1	-0,34805	1
Gtf3c1	-0,34806	1
Zdhhc3	-0,34808	1
Poc1a	-0,34823	1
Zxdb	-0,34818	1
Cep89	-0,34835	1
Vps26b	-0,3485	1
Ppm1h	-0,34901	1
Chpf2	-0,34903	1
Slc4a7	-0,34898	1
Ogfod1	-0,34914	1

Rpp40	-0,34918	1
Gm12924	-0,34942	1
Slfn4	-0,34955	1
Ulk3	-0,34984	1
Atp5g3	-0,34994	1
St3gal3	-0,35003	1
Cisd2	-0,35033	1
Map2k4	-0,35051	1
Tmem259	-0,35051	1
Znrf2	-0,35063	1
Acin1	-0,35077	1
Batf2	-0,35092	1
Gm38009	-0,35127	1
Phf10	-0,35149	1
Gmeb1	-0,35161	1
Cdk12	-0,3516	1
Pcmtd1	-0,35172	1
Il4ra	-0,35194	1
Vrk1	-0,35188	1
Hspa4l	-0,35189	1
Gm16638	-0,35204	1
Gpr180	-0,35197	1
Mcu	-0,35223	1
Zmynd11	-0,35229	1
Gm37706	-0,3526	1
Wasf2	-0,3527	1
Ccne1	-0,35279	1
Six4	-0,35305	1
Tsc22d4	-0,35296	1
Klhl21	-0,35295	1
Uxs1	-0,35309	1
Scrib	-0,35325	1
Pias2	-0,35318	1
Gm8522	-0,35326	1
Zmym5	-0,35328	1
Zc3h18	-0,35338	1
Gm11764	-0,35355	1
Stx4a	-0,35362	1
Pdzd8	-0,35366	1
Hsf1	-0,35384	1
Dmrta2	-0,3541	1
Ddx58	-0,35413	1
Zbtb41	-0,35413	1
Gtf2h1	-0,35432	1
Pskh1	-0,35433	1
Myo9a	-0,35435	1
Gga1	-0,35436	1
A430110C17Rik	-0,35452	1
Exd1	-0,3546	1
Mfn1	-0,35468	1
Safb	-0,35474	1
Dhx16	-0,35477	1

Mpv17	-0,35478	1
Pik3c2a	-0,35484	1
Mfhas1	-0,35489	1
Klf13	-0,35505	1
Scarna17	-0,35524	1
Gm6162	-0,35534	1
Gps2	-0,35575	1
Lat2	-0,35602	1
Gm42979	-0,35615	1
Nr4a2	-0,35622	1
Uty	-0,35654	1
Tmem156	-0,3567	1
Klhl12	-0,35673	1
Sec23ip	-0,35668	1
Smim10l1	-0,35686	1
Hells	-0,35695	1
Abhd11	-0,35688	1
Wdr45	-0,35714	1
Ociad1	-0,35707	1
RP24-325P4.5	-0,35716	1
Btd	-0,35729	1
Trim2	-0,35732	1
Kdelc1	-0,35735	1
Cetn4	-0,35752	1
Gm43692	-0,35749	1
Asb7	-0,35748	1
Eif3b	-0,35745	1
Cdkl2	-0,3576	1
Irgm2	-0,35782	1
Ric1	-0,35785	1
Zfp207	-0,35781	1
Bccip	-0,35806	1
Pex16	-0,35819	1
Cyp20a1	-0,3582	1
5530601H04Rik	-0,3585	1
Gtf3c2	-0,35846	1
Kat5	-0,35858	1
Man2b1	-0,35904	1
Hdhd2	-0,35914	1
9530082P21Rik	-0,35964	1
Fam3a	-0,35991	1
Nelfcd	-0,36007	1
Lrp1	-0,36025	1
Prickle2	-0,36059	1
Stat1	-0,36082	1
Ccni	-0,3609	1
Cenpq	-0,36098	1
Mdh2	-0,36105	1
Ube2v2	-0,36124	1
Gm20342	-0,36122	1
Adgrl2	-0,36155	1
Rbms2	-0,36147	1

Mta1	-0,36165	1
Cpeb3	-0,36155	1
Wapl	-0,36164	1
AA414768	-0,36194	1
Ttc33	-0,36186	1
Oas3	-0,36199	1
Rabif	-0,36215	1
Klhl20	-0,36208	1
Trappc9	-0,36223	1
Rnpep	-0,36217	1
Cdon	-0,36228	1
Arid1a	-0,36285	1
Kti12	-0,36289	1
C330018D20Rik	-0,36328	1
Tulp3	-0,36332	1
Gm13868	-0,36335	1
Zfp110	-0,36354	1
Rpp30	-0,3636	1
Gltscr1l	-0,36382	1
Zfp456	-0,36424	1
Gm28192	-0,36443	1
Gm8550	-0,36453	1
Pkn1	-0,36445	1
Kmt2e	-0,36457	1
As3mt	-0,36478	1
Zfp287	-0,36484	1
Dusp22	-0,36479	1
Zfp451	-0,36475	1
Trim35	-0,36488	1
Tpp2	-0,36496	1
Trim56	-0,36501	1
Cluh	-0,36495	1
Cenpv	-0,36526	1
Atg10	-0,36553	1
Mast2	-0,36572	1
Dgcr2	-0,36573	1
1700088E04Rik	-0,36583	1
Adat1	-0,36575	1
Akna	-0,36584	1
Ppcdc	-0,36593	1
Sdha	-0,36612	1
Slc35a5	-0,36616	1
Lncpint	-0,36642	1
Erlin2	-0,36645	1
D3Ertd751e	-0,36651	1
Agbl3	-0,3665	1
Prkar2b	-0,36661	1
Dennd2a	-0,36663	1
Rai1	-0,36669	1
Gm37558	-0,36696	1
Ncl	-0,36715	1
Mus81	-0,36727	1

Ralgds	-0,36727	1
Dnajc30	-0,36744	1
Malat1	-0,36744	1
Amd2	-0,3675	1
AW549877	-0,36754	1
Gpatch8	-0,36749	1
Lrp4	-0,36773	1
Klhl8	-0,36783	1
Zfp950	-0,36795	1
Rxrb	-0,36785	1
D3Ertd254e	-0,36815	1
Pcid2	-0,36842	1
Usp45	-0,36862	1
Epm2aip1	-0,36864	1
Cog8	-0,36916	1
Zfp687	-0,36954	1
Prkaa1	-0,36952	1
Gm37510	-0,36975	1
Zfp667	-0,36977	1
Sec23b	-0,36996	1
Adck1	-0,37015	1
Uros	-0,37028	1
Gm12089	-0,37036	1
Gnl3l	-0,37043	1
Npr1	-0,37062	1
Polr3e	-0,37061	1
Fhl3	-0,37061	1
Kif13a	-0,37071	1
Mapk7	-0,37074	1
Casp7	-0,37094	1
Ostm1	-0,37091	1
Asxl1	-0,37095	1
Gm43848	-0,37106	1
Rbbp9	-0,37117	1
Iars2	-0,3714	1
Ndufs1	-0,37135	1
Gm16540	-0,37148	1
Otub2	-0,37153	1
Med15	-0,37155	1
Tmem39b	-0,37171	1
Add1	-0,37171	1
Ncbp1	-0,37169	1
Itm2b	-0,37181	1
Saa3	-0,37193	1
Senp2	-0,37186	1
Myo9b	-0,37217	1
Nat10	-0,37222	1
Gm42890	-0,37241	1
Arglu1	-0,37235	1
C1rl	-0,37248	1
Cox5b	-0,37265	1
Cyb5r4	-0,37263	1

Klhl9	-0,37256	1
Cstad	-0,37285	1
Fem1a	-0,37294	1
Cmtr1	-0,373	1
Klhl36	-0,37331	1
Hibch	-0,37329	1
Rad54l2	-0,37341	1
Zfp260	-0,37361	1
Etohd2	-0,37371	1
Utp20	-0,37378	1
Lrrc49	-0,37404	1
Tmem237	-0,37422	1
Serbp1	-0,37418	1
Kdelr1	-0,37417	1
Zfp780b	-0,37443	1
Snapc3	-0,37464	1
Rps6ka1	-0,37462	1
Uchl4	-0,37472	1
1110059E24Rik	-0,37466	1
Nrd1	-0,3752	1
Hs2st1	-0,37539	1
Trps1	-0,37553	1
Slain2	-0,37548	1
Nckipsd	-0,37565	1
Evi5	-0,37586	1
Gm27029	-0,37614	1
Abca3	-0,37624	1
Gm43178	-0,37625	1
Ascc2	-0,3763	1
Zfp317	-0,37626	1
Asah2	-0,37653	1
Unk	-0,37661	1
Atf5	-0,37658	1
Madd	-0,37686	1
Efl1	-0,37688	1
D730045B01Rik	-0,37728	1
Gm16845	-0,37728	1
Agpat1	-0,3773	1
Cdk8	-0,37741	1
Mfsd6	-0,37735	1
Cand1	-0,37742	1
Cpsf7	-0,37772	1
Lair1	-0,37776	1
Zfp846	-0,37799	1
Zfp771	-0,37807	1
Fnip1	-0,37824	1
Tfdp2	-0,3786	1
Fam188a	-0,37862	1
0610009B22Rik	-0,37867	1
Gm17971	-0,37881	1
Gm43513	-0,37892	1
Rcan1	-0,37902	1

Zfp341	-0,37952	1
Mtus2	-0,37945	1
Btbd9	-0,37954	1
Apoo-ps	-0,37957	1
Xpo6	-0,37961	1
Prps2	-0,37956	1
Gm7299	-0,38018	1
Trap1	-0,3802	1
Bok	-0,38033	1
Acat2	-0,38034	1
Spop	-0,38026	1
Gm15903	-0,3804	1
Zdhhc8	-0,38037	1
Naglu	-0,38035	1
Fbxo4	-0,38056	1
Pla2g5	-0,3806	1
Pitpna	-0,38062	1
Hdgfrp2	-0,38071	1
Rif1	-0,38113	1
Cpsf4	-0,38119	1
Gm14328	-0,38149	1
1700066M21Rik	-0,38174	1
Gbf1	-0,38179	1
Ythdf3	-0,38209	1
Was	-0,38234	1
Syvn1	-0,38282	1
Far1	-0,38279	1
Abcf1	-0,38282	1
Cars2	-0,38297	1
Marcksl1	-0,38302	1
Commd4	-0,38312	1
Prdm2	-0,38338	1
Mettl21a	-0,38343	1
Arl6	-0,38348	1
Nop2	-0,38359	1
Gm37490	-0,38403	1
Brca2	-0,38451	1
Homer1	-0,38465	1
Dram2	-0,38474	1
Wdr81	-0,38472	1
Bcl10	-0,38479	1
Pus7	-0,3848	1
Fbxl4	-0,3849	1
Ncbp3	-0,38494	1
9130011E15Rik	-0,38499	1
Ctsl	-0,38507	1
Stub1	-0,38523	1
Gm3650	-0,3853	1
Dtd1	-0,38529	1
Tnrc6a	-0,38545	1
RP24-84O13.9	-0,38557	1
Gnaq	-0,38556	1

Dgkq	-0,38566	1
Nsun5	-0,38576	1
Alg11	-0,38579	1
Tnks2	-0,38601	1
Rab11fip3	-0,38636	1
Rnf111	-0,38637	1
Klf9	-0,38639	1
Aco1	-0,38653	1
Plcg1	-0,38663	1
Golim4	-0,38658	1
Smg6	-0,38675	1
Ercc6l2	-0,38701	1
Hcfc1	-0,38733	1
Gmfb	-0,3873	1
D930015E06Rik	-0,38758	1
Vav3	-0,38774	1
B630019K06Rik	-0,38823	1
Reps1	-0,38823	1
Sptan1	-0,38823	1
Cbfb	-0,38821	1
U2surp	-0,38827	1
Sppl3	-0,38836	1
Endog	-0,38864	1
Ppp3ca	-0,38868	1
Ly6e	-0,38867	1
Gm16523	-0,38885	1
Med20	-0,3889	1
Mthfd1	-0,38888	1
RP24-496O17.7	-0,38917	1
Atp1a3	-0,38957	1
H1fx	-0,38989	1
Tbc1d14	-0,39005	1
Mrip-ps	-0,39015	1
Vamp4	-0,39024	1
Prkci	-0,39028	1
Prkar2a	-0,39047	1
Rprd2	-0,39051	1
Gm10425	-0,39067	1
Def8	-0,39069	1
Usp16	-0,39069	1
Snta1	-0,39077	1
Csf2ra	-0,39088	1
Cdk6	-0,39111	1
Zdhhc17	-0,39126	1
AU022252	-0,39158	1
Gm42535	-0,39167	1
Rad54b	-0,39197	1
Appl1	-0,39198	1
Jagn1	-0,39227	1
Cdc42bpg	-0,39227	1
Fam102b	-0,3923	1
Ice1	-0,39235	1

Ppp1r21	-0,39263	1
Rev3l	-0,39274	1
Prrc2b	-0,39271	1
Trip4	-0,39303	1
Zbtb21	-0,39313	1
Usp19	-0,39324	1
Mrgpre	-0,39329	1
Herpud2	-0,39337	1
Gm37084	-0,39352	1
Nup107	-0,39356	1
Mpv17l	-0,39364	1
Srpk1	-0,39363	1
Swi5	-0,39369	1
Ssr1	-0,39369	1
Olfm1	-0,39379	1
Dcaf7	-0,39376	1
Tvp23b	-0,39392	1
Eme1	-0,39415	1
Ccdc71	-0,39412	1
Tnrc6b	-0,3941	1
Setx	-0,39419	1
Nr4a1	-0,39434	1
Vcp	-0,39429	1
Rpl7a-ps5	-0,39445	1
Lig3	-0,39462	1
Cnot1	-0,39472	1
Cpd	-0,39486	1
Cep170b	-0,39511	1
Adal	-0,39529	1
A430033K04Rik	-0,39549	1
9330151L19Rik	-0,39556	1
Ppargc1b	-0,39556	1
Lrrc59	-0,39582	1
Rab10	-0,39581	1
Phf21a	-0,39585	1
Csnk1a1	-0,39614	1
Alkbh3	-0,3963	1
Ap5s1	-0,39661	1
Tap2	-0,39693	1
Aaas	-0,39693	1
Gm37116	-0,39712	1
Ttc19	-0,39714	1
Pus3	-0,39722	1
Cyb561	-0,39751	1
Gm12940	-0,39757	1
Etv1	-0,39766	1
Ninj1	-0,39789	1
Hgh1	-0,39803	1
Pomt2	-0,39809	1
AW209491	-0,39824	1
Slc22a4	-0,39833	1
Mvd	-0,39858	1

Slx1b	-0,39887	1
Gm23722	-0,39899	1
Nol11	-0,39897	1
Gpatch1	-0,39923	1
Tmem238	-0,3993	1
Atm	-0,39937	1
Arcn1	-0,39969	1
Rpl7l1	-0,39972	1
Rnf5	-0,39978	1
Rmdn1	-0,39979	1
Pccb	-0,3999	1
Fam117a	-0,40011	1
Smurf1	-0,40028	1
Tpk1	-0,40029	1
2310015A10Rik	-0,40052	1
Skil	-0,40049	1
Smarcc1	-0,40047	1
Gm6565	-0,40058	1
Diablo	-0,40058	1
Ube2l3	-0,40055	1
Gpr107	-0,40065	1
Fam185a	-0,40089	1
Acap2	-0,40099	1
Zkscan4	-0,40121	1
C1ra	-0,40156	1
Bpnt1	-0,40184	1
Klc1	-0,40184	1
Sema4g	-0,40187	1
Trabd	-0,40193	1
Plxnb2	-0,40211	1
Tmem140	-0,40217	1
Mier2	-0,40235	1
Ankrd33b	-0,40243	1
Ankrd50	-0,40254	1
Tm4sf5	-0,4026	1
Iba57	-0,40272	1
Rasa2	-0,40273	1
Atrx	-0,40271	1
Zfp418	-0,4028	1
Mertk	-0,40325	1
Zfp770	-0,40326	1
Mtap	-0,4033	1
Plpp5	-0,40337	1
Ttc39a	-0,40365	1
B3glct	-0,4036	1
Zfp12	-0,40373	1
Gm45534	-0,40414	1
Camsap1	-0,40407	1
Tctex1d2	-0,40414	1
Lrrc14	-0,4042	1
Map2k5	-0,40425	1
Fer	-0,40452	1

Pgap2	-0,40448	1
Dhx34	-0,40472	1
Capn7	-0,40476	1
Endov	-0,40489	1
Dcaf6	-0,40493	1
Ipo9	-0,40504	1
Stambp	-0,40511	1
Nmnat1	-0,40522	1
Wwox	-0,40542	1
Stat3	-0,40542	1
Gm19028	-0,40558	1
Ap1g2	-0,40567	1
Vipas39	-0,40593	1
Crot	-0,40606	1
Tcf12	-0,40611	1
Mfsd3	-0,40632	1
Secisbp2	-0,40631	1
Kbtbd7	-0,40644	1
Phtf2	-0,40647	1
Rlf	-0,40717	1
Nab1	-0,40725	1
Acsl1	-0,4075	1
Zfp710	-0,40764	1
Sh3glb1	-0,40761	1
Mfge8	-0,40758	1
Arrb1	-0,40772	1
Cpt2	-0,40791	1
Fcgr4	-0,4082	1
Cyfip1	-0,40824	1
Washc2	-0,40817	1
Kdelr3	-0,40854	1
Nisch	-0,40862	1
Tmem35b	-0,40876	1
Ncoa2	-0,40892	1
Fam206a	-0,40905	1
Hlcs	-0,40907	1
Nop16	-0,40912	1
Cyp4f16	-0,40935	1
Gatb	-0,40953	1
Gm17586	-0,40957	1
Parp2	-0,40955	1
Igf2bp1	-0,41002	1
Gm28417	-0,41032	1
Nup98	-0,41029	1
Polr3b	-0,41041	1
Eya4	-0,41037	1
Slc20a2	-0,41064	1
Slc16a6	-0,41079	1
Ylpm1	-0,41082	1
Ipo7	-0,41079	1
Nmd3	-0,41088	1
Spred3	-0,41097	1

Gm12184	-0,41102	1
Rpusd1	-0,41115	1
Tcirg1	-0,4111	1
Vamp1	-0,41134	1
Phf14	-0,41139	1
Gm37566	-0,41153	1
Gtpbp2	-0,4115	1
Col4a5	-0,41148	1
Snx7	-0,41186	1
Idh3a	-0,41189	1
Men1	-0,41213	1
Klhdc10	-0,41211	1
Cntrl	-0,41224	1
Sorl1	-0,41261	1
Ankrd49	-0,41258	1
Sfpq	-0,41258	1
Gm12906	-0,4127	1
Gm37033	-0,41283	1
Gm42595	-0,41277	1
2510009E07Rik	-0,4129	1
Anapc10	-0,41303	1
Oma1	-0,41298	1
Mast3	-0,41306	1
Gsto2	-0,41322	1
Nagpa	-0,41326	1
Wipi1	-0,41348	1
Rpap1	-0,41372	1
Slc4a8	-0,4138	1
Rlim	-0,41376	1
Pbrm1	-0,41419	1
Zc3hav1l	-0,41432	1
Clec4n	-0,41434	1
Pja1	-0,41432	1
Cpt1a	-0,41428	1
Vasp	-0,41426	1
Atp13a1	-0,41436	1
Ctnnb1	-0,41458	1
Gclc	-0,41471	1
Rab14	-0,41496	1
D230025D16Rik	-0,41557	1
Papd4	-0,41578	1
Caly	-0,41592	1
Zdhhc12	-0,41588	1
Fads2	-0,41607	1
Fryl	-0,41613	1
Habp4	-0,41621	1
Polr1e	-0,41617	1
Vrk2	-0,41619	1
Slc38a2	-0,41631	1
Mrpl3	-0,41645	1
Avl9	-0,41658	1
Slc30a4	-0,4166	1

Clec5a	-0,41684	1
Wdr24	-0,41693	1
Skiv2l2	-0,4169	1
Gm5391	-0,41706	1
Frmd8	-0,41714	1
Kif1c	-0,41722	1
Fbxw7	-0,41734	1
Rpl10a	-0,41756	1
Gm44567	-0,41775	1
Sirt7	-0,41776	1
Cdk5	-0,41801	1
L2hgdh	-0,41812	1
Acy3	-0,41822	1
Ccnf	-0,4182	1
Zfp160	-0,41822	1
Ap4b1	-0,41831	1
Zfp74	-0,41857	1
Sdhaf4	-0,41885	1
Cul5	-0,41884	1
Zfp523	-0,41898	1
Yipf3	-0,41897	1
Gm12833	-0,41924	1
Dopey2	-0,41928	1
Sh3bgrl	-0,41957	1
Nin	-0,41996	1
Zranb3	-0,42009	1
Ankrd46	-0,42015	1
Gm37569	-0,42015	1
Rcbtb1	-0,42018	1
Bre	-0,42032	1
Arhgap18	-0,42046	1
Dcaf8	-0,42045	1
Tram2	-0,42065	1
Gm16253	-0,42056	1
Zfp111	-0,42064	1
Nfx1	-0,42069	1
Rbak	-0,42115	1
Ttc39b	-0,42107	1
Pnpla2	-0,42108	1
Clasp2	-0,4213	1
Sdcbp	-0,4213	1
Gm7285	-0,422	1
Asb3	-0,4221	1
Tmem98	-0,42236	1
Stxbp5	-0,42236	1
Slc39a11	-0,42261	1
Sft2d3	-0,42284	1
Scpep1	-0,42276	1
Slc12a4	-0,42294	1
Gm37407	-0,423	1
Shprh	-0,42313	1
2310010J17Rik	-0,42382	1

Scand1	-0,42397	1
Ficd	-0,42426	1
4831440E17Rik	-0,42447	1
BC037034	-0,42458	1
Farsa	-0,42474	1
Exoc6	-0,42482	1
4930453N24Rik	-0,42478	1
Dmwd	-0,42513	1
Usp49	-0,42527	1
Mia2	-0,42545	1
Uckl1	-0,42564	1
March8	-0,42568	1
Usf3	-0,42585	1
Brat1	-0,42586	1
Ncoa7	-0,42591	1
Adamtsl5	-0,42608	1
Psma8	-0,42608	1
Ptk2	-0,42607	1
Gm44090	-0,42635	1
Nubpl	-0,42632	1
Lancl2	-0,42634	1
Mcm3ap	-0,42689	1
Kyat3	-0,42698	1
Lims1	-0,42698	1
Trim41	-0,42699	1
Fam63a	-0,42715	1
Creg1	-0,42707	1
Uhrf1bp1l	-0,42716	1
Kri1	-0,42739	1
Slc41a3	-0,42742	1
Alpk1	-0,42753	1
Slc24a3	-0,42784	1
Clcn4	-0,42777	1
Fabp5l2	-0,42797	1
Rassf4	-0,4283	1
Gstt1	-0,4284	1
Plin3	-0,42889	1
Prelid3b	-0,42895	1
Crybg3	-0,42924	1
AI846148	-0,42936	1
6430573P05Rik	-0,42942	1
Gdap10	-0,42963	1
Dopey1	-0,42971	1
Mms22l	-0,42969	1
Idh3g	-0,42971	1
Mrps36	-0,4301	1
Dync1li2	-0,43025	1
Mrap	-0,43057	1
Fam91a1	-0,43064	1
Dock9	-0,43068	1
Slc35a1	-0,4307	1
Surf4	-0,43095	1

Zc3hav1	-0,43092	1
AA986860	-0,43113	1
4921511C10Rik	-0,43119	1
Phf8	-0,43124	1
Cln3	-0,43141	1
Htra2	-0,43152	1
Adat3	-0,43164	1
Eif4g1	-0,43161	1
Impdh1	-0,43182	1
Rasal1	-0,43192	1
Stau2	-0,43193	1
Mprip	-0,43189	1
Mif4gd	-0,43195	1
Zmpste24	-0,43207	1
Rela	-0,43214	1
Phykpl	-0,43219	1
Prkdc	-0,43233	1
Tmem205	-0,43231	1
Kif7	-0,4324	1
Anks1	-0,43273	1
Zscan20	-0,43282	1
Mfap3	-0,43279	1
Gm12762	-0,433	1
Brms1	-0,43321	1
Tmem220	-0,43319	1
Trim39	-0,43326	1
Gm5830	-0,43343	1
Tlr6	-0,43364	1
Agl	-0,4336	1
Gm36989	-0,43385	1
Relb	-0,43396	1
Fam135a	-0,43414	1
Wdr13	-0,43413	1
D630045J12Rik	-0,43429	1
Cherp	-0,43428	1
Strn3	-0,43432	1
Zfp54	-0,43438	1
N4bp1	-0,43458	1
Gm42728	-0,435	1
Dus1l	-0,435	1
Ppp4r1	-0,43518	1
Cse1l	-0,43533	1
Pcgf3	-0,43563	1
Oxct1	-0,4356	1
Wdr92	-0,43577	1
Nudt22	-0,43609	1
Kat2a	-0,43625	1
Zfp871	-0,43627	1
Vps33a	-0,43639	1
Dctn4	-0,43639	1
Jmjd4	-0,43652	1
S100pbp	-0,43652	1

Rpl10-ps3	-0,43671	1
Mdfic	-0,43678	1
E330009J07Rik	-0,43693	1
Cpsf3	-0,43698	1
Mbtps1	-0,43698	1
Sap130	-0,43718	1
Ercc4	-0,43721	1
Pkd1l2	-0,43755	1
Lipe	-0,43751	1
Kif1bp	-0,43756	1
Sf1	-0,43783	1
Gaa	-0,43794	1
Ubn2	-0,43785	1
Tsacc	-0,43807	1
Firre	-0,43833	1
Csnk1d	-0,43852	1
Atp6v0d2	-0,43847	1
Akap7	-0,43876	1
Trmt11	-0,43896	1
Arhgap27	-0,43905	1
Abcb7	-0,4392	1
Dusp10	-0,43932	1
Anapc5	-0,43944	1
2610037D02Rik	-0,43952	1
Dmtf1	-0,43958	1
Chac2	-0,43972	1
Dnajc5	-0,43968	1
Rai14	-0,44078	1
Nptxr	-0,44105	1
Gusb	-0,441	1
Ak3	-0,44117	1
Syne1	-0,44183	1
Tefm	-0,44197	1
H2-Ab1	-0,44219	1
2310047D07Rik	-0,44244	1
Ptprj	-0,44236	1
Taz	-0,44236	1
Zdhhc5	-0,44248	1
Ino80b	-0,44306	1
Cep192	-0,44317	1
Iqcg	-0,44334	1
Rp2	-0,44334	1
Ddx5	-0,44362	1
Ints5	-0,4438	1
Pgpep1	-0,44383	1
Zfp266	-0,44393	1
Slc20a1	-0,44386	1
Prim2	-0,44409	1
Insr	-0,44413	1
Ppp1r18	-0,44413	1
Uck2	-0,44435	1
Dnajb9	-0,44484	1

Eif5	-0,44483	1
Becn1	-0,44521	1
Atp2a3	-0,44542	1
Gm42522	-0,44564	1
Fam92a	-0,44573	1
Lrrc24	-0,44581	1
Peli1	-0,44586	1
Metap2	-0,44603	1
Slc26a6	-0,44624	1
Brd7	-0,44638	1
Zfp820	-0,44645	1
Paip1	-0,44672	1
Mlst8	-0,44681	1
Smad1	-0,44698	1
RP23-390D8.2	-0,44714	1
Cfap97	-0,44723	1
Arhgap35	-0,44735	1
Cep78	-0,44775	1
Phf11b	-0,44788	1
Zfp867	-0,44785	1
Gm19705	-0,448	1
Ppip5k1	-0,44796	1
Fabp7	-0,44871	1
Snx27	-0,44885	1
Pip5k1b	-0,44884	1
Ttll1	-0,44897	1
Ly96	-0,44908	1
Tug1	-0,44927	1
Slc46a1	-0,4495	1
Nradd	-0,44974	1
Glt8d1	-0,44971	1
Imp4	-0,44978	1
Slc22a15	-0,44989	1
Fam122a	-0,45006	1
2310035C23Rik	-0,4501	1
Tanc1	-0,45013	1
Mon1a	-0,45054	1
Mef2a	-0,45047	1
Zmiz2	-0,4509	1
Rin3	-0,45115	1
Tubgcp5	-0,45181	1
1700020I14Rik	-0,45192	1
Slc6a8	-0,45199	1
Osbp2	-0,45196	1
Smug1	-0,45208	1
Tgm2	-0,45218	1
Ep300	-0,45242	1
Nlrc5	-0,45248	1
Rsph1	-0,45261	1
Gata3	-0,45263	1
Pecr	-0,45292	1
Taf6l	-0,45344	1

Gm37333	-0,45362	1
Gsap	-0,4538	1
Kmt2b	-0,4539	1
Srbd1	-0,45427	1
Gpr132	-0,45444	1
Ahi1	-0,45458	1
Cnot2	-0,45474	1
Ada	-0,45476	1
Dnajc9	-0,45493	1
7330423F06Rik	-0,45515	1
Abcc10	-0,45526	1
Asb13	-0,45542	1
Cln5	-0,45563	1
Rnf183	-0,4558	1
Kmt2d	-0,4559	1
Tkt	-0,45601	1
Zik1	-0,45618	1
Sppl2a	-0,45654	1
Glg1	-0,457	1
Nfrkb	-0,45721	1
Man2b2	-0,45732	1
Atxn1	-0,45739	1
Tmem214	-0,45751	1
Gm43761	-0,45764	1
Poglut1	-0,45757	1
Tram1	-0,45756	1
Tex10	-0,458	1
Zcchc4	-0,45807	1
Hook3	-0,45809	1
Pogk	-0,45839	1
Gm13864	-0,45848	1
Ddx39b	-0,4585	1
Atg4d	-0,45876	1
Amt	-0,45887	1
Pcyox1l	-0,45898	1
Trim44	-0,45932	1
Slc29a1	-0,45944	1
Ikbkg	-0,45992	1
Hdlbp	-0,45986	1
Hivep1	-0,46013	1
Bptf	-0,46018	1
Sh3bp2	-0,46016	1
Zfp236	-0,46031	1
Nsmaf	-0,46031	1
Nipsnap3b	-0,4605	1
Ssh1	-0,4608	1
Traf7	-0,46076	1
Zfp784	-0,46085	1
Pogz	-0,4609	1
Ezh1	-0,46102	1
Gm43213	-0,46125	1
Mios	-0,46144	1

Smcr8	-0,46139	1
Camta2	-0,46137	1
Wdr41	-0,4617	1
Vash2	-0,46174	1
Parp16	-0,46208	1
Setd5	-0,46206	1
Acad10	-0,4623	1
Tmem199	-0,46226	1
Ash2l	-0,46236	1
Izumo4	-0,46251	1
Gm45762	-0,46273	1
Gm6329	-0,46286	1
Aldh16a1	-0,46306	1
Eif3a	-0,46345	1
Golga2	-0,46346	1
Gm29340	-0,46356	1
Mboat1	-0,46384	1
Gm37349	-0,46444	1
Hus1	-0,46447	1
Wdr76	-0,46459	1
Pum2	-0,4648	1
Pcca	-0,46497	1
Ap3m1	-0,46505	1
Sema4b	-0,46519	1
C230037L18Rik	-0,46518	1
Col20a1	-0,46521	1
Med27	-0,46525	1
Lrrc47	-0,46524	1
Slc25a1	-0,46527	1
Ubxn2b	-0,46547	1
Cul4b	-0,46586	1
Sri	-0,46594	1
Tm9sf4	-0,46598	1
Supv3l1	-0,46609	1
Mlxip	-0,46629	1
5330438D12Rik	-0,46653	1
Znrf3	-0,46672	1
Arhgef1	-0,46705	1
Fyb	-0,46707	1
Cul9	-0,46754	1
Trp53inp2	-0,46751	1
Pms2	-0,4677	1
Spidr	-0,46795	1
Gm43737	-0,46832	1
Manba	-0,4685	1
Babam1	-0,46875	1
Akap8l	-0,46872	1
Lrrc61	-0,46886	1
Gm5898	-0,46914	1
1700003F12Rik	-0,46938	1
Pyurf	-0,46946	1
Dip2a	-0,4695	1

Nudt14	-0,46966	1
Med23	-0,47	1
Smim19	-0,46999	1
Stat6	-0,46995	1
Hmox2	-0,47015	1
Prdm9	-0,47018	1
Eif2ak4	-0,47019	1
Gm8539	-0,47037	1
Hnrnph3	-0,47055	1
Cep63	-0,47068	1
Pcdh7	-0,47072	1
Gem	-0,47094	1
Ttc32	-0,47145	1
Hmgb1	-0,47175	1
Smarca2	-0,4717	1
Cmtm7	-0,47184	1
Creb3l2	-0,47201	1
Zfp282	-0,47243	1
Qsox2	-0,4725	1
Ankrd16	-0,47265	1
Gm6612	-0,47267	1
Ptpn23	-0,47314	1
Serpinb8	-0,47357	1
Hipk2	-0,47389	1
Ccdc17	-0,47404	1
Dpy19l4	-0,4742	1
Ikzf1	-0,47423	1
Selenos	-0,4744	1
Ash1l	-0,47447	1
Xpot	-0,47471	1
Ogt	-0,47476	1
Mavs	-0,47487	1
Prpsap1	-0,47493	1
Ccnl2	-0,47493	1
Trpm7	-0,475	1
Ahcyl2	-0,47506	1
Gm9726	-0,47531	1
Dhx29	-0,47534	1
Gm43547	-0,47548	1
Birc6	-0,47559	1
Slc33a1	-0,47573	1
Rnf6	-0,47575	1
Fbxl20	-0,47591	1
Senp1	-0,47593	1
Gm20633	-0,47593	1
Nt5dc2	-0,47596	1
Itpr1	-0,47643	1
Zfp974	-0,47661	1
Gm15859	-0,47699	1
Plch2	-0,47696	1
Nrbp1	-0,47703	1
Rasal3	-0,4773	1

Ubiad1	-0,47769	1
Hspbap1	-0,47772	1
RP24-233B16.6	-0,47782	1
Zw10	-0,47779	1
Dync1li1	-0,47792	1
Pknox1	-0,47804	1
Tapt1	-0,47809	1
Ptgir	-0,47815	1
Ndst2	-0,47825	1
Dcp1a	-0,47893	1
Rbm5	-0,47891	1
E330011M16Rik	-0,4789	1
Zfp943	-0,47897	1
5430427O19Rik	-0,47961	1
Gm6344	-0,47991	1
RP23-36H21.3	-0,47995	1
Snord72	-0,48002	1
Kifc3	-0,48001	1
Gk	-0,48028	1
Npc1	-0,48031	1
Strn	-0,48055	1
Etv6	-0,48058	1
Huwe1	-0,4806	1
Rragb	-0,48096	1
Atp6ap1	-0,48105	1
Wdr75	-0,48107	1
Tyms	-0,48141	1
Ifi44	-0,48153	1
6430590A07Rik	-0,48199	1
Gpkow	-0,48242	1
Ksr1	-0,48242	1
Vps9d1	-0,48256	1
Lpcat2	-0,48284	1
Snrpc	-0,4831	1
Zmynd8	-0,48334	1
Cutc	-0,48339	1
9030624J02Rik	-0,48355	1
Gm29539	-0,484	1
Mirlet7b	-0,48412	1
Samd1	-0,48411	1
Tm7sf3	-0,48418	1
Gm45185	-0,4843	1
Atp1a1	-0,48431	1
Extl2	-0,48437	1
Atg4c	-0,48463	1
BC025920	-0,48471	1
Scd1	-0,48492	1
Gls	-0,48488	1
Trip12	-0,48515	1
Arl15	-0,4853	1
Lrrk2	-0,4855	1
Tnip3	-0,48559	1

Xbp1	-0,48558	1
Lgalsl	-0,48596	1
Gpr68	-0,48605	1
Cox15	-0,48638	1
Inf2	-0,48657	1
Mroh1	-0,48665	1
Sqstm1	-0,48686	1
Kansl1	-0,48712	1
Spata1	-0,48731	1
Gmeb2	-0,48753	1
Dennd6b	-0,48762	1
Tubgcp3	-0,48781	1
Trim27	-0,48777	1
Zfp995	-0,48809	1
Gm23935	-0,48816	1
Ube2d2a	-0,48817	1
Hif1an	-0,4882	1
Cnot11	-0,48822	1
Jak1	-0,48847	1
2810021J22Rik	-0,48856	1
Fabp3	-0,48867	1
Exoc4	-0,48878	1
Casd1	-0,48951	1
Rusc1	-0,48954	1
Pygb	-0,48963	1
Snora21	-0,48981	1
Mob1a	-0,48976	1
Tnfrsf18	-0,49069	1
Gga3	-0,49096	1
Ptpn5	-0,49099	1
Hsp90ab1	-0,4911	1
Dnm2	-0,49116	1
Jak3	-0,49133	1
Zfp626	-0,49169	1
Gm32856	-0,4918	1
Arid1b	-0,49213	1
Cd53	-0,49219	1
Ube2cbp	-0,49233	1
Lrrc8d	-0,4924	1
Ptpn9	-0,49255	1
Gm6501	-0,49262	1
Dctn1	-0,49273	1
Zfp719	-0,49294	1
Pdss2	-0,49312	1
Eml2	-0,49322	1
Wdyhv1	-0,49344	1
Sp1	-0,49337	1
Dnajc7	-0,49354	1
Xpo5	-0,49393	1
AI987944	-0,49388	1
Notch2	-0,49386	1
Aim1	-0,49399	1

Slco4a1	-0,49421	1
Kank2	-0,49436	1
Plekhb2	-0,49442	1
Slc9a6	-0,49446	1
Bcl7c	-0,49493	1
Arl8a	-0,49503	1
RP23-307F3.6	-0,495	1
Mut	-0,49499	1
Mllt3	-0,49497	1
Dph6	-0,49513	1
Tfg	-0,49506	1
Rasa3	-0,49526	1
Stradb	-0,49532	1
Gk5	-0,49546	1
Smurf2	-0,49555	1
Csk	-0,49605	1
Nmnat3	-0,49642	1
Dennd4b	-0,49646	1
Slc11a2	-0,49654	1
Prpf40b	-0,49663	1
Dars2	-0,49704	1
Slk	-0,49722	1
Camk1d	-0,49734	1
Dnase2a	-0,49733	1
Rnf213	-0,49742	1
Prkacb	-0,49762	1
Vps52	-0,49767	1
Gm38257	-0,4978	1
Exoc7	-0,4979	1
Tmem185b	-0,49811	1
Bmi1	-0,49806	1
Dand5	-0,49848	1
Eri2	-0,49877	1
Mettl15	-0,49894	1
Gnb2	-0,49897	1
Trim30a	-0,49895	1
Hira	-0,49906	1
Gm15421	-0,49906	1
Zkscan5	-0,49936	1
Gm44836	-0,49952	1
Gm15445	-0,49958	1
Cbarp	-0,50009	1
Tfcp2	-0,50015	1
Brpf3	-0,50016	1
Slc1a5	-0,50045	1
Gak	-0,50063	1
Cntrob	-0,50065	1
Zfp46	-0,50072	1
2210408I21Rik	-0,50144	1
Gm9833	-0,50151	1
Antxr2	-0,50164	1
Csde1	-0,50172	1

Twnk	-0,50168	1
Tagap	-0,50168	1
R3hdm2	-0,50205	1
Vapa	-0,50217	1
Fermt3	-0,50254	1
Slc26a11	-0,50262	1
Nek4	-0,50265	1
4930590J08Rik	-0,50279	1
Ppp1r9b	-0,50317	1
Cntln	-0,50319	1
Pan2	-0,50364	1
Ttc26	-0,5036	1
Ap5z1	-0,50419	1
Trp53inp1	-0,50429	1
Gm8210	-0,5045	1
Rasgrp3	-0,50496	1
Xrcc3	-0,50504	1
Limk1	-0,5056	1
Cd63	-0,50573	1
Echdc1	-0,50619	1
Diaph2	-0,50632	1
9930111J21Rik2	-0,50631	1
Ppp1r12b	-0,50643	1
Cdk14	-0,5066	1
Slc35e2	-0,5067	1
Phf19	-0,50673	1
Hk1os	-0,50702	1
Ubxn2a	-0,50784	1
Foxp1	-0,50794	1
Myo7a	-0,50791	1
Cd80	-0,50792	1
Tars	-0,50806	1
Ccdc122	-0,50809	1
Helz	-0,50824	1
Plxdc1	-0,50824	1
Tuft1	-0,50853	1
Plekha8	-0,5085	1
Qser1	-0,50893	1
Il18rap	-0,5089	1
Ampd2	-0,50963	1
Zdhhc14	-0,50959	1
Rsad1	-0,50956	1
Vps41	-0,50966	1
Slc41a2	-0,5098	1
Snrpd3	-0,51003	1
Tada2a	-0,51011	1
Xrcc4	-0,51011	1
Coro7	-0,51025	1
Gm6304	-0,51049	1
Vps13c	-0,51106	1
Rab5b	-0,51112	1
Gphn	-0,51124	1

Nktr	-0,51146	1
Ap5m1	-0,51148	1
Ing4	-0,51153	1
Zfp330	-0,51167	1
Pla2g6	-0,51166	1
Rsl1d1	-0,5119	1
Pqlc3	-0,51189	1
Angpt2	-0,51208	1
Ppp3cb	-0,5125	1
Aldh4a1	-0,51285	1
Mia3	-0,51288	1
Mfsd1	-0,51302	1
Dhx33	-0,51334	1
Rgl2	-0,51353	1
Ypel1	-0,5135	1
Pi4ka	-0,5136	1
Trim21	-0,51384	1
Kcnq1ot1	-0,51388	1
Pkib	-0,514	1
Gm4430	-0,51415	1
Tbc1d23	-0,5144	1
Kdm5d	-0,51441	1
Nucb2	-0,51479	1
Wdfy1	-0,51501	1
Cd109	-0,51511	1
Nectin4	-0,51511	1
Gm17066	-0,51563	1
Ubr2	-0,51585	1
Nlk	-0,51613	1
Mbp	-0,51617	1
Usp35	-0,51629	1
Xiap	-0,51643	1
Rbm10	-0,5164	1
Wac	-0,51646	1
Sec61a1	-0,51655	1
Cep250	-0,51655	1
Cdkl4	-0,5166	1
Ccdc130	-0,5167	1
Ube2j1	-0,51707	1
Gm32175	-0,51709	1
Ribc1	-0,5174	1
Ehmt2	-0,51765	1
Wdr11	-0,51761	1
Spice1	-0,51772	1
Gm42659	-0,51796	1
Eif4ebp2	-0,51809	1
Traf3ip1	-0,51813	1
Phtf1	-0,51844	1
Ascl2	-0,51848	1
Eno3	-0,5188	1
Mpi	-0,51892	1
Bin3	-0,51933	1

Zfp106	-0,51936	1
Atp6v0a2	-0,51982	1
Polr3k	-0,51976	1
Ext2	-0,52022	1
Numbl	-0,52025	1
Slc10a3	-0,52016	1
Strbp	-0,52063	1
Tmem87a	-0,52073	1
Ftsj3	-0,52089	1
Pip4k2c	-0,5216	1
Cdc14a	-0,52161	1
RP24-499N24.6	-0,522	1
Tmcc1	-0,52234	1
Zc4h2	-0,52242	1
Micall2	-0,52248	1
Rab43	-0,5225	1
Nrg4	-0,52251	1
Tab2	-0,52262	1
Wdr7	-0,52277	1
Fsd1l	-0,5228	1
Sacm1l	-0,52285	1
Gm13840	-0,52294	1
Zcchc6	-0,5232	1
Gramd2	-0,52319	1
Jmjd8	-0,52328	1
Mcrs1	-0,52328	1
Nup160	-0,52325	1
Gm29994	-0,52371	1
Kctd3	-0,52394	1
Stat5b	-0,52402	1
Fah	-0,52407	1
Nedd1	-0,52421	1
Impa1	-0,52431	1
Itgav	-0,52436	1
Prtg	-0,52444	1
Tcf4	-0,52484	1
Fam43a	-0,52475	1
Usp25	-0,52495	1
Dcaf11	-0,525	1
Tomm34	-0,52515	1
Mettl8	-0,52544	1
Wdfy4	-0,52581	1
Gm45413	-0,5258	1
Rhoq	-0,52601	1
Phlpp2	-0,52603	1
Gm38345	-0,5262	1
Slc38a10	-0,52672	1
Haus4	-0,52682	1
Itpka	-0,52676	1
Poll	-0,52687	1
Sp110	-0,52693	1
Txlna	-0,52706	1

Rab12	-0,52709	1
Gm37010	-0,52746	1
Limd2	-0,52777	1
Gm19503	-0,52776	1
Zfp827	-0,52793	1
Pias3	-0,52808	1
Gm43800	-0,52806	1
Cdkn3	-0,52818	1
Naa40	-0,52829	1
Slc10a7	-0,52827	1
Tbc1d8	-0,52878	1
Naip6	-0,52912	1
Msr1	-0,52916	1
Trim7	-0,52916	1
Ktn1	-0,52938	1
Rnf113a2	-0,52935	1
Prpf8	-0,52949	1
Pcx	-0,53009	1
Plekhg4	-0,53021	1
Mmachc	-0,53063	1
Jak2	-0,53068	1
Gldc	-0,53138	1
Kat6a	-0,53151	1
Rcc2	-0,53195	1
Msantd3	-0,53222	1
Alg3	-0,53223	1
C2cd3	-0,53217	1
Ncor2	-0,53248	1
Usp8	-0,53285	1
Gm7236	-0,53303	1
Dffb	-0,5333	1
Ubp1	-0,53337	1
Tank	-0,53338	1
Hgsnat	-0,53361	1
Ltn1	-0,5338	1
Trim65	-0,53385	1
Sh3rf1	-0,5341	1
Prune2	-0,53405	1
Zfp579	-0,53432	1
Dcun1d1	-0,53438	1
Gm42748	-0,53437	1
Prepl	-0,53442	1
Slc24a5	-0,53492	1
Cep162	-0,53532	1
Slc7a1	-0,53548	1
Atp2a2	-0,53563	1
Atrn	-0,5356	1
Gm43588	-0,53577	1
Zfand3	-0,5359	1
Acot2	-0,5359	1
Dnajb12	-0,53603	1
Gtf3c3	-0,53605	1

Fktn	-0,53619	1
Trafd1	-0,53655	1
Acaca	-0,53651	1
Zhx3	-0,53648	1
Armc9	-0,53659	1
Zfp318	-0,53679	1
Pstpip2	-0,53684	1
Prox2	-0,53701	1
Cers2	-0,53715	1
6030458C11Rik	-0,53714	1
Ptpn1	-0,53742	1
Gm43362	-0,53743	1
Nkrf	-0,53737	1
Zfp27	-0,53773	1
Gm12276	-0,53819	1
Snrnp200	-0,53854	1
Pde6g	-0,53854	1
Sbno2	-0,53906	1
Vrk3	-0,53926	1
Ubn1	-0,53967	1
Fam35a	-0,53981	1
Sde2	-0,54031	1
Slc25a53	-0,54025	1
Mblac2	-0,54026	1
Chuk	-0,54052	1
F730043M19Rik	-0,54105	1
Filip1l	-0,54107	1
CAAA01194877.2	-0,54111	1
Ick	-0,54123	1
Galnt4	-0,54127	1
Eri1	-0,54193	1
Senp7	-0,54217	1
Txndc9	-0,54259	1
Pop4	-0,54283	1
Ncf1	-0,543	1
Smg7	-0,54313	1
Blvra	-0,5433	1
Taf1c	-0,54327	1
D130007C19Rik	-0,54332	1
Zdhhc24	-0,5433	1
Top2b	-0,54372	1
Zfp512b	-0,54397	1
Atat1	-0,54412	1
Dnajc27	-0,54426	1
Polr3d	-0,54437	1
Ids	-0,54467	1
Aldoc	-0,54485	1
Man2a1	-0,54518	1
Galnt7	-0,54534	1
Sbf2	-0,5454	1
Lamp2	-0,54557	1
Tgfbrap1	-0,54574	1

Pmepa1	-0,54593	1
Gm37670	-0,54605	1
Bivm	-0,54611	1
Slc38a6	-0,5462	1
Pcdhb17	-0,54634	1
Dap	-0,54639	1
Cuedc1	-0,54644	1
RP23-38L16.4	-0,5464	1
Rnaset2a	-0,54678	1
Orai2	-0,54687	1
Gm38082	-0,54704	1
Ankmy2	-0,54711	1
Amacr	-0,54712	1
Zfp358	-0,54722	1
Polr1a	-0,54752	1
Usp26	-0,54768	1
Gm42640	-0,54813	1
Wipf1	-0,54831	1
Miga2	-0,54834	1
Pmm2	-0,54836	1
Fas	-0,54869	1
Sirt6	-0,54879	1
Arpc2	-0,54913	1
Slc2a6	-0,54933	1
Ilvbl	-0,54977	1
Slc39a8	-0,54993	1
Mfsd13b	-0,55005	1
Irak1	-0,55029	1
3110045C21Rik	-0,55043	1
Rfx7	-0,55062	1
Lrrcc1	-0,5506	1
Zfp445	-0,55124	1
Snx32	-0,5512	1
Atp8a1	-0,55153	1
Mbnl1	-0,55166	1
Polm	-0,552	1
DHRSX	-0,55235	1
Srp72	-0,55243	1
1700017B05Rik	-0,5524	1
Gm7488	-0,5525	1
Rpl27-ps3	-0,55263	1
Ttc3	-0,55294	1
Flnc	-0,55321	1
Gm43323	-0,55351	1
Fut10	-0,55364	1
Grk6	-0,55366	1
Nfkbie	-0,55406	1
Tmub2	-0,55425	1
9430034N14Rik	-0,55434	1
Cabin1	-0,55449	1
Trp53cor1	-0,55482	1
Neil1	-0,55489	1

Slc3a2	-0,55526	1
Pdcd6ip	-0,55586	1
Foxo4	-0,55623	1
Zfp945	-0,55654	1
Cacfd1	-0,55684	1
Gm23639	-0,55685	1
Taf1b	-0,55714	1
Dnm1	-0,55709	1
Ptcd1	-0,55724	1
Rrbp1	-0,5573	1
Rcl1	-0,5574	1
Tnf	-0,55755	1
Gfi1	-0,55757	1
Nlrx1	-0,55788	1
Gm5601	-0,55821	1
Car13	-0,55821	1
Synj1	-0,55872	1
Ttc30b	-0,55866	1
Bcar1	-0,5592	1
Magee1	-0,55917	1
Trio	-0,55928	1
Mbtps2	-0,55959	1
Gm28071	-0,5596	1
Frrs1	-0,55978	1
Gm37145	-0,55992	1
Phf11d	-0,56002	1
Srrm1	-0,56015	1
Zbtb8os	-0,56012	1
Uvssa	-0,56016	1
BC048403	-0,5605	1
Gm45445	-0,56055	1
Ccdc66	-0,561	1
Gbas	-0,56129	1
Gm44270	-0,56129	1
Neurl4	-0,56144	1
C77080	-0,56144	1
Bbs10	-0,56145	1
Trappc11	-0,56146	1
Ganc	-0,56196	1
Slc25a36	-0,56213	1
Matn1	-0,56223	1
Hbs1l	-0,5626	1
Zfp219	-0,56284	1
Gda	-0,56354	1
Lrp8os3	-0,5637	1
Fam179b	-0,5642	1
Dclre1c	-0,56418	1
Gm43715	-0,56492	1
Pvr	-0,56505	1
Nfatc3	-0,56528	1
Rere	-0,56543	1
Cx3cr1	-0,56542	1

Pms1	-0,56538	1
Fkbp15	-0,56549	1
Gm38055	-0,56563	1
Slc35d1	-0,56606	1
Macf1	-0,56616	1
Taok2	-0,56621	1
Ovca2	-0,5665	1
Wdr26	-0,56671	1
Syngap1	-0,56688	1
Ttll12	-0,56704	1
Atad3aos	-0,56718	1
Trrap	-0,56729	1
Ubr4	-0,56743	1
Alg1	-0,56751	1
Gm43360	-0,56754	1
Tmem198b	-0,56773	1
Per3	-0,56777	1
Slc4a2	-0,56825	1
Fam3c	-0,56887	1
Ppia	-0,56892	1
2210408F21Rik	-0,56926	1
Card14	-0,56963	1
Ube4b	-0,56976	1
Apaf1	-0,56976	1
4930581F22Rik	-0,5698	1
Epg5	-0,57005	1
Dsel	-0,5701	1
Suox	-0,57039	1
Gab2	-0,57108	1
Rreb1	-0,57156	1
Dmpk	-0,57163	1
Gm16740	-0,57172	1
Mrps2	-0,57269	1
Tmem68	-0,57266	1
Cbfa2t2	-0,57281	1
Rexo2	-0,57346	1
Nf1	-0,57346	1
Oxt	-0,57398	1
Ddx11	-0,57429	1
Smap1	-0,5745	1
Rint1	-0,57476	1
Mpp3	-0,57488	1
Abcd3	-0,57495	1
Cdc23	-0,57511	1
Gm44250	-0,57515	1
Tsnax	-0,57538	1
Sipa1l3	-0,57557	1
Sparc	-0,57574	1
Vps33b	-0,57579	1
Gm26532	-0,57602	1
6330562C20Rik	-0,57621	1
Afg3l1	-0,57645	1

Pfkm	-0,57661	1
Cep70	-0,57668	1
Dok3	-0,57686	1
Mgam	-0,57685	1
Scamp2	-0,57723	1
Katnb1	-0,5773	1
Tnfaip2	-0,57736	1
Gm44667	-0,57755	1
Tbc1d9b	-0,57774	1
Zufsp	-0,57769	1
Pigs	-0,57775	1
Pitpnm1	-0,57873	1
Sfmbt1	-0,57871	1
Dock7	-0,5788	1
Gm42970	-0,57914	1
Fastkd5	-0,57929	1
Dnajc11	-0,5794	1
Parvg	-0,5796	1
Pfkfb4	-0,57969	1
Pacs1	-0,57971	1
Ccdc134	-0,57965	1
Serinc2	-0,5803	1
Fam13a	-0,58027	1
Ice2	-0,58132	1
Gm9732	-0,58157	1
Snord110	-0,58156	1
Trim46	-0,58178	1
Abcc1	-0,58186	1
Prkag2	-0,58201	1
Ehd1	-0,58213	1
Cdk5rap2	-0,58218	1
Nfs1	-0,58253	1
Gm11410	-0,58252	1
Abhd10	-0,58313	1
Myef2	-0,58364	1
Akr1b3	-0,58382	1
Mysm1	-0,58409	1
Gtf2b	-0,58407	1
Cul4a	-0,5844	1
Gm4613	-0,58449	1
Ipo11	-0,58455	1
Gspt2	-0,58469	1
4930589O11Rik	-0,58484	1
Usp24	-0,58486	1
Zdhhc2	-0,5852	1
Tshz3	-0,58532	1
Gcdh	-0,58534	1
Nr6a1	-0,58539	1
Palm	-0,58564	1
Nav1	-0,58582	1
Cldn15	-0,58581	1
Cib1	-0,58617	1

Bcl2l1	-0,58627	1
Cyb5rl	-0,58667	1
Ctnnd1	-0,58694	1
Tnfsf13b	-0,58713	1
Rnasel	-0,58725	1
Hsh2d	-0,58734	1
Srm	-0,58788	1
Ppil3	-0,5879	1
Trim37	-0,58809	1
Luc7l2	-0,5889	1
Glb1	-0,58897	1
Zbtb46	-0,58929	1
Dennd3	-0,58954	1
Gm11613	-0,58958	1
Vdac3-ps1	-0,58971	1
Gm16072	-0,59027	1
Pcdhb15	-0,59064	1
Pank1	-0,59131	1
Slc25a12	-0,5916	1
Mcm9	-0,59163	1
Gm37289	-0,5916	1
Gm18867	-0,59184	1
Foxk1	-0,59201	1
Gm24927	-0,59212	1
Adam15	-0,59219	1
Msrb1	-0,59258	1
Pdpr	-0,59266	1
App	-0,59283	1
Gm45220	-0,59313	1
Zfp566	-0,59321	1
4632427E13Rik	-0,59325	1
Runx2	-0,59362	1
Spcs2	-0,59386	1
Fbxo3	-0,59406	1
Polr2a	-0,59416	1
Gm13196	-0,59432	1
Pabpc1	-0,59454	1
Nphp1	-0,59461	1
Gm18916	-0,5946	1
Arl16	-0,59473	1
Apex2	-0,59466	1
Adck5	-0,59504	1
Arfgef2	-0,59527	1
Zfp94	-0,59535	1
Zfp865	-0,59567	1
Ipo8	-0,59585	1
Ggcx	-0,59583	1
Laptm4b	-0,59602	1
Gm42820	-0,5961	1
Angptl2	-0,59649	1
Pbxip1	-0,59656	1
Sbk1	-0,59662	1

Gli1	-0,59671	1
Lyl1	-0,59712	1
Tcam1	-0,59756	1
Fyco1	-0,59787	1
Gm38366	-0,59788	1
Inpp5k	-0,59828	1
Gm42463	-0,59835	1
Gm45501	-0,59889	1
Ddx21	-0,59903	1
Fancc	-0,59923	1
Dhrs1	-0,59939	1
Slc7a4	-0,59964	1
Scai	-0,5997	1
Gm44075	-0,59992	1
Bcl2l11	-0,60028	1
Mphosph9	-0,6008	1
Mt2	-0,60119	1
Mat2a	-0,60122	1
Tulp4	-0,60132	1
A930001C03Rik	-0,60158	1
Pom121	-0,60219	1
G730013B05Rik	-0,60238	1
Sos2	-0,6028	1
Fnip2	-0,60316	0,99202
Mgat5	-0,60317	1
Scarf1	-0,60326	1
Polr3gl	-0,60338	1
Zfp385a	-0,60346	1
D530018E20Rik	-0,60369	1
Prdm4	-0,60414	1
Tbc1d10b	-0,60432	1
Gfm1	-0,60439	1
Snord65	-0,60439	1
Esyt2	-0,6048	1
Csf1r	-0,60557	1
Myh9	-0,60561	1
Gapvd1	-0,60566	1
Cux1	-0,60599	1
Zfp41	-0,60653	1
Lipt2	-0,60649	1
D2hgdh	-0,6068	1
Fxyd2	-0,6069	1
Xkr8	-0,60709	1
Tpp1	-0,60731	1
Sacs	-0,60735	1
Spen	-0,60746	1
Xpo4	-0,60748	1
Isg20	-0,60769	1
Sgsm1	-0,60791	0,97597
Olfr921	-0,60876	1
Pigg	-0,6089	1
Plekho2	-0,60903	1

Plcb2	-0,61024	1
Gm13397	-0,6112	1
Hspa2	-0,61159	1
Clcn5	-0,61199	1
Gm45053	-0,612	1
Mst1	-0,61217	1
Trim25	-0,61272	1
Gm996	-0,61268	1
Chfr	-0,61277	1
Gm42972	-0,61283	1
Pkd2	-0,6131	1
Il2rg	-0,6137	1
Des	-0,61402	1
Gm8909	-0,61401	1
Gcfc2	-0,61459	1
Vps13b	-0,61489	1
Gm43457	-0,61491	1
Slc35b4	-0,61509	1
Ap3b1	-0,61613	1
Spcs2-ps	-0,61609	1
Rnf220	-0,61647	1
Cish	-0,61652	1
Tmem55a	-0,61744	1
Ofd1	-0,61741	1
Plekho1	-0,61782	0,76115
Mospd1	-0,6182	1
Wbp1l	-0,6186	1
Carm1	-0,61879	1
Acp2	-0,61878	1
Tab3	-0,61888	1
Plekhn1	-0,61915	1
Zfp467	-0,6191	1
Zdhhc20	-0,61952	0,84105
Deptor	-0,6197	1
Iqsec1	-0,61978	1
Dmxl1	-0,62018	1
Pdcd11	-0,62065	0,97533
Psma3	-0,62097	1
Recql	-0,621	1
Gm16053	-0,62097	1
Slc39a9	-0,62141	1
Tst	-0,62154	1
Shmt1	-0,62173	1
Serp1	-0,62188	0,98047
Wdr33	-0,62196	1
Gm16223	-0,62209	1
Fam161a	-0,62231	1
Gm43430	-0,62248	1
Atp6v1a	-0,62256	0,82615
Mfsd7a	-0,62279	1
Gm37519	-0,62279	1
Mospd2	-0,62286	1

Ryk	-0,62299	0,94459
Nod2	-0,62367	1
Rab3gap2	-0,62419	1
Tecpr2	-0,62472	1
Prag1	-0,62499	1
Tpcn1	-0,62514	1
Tnip1	-0,62521	0,97533
Elp2	-0,62543	1
Zfp628	-0,62541	1
Sco2	-0,62552	1
Pitrm1	-0,62561	1
Slc36a4	-0,62626	1
Helq	-0,62638	1
Gmip	-0,62655	1
Crebbp	-0,62715	1
E130308A19Rik	-0,62747	1
Gm43420	-0,62777	1
Tbk1	-0,62884	0,97533
Poc5	-0,62876	1
2610044O15Rik8	-0,62876	1
Moap1	-0,62879	1
Braf	-0,6297	1
Entpd5	-0,63014	1
Abhd4	-0,6303	1
P2rx7	-0,63026	1
Elfn2	-0,63042	1
Hmgxb4	-0,63061	1
Parp11	-0,63061	1
Utp4	-0,63071	1
1110034G24Rik	-0,63094	1
Tubb4a	-0,63114	1
Pofut1	-0,63137	1
Ints10	-0,63157	1
Hist1h2aa	-0,63191	1
Satb2	-0,63199	1
Zscan22	-0,6322	1
Qrsl1	-0,63233	1
Zbtb3	-0,63227	1
Tmem65	-0,63322	0,97217
Ssx2ip	-0,63341	1
Mtcl1	-0,63349	1
Amotl1	-0,63378	0,87237
Ptpdc1	-0,63387	1
Ube2q1	-0,63404	0,81088
Snapin	-0,63416	1
Gm23344	-0,63416	1
Zmiz1	-0,63425	1
Fbxo46	-0,63438	1
Card6	-0,63446	1
2410002F23Rik	-0,63457	0,95696
Arhgef6	-0,63503	0,98117
Ankrd34a	-0,63521	1

Akt1	-0,63534	0,80552
Zfp85	-0,63542	1
2700046G09Rik	-0,63584	1
Natd1	-0,63613	1
Hacd4	-0,63615	0,97597
Msi1	-0,63632	1
Trappc12	-0,63666	1
Stx17	-0,63717	1
Trpt1	-0,63871	1
Tm9sf3	-0,6389	0,8833
Gm11952	-0,6393	1
Slc6a6	-0,6396	1
Marveld1	-0,63974	0,96695
Trim28	-0,63984	0,99338
Mitf	-0,63983	1
Vps4b	-0,64011	0,97533
Gne	-0,64016	1
Gm6377	-0,64054	1
Idh1	-0,64062	0,64463
Svip	-0,64064	1
Scamp1	-0,64081	0,98368
Rnft1	-0,64125	1
Tmem51os1	-0,64117	1
Gm44829	-0,64133	1
Ift140	-0,64145	1
3110001I22Rik	-0,64158	1
Plekha5	-0,64192	1
Rmi1	-0,64186	1
Gtf2h3	-0,64196	1
Srsf5	-0,64255	0,87201
Tnrc6c	-0,64271	1
Ncoa4	-0,64303	1
Itpr2	-0,64347	0,80536
Celf2	-0,64397	0,87771
Fam13c	-0,64409	1
Shox2	-0,64423	1
Evi2a	-0,64438	1
Chd1l	-0,64473	1
Uri1	-0,64487	0,94028
Gcat	-0,6454	1
Ddx59	-0,64545	1
Efcab7	-0,64555	1
Sars2	-0,64567	1
Tecr	-0,64593	1
Faap100	-0,64609	1
Trim16	-0,6461	1
Siglec1	-0,64633	1
Copa	-0,6471	0,83424
Srxn1	-0,64729	1
Numa1	-0,6476	0,85715
Birc3	-0,64758	0,95518
Slc23a2	-0,64783	1

Dock8	-0,64796	1
Rab2b	-0,64807	1
Clptm1l	-0,64872	0,79094
Dst	-0,64888	1
Atic	-0,64923	0,93174
Arih1	-0,64985	0,72997
Slc38a9	-0,64993	0,94028
Hace1	-0,65016	1
Hps1	-0,65045	1
Ubr5	-0,65068	0,87653
Mllt6	-0,65083	0,97217
Noc2l	-0,65087	0,78503
Golga1	-0,65112	1
Gm37968	-0,65134	1
Fech	-0,65143	1
Atxn7l3	-0,65202	0,85435
Clec4e	-0,65246	0,60498
Zfp629	-0,65251	1
Ralgps1	-0,65257	1
Inpp5d	-0,65319	0,90494
Rassf5	-0,65318	0,97845
Paxip1	-0,65329	1
Drg2	-0,65339	1
Psca	-0,65338	1
Eya3	-0,65355	0,94767
Rars2	-0,65354	1
Aldh1b1	-0,65367	1
Spats2	-0,65402	1
Dhodh	-0,65416	1
Lamp1	-0,65454	0,71779
Tdp1	-0,65475	0,94028
Ctns	-0,65478	0,98511
Bloc1s3	-0,65476	1
Itsn1	-0,655	0,83574
Snx20	-0,65538	0,92626
Elmod2	-0,65571	1
Orc3	-0,65591	0,96052
E130309D02Rik	-0,65613	0,87336
Actn1	-0,65621	0,79094
Irak4	-0,6569	1
C1qbp	-0,65736	0,57309
Akap11	-0,6577	0,92358
Arhgap30	-0,6579	0,97539
Arl11	-0,65797	1
1810030O07Rik	-0,65814	0,96352
Gm21975	-0,65865	1
Frmd4a	-0,65898	0,90825
Ank2	-0,65947	1
Ubr1	-0,65981	1
Zscan29	-0,6602	1
Polr3a	-0,66043	1
Tmem186	-0,66048	1

Gm10093	-0,66076	1
Catsperg1	-0,66081	1
Stard9	-0,66103	0,98543
Gm24924	-0,66175	1
Atad1	-0,66181	1
Rsu1	-0,66195	1
Zfp239	-0,6623	1
Atf4	-0,66239	0,61792
Tbcd	-0,6626	0,96055
Reep6	-0,66352	1
2310061I04Rik	-0,66404	0,82013
Eef2k	-0,66396	1
Prob1	-0,6642	1
Lrrk1	-0,66458	1
Gm37678	-0,66478	1
Ngly1	-0,66496	1
Gramd1b	-0,66566	0,78141
Cpt1c	-0,66574	1
Mtmr2	-0,66595	0,64463
Slc30a7	-0,66585	0,92626
Slc7a5	-0,66684	0,77284
Plcd1	-0,66685	1
Yars	-0,66712	0,90996
Ncf4	-0,66726	0,87336
Myh11	-0,66734	1
Pnpla7	-0,66745	0,64665
Ubtf	-0,66739	0,81222
Usp20	-0,66749	0,79809
Mdk	-0,66747	1
Cnpy3	-0,66773	0,83574
Trip10	-0,6679	1
Parn	-0,66842	0,95518
Tpcn2	-0,66863	1
Ide	-0,66878	0,99422
Flcn	-0,66896	0,93133
Mtmr1	-0,66911	1
Gm42571	-0,66921	1
Alg9	-0,66935	1
Ints3	-0,66964	0,59191
Ctdsp2	-0,66976	0,90083
Ccdc92	-0,66986	1
Slc27a4	-0,67016	1
Sdsl	-0,67028	1
Wdr77	-0,67054	0,98002
Tlr2	-0,67122	0,85939
Psd	-0,67124	1
Impact	-0,67232	1
Med1	-0,67245	0,65455
Arhgef12	-0,67242	0,69661
Snap23	-0,67278	0,63802
RP24-91J7.1	-0,67284	1
Araf	-0,67325	0,97533

Tmem25	-0,67329	1
Thg1l	-0,67372	0,8742
Ifi203-ps	-0,67405	1
Fgfrl1	-0,67408	1
Nova1	-0,67461	1
Slc25a30	-0,67554	1
Frmd4b	-0,67588	0,74202
Ago2	-0,67643	0,7302
Dync2h1	-0,67662	0,70871
B4galnt1	-0,6767	0,95518
Trip11	-0,67668	0,97217
Lrch1	-0,67686	1
Airn	-0,67717	1
Pnpla6	-0,6779	1
Lrp12	-0,67801	0,93133
Gm13889	-0,67864	1
Yipf5	-0,6789	0,64665
Cxxc5	-0,67911	1
Srd5a1	-0,67913	1
Zfp873	-0,6791	1
Snora31	-0,67944	1
4932438A13Rik	-0,67951	0,83219
Zfp518a	-0,67977	1
Epn2	-0,67991	0,67411
Ceacam16	-0,68011	1
Camk2b	-0,68011	1
Slc25a37	-0,68039	0,57322
Pigo	-0,68162	1
Clgn	-0,68217	1
Atp6ap2	-0,68269	0,50603
Plxna2	-0,68314	0,7302
Nup88	-0,68393	0,89691
Zer1	-0,68394	1
Zfp93	-0,68456	1
Gm37978	-0,6847	1
Bst2	-0,68558	0,97146
Plpp6	-0,68557	1
Tbc1d22b	-0,68616	1
Phkb	-0,68682	1
Arhgef3	-0,68759	0,96052
Gm43300	-0,68769	1
Cars	-0,68805	0,64131
Nadk	-0,68841	0,63351
Ubqln2	-0,68997	0,94542
9130604C24Rik	-0,69002	1
Nufip2	-0,6903	0,69256
A930024E05Rik	-0,69048	1
Prss50	-0,69083	1
Zfp235	-0,69076	1
Prex1	-0,691	0,79443
Smarcd1	-0,69125	1
Slc17a5	-0,69166	0,70296

Tex9	-0,69178	1
Jade3	-0,69201	1
Sh3pxd2a	-0,6922	0,73291
Lix1l	-0,69226	0,82709
Fam222b	-0,69281	0,98777
Det1	-0,69293	1
Btbd19	-0,69339	0,88019
Marf1	-0,69386	0,97597
B230369F24Rik	-0,6939	1
Bcor	-0,69395	0,88844
Tctn3	-0,69545	1
Rps2-ps10	-0,69661	1
Stk11ip	-0,69679	1
Spire2	-0,69688	1
Taf2	-0,69803	1
Sidt2	-0,69807	0,6488
Islr2	-0,69814	1
Sh3tc1	-0,69863	1
Timm21	-0,69861	1
Dynlt1f	-0,69893	1
Rps6ka3	-0,69929	0,81574
Crebzf	-0,69929	1
Zfp446	-0,69965	1
Nudt5	-0,70069	1
Stx11	-0,70255	1
Ecel1	-0,7025	1
Phka1	-0,70353	0,98408
Gm38077	-0,70377	1
Orc5	-0,70403	0,98797
Tssk6	-0,70425	1
Ncstn	-0,70443	1
Dusp18	-0,70461	0,96052
Atp6v0d1	-0,7049	0,73428
Zfp653	-0,70502	1
B230322F03Rik	-0,70511	1
Sphk2	-0,70544	0,58688
Zfp128	-0,70539	1
Arhgap1	-0,70552	1
Zswim4	-0,70566	1
Cog1	-0,70572	1
Tubgcp6	-0,70624	1
Gm11868	-0,70659	1
Fam65c	-0,70723	0,86114
Inpp4a	-0,70787	0,98755
Gstt3	-0,70798	0,92741
Ints1	-0,708	1
Pkn3	-0,70816	0,84748
Bard1	-0,70923	1
Gid8	-0,70926	0,58245
Gtdc1	-0,7094	1
Gm15690	-0,70942	1
Slc17a7	-0,71002	1

Angel2	-0,71026	0,6649
Timp2	-0,71035	0,72433
Setdb1	-0,71043	0,98583
Acss2	-0,7106	0,98164
Tbc1d5	-0,71067	0,97217
9230112E08Rik	-0,71091	1
Npc1l1	-0,71086	1
Atg2a	-0,71115	0,44402
Msi2	-0,71186	0,89158
Gmpr	-0,71206	0,69297
Trak2	-0,71227	0,71712
Tctn2	-0,71248	1
Fam212b	-0,7127	1
Cpsf6	-0,71303	0,49486
Lrba	-0,71361	0,84772
Gm28187	-0,71379	1
Ttc28	-0,71401	1
Zfp119a	-0,71405	1
Tesk2	-0,71437	1
Golga5	-0,71473	0,67615
E430021H15Rik	-0,71508	1
Gm42876	-0,71509	1
Dock11	-0,71544	0,97248
Son	-0,71568	0,55383
Rbck1	-0,71581	0,6082
Ift122	-0,71605	1
Gm42918	-0,71595	1
Snora57	-0,71635	1
Elf4	-0,71654	1
Micall1	-0,71661	0,67652
Ormdl1	-0,71667	1
Bckdk	-0,71761	0,73679
Gm42636	-0,71791	0,85554
BC005537	-0,71838	0,64629
Eif4g3	-0,71849	0,58417
D6Ertd527e	-0,71869	1
Gm44694	-0,71893	1
Abca2	-0,71895	0,97533
Gm16199	-0,71911	1
Nr1h2	-0,71917	0,9401
Eogt	-0,71918	0,97539
Nek9	-0,71932	0,58274
Galnt1	-0,7194	0,3623
Gsk3b	-0,71995	0,52687
Chst14	-0,71997	1
Ubtd2	-0,72008	1
Gtf2h4	-0,72032	1
Atp6v1d	-0,72042	0,34312
Gm45871	-0,72043	1
Gm14843	-0,72041	1
RP23-476G10.1	-0,72035	1
6720464F23Rik	-0,72035	1

Gm43628	-0,72047	1
Map4k4	-0,72061	0,41202
Zbtb20	-0,7207	0,60093
Cdhr4	-0,72138	1
Tmppe	-0,72149	1
Csnk2a2	-0,72181	0,31717
Pus10	-0,72204	1
Dync2li1	-0,7221	1
Kdm6b	-0,72226	0,77212
Bnip2	-0,72314	0,58029
Gm43499	-0,72306	1
Wdr46-ps	-0,72368	1
Churc1	-0,72414	1
RP23-104D6.2	-0,72419	1
RP24-282C4.10	-0,72431	0,96052
Ppfibp1	-0,7248	0,37654
Hmox1	-0,72485	0,80536
Snx33	-0,72489	1
B230354K17Rik	-0,72487	1
Slc6a12	-0,72524	0,67652
Gm45248	-0,72535	1
Ccdc57	-0,72555	1
Tmem94	-0,72564	0,9873
Gm45853	-0,72606	1
Ddn	-0,72624	1
Herpud1	-0,72656	0,32997
Hoga1	-0,7266	1
Gtpbp8	-0,72693	1
A730011C13Rik	-0,72708	1
Atp10d	-0,72717	1
Tnk2	-0,72725	0,97585
Lrpprc	-0,72767	0,91262
Serpinc1	-0,72824	1
Gpr35	-0,72829	1
Zhx1	-0,7286	0,6459
Txndc16	-0,72896	0,78141
Gm12966	-0,72899	1
Apbb1	-0,72912	1
Rmnd1	-0,72951	1
Gm38036	-0,73018	1
Spi1	-0,73062	0,60852
Gm42611	-0,73081	1
Xrcc5	-0,73104	1
Gm26982	-0,73155	0,94459
Pon3	-0,73196	0,70799
Gm7909	-0,73197	1
Zfp763	-0,73214	1
Socs1	-0,73211	1
C2	-0,73225	1
Rbmx2-ps	-0,73226	1
Isg20l2	-0,7331	1
Car6	-0,73313	1

Nlrc3	-0,73344	1
Depdc1a	-0,73385	1
mt-Rnr1	-0,73414	0,67246
Ttll3	-0,73424	0,92626
Abhd3	-0,73435	1
Mettl25	-0,73444	1
Fat1	-0,73457	1
AW146154	-0,73469	1
Abi3	-0,73496	1
Syk	-0,73584	0,45245
Sh2d3c	-0,73591	1
Gm45358	-0,73588	1
D5Ertd605e	-0,73614	1
Nupl2	-0,73619	1
Srr	-0,73664	0,69661
Magohb	-0,73667	1
Slc12a5	-0,73672	1
Gm9776	-0,73774	1
Paqr3	-0,73766	1
Dennd1a	-0,738	0,86367
Gm38125	-0,73859	1
Flot1	-0,7387	0,96454
F7	-0,73885	1
Ssbp4	-0,73927	0,54053
Zfr2	-0,73955	1
Dbnl	-0,73966	0,71189
Tmem116	-0,7405	0,98555
Itpr3	-0,74084	0,61058
Ldlrad3	-0,74081	0,98555
Tbc1d24	-0,74115	0,71189
RP23-228B2.5	-0,74317	1
Abcc4	-0,74395	0,90083
Rasal2	-0,74442	0,4395
Prkcg	-0,74523	1
Rinl	-0,74539	0,83707
Pfas	-0,74572	0,90233
Sec31a	-0,74593	0,4328
Rfwd3	-0,74611	0,98058
Hmgb1-ps6	-0,74614	1
Lcp1	-0,74623	0,3948
Mdn1	-0,74645	0,45394
Gm5544	-0,74652	1
Gm9008	-0,74668	1
mt-Tp	-0,74713	1
Mlh3	-0,74717	0,90116
Gm37949	-0,74834	1
Slc40a1	-0,74878	1
Fli1	-0,74916	0,52873
Snora73b	-0,74942	0,94591
Tmem17	-0,74968	1
Gm5113	-0,75097	1
4930550C14Rik	-0,75133	1

Zfp672	-0,75152	0,89353
Hacd2	-0,75209	1
Pcyt1a	-0,75235	0,19547
Rnf216	-0,75274	0,76115
Tspoap1	-0,75268	1
Hoxb3	-0,75296	1
Timp1	-0,7541	1
Lrp5	-0,75427	1
Nudt12	-0,75434	1
Itgb3	-0,75433	1
Frg2f1	-0,75453	1
Catip	-0,75485	1
Lpcat3	-0,75494	0,88267
Lin28b	-0,75511	1
Fbxl19	-0,75639	1
Gm44890	-0,75644	1
Tbc1d4	-0,75646	0,60074
Csrnp2	-0,75662	1
Gm37390	-0,7566	1
Gm44291	-0,75676	1
Rab29	-0,75736	0,6622
Scarna9	-0,75747	1
Usp18	-0,75786	1
D330041H03Rik	-0,75847	1
Pafah2	-0,75903	1
Mks1	-0,75952	0,67797
B3galt4	-0,75976	1
Cyp2u1	-0,76008	0,92766
Apoe	-0,76024	1
Ccdc120	-0,76051	1
Gm24959	-0,76056	1
Tmem175	-0,76139	0,84748
Bphl	-0,76155	1
Mib1	-0,76165	0,34671
Traf6	-0,76166	0,97343
Kmt5c	-0,76175	0,47456
Wls	-0,76206	0,40631
Heatr3	-0,76252	0,94767
6330408A02Rik	-0,76257	1
Slc12a7	-0,76313	0,41634
Chil6	-0,76324	1
Cacna1d	-0,76343	0,90825
Tns3	-0,76351	0,30727
Efna2	-0,76432	1
Slc39a13	-0,76441	0,40089
Zbed3	-0,76453	1
Rbfox2	-0,76495	0,42778
Ythdc2	-0,76523	0,85401
Pced1b	-0,76552	1
Dynlt1-ps1	-0,76555	1
Gm37238	-0,76605	1
B130034C11Rik	-0,76614	1

Gm43062	-0,76629	1
Nr2c1	-0,76685	1
Ccdc14	-0,76709	0,87236
Erp27	-0,76753	1
N6amt1	-0,76772	0,8053
Gm11945	-0,76781	1
Dhx57	-0,76854	0,98705
Hpgds	-0,76896	0,36605
Faah	-0,76979	1
Gab3	-0,77005	0,66237
Ralgapa1	-0,77027	0,80552
Rnf128	-0,771	0,30943
Pik3cg	-0,77168	0,82903
9330159M07Rik	-0,77259	1
Bdh2	-0,77279	0,29583
Gm44269	-0,77291	1
Prrc2a	-0,77308	0,78983
Clec4a3	-0,77318	1
Gm44950	-0,7734	1
Gm9920	-0,77355	1
Rnd1	-0,77367	1
Neurl1a	-0,77372	1
Gemin5	-0,77464	0,90083
RP24-460E12.3	-0,7748	1
RP23-63H11.3	-0,775	1
Bms1	-0,7753	0,27851
Grn	-0,77551	0,3134
Actb	-0,77584	0,4553
Gm37660	-0,77597	1
Tdrd3	-0,7769	1
Gm12097	-0,77697	1
Map2k3os	-0,77736	1
Nsun4	-0,77799	0,8053
Stx5a	-0,77841	0,62611
Gabpb2	-0,77852	0,46386
Lekr1	-0,77925	1
Ctla2b	-0,77935	1
Gm26569	-0,77942	1
Srrm2	-0,77948	0,23671
Slc16a12	-0,77962	1
Rfx5	-0,77988	0,24246
A930016O22Rik	-0,78006	1
Ctsh	-0,78034	1
Setd1a	-0,78072	0,35482
Zkscan7	-0,7819	1
Samsn1	-0,78199	0,86022
Rtel1	-0,78223	1
Apbb1ip	-0,7824	0,18251
Pqlc2	-0,78251	1
Top3b	-0,78317	0,89353
Cd200r4	-0,78353	0,81088
Polr3f	-0,78442	0,62475

0610010F05Rik	-0,78484	0,81088
Fam46c	-0,78477	1
Gm13398	-0,78494	1
Zc3h4	-0,78606	0,26485
Kif5c	-0,7865	1
Slc25a15	-0,78776	1
Pml	-0,788	0,59191
Alg6	-0,78803	0,91369
Timm23	-0,78806	1
Slc29a3	-0,78847	0,47127
Gnptab	-0,78884	0,26241
Cc2d2a	-0,78945	1
Abhd1	-0,7898	1
Gca	-0,78986	1
Zfp280c	-0,79006	1
Zfp26	-0,79058	0,69297
Whrn	-0,79083	1
Gm43462	-0,79085	1
Adcy2	-0,79097	0,93133
Gls2	-0,79151	1
Dock6	-0,79162	0,95518
Ccdc94	-0,79169	1
Gm8093	-0,79172	1
Itfg2	-0,79307	0,74253
Gm6257	-0,79373	1
Gabrd	-0,79464	1
Mapkbp1	-0,79483	0,30321
Fam122b	-0,79479	0,69577
Plekhh1	-0,79505	1
Gm37474	-0,79508	0,70621
Pigw	-0,79526	0,90083
Trmo	-0,79541	1
Zfp72	-0,79541	1
Gm23127	-0,79569	1
Cdk5rap1	-0,79607	0,82709
Laptm5	-0,79648	0,13501
Stap2	-0,79694	1
Zbtb38	-0,79744	0,30727
Snora30	-0,79812	1
Gm44834	-0,799	1
Nacc2	-0,79905	0,57309
Gm38335	-0,79935	1
Ivd	-0,79945	0,73812
Bcl9l	-0,80016	0,54879
Gm36963	-0,80046	1
Hacd3	-0,80057	0,45852
Fut8	-0,80082	1
AU040320	-0,80102	0,41912
1810062G17Rik	-0,8012	1
Med24	-0,80149	1
Myh7b	-0,80285	1
Oxsm	-0,80309	1

Kbtbd11	-0,80328	0,15473
Pde8a	-0,80331	0,65182
Myo6	-0,80381	0,64629
Mtbp	-0,80426	1
Nt5e	-0,80451	0,67336
Letmd1	-0,8045	0,74311
Cybb	-0,80508	0,34035
Gm43627	-0,80519	1
Zbtb48	-0,80554	1
Scap	-0,80568	0,48537
Gm43061	-0,80601	1
Tlr13	-0,80634	0,83424
Dpp8	-0,80676	0,28676
Gm44116	-0,80682	1
Wdr6	-0,80686	0,63448
Cad	-0,80778	0,58073
Grap	-0,80807	0,58748
Gm43111	-0,80805	1
2410089E03Rik	-0,80868	1
Cenpt	-0,80944	1
Spred1	-0,81006	0,4278
Zfp40	-0,81086	1
Hmga2	-0,81117	0,16192
Cstf2t	-0,81126	0,45834
Gm16437	-0,81207	1
Ammecr1	-0,81255	0,35554
Zfp691	-0,81282	0,90825
Siah1b	-0,81369	0,76365
Zfp59	-0,81381	1
Tmem14a	-0,8147	1
Gm37255	-0,81492	1
Ticam1	-0,81507	0,49262
Phldb3	-0,81693	0,85586
mt-Nd2	-0,81704	0,41202
Cdan1	-0,81699	1
Ugt1a7c	-0,81726	1
Mthfr	-0,81754	0,63802
Chm	-0,8186	1
RP24-225A16.3	-0,81911	0,96052
Gm10131	-0,81923	1
Ttc7	-0,81937	0,42137
Wbscr27	-0,81977	0,55257
Nr2c2	-0,8199	0,43462
Fsbp	-0,81994	1
Gns	-0,81996	0,15879
L3mbtl3	-0,81996	1
Vwa7	-0,82034	1
Arv1	-0,82039	1
Kdm4d	-0,82065	1
Smarcc2	-0,82076	0,27023
Arfgap1	-0,82175	0,55726
Gm13350	-0,82192	1

Acadsb	-0,82197	0,45799
Gm43681	-0,82211	1
Ifih1	-0,82216	1
D6Wsu163e	-0,82231	0,74005
Zfp983	-0,82307	0,91853
Cep83os	-0,82329	0,79443
Ano8	-0,82382	0,58881
Sla	-0,82499	0,37369
Zbed4	-0,82524	0,86022
Fam213a	-0,82668	0,47938
Cat	-0,82693	0,084862
Uxt	-0,82688	1
Pter	-0,82697	0,74487
Lima1	-0,82792	0,24129
Gm12655	-0,82971	1
Gm45221	-0,83	1
Gm44552	-0,83004	1
Plscr3	-0,83103	0,41029
Gm22980	-0,83157	1
Hdac8	-0,83215	0,77248
A630001G21Rik	-0,8321	1
Washc5	-0,83221	0,76445
5430434F05Rik	-0,83243	1
Nup93	-0,83259	0,20121
Dmrt2	-0,83274	1
Gm12355	-0,83291	1
Depdc5	-0,83324	0,85558
BC017158	-0,83321	1
Slc25a14	-0,83352	0,90825
D030028A08Rik	-0,8336	1
Recql4	-0,8336	1
Tspyl2	-0,83393	1
Lgals2	-0,83405	1
Etfrf1	-0,83433	0,55732
Ermard	-0,83452	0,98677
Agbl5	-0,83459	0,74346
Sptlc1	-0,83489	0,341
Borcs7	-0,83488	0,82936
Srebf2	-0,83509	0,41202
Faap24	-0,83579	1
BC002059	-0,83658	0,32268
Nol4l	-0,83685	0,52544
Srl	-0,83699	1
Kif16b	-0,83742	0,53558
Rnf170	-0,83749	1
Gm16046	-0,83787	1
Dzip3	-0,83951	0,77248
Atg4a	-0,83982	0,3948
Tctex1d4	-0,84029	1
Lypla2	-0,8419	0,26967
Fancm	-0,84217	1
Abcb4	-0,84321	0,12456

Scfd2	-0,84367	0,92867
Zfp959	-0,84404	1
B4galt1	-0,84413	0,055301
Slc38a1	-0,84465	0,078429
Pik3r2	-0,84475	0,35229
Arid2	-0,84498	0,38448
E130208F15Rik	-0,84531	1
Tmem69	-0,84553	0,64629
Fam120aos	-0,84578	1
4930556M19Rik	-0,84603	1
Ikbke	-0,84631	0,90083
Fam188b	-0,84715	1
Armc2	-0,84727	1
Ppip5k2	-0,84742	0,21318
Mical2	-0,84767	0,44165
Fbrsl1	-0,8483	0,60599
Trpv4	-0,84846	0,73428
Zbtb49	-0,84851	1
Lpin3	-0,84887	0,41232
Nudt7	-0,84889	0,74258
Soga1	-0,85018	0,43816
Pde4c	-0,85042	1
Ubxn11	-0,85114	1
Gm9938	-0,85123	1
Gm26620	-0,85158	1
Ubox5	-0,85202	1
Tcea2	-0,85201	1
Gm11722	-0,85209	0,90083
Slc4a11	-0,85279	0,83554
C430042M11Rik	-0,85277	1
Atp13a2	-0,85507	0,18763
Ocrl	-0,85509	0,23493
Gm5578	-0,85591	1
Maged2	-0,85671	1
Nfkbiz	-0,8573	0,41691
Sec16b	-0,85807	0,26485
Lgals3bp	-0,85885	0,52021
Armc5	-0,85878	0,69661
Gm18284	-0,85882	1
Itga4	-0,85915	0,27077
Klhl35	-0,85916	1
Setd1b	-0,8594	0,30943
Eid3	-0,85997	1
Zdhhc9	-0,86007	0,47653
Ank	-0,86061	0,066176
Gm7292	-0,86105	1
Polr1b	-0,86204	0,97539
Rdh1	-0,8621	1
Synrg	-0,863	0,31055
G6pd2	-0,86296	1
mt-Nd1	-0,86338	0,24246
Oprl1	-0,8644	1

Gm8337	-0,86506	1
Scd2	-0,86528	0,16091
Ctbp1	-0,86545	0,14458
Gm42479	-0,86577	1
Il16	-0,86593	0,63351
Plbd2	-0,8667	0,051817
Ppp1r10	-0,86678	0,1889
Xrn1	-0,86716	0,16806
9430092D12Rik	-0,86726	1
Cpsf1	-0,86777	0,28884
9930014A18Rik	-0,86831	0,89006
Nup210	-0,86842	0,5744
Prr14l	-0,86895	0,22096
Gm14673	-0,86964	1
Gm5609	-0,86981	0,87893
Car9	-0,86994	0,37956
Rps6kb2	-0,86994	0,55654
Serinc3	-0,87056	0,2374
F830115B05Rik	-0,87086	1
Phxr4	-0,87117	1
Samd9l	-0,87148	1
Nsf	-0,87252	0,2209
Stac3	-0,87267	1
Mgat4b	-0,87303	0,094685
Mark2	-0,87303	0,27871
Zgpat	-0,87399	0,84797
Dapk1	-0,87416	0,054042
Hif1a	-0,87454	0,11229
RP23-151L20.5	-0,87503	1
Cables2	-0,87578	0,8216
Tmem63b	-0,87641	0,18787
Gtf2ird2	-0,87642	0,74233
Hsd17b7	-0,87685	0,27199
Aldh18a1	-0,87721	0,60852
March4	-0,87727	1
Npc2	-0,87774	0,063801
Snord55	-0,87801	1
Nfat5	-0,87878	0,21509
Uaca	-0,88021	0,17376
Gm20707	-0,88024	1
Slc43a3	-0,88034	0,44718
5730480H06Rik	-0,88038	0,78184
Arpin	-0,88203	0,84984
Neo1	-0,88217	0,2314
Ankrd26	-0,88298	0,58031
Kdm4a	-0,8834	0,044155
Rad51c	-0,88353	0,76488
Foxred2	-0,88513	0,075012
Hnrnpm	-0,88549	0,047667
Igf2r	-0,88564	0,3643
Pigh	-0,88575	0,97539
Pla2g2e	-0,88752	0,8287

Kank3	-0,8876	0,58523
BC065397	-0,88858	1
Nelfa	-0,88899	0,27503
Mbd6	-0,8891	0,50718
5730405O15Rik	-0,88947	1
Gm26225	-0,88966	1
Gm38376	-0,89008	1
1700020D05Rik	-0,89031	1
Dnajc10	-0,89038	0,054094
Rnpc3	-0,89078	0,49377
Klk8	-0,89098	0,98555
Ldb3	-0,89141	0,93492
Gpt2	-0,89185	0,19195
Rdh5	-0,89175	1
Hist1h4h	-0,89224	1
Fam120a	-0,89292	0,15231
Gm45137	-0,89333	1
Slc27a1	-0,89353	0,47127
Epb41l1	-0,89396	0,69297
Scly	-0,89408	0,4637
Kdm1b	-0,89435	1
Gm4017	-0,89452	1
Tbc1d25	-0,89483	0,67652
Gm37900	-0,89481	1
Gm38021	-0,89491	1
Celf5	-0,89512	1
Ppp3cc	-0,89518	1
Traf3ip3	-0,89634	0,92097
Srsf6	-0,8965	0,1252
Il1rap	-0,8967	0,8648
Slc26a2	-0,8971	0,10098
Ivns1abp	-0,89818	0,01252
Asap3	-0,89815	1
3830408C21Rik	-0,89826	1
Efcab11	-0,89832	1
Tfrc	-0,89849	0,12185
Mok	-0,89945	1
1810021B22Rik	-0,90118	0,80486
Ptges	-0,90138	0,61792
Gas6	-0,90163	0,050753
Zfp975	-0,90252	1
Cox10	-0,90402	0,059384
Sass6	-0,90452	0,6474
Dock4	-0,90491	1
Helz2	-0,90633	0,65198
Klf1	-0,90645	1
Gm26740	-0,90689	1
Gm42908	-0,90769	1
Ttc38	-0,90815	0,98583
Exoc8	-0,90923	0,64463
Txk	-0,90997	0,70621
Gm43672	-0,91034	1

Smarcb1	-0,91097	0,25661
Tyk2	-0,91127	0,049748
Dgat2	-0,91151	1
CH25-309J2.1	-0,91203	1
Gm13657	-0,91246	1
Gm42478	-0,91329	1
Gm42715	-0,91431	0,58073
Slc16a7	-0,91486	0,59191
Adamtsl4	-0,91533	0,65198
Pou2f2	-0,91556	0,054552
Lmbr1	-0,91753	0,75076
Zfp607a	-0,91754	0,99256
Acsl4	-0,91902	0,02178
Gm12770	-0,9196	1
Mtx3	-0,91968	0,80612
Plxnd1	-0,91983	0,14311
Ceacam1	-0,91986	1
Tsc2	-0,92047	0,93133
Fblim1	-0,92188	0,15415
Kmt5b	-0,92249	0,21524
Tfec	-0,92344	0,78141
Gm37124	-0,92339	1
Galnt11	-0,92507	1
Ece1	-0,92687	1
Mxra8	-0,92714	0,63351
Mpc1	-0,92793	1
Ampd3	-0,92877	0,27358
Gm42850	-0,92885	1
Snord13	-0,92913	0,15168
Pitpnm2	-0,92933	0,50572
9630010A21Rik	-0,92937	0,74346
Ahnak2	-0,92956	0,12185
1110020A21Rik	-0,93077	1
Treml1	-0,9318	0,13201
Gm15892	-0,93219	1
Agap1	-0,93254	0,20349
Gm42482	-0,93318	0,8287
Gm17259	-0,93407	0,97597
Tlr7	-0,93456	0,13355
Tsga10	-0,93521	0,78141
Atn1	-0,93564	1
Zfp69	-0,93589	1
Amer1	-0,93671	0,75437
Ppp6r3	-0,93692	0,035734
Gm43071	-0,93833	1
Gm10605	-0,93872	1
Csf2rb	-0,93974	0,20335
Calhm2	-0,93984	0,52544
Srd5a3	-0,93984	0,65182
Pigp	-0,94119	0,52544
Dcp1b	-0,94121	1
Lclat1	-0,94194	0,80552

Rpgr	-0,9433	0,82722
D430013B06Rik	-0,94409	1
Cep41	-0,94424	0,92358
C1qtnf6	-0,9452	1
Col7a1	-0,94746	0,95782
Neu3	-0,94785	1
Zfp994	-0,94883	1
9230102O04Rik	-0,94905	1
Dbt	-0,95032	0,26965
Ttpal	-0,95102	0,53755
Ubxn8	-0,95166	0,58031
Arfgef3	-0,95196	0,17376
Slc15a3	-0,95203	0,82443
Camk2n1	-0,95218	1
A130050O07Rik	-0,95368	1
Grik5	-0,95396	1
Gm38200	-0,95438	1
Gm30329	-0,95686	1
Gm16372	-0,95719	1
Gpr183	-0,95759	0,15992
Dennd1c	-0,95795	1
Cyfip2	-0,95998	0,054961
Gm26542	-0,96011	0,76488
Gm16537	-0,96152	1
St7	-0,96159	0,3884
Upk1a	-0,96162	0,87236
Gpr19	-0,96162	1
Mkl2	-0,96187	0,4886
Zfp760	-0,96226	0,65198
Szt2	-0,9623	0,72342
Ecd	-0,96268	0,21883
Gm13341	-0,9628	1
Farp2	-0,96334	1
Pde4b	-0,96377	0,41744
4732491K20Rik	-0,96382	1
Gm43379	-0,96482	1
Pou6f1	-0,96502	0,53755
Crtam	-0,96535	0,98408
Gm43059	-0,96619	1
Asns	-0,96668	0,29389
Lppos	-0,96684	1
Slc37a2	-0,96718	0,024639
Tmem173	-0,96735	0,83424
Gm42632	-0,96758	1
AI606181	-0,96799	0,36821
Alg8	-0,96854	0,5855
Gm22714	-0,96954	1
Gm6088	-0,96986	1
Tmem241	-0,97008	0,41729
Acsf3	-0,97025	0,99081
RP23-3F1.8	-0,97176	0,17213
Map3k7	-0,97244	0,014202

Zfp365	-0,9724	0,71371
Klhl5	-0,97286	0,14593
Pcnx3	-0,97356	0,025936
Pik3r3	-0,97392	0,88267
Rnf25	-0,97441	0,98855
Mgrn1	-0,97462	0,076477
Pcdhb22	-0,97462	1
Tbc1d8b	-0,97534	0,97533
Ppp2r3d	-0,9759	0,25729
Gm44775	-0,97589	1
Sec16a	-0,97782	0,23328
D2Bwg1423e	-0,97787	0,87201
Dido1	-0,9804	0,16657
Cmc4	-0,9806	0,83707
Gas7	-0,98075	0,18884
Ago1	-0,98124	0,17458
Ppm1e	-0,98168	1
Lpar1	-0,98217	0,87871
Ica1	-0,9825	0,2314
Pld2	-0,98338	0,79392
Bcl2l15	-0,98346	0,92112
Ttyh3	-0,98366	0,064914
Arhgef25	-0,98468	0,26752
Gm43200	-0,98585	1
Slc11a1	-0,98767	0,054552
Elp4	-0,98767	1
Ly9	-0,98898	0,35129
Lzts3	-0,98929	1
Gm37106	-0,99044	1
Aldh1l2	-0,99213	0,34633
Snora17	-0,99208	1
Myof	-0,99303	0,02477
Lypla1	-0,99304	0,046556
Plcb4	-0,99325	0,011298
Carmil1	-0,99452	1
Gm20696	-0,9951	0,98116
Inca1	-0,99539	1
Hoxaas3	-0,99642	0,81303
Pcgf6	-0,99663	0,53466
Gm42941	-0,99664	0,95961
Stx1a	-0,99722	1
Plk3	-0,99753	0,20335
Shtn1	-0,99908	0,027083
Trim14	-0,9997	1
Irx5	-1,0001	1
Pspc1	-1,0004	0,27557
Six5	-1,0004	1
Zdhhc13	-1,001	0,68295
Zfp862-ps	-1,0022	0,47653
Calcoco1	-1,0023	0,30201
Got2-ps1	-1,0042	1
Gm43742	-1,0043	0,36461

Tmem80	-1,0043	0,71718
Eml5	-1,0051	0,33273
Mtmr11	-1,0052	1
Tmem2	-1,0053	0,078429
Tmem67	-1,0053	0,95758
Inpp5e	-1,0057	1
Evi5l	-1,0065	0,77112
Cox6a2	-1,0073	0,57309
Dmxl2	-1,0076	0,12194
Gm15708	-1,009	1
Fancf	-1,01	1
Il34	-1,0102	0,51767
Pde4d	-1,0103	0,47653
Gm11716	-1,0113	0,52544
Tbc1d7	-1,0113	0,7109
Setmar	-1,0113	0,97749
Nrp2	-1,012	0,0016428
Zscan26	-1,0125	0,76046
Mapk9	-1,0133	0,19402
Klkb1	-1,0138	1
Acvr1	-1,014	0,14923
Gm15157	-1,0152	1
Nsdhl	-1,016	0,097382
Pam16	-1,016	0,97597
Slc43a2	-1,0163	0,045533
B230312C02Rik	-1,0171	0,32917
Zfhx4	-1,0172	0,092604
Cd82	-1,0174	0,034473
Slc7a11	-1,019	0,08997
Enpp5	-1,0194	0,07573
D930016D06Rik	-1,0197	0,74346
Ccar2	-1,0198	0,8053
Gm30238	-1,02	1
Snord66	-1,0204	1
Gm26132	-1,0206	0,98583
Gm4459	-1,0208	1
Lrif1	-1,021	0,3036
Aak1	-1,0215	0,020398
Gm43024	-1,0216	0,70799
Me1	-1,025	0,012119
Gnat2	-1,0255	1
Gm10132	-1,0272	1
Acot11	-1,0273	1
Lrrc8b	-1,0281	0,18669
Gm25517	-1,0284	0,95111
Naip2	-1,0286	0,095189
Mtus1	-1,0292	1
Kctd12	-1,0305	0,63497
Tmem62	-1,0311	0,97585
Nedd9	-1,0313	0,84824
Sec24d	-1,0328	0,15203
Pcdhb16	-1,033	1

Gm25291	-1,0334	1
Hfe	-1,0351	0,57142
Gm11298	-1,0357	1
Zfp362	-1,0385	0,16798
1110035H17Rik	-1,04	0,97539
RP23-225D5.4	-1,0407	1
Gstm4	-1,0409	0,73266
Gja1	-1,0413	0,64463
9230111E07Rik	-1,0416	1
Hap1	-1,0426	0,20121
1700008J07Rik	-1,0431	1
2900005J15Rik	-1,044	0,58881
Nlrp3	-1,0454	0,22706
Kcne3	-1,0454	1
RP24-282C4.9	-1,0487	0,53662
Gdpd1	-1,0493	0,042202
Tmtc4	-1,0499	0,76488
Abcd1	-1,0511	0,38577
Thada	-1,0516	0,43267
3010003L21Rik	-1,0524	1
Zfp119b	-1,0525	0,85532
Gm44432	-1,0533	1
Gramd1c	-1,0544	0,86435
Wdr35	-1,0559	0,55383
Dph2	-1,057	0,42047
Gm38111	-1,0579	1
Atg4a-ps	-1,0607	0,97742
Iars	-1,0614	0,041562
Mir7078	-1,0619	0,85558
Ppp1cc	-1,0623	1
Pygm	-1,0627	1
Mypop	-1,0635	0,57504
Zfp113	-1,0637	0,19448
Btk	-1,0643	0,16166
BC024978	-1,0648	0,69391
Serinc5	-1,0656	0,61058
Gm22516	-1,0662	1
Cdo1	-1,0663	1
Ppp2r3a	-1,0671	0,52329
Gm43144	-1,0701	1
Tpmt	-1,0706	0,99346
Gm8463	-1,0742	0,67643
Rhobtb1	-1,0752	0,69661
Gm45640	-1,0752	0,88628
Gm15513	-1,0757	0,76488
Pear1	-1,076	0,79443
Pigt	-1,0767	0,1889
Gm20632	-1,0783	0,34066
Fuz	-1,0791	0,69661
Ubc	-1,0792	0,16818
Gm38157	-1,0793	1
Fads6	-1,0794	0,22511

Csf2rb2	-1,0799	0,52021
Lta	-1,083	0,88031
Tanc2	-1,0831	0,37692
Acy1	-1,0836	0,37157
Gm37101	-1,0837	0,90699
RP23-138K22.2	-1,0854	1
Ap5b1	-1,0903	0,35145
Tiam2	-1,0906	0,8066
Mras	-1,0912	0,41202
Tac4	-1,0921	1
Ublcp1	-1,0922	1
Gm10676	-1,0987	1
Sfxn2	-1,0998	0,17908
Rwdd2b	-1,1008	0,69661
Bbs7	-1,1014	0,54617
Arhgef18	-1,1031	0,64463
Iqcf1	-1,1031	0,8287
Gm42481	-1,1031	0,90996
Gm25514	-1,1037	0,4865
Gm42576	-1,1058	0,84961
Elk3	-1,1067	0,058222
Slc9a9	-1,107	0,60852
Creb3l3	-1,1074	1
Zfp182	-1,1077	0,7166
Gpr85	-1,1107	0,97539
Mfap1a	-1,1109	0,91369
Tmem151a	-1,1113	1
Erfe	-1,1113	1
Slc25a10	-1,1114	0,14603
Slc9a8	-1,1117	0,0113
Glyctk	-1,1137	1
Tmigd3	-1,1144	1
Mtr	-1,1157	0,024371
Ccdc136	-1,1168	0,52002
Mzb1	-1,117	0,56454
Ndor1	-1,1186	0,54879
Gm43350	-1,1191	0,64146
Gm23300	-1,1191	1
Zfp462	-1,1202	0,25661
Nkpd1	-1,1211	1
RP23-162P10.2	-1,1213	0,8237
4930529C04Rik	-1,1221	0,39072
Fus	-1,1232	0,12017
3830403N18Rik	-1,1245	1
Gm28438	-1,1248	1
Slc6a9	-1,1261	0,65198
Gm10842	-1,1264	0,89353
Acacb	-1,1264	1
Nckap5l	-1,1265	0,25915
Zfp248	-1,127	0,81265
Gm29488	-1,1278	0,9873
Rbks	-1,129	1

Lamc2	-1,1295	1
Mapkapk2	-1,1297	0,001265
Bbs1	-1,1303	0,8322
Dgkz	-1,1307	0,014055
Ints7	-1,1308	0,044173
Prss44	-1,1308	0,99346
Gm43290	-1,1338	0,32997
Unc93b1	-1,1342	0,010581
Iqce	-1,1354	0,38448
Fam102a	-1,1371	0,00035343
Zfp661	-1,1374	0,52163
A430105I19Rik	-1,1382	0,61284
Slc25a42	-1,1388	0,85401
Hebp2	-1,14	0,17534
Donson	-1,141	0,61995
Gm45630	-1,1414	0,98555
1700007K09Rik	-1,1415	1
Cd33	-1,1422	0,0031271
Tlcd1	-1,1432	0,9016
Gm16536	-1,145	0,26826
Il17rc	-1,1454	0,57309
Gm43359	-1,1456	0,27717
Blnk	-1,147	0,035466
Bend6	-1,149	0,98583
Grtp1	-1,1507	0,2318
Slc45a4	-1,1508	0,71136
Fam208a	-1,1519	0,27077
Slc35d2	-1,1527	0,27879
Aars	-1,1528	0,07281
Crocc	-1,1532	0,85435
Ccdc69	-1,1538	0,98583
mt-Cytb	-1,154	0,027442
Hrc	-1,1544	0,80486
Cdk16	-1,1549	0,14955
Sgsh	-1,1577	0,055942
Tcea3	-1,1579	0,94649
Gm45224	-1,1584	0,97839
Pgap3	-1,1618	0,59191
Lars	-1,1622	0,00073786
Eng	-1,1632	0,086957
Eva1b	-1,1635	0,33273
RP23-444K20.4	-1,1642	0,080829
Tlr3	-1,1661	0,62682
Lcp2	-1,1665	0,0085039
2810029C07Rik	-1,1671	0,84711
Rpl7l1-ps1	-1,1677	0,64665
D230022J07Rik	-1,1689	0,87236
Caskin2	-1,1695	0,73679
Nyap1	-1,1696	0,30201
Gm43445	-1,17	0,97539
Gm15265	-1,1707	1
Rnf123	-1,1708	0,52163

C530005A16Rik	-1,1727	0,85099
Gsn	-1,173	0,001633
Nt5c2	-1,1741	0,46884
C3	-1,1759	1
Lpar2	-1,1762	0,76046
Smco3	-1,1762	1
Myo1d	-1,1763	0,00099048
Nphp3	-1,1765	0,34035
Mterf3	-1,1771	0,41202
Cxcl2	-1,1776	0,07135
Gm4285	-1,1788	1
Neat1	-1,1807	0,030228
4732440D04Rik	-1,1811	0,80744
Gm25596	-1,1821	0,93874
Gm20712	-1,1838	0,36821
B130006D01Rik	-1,1862	0,51767
Slpi	-1,1868	0,014055
Ceacam10	-1,1875	0,77946
Pidd1	-1,1887	0,76324
Bicdl1	-1,1895	0,87871
Cxcr3	-1,1896	1
Atp8b4	-1,1925	0,24938
Hapln3	-1,1929	0,92766
Tbc1d2b	-1,1931	0,0002226
Dock5	-1,194	0,045533
Tsga10ip	-1,1948	1
Plat	-1,1949	0,70905
Celsr3	-1,1955	0,6734
Jade2	-1,1994	0,11815
Fam151b	-1,1999	1
Gm2308	-1,2004	1
Slfn8	-1,2017	0,61199
Gm37206	-1,2052	0,99338
Maats1os	-1,2059	0,82013
Nfatc1	-1,2064	0,0022557
Gm37357	-1,2106	1
Nmb	-1,2107	0,55892
Matn4	-1,213	0,92777
Oscp1	-1,2131	0,90352
Oasl1	-1,214	0,26403
Celf6	-1,2164	0,59191
Col15a1	-1,2169	0,69256
Maml2	-1,219	0,24652
Gm42484	-1,219	0,47653
Acsbg1	-1,2201	0,17261
Dtwd2	-1,2205	0,74233
Ddr2	-1,2207	0,52469
Ntn5	-1,2256	0,54694
Nr0b2	-1,2279	0,77946
Zfp78	-1,2299	0,87893
Ltbp2	-1,2309	0,64906
Tas1r1	-1,2309	0,82013

B230398E01Rik	-1,2316	0,40089
Gm37080	-1,2319	0,61978
Prkar1b	-1,232	0,17458
Ssc5d	-1,2323	1
RP24-175C20.18	-1,2371	0,40826
Ifi47	-1,2381	0,90937
Zfp951	-1,2388	0,53781
Prokr1	-1,239	0,69204
Pycr1	-1,2395	0,27112
Ccdc171	-1,2419	0,80567
Zswim8	-1,2435	0,022162
Ifit2	-1,2467	0,90083
Kctd21	-1,2475	1
9630013D21Rik	-1,2491	0,84269
Snord59a	-1,2494	0,97146
Npl	-1,2513	1
Map3k15	-1,2514	0,81574
Kif5a	-1,2525	0,12322
Snord92	-1,253	0,8287
Gm44623	-1,2554	0,67267
Gm37718	-1,2556	0,56603
Ano7	-1,2565	0,86022
Sh2d2a	-1,2577	0,41835
Gnmt	-1,2661	0,87983
Gdpgp1	-1,2665	0,51643
Tmem8	-1,2689	0,043021
Crtc1	-1,269	0,31055
Tfb2m	-1,2697	0,48398
Gm45495	-1,2713	0,38757
Stab1	-1,2725	0,17261
Zfp658	-1,2727	0,43817
Arg1	-1,2744	0,76488
Gm43668	-1,2747	0,3036
Gm28151	-1,2768	0,72197
Gm44509	-1,2773	0,7626
Poln	-1,2788	0,8287
Zfp866	-1,2864	0,69661
Gm45084	-1,2896	0,6649
Samd10	-1,2926	0,55732
Khnyn	-1,2977	0,43817
Accs	-1,2996	0,27199
Gm43660	-1,3003	0,80486
Vps25	-1,3012	0,8364
Acta2	-1,3033	0,90083
Abcc3	-1,3052	0,78287
Mir155hg	-1,3059	0,60304
Bank1	-1,3061	0,86857
Calml4	-1,3073	0,035473
Slc46a3	-1,3117	0,60093
Fam129c	-1,3143	0,6622
Pde4dip	-1,3149	0,024647
Foxd2	-1,3149	0,66237

Mccc2	-1,3153	0,90671
Hmcn2	-1,3191	0,59876
Gemin8	-1,3216	0,80486
Gm42869	-1,3217	0,60858
Gm20554	-1,322	0,70799
St5	-1,3231	1
4933421A08Rik	-1,3233	0,8053
F630040K05Rik	-1,324	0,27507
Xylb	-1,3243	0,77759
mt-Nd4	-1,3254	0,021822
Gm42566	-1,3277	1
Arhgef10	-1,331	0,097065
Gm43148	-1,3315	0,8287
Rapgef3	-1,3318	0,68186
9130023H24Rik	-1,3353	0,57309
Egr1	-1,3356	0,1271
Nprl3	-1,3359	0,10186
Gm23054	-1,3392	0,9873
Gm42467	-1,3402	0,12456
Lrtm2	-1,3403	0,83707
Zfp811	-1,3469	0,69661
Gm14137	-1,3473	0,74013
Slc22a5	-1,3479	0,13201
Loxl3	-1,3491	0,21506
Gm9951	-1,3492	0,50258
Pars2	-1,3493	0,54024
Egfl7	-1,3501	0,07573
Rhbdd2	-1,3502	0,15416
Gm28404	-1,3553	0,536
A930018M24Rik	-1,3561	0,92403
Gm9207	-1,3573	0,98408
Ddx17	-1,3594	0,00077081
Tlr1	-1,3608	0,17182
Ccdc116	-1,3628	0,81649
RP23-278O17.1	-1,365	0,81649
Nod1	-1,3681	0,064388
Vegfc	-1,37	0,27447
Inpp5b	-1,3715	0,043021
Slc2a4	-1,372	0,37692
Hal	-1,3736	0,61792
Zfp11	-1,3765	0,69661
1700086P04Rik	-1,379	0,61978
Gm43112	-1,3826	0,72633
Prkca	-1,3832	0,025306
Camk2a	-1,3834	0,011389
Gm5914	-1,3844	0,68563
Gm26947	-1,3867	0,26757
Fam69b	-1,3871	0,60891
Adamts6	-1,3908	0,65098
Gm37578	-1,3933	0,88267
Gm24009	-1,398	0,85401
Bdh1	-1,3994	0,15881

Arhgef19	-1,4004	0,57309
Ctnnal1	-1,4008	0,31863
2210417A02Rik	-1,4027	0,98583
Gm43006	-1,406	0,64629
Bcl3	-1,4073	0,0083292
Masp2	-1,4084	0,5735
Idi1	-1,4114	0,7998
Hpdl	-1,4131	0,68295
Fosl2	-1,4143	3,14E-05
Tns4	-1,4152	0,078429
Rnf225	-1,4159	0,79443
Arsg	-1,4171	0,43267
Gm37204	-1,418	0,78287
Nfatc4	-1,4207	0,68186
Meiob	-1,4209	0,64131
Gm37063	-1,4262	0,80536
Ms4a6c	-1,4275	0,26831
Gm44178	-1,4317	0,74013
Gm45289	-1,4368	0,64463
Dnmt3b	-1,4423	0,1889
Zfp459	-1,4442	0,37157
Alg2	-1,4448	0,29493
Gm38020	-1,4462	0,26729
Vps37d	-1,4471	0,85715
Atp8b3	-1,4504	0,64032
Ociad2	-1,4543	0,63448
Aldh7a1	-1,455	0,41202
Ccdc33	-1,4552	0,69297
Plag1	-1,4593	0,41634
Zfp619	-1,4637	0,69482
Gm37121	-1,4642	0,072212
C430049E01Rik	-1,4688	0,52544
Gm15496	-1,4693	0,18986
Jdp2	-1,4705	0,00088797
Olfr933	-1,4768	0,10798
Matk	-1,48	0,64629
Tm4sf19	-1,4857	0,53181
C230035I16Rik	-1,4873	0,85462
Llgl2	-1,489	0,562
D7Bwg0826e	-1,4895	0,56603
Mybpc3	-1,4908	0,049748
Gper1	-1,4922	0,3036
Ccnd2	-1,4938	1,80E-05
Myom1	-1,4944	0,054749
Lgals9	-1,4951	0,48398
Rgs8	-1,4994	0,3884
St18	-1,5013	0,0012271
Galnt15	-1,5019	0,43817
Prss46	-1,5024	0,36495
Tmem150a	-1,5025	0,34029
Gdf9	-1,5066	0,73049
8430408G22Rik	-1,5109	0,56692

RP23-213P10.2	-1,5114	0,83574
Gm43924	-1,5118	0,38939
Zfp169	-1,5188	0,21714
Flywch1	-1,5194	0,0083292
Cnksr1	-1,5204	0,54397
Dixdc1	-1,5217	0,076416
Gm6140	-1,5232	0,51643
Sema4a	-1,524	3,84E-05
Park2	-1,5266	0,69256
Gm43147	-1,5337	0,31994
2010008C14Rik	-1,5362	0,30943
Gm43364	-1,5389	0,72809
Stra8	-1,5391	0,57526
Gm5131	-1,5476	0,58499
Sema6c	-1,5557	0,5192
Olr1	-1,5574	0,057341
Gm23442	-1,5576	0,65751
Rsl1	-1,5599	0,72197
Ctsk	-1,5668	7,40E-06
Krcc1	-1,5679	0,23328
Sh2d6	-1,5685	0,63448
Gm37851	-1,5817	0,75663
Gm37788	-1,5832	0,4391
Trpm2	-1,5832	0,45015
2510016D11Rik	-1,584	0,59191
Spata2l	-1,5899	0,60599
Klhl23	-1,5935	0,55558
Nf2	-1,5961	2,73E-05
Gm42600	-1,598	0,7109
C030013C21Rik	-1,6007	0,14311
Gm15530	-1,6025	0,40206
Zfp52	-1,6026	0,29583
Gm44237	-1,607	0,35554
Piwil2	-1,6116	0,33273
Irf4	-1,6153	0,65887
I830077J02Rik	-1,6163	0,24732
Tubg2	-1,6167	0,37883
Gm21967	-1,6202	0,53558
Nek3	-1,6208	0,52816
Cntnap1	-1,623	0,3481
RP24-226A8.2	-1,626	0,72809
Rrad	-1,6267	0,58274
Arhgap4	-1,627	0,33313
Erbb3	-1,6285	0,099685
Gm13223	-1,631	0,68865
Gm26514	-1,6351	0,44446
2210406H18Rik	-1,6409	0,61341
5430420F09Rik	-1,6416	0,58417
Cyp26b1	-1,6485	0,45245
Gm42486	-1,649	0,53566
Phactr1	-1,65	0,39784
Creb3l1	-1,653	0,28676

Gm20257	-1,6536	0,27207
Gm43727	-1,6542	0,49898
Mrc1	-1,6598	0,6622
9330102E08Rik	-1,6641	0,60391
Med12	-1,6723	0,47568
Klhl41	-1,6795	0,14369
Src	-1,6804	0,00088797
Mmp2	-1,6844	0,47653
Nlrc4	-1,6868	0,35554
Dusp9	-1,6885	0,14611
Apba1	-1,6891	0,48191
Anxa9	-1,6915	0,19402
Nat14	-1,6915	0,28867
Rnft2	-1,7077	0,68471
Pxdn	-1,71	0,024614
Gm19026	-1,7119	0,16806
Ip6k3	-1,7122	0,0011645
Syt8	-1,7197	0,3036
BC037039	-1,7245	0,28453
Ptpn14	-1,7246	0,031369
Rbfox1	-1,7256	0,33273
Lhx1	-1,7311	0,29599
Oas1d	-1,7324	0,16837
Fmo5	-1,7384	0,27077
Gm10033	-1,7414	0,11815
Acot6	-1,7516	0,091517
Gm10167	-1,7554	0,64591
Capn3	-1,7562	0,5246
Gm15634	-1,7587	0,37157
Extl1	-1,7626	0,067744
Slc39a4	-1,7695	0,08362
Gm21816	-1,7731	0,28676
Klhl30	-1,7941	0,037363
AW046200	-1,7947	0,092604
Zfp335os	-1,7954	0,53566
Pus7l	-1,8017	0,092122
Gm45728	-1,8085	0,3575
Grin1	-1,8088	0,57302
Frk	-1,8158	0,26851
Atp6v0c	-1,8222	0,18884
Zfp712	-1,8362	0,40826
Ddr1	-1,8385	0,45799
Tfr2	-1,8655	0,27199
C8g	-1,8662	0,27193
Cd93	-1,8674	0,21011
9930120I10Rik	-1,8775	0,30943
Wnk2	-1,8819	0,00067372
Gm10463	-1,9035	0,27257
Plxnb3	-1,9057	0,21509
Tmem204	-1,9094	0,00165
Slc9b1	-1,9285	0,14222
Gm16712	-1,9295	0,099403

Cpne2	-1,9336	2,20E-05
Tns2	-1,9351	0,19319
Adora2a	-1,9356	0,26826
Tspan10	-1,9609	0,00099957
Rgs20	-1,9609	0,056599
Zfp617	-1,9675	0,13188
Gm13205	-1,9678	0,15168
9030407P20Rik	-1,975	0,10361
Tti1	-1,9767	0,20665
Gm22	-1,9772	0,033707
Lif	-1,9773	0,27828
Nfyc	-1,9831	0,4839
Txlnb	-1,9875	0,26414
Rab11fip4	-1,989	0,14369
Lgr5	-1,9919	0,55383
Trdmt1	-1,994	0,3036
Plekhs1	-1,9965	0,15992
Stc1	-1,9995	0,28512
Gm20219	-2,0012	0,045533
Fendrr	-2,0096	0,20569
Glrp1	-2,0122	0,11309
Rorc	-2,0261	0,18986
Phf11c	-2,0452	0,24739
Hmx3	-2,0568	0,14507
Gm12799	-2,0593	0,18884
Pdpn	-2,0702	0,0021725
Gm15787	-2,0707	0,19988
Gm5532	-2,0754	0,059384
Fzd2	-2,0912	0,18818
Gm29243	-2,0981	0,14592
Fbxo10	-2,0981	0,22096
Celf3	-2,104	0,13501
Gm44901	-2,1092	0,36711
Gm22748	-2,1105	0,0077153
Gm20186	-2,1234	0,17261
Gm2885	-2,127	0,17458
Clec2l	-2,1355	0,16738
Platr3	-2,1478	0,16818
Zbtb45	-2,1523	0,029977
Shisa3	-2,1583	0,15947
Mkx	-2,1647	0,095189
Gm17455	-2,1655	0,12262
2810428J06Rik	-2,1665	0,10567
Pla2g2d	-2,1772	0,0050415
Ahrr	-2,2026	0,09954
Gm24336	-2,2026	0,1889
Cmklr1	-2,2059	0,17788
Cracr2a	-2,2109	0,14603
Robo3	-2,2142	0,0012847
Mlkl	-2,2869	0,064024
Oas2	-2,2889	0,17534
Zfp473	-2,2924	0,10173

Tnfrsf13b	-2,309	0,11815
Slc6a4	-2,3196	0,0061731
Sbk3	-2,3252	0,111
Vamp7-ps	-2,3285	0,18319
Tgfb1i1	-2,3302	0,08362
Skor1	-2,3629	0,07944
Ccl5	-2,3641	0,063969
Gm43154	-2,3651	0,05747
4930461G14Rik	-2,3777	0,071914
Acp5	-2,3803	2,93E-09
Gm42483	-2,3803	0,14105
Rhou	-2,4177	0,078282
Amigo3	-2,4194	0,063881
Adamts7	-2,4444	0,00036759
Gm12663	-2,4923	0,13259
Ehd2	-2,5284	0,011389
Oscar	-2,5715	0,035734
Adh7	-2,6412	0,033173
Chac1	-2,7248	0,012118
Vil1	-2,7259	0,038055
AW047730	-2,7435	0,046556
RP24-131G14.10	-2,7822	0,060772
Gm10069	-2,7999	0,07281
Arl14epl	-2,8229	0,045533
Hsd17b14	-2,8417	0,068547
Gm22767	-2,865	0,035734
Celsr1	-2,883	0,035734
Rap1gap	-2,9068	0,0019943
Met	-2,934	0,00095499
Lctl	-2,9562	0,0087737
Prss42	-2,967	0,013372
Ncmap	-2,9696	0,025466
Prss35	-2,9743	0,0051479
Chd5	-3,015	0,025744
RP23-268C22.3	-3,0336	0,021773
Rab15	-3,0484	0,00073786
Slc30a2	-3,0794	0,035098
Ablim1	-3,5293	0,00068406
Wisp1	-3,5401	0,0075757
Slc1a4	-3,5637	0,0028875
Slc9b2	-3,6296	1,57E-08
Accsl	-3,6764	0,00041588
Acod1	-4,0025	4,90E-06
Scn11a	-4,3635	3,05E-05
Col27a1	-4,7141	3,14E-05
