# Supplementary material for: Bone, dentin and cementum differentially influence the differentiation of osteoclast-like cells
Source: Sci Rep. 2025 Jun 5;15:19857. doi: 10.1038/s41598-025-04874-9 (PMC12141432; doi:10.1038/s41598-025-04874-9)
Supplement: Supplementary file 11 — Supplementary Information 11. [file 41598_2025_4874_MOESM11_ESM.pdf]

Tab. S10:

Transcripts induced in murine macrophage cells stimulated on dentin (n=6), fold of cementum

gene name	regulation of expression	adj.P.Val
Slc9b2	169,4716332	1,00E-07
Olr1	27,57903362	3,95E-05
Slc1a4	26,6285137	0,00031856
Wisp1	18,18655021	0,0022078
Acod1	18,12865516	6,46E-06
Col27a1	17,55974513	8,58E-05
Ablim1	16,96277148	0,00016309
Met	16,15714065	0,00015687
Rab15	15,55277381	0,00018844
Accsl	14,98347136	0,00012484
Scn11a	12,71535644	0,00028595
Ctsk	11,32469272	3,73E-12
Pla2g2d	11,27065925	0,00027516
Slc9b1	10,75622549	0,0042731
Slc30a2	10,38262978	0,031242
Gm29243	10,28236339	0,0073037
Acp5	10,25460488	3,73E-12
Oas1d	9,904608838	0,0080293
Rorc	9,64913756	0,0098535
Gm43154	9,408730002	0,01249
Adh7	9,134970057	0,011657
Rab11fip4	8,477271543	0,020443
Gm19026	8,401809063	0,0092751
Chac1	8,329327391	0,0071948
Ceacam10	8,231184186	0,012947
Lctl	7,986149061	0,006201
RP23-268C22.3	7,656579359	0,020754
Gm16712	7,15623121	0,01414
Gm24336	7,047443468	0,05433
Slc39a4	7,023547984	0,0075393
Arl14epl	7,003616074	0,042179
4930461G14Rik	6,978901646	0,02307
Gm20219	6,959096437	0,0075112
Ociad2	6,881389951	0,15467
Prss35	6,850455885	0,0080293
Adamts7	6,801250362	0,00018844
Slc6a4	6,757548682	0,002078
Zbtb45	6,323397882	0,0092751
Robo3	6,278413288	0,00040616
Gm22767	6,165423517	0,058633
Tmem204	6,082228427	0,000168
Lpar1	6,050691377	0,028491
Rgs20	6,039796745	0,012682
Skor1	6,033102109	0,060953
Calml4	6,029339638	0,00012047
Klhl40	6,021821734	0,17541
Pgap3	5,977739383	0,024612
Pxdn	5,912220186	0,0012399

Rgs8	5,85147364	0,061832
Plat	5,394540208	0,08331
Celf3	5,297457102	0,084259
Pla1a	5,294887384	0,067279
Ccdc33	5,273643325	0,12886
Dixdc1	5,265607549	0,0043877
Gm44775	5,239757339	0,069463
Mras	5,169769651	0,0069374
Nlrc4	5,03119114	0,1344
Gm10463	5,02387302	0,12703
AW047730	5,019000188	0,10531
Plekhs1	5,008574314	0,094277
Chd5	4,990208171	0,09538
Vegfc	4,951617931	0,031907
Gm5532	4,90583938	0,043232
Pars2	4,887172407	0,12886
Arhgap4	4,842996633	0,11683
Rhou	4,819555182	0,12843
Sbk3	4,762767092	0,13396
Ccl5	4,753861921	0,088178
Lin28b	4,729540418	0,48112
Galnt15	4,712524112	0,099541
Clec2l	4,707300652	0,15179
Gja1	4,680296732	0,024771
Ip6k3	4,655706138	0,00013591
Tiam2	4,629319064	0,10156
Poln	4,622905911	0,20916
Hsd17b14	4,622265085	0,22603
Ubox5	4,597979392	0,13125
Lgi4	4,590018601	0,41531
Prokr1	4,581436426	0,1369
Lta	4,56811826	0,15021
Platr3	4,554523104	0,15482
RP23-278O17.1	4,553260497	0,24911
Prss42	4,528081735	0,11325
Zfp119b	4,524630561	0,10685
Smco3	4,4401241	0,44912
9330102E08Rik	4,411899721	0,33214
Psca	4,382639461	0,14562
Sorl1	4,362938084	0,38592
B230377A18Rik	4,360821687	0,24556
Shisa3	4,335805379	0,21878
Oscar	4,306751254	0,11184
Gm15530	4,27789182	0,16583
Gm26569	4,253056439	0,47239
Rap1gap	4,223678432	0,019434
Ddr2	4,222214871	0,073948
Ssc5d	4,199739955	0,52766
Faah	4,17564771	0,32815
Celf6	4,174200791	0,10156
Park2	4,136469659	0,42618
Prss46	4,090564643	0,14859

Nr0b2	4,065125803	0,25941
Amigo3	4,0552757	0,1502
Cyp26b1	4,042926568	0,31228
Dusp9	4,009993783	0,078343
Tspan10	4,003051011	0,0011609
Nek3	3,993628126	0,33516
Ppm1e	3,975675481	0,22307
Oscp1	3,965217492	0,42618
Zfp459	3,919310847	0,14038
Cox6a2	3,911982726	0,028896
4930556M19Rik	3,88307615	0,44424
Gm5544	3,875278515	0,52887
Mkx	3,854116016	0,19797
Prkar1b	3,84131427	0,012214
Ctla2b	3,829882152	0,13964
Celsr1	3,811872858	0,23331
Gm13657	3,783968293	0,28529
Mlkl	3,77532279	0,18461
Myh11	3,76069681	0,5821
Jdp2	3,753925451	2,32E-05
8430408G22Rik	3,737050346	0,37921
Bdh2	3,703527372	1,42E-06
Nod1	3,69916589	0,0069864
Epb41l1	3,677435404	0,020443
Gm22748	3,676415944	0,01882
Arg1	3,66318865	0,42305
Acta2	3,658874673	0,50136
Gm13205	3,648491266	0,22239
Spata2l	3,636372518	0,52228
Gm20554	3,634860508	0,41205
Gm10069	3,628818751	0,41292
9130023H24Rik	3,597513249	0,24839
Kyat3	3,593276615	0,53025
Ehd2	3,561290195	0,077665
Serinc5	3,543315672	0,078343
Gstm4	3,541105926	0,13528
Acot11	3,538897558	0,48797
Arsg	3,52909924	0,2328
Pdpn	3,526165038	0,0089541
Glrp1	3,513965434	0,19933
Csf2rb2	3,513721873	0,063747
RP24-91J7.1	3,500351979	0,47744
Acsbg1	3,46486706	0,019549
Gm22	3,461026529	0,063501
Zfp78	3,460786637	0,50305
Nedd9	3,446184639	0,23171
Foxd2	3,44308071	0,39534
Arhgef19	3,440218027	0,38592
H2-Ab1	3,410066799	0,54463
Fabp7	3,40392677	0,43798
Amigo1	3,402747263	0,32186
Zfp811	3,390739616	0,49297

Srd5a1	3,367784908	0,50309
Gm6088	3,343362996	0,82762
Gm28071	3,34174118	0,54403
Cd93	3,337111753	0,38638
Tbc1d2b	3,318198056	8,65E-08
Gm15496	3,316588446	0,11584
Slc2a4	3,310157814	0,19615
Timp2	3,309010799	3,16E-05
Gm42970	3,298934194	0,72711
Igf2bp1	3,296419841	0,57572
RP23-138K22.2	3,295277587	0,64151
Abhd3	3,29025637	0,63985
Gdf9	3,277509586	0,64406
Gm12663	3,275465607	0,52228
Stra8	3,270474578	0,54909
Gm29462	3,253516793	0,63079
D7Bwg0826e	3,251037055	0,50487
Fosl2	3,240238483	6,02E-07
Pitpnm2	3,237544449	0,015623
Zfp54	3,231715057	0,40423
Gm5131	3,230371304	0,59311
Inca1	3,230147399	0,63668
Gm8463	3,223437475	0,20791
Me1	3,220534172	1,40E-06
Gm43379	3,213844218	0,52313
Tmem151a	3,201393462	0,61483
Zfp882	3,192308327	0,61605
Src	3,176856667	0,0012588
Adora2a	3,171796034	0,51313
Irx5	3,167402038	0,644
Vil1	3,156881266	0,44911
Nt5e	3,152289423	0,0075666
Bok	3,115362406	0,020792
Gm13675	3,114498764	0,45999
Mzb1	3,110615336	0,16888
4933412L11Rik	3,108890923	0,75296
Gm33370	3,098994082	0,64261
Cxxc5	3,098779284	0,53025
Cish	3,086774302	0,76978
Tac4	3,074815828	0,86708
Il34	3,067365319	0,06182
3830408C21Rik	3,066514983	0,69114
Lamc2	3,056329315	0,6938
Tigd5	3,052306841	0,33814
Aldh7a1	3,040692655	0,3705
Myo1d	3,038585738	2,66E-06
Rab42	3,038585738	0,64653
Atp6v0d2	3,037322288	1,88E-06
Adcy2	3,035638506	0,058282
Sgsh	3,034165962	0,0018871
Lif	3,031433133	0,63713
Wnk2	3,028912706	0,004298

Frk	3,022411299	0,50003
Eva1b	3,001950108	0,10072
Rsph1	2,991978898	0,33803
2700046G09Rik	2,974608908	0,48472
Gabrd	2,974196568	0,42618
Apbb1	2,972341746	0,36744
Gm16372	2,971105841	0,83534
Ppp1r26	2,96493402	0,36322
Islr2	2,961852918	0,76396
Lrtm2	2,954471332	0,76899
Soat2	2,942209546	0,79578
B3galt4	2,929389437	0,65409
Gcat	2,928374364	0,043164
Slc24a5	2,925939624	0,83566
Slc4a8	2,92573682	0,55166
Hebp2	2,922493873	0,028281
Gm12799	2,91662519	0,59539
Ap5b1	2,915210378	0,06967
Ccdc80	2,914806272	0,5821
Vps25	2,914806272	0,73623
Rrh	2,913998228	0,81281
Gm45456	2,9087514	0,69114
Matk	2,907340407	0,72091
Ccdc17	2,906735906	0,77603
Fendrr	2,901301044	0,54605
Saa3	2,895675625	0,50487
4831440E17Rik	2,884457487	0,72103
Gm17455	2,883657856	0,50713
Tmem2	2,879463431	0,00043821
Angptl2	2,879263849	0,00011763
Irf4	2,875474413	0,85849
Ccr1	2,874876537	0,7178
Zfp85	2,872685388	0,78947
Rhobtb1	2,870296949	0,31228
Slc47a2	2,86890461	0,76289
C8g	2,862349818	0,61164
Nudt22	2,859771742	0,0012223
Zfp462	2,854820426	0,051327
Mrap	2,854029011	0,74634
Gm11410	2,853237816	0,63985
Shox2	2,842184086	0,10597
Ksr1	2,841199231	0,54403
F630040K05Rik	2,837066564	0,19933
Gm20632	2,830976945	0,077299
Kif5c	2,827447036	0,88516
Olfml3	2,822551687	0,48797
Pmepa1	2,820009456	0,00022712
Sh2d2a	2,819618546	0,27965
Ptpn14	2,819423112	0,098829
9930014A18Rik	2,814151503	0,18273
Six5	2,813566378	0,57886
Klhl23	2,8127864	0,78947

Nfatc4	2,807916433	0,76023
Arhgef15	2,807138021	1
Ifi203-ps	2,802860612	0,83449
Ntn5	2,798977714	0,38302
Zfp93	2,795681481	0,72711
St18	2,795100195	0,0018142
Ddr1	2,794906461	0,83566
Gpr85	2,782342455	0,76289
Acsf3	2,769450975	0,54481
Tsga10ip	2,767340179	0,97151
Rhbdd2	2,766764786	0,11776
Aldh1l2	2,766189514	0,038334
Gm42972	2,765614361	0,95843
Rbks	2,760060709	0,88513
Ms4a6b	2,755855034	0,92562
Gm42850	2,753182033	0,86757
Tns2	2,752228018	0,57587
Arhgap27os1	2,7463205	1
Tas1r1	2,745559164	0,73623
Ptges	2,742515929	0,072485
Prss44	2,742135762	0,83223
Myh7b	2,740045785	0,76899
Gm13889	2,738336988	0,11705
Tfr2	2,736439575	0,68938
Bcl2a1d	2,735301759	0,83534
Oas2	2,732459287	0,74314
Atg4a-ps	2,727917474	0,69476
Gm45629	2,721684805	0,92368
Gm43364	2,720930299	0,92981
9530053A07Rik	2,719233423	0,56894
Gm44434	2,718856484	0,84398
Gm17060	2,716784249	0,69476
Gm44187	2,71508996	0,7894
Camk2a	2,713208655	0,0089194
Notch4	2,71095281	0,7424
Cmklr1	2,706634348	0,75027
Mfsd13b	2,702510082	1
Nfatc1	2,691293998	0,00011483
Gm14248	2,684586703	1
Pqlc2	2,679567205	0,79232
Cdhr4	2,67733933	0,7178
Zgpat	2,666597354	0,19227
Gm44237	2,662903226	0,6143
9130230N09Rik	2,662534095	0,71501
Rnf135	2,660873638	0,50876
Mybpc3	2,6590299	0,08579
Gm24507	2,658292763	1
Grap	2,65755583	0,013089
Gm45640	2,641211806	0,64976
Ncmap	2,635725267	0,71757
2610044O15Rik8	2,632803775	0,88513
Usp27x	2,632438816	0,54896

Gm14137	2,63152664	0,81281
Pced1b	2,625332222	0,77607
Creb3l3	2,623331269	0,90096
Med12	2,61969708	0,79006
F830208F22Rik	2,61969708	1
Lgals9	2,618063337	0,67592
Gm43006	2,615342697	0,77437
Pygm	2,612624884	0,88883
Acad12	2,60611365	0,47814
Chrna1os	2,595837368	0,71757
Gm11298	2,593319574	0,95649
Gsn	2,593139825	7,13E-05
Ms4a6c	2,592960088	0,42988
Ahrr	2,58434737	0,60134
Mfap1a	2,582556657	0,76289
Rcan1	2,581482826	0,00026835
Alg2	2,58040944	0,45547
A930024E05Rik	2,578621457	0,52313
mt-Nd4	2,575049205	0,012145
Lpin3	2,574156915	0,011447
Gpt2	2,567919541	0,0022078
D3Ertd751e	2,559744828	0,44267
Cpt1c	2,554073424	0,12807
Gm4034	2,550181625	0,69476
Stx1a	2,540830261	0,90096
Hrc	2,539773779	0,74314
Acy1	2,533970976	0,1702
Arl11	2,533444106	0,27201
Klhl41	2,527655765	0,43983
BC024978	2,527480567	0,46834
Ptafr	2,52223028	0,42791
mt-Cytb	2,514201014	0,0034572
Mmp2	2,514026749	0,88182
Gm44291	2,508630518	1
Gm5778	2,505502539	1
Dcp1b	2,502205015	0,68571
Ccdc69	2,498911831	0,97367
Msantd3	2,498565433	0,008397
Cracr2a	2,492511222	0,77136
Gm7909	2,490784143	0,84398
Hoxaas3	2,487850856	0,50145
Vwa7	2,486127007	0,45572
Pxylp1	2,483887788	1
Kcne3	2,479587266	1
9030407P20Rik	2,477010527	0,59453
Zfp473	2,475294189	0,72907
Tssk6	2,473236153	1
Rpl7l1-ps1	2,472550522	0,60134
Txlnb	2,470837274	0,90414
Col15a1	2,468954073	0,72711
Cldn15	2,468782944	1
1810024B03Rik	2,468098547	0,73409

Etohd2	2,467585374	0,93298
Il18rap	2,462971613	0,79285
Mtus1	2,462800898	0,87684
A930018M24Rik	2,461094406	1
Atp8b3	2,459389097	0,90828
Tnnc2	2,459048177	1
Ccdc92b	2,457684969	1
C430042M11Rik	2,456322517	0,50779
Fam65c	2,450710381	0,018938
Gm8550	2,450200823	0,98277
Tmem71	2,449012266	1
Extl1	2,448503062	0,37518
Prkca	2,444772126	0,053553
2810428J06Rik	2,44392498	0,72711
Runx2os1	2,442908791	0,66931
Catip	2,437327303	0,48921
Prss50	2,436482736	0,10164
Zfp658	2,433107394	0,54316
St5	2,432601496	1
Gper1	2,42990515	0,6143
Ccnd2	2,421162649	0,00029291
Bdh1	2,418478982	0,35079
Tns4	2,417976126	0,19933
Osbp2	2,415630845	1
6430571L13Rik	2,406105664	1
Tsku	2,402605863	1
Usp18	2,400109122	0,32186
Gm8181	2,394127524	1
Traf3ip3	2,388988651	0,51643
mt-Nd2	2,388657489	0,0054589
Acy3	2,387995305	0,88231
Zfp229	2,387002372	0,94659
Tnfrsf18	2,385679104	0,72711
Gm4924	2,381878792	0,32927
Gm23054	2,379238668	1
RP24-499N24.6	2,371828999	0,36489
Gm15853	2,36805075	1
Trim14	2,36427852	1
Als2cr12	2,363950784	1
RP23-476G10.1	2,361330532	1
4930556M19Rik	2,360348687	1
Zfp975	2,358713185	1
Stab1	2,358059301	0,2487
Caskin2	2,357895858	0,7899
Gm26800	2,356752078	0,13589
Fhit	2,356098738	1
Zfp40	2,354955829	0,55543
2310043L19Rik	2,353487189	1
Gm12059	2,352345547	0,60134
Rpgr	2,348761133	0,51643
Gm13998	2,345669898	0,78867
Dennd5b	2,345344742	0,88394

Erbb3	2,344532048	0,42618
Kctd12	2,344369543	0,47744
Eng	2,342745113	0,069463
2210406H18Rik	2,34095954	1
Gm7292	2,340797283	1
A330074K22Rik	2,335610982	1
Setmar	2,335125356	0,84398
2010008C14Rik	2,333507331	0,71173
Klf1	2,333022143	1
1700086P04Rik	2,330597712	0,91629
A230028O05Rik	2,327369056	1
Cacnb1	2,325595202	1
Bcl3	2,321407829	0,03885
Asap3	2,318513296	1
Ttc7	2,317870556	0,018938
Cpne2	2,317067383	0,011075
Il20rb	2,316746191	0,16382
Gm13398	2,315461871	1
Tmem37	2,315301381	0,43511
Gm23442	2,313536724	1
RP24-175C20.18	2,312735053	0,53226
Aars	2,311452955	0,05433
Pld2	2,310812173	0,59361
Gm9951	2,303934976	0,76289
Zfp14	2,302817371	1
Wdyhv1	2,302657757	0,30477
Hal	2,302498155	0,94659
Serpinc1	2,301859855	0,74146
Gm7815	2,297555959	1
Ttc39a	2,297078245	0,9524
Hfe	2,296441448	0,42988
RP23-228B2.5	2,292306568	1
Fuz	2,290400674	0,68046
Gm43350	2,289924448	0,65258
Nprl3	2,287544803	0,22104
Ccdc63	2,284217455	1
Gm4742	2,28405913	1
Tcea3	2,280894946	1
Klhl30	2,279156513	0,40848
Gm10180	2,278366754	1
4930550C14Rik	2,277419405	0,95064
Csrnp2	2,27710371	1
Gm43924	2,274579721	0,84398
Gm17100	2,273791548	1
Fancc	2,272373526	0,49472
Gm12791	2,272373526	0,89056
Elk3	2,270798982	0,033583
Fam102a	2,266396062	0,00012913
Zfp59	2,259807683	1
Egr1	2,258711484	0,29196
Plcd1	2,256677095	1
Il4ra	2,249025471	0,15014

Myom1	2,248090322	0,26621
Cyp2u1	2,247934501	0,2487
Glb1	2,247467105	0,00050269
Fblim1	2,247155562	0,014798
CH25-309J2.1	2,24622119	1
Gm11764	2,244664768	1
Zfp661	2,243109424	0,57755
Il1rap	2,24139979	0,56
Gm25514	2,241244434	0,51039
D5Ertd605e	2,238605018	1
C030013C21Rik	2,237519104	0,59311
Fam213b	2,236123702	0,8465
mt-Tt	2,233490317	0,59311
Prob1	2,232716382	1
Tm4sf19	2,232406884	1
Fam71e1	2,230705407	1
Gm20712	2,227460734	0,50794
C430049E01Rik	2,224374957	0,98791
Wdr35	2,22206343	0,5301
Gm10676	2,221293455	1
Slc16a7	2,22098554	0,30189
Hoga1	2,220831598	0,98263
Maged2	2,217601287	0,71757
Tagap	2,213608364	0,1696
Tmco4	2,210541777	0,86471
Fam83h	2,209316332	0,96343
Asns	2,20686748	0,12631
Maml2	2,204574147	0,42381
Loxl3	2,204268549	0,53025
Ica1	2,202588519	0,096692
Zfp790	2,201214901	0,83205
Mllt1	2,200299632	0,49447
Ubtd2	2,200147124	1
Slc46a3	2,198775029	0,931
Gm5113	2,198622626	0,90658
Cyfip2	2,198317854	0,0062185
Zfp27	2,197708435	0,6576
Gm15787	2,19177545	1
Ifi44	2,190256753	1
Abi3	2,188435706	0,58224
Nlrc3	2,184192483	0,47239
Jrk	2,18358698	1
Tlr7	2,183435631	0,021106
Fcnaos	2,180410843	1
Mdrl	2,177692118	1
Sec16b	2,176334026	0,023276
1700034H15Rik	2,175579897	0,87464
Ddn	2,174675287	1
Bcl2a1b	2,174524555	0,30477
Sec24d	2,171963713	0,096782
Csf2rb	2,170609194	0,053553
Kdelr3	2,166100228	1

Ttc38	2,165499741	0,82747
Cep41	2,16519956	0,7965
Fbxo10	2,162949527	1
Iqce	2,156661866	0,52766
Gramd1c	2,151883538	0,94659
Gm4459	2,149349359	1
Zfp820	2,148902461	1
Pla2g2e	2,145628046	0,57587
Polm	2,145181921	0,94108
Lhx1	2,145181921	0,99816
Cox5b	2,144735889	0,45999
0610010F05Rik	2,143101233	0,25848
Iqsec3	2,14295269	1
Dmrta2	2,141913175	1
Pter	2,141764714	0,33039
Proser3	2,14028067	1
Cc2d2a	2,140132323	1
Il16	2,13894591	0,29785
Aldh18a1	2,138204736	0,30393
Chchd10	2,137760156	1
Ypel1	2,135390618	1
Cenpt	2,133023707	0,84398
Fam161a	2,132136791	0,10135
Ccdc171	2,130216407	1
Card6	2,129035491	0,78819
Gnmt	2,129035491	1
Sla	2,128592817	0,038097
Hpdl	2,128002728	1
Sp110	2,127265346	0,51632
Slc46a1	2,125938702	0,80563
Stk11ip	2,123435078	0,47758
Gm37108	2,122993568	1
Rps13-ps7	2,12152253	1
Nos1	2,121375483	1
Gm13226	2,120640397	1
Gm3608	2,119024106	1
Klk8	2,116381923	0,85726
Cox10	2,115355297	0,0050978
Zfp629	2,114915467	0,57458
2310061I04Rik	2,112132	0,012682
Gm16537	2,110229633	1
Inpp5b	2,109352196	0,25606
Pkn3	2,108913614	0,087531
Shtn1	2,104532808	0,0098535
BC017158	2,101180353	0,84398
N6amt1	2,097251677	0,24539
Gm37010	2,097251677	1
Neo1	2,09492704	0,05321
Gm5609	2,092459936	0,65409
Dntt	2,092024866	1
RP23-63H11.3	2,091589887	1
Gramd2	2,09014061	0,94921

Slc37a2	2,088837119	0,0054589
Slc10a3	2,088692337	0,43619
C3	2,088547565	1
Clgn	2,087245067	1
1600014C23Rik	2,086377187	1
Clec4n	2,085509668	1
Inhbe	2,084498017	1
Neurl1a	2,083342447	1
Zfp712	2,082764902	1
Aldh5a1	2,08262054	0,2377
Pfkfb4	2,079447123	0,022326
Ercc6l2	2,079447123	0,44194
Unc13b	2,079014759	1
Slc16a12	2,078582485	0,73206
Gm13822	2,078438414	1
Zhx3	2,077286202	0,2428
Nudt7	2,076710336	0,45999
Gldc	2,074696061	1
Gm45250	2,072971095	1
Fam213a	2,071821914	0,13128
Sgsm1	2,071678312	0,0022956
Pdcd1	2,070673371	0,96201
Gm29593	2,068808345	1
C230037L18Rik	2,068378194	1
Stap2	2,066945	0,74314
Siglec1	2,066945	1
Cmc4	2,065083332	0,87135
1700123M08Rik	2,064081591	1
Nat14	2,063223342	1
Slc15a3	2,060650736	0,81393
Itgb3	2,060650736	0,94108
Tgm2	2,059508385	0,0062693
Orai3	2,058509348	0,14811
Mgam	2,057653416	1
Cd82	2,05679784	0,024771
Zfp566	2,056227653	1
Zc3h4	2,052952148	0,012682
Gm20156	2,051956291	1
Lipt2	2,047977691	1
Scarf1	2,046984247	0,4046
Gba2	2,046133107	0,91198
Mcm9	2,044998805	0,70553
Mir7078	2,044998805	1
Pcdhb22	2,044573603	1
Reps2	2,041741186	1
Zfp418	2,040892227	1
Tmem35b	2,03990222	0,76264
Oprl1	2,03990222	1
Gm12151	2,039478078	1
E230016M11Rik	2,038912693	1
Lppos	2,038771371	1
Slc9a3r1	2,037782393	0,88313

Iba57	2,037076274	0,65586
H6pd	2,035100442	0,18866
Ptpn1	2,034818336	0,006201
Plxdc1	2,034113244	0,071379
Filip1l	2,033408397	0,44335
Zfp738	2,033267456	0,86757
Rnaset2a	2,032562902	0,83223
Nt5c3b	2,031576935	0,72864
Gm13341	2,031295318	1
Gm13270	2,030732202	1
Plbd2	2,030450702	0,0036706
Tmem62	2,02988782	1
Gpatch3	2,029747123	0,5301
Trak2	2,02946576	0,064699
Gm9776	2,025109615	0,76899
Gm13868	2,022584532	1
Zscan29	2,018103268	0,65668
RP23-151L20.5	2,016425355	1
Cpsf3	2,014190307	0,10685
Nme6	2,013771511	0,95327
Wdr46-ps	2,013771511	1
Sdr42e1	2,008474298	0,57736
Gpr68	2,007500017	0,21532
Pik3r2	2,007221738	0,12613
Nf2	2,003746499	0,041137
Gne	2,003329875	0,36423
Adap2	2,002496887	0,86757
Pus7l	2,001525505	0,86351
Trpm2	2,000970642	1
Slpi	1,998198629	0,093313
Tfrc	1,996260506	0,042303
Car13	1,99612214	0,72711
Ank	1,993909597	0,0065041
Nrp2	1,989629771	0,0020392
Sult2b1	1,986598049	1
Blnk	1,984946331	0,14562
Dock5	1,984808749	0,22909
Particl	1,984120986	0,81393
Nmb	1,983433461	0,98739
Cep162	1,983295985	0,56206
Gm5251	1,982746175	1
Zfp763	1,982608746	0,78819
Gm37776	1,981921745	1
Txnrd3	1,981097659	0,72711
Tmem150a	1,980411182	1
Chtf18	1,979862171	1
Slc43a2	1,978353177	0,059707
Gatb	1,978353177	0,54403
Tmem140	1,977941833	0,5947
Ctnnal1	1,976023357	0,95327
Ivns1abp	1,973969912	0,0019319
Cd33	1,973833092	0,024864

Gdpgp1	1,970415661	1
Rai14	1,970142523	0,043232
Uaca	1,969596359	0,064983
Nt5dc2	1,968913867	0,54707
Xk	1,968913867	1
Mzf1	1,968504486	1
Plcb4	1,968368044	0,011828
Gm43742	1,967004148	0,52766
Ift122	1,965232496	0,61369
Gm20707	1,962509993	1
Gm9711	1,962509993	1
Sec16a	1,962373967	0,29196
Gm30329	1,959519595	1
Slc11a1	1,958976376	0,069463
Whrn	1,958976376	1
B130006D01Rik	1,958840595	0,94659
F9	1,95789039	1
RP23-225D5.4	1,956805006	1
Zfp786	1,956398142	1
Gspt2	1,95504254	1
C030014I23Rik	1,95422963	1
Wdr77	1,95192822	0,20885
Zfa-ps	1,950170146	1
Kifc2	1,946388903	1
Gm6162	1,944231485	1
Gm20257	1,94342307	1
Gm43547	1,941538075	1
Gm9575	1,94073078	1
Csf1r	1,939117197	0,015727
Arhgef3	1,938445266	0,3048
Mylpf	1,936967839	1
RP24-131G14.10	1,936296653	1
Gm11716	1,936028244	0,74781
Oip5	1,934820864	0,99501
Tst	1,933748269	1
Tnfsf13b	1,933614236	0,91918
Fabp3	1,93254231	0,47104
Naip2	1,930132648	0,21202
6720464F23Rik	1,929463831	1
F7	1,928394206	1
Treml4	1,924655184	1
Gm16638	1,924521782	1
RP24-84O13.9	1,922255357	1
Tmem116	1,921322899	0,62963
Decr2	1,921322899	0,84749
Tmem65	1,92052401	0,071379
Sh3rf1	1,920390894	0,44912
4930589O11Rik	1,919858522	1
mt-Nd1	1,918661226	0,11705
AA414768	1,917730511	1
Il13ra2	1,917597589	1
Gm23639	1,917331772	1

Panx1	1,916534542	0,55554
Cyb561d2	1,916003239	1
Gcdh	1,915073814	1
Pck2	1,914941076	0,48112
1700008J07Rik	1,914675627	1
Numbl	1,91414484	0,42988
Parn	1,9136142	0,21182
Slc43a3	1,912818515	0,42146
Gm42567	1,912685934	1
6720475M21Rik	1,912420797	1
Ctbp1	1,911228139	0,064699
Scap	1,910963205	0,24679
Timp1	1,910565873	1
Fmo5	1,910168623	1
Lmbr1	1,910036225	0,85714
Ticam1	1,909506724	0,275
Mst1	1,909374371	0,71079
Flot1	1,908712747	0,59453
Cass4	1,907919101	0,89517
Pcyt1a	1,907125785	0,0089541
Gm42918	1,906332798	1
Nkain1	1,906068543	1
Zfp365	1,904615792	0,931
Hlcs	1,904615792	0,98277
Tubb4a	1,899869072	1
Slc25a15	1,898947474	0,88182
Oxt	1,898157888	1
Hexim2	1,897763218	1
Zfp11	1,89605393	1
9330162012Rik	1,895265548	1
Ankrd34a	1,895002826	1
Mdk	1,894214881	0,82514
3010003L21Rik	1,893952305	1
Cat	1,891853009	0,017149
Zfp467	1,89145965	0,50413
Armc5	1,89145965	0,70099
Ttll12	1,891066373	0,065366
Zfp862-ps	1,889887032	0,76899
Ccdc88c	1,889363117	1
Trpm1	1,888053964	1
Wbscr27	1,886222674	0,42033
Bbs9	1,885569072	0,72711
Zbtb38	1,885046354	0,098108
Plk3	1,884393159	0,42791
Def6	1,884001352	0,6628
Gm5871	1,883870767	1
Gm23300	1,883870767	1
Slc27a1	1,882956929	0,52766
Rps19-ps3	1,881130583	1
Oasl1	1,880218074	0,83925
Rnasel	1,879827134	0,72711
Upp2	1,878394384	0,84766

Arhgap35	1,877873654	0,90909
Hapln3	1,877353069	1
Tdrd3	1,87579218	0,92979
Gm10842	1,87579218	1
Slc40a1	1,875532158	0,81491
RP23-213P10.2	1,875012222	1
Lamb2	1,873972783	1
Ubxn8	1,873583142	0,7899
Zfp94	1,871376705	1
Uxt	1,871246996	1
Klhl35	1,869950396	1
Ltbp2	1,869691183	1
6330403L08Rik	1,86904331	1
Gm6344	1,866712824	1
Abcd1	1,865160782	0,78044
Tfcp2	1,864514478	0,7614
Gm45733	1,862060567	1
Car9	1,861544366	0,42381
Sh2d6	1,861157309	1
RP24-365A12.2	1,860899315	1
Kif5a	1,85922323	0,72711
Ccdc120	1,858836656	1
Poglut1	1,858192544	0,087559
Nucb2	1,858063748	0,095677
Lars	1,857806184	0,0437
Gm19705	1,856261547	0,98277
Meiob	1,855746953	1
Usp11	1,85497533	1
Sdsl	1,853561521	0,95327
Sec14l2	1,851635336	0,95327
Gstt1	1,851635336	1
Elp4	1,847148685	1
Rasal2	1,846892634	0,11313
2610016A17Rik	1,846124693	1
Pspc1	1,84535707	0,6143
Gtpbp8	1,844973379	0,82126
Zfyve28	1,844589768	1
4930590J08Rik	1,844334071	1
Gns	1,84407841	0,066104
Slc39a13	1,843183874	0,15014
Tm4sf5	1,842545186	1
Washc5	1,841651394	0,77607
Gm42571	1,841396105	1
Tmem8	1,841140851	0,54482
Ints10	1,840630449	0,92368
Bcar1	1,838590256	0,84749
Srl	1,838590256	1
Cog7	1,838207972	0,42618
Zfp974	1,838080561	1
Gm12454	1,836170464	1
Pomt2	1,835915934	1
Rps6kb2	1,835279765	0,67592

Wdr5b	1,834898169	1
Elmod2	1,834516652	0,76289
A430033K04Rik	1,834389497	1
Rasa3	1,833626755	0,19929
Exoc3l4	1,832864329	0,77607
Fah	1,829944634	0,88313
Nsun4	1,829056955	0,68136
Creb3l2	1,82829643	0,38277
Ttc28	1,8276629	1
Fhod3	1,827536221	0,99635
Xrcc5	1,82740955	0,80606
Spsb1	1,826523101	0,88322
Zfp599	1,8263965	1
Hif1a	1,825637081	0,11726
Slc22a4	1,824372084	1
Atp6ap2	1,824245633	0,033614
Nptxr	1,824245633	0,52228
Zfp28	1,824245633	1
Gm43569	1,823613507	1
Tmcc2	1,823360718	0,72311
Eef2k	1,822476232	1
Ophn1	1,82184472	1
Thap7	1,821592176	0,45028
Aldh1b1	1,821339667	0,77136
Greb1	1,821213426	1
Usp20	1,820329982	0,15021
Iars	1,820203811	0,29196
Smap1	1,819446967	0,23074
Ivd	1,818438331	0,69546
Xrcc3	1,818060236	1
AW046200	1,815919186	1
Micall1	1,815415776	0,28219
Pafah2	1,813654941	1
Gm13223	1,813152158	1
Fam129c	1,813026485	1
Gm26532	1,812523877	0,98277
Gm9726	1,812272625	1
Khnyn	1,812021408	1
Gpr183	1,811393518	0,40848
Msi1	1,811267966	0,84398
Tctex1d4	1,810640337	1
Wbp1l	1,810138391	0,19929
Ppfibp1	1,808382673	0,08331
Vamp7-ps	1,807380171	1
Cnksr1	1,807129632	1
Gm44075	1,80575229	1
Gm6377	1,804626154	1
Mrm2	1,804000827	0,8294
Pde4dip	1,803750757	0,59642
Acap1	1,803750757	1
Abhd1	1,80325072	1
Rpsa-ps4	1,801751442	1

Nsdhl	1,80000386	0,42791
Lrpprc	1,799879097	0,63975
Mtcl1	1,799629598	1
Gm5117	1,799255415	1
Slc39a8	1,799255415	1
Nfkb2	1,798756624	1
Tmem220	1,797385664	0,67592
Dnajb12	1,795891262	0,52887
1300002E11Rik	1,795642316	1
Myo6	1,794522485	0,65088
Acvr1	1,793776319	0,54605
Eef1akmt1	1,792409154	1
Gm12966	1,792284918	1
1700112E06Rik	1,791167182	0,92481
Scamp1	1,790670633	0,2677
Hmcn2	1,790174223	1
Fbxl8	1,790050142	0,86757
Zpr1	1,786950907	0,11595
Hsh2d	1,786455528	1
Fam151b	1,785465181	1
Mthfr	1,783733396	0,71135
Zfp579	1,783238908	1
Btbd19	1,782497434	0,51608
Arhgap1	1,782250345	0,77136
Cd109	1,782126813	0,10943
Dnajc30	1,780645098	1
Polr3e	1,780521677	0,64068
Cntln	1,780398265	1
Agap1	1,779904702	0,50794
Eno3	1,77953462	0,54482
Amacr	1,77953462	1
Bcl7c	1,779287941	1
Trim2	1,77805506	0,95843
Plekhh1	1,777931819	1
Gm26225	1,775838026	1
Dcaf11	1,775222675	0,55543
Dclre1c	1,775222675	1
BC037039	1,775222675	1
Slc7a4	1,77509963	0,74314
Zfp788	1,774853566	1
Nkpd1	1,774115578	1
Gm29487	1,77399261	1
Slc7a11	1,773623757	0,48934
Mus81	1,772763398	1
Zdhhc4	1,770675693	0,65668
Npr1	1,770307529	1
A930016O22Rik	1,770307529	1
6330408A02Rik	1,769326134	1
Gm14843	1,769203498	1
Ak3	1,768590445	0,91694
Ap1g2	1,767487485	1
Zfp617	1,767487485	1

Gm10642	1,767487485	1
Gm45728	1,767119985	1
Creb3l1	1,766385213	1
Gm44950	1,765895535	1
Tbx15	1,765405993	1
Apba1	1,76344918	1
Gm5578	1,761616638	1
Srd5a3	1,761372443	1
Anxa9	1,761372443	1
Car6	1,760640061	1
Ino80c	1,760151976	0,21412
Ada	1,760029976	1
C2	1,759907984	1
Ppp1r21	1,758932354	0,93754
Tlr2	1,757957265	0,40495
Ptgir	1,755521907	0,38778
Ehd1	1,755156895	0,14195
Rfxank	1,754913595	1
Gm12097	1,754427097	1
Gm20517	1,753940734	1
Chaf1b	1,752360985	0,93977
Gm8719	1,752360985	1
Hap1	1,752239525	0,78999
Ppp1r10	1,75199663	0,37345
Chm	1,751268147	1
Gm18969	1,751025386	1
Alg9	1,750539966	0,96748
RP23-277D1.1	1,750175989	1
Ccdc85b	1,749327005	1
Xpo5	1,749205755	1
Ddx17	1,748357241	0,41899
Qpctl	1,747145792	1
Il1b	1,745451173	1
Atp6v1d	1,745209219	0,11785
Spred1	1,745209219	0,51481
Elf4	1,745209219	0,94921
Nacc2	1,744362644	0,66931
Acvr2a	1,744362644	1
Irgm1	1,744120841	1
Nfyc	1,743999952	1
Gm15163	1,743274793	1
Kif13a	1,742912327	1
E430018J23Rik	1,742791522	1
Gm26542	1,742670725	1
Ccar2	1,742429156	1
Slc26a2	1,742187621	0,32505
Acot6	1,741946119	1
Shmt1	1,741463216	0,46494
Gm44623	1,741342511	1
Ift20	1,741101127	0,67602
Bckdk	1,740135923	0,54224
Acaa2	1,739894705	0,60134

Pla2g5	1,739412371	0,48797
Nadsyn1	1,739412371	1
Cacna1s	1,739050708	1
Tmem98	1,73868912	1
Nme4	1,738568608	1
Gm45224	1,737243521	1
Nfkbie	1,734355926	1
Atp6v1g2	1,733875127	1
Zfp532	1,732313451	1
Rsad1	1,731833218	1
Sfn	1,731593152	1
Dhrs11	1,731113119	1
Zdhhc12	1,730753181	0,97841
2700033N17Rik	1,730393319	1
Rmnd1	1,727756612	1
Cyba	1,72751711	0,36272
Gm7285	1,727397372	1
BC004004	1,727157921	0,63713
Rapgef3	1,727157921	1
Zfp128	1,727157921	1
Etfrf1	1,726559437	0,80618
Mettl8	1,725363092	0,84749
Grtp1	1,725243503	1
Babam1	1,72500435	0,60005
Dapk1	1,723211759	0,19569
Zfp113	1,722972888	0,89231
Dph6	1,722853465	0,71757
Smim12	1,72273405	0,5476
Klhl5	1,72273405	0,644
Fadd	1,721898377	1
Murc	1,721063109	1
Adamts4	1,721063109	1
Ipo8	1,720466737	0,66495
Dnajc12	1,720228247	1
Polr3d	1,719870572	0,55543
Frmd4a	1,719632164	0,50794
Fads6	1,719274614	0,96052
Ago1	1,718797996	0,72917
Vac14	1,718678862	1
Nupl2	1,717726088	1
Atp6v0c	1,717487977	1
Clec4a3	1,715822125	1
Mmab	1,715108683	1
A930006K02Rik	1,715108683	1
Slc35d2	1,714514375	1
Mapkapk2	1,714276709	0,25145
Srr	1,71356391	0,6875
Rps13-ps5	1,712970136	1
Fbxo32	1,712613971	1
Txk	1,71225788	1
Lgr4	1,712020527	1
RP23-134M7.3	1,711901863	1

Dbnl	1,711308666	0,71314
RP23-3F1.8	1,710715675	0,73206
Gm5898	1,710122888	1
Ap3b1	1,710004356	0,56457
Phactr1	1,708819482	1
Zfp119a	1,70846418	1
Hoxb3	1,708108951	1
Gm38247	1,708108951	1
Gm11868	1,707517069	1
Gm15157	1,70704371	1
Gm8539	1,706925391	1
B3gnt2	1,706688777	1
Acsl4	1,706333918	0,19933
Rbfox1	1,705269784	1
Apbb1ip	1,704324443	0,2328
Dusp18	1,704206313	0,88883
Dennd2a	1,70385197	0,72711
Fam188b	1,703733872	1
Ecd	1,703143505	0,80618
Gm43147	1,702907415	1
Gm8522	1,702435335	1
Zfp41	1,701491566	1
Gm11273	1,701019878	1
Mfsd3	1,699959057	1
Sfxn2	1,699605597	0,9978
Pstpip2	1,698427929	1
Fam120b	1,697721719	1
Trim7	1,696780563	0,86846
Sec22a	1,696192606	1
Sphk2	1,695722387	0,42146
Tfec	1,695017302	1
Bbs7	1,694429956	1
Nt5m	1,693960225	1
Tmem17	1,693960225	1
Zfp759	1,693490625	1
Rbck1	1,693021155	0,51856
Gm26631	1,6924345	1
Def8	1,692317193	0,98277
Habp4	1,692317193	1
Gm9246	1,691496275	1
Mgat5	1,690910149	0,54482
1110034G24Rik	1,690910149	1
Amotl1	1,690324225	0,33246
Akr1b3	1,689855633	1
Zfp951	1,689621385	1
Rsl1	1,689504274	1
Atp8b4	1,689270075	1
2510046G10Rik	1,688333604	1
Ints7	1,688216582	0,76899
Gm9732	1,688216582	1
Pcdhb15	1,687865564	1
Dmxl2	1,687514619	0,77433

Serpinb8	1,686929872	0,56487
Gpr162	1,686462221	1
D17H6S53E	1,685994699	1
Taco1	1,685527307	1
Zfp429	1,685527307	1
Fam35a	1,685176847	1
2210408F21Rik	1,684476148	1
Lat2	1,684125907	0,55543
Natd1	1,683659033	1
Gmpr	1,683075622	0,644
Rpain	1,683075622	1
Ldb3	1,683075622	1
Ttll1	1,682958965	1
Prkaa1	1,682725673	0,71757
Ifi47	1,682609039	1
Sirt6	1,682492414	1
Fry	1,682375797	1
Zfp12	1,681559701	1
Bnip2	1,681326603	0,54403
Tlcd2	1,681210067	1
Trpv4	1,680511016	1
Ncoa4	1,680511016	1
Ccdc116	1,680394536	1
Capn3	1,6801616	1
Batf2	1,679928696	1
Ndufs3	1,678299274	1
Adora2b	1,678066629	1
DHRSX	1,677020123	1
Gm45495	1,676787654	1
Gemin5	1,676671432	1
Myof	1,675858104	0,48128
Diablo	1,674929068	0,8103
Mul1	1,674812975	1
Tmem214	1,673652485	0,98709
Nat8f1	1,673420484	1
Tnfrsf9	1,672608732	0,86143
Ash2l	1,672029151	0,74634
Magee1	1,67156563	1
Mical2	1,671333918	0,88657
Ctsh	1,670638974	1
Zfp318	1,670523178	0,77433
Crtam	1,670175839	1
Gm8242	1,669944319	1
Arhgef25	1,669828572	1
Fus	1,669828572	1
Cd59a	1,669828572	1
Cdk5rap1	1,6695971	1
Nlrx1	1,669018562	0,79006
Flywch1	1,668671536	0,98963
Gm9332	1,668208946	1
Slc25a10	1,667630889	1
9130008F23Rik	1,666937485	1

St7	1,666821946	1
Gm29155	1,666590891	1
Hist1h4h	1,666590891	1
Wipi1	1,665666993	0,74799
Ccdc122	1,665551542	1
Acp2	1,665436099	1
Adamtsl4	1,665205236	1
Scly	1,664974406	1
Asah2	1,664859002	1
E330011M16Rik	1,66462822	1
Lrrc61	1,663820733	0,92401
Frrs1	1,663590095	0,49297
Gm13477	1,663128913	1
Fcgr1	1,662322154	1
Snx20	1,66197652	0,69558
1600010M07Rik	1,661630957	1
Gm42548	1,661630957	1
Stx17	1,661515786	0,91211
Gm9435	1,658984032	1
Hacd4	1,658754064	0,65409
Tspyl2	1,658639092	1
Fcgr4	1,65783451	1
Zfp959	1,65783451	1
Atg10	1,657260048	1
Herpud1	1,657145179	0,37984
Lrrc8b	1,656800622	1
Pigp	1,656570956	1
Gm10033	1,656456135	1
Gm6304	1,656226518	1
Ncf4	1,655882151	0,72711
Efemp2	1,655193632	1
Gm5575	1,65496419	1
Cnpy2	1,65484948	1
Iqcc	1,654390722	1
Gnptab	1,654161391	0,55039
Gm15484	1,652556961	1
Nsf	1,652327884	0,77002
Nmi	1,652098838	1
Abcc4	1,651869825	1
Syk	1,651526364	0,59311
Slc35b4	1,651411893	1
Irak4	1,650954088	1
Ttyh3	1,650839656	0,73206
Rgs16	1,64981013	0,96844
4930430E12Rik	1,649581434	1
Slc38a1	1,649238448	0,42988
Suox	1,648895535	1
Akna	1,648666965	1
Zfp182	1,648552692	1
Arhgef10	1,64832417	1
Ncoa7	1,647867221	1
Poli	1,647296212	1

Evi2a	1,646611264	0,92697
Ccdc136	1,646611264	1
Cd200r4	1,646383011	1
Plpp5	1,646040691	1
Prr14l	1,645584374	0,78947
Gm16845	1,645470315	1
Cul4a	1,645356264	0,9388
Zfp541	1,644672122	1
Zfp455	1,6434186	1
4930453N24Rik	1,643190789	1
Tmem14a	1,643190789	1
Bcl2l15	1,642735263	1
Gm14794	1,642735263	1
Rfx5	1,642621402	0,54707
Rab29	1,642393702	0,95064
Gm45802	1,642166033	1
Gm37121	1,641255676	1
Cdon	1,641141916	1
Kbtbd3	1,641028165	0,93261
Ahi1	1,640914421	0,83566
Xylb	1,640459526	1
Zbtb3	1,639891085	1
Rrp7a	1,639095598	1
Ccni	1,638641207	0,46464
Dgka	1,637732803	1
Phldb3	1,637392282	1
Zfp719	1,637392282	1
Sco2	1,636371142	1
B4galt1	1,636030903	0,41205
Gm38111	1,635690736	1
Angpt2	1,634670657	0,88024
Akap7	1,634557354	1
A430105J06Rik	1,634217493	1
Gipc1	1,633990958	1
Slc2a6	1,632292946	1
Ppp6r3	1,632066678	0,59523
Lcp2	1,631953556	0,81474
Enpp5	1,631727335	0,89616
Rilp	1,631501145	1
Tanc2	1,630822764	1
Zfp760	1,630483679	1
Kctd21	1,629692755	1
Ccdc134	1,629466848	1
Sema4b	1,629240971	1
Zfp189	1,628789312	1
Ick	1,628789312	1
Tmem268	1,628337779	0,81781
Isg20	1,628337779	1
Gm6290	1,628337779	1
Homez	1,627660713	1
Iqcf1	1,627096706	1
Xrra1	1,626194702	1

Dpp3	1,626081987	1
Gpatch1	1,625631204	1
Atg4d	1,625631204	1
Gm11694	1,625405859	1
Cptp	1,625180546	1
Egfl7	1,624955264	1
Gm12013	1,624842634	1
Atg9b	1,624279604	1
Pbxip1	1,623716769	0,54605
Galnt4	1,623604226	1
Rinl	1,62349169	1
Rbmx2-ps	1,623266642	1
Ulk3	1,623041625	1
Trp53inp2	1,622704158	0,89504
Pde8a	1,622366761	1
Rgl1	1,622141869	0,55328
Dennd4b	1,621917008	1
Ammecr1	1,621354992	0,89616
Snx7	1,620905519	1
Galnt10	1,62079317	1
Adgrl2	1,62079317	1
Gm11222	1,62079317	1
Aspscr1	1,620231543	1
D130007C19Rik	1,620006947	1
Plcg1	1,619894661	0,98704
Lrp8os3	1,619670111	1
Parvg	1,619333345	1
Trmt12	1,619333345	1
Gm14056	1,619333345	1
Hmx3	1,61899665	1
Ppp6r2	1,617762697	1
Zfand3	1,617650566	0,66931
B230322F03Rik	1,617538443	1
B3galnt1	1,61720212	1
2810029C07Rik	1,617090028	1
Ampd3	1,616417639	1
Serinc2	1,61574553	0,95327
Slc7a7	1,615521555	1
Lrrc14	1,6150737	1
Gm7102	1,6150737	1
Bcl2l11	1,614737889	0,95902
Rpl3l	1,614178361	1
Tbc1d7	1,614066478	1
1810062G17Rik	1,614066478	1
Arhgap31	1,613954604	1
Tnfsf8	1,613507183	1
Srxn1	1,613283518	1
Nkrf	1,612724494	1
Slc39a11	1,612500938	0,70393
Tnks1bp1	1,612165663	1
Ddb2	1,611830457	1
mt-Rnr1	1,610713609	0,97469

Gm12389	1,610378705	1
3830403N18Rik	1,610378705	1
Enpp1	1,610267086	1
Nmnat1	1,610155475	1
Atp2a3	1,609932275	0,88313
Klhl8	1,609820687	1
Tars	1,609374412	0,94108
Atp8a1	1,609151321	1
Pla2g16	1,608816742	0,89616
Slc9a3r2	1,608705231	1
Atp13a2	1,608147793	0,85849
Slc41a2	1,607590548	0,84398
Inf2	1,607479122	0,64815
Tmem55a	1,607033496	0,92757
D2Bwg1423e	1,606699358	1
Trmt10b	1,606476637	1
Sh3tc1	1,605474777	1
Selenos	1,605363498	0,76329
Gm1848	1,605363498	1
Rpp40	1,605140963	1
RP23-324E2.11	1,605140963	1
Rpsa-ps9	1,60458476	1
AU040320	1,604473542	0,99635
Borcs7	1,604473542	1
Itpa	1,604362333	1
Sppl2b	1,604251131	1
Gmppb	1,60402875	1
Spsb3	1,60402875	1
Piwil2	1,60358408	1
Sh3bgr	1,602584024	1
Ankrd49	1,601695609	1
Slc12a2	1,600807687	1
Gm17586	1,600142068	1
Ahnak2	1,599587597	0,96732
Spg11	1,599255007	1
Fancf	1,598146872	1
1190005I06Rik	1,597703833	1
Fkbp15	1,597482359	0,80618
Crebbp	1,597482359	0,95647
Ktn1	1,597039505	0,644
Reep6	1,59692881	1
Slc25a12	1,596818123	0,92981
Tsc2	1,596486109	1
Prkag2	1,595932905	0,73978
Elp3	1,595711677	1
Gm5914	1,594605997	1
Slc5a6	1,59416394	1
Slc39a1	1,593832477	1
Dzip3	1,593501083	1
Pex11g	1,593280193	1
Tmem127	1,593169759	1
Lima1	1,592838503	0,93193

Gm38157	1,592838503	1
Il7r	1,592728099	1
Cars	1,59195549	0,83925
Wwox	1,591183256	1
Eri2	1,591183256	1
Smarcd1	1,591072968	1
Ptpn21	1,590852413	1
Ptpn7	1,590301161	1
2310001H17Rik	1,590190934	1
Gm44103	1,589089079	1
AI987944	1,588538439	1
Bcar3	1,588208146	0,96545
Hbs1l	1,587987988	0,67986
Birc3	1,586997659	1
Foxp4	1,586557711	1
Hacd3	1,586007947	1
Gm10605	1,586007947	1
Golim4	1,585898017	0,97329
Nrg4	1,585458374	1
Fancg	1,585348482	1
Vrk3	1,584799137	1
Ucp2	1,584689291	1
Gm44178	1,584689291	1
Nudt14	1,584140175	1
Bcat1	1,584030374	1
Ift140	1,583701019	1
Zfp784	1,583481487	1
Zfp386	1,583042513	1
Map2k3os	1,583042513	1
AI839979	1,582713363	1
Slc35d1	1,582603661	1
Tspoap1	1,582164931	1
Gm45871	1,582055268	1
Aldoc	1,581726323	0,81817
Mertk	1,581507064	1
Fabp5	1,581507064	1
Gm2885	1,581178233	1
Gpr35	1,580520776	1
Gm24959	1,580192151	1
Eml5	1,57887833	1
Slc25a33	1,578659466	0,98162
Zfp235	1,578003057	1
Ints3	1,577456257	0,76074
Gm15185	1,57734692	1
Epc1	1,577237591	1
Igsf3	1,576909647	1
Fbxl17	1,576581772	1
Ppp2r3a	1,576363227	1
Tex9	1,576363227	1
Acot2	1,575489348	1
Slc24a3	1,57505259	1
Trim30a	1,574834256	1

Slc1a5	1,573633963	0,78044
Tlr4	1,573197719	1
Tnfaip8l2	1,572761597	1
Smurf1	1,572761597	1
Tyk2	1,571780763	0,89616
2810025M15Rik	1,571562883	0,94108
Echdc1	1,571453954	1
2310074N15Rik	1,571453954	1
Paqr7	1,571018315	1
Slc38a10	1,570800541	0,84398
Pstpip1	1,570691665	0,83566
Pou4f1	1,569712122	1
Fam114a1	1,569168196	1
Gm20302	1,568624458	1
Acat2	1,567972221	1
Slc25a37	1,567754869	0,84398
Gm13712	1,567646204	1
Grn	1,567428897	0,98942
Slc4a2	1,566994374	1
Slc52a2	1,566994374	1
2310068J16Rik	1,566559971	1
Gm38366	1,566125689	1
Tceal8	1,566017137	1
Gaa	1,565583005	1
Lrrc20	1,565365984	1
Zfp169	1,565148993	1
AU022252	1,565040509	1
BC005537	1,564932033	1
Laptm5	1,564172907	0,8294
Zfp3	1,563089081	1
Slc9a4	1,562980739	1
Olfm1	1,562764079	1
Ifi27	1,562764079	1
Mtg2	1,562764079	1
Chrnb2	1,56222256	1
Bank1	1,561897739	1
Fancm	1,561681229	1
Gm21967	1,561464749	1
4921536K21Rik	1,56135652	1
Spag7	1,561248299	1
Zfp626	1,561140085	1
Sod1	1,561031879	0,93494
Gm6257	1,56092368	1
Kmt5b	1,559733989	1
Gm7436	1,559733989	1
Gata3	1,559409685	1
Fkbp11	1,558977385	1
Itpr2	1,558653238	0,93754
Tmem69	1,558545205	1
Cant1	1,558221148	0,95327
Prdm4	1,558113144	1
Acadsb	1,558005148	1

Dok3	1,557789177	1
Gstt3	1,557789177	1
Slc27a4	1,557789177	1
Kdm5d	1,557573237	1
Tbc1d4	1,557465278	1
Gnb1l	1,557249382	1
Gm22716	1,557033516	1
Enox2	1,556817681	1
Zfhx4	1,556709774	1
Sema6b	1,556278222	1
Npc2	1,556062491	0,95642
Cyb5rl	1,555954637	1
Ccdc130	1,555738951	1
Letmd1	1,555415478	1
Qrsl1	1,554984285	1
Plxnb3	1,554445462	1
Tlr3	1,554014538	1
Scamp5	1,553906825	1
Kank3	1,553906825	1
Trafd1	1,55379912	1
Ccr2	1,55347605	1
1110025M09Rik	1,553260708	1
Gemin4	1,55293775	1
Pyurf	1,551754145	1
Abcb4	1,551539042	1
Sh3pxd2a	1,551108923	1
1700001C19Rik	1,551108923	1
Tmem173	1,550786413	1
Blvra	1,550356503	1
Endog	1,550356503	1
Rbm10	1,550249044	1
Tor4a	1,550034149	1
Gm27029	1,54928225	1
Gm43110	1,549174866	1
Gm43681	1,548852757	1
2610002M06Rik	1,548745403	1
Gm6329	1,547779546	1
Marcksl1	1,547672266	1
Cluap1	1,547564994	1
Tmem106c	1,547457728	1
Pcca	1,547135977	1
Synrg	1,546707079	1
Prkar2b	1,546492675	1
Gm8566	1,5462783	1
Ostc	1,546171124	1
Gm5873	1,545956794	1
Tlcd1	1,545635355	1
Setd1b	1,545528223	1
2810021J22Rik	1,545421099	1
Unc93b1	1,545099771	1
Gm12529	1,544564373	1
3110082I17Rik	1,543815128	1

Mon1b	1,543815128	1
Mitf	1,543066246	1
6430590A07Rik	1,542852346	1
Zfp775	1,542531552	1
Stat5b	1,542424636	1
Tldc1	1,542103931	1
Fbxl6	1,541890165	1
Foxo4	1,541783293	1
RP23-110E20.5	1,541355879	1
Pskh1	1,540821779	1
Rnf157	1,540074348	1
Hdhd3	1,540074348	1
Gm26606	1,539967602	1
Chd1l	1,539860864	1
Rdh1	1,538900548	1
Lgalsl	1,538687226	1
L3mbtl3	1,538473933	1
1810030O07Rik	1,53826067	1
Mxra8	1,537834233	1
Sec61a1	1,537727642	1
Gm9333	1,537621059	1
Plekhg3	1,537194799	1
Cep290	1,537194799	1
D6Wsu163e	1,536981713	1
Gm14328	1,53666214	1
Trp53cor1	1,536449128	1
Fech	1,536342633	1
Exoc8	1,536236146	1
Zfp607a	1,536023193	1
Swsap1	1,53581027	1
Gm4943	1,535703819	1
Dnajc11	1,535065271	1
Llph-ps1	1,53485248	1
Rwdd2b	1,534639719	1
Psmb5	1,534320633	1
Trim65	1,534001613	1
Ccdc94	1,53378897	1
Fbxo31	1,53368266	1
Lpcat3	1,533470061	1
Ints5	1,533363772	1
Gm42835	1,533363772	1
1600002H07Rik	1,532832441	1
Gca	1,532832441	1
Phka1	1,53261996	1
Map3k7	1,532301294	1
Arhgap9	1,532301294	1
Gm13421	1,532195087	1
Tlr13	1,531876509	1
Clec1a	1,531876509	1
3110001I22Rik	1,53166416	1
Gm37670	1,531451841	1
Cyp4f13	1,531133418	1

Gab2	1,530602859	1
Akap11	1,53049677	1
9530082P21Rik	1,53049677	1
Ublcp1	1,530178545	1
Ly96	1,529966432	1
Fyb	1,529754348	1
Zfp810	1,529754348	1
Dnase2a	1,529648317	1
Uba7	1,529118274	1
Rela	1,529012287	1
Tbc1d10b	1,527635128	1
Scyl3	1,527211636	1
Zfp764	1,527105781	1
Tubgcp5	1,526576619	1
Adat2	1,526365005	1
1700003F12Rik	1,526047639	1
Lyl1	1,525941865	1
Atp6v0a2	1,525624588	1
Tyw3	1,525518843	1
Rnf31	1,525413106	1
Fam222b	1,525307376	1
Bre	1,525201653	1
Deptor	1,524884529	1
Aldh4a1	1,5244618	1
Mtmr9	1,5244618	1
Dync2h1	1,524356136	1
Traf3ip2	1,52414483	1
Pdzk1ip1	1,52414483	1
Ankrd26	1,523933554	1
Ifi211	1,523933554	1
Zfp772	1,523616694	1
Rtf1	1,52340549	1
Grk6	1,522666508	1
4931414P19Rik	1,522666508	1
Calhm2	1,522138884	1
Gdpd1	1,521505976	1
Nmnat3	1,521505976	1
Tlr1	1,521400517	1
Amer1	1,521295065	1
Cstf2t	1,520978753	1
Mtrf1	1,520978753	1
Ankrd46	1,520557107	1
Foxd2os	1,520346327	1
Actn1	1,520240949	1
Pop7	1,520240949	1
Gm37851	1,520240949	1
Slc26a6	1,519819507	1
Tlr6	1,51887169	1
Vps33a	1,518450628	1
Fem1a	1,517924464	1
Zkscan17	1,517819253	1
Pqlc1	1,517714049	1

Jmjd8	1,517714049	1
Cst7	1,517188141	1
Crtap	1,517188141	1
Ppcdc	1,516662415	1
Mtmr11	1,515821632	1
Zfand1	1,515401415	1
Gm37470	1,515296379	1
Lrch1	1,514876308	1
Ssx2ip	1,513302078	1
Nphp1	1,51288256	1
Pddc1	1,512358325	1
Mblac2	1,510891436	1
Btk	1,510577287	1
Haus7	1,510472586	1
Jmjd4	1,510367891	1
Clec5a	1,509739877	1
Tspan3	1,509425969	1
Cetn4	1,509112125	1
Atp2b4	1,509007525	1
Clec4e	1,508798346	1
Klf8	1,508798346	1
Mkl2	1,508170985	1
Ovca2	1,50806645	1
Xpnpep3	1,507857403	1
Asb7	1,507543885	1
Sec31a	1,507230433	1
Prdm2	1,507021501	1
Stx5a	1,506708157	1
Ppp3cc	1,506708157	1
Zfp994	1,506603723	1
Yif1a	1,506290467	1
Zfp983	1,505872892	1
Ubn1	1,505559787	1
Ssu72	1,505246747	1
Aldh3b1	1,505246747	1
Zfp408	1,505246747	1
Phf11d	1,505246747	1
Wdr6	1,505142415	1
Ndor1	1,504829462	1
Gm42515	1,504829462	1
Glmp	1,504725159	1
2310047D07Rik	1,504620863	1
Tango6	1,504308018	1
D10Jhu81e	1,504099492	1
Parp12	1,503474084	1
Aim1l	1,503474084	1
Ufsp2	1,503369875	1
Gid8	1,503161478	1
Gm9347	1,503161478	1
Shq1	1,502848937	1
Aak1	1,502744771	1
Gm44836	1,502432316	1

Aig1	1,502119927	1
Zfp839	1,501807602	1
Gm12743	1,501703509	1
Lrrc47	1,501599422	1
Tfdp2	1,501495343	1
AW209491	1,501391271	1
Ddx58	1,501391271	1
Tm7sf3	1,500766989	1
Taf1c	1,500662968	1
Rbm45	1,500662968	1
Fan1	1,500662968	1
Noc2l	1,500246953	1
Rnf113a2	1,500142968	1
Crocc	1,499935018	1
Rexo2	1,499727097	1
F8a	1,499727097	1
Zfp667	1,499727097	1
Dtwd2	1,499727097	1
Gm7160	1,498791811	1
Metap2	1,498584048	1
Wdfy1	1,498480178	1
Btd	1,498480178	1
Pfkm	1,497960934	1
Snord13	1,497441871	1
Mnd1-ps	1,49733808	1
Prdm9	1,496611743	1
Abcc3	1,496404283	1
Tulp4	1,496300563	1
Sos2	1,496196851	1
Trim27	1,495989449	1
Cep83os	1,495989449	1
Atf4	1,495782075	1
Inpp5a	1,495782075	1
Adamts6	1,495678399	1
Nudt16	1,49557473	1
Ccdc43	1,495471068	1
Gm44509	1,495471068	1
Smim10l1	1,495056493	1
Zfp446	1,494952867	1
Miga2	1,494952867	1
Rgs12	1,494849249	1
Pogk	1,494745637	1
Tbc1d24	1,494745637	1
Neu3	1,494434846	1
Hook3	1,494227688	1
Ppa2	1,494124119	1
Hoxa4	1,494124119	1
Gm19325	1,494020558	1
Zfp933	1,494020558	1
Prickle2	1,493709917	1
Copa	1,493192327	1
Lrp4	1,492881858	1

Gm13443	1,492571455	1
Ythdc2	1,492364555	1
Tnks2	1,491950841	1
5730480H06Rik	1,49184743	1
Epop	1,49184743	1
Zscan12	1,491744027	1
Foxred2	1,49164063	1
Matn4	1,491020404	1
Ap5s1	1,490917057	1
Lrrc73	1,490917057	1
Inpp5d	1,490503745	1
Cdr2	1,490503745	1
Rhot2	1,490090547	1
Zfp341	1,489987265	1
Pigo	1,489367726	1
Ppip5k1	1,489264494	1
RP24-370M23.1	1,488954842	1
Alkbh2	1,48885164	1
Traf2	1,48885164	1
Gm20223	1,488645255	1
Afg3l1	1,488335732	1
Idh2	1,488232572	1
Txnrd2	1,48771688	1
Tbck	1,487510652	1
Ecel1	1,487304454	1
Gm37578	1,487304454	1
Zbtb22	1,487201365	1
Dcun1d1	1,487098284	1
Eogt	1,486789083	1
Pou6f1	1,486789083	1
Ptgs2os2	1,486170873	1
Txndc9	1,485758876	1
Akap17b	1,485449954	1
Dusp2	1,485346994	1
Sparc	1,485244041	1
Slc2a9	1,484832301	1
Zscan20	1,484729384	1
Zbtb39	1,484523571	1
Stau2	1,484317786	1
Oit3	1,484009163	1
Jade2	1,483906303	1
Tsacc	1,483494934	1
Fam98a	1,483186482	1
Polr3gl	1,483083679	1
Ubiad1	1,482980883	1
Fut8	1,482980883	1
5430427O19Rik	1,48256977	1
Med8	1,482364257	1
Prtg	1,48226151	1
Ighmbp2	1,48205604	1
1110019D14Rik	1,48205604	1
Pdhx	1,481953315	1

Tsg101-ps	1,481645183	1
Kdm1b	1,481337116	1
Fhl3	1,481131773	1
Ypel3	1,480926459	1
Nelfa	1,480618541	1
I830077J02Rik	1,480413298	1
Zadh2	1,480105487	1
Stx11	1,479900315	1
Rfc2	1,47959261	1
Cacfd1	1,479079911	1
Ap4s1	1,478259963	1
Rbm5	1,477952599	1
Phf10	1,477645299	1
Sigirr	1,477645299	1
Cdk16	1,47754288	1
Hoxa1	1,477440468	1
Cnpy3	1,477338064	1
Speg	1,477338064	1
5730409E04Rik	1,477030892	1
Gusb	1,476826146	1
Pid1	1,476314406	1
Samsn1	1,476314406	1
Mgrn1	1,476109759	1
Mrps22	1,475905141	1
Gpat3	1,475700551	1
Dpp8	1,475598267	1
Zfp827	1,475598267	1
Pycr1	1,47549599	1
Trip11	1,47539372	1
Gm16061	1,475291457	1
Abcf2	1,475189201	1
Cyp20a1	1,475086952	1
Ms4a6d	1,474984711	1
Zfp691	1,474780249	1
Zfp106	1,474473609	1
Ugt1a7c	1,474473609	1
Tnrc6c	1,474064854	1
Unc13d	1,473554071	1
Coa4	1,473349807	1
Rab3ip	1,472839271	1
Ints6	1,47243097	1
Zswim4	1,472226862	1
Wdr13	1,471716716	1
Trmt6	1,471614708	1
Snx19	1,470900849	1
Polr3a	1,46977977	1
Zkscan6	1,46947417	1
Dbt	1,469372317	1
Surf4	1,469270472	1
Nars	1,46886316	1
Pgd	1,468659546	1
Coq2	1,468354179	1

Mlh3	1,468252404	1
2310010J17Rik	1,468150636	1
1810021B22Rik	1,468048875	1
Sri	1,467947121	1
Dnmt3b	1,467133344	1
Rgl2	1,46692997	1
Tlk1	1,466726625	1
Nsun3	1,466624963	1
Son	1,466523307	1
Mccc2	1,466015137	1
Ikbkg	1,465811918	1
Slc6a9	1,46571032	1
Zc2hc1a	1,465608728	1
Tmem192	1,464897783	1
Dhodh	1,464897783	1
Cables2	1,464694719	1
Tmem25	1,464694719	1
Ncoa6	1,464593197	1
Mtr	1,464390176	1
Dhx33	1,464288675	1
Pop4	1,463679821	1
Hmox2	1,463476926	1
Trap1	1,463476926	1
Tyw5	1,463375489	1
Qser1	1,463071221	1
Isl2	1,463071221	1
Gm14292	1,463071221	1
Erp29	1,462767015	1
Gm8494	1,462564247	1
1600014C10Rik	1,462057448	1
Krcc1	1,461854778	1
Cryga	1,461753454	1
Uggt2	1,461550826	1
Fam171b	1,461449522	1
Gm22581	1,461145654	1
Hgsnat	1,46094311	1
Trub2	1,460841849	1
Cep70	1,460841849	1
E330009J07Rik	1,460538107	1
Rnf225	1,460538107	1
Fxn	1,460436874	1
Klhl22	1,460234428	1
Smcr8	1,460133216	1
Gtf2ird2	1,459728437	1
Mib1	1,45962726	1
Ier2	1,45952609	1
Ptpn12	1,459323771	1
Gm42869	1,459121479	1
Myo19	1,458919216	1
Ifih1	1,458514774	1
Klhdc1	1,458009379	1
2310009A05Rik	1,457706226	1

Gps2	1,457605189	1
Kdelc1	1,45730212	1
Lrp12	1,457100109	1
Rpl7l1	1,456797145	1
Pank1	1,456696171	1
Hk1os	1,456696171	1
Clstn1	1,456494244	1
E130311K13Rik	1,456090474	1
Cerk	1,455989549	1
Dennd2d	1,455888631	1
Tmem260	1,455686816	1
C1qtnf6	1,455485029	1
Cul5	1,45528327	1
Cib1	1,45528327	1
Gm42600	1,45528327	1
Ikbke	1,455182401	1
Extl2	1,45467816	1
Ermard	1,45467816	1
Rbak	1,4543757	1
Pink1	1,454274894	1
Gm25291	1,454174095	1
Ttc33	1,453871739	1
Zfp46	1,453569446	1
C1ra	1,453468696	1
Zdhhc2	1,453367953	1
Tepsin	1,453267217	1
Sfxn3	1,45296505	1
Gm13604	1,45296505	1
Abca5	1,452360904	1
Rraga	1,452260238	1
Yars	1,452159578	1
Lcp1	1,45195828	1
Ptcd2	1,45175701	1
Bbs10	1,451656385	1
RP23-380K24.3	1,451455157	1
Kdm4a	1,451253956	1
Gas7	1,451052784	1
Rab11fip5	1,450952208	1
Abcd2	1,450952208	1
Mocs3	1,449846334	1
Idi1	1,449846334	1
Atp6v0d1	1,449444407	1
Pik3c3	1,449444407	1
Tbxas1	1,449444407	1
Gm38021	1,449343942	1
Cnbd2	1,449042591	1
Zfp330	1,448841725	1
Gm13391	1,448540478	1
Tnip1	1,448440076	1
Ak1	1,448440076	1
Snord59a	1,448239294	1
Atp6v0b	1,447737459	1

Arl16	1,447737459	1
Msl3	1,447637113	1
Procr	1,447135488	1
Nek4	1,447135488	1
Ncdn	1,447035183	1
Gapvd1	1,446634036	1
Srf	1,446634036	1
Tbc1d8	1,446533766	1
Ddx21	1,446333248	1
Dnajc17	1,446333248	1
Camta2	1,446232999	1
Ggact	1,446032522	1
Fahd2a	1,446032522	1
Rnf183	1,446032522	1
Adck1	1,445932295	1
Eci1	1,445832074	1
Msrb1	1,445531453	1
Pon3	1,445431259	1
Kat6a	1,445130722	1
Rere	1,445030556	1
Fbxw7	1,444830246	1
Fignl2	1,444830246	1
6230400D17Rik	1,444830246	1
Fbxo46	1,444329593	1
Srp72	1,444229483	1
Nfkbiz	1,443929196	1
Itfg2	1,443929196	1
Zfp111	1,443929196	1
Atg2a	1,44362897	1
Cep250	1,44362897	1
Gm15964	1,44362897	1
Vav3	1,443428855	1
Ide	1,443328807	1
Trim21	1,443228767	1
Ube2o	1,443028707	1
Snx32	1,442928687	1
Gm8973	1,442928687	1
Gm2810	1,442728668	1
Cmas	1,442628669	1
Sfmbt1	1,442528678	1
Cenpj	1,442028822	1
Cul7	1,442028822	1
Trim25	1,441928871	1
Rint1	1,441728992	1
Phtf2	1,441529139	1
Dand5	1,441429224	1
Sema4a	1,44102963	1
Rit1	1,44102963	1
Wdr33	1,440829875	1
Slc6a12	1,440730007	1
C130050O18Rik	1,440730007	1
Dock8	1,440630147	1

C1rb	1,440330608	1
Mrgpre	1,440130949	1
Irgm2	1,440130949	1
Rpn1	1,439931319	1
Slx1b	1,439931319	1
Gm37339	1,439931319	1
Eri1	1,439831514	1
Tpm2	1,439631924	1
Psap	1,43953214	1
Gm11918	1,439033323	1
Gm12430	1,438833844	1
Mpnd	1,438135887	1
Zbtb8os	1,438135887	1
Tfg	1,438036206	1
Il2rg	1,437936533	1
Lypla1	1,437637553	1
Nipsnap3b	1,437438268	1
Dock6	1,437338636	1
Ccdc159	1,437139393	1
Mia3	1,436442257	1
Slc36a4	1,436243138	1
Wdr53	1,436243138	1
Gpr180	1,436044046	1
Zc4h2	1,436044046	1
Becn1	1,435944511	1
Srsf5	1,435645945	1
Pld1	1,435347442	1
Gfi1	1,435049001	1
Nubpl	1,434850075	1
Wdr60	1,434750622	1
Cmbl	1,434651176	1
Zfp687	1,434452305	1
Wdr73	1,43435288	1
Sptlc1	1,43415405	1
Prnp	1,433955248	1
Mypop	1,433458363	1
Gucd1	1,433458363	1
Zfp809	1,433359007	1
Gars	1,433160315	1
Ank2	1,432763013	1
Slc17a5	1,432564404	1
Ccdc167	1,432564404	1
Ube2v1	1,432068001	1
Naif1	1,431869488	1
Atp10d	1,431472545	1
Stradb	1,431373326	1
Rtfdc1	1,431373326	1
Mtus2	1,431373326	1
Pced1a	1,431174909	1
Gm7863	1,431075711	1
Prorsd1	1,43097652	1
Gm15903	1,430877336	1

Csde1	1,430778158	1
Mcph1	1,430778158	1
Agpat2	1,430678988	1
Ilvbl	1,430579824	1
Snai2	1,430579824	1
Tuft1	1,430480667	1
Pgm2l1	1,430480667	1
2510016D11Rik	1,430381517	1
Nlrp10	1,430183238	1
Vps26b	1,430084109	1
Gm13445	1,430084109	1
Gsto1	1,429885871	1
Rassf4	1,42968766	1
Mgat2	1,429390396	1
Fut7	1,429390396	1
Ifi203	1,429291322	1
Smarcc2	1,429192254	1
Gm38345	1,429192254	1
Dtx4	1,42899414	1
Haghl	1,42899414	1
Gm12655	1,428895093	1
Cers4	1,428796053	1
Fads1	1,42869702	1
Snx10	1,428597993	1
Parp1	1,428399961	1
Xkr5	1,428399961	1
Rrp9	1,42800398	1
Gm16536	1,42800398	1
Txndc16	1,42780603	1
Clybl	1,427707065	1
Rabep2	1,427707065	1
Tmem68	1,427608108	1
Gm38020	1,427014506	1
Mpv17l	1,426915596	1
Ube2cbp	1,426618908	1
Ss18	1,42642115	1
Wdr41	1,426124565	1
Cd80	1,426124565	1
K230015D01Rik	1,426124565	1
Rnf123	1,426025717	1
Fam210b	1,425729214	1
Taf1b	1,425729214	1
Arhgef12	1,425630394	1
1810055G02Rik	1,425630394	1
Serpinf2	1,425136394	1
Rps2-ps11	1,425037614	1
Wdr45	1,424840076	1
Gphn	1,424741317	1
Slc11a2	1,424148907	1
Rcan3	1,423556743	1
Frg2f1	1,423260753	1
Pdcd11	1,422866196	1

Clptm1	1,422668959	1
Bin3	1,422668959	1
2810006K23Rik	1,422471749	1
Frat1	1,422175985	1
Riox2	1,421781729	1
Eif3a	1,421683182	1
Prune2	1,421584642	1
G730013B05Rik	1,421486108	1
Gm42636	1,421387582	1
Xrcc6	1,421387582	1
Zfp653	1,421387582	1
Gm43721	1,421190549	1
Taf7	1,420698087	1
Il12rb1	1,420599616	1
Snord65	1,420599616	1
Kbtbd11	1,42050115	1
Fzd9	1,420304241	1
Tmem138	1,420205796	1
Plin3	1,420107359	1
Gm20522	1,419910504	1
Slc9a6	1,419812086	1
Plag1	1,419812086	1
Zfp426	1,419615272	1
Emp2	1,419516875	1
Creg1	1,419418485	1
Satb2	1,419221726	1
Mlst8	1,418926638	1
Crnde	1,418631611	1
Crem	1,418336645	1
Nudc-ps1	1,418238337	1
Faap24	1,418140036	1
Serbp1	1,417943453	1
Phf11c	1,417845172	1
Zfp518a	1,41735387	1
Tmem181b-ps	1,41735387	1
Tmem80	1,41735387	1
Ap4m1	1,41725563	1
Snx4	1,41725563	1
Pld3	1,417157397	1
1110059E24Rik	1,41696095	1
Noa1	1,41696095	1
Col7a1	1,41696095	1
Zkscan1	1,41656814	1
Gm7733	1,41656814	1
Cutc	1,416175438	1
Vipas39	1,415979128	1
Itpripl2	1,415782845	1
Eif4g3	1,415488472	1
Fam120aos	1,415488472	1
Endov	1,415292258	1
Spryd4	1,41519416	1
Fcgr2b	1,41489991	1

Specc1l	1,414703776	1
Cyb5r1	1,414703776	1
Ubash3b	1,414409628	1
Ascc2	1,414213562	1
Ofd1	1,413821513	1
Fn3k	1,41362553	1
Gps1	1,413037742	1
Gm45050	1,412156518	1
Tyms	1,412058638	1
Mrps36-ps1	1,411960765	1
Gm20056	1,411862899	1
Snx33	1,411275843	1
Abhd11	1,411275843	1
Tfb2m	1,411080213	1
Rabgap1l	1,410982407	1
Ext1	1,410493483	1
Nsrp1	1,410395718	1
Iqcg	1,409906997	1
Flnb	1,409906997	1
Orc5	1,409711556	1
Zfp707	1,409613846	1
Tmem185b	1,409613846	1
Acot10	1,409516142	1
4732491K20Rik	1,409516142	1
H1fx	1,409320755	1
Fmnl3	1,409223072	1
Mrps6	1,409027726	1
Slc29a3	1,408930063	1
Mcat	1,408734757	1
Tubg2	1,408637114	1
Fars2	1,408344227	1
Timm44	1,407953805	1
Six1	1,407758635	1
Ube2q1	1,407758635	1
Accs	1,40766106	1
Zfp362	1,407563492	1
Sh3kbp1	1,407563492	1
Map2k2	1,40746593	1
Trpv2	1,40746593	1
Mrps2	1,407368375	1
1500011B03Rik	1,407368375	1
Ostm1	1,407075752	1
Zfp52	1,407075752	1
Ctdsp1	1,406685682	1
Elp2	1,406685682	1
Rpusd3	1,406588181	1
Coro1c	1,406100779	1
Ccdc91	1,405710979	1
Naip6	1,405613546	1
Zfp61	1,40551612	1
Lypla2	1,405321287	1
Morc4	1,405126482	1

Snora21	1,404931704	1
Zhx1	1,404834325	1
Zfp672	1,404736952	1
Tars2	1,404736952	1
Dedd	1,404639587	1
Mthfsl	1,404444876	1
Tbc1d20	1,404444876	1
Rufy1	1,40434753	1
Zfp870	1,403860905	1
Phf11b	1,403860905	1
Ly9	1,4037636	1
Umps	1,403666302	1
Ulk1	1,403666302	1
Nyap1	1,403569011	1
Abca2	1,403471726	1
9130221H12Rik	1,403277177	1
Gm43112	1,403179913	1
Rnf216	1,402596468	1
Oxld1	1,402013266	1
Fibp	1,402013266	1
Zbtb20	1,402013266	1
Med1	1,401818919	1
Lims2	1,401527449	1
Apobec3	1,40123604	1
Fam3c	1,40123604	1
Arid1b	1,400750493	1
Tmem57	1,400750493	1
Gpr107	1,400265114	1
Tmem205	1,399488857	1
Gnpda1	1,39929486	1
Ulk2	1,39910089	1
Bphl	1,398616083	1
Slfn10-ps	1,398519141	1
Mars	1,398519141	1
Gm28535	1,39784074	1
Map4k4	1,397743853	1
Ltbr	1,39735637	1
Ercc5	1,39735637	1
Zfp623	1,396872167	1
Nagpa	1,396581725	1
Smarcb1	1,396484925	1
Spi1	1,396484925	1
Osbpl11	1,396388132	1
Tmem175	1,396291345	1
Sergef	1,396097791	1
Txlna	1,396001024	1
Atp6v1b2	1,396001024	1
Nphp3	1,395904264	1
Gm15575	1,395710764	1
Hacl1	1,395420564	1
Afp	1,394937032	1
Ikbkb	1,394840346	1

Six4	1,394743666	1
Hipk2	1,394743666	1
Tnfrsf13b	1,394646993	1
Fdxr	1,394260369	1
Gm9025	1,394067097	1
Zfp90	1,393873851	1
Atp2a2	1,393873851	1
Aldh16a1	1,393777239	1
Cep63	1,393487441	1
Donson	1,393487441	1
Mapre3	1,393390855	1
Lair1	1,393294276	1
Sema6c	1,392714942	1
Itgav	1,392714942	1
Plxna2	1,392714942	1
Mov10	1,392618409	1
Zfp9	1,392521884	1
Rps3a2	1,392232347	1
Runx3	1,39174992	1
Idh1	1,391460544	1
Uchl3	1,391364098	1
Gm12833	1,39126766	1
Slfn8	1,391171228	1
4921511C10Rik	1,390978384	1
Clec4a2	1,390592776	1
Elp6	1,390496391	1
Ralgps1	1,38972555	1
Ap5z1	1,38972555	1
Tns3	1,389532906	1
Gm12689	1,389436594	1
Gm5523	1,389340289	1
Lrrc49	1,389243991	1
Polr3b	1,389243991	1
Sh2d3c	1,389147699	1
Rhod	1,388955136	1
Mfap3l	1,388955136	1
Saysd1	1,388858864	1
Lrrc8d	1,388858864	1
1810044D09Rik	1,388762599	1
Hmgxb4	1,388762599	1
Gm32856	1,388762599	1
Ubac1	1,388185149	1
Cxcl2	1,388088931	1
Pbx1	1,388088931	1
Gm45248	1,387992719	1
BC037034	1,387896514	1
Kif16b	1,387223266	1
Flnc	1,386934831	1
Pik3r4	1,386742574	1
Rasal3	1,386069886	1
Lman2	1,385973814	1
St3gal2	1,385877749	1

Gm11362	1,385589594	1
6430573P05Rik	1,385493555	1
Sidt2	1,385493555	1
Wdr24	1,385301498	1
Qtrtd1	1,38520548	1
Limk1	1,38520548	1
Phxr4	1,38520548	1
Sdf4	1,38520548	1
Slc35a1	1,385013463	1
Tsga10	1,384821473	1
Rnf219	1,384821473	1
Lancl2	1,384629509	1
Cradd	1,384533537	1
Npc1l1	1,384533537	1
Rad9b	1,384053777	1
Tcirg1	1,384053777	1
Setdb2	1,383957845	1
Usp16	1,38386192	1
Gm7799	1,383766001	1
Mettl21a	1,383670089	1
Zfp771	1,383670089	1
E130208F15Rik	1,383574184	1
Ficd	1,383190629	1
Depdc5	1,383094757	1
Gm4032	1,383094757	1
Msr1	1,383094757	1
Gm15634	1,382998891	1
Twnk	1,382903033	1
Gcc1	1,38280718	1
Fabp5l2	1,382328019	1
Ino80b	1,382232207	1
Stard7	1,382136401	1
Cds2	1,382136401	1
Cenpv	1,382040602	1
Gm37101	1,381944809	1
Zfp324	1,381657472	1
Tbc1d8b	1,381657472	1
Ebp	1,381657472	1
D630045J12Rik	1,381657472	1
Apoo-ps	1,381657472	1
Carmil1	1,381465947	1
Klc4	1,381274448	1
Vav2	1,380891531	1
Bcl2l12	1,380795818	1
Gm43351	1,380604412	1
Rab7b	1,380317353	1
Gm5822	1,38022168	1
Csnk2a2	1,38022168	1
Pou2f2	1,379934701	1
Ppil3	1,379743415	1
Ctu1	1,379552155	1
Sec23b	1,378882955	1

Impa2	1,378787381	1
Abcc5	1,378787381	1
Arl6ip4	1,378405153	1
Bcl9l	1,378214079	1
Naa40	1,378023031	1
Erp27	1,377927517	1
Alg1	1,377736509	1
Tbl1xr1	1,377641015	1
Ranbp1	1,377545527	1
Disp1	1,377163643	1
Pnkp	1,377163643	1
Mfsd1	1,3768773	1
Tubgcp6	1,376781866	1
Vps41	1,376591016	1
C230035I16Rik	1,376591016	1
Pkd1l2	1,376400193	1
Abcc1	1,376209397	1
Gstp-ps	1,375827884	1
Mapk9	1,375732522	1
Noc4l	1,375637167	1
Camk2b	1,375541818	1
Zkscan7	1,375446476	1
Madd	1,375351141	1
Fbxo15	1,37516049	1
Tuba1c	1,375065174	1
Fastk	1,375065174	1
Timm8a1	1,374779267	1
Paqr3	1,374683978	1
Araf	1,374588696	1
Rab11fip4os1	1,37449342	1
Slc37a4	1,374302888	1
Gem	1,374302888	1
Adat3	1,374112382	1
Rars2	1,373541024	1
Smim19	1,373350624	1
Syne1	1,373350624	1
Spryd3	1,373255433	1
Zrsr1	1,372779582	1
Suclg1	1,372779582	1
Rreb1	1,37249415	1
Pml	1,37249415	1
Ndufb8	1,372399019	1
Cenpb	1,372113667	1
Ddx1	1,372113667	1
Gm10132	1,371923464	1
Prss36	1,371733289	1
Cfap36	1,371733289	1
Bop1	1,371067881	1
Ccdc166	1,370687793	1
Letm1	1,370402796	1
Rspry1	1,369927933	1
Cblb	1,369738034	1

Nav1	1,369643094	1
Gm6525	1,369548161	1
Exoc7	1,369548161	1
Ddx56	1,369263401	1
Gpr137	1,369073594	1
1110002L01Rik	1,368694059	1
Tasp1	1,368504331	1
Orc3	1,368504331	1
Cyc1	1,368314629	1
Mtmr4	1,368314629	1
Alad	1,367935304	1
Pus3	1,367745681	1
Hps1	1,367366514	1
Pctp	1,36717697	1
Pnpla7	1,36717697	1
Umad1	1,367082208	1
Wdr78	1,366987452	1
Morn2	1,366987452	1
Tmem38a	1,366892703	1
Bms1	1,36679796	1
Smarca2	1,366703224	1
Chrac1	1,366608495	1
Pla2g12a	1,366608495	1
Wipf1	1,366513772	1
Fam122b	1,366419056	1
9330160F10Rik	1,366134947	1
Clptm1l	1,366040257	1
Ccdc93	1,365945573	1
Snora31	1,365850896	1
Slc50a1	1,365850896	1
Mettl22	1,365661562	1
Adck5	1,365661562	1
Mark2	1,365661562	1
Zkscan4	1,365566905	1
Rab12	1,365566905	1
Rab8a	1,365188342	1
Fn3krp	1,3649991	1
Ubr1	1,364904489	1
Exoc4	1,364809884	1
Mapkbp1	1,364715286	1
Hmga2	1,364526109	1
C2cd2	1,364336959	1
Sacs	1,364336959	1
Alkbh3	1,364336959	1
Uck2	1,364336959	1
Gm18284	1,364242394	1
Hdac7	1,364147835	1
Gm17259	1,363958737	1
Zfhx2	1,363864198	1
Pms2	1,363675139	1
Myo9b	1,36358062	1
A430105I19Rik	1,363297101	1

A130010J15Rik	1,363297101	1
Efna1	1,363297101	1
Cyb5r4	1,363202607	1
Ptgr2	1,363202607	1
Srprb	1,363202607	1
Nol4l	1,36301364	1
Mcrs1	1,362919167	1
Zfp628	1,362919167	1
Arhgap30	1,3628247	1
Tcam1	1,362635785	1
Epc2	1,362541338	1
Iqsec1	1,362541338	1
Fgfrl1	1,362446897	1
Ate1	1,362352462	1
AI606181	1,362258035	1
Nfs1	1,362258035	1
Kat5	1,362163613	1
Gtf2h2	1,362069199	1
Mrps35	1,362069199	1
Ormdl1	1,36197479	1
Hgs	1,361880389	1
Zfp950	1,361597223	1
Lrba	1,361502848	1
1700037C18Rik	1,361314116	1
Mustn1	1,361314116	1
Arhgap27	1,361125411	1
4930440I19Rik	1,360842403	1
Pdk3	1,360842403	1
2610306M01Rik	1,360653763	1
Bicdl1	1,360559453	1
Gmppa	1,360465149	1
1700022N22Rik	1,360370852	1
Gm9403	1,360182278	1
Zfp952	1,360088001	1
Pomk	1,35999373	1
AW146154	1,359522474	1
Bptf	1,359522474	1
Enpp4	1,359428242	1
Tpk1	1,358392125	1
Carnmt1	1,358392125	1
Zdhhc6	1,358297971	1
Fbrsl1	1,358203825	1
Rpusd4	1,357921423	1
Gorasp2	1,357921423	1
Wdr4	1,357733189	1
Amdhd2	1,357733189	1
Commd5	1,357639081	1
Ppp1r12b	1,357639081	1
Bbs1	1,357262716	1
Mtmr2	1,357262716	1
Sdhaf4	1,35698051	1
Gm13758	1,356792406	1

Stk4	1,356604327	1
Kiss1r	1,356510298	1
Wtap	1,356322259	1
Nckipsd	1,356134246	1
Lcmt2	1,356134246	1
Gm16199	1,356134246	1
Tbc1d5	1,356040249	1
Tnfrsf1a	1,356040249	1
Wls	1,355946259	1
Taf4b	1,355758298	1
Nudt6	1,355570363	1
Gm5590	1,355476405	1
Galnt1	1,355476405	1
Atp6v1a	1,355382454	1
Map4k3	1,355288509	1
Ift81	1,355194571	1
Pus10	1,355194571	1
D230017M19Rik	1,355100639	1
Arih1	1,354537186	1
Nr1d2	1,35388012	1
Gm42829	1,353692445	1
Cxcl14	1,353598617	1
Dok1	1,353410982	1
Dnajc2	1,353035789	1
Cpne8	1,352379451	1
Ppp3ca	1,352285714	1
Gm7722	1,352098261	1
Polr2f	1,352098261	1
Btrc	1,351910833	1
Lmntd2	1,351910833	1
Fam168a	1,351817129	1
Fndc7	1,35162974	1
Trappc12	1,351536056	1
Nckap5l	1,351442378	1
Rhd	1,351442378	1
Spata13	1,351255041	1
Slc20a2	1,35106773	1
Hsd17b7	1,35106773	1
Mbd6	1,350880445	1
Msto1	1,350693187	1
Sfpq	1,350599567	1
Adrm1	1,350505954	1
RP24-325N9.5	1,350505954	1
Gm23751	1,350037985	1
Fnip2	1,350037985	1
Ndufaf5	1,349944411	1
Snd1	1,349663727	1
Zfp768	1,349570179	1
Zfyve19	1,34919605	1
Limd1	1,349102534	1
Pccb	1,348635052	1
Nxn	1,348541575	1

Galns	1,34835464	1
Ubac2	1,34835464	1
Pemt	1,348167732	1
Gm13433	1,347887417	1
E2f3	1,347887417	1
Eif1ad	1,347887417	1
Psen2	1,347887417	1
Cyb5a	1,347887417	1
Gm11464	1,347793992	1
Hspa2	1,347700573	1
Gm11631	1,347700573	1
Gm9712	1,347607161	1
Serinc3	1,347326963	1
R74862	1,347233577	1
Opa3	1,347233577	1
Mrpl37	1,347233577	1
Foxp1	1,347140197	1
Cdk10	1,346953457	1
Uri1	1,346673395	1
Ftsj3	1,346673395	1
Gzf1	1,346486719	1
Exosc4	1,346486719	1
Arl15	1,346206754	1
Sirt7	1,346113445	1
Dxo	1,346020143	1
Gm11952	1,345646999	1
Snx15	1,345646999	1
A430035B10Rik	1,345646999	1
Stx18	1,345367209	1
Pitpnm1	1,345273958	1
1700086O06Rik	1,344714591	1
Prkch	1,344434994	1
Imp4	1,344434994	1
Vamp4	1,344155456	1
Sbk1	1,344062289	1
Neat1	1,344062289	1
Pelo	1,343782828	1
Rftn1	1,343689688	1
Dhrs1	1,343689688	1
Brdt	1,343224081	1
9330159M07Rik	1,342944795	1
Coq8a	1,342944795	1
Cbarp	1,342572503	1
Zdhhc9	1,341735224	1
Zbtb48	1,341642225	1
Zfp568	1,341642225	1
Apoe	1,341363267	1
Gm10425	1,341363267	1
Mfhas1	1,341084368	1
Pde8b	1,341084368	1
Rnf121	1,340898467	1
Zcchc6	1,340526742	1

Gm10167	1,340248017	1
Pecr	1,340155121	1
Rusc1	1,340155121	1
D830044I16Rik	1,340062232	1
Det1	1,339969349	1
Mbp	1,339505031	1
Gm17530	1,339319349	1
Tpp1	1,339319349	1
Ncf1	1,339319349	1
Dhx57	1,339226518	1
Prmt3	1,339040875	1
Angel2	1,338855257	1
Kif3b	1,338855257	1
D930016D06Rik	1,338762458	1
Gpr19	1,338669665	1
Gpatch4	1,338669665	1
Acad10	1,338298559	1
Tmem51	1,338205798	1
Sdhb	1,338020296	1
Smad1	1,337927555	1
Acaca	1,337927555	1
Ccdc71	1,337556654	1
Slc25a44	1,337556654	1
Senp7	1,337463945	1
Atat1	1,337185856	1
Cybb	1,337093172	1
Chmp3	1,337093172	1
Mrps7	1,337000495	1
Ppp3cb	1,336629852	1
Fndc10	1,336537207	1
Zranb3	1,336166691	1
Abcd3	1,335888872	1
Coa7	1,335703691	1
Zfp316	1,335703691	1
Srsf6	1,335611111	1
Impdh2	1,335425968	1
Trim68	1,335333407	1
Fer	1,335240852	1
Ppp6r1	1,335240852	1
Abcb9	1,335148303	1
Fam212b	1,335055761	1
A730062M13Rik	1,335055761	1
Utp20	1,335055761	1
Ttc32	1,334870696	1
Cdk5rap2	1,334685656	1
Zfp513	1,334685656	1
Ncbp1	1,334593146	1
Poldip2	1,334408145	1
Gm38335	1,334315654	1
Neil1	1,334315654	1
Clpp	1,33403822	1
Ehmt1	1,33403822	1

Hs2st1	1,33403822	1
Parp16	1,333853296	1
Pex16	1,333853296	1
Osbpl7	1,333483524	1
Kifc3	1,333483524	1
Chpt1	1,333298677	1
Gm11613	1,333113855	1
Sfxn1	1,333113855	1
Yipf2	1,333021454	1
Iars2	1,332929059	1
Yeats4	1,332929059	1
Akt1	1,332929059	1
Ptpn23	1,332651913	1
Amfr	1,332467181	1
Phf14	1,332374825	1
Tbcd	1,332374825	1
Coro1b	1,332374825	1
Nr2c1	1,332097794	1
Oas1c	1,332097794	1
Snap23	1,332097794	1
Fbxl19	1,332005464	1
Atp6v1e1	1,332005464	1
RP23-47A1.1	1,331543906	1
Gm36445	1,331543906	1
Gsto2	1,331267048	1
Gclc	1,331174774	1
Cenpo	1,330990247	1
Cul4b	1,330897993	1
A130048G24Rik	1,330805746	1
Calcoco1	1,330805746	1
Rtn4ip1	1,330713504	1
Jagn1	1,330529041	1
Zdhhc24	1,330344603	1
Rnmt	1,330344603	1
Tbc1d16	1,330252394	1
Ubr4	1,330252394	1
Pdzd8	1,330160191	1
Gm38077	1,329699273	1
Plscr3	1,32951495	1
Spats2	1,32951495	1
Lgals3bp	1,329422798	1
Kcnab3	1,329238514	1
Cxcr3	1,329238514	1
Serpini1	1,329238514	1
Usp46	1,329238514	1
Heatr3	1,329054255	1
Isoc2a	1,328962135	1
9530062K07Rik	1,328685814	1
Samd10	1,32859372	1
6030458C11Rik	1,32859372	1
Tpmt	1,328501632	1
Gm5883	1,328501632	1

Zfp26	1,328501632	1
Rsu1	1,328317475	1
Gm43128	1,328225406	1
Stard3	1,328225406	1
Gsk3b	1,328133344	1
Atp5g3	1,328133344	1
Cdk14	1,328041288	1
Eef2kmt	1,327949238	1
D11Wsu47e	1,327765158	1
3300002I08Rik	1,327581104	1
Fbxo25	1,327581104	1
Gm6166	1,327397075	1
Alg3	1,32730507	1
Galnt7	1,32730507	1
Vmac	1,327213071	1
Gm42566	1,326937114	1
Gm26947	1,326753174	1
Ints12	1,326753174	1
Fam136a	1,32656926	1
Slc6a8	1,32656926	1
Dmwd	1,326477312	1
Eif3b	1,326385371	1
Zfp276	1,326201507	1
Marveld1	1,326109585	1
Nfatc3	1,326109585	1
Heatr1	1,32601767	1
Arrb1	1,32601767	1
Jaml	1,32601767	1
Cygb	1,325741961	1
Gm38213	1,32565007	1
Limd2	1,325466309	1
Zfp551	1,325282573	1
Mfsd6	1,325190715	1
Rabif	1,325007017	1
Nfkbia	1,325007017	1
Cog2	1,324915178	1
Yipf5	1,324823345	1
Slain2	1,324731518	1
Irf2bp1	1,324639698	1
Agpat1	1,324639698	1
Gm28530	1,32408891	1
Zfp512b	1,323997135	1
Pitpna	1,323721846	1
Zbed4	1,323630096	1
Wdr59	1,323630096	1
Ccdc32	1,323630096	1
Hemk1	1,323538352	1
Pgpep1	1,32280463	1
Preb	1,32280463	1
Lrp5	1,322621263	1
Tifab	1,322529589	1
Eid2b	1,322529589	1

Selplg	1,322529589	1
Rcn1	1,322437921	1
Cpsf4	1,32234626	1
Gdi2	1,322162957	1
Ccnk	1,321979679	1
Gm43359	1,321796426	1
A530013C23Rik	1,321704809	1
Pomgnt1	1,321704809	1
Exosc1	1,321338406	1
Trim28	1,321338406	1
Vim	1,321338406	1
Trim26	1,321155242	1
Cltb	1,32106367	1
Rb1cc1	1,320972104	1
Dennd1a	1,320697444	1
Vwf	1,320605903	1
Atg4a	1,320605903	1
F830115B05Rik	1,320422841	1
Gm12089	1,320331319	1
Olfr921	1,319690846	1
Dhx16	1,319690846	1
Csnk1d	1,319690846	1
Polg	1,319599375	1
Frmd4b	1,319416453	1
Gas6	1,319416453	1
Tigar	1,319325001	1
Notch2	1,319325001	1
Tef	1,319142116	1
Pdk2	1,319050683	1
Lum	1,318867837	1
Dlst	1,318776423	1
Szt2	1,318685016	1
Brf1	1,31850222	1
Vav1	1,318228073	1
Gm12309	1,317953984	1
Csk	1,317953984	1
Lhfpl2	1,317953984	1
Tmem186	1,317679952	1
Gm4285	1,317405976	1
Itga7	1,317314664	1
Rrp8	1,317223358	1
Nradd	1,316949477	1
Nlrc5	1,316858197	1
Gm8168	1,316584392	1
Svip	1,316493137	1
Zfp282	1,316310644	1
Mgat4b	1,316310644	1
Got2-ps1	1,315945736	1
Cog4	1,315945736	1
Gm43457	1,315672122	1
Runx2	1,315489743	1
Mrpl19	1,31530739	1

Klhl36	1,315125063	1
Scd2	1,315125063	1
Slc12a7	1,315033908	1
Irf3	1,31494276	1
Tmem241	1,314760483	1
Nin	1,314669354	1
Ifngr2	1,314487115	1
Cul9	1,314487115	1
Mir155hg	1,314304901	1
Exosc9	1,314304901	1
Zkscan5	1,314213803	1
Tshz3	1,314213803	1
Phax	1,314213803	1
Zfp563	1,314122712	1
2010320M18Rik	1,314122712	1
Slfn5	1,314122712	1
Derl1	1,314122712	1
Cela1	1,314031627	1
Gm12751	1,313849476	1
8430429K09Rik	1,31375841	1
Gm44901	1,313667351	1
Tspan5	1,313576297	1
Yeats2	1,31348525	1
Atp6v1c1	1,31339421	1
Cxx1b	1,313212147	1
Zfp874b	1,312939101	1
Nup160	1,312848098	1
Exd1	1,312757101	1
Svbp	1,312757101	1
Abr	1,312757101	1
Gm14140	1,312575127	1
Cct3	1,312575127	1
Mtl5	1,312393178	1
Zfp524	1,312393178	1
Supv3l1	1,312302213	1
Prr36	1,312211255	1
Pde4d	1,312211255	1
Rmdn1	1,312211255	1
Ubtf	1,312211255	1
Snord72	1,312120302	1
Kif7	1,312029356	1
Akip1	1,311847483	1
Dnajc10	1,311756556	1
Gnat2	1,31157472	1
D2hgdh	1,31157472	1
Rffl	1,311483812	1
Scfd1	1,311483812	1
4933440N22Rik	1,31139291	1
Arfip2	1,311302014	1
Gm28557	1,311120242	1
Macrod2	1,311029365	1
Gm15824	1,310938494	1

Arl8a	1,31084763	1
Golga2	1,31084763	1
Lrrcc1	1,310756772	1
Akr1b8	1,310756772	1
Dnajc25	1,310211757	1
Gpatch11	1,310211757	1
Tbc1d13	1,310120944	1
Bcl2l1	1,310120944	1
Serp1	1,309666969	1
Cebpe	1,309576193	1
Med24	1,309485423	1
1810011H11Rik	1,30939466	1
Celf4	1,309303902	1
Plekho1	1,309303902	1
Scfd2	1,309031669	1
Eprs	1,308850211	1
Nudt12	1,308668778	1
Yipf3	1,308578071	1
Tmem184c	1,308487371	1
Clcn4	1,308487371	1
Smim13	1,308215307	1
Hps4	1,308124631	1
Cirbp	1,308033962	1
Phf7	1,307852643	1
Tdrkh	1,307127616	1
Dapk3	1,306402992	1
Tmem19	1,306402992	1
Lig3	1,306312442	1
Bloc1s5	1,30604083	1
Synj1	1,30604083	1
Kbtbd8	1,305950306	1
Entpd5	1,305859787	1
Slc41a3	1,305859787	1
Slc4a11	1,305497776	1
Elovl6	1,305497776	1
Adamtsl5	1,305226334	1
Slc29a1	1,304683618	1
RP23-139H6.1	1,304141128	1
Lhpp	1,304050735	1
2210008F06Rik	1,304050735	1
Gm42479	1,303960348	1
Micu1	1,303869968	1
Gm5624	1,303779594	1
9630013D21Rik	1,303598864	1
Gm5909	1,303508509	1
Pigh	1,303418159	1
5430402O13Rik	1,303327816	1
Stac3	1,303327816	1
Puf60	1,30323748	1
Atxn7l3	1,303147149	1
Spata6	1,302966507	1
Lrif1	1,302966507	1

Ubr5	1,302966507	1
BC002059	1,302876196	1
Rab32	1,30278589	1
Atad1	1,302515012	1
Tex264	1,302515012	1
Dcun1d2	1,302334457	1
Gm26514	1,30224419	1
Nrd1	1,302063673	1
Lrrc25	1,301883181	1
Gm8093	1,301792944	1
Idh3g	1,301702714	1
Abcf1	1,30161249	1
Tceanc2	1,301522272	1
Gm43290	1,301161464	1
0610007P14Rik	1,301161464	1
Zfp605	1,301071277	1
Gm5764	1,301071277	1
Pde4b	1,301071277	1
Acacb	1,300981097	1
Exo5	1,300890923	1
Myo18a	1,300890923	1
Tnfaip8l1	1,300620438	1
Evi5l	1,300620438	1
Ipo13	1,30035001	1
Cad	1,300079638	1
Sec61a2	1,299989526	1
Abhd4	1,299989526	1
Flcn	1,299989526	1
Cdc16	1,299989526	1
Vasp	1,299989526	1
Drosha	1,299899421	1
Sacm1l	1,299899421	1
Ap3s2	1,299629143	1
Pde4a	1,299629143	1
Gtf2h3	1,299448988	1
2900005J15Rik	1,299448988	1
Thada	1,299358921	1
Gm5857	1,299268859	1
Tctn3	1,299088755	1
Gm8121	1,298908675	1
Dcaf4	1,298908675	1
Zdhhc14	1,298728621	1
Ankmy2	1,298728621	1
Mtfp1	1,298458586	1
Gm38376	1,298458586	1
Galc	1,298458586	1
Zfp110	1,298458586	1
Smarcd2	1,298458586	1
Fosl1	1,298278594	1
Dennd1c	1,298188607	1
Zfp729b	1,298188607	1
Zer1	1,298188607	1

Aptx	1,298188607	1
2310036O22Rik	1,297828722	1
Maats1os	1,297738767	1
RP23-304C21.3	1,297648818	1
Ube4b	1,297468938	1
Elfn2	1,297289083	1
Tgfb1i1	1,297289083	1
Fbxo18	1,297109253	1
Pip4k2c	1,296749668	1
4932441J04Rik	1,296569912	1
Psmd10	1,296480044	1
Myo1c	1,296480044	1
Il17rc	1,296390182	1
Gm13094	1,296300326	1
Reps1	1,296120633	1
Bco2	1,295940965	1
Atad3aos	1,295940965	1
Keap1	1,295312323	1
Zcchc2	1,295312323	1
Zfp39	1,29486348	1
Cebpz	1,29486348	1
RP23-261O3.5	1,29477373	1
Efl1	1,29477373	1
Gm29759	1,294504518	1
Fbxo4	1,294504518	1
Ddx59	1,294414792	1
Arid2	1,294145654	1
D230025D16Rik	1,294055954	1
Serac1	1,293786891	1
Zbtb12	1,293786891	1
D730003I15Rik	1,293786891	1
Slc39a2	1,293697215	1
Tnfaip2	1,293697215	1
Gm2199	1,293607546	1
Camk2n2	1,293517883	1
Myh9	1,293517883	1
Eya4	1,293248932	1
Ncl	1,293248932	1
Ung	1,292980036	1
Ccdc66	1,292890417	1
Pik3ap1	1,292621596	1
Them4	1,292532001	1
Gm37474	1,292352831	1
A130050O07Rik	1,292084121	1
Nectin4	1,292084121	1
Fam91a1	1,292084121	1
Actr3b	1,291994564	1
Naglu	1,291994564	1
Xrn1	1,291994564	1
Nsmaf	1,291994564	1
Slc16a9	1,291725929	1
Gm18913	1,291636397	1

Impact	1,291636397	1
Cog8	1,291636397	1
Ubxn11	1,29154687	1
Ankrd54	1,29145735	1
Kcnn1	1,291278329	1
Trib1	1,291188827	1
Cttnbp2nl	1,291188827	1
Mbtps2	1,291009843	1
Plekhn1	1,290830883	1
Rnf169	1,290741413	1
Pkn1	1,290741413	1
Tk2	1,290651949	1
Zfp846	1,29056249	1
Commd10	1,29056249	1
Sh3bp5l	1,29056249	1
Gm13368	1,29020472	1
Gls2	1,29020472	1
Inpp4a	1,289847049	1
4933417C20Rik	1,289757647	1
Smarcc1	1,289668251	1
Slc6a13	1,289578861	1
Fmn1	1,289489477	1
Sf3a3	1,289221363	1
Mdfic	1,289132004	1
Dynlt1f	1,288863965	1
Ttc3	1,288863965	1
B230369F24Rik	1,28877463	1
Osbpl10	1,28877463	1
Ykt6	1,288685303	1
Cluh	1,288685303	1
Fastkd5	1,288595981	1
Bcl2l14	1,288328053	1
Mcm3ap	1,28806018	1
Nelfcd	1,287703104	1
Tbc1d9b	1,287703104	1
Plpp6	1,28761385	1
Zmpste24	1,28761385	1
Plekha5	1,287524602	1
Smarca4	1,287524602	1
Arfgap3	1,287435361	1
Rassf5	1,287435361	1
Vps52	1,287346126	1
Ano10	1,287346126	1
Ubtd1	1,287256897	1
B3glct	1,287167674	1
B3galt6	1,286989247	1
Upk1a	1,286989247	1
Pdss2	1,286989247	1
Mrpl17	1,286810845	1
Slamf9	1,286721653	1
9230112E08Rik	1,286543288	1
Aste1	1,286543288	1

Tmub2	1,286364948	1
Rnpepl1	1,286097483	1
Nek9	1,286097483	1
Adprh	1,285740951	1
Traf5	1,285651833	1
Slc9a9	1,285295424	1
Prpf6	1,285295424	1
Pkib	1,285206337	1
Sipa1	1,285117257	1
Gm6155	1,285028182	1
Poc1b	1,285028182	1
Gm6293	1,284939114	1
Armc6	1,284939114	1
Gm12251	1,284850052	1
Tubb2b	1,284671946	1
Dnph1	1,284493865	1
Malsu1	1,284404834	1
Cfap97	1,284315809	1
Cyb561d1	1,28422679	1
Dock11	1,28422679	1
Gm5637	1,28395977	1
Haus2	1,28395977	1
Slc26a9	1,283870776	1
Dapp1	1,283425898	1
Ankrd13c	1,28333694	1
Lamp1	1,28333694	1
D230022J07Rik	1,283247989	1
Gm13450	1,283247989	1
Mdn1	1,283247989	1
Gtf2i	1,282981173	1
Tpm1	1,282892247	1
Tada2a	1,282358818	1
Ttc4	1,282358818	1
Mpv17	1,282269935	1
Cacna1d	1,282181057	1
Srm	1,282181057	1
Zdhhc20	1,282181057	1
Cd200r2	1,281914463	1
Gcfc2	1,281825611	1
Rasgrp3	1,281825611	1
Ppm1h	1,281736764	1
Bet1l	1,281292625	1
Timmdc1	1,281115013	1
Gm16062	1,280671089	1
Lyz2	1,280493562	1
Gm26620	1,280404809	1
Ptpn11	1,280404809	1
Zfp608	1,280316061	1
Hars2	1,280227319	1
Peg12	1,280049854	1
Steap3	1,279961131	1
Mtap	1,2796063	1

9030624J02Rik	1,2796063	1
Mtfr1	1,279428921	1
Scamp2	1,279251567	1
Zbtb32	1,279162899	1
Slc9a8	1,279162899	1
Gm12497	1,278719651	1
Ogfod3	1,278719651	1
Smim3	1,278719651	1
Nrap	1,278542395	1
Gm43672	1,278365164	1
Mlycd	1,278276558	1
Sqstm1	1,278276558	1
Etv6	1,278187957	1
Cog1	1,278099363	1
Ddx51	1,278010775	1
Gas2	1,277922193	1
Flad1	1,277922193	1
Dnajc19-ps	1,277745048	1
Mt2	1,277656484	1
Mad1l1	1,277567927	1
3300005D01Rik	1,277479376	1
Stxbp5	1,277479376	1
Grk4	1,277302292	1
Zfp68	1,277302292	1
Mtg1	1,277302292	1
Slc7a1	1,277213759	1
Tnfrsf14	1,277125233	1
Gm16106	1,277036712	1
Egf	1,276859689	1
Mrpl48-ps	1,27641724	1
Soga1	1,27641724	1
Pih1d1	1,276240304	1
Cpt1a	1,276240304	1
Gm16072	1,276151844	1
Dnmt3a	1,275974944	1
Rbm41	1,275886504	1
Gm10029	1,275886504	1
Mmgt2	1,275798069	1
Ppargc1b	1,275621218	1
Pafah1b1-ps1	1,275179198	1
Bcap29	1,275002433	1
Tpgs1	1,274737331	1
Smco4	1,274737331	1
Mis12	1,274648976	1
Dbp	1,274560627	1
Mtif2	1,274383948	1
Lrrk1	1,274295617	1
Pear1	1,274030663	1
Hnrnpm	1,274030663	1
Fam132a	1,273942357	1
BC052040	1,273942357	1
Aftph	1,273854057	1

Diaph2	1,273765763	1
Stat6	1,273765763	1
Odf2l	1,273677475	1
Nsmce1	1,273677475	1
Larp7	1,273677475	1
Gm43660	1,273500918	1
Prrc1	1,273500918	1
Zfp74	1,273412649	1
Slc39a9	1,273324386	1
Cox7a1	1,273147878	1
Ttll5	1,273147878	1
Xpo4	1,272883162	1
Eif2s2	1,272794935	1
Itsn1	1,272530293	1
Ddx55	1,272442091	1
Atg7	1,272442091	1
Coq10a	1,272442091	1
Mms22l	1,272353895	1
Rnft1	1,272177521	1
Fam3a	1,272177521	1
Snta1	1,272177521	1
Aifm2	1,271560406	1
Arfgef3	1,271119794	1
Cd53	1,271119794	1
Gm42715	1,271031689	1
Os9	1,270943591	1
Akap9	1,270767413	1
Gm43144	1,270679333	1
Inpp1	1,270679333	1
Ttc30b	1,27041513	1
Tal1	1,270327075	1
Pex11b	1,270327075	1
Fli1	1,270239026	1
Larp1b	1,270239026	1
Wrap73	1,270150983	1
Fosb	1,270150983	1
Parp3	1,270150983	1
Churc1	1,269798871	1
Nudt18	1,269798871	1
Ankhd1	1,269798871	1
Commd9	1,269622851	1
Osbpl9	1,269534851	1
Cc2d1a	1,269446857	1
Commd7	1,269270886	1
Stambp	1,269270886	1
Kmt2e	1,26918291	1
Plpp1	1,268919018	1
Tm9sf4	1,268919018	1
Mcoln2	1,268831066	1
Chid1	1,268831066	1
Ctdsp2	1,268743121	1
Mospd1	1,268567248	1

Rpusd1	1,268479321	1
Atn1	1,268039775	1
Sdha	1,267951885	1
Kif2a	1,267864	1
Nfx1	1,267776121	1
Gm5547	1,267600382	1
Comt	1,267600382	1
Gm9796	1,267424668	1
Cd2ap	1,267424668	1
Afmid	1,267248978	1
Tmem198b	1,267248978	1
Polr3f	1,267248978	1
Gm13416	1,267161142	1
Synj2	1,267073312	1
Rcl1	1,266985488	1
Cd63-ps	1,26689767	1
Mvd	1,266809859	1
Wfikkn1	1,266634254	1
Ighd	1,26654646	1
B4galnt1	1,26654646	1
Zbtb25	1,26654646	1
Apex2	1,266370892	1
Ufc1	1,266283117	1
Nr2c2	1,266195348	1
Glud1	1,266107585	1
Rbfox2	1,265932077	1
Gm12186	1,265844333	1
Gm5449	1,265844333	1
Sh3glb2	1,265844333	1
Gm5391	1,265493415	1
Igsf6	1,265493415	1
Plpp2	1,265493415	1
Mcur1	1,2654057	1
Ndufaf7	1,2654057	1
Dnajc8	1,2654057	1
Arl4a	1,265317992	1
Fkrp	1,26523029	1
St3gal3	1,264791871	1
Gm11966	1,264704205	1
Acbd4	1,264704205	1
Lsm4	1,264441244	1
Prkab2	1,264353603	1
Crat	1,264265968	1
0610009O20Rik	1,264090716	1
Inpp5k	1,264090716	1
Ccdc126	1,264003098	1
Rgs19	1,264003098	1
Pdia5	1,263827883	1
Ppcs	1,263477524	1
Them6	1,263477524	1
Htra2	1,263477524	1
P3h1	1,263302381	1

Zswim1	1,263214818	1
Tnf	1,263214818	1
H2-Oa	1,263127262	1
Gm6395	1,263127262	1
Mppe1	1,263127262	1
2310011J03Rik	1,263127262	1
Vdac3-ps1	1,263039712	1
Uhmk1	1,262864629	1
Zufsp	1,262602051	1
Hyal3	1,262514537	1
Pum3	1,262339528	1
Ergic1	1,262339528	1
Kmt2b	1,262077059	1
Herpud2	1,261989581	1
Dnajc27	1,26190211	1
Tbc1d22a	1,26190211	1
Prep	1,261727185	1
D030028A08Rik	1,261639732	1
Dennd3	1,261464844	1
Trib3	1,261377409	1
Lrrc59	1,261377409	1
Nbas	1,261202557	1
Fbxo3	1,261202557	1
Trdmt1	1,26111514	1
Ring1	1,26111514	1
Tmeff1	1,261027729	1
B4gat1	1,260940324	1
Nadk	1,260852926	1
Ap1b1	1,260678147	1
Dus1l	1,260328661	1
Borcs5	1,26006661	1
Tram1	1,26006661	1
Dido1	1,25989194	1
Arhgap21	1,259804614	1
Mtbp	1,259717294	1
Pald1	1,259717294	1
Aim2	1,25962998	1
Trabd	1,25962998	1
Gm13423	1,259542672	1
Gm44829	1,25945537	1
1110037F02Rik	1,259193501	1
Ndufs7	1,259193501	1
Slc25a14	1,259106223	1
Edem2	1,259106223	1
Gm42890	1,259018952	1
Tpm3-rs7	1,259018952	1
Actb	1,258931686	1
Prex1	1,258844427	1
Elk1	1,25849545	1
Zfp212	1,25840822	1
Tbc1d14	1,25840822	1
Zfp322a	1,258146569	1

Fbxo38	1,257884972	1
Ndufa9	1,257797785	1
Stxbp2	1,257710604	1
Parp2	1,257449098	1
3110083C13Rik	1,257361941	1
Net1	1,25727479	1
Ncf2	1,25727479	1
Grik5	1,257100507	1
Usp25	1,257100507	1
Ube2d2a	1,256926248	1
As3mt	1,256752013	1
Zfp69	1,256664905	1
Mtmr1	1,256577802	1
Gdap2	1,256577802	1
Frmd8	1,256490706	1
Rpusd2	1,256316532	1
Lasp1	1,256316532	1
Kdelr2	1,256142381	1
Lyn	1,255968255	1
Gclm	1,255881201	1
Gna15	1,255794153	1
Anapc4	1,255794153	1
Cisd2	1,255794153	1
Gstm1	1,255794153	1
Ints11	1,255794153	1
Map3k4	1,255707111	1
Ptcd1	1,255533045	1
9430092D12Rik	1,255446021	1
Slc25a30	1,255446021	1
Derl2	1,255446021	1
Coa5	1,255446021	1
Sin3b	1,255184985	1
Mrpl36	1,254924004	1
Rnf20	1,254837023	1
Micu2	1,254750047	1
Zfp867	1,254663077	1
Dera	1,254663077	1
Kank2	1,254402205	1
Frmd6	1,254141386	1
Polr1e	1,254141386	1
BC037032	1,254054459	1
Laptm4b	1,254054459	1
Ydjc	1,254054459	1
Prag1	1,253967537	1
Cux1	1,253967537	1
Ppp1r7	1,25353302	1
Fam13a	1,253359256	1
Zfp606	1,253098654	1
Sec61b	1,25292495	1
Rac2	1,25292495	1
Arhgef6	1,252838107	1
Airn	1,252664439	1

Slc25a1	1,252664439	1
Pigm	1,252577614	1
Kif1c	1,252577614	1
Faf2	1,252317174	1
Vps13c	1,252317174	1
Immt	1,252230373	1
Cdk5	1,252143578	1
Rabggta	1,25188323	1
Cyp4f16	1,251796459	1
Dnajc24	1,251796459	1
Dscr3	1,251622935	1
Gm20492	1,251536182	1
Coq7	1,251362694	1
RP23-55A6.4	1,251189231	1
Dlx1	1,251189231	1
Cd63	1,251189231	1
Nudcd1	1,251102508	1
Rilpl2	1,251102508	1
Glrx5	1,251102508	1
Cox15	1,250668984	1
Hira	1,250495616	1
Rpl19-ps9	1,250408942	1
Pigt	1,25023561	1
Elmod3	1,250062303	1
Ndufv1	1,249975658	1
Champ1	1,249975658	1
Agtrap	1,249889019	1
Lzts3	1,249629139	1
Tefm	1,249629139	1
Coq3	1,249629139	1
S100a4	1,249629139	1
9330104G04Rik	1,249542524	1
Hif1an	1,249542524	1
Ubap2	1,249542524	1
Gm3375	1,249369313	1
Ociad1	1,249369313	1
Anks1	1,249282716	1
Mtch2	1,249282716	1
Trim44	1,249196126	1
Sec63	1,249022962	1
Ppm1g	1,249022962	1
Map2k5	1,24893639	1
Supt6	1,248763262	1
Avl9	1,248676708	1
Frmd8os	1,248590159	1
Usp49	1,248503616	1
Chfr	1,248503616	1
Pik3cg	1,248157506	1
Itm2b	1,248157506	1
Sumf2	1,248070993	1
Relb	1,248070993	1
Antxr2	1,247984486	1

Gas2l1	1,247984486	1
Hpgds	1,247984486	1
Wdr7	1,247897985	1
Slc12a4	1,247725002	1
Commd4	1,247638519	1
Fbxl15	1,247465572	1
Nol6	1,247465572	1
Xbp1	1,247379107	1
Ift43	1,247292648	1
mt-Tp	1,246687605	1
Aggf1	1,246687605	1
Dst	1,246601194	1
Pros1	1,246514789	1
Cyb561	1,246428391	1
Eif2ak2	1,246428391	1
Upf3a	1,246428391	1
Plcb2	1,246341998	1
Trmu	1,246255611	1
Apc	1,246255611	1
Brox	1,24616923	1
Dtd1	1,245996486	1
Hagh	1,245996486	1
Hexdc	1,245910123	1
Ubqln2	1,245910123	1
Mok	1,245823766	1
Ogfod1	1,245564732	1
Taok3	1,245392072	1
Ptk2	1,245305751	1
Acss2	1,245046824	1
Tpcn1	1,245046824	1
Gm43378	1,244960526	1
Traf6	1,244960526	1
Rhbdd1	1,244960526	1
Foxk2	1,244874235	1
Kbtbd4	1,24478795	1
Pin1	1,24478795	1
Eftud2	1,24478795	1
A230050P20Rik	1,244701671	1
Mrps18a	1,244701671	1
Mettl18	1,244615398	1
Trim45	1,244529131	1
Baz1a	1,244270365	1
Il10rb	1,244270365	1
Rnf40	1,244184122	1
Kifap3	1,244097885	1
Acsl5	1,244097885	1
Cpsf1	1,244011653	1
Skil	1,244011653	1
Pigw	1,243925428	1
Rad50	1,243925428	1
Fermt3	1,243925428	1
Fryl	1,243925428	1

Slc25a53	1,243752995	1
Nif3l1	1,243752995	1
Ppfia3	1,243666788	1
Polr1a	1,243666788	1
Fuk	1,243408201	1
Dnase1l1	1,243408201	1
Rab14	1,243322018	1
Fig4	1,24323584	1
Zfyve9	1,243063503	1
Dcaf6	1,242805042	1
Rprd2	1,242546635	1
Asxl1	1,242374394	1
Rps11-ps4	1,242202176	1
Arfgef2	1,242202176	1
Bfar	1,242116076	1
Erich1	1,241943894	1
Bmpr2	1,241857812	1
Cog5	1,241857812	1
Arfgap1	1,241771736	1
Tnfaip8	1,241771736	1
Atic	1,241685666	1
Srrm2	1,241599602	1
RP24-282C4.10	1,241513544	1
F2	1,241427492	1
Dph2	1,241427492	1
Ext2	1,241255405	1
Sap130	1,241169371	1
Rpl26-ps4	1,240911304	1
Bbs12	1,240739289	1
Rxrb	1,240739289	1
Ffar4	1,240567298	1
Sephs2	1,240481311	1
Tecr	1,24039533	1
Polr2b	1,24039533	1
Rprd1a	1,240051468	1
Laptm4a	1,239879572	1
Pygb	1,239793633	1
Mier2	1,239621773	1
Tbc1d25	1,239449937	1
Hivep1	1,239278124	1
Mfap3	1,239278124	1
Tvp23b	1,239278124	1
Gm12355	1,239192227	1
Nlk	1,239106336	1
Maf1	1,239106336	1
Foxred1	1,238848698	1
Zfp358	1,23876283	1
Smyd2	1,23876283	1
Sbk2	1,238676969	1
Cipc	1,238676969	1
Ccdc97	1,238505264	1
Kyat1	1,238333582	1

Stub1	1,238333582	1
Tmbim1	1,238333582	1
Gm10399	1,23824775	1
Gm19503	1,23824775	1
Alg8	1,23824775	1
Polr3c	1,238161925	1
Fbxo28	1,238076105	1
Mterf3	1,237990291	1
Cdk6	1,237990291	1
Carm1	1,237904483	1
Ddx41	1,237818681	1
Mllt3	1,237818681	1
Pogz	1,237732885	1
Drg2	1,237475532	1
Cnrip1	1,237475532	1
Tomm34	1,23738976	1
Bcl7b	1,237303994	1
Sass6	1,23704673	1
Itga2b	1,236960988	1
Gm14048	1,236875251	1
Mtf1	1,236875251	1
Ddx42	1,236360956	1
Zfp953	1,23593254	1
Pcgf6	1,235761215	1
Crot	1,235761215	1
Smim4	1,235761215	1
Ttc19	1,235675562	1
Ctsf	1,235504273	1
Atxn7l3b	1,235161766	1
Usf3	1,234990548	1
Nip7	1,234990548	1
Trim37	1,234904948	1
Oxct1	1,234733765	1
Fbxo7	1,234477037	1
Tmem199	1,234391472	1
Pbx3	1,234391472	1
Fdxacb1	1,234305914	1
Zfp428	1,234220361	1
Ddx24	1,234220361	1
C330013E15Rik	1,234134814	1
Zfyve1	1,234134814	1
Dcxr	1,234134814	1
Commd3	1,234134814	1
B630019K06Rik	1,234049273	1
Ube2v2	1,23387821	1
Tbl3	1,23387821	1
2810004N23Rik	1,23387821	1
Exosc5	1,233792687	1
Armc1	1,233792687	1
Esf1	1,23370717	1
Klf9	1,23370717	1
Trim11	1,233621659	1

Mios	1,233536154	1
Gm44269	1,233450654	1
Sel1l	1,233108717	1
Dcakd	1,232852325	1
Gpr65	1,232766874	1
Cdk20	1,232681428	1
Ube2l3	1,232595988	1
Smug1	1,232510554	1
Spef1	1,232425125	1
Scaf11	1,232339703	1
Psma2	1,232254287	1
Zranb2	1,232168876	1
Cd3eap	1,231998073	1
Mrgbp	1,231912681	1
C1d	1,231912681	1
Itpr1	1,231912681	1
Gm14240	1,231827294	1
Cnot2	1,231827294	1
Itgal	1,231741913	1
Mrpl2	1,231656538	1
Helz	1,231656538	1
Ssr1	1,231571169	1
Myo7a	1,231400449	1
Dap	1,231229753	1
Cand1	1,231144413	1
Gm19028	1,23105908	1
Ttyh2	1,23105908	1
2210417A02Rik	1,230973752	1
Gm20604	1,230888431	1
Coq8b	1,230888431	1
Dnm2	1,230803115	1
Gm6123	1,230717805	1
Zfp62	1,230717805	1
Scd1	1,230717805	1
Gbf1	1,230291345	1
2610524H06Rik	1,230120802	1
Mvb12b	1,230035539	1
Cyhr1	1,229865032	1
Wdr61	1,229865032	1
Snip1	1,229694548	1
Coa3	1,229694548	1
Arl2bp	1,229524088	1
Mtrf1l	1,229438867	1
Mef2a	1,229438867	1
C1qbp	1,229438867	1
Mavs	1,229353652	1
Fcgrt	1,229183239	1
Supt5	1,229183239	1
Trim46	1,229098042	1
Mmp19	1,229098042	1
Trappc9	1,22901285	1
Tex10	1,228927664	1

Ubxn6	1,228927664	1
Lancl1	1,228927664	1
Hck	1,228842484	1
Grin1	1,228757311	1
Limk2	1,228672143	1
Fam172a	1,228416674	1
Hddc2	1,228416674	1
Desi1	1,228416674	1
Mesdc2	1,227991011	1
Treml1	1,227905896	1
Slc25a39	1,227905896	1
1190007I07Rik	1,227820787	1
Tgfbrap1	1,227820787	1
Ipo7	1,227820787	1
Lyrm1	1,227650587	1
Mettl15	1,227650587	1
Capn1	1,227650587	1
Gtpbp10	1,227565495	1
Tbc1d2	1,227565495	1
Asb8	1,22748041	1
Tomm70a	1,227225189	1
Ahsa2	1,227140127	1
Cln3	1,227055071	1
Dnajc9	1,226970021	1
Ubxn2a	1,226970021	1
Fut10	1,226884977	1
Ccne1	1,226884977	1
6430548M08Rik	1,22662988	1
Ctdspl	1,22654486	1
Cwc22	1,226374836	1
Mycbp	1,226289833	1
Prelid3b	1,226289833	1
Wapl	1,226289833	1
Wwc2	1,226204836	1
Pidd1	1,226119845	1
Ubr2	1,22603486	1
Slc22a15	1,225949881	1
Tdp1	1,225949881	1
Hhex	1,225864907	1
Gm7769	1,22577994	1
Pcx	1,22577994	1
Phlpp2	1,225525073	1
Ptpa	1,225440129	1
Calu	1,22535519	1
Acly	1,225270258	1
Eif2b3	1,225100411	1
Dok4	1,224930588	1
Hax1	1,224930588	1
Zfp850	1,224930588	1
Lnx2	1,224845685	1
Gm9803	1,224760789	1
Slc25a11	1,224675898	1

Hdlbp	1,224506133	1
Pls3	1,224506133	1
Slc25a46	1,224251531	1
Nup210	1,224166675	1
Gm16124	1,223996981	1
Gm7496	1,223996981	1
Ttll13	1,223996981	1
Thoc3	1,22357285	1
B4galt5	1,223403238	1
Tada2b	1,223148865	1
Il15ra	1,223064086	1
Zfp407	1,222979313	1
Cwc27	1,222979313	1
Snord92	1,222809783	1
Cdc42ep2	1,222301337	1
Riok3	1,222047193	1
Rasa2	1,22196249	1
Clasp2	1,221877793	1
Ndufa4	1,221708416	1
Ccdc12	1,221623737	1
Fam234a	1,221623737	1
Rnf214	1,221539063	1
Shmt2	1,221454396	1
Pla2g6	1,221369734	1
Ice2	1,221285078	1
Hip1	1,221200428	1
Epm2aip1	1,221115784	1
Eif5	1,221031145	1
6330418K02Rik	1,220946513	1
Vars2	1,220946513	1
Ifit2	1,220777265	1
Mthfd1	1,220608041	1
Gas8	1,220354249	1
Hspa9-ps1	1,220354249	1
Ube2r2	1,22010051	1
Ttc26	1,220015942	1
Gm10039	1,220015942	1
Tab1	1,219846824	1
Mthfs	1,21959319	1
Slc3a2	1,21959319	1
Ceacam1	1,219508657	1
Stn1	1,219508657	1
Ccdc6	1,219508657	1
Armc9	1,21942413	1
Samm50	1,21942413	1
Dctn4	1,219339609	1
Il17ra	1,219255094	1
Cndp2	1,219255094	1
Gm16373	1,219170585	1
Ppm1l	1,219170585	1
Chordc1	1,219170585	1
Cdc123	1,219001583	1

Gm5939	1,218832606	1
Map7d1	1,218663651	1
Apeh	1,218579183	1
Ip6k1	1,218579183	1
Agbl3	1,218325813	1
Rnf149	1,218325813	1
Kdelc2	1,218325813	1
Dgcr2	1,218156929	1
Pphln1	1,218072495	1
Plxnd1	1,218072495	1
Alkbh6	1,217988068	1
Dnal1	1,217819231	1
Zmynd19	1,217734821	1
Ppp2r5c	1,217734821	1
Wdr81	1,217650417	1
St3gal5	1,217144116	1
Nipal3	1,216975395	1
Tamm41	1,216975395	1
Rtkn	1,216806698	1
Gmpr2	1,216806698	1
Lace1	1,216722359	1
Tnrc6b	1,216722359	1
Herc6	1,216638025	1
Nudt3	1,216553697	1
Lyrm7	1,216385058	1
Ptpra	1,216385058	1
Tkt	1,216385058	1
4833417C18Rik	1,216300748	1
Mri1	1,216300748	1
Tmem177	1,216216443	1
Hnrnpul1	1,216132144	1
Tnk2	1,216047852	1
Dctn5	1,216047852	1
Prim2	1,215963564	1
1700084J12Rik	1,215795008	1
Msra	1,215710738	1
Ctsc	1,215373718	1
Gm16425	1,215289478	1
Tmem126a	1,215289478	1
Gnl3	1,215289478	1
Pcyox1	1,215205243	1
Rab2a	1,215036792	1
Cdca7l	1,214868364	1
Nrros	1,214784158	1
Poll	1,214784158	1
Pcdh7	1,214784158	1
Des	1,214699959	1
Gm9892	1,214615765	1
Acp1	1,214447395	1
Rab13	1,214279049	1
Mrpl21	1,214279049	1
Utp4	1,214279049	1

Cpq	1,214194884	1
Arl5c	1,214110726	1
Tram2	1,214026573	1
Aurkaip1	1,214026573	1
Mir5128	1,213942426	1
Oas1g	1,213858285	1
Arfgap2	1,213774149	1
Izumo4	1,21369002	1
Vamp1	1,21369002	1
Gm6542	1,213605896	1
Abcb10	1,213521778	1
Fam219a	1,213521778	1
Nploc4	1,21335356	1
Gm16053	1,21326946	1
Psmc3	1,21326946	1
Zfp60	1,213185365	1
Pitpnb	1,213101277	1
Armc2	1,212933117	1
Ddx52	1,212933117	1
Rrm2b	1,212849046	1
Pitpnc1	1,212849046	1
Rbm15b	1,212849046	1
Rptor	1,21276498	1
Fam120a	1,21276498	1
Gpkow	1,212680921	1
Eps15	1,212680921	1
Xab2	1,212596867	1
Braf	1,212596867	1
Kidins220	1,212596867	1
Romo1	1,212512819	1
Cc2d1b	1,212428777	1
E430021H15Rik	1,212344741	1
Spag4	1,21226071	1
Tbc1d22b	1,21226071	1
Wdr83os	1,212176686	1
Slc12a9	1,212176686	1
Brd7	1,212176686	1
Zfp120	1,212092667	1
2900009J06Rik	1,212008654	1
Gm9434	1,212008654	1
Nt5c3	1,211840646	1
Cage1	1,21175665	1
Pik3r3	1,21167266	1
Msi2	1,211588677	1
Aph1c	1,211504699	1
Pus1	1,21133676	1
Aim1	1,21133676	1
Tom1l2	1,211252799	1
Fam167b	1,211252799	1
Tmem238	1,211168845	1
Comtd1	1,211168845	1
Dtnb	1,210833084	1

Dnajc16	1,210749158	1
Ythdf2	1,210749158	1
Phf20	1,210749158	1
Scaf4	1,210665238	1
Dctn1	1,210497416	1
Rnf128	1,210413514	1
Zc3hav1	1,210413514	1
Sppl3	1,210245727	1
Gsap	1,210161842	1
Thap11	1,210161842	1
Ccdc90b	1,209994089	1
Bicd1	1,209910222	1
Nop2	1,20982636	1
Eif2b1	1,209490971	1
1700021F05Rik	1,209490971	1
Ankrd50	1,209490971	1
Nme7	1,209407139	1
Gm9392	1,209071866	1
Abcf3	1,209071866	1
Pacs1	1,208988063	1
Gnl2	1,208401601	1
Sucla2	1,208317843	1
Lrrc75a	1,208234092	1
Lpcat2	1,208150347	1
Gm44116	1,207982873	1
Nop9	1,207647995	1
Ddx18	1,207647995	1
Snap47	1,207480591	1
Pttg1ip	1,207480591	1
Degs1	1,207480591	1
Comp	1,207396898	1
Lpar2	1,207229529	1
2310039H08Rik	1,207229529	1
Psme3	1,207229529	1
Ammecr1l	1,207062183	1
Sh3glb1	1,207062183	1
Gosr1	1,206811208	1
Cpt2	1,206811208	1
Rmi1	1,206727561	1
Cldn12	1,20664392	1
Rpp38	1,206560284	1
Gon4l	1,206560284	1
Gm8172	1,206476655	1
Gtf2ird1	1,206476655	1
Gtpbp3	1,206476655	1
Zfpl1	1,206476655	1
Stk38l	1,206476655	1
Usf2	1,206309413	1
Alg6	1,206058594	1
Pip5k1c	1,206058594	1
Uba5	1,206058594	1
Rnf220	1,206058594	1

Tctex1d2	1,206058594	1
Crybg3	1,205975	1
Ncor2	1,205975	1
Morn1	1,205891411	1
Gm2272	1,205640679	1
Wdsub1	1,205473553	1
Rbpj	1,205473553	1
Zfp141	1,205389999	1
Prpsap1	1,205222908	1
Parp11	1,205139371	1
Tmem237	1,205139371	1
Gm26982	1,205139371	1
Jak2	1,205139371	1
Snrnp200	1,205139371	1
Fes	1,20505584	1
Rps6ka3	1,20505584	1
Trp53inp1	1,20505584	1
Ampd2	1,204972315	1
C130036L24Rik	1,204638272	1
Etfb	1,204638272	1
Gm42481	1,204387801	1
Esrra	1,204137381	1
Cspg4	1,20405392	1
Rwdd3	1,203970464	1
Spn	1,20380357	1
Ell2	1,203636699	1
Tcf25	1,203636699	1
Sdccag8	1,203469851	1
Sugp1	1,203469851	1
Ripk1	1,203386436	1
Gm42535	1,203219622	1
Snhg1	1,203136224	1
Usp54	1,203052832	1
Nt5c2	1,203052832	1
Hgh1	1,202969446	1
Nudt9	1,202886065	1
Wdr26	1,202886065	1
Alg11	1,202719321	1
Pptc7	1,202719321	1
Crebzf	1,202635958	1
Nudt1	1,202385903	1
Wars2	1,202219228	1
Gm16286	1,2021359	1
Twf2	1,20196926	1
Dnajc15	1,201885949	1
Gm15708	1,201802643	1
Clec11a	1,201719343	1
D5Ertd579e	1,201719343	1
Dlat	1,201719343	1
Fam214b	1,20163605	1
Mapkap1	1,201552761	1
Snf8	1,201469479	1

1600020E01Rik	1,201302931	1
Arpc5	1,201302931	1
4930432K21Rik	1,201219666	1
BC065397	1,201136407	1
Mterf4	1,201136407	1
Fancl	1,201053154	1
Gtf2b	1,200803427	1
Strbp	1,200803427	1
Txndc11	1,200720197	1
Arrb2	1,200720197	1
Gatsl3	1,200636972	1
Bcor	1,200636972	1
Mif4gd	1,200387333	1
Ap1s1	1,200304131	1
Spr	1,200220935	1
Senp2	1,200220935	1
Chst14	1,200054561	1
Clasrp	1,199971382	1
Mrpl46	1,199721881	1
Trak1	1,199721881	1
Cryl1	1,199638726	1
Ap4b1	1,199638726	1
Esyt2	1,199638726	1
9030617O03Rik	1,199472432	1
Ppp1r9b	1,199389294	1
Serpinb9	1,199306161	1
Pelp1	1,199306161	1
Ndufb9	1,199306161	1
Gmip	1,199223035	1
Mfsd7a	1,199139914	1
Fxyd2	1,199139914	1
Ngrn	1,199056799	1
Gm42876	1,198973689	1
Acox3	1,198973689	1
Tmem129	1,198890586	1
Alyref2	1,198724396	1
Fam107b	1,198558229	1
Opn3	1,198475154	1
Dtwd1	1,198475154	1
Men1	1,198475154	1
Slc17a7	1,198392085	1
Zfp830	1,198392085	1
Alg5	1,198225964	1
Zcchc7	1,198225964	1
Arhgap18	1,197976826	1
Lrmp	1,197810762	1
Gpr108	1,197727739	1
Gsdmd	1,197478705	1
RP23-359K10.9	1,197395705	1
Txnl4b	1,19731271	1
Tradd	1,19731271	1
Mpi	1,19731271	1

Mrps34	1,19731271	1
Zscan22	1,197146739	1
Glmn	1,197146739	1
Anp32-ps	1,196814866	1
Gm44053	1,196731911	1
Bag5	1,196648963	1
Gm8013	1,196566021	1
Ears2	1,196566021	1
Trip4	1,196483084	1
Rpap3	1,196483084	1
N4bp2l1	1,196483084	1
Txnl4a	1,196317228	1
Top1mt	1,196317228	1
Snapc5	1,196234308	1
Defb25	1,196234308	1
Ppip5k2	1,196068486	1
Srp68	1,196068486	1
Uqcc1	1,195902688	1
Anapc10	1,195819797	1
Wdfy4	1,195819797	1
Zmat1	1,195736912	1
Spcs2	1,195736912	1
Dhx29	1,195736912	1
Pigs	1,195571159	1
Isoc1	1,195571159	1
Zbtb1	1,195488291	1
Alpk1	1,195488291	1
2810030D12Rik	1,195322573	1
Magi2	1,195322573	1
Creld1	1,195322573	1
Ttc9	1,195239722	1
Prpf31	1,195239722	1
Eif2b4	1,195156877	1
Bcl2l13	1,194908377	1
Gpn1	1,194825555	1
Agpat4	1,194825555	1
Psmd6	1,194659929	1
Trim41	1,194494325	1
5330438D12Rik	1,194411532	1
Psmg2	1,194411532	1
Eif2ak4	1,194411532	1
Vps4b	1,194328745	1
Oxsm	1,194245963	1
Lrrc51	1,194163187	1
Eif1-ps1	1,194163187	1
Snapin	1,194163187	1
Tmem167	1,194163187	1
Orc1	1,193997652	1
Uvrag	1,193997652	1
Ythdf1	1,193832141	1
Ttc1	1,193666652	1
Rnaseh2b	1,193418462	1

Capn15	1,193418462	1
Gm7670	1,193335743	1
Polr3k	1,193335743	1
Fam134a	1,193004926	1
Srpk2	1,193004926	1
Ldlrad3	1,192922236	1
Ttc27	1,192922236	1
Gm4705	1,192839552	1
St7l	1,192839552	1
Hsdl2	1,192674201	1
2210016F16Rik	1,192674201	1
Eif4ebp2	1,192674201	1
Gga2	1,192674201	1
A730011C13Rik	1,192343567	1
Jade3	1,192343567	1
Il18	1,192343567	1
Gm28187	1,192260923	1
Slc10a7	1,192260923	1
Rcbtb1	1,192178284	1
Fkbpl	1,192178284	1
Hmox1	1,192178284	1
Slc26a11	1,192178284	1
Pgap2	1,192095652	1
Tor2a	1,191930404	1
4933421A08Rik	1,191765179	1
Sep 07	1,191517384	1
Myg1	1,191352216	1
Pard6a	1,191352216	1
Cnnm3	1,19126964	1
Xpr1	1,19126964	1
Znrf2	1,191187071	1
Canx	1,191187071	1
Ppil1	1,191021949	1
Ddx11	1,19052672	1
Paf1	1,19052672	1
Mogs	1,19052672	1
Zfp202	1,190444201	1
Gramd1b	1,190279182	1
Gm15975	1,190196681	1
Aaas	1,190196681	1
Pnisr	1,190114186	1
Crtc1	1,190031696	1
Sipa1l2	1,190031696	1
2410022M11Rik	1,190031696	1
Mocos	1,190031696	1
Pdia4	1,190031696	1
Actr10	1,190031696	1
Tmem246	1,189949212	1
Cpeb1	1,189866734	1
Cep68	1,189866734	1
Gm10658	1,189701795	1
Ankrd13b	1,189701795	1

Smyd4	1,189371986	1
Ryk	1,189371986	1
Qsox2	1,189289547	1
Larp4b	1,189289547	1
Taz	1,189207115	1
P2rx4	1,189124688	1
Pdcd6ip	1,189124688	1
Heatr5b	1,188959852	1
Tm9sf3	1,188959852	1
Slc38a6	1,188877442	1
Eif2b5	1,188795039	1
Snora73b	1,188630248	1
Cbfa2t2	1,188630248	1
Qdpr	1,188630248	1
Gbas	1,188547861	1
Mib2	1,188547861	1
Por	1,188383105	1
Cnot11	1,188300735	1
Exosc7	1,188218372	1
Ninl	1,187477354	1
Ptrh2	1,187395047	1
Rab11fip3	1,187148161	1
Sptan1	1,187148161	1
Smg9	1,187148161	1
Nxf7	1,186901326	1
Slk	1,186819059	1
Mtdh	1,186819059	1
Specc1	1,186654542	1
Tmem164	1,18624335	1
Mplkip	1,18624335	1
Wrap53	1,186161129	1
Abhd14a	1,186161129	1
Aqr	1,186161129	1
Sypl	1,186078913	1
Slc16a6	1,185996704	1
Scyl1	1,185914499	1
Rabl6	1,185914499	1
Zfp35	1,185832301	1
Ddx46	1,185750108	1
Glyr1	1,185667921	1
Atm	1,185667921	1
Plekhm1	1,18558574	1
Mtmr6	1,18558574	1
D630024D03Rik	1,185503564	1
Ttll3	1,185503564	1
Tmed3	1,185421394	1
Gm7785	1,185257071	1
Tatdn3	1,185174918	1
Pygo2	1,185174918	1
Vwa1	1,185010629	1
Rasgef1a	1,185010629	1
Gm28438	1,184846363	1

Tbp	1,18468212	1
Ddx28	1,184600007	1
Ercc4	1,1845179	1
Rtn3	1,1845179	1
5430403G16Rik	1,184435798	1
Btbd9	1,184435798	1
Gm15453	1,184271612	1
Gm14253	1,184189527	1
Exoc3l2	1,184189527	1
Cdk12	1,184107448	1
Ift27	1,183943308	1
Mtpap	1,18377919	1
Rnf213	1,18377919	1
Gm15513	1,183697139	1
Gm11224	1,183697139	1
Fance	1,183697139	1
Nsmf	1,183615094	1
Sephs1	1,183615094	1
Pomc	1,183451022	1
Lrrc28	1,183122945	1
Vps9d1	1,183122945	1
Atf5	1,183122945	1
Cwf19l2	1,183122945	1
Ing5	1,182958941	1
Eif4ebp1	1,182958941	1
Sdhc	1,182958941	1
Rragb	1,182876947	1
Supt3	1,182794959	1
Dcaf8	1,182712977	1
RP23-187B11.16	1,182467064	1
Pafah1b2	1,182467064	1
Fhod1	1,182385105	1
Samd1	1,182303151	1
Tmem161a	1,18213926	1
Per2	1,182057323	1
Gm45053	1,181647724	1
Zdhhc5	1,181647724	1
Rilpl1	1,181402033	1
B130034C11Rik	1,181320148	1
P2ry6	1,181320148	1
Trmt11	1,181238267	1
Sar1a	1,181156393	1
Hectd1	1,181074524	1
Chd3	1,180910804	1
Setd1a	1,180583431	1
Zfp335os	1,180501603	1
Glt8d1	1,180501603	1
Uso1	1,180419779	1
Rfk	1,180419779	1
BC003331	1,180092543	1
Mrps24	1,180010748	1
Ylpm1	1,179928958	1

Nr1h2	1,179847175	1
Mvk	1,179847175	1
Dpy19l4	1,179438342	1
Slc35c2	1,17919311	1
Stat5a	1,179111377	1
St14	1,179111377	1
Eif3j2	1,179029651	1
Zfp51	1,178947929	1
Zfp260	1,178947929	1
Cwc25	1,178947929	1
Nrbp1	1,178866214	1
Dkc1	1,178866214	1
Zfp945	1,178784504	1
Plekhh2	1,178539408	1
RP23-104D6.2	1,178376039	1
Nvl	1,178294363	1
Gm12704	1,178212693	1
Epn2	1,178212693	1
Gm15625	1,178049369	1
Eif3c	1,178049369	1
RP23-413G8.2	1,177886068	1
Ccdc180	1,177886068	1
Nupr1l	1,177804426	1
Ttl	1,177804426	1
Ctns	1,177804426	1
Add1	1,177804426	1
Tmtc4	1,17772279	1
Gale	1,17772279	1
Gm42486	1,177641159	1
Arhgef18	1,177641159	1
Cct7	1,177559534	1
Rps2-ps10	1,177477914	1
Fads2	1,177477914	1
Dis3l	1,177396301	1
Slc4a1ap	1,17723309	1
Spen	1,17723309	1
Slc36a3os	1,177151494	1
9930021J03Rik	1,177151494	1
Zmym1	1,177069902	1
Cyb5d2	1,177069902	1
Senp1	1,176743595	1
Nosip	1,176662032	1
Rnf170	1,176417377	1
AI597479	1,176417377	1
Hdac8	1,176335837	1
Spata7	1,176335837	1
Impa1	1,176254302	1
Fam19a3	1,17609125	1
Nop16	1,17609125	1
Fam208a	1,176009733	1
Pja1	1,176009733	1
Egln2	1,176009733	1

Dync1li2	1,176009733	1
Snapc4	1,175765214	1
Josd1	1,175602229	1
Rab21	1,175602229	1
Rabgef1	1,175520746	1
Ccdc186	1,175520746	1
Coro7	1,175357795	1
Wdtc1	1,175194867	1
Rps27-ps1	1,175031962	1
Ncstn	1,175031962	1
Dnajc5	1,175031962	1
Pex12	1,174950518	1
Azi2	1,174869079	1
Sec23ip	1,174869079	1
RP23-403D16.3	1,174787646	1
Fam204a	1,174787646	1
Mfsd5	1,174787646	1
Fnbp1	1,174787646	1
Usp30	1,174706219	1
Gm45113	1,174299167	1
G6pd2	1,174217774	1
9130401M01Rik	1,174217774	1
Gtf3c3	1,174055004	1
Megf8	1,173892257	1
Cherp	1,173892257	1
Dtnbp1	1,173810892	1
Hoxa7	1,173485487	1
Lyrm2	1,173485487	1
Gm20633	1,17340415	1
Gk5	1,17340415	1
Dctn6	1,17340415	1
Mmgt1	1,173322819	1
Fbxl4	1,173241493	1
Sema4g	1,173241493	1
Vapb	1,173241493	1
Arid5a	1,173160173	1
Sec24c	1,173160173	1
Fam207a	1,173078859	1
Nectin2	1,17299755	1
Zfp236	1,17259109	1
Acvrl1	1,17259109	1
Prr13	1,17259109	1
Cadps	1,172509815	1
Med20	1,172509815	1
Phkg1	1,172428546	1
Zfp984	1,172347282	1
Tmem97	1,172184772	1
Pex10	1,172184772	1
Ssh1	1,172022284	1
Tmem201	1,171859818	1
Hsd17b11	1,171616162	1
Prpf4	1,171534955	1

Dnajb11	1,171534955	1
Plekhb2	1,171453753	1
Fzd2	1,171372557	1
Mfsd13a	1,171372557	1
Psma7	1,171291366	1
Rab11fip1	1,171210181	1
Ndufc1	1,171129002	1
Itm2c	1,171047828	1
Ap5m1	1,17096666	1
Sh3bgrl	1,17096666	1
Timm21	1,170804341	1
Copg1	1,17072319	1
Appl1	1,17072319	1
Ctsz	1,17072319	1
Stag2	1,170642044	1
Abcb7	1,170560904	1
Dmap1	1,170398641	1
C87436	1,170317518	1
Emc9	1,170236401	1
Wdr36	1,170236401	1
Zbtb7a	1,169993082	1
Chtop	1,169911988	1
Tkfc	1,169830898	1
Vps45	1,169830898	1
Nfic	1,169830898	1
Hdac11	1,169749815	1
Trmt2b	1,169587664	1
Lrp8	1,169587664	1
Fsd2	1,169506597	1
Cops6	1,169425536	1
Dync1li1	1,169425536	1
Fth1	1,169425536	1
Zdhhc3	1,16926343	1
Lgals8	1,169101347	1
Tmem104	1,169020314	1
Fam179b	1,168939287	1
Phf3	1,168939287	1
Zc3h15	1,168858265	1
Rchy1	1,168696238	1
Psmc5	1,168696238	1
U2surp	1,168696238	1
Gm44557	1,168534233	1
Ncbp3	1,168534233	1
Zc3h6	1,16845324	1
Rnpc3	1,168048355	1
Map2k4	1,167967395	1
Eif2ak1	1,167967395	1
Pi4ka	1,167967395	1
Gm43362	1,167805491	1
Palb2	1,167724548	1
Tulp3	1,167724548	1
Galnt3	1,167562678	1

Mrpl58	1,167562678	1
Dhdds	1,167481752	1
Gm12848	1,167400831	1
R3hdm2	1,167319916	1
Lclat1	1,167158102	1
Mgst2	1,167158102	1
N4bp1	1,166996311	1
Ccdc138	1,166834542	1
Was	1,166834542	1
Dnal4	1,166672795	1
Col4a3bp	1,166591931	1
Rxra	1,166430218	1
Pigq	1,16634937	1
Foxo1	1,166026034	1
Exoc3	1,166026034	1
Lipa	1,165945214	1
Pprc1	1,1658644	1
Thtpa	1,165783591	1
Cast	1,165783591	1
Gm45630	1,165702788	1
Gm26759	1,165702788	1
Slc16a13	1,16562199	1
Elac2	1,16562199	1
Atrn	1,16562199	1
Gm6457	1,165460412	1
Gab3	1,165460412	1
Psmd2	1,165460412	1
Kif3a	1,165379631	1
Ap1m1	1,165379631	1
Edil3	1,165298856	1
Tonsl	1,165218086	1
Pigc	1,165137322	1
Cebpd	1,165056564	1
Pex3	1,165056564	1
Ddx27	1,165056564	1
Pex14	1,164975811	1
Zswim7	1,164895064	1
Zfp930	1,164895064	1
Oma1	1,164814322	1
Rgl3	1,164652856	1
Ndufs8	1,164652856	1
Cul2	1,164652856	1
Taf5l	1,164491412	1
mt-Rnr2	1,164410699	1
Hddc3	1,164168591	1
Cdc25b	1,163926534	1
Mak16	1,163765191	1
Sac3d1	1,163361931	1
Nepro	1,163361931	1
Dhrs7	1,163281295	1
Trim12c	1,163120042	1
Brip1os	1,163120042	1

Utp3	1,163120042	1
Zfp84	1,163039423	1
Rnf8	1,163039423	1
Adprm	1,163039423	1
Traf3ip1	1,16295881	1
Msh2	1,16295881	1
Trio	1,16295881	1
Usp45	1,16295881	1
Mon2	1,16295881	1
Gm37718	1,162878203	1
Otx1	1,162878203	1
Kcmf1	1,162797601	1
Plekhg5	1,162636414	1
Pvt1	1,162636414	1
Gm6140	1,162555829	1
Diexf	1,162555829	1
Rad51c	1,162394676	1
Axin1	1,162314108	1
Slc38a9	1,162314108	1
Rab2b	1,162233545	1
Tmem42	1,162072436	1
Zfp30	1,162072436	1
Gm44552	1,161589244	1
Clec7a	1,161589244	1
Ube2d-ps	1,161428225	1
Abca3	1,161428225	1
Ppm1b	1,161347724	1
Rfwd3	1,161186738	1
Fam50a	1,161186738	1
March2	1,161106253	1
Slc22a5	1,161025774	1
Rbl2	1,161025774	1
Vsir	1,160864833	1
Gm10698	1,160784371	1
Lrrc24	1,160703914	1
Fundc2	1,160543018	1
Agpat3	1,160221292	1
Slu7	1,160221292	1
Nf1	1,160140874	1
Tbc1d23	1,159980055	1
Fam160b2	1,159980055	1
Ptdss1	1,159980055	1
Gm45360	1,159899655	1
Tug1	1,159819259	1
Mydgf	1,159819259	1
March9	1,159819259	1
Prpf40a	1,159578107	1
Zmynd11	1,159578107	1
Caly	1,159417366	1
Kctd3	1,159417366	1
Vti1a	1,159417366	1
Slfn9	1,159337004	1

Actr8	1,159337004	1
Cep19	1,159256648	1
Cox6b1	1,159095952	1
Rbmxl1	1,159015612	1
Sec23a	1,159015612	1
Nlrp3	1,15885495	1
Vegfb	1,15885495	1
Hnrnph3	1,158774627	1
Lap3	1,158774627	1
Zfp664	1,158694309	1
Trim24	1,158694309	1
Cbr3	1,158613998	1
Dner	1,158613998	1
Gm8019	1,158453391	1
Ssbp4	1,158453391	1
Myo1e	1,158453391	1
Nfe2l1	1,158373096	1
Lmo2	1,158292806	1
Top2b	1,158212522	1
Dgkq	1,158132244	1
Macrod1	1,158132244	1
Gamt	1,157971704	1
Capn10	1,157971704	1
Ski	1,157971704	1
Fastkd2	1,157891442	1
Neurl2	1,157891442	1
Fbxo9	1,157409989	1
Ankfy1	1,157249549	1
Gm37145	1,15700893	1
Vars	1,15700893	1
Zfx	1,156768362	1
Slc25a16	1,156688184	1
Adprhl2	1,156688184	1
Tsfm	1,156608011	1
Gm11722	1,156608011	1
Tmem126b	1,156367526	1
Pgam5	1,156287376	1
Aagab	1,156287376	1
Mllt6	1,156127091	1
Txnrd1	1,156046958	1
Spout1	1,155886707	1
Mcu	1,155886707	1
Slc30a7	1,155806589	1
Tor1b	1,155806589	1
Ccdc88b	1,155726478	1
Ankrd13a	1,155646372	1
Zbtb4	1,155566271	1
Akr1a1	1,155566271	1
Epg5	1,155486176	1
Cln5	1,155486176	1
Knop1	1,155486176	1
Brd1	1,155486176	1

Atl3	1,155326003	1
Cpd	1,155326003	1
Ddb1	1,155326003	1
Gm37204	1,155245925	1
Mrps9	1,155245925	1
Mcl1	1,155245925	1
Edc4	1,155165852	1
Cdkn3	1,155005723	1
Sorbs1	1,155005723	1
Mettl26	1,154925667	1
Rnf187	1,154925667	1
Tpcn2	1,154845616	1
Ndc1	1,154765571	1
Recql	1,154525469	1
Kif1bp	1,154525469	1
Ago2	1,154365429	1
Gm7128	1,154205411	1
Pxk	1,154205411	1
Ocrl	1,154205411	1
Uvssa	1,153645523	1
Gabarapl2	1,153565561	1
Tbk1	1,153405654	1
Prrc2c	1,153405654	1
Gm10110	1,153325709	1
Usp24	1,153165835	1
Map2k7	1,153165835	1
Pdlim5	1,153165835	1
Mmachc	1,153005984	1
Rcc2	1,152926066	1
Ndufs1	1,152846154	1
Ccdc28b	1,152766248	1
Llph-ps2	1,152766248	1
Ctsl	1,152766248	1
Atg4b	1,152686347	1
Mxd4	1,152686347	1
Gm20689	1,152606451	1
Ubqln4	1,152606451	1
Mprip	1,152606451	1
Zfp456	1,152526562	1
Snrnp35	1,152526562	1
Mto1	1,152526562	1
Selenon	1,152526562	1
Man1a2	1,152526562	1
Otud7b	1,152366799	1
Picalm	1,152366799	1
Rps19-ps9	1,152286925	1
Tbc1d17	1,152047339	1
Tmem55b	1,152047339	1
Sec13	1,151807802	1
Cacna1b	1,151727968	1
Med23	1,151648139	1
Nubp2	1,151568316	1

Gm13578	1,151488498	1
BC025920	1,151408685	1
Naa25	1,151408685	1
Gm13022	1,151328879	1
Cse1l	1,151328879	1
Cmtr1	1,151249077	1
Tmem51os1	1,151089491	1
Armcx5	1,151009707	1
Atp6v0e	1,151009707	1
Lsm10	1,150929928	1
Ndufaf1	1,150929928	1
Camk1	1,150929928	1
Psmd1	1,150929928	1
Tprkb	1,150690623	1
Tstd3	1,150690623	1
Ttpal	1,150610866	1
Suds3	1,150531115	1
Mrps11	1,150451369	1
Galt	1,150291893	1
Gm28417	1,15013244	1
Csad	1,150052722	1
Pofut1	1,150052722	1
Mrpl3	1,149973009	1
Slc13a2	1,149893302	1
Zfp451	1,149893302	1
Dmpk	1,1498136	1
Mfn1	1,149654213	1
Gm5828	1,149574528	1
Baz2b	1,149574528	1
Zdhhc7	1,149494848	1
Fra10ac1	1,149415174	1
Fam46c	1,149096533	1
Zfp523	1,149096533	1
Rap2b	1,149096533	1
Plekha8	1,149016886	1
Dhcr24	1,149016886	1
Mrps36	1,148937245	1
Ppp1r11	1,148937245	1
Ppm1f	1,148777979	1
Dph5	1,148698355	1
Irak2	1,148618736	1
Map4k2	1,148539123	1
Dis3l2	1,148539123	1
Usp38	1,148539123	1
D630023F18Rik	1,148459515	1
Wiz	1,148459515	1
Arpin	1,148061558	1
Pbrm1	1,148061558	1
Hspa4	1,147981983	1
RP23-454I20.1	1,147902414	1
Cyth4	1,14782285	1
Dnm3	1,147663739	1

Gm43106	1,14750465	1
Taf12	1,14750465	1
Acox1	1,14750465	1
Zcrb1	1,14750465	1
Adcy9	1,147425114	1
Itga4	1,147425114	1
Uhrf1bp1l	1,147186538	1
Dnajb9	1,147027516	1
1110038F14Rik	1,146868515	1
Igf2r	1,146709536	1
March6	1,146709536	1
Zfp180	1,146550579	1
Cdpf1	1,146471109	1
Map3k10	1,146391645	1
Kars	1,146391645	1
1700025G04Rik	1,146312186	1
Ece2	1,146232732	1
Abhd6	1,146232732	1
Trappc3	1,146153284	1
Exog	1,146073842	1
Gm15440	1,146073842	1
Egr2	1,145994405	1
2510002D24Rik	1,145994405	1
Kdelr1	1,145994405	1
Eif3g	1,145835547	1
5530601H04Rik	1,145756126	1
Lix1l	1,145756126	1
Nhlrc1	1,145676711	1
Mrpl12	1,145676711	1
Maged1	1,145676711	1
Unk	1,145438499	1
4833418N02Rik	1,145359106	1
Gm27219	1,145359106	1
Usp4	1,145359106	1
Ttc17	1,145279719	1
Copz1	1,145279719	1
Stau1	1,145200337	1
Wbscr22	1,14504159	1
Galk2	1,14480351	1
Dnttip2	1,14480351	1
Tmco1	1,14480351	1
Spg7	1,144724161	1
Timm10	1,144724161	1
Rpl10	1,144565479	1
Aldh3a2	1,144565479	1
Rfx7	1,144565479	1
Secisbp2	1,144327498	1
Idh3a	1,144327498	1
Gm7965	1,144248182	1
Gm8185	1,144089567	1
Dffa	1,144010267	1
RP24-226A8.2	1,143930973	1

Wfs1	1,143851685	1
Ndst2	1,143851685	1
Pias3	1,143851685	1
Pacsin2	1,143693124	1
Ten1	1,143613852	1
Pgp	1,143534586	1
Heatr6	1,143534586	1
Cd200r1	1,143376069	1
Zfp710	1,143296819	1
Hsp90ab1	1,143296819	1
2810402E24Rik	1,142979874	1
Dus3l	1,142900651	1
Polr2m	1,142821434	1
Dpp9	1,142663016	1
Nup93	1,142663016	1
Arpc2	1,142663016	1
Mtfr1l	1,142663016	1
Foxn2	1,142583816	1
Uckl1	1,142583816	1
Camsap1	1,142583816	1
Macf1	1,142583816	1
Shb	1,142425431	1
Lrrk2	1,142425431	1
Shkbp1	1,142425431	1
Sigmar1	1,142346247	1
Dcaf10	1,142346247	1
Uqcrh-ps2	1,142267068	1
Sf3b2	1,142187895	1
Pdpr	1,142108727	1
Otud5	1,142108727	1
Srgap3	1,142108727	1
Eif3d	1,142108727	1
Phf21b	1,142029565	1
4930455G09Rik	1,142029565	1
9130011E15Rik	1,141950408	1
Rbm43	1,141871257	1
G6pdx	1,141712971	1
Gm22516	1,141633836	1
Cdk2ap1	1,141633836	1
Lgals2	1,141554707	1
Psma8	1,141554707	1
Rabep1	1,141475583	1
Poc5	1,141317352	1
Bcas3	1,141317352	1
Gss	1,141238245	1
Prpf19	1,141238245	1
Nans	1,141159143	1
Rab35	1,141080047	1
Ccdc127	1,141080047	1
H2afy	1,14092187	1
Rad18	1,14084279	1
Emc1	1,14084279	1

Alg10b	1,140684647	1
Brap	1,140526525	1
Smarcal1	1,140526525	1
Nudcd3	1,140447473	1
Rab10	1,140447473	1
Zc3hav1l	1,140368426	1
2310033P09Rik	1,140368426	1
Mrpl41	1,140368426	1
Snx17	1,140368426	1
Polr2a	1,140289384	1
Psmb4	1,140210348	1
Aco1	1,140131318	1
Ebag9	1,140131318	1
Adar	1,140131318	1
Cdv3	1,139894259	1
Gm37606	1,139736247	1
Gmeb2	1,139578257	1
Insr	1,13949927	1
Sec11a	1,13949927	1
Tm9sf1	1,139420288	1
Slfn4	1,139341313	1
Gm7236	1,139262342	1
Psd3	1,139104418	1
Zfp703	1,139104418	1
Tmem147	1,139104418	1
Sarnp	1,138946515	1
Tubgcp3	1,138946515	1
Mturn	1,138630776	1
Atg4c	1,138630776	1
Lats1	1,138630776	1
Fbxw9	1,138630776	1
Sap30bp	1,138551855	1
Aven	1,138472939	1
Sft2d3	1,138472939	1
Mbtps1	1,138472939	1
5830487J09Rik	1,138394029	1
Ahcyl2	1,138315124	1
Arhgdib	1,138315124	1
Syvn1	1,13799956	1
Dhx32	1,137920683	1
Tmem67	1,137762944	1
Cnot10	1,137762944	1
Miga1	1,137684083	1
Parvb	1,137605228	1
Rab11fip2	1,137526378	1
Cdc23	1,137526378	1
Dgkz	1,137447533	1
Ntan1	1,137368694	1
Rdh14	1,13728986	1
Mroh2a	1,137211032	1
Snora17	1,13713221	1
Fnta	1,13713221	1

Bbs2	1,137053392	1
Harbi1	1,136895774	1
Sdhaf1	1,136816973	1
Gmfb	1,136738178	1
Eif4g1	1,136738178	1
Uqcrc2	1,136738178	1
Mrc1	1,136501824	1
Dram2	1,136501824	1
Zfp217	1,136501824	1
Farsb	1,136501824	1
Mypopos	1,136344283	1
Rtel1	1,136344283	1
Gtdc1	1,136344283	1
Zfp65	1,13626552	1
Atp5k-ps2	1,136108011	1
Ptp4a2	1,136108011	1
Trmt61a	1,136029265	1
Gcc2	1,136029265	1
Fam175b	1,135950524	1
Golga1	1,135871789	1
Mettl1	1,135871789	1
Trmt2a	1,135871789	1
Vps16	1,135793059	1
Hs6st1	1,135714334	1
Tm7sf2	1,135635615	1
Mfsd11	1,135635615	1
4930427A07Rik	1,135478194	1
Cntnap1	1,135242102	1
Get4	1,135163416	1
Acad9	1,135084735	1
Bcdin3d	1,135084735	1
Cdkl3	1,134770066	1
Ap3m1	1,134770066	1
Hexa	1,134612765	1
Cops5	1,134534122	1
Elf2	1,134455485	1
Galnt6	1,134455485	1
Snap29	1,134455485	1
Zfp248	1,134376853	1
Siae	1,134376853	1
Mboat1	1,134219606	1
Cdan1	1,134140991	1
Gm12669	1,134140991	1
C130023A14Rik	1,133983776	1
Mrps27	1,133983776	1
Ggnbp2	1,133905177	1
Prrc2a	1,133747995	1
Hps6	1,133512264	1
Ccdc86	1,133433697	1
Usp48	1,133433697	1
Tcf4	1,133355136	1
Dhrs13	1,133276581	1

Kat8	1,133198031	1
Mthfd1l	1,133198031	1
Gm13328	1,133119486	1
Tmem267	1,133040947	1
Hyal2	1,132962414	1
Fchsd2	1,132962414	1
Dusp6	1,132962414	1
Zcchc4	1,132805362	1
Hpcal1	1,132726845	1
Scmh1	1,132648333	1
Setx	1,132569827	1
Alas1	1,132569827	1
Exoc2	1,132569827	1
A630001G21Rik	1,132491326	1
Vapa	1,132491326	1
Ddx5	1,13233434	1
Nat10	1,132255855	1
Homer3	1,132255855	1
Mrpl28	1,132177376	1
Ash1l	1,132098902	1
Acap2	1,132020434	1
Gm17745	1,131941971	1
Mfsd9	1,131863513	1
Smim20	1,131863513	1
Ctnna1	1,131863513	1
Surf6	1,131706615	1
Auh	1,131628173	1
Zfp346	1,131392882	1
Gm24890	1,131314463	1
4930431P19Rik	1,131314463	1
Ankrd12	1,131314463	1
Tsr1	1,13115764	1
Rexo1	1,13115764	1
Ccndbp1	1,13115764	1
Grpel1	1,131079237	1
Mpv17l2	1,131000839	1
Eif1	1,131000839	1
Tep1	1,130922447	1
D630029K05Rik	1,130765679	1
Npc1	1,130765679	1
Slc35f6	1,130687303	1
Wasf2	1,130687303	1
Gm2986	1,130530567	1
Lman1	1,130530567	1
Lrrc8a	1,130452207	1
Tjp3	1,130373853	1
Ptpdc1	1,130295504	1
Btbd2	1,130295504	1
Usp34	1,130295504	1
Zfp958	1,130138823	1
Rab5b	1,13006049	1
Ehmt2	1,129982163	1

Ctr9	1,129982163	1
Pfn2	1,129747215	1
Zc3h8	1,129747215	1
Fam206a	1,129590609	1
E2f1	1,129590609	1
Spata24	1,129512315	1
Aldh1l1	1,129434026	1
Lmna	1,129355742	1
Tmbim4	1,129277464	1
Fam65a	1,129199191	1
Pde4c	1,129120923	1
Gm27039	1,129120923	1
Hras	1,129120923	1
Zmiz1	1,129120923	1
Chp1	1,129042661	1
Pcbp1	1,129042661	1
Kctd11	1,128964405	1
Tab3	1,128729668	1
Rab3gap1	1,128729668	1
Mtif3	1,128651433	1
Ltc4s	1,128651433	1
Nos3	1,128573203	1
Pbx2	1,128573203	1
Mysm1	1,128494979	1
Vps33b	1,128182137	1
Ubc	1,128103941	1
Pkmyt1	1,128025749	1
Slc35c1	1,127791207	1
Nceh1	1,127791207	1
Gm43668	1,127713037	1
Cyld	1,127556714	1
Gm24009	1,12747856	1
Trmt5	1,127400412	1
Arel1	1,127322269	1
Gm4994	1,127166	1
Wdpcp	1,127166	1
Lrrc27	1,127166	1
Senp5	1,127009753	1
Bhlhe40	1,126931637	1
Colec12	1,126697322	1
Slc30a6	1,126619228	1
Rbfa	1,126619228	1
Tomm40	1,12654114	1
Trim35	1,12654114	1
Ercc8	1,126463057	1
Pdlim2	1,126463057	1
Zfp287	1,126384979	1
Usp9x	1,126384979	1
Cdk4	1,126306907	1
Xkr8	1,12622884	1
Gm13771	1,12622884	1
Psmb3	1,12622884	1

Camkk2	1,12622884	1
Prox2	1,125994671	1
Trps1	1,125994671	1
Hp1bp3	1,125994671	1
Focad	1,125838586	1
Thap12	1,125838586	1
Klhdc10	1,125838586	1
Ipo11	1,125760552	1
Mtmr10	1,125682523	1
Sdcbp	1,125526481	1
BC005624	1,125448468	1
Brcc3	1,125448468	1
Mmadhc	1,125448468	1
Spast	1,125292458	1
Pgs1	1,125214462	1
Tle6	1,125136471	1
Polr3h	1,125136471	1
Ankle2	1,125136471	1
Mrpl13	1,124902529	1
Rab34	1,124902529	1
Slc25a42	1,124746595	1
Gm13743	1,124590683	1
Crebl2	1,124590683	1
Pisd	1,124590683	1
Rpp30	1,124512735	1
Fntb	1,124434793	1
Tada3	1,124434793	1
Xpo6	1,124434793	1
Lipt1	1,124356856	1
Fam187b	1,124278924	1
Nol9	1,124200997	1
Ipo4	1,124123076	1
Zfp944	1,123967251	1
Erlin2	1,123889346	1
Fth-ps2	1,123811447	1
Eml3	1,123811447	1
Inafm2	1,123733553	1
Arpc1b	1,123733553	1
Cdk5rap3	1,123655664	1
Fam126a	1,123655664	1
Paox	1,123655664	1
Bet1	1,123577781	1
Cops7b	1,123422031	1
Fdft1	1,123344164	1
Hspa9	1,123344164	1
Cope	1,123188446	1
Dctd	1,123110595	1
Xpnpep1	1,123110595	1
Dicer1	1,123110595	1
Spcs2-ps	1,12303275	1
Cep120	1,12303275	1
Ly6e	1,122799246	1

RP23-48A24.3	1,122721422	1
Jmjd7	1,122721422	1
Hjurp	1,122565791	1
Smim15	1,122487983	1
Stx12	1,122410181	1
Brf2	1,122332384	1
Ergic2	1,122254592	1
Cir1	1,122099026	1
Mia2	1,12202125	1
Kin	1,121943481	1
Zfp296	1,121710203	1
Dopey2	1,121710203	1
Fam160b1	1,121710203	1
Spg20	1,121710203	1
Vegfa	1,121710203	1
Ifitm3	1,121632455	1
H2-DMb2	1,121554712	1
Acot13	1,121321515	1
Gm11945	1,121243794	1
Pias1	1,121243794	1
Zfp385a	1,121166078	1
Csrp2bp	1,121166078	1
Serhl	1,120932962	1
Leng9	1,120855268	1
Tnip3	1,120855268	1
Tcf12	1,120777579	1
Ppm1d	1,120699895	1
Mepce	1,120699895	1
Ehd4	1,120699895	1
3830406C13Rik	1,120622217	1
Chst10	1,120544544	1
Nipbl	1,120466876	1
Prepl	1,120311557	1
Fam118a	1,120311557	1
Dnajc14	1,120311557	1
Icam1	1,120311557	1
Git1	1,120311557	1
Tmem185a	1,120233906	1
Yipf1	1,120233906	1
Tlk2	1,12015626	1
Zfp81	1,120078619	1
9130024F11Rik	1,120000984	1
E130307A14Rik	1,119923354	1
Ndufs2	1,11984573	1
Nxpe3	1,119768111	1
Dusp28	1,119690497	1
Pmm2	1,119612889	1
Smad2	1,119612889	1
Rpap2	1,119535286	1
Mndal	1,119224928	1
B230354K17Rik	1,119069781	1
Washc2	1,119069781	1

Nsfl1c	1,118992216	1
Ankrd35	1,118914656	1
2810474O19Rik	1,118837101	1
Zfp995	1,118759552	1
Mettl2	1,118759552	1
Gm42640	1,118682008	1
Pqlc3	1,118682008	1
Rabgap1	1,118682008	1
Zfp512	1,11860447	1
Irak1	1,11860447	1
Mettl25	1,118526937	1
Parp4	1,118449409	1
Gm28192	1,118371887	1
Arhgap10	1,118371887	1
Ppan	1,11829437	1
2300009A05Rik	1,118216858	1
Lss	1,118139352	1
Cox11	1,118061851	1
Thoc7	1,117984356	1
Jak1	1,117906865	1
Kctd7	1,117829381	1
Vcpip1	1,117829381	1
1700017B05Rik	1,117751901	1
Rfesd	1,117674427	1
Tmem251	1,117596959	1
Gm11895	1,117519496	1
9130019O22Rik	1,117519496	1
Plcl2	1,117519496	1
Gstp1	1,117442038	1
Fas	1,117442038	1
Gda	1,117442038	1
Adssl1	1,117364585	1
Tpst1	1,117364585	1
Eif5b	1,117364585	1
Tuba4a	1,117209696	1
Atp6ap1	1,11713226	1
Rlf	1,116977403	1
Snn	1,116822568	1
Ndufab1-ps	1,116822568	1
Arhgap22	1,116822568	1
Narfl	1,116822568	1
Usp5	1,116822568	1
Mettl4	1,116590355	1
Casp8	1,116512962	1
Slc30a4	1,116512962	1
Ric8a	1,116358191	1
Srbd1	1,116280814	1
Zcchc17	1,116280814	1
Wdr12	1,116048714	1
Pip5k1b	1,116048714	1
Ptar1	1,115971358	1
Fbrs	1,115971358	1

Cpsf6	1,115816662	1
P2rx7	1,115661988	1
Mccc1	1,115661988	1
Phlda3	1,115661988	1
Nek6	1,115661988	1
Cog6	1,115584659	1
Mief1	1,115584659	1
Trim16	1,115507335	1
Cep78	1,115352704	1
Dnajc7	1,115352704	1
RP24-282C4.9	1,115275396	1
Ogdh	1,115275396	1
Ccnl2	1,115275396	1
Gm9207	1,115198094	1
Grk2	1,115198094	1
Rhoq	1,115198094	1
Fastkd3	1,115120797	1
Rad23b	1,115120797	1
Snai1	1,114811662	1
Slc35a2	1,114734392	1
Hivep2	1,114734392	1
Gm27046	1,114657127	1
Ppp1r13b	1,114657127	1
Slc7a6	1,114657127	1
S1pr2	1,114657127	1
Gm996	1,114348122	1
Prkar1a	1,114348122	1
Gm19552	1,114270884	1
Twf1	1,114270884	1
Ppp2r5d	1,114270884	1
Tank	1,114193651	1
Mcts1	1,114193651	1
Matr3	1,114193651	1
Gm43859	1,114039201	1
Dap3	1,113961985	1
Zfand2b	1,113730367	1
Spata1	1,113653172	1
Blzf1	1,113653172	1
Rgs10	1,113653172	1
Hcls1	1,113498797	1
Fbxo6	1,113344444	1
Wdr48	1,113344444	1
Eepd1	1,113344444	1
Znfx1	1,113344444	1
Tarsl2	1,113267276	1
Ubxn1	1,113190113	1
Chchd4	1,113112955	1
Gm3724	1,112573	1
Dgkg	1,112495885	1
Arf3	1,112495885	1
Nat9	1,112495885	1
Ppp1cc	1,112418776	1

Vwa8	1,112418776	1
Ndufaf6	1,112418776	1
Ipmk	1,112341671	1
Cacul1	1,112341671	1
Leprotl1	1,112264572	1
Pdgfa	1,112187479	1
Cpsf2	1,112187479	1
Pnpla2	1,11211039	1
Mphosph9	1,112033307	1
Cdk13	1,112033307	1
A630033H20Rik	1,111802091	1
Gm13453	1,111725029	1
Fam43a	1,111725029	1
Gm14857	1,111725029	1
Trpc4ap	1,111725029	1
Gle1	1,111647973	1
Srp19	1,111570922	1
Bod1l	1,111493876	1
Plrg1	1,111339801	1
Orai2	1,111262772	1
Socs6	1,111185748	1
Slc33a1	1,111031715	1
9230114K14Rik	1,110954707	1
Gm20673	1,110723714	1
H1f0	1,110723714	1
Exoc5	1,110723714	1
Trmo	1,110646728	1
Setd5	1,110646728	1
RP24-550H10.6	1,110569746	1
Atp5g2	1,110569746	1
Tmem141	1,110415799	1
Dcaf12	1,110415799	1
Fam192a	1,110338834	1
Tln1	1,110338834	1
Atp5f1	1,110338834	1
Gm13378	1,110261873	1
Hdac4	1,110184919	1
Rap1b	1,110184919	1
Zc3h7b	1,110107969	1
Isca2	1,110107969	1
Ercc2	1,110031025	1
Trim56	1,110031025	1
Gm42463	1,109954086	1
Casp3	1,109877153	1
Gm45568	1,109723302	1
Sdhaf3	1,109723302	1
Mrpl48	1,109723302	1
Zfp553	1,109646384	1
Slc23a2	1,109646384	1
Srebf2	1,109646384	1
Acad8	1,109646384	1
Zbtb6	1,109646384	1

Ywhag	1,109646384	1
Osbpl2	1,109569472	1
Stx8	1,109492565	1
Fhad1	1,109415664	1
Tfam	1,109338768	1
Dennd6a	1,109338768	1
Psmc2	1,109338768	1
Mthfsd	1,109031236	1
Trim13	1,109031236	1
Med13l	1,108954367	1
Klhl2	1,108877502	1
Coq9	1,108877502	1
Ing3	1,108877502	1
Tbcel	1,108800644	1
Phykpl	1,108570099	1
Eri3	1,108570099	1
Gan	1,108570099	1
Sat1	1,108032348	1
Sgpl1	1,107955548	1
Ninj1	1,107955548	1
Acap3	1,107878753	1
Psmb6-ps2	1,107801963	1
Rbm4	1,107801963	1
Atp5o	1,107725179	1
Rbbp5	1,1076484	1
Plch2	1,107494858	1
Nmd3	1,107494858	1
Cdk18	1,107494858	1
Zfp329	1,107418095	1
Gm10146	1,107418095	1
March8	1,107341337	1
Bscl2	1,107264584	1
Skiv2l	1,107111096	1
Atp1a1	1,107111096	1
Mgmt	1,106957628	1
Hacd1	1,106880902	1
Gm15892	1,106804182	1
Rbsn	1,106804182	1
Gm43430	1,106727467	1
Efcab7	1,106574052	1
Fkbp2	1,106574052	1
Capzb	1,106574052	1
Mrpl15	1,106574052	1
Ccpg1	1,106497353	1
Nufip2	1,106497353	1
Sp2	1,106420659	1
Dmrt2	1,106343971	1
Thumpd1	1,106267287	1
Tsn	1,106267287	1
Gm8909	1,106037269	1
Vps36	1,106037269	1
Dars2	1,105960607	1

R3hcc1l	1,105883951	1
Rab7	1,105883951	1
Pigu	1,105807299	1
Got2	1,105807299	1
4930430F08Rik	1,105654013	1
Arap1	1,105654013	1
Cdkl2	1,105577377	1
Cd180	1,105577377	1
Dnajc18	1,105577377	1
Ppp2r3d	1,105270888	1
Per3	1,10519428	1
Gatad2a	1,10519428	1
Tifa	1,10519428	1
Stim2	1,10519428	1
Lmo4	1,105117676	1
Rabl3	1,104964485	1
Lysmd1	1,104964485	1
Sh2b2	1,104811315	1
Srsf9	1,104811315	1
Tsg101	1,104734738	1
Hes6	1,104734738	1
Thoc1	1,1045816	1
B4galt6	1,104505039	1
Mrpl57	1,104505039	1
Kti12	1,104428483	1
Abhd5	1,104428483	1
Ezh1	1,104351932	1
Synpo	1,104198847	1
Usp10	1,104198847	1
Slc30a1	1,104045783	1
Gm3555	1,103969259	1
Gm8822	1,103969259	1
Odc1	1,103969259	1
Hibch	1,103739719	1
Cyp27a1	1,103663216	1
Mpdu1	1,103586719	1
E230020A03Rik	1,103280782	1
Fasn	1,103280782	1
Mast2	1,103204311	1
Pias2	1,103127846	1
Rif1	1,103051385	1
Xylt1	1,10297493	1
Gm5601	1,102745597	1
Mfap1b	1,102745597	1
Rps15a-ps4	1,102669163	1
Hsdl1	1,102592735	1
Trappc5	1,102439893	1
Traf7	1,102439893	1
Tapt1	1,102363481	1
Pafah1b1-ps2	1,102287073	1
Daam1	1,102134274	1
Pramef8	1,102134274	1

Dolpp1	1,102057883	1
Fam129b	1,102057883	1
Chmp5	1,102057883	1
Cnppd1	1,102057883	1
Scpep1	1,101905116	1
Tcf3	1,10182874	1
Las1l	1,101599645	1
Gpr132	1,101523291	1
Aen	1,101523291	1
Dagla	1,101446942	1
Chek2	1,101446942	1
Ubl7	1,101370598	1
Scamp3	1,101294259	1
Aldoa	1,101294259	1
Aifm1	1,101217926	1
Ralgapa1	1,101141598	1
Zfp715	1,100988958	1
Plxna1	1,100912646	1
AI837181	1,100912646	1
Zswim8	1,100836339	1
Herc2	1,100760038	1
Chmp6	1,100683741	1
Phldb1	1,100683741	1
Brat1	1,100531165	1
Anapc15	1,100531165	1
Nol10	1,100531165	1
RP23-349H12.3	1,100454884	1
Ddt	1,100149816	1
Sumo3	1,100149816	1
Ppfia1	1,100073562	1
Zfp780b	1,099997313	1
Abhd17c	1,099997313	1
Usb1	1,099997313	1
Klhdc2	1,099844832	1
Mrpl22	1,099768599	1
Ubp1	1,099768599	1
Grb2	1,099768599	1
Pgm2	1,099692372	1
Tmem5	1,099387514	1
Efr3a	1,099387514	1
Gm6560	1,099311313	1
Fam76a	1,099311313	1
Atp5b	1,099311313	1
Gba	1,099235117	1
Zfp746	1,099158927	1
Nedd4l	1,099158927	1
Zmynd8	1,099158927	1
Etfbkmt	1,099082742	1
Zfp964	1,099082742	1
Gpr179	1,099082742	1
Nek7	1,098854218	1
Hspd1	1,098854218	1

Nubp1	1,098778053	1
Dnaja3	1,098778053	1
Capg	1,098778053	1
Cyfip1	1,098625741	1
Prkacb	1,098549593	1
Arl10	1,09847345	1
Oas3	1,09847345	1
Icam5	1,09816893	1
Prmt9	1,09816893	1
Zfp24	1,098016702	1
Golga4	1,098016702	1
5031439G07Rik	1,098016702	1
Map2k3	1,098016702	1
Adam10	1,097940596	1
Wdr75	1,097940596	1
Zzef1	1,09771231	1
BC005561	1,09771231	1
Mfge8	1,097560145	1
2310057M21Rik	1,097484071	1
Gpat4	1,097179826	1
Ccnf	1,097103778	1
Kdm2a	1,097027735	1
Krtcap2	1,097027735	1
Gm8228	1,096951697	1
Brpf3	1,096951697	1
Mex3d	1,096875665	1
Gm9497	1,096799638	1
Dcaf13	1,096799638	1
Mfn2	1,096723616	1
Nhlrc2	1,0966476	1
Gapdh-ps14	1,096571589	1
Rftn2	1,096495583	1
Tmem209	1,096495583	1
Nat2	1,096419582	1
Coro2a	1,096419582	1
Gm44027	1,096343587	1
Csrp2	1,096343587	1
Tmem63b	1,096191612	1
Slc6a6	1,096115632	1
Faf1	1,095963689	1
Ppil4	1,095887725	1
Timm8b	1,095887725	1
P2ry2	1,095811766	1
Zxdb	1,095735813	1
Dph7	1,095659865	1
Nek8	1,095583922	1
Nsun6	1,095583922	1
Rnf168	1,095583922	1
Ppp4r1	1,095507985	1
Grhpr	1,095507985	1
Fbxl20	1,095507985	1
Maz	1,095280204	1

Gtf3a	1,095280204	1
Sema4d	1,095280204	1
Vps39	1,095204288	1
Utp18	1,095128377	1
Prtn3	1,09497657	1
Gpank1	1,09497657	1
Znhit6	1,09497657	1
Rpsa-ps12	1,094824785	1
Dhx34	1,094824785	1
Naf1	1,094824785	1
2010107E04Rik	1,094824785	1
Cdc14a	1,0947489	1
Gemin7	1,094521277	1
4932438A13Rik	1,094521277	1
Arfrp1	1,094521277	1
St6galnac4	1,094369555	1
Kctd2	1,094293701	1
Cisd1	1,094293701	1
Mta1	1,09414201	1
Rad54l2	1,09414201	1
Aco2	1,09414201	1
Gm14780	1,093990341	1
Gm21816	1,093914514	1
Tmem245	1,093914514	1
Rps6kb1	1,093838692	1
Pik3ca	1,093838692	1
Gm6415	1,093762875	1
C330018D20Rik	1,093535457	1
Casd1	1,093459662	1
Cd151	1,093383872	1
Exoc1	1,093080763	1
Wipf2	1,092853487	1
Il23a	1,092777739	1
Sec11c	1,092777739	1
Paqr4	1,092701996	1
Abracl	1,092701996	1
Wsb2	1,092626258	1
Ccr10	1,092474799	1
Utp11	1,092474799	1
Dusp22	1,092399077	1
Gm43533	1,09232336	1
Ap3d1	1,09232336	1
N4bp3	1,092247649	1
Timm50	1,092247649	1
Ltn1	1,092171942	1
Vps11	1,092171942	1
Ndufa11	1,092096241	1
Gm16437	1,092020546	1
Smpd2	1,091944855	1
Gm32175	1,09186917	1
Cenpq	1,09186917	1
Tmem18	1,09186917	1

8030453O22Rik	1,091642146	1
Gak	1,091642146	1
Mthfd2	1,091642146	1
Cdc42bpg	1,091566482	1
Agl	1,091490823	1
Mrpl10	1,091490823	1
Zfp1	1,091415169	1
Elp5	1,09133952	1
Clcn5	1,091188239	1
Ddx54	1,091188239	1
Tex261	1,091188239	1
Snx12	1,091036979	1
Klf13	1,091036979	1
Zfp956	1,090961356	1
Gm15773	1,090885739	1
Pdss1	1,090734521	1
Flii	1,09065892	1
Immp2l	1,090583324	1
Rft1	1,090507733	1
Palm	1,090507733	1
Ccng1	1,090432147	1
Wdr3	1,090356567	1
Hmgxb3	1,090280992	1
Nifk	1,090280992	1
Sars2	1,090205422	1
Gpatch2l	1,090054298	1
Tbc1d32	1,090054298	1
Vash2	1,089978743	1
Psmc4	1,089978743	1
Rpf2	1,089827651	1
Galk1	1,089752112	1
Bcorl1	1,089676579	1
Pcmtd1	1,089676579	1
Pkd2	1,089601051	1
G3bp1	1,089601051	1
Numa1	1,089450011	1
Pes1	1,089450011	1
Mob1a	1,089374498	1
Gm42484	1,089298991	1
Eral1	1,089298991	1
Bmf	1,089298991	1
Ubxn2b	1,089298991	1
Atxn1	1,089298991	1
Dld	1,089223489	1
Nckap1l	1,089223489	1
Mpp1	1,089072501	1
Rpl31-ps11	1,088997015	1
AC149090.1	1,088997015	1
Ccdc57	1,088921534	1
Ddx10	1,088921534	1
Spa17	1,088846059	1
Usp8	1,088846059	1

Bag2	1,088770588	1
Mtrr	1,088770588	1
Zfp931	1,088695123	1
Zfp280c	1,088695123	1
Erlec1	1,088619663	1
Gm33142	1,088468759	1
Dock9	1,088393315	1
Cbfb	1,088393315	1
Rpl7a-ps5	1,088317876	1
Gm4262	1,08809159	1
Atp6v1h	1,08809159	1
Tcerg1	1,088016172	1
Strn3	1,088016172	1
Rapgef2	1,087940759	1
Rwdd4a	1,087940759	1
Gm35315	1,087865351	1
C77080	1,087865351	1
Abtb2	1,087789948	1
Cep83	1,087714551	1
Naaa	1,087639159	1
Pcyox1l	1,087639159	1
Pola2	1,087488391	1
Jmy	1,087488391	1
Faap100	1,087337643	1
Ist1	1,087337643	1
Dusp8	1,087262278	1
Zfp72	1,087262278	1
Lonp2	1,087262278	1
Fam32a	1,087262278	1
Wac	1,087186917	1
Crtc3	1,087111561	1
4930529C04Rik	1,087036211	1
Tmigd3	1,08658422	1
1600002K03Rik	1,08658422	1
Txndc12	1,086433597	1
Zfp319	1,086282996	1
Esrp2	1,086207703	1
Abca7	1,086207703	1
Nol11	1,086207703	1
Uqcrh	1,086207703	1
Grsf1	1,086132416	1
Gm44834	1,086057133	1
Zfp420	1,085906584	1
Tbc1d15	1,085906584	1
Fam57a	1,085831318	1
Glg1	1,085831318	1
Padi2	1,085756056	1
Cd2bp2	1,085756056	1
Gart	1,085756056	1
Cd276	1,0856808	1
Pom121	1,085605549	1
4632404H12Rik	1,085530303	1

Tyw1	1,085530303	1
Scarb2	1,085379827	1
Llgl1	1,085304597	1
Sap18b	1,085154152	1
Ubald1	1,084928524	1
Gm5453	1,084853325	1
Rpl21-ps5	1,084853325	1
Rras2	1,084778132	1
Snw1	1,08462776	1
Rapgef1	1,084552582	1
Arf1	1,084552582	1
Casp1	1,084477409	1
Syngr2	1,084477409	1
Fbxo36	1,084327079	1
Tnfrsf4	1,084327079	1
Gm9840	1,084251922	1
Zcwpw1	1,08417677	1
Poldip3	1,084026481	1
Leng1	1,083951345	1
Insl6	1,083951345	1
Trp53bp1	1,083951345	1
Tes3-ps	1,083876214	1
Haus4	1,083876214	1
Atp6v1f	1,083876214	1
Sh3bgrl2	1,083801088	1
Gng7	1,083725967	1
Msh5	1,083725967	1
Rpl34-ps1	1,083650851	1
Safb2	1,083650851	1
Rdx	1,083575741	1
Thnsl1	1,083500636	1
Plpp7	1,083425536	1
Rrp1	1,083425536	1
Nedd1	1,083350441	1
RP24-316F13.7	1,083200267	1
Tpr	1,083125188	1
Tmem161b	1,082899982	1
Coq5	1,082824924	1
Cct6a	1,082749871	1
Prdx1	1,082674823	1
Nupr1	1,08259978	1
Golt1b	1,08259978	1
E130308A19Rik	1,08244971	1
Agbl5	1,08244971	1
Tpd52l2	1,08244971	1
Rlim	1,08244971	1
Wdfy3	1,082374683	1
Rab24	1,082374683	1
Rragc	1,082374683	1
Ptgr1	1,082299661	1
Pomt1	1,082224645	1
Adam15	1,082224645	1

Map4k5	1,082149633	1
Fgfr1op2	1,082074627	1
Ufl1	1,082074627	1
Tbrg1	1,082074627	1
Foxc1	1,081999626	1
Siah2	1,081999626	1
2510009E07Rik	1,081999626	1
Nup214	1,08192463	1
Znrf1	1,08192463	1
Stat3	1,08192463	1
Copb2	1,08192463	1
1110012L19Rik	1,081849639	1
Gm4149	1,081774654	1
Crk	1,081774654	1
Ndst1	1,081699673	1
B9d2	1,081624698	1
Zrsr2	1,081624698	1
Mrpl39	1,081474763	1
Ncln	1,081474763	1
1110032A03Rik	1,081399804	1
Zbtb24	1,08132485	1
Stk3	1,08132485	1
Paics	1,081249901	1
Ncoa1	1,081174957	1
Akr1c13	1,081100018	1
Pdcd5-ps	1,080950156	1
Epn1	1,080950156	1
Ifi204	1,080800315	1
Slc39a3	1,080725402	1
Pi4k2a	1,080650494	1
Snx6	1,080575592	1
5730455P16Rik	1,080350916	1
Golph3	1,080276034	1
Bclaf1	1,080201158	1
Glyctk	1,080126287	1
Wdr43	1,07997656	1
Tax1bp1	1,079826854	1
Arl4c	1,079752008	1
Chd1	1,079752008	1
Nop58	1,079752008	1
9230102O04Rik	1,079677168	1
Katnb1	1,079677168	1
Ttc13	1,079677168	1
Mlxip	1,079677168	1
Nop14	1,079677168	1
Cib2	1,079602333	1
Yif1b	1,079602333	1
Rundc1	1,079602333	1
Gm2223	1,079452679	1
4632427E13Rik	1,07937786	1
Ranbp3	1,07937786	1
Gm37900	1,079303045	1

1700123O20Rik	1,079153433	1
Tmem9	1,079153433	1
Gm15703	1,079078634	1
Thoc2	1,079078634	1
Taf1a	1,079003841	1
Srpk1	1,079003841	1
C2cd3	1,078929052	1
Med26	1,078854269	1
Stoml2	1,078854269	1
5830444B04Rik	1,078704719	1
Tmem14c	1,078704719	1
Mbd3	1,07840568	1
Retsat	1,078330933	1
Psmb2	1,078330933	1
Zscan26	1,078181455	1
Ints9	1,078106724	1
Magt1	1,078106724	1
Ids	1,07780785	1
Riok1	1,07780785	1
Dync1h1	1,07780785	1
2410002F23Rik	1,077658445	1
Gm45840	1,07758375	1
Bbip1	1,07758375	1
Gm8762	1,07750906	1
Mrps18c	1,07750906	1
Npm1	1,077434375	1
Dmtf1	1,077135689	1
Tmem156	1,076986376	1
Bmyc	1,076986376	1
Asap1	1,076986376	1
Med17	1,076911728	1
Ccdc191	1,076762446	1
Porcn	1,076687814	1
Sbno2	1,076687814	1
Mdh2	1,076613186	1
D16Ertd472e	1,076463946	1
Ube2j1	1,076389334	1
Ptbp3	1,076389334	1
Gm12589	1,076240125	1
Tspyl1	1,076165528	1
Emc3	1,076090937	1
Mir6236	1,07601635	1
Gm11914	1,07601635	1
Dubr	1,07601635	1
Spop	1,07601635	1
Dhx30	1,075941769	1
Klhl12	1,075792622	1
Trappc11	1,075792622	1
Atf7	1,075643496	1
Igtp	1,075568941	1
Pi4k2b	1,075568941	1
Emd	1,07549439	1

Rpe	1,075419845	1
Calr3	1,075345306	1
Fam129a	1,075270771	1
Rala	1,075270771	1
Setd6	1,075196241	1
Uchl4	1,074972684	1
Sirt5	1,074972684	1
Dlg1	1,074972684	1
Ethe1	1,074898175	1
Atxn10	1,074898175	1
Chkb	1,074749173	1
Fam212a	1,07467468	1
Sfxn5	1,074525708	1
Ap2m1	1,074525708	1
Gm10131	1,074376758	1
Lactb	1,074376758	1
Shc4	1,07430229	1
Psme4	1,07430229	1
Smim1	1,074153371	1
Depdc7	1,074153371	1
Dpagt1	1,074153371	1
March4	1,074078919	1
Mrpl40	1,074004472	1
Cab39l	1,074004472	1
Psme2	1,07393003	1
Flt1	1,07393003	1
Exosc10	1,07393003	1
Nars2	1,073781162	1
D430042O09Rik	1,073781162	1
Scand1	1,073706736	1
Vcp-rs	1,073632315	1
Cops3	1,073557899	1
Gm10136	1,073483488	1
Dock4	1,073334682	1
Slx4	1,073334682	1
Psd	1,073260286	1
Rpl3-ps1	1,073111511	1
Ep400	1,073111511	1
Apool	1,072962757	1
Trip12	1,072962757	1
Urod	1,072739664	1
Gm42747	1,072665309	1
Gtpbp6	1,072590961	1
Tsen2	1,072516617	1
Evi5	1,072516617	1
Gmps	1,072442278	1
Micall2	1,072442278	1
Hyou1	1,072442278	1
Vdac1	1,072367945	1
H13	1,072367945	1
Tmem158	1,072293616	1
Ruvbl1	1,072144975	1

1110059G10Rik	1,072144975	1
Ctnnb1	1,072144975	1
Nipa1	1,072070662	1
Gtf3c6	1,071996355	1
Ptbp2	1,071922052	1
Hnrnpab	1,071847755	1
Fut11	1,071773463	1
Plxnb2	1,071773463	1
Kif5b	1,071773463	1
H2-DMb1	1,071699175	1
Smarce1	1,071624893	1
Ndufb5	1,071624893	1
Ssrp1	1,071550617	1
Gpx4	1,071476345	1
Lsm14b	1,071476345	1
Tcp1	1,071476345	1
Hs1bp3	1,07117931	1
Fgd6	1,07117931	1
Lipe	1,071105064	1
AA986860	1,071030823	1
Spata5	1,071030823	1
Pnkd	1,070956588	1
Klhl26	1,070956588	1
Bri3bp	1,070956588	1
Zfp251	1,070808132	1
Mrpl4	1,070808132	1
Mrpl44	1,070733912	1
Ahcyl1	1,070659697	1
Hmgb1-ps6	1,070585487	1
Rnaseh2a	1,070585487	1
Tnfrsf10b	1,070585487	1
Gadd45gip1	1,070585487	1
Ccdc173	1,070437082	1
Ube2w	1,070362888	1
Gm6563	1,070362888	1
Gm7132	1,070288698	1
A930007I19Rik	1,070214514	1
Mrrf	1,070214514	1
Sppl2a	1,070140335	1
Mapk14	1,070140335	1
Tnfrsf1b	1,070066161	1
Sco1	1,069769517	1
Fam63a	1,069769517	1
Ran	1,069769517	1
Gm5054	1,069695369	1
Ctps	1,069695369	1
Rsbn1l	1,069695369	1
Farp2	1,069547088	1
Maoa	1,069547088	1
Bap1	1,069398827	1
Clp1	1,069324705	1
Gpd1l	1,069324705	1

Zfp654	1,069028266	1
Cep128	1,068954169	1
Naip5	1,068954169	1
Ahsa1	1,06873191	1
Zfp637	1,068657834	1
Elovl5	1,068657834	1
Gm9774	1,068583762	1
Hspbp1	1,068509696	1
Csnk2b	1,068435635	1
Ankrd13d	1,068287529	1
Myo5a	1,068213484	1
Sh3bp5	1,068213484	1
C530005A16Rik	1,068139443	1
Tinf2	1,068139443	1
Ctif	1,068065408	1
Bub3	1,068065408	1
Gm6272	1,067917353	1
Otub2	1,067917353	1
Rgp1	1,067917353	1
Fam96b	1,067917353	1
Gm11652	1,067843333	1
Usp40	1,067843333	1
Dnajc1	1,067843333	1
Vps37d	1,067769318	1
Itgb7	1,067769318	1
Ppp1r18	1,067769318	1
Taf3	1,067621304	1
Tmem94	1,067547305	1
Pknox1	1,067547305	1
Zfp219	1,067473311	1
Pkig	1,067325338	1
Zfp592	1,067177386	1
Trpm4	1,067103417	1
Josd2	1,067103417	1
Xpo7	1,067029454	1
Palld	1,066881542	1
Anapc13	1,066881542	1
Gm16418	1,066807594	1
Ift88	1,066733651	1
Zfp317	1,066659713	1
Gspt1	1,066659713	1
Gins4	1,066585781	1
Hspa13	1,066511853	1
Hspa4l	1,066511853	1
Gngt2	1,066437931	1
Acot9	1,066437931	1
Phf8	1,066437931	1
Mfsd2a	1,066364014	1
Sfswap	1,066364014	1
Trim39	1,066290101	1
Nkiras2	1,066290101	1
Phf20-ps	1,066216194	1

Sf3a1	1,066068396	1
L3hypdh	1,065920617	1
Phyh	1,065920617	1
Ndufa5	1,065920617	1
Nom1	1,065920617	1
Nudt13	1,065846736	1
Csnk1a1	1,065846736	1
Dnaaf3	1,06577286	1
Slc35e2	1,06577286	1
Fdx1	1,065698989	1
Dennd1b	1,065698989	1
Ube3a	1,065698989	1
Plxnc1	1,065625123	1
Synj2bp	1,065625123	1
Prkaca	1,065625123	1
Ddx31	1,065551262	1
Psmb6	1,065477406	1
Aup1	1,065477406	1
Klhl21	1,065255869	1
Trappc13	1,065255869	1
Akap13	1,065255869	1
Fam134b	1,065255869	1
Rpp21	1,065182034	1
Bend6	1,065108203	1
Dusp12	1,065034378	1
Nol8	1,065034378	1
2700062C07Rik	1,064960558	1
Pik3r5	1,064739129	1
Lins1	1,064665329	1
Cdk7	1,064591535	1
Socs3	1,064517746	1
Atrx	1,064517746	1
Alg14	1,064443962	1
Vps37a	1,064296408	1
Ywhae	1,064296408	1
Cgrrf1	1,06422264	1
Pef1	1,06422264	1
Ctps2	1,064148876	1
Rac1	1,064075117	1
Tab2	1,063853872	1
Sdcbp2	1,063780134	1
Fam104a	1,063780134	1
Ghitm	1,063632673	1
Abcd4	1,06355895	1
Psph	1,063485232	1
Paxip1	1,063485232	1
RP24-401G4.1	1,06341152	1
Ciz1	1,06341152	1
Zfp689	1,063337812	1
Trem3	1,06326411	1
Tmem87b	1,063190412	1
Ccz1	1,063190412	1

Nrbf2	1,06311672	1
Gemin8	1,063043033	1
Arl14ep	1,063043033	1
Parg	1,062895674	1
Hsf1	1,062822003	1
Milr1	1,062748336	1
Gga1	1,062748336	1
Arhgef4	1,062601018	1
Sptlc2	1,062601018	1
Htatsf1	1,062306443	1
Clns1a	1,062232812	1
Gm3355	1,062159186	1
Slc41a1	1,062159186	1
Gm7452	1,062085566	1
Ptges3	1,062085566	1
Napa	1,06193834	1
Cdc34b	1,061864734	1
Sft2d2	1,061791134	1
Tpgs2	1,061717539	1
Sars	1,061717539	1
Slc38a7	1,061717539	1
RP24-497N7.2	1,061643949	1
Spcs1	1,061570364	1
2210016L21Rik	1,061423209	1
Spata2	1,061276075	1
Ccnh	1,061276075	1
Smyd3	1,061276075	1
Spink5	1,061202515	1
Srp14	1,061202515	1
Ptges2	1,061128961	1
Tbcc	1,061128961	1
Pex6	1,060981867	1
Cers6	1,060834794	1
Nup188	1,060834794	1
Xrn2	1,060761265	1
Gm37978	1,060540709	1
Gm15050	1,060540709	1
Zfp777	1,0604672	1
Mpeg1	1,0604672	1
Rnf19b	1,060393697	1
Paip1	1,060173217	1
Itpr3	1,060173217	1
Nup62	1,060173217	1
Masp2	1,060099734	1
Nab1	1,059952783	1
Bccip	1,059879316	1
Zfp472	1,059879316	1
Msantd4	1,059658943	1
Rev3l	1,059585495	1
Eif4enif1	1,059585495	1
Pak1ip1	1,059512053	1
Impdh1	1,059438616	1

Utp23	1,059365184	1
4930524J08Rik	1,059218335	1
Gm340	1,059144918	1
Il6st	1,059071506	1
Smc6	1,059071506	1
Zfp239	1,058998099	1
Pick1	1,058924698	1
Sik3	1,058924698	1
Pfas	1,05877791	1
BC060293	1,058704523	1
Lamp2	1,058557766	1
Spice1	1,058411029	1
Xpot	1,058337668	1
Rbbp6	1,058337668	1
Mt1	1,058264312	1
Zfp706	1,058264312	1
Mrps12	1,058190961	1
Ppp2r3c	1,058044275	1
Fam92a	1,057897609	1
4833445I07Rik	1,057750964	1
Pfdn4	1,057750964	1
Capn7	1,057677648	1
Psmg4	1,057604338	1
Drap1	1,057531033	1
Dcun1d5	1,057531033	1
Toe1	1,057457733	1
Apopt1	1,057457733	1
Pa2g4	1,057457733	1
Crybg3	1,057384439	1
Gm8806	1,057311149	1
Lrwd1	1,057311149	1
Bin1	1,057237864	1
Slc25a43	1,057164585	1
Mpp5	1,057164585	1
Psrc1	1,057164585	1
Lyrm4	1,056871517	1
Prmt5	1,056725014	1
Rbbp9	1,056651769	1
Lpcat1	1,056651769	1
RP23-164P21.3	1,056578531	1
Gnpat	1,056578531	1
Gpatch8	1,056432068	1
Tanc1	1,056432068	1
Arglu1	1,056432068	1
Gm13935	1,056358844	1
Zfp871	1,056358844	1
Taf2	1,056285625	1
Rfng	1,056285625	1
Gm11520	1,056212412	1
Usp3	1,056212412	1
Rnf215	1,056139203	1
Prpsap2	1,055992801	1

Cbr4	1,055992801	1
Huwe1	1,055992801	1
Rock1	1,055919608	1
Gm14698	1,05584642	1
Otub1	1,05584642	1
Gabpb2	1,05584642	1
Atraid	1,05584642	1
Ndufb11	1,055773237	1
Cisd3	1,055700059	1
Trp53bp2	1,055700059	1
Stat2	1,055700059	1
Gm3145	1,055553718	1
Zbtb5	1,055480555	1
Phkb	1,055480555	1
Arhgef2	1,055480555	1
Gm12017	1,055407397	1
Cox18	1,055334244	1
Rps3	1,055261097	1
Rps6ka1	1,055187954	1
Tfdp1	1,055041684	1
Tcea1-ps1	1,054968557	1
Psmd4	1,054968557	1
Nol12	1,054749205	1
Rarg	1,054676098	1
Mut	1,054602996	1
Rbm8a	1,054602996	1
Cklf	1,054529899	1
1700061G19Rik	1,054529899	1
Dip2a	1,054456807	1
Snora30	1,05438372	1
Dennd2c	1,05438372	1
Stk35	1,054310638	1
Gdi1	1,054237562	1
Gm14494	1,054091423	1
Als2cl	1,054018362	1
D3Ertd254e	1,054018362	1
Gm12466	1,053945305	1
Sep 06	1,053945305	1
Rrp36	1,053872254	1
Gm38104	1,053799208	1
Efcab2	1,053799208	1
Dtx3l	1,053726166	1
Gm42941	1,05365313	1
Gm12231	1,05365313	1
Ralgapb	1,053434052	1
Nelfe	1,053361036	1
Sf1	1,053215019	1
March11	1,053069023	1
Gm5262	1,052996032	1
Poc1a	1,052996032	1
Cdkl4	1,052996032	1
Wdr82	1,052996032	1

Nup133	1,052850066	1
Gm4879	1,052850066	1
Snx27	1,052850066	1
Fam118b	1,05277709	1
Ptpn4	1,052631155	1
Arhgef7	1,052631155	1
Pak2	1,052631155	1
1700007L15Rik	1,052558194	1
Abtb1	1,052558194	1
Map1s	1,052558194	1
Pan2	1,052485239	1
Arid4a	1,052266404	1
Zc3h14	1,052193469	1
Ehbp1	1,052047614	1
Ngly1	1,051901779	1
Pigk	1,051901779	1
Mrpl45	1,051901779	1
Pithd1	1,051828869	1
Pms1	1,051683065	1
Adal	1,051683065	1
Peli1	1,051537281	1
Zdhhc17	1,051391517	1
Alkbh7	1,051391517	1
Ufd1l	1,051245773	1
Mapk1ip1l	1,051245773	1
1810010D01Rik	1,051172909	1
Map3k20	1,05110005	1
Ankrd33b	1,051027196	1
Gm14593	1,051027196	1
Hyi	1,051027196	1
Hars	1,051027196	1
B230208H11Rik	1,050954347	1
Ap2a1	1,050954347	1
Zfp688	1,050954347	1
Stim1	1,050954347	1
RP23-403E19.1	1,050881502	1
Brms1	1,050881502	1
Olfr95	1,050735829	1
Pkm	1,050735829	1
Rpl35	1,050663001	1
Zbtb41	1,050663001	1
Ppp1r12a	1,050663001	1
Tmem206	1,050590177	1
Rpn2	1,050590177	1
Rfc5	1,050517358	1
1110008L16Rik	1,050444544	1
Lpar6	1,050371735	1
Stambpl1	1,050371735	1
Gm43153	1,050226133	1
Celf5	1,050226133	1
Gm6768	1,050080551	1
Ahcy	1,050080551	1

Tmem41a	1,050080551	1
Tmed1	1,049862215	1
Dpf2	1,049862215	1
Brpf1	1,049862215	1
Tctn2	1,049716684	1
Ube3b	1,049716684	1
Prps2	1,049716684	1
Slc25a3	1,049643925	1
Coq4	1,049571172	1
Vcp	1,049571172	1
Cmss1	1,049425681	1
Tcta	1,049425681	1
Tceal9	1,049352943	1
Snrk	1,049280209	1
BC030499	1,049207481	1
Ibtk	1,049207481	1
Zc3h12a	1,049134758	1
Tbl1x	1,04906204	1
App	1,048989328	1
Top3a	1,04891662	1
Gm44423	1,048843917	1
Map3k11	1,048771219	1
Bbx	1,048698526	1
Zfp943	1,048625839	1
Cmtr2	1,048625839	1
Heca	1,048625839	1
Ccp110	1,048553156	1
Itfg1	1,048553156	1
Slc48a1	1,048407806	1
Ncbp2	1,048262476	1
Birc6	1,048262476	1
Zmat2	1,048262476	1
BC003965	1,048189818	1
Dip2c	1,048044518	1
Hnrnpl	1,047971876	1
Uqcc2	1,047971876	1
Dvl1	1,047971876	1
Ehbp1l1	1,047899238	1
Haus1	1,047826606	1
Rin3	1,047753979	1
4930539J05Rik	1,047681357	1
Tjap1	1,047681357	1
Rnaseh2c	1,047608739	1
Sde2	1,047608739	1
Adsl	1,047536127	1
Zcchc10	1,047536127	1
Htr2b	1,04746352	1
Slc45a4	1,047390918	1
Ndufa13	1,047390918	1
Nit1	1,047173142	1
Uba1	1,047173142	1
AI314180	1,047173142	1

Gm13641	1,046955411	1
Scarb1	1,046882844	1
Mrpl43	1,046882844	1
Dennd6b	1,046810282	1
Atmin	1,046810282	1
Sord	1,046810282	1
Matr3-ps2	1,046665173	1
Gm15696	1,046665173	1
Sdad1	1,046592627	1
Fip1l1	1,046592627	1
1700088E04Rik	1,046520085	1
Gm14270	1,046447548	1
Gmeb1	1,046447548	1
Mon1a	1,046375016	1
Fgf11	1,046375016	1
Neurl4	1,046375016	1
Rpp14	1,046375016	1
Gm4895	1,04630249	1
Acaa1a	1,046157451	1
Nomo1	1,04608494	1
Pdzd11	1,046012433	1
Gpr89	1,046012433	1
Herc1	1,045867435	1
Cd36	1,045649976	1
Twsg1	1,045577499	1
Lamtor3	1,045432562	1
Tmem106b	1,045432562	1
Nt5dc1	1,0453601	1
Lrp10	1,0453601	1
Ccdc28a	1,045287644	1
Zc3h11a	1,045215193	1
Qrich1	1,045215193	1
Cyb5r3	1,045070305	1
Uqcrq	1,045070305	1
Oxa1l	1,044925438	1
Arid3a	1,044853011	1
Eapp	1,04478059	1
Gm14680	1,044708174	1
Qsox1	1,044635763	1
Arcn1	1,044563357	1
Pik3r6	1,04441856	1
Ppp1r8	1,04441856	1
Gstz1	1,044346168	1
Gpd2	1,044273782	1
S100a10	1,044129025	1
Ankib1	1,044056654	1
Twistnb	1,044056654	1
Colgalt1	1,044056654	1
Gm6733	1,043984288	1
5031434O11Rik	1,043911927	1
Usp15	1,043767221	1
Ptrhd1	1,043694875	1

Rpp25l	1,043694875	1
Map4	1,043694875	1
Gm12350	1,043622534	1
Mast3	1,043477867	1
Gm7535	1,043405541	1
Pds5b	1,043405541	1
Exosc2	1,043260904	1
Gm13373	1,043116288	1
Fbf1	1,043116288	1
Sec22b	1,043116288	1
Mks1	1,043043987	1
Pcnx3	1,043043987	1
Isy1	1,043043987	1
Setd7	1,042971691	1
Abhd10	1,0428994	1
Bysl	1,042754834	1
E130309D02Rik	1,042754834	1
Vps37c	1,042538022	1
Zdhhc13	1,042393505	1
Usp7	1,042393505	1
Pon2	1,042321254	1
Fbxw5	1,042249009	1
Rnf113a1	1,042176768	1
Arv1	1,042032302	1
Stxbp1	1,042032302	1
Klhl11	1,041887855	1
Svil	1,041887855	1
Camk2d	1,04181564	1
Psmd3	1,041743429	1
Phf21a	1,041671223	1
Smpd1	1,041599023	1
Haus8	1,041526827	1
E2f4	1,041454636	1
Mapk7	1,041454636	1
Golga5	1,041454636	1
Rbms2	1,041454636	1
Selenok	1,04131027	1
Utp6	1,04131027	1
Pnpt1	1,041165924	1
Tceanc	1,041093758	1
Man2b1	1,041093758	1
Cd81	1,041021598	1
Ubn2	1,040733005	1
Clcc1	1,040588739	1
Fundc1	1,040588739	1
C1rl	1,040444493	1
Hacd2	1,040444493	1
Prkab1	1,040372377	1
Clec16a	1,040300267	1
Wbp2	1,040300267	1
Mlec	1,040300267	1
Tm2d2	1,040228161	1

Ntmt1	1,04015606	1
Smg8	1,04015606	1
Uqcrc1	1,04015606	1
Mrps10	1,040083965	1
D930015E06Rik	1,040083965	1
1110008F13Rik	1,040083965	1
Cetn2	1,040083965	1
Yaf2	1,040011874	1
Pnp	1,039939788	1
Agpat5	1,039939788	1
Chchd1	1,039939788	1
Arsa	1,039795632	1
Rpia	1,039795632	1
Dad1	1,039795632	1
Scaper	1,039579435	1
Mrto4	1,039363283	1
Pofut2	1,039291243	1
Psmd5	1,039291243	1
Cdadc1	1,039219207	1
Wipi2	1,038859103	1
Gpbp1	1,038787098	1
Fam217b	1,038715097	1
Tmed9	1,038715097	1
Atp6v0a1	1,038643101	1
Fam13c	1,038499125	1
Emg1	1,038499125	1
Mrps17	1,038499125	1
Gm7936	1,038427144	1
Dtx3	1,038427144	1
Zcchc9	1,038427144	1
Pi4kb	1,038355168	1
Btbd3	1,038283197	1
Acads	1,038283197	1
Atxn7l1	1,038211231	1
Tox4	1,038211231	1
Chuk	1,037995364	1
Snx24	1,037923418	1
Surf2	1,037851477	1
Rpl31-ps16	1,037779541	1
B230312C02Rik	1,037779541	1
Dcun1d4	1,037635684	1
Fkbp4	1,037635684	1
Gm10863	1,037563763	1
Gm4875	1,037563763	1
Tnnc1	1,037563763	1
Klhl9	1,037491848	1
Ep300	1,037491848	1
Ngdn	1,037491848	1
Ddx39b	1,037419937	1
Cmtm7	1,03727613	1
Skiv2l2	1,037204234	1
Arl6ip5	1,037204234	1

Slc25a51	1,037204234	1
Esyt1	1,037132343	1
Sqrdl	1,037060457	1
Ralgds	1,037060457	1
2200002J24Rik	1,036844828	1
Ccdc92	1,036844828	1
Rasip1	1,036844828	1
Atf2	1,036844828	1
Ifrd1	1,036485547	1
Bad	1,036413706	1
Fam69a	1,03634187	1
Col20a1	1,03634187	1
Znrd1	1,03634187	1
Dnttip1	1,036270039	1
Thap4	1,036198213	1
Gnpda2	1,036126391	1
Rps15a-ps5	1,036054575	1
Ermp1	1,036054575	1
Terf2	1,036054575	1
Nanos1	1,035982764	1
Sesn2	1,035982764	1
Pcgf3	1,035982764	1
Edem1	1,035695568	1
Rwdd2a	1,035552	1
Ptges3l	1,035552	1
Rasal1	1,035480224	1
Psmg1	1,035480224	1
Hexim1	1,035336685	1
Atxn2l	1,035336685	1
Ell	1,035336685	1
Pvr	1,035264924	1
Ywhab	1,035264924	1
Adat1	1,035193167	1
Phlpp1	1,035193167	1
Ptpn6	1,035193167	1
Snx21	1,035121416	1
Pdp2	1,035121416	1
Opa1	1,035049669	1
Gm2756	1,034906191	1
Ndufb4	1,034906191	1
Gfm2	1,034906191	1
1700037H04Rik	1,034906191	1
Tapbpl	1,034834459	1
Aar2	1,034834459	1
Ndufa10	1,034762732	1
Gm8869	1,03469101	1
Tmem87a	1,03469101	1
Znrd1as	1,034547582	1
Trip6	1,034404173	1
Entpd7	1,034260784	1
Gm9169	1,034189097	1
Aldoart1	1,034189097	1

Vps4a	1,034189097	1
Rab33b	1,034117415	1
Rps5	1,034117415	1
Ston1	1,034045738	1
Acot8	1,034045738	1
Ireb2	1,034045738	1
4933408B17Rik	1,033902398	1
Pate2	1,033759079	1
Rel	1,033759079	1
Cfdp1	1,033687427	1
Ybx3	1,033544137	1
Actr1b	1,0334725	1
Swi5	1,033400868	1
Pxmp2	1,03332924	1
Thg1l	1,03332924	1
Jak3	1,033114388	1
Mettl3	1,033114388	1
Vkorc1l1	1,03304278	1
Sra1	1,03304278	1
Lysmd3	1,032971178	1
Gm13186	1,032971178	1
Mr1	1,032827987	1
Rrbp1	1,032827987	1
Cyth1	1,032684817	1
Pxmp4	1,032684817	1
Rpl4	1,032613239	1
Oraov1	1,032541666	1
Tap1	1,032470098	1
Taf6l	1,032326978	1
Mms19	1,032326978	1
Kdm5b	1,032255425	1
Cd68	1,032255425	1
Psmd7	1,032255425	1
Dctn3	1,032112334	1
Gm43737	1,032040795	1
Psma4	1,032040795	1
Nsun5	1,031969262	1
Rbbp4	1,031969262	1
Eif6	1,031897734	1
Zbtb42	1,031683179	1
Csf2ra	1,031611671	1
Hspd1-ps3	1,031611671	1
Osbpl1a	1,031540168	1
Ube2d3	1,031468669	1
Mir763	1,031397176	1
Nup35	1,031325687	1
Dcps	1,031325687	1
Fuca2	1,031182725	1
Gm6565	1,031111251	1
S100pbp	1,031111251	1
Acd	1,030968319	1
Ssr2	1,03089686	1

Pgrmc2	1,030753957	1
Cfap43	1,030682513	1
Ei24	1,030682513	1
Trappc1	1,030611074	1
n-R5-8s1	1,03053964	1
Cox17	1,030468211	1
Cpeb3	1,030396787	1
Zfp445	1,030396787	1
Arid1a	1,030253954	1
Deaf1	1,030182544	1
Gm10320	1,030182544	1
Lin7c	1,030182544	1
Gm11599	1,03011114	1
Chst3	1,03011114	1
Sgta	1,03011114	1
Vamp7	1,030039741	1
Pianp	1,030039741	1
Zfp131	1,030039741	1
Uqcr11	1,029896957	1
Eea1	1,029825572	1
Hdgf	1,029754193	1
Xiap	1,029468724	1
1700030K09Rik	1,029397369	1
Hspa5	1,029397369	1
Xylt2	1,029326019	1
Klhl20	1,029183334	1
Naa38	1,029183334	1
Gm17690	1,029040669	1
Jkamp	1,029040669	1
Slco4a1	1,029040669	1
Pde12	1,028969343	1
Gm38257	1,028826708	1
Rhox5	1,028826708	1
Mpg	1,028826708	1
Rhno1	1,028826708	1
Skap2	1,028826708	1
Pcyt2	1,028612792	1
Gm7488	1,028541496	1
Zfp865	1,028327639	1
Dmxl1	1,028327639	1
Ermap	1,028256363	1
Gm21975	1,028256363	1
Gm37780	1,028113827	1
Gm5244	1,028113827	1
Anapc2	1,028113827	1
Med9	1,028113827	1
Gm12606	1,028042566	1
Apobr	1,028042566	1
Pip5k1a	1,02797131	1
Rpl10a	1,027900059	1
Iffo2	1,027900059	1
Rap2c	1,027900059	1

A930004J17Rik	1,027828812	1
Prkrip1	1,027828812	1
Ltv1	1,027757571	1
Dyrk2	1,027686335	1
Thoc6	1,027615104	1
Usp31	1,027472656	1
0610012G03Rik	1,027187819	1
Ostf1	1,027116623	1
Taf13	1,026974244	1
Snrnp40	1,026974244	1
Uqcrfs1	1,026903062	1
Camsap2	1,026903062	1
Ccdc82	1,026760713	1
Gtf2f2	1,026760713	1
Gm5446	1,026689546	1
Rhog	1,026618383	1
Tnrc6a	1,026547226	1
Rack1	1,026547226	1
Mapre1	1,026547226	1
Hdac10	1,026476074	1
Gm11889	1,026333784	1
Sdhaf2	1,026333784	1
Rbm27	1,026333784	1
Strap	1,026333784	1
Rap1gds1	1,026191514	1
Mphosph8	1,026120386	1
Gm4430	1,025907032	1
Aamp	1,025907032	1
Akt2	1,025835924	1
Ccdc25	1,02562263	1
Hdgfrp2	1,02562263	1
Fam229b	1,025551542	1
Ddrgk1	1,025551542	1
Asl	1,02540938	1
Syf2	1,025338306	1
Lias	1,025338306	1
Sav1	1,025125116	1
Spidr	1,025054062	1
Tmem248	1,024911969	1
Glipr1	1,024911969	1
Cln8	1,02484093	1
Marc2	1,024698867	1
Snrnp27	1,024627842	1
Tut1	1,024485809	1
Usp6nl	1,024485809	1
Spire1	1,024414799	1
Rab18	1,024414799	1
Fdps	1,024343795	1
Smdt1	1,024272795	1
Ubap2l	1,0242018	1
Gm7730	1,02413081	1
Fbxw8	1,02413081	1

Clec4d	1,02413081	1
Tnni3	1,024059826	1
Tnfaip1	1,023988846	1
Nsun2	1,023917871	1
Cltc	1,0238469	1
Eme1	1,02363402	1
Arpc1a	1,02363402	1
Pex11a	1,02363402	1
Sugp2	1,02363402	1
Ccdc107	1,023563069	1
Ppp3r1	1,023492124	1
2810414N06Rik	1,023421183	1
Tmem101	1,023421183	1
Pik3cd	1,023421183	1
Klc3	1,023350247	1
Rab43	1,023350247	1
Dcbld2	1,023208391	1
Cnot6	1,023208391	1
Gm4880	1,023066554	1
Pdxdc1	1,022995643	1
Cdc40	1,022924736	1
Srrm1	1,022924736	1
Serf1	1,022853835	1
Orai1	1,022782939	1
Pnrc1	1,022712047	1
Zdhhc1	1,022570279	1
Tysnd1	1,022428531	1
Egfl8	1,022215945	1
Tdpx-ps1	1,022074245	1
Chchd2	1,022074245	1
Gm6851	1,022003403	1
Gpbp1l1	1,022003403	1
Tmem231	1,021861733	1
Gid4	1,021861733	1
Slc35e1	1,021790905	1
Anapc11	1,021790905	1
Isca1	1,021720083	1
Nrip1	1,021649265	1
Sumf1	1,021649265	1
Cuedc1	1,021578452	1
Fbxw4	1,021507644	1
Shcbp1l	1,021436841	1
Oxr1	1,021436841	1
Mbd5	1,021366043	1
Mcee	1,021366043	1
Hist1h2aa	1,02129525	1
Pdcd6	1,02129525	1
Gm45445	1,021224461	1
Mrpl14	1,021224461	1
Dynlt3	1,021224461	1
Irf9	1,021153678	1
Clta	1,021153678	1

Gm37566	1,021082899	1
Gtf2h1	1,020941357	1
Chchd3	1,020941357	1
Stard5	1,020870593	1
Nsmce2	1,020870593	1
Gm5805	1,020870593	1
B4galt7	1,02072908	1
Slc9a1	1,02072908	1
Pafah1b1	1,02072908	1
0610009B22Rik	1,020658331	1
Rbm26	1,020587587	1
Ankrd40	1,020516848	1
Parp9	1,020446113	1
Gm15920	1,020446113	1
Gm37357	1,02023394	1
Rad17	1,02023394	1
Arnt	1,02023394	1
Mycn	1,02002181	1
Mrps5	1,019880415	1
Ccdc88a	1,019809724	1
Grina	1,019809724	1
Ifitm2	1,019739039	1
Rrp15	1,019597683	1
Brd4	1,019597683	1
Rps12-ps24	1,019527012	1
Ankrd28	1,019456347	1
Tmem86a	1,019385686	1
Prune1	1,019244379	1
Dnali1	1,019173732	1
Llgl2	1,019103091	1
Rhbdd3	1,019103091	1
Arfgef1	1,019103091	1
Fam208b	1,018891197	1
Fam117a	1,018820575	1
Gtf3c2	1,018820575	1
Faap20	1,018749958	1
Abhd12	1,018749958	1
Rab23	1,018608739	1
Klrg2	1,018608739	1
Nmt1	1,018608739	1
Tmem176b	1,018538137	1
Pip4k2a	1,01846754	1
Gm44822	1,01832636	1
Erap1	1,018255777	1
Nfxl1	1,018255777	1
Mga	1,0181852	1
Khdrbs1	1,018044059	1
Rtca	1,018044059	1
Fubp3	1,017902938	1
Nr4a3	1,017832385	1
Nrde2	1,017832385	1
Stt3b	1,017832385	1

Fam173b	1,017761836	1
Wdr1	1,017761836	1
Mboat7	1,017691293	1
Casc3	1,017691293	1
Nudt5	1,017620755	1
Snhg17	1,017550221	1
Slmap	1,017550221	1
Mipol1	1,017479692	1
Zfp560	1,017409168	1
Mapk1	1,017409168	1
Nup155	1,017268135	1
Dhx35	1,017127122	1
Ccdc181	1,017127122	1
Rin2	1,017056622	1
Hnrnpa0	1,016986128	1
Akt1s1	1,016915638	1
Stam2	1,016915638	1
Spag9	1,016845153	1
9430038I01Rik	1,016633728	1
Htatip2	1,016633728	1
Yae1d1	1,016563263	1
Gpsm3	1,016563263	1
Agfg1	1,016563263	1
Gskip	1,016422347	1
Trrap	1,016422347	1
Irf2bp2	1,016422347	1
Zfp748	1,016351897	1
Ptpn22	1,016351897	1
Otulin	1,016351897	1
Stoml1	1,016281451	1
Pdha1	1,016281451	1
Celf2	1,016140574	1
Ppp2r5b	1,015788468	1
Fam53a	1,015788468	1
Atp23	1,015718061	1
Zwint	1,015718061	1
Dgcr6	1,015647659	1
4933434E20Rik	1,015647659	1
Ppm1a	1,015647659	1
Zfp800	1,015577262	1
Gsr	1,01550687	1
Cbx7	1,01550687	1
Ier3ip1	1,01550687	1
Etf1	1,01550687	1
Cdkn2aipnl	1,015436483	1
Camk1d	1,015366101	1
Tmem167b	1,015366101	1
Invs	1,015225351	1
Kmt5c	1,015225351	1
AU019823	1,015154983	1
Arap3	1,015154983	1
Sdc1	1,015154983	1

Zfp729a	1,015014263	1
Pot1a	1,015014263	1
Gm12183	1,01494391	1
Gm37503	1,014873562	1
Ankra2	1,014873562	1
Asrgl1	1,014803218	1
Zeb2	1,014803218	1
Dhrs3	1,01473288	1
Eny2	1,014662547	1
Yipf6	1,014592218	1
Acsl1	1,014521894	1
Ppm1k	1,014451575	1
Prr12	1,014381261	1
Gtf2e1	1,014381261	1
Pycrl	1,014381261	1
Sipa1l1	1,014310952	1
Lamtor1	1,014310952	1
Ankrd17	1,014310952	1
Rab11b	1,014240648	1
Cnst	1,014029765	1
Trappc10	1,01395948	1
Memo1	1,013818925	1
Npepl1	1,013818925	1
Xrcc1	1,013748655	1
Gm2895	1,013678389	1
Zfp277	1,013678389	1
Sympk	1,013678389	1
Nipsnap1	1,013678389	1
Ccs	1,013678389	1
Timm9	1,013608129	1
Tti1	1,013537874	1
Atp13a1	1,013537874	1
Znhit1	1,013537874	1
C2cd2l	1,013467623	1
Pitrm1	1,013397377	1
Prkce	1,013186669	1
Metap1	1,013186669	1
Slc31a2	1,013116443	1
Hint3	1,013116443	1
N4bp2l2	1,013116443	1
Lxn	1,012905793	1
Ppp4r2	1,012905793	1
Il15	1,012835586	1
Ptov1	1,012695187	1
Fam195a	1,012554807	1
Gm4799	1,012554807	1
Mkl1	1,012554807	1
Megf9	1,012414447	1
Havcr2	1,012344274	1
Ppat	1,012344274	1
Gipr	1,012274106	1
Rps2	1,012274106	1

Thop1	1,012274106	1
Wdr11	1,012274106	1
Gm12182	1,012133785	1
Dnmt1	1,012133785	1
Gm5050	1,011993483	1
Ak2	1,011993483	1
Parl	1,01192334	1
Thap3	1,01192334	1
Sec24b	1,011783067	1
Atf7ip	1,011783067	1
Chd9	1,011712938	1
Gls	1,011712938	1
Slc25a4	1,011642814	1
Nub1	1,011642814	1
Zfp868	1,011572695	1
Ldah	1,011572695	1
Lonp1	1,011572695	1
Sep 11	1,01150258	1
0610009L18Rik	1,011432471	1
Gtf2a1	1,011432471	1
Man1b1	1,011222171	1
Tmem263	1,011081996	1
3110043O21Rik	1,011081996	1
Zmiz2	1,011011915	1
C030015A19Rik	1,01094184	1
Zranb1	1,01094184	1
Sla2	1,010871769	1
Stx2	1,010801703	1
Pxn	1,010801703	1
Gm16200	1,010731642	1
Cyp51	1,010731642	1
Ppp2ca	1,010731642	1
Mea1	1,010661586	1
Srek1	1,010661586	1
Abce1	1,010661586	1
Gigyf2	1,010661586	1
Hk2	1,010591535	1
Man2b2	1,010521488	1
Sp3os	1,010521488	1
Sms	1,010521488	1
S100a1	1,010521488	1
Slc30a9	1,010521488	1
Zfp383	1,010451446	1
Reep4	1,010311378	1
Psma1	1,010241351	1
Tspan4	1,010171329	1
Mat2a	1,010171329	1
Adcy7	1,010101311	1
Ap3m2	1,009961291	1
Kdm4d	1,009891289	1
Irf5	1,009891289	1
Zfp384	1,009681309	1

Mzt2	1,009541348	1
Tsnax	1,009471374	1
Morc3	1,009471374	1
Bud31	1,009471374	1
Clcn6	1,009401405	1
Wrb	1,009401405	1
Rab9	1,009401405	1
Map4k1	1,009331441	1
Dnaaf2	1,009331441	1
Wrnip1	1,009261482	1
Asb13	1,009191528	1
Mkks	1,009191528	1
Gse1	1,009121578	1
Bid	1,009051634	1
Gm43137	1,008981694	1
Atox1	1,008911759	1
Zfp207	1,008841829	1
Hectd3	1,008771904	1
Myoz1	1,008701984	1
Rnf6	1,008701984	1
Got1	1,008701984	1
Psen1	1,008632068	1
Zmat5	1,008492252	1
Kdm6b	1,008492252	1
Cct8	1,008492252	1
Mrpl49	1,008422351	1
Api5	1,008422351	1
Mtmr14	1,008282564	1
Pycr2	1,008212677	1
Gm20703	1,008142796	1
Gdpd5	1,008072919	1
Gm16104	1,008003047	1
Brix1	1,00793318	1
Hoxb4	1,007863318	1
Psma6	1,007863318	1
Gm43792	1,007793461	1
Cmip	1,007793461	1
Tec	1,007723608	1
Fndc3b	1,007723608	1
Man2c1	1,00765376	1
Sp100	1,00765376	1
Papola	1,00765376	1
Lsm12	1,007374418	1
Bmp2k	1,007304595	1
Chsy1	1,007304595	1
Pggt1b	1,007234776	1
Prr14	1,007234776	1
Ebpl	1,007234776	1
Firre	1,007095153	1
Trim32	1,007095153	1
Tbrg4	1,007095153	1
Sfr1	1,007095153	1

6030460B20Rik	1,007025349	1
Ptprs	1,007025349	1
Senp6	1,007025349	1
Slc35b1	1,006885756	1
Wdr25	1,006746181	1
Rce1	1,006746181	1
Bmi1	1,006746181	1
Bzw2	1,006746181	1
Cnot1	1,006676401	1
Rps12-ps19	1,006536856	1
Stard9	1,006536856	1
Gpx1	1,006536856	1
4933421O10Rik	1,006467091	1
Anapc5	1,006467091	1
Kansl1l	1,00639733	1
Coasy	1,00639733	1
Ptpre	1,006327574	1
Mafg	1,006327574	1
Hcn2	1,006188077	1
Cryzl1	1,006188077	1
Xrcc4	1,006118336	1
Gm8770	1,0060486	1
B230398E01Rik	1,005909142	1
Dnaja2	1,005909142	1
Tesk2	1,00569999	1
Evl	1,00542119	1
Rnf139	1,005351502	1
Wwp2	1,005351502	1
Klf16	1,005281818	1
Cxxc1	1,005281818	1
Cdc37	1,00521214	1
Gm11249	1,005142466	1
Bag1	1,005072798	1
Stx4a	1,005072798	1
Wnk1	1,005003134	1
Plcd3	1,004933475	1
Rb1	1,00486382	1
Zdhhc16	1,004794171	1
Syngr1	1,004794171	1
Ube2z	1,004654887	1
Serpinf1	1,004585252	1
Adarb1	1,004585252	1
Rpap1	1,004585252	1
Rnf10	1,004515622	1
Gtpbp4	1,004515622	1
Clint1	1,004445996	1
Tmem39a	1,004376376	1
Ube2e1	1,004376376	1
Hspb6	1,00430676	1
Actr3	1,004167543	1
Asah1	1,003958754	1
Ppp2r5a	1,003958754	1

Cda	1,003889167	1
Mical3	1,003889167	1
Fkbp14	1,003819586	1
Cnot8	1,003680436	1
Rpl13a	1,003610869	1
Gpr157	1,003541306	1
Tmem131	1,003471749	1
Gm10250	1,003402196	1
Dhx15	1,003402196	1
Cstf2	1,003332647	1
Clcn7	1,003263104	1
Plod1	1,003263104	1
Cd5l	1,003193566	1
Prpf40b	1,003193566	1
Slc39a6	1,003193566	1
Naa60	1,003124032	1
Rogdi	1,003054503	1
Rbpsuh-rs3	1,002984979	1
Phc1	1,00291546	1
Trp53rka	1,00291546	1
Mrpl55	1,002845945	1
Tirap	1,002776436	1
Snx11	1,002776436	1
Gnl1	1,002776436	1
Ctsa	1,002706931	1
Ppih	1,002498446	1
D330023K18Rik	1,00235948	1
Rasa1	1,00235948	1
RP24-389J11.1	1,002290004	1
Cnot9	1,002220533	1
Cstad	1,002081605	1
Dstyk	1,002012148	1
Slc25a13	1,002012148	1
Rps25-ps1	1,002012148	1
Fmc1	1,00173437	1
9430034N14Rik	1,001664938	1
Sult6b1	1,001526087	1
Peg13	1,001526087	1
Rab3d	1,001526087	1
Epb41l2	1,001526087	1
Fktn	1,001456669	1
Hsd17b12	1,001456669	1
Ctsb	1,001387256	1
Med27	1,001179045	1
Kmt5a	1,001179045	1
Rell1	1,001040261	1
Ppil2	1,001040261	1
Retn	1,000970877	1
Slc25a19	1,000832123	1
Orc2	1,000832123	1
Atp5j	1,000762753	1
Fitm2	1,000554672	1

Kbtbd7	1,000415975	1
Rabepk	1,000415975	1
Rpl23a-ps14	1,000346634	1
Nbeal2	1,000346634	1
Ctso	1,000346634	1
Rbm7	1,000346634	1
Nmt2	1,000277297	1
Msmo1	1,000277297	1
Zfp157	1,000138639	1
Uxs1	1,000138639	1
Gm42418	1	1
Tox2	-0,00014732	1
Ppard	-0,00010846	1
Atp9b	-6,23E-05	1
Fam199x	-0,00010561	1
Sil1	-0,00043825	1
Txnl1	-0,00047411	1
Tmem91	-0,00067774	1
Gm44250	-0,00083405	1
Eef2	-0,00087172	1
2410089E03Rik	-0,0010449	1
Naa30	-0,0011412	1
Washc1	-0,0013428	1
Arhgap12	-0,0012812	1
Lcorl	-0,0013157	1
Bckdha	-0,0014688	1
Phf23	-0,0015095	1
Zfp668	-0,0015568	1
BC031181	-0,0015701	1
Il27	-0,0017864	1
Sirt3	-0,0019697	1
Atg2b	-0,0020695	1
Gm10059	-0,0021547	1
Hcfc1	-0,0021572	1
R3hcc1	-0,0022619	1
Wars	-0,0022912	1
Cenpc1	-0,0023584	1
Pdcd2	-0,0024106	1
Tyrobp	-0,002407	1
Vrk2	-0,0025801	1
Gm44791	-0,0029072	1
Tmc6	-0,0029119	1
Rrs1	-0,0031317	1
Gm43609	-0,0032874	1
Gm43111	-0,0033029	1
Gm7899	-0,0033973	1
Ocel1	-0,0033789	1
Vamp8	-0,0034409	1
CAAA01180111.2	-0,0035976	1
Amhr2	-0,0035702	1
Dnajb14	-0,0036318	1
Serpinb6a	-0,0036458	1

Gm5963	-0,0035767	1
D730045B01Rik	-0,0036573	1
Mtx3	-0,0037383	1
Shc1	-0,0036895	1
Acp6	-0,0036716	1
Ccdc59	-0,0037303	1
BC002163	-0,0038826	1
Acbd6	-0,0039489	1
Fbxo34	-0,0040747	1
Lsg1	-0,0042205	1
Ube2l6	-0,0045051	1
Gga3	-0,0044742	1
Sfi1	-0,0045574	1
Pigv	-0,0046266	1
Chpf	-0,0046023	1
Thoc5	-0,0046342	1
Zfp526	-0,0047216	1
Suclg2	-0,0046515	1
Ddost	-0,0046836	1
Zfp644	-0,0049131	1
B9d1	-0,0050598	1
Ncoa5	-0,0050616	1
Emp3	-0,0054543	1
3110080O07Rik	-0,0057869	1
Gucy2g	-0,00582	1
Nod2	-0,005844	1
Ric1	-0,0058066	1
Cept1	-0,0057902	1
Gm17430	-0,0058667	1
Mpzl1	-0,0058507	1
Cyb5b	-0,0059467	1
Ube2j2	-0,0059297	1
Otud6b	-0,0060449	1
Zfp598	-0,0059695	1
Maml1	-0,0060759	1
Cmc2	-0,0060848	1
Ddx39	-0,0061089	1
Lzts2	-0,0061022	1
2610507B11Rik	-0,0062425	1
Pum2	-0,0063338	1
Psmc1	-0,0063275	1
Srgap2	-0,0062913	1
Ccrl2	-0,0064351	1
Idh3b	-0,0064253	1
Akr1e1	-0,0066061	1
Cops8	-0,0065788	1
Gm20628	-0,0069215	1
Gm12854	-0,0069198	1
Arhgef10l	-0,006926	1
Ik	-0,0068944	1
Gm11687	-0,0071356	1
Acot7	-0,0071896	1

BC017643	-0,0072843	1
Eef1b2	-0,0074097	1
Aff4	-0,007643	1
Fgfbp3	-0,0077523	1
Snx16	-0,0077556	1
Selenoo	-0,0079241	1
Commd6	-0,0079069	1
Lgr5	-0,0080798	1
Nek1	-0,0080792	1
Gm11956	-0,0081906	1
Gm24601	-0,0081957	1
Nfix	-0,0081983	1
Nfat5	-0,0082071	1
Phrf1	-0,0083264	1
Strn4	-0,0084721	1
Lrig2	-0,008588	1
Mrps14	-0,008627	1
Gm28875	-0,00869	1
Gm37063	-0,0089181	1
Tigd2	-0,0089325	1
Rapgef6	-0,0089637	1
Itgb5	-0,0091647	1
Tmem110	-0,0092323	1
Scaf1	-0,0094979	1
Tspo	-0,0096259	1
Ganc	-0,0096903	1
Mief2	-0,0097013	1
Tfpi	-0,0097421	1
Uqcr10	-0,0098208	1
Exosc8	-0,0098706	1
Dph3	-0,0098762	1
AI846148	-0,01002	1
Ube2m	-0,010005	1
RP24-547N4.5	-0,010094	1
Map1lc3b	-0,010095	1
Chd8	-0,010095	1
Shoc2	-0,010234	1
Manba	-0,010251	1
AB124611	-0,010343	1
Usp39	-0,010517	1
Nbn	-0,01058	1
Tbc1d1	-0,010564	1
Pnpo	-0,010715	1
Fam114a2	-0,010788	1
Phf12	-0,011035	1
Emc8	-0,011122	1
Abhd17b	-0,011123	1
Cstb	-0,011145	1
Gm17786	-0,011234	1
Rpl8	-0,011182	1
BC048403	-0,011401	1
Prelid3a	-0,011434	1

Dcaf7	-0,011417	1
Gm14017	-0,011806	1
Gm26912	-0,011847	1
A930005H10Rik	-0,011808	1
Ankrd11	-0,012277	1
Map3k15	-0,012464	1
Gm37033	-0,012492	1
Lims1	-0,012461	1
Ccm2	-0,012515	1
Hipk1	-0,012649	1
BC029722	-0,012684	1
Echs1	-0,012806	1
AW549877	-0,012808	1
Mrps26	-0,012833	1
Rhot1	-0,012994	1
Lcmt1	-0,013086	1
Zfp874a	-0,013174	1
Polr2g	-0,013323	1
Tnpo2	-0,013366	1
Smc1a	-0,013358	1
Ap1g1	-0,013362	1
Nolc1	-0,013485	1
Pigyl	-0,013638	1
Plod3	-0,013609	1
Plaa	-0,013688	1
Gm13204	-0,013821	1
Dsel	-0,01378	1
Irak1bp1	-0,013837	1
Rrn3	-0,013754	1
Tmem70	-0,013909	1
Ncor1	-0,014028	1
Fem1b	-0,014274	1
Sec14l1	-0,014371	1
Pebp1	-0,014355	1
Pnpla6	-0,0145	1
Kdm1a	-0,014529	1
Rai1	-0,014711	1
Zfp821	-0,014726	1
Sgms1	-0,014834	1
Sart1	-0,014778	1
Afdn	-0,014811	1
Acbd3	-0,014848	1
Dgcr14	-0,014991	1
Stxbp4	-0,015096	1
RP23-444K20.4	-0,015059	1
Srsf7	-0,015188	1
March7	-0,015168	1
Zfp799	-0,015696	1
Ppp1ca	-0,015707	1
Luzp1	-0,015701	1
Cep57	-0,015655	1
Eml4	-0,015821	1

Stc1	-0,015927	1
Rae1	-0,015887	1
Chpf2	-0,016096	1
Atf6b	-0,016137	1
Uty	-0,0162	1
Eci2	-0,016168	1
Ldlr	-0,016343	1
Gm15946	-0,016455	1
Soat1	-0,016538	1
Cox6a1	-0,016518	1
Wdr45b	-0,016725	1
Clip1	-0,016837	1
Gmds	-0,016922	1
Gm38125	-0,017116	1
Gm37660	-0,017224	1
Nostrin	-0,017185	1
Mars2	-0,017292	1
Isyna1	-0,017545	1
Tssc1	-0,017547	1
Rab40c	-0,017529	1
Scyl2	-0,017672	1
Cep170b	-0,01769	1
Mlx	-0,017824	1
Klc1	-0,017839	1
Gm7847	-0,017903	1
Pop1	-0,017864	1
Hdac6	-0,01791	1
2810403D21Rik	-0,017871	1
Scnm1	-0,017901	1
Mroh1	-0,017932	1
Tgif1	-0,018162	1
Ube3c	-0,018232	1
Ccdc112	-0,018262	1
Atp1b3	-0,018313	1
Gm37274	-0,01856	1
Hook2	-0,018606	1
Gm2058	-0,018726	1
Atad2	-0,018716	1
Naca	-0,018792	1
Mcoln1	-0,018905	1
Zfand6	-0,018869	1
Cd37	-0,018952	1
Med14	-0,019053	1
Dnajc4	-0,019081	1
Gm6493	-0,019184	1
Zmym6	-0,019264	1
Tagap1	-0,019287	1
Tbce	-0,0194	1
Isg15	-0,019542	1
Ttll4	-0,019527	1
Wdr91	-0,019509	1
Siah1b	-0,019747	1

Ldlrap1	-0,019731	1
Mecp2	-0,0198	1
Traf3	-0,020045	1
Brd9	-0,020022	1
D17Wsu92e	-0,019985	1
Polrmt	-0,020126	1
Cabin1	-0,020052	1
Stk10	-0,020224	1
Gfm1	-0,020439	1
Nup37	-0,020625	1
Gm10012	-0,020762	1
Ribc1	-0,020876	1
Ap3s1	-0,021086	1
Pdcl	-0,021097	1
Sprtn	-0,021248	1
Ankrd39	-0,021264	1
Cars2	-0,021392	1
Rnf34	-0,021402	1
Lsm6	-0,021475	1
Fsd1l	-0,021605	1
Tln2	-0,021737	1
Dcaf15	-0,021735	1
Ccdc51	-0,022049	1
Stard8	-0,022074	1
Pabpc1	-0,022107	1
Ift74	-0,022166	1
Telo2	-0,022174	1
Cxx1a	-0,02227	1
Jtb	-0,02263	1
Slc22a17	-0,022699	1
Galm	-0,022703	1
Cox7a2	-0,02281	1
Manf	-0,022892	1
Ptpmt1	-0,022982	1
Ap4e1	-0,023113	1
Zfp961	-0,023311	1
Gsg2	-0,023423	1
Gm42483	-0,023645	1
Capn2	-0,023569	1
Aatf	-0,023741	1
Slirp	-0,023715	1
Nle1	-0,023836	1
Acsl3	-0,02381	1
Luc7l2	-0,023821	1
Gpalpp1	-0,023887	1
Zfp275	-0,023851	1
Phospho2	-0,023992	1
Gm11633	-0,024134	1
Lemd2	-0,024077	1
Npm3-ps1	-0,024299	1
Gm45109	-0,024359	1
Chmp1a	-0,024574	1

Fam105a	-0,024768	1
Nemf	-0,024802	1
Ogfod2	-0,024875	1
Fam195b	-0,024903	1
Itsn2	-0,024894	1
Gm45454	-0,025039	1
Brca2	-0,024973	1
Fkbp8	-0,024987	1
Shisa5	-0,025299	1
Tuba1a	-0,02537	1
Cyp4v3	-0,02562	1
Zc3h10	-0,025577	1
Gpaa1	-0,025621	1
Mfsd7b	-0,025683	1
Bcl2l2	-0,025814	1
Ralbp1	-0,025807	1
Gm16433	-0,026003	1
Asna1	-0,025975	1
Gm15265	-0,026073	1
Pcmtd2	-0,026118	1
Trappc8	-0,026164	1
Ubr3	-0,02621	1
Rhbdf1	-0,026341	1
Usp42	-0,026262	1
Med15	-0,026486	1
Wwp1	-0,026524	1
Necap2	-0,026497	1
4931406C07Rik	-0,026593	1
Snrpa1	-0,026723	1
Zc3h13	-0,02668	1
Ifnar2	-0,026692	1
Ikzf1	-0,026814	1
Sbno1	-0,026845	1
Ambra1	-0,026988	1
Ddi2	-0,026974	1
Uggt1	-0,027198	1
Cd101	-0,027282	1
Dusp11	-0,0273	1
2010015M23Rik	-0,027354	1
F730043M19Rik	-0,027434	1
Chmp2a	-0,027503	1
Tubb6	-0,027506	1
Efcab14	-0,027491	1
Prmt1	-0,027579	1
Akap8l	-0,027768	1
Rplp1	-0,027845	1
Qtrt1	-0,027908	1
Furin	-0,027927	1
Banf1	-0,027984	1
Zfyve21	-0,028259	1
Pan3	-0,028426	1
Snhg5	-0,028605	1

Gm5422	-0,028669	1
Brms1l	-0,028813	1
Smn1	-0,028828	1
Zfp948	-0,028884	1
1810043G02Rik	-0,028937	1
Elmo1	-0,028942	1
Zfp511	-0,029023	1
Gm6322	-0,029094	1
Cox8a	-0,029194	1
Taf10	-0,029151	1
Fmnl1	-0,029288	1
Pex2	-0,029373	1
Slc37a1	-0,029429	1
Lfng	-0,029523	1
Rbm34	-0,029523	1
Pdap1	-0,029503	1
Ubqln1	-0,029481	1
Gm16253	-0,029649	1
Vrk1	-0,029571	1
Polk	-0,029681	1
Junos	-0,029816	1
Gm11977	-0,029819	1
Pole3	-0,029766	1
Dnm1	-0,029905	1
Casp7	-0,029924	1
St8sia4	-0,029885	1
Pcnx	-0,029913	1
Rnf41	-0,029958	1
Mrps15	-0,030071	1
Aldh6a1	-0,030173	1
Gm44667	-0,030607	1
Cecr5	-0,030629	1
Cln6	-0,030586	1
Vamp5	-0,030844	1
Tmem160	-0,030841	1
Gm45413	-0,030857	1
Fgd3	-0,030876	1
Dhx36	-0,0309	1
Doc2g	-0,031112	1
Dock7	-0,031211	1
Taf1	-0,031281	1
Odf3l1	-0,031409	1
Osgep	-0,031395	1
Fpgt	-0,031483	1
Tsc22d1	-0,031545	1
Gm10762	-0,031925	1
Iqgap1	-0,031943	1
Dhrs7b	-0,032126	1
Rnf32	-0,032213	1
H2afj	-0,032198	1
A130071D04Rik	-0,032332	1
Vps35	-0,032273	1

Slc35a4	-0,032446	1
Fam193b	-0,032372	1
Zmat3	-0,032433	1
Smad4	-0,032382	1
Ptpn18	-0,03238	1
Riiad1	-0,032584	1
Rnft2	-0,032664	1
Med10	-0,032707	1
Eif3l	-0,032762	1
Gm6501	-0,033015	1
Epb41l5	-0,032971	1
Mid1ip1	-0,033003	1
Eid1	-0,033053	1
Ndrg3	-0,033153	1
Cnbp	-0,033155	1
Urgcp	-0,033861	1
Fbxo45	-0,033912	1
Nhej1	-0,034039	1
Coq6	-0,034012	1
Rmnd5b	-0,033973	1
Man2c1os	-0,034189	1
Toporsos	-0,034233	1
Lig4	-0,034349	1
1110051M20Rik	-0,034402	1
Prkar2a	-0,034579	1
Plek	-0,034568	1
Fam185a	-0,034808	1
E4f1	-0,034925	1
Ybey	-0,034999	1
Rbm17	-0,035	1
Sep 09	-0,035283	1
Ddx3x	-0,035283	1
Pgghg	-0,035539	1
Fchsd1	-0,035545	1
Eif4e2	-0,035476	1
Ncoa2	-0,03595	1
B3gnt3	-0,036128	1
Sertad2	-0,036233	1
Far1	-0,036199	1
Mrm1	-0,036282	1
Tbl2	-0,036365	1
Bod1	-0,036398	1
Rufy3	-0,036358	1
Snx9	-0,036455	1
Larp1	-0,036484	1
Surf1	-0,036476	1
Thumpd3	-0,036566	1
Mtx1	-0,036716	1
Zfp787	-0,036747	1
Rbm22	-0,036736	1
Gm42937	-0,03679	1
Rwdd1	-0,036825	1

Lpgat1	-0,03679	1
Cox5a	-0,036791	1
Lekr1	-0,036922	1
Cdc25a	-0,03695	1
Tbc1d10a	-0,037014	1
Sep 08	-0,037005	1
Ubr7	-0,037045	1
Tsen54	-0,03744	1
Fxyd5	-0,037397	1
Parp10	-0,037509	1
Slc7a5	-0,037531	1
Pdk1	-0,037901	1
Fam63b	-0,038128	1
Cd99l2	-0,038224	1
Papss1	-0,038346	1
Ifi30	-0,038283	1
Ndufb7	-0,038415	1
Gm4673	-0,038511	1
Gm5697	-0,038511	1
Baiap2	-0,038548	1
Nras	-0,038535	1
Ubb	-0,038693	1
Actr5	-0,038679	1
Plppr2	-0,038752	1
Idua	-0,03879	1
Pten	-0,038874	1
Aacs	-0,038974	1
Gm42482	-0,038989	1
Fam193a	-0,039051	1
Agap3	-0,039233	1
Arpc4	-0,039332	1
Usp33	-0,039444	1
Cx3cr1	-0,039773	1
Slc22a21	-0,039884	1
Vps13a	-0,040019	1
Psmb7	-0,04009	1
Foxn3	-0,040103	1
Dlgap4	-0,040053	1
Sar1b	-0,040215	1
Slc19a1	-0,040315	1
Anp32b	-0,040687	1
Plekhm2	-0,040674	1
Helz2	-0,040823	1
Slc25a2	-0,040911	1
Rrad	-0,040957	1
Pirb	-0,041022	1
Psme2b	-0,041045	1
Gtf3c1	-0,041201	1
Tti2	-0,041381	1
Selenoh	-0,041742	1
Kcnk6	-0,041777	1
Zkscan8	-0,041752	1

Atp5d	-0,041785	1
Sirt4	-0,042013	1
Arid5b	-0,041978	1
Pgap1	-0,041963	1
Bahd1	-0,042069	1
Tmppe	-0,042212	1
Fam53b	-0,042187	1
Pik3cb	-0,042317	1
Gm12933	-0,0425	1
Sh3bgrl3	-0,042514	1
Cd48	-0,042632	1
Rabac1	-0,042635	1
Gm6921	-0,042941	1
Adam17	-0,042861	1
Dus2	-0,043045	1
Ddx49	-0,042968	1
Dstn	-0,043034	1
Exoc6b	-0,043128	1
Slc35a3	-0,043112	1
Aimp1	-0,043105	1
9230116N13Rik	-0,043158	1
Ifitm6	-0,04323	1
Asnsd1	-0,043191	1
Rubcnl	-0,043438	1
Snx14	-0,043355	1
Mbnl1	-0,043377	1
Zfp580	-0,043454	1
Tpm4	-0,043541	1
Gm14584	-0,043679	1
Gm18867	-0,043682	1
Lst1	-0,043669	1
Sys1	-0,043923	1
Chil6	-0,043994	1
Cmtm3	-0,043961	1
Zbtb14	-0,044029	1
Atp2b1	-0,044043	1
Noxo1	-0,044145	1
Psmd12	-0,044106	1
Polr1c	-0,044156	1
Trappc2l	-0,044229	1
Ndufa7	-0,044559	1
Neu1	-0,044673	1
Prkra	-0,044654	1
Adk	-0,045029	1
Lsm11	-0,045023	1
Tex2	-0,045343	1
Prdx5	-0,045282	1
Utp15	-0,045277	1
Eed	-0,045391	1
Sh3bp2	-0,045461	1
Rps10-ps4	-0,045602	1
Man2a1	-0,045621	1

Gmcl1	-0,045647	1
Srsf3	-0,045731	1
Dusp16	-0,045822	1
Trub1	-0,045839	1
Zkscan3	-0,045797	1
Reep3	-0,045859	1
D330050G23Rik	-0,045992	1
Gm6444	-0,045978	1
Nr4a2	-0,046004	1
Tfip11	-0,046191	1
Kremen1	-0,046321	1
Card11	-0,046361	1
Hnrnpul2	-0,046582	1
Tspan17	-0,046712	1
Ubl3	-0,04674	1
Fpgs	-0,046804	1
Mark3	-0,046766	1
Top3b	-0,046895	1
Hint1	-0,046936	1
Sod2	-0,046872	1
Sun1	-0,047003	1
Rpa1	-0,046993	1
Dopey1	-0,047194	1
Nicn1	-0,047153	1
Trnt1	-0,047269	1
Tmcc1	-0,047377	1
Plekha3	-0,047459	1
Gm15782	-0,047555	1
Kansl3	-0,047604	1
Lpin1	-0,04763	1
Ipo5	-0,047628	1
Nln	-0,047706	1
Socs5	-0,047816	1
Rhof	-0,047914	1
Gm12020	-0,048019	1
Usp14	-0,048078	1
Gm2214	-0,048158	1
Tacc2	-0,048165	1
Etaa1	-0,048415	1
Mvb12a	-0,048359	1
Rubcn	-0,048528	1
Homer1	-0,048576	1
Spata5l1	-0,048661	1
Zfp142	-0,048705	1
Flot2	-0,048656	1
Prdx2	-0,048765	1
Vps13b	-0,048891	1
Tbc1d12	-0,048959	1
Dguok	-0,048973	1
Rcor2	-0,049222	1
Cd72	-0,049185	1
Cep104	-0,049276	1

Cbx8	-0,049638	1
Slc44a1	-0,049597	1
Cdk11b	-0,049592	1
Ube2a	-0,049724	1
Gm28151	-0,049828	1
Ube2e3	-0,049872	1
Nr1h3	-0,049955	1
Gm37494	-0,050154	1
Icmt	-0,050239	1
Zyg11b	-0,050182	1
Ranbp6	-0,050314	1
A930001C03Rik	-0,050408	1
Rpgrip1	-0,050408	1
Tmem258	-0,050365	1
Smim14	-0,050487	1
Apobec1	-0,05068	1
Apbb2	-0,050659	1
Bri3	-0,05079	1
Tcte2	-0,050911	1
Ndufaf3	-0,050894	1
Stk38	-0,050947	1
Epsti1	-0,051193	1
Adnp2	-0,051192	1
Sgpp1	-0,051215	1
Matn1	-0,051444	1
Hells	-0,051355	1
Bivm	-0,051496	1
Fam160a2	-0,051519	1
Gchfr	-0,051642	1
Tmem222	-0,051666	1
Ccdc62	-0,051757	1
Med19	-0,051785	1
Magi1	-0,052009	1
Cxcr4	-0,052238	1
Gm9294	-0,052318	1
Gm37349	-0,052291	1
Mylip	-0,052322	1
Zfpm1	-0,052395	1
Coq10b	-0,052437	1
Tmem50a	-0,052427	1
Alg13	-0,052504	1
Tm9sf2	-0,052548	1
Hus1	-0,05264	1
Fam216a	-0,052777	1
Rnf14	-0,052985	1
Zc3hc1	-0,052992	1
Emc6	-0,053135	1
Trnau1ap	-0,053192	1
Fahd1	-0,053237	1
Unc50	-0,053323	1
Slc35a5	-0,053446	1
Angel1	-0,053447	1

4930568A12Rik	-0,053642	1
Mrpl47	-0,05355	1
Abhd17a	-0,053705	1
Atg16l2	-0,05374	1
Mrpl20	-0,053838	1
Amz2	-0,0538	1
Gm16096	-0,053892	1
Bahcc1	-0,053907	1
Mttp	-0,054118	1
Herc4	-0,054052	1
Rad1	-0,054416	1
Gm25596	-0,054717	1
Cep131	-0,054722	1
Ctdnep1	-0,054752	1
Cysltr1	-0,05495	1
Lage3	-0,054939	1
Mkrn1	-0,054943	1
Gm12517	-0,055223	1
Suz12	-0,055151	1
Fam58b	-0,05535	1
Lacc1	-0,055394	1
Kmt2d	-0,055535	1
Fam78a	-0,055609	1
Snapc2	-0,055709	1
Wdr46	-0,055721	1
Cntrl	-0,055856	1
Commd8	-0,055942	1
Cct2	-0,055876	1
Moap1	-0,056037	1
Dolk	-0,056024	1
Gm17066	-0,056019	1
Prps1	-0,056039	1
Txn2	-0,056026	1
Mospd2	-0,056519	1
Ccl25	-0,056487	1
Reep5	-0,05647	1
Orc6	-0,056715	1
Phtf1	-0,056742	1
Pepd	-0,056949	1
Mipep	-0,056954	1
Bola3	-0,057087	1
Cbr1	-0,05717	1
Mat2b	-0,057363	1
Zfp609	-0,057418	1
Kpna1	-0,057443	1
Uqcrb	-0,057428	1
1110020A21Rik	-0,05764	1
Sh2b3	-0,057595	1
Itgb2	-0,057647	1
Capns1	-0,057829	1
Gm32340	-0,058104	1
Ankrd27	-0,058139	1

Cetn3	-0,058313	1
Gtpbp1	-0,058485	1
Atg5	-0,058499	1
Hipk3	-0,058672	1
Gm20432	-0,058836	1
Phf2	-0,05883	1
Rsl1d1	-0,058896	1
Serinc1	-0,058976	1
Grwd1	-0,059093	1
Gm13803	-0,059256	1
Erp44	-0,05937	1
Nsmce3	-0,059548	1
Rbmx2	-0,059468	1
Ttf1	-0,059587	1
Ino80e	-0,059697	1
Osbp	-0,059829	1
Tgs1	-0,059755	1
Ptp4a3	-0,059781	1
Fcer1g	-0,059821	1
Gm8724	-0,059864	1
Junb	-0,059923	1
Atp5sl	-0,059879	1
Atpif1	-0,059866	1
Dpm2	-0,059984	1
Cox7b	-0,060022	1
Lyrm9	-0,060447	1
Rbm28	-0,060407	1
Gm42467	-0,060609	1
Ppp1r15b	-0,060689	1
Rdh11	-0,060659	1
Zfr2	-0,06086	1
Cdc73	-0,060967	1
Uros	-0,061172	1
Tmem202	-0,061196	1
Ghdc	-0,061172	1
Farsa	-0,061157	1
Oxnad1	-0,061335	1
Amd1	-0,061372	1
AI661453	-0,061646	1
3110040N11Rik	-0,06163	1
Abhd18	-0,06204	1
Tmem218	-0,062017	1
H2-DMa	-0,062001	1
Rars	-0,061951	1
Sf3b5	-0,06203	1
Ptpn9	-0,062107	1
Tnip2	-0,062229	1
Copb1	-0,062256	1
Zfp954	-0,062333	1
Agk	-0,062409	1
Gm10138	-0,062502	1
Mta3	-0,062614	1

Ero1lb	-0,062614	1
Ptbp1	-0,062597	1
Snord55	-0,062706	1
Snora57	-0,062792	1
Pkp4	-0,062758	1
Fcf1	-0,062809	1
Cpox	-0,062759	1
Pdia3	-0,062769	1
Exd2	-0,062957	1
Bst1	-0,063033	1
Gm6368	-0,063307	1
Ubl4a	-0,063337	1
Foxj3	-0,06334	1
Ints1	-0,063448	1
Tmem219	-0,063427	1
Gm37788	-0,063494	1
Itpk1	-0,063461	1
Ctcf	-0,063517	1
Slc31a1	-0,063527	1
Ypel5	-0,063595	1
Trmt44	-0,064016	1
Hcfc2	-0,06402	1
Rps15a-ps3	-0,064143	1
Eloa	-0,064067	1
Gm44935	-0,064236	1
Katnal1	-0,064154	1
Ano6	-0,064176	1
Gm2950	-0,064319	1
Borcs8	-0,064259	1
Sh3pxd2b	-0,064383	1
Tsen34	-0,06472	1
Prkd3	-0,064701	1
Med28	-0,064689	1
Gm45422	-0,064796	1
Cask	-0,064815	1
1810014B01Rik	-0,064886	1
Gfer	-0,064914	1
Igsf8	-0,064912	1
Ndufb3	-0,065032	1
2610008E11Rik	-0,065083	1
Ddx6	-0,065175	1
Mrnip	-0,065267	1
Gm9761	-0,065362	1
Micu3	-0,065423	1
Myo9a	-0,065443	1
Gm14769	-0,065469	1
Ube4a	-0,065479	1
Ikbip	-0,065461	1
Rbm12b1	-0,065571	1
Pinx1	-0,065701	1
Psma3	-0,066076	1
Sat2	-0,066151	1

Sufu	-0,06619	1
Sep 10	-0,066328	1
Cd164	-0,066501	1
Gm7676	-0,06662	1
Chd4	-0,066805	1
Aqp11	-0,0669	1
Ipo9	-0,066881	1
Smndc1	-0,066978	1
Gyg	-0,067046	1
Ebi3	-0,067102	1
Rfc1	-0,067198	1
Nono	-0,067287	1
Hnrnpdl	-0,067331	1
Tmem203	-0,067457	1
Tcea1	-0,067627	1
Smu1	-0,067583	1
Psmf1	-0,067699	1
Cflar	-0,067695	1
Smad5	-0,067775	1
Fuom	-0,067906	1
Hadh	-0,067882	1
Agfg2	-0,067893	1
Gm15846	-0,068152	1
Rps19-ps4	-0,068243	1
Gm15800	-0,06825	1
Ccny	-0,068213	1
Sec61g	-0,068331	1
Gm29340	-0,068292	1
Kdm5c	-0,06828	1
Supt16	-0,068302	1
Scarna9	-0,068369	1
Meaf6	-0,068407	1
Cox4i1	-0,068585	1
Gemin2	-0,068741	1
Cuta	-0,068651	1
Dhx37	-0,068873	1
Malat1	-0,068885	1
Gm10499	-0,068988	1
Gm9794	-0,069018	1
Hmg20a	-0,069203	1
Gcnt1	-0,069378	1
Appbp2	-0,0695	1
Rcc1	-0,069479	1
Fez2	-0,069644	1
Mbd2	-0,069576	1
Lrig3	-0,069663	1
Mettl14	-0,069685	1
Setd3	-0,069689	1
Cstf1	-0,069834	1
Pgls	-0,069798	1
Hint2	-0,069852	1
Mettl5	-0,069851	1

Zfp64	-0,069974	1
Mrpl52	-0,070157	1
Cebpzos	-0,070271	1
Tmed4	-0,070301	1
Usp47	-0,070271	1
Mob3c	-0,070438	1
Slc25a24	-0,070513	1
Adipor2	-0,070451	1
Fbxo30	-0,070619	1
Tomm20	-0,07074	1
Rbm39	-0,070923	1
Xdh	-0,07095	1
Ndufc2	-0,070967	1
Zdhhc21	-0,071018	1
Kmt2a	-0,071036	1
Cct4	-0,071039	1
Nfrkb	-0,071137	1
Arf4	-0,071065	1
Mrs2	-0,071396	1
Fyn	-0,071368	1
Gm4737	-0,071602	1
Tecpr2	-0,071576	1
Bnip1	-0,071613	1
Hk1	-0,071622	1
Yme1l1	-0,071663	1
Esd	-0,071666	1
Fbxw2	-0,071834	1
Gm15420	-0,071873	1
Sgk3	-0,072033	1
Nelfb	-0,071977	1
Idnk	-0,072142	1
Edc3	-0,072086	1
1700066M21Rik	-0,072241	1
Leprot	-0,072202	1
Maea	-0,072293	1
Mark4	-0,072364	1
Nfkbid	-0,072517	1
Ccdc61	-0,072567	1
Gkap1	-0,072797	1
C1galt1	-0,072767	1
Ccdc77	-0,072798	1
Apaf1	-0,072841	1
Gna11	-0,07315	1
Pgm5	-0,073232	1
Map1lc3a	-0,073219	1
Vps50	-0,073253	1
Gatc	-0,073492	1
Nmral1	-0,073602	1
Bin2	-0,073776	1
Uqcc3	-0,073808	1
Fam49b	-0,073844	1
Smg6	-0,073858	1

Samd4b	-0,073974	1
Capza1	-0,074203	1
5031425F14Rik	-0,074333	1
Chmp2b	-0,074253	1
Senp8	-0,074364	1
Card14	-0,074691	1
Mtx2	-0,07469	1
Snord15a	-0,075028	1
Casp2	-0,075065	1
Mdm2	-0,075086	1
Tpd52	-0,075107	1
Dnajb2	-0,075232	1
Dus4l	-0,075319	1
Oxsr1	-0,07535	1
Atxn2	-0,07539	1
Gm13350	-0,07556	1
Meg3	-0,075701	1
Ssh2	-0,075752	1
Zfas1	-0,075754	1
Pgm1	-0,076216	1
Hpf1	-0,076286	1
Gm7308	-0,076372	1
Pja2	-0,076448	1
Ints4	-0,076469	1
Itch	-0,076491	1
Ogfr	-0,076492	1
Aff1	-0,076633	1
Gm43707	-0,076745	1
Kif18a	-0,077035	1
Ube2q2	-0,077045	1
1110004E09Rik	-0,076962	1
Pak1	-0,077101	1
Gm2a	-0,077106	1
Tle3	-0,077208	1
Extl3	-0,077371	1
Fcgr3	-0,077385	1
5430405H02Rik	-0,077514	1
Ercc3	-0,07749	1
Gm6159	-0,077597	1
Ankrd44	-0,07783	1
Cdc5l	-0,077768	1
Pkp2	-0,077898	1
D130019J16Rik	-0,078028	1
Ndufa2	-0,077958	1
Ccpg1os	-0,07817	1
Ssbp1	-0,078336	1
Mrpl1	-0,078268	1
Vps54	-0,078364	1
Slc45a3	-0,078426	1
Mettl21b	-0,078505	1
Lca5	-0,07859	1
Arid4b	-0,078583	1

Sgtb	-0,078731	1
Fam98b	-0,078693	1
Tssc4	-0,078663	1
4931406P16Rik	-0,078838	1
Pold2	-0,078881	1
Thra	-0,078852	1
Cops4	-0,078963	1
Chmp4b	-0,079075	1
Lrp6	-0,079326	1
Wsb1	-0,079334	1
Ddx19a	-0,079271	1
Klc2	-0,079449	1
Papolg	-0,079383	1
Psmc6	-0,079358	1
Zfp397	-0,079479	1
Crnkl1	-0,079482	1
Hnrnpll	-0,079531	1
Hscb	-0,079496	1
Actl6a	-0,079502	1
Arhgap45	-0,079535	1
Peak1os	-0,079577	1
Grk5	-0,079646	1
Hibadh	-0,079601	1
Gabarapl1	-0,079562	1
Sh3gl1	-0,079676	1
Mtfmt	-0,079662	1
Ppic	-0,079719	1
Mtor	-0,079939	1
Echdc3	-0,080059	1
Jarid2	-0,080633	1
Apoa1bp	-0,080645	1
Dusp14	-0,080661	1
Chtf8	-0,080656	1
Cers2	-0,080674	1
Fam102b	-0,080841	1
Dnajc21	-0,080795	1
Xpc	-0,080904	1
Zfp160	-0,080996	1
Abi2	-0,081095	1
Ddx3y	-0,081124	1
Gk	-0,081247	1
Gm42639	-0,081268	1
Mob3a	-0,081257	1
Avpi1	-0,081251	1
Hnrnpk	-0,081302	1
Zfyve16	-0,081368	1
Usp36	-0,081657	1
Timm17a	-0,08183	1
Tada1	-0,08187	1
Stat1	-0,082016	1
Amz1	-0,082009	1
Tmem144	-0,08231	1

Tgfbr1	-0,08227	1
Klhl6	-0,082321	1
Ndufa8	-0,082344	1
Spire2	-0,082517	1
Klhl18	-0,082728	1
Mettl13	-0,08267	1
Eif2b2	-0,082936	1
Fbxo22	-0,082991	1
Trim36	-0,083209	1
Anxa6	-0,083273	1
Usp19	-0,083344	1
Mrpl32	-0,083349	1
Msl1	-0,083323	1
St3gal1	-0,083414	1
Dazap2	-0,083389	1
Rpl36a-ps1	-0,08365	1
Pibf1	-0,083747	1
Cbx3	-0,083757	1
Celsr3	-0,083931	1
Gm12981	-0,08392	1
Gm13835	-0,08397	1
Tsr3	-0,083975	1
Morc2a	-0,08452	1
Slc25a36	-0,084465	1
Plgrkt	-0,08478	1
Mlf2	-0,084841	1
Map3k5	-0,084803	1
Tsc22d4	-0,084811	1
Csnk1e	-0,084772	1
2310035C23Rik	-0,08501	1
Mtch1	-0,084995	1
Aprt	-0,085032	1
Erc1	-0,085542	1
Gm5093	-0,085762	1
Itpka	-0,085838	1
Zfp148	-0,085782	1
Phb2	-0,085767	1
9330111N05Rik	-0,085905	1
Gnl3l	-0,085882	1
Bckdhb	-0,085995	1
Mapk1ip1	-0,086191	1
E030030I06Rik	-0,086342	1
Gm45420	-0,086273	1
Wdr76	-0,086263	1
Naa50	-0,086404	1
Atp5s	-0,086543	1
Ndufaf4	-0,086554	1
Urb2	-0,086723	1
Gramd1a	-0,086693	1
Taok2	-0,086912	1
Ywhaz	-0,086867	1
Ndufb10	-0,087113	1

Sp1	-0,087227	1
Ap1s2	-0,087449	1
Phf19	-0,0875	1
Stap1	-0,087512	1
Tbccd1	-0,087581	1
Zxdc	-0,087626	1
Tshz1	-0,087634	1
Smpd4	-0,087677	1
D330041H03Rik	-0,08793	1
Cdk8	-0,088002	1
Srpr	-0,088012	1
Traf1	-0,088106	1
Il6ra	-0,088053	1
Prr3	-0,088216	1
Dhrs4	-0,088217	1
Tspan31	-0,088158	1
Brd3	-0,088389	1
Rab20	-0,088492	1
Ccdc9	-0,088471	1
Ctdspl2	-0,088575	1
Socs2	-0,088701	1
Mapk8	-0,08871	1
Cdc34	-0,088774	1
Eif4a3	-0,088753	1
Rpl31-ps1	-0,088919	1
Arhgap23	-0,088914	1
Zbtb46	-0,088865	1
Trip13	-0,089233	1
Gm12421	-0,089319	1
Polr2i	-0,089251	1
Mir5136	-0,089463	1
Samhd1	-0,089719	1
Pgm3	-0,08984	1
Tsen15	-0,089918	1
Wdr89	-0,090043	1
Kptn	-0,090134	1
Rnf4	-0,090183	1
Gm43148	-0,090262	1
2310009B15Rik	-0,090324	1
Gm6543	-0,090378	1
Prpf38b	-0,090499	1
Cnp	-0,090677	1
Lrrc58	-0,090736	1
Blmh	-0,090725	1
Ggcx	-0,090945	1
Jpx	-0,091014	1
Cript	-0,091008	1
Vta1	-0,090963	1
Tnpo3	-0,091276	1
Ppp2r5e	-0,091421	1
Shpk	-0,091484	1
Drg1	-0,091606	1

Gm7384	-0,091737	1
Pfkl	-0,091665	1
Flt3l	-0,091711	1
Fam178a	-0,091659	1
Nbr1	-0,091791	1
Gm10237	-0,091861	1
Hmbs	-0,091984	1
Vgll4	-0,092348	1
Atg13	-0,092354	1
Pax6	-0,092464	1
Phkg2	-0,092548	1
Rrp1b	-0,092775	1
Tmed2	-0,09307	1
Eif1ax	-0,093126	1
Gtf2h4	-0,093335	1
Pdik1l	-0,093331	1
Aamdc	-0,09344	1
Atg101	-0,09336	1
Pno1	-0,093541	1
Repin1	-0,093562	1
Cfap74	-0,093824	1
Phf5a	-0,093758	1
Ifngr1	-0,093891	1
Pik3r1	-0,093854	1
Ecsit	-0,093968	1
Utp14b	-0,094176	1
Slc35b2	-0,094258	1
Fbxw17	-0,094434	1
Upf3b	-0,094439	1
Gm12583	-0,094523	1
2610318N02Rik	-0,094451	1
Tor1a	-0,0945	1
Gm12902	-0,094583	1
Tom1l1	-0,094611	1
Mrps21	-0,094758	1
Atp5a1	-0,094762	1
Gm4784	-0,094883	1
Lmtk2	-0,094979	1
Armc7	-0,095321	1
Sntb2	-0,095603	1
Rpl17-ps10	-0,095723	1
Kdm3b	-0,095691	1
Jade1	-0,095738	1
Lsm5	-0,095766	1
C1galt1c1	-0,095794	1
Scai	-0,095917	1
Lrrc42	-0,0959	1
Gm14673	-0,096036	1
Tbc1d30	-0,095982	1
Sart3	-0,095965	1
Clk2	-0,096105	1
Cggbp1	-0,0961	1

Zbtb49	-0,096205	1
Pigx	-0,096208	1
Ndufab1	-0,096542	1
Rnf217	-0,096601	1
Bcl7a	-0,096577	1
Ciapin1	-0,096651	1
Agmo	-0,09682	1
Zmym3	-0,096837	1
2610021A01Rik	-0,096916	1
Uspl1	-0,096887	1
E130317F20Rik	-0,097022	1
Dhfr	-0,096965	1
Nsd2	-0,097135	1
Mff	-0,097165	1
1700047K16Rik	-0,097375	1
Gde1	-0,097406	1
Dact3	-0,097548	1
Arsk	-0,09764	1
Cdk5r1	-0,097574	1
Bax	-0,09758	1
Kxd1	-0,097565	1
Aes	-0,097656	1
Mob4	-0,097725	1
Tgfbr2	-0,097762	1
Mdp1	-0,097801	1
Nipa2	-0,097942	1
Caprin1	-0,097913	1
Rpl14	-0,097873	1
Gm11353	-0,098127	1
Prkdc	-0,098307	1
Pyroxd2	-0,098422	1
Zmym5	-0,098399	1
Selenot	-0,098502	1
Clcnkb	-0,098554	1
Gm43149	-0,098709	1
Rcsd1	-0,098669	1
Tomm22	-0,09871	1
Gm10335	-0,098854	1
Ryr1	-0,09885	1
4930526A20Rik	-0,099044	1
Irf1	-0,099022	1
Cbr2	-0,099112	1
Urb1	-0,099165	1
Cops2	-0,099181	1
Fam149b	-0,099447	1
Pet100	-0,099722	1
Rida	-0,099825	1
Srrd	-0,0998	1
Fto	-0,099773	1
3110070M22Rik	-0,099873	1
Dpy19l1	-0,099985	1
Dpm3	-0,10009	1

Atp5h	-0,10019	1
Fxr2	-0,10044	1
Prcc	-0,1005	1
Tmem135	-0,10053	1
Syt8	-0,10057	1
Hoxc4	-0,10084	1
Prrc2b	-0,10084	1
Elf1	-0,10105	1
Mapk8ip3	-0,10095	1
Trpm7	-0,10103	1
Dusp7	-0,10113	1
Bpgm	-0,10123	1
Dtx2	-0,1013	1
Emc2	-0,10134	1
Gm5865	-0,10137	1
Brwd1	-0,10141	1
Zzz3	-0,10151	1
Mtmr3	-0,10171	1
Ap2s1	-0,10183	1
Ajuba	-0,10194	1
Gm43712	-0,10203	1
Arrdc1	-0,10242	1
Stbd1	-0,10278	1
Ndufv3	-0,10278	1
Cdo1	-0,10287	1
Ttc7b	-0,10294	1
Zcchc8	-0,10291	1
Bcap31	-0,10286	1
Slc2a3	-0,10323	1
Gm12222	-0,10333	1
Zfp7	-0,10335	1
Gm15327	-0,10354	1
Nemp2	-0,10358	1
Leo1	-0,10359	1
Rufy2	-0,10363	1
Rps24-ps2	-0,10369	1
Purb	-0,10373	1
Dhx40	-0,10381	1
Gcsh	-0,10377	1
Sgf29	-0,10379	1
Chd2	-0,10382	1
Foxk1	-0,10423	1
Zmym4	-0,1042	1
Fopnl	-0,10431	1
Nup85	-0,1044	1
Ppig	-0,10435	1
Cdipt	-0,10449	1
Gm11451	-0,10484	1
Mnd1	-0,10505	1
Wdr5	-0,10519	1
Thyn1	-0,10528	1
Gm5297	-0,10545	1

Dpy30	-0,10561	1
Gm5910	-0,10576	1
Gm14277	-0,10581	1
Lsm1	-0,10587	1
Dpp7	-0,10595	1
Fam188a	-0,10587	1
RP24-232D3.1	-0,10621	1
Gtf2a2	-0,1062	1
Gm10073	-0,10621	1
Dnpep	-0,10623	1
Mphosph6	-0,10623	1
Trappc2	-0,10632	1
Nktr	-0,10634	1
Snrnp48	-0,10634	1
Aplp1	-0,10655	1
Gin1	-0,10663	1
Gm12816	-0,10666	1
Wdr55	-0,10667	1
Bcat2	-0,10682	1
Hmgn1	-0,10691	1
Zfp597	-0,10708	1
Inppl1	-0,10716	1
Tmx1	-0,10717	1
Gm6210	-0,1075	1
Gm14403	-0,10761	1
Prpf8	-0,1077	1
1700020I14Rik	-0,1077	1
Mrpl24	-0,10773	1
Gm45380	-0,10782	1
Nudt2	-0,1078	1
Smarca5	-0,1078	1
Ypel4	-0,10813	1
Gm43138	-0,10862	1
Gm10169	-0,10857	1
Pmpca	-0,10873	1
Gm9843	-0,10884	1
Zfp622	-0,1089	1
Ankrd16	-0,10904	1
Cep95	-0,10898	1
Cox6c	-0,10923	1
Spats1	-0,10952	1
Ndufaf8	-0,10948	1
Kri1	-0,10958	1
Trim23	-0,10972	1
D330045A20Rik	-0,10983	1
Gm16379	-0,10991	1
Alg12	-0,11001	1
Myd88	-0,11002	1
Crlf2	-0,11033	1
Ddhd2	-0,11028	1
Ift52	-0,11029	1
Gcn1l1	-0,11043	1

Fam173a	-0,11042	1
Mecr	-0,11054	1
Ppp2r1b	-0,1106	1
Gm43071	-0,1107	1
Gm13840	-0,11067	1
Pigf	-0,11087	1
H2-D1	-0,11106	1
Gm6418	-0,11135	1
Elof1	-0,11134	1
Gm3699	-0,11152	1
Gm13736	-0,11152	1
Zfp516	-0,11168	1
RP24-286J14.3	-0,11189	1
Gm13422	-0,11188	1
Mfsd12	-0,11193	1
Zfp740	-0,11201	1
Id3	-0,11212	1
Znhit2	-0,11223	1
Nisch	-0,11221	1
Ndufv2	-0,11219	1
Lamtor2	-0,11215	1
Ctnnbl1	-0,11231	1
Ap2a2	-0,11235	1
Rpl22	-0,11241	1
Atp6v1g1	-0,1129	1
Fam8a1	-0,11314	1
M1ap	-0,1132	1
RP23-13B8.12	-0,11334	1
Eif2s1	-0,11329	1
Med4	-0,11348	1
Zkscan14	-0,11365	1
Nfia	-0,11366	1
2610020H08Rik	-0,11381	1
Ly6g6d	-0,11388	1
Plcg2	-0,11394	1
Pacs2	-0,11388	1
Ino80	-0,11425	1
Il11ra1	-0,11444	1
Gm9143	-0,11449	1
Rmdn3	-0,11446	1
Tm2d1	-0,11466	1
Sestd1	-0,11493	1
AI662270	-0,11493	1
Unc45a	-0,115	1
Ewsr1	-0,11502	1
Slc25a17	-0,11498	1
Ckap4	-0,11514	1
Rad51d	-0,11523	1
Irf2	-0,11534	1
RP24-310D17.9	-0,11563	1
Nasp	-0,1156	1
Gm10177	-0,11582	1

L2hgdh	-0,11587	1
Acadl	-0,11592	1
Adgrl1	-0,11586	1
Rfx1	-0,11616	1
Gopc	-0,11626	1
Dctn2	-0,11641	1
Fgr	-0,11647	1
Hccs	-0,11653	1
Fyco1	-0,11662	1
Abl1	-0,11668	1
2810428I15Rik	-0,11693	1
Bloc1s6	-0,11701	1
Gnaq	-0,11711	1
Plekho2	-0,11717	1
Zfp414	-0,11731	1
Rdh5	-0,11742	1
Stag1	-0,11736	1
Tes	-0,11739	1
Dirc2	-0,11752	1
D6Ertd527e	-0,11762	1
Gm9703	-0,11772	1
Gm17150	-0,11768	1
Lrch3	-0,11765	1
Lrsam1	-0,11778	1
Mdm4-ps	-0,11778	1
P4hb	-0,11781	1
Ggh	-0,11793	1
Sf3b6	-0,11792	1
Gnal	-0,11798	1
Gm37116	-0,11806	1
Anapc1	-0,11816	1
Gm13776	-0,11833	1
Mknk2	-0,11825	1
B230118H07Rik	-0,11839	1
Bora	-0,1184	1
Cep76	-0,11853	1
Sema5a	-0,11854	1
Fh1	-0,11883	1
Renbp	-0,11875	1
Pmvk	-0,11883	1
Camk2n1	-0,11893	1
Rpl15-ps5	-0,11895	1
Pkd1	-0,11891	1
Fam219b	-0,11887	1
Tarbp1	-0,11896	1
1810022K09Rik	-0,11914	1
Ppme1	-0,11914	1
Tmbim6	-0,11927	1
Mnat1	-0,11929	1
Crry-ps	-0,11959	1
Pld4	-0,1196	1
Lrpap1	-0,11962	1

Actr2	-0,11972	1
Zdhhc8	-0,11976	1
Grpel2	-0,11981	1
Mycbp2	-0,12013	1
Ceacam16	-0,12021	1
Speer9-ps1	-0,12016	1
Timm13	-0,12021	1
Chaf1a	-0,1204	1
Uba2	-0,12044	1
Snrpa	-0,12065	1
Pus7	-0,12069	1
Ndufs6	-0,12097	1
Rnd1	-0,12108	1
Dcaf17	-0,12114	1
Tmco6	-0,12123	1
Ddit3	-0,1214	1
Hexb	-0,12138	1
Pcbp2	-0,12143	1
Psmd14	-0,12136	1
Lmbr1l	-0,12145	1
Apex1	-0,12159	1
Thap6	-0,12171	1
Lrrc40	-0,12165	1
Tmem165	-0,1219	1
Stard10	-0,12202	1
RP24-233B16.6	-0,12214	1
Gm45289	-0,12231	1
4930402H24Rik	-0,12231	1
Cry1	-0,12237	1
Polb	-0,12258	1
Dars	-0,12273	1
Plec	-0,12285	1
Abhd16a	-0,12303	1
Gpnmb	-0,12297	1
Dnajc3	-0,12307	1
Sf3b4	-0,12324	1
Baz1b	-0,12321	1
Ctsd	-0,12321	1
Luc7l	-0,12351	1
Kdm5a	-0,12362	1
Tufm	-0,12381	1
Setdb1	-0,12377	1
Pdhb	-0,12376	1
Akap1	-0,12386	1
Rab22a	-0,12392	1
Cul1	-0,12397	1
Mknk1	-0,12414	1
Ppp1r13l	-0,12434	1
Tmed10	-0,12435	1
Gm3695	-0,12436	1
Wrn	-0,1244	1
Lonrf1	-0,12459	1

Ebna1bp2	-0,12471	1
Frg1	-0,1249	1
Prmt6	-0,12497	1
Taf4	-0,12505	1
Nme1	-0,12508	1
Fam96a	-0,1252	1
Slc37a3	-0,12532	1
5031425E22Rik	-0,12528	1
Klhdc3	-0,12537	1
Cd14	-0,12535	1
Hdac2	-0,12546	1
Gm29650	-0,12561	1
Rfc4	-0,12592	1
Ubap1	-0,12585	1
Dgat1	-0,12596	1
Imp3	-0,12606	1
Vps72	-0,12631	1
Pde6g	-0,12644	1
Pabpc4	-0,12644	1
Pfdn1	-0,12647	1
Lrrfip2	-0,12648	1
Ankrd10	-0,1266	1
Rnd2	-0,12683	1
Cyp2c55	-0,127	1
Ankrd9	-0,12706	1
Gnb5	-0,1273	1
Eif1a	-0,12743	1
Emc10	-0,12754	1
Zfp593	-0,12757	1
Ccdc71l	-0,12773	1
Foxo3	-0,12773	1
Rsrc1	-0,12766	1
Brd8	-0,12783	1
Ubxn4	-0,12775	1
Stk40	-0,12788	1
Myo10	-0,12795	1
Gm29257	-0,12797	1
Ctbs	-0,128	1
Pank3	-0,12804	1
Nfam1	-0,12806	1
Kcnab2	-0,12841	1
Heatr5a	-0,12852	1
Zfyve27	-0,12853	1
Gm15690	-0,12892	1
Ccnd1	-0,12889	1
Mapk3	-0,1291	1
Nfya	-0,12934	1
Tmx3	-0,12935	1
Rab11a	-0,12928	1
Glrx2	-0,12936	1
Slc25a45	-0,12953	1
Golgb1	-0,12959	1

Ube2k	-0,1296	1
Glod4	-0,12964	1
Lmbrd1	-0,12975	1
Gm9920	-0,12989	1
Tango2	-0,12987	1
Bag3	-0,12989	1
Syncrip	-0,12992	1
Wdr92	-0,1301	1
Ttf2	-0,13012	1
Cr1l	-0,13014	1
Crygn	-0,13016	1
Ccnb1	-0,13021	1
Nova1	-0,13044	1
Aebp2	-0,13041	1
Smad7	-0,13068	1
Phpt1	-0,13076	1
Lin37	-0,13093	1
Gm43588	-0,13086	1
5430420F09Rik	-0,13101	1
Cspg5	-0,13113	1
Ticam2	-0,13111	1
Uap1	-0,13111	1
Gltscr1l	-0,13133	1
Hmg20b	-0,13127	1
Trappc4	-0,13151	1
Endod1	-0,13161	1
Slc25a32	-0,13194	1
Mrm3	-0,13202	1
Fam214a	-0,13206	1
Elovl1	-0,13207	1
Borcs6	-0,13212	1
Plagl2	-0,13217	1
Abhd8	-0,13242	1
Hmces	-0,13238	1
AI413582	-0,13265	1
Cs	-0,13265	1
Asxl2	-0,13289	1
Eps8	-0,133	1
C2cd5	-0,13305	1
Alkbh1	-0,13323	1
Ube2h	-0,13326	1
Saal1	-0,13341	1
Socs7	-0,13344	1
Rnf114	-0,1334	1
Cnep1r1	-0,13351	1
Rp2	-0,13347	1
Car5b	-0,13352	1
Adgb	-0,13359	1
Cntd1	-0,13373	1
Ogg1	-0,1337	1
Tmem234	-0,13385	1
Clk1	-0,13386	1

St13	-0,13413	1
Rpl5-ps1	-0,1342	1
Elmsan1	-0,13433	1
Fzr1	-0,1343	1
Smim7	-0,13432	1
Cnpy4	-0,13441	1
C330007P06Rik	-0,13451	1
Glb1l	-0,13472	1
Csgalnact2	-0,13482	1
Max	-0,13483	1
Slc17a9	-0,13488	1
Dlg4	-0,13488	1
Nfe2l2	-0,1349	1
Epm2a	-0,13503	1
Engase	-0,13496	1
Cnih4	-0,13507	1
Lats2	-0,13511	1
Pgam1	-0,13528	1
Alkbh8	-0,13531	1
Uap1l1	-0,13538	1
Gm8116	-0,13579	1
Mlh1	-0,13582	1
Acvr2b	-0,13584	1
Bbs4	-0,13634	1
Mad2l1bp	-0,13651	1
Ccnj	-0,13665	1
Ifrd2	-0,13663	1
Rab28	-0,13673	1
Gm12696	-0,13682	1
Aasdhppt	-0,13691	1
Tiam1	-0,13692	1
C030037D09Rik	-0,13721	1
E330020D12Rik	-0,13721	1
Cdyl	-0,13724	1
Tma16	-0,13735	1
Ahdc1	-0,13739	1
U2af1	-0,13742	1
Aph1b	-0,13742	1
Vmp1	-0,13745	1
Cuedc2	-0,13764	1
Akap8	-0,13759	1
Nr6a1	-0,13774	1
Washc4	-0,13766	1
Slc35e4	-0,13769	1
Smurf2	-0,13812	1
Gm7809	-0,13807	1
Mcfd2	-0,13815	1
Ano8	-0,13835	1
Gm11131	-0,13846	1
Gm6206	-0,13849	1
Caml	-0,13874	1
Ciao1	-0,13868	1

Abcg1	-0,1387	1
Bex3	-0,13871	1
Strada	-0,13877	1
Pdcd10	-0,13893	1
Gm36266	-0,139	1
Gm17491	-0,13907	1
Mvp	-0,13948	1
Tm2d3	-0,13964	1
Ssb	-0,13961	1
Zfp652	-0,13981	1
Tnks	-0,13987	1
Gm10093	-0,14001	1
Foxj2	-0,14012	1
Asb3	-0,14023	1
Kat2a	-0,14029	1
Gm43213	-0,14052	1
Clcn2	-0,14053	1
AI464131	-0,14064	1
4833420G17Rik	-0,14064	1
Gm10080	-0,1407	1
Taf1d	-0,14074	1
Tmem134	-0,14071	1
Ifi27l2a	-0,14079	1
Rnf181	-0,14082	1
Rcor1	-0,14078	1
Itpkc	-0,14095	1
Tmem115	-0,141	1
2210408I21Rik	-0,14127	1
Fbxo11	-0,14133	1
Ptp4a1	-0,14135	1
Prrg4	-0,14164	1
Gm9517	-0,14179	1
Tle1	-0,14177	1
Psmb8	-0,14185	1
Ptdss2	-0,14211	1
Eif4h	-0,1422	1
Gm42820	-0,14235	1
Taf11	-0,14227	1
Mettl6	-0,14237	1
Fam135a	-0,1426	1
Fbxw11	-0,14257	1
Rpl36al	-0,14302	1
Usp2	-0,14297	1
Iqcb1	-0,14309	1
Ccdc174	-0,14305	1
Smpdl3b	-0,14324	1
Mrfap1	-0,14329	1
Snhg3	-0,14352	1
Kat7	-0,14355	1
Hnrnpc	-0,14352	1
Slc4a7	-0,14393	1
Rac3	-0,14407	1

Abcb8	-0,14415	1
Ttc39b	-0,14416	1
Hist1h2ae	-0,14429	1
Tubgcp4	-0,14428	1
Bak1	-0,1444	1
Wnt6	-0,14451	1
Setd2	-0,14448	1
Appl2	-0,14454	1
Gm13612	-0,14462	1
Gm8659	-0,1447	1
Anxa4	-0,14477	1
Mbd1	-0,14485	1
Dynlrb1	-0,14498	1
Fign	-0,14514	1
Cdc27	-0,14523	1
Kcnq1ot1	-0,14536	1
Ints14	-0,14547	1
Gtf3c5	-0,14605	1
Arhgap5	-0,14596	1
Ssr4	-0,14628	1
Col18a1	-0,14626	1
Gm11110	-0,14703	1
Sipa1l3	-0,14707	1
Smap2	-0,14705	1
Casp4	-0,14744	1
AI467606	-0,14738	1
Gm37082	-0,14788	1
Tmem230	-0,14806	1
Purg	-0,14818	1
Smg7	-0,1483	1
Set	-0,14836	1
Gm4366	-0,1484	1
Dync1i2	-0,14844	1
Gm42666	-0,14856	1
Gpr146	-0,14855	1
Gpn3	-0,14875	1
Ndufa3	-0,14895	1
Elac1	-0,14906	1
Rpl10a-ps1	-0,14907	1
Gpsm2	-0,14917	1
Irgq	-0,14931	1
Ppp1r15a	-0,14942	1
Nup88	-0,14937	1
Eml2	-0,14943	1
Psma5	-0,14953	1
Gm5812	-0,14961	1
Gm9013	-0,14978	1
Fam53c	-0,1498	1
Zc3h18	-0,14983	1
Pcbd2	-0,1499	1
Herc3	-0,15004	1
Vdac3	-0,15002	1

Spopl	-0,15003	1
Fnip1	-0,14997	1
4833439L19Rik	-0,15013	1
Gm16380	-0,1502	1
Rbm12	-0,15023	1
Ntpcr	-0,15027	1
Atad3a	-0,15026	1
Commd2	-0,15037	1
Bola2	-0,15051	1
Rpl10a-ps2	-0,15061	1
Gm7079	-0,15071	1
Irf8	-0,15071	1
Pwwp2a	-0,15092	1
2610301B20Rik	-0,15095	1
Zfp595	-0,15113	1
Zbtb44	-0,15114	1
Rps14	-0,15119	1
Pdxk	-0,15127	1
Hdhd2	-0,15139	1
Setd4	-0,15138	1
Gm4540	-0,15145	1
Plekhg2	-0,15166	1
Ifi35	-0,15184	1
Prkcg	-0,15195	1
Rnf166	-0,152	1
Kat6b	-0,15206	1
Eif3i	-0,15207	1
Gm10313	-0,15241	1
Hadha	-0,15237	1
Rnf13	-0,15254	1
Cry2	-0,15273	1
Gm12912	-0,15276	1
Gm16201	-0,15277	1
Gm14586	-0,15285	1
Mblac1	-0,15298	1
Maip1	-0,15333	1
Rbm8a2	-0,15364	1
Lrrc1	-0,15391	1
Gm14822	-0,15402	1
Fndc3a	-0,15397	1
Brk1	-0,15397	1
Mrpl54	-0,15415	1
Immp1l	-0,15405	1
Rc3h2	-0,15418	1
Snrnp70	-0,15429	1
Prdx6	-0,15427	1
Ranbp10	-0,15436	1
Cep152	-0,15455	1
Eya3	-0,15462	1
Aip	-0,15465	1
Sbf2	-0,15481	1
Rpl26	-0,15493	1

Snx2	-0,15514	1
Mir142hg	-0,15522	1
Gm13487	-0,15549	1
Gm17018	-0,15552	1
Slc25a28	-0,15565	1
Abhd14b	-0,15576	1
Mre11a	-0,15577	1
Rpl31-ps14	-0,15592	1
Naa15	-0,15597	1
Trmt10c	-0,15607	1
A930015D03Rik	-0,15625	1
Tnnt1	-0,15632	1
Ddx19b	-0,15631	1
Glo1	-0,15638	1
Dennd5a	-0,15661	1
Tprgl	-0,15665	1
Hk3	-0,15675	1
Arl8b	-0,15682	1
Gm44771	-0,15707	1
Gna13	-0,15706	1
Prkag1	-0,1572	1
Cbl	-0,15736	1
Rpf1	-0,15744	1
Anxa7	-0,15749	1
Brd2	-0,15764	1
Fam111a	-0,15763	1
Mapk12	-0,15773	1
Pcdhb16	-0,15786	1
Gm37893	-0,15805	1
Mdh1	-0,15834	1
Nap1l4	-0,15839	1
Vdac2	-0,15843	1
Hist2h2be	-0,15852	1
Mfsd14b	-0,15846	1
Tmem184b	-0,15851	1
Myl12b	-0,15846	1
Mapk11	-0,15869	1
Lpxn	-0,15865	1
Gm20696	-0,15879	1
Rps25	-0,15902	1
Nr2f6	-0,15912	1
Cpped1	-0,15908	1
Anks3	-0,15927	1
Vma21	-0,15944	1
2010204K13Rik	-0,15955	1
Igf1	-0,15952	1
6330562C20Rik	-0,15994	1
Park7	-0,15988	1
Tmem229b	-0,16052	1
Fbxl18	-0,16045	1
Rbm33	-0,16047	1
Minpp1	-0,16062	1

Ndrg2	-0,16057	1
Calcrl	-0,16069	1
Hsd17b4	-0,16072	1
Rnf141	-0,16065	1
Tubg1	-0,16076	1
Clcn3	-0,16082	1
Fcho2	-0,16091	1
March5	-0,16086	1
Catsper2	-0,16104	1
Gm6265	-0,16097	1
Gm28686	-0,16107	1
Zbtb26	-0,16121	1
Maml3	-0,16122	1
Nae1	-0,16126	1
9130604C24Rik	-0,1614	1
Dusp19	-0,16138	1
Mirlet7b	-0,16146	1
Lrrc8c	-0,16155	1
Gm43062	-0,16158	1
Tsta3	-0,16163	1
Iws1	-0,16163	1
Gm42478	-0,16183	1
Scaf8	-0,16184	1
Mybbp1a	-0,1618	1
D1Ertd622e	-0,16194	1
Ptprj	-0,16212	1
Rabggtb	-0,16215	1
Atf1	-0,16219	1
Ice1	-0,16256	1
Srgn	-0,16267	1
Kdm2b	-0,16313	1
Kctd9	-0,16324	1
Nupl1	-0,16343	1
Snx1	-0,16343	1
Tcea2	-0,16353	1
Nfatc2	-0,16361	1
Srek1ip1	-0,16361	1
Angptl6	-0,1638	1
Ccdc106	-0,16386	1
Prkcsh	-0,16398	1
Olfr933	-0,16424	1
Atxn7	-0,16434	1
Sf3b3	-0,16429	1
Zw10	-0,1643	1
Dhx38	-0,16436	1
Rab27a	-0,16451	1
Eid3	-0,16461	1
Pex19	-0,16474	1
Rab8b	-0,16483	1
Tmem183a	-0,16482	1
Tesk1	-0,16487	1
Gm12230	-0,16512	1

Susd3	-0,16513	1
Cbll1	-0,16507	1
Sec62	-0,16529	1
Eif4a1	-0,16541	1
Exoc6	-0,16568	1
Cfl1	-0,16568	1
Alox5	-0,16574	1
Pnrc2	-0,16578	1
Tomm5	-0,16597	1
Impad1	-0,16596	1
Tipin	-0,16632	1
Vsig10	-0,16644	1
Atg3	-0,16643	1
Gm11598	-0,16648	1
Nudt19	-0,16653	1
Ift22	-0,16664	1
Pla2g15	-0,16673	1
Btg2	-0,16691	1
Dip2b	-0,16689	1
Arl2	-0,16703	1
Med13	-0,16706	1
Gm4342	-0,16716	1
Cenpp	-0,16718	1
Ppm1m	-0,16722	1
Slc20a1	-0,16738	1
Rtcb	-0,16736	1
Gnb4	-0,16766	1
Tmem60	-0,16766	1
Zfat	-0,16777	1
Zbtb33	-0,16802	1
Mrps25	-0,16798	1
Rnf138	-0,16796	1
Layn	-0,16813	1
Exosc3	-0,16817	1
Asb1	-0,16818	1
Gm16523	-0,16829	1
Praf2	-0,16829	1
Slx4ip	-0,16829	1
Cep97	-0,16836	1
Mutyh	-0,16862	1
Rsl24d1	-0,16857	1
Ppa1	-0,16864	1
Mrpl16	-0,16878	1
Gm9833	-0,16903	1
Nsmce4a	-0,169	1
Stx16	-0,16918	1
Tmem106a	-0,16931	1
Gm9835	-0,16946	1
Slc5a3	-0,16947	1
Nagk	-0,16962	1
Sltm	-0,16958	1
Gm7380	-0,16966	1

Nt5dc3	-0,16969	1
Fam175a	-0,16979	1
Urm1	-0,16981	1
Rps12	-0,17	1
Rmi2	-0,17008	1
Espl1	-0,17029	1
Bcl10	-0,17031	1
Supt4a	-0,17035	1
Mrpl18	-0,17063	1
2410131K14Rik	-0,17067	1
Psip1	-0,17065	1
Man2a2	-0,17084	1
Ppt1	-0,1708	1
Gpatch2	-0,17088	1
Trit1	-0,17102	1
Gfap	-0,17119	1
Atxn3	-0,17126	1
Gm11221	-0,17142	1
Uba3	-0,17146	1
Txn1	-0,17149	1
Tmem30a	-0,17155	1
Ganab	-0,17177	1
Zbtb40	-0,17196	1
Zfp410	-0,17198	1
Mrpl11	-0,17273	1
Plscr4	-0,17283	1
Cxcl16	-0,1729	1
Galnt11	-0,17354	1
Nacc1	-0,17348	1
Rbbp7	-0,17353	1
Gm8722	-0,17369	1
Ndufb6	-0,17383	1
Gm5944	-0,17391	1
Crbn	-0,17393	1
Rras	-0,17385	1
Mink1	-0,17405	1
Csnk2a1	-0,17396	1
Naa35	-0,17412	1
Recql5	-0,17428	1
Ppp1cb	-0,17467	1
Msn	-0,17467	1
BC029214	-0,1749	1
Prdx3	-0,17486	1
Psmb10	-0,17506	1
Stx6	-0,1752	1
Hoxc6	-0,17531	1
Tcf7l2	-0,17532	1
Nhp2	-0,17539	1
Sae1	-0,17548	1
Hcst	-0,1756	1
Gm42893	-0,17581	1
Rpsa-ps10	-0,17592	1

Rbm6-ps1	-0,176	1
Tubd1	-0,17606	1
Rpl31-ps17	-0,17617	1
Aasdh	-0,17648	1
Mphosph10	-0,17664	1
Gm43462	-0,17672	1
Spcs3	-0,1767	1
Gm7967	-0,17676	1
Arf5	-0,17695	1
Zfp873	-0,17711	1
Gm12834	-0,17711	1
Klhl24	-0,17706	1
Manea	-0,17723	1
Tpm3	-0,1772	1
Cspp1	-0,17733	1
Tmem29	-0,17738	1
Sugt1	-0,17742	1
Wdr18	-0,1775	1
Gm10557	-0,1776	1
Ift46	-0,1776	1
Klra2	-0,17765	1
Kazald1	-0,17785	1
Ptpro	-0,178	1
Tspan14	-0,17797	1
Glce	-0,17807	1
Nfyb	-0,17813	1
Lsm8	-0,1782	1
Patz1	-0,17824	1
Cdnf	-0,17826	1
Nfu1	-0,17832	1
Scoc	-0,1784	1
Ippk	-0,1785	1
Mdm4	-0,17871	1
Nsd3	-0,17878	1
Fxr1	-0,17882	1
Aplf	-0,17892	1
Azin2	-0,179	1
Akap10	-0,17909	1
Bcl9	-0,17937	1
Osbpl8	-0,1795	1
Rab6a	-0,17947	1
Samd9l	-0,17959	1
Eif2d	-0,17963	1
Tmem123	-0,17958	1
Nkiras1	-0,17972	1
Prosc	-0,17966	1
Xlr	-0,17983	1
Tcp11l2	-0,17994	1
Slc25a23	-0,18001	1
Msh6	-0,18005	1
Zfp292	-0,18017	1
Gm5786	-0,18031	1

Rsf1	-0,1803	1
Polr2c	-0,18048	1
Rest	-0,18048	1
Gatad2b	-0,1806	1
Wdr83	-0,18076	1
Fkbp5	-0,18079	1
Tle4	-0,18094	1
Fbxo42	-0,18118	1
Gm16973	-0,18128	1
Guf1	-0,1817	1
Arl5a	-0,18176	1
Gm43024	-0,18204	1
Mcmbp	-0,18209	1
Nxf1	-0,18243	1
Prickle3	-0,18254	1
Tubgcp2	-0,18252	1
Hn1	-0,18251	1
Edf1	-0,18251	1
Sirt2	-0,18264	1
Eef1e1	-0,18283	1
Nptn	-0,18277	1
Gm6863	-0,18288	1
Kctd20	-0,18299	1
Hsp90b1	-0,183	1
Rp9	-0,18318	1
Myef2	-0,18327	1
Ubl5	-0,1838	1
Ndufaf2	-0,18402	1
Tmem259	-0,18405	1
Srsf11	-0,18414	1
Acin1	-0,18416	1
Pign	-0,18426	1
Eif2s3y	-0,18428	1
Clk4	-0,18427	1
Rbm15	-0,18436	1
Efna2	-0,18467	1
Gm44609	-0,18484	1
Arl6ip1	-0,1848	1
Cab39	-0,18492	1
Rnpep	-0,18541	1
Dusp3	-0,18557	1
Ddhd1	-0,18581	1
Uhrf1bp1	-0,18595	1
Zswim6	-0,18606	1
Gm5745	-0,18619	1
2310022B05Rik	-0,18618	1
Fam168b	-0,18616	1
Ncaph	-0,1863	1
Zik1	-0,18648	1
Usp32	-0,1865	1
Cyth2	-0,18662	1
Vti1b	-0,18666	1

Ppp4r1l-ps	-0,18684	1
Gabarap	-0,18678	1
Stard4	-0,18691	1
Lrrc57	-0,18698	1
Kmt2c	-0,18696	1
Ece1	-0,18713	1
AC168977.1	-0,18713	1
Tnfrsf11a	-0,18711	1
Rps21	-0,18716	1
Zfp281	-0,18743	1
Zbed5	-0,18745	1
Bicd2	-0,18746	1
Gm5735	-0,18791	1
Armc8	-0,18791	1
Ssh3	-0,18792	1
Rprd1b	-0,188	1
Atad2b	-0,18799	1
Lmtk3	-0,1882	1
Bag6	-0,18821	1
BC055324	-0,18829	1
RP23-193N1.2	-0,18837	1
Gm44890	-0,18835	1
Vcl	-0,18843	1
Cenph	-0,18853	1
Gm14165	-0,18908	1
Kantr	-0,18919	1
Proser1	-0,18934	1
Qars	-0,1893	1
Pex26	-0,18939	1
Piezo1	-0,18937	1
Gm8129	-0,18981	1
Ints13	-0,1898	1
Vps26a	-0,18984	1
Irf7	-0,18989	1
Ppox	-0,18989	1
Gm11474	-0,18994	1
Marf1	-0,1899	1
Rpl17	-0,19	1
Tax1bp3	-0,19013	1
Gm4707	-0,19019	1
Gm6222	-0,19023	1
Ubald2	-0,19026	1
Mb21d2	-0,19051	1
2410015M20Rik	-0,19055	1
Rps8	-0,19048	1
Rab31	-0,19056	1
Gm12254	-0,19066	1
Mrpl33	-0,19072	1
Dcun1d3	-0,19096	1
Csnk1g3	-0,191	1
Fen1	-0,19122	1
Snapc3	-0,19117	1

Yy1	-0,19129	1
Rbbp8	-0,19158	1
Cenpx	-0,19179	1
Mgat4a	-0,19195	1
Cerkl	-0,19196	1
Cd320	-0,19201	1
Pdrg1	-0,192	1
Dnlz	-0,19212	1
Gm12543	-0,19218	1
Ppib	-0,1922	1
Sgk1	-0,19227	1
Ift57	-0,19234	1
Erbin	-0,19233	1
Zfp866	-0,19276	1
Nckap1	-0,19275	1
Trappc6b	-0,1928	1
Selenof	-0,1928	1
Zfp266	-0,19276	1
Cmpk1	-0,19277	1
Zfp639	-0,19283	1
Arhgef11	-0,19294	1
2700049A03Rik	-0,19292	1
Mitd1	-0,19295	1
Arih2	-0,19286	1
Trem2	-0,1929	1
Golm1	-0,19331	1
Upf2	-0,19327	1
Selenom	-0,19337	1
D530018E20Rik	-0,19353	1
Ddx50	-0,19364	1
Neurl3	-0,19375	1
Ncs1	-0,1938	1
Wdr37	-0,19391	1
Fsbp	-0,19403	1
Crcp	-0,19399	1
Tmlhe	-0,19423	1
Ascl2	-0,19431	1
Bola1	-0,19442	1
Tcn2	-0,19446	1
Ubfd1	-0,1946	1
Ankzf1	-0,19464	1
Gtf2f1	-0,19477	1
Rab5c	-0,19514	1
Mbip	-0,19515	1
Lysmd4	-0,1952	1
Dbi	-0,19528	1
Ctu2	-0,1955	1
Tfe3	-0,19577	1
Ap1ar	-0,19602	1
Rbms1	-0,19604	1
Inpp5f	-0,1961	1
Ckap5	-0,19653	1

Gm15151	-0,19673	1
Eef1g	-0,19674	1
Hmgcl	-0,19665	1
Cops9	-0,19675	1
Npl	-0,19686	1
Papd7	-0,19693	1
Rab1b	-0,19688	1
Med7	-0,19704	1
Dhdh	-0,19696	1
Nampt	-0,19774	1
Gm36963	-0,19776	1
Thumpd2	-0,198	1
Cap1	-0,19811	1
Stard3nl	-0,19817	1
Gm5867	-0,19835	1
Jmjd1c	-0,19844	1
Aplp2	-0,19843	1
Gm43300	-0,19874	1
Zbtb43	-0,19872	1
Zfp770	-0,19884	1
Gdpd3	-0,19879	1
Trpt1	-0,19893	1
Gm13864	-0,19906	1
1110006O24Rik	-0,19914	1
Meis2	-0,19905	1
Cacybp	-0,19906	1
Pole4	-0,19936	1
Cep295	-0,19954	1
Zswim3	-0,19969	1
Use1	-0,19973	1
Zfp263	-0,19979	1
Lin7b	-0,19992	1
Klhl25	-0,20023	1
Tjp2	-0,20049	1
Cct5	-0,20052	1
9530068E07Rik	-0,20062	1
Msl2	-0,20062	1
Rpl30-ps5	-0,20073	1
Plcb3	-0,2007	1
Cdkal1	-0,20081	1
Gm45292	-0,20102	1
Gm12989	-0,20099	1
2700060E02Rik	-0,20105	1
Ssr3	-0,20098	1
Chd3os	-0,20105	1
Ppp4c	-0,20131	1
Wdr19	-0,20134	1
Acbd5	-0,2014	1
Cactin	-0,20139	1
Nup98	-0,20155	1
Gm17807	-0,20156	1
Rnh1	-0,20172	1

Papd4	-0,20175	1
Casz1	-0,20192	1
Gm7860	-0,20203	1
Cnnm2	-0,20245	1
Pqbp1	-0,20259	1
Rps16-ps2	-0,20275	1
Dock10	-0,20314	1
Aktip	-0,20307	1
Ranbp2	-0,20324	1
Gnai2	-0,20331	1
Nde1	-0,2034	1
Card9	-0,20389	1
Mospd3	-0,20388	1
Pbdc1	-0,20391	1
Mrps16	-0,20386	1
Tmem243	-0,20405	1
1110004F10Rik	-0,20411	1
Sdf2l1	-0,20432	1
Tor3a	-0,20455	1
Mtss1	-0,20452	1
Ppp1r14b	-0,20459	1
Eif3h	-0,20468	1
Pdcd4	-0,20489	1
Slc8a1	-0,20499	1
Rnf5	-0,20541	1
Midn	-0,20536	1
Vps51	-0,2055	1
Rad54b	-0,20571	1
Gm13862	-0,20582	1
Grasp	-0,20581	1
Rps6kc1	-0,20594	1
Adipor1	-0,20604	1
Mgme1	-0,20608	1
Gm23935	-0,20638	1
Sssca1	-0,20637	1
Ascc1	-0,20635	1
Rps20	-0,2064	1
C5ar1	-0,2066	1
Usp22	-0,20681	1
Ago3	-0,20687	1
Sh2b1	-0,20691	1
Daxx	-0,20701	1
Krr1	-0,20705	1
Psmg3	-0,20709	1
Naa10	-0,20719	1
Gpt	-0,20745	1
Gm23502	-0,20753	1
Guca1a	-0,20773	1
Gm33080	-0,20778	1
Pdcd5	-0,20777	1
Ctbp2	-0,20779	1
Mier3	-0,20777	1

Rpsa-ps2	-0,20794	1
RP23-2N7.4	-0,20789	1
Nus1	-0,20806	1
Mettl9	-0,20822	1
Ndufs4	-0,20835	1
1700020D05Rik	-0,20858	1
Gm7867	-0,20861	1
Rps26	-0,20858	1
Ankrd24	-0,2087	1
Mpc2	-0,20871	1
Nlgn2	-0,20888	1
Serpinb6b	-0,2089	1
Tmem26	-0,20886	1
Agrn	-0,20892	1
Cep164	-0,20904	1
Mfsd8	-0,20934	1
Rbm38	-0,20939	1
Apip	-0,20956	1
Ppm1j	-0,20965	1
Safb	-0,20967	1
Rnf38	-0,20979	1
Nedd8	-0,20977	1
Polr2j	-0,20987	1
Sepsecs	-0,20997	1
Parp14	-0,21017	1
Fbxo33	-0,21029	1
Arfip1	-0,21026	1
Cdca7	-0,21049	1
Nkap	-0,21048	1
Rps19	-0,21049	1
Mfsd10	-0,21056	1
Utrn	-0,21081	1
Smc3	-0,21076	1
Fam19a2	-0,21087	1
Ythdc1	-0,21086	1
Gm15753	-0,21119	1
Slc35f5	-0,21118	1
Mpst	-0,21119	1
Birc2	-0,21128	1
Gm14681	-0,21127	1
Gm15393	-0,21151	1
Rabl2	-0,21154	1
Akirin1	-0,21168	1
Etv1	-0,21177	1
Gm18889	-0,21194	1
Gm8738	-0,21222	1
Dbf4	-0,21217	1
Dpys	-0,21227	1
Sgsm3	-0,2123	1
Kif1b	-0,21226	1
Pwwp2b	-0,21245	1
Anp32b-ps1	-0,21242	1

Nrbp2	-0,21249	1
Sec22c	-0,21254	1
1810032O08Rik	-0,21248	1
Ilk	-0,2126	1
Gm9009	-0,21277	1
Utp14a	-0,21285	1
Itgb1	-0,21292	1
Bloc1s2	-0,21309	1
Cwc15	-0,21315	1
Atp5c1	-0,21348	1
Creld2	-0,21358	1
Sec31b	-0,21394	1
Mrpl38	-0,21392	1
Gm37238	-0,21401	1
Odf2	-0,214	1
Kcnn4	-0,21395	1
Rab3gap2	-0,21425	1
Csnk1g1	-0,21434	1
Taok1	-0,2144	1
Nap1l1	-0,21447	1
Rpsa-ps1	-0,21455	1
Nudcd2	-0,21468	1
Minos1	-0,21475	1
Akr1b7	-0,21495	1
Unc119b	-0,21497	1
Ccser2	-0,21524	1
Gm12504	-0,21533	1
Taldo1	-0,21533	1
Cdc42se2	-0,21527	1
Otud3	-0,21543	1
Dnm1l	-0,21542	1
Tmem242	-0,21557	1
Gabpa	-0,21574	1
Dazap1	-0,21576	1
Sapcd2	-0,2161	1
Fkbp3	-0,21617	1
Gm10602	-0,21632	1
Gm15148	-0,21632	1
Itgb1bp1	-0,21631	1
Rps27l	-0,21627	1
Snrpd3	-0,2165	1
Asb6	-0,21656	1
Capn5	-0,2167	1
Gm4332	-0,21669	1
H60c	-0,21682	1
Lrrc41	-0,21685	1
Tatdn1	-0,21677	1
Guk1	-0,21687	1
Eif5a	-0,21689	1
Lztfl1	-0,21709	1
Ftsj1	-0,21728	1
Gnptg	-0,21737	1

Gla	-0,21737	1
Nat6	-0,21752	1
Rpl37rt	-0,21747	1
Abl2	-0,21752	1
Il13ra1	-0,21774	1
Rps19-ps7	-0,21791	1
Prrg2	-0,21791	1
Tube1	-0,21798	1
Tecpr1	-0,21798	1
Dda1	-0,21815	1
Emc7	-0,21819	1
Cpne3	-0,2182	1
Gm38248	-0,21835	1
Arl6	-0,21847	1
Mocs2	-0,2185	1
Kitl	-0,21877	1
Bard1	-0,21911	1
Gm5321	-0,21921	1
9230111E07Rik	-0,21924	1
Unc119	-0,21924	1
Spred3	-0,21933	1
Rpl26-ps2	-0,21944	1
Gm20699	-0,21955	1
Gm42611	-0,21955	1
Mfsd4b4	-0,22013	1
Rer1	-0,22023	1
Gm29284	-0,22026	1
Ago4	-0,22036	1
Tmem59	-0,22039	1
Camk2g	-0,2206	1
Gm21057	-0,22074	1
Garnl3	-0,22105	1
Socs1	-0,22113	1
Mcf2l	-0,2212	1
Gm12577	-0,22137	1
Lamtor5	-0,22137	1
Atp5k	-0,2214	1
Sccpdh	-0,22155	1
Timm29	-0,22172	1
Gm13015	-0,22184	1
Pex7	-0,22187	1
Coil	-0,22199	1
Denr	-0,22207	1
A930029G22Rik	-0,22219	1
Gm8250	-0,22224	1
Rhobtb2	-0,22239	1
Ccdc47	-0,22244	1
Pcnt	-0,22261	1
Tmem181a	-0,22279	1
Gm43756	-0,22287	1
Prps1l3	-0,22287	1
Snx5	-0,22285	1

Nenf	-0,223	1
Traf4	-0,2234	1
Gnpnat1	-0,22366	1
Gm45286	-0,22384	1
Mical1	-0,22397	1
Zbtb2	-0,22398	1
Sort1	-0,22403	1
Lpin2	-0,224	1
Hebp1	-0,22407	1
Ufm1	-0,22413	1
Ormdl2	-0,2242	1
Rab3a	-0,22417	1
Txlng	-0,22432	1
Ikzf5	-0,22445	1
Pcnx4	-0,22449	1
Pde6d	-0,22457	1
Foxm1	-0,22456	1
Gm37289	-0,22471	1
Npat	-0,22469	1
Hdc	-0,22495	1
Gm14650	-0,22504	1
Adi1	-0,22503	1
Rps10-ps2	-0,22513	1
Tspyl3	-0,2253	1
Adck2	-0,22534	1
Mrpl9	-0,22544	1
Lamtor4	-0,22562	1
RP23-162P10.2	-0,22576	1
Kdsr	-0,22602	1
Fbxl5	-0,22598	1
Chmp7	-0,22621	1
Csnk1g2	-0,22637	1
Pfdn6	-0,22655	1
Gm43466	-0,22663	1
Ccdc50	-0,22687	1
Phb	-0,22719	1
Rnf185	-0,22725	1
Znhit3	-0,22734	1
Bzw1	-0,22744	1
Tmem9b	-0,22739	1
Vma21-ps	-0,22751	1
Atg14	-0,22749	1
Apitd1	-0,22775	1
Gm13196	-0,22801	1
Nt5c	-0,22803	1
Bag4	-0,22808	1
Ppp2r2a	-0,22808	1
Alcam	-0,22816	1
L3mbtl2	-0,22835	1
Gm11427	-0,22845	1
Gm37333	-0,22862	1
Zfp335	-0,22857	1

Atp1a3	-0,22871	1
Gm45212	-0,22884	1
0610038B21Rik	-0,22898	1
Cers5	-0,22923	1
Pnpla8	-0,2293	1
Strn	-0,22952	1
Txndc5	-0,22957	1
Fat1	-0,22965	1
Slc29a2	-0,22981	1
Mnt	-0,23005	1
Ppp2r1a	-0,23025	1
Gm16556	-0,23027	1
Snrpd2	-0,23029	1
Trip10	-0,23046	1
Hprt	-0,23066	1
Elmo2	-0,23079	1
Alkbh5	-0,23079	1
Aldh9a1	-0,2308	1
Ptk2b	-0,23097	1
Ahctf1	-0,2313	1
Gm44024	-0,23143	1
Pex13	-0,23165	1
Gm38055	-0,23188	1
Ahnak	-0,23188	1
Fbxo8	-0,23232	1
Gm2788	-0,23245	1
Rpl38	-0,23238	1
Trim33	-0,23254	1
Aatk	-0,23258	1
Fam134c	-0,23271	1
Clip2	-0,23268	1
Arhgef1	-0,2328	1
Igbp1	-0,23279	1
Litaf	-0,23276	1
Tnpo1	-0,23289	1
Osgepl1	-0,23298	1
Rps10-ps1	-0,23304	1
Cotl1	-0,23322	1
Gm45342	-0,2333	1
Clpb	-0,23341	1
Larp4	-0,23339	1
Ctc1	-0,23354	1
Rpl38-ps2	-0,23346	1
Dync2li1	-0,23374	1
Gm5617	-0,23401	1
Mtf2	-0,23434	1
Zfp91	-0,23465	1
Gm26530	-0,23478	1
A430005L14Rik	-0,23476	1
Nfatc2ip	-0,23489	1
Ugp2	-0,23487	1
Tob2	-0,23487	1

Pias4	-0,23505	1
Plekha2	-0,23522	1
Gm19967	-0,23529	1
Gas2l3	-0,23546	1
Vwa5a	-0,23547	1
Eif2a	-0,2355	1
Nfkbib	-0,23558	1
Dock2	-0,23558	1
Gmfg	-0,23568	1
Smim8	-0,23566	1
RP24-550H10.4	-0,23583	1
Mllt11	-0,23583	1
Gm10268	-0,23604	1
Gm14567	-0,23643	1
Helq	-0,23652	1
Jrkl	-0,23646	1
Siah1a	-0,23646	1
Tubb5	-0,23646	1
Polr1b	-0,23668	1
Ndfip2	-0,23668	1
Timm17b	-0,237	1
Gm5436	-0,23708	1
Srp9	-0,23709	1
Gm7123	-0,23722	1
Rps15a-ps7	-0,23773	1
Naga	-0,23778	1
Rnf146	-0,23787	1
Hat1	-0,23792	1
Tmem50b	-0,23809	1
Gm42595	-0,23832	1
Cited2	-0,23841	1
Klhdc4	-0,23835	1
Knstrn	-0,23851	1
Skp1a	-0,23871	1
Slc2a8	-0,2388	1
Wbp11	-0,23881	1
Nudt16l1	-0,23891	1
Gosr2	-0,23889	1
Gna12	-0,2391	1
Gen1	-0,23916	1
Sptbn1	-0,2392	1
Ccdc14	-0,23926	1
Actr6	-0,23943	1
Mob3b	-0,2395	1
Tmem39b	-0,23953	1
Gm3650	-0,23967	1
Lyar	-0,2397	1
Pam	-0,23969	1
Nob1	-0,23981	1
Mss51	-0,24001	1
Nthl1	-0,23995	1
Gm37125	-0,24042	1

Gm2962	-0,24043	1
Tfpt	-0,24045	1
Ambp	-0,24064	1
Zfp692	-0,24062	1
Pomp	-0,24065	1
Gm5845	-0,24067	1
Gm38380	-0,241	1
Pura	-0,24113	1
Psmd8	-0,24124	1
Pdia6	-0,24116	1
Gm15421	-0,24132	1
Gm16177	-0,24129	1
Cep135	-0,24141	1
Kcnc3	-0,24176	1
Hilpda	-0,24175	1
Gm37677	-0,24191	1
C330006A16Rik	-0,2419	1
Lpp	-0,24249	1
Cramp1l	-0,24291	1
Chml	-0,24303	1
Mex3c	-0,24321	1
Gm37305	-0,24328	1
Nrf1	-0,24337	1
Nfkbil1	-0,24347	1
RP23-38L16.4	-0,24358	1
Elob	-0,24357	1
Gm10051	-0,24378	1
Adamts10	-0,2438	1
Gm43728	-0,24405	1
Alkbh4	-0,24408	1
Cdc37l1	-0,24437	1
BC022687	-0,24447	1
Armc10	-0,24474	1
Hsbp1	-0,24489	1
Tns1	-0,24508	1
Abhd13	-0,24524	1
Vhl	-0,24531	1
Gm38305	-0,24558	1
Plekhj1	-0,24564	1
G6pc3	-0,24569	1
Gm37968	-0,24577	1
Fam84b	-0,2459	1
Thrap3	-0,2459	1
Ythdf3	-0,2459	1
Hoxb8	-0,24598	1
Slc7a8	-0,246	1
Sos1	-0,24611	1
Pcm1	-0,24606	1
Cox4i2	-0,2462	1
Zcchc14	-0,24617	1
Gorab	-0,24634	1
Lrrc45	-0,24635	1

Fnbp1l	-0,24649	1
Gm7776	-0,24665	1
Ggnbp1	-0,24658	1
Ubxn7	-0,24661	1
Gm6028	-0,24684	1
Mob2	-0,24685	1
Ggps1	-0,24703	1
2210013O21Rik	-0,24719	1
Slf1	-0,2473	1
Snrpc	-0,24742	1
Rnf44	-0,24743	1
Gipc2	-0,24775	1
Rcc1l	-0,24773	1
Cdip1	-0,24784	1
Tstd2	-0,24781	1
Hspb7	-0,24793	1
Rps15	-0,24791	1
n-R5s151	-0,24847	1
Snord71	-0,24851	1
Dgkd	-0,24861	1
Tmem43	-0,24862	1
Mum1	-0,24893	1
Smpdl3a	-0,24889	1
Stip1	-0,24886	1
A430018G15Rik	-0,24906	1
Ggta1	-0,24907	1
Zfr	-0,24905	1
9330151L19Rik	-0,2493	1
Lnpep	-0,24928	1
Chchd7	-0,24936	1
2010315B03Rik	-0,24959	1
Tbca	-0,24957	1
mt-Nd5	-0,24972	1
Dusp10	-0,24976	1
Bend4	-0,25023	1
Edrf1	-0,25036	1
Rps27	-0,25036	1
Scrib	-0,25037	1
4930412F12Rik	-0,2508	1
Mbnl2	-0,25082	1
Wdr44	-0,2511	1
Top1	-0,25152	1
Vps18	-0,2516	1
Trmt61b	-0,25187	1
Cd44	-0,25202	1
Gm13461	-0,25216	1
Rap2a	-0,25225	1
Dexi	-0,25244	1
Pum1	-0,25247	1
Fnbp4	-0,25257	1
Slfn2	-0,25268	1
Bsg	-0,25284	1

Frs2	-0,25289	1
Prmt7	-0,25318	1
Tmed5	-0,25321	1
Meis3	-0,25334	1
0610037L13Rik	-0,25334	1
Stac2	-0,25361	1
Nprl2	-0,25356	1
Taf8	-0,25366	1
Bbof1	-0,2539	1
Ptprc	-0,25392	1
Gm8276	-0,25397	1
Adap1	-0,2543	1
Gm43323	-0,25439	1
Mdc1	-0,25441	1
Adss	-0,25439	1
Il1rl1	-0,25448	1
Med25	-0,25456	1
Xpa	-0,25462	1
Etfdh	-0,25499	1
Cby1	-0,25509	1
Tprn	-0,25513	1
Gm4204	-0,25506	1
Gm38377	-0,25521	1
Cenpl	-0,25531	1
Gm45718	-0,25542	1
Ripk3	-0,25542	1
Fam98c	-0,25543	1
Naxd	-0,25548	1
Gm37254	-0,25555	1
Otud4	-0,25572	1
Gm5239	-0,25576	1
Zfp382	-0,25588	1
Med29	-0,25599	1
Gm29736	-0,25603	1
Cbx5	-0,25615	1
Ppp4r3b	-0,25608	1
Tchp	-0,25616	1
Actr1a	-0,25622	1
Chchd5	-0,25618	1
Ier3	-0,25617	1
Gm35106	-0,25642	1
Mllt10	-0,25653	1
Gm16020	-0,25656	1
Prkci	-0,25676	1
Dnmbp	-0,25676	1
Cstf3	-0,25677	1
Fbxl12	-0,25685	1
Kcnk13	-0,25689	1
Tnfrsf22	-0,25722	1
Khsrp	-0,25741	1
AU020206	-0,25764	1
Fuca1	-0,25771	1

Afg3l2	-0,25774	1
Eef1d	-0,25786	1
Gm15535	-0,25804	1
Tmem120a	-0,25796	1
Gm16399	-0,25823	1
Gm42659	-0,25834	1
Zfp369	-0,2583	1
Cdk9	-0,25854	1
Slc35b3	-0,25854	1
Abi1	-0,25852	1
1700096K18Rik	-0,25857	1
Cdk2ap2	-0,25856	1
Cdc26	-0,25883	1
Coa6	-0,25886	1
2610203C22Rik	-0,25902	1
Pla2g4a	-0,25901	1
RP23-269H21.1	-0,25918	1
Syngap1	-0,25937	1
Gm7407	-0,2598	1
Snx8	-0,25984	1
Gm43692	-0,2599	1
Olfr920	-0,25997	1
Glrx3	-0,26004	1
Ttc14	-0,26016	1
Gm45222	-0,26041	1
Ccdc115	-0,26068	1
Gm14853	-0,26076	1
Rbx1	-0,26077	1
Lmf1	-0,26095	1
Myo1g	-0,26089	1
Prcp	-0,26103	1
Hnrnpa2b1	-0,26099	1
Tmem189	-0,26142	1
Dnajc13	-0,26155	1
Metrn	-0,26196	1
Pigl	-0,26221	1
Stk11	-0,26217	1
Copg2	-0,26263	1
Gm38162	-0,26284	1
Dnaja1	-0,26278	1
Atp5e	-0,2629	1
Mob1b	-0,26308	1
Gm8927	-0,26327	1
Fkbp7	-0,26349	1
Mau2	-0,26359	1
Zfp398	-0,26371	1
Gm24920	-0,26379	1
Rad23a	-0,26376	1
Rbm6	-0,26399	1
Txndc15	-0,2642	1
Gm15459	-0,2643	1
Mta2	-0,26426	1

Klf7	-0,26438	1
Ola1	-0,26463	1
Psmd13	-0,26458	1
Pola1	-0,26467	1
Rplp0	-0,26474	1
Mrpl23-ps1	-0,26476	1
Gm8357	-0,26486	1
Hadhb	-0,26494	1
Rps15a-ps6	-0,26498	1
Gm9828	-0,265	1
Eif4e3	-0,26496	1
Nit2	-0,26564	1
Sgcb	-0,26583	1
Lcor	-0,266	1
Zfp422	-0,26661	1
Gm12988	-0,26679	1
Gm37678	-0,2669	1
Rpl22l1	-0,267	1
Phf6	-0,26709	1
Bcs1l	-0,26727	1
Mzt1	-0,26729	1
Bdp1	-0,26731	1
Vps8	-0,26778	1
Rfxap	-0,26776	1
Slc35g1	-0,26779	1
Mrpl34	-0,26783	1
Rpl32	-0,2679	1
Snrpb	-0,26789	1
Gm10941	-0,26796	1
Orc4	-0,26798	1
Yod1	-0,26809	1
Ets2	-0,26805	1
E130102H24Rik	-0,26824	1
Katna1	-0,26824	1
Gnb1	-0,26818	1
Car11	-0,26831	1
Dhcr7	-0,26829	1
Rfwd2	-0,26843	1
Dyrk1a	-0,26854	1
Kctd18	-0,26864	1
Pdp1	-0,26869	1
Sash1	-0,26883	1
Gm13611	-0,26904	1
Dctpp1	-0,26905	1
Zbtb18	-0,26939	1
Dym	-0,26964	1
Rnf11	-0,26961	1
Rpph1	-0,26973	1
Col4a5	-0,26974	1
Ube2i	-0,26992	1
Pabpc1l	-0,27012	1
Gm5830	-0,2702	1

Trim8	-0,27029	1
Trmt10a	-0,27061	1
G3bp2	-0,27059	1
Tmub1	-0,27088	1
Kiz	-0,27093	1
Mtpn	-0,27088	1
Hyls1	-0,2711	1
Ttc9c	-0,27118	1
Gm43445	-0,27139	1
Riox1	-0,27139	1
Dcp1a	-0,27149	1
Gm11478	-0,27196	1
Usp12	-0,27278	1
Gm2383	-0,27279	1
Sub1	-0,27293	1
Rps15a-ps1	-0,27313	1
Zbtb21	-0,27308	1
Hotairm1	-0,27318	1
Pdpk1	-0,27318	1
Apoo	-0,2733	1
Gm5121	-0,27341	1
Zbtb8a	-0,27367	1
Cnr2	-0,27372	1
Ltbp4	-0,27384	1
Zfp646	-0,27388	1
Uck1	-0,27399	1
Scp2	-0,27397	1
Gm10060	-0,27405	1
Tfap4	-0,27411	1
Gm13005	-0,27433	1
Ctdp1	-0,27428	1
Rnf103	-0,2749	1
Aph1a	-0,27493	1
Enho	-0,27497	1
Rcbtb2	-0,27527	1
Iah1	-0,27533	1
Ogt	-0,27546	1
Rpl37a	-0,27556	1
Rnf111	-0,27567	1
Rpl36-ps3	-0,27581	1
Ppp2r2d	-0,27603	1
Fam234b	-0,27605	1
M6pr	-0,27617	1
Ppie	-0,27618	1
Ube2g1	-0,27651	1
Pstk	-0,27658	1
Polr3g	-0,27658	1
Sirt1	-0,27658	1
Gfod1	-0,27665	1
Atp2c1	-0,27667	1
Zfp607b	-0,27676	1
Wdr47	-0,27688	1

Cep44	-0,27696	1
Prpf18	-0,27706	1
Ing2	-0,27731	1
Pcsk4	-0,2774	1
Ppt2	-0,27743	1
Tmem120b	-0,27748	1
Kif3c	-0,27747	1
Me2	-0,27757	1
Gm43627	-0,27774	1
Rbm14	-0,27771	1
Rpl23a-ps3	-0,27796	1
Ndufa6	-0,2781	1
Zfp280b	-0,27817	1
Siva1	-0,27844	1
Atp11c	-0,2787	1
Hmgb3	-0,27898	1
Ccdc15	-0,27909	1
Chd6	-0,27919	1
RP23-371B13.3	-0,27931	1
Gng2	-0,27955	1
Usp28	-0,27968	1
Gm37760	-0,27967	1
Srebf1	-0,27976	1
A530072M11Rik	-0,28013	1
Rexo4	-0,28018	1
Cd200r3	-0,2803	1
Sharpin	-0,28028	1
Isg20l2	-0,28048	1
Ppp4r3a	-0,28064	1
Arhgef40	-0,28078	1
Nol7	-0,28082	1
Mrpl23	-0,281	1
Btbd10	-0,28102	1
Mettl16	-0,28103	1
Ephx1	-0,28129	1
Ttbk2	-0,28135	1
Gm12882	-0,2815	1
Rcor3	-0,28147	1
Gm37080	-0,28171	1
Atp5j2	-0,28167	1
Zcchc11	-0,28206	1
Ndel1	-0,28226	1
Uprt	-0,28238	1
Plekhm3	-0,28244	1
RP24-84C23.4	-0,28265	1
Klkb1	-0,28303	1
Mrps33	-0,28301	1
Ccdc22	-0,28307	1
Mpp6	-0,28308	1
Kdm7a	-0,28323	1
Zyx	-0,28334	1
Creb3l4	-0,28341	1

RP23-114G13.1	-0,28353	1
Snrnp25	-0,28348	1
Timm10b	-0,28371	1
Xndc1	-0,28391	1
Spaca9	-0,28398	1
Catsperg1	-0,28404	1
Nup43	-0,28395	1
Gm9800	-0,28414	1
Primpol	-0,28413	1
Cnot6l	-0,28416	1
Rpl31-ps10	-0,28432	1
Map3k3	-0,28444	1
Eda2r	-0,28454	1
Gys1	-0,28479	1
U2af2	-0,28495	1
Esco1	-0,28514	1
Desi2	-0,28543	1
Hmgb1-rs16	-0,28552	1
Slc25a5	-0,28559	1
Gar1	-0,28564	1
Malt1	-0,28559	1
Tiprl	-0,28556	1
Polr2e	-0,28563	1
Abcc10	-0,28571	1
Gm5611	-0,28574	1
Sike1	-0,28567	1
Epb41l4aos	-0,28572	1
Rpgrip1l	-0,28584	1
Gm6209	-0,28606	1
Trp53	-0,28624	1
Pold4	-0,28616	1
Mmd	-0,28667	1
Gm43793	-0,28689	1
Gm10358	-0,28745	1
Gpsm1	-0,28741	1
Rpl34	-0,2876	1
Erh	-0,28771	1
Trim3	-0,28778	1
Atp6v0e2	-0,28804	1
Fam46a	-0,28813	1
Glrx	-0,28812	1
Fam69b	-0,28833	1
Ergic3	-0,28828	1
Cd300lb	-0,28833	1
4933404O12Rik	-0,28837	1
Prpf4b	-0,28843	1
Tmem191c	-0,28844	1
Mrpl50	-0,28838	1
Acadvl	-0,28837	1
Fam131a	-0,28854	1
Gm42480	-0,28848	1
Ddx23	-0,28845	1

Kirrel3	-0,2888	1
Gm4468	-0,28897	1
Snupn	-0,28936	1
9930022D16Rik	-0,2895	1
Gm26740	-0,28957	1
Vkorc1	-0,28974	1
Nck1	-0,28994	1
Med21	-0,29009	1
Gon7	-0,29005	1
Eif2ak3	-0,29015	1
Hmgcs1	-0,2902	1
Gm15210	-0,29042	1
Paip2b	-0,29061	1
Gm15417	-0,2907	1
Rps15-ps2	-0,29098	1
Wbp4	-0,29124	1
Smad6	-0,29127	1
Zfp143	-0,29127	1
H2afx	-0,29159	1
Gm7114	-0,29186	1
Gt(ROSA)26Sor	-0,29198	1
Gm6987	-0,29207	1
Arl4d	-0,29225	1
Gm12312	-0,2923	1
Snrpd1	-0,29227	1
Plekha7	-0,29249	1
9930120I10Rik	-0,29264	1
Rfc3	-0,29258	1
Trappc6a	-0,29256	1
Secisbp2l	-0,29275	1
Topors	-0,29283	1
Zbed3	-0,29322	1
Pcmt1	-0,29325	1
Zfc3h1	-0,29331	1
Psmb9	-0,29337	1
Ccdc137	-0,2937	1
Psmb1	-0,29379	1
Sik2	-0,29388	1
6430531B16Rik	-0,29392	1
Rnf126	-0,29391	1
Gm7224	-0,29423	1
Gm21399	-0,29424	1
Ssna1	-0,29432	1
1810013L24Rik	-0,29433	1
Rps18	-0,29466	1
Ints2	-0,29499	1
Cdc45	-0,29506	1
Dr1	-0,29515	1
Tmem33	-0,29519	1
Fyttd1	-0,29519	1
Nudc	-0,29527	1
Mir22hg	-0,29533	1

Cst3	-0,29546	1
Prss53	-0,29583	1
Btf3l4	-0,29585	1
Git2	-0,29585	1
Rnf25	-0,29594	1
Gm37569	-0,29592	1
Rttn	-0,29609	1
Gm43499	-0,29613	1
Srsf10	-0,29626	1
Gm10031	-0,29658	1
Bst2	-0,29679	1
Ripk2	-0,29698	1
Calr	-0,29721	1
Ppid	-0,29749	1
Gm23722	-0,29767	1
Cnot7	-0,29792	1
Aimp2	-0,29799	1
Golph3l	-0,29814	1
Phf1	-0,2981	1
Gm18860	-0,29829	1
Rplp2	-0,29834	1
Slc38a2	-0,29841	1
Trp53i13	-0,2985	1
Tatdn2	-0,29848	1
Helb	-0,2986	1
Sdf2	-0,29863	1
Nucks1	-0,29859	1
Cul3	-0,29894	1
Prim1	-0,29921	1
Rps29	-0,29925	1
Ssbp3	-0,29963	1
Slc25a22	-0,29969	1
Timm22	-0,29973	1
Tgoln1	-0,29978	1
Blcap	-0,29987	1
Fgd4	-0,30002	1
Gm37199	-0,30021	1
Ndrg4	-0,30029	1
Mgea5	-0,30029	1
Slc39a10	-0,30039	1
Smchd1	-0,30063	1
Gm7117	-0,3007	1
Mef2d	-0,30085	1
Nudt4	-0,30115	1
Clk3	-0,30112	1
Sumo1	-0,3012	1
Eif3k	-0,30116	1
Casp6	-0,3013	1
Vps53	-0,30143	1
Gm5869	-0,30149	1
Zfp444	-0,30162	1
Gm9625	-0,30159	1

Dynll2	-0,30169	1
Tpp2	-0,30178	1
Eif3e	-0,30194	1
Ndufb2	-0,30199	1
Kif21b	-0,30204	1
Tbkbp1	-0,302	1
Eif4b	-0,30201	1
1110003F10Rik	-0,30223	1
Mapk8ip1	-0,30252	1
Thap2	-0,3026	1
Aga	-0,30261	1
Cpeb2	-0,30291	1
Rps12-ps26	-0,3033	1
Mrps23	-0,30351	1
Vprbp	-0,30361	1
Mrps31	-0,30369	1
Ilkap	-0,30391	1
Mast4	-0,3041	1
Gm5069	-0,30442	1
Bcl2	-0,30444	1
Wdr74	-0,30449	1
Nudt21	-0,30452	1
Wbp1	-0,30448	1
Gm45873	-0,30464	1
Fam109a	-0,30469	1
Gm13436	-0,30504	1
Osgin1	-0,30504	1
Gm12459	-0,30511	1
Baat	-0,30529	1
Gm5614	-0,3054	1
Pygl	-0,30536	1
Dag1	-0,30559	1
Ube2e2	-0,30574	1
Gm6030	-0,30578	1
Mrpl35	-0,30577	1
Ppif	-0,30633	1
Agps	-0,3064	1
Fads3	-0,30697	1
Gm7783	-0,30704	1
Klhl7	-0,30719	1
Tomm7	-0,30717	1
Tbc1d19	-0,30728	1
Emsy	-0,30798	1
RP23-390D8.2	-0,30837	1
Pcid2	-0,30856	1
Gm14325	-0,30892	1
Rrnad1	-0,30964	1
Gm5879	-0,30975	1
Strip1	-0,31002	1
Fbxo2	-0,31016	1
Flrt2	-0,3102	1
Gm8186	-0,31032	1

Dcaf5	-0,31063	1
Mif	-0,31082	1
Cpsf7	-0,31082	1
Ap2b1	-0,31091	1
Zfp942	-0,31112	1
Gtf3c4	-0,31107	1
Pgrmc1	-0,31133	1
Eif4e	-0,31154	1
Snord35a	-0,31157	1
Dek	-0,31166	1
Bmpr1a	-0,31193	1
Slc36a1	-0,31194	1
Gm5380	-0,31196	1
Gm12770	-0,31207	1
1600012H06Rik	-0,31233	1
Rgs11	-0,3124	1
Itga5	-0,31256	1
Lman2l	-0,3126	1
Rpl13-ps3	-0,31271	1
Srrt	-0,31271	1
Ccdc114	-0,31338	1
RP23-447C2.2	-0,3135	1
Casp9	-0,31353	1
1810058I24Rik	-0,31355	1
Tmem109	-0,31349	1
Anapc16	-0,31374	1
Fam122a	-0,31408	1
C920009B18Rik	-0,31443	1
Golga3	-0,3147	1
Noc3l	-0,31485	1
Ndufa1	-0,31501	1
Acad11	-0,31515	1
Ccnt1	-0,3151	1
Prpf3	-0,31554	1
Inip	-0,31553	1
Gm44254	-0,31573	1
Nucb1	-0,31571	1
Caap1	-0,31577	1
Naa20	-0,31616	1
Tpst2	-0,31647	1
Lrfn4	-0,31659	1
Mesdc1	-0,31661	1
Kpnb1	-0,31656	1
Zfp638	-0,31661	1
Gxylt1	-0,31673	1
Oas1b	-0,31682	1
Cycs	-0,31705	1
Mcm4	-0,31739	1
Gm20667	-0,31751	1
Fcrl1	-0,31803	1
RP23-184H3.5	-0,31797	1
Dhps	-0,31807	1

Gm4604	-0,31816	1
Swt1	-0,31831	1
Rpl27a	-0,3183	1
Rps9	-0,31837	1
Dlg3	-0,31847	1
Gltscr2	-0,31854	1
Ftx	-0,31873	1
2610001J05Rik	-0,31874	1
Rora	-0,31875	1
Pyroxd1	-0,31891	1
Snrpe	-0,31896	1
Gm5580	-0,31914	1
Narf	-0,3191	1
Hace1	-0,31917	1
Kansl1	-0,31933	1
Actn4	-0,31927	1
Zfp449	-0,31943	1
Sdccag3	-0,31938	1
Gm5687	-0,31943	1
Ube2b	-0,31943	1
4930448A20Rik	-0,31966	1
Fis1	-0,31971	1
4921524J17Rik	-0,31983	1
Cep192	-0,31999	1
Alox5ap	-0,32002	1
Cox14	-0,3208	1
Calm2	-0,32084	1
Gm11737	-0,321	1
Gm7504	-0,32104	1
Snhg15	-0,32153	1
Slc12a6	-0,3217	1
Sp3	-0,32184	1
Rap1a	-0,32192	1
Azin1	-0,32197	1
Cep350	-0,32202	1
Gripap1	-0,3221	1
Txndc17	-0,32244	1
Gm26935	-0,32251	1
Gm14173	-0,32257	1
Mtmr12	-0,32259	1
Capza2	-0,3227	1
Gm26917	-0,32304	1
Kbtbd2	-0,32311	1
Gemin6	-0,32334	1
Gm44190	-0,32336	1
Ric8b	-0,32341	1
Tusc2	-0,32343	1
Snhg18	-0,32356	1
Gm37558	-0,32367	1
Hdac3	-0,32393	1
Pcnp	-0,32402	1
Hspa14	-0,3242	1

B3galnt2	-0,32452	1
Cebpa	-0,32453	1
Arpc5l	-0,32455	1
Rangap1	-0,32517	1
Cwf19l1	-0,32546	1
Etnk1	-0,32565	1
Gm43360	-0,32579	1
Gm4950	-0,32579	1
R3hdm4	-0,3261	1
Zbtb7b	-0,32608	1
Gm7618	-0,32616	1
Tmem128	-0,32616	1
Fam45a	-0,32636	1
C130083A15Rik	-0,32648	1
Slc25a20	-0,32669	1
Mrip-ps	-0,32677	1
Il1rn	-0,32685	1
Clspn	-0,32675	1
2410006H16Rik	-0,32686	1
Gm11688	-0,32716	1
Tmf1	-0,3273	1
Gatad1	-0,32733	1
Dzip1	-0,32744	1
AK157302	-0,32754	1
Triobp	-0,32774	1
Usp37	-0,32775	1
Vat1	-0,3277	1
BC049715	-0,32782	1
Ech1	-0,32783	1
Yrdc	-0,32806	1
Cfp	-0,3284	1
Cdyl2	-0,32842	1
Gm13360	-0,3285	1
Ikbkap	-0,32866	1
Zfand5	-0,32877	1
Plp2	-0,3289	1
Phc3	-0,32912	1
Hist1h3d	-0,32921	1
Gm45501	-0,32929	1
Stk25	-0,3293	1
Snx30	-0,33009	1
Trim47	-0,3303	1
Hnrnpf	-0,33025	1
Tbcb	-0,33026	1
Gm10175	-0,33026	1
1700109H08Rik	-0,33033	1
Phactr4	-0,33027	1
Agtpbp1	-0,33035	1
Gm12762	-0,33044	1
Ccdc124	-0,33041	1
Smad3	-0,3307	1
Arhgef17	-0,33086	1

Tmem41b	-0,33086	1
Pih1d2	-0,33099	1
Ccar1	-0,33139	1
Gm6394	-0,33176	1
Cbx6	-0,33185	1
Gm9530	-0,33202	1
Cd83	-0,33198	1
Cdc42	-0,33202	1
Gm24876	-0,33214	1
Anxa5	-0,33214	1
Btbd7	-0,33229	1
Wdr90	-0,33251	1
Nabp1	-0,3325	1
Aldh2	-0,33254	1
Gm42511	-0,33309	1
Rps26-ps1	-0,33311	1
Kdm4b	-0,33326	1
Gm15501	-0,33345	1
Pip4k2b	-0,33401	1
1110038B12Rik	-0,33396	1
Washc3	-0,33441	1
Rab1a	-0,33451	1
Gm19777	-0,33461	1
Arhgap15	-0,3346	1
Gm19566	-0,33475	1
Uchl5	-0,33485	1
Snrpb2	-0,33517	1
Anp32e	-0,33517	1
Gm15216	-0,33527	1
Mrps30	-0,3356	1
Gm1943	-0,33586	1
Rps13	-0,33594	1
Phf20l1	-0,33613	1
Tagln2	-0,33618	1
Hist4h4	-0,33634	1
Hmgcr	-0,33635	1
Gm38200	-0,33664	1
Sesn1	-0,33655	1
Ecm1	-0,33657	1
Lsm2	-0,33658	1
Olfr460	-0,33681	1
Esr1	-0,33691	1
Clock	-0,33694	1
Cdc14b	-0,33687	1
Oat	-0,33728	1
Rhbdf2	-0,33745	1
Rpl23	-0,33749	1
Itgam	-0,33769	1
Fam110a	-0,33775	1
Gm20568	-0,33767	1
Camkmt	-0,33798	1
Gm4117	-0,33864	1

Gm27477	-0,33877	1
Cdkn1a	-0,33884	1
Tacc1	-0,33887	1
Gm12096	-0,33904	1
Als2	-0,33913	1
Mrps36-ps2	-0,33923	1
Rps23-ps2	-0,33937	1
Cd74	-0,33947	1
Gm3511	-0,3396	1
Nxt1	-0,33973	1
Ndufa12	-0,33965	1
Lztr1	-0,33976	1
Sap30l	-0,3402	1
Zmym2	-0,34048	1
Klhl42	-0,34076	1
Tollip	-0,3408	1
Rheb	-0,34105	1
Rps19-ps5	-0,34113	1
Slc16a3	-0,34116	1
Atf6	-0,34138	1
Cep72	-0,34147	1
Fam220a	-0,34148	1
Gm19196	-0,3416	1
Gm23969	-0,34175	1
Cav2	-0,34203	1
RP23-316F10.2	-0,34225	1
Gm13009	-0,34227	1
Arhgdig	-0,34233	1
Gm11605	-0,34241	1
Trove2	-0,34238	1
Hoxa5	-0,34255	1
Chd7	-0,34265	1
Gm6794	-0,34299	1
Morf4l2	-0,34303	1
Gm10126	-0,34388	1
Hoxa3	-0,34455	1
Polr1d	-0,34454	1
D130017N08Rik	-0,34457	1
Syap1	-0,34478	1
4930581F22Rik	-0,34516	1
Nbeal1	-0,34519	1
Rhpn2	-0,34527	1
Snrpert	-0,34542	1
Lrp1	-0,34538	1
Ttc12	-0,34555	1
Rps27a-ps1	-0,34573	1
Gm20072	-0,34601	1
Bud13	-0,34595	1
Gm5566	-0,34623	1
Gm12924	-0,34646	1
Rnf115	-0,34664	1
Akt3	-0,34658	1

Themis2	-0,34684	1
2410004B18Rik	-0,34687	1
Tbpl1	-0,34692	1
Lrrfip1	-0,34695	1
Vps13d	-0,34723	1
Gm9173	-0,34733	1
Hikeshi	-0,34733	1
Rbm19	-0,34738	1
C630004M23Rik	-0,34764	1
Gm44432	-0,34771	1
Faim	-0,34868	1
Arpp19	-0,34876	1
Pou2f1	-0,34914	1
Gm12479	-0,34936	1
Simc1	-0,34935	1
Brca1	-0,34961	1
Rock2	-0,34957	1
E2f2	-0,3497	1
Vezf1	-0,34965	1
Ginm1	-0,34977	1
Psmd11	-0,34976	1
Snhg4	-0,35018	1
Gm15131	-0,35032	1
Bpnt1	-0,35035	1
Ralgapa2	-0,35039	1
Ado	-0,35126	1
Tcof1	-0,35127	1
2310022A10Rik	-0,35141	1
Ing1	-0,35152	1
Gm3571	-0,35155	1
Aarsd1	-0,35159	1
Man1c1	-0,35162	1
Gm20768	-0,35157	1
Gm37589	-0,35174	1
Rpl18a	-0,35195	1
Acvr1b	-0,35209	1
Fkbp1a	-0,3521	1
Ifi207	-0,35224	1
Sptbn4	-0,35226	1
Zgrf1	-0,35236	1
Nup54	-0,35239	1
Rnf26	-0,35256	1
Gm37124	-0,35291	1
Zc3h12c	-0,35346	1
Stk17b	-0,3536	1
Hbp1	-0,35389	1
Gm12396	-0,35399	1
Rpl35a	-0,35401	1
Ptms	-0,35417	1
Ss18l2	-0,35433	1
RP23-114G13.7	-0,35451	1
Prdx4	-0,35486	1

Gins1	-0,35504	1
Ercc6	-0,35544	1
Pym1	-0,3556	1
Wasl	-0,35559	1
Gm38340	-0,35575	1
Dohh	-0,35571	1
Abcb1b	-0,35604	1
Kdm6a	-0,35637	1
Hnrnpu	-0,35659	1
Gm7701	-0,35666	1
Rps27a	-0,35675	1
Ppp5c	-0,35668	1
Tmem38b	-0,35704	1
Arpc3	-0,35695	1
Arhgap27os2	-0,35721	1
Gm6807	-0,35736	1
Zfp938	-0,35752	1
Ptrh1	-0,35772	1
Hip1r	-0,35807	1
Mrpl30	-0,3582	1
Nfkb1	-0,35834	1
Ndufa4l2	-0,35869	1
Kras	-0,35869	1
Gm11423	-0,35883	1
Pank2	-0,35882	1
RP23-453B15.7	-0,35897	1
Rps19bp1	-0,35912	1
Mfng	-0,35944	1
Tfcp2l1	-0,35951	1
Kctd13	-0,35954	1
Anapc7	-0,35946	1
Uqcrh-ps1	-0,35967	1
Gm12906	-0,35991	1
Snx18	-0,36012	1
Myl6	-0,36052	1
Rnase4	-0,36061	1
Pim2	-0,36068	1
Cep85	-0,36065	1
Ogfrl1	-0,36069	1
Casp8ap2	-0,36113	1
Zbtb11	-0,36115	1
Atg12	-0,3613	1
Gm15032	-0,36154	1
Arvcf	-0,36184	1
Cdt1	-0,36175	1
Gm5384	-0,3621	1
Tmem208	-0,36223	1
Slc9a5	-0,36232	1
Ptpn5	-0,36254	1
Zfp53	-0,3625	1
Gorasp1	-0,36271	1
A430046D13Rik	-0,36273	1

Gm29539	-0,36308	1
Nqo2	-0,36322	1
Prpf38a	-0,36332	1
Gm4217	-0,36341	1
Gng5	-0,36349	1
Cnot3	-0,36346	1
Dalrd3	-0,36364	1
Gm7895	-0,36387	1
RP24-325P4.5	-0,36428	1
RP24-82M14.1	-0,36456	1
Rps11	-0,36471	1
Ppp6c	-0,36467	1
Rpl36	-0,36498	1
Mrpl51	-0,36513	1
Gm14620	-0,36522	1
Armt1	-0,36523	1
Eefsec	-0,36536	1
Tmsb4x	-0,36588	1
Plekhh3	-0,36629	1
Ncoa3	-0,36625	1
Tbx6	-0,36645	1
Dnmt3l	-0,36731	1
Tcp11l1	-0,3675	1
Dfna5	-0,36784	1
Tnfrsf23	-0,36808	1
Creb3	-0,36823	1
Gm37702	-0,36839	1
7330423F06Rik	-0,36841	1
Tcaf1	-0,36852	1
Kctd10	-0,36853	1
Slc44a2	-0,36859	1
2310015A10Rik	-0,36871	1
Gm8731	-0,36868	1
Gm4734	-0,36894	1
Patl1	-0,36887	1
Gm11346	-0,36916	1
Rpsa	-0,3693	1
Manbal	-0,36952	1
Map2k1	-0,36982	1
H2afz	-0,36994	1
Gm43061	-0,37	1
Ppia	-0,37022	1
Kpna6	-0,37034	1
Napg	-0,37063	1
RP24-183O8.6	-0,37079	1
Mettl23	-0,37085	1
Rps12-ps9	-0,37093	1
Mlf1	-0,371	1
Atpaf2	-0,37104	1
Hspb11	-0,37097	1
Nmrk1	-0,37119	1
A830080D01Rik	-0,37118	1

Polr2h	-0,37142	1
Rps6-ps1	-0,37149	1
Rpa2	-0,37164	1
Rbm25	-0,37161	1
Gm44699	-0,37173	1
Srsf2	-0,37187	1
Usf1	-0,37198	1
Rps6	-0,37196	1
Gm28809	-0,37244	1
Sin3a	-0,37239	1
Ube2c	-0,37246	1
Tmem11	-0,3725	1
Fem1c	-0,3727	1
Itpripl1	-0,37281	1
Irf2bpl	-0,37277	1
Cracr2b	-0,37289	1
Spred2	-0,37285	1
Spryd7	-0,3729	1
Stk16	-0,37376	1
2310034G01Rik	-0,37431	1
Supt7l	-0,37429	1
Pds5a	-0,37438	1
Usp35	-0,37447	1
RP23-354J5.3	-0,37464	1
Mad2l2	-0,37468	1
Etfa	-0,37494	1
2900076A07Rik	-0,37519	1
Saraf	-0,37523	1
B3gntl1	-0,37563	1
Atp13a3	-0,37558	1
D430013B06Rik	-0,37579	1
Gm6905	-0,37589	1
Pold3	-0,37596	1
Gnas	-0,37615	1
Rpl22-ps1	-0,37638	1
Phc2	-0,37646	1
Gm42728	-0,37666	1
Maff	-0,37691	1
Pwp2	-0,37687	1
Ighm	-0,37732	1
Nnt	-0,37742	1
Gm43420	-0,37781	1
Tmem171	-0,37791	1
Tmem159	-0,37817	1
Atl2	-0,37819	1
Tubb2a	-0,37858	1
Pank4	-0,3787	1
1700084E18Rik	-0,37883	1
Tmem143	-0,37877	1
Lta4h	-0,37876	1
Wdr34	-0,37903	1
Ube2g2	-0,3792	1

Rab3il1	-0,37961	1
RP23-288C18.3	-0,37967	1
Icam4	-0,37967	1
Pikfyve	-0,37969	1
Tra2b	-0,37976	1
Crlf3	-0,37998	1
Gm23100	-0,38022	1
Cdkn1b	-0,38051	1
Hmx2	-0,38066	1
Gm26881	-0,3809	1
Rsrc2	-0,38088	1
mt-Tv	-0,38121	1
Scrn3	-0,38122	1
Slc25a26	-0,38161	1
St3gal4	-0,38162	1
8030462N17Rik	-0,38177	1
Clasp1	-0,3821	1
Srfbp1	-0,38244	1
3110002H16Rik	-0,38267	1
Crls1	-0,38297	1
Hn1l	-0,3831	1
Lsm3	-0,38316	1
Hist1h2al	-0,38328	1
Gm15446	-0,38342	1
Pcgf1	-0,38367	1
Uba52	-0,38365	1
Gm36378	-0,38377	1
Rpl21	-0,38377	1
D8Ertd738e	-0,38379	1
Gm12990	-0,38391	1
Gpr137b	-0,38391	1
Mrpl42	-0,38387	1
Creb1	-0,38424	1
Rad21	-0,38444	1
Ccnc	-0,38477	1
Gm42690	-0,38497	1
Hivep3	-0,38504	1
Selenoi	-0,38506	1
Gm6297	-0,38525	1
Lmbrd2	-0,38556	1
Nr1d1	-0,38569	1
Gm7658	-0,38566	1
Zfp747	-0,3859	1
Tcaim	-0,38611	1
Gm7565	-0,38652	1
Lyst	-0,38673	1
Cdk19	-0,3869	1
Gm5644	-0,38716	1
B230219D22Rik	-0,38719	1
Tgds	-0,38725	1
Rasgrp4	-0,3874	1
Raly	-0,38752	1

Osgin2	-0,38748	1
Stk19	-0,38775	1
Gm15013	-0,38798	1
Gm20430	-0,38795	1
Gch1	-0,38842	1
Fkbp1b	-0,38847	1
Hltf	-0,38848	1
Rps3a1	-0,38864	1
Noct	-0,38876	1
Stt3a	-0,38883	1
Rbm48	-0,38891	1
D10Wsu102e	-0,38892	1
Selenow	-0,3891	1
Sbf1	-0,38943	1
Cep170	-0,38938	1
Psmd9	-0,38973	1
Sun2	-0,38972	1
Cdk17	-0,38976	1
Smarcad1	-0,38995	1
Gm11989	-0,38998	1
Zfp709	-0,39019	1
Elk4	-0,39029	1
Rps13-ps2	-0,39032	1
Wdcp	-0,39043	1
Cd302	-0,3906	1
Rps10	-0,39059	1
Gm15772	-0,39076	1
Gm10269	-0,39105	1
Gm22980	-0,39121	1
Eps15l1	-0,39143	1
Trmt1	-0,39157	1
Dffb	-0,39167	1
Gm11517	-0,39172	1
1700001G11Rik	-0,39165	1
Gm6808	-0,3918	1
Apol11b	-0,39184	1
Gm12582	-0,39248	1
Pdcd2l	-0,39264	1
Rps18-ps1	-0,3928	1
Mir17hg	-0,39294	1
Ptch1	-0,393	1
Rpl27a-ps2	-0,39307	1
Gm6419	-0,39332	1
Mex3a	-0,3933	1
Bmt2	-0,39333	1
Fam71f2	-0,39353	1
Nova2	-0,3935	1
Chst12	-0,39355	1
Gm14303	-0,39376	1
Cfl2	-0,39391	1
Pcf11	-0,39436	1
Gm15500	-0,39488	1

Baz2a	-0,39488	1
Gm4978	-0,39496	1
6720427I07Rik	-0,3951	1
Gm4374	-0,39517	1
0610030E20Rik	-0,39529	1
Dyrk3	-0,39555	1
Hnrnpd	-0,39568	1
Sh3bp1	-0,39583	1
Commd1	-0,39607	1
Hoxb7	-0,3968	1
Gm16223	-0,39691	1
Mir124-2hg	-0,39712	1
AA474408	-0,3972	1
3110009E18Rik	-0,3974	1
Cnnm4	-0,39755	1
F10	-0,39765	1
Pi16	-0,39802	1
Ube2f	-0,39797	1
Slc8b1	-0,39818	1
Nrm	-0,39827	1
Gm20342	-0,39885	1
Grhl1	-0,39914	1
Gm20274	-0,39935	1
Gpr155	-0,3993	1
Polr2k	-0,39934	1
Mettl10	-0,39942	1
Cdca4	-0,3994	1
Asph	-0,39983	1
2010016I18Rik	-0,40004	1
Gm8318	-0,40003	1
Chic2	-0,40007	1
Jsrp1	-0,40023	1
Glipr2	-0,4002	1
1110065P20Rik	-0,40016	1
Zfp503	-0,40025	1
Dpm1	-0,40029	1
Upf1	-0,40033	1
Gm6198	-0,4004	1
Vps37b	-0,40096	1
Gm6649	-0,40117	1
Epb41	-0,40119	1
Gltp	-0,40115	1
Mybl2	-0,40131	1
Rpl36-ps2	-0,40156	1
Lyz1	-0,4017	1
Rnf167	-0,40184	1
2510039O18Rik	-0,40194	1
Rpl15	-0,40229	1
Vbp1	-0,4025	1
Fam210a	-0,40261	1
Napepld	-0,40267	1
S100a6	-0,40268	1

Gm43655	-0,40277	1
Hsph1	-0,40294	1
Nufip1	-0,40304	1
Gm26890	-0,4031	1
Cdc42bpb	-0,40318	1
Sqle	-0,40328	1
Ttc37	-0,40341	1
Gm45836	-0,40339	1
Zbtb17	-0,40374	1
Gm19739	-0,40369	1
Gm8805	-0,40384	1
5430416N02Rik	-0,40405	1
Gm31166	-0,40407	1
Slc39a14	-0,40424	1
Rps15a	-0,40432	1
Gm8599	-0,40445	1
Naa16	-0,40458	1
Rpl28	-0,40495	1
Rpl14-ps1	-0,40502	1
Cd47	-0,40503	1
Mgat1	-0,40515	1
Rbm18	-0,40515	1
Gtf2e2	-0,40527	1
Gm8444	-0,4057	1
Lig1	-0,40567	1
Mocs1	-0,40579	1
Fam20c	-0,40644	1
Rnf7	-0,40669	1
Tcf19	-0,40675	1
Gm13890	-0,40693	1
Dgat2	-0,40695	1
Gm20442	-0,40697	1
Serf2	-0,40698	1
Timeless	-0,40771	1
Gm12857	-0,40783	1
Gm14706	-0,408	1
2900026A02Rik	-0,40835	1
Zfp651	-0,40869	1
Pfdn2	-0,40883	1
CAAA01194877.2	-0,40912	1
Anxa3	-0,40931	1
Spg21	-0,4096	1
Gabpb1	-0,40973	1
Diaph3	-0,40985	1
Fam198b	-0,41014	1
Gm11944	-0,41013	1
Mcub	-0,41007	1
Rgs14	-0,41027	1
Ctxn1	-0,41083	1
Rpl7a	-0,41091	1
Bcas2	-0,41085	1
Ube2s	-0,41102	1

Kat2b	-0,41124	1
Gas5	-0,41116	1
Dnaaf5	-0,41125	1
Gm5905	-0,41163	1
Gm10288	-0,41169	1
Ywhaq	-0,41167	1
Atpaf1	-0,41191	1
Nectin3	-0,41188	1
Arl13b	-0,41198	1
Metap1d	-0,41236	1
Paip2	-0,41247	1
Dot1l	-0,41246	1
Rad52	-0,41274	1
Gm17511	-0,41293	1
Pcbp4	-0,41287	1
Msh3	-0,41318	1
Eaf1	-0,41343	1
Ilf3	-0,41336	1
Arl5b	-0,41354	1
Hoxb5	-0,4136	1
Lemd3	-0,41381	1
Nop56	-0,41415	1
Gm38375	-0,41428	1
D130020L05Rik	-0,41437	1
RP24-366E11.4	-0,41448	1
Rdh10	-0,41452	1
Acyp1	-0,41469	1
Taf15	-0,41491	1
Map3k2	-0,41502	1
Cdc42se1	-0,41514	1
Enkd1	-0,41519	1
Gm10501	-0,41538	1
Tmem216	-0,41542	1
Ccdc84	-0,41561	1
Jmjd6	-0,41577	1
Gm11488	-0,41591	1
Brwd3	-0,4159	1
Gm1976	-0,41599	1
Cnot4	-0,41601	1
Fiz1	-0,41614	1
Gm14541	-0,41661	1
Pik3ip1	-0,41674	1
Ldha	-0,41694	1
Bach1	-0,41699	1
Rpl13	-0,41701	1
Pafah1b3	-0,41702	1
Ovgp1	-0,41714	1
Alyref	-0,41772	1
Gm24339	-0,41824	1
Gm15694	-0,41845	1
Lsm14a	-0,41839	1
Gm10478	-0,41866	1

Dhrs9	-0,41873	1
Zfp574	-0,41874	1
Pcdhb17	-0,41926	1
Gm13498	-0,4196	1
Gm43223	-0,41964	1
H2-M3	-0,42006	1
Mkln1	-0,42014	1
Mgarp	-0,42042	1
Gm37206	-0,4208	1
Gm12967	-0,42079	1
S100a13	-0,4208	1
Vamp3	-0,42079	1
Med11	-0,42088	1
Pwp1	-0,42111	1
Chac2	-0,42118	1
Car12	-0,42116	1
Pam16	-0,42159	1
Lmf2	-0,42161	1
Tpd52-ps	-0,42169	1
Atxn1l	-0,42192	1
Gm9008	-0,422	1
Rpl10-ps3	-0,42197	1
Paxbp1	-0,4226	1
Hsf2	-0,42334	1
Psme1	-0,42341	1
Mier1	-0,42353	1
Kif13b	-0,42367	1
Ulk4	-0,42386	1
Cox7a2l	-0,42401	1
1810037I17Rik	-0,42408	1
Pag1	-0,42406	1
Gm5619	-0,42461	1
Zc3h3	-0,42457	1
Napsa	-0,42485	1
Xpo1	-0,42499	1
Uhrf2	-0,42497	1
Rps11-ps1	-0,42526	1
Gm5576	-0,42551	1
Dock1	-0,42556	1
Abhd2	-0,42557	1
Sgsm2	-0,42578	1
Gm11808	-0,42614	1
Psat1	-0,4261	1
Scp2-ps2	-0,42621	1
Gm12501	-0,42621	1
Syt11	-0,42616	1
Eif2s3x	-0,42648	1
Gm1947	-0,42665	1
Rpl3	-0,42688	1
Tmpo	-0,42704	1
Rpl37	-0,42712	1
Gtf2h5	-0,42766	1

Fam83a	-0,42783	1
Usmg5	-0,42785	1
Tctn1	-0,42813	1
Dhx8	-0,42815	1
Eif4g2	-0,42809	1
Eif3j1	-0,42851	1
Hdac9	-0,42863	1
Kif24	-0,42872	1
Rsbn1	-0,42879	1
Gm7846	-0,42886	1
Gm10275	-0,42894	1
Scarna2	-0,42928	1
Gm44957	-0,42939	1
Gm9385	-0,42936	1
Cbx1	-0,42975	1
Snu13	-0,42987	1
Calr-ps	-0,4299	1
Med18	-0,43048	1
Gm45221	-0,43061	1
E330037G11Rik	-0,43082	1
Trmt112	-0,43088	1
Mapkapk5	-0,4313	1
Arl6ip6	-0,43147	1
Slc15a4	-0,43148	1
Dusp4	-0,43195	1
Efnb1	-0,43212	1
Oard1	-0,43216	1
Dnajc22	-0,4326	1
Tet3	-0,43304	1
Pkn2	-0,43305	1
Rnf2	-0,43307	1
Mir99ahg	-0,43329	1
Mastl	-0,43342	1
Lck	-0,43344	1
2500002B13Rik	-0,4335	1
Mkrn2	-0,43359	1
Tma7-ps	-0,43368	1
Gm8995	-0,43376	1
Gm12346	-0,43389	1
E2f5	-0,43448	1
Cox19	-0,43469	1
Gm37962	-0,435	1
Szrd1	-0,43502	1
Gm10086	-0,43515	1
Scrn2	-0,43536	1
Gatm	-0,43565	1
Rpl10-ps2	-0,43587	1
Sema4c	-0,43643	1
Gm30238	-0,43653	1
Diaph1	-0,4373	1
Vcpkmt	-0,43768	1
Tubb4b	-0,43821	1

C920021L13Rik	-0,43842	1
Cmc1	-0,43853	1
Pdcl3	-0,43862	1
Anp32a	-0,43865	1
Fcor	-0,43882	1
Rpl36a-ps2	-0,43882	1
Gm15427	-0,43947	1
S100a11	-0,4395	1
Scel	-0,4398	1
Arhgdia	-0,43999	1
Rpl18-ps1	-0,4402	1
St6galnac6	-0,44034	1
Plekhf2	-0,44059	1
Atp5l	-0,44093	1
Mpc1	-0,44088	1
Dbr1	-0,44214	1
Tcf20	-0,4421	1
Gm13408	-0,4422	1
Gm15445	-0,44218	1
Gm45110	-0,44274	1
B2m	-0,44275	1
Slbp	-0,44283	1
Kctd5	-0,44278	1
Tk1	-0,4431	1
Gm18737	-0,4434	1
Gm5276	-0,44345	1
Dgcr8	-0,44347	1
5730508B09Rik	-0,44379	1
Aurka	-0,44382	1
Rgs1	-0,44417	1
Gm23127	-0,44422	1
Insig2	-0,44449	1
Ccnl1	-0,44473	1
Sik1	-0,44486	1
Hyal1	-0,44491	1
Sft2d1	-0,44495	1
Hypk	-0,445	1
Slc13a3	-0,44509	1
Gm31274	-0,44518	1
Spty2d1	-0,44568	1
Gm14776	-0,44596	1
Klhl15	-0,44607	1
Gm3940	-0,44619	1
Gm9762	-0,44649	1
Smg5	-0,44666	1
Gm8213	-0,44673	1
Gm11964	-0,44692	1
Fam20b	-0,44693	1
Rps2-ps5	-0,447	1
Inafm1	-0,44705	1
Myl12a	-0,44703	1
Prkcd	-0,44702	1

Ccnyl1	-0,44759	1
Pou5f2	-0,44756	1
Runx1	-0,4478	1
Atp11a	-0,4479	1
Eif3s6-ps1	-0,44813	1
Sptssa	-0,44822	1
Cic	-0,44846	1
Bsn	-0,44867	1
Prr11	-0,45014	1
Gm13039	-0,45034	1
Trp53rkb	-0,45025	1
Cd40	-0,45042	1
Jam2	-0,45046	1
Higd2a	-0,45047	1
Copz2	-0,4506	1
Tsr2	-0,45084	1
Lamc1	-0,45085	1
RP23-123D6.12	-0,45104	1
Zfp146	-0,45118	1
Htt	-0,45131	1
Nabp2	-0,45139	1
Ccdc34	-0,45139	1
Hmbox1	-0,45146	1
Ppp1r12c	-0,4515	1
BC030336	-0,45212	1
Gm13777	-0,45234	1
Arl1	-0,45238	1
Adgre1	-0,45313	1
Spns1	-0,45355	1
RP23-255F14.4	-0,45366	1
Med30	-0,45452	1
Tmie	-0,45453	1
Ptcd3	-0,45464	1
Gm12693	-0,45474	1
Gm26799	-0,4553	1
Pltp	-0,45534	1
Irx2	-0,45548	1
1110035H17Rik	-0,45638	1
Alox8	-0,45675	1
Gm12184	-0,45706	1
Sf3a2	-0,45716	1
Usp21	-0,45751	1
Entpd6	-0,45822	1
Gm6181	-0,45823	1
Ptma	-0,45845	1
Stx7	-0,45868	1
Ranbp9	-0,45885	1
Edem3	-0,45882	1
Gm10161	-0,45894	1
Nup50	-0,45922	1
6430511E19Rik	-0,45932	1
Gm8894	-0,45939	1

Rpl28-ps1	-0,45956	1
Tuba1b	-0,45962	1
Gm44153	-0,45973	1
Cox16	-0,45965	1
Hdac5	-0,45982	1
Gm14005	-0,45988	1
AI480526	-0,46008	1
Gm28659	-0,4601	1
Shprh	-0,46025	1
Gm14813	-0,46051	1
Myadm	-0,46058	1
Slc30a5	-0,46089	1
Pnn	-0,46107	1
Parpbp	-0,46124	1
Gm38220	-0,46118	1
Rps24	-0,46116	1
Gm14126	-0,46127	1
P3h3	-0,46149	1
Rngtt	-0,4616	1
5830432E09Rik	-0,46192	1
Relt	-0,46215	1
Ak6	-0,46224	1
Gm8919	-0,46254	1
Dynlt1-ps1	-0,46258	1
Bloc1s4	-0,46294	1
Prelid2	-0,46294	1
Gtpbp2	-0,46305	1
Tapbp	-0,46332	1
Ctnnd1	-0,46354	1
Lnpk	-0,46393	1
Coro1a	-0,46414	1
Gfpt1	-0,46413	1
Chmp1b	-0,46462	1
2900055J20Rik	-0,46475	1
Cinp	-0,46494	1
Pik3c2a	-0,46496	1
Gm43059	-0,46499	1
Gm9134	-0,46515	1
Rps6ka4	-0,46537	1
Akr7a5	-0,46553	1
Gm26129	-0,46558	1
Ints6l	-0,4658	1
Ercc1	-0,46586	1
Ip6k2	-0,46604	1
Gm6085	-0,46602	1
Mbtd1	-0,46607	1
Gm19287	-0,46621	1
Gm15542	-0,46658	1
Nhlrc3	-0,46692	1
Papd5	-0,46728	1
Gltscr1	-0,46739	1
Gm7972	-0,46737	1

Pcgf2	-0,46776	1
Kansl2	-0,4679	1
1110008P14Rik	-0,46804	1
Slc16a10	-0,46813	1
Gm17827	-0,46812	1
Rara	-0,46836	1
Peak1	-0,46865	1
2810408I11Rik	-0,469	1
Banp	-0,46935	1
Stk24	-0,46955	1
Gm15829	-0,47024	1
Gm20186	-0,47049	1
Aida	-0,47056	1
Igip	-0,47068	1
Vps29	-0,47105	1
RP23-312A24.1	-0,47111	1
Fcna	-0,47123	1
Uba6	-0,47127	1
Tmem8b	-0,47158	1
Apmap	-0,47184	1
Spata33	-0,4719	1
Gm43761	-0,472	1
Ldb1	-0,47223	1
Jund	-0,47216	1
Gstcd	-0,47226	1
Smc5	-0,47258	1
Incenp	-0,47269	1
5830408C22Rik	-0,4728	1
Gnb2	-0,47278	1
Hnrnph1	-0,47292	1
Acaa1b	-0,47297	1
Tmtc3	-0,47306	1
Gm8451	-0,47309	1
Fbl	-0,47315	1
Stil	-0,47329	1
Lncpint	-0,47362	1
Gm37642	-0,47359	1
Rpl19-ps1	-0,4736	1
Myo1f	-0,47364	1
C630043F03Rik	-0,47369	1
Gm11560	-0,47391	1
Gm12428	-0,47387	1
Gm9165	-0,47443	1
Tardbp	-0,47461	1
Rps11-ps2	-0,47495	1
Rpsa-ps11	-0,47531	1
Rtn4	-0,47527	1
Gm6654	-0,47545	1
Gm43200	-0,47546	1
Ifi202b	-0,47584	1
Gm5525	-0,47579	1
Ddx47	-0,47584	1

Zfp58	-0,47631	1
Xrcc2	-0,47674	1
Rpl36a	-0,4776	1
Gm15730	-0,47755	1
Emilin2	-0,47779	1
Gm15727	-0,47823	1
Gm8730	-0,47827	1
Arhgap25	-0,47826	1
Bbs5	-0,47843	1
Gm9701	-0,47851	1
Suv39h1	-0,47849	1
Chchd6	-0,47853	1
Rps27rt	-0,47848	1
Gm11772	-0,47897	1
Gm45806	-0,47909	1
Rpl18-ps2	-0,47952	1
Triap1	-0,47949	1
Rps6ka5	-0,4801	1
Tmx4	-0,48014	1
Cmtm4	-0,48022	1
Gm7887	-0,48016	1
Gm9825	-0,48046	1
Npepps	-0,48113	1
Rev1	-0,48117	1
Klf6	-0,48181	1
Enoph1	-0,4824	1
Intu	-0,48251	1
Tmem63a	-0,48273	1
Arl3	-0,48321	1
Samd8	-0,48342	1
Lgals1	-0,48361	1
Tnfrsf26	-0,48389	1
Ttc5	-0,48411	1
Gm13038	-0,4842	1
Smox	-0,48478	1
Clic1	-0,48503	1
Gm14336	-0,48513	1
Ndufs5	-0,48535	1
Gm12176	-0,48534	1
Tfeb	-0,48596	1
Adamtsl1	-0,48641	1
Gm13886	-0,48653	1
1700124L16Rik	-0,48734	1
Gm12338	-0,48752	1
Melk	-0,48792	1
Rbpms	-0,48809	1
Thap1	-0,48811	1
Arf6	-0,48845	1
Yipf4	-0,48858	1
Rpl11	-0,48891	1
Rps12-ps4	-0,48892	1
Celf1	-0,48893	1

Rpl31-ps8	-0,48902	1
Rassf8	-0,48907	1
Gm11930	-0,48921	1
Gm6682	-0,48924	1
Gm17971	-0,48925	1
B230217C12Rik	-0,48971	1
Stc2	-0,48992	1
Tmem168	-0,48989	1
Lactb2	-0,4901	1
Acot1	-0,49025	1
Nr3c1	-0,49033	1
Gm6142	-0,49044	1
Terf2ip	-0,49062	1
Sem1	-0,49074	1
Gm7432	-0,49131	1
Trf	-0,49126	1
4933433G15Rik	-0,49144	1
Gm12258	-0,49159	1
Ube2d1	-0,49171	1
Sc5d	-0,49173	1
Gm7589	-0,49191	1
Cryz	-0,49191	1
Gm14539	-0,4921	1
Pmm1	-0,49216	1
Ssfa2	-0,49226	1
C030034I22Rik	-0,49247	1
Cd52	-0,49292	1
Gm12115	-0,49301	1
BC085271	-0,49297	1
Gm7666	-0,49309	1
Nup153	-0,49384	1
mt-Tc	-0,49389	1
Knl1	-0,49413	1
Tm6sf1	-0,49432	1
Gm8254	-0,49434	1
Snx3	-0,49453	1
Hspbap1	-0,49495	1
Gm37234	-0,49488	1
Prelid1	-0,49542	1
RP24-225A16.3	-0,49549	1
Mrpl27	-0,49559	1
Gm13397	-0,49571	1
Mthfd2l	-0,49579	1
Rpl21-ps6	-0,4959	1
Rpl41	-0,49603	1
Rps8-ps3	-0,49613	1
Rps4x-ps	-0,49626	1
Tmed8	-0,49662	1
Gm20045	-0,49678	1
Gm10704	-0,4968	1
Hic2	-0,49689	1
Clpx	-0,49744	1

RP23-43M12.2	-0,49756	1
Rpa3	-0,49778	1
Whamm	-0,49804	1
Ncaph2	-0,49813	1
Pdlim7	-0,49817	1
RP23-359K10.8	-0,49854	1
Flna	-0,49858	1
1700056N10Rik	-0,49871	1
Ccne2	-0,49886	1
Rpl39	-0,49927	1
Ticrr	-0,49961	1
Gm6640	-0,50024	1
Ndfip1	-0,50041	1
Tmod3	-0,50102	1
4933427D14Rik	-0,50098	1
Tslp	-0,50129	1
Ggct	-0,50141	1
Stx3	-0,50155	1
Acadm	-0,50164	1
Ckb	-0,50178	1
Gm7312	-0,50255	1
Gm15198	-0,50278	1
Tmem261	-0,50303	1
Rab26os	-0,50307	1
Gm8606	-0,50319	1
Blvrb	-0,50341	1
Ybx1	-0,5035	1
Nrtn	-0,50386	1
Ccnt2	-0,50407	1
RP23-65M10.2	-0,50432	1
Gm15268	-0,50473	1
Crip2	-0,50497	1
Jag1	-0,50526	1
Dpysl2	-0,50535	1
Ppdpf	-0,50548	1
Rangrf	-0,50549	1
Gm2000	-0,5058	1
Gm43162	-0,50583	1
Slc16a1	-0,50665	1
Gm14427	-0,50685	1
Plxna3	-0,50689	1
Gm9531	-0,50748	1
1110046J04Rik	-0,50746	1
Eif3f	-0,50778	1
Gm6285	-0,50784	1
Gm37297	-0,50781	1
Akr1b10	-0,50793	1
Rpl3-ps2	-0,5081	1
Fbxl3	-0,50819	1
Pde2a	-0,50823	1
Tpra1	-0,50842	1
Gm7808	-0,50864	1

Abt1	-0,50903	1
Rpl31	-0,50912	1
Kif9	-0,50912	1
Gm11686	-0,50919	1
Rps7	-0,5093	1
Sf3b1	-0,50937	1
Abca1	-0,50936	1
Sp4	-0,50993	1
Raph1	-0,50988	1
Ccnd3	-0,51007	1
Rpl9-ps6	-0,51041	1
Rpl5	-0,51044	1
Cenpu	-0,51073	1
Gng12	-0,51087	1
Prmt2	-0,51211	1
Gm42547	-0,51243	1
Thbs3	-0,51272	1
Pax3	-0,51323	1
Ccl9	-0,5136	1
Usp26	-0,51369	1
Gm5599	-0,51378	1
Snrpg	-0,51389	1
Lilr4b	-0,51408	1
Rps24-ps3	-0,51489	1
Gm12716	-0,51501	1
Cbx4	-0,51526	1
Snx29	-0,51532	1
Rhoa	-0,51536	1
Cenpm	-0,5155	1
Ralb	-0,51588	1
Mien1	-0,51608	1
Gm7638	-0,51618	1
Gpi1	-0,51676	1
Gm15506	-0,51693	1
Atp7a	-0,51711	1
Cdc6	-0,51714	1
Ugcg	-0,51757	1
St6gal1	-0,51786	1
Atg16l1	-0,51816	1
Cebpg	-0,51893	1
Btaf1	-0,51906	1
Cacna1a	-0,51919	1
Rapgef5	-0,5195	1
Magoh	-0,5197	1
Ascc3	-0,5197	1
Gm11675	-0,51995	1
Ccnb2	-0,52018	1
Adam8	-0,52112	1
Ankle1	-0,52115	1
Wdr20	-0,52143	1
Kpna3	-0,52173	1
Lars2	-0,52165	1

Arhgap26	-0,52189	1
Ccl3	-0,52209	1
Gm13827	-0,52224	1
Gm14513	-0,5222	1
Rad9a	-0,5223	1
Gm7094	-0,52311	1
Zfp87	-0,52321	1
Gm44567	-0,52318	1
Prkx	-0,52344	1
Gm4017	-0,52356	1
Rdm1	-0,5237	1
Gm18916	-0,52377	1
Gm10443	-0,52413	1
Gm13392	-0,52417	1
9630010A21Rik	-0,52428	1
Cep89	-0,5243	1
Gm10076	-0,5244	1
Ptger4	-0,52485	1
Kif2c	-0,52495	1
Ywhah	-0,52534	1
Cbwd1	-0,52645	1
Gm11450	-0,52662	1
Glul	-0,52711	1
Cds1	-0,52714	1
Gm11942	-0,52721	1
Haus3	-0,52726	1
Zfp655	-0,52781	1
Clmp	-0,52782	1
Ctnnbip1	-0,52814	1
Gm5139	-0,5281	1
Zeb2os	-0,52817	1
RP23-128C4.4	-0,52821	1
Spin1	-0,52867	1
Pex5	-0,52885	1
Cd300a	-0,52914	1
E2f6	-0,5292	1
Zscan2	-0,52964	1
Pop5	-0,52986	1
Fam171a2	-0,5302	1
Gnai3	-0,53036	1
Gm3362	-0,53038	1
Cyth3	-0,5308	1
9430060I03Rik	-0,53122	1
Snord87	-0,53131	1
Rrm2	-0,53192	1
Osbpl3	-0,53241	1
Fgfr1op	-0,53265	1
a	-0,53308	1
Erlin1	-0,53359	1
Rps15a-ps8	-0,53387	1
Tdp2	-0,53402	1
Dck	-0,53411	1

Rab10os	-0,5343	1
Crebrf	-0,53445	1
H2-Q7	-0,53457	1
Gm6305	-0,5347	1
Gm22714	-0,5349	1
Pcsk7	-0,53498	1
Angptl4	-0,53531	1
Gm37949	-0,53542	1
Add3	-0,53572	1
Rassf7	-0,53567	1
Gm25007	-0,53569	1
Mettl7a1	-0,53601	1
Phtf1os	-0,53597	1
Cdca5	-0,53611	1
Amt	-0,53641	1
Gm36189	-0,53701	1
RP24-282C4.13	-0,53702	1
Fbxo21	-0,53719	1
Rps12-ps10	-0,53721	1
Topbp1	-0,53731	1
Sp140	-0,53726	1
Pfn1	-0,53729	1
Slc2a4rg-ps	-0,53743	1
H2-K1	-0,5375	1
Pex1	-0,53811	1
Tmem132a	-0,53843	1
Gm4525	-0,53845	1
Hnrnpr	-0,53867	1
Efhd2	-0,53901	1
Acer3	-0,53942	1
Gm10689	-0,53949	1
Hsd3b7	-0,53983	1
RP24-275P22.2	-0,54008	1
Cmtm6	-0,54028	1
Phka2	-0,54124	1
3110031N09Rik	-0,5414	1
Gm9354	-0,54144	1
Lin52	-0,54185	1
9330020H09Rik	-0,54197	1
Tspyl4	-0,54197	1
Emp1	-0,54208	1
Calm3	-0,54246	1
Hinfp	-0,54297	1
Hspe1	-0,54323	1
Tsc1	-0,54375	1
Sbds	-0,54407	1
Gm10260	-0,54504	1
Gm6378	-0,54539	1
Gsk3a	-0,54552	1
Gm43961	-0,54595	1
Rcn2	-0,54733	1
Icosl	-0,54727	1

Plk1	-0,54748	1
Klf3	-0,54762	1
Gm26244	-0,54771	1
Rn7s6	-0,54774	1
Sertad1	-0,54854	1
Ift80	-0,54892	1
Gm9320	-0,54908	1
Gm10916	-0,54918	1
Gm11334	-0,55005	1
Gm9938	-0,55032	1
Seh1l	-0,55053	1
Map3k9	-0,55069	1
Nexn	-0,55077	1
Gm15859	-0,55132	1
Gm19726	-0,55125	1
Gm42872	-0,55147	1
Wee1	-0,55159	1
Lgmn	-0,5516	1
Gm12038	-0,55162	1
Gm16580	-0,55174	1
Ppp1r16a	-0,55183	1
Pde7a	-0,55218	1
Elavl1	-0,55261	1
Ints8	-0,55272	1
Gm44090	-0,5528	1
Kpna4	-0,55294	1
Susd6	-0,55296	1
Ern1	-0,55346	1
Gm11539	-0,55406	1
Mpp3	-0,55452	1
Cdc42ep4	-0,55465	1
Mybl1	-0,5548	1
Recql4	-0,55488	1
Rpl7-ps7	-0,555	1
Ino80d	-0,55535	1
Tmem81	-0,5556	1
RP23-246F14.1	-0,55572	1
Gm43571	-0,55585	1
Rrp12	-0,55607	1
Srsf4	-0,55616	1
Akirin2	-0,55644	1
Gm6548	-0,55669	1
Gm5881	-0,55683	1
Card19	-0,55702	1
Gm2991	-0,55703	1
Gm8930	-0,55714	1
Gm44419	-0,55723	1
Npm3	-0,55732	1
Ero1l	-0,55742	1
Gm12522	-0,55757	1
Rpl31-ps13	-0,55773	1
Gm15720	-0,55862	1

Magohb	-0,55865	1
Cenpa	-0,55956	1
Cystm1	-0,56013	1
Oaz2	-0,56033	1
Fubp1	-0,56066	1
Gm44694	-0,5608	1
Npy	-0,56135	1
Gm37675	-0,56162	1
Krit1	-0,56168	1
Cpeb4	-0,56181	1
Stxbp3	-0,5618	1
Hsd17b10	-0,56235	1
H2-Q5	-0,56237	1
Aoc2	-0,56334	1
Dynll1	-0,56353	1
Pim3	-0,56365	1
9430015G10Rik	-0,56361	1
Prr7	-0,56372	1
Gstt2	-0,56398	1
Gm6743	-0,56405	1
Adpgk	-0,56433	1
Aaed1	-0,5645	1
C78859	-0,56447	1
Piga	-0,56464	1
Itpkb	-0,56473	1
Dqx1	-0,56517	1
Stam	-0,56533	1
Cenpn	-0,56565	1
Fanca	-0,56568	1
Gm37738	-0,56568	1
Gm15472	-0,56581	1
Rita1	-0,56613	1
Gm45223	-0,56619	1
Gm4997	-0,56645	1
Clic4	-0,56673	1
Gm4602	-0,56667	1
Snhg12	-0,56715	1
Cdca3	-0,56716	1
Tor1aip1	-0,56732	1
Rpl17-ps8	-0,56728	1
D030056L22Rik	-0,56856	1
Hsf2bp	-0,56879	1
Prdm15	-0,56888	1
Polg2	-0,56907	1
Gm7287	-0,56952	1
Pak4	-0,5696	1
Erfe	-0,56963	1
Qk	-0,56996	1
Gm12726	-0,57051	1
Pcif1	-0,57072	1
Plekhg4	-0,57082	1
Gm15159	-0,57105	1

Lmln	-0,57154	1
Gm45749	-0,57146	1
Slc7a6os	-0,57164	1
BC051226	-0,57287	1
Gm19353	-0,57298	1
Gm12732	-0,57301	1
Car2	-0,57327	1
Map3k1	-0,57336	1
Rny1	-0,57355	1
Chek1	-0,57372	1
Atp11b	-0,57421	1
Rps23	-0,5749	1
Sowahc	-0,57548	1
Morf4l1	-0,57553	1
Rpl30	-0,57556	1
Arid3b	-0,57592	1
3110062M04Rik	-0,57609	1
Leng8	-0,57607	1
Gm43773	-0,57614	1
Rps17	-0,57634	1
Metrnl	-0,57681	1
Tmem176a	-0,57722	1
Creb5	-0,57805	1
Depdc1b	-0,5785	1
Gm13622	-0,57946	1
2810405F17Rik	-0,57967	1
Gm43715	-0,57984	1
Gm12267	-0,58001	1
Tmem170	-0,58018	1
Eloc	-0,58112	1
Gm6204	-0,58116	1
Gm45546	-0,58155	1
Tfb1m	-0,58169	1
Lat	-0,58195	1
Gm12074	-0,58215	1
Cdkn2aip	-0,5827	1
Ccdc50-ps	-0,58274	1
Spdl1	-0,58282	1
Ss18l1	-0,58286	1
Gm43578	-0,58289	1
Ddx43	-0,5832	1
Miip	-0,5833	1
Smg1	-0,58353	1
Rgs3	-0,5837	1
Gm22009	-0,58394	1
Cdk2	-0,58451	1
Hist1h4i	-0,58492	1
RP24-454N4.2	-0,585	1
6030400A10Rik	-0,58506	1
Rpl38-ps1	-0,58533	1
Gm38036	-0,58569	1
Med6	-0,5862	1

Rdh13	-0,58678	1
Pfdn5	-0,587	1
Gm8662	-0,58705	1
Gm24951	-0,58714	1
Mtm1	-0,58716	1
Gm6450	-0,58742	1
Fgf13	-0,5883	1
Gm13815	-0,58852	1
Rps12-ps5	-0,58853	1
Smyd5	-0,58883	1
1500002F19Rik	-0,58948	1
Nav2	-0,5899	1
Hpse	-0,58988	1
Tnni2	-0,59029	1
Gm45884	-0,59062	1
Acat1	-0,59076	1
Cebpb	-0,59084	1
Camta1	-0,59086	1
Dusp5	-0,59131	1
Kif14	-0,59142	1
Srcap	-0,59152	1
Gm9396	-0,59194	1
RP23-426K2.3	-0,592	1
Bhlhe41	-0,59226	1
Gm9484	-0,59236	1
Rab5a	-0,59281	1
Ropn1l	-0,59288	1
Tmem256	-0,59326	1
Smagp	-0,59358	1
Gm6433	-0,59375	1
Asf1a	-0,59398	1
Efcab11	-0,59435	1
Gm42576	-0,59484	1
Gm8818	-0,59522	1
Snapc1	-0,59544	1
Gm7351	-0,5956	1
Gm8508	-0,59581	1
Gm37407	-0,59591	1
Gm43336	-0,59653	1
Gm5075	-0,59658	1
Ensa	-0,59671	1
Rbm4b	-0,59669	1
Ppwd1	-0,59691	1
Ruvbl2	-0,59723	1
RP23-331E5.10	-0,59792	1
RP23-58B7.2	-0,59815	1
Tspan13	-0,59828	1
Hmgn5	-0,59866	1
Man1a	-0,59866	1
Gm24991	-0,59883	1
Stom	-0,59929	1
Gm8574	-0,59926	1

Nuak2	-0,59988	1
Cnksr3	-0,60048	1
Rpl18a-ps1	-0,60049	1
Polh	-0,60046	1
Gm9409	-0,60055	1
Gm2225	-0,60125	1
Gm8304	-0,60186	1
Coprs	-0,60213	1
Gm27605	-0,6021	1
Rpl36-ps10	-0,60238	1
Cnih1	-0,60264	1
Dclre1b	-0,60294	1
Gm9727	-0,60293	1
Gm20594	-0,60394	1
Gm10736	-0,60451	1
Syce2	-0,6046	1
Bsdc1	-0,60473	1
Nectin1	-0,60475	1
Fam133b	-0,60555	1
Dnajb5	-0,60553	1
Ifnar1	-0,60592	1
Fmr1	-0,60631	1
Map3k14	-0,60643	1
0610040B10Rik	-0,60651	1
Rasa4	-0,60693	1
mt-Co1	-0,60715	1
Gm6977	-0,60715	1
Gm23458	-0,60713	1
Tmed7	-0,60739	1
Gm5070	-0,6076	1
Mef2c	-0,6084	1
Med22	-0,60894	1
Gm15464	-0,60897	1
Rpl18	-0,61029	1
Phip	-0,61043	1
Rbm12b2	-0,61068	1
Pmpcb	-0,61103	1
H2-Ob	-0,61116	1
Usp53	-0,61167	1
Rpl30-ps11	-0,61181	1
Gm43010	-0,61207	1
1190002N15Rik	-0,61336	1
5430434F05Rik	-0,61374	1
Plaur	-0,61376	1
Hist3h2a	-0,61386	1
Gm12341	-0,61393	1
Gm43813	-0,614	1
Zbtb37	-0,61409	1
Llph	-0,61453	1
Dgke	-0,61459	1
Rps18-ps3	-0,61457	1
Gm43387	-0,61507	1

R3hdm1	-0,61519	1
Gm10240	-0,61516	1
Mcts2	-0,6153	1
Necap1	-0,6154	1
Mis18bp1	-0,61545	1
Ugdh	-0,61557	1
Gm24927	-0,61641	1
Shcbp1	-0,61669	1
RP23-168F21.4	-0,61693	1
Gm8268	-0,61706	1
Tmem44	-0,61741	1
Iffo1	-0,61805	1
Napb	-0,61835	1
Neurl1b	-0,61838	1
Gm17994	-0,61855	1
Fam76b	-0,61957	1
Slc25a38	-0,61956	1
Col11a2	-0,61991	1
Gm37598	-0,62015	1
Gm43247	-0,62014	1
Ctss	-0,62058	1
Fam117b	-0,62063	1
Tmem79	-0,62082	1
Snhg9	-0,62095	1
Fam103a1	-0,62123	1
Mapre2	-0,62151	1
Traip	-0,62146	1
Gm8394	-0,62196	1
Nop10	-0,62234	1
Gm12844	-0,62307	1
Gm4258	-0,62308	1
Nr2c2ap	-0,62335	1
Gm6451	-0,62342	1
Fos	-0,62338	1
Gm25517	-0,62351	1
Dse	-0,62361	1
Gm11343	-0,62383	1
Carf	-0,62423	1
Rpl21-ps12	-0,62427	1
Fbxl12os	-0,62481	1
Gm43878	-0,62555	1
Mir703	-0,62596	1
Tnfaip3	-0,62654	1
Ipp	-0,62663	1
Gm13680	-0,62676	1
Dis3	-0,62721	1
Smim11	-0,62777	1
Gm26930	-0,6278	1
Pigb	-0,6279	1
Gm9378	-0,62808	1
Pradc1	-0,62818	1
Arhgap17	-0,6284	1

Bcl6	-0,62846	1
Cops7a	-0,62861	1
Rps19-ps12	-0,62899	1
Gm43088	-0,62942	1
Zfp955a	-0,62984	1
4921531C22Rik	-0,63002	1
Anpep	-0,63016	1
Sapcd1	-0,63135	1
Gm45084	-0,63154	1
Atp9a	-0,63181	1
Iqsec2	-0,63205	1
Yars2	-0,63199	1
Gm44152	-0,63208	1
Bloc1s1	-0,63256	1
Arrdc4	-0,63301	1
Uevld	-0,63343	1
Col4a6	-0,63342	1
Gm12468	-0,63404	1
Gm37522	-0,63491	1
Zfp36	-0,63511	1
Usp1	-0,63551	1
Trmt1l	-0,63546	1
Gm12481	-0,6357	1
Id2	-0,63651	1
Clu	-0,63667	1
Btbd6	-0,63726	1
Ptpru	-0,63731	1
Dvl2	-0,63843	1
Ufsp1	-0,63869	1
Gm45856	-0,63877	1
Aunip	-0,6393	1
Lmnb2	-0,63947	1
2810403A07Rik	-0,63957	1
4930522L14Rik	-0,63977	1
Gm45890	-0,63976	1
Depdc1a	-0,64002	1
Gm25636	-0,64008	1
Rps28	-0,6415	1
Mfsd14a	-0,64197	1
Gm13268	-0,64221	1
Dcp2	-0,64228	0,96732
Lin9	-0,64303	1
Ercc6l	-0,64301	1
Tbc1d31	-0,64326	1
Cbfa2t3	-0,64327	1
Gm44916	-0,64328	1
Hist1h4n	-0,64374	1
Skp2	-0,64403	1
Rnf19a	-0,6444	1
Hnrnpa3	-0,64441	1
Gm13532	-0,64444	1
Hps3	-0,64502	1

Gm45167	-0,64528	1
Rpl12-ps1	-0,6454	1
Gm12944	-0,64539	1
Eif4a2	-0,64546	1
Gm15564	-0,6455	1
Katnbl1	-0,64565	0,9208
Gm10327	-0,64563	1
Ppp1r37	-0,64568	1
Rmnd5a	-0,646	1
Pgk1	-0,64661	1
Ttc21b	-0,64698	1
Gm9790	-0,64795	1
Gm12943	-0,64803	1
Gm44198	-0,64842	1
Socs4	-0,64875	1
Gm24924	-0,64876	1
Tsc22d2	-0,64909	0,98263
Irak3	-0,65014	1
Gm2796	-0,65072	1
Btg1	-0,65068	1
Hirip3	-0,65097	1
Rpl19-ps11	-0,65134	1
Tmx2	-0,65143	1
Eif1b	-0,65176	0,88513
Rad54l	-0,65299	1
Rhebl1	-0,654	0,97069
Gm14121	-0,65403	1
Gm8430	-0,65405	1
Rin1	-0,65451	1
Svep1	-0,65453	1
Rrm1	-0,65527	1
Ifi213	-0,65529	1
Ptgs2	-0,65548	1
Gm42783	-0,6555	1
Gm38262	-0,65581	1
4930447F24Rik	-0,65588	1
Gm43096	-0,65635	1
Rpl23a-ps5	-0,65646	1
Csrp1	-0,65665	1
9930012K11Rik	-0,65702	1
Tnfsf9	-0,65697	1
Rgs2	-0,6574	0,8865
Spry2	-0,65813	1
Gm36964	-0,65835	1
Pcna	-0,65887	1
Gm5451	-0,65991	1
Tmco3	-0,6602	1
Gm6136	-0,66017	1
Rps3a3	-0,66038	1
Gins3	-0,66085	1
Dennd4c	-0,66166	1
Ing4	-0,66166	1

Gm7434	-0,66168	1
Mis18a	-0,66182	1
Tial1	-0,66207	0,96329
Mcm2	-0,66248	1
Fam126b	-0,66305	0,96759
Fbxo44	-0,66352	1
Gm14138	-0,66355	1
Enc1	-0,66381	0,95327
Tmem170b	-0,66406	1
B4galt3	-0,66433	0,90696
Nab2	-0,66444	1
Il10ra	-0,66449	1
Gm30074	-0,66478	1
Cdk1	-0,66514	0,77002
Gm5787	-0,66533	1
Rnps1	-0,66536	1
6820402A03Rik	-0,66583	1
Cd300ld	-0,66636	1
Inpp5e	-0,66693	1
Asf1b	-0,66742	1
D830050J10Rik	-0,66784	1
Gm44168	-0,66793	1
Gm7332	-0,66805	1
mt-Tq	-0,66853	1
Gprasp1	-0,66865	1
Dusp1	-0,66898	1
Gm37696	-0,66897	1
Gm44951	-0,66955	1
RP23-243B24.1	-0,66991	1
C130071C03Rik	-0,67015	1
Gm37726	-0,67217	1
Gm26520	-0,67264	1
2900052L18Rik	-0,6727	1
Kcnb1	-0,67267	1
Parp6	-0,67303	1
Ptpn2	-0,67299	1
Gm10074	-0,67297	1
Txn-ps1	-0,67313	1
H60b	-0,67398	0,8641
Rab19	-0,67411	1
Sap18	-0,67448	1
Gm43513	-0,67495	1
Pigg	-0,67534	1
Uimc1	-0,67537	0,96379
H3f3a	-0,67541	1
Cadm1	-0,67565	0,56206
Golga7	-0,67611	1
Gm13573	-0,67697	1
Gm43309	-0,67718	1
Dtymk	-0,67775	0,96638
Rpl39-ps	-0,67822	1
Dnajb6	-0,67831	0,8103

Cldnd1	-0,67835	1
Dennd4a	-0,67895	0,83944
Gm16740	-0,67923	1
Mxi1	-0,67939	0,73206
Dram1	-0,68015	1
Tob1	-0,68026	1
Dab2	-0,6804	0,83797
Hist1h2bc	-0,68117	1
Zfp949	-0,68147	1
1700001P01Rik	-0,68158	1
Snord66	-0,68403	1
Cdkn2c	-0,68415	1
Gm37914	-0,68426	1
Gm45828	-0,68452	1
Vezt	-0,6848	1
Cfap20	-0,68482	1
Dut	-0,68496	0,83566
Hnrnph2	-0,685	1
3110045C21Rik	-0,68507	1
Polr2d	-0,68529	1
Gm37452	-0,68533	1
Gm7964	-0,68528	1
Entpd1	-0,68581	1
Zbtb34	-0,68598	1
Ddias	-0,68598	1
5430421F17Rik	-0,68642	1
Gm7327	-0,68676	1
Rictor	-0,687	0,77314
Pde1b	-0,68723	1
Rep15	-0,68771	1
E230029C05Rik	-0,68777	1
Gm5586	-0,68825	1
Ccsap	-0,68858	1
Dpy19l3	-0,68864	1
Znrf3	-0,68899	1
Ifitm5	-0,68902	1
Gm13181	-0,68916	1
Prkd2	-0,68968	1
Fam13b	-0,68977	1
G430095P16Rik	-0,68993	1
Rbm47	-0,69017	0,81105
Crybb3	-0,69016	1
Dnajb1	-0,69034	1
Gm13215	-0,69104	1
Gm43696	-0,691	1
Swap70	-0,6915	0,87123
Gm20620	-0,69174	1
Tnrc18	-0,69269	0,59642
Fgd2	-0,69266	1
Kif22	-0,69358	1
Zc3h7a	-0,69381	0,8294
H2-Q4	-0,6944	1

Vdr	-0,69492	1
Carhsp1	-0,69502	0,71757
Rpl19	-0,69504	1
E230032D23Rik	-0,69595	1
Daglb	-0,69652	0,72711
Snord49b	-0,6966	1
4930579K19Rik	-0,69656	1
Gm12166	-0,697	1
Ppp2cb	-0,69752	1
Gm7424	-0,6989	1
Kdm3a	-0,69905	1
Ptgs1	-0,69964	1
Lgals3	-0,69999	0,5001
Amn1	-0,70093	1
A530017D24Rik	-0,70134	1
Gm561	-0,7018	1
Pcgf5	-0,70275	1
Tarbp2	-0,70304	1
Mynn	-0,70439	0,9978
Vamp2	-0,70455	1
Lmnb1	-0,70465	0,87204
Zfp932	-0,7048	1
Lsr	-0,70579	1
C130089K02Rik	-0,70589	1
5730405O15Rik	-0,70648	1
Cdca8	-0,70685	0,55536
Tra2a	-0,70697	0,71143
Ncapd2	-0,70738	0,96617
BC030867	-0,70763	1
Jun	-0,70769	1
Zfp940	-0,70832	1
Plekha1	-0,70841	0,64068
Gm26384	-0,70839	0,83534
0610039K10Rik	-0,70935	1
Igf2bp2	-0,7096	0,67986
Gm9645	-0,70996	1
Plekhf1	-0,71032	1
Tpt1-ps5	-0,71075	1
Gm13456	-0,71113	1
Snx25	-0,71109	1
Hmga1-rs1	-0,71117	1
Gm12165	-0,71123	1
Erf	-0,71205	0,70444
Kcnj2	-0,71214	1
Gm44851	-0,71211	1
Cd300lf	-0,71291	1
Ift172	-0,71327	0,9524
B230307C23Rik	-0,71395	1
Gm45033	-0,71532	1
2610203C20Rik	-0,71585	1
Gm38399	-0,71608	1
Rpl29	-0,71605	1

Rpl9	-0,7162	0,931
Dimt1	-0,7162	1
Fam72a	-0,71625	1
Gins2	-0,71639	1
Rpl23a	-0,71712	1
Med31	-0,71758	1
Pim1	-0,71929	0,71757
Sec24a	-0,71925	0,88024
Nup107	-0,71959	1
Osm	-0,71984	1
Zfand2a	-0,72012	0,72936
Msantd2	-0,72022	1
Rny3	-0,72076	0,93193
Gm17249	-0,72102	1
Gm6245	-0,72143	1
Yipf7	-0,72163	1
9130230L23Rik	-0,72183	1
Rps12-ps1	-0,72196	1
Asb11	-0,72243	1
Pts	-0,72251	1
Igfbp4	-0,72266	0,46834
Rpl6	-0,72354	0,94387
Supt20	-0,72472	0,88883
RP23-6C18.6	-0,72503	1
AI506816	-0,72506	1
Hps5	-0,7253	0,98942
Gm42967	-0,72528	1
Sep 02	-0,72649	1
Gm29019	-0,72676	1
Gm45762	-0,72698	1
Hbegf	-0,7284	1
Gm22299	-0,72877	1
Chka	-0,72904	0,84749
C330027C09Rik	-0,72939	1
Nxt2	-0,72958	0,84398
Pfkp	-0,73003	0,58262
2010111I01Rik	-0,7303	0,76327
Dok2	-0,73039	0,52623
Gli1	-0,73062	1
C730045M19Rik	-0,73073	1
Pard6b	-0,73092	1
Tor1aip2	-0,73188	0,50413
Mdm1	-0,73261	1
Gm29488	-0,7326	1
Gm45853	-0,73283	1
Terf1	-0,73319	1
Grcc10	-0,73324	1
Gm20900	-0,73355	1
Gm13602	-0,73358	1
Gm7027	-0,73474	1
Gm4617	-0,73482	1
Nusap1	-0,73534	0,74256

2410080I02Rik	-0,73536	1
E2f8	-0,73544	1
Gm37423	-0,73561	1
Gm10036	-0,7361	1
Gm4890	-0,73612	1
Mcm5	-0,73626	0,9217
1700052K11Rik	-0,73677	1
Rpl21-ps1	-0,73682	1
Tmsb10	-0,73689	1
H2-K2	-0,7371	1
Gm4613	-0,73721	1
Emc4	-0,73752	1
Gm12276	-0,73784	1
Phf13	-0,73824	0,90232
Plk4	-0,73869	1
Cenpe	-0,74074	0,47351
Cdc42ep3	-0,74082	1
F11r	-0,74092	1
1700003G18Rik	-0,7415	1
Ccdc163	-0,74157	0,96096
Oaz1	-0,74206	1
Gpam	-0,74248	1
Blm	-0,74326	0,88313
Gm37399	-0,74361	1
Gm6177	-0,74392	1
Gm5841	-0,74426	1
Nkapl	-0,74511	1
4732440D04Rik	-0,74528	1
Gm16585	-0,74528	1
Eif3m	-0,7454	1
Asb10	-0,74629	1
1700012D14Rik	-0,74668	1
Bend3	-0,74891	1
Gm26826	-0,74911	1
Mxd1	-0,74976	0,77002
E130201H02Rik	-0,74984	1
Dbndd2	-0,75057	1
C130026I21Rik	-0,75096	0,71334
Spink10	-0,75142	0,78947
Rab4a	-0,75185	1
Gm44884	-0,75179	1
Mmaa	-0,75221	1
Cenpi	-0,75231	1
Gm37009	-0,75244	1
Adrb2	-0,75469	1
Gm7990	-0,75494	1
Pcdhgc4	-0,75534	1
Gm38009	-0,75552	1
Suco	-0,75592	0,26947
Nsa2	-0,75618	1
Rpl36a-ps3	-0,75719	1
Gm16238	-0,7572	1

Per1	-0,75764	0,70014
Gm8326	-0,75789	1
Fanci	-0,75818	1
Sertad3	-0,75827	0,75886
Hcar2	-0,75861	1
Gm26397	-0,75959	1
9330162G02Rik	-0,75973	1
RP24-418P10.4	-0,76043	1
Dpep2	-0,76079	0,87663
6330403N20Rik	-0,76105	1
Ak4	-0,7613	0,47814
Hmgb1	-0,76135	1
Tmem64	-0,76148	0,33814
Gm2735	-0,7619	1
Taf6	-0,76252	0,56167
Gm11531	-0,76277	0,96638
Bcl6b	-0,76364	1
Hoxb6	-0,76377	1
Syne3	-0,76464	0,56731
Cntrob	-0,76472	1
Dclre1a	-0,76478	1
Gm4859	-0,76528	1
Snord118	-0,76536	1
Dedd2	-0,7661	0,5001
Gm38082	-0,76609	0,85958
Myl6b	-0,76755	1
Gadd45a	-0,7678	1
4930520O04Rik	-0,76861	1
Ilf2	-0,76929	0,55536
Gigyf1	-0,76953	0,79487
Rpl27	-0,76951	1
Btbd1	-0,76977	0,50071
Tusc3	-0,76993	0,54403
Gm13340	-0,76993	1
Gm4987	-0,76997	1
Rps6-ps3	-0,77025	1
Cdc25c	-0,77131	1
Atxn7l2	-0,77151	1
Gm10784	-0,77241	1
Proscos	-0,77253	1
Eno1b	-0,77286	1
Haus6	-0,77304	0,60141
Gm37621	-0,77413	1
Gm17034	-0,77436	1
Gm4866	-0,77457	1
Gm6023	-0,77542	0,78947
B130021K23Rik	-0,77569	1
AA465934	-0,7759	1
Gm8825	-0,776	1
Zfp652os	-0,77627	1
4930503L19Rik	-0,77686	0,57587
Gm9506	-0,77758	1

Mbd4	-0,7777	0,76396
Gpcpd1	-0,77806	0,74314
Gm25541	-0,77819	1
H2afv	-0,77894	0,52313
Gm37084	-0,77925	0,73623
Letm2	-0,77934	0,97223
Tpt1	-0,77987	0,59942
Dna2	-0,78123	0,7894
Trmt112-ps2	-0,78118	1
Apba3	-0,78228	1
Cd274	-0,78234	1
Trmt13	-0,78263	0,64761
Tex30	-0,78303	0,82002
Selenop	-0,78467	1
RP23-308G10.5	-0,78497	1
Gm11970	-0,78509	0,70318
Yjefn3	-0,78537	1
Gm2308	-0,78577	1
Ncapg2	-0,78672	0,77607
Smtn	-0,78752	1
Gm43201	-0,78831	1
Eef1a1	-0,78855	1
Tnfsf12	-0,78961	0,73623
Lzic	-0,7898	0,94921
N4bp2	-0,7902	0,83534
Prdm10	-0,79067	1
Hmga1	-0,79067	1
Gm43788	-0,79081	1
Gm42986	-0,79086	1
Mapk6	-0,7916	0,3622
Sclt1	-0,79192	1
Syne2	-0,79224	1
Rccd1	-0,79313	1
Tia1	-0,79366	0,78044
Efr3b	-0,79382	0,7424
Gm26132	-0,79428	1
Insig1	-0,79501	0,81281
Sag	-0,79521	1
Hdac1	-0,79563	1
Gm11878	-0,79567	1
mt-Tl1	-0,79583	1
Prc1	-0,79607	0,36557
Ltb	-0,79628	0,83328
Gm43774	-0,7964	1
Unc5b	-0,79653	1
Gm43524	-0,7968	1
Ezh2	-0,79714	0,28529
4930518I15Rik	-0,79778	1
C3ar1	-0,79848	0,99816
Dhx9	-0,79867	0,83925
Gm36936	-0,79879	1
Gm26782	-0,7989	1

RP23-36H21.3	-0,79965	1
AW554918	-0,79973	0,75617
Gm43178	-0,80088	1
Gpr137b-ps	-0,80095	0,58551
1500011K16Rik	-0,80115	0,83223
Gm10923	-0,80146	1
Gm12174	-0,80146	1
Gm44510	-0,80148	1
Pdcd7	-0,80172	0,55554
Itgb3bp	-0,80186	1
Gm24916	-0,8021	0,98709
Gm26652	-0,80281	0,94222
9930111J21Rik2	-0,80307	1
Gm7832	-0,80392	1
Psmc3ip	-0,80452	1
RP23-440I21.3	-0,80642	1
4933439C10Rik	-0,80655	0,97037
Tpx2	-0,80694	0,37554
Bnip3l	-0,80687	0,65354
Wdr70	-0,80812	0,59642
Gm45902	-0,80824	1
Cfap126	-0,80921	1
Epha2	-0,81098	0,90096
Kdm4c	-0,81108	1
Gm6526	-0,81123	1
Numb	-0,81129	0,68571
Klhl28	-0,81204	0,79349
Dnajc19	-0,81202	1
Gm6913	-0,81213	1
Tet2	-0,81216	0,55939
Nrp1	-0,81243	1
Rassf2	-0,8129	0,22603
Gm14830	-0,81351	1
Gm8337	-0,81455	1
Rpl13a-ps1	-0,81519	0,56521
AC133103.1	-0,81524	0,5821
Cks1b	-0,81567	0,12416
Gm43133	-0,81602	1
Top2a	-0,81612	0,28147
RP23-88C11.5	-0,81679	1
Clec12a	-0,81907	0,22104
Ccdc18	-0,81942	1
Btf3	-0,81987	1
Rnf145	-0,82043	0,089229
Bub1b	-0,82076	0,35995
Gm5835	-0,82109	1
Clec10a	-0,82177	0,6143
Mogat1	-0,82224	1
Exoc3l	-0,82249	1
Gm18943	-0,82363	1
Gm38120	-0,82383	0,98369
Rsrp1	-0,82407	0,55536

Ypel2	-0,82535	0,78947
5830454E08Rik	-0,82537	1
Usp50	-0,82543	1
Pmf1	-0,82573	0,41706
Ftl1	-0,82612	1
Rnf24	-0,82674	0,64907
Calm1	-0,82689	0,038839
Hsp90aa1	-0,82694	0,36925
Ppp1r35	-0,82735	1
Gm17039	-0,82995	1
Eldr	-0,83008	1
Bbc3	-0,8302	0,41205
Gm4968	-0,83063	1
Mcm6	-0,83075	0,37676
Gm6524	-0,83078	1
Atp8b2	-0,83117	0,50876
Gm43544	-0,83142	1
D930030I03Rik	-0,83157	1
Gm16439	-0,83157	1
RP23-307F3.6	-0,83342	1
Tgfb1	-0,83411	0,40598
Gm22973	-0,83432	1
Nuf2	-0,83482	1
1700120C14Rik	-0,83513	1
Gm16540	-0,83663	1
Arf2	-0,83716	0,10072
Ndc80	-0,83751	0,86757
Gm45716	-0,83752	1
Zbtb11os1	-0,83814	1
Gm37699	-0,8391	1
Sh2d5	-0,84085	0,78947
Alms1	-0,84218	1
Serpine1	-0,84301	0,20711
B330016D10Rik	-0,84407	1
Gm8423	-0,84494	1
Aurkb	-0,84596	0,72711
Cdca2	-0,8465	0,57901
Rnf130	-0,84724	1
Gm6564	-0,84734	1
Snx13	-0,8481	0,53025
Atad5	-0,84808	1
Rnf144b	-0,84833	0,7894
Gm42635	-0,84847	0,8294
Star	-0,84854	0,90307
Gm35931	-0,8489	1
Rpl21-ps14	-0,84996	1
Gm26461	-0,85063	1
A630072M18Rik	-0,85123	1
Tma7	-0,85178	1
Dgkh	-0,85229	0,99864
Dnah8	-0,8527	1
2610528A11Rik	-0,85326	1

B3gat3	-0,85509	0,36562
St3gal6	-0,8557	0,12613
Aspm	-0,85727	0,62373
Gm43411	-0,85726	1
H2-T23	-0,85739	1
Zfp280d	-0,85763	0,36421
H3f3b	-0,85773	0,50876
Rhoh	-0,85803	1
Ncapg	-0,85891	0,60936
Pth1r	-0,8593	1
Cd9	-0,86016	1
Gm26698	-0,86032	1
Gm6598	-0,86044	1
RP23-162P10.8	-0,8606	0,60488
Xxylt1	-0,86068	1
Gm9381	-0,86087	1
Gm9769	-0,86097	1
Hist2h2ac	-0,86097	1
Rpl27-ps3	-0,86123	1
Gm6266	-0,86132	1
Gm19898	-0,86154	0,97037
Ppfia4	-0,86179	0,72711
H2-T22	-0,86182	0,78395
Spaca6	-0,86214	0,72206
Gm42724	-0,8634	0,98709
Gm5776	-0,86363	1
Snhg8	-0,86378	0,73206
Smc4	-0,86405	0,13201
Ube2n	-0,86431	0,72936
1810026B05Rik	-0,86455	0,43983
A430110C17Rik	-0,86537	1
2810002D19Rik	-0,86546	1
Gm1840	-0,8663	0,71431
Gm38115	-0,86713	1
Mad2l1	-0,86716	0,8103
Cep57l1	-0,8679	0,71143
Kif20a	-0,86853	0,70665
B430305J03Rik	-0,86931	1
Kif4	-0,86996	0,98095
B930086L07Rik	-0,87034	0,60975
Dnajb4	-0,87096	0,1096
Gm45133	-0,87112	1
Gm37706	-0,87154	1
Slc19a2	-0,87219	1
Gm37255	-0,87218	1
Scarna17	-0,87305	1
Gm14006	-0,87353	1
Gm8203	-0,87357	1
Gm8618	-0,87469	0,57651
Rassf1	-0,87482	0,13396
Ankrd52	-0,87521	1
Gm37728	-0,87538	1

Kif11	-0,87687	0,62373
2610037D02Rik	-0,87689	1
Anxa1	-0,87698	1
Zfp619	-0,87716	1
Fam162a	-0,87745	0,08331
Gm12240	-0,87754	1
Gm17251	-0,87851	1
Sdc4	-0,8786	0,15331
Gm12248	-0,87869	1
Bnip3	-0,87948	0,11091
Gm10263	-0,87946	1
Psenen	-0,87997	0,89504
Rpl35a-ps4	-0,88036	1
Gm26710	-0,88039	1
Gm19620	-0,88056	1
Gm37060	-0,88123	0,4825
Gm3550	-0,88156	0,89288
Gm8925	-0,88155	1
Gm7206	-0,88467	1
Gm13349	-0,88481	1
Cped1	-0,88619	1
Snhg20	-0,88676	0,27566
Pgf	-0,88701	1
Gm16046	-0,88741	1
Zdhhc18	-0,88748	0,28223
Taf5	-0,88749	0,65668
Lbr	-0,8881	0,040201
2700097O09Rik	-0,88814	0,89612
Rhoc	-0,89076	0,020792
Cox20	-0,89112	0,30021
Khk	-0,89156	0,11313
Srsf1	-0,89229	0,32557
Prpf39	-0,89247	0,22039
Ly86	-0,89275	0,22239
RP24-460E12.3	-0,89491	1
Gm13464	-0,89516	1
Selenbp1	-0,89534	0,4493
Gm14094	-0,89605	1
P4ha2	-0,89673	0,66833
Kif23	-0,89949	0,038839
H2-Q6	-0,90009	1
RP23-70B19.5	-0,90021	0,98709
Zg16	-0,90044	1
Zfyve26	-0,90052	0,36925
Hist1h1e	-0,90148	0,24839
Gm43637	-0,90207	1
Cdkn2d	-0,90218	0,087239
Gramd4	-0,90254	0,5821
BC024386	-0,90285	1
Map3k8	-0,90322	0,30045
Zfp326	-0,90364	0,11583
Gm1862	-0,9042	1

Gm3283	-0,90425	1
Timm23	-0,90439	1
Gm7363	-0,9045	1
Gm12618	-0,9048	1
Plin2	-0,90607	0,10422
Tacc3	-0,90751	0,46137
Rad51	-0,90758	0,83586
Gm15832	-0,90792	0,68183
Gm6134	-0,90811	0,90307
Tspan32	-0,90816	1
Higd1a	-0,90848	0,61599
Gm13140	-0,90853	1
RP24-282C4.3	-0,90861	1
Gm26610	-0,90879	1
Gm42748	-0,90925	0,72711
Gm16288	-0,90964	1
Gm38062	-0,9097	1
Gm42559	-0,91046	1
1500015A07Rik	-0,91075	0,73374
Plau	-0,91147	0,23171
Cenpf	-0,91414	0,15485
Gm26737	-0,91475	1
Arhgap11a	-0,91496	0,22812
Gm15596	-0,91587	1
Lgals4	-0,91612	1
Fbxl14	-0,91618	1
RP24-378K7.3	-0,91633	1
Zfand4	-0,91733	1
Rasd1	-0,91781	1
Rassf3	-0,91808	0,36156
Gm45185	-0,91833	1
Gm43848	-0,91873	0,64653
Ptrf	-0,91905	1
Gm7299	-0,91939	1
Arhgef39	-0,91998	0,25042
RP24-496O17.7	-0,92088	1
Raf1	-0,92148	0,59642
Hmgb1-ps8	-0,92273	1
Nr4a1	-0,92362	0,9978
Spag5	-0,92373	0,63975
Gm5045	-0,92383	1
Spsb2	-0,92556	0,50487
Etv5	-0,92599	0,59311
Gnrh1	-0,9263	1
Fbxo5	-0,92733	0,72711
Fzd5	-0,92816	0,36159
Gm6472	-0,9294	0,66234
Zcchc24	-0,93084	0,46834
Krtcap3	-0,93183	1
Actg1	-0,93259	1
Gm38067	-0,93384	0,87905
Hcfc1r1	-0,93418	0,015346

Adh5	-0,93512	0,097225
Gm15207	-0,93543	1
Kctd6	-0,93608	0,46834
Gdap10	-0,93642	0,72917
Iscu	-0,9365	0,36423
Ckap2l	-0,93681	0,29958
Rps13-ps1	-0,93681	1
Gm8624	-0,93716	1
Dtl	-0,94042	0,38929
Gm13050	-0,94131	0,89638
H2-T10	-0,94142	1
Gm2076	-0,9415	1
Gm11281	-0,94218	1
Gm5362	-0,94245	1
Spc25	-0,94271	0,23407
Spc24	-0,94342	0,49447
Ppp1r2	-0,94553	0,18853
Mgst3	-0,94559	0,82395
AA386476	-0,94607	1
RP24-351I17.3	-0,94632	1
Gm12164	-0,94666	1
Rpl6l	-0,9475	0,88182
Ybx1-ps2	-0,9476	1
Tgif2	-0,94812	0,28189
Eno2	-0,94907	0,11705
Ccna2	-0,95031	0,1888
Rmrp	-0,95059	1
Gm45184	-0,95107	0,49575
Gm5801	-0,95118	1
Rusc2	-0,95144	0,65088
Egln1	-0,95159	0,02137
Gm2531	-0,95156	0,61599
RP23-325K4.10	-0,95295	0,22307
4930589L23Rik	-0,95336	1
Gm43328	-0,95348	1
Rps6-ps4	-0,9536	1
Gm37334	-0,95358	1
mt-Ti	-0,95399	1
Gm13567	-0,95494	1
Rgs9bp	-0,95519	1
Lonrf3	-0,95547	0,14975
Gm26497	-0,95598	1
Gbe1	-0,95614	0,17265
Gm43484	-0,95663	0,54605
Gm44270	-0,95656	1
Ccl6	-0,95663	1
Mrps18b	-0,95736	1
Snhg6	-0,9576	1
Mettl17	-0,95821	0,42618
2810013P06Rik	-0,95842	0,52766
Wdfy2	-0,95963	0,85458
Snord110	-0,95973	1

Gm9844	-0,95979	1
Gm26533	-0,96024	1
Gm29994	-0,9605	1
Tomm40l	-0,96056	0,39489
Myc	-0,96089	1
Gm23346	-0,96098	0,86356
Gm38043	-0,9611	0,89877
RP23-23P9.3	-0,96136	1
Pole2	-0,96181	1
Gm15289	-0,96229	1
Gm15950	-0,96278	0,84825
Gm37183	-0,96316	0,60134
Cdc20	-0,96347	0,10367
Uhrf1	-0,96361	0,83961
Gm12643	-0,9649	1
Mcm3	-0,96514	0,15014
Tnfrsf17	-0,96608	0,81105
3110056K07Rik	-0,96736	1
Ormdl3	-0,96746	0,17162
Gm37354	-0,96793	1
Zfp36l1	-0,96864	0,15975
6030442K20Rik	-0,96876	1
Bvht	-0,97003	0,96844
Gm34121	-0,9707	1
A830073O21Rik	-0,97173	1
Fau	-0,97303	0,58035
Cchcr1	-0,97393	1
Gm43628	-0,9745	1
Gm26202	-0,97474	1
Slc25a40	-0,97559	0,097693
Dsn1	-0,97564	0,65521
RP23-149L23.1	-0,97634	1
Mtfr2	-0,97857	1
Hmmr	-0,97939	0,22104
Rbl1	-0,97977	0,34819
Gm43795	-0,98004	0,73206
Pin4	-0,98215	1
1700007K09Rik	-0,98265	1
RP23-115A18.3	-0,9827	0,92641
Gm7336	-0,98278	1
Klf2	-0,98286	0,52766
Gm5445	-0,98355	0,7899
Ddx20	-0,98391	0,11705
4930404I05Rik	-0,98518	1
Klf4	-0,98681	0,052132
Pmp22	-0,98688	0,019275
AV356131	-0,98686	0,10072
Gm43329	-0,98686	0,75886
Ankrd55	-0,98687	1
Hist1h1d	-0,98744	1
RP24-365N15.9	-0,98754	1
Itga11	-0,98803	1

Gm45137	-0,98816	1
Chst1	-0,98975	0,56731
Arsb	-0,98988	0,27438
Cytip	-0,99187	1
Fam174a	-0,9923	0,045485
Gm38299	-0,99228	1
Gm38192	-0,99319	1
D830025C05Rik	-0,99325	0,92834
Tpi1	-0,99397	0,010493
Otud1	-0,9961	1
Gm43343	-0,99621	0,77437
6430710M23Rik	-0,99619	1
Prr18	-0,99658	0,42618
Gm44913	-0,99677	0,48921
E330034L11Rik	-0,99712	0,24739
Gm43294	-0,99707	1
Gpn2	-0,99723	0,89231
Cldn11	-0,99789	0,098166
Gm11448	-0,99815	1
Gm13092	-0,99931	1
Ccdc117	-1,0011	0,05206
RP24-240E7.1	-1,002	0,57651
Sgol2a	-1,0021	0,099541
Gm43560	-1,0021	1
Gm17494	-1,0032	0,65258
AA914427	-1,0033	1
Pold1	-1,004	0,31484
Gm7561	-1,0058	0,99275
Arrdc3	-1,0064	0,27821
4933437G19Rik	-1,0065	1
2700038G22Rik	-1,0082	1
RP24-282C4.4	-1,0092	1
Gm37219	-1,0094	1
4931440P22Rik	-1,0096	0,53025
Gm8885	-1,0098	1
4930563E22Rik	-1,0123	1
Psd2	-1,0131	1
P4ha1	-1,0138	0,0092751
Ulbp1	-1,0139	0,037646
Gm26670	-1,014	1
Gm7600	-1,0161	1
Gm42979	-1,0183	0,51571
Nup205	-1,0185	0,30189
Lilrb4a	-1,0189	0,40376
Gm11826	-1,0207	0,68707
Gm5100	-1,022	1
Gm38387	-1,0241	1
Gm10717	-1,0252	1
Bloc1s3	-1,0255	0,83888
Gm37486	-1,0262	1
Kif21a	-1,0266	1
Glt1d1	-1,0278	0,997

Zfp773	-1,028	0,472
Gm14013	-1,0294	1
Map3k12	-1,0307	0,32186
Arrdc2	-1,0315	0,20916
Gm16754	-1,0319	0,12643
Amd2	-1,0325	0,94415
Alpk2	-1,0332	1
Jup	-1,035	1
Ccl4	-1,0372	0,012307
Gm14633	-1,0374	1
Ap1s3	-1,0375	0,052379
Gm37105	-1,038	0,92368
1700029J07Rik	-1,0399	0,9978
Slc25a25	-1,0406	0,19929
Pabpn1	-1,0412	0,6061
Gm21781	-1,0423	0,71135
Gm43775	-1,0426	0,45627
Snord104	-1,0433	0,064983
mt-Nd6	-1,0433	0,29464
Fancb	-1,0437	1
Gm16310	-1,044	0,7377
Senp3	-1,0442	0,20218
Pmaip1	-1,0446	0,096692
Brip1	-1,0452	0,65258
Gm42786	-1,0458	1
Gm43794	-1,0474	0,46834
Nsa2-ps2	-1,0476	1
Gm11625	-1,0495	1
Zfp101	-1,0504	0,56432
Arhgap39	-1,0506	0,56497
Mb21d1	-1,0514	0,0022078
Hist1h2be	-1,0551	0,82126
4833412K13Rik	-1,0555	0,11683
Slc27a3	-1,0555	0,46062
Nadk2	-1,0563	0,18781
Gm37902	-1,0571	1
Gm37963	-1,0585	1
Gm15779	-1,0587	0,86407
Gm37352	-1,059	0,88313
Tdrd7	-1,0602	0,84763
C130013H08Rik	-1,0604	1
Kifc1	-1,061	0,25347
Pclaf	-1,0621	0,034003
Tpt1-ps3	-1,0629	0,98263
Pea15a	-1,0636	0,053019
Racgap1	-1,0651	0,028673
Gm45729	-1,0655	1
Ccdc58	-1,0665	0,072689
Rplp1-ps1	-1,0672	1
Cdh23	-1,0686	1
B230317F23Rik	-1,0687	1
Cdc7	-1,0689	0,5821

Nfil3	-1,069	0,2328
Wdr54	-1,0691	0,64308
Ska3	-1,0702	0,48128
Gm14323	-1,0704	1
Gm11516	-1,0709	1
Gm37383	-1,0716	1
Gm45311	-1,0718	1
Luc7l3	-1,0727	0,031586
Ska2	-1,0727	0,42498
E2f7	-1,0743	0,88313
Gm5837	-1,0746	0,9392
Gm15441	-1,0746	1
Hist1h1c	-1,0756	0,060635
B230216N24Rik	-1,0762	0,37318
Fam83d	-1,0765	0,81271
5033430I15Rik	-1,0769	1
Slc12a5	-1,0793	0,53025
Gm43868	-1,0796	1
Fignl1	-1,0815	0,52766
Rasgef1b	-1,0826	0,071379
Pttg1	-1,0835	0,023228
Mcm10	-1,0835	0,73988
Gm12758	-1,0836	1
Gm7353	-1,0842	1
Rpl36-ps4	-1,0858	0,78025
Gm10616	-1,0882	0,48112
Gm10343	-1,0884	0,64761
Gm45051	-1,0885	1
Gm15834	-1,0891	0,48102
Gm14585	-1,0893	1
Hist2h3c2	-1,0895	0,81268
Mafk	-1,0898	0,051479
Tmem240	-1,09	0,86757
Acrbp	-1,0903	0,46371
Gm4832	-1,0903	1
Gm15937	-1,0925	1
Ntrk3	-1,0929	1
Ncapd3	-1,0935	0,05433
Rpl30-ps3	-1,094	0,20049
Gm37906	-1,0947	0,50713
Cox7c	-1,0951	0,87961
Gm14130	-1,0964	1
Rps19-ps8	-1,097	1
Gm5277	-1,0979	0,65521
4930532G15Rik	-1,0983	1
Bub1	-1,0984	0,11477
RP23-356D13.9	-1,0984	1
A130014A01Rik	-1,0994	0,43702
Rpl17-ps4	-1,1005	0,97852
Fam26f	-1,1006	1
Mcm7	-1,1033	0,033583
Rpl31-ps22	-1,104	1

Tmcc3	-1,1069	1
Gm37959	-1,1077	0,53025
Nek2	-1,109	0,61516
Rhob	-1,1097	0,50794
Gm38297	-1,1099	1
Klf11	-1,1133	0,29196
RP24-550H10.3	-1,1136	0,52136
Ptchd1	-1,114	0,008654
Gm28404	-1,114	0,5821
Rc3h1	-1,1143	0,017752
Rpl27a-ps1	-1,1147	0,87765
L1cam	-1,115	0,71135
Spp1	-1,1172	0,17188
Olfr912	-1,1173	1
Pdgfb	-1,1177	0,3048
Csrnp1	-1,1187	0,013644
S100a3	-1,1189	0,94387
Taco1os	-1,1197	1
Gm45343	-1,1207	0,23074
Gm43447	-1,1226	1
Rpl28-ps3	-1,1227	0,86648
Gm4963	-1,1289	1
Cenpw	-1,1328	0,012679
Rpl23a-ps2	-1,134	1
Snord7	-1,1348	1
Nudt8	-1,136	0,96096
Hba-ps4	-1,1379	1
Rps4x	-1,1406	0,82085
mt-Ts2	-1,141	1
Etv3	-1,1421	0,012854
Gm12778	-1,1425	0,84398
Lockd	-1,1428	0,097225
Hnrnpa1	-1,1466	1
Gm5900	-1,1466	1
Fastkd1	-1,1471	0,11785
Gm37140	-1,1474	1
Arntl	-1,1488	0,02176
Dhx58	-1,1489	0,063099
Cit	-1,149	0,24847
Aif1	-1,1493	0,48797
Gm37733	-1,1497	0,72103
Wdr62	-1,15	0,99501
Gm7266	-1,1502	0,7213
Gm37490	-1,1508	0,85844
Gm12940	-1,1509	1
4931428F04Rik	-1,1514	0,71496
Pot1b	-1,1525	1
Gm38190	-1,1532	0,92981
Birc5	-1,1536	0,0019319
Gm42522	-1,1538	1
Gm7984	-1,1539	1
Cenpk	-1,1542	0,3114

Cd300c2	-1,1549	0,0075112
Gm6969	-1,1559	1
Hs3st3b1	-1,156	0,87765
Pcna-ps2	-1,1567	0,6684
RP24-282K24.4	-1,1574	1
Gm44093	-1,1575	1
Anln	-1,1592	0,096692
Gm9568	-1,1605	1
Cks1brt	-1,1617	1
Gm42731	-1,1619	1
Gm9256	-1,162	0,99864
Lin54	-1,1636	0,051202
A630081D01Rik	-1,1641	1
2900093K20Rik	-1,1665	0,057675
Gm44126	-1,1672	1
Ano7	-1,169	0,83449
Hspa8	-1,1697	0,72711
Snord83b	-1,1697	0,9457
Wdhd1	-1,1713	0,071345
Fam64a	-1,1721	0,043959
Zfp36l2	-1,1722	0,068048
Smc2	-1,1725	0,00187
Oaz1-ps	-1,1727	0,77314
Polr2l	-1,1731	0,079663
Rps16	-1,1736	0,64261
Gm14040	-1,1745	1
Sgol1	-1,1747	0,21553
Gsg1	-1,1766	0,28147
Gm44777	-1,1769	1
Gm38319	-1,1779	1
Ank3	-1,183	1
Adamts1	-1,1838	0,41292
Lsp1	-1,1845	0,0045877
Tsc22d3	-1,1845	0,061498
Gatsl2	-1,1855	0,00089493
Sirpa	-1,1871	0,00089358
Zfp174	-1,1892	0,87905
Slfn3	-1,1893	0,77002
Zwilch	-1,1896	0,36421
Gng8	-1,1937	1
Ttk	-1,194	0,048201
Gm5124	-1,195	1
Gm4754	-1,1966	1
Gm37510	-1,1966	1
Zfp367	-1,197	0,022012
RP24-324J2.1	-1,1974	1
Gm37303	-1,1983	0,9978
Zic2	-1,1994	0,30393
Dkkl1	-1,1996	1
Gm10801	-1,2006	1
Gm43482	-1,2011	0,15482
Gm37465	-1,2013	0,73206

Gm43727	-1,2022	0,83205
Zscan21	-1,2034	0,2328
Gm42851	-1,2047	1
9930104L06Rik	-1,2063	0,78044
Gm5257	-1,2065	1
Mki67	-1,2069	0,01491
Tnnt3	-1,2073	0,50309
Kif15	-1,2082	0,064983
Gm5921	-1,2094	1
Tmod1	-1,2114	0,12416
Nemp1	-1,2142	0,012682
Gm7099	-1,2148	0,66234
Gm37747	-1,2163	1
Gm7514	-1,2164	1
Gm37519	-1,2167	1
Pfkfb2	-1,2173	0,42696
Gm15785	-1,2223	1
Gm43800	-1,2252	1
Gm12005	-1,2255	0,93298
A730071L15Rik	-1,2261	1
Gm8667	-1,2268	0,77603
Frat2	-1,2276	0,0085486
Gm28578	-1,2277	0,61302
Cd9-ps	-1,2279	1
Abcg4	-1,2294	0,97852
Gm25008	-1,2302	0,84825
Dlgap5	-1,2317	0,42231
Gm37106	-1,2335	0,8294
Gm5302	-1,2359	0,98791
Nrxn3	-1,238	0,72206
Cox20-ps	-1,2382	0,0073895
4930579G24Rik	-1,2404	0,67986
Dcstamp	-1,2406	0,041367
Slc22a13b-ps	-1,2433	1
Ttc25	-1,2442	0,89832
Gm16630	-1,2457	1
4932416K20Rik	-1,2476	0,63862
Gm37795	-1,2482	0,39516
Ska1	-1,2505	0,0030276
Gm17108	-1,2505	0,76289
Anxa2	-1,2513	0,0018282
Gm10388	-1,2548	0,36621
RP23-402A24.3	-1,2551	0,7965
Xirp1	-1,2552	0,77607
RP23-38L16.3	-1,2556	0,1655
Gm27043	-1,2561	1
Trim59	-1,2567	0,099593
Gmnn	-1,2587	0,053019
Gm25857	-1,2609	0,88313
Gm45153	-1,2642	0,43619
Gm45407	-1,2683	0,47239
Gm11759	-1,272	0,78044

S100a8	-1,2727	0,67047
Mrps28	-1,2754	0,78584
Ube2t	-1,2757	0,018938
Gm24631	-1,2759	0,89612
Gm14057	-1,2768	1
RP23-442M18.5	-1,2776	0,76329
Zmynd10	-1,2814	0,70665
Fancd2	-1,2815	0,13964
A430027C01Rik	-1,2819	0,83534
B930036N10Rik	-1,2828	0,78044
Ost4	-1,2829	0,154
Tmem107	-1,2841	0,055958
Gm28424	-1,2849	1
Hlx	-1,2853	0,70665
Kntc1	-1,2868	0,61483
Gm45855	-1,2874	1
Hist1h2bg	-1,2891	1
Gm24276	-1,292	0,0054589
Tiparp	-1,2929	0,0054589
Troap	-1,293	0,10925
Ang	-1,2949	0,089229
Ezr	-1,2964	0,00028352
Adgre5	-1,2966	0,70393
Rps19-ps6	-1,2981	0,57651
Rpl30-ps9	-1,2981	0,74533
Lhx5	-1,299	0,84398
Adam9	-1,3018	0,38872
Gm45534	-1,3024	0,71813
Tnfrsf12a	-1,3046	0,0080108
RP24-295J1.1	-1,3071	1
Cfh	-1,309	0,0076129
C230096K16Rik	-1,311	0,33679
Gm45266	-1,3111	0,76329
Sdc3	-1,3138	9,36E-05
Rpl35a-ps5	-1,3151	0,1127
Gm36930	-1,3159	0,70728
2610020C07Rik	-1,3163	0,9978
Rpl36-ps8	-1,3164	0,98277
Gm45358	-1,3169	0,78947
Hist1h2bp	-1,3177	1
Dleu2	-1,319	0,038097
Rasl2-9	-1,3195	0,78947
Hmgb1-ps5	-1,3234	0,73777
RP23-159E10.1	-1,3238	1
Gm19272	-1,3292	1
Gm14239	-1,3302	0,10771
Gm12380	-1,3308	0,80618
Gadd45b	-1,333	0,0098535
Ier5	-1,3334	0,00041714
Bloc1s6os	-1,3359	0,68034
Snrpf	-1,3361	0,23047
Ifitm1	-1,3366	0,79006

Rhov	-1,3403	0,38458
Cog3	-1,3407	0,56894
Gm15798	-1,3416	0,6631
Polq	-1,3417	0,46834
Rps19-ps11	-1,3424	0,33803
C330011M18Rik	-1,3428	1
mt-Ta	-1,3431	1
Vaultrc5	-1,3434	0,0012318
Basp1	-1,3446	0,005896
Fn1	-1,3451	0,042179
Rybp	-1,3475	0,92481
Cks2	-1,3484	1
Gm5312	-1,3487	0,54707
A430010J10Rik	-1,3493	1
Paqr5	-1,3506	0,94659
Unc13a	-1,3553	0,039629
Gm11263	-1,3554	0,94354
Gm15644	-1,3557	0,42696
BC028528	-1,3585	0,029362
Iqgap3	-1,3596	0,019434
G2e3	-1,3601	0,0010832
Rbm3	-1,3607	0,56824
Rpl12	-1,3635	0,017975
Pfkfb3	-1,3644	0,0054589
Rnf122	-1,3669	0,32557
5930420M18Rik	-1,3686	1
Eno1	-1,3704	0,019275
Gm2011	-1,3716	0,52766
Snord89	-1,3717	0,10156
Gm28555	-1,3719	0,0062023
Rnu11	-1,3747	0,4277
Tspan33	-1,3758	0,96844
Gm23344	-1,3768	0,644
Hist1h1a	-1,378	1
Gm11363	-1,3816	0,32692
Sap30	-1,3836	0,0069674
Gm15728	-1,3842	0,13964
Gm28791	-1,3854	0,15021
Ddah2	-1,3857	0,22239
Pif1	-1,3879	0,014798
Gm8292	-1,3897	0,0079839
Gm2467	-1,39	0,98162
Gm42819	-1,3914	0,10072
Gm2830	-1,3918	0,031586
Gtse1	-1,3928	0,020534
Gm37065	-1,3929	0,81016
Gm15541	-1,395	1
Gm43714	-1,3981	0,95327
Gm38022	-1,3986	0,64761
Gm6612	-1,4	0,94354
Plk2	-1,4024	0,1344
RP24-131G14.13	-1,4081	0,02413

Ddit4	-1,4101	0,0022078
Gm8210	-1,413	0,9978
Ckap2	-1,4149	0,019434
Atp5g1	-1,4167	0,0060825
Gm15503	-1,4197	0,1096
S1pr1	-1,4242	0,012947
Pask	-1,4242	0,70403
Ect2	-1,4251	0,08353
Kif20b	-1,427	0,0080293
Itga6	-1,4275	0,0098535
2810001G20Rik	-1,4306	0,275
Rpl9-ps7	-1,4342	0,64318
AV099323	-1,4349	0,99996
Gm37390	-1,4385	0,55536
Gm42549	-1,4446	0,8294
RP24-75M13.2	-1,4449	0,78947
A830008E24Rik	-1,4469	0,91115
Rps11-ps3	-1,4504	0,55536
Gm10382	-1,4514	0,29232
Stamos	-1,4518	0,56432
Ier5l	-1,4524	0,040882
Gm42670	-1,4544	0,7959
Lpl	-1,455	7,24E-06
D130051D11Rik	-1,4577	0,043232
Atf3	-1,4595	0,0015748
Neil3	-1,4599	0,28639
Gm7634	-1,4605	0,59311
Dpf1	-1,4606	0,28529
4930426I24Rik	-1,461	0,98709
Gm28731	-1,4638	0,87765
9330175E14Rik	-1,4655	0,15014
Gm43275	-1,4699	0,37627
Gm45220	-1,4725	0,39328
0610005C13Rik	-1,4751	0,76247
Gm12090	-1,4775	0,81393
Errfi1	-1,4778	0,013691
Rbmx	-1,4781	0,39084
Gm29228	-1,479	0,61066
Gm42466	-1,4808	0,80548
Gm42508	-1,4819	0,44486
Gm42743	-1,4836	0,26947
Gm13383	-1,4848	0,84398
RP24-323H7.5	-1,4865	0,94387
Rad51ap1	-1,4875	0,034003
Mir3091	-1,489	0,92545
Gm12604	-1,4916	0,2225
Gm6341	-1,4925	0,001019
Ciart	-1,4925	0,0098535
Mkln1os	-1,4954	0,6369
Gm10636	-1,4964	0,74314
Kifc5b	-1,5153	0,11583
Gm43511	-1,5203	0,11901

2700099C18Rik	-1,5228	0,55268
Suv39h2	-1,5241	0,02413
Lbp	-1,5251	0,86846
RP24-511J14.2	-1,5276	0,1163
Gm38355	-1,5282	0,54482
Ccdc36	-1,5303	0,036614
Rps12-ps23	-1,5331	0,63975
Gm14326	-1,5341	1
Phlda1	-1,5345	0,013698
1700031P21Rik	-1,5361	0,41319
Gm10657	-1,5381	0,73623
Notch1	-1,5424	0,78819
A330023F24Rik	-1,5431	0,012214
4930578M01Rik	-1,5442	0,98709
1810041H14Rik	-1,5445	0,75429
Rdh12	-1,5473	0,83566
Gm12280	-1,5495	0,33768
Gm43684	-1,5505	1
Lsm7	-1,5508	0,1046
Arhgap19	-1,552	0,76329
Wfdc17	-1,5529	0,030949
1600029O15Rik	-1,5539	0,59206
Mmp9	-1,5588	0,048201
Gm26549	-1,5589	0,76289
Rflnb	-1,5615	0,67592
Gm15877	-1,5616	0,68245
Gm28727	-1,5639	0,018913
9530085L11Rik	-1,5646	0,35079
Chrnb1	-1,5691	0,087531
Atp1b4	-1,5712	1
C530043K16Rik	-1,5761	0,0062693
Gdf15	-1,5766	0,01364
1700054M17Rik	-1,577	0,77282
Pou6f2	-1,5785	1
Teddm2	-1,5841	0,81105
Wwc1	-1,5842	0,0009974
Rnf152	-1,5843	0,53276
Gm42798	-1,585	0,22565
Gm15007	-1,5889	0,65409
Clec3b	-1,5897	0,88313
Adcy6	-1,5943	0,0089541
RP23-182J19.2	-1,5946	0,65409
Pbk	-1,5951	0,0015755
Gm12034	-1,5964	0,44342
Sdhd	-1,5972	0,22239
Gm14537	-1,5999	0,52766
9530078K11Rik	-1,601	0,46008
Gm6745	-1,6034	0,72711
Gm527	-1,6035	0,40366
Caprin2	-1,6039	0,82653
Gm43182	-1,6054	0,89612
A530041M06Rik	-1,6068	0,063747

Fam161b	-1,6079	0,54882
Hist1h2an	-1,6134	0,93298
Gm11205	-1,6142	0,012214
Gm43581	-1,6164	0,0073895
Gm14286	-1,6189	0,017149
5330406M23Rik	-1,6224	0,050214
Gm20091	-1,6243	0,57651
Xaf1	-1,6255	0,39535
Ttc30a1	-1,6278	0,6143
5330426L24Rik	-1,6313	0,72711
Slc2a1	-1,6317	2,48E-05
Gm44545	-1,6351	0,64907
Gm43501	-1,6378	0,75646
Gm13413	-1,6455	0,87961
Cbx2	-1,6465	0,026766
Gm11604	-1,6494	0,42618
Gm16045	-1,6503	0,02137
Rpl30-ps1	-1,6504	0,18282
Gm38235	-1,653	0,83566
Atr	-1,6533	0,0075159
Gm11470	-1,6543	0,019434
Gm42856	-1,6575	0,82395
Gm43566	-1,6576	0,017659
Tap2	-1,6621	0,22239
Gm6919	-1,666	0,24419
Gm15610	-1,6696	0,7899
Itgax	-1,6721	0,0019907
Ptgs2os	-1,677	0,46928
Snord82	-1,6777	0,63029
Gm37472	-1,6783	0,33803
Gzmm	-1,6799	0,45475
Gm43273	-1,6816	0,90512
Gm3531	-1,6848	0,86691
Gm3617	-1,6872	0,53025
Pole	-1,6899	0,27033
Gm37420	-1,6952	0,42696
Atp5l-ps1	-1,6964	0,020792
Gm6576	-1,6979	0,58262
Gm23301	-1,6979	0,64653
Gm26601	-1,6984	0,21412
Lrrc17	-1,699	0,0066386
Rps8-ps4	-1,7002	0,13128
Crip1	-1,7018	1,00E-07
Fcrl5	-1,7018	0,82085
4930558J18Rik	-1,7033	0,67592
Gm42432	-1,7039	0,34434
Gm11810	-1,704	0,12916
Gm7856	-1,7064	0,56206
Smarca5-ps	-1,7071	0,50136
Gm44292	-1,7148	0,11313
Sez6	-1,7149	0,0089942
Snhg11	-1,7164	0,62956

Zic5	-1,717	0,4574
Gm8330	-1,7182	0,33395
Hba-a1	-1,7186	0,95649
Rgmb	-1,7235	0,24839
H2-Q10	-1,7253	0,48921
Gm9521	-1,7324	0,60134
2700029L08Rik	-1,7354	0,39474
Gm37531	-1,7383	0,33512
Gm12444	-1,7402	0,22136
2810454H06Rik	-1,7436	0,095677
RP23-226H21.3	-1,75	0,41292
Gm42908	-1,7547	0,4046
Gm44283	-1,758	0,67586
Gm45809	-1,7604	0,49297
Mxd3	-1,7609	0,83449
4833421G17Rik	-1,763	0,18426
Tstd1	-1,7652	0,015811
Gm14279	-1,7655	0,026699
Vsig8	-1,7707	0,54482
Gm26830	-1,7734	0,7462
Efna3	-1,7747	0,28639
A330035P11Rik	-1,7789	0,48813
Hmgb2	-1,7797	0,00068298
Gm42632	-1,7799	0,41205
Gm43817	-1,7827	0,020815
Lgals7	-1,783	0,32927
Nsl1	-1,7836	0,0054603
Mafb	-1,7849	2,82E-06
Gm36989	-1,7859	0,23594
Cpne9	-1,7864	0,275
Ccng2	-1,7865	0,00072708
Crkl	-1,7898	0,13396
Gm6382	-1,7923	0,56542
Hus1b	-1,7925	0,57989
Hist1h1b	-1,7929	0,46834
Gm6520	-1,7933	0,36845
4932422M17Rik	-1,7953	0,020882
RP23-136K21.4	-1,8105	0,81817
Gm2367	-1,8127	0,28705
Gm27003	-1,8162	0,59311
Gm26664	-1,8205	0,22239
Rpl7	-1,824	0,0055303
Gm43331	-1,8241	0,4046
Dnd1	-1,8256	0,66311
Fth-ps3	-1,8333	0,00017112
Gm27010	-1,8389	0,22909
Gm6946	-1,8406	0,38707
Ppp1r18os	-1,8487	0,51883
Gm17541	-1,8502	0,64653
Gm5112	-1,8631	0,093313
Gm42633	-1,8637	0,49297
Rnaseh1	-1,8645	0,51608

Gm23849	-1,8672	0,41704
Klf10	-1,8673	5,69E-06
RP23-356D13.11	-1,868	0,41292
Arc	-1,8744	0,032708
Gm42671	-1,8775	0,41205
Gm45477	-1,8802	0,34763
Gm29170	-1,8821	0,0042024
Tspan15	-1,8846	0,47973
Etv4	-1,8896	0,37756
Gm37482	-1,8927	0,28031
Dtd2	-1,8966	0,00048142
Gm42551	-1,9003	0,30515
Gm44013	-1,9021	0,28424
Gm14034	-1,9035	0,48128
Hist1h4a	-1,9058	0,65088
B3gnt6	-1,9083	0,53394
Gm15796	-1,9111	0,36421
D430001F17Rik	-1,916	0,071379
Gm14439	-1,9184	0,69546
RP23-205H11.3	-1,9216	0,042374
C730034F03Rik	-1,9224	4,02E-05
Cep55	-1,9236	0,0035476
RP23-356P21.1	-1,9263	0,002955
Gm9722	-1,9316	0,1369
Hbb-bh3	-1,9338	0,7424
Gm26730	-1,9355	0,46928
Gfod2	-1,9362	0,00072627
Dynlt1b	-1,9455	0,58386
Gm44258	-1,9537	0,0040511
Rpl32-ps	-1,9606	0,22307
Gm7784	-1,9638	0,42937
Kif18b	-1,9652	0,40465
Gm22513	-1,9732	0,34577
Acoxl	-1,9732	0,55536
RP24-174I4.1	-1,9743	0,16672
Gm29666	-1,9777	0,24635
Gm11491	-1,9853	0,0065356
Gm16755	-1,9869	0,54605
Gm45203	-1,9941	0,019434
Gm5577	-1,9974	0,2564
Gm42895	-2,0155	0,20108
RP23-320D23.6	-2,0184	0,0089942
Gm14636	-2,0253	9,36E-05
Gm44044	-2,0349	0,23131
RP24-122E11.4	-2,035	0,57448
Gm44639	-2,0367	0,14859
2900060B14Rik	-2,0367	0,22191
Ankdd1a	-2,0426	0,13758
Gm26870	-2,0434	1
Gm43011	-2,0442	0,29464
Ndrg1	-2,0485	2,03E-08
Esco2	-2,0602	0,15014

RP24-175C20.10	-2,0684	0,0018282
Gm44164	-2,0937	0,22307
Gm8317	-2,0949	0,032937
Sep 01	-2,0959	0,50794
Gm13992	-2,1022	0,22383
Cxcl10	-2,1034	0,23335
Gm38365	-2,1054	0,0064666
Gm8623	-2,1078	0,077058
RP24-547N4.7	-2,1131	0,14935
Gm37785	-2,1197	0,091026
Crtc2	-2,1333	0,28705
RP23-350F7.3	-2,1418	0,016945
Car7	-2,1426	0,0065041
Hes7	-2,1658	0,32
Gm45251	-2,1676	0,2428
4930509H03Rik	-2,1797	0,060953
Gm42793	-2,1832	0,48046
Hist1h4d	-2,189	0,15026
Gm37584	-2,1963	0,39534
RP24-93F20.12	-2,1964	0,1266
Gadd45g	-2,2316	0,00012047
Gm9442	-2,2377	0,30961
Gm26983	-2,2556	0,024612
A330069E16Rik	-2,266	0,0079839
4930542C12Rik	-2,2729	0,31113
Gm26656	-2,273	0,097225
BC055308	-2,2767	0,00027516
Gm45833	-2,2829	0,0068529
Gm16181	-2,2853	0,30206
RP23-40D21.1	-2,2991	0,0044878
Gm10575	-2,3113	0,32927
Olfr286	-2,3214	0,24541
Gm43421	-2,3216	0,4025
Gm26226	-2,3249	0,41051
Gm45206	-2,3271	0,1266
RP23-366E4.9	-2,3379	0,13327
Tsix	-2,3401	0,079481
Lrrc2	-2,3423	0,086588
Exo1	-2,3561	0,1749
Egln3	-2,4226	0,028673
Gm28373	-2,4269	0,2328
Kcnd1	-2,4327	0,15021
Dvl3	-2,4343	0,064983
Gm8649	-2,4349	4,87E-08
Gm4607	-2,446	0,0099248
Gad2	-2,4724	0,154
Gm26594	-2,4903	0,1006
Gm26847	-2,493	0,20791
Gm10827	-2,5055	4,10E-05
Fzd7	-2,5056	0,0015748
Zp1	-2,508	0,24541
Gm12469	-2,5117	0,051479

RP23-440L7.5	-2,5293	0,11867
Gm28041	-2,5354	0,10834
Gm26810	-2,5403	0,039745
Med16	-2,5423	1,84E-06
Gm44130	-2,5803	0,084259
Mcm8	-2,5943	0,0048431
4632415L05Rik	-2,6023	1,13E-05
Txnip	-2,6264	5,50E-07
Gm37653	-2,6285	0,0030911
Gm45698	-2,6335	0,019533
AY074887	-2,6413	0,018431
2310058D17Rik	-2,6419	0,0042132
Bc1-ps1	-2,6429	0,041367
Gm29438	-2,6777	0,0010196
Gm44953	-2,7302	0,071379
2810433D01Rik	-2,7567	0,14307
Id1	-2,7707	1,03E-08
Hist2h4	-2,7739	0,067279
mt-Tm	-2,8223	0,00072708
Rpl30-ps2	-2,8249	0,00014717
Gm43920	-2,8582	0,033587
Ankrd37	-2,9123	3,47E-06
RP23-451J19.1	-2,9201	0,039274
Aloxe3	-2,936	0,016242
Thap8	-2,9681	0,0053822
Adm	-3,0015	7,80E-07
Gm18709	-3,0138	0,0016782
Gapdh	-3,0736	0,00025696
1500004A13Rik	-3,1795	0,00038299
4921507G05Rik	-3,1987	0,038839
Gm27248	-3,2159	0,026766
Rgcc	-3,2168	5,64E-06
1700030M09Rik	-3,2388	0,010821
Sit1	-3,2861	0,0098835
Gm23037	-3,3518	0,02137
Slc16a5	-3,3532	0,025669
Gm10800	-3,4333	1
Gm26772	-3,4622	0,0021405
Rn7sk	-3,5499	0,0013995
Gm37052	-3,6305	0,023871
Gm44652	-3,691	6,16E-05
Hspa1a	-3,8628	0,01414
4930578M07Rik	-4,0497	0,00071007
Gm29358	-5,5861	0,00012913
Hspa1b	-5,9773	0,00063606
