# Supplementary material for: Bone, dentin and cementum differentially influence the differentiation of osteoclast-like cells
Source: Sci Rep. 2025 Jun 5;15:19857. doi: 10.1038/s41598-025-04874-9 (PMC12141432; doi:10.1038/s41598-025-04874-9)
Supplement: Supplementary file 12 — Supplementary Information 12. [file 41598_2025_4874_MOESM12_ESM.pdf]

Tab. S11:
Significant transcripts (P<0.05) induced in murine macrophage cells stimulated on cementum (n=6), fold of stimulation control

gene name	regulation of expression	adj.P.Val
Hspa1b	226,9609053	3,02E-06
Gm29358	125,7574449	8,63E-07
Rn7sk	72,41416366	5,14E-08
mt-Tm	71,88406054	2,90E-10
Hspa1a	63,71229937	9,56E-06
Gm23037	60,94348092	2,50E-06
4930578M07Rik	58,14567581	4,37E-07
mt-Tc	42,42668343	2,58E-06
Gm44652	36,63879117	4,23E-08
4921507G05Rik	35,69870908	2,76E-05
mt-Ta	35,30987968	7,67E-05
Slc16a5	33,76835509	4,13E-05
RP23-451J19.1	31,45461939	1,12E-05
Aloxe3	29,24463342	3,18E-06
Hist1h2bg	26,47575646	0,00013994
Gm18709	26,29287505	3,04E-07
mt-Ti	25,11191247	9,58E-07
Gm37052	25,06322223	0,0005292
Gapdh	23,84310137	6,86E-08
1700054M17Rik	23,06766386	2,19E-05
Hist1h2an	22,4866992	0,00022689
Hist2h4	21,47222814	9,63E-05
Gm42793	21,0376259	0,00049873
Sit1	20,84746412	7,48E-05
Mcm8	20,49499879	4,01E-07
Gm28373	20,23386473	0,00024645
Snord83b	20,16386087	6,78E-07
Hist1h4d	19,89177345	1,73E-05
Rasd1	19,85595712	7,37E-05
AY074887	19,79274768	5,62E-06
Fzd7	19,63832529	3,47E-08
Adm	19,2595221	5,01E-10
RP23-440L7.5	18,52626307	0,00015941
1500004A13Rik	18,1387106	1,54E-06
Gm45698	17,94611928	1,03E-05
Gm22513	17,92622743	4,81E-05
Crtc2	17,66350815	0,00013527
Gm26810	17,19002812	2,80E-05
Gadd45g	16,91346096	2,90E-10
Hist1h1a	16,83626246	0,002006
Gm24991	16,57802226	0,00037868
mt-Tq	16,44754096	1,97E-06
Gm26226	16,39972821	0,0021342
Hist1h2be	16,10570602	2,33E-08
Gm24631	15,87076758	1,03E-05
mt-Tl1	14,26788908	7,21E-10
4933437G19Rik	14,21655496	9,92E-05
Gm8623	13,84410612	1,32E-05

Atp1b4	13,67624246	0,0035661
Dynlt1b	13,62137087	0,0021494
4632415L05Rik	13,547925	6,98E-09
AV099323	13,3985038	0,00097343
2310058D17Rik	13,25532373	1,08E-05
D430001F17Rik	13,13186574	2,45E-06
Hspa8	13,09006177	5,95E-07
Rgcc	13,05472353	1,90E-07
Acoxl	12,89911338	0,0028378
Rpl7	12,8812438	1,10E-08
Snord66	12,8741029	0,0010652
Thap8	12,83845774	8,85E-05
Gm26772	12,82778346	0,00036245
Zp1	12,79226655	0,0054145
Gm43714	12,51945626	0,00070892
Gm26594	12,47787191	0,00076807
Gm26202	12,45713156	6,38E-05
Rpl30-ps2	12,23125046	6,98E-07
Gm4607	12,15939885	1,20E-05
Gm27248	11,96708607	0,0035196
Gm25008	11,88936699	2,66E-05
Hist1h1d	11,79824199	4,36E-05
Hist1h4a	11,59314428	0,0056036
Id1	11,39240156	7,38E-11
Hist1h1b	11,32626276	0,00058185
1700030M09Rik	11,30116815	0,0021079
Gdf15	11,09547865	7,85E-09
RP24-174I4.1	11,03259297	0,000149
Gm37653	11,0134915	1,64E-05
Ankrd37	10,91318268	6,86E-08
4930578M01Rik	10,86487748	0,0066833
Esco2	10,84305963	0,00020846
Rybp	10,83179173	0,00065964
Gm23969	10,76293767	0,00016273
5930420M18Rik	10,42156473	0,0032805
Snord82	10,38910882	0,0014466
Mkln1os	10,380471	0,00022808
RP23-320D23.6	10,35173008	9,20E-07
Gm29170	10,20638455	4,42E-08
Med16	10,15768686	5,42E-09
Gm12469	9,995647326	0,0010072
Gm8649	9,992876328	7,38E-11
Lrrc2	9,949334303	0,00084152
mt-Ts2	9,895002008	0,0017262
Gm20594	9,892944614	0,0086979
Gm43011	9,884719315	0,0018068
Gm43273	9,729057156	0,01767
Gm26225	9,634434574	0,0002857
RP23-366E4.9	9,501794466	0,0026041
Gm42670	9,432237204	0,0014419
Gm44292	9,374878545	1,34E-05
RP24-295J1.1	9,365785519	0,0029655

Hist1h3d	9,335323387	0,00096638
Gm42743	9,255429884	9,05E-06
Kcnd1	9,167955236	0,0062396
Trf	9,100841823	0,0012493
Gm5112	9,067469579	4,98E-05
Gm19272	8,997349094	0,013413
RP24-282C4.3	8,963113714	0,0014875
Snord89	8,775613578	4,52E-07
Kif18b	8,743433926	0,0059789
Efna3	8,672211849	0,00048523
Gm26656	8,590844746	0,0021653
Hist4h4	8,545707869	0,0039379
Gm14057	8,454972135	0,0096861
Hba-a1	8,426888288	0,033302
Nsl1	8,378546566	1,55E-07
Wwc1	8,236891586	7,21E-10
Hist1h4n	8,224340526	0,0028672
Rnf122	8,210670271	9,41E-06
Gm23301	8,210670271	0,0063144
4932422M17Rik	8,188504553	3,10E-06
Gm26461	8,165832642	0,003761
Lbp	8,160740119	0,0091332
Crkl	8,074091856	0,00013265
Tsix	8,071853553	0,0029616
Gm7099	7,994456744	3,88E-05
Gm3531	7,911218948	0,027329
Olfr286	7,896974326	0,017212
Gm11491	7,823423156	4,11E-06
Lgals7	7,795275438	0,0020277
4930589L23Rik	7,768305834	0,0018853
Gm26847	7,747334333	0,025663
Gm43566	7,711438462	5,47E-07
Ier5l	7,650743734	2,32E-07
Sep 01	7,646502437	0,035261
Gm42856	7,631676404	0,022407
Rbm3	7,61160133	0,00022803
4930542C12Rik	7,562168522	0,025163
Gm8203	7,458057913	0,031472
Gm44130	7,425560869	0,014291
Hist2h2ac	7,39987038	0,012597
Gm8210	7,39987038	0,015199
9530085L11Rik	7,369159091	0,00034143
Gm26983	7,355380626	0,00066456
Gm42432	7,354361025	0,001626
Gm37696	7,34722777	0,010189
Gm43878	7,339592674	0,015673
Gm45251	7,314706612	0,014411
Gm36989	7,302548324	0,0011767
Gm10382	7,275770315	6,24E-05
Snord7	7,262166464	0,01336
Gm5577	7,252608633	0,0060478
B430305J03Rik	7,180578108	0,018351

Gzmm	7,141860726	0,003852
Myc	7,017221976	0,002916
4930509H03Rik	6,969233537	0,0018666
Dpf1	6,957649484	9,37E-05
Gm8317	6,928293272	0,0004627
Mapkapk5	6,876621788	0,015295
5330426L24Rik	6,849981064	0,019062
Gm11516	6,742109226	0,043974
Gm42671	6,695538076	0,014271
Snord59a	6,664053579	0,0026474
RP24-282K24.4	6,652054553	0,0062396
Gm13383	6,641918407	0,018351
mt-Tv	6,640537402	0,00017251
Gm45546	6,639156685	0,012624
2900060B14Rik	6,61939792	0,010797
Lsm7	6,611602545	3,04E-05
C230096K16Rik	6,596039325	2,66E-05
Gm42731	6,590098368	0,036154
RP23-350F7.3	6,586901608	0,00038116
Rpl32-ps	6,580968881	0,0073198
Gm43800	6,564567665	0,0060478
Dvl3	6,537323088	0,011278
Rpl12	6,515157249	6,88E-08
Rpl7a	6,488567476	9,58E-07
Snord87	6,476435495	3,26E-05
Gm44258	6,445087745	9,91E-06
RP23-205H11.3	6,39923745	0,0003536
B3gnt6	6,363409653	0,039562
Gm10657	6,336999932	0,01894
Rhov	6,326905291	0,00011525
Gm10827	6,316388897	8,47E-06
Ang	6,29715422	5,76E-07
Hes7	6,293227076	0,03559
Ccng2	6,263200223	3,07E-07
Ost4	6,252356322	3,32E-06
Rnf152	6,245426081	0,0066444
Ndrg1	6,228565782	7,79E-11
Sdhd	6,224249965	0,00084368
Gm23849	6,217781846	0,019642
Gm2011	6,189401881	0,00090837
Gm17108	6,133029966	0,0018082
Tnfsf9	6,110116975	0,0001793
Gm44639	6,031847692	0,0093486
RP23-40D21.1	6,02432666	0,00038116
Gm11810	6,014313203	0,00076539
Gadd45b	5,996414032	5,14E-08
Gm42908	5,991013149	0,011641
Txnip	5,970285818	5,75E-07
Stamos	5,955406558	0,0045057
Gm29228	5,912630004	0,0073306
Dnali1	5,827996456	0,011256
Gmnn	5,790147097	5,15E-07

Gm37785	5,78413009	0,0081271
Gm42895	5,75015163	0,016703
Gm15937	5,748956044	0,014288
Tap2	5,746963953	0,0028611
Gm14279	5,701335893	0,00011434
Insig1	5,672166964	7,58E-08
Gm10636	5,656854249	0,025427
Ccdc36	5,641191842	1,43E-05
Gm43331	5,629473501	0,02387
Ppp1r18os	5,592913431	0,049944
Sap30	5,589038062	9,51E-08
4930589O11Rik	5,578587974	0,014983
Gm9521	5,569315411	0,045255
Hist2h3c2	5,520504056	0,00044972
Gm43920	5,515531818	0,049769
5033430I15Rik	5,467570812	0,025617
Gm14537	5,457347836	0,017111
Tsc22d3	5,448654454	3,75E-07
RP24-75M13.2	5,440729112	0,025721
Gm28727	5,429050863	9,73E-06
Eno1	5,426041192	5,97E-07
Rbmx	5,410642798	0,0024132
Atp5l-ps1	5,399029127	7,26E-05
Gm2367	5,398654908	0,015272
4833421G17Rik	5,35913441	0,0047073
Itga6	5,297089923	4,91E-07
A330069E16Rik	5,268893431	0,0019055
Polq	5,26305328	0,001205
2700029L08Rik	5,261958973	0,020717
4930412F12Rik	5,251392392	0,038293
Rpl30-ps1	5,238304771	0,0028968
Ciart	5,20644996	2,44E-06
Gm16045	5,173712907	6,76E-05
BC055308	5,172637175	8,33E-05
Cep55	5,156170606	7,37E-05
Slc25a2	5,148670697	0,011854
Gm29438	5,148313831	0,0027466
Cxcl10	5,137975461	0,049158
Zscan21	5,135483105	1,97E-05
Mafk	5,12446005	3,80E-08
Wdfy2	5,121619234	9,37E-05
Atr	5,101070359	1,59E-05
1810026B05Rik	5,084126757	3,17E-08
Proscos	5,06232454	0,0008372
Tstd1	5,051458517	0,00016955
Rgmb	5,033981798	0,011048
Eno1b	5,026311209	0,00013527
RP23-356D13.9	5,010310454	0,027291
Rpl35a-ps4	5,004410031	0,0025259
Hnrnpa1	4,996438164	0,043222
Pabpn1	4,977426458	7,26E-05
Gm12280	4,954021054	0,0066924

Cpne9	4,950931539	0,0191
D830025C05Rik	4,937908146	0,00073383
Gm28659	4,930042214	0,020892
mt-Nd6	4,912985564	3,02E-06
Gm12604	4,902779913	0,0015936
Gm45833	4,893273766	0,0036024
Gm26664	4,892934602	0,019403
mt-Tp	4,875330423	7,62E-06
Gm7327	4,871276923	8,08E-05
Gm43328	4,868576462	0,0015624
Gm26610	4,857452877	0,013351
Ccl6	4,854087114	0,048061
Arc	4,847698588	0,0021313
Gm38299	4,821894212	0,0061551
Ccdc18	4,804878665	0,00019661
Ect2	4,785268911	0,00010499
Pbk	4,759466937	1,48E-06
Gm45051	4,743329204	0,03559
RP24-175C20.10	4,742014256	0,00028187
Adam9	4,737086447	0,0011814
Lockd	4,730524001	1,77E-06
Gm5830	4,717426369	0,013527
Pgam1	4,704691094	0,029837
0610039K10Rik	4,70175707	0,009164
Kif20b	4,698824876	1,81E-06
RP23-226H21.3	4,695569028	0,042263
Snrpf	4,693291276	0,0005292
Klf10	4,689714184	1,24E-07
Hmgb1-ps5	4,661518524	0,021394
Gm6520	4,658288525	0,045297
Gm12758	4,637990934	0,018062
Gm38365	4,621624347	0,0016664
Gm45220	4,615861704	0,0073263
Mrps28	4,615541768	0,017744
Gm5362	4,60371971	0,039903
Phlda1	4,603081543	3,13E-05
Rnu11	4,601167573	0,004636
2700038G22Rik	4,598935614	0,022243
B930036N10Rik	4,595111918	0,020505
Tnfrsf12a	4,577309993	3,47E-07
Pfkfb3	4,564636572	6,78E-07
Hmgb2	4,546952711	1,10E-05
Zfp36l2	4,541283175	1,91E-06
3110056K07Rik	4,540968409	0,0061032
Fam72a	4,539394902	0,018503
4932416K20Rik	4,521182017	0,0063839
1700031P21Rik	4,480003937	0,018712
Snord15a	4,477830752	0,0083162
2900093K20Rik	4,461720066	1,48E-06
Sez6	4,448749914	0,00019848
Aif1	4,42875135	0,00050512
Wfdc17	4,419551589	0,00020226

Mrps18b	4,415570963	0,03267
Ttk	4,409759573	1,72E-06
Ltb	4,398769486	2,55E-06
4930579G24Rik	4,392675722	0,010189
Gm6341	4,386590399	6,54E-07
Rpl9-ps7	4,377478218	0,035441
Slc25a25	4,372626131	2,85E-06
Gm7336	4,368082183	0,004179
Errfi1	4,365358081	2,98E-05
Gm22980	4,361426267	0,003957
Hyal1	4,344830814	6,75E-07
Gm11759	4,327398556	0,027755
L1cam	4,322302368	0,0025259
Snord49b	4,312426894	4,49E-05
n-R5s151	4,310633779	0,046503
Gm14636	4,282638786	2,83E-05
Gm37472	4,279671316	0,03624
Lrrc17	4,274631334	0,00016041
P4ha2	4,266638854	1,67E-05
Zwilch	4,258070983	0,00049873
Frat2	4,255120536	2,39E-07
Amd2	4,237167027	0,0069134
Btf3	4,234231064	0,0012251
Zfp773	4,20673226	7,86E-05
Gm45185	4,20352601	0,011897
Icosl	4,199157788	0,0025545
RP23-356P21.1	4,183469741	0,0005767
4930522L14Rik	4,177384674	0,0066447
Rps19-ps11	4,152557082	0,0039814
Dtd2	4,151693672	7,76E-05
Iqgap3	4,146516979	1,59E-05
Klf11	4,125016814	8,86E-05
Ncapd3	4,110175509	6,62E-07
Kcnj2	4,096523242	0,0019055
Zfp36l1	4,094536078	6,54E-07
Gm15542	4,094536078	0,042102
Gsg1	4,07330544	0,00028844
Suv39h2	4,070765172	0,00026291
Ckap2	4,066253052	5,36E-05
Fancd2	4,06597121	0,00028612
Rps17	4,06315387	4,03E-05
Gm43149	4,037885496	0,012604
Adcy6	4,03760562	0,00015386
Arhgap26	4,035926777	0,0010081
Cit	4,034248631	0,00015476
Gm28555	4,015000034	4,11E-06
Rad51ap1	4,015000034	0,00038121
Tmem240	4,008048594	0,013184
Gm42666	3,995843278	0,013937
H2-DMb2	3,991967569	0,026629
Gm29019	3,98367513	0,0018388
2810454H06Rik	3,971544037	0,014231

Tnfrsf17	3,945477739	0,0013254
Atf3	3,941650881	3,64E-06
Gm43817	3,940011935	0,0031888
4931428F04Rik	3,930464992	0,010459
Rny1	3,918767553	0,00039928
Cenpk	3,912796285	0,0004325
Cd274	3,909001125	0,00066456
C730045M19Rik	3,903585854	0,023522
Gm45153	3,894937019	0,0063448
H2-T23	3,890350122	0,0043451
Gm3550	3,884422155	0,0005022
Rpl17-ps8	3,87608444	8,36E-05
D130051D11Rik	3,870983076	0,00061167
Ddah2	3,870983076	0,0051158
G2e3	3,855184752	5,95E-07
Ddit4	3,85491754	4,13E-06
Crip1	3,852513467	6,47E-09
Cdkn2c	3,848510011	9,50E-05
Bub1	3,837854454	1,40E-05
Cfh	3,823516235	4,22E-06
Gm15564	3,819278171	0,011024
A130014A01Rik	3,810287874	0,00084152
Tmem107	3,806064472	0,00010147
Selenbp1	3,796315776	7,97E-06
5330406M23Rik	3,778726234	0,0042473
Gm43581	3,762521954	0,00037789
2810013P06Rik	3,754706139	0,00013841
Ezr	3,749764522	5,32E-08
Arrdc2	3,742493991	2,46E-05
Basp1	3,730063006	9,07E-06
Usp50	3,729804467	0,017211
Kifc5b	3,725928527	0,0064826
Gm14005	3,724379279	0,0030261
H3f3a	3,723863007	3,18E-06
Gm15728	3,708151017	0,0032507
Gm44090	3,699422306	0,0042691
Gm24920	3,69685895	0,044022
Gm28578	3,695834105	0,019678
Gm15950	3,684324159	0,0039912
Dlgap5	3,684324159	0,0063839
Gm5312	3,680495481	0,039235
Gm10036	3,67922014	0,00017535
Selenop	3,667761934	0,0069435
Slc2a1	3,664458434	9,58E-07
Rpl35a-ps5	3,657606823	0,0011432
Gm7114	3,654059181	0,031472
Oaz1	3,642426852	0,00016058
Tob1	3,639398426	0,00040604
Ly86	3,637380874	7,16E-07
Zic2	3,631083232	0,0020826
Arf2	3,620527728	7,88E-09
S1pr1	3,612506041	0,00015403

Gm6136	3,61150458	0,0014018
Nek2	3,602254222	0,0071734
Nadk2	3,59352569	5,36E-05
Rasgef1b	3,580100284	9,32E-06
Rpl39-ps	3,570930363	4,65E-05
Ftl1	3,563265542	0,017321
Pmaip1	3,561537054	1,03E-05
Gm13092	3,56079653	0,031963
Rpl13a-ps1	3,556356619	6,61E-06
Gm45203	3,5474934	0,025602
Gtse1	3,544543904	0,0002623
Ifi213	3,541596861	1,40E-05
Snhg20	3,533260206	3,02E-06
C730034F03Rik	3,533015307	5,84E-05
Mtfr2	3,521524207	0,027587
1500015A07Rik	3,517133265	0,00070418
Hist1h2al	3,516889484	0,0097619
Dpep2	3,513965434	1,74E-05
Gm15503	3,499866761	0,0043856
Gm13567	3,487999903	0,035466
Ccdc117	3,485824654	1,05E-06
Uhrf1	3,485583043	0,0057994
Gm44270	3,475450507	0,034998
Tk1	3,471598246	0,0072795
Rps28	3,46486706	0,00011147
Maff	3,459347636	4,25E-05
Atp5g1	3,457190256	7,95E-05
Luc7l3	3,445468099	3,17E-06
Gm14094	3,437357724	0,033302
Pea15a	3,434737869	6,83E-06
Pclaf	3,430455139	2,52E-06
Cbx2	3,429504147	0,0072601
Gm16585	3,424990532	0,049116
Gm43637	3,416217903	0,032523
Rps8-ps4	3,414560746	0,045393
Kifc1	3,41361416	0,0002928
Pigb	3,412431296	0,025582
Sdc3	3,408885164	8,91E-08
Cox20-ps	3,407940152	7,85E-06
Map3k12	3,397797796	0,00033984
AV356131	3,391209704	4,63E-06
Eno2	3,388390148	3,18E-06
Prdm10	3,381117141	0,00071671
Unc13a	3,37854015	0,00076779
Gm13408	3,373625894	0,01894
Gm38043	3,367318066	0,01127
Lzic	3,361022033	0,00030291
Nfil3	3,356365895	0,0003536
Igfbp4	3,339194201	3,58E-08
Crip2	3,336417892	0,0015705
RP24-550H10.3	3,33387497	0,0081661
C030034I22Rik	3,332719737	1,45E-05

Gadd45a	3,331564904	0,0010081
Tgif2	3,324183446	4,98E-05
RP23-43M12.2	3,313141912	0,00054519
Ypel2	3,311305226	0,0002623
Gm37733	3,304655768	0,039595
Gm10343	3,30351066	0,013527
Arsb	3,302137054	0,00015176
Kif11	3,301908175	0,0002228
Anln	3,29984898	0,00028748
Itgam	3,29984898	0,0002946
Rsrp1	3,297791069	3,05E-05
Rab5a	3,297105384	0,0048394
Rccd1	3,295734442	0,0025379
RP23-325K4.10	3,2868372	3,27E-05
RP23-288C18.3	3,280919053	0,0015953
Rpl19	3,278872947	6,55E-05
Vamp2	3,272742284	7,37E-05
9330020H09Rik	3,270927993	0,0024725
Gbe1	3,265717508	1,79E-05
Hmgb1	3,257804431	0,0057573
9330151L19Rik	3,257352834	0,012512
RP23-454I20.1	3,253967857	0,030384
Ube2s	3,253967857	0,030542
Gm26737	3,249910527	0,027369
Car7	3,249685268	0,042615
Itgax	3,246533284	0,0010457
Spsb2	3,244733522	0,0004225
Fbxo5	3,244733522	0,0024132
Gm24916	3,241361659	0,0011207
Gm13215	3,23687129	0,0062631
Kif15	3,234852655	0,00040242
2900055J20Rik	3,23462844	0,028272
Hmmr	3,221203933	7,37E-05
Lilrb4a	3,216295586	0,0010793
Lonrf3	3,212062578	1,59E-05
Gm19566	3,202059243	0,034958
Zfp326	3,200284136	1,61E-06
Gm6987	3,194743269	0,0037915
Ska1	3,186118665	8,93E-06
Rps12-ps10	3,181925373	0,00084441
Tiparp	3,181704826	3,79E-05
Rps15a-ps6	3,179059459	0,0015913
Gm13456	3,174875458	0,0090767
Gm14427	3,168060749	0,0060396
Gm12902	3,163014128	0,012903
Mad2l1	3,158413366	0,0019205
Gm9320	3,151415544	0,0001993
Rpl27-ps3	3,151197112	0,0096321
Fn1	3,149886839	0,0020777
Anxa2	3,14792245	5,09E-06
Gdap10	3,14792245	0,004603
Senp3	3,13964186	0,00041401

Tmod1	3,13964186	0,0021653
Gm24276	3,138118866	5,13E-05
Map3k8	3,137683861	3,88E-05
Gm26520	3,129647127	0,0001993
Gm16754	3,128345814	0,00010068
Snord104	3,126178161	3,09E-05
Gm15453	3,123795478	0,011546
Zdhhc18	3,113635362	2,27E-05
Spc24	3,112556445	0,00080763
Ccdc58	3,110184144	7,37E-05
Msantd2	3,099638567	0,00096638
RP23-2N7.4	3,097920239	0,01785
Fth-ps3	3,09448644	0,0010076
Hist1h1e	3,089985354	2,76E-05
Dennd4c	3,089342876	0,0001458
Fam103a1	3,087630256	0,0054669
8030462N17Rik	3,08656035	0,0063016
Nemp1	3,085704693	7,34E-05
Golga7	3,068428571	0,00061665
Sgol1	3,06523992	0,0056463
Tma7-ps	3,064390173	0,0085532
Ormdl3	3,062691386	7,37E-05
H2-Q4	3,06099354	0,011851
Gm5445	3,057176827	0,020343
Pcgf5	3,052095278	0,044178
Ncapg	3,049769059	0,00037826
Tma7	3,039849713	0,021801
Gm42819	3,035848928	0,014288
Cldn11	3,026604156	4,69E-05
Rassf7	3,025974857	0,0097619
Lpl	3,023458968	6,98E-07
Rhob	3,022830323	0,020683
Gm10175	3,016132901	0,00038273
Mcm7	3,010076181	6,43E-05
Gm26244	3,009450318	0,0056981
Gm12577	3,008407503	0,0074164
Wdhd1	3,005281225	0,00076539
Bnip3l	2,995506575	0,00016495
Gm24339	2,995506575	0,023206
Mki67	2,992393703	0,00012501
Cdc7	2,989284066	0,021556
Tomm40l	2,988869692	0,00095157
Mafb	2,983074483	3,01E-05
H2-Ob	2,981214124	0,00014391
Nup205	2,976671465	0,0014574
Cox20	2,975640008	6,81E-05
Rpl10a-ps2	2,974608908	0,0025143
RP23-162P10.8	2,970693987	0,00054555
Tgfb1	2,969458767	3,05E-05
Gm10240	2,968635572	0,0056897
4931440P22Rik	2,965345075	0,0077781
Sdc4	2,963701195	7,60E-06

Tia1	2,962674232	0,0004225
Zfp101	2,958159822	0,01702
Hsp90aa1	2,956929814	2,08E-05
Chrnb1	2,953242857	0,047733
Jsrp1	2,953038161	0,02573
2410080I02Rik	2,948742819	0,019216
Gm12882	2,943841506	0,012597
Csrnp1	2,927562559	3,05E-05
Zfp367	2,924723013	0,00029442
1700084E18Rik	2,923506907	0,011158
Ptchd1	2,922493873	1,34E-05
Cdca2	2,920873747	0,00040682
Aunip	2,919861625	0,025721
Ccna2	2,91925452	0,00012861
Sp4	2,91258469	0,0014149
Tnfaip3	2,910163077	0,015912
Gm11531	2,907541936	0,001103
Gm7799	2,904721808	0,027898
Mturn	2,898085182	0,01268
Gm28404	2,892065045	0,042715
Arhgap39	2,891864589	0,021842
Morf4l1	2,886457533	0,00046394
Tmsb10	2,883457983	0,019547
Gm12943	2,880860894	0,014701
Mgst3	2,868506923	0,026494
Gm5525	2,865128808	0,015685
Hoxb8	2,860961335	0,00018814
Bard1	2,854820426	0,035725
Higd1a	2,852249131	0,0039121
Spdl1	2,848692699	0,0050344
E230032D23Rik	2,846916146	0,011851
Fam174a	2,844746308	2,42E-05
Numb	2,841790103	0,00053285
RP23-58B7.2	2,839821007	0,002916
Hnrnpa3	2,838246712	0,00047419
Nup98	2,830976945	0,0062637
Rps12-ps4	2,829603679	0,0012493
Rpl28-ps1	2,829603679	0,0026716
Dgkh	2,821964815	0,01988
Tpi1	2,820009456	1,91E-06
Atad5	2,817860125	0,04299
Dtl	2,817664813	0,0013125
Ube2t	2,813566378	0,0012975
Acrbp	2,812591439	0,024296
Mmp9	2,812201558	0,043356
RP24-454N4.2	2,8108374	0,0003536
Pif1	2,810447763	0,003766
Gm11826	2,809863407	0,035261
Smc2	2,806943452	1,08E-05
Gm8304	2,806359825	0,038637
Blm	2,796069071	0,0004244
Nat6	2,795487705	0,00066456

Gm14286	2,794131655	0,025944
Rpl30	2,793357065	0,022586
Rps12-ps9	2,791034582	0,0039039
Polr2l	2,790067451	0,0028347
Lsp1	2,788907335	4,98E-05
BC028528	2,788520738	0,0059077
Raf1	2,781378336	0,0063448
Insig2	2,779643763	7,17E-05
Gm7638	2,775408248	0,0063016
Gm4987	2,774061938	0,028933
H2-T22	2,771947628	0,0069832
Mettl7a1	2,771563381	0,015868
Tra2a	2,769067074	1,20E-05
Rps27rt	2,764464413	0,00024409
Gm5075	2,763314944	0,029217
Tpt1	2,762357418	0,00014509
3110062M04Rik	2,761208824	4,81E-05
AW554918	2,75891307	0,0011794
Selenow	2,755473018	3,61E-05
RP24-310D17.9	2,75032098	0,014049
Tbc1d31	2,7463205	0,0004244
Tsc22d2	2,744798038	6,83E-06
Gm19967	2,740425662	0,032373
Gm29736	2,740235717	0,020117
Rc3h1	2,737388117	0,00013542
Nuf2	2,734733028	0,033302
Gm13611	2,73321699	0,043536
Fau	2,732080514	0,014586
Rbl1	2,726783201	0,0035333
Asb10	2,725838333	0,019234
Gm14513	2,725082675	0,044093
Ccdc50-ps	2,722062137	0,0049109
Pmf1	2,721118906	0,00013634
Gm4617	2,720741705	0,01438
Tpx2	2,717160902	4,49E-05
Ip6k2	2,715842847	8,89E-05
Hist1h2bc	2,713020596	0,00013994
Mcm3	2,712080498	0,00038273
Gm15832	2,71170455	0,01091
Supt20	2,70869884	0,0003442
Aurkb	2,708323361	0,0042412
Rpl31-ps13	2,703821666	0,005539
Fam64a	2,690547916	0,0021658
Med22	2,686634379	0,00039646
Gatsl2	2,685889589	1,54E-05
Mxd1	2,684586703	0,0003536
Gm8430	2,681239327	0,00072199
Kif4	2,680310242	0,040775
Clec12a	2,679010062	1,47E-05
Cenpw	2,67566964	0,00018814
Zfp280d	2,671592557	0,00031595
Car12	2,667336794	0,00086777

Gm26826	2,666782195	0,012624
Ncapg2	2,665303827	0,0016633
Rassf1	2,663826278	3,31E-05
Parp6	2,657003264	0,00051938
Rpl9	2,656450813	0,00049873
Piga	2,655162208	0,0001641
Top2a	2,653322427	3,88E-05
Slbp	2,648912153	0,042485
Gm6472	2,648361385	0,018528
4933439C10Rik	2,645425886	0,013401
Pim1	2,641944207	6,46E-05
Gm6807	2,640113586	0,0060171
H2afz	2,633351308	0,0041079
Kctd6	2,63152664	0,006225
Zc3h12c	2,630250125	0,0024132
Tmx2	2,628245424	0,0087083
Tacc3	2,623331269	0,0035181
Pi16	2,62314944	0,0024165
Rab26os	2,616067925	0,00085078
Bsdc1	2,611357538	0,043459
Lin54	2,607559187	0,0035709
Gm42724	2,60070002	0,047733
Dedd2	2,597997423	7,86E-05
RP23-354J5.3	2,596737173	0,0193
RP24-275P22.2	2,592960088	0,037541
Racgap1	2,592780364	0,00035607
Rpl11	2,590983809	0,022003
Plin2	2,58972696	8,00E-05
H2-K2	2,58972696	0,016454
Ift80	2,586677152	0,0035333
Trim59	2,585601607	0,022003
Cd74	2,583810026	0,00010699
Gfod2	2,572729895	0,030999
Cox4i2	2,567207661	0,047664
Rpl23a-ps3	2,564717636	0,018961
C530043K16Rik	2,559922262	0,023291
Rpl6	2,557262041	0,0015374
Rpl10-ps3	2,556553115	0,02704
Zfp932	2,552834481	0,010762
Dnajb6	2,547178388	3,85E-05
Hsf2bp	2,542944543	0,04689
Hist1h1c	2,542415808	0,001483
mt-Co1	2,540830261	7,86E-05
Ndc80	2,531864155	0,023206
Iscu	2,528707206	0,0053018
Car11	2,527130209	0,030478
Socs4	2,526779898	0,0030261
Slc25a38	2,525029075	0,0042533
Rpl21-ps6	2,524154118	0,01739
Smc4	2,522754818	7,06E-05
Spag5	2,51559557	0,02584
4921524J17Rik	2,515246858	0,0029136

Tmem176a	2,514898195	0,00097281
Cdc6	2,507935074	0,012167
Prim1	2,503419387	0,041036
Zeb2os	2,501337968	0,021083
Rnf24	2,496834159	0,0050434
Asf1b	2,493548044	0,026629
Mettl17	2,490611501	0,014349
Rdh13	2,490266253	0,00034115
Gm43482	2,486816403	0,037272
Plau	2,486471681	0,0013066
Eif4a2	2,482338742	0,013174
Gins2	2,480962622	0,024956
Gm11539	2,479587266	0,00017289
Khk	2,475980582	0,00016273
Ppfia4	2,474436466	0,018351
Zfyve26	2,473236153	0,0038928
Calr-ps	2,470494767	7,08E-05
Pmp22	2,468098547	8,00E-05
Ier5	2,468098547	0,00031718
BC051226	2,462630196	0,0088865
Birc5	2,461947505	0,00012193
Rpl21	2,461947505	0,0047073
Rps18-ps3	2,46160623	0,03596
Jun	2,455471368	0,0043637
C330027C09Rik	2,455130991	0,0084119
Sowahc	2,453599878	0,0026736
Ywhah	2,450370664	0,0077246
Gramd4	2,450200823	0,019475
Ptgs2	2,44833335	0,0046811
Gm2531	2,44833335	0,043506
Rnf19a	2,446806477	0,0023019
Mef2c	2,443416833	4,86E-05
Gm42635	2,436820527	0,032028
Mettl21b	2,434963253	0,028705
Rps27a	2,42990515	0,037023
B3gat3	2,428221451	0,001761
Gm12844	2,416300693	0,037864
Bbc3	2,413455122	0,0013868
Erf	2,412618827	0,00024431
Mdm4-ps	2,406439245	0,030918
C030037D09Rik	2,406439245	0,049116
Klf4	2,399443763	0,00076863
Hist1h4i	2,395621524	0,0018403
H2-Q7	2,387498787	0,0043629
Dnajb4	2,385679104	0,00018359
Hist3h2a	2,385183067	0,0011814
Tmed7	2,384521846	0,013571
Pde1b	2,384191304	0,0067037
Gm7308	2,376601471	0,030814
Smagp	2,375777946	0,00035506
Arhgap15	2,373638118	0,0097923
Gm12481	2,369856987	0,0028968

Pltp	2,367722491	0,00030874
Sgol2a	2,362640295	0,004049
Rrm1	2,359040194	0,0012669
P4ha1	2,356915441	0,00013088
Kdm4b	2,356752078	0,036238
Rpl36-ps10	2,354139805	0,0042739
N4bp2	2,353487189	0,018183
Rpl30-ps3	2,353324064	0,039447
Rpl31-ps8	2,351856441	0,029653
Dram1	2,350878535	0,0098813
Adh5	2,350389735	0,0010081
Rps23	2,347784514	0,023087
Lig1	2,347296357	8,39E-05
Prr11	2,342258004	0,027291
Cdkal1	2,340797283	0,02747
Carhsp1	2,339337473	0,00022245
Ppwd1	2,337878573	0,0031071
Kdm3a	2,334801662	0,009777
Kif18a	2,334639831	0,021842
Eif1b	2,334154407	0,00010125
Ppp1r2	2,333345591	0,004636
Bub1b	2,332537055	0,0013245
Gm43309	2,332537055	0,015265
Rpl31	2,33124398	0,00030585
Crebrf	2,330920824	0,00036494
Prc1	2,330597712	0,00065964
Usp53	2,330597712	0,0025259
Clu	2,330274645	0,001294
Jund	2,328498576	0,00037066
Srfbp1	2,328337183	0,0031772
Trmt112	2,327853069	0,0063004
5730508B09Rik	2,327369056	0,032028
Pgk1	2,32720774	0,033482
Kpna4	2,323017464	0,0010793
Rdm1	2,320442583	0,0032179
Gm12166	2,312254183	0,016572
Nxt2	2,301859855	0,0051661
Gm6023	2,296759825	0,013413
Gm8618	2,2956457	0,02584
Gm18889	2,294532116	0,029275
Trp53rkb	2,294373077	0,033251
Spink10	2,29326011	0,0071875
Dvl2	2,291512253	0,0010205
Stom	2,286435149	0,0050784
Mcm5	2,286435149	0,011897
Tmem64	2,284217455	0,00020846
Klf6	2,278840577	0,0037216
Hspe1	2,27599912	0,00086777
Zfp87	2,275368167	0,0074164
Mapre2	2,271586117	0,00042395
Nr2c2ap	2,270012119	0,0043929
Gm2000	2,263256347	0,018307

Per1	2,261061134	0,0050784
Gm19353	2,259964327	0,0029179
Gm5881	2,259181218	0,03559
2010111I01Rik	2,258554927	0,0036399
Pold1	2,253707053	0,046964
Uba52	2,252926113	0,04587
Oaz2	2,250116969	0,0003595
Arhgap11a	2,248869585	0,0083369
Hcfc1r1	2,242487588	0,00013499
Etv3	2,241710536	0,0054405
Dut	2,240467812	0,0012155
Rpl36	2,240157239	0,034679
Pcif1	2,239846709	0,0017686
Rpl5	2,239380994	0,0077945
Rbm4b	2,238139562	0,036605
Ezh2	2,237829311	0,00080789
B230219D22Rik	2,236898818	0,0019958
Wdr70	2,235038991	0,013696
Ccdc163	2,235038991	0,024848
Bcat2	2,233954806	0,032602
Gm15501	2,231788015	0,024001
Gm23935	2,230396187	0,040084
Polg2	2,225917311	0,033205
E330034L11Rik	2,221909414	0,036238
B4galt3	2,220677667	0,00096339
Rps3a1	2,216218307	0,018939
Tusc3	2,212534575	0,0037357
Sirpa	2,211614599	0,00090393
Cdkn2d	2,209010077	0,0012892
Tex30	2,207020454	0,032041
Mcm6	2,200909769	0,0063448
Gm6142	2,198470235	0,0063967
Metap1d	2,197099185	0,0182
Swap70	2,196946899	0,0035284
Rpl13	2,194055469	0,0079885
Necap1	2,192839164	0,0031812
Atp11b	2,190256753	0,0039912
Sik2	2,182527754	0,035574
Tmem170b	2,180713133	0,034993
1500011K16Rik	2,180259714	0,049944
H2afv	2,179051056	0,0050531
Nucb1	2,178748997	0,0005292
Rps27	2,178446979	0,04168
Hist2h2be	2,177994031	0,00655
Cenpx	2,177541177	0,028702
Calm3	2,176786629	0,0013254
AC133103.1	2,170609194	0,021596
Pttg1	2,16925552	0,0086124
Cenpf	2,16790269	0,0076834
Ero1l	2,167301703	0,0024819
Mb21d1	2,167001272	0,0004225
Csrp1	2,166550703	0,011849

Raly	2,165349645	0,027039
Adgre1	2,164299266	0,00062306
Gins3	2,163999251	0,0072601
Slc16a10	2,162649699	0,0043065
Pfkp	2,162499801	0,0020277
Uimc1	2,161450804	0,0047297
Gon7	2,160252574	0,004704
Upf2	2,159803408	0,0036456
Ckap2l	2,157409437	0,034993
Gm9790	2,156512383	0,01179
Man1a	2,15248025	0,04929
Lmnb1	2,14860458	0,0086606
Osbpl3	2,147115797	0,010189
Trmt13	2,146966976	0,017869
Gm10076	2,145479327	0,00771
Snx13	2,141616263	0,035672
Rpl26	2,141319393	0,02542
Ubb	2,13746382	0,004049
Fzd5	2,135982756	0,047664
Lbr	2,135242609	0,00066456
Emc10	2,130954811	0,010153
Mif	2,128002728	0,021747
Rab10os	2,126233441	0,0033485
Dusp4	2,125791349	0,0046974
Prelid1	2,124907441	0,0023588
Chchd6	2,122993568	0,023637
Skp2	2,121816656	0,03266
Dnajb1	2,115501927	0,03768
Fbxo33	2,110083368	0,024283
H60b	2,108475123	0,0034633
Mapk6	2,107160198	0,0042139
Cdc20	2,103949388	0,016454
Plekha1	2,103803558	0,0023594
Tmx4	2,103657739	0,00049873
Vezt	2,090285493	0,03266
Mynn	2,089995738	0,031478
Srsf1	2,087679143	0,028418
Rpl17	2,08666644	0,040534
Rpsa	2,086377187	0,0049735
Atp9a	2,08478701	0,01291
G6pc3	2,083631279	0,021129
Arhgap27os2	2,083486858	0,046587
RP24-84C23.4	2,079735416	0,048447
Pcna	2,07915887	0,038355
Fam76b	2,078294352	0,02172
Ak4	2,067518158	0,0059086
St6galnac6	2,067374854	0,008649
Gt(ROSA)26Sor	2,058794738	0,021556
Psat1	2,058081337	0,00084757
Syt11	2,057796046	0,01268
Rpl18	2,057796046	0,018712
C330006A16Rik	2,054518042	0,014558

Calm1	2,054233245	0,00024409
Gm6085	2,053948488	0,0050344
C920021L13Rik	2,052525293	0,035083
Gm16580	2,051956291	0,020505
Cenpe	2,05124526	0,0042989
Rps24	2,050676613	0,0075506
Inafm1	2,047977691	0,015968
Chka	2,047126138	0,033482
Gys1	2,044715327	0,0053968
Cbx5	2,043015288	0,0051344
Pnn	2,041599669	0,003761
Coprs	2,040750768	0,028418
Vps51	2,039760829	0,038355
Gpr137b-ps	2,034677298	0,039162
Gm15464	2,024828894	0,041837
Ulbp1	2,021743538	0,020419
D030056L22Rik	2,0120972	0,016091
Ctdspl2	2,010284933	0,041965
Sec24a	2,00861352	0,038746
Tor1aip2	2,004024297	0,0057994
Nnt	2,002358089	0,018528
Rell1	2,000693267	0,019642
Sh3bgrl2	1,999861375	0,01247
Gm14620	1,996260506	0,016702
Egln1	1,994739013	0,0065599
Tcf20	1,994462503	0,016821
Cnih1	1,991975634	0,016322
Ap1s3	1,984396063	0,047543
Gm15210	1,982883613	0,01958
Nusap1	1,981097659	0,037288
BC055324	1,975886394	0,045186
Gm10275	1,969323334	0,046793
Rsrc2	1,96686781	0,0058497
4930503L19Rik	1,964279191	0,043504
Tial1	1,962918128	0,017954
Rpl15	1,961422048	0,048648
Asf1a	1,95626254	0,031127
Cd300c2	1,955584669	0,043459
Gm9385	1,95504254	0,01251
Ptrh1	1,953281662	0,037804
Dtymk	1,949629519	0,036655
Sf3b1	1,947468509	0,0072795
Rps15a	1,943153673	0,0096098
Fmr1	1,942884313	0,022363
Syne3	1,931203238	0,044146
Fam162a	1,928527877	0,013463
Gm24951	1,927325173	0,041999
Gm10086	1,924255005	0,033351
Gnas	1,922521857	0,041802
Anp32a	1,918661226	0,0076625
Dcp2	1,917996383	0,013465
Cln6	1,917331772	0,021046

Rasa4	1,915206561	0,03175
Rpl14-ps1	1,904615792	0,03734
Fgfr1op	1,901449996	0,025892
Fem1c	1,901318202	0,036739
Pfdn5	1,892115293	0,0070043
Mxi1	1,890542131	0,016244
Fam126b	1,885961206	0,037457
Clic4	1,883479068	0,041213
Zfand5	1,882826417	0,036739
Nop56	1,879436276	0,049904
Nop10	1,878134001	0,016327
Dennd4a	1,875012222	0,037381
Igf1	1,871506424	0,036433
D8Ertd738e	1,866454061	0,029228
Kpna3	1,862964264	0,030892
Rbx1	1,853946998	0,017665
Gtf2h5	1,853818497	0,012019
Usp1	1,853433046	0,04299
Cd47	1,850095829	0,019139
Rnf145	1,848557603	0,012831
Rhoc	1,846124693	0,01093
Ift57	1,843056119	0,047531
Qk	1,842417475	0,037999
Gm4997	1,840758036	0,039916
Mrpl42	1,835534206	0,042745
Arrdc4	1,829310534	0,043869
Clic1	1,827282888	0,038326
Phip	1,826902955	0,025257
Nr3c1	1,8229816	0,01894
Tspan13	1,821339667	0,018307
Sun2	1,814283614	0,017261
Cks1b	1,811142423	0,029653
Cdca8	1,80625302	0,037603
Rps8	1,805376833	0,014417
Napsa	1,803375716	0,02419
Kif23	1,803125733	0,042296
Ddx47	1,78967795	0,039266
Rpl37a	1,787942078	0,027329
Cox7a2l	1,786455528	0,039562
Agtpbp1	1,780521677	0,04587
Ybx1	1,778424835	0,047995
Vat1	1,771903457	0,038637
Higd2a	1,768835641	0,040084
Uhrf2	1,759298152	0,037127
Calr	1,751753769	0,016454
Lgals1	1,74956953	0,024848
Cic	1,747509138	0,032721
Atg12	1,736641542	0,032078
Golga2	-0,80398	0,027503
Rtf1	-0,81432	0,049328
Atp6v1e1	-0,81546	0,042539
Sec61a1	-0,82035	0,041551

Abr	-0,82976	0,022575
Hp1bp3	-0,83065	0,022152
Rap1b	-0,83698	0,029375
Zdhhc20	-0,84069	0,027898
Mfsd1	-0,84255	0,018307
Chmp3	-0,84807	0,044541
Tm9sf4	-0,85077	0,0098131
Atp6v1c1	-0,85281	0,014201
Wdfy4	-0,85426	0,043917
Uck2	-0,85443	0,021596
Ube2q1	-0,85878	0,02685
Sod1	-0,85949	0,034478
Psma2	-0,86855	0,02848
Surf4	-0,87312	0,013279
Gusb	-0,87349	0,013955
Irf5	-0,8821	0,031102
Hook3	-0,88672	0,036655
Ube2l3	-0,88749	0,04974
Bcl2l1	-0,88772	0,01958
Sde2	-0,88934	0,015625
Zfand3	-0,88944	0,037864
Csnk2a2	-0,89078	0,014201
Atxn7l3b	-0,89127	0,017159
Itpr2	-0,89264	0,027691
Plbd2	-0,89485	0,016576
Ddx21	-0,89541	0,018712
Psmd6	-0,89622	0,046793
Ktn1	-0,89683	0,014745
Hnrnpm	-0,89865	0,01702
Msr1	-0,90155	0,023896
Hectd1	-0,90326	0,011696
Mib1	-0,90353	0,048272
Cd109	-0,90733	0,021649
Foxp1	-0,90819	0,013527
Ostc	-0,90873	0,037063
Sh3bp5	-0,9112	0,015353
Tpm1	-0,91488	0,003094
Cars	-0,91864	0,044234
2810025M15Rik	-0,9227	0,021649
Wdr61	-0,92305	0,017869
Lhfpl2	-0,9247	0,0040801
Cux1	-0,92662	0,011641
Ptpra	-0,92678	0,039038
Gramd1b	-0,92783	0,025018
Mrpl37	-0,93086	0,025864
Tyk2	-0,93111	0,024033
Strn3	-0,93159	0,0028935
Usp25	-0,93474	0,046804
U2surp	-0,93584	0,011851
Arhgap27	-0,93759	0,030384
Taok3	-0,93778	0,010536
Arhgef12	-0,93781	0,025721

Sel1l	-0,93936	0,004843
Becn1	-0,94051	0,014701
Eif3b	-0,94155	0,013798
Ncbp1	-0,94286	0,019449
Abcd3	-0,94336	0,038355
Tbc1d10b	-0,94616	0,02178
Cant1	-0,94961	0,012919
Csnk1d	-0,95288	0,011757
Tgm2	-0,95287	0,014701
Tnfrsf1a	-0,9533	0,018521
Msantd4	-0,95508	0,028059
Ppp6r1	-0,95547	0,037541
Pstpip1	-0,95629	0,006927
Kif3b	-0,95713	0,033736
Ppm1l	-0,96118	0,024044
Mtmr2	-0,96199	0,0071097
Zcchc2	-0,96295	0,033371
Tpcn1	-0,9688	0,028977
Zfp217	-0,97022	0,041663
Myof	-0,97032	0,015625
Gab2	-0,97162	0,010492
Cerk	-0,97278	0,011329
Galnt1	-0,97686	0,0068016
Prorsd1	-0,97725	0,011702
Idh1	-0,9788	0,0011639
Aig1	-0,97892	0,019654
Arid1b	-0,9804	0,016454
H2afy	-0,98064	0,01615
Abcc1	-0,98076	0,010232
Brpf1	-0,98199	0,02704
Bid	-0,98215	0,025201
Nf2	-0,98687	0,024925
Mbp	-0,98881	0,0042059
Notch2	-0,98939	0,026249
Slc9a8	-0,99183	0,038175
Pgd	-0,99211	0,0022665
Hbs1l	-0,99212	0,0044088
Chordc1	-0,99301	0,0046974
Smap1	-0,99298	0,038355
Mgrn1	-0,99303	0,046609
Tcirg1	-0,99353	0,0057512
Haus2	-0,99391	0,024099
Spata13	-0,99466	0,01707
Map3k7	-0,99546	0,0057568
Ythdf2	-0,99748	0,016134
Foxred2	-1,0038	0,011851
Zfp330	-1,0046	0,023755
Zfp809	-1,0068	0,0052513
Pnpla7	-1,0076	0,0079758
Atp6ap2	-1,0091	0,0013357
Zmiz1	-1,0093	0,032462
Gnl3	-1,0145	0,014735

Scfd1	-1,017	0,048378
Fth1	-1,019	0,00044509
Phf20	-1,0248	0,012042
Mrpl17	-1,0286	0,019642
Smim12	-1,0295	0,040783
Mtmr4	-1,0317	0,041036
Nip7	-1,032	0,0069682
Trim25	-1,0323	0,0096155
Tnfaip2	-1,0361	0,0059077
Rin2	-1,0393	0,016273
Klf9	-1,0416	0,00076777
Trafd1	-1,0421	0,010119
Creg1	-1,0428	0,0040542
Slc17a5	-1,0497	0,011557
Faf2	-1,055	0,025516
St8sia4	-1,0555	0,024925
Gpr107	-1,0561	0,0019433
Nsf	-1,0615	0,023014
Rgl1	-1,0619	0,0011231
Bcl2l11	-1,0653	0,021048
Tns3	-1,0681	0,0034847
BC003965	-1,0681	0,039886
Glb1	-1,0688	0,0023874
Adprh	-1,0694	0,0051165
Fastkd3	-1,0726	0,04063
2810004N23Rik	-1,075	0,0062914
Ptpn1	-1,0758	0,0015936
Prmt3	-1,0836	0,030918
Phf10	-1,0842	0,0023445
Ncf4	-1,0862	0,022069
Itpripl2	-1,0866	0,0031772
Snx4	-1,0878	0,016572
Nucb2	-1,0883	0,0083157
Rere	-1,0896	0,00012236
Cmas	-1,0899	0,0022936
Slc39a9	-1,0906	0,026249
Pogz	-1,0947	0,0086365
0610007P14Rik	-1,0993	0,014271
Mdn1	-1,0998	0,0066849
Ryk	-1,103	0,0014452
Alkbh3	-1,1041	0,02419
Nagpa	-1,1044	0,033409
Abcb4	-1,1048	0,0014856
Rasgrp3	-1,1084	0,0046328
Ndufaf7	-1,1084	0,0296
Pde4a	-1,1135	0,035181
Kat6a	-1,1141	0,0096321
Fbxo25	-1,117	0,039916
Mdm2	-1,118	0,0059453
Mlycd	-1,1181	0,048525
Amotl1	-1,1189	0,00098233
Aptx	-1,1208	0,047116

Htra2	-1,1216	0,01336
Zc3h10	-1,1225	0,043295
Stard7	-1,1308	0,0029036
Rbm5	-1,131	0,0037554
Mrps35	-1,1322	0,038355
Tubgcp3	-1,1329	0,027148
Laptm5	-1,1361	0,00016771
Ptpn12	-1,137	0,0019615
Arhgap18	-1,139	0,0058601
Slc26a2	-1,1397	0,0050344
Xpot	-1,1402	0,0069486
Bag5	-1,1412	0,0219
Ppan	-1,1429	0,0021313
Rpl7l1	-1,1451	0,009777
Aak1	-1,1465	0,0037915
Csf1r	-1,1474	0,00042504
Eno3	-1,1532	0,035962
March9	-1,1536	0,023896
Sptlc1	-1,1537	0,023502
Hacd3	-1,1549	0,021046
Nfs1	-1,1641	0,004625
Cyba	-1,1661	0,0013432
Mier2	-1,1675	0,00026419
Lrrc20	-1,1713	0,04531
mt-Nd1	-1,1728	0,0038394
Ncoa6	-1,1739	0,0054125
Mettl21a	-1,1753	0,01767
Gid8	-1,1774	0,004704
Ammecr1	-1,1783	0,016585
Clptm1l	-1,186	0,00045593
Zfp830	-1,1862	0,032462
Sema4a	-1,1864	0,0021492
Fkbp15	-1,1881	0,0016172
Ufsp2	-1,1899	0,0023588
Zfp106	-1,1926	0,00017251
Nudt16	-1,2005	0,0098312
Yif1a	-1,2026	0,038076
Exosc4	-1,2045	0,019642
Nif3l1	-1,2076	0,037603
Coro1c	-1,208	0,0002279
Atxn7l3	-1,2083	0,0026934
Zfp180	-1,2091	0,014161
Son	-1,2098	0,00078781
Mks1	-1,2098	0,037541
Ap5s1	-1,2121	0,024017
Knop1	-1,2134	0,0014574
Mapkapk2	-1,2159	0,00013604
Spryd3	-1,2169	0,038355
Cul4a	-1,2196	0,023291
Bcar3	-1,2202	0,0050784
Trip11	-1,2224	0,025792
Diablo	-1,2229	0,025357

Rbck1	-1,223	0,0032179
Lcp2	-1,2241	0,0019639
Ehd1	-1,2248	0,00015955
Ubn1	-1,2299	0,00043129
Sh3pxd2a	-1,2306	0,0047151
Dnajc8	-1,2315	0,00030933
Ranbp1	-1,2331	0,02538
Slc16a6	-1,2352	0,0054043
Polr1a	-1,2354	0,0063115
Znhit6	-1,2367	0,036655
Ppip5k2	-1,2385	0,0042739
Wipf1	-1,2385	0,0052898
Srr	-1,2404	0,023124
Tbc1d13	-1,2432	0,0052898
Ints7	-1,2432	0,018393
Syk	-1,2438	0,0015193
Slc35c1	-1,2471	0,0067471
Rnmt	-1,248	0,00072199
Rtfdc1	-1,2481	0,0032082
Cbr3	-1,2483	0,0035426
Pin1	-1,2488	0,023206
Pigc	-1,2523	0,006756
Inf2	-1,2581	7,48E-05
Sh3bp5l	-1,2583	0,0082578
Med1	-1,2601	0,0001971
Mgat5	-1,2628	0,0026934
Wdr6	-1,2631	0,047649
Gdpd1	-1,2648	0,0057579
Ltbr	-1,2649	0,011263
Car9	-1,2651	0,031039
Atad1	-1,2658	0,01905
Blvra	-1,2662	0,0013254
Tmem55a	-1,2669	0,0037031
Rasa3	-1,2682	0,0021664
Rrp9	-1,2692	0,024283
Gsk3b	-1,2709	0,0002857
Arhgap30	-1,2711	0,0045236
Zfp362	-1,2729	0,039235
Slc43a3	-1,2749	0,038761
Cul5	-1,2762	0,00023473
Fnip2	-1,2765	2,31E-05
Stx17	-1,2801	0,022846
Thap7	-1,2803	0,018301
Kmt2e	-1,2831	0,0005556
Enpp5	-1,2835	0,011005
Gns	-1,2845	7,95E-05
Ssu72	-1,2853	0,00022245
RP23-444K20.4	-1,2856	0,03838
Polr3f	-1,287	0,021842
Noc2l	-1,2907	9,37E-05
Pdcd11	-1,2917	0,00038391
mt-Nd4	-1,294	0,010829

Mrps6	-1,2968	0,00038116
Lnx2	-1,2971	0,009777
mt-Cytb	-1,2973	0,0023325
Dtx4	-1,2991	0,0001324
Asb7	-1,3004	0,022166
Gpatch4	-1,3006	0,0057573
Atp6v1d	-1,3009	1,78E-05
Acsl4	-1,3018	1,64E-05
Slc12a2	-1,303	0,024424
Prkca	-1,3034	0,04063
Ftsj3	-1,3057	0,00036245
Med8	-1,3058	0,012019
Fli1	-1,3061	0,0031071
Fam102a	-1,3081	1,60E-05
Zfp26	-1,3097	0,041003
Nsrp1	-1,31	0,0028637
Ubash3b	-1,311	0,0007513
Ptpro	-1,312	0,032133
Ift20	-1,3133	0,0099012
Trim11	-1,3139	0,00010067
Ivns1abp	-1,3154	3,02E-06
Rundc1	-1,3166	0,035181
Prtg	-1,3171	0,048031
Poglut1	-1,3222	0,00050507
Cmtr2	-1,3222	0,004603
Actn1	-1,3235	9,37E-05
Nelfa	-1,3241	0,010221
Zbtb6	-1,3264	0,012792
Cd33	-1,3272	0,00013527
Arih1	-1,3285	1,62E-05
Ncdn	-1,3285	0,042679
Orai3	-1,3306	0,015968
Epc1	-1,3311	0,035466
Fam3c	-1,3317	0,0020958
Twnk	-1,333	0,011297
Gm42636	-1,3343	0,026409
Ppp1r7	-1,3376	0,023448
Slc35f6	-1,3387	0,0019864
Ppp6r3	-1,341	7,76E-05
Ddx28	-1,3416	0,036238
Tars	-1,3418	0,0011712
Rlf	-1,3422	0,0049064
Dedd	-1,3437	0,046587
Ints3	-1,345	3,41E-05
Ttll12	-1,3467	0,00032975
Polr3d	-1,3482	0,0045723
Pcyt1a	-1,3519	3,97E-06
Slc25a33	-1,352	0,0012167
Wfs1	-1,353	0,042558
Slc41a2	-1,3532	0,0010463
Lrrc8d	-1,3541	0,00089359
Tshz3	-1,3553	0,041445

Ppil3	-1,3559	0,031127
Plxdc1	-1,3564	0,0040324
Zfp318	-1,3567	0,0066094
Rasal2	-1,358	0,00036444
Pms2	-1,3588	0,049042
Clec7a	-1,3607	0,014828
Slc23a2	-1,3618	0,045094
Trim68	-1,3636	0,035083
Gzf1	-1,366	0,015228
Clec5a	-1,3664	0,0061879
Bckdk	-1,3665	0,0034846
Irak4	-1,3679	0,017568
Nfatc3	-1,3687	4,25E-05
Slc1a5	-1,3697	2,01E-05
Cep19	-1,3701	0,028428
Slc11a1	-1,371	0,0012161
1810030O07Rik	-1,3712	0,0032015
Pid1	-1,3722	0,0033629
Zfp839	-1,3722	0,020591
BC005537	-1,3736	0,00028612
Elp2	-1,3748	0,0023109
Etfrf1	-1,3755	0,024848
Hps6	-1,376	0,035901
Kdm4a	-1,3792	2,59E-05
2210016F16Rik	-1,3811	0,038355
Nfatc1	-1,3813	0,0001641
Bet1l	-1,382	0,00083994
Cat	-1,3833	1,02E-05
Tbl3	-1,3865	0,018321
Maf1	-1,3891	0,00029254
Trim27	-1,3922	0,00034125
Apbb1ip	-1,3926	1,03E-05
Spi1	-1,3949	0,00028736
Ica1	-1,3968	0,018301
Naa40	-1,3971	0,0081005
Trappc12	-1,3982	0,0068305
Zhx1	-1,3988	0,0025675
Aldh3b1	-1,3999	0,0063448
BC002059	-1,4006	0,0023184
Hhex	-1,402	0,028702
4632427E13Rik	-1,403	0,0027045
Rit1	-1,4031	0,0030769
Lsm10	-1,4043	0,028776
Pknox1	-1,4054	0,021596
Zadh2	-1,4116	0,0090214
Flywch1	-1,4171	0,018636
Rela	-1,4201	0,0003129
Dbnl	-1,4209	0,0048116
Lrp12	-1,4209	0,0053647
Rnf157	-1,4248	0,0013245
Cog8	-1,4252	0,018381
Arhgap31	-1,4297	0,040534

Pde8a	-1,4311	0,020601
Zfhx4	-1,4342	0,0023313
Uri1	-1,4351	0,00056528
Nudt22	-1,4356	0,0041901
Bag2	-1,4356	0,018961
Mfsd6	-1,4375	0,0010886
Utp20	-1,4434	0,0077762
Etv6	-1,444	0,0075223
Eif1ad	-1,448	0,0018622
Camk2a	-1,4511	0,011851
Kmt5b	-1,4519	0,0047073
Metap2	-1,4527	0,00018232
Fblim1	-1,4575	0,0014574
Tgfbr2	-1,462	0,0010229
Slc38a1	-1,469	7,70E-06
Zscan12	-1,4703	0,0051914
Iars	-1,4724	0,0011814
Rfx5	-1,4748	4,41E-05
Zkscan6	-1,4754	0,0044995
1700017B05Rik	-1,4756	0,00015476
Gmpr	-1,4774	0,00077364
Ahnak2	-1,4783	0,00023722
Fam65c	-1,4789	0,0068899
Dhodh	-1,479	0,032077
Cx3cr1	-1,4791	0,00062139
AU040320	-1,4795	0,0012135
Gtf2b	-1,4796	0,011546
Lrrc8b	-1,4803	0,015272
Fgfrl1	-1,4827	0,035261
Stat5b	-1,4835	0,016375
Tmem241	-1,4846	0,041208
Champ1	-1,4853	4,69E-05
Ankrd26	-1,4856	0,037063
Timp2	-1,4871	0,00041078
Dennd2a	-1,4873	0,0059856
Trmt6	-1,4877	0,0011806
Wbp1l	-1,4889	0,00028612
Aars	-1,4889	0,010189
AW209491	-1,4902	0,012936
Sec16b	-1,4949	0,0010166
Treml1	-1,4956	0,0004193
Cyfip2	-1,4957	0,00012342
Tk2	-1,4971	5,59E-05
Golim4	-1,4975	0,0003676
Jagn1	-1,498	0,0024581
Soga1	-1,5029	0,004875
Gstt3	-1,5029	0,012919
Wdr77	-1,5038	0,0041976
Xbp1	-1,504	1,96E-05
Zfp775	-1,5059	0,041445
mt-Nd2	-1,5089	0,0001793
Zfp113	-1,5095	0,022161

Hmga2	-1,5123	3,89E-06
Ighmbp2	-1,5124	0,019199
Lrif1	-1,5128	0,032384
Slc7a11	-1,5199	0,0020777
Fam136a	-1,52	0,0039305
Btbd19	-1,5221	0,0043995
Fus	-1,5226	0,0023082
Zbtb22	-1,5244	0,0013573
Dlx1	-1,5257	0,021747
Surf2	-1,5271	0,011851
5730409E04Rik	-1,5286	0,019526
Rab12	-1,5291	0,00013499
Pmepa1	-1,5307	0,0003536
Keap1	-1,535	9,13E-05
Usp20	-1,5353	6,64E-05
Myo1d	-1,5385	1,21E-05
Tada2b	-1,5386	0,032235
2310011J03Rik	-1,5388	0,0048285
Lrrc47	-1,5413	6,10E-06
Nsun4	-1,5424	0,023849
Tmem220	-1,5427	0,012208
Vac14	-1,5437	0,047733
Cdc42ep2	-1,5449	0,024283
Polr3a	-1,5553	0,029986
Uaca	-1,5603	0,00013265
Trak2	-1,561	0,00055718
Dpp3	-1,5651	0,042876
3110001I22Rik	-1,5669	0,031356
Mcat	-1,5675	0,029653
Prkch	-1,5679	0,00024761
Bnip2	-1,5708	2,36E-05
Nop9	-1,5711	0,020429
N6amt1	-1,5714	0,015912
Fosl1	-1,5721	0,011784
Scap	-1,5747	0,0024888
Frmd8	-1,5773	1,89E-05
Bok	-1,5774	0,033205
Ddx17	-1,58	3,00E-05
Gm20604	-1,5819	0,0196
Snip1	-1,5832	0,0085532
Cenpb	-1,5912	0,0043856
Plcb4	-1,5913	1,61E-06
Zpr1	-1,5913	3,10E-06
Airn	-1,5917	0,013824
Cd3eap	-1,594	0,0026066
Myom1	-1,5956	0,042326
Pitpnm2	-1,5982	0,02584
Tmem175	-1,5995	0,018049
Mgat2	-1,6003	0,00094881
Tmem177	-1,6027	0,025675
Ppfibp1	-1,6034	7,34E-06
Cox10	-1,6038	1,00E-05

Pla2g5	-1,6042	0,0001993
Cyth4	-1,605	1,02E-05
Bms1	-1,6056	4,13E-06
Nod1	-1,6062	0,02747
Ovca2	-1,6101	0,0023985
Mrps2	-1,6133	0,00061557
Dhx33	-1,6137	0,0068583
Marveld1	-1,6141	5,46E-05
AI987944	-1,6141	0,011024
Taf1b	-1,6211	0,0063448
Acvr1	-1,6213	0,0044655
Dph2	-1,6235	0,046433
Ccnd2	-1,6261	1,05E-05
Erlin2	-1,6283	0,010326
Paqr7	-1,6386	0,0032015
Frat1	-1,6386	0,025126
Pkn3	-1,639	0,0018433
Rnasel	-1,6406	0,030478
Ank	-1,6417	1,68E-07
Arhgef10	-1,6424	0,031127
Irf2bp1	-1,6429	0,00041609
Fem1a	-1,6433	1,99E-05
Wbscr27	-1,6451	0,0058265
Grap	-1,6471	0,0047414
Rab43	-1,6477	0,039369
Dcaf11	-1,6481	0,002515
Nrp2	-1,6517	2,42E-08
Mst1	-1,652	0,032462
Pik3r2	-1,6545	0,0013549
Dock5	-1,6576	0,0032395
Il4ra	-1,6586	0,012222
Det1	-1,6594	0,036811
1600002H07Rik	-1,6604	0,0075763
Fastkd2	-1,6621	0,023671
Hgh1	-1,6639	0,015968
Dapk1	-1,6668	1,16E-06
Ptgir	-1,6724	0,00012759
Rars2	-1,6754	0,0062637
Elf4	-1,6771	0,026866
Snx20	-1,6795	0,00013841
Isl2	-1,682	0,047096
Cpsf3	-1,6828	0,00053285
Frrs1	-1,6835	1,84E-06
Alkbh2	-1,6902	0,011192
Ccdc166	-1,6921	0,049641
Lat2	-1,6937	3,03E-06
Lcmt2	-1,6941	0,032721
Prr14l	-1,6977	0,00026988
Cpne2	-1,7049	0,00044509
Shtn1	-1,7069	2,01E-05
Slc39a13	-1,7082	7,08E-05
E130311K13Rik	-1,7082	0,025201

Fam161a	-1,7086	0,0018433
Lysmd4	-1,7096	0,0017644
Fbxw7	-1,7138	0,0015913
Tlr6	-1,7143	0,0041344
C330018D20Rik	-1,7162	0,03714
Oit3	-1,7182	0,038091
Bcdin3d	-1,7186	0,034993
Cstf2t	-1,729	0,00051975
Srf	-1,7305	1,03E-05
Msi1	-1,7313	0,025094
Elk3	-1,733	0,0013814
Bdh2	-1,7332	2,32E-05
Tmem65	-1,7339	4,81E-05
Trim45	-1,7359	0,049332
Dnajc11	-1,7414	0,0026934
Prss50	-1,7458	0,013527
Selenos	-1,748	1,11E-06
Iqce	-1,7564	0,047323
Slc39a11	-1,7646	6,98E-07
Zfp462	-1,772	0,021386
Scamp1	-1,7724	1,00E-05
Jade2	-1,7756	0,010969
Nfkbie	-1,7757	0,039595
Tuba1c	-1,7807	0,0002501
Pde4b	-1,7824	0,011851
Zfp46	-1,7878	0,013932
Tmem181b-ps	-1,7894	0,046384
Ctbp1	-1,7916	4,76E-06
Pde4dip	-1,7918	0,0011785
Hck	-1,7931	0,018228
Extl2	-1,7991	0,011052
Traf6	-1,8005	0,028059
Gsn	-1,8016	2,71E-07
Igsf3	-1,8019	0,0069726
Zfp653	-1,8058	0,035083
Wdr24	-1,8082	0,0069832
Me1	-1,8088	1,06E-06
Sdsl	-1,8117	0,047418
Sh3tc1	-1,8132	0,0067421
Trpv4	-1,8147	0,022194
Prag1	-1,8154	0,0028968
Tspan10	-1,8211	0,0041913
4931414P19Rik	-1,8229	0,028657
Ivd	-1,8235	0,0062804
0610010F05Rik	-1,8243	0,0086606
P2ry6	-1,8322	0,00017183
Arhgap35	-1,8342	0,041883
Cdk5rap1	-1,8359	0,02094
Fam120b	-1,8389	0,019131
Ticam1	-1,8419	0,00066456
Sema4b	-1,8437	0,012882
Tlr7	-1,8447	0,00013542

Wdr73	-1,8455	0,0048341
Slc25a15	-1,846	0,043356
Mir22hg	-1,8521	5,36E-05
Zfp764	-1,8593	0,022554
Tmem140	-1,8653	0,020455
Chst14	-1,8677	0,025721
Zdhhc12	-1,8694	0,01194
Msantd3	-1,8714	0,00048583
Fbxo32	-1,8829	0,02303
Rab7b	-1,8844	1,30E-05
Gpt2	-1,8867	0,00010067
Scly	-1,8871	0,0056131
Hemk1	-1,8872	0,020421
Sphk2	-1,8877	6,98E-07
Cptp	-1,8903	0,049204
Sgsm1	-1,8934	5,32E-08
Bmf	-1,8943	0,011069
Arhgef3	-1,895	0,0017234
Ecd	-1,8992	0,00061417
Zbtb38	-1,8993	1,95E-06
Setd1b	-1,9035	9,93E-05
Tmem116	-1,9037	0,011897
Il1rap	-1,9048	0,044541
Dido1	-1,9055	0,00047157
Tbc1d25	-1,9115	0,020461
Pomk	-1,9128	0,011069
Rftn1	-1,9147	0,00072327
Klhl5	-1,9153	3,90E-05
Ino80c	-1,9166	2,58E-06
Tbc1d2b	-1,9217	4,48E-08
AI467606	-1,9238	0,0016867
Suox	-1,9245	0,014917
Robo3	-1,9288	0,0079853
Eogt	-1,929	0,0062631
Lars	-1,9292	6,86E-08
Gja1	-1,9308	0,043099
Sfxn2	-1,933	0,0050344
Gcc1	-1,9388	2,79E-05
Pfkfb4	-1,9414	2,66E-05
Ptpn14	-1,9468	0,022756
Prkar1b	-1,9476	0,012955
Mthfr	-1,9494	0,0028612
Polr3e	-1,9506	0,0013104
Sec16a	-1,9543	0,0014609
4930432K21Rik	-1,9626	0,0034716
Ankrd49	-1,9696	0,00083819
Pspc1	-1,9727	0,0022481
Zfp276	-1,9756	0,02788
Zfp65	-1,9768	0,0057579
Tmem37	-1,9812	0,038355
Zfp628	-1,9817	0,015526
Slc22a4	-1,9844	0,049931

Gtpbp8	-1,9867	0,015161
Gnptab	-1,992	2,01E-07
Gm26532	-1,993	0,027452
Ttc7	-1,9936	0,00014862
Slc39a1	-1,9939	1,87E-05
Lyl1	-2,0016	0,0001534
Rwdd3	-2,0101	0,0035029
Armc5	-2,0113	0,0082547
Tmem8	-2,0209	0,0015061
Mrm2	-2,034	0,0062631
Arl11	-2,0387	0,027133
Evi2a	-2,0393	0,00012236
Prss35	-2,0543	0,047813
Zhx3	-2,0548	0,0025196
Calml4	-2,0567	0,0016172
Ctu1	-2,0583	0,0017156
Pomt2	-2,0591	0,049274
6330408A02Rik	-2,0611	0,041668
Zfp324	-2,0647	0,039562
Fbxo46	-2,0662	0,0020991
Pskh1	-2,067	0,0010229
Eng	-2,0682	0,0017409
Serinc5	-2,0685	0,03596
Sdr42e1	-2,0696	0,010872
Olfm1	-2,0713	3,00E-08
Tlr4	-2,0732	0,0020756
Zfp189	-2,0744	0,018521
Tmem185b	-2,0798	0,0010793
Pla2g2d	-2,0816	0,011954
Csf2rb	-2,0846	0,00013298
Pomgnt1	-2,0863	0,0018068
Il34	-2,0877	0,016458
Tlr13	-2,0894	0,0083787
Snord13	-2,0975	6,97E-06
Gm15496	-2,1056	0,035902
Pctp	-2,1061	0,0060925
Zfp35	-2,1065	0,016218
A430033K04Rik	-2,1086	0,038761
Nrros	-2,1104	1,78E-06
Ampd3	-2,1113	0,00066834
Pter	-2,1154	0,0052513
Frmd4a	-2,1161	2,32E-05
Zfp768	-2,1188	2,62E-05
Trmo	-2,1192	0,044307
Ints5	-2,1242	0,00024645
Bcl3	-2,1278	0,0005292
Rcan1	-2,133	8,78E-07
Cyp2u1	-2,1345	0,0057994
Egr1	-2,1378	0,00049174
St18	-2,1445	7,37E-05
Cxcl2	-2,1467	0,00090622
Nt5e	-2,1615	0,0015483

Pkd2	-2,162	0,027306
Ptpn7	-2,1657	0,00056973
Acy1	-2,1658	0,012562
Gpr183	-2,1782	2,32E-05
Mul1	-2,1786	0,001598
Tepsin	-2,1924	0,00016884
Zfp810	-2,1928	0,0061032
Zfp729b	-2,1945	0,0039647
Nprl3	-2,1948	0,0055092
Gtf2h3	-2,1979	0,040679
Zfp61	-2,2028	0,003349
Mboat1	-2,2048	0,011204
Zfp518a	-2,2053	0,0016508
Zfp763	-2,2084	0,016327
Atp6v0d2	-2,2104	4,27E-08
Naip2	-2,2251	7,76E-05
Exoc8	-2,2262	0,0072795
Nkrf	-2,2263	0,016682
9130019O22Rik	-2,2276	0,03873
Tagap	-2,2302	0,0013184
Pafah2	-2,2341	0,036739
Birc3	-2,2471	9,06E-06
Depdc5	-2,2476	0,010616
Trib1	-2,2527	3,05E-05
Gpatch3	-2,2561	0,01054
Gmppb	-2,2584	0,00111
Mettl18	-2,2604	0,016129
Gm38020	-2,2608	0,03266
Gstm4	-2,2694	0,049332
Nmnat3	-2,2724	0,0063415
Tmem260	-2,2755	0,020455
1810055G02Rik	-2,2888	0,032545
Endov	-2,2895	0,011897
Gpr180	-2,2905	0,028428
Slc35d2	-2,2914	0,0074164
Cog1	-2,2955	0,028776
Mybpc3	-2,2994	0,0050942
Nlrp3	-2,3151	5,00E-05
Hebp2	-2,3158	0,0024132
Slc43a2	-2,3164	5,32E-08
Gm42640	-2,3166	0,015968
Slc9b1	-2,3193	0,046587
Zfp90	-2,3254	0,0084119
Endog	-2,3389	0,0066466
Cttnbp2nl	-2,3397	5,49E-07
Arhgef18	-2,3457	0,029254
Zfp12	-2,3458	0,0035045
Mtg2	-2,3473	0,0072795
Mdk	-2,3492	0,0084119
Gm43024	-2,3566	0,023291
Lima1	-2,3584	6,62E-07
Tmem98	-2,3643	0,036646

Mlh3	-2,372	0,0015624
Inpp5b	-2,3727	0,0014609
Gm17586	-2,3768	0,036757
Zc3h4	-2,3771	3,75E-07
Tmem2	-2,3826	7,16E-06
Tlr1	-2,3858	0,0066201
Zfp974	-2,3927	0,01894
Dusp9	-2,3935	0,027995
Gm16712	-2,3992	0,032658
I830077J02Rik	-2,4036	0,037063
Wnk2	-2,4076	0,00023678
Snx19	-2,4208	0,003761
Ubiad1	-2,4214	0,00068792
Mfsd9	-2,4223	0,011466
Oscar	-2,4429	0,02542
Tns4	-2,4461	0,0054043
Srxn1	-2,4483	7,62E-06
Srl	-2,4566	0,02848
Zfp790	-2,4566	0,038293
Sgsh	-2,4619	4,13E-05
Sbk3	-2,4628	0,042296
Slc46a1	-2,466	0,025224
Zfp738	-2,4684	0,023755
Commd5	-2,4709	0,00063186
Tigd5	-2,4768	0,044996
Prss46	-2,4784	0,045094
Skor1	-2,4845	0,045178
Jrk	-2,49	0,04587
Ubxn8	-2,4923	0,0040593
Bdh1	-2,4929	0,012012
Jdp2	-2,4946	4,63E-06
Ehd2	-2,5048	0,016043
Gm20712	-2,5055	0,010801
Oas1d	-2,5069	0,028418
Il20rb	-2,5211	0,0019866
Gm25514	-2,5221	0,013413
Gm37642	-2,5228	0,015868
Nupl2	-2,5248	0,01785
Ccdc130	-2,5253	9,92E-05
Swsap1	-2,5278	0,028001
RP23-268C22.3	-2,5303	0,035141
Card6	-2,5397	0,022085
Gipc1	-2,549	0,0018427
D6Wsu163e	-2,5546	0,0018082
Nmb	-2,5598	0,02728
Aldh1b1	-2,5611	0,0014466
Limk1	-2,5634	0,010512
Lctl	-2,5654	0,014021
Sh3rf1	-2,5684	0,00040682
Acsbg1	-2,5688	0,0028612
Dmrt2	-2,5692	0,033351
Gm42463	-2,5772	0,036083

Tmem51	-2,5834	0,00061583
Acot6	-2,5862	0,012903
Sec22a	-2,5872	0,0009108
Gm22748	-2,6058	0,0036873
Gm9951	-2,6073	0,016702
Angptl2	-2,6088	1,39E-07
Zfp870	-2,613	0,0025309
Plekhs1	-2,6176	0,023484
Fosl2	-2,6362	1,60E-10
Maml2	-2,6418	0,0040324
3110082I17Rik	-2,648	0,0010047
Cass4	-2,6513	0,010104
Tspoap1	-2,6539	0,0063448
Irgm2	-2,6667	0,039595
Gm43350	-2,6684	0,021084
Lpin3	-2,6687	7,49E-05
Rpl7l1-ps1	-2,6754	0,019335
F630040K05Rik	-2,6796	0,0099732
Zfp658	-2,6877	0,024017
Erbb3	-2,6923	0,0095732
Gm4262	-2,6944	0,020701
Col7a1	-2,7072	0,024426
BC024978	-2,7169	0,011765
Fut7	-2,7192	0,02685
Numbl	-2,7217	0,00036795
Lrrc14	-2,7345	0,00061557
Gm13205	-2,7404	0,028476
Fam222b	-2,7452	0,00020766
Hdhd3	-2,7464	0,0089466
Tctn2	-2,7517	0,029837
Zfp3	-2,7657	0,0020277
Dnmt3b	-2,7753	0,0052739
Zfp94	-2,7794	0,0079885
Elmod3	-2,7805	0,011818
Ctla2b	-2,7846	0,01924
AW047730	-2,7884	0,022161
Zfp951	-2,8024	0,020898
Ubox5	-2,8091	0,020898
Rab11fip4	-2,818	0,026249
Fmo5	-2,8287	0,021338
Rinl	-2,8296	0,00020244
D17H6S53E	-2,8335	0,0069832
Gm37121	-2,8919	0,0013122
Zfp862-ps	-2,9087	0,0022986
Zfp28	-2,9164	0,025944
2010008C14Rik	-2,9422	0,016068
Lpar1	-2,9474	0,011069
Src	-2,9544	5,48E-05
Zfp408	-2,9662	0,00028674
Gm5532	-2,9679	0,015519
Particl	-2,9807	0,0063448
Rgs8	-2,9916	0,021375

Fastkd5	-2,9951	0,0055366
Spred1	-2,9987	1,77E-09
Rgs20	-3,0008	0,0063448
Scn11a	-3,0238	0,0021653
Slc10a3	-3,0338	0,00079056
Csf2rb2	-3,0442	0,0061809
Gm43154	-3,0485	0,014649
Celsr1	-3,0495	0,014071
Ppp1r10	-3,0832	1,08E-08
Mir763	-3,1182	0,0069682
Nedd9	-3,1558	0,011298
Accsl	-3,1834	0,0012413
Hoxb3	-3,204	0,0037915
RP24-175C20.18	-3,2082	0,003761
Ip6k3	-3,2123	3,51E-05
Pus7l	-3,2276	0,004387
Wdr35	-3,2282	0,0040542
Gper1	-3,2283	0,0093926
Adamts7	-3,2386	0,00022106
Zfp27	-3,2427	0,0047919
Gm19026	-3,258	0,0075205
Gramd1c	-3,2605	0,0092359
Pxdn	-3,264	0,00049873
Pdpn	-3,2776	0,00031149
Sla	-3,2982	4,44E-07
Zfp40	-3,3149	0,0017008
Zfp719	-3,3481	0,0022584
Oasl1	-3,364	0,0011139
Gdpgp1	-3,4111	0,0063448
Zfp111	-3,424	0,0019607
Shisa3	-3,4246	0,015066
Ablim1	-3,4519	0,0010457
Zfp691	-3,463	0,00076539
9930014A18Rik	-3,4781	0,0015855
Olr1	-3,4821	0,0007818
Gm22	-3,4873	0,0020443
Acp5	-3,5701	1,18E-10
Slc6a4	-3,594	0,00077723
Rbak	-3,6639	0,00061557
Ap5b1	-3,6657	0,00076539
Slc1a4	-3,7009	0,0032062
Ppp1r26	-3,7031	0,010659
Filip1l	-3,7136	0,0003536
Krcc1	-3,7299	0,0097336
Zfp41	-3,7487	0,0015913
Met	-3,7528	0,00038059
Rap1gap	-3,7924	0,00083436
Gm20219	-3,8091	0,0013254
Gm20632	-3,871	0,00032617
Epb41l1	-3,9032	0,0004244
Usp27x	-3,9127	0,014332
Wisp1	-3,9597	0,0028002

Chac1	-3,9741	0,0022118
Mras	-4,0627	0,00043777
Zbtb45	-4,1862	0,00054555
Col27a1	-4,4785	0,00010125
Tmem204	-4,5627	1,77E-05
Rab15	-4,607	0,00015403
Vegfc	-4,6292	0,00068267
Ctsk	-4,8202	7,38E-11
Acod1	-5,3988	2,31E-05
Slc9b2	-7,7212	2,59E-07
