# Supplementary material for: Bone, dentin and cementum differentially influence the differentiation of osteoclast-like cells
Source: Sci Rep. 2025 Jun 5;15:19857. doi: 10.1038/s41598-025-04874-9 (PMC12141432; doi:10.1038/s41598-025-04874-9)
Supplement: Supplementary file 13 — Supplementary Information 13. [file 41598_2025_4874_MOESM13_ESM.pdf]

Tab. S12:
Significant transcripts (P<0.05) induced in murine macrophage cells stimulated on cementum (n=6), fold of negative control

gene name	regulation of expression	adj.P.Val
Gm29358	111,4381961	2,80E-06
4921507G05Rik	47,69611983	1,92E-05
4930578M07Rik	44,191592	3,50E-06
Hspa1b	43,88633838	0,0010808
RP23-451J19.1	33,7566539	1,85E-05
Slc16a5	27,01146531	0,00031078
mt-Ti	25,33043592	2,24E-06
RP23-440L7.5	24,18934836	0,00010405
AY074887	22,53975611	6,38E-06
1500004A13Rik	21,26486808	1,28E-06
Zp1	21,13848387	0,0011315
mt-Ta	20,84168477	0,002093
Tsix	20,66618235	2,06E-05
1700030M09Rik	19,23817445	0,00025871
Rpl30-ps2	19,13709578	5,38E-08
Gm44652	18,62798861	4,72E-06
Gm18709	18,02715716	7,41E-06
Rn7sk	17,66228385	0,00011858
mt-Ts2	17,63659317	0,00015422
Gm23037	17,15788712	0,0020706
mt-Tm	16,79197478	1,89E-06
Fzd7	16,36906486	2,56E-07
RP24-174I4.1	16,10347345	2,90E-05
Adm	14,43901077	1,08E-08
Lbp	14,22246869	0,0013158
Gm12469	14,06853306	0,000327
mt-Tc	13,93750097	0,0024601
RP24-295J1.1	13,82780252	0,00087559
Gm27248	13,37252504	0,0059334
Gm28373	13,2076327	0,0059334
Gm8649	12,8973253	9,04E-12
Thap8	12,426947	0,000309
Gm8885	12,41489367	0,0011315
Mcm8	12,35394579	2,80E-05
Gm8317	12,32144882	1,44E-05
Med16	12,05282979	2,06E-09
RP23-136K21.4	11,97787436	0,019581
Gapdh	11,21766112	3,38E-05
Hist1h2bg	11,15717595	0,020931
Gm8623	11,04177344	0,00019649
Gm43878	10,9003307	0,0053384
Rgcc	10,86412441	2,25E-06
Aloxe3	10,60814136	0,0033715
Gdf15	10,56191843	2,11E-08
Gm10827	10,53997836	1,27E-07
Gm37052	10,49696242	0,042873
Gm9521	10,25958166	0,0059779
Gm26226	10,17883116	0,036581

Acoxl	10,09871622	0,02077
Gm43714	10,0540161	0,0059343
2310058D17Rik	9,714905754	0,00019461
Gm16181	9,694053201	0,020667
Gm42670	9,662523381	0,0035897
Hes7	9,59311931	0,014239
Gm26656	9,563243432	0,0027026
RP24-175C20.10	9,528836055	9,48E-07
4632415L05Rik	9,397649753	2,55E-07
mt-Tl1	9,382679594	7,65E-08
Gm26810	9,292713955	0,0038361
Gm10636	9,270839598	0,0042873
1700054M17Rik	9,199784071	0,010969
Gm14279	9,112203711	3,43E-06
Car7	8,978659071	1,79E-05
Rpl30-ps1	8,828081815	0,00011502
RP23-350F7.3	8,747070974	0,00010506
Ankrd37	8,719829947	1,72E-06
4930542C12Rik	8,61589101	0,035356
RP24-93F20.12	8,564090255	0,0056622
D430001F17Rik	8,546300233	0,00022005
Gm4607	8,466114497	0,00055583
Gm7351	8,282693483	0,022688
Rnf122	8,211808591	2,71E-05
Hist1h2be	8,181129286	2,32E-05
Gm45833	8,139837537	0,00013568
Gm43182	8,133633586	0,046427
Trf	8,100999958	0,006639
Rasd1	8,089777368	0,02235
Snord82	8,027217564	0,015401
Gm26730	7,896426968	0,02863
Gm45251	7,840251859	0,02235
Gm26983	7,757544137	0,0012231
Gad2	7,707697756	0,041582
Gm26772	7,648622792	0,016412
Gm11810	7,525565523	0,0003191
Hist2h4	7,522957813	0,049501
Gm24631	7,521914981	0,0047847
Mafb	7,482394333	1,52E-10
Kcnd1	7,4044881	0,041069
Lrrc2	7,37171349	0,01371
Bc1-ps1	7,315213646	0,021411
Id1	7,286873774	1,08E-08
Hlx	7,269217134	0,001025
A830008E24Rik	7,033291441	0,035187
Gm13383	6,983740729	0,033777
Gm25008	6,969233537	0,0033581
Gadd45g	6,779129357	3,43E-06
S1pr1	6,770676549	1,17E-07
Gm15877	6,76692313	0,023304
Cbx2	6,643760193	1,87E-05
Rybp	6,643299698	0,026915

Egln3	6,639156685	0,010254
Gm29228	6,605647571	0,0097988
Gm5112	6,568664133	0,002011
Gm8818	6,558655054	0,024293
Gm6564	6,520126703	0,0083375
Gm42432	6,491716512	0,0097932
Notch1	6,481375413	0,049375
Hist1h1b	6,319892418	0,039342
Gm26664	6,309825029	0,0089775
Efna3	6,235477315	0,013415
Plk2	6,14664856	6,15E-05
Xaf1	6,142815277	0,011835
Gfod2	6,124958207	5,12E-06
Lrrc17	6,042309149	1,03E-05
Arc	6,029339638	0,00083307
4933437G19Rik	5,957883857	0,04164
Sez6	5,94798084	2,50E-05
Gm12280	5,906485714	0,0051774
Socs2	5,902393069	0,03478
Stamos	5,839318485	0,013454
Gm12604	5,827592504	0,0010735
RP24-316F13.7	5,794161913	0,038725
Wwc1	5,786135064	1,44E-07
Gm10382	5,751347465	0,0016261
RP23-226H21.3	5,74218575	0,037753
mt-Tv	5,725890179	0,0021547
Gm7099	5,695805966	0,0025378
Lgals7	5,665879817	0,031133
Snord89	5,647843059	5,47E-05
Gm45051	5,643147269	0,038307
Gm16045	5,634939028	8,23E-05
Gm42851	5,631034539	0,038809
Gm11343	5,614276043	0,033777
Gm12778	5,605721224	0,0043699
Esco2	5,600672232	0,037423
Wfdc17	5,587488667	4,81E-05
Fam72a	5,446766421	0,01464
Dtd2	5,432062203	8,37E-06
Jup	5,427545819	0,036112
Aif1	5,411017848	0,00021092
Ciart	5,404270921	4,02E-06
Gm15542	5,404270921	0,021732
Rhov	5,363222104	0,0018058
Selenop	5,338372585	0,00058196
Txnip	5,33356439	3,50E-06
Gm43566	5,328391177	0,00011345
1700031P21Rik	5,29525441	0,015937
Rhob	5,2634181	0,00019649
Atr	5,253941002	2,94E-05
Atf3	5,243390521	1,61E-07
Ang	5,235763747	1,85E-05
Atp5l-ps1	5,212227301	0,0002975

C730034F03Rik	5,183404563	8,96E-07
Otud1	5,143676819	0,0050181
Gm6341	5,092238532	2,07E-07
Ndrg1	5,083421996	2,06E-09
Tstd1	5,054260417	0,0005591
Gm5312	5,040965218	0,011523
Arhgap26	5,024917813	0,00024874
Nsl1	4,999209546	0,00015392
Tnfrsf12a	4,968464304	2,56E-07
Gm28555	4,931067494	5,88E-07
Arhgap39	4,915710657	0,00021298
Ier5l	4,892934602	0,00010196
A330069E16Rik	4,880740337	0,0075145
Zfp36l1	4,850723683	1,27E-07
Ccng2	4,849378963	1,70E-05
Zfp36l2	4,846354706	2,31E-06
Ccdc36	4,846354706	0,00025107
Gm11759	4,844675379	0,034754
Slc25a2	4,818219103	0,042237
Gm15728	4,816549525	0,00060768
9530085L11Rik	4,741685576	0,025137
C3ar1	4,740042518	2,52E-05
Gm29170	4,685165465	0,00056312
Crkl	4,678026389	0,027441
mt-Nd6	4,673812958	1,70E-05
RP24-511J14.2	4,562106102	0,0038745
Gm19566	4,476899711	0,006836
Gm14094	4,405787767	0,013881
Gm16585	4,348144845	0,025118
Rpl27a-ps1	4,287688209	0,028248
0610039K10Rik	4,27700235	0,040982
4932422M17Rik	4,259251735	0,0054849
Gm10343	4,241868802	0,0041431
Gm28578	4,220751816	0,020931
Phlda1	4,21519683	0,00029118
Gadd45b	4,199157788	8,40E-06
Gm45167	4,188402239	0,00025874
Cfh	4,159759154	3,99E-06
Klf10	4,139050928	1,86E-06
Mcm10	4,107042852	0,011218
Rpl35a-ps5	4,099647876	0,0008589
Klf11	4,089714122	0,00035847
Errfi1	4,084048503	0,00019649
Hist2h3c2	4,069072541	0,020748
Polr2l	4,064562296	3,95E-05
Igf1	4,052465771	1,56E-08
Gm29019	4,001663899	0,0053627
Rasgef1b	3,99916831	5,12E-06
Gtse1	3,996674277	0,00016061
Insig1	3,986990036	2,38E-05
Kctd6	3,958626626	5,75E-05
Kif20b	3,909001125	8,23E-05

Ddit4	3,897097429	8,12E-06
2900093K20Rik	3,847709819	3,73E-05
Mafk	3,826432632	5,76E-06
Pmaip1	3,824841595	8,40E-06
Fth-ps3	3,819013448	0,00017026
Gmnn	3,818748743	0,00018624
Gm37733	3,813722839	0,038345
Sap30	3,806592141	3,95E-05
Tma7	3,80131874	0,0078754
Adamts1	3,789480282	0,0075533
Crip1	3,748984862	1,88E-08
Map3k12	3,745348589	0,00029612
Gm10388	3,727995194	0,01535
Cped1	3,695577938	0,031296
Tnfsf9	3,685090372	0,038731
Ect2	3,682536947	0,0059334
Rny1	3,664204442	0,0031138
Zic2	3,65659286	0,0062459
Cenpu	3,642174387	0,028432
Spdl1	3,619524044	0,00072719
Slc25a25	3,61150458	0,00014115
D830025C05Rik	3,60825175	0,035505
Gm44913	3,601505232	0,0012641
Gm11363	3,590786808	0,049375
Rad51ap1	3,558082593	0,0047927
Bvht	3,552660923	0,037661
Rpl30-ps3	3,541351385	0,00058446
Itga6	3,540124259	0,00048787
Nfil3	3,521524207	0,00058196
Gm15950	3,519815968	0,016938
Gm26737	3,508610988	0,042873
Cep55	3,500594613	0,011371
Ezr	3,499866761	4,50E-07
Gm7327	3,483892238	0,010581
Tmod1	3,481960894	0,0019782
Atp5g1	3,466068099	0,00025107
1500015A07Rik	3,454315841	0,0028718
Csrnp1	3,449291365	4,02E-06
Unc13a	3,411721775	0,002421
Gm26782	3,405578766	0,0044087
Fn1	3,405342718	0,0023462
2810013P06Rik	3,402275575	0,0016838
Asb10	3,391679859	0,0046205
Pbk	3,376199136	0,0008003
Ccl4	3,362420133	5,88E-07
2610528A11Rik	3,324413869	0,04095
Brip1	3,322570934	0,024627
Pea15a	3,300764019	3,69E-05
Ier5	3,293222527	3,43E-06
Id2	3,289116251	1,57E-05
Hist1h1c	3,266849516	6,15E-05
Ppfia4	3,258707811	0,0016611

Iqgap3	3,25825609	0,0016895
Sdc3	3,256224118	5,11E-07
Zfp101	3,253291284	0,02034
D130051D11Rik	3,242709983	0,013771
Pmp22	3,235525393	7,12E-07
Zwilch	3,231267077	0,027011
Slc2a1	3,215403963	2,12E-05
Tnfrsf17	3,211172129	0,031361
Gadd45a	3,209614436	0,00506
Pgf	3,189875241	0,03544
Atad5	3,182366512	0,042873
Gm13215	3,168719597	0,021605
Tgif2	3,167621593	0,0002975
Gm24276	3,131383053	0,00016697
Nuf2	3,11752256	0,028523
Cenpk	3,115578354	0,019993
Sgk1	3,084421651	0,015937
Jun	3,080148697	0,00030996
Ckap2	3,07332428	0,0055238
Ska1	3,065027462	5,75E-05
Ormdl3	3,063328321	0,00019649
5830432E09Rik	3,055058488	0,04583
Sertad1	3,026604156	0,00084416
Nadk2	3,026394375	0,0024461
Mgst3	3,016132901	0,042478
Basp1	3,012789757	0,0004514
RP23-356P21.1	3,010910867	0,047098
Hmgb2	3,000701892	0,0061673
Dusp1	3,000285935	0,0012167
Sec24a	2,974608908	0,00014115
Sep 02	2,974608908	0,011043
Lin54	2,972135727	0,0015639
Hpse	2,962674232	0,010389
Tob1	2,959800629	0,014866
Pim1	2,953652292	1,87E-05
Sgol1	2,953447567	0,022796
Klf4	2,951605686	3,73E-05
Aurkb	2,949969421	0,0044033
Spsb2	2,948334065	0,003966
Adh5	2,938133604	3,18E-05
Kifc1	2,932640036	0,0075145
Tiparp	2,929795565	0,00032952
Gm26826	2,926548119	0,01233
Rps15a-ps6	2,922696452	0,014537
4930430E12Rik	2,921076214	0,032818
Lockd	2,899089756	0,0040445
Zfp326	2,895474919	2,76E-05
Ltb	2,891263305	0,0034714
Kif11	2,889259929	0,0054353
Frat2	2,889059667	0,00047491
Mcm3	2,887057818	0,00042368
Rad51	2,883258124	0,040955

Maff	2,876271774	0,0025378
Gsg1	2,867711715	0,046708
Lonrf3	2,865724657	0,00033209
Ccrl2	2,851260788	0,028079
Lilrb4a	2,846916146	0,01733
Nemp1	2,845929651	0,0008968
BC028528	2,835297262	0,011916
Gm29736	2,83215456	0,037494
2010111I01Rik	2,82392153	0,00014731
Crip2	2,81590761	0,033835
Gm26520	2,801500985	0,0032787
Gm45184	2,796456716	0,010254
RP23-325K4.10	2,791615022	0,0012641
AV356131	2,789487333	0,0004855
Fau	2,775600631	0,035187
Iscu	2,775023521	0,0038745
Mad2l1	2,756237103	0,02725
Spc24	2,747082047	0,013881
Sdc4	2,727350278	8,01E-05
Ttk	2,721873465	0,0087195
Hmmr	2,707947933	0,0041248
Sirpa	2,704383968	2,57E-05
Rbm48	2,696709281	0,0032743
Snord49b	2,681982828	0,0437
Cox20	2,66881629	0,0014964
Haus3	2,665303827	0,0045466
Rpl39-ps	2,66474965	0,008987
Dusp5	2,662903226	0,013395
Ly86	2,661058082	0,00063088
Rdh13	2,64964669	0,00027494
Rnf19a	2,646709766	0,0019944
Ypel2	2,645609259	0,017274
Cox20-ps	2,641211806	0,0023684
H60b	2,634264116	7,35E-05
Gm6472	2,627152595	0,049623
Cdc6	2,620968473	0,020671
Gm45223	2,593499335	0,010248
Suco	2,590624647	8,40E-06
Rc3h1	2,586856453	0,00086178
Ube2t	2,562230025	0,013897
Cenpw	2,555312969	0,0012475
Arrdc2	2,549297952	0,018948
Cldn11	2,545766324	0,0021547
Jund	2,536958641	0,00013848
Hcfc1r1	2,529583741	1,70E-05
Rps6-ps1	2,527830975	0,044487
Phf13	2,520482609	0,0061288
Ccdc117	2,518736149	0,00086499
Dedd2	2,518037904	0,00044681
H2-Ob	2,514723882	0,0080381
Lsp1	2,51124015	0,001122
3110062M04Rik	2,51106609	0,00084416

Zfp36	2,509152227	0,041064
Bub1	2,498392251	0,025195
Rassf3	2,493029579	0,0090827
Cd300c2	2,486471681	0,0016386
Map3k8	2,485437801	0,0066681
G2e3	2,478899874	0,0040879
Mynn	2,472379144	0,0065216
Snord104	2,470837274	0,0053014
Zdhhc18	2,468269628	0,0043591
Tsc22d3	2,46160623	0,022688
Pfkfb3	2,45496082	0,010014
Snx13	2,454450378	0,013656
Necap1	2,449521577	0,0008589
Hist3h2a	2,443247474	0,0019255
Insig2	2,443078127	0,00207
Tmem171	2,441385301	0,04994
Fzd5	2,439017315	0,021246
Gins3	2,435469642	0,0023217
C030034I22Rik	2,433782088	0,00872
Zfp367	2,43327605	0,01404
Ap1s3	2,430747437	0,004643
Rell1	2,414626421	0,0014066
Il10ra	2,407607139	0,0020906
Tex30	2,395953651	0,027011
H3f3a	2,395455478	0,015514
Tbc1d10a	2,389485479	0,0041518
Fam174a	2,378579095	0,0016604
Bbc3	2,371500216	0,0042038
Mxd1	2,3690358	0,0091449
Kif18a	2,363950784	0,049333
Smc2	2,353160949	0,0013763
Hist1h1e	2,344369543	0,010254
Clec12a	2,340797283	0,00082093
Carhsp1	2,334478012	0,00071672
Hist1h4i	2,330274645	0,0069063
Arsb	2,327530382	0,045123
Dennd4c	2,32720774	0,037472
Zfand2a	2,324144872	0,0031138
Ppwd1	2,321407829	0,010014
Hist1h2bc	2,319477739	0,0054353
Fem1c	2,310171569	0,002011
Smagp	2,309210996	0,0019088
Dqx1	2,304733597	0,025837
Sowahc	2,304414115	0,020931
4921524J17Rik	2,290559438	0,033303
Pclaf	2,289765728	0,01233
Ddx20	2,289765728	0,014906
Gm16754	2,281369294	0,040955
P4ha1	2,279472493	0,00059042
Rps3a1	2,27253104	0,032484
Med22	2,271271231	0,018631
Tspan13	2,267810354	0,00014134

Ptger4	2,264982651	0,0036584
Cbx4	2,260277646	0,020931
Etv3	2,255113428	0,01181
Mcm6	2,244664768	0,01233
Ranbp9	2,242953949	0,011917
Ccna2	2,242643031	0,03246
Spty2d1	2,239225777	0,0023179
Dnajb6	2,237674202	0,0014964
Mki67	2,237053873	0,038809
Prc1	2,233799966	0,005061
Rgs2	2,233645136	0,00090905
RP24-454N4.2	2,226534552	0,044487
Mkrn2	2,22206343	0,041582
Pcif1	2,219292768	0,0053384
Cd83	2,209316332	0,0015993
Lbr	2,208397694	0,0007815
Tgfb1	2,204726961	0,02235
Tor1aip2	2,204421342	0,0015939
Bub1b	2,200147124	0,01032
Rcbtb2	2,19542462	0,030849
Arl4c	2,192687173	0,0037131
Mcm7	2,191927377	0,038024
Supt7l	2,181317839	0,046816
Per1	2,177541177	0,020485
Dyrk3	2,176334026	0,016938
Eno2	2,169105163	0,016412
Haus6	2,158306865	0,028845
Gon7	2,157409437	0,012268
Lpl	2,15636291	0,0021218
Rgs1	2,151137881	0,034721
Rrm1	2,144141325	0,022046
Sertad3	2,141913175	0,040104
Plau	2,139094176	0,029994
B3gat3	2,137315667	0,037753
Birc5	2,134798644	0,0066964
Racgap1	2,133910991	0,033608
Calm1	2,131693472	0,00018233
Smc4	2,130954811	0,0055923
Csrp1	2,130068757	0,038217
Ppp1r2	2,125496672	0,038549
Ppp1r15a	2,124612886	0,033777
Rpl18	2,122993568	0,024928
Tmem64	2,119317886	0,003115
Mb21d1	2,112571251	0,0021547
Klf6	2,110522193	0,026327
Tpi1	2,106138045	0,0031892
Plekhf2	2,102637285	0,042873
D030056L22Rik	2,097687834	0,018374
Rassf2	2,085076043	0,0090827
Mapre2	2,08478701	0,0077624
St3gal6	2,075559082	0,0090827
Gm9385	2,075415221	0,0078868

Lpar6	2,073689657	0,040925
Rassf1	2,070960447	0,013854
Eif1b	2,066515235	0,003983
Plin2	2,063652422	0,019581
Erf	2,05537267	0,016341
Arf2	2,054518042	0,004368
Usp1	2,048829598	0,015401
Dnajb4	2,046558633	0,014239
Ubb	2,043440165	0,024302
Kpna4	2,042590499	0,027011
Anxa2	2,040892227	0,028817
Khk	2,029325093	0,023674
H2afv	2,026654272	0,037
Ptchd1	2,02384668	0,04164
Snapc1	2,017403968	0,012041
Gatsl2	2,015586921	0,025137
Sumo1	2,01042428	0,046966
Ezh2	2,006804391	0,022796
Dab2	2,000970642	0,022013
Dok2	1,992389896	0,020483
Cenpe	1,982471327	0,024001
Gm14620	1,976023357	0,047714
Mef2c	1,969323334	0,01404
Fmr1	1,95422963	0,04862
Chmp1b	1,946928631	0,021035
Bnip3	1,942480344	0,044168
Cxcr4	1,940596264	0,036869
B4galt3	1,93522324	0,039563
Calr-ps	1,931337103	0,032023
Tsc22d2	1,928260544	0,040468
Cdkn2d	1,926523788	0,04164
Litaf	1,912685934	0,0044649
Rhoc	1,912420797	0,0088266
Metrnl	1,899605713	0,035356
Igfbp4	1,876052238	0,032402
Napsa	1,864772973	0,023304
Cadm1	1,814283614	0,026474
Idh1	-0,82732	0,040955
Gm26917	-0,86368	0,048461
Ubr4	-0,87108	0,049282
Rgl1	-0,88006	0,041069
Zdhhc20	-0,8878	0,033161
Hnrnpm	-0,89604	0,036953
Pcyt1a	-0,89691	0,01908
Rere	-0,90012	0,010254
Tpm1	-0,90829	0,0076907
Abr	-0,90973	0,011835
Cant1	-0,91532	0,046708
Stub1	-0,92086	0,024905
Kdm4a	-0,92515	0,03478
Vim	-0,93561	0,021499
Sec61a1	-0,93713	0,01361

Gsk3b	-0,94459	0,045137
Pde8b	-0,9458	0,022139
Creg1	-0,94998	0,029994
Cux1	-0,95689	0,015128
Ssu72	-0,95913	0,037066
Zfp809	-0,96419	0,02235
Csnk2a2	-0,96535	0,010389
Clptm1l	-0,97363	0,02235
Cd109	-0,97967	0,020671
Srrm2	-0,98895	0,011988
Itpr2	-0,98933	0,019581
Dync1li2	-0,99161	0,02725
Lair1	-0,99872	0,043884
Ccni	-1,0018	0,0097071
Mapkapk2	-1,007	0,0076631
Zfp106	-1,0105	0,0071908
Akap9	-1,0236	0,013395
Kbtbd11	-1,0256	0,0051574
Ppp6r3	-1,0277	0,013486
Son	-1,0337	0,018374
Galnt1	-1,0386	0,0083205
Rab12	-1,0393	0,046708
Arih1	-1,0456	0,0031892
Eif4g3	-1,0498	0,039283
Fem1a	-1,0511	0,025195
Acsl4	-1,0541	0,0021102
2810025M15Rik	-1,0544	0,0071908
Lat2	-1,0546	0,016791
Pbxip1	-1,0564	0,003983
Slc1a5	-1,0576	0,004643
Prkag2	-1,0622	0,014681
Macf1	-1,0628	0,011418
Ppp1r12b	-1,0637	0,0055938
Aldoc	-1,0654	0,0041248
Scamp1	-1,0664	0,028911
Mitf	-1,08	0,032541
Cd33	-1,0952	0,0069063
Lcp2	-1,0967	0,018856
Lrrc47	-1,0982	0,0046878
Atp2b4	-1,0986	0,029797
Esyt2	-1,1023	0,01908
BC005537	-1,1033	0,015304
Iars2	-1,1096	0,049501
Tyk2	-1,1163	0,007415
Slc38a1	-1,1178	0,0023462
Idh2	-1,1209	0,018374
Sod1	-1,1225	0,0013006
Cacfd1	-1,1251	0,0054353
Smap1	-1,1281	0,02988
Med1	-1,1344	0,0031344
Metap2	-1,1365	0,014252
Dnajc10	-1,1406	0,0055938

Fryl	-1,1492	0,038809
Slc25a37	-1,1516	0,0010126
Pstpip1	-1,1543	0,00075927
Rfx5	-1,156	0,0054996
Ppm1l	-1,1561	0,0078977
Gns	-1,1591	0,0011621
Sec31a	-1,1594	0,0070753
Cyth4	-1,1634	0,0044649
Cyba	-1,1783	0,002563
Srxn1	-1,179	0,043655
Ints3	-1,1809	0,0010808
Birc3	-1,1814	0,028845
Slc37a2	-1,1839	0,0024601
Syne1	-1,1841	0,022909
Sema4a	-1,186	0,0059334
Atp6ap2	-1,1861	0,00013848
Zbtb38	-1,1878	0,0059334
Kcnq1ot1	-1,1879	0,022568
Noc2l	-1,1886	0,001064
Olfm1	-1,1974	0,0016261
Arrb1	-1,2003	0,022954
Ehd1	-1,2006	0,00061333
Tfrc	-1,201	0,014752
Prr14l	-1,201	0,033835
Trim27	-1,2034	0,0074952
Sphk2	-1,2104	0,0024805
Gm6329	-1,2142	0,046689
Cnpy3	-1,2149	0,01662
Golim4	-1,2212	0,014711
Zc3h4	-1,2253	0,0050428
AU040320	-1,2347	0,024697
Pecr	-1,236	0,037423
Abcb4	-1,2369	0,00057688
Inf2	-1,2382	0,00025207
Pfkfb4	-1,2388	0,01597
Sptlc1	-1,2408	0,037494
Dpp8	-1,2414	0,013395
Foxred2	-1,2468	0,0025172
Plbd2	-1,2472	0,00022148
Zpr1	-1,2501	0,00057787
Ppfibp1	-1,2515	0,0012475
Mrps6	-1,2525	0,0016369
Tlr7	-1,2529	0,025186
Frmd8	-1,2549	0,00207
Arhgef12	-1,2613	0,0019255
Flnb	-1,2634	0,0028707
Csf1r	-1,2649	0,00019084
Rasa3	-1,2682	0,0066321
Bre	-1,278	0,0055238
Frrs1	-1,285	0,00053542
Nav1	-1,2863	0,0028709
Plxdc1	-1,2863	0,024752

Nudt22	-1,2887	0,036035
Rbck1	-1,2919	0,0055938
Ctbp1	-1,295	0,0019088
Apbb1ip	-1,3124	9,03E-05
Neil1	-1,317	0,048715
Uvssa	-1,3192	0,049542
Ivns1abp	-1,3225	7,02E-06
Gnptab	-1,3284	0,00042122
Gpatch4	-1,332	0,015343
Rnf157	-1,3404	0,0090015
Actn1	-1,3463	0,00020139
mt-Nd4	-1,3466	0,014224
Setd1b	-1,3473	0,013395
Pla2g5	-1,3482	0,0059612
mt-Cytb	-1,3529	0,0028087
Slc11a1	-1,3705	0,0043699
Dbnl	-1,3753	0,022232
Myof	-1,3783	0,00022785
Pmepa1	-1,3791	0,0048026
Tmem2	-1,3791	0,0075533
Clec5a	-1,3809	0,017121
Frmd4a	-1,3876	0,0085952
Ino80c	-1,3879	0,00083369
Nagpa	-1,3943	0,011043
Cat	-1,3974	2,64E-05
Dph6	-1,4034	0,033512
Cpne2	-1,4047	0,01181
Tmem65	-1,405	0,0027231
Amotl1	-1,4078	5,86E-05
Fam102a	-1,4129	1,17E-05
Cyfip2	-1,4365	0,0008148
Zbtb20	-1,4368	0,00082524
Ahnak2	-1,4465	0,0010555
Ash2l	-1,4485	0,010054
Bnip2	-1,4507	0,00025107
Gmpr	-1,4509	0,0041504
Slc7a11	-1,455	0,013839
Nf2	-1,4571	0,00027494
Gpr68	-1,4708	0,032402
Aldh3b1	-1,4741	0,013296
Cox10	-1,4778	0,00014412
Lars	-1,4801	2,32E-05
Rcan1	-1,484	0,0004406
Slc39a11	-1,4869	4,19E-05
Rai14	-1,4932	0,001278
Slc43a2	-1,496	0,00019649
Ttll12	-1,5096	0,00029324
Mdn1	-1,521	0,00035079
Acvr1	-1,5219	0,022781
Pkn3	-1,5255	0,013395
Spink5	-1,5279	2,57E-05
Wbscr27	-1,5307	0,030849

Cpsf3	-1,5348	0,005265
Eng	-1,5515	0,038487
Fkbp15	-1,5517	0,00015135
Ammecr1	-1,5571	0,0049148
Fam213a	-1,5613	0,02235
Nucb2	-1,5654	0,00038972
Neat1	-1,5672	0,0005591
Lyl1	-1,5676	0,0069063
Zhx3	-1,5676	0,044391
Slc43a3	-1,5679	0,03544
Lrrc20	-1,5689	0,017125
Glb1	-1,5783	2,24E-05
Numbl	-1,5783	0,038487
Gstt3	-1,5842	0,028845
6030458C11Rik	-1,5894	0,0065275
Acaa2	-1,6006	0,00872
Snord13	-1,6052	0,00065522
Btbd19	-1,6242	0,010014
Zfp113	-1,6343	0,040104
Ampd3	-1,6347	0,017202
Fam65c	-1,641	0,01181
Tmem214	-1,6448	0,030505
Marveld1	-1,6472	0,00016697
Bckdk	-1,6568	0,0019043
Gne	-1,658	0,038487
Nfatc1	-1,6719	2,37E-05
Dync2h1	-1,6758	0,00011347
Nsf	-1,689	0,00035847
Rasal2	-1,6943	8,01E-05
Sgsm1	-1,6966	1,45E-06
Usp20	-1,7019	6,15E-05
Spred1	-1,7128	4,80E-05
Nrp2	-1,7146	1,88E-08
Dennd2a	-1,7182	0,0060763
Gm16845	-1,7203	0,03608
0610010F05Rik	-1,7232	0,041069
Elk3	-1,7272	0,0056581
Prss50	-1,7298	0,048486
Ccnd2	-1,743	1,85E-05
Gpt2	-1,7507	0,0010442
Dcaf11	-1,7529	0,0075533
Tmem241	-1,756	0,041064
Pik3r2	-1,7621	0,0037445
Sec24d	-1,7666	0,0097071
Plcb4	-1,7725	8,56E-07
Ttc7	-1,7833	0,0023179
Lima1	-1,8038	6,42E-05
Hmga2	-1,8124	3,95E-07
Ivd	-1,8291	0,027377
Pde4dip	-1,8301	0,0034711
Slc39a13	-1,8312	0,00015569
Timp2	-1,8333	0,00011858

Ank	-1,8754	1,88E-08
Agap1	-1,8831	0,0022146
Grap	-1,9015	0,0066681
Airn	-1,9102	0,015094
Nudt14	-1,9118	0,022139
Sec16a	-1,9144	0,0066681
Dock5	-1,9188	0,00471
Treml1	-1,9263	9,34E-05
Hck	-1,9319	0,038487
Fblim1	-1,9371	0,00029922
Slc25a10	-1,9449	0,014752
Lpin3	-1,971	0,003115
Klhl5	-1,9715	0,00010617
Ddx17	-1,9764	1,66E-06
Arhgef10	-1,9937	0,0288
Bdh2	-2,0007	2,57E-05
Nprl3	-2,0077	0,028911
Pter	-2,0083	0,025137
Zfhx4	-2,0118	0,00027494
Tbc1d2b	-2,0274	7,65E-08
Fosl2	-2,0286	5,38E-08
Sec16b	-2,0373	0,00018624
Hebp2	-2,0422	0,021246
Myom1	-2,0469	0,028845
Sfxn2	-2,05	0,01378
Tmem204	-2,0726	0,0089083
Naip2	-2,0806	0,00084416
Pctp	-2,0841	0,021393
Slc16a7	-2,09	0,022796
Cyp2u1	-2,1012	0,020671
Tpm2	-2,115	0,01233
Pitpnm2	-2,1448	0,014239
Gsn	-2,1524	1,88E-08
Shtn1	-2,1601	7,02E-06
Gm42480	-2,1666	0,022781
Me1	-2,1953	3,36E-07
Prkar1b	-2,1963	0,0232
Serinc2	-2,2004	0,00038972
Nod1	-2,2155	0,011746
Gm22748	-2,2274	0,030743
Mical2	-2,2617	0,0016611
Hfe	-2,2659	0,03478
Aldh1l2	-2,2904	0,0089922
Angptl2	-2,3279	2,09E-06
Zfp462	-2,344	0,01233
Ap5b1	-2,3472	0,022812
Lctl	-2,362	0,047996
Myo1d	-2,3669	1,27E-07
Olfr933	-2,3784	0,03141
Tspan10	-2,3785	0,0031138
Bdh1	-2,382	0,038487
Atp6v0d2	-2,3925	7,65E-08

B3glct	-2,408	0,0042237
Cradd	-2,4093	0,011418
Prkca	-2,4143	0,003391
Wdr35	-2,4474	0,038095
Sec14l2	-2,4554	0,028911
Tmem116	-2,456	0,011636
Emp2	-2,4613	0,0025649
Jdp2	-2,4801	2,15E-05
Sgsh	-2,483	0,00021775
St18	-2,5028	0,00016116
Serinc5	-2,5077	0,037494
Ptges	-2,5109	0,012717
Acy1	-2,5225	0,020979
Sla	-2,5387	1,53E-05
Txk	-2,5775	0,021212
RP24-175C20.18	-2,5852	0,038809
Ip6k3	-2,5865	0,00079658
Camk2a	-2,6016	0,00057286
Calml4	-2,6316	0,0015064
F630040K05Rik	-2,6372	0,029711
Gm38020	-2,6459	0,032725
Slc6a4	-2,6522	0,015343
Gm11716	-2,6693	0,01517
Iqce	-2,6953	0,018948
Ehd2	-2,7205	0,026915
Zfp862-ps	-2,7425	0,010014
Anxa9	-2,7432	0,047345
Ccpg1os	-2,8014	0,034878
9930014A18Rik	-2,8132	0,015465
Adh7	-2,8801	0,049407
Ccdc122	-2,8928	0,023784
Rap1gap	-2,8989	0,013177
Gm36963	-2,9115	0,021499
Zfp169	-2,9202	0,023862
Msantd3	-2,9227	8,18E-05
Epb41l1	-2,9247	0,0061416
Gper1	-2,9488	0,036501
Gm42640	-2,949	0,016175
Tiam2	-2,9494	0,02951
Extl1	-2,9607	0,014224
Enpp5	-2,9655	3,79E-05
Accsl	-2,98	0,0049208
Gja1	-2,9918	0,016887
Il20rb	-3,0491	0,0028709
Plat	-3,0557	0,032733
BC024978	-3,1411	0,0075533
Chac1	-3,1819	0,023391
Gm43154	-3,2037	0,019169
Rgs8	-3,2078	0,024752
Slc35d2	-3,2347	0,0036221
Scn11a	-3,24	0,0021218
Ddr2	-3,2435	0,0078754

Vegfc	-3,2453	0,022464
Src	-3,2891	9,79E-05
Dixdc1	-3,2985	0,003983
Rgs16	-3,3146	7,38E-05
Rhbdd2	-3,3251	0,0059334
Robo3	-3,3342	0,0017713
Wnk2	-3,3518	0,00022874
Olr1	-3,3591	0,002629
Gm25514	-3,5178	0,0070753
Pdpn	-3,58	0,00075927
Ablim1	-3,6681	0,0010808
Bok	-3,6765	0,00075108
Gm19026	-3,6822	0,0059779
Gm15496	-3,6882	0,0085253
Wisp1	-3,7563	0,0088431
Adamts7	-3,8387	0,0004291
Nt5e	-3,8745	0,00019084
Acsbg1	-3,9445	0,0013411
Met	-3,9691	0,00039274
Pxdn	-4,0517	0,00040273
Col27a1	-4,1156	0,00058604
Slc1a4	-4,1251	0,0027026
Rab15	-4,244	0,00084416
Acp5	-4,3574	5,32E-10
Il34	-4,4836	0,0034816
Mras	-4,4869	0,00034979
Acod1	-4,6299	0,00012991
Ctsk	-5,424	1,53E-09
Slc9b2	-6,1215	2,70E-06
