# Supplementary material for: Bone, dentin and cementum differentially influence the differentiation of osteoclast-like cells
Source: Sci Rep. 2025 Jun 5;15:19857. doi: 10.1038/s41598-025-04874-9 (PMC12141432; doi:10.1038/s41598-025-04874-9)
Supplement: Supplementary file 14 — Supplementary Information 14. [file 41598_2025_4874_MOESM14_ESM.pdf]

Tab. S13:
Significant transcripts (P<0.05) induced in murine macrophage cells stimulated on bone (n=6), fold of stimulation control

gene name	regulation of expression	adj.P.Val
mt-Tc	47,01339626	4,55E-05
Hspa1b	33,25962506	1,10E-05
Hspa1a	26,32205096	0,00056436
Snord66	26,11487694	0,0020185
mt-Tm	25,22530836	1,21E-07
Snord83b	21,06097031	4,55E-05
Rasd1	20,80560016	0,00042982
Gm26225	17,84935454	0,0022741
mt-Tq	17,64026098	5,21E-05
Gm24631	16,20424615	0,00027129
Snord59a	15,84328945	0,0012494
Hist1h2an	14,8028186	0,0050484
Crtc2	13,81151812	0,0021124
mt-Tl1	13,79142858	8,84E-08
mt-Ta	13,12003806	0,0034301
Gm42793	12,29670602	0,011988
Lgr5	11,6778275	0,036865
Gm42908	11,23945363	0,012768
Rn7sk	10,90486496	1,10E-05
Gm23037	10,62359391	0,00019041
Gm8210	10,49769004	0,039416
Aloxe3	10,22550366	0,00067545
mt-Ti	10,18588903	0,00015586
Gm22513	9,886089723	0,0024583
Gm36989	9,864185975	0,004075
Gm26461	9,538748536	0,015874
Gm43800	9,466295776	0,012661
Gm24991	9,440740342	0,012445
Gm20186	9,380078523	0,0090065
Hspa8	9,213185112	4,92E-05
Gm26202	9,049261122	0,0021314
Gm18709	8,894416725	4,95E-05
Gm44292	8,837265328	0,0003632
4930589O11Rik	8,366939481	0,02083
mt-Tp	8,183397891	2,05E-05
Gdf15	8,148305096	1,26E-06
Kif18b	7,774770017	0,048572
1700054M17Rik	7,766152301	0,0034301
Gm22980	7,761309034	0,0039193
mt-Ts2	7,643323007	0,02078
Rpl7	7,60105672	5,95E-06
Tap2	7,567412035	0,0058505
Gm23969	7,502128633	0,0058505
Gm26810	7,445145275	0,0039193
Gm28404	7,398844611	0,0034301
Gm26594	7,322823381	0,022148
RP24-282K24.4	7,141365707	0,030492
Mcm8	7,114685575	0,00019121

Hist1h4n	6,988583167	0,03058
Gm45220	6,963439102	0,012661
Gm25008	6,944640438	0,0022233
Snord87	6,835277927	0,00065785
Gm45698	6,767861289	0,0027161
Gm8337	6,725772592	0,036865
Hist2h4	6,621233462	0,0133
Tnfsf9	6,495767519	0,0020861
Rbfox1	6,462534154	0,028725
RP23-320D23.6	6,401011941	0,00018781
Gm5830	6,370470815	0,026566
Lgals7	6,351951954	0,025321
4930589L23Rik	6,316826731	0,024686
Gm42743	6,234612954	0,00081241
Gm43714	5,97483967	0,03985
Gm45185	5,880341737	0,017912
Rpl7a	5,804613466	5,08E-05
Gm26772	5,738604705	0,031382
Adam9	5,552740596	0,0056723
Gm42576	5,510182109	0,017908
Gm29358	5,486933154	0,00043436
Snord15a	5,480091569	0,028214
Amd2	5,46643398	0,017751
Rbm3	5,438466847	0,0085734
mt-Tv	5,408018174	0,0056051
Hist1h1d	5,376994515	0,0077135
Sdhd	5,35579225	0,01157
Gm28151	5,338372585	0,046439
Snord89	5,293052633	0,00020982
Gm11491	5,229597817	0,00061649
Gm44270	5,128368746	0,034412
Kcnj2	5,092238532	0,0058096
Gm29170	4,992283971	4,56E-05
4930578M07Rik	4,980187294	0,0015948
Gm44090	4,97122017	0,0066021
2310058D17Rik	4,925260392	0,0080064
Fzd7	4,88784996	0,00012011
Gm3550	4,856779538	0,0015948
1110035H17Rik	4,853414241	0,049107
Ost4	4,739385454	0,0003708
4833421G17Rik	4,715791717	0,048987
Icosl	4,705343348	0,011332
4921507G05Rik	4,705343348	0,045418
Hist1h2be	4,691339797	0,00011336
RP23-205H11.3	4,688739087	0,012862
Bard1	4,667338167	0,016145
Gm38365	4,629639955	0,012661
Rpl27-ps3	4,622265085	0,0079281
Slc16a5	4,590654956	0,043814
Gm43566	4,585884437	0,00025606
Mkln1os	4,569068273	0,029705
Hmgb1	4,517736102	0,0064443

Snord49b	4,487773922	0,00061515
Gadd45g	4,466361422	2,50E-05
Socs1	4,439816345	0,021936
Hist1h4d	4,352366359	0,02342
Rpl35a-ps4	4,329198645	0,0345
Gm44652	4,328298507	0,00043436
4632415L05Rik	4,286796702	0,00033584
9330151L19Rik	4,285014244	0,02057
Gdap10	4,239811134	0,0058505
L1cam	4,234231064	0,022012
D430001F17Rik	4,186370506	0,0044358
A130014A01Rik	4,090848189	0,0058229
Gm4607	3,978707938	0,01444
4930529C04Rik	3,937827734	0,0065559
Tob1	3,911440448	0,0027161
Nup98	3,762000394	0,0059512
Slc12a5	3,750284386	0,0484
Hyal1	3,741715842	5,94E-05
Cd274	3,731614619	0,0070719
Gm7099	3,710722203	0,017946
Gm43328	3,692249385	0,043814
Eno1b	3,674887283	0,010209
Gm37653	3,652033502	0,01917
Pabpn1	3,580596625	0,0066296
Ier5l	3,55611012	0,0010853
Gm26244	3,485583043	0,011988
Rpl10-ps3	3,460306904	0,0080064
RP24-225A16.3	3,456711021	0,016249
Gm13456	3,446662415	0,03058
Gm28727	3,444990489	0,0035031
1810026B05Rik	3,440218027	4,55E-05
Cit	3,407231564	0,0064844
Mterf3	3,40487067	0,048782
D830025C05Rik	3,40416272	0,045087
Gm12902	3,355435442	0,0484
Nat6	3,351483892	0,00043436
Zfp773	3,329025681	0,0063649
Ckap2	3,320268701	0,0034115
Insig1	3,311534756	0,00032407
Gm24916	3,287520749	0,0080135
Wwc1	3,280009515	8,64E-05
Zscan21	3,236646935	0,0074115
Gm15503	3,209614436	0,044617
Btf3	3,207612799	0,043814
Id1	3,194743269	0,00025558
Gm23935	3,128345814	0,0053748
Rpl12	3,11752256	0,0013263
Morf4l1	3,107598241	0,0021292
C230096K16Rik	3,095773668	0,027795
Gmnn	3,085490815	0,0026753
Rps12-ps9	3,061205719	0,0077888
Rpl28-ps1	3,055693835	0,0080064

Gm6136	3,04998046	0,032682
Hnrnpa3	3,049769059	0,0021091
Gm7638	3,044277768	0,018897
Ltb	3,019689057	0,0018327
Msantd2	3,014251926	0,010209
BC055308	3,001742036	0,03058
Ncapd3	2,99675263	0,00043902
RP23-58B7.2	2,994261038	0,0077693
Rps12-ps10	2,994053498	0,0123
Gm10175	2,983901681	0,0034301
Mafk	2,979767981	0,00033584
Rpl19	2,958159822	0,0021292
Tsc22d3	2,943229415	0,0027602
Bst2	2,931014286	0,0011128
Dennd4c	2,925939624	0,0031028
H2afz	2,911979098	0,010209
Eno1	2,90552728	0,0031555
Itgam	2,897482605	0,0080064
Man2b2	2,88885942	0,039416
Rpl13a-ps1	2,862746652	0,0010853
Gadd45b	2,861159648	0,0023695
Rpl31-ps13	2,834904233	0,023181
Rpl39-ps	2,80616531	0,0061956
Gm9320	2,805192936	0,0064443
Nsl1	2,80091849	0,0081476
Ly86	2,795681481	0,00040412
Rsrp1	2,79296985	0,0020261
Rps12-ps4	2,754136378	0,011047
Wdhd1	2,725649399	0,016145
Maff	2,71396102	0,0065631
Sp4	2,713584812	0,02078
Bub1	2,69148055	0,0071014
G2e3	2,691107458	0,0010071
Trmt112	2,651667715	0,0076742
Gm26520	2,651483921	0,010436
Gapdh	2,648728551	0,036327
Raf1	2,647994269	0,048097
Rps28	2,64469252	0,016189
Adcy6	2,62314944	0,046274
Cmtm7	2,619333938	0,0057965
Senp3	2,612262722	0,020114
Zc3h12c	2,608101471	0,016637
Rpl17-ps8	2,602864121	0,03058
Rps17	2,593139825	0,025141
Snhg20	2,578085303	0,0023779
Pigt	2,577727929	0,0345
Stom	2,561164645	0,0052592
Iqgap3	2,556198726	0,02083
Zfp36l2	2,542239587	0,011971
H3f3a	2,50689227	0,0057965
Sh3bgrl2	2,501164594	0,0015076
Anln	2,498565433	0,03658

N4bp2	2,491647533	0,047424
Tnfrsf12a	2,485954687	0,0063006
AW554918	2,483027087	0,026566
Kif11	2,481822607	0,0345
Tbc1d31	2,478384454	0,011988
Rnf24	2,472550522	0,028185
Gm15501	2,470494767	0,024954
Lzic	2,467072307	0,045087
Gm8430	2,466730322	0,010702
Tra2a	2,453089719	0,000844
Gt(ROSA)26Sor	2,424185334	0,011988
Tia1	2,414626421	0,030239
Cln6	2,403438687	0,0016282
Selenbp1	2,392468616	0,019172
Rps27rt	2,385017745	0,011098
RP23-325K4.10	2,382869594	0,016688
Ift80	2,379898425	0,048095
Luc7l3	2,375283969	0,0065559
Pabpc1	2,372651155	0,021282
Basp1	2,367886615	0,021282
Ogt	2,365589918	0,038317
Pi16	2,351856441	0,046274
Fbxo33	2,332860436	0,028214
St3gal4	2,315301381	0,048782
Top2a	2,312254183	0,0030739
Cbx5	2,307131123	0,0031555
Blm	2,305852132	0,033133
Tpt1	2,302019413	0,013485
Vamp2	2,262628926	0,045087
Ifi213	2,247934501	0,035977
Mxd1	2,244353613	0,027709
Pclaf	2,24031252	0,012514
Ier3	2,237519104	0,0018553
Pmf1	2,226688889	0,01989
Anapc5	2,21898513	0,0034301
mt-Rnr1	2,211921215	0,02078
Fam76b	2,207632455	0,040576
Nemp1	2,194816003	0,045875
Arf2	2,186464614	0,0014205
Tsc22d2	2,183889711	0,004355
Tmem259	2,177239326	0,02078
Selenow	2,151137881	0,016724
Gbe1	2,143546925	0,045087
Smg6	2,133171562	0,032513
Piga	2,101908693	0,039915
Neurl1b	2,093330347	0,028214
Cd74	2,092024866	0,023466
Ip6k2	2,091010056	0,036865
Hsp90aa1	2,083920152	0,036249
Rdh13	2,079591264	0,03658
Pabpc4	2,068234829	0,037502
mt-Co1	2,021743538	0,028725

Zfp326	1,998614186	0,03967
Igfbp4	1,978764607	0,011773
Dnajb4	1,969050347	0,03985
Atp6ap1	1,929597576	0,028237
Lig1	1,92612322	0,031498
Ezr	1,924788596	0,0496
Oaz2	1,918528239	0,045087
Slc31a1	1,874622365	0,033365
Mtdh	-0,83275	0,03058
Ckap4	-0,90247	0,049575
Cmas	-0,95921	0,048849
2810025M15Rik	-0,97244	0,036865
Chchd4	-0,98811	0,043814
Dtx4	-1,0173	0,028214
Xbp1	-1,0185	0,042549
Gm26917	-1,0284	0,0068458
Ppan	-1,0445	0,028922
Mrps6	-1,0524	0,033308
Trim11	-1,0533	0,017806
Coro1c	-1,0579	0,0098944
Lrrc47	-1,076	0,01548
Rgs2	-1,0841	0,0066296
Tnfrsf1a	-1,088	0,011705
Mrpl17	-1,0967	0,031498
Zbtb38	-1,1018	0,047134
Grpel1	-1,1104	0,028072
Frrs1	-1,1238	0,011813
Bcar3	-1,1271	0,048097
Scamp1	-1,1316	0,040576
Slc25a33	-1,1342	0,046423
Rnmt	-1,1438	0,011904
Cd63-ps	-1,1535	0,023271
Frmd8	-1,1602	0,014398
Tk2	-1,1673	0,01445
Kcnn4	-1,1737	0,0024583
Tubb6	-1,1766	0,024866
Sphk2	-1,1823	0,01247
BC003965	-1,1847	0,0484
Ptgir	-1,1943	0,049107
Dnajc8	-1,2017	0,0024583
Gnptab	-1,2031	0,0086175
Maf1	-1,2139	0,010854
Psma7	-1,2169	0,004957
Fosl2	-1,222	0,0040886
Pla2g5	-1,2236	0,034412
Bet1l	-1,2336	0,016819
Rnf157	-1,2477	0,02892
Champ1	-1,2654	0,0038989
Nsrp1	-1,2694	0,017566
Fem1a	-1,2703	0,0070719
Exosc4	-1,2706	0,039838
Knop1	-1,2735	0,0034074

Selenos	-1,2736	0,0023795
Cyth4	-1,2778	0,0030739
Ntmt1	-1,2781	0,037009
Sgsm1	-1,2855	0,0008647
Slc43a2	-1,3002	0,0076818
Mgat2	-1,3139	0,041522
Lat2	-1,3377	0,001439
Slc39a11	-1,342	0,0008647
Ubash3b	-1,344	0,0026606
Pigc	-1,3529	0,010696
Zbtb22	-1,3563	0,024424
Pfkfb4	-1,3617	0,022031
Zc3h10	-1,3757	0,020515
Ltbr	-1,3833	0,015369
Zpr1	-1,3862	0,00033584
Slc35f6	-1,3961	0,0052592
Zkscan6	-1,4002	0,031152
Lyl1	-1,4045	0,049107
Mdm2	-1,4101	0,0008647
Srf	-1,417	0,0019979
Keap1	-1,4247	0,0018553
Cbr3	-1,4346	0,0023695
Tgfbr2	-1,4365	0,0059926
Cenpb	-1,4393	0,046274
Paqr7	-1,4447	0,044687
Susd3	-1,4496	0,0018553
Zscan12	-1,4525	0,02342
Frmd4a	-1,4571	0,021936
Cd3eap	-1,4601	0,028725
Prkch	-1,4959	0,0027161
Lima1	-1,5305	0,0050027
2310011J03Rik	-1,5412	0,019015
Mrps35	-1,5482	0,0048132
Ankrd49	-1,557	0,045087
Polr3e	-1,58	0,048782
Eif1ad	-1,5896	0,0024583
Zc3h4	-1,591	0,0023328
Birc3	-1,5996	0,0077888
Sac3d1	-1,6489	0,016819
Alkbh2	-1,6489	0,049107
Ddx28	-1,6615	0,01752
Slc39a1	-1,6687	0,0019979
Igsf3	-1,6773	0,049508
Olfm1	-1,6775	2,27E-05
Cmtr2	-1,6775	0,00085025
Ints5	-1,6804	0,02057
Clec7a	-1,6855	0,0055685
Fosl1	-1,6896	0,021714
4930432K21Rik	-1,7095	0,049329
Pskh1	-1,7127	0,033215
Ino80c	-1,7137	0,0001722
P2ry6	-1,7248	0,0023695

Cd200r2	-1,7273	0,023271
Rab7b	-1,7323	0,00035579
Rcan1	-1,754	0,00025558
Rwdd3	-1,7673	0,046274
Atp6v0d2	-1,7719	3,77E-05
Tlr4	-1,783	0,038791
Dpp3	-1,7874	0,049107
Gcc1	-1,7974	0,00062809
Srxn1	-1,8011	0,0042504
Nop9	-1,8035	0,020571
Irf2bp1	-1,8085	0,00059348
Rftn1	-1,8165	0,0065631
Lpin3	-1,8198	0,034412
Tepsin	-1,8671	0,0073675
Mcat	-1,8789	0,021282
Mir22hg	-1,911	0,00024244
Znhit2	-1,9365	0,028214
Hps6	-1,9393	0,004355
Wdr73	-1,9427	0,011556
Ubiad1	-1,9437	0,032749
Pomgnt1	-1,9708	0,014484
Nrros	-1,9721	4,95E-05
Hemk1	-1,9987	0,043814
Ccdc130	-2,0086	0,0095361
Angptl2	-2,0123	0,00012879
Lcmt2	-2,0164	0,027225
Ptpn7	-2,0183	0,0065559
Ccdc166	-2,0222	0,043814
Sh3rf1	-2,0343	0,025321
Mul1	-2,0372	0,013993
Lysmd4	-2,0494	0,000844
Fam222b	-2,0524	0,026498
Rinl	-2,0842	0,029757
Trim45	-2,0842	0,042372
Zfp768	-2,0848	0,00025558
Ppcs	-2,094	0,018371
Tuba1c	-2,0941	0,00014419
Ctu1	-2,0948	0,0063649
Mrm2	-2,1235	0,016637
Snx19	-2,1371	0,043993
Cttnbp2nl	-2,1378	3,12E-05
Dcstamp	-2,1825	0,000844
Spred1	-2,1887	1,10E-05
Numbl	-2,2014	0,018509
Trib1	-2,2034	0,00031075
Gpatch3	-2,2111	0,043814
Gmppb	-2,2135	0,0065055
Ppp1r10	-2,2165	4,55E-05
Lrrc14	-2,3303	0,016145
AI467606	-2,3307	0,00074579
Zfp61	-2,3668	0,0065631
Trp53rka	-2,4046	0,0027076

Zfp35	-2,4142	0,017726
Mettl18	-2,4172	0,032685
Sla	-2,4732	0,00033584
Il20rb	-2,5016	0,0091506
Slc10a3	-2,5136	0,024686
Sec22a	-2,5606	0,0051
Gipc1	-2,5698	0,0073819
9130019O22Rik	-2,5904	0,043814
Zfp870	-2,6197	0,010436
Zfp90	-2,6246	0,01052
Dlg3	-2,6432	0,011332
Zfp691	-2,6502	0,043894
Tmem204	-2,6533	0,043814
Zfp3	-2,6863	0,011813
Mfsd9	-2,688	0,016819
Tmem51	-2,7229	0,0018327
Gm20632	-2,7926	0,039833
3110082I17Rik	-2,8143	0,0023795
Particl	-2,8485	0,0345
Zfp719	-2,8552	0,037406
D17H6S53E	-2,8942	0,021936
Mir763	-2,9288	0,042142
Commd5	-2,9542	0,00034541
Mras	-2,9716	0,043082
Zfp408	-2,999	0,0014727
Zfp111	-3,0033	0,027065
Epb41l1	-3,0093	0,028185
Zfp41	-3,1422	0,033199
Filip1l	-3,1725	0,0093122
Rbak	-3,2428	0,010357
Ctsk	-3,2534	1,26E-06
Gm11205	-3,5408	0,0020051
Usp27x	-3,9797	0,042549
Slc9b2	-4,0916	0,0073819
