# Supplementary material for: Bone, dentin and cementum differentially influence the differentiation of osteoclast-like cells
Source: Sci Rep. 2025 Jun 5;15:19857. doi: 10.1038/s41598-025-04874-9 (PMC12141432; doi:10.1038/s41598-025-04874-9)
Supplement: Supplementary file 15 — Supplementary Information 15. [file 41598_2025_4874_MOESM15_ESM.pdf]

Tab. S14:
Significant transcripts (P<0.05) induced in murine macrophage cells stimulated on dentin (n=6), fold of stimulation control

gene name	regulation of expression	adj.P.Val
mt-Tc	30,1293992	0,00047184
Gm26225	17,10919528	0,011176
mt-Ta	13,91819287	0,016728
mt-Ti	12,96275083	0,00049497
Gm24991	10,94651703	0,034639
Ccdc33	10,88296682	0,031686
Hist1h2bg	10,83404438	0,029431
4930589O11Rik	10,71009966	0,029431
Rasd1	10,51006728	0,01782
mt-Tq	10,34742581	0,00061947
mt-Tm	10,16402554	4,63E-05
Snord59a	9,65114425	0,022907
Snord83b	8,963113714	0,00049497
Gm23969	8,492562909	0,018926
mt-Tl1	8,218641822	2,36E-06
Hist1h2be	7,751094276	5,04E-05
1700054M17Rik	7,731240943	0,019133
4933437G19Rik	7,076323483	0,033185
Gm24631	6,553656236	0,010145
Gm26202	6,338757165	0,023307
Rn7sk	6,182969976	0,00049497
mt-Tp	6,078435319	0,00032
Gm23037	5,969458218	0,0047342
Hist1h1d	5,95045505	0,022907
Hspa8	5,818712641	0,0012033
mt-Tv	5,098595905	0,029431
Gm25008	5,068293247	0,025215
Rpl7a	4,880402041	0,00061947
Snord87	4,480935624	0,017151
Gdf15	3,719993241	0,0013043
Rpl7	3,638137325	0,0041586
Hspa1b	3,602503919	0,031023
Gadd45g	3,601255603	0,00059897
4930578M07Rik	3,511043815	0,039732
Fzd7	3,458148925	0,0064051
Mcm8	3,393796359	0,045372
Snord89	3,391444773	0,024472
Insig1	3,269114708	0,0012033
Gm18709	3,255321426	0,035021
Hyal1	3,19186581	0,0012033
Ier5l	2,795681481	0,034639
1810026B05Rik	2,792195582	0,0021695
Gm29170	2,768875144	0,025215
Wwc1	2,747082047	0,0033953
Maff	2,664010927	0,031602
Ltb	2,532917346	0,043218
Rpl12	2,53221517	0,045372
Mafk	2,407607139	0,022907

C030034I22Rik	2,368871596	0,045314
Sh3bgrl2	2,167451934	0,045629
Arf2	2,0265138	0,022907
Igfbp4	2,023566134	0,024428
Hmga2	-1,064	0,035021
Gm26917	-1,1177	0,0059982
Bms1	-1,1548	0,024472
Champ1	-1,1635	0,038501
Xbp1	-1,1851	0,020261
Mdm2	-1,1931	0,029431
Frmd8	-1,248	0,019679
Gnptab	-1,2659	0,016928
1700017B05Rik	-1,315	0,017151
Slc43a2	-1,3321	0,019679
Cyth4	-1,4061	0,0021695
Rab7b	-1,4195	0,019679
Olfm1	-1,4273	0,00082689
Gcc1	-1,4712	0,029431
Snord13	-1,515	0,02024
Cx3cr1	-1,5189	0,0070612
Tgfbr2	-1,5598	0,0066269
Birc3	-1,5808	0,029431
P2ry6	-1,5918	0,022056
Zfp768	-1,6864	0,016929
Lima1	-1,6868	0,0046683
Srxn1	-1,7583	0,019679
Nrros	-1,8298	0,00049497
Trib1	-1,884	0,0081259
Lysmd4	-1,9048	0,0075008
Cttnbp2nl	-1,971	0,00034365
AI467606	-2,0712	0,011531
Nlrp3	-2,1025	0,0041506
Mir22hg	-2,1475	0,00010988
Spred1	-2,1953	4,34E-05
Sla	-2,2083	0,0052152
Ppp1r10	-2,2742	9,21E-05
Gm37642	-2,9964	0,039732
Rbak	-3,1235	0,045372
Gm11205	-3,5714	0,0057795
