# Supplementary material for: Bone, dentin and cementum differentially influence the differentiation of osteoclast-like cells
Source: Sci Rep. 2025 Jun 5;15:19857. doi: 10.1038/s41598-025-04874-9 (PMC12141432; doi:10.1038/s41598-025-04874-9)
Supplement: Supplementary file 16 — Supplementary Information 16. [file 41598_2025_4874_MOESM16_ESM.pdf]

Tab. S15:
Significant transcripts (P<0.05) induced in murine macrophage cells stimulated on dentin (n=6), fold of cementum

gene name	regulation of expression	adj.P.Val
Slc9b2	169,4716332	1,00E-07
Olr1	27,57903362	3,95E-05
Slc1a4	26,6285137	0,00031856
Wisp1	18,18655021	0,0022078
Acod1	18,12865516	6,46E-06
Col27a1	17,55974513	8,58E-05
Ablim1	16,96277148	0,00016309
Met	16,15714065	0,00015687
Rab15	15,55277381	0,00018844
Accsl	14,98347136	0,00012484
Scn11a	12,71535644	0,00028595
Ctsk	11,32469272	3,73E-12
Pla2g2d	11,27065925	0,00027516
Slc9b1	10,75622549	0,0042731
Slc30a2	10,38262978	0,031242
Gm29243	10,28236339	0,0073037
Acp5	10,25460488	3,73E-12
Oas1d	9,904608838	0,0080293
Rorc	9,64913756	0,0098535
Gm43154	9,408730002	0,01249
Adh7	9,134970057	0,011657
Rab11fip4	8,477271543	0,020443
Gm19026	8,401809063	0,0092751
Chac1	8,329327391	0,0071948
Ceacam10	8,231184186	0,012947
Lctl	7,986149061	0,006201
RP23-268C22.3	7,656579359	0,020754
Gm16712	7,15623121	0,01414
Slc39a4	7,023547984	0,0075393
Arl14epl	7,003616074	0,042179
4930461G14Rik	6,978901646	0,02307
Gm20219	6,959096437	0,0075112
Prss35	6,850455885	0,0080293
Adamts7	6,801250362	0,00018844
Slc6a4	6,757548682	0,002078
Zbtb45	6,323397882	0,0092751
Robo3	6,278413288	0,00040616
Tmem204	6,082228427	0,000168
Lpar1	6,050691377	0,028491
Rgs20	6,039796745	0,012682
Calml4	6,029339638	0,00012047
Pgap3	5,977739383	0,024612
Pxdn	5,912220186	0,0012399
Dixdc1	5,265607549	0,0043877
Mras	5,169769651	0,0069374
Vegfc	4,951617931	0,031907
Gm5532	4,90583938	0,043232
Gja1	4,680296732	0,024771

Ip6k3	4,655706138	0,00013591
Rap1gap	4,223678432	0,019434
Tspan10	4,003051011	0,0011609
Cox6a2	3,911982726	0,028896
Prkar1b	3,84131427	0,012214
Jdp2	3,753925451	2,32E-05
Bdh2	3,703527372	1,42E-06
Nod1	3,69916589	0,0069864
Epb41l1	3,677435404	0,020443
Gm22748	3,676415944	0,01882
Pdpn	3,526165038	0,0089541
Acsbg1	3,46486706	0,019549
Tbc1d2b	3,318198056	8,65E-08
Timp2	3,309010799	3,16E-05
Fosl2	3,240238483	6,02E-07
Pitpnm2	3,237544449	0,015623
Me1	3,220534172	1,40E-06
Src	3,176856667	0,0012588
Nt5e	3,152289423	0,0075666
Bok	3,115362406	0,020792
Myo1d	3,038585738	2,66E-06
Atp6v0d2	3,037322288	1,88E-06
Sgsh	3,034165962	0,0018871
Wnk2	3,028912706	0,004298
Gcat	2,928374364	0,043164
Hebp2	2,922493873	0,028281
Tmem2	2,879463431	0,00043821
Angptl2	2,879263849	0,00011763
Nudt22	2,859771742	0,0012223
Pmepa1	2,820009456	0,00022712
St18	2,795100195	0,0018142
Aldh1l2	2,766189514	0,038334
Camk2a	2,713208655	0,0089194
Nfatc1	2,691293998	0,00011483
Grap	2,65755583	0,013089
Gsn	2,593139825	7,13E-05
Rcan1	2,581482826	0,00026835
mt-Nd4	2,575049205	0,012145
Lpin3	2,574156915	0,011447
Gpt2	2,567919541	0,0022078
mt-Cytb	2,514201014	0,0034572
Msantd3	2,498565433	0,008397
Fam65c	2,450710381	0,018938
Ccnd2	2,421162649	0,00029291
mt-Nd2	2,388657489	0,0054589
Bcl3	2,321407829	0,03885
Ttc7	2,317870556	0,018938
Cpne2	2,317067383	0,011075
Elk3	2,270798982	0,033583
Fam102a	2,266396062	0,00012913
Glb1	2,247467105	0,00050269
Fblim1	2,247155562	0,014798

Cyfip2	2,198317854	0,0062185
Tlr7	2,183435631	0,021106
Sec16b	2,176334026	0,023276
Sla	2,128592817	0,038097
Cox10	2,115355297	0,0050978
2310061I04Rik	2,112132	0,012682
Shtn1	2,104532808	0,0098535
Slc37a2	2,088837119	0,0054589
Pfkfb4	2,079447123	0,022326
Sgsm1	2,071678312	0,0022956
Tgm2	2,059508385	0,0062693
Cd82	2,05679784	0,024771
Zc3h4	2,052952148	0,012682
Ptpn1	2,034818336	0,006201
Plbd2	2,030450702	0,0036706
Nf2	2,003746499	0,041137
Tfrc	1,996260506	0,042303
Ank	1,993909597	0,0065041
Nrp2	1,989629771	0,0020392
Ivns1abp	1,973969912	0,0019319
Cd33	1,973833092	0,024864
Rai14	1,970142523	0,043232
Plcb4	1,968368044	0,011828
Csf1r	1,939117197	0,015727
Pcyt1a	1,907125785	0,0089541
Cat	1,891853009	0,017149
Lars	1,857806184	0,0437
Atp6ap2	1,824245633	0,033614
Calm1	-0,82689	0,038839
Lbr	-0,8881	0,040201
Rhoc	-0,89076	0,020792
Kif23	-0,89949	0,038839
Hcfc1r1	-0,93418	0,015346
Egln1	-0,95159	0,02137
Pmp22	-0,98688	0,019275
Fam174a	-0,9923	0,045485
Tpi1	-0,99397	0,010493
P4ha1	-1,0138	0,0092751
Ulbp1	-1,0139	0,037646
Ccl4	-1,0372	0,012307
Mb21d1	-1,0514	0,0022078
Pclaf	-1,0621	0,034003
Racgap1	-1,0651	0,028673
Luc7l3	-1,0727	0,031586
Pttg1	-1,0835	0,023228
Mcm7	-1,1033	0,033583
Ptchd1	-1,114	0,008654
Rc3h1	-1,1143	0,017752
Csrnp1	-1,1187	0,013644
Cenpw	-1,1328	0,012679
Etv3	-1,1421	0,012854
Arntl	-1,1488	0,02176

Birc5	-1,1536	0,0019319
Cd300c2	-1,1549	0,0075112
Fam64a	-1,1721	0,043959
Smc2	-1,1725	0,00187
Lsp1	-1,1845	0,0045877
Gatsl2	-1,1855	0,00089493
Sirpa	-1,1871	0,00089358
Ttk	-1,194	0,048201
Zfp367	-1,197	0,022012
Mki67	-1,2069	0,01491
Nemp1	-1,2142	0,012682
Frat2	-1,2276	0,0085486
Cox20-ps	-1,2382	0,0073895
Dcstamp	-1,2406	0,041367
Ska1	-1,2505	0,0030276
Anxa2	-1,2513	0,0018282
Ube2t	-1,2757	0,018938
Gm24276	-1,292	0,0054589
Tiparp	-1,2929	0,0054589
Ezr	-1,2964	0,00028352
Tnfrsf12a	-1,3046	0,0080108
Cfh	-1,309	0,0076129
Sdc3	-1,3138	9,36E-05
Dleu2	-1,319	0,038097
Gadd45b	-1,333	0,0098535
Ier5	-1,3334	0,00041714
Vaultrc5	-1,3434	0,0012318
Basp1	-1,3446	0,005896
Fn1	-1,3451	0,042179
Unc13a	-1,3553	0,039629
BC028528	-1,3585	0,029362
Iqgap3	-1,3596	0,019434
G2e3	-1,3601	0,0010832
Rpl12	-1,3635	0,017975
Pfkfb3	-1,3644	0,0054589
Eno1	-1,3704	0,019275
Gm28555	-1,3719	0,0062023
Sap30	-1,3836	0,0069674
Pif1	-1,3879	0,014798
Gm8292	-1,3897	0,0079839
Gm2830	-1,3918	0,031586
Gtse1	-1,3928	0,020534
RP24-131G14.13	-1,4081	0,02413
Ddit4	-1,4101	0,0022078
Ckap2	-1,4149	0,019434
Atp5g1	-1,4167	0,0060825
S1pr1	-1,4242	0,012947
Kif20b	-1,427	0,0080293
Itga6	-1,4275	0,0098535
Ier5l	-1,4524	0,040882
Lpl	-1,455	7,24E-06
D130051D11Rik	-1,4577	0,043232

Atf3	-1,4595	0,0015748
Errfi1	-1,4778	0,013691
Rad51ap1	-1,4875	0,034003
Gm6341	-1,4925	0,001019
Ciart	-1,4925	0,0098535
Suv39h2	-1,5241	0,02413
Ccdc36	-1,5303	0,036614
Phlda1	-1,5345	0,013698
A330023F24Rik	-1,5431	0,012214
Wfdc17	-1,5529	0,030949
Mmp9	-1,5588	0,048201
Gm28727	-1,5639	0,018913
C530043K16Rik	-1,5761	0,0062693
Gdf15	-1,5766	0,01364
Wwc1	-1,5842	0,0009974
Adcy6	-1,5943	0,0089541
Pbk	-1,5951	0,0015755
Gm11205	-1,6142	0,012214
Gm43581	-1,6164	0,0073895
Gm14286	-1,6189	0,017149
Slc2a1	-1,6317	2,48E-05
Cbx2	-1,6465	0,026766
Gm16045	-1,6503	0,02137
Atr	-1,6533	0,0075159
Gm11470	-1,6543	0,019434
Gm43566	-1,6576	0,017659
Itgax	-1,6721	0,0019907
Atp5l-ps1	-1,6964	0,020792
Lrrc17	-1,699	0,0066386
Crip1	-1,7018	1,00E-07
Sez6	-1,7149	0,0089942
Tstd1	-1,7652	0,015811
Gm14279	-1,7655	0,026699
Hmgb2	-1,7797	0,00068298
Gm43817	-1,7827	0,020815
Nsl1	-1,7836	0,0054603
Mafb	-1,7849	2,82E-06
Ccng2	-1,7865	0,00072708
4932422M17Rik	-1,7953	0,020882
Rpl7	-1,824	0,0055303
Fth-ps3	-1,8333	0,00017112
Klf10	-1,8673	5,69E-06
Arc	-1,8744	0,032708
Gm29170	-1,8821	0,0042024
Dtd2	-1,8966	0,00048142
RP23-205H11.3	-1,9216	0,042374
C730034F03Rik	-1,9224	4,02E-05
Cep55	-1,9236	0,0035476
RP23-356P21.1	-1,9263	0,002955
Gfod2	-1,9362	0,00072627
Gm44258	-1,9537	0,0040511
Gm11491	-1,9853	0,0065356

Gm45203	-1,9941	0,019434
RP23-320D23.6	-2,0184	0,0089942
Gm14636	-2,0253	9,36E-05
Ndrg1	-2,0485	2,03E-08
RP24-175C20.10	-2,0684	0,0018282
Gm8317	-2,0949	0,032937
Gm38365	-2,1054	0,0064666
RP23-350F7.3	-2,1418	0,016945
Car7	-2,1426	0,0065041
Gadd45g	-2,2316	0,00012047
Gm26983	-2,2556	0,024612
A330069E16Rik	-2,266	0,0079839
BC055308	-2,2767	0,00027516
Gm45833	-2,2829	0,0068529
RP23-40D21.1	-2,2991	0,0044878
Egln3	-2,4226	0,028673
Gm8649	-2,4349	4,87E-08
Gm4607	-2,446	0,0099248
Gm10827	-2,5055	4,10E-05
Fzd7	-2,5056	0,0015748
Gm26810	-2,5403	0,039745
Med16	-2,5423	1,84E-06
Mcm8	-2,5943	0,0048431
4632415L05Rik	-2,6023	1,13E-05
Txnip	-2,6264	5,50E-07
Gm37653	-2,6285	0,0030911
Gm45698	-2,6335	0,019533
AY074887	-2,6413	0,018431
2310058D17Rik	-2,6419	0,0042132
Bc1-ps1	-2,6429	0,041367
Gm29438	-2,6777	0,0010196
Id1	-2,7707	1,03E-08
mt-Tm	-2,8223	0,00072708
Rpl30-ps2	-2,8249	0,00014717
Gm43920	-2,8582	0,033587
Ankrd37	-2,9123	3,47E-06
RP23-451J19.1	-2,9201	0,039274
Aloxe3	-2,936	0,016242
Thap8	-2,9681	0,0053822
Adm	-3,0015	7,80E-07
Gm18709	-3,0138	0,0016782
Gapdh	-3,0736	0,00025696
1500004A13Rik	-3,1795	0,00038299
4921507G05Rik	-3,1987	0,038839
Gm27248	-3,2159	0,026766
Rgcc	-3,2168	5,64E-06
1700030M09Rik	-3,2388	0,010821
Sit1	-3,2861	0,0098835
Gm23037	-3,3518	0,02137
Slc16a5	-3,3532	0,025669
Gm26772	-3,4622	0,0021405
Rn7sk	-3,5499	0,0013995

Gm37052	-3,6305	0,023871
Gm44652	-3,691	6,16E-05
Hspa1a	-3,8628	0,01414
4930578M07Rik	-4,0497	0,00071007
Gm29358	-5,5861	0,00012913
Hspa1b	-5,9773	0,00063606
