# Supplementary material for: Bone, dentin and cementum differentially influence the differentiation of osteoclast-like cells
Source: Sci Rep. 2025 Jun 5;15:19857. doi: 10.1038/s41598-025-04874-9 (PMC12141432; doi:10.1038/s41598-025-04874-9)
Supplement: Supplementary file 17 — Supplementary Information 17. [file 41598_2025_4874_MOESM17_ESM.pdf]

Tab. S16:
Significant transcripts (P<0.05) induced in murine macrophage cells stimulated on cementum (n=6), fold of bone

gene name	regulation of expression	adj.P.Val
Gm29358	22,9194	0,0030048
Gad2	11,9249	0,0060419
4930578M07Rik	11,6754	0,0051452
Adm	10,5546	6,65E-08
Rpl30-ps2	9,6318	1,25E-05
Ankrd37	9,5885	3,30E-07
Gm15610	9,0405	0,025306
Gapdh	9,0017	0,00018124
Sit1	8,9154	0,018962
RP23-451J19.1	8,6818	0,024694
Gm44652	8,4649	0,001163
Gm12469	7,8598	0,010843
Gm8317	7,5329	0,00060105
1500004A13Rik	7,5047	0,0016428
Rgcc	7,4426	4,38E-05
Dvl3	6,9649	0,019829
AY074887	6,8642	0,012386
Gm9381	6,7300	0,040865
Itgax	6,7295	8,08E-08
Rn7sk	6,6405	0,032117
Gm8649	6,2745	6,08E-09
Thap8	6,2186	0,02178
Gm3695	5,8547	0,029977
Egln3	5,5995	0,024774
Gm26983	5,5840	0,010482
Gm45833	5,4771	0,002723
RP23-350F7.3	5,3837	0,0040633
Gm10827	5,0585	0,0001613
Gm8623	4,9561	0,035734
Gm11810	4,8006	0,0092825
Med16	4,6803	3,05E-05
Rpl27a-ps1	4,1609	0,027083
Rpl35a-ps5	4,1345	0,00045887
Fzd7	4,0175	0,027583
Dcstamp	3,9417	2,20E-05
Fth-ps3	3,9367	3,84E-05
Rpl30-ps1	3,9250	0,044421
Car7	3,8976	0,020335
Crip1	3,8882	4,63E-09
Tstd1	3,8440	0,0051479
Gadd45g	3,7866	0,001542
Rhob	3,7685	0,0043863
Gm2830	3,7438	0,00050932
RP24-175C20.10	3,7386	0,0066208
Gm8292	3,6493	9,23E-05
Rhov	3,5881	0,033707
Id1	3,5660	5,65E-05
4932422M17Rik	3,5554	0,01937

Gm12182	3,5179	0,045533
Gfod2	3,5110	0,0022557
Mafb	3,4603	3,78E-06
Plk2	3,4448	0,014629
Hist1h2be	3,4331	0,040518
Atp5l-ps1	3,4141	0,014055
Lpl	3,3648	9,96E-08
Ndrg1	3,3309	9,74E-07
Spsb2	3,2736	0,00084546
Gm44258	3,2727	0,02178
Phlda1	3,2034	0,0047153
Gm10260	3,1952	0,016037
Gm14138	3,1744	0,038969
Slc2a1	3,1698	2,13E-05
4632415L05Rik	3,1604	0,01508
Klf10	3,1162	9,95E-05
Gm16045	3,1143	0,025892
Txnip	3,0827	0,0031271
D130051D11Rik	3,0703	0,014055
Gm5905	3,0354	0,030228
Hmgb2	3,0153	0,0043498
Gm11205	2,9972	0,017401
Wfdc17	2,9972	0,027717
2900093K20Rik	2,9963	0,00088797
Nsl1	2,9914	0,025936
Ccng2	2,9789	0,0057048
C730034F03Rik	2,9559	0,0016428
Gsg1	2,8253	0,034283
5830454E08Rik	2,8171	0,033554
Errfi1	2,8104	0,014202
Itga6	2,7357	0,008444
Ang	2,7340	0,029087
Gm6341	2,7268	0,00165
Tnfsf12	2,6838	0,0016035
Coro1a	2,6343	0,0050415
Pop5	2,6317	0,029977
Dtd2	2,6252	0,036873
Cfh	2,6030	0,0034038
Sap30	2,5788	0,0097773
S100a13	2,5626	0,013691
Nsa2	2,5578	0,046282
Gm44913	2,5546	0,045533
Kif20b	2,5498	0,019226
Hbegf	2,5256	0,01252
Ap1s3	2,5221	0,0013752
Rps23-ps2	2,5121	0,03505
Wwc1	2,5112	0,01508
Ciart	2,5008	0,045533
Fam109a	2,4755	0,02477
Rasgef1b	2,4748	0,0065657
Gm15772	2,4494	0,0043863
Sirpa	2,4409	0,0002226

Gm1840	2,4358	0,045533
Atf3	2,4247	0,013308
Polr2l	2,4205	0,034473
Lockd	2,4180	0,033707
Cenpw	2,4145	0,0023164
Tnni2	2,3911	0,017401
Gm5881	2,3883	0,031862
F10	2,3748	0,0050976
Csrnp1	2,3733	0,0033135
Hist1h1c	2,3595	0,013691
Cd200r2	2,3427	0,043091
Ttk	2,3390	0,035734
Rpl36-ps10	2,3230	0,010482
Gm26881	2,3001	0,049512
B3gat3	2,2961	0,0092825
Gm6023	2,2942	0,029087
Rgs2	2,2773	0,00075647
Eno2	2,2575	0,010509
Ezh2	2,2411	0,001265
Cox20-ps	2,2403	0,019184
Cox20	2,2391	0,017384
Pmaip1	2,2375	0,033554
Gm10076	2,2330	0,010044
Lyz1	2,2289	0,00092086
Gm6204	2,1963	0,029252
Sowahc	2,1851	0,030775
Hcfc1r1	2,1839	0,00067372
Sdc3	2,1437	0,0034038
Fam162a	2,1422	0,0032057
AV356131	2,1415	0,029977
Frat2	2,1369	0,042201
Nr2c2ap	2,0959	0,03505
Khk	2,0849	0,013308
Ccl9	2,0832	0,0078764
Birc5	2,0806	0,010482
Rps9	2,0524	0,049748
Taf6	2,0347	0,035466
H2afv	2,0323	0,044652
Arrdc4	2,0289	0,011032
Rpl41	2,0229	0,036873
Ulbp1	2,0209	0,041243
Calm3	2,0181	0,015453
Manbal	2,0161	0,040495
Tusc2	1,9903	0,035734
Ezr	1,9481	0,047667
Calr-ps	1,9461	0,033554
Pltp	1,9289	0,049279
Calm1	1,7737	0,045533
Kdm4a	-0,8834	0,044155
Hnrnpm	-0,88549	0,047667
Ivns1abp	-0,89818	0,01252
Tyk2	-0,91127	0,049748

Acsl4	-0,91902	0,02178
Ppp6r3	-0,93692	0,035734
Slc37a2	-0,96718	0,024639
Map3k7	-0,97244	0,014202
Pcnx3	-0,97356	0,025936
Myof	-0,99303	0,02477
Lypla1	-0,99304	0,046556
Plcb4	-0,99325	0,011298
Shtn1	-0,99908	0,027083
Nrp2	-1,012	0,0016428
Slc43a2	-1,0163	0,045533
Cd82	-1,0174	0,034473
Aak1	-1,0215	0,020398
Me1	-1,025	0,012119
Gdpd1	-1,0493	0,042202
Iars	-1,0614	0,041562
Slc9a8	-1,1117	0,0113
Mtr	-1,1157	0,024371
Mapkapk2	-1,1297	0,001265
Dgkz	-1,1307	0,014055
Ints7	-1,1308	0,044173
Unc93b1	-1,1342	0,010581
Fam102a	-1,1371	0,00035343
Cd33	-1,1422	0,0031271
Blnk	-1,147	0,035466
mt-Cytb	-1,154	0,027442
Lars	-1,1622	0,00073786
Lcp2	-1,1665	0,0085039
Gsn	-1,173	0,001633
Myo1d	-1,1763	0,00099048
Neat1	-1,1807	0,030228
Slpi	-1,1868	0,014055
Tbc1d2b	-1,1931	0,0002226
Dock5	-1,194	0,045533
Nfatc1	-1,2064	0,0022557
Zswim8	-1,2435	0,022162
Tmem8	-1,2689	0,043021
Calml4	-1,3073	0,035473
Pde4dip	-1,3149	0,024647
mt-Nd4	-1,3254	0,021822
Ddx17	-1,3594	0,00077081
Inpp5b	-1,3715	0,043021
Prkca	-1,3832	0,025306
Camk2a	-1,3834	0,011389
Bcl3	-1,4073	0,0083292
Fosl2	-1,4143	3,14E-05
Jdp2	-1,4705	0,00088797
Mybpc3	-1,4908	0,049748
Ccnd2	-1,4938	1,80E-05
St18	-1,5013	0,0012271
Flywch1	-1,5194	0,0083292
Sema4a	-1,524	3,84E-05

Ctsk	-1,5668	7,40E-06
Nf2	-1,5961	2,73E-05
Src	-1,6804	0,00088797
Pxdn	-1,71	0,024614
Ip6k3	-1,7122	0,0011645
Ptpn14	-1,7246	0,031369
Klhl30	-1,7941	0,037363
Wnk2	-1,8819	0,00067372
Tmem204	-1,9094	0,00165
Cpne2	-1,9336	2,20E-05
Tspan10	-1,9609	0,00099957
Gm22	-1,9772	0,033707
Gm20219	-2,0012	0,045533
Pdpn	-2,0702	0,0021725
Gm22748	-2,1105	0,0077153
Zbtb45	-2,1523	0,029977
Pla2g2d	-2,1772	0,0050415
Robo3	-2,2142	0,0012847
Slc6a4	-2,3196	0,0061731
Acp5	-2,3803	2,93E-09
Adamts7	-2,4444	0,00036759
Ehd2	-2,5284	0,011389
Oscar	-2,5715	0,035734
Adh7	-2,6412	0,033173
Chac1	-2,7248	0,012118
Vil1	-2,7259	0,038055
AW047730	-2,7435	0,046556
Arl14epl	-2,8229	0,045533
Gm22767	-2,865	0,035734
Celsr1	-2,883	0,035734
Rap1gap	-2,9068	0,0019943
Met	-2,934	0,00095499
Lctl	-2,9562	0,0087737
Prss42	-2,967	0,013372
Ncmap	-2,9696	0,025466
Prss35	-2,9743	0,0051479
Chd5	-3,015	0,025744
RP23-268C22.3	-3,0336	0,021773
Rab15	-3,0484	0,00073786
Slc30a2	-3,0794	0,035098
Ablim1	-3,5293	0,00068406
Wisp1	-3,5401	0,0075757
Slc1a4	-3,5637	0,0028875
Slc9b2	-3,6296	1,57E-08
Accsl	-3,6764	0,00041588
Acod1	-4,0025	4,90E-06
Scn11a	-4,3635	3,05E-05
Col27a1	-4,7141	3,14E-05
