# Supplementary material for: Bone, dentin and cementum differentially influence the differentiation of osteoclast-like cells
Source: Sci Rep. 2025 Jun 5;15:19857. doi: 10.1038/s41598-025-04874-9 (PMC12141432; doi:10.1038/s41598-025-04874-9)
Supplement: Supplementary file 18 — Supplementary Information 18. [file 41598_2025_4874_MOESM18_ESM.pdf]

Tab. S17:
Significant transcripts (P<0.05) induced in murine macrophage cells stimulated on dentine (n=6), fold of bone

gene name	regulation of expression	adj.P.Val
Ctsk	3,82272124	9,65E-05
