# Supplementary material for: Bone, dentin and cementum differentially influence the differentiation of osteoclast-like cells
Source: Sci Rep. 2025 Jun 5;15:19857. doi: 10.1038/s41598-025-04874-9 (PMC12141432; doi:10.1038/s41598-025-04874-9)
Supplement: Supplementary file 19 — Supplementary Information 19. [file 41598_2025_4874_MOESM19_ESM.pdf]

Tab. S18:

Significant transcripts (P<0.05) induced in murine macrophage cells stimulated on polystyrene (n=6), fold of negative control

gene name	regulation of expression	adj.P.Val
Trib1	5,913859628	0,001392
Cxcl2	5,151169454	0,030839
Egr1	4,966742664	0,023829
Tmem185b	4,569384987	0,038583
Ppp1r10	4,511477529	0,0019331
C3ar1	4,211400264	0,035008
Lysmd4	3,875547138	0,031957
Mir22hg	3,702757325	0,0077196
Nrros	3,419297615	0,0077196
Cttnbp2nl	3,415270858	0,014421
Tuba1c	3,321189403	0,032407
Cmtr2	2,995298949	0,037273
Champ1	2,93609775	0,0066202
Mdm2	2,532917346	0,032407
Mafb	2,508456639	0,030987
Spink5	-1,3087	0,030839
1810026B05Rik	-1,4835	0,03978
Rpl12	-1,6753	0,0451
Rpl7	-2,2194	0,016219
Hspa8	-2,6946	0,03978
Gm45698	-3,7689	0,032407
Snord83b	-3,7928	0,030987
Gm23969	-4,172	0,031957
