# Supplementary material for: Bone, dentin and cementum differentially influence the differentiation of osteoclast-like cells
Source: Sci Rep. 2025 Jun 5;15:19857. doi: 10.1038/s41598-025-04874-9 (PMC12141432; doi:10.1038/s41598-025-04874-9)
Supplement: Supplementary file 20 — Supplementary Information 20. [file 41598_2025_4874_MOESM20_ESM.pdf]

Tab. S19:
Significant transcripts (P<0.05) induced in murine macrophage cells stimulated on dentin (n=6), fold of negative control

gene name	regulation of expression	adj.P.Val
mt-Ti	13,07555247	0,0025879
mt-Tl1	5,404645529	0,00046663
Igf1	3,628315724	4,18E-06
Gdf15	3,541105926	0,011022
Arl4c	2,367558379	0,049241
Gm26917	-1,1867	0,007978
Hmga2	-1,364	0,0049431
Spink5	-1,4422	0,0031561
Treml1	-1,6301	0,043237
Vaultrc5	-1,7788	0,032545
Gm11205	-3,8507	0,027043
