# Supplementary material for: Bone, dentin and cementum differentially influence the differentiation of osteoclast-like cells
Source: Sci Rep. 2025 Jun 5;15:19857. doi: 10.1038/s41598-025-04874-9 (PMC12141432; doi:10.1038/s41598-025-04874-9)
Supplement: Supplementary file 21 — Supplementary Information 21. [file 41598_2025_4874_MOESM21_ESM.pdf]

Tab. S20:

Significant transcripts (P<0.05) induced in murine macrophage cells stimulated on bone (n=6), fold of negative control

gene name	regulation of expression	adj.P.Val
mt-Tc	15,4442724	0,039238
mt-Ts2	13,62420365	0,0090394
mt-Ti	10,27452647	0,0007572
mt-Tl1	9,069355302	4,11E-06
Gdf15	7,756468787	4,11E-06
Gm18709	6,098269886	0,0085007
mt-Tm	5,892173858	0,0097563
Gm29358	4,862168866	0,010814
Fzd7	4,074434957	0,008065
Gm45167	4,011383782	0,010814
S1pr1	3,753665258	0,0007572
Lrrc17	3,575388468	0,023749
Igf1	2,972547781	3,35E-05
Zfp36l2	2,713208655	0,040849
Tnfrsf12a	2,6983921	0,012785
Zfp36l1	2,465362855	0,03277
Gm26917	-1,0975	0,014849
2810025M15Rik	-1,1041	0,046754
Ppic	-1,3434	0,034765
Angptl2	-1,7314	0,0074793
Spink5	-1,9106	4,11E-06
Atp6v0d2	-1,954	4,84E-05
Acp5	-1,9771	0,0097563
Msantd3	-2,3904	0,020257
Itgax	-2,6087	0,0013074
Il20rb	-3,0296	0,041429
Nt5e	-3,0699	0,046754
Rgs16	-3,0972	0,0035645
Bok	-3,2962	0,039238
Gm11205	-3,8202	0,012339
Ctsk	-3,8572	5,41E-06
